# 5‐Lipoxygenase Gene Variants Are Not Associated With Atherosclerosis or Incident Coronary Heart Disease in the Multi‐Ethnic Study of Atherosclerosis Cohort

**DOI:** 10.1161/JAHA.115.002814

**Published:** 2016-03-29

**Authors:** Michael Y. Tsai, Jing Cao, Brian T. Steffen, Natalie L. Weir, Stephen S. Rich, Shuang Liang, Weihua Guan

**Affiliations:** ^1^ Department of Laboratory Medicine and PathologyUniversity of MinnesotaMN; ^2^ Center for Public Health GenomicsUniversity of VirginiaCharlottesvilleVA; ^3^ Division of BiostatisticsSchool of Public HealthUniversity of MinnesotaMinneapolisMN

**Keywords:** *ALOX5*, coronary heart disease, fatty acids, intima‐media thickness, Cardiovascular Disease, Atherosclerosis, Genetic, Association Studies

## Abstract

**Background:**

The arachidonate 5‐lipoxygenase enzyme plays a crucial role in mediating inflammation to maintain homeostasis, yet certain allelic variants of the 5‐lipoxygenase gene, *ALOX5*, may increase risk of atherosclerosis and coronary heart disease (CHD). Further, relations between *ALOX5* and disease outcomes may be enhanced or attenuated depending on the bioavailability of 5‐lipoxygenase enzyme substrates. By using a candidate gene approach in 6153 Multi‐Ethnic Study of Atherosclerosis (MESA) participants, associations were determined among 1348 *ALOX5* single nucleotide polymorphisms (SNPs) and carotid intima‐media thickness (cIMT) as well as incident CHD, and interactions with plasma concentrations of arachidonic acid, eicosapentaenoic acid, or docosahexaenoic acid were tested.

**Methods and Results:**

Multivariable linear regression was used to test for associations between cIMT and *ALOX5* SNPs, and Cox regression was used for incident CHD. Bonferroni correction was used for multiple hypothesis testing. No significant associations between *ALOX5* SNPs and cIMT or CHD events were observed. Levels of arachidonic acid, eicosapentaenoic acid, or docosahexaenoic acid concentrations did not modify the relations of *ALOX5* with either outcome.

**Conclusions:**

*ALOX5* gene variants do not appear to be related to clinical CHD events or subclinical atherosclerosis regardless of bioavailable enzyme substrate levels in this multiethnic cohort. Further studies that directly examine protein expression or enzyme activity may better define the arachidonate 5‐lipoxygenase pathway in disease development and progression.

## Introduction

Arachidonate 5‐lipoxygenase (5‐LOX) is a well‐characterized enzyme that mediates the conversion of arachidonic acid (AA) to leukotriene B4—a crucial step in the acute phase immune response in maintaining homeostasis.^1^ However, recent studies have indicated that 5‐LOX activity may also promote pathophysiological events including atherogenesis and cardiovascular events.^2^ More specifically, single nucleotide polymorphisms (SNPs) in the gene encoding 5‐LOX (*ALOX5*) have been shown to increase inflammatory leukotriene production, which may, in turn, have implications for promoting inappropriate or excessive vascular inflammation resulting in atherosclerosis and eventual coronary heart disease (CHD).^3^, ^4^, ^5^, ^6^, ^7^

To date, a number of well‐powered case‐control studies have examined associations of *ALOX5* gene variants with metrics of atherosclerosis and CHD, yet findings have so far been inconclusive. Positive associations between *ALOX5* and carotid intima‐medial thickness (cIMT) as well as coronary artery stenosis (>80%) were reported by Dwyer et al^8^ and Carlson et al,^9^ respectively. In one of the largest case‐control studies,^9^ a variant in *ALOX5* was shown to associate with the presence of coronary stenosis (>50%) in 3770 individuals; however, investigators were unable to replicate this result in a larger cohort of 13 152 participants of the Atherosclerosis Risk in Communities (ARIC) study.^10^ In the context of CHD event outcomes, null findings between *ALOX5* and myocardial infarction (MI) were previously reported, although a follow‐up interaction analysis revealed that certain *ALOX5* alleles were related to MI but only in those with a high intake of AA.^11^ In total, evidence remains equivocal as to whether *ALOX5* influences atherosclerosis or CHD, and further studies are needed.

To more thoroughly assess whether *ALOX5* is related to atherosclerosis and CHD, a number of interaction terms must be considered. Specifically, levels of polyunsaturated fatty acids (PUFAs) such as AA and the marine omega‐3 eicosapentaenoic acid (EPA) and docosahexaenoic acid (DHA) may modify the relation of *ALOX5* and CHD outcomes as suggested by previous studies.^11^, ^12^ Finally, race/ethnicity remains an important consideration in genetic studies, but whether it may uncover or otherwise modify putative associations of *ALOX5* gene variants and atherosclerosis or CHD outcomes has yet to be examined.

Overall, whether *ALOX5* variants associate with atherosclerosis or CHD is unclear, and potential interactions with PUFAs and race/ethnicity have not been collectively studied in a large cohort population. The present analysis examines common *ALOX5* gene SNPs and their cross‐sectional association with subclinical atherosclerosis determined by common and internal cIMT values in 6153 participants of the Multi‐Ethnic Study of Atherosclerosis (MESA). In addition, their associations with incident CHD during a median 8.5‐year study period were also determined. and modifying influences of plasma phospholipid levels of AA, EPA, and DHA as well as race/ethnicity among black, white, Chinese American, and Hispanic participants were tested.

## Methods

### Study Population

The design of the MESA study has been described previously,^13^ and information about the MESA protocol is available at www.mesa-nhlbi.org. Briefly, 6814 men and women between the ages of 45 and 84 years without clinical evidence of cardiovascular disease were recruited from 6 communities in the United States. Institutional review board approval was obtained at all MESA sites, and all participants gave informed consent.

The current study excluded participants with missing covariates. The remaining study population contains 6153 individuals of the following races/ethnicities: white (n=2477), black (n=1540), Hispanic (n=1399), and Chinese American (n=767). All study participants gave informed consent and were followed for a median follow‐up period of 8.5 years. Age, race/ethnicity, sex, and baseline measurements including hypertension (taking hypertension medication or with systolic blood pressure >140 mm Hg), diabetes (treated or untreated diabetes mellitus as determined by having fasting plasma glucose levels >126 mg/dL according to 2003 American Diabetes Association fasting criteria algorithm),^14^ lipid‐lowering medication, and smoking status (former and current) were recorded. All related laboratory measurements and imaging data were obtained at baseline as well.

### Genotyping

Participants in the original MESA cohort who consented to genetic analyses were genotyped in 2009 with use of the Affymetrix Human SNP array 6.0. Genotype quality control for these data included filtering on an SNP level call rate <95%, an individual level call rate <95%, and heterozygosity >53%, as described previously.^15^ The cleaned genotypic data were deposited with MESA phenotypic data into dbGaP as the MESA SHARe (SNP Health Association Resource) project (study accession phs000209.v2.p1) with 897 981 SNPs passing study‐specific quality control. IMPUTE version 2.2.2 was used to perform imputation for the MESA SHARe participants by using the cosmopolitan 1000 Genomes Phase 1 v3 March 2012 reference set. All 1348 SNPs (35 genotyped, remainder imputed) in the *ALOX5* gene were included in the analysis. SNPs with minor allele frequencies <0.01 and imputation quality <0.3 in a single race group were excluded.

### Laboratory Measurements

Fasting plasma triglyceride, total cholesterol, and high‐density lipoprotein cholesterol (HDL‐C) concentrations were measured as described previously.^16^ Low‐density lipoprotein‐cholesterol (LDL‐C) was calculated based on the Friedewald formula in participants with triglycerides <400 mg/dL. Plasma phospholipid AA (20:4, n‐6), EPA (20:5, n‐3), and DHA (22:6, n‐3) were measured by using gas chromatography flame‐ionization as described previously.^17^ The fatty acids were assayed in 2 batches in 2008 and then in 2012. A shift in fatty acid levels was observed between batches, and assay batch was therefore included as a covariate in the analysis. In terms of reproducibility, correlations of variance for fatty acids were as follows: AA (10.7%), EPA (7.6%), and DHA (8.5%).

### Subclinical Atherosclerosis

The cIMT was assessed at visit 1 by using ultrasonography. Both common and internal cIMT values were included in the analysis. Trained technicians performed B‐mode ultrasonography of the right and left near and far walls of the internal carotid and common carotid arteries by using the Logiq 700 ultrasound device (General Electric Medical Systems) as described previously.^18^ Maximal IMT values of the internal and common carotids were measured as the mean of the maximum IMT values of the near and far walls of the right and left sides. Reproducibility of IMT measurements has previously been assessed by blinded replicate readings, performed by 2 readers. One reader reread 66 studies for an interreader correlation coefficient of 0.84 (n=66), and a second reader reread 48 studies, with a correlation coefficient of 0.86. The rescan and the reread coefficients of variation were 7.07% and 3.48%, respectively.^19^, ^20^

### Incident Coronary Heart Disease

Incident CHD was defined as the first occurrence of MI (n=150), resuscitated cardiac arrest (n=19), CHD death (n=70), or definite angina (n=158). Definite angina was defined as symptoms of typical chest pain and physician diagnosis of angina followed by percutaneous coronary intervention or coronary artery bypass graft surgery, evidence of ischemia on stress tests or resting ECG, or ≥70% obstruction on coronary angiography.

### Statistical Analysis

The fatty acid measures (EPA and DHA) were log‐transformed. Allele frequencies were assessed for each SNP and tested for Hardy‐Weinberg equilibrium separately. SNPs that did not satisfy Hardy‐Weinberg equilibrium were excluded. Multivariable linear regression was used to test for associations between cIMT and *ALOX5* SNPs separately assuming an additive model, with or without SNP–fatty acid interaction. Robust standard error was used when assessing the significance of interactions. Assumptions of linear model were examined and confirmed by using residual plots. Cox proportional hazard regression was used to test for CHD–SNP associations. The proportional hazards assumption was examined by using the scaled Schoenfeld residuals.^21^

Age, sex, body mass index, smoking, systolic blood pressure, hypertension and lipid‐lowering medication use, diabetes, cholesterol LDL, HDL, and (log‐transformed) triglycerides, and batch of fatty acid measure (described earlier) were adjusted. All analyses were performed based on race/ethnicity, a subsequent meta‐analysis was carried out to combine the association results, and the heterogeneity of effect size was further tested. Bonferroni correction was used to account for multiple testing of 1348 SNPs and in 4 race groups.

## Results

Baseline characteristics for 6153 MESA participants, stratified by race/ethnicity, are shown in Table 1. Associations of 1348 *ALOX5* SNPs with common cIMT, internal cIMT, and CHD events were determined. No significant associations in the cohort or within each race/ethnic group were detected. We then tested interactions among *ALOX5* SNPs, the aforementioned outcome variables, and plasma levels of PUFAs AA, EPA, DHA, or their ratios (DHA+EPA):AA, EPA:AA, and DHA:AA. No significant interactions were observed after accounting for multiple testing.

**Table 1:** Characteristics of MESA Participants Across 4 Ethnic Groups at Baseline

	Whites	Blacks	Hispanics	Chinese Americans
n	2447	1540	1399	767
Age, y	63 (54–71)	63 (53–70)	61 (53–69)	62 (53–71)
Sex (male), n	1169 (47.8%)	708 (46.0%)	677 (48.4%)	377 (49.2%)
BMI, n	27 (24–30)	29 (26–34)	29 (26–32)	24 (22–26)
Smoker, n	1373 (56.1%)	850 (55.2%)	644 (46.0%)	191 (24.9%)
Diabetes, n	423 (17.3%)	500 (32.5%)	461 (33.0%)	238 (31.0%)
Hypertension, n	949 (38.8%)	916 (59.5%)	577 (41.2%)	287 (37.4%)
LDL‐C, mg/dL	115 (96–137)	115 (95–136)	119 (97–139)	113 (96–131)
HDL‐C, mg/dL	52 (41–61)	52 (41–61)	48 (39–54)	49 (40–56)
Triglycerides, mg/dL	114 (77–164)	90 (66–123)	136 (97–193)	124 (87–173)
AA (% total)	11.3 (9.7–12.8)	13.2 (11.6–14.8)	10.9 (9.3–12.8)	10.3 (8.9–11.8)
EPA (% total)	0.7 (0.5–1.0)	0.8 (0.5–1.0)	0.5 (0.4–0.7)	0.9 (0.6–1.5)
DHA (% total)	3.2 (2.5–4.2)	4.1 (3.3–5.0)	3.0 (2.3–3.8)	5.0 (4.1–5.9)
Common cIMT, mm	0.8 (0.7–1.0)	0.9 (0.8–1.0)	0.8 (0.7–0.9)	0.8 (0.7–0.9)
Internal cIMT, mm	0.9 (0.7–1.4)	0.9 (0.7–1.3)	0.8 (0.7–1.2)	0.7 (0.6–0.9)
CHD events, n	163 (6.7%)	82 (5.3%)	75 (5.4%)	25 (3.3%)

Median (IQR) values are shown for continuous variables and as counts (%) for categorical variables. Smoker (former and current), diabetes (treated or untreated), hypertensive (systolic blood pressure ≥140 mm Hg or taking hypertension medication). AA indicates arachidonic acid; BMI, body mass index; CHD, coronary heart disease; cIMT, carotid intima‐media thickness; DHA, docosahexaenoic acid; EPA, eicosapentaenoic acid; HDL‐C, high‐density lipoprotein cholesterol; LDL‐C, low‐density lipoprotein cholesterol; MESA, Multi‐Ethnic Study of Atherosclerosis.

Associations for all 1348 SNPs are reported in Supplementary Material. A sample of 7 common SNPs in the *ALOX5* gene that were presented by Assimes et al^10^ was selected for presentation to directly compare results. The allele frequencies of these 7 *ALOX5* SNPs are shown in Table 2. All minor allele frequencies exceeded 1%, with the exception of rs2228064 and rs2228065 in whites and rs41526545 in Chinese Americans.

**Table 2:** Summary of 7 Previously Reported *ALOX5* SNP Allele Frequencies

SNP	Major→Minor Allele	Location	Minor Allele Frequency (%)				
			White	Black	Hispanic	Chinese American	Entire Cohort
rs12762303	T→C	Promotor	16	18	15	20	17
rs2228064	G→A	Thr→Thr in exon 2	0.2	28	7	6	10
rs41526545	A→G	Intron	15	5	9	0.3	10
rs2029253	A→G	Intron	40	29	50	62	42
rs28395866	T→C	Intron	4	26	8	3	10
rs2228065	G→A	Glu→Lys in exon 6		9	1	3	5
rs2229136	A→G	3′ UTR	6	16	6	4	10

Main and interaction effects are shown in Table 3 with SE and *P*‐values specified. Regression coefficients are presented for associations between common or internal IMT outcomes and the specified fatty acid (EPA, DHA, or AA) as well as associations of the specified fatty acid with *ALOX5* variants. Interactions of the *ALOX5* variants, levels of specified fatty acid, and IMT are further indicated. No significant interactions were identified after corrections for multiple testing.

**Table 3:** Main and Interaction Effects With Subclinical Atherosclerosis are Shown for 6153 Participants of the MESA

EPA	SNP ID	EPA			SNP			Interaction		
		β	SE	*P* Value	β	SE	*P* Value	β	SE	*P* Value
Common IMT	rs12762303	0.00097	0.0041	0.813	−0.0049	0.0046	0.282	−0.0087	0.006	0.149
	rs2228064	−0.0021	0.0037	0.566	0.0068	0.0057	0.235	−0.00026	0.0083	0.975
	rs41526545	−0.0013	0.0037	0.724	−0.0098	0.0055	0.075	−0.0048	0.0079	0.544
	rs2029253	−0.0053	0.0053	0.313	−0.0021	0.0033	0.526	0.0036	0.0044	0.421
	rs28395866	−0.0033	0.0037	0.384	0.0094	0.0051	0.064	0.0049	0.0069	0.474
	rs2228065	−0.0016	0.0046	0.723	0.02	0.0096	0.037	0.0045	0.015	0.759
	rs2229136	−0.0025	0.0037	0.508	0.011	0.0065	0.094	0.0022	0.0085	0.796
Internal IMT	rs12762303	−0.022	0.014	0.11	−0.018	0.015	0.232	−0.0095	0.019	0.626
	rs2228064	−0.026	0.012	0.032	−0.019	0.021	0.354	0.0027	0.03	0.929
	rs41526545	−0.026	0.012	0.037	0.014	0.019	0.479	−0.00067	0.027	0.98
	rs2029253	−0.024	0.018	0.18	0.0086	0.012	0.46	−0.0014	0.016	0.933
	rs28395866	−0.031	0.012	0.012	−0.0062	0.019	0.747	0.027	0.028	0.34
	rs2228065	−0.022	0.015	0.142	0.069	0.038	0.074	0.014	0.047	0.768
	rs2229136	−0.026	0.012	0.034	0.032	0.022	0.14	0.0045	0.031	0.882

DHA	SNP ID	DHA			SNP			Interaction		
		β	SE	*P* Value	β	SE	*P* Value	β	SE	*P* Value
Common IMT	rs12762303	−0.008	0.0069	0.243	0.0088	0.014	0.523	−0.0084	0.0098	0.393
	rs2228064	−0.013	0.0062	0.034	−0.002	0.018	0.914	0.0075	0.013	0.569
	rs41526545	−0.011	0.0063	0.087	−0.0041	0.016	0.804	−0.004	0.013	0.757
	rs2029253	−0.0067	0.0084	0.425	0.0031	0.0095	0.742	−0.0053	0.007	0.451
	rs28395866	−0.015	0.0062	0.013	−0.02	0.018	0.255	0.022	0.012	0.078
	rs2228065	−0.0085	0.008	0.287	0.047	0.043	0.28	−0.019	0.028	0.501
	rs2229136	−0.011	0.0062	0.073	0.012	0.019	0.512	−0.0013	0.014	0.923
Internal IMT	rs12762303	−0.061	0.023	0.0066	0.049	0.049	0.312	−0.049	0.035	0.17
	rs2228064	−0.078	0.021	0.00017	−0.027	0.065	0.671	0.012	0.047	0.801
	rs41526545	−0.066	0.021	0.0014	0.092	0.065	0.157	−0.069	0.05	0.172
	rs2029253	−0.078	0.029	0.0072	0.0066	0.034	0.847	0.00059	0.025	0.982
	rs28395866	−0.083	0.021	0.000063	−0.053	0.058	0.36	0.033	0.042	0.429
	rs2228065	−0.048	0.025	0.051	0.189	0.157	0.229	−0.085	0.103	0.409
	rs2229136	−0.082	0.021	0.000092	0.015	0.063	0.818	0.015	0.047	0.751

AA	SNP ID	AA			SNP			Interaction		
		Beta	SE	*P* Value	Beta	SE	*P* Value	Beta	SE	*P* Value
Common IMT	rs12762303	0.031	0.012	0.0066	−0.057	0.048	0.238	0.022	0.02	0.265
	rs2228064	0.034	0.011	0.0025	−0.044	0.059	0.451	0.019	0.023	0.408
	rs41526545	0.042	0.011	0.000083	0.039	0.065	0.553	−0.019	0.027	0.469
	rs2029253	0.04	0.016	0.013	0.0042	0.032	0.896	−0.0026	0.013	0.844
	rs28395866	0.029	0.011	0.009	−0.103	0.055	0.06	0.043	0.022	0.047
	rs2228065	0.042	0.013	0.001	0.043	0.111	0.701	−0.01	0.044	0.813
	rs2229136	0.037	0.011	0.00087	−0.0033	0.066	0.96	0.0046	0.027	0.863
Internal IMT	rs12762303	0.04	0.038	0.301	−0.404	0.149	0.0066	0.16	0.061	0.0094
	rs2228064	0.101	0.037	0.0058	−0.012	0.19	0.951	−0.0063	0.076	0.934
	rs41526545	0.069	0.035	0.048	−0.349	0.206	0.091	0.149	0.086	0.083
	rs2029253	0.106	0.052	0.039	0.041	0.109	0.708	−0.012	0.044	0.792
	rs28395866	0.096	0.036	0.0082	−0.08	0.188	0.67	0.023	0.075	0.762
	rs2228065	0.08	0.042	0.056	−0.052	0.418	0.901	0.044	0.168	0.793
	rs2229136	0.106	0.036	0.0034	0.307	0.201	0.127	−0.113	0.081	0.16

Regression coefficients are shown for associations between (1) IMT outcomes (common or internal) and the specified fatty acid (arachidonic acid [AA], EPA, docosahexaenoic acid [DHA]); (2) the specified fatty acid and *ALOX5* variants (SNP). Interactions of 7 selected *ALOX5* variants, levels of fatty acids, and IMT are further indicated. SE and *P*‐values are specified. EPA indicates eicosapentaenoic acid; IMT, intima‐media thickness; MESA, Multi‐Ethnic Study of Atherosclerosis; SNP, single nucleotide polymorphism.

The marginal associations between 7 *ALOX5* polymorphisms and CHD risk with plasma fatty acids serving as modifying variables are shown in Table 4. In the MESA cohort, only EPA was significantly associated with CHD risk, so only log‐transformed EPA was considered as a modifying variable. Hazard ratios are indicated for associations between EPA and incident CHD as well as EPA and *ALOX5* variants. The interactions of *ALOX5* variants, EPA, and incident CHD are further specified. No associations were found among single SNPs and CHD, including rs12762303, which was previously reported to be tightly linked to promoter region SP1 tandem repeats variation in European Americans.^10^ Associations for all 1348 SNPs are reported in Supplementary Material.

**Table 4:** Main Effect and Interaction Associations With Incident CHD in 6153 Participants of the MESA

SNP ID	EPA			SNP			Interaction			
	β	SE	*P* Value	β	SE	*P* Value	β	SE	*P* Value	RERI
rs12762303	0.753	0.087	0.014	0.891	0.125	0.413	0.908	0.171	0.606	−0.035
rs2228064	0.7	0.076	0.001	0.922	0.179	0.675	1.34	0.342	0.256	0.241
rs41526545	0.716	0.082	0.0036	0.975	0.152	0.871	1.11	0.247	0.644	0.083
rs2029253	0.895	0.134	0.46	0.975	0.099	0.802	0.791	0.098	0.06	−0.18
rs28395866	0.715	0.078	0.0021	0.984	0.158	0.92	1.14	0.281	0.596	0.103
rs2228065	0.681	0.111	0.019	1.31	0.419	0.392	1.57	0.675	0.294	0.41
rs2229136	0.68	0.074	0.0004	1.22	0.191	0.206	1.48	0.356	0.1	0.331

Hazard ratios are shown for associations between (1) incident CHD and EPA and (2) EPA and *ALOX5* SNPs. The interactions of *ALOX5* variants, EPA, and CHD are shown in the last column. CHD indicates coronary heart disease; EPA, eicosapentaenoic acid; MESA, Multi‐Ethnic Study of Atherosclerosis; RERI, relative excess risk as a result of interaction; SNP, single nucleotide polymorphism.

## Discussion

In the present study of 6153 apparently healthy MESA participants at baseline, associations among *ALOX5* SNPs and subclinical atherosclerosis as well as CHD events were examined. Modifying influences of race/ethnicity and plasma PUFAs were also tested. Our results showed no significant associations between 1348 *ALOX5* SNPs and the presence of subclinical atherosclerosis or the occurrence of clinical CHD events. The null findings persisted after including plasma levels of AA, EPA, and DHA as modifying variables.

The LOX‐5 pathway generates key players in the inflammatory response including the series‐4 leukotrienes, which have been shown to mediate chemotaxis and vascular permeability and maintain the overall inflammatory environment.^22^, ^23^ As these processes are also hallmarks of atherogenesis, LOX‐5 activity and *ALOX5* genotypes have been proposed as potential contributors to disease. Human tissue studies as well as those using rodent models of atherosclerosis support a role for the LOX‐5 leukotriene pathway for inducing atherosclerosis.^4^, ^6^, ^7^, ^24^, ^25^, ^26^, ^27^ Corresponding case‐control studies in human subjects have reported positive associations between *ALOX5* and atherosclerosis, though null findings have also been shown. In a cohort of 470 healthy, middle‐aged individuals from the Los Angeles Atherosclerosis Study, participants with 2 copies of variant alleles (deletion or insertion of Sp1 motifs) of a tandem SP1 binding motif polymorphism in the *ALOX5* promoter region showed an increase in cIMT compared with those who carried the wild‐type allele.^8^ This association was enhanced by dietary intake of AA and blunted by intake of long‐chain omega‐3 fatty acids EPA (20:5, n‐3) and DHA (22:6, n‐3). A follow‐up study in a larger case‐control population of Costa Rican individuals (1885 pairs) showed that the variant alleles of the *ALOX5* SP1 motifs were not associated with the occurrence of MI.^11^ However, once dietary PUFA intake was included as a modifying variable, a significant MI risk was observed in subjects with high dietary AA intake, while EPA or DHA showed no modifying effect on the *ALOX5*–MI association.

In contrast to these results, subsequent studies have been unable to demonstrate a relationship between *ALOX5* SNPs and atherosclerosis or CHD. These include a case‐control study of MI in the United Kingdom^28^ and 2 case‐control studies of coronary artery diseases in the United States.^10^ In the latter, Assimes et al genotyped 7 SNPs in *ALOX5* in 1552 cases with clinically significant coronary artery disease and 1583 controls from the Atherosclerotic Disease, Vascular Function, and Genetic Epidemiology cohort and a subcohort of 479 individuals in the Coronary Artery Risk Development in Young Adults study. Although a nominally significant association was detected between SNP rs12762303 and coronary artery disease, the association could not be replicated in the multicenter ARIC cohort, with or without considering gene–dietary fatty acids interaction. Our results agree with this latter finding from ARIC that rs12762303 does not significantly associate with cIMT or CHD risk after adjustment for known risk factors of CHD.

A notable and intriguing aspect of 2 previous studies of *ALOX5* is the purported nutrition–genetics interactions between gene variants and long‐chain omega‐6 and omega‐3 fatty acids. Dwyer et al^8^ reported that the observed association between *ALOX5* SP1 tandem variation and atherosclerosis was modified by dietary intakes of AA, EPA, and DHA. Increased AA intake enhanced the atherogenic effect of the non–wild‐type *ALOX5* genotype, while increased EPA and DHA intake rendered it nonsignificant. In a follow‐up study in a Costa Rican population from the same author group, an *ALOX5* gene–diet interaction was found with AA but not with EPA or DHA. To avoid relying on an estimate of fatty acid intake from dietary questionnaires, we determined the phospholipid fatty acid composition in all of 6153 MESA participants. Using this objective measure shown to relate to bioavailable fatty acids,^17^ we showed no significant interaction with the *ALOX5* SNPs on their association with subclinical atherosclerosis or clinical CHD outcomes. These findings are in agreement with those from the ARIC cohort.^10^

### Strengths and Limitations

The present analysis included a large multiethnic population, and quantified fatty acids were available in all study participants. However, it must be acknowledged that only *ALOX5* genotyping was performed and no corresponding evidence of LOX‐5 protein expression or enzymatic activity was measured. In addition, we did not use an autosomal recessive model for assessing the effect of SP1 tandem variants but instead used a logistic additive model to test the impact of these SNPs. A final limitation of the present analysis is that the vast majority (97.4%) of the 1348 *ALOX5* SNPs were imputed but were subsequently filtered based on imputation quality.

## Conclusions

While previous studies using cell culture, animal models, and human case‐control designs have supported a role for LOX‐5 pathways in the development of atherosclerosis, we found that *ALOX5* SNPs were not associated with either the presence of atherosclerosis or CHD incidence over time—even after considering interactions with plasma fatty acids and race/ethnicity. LOX‐5 protein expression and enzyme activity likely contribute to disease pathogenesis, but *ALOX5* SNPs do not appear to be related to these disease outcomes.

## Sources of Funding

MESA and the MESA SHARe project are conducted and supported by the National Heart, Lung, and Blood Institute in collaboration with MESA investigators. Support for MESA is provided by contracts N01‐HC‐95159, N01‐HC‐95160, N01‐HC‐95161, N01‐HC‐95162, N01‐HC‐95163, N01‐HC‐95164, N01‐HC‐95165, N01‐HC‐95166, N01‐HC‐95167, N01‐HC‐95168, N01‐HC‐95169, UL1‐TR‐001079, UL1‐TR‐000040, and DK063491. Funding for SHARe genotyping was provided by National Heart, Lung, and Blood Institute Contract N02‐HL‐64278. Genotyping was performed at Affymetrix and the Broad Institute of Harvard and MIT using the Affymetrix Genome‐Wide Human SNP Array 6.0.

## Disclosures

None.

## Supporting information


**Table S1A.** Main and Interaction Effects of ALOX5 SNPs and Levels of EPA on Common IMT in All Race/Ethnicity Groups. SNPs with minor allele frequency of <1% were excluded. SE indicates standard error
**Table S1B.** Main and Interaction Effects of ALOX5 SNPs and Levels of EPA on Common IMT in Caucasians
**Table S1C.** Main and Interaction Effects of ALOX5 SNPs and Levels of EPA on Common IMT in Blacks
**Table S1D.** Main and Interaction Effects of ALOX5 SNPs and Levels of EPA on Common IMT in Hispanics
**Table S1E.** Main and Interaction Effects of ALOX5 SNPs and Levels of EPA on Common IMT in Chinese
**Table S2A.** Main and Interaction Effects of ALOX5 SNPs and Levels of DHA on Common IMT in All Race/Ethnicity Groups. SNPs with minor allele frequency of <1% were excluded. SE indicates standard error
**Table S2B.** Main and Interaction Effects of ALOX5 SNPs and Levels of DHA on Common IMT in Caucasians
**Table S2C.** Main and Interaction Effects of ALOX5 SNPs and Levels of DHA on Common IMT in Blacks
**Table S2D.** Main and Interaction Effects of ALOX5 SNPs and Levels of DHA on Common IMT in Hispanics
**Table S2E.** Main and Interaction Effects of ALOX5 SNPs and Levels of DHA on Common IMT in Chinese
**Table S3A.** Main and Interaction Effects of ALOX5 SNPs and Levels of AA on Common IMT in All Race/Ethnicity Groups. SNPs with minor allele frequency of <1% were excluded. SE indicates standard error
**Table S3B.** Main and Interaction Effects of ALOX5 SNPs and Levels of AA on Common IMT in Caucasians
**Table S3C.** Main and Interaction Effects of ALOX5 SNPs and Levels of AA on Common IMT in Blacks
**Table S3D.** Main and Interaction Effects of ALOX5 SNPs and Levels of AA on Common IMT in Hispanics
**Table S3E.** Main and Interaction Effects of ALOX5 SNPs and Levels of AA on Common IMT in Chinese

**Table S4A.** Main and Interaction Effects of ALOX5 SNPs and Levels of EPA on Internal IMT in All Race/Ethnicity Groups. SNPs with minor allele frequency of <1% were excluded. SE indicates standard error
**Table S4B.** Main and Interaction Effects of ALOX5 SNPs and Levels of EPA on Internal IMT in Caucasians
**Table S4C.** Main and Interaction Effects of ALOX5 SNPs and Levels of EPA on Internal IMT in Blacks
**Table S4D.** Main and Interaction Effects of ALOX5 SNPs and Levels of EPA on Internal IMT in Hispanics
**Table S4E.** Main and Interaction Effects of ALOX5 SNPs and Levels of EPA on Internal IMT in Chinese
**Table S5A.** Main and Interaction Effects of ALOX5 SNPs and Levels of DHA on Internal IMT in All Race/Ethnicity Groups. SNPs with minor allele frequency of <1% were excluded. SE indicates standard error
**Table S5B.** Main and Interaction Effects of ALOX5 SNPs and Levels of DHA on Internal IMT in Caucasians
**Table S5C.** Main and Interaction Effects of ALOX5 SNPs and Levels of DHA on Internal IMT in Blacks
**Table S5D.** Main and Interaction Effects of ALOX5 SNPs and Levels of DHA on Internal IMT in Hispanics
**Table S5E.** Main and Interaction Effects of ALOX5 SNPs and Levels of DHA on Internal IMT in Chinese
**Table S6A.** Main and Interaction Effects of ALOX5 SNPs and Levels of AA on Internal IMT in All Race/Ethnicity Groups. SNPs with minor allele frequency of <1% were excluded. SE indicates standard error
**Table S6B.** Main and Interaction Effects of ALOX5 SNPs and Levels of AA on Internal IMT in Caucasians
**Table S6C.** Main and Interaction Effects of ALOX5 SNPs and Levels of AA on Internal IMT in Blacks
**Table S6D.** Main and Interaction Effects of ALOX5 SNPs and Levels of AA on Internal IMT in Hispanics
**Table S6E.** Main and Interaction Effects of ALOX5 SNPs and Levels of AA on Internal IMT in Chinese

**Table S7A.** Main and Interaction Effects of ALOX5 SNPs and Levels of EPA on CHD Risk in All Race/Ethnicity Groups. SNPs with minor allele frequency of <1% were excluded. HR indicates hazard ratio; SE, standard error
**Table S7B.** Main and Interaction Effects of ALOX5 SNPs and Levels of EPA on CHD Risk in Caucasians
**Table S7C.** Main and Interaction Effects of ALOX5 SNPs and Levels of EPA on CHD Risk in Blacks
**Table S7D.** Main and Interaction Effects of ALOX5 SNPs and Levels of EPA on CHD Risk in Hispanics
**Table S7E.** Main and Interaction Effects of ALOX5 SNPs and Levels of EPA on CHD Risk in Chinese
**Table S8A.** Main and Interaction Effects of ALOX5 SNPs and Levels of DHA on CHD Risk in All Race/Ethnicity Groups. SNPs with minor allele frequency of <1% were excluded. HR indicates hazard ratio; SE, standard error
**Table S8B.** Main and Interaction Effects of ALOX5 SNPs and Levels of DHA on CHD Risk in Caucasians
**Table S8C.** Main and Interaction Effects of ALOX5 SNPs and Levels of DHA on CHD Risk in Blacks
**Table S8D.** Main and Interaction Effects of ALOX5 SNPs and Levels of DHA on CHD Risk in Hispanics
**Table S8E.** Main and Interaction Effects of ALOX5 SNPs and Levels of DHA on CHD Risk in Chinese
