# Supplementary material for: 5‐Lipoxygenase Gene Variants Are Not Associated With Atherosclerosis or Incident Coronary Heart Disease in the Multi‐Ethnic Study of Atherosclerosis Cohort
Source: J Am Heart Assoc. 2016 Mar 29;5(3):e002814. doi: 10.1161/JAHA.115.002814 (PMC4943258; doi:10.1161/JAHA.115.002814)
Supplement: Supplementary file 2 — Table S4A. Main and Interaction Effects of ALOX5 SNPs and Levels of EPA on Internal IMT in All Race/Ethnicity Groups. SNPs with minor allele frequency of <1% were excluded. SE indicates standard error Table S4B. Main and Interaction Effects of ALOX5 SNPs and Levels of EPA on Internal IMT in Caucasians Table S4C. Main and Interaction Effects of ALOX5 SNPs and Levels of EPA on Internal IMT in Blacks Table S4D. Main and Interaction Effects of ALOX5 SNPs and Levels of EPA on Internal IMT in Hispanics Table S4E. Main and Interaction Effects of ALOX5 SNPs and Levels of EPA on Internal IMT in Chinese Table S5A. Main and Interaction Effects of ALOX5 SNPs and Levels of DHA on Internal IMT in All Race/Ethnicity Groups. SNPs with minor allele frequency of <1% were excluded. SE indicates standard error Table S5B. Main and Interaction Effects of ALOX5 SNPs and Levels of DHA on Internal IMT in Caucasians Table S5C. Main and Interaction Effects of ALOX5 SNPs and Levels of DHA on Internal IMT in Blacks Table S5D. Main and Interaction Effects of ALOX5 SNPs and Levels of DHA on Internal IMT in Hispanics Table S5E. Main and Interaction Effects of ALOX5 SNPs and Levels of DHA on Internal IMT in Chinese Table S6A. Main and Interaction Effects of ALOX5 SNPs and Levels of AA on Internal IMT in All Race/Ethnicity Groups. SNPs with minor allele frequency of <1% were excluded. SE indicates standard error Table S6B. Main and Interaction Effects of ALOX5 SNPs and Levels of AA on Internal IMT in Caucasians Table S6C. Main and Interaction Effects of ALOX5 SNPs and Levels of AA on Internal IMT in Blacks Table S6D. Main and Interaction Effects of ALOX5 SNPs and Levels of AA on Internal IMT in Hispanics Table S6E. Main and Interaction Effects of ALOX5 SNPs and Levels of AA on Internal IMT in Chinese [file JAH3-5-e002814-s002.docx]

Supplemental table S16.

| phen | covar(fa) | rs_id | maf | beta_FA | se_FA | p_FA | beta_SNP | se_SNP | p_SNP | beta_inter | se_inter | p_inter |
| --- | --- | --- | --- | --- | --- | --- | --- | --- | --- | --- | --- | --- |
| InternalIMT | epa | rs76035182 | 0.024 | -0.016 | 0.014 | 0.26 | -8.80E-03 | 0.038 | 0.816 | -0.078 | 0.056 | 0.166 |
| InternalIMT | epa | rs12359547 | 0.069 | -0.025 | 0.012 | 0.041 | -3.40E-03 | 0.023 | 0.883 | -5.30E-03 | 0.03 | 0.859 |
| InternalIMT | epa | rs74595484 | 0.039 | -0.023 | 0.014 | 0.099 | 0.024 | 0.036 | 0.499 | 0.035 | 0.042 | 0.403 |
| InternalIMT | epa | rs1864415 | 0.701 | -0.016 | 0.028 | 0.553 | -0.01 | 0.012 | 0.404 | -6.60E-03 | 0.017 | 0.701 |
| InternalIMT | epa | rs72788101 | 0.073 | -0.028 | 0.012 | 0.021 | 0.011 | 0.023 | 0.633 | 0.017 | 0.03 | 0.581 |
| InternalIMT | epa | rs11239481 | 0.14 | -0.022 | 0.015 | 0.133 | 0.015 | 0.018 | 0.401 | 0.01 | 0.025 | 0.691 |
| InternalIMT | epa | rs72788102 | 0.073 | -0.028 | 0.012 | 0.021 | 0.011 | 0.023 | 0.632 | 0.017 | 0.03 | 0.58 |
| InternalIMT | epa | rs72789705 | 0.073 | -0.028 | 0.012 | 0.021 | 0.011 | 0.023 | 0.632 | 0.017 | 0.03 | 0.579 |
| InternalIMT | epa | rs141529858 | 0.005 | . | . | . | . | . | . | . | . | . |
| InternalIMT | epa | rs145376292 | 0 | . | . | . | . | . | . | . | . | . |
| InternalIMT | epa | rs190418301 | 0.001 | . | . | . | . | . | . | . | . | . |
| InternalIMT | epa | rs11239482 | 0.073 | -0.028 | 0.012 | 0.021 | 0.011 | 0.023 | 0.631 | 0.017 | 0.03 | 0.578 |
| InternalIMT | epa | rs61855872 | 0.01 | -0.022 | 0.014 | 0.104 | 0.159 | 0.097 | 0.103 | 0.112 | 0.121 | 0.357 |
| InternalIMT | epa | rs148991144 | 0.007 | . | . | . | . | . | . | . | . | . |
| InternalIMT | epa | rs191932221 | 0.005 | . | . | . | . | . | . | . | . | . |
| InternalIMT | epa | rs55978138 | 0.166 | -0.021 | 0.014 | 0.131 | -0.019 | 0.015 | 0.19 | -0.013 | 0.02 | 0.511 |
| InternalIMT | epa | rs73281167 | 0.015 | -0.02 | 0.013 | 0.13 | -0.014 | 0.062 | 0.824 | 0.018 | 0.095 | 0.848 |
| InternalIMT | epa | rs12768010 | 0.086 | -0.02 | 0.013 | 0.126 | -0.038 | 0.018 | 0.035 | -0.028 | 0.023 | 0.218 |
| InternalIMT | epa | rs11239484 | 0.701 | -0.017 | 0.028 | 0.545 | -0.01 | 0.012 | 0.415 | -6.30E-03 | 0.017 | 0.71 |
| InternalIMT | epa | rs35906360 | 0.166 | -0.021 | 0.014 | 0.123 | -0.017 | 0.015 | 0.239 | -0.012 | 0.02 | 0.55 |
| InternalIMT | epa | rs186250512 | 0.003 | . | . | . | . | . | . | . | . | . |
| InternalIMT | epa | rs150810743 | 0.002 | . | . | . | . | . | . | . | . | . |
| InternalIMT | epa | rs55965812 | 0.161 | -0.018 | 0.014 | 0.187 | -0.025 | 0.015 | 0.099 | -0.021 | 0.02 | 0.299 |
| InternalIMT | epa | rs56180358 | 0.167 | -0.021 | 0.014 | 0.123 | -0.017 | 0.015 | 0.239 | -0.012 | 0.02 | 0.551 |
| InternalIMT | epa | rs55864145 | 0.086 | -0.02 | 0.013 | 0.126 | -0.038 | 0.018 | 0.035 | -0.028 | 0.023 | 0.219 |
| InternalIMT | epa | rs55986122 | 0.166 | -0.021 | 0.014 | 0.123 | -0.017 | 0.015 | 0.24 | -0.012 | 0.02 | 0.552 |
| InternalIMT | epa | rs7477350 | 0.701 | -0.017 | 0.028 | 0.543 | -0.01 | 0.012 | 0.418 | -6.30E-03 | 0.017 | 0.712 |
| InternalIMT | epa | rs2099170 | 0.166 | -0.021 | 0.014 | 0.123 | -0.017 | 0.015 | 0.245 | -0.012 | 0.02 | 0.549 |
| InternalIMT | epa | rs2082108 | 0.167 | -0.021 | 0.014 | 0.122 | -0.017 | 0.015 | 0.242 | -0.012 | 0.02 | 0.553 |
| InternalIMT | epa | rs55902404 | 0.007 | . | . | . | . | . | . | . | . | . |
| InternalIMT | epa | rs58402662 | 0.007 | . | . | . | . | . | . | . | . | . |
| InternalIMT | epa | rs11511618 | 0.14 | -0.023 | 0.015 | 0.132 | 0.013 | 0.018 | 0.473 | 0.01 | 0.025 | 0.679 |
| InternalIMT | epa | rs1816492 | 0.7 | -0.016 | 0.028 | 0.557 | -9.50E-03 | 0.012 | 0.441 | -6.70E-03 | 0.017 | 0.694 |
| InternalIMT | epa | rs146611093 | 0.007 | . | . | . | . | . | . | . | . | . |
| InternalIMT | epa | rs186897689 | 0.002 | . | . | . | . | . | . | . | . | . |
| InternalIMT | epa | rs10793616 | 0.701 | -0.017 | 0.028 | 0.541 | -1.00E-02 | 0.012 | 0.42 | -6.20E-03 | 0.017 | 0.715 |
| InternalIMT | epa | rs116700479 | 0.019 | -0.015 | 0.014 | 0.276 | -9.40E-03 | 0.042 | 0.823 | -0.118 | 0.059 | 0.047 |
| InternalIMT | epa | rs192535936 | 0.002 | . | . | . | . | . | . | . | . | . |
| InternalIMT | epa | rs113363297 | 0.662 | -0.018 | 0.026 | 0.484 | -0.01 | 0.013 | 0.42 | -5.30E-03 | 0.017 | 0.758 |
| InternalIMT | epa | rs12354994 | 0.158 | -0.022 | 0.014 | 0.119 | -0.016 | 0.015 | 0.307 | -0.012 | 0.02 | 0.559 |
| InternalIMT | epa | rs148068329 | 0.02 | -0.027 | 0.012 | 0.023 | -0.064 | 0.041 | 0.115 | 0.02 | 0.043 | 0.63 |
| InternalIMT | epa | rs12356109 | 0.171 | -0.021 | 0.014 | 0.129 | -0.021 | 0.015 | 0.155 | -0.011 | 0.019 | 0.558 |
| InternalIMT | epa | rs55908141 | 0.007 | . | . | . | . | . | . | . | . | . |
| InternalIMT | epa | rs12354712 | 0.16 | -0.019 | 0.014 | 0.177 | -0.025 | 0.015 | 0.097 | -0.019 | 0.02 | 0.326 |
| InternalIMT | epa | rs147282684 | 0.003 | . | . | . | . | . | . | . | . | . |
| InternalIMT | epa | rs12356140 | 0.168 | -0.023 | 0.014 | 0.094 | -0.017 | 0.015 | 0.268 | -6.40E-03 | 0.02 | 0.744 |
| InternalIMT | epa | rs185476187 | 0.002 | . | . | . | . | . | . | . | . | . |
| InternalIMT | epa | rs145720057 | 0.026 | -0.024 | 0.014 | 0.078 | 0.089 | 0.052 | 0.09 | 0.101 | 0.074 | 0.174 |
| InternalIMT | epa | rs12354742 | 0.166 | -0.022 | 0.014 | 0.116 | -0.018 | 0.015 | 0.236 | -0.011 | 0.02 | 0.591 |
| InternalIMT | epa | rs180913842 | 0.007 | . | . | . | . | . | . | . | . | . |
| InternalIMT | epa | rs142976914 | 0.003 | . | . | . | . | . | . | . | . | . |
| InternalIMT | epa | rs11511619 | 0.174 | -0.021 | 0.014 | 0.144 | -0.022 | 0.015 | 0.133 | -0.013 | 0.019 | 0.503 |
| InternalIMT | epa | rs146702870 | 0.001 | . | . | . | . | . | . | . | . | . |
| InternalIMT | epa | rs150287496 | 0.005 | . | . | . | . | . | . | . | . | . |
| InternalIMT | epa | rs190917973 | 0.005 | . | . | . | . | . | . | . | . | . |
| InternalIMT | epa | rs11517281 | 0.166 | -0.022 | 0.014 | 0.116 | -0.018 | 0.015 | 0.232 | -0.011 | 0.019 | 0.588 |
| InternalIMT | epa | rs12359299 | 0.168 | -0.021 | 0.014 | 0.124 | -0.019 | 0.015 | 0.204 | -0.011 | 0.02 | 0.558 |
| InternalIMT | epa | rs182307932 | 0.024 | -0.019 | 0.013 | 0.141 | -0.078 | 0.05 | 0.118 | 5.80E-04 | 0.085 | 0.995 |
| InternalIMT | epa | rs61855873 | 0.025 | -0.024 | 0.014 | 0.091 | 0.125 | 0.062 | 0.044 | 0.083 | 0.082 | 0.315 |
| InternalIMT | epa | rs12359302 | 0.164 | -0.022 | 0.014 | 0.118 | -0.016 | 0.015 | 0.292 | -0.011 | 0.02 | 0.563 |
| InternalIMT | epa | rs143208140 | 0.021 | -0.027 | 0.012 | 0.022 | -0.044 | 0.04 | 0.27 | 0.024 | 0.042 | 0.577 |
| InternalIMT | epa | rs9988764 | 0.682 | -0.022 | 0.027 | 0.418 | -0.011 | 0.012 | 0.356 | -2.90E-03 | 0.017 | 0.864 |
| InternalIMT | epa | rs192875767 | 0.006 | . | . | . | . | . | . | . | . | . |
| InternalIMT | epa | rs147268846 | 0.012 | -0.024 | 0.014 | 0.085 | 0.19 | 0.12 | 0.115 | 0.206 | 0.16 | 0.198 |
| InternalIMT | epa | rs11239485 | 0.105 | -0.024 | 0.013 | 0.068 | -0.026 | 0.021 | 0.213 | -6.20E-03 | 0.028 | 0.824 |
| InternalIMT | epa | rs151317885 | 0.424 | -0.02 | 0.019 | 0.271 | 5.40E-03 | 0.012 | 0.65 | -6.00E-03 | 0.016 | 0.71 |
| InternalIMT | epa | rs11499085 | 0.106 | -0.021 | 0.014 | 0.128 | -0.016 | 0.02 | 0.442 | 0.011 | 0.029 | 0.703 |
| InternalIMT | epa | rs188130606 | 0.028 | -0.019 | 0.014 | 0.163 | 8.30E-03 | 0.04 | 0.838 | -0.01 | 0.051 | 0.838 |
| InternalIMT | epa | rs11511620 | 0.16 | -0.019 | 0.014 | 0.17 | -0.025 | 0.015 | 0.101 | -0.019 | 0.02 | 0.348 |
| InternalIMT | epa | rs181498390 | 0.017 | -0.026 | 0.012 | 0.024 | -0.025 | 0.05 | 0.615 | 0.014 | 0.075 | 0.851 |
| InternalIMT | epa | rs186280701 | 0.007 | . | . | . | . | . | . | . | . | . |
| InternalIMT | epa | rs150059185 | 0.028 | -0.023 | 0.012 | 0.057 | -0.031 | 0.04 | 0.436 | -0.057 | 0.059 | 0.34 |

| InternalIMT | epa | rs183338707 | 0.003 | . | . | . | . | . | . | . | . | . |
| --- | --- | --- | --- | --- | --- | --- | --- | --- | --- | --- | --- | --- |
| InternalIMT | epa | rs11499038 | 0.09 | -0.029 | 0.012 | 0.016 | 3.00E-03 | 0.022 | 0.891 | 0.02 | 0.031 | 0.534 |
| InternalIMT | epa | rs190699595 | 0.006 | . | . | . | . | . | . | . | . | . |
| InternalIMT | epa | rs11523408 | 0.164 | -0.022 | 0.014 | 0.121 | -0.018 | 0.015 | 0.239 | -0.011 | 0.02 | 0.559 |
| InternalIMT | epa | rs188049317 | 0.002 | . | . | . | . | . | . | . | . | . |
| InternalIMT | epa | rs144948634 | 0.395 | -0.015 | 0.018 | 0.396 | 9.00E-03 | 0.012 | 0.456 | -0.013 | 0.017 | 0.445 |
| InternalIMT | epa | rs143833576 | 0.003 | . | . | . | . | . | . | . | . | . |
| InternalIMT | epa | rs151239879 | 0.165 | -0.021 | 0.014 | 0.126 | -0.018 | 0.015 | 0.222 | -0.012 | 0.02 | 0.537 |
| InternalIMT | epa | rs7073743 | 0.407 | -0.02 | 0.018 | 0.264 | 0.01 | 0.012 | 0.397 | -6.50E-03 | 0.016 | 0.693 |
| InternalIMT | epa | rs142563349 | 0.03 | -0.019 | 0.014 | 0.165 | -1.90E-03 | 0.04 | 0.961 | -0.01 | 0.051 | 0.841 |
| InternalIMT | epa | rs187246403 | 0.007 | . | . | . | . | . | . | . | . | . |
| InternalIMT | epa | rs150423942 | 0.162 | -0.021 | 0.014 | 0.129 | -0.018 | 0.015 | 0.243 | -0.013 | 0.02 | 0.513 |
| InternalIMT | epa | rs184706102 | 0.002 | . | . | . | . | . | . | . | . | . |
| InternalIMT | epa | rs189626288 | 0.02 | -0.022 | 0.014 | 0.113 | 0.094 | 0.073 | 0.199 | 0.063 | 0.093 | 0.498 |
| InternalIMT | epa | rs140749834 | 0.048 | -0.018 | 0.013 | 0.185 | -0.043 | 0.033 | 0.197 | -0.017 | 0.056 | 0.755 |
| InternalIMT | epa | rs7078822 | 0.133 | -0.016 | 0.014 | 0.243 | -0.04 | 0.017 | 0.018 | -0.031 | 0.022 | 0.169 |
| InternalIMT | epa | rs186227617 | 0.164 | -0.021 | 0.014 | 0.124 | -0.02 | 0.015 | 0.19 | -0.012 | 0.02 | 0.547 |
| InternalIMT | epa | rs188984368 | 0.022 | -0.02 | 0.012 | 0.099 | -7.80E-03 | 0.057 | 0.891 | -0.139 | 0.098 | 0.157 |
| InternalIMT | epa | rs182078109 | 0.031 | -0.019 | 0.014 | 0.17 | 0.049 | 0.057 | 0.388 | -8.00E-03 | 0.077 | 0.917 |
| InternalIMT | epa | rs141456274 | 0.005 | . | . | . | . | . | . | . | . | . |
| InternalIMT | epa | rs146976512 | 0.166 | -0.022 | 0.014 | 0.114 | -0.018 | 0.015 | 0.237 | -0.01 | 0.02 | 0.604 |
| InternalIMT | epa | rs138036816 | 0.1 | -0.022 | 0.014 | 0.115 | -9.40E-03 | 0.022 | 0.667 | 0.014 | 0.032 | 0.666 |
| InternalIMT | epa | rs114879777 | 0.019 | -0.015 | 0.014 | 0.269 | -0.014 | 0.043 | 0.75 | -0.112 | 0.061 | 0.064 |
| InternalIMT | epa | rs149366475 | 0.101 | -0.022 | 0.014 | 0.119 | -0.013 | 0.022 | 0.56 | 0.013 | 0.032 | 0.685 |
| InternalIMT | epa | rs182463043 | 0.002 | . | . | . | . | . | . | . | . | . |
| InternalIMT | epa | rs144754632 | 0.019 | -0.027 | 0.012 | 0.022 | -0.057 | 0.04 | 0.153 | 0.025 | 0.042 | 0.559 |
| InternalIMT | epa | rs55717607 | 0.166 | -0.022 | 0.014 | 0.114 | -0.017 | 0.015 | 0.238 | -0.01 | 0.019 | 0.602 |
| InternalIMT | epa | rs187537973 | 0 | . | . | . | . | . | . | . | . | . |
| InternalIMT | epa | rs148085971 | 0.002 | . | . | . | . | . | . | . | . | . |
| InternalIMT | epa | rs11239491 | 0.166 | -0.022 | 0.014 | 0.113 | -0.017 | 0.015 | 0.239 | -0.01 | 0.019 | 0.604 |
| InternalIMT | epa | rs184412184 | 0.005 | . | . | . | . | . | . | . | . | . |
| InternalIMT | epa | rs4948998 | 0.716 | -0.02 | 0.028 | 0.479 | -8.50E-03 | 0.012 | 0.496 | -4.30E-03 | 0.017 | 0.802 |
| InternalIMT | epa | rs7076696 | 0.716 | -0.019 | 0.028 | 0.496 | -8.90E-03 | 0.012 | 0.475 | -4.70E-03 | 0.017 | 0.78 |
| InternalIMT | epa | rs11239492 | 0.172 | -0.021 | 0.014 | 0.125 | -0.02 | 0.015 | 0.163 | -0.011 | 0.019 | 0.577 |
| InternalIMT | epa | rs79462470 | 0.007 | . | . | . | . | . | . | . | . | . |
| InternalIMT | epa | rs187804654 | 0.003 | . | . | . | . | . | . | . | . | . |
| InternalIMT | epa | rs7093687 | 0.161 | -0.019 | 0.014 | 0.173 | -0.025 | 0.015 | 0.099 | -0.019 | 0.02 | 0.338 |
| InternalIMT | epa | rs7081004 | 0.161 | -0.019 | 0.014 | 0.174 | -0.025 | 0.015 | 0.1 | -0.019 | 0.02 | 0.336 |
| InternalIMT | epa | rs7084793 | 0.166 | -0.022 | 0.014 | 0.113 | -0.017 | 0.015 | 0.239 | -1.00E-02 | 0.019 | 0.609 |
| InternalIMT | epa | rs61556080 | 0.007 | . | . | . | . | . | . | . | . | . |
| InternalIMT | epa | rs115552064 | 0.007 | . | . | . | . | . | . | . | . | . |
| InternalIMT | epa | rs7921503 | 0.166 | -0.022 | 0.014 | 0.113 | -0.018 | 0.015 | 0.236 | -0.01 | 0.019 | 0.608 |
| InternalIMT | epa | rs7909740 | 0.166 | -0.022 | 0.014 | 0.112 | -0.017 | 0.015 | 0.238 | -9.80E-03 | 0.019 | 0.613 |
| InternalIMT | epa | rs7909768 | 0.166 | -0.022 | 0.014 | 0.112 | -0.017 | 0.015 | 0.237 | -9.80E-03 | 0.019 | 0.613 |
| InternalIMT | epa | rs144168004 | 0 | . | . | . | . | . | . | . | . | . |
| InternalIMT | epa | rs75850448 | 0.007 | . | . | . | . | . | . | . | . | . |
| InternalIMT | epa | rs147777574 | 0.006 | . | . | . | . | . | . | . | . | . |
| InternalIMT | epa | rs7913488 | 0.166 | -0.022 | 0.014 | 0.112 | -0.017 | 0.015 | 0.237 | -9.80E-03 | 0.019 | 0.614 |
| InternalIMT | epa | rs74390874 | 0.049 | -0.021 | 0.012 | 0.081 | 0.063 | 0.032 | 0.048 | -0.05 | 0.048 | 0.296 |
| InternalIMT | epa | rs141391142 | 0.006 | . | . | . | . | . | . | . | . | . |
| InternalIMT | epa | rs115294747 | 0.007 | . | . | . | . | . | . | . | . | . |
| InternalIMT | epa | rs148031781 | 0.002 | . | . | . | . | . | . | . | . | . |
| InternalIMT | epa | rs181340128 | 0.018 | -0.022 | 0.014 | 0.108 | 0.063 | 0.082 | 0.442 | 0.077 | 0.104 | 0.46 |
| InternalIMT | epa | rs189930007 | 0.001 | . | . | . | . | . | . | . | . | . |
| InternalIMT | epa | rs12242128 | 0.1 | -0.022 | 0.014 | 0.116 | -0.01 | 0.022 | 0.649 | 0.014 | 0.032 | 0.675 |
| InternalIMT | epa | rs78421750 | 0.006 | . | . | . | . | . | . | . | . | . |
| InternalIMT | epa | rs114370747 | 0.003 | . | . | . | . | . | . | . | . | . |
| InternalIMT | epa | rs12264166 | 0.1 | -0.022 | 0.014 | 0.117 | -1.00E-02 | 0.022 | 0.652 | 0.013 | 0.032 | 0.676 |
| InternalIMT | epa | rs182964822 | 0.001 | . | . | . | . | . | . | . | . | . |
| InternalIMT | epa | rs67491417 | 0.159 | -0.023 | 0.014 | 0.097 | -0.011 | 0.015 | 0.461 | -8.20E-03 | 0.02 | 0.689 |
| InternalIMT | epa | rs72789720 | 0.015 | -0.023 | 0.014 | 0.089 | 0.037 | 0.055 | 0.5 | 0.127 | 0.079 | 0.105 |
| InternalIMT | epa | rs6413416 | 0.167 | -0.022 | 0.014 | 0.11 | -0.018 | 0.015 | 0.225 | -9.40E-03 | 0.019 | 0.627 |
| InternalIMT | epa | rs4986832 | 0.167 | -0.022 | 0.014 | 0.11 | -0.018 | 0.015 | 0.234 | -9.50E-03 | 0.019 | 0.625 |
| InternalIMT | epa | rs144866316 | 0.005 | . | . | . | . | . | . | . | . | . |
| InternalIMT | epa | rs187769540 | 0.002 | . | . | . | . | . | . | . | . | . |
| InternalIMT | epa | rs6593482 | 0.167 | -0.021 | 0.014 | 0.123 | -0.019 | 0.015 | 0.191 | -0.011 | 0.019 | 0.565 |
| InternalIMT | epa | rs7913948 | 0.167 | -0.022 | 0.014 | 0.115 | -0.017 | 0.015 | 0.238 | -0.01 | 0.019 | 0.596 |
| InternalIMT | epa | rs77185151 | 0.019 | -0.027 | 0.012 | 0.022 | -0.057 | 0.04 | 0.153 | 0.024 | 0.042 | 0.567 |
| InternalIMT | epa | rs12762303 | 0.167 | -0.022 | 0.014 | 0.11 | -0.018 | 0.015 | 0.232 | -9.50E-03 | 0.019 | 0.626 |
| InternalIMT | epa | rs73283136 | 0.005 | . | . | . | . | . | . | . | . | . |
| InternalIMT | epa | rs187180218 | 0.002 | . | . | . | . | . | . | . | . | . |
| InternalIMT | epa | rs78137087 | 0.166 | -0.022 | 0.014 | 0.112 | -0.018 | 0.015 | 0.222 | -9.80E-03 | 0.019 | 0.613 |
| InternalIMT | epa | rs72561472 | 0.165 | -0.023 | 0.014 | 0.106 | -0.019 | 0.015 | 0.201 | -8.10E-03 | 0.02 | 0.683 |
| InternalIMT | epa | rs4987106 | 0.162 | -0.021 | 0.014 | 0.122 | -0.021 | 0.015 | 0.17 | -0.012 | 0.02 | 0.558 |

| InternalIMT | epa | rs4987105 | 0.158 | -0.019 | 0.014 | 0.182 | -0.026 | 0.015 | 0.091 | -0.02 | 0.02 | 0.306 |
| --- | --- | --- | --- | --- | --- | --- | --- | --- | --- | --- | --- | --- |
| InternalIMT | epa | rs111916157 | 0.182 | -0.023 | 0.014 | 0.105 | -9.80E-03 | 0.015 | 0.505 | -7.70E-03 | 0.019 | 0.693 |
| InternalIMT | epa | rs111528152 | 0.171 | -0.023 | 0.014 | 0.097 | -0.021 | 0.015 | 0.167 | -6.40E-03 | 0.02 | 0.751 |
| InternalIMT | epa | rs12763738 | 0.166 | -0.022 | 0.014 | 0.108 | -0.017 | 0.015 | 0.241 | -9.10E-03 | 0.02 | 0.643 |
| InternalIMT | epa | rs191675886 | 0.007 | . | . | . | . | . | . | . | . | . |
| InternalIMT | epa | rs66489870 | 0.167 | -0.022 | 0.014 | 0.11 | -0.018 | 0.015 | 0.232 | -9.50E-03 | 0.019 | 0.626 |
| InternalIMT | epa | rs71494775 | 0.167 | -0.022 | 0.014 | 0.11 | -0.018 | 0.015 | 0.232 | -9.50E-03 | 0.019 | 0.626 |
| InternalIMT | epa | rs61855875 | 0.024 | -0.024 | 0.014 | 0.085 | 0.11 | 0.065 | 0.088 | 0.095 | 0.088 | 0.28 |
| InternalIMT | epa | rs116356837 | 0.007 | . | . | . | . | . | . | . | . | . |
| InternalIMT | epa | rs1864414 | 0.166 | -0.022 | 0.014 | 0.116 | -0.017 | 0.015 | 0.244 | -0.01 | 0.019 | 0.591 |
| InternalIMT | epa | rs1864413 | 0.167 | -0.022 | 0.014 | 0.11 | -0.018 | 0.015 | 0.229 | -9.30E-03 | 0.019 | 0.631 |
| InternalIMT | epa | rs139497212 | 0 | . | . | . | . | . | . | . | . | . |
| InternalIMT | epa | rs892690 | 0.328 | -0.024 | 0.017 | 0.148 | 0.012 | 0.013 | 0.362 | -1.80E-03 | 0.017 | 0.917 |
| InternalIMT | epa | rs1864412 | 0.167 | -0.022 | 0.014 | 0.11 | -0.018 | 0.015 | 0.233 | -9.40E-03 | 0.019 | 0.628 |
| InternalIMT | epa | rs77827771 | 0.031 | -0.019 | 0.014 | 0.17 | 0.026 | 0.04 | 0.527 | -0.015 | 0.052 | 0.772 |
| InternalIMT | epa | rs145294963 | 0.004 | . | . | . | . | . | . | . | . | . |
| InternalIMT | epa | rs149081327 | 0.008 | . | . | . | . | . | . | . | . | . |
| InternalIMT | epa | rs67008983 | 0.167 | -0.022 | 0.014 | 0.109 | -0.018 | 0.015 | 0.235 | -9.30E-03 | 0.019 | 0.632 |
| InternalIMT | epa | rs56150414 | 0.17 | -0.022 | 0.014 | 0.12 | -0.017 | 0.015 | 0.26 | -0.011 | 0.019 | 0.575 |
| InternalIMT | epa | rs55787481 | 0.167 | -0.022 | 0.014 | 0.115 | -0.018 | 0.015 | 0.22 | -0.01 | 0.019 | 0.599 |
| InternalIMT | epa | rs141270101 | 0.001 | . | . | . | . | . | . | . | . | . |
| InternalIMT | epa | rs114980075 | 0.008 | . | . | . | . | . | . | . | . | . |
| InternalIMT | epa | rs12783095 | 0.166 | -0.023 | 0.014 | 0.102 | -0.019 | 0.015 | 0.208 | -8.10E-03 | 0.02 | 0.677 |
| InternalIMT | epa | rs182342688 | 0.003 | . | . | . | . | . | . | . | . | . |
| InternalIMT | epa | rs17157728 | 0.166 | -0.023 | 0.014 | 0.102 | -0.019 | 0.015 | 0.209 | -8.10E-03 | 0.02 | 0.678 |
| InternalIMT | epa | rs114835563 | 0.028 | -0.019 | 0.014 | 0.16 | 0.011 | 0.041 | 0.795 | -8.30E-03 | 0.052 | 0.873 |
| InternalIMT | epa | rs3824612 | 0.328 | -0.024 | 0.017 | 0.149 | 0.012 | 0.013 | 0.347 | -1.80E-03 | 0.017 | 0.918 |
| InternalIMT | epa | rs17157733 | 0.165 | -0.023 | 0.014 | 0.101 | -0.019 | 0.015 | 0.209 | -8.00E-03 | 0.02 | 0.683 |
| InternalIMT | epa | rs74128478 | 0.007 | . | . | . | . | . | . | . | . | . |
| InternalIMT | epa | rs184973589 | 0.001 | . | . | . | . | . | . | . | . | . |
| InternalIMT | epa | rs150479461 | 0 | . | . | . | . | . | . | . | . | . |
| InternalIMT | epa | rs138310407 | 0.003 | . | . | . | . | . | . | . | . | . |
| InternalIMT | epa | rs187426603 | 0.001 | . | . | . | . | . | . | . | . | . |
| InternalIMT | epa | rs190843143 | 0.002 | . | . | . | . | . | . | . | . | . |
| InternalIMT | epa | rs191883449 | 0.001 | . | . | . | . | . | . | . | . | . |
| InternalIMT | epa | rs58238740 | 0.163 | -0.023 | 0.014 | 0.089 | -0.017 | 0.015 | 0.248 | -5.90E-03 | 0.02 | 0.765 |
| InternalIMT | epa | rs3780894 | 0.163 | -0.023 | 0.014 | 0.089 | -0.017 | 0.015 | 0.249 | -5.90E-03 | 0.02 | 0.766 |
| InternalIMT | epa | rs149646626 | 0.018 | -0.017 | 0.013 | 0.208 | 0.026 | 0.062 | 0.68 | -0.079 | 0.121 | 0.517 |
| InternalIMT | epa | rs79615212 | 0.016 | -0.021 | 0.014 | 0.121 | -0.032 | 0.065 | 0.626 | 0.042 | 0.072 | 0.562 |
| InternalIMT | epa | rs3780895 | 0.163 | -0.023 | 0.014 | 0.089 | -0.017 | 0.015 | 0.25 | -6.00E-03 | 0.02 | 0.764 |
| InternalIMT | epa | rs3780896 | 0.163 | -0.024 | 0.014 | 0.089 | -0.017 | 0.015 | 0.249 | -5.90E-03 | 0.02 | 0.766 |
| InternalIMT | epa | rs142747163 | 0.005 | . | . | . | . | . | . | . | . | . |
| InternalIMT | epa | rs189417584 | 0.007 | . | . | . | . | . | . | . | . | . |
| InternalIMT | epa | rs3780897 | 0.162 | -0.024 | 0.014 | 0.088 | -0.014 | 0.015 | 0.345 | -5.90E-03 | 0.02 | 0.765 |
| InternalIMT | epa | rs117509491 | 0.009 | . | . | . | . | . | . | . | . | . |
| InternalIMT | epa | rs117811514 | 0.009 | . | . | . | . | . | . | . | . | . |
| InternalIMT | epa | rs116500932 | 0.028 | -0.019 | 0.014 | 0.16 | 0.011 | 0.041 | 0.795 | -8.20E-03 | 0.052 | 0.874 |
| InternalIMT | epa | rs77584168 | 0.004 | . | . | . | . | . | . | . | . | . |
| InternalIMT | epa | rs2310776 | 0.163 | -0.024 | 0.014 | 0.089 | -0.017 | 0.015 | 0.249 | -5.90E-03 | 0.02 | 0.767 |
| InternalIMT | epa | rs17157736 | 0.163 | -0.024 | 0.014 | 0.089 | -0.017 | 0.015 | 0.249 | -5.90E-03 | 0.02 | 0.767 |
| InternalIMT | epa | rs72789735 | 0.065 | -0.026 | 0.012 | 0.034 | -9.50E-03 | 0.024 | 0.69 | -1.50E-04 | 0.032 | 0.996 |
| InternalIMT | epa | rs183747187 | 0.002 | . | . | . | . | . | . | . | . | . |
| InternalIMT | epa | rs7077891 | 0.163 | -0.024 | 0.014 | 0.089 | -0.017 | 0.015 | 0.25 | -5.90E-03 | 0.02 | 0.767 |
| InternalIMT | epa | rs145063305 | 0.003 | . | . | . | . | . | . | . | . | . |
| InternalIMT | epa | rs3824613 | 0.164 | -0.023 | 0.014 | 0.094 | -0.016 | 0.015 | 0.282 | -7.20E-03 | 0.02 | 0.72 |
| InternalIMT | epa | rs145543199 | 0.009 | . | . | . | . | . | . | . | . | . |
| InternalIMT | epa | rs140361993 | 0.003 | . | . | . | . | . | . | . | . | . |
| InternalIMT | epa | rs11239496 | 0.065 | -0.026 | 0.012 | 0.034 | -9.50E-03 | 0.024 | 0.691 | -1.50E-04 | 0.032 | 0.996 |
| InternalIMT | epa | rs11239497 | 0.158 | -0.02 | 0.014 | 0.14 | -0.025 | 0.015 | 0.104 | -0.015 | 0.02 | 0.455 |
| InternalIMT | epa | rs10900212 | 0.184 | -0.027 | 0.014 | 0.053 | -0.011 | 0.014 | 0.441 | 4.60E-03 | 0.019 | 0.804 |
| InternalIMT | epa | rs186487136 | 0 | . | . | . | . | . | . | . | . | . |
| InternalIMT | epa | rs12774369 | 0.163 | -0.023 | 0.014 | 0.089 | -0.018 | 0.015 | 0.243 | -6.00E-03 | 0.02 | 0.762 |
| InternalIMT | epa | rs60842538 | 0.163 | -0.023 | 0.014 | 0.09 | -0.018 | 0.015 | 0.235 | -6.20E-03 | 0.02 | 0.755 |
| InternalIMT | epa | rs3780898 | 0.157 | -0.02 | 0.014 | 0.145 | -0.026 | 0.015 | 0.09 | -0.016 | 0.02 | 0.438 |
| InternalIMT | epa | rs3780899 | 0.163 | -0.023 | 0.014 | 0.091 | -0.018 | 0.015 | 0.223 | -6.50E-03 | 0.02 | 0.745 |
| InternalIMT | epa | rs12252472 | 0.09 | -0.018 | 0.014 | 0.184 | -0.019 | 0.023 | 0.415 | -5.70E-03 | 0.036 | 0.874 |
| InternalIMT | epa | rs185411011 | 0.002 | . | . | . | . | . | . | . | . | . |
| InternalIMT | epa | rs28395865 | 0.155 | -0.02 | 0.014 | 0.145 | -0.027 | 0.015 | 0.083 | -0.016 | 0.02 | 0.436 |
| InternalIMT | epa | rs191004074 | 0.03 | -0.011 | 0.014 | 0.417 | -0.061 | 0.051 | 0.229 | -0.132 | 0.078 | 0.09 |
| InternalIMT | epa | rs182401295 | 0.003 | . | . | . | . | . | . | . | . | . |
| InternalIMT | epa | rs2228064 | 0.093 | -0.026 | 0.012 | 0.032 | -0.019 | 0.021 | 0.354 | 2.70E-03 | 0.03 | 0.929 |
| InternalIMT | epa | rs188284159 | 0.001 | . | . | . | . | . | . | . | . | . |
| InternalIMT | epa | rs2303887 | 0.013 | -0.027 | 0.012 | 0.025 | -0.126 | 0.041 | 2.40E-03 | 5.50E-03 | 0.044 | 0.901 |
| InternalIMT | epa | rs2303886 | 0.016 | -0.028 | 0.012 | 0.017 | -0.08 | 0.042 | 0.057 | 0.046 | 0.047 | 0.322 |

| InternalIMT | epa | rs192621006 | 0.003 | . | . | . | . | . | . | . | . | . |
| --- | --- | --- | --- | --- | --- | --- | --- | --- | --- | --- | --- | --- |
| InternalIMT | epa | rs2303885 | 0.252 | -0.022 | 0.014 | 0.117 | -0.02 | 0.013 | 0.107 | -6.70E-03 | 0.017 | 0.692 |
| InternalIMT | epa | rs114466168 | 0.028 | -0.019 | 0.014 | 0.177 | 0.011 | 0.041 | 0.794 | -0.021 | 0.052 | 0.688 |
| InternalIMT | epa | rs11239501 | 0.088 | -0.026 | 0.012 | 0.033 | 0.023 | 0.02 | 0.258 | 2.20E-03 | 0.028 | 0.937 |
| InternalIMT | epa | rs745986 | 0.18 | -0.021 | 0.014 | 0.132 | -0.017 | 0.014 | 0.221 | -0.012 | 0.019 | 0.521 |
| InternalIMT | epa | rs75262268 | 0.002 | . | . | . | . | . | . | . | . | . |
| InternalIMT | epa | rs71494776 | 0.061 | -0.024 | 0.013 | 0.06 | -0.061 | 0.021 | 3.20E-03 | -0.01 | 0.025 | 0.684 |
| InternalIMT | epa | rs74386871 | 0.02 | -0.029 | 0.012 | 0.016 | -0.079 | 0.042 | 0.062 | 0.044 | 0.046 | 0.341 |
| InternalIMT | epa | rs76502040 | 0.007 | . | . | . | . | . | . | . | . | . |
| InternalIMT | epa | rs142213734 | 0.001 | . | . | . | . | . | . | . | . | . |
| InternalIMT | epa | rs4948671 | 0.714 | -0.022 | 0.027 | 0.422 | -7.30E-03 | 0.012 | 0.555 | -2.70E-03 | 0.017 | 0.873 |
| InternalIMT | epa | rs146286085 | 0.001 | . | . | . | . | . | . | . | . | . |
| InternalIMT | epa | rs144080623 | 0 | . | . | . | . | . | . | . | . | . |
| InternalIMT | epa | rs115074014 | 0.044 | -0.018 | 0.013 | 0.187 | -0.045 | 0.034 | 0.178 | -0.02 | 0.06 | 0.734 |
| InternalIMT | epa | rs17157756 | 0.044 | -0.018 | 0.013 | 0.187 | -0.045 | 0.034 | 0.178 | -0.02 | 0.06 | 0.734 |
| InternalIMT | epa | rs190459780 | 0.001 | . | . | . | . | . | . | . | . | . |
| InternalIMT | epa | rs12358647 | 0.208 | -0.028 | 0.014 | 0.047 | -6.60E-03 | 0.013 | 0.627 | 6.90E-03 | 0.018 | 0.699 |
| InternalIMT | epa | rs147080556 | 0.013 | -0.023 | 0.012 | 0.053 | -0.185 | 0.046 | 6.50E-05 | -0.121 | 0.066 | 0.068 |
| InternalIMT | epa | rs187543349 | 0.005 | . | . | . | . | . | . | . | . | . |
| InternalIMT | epa | rs114375595 | 0.01 | -0.03 | 0.012 | 0.01 | 0.154 | 0.087 | 0.077 | 0.26 | 0.121 | 0.032 |
| InternalIMT | epa | rs117211200 | 0.006 | . | . | . | . | . | . | . | . | . |
| InternalIMT | epa | rs192963047 | 0.001 | . | . | . | . | . | . | . | . | . |
| InternalIMT | epa | rs11239505 | 0.209 | -0.028 | 0.014 | 0.054 | -6.60E-03 | 0.013 | 0.622 | 4.90E-03 | 0.018 | 0.781 |
| InternalIMT | epa | rs184434116 | 0.002 | . | . | . | . | . | . | . | . | . |
| InternalIMT | epa | rs4948999 | 0.715 | -0.022 | 0.027 | 0.419 | -8.50E-03 | 0.012 | 0.493 | -2.50E-03 | 0.017 | 0.883 |
| InternalIMT | epa | rs11239506 | 0.102 | -0.027 | 0.012 | 0.033 | 0.01 | 0.018 | 0.572 | 3.70E-03 | 0.026 | 0.886 |
| InternalIMT | epa | rs190706620 | 0.004 | . | . | . | . | . | . | . | . | . |
| InternalIMT | epa | rs190770429 | 0.002 | . | . | . | . | . | . | . | . | . |
| InternalIMT | epa | rs181327133 | 0.005 | . | . | . | . | . | . | . | . | . |
| InternalIMT | epa | rs149251697 | 0.023 | -0.024 | 0.014 | 0.079 | 0.071 | 0.052 | 0.17 | 0.08 | 0.064 | 0.217 |
| InternalIMT | epa | rs118081602 | 0.013 | -0.027 | 0.012 | 0.025 | -0.125 | 0.041 | 2.50E-03 | 5.90E-03 | 0.044 | 0.893 |
| InternalIMT | epa | rs188233691 | 0.002 | . | . | . | . | . | . | . | . | . |
| InternalIMT | epa | rs183778794 | 0.008 | . | . | . | . | . | . | . | . | . |
| InternalIMT | epa | rs188040078 | 0.006 | . | . | . | . | . | . | . | . | . |
| InternalIMT | epa | rs184675372 | 0.002 | . | . | . | . | . | . | . | . | . |
| InternalIMT | epa | rs150326891 | 0.069 | -0.024 | 0.012 | 0.054 | 0.017 | 0.023 | 0.452 | -0.018 | 0.031 | 0.569 |
| InternalIMT | epa | rs185520021 | 0.006 | . | . | . | . | . | . | . | . | . |
| InternalIMT | epa | rs7907779 | 0.102 | -0.027 | 0.012 | 0.034 | 0.01 | 0.018 | 0.576 | 3.50E-03 | 0.026 | 0.892 |
| InternalIMT | epa | rs184962860 | 0.001 | . | . | . | . | . | . | . | . | . |
| InternalIMT | epa | rs149762784 | 0.037 | -0.022 | 0.012 | 0.08 | -0.04 | 0.044 | 0.361 | -0.057 | 0.057 | 0.315 |
| InternalIMT | epa | rs149203427 | 0.013 | -0.019 | 0.013 | 0.16 | 0.086 | 0.086 | 0.318 | -0.019 | 0.123 | 0.878 |
| InternalIMT | epa | rs60153083 | 0.016 | -0.02 | 0.013 | 0.127 | -0.016 | 0.062 | 0.796 | 0.025 | 0.094 | 0.794 |
| InternalIMT | epa | rs112001069 | 0.076 | -0.021 | 0.013 | 0.094 | -0.05 | 0.019 | 8.30E-03 | -0.022 | 0.024 | 0.364 |
| InternalIMT | epa | rs182355382 | 0.031 | -0.029 | 0.012 | 0.018 | 0.043 | 0.045 | 0.333 | 0.046 | 0.059 | 0.438 |
| InternalIMT | epa | rs186740974 | 0.125 | -0.014 | 0.015 | 0.342 | -0.022 | 0.024 | 0.371 | -0.046 | 0.033 | 0.164 |
| InternalIMT | epa | rs191874916 | 0.036 | -0.023 | 0.012 | 0.053 | 0.016 | 0.037 | 0.656 | -0.041 | 0.058 | 0.477 |
| InternalIMT | epa | rs182989412 | 0.146 | -0.028 | 0.014 | 0.055 | 0.043 | 0.023 | 0.063 | 8.40E-03 | 0.032 | 0.792 |
| InternalIMT | epa | rs188819476 | 0.002 | . | . | . | . | . | . | . | . | . |
| InternalIMT | epa | rs141217183 | 0.093 | -0.025 | 0.013 | 0.065 | -0.046 | 0.019 | 0.017 | -3.80E-03 | 0.023 | 0.871 |
| InternalIMT | epa | rs192922652 | 0.077 | -0.022 | 0.013 | 0.09 | -0.052 | 0.019 | 6.00E-03 | -0.018 | 0.023 | 0.434 |
| InternalIMT | epa | rs185092343 | 0.02 | -0.03 | 0.012 | 0.01 | 0.193 | 0.077 | 0.012 | 0.117 | 0.103 | 0.259 |
| InternalIMT | epa | rs145193311 | 0.08 | -0.02 | 0.013 | 0.112 | -0.047 | 0.019 | 0.012 | -0.026 | 0.023 | 0.26 |
| InternalIMT | epa | rs55789336 | 0.104 | -0.028 | 0.013 | 0.025 | 0.015 | 0.019 | 0.415 | 0.011 | 0.026 | 0.676 |
| InternalIMT | epa | rs55837212 | 0.104 | -0.027 | 0.013 | 0.031 | 0.011 | 0.018 | 0.561 | 6.00E-03 | 0.026 | 0.82 |
| InternalIMT | epa | rs75408574 | 0.36 | -0.023 | 0.017 | 0.181 | 4.80E-03 | 0.012 | 0.702 | -3.40E-03 | 0.017 | 0.843 |
| InternalIMT | epa | rs149306926 | 0.11 | -0.025 | 0.013 | 0.053 | 9.50E-03 | 0.018 | 0.601 | -6.10E-03 | 0.026 | 0.81 |
| InternalIMT | epa | rs35990892 | 0.674 | -2.70E-03 | 0.027 | 0.921 | -0.024 | 0.013 | 0.059 | -0.017 | 0.017 | 0.321 |
| InternalIMT | epa | rs192356207 | 0.05 | -0.028 | 0.012 | 0.022 | 0.031 | 0.031 | 0.316 | 0.017 | 0.042 | 0.69 |
| InternalIMT | epa | rs183741163 | 0.102 | -0.027 | 0.013 | 0.035 | 0.019 | 0.022 | 0.387 | 6.30E-03 | 0.03 | 0.832 |
| InternalIMT | epa | rs34737297 | 0.555 | -0.031 | 0.024 | 0.191 | 4.10E-03 | 0.013 | 0.757 | 5.00E-03 | 0.018 | 0.785 |
| InternalIMT | epa | rs181346933 | 0.03 | -0.012 | 0.014 | 0.411 | -0.07 | 0.038 | 0.068 | -0.128 | 0.061 | 0.035 |
| InternalIMT | epa | rs36093253 | 0.361 | -0.022 | 0.018 | 0.219 | -3.40E-03 | 0.013 | 0.796 | -4.90E-03 | 0.017 | 0.778 |
| InternalIMT | epa | rs186307855 | 0.099 | -0.015 | 0.014 | 0.294 | -0.022 | 0.023 | 0.345 | -0.025 | 0.035 | 0.476 |
| InternalIMT | epa | rs189681865 | 0.034 | -0.026 | 0.014 | 0.066 | 4.40E-03 | 0.036 | 0.903 | 0.081 | 0.042 | 0.051 |
| InternalIMT | epa | rs34942971 | 0.214 | -0.024 | 0.015 | 0.103 | -9.70E-03 | 0.014 | 0.475 | -3.90E-03 | 0.018 | 0.829 |
| InternalIMT | epa | rs181816554 | 0.033 | -0.018 | 0.014 | 0.188 | 0.029 | 0.054 | 0.589 | -0.025 | 0.076 | 0.746 |
| InternalIMT | epa | rs186846881 | 0.094 | -0.019 | 0.014 | 0.166 | -9.30E-03 | 0.023 | 0.681 | -1.20E-03 | 0.035 | 0.972 |
| InternalIMT | epa | rs191178220 | 0.016 | -0.025 | 0.012 | 0.036 | 0.068 | 0.057 | 0.229 | -0.032 | 0.088 | 0.715 |
| InternalIMT | epa | rs183305228 | 0.016 | -0.021 | 0.013 | 0.125 | -0.013 | 0.061 | 0.829 | 0.028 | 0.092 | 0.756 |
| InternalIMT | epa | rs139912761 | 0.121 | -0.031 | 0.013 | 0.017 | 0.022 | 0.018 | 0.214 | 0.02 | 0.024 | 0.411 |
| InternalIMT | epa | rs185499901 | 0.018 | -0.02 | 0.014 | 0.151 | 0.113 | 0.076 | 0.134 | -0.016 | 0.13 | 0.904 |
| InternalIMT | epa | rs189816197 | 0.087 | -0.021 | 0.013 | 0.108 | -0.048 | 0.019 | 0.011 | -0.024 | 0.024 | 0.315 |
| InternalIMT | epa | rs145656938 | 0.026 | -0.029 | 0.012 | 0.016 | 0.019 | 0.045 | 0.677 | 0.064 | 0.051 | 0.204 |
| InternalIMT | epa | rs35745197 | 0.076 | -0.022 | 0.013 | 0.09 | -0.05 | 0.019 | 8.90E-03 | -0.02 | 0.024 | 0.405 |

| InternalIMT | epa | rs192856637 | 0.005 | . | . | . | . | . | . | . | . | . |
| --- | --- | --- | --- | --- | --- | --- | --- | --- | --- | --- | --- | --- |
| InternalIMT | epa | rs2310775 | 0.415 | -0.021 | 0.018 | 0.246 | 4.60E-03 | 0.012 | 0.696 | -5.20E-03 | 0.016 | 0.748 |
| InternalIMT | epa | rs11239507 | 0.506 | -0.019 | 0.019 | 0.326 | -3.50E-03 | 0.011 | 0.753 | -6.80E-03 | 0.015 | 0.653 |
| InternalIMT | epa | rs74648467 | 0.062 | -0.024 | 0.013 | 0.051 | -0.063 | 0.021 | 2.50E-03 | -4.10E-03 | 0.025 | 0.869 |
| InternalIMT | epa | rs145815934 | 0.015 | -0.019 | 0.014 | 0.17 | 0.035 | 0.065 | 0.587 | -0.026 | 0.083 | 0.756 |
| InternalIMT | epa | rs145390687 | 0.02 | -0.023 | 0.014 | 0.089 | 0.149 | 0.083 | 0.074 | 0.12 | 0.11 | 0.274 |
| InternalIMT | epa | rs184354913 | 0.034 | -0.019 | 0.014 | 0.17 | 0.033 | 0.043 | 0.449 | -9.00E-03 | 0.067 | 0.893 |
| InternalIMT | epa | rs181244920 | 0.006 | . | . | . | . | . | . | . | . | . |
| InternalIMT | epa | rs11499087 | 0.055 | -0.017 | 0.013 | 0.201 | -0.058 | 0.029 | 0.049 | -0.022 | 0.05 | 0.669 |
| InternalIMT | epa | rs148263794 | 0.08 | -0.021 | 0.013 | 0.111 | -0.051 | 0.018 | 5.60E-03 | -0.025 | 0.024 | 0.28 |
| InternalIMT | epa | rs150758903 | 0.036 | -0.024 | 0.014 | 0.089 | -3.30E-03 | 0.038 | 0.93 | 0.055 | 0.044 | 0.213 |
| InternalIMT | epa | rs147654382 | 0.179 | -0.029 | 0.014 | 0.037 | -1.80E-03 | 0.015 | 0.904 | 0.01 | 0.02 | 0.612 |
| InternalIMT | epa | rs147495166 | 0.025 | -0.017 | 0.014 | 0.223 | -1.60E-03 | 0.04 | 0.969 | -0.055 | 0.047 | 0.241 |
| InternalIMT | epa | rs35198778 | 0.106 | -0.023 | 0.014 | 0.092 | -0.025 | 0.021 | 0.233 | -0.011 | 0.025 | 0.661 |
| InternalIMT | epa | rs145207659 | 0.049 | -0.023 | 0.012 | 0.057 | 0.079 | 0.033 | 0.016 | -0.028 | 0.049 | 0.576 |
| InternalIMT | epa | rs186466464 | 0.01 | -0.022 | 0.013 | 0.101 | 0.065 | 0.104 | 0.535 | 0.088 | 0.115 | 0.447 |
| InternalIMT | epa | rs12778413 | 0.162 | -0.026 | 0.014 | 0.061 | 2.00E-03 | 0.017 | 0.905 | 1.60E-03 | 0.022 | 0.944 |
| InternalIMT | epa | rs7895552 | 0.64 | -0.016 | 0.026 | 0.541 | -0.011 | 0.013 | 0.378 | -7.70E-03 | 0.017 | 0.651 |
| InternalIMT | epa | rs12783895 | 0.078 | -0.021 | 0.013 | 0.097 | -0.05 | 0.019 | 7.90E-03 | -0.022 | 0.024 | 0.356 |
| InternalIMT | epa | rs34929604 | 0.079 | -0.021 | 0.013 | 0.106 | -0.048 | 0.019 | 9.20E-03 | -0.025 | 0.024 | 0.292 |
| InternalIMT | epa | rs12761961 | 0.075 | -0.022 | 0.013 | 0.081 | -0.047 | 0.02 | 0.016 | -0.017 | 0.024 | 0.486 |
| InternalIMT | epa | rs35536930 | 0.206 | -0.027 | 0.014 | 0.055 | -6.50E-03 | 0.013 | 0.632 | 4.50E-03 | 0.018 | 0.802 |
| InternalIMT | epa | rs180951060 | 0.002 | . | . | . | . | . | . | . | . | . |
| InternalIMT | epa | rs148892128 | 0.015 | -0.019 | 0.014 | 0.17 | 0.035 | 0.065 | 0.586 | -0.026 | 0.083 | 0.757 |
| InternalIMT | epa | rs11239509 | 0.084 | -0.015 | 0.014 | 0.277 | -0.035 | 0.023 | 0.123 | -0.027 | 0.035 | 0.436 |
| InternalIMT | epa | rs41526545 | 0.093 | -0.026 | 0.012 | 0.037 | 0.014 | 0.019 | 0.479 | -6.70E-04 | 0.027 | 0.98 |
| InternalIMT | epa | rs2029253 | 0.421 | -0.024 | 0.018 | 0.18 | 8.60E-03 | 0.012 | 0.46 | -1.40E-03 | 0.016 | 0.933 |
| InternalIMT | epa | rs17444064 | 0.092 | -0.026 | 0.012 | 0.038 | 0.019 | 0.019 | 0.317 | -1.10E-03 | 0.027 | 0.966 |
| InternalIMT | epa | rs71494779 | 0.08 | -0.02 | 0.013 | 0.112 | -0.048 | 0.018 | 9.50E-03 | -0.027 | 0.023 | 0.256 |
| InternalIMT | epa | rs74991420 | 0.012 | -0.027 | 0.012 | 0.024 | -0.129 | 0.047 | 5.80E-03 | 5.50E-03 | 0.05 | 0.911 |
| InternalIMT | epa | rs182736551 | 0.012 | -0.027 | 0.012 | 0.024 | -0.129 | 0.047 | 5.80E-03 | 5.50E-03 | 0.05 | 0.911 |
| InternalIMT | epa | rs78702074 | 0.013 | -0.026 | 0.012 | 0.026 | -0.13 | 0.042 | 1.90E-03 | 3.30E-03 | 0.044 | 0.94 |
| InternalIMT | epa | rs187791466 | 0.004 | . | . | . | . | . | . | . | . | . |
| InternalIMT | epa | rs35300270 | 0.082 | -0.02 | 0.013 | 0.12 | -0.048 | 0.018 | 8.20E-03 | -0.027 | 0.023 | 0.243 |
| InternalIMT | epa | rs6593484 | 0.102 | -0.027 | 0.013 | 0.034 | 9.40E-03 | 0.018 | 0.61 | 3.50E-03 | 0.026 | 0.893 |
| InternalIMT | epa | rs12244601 | 0.023 | -0.023 | 0.014 | 0.095 | 6.00E-03 | 0.043 | 0.889 | 0.064 | 0.049 | 0.191 |
| InternalIMT | epa | rs184429574 | 0.005 | . | . | . | . | . | . | . | . | . |
| InternalIMT | epa | rs7080897 | 0.08 | -0.02 | 0.013 | 0.112 | -0.047 | 0.018 | 9.60E-03 | -0.027 | 0.023 | 0.256 |
| InternalIMT | epa | rs7097429 | 0.083 | -0.02 | 0.013 | 0.123 | -0.046 | 0.018 | 0.011 | -0.029 | 0.024 | 0.226 |
| InternalIMT | epa | rs77979229 | 0.003 | . | . | . | . | . | . | . | . | . |
| InternalIMT | epa | rs11239512 | 0.103 | -0.026 | 0.013 | 0.035 | 9.30E-03 | 0.018 | 0.612 | 2.90E-03 | 0.026 | 0.911 |
| InternalIMT | epa | rs60146827 | 0.08 | -0.02 | 0.013 | 0.113 | -0.048 | 0.018 | 9.10E-03 | -0.027 | 0.023 | 0.255 |
| InternalIMT | epa | rs79032236 | 0.034 | -0.021 | 0.014 | 0.128 | 0.044 | 0.04 | 0.268 | 0.013 | 0.052 | 0.8 |
| InternalIMT | epa | rs140854206 | 0.007 | . | . | . | . | . | . | . | . | . |
| InternalIMT | epa | rs184049550 | 0.004 | . | . | . | . | . | . | . | . | . |
| InternalIMT | epa | rs114433607 | 0.01 | -0.019 | 0.013 | 0.15 | -0.039 | 0.072 | 0.59 | -0.023 | 0.117 | 0.848 |
| InternalIMT | epa | rs117653793 | 0.013 | -0.027 | 0.012 | 0.025 | -0.124 | 0.042 | 3.00E-03 | 7.30E-03 | 0.044 | 0.869 |
| InternalIMT | epa | rs117039533 | 0.013 | -0.027 | 0.012 | 0.025 | -0.124 | 0.042 | 2.90E-03 | 6.90E-03 | 0.044 | 0.875 |
| InternalIMT | epa | rs191660761 | 0.003 | . | . | . | . | . | . | . | . | . |
| InternalIMT | epa | rs59514896 | 0.064 | -0.024 | 0.013 | 0.061 | -0.06 | 0.02 | 3.00E-03 | -0.01 | 0.025 | 0.677 |
| InternalIMT | epa | rs184661876 | 0.002 | . | . | . | . | . | . | . | . | . |
| InternalIMT | epa | rs61854073 | 0.027 | -0.024 | 0.014 | 0.089 | 0.098 | 0.061 | 0.109 | 0.078 | 0.09 | 0.389 |
| InternalIMT | epa | rs2018518 | 0.299 | -0.023 | 0.016 | 0.151 | -6.70E-05 | 0.013 | 0.996 | -3.70E-03 | 0.018 | 0.834 |
| InternalIMT | epa | rs934187 | 0.718 | -0.022 | 0.027 | 0.427 | -8.80E-03 | 0.012 | 0.472 | -2.70E-03 | 0.017 | 0.871 |
| InternalIMT | epa | rs7918542 | 0.214 | -0.025 | 0.014 | 0.08 | -0.01 | 0.013 | 0.445 | -4.20E-04 | 0.017 | 0.98 |
| InternalIMT | epa | rs144311920 | 0.002 | . | . | . | . | . | . | . | . | . |
| InternalIMT | epa | rs7099684 | 0.84 | -0.028 | 0.038 | 0.459 | 6.60E-03 | 0.015 | 0.653 | 1.30E-03 | 0.021 | 0.951 |
| InternalIMT | epa | rs150829171 | 0.002 | . | . | . | . | . | . | . | . | . |
| InternalIMT | epa | rs186060697 | 0.002 | . | . | . | . | . | . | . | . | . |
| InternalIMT | epa | rs11239513 | 0.097 | -0.018 | 0.014 | 0.197 | -0.01 | 0.023 | 0.653 | -8.90E-03 | 0.035 | 0.801 |
| InternalIMT | epa | rs7917687 | 0.081 | -0.02 | 0.013 | 0.116 | -0.049 | 0.018 | 6.50E-03 | -0.027 | 0.023 | 0.241 |
| InternalIMT | epa | rs61854076 | 0.01 | . | . | . | . | . | . | . | . | . |
| InternalIMT | epa | rs71494780 | 0.018 | -0.019 | 0.014 | 0.162 | -0.034 | 0.042 | 0.423 | -0.029 | 0.066 | 0.664 |
| InternalIMT | epa | rs7919239 | 0.218 | -0.027 | 0.014 | 0.058 | -9.90E-03 | 0.013 | 0.432 | 3.60E-03 | 0.017 | 0.826 |
| InternalIMT | epa | rs78334475 | 0.034 | -0.031 | 0.012 | 0.013 | -0.096 | 0.031 | 1.70E-03 | 0.056 | 0.032 | 0.077 |
| InternalIMT | epa | rs3780900 | 0.026 | -0.023 | 0.012 | 0.05 | -5.80E-03 | 0.045 | 0.897 | -0.059 | 0.071 | 0.406 |
| InternalIMT | epa | rs186729292 | 0.001 | . | . | . | . | . | . | . | . | . |
| InternalIMT | epa | rs12267438 | 0.026 | -0.023 | 0.014 | 0.096 | 6.50E-03 | 0.041 | 0.872 | 0.057 | 0.049 | 0.245 |
| InternalIMT | epa | rs1369214 | 0.568 | -0.021 | 0.021 | 0.32 | -0.015 | 0.011 | 0.184 | -4.10E-03 | 0.015 | 0.79 |
| InternalIMT | epa | rs9282589 | 0.041 | -0.021 | 0.014 | 0.122 | -0.062 | 0.034 | 0.071 | -0.016 | 0.043 | 0.709 |
| InternalIMT | epa | rs114522915 | 0.025 | -0.019 | 0.014 | 0.177 | 0.024 | 0.042 | 0.564 | -0.024 | 0.051 | 0.635 |
| InternalIMT | epa | rs2115819 | 0.477 | -0.019 | 0.02 | 0.333 | -9.40E-03 | 0.011 | 0.414 | -6.60E-03 | 0.016 | 0.675 |
| InternalIMT | epa | rs117664603 | 0.013 | -0.027 | 0.012 | 0.025 | -0.124 | 0.041 | 2.70E-03 | 5.50E-03 | 0.044 | 0.9 |
| InternalIMT | epa | rs184979202 | 0.001 | . | . | . | . | . | . | . | . | . |

| InternalIMT | epa | rs190714671 | 0.002 | . | . | . | . | . | . | . | . | . |
| --- | --- | --- | --- | --- | --- | --- | --- | --- | --- | --- | --- | --- |
| InternalIMT | epa | rs141538996 | 0 | . | . | . | . | . | . | . | . | . |
| InternalIMT | epa | rs150881741 | 0.002 | . | . | . | . | . | . | . | . | . |
| InternalIMT | epa | rs139076633 | 0.003 | . | . | . | . | . | . | . | . | . |
| InternalIMT | epa | rs12243649 | 0.059 | -0.03 | 0.012 | 0.016 | 2.10E-03 | 0.024 | 0.93 | 0.029 | 0.028 | 0.309 |
| InternalIMT | epa | rs140348708 | 0.001 | . | . | . | . | . | . | . | . | . |
| InternalIMT | epa | rs79023256 | 0.013 | -0.027 | 0.012 | 0.025 | -0.125 | 0.041 | 2.70E-03 | 5.20E-03 | 0.044 | 0.905 |
| InternalIMT | epa | rs11239515 | 0.094 | -0.024 | 0.012 | 0.053 | 3.60E-03 | 0.019 | 0.851 | -0.011 | 0.027 | 0.698 |
| InternalIMT | epa | rs55866086 | 0.157 | -0.028 | 0.013 | 0.034 | 1.50E-03 | 0.016 | 0.921 | 8.30E-03 | 0.02 | 0.684 |
| InternalIMT | epa | rs181340150 | 0.003 | . | . | . | . | . | . | . | . | . |
| InternalIMT | epa | rs11239516 | 0.12 | -0.029 | 0.013 | 0.025 | 9.40E-03 | 0.018 | 0.594 | 0.012 | 0.024 | 0.605 |
| InternalIMT | epa | rs11239517 | 0.12 | -0.029 | 0.013 | 0.025 | 9.50E-03 | 0.018 | 0.593 | 0.012 | 0.024 | 0.606 |
| InternalIMT | epa | rs11239519 | 0.106 | -0.029 | 0.013 | 0.023 | 6.80E-03 | 0.019 | 0.718 | 0.014 | 0.026 | 0.58 |
| InternalIMT | epa | rs7909514 | 0.709 | -0.027 | 0.027 | 0.313 | -0.011 | 0.012 | 0.384 | 7.20E-04 | 0.017 | 0.965 |
| InternalIMT | epa | rs7894352 | 0.835 | -0.041 | 0.036 | 0.257 | 7.50E-03 | 0.015 | 0.615 | 8.90E-03 | 0.02 | 0.664 |
| InternalIMT | epa | rs189384146 | 0.002 | . | . | . | . | . | . | . | . | . |
| InternalIMT | epa | rs11239520 | 0.105 | -0.024 | 0.012 | 0.05 | 9.50E-04 | 0.019 | 0.96 | -7.60E-03 | 0.027 | 0.775 |
| InternalIMT | epa | rs12221233 | 0.406 | -0.024 | 0.018 | 0.176 | 4.70E-03 | 0.012 | 0.693 | -1.60E-03 | 0.016 | 0.921 |
| InternalIMT | epa | rs74523293 | 0.032 | -0.026 | 0.012 | 0.028 | -0.02 | 0.032 | 0.541 | 7.20E-03 | 0.039 | 0.854 |
| InternalIMT | epa | rs79376040 | 0.032 | -0.026 | 0.012 | 0.028 | -0.02 | 0.032 | 0.541 | 7.30E-03 | 0.039 | 0.853 |
| InternalIMT | epa | rs4949000 | 0.406 | -0.024 | 0.018 | 0.175 | 4.70E-03 | 0.012 | 0.697 | -1.60E-03 | 0.016 | 0.922 |
| InternalIMT | epa | rs7089406 | 0.025 | -0.016 | 0.014 | 0.247 | -0.057 | 0.034 | 0.093 | -0.062 | 0.044 | 0.156 |
| InternalIMT | epa | rs138211056 | 0 | . | . | . | . | . | . | . | . | . |
| InternalIMT | epa | rs10900213 | 0.537 | -0.026 | 0.022 | 0.22 | 7.80E-03 | 0.012 | 0.515 | 7.10E-04 | 0.017 | 0.966 |
| InternalIMT | epa | rs145152827 | 0.003 | . | . | . | . | . | . | . | . | . |
| InternalIMT | epa | rs74443325 | 0.012 | -0.027 | 0.012 | 0.024 | -0.143 | 0.04 | 2.90E-04 | 6.00E-03 | 0.036 | 0.87 |
| InternalIMT | epa | rs188299446 | 0.011 | -0.015 | 0.014 | 0.279 | 0.056 | 0.085 | 0.506 | -0.227 | 0.122 | 0.063 |
| InternalIMT | epa | rs192699586 | 0.003 | . | . | . | . | . | . | . | . | . |
| InternalIMT | epa | rs72796464 | 0.086 | -0.025 | 0.012 | 0.046 | 0.013 | 0.02 | 0.51 | -8.30E-03 | 0.028 | 0.771 |
| InternalIMT | epa | rs4949001 | 0.266 | -0.027 | 0.016 | 0.088 | -0.011 | 0.014 | 0.41 | 2.10E-03 | 0.018 | 0.911 |
| InternalIMT | epa | rs147174641 | 0.008 | . | . | . | . | . | . | . | . | . |
| InternalIMT | epa | rs190835238 | 0.001 | . | . | . | . | . | . | . | . | . |
| InternalIMT | epa | rs76698944 | 0.022 | -0.023 | 0.014 | 0.087 | 0.05 | 0.051 | 0.324 | 0.089 | 0.065 | 0.171 |
| InternalIMT | epa | rs56071381 | 0.11 | -0.024 | 0.013 | 0.053 | -1.50E-03 | 0.019 | 0.934 | -6.90E-03 | 0.025 | 0.786 |
| InternalIMT | epa | rs78130427 | 0.047 | -0.024 | 0.012 | 0.052 | -0.079 | 0.024 | 9.60E-04 | 3.50E-03 | 0.026 | 0.891 |
| InternalIMT | epa | rs10900215 | 0.148 | -0.029 | 0.013 | 0.028 | 5.90E-03 | 0.017 | 0.728 | 1.00E-02 | 0.023 | 0.662 |
| InternalIMT | epa | rs41491445 | 0.016 | -0.02 | 0.013 | 0.135 | -0.026 | 0.059 | 0.656 | 0.013 | 0.09 | 0.886 |
| InternalIMT | epa | rs144995222 | 0.002 | . | . | . | . | . | . | . | . | . |
| InternalIMT | epa | rs149097803 | 0.003 | . | . | . | . | . | . | . | . | . |
| InternalIMT | epa | rs139429646 | 0.005 | . | . | . | . | . | . | . | . | . |
| InternalIMT | epa | rs7075620 | 0.681 | -0.02 | 0.026 | 0.449 | -0.012 | 0.012 | 0.341 | -4.10E-03 | 0.017 | 0.807 |
| InternalIMT | epa | rs148299213 | 0.017 | -0.019 | 0.013 | 0.149 | -0.029 | 0.059 | 0.616 | -0.011 | 0.091 | 0.903 |
| InternalIMT | epa | rs4949002 | 0.245 | -0.021 | 0.015 | 0.182 | 0.03 | 0.015 | 0.048 | -8.50E-03 | 0.021 | 0.686 |
| InternalIMT | epa | rs150496007 | 0.021 | -0.02 | 0.014 | 0.155 | -0.042 | 0.049 | 0.393 | -5.00E-03 | 0.064 | 0.937 |
| InternalIMT | epa | rs147303455 | 0.012 | -0.016 | 0.014 | 0.23 | -0.013 | 0.058 | 0.828 | -0.111 | 0.082 | 0.174 |
| InternalIMT | epa | rs189518433 | 0.005 | . | . | . | . | . | . | . | . | . |
| InternalIMT | epa | rs186281355 | 0.005 | . | . | . | . | . | . | . | . | . |
| InternalIMT | epa | rs115297114 | 0.026 | -0.018 | 0.014 | 0.183 | 0.021 | 0.042 | 0.618 | -0.026 | 0.048 | 0.587 |
| InternalIMT | epa | rs11239523 | 0.105 | -0.025 | 0.012 | 0.041 | 4.80E-03 | 0.019 | 0.803 | -3.00E-03 | 0.026 | 0.911 |
| InternalIMT | epa | rs143704897 | 0.005 | . | . | . | . | . | . | . | . | . |
| InternalIMT | epa | rs188024096 | 0.003 | . | . | . | . | . | . | . | . | . |
| InternalIMT | epa | rs11239524 | 0.107 | -0.024 | 0.013 | 0.058 | -5.70E-03 | 0.02 | 0.772 | -8.90E-03 | 0.027 | 0.742 |
| InternalIMT | epa | rs191656573 | 0.004 | . | . | . | . | . | . | . | . | . |
| InternalIMT | epa | rs193295875 | 0.002 | . | . | . | . | . | . | . | . | . |
| InternalIMT | epa | rs190458228 | 0.002 | . | . | . | . | . | . | . | . | . |
| InternalIMT | epa | rs139253993 | 0.002 | . | . | . | . | . | . | . | . | . |
| InternalIMT | epa | rs4948672 | 0.588 | -9.20E-03 | 0.025 | 0.71 | -2.40E-03 | 0.013 | 0.855 | -0.014 | 0.018 | 0.446 |
| InternalIMT | epa | rs180954921 | 0.002 | . | . | . | . | . | . | . | . | . |
| InternalIMT | epa | rs12264801 | 0.425 | -0.027 | 0.02 | 0.193 | 6.20E-03 | 0.014 | 0.655 | 9.60E-04 | 0.019 | 0.959 |
| InternalIMT | epa | rs73283184 | 0.02 | -0.019 | 0.013 | 0.17 | -0.051 | 0.047 | 0.283 | -0.028 | 0.079 | 0.721 |
| InternalIMT | epa | rs142746187 | 0.009 | . | . | . | . | . | . | . | . | . |
| InternalIMT | epa | rs58704739 | 0.019 | -0.02 | 0.013 | 0.146 | -0.044 | 0.048 | 0.367 | 2.30E-03 | 0.077 | 0.976 |
| InternalIMT | epa | rs61272335 | 0.014 | -0.021 | 0.013 | 0.125 | -0.025 | 0.061 | 0.687 | 0.036 | 0.097 | 0.712 |
| InternalIMT | epa | rs60959489 | 0.035 | -0.017 | 0.014 | 0.223 | -0.054 | 0.033 | 0.102 | -0.031 | 0.046 | 0.507 |
| InternalIMT | epa | rs58502629 | 0.014 | -0.021 | 0.013 | 0.125 | -0.024 | 0.061 | 0.691 | 0.036 | 0.097 | 0.713 |
| InternalIMT | epa | rs10900216 | 0.735 | -0.043 | 0.028 | 0.125 | 0.022 | 0.012 | 0.074 | 0.011 | 0.017 | 0.515 |
| InternalIMT | epa | rs181118141 | 0.002 | . | . | . | . | . | . | . | . | . |
| InternalIMT | epa | rs137882665 | 0.01 | . | . | . | . | . | . | . | . | . |
| InternalIMT | epa | rs149466736 | 0.003 | . | . | . | . | . | . | . | . | . |
| InternalIMT | epa | rs2099171 | 0.656 | -0.036 | 0.023 | 0.11 | 0.024 | 0.011 | 0.031 | 8.30E-03 | 0.015 | 0.589 |
| InternalIMT | epa | rs141852280 | 0.006 | . | . | . | . | . | . | . | . | . |
| InternalIMT | epa | rs191539257 | 0.002 | . | . | . | . | . | . | . | . | . |
| InternalIMT | epa | rs17157771 | 0.034 | -0.026 | 0.012 | 0.027 | -0.047 | 0.029 | 0.109 | 0.013 | 0.036 | 0.705 |
| InternalIMT | epa | rs111339241 | 0.032 | -0.026 | 0.012 | 0.027 | -0.048 | 0.03 | 0.112 | 0.014 | 0.036 | 0.687 |

| InternalIMT | epa | rs116983255 | 0.008 | . | . | . | . | . | . | . | . | . |
| --- | --- | --- | --- | --- | --- | --- | --- | --- | --- | --- | --- | --- |
| InternalIMT | epa | rs142324341 | 0.011 | -0.046 | 0.016 | 3.30E-03 | -0.058 | 0.082 | 0.475 | 0.126 | 0.129 | 0.331 |
| InternalIMT | epa | rs187191219 | 0.01 | . | . | . | . | . | . | . | . | . |
| InternalIMT | epa | rs151283973 | 0.002 | . | . | . | . | . | . | . | . | . |
| InternalIMT | epa | rs139316218 | 0.003 | . | . | . | . | . | . | . | . | . |
| InternalIMT | epa | rs6593485 | 0.732 | -0.045 | 0.028 | 0.107 | 0.013 | 0.012 | 0.291 | 0.013 | 0.017 | 0.459 |
| InternalIMT | epa | rs6593486 | 0.732 | -0.045 | 0.028 | 0.106 | 0.013 | 0.012 | 0.306 | 0.013 | 0.017 | 0.454 |
| InternalIMT | epa | rs145196618 | 0.014 | -0.021 | 0.013 | 0.12 | 0.028 | 0.061 | 0.647 | 0.04 | 0.087 | 0.645 |
| InternalIMT | epa | rs142598335 | 0.004 | . | . | . | . | . | . | . | . | . |
| InternalIMT | epa | rs892691 | 0.726 | -0.037 | 0.028 | 0.178 | 8.90E-03 | 0.012 | 0.467 | 7.60E-03 | 0.017 | 0.653 |
| InternalIMT | epa | rs7080713 | 0.671 | -0.031 | 0.023 | 0.192 | 0.021 | 0.012 | 0.067 | 3.80E-03 | 0.016 | 0.81 |
| InternalIMT | epa | rs7077173 | 0.08 | -0.032 | 0.012 | 7.50E-03 | -7.10E-03 | 0.018 | 0.692 | 0.044 | 0.025 | 0.078 |
| InternalIMT | epa | rs7080977 | 0.048 | -0.03 | 0.012 | 0.015 | -0.02 | 0.026 | 0.442 | 0.04 | 0.032 | 0.213 |
| InternalIMT | epa | rs3780901 | 0.671 | -0.033 | 0.023 | 0.158 | 0.022 | 0.012 | 0.052 | 5.60E-03 | 0.016 | 0.72 |
| InternalIMT | epa | rs61854091 | 0.051 | -0.021 | 0.014 | 0.134 | 0.073 | 0.037 | 0.049 | 0.011 | 0.057 | 0.845 |
| InternalIMT | epa | rs77550209 | 0.005 | . | . | . | . | . | . | . | . | . |
| InternalIMT | epa | rs73283191 | 0.061 | -0.03 | 0.012 | 0.015 | -0.038 | 0.023 | 0.088 | 0.034 | 0.027 | 0.211 |
| InternalIMT | epa | rs191866099 | 0.007 | . | . | . | . | . | . | . | . | . |
| InternalIMT | epa | rs147998395 | 0.002 | . | . | . | . | . | . | . | . | . |
| InternalIMT | epa | rs7090328 | 0.783 | -0.056 | 0.029 | 0.054 | 0.014 | 0.013 | 0.275 | 0.019 | 0.018 | 0.285 |
| InternalIMT | epa | rs79814535 | 0.012 | -0.026 | 0.012 | 0.028 | -0.147 | 0.038 | 9.50E-05 | -0.018 | 0.035 | 0.609 |
| InternalIMT | epa | rs7073082 | 0.783 | -0.056 | 0.029 | 0.054 | 0.014 | 0.013 | 0.275 | 0.019 | 0.018 | 0.285 |
| InternalIMT | epa | rs11239526 | 0.036 | -0.024 | 0.012 | 0.044 | 0.04 | 0.031 | 0.186 | -0.021 | 0.042 | 0.624 |
| InternalIMT | epa | rs141839841 | 0.002 | . | . | . | . | . | . | . | . | . |
| InternalIMT | epa | rs75697371 | 0.01 | . | . | . | . | . | . | . | . | . |
| InternalIMT | epa | rs75972854 | 0.01 | . | . | . | . | . | . | . | . | . |
| InternalIMT | epa | rs139291975 | 0.001 | . | . | . | . | . | . | . | . | . |
| InternalIMT | epa | rs144064711 | 0.006 | . | . | . | . | . | . | . | . | . |
| InternalIMT | epa | rs3780902 | 0.062 | -0.03 | 0.012 | 0.015 | -0.036 | 0.022 | 0.105 | 0.032 | 0.027 | 0.237 |
| InternalIMT | epa | rs190449801 | 0.002 | . | . | . | . | . | . | . | . | . |
| InternalIMT | epa | rs73283196 | 0.061 | -0.03 | 0.012 | 0.015 | -0.038 | 0.023 | 0.089 | 0.033 | 0.027 | 0.217 |
| InternalIMT | epa | rs28395867 | 0.044 | -0.021 | 0.013 | 0.11 | -0.023 | 0.036 | 0.517 | 0.022 | 0.064 | 0.735 |
| InternalIMT | epa | rs28395866 | 0.102 | -0.031 | 0.012 | 0.012 | -6.20E-03 | 0.019 | 0.747 | 0.027 | 0.028 | 0.34 |
| InternalIMT | epa | rs55661611 | 0.047 | -0.03 | 0.012 | 0.014 | -0.019 | 0.026 | 0.467 | 0.044 | 0.033 | 0.184 |
| InternalIMT | epa | rs55684518 | 0.02 | -0.024 | 0.014 | 0.084 | -0.059 | 0.047 | 0.212 | 0.109 | 0.07 | 0.118 |
| InternalIMT | epa | rs78552493 | 0.047 | -0.014 | 0.014 | 0.305 | -0.063 | 0.026 | 0.017 | -0.052 | 0.037 | 0.162 |
| InternalIMT | epa | rs147665434 | 0.004 | . | . | . | . | . | . | . | . | . |
| InternalIMT | epa | rs34445001 | 0.01 | . | . | . | . | . | . | . | . | . |
| InternalIMT | epa | rs184019449 | 0.003 | . | . | . | . | . | . | . | . | . |
| InternalIMT | epa | rs3780903 | 0.714 | -0.02 | 0.026 | 0.451 | 0.015 | 0.012 | 0.21 | -4.00E-03 | 0.017 | 0.81 |
| InternalIMT | epa | rs17157784 | 0.061 | -0.028 | 0.012 | 0.018 | -0.024 | 0.025 | 0.336 | 0.018 | 0.037 | 0.622 |
| InternalIMT | epa | rs3780904 | 0.013 | -0.026 | 0.012 | 0.026 | -0.093 | 0.04 | 0.021 | 4.30E-03 | 0.039 | 0.91 |
| InternalIMT | epa | rs192064730 | 0 | . | . | . | . | . | . | . | . | . |
| InternalIMT | epa | rs12774534 | 0.01 | . | . | . | . | . | . | . | . | . |
| InternalIMT | epa | rs79179915 | 0.011 | -0.026 | 0.012 | 0.027 | -0.149 | 0.038 | 7.40E-05 | -0.014 | 0.035 | 0.686 |
| InternalIMT | epa | rs117811571 | 0.011 | -0.016 | 0.014 | 0.256 | 0.085 | 0.088 | 0.338 | -0.196 | 0.123 | 0.11 |
| InternalIMT | epa | rs72796477 | 0.01 | -0.021 | 0.013 | 0.117 | 0.089 | 0.09 | 0.323 | 0.091 | 0.112 | 0.415 |
| InternalIMT | epa | rs55729342 | 0.065 | -0.027 | 0.012 | 0.026 | -0.022 | 0.024 | 0.352 | 7.30E-03 | 0.035 | 0.837 |
| InternalIMT | epa | rs59317220 | 0.064 | -0.027 | 0.012 | 0.024 | -0.02 | 0.025 | 0.412 | 8.80E-03 | 0.036 | 0.807 |
| InternalIMT | epa | rs3780905 | 0.104 | -0.026 | 0.013 | 0.05 | -0.082 | 0.015 | 4.80E-08 | 0.019 | 0.02 | 0.336 |
| InternalIMT | epa | rs3780906 | 0.714 | -0.019 | 0.026 | 0.467 | 0.015 | 0.012 | 0.22 | -4.40E-03 | 0.017 | 0.793 |
| InternalIMT | epa | rs149047975 | 0.036 | -0.021 | 0.014 | 0.13 | -0.087 | 0.036 | 0.016 | -0.033 | 0.045 | 0.465 |
| InternalIMT | epa | rs3824614 | 0.011 | -0.026 | 0.012 | 0.026 | -0.156 | 0.037 | 3.00E-05 | -0.014 | 0.035 | 0.691 |
| InternalIMT | epa | rs140254404 | 0.003 | . | . | . | . | . | . | . | . | . |
| InternalIMT | epa | rs145262684 | 0.014 | -0.018 | 0.014 | 0.187 | 0.149 | 0.084 | 0.076 | -0.068 | 0.106 | 0.52 |
| InternalIMT | epa | rs76735482 | 0.011 | -0.026 | 0.012 | 0.026 | -0.158 | 0.037 | 2.00E-05 | -0.014 | 0.035 | 0.696 |
| InternalIMT | epa | rs73283198 | 0.034 | -0.027 | 0.012 | 0.023 | -0.053 | 0.031 | 0.088 | 0.029 | 0.04 | 0.47 |
| InternalIMT | epa | rs74911239 | 0.035 | -0.018 | 0.014 | 0.208 | 0.021 | 0.039 | 0.583 | -0.028 | 0.056 | 0.625 |
| InternalIMT | epa | rs150551647 | 0.006 | . | . | . | . | . | . | . | . | . |
| InternalIMT | epa | rs115200794 | 0.051 | -0.02 | 0.013 | 0.125 | -0.019 | 0.033 | 0.553 | 0.01 | 0.056 | 0.852 |
| InternalIMT | epa | rs74130823 | 0.168 | -0.034 | 0.013 | 7.90E-03 | -3.60E-03 | 0.016 | 0.824 | 0.027 | 0.023 | 0.235 |
| InternalIMT | epa | rs2279435 | 0.62 | -0.034 | 0.024 | 0.159 | 7.60E-03 | 0.012 | 0.527 | 6.60E-03 | 0.016 | 0.688 |
| InternalIMT | epa | rs147279673 | 0.014 | -0.027 | 0.012 | 0.022 | 0.076 | 0.072 | 0.291 | 0.06 | 0.101 | 0.556 |
| InternalIMT | epa | rs183525146 | 0.004 | . | . | . | . | . | . | . | . | . |
| InternalIMT | epa | rs3740107 | 0.745 | -0.076 | 0.029 | 9.30E-03 | 0.023 | 0.013 | 0.075 | 0.034 | 0.018 | 0.061 |
| InternalIMT | epa | rs140697247 | 0.001 | . | . | . | . | . | . | . | . | . |
| InternalIMT | epa | rs1565096 | 0.81 | -0.067 | 0.033 | 0.043 | 0.011 | 0.014 | 0.431 | 0.025 | 0.02 | 0.196 |
| InternalIMT | epa | rs1565097 | 0.767 | -0.057 | 0.032 | 0.076 | 0.012 | 0.013 | 0.359 | 0.02 | 0.019 | 0.29 |
| InternalIMT | epa | rs144584548 | 0.02 | -0.024 | 0.014 | 0.081 | -0.049 | 0.047 | 0.296 | 0.111 | 0.07 | 0.112 |
| InternalIMT | epa | rs75811053 | 0.011 | -0.027 | 0.012 | 0.024 | -0.171 | 0.037 | 4.40E-06 | -0.012 | 0.035 | 0.742 |
| InternalIMT | epa | rs147462967 | 0.01 | . | . | . | . | . | . | . | . | . |
| InternalIMT | epa | rs74130824 | 0.049 | -0.026 | 0.012 | 0.028 | -0.059 | 0.028 | 0.035 | 1.60E-03 | 0.043 | 0.971 |
| InternalIMT | epa | rs116883184 | 0.018 | -0.02 | 0.014 | 0.149 | 0.129 | 0.075 | 0.083 | 9.00E-03 | 0.094 | 0.924 |
| InternalIMT | epa | rs117258049 | 0.018 | -0.02 | 0.014 | 0.149 | 0.129 | 0.075 | 0.083 | 8.90E-03 | 0.094 | 0.925 |

| InternalIMT | epa | rs3780908 | 0.128 | -0.031 | 0.013 | 0.015 | 1.20E-03 | 0.017 | 0.945 | 0.023 | 0.024 | 0.334 |
| --- | --- | --- | --- | --- | --- | --- | --- | --- | --- | --- | --- | --- |
| InternalIMT | epa | rs180938522 | 0.002 | . | . | . | . | . | . | . | . | . |
| InternalIMT | epa | rs76687372 | 0.008 | . | . | . | . | . | . | . | . | . |
| InternalIMT | epa | rs3780909 | 0.542 | -0.026 | 0.021 | 0.209 | 1.20E-03 | 0.011 | 0.913 | 1.80E-04 | 0.015 | 0.991 |
| InternalIMT | epa | rs78281156 | 0.011 | -0.027 | 0.012 | 0.024 | -0.171 | 0.037 | 4.00E-06 | -0.011 | 0.035 | 0.749 |
| InternalIMT | epa | rs187840631 | 0.002 | . | . | . | . | . | . | . | . | . |
| InternalIMT | epa | rs76868242 | 0.011 | -0.027 | 0.012 | 0.024 | -0.171 | 0.037 | 4.00E-06 | -0.011 | 0.035 | 0.75 |
| InternalIMT | epa | rs192794192 | 0.001 | . | . | . | . | . | . | . | . | . |
| InternalIMT | epa | rs12781248 | 0.01 | -0.018 | 0.013 | 0.185 | -0.016 | 0.061 | 0.792 | -0.099 | 0.083 | 0.233 |
| InternalIMT | epa | rs12762375 | 0.01 | . | . | . | . | . | . | . | . | . |
| InternalIMT | epa | rs192256986 | 0.006 | . | . | . | . | . | . | . | . | . |
| InternalIMT | epa | rs184411030 | 0.012 | -0.023 | 0.013 | 0.084 | 0.097 | 0.059 | 0.1 | 0.109 | 0.077 | 0.155 |
| InternalIMT | epa | rs189601483 | 0.011 | -0.027 | 0.012 | 0.023 | -0.167 | 0.039 | 1.50E-05 | -3.00E-03 | 0.036 | 0.933 |
| InternalIMT | epa | rs143254579 | 0.037 | -0.024 | 0.012 | 0.046 | 0.032 | 0.03 | 0.283 | -0.022 | 0.042 | 0.605 |
| InternalIMT | epa | rs12253903 | 0.028 | -0.016 | 0.014 | 0.233 | -0.041 | 0.043 | 0.341 | -0.082 | 0.072 | 0.255 |
| InternalIMT | epa | rs35489926 | 0.009 | . | . | . | . | . | . | . | . | . |
| InternalIMT | epa | rs71494783 | 0.048 | -0.027 | 0.012 | 0.025 | -0.012 | 0.027 | 0.663 | 0.013 | 0.032 | 0.679 |
| InternalIMT | epa | rs180711602 | 0.003 | . | . | . | . | . | . | . | . | . |
| InternalIMT | epa | rs117312402 | 0.011 | -0.027 | 0.012 | 0.024 | -0.171 | 0.037 | 3.90E-06 | -0.011 | 0.035 | 0.756 |
| InternalIMT | epa | rs74831694 | 0.008 | . | . | . | . | . | . | . | . | . |
| InternalIMT | epa | rs140301943 | 0.003 | . | . | . | . | . | . | . | . | . |
| InternalIMT | epa | rs78553679 | 0.016 | -0.018 | 0.014 | 0.176 | 0.065 | 0.064 | 0.316 | -0.031 | 0.088 | 0.722 |
| InternalIMT | epa | rs78334250 | 0.011 | -0.027 | 0.012 | 0.024 | -0.171 | 0.037 | 3.80E-06 | -0.011 | 0.035 | 0.756 |
| InternalIMT | epa | rs76000028 | 0.011 | -0.027 | 0.012 | 0.024 | -0.171 | 0.037 | 3.90E-06 | -0.011 | 0.035 | 0.756 |
| InternalIMT | epa | rs145195254 | 0.002 | . | . | . | . | . | . | . | . | . |
| InternalIMT | epa | rs137888527 | 0.002 | . | . | . | . | . | . | . | . | . |
| InternalIMT | epa | rs149109592 | 0.005 | . | . | . | . | . | . | . | . | . |
| InternalIMT | epa | rs80280010 | 0.035 | -0.025 | 0.012 | 0.035 | 0.039 | 0.031 | 0.201 | -5.00E-03 | 0.043 | 0.907 |
| InternalIMT | epa | rs189995374 | 0.002 | . | . | . | . | . | . | . | . | . |
| InternalIMT | epa | rs75467770 | 0.012 | -0.026 | 0.012 | 0.028 | -0.173 | 0.036 | 1.90E-06 | -0.014 | 0.034 | 0.688 |
| InternalIMT | epa | rs1487562 | 0.167 | -0.031 | 0.013 | 0.015 | -2.20E-03 | 0.016 | 0.888 | 0.017 | 0.023 | 0.444 |
| InternalIMT | epa | rs12266963 | 0.048 | -0.027 | 0.012 | 0.025 | -0.012 | 0.027 | 0.653 | 0.013 | 0.032 | 0.679 |
| InternalIMT | epa | rs7099874 | 0.764 | -0.065 | 0.03 | 0.031 | 5.90E-03 | 0.013 | 0.644 | 0.025 | 0.019 | 0.173 |
| InternalIMT | epa | rs75687019 | 0.011 | -0.027 | 0.012 | 0.024 | -0.171 | 0.037 | 3.80E-06 | -0.011 | 0.035 | 0.758 |
| InternalIMT | epa | rs148240462 | 0.038 | -0.019 | 0.014 | 0.181 | 0.041 | 0.039 | 0.3 | -0.01 | 0.056 | 0.857 |
| InternalIMT | epa | rs141278174 | 0.002 | . | . | . | . | . | . | . | . | . |
| InternalIMT | epa | rs76404852 | 0.011 | -0.027 | 0.012 | 0.024 | -0.171 | 0.037 | 3.80E-06 | -0.011 | 0.035 | 0.759 |
| InternalIMT | epa | rs139112240 | 0.008 | . | . | . | . | . | . | . | . | . |
| InternalIMT | epa | rs12783037 | 0.054 | -0.029 | 0.012 | 0.018 | 8.00E-03 | 0.027 | 0.769 | 0.027 | 0.032 | 0.405 |
| InternalIMT | epa | rs72796481 | 0.009 | . | . | . | . | . | . | . | . | . |
| InternalIMT | epa | rs76798749 | 0.017 | -0.028 | 0.012 | 0.018 | -0.05 | 0.045 | 0.265 | 0.036 | 0.046 | 0.43 |
| InternalIMT | epa | rs187732548 | 0.001 | . | . | . | . | . | . | . | . | . |
| InternalIMT | epa | rs75675474 | 0.017 | -0.028 | 0.012 | 0.018 | -0.05 | 0.045 | 0.265 | 0.036 | 0.046 | 0.43 |
| InternalIMT | epa | rs76552293 | 0.017 | -0.028 | 0.012 | 0.018 | -0.05 | 0.045 | 0.265 | 0.036 | 0.046 | 0.429 |
| InternalIMT | epa | rs147445993 | 0.005 | . | . | . | . | . | . | . | . | . |
| InternalIMT | epa | rs115902283 | 0.017 | -0.028 | 0.012 | 0.017 | -0.051 | 0.045 | 0.255 | 0.038 | 0.046 | 0.406 |
| InternalIMT | epa | rs4540799 | 0.762 | -0.063 | 0.03 | 0.036 | 6.30E-03 | 0.013 | 0.62 | 0.024 | 0.019 | 0.195 |
| InternalIMT | epa | rs12765320 | 0.211 | -0.032 | 0.013 | 0.017 | -2.30E-03 | 0.015 | 0.878 | 0.016 | 0.02 | 0.429 |
| InternalIMT | epa | rs12782256 | 0.009 | . | . | . | . | . | . | . | . | . |
| InternalIMT | epa | rs191727256 | 0.002 | . | . | . | . | . | . | . | . | . |
| InternalIMT | epa | rs2082111 | 0.123 | -0.031 | 0.013 | 0.016 | 6.40E-03 | 0.018 | 0.714 | 0.021 | 0.024 | 0.387 |
| InternalIMT | epa | rs2082110 | 0.175 | -0.034 | 0.013 | 9.60E-03 | -7.30E-03 | 0.016 | 0.638 | 0.023 | 0.021 | 0.276 |
| InternalIMT | epa | rs2082109 | 0.175 | -0.034 | 0.013 | 9.60E-03 | -7.30E-03 | 0.016 | 0.638 | 0.023 | 0.021 | 0.277 |
| InternalIMT | epa | rs117316662 | 0.011 | -0.027 | 0.012 | 0.024 | -0.171 | 0.037 | 3.60E-06 | -0.01 | 0.035 | 0.769 |
| InternalIMT | epa | rs12247163 | 0.214 | -0.035 | 0.013 | 8.60E-03 | 1.80E-03 | 0.015 | 0.903 | 0.023 | 0.019 | 0.235 |
| InternalIMT | epa | rs12355888 | 0.082 | -0.025 | 0.012 | 0.039 | 0.013 | 0.021 | 0.547 | -4.40E-03 | 0.03 | 0.884 |
| InternalIMT | epa | rs143772290 | 0.011 | -0.023 | 0.013 | 0.082 | 0.151 | 0.08 | 0.061 | 0.184 | 0.112 | 0.101 |
| InternalIMT | epa | rs146849452 | 0.005 | . | . | . | . | . | . | . | . | . |
| InternalIMT | epa | rs140267121 | 0.004 | . | . | . | . | . | . | . | . | . |
| InternalIMT | epa | rs7080474 | 0.202 | -0.035 | 0.013 | 6.70E-03 | 0.013 | 0.015 | 0.396 | 0.026 | 0.02 | 0.211 |
| InternalIMT | epa | rs144066203 | 0.002 | . | . | . | . | . | . | . | . | . |
| InternalIMT | epa | rs12765209 | 0.012 | -0.026 | 0.012 | 0.025 | -0.129 | 0.043 | 2.40E-03 | -6.00E-03 | 0.036 | 0.867 |
| InternalIMT | epa | rs150930735 | 0.002 | . | . | . | . | . | . | . | . | . |
| InternalIMT | epa | rs7068039 | 0.216 | -0.036 | 0.013 | 7.70E-03 | 2.60E-03 | 0.014 | 0.859 | 0.024 | 0.019 | 0.211 |
| InternalIMT | epa | rs117622652 | 0.011 | -0.027 | 0.012 | 0.024 | -0.172 | 0.037 | 3.50E-06 | -0.01 | 0.035 | 0.774 |
| InternalIMT | epa | rs190191514 | 0.001 | . | . | . | . | . | . | . | . | . |
| InternalIMT | epa | rs74130835 | 0.04 | -0.019 | 0.013 | 0.164 | -0.047 | 0.037 | 0.204 | -7.00E-03 | 0.067 | 0.917 |
| InternalIMT | epa | rs1811604 | 0.039 | -0.02 | 0.013 | 0.142 | -0.047 | 0.038 | 0.224 | 7.30E-03 | 0.069 | 0.916 |
| InternalIMT | epa | rs146971256 | 0 | . | . | . | . | . | . | . | . | . |
| InternalIMT | epa | rs74336000 | 0.011 | -0.023 | 0.013 | 0.081 | 0.151 | 0.08 | 0.06 | 0.188 | 0.112 | 0.093 |
| InternalIMT | epa | rs186046492 | 0.007 | . | . | . | . | . | . | . | . | . |
| InternalIMT | epa | rs34971823 | 0.011 | -0.018 | 0.013 | 0.173 | -1.60E-03 | 0.064 | 0.98 | -0.07 | 0.081 | 0.384 |
| InternalIMT | epa | rs75227446 | 0.011 | -0.027 | 0.012 | 0.024 | -0.17 | 0.037 | 4.50E-06 | -8.00E-03 | 0.035 | 0.82 |
| InternalIMT | epa | rs190457599 | 0.001 | . | . | . | . | . | . | . | . | . |

| InternalIMT | epa | rs79591917 | 0.01 | -0.027 | 0.012 | 0.024 | -0.172 | 0.037 | 3.20E-06 | -9.60E-03 | 0.035 | 0.784 |
| --- | --- | --- | --- | --- | --- | --- | --- | --- | --- | --- | --- | --- |
| InternalIMT | epa | rs702365 | 0.545 | -0.029 | 0.021 | 0.164 | 1.90E-03 | 0.011 | 0.865 | 2.90E-03 | 0.015 | 0.852 |
| InternalIMT | epa | rs79643836 | 0.011 | -0.026 | 0.012 | 0.026 | -0.179 | 0.037 | 1.10E-06 | -0.023 | 0.035 | 0.51 |
| InternalIMT | epa | rs702366 | 0.546 | -0.028 | 0.021 | 0.181 | 2.40E-03 | 0.011 | 0.829 | 1.80E-03 | 0.015 | 0.905 |
| InternalIMT | epa | rs1059696 | 0.151 | -0.033 | 0.013 | 0.012 | 0.012 | 0.017 | 0.467 | 0.025 | 0.023 | 0.287 |
| InternalIMT | epa | rs901681 | 0.546 | -0.029 | 0.021 | 0.159 | 3.10E-03 | 0.011 | 0.783 | 3.10E-03 | 0.015 | 0.839 |
| InternalIMT | epa | rs61854092 | 0.15 | -0.033 | 0.013 | 0.012 | 0.013 | 0.017 | 0.463 | 0.024 | 0.023 | 0.298 |
| InternalIMT | epa | rs148080753 | 0.001 | . | . | . | . | . | . | . | . | . |
| InternalIMT | epa | rs61854093 | 0.186 | -0.032 | 0.013 | 0.012 | 3.30E-03 | 0.015 | 0.829 | 0.019 | 0.021 | 0.369 |
| InternalIMT | epa | rs141822774 | 0.001 | . | . | . | . | . | . | . | . | . |
| InternalIMT | epa | rs74130836 | 0.04 | -0.019 | 0.013 | 0.164 | -0.048 | 0.037 | 0.201 | -7.20E-03 | 0.067 | 0.915 |
| InternalIMT | epa | rs150620963 | 0.013 | -0.021 | 0.014 | 0.12 | 0.025 | 0.069 | 0.714 | 0.057 | 0.094 | 0.546 |
| InternalIMT | epa | rs3780913 | 0.753 | -0.066 | 0.029 | 0.022 | 4.90E-03 | 0.013 | 0.696 | 0.027 | 0.018 | 0.143 |
| InternalIMT | epa | rs142255723 | 0.002 | . | . | . | . | . | . | . | . | . |
| InternalIMT | epa | rs3780914 | 0.542 | -0.028 | 0.021 | 0.173 | 2.90E-03 | 0.011 | 0.799 | 2.20E-03 | 0.015 | 0.887 |
| InternalIMT | epa | rs182656507 | 0.002 | . | . | . | . | . | . | . | . | . |
| InternalIMT | epa | rs2291427 | 0.735 | -0.053 | 0.027 | 0.053 | 9.90E-03 | 0.012 | 0.429 | 0.019 | 0.018 | 0.294 |
| InternalIMT | epa | rs10751382 | 0.733 | -0.053 | 0.027 | 0.054 | 7.40E-03 | 0.012 | 0.55 | 0.018 | 0.018 | 0.303 |
| InternalIMT | epa | rs117610108 | 0.01 | -0.027 | 0.012 | 0.023 | -0.174 | 0.037 | 2.40E-06 | -7.90E-03 | 0.035 | 0.821 |
| InternalIMT | epa | rs144966941 | 0.023 | -0.025 | 0.012 | 0.031 | 0.071 | 0.048 | 0.141 | -8.70E-04 | 0.067 | 0.99 |
| InternalIMT | epa | rs41502051 | 0.03 | -0.022 | 0.012 | 0.062 | 4.60E-03 | 0.033 | 0.891 | -0.067 | 0.05 | 0.18 |
| InternalIMT | epa | rs41426046 | 0.011 | -0.027 | 0.012 | 0.024 | -0.179 | 0.037 | 1.00E-06 | -7.20E-03 | 0.035 | 0.836 |
| InternalIMT | epa | rs41519248 | 0.01 | -0.027 | 0.012 | 0.023 | -0.175 | 0.037 | 2.20E-06 | -7.30E-03 | 0.035 | 0.835 |
| InternalIMT | epa | rs189108245 | 0.004 | . | . | . | . | . | . | . | . | . |
| InternalIMT | epa | rs7896431 | 0.532 | -0.032 | 0.021 | 0.121 | -1.20E-04 | 0.011 | 0.992 | 5.80E-03 | 0.016 | 0.712 |
| InternalIMT | epa | rs183724547 | 0.003 | . | . | . | . | . | . | . | . | . |
| InternalIMT | epa | rs142044990 | 0.008 | . | . | . | . | . | . | . | . | . |
| InternalIMT | epa | rs188140929 | 0.007 | . | . | . | . | . | . | . | . | . |
| InternalIMT | epa | rs76819096 | 0.014 | -0.019 | 0.013 | 0.159 | 0.029 | 0.069 | 0.676 | -0.047 | 0.121 | 0.7 |
| InternalIMT | epa | rs10751383 | 0.601 | -0.034 | 0.022 | 0.124 | 0.011 | 0.011 | 0.315 | 6.70E-03 | 0.015 | 0.66 |
| InternalIMT | epa | rs117819321 | 0.014 | -0.02 | 0.014 | 0.146 | 0.106 | 0.086 | 0.216 | -4.50E-03 | 0.185 | 0.98 |
| InternalIMT | epa | rs2242332 | 0.702 | -0.045 | 0.027 | 0.099 | -2.30E-05 | 0.012 | 0.998 | 0.014 | 0.017 | 0.432 |
| InternalIMT | epa | rs2242333 | 0.096 | -0.028 | 0.013 | 0.031 | -0.077 | 0.015 | 4.20E-07 | 0.03 | 0.02 | 0.127 |
| InternalIMT | epa | rs2242334 | 0.703 | -0.045 | 0.028 | 0.103 | -2.10E-04 | 0.012 | 0.986 | 0.013 | 0.017 | 0.443 |
| InternalIMT | epa | rs189465041 | 0.001 | . | . | . | . | . | . | . | . | . |
| InternalIMT | epa | rs28395868 | 0.025 | -0.021 | 0.014 | 0.117 | 0.027 | 0.049 | 0.576 | 0.037 | 0.073 | 0.607 |
| InternalIMT | epa | rs56067550 | 0.02 | -0.024 | 0.014 | 0.081 | -0.047 | 0.048 | 0.318 | 0.113 | 0.071 | 0.11 |
| InternalIMT | epa | rs1051713 | 0.154 | -0.03 | 0.013 | 0.026 | 2.40E-03 | 0.016 | 0.885 | 0.014 | 0.022 | 0.53 |
| InternalIMT | epa | rs115074210 | 0.016 | -0.02 | 0.013 | 0.134 | 0.039 | 0.066 | 0.56 | 6.70E-03 | 0.114 | 0.953 |
| InternalIMT | epa | rs7900977 | 0.802 | -0.058 | 0.033 | 0.075 | 0.012 | 0.013 | 0.371 | 0.02 | 0.019 | 0.3 |
| InternalIMT | epa | rs147613891 | 0.003 | . | . | . | . | . | . | . | . | . |
| InternalIMT | epa | rs2229136 | 0.084 | -0.026 | 0.012 | 0.034 | 0.032 | 0.022 | 0.14 | 4.50E-03 | 0.031 | 0.882 |
| InternalIMT | epa | rs185482841 | 0.003 | . | . | . | . | . | . | . | . | . |
| InternalIMT | epa | rs2288619 | 0.083 | -0.026 | 0.012 | 0.034 | 0.028 | 0.022 | 0.199 | 3.60E-03 | 0.031 | 0.906 |
| InternalIMT | epa | rs189891524 | 0.001 | . | . | . | . | . | . | . | . | . |
| InternalIMT | epa | rs56145077 | 0.143 | -0.029 | 0.013 | 0.028 | 0.011 | 0.016 | 0.495 | 0.01 | 0.022 | 0.653 |
| InternalIMT | epa | rs143216970 | 0.005 | . | . | . | . | . | . | . | . | . |
| InternalIMT | epa | rs142246478 | 0.001 | . | . | . | . | . | . | . | . | . |
| InternalIMT | epa | rs13134 | 0.012 | -0.019 | 0.013 | 0.166 | -5.10E-03 | 0.057 | 0.928 | -0.049 | 0.073 | 0.5 |
| InternalIMT | epa | rs41314980 | 0.027 | -0.024 | 0.014 | 0.082 | 0.077 | 0.052 | 0.136 | 0.094 | 0.077 | 0.218 |
| InternalIMT | epa | rs41304611 | 0.013 | -0.019 | 0.014 | 0.174 | -0.017 | 0.065 | 0.792 | -0.047 | 0.13 | 0.719 |
| InternalIMT | epa | rs193299617 | 0.001 | . | . | . | . | . | . | . | . | . |
| InternalIMT | epa | rs12772724 | 0.012 | -0.019 | 0.013 | 0.166 | -5.10E-03 | 0.057 | 0.929 | -0.049 | 0.073 | 0.5 |
| InternalIMT | epa | rs139489537 | 0.033 | -0.026 | 0.012 | 0.028 | 0.054 | 0.031 | 0.086 | 8.40E-03 | 0.043 | 0.845 |
| InternalIMT | epa | rs12776062 | 0.009 | . | . | . | . | . | . | . | . | . |
| InternalIMT | epa | rs752059 | 0.144 | -0.03 | 0.013 | 0.022 | 8.70E-03 | 0.016 | 0.591 | 0.014 | 0.022 | 0.526 |
| InternalIMT | epa | rs147649982 | 0.03 | -0.029 | 0.012 | 0.014 | 0.054 | 0.056 | 0.34 | 0.061 | 0.07 | 0.384 |
| InternalIMT | epa | rs17157798 | 0.07 | -0.025 | 0.012 | 0.045 | 0.025 | 0.023 | 0.279 | -7.90E-03 | 0.033 | 0.81 |
| InternalIMT | epa | rs142298619 | 0.003 | . | . | . | . | . | . | . | . | . |
| InternalIMT | epa | rs4948673 | 0.793 | -0.059 | 0.032 | 0.066 | 8.20E-03 | 0.013 | 0.529 | 0.021 | 0.019 | 0.279 |
| InternalIMT | epa | rs74231484 | 0.102 | -0.028 | 0.013 | 0.033 | -0.075 | 0.015 | 8.00E-07 | 0.026 | 0.019 | 0.174 |
| InternalIMT | epa | rs72796489 | 0.002 | . | . | . | . | . | . | . | . | . |
| InternalIMT | epa | rs113447578 | 0.016 | -0.027 | 0.012 | 0.02 | -0.092 | 0.037 | 0.014 | 0.059 | 0.044 | 0.184 |
| InternalIMT | epa | rs34063374 | 0.013 | -0.018 | 0.014 | 0.173 | -8.00E-03 | 0.056 | 0.886 | -0.06 | 0.072 | 0.402 |
| InternalIMT | epa | rs11239527 | 0.013 | -0.019 | 0.014 | 0.157 | 0.089 | 0.072 | 0.217 | -0.019 | 0.108 | 0.862 |
| InternalIMT | epa | rs34371883 | 0.011 | -0.019 | 0.013 | 0.167 | -0.033 | 0.065 | 0.617 | -0.065 | 0.082 | 0.429 |
| InternalIMT | epa | rs118168828 | 0.007 | . | . | . | . | . | . | . | . | . |
| InternalIMT | epa | rs34349618 | 0.009 | . | . | . | . | . | . | . | . | . |
| InternalIMT | epa | rs80041679 | 0.033 | -0.026 | 0.012 | 0.028 | 0.054 | 0.031 | 0.085 | 8.80E-03 | 0.043 | 0.837 |
| InternalIMT | epa | rs149640607 | 0.003 | . | . | . | . | . | . | . | . | . |
| InternalIMT | epa | rs4427523 | 0.888 | -0.052 | 0.057 | 0.358 | -0.038 | 0.021 | 0.069 | 0.015 | 0.031 | 0.641 |
| InternalIMT | epa | rs185880109 | 0.002 | . | . | . | . | . | . | . | . | . |
| InternalIMT | epa | rs191523220 | 0.003 | . | . | . | . | . | . | . | . | . |
| InternalIMT | epa | rs144315284 | 0.006 | . | . | . | . | . | . | . | . | . |

| InternalIMT | epa | rs4948674 | 0.341 | -0.027 | 0.015 | 0.068 | 0.041 | 0.012 | 5.50E-04 | 3.10E-03 | 0.016 | 0.843 |
| --- | --- | --- | --- | --- | --- | --- | --- | --- | --- | --- | --- | --- |
| InternalIMT | epa | rs182984298 | 0.006 | . | . | . | . | . | . | . | . | . |
| InternalIMT | epa | rs56749023 | 0.025 | -0.018 | 0.013 | 0.179 | -0.021 | 0.043 | 0.627 | -0.037 | 0.07 | 0.596 |
| InternalIMT | epa | rs112508957 | 0.015 | -0.021 | 0.013 | 0.113 | 0.064 | 0.047 | 0.171 | 0.037 | 0.07 | 0.598 |
| InternalIMT | epa | rs7089063 | 0.229 | -0.026 | 0.014 | 0.074 | -2.70E-03 | 0.013 | 0.839 | -4.30E-04 | 0.018 | 0.981 |
| InternalIMT | epa | rs145000779 | 0.001 | . | . | . | . | . | . | . | . | . |
| InternalIMT | epa | rs80201572 | 0.034 | -0.026 | 0.012 | 0.028 | 0.053 | 0.031 | 0.089 | 8.90E-03 | 0.043 | 0.835 |
| InternalIMT | epa | rs140568227 | 0.005 | . | . | . | . | . | . | . | . | . |
| InternalIMT | epa | rs149756228 | 0.01 | . | . | . | . | . | . | . | . | . |
| InternalIMT | epa | rs112380685 | 0.007 | . | . | . | . | . | . | . | . | . |
| InternalIMT | epa | rs60481109 | 0.023 | -0.018 | 0.013 | 0.179 | -0.021 | 0.044 | 0.643 | -0.039 | 0.072 | 0.586 |
| InternalIMT | epa | rs138221843 | 0.003 | . | . | . | . | . | . | . | . | . |
| InternalIMT | epa | rs150283194 | 0.003 | . | . | . | . | . | . | . | . | . |
| InternalIMT | epa | rs138879661 | 0.004 | . | . | . | . | . | . | . | . | . |
| InternalIMT | epa | rs34960345 | 0.056 | -0.025 | 0.012 | 0.044 | 0.029 | 0.024 | 0.234 | -0.01 | 0.034 | 0.765 |
| InternalIMT | epa | rs147514068 | 0.004 | . | . | . | . | . | . | . | . | . |
| InternalIMT | epa | rs35410535 | 0.078 | -0.024 | 0.012 | 0.054 | 0.021 | 0.022 | 0.331 | -0.013 | 0.031 | 0.673 |
| InternalIMT | epa | rs191613635 | 0.003 | . | . | . | . | . | . | . | . | . |
| InternalIMT | epa | rs17157800 | 0.007 | . | . | . | . | . | . | . | . | . |
| InternalIMT | epa | rs17523413 | 0.045 | -0.025 | 0.012 | 0.037 | 0.043 | 0.027 | 0.118 | -2.80E-03 | 0.037 | 0.939 |
| InternalIMT | epa | rs7897036 | 0.035 | -0.021 | 0.012 | 0.082 | -0.034 | 0.033 | 0.301 | -0.07 | 0.042 | 0.097 |
| InternalIMT | epa | rs76334323 | 0.01 | -0.027 | 0.012 | 0.023 | -0.18 | 0.037 | 1.10E-06 | -1.70E-03 | 0.035 | 0.96 |
| InternalIMT | epa | rs143535991 | 0.001 | . | . | . | . | . | . | . | . | . |
| InternalIMT | epa | rs140344979 | 0.005 | . | . | . | . | . | . | . | . | . |
| InternalIMT | epa | rs36047811 | 0.078 | -0.024 | 0.012 | 0.054 | 0.021 | 0.022 | 0.331 | -0.013 | 0.031 | 0.676 |
| InternalIMT | epa | rs76493570 | 0.054 | -0.024 | 0.012 | 0.046 | 0.03 | 0.025 | 0.231 | -0.013 | 0.034 | 0.704 |
| InternalIMT | epa | rs192998920 | 0.003 | . | . | . | . | . | . | . | . | . |
| InternalIMT | epa | rs116840852 | 0.016 | -0.019 | 0.014 | 0.171 | 0.065 | 0.064 | 0.311 | -0.024 | 0.087 | 0.781 |
| InternalIMT | epa | rs184824101 | 0.001 | . | . | . | . | . | . | . | . | . |
| InternalIMT | epa | rs41301621 | 0.005 | . | . | . | . | . | . | . | . | . |
| InternalIMT | epa | rs3740109 | 0.078 | -0.024 | 0.012 | 0.054 | 0.021 | 0.022 | 0.33 | -0.013 | 0.031 | 0.683 |
| InternalIMT | epa | rs7899415 | 0.033 | -0.021 | 0.012 | 0.078 | -3.20E-03 | 0.034 | 0.926 | -0.069 | 0.043 | 0.115 |
| InternalIMT | epa | rs184395284 | 0.002 | . | . | . | . | . | . | . | . | . |
| InternalIMT | epa | rs139209589 | 0.004 | . | . | . | . | . | . | . | . | . |
| InternalIMT | epa | rs149924163 | 0.003 | . | . | . | . | . | . | . | . | . |
| InternalIMT | epa | rs3740110 | 0.01 | -0.027 | 0.012 | 0.023 | -0.179 | 0.037 | 1.10E-06 | -1.80E-03 | 0.035 | 0.96 |
| InternalIMT | epa | rs144335113 | 0.003 | . | . | . | . | . | . | . | . | . |
| InternalIMT | epa | rs74743385 | 0.015 | -0.021 | 0.013 | 0.113 | 0.064 | 0.047 | 0.172 | 0.037 | 0.07 | 0.596 |
| InternalIMT | epa | rs146545829 | 0.003 | . | . | . | . | . | . | . | . | . |
| InternalIMT | epa | rs181842689 | 0 | . | . | . | . | . | . | . | . | . |
| InternalIMT | epa | rs3740111 | 0.056 | -0.025 | 0.012 | 0.044 | 0.029 | 0.024 | 0.234 | -9.50E-03 | 0.034 | 0.777 |
| InternalIMT | epa | rs186261599 | 0.003 | . | . | . | . | . | . | . | . | . |
| InternalIMT | epa | rs73285348 | 0.009 | . | . | . | . | . | . | . | . | . |
| InternalIMT | epa | rs186356901 | 0.003 | . | . | . | . | . | . | . | . | . |
| InternalIMT | epa | rs3824618 | 0.033 | -0.026 | 0.012 | 0.028 | 0.054 | 0.031 | 0.087 | 9.80E-03 | 0.043 | 0.819 |
| InternalIMT | epa | rs3802548 | 0.219 | -0.031 | 0.014 | 0.028 | 6.60E-03 | 0.014 | 0.63 | 0.011 | 0.018 | 0.534 |
| InternalIMT | epa | rs3802547 | 0.01 | . | . | . | . | . | . | . | . | . |
| InternalIMT | epa | rs77677521 | 0.016 | -0.019 | 0.014 | 0.171 | 0.065 | 0.064 | 0.31 | -0.024 | 0.087 | 0.781 |
| InternalIMT | epa | rs3824616 | 0.078 | -0.024 | 0.012 | 0.053 | 0.021 | 0.022 | 0.33 | -0.012 | 0.031 | 0.692 |
| InternalIMT | epa | rs191073818 | 0.003 | . | . | . | . | . | . | . | . | . |
| InternalIMT | epa | rs147078004 | 0.002 | . | . | . | . | . | . | . | . | . |
| InternalIMT | epa | rs7908745 | 0.31 | -0.028 | 0.015 | 0.069 | 3.50E-03 | 0.012 | 0.772 | 3.70E-03 | 0.017 | 0.822 |
| InternalIMT | epa | rs17157811 | 0.007 | . | . | . | . | . | . | . | . | . |
| InternalIMT | epa | rs138506087 | 0.006 | . | . | . | . | . | . | . | . | . |
| InternalIMT | epa | rs77241269 | 0.025 | -0.018 | 0.013 | 0.175 | -0.018 | 0.043 | 0.669 | -0.033 | 0.07 | 0.637 |
| InternalIMT | epa | rs138374938 | 0.001 | . | . | . | . | . | . | . | . | . |
| InternalIMT | epa | rs2279433 | 0.056 | -0.025 | 0.012 | 0.042 | 0.027 | 0.024 | 0.268 | -7.70E-03 | 0.033 | 0.815 |
| InternalIMT | epa | rs73285358 | 0.025 | -0.018 | 0.013 | 0.175 | -0.018 | 0.043 | 0.673 | -0.033 | 0.07 | 0.641 |
| InternalIMT | epa | rs11239528 | 0.218 | -0.031 | 0.014 | 0.027 | 7.10E-03 | 0.014 | 0.604 | 0.012 | 0.018 | 0.524 |
| InternalIMT | epa | rs77453386 | 0.01 | -0.027 | 0.012 | 0.023 | -0.178 | 0.037 | 1.30E-06 | -1.80E-03 | 0.035 | 0.959 |
| InternalIMT | epa | rs2279434 | 0.061 | -0.024 | 0.012 | 0.057 | 0.022 | 0.024 | 0.359 | -0.018 | 0.032 | 0.58 |
| InternalIMT | epa | rs149236525 | 0.005 | . | . | . | . | . | . | . | . | . |
| InternalIMT | epa | rs12573609 | 0.05 | -0.019 | 0.012 | 0.135 | -0.063 | 0.025 | 0.01 | -0.064 | 0.045 | 0.16 |
| InternalIMT | epa | rs191716655 | 0.002 | . | . | . | . | . | . | . | . | . |
| InternalIMT | epa | rs17523660 | 0.009 | . | . | . | . | . | . | . | . | . |
| InternalIMT | epa | rs142671360 | 0.003 | . | . | . | . | . | . | . | . | . |
| InternalIMT | epa | rs3764990 | 0.044 | -0.025 | 0.012 | 0.036 | 0.044 | 0.027 | 0.109 | -2.10E-03 | 0.037 | 0.955 |
| InternalIMT | epa | rs113682861 | 0.004 | . | . | . | . | . | . | . | . | . |
| InternalIMT | epa | rs117658270 | 0.01 | -0.027 | 0.012 | 0.023 | -0.178 | 0.037 | 1.40E-06 | -1.80E-03 | 0.035 | 0.959 |
| InternalIMT | epa | rs187309063 | 0 | . | . | . | . | . | . | . | . | . |
| InternalIMT | epa | rs73285360 | 0.025 | -0.018 | 0.013 | 0.174 | -0.018 | 0.043 | 0.676 | -0.032 | 0.07 | 0.643 |
| InternalIMT | epa | rs80133289 | 0.015 | -0.027 | 0.012 | 0.023 | -0.18 | 0.048 | 2.10E-04 | 0.078 | 0.06 | 0.189 |
| InternalIMT | epa | rs186693257 | 0.001 | . | . | . | . | . | . | . | . | . |
| InternalIMT | epa | rs35735968 | 0.062 | -0.025 | 0.012 | 0.043 | 0.023 | 0.024 | 0.326 | -7.20E-03 | 0.032 | 0.824 |

| InternalIMT | epa | rs17157825 | 0.034 | -0.026 | 0.012 | 0.027 | 0.057 | 0.032 | 0.072 | 0.012 | 0.043 | 0.773 |
| --- | --- | --- | --- | --- | --- | --- | --- | --- | --- | --- | --- | --- |
| InternalIMT | epa | rs2291428 | 0.213 | -0.029 | 0.014 | 0.034 | 9.90E-03 | 0.014 | 0.474 | 9.60E-03 | 0.019 | 0.61 |
| InternalIMT | epa | rs2291429 | 0.222 | -0.03 | 0.014 | 0.033 | 6.30E-03 | 0.014 | 0.647 | 9.70E-03 | 0.018 | 0.598 |
| InternalIMT | epa | rs145714792 | 0.005 | . | . | . | . | . | . | . | . | . |
| InternalIMT | epa | rs12769244 | 0.056 | -0.025 | 0.012 | 0.043 | 0.027 | 0.024 | 0.269 | -8.70E-03 | 0.033 | 0.79 |
| InternalIMT | epa | rs12771275 | 0.056 | -0.025 | 0.012 | 0.043 | 0.027 | 0.024 | 0.269 | -8.70E-03 | 0.033 | 0.79 |
| InternalIMT | epa | rs146266174 | 0.001 | . | . | . | . | . | . | . | . | . |
| InternalIMT | epa | rs41301623 | 0.003 | . | . | . | . | . | . | . | . | . |
| InternalIMT | epa | rs2306814 | 0.011 | -0.027 | 0.012 | 0.024 | -0.176 | 0.037 | 2.10E-06 | -6.00E-03 | 0.035 | 0.865 |
| InternalIMT | epa | rs12244834 | 0.027 | -0.028 | 0.012 | 0.018 | 0.038 | 0.04 | 0.342 | 0.037 | 0.053 | 0.491 |
| InternalIMT | epa | rs184006011 | 0.002 | . | . | . | . | . | . | . | . | . |
| InternalIMT | epa | rs78829066 | 0.01 | -0.027 | 0.012 | 0.022 | -0.179 | 0.037 | 1.70E-06 | -2.10E-03 | 0.036 | 0.953 |
| InternalIMT | epa | rs34731408 | 0.056 | -0.025 | 0.012 | 0.043 | 0.027 | 0.024 | 0.268 | -8.70E-03 | 0.033 | 0.791 |
| InternalIMT | epa | rs17451516 | 0.012 | -0.019 | 0.013 | 0.165 | -3.30E-03 | 0.057 | 0.953 | -0.047 | 0.072 | 0.518 |
| InternalIMT | epa | rs139910133 | 0.003 | . | . | . | . | . | . | . | . | . |
| InternalIMT | epa | rs10900218 | 0.233 | -0.03 | 0.014 | 0.039 | 7.60E-03 | 0.013 | 0.568 | 8.20E-03 | 0.019 | 0.657 |
| InternalIMT | epa | rs146023999 | 0.009 | . | . | . | . | . | . | . | . | . |
| InternalIMT | epa | rs187296958 | 0.003 | . | . | . | . | . | . | . | . | . |
| InternalIMT | epa | rs142342833 | 0.015 | -0.017 | 0.025 | 0.485 | -0.124 | 0.081 | 0.126 | 0.149 | 0.109 | 0.169 |
| InternalIMT | epa | rs143489255 | 0.006 | . | . | . | . | . | . | . | . | . |
| InternalIMT | epa | rs138532518 | 0.006 | . | . | . | . | . | . | . | . | . |
| InternalIMT | epa | rs183768079 | 0.005 | . | . | . | . | . | . | . | . | . |
| InternalIMT | epa | rs117892272 | 0.012 | -3.50E-03 | 0.022 | 0.873 | -0.109 | 0.173 | 0.531 | -0.48 | 0.45 | 0.286 |
| InternalIMT | epa | rs192993943 | 0.003 | . | . | . | . | . | . | . | . | . |
| InternalIMT | epa | rs182989043 | 0.004 | . | . | . | . | . | . | . | . | . |
| InternalIMT | epa | rs189703435 | 0.007 | . | . | . | . | . | . | . | . | . |
| InternalIMT | epa | rs188794040 | 0.007 | . | . | . | . | . | . | . | . | . |
| InternalIMT | epa | rs143920953 | 0.003 | . | . | . | . | . | . | . | . | . |
| InternalIMT | epa | rs191029121 | 0.006 | . | . | . | . | . | . | . | . | . |
| InternalIMT | epa | rs116875448 | 0.006 | . | . | . | . | . | . | . | . | . |
| InternalIMT | epa | rs190167877 | 0.002 | . | . | . | . | . | . | . | . | . |
| InternalIMT | epa | rs184993418 | 0.003 | . | . | . | . | . | . | . | . | . |
| InternalIMT | epa | rs143241158 | 0.01 | . | . | . | . | . | . | . | . | . |
| InternalIMT | epa | rs11239500 | 0.038 | -0.028 | 0.015 | 0.066 | 0.074 | 0.042 | 0.077 | 0.096 | 0.049 | 0.052 |
| InternalIMT | epa | rs140568994 | 0.001 | . | . | . | . | . | . | . | . | . |
| InternalIMT | epa | rs187530317 | 0.003 | . | . | . | . | . | . | . | . | . |
| InternalIMT | epa | rs145189326 | 0.002 | . | . | . | . | . | . | . | . | . |
| InternalIMT | epa | rs186203332 | 0.009 | . | . | . | . | . | . | . | . | . |
| InternalIMT | epa | rs189921909 | 0.004 | . | . | . | . | . | . | . | . | . |
| InternalIMT | epa | rs187260989 | 0.01 | -8.00E-03 | 0.024 | 0.741 | -0.038 | 0.153 | 0.804 | -0.325 | 0.254 | 0.2 |
| InternalIMT | epa | rs114376586 | 0.037 | -0.027 | 0.015 | 0.074 | 0.069 | 0.042 | 0.096 | 0.085 | 0.048 | 0.076 |
| InternalIMT | epa | rs151070808 | 0.019 | -7.40E-03 | 0.025 | 0.769 | -0.049 | 0.084 | 0.559 | -0.242 | 0.145 | 0.096 |
| InternalIMT | epa | rs140927225 | 0.038 | -0.027 | 0.015 | 0.068 | 0.07 | 0.042 | 0.099 | 0.093 | 0.049 | 0.056 |
| InternalIMT | epa | rs191555615 | 0.012 | -1.70E-03 | 0.022 | 0.939 | -0.13 | 0.205 | 0.525 | -0.569 | 0.488 | 0.244 |
| InternalIMT | epa | rs80278898 | 0.012 | -0.022 | 0.015 | 0.139 | -0.143 | 0.098 | 0.142 | 0.137 | 0.158 | 0.385 |
| InternalIMT | epa | rs190849503 | 0.004 | . | . | . | . | . | . | . | . | . |
| InternalIMT | epa | rs150080315 | 0.006 | . | . | . | . | . | . | . | . | . |
| InternalIMT | epa | rs143623885 | 0.011 | -0.016 | 0.025 | 0.506 | -0.125 | 0.094 | 0.182 | 0.179 | 0.13 | 0.17 |
| InternalIMT | epa | rs187760040 | 0.002 | . | . | . | . | . | . | . | . | . |
| InternalIMT | epa | rs143917143 | 0.01 | -0.011 | 0.017 | 0.506 | -0.139 | 0.086 | 0.107 | -0.098 | 0.12 | 0.414 |
| InternalIMT | epa | rs149851136 | 0.001 | . | . | . | . | . | . | . | . | . |
| InternalIMT | epa | rs181612054 | 0.001 | . | . | . | . | . | . | . | . | . |
| InternalIMT | epa | rs191006548 | 0.001 | . | . | . | . | . | . | . | . | . |
| InternalIMT | epa | rs117377631 | 0.012 | -0.017 | 0.015 | 0.256 | 0.111 | 0.118 | 0.349 | -0.073 | 0.141 | 0.606 |
| InternalIMT | epa | rs182107634 | 0.002 | . | . | . | . | . | . | . | . | . |
| InternalIMT | epa | rs190211867 | 0.002 | . | . | . | . | . | . | . | . | . |
| InternalIMT | epa | rs148293457 | 0.031 | -7.80E-03 | 0.026 | 0.765 | 4.40E-03 | 0.062 | 0.944 | -0.11 | 0.082 | 0.18 |
| InternalIMT | epa | rs191811733 | 0.006 | . | . | . | . | . | . | . | . | . |
| InternalIMT | epa | rs146109272 | 0.008 | . | . | . | . | . | . | . | . | . |
| InternalIMT | epa | rs148397915 | 0.006 | . | . | . | . | . | . | . | . | . |
| InternalIMT | epa | rs142233290 | 0.007 | . | . | . | . | . | . | . | . | . |
| InternalIMT | epa | rs80226874 | 0.022 | -0.015 | 0.025 | 0.563 | 0.108 | 0.138 | 0.435 | 0.025 | 0.215 | 0.906 |
| InternalIMT | epa | rs144496203 | 0.004 | . | . | . | . | . | . | . | . | . |
| InternalIMT | epa | rs191062130 | 0.001 | . | . | . | . | . | . | . | . | . |
| InternalIMT | epa | rs185807817 | 0.004 | . | . | . | . | . | . | . | . | . |
| InternalIMT | epa | rs117846213 | 0.012 | -0.032 | 0.019 | 0.098 | -0.12 | 0.069 | 0.08 | -0.019 | 0.097 | 0.847 |
| InternalIMT | epa | rs190545473 | 0.002 | . | . | . | . | . | . | . | . | . |
| InternalIMT | epa | rs192364918 | 0.01 | -0.019 | 0.025 | 0.438 | -0.028 | 0.347 | 0.935 | 0.309 | 0.438 | 0.48 |
| InternalIMT | epa | rs182939241 | 0.003 | . | . | . | . | . | . | . | . | . |
| InternalIMT | epa | rs12571125 | 0.011 | -0.031 | 0.019 | 0.106 | -0.167 | 0.087 | 0.056 | 0.026 | 0.102 | 0.801 |
| InternalIMT | epa | rs192418941 | 0.002 | . | . | . | . | . | . | . | . | . |
| InternalIMT | epa | rs141270243 | 0.015 | -0.01 | 0.025 | 0.674 | -0.203 | 0.088 | 0.022 | -0.095 | 0.144 | 0.508 |
| InternalIMT | epa | rs182615502 | 0.006 | . | . | . | . | . | . | . | . | . |
| InternalIMT | epa | rs146669856 | 0.01 | -0.024 | 0.014 | 0.099 | 0.155 | 0.099 | 0.116 | 0.193 | 0.158 | 0.223 |

| InternalIMT | epa | rs78290029 | 0.053 | -0.022 | 0.015 | 0.147 | 0.08 | 0.037 | 0.03 | 7.00E-03 | 0.047 | 0.881 |
| --- | --- | --- | --- | --- | --- | --- | --- | --- | --- | --- | --- | --- |
| InternalIMT | epa | rs139955565 | 0.004 | . | . | . | . | . | . | . | . | . |
| InternalIMT | epa | rs12267314 | 0.047 | -0.023 | 0.015 | 0.133 | 0.068 | 0.038 | 0.077 | 0.02 | 0.047 | 0.669 |
| InternalIMT | epa | rs74477197 | 0.004 | . | . | . | . | . | . | . | . | . |
| InternalIMT | epa | rs188147236 | 0.005 | . | . | . | . | . | . | . | . | . |
| InternalIMT | epa | rs2228065 | 0.046 | -0.022 | 0.015 | 0.142 | 0.069 | 0.038 | 0.074 | 0.014 | 0.047 | 0.768 |
| InternalIMT | epa | rs116961353 | 0.01 | . | . | . | . | . | . | . | . | . |
| InternalIMT | epa | rs190222272 | 0.007 | . | . | . | . | . | . | . | . | . |
| InternalIMT | epa | rs182719940 | 0.006 | . | . | . | . | . | . | . | . | . |
| InternalIMT | epa | rs184477413 | 0.004 | . | . | . | . | . | . | . | . | . |
| InternalIMT | epa | rs12265175 | 0.048 | -0.023 | 0.015 | 0.134 | 0.074 | 0.038 | 0.054 | 0.018 | 0.047 | 0.694 |
| InternalIMT | epa | rs182928276 | 0.003 | . | . | . | . | . | . | . | . | . |
| InternalIMT | epa | rs144409895 | 0.011 | -0.02 | 0.025 | 0.412 | -0.033 | 0.329 | 0.921 | 0.392 | 0.441 | 0.374 |
| InternalIMT | epa | rs58998447 | 0.031 | -0.017 | 0.015 | 0.265 | -0.095 | 0.036 | 9.10E-03 | 2.90E-03 | 0.042 | 0.946 |
| InternalIMT | epa | rs188668832 | 0.005 | . | . | . | . | . | . | . | . | . |
| InternalIMT | epa | rs148641686 | 0.002 | . | . | . | . | . | . | . | . | . |
| InternalIMT | epa | rs75468791 | 0.054 | -0.025 | 0.026 | 0.336 | 0.05 | 0.08 | 0.536 | 0.133 | 0.128 | 0.3 |
| InternalIMT | epa | rs147327397 | 0.003 | . | . | . | . | . | . | . | . | . |
| InternalIMT | epa | rs77354597 | 0.007 | . | . | . | . | . | . | . | . | . |
| InternalIMT | epa | rs41365544 | 0.045 | -0.022 | 0.015 | 0.145 | 0.061 | 0.04 | 0.127 | 0.015 | 0.051 | 0.77 |
| InternalIMT | epa | rs187564188 | 0.016 | -3.30E-03 | 0.025 | 0.897 | 0.06 | 0.289 | 0.835 | -0.423 | 0.33 | 0.2 |
| InternalIMT | epa | rs183511984 | 0.002 | . | . | . | . | . | . | . | . | . |
| InternalIMT | epa | rs12262783 | 0.038 | -0.022 | 0.015 | 0.129 | 0.096 | 0.047 | 0.04 | 0.025 | 0.06 | 0.681 |
| InternalIMT | epa | rs188817687 | 0.002 | . | . | . | . | . | . | . | . | . |
| InternalIMT | epa | rs3815968 | 0.005 | . | . | . | . | . | . | . | . | . |
| InternalIMT | epa | rs187822300 | 0.001 | . | . | . | . | . | . | . | . | . |
| InternalIMT | epa | rs188902172 | 0.008 | . | . | . | . | . | . | . | . | . |
| InternalIMT | epa | rs181190448 | 0.006 | . | . | . | . | . | . | . | . | . |
| InternalIMT | epa | rs182699093 | 0.001 | . | . | . | . | . | . | . | . | . |
| InternalIMT | epa | rs182631303 | 0.005 | . | . | . | . | . | . | . | . | . |
| InternalIMT | epa | rs187014172 | 0.003 | . | . | . | . | . | . | . | . | . |
| InternalIMT | epa | rs142575406 | 0.009 | . | . | . | . | . | . | . | . | . |
| InternalIMT | epa | rs180711033 | 0.007 | . | . | . | . | . | . | . | . | . |
| InternalIMT | epa | rs137894823 | 0.002 | . | . | . | . | . | . | . | . | . |
| InternalIMT | epa | rs189053273 | 0.001 | . | . | . | . | . | . | . | . | . |
| InternalIMT | epa | rs143499388 | 0.002 | . | . | . | . | . | . | . | . | . |
| InternalIMT | epa | rs182936001 | 0.003 | . | . | . | . | . | . | . | . | . |
| InternalIMT | epa | rs148456406 | 0.003 | . | . | . | . | . | . | . | . | . |
| InternalIMT | epa | rs182499531 | 0.005 | . | . | . | . | . | . | . | . | . |
| InternalIMT | epa | rs74884915 | 0.016 | 7.20E-04 | 0.019 | 0.97 | -0.068 | 0.055 | 0.219 | -0.066 | 0.072 | 0.355 |
| InternalIMT | epa | rs184046374 | 0.001 | . | . | . | . | . | . | . | . | . |
| InternalIMT | epa | rs74333993 | 0.019 | -9.30E-03 | 0.019 | 0.628 | 0.076 | 0.073 | 0.292 | 0.106 | 0.08 | 0.186 |
| InternalIMT | epa | rs138889719 | 0.001 | . | . | . | . | . | . | . | . | . |
| InternalIMT | epa | rs75330330 | 0.013 | -5.20E-03 | 0.019 | 0.784 | 0.097 | 0.084 | 0.248 | 0.068 | 0.111 | 0.541 |
| InternalIMT | epa | rs193051693 | 0 | . | . | . | . | . | . | . | . | . |
| InternalIMT | epa | rs145929633 | 0 | . | . | . | . | . | . | . | . | . |
| InternalIMT | epa | rs184076904 | 0.003 | . | . | . | . | . | . | . | . | . |
| InternalIMT | epa | rs141802494 | 0.011 | -6.30E-03 | 0.019 | 0.741 | 0.161 | 0.105 | 0.125 | 0.178 | 0.124 | 0.149 |
| InternalIMT | epa | rs149519759 | 0.001 | . | . | . | . | . | . | . | . | . |
| InternalIMT | epa | rs145467260 | 0.008 | . | . | . | . | . | . | . | . | . |
| InternalIMT | epa | rs79620914 | 0.017 | 1.70E-03 | 0.019 | 0.928 | -0.05 | 0.059 | 0.392 | -0.11 | 0.067 | 0.101 |
| InternalIMT | epa | rs140538633 | 0.011 | -1.00E-03 | 0.019 | 0.958 | -0.096 | 0.057 | 0.092 | -0.036 | 0.067 | 0.593 |
| InternalIMT | epa | rs140862301 | 0.005 | . | . | . | . | . | . | . | . | . |
| InternalIMT | epa | rs114615847 | 0.006 | . | . | . | . | . | . | . | . | . |
| InternalIMT | epa | rs141800259 | 0.016 | -6.60E-03 | 0.019 | 0.728 | 0.094 | 0.085 | 0.267 | 0.111 | 0.11 | 0.309 |
| InternalIMT | epa | rs149779059 | 0.011 | -7.70E-03 | 0.019 | 0.682 | 0.122 | 0.085 | 0.149 | 0.154 | 0.139 | 0.27 |
| InternalIMT | epa | rs116539253 | 0.004 | . | . | . | . | . | . | . | . | . |
| InternalIMT | epa | rs184979550 | 0.001 | . | . | . | . | . | . | . | . | . |
| InternalIMT | epa | rs190128940 | 0.003 | . | . | . | . | . | . | . | . | . |
| InternalIMT | epa | rs146594816 | 0.002 | . | . | . | . | . | . | . | . | . |
| InternalIMT | epa | rs141370766 | 0.013 | -5.20E-03 | 0.019 | 0.783 | 0.1 | 0.085 | 0.239 | 0.069 | 0.113 | 0.538 |
| InternalIMT | epa | rs144670033 | 0.002 | . | . | . | . | . | . | . | . | . |
| InternalIMT | epa | rs187152174 | 0.005 | . | . | . | . | . | . | . | . | . |
| InternalIMT | epa | rs184180263 | 0.003 | . | . | . | . | . | . | . | . | . |
| InternalIMT | epa | rs140075861 | 0.007 | . | . | . | . | . | . | . | . | . |
| InternalIMT | epa | rs186483129 | 0 | . | . | . | . | . | . | . | . | . |
| InternalIMT | epa | rs186634282 | 0.008 | . | . | . | . | . | . | . | . | . |
| InternalIMT | epa | rs9988663 | 0.019 | -9.30E-03 | 0.019 | 0.626 | 0.077 | 0.073 | 0.288 | 0.107 | 0.08 | 0.185 |
| InternalIMT | epa | rs138828529 | 0.013 | -3.20E-03 | 0.019 | 0.861 | 0.048 | 0.14 | 0.732 | -0.017 | 0.225 | 0.941 |
| InternalIMT | epa | rs188777615 | 0.003 | . | . | . | . | . | . | . | . | . |
| InternalIMT | epa | rs192321882 | 0.005 | . | . | . | . | . | . | . | . | . |
| InternalIMT | epa | rs150812270 | 0.016 | -9.80E-04 | 0.019 | 0.959 | -8.20E-04 | 0.071 | 0.991 | -0.08 | 0.12 | 0.509 |
| InternalIMT | epa | rs185906603 | 0.001 | . | . | . | . | . | . | . | . | . |
| InternalIMT | epa | rs191070862 | 0.004 | . | . | . | . | . | . | . | . | . |

| InternalIMT | epa | rs182904978 | 0 | . | . | . | . | . | . | . | . | . |
| --- | --- | --- | --- | --- | --- | --- | --- | --- | --- | --- | --- | --- |
| InternalIMT | epa | rs192679655 | 0.001 | . | . | . | . | . | . | . | . | . |
| InternalIMT | epa | rs189673086 | 0.005 | . | . | . | . | . | . | . | . | . |
| InternalIMT | epa | rs181141892 | 0.002 | . | . | . | . | . | . | . | . | . |
| InternalIMT | epa | rs186404123 | 0 | . | . | . | . | . | . | . | . | . |
| InternalIMT | epa | rs192117287 | 0.003 | . | . | . | . | . | . | . | . | . |
| InternalIMT | epa | rs182748835 | 0.001 | . | . | . | . | . | . | . | . | . |
| InternalIMT | epa | rs141854549 | 0.011 | -1.00E-03 | 0.019 | 0.957 | -0.094 | 0.057 | 0.098 | -0.034 | 0.067 | 0.606 |
| InternalIMT | epa | rs140163397 | 0.003 | . | . | . | . | . | . | . | . | . |
| InternalIMT | epa | rs146347300 | 0.005 | . | . | . | . | . | . | . | . | . |
| InternalIMT | epa | rs139362377 | 0.011 | -1.00E-03 | 0.019 | 0.957 | -0.094 | 0.057 | 0.099 | -0.034 | 0.067 | 0.607 |
| InternalIMT | epa | rs185087671 | 0.001 | . | . | . | . | . | . | . | . | . |
| InternalIMT | epa | rs190466574 | 0.003 | . | . | . | . | . | . | . | . | . |
| InternalIMT | epa | rs147012778 | 0.003 | . | . | . | . | . | . | . | . | . |
| InternalIMT | epa | rs138236960 | 0.006 | . | . | . | . | . | . | . | . | . |
| InternalIMT | epa | rs116310311 | 0.011 | -1.00E-03 | 0.019 | 0.956 | -0.094 | 0.057 | 0.1 | -0.034 | 0.067 | 0.609 |
| InternalIMT | epa | rs142868356 | 0.008 | . | . | . | . | . | . | . | . | . |
| InternalIMT | epa | rs144956240 | 0.002 | . | . | . | . | . | . | . | . | . |
| InternalIMT | epa | rs188527776 | 0.002 | . | . | . | . | . | . | . | . | . |
| InternalIMT | epa | rs183971165 | 0.005 | . | . | . | . | . | . | . | . | . |
| InternalIMT | epa | rs76748216 | 0.006 | . | . | . | . | . | . | . | . | . |
| InternalIMT | epa | rs148554990 | 0.001 | . | . | . | . | . | . | . | . | . |
| InternalIMT | epa | rs114446477 | 0.017 | 1.50E-03 | 0.019 | 0.938 | -0.047 | 0.059 | 0.428 | -0.104 | 0.066 | 0.117 |
| InternalIMT | epa | rs185223933 | 0.004 | . | . | . | . | . | . | . | . | . |
| InternalIMT | epa | rs76869726 | 0.006 | . | . | . | . | . | . | . | . | . |
| InternalIMT | epa | rs151200830 | 0.011 | -1.10E-03 | 0.019 | 0.955 | -0.089 | 0.057 | 0.119 | -0.033 | 0.067 | 0.623 |
| InternalIMT | epa | rs17157731 | 0.011 | -1.10E-03 | 0.019 | 0.955 | -0.088 | 0.057 | 0.121 | -0.033 | 0.067 | 0.627 |
| InternalIMT | epa | rs181243469 | 0 | . | . | . | . | . | . | . | . | . |
| InternalIMT | epa | rs186890707 | 0 | . | . | . | . | . | . | . | . | . |
| InternalIMT | epa | rs184063360 | 0 | . | . | . | . | . | . | . | . | . |
| InternalIMT | epa | rs188550237 | 0.004 | . | . | . | . | . | . | . | . | . |
| InternalIMT | epa | rs140931068 | 0.01 | -4.80E-03 | 0.019 | 0.801 | 0.167 | 0.12 | 0.166 | 0.046 | 0.175 | 0.795 |
| InternalIMT | epa | rs74867877 | 0.004 | . | . | . | . | . | . | . | . | . |
| InternalIMT | epa | rs11239495 | 0.036 | -0.013 | 0.02 | 0.507 | 0.096 | 0.056 | 0.086 | 0.112 | 0.066 | 0.091 |
| InternalIMT | epa | rs149745800 | 0 | . | . | . | . | . | . | . | . | . |
| InternalIMT | epa | rs145044458 | 0.01 | -1.10E-03 | 0.019 | 0.954 | -0.08 | 0.063 | 0.207 | -0.041 | 0.073 | 0.576 |
| InternalIMT | epa | rs188067427 | 0.001 | . | . | . | . | . | . | . | . | . |
| InternalIMT | epa | rs146827800 | 0.001 | . | . | . | . | . | . | . | . | . |
| InternalIMT | epa | rs140619817 | 0.01 | -1.30E-03 | 0.019 | 0.947 | -0.077 | 0.064 | 0.229 | -0.035 | 0.073 | 0.632 |
| InternalIMT | epa | rs892689 | 0.997 | . | . | . | . | . | . | . | . | . |
| InternalIMT | epa | rs143642005 | 0.004 | . | . | . | . | . | . | . | . | . |
| InternalIMT | epa | rs181929304 | 0.004 | . | . | . | . | . | . | . | . | . |
| InternalIMT | epa | rs191086173 | 0.001 | . | . | . | . | . | . | . | . | . |
| InternalIMT | epa | rs114613116 | 0.012 | -1.50E-03 | 0.019 | 0.936 | -0.08 | 0.057 | 0.163 | -0.018 | 0.067 | 0.787 |
| InternalIMT | epa | rs141076768 | 0.002 | . | . | . | . | . | . | . | . | . |
| InternalIMT | epa | rs187316531 | 0 | . | . | . | . | . | . | . | . | . |
| InternalIMT | epa | rs11239499 | 0.046 | -0.011 | 0.02 | 0.589 | 0.045 | 0.043 | 0.293 | 0.061 | 0.051 | 0.229 |
| InternalIMT | epa | rs192726371 | 0.002 | . | . | . | . | . | . | . | . | . |
| InternalIMT | epa | rs74128481 | 0.012 | -8.60E-03 | 0.019 | 0.647 | 0.147 | 0.102 | 0.151 | 0.155 | 0.148 | 0.296 |
| InternalIMT | epa | rs74128482 | 0.008 | . | . | . | . | . | . | . | . | . |
| InternalIMT | epa | rs11239502 | 0.033 | -0.012 | 0.019 | 0.523 | 0.095 | 0.056 | 0.093 | 0.108 | 0.067 | 0.108 |
| InternalIMT | epa | rs116330285 | 0.002 | . | . | . | . | . | . | . | . | . |
| InternalIMT | epa | rs189926729 | 0.002 | . | . | . | . | . | . | . | . | . |
| InternalIMT | epa | rs188975779 | 0.008 | . | . | . | . | . | . | . | . | . |
| InternalIMT | epa | rs143846038 | 0.002 | . | . | . | . | . | . | . | . | . |
| InternalIMT | epa | rs74128483 | 0.012 | -8.60E-03 | 0.019 | 0.646 | 0.147 | 0.102 | 0.151 | 0.156 | 0.149 | 0.295 |
| InternalIMT | epa | rs139292055 | 0.045 | -0.011 | 0.02 | 0.571 | 0.047 | 0.043 | 0.274 | 0.067 | 0.051 | 0.188 |
| InternalIMT | epa | rs11239503 | 0.045 | -0.011 | 0.02 | 0.57 | 0.047 | 0.043 | 0.274 | 0.067 | 0.051 | 0.188 |
| InternalIMT | epa | rs58838686 | 0.008 | . | . | . | . | . | . | . | . | . |
| InternalIMT | epa | rs185825373 | 0.005 | . | . | . | . | . | . | . | . | . |
| InternalIMT | epa | rs11239504 | 0.045 | -0.011 | 0.02 | 0.57 | 0.047 | 0.043 | 0.273 | 0.067 | 0.051 | 0.187 |
| InternalIMT | epa | rs150012354 | 0.003 | . | . | . | . | . | . | . | . | . |
| InternalIMT | epa | rs187763096 | 0.005 | . | . | . | . | . | . | . | . | . |
| InternalIMT | epa | rs193043982 | 0.005 | . | . | . | . | . | . | . | . | . |
| InternalIMT | epa | rs79186884 | 0.014 | -9.30E-03 | 0.019 | 0.624 | 0.122 | 0.075 | 0.105 | 0.159 | 0.121 | 0.188 |
| InternalIMT | epa | rs74128484 | 0.008 | . | . | . | . | . | . | . | . | . |
| InternalIMT | epa | rs58461537 | 0.045 | -0.011 | 0.02 | 0.569 | 0.047 | 0.043 | 0.272 | 0.067 | 0.051 | 0.186 |
| InternalIMT | epa | rs188527474 | 0.005 | . | . | . | . | . | . | . | . | . |
| InternalIMT | epa | rs141659976 | 0.005 | . | . | . | . | . | . | . | . | . |
| InternalIMT | epa | rs115846407 | 0.045 | -0.011 | 0.02 | 0.569 | 0.048 | 0.043 | 0.271 | 0.068 | 0.051 | 0.185 |
| InternalIMT | epa | rs138378816 | 0.045 | -0.011 | 0.02 | 0.569 | 0.048 | 0.043 | 0.272 | 0.068 | 0.051 | 0.185 |
| InternalIMT | epa | rs146188136 | 0.033 | -0.012 | 0.019 | 0.522 | 0.095 | 0.056 | 0.092 | 0.109 | 0.067 | 0.106 |
| InternalIMT | epa | rs139383871 | 0.008 | . | . | . | . | . | . | . | . | . |
| InternalIMT | epa | rs182953869 | 0.004 | . | . | . | . | . | . | . | . | . |

| InternalIMT | epa | rs192430299 | 0.012 | -8.80E-03 | 0.019 | 0.642 | 0.198 | 0.099 | 0.046 | 0.168 | 0.159 | 0.289 |
| --- | --- | --- | --- | --- | --- | --- | --- | --- | --- | --- | --- | --- |
| InternalIMT | epa | rs144152844 | 0.045 | -0.011 | 0.02 | 0.568 | 0.048 | 0.043 | 0.271 | 0.068 | 0.051 | 0.184 |
| InternalIMT | epa | rs145987786 | 0.033 | 6.70E-03 | 0.02 | 0.736 | -8.50E-03 | 0.044 | 0.847 | -0.141 | 0.062 | 0.023 |
| InternalIMT | epa | rs181132926 | 0.002 | . | . | . | . | . | . | . | . | . |
| InternalIMT | epa | rs147882253 | 0.045 | -0.011 | 0.02 | 0.568 | 0.048 | 0.043 | 0.27 | 0.068 | 0.051 | 0.184 |
| InternalIMT | epa | rs141807621 | 0.015 | -2.10E-03 | 0.019 | 0.91 | 2.10E-04 | 0.123 | 0.999 | -0.064 | 0.196 | 0.743 |
| InternalIMT | epa | rs147116052 | 0.045 | -0.011 | 0.02 | 0.568 | 0.048 | 0.043 | 0.27 | 0.068 | 0.051 | 0.184 |
| InternalIMT | epa | rs147716618 | 0.045 | -0.011 | 0.02 | 0.568 | 0.048 | 0.043 | 0.27 | 0.068 | 0.051 | 0.184 |
| InternalIMT | epa | rs187198855 | 0.029 | 4.00E-03 | 0.02 | 0.84 | 0.029 | 0.055 | 0.606 | -0.135 | 0.075 | 0.074 |
| InternalIMT | epa | rs189409638 | 0.011 | -0.011 | 0.019 | 0.568 | 0.18 | 0.103 | 0.082 | 0.25 | 0.147 | 0.09 |
| InternalIMT | epa | rs182647339 | 0.001 | . | . | . | . | . | . | . | . | . |
| InternalIMT | epa | rs187995988 | 0.004 | . | . | . | . | . | . | . | . | . |
| InternalIMT | epa | rs141034184 | 0.048 | -0.014 | 0.02 | 0.481 | 0.035 | 0.043 | 0.41 | 0.093 | 0.051 | 0.069 |
| InternalIMT | epa | rs187310430 | 0.005 | . | . | . | . | . | . | . | . | . |
| InternalIMT | epa | rs192608488 | 0.045 | -0.012 | 0.02 | 0.566 | 0.048 | 0.043 | 0.266 | 0.068 | 0.051 | 0.18 |
| InternalIMT | epa | rs148840366 | 0.006 | . | . | . | . | . | . | . | . | . |
| InternalIMT | epa | rs186785022 | 0.012 | -8.70E-03 | 0.019 | 0.645 | 0.148 | 0.102 | 0.148 | 0.158 | 0.149 | 0.289 |
| InternalIMT | epa | rs192348728 | 0.008 | . | . | . | . | . | . | . | . | . |
| InternalIMT | epa | rs189656631 | 0.045 | -0.012 | 0.02 | 0.565 | 0.048 | 0.043 | 0.266 | 0.068 | 0.051 | 0.18 |
| InternalIMT | epa | rs185289750 | 0.004 | . | . | . | . | . | . | . | . | . |
| InternalIMT | epa | rs142382395 | 0.045 | -0.012 | 0.02 | 0.565 | 0.048 | 0.043 | 0.266 | 0.068 | 0.051 | 0.18 |
| InternalIMT | epa | rs144866696 | 0.012 | -8.70E-03 | 0.019 | 0.645 | 0.148 | 0.102 | 0.148 | 0.158 | 0.149 | 0.288 |
| InternalIMT | epa | rs141371289 | 0.045 | -0.012 | 0.02 | 0.565 | 0.048 | 0.043 | 0.265 | 0.068 | 0.051 | 0.18 |
| InternalIMT | epa | rs114750008 | 0.045 | -0.012 | 0.02 | 0.565 | 0.048 | 0.043 | 0.265 | 0.068 | 0.051 | 0.18 |
| InternalIMT | epa | rs189063597 | 0.001 | . | . | . | . | . | . | . | . | . |
| InternalIMT | epa | rs139151571 | 0.041 | -0.01 | 0.02 | 0.615 | 0.043 | 0.045 | 0.343 | 0.059 | 0.054 | 0.271 |
| InternalIMT | epa | rs139846368 | 0.046 | -0.012 | 0.02 | 0.551 | 0.047 | 0.043 | 0.283 | 0.07 | 0.051 | 0.167 |
| InternalIMT | epa | rs182057653 | 0.012 | -7.10E-04 | 0.019 | 0.971 | -0.045 | 0.075 | 0.547 | -0.052 | 0.089 | 0.558 |
| InternalIMT | epa | rs148785420 | 0.045 | -0.012 | 0.02 | 0.565 | 0.048 | 0.043 | 0.265 | 0.068 | 0.051 | 0.18 |
| InternalIMT | epa | rs138383251 | 0.007 | . | . | . | . | . | . | . | . | . |
| InternalIMT | epa | rs116526705 | 0.012 | -2.30E-03 | 0.019 | 0.906 | -0.068 | 0.058 | 0.239 | 2.20E-03 | 0.068 | 0.974 |
| InternalIMT | epa | rs185946436 | 0 | . | . | . | . | . | . | . | . | . |
| InternalIMT | epa | rs190455427 | 0.004 | . | . | . | . | . | . | . | . | . |
| InternalIMT | epa | rs9282584 | 0.045 | -0.012 | 0.02 | 0.565 | 0.048 | 0.043 | 0.265 | 0.068 | 0.051 | 0.179 |
| InternalIMT | epa | rs17153289 | 0.045 | -0.012 | 0.02 | 0.564 | 0.048 | 0.043 | 0.264 | 0.068 | 0.051 | 0.179 |
| InternalIMT | epa | rs138359103 | 0.005 | . | . | . | . | . | . | . | . | . |
| InternalIMT | epa | rs150605268 | 0.002 | . | . | . | . | . | . | . | . | . |
| InternalIMT | epa | rs79242910 | 0.046 | -0.011 | 0.02 | 0.569 | 0.044 | 0.042 | 0.29 | 0.065 | 0.048 | 0.174 |
| InternalIMT | epa | rs139716904 | 0.013 | -7.70E-04 | 0.019 | 0.968 | -0.108 | 0.069 | 0.116 | -0.086 | 0.087 | 0.327 |
| InternalIMT | epa | rs76238498 | 0.034 | 6.50E-03 | 0.02 | 0.745 | -2.60E-03 | 0.044 | 0.952 | -0.135 | 0.061 | 0.026 |
| InternalIMT | epa | rs184140639 | 0 | . | . | . | . | . | . | . | . | . |
| InternalIMT | epa | rs188517278 | 0.006 | . | . | . | . | . | . | . | . | . |
| InternalIMT | epa | rs139678913 | 0.002 | . | . | . | . | . | . | . | . | . |
| InternalIMT | epa | rs116697955 | 0.004 | . | . | . | . | . | . | . | . | . |
| InternalIMT | epa | rs79267994 | 0.007 | . | . | . | . | . | . | . | . | . |
| InternalIMT | epa | rs11239510 | 0.013 | 7.30E-04 | 0.019 | 0.969 | -0.107 | 0.069 | 0.122 | -0.195 | 0.097 | 0.046 |
| InternalIMT | epa | rs75831645 | 0.012 | -2.30E-03 | 0.019 | 0.903 | -0.067 | 0.058 | 0.244 | 3.80E-03 | 0.068 | 0.955 |
| InternalIMT | epa | rs78452276 | 0.031 | 1.80E-03 | 0.019 | 0.925 | -5.40E-03 | 0.044 | 0.902 | -0.087 | 0.072 | 0.23 |
| InternalIMT | epa | rs144366924 | 0 | . | . | . | . | . | . | . | . | . |
| InternalIMT | epa | rs77519841 | 0.033 | -0.013 | 0.019 | 0.517 | 0.095 | 0.056 | 0.089 | 0.11 | 0.067 | 0.1 |
| InternalIMT | epa | rs76944454 | 0.045 | -0.012 | 0.02 | 0.562 | 0.048 | 0.043 | 0.263 | 0.069 | 0.051 | 0.173 |
| InternalIMT | epa | rs76443855 | 0.034 | 6.50E-03 | 0.02 | 0.744 | -2.90E-03 | 0.044 | 0.948 | -0.136 | 0.061 | 0.026 |
| InternalIMT | epa | rs146613174 | 0.001 | . | . | . | . | . | . | . | . | . |
| InternalIMT | epa | rs181424761 | 0.001 | . | . | . | . | . | . | . | . | . |
| InternalIMT | epa | rs142119241 | 0.001 | . | . | . | . | . | . | . | . | . |
| InternalIMT | epa | rs140137954 | 0.012 | -2.30E-03 | 0.019 | 0.905 | -0.068 | 0.058 | 0.242 | 2.80E-03 | 0.068 | 0.966 |
| InternalIMT | epa | rs187319549 | 0.002 | . | . | . | . | . | . | . | . | . |
| InternalIMT | epa | rs12241545 | 0.021 | -1.50E-03 | 0.019 | 0.938 | -0.119 | 0.071 | 0.093 | -0.041 | 0.121 | 0.734 |
| InternalIMT | epa | rs10128228 | 0.045 | -0.012 | 0.02 | 0.561 | 0.048 | 0.043 | 0.262 | 0.069 | 0.051 | 0.172 |
| InternalIMT | epa | rs58580249 | 0.006 | . | . | . | . | . | . | . | . | . |
| InternalIMT | epa | rs193148863 | 0.001 | . | . | . | . | . | . | . | . | . |
| InternalIMT | epa | rs185638946 | 0.005 | . | . | . | . | . | . | . | . | . |
| InternalIMT | epa | rs117752921 | 0.001 | . | . | . | . | . | . | . | . | . |
| InternalIMT | epa | rs180841236 | 0.001 | . | . | . | . | . | . | . | . | . |
| InternalIMT | epa | rs73283155 | 0.006 | . | . | . | . | . | . | . | . | . |
| InternalIMT | epa | rs76560599 | 0.038 | -8.40E-03 | 0.02 | 0.672 | 0.029 | 0.046 | 0.534 | 0.05 | 0.055 | 0.367 |
| InternalIMT | epa | rs76273620 | 0.038 | -8.40E-03 | 0.02 | 0.672 | 0.029 | 0.046 | 0.534 | 0.05 | 0.055 | 0.367 |
| InternalIMT | epa | rs141432923 | 0.012 | -7.80E-03 | 0.018 | 0.658 | 0.107 | 0.166 | 0.518 | 0.236 | 0.358 | 0.51 |
| InternalIMT | epa | rs113529322 | 0.012 | -8.70E-03 | 0.019 | 0.644 | 0.149 | 0.102 | 0.146 | 0.159 | 0.149 | 0.286 |
| InternalIMT | epa | rs10128306 | 0.045 | -0.012 | 0.02 | 0.56 | 0.049 | 0.043 | 0.259 | 0.07 | 0.051 | 0.172 |
| InternalIMT | epa | rs149691688 | 0.001 | . | . | . | . | . | . | . | . | . |
| InternalIMT | epa | rs75872495 | 0.045 | -0.012 | 0.02 | 0.56 | 0.049 | 0.043 | 0.256 | 0.069 | 0.051 | 0.174 |
| InternalIMT | epa | rs191873960 | 0.004 | . | . | . | . | . | . | . | . | . |
| InternalIMT | epa | rs80155622 | 0.045 | -0.012 | 0.02 | 0.56 | 0.049 | 0.043 | 0.257 | 0.069 | 0.051 | 0.174 |

| InternalIMT | epa | rs113914656 | 0.005 | . | . | . | . | . | . | . | . | . |
| --- | --- | --- | --- | --- | --- | --- | --- | --- | --- | --- | --- | --- |
| InternalIMT | epa | rs12242840 | 0.045 | -0.012 | 0.02 | 0.561 | 0.049 | 0.043 | 0.256 | 0.069 | 0.051 | 0.176 |
| InternalIMT | epa | rs147824078 | 0.006 | . | . | . | . | . | . | . | . | . |
| InternalIMT | epa | rs59051493 | 0.045 | -0.012 | 0.02 | 0.56 | 0.049 | 0.043 | 0.253 | 0.069 | 0.051 | 0.174 |
| InternalIMT | epa | rs9282588 | 0.005 | . | . | . | . | . | . | . | . | . |
| InternalIMT | epa | rs150215207 | 0.006 | . | . | . | . | . | . | . | . | . |
| InternalIMT | epa | rs138772243 | 0.002 | . | . | . | . | . | . | . | . | . |
| InternalIMT | epa | rs149383244 | 0.014 | -2.00E-04 | 0.019 | 0.992 | -0.025 | 0.067 | 0.71 | -0.078 | 0.103 | 0.453 |
| InternalIMT | epa | rs17522720 | 0.011 | -4.30E-03 | 0.019 | 0.821 | 0.014 | 0.078 | 0.858 | 0.037 | 0.118 | 0.754 |
| InternalIMT | epa | rs79245105 | 0.045 | -0.012 | 0.02 | 0.558 | 0.05 | 0.043 | 0.252 | 0.07 | 0.051 | 0.173 |
| InternalIMT | epa | rs138222133 | 0.002 | . | . | . | . | . | . | . | . | . |
| InternalIMT | epa | rs12247314 | 0.05 | -0.012 | 0.02 | 0.558 | 0.048 | 0.042 | 0.248 | 0.066 | 0.05 | 0.19 |
| InternalIMT | epa | rs12263894 | 0.049 | -0.012 | 0.02 | 0.563 | 0.05 | 0.042 | 0.238 | 0.065 | 0.051 | 0.204 |
| InternalIMT | epa | rs149907880 | 0.005 | . | . | . | . | . | . | . | . | . |
| InternalIMT | epa | rs188153885 | 0.005 | . | . | . | . | . | . | . | . | . |
| InternalIMT | epa | rs73283159 | 0.003 | . | . | . | . | . | . | . | . | . |
| InternalIMT | epa | rs140476687 | 0.002 | . | . | . | . | . | . | . | . | . |
| InternalIMT | epa | rs186603148 | 0 | . | . | . | . | . | . | . | . | . |
| InternalIMT | epa | rs183296063 | 0.002 | . | . | . | . | . | . | . | . | . |
| InternalIMT | epa | rs145733694 | 0.006 | . | . | . | . | . | . | . | . | . |
| InternalIMT | epa | rs189807449 | 0 | . | . | . | . | . | . | . | . | . |
| InternalIMT | epa | rs12253223 | 0.038 | -0.013 | 0.02 | 0.51 | 0.091 | 0.054 | 0.091 | 0.105 | 0.065 | 0.108 |
| InternalIMT | epa | rs182113637 | 0 | . | . | . | . | . | . | . | . | . |
| InternalIMT | epa | rs142792099 | 0.01 | -6.30E-03 | 0.019 | 0.737 | 0.175 | 0.109 | 0.108 | 0.196 | 0.128 | 0.124 |
| InternalIMT | epa | rs12249910 | 0.038 | -0.013 | 0.02 | 0.51 | 0.091 | 0.054 | 0.092 | 0.105 | 0.065 | 0.108 |
| InternalIMT | epa | rs149436339 | 0.001 | . | . | . | . | . | . | . | . | . |
| InternalIMT | epa | rs77632210 | 0 | . | . | . | . | . | . | . | . | . |
| InternalIMT | epa | rs74130818 | 0.008 | . | . | . | . | . | . | . | . | . |
| InternalIMT | epa | rs187583648 | 0 | . | . | . | . | . | . | . | . | . |
| InternalIMT | epa | rs191295987 | 0.004 | . | . | . | . | . | . | . | . | . |
| InternalIMT | epa | rs184200686 | 0.006 | . | . | . | . | . | . | . | . | . |
| InternalIMT | epa | rs76117263 | 0.033 | 6.60E-03 | 0.02 | 0.738 | 3.10E-03 | 0.045 | 0.946 | -0.14 | 0.062 | 0.023 |
| InternalIMT | epa | rs185393149 | 0.001 | . | . | . | . | . | . | . | . | . |
| InternalIMT | epa | rs144760014 | 0.003 | . | . | . | . | . | . | . | . | . |
| InternalIMT | epa | rs7922292 | 0.005 | . | . | . | . | . | . | . | . | . |
| InternalIMT | epa | rs115372844 | 0.006 | . | . | . | . | . | . | . | . | . |
| InternalIMT | epa | rs111460364 | 0.033 | 6.70E-03 | 0.02 | 0.735 | 3.20E-03 | 0.045 | 0.943 | -0.141 | 0.062 | 0.023 |
| InternalIMT | epa | rs182052255 | 0.001 | . | . | . | . | . | . | . | . | . |
| InternalIMT | epa | rs180983889 | 0.004 | . | . | . | . | . | . | . | . | . |
| InternalIMT | epa | rs139175327 | 0.006 | . | . | . | . | . | . | . | . | . |
| InternalIMT | epa | rs149959620 | 0.011 | -8.80E-04 | 0.019 | 0.963 | -0.094 | 0.071 | 0.186 | -0.095 | 0.094 | 0.311 |
| InternalIMT | epa | rs114653117 | 0.005 | . | . | . | . | . | . | . | . | . |
| InternalIMT | epa | rs144270018 | 0.003 | . | . | . | . | . | . | . | . | . |
| InternalIMT | epa | rs182723204 | 0.005 | . | . | . | . | . | . | . | . | . |
| InternalIMT | epa | rs57204067 | 0.008 | . | . | . | . | . | . | . | . | . |
| InternalIMT | epa | rs144956226 | 0.038 | -0.013 | 0.02 | 0.504 | 0.09 | 0.055 | 0.102 | 0.109 | 0.066 | 0.097 |
| InternalIMT | epa | rs187842704 | 0.01 | 6.40E-04 | 0.019 | 0.973 | 0.094 | 0.092 | 0.307 | -0.168 | 0.103 | 0.104 |
| InternalIMT | epa | rs184620511 | 0.008 | . | . | . | . | . | . | . | . | . |
| InternalIMT | epa | rs144031303 | 0.012 | -2.50E-03 | 0.019 | 0.898 | -0.062 | 0.06 | 0.296 | 4.50E-03 | 0.071 | 0.949 |
| InternalIMT | epa | rs187526473 | 0 | . | . | . | . | . | . | . | . | . |
| InternalIMT | epa | rs193002805 | 0.002 | . | . | . | . | . | . | . | . | . |
| InternalIMT | epa | rs138707426 | 0.014 | 6.10E-06 | 0.019 | 1 | -0.02 | 0.066 | 0.765 | -0.088 | 0.101 | 0.382 |
| InternalIMT | epa | rs142754243 | 0.03 | -2.10E-03 | 0.019 | 0.912 | -4.10E-03 | 0.06 | 0.945 | -0.021 | 0.092 | 0.82 |
| InternalIMT | epa | rs181600331 | 0.005 | . | . | . | . | . | . | . | . | . |
| InternalIMT | epa | rs144421264 | 0.018 | -1.70E-03 | 0.019 | 0.929 | -0.013 | 0.076 | 0.861 | -0.044 | 0.124 | 0.722 |
| InternalIMT | epa | rs184414519 | 0.004 | . | . | . | . | . | . | . | . | . |
| InternalIMT | epa | rs150596349 | 0.031 | 7.90E-03 | 0.02 | 0.69 | 1.60E-03 | 0.048 | 0.974 | -0.165 | 0.064 | 0.01 |
| InternalIMT | epa | rs190753747 | 0.001 | . | . | . | . | . | . | . | . | . |
| InternalIMT | epa | rs139560940 | 0.004 | . | . | . | . | . | . | . | . | . |
| InternalIMT | epa | rs75640804 | 0.033 | -0.013 | 0.019 | 0.503 | 0.096 | 0.057 | 0.092 | 0.114 | 0.065 | 0.081 |
| InternalIMT | epa | rs73283175 | 0.052 | 1.90E-03 | 0.02 | 0.925 | -0.029 | 0.04 | 0.467 | -0.041 | 0.055 | 0.461 |
| InternalIMT | epa | rs143337229 | 0.002 | . | . | . | . | . | . | . | . | . |
| InternalIMT | epa | rs73283177 | 0.03 | -2.20E-03 | 0.019 | 0.909 | -9.90E-04 | 0.062 | 0.987 | -0.02 | 0.094 | 0.828 |
| InternalIMT | epa | rs76053611 | 0.029 | 7.40E-03 | 0.02 | 0.706 | -5.40E-03 | 0.049 | 0.912 | -0.163 | 0.066 | 0.014 |
| InternalIMT | epa | rs116325983 | 0.004 | . | . | . | . | . | . | . | . | . |
| InternalIMT | epa | rs115451825 | 0.005 | . | . | . | . | . | . | . | . | . |
| InternalIMT | epa | rs183845246 | 0.001 | . | . | . | . | . | . | . | . | . |
| InternalIMT | epa | rs79756542 | 0.033 | 7.40E-03 | 0.02 | 0.708 | 4.70E-04 | 0.046 | 0.992 | -0.152 | 0.063 | 0.015 |
| InternalIMT | epa | rs187242860 | 0.002 | . | . | . | . | . | . | . | . | . |
| InternalIMT | epa | rs57318980 | 0.03 | -1.80E-03 | 0.019 | 0.924 | 6.40E-03 | 0.064 | 0.92 | -0.029 | 0.098 | 0.769 |
| InternalIMT | epa | rs59969732 | 0.027 | -4.10E-03 | 0.019 | 0.83 | 0.016 | 0.067 | 0.814 | 0.015 | 0.099 | 0.876 |
| InternalIMT | epa | rs143811488 | 0.006 | . | . | . | . | . | . | . | . | . |
| InternalIMT | epa | rs189067644 | 0.006 | . | . | . | . | . | . | . | . | . |
| InternalIMT | epa | rs60449178 | 0.025 | -4.90E-03 | 0.019 | 0.799 | 4.80E-03 | 0.07 | 0.946 | 0.038 | 0.11 | 0.729 |

| InternalIMT | epa | rs59243389 | 0.025 | -4.90E-03 | 0.019 | 0.799 | 4.80E-03 | 0.07 | 0.945 | 0.038 | 0.11 | 0.729 |
| --- | --- | --- | --- | --- | --- | --- | --- | --- | --- | --- | --- | --- |
| InternalIMT | epa | rs184946540 | 0.027 | 3.00E-03 | 0.019 | 0.877 | -0.064 | 0.058 | 0.268 | -0.101 | 0.092 | 0.269 |
| InternalIMT | epa | rs188685881 | 0.001 | . | . | . | . | . | . | . | . | . |
| InternalIMT | epa | rs150335797 | 0.004 | . | . | . | . | . | . | . | . | . |
| InternalIMT | epa | rs143879260 | 0.004 | . | . | . | . | . | . | . | . | . |
| InternalIMT | epa | rs186044491 | 0.006 | . | . | . | . | . | . | . | . | . |
| InternalIMT | epa | rs11819084 | 0.018 | 1.10E-03 | 0.019 | 0.956 | 0.016 | 0.06 | 0.795 | -0.103 | 0.08 | 0.196 |
| InternalIMT | epa | rs146915670 | 0.006 | . | . | . | . | . | . | . | . | . |
| InternalIMT | epa | rs113995695 | 0.017 | 5.10E-04 | 0.019 | 0.979 | 0.021 | 0.061 | 0.728 | -0.093 | 0.08 | 0.25 |
| InternalIMT | epa | rs143489451 | 0.003 | . | . | . | . | . | . | . | . | . |
| InternalIMT | epa | rs182653839 | 0 | . | . | . | . | . | . | . | . | . |
| InternalIMT | epa | rs141692564 | 0.004 | . | . | . | . | . | . | . | . | . |
| InternalIMT | epa | rs146281742 | 0.003 | . | . | . | . | . | . | . | . | . |
| InternalIMT | epa | rs192914353 | 0.003 | . | . | . | . | . | . | . | . | . |
| InternalIMT | epa | rs185182848 | 0.002 | . | . | . | . | . | . | . | . | . |
| InternalIMT | epa | rs28395858 | 0.017 | 2.40E-04 | 0.019 | 0.99 | 0.024 | 0.061 | 0.701 | -0.087 | 0.082 | 0.288 |
| InternalIMT | epa | rs41429645 | 0.005 | . | . | . | . | . | . | . | . | . |
| InternalIMT | epa | rs28395874 | 0.017 | 5.10E-05 | 0.019 | 0.998 | 0.025 | 0.061 | 0.682 | -0.083 | 0.082 | 0.313 |
| InternalIMT | epa | rs189663634 | 0.001 | . | . | . | . | . | . | . | . | . |
| InternalIMT | epa | rs77666700 | 0.006 | . | . | . | . | . | . | . | . | . |
| InternalIMT | epa | rs146711717 | 0.002 | . | . | . | . | . | . | . | . | . |
| InternalIMT | epa | rs144904251 | 0.001 | . | . | . | . | . | . | . | . | . |
| InternalIMT | epa | rs189893153 | 0.001 | . | . | . | . | . | . | . | . | . |
| InternalIMT | epa | rs150447740 | 0.006 | . | . | . | . | . | . | . | . | . |
| InternalIMT | epa | rs143099649 | 0.001 | . | . | . | . | . | . | . | . | . |
| InternalIMT | epa | rs191288436 | 0.007 | . | . | . | . | . | . | . | . | . |
| InternalIMT | epa | rs188577862 | 0.004 | . | . | . | . | . | . | . | . | . |
| InternalIMT | epa | rs192695334 | 0.004 | . | . | . | . | . | . | . | . | . |
| InternalIMT | epa | rs138612906 | 0.022 | 1.50E-04 | 0.02 | 0.994 | 6.20E-04 | 0.052 | 0.99 | -0.061 | 0.074 | 0.412 |
| InternalIMT | epa | rs191290051 | 0.022 | -7.40E-03 | 0.019 | 0.695 | 0.128 | 0.084 | 0.126 | 0.092 | 0.125 | 0.461 |
| InternalIMT | epa | rs189631459 | 0.001 | . | . | . | . | . | . | . | . | . |
| InternalIMT | epa | rs114620874 | 0.005 | . | . | . | . | . | . | . | . | . |
| InternalIMT | epa | rs78246652 | 0.013 | -6.70E-03 | 0.019 | 0.722 | 0.188 | 0.103 | 0.069 | 0.155 | 0.147 | 0.292 |
| InternalIMT | epa | rs79432451 | 0.005 | . | . | . | . | . | . | . | . | . |
| InternalIMT | epa | rs186022585 | 0.001 | . | . | . | . | . | . | . | . | . |
| InternalIMT | epa | rs142978891 | 0.005 | . | . | . | . | . | . | . | . | . |
| InternalIMT | epa | rs151086840 | 0.007 | . | . | . | . | . | . | . | . | . |
| InternalIMT | epa | rs183270259 | 0.006 | . | . | . | . | . | . | . | . | . |
| InternalIMT | epa | rs188544289 | 0.006 | . | . | . | . | . | . | . | . | . |
| InternalIMT | epa | rs188500808 | 0.001 | . | . | . | . | . | . | . | . | . |
| InternalIMT | epa | rs189424589 | 0.002 | . | . | . | . | . | . | . | . | . |
| InternalIMT | epa | rs78622303 | 0.025 | -1.50E-03 | 0.019 | 0.938 | 0.055 | 0.069 | 0.42 | -0.069 | 0.122 | 0.571 |
| InternalIMT | epa | rs143210835 | 0.008 | . | . | . | . | . | . | . | . | . |
| InternalIMT | epa | rs114761840 | 0.004 | . | . | . | . | . | . | . | . | . |
| InternalIMT | epa | rs185805402 | 0.003 | . | . | . | . | . | . | . | . | . |
| InternalIMT | epa | rs139273744 | 0.002 | . | . | . | . | . | . | . | . | . |
| InternalIMT | epa | rs184654150 | 0.004 | . | . | . | . | . | . | . | . | . |
| InternalIMT | epa | rs189069710 | 0 | . | . | . | . | . | . | . | . | . |
| InternalIMT | epa | rs114752115 | 0.018 | -6.30E-03 | 0.019 | 0.739 | 0.111 | 0.085 | 0.19 | 0.068 | 0.107 | 0.522 |
| InternalIMT | epa | rs150162515 | 0.001 | . | . | . | . | . | . | . | . | . |
| InternalIMT | epa | rs180982454 | 0.004 | . | . | . | . | . | . | . | . | . |
| InternalIMT | epa | rs184932088 | 0.001 | . | . | . | . | . | . | . | . | . |
| InternalIMT | epa | rs181109625 | 0 | . | . | . | . | . | . | . | . | . |
| InternalIMT | epa | rs138121134 | 0.001 | . | . | . | . | . | . | . | . | . |
| InternalIMT | epa | rs183253861 | 0.003 | . | . | . | . | . | . | . | . | . |
| InternalIMT | epa | rs147073946 | 0.001 | . | . | . | . | . | . | . | . | . |
| InternalIMT | epa | rs139938489 | 0.005 | . | . | . | . | . | . | . | . | . |
| InternalIMT | epa | rs146362560 | 0.008 | . | . | . | . | . | . | . | . | . |
| InternalIMT | epa | rs193155956 | 0.004 | . | . | . | . | . | . | . | . | . |
| InternalIMT | epa | rs77663092 | 0.004 | . | . | . | . | . | . | . | . | . |
| InternalIMT | epa | rs73285319 | 0.021 | 5.20E-04 | 0.019 | 0.978 | -0.078 | 0.074 | 0.291 | -0.104 | 0.103 | 0.312 |
| InternalIMT | epa | rs184019276 | 0.007 | . | . | . | . | . | . | . | . | . |
| InternalIMT | epa | rs28395863 | 0.016 | -1.60E-03 | 0.019 | 0.934 | 0.017 | 0.068 | 0.802 | -0.044 | 0.093 | 0.635 |
| InternalIMT | epa | rs182121645 | 0.011 | -5.00E-03 | 0.019 | 0.79 | 0.03 | 0.131 | 0.817 | 0.085 | 0.15 | 0.573 |
| InternalIMT | epa | rs73285327 | 0.016 | -7.80E-04 | 0.019 | 0.967 | -0.052 | 0.091 | 0.569 | -0.087 | 0.114 | 0.445 |
| InternalIMT | epa | rs149589584 | 0.001 | . | . | . | . | . | . | . | . | . |
| InternalIMT | epa | rs148310335 | 0.005 | . | . | . | . | . | . | . | . | . |
| InternalIMT | epa | rs187246093 | 0.007 | . | . | . | . | . | . | . | . | . |
| InternalIMT | epa | rs184668894 | 0 | . | . | . | . | . | . | . | . | . |
| InternalIMT | epa | rs114269961 | 0.001 | . | . | . | . | . | . | . | . | . |
| InternalIMT | epa | rs149694374 | 0.02 | -1.00E-03 | 0.019 | 0.958 | -0.019 | 0.057 | 0.745 | -0.04 | 0.085 | 0.636 |
| InternalIMT | epa | rs145284227 | 0.004 | . | . | . | . | . | . | . | . | . |
| InternalIMT | epa | rs189983820 | 0.004 | . | . | . | . | . | . | . | . | . |
| InternalIMT | epa | rs181200444 | 0 | . | . | . | . | . | . | . | . | . |

| InternalIMT | epa | rs185486385 | 0 | . | . | . | . | . | . | . | . | . |
| --- | --- | --- | --- | --- | --- | --- | --- | --- | --- | --- | --- | --- |
| InternalIMT | epa | rs151198241 | 0.006 | . | . | . | . | . | . | . | . | . |
| InternalIMT | epa | rs189959341 | 0.006 | . | . | . | . | . | . | . | . | . |
| InternalIMT | epa | rs140549257 | 0.005 | . | . | . | . | . | . | . | . | . |
| InternalIMT | epa | rs186453139 | 0.016 | 1.60E-03 | 0.019 | 0.932 | -0.111 | 0.069 | 0.105 | -0.152 | 0.095 | 0.109 |
| InternalIMT | epa | rs180922711 | 0.027 | 5.20E-03 | 0.019 | 0.791 | -0.092 | 0.06 | 0.127 | -0.149 | 0.087 | 0.087 |
| InternalIMT | epa | rs56659903 | 0.004 | . | . | . | . | . | . | . | . | . |
| InternalIMT | epa | rs141202128 | 0.004 | . | . | . | . | . | . | . | . | . |
| InternalIMT | epa | rs59085314 | 0.033 | -4.30E-03 | 0.019 | 0.821 | 0.021 | 0.049 | 0.667 | 0.014 | 0.078 | 0.858 |
| InternalIMT | epa | rs145564996 | 0.004 | . | . | . | . | . | . | . | . | . |
| InternalIMT | epa | rs114961009 | 0.006 | . | . | . | . | . | . | . | . | . |
| InternalIMT | epa | rs182294189 | 0.003 | . | . | . | . | . | . | . | . | . |
| InternalIMT | epa | rs187586636 | 0.005 | . | . | . | . | . | . | . | . | . |
| InternalIMT | epa | rs192254907 | 0.003 | . | . | . | . | . | . | . | . | . |
| InternalIMT | epa | rs149145663 | 0.001 | . | . | . | . | . | . | . | . | . |
| InternalIMT | epa | rs180713064 | 0.002 | . | . | . | . | . | . | . | . | . |
| InternalIMT | epa | rs183009023 | 0.004 | . | . | . | . | . | . | . | . | . |
| InternalIMT | epa | rs143385347 | 0.01 | -4.70E-03 | 0.019 | 0.804 | 0.038 | 0.128 | 0.767 | 0.067 | 0.147 | 0.649 |
| InternalIMT | epa | rs185786329 | 0.004 | . | . | . | . | . | . | . | . | . |
| InternalIMT | epa | rs192341030 | 0.003 | . | . | . | . | . | . | . | . | . |
| InternalIMT | epa | rs74661429 | 0.009 | . | . | . | . | . | . | . | . | . |
| InternalIMT | epa | rs188034853 | 0.001 | . | . | . | . | . | . | . | . | . |
| InternalIMT | epa | rs116808430 | 0.016 | 1.10E-03 | 0.019 | 0.953 | -0.107 | 0.067 | 0.112 | -0.125 | 0.093 | 0.179 |
| InternalIMT | epa | rs148469010 | 0 | . | . | . | . | . | . | . | . | . |
| InternalIMT | epa | rs78621663 | 0.006 | . | . | . | . | . | . | . | . | . |
| InternalIMT | epa | rs192654886 | 0.001 | . | . | . | . | . | . | . | . | . |
| InternalIMT | epa | rs145996035 | 0.005 | . | . | . | . | . | . | . | . | . |
| InternalIMT | epa | rs139942226 | 0 | . | . | . | . | . | . | . | . | . |
| InternalIMT | epa | rs113645632 | 0.006 | . | . | . | . | . | . | . | . | . |
| InternalIMT | epa | rs74940661 | 0.016 | -2.40E-03 | 0.019 | 0.9 | -2.80E-03 | 0.066 | 0.966 | -0.025 | 0.103 | 0.805 |
| InternalIMT | epa | rs115355800 | 0.013 | -8.80E-03 | 0.019 | 0.639 | 0.213 | 0.106 | 0.044 | 0.242 | 0.145 | 0.096 |
| InternalIMT | epa | rs150285898 | 0.005 | . | . | . | . | . | . | . | . | . |
| InternalIMT | epa | rs188117932 | 0.004 | . | . | . | . | . | . | . | . | . |
| InternalIMT | epa | rs139956566 | 0 | . | . | . | . | . | . | . | . | . |
| InternalIMT | epa | rs147673885 | 0 | . | . | . | . | . | . | . | . | . |
| InternalIMT | epa | rs189127928 | 0.005 | . | . | . | . | . | . | . | . | . |
| InternalIMT | epa | rs187558772 | 0.002 | . | . | . | . | . | . | . | . | . |
| InternalIMT | epa | rs181948587 | 0 | . | . | . | . | . | . | . | . | . |
| InternalIMT | epa | rs189983269 | 0.002 | . | . | . | . | . | . | . | . | . |
| InternalIMT | epa | rs187250069 | 0.002 | . | . | . | . | . | . | . | . | . |
| InternalIMT | epa | rs191485859 | 0.001 | . | . | . | . | . | . | . | . | . |
| InternalIMT | epa | rs185499555 | 0.002 | . | . | . | . | . | . | . | . | . |
| InternalIMT | epa | rs180862110 | 0.003 | . | . | . | . | . | . | . | . | . |
| InternalIMT | epa | rs190472603 | 0.005 | . | . | . | . | . | . | . | . | . |
| InternalIMT | epa | rs189226167 | 0.005 | . | . | . | . | . | . | . | . | . |
| InternalIMT | epa | rs181623256 | 0.002 | . | . | . | . | . | . | . | . | . |
| InternalIMT | epa | rs186079557 | 0.001 | . | . | . | . | . | . | . | . | . |
| InternalIMT | epa | rs147760994 | 0.002 | . | . | . | . | . | . | . | . | . |
| InternalIMT | epa | rs189176167 | 0 | . | . | . | . | . | . | . | . | . |
| InternalIMT | epa | rs117901000 | 0.001 | . | . | . | . | . | . | . | . | . |
| InternalIMT | epa | rs141984888 | 0 | . | . | . | . | . | . | . | . | . |
| InternalIMT | epa | rs192365061 | 0.007 | . | . | . | . | . | . | . | . | . |
| InternalIMT | epa | rs185976038 | 0 | . | . | . | . | . | . | . | . | . |
| InternalIMT | epa | rs184697303 | 0.002 | . | . | . | . | . | . | . | . | . |
| InternalIMT | epa | rs143383132 | 0.002 | . | . | . | . | . | . | . | . | . |
| InternalIMT | epa | rs141873881 | 0.004 | . | . | . | . | . | . | . | . | . |
| InternalIMT | epa | rs185507255 | 0 | . | . | . | . | . | . | . | . | . |
| InternalIMT | epa | rs191379425 | 0.001 | . | . | . | . | . | . | . | . | . |
| InternalIMT | epa | rs184199584 | 0.002 | . | . | . | . | . | . | . | . | . |
| InternalIMT | epa | rs183181360 | 0.002 | . | . | . | . | . | . | . | . | . |
| InternalIMT | epa | rs184495673 | 0.001 | . | . | . | . | . | . | . | . | . |
| InternalIMT | epa | rs182946297 | 0.001 | . | . | . | . | . | . | . | . | . |
| InternalIMT | epa | rs187743615 | 0 | . | . | . | . | . | . | . | . | . |
| InternalIMT | epa | rs183259862 | 0.001 | . | . | . | . | . | . | . | . | . |
| InternalIMT | epa | rs112433566 | 0 | . | . | . | . | . | . | . | . | . |
| InternalIMT | epa | rs182351085 | 0.001 | . | . | . | . | . | . | . | . | . |
| InternalIMT | epa | rs146248508 | 0.001 | . | . | . | . | . | . | . | . | . |
| InternalIMT | epa | rs188528439 | 0.001 | . | . | . | . | . | . | . | . | . |
| InternalIMT | epa | rs146167018 | 0.005 | . | . | . | . | . | . | . | . | . |
| InternalIMT | epa | rs192440980 | 0.004 | . | . | . | . | . | . | . | . | . |
| InternalIMT | epa | rs185601882 | 0.001 | . | . | . | . | . | . | . | . | . |
| InternalIMT | epa | rs117272563 | 0.002 | . | . | . | . | . | . | . | . | . |
| InternalIMT | epa | rs10430734 | 0.003 | . | . | . | . | . | . | . | . | . |
| InternalIMT | epa | rs181320952 | 0.001 | . | . | . | . | . | . | . | . | . |

| InternalIMT | epa | rs185480660 | 0.001 | . | . | . | . | . | . | . | . | . |
| --- | --- | --- | --- | --- | --- | --- | --- | --- | --- | --- | --- | --- |
| InternalIMT | epa | rs188077917 | 0.001 | . | . | . | . | . | . | . | . | . |
| InternalIMT | epa | rs187562953 | 0.002 | . | . | . | . | . | . | . | . | . |
| InternalIMT | epa | rs182044631 | 0 | . | . | . | . | . | . | . | . | . |
| InternalIMT | epa | rs145455596 | 0.001 | . | . | . | . | . | . | . | . | . |
| InternalIMT | epa | rs181475877 | 0.004 | . | . | . | . | . | . | . | . | . |
| InternalIMT | epa | rs149413085 | 0.002 | . | . | . | . | . | . | . | . | . |
| InternalIMT | epa | rs12253656 | 0 | . | . | . | . | . | . | . | . | . |
| InternalIMT | epa | rs182718239 | 0 | . | . | . | . | . | . | . | . | . |
| InternalIMT | epa | rs192435695 | 0.002 | . | . | . | . | . | . | . | . | . |
| InternalIMT | epa | rs12248277 | 0 | . | . | . | . | . | . | . | . | . |
| InternalIMT | epa | rs143521710 | 0.001 | . | . | . | . | . | . | . | . | . |
| InternalIMT | epa | rs144454454 | 0 | . | . | . | . | . | . | . | . | . |
| InternalIMT | epa | rs190441610 | 0.004 | . | . | . | . | . | . | . | . | . |
| InternalIMT | epa | rs114690462 | 0 | . | . | . | . | . | . | . | . | . |
| InternalIMT | epa | rs184541357 | 0.001 | . | . | . | . | . | . | . | . | . |
| InternalIMT | epa | rs142657653 | 0 | . | . | . | . | . | . | . | . | . |
| InternalIMT | epa | rsurf1 | 2.145 | . | . | . | . | . | . | . | . | . |
| InternalIMT | epa | rsten1 | 2.303 | . | . | . | . | . | . | . | . | . |
| InternalIMT | epa | chr10:45852312:D | 0.086 | -0.02 | 0.013 | 0.125 | -0.038 | 0.018 | 0.036 | -0.028 | 0.023 | 0.219 |
| InternalIMT | epa | chr10:45853232:I | 0.1 | -0.023 | 0.013 | 0.084 | -0.023 | 0.018 | 0.213 | -0.013 | 0.024 | 0.579 |
| InternalIMT | epa | chr10:45853234:I | 0.168 | -0.022 | 0.014 | 0.105 | -0.017 | 0.015 | 0.252 | -8.60E-03 | 0.02 | 0.66 |
| InternalIMT | epa | chr10:45854796:D | 0.013 | -0.027 | 0.012 | 0.025 | -0.127 | 0.043 | 2.80E-03 | 3.80E-03 | 0.044 | 0.932 |
| InternalIMT | epa | chr10:45854994:I | 0.019 | -0.027 | 0.012 | 0.022 | -0.055 | 0.041 | 0.182 | 0.026 | 0.043 | 0.55 |
| InternalIMT | epa | chr10:45858290:D | 0.045 | -0.026 | 0.013 | 0.041 | -8.20E-04 | 0.042 | 0.985 | -1.40E-03 | 0.056 | 0.981 |
| InternalIMT | epa | chr10:45858820:I | 0.015 | -0.022 | 0.012 | 0.059 | -0.01 | 0.056 | 0.856 | -0.128 | 0.095 | 0.176 |
| InternalIMT | epa | chr10:45860888:D | 0.081 | -0.024 | 0.013 | 0.068 | -0.028 | 0.026 | 0.293 | -0.01 | 0.037 | 0.779 |
| InternalIMT | epa | chr10:45863913:I | 0.165 | -0.021 | 0.014 | 0.128 | -0.017 | 0.015 | 0.244 | -0.013 | 0.02 | 0.523 |
| InternalIMT | epa | chr10:45866601:D | 0.036 | -0.024 | 0.014 | 0.09 | 0.028 | 0.037 | 0.455 | 0.045 | 0.043 | 0.294 |
| InternalIMT | epa | chr10:45867197:I | 0.006 | . | . | . | . | . | . | . | . | . |
| InternalIMT | epa | chr10:45867538:D | 0.171 | -0.022 | 0.014 | 0.12 | -0.018 | 0.015 | 0.219 | -0.01 | 0.02 | 0.592 |
| InternalIMT | epa | chr10:45868356:D | 0.161 | -0.022 | 0.014 | 0.109 | -0.019 | 0.015 | 0.205 | -9.80E-03 | 0.02 | 0.617 |
| InternalIMT | epa | chr10:45871158:I | 0.167 | -0.022 | 0.014 | 0.109 | -0.018 | 0.015 | 0.234 | -9.30E-03 | 0.019 | 0.631 |
| InternalIMT | epa | chr10:45871637:D | 0.084 | -0.024 | 0.013 | 0.06 | -0.039 | 0.022 | 0.081 | -2.30E-03 | 0.028 | 0.934 |
| InternalIMT | epa | chr10:45871638:I | 0.132 | -0.024 | 0.014 | 0.081 | -0.022 | 0.017 | 0.205 | -5.90E-03 | 0.022 | 0.789 |
| InternalIMT | epa | chr10:45873629:I | 0.158 | -0.02 | 0.014 | 0.142 | -0.025 | 0.015 | 0.104 | -0.015 | 0.02 | 0.451 |
| InternalIMT | epa | chr10:45873701:D | 0.019 | -0.025 | 0.014 | 0.07 | 0.054 | 0.054 | 0.319 | 0.114 | 0.064 | 0.077 |
| InternalIMT | epa | chr10:45874997:I | 0.021 | -0.022 | 0.014 | 0.108 | 0.018 | 0.047 | 0.704 | 0.057 | 0.064 | 0.374 |
| InternalIMT | epa | chr10:45882030:I | 0.045 | -0.022 | 0.014 | 0.112 | -5.00E-03 | 0.036 | 0.89 | 0.03 | 0.044 | 0.493 |
| InternalIMT | epa | chr10:45883774:D | 0.102 | -0.027 | 0.012 | 0.033 | 0.01 | 0.018 | 0.573 | 3.70E-03 | 0.026 | 0.887 |
| InternalIMT | epa | chr10:45885169:D | 0.067 | -0.021 | 0.013 | 0.097 | -0.042 | 0.021 | 0.042 | -0.028 | 0.027 | 0.295 |
| InternalIMT | epa | chr10:45886451:D | 0.67 | -0.033 | 0.029 | 0.247 | 0.01 | 0.013 | 0.445 | 5.40E-03 | 0.019 | 0.778 |
| InternalIMT | epa | chr10:45891800:I | 0.685 | -0.02 | 0.027 | 0.466 | -7.70E-03 | 0.013 | 0.538 | -4.40E-03 | 0.017 | 0.795 |
| InternalIMT | epa | chr10:45904771:I | 0.685 | -0.026 | 0.026 | 0.315 | -7.30E-03 | 0.012 | 0.551 | 1.90E-04 | 0.017 | 0.991 |
| InternalIMT | epa | chr10:45904959:I | 0.113 | -0.033 | 0.014 | 0.014 | 9.70E-03 | 0.022 | 0.663 | 0.034 | 0.03 | 0.266 |
| InternalIMT | epa | chr10:45911028:I | 0.077 | -0.028 | 0.013 | 0.029 | -1.10E-03 | 0.028 | 0.97 | 0.014 | 0.041 | 0.725 |
| InternalIMT | epa | chr10:45911031:I | 0.355 | -0.015 | 0.018 | 0.402 | 8.30E-03 | 0.014 | 0.552 | -0.015 | 0.02 | 0.461 |
| InternalIMT | epa | chr10:45912154:D | 0.103 | -0.024 | 0.012 | 0.048 | 3.30E-03 | 0.019 | 0.862 | -7.20E-03 | 0.027 | 0.788 |
| InternalIMT | epa | chr10:45913885:I | 0.011 | -0.027 | 0.012 | 0.023 | -0.157 | 0.042 | 1.60E-04 | -2.60E-03 | 0.037 | 0.945 |
| InternalIMT | epa | chr10:45914753:D | 0.245 | -0.034 | 0.015 | 0.023 | 0.03 | 0.015 | 0.043 | 0.016 | 0.02 | 0.436 |
| InternalIMT | epa | chr10:45919621 | 0.003 | . | . | . | . | . | . | . | . | . |
| InternalIMT | epa | chr10:45921739:I | 0.066 | -0.027 | 0.012 | 0.024 | -0.021 | 0.024 | 0.37 | 9.00E-03 | 0.035 | 0.799 |
| InternalIMT | epa | chr10:45923527:I | 0.057 | -0.017 | 0.013 | 0.173 | -0.033 | 0.031 | 0.293 | -0.077 | 0.048 | 0.108 |
| InternalIMT | epa | chr10:45927001:I | 0.019 | -0.03 | 0.012 | 0.01 | 0.108 | 0.069 | 0.121 | 0.145 | 0.096 | 0.132 |
| InternalIMT | epa | chr10:45930354:I | 0.017 | -0.028 | 0.012 | 0.018 | -0.05 | 0.045 | 0.265 | 0.036 | 0.046 | 0.43 |
| InternalIMT | epa | chr10:45932864:I | 0.04 | -0.021 | 0.012 | 0.079 | -0.02 | 0.037 | 0.588 | -0.051 | 0.044 | 0.245 |
| InternalIMT | epa | chr10:45936163:I | 0.029 | -0.027 | 0.012 | 0.023 | -0.051 | 0.036 | 0.151 | 0.03 | 0.047 | 0.529 |
| InternalIMT | epa | chr10:45938095:D | 0.144 | -0.03 | 0.013 | 0.021 | 9.40E-03 | 0.016 | 0.561 | 0.015 | 0.023 | 0.511 |
| InternalIMT | epa | chr10:45938886 | 0 | . | . | . | . | . | . | . | . | . |
| InternalIMT | epa | chr10:45939320 | 0.001 | . | . | . | . | . | . | . | . | . |
| InternalIMT | epa | chr10:45939659 | 0.002 | . | . | . | . | . | . | . | . | . |
| InternalIMT | epa | chr10:45941150:D | 0.012 | -0.026 | 0.012 | 0.026 | -0.167 | 0.036 | 4.50E-06 | -0.013 | 0.035 | 0.708 |
| InternalIMT | epa | chr10:45947128:D | 0.058 | -0.024 | 0.012 | 0.047 | 0.031 | 0.024 | 0.208 | -0.012 | 0.033 | 0.721 |
| InternalIMT | epa | chr10:45950990:I | 0.047 | -0.024 | 0.012 | 0.053 | -0.036 | 0.032 | 0.251 | -0.02 | 0.036 | 0.571 |
| InternalIMT | epa | chr10:45951854:I | 0.03 | -0.016 | 0.014 | 0.261 | -0.013 | 0.036 | 0.715 | -0.074 | 0.053 | 0.162 |
| InternalIMT | epa | chr10:45952670:I | 0.049 | -0.023 | 0.012 | 0.064 | -0.049 | 0.023 | 0.037 | -0.032 | 0.032 | 0.323 |
| InternalIMT | epa | chr10:45956741 | 0.001 | . | . | . | . | . | . | . | . | . |
| InternalIMT | epa | chr10:45957247:I | 0.219 | -0.031 | 0.014 | 0.026 | 7.30E-03 | 0.014 | 0.592 | 0.013 | 0.018 | 0.498 |
| InternalIMT | epa | chr10:45958082:I | 0.216 | -0.03 | 0.014 | 0.034 | 6.60E-03 | 0.014 | 0.631 | 9.60E-03 | 0.019 | 0.608 |
| InternalIMT | epa | chr10:45905041:D | 0.053 | -0.019 | 0.015 | 0.224 | 7.20E-03 | 0.033 | 0.828 | -6.90E-03 | 0.041 | 0.865 |
| InternalIMT | epa | chr10:45938871 | 0.003 | . | . | . | . | . | . | . | . | . |
| InternalIMT | epa | chr10:45939160 | 0.004 | . | . | . | . | . | . | . | . | . |
| InternalIMT | epa | chr10:45869910 | 0.008 | . | . | . | . | . | . | . | . | . |
| InternalIMT | epa | chr10:45876214:D | 0.012 | -1.10E-03 | 0.019 | 0.954 | -0.087 | 0.057 | 0.127 | -0.031 | 0.067 | 0.639 |

| InternalIMT | epa | chr10:45880237:I | 0.056 | 3.80E-03 | 0.02 | 0.849 | 0.027 | 0.04 | 0.506 | -0.067 | 0.049 | 0.176 |
| --- | --- | --- | --- | --- | --- | --- | --- | --- | --- | --- | --- | --- |
| InternalIMT | epa | chr10:45884371:I | 0.045 | -0.011 | 0.02 | 0.569 | 0.048 | 0.043 | 0.271 | 0.068 | 0.051 | 0.185 |
| InternalIMT | epa | chr10:45891535:D | 0.012 | -8.70E-03 | 0.019 | 0.644 | 0.149 | 0.102 | 0.147 | 0.159 | 0.149 | 0.287 |
| InternalIMT | epa | chr10:45893516:D | 0.045 | -0.012 | 0.02 | 0.562 | 0.048 | 0.043 | 0.263 | 0.069 | 0.051 | 0.174 |
| InternalIMT | epa | chr10:45893782:D | 0.01 | 1.70E-04 | 0.019 | 0.993 | 0.078 | 0.092 | 0.395 | -0.15 | 0.102 | 0.141 |
| InternalIMT | epa | chr10:45895254:I | 0.012 | -8.70E-03 | 0.019 | 0.644 | 0.149 | 0.102 | 0.146 | 0.159 | 0.149 | 0.286 |
| InternalIMT | epa | chr10:45895848:D | 0.035 | -7.10E-03 | 0.02 | 0.72 | 0.014 | 0.045 | 0.752 | 0.047 | 0.053 | 0.375 |
| InternalIMT | epa | chr10:45896316:D | 0.045 | -0.012 | 0.02 | 0.56 | 0.048 | 0.043 | 0.261 | 0.07 | 0.051 | 0.171 |
| InternalIMT | epa | chr10:45896317:D | 0.045 | -0.012 | 0.02 | 0.56 | 0.048 | 0.043 | 0.261 | 0.07 | 0.051 | 0.171 |
| InternalIMT | epa | chr10:45902683:I | 0.013 | -2.70E-03 | 0.019 | 0.89 | -0.065 | 0.056 | 0.246 | 0.012 | 0.067 | 0.857 |
| InternalIMT | epa | chr10:45905003:D | 0.006 | . | . | . | . | . | . | . | . | . |
| InternalIMT | epa | chr10:45927476:D | 0.021 | -4.40E-04 | 0.019 | 0.982 | -0.012 | 0.056 | 0.829 | -0.053 | 0.082 | 0.52 |
| InternalIMT | epa | chr10:45935882 | 0.001 | . | . | . | . | . | . | . | . | . |
| InternalIMT | epa | chr10:45936100 | 0.001 | . | . | . | . | . | . | . | . | . |
| InternalIMT | epa | chr10:45939553 | 0.003 | . | . | . | . | . | . | . | . | . |
| InternalIMT | epa | chr10:45939631 | 0 | . | . | . | . | . | . | . | . | . |
| InternalIMT | epa | chr10:45944193:D | 0.008 | . | . | . | . | . | . | . | . | . |
| InternalIMT | epa | chr10:45954030 | 0 | . | . | . | . | . | . | . | . | . |

Supplemental table S17.

| phen | covar(fa) | rs_id | maf | beta_FA | se_FA | p_FA | beta_SNP | se_SNP | p_SNP | beta_inter | se_inter | p_inter |
| --- | --- | --- | --- | --- | --- | --- | --- | --- | --- | --- | --- | --- |
| InternalIMT | epa | rs76035182 | 0.001 | . | . | . | . | . | . | . | . | . |
| InternalIMT | epa | rs12359547 | 0.104 | -0.049 | 0.021 | 0.021 | -0.035 | 0.029 | 0.227 | -0.026 | 0.042 | 0.538 |
| InternalIMT | epa | rs74595484 | 0 | . | . | . | . | . | . | . | . | . |
| InternalIMT | epa | rs1864415 | 0.605 | -0.028 | 0.039 | 0.472 | 9.40E-03 | 0.019 | 0.611 | -0.023 | 0.028 | 0.413 |
| InternalIMT | epa | rs72788101 | 0.104 | -0.049 | 0.021 | 0.021 | -0.035 | 0.029 | 0.227 | -0.026 | 0.042 | 0.537 |
| InternalIMT | epa | rs11239481 | 0.183 | -0.059 | 0.022 | 8.30E-03 | 0.018 | 0.024 | 0.453 | 0.012 | 0.035 | 0.74 |
| InternalIMT | epa | rs72788102 | 0.104 | -0.049 | 0.021 | 0.021 | -0.035 | 0.029 | 0.227 | -0.026 | 0.042 | 0.537 |
| InternalIMT | epa | rs72789705 | 0.104 | -0.049 | 0.021 | 0.021 | -0.035 | 0.029 | 0.227 | -0.026 | 0.042 | 0.537 |
| InternalIMT | epa | rs141529858 | 0.008 | . | . | . | . | . | . | . | . | . |
| InternalIMT | epa | rs145376292 | 0 | . | . | . | . | . | . | . | . | . |
| InternalIMT | epa | rs190418301 | 0.001 | . | . | . | . | . | . | . | . | . |
| InternalIMT | epa | rs11239482 | 0.104 | -0.049 | 0.021 | 0.021 | -0.035 | 0.029 | 0.227 | -0.026 | 0.042 | 0.537 |
| InternalIMT | epa | rs61855872 | 0.014 | -0.062 | 0.02 | 1.90E-03 | 0.106 | 0.121 | 0.381 | 0.293 | 0.174 | 0.094 |
| InternalIMT | epa | rs148991144 | 0.011 | -0.053 | 0.02 | 7.70E-03 | -7.10E-03 | 0.076 | 0.925 | -0.108 | 0.1 | 0.284 |
| InternalIMT | epa | rs191932221 | 0.007 | . | . | . | . | . | . | . | . | . |
| InternalIMT | epa | rs55978138 | 0.16 | -0.047 | 0.023 | 0.038 | -0.029 | 0.025 | 0.238 | -0.023 | 0.038 | 0.541 |
| InternalIMT | epa | rs73281167 | 0 | . | . | . | . | . | . | . | . | . |
| InternalIMT | epa | rs12768010 | 0.055 | -0.054 | 0.021 | 9.50E-03 | -0.013 | 0.038 | 0.734 | -0.01 | 0.06 | 0.861 |
| InternalIMT | epa | rs11239484 | 0.605 | -0.028 | 0.039 | 0.468 | 9.70E-03 | 0.019 | 0.602 | -0.022 | 0.028 | 0.417 |
| InternalIMT | epa | rs35906360 | 0.16 | -0.047 | 0.023 | 0.038 | -0.029 | 0.025 | 0.237 | -0.023 | 0.038 | 0.542 |
| InternalIMT | epa | rs186250512 | 0.004 | . | . | . | . | . | . | . | . | . |
| InternalIMT | epa | rs150810743 | 0 | . | . | . | . | . | . | . | . | . |
| InternalIMT | epa | rs55965812 | 0.16 | -0.047 | 0.023 | 0.038 | -0.029 | 0.025 | 0.238 | -0.023 | 0.038 | 0.541 |
| InternalIMT | epa | rs56180358 | 0.16 | -0.047 | 0.023 | 0.038 | -0.029 | 0.025 | 0.237 | -0.023 | 0.038 | 0.542 |
| InternalIMT | epa | rs55864145 | 0.055 | -0.054 | 0.021 | 9.50E-03 | -0.013 | 0.038 | 0.734 | -0.01 | 0.06 | 0.861 |
| InternalIMT | epa | rs55986122 | 0.16 | -0.047 | 0.023 | 0.038 | -0.029 | 0.025 | 0.237 | -0.023 | 0.038 | 0.541 |
| InternalIMT | epa | rs7477350 | 0.605 | -0.028 | 0.039 | 0.467 | 9.80E-03 | 0.019 | 0.597 | -0.022 | 0.028 | 0.418 |
| InternalIMT | epa | rs2099170 | 0.159 | -0.047 | 0.023 | 0.038 | -0.029 | 0.025 | 0.233 | -0.023 | 0.038 | 0.542 |
| InternalIMT | epa | rs2082108 | 0.16 | -0.047 | 0.023 | 0.038 | -0.029 | 0.025 | 0.237 | -0.023 | 0.038 | 0.541 |
| InternalIMT | epa | rs55902404 | 0 | . | . | . | . | . | . | . | . | . |
| InternalIMT | epa | rs58402662 | 0 | . | . | . | . | . | . | . | . | . |
| InternalIMT | epa | rs11511618 | 0.182 | -0.059 | 0.022 | 8.50E-03 | 0.016 | 0.023 | 0.503 | 0.011 | 0.035 | 0.748 |
| InternalIMT | epa | rs1816492 | 0.605 | -0.028 | 0.039 | 0.466 | 9.80E-03 | 0.019 | 0.595 | -0.022 | 0.028 | 0.419 |
| InternalIMT | epa | rs146611093 | 0.01 | -0.05 | 0.02 | 0.013 | -0.218 | 0.082 | 8.00E-03 | -0.269 | 0.109 | 0.013 |
| InternalIMT | epa | rs186897689 | 0 | . | . | . | . | . | . | . | . | . |
| InternalIMT | epa | rs10793616 | 0.605 | -0.028 | 0.039 | 0.466 | 9.90E-03 | 0.019 | 0.594 | -0.022 | 0.028 | 0.42 |
| InternalIMT | epa | rs116700479 | 0.001 | . | . | . | . | . | . | . | . | . |
| InternalIMT | epa | rs192535936 | 0 | . | . | . | . | . | . | . | . | . |
| InternalIMT | epa | rs113363297 | 0.57 | -0.026 | 0.038 | 0.496 | 4.90E-03 | 0.019 | 0.794 | -0.026 | 0.028 | 0.359 |
| InternalIMT | epa | rs12354994 | 0.152 | -0.045 | 0.023 | 0.045 | -0.031 | 0.025 | 0.216 | -0.03 | 0.039 | 0.443 |
| InternalIMT | epa | rs148068329 | 0.001 | . | . | . | . | . | . | . | . | . |
| InternalIMT | epa | rs12356109 | 0.16 | -0.047 | 0.023 | 0.038 | -0.029 | 0.025 | 0.237 | -0.023 | 0.038 | 0.54 |
| InternalIMT | epa | rs55908141 | 0 | . | . | . | . | . | . | . | . | . |
| InternalIMT | epa | rs12354712 | 0.159 | -0.047 | 0.023 | 0.038 | -0.029 | 0.025 | 0.244 | -0.023 | 0.038 | 0.534 |
| InternalIMT | epa | rs147282684 | 0.004 | . | . | . | . | . | . | . | . | . |
| InternalIMT | epa | rs12356140 | 0.16 | -0.047 | 0.023 | 0.039 | -0.029 | 0.025 | 0.244 | -0.024 | 0.038 | 0.528 |
| InternalIMT | epa | rs185476187 | 0 | . | . | . | . | . | . | . | . | . |
| InternalIMT | epa | rs145720057 | 0.039 | -0.064 | 0.02 | 1.50E-03 | 0.053 | 0.06 | 0.377 | 0.13 | 0.093 | 0.165 |
| InternalIMT | epa | rs12354742 | 0.159 | -0.047 | 0.023 | 0.038 | -0.029 | 0.025 | 0.243 | -0.023 | 0.038 | 0.534 |
| InternalIMT | epa | rs180913842 | 0 | . | . | . | . | . | . | . | . | . |
| InternalIMT | epa | rs142976914 | 0.006 | . | . | . | . | . | . | . | . | . |
| InternalIMT | epa | rs11511619 | 0.163 | -0.044 | 0.023 | 0.053 | -0.033 | 0.025 | 0.185 | -0.031 | 0.038 | 0.408 |
| InternalIMT | epa | rs146702870 | 0 | . | . | . | . | . | . | . | . | . |
| InternalIMT | epa | rs150287496 | 0.002 | . | . | . | . | . | . | . | . | . |
| InternalIMT | epa | rs190917973 | 0.006 | . | . | . | . | . | . | . | . | . |
| InternalIMT | epa | rs11517281 | 0.16 | -0.047 | 0.023 | 0.038 | -0.029 | 0.025 | 0.237 | -0.023 | 0.038 | 0.54 |
| InternalIMT | epa | rs12359299 | 0.16 | -0.047 | 0.023 | 0.038 | -0.029 | 0.025 | 0.242 | -0.023 | 0.038 | 0.538 |
| InternalIMT | epa | rs182307932 | 0 | . | . | . | . | . | . | . | . | . |
| InternalIMT | epa | rs61855873 | 0.035 | -0.064 | 0.021 | 1.80E-03 | 0.102 | 0.084 | 0.225 | 0.167 | 0.129 | 0.193 |
| InternalIMT | epa | rs12359302 | 0.156 | -0.046 | 0.022 | 0.04 | -0.032 | 0.025 | 0.188 | -0.026 | 0.038 | 0.501 |
| InternalIMT | epa | rs143208140 | 0.003 | . | . | . | . | . | . | . | . | . |
| InternalIMT | epa | rs9988764 | 0.594 | -0.034 | 0.038 | 0.377 | 9.80E-03 | 0.019 | 0.599 | -0.018 | 0.028 | 0.519 |
| InternalIMT | epa | rs192875767 | 0.008 | . | . | . | . | . | . | . | . | . |
| InternalIMT | epa | rs147268846 | 0.015 | -0.067 | 0.02 | 8.20E-04 | 0.235 | 0.144 | 0.104 | 0.461 | 0.183 | 0.012 |
| InternalIMT | epa | rs11239485 | 0.111 | -0.048 | 0.022 | 0.027 | -0.053 | 0.032 | 0.106 | -0.027 | 0.047 | 0.576 |
| InternalIMT | epa | rs151317885 | 0.435 | -0.047 | 0.032 | 0.14 | 0.027 | 0.019 | 0.161 | -8.20E-03 | 0.029 | 0.777 |
| InternalIMT | epa | rs11499085 | 0 | . | . | . | . | . | . | . | . | . |
| InternalIMT | epa | rs188130606 | 0 | . | . | . | . | . | . | . | . | . |
| InternalIMT | epa | rs11511620 | 0.16 | -0.047 | 0.023 | 0.038 | -0.029 | 0.025 | 0.237 | -0.023 | 0.038 | 0.539 |
| InternalIMT | epa | rs181498390 | 0 | . | . | . | . | . | . | . | . | . |
| InternalIMT | epa | rs186280701 | 0 | . | . | . | . | . | . | . | . | . |

| InternalIMT | epa | rs150059185 | 0.011 | -0.056 | 0.02 | 4.70E-03 | 0.158 | 0.128 | 0.217 | 0.064 | 0.166 | 0.699 |
| --- | --- | --- | --- | --- | --- | --- | --- | --- | --- | --- | --- | --- |
| InternalIMT | epa | rs183338707 | 0.004 | . | . | . | . | . | . | . | . | . |
| InternalIMT | epa | rs11499038 | 0.002 | . | . | . | . | . | . | . | . | . |
| InternalIMT | epa | rs190699595 | 0.008 | . | . | . | . | . | . | . | . | . |
| InternalIMT | epa | rs11523408 | 0.16 | -0.047 | 0.023 | 0.038 | -0.029 | 0.025 | 0.237 | -0.023 | 0.038 | 0.54 |
| InternalIMT | epa | rs188049317 | 0 | . | . | . | . | . | . | . | . | . |
| InternalIMT | epa | rs144948634 | 0.427 | -0.04 | 0.032 | 0.218 | 0.022 | 0.019 | 0.253 | -0.017 | 0.03 | 0.57 |
| InternalIMT | epa | rs143833576 | 0.001 | . | . | . | . | . | . | . | . | . |
| InternalIMT | epa | rs151239879 | 0.16 | -0.047 | 0.023 | 0.037 | -0.029 | 0.025 | 0.238 | -0.023 | 0.038 | 0.548 |
| InternalIMT | epa | rs7073743 | 0.438 | -0.051 | 0.032 | 0.12 | 0.026 | 0.019 | 0.159 | -4.10E-03 | 0.029 | 0.888 |
| InternalIMT | epa | rs142563349 | 0 | . | . | . | . | . | . | . | . | . |
| InternalIMT | epa | rs187246403 | 0 | . | . | . | . | . | . | . | . | . |
| InternalIMT | epa | rs150423942 | 0.158 | -0.047 | 0.022 | 0.039 | -0.03 | 0.025 | 0.223 | -0.024 | 0.038 | 0.528 |
| InternalIMT | epa | rs184706102 | 0.002 | . | . | . | . | . | . | . | . | . |
| InternalIMT | epa | rs189626288 | 0.03 | -0.059 | 0.02 | 3.90E-03 | 0.134 | 0.083 | 0.108 | 0.11 | 0.105 | 0.293 |
| InternalIMT | epa | rs140749834 | 0 | . | . | . | . | . | . | . | . | . |
| InternalIMT | epa | rs7078822 | 0.131 | -0.043 | 0.022 | 0.056 | -0.056 | 0.027 | 0.038 | -0.037 | 0.042 | 0.384 |
| InternalIMT | epa | rs186227617 | 0.159 | -0.047 | 0.023 | 0.038 | -0.03 | 0.025 | 0.228 | -0.023 | 0.038 | 0.535 |
| InternalIMT | epa | rs188984368 | 0.024 | -0.049 | 0.021 | 0.019 | 0.017 | 0.096 | 0.862 | -0.116 | 0.133 | 0.382 |
| InternalIMT | epa | rs182078109 | 0.027 | -0.046 | 0.021 | 0.028 | 0.026 | 0.094 | 0.78 | -0.15 | 0.129 | 0.246 |
| InternalIMT | epa | rs141456274 | 0.002 | . | . | . | . | . | . | . | . | . |
| InternalIMT | epa | rs146976512 | 0.16 | -0.047 | 0.023 | 0.038 | -0.029 | 0.025 | 0.237 | -0.023 | 0.038 | 0.539 |
| InternalIMT | epa | rs138036816 | 0 | . | . | . | . | . | . | . | . | . |
| InternalIMT | epa | rs114879777 | 0.001 | . | . | . | . | . | . | . | . | . |
| InternalIMT | epa | rs149366475 | 0 | . | . | . | . | . | . | . | . | . |
| InternalIMT | epa | rs182463043 | 0 | . | . | . | . | . | . | . | . | . |
| InternalIMT | epa | rs144754632 | 0.001 | . | . | . | . | . | . | . | . | . |
| InternalIMT | epa | rs55717607 | 0.16 | -0.047 | 0.023 | 0.038 | -0.029 | 0.025 | 0.236 | -0.023 | 0.038 | 0.539 |
| InternalIMT | epa | rs187537973 | 0 | . | . | . | . | . | . | . | . | . |
| InternalIMT | epa | rs148085971 | 0 | . | . | . | . | . | . | . | . | . |
| InternalIMT | epa | rs11239491 | 0.16 | -0.047 | 0.023 | 0.038 | -0.029 | 0.025 | 0.236 | -0.023 | 0.038 | 0.539 |
| InternalIMT | epa | rs184412184 | 0.008 | . | . | . | . | . | . | . | . | . |
| InternalIMT | epa | rs4948998 | 0.606 | -0.028 | 0.039 | 0.471 | 0.01 | 0.019 | 0.575 | -0.023 | 0.028 | 0.415 |
| InternalIMT | epa | rs7076696 | 0.607 | -0.028 | 0.039 | 0.47 | 0.01 | 0.019 | 0.578 | -0.022 | 0.028 | 0.416 |
| InternalIMT | epa | rs11239492 | 0.16 | -0.047 | 0.023 | 0.038 | -0.029 | 0.025 | 0.237 | -0.023 | 0.038 | 0.536 |
| InternalIMT | epa | rs79462470 | 0 | . | . | . | . | . | . | . | . | . |
| InternalIMT | epa | rs187804654 | 0.003 | . | . | . | . | . | . | . | . | . |
| InternalIMT | epa | rs7093687 | 0.16 | -0.047 | 0.023 | 0.038 | -0.029 | 0.025 | 0.236 | -0.023 | 0.038 | 0.537 |
| InternalIMT | epa | rs7081004 | 0.16 | -0.047 | 0.023 | 0.038 | -0.029 | 0.025 | 0.237 | -0.023 | 0.038 | 0.537 |
| InternalIMT | epa | rs7084793 | 0.16 | -0.047 | 0.023 | 0.038 | -0.029 | 0.025 | 0.236 | -0.023 | 0.038 | 0.538 |
| InternalIMT | epa | rs61556080 | 0 | . | . | . | . | . | . | . | . | . |
| InternalIMT | epa | rs115552064 | . | . | . | . | . | . | . | . | . | . |
| InternalIMT | epa | rs7921503 | 0.16 | -0.047 | 0.023 | 0.038 | -0.029 | 0.025 | 0.236 | -0.023 | 0.038 | 0.537 |
| InternalIMT | epa | rs7909740 | 0.16 | -0.047 | 0.023 | 0.038 | -0.029 | 0.025 | 0.236 | -0.023 | 0.038 | 0.538 |
| InternalIMT | epa | rs7909768 | 0.16 | -0.047 | 0.023 | 0.038 | -0.029 | 0.025 | 0.236 | -0.023 | 0.038 | 0.538 |
| InternalIMT | epa | rs144168004 | 0 | . | . | . | . | . | . | . | . | . |
| InternalIMT | epa | rs75850448 | 0 | . | . | . | . | . | . | . | . | . |
| InternalIMT | epa | rs147777574 | 0 | . | . | . | . | . | . | . | . | . |
| InternalIMT | epa | rs7913488 | 0.16 | -0.047 | 0.023 | 0.038 | -0.029 | 0.025 | 0.236 | -0.023 | 0.038 | 0.538 |
| InternalIMT | epa | rs74390874 | 0.071 | -0.045 | 0.021 | 0.035 | 0.039 | 0.04 | 0.332 | -0.067 | 0.063 | 0.285 |
| InternalIMT | epa | rs141391142 | 0.009 | . | . | . | . | . | . | . | . | . |
| InternalIMT | epa | rs115294747 | 0.001 | . | . | . | . | . | . | . | . | . |
| InternalIMT | epa | rs148031781 | 0.003 | . | . | . | . | . | . | . | . | . |
| InternalIMT | epa | rs181340128 | 0.021 | -0.064 | 0.02 | 1.60E-03 | 0.031 | 0.106 | 0.772 | 0.215 | 0.147 | 0.142 |
| InternalIMT | epa | rs189930007 | 0.002 | . | . | . | . | . | . | . | . | . |
| InternalIMT | epa | rs12242128 | 0 | . | . | . | . | . | . | . | . | . |
| InternalIMT | epa | rs78421750 | 0.002 | . | . | . | . | . | . | . | . | . |
| InternalIMT | epa | rs114370747 | 0.002 | . | . | . | . | . | . | . | . | . |
| InternalIMT | epa | rs12264166 | 0 | . | . | . | . | . | . | . | . | . |
| InternalIMT | epa | rs182964822 | 0.001 | . | . | . | . | . | . | . | . | . |
| InternalIMT | epa | rs67491417 | 0.15 | -0.046 | 0.022 | 0.038 | -0.028 | 0.026 | 0.278 | -0.028 | 0.041 | 0.497 |
| InternalIMT | epa | rs72789720 | 0.023 | -0.062 | 0.02 | 2.00E-03 | 0.051 | 0.066 | 0.435 | 0.203 | 0.117 | 0.082 |
| InternalIMT | epa | rs6413416 | 0.16 | -0.047 | 0.023 | 0.038 | -0.03 | 0.025 | 0.226 | -0.023 | 0.038 | 0.543 |
| InternalIMT | epa | rs4986832 | 0.16 | -0.047 | 0.023 | 0.038 | -0.029 | 0.025 | 0.236 | -0.023 | 0.038 | 0.536 |
| InternalIMT | epa | rs144866316 | 0.005 | . | . | . | . | . | . | . | . | . |
| InternalIMT | epa | rs187769540 | 0.001 | . | . | . | . | . | . | . | . | . |
| InternalIMT | epa | rs6593482 | 0.16 | -0.046 | 0.023 | 0.044 | -0.033 | 0.025 | 0.179 | -0.026 | 0.037 | 0.493 |
| InternalIMT | epa | rs7913948 | 0.16 | -0.047 | 0.023 | 0.038 | -0.029 | 0.025 | 0.235 | -0.023 | 0.038 | 0.537 |
| InternalIMT | epa | rs77185151 | 0.001 | . | . | . | . | . | . | . | . | . |
| InternalIMT | epa | rs12762303 | 0.16 | -0.047 | 0.023 | 0.038 | -0.029 | 0.025 | 0.236 | -0.023 | 0.038 | 0.535 |
| InternalIMT | epa | rs73283136 | 0.003 | . | . | . | . | . | . | . | . | . |
| InternalIMT | epa | rs187180218 | 0 | . | . | . | . | . | . | . | . | . |
| InternalIMT | epa | rs78137087 | 0.159 | -0.046 | 0.023 | 0.04 | -0.03 | 0.025 | 0.224 | -0.025 | 0.038 | 0.512 |

| InternalIMT | epa | rs72561472 | 0.158 | -0.045 | 0.023 | 0.047 | -0.029 | 0.025 | 0.249 | -0.029 | 0.038 | 0.448 |
| --- | --- | --- | --- | --- | --- | --- | --- | --- | --- | --- | --- | --- |
| InternalIMT | epa | rs4987106 | 0.152 | -0.046 | 0.022 | 0.039 | -0.033 | 0.025 | 0.194 | -0.025 | 0.039 | 0.511 |
| InternalIMT | epa | rs4987105 | 0.155 | -0.046 | 0.022 | 0.042 | -0.029 | 0.025 | 0.249 | -0.028 | 0.038 | 0.467 |
| InternalIMT | epa | rs111916157 | 0.188 | -0.048 | 0.023 | 0.039 | -0.017 | 0.025 | 0.485 | -0.017 | 0.036 | 0.631 |
| InternalIMT | epa | rs111528152 | 0.16 | -0.047 | 0.023 | 0.038 | -0.029 | 0.025 | 0.234 | -0.023 | 0.038 | 0.539 |
| InternalIMT | epa | rs12763738 | 0.16 | -0.047 | 0.023 | 0.038 | -0.029 | 0.025 | 0.236 | -0.023 | 0.038 | 0.535 |
| InternalIMT | epa | rs191675886 | 0.006 | . | . | . | . | . | . | . | . | . |
| InternalIMT | epa | rs66489870 | 0.16 | -0.047 | 0.023 | 0.038 | -0.029 | 0.025 | 0.236 | -0.023 | 0.038 | 0.535 |
| InternalIMT | epa | rs71494775 | 0.16 | -0.047 | 0.023 | 0.038 | -0.029 | 0.025 | 0.236 | -0.023 | 0.038 | 0.535 |
| InternalIMT | epa | rs61855875 | 0.033 | -0.064 | 0.02 | 1.60E-03 | 0.099 | 0.086 | 0.245 | 0.178 | 0.133 | 0.179 |
| InternalIMT | epa | rs116356837 | 0 | . | . | . | . | . | . | . | . | . |
| InternalIMT | epa | rs1864414 | 0.16 | -0.047 | 0.023 | 0.038 | -0.029 | 0.025 | 0.236 | -0.023 | 0.038 | 0.535 |
| InternalIMT | epa | rs1864413 | 0.16 | -0.046 | 0.023 | 0.039 | -0.03 | 0.025 | 0.23 | -0.024 | 0.038 | 0.523 |
| InternalIMT | epa | rs139497212 | 0 | . | . | . | . | . | . | . | . | . |
| InternalIMT | epa | rs892690 | 0.358 | -0.04 | 0.03 | 0.172 | 0.02 | 0.02 | 0.312 | -0.019 | 0.031 | 0.536 |
| InternalIMT | epa | rs1864412 | 0.16 | -0.047 | 0.023 | 0.038 | -0.029 | 0.025 | 0.236 | -0.024 | 0.038 | 0.533 |
| InternalIMT | epa | rs77827771 | 0.004 | . | . | . | . | . | . | . | . | . |
| InternalIMT | epa | rs145294963 | 0.004 | . | . | . | . | . | . | . | . | . |
| InternalIMT | epa | rs149081327 | 0.012 | -0.044 | 0.02 | 0.027 | 2.60E-04 | 0.102 | 0.998 | -0.437 | 0.243 | 0.073 |
| InternalIMT | epa | rs67008983 | 0.16 | -0.047 | 0.023 | 0.039 | -0.029 | 0.025 | 0.236 | -0.024 | 0.038 | 0.527 |
| InternalIMT | epa | rs56150414 | 0.168 | -0.046 | 0.023 | 0.043 | -0.029 | 0.024 | 0.242 | -0.024 | 0.037 | 0.528 |
| InternalIMT | epa | rs55787481 | 0.16 | -0.046 | 0.023 | 0.04 | -0.029 | 0.025 | 0.232 | -0.025 | 0.038 | 0.516 |
| InternalIMT | epa | rs141270101 | 0.001 | . | . | . | . | . | . | . | . | . |
| InternalIMT | epa | rs114980075 | 0.002 | . | . | . | . | . | . | . | . | . |
| InternalIMT | epa | rs12783095 | 0.159 | -0.046 | 0.022 | 0.041 | -0.03 | 0.025 | 0.229 | -0.026 | 0.038 | 0.495 |
| InternalIMT | epa | rs182342688 | 0.005 | . | . | . | . | . | . | . | . | . |
| InternalIMT | epa | rs17157728 | 0.159 | -0.046 | 0.022 | 0.041 | -0.03 | 0.025 | 0.229 | -0.026 | 0.038 | 0.494 |
| InternalIMT | epa | rs114835563 | 0 | . | . | . | . | . | . | . | . | . |
| InternalIMT | epa | rs3824612 | 0.358 | -0.041 | 0.03 | 0.166 | 0.021 | 0.02 | 0.288 | -0.018 | 0.031 | 0.556 |
| InternalIMT | epa | rs17157733 | 0.158 | -0.046 | 0.022 | 0.041 | -0.03 | 0.025 | 0.221 | -0.026 | 0.038 | 0.489 |
| InternalIMT | epa | rs74128478 | 0 | . | . | . | . | . | . | . | . | . |
| InternalIMT | epa | rs184973589 | 0.001 | . | . | . | . | . | . | . | . | . |
| InternalIMT | epa | rs150479461 | 0 | . | . | . | . | . | . | . | . | . |
| InternalIMT | epa | rs138310407 | 0.004 | . | . | . | . | . | . | . | . | . |
| InternalIMT | epa | rs187426603 | 0.001 | . | . | . | . | . | . | . | . | . |
| InternalIMT | epa | rs190843143 | 0.003 | . | . | . | . | . | . | . | . | . |
| InternalIMT | epa | rs191883449 | 0.002 | . | . | . | . | . | . | . | . | . |
| InternalIMT | epa | rs58238740 | 0.158 | -0.046 | 0.022 | 0.041 | -0.03 | 0.025 | 0.229 | -0.026 | 0.038 | 0.485 |
| InternalIMT | epa | rs3780894 | 0.158 | -0.046 | 0.022 | 0.041 | -0.03 | 0.025 | 0.229 | -0.026 | 0.038 | 0.484 |
| InternalIMT | epa | rs149646626 | 0.027 | -0.048 | 0.019 | 0.014 | 0.056 | 0.081 | 0.49 | -0.126 | 0.169 | 0.457 |
| InternalIMT | epa | rs79615212 | 0.023 | -0.06 | 0.02 | 3.50E-03 | -0.064 | 0.075 | 0.388 | 0.091 | 0.088 | 0.299 |
| InternalIMT | epa | rs3780895 | 0.158 | -0.046 | 0.022 | 0.041 | -0.03 | 0.025 | 0.23 | -0.027 | 0.038 | 0.483 |
| InternalIMT | epa | rs3780896 | 0.158 | -0.046 | 0.022 | 0.041 | -0.03 | 0.025 | 0.229 | -0.027 | 0.038 | 0.484 |
| InternalIMT | epa | rs142747163 | 0.006 | . | . | . | . | . | . | . | . | . |
| InternalIMT | epa | rs189417584 | 0.005 | . | . | . | . | . | . | . | . | . |
| InternalIMT | epa | rs3780897 | 0.158 | -0.046 | 0.022 | 0.042 | -0.029 | 0.025 | 0.233 | -0.027 | 0.038 | 0.473 |
| InternalIMT | epa | rs117509491 | 0.004 | . | . | . | . | . | . | . | . | . |
| InternalIMT | epa | rs117811514 | 0.004 | . | . | . | . | . | . | . | . | . |
| InternalIMT | epa | rs116500932 | 0 | . | . | . | . | . | . | . | . | . |
| InternalIMT | epa | rs77584168 | 0.001 | . | . | . | . | . | . | . | . | . |
| InternalIMT | epa | rs2310776 | 0.158 | -0.046 | 0.022 | 0.041 | -0.03 | 0.025 | 0.229 | -0.027 | 0.038 | 0.483 |
| InternalIMT | epa | rs17157736 | 0.158 | -0.046 | 0.022 | 0.041 | -0.03 | 0.025 | 0.229 | -0.027 | 0.038 | 0.483 |
| InternalIMT | epa | rs72789735 | 0.105 | -0.049 | 0.021 | 0.021 | -0.035 | 0.029 | 0.226 | -0.026 | 0.042 | 0.535 |
| InternalIMT | epa | rs183747187 | 0.002 | . | . | . | . | . | . | . | . | . |
| InternalIMT | epa | rs7077891 | 0.158 | -0.046 | 0.022 | 0.041 | -0.03 | 0.025 | 0.229 | -0.027 | 0.038 | 0.482 |
| InternalIMT | epa | rs145063305 | 0.006 | . | . | . | . | . | . | . | . | . |
| InternalIMT | epa | rs3824613 | 0.159 | -0.046 | 0.022 | 0.041 | -0.03 | 0.025 | 0.231 | -0.027 | 0.038 | 0.481 |
| InternalIMT | epa | rs145543199 | 0.014 | -0.061 | 0.02 | 2.20E-03 | 0.044 | 0.089 | 0.622 | 0.265 | 0.151 | 0.079 |
| InternalIMT | epa | rs140361993 | 0.005 | . | . | . | . | . | . | . | . | . |
| InternalIMT | epa | rs11239496 | 0.105 | -0.049 | 0.021 | 0.021 | -0.035 | 0.029 | 0.226 | -0.026 | 0.042 | 0.534 |
| InternalIMT | epa | rs11239497 | 0.158 | -0.046 | 0.022 | 0.042 | -0.03 | 0.025 | 0.228 | -0.027 | 0.038 | 0.479 |
| InternalIMT | epa | rs10900212 | 0.158 | -0.046 | 0.022 | 0.042 | -0.03 | 0.025 | 0.227 | -0.027 | 0.038 | 0.48 |
| InternalIMT | epa | rs186487136 | 0 | . | . | . | . | . | . | . | . | . |
| InternalIMT | epa | rs12774369 | 0.158 | -0.045 | 0.022 | 0.043 | -0.03 | 0.025 | 0.219 | -0.028 | 0.038 | 0.467 |
| InternalIMT | epa | rs60842538 | 0.158 | -0.045 | 0.022 | 0.045 | -0.031 | 0.025 | 0.208 | -0.029 | 0.038 | 0.45 |
| InternalIMT | epa | rs3780898 | 0.158 | -0.045 | 0.022 | 0.047 | -0.032 | 0.025 | 0.195 | -0.03 | 0.038 | 0.429 |
| InternalIMT | epa | rs3780899 | 0.159 | -0.044 | 0.022 | 0.048 | -0.032 | 0.025 | 0.19 | -0.031 | 0.038 | 0.422 |
| InternalIMT | epa | rs12252472 | 0 | . | . | . | . | . | . | . | . | . |
| InternalIMT | epa | rs185411011 | 0.003 | . | . | . | . | . | . | . | . | . |
| InternalIMT | epa | rs28395865 | 0.156 | -0.044 | 0.022 | 0.051 | -0.033 | 0.025 | 0.189 | -0.033 | 0.039 | 0.392 |
| InternalIMT | epa | rs191004074 | 0.024 | -0.044 | 0.02 | 0.032 | -0.059 | 0.083 | 0.477 | -0.19 | 0.149 | 0.203 |
| InternalIMT | epa | rs182401295 | 0.004 | . | . | . | . | . | . | . | . | . |
| InternalIMT | epa | rs2228064 | 0.002 | . | . | . | . | . | . | . | . | . |

| InternalIMT | epa | rs188284159 | 0.001 | . | . | . | . | . | . | . | . | . |
| --- | --- | --- | --- | --- | --- | --- | --- | --- | --- | --- | --- | --- |
| InternalIMT | epa | rs2303887 | 0.001 | . | . | . | . | . | . | . | . | . |
| InternalIMT | epa | rs2303886 | 0.001 | . | . | . | . | . | . | . | . | . |
| InternalIMT | epa | rs192621006 | 0.003 | . | . | . | . | . | . | . | . | . |
| InternalIMT | epa | rs2303885 | 0.154 | -0.042 | 0.022 | 0.062 | -0.043 | 0.024 | 0.074 | -0.041 | 0.036 | 0.259 |
| InternalIMT | epa | rs114466168 | 0 | . | . | . | . | . | . | . | . | . |
| InternalIMT | epa | rs11239501 | 0.149 | -0.046 | 0.022 | 0.031 | -0.016 | 0.024 | 0.512 | -0.027 | 0.037 | 0.475 |
| InternalIMT | epa | rs745986 | 0.204 | -0.039 | 0.023 | 0.084 | -0.029 | 0.021 | 0.168 | -0.037 | 0.033 | 0.258 |
| InternalIMT | epa | rs75262268 | 0 | . | . | . | . | . | . | . | . | . |
| InternalIMT | epa | rs71494776 | 0.05 | -0.049 | 0.021 | 0.017 | -0.054 | 0.038 | 0.154 | -0.056 | 0.057 | 0.327 |
| InternalIMT | epa | rs74386871 | 0.011 | -0.06 | 0.02 | 2.40E-03 | 0.295 | 0.163 | 0.071 | 0.273 | 0.214 | 0.202 |
| InternalIMT | epa | rs76502040 | 0.01 | -0.057 | 0.02 | 4.10E-03 | 0.131 | 0.142 | 0.353 | 0.129 | 0.215 | 0.548 |
| InternalIMT | epa | rs142213734 | 0.001 | . | . | . | . | . | . | . | . | . |
| InternalIMT | epa | rs4948671 | 0.604 | -0.033 | 0.038 | 0.384 | 0.015 | 0.018 | 0.411 | -0.019 | 0.027 | 0.495 |
| InternalIMT | epa | rs146286085 | 0.002 | . | . | . | . | . | . | . | . | . |
| InternalIMT | epa | rs144080623 | 0 | . | . | . | . | . | . | . | . | . |
| InternalIMT | epa | rs115074014 | 0 | . | . | . | . | . | . | . | . | . |
| InternalIMT | epa | rs17157756 | 0 | . | . | . | . | . | . | . | . | . |
| InternalIMT | epa | rs190459780 | 0.001 | . | . | . | . | . | . | . | . | . |
| InternalIMT | epa | rs12358647 | 0.204 | -0.039 | 0.023 | 0.088 | -0.029 | 0.021 | 0.171 | -0.038 | 0.033 | 0.242 |
| InternalIMT | epa | rs147080556 | 0.011 | -0.054 | 0.02 | 7.00E-03 | -0.165 | 0.065 | 0.012 | -0.032 | 0.077 | 0.682 |
| InternalIMT | epa | rs187543349 | 0.004 | . | . | . | . | . | . | . | . | . |
| InternalIMT | epa | rs114375595 | 0.004 | . | . | . | . | . | . | . | . | . |
| InternalIMT | epa | rs117211200 | 0.01 | . | . | . | . | . | . | . | . | . |
| InternalIMT | epa | rs192963047 | 0.001 | . | . | . | . | . | . | . | . | . |
| InternalIMT | epa | rs11239505 | 0.204 | -0.039 | 0.023 | 0.089 | -0.028 | 0.021 | 0.178 | -0.038 | 0.033 | 0.238 |
| InternalIMT | epa | rs184434116 | 0.003 | . | . | . | . | . | . | . | . | . |
| InternalIMT | epa | rs4948999 | 0.604 | -0.032 | 0.038 | 0.39 | 0.016 | 0.018 | 0.397 | -0.019 | 0.027 | 0.486 |
| InternalIMT | epa | rs11239506 | 0.154 | -0.045 | 0.022 | 0.035 | -0.016 | 0.023 | 0.486 | -0.029 | 0.037 | 0.421 |
| InternalIMT | epa | rs190706620 | 0.006 | . | . | . | . | . | . | . | . | . |
| InternalIMT | epa | rs190770429 | 0.002 | . | . | . | . | . | . | . | . | . |
| InternalIMT | epa | rs181327133 | 0.006 | . | . | . | . | . | . | . | . | . |
| InternalIMT | epa | rs149251697 | 0.008 | . | . | . | . | . | . | . | . | . |
| InternalIMT | epa | rs118081602 | 0.001 | . | . | . | . | . | . | . | . | . |
| InternalIMT | epa | rs188233691 | 0.001 | . | . | . | . | . | . | . | . | . |
| InternalIMT | epa | rs183778794 | 0.005 | . | . | . | . | . | . | . | . | . |
| InternalIMT | epa | rs188040078 | 0.007 | . | . | . | . | . | . | . | . | . |
| InternalIMT | epa | rs184675372 | 0.003 | . | . | . | . | . | . | . | . | . |
| InternalIMT | epa | rs150326891 | 0.116 | -0.038 | 0.021 | 0.076 | -0.029 | 0.026 | 0.28 | -0.068 | 0.042 | 0.104 |
| InternalIMT | epa | rs185520021 | 0.002 | . | . | . | . | . | . | . | . | . |
| InternalIMT | epa | rs7907779 | 0.154 | -0.045 | 0.022 | 0.036 | -0.016 | 0.023 | 0.491 | -0.03 | 0.037 | 0.413 |
| InternalIMT | epa | rs184962860 | 0.001 | . | . | . | . | . | . | . | . | . |
| InternalIMT | epa | rs149762784 | 0.025 | -0.049 | 0.02 | 0.016 | -0.014 | 0.094 | 0.882 | -0.105 | 0.132 | 0.426 |
| InternalIMT | epa | rs149203427 | 0.021 | -0.055 | 0.02 | 6.70E-03 | 0.082 | 0.092 | 0.37 | 0.027 | 0.131 | 0.834 |
| InternalIMT | epa | rs60153083 | 0 | . | . | . | . | . | . | . | . | . |
| InternalIMT | epa | rs112001069 | 0.05 | -0.049 | 0.021 | 0.017 | -0.054 | 0.038 | 0.16 | -0.057 | 0.057 | 0.315 |
| InternalIMT | epa | rs182355382 | 0.021 | -0.055 | 0.02 | 6.20E-03 | 0.081 | 0.089 | 0.363 | -0.016 | 0.132 | 0.901 |
| InternalIMT | epa | rs186740974 | 0.117 | -0.052 | 0.024 | 0.029 | -7.40E-03 | 0.038 | 0.847 | -9.90E-03 | 0.055 | 0.858 |
| InternalIMT | epa | rs191874916 | 0.014 | -0.052 | 0.02 | 8.40E-03 | 0.095 | 0.124 | 0.441 | -0.093 | 0.202 | 0.646 |
| InternalIMT | epa | rs182989412 | 0.152 | -0.058 | 0.024 | 0.018 | 0.027 | 0.038 | 0.465 | 0.011 | 0.059 | 0.85 |
| InternalIMT | epa | rs188819476 | 0.002 | . | . | . | . | . | . | . | . | . |
| InternalIMT | epa | rs141217183 | 0.078 | -0.06 | 0.022 | 6.80E-03 | -0.047 | 0.038 | 0.213 | 0.033 | 0.052 | 0.528 |
| InternalIMT | epa | rs192922652 | 0.045 | -0.05 | 0.021 | 0.015 | -0.071 | 0.043 | 0.101 | -0.051 | 0.062 | 0.406 |
| InternalIMT | epa | rs185092343 | 0.024 | -0.056 | 0.02 | 6.50E-03 | 0.148 | 0.109 | 0.175 | 0.016 | 0.154 | 0.916 |
| InternalIMT | epa | rs145193311 | 0.051 | -0.047 | 0.021 | 0.024 | -0.07 | 0.042 | 0.092 | -0.085 | 0.06 | 0.159 |
| InternalIMT | epa | rs55789336 | 0.155 | -0.048 | 0.022 | 0.026 | -9.30E-03 | 0.024 | 0.697 | -0.021 | 0.036 | 0.567 |
| InternalIMT | epa | rs55837212 | 0.152 | -0.046 | 0.022 | 0.034 | -0.015 | 0.023 | 0.504 | -0.029 | 0.037 | 0.43 |
| InternalIMT | epa | rs75408574 | 0.366 | -0.065 | 0.03 | 0.032 | 0.038 | 0.02 | 0.054 | 0.015 | 0.031 | 0.634 |
| InternalIMT | epa | rs149306926 | 0.161 | -0.04 | 0.022 | 0.07 | -0.027 | 0.023 | 0.247 | -0.045 | 0.037 | 0.219 |
| InternalIMT | epa | rs35990892 | 0.597 | -0.023 | 0.039 | 0.561 | -4.80E-04 | 0.019 | 0.98 | -0.027 | 0.028 | 0.346 |
| InternalIMT | epa | rs192356207 | 0.019 | -0.055 | 0.02 | 7.10E-03 | -1.90E-03 | 0.075 | 0.98 | 4.40E-04 | 0.115 | 0.997 |
| InternalIMT | epa | rs183741163 | 0.129 | -0.044 | 0.021 | 0.042 | -8.50E-03 | 0.027 | 0.753 | -0.043 | 0.043 | 0.313 |
| InternalIMT | epa | rs34737297 | 0.48 | -0.058 | 0.036 | 0.107 | 0.022 | 0.021 | 0.297 | 3.50E-03 | 0.031 | 0.91 |
| InternalIMT | epa | rs181346933 | 0.026 | -0.044 | 0.021 | 0.035 | -0.075 | 0.068 | 0.267 | -0.164 | 0.094 | 0.081 |
| InternalIMT | epa | rs36093253 | 0.343 | -0.069 | 0.03 | 0.021 | 0.028 | 0.021 | 0.18 | 0.022 | 0.033 | 0.504 |
| InternalIMT | epa | rs186307855 | 0.02 | -0.051 | 0.02 | 0.012 | 0.123 | 0.09 | 0.171 | -0.091 | 0.119 | 0.445 |
| InternalIMT | epa | rs189681865 | 0.003 | . | . | . | . | . | . | . | . | . |
| InternalIMT | epa | rs34942971 | 0.207 | -0.035 | 0.023 | 0.128 | -0.025 | 0.021 | 0.235 | -0.045 | 0.033 | 0.176 |
| InternalIMT | epa | rs181816554 | 0.019 | -0.065 | 0.02 | 1.20E-03 | 0.165 | 0.144 | 0.251 | 0.259 | 0.188 | 0.17 |
| InternalIMT | epa | rs186846881 | 0.004 | . | . | . | . | . | . | . | . | . |
| InternalIMT | epa | rs191178220 | 0.024 | -0.053 | 0.02 | 8.60E-03 | 0.091 | 0.072 | 0.208 | -0.04 | 0.116 | 0.731 |
| InternalIMT | epa | rs183305228 | 0 | . | . | . | . | . | . | . | . | . |
| InternalIMT | epa | rs139912761 | 0.15 | -0.045 | 0.022 | 0.04 | -0.019 | 0.024 | 0.438 | -0.033 | 0.038 | 0.385 |

| InternalIMT | epa | rs185499901 | 0.019 | -0.048 | 0.02 | 0.014 | 0.094 | 0.135 | 0.486 | -0.152 | 0.266 | 0.568 |
| --- | --- | --- | --- | --- | --- | --- | --- | --- | --- | --- | --- | --- |
| InternalIMT | epa | rs189816197 | 0.061 | -0.05 | 0.021 | 0.018 | -0.048 | 0.038 | 0.199 | -0.05 | 0.056 | 0.369 |
| InternalIMT | epa | rs145656938 | 0.014 | -0.062 | 0.02 | 2.00E-03 | 0.084 | 0.148 | 0.573 | 0.274 | 0.171 | 0.11 |
| InternalIMT | epa | rs35745197 | 0.05 | -0.049 | 0.021 | 0.017 | -0.055 | 0.038 | 0.147 | -0.056 | 0.057 | 0.325 |
| InternalIMT | epa | rs192856637 | 0.006 | . | . | . | . | . | . | . | . | . |
| InternalIMT | epa | rs2310775 | 0.401 | -0.061 | 0.031 | 0.049 | 0.033 | 0.019 | 0.078 | 8.90E-03 | 0.03 | 0.763 |
| InternalIMT | epa | rs11239507 | 0.403 | -0.062 | 0.031 | 0.044 | 0.035 | 0.019 | 0.059 | 0.01 | 0.029 | 0.723 |
| InternalIMT | epa | rs74648467 | 0.049 | -0.049 | 0.021 | 0.017 | -0.055 | 0.038 | 0.153 | -0.057 | 0.058 | 0.322 |
| InternalIMT | epa | rs145815934 | 0.022 | -0.055 | 0.02 | 6.70E-03 | -8.50E-03 | 0.07 | 0.904 | 0.011 | 0.095 | 0.912 |
| InternalIMT | epa | rs145390687 | 0.029 | -0.06 | 0.02 | 3.20E-03 | 0.178 | 0.089 | 0.046 | 0.152 | 0.123 | 0.218 |
| InternalIMT | epa | rs184354913 | 0.049 | -0.053 | 0.02 | 8.20E-03 | 0.043 | 0.053 | 0.415 | -0.011 | 0.092 | 0.901 |
| InternalIMT | epa | rs181244920 | 0.007 | . | . | . | . | . | . | . | . | . |
| InternalIMT | epa | rs11499087 | 0 | . | . | . | . | . | . | . | . | . |
| InternalIMT | epa | rs148263794 | 0.05 | -0.049 | 0.021 | 0.017 | -0.055 | 0.038 | 0.146 | -0.056 | 0.057 | 0.327 |
| InternalIMT | epa | rs150758903 | 0.01 | -0.055 | 0.02 | 5.70E-03 | -0.08 | 0.125 | 0.52 | 0.035 | 0.147 | 0.811 |
| InternalIMT | epa | rs147654382 | 0.183 | -0.04 | 0.022 | 0.075 | -0.023 | 0.022 | 0.29 | -0.04 | 0.034 | 0.24 |
| InternalIMT | epa | rs147495166 | 0 | . | . | . | . | . | . | . | . | . |
| InternalIMT | epa | rs35198778 | 0.092 | -0.043 | 0.022 | 0.053 | -0.046 | 0.032 | 0.142 | -0.058 | 0.045 | 0.199 |
| InternalIMT | epa | rs145207659 | 0.071 | -0.049 | 0.021 | 0.021 | 0.067 | 0.042 | 0.116 | -0.033 | 0.067 | 0.622 |
| InternalIMT | epa | rs186466464 | 0.003 | . | . | . | . | . | . | . | . | . |
| InternalIMT | epa | rs12778413 | 0.157 | -0.04 | 0.022 | 0.071 | -0.012 | 0.027 | 0.657 | -0.049 | 0.041 | 0.236 |
| InternalIMT | epa | rs7895552 | 0.531 | -0.033 | 0.036 | 0.355 | 0.024 | 0.02 | 0.217 | -0.021 | 0.029 | 0.465 |
| InternalIMT | epa | rs12783895 | 0.05 | -0.049 | 0.021 | 0.017 | -0.056 | 0.038 | 0.143 | -0.057 | 0.057 | 0.321 |
| InternalIMT | epa | rs34929604 | 0.05 | -0.049 | 0.021 | 0.017 | -0.056 | 0.038 | 0.143 | -0.057 | 0.057 | 0.321 |
| InternalIMT | epa | rs12761961 | 0.049 | -0.049 | 0.021 | 0.017 | -0.054 | 0.038 | 0.159 | -0.059 | 0.057 | 0.305 |
| InternalIMT | epa | rs35536930 | 0.198 | -0.037 | 0.023 | 0.103 | -0.03 | 0.021 | 0.158 | -0.044 | 0.033 | 0.183 |
| InternalIMT | epa | rs180951060 | 0 | . | . | . | . | . | . | . | . | . |
| InternalIMT | epa | rs148892128 | 0.022 | -0.055 | 0.02 | 6.70E-03 | -8.40E-03 | 0.07 | 0.905 | 0.011 | 0.095 | 0.911 |
| InternalIMT | epa | rs11239509 | 0.001 | . | . | . | . | . | . | . | . | . |
| InternalIMT | epa | rs41526545 | 0.154 | -0.045 | 0.022 | 0.038 | -0.015 | 0.023 | 0.508 | -0.031 | 0.037 | 0.389 |
| InternalIMT | epa | rs2029253 | 0.401 | -0.062 | 0.031 | 0.045 | 0.033 | 0.019 | 0.084 | 0.01 | 0.03 | 0.731 |
| InternalIMT | epa | rs17444064 | 0.154 | -0.045 | 0.022 | 0.039 | -0.015 | 0.023 | 0.517 | -0.032 | 0.037 | 0.379 |
| InternalIMT | epa | rs71494779 | 0.05 | -0.049 | 0.021 | 0.017 | -0.055 | 0.038 | 0.146 | -0.057 | 0.057 | 0.319 |
| InternalIMT | epa | rs74991420 | 0.001 | . | . | . | . | . | . | . | . | . |
| InternalIMT | epa | rs182736551 | 0.001 | . | . | . | . | . | . | . | . | . |
| InternalIMT | epa | rs78702074 | 0.001 | . | . | . | . | . | . | . | . | . |
| InternalIMT | epa | rs187791466 | 0.005 | . | . | . | . | . | . | . | . | . |
| InternalIMT | epa | rs35300270 | 0.054 | -0.049 | 0.021 | 0.019 | -0.053 | 0.037 | 0.156 | -0.053 | 0.055 | 0.333 |
| InternalIMT | epa | rs6593484 | 0.155 | -0.045 | 0.022 | 0.039 | -0.015 | 0.023 | 0.511 | -0.032 | 0.037 | 0.383 |
| InternalIMT | epa | rs12244601 | 0.004 | . | . | . | . | . | . | . | . | . |
| InternalIMT | epa | rs184429574 | 0.005 | . | . | . | . | . | . | . | . | . |
| InternalIMT | epa | rs7080897 | 0.05 | -0.049 | 0.021 | 0.017 | -0.055 | 0.038 | 0.146 | -0.057 | 0.057 | 0.318 |
| InternalIMT | epa | rs7097429 | 0.054 | -0.049 | 0.021 | 0.018 | -0.042 | 0.037 | 0.254 | -0.057 | 0.056 | 0.307 |
| InternalIMT | epa | rs77979229 | 0.001 | . | . | . | . | . | . | . | . | . |
| InternalIMT | epa | rs11239512 | 0.155 | -0.045 | 0.022 | 0.039 | -0.015 | 0.023 | 0.513 | -0.032 | 0.037 | 0.379 |
| InternalIMT | epa | rs60146827 | 0.05 | -0.049 | 0.021 | 0.017 | -0.055 | 0.038 | 0.146 | -0.057 | 0.057 | 0.318 |
| InternalIMT | epa | rs79032236 | 0 | . | . | . | . | . | . | . | . | . |
| InternalIMT | epa | rs140854206 | 0.009 | . | . | . | . | . | . | . | . | . |
| InternalIMT | epa | rs184049550 | 0 | . | . | . | . | . | . | . | . | . |
| InternalIMT | epa | rs114433607 | 0 | . | . | . | . | . | . | . | . | . |
| InternalIMT | epa | rs117653793 | 0.001 | . | . | . | . | . | . | . | . | . |
| InternalIMT | epa | rs117039533 | 0.001 | . | . | . | . | . | . | . | . | . |
| InternalIMT | epa | rs191660761 | 0.006 | . | . | . | . | . | . | . | . | . |
| InternalIMT | epa | rs59514896 | 0.05 | -0.049 | 0.021 | 0.017 | -0.055 | 0.038 | 0.153 | -0.057 | 0.057 | 0.317 |
| InternalIMT | epa | rs184661876 | 0.002 | . | . | . | . | . | . | . | . | . |
| InternalIMT | epa | rs61854073 | 0.037 | -0.066 | 0.02 | 1.20E-03 | 0.138 | 0.079 | 0.082 | 0.183 | 0.129 | 0.156 |
| InternalIMT | epa | rs2018518 | 0.315 | -0.054 | 0.028 | 0.057 | 0.025 | 0.021 | 0.234 | -5.00E-04 | 0.033 | 0.988 |
| InternalIMT | epa | rs934187 | 0.607 | -0.033 | 0.038 | 0.376 | 0.015 | 0.018 | 0.414 | -0.018 | 0.027 | 0.506 |
| InternalIMT | epa | rs7918542 | 0.205 | -0.038 | 0.023 | 0.1 | -0.027 | 0.021 | 0.188 | -0.042 | 0.033 | 0.199 |
| InternalIMT | epa | rs144311920 | 0 | . | . | . | . | . | . | . | . | . |
| InternalIMT | epa | rs7099684 | 0.79 | -0.027 | 0.049 | 0.576 | 0.027 | 0.021 | 0.195 | -0.017 | 0.03 | 0.57 |
| InternalIMT | epa | rs150829171 | 0.002 | . | . | . | . | . | . | . | . | . |
| InternalIMT | epa | rs186060697 | 0 | . | . | . | . | . | . | . | . | . |
| InternalIMT | epa | rs11239513 | 0.009 | . | . | . | . | . | . | . | . | . |
| InternalIMT | epa | rs7917687 | 0.05 | -0.049 | 0.021 | 0.017 | -0.055 | 0.038 | 0.148 | -0.057 | 0.057 | 0.318 |
| InternalIMT | epa | rs61854076 | 0.014 | -0.062 | 0.02 | 1.90E-03 | 0.115 | 0.122 | 0.348 | 0.296 | 0.175 | 0.09 |
| InternalIMT | epa | rs71494780 | 0.019 | -0.052 | 0.02 | 8.70E-03 | -0.067 | 0.046 | 0.149 | -0.081 | 0.092 | 0.378 |
| InternalIMT | epa | rs7919239 | 0.205 | -0.037 | 0.023 | 0.106 | -0.026 | 0.021 | 0.221 | -0.044 | 0.032 | 0.18 |
| InternalIMT | epa | rs78334475 | 0.003 | . | . | . | . | . | . | . | . | . |
| InternalIMT | epa | rs3780900 | 0.011 | -0.052 | 0.02 | 8.40E-03 | 0.164 | 0.138 | 0.233 | -0.108 | 0.232 | 0.641 |
| InternalIMT | epa | rs186729292 | 0.001 | . | . | . | . | . | . | . | . | . |
| InternalIMT | epa | rs12267438 | 0.001 | . | . | . | . | . | . | . | . | . |
| InternalIMT | epa | rs1369214 | 0.454 | -0.058 | 0.033 | 0.077 | 0.025 | 0.018 | 0.173 | 3.80E-03 | 0.028 | 0.893 |

| InternalIMT | epa | rs9282589 | 0.011 | -0.058 | 0.02 | 3.20E-03 | 0.027 | 0.117 | 0.818 | 0.145 | 0.16 | 0.366 |
| --- | --- | --- | --- | --- | --- | --- | --- | --- | --- | --- | --- | --- |
| InternalIMT | epa | rs114522915 | 0 | . | . | . | . | . | . | . | . | . |
| InternalIMT | epa | rs2115819 | 0.452 | -0.057 | 0.033 | 0.08 | 0.022 | 0.018 | 0.233 | 3.10E-03 | 0.029 | 0.912 |
| InternalIMT | epa | rs117664603 | 0.001 | . | . | . | . | . | . | . | . | . |
| InternalIMT | epa | rs184979202 | 0.001 | . | . | . | . | . | . | . | . | . |
| InternalIMT | epa | rs190714671 | 0.001 | . | . | . | . | . | . | . | . | . |
| InternalIMT | epa | rs141538996 | 0 | . | . | . | . | . | . | . | . | . |
| InternalIMT | epa | rs150881741 | 0.003 | . | . | . | . | . | . | . | . | . |
| InternalIMT | epa | rs139076633 | 0 | . | . | . | . | . | . | . | . | . |
| InternalIMT | epa | rs12243649 | 0.002 | . | . | . | . | . | . | . | . | . |
| InternalIMT | epa | rs140348708 | 0.001 | . | . | . | . | . | . | . | . | . |
| InternalIMT | epa | rs79023256 | 0.001 | . | . | . | . | . | . | . | . | . |
| InternalIMT | epa | rs11239515 | 0.146 | -0.043 | 0.021 | 0.043 | -0.02 | 0.024 | 0.409 | -0.037 | 0.038 | 0.318 |
| InternalIMT | epa | rs55866086 | 0.148 | -0.044 | 0.022 | 0.041 | -0.016 | 0.024 | 0.518 | -0.034 | 0.037 | 0.359 |
| InternalIMT | epa | rs181340150 | 0.005 | . | . | . | . | . | . | . | . | . |
| InternalIMT | epa | rs11239516 | 0.146 | -0.043 | 0.021 | 0.043 | -0.02 | 0.024 | 0.408 | -0.038 | 0.038 | 0.318 |
| InternalIMT | epa | rs11239517 | 0.146 | -0.043 | 0.021 | 0.043 | -0.02 | 0.024 | 0.407 | -0.038 | 0.038 | 0.318 |
| InternalIMT | epa | rs11239519 | 0.135 | -0.052 | 0.021 | 0.014 | -3.20E-03 | 0.026 | 0.902 | -9.30E-03 | 0.04 | 0.814 |
| InternalIMT | epa | rs7909514 | 0.601 | -0.033 | 0.038 | 0.377 | 0.013 | 0.018 | 0.489 | -0.018 | 0.027 | 0.504 |
| InternalIMT | epa | rs7894352 | 0.782 | -0.04 | 0.048 | 0.403 | 0.029 | 0.021 | 0.167 | -9.30E-03 | 0.03 | 0.755 |
| InternalIMT | epa | rs189384146 | 0.004 | . | . | . | . | . | . | . | . | . |
| InternalIMT | epa | rs11239520 | 0.146 | -0.043 | 0.021 | 0.044 | -0.02 | 0.024 | 0.408 | -0.038 | 0.038 | 0.316 |
| InternalIMT | epa | rs12221233 | 0.404 | -0.065 | 0.031 | 0.036 | 0.035 | 0.019 | 0.061 | 0.014 | 0.03 | 0.634 |
| InternalIMT | epa | rs74523293 | 0.001 | . | . | . | . | . | . | . | . | . |
| InternalIMT | epa | rs79376040 | 0.001 | . | . | . | . | . | . | . | . | . |
| InternalIMT | epa | rs4949000 | 0.404 | -0.065 | 0.031 | 0.036 | 0.036 | 0.019 | 0.06 | 0.014 | 0.03 | 0.632 |
| InternalIMT | epa | rs7089406 | 0.018 | -0.054 | 0.02 | 7.50E-03 | -0.037 | 0.057 | 0.514 | -7.30E-03 | 0.066 | 0.912 |
| InternalIMT | epa | rs138211056 | 0 | . | . | . | . | . | . | . | . | . |
| InternalIMT | epa | rs10900213 | 0.548 | -0.044 | 0.035 | 0.212 | 0.022 | 0.018 | 0.24 | -9.90E-03 | 0.028 | 0.722 |
| InternalIMT | epa | rs145152827 | 0 | . | . | . | . | . | . | . | . | . |
| InternalIMT | epa | rs74443325 | 0.001 | . | . | . | . | . | . | . | . | . |
| InternalIMT | epa | rs188299446 | 0.016 | -0.043 | 0.02 | 0.032 | 0.021 | 0.096 | 0.829 | -0.364 | 0.161 | 0.024 |
| InternalIMT | epa | rs192699586 | 0.002 | . | . | . | . | . | . | . | . | . |
| InternalIMT | epa | rs72796464 | 0.145 | -0.043 | 0.021 | 0.044 | -0.02 | 0.024 | 0.409 | -0.038 | 0.038 | 0.311 |
| InternalIMT | epa | rs4949001 | 0.268 | -0.056 | 0.028 | 0.041 | 8.60E-03 | 0.024 | 0.721 | 2.60E-03 | 0.036 | 0.943 |
| InternalIMT | epa | rs147174641 | 0.013 | -0.051 | 0.02 | 9.60E-03 | -0.022 | 0.134 | 0.868 | -0.162 | 0.215 | 0.45 |
| InternalIMT | epa | rs190835238 | 0.001 | . | . | . | . | . | . | . | . | . |
| InternalIMT | epa | rs76698944 | 0.03 | -0.057 | 0.02 | 5.40E-03 | 0.014 | 0.056 | 0.798 | 0.035 | 0.073 | 0.638 |
| InternalIMT | epa | rs56071381 | 0.142 | -0.044 | 0.021 | 0.038 | -0.02 | 0.024 | 0.418 | -0.034 | 0.037 | 0.356 |
| InternalIMT | epa | rs78130427 | 0.031 | -0.052 | 0.02 | 9.90E-03 | -0.043 | 0.055 | 0.44 | -0.036 | 0.072 | 0.618 |
| InternalIMT | epa | rs10900215 | 0.146 | -0.043 | 0.021 | 0.046 | -0.022 | 0.024 | 0.354 | -0.039 | 0.037 | 0.3 |
| InternalIMT | epa | rs41491445 | 0 | . | . | . | . | . | . | . | . | . |
| InternalIMT | epa | rs144995222 | . | . | . | . | . | . | . | . | . | . |
| InternalIMT | epa | rs149097803 | 0.003 | . | . | . | . | . | . | . | . | . |
| InternalIMT | epa | rs139429646 | 0.007 | . | . | . | . | . | . | . | . | . |
| InternalIMT | epa | rs7075620 | 0.576 | -0.037 | 0.037 | 0.321 | 0.018 | 0.019 | 0.33 | -0.016 | 0.028 | 0.571 |
| InternalIMT | epa | rs148299213 | 0 | . | . | . | . | . | . | . | . | . |
| InternalIMT | epa | rs4949002 | 0.31 | -0.061 | 0.029 | 0.035 | 0.034 | 0.022 | 0.121 | 0.011 | 0.034 | 0.746 |
| InternalIMT | epa | rs150496007 | 0.011 | -0.055 | 0.02 | 5.90E-03 | 0.134 | 0.143 | 0.35 | -0.025 | 0.216 | 0.908 |
| InternalIMT | epa | rs147303455 | 0.004 | . | . | . | . | . | . | . | . | . |
| InternalIMT | epa | rs189518433 | 0.005 | . | . | . | . | . | . | . | . | . |
| InternalIMT | epa | rs186281355 | 0.005 | . | . | . | . | . | . | . | . | . |
| InternalIMT | epa | rs115297114 | 0 | . | . | . | . | . | . | . | . | . |
| InternalIMT | epa | rs11239523 | 0.146 | -0.046 | 0.021 | 0.032 | -0.017 | 0.024 | 0.476 | -0.029 | 0.037 | 0.444 |
| InternalIMT | epa | rs143704897 | 0.004 | . | . | . | . | . | . | . | . | . |
| InternalIMT | epa | rs188024096 | 0.004 | . | . | . | . | . | . | . | . | . |
| InternalIMT | epa | rs11239524 | 0.134 | -0.043 | 0.021 | 0.045 | -0.024 | 0.027 | 0.367 | -0.042 | 0.041 | 0.306 |
| InternalIMT | epa | rs191656573 | 0.005 | . | . | . | . | . | . | . | . | . |
| InternalIMT | epa | rs193295875 | 0 | . | . | . | . | . | . | . | . | . |
| InternalIMT | epa | rs190458228 | 0.002 | . | . | . | . | . | . | . | . | . |
| InternalIMT | epa | rs139253993 | 0.002 | . | . | . | . | . | . | . | . | . |
| InternalIMT | epa | rs4948672 | 0.565 | -0.064 | 0.038 | 0.094 | 0.052 | 0.02 | 9.30E-03 | 8.90E-03 | 0.031 | 0.775 |
| InternalIMT | epa | rs180954921 | 0.004 | . | . | . | . | . | . | . | . | . |
| InternalIMT | epa | rs12264801 | 0.431 | -0.021 | 0.032 | 0.501 | -9.40E-03 | 0.02 | 0.644 | -0.038 | 0.03 | 0.212 |
| InternalIMT | epa | rs73283184 | 0 | . | . | . | . | . | . | . | . | . |
| InternalIMT | epa | rs142746187 | 0.002 | . | . | . | . | . | . | . | . | . |
| InternalIMT | epa | rs58704739 | 0 | . | . | . | . | . | . | . | . | . |
| InternalIMT | epa | rs61272335 | 0 | . | . | . | . | . | . | . | . | . |
| InternalIMT | epa | rs60959489 | 0 | . | . | . | . | . | . | . | . | . |
| InternalIMT | epa | rs58502629 | 0 | . | . | . | . | . | . | . | . | . |
| InternalIMT | epa | rs10900216 | 0.695 | -0.048 | 0.038 | 0.202 | 0.047 | 0.018 | 0.01 | -3.40E-03 | 0.027 | 0.9 |
| InternalIMT | epa | rs181118141 | 0 | . | . | . | . | . | . | . | . | . |
| InternalIMT | epa | rs137882665 | 0.004 | . | . | . | . | . | . | . | . | . |

| InternalIMT | epa | rs149466736 | 0.004 | . | . | . | . | . | . | . | . | . |
| --- | --- | --- | --- | --- | --- | --- | --- | --- | --- | --- | --- | --- |
| InternalIMT | epa | rs2099171 | 0.662 | -0.046 | 0.036 | 0.196 | 0.058 | 0.018 | 1.10E-03 | -5.00E-03 | 0.027 | 0.853 |
| InternalIMT | epa | rs141852280 | 0.009 | . | . | . | . | . | . | . | . | . |
| InternalIMT | epa | rs191539257 | 0.002 | . | . | . | . | . | . | . | . | . |
| InternalIMT | epa | rs17157771 | 0.033 | -0.055 | 0.02 | 7.50E-03 | -0.098 | 0.041 | 0.018 | 5.10E-03 | 0.051 | 0.919 |
| InternalIMT | epa | rs111339241 | 0.032 | -0.054 | 0.02 | 7.80E-03 | -0.095 | 0.043 | 0.027 | 3.00E-03 | 0.052 | 0.954 |
| InternalIMT | epa | rs116983255 | 0.013 | -0.054 | 0.02 | 6.50E-03 | -0.119 | 0.066 | 0.071 | -0.012 | 0.081 | 0.882 |
| InternalIMT | epa | rs142324341 | 0.003 | . | . | . | . | . | . | . | . | . |
| InternalIMT | epa | rs187191219 | 0.009 | . | . | . | . | . | . | . | . | . |
| InternalIMT | epa | rs151283973 | . | . | . | . | . | . | . | . | . | . |
| InternalIMT | epa | rs139316218 | 0.005 | . | . | . | . | . | . | . | . | . |
| InternalIMT | epa | rs6593485 | 0.696 | -0.041 | 0.038 | 0.291 | 0.045 | 0.018 | 0.014 | -9.10E-03 | 0.027 | 0.738 |
| InternalIMT | epa | rs6593486 | 0.696 | -0.041 | 0.038 | 0.29 | 0.045 | 0.018 | 0.014 | -9.10E-03 | 0.027 | 0.739 |
| InternalIMT | epa | rs145196618 | 0.002 | . | . | . | . | . | . | . | . | . |
| InternalIMT | epa | rs142598335 | 0.006 | . | . | . | . | . | . | . | . | . |
| InternalIMT | epa | rs892691 | 0.704 | -0.049 | 0.039 | 0.211 | 0.051 | 0.019 | 6.30E-03 | -3.50E-03 | 0.027 | 0.899 |
| InternalIMT | epa | rs7080713 | 0.663 | -0.043 | 0.036 | 0.227 | 0.055 | 0.018 | 1.80E-03 | -7.30E-03 | 0.027 | 0.788 |
| InternalIMT | epa | rs7077173 | 0.057 | -0.054 | 0.02 | 7.50E-03 | -0.037 | 0.031 | 0.232 | -6.10E-03 | 0.043 | 0.886 |
| InternalIMT | epa | rs7080977 | 0.032 | -0.055 | 0.02 | 7.50E-03 | -0.092 | 0.043 | 0.031 | 5.20E-03 | 0.052 | 0.921 |
| InternalIMT | epa | rs3780901 | 0.663 | -0.044 | 0.036 | 0.217 | 0.056 | 0.018 | 1.70E-03 | -6.60E-03 | 0.027 | 0.807 |
| InternalIMT | epa | rs61854091 | 0.073 | -0.063 | 0.021 | 2.70E-03 | 0.081 | 0.044 | 0.068 | 0.06 | 0.072 | 0.406 |
| InternalIMT | epa | rs77550209 | 0.003 | . | . | . | . | . | . | . | . | . |
| InternalIMT | epa | rs73283191 | 0.033 | -0.054 | 0.02 | 8.50E-03 | -0.09 | 0.041 | 0.028 | -7.30E-04 | 0.049 | 0.988 |
| InternalIMT | epa | rs191866099 | 0.01 | -0.058 | 0.02 | 4.00E-03 | 0.046 | 0.165 | 0.78 | 0.143 | 0.223 | 0.521 |
| InternalIMT | epa | rs147998395 | . | . | . | . | . | . | . | . | . | . |
| InternalIMT | epa | rs7090328 | 0.699 | -0.046 | 0.038 | 0.227 | 0.046 | 0.018 | 0.011 | -5.20E-03 | 0.027 | 0.847 |
| InternalIMT | epa | rs79814535 | 0.001 | . | . | . | . | . | . | . | . | . |
| InternalIMT | epa | rs7073082 | 0.699 | -0.046 | 0.038 | 0.226 | 0.046 | 0.018 | 0.012 | -5.20E-03 | 0.027 | 0.849 |
| InternalIMT | epa | rs11239526 | 0.052 | -0.053 | 0.021 | 0.01 | 0.034 | 0.041 | 0.404 | -0.011 | 0.057 | 0.843 |
| InternalIMT | epa | rs141839841 | . | . | . | . | . | . | . | . | . | . |
| InternalIMT | epa | rs75697371 | 0.014 | -0.052 | 0.02 | 9.60E-03 | -0.047 | 0.079 | 0.546 | -0.122 | 0.109 | 0.262 |
| InternalIMT | epa | rs75972854 | 0.016 | -0.06 | 0.02 | 2.50E-03 | 0.099 | 0.161 | 0.539 | 0.275 | 0.218 | 0.209 |
| InternalIMT | epa | rs139291975 | 0.001 | . | . | . | . | . | . | . | . | . |
| InternalIMT | epa | rs144064711 | 0.009 | . | . | . | . | . | . | . | . | . |
| InternalIMT | epa | rs3780902 | 0.033 | -0.054 | 0.02 | 8.90E-03 | -0.095 | 0.04 | 0.018 | -4.00E-03 | 0.048 | 0.932 |
| InternalIMT | epa | rs190449801 | 0 | . | . | . | . | . | . | . | . | . |
| InternalIMT | epa | rs73283196 | 0.033 | -0.054 | 0.02 | 8.70E-03 | -0.095 | 0.041 | 0.02 | -2.20E-03 | 0.048 | 0.964 |
| InternalIMT | epa | rs28395867 | 0.005 | . | . | . | . | . | . | . | . | . |
| InternalIMT | epa | rs28395866 | 0.036 | -0.056 | 0.02 | 6.00E-03 | -0.086 | 0.041 | 0.037 | 0.024 | 0.055 | 0.665 |
| InternalIMT | epa | rs55661611 | 0.032 | -0.054 | 0.02 | 7.70E-03 | -0.098 | 0.042 | 0.02 | 3.20E-03 | 0.051 | 0.95 |
| InternalIMT | epa | rs55684518 | 0.03 | -0.064 | 0.02 | 1.50E-03 | -0.114 | 0.053 | 0.031 | 0.166 | 0.085 | 0.05 |
| InternalIMT | epa | rs78552493 | 0.057 | -0.053 | 0.021 | 0.013 | -0.059 | 0.034 | 0.083 | -0.012 | 0.045 | 0.789 |
| InternalIMT | epa | rs147665434 | 0.007 | . | . | . | . | . | . | . | . | . |
| InternalIMT | epa | rs34445001 | 0.014 | -0.052 | 0.02 | 9.60E-03 | -0.048 | 0.078 | 0.541 | -0.124 | 0.109 | 0.259 |
| InternalIMT | epa | rs184019449 | 0.004 | . | . | . | . | . | . | . | . | . |
| InternalIMT | epa | rs3780903 | 0.719 | -0.038 | 0.041 | 0.353 | 0.046 | 0.019 | 0.016 | -0.01 | 0.028 | 0.713 |
| InternalIMT | epa | rs17157784 | 0.005 | . | . | . | . | . | . | . | . | . |
| InternalIMT | epa | rs3780904 | 0.001 | . | . | . | . | . | . | . | . | . |
| InternalIMT | epa | rs192064730 | 0 | . | . | . | . | . | . | . | . | . |
| InternalIMT | epa | rs12774534 | 0.014 | -0.051 | 0.02 | 9.70E-03 | -0.048 | 0.078 | 0.537 | -0.125 | 0.109 | 0.255 |
| InternalIMT | epa | rs79179915 | 0 | . | . | . | . | . | . | . | . | . |
| InternalIMT | epa | rs117811571 | 0.016 | -0.044 | 0.02 | 0.028 | 0.06 | 0.1 | 0.549 | -0.34 | 0.163 | 0.037 |
| InternalIMT | epa | rs72796477 | 0.015 | -0.057 | 0.02 | 4.30E-03 | 0.108 | 0.098 | 0.269 | 0.126 | 0.122 | 0.301 |
| InternalIMT | epa | rs55729342 | 0.005 | . | . | . | . | . | . | . | . | . |
| InternalIMT | epa | rs59317220 | 0.004 | . | . | . | . | . | . | . | . | . |
| InternalIMT | epa | rs3780905 | 0.042 | -0.051 | 0.021 | 0.015 | -0.075 | 0.04 | 0.058 | -0.028 | 0.061 | 0.646 |
| InternalIMT | epa | rs3780906 | 0.718 | -0.039 | 0.041 | 0.35 | 0.045 | 0.019 | 0.018 | -0.01 | 0.028 | 0.716 |
| InternalIMT | epa | rs149047975 | 0.006 | . | . | . | . | . | . | . | . | . |
| InternalIMT | epa | rs3824614 | 0.001 | . | . | . | . | . | . | . | . | . |
| InternalIMT | epa | rs140254404 | 0.003 | . | . | . | . | . | . | . | . | . |
| InternalIMT | epa | rs145262684 | 0.02 | -0.049 | 0.02 | 0.017 | 0.14 | 0.096 | 0.146 | -0.167 | 0.135 | 0.215 |
| InternalIMT | epa | rs76735482 | 0.001 | . | . | . | . | . | . | . | . | . |
| InternalIMT | epa | rs73283198 | 0.032 | -0.054 | 0.02 | 7.60E-03 | -0.097 | 0.042 | 0.021 | 6.00E-03 | 0.051 | 0.907 |
| InternalIMT | epa | rs74911239 | 0.052 | -0.061 | 0.022 | 4.80E-03 | -0.039 | 0.043 | 0.367 | 0.052 | 0.059 | 0.382 |
| InternalIMT | epa | rs150551647 | 0.008 | . | . | . | . | . | . | . | . | . |
| InternalIMT | epa | rs115200794 | 0.004 | . | . | . | . | . | . | . | . | . |
| InternalIMT | epa | rs74130823 | 0.177 | -0.047 | 0.023 | 0.037 | -0.032 | 0.023 | 0.17 | -0.019 | 0.035 | 0.586 |
| InternalIMT | epa | rs2279435 | 0.563 | -0.09 | 0.036 | 0.012 | 0.043 | 0.019 | 0.019 | 0.032 | 0.028 | 0.24 |
| InternalIMT | epa | rs147279673 | 0.02 | -0.055 | 0.02 | 7.40E-03 | 0.025 | 0.087 | 0.773 | -8.60E-04 | 0.131 | 0.995 |
| InternalIMT | epa | rs183525146 | 0.005 | . | . | . | . | . | . | . | . | . |
| InternalIMT | epa | rs3740107 | 0.727 | -0.066 | 0.044 | 0.133 | 0.025 | 0.02 | 0.213 | 7.70E-03 | 0.029 | 0.79 |
| InternalIMT | epa | rs140697247 | 0.002 | . | . | . | . | . | . | . | . | . |
| InternalIMT | epa | rs1565096 | 0.744 | -0.054 | 0.044 | 0.219 | 0.027 | 0.019 | 0.166 | -3.40E-04 | 0.029 | 0.991 |

| InternalIMT | epa | rs1565097 | 0.741 | -0.046 | 0.044 | 0.294 | 0.027 | 0.02 | 0.17 | -5.30E-03 | 0.029 | 0.854 |
| --- | --- | --- | --- | --- | --- | --- | --- | --- | --- | --- | --- | --- |
| InternalIMT | epa | rs144584548 | 0.03 | -0.064 | 0.02 | 1.50E-03 | -0.102 | 0.053 | 0.054 | 0.166 | 0.085 | 0.051 |
| InternalIMT | epa | rs75811053 | 0.001 | . | . | . | . | . | . | . | . | . |
| InternalIMT | epa | rs147462967 | 0.01 | -0.053 | 0.02 | 7.00E-03 | 0.053 | 0.149 | 0.724 | -0.054 | 0.201 | 0.79 |
| InternalIMT | epa | rs74130824 | 0.004 | . | . | . | . | . | . | . | . | . |
| InternalIMT | epa | rs116883184 | 0.025 | -0.051 | 0.021 | 0.013 | 0.104 | 0.086 | 0.226 | -0.072 | 0.118 | 0.543 |
| InternalIMT | epa | rs117258049 | 0.025 | -0.051 | 0.021 | 0.013 | 0.104 | 0.086 | 0.226 | -0.072 | 0.118 | 0.543 |
| InternalIMT | epa | rs3780908 | 0.171 | -0.041 | 0.022 | 0.065 | -0.035 | 0.023 | 0.125 | -0.036 | 0.035 | 0.304 |
| InternalIMT | epa | rs180938522 | 0.004 | . | . | . | . | . | . | . | . | . |
| InternalIMT | epa | rs76687372 | 0 | . | . | . | . | . | . | . | . | . |
| InternalIMT | epa | rs3780909 | 0.504 | -0.074 | 0.032 | 0.02 | 0.036 | 0.018 | 0.041 | 0.019 | 0.027 | 0.477 |
| InternalIMT | epa | rs78281156 | 0.001 | . | . | . | . | . | . | . | . | . |
| InternalIMT | epa | rs187840631 | 0.002 | . | . | . | . | . | . | . | . | . |
| InternalIMT | epa | rs76868242 | 0.001 | . | . | . | . | . | . | . | . | . |
| InternalIMT | epa | rs192794192 | 0.001 | . | . | . | . | . | . | . | . | . |
| InternalIMT | epa | rs12781248 | 0.015 | -0.052 | 0.02 | 9.10E-03 | -0.036 | 0.075 | 0.636 | -0.1 | 0.108 | 0.357 |
| InternalIMT | epa | rs12762375 | 0.014 | -0.052 | 0.02 | 9.40E-03 | -0.044 | 0.078 | 0.577 | -0.118 | 0.11 | 0.283 |
| InternalIMT | epa | rs192256986 | 0.006 | . | . | . | . | . | . | . | . | . |
| InternalIMT | epa | rs184411030 | 0 | . | . | . | . | . | . | . | . | . |
| InternalIMT | epa | rs189601483 | 0.001 | . | . | . | . | . | . | . | . | . |
| InternalIMT | epa | rs143254579 | 0.05 | -0.055 | 0.021 | 7.80E-03 | 0.034 | 0.043 | 0.435 | 9.60E-03 | 0.062 | 0.878 |
| InternalIMT | epa | rs12253903 | 0 | . | . | . | . | . | . | . | . | . |
| InternalIMT | epa | rs35489926 | 0.014 | -0.052 | 0.02 | 9.40E-03 | -0.044 | 0.078 | 0.573 | -0.119 | 0.11 | 0.28 |
| InternalIMT | epa | rs71494783 | 0.015 | -0.053 | 0.02 | 8.10E-03 | -9.50E-03 | 0.075 | 0.899 | -0.056 | 0.102 | 0.584 |
| InternalIMT | epa | rs180711602 | 0.004 | . | . | . | . | . | . | . | . | . |
| InternalIMT | epa | rs117312402 | 0.001 | . | . | . | . | . | . | . | . | . |
| InternalIMT | epa | rs74831694 | 0.001 | . | . | . | . | . | . | . | . | . |
| InternalIMT | epa | rs140301943 | 0.004 | . | . | . | . | . | . | . | . | . |
| InternalIMT | epa | rs78553679 | 0.023 | -0.056 | 0.021 | 6.20E-03 | 0.021 | 0.07 | 0.767 | 0.03 | 0.093 | 0.746 |
| InternalIMT | epa | rs78334250 | 0.001 | . | . | . | . | . | . | . | . | . |
| InternalIMT | epa | rs76000028 | 0.001 | . | . | . | . | . | . | . | . | . |
| InternalIMT | epa | rs145195254 | 0 | . | . | . | . | . | . | . | . | . |
| InternalIMT | epa | rs137888527 | 0 | . | . | . | . | . | . | . | . | . |
| InternalIMT | epa | rs149109592 | 0.005 | . | . | . | . | . | . | . | . | . |
| InternalIMT | epa | rs80280010 | 0.049 | -0.055 | 0.021 | 7.70E-03 | 0.034 | 0.043 | 0.424 | 0.01 | 0.062 | 0.87 |
| InternalIMT | epa | rs189995374 | 0.001 | . | . | . | . | . | . | . | . | . |
| InternalIMT | epa | rs75467770 | 0.001 | . | . | . | . | . | . | . | . | . |
| InternalIMT | epa | rs1487562 | 0.178 | -0.04 | 0.023 | 0.076 | -0.033 | 0.023 | 0.145 | -0.039 | 0.035 | 0.269 |
| InternalIMT | epa | rs12266963 | 0.015 | -0.053 | 0.02 | 8.10E-03 | -9.00E-03 | 0.075 | 0.904 | -0.056 | 0.102 | 0.585 |
| InternalIMT | epa | rs7099874 | 0.692 | -0.054 | 0.04 | 0.176 | 0.02 | 0.019 | 0.294 | -2.60E-04 | 0.028 | 0.993 |
| InternalIMT | epa | rs75687019 | 0.001 | . | . | . | . | . | . | . | . | . |
| InternalIMT | epa | rs148240462 | 0.056 | -0.063 | 0.022 | 4.10E-03 | -0.016 | 0.046 | 0.724 | 0.067 | 0.061 | 0.268 |
| InternalIMT | epa | rs141278174 | 0.003 | . | . | . | . | . | . | . | . | . |
| InternalIMT | epa | rs76404852 | 0.001 | . | . | . | . | . | . | . | . | . |
| InternalIMT | epa | rs139112240 | 0.012 | -0.055 | 0.02 | 5.50E-03 | 0.093 | 0.157 | 0.554 | 1.80E-03 | 0.198 | 0.993 |
| InternalIMT | epa | rs12783037 | 0.015 | -0.053 | 0.02 | 8.10E-03 | -9.10E-03 | 0.075 | 0.903 | -0.055 | 0.102 | 0.586 |
| InternalIMT | epa | rs72796481 | 0.013 | -0.055 | 0.02 | 5.80E-03 | 0.115 | 0.104 | 0.267 | -0.031 | 0.3 | 0.918 |
| InternalIMT | epa | rs76798749 | 0.001 | . | . | . | . | . | . | . | . | . |
| InternalIMT | epa | rs187732548 | 0.001 | . | . | . | . | . | . | . | . | . |
| InternalIMT | epa | rs75675474 | 0.001 | . | . | . | . | . | . | . | . | . |
| InternalIMT | epa | rs76552293 | 0.001 | . | . | . | . | . | . | . | . | . |
| InternalIMT | epa | rs147445993 | 0.005 | . | . | . | . | . | . | . | . | . |
| InternalIMT | epa | rs115902283 | 0.001 | . | . | . | . | . | . | . | . | . |
| InternalIMT | epa | rs4540799 | 0.692 | -0.053 | 0.04 | 0.185 | 0.019 | 0.019 | 0.317 | -1.00E-03 | 0.028 | 0.97 |
| InternalIMT | epa | rs12765320 | 0.179 | -0.033 | 0.023 | 0.15 | -0.037 | 0.024 | 0.115 | -0.06 | 0.037 | 0.107 |
| InternalIMT | epa | rs12782256 | 0.014 | -0.052 | 0.02 | 9.30E-03 | -0.044 | 0.078 | 0.578 | -0.119 | 0.11 | 0.282 |
| InternalIMT | epa | rs191727256 | 0 | . | . | . | . | . | . | . | . | . |
| InternalIMT | epa | rs2082111 | 0.165 | -0.043 | 0.022 | 0.051 | -0.028 | 0.023 | 0.23 | -0.033 | 0.036 | 0.358 |
| InternalIMT | epa | rs2082110 | 0.166 | -0.042 | 0.022 | 0.058 | -0.028 | 0.023 | 0.232 | -0.036 | 0.036 | 0.319 |
| InternalIMT | epa | rs2082109 | 0.166 | -0.042 | 0.022 | 0.058 | -0.028 | 0.023 | 0.232 | -0.036 | 0.036 | 0.319 |
| InternalIMT | epa | rs117316662 | 0.001 | . | . | . | . | . | . | . | . | . |
| InternalIMT | epa | rs12247163 | 0.181 | -0.04 | 0.022 | 0.073 | -0.027 | 0.022 | 0.235 | -0.038 | 0.035 | 0.275 |
| InternalIMT | epa | rs12355888 | 0.139 | -0.043 | 0.021 | 0.044 | -0.015 | 0.025 | 0.54 | -0.041 | 0.04 | 0.31 |
| InternalIMT | epa | rs143772290 | 0 | . | . | . | . | . | . | . | . | . |
| InternalIMT | epa | rs146849452 | 0.005 | . | . | . | . | . | . | . | . | . |
| InternalIMT | epa | rs140267121 | 0.006 | . | . | . | . | . | . | . | . | . |
| InternalIMT | epa | rs7080474 | 0.18 | -0.041 | 0.022 | 0.068 | -0.026 | 0.022 | 0.241 | -0.037 | 0.035 | 0.282 |
| InternalIMT | epa | rs144066203 | 0 | . | . | . | . | . | . | . | . | . |
| InternalIMT | epa | rs12765209 | 0.001 | . | . | . | . | . | . | . | . | . |
| InternalIMT | epa | rs150930735 | 0 | . | . | . | . | . | . | . | . | . |
| InternalIMT | epa | rs7068039 | 0.181 | -0.04 | 0.022 | 0.074 | -0.027 | 0.022 | 0.231 | -0.038 | 0.035 | 0.272 |
| InternalIMT | epa | rs117622652 | 0.001 | . | . | . | . | . | . | . | . | . |
| InternalIMT | epa | rs190191514 | 0.002 | . | . | . | . | . | . | . | . | . |

| InternalIMT | epa | rs74130835 | 0 | . | . | . | . | . | . | . | . | . |
| --- | --- | --- | --- | --- | --- | --- | --- | --- | --- | --- | --- | --- |
| InternalIMT | epa | rs1811604 | 0.001 | . | . | . | . | . | . | . | . | . |
| InternalIMT | epa | rs146971256 | 0 | . | . | . | . | . | . | . | . | . |
| InternalIMT | epa | rs74336000 | 0 | . | . | . | . | . | . | . | . | . |
| InternalIMT | epa | rs186046492 | 0.009 | . | . | . | . | . | . | . | . | . |
| InternalIMT | epa | rs34971823 | 0.014 | -0.052 | 0.02 | 9.40E-03 | -0.044 | 0.078 | 0.575 | -0.119 | 0.11 | 0.276 |
| InternalIMT | epa | rs75227446 | 0.001 | . | . | . | . | . | . | . | . | . |
| InternalIMT | epa | rs190457599 | 0.001 | . | . | . | . | . | . | . | . | . |
| InternalIMT | epa | rs79591917 | 0.001 | . | . | . | . | . | . | . | . | . |
| InternalIMT | epa | rs702365 | 0.51 | -0.079 | 0.033 | 0.015 | 0.031 | 0.018 | 0.083 | 0.024 | 0.027 | 0.376 |
| InternalIMT | epa | rs79643836 | 0.001 | . | . | . | . | . | . | . | . | . |
| InternalIMT | epa | rs702366 | 0.511 | -0.075 | 0.033 | 0.022 | 0.031 | 0.018 | 0.082 | 0.02 | 0.027 | 0.468 |
| InternalIMT | epa | rs1059696 | 0.166 | -0.045 | 0.022 | 0.042 | -0.026 | 0.023 | 0.256 | -0.028 | 0.036 | 0.444 |
| InternalIMT | epa | rs901681 | 0.515 | -0.076 | 0.033 | 0.02 | 0.033 | 0.018 | 0.062 | 0.021 | 0.027 | 0.443 |
| InternalIMT | epa | rs61854092 | 0.165 | -0.044 | 0.022 | 0.043 | -0.027 | 0.023 | 0.251 | -0.029 | 0.036 | 0.424 |
| InternalIMT | epa | rs148080753 | 0.001 | . | . | . | . | . | . | . | . | . |
| InternalIMT | epa | rs61854093 | 0.166 | -0.044 | 0.022 | 0.045 | -0.026 | 0.023 | 0.255 | -0.03 | 0.036 | 0.41 |
| InternalIMT | epa | rs141822774 | 0.001 | . | . | . | . | . | . | . | . | . |
| InternalIMT | epa | rs74130836 | 0 | . | . | . | . | . | . | . | . | . |
| InternalIMT | epa | rs150620963 | 0.019 | -0.057 | 0.02 | 5.00E-03 | 0.018 | 0.078 | 0.822 | 0.051 | 0.112 | 0.652 |
| InternalIMT | epa | rs3780913 | 0.678 | -0.058 | 0.039 | 0.139 | 0.019 | 0.019 | 0.32 | 2.50E-03 | 0.028 | 0.928 |
| InternalIMT | epa | rs142255723 | 0.002 | . | . | . | . | . | . | . | . | . |
| InternalIMT | epa | rs3780914 | 0.511 | -0.075 | 0.033 | 0.022 | 0.031 | 0.018 | 0.08 | 0.02 | 0.027 | 0.472 |
| InternalIMT | epa | rs182656507 | 0.002 | . | . | . | . | . | . | . | . | . |
| InternalIMT | epa | rs2291427 | 0.677 | -0.058 | 0.039 | 0.144 | 0.019 | 0.019 | 0.308 | 1.90E-03 | 0.028 | 0.946 |
| InternalIMT | epa | rs10751382 | 0.677 | -0.058 | 0.039 | 0.138 | 0.018 | 0.019 | 0.33 | 2.50E-03 | 0.028 | 0.927 |
| InternalIMT | epa | rs117610108 | 0.001 | . | . | . | . | . | . | . | . | . |
| InternalIMT | epa | rs144966941 | 0.031 | -0.05 | 0.02 | 0.013 | 0.025 | 0.06 | 0.673 | -0.07 | 0.092 | 0.445 |
| InternalIMT | epa | rs41502051 | 0.052 | -0.049 | 0.021 | 0.02 | -0.014 | 0.04 | 0.716 | -0.064 | 0.063 | 0.31 |
| InternalIMT | epa | rs41426046 | 0.001 | . | . | . | . | . | . | . | . | . |
| InternalIMT | epa | rs41519248 | 0.001 | . | . | . | . | . | . | . | . | . |
| InternalIMT | epa | rs189108245 | 0.008 | . | . | . | . | . | . | . | . | . |
| InternalIMT | epa | rs7896431 | 0.476 | -0.068 | 0.033 | 0.038 | 0.026 | 0.019 | 0.169 | 0.014 | 0.029 | 0.619 |
| InternalIMT | epa | rs183724547 | 0.001 | . | . | . | . | . | . | . | . | . |
| InternalIMT | epa | rs142044990 | 0.012 | -0.055 | 0.02 | 5.00E-03 | 0.082 | 0.158 | 0.603 | 0.03 | 0.198 | 0.882 |
| InternalIMT | epa | rs188140929 | 0.002 | . | . | . | . | . | . | . | . | . |
| InternalIMT | epa | rs76819096 | 0 | . | . | . | . | . | . | . | . | . |
| InternalIMT | epa | rs10751383 | 0.56 | -0.077 | 0.035 | 0.027 | 0.038 | 0.018 | 0.03 | 0.021 | 0.027 | 0.435 |
| InternalIMT | epa | rs117819321 | 0.019 | -0.054 | 0.02 | 6.90E-03 | 0.217 | 0.124 | 0.08 | -0.012 | 0.282 | 0.967 |
| InternalIMT | epa | rs2242332 | 0.675 | -0.053 | 0.04 | 0.183 | 0.016 | 0.019 | 0.381 | -1.50E-03 | 0.028 | 0.958 |
| InternalIMT | epa | rs2242333 | 0.046 | -0.049 | 0.021 | 0.019 | -0.086 | 0.037 | 0.021 | -0.039 | 0.058 | 0.504 |
| InternalIMT | epa | rs2242334 | 0.677 | -0.051 | 0.04 | 0.201 | 0.016 | 0.019 | 0.394 | -2.90E-03 | 0.028 | 0.917 |
| InternalIMT | epa | rs189465041 | 0.001 | . | . | . | . | . | . | . | . | . |
| InternalIMT | epa | rs28395868 | 0.041 | -0.057 | 0.02 | 4.80E-03 | 0.029 | 0.054 | 0.598 | 0.029 | 0.084 | 0.728 |
| InternalIMT | epa | rs56067550 | 0.03 | -0.065 | 0.02 | 1.40E-03 | -0.1 | 0.054 | 0.062 | 0.167 | 0.086 | 0.052 |
| InternalIMT | epa | rs1051713 | 0.165 | -0.041 | 0.022 | 0.065 | -0.031 | 0.023 | 0.176 | -0.038 | 0.036 | 0.291 |
| InternalIMT | epa | rs115074210 | 0 | . | . | . | . | . | . | . | . | . |
| InternalIMT | epa | rs7900977 | 0.74 | -0.045 | 0.044 | 0.303 | 0.026 | 0.019 | 0.186 | -6.10E-03 | 0.028 | 0.829 |
| InternalIMT | epa | rs147613891 | 0.004 | . | . | . | . | . | . | . | . | . |
| InternalIMT | epa | rs2229136 | 0.063 | -0.054 | 0.021 | 9.30E-03 | 0.027 | 0.039 | 0.489 | 2.40E-03 | 0.056 | 0.966 |
| InternalIMT | epa | rs185482841 | 0 | . | . | . | . | . | . | . | . | . |
| InternalIMT | epa | rs2288619 | 0.063 | -0.054 | 0.021 | 9.30E-03 | 0.027 | 0.039 | 0.488 | 2.40E-03 | 0.056 | 0.966 |
| InternalIMT | epa | rs189891524 | 0.001 | . | . | . | . | . | . | . | . | . |
| InternalIMT | epa | rs56145077 | 0.164 | -0.042 | 0.022 | 0.058 | -0.031 | 0.023 | 0.178 | -0.036 | 0.037 | 0.322 |
| InternalIMT | epa | rs143216970 | 0 | . | . | . | . | . | . | . | . | . |
| InternalIMT | epa | rs142246478 | 0.002 | . | . | . | . | . | . | . | . | . |
| InternalIMT | epa | rs13134 | 0.014 | -0.052 | 0.02 | 9.50E-03 | -0.045 | 0.078 | 0.564 | -0.121 | 0.105 | 0.25 |
| InternalIMT | epa | rs41314980 | 0.034 | -0.07 | 0.021 | 6.60E-04 | 0.091 | 0.062 | 0.142 | 0.241 | 0.097 | 0.013 |
| InternalIMT | epa | rs41304611 | 0.02 | -0.058 | 0.021 | 4.50E-03 | -0.089 | 0.072 | 0.215 | 0.064 | 0.106 | 0.546 |
| InternalIMT | epa | rs193299617 | 0 | . | . | . | . | . | . | . | . | . |
| InternalIMT | epa | rs12772724 | 0.014 | -0.052 | 0.02 | 9.50E-03 | -0.045 | 0.078 | 0.565 | -0.121 | 0.105 | 0.25 |
| InternalIMT | epa | rs139489537 | 0.048 | -0.058 | 0.021 | 4.50E-03 | 0.055 | 0.044 | 0.216 | 0.05 | 0.064 | 0.44 |
| InternalIMT | epa | rs12776062 | 0.014 | -0.052 | 0.02 | 9.50E-03 | -0.045 | 0.078 | 0.566 | -0.122 | 0.105 | 0.247 |
| InternalIMT | epa | rs752059 | 0.164 | -0.041 | 0.022 | 0.061 | -0.032 | 0.023 | 0.172 | -0.037 | 0.037 | 0.31 |
| InternalIMT | epa | rs147649982 | 0.035 | -0.061 | 0.02 | 3.00E-03 | 0.036 | 0.084 | 0.665 | 0.093 | 0.111 | 0.401 |
| InternalIMT | epa | rs17157798 | 0.062 | -0.055 | 0.021 | 8.30E-03 | 0.032 | 0.039 | 0.406 | 0.01 | 0.056 | 0.856 |
| InternalIMT | epa | rs142298619 | 0.001 | . | . | . | . | . | . | . | . | . |
| InternalIMT | epa | rs4948673 | 0.72 | -0.053 | 0.043 | 0.213 | 0.022 | 0.019 | 0.254 | -1.10E-03 | 0.028 | 0.97 |
| InternalIMT | epa | rs74231484 | 0.041 | -0.048 | 0.021 | 0.021 | -0.086 | 0.038 | 0.023 | -0.053 | 0.059 | 0.367 |
| InternalIMT | epa | rs72796489 | 0.004 | . | . | . | . | . | . | . | . | . |
| InternalIMT | epa | rs113447578 | 0.021 | -0.055 | 0.02 | 6.50E-03 | -0.096 | 0.054 | 0.077 | 0.029 | 0.066 | 0.661 |
| InternalIMT | epa | rs34063374 | 0.014 | -0.051 | 0.02 | 1.00E-02 | -0.038 | 0.078 | 0.629 | -0.129 | 0.106 | 0.221 |
| InternalIMT | epa | rs11239527 | 0.019 | -0.057 | 0.02 | 4.90E-03 | 0.139 | 0.088 | 0.115 | 0.044 | 0.131 | 0.739 |

| InternalIMT | epa | rs34371883 | 0.014 | -0.052 | 0.02 | 9.50E-03 | -0.045 | 0.078 | 0.567 | -0.121 | 0.105 | 0.251 |
| --- | --- | --- | --- | --- | --- | --- | --- | --- | --- | --- | --- | --- |
| InternalIMT | epa | rs118168828 | 0.012 | -0.06 | 0.02 | 2.70E-03 | 0.058 | 0.11 | 0.6 | 0.247 | 0.145 | 0.089 |
| InternalIMT | epa | rs34349618 | 0.014 | -0.052 | 0.02 | 9.50E-03 | -0.045 | 0.078 | 0.567 | -0.121 | 0.105 | 0.251 |
| InternalIMT | epa | rs80041679 | 0.048 | -0.058 | 0.021 | 4.50E-03 | 0.055 | 0.044 | 0.212 | 0.05 | 0.064 | 0.437 |
| InternalIMT | epa | rs149640607 | 0 | . | . | . | . | . | . | . | . | . |
| InternalIMT | epa | rs4427523 | 0.834 | -0.086 | 0.076 | 0.258 | -0.012 | 0.028 | 0.675 | 0.019 | 0.044 | 0.671 |
| InternalIMT | epa | rs185880109 | 0.002 | . | . | . | . | . | . | . | . | . |
| InternalIMT | epa | rs191523220 | 0.004 | . | . | . | . | . | . | . | . | . |
| InternalIMT | epa | rs144315284 | 0 | . | . | . | . | . | . | . | . | . |
| InternalIMT | epa | rs4948674 | 0.404 | -0.079 | 0.026 | 3.00E-03 | 0.053 | 0.018 | 3.80E-03 | 0.032 | 0.026 | 0.223 |
| InternalIMT | epa | rs182984298 | 0.01 | -0.055 | 0.02 | 5.80E-03 | -0.074 | 0.102 | 0.468 | 0.012 | 0.15 | 0.934 |
| InternalIMT | epa | rs56749023 | 0 | . | . | . | . | . | . | . | . | . |
| InternalIMT | epa | rs112508957 | 0 | . | . | . | . | . | . | . | . | . |
| InternalIMT | epa | rs7089063 | 0.224 | -0.036 | 0.024 | 0.124 | -0.022 | 0.02 | 0.288 | -0.04 | 0.032 | 0.204 |
| InternalIMT | epa | rs145000779 | 0.001 | . | . | . | . | . | . | . | . | . |
| InternalIMT | epa | rs80201572 | 0.048 | -0.058 | 0.021 | 4.50E-03 | 0.055 | 0.044 | 0.212 | 0.05 | 0.064 | 0.44 |
| InternalIMT | epa | rs140568227 | 0.009 | . | . | . | . | . | . | . | . | . |
| InternalIMT | epa | rs149756228 | 0.014 | -0.057 | 0.02 | 5.00E-03 | 0.037 | 0.092 | 0.686 | 0.058 | 0.12 | 0.626 |
| InternalIMT | epa | rs112380685 | 0.009 | . | . | . | . | . | . | . | . | . |
| InternalIMT | epa | rs60481109 | 0 | . | . | . | . | . | . | . | . | . |
| InternalIMT | epa | rs138221843 | 0.004 | . | . | . | . | . | . | . | . | . |
| InternalIMT | epa | rs150283194 | 0 | . | . | . | . | . | . | . | . | . |
| InternalIMT | epa | rs138879661 | 0.003 | . | . | . | . | . | . | . | . | . |
| InternalIMT | epa | rs34960345 | 0.062 | -0.055 | 0.021 | 8.40E-03 | 0.035 | 0.039 | 0.37 | 9.80E-03 | 0.056 | 0.86 |
| InternalIMT | epa | rs147514068 | 0.004 | . | . | . | . | . | . | . | . | . |
| InternalIMT | epa | rs35410535 | 0.063 | -0.055 | 0.021 | 8.30E-03 | 0.033 | 0.039 | 0.398 | 0.01 | 0.056 | 0.855 |
| InternalIMT | epa | rs191613635 | 0.005 | . | . | . | . | . | . | . | . | . |
| InternalIMT | epa | rs17157800 | 0.009 | . | . | . | . | . | . | . | . | . |
| InternalIMT | epa | rs17523413 | 0.062 | -0.055 | 0.021 | 8.40E-03 | 0.035 | 0.039 | 0.371 | 9.70E-03 | 0.056 | 0.861 |
| InternalIMT | epa | rs7897036 | 0.058 | -0.037 | 0.02 | 0.07 | -0.066 | 0.041 | 0.11 | -0.134 | 0.061 | 0.029 |
| InternalIMT | epa | rs76334323 | 0.001 | . | . | . | . | . | . | . | . | . |
| InternalIMT | epa | rs143535991 | 0.001 | . | . | . | . | . | . | . | . | . |
| InternalIMT | epa | rs140344979 | 0.007 | . | . | . | . | . | . | . | . | . |
| InternalIMT | epa | rs36047811 | 0.063 | -0.055 | 0.021 | 8.30E-03 | 0.033 | 0.039 | 0.398 | 0.01 | 0.056 | 0.854 |
| InternalIMT | epa | rs76493570 | 0.062 | -0.055 | 0.021 | 8.40E-03 | 0.035 | 0.039 | 0.369 | 9.80E-03 | 0.056 | 0.861 |
| InternalIMT | epa | rs192998920 | 0.003 | . | . | . | . | . | . | . | . | . |
| InternalIMT | epa | rs116840852 | 0.023 | -0.056 | 0.021 | 6.20E-03 | 0.014 | 0.07 | 0.837 | 0.03 | 0.092 | 0.744 |
| InternalIMT | epa | rs184824101 | 0.001 | . | . | . | . | . | . | . | . | . |
| InternalIMT | epa | rs41301621 | 0.008 | . | . | . | . | . | . | . | . | . |
| InternalIMT | epa | rs3740109 | 0.063 | -0.055 | 0.021 | 8.30E-03 | 0.033 | 0.039 | 0.396 | 0.01 | 0.056 | 0.852 |
| InternalIMT | epa | rs7899415 | 0.02 | -0.052 | 0.02 | 9.60E-03 | 0.222 | 0.112 | 0.048 | -0.054 | 0.171 | 0.754 |
| InternalIMT | epa | rs184395284 | 0.001 | . | . | . | . | . | . | . | . | . |
| InternalIMT | epa | rs139209589 | 0.006 | . | . | . | . | . | . | . | . | . |
| InternalIMT | epa | rs149924163 | 0 | . | . | . | . | . | . | . | . | . |
| InternalIMT | epa | rs3740110 | 0.001 | . | . | . | . | . | . | . | . | . |
| InternalIMT | epa | rs144335113 | 0 | . | . | . | . | . | . | . | . | . |
| InternalIMT | epa | rs74743385 | 0 | . | . | . | . | . | . | . | . | . |
| InternalIMT | epa | rs146545829 | 0 | . | . | . | . | . | . | . | . | . |
| InternalIMT | epa | rs181842689 | 0 | . | . | . | . | . | . | . | . | . |
| InternalIMT | epa | rs3740111 | 0.062 | -0.055 | 0.021 | 8.40E-03 | 0.035 | 0.039 | 0.367 | 0.01 | 0.056 | 0.857 |
| InternalIMT | epa | rs186261599 | 0 | . | . | . | . | . | . | . | . | . |
| InternalIMT | epa | rs73285348 | 0.012 | -0.055 | 0.02 | 6.50E-03 | -0.136 | 0.07 | 0.054 | -7.20E-03 | 0.082 | 0.931 |
| InternalIMT | epa | rs186356901 | 0 | . | . | . | . | . | . | . | . | . |
| InternalIMT | epa | rs3824618 | 0.048 | -0.058 | 0.021 | 4.50E-03 | 0.055 | 0.044 | 0.212 | 0.05 | 0.064 | 0.44 |
| InternalIMT | epa | rs3802548 | 0.231 | -0.04 | 0.023 | 0.086 | -0.032 | 0.02 | 0.111 | -0.029 | 0.029 | 0.314 |
| InternalIMT | epa | rs3802547 | 0.001 | . | . | . | . | . | . | . | . | . |
| InternalIMT | epa | rs77677521 | 0.023 | -0.056 | 0.021 | 6.20E-03 | 0.014 | 0.07 | 0.836 | 0.03 | 0.092 | 0.744 |
| InternalIMT | epa | rs3824616 | 0.063 | -0.055 | 0.021 | 8.30E-03 | 0.033 | 0.039 | 0.394 | 0.011 | 0.056 | 0.849 |
| InternalIMT | epa | rs191073818 | 0 | . | . | . | . | . | . | . | . | . |
| InternalIMT | epa | rs147078004 | 0.002 | . | . | . | . | . | . | . | . | . |
| InternalIMT | epa | rs7908745 | 0.297 | -0.036 | 0.025 | 0.143 | -0.019 | 0.018 | 0.305 | -0.03 | 0.029 | 0.293 |
| InternalIMT | epa | rs17157811 | 0.009 | . | . | . | . | . | . | . | . | . |
| InternalIMT | epa | rs138506087 | 0.009 | . | . | . | . | . | . | . | . | . |
| InternalIMT | epa | rs77241269 | 0 | . | . | . | . | . | . | . | . | . |
| InternalIMT | epa | rs138374938 | 0.002 | . | . | . | . | . | . | . | . | . |
| InternalIMT | epa | rs2279433 | 0.062 | -0.055 | 0.021 | 8.40E-03 | 0.037 | 0.039 | 0.346 | 0.011 | 0.056 | 0.85 |
| InternalIMT | epa | rs73285358 | 0 | . | . | . | . | . | . | . | . | . |
| InternalIMT | epa | rs11239528 | 0.231 | -0.041 | 0.023 | 0.081 | -0.032 | 0.02 | 0.115 | -0.028 | 0.029 | 0.336 |
| InternalIMT | epa | rs77453386 | 0.001 | . | . | . | . | . | . | . | . | . |
| InternalIMT | epa | rs2279434 | 0.069 | -0.052 | 0.021 | 0.015 | 0.017 | 0.037 | 0.641 | -0.018 | 0.053 | 0.726 |
| InternalIMT | epa | rs149236525 | 0.006 | . | . | . | . | . | . | . | . | . |
| InternalIMT | epa | rs12573609 | 0.052 | -0.051 | 0.021 | 0.015 | -0.012 | 0.043 | 0.786 | -0.04 | 0.064 | 0.539 |
| InternalIMT | epa | rs191716655 | 0 | . | . | . | . | . | . | . | . | . |

| InternalIMT | epa | rs17523660 | 0.014 | -0.052 | 0.02 | 9.30E-03 | -0.043 | 0.078 | 0.581 | -0.117 | 0.105 | 0.264 |
| --- | --- | --- | --- | --- | --- | --- | --- | --- | --- | --- | --- | --- |
| InternalIMT | epa | rs142671360 | 0.004 | . | . | . | . | . | . | . | . | . |
| InternalIMT | epa | rs3764990 | 0.062 | -0.055 | 0.021 | 8.40E-03 | 0.037 | 0.039 | 0.34 | 0.01 | 0.056 | 0.853 |
| InternalIMT | epa | rs113682861 | 0.004 | . | . | . | . | . | . | . | . | . |
| InternalIMT | epa | rs117658270 | 0.001 | . | . | . | . | . | . | . | . | . |
| InternalIMT | epa | rs187309063 | 0 | . | . | . | . | . | . | . | . | . |
| InternalIMT | epa | rs73285360 | 0 | . | . | . | . | . | . | . | . | . |
| InternalIMT | epa | rs80133289 | 0.001 | . | . | . | . | . | . | . | . | . |
| InternalIMT | epa | rs186693257 | 0.001 | . | . | . | . | . | . | . | . | . |
| InternalIMT | epa | rs35735968 | 0.063 | -0.055 | 0.021 | 8.50E-03 | 0.038 | 0.039 | 0.334 | 0.01 | 0.056 | 0.852 |
| InternalIMT | epa | rs17157825 | 0.048 | -0.059 | 0.021 | 4.30E-03 | 0.061 | 0.045 | 0.17 | 0.054 | 0.065 | 0.407 |
| InternalIMT | epa | rs2291428 | 0.229 | -0.041 | 0.023 | 0.081 | -0.033 | 0.02 | 0.103 | -0.029 | 0.029 | 0.334 |
| InternalIMT | epa | rs2291429 | 0.231 | -0.041 | 0.023 | 0.081 | -0.032 | 0.02 | 0.112 | -0.028 | 0.029 | 0.34 |
| InternalIMT | epa | rs145714792 | 0.007 | . | . | . | . | . | . | . | . | . |
| InternalIMT | epa | rs12769244 | 0.062 | -0.055 | 0.021 | 8.40E-03 | 0.037 | 0.039 | 0.339 | 0.011 | 0.056 | 0.849 |
| InternalIMT | epa | rs12771275 | 0.062 | -0.055 | 0.021 | 8.40E-03 | 0.037 | 0.039 | 0.339 | 0.011 | 0.056 | 0.849 |
| InternalIMT | epa | rs146266174 | 0.002 | . | . | . | . | . | . | . | . | . |
| InternalIMT | epa | rs41301623 | 0.004 | . | . | . | . | . | . | . | . | . |
| InternalIMT | epa | rs2306814 | 0.001 | . | . | . | . | . | . | . | . | . |
| InternalIMT | epa | rs12244834 | 0 | . | . | . | . | . | . | . | . | . |
| InternalIMT | epa | rs184006011 | 0.004 | . | . | . | . | . | . | . | . | . |
| InternalIMT | epa | rs78829066 | 0.001 | . | . | . | . | . | . | . | . | . |
| InternalIMT | epa | rs34731408 | 0.062 | -0.055 | 0.021 | 8.40E-03 | 0.037 | 0.039 | 0.339 | 0.011 | 0.056 | 0.849 |
| InternalIMT | epa | rs17451516 | 0.014 | -0.052 | 0.02 | 9.30E-03 | -0.043 | 0.078 | 0.58 | -0.116 | 0.105 | 0.268 |
| InternalIMT | epa | rs139910133 | . | . | . | . | . | . | . | . | . | . |
| InternalIMT | epa | rs10900218 | 0.229 | -0.042 | 0.024 | 0.073 | -0.012 | 0.021 | 0.555 | -0.027 | 0.032 | 0.393 |
| InternalIMT | epa | rs146023999 | . | . | . | . | . | . | . | . | . | . |
| InternalIMT | epa | rs187296958 | . | . | . | . | . | . | . | . | . | . |
| InternalIMT | epa | rs142342833 | . | . | . | . | . | . | . | . | . | . |
| InternalIMT | epa | rs143489255 | . | . | . | . | . | . | . | . | . | . |
| InternalIMT | epa | rs138532518 | . | . | . | . | . | . | . | . | . | . |
| InternalIMT | epa | rs183768079 | . | . | . | . | . | . | . | . | . | . |
| InternalIMT | epa | rs117892272 | . | . | . | . | . | . | . | . | . | . |
| InternalIMT | epa | rs192993943 | . | . | . | . | . | . | . | . | . | . |
| InternalIMT | epa | rs182989043 | . | . | . | . | . | . | . | . | . | . |
| InternalIMT | epa | rs189703435 | . | . | . | . | . | . | . | . | . | . |
| InternalIMT | epa | rs188794040 | . | . | . | . | . | . | . | . | . | . |
| InternalIMT | epa | rs143920953 | . | . | . | . | . | . | . | . | . | . |
| InternalIMT | epa | rs191029121 | . | . | . | . | . | . | . | . | . | . |
| InternalIMT | epa | rs116875448 | . | . | . | . | . | . | . | . | . | . |
| InternalIMT | epa | rs190167877 | . | . | . | . | . | . | . | . | . | . |
| InternalIMT | epa | rs184993418 | . | . | . | . | . | . | . | . | . | . |
| InternalIMT | epa | rs143241158 | . | . | . | . | . | . | . | . | . | . |
| InternalIMT | epa | rs11239500 | . | . | . | . | . | . | . | . | . | . |
| InternalIMT | epa | rs140568994 | . | . | . | . | . | . | . | . | . | . |
| InternalIMT | epa | rs187530317 | . | . | . | . | . | . | . | . | . | . |
| InternalIMT | epa | rs145189326 | . | . | . | . | . | . | . | . | . | . |
| InternalIMT | epa | rs186203332 | . | . | . | . | . | . | . | . | . | . |
| InternalIMT | epa | rs189921909 | . | . | . | . | . | . | . | . | . | . |
| InternalIMT | epa | rs187260989 | . | . | . | . | . | . | . | . | . | . |
| InternalIMT | epa | rs114376586 | . | . | . | . | . | . | . | . | . | . |
| InternalIMT | epa | rs151070808 | . | . | . | . | . | . | . | . | . | . |
| InternalIMT | epa | rs140927225 | . | . | . | . | . | . | . | . | . | . |
| InternalIMT | epa | rs191555615 | . | . | . | . | . | . | . | . | . | . |
| InternalIMT | epa | rs80278898 | . | . | . | . | . | . | . | . | . | . |
| InternalIMT | epa | rs190849503 | . | . | . | . | . | . | . | . | . | . |
| InternalIMT | epa | rs150080315 | . | . | . | . | . | . | . | . | . | . |
| InternalIMT | epa | rs143623885 | . | . | . | . | . | . | . | . | . | . |
| InternalIMT | epa | rs187760040 | . | . | . | . | . | . | . | . | . | . |
| InternalIMT | epa | rs143917143 | . | . | . | . | . | . | . | . | . | . |
| InternalIMT | epa | rs149851136 | . | . | . | . | . | . | . | . | . | . |
| InternalIMT | epa | rs181612054 | . | . | . | . | . | . | . | . | . | . |
| InternalIMT | epa | rs191006548 | . | . | . | . | . | . | . | . | . | . |
| InternalIMT | epa | rs117377631 | . | . | . | . | . | . | . | . | . | . |
| InternalIMT | epa | rs182107634 | . | . | . | . | . | . | . | . | . | . |
| InternalIMT | epa | rs190211867 | . | . | . | . | . | . | . | . | . | . |
| InternalIMT | epa | rs148293457 | . | . | . | . | . | . | . | . | . | . |
| InternalIMT | epa | rs191811733 | . | . | . | . | . | . | . | . | . | . |
| InternalIMT | epa | rs146109272 | . | . | . | . | . | . | . | . | . | . |
| InternalIMT | epa | rs148397915 | . | . | . | . | . | . | . | . | . | . |
| InternalIMT | epa | rs142233290 | . | . | . | . | . | . | . | . | . | . |
| InternalIMT | epa | rs80226874 | . | . | . | . | . | . | . | . | . | . |
| InternalIMT | epa | rs144496203 | . | . | . | . | . | . | . | . | . | . |

| InternalIMT | epa | rs191062130 | . | . | . | . | . | . | . | . | . | . |
| --- | --- | --- | --- | --- | --- | --- | --- | --- | --- | --- | --- | --- |
| InternalIMT | epa | rs185807817 | . | . | . | . | . | . | . | . | . | . |
| InternalIMT | epa | rs117846213 | . | . | . | . | . | . | . | . | . | . |
| InternalIMT | epa | rs190545473 | . | . | . | . | . | . | . | . | . | . |
| InternalIMT | epa | rs192364918 | . | . | . | . | . | . | . | . | . | . |
| InternalIMT | epa | rs182939241 | . | . | . | . | . | . | . | . | . | . |
| InternalIMT | epa | rs12571125 | . | . | . | . | . | . | . | . | . | . |
| InternalIMT | epa | rs192418941 | . | . | . | . | . | . | . | . | . | . |
| InternalIMT | epa | rs141270243 | . | . | . | . | . | . | . | . | . | . |
| InternalIMT | epa | rs182615502 | . | . | . | . | . | . | . | . | . | . |
| InternalIMT | epa | rs146669856 | . | . | . | . | . | . | . | . | . | . |
| InternalIMT | epa | rs78290029 | . | . | . | . | . | . | . | . | . | . |
| InternalIMT | epa | rs139955565 | . | . | . | . | . | . | . | . | . | . |
| InternalIMT | epa | rs12267314 | . | . | . | . | . | . | . | . | . | . |
| InternalIMT | epa | rs74477197 | . | . | . | . | . | . | . | . | . | . |
| InternalIMT | epa | rs188147236 | . | . | . | . | . | . | . | . | . | . |
| InternalIMT | epa | rs2228065 | . | . | . | . | . | . | . | . | . | . |
| InternalIMT | epa | rs116961353 | . | . | . | . | . | . | . | . | . | . |
| InternalIMT | epa | rs190222272 | . | . | . | . | . | . | . | . | . | . |
| InternalIMT | epa | rs182719940 | . | . | . | . | . | . | . | . | . | . |
| InternalIMT | epa | rs184477413 | . | . | . | . | . | . | . | . | . | . |
| InternalIMT | epa | rs12265175 | . | . | . | . | . | . | . | . | . | . |
| InternalIMT | epa | rs182928276 | . | . | . | . | . | . | . | . | . | . |
| InternalIMT | epa | rs144409895 | . | . | . | . | . | . | . | . | . | . |
| InternalIMT | epa | rs58998447 | . | . | . | . | . | . | . | . | . | . |
| InternalIMT | epa | rs188668832 | . | . | . | . | . | . | . | . | . | . |
| InternalIMT | epa | rs148641686 | . | . | . | . | . | . | . | . | . | . |
| InternalIMT | epa | rs75468791 | . | . | . | . | . | . | . | . | . | . |
| InternalIMT | epa | rs147327397 | . | . | . | . | . | . | . | . | . | . |
| InternalIMT | epa | rs77354597 | . | . | . | . | . | . | . | . | . | . |
| InternalIMT | epa | rs41365544 | . | . | . | . | . | . | . | . | . | . |
| InternalIMT | epa | rs187564188 | . | . | . | . | . | . | . | . | . | . |
| InternalIMT | epa | rs183511984 | . | . | . | . | . | . | . | . | . | . |
| InternalIMT | epa | rs12262783 | . | . | . | . | . | . | . | . | . | . |
| InternalIMT | epa | rs188817687 | . | . | . | . | . | . | . | . | . | . |
| InternalIMT | epa | rs3815968 | . | . | . | . | . | . | . | . | . | . |
| InternalIMT | epa | rs187822300 | . | . | . | . | . | . | . | . | . | . |
| InternalIMT | epa | rs188902172 | . | . | . | . | . | . | . | . | . | . |
| InternalIMT | epa | rs181190448 | . | . | . | . | . | . | . | . | . | . |
| InternalIMT | epa | rs182699093 | . | . | . | . | . | . | . | . | . | . |
| InternalIMT | epa | rs182631303 | . | . | . | . | . | . | . | . | . | . |
| InternalIMT | epa | rs187014172 | . | . | . | . | . | . | . | . | . | . |
| InternalIMT | epa | rs142575406 | . | . | . | . | . | . | . | . | . | . |
| InternalIMT | epa | rs180711033 | . | . | . | . | . | . | . | . | . | . |
| InternalIMT | epa | rs137894823 | . | . | . | . | . | . | . | . | . | . |
| InternalIMT | epa | rs189053273 | . | . | . | . | . | . | . | . | . | . |
| InternalIMT | epa | rs143499388 | . | . | . | . | . | . | . | . | . | . |
| InternalIMT | epa | rs182936001 | . | . | . | . | . | . | . | . | . | . |
| InternalIMT | epa | rs148456406 | . | . | . | . | . | . | . | . | . | . |
| InternalIMT | epa | rs182499531 | . | . | . | . | . | . | . | . | . | . |
| InternalIMT | epa | rs74884915 | . | . | . | . | . | . | . | . | . | . |
| InternalIMT | epa | rs184046374 | . | . | . | . | . | . | . | . | . | . |
| InternalIMT | epa | rs74333993 | . | . | . | . | . | . | . | . | . | . |
| InternalIMT | epa | rs138889719 | . | . | . | . | . | . | . | . | . | . |
| InternalIMT | epa | rs75330330 | . | . | . | . | . | . | . | . | . | . |
| InternalIMT | epa | rs193051693 | . | . | . | . | . | . | . | . | . | . |
| InternalIMT | epa | rs145929633 | . | . | . | . | . | . | . | . | . | . |
| InternalIMT | epa | rs184076904 | . | . | . | . | . | . | . | . | . | . |
| InternalIMT | epa | rs141802494 | . | . | . | . | . | . | . | . | . | . |
| InternalIMT | epa | rs149519759 | . | . | . | . | . | . | . | . | . | . |
| InternalIMT | epa | rs145467260 | . | . | . | . | . | . | . | . | . | . |
| InternalIMT | epa | rs79620914 | . | . | . | . | . | . | . | . | . | . |
| InternalIMT | epa | rs140538633 | . | . | . | . | . | . | . | . | . | . |
| InternalIMT | epa | rs140862301 | . | . | . | . | . | . | . | . | . | . |
| InternalIMT | epa | rs114615847 | . | . | . | . | . | . | . | . | . | . |
| InternalIMT | epa | rs141800259 | . | . | . | . | . | . | . | . | . | . |
| InternalIMT | epa | rs149779059 | . | . | . | . | . | . | . | . | . | . |
| InternalIMT | epa | rs116539253 | . | . | . | . | . | . | . | . | . | . |
| InternalIMT | epa | rs184979550 | . | . | . | . | . | . | . | . | . | . |
| InternalIMT | epa | rs190128940 | . | . | . | . | . | . | . | . | . | . |
| InternalIMT | epa | rs146594816 | . | . | . | . | . | . | . | . | . | . |
| InternalIMT | epa | rs141370766 | . | . | . | . | . | . | . | . | . | . |
| InternalIMT | epa | rs144670033 | . | . | . | . | . | . | . | . | . | . |

| InternalIMT | epa | rs187152174 | . | . | . | . | . | . | . | . | . | . |
| --- | --- | --- | --- | --- | --- | --- | --- | --- | --- | --- | --- | --- |
| InternalIMT | epa | rs184180263 | . | . | . | . | . | . | . | . | . | . |
| InternalIMT | epa | rs140075861 | . | . | . | . | . | . | . | . | . | . |
| InternalIMT | epa | rs186483129 | . | . | . | . | . | . | . | . | . | . |
| InternalIMT | epa | rs186634282 | . | . | . | . | . | . | . | . | . | . |
| InternalIMT | epa | rs9988663 | . | . | . | . | . | . | . | . | . | . |
| InternalIMT | epa | rs138828529 | . | . | . | . | . | . | . | . | . | . |
| InternalIMT | epa | rs188777615 | . | . | . | . | . | . | . | . | . | . |
| InternalIMT | epa | rs192321882 | . | . | . | . | . | . | . | . | . | . |
| InternalIMT | epa | rs150812270 | . | . | . | . | . | . | . | . | . | . |
| InternalIMT | epa | rs185906603 | . | . | . | . | . | . | . | . | . | . |
| InternalIMT | epa | rs191070862 | . | . | . | . | . | . | . | . | . | . |
| InternalIMT | epa | rs182904978 | . | . | . | . | . | . | . | . | . | . |
| InternalIMT | epa | rs192679655 | . | . | . | . | . | . | . | . | . | . |
| InternalIMT | epa | rs189673086 | . | . | . | . | . | . | . | . | . | . |
| InternalIMT | epa | rs181141892 | . | . | . | . | . | . | . | . | . | . |
| InternalIMT | epa | rs186404123 | . | . | . | . | . | . | . | . | . | . |
| InternalIMT | epa | rs192117287 | . | . | . | . | . | . | . | . | . | . |
| InternalIMT | epa | rs182748835 | . | . | . | . | . | . | . | . | . | . |
| InternalIMT | epa | rs141854549 | . | . | . | . | . | . | . | . | . | . |
| InternalIMT | epa | rs140163397 | . | . | . | . | . | . | . | . | . | . |
| InternalIMT | epa | rs146347300 | . | . | . | . | . | . | . | . | . | . |
| InternalIMT | epa | rs139362377 | . | . | . | . | . | . | . | . | . | . |
| InternalIMT | epa | rs185087671 | . | . | . | . | . | . | . | . | . | . |
| InternalIMT | epa | rs190466574 | . | . | . | . | . | . | . | . | . | . |
| InternalIMT | epa | rs147012778 | . | . | . | . | . | . | . | . | . | . |
| InternalIMT | epa | rs138236960 | . | . | . | . | . | . | . | . | . | . |
| InternalIMT | epa | rs116310311 | . | . | . | . | . | . | . | . | . | . |
| InternalIMT | epa | rs142868356 | . | . | . | . | . | . | . | . | . | . |
| InternalIMT | epa | rs144956240 | . | . | . | . | . | . | . | . | . | . |
| InternalIMT | epa | rs188527776 | . | . | . | . | . | . | . | . | . | . |
| InternalIMT | epa | rs183971165 | . | . | . | . | . | . | . | . | . | . |
| InternalIMT | epa | rs76748216 | . | . | . | . | . | . | . | . | . | . |
| InternalIMT | epa | rs148554990 | . | . | . | . | . | . | . | . | . | . |
| InternalIMT | epa | rs114446477 | . | . | . | . | . | . | . | . | . | . |
| InternalIMT | epa | rs185223933 | . | . | . | . | . | . | . | . | . | . |
| InternalIMT | epa | rs76869726 | . | . | . | . | . | . | . | . | . | . |
| InternalIMT | epa | rs151200830 | . | . | . | . | . | . | . | . | . | . |
| InternalIMT | epa | rs17157731 | . | . | . | . | . | . | . | . | . | . |
| InternalIMT | epa | rs181243469 | . | . | . | . | . | . | . | . | . | . |
| InternalIMT | epa | rs186890707 | . | . | . | . | . | . | . | . | . | . |
| InternalIMT | epa | rs184063360 | . | . | . | . | . | . | . | . | . | . |
| InternalIMT | epa | rs188550237 | . | . | . | . | . | . | . | . | . | . |
| InternalIMT | epa | rs140931068 | . | . | . | . | . | . | . | . | . | . |
| InternalIMT | epa | rs74867877 | . | . | . | . | . | . | . | . | . | . |
| InternalIMT | epa | rs11239495 | . | . | . | . | . | . | . | . | . | . |
| InternalIMT | epa | rs149745800 | . | . | . | . | . | . | . | . | . | . |
| InternalIMT | epa | rs145044458 | . | . | . | . | . | . | . | . | . | . |
| InternalIMT | epa | rs188067427 | . | . | . | . | . | . | . | . | . | . |
| InternalIMT | epa | rs146827800 | . | . | . | . | . | . | . | . | . | . |
| InternalIMT | epa | rs140619817 | . | . | . | . | . | . | . | . | . | . |
| InternalIMT | epa | rs892689 | . | . | . | . | . | . | . | . | . | . |
| InternalIMT | epa | rs143642005 | . | . | . | . | . | . | . | . | . | . |
| InternalIMT | epa | rs181929304 | . | . | . | . | . | . | . | . | . | . |
| InternalIMT | epa | rs191086173 | . | . | . | . | . | . | . | . | . | . |
| InternalIMT | epa | rs114613116 | . | . | . | . | . | . | . | . | . | . |
| InternalIMT | epa | rs141076768 | . | . | . | . | . | . | . | . | . | . |
| InternalIMT | epa | rs187316531 | . | . | . | . | . | . | . | . | . | . |
| InternalIMT | epa | rs11239499 | . | . | . | . | . | . | . | . | . | . |
| InternalIMT | epa | rs192726371 | . | . | . | . | . | . | . | . | . | . |
| InternalIMT | epa | rs74128481 | . | . | . | . | . | . | . | . | . | . |
| InternalIMT | epa | rs74128482 | . | . | . | . | . | . | . | . | . | . |
| InternalIMT | epa | rs11239502 | . | . | . | . | . | . | . | . | . | . |
| InternalIMT | epa | rs116330285 | . | . | . | . | . | . | . | . | . | . |
| InternalIMT | epa | rs189926729 | . | . | . | . | . | . | . | . | . | . |
| InternalIMT | epa | rs188975779 | . | . | . | . | . | . | . | . | . | . |
| InternalIMT | epa | rs143846038 | . | . | . | . | . | . | . | . | . | . |
| InternalIMT | epa | rs74128483 | . | . | . | . | . | . | . | . | . | . |
| InternalIMT | epa | rs139292055 | . | . | . | . | . | . | . | . | . | . |
| InternalIMT | epa | rs11239503 | . | . | . | . | . | . | . | . | . | . |
| InternalIMT | epa | rs58838686 | . | . | . | . | . | . | . | . | . | . |
| InternalIMT | epa | rs185825373 | . | . | . | . | . | . | . | . | . | . |
| InternalIMT | epa | rs11239504 | . | . | . | . | . | . | . | . | . | . |

| InternalIMT | epa | rs150012354 | . | . | . | . | . | . | . | . | . | . |
| --- | --- | --- | --- | --- | --- | --- | --- | --- | --- | --- | --- | --- |
| InternalIMT | epa | rs187763096 | . | . | . | . | . | . | . | . | . | . |
| InternalIMT | epa | rs193043982 | . | . | . | . | . | . | . | . | . | . |
| InternalIMT | epa | rs79186884 | . | . | . | . | . | . | . | . | . | . |
| InternalIMT | epa | rs74128484 | . | . | . | . | . | . | . | . | . | . |
| InternalIMT | epa | rs58461537 | . | . | . | . | . | . | . | . | . | . |
| InternalIMT | epa | rs188527474 | . | . | . | . | . | . | . | . | . | . |
| InternalIMT | epa | rs141659976 | . | . | . | . | . | . | . | . | . | . |
| InternalIMT | epa | rs115846407 | . | . | . | . | . | . | . | . | . | . |
| InternalIMT | epa | rs138378816 | . | . | . | . | . | . | . | . | . | . |
| InternalIMT | epa | rs146188136 | . | . | . | . | . | . | . | . | . | . |
| InternalIMT | epa | rs139383871 | . | . | . | . | . | . | . | . | . | . |
| InternalIMT | epa | rs182953869 | . | . | . | . | . | . | . | . | . | . |
| InternalIMT | epa | rs192430299 | . | . | . | . | . | . | . | . | . | . |
| InternalIMT | epa | rs144152844 | . | . | . | . | . | . | . | . | . | . |
| InternalIMT | epa | rs145987786 | . | . | . | . | . | . | . | . | . | . |
| InternalIMT | epa | rs181132926 | . | . | . | . | . | . | . | . | . | . |
| InternalIMT | epa | rs147882253 | . | . | . | . | . | . | . | . | . | . |
| InternalIMT | epa | rs141807621 | . | . | . | . | . | . | . | . | . | . |
| InternalIMT | epa | rs147116052 | . | . | . | . | . | . | . | . | . | . |
| InternalIMT | epa | rs147716618 | . | . | . | . | . | . | . | . | . | . |
| InternalIMT | epa | rs187198855 | . | . | . | . | . | . | . | . | . | . |
| InternalIMT | epa | rs189409638 | . | . | . | . | . | . | . | . | . | . |
| InternalIMT | epa | rs182647339 | . | . | . | . | . | . | . | . | . | . |
| InternalIMT | epa | rs187995988 | . | . | . | . | . | . | . | . | . | . |
| InternalIMT | epa | rs141034184 | . | . | . | . | . | . | . | . | . | . |
| InternalIMT | epa | rs187310430 | . | . | . | . | . | . | . | . | . | . |
| InternalIMT | epa | rs192608488 | . | . | . | . | . | . | . | . | . | . |
| InternalIMT | epa | rs148840366 | . | . | . | . | . | . | . | . | . | . |
| InternalIMT | epa | rs186785022 | . | . | . | . | . | . | . | . | . | . |
| InternalIMT | epa | rs192348728 | . | . | . | . | . | . | . | . | . | . |
| InternalIMT | epa | rs189656631 | . | . | . | . | . | . | . | . | . | . |
| InternalIMT | epa | rs185289750 | . | . | . | . | . | . | . | . | . | . |
| InternalIMT | epa | rs142382395 | . | . | . | . | . | . | . | . | . | . |
| InternalIMT | epa | rs144866696 | . | . | . | . | . | . | . | . | . | . |
| InternalIMT | epa | rs141371289 | . | . | . | . | . | . | . | . | . | . |
| InternalIMT | epa | rs114750008 | . | . | . | . | . | . | . | . | . | . |
| InternalIMT | epa | rs189063597 | . | . | . | . | . | . | . | . | . | . |
| InternalIMT | epa | rs139151571 | . | . | . | . | . | . | . | . | . | . |
| InternalIMT | epa | rs139846368 | . | . | . | . | . | . | . | . | . | . |
| InternalIMT | epa | rs182057653 | . | . | . | . | . | . | . | . | . | . |
| InternalIMT | epa | rs148785420 | . | . | . | . | . | . | . | . | . | . |
| InternalIMT | epa | rs138383251 | . | . | . | . | . | . | . | . | . | . |
| InternalIMT | epa | rs116526705 | . | . | . | . | . | . | . | . | . | . |
| InternalIMT | epa | rs185946436 | . | . | . | . | . | . | . | . | . | . |
| InternalIMT | epa | rs190455427 | . | . | . | . | . | . | . | . | . | . |
| InternalIMT | epa | rs9282584 | . | . | . | . | . | . | . | . | . | . |
| InternalIMT | epa | rs17153289 | . | . | . | . | . | . | . | . | . | . |
| InternalIMT | epa | rs138359103 | . | . | . | . | . | . | . | . | . | . |
| InternalIMT | epa | rs150605268 | . | . | . | . | . | . | . | . | . | . |
| InternalIMT | epa | rs79242910 | . | . | . | . | . | . | . | . | . | . |
| InternalIMT | epa | rs139716904 | . | . | . | . | . | . | . | . | . | . |
| InternalIMT | epa | rs76238498 | . | . | . | . | . | . | . | . | . | . |
| InternalIMT | epa | rs184140639 | . | . | . | . | . | . | . | . | . | . |
| InternalIMT | epa | rs188517278 | . | . | . | . | . | . | . | . | . | . |
| InternalIMT | epa | rs139678913 | . | . | . | . | . | . | . | . | . | . |
| InternalIMT | epa | rs116697955 | . | . | . | . | . | . | . | . | . | . |
| InternalIMT | epa | rs79267994 | . | . | . | . | . | . | . | . | . | . |
| InternalIMT | epa | rs11239510 | . | . | . | . | . | . | . | . | . | . |
| InternalIMT | epa | rs75831645 | . | . | . | . | . | . | . | . | . | . |
| InternalIMT | epa | rs78452276 | . | . | . | . | . | . | . | . | . | . |
| InternalIMT | epa | rs144366924 | . | . | . | . | . | . | . | . | . | . |
| InternalIMT | epa | rs77519841 | . | . | . | . | . | . | . | . | . | . |
| InternalIMT | epa | rs76944454 | . | . | . | . | . | . | . | . | . | . |
| InternalIMT | epa | rs76443855 | . | . | . | . | . | . | . | . | . | . |
| InternalIMT | epa | rs146613174 | . | . | . | . | . | . | . | . | . | . |
| InternalIMT | epa | rs181424761 | . | . | . | . | . | . | . | . | . | . |
| InternalIMT | epa | rs142119241 | . | . | . | . | . | . | . | . | . | . |
| InternalIMT | epa | rs140137954 | . | . | . | . | . | . | . | . | . | . |
| InternalIMT | epa | rs187319549 | . | . | . | . | . | . | . | . | . | . |
| InternalIMT | epa | rs12241545 | . | . | . | . | . | . | . | . | . | . |
| InternalIMT | epa | rs10128228 | . | . | . | . | . | . | . | . | . | . |
| InternalIMT | epa | rs58580249 | . | . | . | . | . | . | . | . | . | . |

| InternalIMT | epa | rs193148863 | . | . | . | . | . | . | . | . | . | . |
| --- | --- | --- | --- | --- | --- | --- | --- | --- | --- | --- | --- | --- |
| InternalIMT | epa | rs185638946 | . | . | . | . | . | . | . | . | . | . |
| InternalIMT | epa | rs117752921 | . | . | . | . | . | . | . | . | . | . |
| InternalIMT | epa | rs180841236 | . | . | . | . | . | . | . | . | . | . |
| InternalIMT | epa | rs73283155 | . | . | . | . | . | . | . | . | . | . |
| InternalIMT | epa | rs76560599 | . | . | . | . | . | . | . | . | . | . |
| InternalIMT | epa | rs76273620 | . | . | . | . | . | . | . | . | . | . |
| InternalIMT | epa | rs141432923 | . | . | . | . | . | . | . | . | . | . |
| InternalIMT | epa | rs113529322 | . | . | . | . | . | . | . | . | . | . |
| InternalIMT | epa | rs10128306 | . | . | . | . | . | . | . | . | . | . |
| InternalIMT | epa | rs149691688 | . | . | . | . | . | . | . | . | . | . |
| InternalIMT | epa | rs75872495 | . | . | . | . | . | . | . | . | . | . |
| InternalIMT | epa | rs191873960 | . | . | . | . | . | . | . | . | . | . |
| InternalIMT | epa | rs80155622 | . | . | . | . | . | . | . | . | . | . |
| InternalIMT | epa | rs113914656 | . | . | . | . | . | . | . | . | . | . |
| InternalIMT | epa | rs12242840 | . | . | . | . | . | . | . | . | . | . |
| InternalIMT | epa | rs147824078 | . | . | . | . | . | . | . | . | . | . |
| InternalIMT | epa | rs59051493 | . | . | . | . | . | . | . | . | . | . |
| InternalIMT | epa | rs9282588 | . | . | . | . | . | . | . | . | . | . |
| InternalIMT | epa | rs150215207 | . | . | . | . | . | . | . | . | . | . |
| InternalIMT | epa | rs138772243 | . | . | . | . | . | . | . | . | . | . |
| InternalIMT | epa | rs149383244 | . | . | . | . | . | . | . | . | . | . |
| InternalIMT | epa | rs17522720 | . | . | . | . | . | . | . | . | . | . |
| InternalIMT | epa | rs79245105 | . | . | . | . | . | . | . | . | . | . |
| InternalIMT | epa | rs138222133 | . | . | . | . | . | . | . | . | . | . |
| InternalIMT | epa | rs12247314 | . | . | . | . | . | . | . | . | . | . |
| InternalIMT | epa | rs12263894 | . | . | . | . | . | . | . | . | . | . |
| InternalIMT | epa | rs149907880 | . | . | . | . | . | . | . | . | . | . |
| InternalIMT | epa | rs188153885 | . | . | . | . | . | . | . | . | . | . |
| InternalIMT | epa | rs73283159 | . | . | . | . | . | . | . | . | . | . |
| InternalIMT | epa | rs140476687 | . | . | . | . | . | . | . | . | . | . |
| InternalIMT | epa | rs186603148 | . | . | . | . | . | . | . | . | . | . |
| InternalIMT | epa | rs183296063 | . | . | . | . | . | . | . | . | . | . |
| InternalIMT | epa | rs145733694 | . | . | . | . | . | . | . | . | . | . |
| InternalIMT | epa | rs189807449 | . | . | . | . | . | . | . | . | . | . |
| InternalIMT | epa | rs12253223 | . | . | . | . | . | . | . | . | . | . |
| InternalIMT | epa | rs182113637 | . | . | . | . | . | . | . | . | . | . |
| InternalIMT | epa | rs142792099 | . | . | . | . | . | . | . | . | . | . |
| InternalIMT | epa | rs12249910 | . | . | . | . | . | . | . | . | . | . |
| InternalIMT | epa | rs149436339 | . | . | . | . | . | . | . | . | . | . |
| InternalIMT | epa | rs77632210 | . | . | . | . | . | . | . | . | . | . |
| InternalIMT | epa | rs74130818 | . | . | . | . | . | . | . | . | . | . |
| InternalIMT | epa | rs187583648 | . | . | . | . | . | . | . | . | . | . |
| InternalIMT | epa | rs191295987 | . | . | . | . | . | . | . | . | . | . |
| InternalIMT | epa | rs184200686 | . | . | . | . | . | . | . | . | . | . |
| InternalIMT | epa | rs76117263 | . | . | . | . | . | . | . | . | . | . |
| InternalIMT | epa | rs185393149 | . | . | . | . | . | . | . | . | . | . |
| InternalIMT | epa | rs144760014 | . | . | . | . | . | . | . | . | . | . |
| InternalIMT | epa | rs7922292 | . | . | . | . | . | . | . | . | . | . |
| InternalIMT | epa | rs115372844 | . | . | . | . | . | . | . | . | . | . |
| InternalIMT | epa | rs111460364 | . | . | . | . | . | . | . | . | . | . |
| InternalIMT | epa | rs182052255 | . | . | . | . | . | . | . | . | . | . |
| InternalIMT | epa | rs180983889 | . | . | . | . | . | . | . | . | . | . |
| InternalIMT | epa | rs139175327 | . | . | . | . | . | . | . | . | . | . |
| InternalIMT | epa | rs149959620 | . | . | . | . | . | . | . | . | . | . |
| InternalIMT | epa | rs114653117 | . | . | . | . | . | . | . | . | . | . |
| InternalIMT | epa | rs144270018 | . | . | . | . | . | . | . | . | . | . |
| InternalIMT | epa | rs182723204 | . | . | . | . | . | . | . | . | . | . |
| InternalIMT | epa | rs57204067 | . | . | . | . | . | . | . | . | . | . |
| InternalIMT | epa | rs144956226 | . | . | . | . | . | . | . | . | . | . |
| InternalIMT | epa | rs187842704 | . | . | . | . | . | . | . | . | . | . |
| InternalIMT | epa | rs184620511 | . | . | . | . | . | . | . | . | . | . |
| InternalIMT | epa | rs144031303 | . | . | . | . | . | . | . | . | . | . |
| InternalIMT | epa | rs187526473 | . | . | . | . | . | . | . | . | . | . |
| InternalIMT | epa | rs193002805 | . | . | . | . | . | . | . | . | . | . |
| InternalIMT | epa | rs138707426 | . | . | . | . | . | . | . | . | . | . |
| InternalIMT | epa | rs142754243 | . | . | . | . | . | . | . | . | . | . |
| InternalIMT | epa | rs181600331 | . | . | . | . | . | . | . | . | . | . |
| InternalIMT | epa | rs144421264 | . | . | . | . | . | . | . | . | . | . |
| InternalIMT | epa | rs184414519 | . | . | . | . | . | . | . | . | . | . |
| InternalIMT | epa | rs150596349 | . | . | . | . | . | . | . | . | . | . |
| InternalIMT | epa | rs190753747 | . | . | . | . | . | . | . | . | . | . |
| InternalIMT | epa | rs139560940 | . | . | . | . | . | . | . | . | . | . |

| InternalIMT | epa | rs75640804 | . | . | . | . | . | . | . | . | . | . |
| --- | --- | --- | --- | --- | --- | --- | --- | --- | --- | --- | --- | --- |
| InternalIMT | epa | rs73283175 | . | . | . | . | . | . | . | . | . | . |
| InternalIMT | epa | rs143337229 | . | . | . | . | . | . | . | . | . | . |
| InternalIMT | epa | rs73283177 | . | . | . | . | . | . | . | . | . | . |
| InternalIMT | epa | rs76053611 | . | . | . | . | . | . | . | . | . | . |
| InternalIMT | epa | rs116325983 | . | . | . | . | . | . | . | . | . | . |
| InternalIMT | epa | rs115451825 | . | . | . | . | . | . | . | . | . | . |
| InternalIMT | epa | rs183845246 | . | . | . | . | . | . | . | . | . | . |
| InternalIMT | epa | rs79756542 | . | . | . | . | . | . | . | . | . | . |
| InternalIMT | epa | rs187242860 | . | . | . | . | . | . | . | . | . | . |
| InternalIMT | epa | rs57318980 | . | . | . | . | . | . | . | . | . | . |
| InternalIMT | epa | rs59969732 | . | . | . | . | . | . | . | . | . | . |
| InternalIMT | epa | rs143811488 | . | . | . | . | . | . | . | . | . | . |
| InternalIMT | epa | rs189067644 | . | . | . | . | . | . | . | . | . | . |
| InternalIMT | epa | rs60449178 | . | . | . | . | . | . | . | . | . | . |
| InternalIMT | epa | rs59243389 | . | . | . | . | . | . | . | . | . | . |
| InternalIMT | epa | rs184946540 | . | . | . | . | . | . | . | . | . | . |
| InternalIMT | epa | rs188685881 | . | . | . | . | . | . | . | . | . | . |
| InternalIMT | epa | rs150335797 | . | . | . | . | . | . | . | . | . | . |
| InternalIMT | epa | rs143879260 | . | . | . | . | . | . | . | . | . | . |
| InternalIMT | epa | rs186044491 | . | . | . | . | . | . | . | . | . | . |
| InternalIMT | epa | rs11819084 | . | . | . | . | . | . | . | . | . | . |
| InternalIMT | epa | rs146915670 | . | . | . | . | . | . | . | . | . | . |
| InternalIMT | epa | rs113995695 | . | . | . | . | . | . | . | . | . | . |
| InternalIMT | epa | rs143489451 | . | . | . | . | . | . | . | . | . | . |
| InternalIMT | epa | rs182653839 | . | . | . | . | . | . | . | . | . | . |
| InternalIMT | epa | rs141692564 | . | . | . | . | . | . | . | . | . | . |
| InternalIMT | epa | rs146281742 | . | . | . | . | . | . | . | . | . | . |
| InternalIMT | epa | rs192914353 | . | . | . | . | . | . | . | . | . | . |
| InternalIMT | epa | rs185182848 | . | . | . | . | . | . | . | . | . | . |
| InternalIMT | epa | rs28395858 | . | . | . | . | . | . | . | . | . | . |
| InternalIMT | epa | rs41429645 | . | . | . | . | . | . | . | . | . | . |
| InternalIMT | epa | rs28395874 | . | . | . | . | . | . | . | . | . | . |
| InternalIMT | epa | rs189663634 | . | . | . | . | . | . | . | . | . | . |
| InternalIMT | epa | rs77666700 | . | . | . | . | . | . | . | . | . | . |
| InternalIMT | epa | rs146711717 | . | . | . | . | . | . | . | . | . | . |
| InternalIMT | epa | rs144904251 | . | . | . | . | . | . | . | . | . | . |
| InternalIMT | epa | rs189893153 | . | . | . | . | . | . | . | . | . | . |
| InternalIMT | epa | rs150447740 | . | . | . | . | . | . | . | . | . | . |
| InternalIMT | epa | rs143099649 | . | . | . | . | . | . | . | . | . | . |
| InternalIMT | epa | rs191288436 | . | . | . | . | . | . | . | . | . | . |
| InternalIMT | epa | rs188577862 | . | . | . | . | . | . | . | . | . | . |
| InternalIMT | epa | rs192695334 | . | . | . | . | . | . | . | . | . | . |
| InternalIMT | epa | rs138612906 | . | . | . | . | . | . | . | . | . | . |
| InternalIMT | epa | rs191290051 | . | . | . | . | . | . | . | . | . | . |
| InternalIMT | epa | rs189631459 | . | . | . | . | . | . | . | . | . | . |
| InternalIMT | epa | rs114620874 | . | . | . | . | . | . | . | . | . | . |
| InternalIMT | epa | rs78246652 | . | . | . | . | . | . | . | . | . | . |
| InternalIMT | epa | rs79432451 | . | . | . | . | . | . | . | . | . | . |
| InternalIMT | epa | rs186022585 | . | . | . | . | . | . | . | . | . | . |
| InternalIMT | epa | rs142978891 | . | . | . | . | . | . | . | . | . | . |
| InternalIMT | epa | rs151086840 | . | . | . | . | . | . | . | . | . | . |
| InternalIMT | epa | rs183270259 | . | . | . | . | . | . | . | . | . | . |
| InternalIMT | epa | rs188544289 | . | . | . | . | . | . | . | . | . | . |
| InternalIMT | epa | rs188500808 | . | . | . | . | . | . | . | . | . | . |
| InternalIMT | epa | rs189424589 | . | . | . | . | . | . | . | . | . | . |
| InternalIMT | epa | rs78622303 | . | . | . | . | . | . | . | . | . | . |
| InternalIMT | epa | rs143210835 | . | . | . | . | . | . | . | . | . | . |
| InternalIMT | epa | rs114761840 | . | . | . | . | . | . | . | . | . | . |
| InternalIMT | epa | rs185805402 | . | . | . | . | . | . | . | . | . | . |
| InternalIMT | epa | rs139273744 | . | . | . | . | . | . | . | . | . | . |
| InternalIMT | epa | rs184654150 | . | . | . | . | . | . | . | . | . | . |
| InternalIMT | epa | rs189069710 | . | . | . | . | . | . | . | . | . | . |
| InternalIMT | epa | rs114752115 | . | . | . | . | . | . | . | . | . | . |
| InternalIMT | epa | rs150162515 | . | . | . | . | . | . | . | . | . | . |
| InternalIMT | epa | rs180982454 | . | . | . | . | . | . | . | . | . | . |
| InternalIMT | epa | rs184932088 | . | . | . | . | . | . | . | . | . | . |
| InternalIMT | epa | rs181109625 | . | . | . | . | . | . | . | . | . | . |
| InternalIMT | epa | rs138121134 | . | . | . | . | . | . | . | . | . | . |
| InternalIMT | epa | rs183253861 | . | . | . | . | . | . | . | . | . | . |
| InternalIMT | epa | rs147073946 | . | . | . | . | . | . | . | . | . | . |
| InternalIMT | epa | rs139938489 | . | . | . | . | . | . | . | . | . | . |
| InternalIMT | epa | rs146362560 | . | . | . | . | . | . | . | . | . | . |

| InternalIMT | epa | rs193155956 | . | . | . | . | . | . | . | . | . | . |
| --- | --- | --- | --- | --- | --- | --- | --- | --- | --- | --- | --- | --- |
| InternalIMT | epa | rs77663092 | . | . | . | . | . | . | . | . | . | . |
| InternalIMT | epa | rs73285319 | . | . | . | . | . | . | . | . | . | . |
| InternalIMT | epa | rs184019276 | . | . | . | . | . | . | . | . | . | . |
| InternalIMT | epa | rs28395863 | . | . | . | . | . | . | . | . | . | . |
| InternalIMT | epa | rs182121645 | . | . | . | . | . | . | . | . | . | . |
| InternalIMT | epa | rs73285327 | . | . | . | . | . | . | . | . | . | . |
| InternalIMT | epa | rs149589584 | . | . | . | . | . | . | . | . | . | . |
| InternalIMT | epa | rs148310335 | . | . | . | . | . | . | . | . | . | . |
| InternalIMT | epa | rs187246093 | . | . | . | . | . | . | . | . | . | . |
| InternalIMT | epa | rs184668894 | . | . | . | . | . | . | . | . | . | . |
| InternalIMT | epa | rs114269961 | . | . | . | . | . | . | . | . | . | . |
| InternalIMT | epa | rs149694374 | . | . | . | . | . | . | . | . | . | . |
| InternalIMT | epa | rs145284227 | . | . | . | . | . | . | . | . | . | . |
| InternalIMT | epa | rs189983820 | . | . | . | . | . | . | . | . | . | . |
| InternalIMT | epa | rs181200444 | . | . | . | . | . | . | . | . | . | . |
| InternalIMT | epa | rs185486385 | . | . | . | . | . | . | . | . | . | . |
| InternalIMT | epa | rs151198241 | . | . | . | . | . | . | . | . | . | . |
| InternalIMT | epa | rs189959341 | . | . | . | . | . | . | . | . | . | . |
| InternalIMT | epa | rs140549257 | . | . | . | . | . | . | . | . | . | . |
| InternalIMT | epa | rs186453139 | . | . | . | . | . | . | . | . | . | . |
| InternalIMT | epa | rs180922711 | . | . | . | . | . | . | . | . | . | . |
| InternalIMT | epa | rs56659903 | . | . | . | . | . | . | . | . | . | . |
| InternalIMT | epa | rs141202128 | . | . | . | . | . | . | . | . | . | . |
| InternalIMT | epa | rs59085314 | . | . | . | . | . | . | . | . | . | . |
| InternalIMT | epa | rs145564996 | . | . | . | . | . | . | . | . | . | . |
| InternalIMT | epa | rs114961009 | . | . | . | . | . | . | . | . | . | . |
| InternalIMT | epa | rs182294189 | . | . | . | . | . | . | . | . | . | . |
| InternalIMT | epa | rs187586636 | . | . | . | . | . | . | . | . | . | . |
| InternalIMT | epa | rs192254907 | . | . | . | . | . | . | . | . | . | . |
| InternalIMT | epa | rs149145663 | . | . | . | . | . | . | . | . | . | . |
| InternalIMT | epa | rs180713064 | . | . | . | . | . | . | . | . | . | . |
| InternalIMT | epa | rs183009023 | . | . | . | . | . | . | . | . | . | . |
| InternalIMT | epa | rs143385347 | . | . | . | . | . | . | . | . | . | . |
| InternalIMT | epa | rs185786329 | . | . | . | . | . | . | . | . | . | . |
| InternalIMT | epa | rs192341030 | . | . | . | . | . | . | . | . | . | . |
| InternalIMT | epa | rs74661429 | . | . | . | . | . | . | . | . | . | . |
| InternalIMT | epa | rs188034853 | . | . | . | . | . | . | . | . | . | . |
| InternalIMT | epa | rs116808430 | . | . | . | . | . | . | . | . | . | . |
| InternalIMT | epa | rs148469010 | . | . | . | . | . | . | . | . | . | . |
| InternalIMT | epa | rs78621663 | . | . | . | . | . | . | . | . | . | . |
| InternalIMT | epa | rs192654886 | . | . | . | . | . | . | . | . | . | . |
| InternalIMT | epa | rs145996035 | . | . | . | . | . | . | . | . | . | . |
| InternalIMT | epa | rs139942226 | . | . | . | . | . | . | . | . | . | . |
| InternalIMT | epa | rs113645632 | . | . | . | . | . | . | . | . | . | . |
| InternalIMT | epa | rs74940661 | . | . | . | . | . | . | . | . | . | . |
| InternalIMT | epa | rs115355800 | . | . | . | . | . | . | . | . | . | . |
| InternalIMT | epa | rs150285898 | . | . | . | . | . | . | . | . | . | . |
| InternalIMT | epa | rs188117932 | . | . | . | . | . | . | . | . | . | . |
| InternalIMT | epa | rs139956566 | . | . | . | . | . | . | . | . | . | . |
| InternalIMT | epa | rs147673885 | . | . | . | . | . | . | . | . | . | . |
| InternalIMT | epa | rs189127928 | . | . | . | . | . | . | . | . | . | . |
| InternalIMT | epa | rs187558772 | . | . | . | . | . | . | . | . | . | . |
| InternalIMT | epa | rs181948587 | . | . | . | . | . | . | . | . | . | . |
| InternalIMT | epa | rs189983269 | . | . | . | . | . | . | . | . | . | . |
| InternalIMT | epa | rs187250069 | . | . | . | . | . | . | . | . | . | . |
| InternalIMT | epa | rs191485859 | . | . | . | . | . | . | . | . | . | . |
| InternalIMT | epa | rs185499555 | . | . | . | . | . | . | . | . | . | . |
| InternalIMT | epa | rs180862110 | . | . | . | . | . | . | . | . | . | . |
| InternalIMT | epa | rs190472603 | . | . | . | . | . | . | . | . | . | . |
| InternalIMT | epa | rs189226167 | . | . | . | . | . | . | . | . | . | . |
| InternalIMT | epa | rs181623256 | . | . | . | . | . | . | . | . | . | . |
| InternalIMT | epa | rs186079557 | . | . | . | . | . | . | . | . | . | . |
| InternalIMT | epa | rs147760994 | . | . | . | . | . | . | . | . | . | . |
| InternalIMT | epa | rs189176167 | . | . | . | . | . | . | . | . | . | . |
| InternalIMT | epa | rs117901000 | . | . | . | . | . | . | . | . | . | . |
| InternalIMT | epa | rs141984888 | . | . | . | . | . | . | . | . | . | . |
| InternalIMT | epa | rs192365061 | . | . | . | . | . | . | . | . | . | . |
| InternalIMT | epa | rs185976038 | . | . | . | . | . | . | . | . | . | . |
| InternalIMT | epa | rs184697303 | . | . | . | . | . | . | . | . | . | . |
| InternalIMT | epa | rs143383132 | . | . | . | . | . | . | . | . | . | . |
| InternalIMT | epa | rs141873881 | . | . | . | . | . | . | . | . | . | . |
| InternalIMT | epa | rs185507255 | . | . | . | . | . | . | . | . | . | . |

| InternalIMT | epa | rs191379425 | . | . | . | . | . | . | . | . | . | . |
| --- | --- | --- | --- | --- | --- | --- | --- | --- | --- | --- | --- | --- |
| InternalIMT | epa | rs184199584 | . | . | . | . | . | . | . | . | . | . |
| InternalIMT | epa | rs183181360 | . | . | . | . | . | . | . | . | . | . |
| InternalIMT | epa | rs184495673 | . | . | . | . | . | . | . | . | . | . |
| InternalIMT | epa | rs182946297 | . | . | . | . | . | . | . | . | . | . |
| InternalIMT | epa | rs187743615 | . | . | . | . | . | . | . | . | . | . |
| InternalIMT | epa | rs183259862 | . | . | . | . | . | . | . | . | . | . |
| InternalIMT | epa | rs112433566 | . | . | . | . | . | . | . | . | . | . |
| InternalIMT | epa | rs182351085 | . | . | . | . | . | . | . | . | . | . |
| InternalIMT | epa | rs146248508 | . | . | . | . | . | . | . | . | . | . |
| InternalIMT | epa | rs188528439 | . | . | . | . | . | . | . | . | . | . |
| InternalIMT | epa | rs146167018 | . | . | . | . | . | . | . | . | . | . |
| InternalIMT | epa | rs192440980 | . | . | . | . | . | . | . | . | . | . |
| InternalIMT | epa | rs185601882 | . | . | . | . | . | . | . | . | . | . |
| InternalIMT | epa | rs117272563 | . | . | . | . | . | . | . | . | . | . |
| InternalIMT | epa | rs10430734 | . | . | . | . | . | . | . | . | . | . |
| InternalIMT | epa | rs181320952 | . | . | . | . | . | . | . | . | . | . |
| InternalIMT | epa | rs185480660 | . | . | . | . | . | . | . | . | . | . |
| InternalIMT | epa | rs188077917 | . | . | . | . | . | . | . | . | . | . |
| InternalIMT | epa | rs187562953 | . | . | . | . | . | . | . | . | . | . |
| InternalIMT | epa | rs182044631 | . | . | . | . | . | . | . | . | . | . |
| InternalIMT | epa | rs145455596 | . | . | . | . | . | . | . | . | . | . |
| InternalIMT | epa | rs181475877 | . | . | . | . | . | . | . | . | . | . |
| InternalIMT | epa | rs149413085 | . | . | . | . | . | . | . | . | . | . |
| InternalIMT | epa | rs12253656 | . | . | . | . | . | . | . | . | . | . |
| InternalIMT | epa | rs182718239 | . | . | . | . | . | . | . | . | . | . |
| InternalIMT | epa | rs192435695 | . | . | . | . | . | . | . | . | . | . |
| InternalIMT | epa | rs12248277 | . | . | . | . | . | . | . | . | . | . |
| InternalIMT | epa | rs143521710 | . | . | . | . | . | . | . | . | . | . |
| InternalIMT | epa | rs144454454 | . | . | . | . | . | . | . | . | . | . |
| InternalIMT | epa | rs190441610 | . | . | . | . | . | . | . | . | . | . |
| InternalIMT | epa | rs114690462 | . | . | . | . | . | . | . | . | . | . |
| InternalIMT | epa | rs184541357 | . | . | . | . | . | . | . | . | . | . |
| InternalIMT | epa | rs142657653 | . | . | . | . | . | . | . | . | . | . |
| InternalIMT | epa | rsurf1 | 2.019 | . | . | . | . | . | . | . | . | . |
| InternalIMT | epa | rsten1 | 2.195 | . | . | . | . | . | . | . | . | . |
| InternalIMT | epa | chr10:45852312:D | 0.055 | -0.054 | 0.021 | 9.60E-03 | -0.012 | 0.038 | 0.755 | -0.011 | 0.06 | 0.849 |
| InternalIMT | epa | chr10:45853232:I | 0.071 | -0.052 | 0.021 | 0.014 | -0.015 | 0.04 | 0.709 | -0.018 | 0.063 | 0.773 |
| InternalIMT | epa | chr10:45853234:I | 0.157 | -0.048 | 0.022 | 0.031 | -0.031 | 0.025 | 0.213 | -0.018 | 0.037 | 0.629 |
| InternalIMT | epa | chr10:45854796:D | 0.001 | . | . | . | . | . | . | . | . | . |
| InternalIMT | epa | chr10:45854994:I | 0.001 | . | . | . | . | . | . | . | . | . |
| InternalIMT | epa | chr10:45858290:D | 0.04 | -0.056 | 0.021 | 7.30E-03 | 0.032 | 0.061 | 0.606 | 0.013 | 0.093 | 0.891 |
| InternalIMT | epa | chr10:45858820:I | 0.015 | -0.044 | 0.02 | 0.029 | -0.057 | 0.11 | 0.6 | -0.372 | 0.217 | 0.087 |
| InternalIMT | epa | chr10:45860888:D | 0.067 | -0.05 | 0.022 | 0.02 | -0.024 | 0.05 | 0.626 | -0.038 | 0.076 | 0.623 |
| InternalIMT | epa | chr10:45863913:I | 0.159 | -0.047 | 0.023 | 0.038 | -0.029 | 0.025 | 0.24 | -0.023 | 0.038 | 0.534 |
| InternalIMT | epa | chr10:45866601:D | 0 | . | . | . | . | . | . | . | . | . |
| InternalIMT | epa | chr10:45867197:I | 0.008 | . | . | . | . | . | . | . | . | . |
| InternalIMT | epa | chr10:45867538:D | 0.167 | -0.046 | 0.023 | 0.046 | -0.028 | 0.025 | 0.262 | -0.026 | 0.037 | 0.492 |
| InternalIMT | epa | chr10:45868356:D | 0.151 | -0.051 | 0.022 | 0.024 | -0.026 | 0.026 | 0.314 | -0.012 | 0.038 | 0.751 |
| InternalIMT | epa | chr10:45871158:I | 0.16 | -0.047 | 0.023 | 0.039 | -0.029 | 0.025 | 0.236 | -0.024 | 0.038 | 0.529 |
| InternalIMT | epa | chr10:45871637:D | 0.061 | -0.052 | 0.021 | 0.014 | -0.134 | 0.045 | 2.90E-03 | 1.80E-03 | 0.057 | 0.975 |
| InternalIMT | epa | chr10:45871638:I | 0.115 | -0.048 | 0.022 | 0.029 | -0.051 | 0.031 | 0.095 | -0.025 | 0.045 | 0.585 |
| InternalIMT | epa | chr10:45873629:I | 0.159 | -0.046 | 0.022 | 0.041 | -0.03 | 0.025 | 0.229 | -0.026 | 0.038 | 0.486 |
| InternalIMT | epa | chr10:45873701:D | 0.003 | . | . | . | . | . | . | . | . | . |
| InternalIMT | epa | chr10:45874997:I | 0.03 | -0.057 | 0.02 | 5.50E-03 | 0.023 | 0.054 | 0.67 | 0.031 | 0.07 | 0.661 |
| InternalIMT | epa | chr10:45882030:I | 0.021 | -0.052 | 0.02 | 0.011 | -0.062 | 0.099 | 0.534 | -0.067 | 0.143 | 0.638 |
| InternalIMT | epa | chr10:45883774:D | 0.154 | -0.045 | 0.022 | 0.036 | -0.016 | 0.023 | 0.487 | -0.03 | 0.037 | 0.42 |
| InternalIMT | epa | chr10:45885169:D | 0.035 | -0.052 | 0.02 | 0.011 | -0.011 | 0.053 | 0.836 | -0.059 | 0.086 | 0.489 |
| InternalIMT | epa | chr10:45886451:D | 0.588 | -0.042 | 0.041 | 0.304 | 0.037 | 0.021 | 0.072 | -0.011 | 0.032 | 0.724 |
| InternalIMT | epa | chr10:45891800:I | 0.592 | -0.029 | 0.037 | 0.433 | 0.013 | 0.018 | 0.478 | -0.022 | 0.027 | 0.414 |
| InternalIMT | epa | chr10:45904771:I | 0.573 | -0.034 | 0.037 | 0.356 | 0.016 | 0.018 | 0.376 | -0.018 | 0.028 | 0.509 |
| InternalIMT | epa | chr10:45904959:I | 0.103 | -0.054 | 0.021 | 0.011 | 0.017 | 0.037 | 0.643 | -1.80E-03 | 0.061 | 0.976 |
| InternalIMT | epa | chr10:45911028:I | 0.034 | -0.053 | 0.021 | 0.012 | -0.076 | 0.054 | 0.157 | -0.024 | 0.088 | 0.783 |
| InternalIMT | epa | chr10:45911031:I | 0.363 | -0.051 | 0.031 | 0.095 | 0.025 | 0.021 | 0.226 | -3.60E-03 | 0.034 | 0.915 |
| InternalIMT | epa | chr10:45912154:D | 0.145 | -0.043 | 0.021 | 0.043 | -0.02 | 0.024 | 0.399 | -0.038 | 0.038 | 0.317 |
| InternalIMT | epa | chr10:45913885:I | 0.001 | . | . | . | . | . | . | . | . | . |
| InternalIMT | epa | chr10:45914753:D | 0.243 | -0.031 | 0.025 | 0.209 | -6.20E-03 | 0.022 | 0.776 | -0.049 | 0.033 | 0.134 |
| InternalIMT | epa | chr10:45919621 | 0.003 | . | . | . | . | . | . | . | . | . |
| InternalIMT | epa | chr10:45921739:I | 0.004 | . | . | . | . | . | . | . | . | . |
| InternalIMT | epa | chr10:45923527:I | 0.052 | -0.048 | 0.021 | 0.024 | 0.078 | 0.054 | 0.145 | -0.072 | 0.09 | 0.427 |
| InternalIMT | epa | chr10:45927001:I | 0.014 | -0.056 | 0.02 | 6.20E-03 | -0.137 | 0.119 | 0.248 | 0.022 | 0.145 | 0.877 |
| InternalIMT | epa | chr10:45930354:I | 0.001 | . | . | . | . | . | . | . | . | . |
| InternalIMT | epa | chr10:45932864:I | 0.027 | -0.047 | 0.021 | 0.024 | 0.043 | 0.072 | 0.548 | -0.165 | 0.138 | 0.231 |

| InternalIMT | epa | chr10:45936163:I | 0.026 | -0.054 | 0.02 | 7.00E-03 | -0.087 | 0.051 | 0.091 | 0.012 | 0.068 | 0.863 |
| --- | --- | --- | --- | --- | --- | --- | --- | --- | --- | --- | --- | --- |
| InternalIMT | epa | chr10:45938095:D | 0.164 | -0.042 | 0.022 | 0.055 | -0.031 | 0.023 | 0.184 | -0.035 | 0.037 | 0.335 |
| InternalIMT | epa | chr10:45938886 | 0 | . | . | . | . | . | . | . | . | . |
| InternalIMT | epa | chr10:45939320 | 0.001 | . | . | . | . | . | . | . | . | . |
| InternalIMT | epa | chr10:45939659 | 0.001 | . | . | . | . | . | . | . | . | . |
| InternalIMT | epa | chr10:45941150:D | 0.001 | . | . | . | . | . | . | . | . | . |
| InternalIMT | epa | chr10:45947128:D | 0.064 | -0.056 | 0.021 | 7.90E-03 | 0.04 | 0.039 | 0.305 | 0.016 | 0.055 | 0.769 |
| InternalIMT | epa | chr10:45950990:I | 0.044 | -0.043 | 0.021 | 0.038 | -0.016 | 0.053 | 0.768 | -0.138 | 0.083 | 0.097 |
| InternalIMT | epa | chr10:45951854:I | 0.048 | -0.049 | 0.021 | 0.019 | -0.012 | 0.041 | 0.764 | -0.064 | 0.065 | 0.327 |
| InternalIMT | epa | chr10:45952670:I | 0.061 | -0.053 | 0.021 | 0.014 | -0.071 | 0.033 | 0.031 | -0.014 | 0.045 | 0.761 |
| InternalIMT | epa | chr10:45956741 | 0.001 | . | . | . | . | . | . | . | . | . |
| InternalIMT | epa | chr10:45957247:I | 0.233 | -0.042 | 0.023 | 0.074 | -0.031 | 0.02 | 0.122 | -0.025 | 0.029 | 0.378 |
| InternalIMT | epa | chr10:45958082:I | 0.232 | -0.044 | 0.023 | 0.061 | -0.028 | 0.02 | 0.167 | -0.021 | 0.029 | 0.484 |
| InternalIMT | epa | chr10:45905041:D | . | . | . | . | . | . | . | . | . | . |
| InternalIMT | epa | chr10:45938871 | . | . | . | . | . | . | . | . | . | . |
| InternalIMT | epa | chr10:45939160 | . | . | . | . | . | . | . | . | . | . |
| InternalIMT | epa | chr10:45869910 | . | . | . | . | . | . | . | . | . | . |
| InternalIMT | epa | chr10:45876214:D | . | . | . | . | . | . | . | . | . | . |
| InternalIMT | epa | chr10:45880237:I | . | . | . | . | . | . | . | . | . | . |
| InternalIMT | epa | chr10:45884371:I | . | . | . | . | . | . | . | . | . | . |
| InternalIMT | epa | chr10:45891535:D | . | . | . | . | . | . | . | . | . | . |
| InternalIMT | epa | chr10:45893516:D | . | . | . | . | . | . | . | . | . | . |
| InternalIMT | epa | chr10:45893782:D | . | . | . | . | . | . | . | . | . | . |
| InternalIMT | epa | chr10:45895254:I | . | . | . | . | . | . | . | . | . | . |
| InternalIMT | epa | chr10:45895848:D | . | . | . | . | . | . | . | . | . | . |
| InternalIMT | epa | chr10:45896316:D | . | . | . | . | . | . | . | . | . | . |
| InternalIMT | epa | chr10:45896317:D | . | . | . | . | . | . | . | . | . | . |
| InternalIMT | epa | chr10:45902683:I | . | . | . | . | . | . | . | . | . | . |
| InternalIMT | epa | chr10:45905003:D | . | . | . | . | . | . | . | . | . | . |
| InternalIMT | epa | chr10:45927476:D | . | . | . | . | . | . | . | . | . | . |
| InternalIMT | epa | chr10:45935882 | . | . | . | . | . | . | . | . | . | . |
| InternalIMT | epa | chr10:45936100 | . | . | . | . | . | . | . | . | . | . |
| InternalIMT | epa | chr10:45939553 | . | . | . | . | . | . | . | . | . | . |
| InternalIMT | epa | chr10:45939631 | . | . | . | . | . | . | . | . | . | . |
| InternalIMT | epa | chr10:45944193:D | . | . | . | . | . | . | . | . | . | . |
| InternalIMT | epa | chr10:45954030 | . | . | . | . | . | . | . | . | . | . |

Supplemental table S18.

| phen | covar(fa) | rs_id | maf | beta_FA | se_FA | p_FA | beta_SNP | se_SNP | p_SNP | beta_inter | se_inter | p_inter |
| --- | --- | --- | --- | --- | --- | --- | --- | --- | --- | --- | --- | --- |
| InternalIMT | epa | rs76035182 | 0.068 | -0.013 | 0.032 | 0.687 | 0.022 | 0.046 | 0.63 | -0.113 | 0.071 | 0.113 |
| InternalIMT | epa | rs12359547 | 0.052 | -0.038 | 0.03 | 0.2 | 0.078 | 0.068 | 0.249 | 0.08 | 0.083 | 0.338 |
| InternalIMT | epa | rs74595484 | 0.12 | -0.029 | 0.033 | 0.385 | 0.033 | 0.04 | 0.408 | -9.10E-03 | 0.052 | 0.863 |
| InternalIMT | epa | rs1864415 | 0.749 | 4.40E-03 | 0.071 | 0.951 | 0.03 | 0.024 | 0.222 | -0.024 | 0.041 | 0.565 |
| InternalIMT | epa | rs72788101 | 0.064 | -0.051 | 0.03 | 0.086 | 0.149 | 0.065 | 0.022 | 0.176 | 0.084 | 0.037 |
| InternalIMT | epa | rs11239481 | 0.081 | -0.039 | 0.031 | 0.208 | 0.012 | 0.038 | 0.745 | 0.042 | 0.063 | 0.508 |
| InternalIMT | epa | rs72788102 | 0.064 | -0.051 | 0.03 | 0.086 | 0.149 | 0.065 | 0.022 | 0.176 | 0.084 | 0.037 |
| InternalIMT | epa | rs72789705 | 0.064 | -0.051 | 0.03 | 0.086 | 0.149 | 0.065 | 0.022 | 0.176 | 0.084 | 0.037 |
| InternalIMT | epa | rs141529858 | 0.002 | . | . | . | . | . | . | . | . | . |
| InternalIMT | epa | rs145376292 | 0 | . | . | . | . | . | . | . | . | . |
| InternalIMT | epa | rs190418301 | 0 | . | . | . | . | . | . | . | . | . |
| InternalIMT | epa | rs11239482 | 0.064 | -0.051 | 0.03 | 0.086 | 0.149 | 0.065 | 0.022 | 0.176 | 0.084 | 0.037 |
| InternalIMT | epa | rs61855872 | 0.004 | . | . | . | . | . | . | . | . | . |
| InternalIMT | epa | rs148991144 | 0.003 | . | . | . | . | . | . | . | . | . |
| InternalIMT | epa | rs191932221 | 0.002 | . | . | . | . | . | . | . | . | . |
| InternalIMT | epa | rs55978138 | 0.179 | -0.029 | 0.036 | 0.419 | 0.048 | 0.03 | 0.102 | -5.90E-03 | 0.044 | 0.894 |
| InternalIMT | epa | rs73281167 | 0.045 | -0.033 | 0.029 | 0.259 | -0.027 | 0.067 | 0.683 | 0.013 | 0.107 | 0.904 |
| InternalIMT | epa | rs12768010 | 0.092 | -0.011 | 0.033 | 0.733 | 4.90E-03 | 0.035 | 0.888 | -0.09 | 0.052 | 0.082 |
| InternalIMT | epa | rs11239484 | 0.749 | 4.40E-03 | 0.071 | 0.951 | 0.03 | 0.024 | 0.222 | -0.024 | 0.041 | 0.565 |
| InternalIMT | epa | rs35906360 | 0.18 | -0.033 | 0.036 | 0.356 | 0.052 | 0.03 | 0.076 | 5.40E-03 | 0.044 | 0.903 |
| InternalIMT | epa | rs186250512 | 0.001 | . | . | . | . | . | . | . | . | . |
| InternalIMT | epa | rs150810743 | 0.007 | . | . | . | . | . | . | . | . | . |
| InternalIMT | epa | rs55965812 | 0.161 | -0.019 | 0.036 | 0.599 | 0.026 | 0.03 | 0.39 | -0.033 | 0.045 | 0.471 |
| InternalIMT | epa | rs56180358 | 0.181 | -0.033 | 0.036 | 0.355 | 0.052 | 0.03 | 0.075 | 5.50E-03 | 0.044 | 0.901 |
| InternalIMT | epa | rs55864145 | 0.092 | -0.011 | 0.033 | 0.731 | 5.10E-03 | 0.035 | 0.884 | -0.089 | 0.052 | 0.083 |
| InternalIMT | epa | rs55986122 | 0.181 | -0.033 | 0.036 | 0.355 | 0.053 | 0.03 | 0.075 | 5.60E-03 | 0.044 | 0.9 |
| InternalIMT | epa | rs7477350 | 0.749 | 4.30E-03 | 0.071 | 0.951 | 0.03 | 0.024 | 0.221 | -0.023 | 0.041 | 0.566 |
| InternalIMT | epa | rs2099170 | 0.18 | -0.034 | 0.036 | 0.352 | 0.053 | 0.03 | 0.072 | 6.10E-03 | 0.044 | 0.891 |
| InternalIMT | epa | rs2082108 | 0.181 | -0.033 | 0.036 | 0.353 | 0.053 | 0.03 | 0.074 | 5.80E-03 | 0.044 | 0.896 |
| InternalIMT | epa | rs55902404 | 0.02 | -0.045 | 0.028 | 0.118 | 0.298 | 0.112 | 8.10E-03 | 0.374 | 0.162 | 0.021 |
| InternalIMT | epa | rs58402662 | 0.02 | -0.045 | 0.028 | 0.118 | 0.298 | 0.112 | 8.00E-03 | 0.375 | 0.162 | 0.021 |
| InternalIMT | epa | rs11511618 | 0.084 | -0.039 | 0.031 | 0.208 | 4.30E-03 | 0.038 | 0.911 | 0.043 | 0.063 | 0.499 |
| InternalIMT | epa | rs1816492 | 0.747 | 4.10E-03 | 0.071 | 0.954 | 0.031 | 0.024 | 0.197 | -0.023 | 0.041 | 0.569 |
| InternalIMT | epa | rs146611093 | 0.002 | . | . | . | . | . | . | . | . | . |
| InternalIMT | epa | rs186897689 | 0.006 | . | . | . | . | . | . | . | . | . |
| InternalIMT | epa | rs10793616 | 0.749 | 4.30E-03 | 0.071 | 0.952 | 0.03 | 0.024 | 0.221 | -0.023 | 0.041 | 0.566 |
| InternalIMT | epa | rs116700479 | 0.052 | -9.30E-03 | 0.031 | 0.769 | 0.018 | 0.052 | 0.724 | -0.177 | 0.072 | 0.014 |
| InternalIMT | epa | rs192535936 | 0.004 | . | . | . | . | . | . | . | . | . |
| InternalIMT | epa | rs113363297 | 0.723 | -0.01 | 0.068 | 0.88 | 0.031 | 0.025 | 0.213 | -0.014 | 0.04 | 0.721 |
| InternalIMT | epa | rs12354994 | 0.172 | -0.034 | 0.036 | 0.338 | 0.062 | 0.031 | 0.047 | 8.30E-03 | 0.047 | 0.858 |
| InternalIMT | epa | rs148068329 | 0.025 | -0.033 | 0.029 | 0.252 | 0.105 | 0.078 | 0.178 | 0.072 | 0.118 | 0.542 |
| InternalIMT | epa | rs12356109 | 0.198 | -0.029 | 0.037 | 0.438 | 0.042 | 0.028 | 0.133 | -5.80E-03 | 0.042 | 0.891 |
| InternalIMT | epa | rs55908141 | 0.02 | -0.044 | 0.028 | 0.119 | 0.297 | 0.112 | 8.20E-03 | 0.373 | 0.162 | 0.021 |
| InternalIMT | epa | rs12354712 | 0.161 | -0.019 | 0.036 | 0.595 | 0.027 | 0.03 | 0.381 | -0.032 | 0.045 | 0.478 |
| InternalIMT | epa | rs147282684 | 0.002 | . | . | . | . | . | . | . | . | . |
| InternalIMT | epa | rs12356140 | 0.191 | -0.039 | 0.036 | 0.281 | 0.058 | 0.03 | 0.052 | 0.019 | 0.044 | 0.664 |
| InternalIMT | epa | rs185476187 | 0.004 | . | . | . | . | . | . | . | . | . |
| InternalIMT | epa | rs145720057 | 0.012 | -0.031 | 0.029 | 0.275 | 0.067 | 0.147 | 0.649 | 8.10E-03 | 0.177 | 0.963 |
| InternalIMT | epa | rs12354742 | 0.181 | -0.034 | 0.036 | 0.352 | 0.053 | 0.03 | 0.073 | 6.00E-03 | 0.044 | 0.891 |
| InternalIMT | epa | rs180913842 | 0.02 | -0.044 | 0.028 | 0.119 | 0.296 | 0.112 | 8.20E-03 | 0.372 | 0.162 | 0.021 |
| InternalIMT | epa | rs142976914 | 0.002 | . | . | . | . | . | . | . | . | . |
| InternalIMT | epa | rs11511619 | 0.201 | -0.03 | 0.038 | 0.426 | 0.04 | 0.028 | 0.155 | -3.60E-03 | 0.042 | 0.933 |
| InternalIMT | epa | rs146702870 | 0.003 | . | . | . | . | . | . | . | . | . |
| InternalIMT | epa | rs150287496 | 0.001 | . | . | . | . | . | . | . | . | . |
| InternalIMT | epa | rs190917973 | 0.002 | . | . | . | . | . | . | . | . | . |
| InternalIMT | epa | rs11517281 | 0.181 | -0.034 | 0.036 | 0.351 | 0.053 | 0.029 | 0.073 | 6.10E-03 | 0.044 | 0.89 |
| InternalIMT | epa | rs12359299 | 0.181 | -0.034 | 0.036 | 0.351 | 0.053 | 0.029 | 0.073 | 6.20E-03 | 0.044 | 0.889 |
| InternalIMT | epa | rs182307932 | 0.071 | -0.031 | 0.027 | 0.262 | -0.057 | 0.058 | 0.32 | -7.80E-03 | 0.104 | 0.94 |
| InternalIMT | epa | rs61855873 | 0.011 | -0.035 | 0.029 | 0.233 | -0.027 | 0.139 | 0.844 | 0.139 | 0.142 | 0.327 |
| InternalIMT | epa | rs12359302 | 0.18 | -0.034 | 0.036 | 0.341 | 0.063 | 0.03 | 0.035 | 7.70E-03 | 0.045 | 0.862 |
| InternalIMT | epa | rs143208140 | 0.028 | -0.034 | 0.029 | 0.241 | 0.096 | 0.073 | 0.187 | 0.081 | 0.107 | 0.451 |
| InternalIMT | epa | rs9988764 | 0.712 | -0.015 | 0.065 | 0.811 | 0.032 | 0.024 | 0.195 | -0.01 | 0.04 | 0.795 |
| InternalIMT | epa | rs192875767 | 0.002 | . | . | . | . | . | . | . | . | . |
| InternalIMT | epa | rs147268846 | 0.005 | . | . | . | . | . | . | . | . | . |
| InternalIMT | epa | rs11239485 | 0.109 | -0.036 | 0.034 | 0.292 | 0.046 | 0.042 | 0.271 | 0.017 | 0.059 | 0.773 |
| InternalIMT | epa | rs151317885 | 0.24 | -0.032 | 0.035 | 0.36 | -0.014 | 0.028 | 0.617 | 1.20E-04 | 0.042 | 0.998 |
| InternalIMT | epa | rs11499085 | 0.322 | -0.019 | 0.036 | 0.61 | 5.80E-04 | 0.027 | 0.983 | -0.02 | 0.041 | 0.622 |
| InternalIMT | epa | rs188130606 | 0.086 | -0.022 | 0.032 | 0.488 | 0.026 | 0.048 | 0.59 | -0.05 | 0.065 | 0.441 |
| InternalIMT | epa | rs11511620 | 0.161 | -0.019 | 0.036 | 0.592 | 0.027 | 0.03 | 0.378 | -0.032 | 0.045 | 0.482 |
| InternalIMT | epa | rs181498390 | 0.057 | -0.031 | 0.03 | 0.303 | -0.051 | 0.055 | 0.353 | -9.10E-03 | 0.086 | 0.916 |
| InternalIMT | epa | rs186280701 | 0.02 | -0.044 | 0.028 | 0.119 | 0.294 | 0.111 | 8.40E-03 | 0.37 | 0.161 | 0.022 |

| InternalIMT | epa | rs150059185 | 0.034 | -0.024 | 0.029 | 0.407 | -0.059 | 0.083 | 0.477 | -0.143 | 0.156 | 0.358 |
| --- | --- | --- | --- | --- | --- | --- | --- | --- | --- | --- | --- | --- |
| InternalIMT | epa | rs183338707 | 0.001 | . | . | . | . | . | . | . | . | . |
| InternalIMT | epa | rs11499038 | 0.306 | -0.025 | 0.035 | 0.475 | 6.60E-03 | 0.028 | 0.814 | -0.01 | 0.043 | 0.812 |
| InternalIMT | epa | rs190699595 | 0.004 | . | . | . | . | . | . | . | . | . |
| InternalIMT | epa | rs11523408 | 0.175 | -0.032 | 0.036 | 0.366 | 0.052 | 0.03 | 0.084 | 2.90E-03 | 0.045 | 0.948 |
| InternalIMT | epa | rs188049317 | 0.007 | . | . | . | . | . | . | . | . | . |
| InternalIMT | epa | rs144948634 | 0.214 | -0.029 | 0.034 | 0.393 | -0.036 | 0.029 | 0.214 | -9.20E-03 | 0.044 | 0.833 |
| InternalIMT | epa | rs143833576 | . | . | . | . | . | . | . | . | . | . |
| InternalIMT | epa | rs151239879 | 0.175 | -0.031 | 0.036 | 0.381 | 0.052 | 0.03 | 0.085 | 7.50E-04 | 0.045 | 0.987 |
| InternalIMT | epa | rs7073743 | 0.212 | -0.027 | 0.034 | 0.42 | -0.033 | 0.028 | 0.236 | -0.014 | 0.043 | 0.748 |
| InternalIMT | epa | rs142563349 | 0.092 | -0.022 | 0.032 | 0.491 | 0.014 | 0.048 | 0.765 | -0.05 | 0.066 | 0.45 |
| InternalIMT | epa | rs187246403 | 0.02 | -0.044 | 0.028 | 0.119 | 0.293 | 0.111 | 8.50E-03 | 0.368 | 0.161 | 0.022 |
| InternalIMT | epa | rs150423942 | 0.178 | -0.032 | 0.036 | 0.369 | 0.054 | 0.03 | 0.069 | 3.00E-03 | 0.045 | 0.947 |
| InternalIMT | epa | rs184706102 | . | . | . | . | . | . | . | . | . | . |
| InternalIMT | epa | rs189626288 | 0.007 | . | . | . | . | . | . | . | . | . |
| InternalIMT | epa | rs140749834 | 0.142 | -0.023 | 0.029 | 0.424 | -0.037 | 0.037 | 0.317 | -0.029 | 0.068 | 0.665 |
| InternalIMT | epa | rs7078822 | 0.136 | -0.023 | 0.036 | 0.523 | 0.014 | 0.035 | 0.698 | -0.026 | 0.051 | 0.617 |
| InternalIMT | epa | rs186227617 | 0.177 | -0.034 | 0.036 | 0.346 | 0.05 | 0.03 | 0.102 | 7.30E-03 | 0.046 | 0.874 |
| InternalIMT | epa | rs188984368 | 0.018 | -0.025 | 0.029 | 0.387 | -0.04 | 0.087 | 0.648 | -0.184 | 0.158 | 0.245 |
| InternalIMT | epa | rs182078109 | 0.04 | -0.04 | 0.031 | 0.196 | 0.099 | 0.079 | 0.212 | 0.082 | 0.11 | 0.458 |
| InternalIMT | epa | rs141456274 | 0.001 | . | . | . | . | . | . | . | . | . |
| InternalIMT | epa | rs146976512 | 0.181 | -0.034 | 0.036 | 0.34 | 0.054 | 0.03 | 0.071 | 8.20E-03 | 0.045 | 0.854 |
| InternalIMT | epa | rs138036816 | 0.305 | -0.025 | 0.035 | 0.476 | 6.60E-03 | 0.028 | 0.815 | -0.01 | 0.043 | 0.809 |
| InternalIMT | epa | rs114879777 | 0.051 | -9.40E-03 | 0.032 | 0.767 | 0.011 | 0.052 | 0.83 | -0.178 | 0.074 | 0.017 |
| InternalIMT | epa | rs149366475 | 0.307 | -0.025 | 0.035 | 0.474 | 3.30E-03 | 0.028 | 0.907 | -0.01 | 0.043 | 0.817 |
| InternalIMT | epa | rs182463043 | 0.007 | . | . | . | . | . | . | . | . | . |
| InternalIMT | epa | rs144754632 | 0.025 | -0.033 | 0.029 | 0.252 | 0.105 | 0.078 | 0.178 | 0.074 | 0.118 | 0.533 |
| InternalIMT | epa | rs55717607 | 0.181 | -0.034 | 0.036 | 0.345 | 0.053 | 0.029 | 0.069 | 7.00E-03 | 0.044 | 0.873 |
| InternalIMT | epa | rs187537973 | 0.001 | . | . | . | . | . | . | . | . | . |
| InternalIMT | epa | rs148085971 | 0.007 | . | . | . | . | . | . | . | . | . |
| InternalIMT | epa | rs11239491 | 0.181 | -0.034 | 0.036 | 0.344 | 0.054 | 0.029 | 0.069 | 7.10E-03 | 0.044 | 0.871 |
| InternalIMT | epa | rs184412184 | 0.002 | . | . | . | . | . | . | . | . | . |
| InternalIMT | epa | rs4948998 | 0.798 | 4.50E-03 | 0.076 | 0.953 | 0.029 | 0.025 | 0.249 | -0.022 | 0.042 | 0.605 |
| InternalIMT | epa | rs7076696 | 0.798 | 5.50E-03 | 0.077 | 0.943 | 0.029 | 0.025 | 0.242 | -0.022 | 0.042 | 0.596 |
| InternalIMT | epa | rs11239492 | 0.198 | -0.029 | 0.037 | 0.431 | 0.043 | 0.028 | 0.125 | -4.90E-03 | 0.042 | 0.908 |
| InternalIMT | epa | rs79462470 | 0.02 | -0.044 | 0.028 | 0.12 | 0.289 | 0.11 | 8.90E-03 | 0.364 | 0.16 | 0.023 |
| InternalIMT | epa | rs187804654 | . | . | . | . | . | . | . | . | . | . |
| InternalIMT | epa | rs7093687 | 0.161 | -0.019 | 0.036 | 0.59 | 0.027 | 0.03 | 0.373 | -0.032 | 0.045 | 0.477 |
| InternalIMT | epa | rs7081004 | 0.162 | -0.02 | 0.036 | 0.583 | 0.027 | 0.03 | 0.364 | -0.03 | 0.045 | 0.499 |
| InternalIMT | epa | rs7084793 | 0.181 | -0.034 | 0.036 | 0.342 | 0.054 | 0.029 | 0.068 | 7.50E-03 | 0.044 | 0.864 |
| InternalIMT | epa | rs61556080 | 0.02 | -0.044 | 0.028 | 0.12 | 0.289 | 0.11 | 9.00E-03 | 0.363 | 0.16 | 0.024 |
| InternalIMT | epa | rs115552064 | 0.019 | -0.039 | 0.028 | 0.167 | 0.202 | 0.099 | 0.042 | 0.16 | 0.165 | 0.332 |
| InternalIMT | epa | rs7921503 | 0.18 | -0.034 | 0.036 | 0.341 | 0.054 | 0.03 | 0.069 | 7.90E-03 | 0.044 | 0.858 |
| InternalIMT | epa | rs7909740 | 0.181 | -0.034 | 0.036 | 0.34 | 0.054 | 0.029 | 0.068 | 7.90E-03 | 0.044 | 0.857 |
| InternalIMT | epa | rs7909768 | 0.181 | -0.034 | 0.036 | 0.339 | 0.054 | 0.029 | 0.068 | 7.90E-03 | 0.044 | 0.857 |
| InternalIMT | epa | rs144168004 | 0.001 | . | . | . | . | . | . | . | . | . |
| InternalIMT | epa | rs75850448 | 0.02 | -0.044 | 0.028 | 0.12 | 0.289 | 0.11 | 8.90E-03 | 0.363 | 0.16 | 0.024 |
| InternalIMT | epa | rs147777574 | 0.016 | -0.019 | 0.028 | 0.51 | -0.09 | 0.076 | 0.237 | -0.476 | 0.176 | 7.00E-03 |
| InternalIMT | epa | rs7913488 | 0.181 | -0.034 | 0.036 | 0.339 | 0.054 | 0.029 | 0.068 | 8.00E-03 | 0.044 | 0.855 |
| InternalIMT | epa | rs74390874 | 0.019 | -0.03 | 0.029 | 0.303 | -0.099 | 0.136 | 0.467 | -0.073 | 0.236 | 0.756 |
| InternalIMT | epa | rs141391142 | 0.002 | . | . | . | . | . | . | . | . | . |
| InternalIMT | epa | rs115294747 | 0.017 | -0.027 | 0.029 | 0.346 | -0.056 | 0.068 | 0.41 | -0.09 | 0.101 | 0.371 |
| InternalIMT | epa | rs148031781 | 0.001 | . | . | . | . | . | . | . | . | . |
| InternalIMT | epa | rs181340128 | 0.007 | . | . | . | . | . | . | . | . | . |
| InternalIMT | epa | rs189930007 | 0 | . | . | . | . | . | . | . | . | . |
| InternalIMT | epa | rs12242128 | 0.304 | -0.025 | 0.035 | 0.481 | 5.20E-03 | 0.028 | 0.853 | -0.011 | 0.043 | 0.801 |
| InternalIMT | epa | rs78421750 | 0.016 | -0.026 | 0.029 | 0.379 | -0.148 | 0.085 | 0.083 | -0.183 | 0.128 | 0.152 |
| InternalIMT | epa | rs114370747 | 0.007 | . | . | . | . | . | . | . | . | . |
| InternalIMT | epa | rs12264166 | 0.304 | -0.025 | 0.035 | 0.481 | 5.20E-03 | 0.028 | 0.852 | -0.011 | 0.043 | 0.8 |
| InternalIMT | epa | rs182964822 | 0 | . | . | . | . | . | . | . | . | . |
| InternalIMT | epa | rs67491417 | 0.178 | -0.036 | 0.036 | 0.316 | 0.063 | 0.03 | 0.035 | 0.012 | 0.045 | 0.791 |
| InternalIMT | epa | rs72789720 | 0.006 | . | . | . | . | . | . | . | . | . |
| InternalIMT | epa | rs6413416 | 0.182 | -0.035 | 0.036 | 0.332 | 0.053 | 0.029 | 0.069 | 9.10E-03 | 0.044 | 0.834 |
| InternalIMT | epa | rs4986832 | 0.182 | -0.035 | 0.036 | 0.333 | 0.053 | 0.029 | 0.068 | 8.90E-03 | 0.044 | 0.838 |
| InternalIMT | epa | rs144866316 | . | . | . | . | . | . | . | . | . | . |
| InternalIMT | epa | rs187769540 | 0.001 | . | . | . | . | . | . | . | . | . |
| InternalIMT | epa | rs6593482 | 0.182 | -0.035 | 0.036 | 0.331 | 0.053 | 0.029 | 0.069 | 9.90E-03 | 0.044 | 0.82 |
| InternalIMT | epa | rs7913948 | 0.183 | -0.035 | 0.036 | 0.335 | 0.051 | 0.029 | 0.079 | 8.60E-03 | 0.044 | 0.844 |
| InternalIMT | epa | rs77185151 | 0.025 | -0.033 | 0.029 | 0.251 | 0.105 | 0.078 | 0.176 | 0.075 | 0.118 | 0.528 |
| InternalIMT | epa | rs12762303 | 0.182 | -0.035 | 0.036 | 0.333 | 0.053 | 0.029 | 0.069 | 8.90E-03 | 0.044 | 0.838 |
| InternalIMT | epa | rs73283136 | 0.008 | . | . | . | . | . | . | . | . | . |
| InternalIMT | epa | rs187180218 | 0.007 | . | . | . | . | . | . | . | . | . |
| InternalIMT | epa | rs78137087 | 0.182 | -0.035 | 0.036 | 0.332 | 0.054 | 0.029 | 0.068 | 9.10E-03 | 0.044 | 0.836 |

| InternalIMT | epa | rs72561472 | 0.181 | -0.041 | 0.037 | 0.268 | 0.05 | 0.03 | 0.1 | 0.022 | 0.045 | 0.634 |
| --- | --- | --- | --- | --- | --- | --- | --- | --- | --- | --- | --- | --- |
| InternalIMT | epa | rs4987106 | 0.18 | -0.036 | 0.036 | 0.311 | 0.052 | 0.03 | 0.079 | 0.013 | 0.044 | 0.776 |
| InternalIMT | epa | rs4987105 | 0.159 | -0.021 | 0.036 | 0.565 | 0.028 | 0.031 | 0.368 | -0.028 | 0.045 | 0.532 |
| InternalIMT | epa | rs111916157 | 0.187 | -0.037 | 0.036 | 0.306 | 0.051 | 0.029 | 0.081 | 0.013 | 0.044 | 0.763 |
| InternalIMT | epa | rs111528152 | 0.184 | -0.04 | 0.036 | 0.27 | 0.05 | 0.03 | 0.097 | 0.021 | 0.046 | 0.646 |
| InternalIMT | epa | rs12763738 | 0.178 | -0.036 | 0.036 | 0.315 | 0.057 | 0.03 | 0.058 | 0.012 | 0.045 | 0.781 |
| InternalIMT | epa | rs191675886 | 0.011 | -0.03 | 0.029 | 0.298 | 0.044 | 0.153 | 0.774 | -0.065 | 0.232 | 0.78 |
| InternalIMT | epa | rs66489870 | 0.182 | -0.035 | 0.036 | 0.333 | 0.053 | 0.029 | 0.068 | 8.90E-03 | 0.044 | 0.838 |
| InternalIMT | epa | rs71494775 | 0.182 | -0.035 | 0.036 | 0.333 | 0.053 | 0.029 | 0.068 | 8.90E-03 | 0.044 | 0.838 |
| InternalIMT | epa | rs61855875 | 0.01 | -0.034 | 0.029 | 0.235 | -0.061 | 0.148 | 0.681 | 0.158 | 0.167 | 0.345 |
| InternalIMT | epa | rs116356837 | 0.019 | -0.04 | 0.028 | 0.163 | 0.2 | 0.099 | 0.044 | 0.17 | 0.166 | 0.304 |
| InternalIMT | epa | rs1864414 | 0.179 | -0.034 | 0.036 | 0.342 | 0.055 | 0.03 | 0.061 | 7.50E-03 | 0.044 | 0.865 |
| InternalIMT | epa | rs1864413 | 0.182 | -0.035 | 0.036 | 0.331 | 0.054 | 0.029 | 0.066 | 9.40E-03 | 0.044 | 0.83 |
| InternalIMT | epa | rs139497212 | 0 | . | . | . | . | . | . | . | . | . |
| InternalIMT | epa | rs892690 | 0.104 | -0.041 | 0.031 | 0.189 | -9.20E-03 | 0.041 | 0.823 | 0.053 | 0.054 | 0.33 |
| InternalIMT | epa | rs1864412 | 0.182 | -0.035 | 0.036 | 0.333 | 0.054 | 0.029 | 0.068 | 9.00E-03 | 0.044 | 0.838 |
| InternalIMT | epa | rs77827771 | 0.087 | -0.022 | 0.032 | 0.485 | 0.027 | 0.049 | 0.585 | -0.05 | 0.067 | 0.457 |
| InternalIMT | epa | rs145294963 | . | . | . | . | . | . | . | . | . | . |
| InternalIMT | epa | rs149081327 | 0.003 | . | . | . | . | . | . | . | . | . |
| InternalIMT | epa | rs67008983 | 0.182 | -0.035 | 0.036 | 0.334 | 0.054 | 0.029 | 0.066 | 9.00E-03 | 0.044 | 0.837 |
| InternalIMT | epa | rs56150414 | 0.182 | -0.035 | 0.036 | 0.33 | 0.053 | 0.029 | 0.07 | 9.70E-03 | 0.044 | 0.825 |
| InternalIMT | epa | rs55787481 | 0.182 | -0.033 | 0.036 | 0.356 | 0.053 | 0.03 | 0.073 | 5.50E-03 | 0.044 | 0.902 |
| InternalIMT | epa | rs141270101 | 0.001 | . | . | . | . | . | . | . | . | . |
| InternalIMT | epa | rs114980075 | 0.022 | -0.032 | 0.029 | 0.258 | 0.038 | 0.082 | 0.641 | 8.00E-03 | 0.127 | 0.95 |
| InternalIMT | epa | rs12783095 | 0.181 | -0.035 | 0.036 | 0.331 | 0.056 | 0.03 | 0.059 | 9.60E-03 | 0.044 | 0.828 |
| InternalIMT | epa | rs182342688 | 0.001 | . | . | . | . | . | . | . | . | . |
| InternalIMT | epa | rs17157728 | 0.181 | -0.035 | 0.036 | 0.331 | 0.056 | 0.03 | 0.059 | 9.60E-03 | 0.044 | 0.828 |
| InternalIMT | epa | rs114835563 | 0.086 | -0.022 | 0.032 | 0.488 | 0.028 | 0.049 | 0.57 | -0.051 | 0.067 | 0.446 |
| InternalIMT | epa | rs3824612 | 0.104 | -0.041 | 0.031 | 0.191 | -0.01 | 0.041 | 0.804 | 0.052 | 0.054 | 0.34 |
| InternalIMT | epa | rs17157733 | 0.181 | -0.035 | 0.036 | 0.331 | 0.056 | 0.03 | 0.057 | 9.60E-03 | 0.044 | 0.829 |
| InternalIMT | epa | rs74128478 | 0.019 | -0.044 | 0.028 | 0.12 | 0.291 | 0.112 | 9.50E-03 | 0.366 | 0.162 | 0.024 |
| InternalIMT | epa | rs184973589 | 0.001 | . | . | . | . | . | . | . | . | . |
| InternalIMT | epa | rs150479461 | 0 | . | . | . | . | . | . | . | . | . |
| InternalIMT | epa | rs138310407 | . | . | . | . | . | . | . | . | . | . |
| InternalIMT | epa | rs187426603 | . | . | . | . | . | . | . | . | . | . |
| InternalIMT | epa | rs190843143 | 0.001 | . | . | . | . | . | . | . | . | . |
| InternalIMT | epa | rs191883449 | 0 | . | . | . | . | . | . | . | . | . |
| InternalIMT | epa | rs58238740 | 0.173 | -0.038 | 0.035 | 0.272 | 0.064 | 0.031 | 0.041 | 0.02 | 0.047 | 0.669 |
| InternalIMT | epa | rs3780894 | 0.173 | -0.038 | 0.035 | 0.272 | 0.064 | 0.031 | 0.041 | 0.02 | 0.047 | 0.669 |
| InternalIMT | epa | rs149646626 | 0.006 | . | . | . | . | . | . | . | . | . |
| InternalIMT | epa | rs79615212 | 0.006 | . | . | . | . | . | . | . | . | . |
| InternalIMT | epa | rs3780895 | 0.173 | -0.038 | 0.035 | 0.273 | 0.064 | 0.031 | 0.041 | 0.02 | 0.047 | 0.671 |
| InternalIMT | epa | rs3780896 | 0.173 | -0.038 | 0.035 | 0.272 | 0.064 | 0.031 | 0.041 | 0.02 | 0.047 | 0.668 |
| InternalIMT | epa | rs142747163 | 0.003 | . | . | . | . | . | . | . | . | . |
| InternalIMT | epa | rs189417584 | 0.01 | -0.03 | 0.029 | 0.291 | -0.071 | 0.077 | 0.362 | -0.084 | 0.104 | 0.419 |
| InternalIMT | epa | rs3780897 | 0.17 | -0.039 | 0.035 | 0.267 | 0.076 | 0.032 | 0.017 | 0.022 | 0.048 | 0.646 |
| InternalIMT | epa | rs117509491 | 0.002 | . | . | . | . | . | . | . | . | . |
| InternalIMT | epa | rs117811514 | 0.002 | . | . | . | . | . | . | . | . | . |
| InternalIMT | epa | rs116500932 | 0.086 | -0.022 | 0.032 | 0.488 | 0.028 | 0.049 | 0.567 | -0.051 | 0.067 | 0.446 |
| InternalIMT | epa | rs77584168 | . | . | . | . | . | . | . | . | . | . |
| InternalIMT | epa | rs2310776 | 0.173 | -0.038 | 0.035 | 0.272 | 0.064 | 0.031 | 0.04 | 0.02 | 0.047 | 0.669 |
| InternalIMT | epa | rs17157736 | 0.173 | -0.038 | 0.035 | 0.272 | 0.064 | 0.031 | 0.04 | 0.02 | 0.047 | 0.669 |
| InternalIMT | epa | rs72789735 | 0.036 | -0.041 | 0.029 | 0.163 | 0.058 | 0.091 | 0.521 | 0.149 | 0.132 | 0.26 |
| InternalIMT | epa | rs183747187 | 0.001 | . | . | . | . | . | . | . | . | . |
| InternalIMT | epa | rs7077891 | 0.173 | -0.038 | 0.035 | 0.272 | 0.064 | 0.031 | 0.04 | 0.02 | 0.047 | 0.669 |
| InternalIMT | epa | rs145063305 | 0.002 | . | . | . | . | . | . | . | . | . |
| InternalIMT | epa | rs3824613 | 0.174 | -0.036 | 0.035 | 0.306 | 0.066 | 0.031 | 0.034 | 0.015 | 0.047 | 0.76 |
| InternalIMT | epa | rs145543199 | 0.004 | . | . | . | . | . | . | . | . | . |
| InternalIMT | epa | rs140361993 | 0.001 | . | . | . | . | . | . | . | . | . |
| InternalIMT | epa | rs11239496 | 0.036 | -0.041 | 0.029 | 0.162 | 0.058 | 0.091 | 0.521 | 0.149 | 0.132 | 0.259 |
| InternalIMT | epa | rs11239497 | 0.154 | -0.024 | 0.035 | 0.493 | 0.036 | 0.032 | 0.256 | -0.021 | 0.049 | 0.671 |
| InternalIMT | epa | rs10900212 | 0.245 | -0.046 | 0.04 | 0.251 | 0.06 | 0.026 | 0.022 | 0.025 | 0.041 | 0.539 |
| InternalIMT | epa | rs186487136 | . | . | . | . | . | . | . | . | . | . |
| InternalIMT | epa | rs12774369 | 0.173 | -0.039 | 0.035 | 0.266 | 0.064 | 0.031 | 0.041 | 0.021 | 0.047 | 0.652 |
| InternalIMT | epa | rs60842538 | 0.173 | -0.039 | 0.035 | 0.259 | 0.064 | 0.032 | 0.041 | 0.023 | 0.048 | 0.632 |
| InternalIMT | epa | rs3780898 | 0.153 | -0.025 | 0.035 | 0.469 | 0.035 | 0.032 | 0.272 | -0.018 | 0.049 | 0.722 |
| InternalIMT | epa | rs3780899 | 0.172 | -0.04 | 0.035 | 0.246 | 0.064 | 0.032 | 0.043 | 0.025 | 0.048 | 0.598 |
| InternalIMT | epa | rs12252472 | 0.273 | -0.016 | 0.033 | 0.626 | -6.20E-04 | 0.029 | 0.983 | -0.029 | 0.046 | 0.528 |
| InternalIMT | epa | rs185411011 | 0.001 | . | . | . | . | . | . | . | . | . |
| InternalIMT | epa | rs28395865 | 0.152 | -0.026 | 0.035 | 0.461 | 0.034 | 0.032 | 0.289 | -0.017 | 0.049 | 0.732 |
| InternalIMT | epa | rs191004074 | 0.034 | -0.021 | 0.03 | 0.469 | 6.00E-03 | 0.099 | 0.951 | -0.145 | 0.147 | 0.323 |
| InternalIMT | epa | rs182401295 | . | . | . | . | . | . | . | . | . | . |
| InternalIMT | epa | rs2228064 | 0.277 | -0.013 | 0.033 | 0.698 | 1.90E-03 | 0.029 | 0.948 | -0.035 | 0.046 | 0.447 |

| InternalIMT | epa | rs188284159 | . | . | . | . | . | . | . | . | . | . |
| --- | --- | --- | --- | --- | --- | --- | --- | --- | --- | --- | --- | --- |
| InternalIMT | epa | rs2303887 | 0.003 | . | . | . | . | . | . | . | . | . |
| InternalIMT | epa | rs2303886 | 0.014 | -0.037 | 0.029 | 0.202 | 0.129 | 0.116 | 0.268 | 0.127 | 0.179 | 0.477 |
| InternalIMT | epa | rs192621006 | . | . | . | . | . | . | . | . | . | . |
| InternalIMT | epa | rs2303885 | 0.491 | -0.013 | 0.042 | 0.753 | 0.023 | 0.022 | 0.295 | -0.017 | 0.034 | 0.625 |
| InternalIMT | epa | rs114466168 | 0.086 | -0.019 | 0.031 | 0.556 | 0.027 | 0.049 | 0.577 | -0.068 | 0.067 | 0.312 |
| InternalIMT | epa | rs11239501 | 0.038 | -0.041 | 0.029 | 0.156 | 0.131 | 0.097 | 0.177 | 0.204 | 0.138 | 0.141 |
| InternalIMT | epa | rs745986 | 0.156 | -0.03 | 0.035 | 0.379 | 0.02 | 0.032 | 0.527 | -2.70E-03 | 0.048 | 0.955 |
| InternalIMT | epa | rs75262268 | 0.004 | . | . | . | . | . | . | . | . | . |
| InternalIMT | epa | rs71494776 | 0.022 | -0.036 | 0.029 | 0.223 | 1.90E-03 | 0.074 | 0.98 | 0.1 | 0.112 | 0.369 |
| InternalIMT | epa | rs74386871 | 0.006 | . | . | . | . | . | . | . | . | . |
| InternalIMT | epa | rs76502040 | 0.004 | . | . | . | . | . | . | . | . | . |
| InternalIMT | epa | rs142213734 | 0.001 | . | . | . | . | . | . | . | . | . |
| InternalIMT | epa | rs4948671 | 0.796 | 0.013 | 0.077 | 0.864 | 0.029 | 0.025 | 0.244 | -0.027 | 0.043 | 0.521 |
| InternalIMT | epa | rs146286085 | 0 | . | . | . | . | . | . | . | . | . |
| InternalIMT | epa | rs144080623 | 0 | . | . | . | . | . | . | . | . | . |
| InternalIMT | epa | rs115074014 | 0.133 | -0.021 | 0.029 | 0.455 | -0.028 | 0.038 | 0.47 | -0.039 | 0.07 | 0.571 |
| InternalIMT | epa | rs17157756 | 0.133 | -0.021 | 0.029 | 0.455 | -0.028 | 0.038 | 0.47 | -0.039 | 0.07 | 0.571 |
| InternalIMT | epa | rs190459780 | 0.001 | . | . | . | . | . | . | . | . | . |
| InternalIMT | epa | rs12358647 | 0.25 | -0.055 | 0.04 | 0.17 | 0.042 | 0.027 | 0.119 | 0.043 | 0.042 | 0.302 |
| InternalIMT | epa | rs147080556 | 0.009 | . | . | . | . | . | . | . | . | . |
| InternalIMT | epa | rs187543349 | 0.002 | . | . | . | . | . | . | . | . | . |
| InternalIMT | epa | rs114375595 | 0.024 | -0.036 | 0.029 | 0.211 | 0.136 | 0.082 | 0.1 | 0.164 | 0.116 | 0.159 |
| InternalIMT | epa | rs117211200 | 0.003 | . | . | . | . | . | . | . | . | . |
| InternalIMT | epa | rs192963047 | . | . | . | . | . | . | . | . | . | . |
| InternalIMT | epa | rs11239505 | 0.254 | -0.048 | 0.04 | 0.229 | 0.042 | 0.027 | 0.115 | 0.03 | 0.041 | 0.467 |
| InternalIMT | epa | rs184434116 | 0.001 | . | . | . | . | . | . | . | . | . |
| InternalIMT | epa | rs4948999 | 0.801 | 0.011 | 0.078 | 0.884 | 0.023 | 0.025 | 0.37 | -0.026 | 0.043 | 0.542 |
| InternalIMT | epa | rs11239506 | 0.078 | -0.045 | 0.031 | 0.141 | 0.017 | 0.053 | 0.745 | 0.085 | 0.074 | 0.248 |
| InternalIMT | epa | rs190706620 | 0.001 | . | . | . | . | . | . | . | . | . |
| InternalIMT | epa | rs190770429 | 0.001 | . | . | . | . | . | . | . | . | . |
| InternalIMT | epa | rs181327133 | 0.003 | . | . | . | . | . | . | . | . | . |
| InternalIMT | epa | rs149251697 | 0.057 | -0.043 | 0.031 | 0.166 | 0.074 | 0.051 | 0.149 | 0.095 | 0.068 | 0.159 |
| InternalIMT | epa | rs118081602 | 0.003 | . | . | . | . | . | . | . | . | . |
| InternalIMT | epa | rs188233691 | . | . | . | . | . | . | . | . | . | . |
| InternalIMT | epa | rs183778794 | 0.006 | . | . | . | . | . | . | . | . | . |
| InternalIMT | epa | rs188040078 | 0.003 | . | . | . | . | . | . | . | . | . |
| InternalIMT | epa | rs184675372 | 0.001 | . | . | . | . | . | . | . | . | . |
| InternalIMT | epa | rs150326891 | 0.03 | -0.041 | 0.029 | 0.156 | 0.218 | 0.115 | 0.059 | 0.264 | 0.177 | 0.136 |
| InternalIMT | epa | rs185520021 | . | . | . | . | . | . | . | . | . | . |
| InternalIMT | epa | rs7907779 | 0.078 | -0.045 | 0.031 | 0.142 | 0.017 | 0.053 | 0.754 | 0.085 | 0.074 | 0.249 |
| InternalIMT | epa | rs184962860 | . | . | . | . | . | . | . | . | . | . |
| InternalIMT | epa | rs149762784 | 0.034 | -0.024 | 0.031 | 0.441 | 0.063 | 0.087 | 0.467 | -0.091 | 0.087 | 0.293 |
| InternalIMT | epa | rs149203427 | 0.005 | . | . | . | . | . | . | . | . | . |
| InternalIMT | epa | rs60153083 | 0.045 | -0.034 | 0.029 | 0.248 | -0.023 | 0.067 | 0.729 | 0.024 | 0.107 | 0.825 |
| InternalIMT | epa | rs112001069 | 0.073 | -0.017 | 0.032 | 0.605 | 0.017 | 0.041 | 0.678 | -0.099 | 0.068 | 0.144 |
| InternalIMT | epa | rs182355382 | 0.057 | -0.038 | 0.031 | 0.227 | 0.036 | 0.058 | 0.535 | 0.056 | 0.076 | 0.461 |
| InternalIMT | epa | rs186740974 | 0.157 | -4.80E-03 | 0.039 | 0.901 | -0.07 | 0.045 | 0.12 | -0.084 | 0.067 | 0.21 |
| InternalIMT | epa | rs191874916 | 0.06 | -0.03 | 0.03 | 0.311 | 0.071 | 0.063 | 0.263 | -9.60E-03 | 0.119 | 0.935 |
| InternalIMT | epa | rs182989412 | 0.126 | -0.027 | 0.035 | 0.44 | -0.011 | 0.042 | 0.788 | -0.016 | 0.059 | 0.785 |
| InternalIMT | epa | rs188819476 | 0.003 | . | . | . | . | . | . | . | . | . |
| InternalIMT | epa | rs141217183 | 0.083 | -0.014 | 0.033 | 0.668 | 6.80E-03 | 0.041 | 0.867 | -0.1 | 0.067 | 0.134 |
| InternalIMT | epa | rs192922652 | 0.081 | -0.014 | 0.032 | 0.66 | 0.016 | 0.038 | 0.677 | -0.1 | 0.062 | 0.104 |
| InternalIMT | epa | rs185092343 | 0.025 | -0.041 | 0.029 | 0.156 | 0.147 | 0.141 | 0.295 | 0.187 | 0.199 | 0.347 |
| InternalIMT | epa | rs145193311 | 0.082 | -0.015 | 0.032 | 0.65 | 0.015 | 0.038 | 0.692 | -0.095 | 0.06 | 0.115 |
| InternalIMT | epa | rs55789336 | 0.084 | -0.046 | 0.03 | 0.131 | 0.023 | 0.052 | 0.664 | 0.087 | 0.073 | 0.234 |
| InternalIMT | epa | rs55837212 | 0.083 | -0.049 | 0.031 | 0.11 | 0.027 | 0.054 | 0.619 | 0.106 | 0.074 | 0.151 |
| InternalIMT | epa | rs75408574 | 0.208 | -0.033 | 0.035 | 0.342 | -0.04 | 0.03 | 0.181 | -5.90E-04 | 0.046 | 0.99 |
| InternalIMT | epa | rs149306926 | 0.089 | -0.049 | 0.031 | 0.119 | 0.023 | 0.051 | 0.647 | 0.096 | 0.07 | 0.171 |
| InternalIMT | epa | rs35990892 | 0.683 | 0.016 | 0.06 | 0.794 | -0.021 | 0.025 | 0.396 | -0.036 | 0.038 | 0.337 |
| InternalIMT | epa | rs192356207 | 0.128 | -0.043 | 0.035 | 0.222 | 0.05 | 0.039 | 0.207 | 0.034 | 0.054 | 0.529 |
| InternalIMT | epa | rs183741163 | 0.097 | -0.048 | 0.033 | 0.145 | 0.037 | 0.058 | 0.515 | 0.081 | 0.079 | 0.301 |
| InternalIMT | epa | rs34737297 | 0.591 | -0.033 | 0.054 | 0.545 | 0.058 | 0.026 | 0.029 | 3.90E-03 | 0.04 | 0.923 |
| InternalIMT | epa | rs181346933 | 0.042 | -0.015 | 0.029 | 0.608 | -0.089 | 0.059 | 0.131 | -0.27 | 0.123 | 0.029 |
| InternalIMT | epa | rs36093253 | 0.24 | -0.024 | 0.038 | 0.516 | -0.032 | 0.03 | 0.279 | -0.019 | 0.045 | 0.676 |
| InternalIMT | epa | rs186307855 | 0.262 | -2.70E-03 | 0.033 | 0.935 | -0.012 | 0.03 | 0.695 | -0.058 | 0.047 | 0.219 |
| InternalIMT | epa | rs189681865 | 0.096 | -0.04 | 0.034 | 0.239 | 0.022 | 0.038 | 0.567 | 0.037 | 0.053 | 0.491 |
| InternalIMT | epa | rs34942971 | 0.259 | -0.036 | 0.04 | 0.377 | 0.027 | 0.027 | 0.33 | 6.60E-03 | 0.04 | 0.868 |
| InternalIMT | epa | rs181816554 | 0.064 | -0.015 | 0.032 | 0.64 | -0.029 | 0.057 | 0.605 | -0.13 | 0.086 | 0.132 |
| InternalIMT | epa | rs186846881 | 0.278 | -0.013 | 0.034 | 0.701 | 6.20E-03 | 0.029 | 0.831 | -0.034 | 0.045 | 0.447 |
| InternalIMT | epa | rs191178220 | 0.008 | . | . | . | . | . | . | . | . | . |
| InternalIMT | epa | rs183305228 | 0.046 | -0.035 | 0.03 | 0.244 | -0.019 | 0.066 | 0.77 | 0.029 | 0.104 | 0.784 |
| InternalIMT | epa | rs139912761 | 0.149 | -0.056 | 0.035 | 0.115 | 0.035 | 0.037 | 0.35 | 0.077 | 0.052 | 0.138 |

| InternalIMT | epa | rs185499901 | 0.018 | -0.038 | 0.03 | 0.21 | 0.081 | 0.099 | 0.418 | 0.11 | 0.128 | 0.389 |
| --- | --- | --- | --- | --- | --- | --- | --- | --- | --- | --- | --- | --- |
| InternalIMT | epa | rs189816197 | 0.086 | -0.018 | 0.032 | 0.568 | 6.10E-03 | 0.038 | 0.874 | -0.073 | 0.058 | 0.213 |
| InternalIMT | epa | rs145656938 | 0.058 | -0.03 | 0.032 | 0.35 | 0.036 | 0.052 | 0.488 | -0.017 | 0.075 | 0.823 |
| InternalIMT | epa | rs35745197 | 0.072 | -0.019 | 0.032 | 0.549 | 0.016 | 0.041 | 0.697 | -0.083 | 0.067 | 0.213 |
| InternalIMT | epa | rs192856637 | . | . | . | . | . | . | . | . | . | . |
| InternalIMT | epa | rs2310775 | 0.267 | -0.028 | 0.038 | 0.461 | -0.026 | 0.028 | 0.349 | -9.70E-03 | 0.043 | 0.82 |
| InternalIMT | epa | rs11239507 | 0.538 | 6.50E-03 | 0.042 | 0.877 | -0.018 | 0.022 | 0.404 | -0.038 | 0.032 | 0.234 |
| InternalIMT | epa | rs74648467 | 0.027 | -0.039 | 0.029 | 0.186 | -6.20E-03 | 0.082 | 0.94 | 0.146 | 0.13 | 0.261 |
| InternalIMT | epa | rs145815934 | 0.005 | . | . | . | . | . | . | . | . | . |
| InternalIMT | epa | rs145390687 | 0.012 | -0.038 | 0.029 | 0.191 | 0.031 | 0.334 | 0.926 | 0.427 | 0.494 | 0.387 |
| InternalIMT | epa | rs184354913 | 0.012 | -0.034 | 0.029 | 0.244 | -0.045 | 0.101 | 0.654 | 0.095 | 0.131 | 0.469 |
| InternalIMT | epa | rs181244920 | 0.003 | . | . | . | . | . | . | . | . | . |
| InternalIMT | epa | rs11499087 | 0.167 | -0.021 | 0.029 | 0.468 | -0.046 | 0.034 | 0.167 | -0.036 | 0.059 | 0.543 |
| InternalIMT | epa | rs148263794 | 0.079 | -0.016 | 0.032 | 0.616 | 0.012 | 0.038 | 0.743 | -0.091 | 0.061 | 0.139 |
| InternalIMT | epa | rs150758903 | 0.089 | -0.034 | 0.033 | 0.31 | 0.018 | 0.04 | 0.651 | 9.50E-03 | 0.056 | 0.867 |
| InternalIMT | epa | rs147654382 | 0.192 | -0.068 | 0.037 | 0.065 | 0.084 | 0.034 | 0.014 | 0.096 | 0.052 | 0.065 |
| InternalIMT | epa | rs147495166 | 0.077 | -0.013 | 0.031 | 0.69 | 0.019 | 0.048 | 0.691 | -0.112 | 0.06 | 0.063 |
| InternalIMT | epa | rs35198778 | 0.14 | -0.037 | 0.035 | 0.29 | 0.083 | 0.041 | 0.044 | 0.021 | 0.058 | 0.716 |
| InternalIMT | epa | rs145207659 | 0.02 | -0.028 | 0.029 | 0.335 | -0.108 | 0.136 | 0.426 | -0.126 | 0.24 | 0.6 |
| InternalIMT | epa | rs186466464 | 0.024 | -0.028 | 0.03 | 0.351 | 0.059 | 0.097 | 0.539 | -0.079 | 0.134 | 0.557 |
| InternalIMT | epa | rs12778413 | 0.199 | -0.041 | 0.038 | 0.272 | 0.052 | 0.032 | 0.099 | 0.023 | 0.048 | 0.638 |
| InternalIMT | epa | rs7895552 | 0.724 | 0.016 | 0.07 | 0.816 | 0.014 | 0.026 | 0.579 | -0.033 | 0.042 | 0.436 |
| InternalIMT | epa | rs12783895 | 0.076 | -0.018 | 0.032 | 0.566 | 0.016 | 0.039 | 0.687 | -0.082 | 0.063 | 0.192 |
| InternalIMT | epa | rs34929604 | 0.081 | -0.015 | 0.032 | 0.631 | 0.015 | 0.038 | 0.685 | -0.093 | 0.061 | 0.13 |
| InternalIMT | epa | rs12761961 | 0.072 | -0.022 | 0.032 | 0.505 | 0.024 | 0.043 | 0.57 | -0.066 | 0.069 | 0.339 |
| InternalIMT | epa | rs35536930 | 0.252 | -0.052 | 0.04 | 0.198 | 0.044 | 0.027 | 0.104 | 0.036 | 0.041 | 0.377 |
| InternalIMT | epa | rs180951060 | 0.006 | . | . | . | . | . | . | . | . | . |
| InternalIMT | epa | rs148892128 | 0.005 | . | . | . | . | . | . | . | . | . |
| InternalIMT | epa | rs11239509 | 0.255 | -1.60E-03 | 0.032 | 0.961 | -0.022 | 0.028 | 0.424 | -0.061 | 0.044 | 0.164 |
| InternalIMT | epa | rs41526545 | 0.045 | -0.041 | 0.029 | 0.161 | 0.048 | 0.084 | 0.571 | 0.137 | 0.123 | 0.266 |
| InternalIMT | epa | rs2029253 | 0.286 | -0.038 | 0.037 | 0.309 | -3.90E-03 | 0.026 | 0.883 | 0.012 | 0.04 | 0.759 |
| InternalIMT | epa | rs17444064 | 0.041 | -0.041 | 0.029 | 0.162 | 0.106 | 0.094 | 0.258 | 0.178 | 0.132 | 0.178 |
| InternalIMT | epa | rs71494779 | 0.084 | -0.014 | 0.032 | 0.672 | 0.013 | 0.037 | 0.722 | -0.099 | 0.059 | 0.096 |
| InternalIMT | epa | rs74991420 | 0.003 | . | . | . | . | . | . | . | . | . |
| InternalIMT | epa | rs182736551 | 0.003 | . | . | . | . | . | . | . | . | . |
| InternalIMT | epa | rs78702074 | 0.003 | . | . | . | . | . | . | . | . | . |
| InternalIMT | epa | rs187791466 | . | . | . | . | . | . | . | . | . | . |
| InternalIMT | epa | rs35300270 | 0.085 | -0.013 | 0.032 | 0.684 | 0.01 | 0.037 | 0.785 | -0.1 | 0.059 | 0.091 |
| InternalIMT | epa | rs6593484 | 0.078 | -0.045 | 0.03 | 0.143 | 0.011 | 0.054 | 0.832 | 0.084 | 0.074 | 0.259 |
| InternalIMT | epa | rs12244601 | 0.064 | -0.037 | 0.032 | 0.245 | 0.038 | 0.047 | 0.419 | 0.039 | 0.065 | 0.551 |
| InternalIMT | epa | rs184429574 | 0.006 | . | . | . | . | . | . | . | . | . |
| InternalIMT | epa | rs7080897 | 0.084 | -0.014 | 0.032 | 0.672 | 0.013 | 0.037 | 0.72 | -0.099 | 0.059 | 0.096 |
| InternalIMT | epa | rs7097429 | 0.085 | -0.014 | 0.032 | 0.668 | 0.013 | 0.037 | 0.716 | -0.096 | 0.059 | 0.102 |
| InternalIMT | epa | rs77979229 | . | . | . | . | . | . | . | . | . | . |
| InternalIMT | epa | rs11239512 | 0.079 | -0.044 | 0.031 | 0.145 | 0.011 | 0.052 | 0.834 | 0.081 | 0.072 | 0.265 |
| InternalIMT | epa | rs60146827 | 0.084 | -0.014 | 0.032 | 0.672 | 0.013 | 0.037 | 0.721 | -0.099 | 0.059 | 0.096 |
| InternalIMT | epa | rs79032236 | 0.105 | -0.028 | 0.032 | 0.392 | 0.073 | 0.048 | 0.129 | -0.015 | 0.069 | 0.83 |
| InternalIMT | epa | rs140854206 | . | . | . | . | . | . | . | . | . | . |
| InternalIMT | epa | rs184049550 | 0.012 | -0.031 | 0.029 | 0.283 | -0.092 | 0.078 | 0.24 | -9.20E-03 | 0.105 | 0.93 |
| InternalIMT | epa | rs114433607 | 0.029 | -0.028 | 0.029 | 0.33 | -0.078 | 0.074 | 0.292 | -0.058 | 0.129 | 0.654 |
| InternalIMT | epa | rs117653793 | 0.003 | . | . | . | . | . | . | . | . | . |
| InternalIMT | epa | rs117039533 | 0.003 | . | . | . | . | . | . | . | . | . |
| InternalIMT | epa | rs191660761 | 0.002 | . | . | . | . | . | . | . | . | . |
| InternalIMT | epa | rs59514896 | 0.03 | -0.035 | 0.03 | 0.233 | 0.032 | 0.066 | 0.629 | 0.068 | 0.103 | 0.508 |
| InternalIMT | epa | rs184661876 | 0.001 | . | . | . | . | . | . | . | . | . |
| InternalIMT | epa | rs61854073 | 0.011 | -0.034 | 0.029 | 0.235 | -0.172 | 0.118 | 0.144 | 0.136 | 0.142 | 0.34 |
| InternalIMT | epa | rs2018518 | 0.09 | -0.039 | 0.031 | 0.216 | -0.059 | 0.042 | 0.161 | 0.047 | 0.058 | 0.419 |
| InternalIMT | epa | rs934187 | 0.802 | 9.00E-03 | 0.079 | 0.91 | 0.019 | 0.025 | 0.456 | -0.025 | 0.043 | 0.568 |
| InternalIMT | epa | rs7918542 | 0.269 | -0.037 | 0.041 | 0.367 | 0.03 | 0.026 | 0.237 | 8.20E-03 | 0.039 | 0.833 |
| InternalIMT | epa | rs144311920 | 0.004 | . | . | . | . | . | . | . | . | . |
| InternalIMT | epa | rs7099684 | 0.883 | 5.90E-03 | 0.121 | 0.961 | 0.04 | 0.034 | 0.243 | -0.02 | 0.063 | 0.75 |
| InternalIMT | epa | rs150829171 | 0.001 | . | . | . | . | . | . | . | . | . |
| InternalIMT | epa | rs186060697 | 0.006 | . | . | . | . | . | . | . | . | . |
| InternalIMT | epa | rs11239513 | 0.277 | -0.015 | 0.033 | 0.64 | 1.10E-04 | 0.029 | 0.997 | -0.03 | 0.046 | 0.516 |
| InternalIMT | epa | rs7917687 | 0.084 | -0.014 | 0.032 | 0.673 | 0.013 | 0.037 | 0.728 | -0.099 | 0.059 | 0.097 |
| InternalIMT | epa | rs61854076 | 0.004 | . | . | . | . | . | . | . | . | . |
| InternalIMT | epa | rs71494780 | 0.007 | . | . | . | . | . | . | . | . | . |
| InternalIMT | epa | rs7919239 | 0.257 | -0.047 | 0.04 | 0.236 | 0.038 | 0.026 | 0.141 | 0.028 | 0.04 | 0.487 |
| InternalIMT | epa | rs78334475 | 0.004 | . | . | . | . | . | . | . | . | . |
| InternalIMT | epa | rs3780900 | 0.035 | -0.025 | 0.029 | 0.377 | -0.047 | 0.082 | 0.57 | -0.112 | 0.158 | 0.478 |
| InternalIMT | epa | rs186729292 | 0.001 | . | . | . | . | . | . | . | . | . |
| InternalIMT | epa | rs12267438 | 0.073 | -0.039 | 0.032 | 0.227 | 0.035 | 0.042 | 0.398 | 0.042 | 0.059 | 0.478 |
| InternalIMT | epa | rs1369214 | 0.564 | -0.016 | 0.044 | 0.716 | -0.012 | 0.023 | 0.61 | -0.015 | 0.034 | 0.654 |

| InternalIMT | epa | rs9282589 | 0.005 | . | . | . | . | . | . | . | . | . |
| --- | --- | --- | --- | --- | --- | --- | --- | --- | --- | --- | --- | --- |
| InternalIMT | epa | rs114522915 | 0.078 | -0.019 | 0.031 | 0.537 | 0.045 | 0.051 | 0.374 | -0.067 | 0.067 | 0.321 |
| InternalIMT | epa | rs2115819 | 0.292 | -0.035 | 0.038 | 0.358 | -0.024 | 0.028 | 0.39 | 4.90E-03 | 0.041 | 0.905 |
| InternalIMT | epa | rs117664603 | 0.003 | . | . | . | . | . | . | . | . | . |
| InternalIMT | epa | rs184979202 | 0.001 | . | . | . | . | . | . | . | . | . |
| InternalIMT | epa | rs190714671 | . | . | . | . | . | . | . | . | . | . |
| InternalIMT | epa | rs141538996 | . | . | . | . | . | . | . | . | . | . |
| InternalIMT | epa | rs150881741 | 0.001 | . | . | . | . | . | . | . | . | . |
| InternalIMT | epa | rs139076633 | . | . | . | . | . | . | . | . | . | . |
| InternalIMT | epa | rs12243649 | 0.156 | -0.027 | 0.036 | 0.449 | 0.063 | 0.032 | 0.048 | -0.017 | 0.047 | 0.717 |
| InternalIMT | epa | rs140348708 | . | . | . | . | . | . | . | . | . | . |
| InternalIMT | epa | rs79023256 | 0.003 | . | . | . | . | . | . | . | . | . |
| InternalIMT | epa | rs11239515 | 0.071 | -0.038 | 0.03 | 0.21 | 8.60E-03 | 0.057 | 0.879 | 0.046 | 0.084 | 0.581 |
| InternalIMT | epa | rs55866086 | 0.24 | -0.036 | 0.04 | 0.361 | 0.041 | 0.029 | 0.153 | 8.60E-03 | 0.043 | 0.84 |
| InternalIMT | epa | rs181340150 | 0.002 | . | . | . | . | . | . | . | . | . |
| InternalIMT | epa | rs11239516 | 0.158 | -0.048 | 0.035 | 0.176 | 0.016 | 0.036 | 0.651 | 0.051 | 0.05 | 0.309 |
| InternalIMT | epa | rs11239517 | 0.158 | -0.048 | 0.035 | 0.176 | 0.016 | 0.036 | 0.652 | 0.051 | 0.05 | 0.31 |
| InternalIMT | epa | rs11239519 | 0.128 | -0.039 | 0.034 | 0.255 | -0.015 | 0.039 | 0.706 | 0.028 | 0.057 | 0.628 |
| InternalIMT | epa | rs7909514 | 0.788 | -0.022 | 0.066 | 0.742 | 0.017 | 0.025 | 0.48 | -5.80E-03 | 0.038 | 0.878 |
| InternalIMT | epa | rs7894352 | 0.885 | -0.013 | 0.109 | 0.904 | 0.052 | 0.033 | 0.117 | -9.00E-03 | 0.057 | 0.876 |
| InternalIMT | epa | rs189384146 | 0.001 | . | . | . | . | . | . | . | . | . |
| InternalIMT | epa | rs11239520 | 0.107 | -0.038 | 0.031 | 0.223 | -9.10E-03 | 0.049 | 0.854 | 0.031 | 0.069 | 0.654 |
| InternalIMT | epa | rs12221233 | 0.234 | -0.035 | 0.035 | 0.325 | -0.036 | 0.03 | 0.237 | 4.30E-03 | 0.045 | 0.924 |
| InternalIMT | epa | rs74523293 | 0.066 | -0.023 | 0.031 | 0.454 | 0.099 | 0.052 | 0.056 | -0.061 | 0.08 | 0.444 |
| InternalIMT | epa | rs79376040 | 0.066 | -0.023 | 0.031 | 0.454 | 0.099 | 0.052 | 0.056 | -0.061 | 0.08 | 0.445 |
| InternalIMT | epa | rs4949000 | 0.234 | -0.035 | 0.035 | 0.322 | -0.035 | 0.03 | 0.239 | 4.70E-03 | 0.045 | 0.916 |
| InternalIMT | epa | rs7089406 | 0.039 | -0.019 | 0.029 | 0.522 | -0.085 | 0.046 | 0.064 | -0.147 | 0.073 | 0.046 |
| InternalIMT | epa | rs138211056 | 0 | . | . | . | . | . | . | . | . | . |
| InternalIMT | epa | rs10900213 | 0.434 | -0.05 | 0.049 | 0.309 | -0.019 | 0.026 | 0.469 | 0.021 | 0.041 | 0.613 |
| InternalIMT | epa | rs145152827 | . | . | . | . | . | . | . | . | . | . |
| InternalIMT | epa | rs74443325 | 0.003 | . | . | . | . | . | . | . | . | . |
| InternalIMT | epa | rs188299446 | 0.004 | . | . | . | . | . | . | . | . | . |
| InternalIMT | epa | rs192699586 | . | . | . | . | . | . | . | . | . | . |
| InternalIMT | epa | rs72796464 | 0.041 | -0.041 | 0.029 | 0.163 | 0.106 | 0.097 | 0.278 | 0.183 | 0.137 | 0.183 |
| InternalIMT | epa | rs4949001 | 0.077 | -0.045 | 0.031 | 0.147 | -0.043 | 0.044 | 0.336 | 0.093 | 0.059 | 0.113 |
| InternalIMT | epa | rs147174641 | 0.003 | . | . | . | . | . | . | . | . | . |
| InternalIMT | epa | rs190835238 | 0.001 | . | . | . | . | . | . | . | . | . |
| InternalIMT | epa | rs76698944 | 0.013 | -0.037 | 0.029 | 0.198 | 0.15 | 0.181 | 0.408 | 0.257 | 0.249 | 0.301 |
| InternalIMT | epa | rs56071381 | 0.129 | -0.034 | 0.033 | 0.3 | -0.019 | 0.041 | 0.638 | 7.60E-03 | 0.059 | 0.897 |
| InternalIMT | epa | rs78130427 | 0.019 | -0.031 | 0.029 | 0.295 | -0.121 | 0.087 | 0.162 | -0.01 | 0.163 | 0.95 |
| InternalIMT | epa | rs10900215 | 0.249 | -0.045 | 0.039 | 0.252 | 9.30E-05 | 0.03 | 0.998 | 0.027 | 0.044 | 0.546 |
| InternalIMT | epa | rs41491445 | 0.046 | -0.034 | 0.03 | 0.256 | -0.032 | 0.064 | 0.621 | 0.018 | 0.102 | 0.857 |
| InternalIMT | epa | rs144995222 | 0.006 | . | . | . | . | . | . | . | . | . |
| InternalIMT | epa | rs149097803 | 0.002 | . | . | . | . | . | . | . | . | . |
| InternalIMT | epa | rs139429646 | 0.002 | . | . | . | . | . | . | . | . | . |
| InternalIMT | epa | rs7075620 | 0.764 | 0.029 | 0.072 | 0.689 | 2.10E-03 | 0.027 | 0.938 | -0.04 | 0.042 | 0.345 |
| InternalIMT | epa | rs148299213 | 0.049 | -0.031 | 0.03 | 0.296 | -0.036 | 0.064 | 0.57 | -9.90E-03 | 0.103 | 0.924 |
| InternalIMT | epa | rs4949002 | 0.084 | -0.039 | 0.031 | 0.203 | -0.056 | 0.046 | 0.219 | 0.054 | 0.063 | 0.389 |
| InternalIMT | epa | rs150496007 | 0.023 | -0.029 | 0.029 | 0.319 | -0.023 | 0.068 | 0.735 | -0.038 | 0.101 | 0.703 |
| InternalIMT | epa | rs147303455 | 0.028 | -0.021 | 0.03 | 0.483 | -0.028 | 0.061 | 0.641 | -0.125 | 0.078 | 0.108 |
| InternalIMT | epa | rs189518433 | . | . | . | . | . | . | . | . | . | . |
| InternalIMT | epa | rs186281355 | . | . | . | . | . | . | . | . | . | . |
| InternalIMT | epa | rs115297114 | 0.077 | -0.015 | 0.032 | 0.628 | 0.047 | 0.052 | 0.364 | -0.093 | 0.065 | 0.149 |
| InternalIMT | epa | rs11239523 | 0.106 | -0.038 | 0.031 | 0.223 | -6.10E-03 | 0.049 | 0.901 | 0.03 | 0.068 | 0.661 |
| InternalIMT | epa | rs143704897 | 0.007 | . | . | . | . | . | . | . | . | . |
| InternalIMT | epa | rs188024096 | 0.001 | . | . | . | . | . | . | . | . | . |
| InternalIMT | epa | rs11239524 | 0.133 | -0.032 | 0.034 | 0.343 | -0.019 | 0.041 | 0.643 | 1.20E-03 | 0.059 | 0.984 |
| InternalIMT | epa | rs191656573 | 0.002 | . | . | . | . | . | . | . | . | . |
| InternalIMT | epa | rs193295875 | 0.001 | . | . | . | . | . | . | . | . | . |
| InternalIMT | epa | rs190458228 | . | . | . | . | . | . | . | . | . | . |
| InternalIMT | epa | rs139253993 | 0.001 | . | . | . | . | . | . | . | . | . |
| InternalIMT | epa | rs4948672 | 0.497 | 0.054 | 0.054 | 0.318 | -0.036 | 0.027 | 0.174 | -0.083 | 0.041 | 0.045 |
| InternalIMT | epa | rs180954921 | 0.001 | . | . | . | . | . | . | . | . | . |
| InternalIMT | epa | rs12264801 | 0.383 | -0.076 | 0.049 | 0.12 | 0.072 | 0.031 | 0.021 | 0.062 | 0.049 | 0.208 |
| InternalIMT | epa | rs73283184 | 0.057 | -0.027 | 0.03 | 0.38 | -0.049 | 0.052 | 0.35 | -0.044 | 0.09 | 0.622 |
| InternalIMT | epa | rs142746187 | 0.022 | -0.056 | 0.028 | 0.048 | 0.271 | 0.124 | 0.029 | 0.543 | 0.169 | 1.40E-03 |
| InternalIMT | epa | rs58704739 | 0.053 | -0.03 | 0.03 | 0.329 | -0.042 | 0.053 | 0.426 | -0.019 | 0.089 | 0.834 |
| InternalIMT | epa | rs61272335 | 0.04 | -0.034 | 0.029 | 0.251 | -0.04 | 0.067 | 0.548 | 0.027 | 0.113 | 0.814 |
| InternalIMT | epa | rs60959489 | 0.103 | -0.018 | 0.034 | 0.595 | -0.036 | 0.038 | 0.34 | -0.055 | 0.059 | 0.348 |
| InternalIMT | epa | rs58502629 | 0.04 | -0.034 | 0.029 | 0.251 | -0.04 | 0.067 | 0.553 | 0.026 | 0.112 | 0.815 |
| InternalIMT | epa | rs10900216 | 0.744 | -0.037 | 0.081 | 0.653 | 0.021 | 0.027 | 0.433 | 4.00E-03 | 0.047 | 0.932 |
| InternalIMT | epa | rs181118141 | 0.004 | . | . | . | . | . | . | . | . | . |
| InternalIMT | epa | rs137882665 | 0.019 | -0.023 | 0.029 | 0.417 | -0.014 | 0.061 | 0.817 | -0.188 | 0.108 | 0.082 |

| InternalIMT | epa | rs149466736 | 0.002 | . | . | . | . | . | . | . | . | . |
| --- | --- | --- | --- | --- | --- | --- | --- | --- | --- | --- | --- | --- |
| InternalIMT | epa | rs2099171 | 0.553 | -0.051 | 0.049 | 0.295 | 0.016 | 0.022 | 0.48 | 0.018 | 0.033 | 0.588 |
| InternalIMT | epa | rs141852280 | 0.002 | . | . | . | . | . | . | . | . | . |
| InternalIMT | epa | rs191539257 | . | . | . | . | . | . | . | . | . | . |
| InternalIMT | epa | rs17157771 | 0.045 | -0.035 | 0.029 | 0.22 | -0.022 | 0.066 | 0.736 | 0.059 | 0.115 | 0.609 |
| InternalIMT | epa | rs111339241 | 0.041 | -0.034 | 0.029 | 0.238 | -0.029 | 0.067 | 0.668 | 0.047 | 0.116 | 0.684 |
| InternalIMT | epa | rs116983255 | 0.004 | . | . | . | . | . | . | . | . | . |
| InternalIMT | epa | rs142324341 | . | . | . | . | . | . | . | . | . | . |
| InternalIMT | epa | rs187191219 | 0.015 | -0.027 | 0.029 | 0.345 | -0.015 | 0.065 | 0.814 | -0.127 | 0.117 | 0.275 |
| InternalIMT | epa | rs151283973 | 0.005 | . | . | . | . | . | . | . | . | . |
| InternalIMT | epa | rs139316218 | 0.001 | . | . | . | . | . | . | . | . | . |
| InternalIMT | epa | rs6593485 | 0.697 | -0.05 | 0.063 | 0.424 | 0.015 | 0.023 | 0.52 | 0.014 | 0.039 | 0.715 |
| InternalIMT | epa | rs6593486 | 0.697 | -0.05 | 0.063 | 0.426 | 0.015 | 0.023 | 0.525 | 0.014 | 0.038 | 0.718 |
| InternalIMT | epa | rs145196618 | 0.036 | -0.034 | 0.029 | 0.252 | 0.069 | 0.066 | 0.299 | 0.039 | 0.102 | 0.703 |
| InternalIMT | epa | rs142598335 | 0.001 | . | . | . | . | . | . | . | . | . |
| InternalIMT | epa | rs892691 | 0.663 | -0.028 | 0.058 | 0.637 | -0.012 | 0.023 | 0.596 | -3.30E-03 | 0.036 | 0.926 |
| InternalIMT | epa | rs7080713 | 0.597 | -0.028 | 0.054 | 0.598 | -3.70E-03 | 0.022 | 0.87 | -2.70E-03 | 0.035 | 0.939 |
| InternalIMT | epa | rs7077173 | 0.135 | -0.051 | 0.031 | 0.103 | 0.024 | 0.032 | 0.452 | 0.08 | 0.054 | 0.138 |
| InternalIMT | epa | rs7080977 | 0.088 | -0.044 | 0.03 | 0.147 | 0.038 | 0.046 | 0.412 | 0.078 | 0.074 | 0.287 |
| InternalIMT | epa | rs3780901 | 0.599 | -0.04 | 0.054 | 0.462 | 8.30E-04 | 0.023 | 0.971 | 6.70E-03 | 0.035 | 0.849 |
| InternalIMT | epa | rs61854091 | 0.021 | -0.029 | 0.028 | 0.311 | -7.20E-04 | 0.108 | 0.995 | -0.063 | 0.155 | 0.683 |
| InternalIMT | epa | rs77550209 | 0.011 | -0.029 | 0.029 | 0.318 | -0.139 | 0.071 | 0.053 | -0.084 | 0.088 | 0.341 |
| InternalIMT | epa | rs73283191 | 0.097 | -0.044 | 0.03 | 0.153 | 0.057 | 0.045 | 0.201 | 0.07 | 0.073 | 0.341 |
| InternalIMT | epa | rs191866099 | 0.003 | . | . | . | . | . | . | . | . | . |
| InternalIMT | epa | rs147998395 | 0.005 | . | . | . | . | . | . | . | . | . |
| InternalIMT | epa | rs7090328 | 0.861 | -0.108 | 0.09 | 0.229 | 0.039 | 0.029 | 0.179 | 0.045 | 0.052 | 0.381 |
| InternalIMT | epa | rs79814535 | 0.003 | . | . | . | . | . | . | . | . | . |
| InternalIMT | epa | rs7073082 | 0.861 | -0.108 | 0.09 | 0.231 | 0.039 | 0.029 | 0.178 | 0.045 | 0.052 | 0.384 |
| InternalIMT | epa | rs11239526 | 0.012 | -0.033 | 0.029 | 0.254 | -0.199 | 0.086 | 0.02 | 0.069 | 0.156 | 0.659 |
| InternalIMT | epa | rs141839841 | 0.005 | . | . | . | . | . | . | . | . | . |
| InternalIMT | epa | rs75697371 | 0.005 | . | . | . | . | . | . | . | . | . |
| InternalIMT | epa | rs75972854 | 0.005 | . | . | . | . | . | . | . | . | . |
| InternalIMT | epa | rs139291975 | . | . | . | . | . | . | . | . | . | . |
| InternalIMT | epa | rs144064711 | 0.002 | . | . | . | . | . | . | . | . | . |
| InternalIMT | epa | rs3780902 | 0.098 | -0.044 | 0.03 | 0.153 | 0.064 | 0.044 | 0.15 | 0.067 | 0.072 | 0.353 |
| InternalIMT | epa | rs190449801 | 0.004 | . | . | . | . | . | . | . | . | . |
| InternalIMT | epa | rs73283196 | 0.096 | -0.043 | 0.03 | 0.154 | 0.059 | 0.045 | 0.185 | 0.069 | 0.073 | 0.348 |
| InternalIMT | epa | rs28395867 | 0.118 | -0.041 | 0.028 | 0.142 | 0.01 | 0.043 | 0.808 | 0.044 | 0.079 | 0.576 |
| InternalIMT | epa | rs28395866 | 0.264 | -0.052 | 0.032 | 0.103 | 0.036 | 0.029 | 0.217 | 0.039 | 0.046 | 0.392 |
| InternalIMT | epa | rs55661611 | 0.088 | -0.043 | 0.03 | 0.149 | 0.041 | 0.046 | 0.382 | 0.078 | 0.075 | 0.299 |
| InternalIMT | epa | rs55684518 | 0.008 | . | . | . | . | . | . | . | . | . |
| InternalIMT | epa | rs78552493 | 0.041 | -0.019 | 0.029 | 0.502 | -0.087 | 0.052 | 0.096 | -0.191 | 0.116 | 0.1 |
| InternalIMT | epa | rs147665434 | 0.001 | . | . | . | . | . | . | . | . | . |
| InternalIMT | epa | rs34445001 | 0.005 | . | . | . | . | . | . | . | . | . |
| InternalIMT | epa | rs184019449 | 0.001 | . | . | . | . | . | . | . | . | . |
| InternalIMT | epa | rs3780903 | 0.645 | 0.013 | 0.059 | 0.823 | -0.022 | 0.024 | 0.366 | -0.035 | 0.036 | 0.339 |
| InternalIMT | epa | rs17157784 | 0.159 | -0.042 | 0.029 | 0.149 | 0.029 | 0.034 | 0.393 | 0.032 | 0.059 | 0.586 |
| InternalIMT | epa | rs3780904 | 0.01 | . | . | . | . | . | . | . | . | . |
| InternalIMT | epa | rs192064730 | 0 | . | . | . | . | . | . | . | . | . |
| InternalIMT | epa | rs12774534 | 0.005 | . | . | . | . | . | . | . | . | . |
| InternalIMT | epa | rs79179915 | 0.003 | . | . | . | . | . | . | . | . | . |
| InternalIMT | epa | rs117811571 | 0.004 | . | . | . | . | . | . | . | . | . |
| InternalIMT | epa | rs72796477 | 0.004 | . | . | . | . | . | . | . | . | . |
| InternalIMT | epa | rs55729342 | 0.172 | -0.037 | 0.03 | 0.217 | 0.036 | 0.034 | 0.286 | 0.014 | 0.058 | 0.813 |
| InternalIMT | epa | rs59317220 | 0.172 | -0.037 | 0.03 | 0.218 | 0.036 | 0.034 | 0.286 | 0.013 | 0.057 | 0.815 |
| InternalIMT | epa | rs3780905 | 0.018 | -0.037 | 0.029 | 0.206 | -0.139 | 0.085 | 0.1 | 0.143 | 0.122 | 0.24 |
| InternalIMT | epa | rs3780906 | 0.645 | 0.015 | 0.059 | 0.8 | -0.022 | 0.024 | 0.351 | -0.036 | 0.036 | 0.321 |
| InternalIMT | epa | rs149047975 | 0.003 | . | . | . | . | . | . | . | . | . |
| InternalIMT | epa | rs3824614 | 0.002 | . | . | . | . | . | . | . | . | . |
| InternalIMT | epa | rs140254404 | . | . | . | . | . | . | . | . | . | . |
| InternalIMT | epa | rs145262684 | 0.005 | . | . | . | . | . | . | . | . | . |
| InternalIMT | epa | rs76735482 | 0.002 | . | . | . | . | . | . | . | . | . |
| InternalIMT | epa | rs73283198 | 0.045 | -0.033 | 0.029 | 0.251 | -0.036 | 0.065 | 0.578 | 0.034 | 0.111 | 0.76 |
| InternalIMT | epa | rs74911239 | 0.013 | -0.035 | 0.029 | 0.222 | -0.083 | 0.109 | 0.446 | 0.192 | 0.21 | 0.359 |
| InternalIMT | epa | rs150551647 | 0.004 | . | . | . | . | . | . | . | . | . |
| InternalIMT | epa | rs115200794 | 0.143 | -0.036 | 0.028 | 0.206 | 0.011 | 0.037 | 0.769 | 0.018 | 0.067 | 0.793 |
| InternalIMT | epa | rs74130823 | 0.245 | -0.08 | 0.029 | 6.40E-03 | 0.032 | 0.033 | 0.332 | 0.117 | 0.054 | 0.032 |
| InternalIMT | epa | rs2279435 | 0.604 | 0.044 | 0.059 | 0.454 | -0.034 | 0.025 | 0.187 | -0.06 | 0.039 | 0.122 |
| InternalIMT | epa | rs147279673 | 0.007 | . | . | . | . | . | . | . | . | . |
| InternalIMT | epa | rs183525146 | 0.002 | . | . | . | . | . | . | . | . | . |
| InternalIMT | epa | rs3740107 | 0.769 | 0.018 | 0.079 | 0.818 | 1.40E-03 | 0.026 | 0.957 | -0.031 | 0.043 | 0.47 |
| InternalIMT | epa | rs140697247 | 0 | . | . | . | . | . | . | . | . | . |
| InternalIMT | epa | rs1565096 | 0.903 | 0.036 | 0.103 | 0.726 | 9.80E-03 | 0.035 | 0.78 | -0.037 | 0.057 | 0.514 |

| InternalIMT | epa | rs1565097 | 0.766 | 1.50E-03 | 0.077 | 0.984 | 4.20E-03 | 0.026 | 0.871 | -0.021 | 0.043 | 0.623 |
| --- | --- | --- | --- | --- | --- | --- | --- | --- | --- | --- | --- | --- |
| InternalIMT | epa | rs144584548 | 0.008 | . | . | . | . | . | . | . | . | . |
| InternalIMT | epa | rs75811053 | 0.001 | . | . | . | . | . | . | . | . | . |
| InternalIMT | epa | rs147462967 | 0.009 | . | . | . | . | . | . | . | . | . |
| InternalIMT | epa | rs74130824 | 0.121 | -0.027 | 0.028 | 0.345 | -0.016 | 0.042 | 0.695 | -0.021 | 0.08 | 0.79 |
| InternalIMT | epa | rs116883184 | 0.006 | . | . | . | . | . | . | . | . | . |
| InternalIMT | epa | rs117258049 | 0.006 | . | . | . | . | . | . | . | . | . |
| InternalIMT | epa | rs3780908 | 0.129 | -0.056 | 0.031 | 0.074 | 0.063 | 0.045 | 0.164 | 0.12 | 0.069 | 0.082 |
| InternalIMT | epa | rs180938522 | 0.001 | . | . | . | . | . | . | . | . | . |
| InternalIMT | epa | rs76687372 | 0.024 | -0.038 | 0.029 | 0.184 | 0.157 | 0.097 | 0.107 | 0.192 | 0.138 | 0.163 |
| InternalIMT | epa | rs3780909 | 0.483 | 0.032 | 0.044 | 0.469 | -0.042 | 0.023 | 0.068 | -0.064 | 0.032 | 0.045 |
| InternalIMT | epa | rs78281156 | 0.001 | . | . | . | . | . | . | . | . | . |
| InternalIMT | epa | rs187840631 | . | . | . | . | . | . | . | . | . | . |
| InternalIMT | epa | rs76868242 | 0.001 | . | . | . | . | . | . | . | . | . |
| InternalIMT | epa | rs192794192 | 0 | . | . | . | . | . | . | . | . | . |
| InternalIMT | epa | rs12781248 | 0.005 | . | . | . | . | . | . | . | . | . |
| InternalIMT | epa | rs12762375 | 0.005 | . | . | . | . | . | . | . | . | . |
| InternalIMT | epa | rs192256986 | . | . | . | . | . | . | . | . | . | . |
| InternalIMT | epa | rs184411030 | 0.033 | -0.041 | 0.029 | 0.159 | 0.192 | 0.075 | 0.011 | 0.13 | 0.115 | 0.26 |
| InternalIMT | epa | rs189601483 | 0.001 | . | . | . | . | . | . | . | . | . |
| InternalIMT | epa | rs143254579 | 0.015 | -0.028 | 0.029 | 0.334 | -0.129 | 0.074 | 0.083 | -0.106 | 0.13 | 0.416 |
| InternalIMT | epa | rs12253903 | 0.081 | -0.016 | 0.031 | 0.591 | -0.051 | 0.047 | 0.275 | -0.116 | 0.084 | 0.166 |
| InternalIMT | epa | rs35489926 | 0.005 | . | . | . | . | . | . | . | . | . |
| InternalIMT | epa | rs71494783 | 0.095 | -0.033 | 0.032 | 0.297 | 0.055 | 0.042 | 0.186 | 8.10E-03 | 0.059 | 0.892 |
| InternalIMT | epa | rs180711602 | 0.001 | . | . | . | . | . | . | . | . | . |
| InternalIMT | epa | rs117312402 | 0.001 | . | . | . | . | . | . | . | . | . |
| InternalIMT | epa | rs74831694 | 0.001 | . | . | . | . | . | . | . | . | . |
| InternalIMT | epa | rs140301943 | . | . | . | . | . | . | . | . | . | . |
| InternalIMT | epa | rs78553679 | 0.006 | . | . | . | . | . | . | . | . | . |
| InternalIMT | epa | rs78334250 | 0.001 | . | . | . | . | . | . | . | . | . |
| InternalIMT | epa | rs76000028 | 0.001 | . | . | . | . | . | . | . | . | . |
| InternalIMT | epa | rs145195254 | 0.005 | . | . | . | . | . | . | . | . | . |
| InternalIMT | epa | rs137888527 | 0.005 | . | . | . | . | . | . | . | . | . |
| InternalIMT | epa | rs149109592 | . | . | . | . | . | . | . | . | . | . |
| InternalIMT | epa | rs80280010 | 0.013 | -0.033 | 0.029 | 0.253 | -0.184 | 0.078 | 0.018 | 0.045 | 0.137 | 0.744 |
| InternalIMT | epa | rs189995374 | . | . | . | . | . | . | . | . | . | . |
| InternalIMT | epa | rs75467770 | 0.002 | . | . | . | . | . | . | . | . | . |
| InternalIMT | epa | rs1487562 | 0.242 | -0.059 | 0.03 | 0.05 | 0.038 | 0.032 | 0.237 | 0.065 | 0.052 | 0.209 |
| InternalIMT | epa | rs12266963 | 0.095 | -0.033 | 0.032 | 0.297 | 0.055 | 0.042 | 0.191 | 7.90E-03 | 0.059 | 0.894 |
| InternalIMT | epa | rs7099874 | 0.857 | 9.40E-03 | 0.083 | 0.911 | 0.036 | 0.031 | 0.245 | -0.023 | 0.049 | 0.633 |
| InternalIMT | epa | rs75687019 | 0.001 | . | . | . | . | . | . | . | . | . |
| InternalIMT | epa | rs148240462 | 0.014 | -0.039 | 0.029 | 0.186 | -0.032 | 0.098 | 0.743 | 0.259 | 0.186 | 0.164 |
| InternalIMT | epa | rs141278174 | 0.001 | . | . | . | . | . | . | . | . | . |
| InternalIMT | epa | rs76404852 | 0.001 | . | . | . | . | . | . | . | . | . |
| InternalIMT | epa | rs139112240 | 0.004 | . | . | . | . | . | . | . | . | . |
| InternalIMT | epa | rs12783037 | 0.118 | -0.04 | 0.033 | 0.22 | 0.078 | 0.039 | 0.048 | 0.04 | 0.057 | 0.488 |
| InternalIMT | epa | rs72796481 | 0.003 | . | . | . | . | . | . | . | . | . |
| InternalIMT | epa | rs76798749 | 0.025 | -0.038 | 0.029 | 0.189 | 0.161 | 0.094 | 0.087 | 0.173 | 0.138 | 0.21 |
| InternalIMT | epa | rs187732548 | 0.001 | . | . | . | . | . | . | . | . | . |
| InternalIMT | epa | rs75675474 | 0.025 | -0.038 | 0.029 | 0.189 | 0.161 | 0.094 | 0.087 | 0.173 | 0.138 | 0.21 |
| InternalIMT | epa | rs76552293 | 0.025 | -0.038 | 0.029 | 0.188 | 0.161 | 0.094 | 0.087 | 0.174 | 0.138 | 0.209 |
| InternalIMT | epa | rs147445993 | 0.008 | . | . | . | . | . | . | . | . | . |
| InternalIMT | epa | rs115902283 | 0.025 | -0.038 | 0.029 | 0.188 | 0.161 | 0.094 | 0.088 | 0.174 | 0.138 | 0.208 |
| InternalIMT | epa | rs4540799 | 0.855 | 0.01 | 0.084 | 0.904 | 0.036 | 0.03 | 0.234 | -0.024 | 0.049 | 0.627 |
| InternalIMT | epa | rs12765320 | 0.35 | -0.058 | 0.034 | 0.091 | 0.052 | 0.026 | 0.049 | 0.042 | 0.04 | 0.287 |
| InternalIMT | epa | rs12782256 | 0.005 | . | . | . | . | . | . | . | . | . |
| InternalIMT | epa | rs191727256 | 0.007 | . | . | . | . | . | . | . | . | . |
| InternalIMT | epa | rs2082111 | 0.113 | -0.05 | 0.031 | 0.102 | 0.076 | 0.047 | 0.109 | 0.11 | 0.072 | 0.126 |
| InternalIMT | epa | rs2082110 | 0.257 | -0.057 | 0.031 | 0.065 | 0.042 | 0.031 | 0.18 | 0.059 | 0.049 | 0.234 |
| InternalIMT | epa | rs2082109 | 0.257 | -0.057 | 0.031 | 0.065 | 0.042 | 0.031 | 0.18 | 0.059 | 0.049 | 0.234 |
| InternalIMT | epa | rs117316662 | 0.001 | . | . | . | . | . | . | . | . | . |
| InternalIMT | epa | rs12247163 | 0.357 | -0.063 | 0.034 | 0.067 | 0.056 | 0.026 | 0.033 | 0.049 | 0.039 | 0.208 |
| InternalIMT | epa | rs12355888 | 0.037 | -0.043 | 0.029 | 0.128 | 0.124 | 0.091 | 0.174 | 0.252 | 0.133 | 0.057 |
| InternalIMT | epa | rs143772290 | 0.033 | -0.042 | 0.03 | 0.152 | 0.157 | 0.08 | 0.051 | 0.184 | 0.112 | 0.102 |
| InternalIMT | epa | rs146849452 | 0.009 | . | . | . | . | . | . | . | . | . |
| InternalIMT | epa | rs140267121 | 0.003 | . | . | . | . | . | . | . | . | . |
| InternalIMT | epa | rs7080474 | 0.352 | -0.071 | 0.034 | 0.039 | 0.057 | 0.027 | 0.032 | 0.062 | 0.039 | 0.118 |
| InternalIMT | epa | rs144066203 | 0.007 | . | . | . | . | . | . | . | . | . |
| InternalIMT | epa | rs12765209 | 0.005 | . | . | . | . | . | . | . | . | . |
| InternalIMT | epa | rs150930735 | 0.007 | . | . | . | . | . | . | . | . | . |
| InternalIMT | epa | rs7068039 | 0.365 | -0.069 | 0.035 | 0.047 | 0.061 | 0.026 | 0.02 | 0.056 | 0.039 | 0.149 |
| InternalIMT | epa | rs117622652 | 0.001 | . | . | . | . | . | . | . | . | . |
| InternalIMT | epa | rs190191514 | . | . | . | . | . | . | . | . | . | . |

| InternalIMT | epa | rs74130835 | 0.118 | -0.027 | 0.028 | 0.346 | -0.019 | 0.042 | 0.654 | -0.021 | 0.081 | 0.793 |
| --- | --- | --- | --- | --- | --- | --- | --- | --- | --- | --- | --- | --- |
| InternalIMT | epa | rs1811604 | 0.114 | -0.032 | 0.028 | 0.26 | -0.017 | 0.044 | 0.701 | 2.80E-03 | 0.084 | 0.974 |
| InternalIMT | epa | rs146971256 | . | . | . | . | . | . | . | . | . | . |
| InternalIMT | epa | rs74336000 | 0.033 | -0.043 | 0.03 | 0.151 | 0.157 | 0.08 | 0.052 | 0.186 | 0.112 | 0.099 |
| InternalIMT | epa | rs186046492 | 0.003 | . | . | . | . | . | . | . | . | . |
| InternalIMT | epa | rs34971823 | 0.009 | . | . | . | . | . | . | . | . | . |
| InternalIMT | epa | rs75227446 | 0.001 | . | . | . | . | . | . | . | . | . |
| InternalIMT | epa | rs190457599 | 0.001 | . | . | . | . | . | . | . | . | . |
| InternalIMT | epa | rs79591917 | 0.001 | . | . | . | . | . | . | . | . | . |
| InternalIMT | epa | rs702365 | 0.492 | 0.025 | 0.044 | 0.569 | -0.036 | 0.023 | 0.112 | -0.056 | 0.032 | 0.08 |
| InternalIMT | epa | rs79643836 | 0.004 | . | . | . | . | . | . | . | . | . |
| InternalIMT | epa | rs702366 | 0.493 | 0.025 | 0.044 | 0.567 | -0.038 | 0.023 | 0.101 | -0.056 | 0.032 | 0.079 |
| InternalIMT | epa | rs1059696 | 0.197 | -0.052 | 0.035 | 0.14 | 0.069 | 0.035 | 0.049 | 0.06 | 0.054 | 0.264 |
| InternalIMT | epa | rs901681 | 0.489 | 0.019 | 0.044 | 0.663 | -0.042 | 0.023 | 0.07 | -0.051 | 0.032 | 0.114 |
| InternalIMT | epa | rs61854092 | 0.196 | -0.052 | 0.035 | 0.14 | 0.07 | 0.035 | 0.047 | 0.061 | 0.054 | 0.263 |
| InternalIMT | epa | rs148080753 | . | . | . | . | . | . | . | . | . | . |
| InternalIMT | epa | rs61854093 | 0.315 | -0.047 | 0.032 | 0.144 | 0.038 | 0.027 | 0.162 | 0.028 | 0.041 | 0.494 |
| InternalIMT | epa | rs141822774 | 0 | . | . | . | . | . | . | . | . | . |
| InternalIMT | epa | rs74130836 | 0.118 | -0.026 | 0.028 | 0.35 | -0.019 | 0.042 | 0.648 | -0.022 | 0.081 | 0.785 |
| InternalIMT | epa | rs150620963 | 0.006 | . | . | . | . | . | . | . | . | . |
| InternalIMT | epa | rs3780913 | 0.849 | 0.02 | 0.081 | 0.808 | 0.029 | 0.03 | 0.34 | -0.03 | 0.048 | 0.535 |
| InternalIMT | epa | rs142255723 | 0.001 | . | . | . | . | . | . | . | . | . |
| InternalIMT | epa | rs3780914 | 0.479 | 0.021 | 0.044 | 0.637 | -0.033 | 0.023 | 0.15 | -0.053 | 0.032 | 0.102 |
| InternalIMT | epa | rs182656507 | 0.001 | . | . | . | . | . | . | . | . | . |
| InternalIMT | epa | rs2291427 | 0.821 | 0.059 | 0.077 | 0.448 | 8.00E-03 | 0.03 | 0.791 | -0.055 | 0.048 | 0.246 |
| InternalIMT | epa | rs10751382 | 0.816 | 0.055 | 0.074 | 0.456 | -2.20E-03 | 0.03 | 0.941 | -0.054 | 0.046 | 0.241 |
| InternalIMT | epa | rs117610108 | 0.001 | . | . | . | . | . | . | . | . | . |
| InternalIMT | epa | rs144966941 | 0.009 | . | . | . | . | . | . | . | . | . |
| InternalIMT | epa | rs41502051 | 0.014 | -0.032 | 0.029 | 0.259 | -0.101 | 0.092 | 0.277 | 0.06 | 0.131 | 0.645 |
| InternalIMT | epa | rs41426046 | 0.001 | . | . | . | . | . | . | . | . | . |
| InternalIMT | epa | rs41519248 | 0.001 | . | . | . | . | . | . | . | . | . |
| InternalIMT | epa | rs189108245 | 0.001 | . | . | . | . | . | . | . | . | . |
| InternalIMT | epa | rs7896431 | 0.497 | 9.10E-03 | 0.044 | 0.835 | -0.021 | 0.023 | 0.359 | -0.04 | 0.032 | 0.214 |
| InternalIMT | epa | rs183724547 | 0.009 | . | . | . | . | . | . | . | . | . |
| InternalIMT | epa | rs142044990 | 0.004 | . | . | . | . | . | . | . | . | . |
| InternalIMT | epa | rs188140929 | . | . | . | . | . | . | . | . | . | . |
| InternalIMT | epa | rs76819096 | 0.041 | -0.023 | 0.029 | 0.438 | -9.40E-03 | 0.074 | 0.899 | -0.125 | 0.133 | 0.347 |
| InternalIMT | epa | rs10751383 | 0.58 | 0.013 | 0.051 | 0.792 | -0.032 | 0.023 | 0.158 | -0.038 | 0.032 | 0.241 |
| InternalIMT | epa | rs117819321 | 0.005 | . | . | . | . | . | . | . | . | . |
| InternalIMT | epa | rs2242332 | 0.681 | 0.039 | 0.061 | 0.518 | -4.00E-03 | 0.024 | 0.866 | -0.052 | 0.038 | 0.169 |
| InternalIMT | epa | rs2242333 | 0.02 | -0.035 | 0.029 | 0.228 | -0.133 | 0.077 | 0.083 | 0.09 | 0.114 | 0.43 |
| InternalIMT | epa | rs2242334 | 0.681 | 0.037 | 0.061 | 0.549 | -2.80E-03 | 0.024 | 0.908 | -0.05 | 0.038 | 0.19 |
| InternalIMT | epa | rs189465041 | . | . | . | . | . | . | . | . | . | . |
| InternalIMT | epa | rs28395868 | 0.009 | . | . | . | . | . | . | . | . | . |
| InternalIMT | epa | rs56067550 | 0.008 | . | . | . | . | . | . | . | . | . |
| InternalIMT | epa | rs1051713 | 0.182 | -0.037 | 0.035 | 0.292 | 0.081 | 0.035 | 0.022 | 0.018 | 0.055 | 0.749 |
| InternalIMT | epa | rs115074210 | 0.048 | -0.026 | 0.029 | 0.377 | 1.40E-03 | 0.074 | 0.985 | -0.065 | 0.128 | 0.615 |
| InternalIMT | epa | rs7900977 | 0.893 | 0.08 | 0.096 | 0.404 | 0.024 | 0.035 | 0.482 | -0.062 | 0.054 | 0.252 |
| InternalIMT | epa | rs147613891 | 0.001 | . | . | . | . | . | . | . | . | . |
| InternalIMT | epa | rs2229136 | 0.161 | -0.038 | 0.034 | 0.264 | 7.00E-03 | 0.035 | 0.844 | 0.023 | 0.054 | 0.677 |
| InternalIMT | epa | rs185482841 | 0.009 | . | . | . | . | . | . | . | . | . |
| InternalIMT | epa | rs2288619 | 0.156 | -0.038 | 0.034 | 0.268 | -3.50E-03 | 0.035 | 0.92 | 0.02 | 0.054 | 0.708 |
| InternalIMT | epa | rs189891524 | . | . | . | . | . | . | . | . | . | . |
| InternalIMT | epa | rs56145077 | 0.176 | -0.035 | 0.035 | 0.306 | 0.07 | 0.034 | 0.039 | 0.013 | 0.055 | 0.81 |
| InternalIMT | epa | rs143216970 | 0.013 | -0.029 | 0.029 | 0.313 | -0.019 | 0.073 | 0.801 | -0.068 | 0.11 | 0.537 |
| InternalIMT | epa | rs142246478 | 0 | . | . | . | . | . | . | . | . | . |
| InternalIMT | epa | rs13134 | 0.012 | -0.035 | 0.029 | 0.216 | 0.044 | 0.1 | 0.664 | 0.227 | 0.146 | 0.121 |
| InternalIMT | epa | rs41314980 | 0.012 | -0.035 | 0.028 | 0.225 | -0.02 | 0.15 | 0.895 | 0.14 | 0.223 | 0.529 |
| InternalIMT | epa | rs41304611 | 0.005 | . | . | . | . | . | . | . | . | . |
| InternalIMT | epa | rs193299617 | . | . | . | . | . | . | . | . | . | . |
| InternalIMT | epa | rs12772724 | 0.012 | -0.035 | 0.029 | 0.216 | 0.044 | 0.1 | 0.664 | 0.227 | 0.146 | 0.121 |
| InternalIMT | epa | rs139489537 | 0.011 | -0.033 | 0.029 | 0.251 | -0.146 | 0.084 | 0.082 | 0.088 | 0.133 | 0.511 |
| InternalIMT | epa | rs12776062 | 0.005 | . | . | . | . | . | . | . | . | . |
| InternalIMT | epa | rs752059 | 0.181 | -0.042 | 0.034 | 0.226 | 0.065 | 0.034 | 0.055 | 0.031 | 0.055 | 0.569 |
| InternalIMT | epa | rs147649982 | 0.013 | -0.038 | 0.026 | 0.147 | 0.084 | 0.213 | 0.692 | 0.2 | 0.407 | 0.624 |
| InternalIMT | epa | rs17157798 | 0.113 | -0.033 | 0.032 | 0.315 | -0.025 | 0.04 | 0.537 | 4.40E-03 | 0.065 | 0.946 |
| InternalIMT | epa | rs142298619 | 0.009 | . | . | . | . | . | . | . | . | . |
| InternalIMT | epa | rs4948673 | 0.893 | 0.066 | 0.093 | 0.482 | 0.037 | 0.035 | 0.291 | -0.054 | 0.053 | 0.308 |
| InternalIMT | epa | rs74231484 | 0.045 | -0.03 | 0.03 | 0.313 | -0.065 | 0.062 | 0.293 | -0.021 | 0.087 | 0.813 |
| InternalIMT | epa | rs72796489 | 0.001 | . | . | . | . | . | . | . | . | . |
| InternalIMT | epa | rs113447578 | 0.006 | . | . | . | . | . | . | . | . | . |
| InternalIMT | epa | rs34063374 | 0.014 | -0.036 | 0.029 | 0.215 | 0.044 | 0.096 | 0.646 | 0.211 | 0.144 | 0.142 |
| InternalIMT | epa | rs11239527 | 0.006 | . | . | . | . | . | . | . | . | . |

| InternalIMT | epa | rs34371883 | 0.007 | . | . | . | . | . | . | . | . | . |
| --- | --- | --- | --- | --- | --- | --- | --- | --- | --- | --- | --- | --- |
| InternalIMT | epa | rs118168828 | 0.004 | . | . | . | . | . | . | . | . | . |
| InternalIMT | epa | rs34349618 | 0.005 | . | . | . | . | . | . | . | . | . |
| InternalIMT | epa | rs80041679 | 0.011 | -0.033 | 0.029 | 0.25 | -0.144 | 0.083 | 0.083 | 0.09 | 0.131 | 0.492 |
| InternalIMT | epa | rs149640607 | 0.01 | -0.036 | 0.029 | 0.205 | 0.064 | 0.158 | 0.687 | 0.232 | 0.23 | 0.313 |
| InternalIMT | epa | rs4427523 | 0.932 | 0.063 | 0.157 | 0.689 | 0.022 | 0.054 | 0.676 | -0.051 | 0.085 | 0.554 |
| InternalIMT | epa | rs185880109 | . | . | . | . | . | . | . | . | . | . |
| InternalIMT | epa | rs191523220 | 0.001 | . | . | . | . | . | . | . | . | . |
| InternalIMT | epa | rs144315284 | 0.02 | -0.034 | 0.029 | 0.248 | -0.059 | 0.088 | 0.505 | 0.051 | 0.109 | 0.644 |
| InternalIMT | epa | rs4948674 | 0.329 | -5.90E-03 | 0.036 | 0.871 | -4.60E-03 | 0.024 | 0.846 | -0.037 | 0.037 | 0.323 |
| InternalIMT | epa | rs182984298 | 0.002 | . | . | . | . | . | . | . | . | . |
| InternalIMT | epa | rs56749023 | 0.071 | -0.024 | 0.03 | 0.436 | -0.031 | 0.047 | 0.512 | -0.059 | 0.078 | 0.451 |
| InternalIMT | epa | rs112508957 | 0.045 | -0.033 | 0.029 | 0.264 | 0.128 | 0.057 | 0.026 | 0.013 | 0.097 | 0.896 |
| InternalIMT | epa | rs7089063 | 0.313 | -0.029 | 0.041 | 0.475 | 0.015 | 0.027 | 0.575 | -3.40E-03 | 0.044 | 0.939 |
| InternalIMT | epa | rs145000779 | . | . | . | . | . | . | . | . | . | . |
| InternalIMT | epa | rs80201572 | 0.013 | -0.032 | 0.029 | 0.259 | -0.144 | 0.077 | 0.062 | 0.035 | 0.123 | 0.773 |
| InternalIMT | epa | rs140568227 | 0.003 | . | . | . | . | . | . | . | . | . |
| InternalIMT | epa | rs149756228 | 0.004 | . | . | . | . | . | . | . | . | . |
| InternalIMT | epa | rs112380685 | 0.003 | . | . | . | . | . | . | . | . | . |
| InternalIMT | epa | rs60481109 | 0.067 | -0.023 | 0.03 | 0.442 | -0.032 | 0.048 | 0.509 | -0.064 | 0.079 | 0.418 |
| InternalIMT | epa | rs138221843 | . | . | . | . | . | . | . | . | . | . |
| InternalIMT | epa | rs150283194 | 0.009 | . | . | . | . | . | . | . | . | . |
| InternalIMT | epa | rs138879661 | 0.008 | . | . | . | . | . | . | . | . | . |
| InternalIMT | epa | rs34960345 | 0.067 | -0.035 | 0.03 | 0.248 | -7.50E-03 | 0.044 | 0.867 | 0.035 | 0.07 | 0.623 |
| InternalIMT | epa | rs147514068 | . | . | . | . | . | . | . | . | . | . |
| InternalIMT | epa | rs35410535 | 0.138 | -0.027 | 0.033 | 0.408 | -0.02 | 0.035 | 0.566 | -0.017 | 0.057 | 0.758 |
| InternalIMT | epa | rs191613635 | 0.001 | . | . | . | . | . | . | . | . | . |
| InternalIMT | epa | rs17157800 | 0.004 | . | . | . | . | . | . | . | . | . |
| InternalIMT | epa | rs17523413 | 0.027 | -0.037 | 0.029 | 0.209 | -0.037 | 0.063 | 0.555 | 0.136 | 0.091 | 0.134 |
| InternalIMT | epa | rs7897036 | 0.013 | -0.034 | 0.028 | 0.226 | 0.231 | 0.15 | 0.122 | 0.147 | 0.307 | 0.632 |
| InternalIMT | epa | rs76334323 | 0.001 | . | . | . | . | . | . | . | . | . |
| InternalIMT | epa | rs143535991 | 0 | . | . | . | . | . | . | . | . | . |
| InternalIMT | epa | rs140344979 | 0.002 | . | . | . | . | . | . | . | . | . |
| InternalIMT | epa | rs36047811 | 0.138 | -0.027 | 0.033 | 0.406 | -0.02 | 0.035 | 0.567 | -0.017 | 0.057 | 0.764 |
| InternalIMT | epa | rs76493570 | 0.057 | -0.032 | 0.03 | 0.291 | -0.025 | 0.045 | 0.582 | 5.30E-03 | 0.071 | 0.94 |
| InternalIMT | epa | rs192998920 | . | . | . | . | . | . | . | . | . | . |
| InternalIMT | epa | rs116840852 | 0.007 | . | . | . | . | . | . | . | . | . |
| InternalIMT | epa | rs184824101 | 0 | . | . | . | . | . | . | . | . | . |
| InternalIMT | epa | rs41301621 | 0.002 | . | . | . | . | . | . | . | . | . |
| InternalIMT | epa | rs3740109 | 0.138 | -0.028 | 0.033 | 0.404 | -0.02 | 0.035 | 0.567 | -0.016 | 0.057 | 0.772 |
| InternalIMT | epa | rs7899415 | 0.032 | -0.028 | 0.028 | 0.331 | -0.037 | 0.081 | 0.651 | -0.057 | 0.14 | 0.685 |
| InternalIMT | epa | rs184395284 | 0.001 | . | . | . | . | . | . | . | . | . |
| InternalIMT | epa | rs139209589 | 0.002 | . | . | . | . | . | . | . | . | . |
| InternalIMT | epa | rs149924163 | 0.009 | . | . | . | . | . | . | . | . | . |
| InternalIMT | epa | rs3740110 | 0.001 | . | . | . | . | . | . | . | . | . |
| InternalIMT | epa | rs144335113 | 0.009 | . | . | . | . | . | . | . | . | . |
| InternalIMT | epa | rs74743385 | 0.045 | -0.033 | 0.029 | 0.264 | 0.128 | 0.057 | 0.026 | 0.013 | 0.097 | 0.895 |
| InternalIMT | epa | rs146545829 | 0.009 | . | . | . | . | . | . | . | . | . |
| InternalIMT | epa | rs181842689 | . | . | . | . | . | . | . | . | . | . |
| InternalIMT | epa | rs3740111 | 0.067 | -0.035 | 0.03 | 0.245 | -7.60E-03 | 0.044 | 0.865 | 0.037 | 0.07 | 0.601 |
| InternalIMT | epa | rs186261599 | 0.009 | . | . | . | . | . | . | . | . | . |
| InternalIMT | epa | rs73285348 | 0.004 | . | . | . | . | . | . | . | . | . |
| InternalIMT | epa | rs186356901 | 0.009 | . | . | . | . | . | . | . | . | . |
| InternalIMT | epa | rs3824618 | 0.011 | -0.033 | 0.029 | 0.245 | -0.144 | 0.083 | 0.081 | 0.099 | 0.127 | 0.436 |
| InternalIMT | epa | rs3802548 | 0.201 | -0.047 | 0.036 | 0.187 | 0.054 | 0.032 | 0.091 | 0.042 | 0.053 | 0.428 |
| InternalIMT | epa | rs3802547 | 0.001 | . | . | . | . | . | . | . | . | . |
| InternalIMT | epa | rs77677521 | 0.007 | . | . | . | . | . | . | . | . | . |
| InternalIMT | epa | rs3824616 | 0.138 | -0.028 | 0.033 | 0.4 | -0.02 | 0.035 | 0.566 | -0.016 | 0.057 | 0.784 |
| InternalIMT | epa | rs191073818 | 0.009 | . | . | . | . | . | . | . | . | . |
| InternalIMT | epa | rs147078004 | . | . | . | . | . | . | . | . | . | . |
| InternalIMT | epa | rs7908745 | 0.342 | -0.045 | 0.043 | 0.292 | 0.028 | 0.027 | 0.293 | 0.022 | 0.045 | 0.619 |
| InternalIMT | epa | rs17157811 | 0.004 | . | . | . | . | . | . | . | . | . |
| InternalIMT | epa | rs138506087 | 0.002 | . | . | . | . | . | . | . | . | . |
| InternalIMT | epa | rs77241269 | 0.071 | -0.024 | 0.03 | 0.43 | -0.03 | 0.047 | 0.525 | -0.056 | 0.078 | 0.474 |
| InternalIMT | epa | rs138374938 | 0.001 | . | . | . | . | . | . | . | . | . |
| InternalIMT | epa | rs2279433 | 0.068 | -0.037 | 0.031 | 0.22 | -7.80E-03 | 0.043 | 0.855 | 0.053 | 0.065 | 0.417 |
| InternalIMT | epa | rs73285358 | 0.071 | -0.024 | 0.03 | 0.429 | -0.03 | 0.047 | 0.527 | -0.056 | 0.078 | 0.476 |
| InternalIMT | epa | rs11239528 | 0.198 | -0.046 | 0.036 | 0.2 | 0.056 | 0.032 | 0.084 | 0.039 | 0.054 | 0.474 |
| InternalIMT | epa | rs77453386 | 0.001 | . | . | . | . | . | . | . | . | . |
| InternalIMT | epa | rs2279434 | 0.071 | -0.037 | 0.031 | 0.232 | -4.00E-03 | 0.042 | 0.925 | 0.044 | 0.064 | 0.493 |
| InternalIMT | epa | rs149236525 | 0.003 | . | . | . | . | . | . | . | . | . |
| InternalIMT | epa | rs12573609 | 0.018 | -0.027 | 0.029 | 0.351 | -0.159 | 0.096 | 0.1 | -0.116 | 0.133 | 0.383 |
| InternalIMT | epa | rs191716655 | 0.006 | . | . | . | . | . | . | . | . | . |

| InternalIMT | epa | rs17523660 | 0.005 | . | . | . | . | . | . | . | . | . |
| --- | --- | --- | --- | --- | --- | --- | --- | --- | --- | --- | --- | --- |
| InternalIMT | epa | rs142671360 | 0.001 | . | . | . | . | . | . | . | . | . |
| InternalIMT | epa | rs3764990 | 0.025 | -0.038 | 0.029 | 0.19 | -0.023 | 0.065 | 0.725 | 0.186 | 0.089 | 0.037 |
| InternalIMT | epa | rs113682861 | 0.001 | . | . | . | . | . | . | . | . | . |
| InternalIMT | epa | rs117658270 | 0.001 | . | . | . | . | . | . | . | . | . |
| InternalIMT | epa | rs187309063 | . | . | . | . | . | . | . | . | . | . |
| InternalIMT | epa | rs73285360 | 0.072 | -0.024 | 0.03 | 0.429 | -0.03 | 0.047 | 0.528 | -0.055 | 0.078 | 0.477 |
| InternalIMT | epa | rs80133289 | 0.002 | . | . | . | . | . | . | . | . | . |
| InternalIMT | epa | rs186693257 | . | . | . | . | . | . | . | . | . | . |
| InternalIMT | epa | rs35735968 | 0.081 | -0.037 | 0.031 | 0.237 | -0.017 | 0.041 | 0.674 | 0.038 | 0.064 | 0.557 |
| InternalIMT | epa | rs17157825 | 0.011 | -0.034 | 0.029 | 0.233 | -0.124 | 0.085 | 0.143 | 0.122 | 0.126 | 0.332 |
| InternalIMT | epa | rs2291428 | 0.183 | -0.04 | 0.035 | 0.256 | 0.072 | 0.033 | 0.032 | 0.025 | 0.056 | 0.658 |
| InternalIMT | epa | rs2291429 | 0.21 | -0.04 | 0.036 | 0.267 | 0.05 | 0.032 | 0.12 | 0.023 | 0.054 | 0.665 |
| InternalIMT | epa | rs145714792 | 0.002 | . | . | . | . | . | . | . | . | . |
| InternalIMT | epa | rs12769244 | 0.068 | -0.037 | 0.031 | 0.225 | -8.10E-03 | 0.042 | 0.849 | 0.05 | 0.065 | 0.441 |
| InternalIMT | epa | rs12771275 | 0.068 | -0.037 | 0.031 | 0.225 | -8.10E-03 | 0.042 | 0.848 | 0.05 | 0.065 | 0.441 |
| InternalIMT | epa | rs146266174 | 0.001 | . | . | . | . | . | . | . | . | . |
| InternalIMT | epa | rs41301623 | 0.001 | . | . | . | . | . | . | . | . | . |
| InternalIMT | epa | rs2306814 | 0.001 | . | . | . | . | . | . | . | . | . |
| InternalIMT | epa | rs12244834 | 0.08 | -0.028 | 0.031 | 0.373 | 0.048 | 0.047 | 0.303 | -0.024 | 0.073 | 0.741 |
| InternalIMT | epa | rs184006011 | 0.001 | . | . | . | . | . | . | . | . | . |
| InternalIMT | epa | rs78829066 | 0.001 | . | . | . | . | . | . | . | . | . |
| InternalIMT | epa | rs34731408 | 0.068 | -0.037 | 0.031 | 0.225 | -8.10E-03 | 0.042 | 0.848 | 0.05 | 0.065 | 0.441 |
| InternalIMT | epa | rs17451516 | 0.012 | -0.035 | 0.029 | 0.216 | 0.047 | 0.1 | 0.64 | 0.224 | 0.147 | 0.128 |
| InternalIMT | epa | rs139910133 | 0.009 | . | . | . | . | . | . | . | . | . |
| InternalIMT | epa | rs10900218 | 0.319 | -0.039 | 0.041 | 0.347 | 0.031 | 0.028 | 0.267 | 0.014 | 0.046 | 0.769 |
| InternalIMT | epa | rs146023999 | . | . | . | . | . | . | . | . | . | . |
| InternalIMT | epa | rs187296958 | 0 | . | . | . | . | . | . | . | . | . |
| InternalIMT | epa | rs142342833 | . | . | . | . | . | . | . | . | . | . |
| InternalIMT | epa | rs143489255 | . | . | . | . | . | . | . | . | . | . |
| InternalIMT | epa | rs138532518 | . | . | . | . | . | . | . | . | . | . |
| InternalIMT | epa | rs183768079 | 0.007 | . | . | . | . | . | . | . | . | . |
| InternalIMT | epa | rs117892272 | . | . | . | . | . | . | . | . | . | . |
| InternalIMT | epa | rs192993943 | . | . | . | . | . | . | . | . | . | . |
| InternalIMT | epa | rs182989043 | 0.001 | . | . | . | . | . | . | . | . | . |
| InternalIMT | epa | rs189703435 | 0.001 | . | . | . | . | . | . | . | . | . |
| InternalIMT | epa | rs188794040 | 0 | . | . | . | . | . | . | . | . | . |
| InternalIMT | epa | rs143920953 | . | . | . | . | . | . | . | . | . | . |
| InternalIMT | epa | rs191029121 | . | . | . | . | . | . | . | . | . | . |
| InternalIMT | epa | rs116875448 | . | . | . | . | . | . | . | . | . | . |
| InternalIMT | epa | rs190167877 | 0.003 | . | . | . | . | . | . | . | . | . |
| InternalIMT | epa | rs184993418 | . | . | . | . | . | . | . | . | . | . |
| InternalIMT | epa | rs143241158 | . | . | . | . | . | . | . | . | . | . |
| InternalIMT | epa | rs11239500 | 0.074 | -0.039 | 0.032 | 0.228 | 0.034 | 0.042 | 0.413 | 0.042 | 0.059 | 0.481 |
| InternalIMT | epa | rs140568994 | . | . | . | . | . | . | . | . | . | . |
| InternalIMT | epa | rs187530317 | 0.001 | . | . | . | . | . | . | . | . | . |
| InternalIMT | epa | rs145189326 | 0.001 | . | . | . | . | . | . | . | . | . |
| InternalIMT | epa | rs186203332 | . | . | . | . | . | . | . | . | . | . |
| InternalIMT | epa | rs189921909 | . | . | . | . | . | . | . | . | . | . |
| InternalIMT | epa | rs187260989 | . | . | . | . | . | . | . | . | . | . |
| InternalIMT | epa | rs114376586 | 0.073 | -0.039 | 0.032 | 0.226 | 0.034 | 0.042 | 0.413 | 0.043 | 0.059 | 0.467 |
| InternalIMT | epa | rs151070808 | . | . | . | . | . | . | . | . | . | . |
| InternalIMT | epa | rs140927225 | 0.074 | -0.04 | 0.032 | 0.212 | 0.039 | 0.042 | 0.358 | 0.048 | 0.058 | 0.413 |
| InternalIMT | epa | rs191555615 | . | . | . | . | . | . | . | . | . | . |
| InternalIMT | epa | rs80278898 | 0.001 | . | . | . | . | . | . | . | . | . |
| InternalIMT | epa | rs190849503 | . | . | . | . | . | . | . | . | . | . |
| InternalIMT | epa | rs150080315 | 0.007 | . | . | . | . | . | . | . | . | . |
| InternalIMT | epa | rs143623885 | . | . | . | . | . | . | . | . | . | . |
| InternalIMT | epa | rs187760040 | . | . | . | . | . | . | . | . | . | . |
| InternalIMT | epa | rs143917143 | . | . | . | . | . | . | . | . | . | . |
| InternalIMT | epa | rs149851136 | 0.002 | . | . | . | . | . | . | . | . | . |
| InternalIMT | epa | rs181612054 | . | . | . | . | . | . | . | . | . | . |
| InternalIMT | epa | rs191006548 | . | . | . | . | . | . | . | . | . | . |
| InternalIMT | epa | rs117377631 | 0.003 | . | . | . | . | . | . | . | . | . |
| InternalIMT | epa | rs182107634 | . | . | . | . | . | . | . | . | . | . |
| InternalIMT | epa | rs190211867 | . | . | . | . | . | . | . | . | . | . |
| InternalIMT | epa | rs148293457 | . | . | . | . | . | . | . | . | . | . |
| InternalIMT | epa | rs191811733 | . | . | . | . | . | . | . | . | . | . |
| InternalIMT | epa | rs146109272 | . | . | . | . | . | . | . | . | . | . |
| InternalIMT | epa | rs148397915 | . | . | . | . | . | . | . | . | . | . |
| InternalIMT | epa | rs142233290 | . | . | . | . | . | . | . | . | . | . |
| InternalIMT | epa | rs80226874 | . | . | . | . | . | . | . | . | . | . |
| InternalIMT | epa | rs144496203 | 0 | . | . | . | . | . | . | . | . | . |

| InternalIMT | epa | rs191062130 | . | . | . | . | . | . | . | . | . | . |
| --- | --- | --- | --- | --- | --- | --- | --- | --- | --- | --- | --- | --- |
| InternalIMT | epa | rs185807817 | . | . | . | . | . | . | . | . | . | . |
| InternalIMT | epa | rs117846213 | 0.001 | . | . | . | . | . | . | . | . | . |
| InternalIMT | epa | rs190545473 | . | . | . | . | . | . | . | . | . | . |
| InternalIMT | epa | rs192364918 | . | . | . | . | . | . | . | . | . | . |
| InternalIMT | epa | rs182939241 | . | . | . | . | . | . | . | . | . | . |
| InternalIMT | epa | rs12571125 | 0.001 | . | . | . | . | . | . | . | . | . |
| InternalIMT | epa | rs192418941 | 0 | . | . | . | . | . | . | . | . | . |
| InternalIMT | epa | rs141270243 | . | . | . | . | . | . | . | . | . | . |
| InternalIMT | epa | rs182615502 | . | . | . | . | . | . | . | . | . | . |
| InternalIMT | epa | rs146669856 | 0.02 | -0.04 | 0.029 | 0.162 | 0.155 | 0.107 | 0.146 | 0.17 | 0.17 | 0.32 |
| InternalIMT | epa | rs78290029 | 0.098 | -0.026 | 0.032 | 0.421 | 0.071 | 0.042 | 0.091 | -0.028 | 0.061 | 0.645 |
| InternalIMT | epa | rs139955565 | . | . | . | . | . | . | . | . | . | . |
| InternalIMT | epa | rs12267314 | 0.087 | -0.03 | 0.032 | 0.343 | 0.058 | 0.043 | 0.177 | -8.50E-03 | 0.061 | 0.889 |
| InternalIMT | epa | rs74477197 | . | . | . | . | . | . | . | . | . | . |
| InternalIMT | epa | rs188147236 | . | . | . | . | . | . | . | . | . | . |
| InternalIMT | epa | rs2228065 | 0.086 | -0.03 | 0.031 | 0.341 | 0.057 | 0.044 | 0.19 | -9.20E-03 | 0.061 | 0.881 |
| InternalIMT | epa | rs116961353 | . | . | . | . | . | . | . | . | . | . |
| InternalIMT | epa | rs190222272 | . | . | . | . | . | . | . | . | . | . |
| InternalIMT | epa | rs182719940 | . | . | . | . | . | . | . | . | . | . |
| InternalIMT | epa | rs184477413 | . | . | . | . | . | . | . | . | . | . |
| InternalIMT | epa | rs12265175 | 0.089 | -0.032 | 0.032 | 0.319 | 0.065 | 0.044 | 0.136 | -2.20E-04 | 0.061 | 0.997 |
| InternalIMT | epa | rs182928276 | . | . | . | . | . | . | . | . | . | . |
| InternalIMT | epa | rs144409895 | . | . | . | . | . | . | . | . | . | . |
| InternalIMT | epa | rs58998447 | 0.003 | . | . | . | . | . | . | . | . | . |
| InternalIMT | epa | rs188668832 | . | . | . | . | . | . | . | . | . | . |
| InternalIMT | epa | rs148641686 | . | . | . | . | . | . | . | . | . | . |
| InternalIMT | epa | rs75468791 | . | . | . | . | . | . | . | . | . | . |
| InternalIMT | epa | rs147327397 | . | . | . | . | . | . | . | . | . | . |
| InternalIMT | epa | rs77354597 | 0.002 | . | . | . | . | . | . | . | . | . |
| InternalIMT | epa | rs41365544 | 0.082 | -0.028 | 0.032 | 0.381 | 0.046 | 0.046 | 0.321 | -0.025 | 0.069 | 0.724 |
| InternalIMT | epa | rs187564188 | . | . | . | . | . | . | . | . | . | . |
| InternalIMT | epa | rs183511984 | 0 | . | . | . | . | . | . | . | . | . |
| InternalIMT | epa | rs12262783 | 0.08 | -0.026 | 0.031 | 0.407 | 0.052 | 0.047 | 0.265 | -0.036 | 0.071 | 0.608 |
| InternalIMT | epa | rs188817687 | 0.002 | . | . | . | . | . | . | . | . | . |
| InternalIMT | epa | rs3815968 | . | . | . | . | . | . | . | . | . | . |
| InternalIMT | epa | rs187822300 | . | . | . | . | . | . | . | . | . | . |
| InternalIMT | epa | rs188902172 | 0.002 | . | . | . | . | . | . | . | . | . |
| InternalIMT | epa | rs181190448 | . | . | . | . | . | . | . | . | . | . |
| InternalIMT | epa | rs182699093 | . | . | . | . | . | . | . | . | . | . |
| InternalIMT | epa | rs182631303 | 0.001 | . | . | . | . | . | . | . | . | . |
| InternalIMT | epa | rs187014172 | . | . | . | . | . | . | . | . | . | . |
| InternalIMT | epa | rs142575406 | . | . | . | . | . | . | . | . | . | . |
| InternalIMT | epa | rs180711033 | . | . | . | . | . | . | . | . | . | . |
| InternalIMT | epa | rs137894823 | 0.004 | . | . | . | . | . | . | . | . | . |
| InternalIMT | epa | rs189053273 | 0.001 | . | . | . | . | . | . | . | . | . |
| InternalIMT | epa | rs143499388 | 0.002 | . | . | . | . | . | . | . | . | . |
| InternalIMT | epa | rs182936001 | 0.006 | . | . | . | . | . | . | . | . | . |
| InternalIMT | epa | rs148456406 | 0.006 | . | . | . | . | . | . | . | . | . |
| InternalIMT | epa | rs182499531 | 0.008 | . | . | . | . | . | . | . | . | . |
| InternalIMT | epa | rs74884915 | 0.026 | -0.024 | 0.03 | 0.416 | -0.039 | 0.063 | 0.538 | -0.105 | 0.094 | 0.264 |
| InternalIMT | epa | rs184046374 | 0.001 | . | . | . | . | . | . | . | . | . |
| InternalIMT | epa | rs74333993 | 0.032 | -0.037 | 0.03 | 0.22 | 0.025 | 0.063 | 0.69 | 0.065 | 0.076 | 0.391 |
| InternalIMT | epa | rs138889719 | 0.001 | . | . | . | . | . | . | . | . | . |
| InternalIMT | epa | rs75330330 | 0.022 | -0.035 | 0.029 | 0.229 | 0.116 | 0.08 | 0.148 | 0.124 | 0.115 | 0.277 |
| InternalIMT | epa | rs193051693 | 0 | . | . | . | . | . | . | . | . | . |
| InternalIMT | epa | rs145929633 | 0 | . | . | . | . | . | . | . | . | . |
| InternalIMT | epa | rs184076904 | 0.004 | . | . | . | . | . | . | . | . | . |
| InternalIMT | epa | rs141802494 | 0.018 | -0.035 | 0.029 | 0.225 | 0.14 | 0.097 | 0.147 | 0.161 | 0.125 | 0.197 |
| InternalIMT | epa | rs149519759 | 0.002 | . | . | . | . | . | . | . | . | . |
| InternalIMT | epa | rs145467260 | 0.013 | -0.026 | 0.029 | 0.372 | -0.032 | 0.095 | 0.737 | -0.157 | 0.132 | 0.234 |
| InternalIMT | epa | rs79620914 | 0.027 | -0.025 | 0.029 | 0.39 | -0.096 | 0.055 | 0.082 | -0.08 | 0.079 | 0.309 |
| InternalIMT | epa | rs140538633 | 0.017 | -0.027 | 0.029 | 0.355 | -0.061 | 0.067 | 0.363 | -0.102 | 0.1 | 0.309 |
| InternalIMT | epa | rs140862301 | 0.008 | . | . | . | . | . | . | . | . | . |
| InternalIMT | epa | rs114615847 | 0.009 | . | . | . | . | . | . | . | . | . |
| InternalIMT | epa | rs141800259 | 0.026 | -0.037 | 0.029 | 0.208 | 0.104 | 0.082 | 0.207 | 0.141 | 0.117 | 0.226 |
| InternalIMT | epa | rs149779059 | 0.019 | -0.038 | 0.029 | 0.178 | 0.154 | 0.093 | 0.097 | 0.155 | 0.158 | 0.324 |
| InternalIMT | epa | rs116539253 | 0.007 | . | . | . | . | . | . | . | . | . |
| InternalIMT | epa | rs184979550 | 0.001 | . | . | . | . | . | . | . | . | . |
| InternalIMT | epa | rs190128940 | 0.004 | . | . | . | . | . | . | . | . | . |
| InternalIMT | epa | rs146594816 | 0.002 | . | . | . | . | . | . | . | . | . |
| InternalIMT | epa | rs141370766 | 0.022 | -0.035 | 0.029 | 0.229 | 0.119 | 0.081 | 0.142 | 0.126 | 0.115 | 0.274 |
| InternalIMT | epa | rs144670033 | 0.002 | . | . | . | . | . | . | . | . | . |

| InternalIMT | epa | rs187152174 | 0.007 | . | . | . | . | . | . | . | . | . |
| --- | --- | --- | --- | --- | --- | --- | --- | --- | --- | --- | --- | --- |
| InternalIMT | epa | rs184180263 | 0.005 | . | . | . | . | . | . | . | . | . |
| InternalIMT | epa | rs140075861 | 0.01 | -0.029 | 0.029 | 0.319 | -0.024 | 0.079 | 0.762 | -0.115 | 0.131 | 0.377 |
| InternalIMT | epa | rs186483129 | 0 | . | . | . | . | . | . | . | . | . |
| InternalIMT | epa | rs186634282 | 0.012 | -0.044 | 0.028 | 0.114 | 0.411 | 0.142 | 3.80E-03 | 0.56 | 0.209 | 7.40E-03 |
| InternalIMT | epa | rs9988663 | 0.032 | -0.037 | 0.03 | 0.219 | 0.026 | 0.063 | 0.684 | 0.065 | 0.076 | 0.389 |
| InternalIMT | epa | rs138828529 | 0.02 | -0.03 | 0.029 | 0.295 | -0.02 | 0.116 | 0.861 | -0.056 | 0.193 | 0.77 |
| InternalIMT | epa | rs188777615 | 0.004 | . | . | . | . | . | . | . | . | . |
| InternalIMT | epa | rs192321882 | 0.008 | . | . | . | . | . | . | . | . | . |
| InternalIMT | epa | rs150812270 | 0.027 | -0.027 | 0.029 | 0.352 | -0.012 | 0.076 | 0.874 | -0.074 | 0.126 | 0.557 |
| InternalIMT | epa | rs185906603 | 0.001 | . | . | . | . | . | . | . | . | . |
| InternalIMT | epa | rs191070862 | 0.004 | . | . | . | . | . | . | . | . | . |
| InternalIMT | epa | rs182904978 | 0 | . | . | . | . | . | . | . | . | . |
| InternalIMT | epa | rs192679655 | 0.001 | . | . | . | . | . | . | . | . | . |
| InternalIMT | epa | rs189673086 | 0.007 | . | . | . | . | . | . | . | . | . |
| InternalIMT | epa | rs181141892 | 0.002 | . | . | . | . | . | . | . | . | . |
| InternalIMT | epa | rs186404123 | 0 | . | . | . | . | . | . | . | . | . |
| InternalIMT | epa | rs192117287 | 0.005 | . | . | . | . | . | . | . | . | . |
| InternalIMT | epa | rs182748835 | 0.001 | . | . | . | . | . | . | . | . | . |
| InternalIMT | epa | rs141854549 | 0.017 | -0.027 | 0.029 | 0.354 | -0.06 | 0.067 | 0.369 | -0.102 | 0.1 | 0.308 |
| InternalIMT | epa | rs140163397 | 0.005 | . | . | . | . | . | . | . | . | . |
| InternalIMT | epa | rs146347300 | 0.008 | . | . | . | . | . | . | . | . | . |
| InternalIMT | epa | rs139362377 | 0.017 | -0.027 | 0.029 | 0.354 | -0.06 | 0.067 | 0.369 | -0.102 | 0.1 | 0.308 |
| InternalIMT | epa | rs185087671 | 0.001 | . | . | . | . | . | . | . | . | . |
| InternalIMT | epa | rs190466574 | 0.005 | . | . | . | . | . | . | . | . | . |
| InternalIMT | epa | rs147012778 | 0.005 | . | . | . | . | . | . | . | . | . |
| InternalIMT | epa | rs138236960 | 0.009 | . | . | . | . | . | . | . | . | . |
| InternalIMT | epa | rs116310311 | 0.017 | -0.027 | 0.029 | 0.354 | -0.06 | 0.067 | 0.37 | -0.102 | 0.1 | 0.309 |
| InternalIMT | epa | rs142868356 | 0.012 | -0.036 | 0.028 | 0.199 | 0.228 | 0.156 | 0.144 | 0.249 | 0.28 | 0.374 |
| InternalIMT | epa | rs144956240 | 0.003 | . | . | . | . | . | . | . | . | . |
| InternalIMT | epa | rs188527776 | 0.002 | . | . | . | . | . | . | . | . | . |
| InternalIMT | epa | rs183971165 | 0.007 | . | . | . | . | . | . | . | . | . |
| InternalIMT | epa | rs76748216 | 0.009 | . | . | . | . | . | . | . | . | . |
| InternalIMT | epa | rs148554990 | 0.001 | . | . | . | . | . | . | . | . | . |
| InternalIMT | epa | rs114446477 | 0.027 | -0.025 | 0.029 | 0.387 | -0.102 | 0.055 | 0.065 | -0.077 | 0.079 | 0.33 |
| InternalIMT | epa | rs185223933 | 0.006 | . | . | . | . | . | . | . | . | . |
| InternalIMT | epa | rs76869726 | 0.009 | . | . | . | . | . | . | . | . | . |
| InternalIMT | epa | rs151200830 | 0.017 | -0.027 | 0.029 | 0.356 | -0.056 | 0.067 | 0.403 | -0.103 | 0.101 | 0.305 |
| InternalIMT | epa | rs17157731 | 0.017 | -0.027 | 0.029 | 0.356 | -0.056 | 0.067 | 0.403 | -0.103 | 0.101 | 0.306 |
| InternalIMT | epa | rs181243469 | 0 | . | . | . | . | . | . | . | . | . |
| InternalIMT | epa | rs186890707 | 0 | . | . | . | . | . | . | . | . | . |
| InternalIMT | epa | rs184063360 | 0 | . | . | . | . | . | . | . | . | . |
| InternalIMT | epa | rs188550237 | 0.007 | . | . | . | . | . | . | . | . | . |
| InternalIMT | epa | rs140931068 | 0.017 | -0.033 | 0.029 | 0.257 | 0.25 | 0.133 | 0.059 | 0.054 | 0.186 | 0.771 |
| InternalIMT | epa | rs74867877 | 0.007 | . | . | . | . | . | . | . | . | . |
| InternalIMT | epa | rs11239495 | 0.055 | -0.041 | 0.031 | 0.179 | 0.065 | 0.051 | 0.205 | 0.087 | 0.068 | 0.198 |
| InternalIMT | epa | rs149745800 | 0 | . | . | . | . | . | . | . | . | . |
| InternalIMT | epa | rs145044458 | 0.015 | -0.027 | 0.029 | 0.352 | -0.043 | 0.076 | 0.57 | -0.111 | 0.106 | 0.296 |
| InternalIMT | epa | rs188067427 | 0.002 | . | . | . | . | . | . | . | . | . |
| InternalIMT | epa | rs146827800 | 0.001 | . | . | . | . | . | . | . | . | . |
| InternalIMT | epa | rs140619817 | 0.015 | -0.027 | 0.029 | 0.349 | -0.04 | 0.076 | 0.594 | -0.106 | 0.106 | 0.32 |
| InternalIMT | epa | rs892689 | 0.997 | . | . | . | . | . | . | . | . | . |
| InternalIMT | epa | rs143642005 | 0.008 | . | . | . | . | . | . | . | . | . |
| InternalIMT | epa | rs181929304 | 0.007 | . | . | . | . | . | . | . | . | . |
| InternalIMT | epa | rs191086173 | 0.002 | . | . | . | . | . | . | . | . | . |
| InternalIMT | epa | rs114613116 | 0.018 | -0.027 | 0.029 | 0.349 | -0.05 | 0.067 | 0.456 | -0.089 | 0.101 | 0.376 |
| InternalIMT | epa | rs141076768 | 0.002 | . | . | . | . | . | . | . | . | . |
| InternalIMT | epa | rs187316531 | 0 | . | . | . | . | . | . | . | . | . |
| InternalIMT | epa | rs11239499 | 0.075 | -0.037 | 0.032 | 0.248 | 0.037 | 0.042 | 0.38 | 0.032 | 0.059 | 0.59 |
| InternalIMT | epa | rs192726371 | 0.003 | . | . | . | . | . | . | . | . | . |
| InternalIMT | epa | rs74128481 | 0.019 | -0.041 | 0.029 | 0.153 | 0.185 | 0.106 | 0.081 | 0.175 | 0.15 | 0.244 |
| InternalIMT | epa | rs74128482 | 0.012 | -0.044 | 0.028 | 0.118 | 0.434 | 0.143 | 2.40E-03 | 0.586 | 0.212 | 5.90E-03 |
| InternalIMT | epa | rs11239502 | 0.055 | -0.042 | 0.031 | 0.172 | 0.067 | 0.051 | 0.193 | 0.093 | 0.067 | 0.17 |
| InternalIMT | epa | rs116330285 | 0.002 | . | . | . | . | . | . | . | . | . |
| InternalIMT | epa | rs189926729 | 0.003 | . | . | . | . | . | . | . | . | . |
| InternalIMT | epa | rs188975779 | 0.012 | -0.036 | 0.028 | 0.196 | 0.241 | 0.157 | 0.124 | 0.267 | 0.281 | 0.342 |
| InternalIMT | epa | rs143846038 | 0.002 | . | . | . | . | . | . | . | . | . |
| InternalIMT | epa | rs74128483 | 0.019 | -0.041 | 0.029 | 0.152 | 0.186 | 0.106 | 0.081 | 0.176 | 0.15 | 0.243 |
| InternalIMT | epa | rs139292055 | 0.073 | -0.039 | 0.032 | 0.227 | 0.034 | 0.042 | 0.418 | 0.042 | 0.059 | 0.473 |
| InternalIMT | epa | rs11239503 | 0.073 | -0.039 | 0.032 | 0.227 | 0.034 | 0.042 | 0.417 | 0.042 | 0.059 | 0.473 |
| InternalIMT | epa | rs58838686 | 0.012 | -0.044 | 0.028 | 0.118 | 0.435 | 0.143 | 2.40E-03 | 0.588 | 0.212 | 5.60E-03 |
| InternalIMT | epa | rs185825373 | 0.008 | . | . | . | . | . | . | . | . | . |
| InternalIMT | epa | rs11239504 | 0.073 | -0.039 | 0.032 | 0.227 | 0.034 | 0.042 | 0.417 | 0.042 | 0.059 | 0.472 |

| InternalIMT | epa | rs150012354 | 0.006 | . | . | . | . | . | . | . | . | . |
| --- | --- | --- | --- | --- | --- | --- | --- | --- | --- | --- | --- | --- |
| InternalIMT | epa | rs187763096 | 0.01 | . | . | . | . | . | . | . | . | . |
| InternalIMT | epa | rs193043982 | 0.008 | . | . | . | . | . | . | . | . | . |
| InternalIMT | epa | rs79186884 | 0.023 | -0.041 | 0.029 | 0.151 | 0.148 | 0.083 | 0.073 | 0.166 | 0.139 | 0.232 |
| InternalIMT | epa | rs74128484 | 0.012 | -0.044 | 0.028 | 0.118 | 0.436 | 0.143 | 2.30E-03 | 0.589 | 0.212 | 5.50E-03 |
| InternalIMT | epa | rs58461537 | 0.073 | -0.039 | 0.032 | 0.226 | 0.034 | 0.042 | 0.416 | 0.043 | 0.059 | 0.471 |
| InternalIMT | epa | rs188527474 | 0.008 | . | . | . | . | . | . | . | . | . |
| InternalIMT | epa | rs141659976 | 0.008 | . | . | . | . | . | . | . | . | . |
| InternalIMT | epa | rs115846407 | 0.073 | -0.039 | 0.032 | 0.226 | 0.034 | 0.042 | 0.415 | 0.043 | 0.059 | 0.47 |
| InternalIMT | epa | rs138378816 | 0.073 | -0.039 | 0.032 | 0.226 | 0.034 | 0.042 | 0.415 | 0.043 | 0.059 | 0.47 |
| InternalIMT | epa | rs146188136 | 0.055 | -0.042 | 0.031 | 0.172 | 0.067 | 0.051 | 0.191 | 0.093 | 0.067 | 0.168 |
| InternalIMT | epa | rs139383871 | 0.014 | -0.029 | 0.029 | 0.312 | -0.142 | 0.089 | 0.111 | -0.142 | 0.199 | 0.475 |
| InternalIMT | epa | rs182953869 | 0.004 | . | . | . | . | . | . | . | . | . |
| InternalIMT | epa | rs192430299 | 0.019 | -0.041 | 0.028 | 0.153 | 0.224 | 0.105 | 0.032 | 0.209 | 0.177 | 0.239 |
| InternalIMT | epa | rs144152844 | 0.073 | -0.039 | 0.032 | 0.226 | 0.034 | 0.042 | 0.415 | 0.043 | 0.059 | 0.468 |
| InternalIMT | epa | rs145987786 | 0.051 | -8.70E-03 | 0.031 | 0.781 | 0.011 | 0.051 | 0.829 | -0.188 | 0.073 | 0.011 |
| InternalIMT | epa | rs181132926 | 0.003 | . | . | . | . | . | . | . | . | . |
| InternalIMT | epa | rs147882253 | 0.073 | -0.039 | 0.032 | 0.226 | 0.034 | 0.042 | 0.414 | 0.043 | 0.059 | 0.468 |
| InternalIMT | epa | rs141807621 | 0.023 | -0.03 | 0.029 | 0.301 | -0.049 | 0.105 | 0.641 | -0.071 | 0.18 | 0.694 |
| InternalIMT | epa | rs147116052 | 0.073 | -0.039 | 0.032 | 0.226 | 0.034 | 0.042 | 0.414 | 0.043 | 0.059 | 0.468 |
| InternalIMT | epa | rs147716618 | 0.073 | -0.039 | 0.032 | 0.226 | 0.034 | 0.042 | 0.414 | 0.043 | 0.059 | 0.468 |
| InternalIMT | epa | rs187198855 | 0.046 | -0.012 | 0.031 | 0.699 | 0.041 | 0.063 | 0.517 | -0.194 | 0.09 | 0.032 |
| InternalIMT | epa | rs189409638 | 0.016 | -0.044 | 0.028 | 0.122 | 0.233 | 0.12 | 0.052 | 0.322 | 0.187 | 0.085 |
| InternalIMT | epa | rs182647339 | 0.001 | . | . | . | . | . | . | . | . | . |
| InternalIMT | epa | rs187995988 | 0.004 | . | . | . | . | . | . | . | . | . |
| InternalIMT | epa | rs141034184 | 0.079 | -0.045 | 0.032 | 0.16 | 0.024 | 0.042 | 0.576 | 0.079 | 0.06 | 0.193 |
| InternalIMT | epa | rs187310430 | 0.008 | . | . | . | . | . | . | . | . | . |
| InternalIMT | epa | rs192608488 | 0.073 | -0.039 | 0.032 | 0.224 | 0.035 | 0.042 | 0.408 | 0.044 | 0.059 | 0.46 |
| InternalIMT | epa | rs148840366 | 0.009 | . | . | . | . | . | . | . | . | . |
| InternalIMT | epa | rs186785022 | 0.019 | -0.041 | 0.029 | 0.151 | 0.186 | 0.106 | 0.08 | 0.178 | 0.151 | 0.237 |
| InternalIMT | epa | rs192348728 | 0.014 | -0.036 | 0.029 | 0.206 | 0.023 | 0.094 | 0.806 | 0.118 | 0.155 | 0.448 |
| InternalIMT | epa | rs189656631 | 0.073 | -0.039 | 0.032 | 0.224 | 0.035 | 0.042 | 0.408 | 0.044 | 0.059 | 0.46 |
| InternalIMT | epa | rs185289750 | 0.004 | . | . | . | . | . | . | . | . | . |
| InternalIMT | epa | rs142382395 | 0.073 | -0.039 | 0.032 | 0.224 | 0.035 | 0.042 | 0.408 | 0.044 | 0.059 | 0.46 |
| InternalIMT | epa | rs144866696 | 0.019 | -0.041 | 0.029 | 0.151 | 0.186 | 0.106 | 0.08 | 0.178 | 0.151 | 0.237 |
| InternalIMT | epa | rs141371289 | 0.073 | -0.039 | 0.032 | 0.224 | 0.035 | 0.042 | 0.408 | 0.044 | 0.059 | 0.46 |
| InternalIMT | epa | rs114750008 | 0.073 | -0.039 | 0.032 | 0.224 | 0.035 | 0.042 | 0.408 | 0.044 | 0.059 | 0.46 |
| InternalIMT | epa | rs189063597 | 0.001 | . | . | . | . | . | . | . | . | . |
| InternalIMT | epa | rs139151571 | 0.067 | -0.036 | 0.032 | 0.252 | 0.028 | 0.044 | 0.524 | 0.031 | 0.062 | 0.616 |
| InternalIMT | epa | rs139846368 | 0.075 | -0.039 | 0.032 | 0.217 | 0.035 | 0.042 | 0.414 | 0.045 | 0.059 | 0.447 |
| InternalIMT | epa | rs182057653 | 0.019 | -0.027 | 0.03 | 0.359 | -3.10E-03 | 0.081 | 0.97 | -0.062 | 0.093 | 0.503 |
| InternalIMT | epa | rs148785420 | 0.073 | -0.039 | 0.032 | 0.224 | 0.035 | 0.042 | 0.408 | 0.044 | 0.059 | 0.46 |
| InternalIMT | epa | rs138383251 | 0.011 | -0.029 | 0.029 | 0.312 | -0.013 | 0.079 | 0.868 | -0.098 | 0.132 | 0.456 |
| InternalIMT | epa | rs116526705 | 0.018 | -0.028 | 0.029 | 0.332 | -0.042 | 0.068 | 0.541 | -0.067 | 0.102 | 0.51 |
| InternalIMT | epa | rs185946436 | 0 | . | . | . | . | . | . | . | . | . |
| InternalIMT | epa | rs190455427 | 0.004 | . | . | . | . | . | . | . | . | . |
| InternalIMT | epa | rs9282584 | 0.073 | -0.039 | 0.032 | 0.224 | 0.035 | 0.042 | 0.407 | 0.044 | 0.059 | 0.458 |
| InternalIMT | epa | rs17153289 | 0.073 | -0.039 | 0.032 | 0.224 | 0.035 | 0.042 | 0.407 | 0.044 | 0.059 | 0.458 |
| InternalIMT | epa | rs138359103 | 0.008 | . | . | . | . | . | . | . | . | . |
| InternalIMT | epa | rs150605268 | 0.003 | . | . | . | . | . | . | . | . | . |
| InternalIMT | epa | rs79242910 | 0.075 | -0.039 | 0.032 | 0.222 | 0.036 | 0.041 | 0.385 | 0.044 | 0.057 | 0.442 |
| InternalIMT | epa | rs139716904 | 0.021 | -0.03 | 0.029 | 0.295 | -0.136 | 0.089 | 0.125 | -0.075 | 0.127 | 0.554 |
| InternalIMT | epa | rs76238498 | 0.053 | -8.90E-03 | 0.031 | 0.776 | 0.017 | 0.051 | 0.729 | -0.182 | 0.072 | 0.012 |
| InternalIMT | epa | rs184140639 | 0 | . | . | . | . | . | . | . | . | . |
| InternalIMT | epa | rs188517278 | 0.009 | . | . | . | . | . | . | . | . | . |
| InternalIMT | epa | rs139678913 | 0.004 | . | . | . | . | . | . | . | . | . |
| InternalIMT | epa | rs116697955 | 0.004 | . | . | . | . | . | . | . | . | . |
| InternalIMT | epa | rs79267994 | 0.012 | -0.044 | 0.028 | 0.119 | 0.438 | 0.143 | 2.20E-03 | 0.593 | 0.21 | 4.90E-03 |
| InternalIMT | epa | rs11239510 | 0.022 | -0.025 | 0.029 | 0.388 | -0.124 | 0.066 | 0.062 | -0.204 | 0.108 | 0.058 |
| InternalIMT | epa | rs75831645 | 0.018 | -0.028 | 0.029 | 0.331 | -0.042 | 0.068 | 0.544 | -0.065 | 0.102 | 0.521 |
| InternalIMT | epa | rs78452276 | 0.052 | -0.022 | 0.03 | 0.465 | -0.011 | 0.048 | 0.815 | -0.085 | 0.079 | 0.285 |
| InternalIMT | epa | rs144366924 | 0 | . | . | . | . | . | . | . | . | . |
| InternalIMT | epa | rs77519841 | 0.056 | -0.042 | 0.031 | 0.171 | 0.067 | 0.051 | 0.189 | 0.094 | 0.067 | 0.165 |
| InternalIMT | epa | rs76944454 | 0.073 | -0.039 | 0.032 | 0.223 | 0.035 | 0.042 | 0.407 | 0.044 | 0.059 | 0.455 |
| InternalIMT | epa | rs76443855 | 0.053 | -8.90E-03 | 0.031 | 0.778 | 0.017 | 0.05 | 0.736 | -0.183 | 0.072 | 0.011 |
| InternalIMT | epa | rs146613174 | 0.002 | . | . | . | . | . | . | . | . | . |
| InternalIMT | epa | rs181424761 | 0.002 | . | . | . | . | . | . | . | . | . |
| InternalIMT | epa | rs142119241 | 0.001 | . | . | . | . | . | . | . | . | . |
| InternalIMT | epa | rs140137954 | 0.018 | -0.028 | 0.029 | 0.332 | -0.042 | 0.068 | 0.543 | -0.066 | 0.102 | 0.514 |
| InternalIMT | epa | rs187319549 | 0.002 | . | . | . | . | . | . | . | . | . |
| InternalIMT | epa | rs12241545 | 0.035 | -0.032 | 0.029 | 0.273 | -0.128 | 0.076 | 0.09 | -0.027 | 0.141 | 0.847 |
| InternalIMT | epa | rs10128228 | 0.073 | -0.039 | 0.032 | 0.223 | 0.035 | 0.042 | 0.406 | 0.044 | 0.059 | 0.454 |
| InternalIMT | epa | rs58580249 | 0.009 | . | . | . | . | . | . | . | . | . |

| InternalIMT | epa | rs193148863 | 0.001 | . | . | . | . | . | . | . | . | . |
| --- | --- | --- | --- | --- | --- | --- | --- | --- | --- | --- | --- | --- |
| InternalIMT | epa | rs185638946 | 0.005 | . | . | . | . | . | . | . | . | . |
| InternalIMT | epa | rs117752921 | 0.001 | . | . | . | . | . | . | . | . | . |
| InternalIMT | epa | rs180841236 | 0.002 | . | . | . | . | . | . | . | . | . |
| InternalIMT | epa | rs73283155 | 0.009 | . | . | . | . | . | . | . | . | . |
| InternalIMT | epa | rs76560599 | 0.061 | -0.034 | 0.032 | 0.282 | 0.01 | 0.045 | 0.821 | 0.018 | 0.064 | 0.778 |
| InternalIMT | epa | rs76273620 | 0.061 | -0.034 | 0.032 | 0.282 | 0.01 | 0.045 | 0.821 | 0.018 | 0.064 | 0.778 |
| InternalIMT | epa | rs141432923 | 0.02 | -0.039 | 0.026 | 0.129 | 0.128 | 0.179 | 0.475 | 0.251 | 0.392 | 0.522 |
| InternalIMT | epa | rs113529322 | 0.019 | -0.041 | 0.029 | 0.151 | 0.186 | 0.106 | 0.08 | 0.179 | 0.151 | 0.236 |
| InternalIMT | epa | rs10128306 | 0.073 | -0.039 | 0.032 | 0.223 | 0.035 | 0.042 | 0.404 | 0.044 | 0.059 | 0.452 |
| InternalIMT | epa | rs149691688 | 0.002 | . | . | . | . | . | . | . | . | . |
| InternalIMT | epa | rs75872495 | 0.073 | -0.039 | 0.032 | 0.223 | 0.035 | 0.042 | 0.4 | 0.044 | 0.059 | 0.455 |
| InternalIMT | epa | rs191873960 | 0.007 | . | . | . | . | . | . | . | . | . |
| InternalIMT | epa | rs80155622 | 0.073 | -0.039 | 0.032 | 0.222 | 0.035 | 0.042 | 0.404 | 0.044 | 0.059 | 0.451 |
| InternalIMT | epa | rs113914656 | 0.008 | . | . | . | . | . | . | . | . | . |
| InternalIMT | epa | rs12242840 | 0.073 | -0.039 | 0.032 | 0.223 | 0.035 | 0.042 | 0.405 | 0.044 | 0.059 | 0.452 |
| InternalIMT | epa | rs147824078 | 0.009 | . | . | . | . | . | . | . | . | . |
| InternalIMT | epa | rs59051493 | 0.073 | -0.039 | 0.032 | 0.223 | 0.035 | 0.042 | 0.405 | 0.044 | 0.059 | 0.453 |
| InternalIMT | epa | rs9282588 | 0.008 | . | . | . | . | . | . | . | . | . |
| InternalIMT | epa | rs150215207 | 0.009 | . | . | . | . | . | . | . | . | . |
| InternalIMT | epa | rs138772243 | 0.003 | . | . | . | . | . | . | . | . | . |
| InternalIMT | epa | rs149383244 | 0.025 | -0.028 | 0.029 | 0.339 | -0.018 | 0.071 | 0.803 | -0.051 | 0.114 | 0.658 |
| InternalIMT | epa | rs17522720 | 0.016 | -0.035 | 0.029 | 0.218 | 0.022 | 0.087 | 0.803 | 0.088 | 0.142 | 0.536 |
| InternalIMT | epa | rs79245105 | 0.073 | -0.039 | 0.032 | 0.223 | 0.035 | 0.042 | 0.406 | 0.044 | 0.059 | 0.452 |
| InternalIMT | epa | rs138222133 | 0.004 | . | . | . | . | . | . | . | . | . |
| InternalIMT | epa | rs12247314 | 0.082 | -0.039 | 0.032 | 0.231 | 0.041 | 0.041 | 0.315 | 0.04 | 0.058 | 0.49 |
| InternalIMT | epa | rs12263894 | 0.08 | -0.039 | 0.032 | 0.225 | 0.044 | 0.042 | 0.294 | 0.044 | 0.058 | 0.454 |
| InternalIMT | epa | rs149907880 | 0.008 | . | . | . | . | . | . | . | . | . |
| InternalIMT | epa | rs188153885 | 0.007 | . | . | . | . | . | . | . | . | . |
| InternalIMT | epa | rs73283159 | 0.005 | . | . | . | . | . | . | . | . | . |
| InternalIMT | epa | rs140476687 | 0.002 | . | . | . | . | . | . | . | . | . |
| InternalIMT | epa | rs186603148 | 0 | . | . | . | . | . | . | . | . | . |
| InternalIMT | epa | rs183296063 | 0.003 | . | . | . | . | . | . | . | . | . |
| InternalIMT | epa | rs145733694 | 0.009 | . | . | . | . | . | . | . | . | . |
| InternalIMT | epa | rs189807449 | 0 | . | . | . | . | . | . | . | . | . |
| InternalIMT | epa | rs12253223 | 0.064 | -0.042 | 0.031 | 0.179 | 0.07 | 0.049 | 0.154 | 0.084 | 0.066 | 0.204 |
| InternalIMT | epa | rs182113637 | 0 | . | . | . | . | . | . | . | . | . |
| InternalIMT | epa | rs142792099 | 0.018 | -0.035 | 0.029 | 0.222 | 0.149 | 0.099 | 0.132 | 0.175 | 0.126 | 0.165 |
| InternalIMT | epa | rs12249910 | 0.064 | -0.042 | 0.031 | 0.179 | 0.07 | 0.049 | 0.157 | 0.084 | 0.066 | 0.206 |
| InternalIMT | epa | rs149436339 | 0.001 | . | . | . | . | . | . | . | . | . |
| InternalIMT | epa | rs77632210 | 0 | . | . | . | . | . | . | . | . | . |
| InternalIMT | epa | rs74130818 | 0.012 | -0.044 | 0.028 | 0.116 | 0.443 | 0.143 | 2.10E-03 | 0.615 | 0.21 | 3.40E-03 |
| InternalIMT | epa | rs187583648 | 0 | . | . | . | . | . | . | . | . | . |
| InternalIMT | epa | rs191295987 | 0.008 | . | . | . | . | . | . | . | . | . |
| InternalIMT | epa | rs184200686 | 0.01 | . | . | . | . | . | . | . | . | . |
| InternalIMT | epa | rs76117263 | 0.052 | -8.80E-03 | 0.031 | 0.778 | 0.026 | 0.052 | 0.615 | -0.187 | 0.072 | 9.60E-03 |
| InternalIMT | epa | rs185393149 | 0.001 | . | . | . | . | . | . | . | . | . |
| InternalIMT | epa | rs144760014 | 0.006 | . | . | . | . | . | . | . | . | . |
| InternalIMT | epa | rs7922292 | 0.008 | . | . | . | . | . | . | . | . | . |
| InternalIMT | epa | rs115372844 | 0.009 | . | . | . | . | . | . | . | . | . |
| InternalIMT | epa | rs111460364 | 0.052 | -8.70E-03 | 0.031 | 0.78 | 0.026 | 0.052 | 0.614 | -0.188 | 0.072 | 9.50E-03 |
| InternalIMT | epa | rs182052255 | 0.002 | . | . | . | . | . | . | . | . | . |
| InternalIMT | epa | rs180983889 | 0.006 | . | . | . | . | . | . | . | . | . |
| InternalIMT | epa | rs139175327 | 0.009 | . | . | . | . | . | . | . | . | . |
| InternalIMT | epa | rs149959620 | 0.018 | -0.03 | 0.029 | 0.298 | -0.115 | 0.093 | 0.215 | -0.079 | 0.143 | 0.581 |
| InternalIMT | epa | rs114653117 | 0.009 | . | . | . | . | . | . | . | . | . |
| InternalIMT | epa | rs144270018 | 0.005 | . | . | . | . | . | . | . | . | . |
| InternalIMT | epa | rs182723204 | 0.007 | . | . | . | . | . | . | . | . | . |
| InternalIMT | epa | rs57204067 | 0.012 | -0.044 | 0.028 | 0.113 | 0.445 | 0.144 | 2.00E-03 | 0.627 | 0.209 | 2.70E-03 |
| InternalIMT | epa | rs144956226 | 0.064 | -0.042 | 0.031 | 0.182 | 0.064 | 0.05 | 0.197 | 0.082 | 0.066 | 0.217 |
| InternalIMT | epa | rs187842704 | 0.019 | -0.023 | 0.029 | 0.419 | 0.074 | 0.093 | 0.426 | -0.153 | 0.105 | 0.147 |
| InternalIMT | epa | rs184620511 | 0.013 | -0.023 | 0.029 | 0.432 | 0.061 | 0.144 | 0.67 | -0.266 | 0.179 | 0.138 |
| InternalIMT | epa | rs144031303 | 0.019 | -0.029 | 0.029 | 0.326 | -0.032 | 0.071 | 0.648 | -0.053 | 0.102 | 0.6 |
| InternalIMT | epa | rs187526473 | 0 | . | . | . | . | . | . | . | . | . |
| InternalIMT | epa | rs193002805 | 0.002 | . | . | . | . | . | . | . | . | . |
| InternalIMT | epa | rs138707426 | 0.024 | -0.027 | 0.029 | 0.348 | -0.014 | 0.07 | 0.839 | -0.06 | 0.112 | 0.59 |
| InternalIMT | epa | rs142754243 | 0.049 | -0.031 | 0.03 | 0.296 | -0.035 | 0.064 | 0.584 | -9.80E-03 | 0.104 | 0.925 |
| InternalIMT | epa | rs181600331 | 0.008 | . | . | . | . | . | . | . | . | . |
| InternalIMT | epa | rs144421264 | 0.029 | -0.028 | 0.029 | 0.332 | -0.079 | 0.079 | 0.316 | -0.06 | 0.137 | 0.661 |
| InternalIMT | epa | rs184414519 | 0.006 | . | . | . | . | . | . | . | . | . |
| InternalIMT | epa | rs150596349 | 0.048 | -6.90E-03 | 0.031 | 0.823 | 0.028 | 0.055 | 0.61 | -0.211 | 0.075 | 5.00E-03 |
| InternalIMT | epa | rs190753747 | 0.001 | . | . | . | . | . | . | . | . | . |
| InternalIMT | epa | rs139560940 | 0.004 | . | . | . | . | . | . | . | . | . |

| InternalIMT | epa | rs75640804 | 0.057 | -0.042 | 0.031 | 0.171 | 0.06 | 0.051 | 0.239 | 0.089 | 0.064 | 0.165 |
| --- | --- | --- | --- | --- | --- | --- | --- | --- | --- | --- | --- | --- |
| InternalIMT | epa | rs73283175 | 0.082 | -0.024 | 0.032 | 0.468 | -0.035 | 0.043 | 0.416 | -0.043 | 0.066 | 0.51 |
| InternalIMT | epa | rs143337229 | 0.003 | . | . | . | . | . | . | . | . | . |
| InternalIMT | epa | rs73283177 | 0.048 | -0.031 | 0.03 | 0.296 | -0.032 | 0.066 | 0.626 | -9.30E-03 | 0.107 | 0.931 |
| InternalIMT | epa | rs76053611 | 0.045 | -7.80E-03 | 0.031 | 0.8 | 0.021 | 0.056 | 0.704 | -0.208 | 0.076 | 6.60E-03 |
| InternalIMT | epa | rs116325983 | 0.004 | . | . | . | . | . | . | . | . | . |
| InternalIMT | epa | rs115451825 | 0.008 | . | . | . | . | . | . | . | . | . |
| InternalIMT | epa | rs183845246 | 0.002 | . | . | . | . | . | . | . | . | . |
| InternalIMT | epa | rs79756542 | 0.051 | -7.60E-03 | 0.031 | 0.807 | 0.026 | 0.053 | 0.628 | -0.198 | 0.073 | 7.10E-03 |
| InternalIMT | epa | rs187242860 | 0.002 | . | . | . | . | . | . | . | . | . |
| InternalIMT | epa | rs57318980 | 0.048 | -0.03 | 0.03 | 0.313 | -0.025 | 0.068 | 0.711 | -0.02 | 0.111 | 0.858 |
| InternalIMT | epa | rs59969732 | 0.043 | -0.034 | 0.03 | 0.252 | -0.014 | 0.072 | 0.851 | 0.026 | 0.112 | 0.818 |
| InternalIMT | epa | rs143811488 | 0.009 | . | . | . | . | . | . | . | . | . |
| InternalIMT | epa | rs189067644 | 0.01 | . | . | . | . | . | . | . | . | . |
| InternalIMT | epa | rs60449178 | 0.038 | -0.034 | 0.029 | 0.246 | -0.041 | 0.076 | 0.585 | 0.029 | 0.125 | 0.816 |
| InternalIMT | epa | rs59243389 | 0.038 | -0.034 | 0.029 | 0.246 | -0.041 | 0.076 | 0.586 | 0.029 | 0.125 | 0.816 |
| InternalIMT | epa | rs184946540 | 0.043 | -0.018 | 0.03 | 0.558 | -0.118 | 0.052 | 0.023 | -0.169 | 0.088 | 0.054 |
| InternalIMT | epa | rs188685881 | 0.001 | . | . | . | . | . | . | . | . | . |
| InternalIMT | epa | rs150335797 | 0.006 | . | . | . | . | . | . | . | . | . |
| InternalIMT | epa | rs143879260 | 0.006 | . | . | . | . | . | . | . | . | . |
| InternalIMT | epa | rs186044491 | 0.009 | . | . | . | . | . | . | . | . | . |
| InternalIMT | epa | rs11819084 | 0.03 | -0.026 | 0.03 | 0.392 | 0.033 | 0.066 | 0.614 | -0.068 | 0.091 | 0.454 |
| InternalIMT | epa | rs146915670 | 0.006 | . | . | . | . | . | . | . | . | . |
| InternalIMT | epa | rs113995695 | 0.029 | -0.027 | 0.03 | 0.371 | 0.039 | 0.066 | 0.56 | -0.057 | 0.092 | 0.536 |
| InternalIMT | epa | rs143489451 | 0.005 | . | . | . | . | . | . | . | . | . |
| InternalIMT | epa | rs182653839 | 0 | . | . | . | . | . | . | . | . | . |
| InternalIMT | epa | rs141692564 | 0.007 | . | . | . | . | . | . | . | . | . |
| InternalIMT | epa | rs146281742 | 0.005 | . | . | . | . | . | . | . | . | . |
| InternalIMT | epa | rs192914353 | 0.005 | . | . | . | . | . | . | . | . | . |
| InternalIMT | epa | rs185182848 | 0.003 | . | . | . | . | . | . | . | . | . |
| InternalIMT | epa | rs28395858 | 0.029 | -0.028 | 0.03 | 0.36 | 0.042 | 0.067 | 0.526 | -0.05 | 0.093 | 0.589 |
| InternalIMT | epa | rs41429645 | 0.001 | . | . | . | . | . | . | . | . | . |
| InternalIMT | epa | rs28395874 | 0.029 | -0.028 | 0.03 | 0.353 | 0.044 | 0.067 | 0.51 | -0.046 | 0.093 | 0.621 |
| InternalIMT | epa | rs189663634 | 0.001 | . | . | . | . | . | . | . | . | . |
| InternalIMT | epa | rs77666700 | 0.01 | . | . | . | . | . | . | . | . | . |
| InternalIMT | epa | rs146711717 | 0.003 | . | . | . | . | . | . | . | . | . |
| InternalIMT | epa | rs144904251 | 0.001 | . | . | . | . | . | . | . | . | . |
| InternalIMT | epa | rs189893153 | 0.001 | . | . | . | . | . | . | . | . | . |
| InternalIMT | epa | rs150447740 | 0.009 | . | . | . | . | . | . | . | . | . |
| InternalIMT | epa | rs143099649 | 0.001 | . | . | . | . | . | . | . | . | . |
| InternalIMT | epa | rs191288436 | 0.01 | -0.029 | 0.028 | 0.299 | -0.014 | 0.112 | 0.897 | -0.137 | 0.209 | 0.514 |
| InternalIMT | epa | rs188577862 | 0.006 | . | . | . | . | . | . | . | . | . |
| InternalIMT | epa | rs192695334 | 0.006 | . | . | . | . | . | . | . | . | . |
| InternalIMT | epa | rs138612906 | 0.036 | -0.027 | 0.031 | 0.373 | 0.032 | 0.058 | 0.58 | -0.047 | 0.085 | 0.583 |
| InternalIMT | epa | rs191290051 | 0.037 | -0.037 | 0.029 | 0.205 | 0.088 | 0.084 | 0.296 | 0.1 | 0.128 | 0.434 |
| InternalIMT | epa | rs189631459 | 0.001 | . | . | . | . | . | . | . | . | . |
| InternalIMT | epa | rs114620874 | 0.007 | . | . | . | . | . | . | . | . | . |
| InternalIMT | epa | rs78246652 | 0.023 | -0.037 | 0.029 | 0.197 | 0.16 | 0.097 | 0.1 | 0.173 | 0.141 | 0.222 |
| InternalIMT | epa | rs79432451 | 0.007 | . | . | . | . | . | . | . | . | . |
| InternalIMT | epa | rs186022585 | 0.001 | . | . | . | . | . | . | . | . | . |
| InternalIMT | epa | rs142978891 | 0.009 | . | . | . | . | . | . | . | . | . |
| InternalIMT | epa | rs151086840 | 0.011 | -0.032 | 0.028 | 0.264 | -0.137 | 0.131 | 0.294 | -0.024 | 0.197 | 0.904 |
| InternalIMT | epa | rs183270259 | 0.01 | . | . | . | . | . | . | . | . | . |
| InternalIMT | epa | rs188544289 | 0.01 | . | . | . | . | . | . | . | . | . |
| InternalIMT | epa | rs188500808 | 0.001 | . | . | . | . | . | . | . | . | . |
| InternalIMT | epa | rs189424589 | 0.003 | . | . | . | . | . | . | . | . | . |
| InternalIMT | epa | rs78622303 | 0.04 | -0.022 | 0.029 | 0.45 | -6.10E-03 | 0.074 | 0.934 | -0.132 | 0.135 | 0.328 |
| InternalIMT | epa | rs143210835 | 0.013 | -0.024 | 0.028 | 0.397 | 0.047 | 0.146 | 0.747 | -0.422 | 0.274 | 0.124 |
| InternalIMT | epa | rs114761840 | 0.007 | . | . | . | . | . | . | . | . | . |
| InternalIMT | epa | rs185805402 | 0.003 | . | . | . | . | . | . | . | . | . |
| InternalIMT | epa | rs139273744 | 0.002 | . | . | . | . | . | . | . | . | . |
| InternalIMT | epa | rs184654150 | 0.006 | . | . | . | . | . | . | . | . | . |
| InternalIMT | epa | rs189069710 | 0 | . | . | . | . | . | . | . | . | . |
| InternalIMT | epa | rs114752115 | 0.031 | -0.037 | 0.03 | 0.216 | 0.09 | 0.082 | 0.273 | 0.082 | 0.103 | 0.426 |
| InternalIMT | epa | rs150162515 | 0.002 | . | . | . | . | . | . | . | . | . |
| InternalIMT | epa | rs180982454 | 0.008 | . | . | . | . | . | . | . | . | . |
| InternalIMT | epa | rs184932088 | 0.001 | . | . | . | . | . | . | . | . | . |
| InternalIMT | epa | rs181109625 | 0 | . | . | . | . | . | . | . | . | . |
| InternalIMT | epa | rs138121134 | 0.001 | . | . | . | . | . | . | . | . | . |
| InternalIMT | epa | rs183253861 | 0.004 | . | . | . | . | . | . | . | . | . |
| InternalIMT | epa | rs147073946 | 0.001 | . | . | . | . | . | . | . | . | . |
| InternalIMT | epa | rs139938489 | 0.008 | . | . | . | . | . | . | . | . | . |
| InternalIMT | epa | rs146362560 | 0.013 | -0.027 | 0.029 | 0.359 | 0.096 | 0.108 | 0.373 | -0.157 | 0.112 | 0.162 |

| InternalIMT | epa | rs193155956 | 0.007 | . | . | . | . | . | . | . | . | . |
| --- | --- | --- | --- | --- | --- | --- | --- | --- | --- | --- | --- | --- |
| InternalIMT | epa | rs77663092 | 0.007 | . | . | . | . | . | . | . | . | . |
| InternalIMT | epa | rs73285319 | 0.033 | -0.028 | 0.029 | 0.346 | -0.064 | 0.08 | 0.428 | -0.088 | 0.122 | 0.475 |
| InternalIMT | epa | rs184019276 | 0.007 | . | . | . | . | . | . | . | . | . |
| InternalIMT | epa | rs28395863 | 0.027 | -0.031 | 0.03 | 0.296 | 0.037 | 0.074 | 0.618 | -0.01 | 0.104 | 0.921 |
| InternalIMT | epa | rs182121645 | 0.017 | -0.032 | 0.028 | 0.256 | 9.00E-03 | 0.135 | 0.947 | 0.032 | 0.182 | 0.859 |
| InternalIMT | epa | rs73285327 | 0.025 | -0.027 | 0.029 | 0.353 | -0.047 | 0.095 | 0.619 | -0.118 | 0.131 | 0.367 |
| InternalIMT | epa | rs149589584 | 0.001 | . | . | . | . | . | . | . | . | . |
| InternalIMT | epa | rs148310335 | 0.005 | . | . | . | . | . | . | . | . | . |
| InternalIMT | epa | rs187246093 | 0.01 | -0.031 | 0.029 | 0.277 | -0.044 | 0.098 | 0.652 | -0.016 | 0.153 | 0.914 |
| InternalIMT | epa | rs184668894 | 0 | . | . | . | . | . | . | . | . | . |
| InternalIMT | epa | rs114269961 | 0.001 | . | . | . | . | . | . | . | . | . |
| InternalIMT | epa | rs149694374 | 0.034 | -0.031 | 0.03 | 0.3 | -7.70E-03 | 0.062 | 0.902 | -4.50E-03 | 0.094 | 0.962 |
| InternalIMT | epa | rs145284227 | 0.004 | . | . | . | . | . | . | . | . | . |
| InternalIMT | epa | rs189983820 | 0.004 | . | . | . | . | . | . | . | . | . |
| InternalIMT | epa | rs181200444 | 0 | . | . | . | . | . | . | . | . | . |
| InternalIMT | epa | rs185486385 | 0 | . | . | . | . | . | . | . | . | . |
| InternalIMT | epa | rs151198241 | 0.01 | -0.032 | 0.029 | 0.267 | -0.155 | 0.115 | 0.18 | -0.022 | 0.172 | 0.898 |
| InternalIMT | epa | rs189959341 | 0.01 | -0.032 | 0.029 | 0.267 | -0.155 | 0.115 | 0.18 | -0.022 | 0.172 | 0.898 |
| InternalIMT | epa | rs140549257 | 0.007 | . | . | . | . | . | . | . | . | . |
| InternalIMT | epa | rs186453139 | 0.027 | -0.026 | 0.029 | 0.381 | -0.097 | 0.069 | 0.161 | -0.147 | 0.106 | 0.165 |
| InternalIMT | epa | rs180922711 | 0.044 | -0.019 | 0.03 | 0.54 | -0.1 | 0.06 | 0.092 | -0.17 | 0.092 | 0.066 |
| InternalIMT | epa | rs56659903 | 0.005 | . | . | . | . | . | . | . | . | . |
| InternalIMT | epa | rs141202128 | 0.006 | . | . | . | . | . | . | . | . | . |
| InternalIMT | epa | rs59085314 | 0.056 | -0.033 | 0.029 | 0.262 | 7.90E-03 | 0.053 | 0.881 | 0.015 | 0.084 | 0.854 |
| InternalIMT | epa | rs145564996 | 0.004 | . | . | . | . | . | . | . | . | . |
| InternalIMT | epa | rs114961009 | 0.01 | -0.032 | 0.029 | 0.268 | -0.154 | 0.116 | 0.182 | -0.026 | 0.173 | 0.881 |
| InternalIMT | epa | rs182294189 | 0.004 | . | . | . | . | . | . | . | . | . |
| InternalIMT | epa | rs187586636 | 0.008 | . | . | . | . | . | . | . | . | . |
| InternalIMT | epa | rs192254907 | 0.002 | . | . | . | . | . | . | . | . | . |
| InternalIMT | epa | rs149145663 | 0.001 | . | . | . | . | . | . | . | . | . |
| InternalIMT | epa | rs180713064 | 0.001 | . | . | . | . | . | . | . | . | . |
| InternalIMT | epa | rs183009023 | 0.007 | . | . | . | . | . | . | . | . | . |
| InternalIMT | epa | rs143385347 | 0.017 | -0.031 | 0.029 | 0.271 | 0.017 | 0.131 | 0.899 | -1.80E-04 | 0.177 | 0.999 |
| InternalIMT | epa | rs185786329 | 0.007 | . | . | . | . | . | . | . | . | . |
| InternalIMT | epa | rs192341030 | 0.004 | . | . | . | . | . | . | . | . | . |
| InternalIMT | epa | rs74661429 | 0.017 | -0.021 | 0.029 | 0.474 | 0.087 | 0.096 | 0.367 | -0.268 | 0.136 | 0.049 |
| InternalIMT | epa | rs188034853 | 0.002 | . | . | . | . | . | . | . | . | . |
| InternalIMT | epa | rs116808430 | 0.027 | -0.027 | 0.029 | 0.357 | -0.091 | 0.069 | 0.188 | -0.106 | 0.104 | 0.31 |
| InternalIMT | epa | rs148469010 | 0 | . | . | . | . | . | . | . | . | . |
| InternalIMT | epa | rs78621663 | 0.01 | . | . | . | . | . | . | . | . | . |
| InternalIMT | epa | rs192654886 | 0.001 | . | . | . | . | . | . | . | . | . |
| InternalIMT | epa | rs145996035 | 0.008 | . | . | . | . | . | . | . | . | . |
| InternalIMT | epa | rs139942226 | 0.001 | . | . | . | . | . | . | . | . | . |
| InternalIMT | epa | rs113645632 | 0.01 | -0.034 | 0.028 | 0.228 | 0.288 | 0.204 | 0.159 | 0.344 | 0.313 | 0.273 |
| InternalIMT | epa | rs74940661 | 0.027 | -0.033 | 0.029 | 0.258 | 0.014 | 0.071 | 0.841 | 0.026 | 0.115 | 0.825 |
| InternalIMT | epa | rs115355800 | 0.022 | -0.039 | 0.029 | 0.176 | 0.171 | 0.104 | 0.099 | 0.217 | 0.146 | 0.136 |
| InternalIMT | epa | rs150285898 | 0.008 | . | . | . | . | . | . | . | . | . |
| InternalIMT | epa | rs188117932 | 0.004 | . | . | . | . | . | . | . | . | . |
| InternalIMT | epa | rs139956566 | . | . | . | . | . | . | . | . | . | . |
| InternalIMT | epa | rs147673885 | . | . | . | . | . | . | . | . | . | . |
| InternalIMT | epa | rs189127928 | . | . | . | . | . | . | . | . | . | . |
| InternalIMT | epa | rs187558772 | . | . | . | . | . | . | . | . | . | . |
| InternalIMT | epa | rs181948587 | . | . | . | . | . | . | . | . | . | . |
| InternalIMT | epa | rs189983269 | . | . | . | . | . | . | . | . | . | . |
| InternalIMT | epa | rs187250069 | . | . | . | . | . | . | . | . | . | . |
| InternalIMT | epa | rs191485859 | . | . | . | . | . | . | . | . | . | . |
| InternalIMT | epa | rs185499555 | . | . | . | . | . | . | . | . | . | . |
| InternalIMT | epa | rs180862110 | . | . | . | . | . | . | . | . | . | . |
| InternalIMT | epa | rs190472603 | . | . | . | . | . | . | . | . | . | . |
| InternalIMT | epa | rs189226167 | . | . | . | . | . | . | . | . | . | . |
| InternalIMT | epa | rs181623256 | . | . | . | . | . | . | . | . | . | . |
| InternalIMT | epa | rs186079557 | . | . | . | . | . | . | . | . | . | . |
| InternalIMT | epa | rs147760994 | . | . | . | . | . | . | . | . | . | . |
| InternalIMT | epa | rs189176167 | . | . | . | . | . | . | . | . | . | . |
| InternalIMT | epa | rs117901000 | . | . | . | . | . | . | . | . | . | . |
| InternalIMT | epa | rs141984888 | . | . | . | . | . | . | . | . | . | . |
| InternalIMT | epa | rs192365061 | . | . | . | . | . | . | . | . | . | . |
| InternalIMT | epa | rs185976038 | . | . | . | . | . | . | . | . | . | . |
| InternalIMT | epa | rs184697303 | . | . | . | . | . | . | . | . | . | . |
| InternalIMT | epa | rs143383132 | . | . | . | . | . | . | . | . | . | . |
| InternalIMT | epa | rs141873881 | . | . | . | . | . | . | . | . | . | . |
| InternalIMT | epa | rs185507255 | . | . | . | . | . | . | . | . | . | . |

| InternalIMT | epa | rs191379425 | . | . | . | . | . | . | . | . | . | . |
| --- | --- | --- | --- | --- | --- | --- | --- | --- | --- | --- | --- | --- |
| InternalIMT | epa | rs184199584 | . | . | . | . | . | . | . | . | . | . |
| InternalIMT | epa | rs183181360 | . | . | . | . | . | . | . | . | . | . |
| InternalIMT | epa | rs184495673 | . | . | . | . | . | . | . | . | . | . |
| InternalIMT | epa | rs182946297 | . | . | . | . | . | . | . | . | . | . |
| InternalIMT | epa | rs187743615 | . | . | . | . | . | . | . | . | . | . |
| InternalIMT | epa | rs183259862 | . | . | . | . | . | . | . | . | . | . |
| InternalIMT | epa | rs112433566 | . | . | . | . | . | . | . | . | . | . |
| InternalIMT | epa | rs182351085 | . | . | . | . | . | . | . | . | . | . |
| InternalIMT | epa | rs146248508 | . | . | . | . | . | . | . | . | . | . |
| InternalIMT | epa | rs188528439 | . | . | . | . | . | . | . | . | . | . |
| InternalIMT | epa | rs146167018 | . | . | . | . | . | . | . | . | . | . |
| InternalIMT | epa | rs192440980 | . | . | . | . | . | . | . | . | . | . |
| InternalIMT | epa | rs185601882 | . | . | . | . | . | . | . | . | . | . |
| InternalIMT | epa | rs117272563 | . | . | . | . | . | . | . | . | . | . |
| InternalIMT | epa | rs10430734 | . | . | . | . | . | . | . | . | . | . |
| InternalIMT | epa | rs181320952 | . | . | . | . | . | . | . | . | . | . |
| InternalIMT | epa | rs185480660 | . | . | . | . | . | . | . | . | . | . |
| InternalIMT | epa | rs188077917 | . | . | . | . | . | . | . | . | . | . |
| InternalIMT | epa | rs187562953 | . | . | . | . | . | . | . | . | . | . |
| InternalIMT | epa | rs182044631 | . | . | . | . | . | . | . | . | . | . |
| InternalIMT | epa | rs145455596 | . | . | . | . | . | . | . | . | . | . |
| InternalIMT | epa | rs181475877 | . | . | . | . | . | . | . | . | . | . |
| InternalIMT | epa | rs149413085 | . | . | . | . | . | . | . | . | . | . |
| InternalIMT | epa | rs12253656 | . | . | . | . | . | . | . | . | . | . |
| InternalIMT | epa | rs182718239 | . | . | . | . | . | . | . | . | . | . |
| InternalIMT | epa | rs192435695 | . | . | . | . | . | . | . | . | . | . |
| InternalIMT | epa | rs12248277 | . | . | . | . | . | . | . | . | . | . |
| InternalIMT | epa | rs143521710 | . | . | . | . | . | . | . | . | . | . |
| InternalIMT | epa | rs144454454 | . | . | . | . | . | . | . | . | . | . |
| InternalIMT | epa | rs190441610 | . | . | . | . | . | . | . | . | . | . |
| InternalIMT | epa | rs114690462 | . | . | . | . | . | . | . | . | . | . |
| InternalIMT | epa | rs184541357 | . | . | . | . | . | . | . | . | . | . |
| InternalIMT | epa | rs142657653 | . | . | . | . | . | . | . | . | . | . |
| InternalIMT | epa | rsurf1 | 2.117 | . | . | . | . | . | . | . | . | . |
| InternalIMT | epa | rsten1 | 2.28 | . | . | . | . | . | . | . | . | . |
| InternalIMT | epa | chr10:45852312:D | 0.092 | -0.011 | 0.033 | 0.734 | 4.80E-03 | 0.035 | 0.89 | -0.09 | 0.052 | 0.081 |
| InternalIMT | epa | chr10:45853232:I | 0.108 | -0.026 | 0.033 | 0.438 | 0.066 | 0.036 | 0.065 | -0.025 | 0.054 | 0.641 |
| InternalIMT | epa | chr10:45853234:I | 0.198 | -0.032 | 0.037 | 0.38 | 0.049 | 0.029 | 0.096 | 3.50E-03 | 0.045 | 0.937 |
| InternalIMT | epa | chr10:45854796:D | 0.003 | . | . | . | . | . | . | . | . | . |
| InternalIMT | epa | chr10:45854994:I | 0.025 | -0.033 | 0.029 | 0.253 | 0.105 | 0.078 | 0.179 | 0.072 | 0.118 | 0.542 |
| InternalIMT | epa | chr10:45858290:D | 0.052 | -0.038 | 0.032 | 0.23 | 0.058 | 0.078 | 0.457 | 0.051 | 0.095 | 0.596 |
| InternalIMT | epa | chr10:45858820:I | 0.022 | -0.027 | 0.029 | 0.361 | -0.053 | 0.064 | 0.409 | -0.093 | 0.107 | 0.385 |
| InternalIMT | epa | chr10:45860888:D | 0.122 | -0.015 | 0.035 | 0.663 | -0.02 | 0.04 | 0.61 | -0.061 | 0.066 | 0.358 |
| InternalIMT | epa | chr10:45863913:I | 0.179 | -0.031 | 0.036 | 0.397 | 0.054 | 0.03 | 0.072 | -7.90E-04 | 0.045 | 0.986 |
| InternalIMT | epa | chr10:45866601:D | 0.111 | -0.032 | 0.033 | 0.343 | 0.034 | 0.041 | 0.402 | 6.70E-04 | 0.053 | 0.99 |
| InternalIMT | epa | chr10:45867197:I | 0.002 | . | . | . | . | . | . | . | . | . |
| InternalIMT | epa | chr10:45867538:D | 0.182 | -0.034 | 0.036 | 0.342 | 0.05 | 0.03 | 0.093 | 7.20E-03 | 0.044 | 0.871 |
| InternalIMT | epa | chr10:45868356:D | 0.18 | -0.031 | 0.036 | 0.387 | 0.044 | 0.029 | 0.129 | 3.80E-04 | 0.044 | 0.993 |
| InternalIMT | epa | chr10:45871158:I | 0.182 | -0.035 | 0.036 | 0.333 | 0.054 | 0.029 | 0.066 | 9.00E-03 | 0.044 | 0.837 |
| InternalIMT | epa | chr10:45871637:D | 0.127 | -0.025 | 0.035 | 0.47 | 0.061 | 0.037 | 0.093 | -0.023 | 0.052 | 0.65 |
| InternalIMT | epa | chr10:45871638:I | 0.164 | -0.034 | 0.036 | 0.345 | 0.077 | 0.031 | 0.015 | 5.50E-03 | 0.047 | 0.906 |
| InternalIMT | epa | chr10:45873629:I | 0.154 | -0.024 | 0.035 | 0.496 | 0.036 | 0.032 | 0.262 | -0.021 | 0.049 | 0.667 |
| InternalIMT | epa | chr10:45873701:D | 0.055 | -0.041 | 0.031 | 0.187 | 0.057 | 0.05 | 0.261 | 0.081 | 0.067 | 0.231 |
| InternalIMT | epa | chr10:45874997:I | 0.011 | -0.035 | 0.028 | 0.225 | -0.129 | 0.159 | 0.416 | 0.197 | 0.291 | 0.5 |
| InternalIMT | epa | chr10:45882030:I | 0.096 | -0.033 | 0.032 | 0.312 | 0.011 | 0.04 | 0.786 | 6.20E-03 | 0.057 | 0.913 |
| InternalIMT | epa | chr10:45883774:D | 0.078 | -0.045 | 0.031 | 0.142 | 0.017 | 0.053 | 0.747 | 0.085 | 0.074 | 0.248 |
| InternalIMT | epa | chr10:45885169:D | 0.075 | -9.70E-03 | 0.032 | 0.763 | 6.80E-03 | 0.039 | 0.862 | -0.134 | 0.063 | 0.033 |
| InternalIMT | epa | chr10:45886451:D | 0.736 | 0.048 | 0.081 | 0.553 | 2.60E-03 | 0.029 | 0.928 | -0.054 | 0.047 | 0.257 |
| InternalIMT | epa | chr10:45891800:I | 0.768 | 2.90E-03 | 0.075 | 0.969 | 0.016 | 0.026 | 0.536 | -0.022 | 0.043 | 0.606 |
| InternalIMT | epa | chr10:45904771:I | 0.772 | -0.028 | 0.069 | 0.682 | 0.026 | 0.025 | 0.301 | -1.20E-03 | 0.04 | 0.976 |
| InternalIMT | epa | chr10:45904959:I | 0.156 | -0.039 | 0.037 | 0.287 | 7.60E-04 | 0.037 | 0.984 | 0.025 | 0.057 | 0.66 |
| InternalIMT | epa | chr10:45911028:I | 0.161 | -0.021 | 0.033 | 0.532 | 0.02 | 0.038 | 0.598 | -0.037 | 0.059 | 0.531 |
| InternalIMT | epa | chr10:45911031:I | 0.259 | -0.039 | 0.036 | 0.276 | -0.034 | 0.034 | 0.32 | 0.014 | 0.051 | 0.779 |
| InternalIMT | epa | chr10:45912154:D | 0.104 | -0.038 | 0.031 | 0.218 | -5.10E-03 | 0.049 | 0.917 | 0.033 | 0.068 | 0.635 |
| InternalIMT | epa | chr10:45913885:I | 0.003 | . | . | . | . | . | . | . | . | . |
| InternalIMT | epa | chr10:45914753:D | 0.351 | -0.078 | 0.046 | 0.086 | 0.075 | 0.031 | 0.015 | 0.073 | 0.05 | 0.141 |
| InternalIMT | epa | chr10:45919621 | . | . | . | . | . | . | . | . | . | . |
| InternalIMT | epa | chr10:45921739:I | 0.178 | -0.036 | 0.03 | 0.234 | 0.032 | 0.033 | 0.33 | 0.01 | 0.056 | 0.857 |
| InternalIMT | epa | chr10:45923527:I | 0.066 | -0.03 | 0.031 | 0.338 | -0.096 | 0.048 | 0.048 | -0.022 | 0.073 | 0.766 |
| InternalIMT | epa | chr10:45927001:I | 0.04 | -0.045 | 0.029 | 0.127 | 0.101 | 0.083 | 0.221 | 0.183 | 0.121 | 0.13 |
| InternalIMT | epa | chr10:45930354:I | 0.025 | -0.038 | 0.029 | 0.189 | 0.161 | 0.094 | 0.087 | 0.173 | 0.138 | 0.21 |
| InternalIMT | epa | chr10:45932864:I | 0.043 | -3.50E-03 | 0.03 | 0.908 | 0.068 | 0.101 | 0.5 | -0.343 | 0.174 | 0.049 |

| InternalIMT | epa | chr10:45936163:I | 0.038 | -0.027 | 0.029 | 0.365 | -0.041 | 0.07 | 0.554 | -0.076 | 0.107 | 0.48 |
| --- | --- | --- | --- | --- | --- | --- | --- | --- | --- | --- | --- | --- |
| InternalIMT | epa | chr10:45938095:D | 0.179 | -0.042 | 0.034 | 0.225 | 0.067 | 0.034 | 0.048 | 0.032 | 0.055 | 0.564 |
| InternalIMT | epa | chr10:45938886 | . | . | . | . | . | . | . | . | . | . |
| InternalIMT | epa | chr10:45939320 | 0.001 | . | . | . | . | . | . | . | . | . |
| InternalIMT | epa | chr10:45939659 | 0.003 | . | . | . | . | . | . | . | . | . |
| InternalIMT | epa | chr10:45941150:D | 0.008 | . | . | . | . | . | . | . | . | . |
| InternalIMT | epa | chr10:45947128:D | 0.067 | -0.035 | 0.03 | 0.248 | -8.20E-03 | 0.044 | 0.853 | 0.035 | 0.07 | 0.617 |
| InternalIMT | epa | chr10:45950990:I | 0.039 | -0.026 | 0.03 | 0.387 | 0.058 | 0.084 | 0.487 | -0.07 | 0.12 | 0.559 |
| InternalIMT | epa | chr10:45951854:I | 0.012 | -0.031 | 0.029 | 0.272 | -0.1 | 0.098 | 0.305 | 0.04 | 0.151 | 0.791 |
| InternalIMT | epa | chr10:45952670:I | 0.061 | -0.019 | 0.03 | 0.527 | -0.07 | 0.04 | 0.077 | -0.091 | 0.067 | 0.177 |
| InternalIMT | epa | chr10:45956741 | . | . | . | . | . | . | . | . | . | . |
| InternalIMT | epa | chr10:45957247:I | 0.198 | -0.046 | 0.036 | 0.2 | 0.056 | 0.032 | 0.085 | 0.039 | 0.054 | 0.475 |
| InternalIMT | epa | chr10:45958082:I | 0.188 | -0.042 | 0.036 | 0.235 | 0.057 | 0.034 | 0.099 | 0.033 | 0.058 | 0.573 |
| InternalIMT | epa | chr10:45905041:D | 0.068 | -0.023 | 0.031 | 0.462 | 0.099 | 0.052 | 0.057 | -0.06 | 0.08 | 0.455 |
| InternalIMT | epa | chr10:45938871 | . | . | . | . | . | . | . | . | . | . |
| InternalIMT | epa | chr10:45939160 | . | . | . | . | . | . | . | . | . | . |
| InternalIMT | epa | chr10:45869910 | 0.015 | -0.031 | 0.029 | 0.281 | -0.025 | 0.105 | 0.814 | -2.80E-03 | 0.118 | 0.981 |
| InternalIMT | epa | chr10:45876214:D | 0.017 | -0.027 | 0.029 | 0.357 | -0.056 | 0.067 | 0.405 | -0.102 | 0.101 | 0.309 |
| InternalIMT | epa | chr10:45880237:I | 0.094 | -0.013 | 0.032 | 0.681 | 0.031 | 0.046 | 0.498 | -0.087 | 0.06 | 0.147 |
| InternalIMT | epa | chr10:45884371:I | 0.073 | -0.039 | 0.032 | 0.226 | 0.034 | 0.042 | 0.415 | 0.043 | 0.059 | 0.469 |
| InternalIMT | epa | chr10:45891535:D | 0.019 | -0.041 | 0.029 | 0.151 | 0.186 | 0.106 | 0.08 | 0.179 | 0.151 | 0.236 |
| InternalIMT | epa | chr10:45893516:D | 0.073 | -0.039 | 0.032 | 0.223 | 0.035 | 0.042 | 0.407 | 0.044 | 0.059 | 0.456 |
| InternalIMT | epa | chr10:45893782:D | 0.018 | -0.025 | 0.029 | 0.398 | 0.056 | 0.092 | 0.543 | -0.137 | 0.104 | 0.188 |
| InternalIMT | epa | chr10:45895254:I | 0.019 | -0.041 | 0.029 | 0.151 | 0.186 | 0.106 | 0.08 | 0.179 | 0.151 | 0.235 |
| InternalIMT | epa | chr10:45895848:D | 0.057 | -0.036 | 0.031 | 0.247 | 0.021 | 0.047 | 0.656 | 0.039 | 0.067 | 0.562 |
| InternalIMT | epa | chr10:45896316:D | 0.073 | -0.039 | 0.032 | 0.223 | 0.035 | 0.042 | 0.405 | 0.044 | 0.059 | 0.454 |
| InternalIMT | epa | chr10:45896317:D | 0.073 | -0.039 | 0.032 | 0.223 | 0.035 | 0.042 | 0.405 | 0.044 | 0.059 | 0.454 |
| InternalIMT | epa | chr10:45902683:I | 0.019 | -0.029 | 0.029 | 0.326 | -0.042 | 0.065 | 0.52 | -0.055 | 0.099 | 0.582 |
| InternalIMT | epa | chr10:45905003:D | 0.01 | . | . | . | . | . | . | . | . | . |
| InternalIMT | epa | chr10:45927476:D | 0.036 | -0.03 | 0.03 | 0.319 | -3.80E-03 | 0.06 | 0.95 | -0.016 | 0.091 | 0.865 |
| InternalIMT | epa | chr10:45935882 | 0.001 | . | . | . | . | . | . | . | . | . |
| InternalIMT | epa | chr10:45936100 | 0.001 | . | . | . | . | . | . | . | . | . |
| InternalIMT | epa | chr10:45939553 | 0.001 | . | . | . | . | . | . | . | . | . |
| InternalIMT | epa | chr10:45939631 | 0 | . | . | . | . | . | . | . | . | . |
| InternalIMT | epa | chr10:45944193:D | 0.012 | -0.028 | 0.029 | 0.327 | 3.00E-03 | 0.078 | 0.969 | -0.103 | 0.117 | 0.379 |
| InternalIMT | epa | chr10:45954030 | 0 | . | . | . | . | . | . | . | . | . |

Supplemental table S19.

| phen | covar(fa) | rs_id | maf | beta_FA | se_FA | p_FA | beta_SNP | se_SNP | p_SNP | beta_inter | se_inter | p_inter |
| --- | --- | --- | --- | --- | --- | --- | --- | --- | --- | --- | --- | --- |
| InternalIMT | epa | rs76035182 | 0.016 | 0.027 | 0.027 | 0.318 | -0.024 | 0.078 | 0.762 | -0.026 | 0.118 | 0.824 |
| InternalIMT | epa | rs12359547 | 0.066 | 0.037 | 0.03 | 0.22 | -0.104 | 0.051 | 0.04 | -0.052 | 0.054 | 0.337 |
| InternalIMT | epa | rs74595484 | 0.017 | 0.013 | 0.027 | 0.613 | 0.079 | 0.127 | 0.533 | 0.17 | 0.12 | 0.159 |
| InternalIMT | epa | rs1864415 | 0.722 | 0.046 | 0.074 | 0.537 | -0.02 | 0.038 | 0.601 | -0.013 | 0.045 | 0.773 |
| InternalIMT | epa | rs72788101 | 0.069 | 0.037 | 0.03 | 0.214 | -0.11 | 0.05 | 0.028 | -0.053 | 0.054 | 0.326 |
| InternalIMT | epa | rs11239481 | 0.129 | 0.026 | 0.03 | 0.39 | -0.026 | 0.048 | 0.589 | 3.30E-03 | 0.054 | 0.951 |
| InternalIMT | epa | rs72788102 | 0.069 | 0.037 | 0.03 | 0.214 | -0.11 | 0.05 | 0.028 | -0.053 | 0.054 | 0.327 |
| InternalIMT | epa | rs72789705 | 0.069 | 0.037 | 0.03 | 0.215 | -0.109 | 0.05 | 0.028 | -0.052 | 0.054 | 0.328 |
| InternalIMT | epa | rs141529858 | 0.005 | . | . | . | . | . | . | . | . | . |
| InternalIMT | epa | rs145376292 | 0 | . | . | . | . | . | . | . | . | . |
| InternalIMT | epa | rs190418301 | 0.001 | . | . | . | . | . | . | . | . | . |
| InternalIMT | epa | rs11239482 | 0.069 | 0.037 | 0.03 | 0.215 | -0.109 | 0.05 | 0.028 | -0.052 | 0.054 | 0.329 |
| InternalIMT | epa | rs61855872 | 0.011 | 0.024 | 0.027 | 0.367 | 0.352 | 0.262 | 0.18 | 0.069 | 0.284 | 0.808 |
| InternalIMT | epa | rs148991144 | 0.005 | . | . | . | . | . | . | . | . | . |
| InternalIMT | epa | rs191932221 | 0.005 | . | . | . | . | . | . | . | . | . |
| InternalIMT | epa | rs55978138 | 0.144 | 0.042 | 0.032 | 0.193 | -0.101 | 0.041 | 0.015 | -0.055 | 0.05 | 0.275 |
| InternalIMT | epa | rs73281167 | 0.01 | . | . | . | . | . | . | . | . | . |
| InternalIMT | epa | rs12768010 | 0.072 | 0.029 | 0.028 | 0.308 | -0.051 | 0.054 | 0.347 | -0.028 | 0.071 | 0.697 |
| InternalIMT | epa | rs11239484 | 0.722 | 0.044 | 0.074 | 0.547 | -0.019 | 0.038 | 0.614 | -0.012 | 0.045 | 0.787 |
| InternalIMT | epa | rs35906360 | 0.144 | 0.044 | 0.033 | 0.183 | -0.096 | 0.04 | 0.016 | -0.056 | 0.049 | 0.251 |
| InternalIMT | epa | rs186250512 | 0.004 | . | . | . | . | . | . | . | . | . |
| InternalIMT | epa | rs150810743 | 0.001 | . | . | . | . | . | . | . | . | . |
| InternalIMT | epa | rs55965812 | 0.14 | 0.041 | 0.032 | 0.203 | -0.091 | 0.041 | 0.028 | -0.052 | 0.05 | 0.303 |
| InternalIMT | epa | rs56180358 | 0.144 | 0.043 | 0.033 | 0.184 | -0.096 | 0.04 | 0.016 | -0.056 | 0.048 | 0.252 |
| InternalIMT | epa | rs55864145 | 0.073 | 0.029 | 0.028 | 0.308 | -0.051 | 0.054 | 0.347 | -0.027 | 0.071 | 0.699 |
| InternalIMT | epa | rs55986122 | 0.144 | 0.043 | 0.033 | 0.184 | -0.096 | 0.04 | 0.016 | -0.055 | 0.048 | 0.253 |
| InternalIMT | epa | rs7477350 | 0.722 | 0.044 | 0.074 | 0.548 | -0.019 | 0.038 | 0.615 | -0.012 | 0.045 | 0.787 |
| InternalIMT | epa | rs2099170 | 0.143 | 0.044 | 0.033 | 0.182 | -0.096 | 0.04 | 0.017 | -0.056 | 0.049 | 0.248 |
| InternalIMT | epa | rs2082108 | 0.144 | 0.044 | 0.033 | 0.183 | -0.096 | 0.04 | 0.016 | -0.056 | 0.048 | 0.25 |
| InternalIMT | epa | rs55902404 | 0.004 | . | . | . | . | . | . | . | . | . |
| InternalIMT | epa | rs58402662 | 0.004 | . | . | . | . | . | . | . | . | . |
| InternalIMT | epa | rs11511618 | 0.129 | 0.024 | 0.03 | 0.423 | -0.021 | 0.049 | 0.671 | 0.011 | 0.054 | 0.847 |
| InternalIMT | epa | rs1816492 | 0.721 | 0.048 | 0.074 | 0.52 | -0.019 | 0.038 | 0.618 | -0.014 | 0.046 | 0.752 |
| InternalIMT | epa | rs146611093 | 0.006 | . | . | . | . | . | . | . | . | . |
| InternalIMT | epa | rs186897689 | 0.001 | . | . | . | . | . | . | . | . | . |
| InternalIMT | epa | rs10793616 | 0.722 | 0.044 | 0.074 | 0.547 | -0.019 | 0.038 | 0.615 | -0.012 | 0.045 | 0.787 |
| InternalIMT | epa | rs116700479 | 0.013 | 0.026 | 0.027 | 0.334 | -1.00E-03 | 0.088 | 0.991 | -1.70E-04 | 0.141 | 0.999 |
| InternalIMT | epa | rs192535936 | . | . | . | . | . | . | . | . | . | . |
| InternalIMT | epa | rs113363297 | 0.653 | 9.50E-03 | 0.068 | 0.889 | 5.90E-03 | 0.039 | 0.881 | 0.012 | 0.046 | 0.792 |
| InternalIMT | epa | rs12354994 | 0.137 | 0.04 | 0.032 | 0.217 | -0.088 | 0.042 | 0.036 | -0.048 | 0.051 | 0.346 |
| InternalIMT | epa | rs148068329 | 0.025 | 0.018 | 0.027 | 0.513 | 0.036 | 0.156 | 0.815 | 0.126 | 0.13 | 0.334 |
| InternalIMT | epa | rs12356109 | 0.149 | 0.042 | 0.034 | 0.21 | -0.099 | 0.04 | 0.013 | -0.047 | 0.048 | 0.324 |
| InternalIMT | epa | rs55908141 | 0.004 | . | . | . | . | . | . | . | . | . |
| InternalIMT | epa | rs12354712 | 0.14 | 0.04 | 0.032 | 0.215 | -0.09 | 0.041 | 0.03 | -0.048 | 0.05 | 0.336 |
| InternalIMT | epa | rs147282684 | 0.003 | . | . | . | . | . | . | . | . | . |
| InternalIMT | epa | rs12356140 | 0.145 | 0.042 | 0.033 | 0.198 | -0.097 | 0.04 | 0.016 | -0.051 | 0.049 | 0.291 |
| InternalIMT | epa | rs185476187 | 0.001 | . | . | . | . | . | . | . | . | . |
| InternalIMT | epa | rs145720057 | 0.018 | 0.019 | 0.027 | 0.49 | 0.243 | 0.165 | 0.141 | 0.245 | 0.185 | 0.186 |
| InternalIMT | epa | rs12354742 | 0.144 | 0.042 | 0.033 | 0.195 | -0.095 | 0.04 | 0.017 | -0.052 | 0.048 | 0.281 |
| InternalIMT | epa | rs180913842 | 0.004 | . | . | . | . | . | . | . | . | . |
| InternalIMT | epa | rs142976914 | 0.002 | . | . | . | . | . | . | . | . | . |
| InternalIMT | epa | rs11511619 | 0.151 | 0.042 | 0.034 | 0.212 | -0.097 | 0.04 | 0.014 | -0.047 | 0.048 | 0.33 |
| InternalIMT | epa | rs146702870 | 0.002 | . | . | . | . | . | . | . | . | . |
| InternalIMT | epa | rs150287496 | . | . | . | . | . | . | . | . | . | . |
| InternalIMT | epa | rs190917973 | 0.004 | . | . | . | . | . | . | . | . | . |
| InternalIMT | epa | rs11517281 | 0.144 | 0.042 | 0.033 | 0.196 | -0.095 | 0.04 | 0.018 | -0.052 | 0.048 | 0.286 |
| InternalIMT | epa | rs12359299 | 0.144 | 0.042 | 0.033 | 0.196 | -0.095 | 0.04 | 0.018 | -0.052 | 0.048 | 0.285 |
| InternalIMT | epa | rs182307932 | 0.014 | 0.028 | 0.027 | 0.299 | -0.107 | 0.081 | 0.189 | -0.018 | 0.084 | 0.833 |
| InternalIMT | epa | rs61855873 | 0.026 | 0.025 | 0.028 | 0.377 | 0.176 | 0.137 | 0.198 | 0.024 | 0.155 | 0.877 |
| InternalIMT | epa | rs12359302 | 0.143 | 0.043 | 0.033 | 0.189 | -0.092 | 0.04 | 0.023 | -0.054 | 0.049 | 0.262 |
| InternalIMT | epa | rs143208140 | 0.027 | 0.018 | 0.027 | 0.511 | 0.063 | 0.155 | 0.686 | 0.132 | 0.131 | 0.313 |
| InternalIMT | epa | rs9988764 | 0.708 | 0.056 | 0.076 | 0.457 | -0.033 | 0.038 | 0.393 | -0.021 | 0.046 | 0.65 |
| InternalIMT | epa | rs192875767 | 0.003 | . | . | . | . | . | . | . | . | . |
| InternalIMT | epa | rs147268846 | 0.014 | 0.031 | 0.027 | 0.254 | -0.075 | 0.291 | 0.796 | -0.256 | 0.307 | 0.404 |
| InternalIMT | epa | rs11239485 | 0.094 | 0.033 | 0.032 | 0.303 | -0.097 | 0.054 | 0.073 | -0.03 | 0.059 | 0.61 |
| InternalIMT | epa | rs151317885 | 0.476 | 0.024 | 0.043 | 0.58 | 0.046 | 0.034 | 0.168 | 4.60E-03 | 0.039 | 0.907 |
| InternalIMT | epa | rs11499085 | 0.055 | 0.014 | 0.028 | 0.605 | -1.80E-03 | 0.053 | 0.972 | 0.082 | 0.053 | 0.119 |
| InternalIMT | epa | rs188130606 | 0.012 | 0.025 | 0.027 | 0.366 | -0.057 | 0.072 | 0.432 | 0.05 | 0.075 | 0.511 |
| InternalIMT | epa | rs11511620 | 0.141 | 0.04 | 0.032 | 0.218 | -0.089 | 0.041 | 0.031 | -0.047 | 0.05 | 0.344 |
| InternalIMT | epa | rs181498390 | 0.012 | 0.021 | 0.026 | 0.42 | 0.178 | 0.167 | 0.284 | 0.187 | 0.21 | 0.373 |
| InternalIMT | epa | rs186280701 | 0.004 | . | . | . | . | . | . | . | . | . |

| InternalIMT | epa | rs150059185 | 0.02 | 0.025 | 0.027 | 0.34 | 0.074 | 0.279 | 0.792 | 4.90E-03 | 0.344 | 0.989 |
| --- | --- | --- | --- | --- | --- | --- | --- | --- | --- | --- | --- | --- |
| InternalIMT | epa | rs183338707 | . | . | . | . | . | . | . | . | . | . |
| InternalIMT | epa | rs11499038 | 0.052 | 0.014 | 0.028 | 0.621 | 0.02 | 0.063 | 0.748 | 0.09 | 0.068 | 0.187 |
| InternalIMT | epa | rs190699595 | 0.004 | . | . | . | . | . | . | . | . | . |
| InternalIMT | epa | rs11523408 | 0.143 | 0.04 | 0.033 | 0.215 | -0.089 | 0.04 | 0.027 | -0.047 | 0.049 | 0.337 |
| InternalIMT | epa | rs188049317 | 0.001 | . | . | . | . | . | . | . | . | . |
| InternalIMT | epa | rs144948634 | 0.435 | 0.045 | 0.041 | 0.265 | 0.04 | 0.034 | 0.233 | -0.022 | 0.04 | 0.577 |
| InternalIMT | epa | rs143833576 | 0.001 | . | . | . | . | . | . | . | . | . |
| InternalIMT | epa | rs151239879 | 0.144 | 0.041 | 0.033 | 0.214 | -0.09 | 0.04 | 0.026 | -0.047 | 0.049 | 0.334 |
| InternalIMT | epa | rs7073743 | 0.455 | 0.036 | 0.042 | 0.393 | 0.04 | 0.035 | 0.246 | -0.01 | 0.04 | 0.797 |
| InternalIMT | epa | rs142563349 | 0.013 | 0.025 | 0.027 | 0.367 | -0.072 | 0.07 | 0.302 | 0.057 | 0.074 | 0.438 |
| InternalIMT | epa | rs187246403 | 0.004 | . | . | . | . | . | . | . | . | . |
| InternalIMT | epa | rs150423942 | 0.134 | 0.042 | 0.033 | 0.197 | -0.096 | 0.041 | 0.018 | -0.051 | 0.05 | 0.313 |
| InternalIMT | epa | rs184706102 | . | . | . | . | . | . | . | . | . | . |
| InternalIMT | epa | rs189626288 | 0.014 | 0.025 | 0.027 | 0.357 | 0.019 | 0.183 | 0.917 | 0.023 | 0.213 | 0.914 |
| InternalIMT | epa | rs140749834 | 0.027 | 0.027 | 0.028 | 0.333 | 0.014 | 0.097 | 0.883 | -0.015 | 0.088 | 0.862 |
| InternalIMT | epa | rs7078822 | 0.115 | 0.043 | 0.032 | 0.18 | -0.096 | 0.046 | 0.038 | -0.065 | 0.055 | 0.236 |
| InternalIMT | epa | rs186227617 | 0.143 | 0.041 | 0.033 | 0.21 | -0.092 | 0.04 | 0.022 | -0.048 | 0.049 | 0.32 |
| InternalIMT | epa | rs188984368 | 0.022 | 0.032 | 0.028 | 0.24 | -0.132 | 0.119 | 0.267 | -0.164 | 0.17 | 0.336 |
| InternalIMT | epa | rs182078109 | 0.027 | 0.029 | 0.027 | 0.276 | -0.049 | 0.176 | 0.779 | -0.063 | 0.224 | 0.778 |
| InternalIMT | epa | rs141456274 | 0.006 | . | . | . | . | . | . | . | . | . |
| InternalIMT | epa | rs146976512 | 0.144 | 0.042 | 0.033 | 0.198 | -0.095 | 0.04 | 0.017 | -0.051 | 0.048 | 0.287 |
| InternalIMT | epa | rs138036816 | 0.05 | 0.014 | 0.028 | 0.616 | 0.022 | 0.063 | 0.729 | 0.089 | 0.068 | 0.191 |
| InternalIMT | epa | rs114879777 | 0.013 | 0.026 | 0.027 | 0.344 | 5.40E-03 | 0.093 | 0.954 | 0.018 | 0.139 | 0.895 |
| InternalIMT | epa | rs149366475 | 0.052 | 0.016 | 0.028 | 0.57 | 0.01 | 0.062 | 0.867 | 0.075 | 0.067 | 0.264 |
| InternalIMT | epa | rs182463043 | 0.001 | . | . | . | . | . | . | . | . | . |
| InternalIMT | epa | rs144754632 | 0.025 | 0.018 | 0.027 | 0.515 | 0.039 | 0.156 | 0.804 | 0.128 | 0.13 | 0.328 |
| InternalIMT | epa | rs55717607 | 0.145 | 0.042 | 0.033 | 0.2 | -0.094 | 0.04 | 0.018 | -0.05 | 0.048 | 0.296 |
| InternalIMT | epa | rs187537973 | . | . | . | . | . | . | . | . | . | . |
| InternalIMT | epa | rs148085971 | 0.001 | . | . | . | . | . | . | . | . | . |
| InternalIMT | epa | rs11239491 | 0.145 | 0.042 | 0.033 | 0.2 | -0.094 | 0.04 | 0.018 | -0.05 | 0.048 | 0.297 |
| InternalIMT | epa | rs184412184 | 0.002 | . | . | . | . | . | . | . | . | . |
| InternalIMT | epa | rs4948998 | 0.733 | 0.028 | 0.073 | 0.702 | -6.20E-03 | 0.038 | 0.872 | -1.20E-03 | 0.045 | 0.978 |
| InternalIMT | epa | rs7076696 | 0.733 | 0.028 | 0.073 | 0.701 | -6.30E-03 | 0.038 | 0.869 | -1.30E-03 | 0.045 | 0.978 |
| InternalIMT | epa | rs11239492 | 0.15 | 0.042 | 0.034 | 0.216 | -0.098 | 0.039 | 0.013 | -0.046 | 0.048 | 0.34 |
| InternalIMT | epa | rs79462470 | 0.004 | . | . | . | . | . | . | . | . | . |
| InternalIMT | epa | rs187804654 | 0.004 | . | . | . | . | . | . | . | . | . |
| InternalIMT | epa | rs7093687 | 0.141 | 0.039 | 0.032 | 0.23 | -0.086 | 0.041 | 0.034 | -0.044 | 0.049 | 0.376 |
| InternalIMT | epa | rs7081004 | 0.141 | 0.04 | 0.032 | 0.222 | -0.088 | 0.041 | 0.032 | -0.046 | 0.05 | 0.354 |
| InternalIMT | epa | rs7084793 | 0.145 | 0.042 | 0.033 | 0.201 | -0.094 | 0.04 | 0.018 | -0.05 | 0.048 | 0.298 |
| InternalIMT | epa | rs61556080 | 0.004 | . | . | . | . | . | . | . | . | . |
| InternalIMT | epa | rs115552064 | 0.004 | . | . | . | . | . | . | . | . | . |
| InternalIMT | epa | rs7921503 | 0.145 | 0.042 | 0.033 | 0.198 | -0.095 | 0.04 | 0.017 | -0.051 | 0.048 | 0.291 |
| InternalIMT | epa | rs7909740 | 0.145 | 0.042 | 0.033 | 0.201 | -0.094 | 0.04 | 0.018 | -0.05 | 0.048 | 0.298 |
| InternalIMT | epa | rs7909768 | 0.145 | 0.042 | 0.033 | 0.201 | -0.094 | 0.04 | 0.018 | -0.05 | 0.048 | 0.297 |
| InternalIMT | epa | rs144168004 | . | . | . | . | . | . | . | . | . | . |
| InternalIMT | epa | rs75850448 | 0.004 | . | . | . | . | . | . | . | . | . |
| InternalIMT | epa | rs147777574 | 0.003 | . | . | . | . | . | . | . | . | . |
| InternalIMT | epa | rs7913488 | 0.145 | 0.042 | 0.033 | 0.201 | -0.094 | 0.04 | 0.018 | -0.05 | 0.048 | 0.297 |
| InternalIMT | epa | rs74390874 | 0.042 | 0.037 | 0.028 | 0.186 | 0.04 | 0.093 | 0.665 | -0.158 | 0.123 | 0.201 |
| InternalIMT | epa | rs141391142 | 0.004 | . | . | . | . | . | . | . | . | . |
| InternalIMT | epa | rs115294747 | 0.006 | . | . | . | . | . | . | . | . | . |
| InternalIMT | epa | rs148031781 | 0.001 | . | . | . | . | . | . | . | . | . |
| InternalIMT | epa | rs181340128 | 0.025 | 0.029 | 0.027 | 0.288 | 0.023 | 0.17 | 0.893 | -0.071 | 0.178 | 0.689 |
| InternalIMT | epa | rs189930007 | 0.001 | . | . | . | . | . | . | . | . | . |
| InternalIMT | epa | rs12242128 | 0.049 | 0.014 | 0.028 | 0.62 | 0.025 | 0.063 | 0.696 | 0.09 | 0.068 | 0.188 |
| InternalIMT | epa | rs78421750 | 0.003 | . | . | . | . | . | . | . | . | . |
| InternalIMT | epa | rs114370747 | 0.002 | . | . | . | . | . | . | . | . | . |
| InternalIMT | epa | rs12264166 | 0.049 | 0.014 | 0.028 | 0.621 | 0.025 | 0.063 | 0.691 | 0.09 | 0.068 | 0.188 |
| InternalIMT | epa | rs182964822 | 0.001 | . | . | . | . | . | . | . | . | . |
| InternalIMT | epa | rs67491417 | 0.139 | 0.044 | 0.032 | 0.176 | -0.095 | 0.041 | 0.02 | -0.06 | 0.049 | 0.219 |
| InternalIMT | epa | rs72789720 | 0.008 | . | . | . | . | . | . | . | . | . |
| InternalIMT | epa | rs6413416 | 0.145 | 0.042 | 0.033 | 0.2 | -0.095 | 0.039 | 0.017 | -0.05 | 0.048 | 0.295 |
| InternalIMT | epa | rs4986832 | 0.145 | 0.042 | 0.033 | 0.2 | -0.094 | 0.039 | 0.017 | -0.05 | 0.048 | 0.297 |
| InternalIMT | epa | rs144866316 | 0.004 | . | . | . | . | . | . | . | . | . |
| InternalIMT | epa | rs187769540 | 0.007 | . | . | . | . | . | . | . | . | . |
| InternalIMT | epa | rs6593482 | 0.146 | 0.045 | 0.033 | 0.173 | -0.1 | 0.04 | 0.011 | -0.059 | 0.048 | 0.218 |
| InternalIMT | epa | rs7913948 | 0.146 | 0.042 | 0.033 | 0.203 | -0.088 | 0.04 | 0.026 | -0.049 | 0.048 | 0.303 |
| InternalIMT | epa | rs77185151 | 0.025 | 0.018 | 0.027 | 0.51 | 0.037 | 0.156 | 0.814 | 0.124 | 0.13 | 0.34 |
| InternalIMT | epa | rs12762303 | 0.145 | 0.042 | 0.033 | 0.2 | -0.095 | 0.039 | 0.016 | -0.05 | 0.048 | 0.296 |
| InternalIMT | epa | rs73283136 | 0.003 | . | . | . | . | . | . | . | . | . |
| InternalIMT | epa | rs187180218 | 0.001 | . | . | . | . | . | . | . | . | . |
| InternalIMT | epa | rs78137087 | 0.145 | 0.042 | 0.033 | 0.199 | -0.096 | 0.039 | 0.015 | -0.05 | 0.048 | 0.292 |

| InternalIMT | epa | rs72561472 | 0.146 | 0.042 | 0.033 | 0.208 | -0.093 | 0.041 | 0.023 | -0.048 | 0.049 | 0.327 |
| --- | --- | --- | --- | --- | --- | --- | --- | --- | --- | --- | --- | --- |
| InternalIMT | epa | rs4987106 | 0.142 | 0.043 | 0.033 | 0.188 | -0.099 | 0.04 | 0.013 | -0.054 | 0.048 | 0.261 |
| InternalIMT | epa | rs4987105 | 0.139 | 0.04 | 0.032 | 0.213 | -0.092 | 0.041 | 0.026 | -0.049 | 0.05 | 0.328 |
| InternalIMT | epa | rs111916157 | 0.158 | 0.041 | 0.033 | 0.22 | -0.083 | 0.038 | 0.031 | -0.042 | 0.048 | 0.38 |
| InternalIMT | epa | rs111528152 | 0.162 | 0.051 | 0.035 | 0.143 | -0.112 | 0.04 | 5.00E-03 | -0.069 | 0.049 | 0.157 |
| InternalIMT | epa | rs12763738 | 0.145 | 0.042 | 0.033 | 0.201 | -0.095 | 0.04 | 0.017 | -0.05 | 0.048 | 0.297 |
| InternalIMT | epa | rs191675886 | 0.004 | . | . | . | . | . | . | . | . | . |
| InternalIMT | epa | rs66489870 | 0.145 | 0.042 | 0.033 | 0.201 | -0.095 | 0.039 | 0.016 | -0.05 | 0.048 | 0.297 |
| InternalIMT | epa | rs71494775 | 0.145 | 0.042 | 0.033 | 0.201 | -0.095 | 0.039 | 0.016 | -0.05 | 0.048 | 0.297 |
| InternalIMT | epa | rs61855875 | 0.024 | 0.025 | 0.028 | 0.377 | 0.138 | 0.135 | 0.305 | 0.025 | 0.152 | 0.871 |
| InternalIMT | epa | rs116356837 | 0.004 | . | . | . | . | . | . | . | . | . |
| InternalIMT | epa | rs1864414 | 0.145 | 0.042 | 0.033 | 0.196 | -0.094 | 0.04 | 0.018 | -0.051 | 0.048 | 0.285 |
| InternalIMT | epa | rs1864413 | 0.145 | 0.042 | 0.033 | 0.205 | -0.095 | 0.039 | 0.016 | -0.049 | 0.048 | 0.308 |
| InternalIMT | epa | rs139497212 | 0 | . | . | . | . | . | . | . | . | . |
| InternalIMT | epa | rs892690 | 0.424 | 0.05 | 0.042 | 0.239 | 0.02 | 0.035 | 0.564 | -0.027 | 0.04 | 0.508 |
| InternalIMT | epa | rs1864412 | 0.145 | 0.042 | 0.033 | 0.201 | -0.095 | 0.039 | 0.016 | -0.05 | 0.048 | 0.299 |
| InternalIMT | epa | rs77827771 | 0.016 | 0.023 | 0.027 | 0.4 | 0.03 | 0.087 | 0.729 | 0.049 | 0.085 | 0.564 |
| InternalIMT | epa | rs145294963 | . | . | . | . | . | . | . | . | . | . |
| InternalIMT | epa | rs149081327 | 0.008 | . | . | . | . | . | . | . | . | . |
| InternalIMT | epa | rs67008983 | 0.145 | 0.042 | 0.033 | 0.204 | -0.095 | 0.039 | 0.016 | -0.049 | 0.048 | 0.306 |
| InternalIMT | epa | rs56150414 | 0.147 | 0.045 | 0.032 | 0.17 | -0.099 | 0.04 | 0.012 | -0.058 | 0.048 | 0.226 |
| InternalIMT | epa | rs55787481 | 0.145 | 0.041 | 0.033 | 0.211 | -0.093 | 0.04 | 0.019 | -0.047 | 0.048 | 0.328 |
| InternalIMT | epa | rs141270101 | 0.001 | . | . | . | . | . | . | . | . | . |
| InternalIMT | epa | rs114980075 | 0.003 | . | . | . | . | . | . | . | . | . |
| InternalIMT | epa | rs12783095 | 0.144 | 0.042 | 0.033 | 0.204 | -0.104 | 0.039 | 8.50E-03 | -0.05 | 0.048 | 0.302 |
| InternalIMT | epa | rs182342688 | . | . | . | . | . | . | . | . | . | . |
| InternalIMT | epa | rs17157728 | 0.144 | 0.041 | 0.033 | 0.204 | -0.104 | 0.039 | 8.50E-03 | -0.049 | 0.048 | 0.303 |
| InternalIMT | epa | rs114835563 | 0.012 | 0.024 | 0.027 | 0.377 | -0.058 | 0.073 | 0.431 | 0.062 | 0.076 | 0.414 |
| InternalIMT | epa | rs3824612 | 0.424 | 0.05 | 0.042 | 0.231 | 0.02 | 0.035 | 0.562 | -0.027 | 0.04 | 0.493 |
| InternalIMT | epa | rs17157733 | 0.144 | 0.041 | 0.033 | 0.206 | -0.104 | 0.04 | 8.40E-03 | -0.049 | 0.048 | 0.309 |
| InternalIMT | epa | rs74128478 | 0.004 | . | . | . | . | . | . | . | . | . |
| InternalIMT | epa | rs184973589 | 0.002 | . | . | . | . | . | . | . | . | . |
| InternalIMT | epa | rs150479461 | 0 | . | . | . | . | . | . | . | . | . |
| InternalIMT | epa | rs138310407 | 0.001 | . | . | . | . | . | . | . | . | . |
| InternalIMT | epa | rs187426603 | . | . | . | . | . | . | . | . | . | . |
| InternalIMT | epa | rs190843143 | 0.002 | . | . | . | . | . | . | . | . | . |
| InternalIMT | epa | rs191883449 | 0.001 | . | . | . | . | . | . | . | . | . |
| InternalIMT | epa | rs58238740 | 0.143 | 0.041 | 0.033 | 0.208 | -0.105 | 0.04 | 7.80E-03 | -0.049 | 0.048 | 0.31 |
| InternalIMT | epa | rs3780894 | 0.143 | 0.041 | 0.033 | 0.208 | -0.105 | 0.04 | 7.80E-03 | -0.049 | 0.048 | 0.311 |
| InternalIMT | epa | rs149646626 | 0.018 | 0.031 | 0.027 | 0.266 | -0.162 | 0.109 | 0.137 | -0.099 | 0.128 | 0.44 |
| InternalIMT | epa | rs79615212 | 0.015 | 0.019 | 0.027 | 0.479 | 0.178 | 0.228 | 0.434 | 0.245 | 0.229 | 0.285 |
| InternalIMT | epa | rs3780895 | 0.143 | 0.041 | 0.033 | 0.208 | -0.106 | 0.04 | 7.60E-03 | -0.049 | 0.048 | 0.309 |
| InternalIMT | epa | rs3780896 | 0.143 | 0.041 | 0.033 | 0.208 | -0.105 | 0.04 | 7.80E-03 | -0.049 | 0.048 | 0.311 |
| InternalIMT | epa | rs142747163 | 0.005 | . | . | . | . | . | . | . | . | . |
| InternalIMT | epa | rs189417584 | 0.005 | . | . | . | . | . | . | . | . | . |
| InternalIMT | epa | rs3780897 | 0.141 | 0.041 | 0.032 | 0.212 | -0.104 | 0.04 | 9.10E-03 | -0.049 | 0.048 | 0.315 |
| InternalIMT | epa | rs117509491 | 0.025 | 0.033 | 0.027 | 0.229 | 0.114 | 0.149 | 0.444 | -0.108 | 0.162 | 0.508 |
| InternalIMT | epa | rs117811514 | 0.025 | 0.033 | 0.027 | 0.229 | 0.114 | 0.149 | 0.444 | -0.108 | 0.162 | 0.508 |
| InternalIMT | epa | rs116500932 | 0.012 | 0.024 | 0.027 | 0.377 | -0.057 | 0.073 | 0.435 | 0.063 | 0.076 | 0.411 |
| InternalIMT | epa | rs77584168 | . | . | . | . | . | . | . | . | . | . |
| InternalIMT | epa | rs2310776 | 0.143 | 0.041 | 0.033 | 0.209 | -0.106 | 0.04 | 7.80E-03 | -0.049 | 0.048 | 0.312 |
| InternalIMT | epa | rs17157736 | 0.143 | 0.041 | 0.033 | 0.209 | -0.106 | 0.04 | 7.80E-03 | -0.049 | 0.048 | 0.312 |
| InternalIMT | epa | rs72789735 | 0.063 | 0.034 | 0.03 | 0.251 | -0.102 | 0.054 | 0.06 | -0.04 | 0.058 | 0.496 |
| InternalIMT | epa | rs183747187 | 0.001 | . | . | . | . | . | . | . | . | . |
| InternalIMT | epa | rs7077891 | 0.143 | 0.041 | 0.033 | 0.209 | -0.106 | 0.04 | 7.70E-03 | -0.049 | 0.048 | 0.313 |
| InternalIMT | epa | rs145063305 | 0.002 | . | . | . | . | . | . | . | . | . |
| InternalIMT | epa | rs3824613 | 0.143 | 0.04 | 0.033 | 0.218 | -0.104 | 0.04 | 8.50E-03 | -0.046 | 0.048 | 0.337 |
| InternalIMT | epa | rs145543199 | 0.006 | . | . | . | . | . | . | . | . | . |
| InternalIMT | epa | rs140361993 | 0.003 | . | . | . | . | . | . | . | . | . |
| InternalIMT | epa | rs11239496 | 0.063 | 0.034 | 0.03 | 0.251 | -0.102 | 0.054 | 0.06 | -0.04 | 0.058 | 0.496 |
| InternalIMT | epa | rs11239497 | 0.139 | 0.039 | 0.032 | 0.231 | -0.1 | 0.041 | 0.015 | -0.045 | 0.05 | 0.369 |
| InternalIMT | epa | rs10900212 | 0.156 | 0.028 | 0.032 | 0.386 | -0.069 | 0.041 | 0.091 | -1.40E-03 | 0.048 | 0.976 |
| InternalIMT | epa | rs186487136 | . | . | . | . | . | . | . | . | . | . |
| InternalIMT | epa | rs12774369 | 0.143 | 0.041 | 0.033 | 0.205 | -0.106 | 0.04 | 7.50E-03 | -0.05 | 0.048 | 0.303 |
| InternalIMT | epa | rs60842538 | 0.142 | 0.042 | 0.033 | 0.201 | -0.107 | 0.04 | 7.30E-03 | -0.051 | 0.048 | 0.292 |
| InternalIMT | epa | rs3780898 | 0.138 | 0.04 | 0.032 | 0.217 | -0.103 | 0.041 | 0.013 | -0.049 | 0.05 | 0.331 |
| InternalIMT | epa | rs3780899 | 0.142 | 0.042 | 0.033 | 0.195 | -0.108 | 0.04 | 6.90E-03 | -0.053 | 0.049 | 0.274 |
| InternalIMT | epa | rs12252472 | 0.045 | 0.025 | 0.029 | 0.391 | -0.032 | 0.05 | 0.521 | 0.027 | 0.056 | 0.627 |
| InternalIMT | epa | rs185411011 | . | . | . | . | . | . | . | . | . | . |
| InternalIMT | epa | rs28395865 | 0.137 | 0.041 | 0.032 | 0.202 | -0.108 | 0.042 | 9.80E-03 | -0.054 | 0.051 | 0.287 |
| InternalIMT | epa | rs191004074 | 0.038 | 0.035 | 0.029 | 0.231 | -0.121 | 0.131 | 0.356 | -0.138 | 0.18 | 0.443 |
| InternalIMT | epa | rs182401295 | 0.002 | . | . | . | . | . | . | . | . | . |
| InternalIMT | epa | rs2228064 | 0.068 | 0.016 | 0.03 | 0.59 | -0.024 | 0.05 | 0.63 | 0.06 | 0.051 | 0.24 |

| InternalIMT | epa | rs188284159 | . | . | . | . | . | . | . | . | . | . |
| --- | --- | --- | --- | --- | --- | --- | --- | --- | --- | --- | --- | --- |
| InternalIMT | epa | rs2303887 | 0.022 | 0.018 | 0.027 | 0.515 | 0.059 | 0.151 | 0.698 | 0.162 | 0.124 | 0.192 |
| InternalIMT | epa | rs2303886 | 0.024 | 0.015 | 0.027 | 0.577 | 0.083 | 0.138 | 0.544 | 0.192 | 0.113 | 0.09 |
| InternalIMT | epa | rs192621006 | . | . | . | . | . | . | . | . | . | . |
| InternalIMT | epa | rs2303885 | 0.191 | 0.035 | 0.034 | 0.308 | -0.067 | 0.033 | 0.041 | -0.014 | 0.039 | 0.72 |
| InternalIMT | epa | rs114466168 | 0.012 | 0.024 | 0.027 | 0.377 | -0.06 | 0.073 | 0.412 | 0.063 | 0.076 | 0.402 |
| InternalIMT | epa | rs11239501 | 0.083 | 0.025 | 0.03 | 0.408 | -0.029 | 0.05 | 0.566 | 0.01 | 0.055 | 0.852 |
| InternalIMT | epa | rs745986 | 0.156 | 0.031 | 0.033 | 0.349 | -0.06 | 0.039 | 0.123 | -0.015 | 0.046 | 0.751 |
| InternalIMT | epa | rs75262268 | . | . | . | . | . | . | . | . | . | . |
| InternalIMT | epa | rs71494776 | 0.051 | 0.028 | 0.027 | 0.314 | -0.076 | 0.075 | 0.306 | -0.042 | 0.094 | 0.653 |
| InternalIMT | epa | rs74386871 | 0.03 | 0.017 | 0.028 | 0.529 | -0.038 | 0.128 | 0.766 | 0.096 | 0.109 | 0.379 |
| InternalIMT | epa | rs76502040 | 0.006 | . | . | . | . | . | . | . | . | . |
| InternalIMT | epa | rs142213734 | 0.001 | . | . | . | . | . | . | . | . | . |
| InternalIMT | epa | rs4948671 | 0.731 | 0.043 | 0.072 | 0.552 | -0.018 | 0.037 | 0.623 | -0.011 | 0.044 | 0.803 |
| InternalIMT | epa | rs146286085 | 0.001 | . | . | . | . | . | . | . | . | . |
| InternalIMT | epa | rs144080623 | . | . | . | . | . | . | . | . | . | . |
| InternalIMT | epa | rs115074014 | 0.023 | 0.028 | 0.028 | 0.304 | -0.064 | 0.072 | 0.371 | -0.015 | 0.078 | 0.848 |
| InternalIMT | epa | rs17157756 | 0.023 | 0.028 | 0.028 | 0.304 | -0.064 | 0.072 | 0.371 | -0.015 | 0.078 | 0.848 |
| InternalIMT | epa | rs190459780 | . | . | . | . | . | . | . | . | . | . |
| InternalIMT | epa | rs12358647 | 0.174 | 0.017 | 0.033 | 0.611 | -0.032 | 0.039 | 0.409 | 0.025 | 0.045 | 0.574 |
| InternalIMT | epa | rs147080556 | 0.024 | 0.034 | 0.028 | 0.233 | -0.117 | 0.11 | 0.288 | -0.132 | 0.145 | 0.362 |
| InternalIMT | epa | rs187543349 | 0.01 | 0.024 | 0.027 | 0.379 | -0.051 | 0.285 | 0.857 | 0.039 | 0.285 | 0.891 |
| InternalIMT | epa | rs114375595 | 0.006 | . | . | . | . | . | . | . | . | . |
| InternalIMT | epa | rs117211200 | 0.005 | . | . | . | . | . | . | . | . | . |
| InternalIMT | epa | rs192963047 | . | . | . | . | . | . | . | . | . | . |
| InternalIMT | epa | rs11239505 | 0.175 | 0.016 | 0.033 | 0.631 | -0.034 | 0.039 | 0.388 | 0.027 | 0.045 | 0.546 |
| InternalIMT | epa | rs184434116 | 0 | . | . | . | . | . | . | . | . | . |
| InternalIMT | epa | rs4948999 | 0.733 | 0.04 | 0.074 | 0.588 | -0.017 | 0.038 | 0.644 | -8.90E-03 | 0.045 | 0.841 |
| InternalIMT | epa | rs11239506 | 0.092 | 0.028 | 0.031 | 0.37 | -0.046 | 0.046 | 0.326 | 2.90E-04 | 0.05 | 0.995 |
| InternalIMT | epa | rs190706620 | 0.003 | . | . | . | . | . | . | . | . | . |
| InternalIMT | epa | rs190770429 | . | . | . | . | . | . | . | . | . | . |
| InternalIMT | epa | rs181327133 | 0.005 | . | . | . | . | . | . | . | . | . |
| InternalIMT | epa | rs149251697 | 0.011 | 0.011 | 0.026 | 0.66 | 0.335 | 0.303 | 0.269 | 0.34 | 0.281 | 0.227 |
| InternalIMT | epa | rs118081602 | 0.022 | 0.018 | 0.027 | 0.515 | 0.059 | 0.152 | 0.698 | 0.162 | 0.124 | 0.194 |
| InternalIMT | epa | rs188233691 | 0.002 | . | . | . | . | . | . | . | . | . |
| InternalIMT | epa | rs183778794 | 0.003 | . | . | . | . | . | . | . | . | . |
| InternalIMT | epa | rs188040078 | 0.007 | . | . | . | . | . | . | . | . | . |
| InternalIMT | epa | rs184675372 | 0 | . | . | . | . | . | . | . | . | . |
| InternalIMT | epa | rs150326891 | 0.067 | 0.027 | 0.03 | 0.375 | -0.059 | 0.056 | 0.294 | 4.30E-03 | 0.059 | 0.941 |
| InternalIMT | epa | rs185520021 | 0.014 | 0.019 | 0.027 | 0.474 | 0.115 | 0.301 | 0.703 | 0.294 | 0.312 | 0.346 |
| InternalIMT | epa | rs7907779 | 0.092 | 0.028 | 0.031 | 0.369 | -0.046 | 0.046 | 0.32 | 1.70E-04 | 0.05 | 0.997 |
| InternalIMT | epa | rs184962860 | 0.001 | . | . | . | . | . | . | . | . | . |
| InternalIMT | epa | rs149762784 | 0.066 | 0.041 | 0.031 | 0.191 | -0.158 | 0.085 | 0.063 | -0.125 | 0.103 | 0.226 |
| InternalIMT | epa | rs149203427 | 0.008 | . | . | . | . | . | . | . | . | . |
| InternalIMT | epa | rs60153083 | 0.01 | 0.021 | 0.026 | 0.419 | 0.23 | 0.181 | 0.204 | 0.203 | 0.225 | 0.368 |
| InternalIMT | epa | rs112001069 | 0.062 | 0.026 | 0.028 | 0.357 | -0.065 | 0.063 | 0.308 | -0.018 | 0.081 | 0.825 |
| InternalIMT | epa | rs182355382 | 0.032 | 0.023 | 0.028 | 0.418 | 0.055 | 0.178 | 0.758 | 0.046 | 0.189 | 0.806 |
| InternalIMT | epa | rs186740974 | 0.111 | 0.028 | 0.033 | 0.389 | 0.019 | 0.074 | 0.799 | -0.011 | 0.089 | 0.905 |
| InternalIMT | epa | rs191874916 | 0.026 | 0.027 | 0.027 | 0.309 | 0.03 | 0.232 | 0.897 | -0.032 | 0.298 | 0.915 |
| InternalIMT | epa | rs182989412 | 0.168 | 2.20E-03 | 0.034 | 0.948 | 0.126 | 0.06 | 0.037 | 0.079 | 0.071 | 0.267 |
| InternalIMT | epa | rs188819476 | 0.001 | . | . | . | . | . | . | . | . | . |
| InternalIMT | epa | rs141217183 | 0.08 | 0.016 | 0.029 | 0.592 | -0.022 | 0.062 | 0.719 | 0.054 | 0.072 | 0.455 |
| InternalIMT | epa | rs192922652 | 0.064 | 0.022 | 0.028 | 0.438 | -0.064 | 0.057 | 0.267 | 0.012 | 0.07 | 0.867 |
| InternalIMT | epa | rs185092343 | 0.014 | 0.019 | 0.028 | 0.484 | 0.143 | 0.221 | 0.517 | 0.235 | 0.267 | 0.377 |
| InternalIMT | epa | rs145193311 | 0.065 | 0.022 | 0.028 | 0.431 | -0.04 | 0.058 | 0.487 | 0.016 | 0.07 | 0.818 |
| InternalIMT | epa | rs55789336 | 0.094 | 0.029 | 0.031 | 0.357 | -0.051 | 0.047 | 0.28 | -3.90E-03 | 0.051 | 0.94 |
| InternalIMT | epa | rs55837212 | 0.094 | 0.028 | 0.031 | 0.366 | -0.039 | 0.047 | 0.412 | -2.40E-03 | 0.051 | 0.963 |
| InternalIMT | epa | rs75408574 | 0.406 | 0.06 | 0.041 | 0.141 | 0.016 | 0.037 | 0.664 | -0.042 | 0.042 | 0.317 |
| InternalIMT | epa | rs149306926 | 0.099 | 0.027 | 0.032 | 0.395 | -0.025 | 0.05 | 0.615 | 1.00E-03 | 0.053 | 0.984 |
| InternalIMT | epa | rs35990892 | 0.706 | 0.048 | 0.075 | 0.525 | -7.10E-03 | 0.039 | 0.857 | -0.015 | 0.045 | 0.744 |
| InternalIMT | epa | rs192356207 | 0.036 | 0.021 | 0.026 | 0.422 | 0.089 | 0.117 | 0.444 | 0.047 | 0.129 | 0.718 |
| InternalIMT | epa | rs183741163 | 0.092 | 0.018 | 0.03 | 0.555 | -0.012 | 0.062 | 0.843 | 0.044 | 0.07 | 0.532 |
| InternalIMT | epa | rs34737297 | 0.558 | 0.046 | 0.06 | 0.446 | 3.60E-03 | 0.041 | 0.93 | -0.017 | 0.049 | 0.732 |
| InternalIMT | epa | rs181346933 | 0.022 | 0.024 | 0.028 | 0.405 | -0.077 | 0.099 | 0.437 | 0.082 | 0.103 | 0.425 |
| InternalIMT | epa | rs36093253 | 0.408 | 0.049 | 0.041 | 0.228 | 0.026 | 0.039 | 0.504 | -0.028 | 0.044 | 0.528 |
| InternalIMT | epa | rs186307855 | 0.059 | 0.023 | 0.029 | 0.422 | -0.056 | 0.047 | 0.231 | 0.025 | 0.052 | 0.627 |
| InternalIMT | epa | rs189681865 | 0.019 | 0.011 | 0.026 | 0.689 | 0.058 | 0.155 | 0.709 | 0.219 | 0.126 | 0.083 |
| InternalIMT | epa | rs34942971 | 0.176 | 0.012 | 0.033 | 0.726 | -0.015 | 0.042 | 0.726 | 0.036 | 0.048 | 0.448 |
| InternalIMT | epa | rs181816554 | 0.025 | 0.027 | 0.026 | 0.315 | 0.104 | 0.198 | 0.599 | -0.046 | 0.26 | 0.859 |
| InternalIMT | epa | rs186846881 | 0.049 | 0.018 | 0.028 | 0.515 | 0.01 | 0.06 | 0.861 | 0.066 | 0.065 | 0.311 |
| InternalIMT | epa | rs191178220 | 0.016 | 0.028 | 0.027 | 0.304 | -0.071 | 0.163 | 0.663 | -0.087 | 0.221 | 0.693 |
| InternalIMT | epa | rs183305228 | 0.01 | 0.021 | 0.026 | 0.419 | 0.226 | 0.181 | 0.211 | 0.201 | 0.225 | 0.372 |
| InternalIMT | epa | rs139912761 | 0.102 | 0.013 | 0.03 | 0.675 | 4.50E-03 | 0.049 | 0.927 | 0.054 | 0.053 | 0.302 |

| InternalIMT | epa | rs185499901 | 0.016 | 0.022 | 0.026 | 0.41 | 0.105 | 0.237 | 0.659 | 0.101 | 0.282 | 0.72 |
| --- | --- | --- | --- | --- | --- | --- | --- | --- | --- | --- | --- | --- |
| InternalIMT | epa | rs189816197 | 0.072 | 0.031 | 0.029 | 0.286 | -0.084 | 0.063 | 0.184 | -0.045 | 0.079 | 0.567 |
| InternalIMT | epa | rs145656938 | 0.022 | 0.023 | 0.028 | 0.409 | -0.043 | 0.142 | 0.761 | 0.062 | 0.104 | 0.554 |
| InternalIMT | epa | rs35745197 | 0.062 | 0.026 | 0.028 | 0.358 | -0.065 | 0.064 | 0.313 | -0.018 | 0.082 | 0.83 |
| InternalIMT | epa | rs192856637 | 0.004 | . | . | . | . | . | . | . | . | . |
| InternalIMT | epa | rs2310775 | 0.492 | 0.065 | 0.041 | 0.111 | 0.011 | 0.031 | 0.727 | -0.041 | 0.036 | 0.256 |
| InternalIMT | epa | rs11239507 | 0.558 | 0.062 | 0.049 | 0.204 | 2.20E-03 | 0.033 | 0.947 | -0.032 | 0.037 | 0.38 |
| InternalIMT | epa | rs74648467 | 0.053 | 0.025 | 0.027 | 0.357 | -0.064 | 0.075 | 0.393 | -0.02 | 0.093 | 0.831 |
| InternalIMT | epa | rs145815934 | 0.012 | 0.03 | 0.027 | 0.268 | -0.158 | 0.173 | 0.359 | -0.177 | 0.244 | 0.468 |
| InternalIMT | epa | rs145390687 | 0.015 | 0.025 | 0.027 | 0.358 | 0.114 | 0.178 | 0.521 | 0.038 | 0.236 | 0.871 |
| InternalIMT | epa | rs184354913 | 0.033 | 0.026 | 0.028 | 0.349 | -0.036 | 0.11 | 0.745 | 3.50E-03 | 0.123 | 0.977 |
| InternalIMT | epa | rs181244920 | 0.007 | . | . | . | . | . | . | . | . | . |
| InternalIMT | epa | rs11499087 | 0.028 | 0.027 | 0.028 | 0.337 | -0.044 | 0.065 | 0.502 | 0.011 | 0.071 | 0.876 |
| InternalIMT | epa | rs148263794 | 0.064 | 0.027 | 0.028 | 0.327 | -0.078 | 0.059 | 0.186 | -0.031 | 0.077 | 0.685 |
| InternalIMT | epa | rs150758903 | 0.021 | 0.011 | 0.026 | 0.67 | 0.111 | 0.169 | 0.511 | 0.234 | 0.154 | 0.129 |
| InternalIMT | epa | rs147654382 | 0.152 | 0.032 | 0.034 | 0.334 | -0.068 | 0.042 | 0.107 | -0.015 | 0.048 | 0.75 |
| InternalIMT | epa | rs147495166 | 0.011 | 0.026 | 0.027 | 0.348 | -0.091 | 0.067 | 0.175 | 0.037 | 0.067 | 0.58 |
| InternalIMT | epa | rs35198778 | 0.081 | 0.031 | 0.032 | 0.333 | -0.116 | 0.058 | 0.046 | -0.015 | 0.064 | 0.82 |
| InternalIMT | epa | rs145207659 | 0.043 | 0.035 | 0.028 | 0.213 | 0.051 | 0.094 | 0.589 | -0.128 | 0.124 | 0.301 |
| InternalIMT | epa | rs186466464 | 0.007 | . | . | . | . | . | . | . | . | . |
| InternalIMT | epa | rs12778413 | 0.146 | 0.041 | 0.034 | 0.237 | -0.106 | 0.047 | 0.023 | -0.044 | 0.054 | 0.417 |
| InternalIMT | epa | rs7895552 | 0.632 | 0.047 | 0.066 | 0.476 | -0.025 | 0.036 | 0.492 | -0.016 | 0.045 | 0.727 |
| InternalIMT | epa | rs12783895 | 0.064 | 0.027 | 0.028 | 0.329 | -0.077 | 0.059 | 0.193 | -0.03 | 0.077 | 0.696 |
| InternalIMT | epa | rs34929604 | 0.065 | 0.027 | 0.028 | 0.328 | -0.079 | 0.058 | 0.172 | -0.031 | 0.075 | 0.684 |
| InternalIMT | epa | rs12761961 | 0.063 | 0.026 | 0.028 | 0.357 | -0.062 | 0.064 | 0.335 | -0.018 | 0.082 | 0.83 |
| InternalIMT | epa | rs35536930 | 0.173 | 0.015 | 0.033 | 0.638 | -0.029 | 0.04 | 0.468 | 0.029 | 0.046 | 0.528 |
| InternalIMT | epa | rs180951060 | 0.001 | . | . | . | . | . | . | . | . | . |
| InternalIMT | epa | rs148892128 | 0.012 | 0.03 | 0.027 | 0.268 | -0.158 | 0.173 | 0.36 | -0.177 | 0.244 | 0.469 |
| InternalIMT | epa | rs11239509 | 0.043 | 0.026 | 0.029 | 0.369 | -0.05 | 0.047 | 0.29 | 0.024 | 0.053 | 0.658 |
| InternalIMT | epa | rs41526545 | 0.088 | 0.027 | 0.03 | 0.377 | -0.043 | 0.049 | 0.376 | 3.10E-03 | 0.053 | 0.953 |
| InternalIMT | epa | rs2029253 | 0.496 | 0.066 | 0.041 | 0.11 | 0.012 | 0.031 | 0.701 | -0.041 | 0.036 | 0.253 |
| InternalIMT | epa | rs17444064 | 0.086 | 0.026 | 0.03 | 0.397 | -0.037 | 0.05 | 0.461 | 8.00E-03 | 0.054 | 0.882 |
| InternalIMT | epa | rs71494779 | 0.066 | 0.028 | 0.028 | 0.325 | -0.08 | 0.057 | 0.163 | -0.032 | 0.075 | 0.668 |
| InternalIMT | epa | rs74991420 | 0.022 | 0.018 | 0.027 | 0.512 | 0.047 | 0.154 | 0.761 | 0.155 | 0.126 | 0.217 |
| InternalIMT | epa | rs182736551 | 0.022 | 0.018 | 0.027 | 0.512 | 0.047 | 0.154 | 0.761 | 0.155 | 0.126 | 0.217 |
| InternalIMT | epa | rs78702074 | 0.021 | 0.018 | 0.027 | 0.512 | 0.049 | 0.156 | 0.754 | 0.156 | 0.127 | 0.218 |
| InternalIMT | epa | rs187791466 | 0.002 | . | . | . | . | . | . | . | . | . |
| InternalIMT | epa | rs35300270 | 0.068 | 0.029 | 0.028 | 0.301 | -0.091 | 0.057 | 0.111 | -0.043 | 0.075 | 0.565 |
| InternalIMT | epa | rs6593484 | 0.093 | 0.028 | 0.031 | 0.377 | -0.047 | 0.047 | 0.319 | 2.80E-03 | 0.051 | 0.956 |
| InternalIMT | epa | rs12244601 | 0.013 | 0.02 | 0.027 | 0.45 | -8.30E-04 | 0.148 | 0.996 | 0.132 | 0.102 | 0.193 |
| InternalIMT | epa | rs184429574 | 0.004 | . | . | . | . | . | . | . | . | . |
| InternalIMT | epa | rs7080897 | 0.066 | 0.028 | 0.028 | 0.325 | -0.08 | 0.057 | 0.163 | -0.032 | 0.075 | 0.668 |
| InternalIMT | epa | rs7097429 | 0.07 | 0.027 | 0.028 | 0.328 | -0.082 | 0.057 | 0.148 | -0.034 | 0.072 | 0.637 |
| InternalIMT | epa | rs77979229 | 0.001 | . | . | . | . | . | . | . | . | . |
| InternalIMT | epa | rs11239512 | 0.093 | 0.028 | 0.031 | 0.369 | -0.048 | 0.046 | 0.297 | 8.90E-04 | 0.05 | 0.986 |
| InternalIMT | epa | rs60146827 | 0.066 | 0.028 | 0.028 | 0.325 | -0.08 | 0.057 | 0.161 | -0.032 | 0.075 | 0.668 |
| InternalIMT | epa | rs79032236 | 0.017 | 0.024 | 0.027 | 0.381 | -0.028 | 0.075 | 0.713 | 0.048 | 0.076 | 0.524 |
| InternalIMT | epa | rs140854206 | 0.005 | . | . | . | . | . | . | . | . | . |
| InternalIMT | epa | rs184049550 | 0.002 | . | . | . | . | . | . | . | . | . |
| InternalIMT | epa | rs114433607 | 0.007 | . | . | . | . | . | . | . | . | . |
| InternalIMT | epa | rs117653793 | 0.022 | 0.018 | 0.027 | 0.517 | 0.062 | 0.153 | 0.685 | 0.165 | 0.125 | 0.186 |
| InternalIMT | epa | rs117039533 | 0.022 | 0.018 | 0.027 | 0.516 | 0.061 | 0.153 | 0.692 | 0.164 | 0.125 | 0.189 |
| InternalIMT | epa | rs191660761 | 0.002 | . | . | . | . | . | . | . | . | . |
| InternalIMT | epa | rs59514896 | 0.053 | 0.028 | 0.027 | 0.307 | -0.089 | 0.074 | 0.231 | -0.05 | 0.093 | 0.59 |
| InternalIMT | epa | rs184661876 | 0.001 | . | . | . | . | . | . | . | . | . |
| InternalIMT | epa | rs61854073 | 0.028 | 0.032 | 0.028 | 0.256 | 9.20E-03 | 0.112 | 0.935 | -0.129 | 0.136 | 0.343 |
| InternalIMT | epa | rs2018518 | 0.391 | 0.061 | 0.039 | 0.118 | 4.40E-03 | 0.035 | 0.899 | -0.045 | 0.039 | 0.251 |
| InternalIMT | epa | rs934187 | 0.734 | 0.042 | 0.073 | 0.563 | -0.016 | 0.037 | 0.663 | -0.011 | 0.044 | 0.813 |
| InternalIMT | epa | rs7918542 | 0.179 | 0.017 | 0.033 | 0.614 | -0.037 | 0.039 | 0.337 | 0.026 | 0.045 | 0.569 |
| InternalIMT | epa | rs144311920 | 0.002 | . | . | . | . | . | . | . | . | . |
| InternalIMT | epa | rs7099684 | 0.864 | 0.071 | 0.117 | 0.543 | -0.052 | 0.052 | 0.315 | -0.025 | 0.063 | 0.695 |
| InternalIMT | epa | rs150829171 | . | . | . | . | . | . | . | . | . | . |
| InternalIMT | epa | rs186060697 | 0.001 | . | . | . | . | . | . | . | . | . |
| InternalIMT | epa | rs11239513 | 0.054 | 0.022 | 0.029 | 0.446 | -3.80E-03 | 0.052 | 0.942 | 0.034 | 0.057 | 0.548 |
| InternalIMT | epa | rs7917687 | 0.067 | 0.028 | 0.028 | 0.326 | -0.081 | 0.057 | 0.155 | -0.032 | 0.075 | 0.668 |
| InternalIMT | epa | rs61854076 | 0.01 | 0.027 | 0.027 | 0.325 | 0.258 | 0.261 | 0.323 | -0.023 | 0.282 | 0.935 |
| InternalIMT | epa | rs71494780 | 0.029 | 0.03 | 0.027 | 0.258 | -0.078 | 0.12 | 0.519 | -0.093 | 0.145 | 0.521 |
| InternalIMT | epa | rs7919239 | 0.187 | 0.019 | 0.033 | 0.552 | -0.042 | 0.037 | 0.256 | 0.017 | 0.043 | 0.684 |
| InternalIMT | epa | rs78334475 | 0.047 | 0.019 | 0.028 | 0.48 | -0.018 | 0.091 | 0.839 | 0.067 | 0.1 | 0.5 |
| InternalIMT | epa | rs3780900 | 0.02 | 0.025 | 0.026 | 0.341 | 0.079 | 0.278 | 0.777 | 0.013 | 0.349 | 0.971 |
| InternalIMT | epa | rs186729292 | 0.001 | . | . | . | . | . | . | . | . | . |
| InternalIMT | epa | rs12267438 | 0.015 | 0.015 | 0.026 | 0.564 | 0.07 | 0.154 | 0.648 | 0.203 | 0.134 | 0.13 |
| InternalIMT | epa | rs1369214 | 0.61 | 0.059 | 0.052 | 0.252 | 5.60E-04 | 0.033 | 0.986 | -0.027 | 0.038 | 0.48 |

| InternalIMT | epa | rs9282589 | 0.134 | 0.051 | 0.031 | 0.099 | -0.078 | 0.041 | 0.054 | -0.095 | 0.05 | 0.057 |
| --- | --- | --- | --- | --- | --- | --- | --- | --- | --- | --- | --- | --- |
| InternalIMT | epa | rs114522915 | 0.011 | 0.025 | 0.027 | 0.356 | -0.078 | 0.068 | 0.25 | 0.053 | 0.076 | 0.488 |
| InternalIMT | epa | rs2115819 | 0.542 | 0.076 | 0.044 | 0.082 | 2.10E-03 | 0.031 | 0.945 | -0.047 | 0.037 | 0.209 |
| InternalIMT | epa | rs117664603 | 0.022 | 0.018 | 0.027 | 0.514 | 0.059 | 0.154 | 0.701 | 0.162 | 0.127 | 0.202 |
| InternalIMT | epa | rs184979202 | . | . | . | . | . | . | . | . | . | . |
| InternalIMT | epa | rs190714671 | 0.002 | . | . | . | . | . | . | . | . | . |
| InternalIMT | epa | rs141538996 | . | . | . | . | . | . | . | . | . | . |
| InternalIMT | epa | rs150881741 | . | . | . | . | . | . | . | . | . | . |
| InternalIMT | epa | rs139076633 | . | . | . | . | . | . | . | . | . | . |
| InternalIMT | epa | rs12243649 | 0.054 | 3.50E-03 | 0.027 | 0.898 | 0.037 | 0.084 | 0.662 | 0.159 | 0.081 | 0.049 |
| InternalIMT | epa | rs140348708 | 0.001 | . | . | . | . | . | . | . | . | . |
| InternalIMT | epa | rs79023256 | 0.022 | 0.018 | 0.027 | 0.514 | 0.059 | 0.154 | 0.7 | 0.162 | 0.127 | 0.202 |
| InternalIMT | epa | rs11239515 | 0.081 | 0.031 | 0.03 | 0.306 | -0.072 | 0.049 | 0.145 | -0.016 | 0.054 | 0.765 |
| InternalIMT | epa | rs55866086 | 0.137 | 4.80E-03 | 0.032 | 0.881 | -0.029 | 0.044 | 0.503 | 0.067 | 0.048 | 0.161 |
| InternalIMT | epa | rs181340150 | 0.001 | . | . | . | . | . | . | . | . | . |
| InternalIMT | epa | rs11239516 | 0.097 | 0.015 | 0.03 | 0.62 | -0.021 | 0.05 | 0.667 | 0.053 | 0.054 | 0.323 |
| InternalIMT | epa | rs11239517 | 0.096 | 0.015 | 0.03 | 0.622 | -0.021 | 0.05 | 0.674 | 0.053 | 0.054 | 0.321 |
| InternalIMT | epa | rs11239519 | 0.089 | 0.024 | 0.031 | 0.437 | -0.051 | 0.049 | 0.298 | 0.022 | 0.053 | 0.68 |
| InternalIMT | epa | rs7909514 | 0.721 | 0.042 | 0.073 | 0.565 | -0.02 | 0.038 | 0.594 | -0.01 | 0.046 | 0.818 |
| InternalIMT | epa | rs7894352 | 0.853 | 0.057 | 0.115 | 0.623 | -0.056 | 0.054 | 0.305 | -0.017 | 0.064 | 0.791 |
| InternalIMT | epa | rs189384146 | 0.002 | . | . | . | . | . | . | . | . | . |
| InternalIMT | epa | rs11239520 | 0.088 | 0.029 | 0.029 | 0.322 | -0.052 | 0.053 | 0.332 | -1.90E-03 | 0.06 | 0.975 |
| InternalIMT | epa | rs12221233 | 0.482 | 0.069 | 0.045 | 0.123 | 6.40E-03 | 0.034 | 0.85 | -0.045 | 0.039 | 0.241 |
| InternalIMT | epa | rs74523293 | 0.038 | 0.018 | 0.028 | 0.528 | -0.048 | 0.079 | 0.545 | 0.077 | 0.077 | 0.317 |
| InternalIMT | epa | rs79376040 | 0.038 | 0.018 | 0.028 | 0.528 | -0.048 | 0.079 | 0.545 | 0.077 | 0.077 | 0.316 |
| InternalIMT | epa | rs4949000 | 0.482 | 0.07 | 0.045 | 0.119 | 5.30E-03 | 0.034 | 0.875 | -0.046 | 0.039 | 0.233 |
| InternalIMT | epa | rs7089406 | 0.023 | 0.025 | 0.027 | 0.349 | 0.018 | 0.126 | 0.886 | 0.028 | 0.144 | 0.847 |
| InternalIMT | epa | rs138211056 | 0.001 | . | . | . | . | . | . | . | . | . |
| InternalIMT | epa | rs10900213 | 0.586 | 0.062 | 0.054 | 0.252 | 1.10E-03 | 0.035 | 0.975 | -0.03 | 0.042 | 0.466 |
| InternalIMT | epa | rs145152827 | . | . | . | . | . | . | . | . | . | . |
| InternalIMT | epa | rs74443325 | 0.022 | 0.018 | 0.027 | 0.5 | 0.034 | 0.159 | 0.831 | 0.142 | 0.131 | 0.279 |
| InternalIMT | epa | rs188299446 | 0.011 | 0.019 | 0.027 | 0.466 | 0.643 | 0.334 | 0.054 | 0.463 | 0.27 | 0.087 |
| InternalIMT | epa | rs192699586 | 0.005 | . | . | . | . | . | . | . | . | . |
| InternalIMT | epa | rs72796464 | 0.076 | 0.029 | 0.03 | 0.341 | -0.057 | 0.052 | 0.27 | -6.30E-03 | 0.057 | 0.912 |
| InternalIMT | epa | rs4949001 | 0.366 | 0.054 | 0.039 | 0.166 | -5.40E-03 | 0.035 | 0.876 | -0.038 | 0.041 | 0.346 |
| InternalIMT | epa | rs147174641 | 0.005 | . | . | . | . | . | . | . | . | . |
| InternalIMT | epa | rs190835238 | 0.001 | . | . | . | . | . | . | . | . | . |
| InternalIMT | epa | rs76698944 | 0.017 | 0.018 | 0.027 | 0.501 | 0.134 | 0.166 | 0.42 | 0.335 | 0.198 | 0.09 |
| InternalIMT | epa | rs56071381 | 0.092 | 0.027 | 0.03 | 0.364 | -0.049 | 0.052 | 0.342 | 7.10E-03 | 0.058 | 0.902 |
| InternalIMT | epa | rs78130427 | 0.023 | 0.022 | 0.027 | 0.425 | -0.031 | 0.082 | 0.703 | 0.104 | 0.104 | 0.314 |
| InternalIMT | epa | rs10900215 | 0.118 | 5.30E-03 | 0.031 | 0.862 | 0.019 | 0.057 | 0.742 | 0.071 | 0.064 | 0.261 |
| InternalIMT | epa | rs41491445 | 0.01 | 0.022 | 0.026 | 0.408 | 0.205 | 0.178 | 0.251 | 0.171 | 0.223 | 0.444 |
| InternalIMT | epa | rs144995222 | 0.002 | . | . | . | . | . | . | . | . | . |
| InternalIMT | epa | rs149097803 | 0.002 | . | . | . | . | . | . | . | . | . |
| InternalIMT | epa | rs139429646 | 0.005 | . | . | . | . | . | . | . | . | . |
| InternalIMT | epa | rs7075620 | 0.675 | 0.018 | 0.07 | 0.79 | -8.20E-03 | 0.038 | 0.83 | 5.70E-03 | 0.045 | 0.899 |
| InternalIMT | epa | rs148299213 | 0.01 | 0.022 | 0.026 | 0.407 | 0.202 | 0.18 | 0.261 | 0.164 | 0.226 | 0.467 |
| InternalIMT | epa | rs4949002 | 0.313 | 0.062 | 0.038 | 0.102 | 0.022 | 0.04 | 0.593 | -0.057 | 0.047 | 0.22 |
| InternalIMT | epa | rs150496007 | 0.036 | 0.035 | 0.029 | 0.231 | -0.127 | 0.076 | 0.094 | -0.104 | 0.097 | 0.286 |
| InternalIMT | epa | rs147303455 | 0.008 | . | . | . | . | . | . | . | . | . |
| InternalIMT | epa | rs189518433 | . | . | . | . | . | . | . | . | . | . |
| InternalIMT | epa | rs186281355 | . | . | . | . | . | . | . | . | . | . |
| InternalIMT | epa | rs115297114 | 0.014 | 0.024 | 0.027 | 0.388 | -0.059 | 0.071 | 0.408 | 0.065 | 0.069 | 0.343 |
| InternalIMT | epa | rs11239523 | 0.087 | 0.026 | 0.029 | 0.367 | -0.039 | 0.053 | 0.46 | 8.10E-03 | 0.059 | 0.892 |
| InternalIMT | epa | rs143704897 | 0.005 | . | . | . | . | . | . | . | . | . |
| InternalIMT | epa | rs188024096 | 0.002 | . | . | . | . | . | . | . | . | . |
| InternalIMT | epa | rs11239524 | 0.087 | 0.025 | 0.03 | 0.396 | -0.057 | 0.055 | 0.296 | 0.019 | 0.061 | 0.755 |
| InternalIMT | epa | rs191656573 | 0.004 | . | . | . | . | . | . | . | . | . |
| InternalIMT | epa | rs193295875 | . | . | . | . | . | . | . | . | . | . |
| InternalIMT | epa | rs190458228 | . | . | . | . | . | . | . | . | . | . |
| InternalIMT | epa | rs139253993 | 0.002 | . | . | . | . | . | . | . | . | . |
| InternalIMT | epa | rs4948672 | 0.604 | 6.40E-03 | 0.059 | 0.914 | 0.016 | 0.038 | 0.685 | 0.017 | 0.045 | 0.698 |
| InternalIMT | epa | rs180954921 | 0.002 | . | . | . | . | . | . | . | . | . |
| InternalIMT | epa | rs12264801 | 0.447 | -0.017 | 0.045 | 0.708 | 0.019 | 0.039 | 0.632 | 0.045 | 0.043 | 0.293 |
| InternalIMT | epa | rs73283184 | 0.014 | 0.021 | 0.026 | 0.419 | 0.119 | 0.138 | 0.388 | 0.16 | 0.195 | 0.412 |
| InternalIMT | epa | rs142746187 | 0.007 | . | . | . | . | . | . | . | . | . |
| InternalIMT | epa | rs58704739 | 0.014 | 0.02 | 0.026 | 0.454 | 0.14 | 0.134 | 0.296 | 0.223 | 0.18 | 0.217 |
| InternalIMT | epa | rs61272335 | 0.011 | 0.02 | 0.026 | 0.447 | 0.213 | 0.146 | 0.145 | 0.215 | 0.194 | 0.267 |
| InternalIMT | epa | rs60959489 | 0.021 | 0.021 | 0.027 | 0.436 | 0.041 | 0.109 | 0.706 | 0.134 | 0.142 | 0.345 |
| InternalIMT | epa | rs58502629 | 0.011 | 0.02 | 0.026 | 0.448 | 0.213 | 0.146 | 0.145 | 0.215 | 0.193 | 0.266 |
| InternalIMT | epa | rs10900216 | 0.739 | -5.60E-04 | 0.072 | 0.994 | -3.10E-03 | 0.039 | 0.937 | 0.018 | 0.045 | 0.691 |
| InternalIMT | epa | rs181118141 | 0.001 | . | . | . | . | . | . | . | . | . |
| InternalIMT | epa | rs137882665 | 0.01 | 0.031 | 0.027 | 0.245 | -0.206 | 0.099 | 0.037 | -0.128 | 0.093 | 0.172 |

| InternalIMT | epa | rs149466736 | 0.004 | . | . | . | . | . | . | . | . | . |
| --- | --- | --- | --- | --- | --- | --- | --- | --- | --- | --- | --- | --- |
| InternalIMT | epa | rs2099171 | 0.675 | 0.027 | 0.058 | 0.641 | -8.20E-03 | 0.032 | 0.801 | -7.60E-04 | 0.037 | 0.983 |
| InternalIMT | epa | rs141852280 | 0.005 | . | . | . | . | . | . | . | . | . |
| InternalIMT | epa | rs191539257 | . | . | . | . | . | . | . | . | . | . |
| InternalIMT | epa | rs17157771 | 0.027 | 0.021 | 0.026 | 0.416 | 0.042 | 0.121 | 0.728 | 0.077 | 0.134 | 0.568 |
| InternalIMT | epa | rs111339241 | 0.025 | 0.02 | 0.026 | 0.433 | 0.042 | 0.131 | 0.747 | 0.097 | 0.145 | 0.506 |
| InternalIMT | epa | rs116983255 | 0.006 | . | . | . | . | . | . | . | . | . |
| InternalIMT | epa | rs142324341 | . | . | . | . | . | . | . | . | . | . |
| InternalIMT | epa | rs187191219 | 0.007 | . | . | . | . | . | . | . | . | . |
| InternalIMT | epa | rs151283973 | 0 | . | . | . | . | . | . | . | . | . |
| InternalIMT | epa | rs139316218 | 0.003 | . | . | . | . | . | . | . | . | . |
| InternalIMT | epa | rs6593485 | 0.748 | -0.052 | 0.072 | 0.474 | 9.00E-03 | 0.04 | 0.822 | 0.051 | 0.044 | 0.251 |
| InternalIMT | epa | rs6593486 | 0.751 | -0.051 | 0.073 | 0.486 | 7.30E-03 | 0.04 | 0.854 | 0.05 | 0.044 | 0.259 |
| InternalIMT | epa | rs145196618 | 0.011 | 0.031 | 0.026 | 0.246 | -0.091 | 0.178 | 0.612 | -0.153 | 0.153 | 0.318 |
| InternalIMT | epa | rs142598335 | . | . | . | . | . | . | . | . | . | . |
| InternalIMT | epa | rs892691 | 0.746 | -0.043 | 0.066 | 0.512 | 0.013 | 0.038 | 0.734 | 0.046 | 0.041 | 0.263 |
| InternalIMT | epa | rs7080713 | 0.689 | 5.50E-03 | 0.059 | 0.926 | 6.40E-03 | 0.035 | 0.856 | 0.015 | 0.038 | 0.69 |
| InternalIMT | epa | rs7077173 | 0.068 | 0.022 | 0.028 | 0.42 | -0.047 | 0.046 | 0.312 | 0.029 | 0.052 | 0.576 |
| InternalIMT | epa | rs7080977 | 0.04 | 0.02 | 0.027 | 0.462 | -0.021 | 0.072 | 0.776 | 0.055 | 0.073 | 0.453 |
| InternalIMT | epa | rs3780901 | 0.689 | 5.90E-03 | 0.059 | 0.922 | 8.60E-03 | 0.035 | 0.809 | 0.015 | 0.038 | 0.696 |
| InternalIMT | epa | rs61854091 | 0.046 | 0.033 | 0.028 | 0.246 | -5.50E-03 | 0.084 | 0.948 | -0.082 | 0.108 | 0.447 |
| InternalIMT | epa | rs77550209 | 0.003 | . | . | . | . | . | . | . | . | . |
| InternalIMT | epa | rs73283191 | 0.062 | 0.012 | 0.028 | 0.68 | -0.023 | 0.066 | 0.73 | 0.078 | 0.064 | 0.223 |
| InternalIMT | epa | rs191866099 | . | . | . | . | . | . | . | . | . | . |
| InternalIMT | epa | rs147998395 | 0 | . | . | . | . | . | . | . | . | . |
| InternalIMT | epa | rs7090328 | 0.789 | -5.80E-04 | 0.087 | 0.995 | -0.024 | 0.044 | 0.578 | 0.017 | 0.05 | 0.741 |
| InternalIMT | epa | rs79814535 | 0.021 | 0.019 | 0.027 | 0.47 | -0.021 | 0.165 | 0.899 | 0.092 | 0.138 | 0.506 |
| InternalIMT | epa | rs7073082 | 0.789 | -7.20E-04 | 0.087 | 0.993 | -0.024 | 0.044 | 0.58 | 0.017 | 0.05 | 0.739 |
| InternalIMT | epa | rs11239526 | 0.033 | 0.029 | 0.028 | 0.293 | 0.074 | 0.103 | 0.47 | -0.045 | 0.126 | 0.72 |
| InternalIMT | epa | rs141839841 | 0 | . | . | . | . | . | . | . | . | . |
| InternalIMT | epa | rs75697371 | 0.006 | . | . | . | . | . | . | . | . | . |
| InternalIMT | epa | rs75972854 | 0.005 | . | . | . | . | . | . | . | . | . |
| InternalIMT | epa | rs139291975 | . | . | . | . | . | . | . | . | . | . |
| InternalIMT | epa | rs144064711 | 0.004 | . | . | . | . | . | . | . | . | . |
| InternalIMT | epa | rs3780902 | 0.062 | 0.013 | 0.028 | 0.649 | -0.026 | 0.064 | 0.686 | 0.071 | 0.063 | 0.254 |
| InternalIMT | epa | rs190449801 | 0.001 | . | . | . | . | . | . | . | . | . |
| InternalIMT | epa | rs73283196 | 0.061 | 0.012 | 0.028 | 0.677 | -0.022 | 0.066 | 0.736 | 0.079 | 0.064 | 0.221 |
| InternalIMT | epa | rs28395867 | 0.031 | 0.038 | 0.027 | 0.159 | -0.109 | 0.071 | 0.124 | -0.146 | 0.102 | 0.155 |
| InternalIMT | epa | rs28395866 | 0.079 | 0.035 | 0.028 | 0.214 | -0.061 | 0.049 | 0.213 | -0.024 | 0.055 | 0.662 |
| InternalIMT | epa | rs55661611 | 0.04 | 0.02 | 0.027 | 0.477 | -0.021 | 0.073 | 0.777 | 0.062 | 0.074 | 0.406 |
| InternalIMT | epa | rs55684518 | 0.016 | 0.025 | 0.027 | 0.343 | 0.037 | 0.173 | 0.831 | 0.031 | 0.179 | 0.86 |
| InternalIMT | epa | rs78552493 | 0.038 | 0.028 | 0.028 | 0.307 | -0.065 | 0.07 | 0.349 | -9.90E-03 | 0.084 | 0.906 |
| InternalIMT | epa | rs147665434 | 0.001 | . | . | . | . | . | . | . | . | . |
| InternalIMT | epa | rs34445001 | 0.006 | . | . | . | . | . | . | . | . | . |
| InternalIMT | epa | rs184019449 | 0.003 | . | . | . | . | . | . | . | . | . |
| InternalIMT | epa | rs3780903 | 0.731 | -9.10E-03 | 0.065 | 0.888 | 0.015 | 0.037 | 0.694 | 0.025 | 0.04 | 0.54 |
| InternalIMT | epa | rs17157784 | 0.058 | 0.034 | 0.028 | 0.214 | -0.113 | 0.064 | 0.08 | -0.062 | 0.069 | 0.371 |
| InternalIMT | epa | rs3780904 | 0.021 | 0.019 | 0.027 | 0.473 | -0.016 | 0.157 | 0.919 | 0.1 | 0.131 | 0.446 |
| InternalIMT | epa | rs192064730 | . | . | . | . | . | . | . | . | . | . |
| InternalIMT | epa | rs12774534 | 0.006 | . | . | . | . | . | . | . | . | . |
| InternalIMT | epa | rs79179915 | 0.02 | 0.02 | 0.027 | 0.468 | -0.024 | 0.171 | 0.887 | 0.092 | 0.142 | 0.518 |
| InternalIMT | epa | rs117811571 | 0.011 | 0.019 | 0.027 | 0.466 | 0.614 | 0.336 | 0.068 | 0.453 | 0.267 | 0.09 |
| InternalIMT | epa | rs72796477 | 0.009 | . | . | . | . | . | . | . | . | . |
| InternalIMT | epa | rs55729342 | 0.058 | 0.035 | 0.028 | 0.209 | -0.111 | 0.066 | 0.096 | -0.07 | 0.069 | 0.313 |
| InternalIMT | epa | rs59317220 | 0.058 | 0.035 | 0.028 | 0.209 | -0.111 | 0.066 | 0.095 | -0.07 | 0.069 | 0.314 |
| InternalIMT | epa | rs3780905 | 0.057 | 0.011 | 0.028 | 0.69 | 0.015 | 0.084 | 0.86 | 0.133 | 0.088 | 0.131 |
| InternalIMT | epa | rs3780906 | 0.731 | -0.011 | 0.065 | 0.869 | 0.016 | 0.037 | 0.666 | 0.026 | 0.041 | 0.524 |
| InternalIMT | epa | rs149047975 | 0.125 | 0.049 | 0.03 | 0.106 | -0.08 | 0.042 | 0.059 | -0.098 | 0.052 | 0.06 |
| InternalIMT | epa | rs3824614 | 0.02 | 0.02 | 0.027 | 0.465 | -0.034 | 0.172 | 0.843 | 0.084 | 0.143 | 0.554 |
| InternalIMT | epa | rs140254404 | . | . | . | . | . | . | . | . | . | . |
| InternalIMT | epa | rs145262684 | 0.013 | 0.017 | 0.027 | 0.515 | 0.569 | 0.297 | 0.056 | 0.462 | 0.239 | 0.053 |
| InternalIMT | epa | rs76735482 | 0.02 | 0.02 | 0.027 | 0.464 | -0.039 | 0.171 | 0.821 | 0.081 | 0.143 | 0.572 |
| InternalIMT | epa | rs73283198 | 0.027 | 0.019 | 0.026 | 0.47 | 0.024 | 0.126 | 0.851 | 0.121 | 0.145 | 0.405 |
| InternalIMT | epa | rs74911239 | 0.029 | 0.029 | 0.027 | 0.281 | 0.285 | 0.141 | 0.043 | -0.031 | 0.18 | 0.862 |
| InternalIMT | epa | rs150551647 | 0.005 | . | . | . | . | . | . | . | . | . |
| InternalIMT | epa | rs115200794 | 0.034 | 0.038 | 0.027 | 0.167 | -0.099 | 0.076 | 0.189 | -0.133 | 0.101 | 0.187 |
| InternalIMT | epa | rs74130823 | 0.139 | 0.037 | 0.033 | 0.261 | -0.076 | 0.037 | 0.039 | -0.015 | 0.041 | 0.709 |
| InternalIMT | epa | rs2279435 | 0.662 | 0.035 | 0.059 | 0.552 | 0.014 | 0.035 | 0.688 | -7.00E-03 | 0.04 | 0.862 |
| InternalIMT | epa | rs147279673 | 0.015 | 0.013 | 0.027 | 0.622 | 0.454 | 0.242 | 0.06 | 0.44 | 0.263 | 0.094 |
| InternalIMT | epa | rs183525146 | 0.004 | . | . | . | . | . | . | . | . | . |
| InternalIMT | epa | rs3740107 | 0.754 | -0.083 | 0.076 | 0.272 | 0.022 | 0.043 | 0.61 | 0.072 | 0.047 | 0.122 |
| InternalIMT | epa | rs140697247 | . | . | . | . | . | . | . | . | . | . |
| InternalIMT | epa | rs1565096 | 0.825 | -0.025 | 0.102 | 0.808 | -0.019 | 0.052 | 0.707 | 0.031 | 0.058 | 0.59 |

| InternalIMT | epa | rs1565097 | 0.793 | -0.075 | 0.089 | 0.401 | 0.012 | 0.045 | 0.795 | 0.063 | 0.052 | 0.23 |
| --- | --- | --- | --- | --- | --- | --- | --- | --- | --- | --- | --- | --- |
| InternalIMT | epa | rs144584548 | 0.016 | 0.025 | 0.027 | 0.344 | 0.039 | 0.173 | 0.821 | 0.035 | 0.179 | 0.845 |
| InternalIMT | epa | rs75811053 | 0.02 | 0.02 | 0.027 | 0.453 | -0.064 | 0.169 | 0.705 | 0.057 | 0.141 | 0.688 |
| InternalIMT | epa | rs147462967 | 0.009 | . | . | . | . | . | . | . | . | . |
| InternalIMT | epa | rs74130824 | 0.051 | 0.031 | 0.028 | 0.266 | -0.123 | 0.063 | 0.05 | -0.05 | 0.066 | 0.452 |
| InternalIMT | epa | rs116883184 | 0.017 | 0.012 | 0.027 | 0.664 | 0.511 | 0.228 | 0.025 | 0.508 | 0.202 | 0.012 |
| InternalIMT | epa | rs117258049 | 0.017 | 0.012 | 0.027 | 0.664 | 0.511 | 0.228 | 0.025 | 0.508 | 0.202 | 0.012 |
| InternalIMT | epa | rs3780908 | 0.107 | 0.015 | 0.03 | 0.619 | -0.053 | 0.043 | 0.213 | 0.054 | 0.047 | 0.253 |
| InternalIMT | epa | rs180938522 | 0.002 | . | . | . | . | . | . | . | . | . |
| InternalIMT | epa | rs76687372 | 0.003 | . | . | . | . | . | . | . | . | . |
| InternalIMT | epa | rs3780909 | 0.606 | 0.021 | 0.055 | 0.706 | 0.01 | 0.035 | 0.766 | 4.80E-03 | 0.04 | 0.904 |
| InternalIMT | epa | rs78281156 | 0.02 | 0.02 | 0.027 | 0.453 | -0.066 | 0.169 | 0.697 | 0.056 | 0.141 | 0.694 |
| InternalIMT | epa | rs187840631 | . | . | . | . | . | . | . | . | . | . |
| InternalIMT | epa | rs76868242 | 0.02 | 0.02 | 0.027 | 0.453 | -0.066 | 0.169 | 0.696 | 0.055 | 0.141 | 0.695 |
| InternalIMT | epa | rs192794192 | 0.001 | . | . | . | . | . | . | . | . | . |
| InternalIMT | epa | rs12781248 | 0.007 | . | . | . | . | . | . | . | . | . |
| InternalIMT | epa | rs12762375 | 0.006 | . | . | . | . | . | . | . | . | . |
| InternalIMT | epa | rs192256986 | 0.004 | . | . | . | . | . | . | . | . | . |
| InternalIMT | epa | rs184411030 | 0.01 | . | . | . | . | . | . | . | . | . |
| InternalIMT | epa | rs189601483 | 0.02 | 0.02 | 0.027 | 0.452 | -0.066 | 0.169 | 0.697 | 0.055 | 0.141 | 0.695 |
| InternalIMT | epa | rs143254579 | 0.033 | 0.029 | 0.028 | 0.298 | 0.074 | 0.099 | 0.458 | -0.037 | 0.121 | 0.76 |
| InternalIMT | epa | rs12253903 | 0.017 | 0.018 | 0.026 | 0.501 | 0.175 | 0.1 | 0.081 | 0.146 | 0.135 | 0.281 |
| InternalIMT | epa | rs35489926 | 0.006 | . | . | . | . | . | . | . | . | . |
| InternalIMT | epa | rs71494783 | 0.039 | 0.012 | 0.027 | 0.648 | 0.07 | 0.141 | 0.619 | 0.145 | 0.133 | 0.278 |
| InternalIMT | epa | rs180711602 | 0.002 | . | . | . | . | . | . | . | . | . |
| InternalIMT | epa | rs117312402 | 0.02 | 0.02 | 0.027 | 0.453 | -0.067 | 0.169 | 0.694 | 0.055 | 0.141 | 0.696 |
| InternalIMT | epa | rs74831694 | 0.004 | . | . | . | . | . | . | . | . | . |
| InternalIMT | epa | rs140301943 | 0.002 | . | . | . | . | . | . | . | . | . |
| InternalIMT | epa | rs78553679 | 0.013 | 0.034 | 0.027 | 0.21 | -0.132 | 0.176 | 0.454 | -0.317 | 0.328 | 0.334 |
| InternalIMT | epa | rs78334250 | 0.02 | 0.02 | 0.027 | 0.453 | -0.067 | 0.169 | 0.694 | 0.055 | 0.141 | 0.696 |
| InternalIMT | epa | rs76000028 | 0.02 | 0.02 | 0.027 | 0.453 | -0.067 | 0.169 | 0.694 | 0.055 | 0.141 | 0.696 |
| InternalIMT | epa | rs145195254 | 0.001 | . | . | . | . | . | . | . | . | . |
| InternalIMT | epa | rs137888527 | 0.001 | . | . | . | . | . | . | . | . | . |
| InternalIMT | epa | rs149109592 | 0.005 | . | . | . | . | . | . | . | . | . |
| InternalIMT | epa | rs80280010 | 0.032 | 0.028 | 0.028 | 0.305 | 0.077 | 0.101 | 0.447 | -0.032 | 0.122 | 0.796 |
| InternalIMT | epa | rs189995374 | . | . | . | . | . | . | . | . | . | . |
| InternalIMT | epa | rs75467770 | 0.021 | 0.02 | 0.027 | 0.455 | -0.06 | 0.169 | 0.723 | 0.061 | 0.141 | 0.666 |
| InternalIMT | epa | rs1487562 | 0.139 | 0.03 | 0.031 | 0.334 | -0.085 | 0.037 | 0.021 | 8.90E-03 | 0.041 | 0.829 |
| InternalIMT | epa | rs12266963 | 0.039 | 0.012 | 0.027 | 0.646 | 0.068 | 0.141 | 0.627 | 0.144 | 0.133 | 0.281 |
| InternalIMT | epa | rs7099874 | 0.787 | -0.027 | 0.094 | 0.778 | -0.029 | 0.049 | 0.563 | 0.034 | 0.056 | 0.544 |
| InternalIMT | epa | rs75687019 | 0.02 | 0.02 | 0.027 | 0.453 | -0.067 | 0.169 | 0.693 | 0.055 | 0.141 | 0.697 |
| InternalIMT | epa | rs148240462 | 0.031 | 0.031 | 0.027 | 0.253 | 0.243 | 0.142 | 0.086 | -0.062 | 0.179 | 0.729 |
| InternalIMT | epa | rs141278174 | . | . | . | . | . | . | . | . | . | . |
| InternalIMT | epa | rs76404852 | 0.02 | 0.02 | 0.027 | 0.453 | -0.067 | 0.169 | 0.692 | 0.055 | 0.141 | 0.697 |
| InternalIMT | epa | rs139112240 | 0.006 | . | . | . | . | . | . | . | . | . |
| InternalIMT | epa | rs12783037 | 0.042 | 0.01 | 0.026 | 0.695 | 0.111 | 0.144 | 0.442 | 0.168 | 0.136 | 0.216 |
| InternalIMT | epa | rs72796481 | 0.006 | . | . | . | . | . | . | . | . | . |
| InternalIMT | epa | rs76798749 | 0.023 | 0.019 | 0.027 | 0.48 | 0.066 | 0.211 | 0.753 | 0.154 | 0.183 | 0.401 |
| InternalIMT | epa | rs187732548 | . | . | . | . | . | . | . | . | . | . |
| InternalIMT | epa | rs75675474 | 0.023 | 0.019 | 0.027 | 0.48 | 0.066 | 0.211 | 0.753 | 0.154 | 0.183 | 0.401 |
| InternalIMT | epa | rs76552293 | 0.023 | 0.019 | 0.027 | 0.48 | 0.067 | 0.211 | 0.752 | 0.154 | 0.183 | 0.4 |
| InternalIMT | epa | rs147445993 | 0.002 | . | . | . | . | . | . | . | . | . |
| InternalIMT | epa | rs115902283 | 0.023 | 0.018 | 0.027 | 0.488 | 0.068 | 0.211 | 0.747 | 0.158 | 0.183 | 0.386 |
| InternalIMT | epa | rs4540799 | 0.786 | -0.029 | 0.094 | 0.761 | -0.028 | 0.049 | 0.575 | 0.035 | 0.056 | 0.53 |
| InternalIMT | epa | rs12765320 | 0.172 | 9.40E-03 | 0.032 | 0.768 | -0.057 | 0.038 | 0.132 | 0.044 | 0.04 | 0.271 |
| InternalIMT | epa | rs12782256 | 0.006 | . | . | . | . | . | . | . | . | . |
| InternalIMT | epa | rs191727256 | 0 | . | . | . | . | . | . | . | . | . |
| InternalIMT | epa | rs2082111 | 0.111 | 0.019 | 0.03 | 0.515 | -0.062 | 0.042 | 0.134 | 0.037 | 0.045 | 0.415 |
| InternalIMT | epa | rs2082110 | 0.157 | 0.014 | 0.031 | 0.655 | -0.066 | 0.038 | 0.08 | 0.038 | 0.041 | 0.354 |
| InternalIMT | epa | rs2082109 | 0.157 | 0.014 | 0.031 | 0.655 | -0.066 | 0.038 | 0.08 | 0.038 | 0.041 | 0.354 |
| InternalIMT | epa | rs117316662 | 0.02 | 0.02 | 0.027 | 0.453 | -0.066 | 0.169 | 0.697 | 0.056 | 0.141 | 0.69 |
| InternalIMT | epa | rs12247163 | 0.177 | 6.00E-03 | 0.032 | 0.854 | -0.043 | 0.045 | 0.337 | 0.051 | 0.048 | 0.286 |
| InternalIMT | epa | rs12355888 | 0.075 | 0.03 | 0.029 | 0.304 | -0.081 | 0.05 | 0.11 | -0.013 | 0.055 | 0.811 |
| InternalIMT | epa | rs143772290 | 0.005 | . | . | . | . | . | . | . | . | . |
| InternalIMT | epa | rs146849452 | 0.003 | . | . | . | . | . | . | . | . | . |
| InternalIMT | epa | rs140267121 | 0.003 | . | . | . | . | . | . | . | . | . |
| InternalIMT | epa | rs7080474 | 0.155 | 0.015 | 0.031 | 0.633 | -0.04 | 0.045 | 0.368 | 0.039 | 0.048 | 0.425 |
| InternalIMT | epa | rs144066203 | 0.001 | . | . | . | . | . | . | . | . | . |
| InternalIMT | epa | rs12765209 | 0.021 | 0.021 | 0.027 | 0.446 | -0.079 | 0.169 | 0.64 | 0.042 | 0.141 | 0.765 |
| InternalIMT | epa | rs150930735 | 0.001 | . | . | . | . | . | . | . | . | . |
| InternalIMT | epa | rs7068039 | 0.177 | 7.00E-03 | 0.032 | 0.829 | -0.046 | 0.044 | 0.302 | 0.048 | 0.047 | 0.305 |
| InternalIMT | epa | rs117622652 | 0.02 | 0.02 | 0.027 | 0.454 | -0.065 | 0.169 | 0.7 | 0.057 | 0.141 | 0.687 |
| InternalIMT | epa | rs190191514 | 0.001 | . | . | . | . | . | . | . | . | . |

| InternalIMT | epa | rs74130835 | 0.023 | 0.031 | 0.027 | 0.261 | -0.13 | 0.076 | 0.086 | -0.05 | 0.089 | 0.572 |
| --- | --- | --- | --- | --- | --- | --- | --- | --- | --- | --- | --- | --- |
| InternalIMT | epa | rs1811604 | 0.022 | 0.03 | 0.027 | 0.272 | -0.132 | 0.076 | 0.082 | -0.031 | 0.088 | 0.727 |
| InternalIMT | epa | rs146971256 | . | . | . | . | . | . | . | . | . | . |
| InternalIMT | epa | rs74336000 | 0.005 | . | . | . | . | . | . | . | . | . |
| InternalIMT | epa | rs186046492 | 0.005 | . | . | . | . | . | . | . | . | . |
| InternalIMT | epa | rs34971823 | 0.006 | . | . | . | . | . | . | . | . | . |
| InternalIMT | epa | rs75227446 | 0.02 | 0.02 | 0.027 | 0.457 | -0.058 | 0.169 | 0.73 | 0.063 | 0.141 | 0.654 |
| InternalIMT | epa | rs190457599 | 0.003 | . | . | . | . | . | . | . | . | . |
| InternalIMT | epa | rs79591917 | 0.02 | 0.02 | 0.027 | 0.454 | -0.064 | 0.169 | 0.705 | 0.058 | 0.141 | 0.68 |
| InternalIMT | epa | rs702365 | 0.609 | 0.038 | 0.056 | 0.502 | 1.50E-03 | 0.035 | 0.965 | -9.90E-03 | 0.04 | 0.805 |
| InternalIMT | epa | rs79643836 | 0.021 | 0.02 | 0.027 | 0.454 | -0.061 | 0.169 | 0.716 | 0.055 | 0.141 | 0.694 |
| InternalIMT | epa | rs702366 | 0.61 | 0.029 | 0.056 | 0.6 | 8.80E-03 | 0.035 | 0.801 | -2.70E-03 | 0.04 | 0.946 |
| InternalIMT | epa | rs1059696 | 0.124 | 0.012 | 0.031 | 0.704 | -0.043 | 0.052 | 0.412 | 0.055 | 0.056 | 0.327 |
| InternalIMT | epa | rs901681 | 0.61 | 0.03 | 0.056 | 0.586 | 0.01 | 0.035 | 0.775 | -3.80E-03 | 0.04 | 0.925 |
| InternalIMT | epa | rs61854092 | 0.122 | 0.012 | 0.031 | 0.709 | -0.042 | 0.052 | 0.423 | 0.056 | 0.056 | 0.317 |
| InternalIMT | epa | rs148080753 | 0.001 | . | . | . | . | . | . | . | . | . |
| InternalIMT | epa | rs61854093 | 0.145 | 0.016 | 0.032 | 0.621 | -0.055 | 0.047 | 0.243 | 0.042 | 0.051 | 0.413 |
| InternalIMT | epa | rs141822774 | 0.002 | . | . | . | . | . | . | . | . | . |
| InternalIMT | epa | rs74130836 | 0.023 | 0.03 | 0.027 | 0.263 | -0.129 | 0.075 | 0.086 | -0.048 | 0.088 | 0.589 |
| InternalIMT | epa | rs150620963 | 0.01 | . | . | . | . | . | . | . | . | . |
| InternalIMT | epa | rs3780913 | 0.781 | -0.033 | 0.091 | 0.716 | -0.029 | 0.048 | 0.551 | 0.038 | 0.055 | 0.486 |
| InternalIMT | epa | rs142255723 | 0.002 | . | . | . | . | . | . | . | . | . |
| InternalIMT | epa | rs3780914 | 0.607 | 0.037 | 0.054 | 0.486 | 2.60E-03 | 0.034 | 0.938 | -9.80E-03 | 0.039 | 0.801 |
| InternalIMT | epa | rs182656507 | 0.002 | . | . | . | . | . | . | . | . | . |
| InternalIMT | epa | rs2291427 | 0.756 | 0.019 | 0.085 | 0.82 | -0.038 | 0.046 | 0.408 | 6.30E-03 | 0.052 | 0.904 |
| InternalIMT | epa | rs10751382 | 0.756 | 0.018 | 0.085 | 0.832 | -0.037 | 0.046 | 0.424 | 7.10E-03 | 0.052 | 0.891 |
| InternalIMT | epa | rs117610108 | 0.02 | 0.02 | 0.027 | 0.457 | -0.059 | 0.169 | 0.727 | 0.063 | 0.141 | 0.652 |
| InternalIMT | epa | rs144966941 | 0.037 | 0.02 | 0.027 | 0.45 | 0.207 | 0.132 | 0.118 | 0.13 | 0.161 | 0.422 |
| InternalIMT | epa | rs41502051 | 0.022 | 0.032 | 0.027 | 0.245 | -0.123 | 0.099 | 0.216 | -0.125 | 0.128 | 0.326 |
| InternalIMT | epa | rs41426046 | 0.02 | 0.02 | 0.027 | 0.457 | -0.059 | 0.169 | 0.727 | 0.064 | 0.141 | 0.652 |
| InternalIMT | epa | rs41519248 | 0.02 | 0.02 | 0.027 | 0.458 | -0.057 | 0.169 | 0.737 | 0.066 | 0.141 | 0.64 |
| InternalIMT | epa | rs189108245 | 0.002 | . | . | . | . | . | . | . | . | . |
| InternalIMT | epa | rs7896431 | 0.591 | 0.033 | 0.054 | 0.533 | -1.10E-05 | 0.035 | 1 | -6.40E-03 | 0.04 | 0.873 |
| InternalIMT | epa | rs183724547 | 0.003 | . | . | . | . | . | . | . | . | . |
| InternalIMT | epa | rs142044990 | 0.006 | . | . | . | . | . | . | . | . | . |
| InternalIMT | epa | rs188140929 | . | . | . | . | . | . | . | . | . | . |
| InternalIMT | epa | rs76819096 | 0.008 | . | . | . | . | . | . | . | . | . |
| InternalIMT | epa | rs10751383 | 0.654 | 0.038 | 0.056 | 0.5 | 0.014 | 0.035 | 0.687 | -9.50E-03 | 0.04 | 0.81 |
| InternalIMT | epa | rs117819321 | 0.016 | 0.035 | 0.028 | 0.205 | -0.265 | 0.092 | 4.00E-03 | -0.197 | 0.106 | 0.063 |
| InternalIMT | epa | rs2242332 | 0.75 | -0.037 | 0.076 | 0.624 | -0.022 | 0.039 | 0.575 | 0.042 | 0.046 | 0.361 |
| InternalIMT | epa | rs2242333 | 0.06 | 0.014 | 0.028 | 0.612 | 3.30E-03 | 0.079 | 0.967 | 0.1 | 0.082 | 0.224 |
| InternalIMT | epa | rs2242334 | 0.752 | -0.03 | 0.078 | 0.702 | -0.028 | 0.04 | 0.487 | 0.037 | 0.047 | 0.43 |
| InternalIMT | epa | rs189465041 | 0.002 | . | . | . | . | . | . | . | . | . |
| InternalIMT | epa | rs28395868 | 0.014 | 0.022 | 0.027 | 0.406 | 0.178 | 0.221 | 0.42 | 0.26 | 0.256 | 0.309 |
| InternalIMT | epa | rs56067550 | 0.016 | 0.025 | 0.027 | 0.344 | 0.036 | 0.173 | 0.837 | 0.034 | 0.182 | 0.851 |
| InternalIMT | epa | rs1051713 | 0.134 | -2.80E-03 | 0.031 | 0.929 | -0.028 | 0.052 | 0.597 | 0.088 | 0.056 | 0.115 |
| InternalIMT | epa | rs115074210 | 0.009 | . | . | . | . | . | . | . | . | . |
| InternalIMT | epa | rs7900977 | 0.822 | -0.014 | 0.107 | 0.898 | -0.031 | 0.052 | 0.544 | 0.025 | 0.06 | 0.682 |
| InternalIMT | epa | rs147613891 | 0.004 | . | . | . | . | . | . | . | . | . |
| InternalIMT | epa | rs2229136 | 0.064 | 0.021 | 0.028 | 0.439 | 0.098 | 0.066 | 0.138 | 6.00E-03 | 0.082 | 0.942 |
| InternalIMT | epa | rs185482841 | 0.001 | . | . | . | . | . | . | . | . | . |
| InternalIMT | epa | rs2288619 | 0.063 | 0.021 | 0.028 | 0.444 | 0.099 | 0.066 | 0.134 | 7.40E-03 | 0.082 | 0.928 |
| InternalIMT | epa | rs189891524 | . | . | . | . | . | . | . | . | . | . |
| InternalIMT | epa | rs56145077 | 0.115 | 0.012 | 0.03 | 0.689 | -0.045 | 0.041 | 0.278 | 0.058 | 0.045 | 0.201 |
| InternalIMT | epa | rs143216970 | 0.003 | . | . | . | . | . | . | . | . | . |
| InternalIMT | epa | rs142246478 | 0.001 | . | . | . | . | . | . | . | . | . |
| InternalIMT | epa | rs13134 | 0.008 | . | . | . | . | . | . | . | . | . |
| InternalIMT | epa | rs41314980 | 0.031 | 0.033 | 0.028 | 0.236 | 0.027 | 0.114 | 0.815 | -0.123 | 0.129 | 0.34 |
| InternalIMT | epa | rs41304611 | 0.009 | . | . | . | . | . | . | . | . | . |
| InternalIMT | epa | rs193299617 | . | . | . | . | . | . | . | . | . | . |
| InternalIMT | epa | rs12772724 | 0.008 | . | . | . | . | . | . | . | . | . |
| InternalIMT | epa | rs139489537 | 0.03 | 0.029 | 0.027 | 0.293 | 0.089 | 0.104 | 0.39 | -0.04 | 0.123 | 0.747 |
| InternalIMT | epa | rs12776062 | 0.006 | . | . | . | . | . | . | . | . | . |
| InternalIMT | epa | rs752059 | 0.116 | 0.012 | 0.03 | 0.697 | -0.046 | 0.041 | 0.26 | 0.059 | 0.045 | 0.19 |
| InternalIMT | epa | rs147649982 | 0.049 | 0.031 | 0.028 | 0.267 | -0.031 | 0.082 | 0.709 | -0.054 | 0.091 | 0.555 |
| InternalIMT | epa | rs17157798 | 0.056 | 0.025 | 0.028 | 0.365 | 0.086 | 0.067 | 0.203 | -0.018 | 0.085 | 0.827 |
| InternalIMT | epa | rs142298619 | 0.001 | . | . | . | . | . | . | . | . | . |
| InternalIMT | epa | rs4948673 | 0.819 | 6.10E-03 | 0.106 | 0.954 | -0.041 | 0.052 | 0.431 | 0.012 | 0.06 | 0.836 |
| InternalIMT | epa | rs74231484 | 0.066 | 0.011 | 0.028 | 0.686 | 2.90E-03 | 0.071 | 0.968 | 0.104 | 0.072 | 0.149 |
| InternalIMT | epa | rs72796489 | 0.002 | . | . | . | . | . | . | . | . | . |
| InternalIMT | epa | rs113447578 | 0.014 | 0.02 | 0.027 | 0.455 | -0.046 | 0.102 | 0.651 | 0.167 | 0.101 | 0.097 |
| InternalIMT | epa | rs34063374 | 0.008 | . | . | . | . | . | . | . | . | . |
| InternalIMT | epa | rs11239527 | 0.011 | 0.032 | 0.027 | 0.237 | -0.255 | 0.171 | 0.136 | -0.345 | 0.251 | 0.169 |

| InternalIMT | epa | rs34371883 | 0.008 | . | . | . | . | . | . | . | . | . |
| --- | --- | --- | --- | --- | --- | --- | --- | --- | --- | --- | --- | --- |
| InternalIMT | epa | rs118168828 | 0.006 | . | . | . | . | . | . | . | . | . |
| InternalIMT | epa | rs34349618 | 0.006 | . | . | . | . | . | . | . | . | . |
| InternalIMT | epa | rs80041679 | 0.03 | 0.029 | 0.027 | 0.294 | 0.089 | 0.104 | 0.391 | -0.038 | 0.123 | 0.755 |
| InternalIMT | epa | rs149640607 | 0.001 | . | . | . | . | . | . | . | . | . |
| InternalIMT | epa | rs4427523 | 0.896 | -0.077 | 0.133 | 0.561 | 0.012 | 0.066 | 0.862 | 0.057 | 0.075 | 0.445 |
| InternalIMT | epa | rs185880109 | . | . | . | . | . | . | . | . | . | . |
| InternalIMT | epa | rs191523220 | 0.001 | . | . | . | . | . | . | . | . | . |
| InternalIMT | epa | rs144315284 | 0.003 | . | . | . | . | . | . | . | . | . |
| InternalIMT | epa | rs4948674 | 0.345 | 0.04 | 0.039 | 0.307 | -6.00E-03 | 0.032 | 0.853 | -0.022 | 0.037 | 0.558 |
| InternalIMT | epa | rs182984298 | 0.003 | . | . | . | . | . | . | . | . | . |
| InternalIMT | epa | rs56749023 | 0.016 | 0.017 | 0.026 | 0.51 | 0.191 | 0.098 | 0.051 | 0.188 | 0.156 | 0.228 |
| InternalIMT | epa | rs112508957 | 0.01 | 0.028 | 0.027 | 0.301 | -0.168 | 0.07 | 0.016 | 0.014 | 0.077 | 0.859 |
| InternalIMT | epa | rs7089063 | 0.195 | 2.80E-03 | 0.032 | 0.929 | -9.40E-03 | 0.035 | 0.785 | 0.051 | 0.038 | 0.183 |
| InternalIMT | epa | rs145000779 | . | . | . | . | . | . | . | . | . | . |
| InternalIMT | epa | rs80201572 | 0.03 | 0.029 | 0.027 | 0.294 | 0.088 | 0.104 | 0.397 | -0.038 | 0.122 | 0.755 |
| InternalIMT | epa | rs140568227 | 0.002 | . | . | . | . | . | . | . | . | . |
| InternalIMT | epa | rs149756228 | 0.009 | . | . | . | . | . | . | . | . | . |
| InternalIMT | epa | rs112380685 | 0.008 | . | . | . | . | . | . | . | . | . |
| InternalIMT | epa | rs60481109 | 0.015 | 0.017 | 0.026 | 0.507 | 0.194 | 0.098 | 0.049 | 0.194 | 0.158 | 0.218 |
| InternalIMT | epa | rs138221843 | 0.002 | . | . | . | . | . | . | . | . | . |
| InternalIMT | epa | rs150283194 | 0.001 | . | . | . | . | . | . | . | . | . |
| InternalIMT | epa | rs138879661 | 0.004 | . | . | . | . | . | . | . | . | . |
| InternalIMT | epa | rs34960345 | 0.045 | 0.035 | 0.028 | 0.212 | 0.019 | 0.072 | 0.795 | -0.081 | 0.082 | 0.32 |
| InternalIMT | epa | rs147514068 | 0.004 | . | . | . | . | . | . | . | . | . |
| InternalIMT | epa | rs35410535 | 0.06 | 0.025 | 0.028 | 0.367 | 0.075 | 0.064 | 0.245 | -0.016 | 0.08 | 0.844 |
| InternalIMT | epa | rs191613635 | 0.001 | . | . | . | . | . | . | . | . | . |
| InternalIMT | epa | rs17157800 | 0.005 | . | . | . | . | . | . | . | . | . |
| InternalIMT | epa | rs17523413 | 0.039 | 0.03 | 0.028 | 0.276 | 0.069 | 0.088 | 0.434 | -0.046 | 0.102 | 0.649 |
| InternalIMT | epa | rs7897036 | 0.037 | 0.036 | 0.028 | 0.209 | -0.178 | 0.067 | 8.30E-03 | -0.073 | 0.065 | 0.262 |
| InternalIMT | epa | rs76334323 | 0.02 | 0.02 | 0.027 | 0.463 | -0.046 | 0.169 | 0.787 | 0.081 | 0.141 | 0.564 |
| InternalIMT | epa | rs143535991 | 0.001 | . | . | . | . | . | . | . | . | . |
| InternalIMT | epa | rs140344979 | 0.003 | . | . | . | . | . | . | . | . | . |
| InternalIMT | epa | rs36047811 | 0.06 | 0.025 | 0.028 | 0.367 | 0.075 | 0.064 | 0.245 | -0.016 | 0.08 | 0.844 |
| InternalIMT | epa | rs76493570 | 0.043 | 0.032 | 0.028 | 0.243 | 0.038 | 0.078 | 0.629 | -0.065 | 0.09 | 0.474 |
| InternalIMT | epa | rs192998920 | . | . | . | . | . | . | . | . | . | . |
| InternalIMT | epa | rs116840852 | 0.013 | 0.033 | 0.027 | 0.226 | -0.086 | 0.185 | 0.644 | -0.274 | 0.329 | 0.406 |
| InternalIMT | epa | rs184824101 | 0 | . | . | . | . | . | . | . | . | . |
| InternalIMT | epa | rs41301621 | 0.003 | . | . | . | . | . | . | . | . | . |
| InternalIMT | epa | rs3740109 | 0.06 | 0.025 | 0.028 | 0.367 | 0.075 | 0.064 | 0.245 | -0.016 | 0.08 | 0.844 |
| InternalIMT | epa | rs7899415 | 0.025 | 0.028 | 0.028 | 0.307 | 0.046 | 0.111 | 0.679 | -0.042 | 0.118 | 0.723 |
| InternalIMT | epa | rs184395284 | 0.003 | . | . | . | . | . | . | . | . | . |
| InternalIMT | epa | rs139209589 | 0.004 | . | . | . | . | . | . | . | . | . |
| InternalIMT | epa | rs149924163 | 0.001 | . | . | . | . | . | . | . | . | . |
| InternalIMT | epa | rs3740110 | 0.02 | 0.02 | 0.027 | 0.463 | -0.044 | 0.169 | 0.795 | 0.083 | 0.142 | 0.559 |
| InternalIMT | epa | rs144335113 | 0.001 | . | . | . | . | . | . | . | . | . |
| InternalIMT | epa | rs74743385 | 0.01 | 0.028 | 0.027 | 0.301 | -0.168 | 0.07 | 0.016 | 0.014 | 0.077 | 0.857 |
| InternalIMT | epa | rs146545829 | 0.001 | . | . | . | . | . | . | . | . | . |
| InternalIMT | epa | rs181842689 | 0.001 | . | . | . | . | . | . | . | . | . |
| InternalIMT | epa | rs3740111 | 0.045 | 0.035 | 0.028 | 0.212 | 0.019 | 0.072 | 0.796 | -0.081 | 0.082 | 0.319 |
| InternalIMT | epa | rs186261599 | 0.001 | . | . | . | . | . | . | . | . | . |
| InternalIMT | epa | rs73285348 | 0.008 | . | . | . | . | . | . | . | . | . |
| InternalIMT | epa | rs186356901 | 0.001 | . | . | . | . | . | . | . | . | . |
| InternalIMT | epa | rs3824618 | 0.03 | 0.029 | 0.027 | 0.294 | 0.088 | 0.104 | 0.399 | -0.038 | 0.122 | 0.753 |
| InternalIMT | epa | rs3802548 | 0.304 | 0.028 | 0.035 | 0.424 | -0.025 | 0.03 | 0.411 | -3.70E-03 | 0.035 | 0.916 |
| InternalIMT | epa | rs3802547 | 0.02 | 0.02 | 0.027 | 0.463 | -0.043 | 0.17 | 0.8 | 0.083 | 0.142 | 0.558 |
| InternalIMT | epa | rs77677521 | 0.013 | 0.033 | 0.027 | 0.226 | -0.085 | 0.185 | 0.645 | -0.274 | 0.329 | 0.406 |
| InternalIMT | epa | rs3824616 | 0.06 | 0.025 | 0.028 | 0.368 | 0.075 | 0.064 | 0.245 | -0.016 | 0.08 | 0.844 |
| InternalIMT | epa | rs191073818 | 0.001 | . | . | . | . | . | . | . | . | . |
| InternalIMT | epa | rs147078004 | . | . | . | . | . | . | . | . | . | . |
| InternalIMT | epa | rs7908745 | 0.388 | 0.023 | 0.037 | 0.54 | -0.012 | 0.03 | 0.684 | 3.50E-03 | 0.032 | 0.913 |
| InternalIMT | epa | rs17157811 | 0.005 | . | . | . | . | . | . | . | . | . |
| InternalIMT | epa | rs138506087 | 0.006 | . | . | . | . | . | . | . | . | . |
| InternalIMT | epa | rs77241269 | 0.016 | 0.017 | 0.026 | 0.518 | 0.194 | 0.095 | 0.042 | 0.185 | 0.149 | 0.215 |
| InternalIMT | epa | rs138374938 | 0.001 | . | . | . | . | . | . | . | . | . |
| InternalIMT | epa | rs2279433 | 0.045 | 0.036 | 0.028 | 0.195 | 7.70E-03 | 0.068 | 0.909 | -0.09 | 0.076 | 0.239 |
| InternalIMT | epa | rs73285358 | 0.016 | 0.017 | 0.026 | 0.518 | 0.194 | 0.095 | 0.041 | 0.184 | 0.148 | 0.214 |
| InternalIMT | epa | rs11239528 | 0.303 | 0.025 | 0.035 | 0.473 | -0.022 | 0.03 | 0.461 | 7.00E-04 | 0.035 | 0.984 |
| InternalIMT | epa | rs77453386 | 0.02 | 0.02 | 0.027 | 0.463 | -0.041 | 0.168 | 0.807 | 0.085 | 0.141 | 0.548 |
| InternalIMT | epa | rs2279434 | 0.05 | 0.038 | 0.028 | 0.183 | 6.00E-03 | 0.066 | 0.927 | -0.093 | 0.074 | 0.212 |
| InternalIMT | epa | rs149236525 | 0.005 | . | . | . | . | . | . | . | . | . |
| InternalIMT | epa | rs12573609 | 0.037 | 0.028 | 0.028 | 0.323 | -0.074 | 0.092 | 0.423 | -0.031 | 0.117 | 0.793 |
| InternalIMT | epa | rs191716655 | . | . | . | . | . | . | . | . | . | . |

| InternalIMT | epa | rs17523660 | 0.006 | . | . | . | . | . | . | . | . | . |
| --- | --- | --- | --- | --- | --- | --- | --- | --- | --- | --- | --- | --- |
| InternalIMT | epa | rs142671360 | 0.004 | . | . | . | . | . | . | . | . | . |
| InternalIMT | epa | rs3764990 | 0.038 | 0.031 | 0.028 | 0.257 | 0.056 | 0.088 | 0.527 | -0.061 | 0.101 | 0.547 |
| InternalIMT | epa | rs113682861 | 0.005 | . | . | . | . | . | . | . | . | . |
| InternalIMT | epa | rs117658270 | 0.02 | 0.02 | 0.027 | 0.463 | -0.041 | 0.168 | 0.807 | 0.085 | 0.141 | 0.548 |
| InternalIMT | epa | rs187309063 | . | . | . | . | . | . | . | . | . | . |
| InternalIMT | epa | rs73285360 | 0.016 | 0.017 | 0.026 | 0.518 | 0.194 | 0.095 | 0.041 | 0.184 | 0.147 | 0.213 |
| InternalIMT | epa | rs80133289 | 0.007 | . | . | . | . | . | . | . | . | . |
| InternalIMT | epa | rs186693257 | . | . | . | . | . | . | . | . | . | . |
| InternalIMT | epa | rs35735968 | 0.049 | 0.034 | 0.028 | 0.23 | 0.019 | 0.069 | 0.778 | -0.064 | 0.077 | 0.407 |
| InternalIMT | epa | rs17157825 | 0.031 | 0.03 | 0.028 | 0.28 | 0.069 | 0.104 | 0.503 | -0.054 | 0.122 | 0.66 |
| InternalIMT | epa | rs2291428 | 0.299 | 0.027 | 0.034 | 0.439 | -0.024 | 0.03 | 0.43 | -2.40E-03 | 0.036 | 0.946 |
| InternalIMT | epa | rs2291429 | 0.304 | 0.021 | 0.035 | 0.543 | -0.017 | 0.03 | 0.584 | 6.30E-03 | 0.035 | 0.857 |
| InternalIMT | epa | rs145714792 | 0.003 | . | . | . | . | . | . | . | . | . |
| InternalIMT | epa | rs12769244 | 0.044 | 0.037 | 0.028 | 0.191 | 5.60E-03 | 0.067 | 0.934 | -0.092 | 0.075 | 0.22 |
| InternalIMT | epa | rs12771275 | 0.044 | 0.037 | 0.028 | 0.191 | 5.60E-03 | 0.067 | 0.934 | -0.092 | 0.075 | 0.22 |
| InternalIMT | epa | rs146266174 | 0 | . | . | . | . | . | . | . | . | . |
| InternalIMT | epa | rs41301623 | 0.003 | . | . | . | . | . | . | . | . | . |
| InternalIMT | epa | rs2306814 | 0.021 | 0.019 | 0.027 | 0.481 | 0.014 | 0.161 | 0.931 | 0.132 | 0.135 | 0.33 |
| InternalIMT | epa | rs12244834 | 0.012 | 0.015 | 0.026 | 0.572 | 0.338 | 0.293 | 0.249 | 0.446 | 0.308 | 0.148 |
| InternalIMT | epa | rs184006011 | 0.002 | . | . | . | . | . | . | . | . | . |
| InternalIMT | epa | rs78829066 | 0.02 | 0.02 | 0.027 | 0.463 | -0.042 | 0.168 | 0.801 | 0.084 | 0.141 | 0.553 |
| InternalIMT | epa | rs34731408 | 0.044 | 0.037 | 0.028 | 0.192 | 5.90E-03 | 0.067 | 0.93 | -0.092 | 0.075 | 0.22 |
| InternalIMT | epa | rs17451516 | 0.008 | . | . | . | . | . | . | . | . | . |
| InternalIMT | epa | rs139910133 | 0.002 | . | . | . | . | . | . | . | . | . |
| InternalIMT | epa | rs10900218 | 0.197 | 5.30E-04 | 0.031 | 0.987 | 1.00E-03 | 0.034 | 0.977 | 0.055 | 0.038 | 0.153 |
| InternalIMT | epa | rs146023999 | 0.001 | . | . | . | . | . | . | . | . | . |
| InternalIMT | epa | rs187296958 | . | . | . | . | . | . | . | . | . | . |
| InternalIMT | epa | rs142342833 | . | . | . | . | . | . | . | . | . | . |
| InternalIMT | epa | rs143489255 | . | . | . | . | . | . | . | . | . | . |
| InternalIMT | epa | rs138532518 | . | . | . | . | . | . | . | . | . | . |
| InternalIMT | epa | rs183768079 | 0.001 | . | . | . | . | . | . | . | . | . |
| InternalIMT | epa | rs117892272 | . | . | . | . | . | . | . | . | . | . |
| InternalIMT | epa | rs192993943 | . | . | . | . | . | . | . | . | . | . |
| InternalIMT | epa | rs182989043 | 0.001 | . | . | . | . | . | . | . | . | . |
| InternalIMT | epa | rs189703435 | . | . | . | . | . | . | . | . | . | . |
| InternalIMT | epa | rs188794040 | . | . | . | . | . | . | . | . | . | . |
| InternalIMT | epa | rs143920953 | . | . | . | . | . | . | . | . | . | . |
| InternalIMT | epa | rs191029121 | . | . | . | . | . | . | . | . | . | . |
| InternalIMT | epa | rs116875448 | . | . | . | . | . | . | . | . | . | . |
| InternalIMT | epa | rs190167877 | 0.001 | . | . | . | . | . | . | . | . | . |
| InternalIMT | epa | rs184993418 | . | . | . | . | . | . | . | . | . | . |
| InternalIMT | epa | rs143241158 | . | . | . | . | . | . | . | . | . | . |
| InternalIMT | epa | rs11239500 | 0.015 | 0.013 | 0.026 | 0.605 | 0.13 | 0.18 | 0.469 | 0.216 | 0.15 | 0.15 |
| InternalIMT | epa | rs140568994 | . | . | . | . | . | . | . | . | . | . |
| InternalIMT | epa | rs187530317 | . | . | . | . | . | . | . | . | . | . |
| InternalIMT | epa | rs145189326 | . | . | . | . | . | . | . | . | . | . |
| InternalIMT | epa | rs186203332 | . | . | . | . | . | . | . | . | . | . |
| InternalIMT | epa | rs189921909 | . | . | . | . | . | . | . | . | . | . |
| InternalIMT | epa | rs187260989 | . | . | . | . | . | . | . | . | . | . |
| InternalIMT | epa | rs114376586 | 0.013 | 0.014 | 0.026 | 0.602 | 0.142 | 0.181 | 0.432 | 0.221 | 0.15 | 0.142 |
| InternalIMT | epa | rs151070808 | . | . | . | . | . | . | . | . | . | . |
| InternalIMT | epa | rs140927225 | 0.015 | 0.013 | 0.026 | 0.61 | 0.139 | 0.179 | 0.438 | 0.222 | 0.151 | 0.141 |
| InternalIMT | epa | rs191555615 | . | . | . | . | . | . | . | . | . | . |
| InternalIMT | epa | rs80278898 | 0.005 | . | . | . | . | . | . | . | . | . |
| InternalIMT | epa | rs190849503 | . | . | . | . | . | . | . | . | . | . |
| InternalIMT | epa | rs150080315 | 0.003 | . | . | . | . | . | . | . | . | . |
| InternalIMT | epa | rs143623885 | . | . | . | . | . | . | . | . | . | . |
| InternalIMT | epa | rs187760040 | . | . | . | . | . | . | . | . | . | . |
| InternalIMT | epa | rs143917143 | 0.005 | . | . | . | . | . | . | . | . | . |
| InternalIMT | epa | rs149851136 | 0.001 | . | . | . | . | . | . | . | . | . |
| InternalIMT | epa | rs181612054 | . | . | . | . | . | . | . | . | . | . |
| InternalIMT | epa | rs191006548 | . | . | . | . | . | . | . | . | . | . |
| InternalIMT | epa | rs117377631 | 0.026 | 0.033 | 0.027 | 0.218 | 0.101 | 0.147 | 0.495 | -0.123 | 0.162 | 0.446 |
| InternalIMT | epa | rs182107634 | . | . | . | . | . | . | . | . | . | . |
| InternalIMT | epa | rs190211867 | . | . | . | . | . | . | . | . | . | . |
| InternalIMT | epa | rs148293457 | . | . | . | . | . | . | . | . | . | . |
| InternalIMT | epa | rs191811733 | . | . | . | . | . | . | . | . | . | . |
| InternalIMT | epa | rs146109272 | . | . | . | . | . | . | . | . | . | . |
| InternalIMT | epa | rs148397915 | . | . | . | . | . | . | . | . | . | . |
| InternalIMT | epa | rs142233290 | . | . | . | . | . | . | . | . | . | . |
| InternalIMT | epa | rs80226874 | . | . | . | . | . | . | . | . | . | . |
| InternalIMT | epa | rs144496203 | . | . | . | . | . | . | . | . | . | . |

| InternalIMT | epa | rs191062130 | . | . | . | . | . | . | . | . | . | . |
| --- | --- | --- | --- | --- | --- | --- | --- | --- | --- | --- | --- | --- |
| InternalIMT | epa | rs185807817 | . | . | . | . | . | . | . | . | . | . |
| InternalIMT | epa | rs117846213 | . | . | . | . | . | . | . | . | . | . |
| InternalIMT | epa | rs190545473 | . | . | . | . | . | . | . | . | . | . |
| InternalIMT | epa | rs192364918 | . | . | . | . | . | . | . | . | . | . |
| InternalIMT | epa | rs182939241 | . | . | . | . | . | . | . | . | . | . |
| InternalIMT | epa | rs12571125 | . | . | . | . | . | . | . | . | . | . |
| InternalIMT | epa | rs192418941 | . | . | . | . | . | . | . | . | . | . |
| InternalIMT | epa | rs141270243 | . | . | . | . | . | . | . | . | . | . |
| InternalIMT | epa | rs182615502 | . | . | . | . | . | . | . | . | . | . |
| InternalIMT | epa | rs146669856 | 0.004 | . | . | . | . | . | . | . | . | . |
| InternalIMT | epa | rs78290029 | 0.015 | 0.01 | 0.026 | 0.688 | 0.237 | 0.214 | 0.269 | 0.333 | 0.223 | 0.136 |
| InternalIMT | epa | rs139955565 | . | . | . | . | . | . | . | . | . | . |
| InternalIMT | epa | rs12267314 | 0.013 | 0.015 | 0.026 | 0.565 | 0.179 | 0.249 | 0.473 | 0.274 | 0.251 | 0.276 |
| InternalIMT | epa | rs74477197 | . | . | . | . | . | . | . | . | . | . |
| InternalIMT | epa | rs188147236 | . | . | . | . | . | . | . | . | . | . |
| InternalIMT | epa | rs2228065 | 0.012 | 0.016 | 0.026 | 0.54 | 0.201 | 0.254 | 0.429 | 0.257 | 0.258 | 0.319 |
| InternalIMT | epa | rs116961353 | 0.007 | . | . | . | . | . | . | . | . | . |
| InternalIMT | epa | rs190222272 | . | . | . | . | . | . | . | . | . | . |
| InternalIMT | epa | rs182719940 | . | . | . | . | . | . | . | . | . | . |
| InternalIMT | epa | rs184477413 | . | . | . | . | . | . | . | . | . | . |
| InternalIMT | epa | rs12265175 | 0.013 | 0.017 | 0.026 | 0.512 | 0.153 | 0.25 | 0.541 | 0.221 | 0.253 | 0.382 |
| InternalIMT | epa | rs182928276 | . | . | . | . | . | . | . | . | . | . |
| InternalIMT | epa | rs144409895 | . | . | . | . | . | . | . | . | . | . |
| InternalIMT | epa | rs58998447 | 0.003 | . | . | . | . | . | . | . | . | . |
| InternalIMT | epa | rs188668832 | . | . | . | . | . | . | . | . | . | . |
| InternalIMT | epa | rs148641686 | . | . | . | . | . | . | . | . | . | . |
| InternalIMT | epa | rs75468791 | . | . | . | . | . | . | . | . | . | . |
| InternalIMT | epa | rs147327397 | . | . | . | . | . | . | . | . | . | . |
| InternalIMT | epa | rs77354597 | 0.001 | . | . | . | . | . | . | . | . | . |
| InternalIMT | epa | rs41365544 | 0.012 | 0.015 | 0.026 | 0.564 | 0.304 | 0.294 | 0.301 | 0.403 | 0.308 | 0.191 |
| InternalIMT | epa | rs187564188 | . | . | . | . | . | . | . | . | . | . |
| InternalIMT | epa | rs183511984 | . | . | . | . | . | . | . | . | . | . |
| InternalIMT | epa | rs12262783 | 0.011 | 0.015 | 0.026 | 0.566 | 0.326 | 0.311 | 0.295 | 0.422 | 0.319 | 0.186 |
| InternalIMT | epa | rs188817687 | 0.001 | . | . | . | . | . | . | . | . | . |
| InternalIMT | epa | rs3815968 | . | . | . | . | . | . | . | . | . | . |
| InternalIMT | epa | rs187822300 | . | . | . | . | . | . | . | . | . | . |
| InternalIMT | epa | rs188902172 | 0.001 | . | . | . | . | . | . | . | . | . |
| InternalIMT | epa | rs181190448 | . | . | . | . | . | . | . | . | . | . |
| InternalIMT | epa | rs182699093 | . | . | . | . | . | . | . | . | . | . |
| InternalIMT | epa | rs182631303 | . | . | . | . | . | . | . | . | . | . |
| InternalIMT | epa | rs187014172 | 0.001 | . | . | . | . | . | . | . | . | . |
| InternalIMT | epa | rs142575406 | . | . | . | . | . | . | . | . | . | . |
| InternalIMT | epa | rs180711033 | . | . | . | . | . | . | . | . | . | . |
| InternalIMT | epa | rs137894823 | 0.001 | . | . | . | . | . | . | . | . | . |
| InternalIMT | epa | rs189053273 | 0 | . | . | . | . | . | . | . | . | . |
| InternalIMT | epa | rs143499388 | . | . | . | . | . | . | . | . | . | . |
| InternalIMT | epa | rs182936001 | 0.001 | . | . | . | . | . | . | . | . | . |
| InternalIMT | epa | rs148456406 | 0 | . | . | . | . | . | . | . | . | . |
| InternalIMT | epa | rs182499531 | 0.001 | . | . | . | . | . | . | . | . | . |
| InternalIMT | epa | rs74884915 | 0.005 | . | . | . | . | . | . | . | . | . |
| InternalIMT | epa | rs184046374 | . | . | . | . | . | . | . | . | . | . |
| InternalIMT | epa | rs74333993 | 0.004 | . | . | . | . | . | . | . | . | . |
| InternalIMT | epa | rs138889719 | . | . | . | . | . | . | . | . | . | . |
| InternalIMT | epa | rs75330330 | 0.003 | . | . | . | . | . | . | . | . | . |
| InternalIMT | epa | rs193051693 | . | . | . | . | . | . | . | . | . | . |
| InternalIMT | epa | rs145929633 | . | . | . | . | . | . | . | . | . | . |
| InternalIMT | epa | rs184076904 | 0.001 | . | . | . | . | . | . | . | . | . |
| InternalIMT | epa | rs141802494 | 0.002 | . | . | . | . | . | . | . | . | . |
| InternalIMT | epa | rs149519759 | 0 | . | . | . | . | . | . | . | . | . |
| InternalIMT | epa | rs145467260 | 0.002 | . | . | . | . | . | . | . | . | . |
| InternalIMT | epa | rs79620914 | 0.006 | . | . | . | . | . | . | . | . | . |
| InternalIMT | epa | rs140538633 | 0.005 | . | . | . | . | . | . | . | . | . |
| InternalIMT | epa | rs140862301 | 0.001 | . | . | . | . | . | . | . | . | . |
| InternalIMT | epa | rs114615847 | 0.003 | . | . | . | . | . | . | . | . | . |
| InternalIMT | epa | rs141800259 | 0.004 | . | . | . | . | . | . | . | . | . |
| InternalIMT | epa | rs149779059 | 0.003 | . | . | . | . | . | . | . | . | . |
| InternalIMT | epa | rs116539253 | 0.001 | . | . | . | . | . | . | . | . | . |
| InternalIMT | epa | rs184979550 | 0 | . | . | . | . | . | . | . | . | . |
| InternalIMT | epa | rs190128940 | 0.001 | . | . | . | . | . | . | . | . | . |
| InternalIMT | epa | rs146594816 | . | . | . | . | . | . | . | . | . | . |
| InternalIMT | epa | rs141370766 | 0.003 | . | . | . | . | . | . | . | . | . |
| InternalIMT | epa | rs144670033 | . | . | . | . | . | . | . | . | . | . |

| InternalIMT | epa | rs187152174 | 0.003 | . | . | . | . | . | . | . | . | . |
| --- | --- | --- | --- | --- | --- | --- | --- | --- | --- | --- | --- | --- |
| InternalIMT | epa | rs184180263 | 0.002 | . | . | . | . | . | . | . | . | . |
| InternalIMT | epa | rs140075861 | 0.002 | . | . | . | . | . | . | . | . | . |
| InternalIMT | epa | rs186483129 | . | . | . | . | . | . | . | . | . | . |
| InternalIMT | epa | rs186634282 | 0.003 | . | . | . | . | . | . | . | . | . |
| InternalIMT | epa | rs9988663 | 0.004 | . | . | . | . | . | . | . | . | . |
| InternalIMT | epa | rs138828529 | 0.004 | . | . | . | . | . | . | . | . | . |
| InternalIMT | epa | rs188777615 | 0.001 | . | . | . | . | . | . | . | . | . |
| InternalIMT | epa | rs192321882 | 0.001 | . | . | . | . | . | . | . | . | . |
| InternalIMT | epa | rs150812270 | 0.003 | . | . | . | . | . | . | . | . | . |
| InternalIMT | epa | rs185906603 | . | . | . | . | . | . | . | . | . | . |
| InternalIMT | epa | rs191070862 | . | . | . | . | . | . | . | . | . | . |
| InternalIMT | epa | rs182904978 | . | . | . | . | . | . | . | . | . | . |
| InternalIMT | epa | rs192679655 | . | . | . | . | . | . | . | . | . | . |
| InternalIMT | epa | rs189673086 | 0.003 | . | . | . | . | . | . | . | . | . |
| InternalIMT | epa | rs181141892 | 0.001 | . | . | . | . | . | . | . | . | . |
| InternalIMT | epa | rs186404123 | . | . | . | . | . | . | . | . | . | . |
| InternalIMT | epa | rs192117287 | 0.001 | . | . | . | . | . | . | . | . | . |
| InternalIMT | epa | rs182748835 | 0 | . | . | . | . | . | . | . | . | . |
| InternalIMT | epa | rs141854549 | 0.005 | . | . | . | . | . | . | . | . | . |
| InternalIMT | epa | rs140163397 | 0.001 | . | . | . | . | . | . | . | . | . |
| InternalIMT | epa | rs146347300 | 0.001 | . | . | . | . | . | . | . | . | . |
| InternalIMT | epa | rs139362377 | 0.005 | . | . | . | . | . | . | . | . | . |
| InternalIMT | epa | rs185087671 | 0 | . | . | . | . | . | . | . | . | . |
| InternalIMT | epa | rs190466574 | 0 | . | . | . | . | . | . | . | . | . |
| InternalIMT | epa | rs147012778 | 0.001 | . | . | . | . | . | . | . | . | . |
| InternalIMT | epa | rs138236960 | 0.003 | . | . | . | . | . | . | . | . | . |
| InternalIMT | epa | rs116310311 | 0.005 | . | . | . | . | . | . | . | . | . |
| InternalIMT | epa | rs142868356 | 0.003 | . | . | . | . | . | . | . | . | . |
| InternalIMT | epa | rs144956240 | 0.001 | . | . | . | . | . | . | . | . | . |
| InternalIMT | epa | rs188527776 | . | . | . | . | . | . | . | . | . | . |
| InternalIMT | epa | rs183971165 | 0.003 | . | . | . | . | . | . | . | . | . |
| InternalIMT | epa | rs76748216 | 0.003 | . | . | . | . | . | . | . | . | . |
| InternalIMT | epa | rs148554990 | . | . | . | . | . | . | . | . | . | . |
| InternalIMT | epa | rs114446477 | 0.007 | . | . | . | . | . | . | . | . | . |
| InternalIMT | epa | rs185223933 | 0.002 | . | . | . | . | . | . | . | . | . |
| InternalIMT | epa | rs76869726 | 0.003 | . | . | . | . | . | . | . | . | . |
| InternalIMT | epa | rs151200830 | 0.005 | . | . | . | . | . | . | . | . | . |
| InternalIMT | epa | rs17157731 | 0.005 | . | . | . | . | . | . | . | . | . |
| InternalIMT | epa | rs181243469 | . | . | . | . | . | . | . | . | . | . |
| InternalIMT | epa | rs186890707 | . | . | . | . | . | . | . | . | . | . |
| InternalIMT | epa | rs184063360 | . | . | . | . | . | . | . | . | . | . |
| InternalIMT | epa | rs188550237 | 0.001 | . | . | . | . | . | . | . | . | . |
| InternalIMT | epa | rs140931068 | 0.003 | . | . | . | . | . | . | . | . | . |
| InternalIMT | epa | rs74867877 | 0.001 | . | . | . | . | . | . | . | . | . |
| InternalIMT | epa | rs11239495 | 0.015 | 0.012 | 0.026 | 0.631 | 0.307 | 0.254 | 0.228 | 0.304 | 0.252 | 0.228 |
| InternalIMT | epa | rs149745800 | . | . | . | . | . | . | . | . | . | . |
| InternalIMT | epa | rs145044458 | 0.005 | . | . | . | . | . | . | . | . | . |
| InternalIMT | epa | rs188067427 | 0 | . | . | . | . | . | . | . | . | . |
| InternalIMT | epa | rs146827800 | . | . | . | . | . | . | . | . | . | . |
| InternalIMT | epa | rs140619817 | 0.005 | . | . | . | . | . | . | . | . | . |
| InternalIMT | epa | rs892689 | 0.998 | . | . | . | . | . | . | . | . | . |
| InternalIMT | epa | rs143642005 | 0.001 | . | . | . | . | . | . | . | . | . |
| InternalIMT | epa | rs181929304 | 0.001 | . | . | . | . | . | . | . | . | . |
| InternalIMT | epa | rs191086173 | 0.001 | . | . | . | . | . | . | . | . | . |
| InternalIMT | epa | rs114613116 | 0.005 | . | . | . | . | . | . | . | . | . |
| InternalIMT | epa | rs141076768 | . | . | . | . | . | . | . | . | . | . |
| InternalIMT | epa | rs187316531 | . | . | . | . | . | . | . | . | . | . |
| InternalIMT | epa | rs11239499 | 0.014 | 0.014 | 0.026 | 0.588 | 0.114 | 0.18 | 0.526 | 0.218 | 0.151 | 0.148 |
| InternalIMT | epa | rs192726371 | 0.001 | . | . | . | . | . | . | . | . | . |
| InternalIMT | epa | rs74128481 | 0.004 | . | . | . | . | . | . | . | . | . |
| InternalIMT | epa | rs74128482 | 0.003 | . | . | . | . | . | . | . | . | . |
| InternalIMT | epa | rs11239502 | 0.008 | . | . | . | . | . | . | . | . | . |
| InternalIMT | epa | rs116330285 | . | . | . | . | . | . | . | . | . | . |
| InternalIMT | epa | rs189926729 | 0.001 | . | . | . | . | . | . | . | . | . |
| InternalIMT | epa | rs188975779 | 0.003 | . | . | . | . | . | . | . | . | . |
| InternalIMT | epa | rs143846038 | . | . | . | . | . | . | . | . | . | . |
| InternalIMT | epa | rs74128483 | 0.004 | . | . | . | . | . | . | . | . | . |
| InternalIMT | epa | rs139292055 | 0.013 | 0.014 | 0.026 | 0.6 | 0.142 | 0.182 | 0.434 | 0.221 | 0.151 | 0.144 |
| InternalIMT | epa | rs11239503 | 0.013 | 0.014 | 0.026 | 0.6 | 0.142 | 0.182 | 0.433 | 0.221 | 0.151 | 0.144 |
| InternalIMT | epa | rs58838686 | 0.003 | . | . | . | . | . | . | . | . | . |
| InternalIMT | epa | rs185825373 | 0.001 | . | . | . | . | . | . | . | . | . |
| InternalIMT | epa | rs11239504 | 0.013 | 0.014 | 0.026 | 0.6 | 0.142 | 0.182 | 0.433 | 0.221 | 0.151 | 0.144 |

| InternalIMT | epa | rs150012354 | 0 | . | . | . | . | . | . | . | . | . |
| --- | --- | --- | --- | --- | --- | --- | --- | --- | --- | --- | --- | --- |
| InternalIMT | epa | rs187763096 | 0.001 | . | . | . | . | . | . | . | . | . |
| InternalIMT | epa | rs193043982 | 0.002 | . | . | . | . | . | . | . | . | . |
| InternalIMT | epa | rs79186884 | 0.004 | . | . | . | . | . | . | . | . | . |
| InternalIMT | epa | rs74128484 | 0.003 | . | . | . | . | . | . | . | . | . |
| InternalIMT | epa | rs58461537 | 0.013 | 0.014 | 0.026 | 0.601 | 0.142 | 0.181 | 0.432 | 0.221 | 0.151 | 0.143 |
| InternalIMT | epa | rs188527474 | 0.002 | . | . | . | . | . | . | . | . | . |
| InternalIMT | epa | rs141659976 | 0.002 | . | . | . | . | . | . | . | . | . |
| InternalIMT | epa | rs115846407 | 0.013 | 0.014 | 0.026 | 0.601 | 0.142 | 0.181 | 0.431 | 0.221 | 0.151 | 0.143 |
| InternalIMT | epa | rs138378816 | 0.013 | 0.014 | 0.026 | 0.601 | 0.142 | 0.181 | 0.431 | 0.221 | 0.151 | 0.143 |
| InternalIMT | epa | rs146188136 | 0.008 | . | . | . | . | . | . | . | . | . |
| InternalIMT | epa | rs139383871 | 0.002 | . | . | . | . | . | . | . | . | . |
| InternalIMT | epa | rs182953869 | . | . | . | . | . | . | . | . | . | . |
| InternalIMT | epa | rs192430299 | 0.004 | . | . | . | . | . | . | . | . | . |
| InternalIMT | epa | rs144152844 | 0.013 | 0.014 | 0.026 | 0.601 | 0.142 | 0.181 | 0.431 | 0.221 | 0.151 | 0.142 |
| InternalIMT | epa | rs145987786 | 0.013 | 0.026 | 0.027 | 0.34 | -0.088 | 0.087 | 0.312 | 0.041 | 0.128 | 0.751 |
| InternalIMT | epa | rs181132926 | 0.001 | . | . | . | . | . | . | . | . | . |
| InternalIMT | epa | rs147882253 | 0.013 | 0.014 | 0.026 | 0.601 | 0.142 | 0.18 | 0.43 | 0.221 | 0.15 | 0.142 |
| InternalIMT | epa | rs141807621 | 0.005 | . | . | . | . | . | . | . | . | . |
| InternalIMT | epa | rs147116052 | 0.013 | 0.014 | 0.026 | 0.601 | 0.142 | 0.18 | 0.43 | 0.221 | 0.15 | 0.142 |
| InternalIMT | epa | rs147716618 | 0.013 | 0.014 | 0.026 | 0.601 | 0.142 | 0.18 | 0.43 | 0.221 | 0.15 | 0.142 |
| InternalIMT | epa | rs187198855 | 0.01 | 0.024 | 0.027 | 0.381 | -0.015 | 0.111 | 0.891 | 0.125 | 0.151 | 0.406 |
| InternalIMT | epa | rs189409638 | 0.005 | . | . | . | . | . | . | . | . | . |
| InternalIMT | epa | rs182647339 | . | . | . | . | . | . | . | . | . | . |
| InternalIMT | epa | rs187995988 | . | . | . | . | . | . | . | . | . | . |
| InternalIMT | epa | rs141034184 | 0.015 | 0.013 | 0.026 | 0.618 | 0.117 | 0.167 | 0.482 | 0.221 | 0.142 | 0.119 |
| InternalIMT | epa | rs187310430 | 0.002 | . | . | . | . | . | . | . | . | . |
| InternalIMT | epa | rs192608488 | 0.013 | 0.014 | 0.026 | 0.601 | 0.141 | 0.179 | 0.429 | 0.22 | 0.149 | 0.14 |
| InternalIMT | epa | rs148840366 | 0.003 | . | . | . | . | . | . | . | . | . |
| InternalIMT | epa | rs186785022 | 0.004 | . | . | . | . | . | . | . | . | . |
| InternalIMT | epa | rs192348728 | 0.002 | . | . | . | . | . | . | . | . | . |
| InternalIMT | epa | rs189656631 | 0.013 | 0.014 | 0.026 | 0.601 | 0.141 | 0.179 | 0.429 | 0.22 | 0.149 | 0.14 |
| InternalIMT | epa | rs185289750 | . | . | . | . | . | . | . | . | . | . |
| InternalIMT | epa | rs142382395 | 0.013 | 0.014 | 0.026 | 0.601 | 0.141 | 0.178 | 0.429 | 0.22 | 0.149 | 0.14 |
| InternalIMT | epa | rs144866696 | 0.004 | . | . | . | . | . | . | . | . | . |
| InternalIMT | epa | rs141371289 | 0.013 | 0.014 | 0.026 | 0.601 | 0.141 | 0.178 | 0.429 | 0.22 | 0.149 | 0.14 |
| InternalIMT | epa | rs114750008 | 0.013 | 0.014 | 0.026 | 0.601 | 0.141 | 0.178 | 0.429 | 0.22 | 0.149 | 0.14 |
| InternalIMT | epa | rs189063597 | 0.002 | . | . | . | . | . | . | . | . | . |
| InternalIMT | epa | rs139151571 | 0.013 | 0.014 | 0.026 | 0.592 | 0.145 | 0.182 | 0.427 | 0.22 | 0.152 | 0.146 |
| InternalIMT | epa | rs139846368 | 0.014 | 0.013 | 0.026 | 0.614 | 0.134 | 0.177 | 0.451 | 0.225 | 0.149 | 0.131 |
| InternalIMT | epa | rs182057653 | 0.004 | . | . | . | . | . | . | . | . | . |
| InternalIMT | epa | rs148785420 | 0.013 | 0.014 | 0.026 | 0.601 | 0.141 | 0.178 | 0.428 | 0.22 | 0.149 | 0.14 |
| InternalIMT | epa | rs138383251 | 0.002 | . | . | . | . | . | . | . | . | . |
| InternalIMT | epa | rs116526705 | 0.006 | . | . | . | . | . | . | . | . | . |
| InternalIMT | epa | rs185946436 | . | . | . | . | . | . | . | . | . | . |
| InternalIMT | epa | rs190455427 | . | . | . | . | . | . | . | . | . | . |
| InternalIMT | epa | rs9282584 | 0.013 | 0.014 | 0.026 | 0.601 | 0.141 | 0.178 | 0.428 | 0.22 | 0.148 | 0.139 |
| InternalIMT | epa | rs17153289 | 0.013 | 0.014 | 0.026 | 0.602 | 0.141 | 0.178 | 0.428 | 0.22 | 0.148 | 0.139 |
| InternalIMT | epa | rs138359103 | 0.001 | . | . | . | . | . | . | . | . | . |
| InternalIMT | epa | rs150605268 | 0.001 | . | . | . | . | . | . | . | . | . |
| InternalIMT | epa | rs79242910 | 0.015 | 0.015 | 0.026 | 0.573 | 0.108 | 0.168 | 0.521 | 0.185 | 0.14 | 0.184 |
| InternalIMT | epa | rs139716904 | 0.004 | . | . | . | . | . | . | . | . | . |
| InternalIMT | epa | rs76238498 | 0.013 | 0.025 | 0.027 | 0.346 | -0.076 | 0.086 | 0.378 | 0.047 | 0.126 | 0.709 |
| InternalIMT | epa | rs184140639 | . | . | . | . | . | . | . | . | . | . |
| InternalIMT | epa | rs188517278 | 0.002 | . | . | . | . | . | . | . | . | . |
| InternalIMT | epa | rs139678913 | 0.001 | . | . | . | . | . | . | . | . | . |
| InternalIMT | epa | rs116697955 | . | . | . | . | . | . | . | . | . | . |
| InternalIMT | epa | rs79267994 | 0.003 | . | . | . | . | . | . | . | . | . |
| InternalIMT | epa | rs11239510 | 0.003 | . | . | . | . | . | . | . | . | . |
| InternalIMT | epa | rs75831645 | 0.006 | . | . | . | . | . | . | . | . | . |
| InternalIMT | epa | rs78452276 | 0.008 | . | . | . | . | . | . | . | . | . |
| InternalIMT | epa | rs144366924 | . | . | . | . | . | . | . | . | . | . |
| InternalIMT | epa | rs77519841 | 0.008 | . | . | . | . | . | . | . | . | . |
| InternalIMT | epa | rs76944454 | 0.013 | 0.013 | 0.026 | 0.605 | 0.141 | 0.178 | 0.428 | 0.222 | 0.148 | 0.134 |
| InternalIMT | epa | rs76443855 | 0.013 | 0.025 | 0.027 | 0.347 | -0.075 | 0.086 | 0.383 | 0.049 | 0.126 | 0.697 |
| InternalIMT | epa | rs146613174 | 0.001 | . | . | . | . | . | . | . | . | . |
| InternalIMT | epa | rs181424761 | 0 | . | . | . | . | . | . | . | . | . |
| InternalIMT | epa | rs142119241 | 0 | . | . | . | . | . | . | . | . | . |
| InternalIMT | epa | rs140137954 | 0.006 | . | . | . | . | . | . | . | . | . |
| InternalIMT | epa | rs187319549 | . | . | . | . | . | . | . | . | . | . |
| InternalIMT | epa | rs12241545 | 0.006 | . | . | . | . | . | . | . | . | . |
| InternalIMT | epa | rs10128228 | 0.013 | 0.013 | 0.026 | 0.606 | 0.141 | 0.177 | 0.426 | 0.223 | 0.148 | 0.133 |
| InternalIMT | epa | rs58580249 | 0.003 | . | . | . | . | . | . | . | . | . |

| InternalIMT | epa | rs193148863 | . | . | . | . | . | . | . | . | . | . |
| --- | --- | --- | --- | --- | --- | --- | --- | --- | --- | --- | --- | --- |
| InternalIMT | epa | rs185638946 | . | . | . | . | . | . | . | . | . | . |
| InternalIMT | epa | rs117752921 | 0 | . | . | . | . | . | . | . | . | . |
| InternalIMT | epa | rs180841236 | 0 | . | . | . | . | . | . | . | . | . |
| InternalIMT | epa | rs73283155 | 0.003 | . | . | . | . | . | . | . | . | . |
| InternalIMT | epa | rs76560599 | 0.012 | 0.014 | 0.026 | 0.595 | 0.155 | 0.187 | 0.408 | 0.229 | 0.156 | 0.141 |
| InternalIMT | epa | rs76273620 | 0.012 | 0.014 | 0.026 | 0.595 | 0.155 | 0.187 | 0.408 | 0.229 | 0.156 | 0.141 |
| InternalIMT | epa | rs141432923 | 0.003 | . | . | . | . | . | . | . | . | . |
| InternalIMT | epa | rs113529322 | 0.004 | . | . | . | . | . | . | . | . | . |
| InternalIMT | epa | rs10128306 | 0.013 | 0.013 | 0.026 | 0.607 | 0.141 | 0.177 | 0.425 | 0.224 | 0.148 | 0.13 |
| InternalIMT | epa | rs149691688 | 0.001 | . | . | . | . | . | . | . | . | . |
| InternalIMT | epa | rs75872495 | 0.013 | 0.013 | 0.026 | 0.607 | 0.14 | 0.176 | 0.426 | 0.223 | 0.146 | 0.127 |
| InternalIMT | epa | rs191873960 | 0.001 | . | . | . | . | . | . | . | . | . |
| InternalIMT | epa | rs80155622 | 0.013 | 0.013 | 0.026 | 0.608 | 0.142 | 0.177 | 0.422 | 0.224 | 0.148 | 0.129 |
| InternalIMT | epa | rs113914656 | 0.001 | . | . | . | . | . | . | . | . | . |
| InternalIMT | epa | rs12242840 | 0.013 | 0.013 | 0.026 | 0.607 | 0.145 | 0.178 | 0.415 | 0.224 | 0.148 | 0.132 |
| InternalIMT | epa | rs147824078 | 0.002 | . | . | . | . | . | . | . | . | . |
| InternalIMT | epa | rs59051493 | 0.013 | 0.013 | 0.026 | 0.612 | 0.148 | 0.178 | 0.406 | 0.227 | 0.149 | 0.129 |
| InternalIMT | epa | rs9282588 | 0.001 | . | . | . | . | . | . | . | . | . |
| InternalIMT | epa | rs150215207 | 0.003 | . | . | . | . | . | . | . | . | . |
| InternalIMT | epa | rs138772243 | 0.001 | . | . | . | . | . | . | . | . | . |
| InternalIMT | epa | rs149383244 | 0.002 | . | . | . | . | . | . | . | . | . |
| InternalIMT | epa | rs17522720 | 0.006 | . | . | . | . | . | . | . | . | . |
| InternalIMT | epa | rs79245105 | 0.013 | 0.013 | 0.026 | 0.615 | 0.15 | 0.179 | 0.401 | 0.23 | 0.15 | 0.127 |
| InternalIMT | epa | rs138222133 | 0.001 | . | . | . | . | . | . | . | . | . |
| InternalIMT | epa | rs12247314 | 0.015 | 0.014 | 0.026 | 0.596 | 0.112 | 0.171 | 0.513 | 0.212 | 0.144 | 0.141 |
| InternalIMT | epa | rs12263894 | 0.014 | 0.014 | 0.026 | 0.581 | 0.122 | 0.174 | 0.484 | 0.203 | 0.147 | 0.167 |
| InternalIMT | epa | rs149907880 | 0.002 | . | . | . | . | . | . | . | . | . |
| InternalIMT | epa | rs188153885 | 0.002 | . | . | . | . | . | . | . | . | . |
| InternalIMT | epa | rs73283159 | 0.002 | . | . | . | . | . | . | . | . | . |
| InternalIMT | epa | rs140476687 | . | . | . | . | . | . | . | . | . | . |
| InternalIMT | epa | rs186603148 | . | . | . | . | . | . | . | . | . | . |
| InternalIMT | epa | rs183296063 | 0.001 | . | . | . | . | . | . | . | . | . |
| InternalIMT | epa | rs145733694 | 0.003 | . | . | . | . | . | . | . | . | . |
| InternalIMT | epa | rs189807449 | . | . | . | . | . | . | . | . | . | . |
| InternalIMT | epa | rs12253223 | 0.009 | . | . | . | . | . | . | . | . | . |
| InternalIMT | epa | rs182113637 | . | . | . | . | . | . | . | . | . | . |
| InternalIMT | epa | rs142792099 | 0.002 | . | . | . | . | . | . | . | . | . |
| InternalIMT | epa | rs12249910 | 0.009 | . | . | . | . | . | . | . | . | . |
| InternalIMT | epa | rs149436339 | 0 | . | . | . | . | . | . | . | . | . |
| InternalIMT | epa | rs77632210 | . | . | . | . | . | . | . | . | . | . |
| InternalIMT | epa | rs74130818 | 0.003 | . | . | . | . | . | . | . | . | . |
| InternalIMT | epa | rs187583648 | . | . | . | . | . | . | . | . | . | . |
| InternalIMT | epa | rs191295987 | 0.001 | . | . | . | . | . | . | . | . | . |
| InternalIMT | epa | rs184200686 | 0.002 | . | . | . | . | . | . | . | . | . |
| InternalIMT | epa | rs76117263 | 0.013 | 0.025 | 0.027 | 0.352 | -0.08 | 0.086 | 0.35 | 0.059 | 0.129 | 0.647 |
| InternalIMT | epa | rs185393149 | 0.001 | . | . | . | . | . | . | . | . | . |
| InternalIMT | epa | rs144760014 | 0 | . | . | . | . | . | . | . | . | . |
| InternalIMT | epa | rs7922292 | 0.002 | . | . | . | . | . | . | . | . | . |
| InternalIMT | epa | rs115372844 | 0.003 | . | . | . | . | . | . | . | . | . |
| InternalIMT | epa | rs111460364 | 0.013 | 0.025 | 0.027 | 0.352 | -0.08 | 0.086 | 0.349 | 0.061 | 0.129 | 0.638 |
| InternalIMT | epa | rs182052255 | 0 | . | . | . | . | . | . | . | . | . |
| InternalIMT | epa | rs180983889 | 0.002 | . | . | . | . | . | . | . | . | . |
| InternalIMT | epa | rs139175327 | 0.002 | . | . | . | . | . | . | . | . | . |
| InternalIMT | epa | rs149959620 | 0.004 | . | . | . | . | . | . | . | . | . |
| InternalIMT | epa | rs114653117 | 0.002 | . | . | . | . | . | . | . | . | . |
| InternalIMT | epa | rs144270018 | 0 | . | . | . | . | . | . | . | . | . |
| InternalIMT | epa | rs182723204 | 0.002 | . | . | . | . | . | . | . | . | . |
| InternalIMT | epa | rs57204067 | 0.003 | . | . | . | . | . | . | . | . | . |
| InternalIMT | epa | rs144956226 | 0.009 | . | . | . | . | . | . | . | . | . |
| InternalIMT | epa | rs187842704 | 0.001 | . | . | . | . | . | . | . | . | . |
| InternalIMT | epa | rs184620511 | 0.003 | . | . | . | . | . | . | . | . | . |
| InternalIMT | epa | rs144031303 | 0.005 | . | . | . | . | . | . | . | . | . |
| InternalIMT | epa | rs187526473 | . | . | . | . | . | . | . | . | . | . |
| InternalIMT | epa | rs193002805 | . | . | . | . | . | . | . | . | . | . |
| InternalIMT | epa | rs138707426 | 0.002 | . | . | . | . | . | . | . | . | . |
| InternalIMT | epa | rs142754243 | 0.01 | 0.022 | 0.026 | 0.406 | 0.202 | 0.18 | 0.263 | 0.162 | 0.227 | 0.475 |
| InternalIMT | epa | rs181600331 | 0.002 | . | . | . | . | . | . | . | . | . |
| InternalIMT | epa | rs144421264 | 0.006 | . | . | . | . | . | . | . | . | . |
| InternalIMT | epa | rs184414519 | 0.002 | . | . | . | . | . | . | . | . | . |
| InternalIMT | epa | rs150596349 | 0.012 | 0.025 | 0.027 | 0.352 | -0.107 | 0.091 | 0.237 | 0.08 | 0.122 | 0.508 |
| InternalIMT | epa | rs190753747 | . | . | . | . | . | . | . | . | . | . |
| InternalIMT | epa | rs139560940 | . | . | . | . | . | . | . | . | . | . |

| InternalIMT | epa | rs75640804 | 0.008 | . | . | . | . | . | . | . | . | . |
| --- | --- | --- | --- | --- | --- | --- | --- | --- | --- | --- | --- | --- |
| InternalIMT | epa | rs73283175 | 0.018 | 0.021 | 0.027 | 0.439 | 0.049 | 0.103 | 0.63 | 0.119 | 0.115 | 0.298 |
| InternalIMT | epa | rs143337229 | 0.001 | . | . | . | . | . | . | . | . | . |
| InternalIMT | epa | rs73283177 | 0.01 | 0.022 | 0.026 | 0.405 | 0.2 | 0.182 | 0.272 | 0.155 | 0.229 | 0.499 |
| InternalIMT | epa | rs76053611 | 0.011 | 0.025 | 0.027 | 0.36 | -0.103 | 0.09 | 0.252 | 0.108 | 0.126 | 0.391 |
| InternalIMT | epa | rs116325983 | . | . | . | . | . | . | . | . | . | . |
| InternalIMT | epa | rs115451825 | 0.001 | . | . | . | . | . | . | . | . | . |
| InternalIMT | epa | rs183845246 | 0 | . | . | . | . | . | . | . | . | . |
| InternalIMT | epa | rs79756542 | 0.013 | 0.025 | 0.027 | 0.366 | -0.088 | 0.084 | 0.298 | 0.104 | 0.114 | 0.363 |
| InternalIMT | epa | rs187242860 | . | . | . | . | . | . | . | . | . | . |
| InternalIMT | epa | rs57318980 | 0.01 | 0.022 | 0.026 | 0.4 | 0.193 | 0.183 | 0.292 | 0.136 | 0.233 | 0.558 |
| InternalIMT | epa | rs59969732 | 0.01 | 0.022 | 0.026 | 0.413 | 0.193 | 0.181 | 0.287 | 0.171 | 0.228 | 0.453 |
| InternalIMT | epa | rs143811488 | 0.003 | . | . | . | . | . | . | . | . | . |
| InternalIMT | epa | rs189067644 | 0.002 | . | . | . | . | . | . | . | . | . |
| InternalIMT | epa | rs60449178 | 0.01 | . | . | . | . | . | . | . | . | . |
| InternalIMT | epa | rs59243389 | 0.01 | . | . | . | . | . | . | . | . | . |
| InternalIMT | epa | rs184946540 | 0.009 | . | . | . | . | . | . | . | . | . |
| InternalIMT | epa | rs188685881 | 0 | . | . | . | . | . | . | . | . | . |
| InternalIMT | epa | rs150335797 | 0.002 | . | . | . | . | . | . | . | . | . |
| InternalIMT | epa | rs143879260 | 0.002 | . | . | . | . | . | . | . | . | . |
| InternalIMT | epa | rs186044491 | 0.003 | . | . | . | . | . | . | . | . | . |
| InternalIMT | epa | rs11819084 | 0.004 | . | . | . | . | . | . | . | . | . |
| InternalIMT | epa | rs146915670 | . | . | . | . | . | . | . | . | . | . |
| InternalIMT | epa | rs113995695 | 0.004 | . | . | . | . | . | . | . | . | . |
| InternalIMT | epa | rs143489451 | 0.001 | . | . | . | . | . | . | . | . | . |
| InternalIMT | epa | rs182653839 | . | . | . | . | . | . | . | . | . | . |
| InternalIMT | epa | rs141692564 | 0.002 | . | . | . | . | . | . | . | . | . |
| InternalIMT | epa | rs146281742 | 0.001 | . | . | . | . | . | . | . | . | . |
| InternalIMT | epa | rs192914353 | 0.001 | . | . | . | . | . | . | . | . | . |
| InternalIMT | epa | rs185182848 | 0 | . | . | . | . | . | . | . | . | . |
| InternalIMT | epa | rs28395858 | 0.004 | . | . | . | . | . | . | . | . | . |
| InternalIMT | epa | rs41429645 | 0.01 | . | . | . | . | . | . | . | . | . |
| InternalIMT | epa | rs28395874 | 0.004 | . | . | . | . | . | . | . | . | . |
| InternalIMT | epa | rs189663634 | . | . | . | . | . | . | . | . | . | . |
| InternalIMT | epa | rs77666700 | 0.003 | . | . | . | . | . | . | . | . | . |
| InternalIMT | epa | rs146711717 | 0.001 | . | . | . | . | . | . | . | . | . |
| InternalIMT | epa | rs144904251 | . | . | . | . | . | . | . | . | . | . |
| InternalIMT | epa | rs189893153 | . | . | . | . | . | . | . | . | . | . |
| InternalIMT | epa | rs150447740 | 0.002 | . | . | . | . | . | . | . | . | . |
| InternalIMT | epa | rs143099649 | . | . | . | . | . | . | . | . | . | . |
| InternalIMT | epa | rs191288436 | 0.003 | . | . | . | . | . | . | . | . | . |
| InternalIMT | epa | rs188577862 | 0.001 | . | . | . | . | . | . | . | . | . |
| InternalIMT | epa | rs192695334 | 0.001 | . | . | . | . | . | . | . | . | . |
| InternalIMT | epa | rs138612906 | 0.007 | . | . | . | . | . | . | . | . | . |
| InternalIMT | epa | rs191290051 | 0.006 | . | . | . | . | . | . | . | . | . |
| InternalIMT | epa | rs189631459 | 0 | . | . | . | . | . | . | . | . | . |
| InternalIMT | epa | rs114620874 | 0.002 | . | . | . | . | . | . | . | . | . |
| InternalIMT | epa | rs78246652 | 0.003 | . | . | . | . | . | . | . | . | . |
| InternalIMT | epa | rs79432451 | 0.002 | . | . | . | . | . | . | . | . | . |
| InternalIMT | epa | rs186022585 | . | . | . | . | . | . | . | . | . | . |
| InternalIMT | epa | rs142978891 | 0.002 | . | . | . | . | . | . | . | . | . |
| InternalIMT | epa | rs151086840 | 0.003 | . | . | . | . | . | . | . | . | . |
| InternalIMT | epa | rs183270259 | 0.002 | . | . | . | . | . | . | . | . | . |
| InternalIMT | epa | rs188544289 | 0.002 | . | . | . | . | . | . | . | . | . |
| InternalIMT | epa | rs188500808 | 0 | . | . | . | . | . | . | . | . | . |
| InternalIMT | epa | rs189424589 | 0 | . | . | . | . | . | . | . | . | . |
| InternalIMT | epa | rs78622303 | 0.008 | . | . | . | . | . | . | . | . | . |
| InternalIMT | epa | rs143210835 | 0.002 | . | . | . | . | . | . | . | . | . |
| InternalIMT | epa | rs114761840 | 0.001 | . | . | . | . | . | . | . | . | . |
| InternalIMT | epa | rs185805402 | . | . | . | . | . | . | . | . | . | . |
| InternalIMT | epa | rs139273744 | 0.001 | . | . | . | . | . | . | . | . | . |
| InternalIMT | epa | rs184654150 | 0.001 | . | . | . | . | . | . | . | . | . |
| InternalIMT | epa | rs189069710 | . | . | . | . | . | . | . | . | . | . |
| InternalIMT | epa | rs114752115 | 0.004 | . | . | . | . | . | . | . | . | . |
| InternalIMT | epa | rs150162515 | 0 | . | . | . | . | . | . | . | . | . |
| InternalIMT | epa | rs180982454 | 0.001 | . | . | . | . | . | . | . | . | . |
| InternalIMT | epa | rs184932088 | . | . | . | . | . | . | . | . | . | . |
| InternalIMT | epa | rs181109625 | . | . | . | . | . | . | . | . | . | . |
| InternalIMT | epa | rs138121134 | . | . | . | . | . | . | . | . | . | . |
| InternalIMT | epa | rs183253861 | 0 | . | . | . | . | . | . | . | . | . |
| InternalIMT | epa | rs147073946 | . | . | . | . | . | . | . | . | . | . |
| InternalIMT | epa | rs139938489 | 0.002 | . | . | . | . | . | . | . | . | . |
| InternalIMT | epa | rs146362560 | 0.003 | . | . | . | . | . | . | . | . | . |

| InternalIMT | epa | rs193155956 | 0.001 | . | . | . | . | . | . | . | . | . |
| --- | --- | --- | --- | --- | --- | --- | --- | --- | --- | --- | --- | --- |
| InternalIMT | epa | rs77663092 | 0.001 | . | . | . | . | . | . | . | . | . |
| InternalIMT | epa | rs73285319 | 0.007 | . | . | . | . | . | . | . | . | . |
| InternalIMT | epa | rs184019276 | . | . | . | . | . | . | . | . | . | . |
| InternalIMT | epa | rs28395863 | 0.004 | . | . | . | . | . | . | . | . | . |
| InternalIMT | epa | rs182121645 | 0.004 | . | . | . | . | . | . | . | . | . |
| InternalIMT | epa | rs73285327 | 0.006 | . | . | . | . | . | . | . | . | . |
| InternalIMT | epa | rs149589584 | . | . | . | . | . | . | . | . | . | . |
| InternalIMT | epa | rs148310335 | . | . | . | . | . | . | . | . | . | . |
| InternalIMT | epa | rs187246093 | 0.003 | . | . | . | . | . | . | . | . | . |
| InternalIMT | epa | rs184668894 | . | . | . | . | . | . | . | . | . | . |
| InternalIMT | epa | rs114269961 | . | . | . | . | . | . | . | . | . | . |
| InternalIMT | epa | rs149694374 | 0.005 | . | . | . | . | . | . | . | . | . |
| InternalIMT | epa | rs145284227 | . | . | . | . | . | . | . | . | . | . |
| InternalIMT | epa | rs189983820 | . | . | . | . | . | . | . | . | . | . |
| InternalIMT | epa | rs181200444 | . | . | . | . | . | . | . | . | . | . |
| InternalIMT | epa | rs185486385 | . | . | . | . | . | . | . | . | . | . |
| InternalIMT | epa | rs151198241 | 0.002 | . | . | . | . | . | . | . | . | . |
| InternalIMT | epa | rs189959341 | 0.002 | . | . | . | . | . | . | . | . | . |
| InternalIMT | epa | rs140549257 | 0.002 | . | . | . | . | . | . | . | . | . |
| InternalIMT | epa | rs186453139 | 0.005 | . | . | . | . | . | . | . | . | . |
| InternalIMT | epa | rs180922711 | 0.009 | . | . | . | . | . | . | . | . | . |
| InternalIMT | epa | rs56659903 | 0.002 | . | . | . | . | . | . | . | . | . |
| InternalIMT | epa | rs141202128 | 0.002 | . | . | . | . | . | . | . | . | . |
| InternalIMT | epa | rs59085314 | 0.008 | . | . | . | . | . | . | . | . | . |
| InternalIMT | epa | rs145564996 | . | . | . | . | . | . | . | . | . | . |
| InternalIMT | epa | rs114961009 | 0.002 | . | . | . | . | . | . | . | . | . |
| InternalIMT | epa | rs182294189 | 0.001 | . | . | . | . | . | . | . | . | . |
| InternalIMT | epa | rs187586636 | 0.002 | . | . | . | . | . | . | . | . | . |
| InternalIMT | epa | rs192254907 | 0.005 | . | . | . | . | . | . | . | . | . |
| InternalIMT | epa | rs149145663 | . | . | . | . | . | . | . | . | . | . |
| InternalIMT | epa | rs180713064 | 0.002 | . | . | . | . | . | . | . | . | . |
| InternalIMT | epa | rs183009023 | 0.001 | . | . | . | . | . | . | . | . | . |
| InternalIMT | epa | rs143385347 | 0.004 | . | . | . | . | . | . | . | . | . |
| InternalIMT | epa | rs185786329 | 0.001 | . | . | . | . | . | . | . | . | . |
| InternalIMT | epa | rs192341030 | 0.001 | . | . | . | . | . | . | . | . | . |
| InternalIMT | epa | rs74661429 | 0.002 | . | . | . | . | . | . | . | . | . |
| InternalIMT | epa | rs188034853 | 0 | . | . | . | . | . | . | . | . | . |
| InternalIMT | epa | rs116808430 | 0.005 | . | . | . | . | . | . | . | . | . |
| InternalIMT | epa | rs148469010 | . | . | . | . | . | . | . | . | . | . |
| InternalIMT | epa | rs78621663 | 0.002 | . | . | . | . | . | . | . | . | . |
| InternalIMT | epa | rs192654886 | . | . | . | . | . | . | . | . | . | . |
| InternalIMT | epa | rs145996035 | 0.002 | . | . | . | . | . | . | . | . | . |
| InternalIMT | epa | rs139942226 | 0 | . | . | . | . | . | . | . | . | . |
| InternalIMT | epa | rs113645632 | 0.002 | . | . | . | . | . | . | . | . | . |
| InternalIMT | epa | rs74940661 | 0.005 | . | . | . | . | . | . | . | . | . |
| InternalIMT | epa | rs115355800 | 0.003 | . | . | . | . | . | . | . | . | . |
| InternalIMT | epa | rs150285898 | 0.001 | . | . | . | . | . | . | . | . | . |
| InternalIMT | epa | rs188117932 | . | . | . | . | . | . | . | . | . | . |
| InternalIMT | epa | rs139956566 | 0 | . | . | . | . | . | . | . | . | . |
| InternalIMT | epa | rs147673885 | 0 | . | . | . | . | . | . | . | . | . |
| InternalIMT | epa | rs189127928 | 0.005 | . | . | . | . | . | . | . | . | . |
| InternalIMT | epa | rs187558772 | 0.002 | . | . | . | . | . | . | . | . | . |
| InternalIMT | epa | rs181948587 | 0 | . | . | . | . | . | . | . | . | . |
| InternalIMT | epa | rs189983269 | 0.002 | . | . | . | . | . | . | . | . | . |
| InternalIMT | epa | rs187250069 | 0.002 | . | . | . | . | . | . | . | . | . |
| InternalIMT | epa | rs191485859 | 0.001 | . | . | . | . | . | . | . | . | . |
| InternalIMT | epa | rs185499555 | 0.002 | . | . | . | . | . | . | . | . | . |
| InternalIMT | epa | rs180862110 | 0.003 | . | . | . | . | . | . | . | . | . |
| InternalIMT | epa | rs190472603 | 0.005 | . | . | . | . | . | . | . | . | . |
| InternalIMT | epa | rs189226167 | 0.005 | . | . | . | . | . | . | . | . | . |
| InternalIMT | epa | rs181623256 | 0.002 | . | . | . | . | . | . | . | . | . |
| InternalIMT | epa | rs186079557 | 0.001 | . | . | . | . | . | . | . | . | . |
| InternalIMT | epa | rs147760994 | 0.002 | . | . | . | . | . | . | . | . | . |
| InternalIMT | epa | rs189176167 | 0 | . | . | . | . | . | . | . | . | . |
| InternalIMT | epa | rs117901000 | 0.001 | . | . | . | . | . | . | . | . | . |
| InternalIMT | epa | rs141984888 | 0 | . | . | . | . | . | . | . | . | . |
| InternalIMT | epa | rs192365061 | 0.007 | . | . | . | . | . | . | . | . | . |
| InternalIMT | epa | rs185976038 | 0 | . | . | . | . | . | . | . | . | . |
| InternalIMT | epa | rs184697303 | 0.002 | . | . | . | . | . | . | . | . | . |
| InternalIMT | epa | rs143383132 | 0.002 | . | . | . | . | . | . | . | . | . |
| InternalIMT | epa | rs141873881 | 0.004 | . | . | . | . | . | . | . | . | . |
| InternalIMT | epa | rs185507255 | 0 | . | . | . | . | . | . | . | . | . |

| InternalIMT | epa | rs191379425 | 0.001 | . | . | . | . | . | . | . | . | . |
| --- | --- | --- | --- | --- | --- | --- | --- | --- | --- | --- | --- | --- |
| InternalIMT | epa | rs184199584 | 0.002 | . | . | . | . | . | . | . | . | . |
| InternalIMT | epa | rs183181360 | 0.002 | . | . | . | . | . | . | . | . | . |
| InternalIMT | epa | rs184495673 | 0.001 | . | . | . | . | . | . | . | . | . |
| InternalIMT | epa | rs182946297 | 0.001 | . | . | . | . | . | . | . | . | . |
| InternalIMT | epa | rs187743615 | 0 | . | . | . | . | . | . | . | . | . |
| InternalIMT | epa | rs183259862 | 0.001 | . | . | . | . | . | . | . | . | . |
| InternalIMT | epa | rs112433566 | 0 | . | . | . | . | . | . | . | . | . |
| InternalIMT | epa | rs182351085 | 0.001 | . | . | . | . | . | . | . | . | . |
| InternalIMT | epa | rs146248508 | 0.001 | . | . | . | . | . | . | . | . | . |
| InternalIMT | epa | rs188528439 | 0.001 | . | . | . | . | . | . | . | . | . |
| InternalIMT | epa | rs146167018 | 0.005 | . | . | . | . | . | . | . | . | . |
| InternalIMT | epa | rs192440980 | 0.004 | . | . | . | . | . | . | . | . | . |
| InternalIMT | epa | rs185601882 | 0.001 | . | . | . | . | . | . | . | . | . |
| InternalIMT | epa | rs117272563 | 0.002 | . | . | . | . | . | . | . | . | . |
| InternalIMT | epa | rs10430734 | 0.003 | . | . | . | . | . | . | . | . | . |
| InternalIMT | epa | rs181320952 | 0.001 | . | . | . | . | . | . | . | . | . |
| InternalIMT | epa | rs185480660 | 0.001 | . | . | . | . | . | . | . | . | . |
| InternalIMT | epa | rs188077917 | 0.001 | . | . | . | . | . | . | . | . | . |
| InternalIMT | epa | rs187562953 | 0.002 | . | . | . | . | . | . | . | . | . |
| InternalIMT | epa | rs182044631 | 0 | . | . | . | . | . | . | . | . | . |
| InternalIMT | epa | rs145455596 | 0.001 | . | . | . | . | . | . | . | . | . |
| InternalIMT | epa | rs181475877 | 0.004 | . | . | . | . | . | . | . | . | . |
| InternalIMT | epa | rs149413085 | 0.002 | . | . | . | . | . | . | . | . | . |
| InternalIMT | epa | rs12253656 | 0 | . | . | . | . | . | . | . | . | . |
| InternalIMT | epa | rs182718239 | 0 | . | . | . | . | . | . | . | . | . |
| InternalIMT | epa | rs192435695 | 0.002 | . | . | . | . | . | . | . | . | . |
| InternalIMT | epa | rs12248277 | 0 | . | . | . | . | . | . | . | . | . |
| InternalIMT | epa | rs143521710 | 0.001 | . | . | . | . | . | . | . | . | . |
| InternalIMT | epa | rs144454454 | 0 | . | . | . | . | . | . | . | . | . |
| InternalIMT | epa | rs190441610 | 0.004 | . | . | . | . | . | . | . | . | . |
| InternalIMT | epa | rs114690462 | 0 | . | . | . | . | . | . | . | . | . |
| InternalIMT | epa | rs184541357 | 0.001 | . | . | . | . | . | . | . | . | . |
| InternalIMT | epa | rs142657653 | 0 | . | . | . | . | . | . | . | . | . |
| InternalIMT | epa | rsurf1 | 2.224 | . | . | . | . | . | . | . | . | . |
| InternalIMT | epa | rsten1 | 2.372 | . | . | . | . | . | . | . | . | . |
| InternalIMT | epa | chr10:45852312:D | 0.072 | 0.029 | 0.028 | 0.306 | -0.051 | 0.054 | 0.344 | -0.028 | 0.071 | 0.689 |
| InternalIMT | epa | chr10:45853232:I | 0.086 | 0.034 | 0.029 | 0.242 | -0.11 | 0.055 | 0.046 | -0.063 | 0.071 | 0.369 |
| InternalIMT | epa | chr10:45853234:I | 0.141 | 0.041 | 0.033 | 0.214 | -0.091 | 0.041 | 0.025 | -0.047 | 0.049 | 0.338 |
| InternalIMT | epa | chr10:45854796:D | 0.022 | 0.017 | 0.027 | 0.521 | 0.057 | 0.147 | 0.7 | 0.162 | 0.121 | 0.178 |
| InternalIMT | epa | chr10:45854994:I | 0.025 | 0.018 | 0.027 | 0.514 | 0.037 | 0.155 | 0.812 | 0.126 | 0.13 | 0.332 |
| InternalIMT | epa | chr10:45858290:D | 0.045 | 0.033 | 0.028 | 0.231 | -0.098 | 0.154 | 0.526 | -0.085 | 0.185 | 0.645 |
| InternalIMT | epa | chr10:45858820:I | 0.012 | 0.024 | 0.027 | 0.38 | 0.025 | 0.197 | 0.899 | 0.1 | 0.225 | 0.656 |
| InternalIMT | epa | chr10:45860888:D | 0.072 | 0.011 | 0.03 | 0.717 | -0.02 | 0.077 | 0.796 | 0.097 | 0.087 | 0.265 |
| InternalIMT | epa | chr10:45863913:I | 0.144 | 0.042 | 0.033 | 0.2 | -0.093 | 0.04 | 0.02 | -0.05 | 0.048 | 0.3 |
| InternalIMT | epa | chr10:45866601:D | 0.016 | 0.012 | 0.027 | 0.641 | 0.105 | 0.139 | 0.451 | 0.192 | 0.132 | 0.146 |
| InternalIMT | epa | chr10:45867197:I | 0.005 | . | . | . | . | . | . | . | . | . |
| InternalIMT | epa | chr10:45867538:D | 0.15 | 0.043 | 0.033 | 0.19 | -0.099 | 0.04 | 0.014 | -0.053 | 0.049 | 0.279 |
| InternalIMT | epa | chr10:45868356:D | 0.14 | 0.044 | 0.033 | 0.181 | -0.096 | 0.041 | 0.018 | -0.058 | 0.049 | 0.241 |
| InternalIMT | epa | chr10:45871158:I | 0.145 | 0.042 | 0.033 | 0.203 | -0.095 | 0.039 | 0.016 | -0.049 | 0.048 | 0.304 |
| InternalIMT | epa | chr10:45871637:D | 0.066 | 0.036 | 0.031 | 0.242 | -0.129 | 0.056 | 0.022 | -0.037 | 0.078 | 0.639 |
| InternalIMT | epa | chr10:45871638:I | 0.111 | 0.041 | 0.033 | 0.204 | -0.132 | 0.044 | 2.80E-03 | -0.054 | 0.053 | 0.313 |
| InternalIMT | epa | chr10:45873629:I | 0.139 | 0.039 | 0.032 | 0.229 | -0.1 | 0.041 | 0.015 | -0.045 | 0.05 | 0.365 |
| InternalIMT | epa | chr10:45873701:D | 0.009 | . | . | . | . | . | . | . | . | . |
| InternalIMT | epa | chr10:45874997:I | 0.016 | 0.02 | 0.027 | 0.46 | 0.124 | 0.162 | 0.447 | 0.293 | 0.203 | 0.149 |
| InternalIMT | epa | chr10:45882030:I | 0.028 | 9.70E-03 | 0.027 | 0.717 | 0.106 | 0.149 | 0.475 | 0.186 | 0.131 | 0.156 |
| InternalIMT | epa | chr10:45883774:D | 0.092 | 0.028 | 0.031 | 0.37 | -0.046 | 0.046 | 0.325 | 2.70E-04 | 0.05 | 0.996 |
| InternalIMT | epa | chr10:45885169:D | 0.054 | 0.029 | 0.028 | 0.297 | -0.108 | 0.068 | 0.111 | -0.061 | 0.092 | 0.506 |
| InternalIMT | epa | chr10:45886451:D | 0.701 | 0.014 | 0.077 | 0.856 | 0.014 | 0.041 | 0.734 | 8.30E-03 | 0.05 | 0.869 |
| InternalIMT | epa | chr10:45891800:I | 0.669 | 0.011 | 0.069 | 0.878 | 4.50E-03 | 0.038 | 0.907 | 0.011 | 0.046 | 0.812 |
| InternalIMT | epa | chr10:45904771:I | 0.683 | 0.028 | 0.069 | 0.683 | -6.30E-03 | 0.038 | 0.868 | -1.20E-03 | 0.044 | 0.978 |
| InternalIMT | epa | chr10:45904959:I | 0.083 | 2.30E-03 | 0.029 | 0.937 | 0.05 | 0.079 | 0.53 | 0.127 | 0.089 | 0.152 |
| InternalIMT | epa | chr10:45911028:I | 0.045 | 0.011 | 0.029 | 0.71 | 0.117 | 0.121 | 0.331 | 0.117 | 0.135 | 0.385 |
| InternalIMT | epa | chr10:45911031:I | 0.411 | 0.092 | 0.048 | 0.059 | -0.015 | 0.039 | 0.692 | -0.079 | 0.045 | 0.084 |
| InternalIMT | epa | chr10:45912154:D | 0.086 | 0.027 | 0.029 | 0.359 | -0.04 | 0.054 | 0.466 | 6.20E-03 | 0.061 | 0.92 |
| InternalIMT | epa | chr10:45913885:I | 0.021 | 0.018 | 0.027 | 0.5 | 0.026 | 0.166 | 0.875 | 0.144 | 0.137 | 0.292 |
| InternalIMT | epa | chr10:45914753:D | 0.181 | -1.30E-03 | 0.033 | 0.969 | 0.021 | 0.048 | 0.655 | 0.065 | 0.054 | 0.228 |
| InternalIMT | epa | chr10:45919621 | . | . | . | . | . | . | . | . | . | . |
| InternalIMT | epa | chr10:45921739:I | 0.06 | 0.034 | 0.028 | 0.224 | -0.107 | 0.064 | 0.091 | -0.059 | 0.067 | 0.383 |
| InternalIMT | epa | chr10:45923527:I | 0.054 | 0.041 | 0.029 | 0.155 | -0.069 | 0.089 | 0.436 | -0.159 | 0.118 | 0.18 |
| InternalIMT | epa | chr10:45927001:I | 0.011 | 0.018 | 0.027 | 0.521 | 0.617 | 0.44 | 0.161 | 0.363 | 0.681 | 0.594 |
| InternalIMT | epa | chr10:45930354:I | 0.023 | 0.019 | 0.027 | 0.48 | 0.066 | 0.211 | 0.753 | 0.153 | 0.183 | 0.401 |
| InternalIMT | epa | chr10:45932864:I | 0.046 | 4.40E-03 | 0.028 | 0.873 | 0.194 | 0.179 | 0.278 | 0.262 | 0.175 | 0.135 |

| InternalIMT | epa | chr10:45936163:I | 0.025 | 0.017 | 0.026 | 0.52 | 7.10E-03 | 0.146 | 0.961 | 0.17 | 0.166 | 0.304 |
| --- | --- | --- | --- | --- | --- | --- | --- | --- | --- | --- | --- | --- |
| InternalIMT | epa | chr10:45938095:D | 0.116 | 0.012 | 0.03 | 0.687 | -0.047 | 0.041 | 0.251 | 0.058 | 0.045 | 0.2 |
| InternalIMT | epa | chr10:45938886 | . | . | . | . | . | . | . | . | . | . |
| InternalIMT | epa | chr10:45939320 | . | . | . | . | . | . | . | . | . | . |
| InternalIMT | epa | chr10:45939659 | 0.002 | . | . | . | . | . | . | . | . | . |
| InternalIMT | epa | chr10:45941150:D | 0.021 | 0.02 | 0.027 | 0.465 | -0.034 | 0.154 | 0.825 | 0.089 | 0.129 | 0.489 |
| InternalIMT | epa | chr10:45947128:D | 0.046 | 0.037 | 0.028 | 0.189 | 0.016 | 0.071 | 0.825 | -0.093 | 0.081 | 0.249 |
| InternalIMT | epa | chr10:45950990:I | 0.06 | 0.013 | 0.029 | 0.661 | -0.02 | 0.099 | 0.844 | 0.088 | 0.096 | 0.364 |
| InternalIMT | epa | chr10:45951854:I | 0.02 | 0.03 | 0.027 | 0.272 | -0.119 | 0.104 | 0.254 | -0.093 | 0.129 | 0.474 |
| InternalIMT | epa | chr10:45952670:I | 0.042 | 0.025 | 0.027 | 0.357 | -0.031 | 0.075 | 0.676 | 0.034 | 0.087 | 0.693 |
| InternalIMT | epa | chr10:45956741 | . | . | . | . | . | . | . | . | . | . |
| InternalIMT | epa | chr10:45957247:I | 0.304 | 0.025 | 0.035 | 0.473 | -0.022 | 0.03 | 0.46 | 5.70E-04 | 0.036 | 0.987 |
| InternalIMT | epa | chr10:45958082:I | 0.302 | 0.032 | 0.036 | 0.367 | -0.03 | 0.03 | 0.311 | -0.011 | 0.035 | 0.757 |
| InternalIMT | epa | chr10:45905041:D | 0.039 | 0.018 | 0.028 | 0.527 | -0.052 | 0.079 | 0.506 | 0.072 | 0.077 | 0.354 |
| InternalIMT | epa | chr10:45938871 | . | . | . | . | . | . | . | . | . | . |
| InternalIMT | epa | chr10:45939160 | . | . | . | . | . | . | . | . | . | . |
| InternalIMT | epa | chr10:45869910 | 0.001 | . | . | . | . | . | . | . | . | . |
| InternalIMT | epa | chr10:45876214:D | 0.005 | . | . | . | . | . | . | . | . | . |
| InternalIMT | epa | chr10:45880237:I | 0.014 | 0.025 | 0.027 | 0.371 | -0.085 | 0.071 | 0.228 | 0.06 | 0.074 | 0.412 |
| InternalIMT | epa | chr10:45884371:I | 0.013 | 0.014 | 0.026 | 0.601 | 0.142 | 0.181 | 0.431 | 0.221 | 0.151 | 0.142 |
| InternalIMT | epa | chr10:45891535:D | 0.004 | . | . | . | . | . | . | . | . | . |
| InternalIMT | epa | chr10:45893516:D | 0.013 | 0.013 | 0.026 | 0.605 | 0.141 | 0.178 | 0.428 | 0.222 | 0.148 | 0.135 |
| InternalIMT | epa | chr10:45893782:D | 0.001 | . | . | . | . | . | . | . | . | . |
| InternalIMT | epa | chr10:45895254:I | 0.004 | . | . | . | . | . | . | . | . | . |
| InternalIMT | epa | chr10:45895848:D | 0.011 | 0.021 | 0.027 | 0.437 | 4.70E-03 | 0.142 | 0.973 | 0.131 | 0.098 | 0.18 |
| InternalIMT | epa | chr10:45896316:D | 0.013 | 0.013 | 0.026 | 0.606 | 0.141 | 0.177 | 0.425 | 0.223 | 0.148 | 0.132 |
| InternalIMT | epa | chr10:45896317:D | 0.013 | 0.013 | 0.026 | 0.606 | 0.141 | 0.177 | 0.425 | 0.223 | 0.148 | 0.132 |
| InternalIMT | epa | chr10:45902683:I | 0.006 | . | . | . | . | . | . | . | . | . |
| InternalIMT | epa | chr10:45905003:D | 0.002 | . | . | . | . | . | . | . | . | . |
| InternalIMT | epa | chr10:45927476:D | 0.005 | . | . | . | . | . | . | . | . | . |
| InternalIMT | epa | chr10:45935882 | . | . | . | . | . | . | . | . | . | . |
| InternalIMT | epa | chr10:45936100 | . | . | . | . | . | . | . | . | . | . |
| InternalIMT | epa | chr10:45939553 | 0.004 | . | . | . | . | . | . | . | . | . |
| InternalIMT | epa | chr10:45939631 | . | . | . | . | . | . | . | . | . | . |
| InternalIMT | epa | chr10:45944193:D | 0.003 | . | . | . | . | . | . | . | . | . |
| InternalIMT | epa | chr10:45954030 | . | . | . | . | . | . | . | . | . | . |

Supplemental table S20.

| phen | covar(fa) | rs_id | maf | beta_FA | se_FA | p_FA | beta_SNP | se_SNP | p_SNP | beta_inter | se_inter | p_inter |
| --- | --- | --- | --- | --- | --- | --- | --- | --- | --- | --- | --- | --- |
| InternalIMT | epa | rs76035182 | . | . | . | . | . | . | . | . | . | . |
| InternalIMT | epa | rs12359547 | 0.001 | . | . | . | . | . | . | . | . | . |
| InternalIMT | epa | rs74595484 | . | . | . | . | . | . | . | . | . | . |
| InternalIMT | epa | rs1864415 | 0.873 | 5.30E-04 | 0.081 | 0.995 | 0.016 | 0.032 | 0.612 | -8.20E-03 | 0.047 | 0.86 |
| InternalIMT | epa | rs72788101 | 0.001 | . | . | . | . | . | . | . | . | . |
| InternalIMT | epa | rs11239481 | . | . | . | . | . | . | . | . | . | . |
| InternalIMT | epa | rs72788102 | 0.001 | . | . | . | . | . | . | . | . | . |
| InternalIMT | epa | rs72789705 | 0.001 | . | . | . | . | . | . | . | . | . |
| InternalIMT | epa | rs141529858 | . | . | . | . | . | . | . | . | . | . |
| InternalIMT | epa | rs145376292 | . | . | . | . | . | . | . | . | . | . |
| InternalIMT | epa | rs190418301 | . | . | . | . | . | . | . | . | . | . |
| InternalIMT | epa | rs11239482 | 0.001 | . | . | . | . | . | . | . | . | . |
| InternalIMT | epa | rs61855872 | . | . | . | . | . | . | . | . | . | . |
| InternalIMT | epa | rs148991144 | . | . | . | . | . | . | . | . | . | . |
| InternalIMT | epa | rs191932221 | . | . | . | . | . | . | . | . | . | . |
| InternalIMT | epa | rs55978138 | 0.201 | -2.20E-03 | 0.031 | 0.942 | 4.90E-03 | 0.033 | 0.883 | -0.03 | 0.035 | 0.4 |
| InternalIMT | epa | rs73281167 | . | . | . | . | . | . | . | . | . | . |
| InternalIMT | epa | rs12768010 | 0.2 | -1.10E-03 | 0.03 | 0.97 | 6.90E-03 | 0.034 | 0.838 | -0.033 | 0.035 | 0.352 |
| InternalIMT | epa | rs11239484 | 0.873 | -2.70E-05 | 0.081 | 1 | 0.016 | 0.032 | 0.616 | -7.90E-03 | 0.047 | 0.865 |
| InternalIMT | epa | rs35906360 | 0.201 | -2.20E-03 | 0.031 | 0.942 | 4.90E-03 | 0.033 | 0.884 | -0.03 | 0.035 | 0.4 |
| InternalIMT | epa | rs186250512 | . | . | . | . | . | . | . | . | . | . |
| InternalIMT | epa | rs150810743 | . | . | . | . | . | . | . | . | . | . |
| InternalIMT | epa | rs55965812 | 0.201 | -2.20E-03 | 0.031 | 0.942 | 4.90E-03 | 0.033 | 0.884 | -0.03 | 0.035 | 0.4 |
| InternalIMT | epa | rs56180358 | 0.203 | -2.00E-03 | 0.031 | 0.948 | 5.20E-03 | 0.033 | 0.876 | -0.03 | 0.035 | 0.397 |
| InternalIMT | epa | rs55864145 | 0.2 | -1.10E-03 | 0.03 | 0.97 | 6.90E-03 | 0.034 | 0.838 | -0.033 | 0.035 | 0.352 |
| InternalIMT | epa | rs55986122 | 0.201 | -2.20E-03 | 0.031 | 0.942 | 4.90E-03 | 0.033 | 0.884 | -0.03 | 0.035 | 0.4 |
| InternalIMT | epa | rs7477350 | 0.873 | -1.80E-04 | 0.081 | 0.998 | 0.016 | 0.032 | 0.616 | -7.80E-03 | 0.047 | 0.867 |
| InternalIMT | epa | rs2099170 | 0.201 | -2.20E-03 | 0.031 | 0.942 | 4.90E-03 | 0.033 | 0.883 | -0.03 | 0.035 | 0.4 |
| InternalIMT | epa | rs2082108 | 0.201 | -2.20E-03 | 0.031 | 0.942 | 4.90E-03 | 0.033 | 0.884 | -0.03 | 0.035 | 0.4 |
| InternalIMT | epa | rs55902404 | . | . | . | . | . | . | . | . | . | . |
| InternalIMT | epa | rs58402662 | . | . | . | . | . | . | . | . | . | . |
| InternalIMT | epa | rs11511618 | . | . | . | . | . | . | . | . | . | . |
| InternalIMT | epa | rs1816492 | 0.873 | -2.80E-04 | 0.081 | 0.997 | 0.016 | 0.032 | 0.617 | -7.80E-03 | 0.047 | 0.868 |
| InternalIMT | epa | rs146611093 | . | . | . | . | . | . | . | . | . | . |
| InternalIMT | epa | rs186897689 | . | . | . | . | . | . | . | . | . | . |
| InternalIMT | epa | rs10793616 | 0.873 | -2.80E-04 | 0.081 | 0.997 | 0.016 | 0.032 | 0.617 | -7.70E-03 | 0.047 | 0.868 |
| InternalIMT | epa | rs116700479 | . | . | . | . | . | . | . | . | . | . |
| InternalIMT | epa | rs192535936 | . | . | . | . | . | . | . | . | . | . |
| InternalIMT | epa | rs113363297 | 0.852 | -0.013 | 0.079 | 0.869 | 0.015 | 0.031 | 0.644 | -5.40E-04 | 0.047 | 0.991 |
| InternalIMT | epa | rs12354994 | 0.186 | -4.50E-03 | 0.03 | 0.883 | -6.50E-04 | 0.033 | 0.984 | -0.025 | 0.036 | 0.479 |
| InternalIMT | epa | rs148068329 | 0.059 | -8.40E-03 | 0.026 | 0.751 | -0.088 | 0.045 | 0.052 | -0.044 | 0.078 | 0.575 |
| InternalIMT | epa | rs12356109 | 0.198 | -3.00E-03 | 0.031 | 0.923 | 1.40E-03 | 0.034 | 0.967 | -0.028 | 0.035 | 0.426 |
| InternalIMT | epa | rs55908141 | . | . | . | . | . | . | . | . | . | . |
| InternalIMT | epa | rs12354712 | 0.196 | -3.90E-03 | 0.03 | 0.898 | -1.60E-04 | 0.034 | 0.996 | -0.026 | 0.035 | 0.466 |
| InternalIMT | epa | rs147282684 | . | . | . | . | . | . | . | . | . | . |
| InternalIMT | epa | rs12356140 | 0.194 | -4.30E-03 | 0.031 | 0.888 | -2.90E-03 | 0.035 | 0.934 | -0.025 | 0.036 | 0.487 |
| InternalIMT | epa | rs185476187 | . | . | . | . | . | . | . | . | . | . |
| InternalIMT | epa | rs145720057 | . | . | . | . | . | . | . | . | . | . |
| InternalIMT | epa | rs12354742 | 0.196 | -3.90E-03 | 0.03 | 0.898 | -1.60E-04 | 0.034 | 0.996 | -0.026 | 0.035 | 0.466 |
| InternalIMT | epa | rs180913842 | . | . | . | . | . | . | . | . | . | . |
| InternalIMT | epa | rs142976914 | 0.001 | . | . | . | . | . | . | . | . | . |
| InternalIMT | epa | rs11511619 | 0.198 | -3.00E-03 | 0.031 | 0.923 | 2.40E-03 | 0.034 | 0.944 | -0.028 | 0.035 | 0.427 |
| InternalIMT | epa | rs146702870 | . | . | . | . | . | . | . | . | . | . |
| InternalIMT | epa | rs150287496 | 0.024 | -0.015 | 0.026 | 0.557 | -0.049 | 0.107 | 0.651 | 0.028 | 0.132 | 0.833 |
| InternalIMT | epa | rs190917973 | . | . | . | . | . | . | . | . | . | . |
| InternalIMT | epa | rs11517281 | 0.197 | -3.00E-03 | 0.031 | 0.923 | 1.40E-03 | 0.034 | 0.967 | -0.028 | 0.035 | 0.426 |
| InternalIMT | epa | rs12359299 | 0.208 | -1.30E-03 | 0.031 | 0.966 | -1.50E-03 | 0.033 | 0.963 | -0.031 | 0.035 | 0.386 |
| InternalIMT | epa | rs182307932 | . | . | . | . | . | . | . | . | . | . |
| InternalIMT | epa | rs61855873 | . | . | . | . | . | . | . | . | . | . |
| InternalIMT | epa | rs12359302 | 0.193 | -3.60E-03 | 0.03 | 0.906 | 3.10E-03 | 0.034 | 0.928 | -0.027 | 0.035 | 0.444 |
| InternalIMT | epa | rs143208140 | 0.054 | -8.90E-03 | 0.026 | 0.733 | -0.075 | 0.046 | 0.105 | -0.04 | 0.077 | 0.603 |
| InternalIMT | epa | rs9988764 | 0.858 | 2.20E-03 | 0.075 | 0.977 | 0.014 | 0.031 | 0.655 | -9.20E-03 | 0.044 | 0.834 |
| InternalIMT | epa | rs192875767 | 0.015 | -0.019 | 0.025 | 0.443 | 0.068 | 0.093 | 0.467 | 0.209 | 0.097 | 0.032 |
| InternalIMT | epa | rs147268846 | . | . | . | . | . | . | . | . | . | . |
| InternalIMT | epa | rs11239485 | 0.1 | -8.70E-03 | 0.028 | 0.761 | -7.80E-03 | 0.055 | 0.886 | -0.031 | 0.063 | 0.625 |
| InternalIMT | epa | rs151317885 | 0.66 | -0.042 | 0.045 | 0.35 | 6.20E-03 | 0.027 | 0.816 | 0.021 | 0.034 | 0.548 |
| InternalIMT | epa | rs11499085 | . | . | . | . | . | . | . | . | . | . |
| InternalIMT | epa | rs188130606 | . | . | . | . | . | . | . | . | . | . |
| InternalIMT | epa | rs11511620 | 0.196 | -5.80E-03 | 0.03 | 0.848 | 1.20E-03 | 0.034 | 0.972 | -0.021 | 0.035 | 0.548 |
| InternalIMT | epa | rs181498390 | 0.002 | . | . | . | . | . | . | . | . | . |
| InternalIMT | epa | rs186280701 | . | . | . | . | . | . | . | . | . | . |

| InternalIMT | epa | rs150059185 | 0.082 | 1.50E-03 | 0.025 | 0.951 | 0.014 | 0.043 | 0.747 | -0.099 | 0.062 | 0.113 |
| --- | --- | --- | --- | --- | --- | --- | --- | --- | --- | --- | --- | --- |
| InternalIMT | epa | rs183338707 | . | . | . | . | . | . | . | . | . | . |
| InternalIMT | epa | rs11499038 | 0.006 | . | . | . | . | . | . | . | . | . |
| InternalIMT | epa | rs190699595 | . | . | . | . | . | . | . | . | . | . |
| InternalIMT | epa | rs11523408 | 0.197 | -3.00E-03 | 0.031 | 0.923 | 1.40E-03 | 0.034 | 0.967 | -0.028 | 0.035 | 0.426 |
| InternalIMT | epa | rs188049317 | . | . | . | . | . | . | . | . | . | . |
| InternalIMT | epa | rs144948634 | 0.584 | -0.048 | 0.041 | 0.242 | 0.022 | 0.025 | 0.392 | 0.029 | 0.036 | 0.418 |
| InternalIMT | epa | rs143833576 | 0.016 | -0.011 | 0.025 | 0.646 | 0.047 | 0.091 | 0.609 | -0.075 | 0.129 | 0.56 |
| InternalIMT | epa | rs151239879 | 0.2 | -2.50E-03 | 0.031 | 0.936 | -1.40E-04 | 0.034 | 0.997 | -0.029 | 0.035 | 0.413 |
| InternalIMT | epa | rs7073743 | 0.61 | -0.049 | 0.041 | 0.234 | 0.024 | 0.025 | 0.338 | 0.028 | 0.034 | 0.398 |
| InternalIMT | epa | rs142563349 | . | . | . | . | . | . | . | . | . | . |
| InternalIMT | epa | rs187246403 | . | . | . | . | . | . | . | . | . | . |
| InternalIMT | epa | rs150423942 | 0.197 | -2.90E-03 | 0.031 | 0.924 | 1.80E-03 | 0.034 | 0.958 | -0.028 | 0.035 | 0.425 |
| InternalIMT | epa | rs184706102 | . | . | . | . | . | . | . | . | . | . |
| InternalIMT | epa | rs189626288 | . | . | . | . | . | . | . | . | . | . |
| InternalIMT | epa | rs140749834 | . | . | . | . | . | . | . | . | . | . |
| InternalIMT | epa | rs7078822 | 0.163 | -2.10E-03 | 0.03 | 0.942 | 0.012 | 0.04 | 0.771 | -0.036 | 0.039 | 0.357 |
| InternalIMT | epa | rs186227617 | 0.188 | -2.50E-03 | 0.03 | 0.935 | -4.40E-03 | 0.035 | 0.899 | -0.031 | 0.035 | 0.384 |
| InternalIMT | epa | rs188984368 | 0.024 | 2.60E-03 | 0.024 | 0.913 | -0.019 | 0.165 | 0.908 | -0.382 | 0.406 | 0.347 |
| InternalIMT | epa | rs182078109 | . | . | . | . | . | . | . | . | . | . |
| InternalIMT | epa | rs141456274 | 0.023 | -0.013 | 0.025 | 0.617 | -0.1 | 0.065 | 0.124 | -0.028 | 0.092 | 0.761 |
| InternalIMT | epa | rs146976512 | 0.198 | -3.00E-03 | 0.031 | 0.923 | 1.40E-03 | 0.034 | 0.967 | -0.028 | 0.035 | 0.427 |
| InternalIMT | epa | rs138036816 | . | . | . | . | . | . | . | . | . | . |
| InternalIMT | epa | rs114879777 | . | . | . | . | . | . | . | . | . | . |
| InternalIMT | epa | rs149366475 | . | . | . | . | . | . | . | . | . | . |
| InternalIMT | epa | rs182463043 | . | . | . | . | . | . | . | . | . | . |
| InternalIMT | epa | rs144754632 | 0.055 | -9.00E-03 | 0.026 | 0.732 | -0.076 | 0.045 | 0.09 | -0.037 | 0.076 | 0.625 |
| InternalIMT | epa | rs55717607 | 0.198 | -3.00E-03 | 0.031 | 0.923 | 1.40E-03 | 0.034 | 0.968 | -0.028 | 0.035 | 0.427 |
| InternalIMT | epa | rs187537973 | . | . | . | . | . | . | . | . | . | . |
| InternalIMT | epa | rs148085971 | . | . | . | . | . | . | . | . | . | . |
| InternalIMT | epa | rs11239491 | 0.198 | -3.00E-03 | 0.031 | 0.923 | 1.40E-03 | 0.034 | 0.968 | -0.028 | 0.035 | 0.427 |
| InternalIMT | epa | rs184412184 | . | . | . | . | . | . | . | . | . | . |
| InternalIMT | epa | rs4948998 | 0.868 | -6.60E-03 | 0.079 | 0.933 | 0.019 | 0.031 | 0.55 | -4.20E-03 | 0.046 | 0.928 |
| InternalIMT | epa | rs7076696 | 0.873 | -2.20E-03 | 0.081 | 0.978 | 0.016 | 0.032 | 0.602 | -6.70E-03 | 0.046 | 0.886 |
| InternalIMT | epa | rs11239492 | 0.197 | -2.90E-03 | 0.031 | 0.924 | 1.70E-03 | 0.034 | 0.959 | -0.028 | 0.035 | 0.425 |
| InternalIMT | epa | rs79462470 | . | . | . | . | . | . | . | . | . | . |
| InternalIMT | epa | rs187804654 | . | . | . | . | . | . | . | . | . | . |
| InternalIMT | epa | rs7093687 | 0.198 | -3.00E-03 | 0.031 | 0.923 | 1.40E-03 | 0.034 | 0.968 | -0.028 | 0.035 | 0.427 |
| InternalIMT | epa | rs7081004 | 0.198 | -3.00E-03 | 0.031 | 0.923 | 1.40E-03 | 0.034 | 0.968 | -0.028 | 0.035 | 0.427 |
| InternalIMT | epa | rs7084793 | 0.198 | -3.00E-03 | 0.031 | 0.923 | 1.40E-03 | 0.034 | 0.968 | -0.028 | 0.035 | 0.427 |
| InternalIMT | epa | rs61556080 | . | . | . | . | . | . | . | . | . | . |
| InternalIMT | epa | rs115552064 | . | . | . | . | . | . | . | . | . | . |
| InternalIMT | epa | rs7921503 | 0.198 | -3.00E-03 | 0.031 | 0.923 | 1.40E-03 | 0.034 | 0.968 | -0.028 | 0.035 | 0.427 |
| InternalIMT | epa | rs7909740 | 0.198 | -3.00E-03 | 0.031 | 0.923 | 1.40E-03 | 0.034 | 0.968 | -0.028 | 0.035 | 0.427 |
| InternalIMT | epa | rs7909768 | 0.198 | -3.00E-03 | 0.031 | 0.923 | 1.40E-03 | 0.034 | 0.968 | -0.028 | 0.035 | 0.427 |
| InternalIMT | epa | rs144168004 | . | . | . | . | . | . | . | . | . | . |
| InternalIMT | epa | rs75850448 | . | . | . | . | . | . | . | . | . | . |
| InternalIMT | epa | rs147777574 | . | . | . | . | . | . | . | . | . | . |
| InternalIMT | epa | rs7913488 | 0.198 | -3.00E-03 | 0.031 | 0.923 | 1.40E-03 | 0.034 | 0.968 | -0.028 | 0.035 | 0.427 |
| InternalIMT | epa | rs74390874 | 0.048 | -0.03 | 0.025 | 0.241 | 0.048 | 0.067 | 0.479 | 0.161 | 0.095 | 0.092 |
| InternalIMT | epa | rs141391142 | . | . | . | . | . | . | . | . | . | . |
| InternalIMT | epa | rs115294747 | . | . | . | . | . | . | . | . | . | . |
| InternalIMT | epa | rs148031781 | . | . | . | . | . | . | . | . | . | . |
| InternalIMT | epa | rs181340128 | . | . | . | . | . | . | . | . | . | . |
| InternalIMT | epa | rs189930007 | . | . | . | . | . | . | . | . | . | . |
| InternalIMT | epa | rs12242128 | . | . | . | . | . | . | . | . | . | . |
| InternalIMT | epa | rs78421750 | . | . | . | . | . | . | . | . | . | . |
| InternalIMT | epa | rs114370747 | . | . | . | . | . | . | . | . | . | . |
| InternalIMT | epa | rs12264166 | . | . | . | . | . | . | . | . | . | . |
| InternalIMT | epa | rs182964822 | . | . | . | . | . | . | . | . | . | . |
| InternalIMT | epa | rs67491417 | 0.188 | -7.40E-03 | 0.031 | 0.809 | 1.30E-03 | 0.035 | 0.971 | -0.018 | 0.037 | 0.64 |
| InternalIMT | epa | rs72789720 | . | . | . | . | . | . | . | . | . | . |
| InternalIMT | epa | rs6413416 | 0.198 | -3.00E-03 | 0.031 | 0.923 | 1.40E-03 | 0.034 | 0.968 | -0.028 | 0.035 | 0.427 |
| InternalIMT | epa | rs4986832 | 0.198 | -3.00E-03 | 0.031 | 0.923 | 1.40E-03 | 0.034 | 0.968 | -0.028 | 0.035 | 0.427 |
| InternalIMT | epa | rs144866316 | . | . | . | . | . | . | . | . | . | . |
| InternalIMT | epa | rs187769540 | . | . | . | . | . | . | . | . | . | . |
| InternalIMT | epa | rs6593482 | 0.198 | -2.70E-03 | 0.031 | 0.929 | 1.70E-03 | 0.034 | 0.96 | -0.028 | 0.035 | 0.42 |
| InternalIMT | epa | rs7913948 | 0.199 | -3.30E-03 | 0.031 | 0.913 | -2.10E-05 | 0.034 | 1 | -0.027 | 0.035 | 0.443 |
| InternalIMT | epa | rs77185151 | 0.055 | -9.00E-03 | 0.026 | 0.732 | -0.076 | 0.045 | 0.09 | -0.037 | 0.076 | 0.625 |
| InternalIMT | epa | rs12762303 | 0.198 | -3.00E-03 | 0.031 | 0.923 | 1.40E-03 | 0.034 | 0.968 | -0.028 | 0.035 | 0.427 |
| InternalIMT | epa | rs73283136 | . | . | . | . | . | . | . | . | . | . |
| InternalIMT | epa | rs187180218 | . | . | . | . | . | . | . | . | . | . |
| InternalIMT | epa | rs78137087 | 0.198 | -3.00E-03 | 0.031 | 0.923 | 1.40E-03 | 0.034 | 0.968 | -0.028 | 0.035 | 0.427 |

| InternalIMT | epa | rs72561472 | 0.193 | -3.30E-03 | 0.031 | 0.914 | 8.80E-04 | 0.034 | 0.979 | -0.028 | 0.036 | 0.442 |
| --- | --- | --- | --- | --- | --- | --- | --- | --- | --- | --- | --- | --- |
| InternalIMT | epa | rs4987106 | 0.193 | -4.70E-04 | 0.031 | 0.988 | -6.90E-04 | 0.033 | 0.983 | -0.036 | 0.037 | 0.343 |
| InternalIMT | epa | rs4987105 | 0.198 | -3.00E-03 | 0.031 | 0.923 | 1.40E-03 | 0.034 | 0.967 | -0.028 | 0.035 | 0.427 |
| InternalIMT | epa | rs111916157 | 0.199 | -3.20E-03 | 0.031 | 0.916 | 8.20E-04 | 0.034 | 0.981 | -0.027 | 0.035 | 0.44 |
| InternalIMT | epa | rs111528152 | 0.198 | -3.00E-03 | 0.031 | 0.923 | 1.40E-03 | 0.034 | 0.968 | -0.028 | 0.035 | 0.427 |
| InternalIMT | epa | rs12763738 | 0.197 | -3.00E-03 | 0.031 | 0.922 | 1.40E-03 | 0.034 | 0.968 | -0.028 | 0.035 | 0.427 |
| InternalIMT | epa | rs191675886 | . | . | . | . | . | . | . | . | . | . |
| InternalIMT | epa | rs66489870 | 0.198 | -3.00E-03 | 0.031 | 0.923 | 1.40E-03 | 0.034 | 0.968 | -0.028 | 0.035 | 0.427 |
| InternalIMT | epa | rs71494775 | 0.198 | -3.00E-03 | 0.031 | 0.923 | 1.40E-03 | 0.034 | 0.968 | -0.028 | 0.035 | 0.427 |
| InternalIMT | epa | rs61855875 | . | . | . | . | . | . | . | . | . | . |
| InternalIMT | epa | rs116356837 | . | . | . | . | . | . | . | . | . | . |
| InternalIMT | epa | rs1864414 | 0.198 | -3.00E-03 | 0.031 | 0.923 | 1.40E-03 | 0.034 | 0.968 | -0.028 | 0.035 | 0.427 |
| InternalIMT | epa | rs1864413 | 0.198 | -2.90E-03 | 0.031 | 0.923 | 1.30E-03 | 0.034 | 0.97 | -0.028 | 0.035 | 0.426 |
| InternalIMT | epa | rs139497212 | . | . | . | . | . | . | . | . | . | . |
| InternalIMT | epa | rs892690 | 0.504 | -0.069 | 0.036 | 0.058 | 0.023 | 0.025 | 0.363 | 0.053 | 0.034 | 0.117 |
| InternalIMT | epa | rs1864412 | 0.198 | -3.00E-03 | 0.031 | 0.923 | 1.40E-03 | 0.034 | 0.968 | -0.028 | 0.035 | 0.427 |
| InternalIMT | epa | rs77827771 | . | . | . | . | . | . | . | . | . | . |
| InternalIMT | epa | rs145294963 | . | . | . | . | . | . | . | . | . | . |
| InternalIMT | epa | rs149081327 | . | . | . | . | . | . | . | . | . | . |
| InternalIMT | epa | rs67008983 | 0.198 | -3.00E-03 | 0.031 | 0.923 | 1.40E-03 | 0.034 | 0.968 | -0.028 | 0.035 | 0.427 |
| InternalIMT | epa | rs56150414 | 0.198 | -2.90E-03 | 0.031 | 0.924 | 1.50E-03 | 0.034 | 0.965 | -0.028 | 0.035 | 0.425 |
| InternalIMT | epa | rs55787481 | 0.199 | -2.90E-03 | 0.031 | 0.924 | 1.10E-03 | 0.034 | 0.974 | -0.028 | 0.035 | 0.428 |
| InternalIMT | epa | rs141270101 | . | . | . | . | . | . | . | . | . | . |
| InternalIMT | epa | rs114980075 | . | . | . | . | . | . | . | . | . | . |
| InternalIMT | epa | rs12783095 | 0.198 | -3.00E-03 | 0.031 | 0.923 | 1.40E-03 | 0.034 | 0.968 | -0.028 | 0.035 | 0.427 |
| InternalIMT | epa | rs182342688 | . | . | . | . | . | . | . | . | . | . |
| InternalIMT | epa | rs17157728 | 0.198 | -3.00E-03 | 0.031 | 0.923 | 1.40E-03 | 0.034 | 0.968 | -0.028 | 0.035 | 0.427 |
| InternalIMT | epa | rs114835563 | . | . | . | . | . | . | . | . | . | . |
| InternalIMT | epa | rs3824612 | 0.504 | -0.069 | 0.036 | 0.058 | 0.022 | 0.025 | 0.369 | 0.053 | 0.034 | 0.117 |
| InternalIMT | epa | rs17157733 | 0.196 | -3.60E-03 | 0.03 | 0.907 | 1.40E-03 | 0.034 | 0.968 | -0.027 | 0.035 | 0.45 |
| InternalIMT | epa | rs74128478 | . | . | . | . | . | . | . | . | . | . |
| InternalIMT | epa | rs184973589 | . | . | . | . | . | . | . | . | . | . |
| InternalIMT | epa | rs150479461 | . | . | . | . | . | . | . | . | . | . |
| InternalIMT | epa | rs138310407 | . | . | . | . | . | . | . | . | . | . |
| InternalIMT | epa | rs187426603 | . | . | . | . | . | . | . | . | . | . |
| InternalIMT | epa | rs190843143 | . | . | . | . | . | . | . | . | . | . |
| InternalIMT | epa | rs191883449 | . | . | . | . | . | . | . | . | . | . |
| InternalIMT | epa | rs58238740 | 0.198 | -3.00E-03 | 0.031 | 0.922 | 1.40E-03 | 0.034 | 0.968 | -0.028 | 0.035 | 0.427 |
| InternalIMT | epa | rs3780894 | 0.198 | -3.00E-03 | 0.031 | 0.922 | 1.40E-03 | 0.034 | 0.968 | -0.028 | 0.035 | 0.427 |
| InternalIMT | epa | rs149646626 | . | . | . | . | . | . | . | . | . | . |
| InternalIMT | epa | rs79615212 | . | . | . | . | . | . | . | . | . | . |
| InternalIMT | epa | rs3780895 | 0.197 | -2.90E-03 | 0.031 | 0.924 | 1.70E-03 | 0.034 | 0.959 | -0.028 | 0.035 | 0.425 |
| InternalIMT | epa | rs3780896 | 0.198 | -3.00E-03 | 0.031 | 0.922 | 1.40E-03 | 0.034 | 0.968 | -0.028 | 0.035 | 0.427 |
| InternalIMT | epa | rs142747163 | 0.003 | . | . | . | . | . | . | . | . | . |
| InternalIMT | epa | rs189417584 | . | . | . | . | . | . | . | . | . | . |
| InternalIMT | epa | rs3780897 | 0.197 | -3.00E-03 | 0.031 | 0.923 | 1.40E-03 | 0.034 | 0.967 | -0.028 | 0.035 | 0.427 |
| InternalIMT | epa | rs117509491 | . | . | . | . | . | . | . | . | . | . |
| InternalIMT | epa | rs117811514 | . | . | . | . | . | . | . | . | . | . |
| InternalIMT | epa | rs116500932 | . | . | . | . | . | . | . | . | . | . |
| InternalIMT | epa | rs77584168 | 0.014 | -0.013 | 0.025 | 0.598 | -0.095 | 0.115 | 0.411 | 3.60E-03 | 0.148 | 0.98 |
| InternalIMT | epa | rs2310776 | 0.198 | -3.00E-03 | 0.031 | 0.922 | 1.40E-03 | 0.034 | 0.968 | -0.028 | 0.035 | 0.427 |
| InternalIMT | epa | rs17157736 | 0.198 | -3.00E-03 | 0.031 | 0.922 | 1.40E-03 | 0.034 | 0.968 | -0.028 | 0.035 | 0.427 |
| InternalIMT | epa | rs72789735 | 0.001 | . | . | . | . | . | . | . | . | . |
| InternalIMT | epa | rs183747187 | . | . | . | . | . | . | . | . | . | . |
| InternalIMT | epa | rs7077891 | 0.198 | -3.00E-03 | 0.031 | 0.922 | 1.40E-03 | 0.034 | 0.968 | -0.028 | 0.035 | 0.427 |
| InternalIMT | epa | rs145063305 | 0.001 | . | . | . | . | . | . | . | . | . |
| InternalIMT | epa | rs3824613 | 0.198 | -3.20E-03 | 0.031 | 0.916 | 1.90E-03 | 0.034 | 0.954 | -0.027 | 0.035 | 0.44 |
| InternalIMT | epa | rs145543199 | . | . | . | . | . | . | . | . | . | . |
| InternalIMT | epa | rs140361993 | . | . | . | . | . | . | . | . | . | . |
| InternalIMT | epa | rs11239496 | 0.001 | . | . | . | . | . | . | . | . | . |
| InternalIMT | epa | rs11239497 | 0.197 | -3.00E-03 | 0.031 | 0.922 | 1.40E-03 | 0.034 | 0.968 | -0.028 | 0.035 | 0.427 |
| InternalIMT | epa | rs10900212 | 0.198 | -2.90E-03 | 0.031 | 0.923 | 1.50E-03 | 0.034 | 0.965 | -0.028 | 0.035 | 0.426 |
| InternalIMT | epa | rs186487136 | . | . | . | . | . | . | . | . | . | . |
| InternalIMT | epa | rs12774369 | 0.197 | -3.00E-03 | 0.031 | 0.923 | 1.50E-03 | 0.034 | 0.965 | -0.028 | 0.035 | 0.427 |
| InternalIMT | epa | rs60842538 | 0.197 | -3.00E-03 | 0.031 | 0.923 | 1.60E-03 | 0.034 | 0.963 | -0.028 | 0.035 | 0.427 |
| InternalIMT | epa | rs3780898 | 0.197 | -3.00E-03 | 0.031 | 0.923 | 1.70E-03 | 0.034 | 0.96 | -0.028 | 0.035 | 0.426 |
| InternalIMT | epa | rs3780899 | 0.197 | -2.90E-03 | 0.031 | 0.923 | 1.80E-03 | 0.034 | 0.958 | -0.028 | 0.035 | 0.426 |
| InternalIMT | epa | rs12252472 | . | . | . | . | . | . | . | . | . | . |
| InternalIMT | epa | rs185411011 | . | . | . | . | . | . | . | . | . | . |
| InternalIMT | epa | rs28395865 | 0.193 | -3.40E-03 | 0.031 | 0.912 | 1.60E-03 | 0.034 | 0.962 | -0.027 | 0.036 | 0.442 |
| InternalIMT | epa | rs191004074 | . | . | . | . | . | . | . | . | . | . |
| InternalIMT | epa | rs182401295 | . | . | . | . | . | . | . | . | . | . |
| InternalIMT | epa | rs2228064 | 0.057 | -9.10E-03 | 0.026 | 0.729 | -0.079 | 0.045 | 0.077 | -0.035 | 0.077 | 0.648 |

| InternalIMT | epa | rs188284159 | . | . | . | . | . | . | . | . | . | . |
| --- | --- | --- | --- | --- | --- | --- | --- | --- | --- | --- | --- | --- |
| InternalIMT | epa | rs2303887 | 0.055 | -9.00E-03 | 0.026 | 0.731 | -0.076 | 0.045 | 0.089 | -0.037 | 0.076 | 0.63 |
| InternalIMT | epa | rs2303886 | 0.055 | -9.00E-03 | 0.026 | 0.731 | -0.076 | 0.045 | 0.089 | -0.037 | 0.076 | 0.63 |
| InternalIMT | epa | rs192621006 | . | . | . | . | . | . | . | . | . | . |
| InternalIMT | epa | rs2303885 | 0.195 | -5.90E-03 | 0.03 | 0.846 | 1.10E-03 | 0.034 | 0.974 | -0.021 | 0.035 | 0.552 |
| InternalIMT | epa | rs114466168 | . | . | . | . | . | . | . | . | . | . |
| InternalIMT | epa | rs11239501 | 0.003 | . | . | . | . | . | . | . | . | . |
| InternalIMT | epa | rs745986 | 0.196 | -4.30E-03 | 0.03 | 0.889 | 9.40E-03 | 0.036 | 0.796 | -0.025 | 0.035 | 0.477 |
| InternalIMT | epa | rs75262268 | . | . | . | . | . | . | . | . | . | . |
| InternalIMT | epa | rs71494776 | 0.192 | -4.80E-03 | 0.03 | 0.873 | 3.50E-03 | 0.035 | 0.918 | -0.024 | 0.035 | 0.495 |
| InternalIMT | epa | rs74386871 | 0.055 | -9.00E-03 | 0.026 | 0.731 | -0.076 | 0.045 | 0.089 | -0.037 | 0.076 | 0.632 |
| InternalIMT | epa | rs76502040 | . | . | . | . | . | . | . | . | . | . |
| InternalIMT | epa | rs142213734 | . | . | . | . | . | . | . | . | . | . |
| InternalIMT | epa | rs4948671 | 0.869 | -0.028 | 0.079 | 0.727 | 0.012 | 0.03 | 0.687 | 7.70E-03 | 0.045 | 0.865 |
| InternalIMT | epa | rs146286085 | . | . | . | . | . | . | . | . | . | . |
| InternalIMT | epa | rs144080623 | . | . | . | . | . | . | . | . | . | . |
| InternalIMT | epa | rs115074014 | . | . | . | . | . | . | . | . | . | . |
| InternalIMT | epa | rs17157756 | . | . | . | . | . | . | . | . | . | . |
| InternalIMT | epa | rs190459780 | . | . | . | . | . | . | . | . | . | . |
| InternalIMT | epa | rs12358647 | 0.195 | -4.80E-03 | 0.03 | 0.873 | 9.80E-03 | 0.037 | 0.79 | -0.024 | 0.035 | 0.501 |
| InternalIMT | epa | rs147080556 | 0.009 | . | . | . | . | . | . | . | . | . |
| InternalIMT | epa | rs187543349 | . | . | . | . | . | . | . | . | . | . |
| InternalIMT | epa | rs114375595 | 0.01 | . | . | . | . | . | . | . | . | . |
| InternalIMT | epa | rs117211200 | . | . | . | . | . | . | . | . | . | . |
| InternalIMT | epa | rs192963047 | . | . | . | . | . | . | . | . | . | . |
| InternalIMT | epa | rs11239505 | 0.196 | -4.20E-03 | 0.03 | 0.89 | 9.70E-03 | 0.036 | 0.788 | -0.025 | 0.035 | 0.476 |
| InternalIMT | epa | rs184434116 | . | . | . | . | . | . | . | . | . | . |
| InternalIMT | epa | rs4948999 | 0.869 | -0.028 | 0.079 | 0.725 | 0.012 | 0.03 | 0.689 | 7.80E-03 | 0.045 | 0.864 |
| InternalIMT | epa | rs11239506 | 0.003 | . | . | . | . | . | . | . | . | . |
| InternalIMT | epa | rs190706620 | . | . | . | . | . | . | . | . | . | . |
| InternalIMT | epa | rs190770429 | . | . | . | . | . | . | . | . | . | . |
| InternalIMT | epa | rs181327133 | . | . | . | . | . | . | . | . | . | . |
| InternalIMT | epa | rs149251697 | . | . | . | . | . | . | . | . | . | . |
| InternalIMT | epa | rs118081602 | 0.055 | -9.00E-03 | 0.026 | 0.731 | -0.076 | 0.045 | 0.089 | -0.037 | 0.076 | 0.632 |
| InternalIMT | epa | rs188233691 | . | . | . | . | . | . | . | . | . | . |
| InternalIMT | epa | rs183778794 | 0.032 | -1.50E-03 | 0.025 | 0.953 | -0.032 | 0.077 | 0.673 | -0.203 | 0.11 | 0.064 |
| InternalIMT | epa | rs188040078 | . | . | . | . | . | . | . | . | . | . |
| InternalIMT | epa | rs184675372 | . | . | . | . | . | . | . | . | . | . |
| InternalIMT | epa | rs150326891 | 0.004 | . | . | . | . | . | . | . | . | . |
| InternalIMT | epa | rs185520021 | . | . | . | . | . | . | . | . | . | . |
| InternalIMT | epa | rs7907779 | 0.003 | . | . | . | . | . | . | . | . | . |
| InternalIMT | epa | rs184962860 | . | . | . | . | . | . | . | . | . | . |
| InternalIMT | epa | rs149762784 | 0.027 | 1.10E-03 | 0.024 | 0.963 | -0.051 | 0.134 | 0.703 | -0.314 | 0.329 | 0.34 |
| InternalIMT | epa | rs149203427 | . | . | . | . | . | . | . | . | . | . |
| InternalIMT | epa | rs60153083 | . | . | . | . | . | . | . | . | . | . |
| InternalIMT | epa | rs112001069 | 0.194 | -7.00E-03 | 0.03 | 0.817 | -2.30E-03 | 0.034 | 0.946 | -0.019 | 0.035 | 0.599 |
| InternalIMT | epa | rs182355382 | 0.012 | -4.80E-03 | 0.023 | 0.833 | 0.027 | 0.189 | 0.888 | -0.387 | 0.484 | 0.425 |
| InternalIMT | epa | rs186740974 | 0.109 | 4.50E-03 | 0.031 | 0.882 | 0.056 | 0.057 | 0.323 | -0.095 | 0.08 | 0.237 |
| InternalIMT | epa | rs191874916 | 0.081 | 1.50E-05 | 0.025 | 1 | 0.013 | 0.043 | 0.757 | -0.09 | 0.063 | 0.15 |
| InternalIMT | epa | rs182989412 | 0.122 | -7.30E-03 | 0.03 | 0.81 | 0.078 | 0.064 | 0.225 | -0.04 | 0.084 | 0.638 |
| InternalIMT | epa | rs188819476 | . | . | . | . | . | . | . | . | . | . |
| InternalIMT | epa | rs141217183 | 0.186 | -2.50E-03 | 0.031 | 0.936 | 6.40E-03 | 0.037 | 0.862 | -0.031 | 0.038 | 0.413 |
| InternalIMT | epa | rs192922652 | 0.199 | -4.90E-03 | 0.031 | 0.873 | 1.90E-03 | 0.034 | 0.955 | -0.023 | 0.035 | 0.509 |
| InternalIMT | epa | rs185092343 | 0.012 | -0.017 | 0.024 | 0.491 | 0.353 | 0.306 | 0.249 | -0.012 | 0.294 | 0.968 |
| InternalIMT | epa | rs145193311 | 0.194 | -5.20E-03 | 0.03 | 0.863 | 3.90E-03 | 0.035 | 0.911 | -0.023 | 0.035 | 0.516 |
| InternalIMT | epa | rs55789336 | 0.004 | . | . | . | . | . | . | . | . | . |
| InternalIMT | epa | rs55837212 | 0.009 | . | . | . | . | . | . | . | . | . |
| InternalIMT | epa | rs75408574 | 0.565 | -0.056 | 0.04 | 0.167 | 8.20E-03 | 0.026 | 0.754 | 0.037 | 0.031 | 0.235 |
| InternalIMT | epa | rs149306926 | 0.01 | -0.011 | 0.025 | 0.664 | 0.213 | 0.309 | 0.492 | -0.105 | 0.265 | 0.692 |
| InternalIMT | epa | rs35990892 | 0.843 | -0.044 | 0.095 | 0.642 | 6.70E-03 | 0.032 | 0.834 | 0.018 | 0.051 | 0.725 |
| InternalIMT | epa | rs192356207 | 0.015 | 3.70E-03 | 0.022 | 0.869 | -0.149 | 0.164 | 0.366 | -0.651 | 0.407 | 0.11 |
| InternalIMT | epa | rs183741163 | 0.049 | -0.013 | 0.028 | 0.646 | 0.044 | 0.116 | 0.703 | -0.012 | 0.07 | 0.862 |
| InternalIMT | epa | rs34737297 | 0.719 | -0.037 | 0.082 | 0.65 | -0.016 | 0.032 | 0.616 | 0.016 | 0.051 | 0.747 |
| InternalIMT | epa | rs181346933 | . | . | . | . | . | . | . | . | . | . |
| InternalIMT | epa | rs36093253 | 0.576 | -0.036 | 0.043 | 0.393 | -2.30E-03 | 0.029 | 0.937 | 0.019 | 0.03 | 0.528 |
| InternalIMT | epa | rs186307855 | . | . | . | . | . | . | . | . | . | . |
| InternalIMT | epa | rs189681865 | . | . | . | . | . | . | . | . | . | . |
| InternalIMT | epa | rs34942971 | 0.214 | -1.60E-03 | 0.031 | 0.958 | 7.10E-03 | 0.035 | 0.841 | -0.03 | 0.036 | 0.402 |
| InternalIMT | epa | rs181816554 | . | . | . | . | . | . | . | . | . | . |
| InternalIMT | epa | rs186846881 | . | . | . | . | . | . | . | . | . | . |
| InternalIMT | epa | rs191178220 | 0.003 | . | . | . | . | . | . | . | . | . |
| InternalIMT | epa | rs183305228 | . | . | . | . | . | . | . | . | . | . |
| InternalIMT | epa | rs139912761 | 0.004 | . | . | . | . | . | . | . | . | . |

| InternalIMT | epa | rs185499901 | . | . | . | . | . | . | . | . | . | . |
| --- | --- | --- | --- | --- | --- | --- | --- | --- | --- | --- | --- | --- |
| InternalIMT | epa | rs189816197 | 0.194 | -5.50E-03 | 0.031 | 0.857 | 4.20E-03 | 0.035 | 0.905 | -0.022 | 0.036 | 0.546 |
| InternalIMT | epa | rs145656938 | 0.009 | . | . | . | . | . | . | . | . | . |
| InternalIMT | epa | rs35745197 | 0.193 | -4.80E-03 | 0.03 | 0.875 | 3.20E-03 | 0.035 | 0.927 | -0.024 | 0.035 | 0.492 |
| InternalIMT | epa | rs192856637 | 0.006 | . | . | . | . | . | . | . | . | . |
| InternalIMT | epa | rs2310775 | 0.617 | -0.052 | 0.041 | 0.208 | 0.014 | 0.026 | 0.579 | 0.03 | 0.033 | 0.359 |
| InternalIMT | epa | rs11239507 | 0.673 | -0.046 | 0.045 | 0.307 | -1.30E-03 | 0.028 | 0.963 | 0.023 | 0.034 | 0.487 |
| InternalIMT | epa | rs74648467 | 0.192 | -4.90E-03 | 0.03 | 0.872 | 3.20E-03 | 0.035 | 0.927 | -0.024 | 0.035 | 0.496 |
| InternalIMT | epa | rs145815934 | . | . | . | . | . | . | . | . | . | . |
| InternalIMT | epa | rs145390687 | . | . | . | . | . | . | . | . | . | . |
| InternalIMT | epa | rs184354913 | . | . | . | . | . | . | . | . | . | . |
| InternalIMT | epa | rs181244920 | . | . | . | . | . | . | . | . | . | . |
| InternalIMT | epa | rs11499087 | . | . | . | . | . | . | . | . | . | . |
| InternalIMT | epa | rs148263794 | 0.207 | -2.00E-03 | 0.031 | 0.948 | 5.60E-03 | 0.034 | 0.869 | -0.03 | 0.036 | 0.405 |
| InternalIMT | epa | rs150758903 | . | . | . | . | . | . | . | . | . | . |
| InternalIMT | epa | rs147654382 | 0.187 | -8.90E-03 | 0.03 | 0.769 | 4.20E-03 | 0.038 | 0.912 | -0.014 | 0.037 | 0.705 |
| InternalIMT | epa | rs147495166 | . | . | . | . | . | . | . | . | . | . |
| InternalIMT | epa | rs35198778 | 0.131 | 2.10E-03 | 0.03 | 0.944 | -0.017 | 0.048 | 0.723 | -0.064 | 0.049 | 0.192 |
| InternalIMT | epa | rs145207659 | 0.048 | -0.03 | 0.025 | 0.243 | 0.049 | 0.068 | 0.469 | 0.16 | 0.096 | 0.098 |
| InternalIMT | epa | rs186466464 | . | . | . | . | . | . | . | . | . | . |
| InternalIMT | epa | rs12778413 | 0.131 | -6.00E-03 | 0.03 | 0.841 | 0.031 | 0.051 | 0.541 | -0.031 | 0.049 | 0.526 |
| InternalIMT | epa | rs7895552 | 0.833 | -0.03 | 0.07 | 0.667 | 0.015 | 0.03 | 0.61 | 9.60E-03 | 0.043 | 0.821 |
| InternalIMT | epa | rs12783895 | 0.193 | -4.80E-03 | 0.03 | 0.874 | 3.00E-03 | 0.034 | 0.93 | -0.024 | 0.035 | 0.492 |
| InternalIMT | epa | rs34929604 | 0.193 | -4.80E-03 | 0.03 | 0.874 | 3.00E-03 | 0.034 | 0.93 | -0.024 | 0.035 | 0.492 |
| InternalIMT | epa | rs12761961 | 0.185 | -6.00E-03 | 0.03 | 0.842 | 6.50E-03 | 0.037 | 0.86 | -0.022 | 0.036 | 0.55 |
| InternalIMT | epa | rs35536930 | 0.197 | -4.30E-03 | 0.03 | 0.887 | 0.01 | 0.036 | 0.781 | -0.025 | 0.035 | 0.48 |
| InternalIMT | epa | rs180951060 | . | . | . | . | . | . | . | . | . | . |
| InternalIMT | epa | rs148892128 | . | . | . | . | . | . | . | . | . | . |
| InternalIMT | epa | rs11239509 | . | . | . | . | . | . | . | . | . | . |
| InternalIMT | epa | rs41526545 | 0.003 | . | . | . | . | . | . | . | . | . |
| InternalIMT | epa | rs2029253 | 0.617 | -0.052 | 0.041 | 0.208 | 0.014 | 0.026 | 0.58 | 0.03 | 0.033 | 0.358 |
| InternalIMT | epa | rs17444064 | 0.003 | . | . | . | . | . | . | . | . | . |
| InternalIMT | epa | rs71494779 | 0.194 | -4.70E-03 | 0.03 | 0.877 | 3.70E-03 | 0.034 | 0.913 | -0.024 | 0.035 | 0.488 |
| InternalIMT | epa | rs74991420 | 0.05 | -8.80E-03 | 0.026 | 0.736 | -0.082 | 0.048 | 0.087 | -0.045 | 0.089 | 0.614 |
| InternalIMT | epa | rs182736551 | 0.05 | -8.80E-03 | 0.026 | 0.736 | -0.082 | 0.048 | 0.087 | -0.045 | 0.089 | 0.614 |
| InternalIMT | epa | rs78702074 | 0.055 | -9.00E-03 | 0.026 | 0.731 | -0.076 | 0.045 | 0.095 | -0.037 | 0.077 | 0.634 |
| InternalIMT | epa | rs187791466 | . | . | . | . | . | . | . | . | . | . |
| InternalIMT | epa | rs35300270 | 0.194 | -4.70E-03 | 0.03 | 0.877 | 3.70E-03 | 0.034 | 0.913 | -0.024 | 0.035 | 0.488 |
| InternalIMT | epa | rs6593484 | 0.003 | . | . | . | . | . | . | . | . | . |
| InternalIMT | epa | rs12244601 | . | . | . | . | . | . | . | . | . | . |
| InternalIMT | epa | rs184429574 | . | . | . | . | . | . | . | . | . | . |
| InternalIMT | epa | rs7080897 | 0.194 | -4.70E-03 | 0.03 | 0.876 | 3.80E-03 | 0.034 | 0.913 | -0.024 | 0.035 | 0.488 |
| InternalIMT | epa | rs7097429 | 0.196 | -3.90E-03 | 0.031 | 0.899 | -4.20E-04 | 0.035 | 0.99 | -0.027 | 0.039 | 0.487 |
| InternalIMT | epa | rs77979229 | 0.016 | -0.011 | 0.025 | 0.669 | 0.038 | 0.091 | 0.675 | -0.096 | 0.119 | 0.419 |
| InternalIMT | epa | rs11239512 | 0.003 | . | . | . | . | . | . | . | . | . |
| InternalIMT | epa | rs60146827 | 0.195 | -4.60E-03 | 0.03 | 0.88 | 3.20E-03 | 0.034 | 0.925 | -0.025 | 0.035 | 0.483 |
| InternalIMT | epa | rs79032236 | . | . | . | . | . | . | . | . | . | . |
| InternalIMT | epa | rs140854206 | . | . | . | . | . | . | . | . | . | . |
| InternalIMT | epa | rs184049550 | . | . | . | . | . | . | . | . | . | . |
| InternalIMT | epa | rs114433607 | . | . | . | . | . | . | . | . | . | . |
| InternalIMT | epa | rs117653793 | 0.055 | -9.00E-03 | 0.026 | 0.731 | -0.076 | 0.045 | 0.096 | -0.037 | 0.077 | 0.635 |
| InternalIMT | epa | rs117039533 | 0.055 | -9.00E-03 | 0.026 | 0.731 | -0.076 | 0.045 | 0.096 | -0.037 | 0.077 | 0.635 |
| InternalIMT | epa | rs191660761 | 0.001 | . | . | . | . | . | . | . | . | . |
| InternalIMT | epa | rs59514896 | 0.197 | -4.30E-03 | 0.03 | 0.888 | 1.60E-03 | 0.034 | 0.961 | -0.025 | 0.035 | 0.473 |
| InternalIMT | epa | rs184661876 | . | . | . | . | . | . | . | . | . | . |
| InternalIMT | epa | rs61854073 | . | . | . | . | . | . | . | . | . | . |
| InternalIMT | epa | rs2018518 | 0.5 | -0.062 | 0.038 | 0.107 | 8.60E-03 | 0.026 | 0.74 | 0.046 | 0.037 | 0.212 |
| InternalIMT | epa | rs934187 | 0.872 | -0.03 | 0.079 | 0.707 | 0.011 | 0.03 | 0.707 | 8.90E-03 | 0.045 | 0.844 |
| InternalIMT | epa | rs7918542 | 0.202 | -3.70E-03 | 0.031 | 0.904 | 7.50E-03 | 0.035 | 0.832 | -0.026 | 0.035 | 0.456 |
| InternalIMT | epa | rs144311920 | . | . | . | . | . | . | . | . | . | . |
| InternalIMT | epa | rs7099684 | 0.871 | -0.031 | 0.079 | 0.692 | 0.01 | 0.03 | 0.731 | 9.80E-03 | 0.045 | 0.829 |
| InternalIMT | epa | rs150829171 | . | . | . | . | . | . | . | . | . | . |
| InternalIMT | epa | rs186060697 | . | . | . | . | . | . | . | . | . | . |
| InternalIMT | epa | rs11239513 | . | . | . | . | . | . | . | . | . | . |
| InternalIMT | epa | rs7917687 | 0.2 | -3.50E-03 | 0.03 | 0.907 | -3.60E-04 | 0.033 | 0.991 | -0.027 | 0.035 | 0.444 |
| InternalIMT | epa | rs61854076 | . | . | . | . | . | . | . | . | . | . |
| InternalIMT | epa | rs71494780 | . | . | . | . | . | . | . | . | . | . |
| InternalIMT | epa | rs7919239 | 0.235 | -5.00E-03 | 0.031 | 0.87 | 8.00E-03 | 0.028 | 0.773 | -0.019 | 0.03 | 0.535 |
| InternalIMT | epa | rs78334475 | 0.166 | -0.037 | 0.033 | 0.256 | 3.30E-03 | 0.037 | 0.929 | 0.062 | 0.043 | 0.153 |
| InternalIMT | epa | rs3780900 | 0.068 | -6.50E-03 | 0.026 | 0.8 | 0.042 | 0.051 | 0.409 | -0.062 | 0.074 | 0.407 |
| InternalIMT | epa | rs186729292 | . | . | . | . | . | . | . | . | . | . |
| InternalIMT | epa | rs12267438 | . | . | . | . | . | . | . | . | . | . |
| InternalIMT | epa | rs1369214 | 0.868 | -0.034 | 0.079 | 0.664 | 1.40E-03 | 0.032 | 0.965 | 0.012 | 0.045 | 0.799 |

| InternalIMT | epa | rs9282589 | . | . | . | . | . | . | . | . | . | . |
| --- | --- | --- | --- | --- | --- | --- | --- | --- | --- | --- | --- | --- |
| InternalIMT | epa | rs114522915 | . | . | . | . | . | . | . | . | . | . |
| InternalIMT | epa | rs2115819 | 0.812 | -0.052 | 0.063 | 0.409 | 0.026 | 0.026 | 0.317 | 0.024 | 0.039 | 0.542 |
| InternalIMT | epa | rs117664603 | 0.055 | -9.00E-03 | 0.026 | 0.731 | -0.077 | 0.045 | 0.086 | -0.037 | 0.076 | 0.629 |
| InternalIMT | epa | rs184979202 | . | . | . | . | . | . | . | . | . | . |
| InternalIMT | epa | rs190714671 | . | . | . | . | . | . | . | . | . | . |
| InternalIMT | epa | rs141538996 | . | . | . | . | . | . | . | . | . | . |
| InternalIMT | epa | rs150881741 | . | . | . | . | . | . | . | . | . | . |
| InternalIMT | epa | rs139076633 | 0.011 | -0.012 | 0.025 | 0.632 | -0.103 | 0.1 | 0.304 | -0.14 | 0.168 | 0.405 |
| InternalIMT | epa | rs12243649 | 0.056 | -8.60E-03 | 0.026 | 0.742 | -0.075 | 0.044 | 0.092 | -0.04 | 0.076 | 0.597 |
| InternalIMT | epa | rs140348708 | . | . | . | . | . | . | . | . | . | . |
| InternalIMT | epa | rs79023256 | 0.055 | -9.00E-03 | 0.026 | 0.732 | -0.077 | 0.045 | 0.085 | -0.037 | 0.076 | 0.626 |
| InternalIMT | epa | rs11239515 | 0.003 | . | . | . | . | . | . | . | . | . |
| InternalIMT | epa | rs55866086 | 0.059 | -8.30E-03 | 0.026 | 0.754 | -0.052 | 0.047 | 0.273 | -0.043 | 0.072 | 0.548 |
| InternalIMT | epa | rs181340150 | . | . | . | . | . | . | . | . | . | . |
| InternalIMT | epa | rs11239516 | 0.003 | . | . | . | . | . | . | . | . | . |
| InternalIMT | epa | rs11239517 | 0.003 | . | . | . | . | . | . | . | . | . |
| InternalIMT | epa | rs11239519 | 0.003 | . | . | . | . | . | . | . | . | . |
| InternalIMT | epa | rs7909514 | 0.872 | -0.034 | 0.083 | 0.682 | 0.011 | 0.032 | 0.738 | 0.011 | 0.047 | 0.811 |
| InternalIMT | epa | rs7894352 | 0.872 | -0.034 | 0.083 | 0.684 | 0.011 | 0.032 | 0.736 | 0.011 | 0.047 | 0.813 |
| InternalIMT | epa | rs189384146 | 0 | . | . | . | . | . | . | . | . | . |
| InternalIMT | epa | rs11239520 | 0.004 | . | . | . | . | . | . | . | . | . |
| InternalIMT | epa | rs12221233 | 0.617 | -0.05 | 0.04 | 0.207 | 0.019 | 0.026 | 0.476 | 0.029 | 0.033 | 0.378 |
| InternalIMT | epa | rs74523293 | 0.049 | -0.012 | 0.026 | 0.645 | -0.091 | 0.041 | 0.026 | -0.012 | 0.057 | 0.831 |
| InternalIMT | epa | rs79376040 | 0.049 | -0.012 | 0.026 | 0.645 | -0.091 | 0.041 | 0.026 | -0.012 | 0.057 | 0.83 |
| InternalIMT | epa | rs4949000 | 0.617 | -0.05 | 0.04 | 0.208 | 0.019 | 0.026 | 0.477 | 0.029 | 0.033 | 0.378 |
| InternalIMT | epa | rs7089406 | . | . | . | . | . | . | . | . | . | . |
| InternalIMT | epa | rs138211056 | . | . | . | . | . | . | . | . | . | . |
| InternalIMT | epa | rs10900213 | 0.621 | -0.049 | 0.04 | 0.222 | 0.023 | 0.026 | 0.369 | 0.028 | 0.033 | 0.395 |
| InternalIMT | epa | rs145152827 | 0.011 | -0.012 | 0.025 | 0.631 | -0.103 | 0.101 | 0.305 | -0.141 | 0.171 | 0.409 |
| InternalIMT | epa | rs74443325 | 0.048 | -0.012 | 0.026 | 0.643 | -0.093 | 0.041 | 0.024 | -0.011 | 0.057 | 0.845 |
| InternalIMT | epa | rs188299446 | . | . | . | . | . | . | . | . | . | . |
| InternalIMT | epa | rs192699586 | . | . | . | . | . | . | . | . | . | . |
| InternalIMT | epa | rs72796464 | 0.003 | . | . | . | . | . | . | . | . | . |
| InternalIMT | epa | rs4949001 | 0.455 | -0.045 | 0.035 | 0.2 | 4.10E-03 | 0.026 | 0.873 | 0.033 | 0.036 | 0.357 |
| InternalIMT | epa | rs147174641 | . | . | . | . | . | . | . | . | . | . |
| InternalIMT | epa | rs190835238 | . | . | . | . | . | . | . | . | . | . |
| InternalIMT | epa | rs76698944 | . | . | . | . | . | . | . | . | . | . |
| InternalIMT | epa | rs56071381 | 0.003 | . | . | . | . | . | . | . | . | . |
| InternalIMT | epa | rs78130427 | 0.2 | -4.10E-03 | 0.03 | 0.893 | -1.20E-03 | 0.034 | 0.972 | -0.025 | 0.035 | 0.475 |
| InternalIMT | epa | rs10900215 | 0.007 | . | . | . | . | . | . | . | . | . |
| InternalIMT | epa | rs41491445 | . | . | . | . | . | . | . | . | . | . |
| InternalIMT | epa | rs144995222 | . | . | . | . | . | . | . | . | . | . |
| InternalIMT | epa | rs149097803 | . | . | . | . | . | . | . | . | . | . |
| InternalIMT | epa | rs139429646 | . | . | . | . | . | . | . | . | . | . |
| InternalIMT | epa | rs7075620 | 0.861 | -0.033 | 0.088 | 0.706 | 0.016 | 0.033 | 0.628 | 0.011 | 0.05 | 0.827 |
| InternalIMT | epa | rs148299213 | . | . | . | . | . | . | . | . | . | . |
| InternalIMT | epa | rs4949002 | 0.238 | -0.014 | 0.028 | 0.625 | 0.021 | 0.034 | 0.542 | -1.90E-03 | 0.042 | 0.964 |
| InternalIMT | epa | rs150496007 | . | . | . | . | . | . | . | . | . | . |
| InternalIMT | epa | rs147303455 | . | . | . | . | . | . | . | . | . | . |
| InternalIMT | epa | rs189518433 | . | . | . | . | . | . | . | . | . | . |
| InternalIMT | epa | rs186281355 | . | . | . | . | . | . | . | . | . | . |
| InternalIMT | epa | rs115297114 | . | . | . | . | . | . | . | . | . | . |
| InternalIMT | epa | rs11239523 | 0.003 | . | . | . | . | . | . | . | . | . |
| InternalIMT | epa | rs143704897 | . | . | . | . | . | . | . | . | . | . |
| InternalIMT | epa | rs188024096 | . | . | . | . | . | . | . | . | . | . |
| InternalIMT | epa | rs11239524 | 0.003 | . | . | . | . | . | . | . | . | . |
| InternalIMT | epa | rs191656573 | . | . | . | . | . | . | . | . | . | . |
| InternalIMT | epa | rs193295875 | 0.011 | -0.011 | 0.025 | 0.658 | -0.121 | 0.081 | 0.137 | -0.096 | 0.118 | 0.417 |
| InternalIMT | epa | rs190458228 | . | . | . | . | . | . | . | . | . | . |
| InternalIMT | epa | rs139253993 | 0.002 | . | . | . | . | . | . | . | . | . |
| InternalIMT | epa | rs4948672 | 0.812 | -0.078 | 0.069 | 0.262 | 7.60E-03 | 0.035 | 0.829 | 0.04 | 0.043 | 0.358 |
| InternalIMT | epa | rs180954921 | . | . | . | . | . | . | . | . | . | . |
| InternalIMT | epa | rs12264801 | 0.447 | 0.049 | 0.046 | 0.291 | -0.02 | 0.032 | 0.526 | -0.067 | 0.04 | 0.089 |
| InternalIMT | epa | rs73283184 | . | . | . | . | . | . | . | . | . | . |
| InternalIMT | epa | rs142746187 | . | . | . | . | . | . | . | . | . | . |
| InternalIMT | epa | rs58704739 | . | . | . | . | . | . | . | . | . | . |
| InternalIMT | epa | rs61272335 | . | . | . | . | . | . | . | . | . | . |
| InternalIMT | epa | rs60959489 | . | . | . | . | . | . | . | . | . | . |
| InternalIMT | epa | rs58502629 | . | . | . | . | . | . | . | . | . | . |
| InternalIMT | epa | rs10900216 | 0.838 | -0.032 | 0.062 | 0.608 | 0.069 | 0.028 | 0.013 | 0.011 | 0.037 | 0.769 |
| InternalIMT | epa | rs181118141 | . | . | . | . | . | . | . | . | . | . |
| InternalIMT | epa | rs137882665 | . | . | . | . | . | . | . | . | . | . |

| InternalIMT | epa | rs149466736 | . | . | . | . | . | . | . | . | . | . |
| --- | --- | --- | --- | --- | --- | --- | --- | --- | --- | --- | --- | --- |
| InternalIMT | epa | rs2099171 | 0.806 | -0.04 | 0.053 | 0.451 | 0.048 | 0.026 | 0.067 | 0.016 | 0.033 | 0.619 |
| InternalIMT | epa | rs141852280 | . | . | . | . | . | . | . | . | . | . |
| InternalIMT | epa | rs191539257 | 0.001 | . | . | . | . | . | . | . | . | . |
| InternalIMT | epa | rs17157771 | 0.029 | -0.01 | 0.025 | 0.682 | 0.043 | 0.065 | 0.503 | -0.046 | 0.054 | 0.393 |
| InternalIMT | epa | rs111339241 | 0.029 | -0.011 | 0.025 | 0.67 | 0.051 | 0.066 | 0.44 | -0.042 | 0.054 | 0.429 |
| InternalIMT | epa | rs116983255 | . | . | . | . | . | . | . | . | . | . |
| InternalIMT | epa | rs142324341 | 0.037 | -0.015 | 0.026 | 0.566 | 0.065 | 0.083 | 0.434 | -2.40E-03 | 0.13 | 0.985 |
| InternalIMT | epa | rs187191219 | . | . | . | . | . | . | . | . | . | . |
| InternalIMT | epa | rs151283973 | . | . | . | . | . | . | . | . | . | . |
| InternalIMT | epa | rs139316218 | . | . | . | . | . | . | . | . | . | . |
| InternalIMT | epa | rs6593485 | 0.886 | 5.60E-03 | 0.098 | 0.955 | 0.054 | 0.035 | 0.126 | -0.011 | 0.054 | 0.845 |
| InternalIMT | epa | rs6593486 | 0.886 | 5.90E-03 | 0.098 | 0.952 | 0.054 | 0.035 | 0.125 | -0.011 | 0.054 | 0.842 |
| InternalIMT | epa | rs145196618 | . | . | . | . | . | . | . | . | . | . |
| InternalIMT | epa | rs142598335 | . | . | . | . | . | . | . | . | . | . |
| InternalIMT | epa | rs892691 | 0.885 | -4.10E-03 | 0.098 | 0.967 | 0.055 | 0.034 | 0.112 | -5.30E-03 | 0.054 | 0.922 |
| InternalIMT | epa | rs7080713 | 0.809 | -0.038 | 0.053 | 0.467 | 0.051 | 0.026 | 0.056 | 0.015 | 0.033 | 0.635 |
| InternalIMT | epa | rs7077173 | 0.065 | -0.015 | 0.025 | 0.535 | 0.055 | 0.043 | 0.197 | 0.01 | 0.059 | 0.864 |
| InternalIMT | epa | rs7080977 | 0.03 | -9.80E-03 | 0.025 | 0.697 | 0.048 | 0.066 | 0.469 | -0.05 | 0.054 | 0.356 |
| InternalIMT | epa | rs3780901 | 0.809 | -0.04 | 0.053 | 0.45 | 0.05 | 0.026 | 0.058 | 0.016 | 0.033 | 0.615 |
| InternalIMT | epa | rs61854091 | . | . | . | . | . | . | . | . | . | . |
| InternalIMT | epa | rs77550209 | . | . | . | . | . | . | . | . | . | . |
| InternalIMT | epa | rs73283191 | 0.077 | -7.40E-03 | 0.027 | 0.782 | -0.034 | 0.036 | 0.357 | -0.031 | 0.04 | 0.441 |
| InternalIMT | epa | rs191866099 | . | . | . | . | . | . | . | . | . | . |
| InternalIMT | epa | rs147998395 | . | . | . | . | . | . | . | . | . | . |
| InternalIMT | epa | rs7090328 | 0.885 | 1.50E-03 | 0.098 | 0.988 | 0.053 | 0.035 | 0.128 | -8.30E-03 | 0.054 | 0.878 |
| InternalIMT | epa | rs79814535 | 0.048 | -0.011 | 0.026 | 0.683 | -0.09 | 0.037 | 0.016 | -0.024 | 0.053 | 0.646 |
| InternalIMT | epa | rs7073082 | 0.885 | 1.20E-03 | 0.098 | 0.99 | 0.053 | 0.035 | 0.127 | -8.20E-03 | 0.054 | 0.88 |
| InternalIMT | epa | rs11239526 | 0.039 | -0.021 | 0.025 | 0.388 | 0.078 | 0.061 | 0.196 | 0.058 | 0.088 | 0.512 |
| InternalIMT | epa | rs141839841 | . | . | . | . | . | . | . | . | . | . |
| InternalIMT | epa | rs75697371 | . | . | . | . | . | . | . | . | . | . |
| InternalIMT | epa | rs75972854 | . | . | . | . | . | . | . | . | . | . |
| InternalIMT | epa | rs139291975 | . | . | . | . | . | . | . | . | . | . |
| InternalIMT | epa | rs144064711 | . | . | . | . | . | . | . | . | . | . |
| InternalIMT | epa | rs3780902 | 0.078 | -7.40E-03 | 0.027 | 0.782 | -0.034 | 0.036 | 0.343 | -0.031 | 0.04 | 0.441 |
| InternalIMT | epa | rs190449801 | . | . | . | . | . | . | . | . | . | . |
| InternalIMT | epa | rs73283196 | 0.077 | -7.60E-03 | 0.027 | 0.777 | -0.034 | 0.036 | 0.353 | -0.031 | 0.041 | 0.45 |
| InternalIMT | epa | rs28395867 | . | . | . | . | . | . | . | . | . | . |
| InternalIMT | epa | rs28395866 | 0.029 | -0.011 | 0.025 | 0.66 | 0.05 | 0.065 | 0.443 | -0.039 | 0.055 | 0.475 |
| InternalIMT | epa | rs55661611 | 0.028 | -0.011 | 0.025 | 0.656 | 0.05 | 0.065 | 0.446 | -0.038 | 0.055 | 0.489 |
| InternalIMT | epa | rs55684518 | . | . | . | . | . | . | . | . | . | . |
| InternalIMT | epa | rs78552493 | . | . | . | . | . | . | . | . | . | . |
| InternalIMT | epa | rs147665434 | . | . | . | . | . | . | . | . | . | . |
| InternalIMT | epa | rs34445001 | . | . | . | . | . | . | . | . | . | . |
| InternalIMT | epa | rs184019449 | . | . | . | . | . | . | . | . | . | . |
| InternalIMT | epa | rs3780903 | 0.808 | -0.038 | 0.054 | 0.477 | 0.051 | 0.027 | 0.057 | 0.016 | 0.033 | 0.643 |
| InternalIMT | epa | rs17157784 | 0.048 | -0.011 | 0.026 | 0.661 | -0.086 | 0.037 | 0.021 | -0.019 | 0.053 | 0.716 |
| InternalIMT | epa | rs3780904 | 0.048 | -0.011 | 0.026 | 0.661 | -0.086 | 0.037 | 0.021 | -0.019 | 0.053 | 0.716 |
| InternalIMT | epa | rs192064730 | . | . | . | . | . | . | . | . | . | . |
| InternalIMT | epa | rs12774534 | . | . | . | . | . | . | . | . | . | . |
| InternalIMT | epa | rs79179915 | 0.047 | -0.011 | 0.026 | 0.662 | -0.087 | 0.038 | 0.022 | -0.02 | 0.053 | 0.709 |
| InternalIMT | epa | rs117811571 | . | . | . | . | . | . | . | . | . | . |
| InternalIMT | epa | rs72796477 | . | . | . | . | . | . | . | . | . | . |
| InternalIMT | epa | rs55729342 | 0.057 | -0.011 | 0.026 | 0.678 | -0.089 | 0.037 | 0.017 | -0.022 | 0.051 | 0.668 |
| InternalIMT | epa | rs59317220 | 0.047 | -0.011 | 0.026 | 0.664 | -0.086 | 0.038 | 0.022 | -0.02 | 0.053 | 0.699 |
| InternalIMT | epa | rs3780905 | 0.562 | -0.038 | 0.036 | 0.299 | 2.00E-03 | 0.023 | 0.932 | 0.021 | 0.032 | 0.504 |
| InternalIMT | epa | rs3780906 | 0.807 | -0.037 | 0.055 | 0.504 | 0.052 | 0.027 | 0.051 | 0.015 | 0.034 | 0.671 |
| InternalIMT | epa | rs149047975 | . | . | . | . | . | . | . | . | . | . |
| InternalIMT | epa | rs3824614 | 0.047 | -0.011 | 0.026 | 0.664 | -0.087 | 0.038 | 0.022 | -0.02 | 0.053 | 0.698 |
| InternalIMT | epa | rs140254404 | . | . | . | . | . | . | . | . | . | . |
| InternalIMT | epa | rs145262684 | . | . | . | . | . | . | . | . | . | . |
| InternalIMT | epa | rs76735482 | 0.047 | -0.011 | 0.026 | 0.665 | -0.086 | 0.038 | 0.023 | -0.021 | 0.053 | 0.692 |
| InternalIMT | epa | rs73283198 | 0.028 | -0.013 | 0.025 | 0.595 | 0.041 | 0.062 | 0.514 | -0.011 | 0.067 | 0.867 |
| InternalIMT | epa | rs74911239 | . | . | . | . | . | . | . | . | . | . |
| InternalIMT | epa | rs150551647 | . | . | . | . | . | . | . | . | . | . |
| InternalIMT | epa | rs115200794 | . | . | . | . | . | . | . | . | . | . |
| InternalIMT | epa | rs74130823 | 0.038 | -0.013 | 0.026 | 0.606 | 0.023 | 0.071 | 0.742 | -8.80E-03 | 0.072 | 0.903 |
| InternalIMT | epa | rs2279435 | 0.754 | -0.103 | 0.088 | 0.242 | 0.028 | 0.028 | 0.323 | 0.058 | 0.052 | 0.265 |
| InternalIMT | epa | rs147279673 | 0.01 | . | . | . | . | . | . | . | . | . |
| InternalIMT | epa | rs183525146 | 0.006 | . | . | . | . | . | . | . | . | . |
| InternalIMT | epa | rs3740107 | 0.737 | -0.152 | 0.077 | 0.049 | 0.055 | 0.028 | 0.045 | 0.095 | 0.047 | 0.043 |
| InternalIMT | epa | rs140697247 | . | . | . | . | . | . | . | . | . | . |
| InternalIMT | epa | rs1565096 | 0.806 | -0.12 | 0.103 | 0.242 | 0.034 | 0.03 | 0.25 | 0.065 | 0.058 | 0.261 |

| InternalIMT | epa | rs1565097 | 0.806 | -0.12 | 0.103 | 0.244 | 0.034 | 0.03 | 0.253 | 0.065 | 0.058 | 0.263 |
| --- | --- | --- | --- | --- | --- | --- | --- | --- | --- | --- | --- | --- |
| InternalIMT | epa | rs144584548 | . | . | . | . | . | . | . | . | . | . |
| InternalIMT | epa | rs75811053 | 0.044 | -0.012 | 0.026 | 0.646 | -0.092 | 0.039 | 0.018 | -0.02 | 0.054 | 0.718 |
| InternalIMT | epa | rs147462967 | . | . | . | . | . | . | . | . | . | . |
| InternalIMT | epa | rs74130824 | 0.044 | -0.012 | 0.026 | 0.646 | -0.092 | 0.039 | 0.018 | -0.02 | 0.054 | 0.715 |
| InternalIMT | epa | rs116883184 | . | . | . | . | . | . | . | . | . | . |
| InternalIMT | epa | rs117258049 | . | . | . | . | . | . | . | . | . | . |
| InternalIMT | epa | rs3780908 | 0.029 | -0.015 | 0.025 | 0.554 | 0.072 | 0.075 | 0.341 | 0.022 | 0.083 | 0.793 |
| InternalIMT | epa | rs180938522 | . | . | . | . | . | . | . | . | . | . |
| InternalIMT | epa | rs76687372 | . | . | . | . | . | . | . | . | . | . |
| InternalIMT | epa | rs3780909 | 0.666 | -0.067 | 0.064 | 0.295 | 0.015 | 0.024 | 0.551 | 0.041 | 0.041 | 0.322 |
| InternalIMT | epa | rs78281156 | 0.044 | -0.012 | 0.026 | 0.646 | -0.092 | 0.039 | 0.018 | -0.02 | 0.054 | 0.718 |
| InternalIMT | epa | rs187840631 | . | . | . | . | . | . | . | . | . | . |
| InternalIMT | epa | rs76868242 | 0.044 | -0.012 | 0.026 | 0.646 | -0.092 | 0.039 | 0.018 | -0.02 | 0.054 | 0.717 |
| InternalIMT | epa | rs192794192 | . | . | . | . | . | . | . | . | . | . |
| InternalIMT | epa | rs12781248 | . | . | . | . | . | . | . | . | . | . |
| InternalIMT | epa | rs12762375 | . | . | . | . | . | . | . | . | . | . |
| InternalIMT | epa | rs192256986 | . | . | . | . | . | . | . | . | . | . |
| InternalIMT | epa | rs184411030 | . | . | . | . | . | . | . | . | . | . |
| InternalIMT | epa | rs189601483 | 0.049 | -0.013 | 0.026 | 0.617 | -0.083 | 0.042 | 0.048 | -8.80E-03 | 0.053 | 0.87 |
| InternalIMT | epa | rs143254579 | 0.045 | -0.021 | 0.025 | 0.408 | 0.05 | 0.058 | 0.386 | 0.05 | 0.086 | 0.561 |
| InternalIMT | epa | rs12253903 | . | . | . | . | . | . | . | . | . | . |
| InternalIMT | epa | rs35489926 | . | . | . | . | . | . | . | . | . | . |
| InternalIMT | epa | rs71494783 | 0.073 | -8.90E-03 | 0.027 | 0.738 | -0.062 | 0.034 | 0.072 | -0.032 | 0.046 | 0.481 |
| InternalIMT | epa | rs180711602 | . | . | . | . | . | . | . | . | . | . |
| InternalIMT | epa | rs117312402 | 0.044 | -0.012 | 0.026 | 0.646 | -0.091 | 0.039 | 0.018 | -0.02 | 0.054 | 0.716 |
| InternalIMT | epa | rs74831694 | 0.053 | -0.036 | 0.025 | 0.156 | 0.089 | 0.095 | 0.347 | 0.252 | 0.16 | 0.115 |
| InternalIMT | epa | rs140301943 | . | . | . | . | . | . | . | . | . | . |
| InternalIMT | epa | rs78553679 | . | . | . | . | . | . | . | . | . | . |
| InternalIMT | epa | rs78334250 | 0.044 | -0.012 | 0.026 | 0.646 | -0.091 | 0.039 | 0.019 | -0.02 | 0.054 | 0.715 |
| InternalIMT | epa | rs76000028 | 0.044 | -0.012 | 0.026 | 0.646 | -0.091 | 0.039 | 0.019 | -0.02 | 0.054 | 0.716 |
| InternalIMT | epa | rs145195254 | . | . | . | . | . | . | . | . | . | . |
| InternalIMT | epa | rs137888527 | . | . | . | . | . | . | . | . | . | . |
| InternalIMT | epa | rs149109592 | . | . | . | . | . | . | . | . | . | . |
| InternalIMT | epa | rs80280010 | 0.038 | -0.021 | 0.025 | 0.398 | 0.076 | 0.059 | 0.204 | 0.053 | 0.086 | 0.537 |
| InternalIMT | epa | rs189995374 | 0.005 | . | . | . | . | . | . | . | . | . |
| InternalIMT | epa | rs75467770 | 0.05 | -9.70E-03 | 0.026 | 0.712 | -0.097 | 0.039 | 0.012 | -0.028 | 0.053 | 0.595 |
| InternalIMT | epa | rs1487562 | 0.029 | -0.015 | 0.025 | 0.553 | 0.073 | 0.076 | 0.334 | 0.023 | 0.084 | 0.789 |
| InternalIMT | epa | rs12266963 | 0.073 | -9.00E-03 | 0.027 | 0.737 | -0.062 | 0.034 | 0.071 | -0.032 | 0.045 | 0.482 |
| InternalIMT | epa | rs7099874 | 0.769 | -0.082 | 0.092 | 0.376 | 0.013 | 0.028 | 0.652 | 0.045 | 0.054 | 0.406 |
| InternalIMT | epa | rs75687019 | 0.044 | -0.012 | 0.026 | 0.646 | -0.091 | 0.039 | 0.019 | -0.02 | 0.054 | 0.715 |
| InternalIMT | epa | rs148240462 | . | . | . | . | . | . | . | . | . | . |
| InternalIMT | epa | rs141278174 | . | . | . | . | . | . | . | . | . | . |
| InternalIMT | epa | rs76404852 | 0.044 | -0.012 | 0.026 | 0.646 | -0.091 | 0.039 | 0.019 | -0.02 | 0.054 | 0.715 |
| InternalIMT | epa | rs139112240 | . | . | . | . | . | . | . | . | . | . |
| InternalIMT | epa | rs12783037 | 0.073 | -9.00E-03 | 0.027 | 0.736 | -0.062 | 0.034 | 0.071 | -0.032 | 0.045 | 0.484 |
| InternalIMT | epa | rs72796481 | . | . | . | . | . | . | . | . | . | . |
| InternalIMT | epa | rs76798749 | 0.044 | -0.012 | 0.026 | 0.646 | -0.091 | 0.039 | 0.019 | -0.02 | 0.054 | 0.714 |
| InternalIMT | epa | rs187732548 | . | . | . | . | . | . | . | . | . | . |
| InternalIMT | epa | rs75675474 | 0.044 | -0.012 | 0.026 | 0.646 | -0.091 | 0.039 | 0.019 | -0.02 | 0.054 | 0.714 |
| InternalIMT | epa | rs76552293 | 0.044 | -0.012 | 0.026 | 0.646 | -0.091 | 0.039 | 0.019 | -0.02 | 0.054 | 0.714 |
| InternalIMT | epa | rs147445993 | 0.001 | . | . | . | . | . | . | . | . | . |
| InternalIMT | epa | rs115902283 | 0.044 | -0.012 | 0.026 | 0.643 | -0.092 | 0.039 | 0.017 | -0.019 | 0.054 | 0.725 |
| InternalIMT | epa | rs4540799 | 0.759 | -0.069 | 0.091 | 0.451 | 0.012 | 0.028 | 0.667 | 0.037 | 0.054 | 0.494 |
| InternalIMT | epa | rs12765320 | 0.102 | -8.80E-03 | 0.028 | 0.753 | -0.023 | 0.033 | 0.482 | -0.025 | 0.042 | 0.557 |
| InternalIMT | epa | rs12782256 | . | . | . | . | . | . | . | . | . | . |
| InternalIMT | epa | rs191727256 | . | . | . | . | . | . | . | . | . | . |
| InternalIMT | epa | rs2082111 | 0.028 | -0.015 | 0.025 | 0.556 | 0.077 | 0.077 | 0.317 | 0.02 | 0.085 | 0.816 |
| InternalIMT | epa | rs2082110 | 0.072 | -0.012 | 0.027 | 0.655 | -0.025 | 0.04 | 0.537 | -0.014 | 0.047 | 0.766 |
| InternalIMT | epa | rs2082109 | 0.072 | -0.012 | 0.027 | 0.655 | -0.025 | 0.04 | 0.536 | -0.014 | 0.047 | 0.767 |
| InternalIMT | epa | rs117316662 | 0.044 | -0.012 | 0.026 | 0.646 | -0.091 | 0.039 | 0.019 | -0.02 | 0.054 | 0.716 |
| InternalIMT | epa | rs12247163 | 0.102 | -9.50E-03 | 0.028 | 0.733 | -0.022 | 0.034 | 0.519 | -0.021 | 0.042 | 0.615 |
| InternalIMT | epa | rs12355888 | 0.002 | . | . | . | . | . | . | . | . | . |
| InternalIMT | epa | rs143772290 | . | . | . | . | . | . | . | . | . | . |
| InternalIMT | epa | rs146849452 | . | . | . | . | . | . | . | . | . | . |
| InternalIMT | epa | rs140267121 | . | . | . | . | . | . | . | . | . | . |
| InternalIMT | epa | rs7080474 | 0.058 | -0.013 | 0.026 | 0.63 | 0.034 | 0.049 | 0.478 | -8.00E-03 | 0.06 | 0.893 |
| InternalIMT | epa | rs144066203 | . | . | . | . | . | . | . | . | . | . |
| InternalIMT | epa | rs12765209 | 0.044 | -0.012 | 0.026 | 0.646 | -0.091 | 0.039 | 0.019 | -0.02 | 0.054 | 0.718 |
| InternalIMT | epa | rs150930735 | . | . | . | . | . | . | . | . | . | . |
| InternalIMT | epa | rs7068039 | 0.102 | -9.50E-03 | 0.028 | 0.732 | -0.022 | 0.034 | 0.518 | -0.021 | 0.042 | 0.616 |
| InternalIMT | epa | rs117622652 | 0.043 | -0.012 | 0.026 | 0.645 | -0.091 | 0.039 | 0.019 | -0.02 | 0.054 | 0.719 |
| InternalIMT | epa | rs190191514 | . | . | . | . | . | . | . | . | . | . |

| InternalIMT | epa | rs74130835 | . | . | . | . | . | . | . | . | . | . |
| --- | --- | --- | --- | --- | --- | --- | --- | --- | --- | --- | --- | --- |
| InternalIMT | epa | rs1811604 | . | . | . | . | . | . | . | . | . | . |
| InternalIMT | epa | rs146971256 | 0.001 | . | . | . | . | . | . | . | . | . |
| InternalIMT | epa | rs74336000 | . | . | . | . | . | . | . | . | . | . |
| InternalIMT | epa | rs186046492 | . | . | . | . | . | . | . | . | . | . |
| InternalIMT | epa | rs34971823 | . | . | . | . | . | . | . | . | . | . |
| InternalIMT | epa | rs75227446 | 0.043 | -0.012 | 0.026 | 0.644 | -0.092 | 0.039 | 0.018 | -0.019 | 0.054 | 0.725 |
| InternalIMT | epa | rs190457599 | . | . | . | . | . | . | . | . | . | . |
| InternalIMT | epa | rs79591917 | 0.043 | -0.012 | 0.026 | 0.644 | -0.092 | 0.039 | 0.018 | -0.019 | 0.054 | 0.725 |
| InternalIMT | epa | rs702365 | 0.647 | -0.069 | 0.062 | 0.262 | 0.019 | 0.024 | 0.431 | 0.044 | 0.041 | 0.282 |
| InternalIMT | epa | rs79643836 | 0.043 | -0.012 | 0.026 | 0.644 | -0.092 | 0.039 | 0.018 | -0.019 | 0.054 | 0.725 |
| InternalIMT | epa | rs702366 | 0.649 | -0.07 | 0.062 | 0.261 | 0.019 | 0.024 | 0.421 | 0.045 | 0.041 | 0.28 |
| InternalIMT | epa | rs1059696 | 0.059 | -0.013 | 0.026 | 0.626 | 0.034 | 0.049 | 0.489 | -6.60E-03 | 0.06 | 0.912 |
| InternalIMT | epa | rs901681 | 0.648 | -0.07 | 0.062 | 0.257 | 0.019 | 0.024 | 0.428 | 0.045 | 0.041 | 0.275 |
| InternalIMT | epa | rs61854092 | 0.058 | -0.012 | 0.026 | 0.631 | 0.034 | 0.049 | 0.485 | -8.30E-03 | 0.06 | 0.89 |
| InternalIMT | epa | rs148080753 | . | . | . | . | . | . | . | . | . | . |
| InternalIMT | epa | rs61854093 | 0.064 | -0.011 | 0.026 | 0.666 | 0.036 | 0.048 | 0.451 | -0.016 | 0.06 | 0.786 |
| InternalIMT | epa | rs141822774 | . | . | . | . | . | . | . | . | . | . |
| InternalIMT | epa | rs74130836 | . | . | . | . | . | . | . | . | . | . |
| InternalIMT | epa | rs150620963 | . | . | . | . | . | . | . | . | . | . |
| InternalIMT | epa | rs3780913 | 0.749 | -0.078 | 0.087 | 0.369 | 0.01 | 0.027 | 0.699 | 0.043 | 0.051 | 0.399 |
| InternalIMT | epa | rs142255723 | . | . | . | . | . | . | . | . | . | . |
| InternalIMT | epa | rs3780914 | 0.647 | -0.07 | 0.062 | 0.257 | 0.018 | 0.024 | 0.446 | 0.045 | 0.041 | 0.275 |
| InternalIMT | epa | rs182656507 | . | . | . | . | . | . | . | . | . | . |
| InternalIMT | epa | rs2291427 | 0.703 | -0.079 | 0.073 | 0.281 | 0.028 | 0.026 | 0.279 | 0.048 | 0.046 | 0.299 |
| InternalIMT | epa | rs10751382 | 0.703 | -0.079 | 0.073 | 0.281 | 0.028 | 0.026 | 0.282 | 0.048 | 0.046 | 0.299 |
| InternalIMT | epa | rs117610108 | 0.043 | -0.012 | 0.026 | 0.641 | -0.094 | 0.039 | 0.016 | -0.018 | 0.055 | 0.748 |
| InternalIMT | epa | rs144966941 | 0.002 | . | . | . | . | . | . | . | . | . |
| InternalIMT | epa | rs41502051 | 0.007 | . | . | . | . | . | . | . | . | . |
| InternalIMT | epa | rs41426046 | 0.047 | -0.012 | 0.026 | 0.636 | -0.098 | 0.039 | 0.011 | -0.013 | 0.054 | 0.818 |
| InternalIMT | epa | rs41519248 | 0.043 | -0.012 | 0.026 | 0.639 | -0.094 | 0.039 | 0.016 | -0.017 | 0.055 | 0.756 |
| InternalIMT | epa | rs189108245 | . | . | . | . | . | . | . | . | . | . |
| InternalIMT | epa | rs7896431 | 0.668 | -0.071 | 0.067 | 0.289 | 0.018 | 0.024 | 0.469 | 0.044 | 0.044 | 0.312 |
| InternalIMT | epa | rs183724547 | . | . | . | . | . | . | . | . | . | . |
| InternalIMT | epa | rs142044990 | . | . | . | . | . | . | . | . | . | . |
| InternalIMT | epa | rs188140929 | 0.021 | -0.018 | 0.025 | 0.466 | 0.127 | 0.081 | 0.116 | 0.069 | 0.096 | 0.473 |
| InternalIMT | epa | rs76819096 | . | . | . | . | . | . | . | . | . | . |
| InternalIMT | epa | rs10751383 | 0.681 | -0.09 | 0.065 | 0.162 | 0.029 | 0.024 | 0.229 | 0.057 | 0.042 | 0.174 |
| InternalIMT | epa | rs117819321 | . | . | . | . | . | . | . | . | . | . |
| InternalIMT | epa | rs2242332 | 0.744 | -0.077 | 0.084 | 0.364 | 7.10E-03 | 0.027 | 0.792 | 0.043 | 0.05 | 0.394 |
| InternalIMT | epa | rs2242333 | 0.476 | -0.061 | 0.04 | 0.127 | 7.20E-04 | 0.022 | 0.974 | 0.052 | 0.033 | 0.119 |
| InternalIMT | epa | rs2242334 | 0.744 | -0.077 | 0.084 | 0.365 | 7.00E-03 | 0.027 | 0.795 | 0.043 | 0.05 | 0.395 |
| InternalIMT | epa | rs189465041 | . | . | . | . | . | . | . | . | . | . |
| InternalIMT | epa | rs28395868 | . | . | . | . | . | . | . | . | . | . |
| InternalIMT | epa | rs56067550 | . | . | . | . | . | . | . | . | . | . |
| InternalIMT | epa | rs1051713 | 0.1 | -9.00E-03 | 0.028 | 0.746 | -0.025 | 0.034 | 0.464 | -0.023 | 0.043 | 0.581 |
| InternalIMT | epa | rs115074210 | . | . | . | . | . | . | . | . | . | . |
| InternalIMT | epa | rs7900977 | 0.782 | -0.107 | 0.093 | 0.25 | 0.024 | 0.029 | 0.412 | 0.059 | 0.054 | 0.27 |
| InternalIMT | epa | rs147613891 | . | . | . | . | . | . | . | . | . | . |
| InternalIMT | epa | rs2229136 | 0.038 | -0.021 | 0.025 | 0.4 | 0.074 | 0.059 | 0.212 | 0.053 | 0.086 | 0.539 |
| InternalIMT | epa | rs185482841 | . | . | . | . | . | . | . | . | . | . |
| InternalIMT | epa | rs2288619 | 0.038 | -0.021 | 0.025 | 0.4 | 0.074 | 0.059 | 0.212 | 0.053 | 0.086 | 0.539 |
| InternalIMT | epa | rs189891524 | 0.003 | . | . | . | . | . | . | . | . | . |
| InternalIMT | epa | rs56145077 | 0.058 | -0.013 | 0.026 | 0.63 | 0.035 | 0.048 | 0.473 | -7.90E-03 | 0.06 | 0.894 |
| InternalIMT | epa | rs143216970 | . | . | . | . | . | . | . | . | . | . |
| InternalIMT | epa | rs142246478 | . | . | . | . | . | . | . | . | . | . |
| InternalIMT | epa | rs13134 | . | . | . | . | . | . | . | . | . | . |
| InternalIMT | epa | rs41314980 | . | . | . | . | . | . | . | . | . | . |
| InternalIMT | epa | rs41304611 | . | . | . | . | . | . | . | . | . | . |
| InternalIMT | epa | rs193299617 | 0.004 | . | . | . | . | . | . | . | . | . |
| InternalIMT | epa | rs12772724 | . | . | . | . | . | . | . | . | . | . |
| InternalIMT | epa | rs139489537 | 0.038 | -0.021 | 0.025 | 0.401 | 0.074 | 0.059 | 0.213 | 0.052 | 0.086 | 0.543 |
| InternalIMT | epa | rs12776062 | . | . | . | . | . | . | . | . | . | . |
| InternalIMT | epa | rs752059 | 0.059 | -0.012 | 0.026 | 0.631 | 0.034 | 0.049 | 0.489 | -8.30E-03 | 0.06 | 0.889 |
| InternalIMT | epa | rs147649982 | 0.015 | -0.017 | 0.026 | 0.507 | 0.049 | 0.125 | 0.695 | 0.098 | 0.148 | 0.507 |
| InternalIMT | epa | rs17157798 | 0.038 | -0.021 | 0.025 | 0.4 | 0.074 | 0.059 | 0.213 | 0.052 | 0.086 | 0.541 |
| InternalIMT | epa | rs142298619 | . | . | . | . | . | . | . | . | . | . |
| InternalIMT | epa | rs4948673 | 0.777 | -0.101 | 0.094 | 0.284 | 0.021 | 0.029 | 0.465 | 0.055 | 0.054 | 0.308 |
| InternalIMT | epa | rs74231484 | 0.476 | -0.061 | 0.04 | 0.129 | 5.10E-04 | 0.022 | 0.982 | 0.052 | 0.033 | 0.12 |
| InternalIMT | epa | rs72796489 | . | . | . | . | . | . | . | . | . | . |
| InternalIMT | epa | rs113447578 | 0.026 | -0.015 | 0.025 | 0.542 | 0.043 | 0.065 | 0.508 | 0.023 | 0.088 | 0.79 |
| InternalIMT | epa | rs34063374 | . | . | . | . | . | . | . | . | . | . |
| InternalIMT | epa | rs11239527 | . | . | . | . | . | . | . | . | . | . |

| InternalIMT | epa | rs34371883 | . | . | . | . | . | . | . | . | . | . |
| --- | --- | --- | --- | --- | --- | --- | --- | --- | --- | --- | --- | --- |
| InternalIMT | epa | rs118168828 | 0.002 | . | . | . | . | . | . | . | . | . |
| InternalIMT | epa | rs34349618 | . | . | . | . | . | . | . | . | . | . |
| InternalIMT | epa | rs80041679 | 0.038 | -0.021 | 0.025 | 0.401 | 0.074 | 0.059 | 0.213 | 0.052 | 0.086 | 0.543 |
| InternalIMT | epa | rs149640607 | . | . | . | . | . | . | . | . | . | . |
| InternalIMT | epa | rs4427523 | 0.957 | 0.199 | 0.325 | 0.542 | -0.109 | 0.113 | 0.336 | -0.11 | 0.168 | 0.512 |
| InternalIMT | epa | rs185880109 | . | . | . | . | . | . | . | . | . | . |
| InternalIMT | epa | rs191523220 | . | . | . | . | . | . | . | . | . | . |
| InternalIMT | epa | rs144315284 | . | . | . | . | . | . | . | . | . | . |
| InternalIMT | epa | rs4948674 | 0.156 | -6.90E-03 | 0.03 | 0.818 | 0.023 | 0.031 | 0.447 | -0.022 | 0.037 | 0.545 |
| InternalIMT | epa | rs182984298 | . | . | . | . | . | . | . | . | . | . |
| InternalIMT | epa | rs56749023 | . | . | . | . | . | . | . | . | . | . |
| InternalIMT | epa | rs112508957 | . | . | . | . | . | . | . | . | . | . |
| InternalIMT | epa | rs7089063 | 0.138 | -0.016 | 0.028 | 0.578 | 6.30E-03 | 0.031 | 0.838 | 5.20E-03 | 0.043 | 0.902 |
| InternalIMT | epa | rs145000779 | . | . | . | . | . | . | . | . | . | . |
| InternalIMT | epa | rs80201572 | 0.038 | -0.021 | 0.025 | 0.4 | 0.074 | 0.059 | 0.214 | 0.053 | 0.085 | 0.539 |
| InternalIMT | epa | rs140568227 | . | . | . | . | . | . | . | . | . | . |
| InternalIMT | epa | rs149756228 | . | . | . | . | . | . | . | . | . | . |
| InternalIMT | epa | rs112380685 | . | . | . | . | . | . | . | . | . | . |
| InternalIMT | epa | rs60481109 | . | . | . | . | . | . | . | . | . | . |
| InternalIMT | epa | rs138221843 | . | . | . | . | . | . | . | . | . | . |
| InternalIMT | epa | rs150283194 | . | . | . | . | . | . | . | . | . | . |
| InternalIMT | epa | rs138879661 | . | . | . | . | . | . | . | . | . | . |
| InternalIMT | epa | rs34960345 | 0.038 | -0.021 | 0.025 | 0.399 | 0.073 | 0.059 | 0.216 | 0.053 | 0.085 | 0.532 |
| InternalIMT | epa | rs147514068 | . | . | . | . | . | . | . | . | . | . |
| InternalIMT | epa | rs35410535 | 0.038 | -0.021 | 0.025 | 0.399 | 0.073 | 0.059 | 0.216 | 0.053 | 0.085 | 0.532 |
| InternalIMT | epa | rs191613635 | . | . | . | . | . | . | . | . | . | . |
| InternalIMT | epa | rs17157800 | . | . | . | . | . | . | . | . | . | . |
| InternalIMT | epa | rs17523413 | 0.038 | -0.021 | 0.025 | 0.4 | 0.074 | 0.059 | 0.214 | 0.053 | 0.085 | 0.538 |
| InternalIMT | epa | rs7897036 | 0.001 | . | . | . | . | . | . | . | . | . |
| InternalIMT | epa | rs76334323 | 0.042 | -0.012 | 0.026 | 0.634 | -0.098 | 0.039 | 0.012 | -0.015 | 0.055 | 0.791 |
| InternalIMT | epa | rs143535991 | . | . | . | . | . | . | . | . | . | . |
| InternalIMT | epa | rs140344979 | . | . | . | . | . | . | . | . | . | . |
| InternalIMT | epa | rs36047811 | 0.038 | -0.021 | 0.025 | 0.399 | 0.073 | 0.059 | 0.216 | 0.053 | 0.085 | 0.532 |
| InternalIMT | epa | rs76493570 | 0.038 | -0.021 | 0.025 | 0.399 | 0.073 | 0.059 | 0.216 | 0.053 | 0.085 | 0.532 |
| InternalIMT | epa | rs192998920 | . | . | . | . | . | . | . | . | . | . |
| InternalIMT | epa | rs116840852 | . | . | . | . | . | . | . | . | . | . |
| InternalIMT | epa | rs184824101 | . | . | . | . | . | . | . | . | . | . |
| InternalIMT | epa | rs41301621 | . | . | . | . | . | . | . | . | . | . |
| InternalIMT | epa | rs3740109 | 0.038 | -0.021 | 0.025 | 0.399 | 0.073 | 0.059 | 0.216 | 0.053 | 0.085 | 0.532 |
| InternalIMT | epa | rs7899415 | 0.089 | -1.60E-03 | 0.028 | 0.956 | 8.30E-03 | 0.043 | 0.847 | -0.071 | 0.053 | 0.176 |
| InternalIMT | epa | rs184395284 | 0.004 | . | . | . | . | . | . | . | . | . |
| InternalIMT | epa | rs139209589 | . | . | . | . | . | . | . | . | . | . |
| InternalIMT | epa | rs149924163 | . | . | . | . | . | . | . | . | . | . |
| InternalIMT | epa | rs3740110 | 0.042 | -0.012 | 0.026 | 0.633 | -0.098 | 0.039 | 0.012 | -0.015 | 0.055 | 0.791 |
| InternalIMT | epa | rs144335113 | . | . | . | . | . | . | . | . | . | . |
| InternalIMT | epa | rs74743385 | . | . | . | . | . | . | . | . | . | . |
| InternalIMT | epa | rs146545829 | . | . | . | . | . | . | . | . | . | . |
| InternalIMT | epa | rs181842689 | . | . | . | . | . | . | . | . | . | . |
| InternalIMT | epa | rs3740111 | 0.038 | -0.021 | 0.025 | 0.399 | 0.073 | 0.059 | 0.216 | 0.053 | 0.085 | 0.532 |
| InternalIMT | epa | rs186261599 | . | . | . | . | . | . | . | . | . | . |
| InternalIMT | epa | rs73285348 | 0.013 | -6.50E-03 | 0.025 | 0.79 | -0.043 | 0.089 | 0.63 | -0.398 | 0.24 | 0.097 |
| InternalIMT | epa | rs186356901 | . | . | . | . | . | . | . | . | . | . |
| InternalIMT | epa | rs3824618 | 0.038 | -0.021 | 0.025 | 0.4 | 0.074 | 0.059 | 0.214 | 0.053 | 0.085 | 0.539 |
| InternalIMT | epa | rs3802548 | 0.065 | -9.90E-03 | 0.026 | 0.708 | 0.023 | 0.047 | 0.624 | -0.028 | 0.055 | 0.612 |
| InternalIMT | epa | rs3802547 | 0.04 | -0.012 | 0.026 | 0.63 | -0.101 | 0.041 | 0.014 | -0.015 | 0.055 | 0.78 |
| InternalIMT | epa | rs77677521 | . | . | . | . | . | . | . | . | . | . |
| InternalIMT | epa | rs3824616 | 0.038 | -0.021 | 0.025 | 0.399 | 0.073 | 0.059 | 0.216 | 0.053 | 0.085 | 0.532 |
| InternalIMT | epa | rs191073818 | . | . | . | . | . | . | . | . | . | . |
| InternalIMT | epa | rs147078004 | . | . | . | . | . | . | . | . | . | . |
| InternalIMT | epa | rs7908745 | 0.145 | -0.013 | 0.028 | 0.642 | 2.10E-03 | 0.031 | 0.945 | -2.30E-03 | 0.04 | 0.953 |
| InternalIMT | epa | rs17157811 | . | . | . | . | . | . | . | . | . | . |
| InternalIMT | epa | rs138506087 | . | . | . | . | . | . | . | . | . | . |
| InternalIMT | epa | rs77241269 | . | . | . | . | . | . | . | . | . | . |
| InternalIMT | epa | rs138374938 | . | . | . | . | . | . | . | . | . | . |
| InternalIMT | epa | rs2279433 | 0.038 | -0.021 | 0.025 | 0.398 | 0.074 | 0.059 | 0.213 | 0.053 | 0.085 | 0.531 |
| InternalIMT | epa | rs73285358 | . | . | . | . | . | . | . | . | . | . |
| InternalIMT | epa | rs11239528 | 0.065 | -9.90E-03 | 0.026 | 0.708 | 0.022 | 0.047 | 0.638 | -0.028 | 0.055 | 0.614 |
| InternalIMT | epa | rs77453386 | 0.042 | -0.012 | 0.026 | 0.633 | -0.098 | 0.039 | 0.012 | -0.014 | 0.055 | 0.795 |
| InternalIMT | epa | rs2279434 | 0.04 | -0.021 | 0.025 | 0.392 | 0.074 | 0.059 | 0.21 | 0.054 | 0.085 | 0.526 |
| InternalIMT | epa | rs149236525 | . | . | . | . | . | . | . | . | . | . |
| InternalIMT | epa | rs12573609 | 0.13 | 9.80E-03 | 0.028 | 0.721 | -0.023 | 0.04 | 0.571 | -0.09 | 0.081 | 0.263 |
| InternalIMT | epa | rs191716655 | . | . | . | . | . | . | . | . | . | . |

| InternalIMT | epa | rs17523660 | . | . | . | . | . | . | . | . | . | . |
| --- | --- | --- | --- | --- | --- | --- | --- | --- | --- | --- | --- | --- |
| InternalIMT | epa | rs142671360 | . | . | . | . | . | . | . | . | . | . |
| InternalIMT | epa | rs3764990 | 0.037 | -0.021 | 0.025 | 0.399 | 0.074 | 0.059 | 0.21 | 0.053 | 0.085 | 0.537 |
| InternalIMT | epa | rs113682861 | . | . | . | . | . | . | . | . | . | . |
| InternalIMT | epa | rs117658270 | 0.042 | -0.012 | 0.026 | 0.633 | -0.098 | 0.039 | 0.012 | -0.014 | 0.055 | 0.795 |
| InternalIMT | epa | rs187309063 | . | . | . | . | . | . | . | . | . | . |
| InternalIMT | epa | rs73285360 | . | . | . | . | . | . | . | . | . | . |
| InternalIMT | epa | rs80133289 | 0.1 | -0.031 | 0.028 | 0.281 | -1.80E-03 | 0.058 | 0.975 | 0.083 | 0.071 | 0.245 |
| InternalIMT | epa | rs186693257 | 0.003 | . | . | . | . | . | . | . | . | . |
| InternalIMT | epa | rs35735968 | 0.05 | -0.023 | 0.025 | 0.371 | 0.078 | 0.058 | 0.178 | 0.054 | 0.081 | 0.51 |
| InternalIMT | epa | rs17157825 | 0.038 | -0.021 | 0.025 | 0.401 | 0.078 | 0.059 | 0.192 | 0.051 | 0.085 | 0.547 |
| InternalIMT | epa | rs2291428 | 0.065 | -9.90E-03 | 0.026 | 0.708 | 0.022 | 0.047 | 0.643 | -0.028 | 0.055 | 0.614 |
| InternalIMT | epa | rs2291429 | 0.065 | -9.90E-03 | 0.026 | 0.707 | 0.022 | 0.047 | 0.647 | -0.028 | 0.055 | 0.617 |
| InternalIMT | epa | rs145714792 | . | . | . | . | . | . | . | . | . | . |
| InternalIMT | epa | rs12769244 | 0.038 | -0.021 | 0.025 | 0.398 | 0.074 | 0.059 | 0.213 | 0.053 | 0.085 | 0.531 |
| InternalIMT | epa | rs12771275 | 0.038 | -0.021 | 0.025 | 0.398 | 0.074 | 0.059 | 0.213 | 0.053 | 0.085 | 0.531 |
| InternalIMT | epa | rs146266174 | . | . | . | . | . | . | . | . | . | . |
| InternalIMT | epa | rs41301623 | . | . | . | . | . | . | . | . | . | . |
| InternalIMT | epa | rs2306814 | 0.043 | -0.013 | 0.026 | 0.619 | -0.1 | 0.039 | 9.80E-03 | -0.011 | 0.054 | 0.845 |
| InternalIMT | epa | rs12244834 | 0.03 | -0.012 | 0.025 | 0.633 | -0.011 | 0.058 | 0.848 | -0.032 | 0.077 | 0.675 |
| InternalIMT | epa | rs184006011 | 0 | . | . | . | . | . | . | . | . | . |
| InternalIMT | epa | rs78829066 | 0.042 | -0.013 | 0.026 | 0.625 | -0.098 | 0.039 | 0.013 | -0.014 | 0.055 | 0.803 |
| InternalIMT | epa | rs34731408 | 0.038 | -0.021 | 0.025 | 0.398 | 0.074 | 0.059 | 0.212 | 0.053 | 0.085 | 0.531 |
| InternalIMT | epa | rs17451516 | . | . | . | . | . | . | . | . | . | . |
| InternalIMT | epa | rs139910133 | . | . | . | . | . | . | . | . | . | . |
| InternalIMT | epa | rs10900218 | 0.138 | -0.016 | 0.028 | 0.57 | 7.80E-03 | 0.031 | 0.804 | 6.10E-03 | 0.043 | 0.887 |
| InternalIMT | epa | rs146023999 | 0.025 | -0.012 | 0.025 | 0.637 | 0.113 | 0.095 | 0.234 | -0.08 | 0.136 | 0.556 |
| InternalIMT | epa | rs187296958 | 0.007 | . | . | . | . | . | . | . | . | . |
| InternalIMT | epa | rs142342833 | 0.015 | -0.017 | 0.025 | 0.485 | -0.124 | 0.081 | 0.126 | 0.149 | 0.109 | 0.169 |
| InternalIMT | epa | rs143489255 | 0.006 | . | . | . | . | . | . | . | . | . |
| InternalIMT | epa | rs138532518 | 0.006 | . | . | . | . | . | . | . | . | . |
| InternalIMT | epa | rs183768079 | 0.007 | . | . | . | . | . | . | . | . | . |
| InternalIMT | epa | rs117892272 | 0.012 | -3.50E-03 | 0.022 | 0.873 | -0.109 | 0.173 | 0.531 | -0.48 | 0.45 | 0.286 |
| InternalIMT | epa | rs192993943 | 0.003 | . | . | . | . | . | . | . | . | . |
| InternalIMT | epa | rs182989043 | 0.015 | -0.017 | 0.025 | 0.503 | 0.078 | 0.159 | 0.623 | 0.085 | 0.109 | 0.439 |
| InternalIMT | epa | rs189703435 | 0.021 | -0.013 | 0.025 | 0.589 | -0.017 | 0.086 | 0.842 | -0.013 | 0.105 | 0.898 |
| InternalIMT | epa | rs188794040 | 0.02 | -0.013 | 0.025 | 0.604 | -0.043 | 0.087 | 0.618 | -0.03 | 0.119 | 0.804 |
| InternalIMT | epa | rs143920953 | 0.003 | . | . | . | . | . | . | . | . | . |
| InternalIMT | epa | rs191029121 | 0.006 | . | . | . | . | . | . | . | . | . |
| InternalIMT | epa | rs116875448 | 0.006 | . | . | . | . | . | . | . | . | . |
| InternalIMT | epa | rs190167877 | 0.002 | . | . | . | . | . | . | . | . | . |
| InternalIMT | epa | rs184993418 | 0.003 | . | . | . | . | . | . | . | . | . |
| InternalIMT | epa | rs143241158 | 0.01 | . | . | . | . | . | . | . | . | . |
| InternalIMT | epa | rs11239500 | 0.007 | . | . | . | . | . | . | . | . | . |
| InternalIMT | epa | rs140568994 | 0.001 | . | . | . | . | . | . | . | . | . |
| InternalIMT | epa | rs187530317 | 0.008 | . | . | . | . | . | . | . | . | . |
| InternalIMT | epa | rs145189326 | 0.003 | . | . | . | . | . | . | . | . | . |
| InternalIMT | epa | rs186203332 | 0.009 | . | . | . | . | . | . | . | . | . |
| InternalIMT | epa | rs189921909 | 0.004 | . | . | . | . | . | . | . | . | . |
| InternalIMT | epa | rs187260989 | 0.01 | -8.00E-03 | 0.024 | 0.741 | -0.038 | 0.153 | 0.804 | -0.325 | 0.254 | 0.2 |
| InternalIMT | epa | rs114376586 | 0.006 | . | . | . | . | . | . | . | . | . |
| InternalIMT | epa | rs151070808 | 0.019 | -7.40E-03 | 0.025 | 0.769 | -0.049 | 0.084 | 0.559 | -0.242 | 0.145 | 0.096 |
| InternalIMT | epa | rs140927225 | 0.006 | . | . | . | . | . | . | . | . | . |
| InternalIMT | epa | rs191555615 | 0.012 | -1.70E-03 | 0.022 | 0.939 | -0.13 | 0.205 | 0.525 | -0.569 | 0.488 | 0.244 |
| InternalIMT | epa | rs80278898 | 0.047 | -0.03 | 0.024 | 0.213 | 0.121 | 0.106 | 0.252 | 0.175 | 0.173 | 0.312 |
| InternalIMT | epa | rs190849503 | 0.004 | . | . | . | . | . | . | . | . | . |
| InternalIMT | epa | rs150080315 | 0.009 | . | . | . | . | . | . | . | . | . |
| InternalIMT | epa | rs143623885 | 0.011 | -0.016 | 0.025 | 0.506 | -0.125 | 0.094 | 0.182 | 0.179 | 0.13 | 0.17 |
| InternalIMT | epa | rs187760040 | 0.002 | . | . | . | . | . | . | . | . | . |
| InternalIMT | epa | rs143917143 | 0.02 | -7.50E-03 | 0.025 | 0.766 | -0.049 | 0.084 | 0.559 | -0.239 | 0.145 | 0.1 |
| InternalIMT | epa | rs149851136 | 0.002 | . | . | . | . | . | . | . | . | . |
| InternalIMT | epa | rs181612054 | 0.001 | . | . | . | . | . | . | . | . | . |
| InternalIMT | epa | rs191006548 | 0.001 | . | . | . | . | . | . | . | . | . |
| InternalIMT | epa | rs117377631 | 0.006 | . | . | . | . | . | . | . | . | . |
| InternalIMT | epa | rs182107634 | 0.002 | . | . | . | . | . | . | . | . | . |
| InternalIMT | epa | rs190211867 | 0.002 | . | . | . | . | . | . | . | . | . |
| InternalIMT | epa | rs148293457 | 0.031 | -7.80E-03 | 0.026 | 0.765 | 4.40E-03 | 0.062 | 0.944 | -0.11 | 0.082 | 0.18 |
| InternalIMT | epa | rs191811733 | 0.006 | . | . | . | . | . | . | . | . | . |
| InternalIMT | epa | rs146109272 | 0.008 | . | . | . | . | . | . | . | . | . |
| InternalIMT | epa | rs148397915 | 0.006 | . | . | . | . | . | . | . | . | . |
| InternalIMT | epa | rs142233290 | 0.007 | . | . | . | . | . | . | . | . | . |
| InternalIMT | epa | rs80226874 | 0.022 | -0.015 | 0.025 | 0.563 | 0.108 | 0.138 | 0.435 | 0.025 | 0.215 | 0.906 |
| InternalIMT | epa | rs144496203 | 0.012 | -0.017 | 0.024 | 0.47 | -0.116 | 0.105 | 0.272 | 0.294 | 0.215 | 0.173 |

| InternalIMT | epa | rs191062130 | 0.001 | . | . | . | . | . | . | . | . | . |
| --- | --- | --- | --- | --- | --- | --- | --- | --- | --- | --- | --- | --- |
| InternalIMT | epa | rs185807817 | 0.004 | . | . | . | . | . | . | . | . | . |
| InternalIMT | epa | rs117846213 | 0.032 | -9.90E-03 | 0.025 | 0.69 | -0.04 | 0.068 | 0.556 | -0.071 | 0.092 | 0.441 |
| InternalIMT | epa | rs190545473 | 0.002 | . | . | . | . | . | . | . | . | . |
| InternalIMT | epa | rs192364918 | 0.01 | -0.019 | 0.025 | 0.438 | -0.028 | 0.347 | 0.935 | 0.309 | 0.438 | 0.48 |
| InternalIMT | epa | rs182939241 | 0.003 | . | . | . | . | . | . | . | . | . |
| InternalIMT | epa | rs12571125 | 0.031 | -0.011 | 0.026 | 0.686 | -0.039 | 0.086 | 0.656 | -0.044 | 0.105 | 0.678 |
| InternalIMT | epa | rs192418941 | 0.005 | . | . | . | . | . | . | . | . | . |
| InternalIMT | epa | rs141270243 | 0.015 | -0.01 | 0.025 | 0.674 | -0.203 | 0.088 | 0.022 | -0.095 | 0.144 | 0.508 |
| InternalIMT | epa | rs182615502 | 0.006 | . | . | . | . | . | . | . | . | . |
| InternalIMT | epa | rs146669856 | 0.001 | . | . | . | . | . | . | . | . | . |
| InternalIMT | epa | rs78290029 | 0.034 | -0.01 | 0.025 | 0.683 | -0.031 | 0.063 | 0.627 | -0.061 | 0.082 | 0.457 |
| InternalIMT | epa | rs139955565 | 0.004 | . | . | . | . | . | . | . | . | . |
| InternalIMT | epa | rs12267314 | 0.03 | -0.012 | 0.025 | 0.643 | -0.017 | 0.061 | 0.779 | -0.04 | 0.081 | 0.622 |
| InternalIMT | epa | rs74477197 | 0.004 | . | . | . | . | . | . | . | . | . |
| InternalIMT | epa | rs188147236 | 0.005 | . | . | . | . | . | . | . | . | . |
| InternalIMT | epa | rs2228065 | 0.03 | -0.012 | 0.025 | 0.641 | -0.014 | 0.06 | 0.811 | -0.038 | 0.08 | 0.631 |
| InternalIMT | epa | rs116961353 | 0.015 | -0.018 | 0.026 | 0.475 | 0.199 | 0.203 | 0.325 | 0.183 | 0.283 | 0.518 |
| InternalIMT | epa | rs190222272 | 0.007 | . | . | . | . | . | . | . | . | . |
| InternalIMT | epa | rs182719940 | 0.006 | . | . | . | . | . | . | . | . | . |
| InternalIMT | epa | rs184477413 | 0.004 | . | . | . | . | . | . | . | . | . |
| InternalIMT | epa | rs12265175 | 0.03 | -0.012 | 0.025 | 0.636 | -0.012 | 0.058 | 0.843 | -0.035 | 0.078 | 0.656 |
| InternalIMT | epa | rs182928276 | 0.003 | . | . | . | . | . | . | . | . | . |
| InternalIMT | epa | rs144409895 | 0.011 | -0.02 | 0.025 | 0.412 | -0.033 | 0.329 | 0.921 | 0.392 | 0.441 | 0.374 |
| InternalIMT | epa | rs58998447 | 0.136 | -7.10E-03 | 0.028 | 0.803 | -0.011 | 0.038 | 0.772 | -0.027 | 0.047 | 0.566 |
| InternalIMT | epa | rs188668832 | 0.005 | . | . | . | . | . | . | . | . | . |
| InternalIMT | epa | rs148641686 | 0.002 | . | . | . | . | . | . | . | . | . |
| InternalIMT | epa | rs75468791 | 0.054 | -0.025 | 0.026 | 0.336 | 0.05 | 0.08 | 0.536 | 0.133 | 0.128 | 0.3 |
| InternalIMT | epa | rs147327397 | 0.003 | . | . | . | . | . | . | . | . | . |
| InternalIMT | epa | rs77354597 | 0.029 | -0.012 | 0.025 | 0.639 | -0.013 | 0.06 | 0.824 | -0.037 | 0.078 | 0.637 |
| InternalIMT | epa | rs41365544 | 0.03 | -0.012 | 0.025 | 0.633 | -0.012 | 0.058 | 0.841 | -0.033 | 0.077 | 0.671 |
| InternalIMT | epa | rs187564188 | 0.016 | -3.30E-03 | 0.025 | 0.897 | 0.06 | 0.289 | 0.835 | -0.423 | 0.33 | 0.2 |
| InternalIMT | epa | rs183511984 | 0.007 | . | . | . | . | . | . | . | . | . |
| InternalIMT | epa | rs12262783 | 0.003 | . | . | . | . | . | . | . | . | . |
| InternalIMT | epa | rs188817687 | 0.003 | . | . | . | . | . | . | . | . | . |
| InternalIMT | epa | rs3815968 | 0.005 | . | . | . | . | . | . | . | . | . |
| InternalIMT | epa | rs187822300 | 0.001 | . | . | . | . | . | . | . | . | . |
| InternalIMT | epa | rs188902172 | 0.032 | -0.012 | 0.025 | 0.636 | -0.022 | 0.059 | 0.711 | -0.032 | 0.078 | 0.68 |
| InternalIMT | epa | rs181190448 | 0.006 | . | . | . | . | . | . | . | . | . |
| InternalIMT | epa | rs182699093 | 0.001 | . | . | . | . | . | . | . | . | . |
| InternalIMT | epa | rs182631303 | 0.014 | -0.01 | 0.025 | 0.681 | 1.90E-03 | 0.091 | 0.983 | -0.176 | 0.173 | 0.31 |
| InternalIMT | epa | rs187014172 | 0.006 | . | . | . | . | . | . | . | . | . |
| InternalIMT | epa | rs142575406 | 0.009 | . | . | . | . | . | . | . | . | . |
| InternalIMT | epa | rs180711033 | 0.007 | . | . | . | . | . | . | . | . | . |
| InternalIMT | epa | rs137894823 | . | . | . | . | . | . | . | . | . | . |
| InternalIMT | epa | rs189053273 | . | . | . | . | . | . | . | . | . | . |
| InternalIMT | epa | rs143499388 | . | . | . | . | . | . | . | . | . | . |
| InternalIMT | epa | rs182936001 | . | . | . | . | . | . | . | . | . | . |
| InternalIMT | epa | rs148456406 | . | . | . | . | . | . | . | . | . | . |
| InternalIMT | epa | rs182499531 | . | . | . | . | . | . | . | . | . | . |
| InternalIMT | epa | rs74884915 | . | . | . | . | . | . | . | . | . | . |
| InternalIMT | epa | rs184046374 | . | . | . | . | . | . | . | . | . | . |
| InternalIMT | epa | rs74333993 | . | . | . | . | . | . | . | . | . | . |
| InternalIMT | epa | rs138889719 | . | . | . | . | . | . | . | . | . | . |
| InternalIMT | epa | rs75330330 | . | . | . | . | . | . | . | . | . | . |
| InternalIMT | epa | rs193051693 | . | . | . | . | . | . | . | . | . | . |
| InternalIMT | epa | rs145929633 | . | . | . | . | . | . | . | . | . | . |
| InternalIMT | epa | rs184076904 | . | . | . | . | . | . | . | . | . | . |
| InternalIMT | epa | rs141802494 | . | . | . | . | . | . | . | . | . | . |
| InternalIMT | epa | rs149519759 | . | . | . | . | . | . | . | . | . | . |
| InternalIMT | epa | rs145467260 | . | . | . | . | . | . | . | . | . | . |
| InternalIMT | epa | rs79620914 | . | . | . | . | . | . | . | . | . | . |
| InternalIMT | epa | rs140538633 | . | . | . | . | . | . | . | . | . | . |
| InternalIMT | epa | rs140862301 | . | . | . | . | . | . | . | . | . | . |
| InternalIMT | epa | rs114615847 | . | . | . | . | . | . | . | . | . | . |
| InternalIMT | epa | rs141800259 | . | . | . | . | . | . | . | . | . | . |
| InternalIMT | epa | rs149779059 | . | . | . | . | . | . | . | . | . | . |
| InternalIMT | epa | rs116539253 | . | . | . | . | . | . | . | . | . | . |
| InternalIMT | epa | rs184979550 | . | . | . | . | . | . | . | . | . | . |
| InternalIMT | epa | rs190128940 | . | . | . | . | . | . | . | . | . | . |
| InternalIMT | epa | rs146594816 | . | . | . | . | . | . | . | . | . | . |
| InternalIMT | epa | rs141370766 | . | . | . | . | . | . | . | . | . | . |
| InternalIMT | epa | rs144670033 | . | . | . | . | . | . | . | . | . | . |

| InternalIMT | epa | rs187152174 | . | . | . | . | . | . | . | . | . | . |
| --- | --- | --- | --- | --- | --- | --- | --- | --- | --- | --- | --- | --- |
| InternalIMT | epa | rs184180263 | . | . | . | . | . | . | . | . | . | . |
| InternalIMT | epa | rs140075861 | . | . | . | . | . | . | . | . | . | . |
| InternalIMT | epa | rs186483129 | . | . | . | . | . | . | . | . | . | . |
| InternalIMT | epa | rs186634282 | . | . | . | . | . | . | . | . | . | . |
| InternalIMT | epa | rs9988663 | . | . | . | . | . | . | . | . | . | . |
| InternalIMT | epa | rs138828529 | . | . | . | . | . | . | . | . | . | . |
| InternalIMT | epa | rs188777615 | . | . | . | . | . | . | . | . | . | . |
| InternalIMT | epa | rs192321882 | . | . | . | . | . | . | . | . | . | . |
| InternalIMT | epa | rs150812270 | . | . | . | . | . | . | . | . | . | . |
| InternalIMT | epa | rs185906603 | . | . | . | . | . | . | . | . | . | . |
| InternalIMT | epa | rs191070862 | . | . | . | . | . | . | . | . | . | . |
| InternalIMT | epa | rs182904978 | . | . | . | . | . | . | . | . | . | . |
| InternalIMT | epa | rs192679655 | . | . | . | . | . | . | . | . | . | . |
| InternalIMT | epa | rs189673086 | . | . | . | . | . | . | . | . | . | . |
| InternalIMT | epa | rs181141892 | . | . | . | . | . | . | . | . | . | . |
| InternalIMT | epa | rs186404123 | . | . | . | . | . | . | . | . | . | . |
| InternalIMT | epa | rs192117287 | . | . | . | . | . | . | . | . | . | . |
| InternalIMT | epa | rs182748835 | . | . | . | . | . | . | . | . | . | . |
| InternalIMT | epa | rs141854549 | . | . | . | . | . | . | . | . | . | . |
| InternalIMT | epa | rs140163397 | . | . | . | . | . | . | . | . | . | . |
| InternalIMT | epa | rs146347300 | . | . | . | . | . | . | . | . | . | . |
| InternalIMT | epa | rs139362377 | . | . | . | . | . | . | . | . | . | . |
| InternalIMT | epa | rs185087671 | . | . | . | . | . | . | . | . | . | . |
| InternalIMT | epa | rs190466574 | . | . | . | . | . | . | . | . | . | . |
| InternalIMT | epa | rs147012778 | . | . | . | . | . | . | . | . | . | . |
| InternalIMT | epa | rs138236960 | . | . | . | . | . | . | . | . | . | . |
| InternalIMT | epa | rs116310311 | . | . | . | . | . | . | . | . | . | . |
| InternalIMT | epa | rs142868356 | . | . | . | . | . | . | . | . | . | . |
| InternalIMT | epa | rs144956240 | . | . | . | . | . | . | . | . | . | . |
| InternalIMT | epa | rs188527776 | . | . | . | . | . | . | . | . | . | . |
| InternalIMT | epa | rs183971165 | . | . | . | . | . | . | . | . | . | . |
| InternalIMT | epa | rs76748216 | . | . | . | . | . | . | . | . | . | . |
| InternalIMT | epa | rs148554990 | . | . | . | . | . | . | . | . | . | . |
| InternalIMT | epa | rs114446477 | . | . | . | . | . | . | . | . | . | . |
| InternalIMT | epa | rs185223933 | . | . | . | . | . | . | . | . | . | . |
| InternalIMT | epa | rs76869726 | . | . | . | . | . | . | . | . | . | . |
| InternalIMT | epa | rs151200830 | . | . | . | . | . | . | . | . | . | . |
| InternalIMT | epa | rs17157731 | . | . | . | . | . | . | . | . | . | . |
| InternalIMT | epa | rs181243469 | . | . | . | . | . | . | . | . | . | . |
| InternalIMT | epa | rs186890707 | . | . | . | . | . | . | . | . | . | . |
| InternalIMT | epa | rs184063360 | . | . | . | . | . | . | . | . | . | . |
| InternalIMT | epa | rs188550237 | . | . | . | . | . | . | . | . | . | . |
| InternalIMT | epa | rs140931068 | . | . | . | . | . | . | . | . | . | . |
| InternalIMT | epa | rs74867877 | . | . | . | . | . | . | . | . | . | . |
| InternalIMT | epa | rs11239495 | . | . | . | . | . | . | . | . | . | . |
| InternalIMT | epa | rs149745800 | . | . | . | . | . | . | . | . | . | . |
| InternalIMT | epa | rs145044458 | . | . | . | . | . | . | . | . | . | . |
| InternalIMT | epa | rs188067427 | . | . | . | . | . | . | . | . | . | . |
| InternalIMT | epa | rs146827800 | . | . | . | . | . | . | . | . | . | . |
| InternalIMT | epa | rs140619817 | . | . | . | . | . | . | . | . | . | . |
| InternalIMT | epa | rs892689 | . | . | . | . | . | . | . | . | . | . |
| InternalIMT | epa | rs143642005 | . | . | . | . | . | . | . | . | . | . |
| InternalIMT | epa | rs181929304 | . | . | . | . | . | . | . | . | . | . |
| InternalIMT | epa | rs191086173 | . | . | . | . | . | . | . | . | . | . |
| InternalIMT | epa | rs114613116 | . | . | . | . | . | . | . | . | . | . |
| InternalIMT | epa | rs141076768 | . | . | . | . | . | . | . | . | . | . |
| InternalIMT | epa | rs187316531 | . | . | . | . | . | . | . | . | . | . |
| InternalIMT | epa | rs11239499 | . | . | . | . | . | . | . | . | . | . |
| InternalIMT | epa | rs192726371 | . | . | . | . | . | . | . | . | . | . |
| InternalIMT | epa | rs74128481 | . | . | . | . | . | . | . | . | . | . |
| InternalIMT | epa | rs74128482 | . | . | . | . | . | . | . | . | . | . |
| InternalIMT | epa | rs11239502 | . | . | . | . | . | . | . | . | . | . |
| InternalIMT | epa | rs116330285 | . | . | . | . | . | . | . | . | . | . |
| InternalIMT | epa | rs189926729 | . | . | . | . | . | . | . | . | . | . |
| InternalIMT | epa | rs188975779 | . | . | . | . | . | . | . | . | . | . |
| InternalIMT | epa | rs143846038 | . | . | . | . | . | . | . | . | . | . |
| InternalIMT | epa | rs74128483 | . | . | . | . | . | . | . | . | . | . |
| InternalIMT | epa | rs139292055 | . | . | . | . | . | . | . | . | . | . |
| InternalIMT | epa | rs11239503 | . | . | . | . | . | . | . | . | . | . |
| InternalIMT | epa | rs58838686 | . | . | . | . | . | . | . | . | . | . |
| InternalIMT | epa | rs185825373 | . | . | . | . | . | . | . | . | . | . |
| InternalIMT | epa | rs11239504 | . | . | . | . | . | . | . | . | . | . |

| InternalIMT | epa | rs150012354 | . | . | . | . | . | . | . | . | . | . |
| --- | --- | --- | --- | --- | --- | --- | --- | --- | --- | --- | --- | --- |
| InternalIMT | epa | rs187763096 | . | . | . | . | . | . | . | . | . | . |
| InternalIMT | epa | rs193043982 | . | . | . | . | . | . | . | . | . | . |
| InternalIMT | epa | rs79186884 | . | . | . | . | . | . | . | . | . | . |
| InternalIMT | epa | rs74128484 | . | . | . | . | . | . | . | . | . | . |
| InternalIMT | epa | rs58461537 | . | . | . | . | . | . | . | . | . | . |
| InternalIMT | epa | rs188527474 | . | . | . | . | . | . | . | . | . | . |
| InternalIMT | epa | rs141659976 | . | . | . | . | . | . | . | . | . | . |
| InternalIMT | epa | rs115846407 | . | . | . | . | . | . | . | . | . | . |
| InternalIMT | epa | rs138378816 | . | . | . | . | . | . | . | . | . | . |
| InternalIMT | epa | rs146188136 | . | . | . | . | . | . | . | . | . | . |
| InternalIMT | epa | rs139383871 | . | . | . | . | . | . | . | . | . | . |
| InternalIMT | epa | rs182953869 | . | . | . | . | . | . | . | . | . | . |
| InternalIMT | epa | rs192430299 | . | . | . | . | . | . | . | . | . | . |
| InternalIMT | epa | rs144152844 | . | . | . | . | . | . | . | . | . | . |
| InternalIMT | epa | rs145987786 | . | . | . | . | . | . | . | . | . | . |
| InternalIMT | epa | rs181132926 | . | . | . | . | . | . | . | . | . | . |
| InternalIMT | epa | rs147882253 | . | . | . | . | . | . | . | . | . | . |
| InternalIMT | epa | rs141807621 | . | . | . | . | . | . | . | . | . | . |
| InternalIMT | epa | rs147116052 | . | . | . | . | . | . | . | . | . | . |
| InternalIMT | epa | rs147716618 | . | . | . | . | . | . | . | . | . | . |
| InternalIMT | epa | rs187198855 | . | . | . | . | . | . | . | . | . | . |
| InternalIMT | epa | rs189409638 | . | . | . | . | . | . | . | . | . | . |
| InternalIMT | epa | rs182647339 | . | . | . | . | . | . | . | . | . | . |
| InternalIMT | epa | rs187995988 | . | . | . | . | . | . | . | . | . | . |
| InternalIMT | epa | rs141034184 | . | . | . | . | . | . | . | . | . | . |
| InternalIMT | epa | rs187310430 | . | . | . | . | . | . | . | . | . | . |
| InternalIMT | epa | rs192608488 | . | . | . | . | . | . | . | . | . | . |
| InternalIMT | epa | rs148840366 | . | . | . | . | . | . | . | . | . | . |
| InternalIMT | epa | rs186785022 | . | . | . | . | . | . | . | . | . | . |
| InternalIMT | epa | rs192348728 | . | . | . | . | . | . | . | . | . | . |
| InternalIMT | epa | rs189656631 | . | . | . | . | . | . | . | . | . | . |
| InternalIMT | epa | rs185289750 | . | . | . | . | . | . | . | . | . | . |
| InternalIMT | epa | rs142382395 | . | . | . | . | . | . | . | . | . | . |
| InternalIMT | epa | rs144866696 | . | . | . | . | . | . | . | . | . | . |
| InternalIMT | epa | rs141371289 | . | . | . | . | . | . | . | . | . | . |
| InternalIMT | epa | rs114750008 | . | . | . | . | . | . | . | . | . | . |
| InternalIMT | epa | rs189063597 | . | . | . | . | . | . | . | . | . | . |
| InternalIMT | epa | rs139151571 | . | . | . | . | . | . | . | . | . | . |
| InternalIMT | epa | rs139846368 | . | . | . | . | . | . | . | . | . | . |
| InternalIMT | epa | rs182057653 | . | . | . | . | . | . | . | . | . | . |
| InternalIMT | epa | rs148785420 | . | . | . | . | . | . | . | . | . | . |
| InternalIMT | epa | rs138383251 | . | . | . | . | . | . | . | . | . | . |
| InternalIMT | epa | rs116526705 | . | . | . | . | . | . | . | . | . | . |
| InternalIMT | epa | rs185946436 | . | . | . | . | . | . | . | . | . | . |
| InternalIMT | epa | rs190455427 | . | . | . | . | . | . | . | . | . | . |
| InternalIMT | epa | rs9282584 | . | . | . | . | . | . | . | . | . | . |
| InternalIMT | epa | rs17153289 | . | . | . | . | . | . | . | . | . | . |
| InternalIMT | epa | rs138359103 | . | . | . | . | . | . | . | . | . | . |
| InternalIMT | epa | rs150605268 | . | . | . | . | . | . | . | . | . | . |
| InternalIMT | epa | rs79242910 | . | . | . | . | . | . | . | . | . | . |
| InternalIMT | epa | rs139716904 | . | . | . | . | . | . | . | . | . | . |
| InternalIMT | epa | rs76238498 | . | . | . | . | . | . | . | . | . | . |
| InternalIMT | epa | rs184140639 | . | . | . | . | . | . | . | . | . | . |
| InternalIMT | epa | rs188517278 | . | . | . | . | . | . | . | . | . | . |
| InternalIMT | epa | rs139678913 | . | . | . | . | . | . | . | . | . | . |
| InternalIMT | epa | rs116697955 | . | . | . | . | . | . | . | . | . | . |
| InternalIMT | epa | rs79267994 | . | . | . | . | . | . | . | . | . | . |
| InternalIMT | epa | rs11239510 | . | . | . | . | . | . | . | . | . | . |
| InternalIMT | epa | rs75831645 | . | . | . | . | . | . | . | . | . | . |
| InternalIMT | epa | rs78452276 | . | . | . | . | . | . | . | . | . | . |
| InternalIMT | epa | rs144366924 | . | . | . | . | . | . | . | . | . | . |
| InternalIMT | epa | rs77519841 | . | . | . | . | . | . | . | . | . | . |
| InternalIMT | epa | rs76944454 | . | . | . | . | . | . | . | . | . | . |
| InternalIMT | epa | rs76443855 | . | . | . | . | . | . | . | . | . | . |
| InternalIMT | epa | rs146613174 | . | . | . | . | . | . | . | . | . | . |
| InternalIMT | epa | rs181424761 | . | . | . | . | . | . | . | . | . | . |
| InternalIMT | epa | rs142119241 | . | . | . | . | . | . | . | . | . | . |
| InternalIMT | epa | rs140137954 | . | . | . | . | . | . | . | . | . | . |
| InternalIMT | epa | rs187319549 | . | . | . | . | . | . | . | . | . | . |
| InternalIMT | epa | rs12241545 | . | . | . | . | . | . | . | . | . | . |
| InternalIMT | epa | rs10128228 | . | . | . | . | . | . | . | . | . | . |
| InternalIMT | epa | rs58580249 | . | . | . | . | . | . | . | . | . | . |

| InternalIMT | epa | rs193148863 | . | . | . | . | . | . | . | . | . | . |
| --- | --- | --- | --- | --- | --- | --- | --- | --- | --- | --- | --- | --- |
| InternalIMT | epa | rs185638946 | . | . | . | . | . | . | . | . | . | . |
| InternalIMT | epa | rs117752921 | . | . | . | . | . | . | . | . | . | . |
| InternalIMT | epa | rs180841236 | . | . | . | . | . | . | . | . | . | . |
| InternalIMT | epa | rs73283155 | . | . | . | . | . | . | . | . | . | . |
| InternalIMT | epa | rs76560599 | . | . | . | . | . | . | . | . | . | . |
| InternalIMT | epa | rs76273620 | . | . | . | . | . | . | . | . | . | . |
| InternalIMT | epa | rs141432923 | . | . | . | . | . | . | . | . | . | . |
| InternalIMT | epa | rs113529322 | . | . | . | . | . | . | . | . | . | . |
| InternalIMT | epa | rs10128306 | . | . | . | . | . | . | . | . | . | . |
| InternalIMT | epa | rs149691688 | . | . | . | . | . | . | . | . | . | . |
| InternalIMT | epa | rs75872495 | . | . | . | . | . | . | . | . | . | . |
| InternalIMT | epa | rs191873960 | . | . | . | . | . | . | . | . | . | . |
| InternalIMT | epa | rs80155622 | . | . | . | . | . | . | . | . | . | . |
| InternalIMT | epa | rs113914656 | . | . | . | . | . | . | . | . | . | . |
| InternalIMT | epa | rs12242840 | . | . | . | . | . | . | . | . | . | . |
| InternalIMT | epa | rs147824078 | . | . | . | . | . | . | . | . | . | . |
| InternalIMT | epa | rs59051493 | . | . | . | . | . | . | . | . | . | . |
| InternalIMT | epa | rs9282588 | . | . | . | . | . | . | . | . | . | . |
| InternalIMT | epa | rs150215207 | . | . | . | . | . | . | . | . | . | . |
| InternalIMT | epa | rs138772243 | . | . | . | . | . | . | . | . | . | . |
| InternalIMT | epa | rs149383244 | . | . | . | . | . | . | . | . | . | . |
| InternalIMT | epa | rs17522720 | . | . | . | . | . | . | . | . | . | . |
| InternalIMT | epa | rs79245105 | . | . | . | . | . | . | . | . | . | . |
| InternalIMT | epa | rs138222133 | . | . | . | . | . | . | . | . | . | . |
| InternalIMT | epa | rs12247314 | . | . | . | . | . | . | . | . | . | . |
| InternalIMT | epa | rs12263894 | . | . | . | . | . | . | . | . | . | . |
| InternalIMT | epa | rs149907880 | . | . | . | . | . | . | . | . | . | . |
| InternalIMT | epa | rs188153885 | . | . | . | . | . | . | . | . | . | . |
| InternalIMT | epa | rs73283159 | . | . | . | . | . | . | . | . | . | . |
| InternalIMT | epa | rs140476687 | . | . | . | . | . | . | . | . | . | . |
| InternalIMT | epa | rs186603148 | . | . | . | . | . | . | . | . | . | . |
| InternalIMT | epa | rs183296063 | . | . | . | . | . | . | . | . | . | . |
| InternalIMT | epa | rs145733694 | . | . | . | . | . | . | . | . | . | . |
| InternalIMT | epa | rs189807449 | . | . | . | . | . | . | . | . | . | . |
| InternalIMT | epa | rs12253223 | . | . | . | . | . | . | . | . | . | . |
| InternalIMT | epa | rs182113637 | . | . | . | . | . | . | . | . | . | . |
| InternalIMT | epa | rs142792099 | . | . | . | . | . | . | . | . | . | . |
| InternalIMT | epa | rs12249910 | . | . | . | . | . | . | . | . | . | . |
| InternalIMT | epa | rs149436339 | . | . | . | . | . | . | . | . | . | . |
| InternalIMT | epa | rs77632210 | . | . | . | . | . | . | . | . | . | . |
| InternalIMT | epa | rs74130818 | . | . | . | . | . | . | . | . | . | . |
| InternalIMT | epa | rs187583648 | . | . | . | . | . | . | . | . | . | . |
| InternalIMT | epa | rs191295987 | . | . | . | . | . | . | . | . | . | . |
| InternalIMT | epa | rs184200686 | . | . | . | . | . | . | . | . | . | . |
| InternalIMT | epa | rs76117263 | . | . | . | . | . | . | . | . | . | . |
| InternalIMT | epa | rs185393149 | . | . | . | . | . | . | . | . | . | . |
| InternalIMT | epa | rs144760014 | . | . | . | . | . | . | . | . | . | . |
| InternalIMT | epa | rs7922292 | . | . | . | . | . | . | . | . | . | . |
| InternalIMT | epa | rs115372844 | . | . | . | . | . | . | . | . | . | . |
| InternalIMT | epa | rs111460364 | . | . | . | . | . | . | . | . | . | . |
| InternalIMT | epa | rs182052255 | . | . | . | . | . | . | . | . | . | . |
| InternalIMT | epa | rs180983889 | . | . | . | . | . | . | . | . | . | . |
| InternalIMT | epa | rs139175327 | . | . | . | . | . | . | . | . | . | . |
| InternalIMT | epa | rs149959620 | . | . | . | . | . | . | . | . | . | . |
| InternalIMT | epa | rs114653117 | . | . | . | . | . | . | . | . | . | . |
| InternalIMT | epa | rs144270018 | . | . | . | . | . | . | . | . | . | . |
| InternalIMT | epa | rs182723204 | . | . | . | . | . | . | . | . | . | . |
| InternalIMT | epa | rs57204067 | . | . | . | . | . | . | . | . | . | . |
| InternalIMT | epa | rs144956226 | . | . | . | . | . | . | . | . | . | . |
| InternalIMT | epa | rs187842704 | . | . | . | . | . | . | . | . | . | . |
| InternalIMT | epa | rs184620511 | . | . | . | . | . | . | . | . | . | . |
| InternalIMT | epa | rs144031303 | . | . | . | . | . | . | . | . | . | . |
| InternalIMT | epa | rs187526473 | . | . | . | . | . | . | . | . | . | . |
| InternalIMT | epa | rs193002805 | . | . | . | . | . | . | . | . | . | . |
| InternalIMT | epa | rs138707426 | . | . | . | . | . | . | . | . | . | . |
| InternalIMT | epa | rs142754243 | . | . | . | . | . | . | . | . | . | . |
| InternalIMT | epa | rs181600331 | . | . | . | . | . | . | . | . | . | . |
| InternalIMT | epa | rs144421264 | . | . | . | . | . | . | . | . | . | . |
| InternalIMT | epa | rs184414519 | . | . | . | . | . | . | . | . | . | . |
| InternalIMT | epa | rs150596349 | . | . | . | . | . | . | . | . | . | . |
| InternalIMT | epa | rs190753747 | . | . | . | . | . | . | . | . | . | . |
| InternalIMT | epa | rs139560940 | . | . | . | . | . | . | . | . | . | . |

| InternalIMT | epa | rs75640804 | . | . | . | . | . | . | . | . | . | . |
| --- | --- | --- | --- | --- | --- | --- | --- | --- | --- | --- | --- | --- |
| InternalIMT | epa | rs73283175 | . | . | . | . | . | . | . | . | . | . |
| InternalIMT | epa | rs143337229 | . | . | . | . | . | . | . | . | . | . |
| InternalIMT | epa | rs73283177 | . | . | . | . | . | . | . | . | . | . |
| InternalIMT | epa | rs76053611 | . | . | . | . | . | . | . | . | . | . |
| InternalIMT | epa | rs116325983 | . | . | . | . | . | . | . | . | . | . |
| InternalIMT | epa | rs115451825 | . | . | . | . | . | . | . | . | . | . |
| InternalIMT | epa | rs183845246 | . | . | . | . | . | . | . | . | . | . |
| InternalIMT | epa | rs79756542 | . | . | . | . | . | . | . | . | . | . |
| InternalIMT | epa | rs187242860 | . | . | . | . | . | . | . | . | . | . |
| InternalIMT | epa | rs57318980 | . | . | . | . | . | . | . | . | . | . |
| InternalIMT | epa | rs59969732 | . | . | . | . | . | . | . | . | . | . |
| InternalIMT | epa | rs143811488 | . | . | . | . | . | . | . | . | . | . |
| InternalIMT | epa | rs189067644 | . | . | . | . | . | . | . | . | . | . |
| InternalIMT | epa | rs60449178 | . | . | . | . | . | . | . | . | . | . |
| InternalIMT | epa | rs59243389 | . | . | . | . | . | . | . | . | . | . |
| InternalIMT | epa | rs184946540 | . | . | . | . | . | . | . | . | . | . |
| InternalIMT | epa | rs188685881 | . | . | . | . | . | . | . | . | . | . |
| InternalIMT | epa | rs150335797 | . | . | . | . | . | . | . | . | . | . |
| InternalIMT | epa | rs143879260 | . | . | . | . | . | . | . | . | . | . |
| InternalIMT | epa | rs186044491 | . | . | . | . | . | . | . | . | . | . |
| InternalIMT | epa | rs11819084 | . | . | . | . | . | . | . | . | . | . |
| InternalIMT | epa | rs146915670 | . | . | . | . | . | . | . | . | . | . |
| InternalIMT | epa | rs113995695 | . | . | . | . | . | . | . | . | . | . |
| InternalIMT | epa | rs143489451 | . | . | . | . | . | . | . | . | . | . |
| InternalIMT | epa | rs182653839 | . | . | . | . | . | . | . | . | . | . |
| InternalIMT | epa | rs141692564 | . | . | . | . | . | . | . | . | . | . |
| InternalIMT | epa | rs146281742 | . | . | . | . | . | . | . | . | . | . |
| InternalIMT | epa | rs192914353 | . | . | . | . | . | . | . | . | . | . |
| InternalIMT | epa | rs185182848 | . | . | . | . | . | . | . | . | . | . |
| InternalIMT | epa | rs28395858 | . | . | . | . | . | . | . | . | . | . |
| InternalIMT | epa | rs41429645 | . | . | . | . | . | . | . | . | . | . |
| InternalIMT | epa | rs28395874 | . | . | . | . | . | . | . | . | . | . |
| InternalIMT | epa | rs189663634 | . | . | . | . | . | . | . | . | . | . |
| InternalIMT | epa | rs77666700 | . | . | . | . | . | . | . | . | . | . |
| InternalIMT | epa | rs146711717 | . | . | . | . | . | . | . | . | . | . |
| InternalIMT | epa | rs144904251 | . | . | . | . | . | . | . | . | . | . |
| InternalIMT | epa | rs189893153 | . | . | . | . | . | . | . | . | . | . |
| InternalIMT | epa | rs150447740 | . | . | . | . | . | . | . | . | . | . |
| InternalIMT | epa | rs143099649 | . | . | . | . | . | . | . | . | . | . |
| InternalIMT | epa | rs191288436 | . | . | . | . | . | . | . | . | . | . |
| InternalIMT | epa | rs188577862 | . | . | . | . | . | . | . | . | . | . |
| InternalIMT | epa | rs192695334 | . | . | . | . | . | . | . | . | . | . |
| InternalIMT | epa | rs138612906 | . | . | . | . | . | . | . | . | . | . |
| InternalIMT | epa | rs191290051 | . | . | . | . | . | . | . | . | . | . |
| InternalIMT | epa | rs189631459 | . | . | . | . | . | . | . | . | . | . |
| InternalIMT | epa | rs114620874 | . | . | . | . | . | . | . | . | . | . |
| InternalIMT | epa | rs78246652 | . | . | . | . | . | . | . | . | . | . |
| InternalIMT | epa | rs79432451 | . | . | . | . | . | . | . | . | . | . |
| InternalIMT | epa | rs186022585 | . | . | . | . | . | . | . | . | . | . |
| InternalIMT | epa | rs142978891 | . | . | . | . | . | . | . | . | . | . |
| InternalIMT | epa | rs151086840 | . | . | . | . | . | . | . | . | . | . |
| InternalIMT | epa | rs183270259 | . | . | . | . | . | . | . | . | . | . |
| InternalIMT | epa | rs188544289 | . | . | . | . | . | . | . | . | . | . |
| InternalIMT | epa | rs188500808 | . | . | . | . | . | . | . | . | . | . |
| InternalIMT | epa | rs189424589 | . | . | . | . | . | . | . | . | . | . |
| InternalIMT | epa | rs78622303 | . | . | . | . | . | . | . | . | . | . |
| InternalIMT | epa | rs143210835 | . | . | . | . | . | . | . | . | . | . |
| InternalIMT | epa | rs114761840 | . | . | . | . | . | . | . | . | . | . |
| InternalIMT | epa | rs185805402 | . | . | . | . | . | . | . | . | . | . |
| InternalIMT | epa | rs139273744 | . | . | . | . | . | . | . | . | . | . |
| InternalIMT | epa | rs184654150 | . | . | . | . | . | . | . | . | . | . |
| InternalIMT | epa | rs189069710 | . | . | . | . | . | . | . | . | . | . |
| InternalIMT | epa | rs114752115 | . | . | . | . | . | . | . | . | . | . |
| InternalIMT | epa | rs150162515 | . | . | . | . | . | . | . | . | . | . |
| InternalIMT | epa | rs180982454 | . | . | . | . | . | . | . | . | . | . |
| InternalIMT | epa | rs184932088 | . | . | . | . | . | . | . | . | . | . |
| InternalIMT | epa | rs181109625 | . | . | . | . | . | . | . | . | . | . |
| InternalIMT | epa | rs138121134 | . | . | . | . | . | . | . | . | . | . |
| InternalIMT | epa | rs183253861 | . | . | . | . | . | . | . | . | . | . |
| InternalIMT | epa | rs147073946 | . | . | . | . | . | . | . | . | . | . |
| InternalIMT | epa | rs139938489 | . | . | . | . | . | . | . | . | . | . |
| InternalIMT | epa | rs146362560 | . | . | . | . | . | . | . | . | . | . |

| InternalIMT | epa | rs193155956 | . | . | . | . | . | . | . | . | . | . |
| --- | --- | --- | --- | --- | --- | --- | --- | --- | --- | --- | --- | --- |
| InternalIMT | epa | rs77663092 | . | . | . | . | . | . | . | . | . | . |
| InternalIMT | epa | rs73285319 | . | . | . | . | . | . | . | . | . | . |
| InternalIMT | epa | rs184019276 | . | . | . | . | . | . | . | . | . | . |
| InternalIMT | epa | rs28395863 | . | . | . | . | . | . | . | . | . | . |
| InternalIMT | epa | rs182121645 | . | . | . | . | . | . | . | . | . | . |
| InternalIMT | epa | rs73285327 | . | . | . | . | . | . | . | . | . | . |
| InternalIMT | epa | rs149589584 | . | . | . | . | . | . | . | . | . | . |
| InternalIMT | epa | rs148310335 | . | . | . | . | . | . | . | . | . | . |
| InternalIMT | epa | rs187246093 | . | . | . | . | . | . | . | . | . | . |
| InternalIMT | epa | rs184668894 | . | . | . | . | . | . | . | . | . | . |
| InternalIMT | epa | rs114269961 | . | . | . | . | . | . | . | . | . | . |
| InternalIMT | epa | rs149694374 | . | . | . | . | . | . | . | . | . | . |
| InternalIMT | epa | rs145284227 | . | . | . | . | . | . | . | . | . | . |
| InternalIMT | epa | rs189983820 | . | . | . | . | . | . | . | . | . | . |
| InternalIMT | epa | rs181200444 | . | . | . | . | . | . | . | . | . | . |
| InternalIMT | epa | rs185486385 | . | . | . | . | . | . | . | . | . | . |
| InternalIMT | epa | rs151198241 | . | . | . | . | . | . | . | . | . | . |
| InternalIMT | epa | rs189959341 | . | . | . | . | . | . | . | . | . | . |
| InternalIMT | epa | rs140549257 | . | . | . | . | . | . | . | . | . | . |
| InternalIMT | epa | rs186453139 | . | . | . | . | . | . | . | . | . | . |
| InternalIMT | epa | rs180922711 | . | . | . | . | . | . | . | . | . | . |
| InternalIMT | epa | rs56659903 | . | . | . | . | . | . | . | . | . | . |
| InternalIMT | epa | rs141202128 | . | . | . | . | . | . | . | . | . | . |
| InternalIMT | epa | rs59085314 | . | . | . | . | . | . | . | . | . | . |
| InternalIMT | epa | rs145564996 | . | . | . | . | . | . | . | . | . | . |
| InternalIMT | epa | rs114961009 | . | . | . | . | . | . | . | . | . | . |
| InternalIMT | epa | rs182294189 | . | . | . | . | . | . | . | . | . | . |
| InternalIMT | epa | rs187586636 | . | . | . | . | . | . | . | . | . | . |
| InternalIMT | epa | rs192254907 | . | . | . | . | . | . | . | . | . | . |
| InternalIMT | epa | rs149145663 | . | . | . | . | . | . | . | . | . | . |
| InternalIMT | epa | rs180713064 | . | . | . | . | . | . | . | . | . | . |
| InternalIMT | epa | rs183009023 | . | . | . | . | . | . | . | . | . | . |
| InternalIMT | epa | rs143385347 | . | . | . | . | . | . | . | . | . | . |
| InternalIMT | epa | rs185786329 | . | . | . | . | . | . | . | . | . | . |
| InternalIMT | epa | rs192341030 | . | . | . | . | . | . | . | . | . | . |
| InternalIMT | epa | rs74661429 | . | . | . | . | . | . | . | . | . | . |
| InternalIMT | epa | rs188034853 | . | . | . | . | . | . | . | . | . | . |
| InternalIMT | epa | rs116808430 | . | . | . | . | . | . | . | . | . | . |
| InternalIMT | epa | rs148469010 | . | . | . | . | . | . | . | . | . | . |
| InternalIMT | epa | rs78621663 | . | . | . | . | . | . | . | . | . | . |
| InternalIMT | epa | rs192654886 | . | . | . | . | . | . | . | . | . | . |
| InternalIMT | epa | rs145996035 | . | . | . | . | . | . | . | . | . | . |
| InternalIMT | epa | rs139942226 | . | . | . | . | . | . | . | . | . | . |
| InternalIMT | epa | rs113645632 | . | . | . | . | . | . | . | . | . | . |
| InternalIMT | epa | rs74940661 | . | . | . | . | . | . | . | . | . | . |
| InternalIMT | epa | rs115355800 | . | . | . | . | . | . | . | . | . | . |
| InternalIMT | epa | rs150285898 | . | . | . | . | . | . | . | . | . | . |
| InternalIMT | epa | rs188117932 | . | . | . | . | . | . | . | . | . | . |
| InternalIMT | epa | rs139956566 | . | . | . | . | . | . | . | . | . | . |
| InternalIMT | epa | rs147673885 | . | . | . | . | . | . | . | . | . | . |
| InternalIMT | epa | rs189127928 | . | . | . | . | . | . | . | . | . | . |
| InternalIMT | epa | rs187558772 | . | . | . | . | . | . | . | . | . | . |
| InternalIMT | epa | rs181948587 | . | . | . | . | . | . | . | . | . | . |
| InternalIMT | epa | rs189983269 | . | . | . | . | . | . | . | . | . | . |
| InternalIMT | epa | rs187250069 | . | . | . | . | . | . | . | . | . | . |
| InternalIMT | epa | rs191485859 | . | . | . | . | . | . | . | . | . | . |
| InternalIMT | epa | rs185499555 | . | . | . | . | . | . | . | . | . | . |
| InternalIMT | epa | rs180862110 | . | . | . | . | . | . | . | . | . | . |
| InternalIMT | epa | rs190472603 | . | . | . | . | . | . | . | . | . | . |
| InternalIMT | epa | rs189226167 | . | . | . | . | . | . | . | . | . | . |
| InternalIMT | epa | rs181623256 | . | . | . | . | . | . | . | . | . | . |
| InternalIMT | epa | rs186079557 | . | . | . | . | . | . | . | . | . | . |
| InternalIMT | epa | rs147760994 | . | . | . | . | . | . | . | . | . | . |
| InternalIMT | epa | rs189176167 | . | . | . | . | . | . | . | . | . | . |
| InternalIMT | epa | rs117901000 | . | . | . | . | . | . | . | . | . | . |
| InternalIMT | epa | rs141984888 | . | . | . | . | . | . | . | . | . | . |
| InternalIMT | epa | rs192365061 | . | . | . | . | . | . | . | . | . | . |
| InternalIMT | epa | rs185976038 | . | . | . | . | . | . | . | . | . | . |
| InternalIMT | epa | rs184697303 | . | . | . | . | . | . | . | . | . | . |
| InternalIMT | epa | rs143383132 | . | . | . | . | . | . | . | . | . | . |
| InternalIMT | epa | rs141873881 | . | . | . | . | . | . | . | . | . | . |
| InternalIMT | epa | rs185507255 | . | . | . | . | . | . | . | . | . | . |

| InternalIMT | epa | rs191379425 | . | . | . | . | . | . | . | . | . | . |
| --- | --- | --- | --- | --- | --- | --- | --- | --- | --- | --- | --- | --- |
| InternalIMT | epa | rs184199584 | . | . | . | . | . | . | . | . | . | . |
| InternalIMT | epa | rs183181360 | . | . | . | . | . | . | . | . | . | . |
| InternalIMT | epa | rs184495673 | . | . | . | . | . | . | . | . | . | . |
| InternalIMT | epa | rs182946297 | . | . | . | . | . | . | . | . | . | . |
| InternalIMT | epa | rs187743615 | . | . | . | . | . | . | . | . | . | . |
| InternalIMT | epa | rs183259862 | . | . | . | . | . | . | . | . | . | . |
| InternalIMT | epa | rs112433566 | . | . | . | . | . | . | . | . | . | . |
| InternalIMT | epa | rs182351085 | . | . | . | . | . | . | . | . | . | . |
| InternalIMT | epa | rs146248508 | . | . | . | . | . | . | . | . | . | . |
| InternalIMT | epa | rs188528439 | . | . | . | . | . | . | . | . | . | . |
| InternalIMT | epa | rs146167018 | . | . | . | . | . | . | . | . | . | . |
| InternalIMT | epa | rs192440980 | . | . | . | . | . | . | . | . | . | . |
| InternalIMT | epa | rs185601882 | . | . | . | . | . | . | . | . | . | . |
| InternalIMT | epa | rs117272563 | . | . | . | . | . | . | . | . | . | . |
| InternalIMT | epa | rs10430734 | . | . | . | . | . | . | . | . | . | . |
| InternalIMT | epa | rs181320952 | . | . | . | . | . | . | . | . | . | . |
| InternalIMT | epa | rs185480660 | . | . | . | . | . | . | . | . | . | . |
| InternalIMT | epa | rs188077917 | . | . | . | . | . | . | . | . | . | . |
| InternalIMT | epa | rs187562953 | . | . | . | . | . | . | . | . | . | . |
| InternalIMT | epa | rs182044631 | . | . | . | . | . | . | . | . | . | . |
| InternalIMT | epa | rs145455596 | . | . | . | . | . | . | . | . | . | . |
| InternalIMT | epa | rs181475877 | . | . | . | . | . | . | . | . | . | . |
| InternalIMT | epa | rs149413085 | . | . | . | . | . | . | . | . | . | . |
| InternalIMT | epa | rs12253656 | . | . | . | . | . | . | . | . | . | . |
| InternalIMT | epa | rs182718239 | . | . | . | . | . | . | . | . | . | . |
| InternalIMT | epa | rs192435695 | . | . | . | . | . | . | . | . | . | . |
| InternalIMT | epa | rs12248277 | . | . | . | . | . | . | . | . | . | . |
| InternalIMT | epa | rs143521710 | . | . | . | . | . | . | . | . | . | . |
| InternalIMT | epa | rs144454454 | . | . | . | . | . | . | . | . | . | . |
| InternalIMT | epa | rs190441610 | . | . | . | . | . | . | . | . | . | . |
| InternalIMT | epa | rs114690462 | . | . | . | . | . | . | . | . | . | . |
| InternalIMT | epa | rs184541357 | . | . | . | . | . | . | . | . | . | . |
| InternalIMT | epa | rs142657653 | . | . | . | . | . | . | . | . | . | . |
| InternalIMT | epa | rsurf1 | 2.462 | . | . | . | . | . | . | . | . | . |
| InternalIMT | epa | rsten1 | 2.569 | . | . | . | . | . | . | . | . | . |
| InternalIMT | epa | chr10:45852312:D | 0.198 | -2.10E-03 | 0.03 | 0.946 | 5.40E-03 | 0.034 | 0.874 | -0.031 | 0.035 | 0.387 |
| InternalIMT | epa | chr10:45853232:I | 0.2 | -1.20E-03 | 0.031 | 0.967 | 6.70E-03 | 0.034 | 0.843 | -0.032 | 0.035 | 0.358 |
| InternalIMT | epa | chr10:45853234:I | 0.194 | -2.30E-03 | 0.031 | 0.94 | 6.20E-03 | 0.033 | 0.853 | -0.03 | 0.036 | 0.399 |
| InternalIMT | epa | chr10:45854796:D | 0.053 | -8.90E-03 | 0.026 | 0.733 | -0.075 | 0.046 | 0.105 | -0.04 | 0.077 | 0.604 |
| InternalIMT | epa | chr10:45854994:I | 0.054 | -8.90E-03 | 0.026 | 0.733 | -0.075 | 0.046 | 0.105 | -0.04 | 0.077 | 0.603 |
| InternalIMT | epa | chr10:45858290:D | 0.048 | -1.80E-04 | 0.027 | 0.995 | -9.30E-03 | 0.123 | 0.94 | -0.152 | 0.132 | 0.25 |
| InternalIMT | epa | chr10:45858820:I | 0.005 | . | . | . | . | . | . | . | . | . |
| InternalIMT | epa | chr10:45860888:D | 0.063 | 1.20E-03 | 0.026 | 0.963 | -0.026 | 0.076 | 0.734 | -0.135 | 0.091 | 0.137 |
| InternalIMT | epa | chr10:45863913:I | 0.195 | -2.50E-03 | 0.03 | 0.935 | 2.20E-04 | 0.033 | 0.995 | -0.03 | 0.035 | 0.404 |
| InternalIMT | epa | chr10:45866601:D | . | . | . | . | . | . | . | . | . | . |
| InternalIMT | epa | chr10:45867197:I | . | . | . | . | . | . | . | . | . | . |
| InternalIMT | epa | chr10:45867538:D | 0.198 | -3.00E-03 | 0.031 | 0.921 | 1.30E-03 | 0.034 | 0.97 | -0.028 | 0.035 | 0.431 |
| InternalIMT | epa | chr10:45868356:D | 0.197 | -2.90E-03 | 0.031 | 0.924 | 1.50E-03 | 0.034 | 0.965 | -0.028 | 0.035 | 0.424 |
| InternalIMT | epa | chr10:45871158:I | 0.198 | -3.00E-03 | 0.031 | 0.923 | 1.40E-03 | 0.034 | 0.968 | -0.028 | 0.035 | 0.427 |
| InternalIMT | epa | chr10:45871637:D | 0.1 | -0.012 | 0.029 | 0.68 | -7.30E-03 | 0.049 | 0.881 | -0.012 | 0.058 | 0.838 |
| InternalIMT | epa | chr10:45871638:I | 0.161 | -6.30E-03 | 0.03 | 0.834 | 3.60E-03 | 0.039 | 0.926 | -0.023 | 0.04 | 0.56 |
| InternalIMT | epa | chr10:45873629:I | 0.198 | -3.00E-03 | 0.031 | 0.922 | 1.40E-03 | 0.034 | 0.968 | -0.028 | 0.035 | 0.427 |
| InternalIMT | epa | chr10:45873701:D | . | . | . | . | . | . | . | . | . | . |
| InternalIMT | epa | chr10:45874997:I | . | . | . | . | . | . | . | . | . | . |
| InternalIMT | epa | chr10:45882030:I | . | . | . | . | . | . | . | . | . | . |
| InternalIMT | epa | chr10:45883774:D | 0.003 | . | . | . | . | . | . | . | . | . |
| InternalIMT | epa | chr10:45885169:D | 0.176 | -9.10E-03 | 0.03 | 0.765 | -1.30E-03 | 0.036 | 0.971 | -0.014 | 0.038 | 0.711 |
| InternalIMT | epa | chr10:45886451:D | 0.744 | -0.102 | 0.067 | 0.128 | 0.014 | 0.029 | 0.631 | 0.058 | 0.041 | 0.153 |
| InternalIMT | epa | chr10:45891800:I | 0.85 | -0.037 | 0.074 | 0.611 | 9.70E-03 | 0.03 | 0.749 | 0.014 | 0.044 | 0.755 |
| InternalIMT | epa | chr10:45904771:I | 0.87 | -0.04 | 0.083 | 0.628 | 9.80E-03 | 0.032 | 0.758 | 0.015 | 0.048 | 0.755 |
| InternalIMT | epa | chr10:45904959:I | 0.11 | -0.02 | 0.031 | 0.519 | 0.011 | 0.059 | 0.853 | 0.026 | 0.063 | 0.678 |
| InternalIMT | epa | chr10:45911028:I | 0.105 | -0.033 | 0.027 | 0.221 | 0.069 | 0.059 | 0.238 | 0.112 | 0.097 | 0.248 |
| InternalIMT | epa | chr10:45911031:I | 0.419 | -0.019 | 0.04 | 0.629 | 0.034 | 0.029 | 0.242 | 4.90E-03 | 0.052 | 0.924 |
| InternalIMT | epa | chr10:45912154:D | 0.003 | . | . | . | . | . | . | . | . | . |
| InternalIMT | epa | chr10:45913885:I | 0.043 | -0.012 | 0.026 | 0.639 | -0.109 | 0.043 | 0.011 | -0.015 | 0.059 | 0.799 |
| InternalIMT | epa | chr10:45914753:D | 0.159 | -0.015 | 0.028 | 0.603 | 0.055 | 0.037 | 0.131 | 2.30E-04 | 0.043 | 0.996 |
| InternalIMT | epa | chr10:45919621 | . | . | . | . | . | . | . | . | . | . |
| InternalIMT | epa | chr10:45921739:I | 0.048 | -0.011 | 0.026 | 0.666 | -0.086 | 0.038 | 0.022 | -0.021 | 0.053 | 0.693 |
| InternalIMT | epa | chr10:45923527:I | 0.062 | -4.50E-03 | 0.029 | 0.878 | -0.074 | 0.068 | 0.274 | -0.088 | 0.093 | 0.345 |
| InternalIMT | epa | chr10:45927001:I | 0.005 | . | . | . | . | . | . | . | . | . |
| InternalIMT | epa | chr10:45930354:I | 0.044 | -0.012 | 0.026 | 0.646 | -0.091 | 0.039 | 0.019 | -0.02 | 0.054 | 0.714 |
| InternalIMT | epa | chr10:45932864:I | 0.065 | -8.60E-03 | 0.027 | 0.747 | -0.102 | 0.041 | 0.013 | -0.038 | 0.055 | 0.488 |

| InternalIMT | epa | chr10:45936163:I | 0.027 | -0.015 | 0.025 | 0.544 | 0.041 | 0.064 | 0.52 | 0.022 | 0.087 | 0.797 |
| --- | --- | --- | --- | --- | --- | --- | --- | --- | --- | --- | --- | --- |
| InternalIMT | epa | chr10:45938095:D | 0.058 | -0.013 | 0.026 | 0.631 | 0.034 | 0.048 | 0.477 | -8.20E-03 | 0.06 | 0.891 |
| InternalIMT | epa | chr10:45938886 | . | . | . | . | . | . | . | . | . | . |
| InternalIMT | epa | chr10:45939320 | . | . | . | . | . | . | . | . | . | . |
| InternalIMT | epa | chr10:45939659 | . | . | . | . | . | . | . | . | . | . |
| InternalIMT | epa | chr10:45941150:D | 0.042 | -0.012 | 0.026 | 0.635 | -0.097 | 0.039 | 0.013 | -0.015 | 0.055 | 0.785 |
| InternalIMT | epa | chr10:45947128:D | 0.038 | -0.021 | 0.025 | 0.401 | 0.073 | 0.059 | 0.22 | 0.052 | 0.085 | 0.541 |
| InternalIMT | epa | chr10:45950990:I | 0.052 | -0.013 | 0.026 | 0.617 | -0.083 | 0.042 | 0.048 | -5.40E-03 | 0.055 | 0.922 |
| InternalIMT | epa | chr10:45951854:I | . | . | . | . | . | . | . | . | . | . |
| InternalIMT | epa | chr10:45952670:I | 0.001 | . | . | . | . | . | . | . | . | . |
| InternalIMT | epa | chr10:45956741 | . | . | . | . | . | . | . | . | . | . |
| InternalIMT | epa | chr10:45957247:I | 0.065 | -9.90E-03 | 0.026 | 0.708 | 0.022 | 0.047 | 0.644 | -0.028 | 0.055 | 0.614 |
| InternalIMT | epa | chr10:45958082:I | 0.065 | -9.70E-03 | 0.026 | 0.712 | 0.022 | 0.047 | 0.634 | -0.029 | 0.055 | 0.603 |
| InternalIMT | epa | chr10:45905041:D | 0.05 | -0.012 | 0.026 | 0.644 | -0.093 | 0.041 | 0.023 | -0.012 | 0.057 | 0.838 |
| InternalIMT | epa | chr10:45938871 | 0.003 | . | . | . | . | . | . | . | . | . |
| InternalIMT | epa | chr10:45939160 | 0.004 | . | . | . | . | . | . | . | . | . |
| InternalIMT | epa | chr10:45869910 | . | . | . | . | . | . | . | . | . | . |
| InternalIMT | epa | chr10:45876214:D | . | . | . | . | . | . | . | . | . | . |
| InternalIMT | epa | chr10:45880237:I | . | . | . | . | . | . | . | . | . | . |
| InternalIMT | epa | chr10:45884371:I | . | . | . | . | . | . | . | . | . | . |
| InternalIMT | epa | chr10:45891535:D | . | . | . | . | . | . | . | . | . | . |
| InternalIMT | epa | chr10:45893516:D | . | . | . | . | . | . | . | . | . | . |
| InternalIMT | epa | chr10:45893782:D | . | . | . | . | . | . | . | . | . | . |
| InternalIMT | epa | chr10:45895254:I | . | . | . | . | . | . | . | . | . | . |
| InternalIMT | epa | chr10:45895848:D | . | . | . | . | . | . | . | . | . | . |
| InternalIMT | epa | chr10:45896316:D | . | . | . | . | . | . | . | . | . | . |
| InternalIMT | epa | chr10:45896317:D | . | . | . | . | . | . | . | . | . | . |
| InternalIMT | epa | chr10:45902683:I | . | . | . | . | . | . | . | . | . | . |
| InternalIMT | epa | chr10:45905003:D | . | . | . | . | . | . | . | . | . | . |
| InternalIMT | epa | chr10:45927476:D | . | . | . | . | . | . | . | . | . | . |
| InternalIMT | epa | chr10:45935882 | . | . | . | . | . | . | . | . | . | . |
| InternalIMT | epa | chr10:45936100 | . | . | . | . | . | . | . | . | . | . |
| InternalIMT | epa | chr10:45939553 | . | . | . | . | . | . | . | . | . | . |
| InternalIMT | epa | chr10:45939631 | . | . | . | . | . | . | . | . | . | . |
| InternalIMT | epa | chr10:45944193:D | . | . | . | . | . | . | . | . | . | . |
| InternalIMT | epa | chr10:45954030 | . | . | . | . | . | . | . | . | . | . |

Supplemental table S21.

| phen | covar(fa) | rs_id | maf | beta_FA | se_FA | p_FA | beta_SNP | se_SNP | p_SNP | beta_inter | se_inter | p_inter |
| --- | --- | --- | --- | --- | --- | --- | --- | --- | --- | --- | --- | --- |
| InternalIMT | dha | rs76035182 | 0.024 | -0.055 | 0.022 | 0.012 | 0.217 | 0.141 | 0.123 | -0.142 | 0.094 | 0.132 |
| InternalIMT | dha | rs12359547 | 0.069 | -0.064 | 0.02 | 1.50E-03 | 0.128 | 0.081 | 0.113 | -0.108 | 0.061 | 0.077 |
| InternalIMT | dha | rs74595484 | 0.039 | -0.059 | 0.022 | 7.40E-03 | 0.087 | 0.122 | 0.474 | -0.046 | 0.083 | 0.579 |
| InternalIMT | dha | rs1864415 | 0.701 | -0.026 | 0.041 | 0.521 | 0.039 | 0.035 | 0.263 | -0.037 | 0.026 | 0.161 |
| InternalIMT | dha | rs72788101 | 0.073 | -0.069 | 0.02 | 7.10E-04 | 0.089 | 0.081 | 0.272 | -0.07 | 0.062 | 0.257 |
| InternalIMT | dha | rs11239481 | 0.14 | -0.064 | 0.024 | 7.10E-03 | -0.01 | 0.052 | 0.841 | 0.017 | 0.041 | 0.675 |
| InternalIMT | dha | rs72788102 | 0.073 | -0.069 | 0.02 | 7.10E-04 | 0.089 | 0.081 | 0.272 | -0.07 | 0.062 | 0.258 |
| InternalIMT | dha | rs72789705 | 0.073 | -0.069 | 0.02 | 7.10E-04 | 0.089 | 0.081 | 0.273 | -0.07 | 0.062 | 0.258 |
| InternalIMT | dha | rs141529858 | 0.005 | . | . | . | . | . | . | . | . | . |
| InternalIMT | dha | rs145376292 | 0 | . | . | . | . | . | . | . | . | . |
| InternalIMT | dha | rs190418301 | 0.001 | . | . | . | . | . | . | . | . | . |
| InternalIMT | dha | rs11239482 | 0.073 | -0.069 | 0.02 | 7.00E-04 | 0.088 | 0.081 | 0.273 | -0.07 | 0.062 | 0.259 |
| InternalIMT | dha | rs61855872 | 0.01 | -0.066 | 0.022 | 2.20E-03 | -0.281 | 0.249 | 0.259 | 0.338 | 0.209 | 0.106 |
| InternalIMT | dha | rs148991144 | 0.007 | . | . | . | . | . | . | . | . | . |
| InternalIMT | dha | rs191932221 | 0.005 | . | . | . | . | . | . | . | . | . |
| InternalIMT | dha | rs55978138 | 0.166 | -0.06 | 0.023 | 7.60E-03 | 0.052 | 0.049 | 0.286 | -0.052 | 0.036 | 0.147 |
| InternalIMT | dha | rs73281167 | 0.015 | -0.058 | 0.022 | 6.80E-03 | 0.046 | 0.173 | 0.792 | -0.043 | 0.122 | 0.727 |
| InternalIMT | dha | rs12768010 | 0.086 | -0.072 | 0.021 | 8.40E-04 | 8.00E-03 | 0.055 | 0.883 | -0.025 | 0.039 | 0.516 |
| InternalIMT | dha | rs11239484 | 0.701 | -0.027 | 0.041 | 0.514 | 0.039 | 0.035 | 0.265 | -0.036 | 0.026 | 0.164 |
| InternalIMT | dha | rs35906360 | 0.166 | -0.06 | 0.023 | 7.50E-03 | 0.054 | 0.049 | 0.269 | -0.052 | 0.036 | 0.146 |
| InternalIMT | dha | rs186250512 | 0.003 | . | . | . | . | . | . | . | . | . |
| InternalIMT | dha | rs150810743 | 0.002 | . | . | . | . | . | . | . | . | . |
| InternalIMT | dha | rs55965812 | 0.161 | -0.057 | 0.023 | 0.012 | 0.064 | 0.049 | 0.192 | -0.064 | 0.036 | 0.077 |
| InternalIMT | dha | rs56180358 | 0.167 | -0.06 | 0.023 | 7.60E-03 | 0.054 | 0.049 | 0.269 | -0.052 | 0.036 | 0.147 |
| InternalIMT | dha | rs55864145 | 0.086 | -0.072 | 0.022 | 8.40E-04 | 7.90E-03 | 0.055 | 0.886 | -0.025 | 0.039 | 0.518 |
| InternalIMT | dha | rs55986122 | 0.166 | -0.06 | 0.023 | 7.50E-03 | 0.054 | 0.049 | 0.27 | -0.052 | 0.036 | 0.147 |
| InternalIMT | dha | rs7477350 | 0.701 | -0.027 | 0.041 | 0.512 | 0.039 | 0.035 | 0.266 | -0.036 | 0.026 | 0.165 |
| InternalIMT | dha | rs2099170 | 0.166 | -0.06 | 0.023 | 7.50E-03 | 0.055 | 0.049 | 0.264 | -0.052 | 0.036 | 0.145 |
| InternalIMT | dha | rs2082108 | 0.167 | -0.061 | 0.023 | 7.50E-03 | 0.054 | 0.049 | 0.273 | -0.051 | 0.036 | 0.149 |
| InternalIMT | dha | rs55902404 | 0.007 | . | . | . | . | . | . | . | . | . |
| InternalIMT | dha | rs58402662 | 0.007 | . | . | . | . | . | . | . | . | . |
| InternalIMT | dha | rs11511618 | 0.14 | -0.064 | 0.024 | 7.30E-03 | -0.012 | 0.052 | 0.823 | 0.016 | 0.041 | 0.693 |
| InternalIMT | dha | rs1816492 | 0.7 | -0.026 | 0.041 | 0.52 | 0.04 | 0.035 | 0.252 | -0.037 | 0.026 | 0.161 |
| InternalIMT | dha | rs146611093 | 0.007 | . | . | . | . | . | . | . | . | . |
| InternalIMT | dha | rs186897689 | 0.002 | . | . | . | . | . | . | . | . | . |
| InternalIMT | dha | rs10793616 | 0.701 | -0.027 | 0.041 | 0.51 | 0.039 | 0.035 | 0.266 | -0.036 | 0.026 | 0.166 |
| InternalIMT | dha | rs116700479 | 0.019 | -0.055 | 0.022 | 0.011 | 0.329 | 0.163 | 0.044 | -0.212 | 0.107 | 0.048 |
| InternalIMT | dha | rs192535936 | 0.002 | . | . | . | . | . | . | . | . | . |
| InternalIMT | dha | rs113363297 | 0.662 | -0.032 | 0.039 | 0.419 | 0.038 | 0.035 | 0.287 | -0.035 | 0.026 | 0.187 |
| InternalIMT | dha | rs12354994 | 0.158 | -0.063 | 0.023 | 5.20E-03 | 0.05 | 0.047 | 0.279 | -0.047 | 0.034 | 0.165 |
| InternalIMT | dha | rs148068329 | 0.02 | -0.081 | 0.02 | 4.20E-05 | -0.215 | 0.114 | 0.06 | 0.111 | 0.078 | 0.153 |
| InternalIMT | dha | rs12356109 | 0.171 | -0.06 | 0.023 | 8.50E-03 | 0.05 | 0.048 | 0.303 | -0.051 | 0.035 | 0.148 |
| InternalIMT | dha | rs55908141 | 0.007 | . | . | . | . | . | . | . | . | . |
| InternalIMT | dha | rs12354712 | 0.16 | -0.057 | 0.023 | 0.011 | 0.063 | 0.049 | 0.204 | -0.063 | 0.036 | 0.082 |
| InternalIMT | dha | rs147282684 | 0.003 | . | . | . | . | . | . | . | . | . |
| InternalIMT | dha | rs12356140 | 0.168 | -0.062 | 0.023 | 6.70E-03 | 0.049 | 0.049 | 0.322 | -0.048 | 0.036 | 0.179 |
| InternalIMT | dha | rs185476187 | 0.002 | . | . | . | . | . | . | . | . | . |
| InternalIMT | dha | rs145720057 | 0.026 | -0.059 | 0.022 | 7.20E-03 | 0.049 | 0.121 | 0.688 | -2.40E-03 | 0.105 | 0.982 |
| InternalIMT | dha | rs12354742 | 0.166 | -0.061 | 0.023 | 7.10E-03 | 0.052 | 0.049 | 0.286 | -0.051 | 0.036 | 0.155 |
| InternalIMT | dha | rs180913842 | 0.007 | . | . | . | . | . | . | . | . | . |
| InternalIMT | dha | rs142976914 | 0.003 | . | . | . | . | . | . | . | . | . |
| InternalIMT | dha | rs11511619 | 0.174 | -0.06 | 0.023 | 9.30E-03 | 0.049 | 0.048 | 0.308 | -0.051 | 0.035 | 0.147 |
| InternalIMT | dha | rs146702870 | 0.001 | . | . | . | . | . | . | . | . | . |
| InternalIMT | dha | rs150287496 | 0.005 | . | . | . | . | . | . | . | . | . |
| InternalIMT | dha | rs190917973 | 0.005 | . | . | . | . | . | . | . | . | . |
| InternalIMT | dha | rs11517281 | 0.166 | -0.061 | 0.023 | 7.20E-03 | 0.052 | 0.049 | 0.284 | -0.051 | 0.036 | 0.153 |
| InternalIMT | dha | rs12359299 | 0.168 | -0.06 | 0.023 | 8.20E-03 | 0.053 | 0.049 | 0.273 | -0.052 | 0.036 | 0.141 |
| InternalIMT | dha | rs182307932 | 0.024 | -0.055 | 0.022 | 0.011 | -7.90E-04 | 0.134 | 0.995 | -0.051 | 0.098 | 0.601 |
| InternalIMT | dha | rs61855873 | 0.025 | -0.068 | 0.022 | 2.20E-03 | -0.122 | 0.158 | 0.44 | 0.183 | 0.131 | 0.162 |
| InternalIMT | dha | rs12359302 | 0.164 | -0.065 | 0.023 | 4.10E-03 | 0.04 | 0.045 | 0.376 | -0.04 | 0.033 | 0.237 |
| InternalIMT | dha | rs143208140 | 0.021 | -0.082 | 0.02 | 4.00E-05 | -0.187 | 0.113 | 0.099 | 0.104 | 0.077 | 0.18 |
| InternalIMT | dha | rs9988764 | 0.682 | -0.031 | 0.04 | 0.449 | 0.034 | 0.036 | 0.336 | -0.035 | 0.026 | 0.192 |
| InternalIMT | dha | rs192875767 | 0.006 | . | . | . | . | . | . | . | . | . |
| InternalIMT | dha | rs147268846 | 0.012 | -0.069 | 0.022 | 1.60E-03 | -0.401 | 0.232 | 0.083 | 0.445 | 0.205 | 0.03 |
| InternalIMT | dha | rs11239485 | 0.105 | -0.061 | 0.022 | 4.90E-03 | 0.077 | 0.07 | 0.275 | -0.08 | 0.053 | 0.129 |
| InternalIMT | dha | rs151317885 | 0.424 | -0.076 | 0.029 | 9.40E-03 | 7.40E-03 | 0.035 | 0.833 | -1.90E-03 | 0.026 | 0.94 |
| InternalIMT | dha | rs11499085 | 0.106 | -0.052 | 0.023 | 0.025 | 0.032 | 0.066 | 0.626 | -0.032 | 0.047 | 0.498 |
| InternalIMT | dha | rs188130606 | 0.028 | -0.059 | 0.022 | 7.40E-03 | 0.103 | 0.134 | 0.445 | -0.057 | 0.093 | 0.539 |
| InternalIMT | dha | rs11511620 | 0.16 | -0.058 | 0.023 | 0.011 | 0.062 | 0.049 | 0.21 | -0.062 | 0.036 | 0.085 |
| InternalIMT | dha | rs181498390 | 0.017 | -0.077 | 0.02 | 1.00E-04 | 0.015 | 0.145 | 0.919 | -0.025 | 0.102 | 0.803 |
| InternalIMT | dha | rs186280701 | 0.007 | . | . | . | . | . | . | . | . | . |
| InternalIMT | dha | rs150059185 | 0.028 | -0.077 | 0.02 | 1.10E-04 | 0.025 | 0.144 | 0.864 | -0.025 | 0.1 | 0.803 |
| InternalIMT | dha | rs183338707 | 0.003 | . | . | . | . | . | . | . | . | . |
| InternalIMT | dha | rs11499038 | 0.09 | -0.077 | 0.021 | 1.80E-04 | 0.024 | 0.07 | 0.727 | -0.013 | 0.049 | 0.795 |
| InternalIMT | dha | rs190699595 | 0.006 | . | . | . | . | . | . | . | . | . |
| InternalIMT | dha | rs11523408 | 0.164 | -0.061 | 0.023 | 7.00E-03 | 0.052 | 0.049 | 0.287 | -0.051 | 0.036 | 0.157 |
| InternalIMT | dha | rs188049317 | 0.002 | . | . | . | . | . | . | . | . | . |
| InternalIMT | dha | rs144948634 | 0.395 | -0.063 | 0.029 | 0.027 | 0.032 | 0.036 | 0.381 | -0.017 | 0.027 | 0.526 |
| InternalIMT | dha | rs143833576 | 0.003 | . | . | . | . | . | . | . | . | . |
| InternalIMT | dha | rs151239879 | 0.165 | -0.061 | 0.023 | 7.40E-03 | 0.053 | 0.049 | 0.282 | -0.052 | 0.036 | 0.151 |
| InternalIMT | dha | rs7073743 | 0.407 | -0.071 | 0.029 | 0.013 | 0.018 | 0.035 | 0.616 | -6.40E-03 | 0.026 | 0.803 |
| InternalIMT | dha | rs142563349 | 0.03 | -0.059 | 0.022 | 7.20E-03 | 0.057 | 0.131 | 0.664 | -0.032 | 0.092 | 0.724 |
| InternalIMT | dha | rs187246403 | 0.007 | . | . | . | . | . | . | . | . | . |
| InternalIMT | dha | rs150423942 | 0.162 | -0.059 | 0.023 | 8.80E-03 | 0.062 | 0.051 | 0.217 | -0.058 | 0.037 | 0.116 |

| InternalIMT | dha | rs184706102 | 0.002 | . | . | . | . | . | . | . | . | . |
| --- | --- | --- | --- | --- | --- | --- | --- | --- | --- | --- | --- | --- |
| InternalIMT | dha | rs189626288 | 0.02 | -0.06 | 0.022 | 5.60E-03 | 0.026 | 0.205 | 0.898 | 0.03 | 0.169 | 0.86 |
| InternalIMT | dha | rs140749834 | 0.048 | -0.051 | 0.022 | 0.021 | 0.088 | 0.108 | 0.414 | -0.085 | 0.076 | 0.266 |
| InternalIMT | dha | rs7078822 | 0.133 | -0.06 | 0.022 | 7.60E-03 | 0.051 | 0.052 | 0.331 | -0.062 | 0.039 | 0.107 |
| InternalIMT | dha | rs186227617 | 0.164 | -0.06 | 0.023 | 8.10E-03 | 0.055 | 0.049 | 0.268 | -0.054 | 0.036 | 0.134 |
| InternalIMT | dha | rs188984368 | 0.022 | -0.072 | 0.02 | 4.00E-04 | 0.185 | 0.145 | 0.202 | -0.117 | 0.107 | 0.272 |
| InternalIMT | dha | rs182078109 | 0.031 | -0.055 | 0.023 | 0.015 | 0.16 | 0.151 | 0.288 | -0.084 | 0.115 | 0.463 |
| InternalIMT | dha | rs141456274 | 0.005 | . | . | . | . | . | . | . | . | . |
| InternalIMT | dha | rs146976512 | 0.166 | -0.061 | 0.023 | 7.10E-03 | 0.052 | 0.049 | 0.291 | -0.05 | 0.036 | 0.158 |
| InternalIMT | dha | rs138036816 | 0.1 | -0.053 | 0.023 | 0.021 | 0.042 | 0.071 | 0.551 | -0.035 | 0.05 | 0.488 |
| InternalIMT | dha | rs114879777 | 0.019 | -0.055 | 0.022 | 0.011 | 0.307 | 0.165 | 0.063 | -0.2 | 0.109 | 0.067 |
| InternalIMT | dha | rs149366475 | 0.101 | -0.053 | 0.023 | 0.022 | 0.034 | 0.071 | 0.632 | -0.031 | 0.05 | 0.536 |
| InternalIMT | dha | rs182463043 | 0.002 | . | . | . | . | . | . | . | . | . |
| InternalIMT | dha | rs144754632 | 0.019 | -0.082 | 0.02 | 3.80E-05 | -0.223 | 0.113 | 0.05 | 0.12 | 0.077 | 0.118 |
| InternalIMT | dha | rs55717607 | 0.166 | -0.061 | 0.023 | 7.00E-03 | 0.051 | 0.049 | 0.292 | -0.05 | 0.036 | 0.159 |
| InternalIMT | dha | rs187537973 | 0 | . | . | . | . | . | . | . | . | . |
| InternalIMT | dha | rs148085971 | 0.002 | . | . | . | . | . | . | . | . | . |
| InternalIMT | dha | rs11239491 | 0.166 | -0.061 | 0.023 | 7.00E-03 | 0.051 | 0.049 | 0.292 | -0.05 | 0.036 | 0.159 |
| InternalIMT | dha | rs184412184 | 0.005 | . | . | . | . | . | . | . | . | . |
| InternalIMT | dha | rs4948998 | 0.716 | -0.027 | 0.042 | 0.521 | 0.04 | 0.035 | 0.26 | -0.036 | 0.026 | 0.176 |
| InternalIMT | dha | rs7076696 | 0.716 | -0.026 | 0.042 | 0.536 | 0.04 | 0.035 | 0.254 | -0.036 | 0.026 | 0.169 |
| InternalIMT | dha | rs11239492 | 0.172 | -0.06 | 0.023 | 8.10E-03 | 0.049 | 0.048 | 0.313 | -0.05 | 0.035 | 0.155 |
| InternalIMT | dha | rs79462470 | 0.007 | . | . | . | . | . | . | . | . | . |
| InternalIMT | dha | rs187804654 | 0.003 | . | . | . | . | . | . | . | . | . |
| InternalIMT | dha | rs7093687 | 0.161 | -0.058 | 0.023 | 0.011 | 0.06 | 0.049 | 0.218 | -0.061 | 0.036 | 0.089 |
| InternalIMT | dha | rs7081004 | 0.161 | -0.057 | 0.023 | 0.011 | 0.062 | 0.049 | 0.209 | -0.062 | 0.036 | 0.084 |
| InternalIMT | dha | rs7084793 | 0.166 | -0.061 | 0.023 | 6.90E-03 | 0.051 | 0.049 | 0.296 | -0.05 | 0.036 | 0.161 |
| InternalIMT | dha | rs61556080 | 0.007 | . | . | . | . | . | . | . | . | . |
| InternalIMT | dha | rs115552064 | 0.007 | . | . | . | . | . | . | . | . | . |
| InternalIMT | dha | rs7921503 | 0.166 | -0.061 | 0.023 | 7.10E-03 | 0.051 | 0.049 | 0.291 | -0.05 | 0.036 | 0.157 |
| InternalIMT | dha | rs7909740 | 0.166 | -0.061 | 0.023 | 6.90E-03 | 0.051 | 0.049 | 0.299 | -0.05 | 0.036 | 0.163 |
| InternalIMT | dha | rs7909768 | 0.166 | -0.061 | 0.023 | 6.80E-03 | 0.051 | 0.049 | 0.299 | -0.05 | 0.036 | 0.163 |
| InternalIMT | dha | rs144168004 | 0 | . | . | . | . | . | . | . | . | . |
| InternalIMT | dha | rs75850448 | 0.007 | . | . | . | . | . | . | . | . | . |
| InternalIMT | dha | rs147777574 | 0.006 | . | . | . | . | . | . | . | . | . |
| InternalIMT | dha | rs7913488 | 0.166 | -0.061 | 0.023 | 6.80E-03 | 0.05 | 0.049 | 0.3 | -0.049 | 0.036 | 0.164 |
| InternalIMT | dha | rs74390874 | 0.049 | -0.08 | 0.02 | 9.40E-05 | 0.028 | 0.091 | 0.762 | 0.041 | 0.07 | 0.562 |
| InternalIMT | dha | rs141391142 | 0.006 | . | . | . | . | . | . | . | . | . |
| InternalIMT | dha | rs115294747 | 0.007 | . | . | . | . | . | . | . | . | . |
| InternalIMT | dha | rs148031781 | 0.002 | . | . | . | . | . | . | . | . | . |
| InternalIMT | dha | rs181340128 | 0.018 | -0.066 | 0.022 | 2.80E-03 | -0.178 | 0.165 | 0.28 | 0.18 | 0.144 | 0.21 |
| InternalIMT | dha | rs189930007 | 0.001 | . | . | . | . | . | . | . | . | . |
| InternalIMT | dha | rs12242128 | 0.1 | -0.053 | 0.023 | 0.022 | 0.042 | 0.071 | 0.558 | -0.035 | 0.051 | 0.491 |
| InternalIMT | dha | rs78421750 | 0.006 | . | . | . | . | . | . | . | . | . |
| InternalIMT | dha | rs114370747 | 0.003 | . | . | . | . | . | . | . | . | . |
| InternalIMT | dha | rs12264166 | 0.1 | -0.053 | 0.023 | 0.022 | 0.042 | 0.071 | 0.553 | -0.035 | 0.051 | 0.487 |
| InternalIMT | dha | rs182964822 | 0.001 | . | . | . | . | . | . | . | . | . |
| InternalIMT | dha | rs67491417 | 0.159 | -0.065 | 0.022 | 4.00E-03 | 0.047 | 0.046 | 0.315 | -0.042 | 0.034 | 0.222 |
| InternalIMT | dha | rs72789720 | 0.015 | -0.062 | 0.022 | 4.80E-03 | -0.086 | 0.133 | 0.52 | 0.068 | 0.113 | 0.546 |
| InternalIMT | dha | rs6413416 | 0.167 | -0.062 | 0.023 | 6.50E-03 | 0.048 | 0.049 | 0.32 | -0.048 | 0.035 | 0.173 |
| InternalIMT | dha | rs4986832 | 0.167 | -0.061 | 0.023 | 6.70E-03 | 0.049 | 0.049 | 0.309 | -0.049 | 0.035 | 0.168 |
| InternalIMT | dha | rs144866316 | 0.005 | . | . | . | . | . | . | . | . | . |
| InternalIMT | dha | rs187769540 | 0.002 | . | . | . | . | . | . | . | . | . |
| InternalIMT | dha | rs6593482 | 0.167 | -0.061 | 0.023 | 7.40E-03 | 0.05 | 0.049 | 0.305 | -0.05 | 0.035 | 0.157 |
| InternalIMT | dha | rs7913948 | 0.167 | -0.06 | 0.023 | 8.10E-03 | 0.056 | 0.049 | 0.253 | -0.053 | 0.035 | 0.133 |
| InternalIMT | dha | rs77185151 | 0.019 | -0.082 | 0.02 | 3.80E-05 | -0.222 | 0.113 | 0.051 | 0.12 | 0.077 | 0.12 |
| InternalIMT | dha | rs12762303 | 0.167 | -0.061 | 0.023 | 6.60E-03 | 0.049 | 0.049 | 0.312 | -0.049 | 0.035 | 0.17 |
| InternalIMT | dha | rs73283136 | 0.005 | . | . | . | . | . | . | . | . | . |
| InternalIMT | dha | rs187180218 | 0.002 | . | . | . | . | . | . | . | . | . |
| InternalIMT | dha | rs78137087 | 0.166 | -0.061 | 0.023 | 6.70E-03 | 0.049 | 0.049 | 0.312 | -0.049 | 0.036 | 0.168 |
| InternalIMT | dha | rs72561472 | 0.165 | -0.062 | 0.023 | 6.90E-03 | 0.046 | 0.049 | 0.349 | -0.048 | 0.036 | 0.18 |
| InternalIMT | dha | rs4987106 | 0.162 | -0.062 | 0.023 | 5.80E-03 | 0.044 | 0.05 | 0.371 | -0.047 | 0.036 | 0.197 |
| InternalIMT | dha | rs4987105 | 0.158 | -0.058 | 0.023 | 0.01 | 0.059 | 0.049 | 0.228 | -0.061 | 0.036 | 0.093 |
| InternalIMT | dha | rs111916157 | 0.182 | -0.062 | 0.023 | 6.60E-03 | 0.048 | 0.048 | 0.315 | -0.043 | 0.035 | 0.219 |
| InternalIMT | dha | rs111528152 | 0.171 | -0.062 | 0.023 | 6.30E-03 | 0.039 | 0.049 | 0.426 | -0.044 | 0.036 | 0.221 |
| InternalIMT | dha | rs12763738 | 0.166 | -0.062 | 0.023 | 6.30E-03 | 0.048 | 0.049 | 0.326 | -0.048 | 0.036 | 0.181 |
| InternalIMT | dha | rs191675886 | 0.007 | . | . | . | . | . | . | . | . | . |
| InternalIMT | dha | rs66489870 | 0.167 | -0.061 | 0.023 | 6.60E-03 | 0.049 | 0.049 | 0.312 | -0.049 | 0.035 | 0.17 |
| InternalIMT | dha | rs71494775 | 0.167 | -0.061 | 0.023 | 6.60E-03 | 0.049 | 0.049 | 0.312 | -0.049 | 0.035 | 0.17 |
| InternalIMT | dha | rs61855875 | 0.024 | -0.067 | 0.022 | 2.50E-03 | -0.119 | 0.164 | 0.466 | 0.164 | 0.137 | 0.229 |
| InternalIMT | dha | rs116356837 | 0.007 | . | . | . | . | . | . | . | . | . |
| InternalIMT | dha | rs1864414 | 0.166 | -0.062 | 0.023 | 6.30E-03 | 0.049 | 0.049 | 0.319 | -0.048 | 0.036 | 0.179 |
| InternalIMT | dha | rs1864413 | 0.167 | -0.061 | 0.023 | 6.70E-03 | 0.049 | 0.049 | 0.314 | -0.049 | 0.035 | 0.17 |
| InternalIMT | dha | rs139497212 | 0 | . | . | . | . | . | . | . | . | . |
| InternalIMT | dha | rs892690 | 0.328 | -0.078 | 0.027 | 3.70E-03 | 5.50E-03 | 0.036 | 0.879 | 2.50E-03 | 0.027 | 0.926 |
| InternalIMT | dha | rs1864412 | 0.167 | -0.061 | 0.023 | 6.60E-03 | 0.049 | 0.049 | 0.313 | -0.049 | 0.035 | 0.171 |
| InternalIMT | dha | rs77827771 | 0.031 | -0.059 | 0.022 | 7.80E-03 | 0.155 | 0.13 | 0.235 | -0.082 | 0.092 | 0.37 |
| InternalIMT | dha | rs145294963 | 0.004 | . | . | . | . | . | . | . | . | . |
| InternalIMT | dha | rs149081327 | 0.008 | . | . | . | . | . | . | . | . | . |
| InternalIMT | dha | rs67008983 | 0.167 | -0.062 | 0.023 | 6.50E-03 | 0.049 | 0.049 | 0.316 | -0.048 | 0.036 | 0.173 |
| InternalIMT | dha | rs56150414 | 0.17 | -0.061 | 0.023 | 7.50E-03 | 0.052 | 0.048 | 0.278 | -0.05 | 0.035 | 0.154 |
| InternalIMT | dha | rs55787481 | 0.167 | -0.061 | 0.023 | 6.80E-03 | 0.049 | 0.049 | 0.309 | -0.049 | 0.036 | 0.167 |
| InternalIMT | dha | rs141270101 | 0.001 | . | . | . | . | . | . | . | . | . |
| InternalIMT | dha | rs114980075 | 0.008 | . | . | . | . | . | . | . | . | . |
| InternalIMT | dha | rs12783095 | 0.166 | -0.062 | 0.023 | 6.30E-03 | 0.047 | 0.049 | 0.338 | -0.048 | 0.036 | 0.178 |
| InternalIMT | dha | rs182342688 | 0.003 | . | . | . | . | . | . | . | . | . |

| InternalIMT | dha | rs17157728 | 0.166 | -0.062 | 0.023 | 6.30E-03 | 0.047 | 0.049 | 0.339 | -0.048 | 0.036 | 0.179 |
| --- | --- | --- | --- | --- | --- | --- | --- | --- | --- | --- | --- | --- |
| InternalIMT | dha | rs114835563 | 0.028 | -0.059 | 0.022 | 7.50E-03 | 0.113 | 0.134 | 0.401 | -0.063 | 0.094 | 0.499 |
| InternalIMT | dha | rs3824612 | 0.328 | -0.078 | 0.027 | 3.70E-03 | 5.60E-03 | 0.036 | 0.876 | 2.70E-03 | 0.027 | 0.921 |
| InternalIMT | dha | rs17157733 | 0.165 | -0.062 | 0.023 | 6.20E-03 | 0.046 | 0.049 | 0.345 | -0.047 | 0.036 | 0.183 |
| InternalIMT | dha | rs74128478 | 0.007 | . | . | . | . | . | . | . | . | . |
| InternalIMT | dha | rs184973589 | 0.001 | . | . | . | . | . | . | . | . | . |
| InternalIMT | dha | rs150479461 | 0 | . | . | . | . | . | . | . | . | . |
| InternalIMT | dha | rs138310407 | 0.003 | . | . | . | . | . | . | . | . | . |
| InternalIMT | dha | rs187426603 | 0.001 | . | . | . | . | . | . | . | . | . |
| InternalIMT | dha | rs190843143 | 0.002 | . | . | . | . | . | . | . | . | . |
| InternalIMT | dha | rs191883449 | 0.001 | . | . | . | . | . | . | . | . | . |
| InternalIMT | dha | rs58238740 | 0.163 | -0.063 | 0.023 | 5.60E-03 | 0.045 | 0.049 | 0.365 | -0.046 | 0.036 | 0.203 |
| InternalIMT | dha | rs3780894 | 0.163 | -0.063 | 0.023 | 5.60E-03 | 0.045 | 0.049 | 0.365 | -0.046 | 0.036 | 0.203 |
| InternalIMT | dha | rs149646626 | 0.018 | -0.051 | 0.022 | 0.018 | 0.304 | 0.223 | 0.171 | -0.224 | 0.172 | 0.195 |
| InternalIMT | dha | rs79615212 | 0.016 | -0.064 | 0.022 | 3.50E-03 | -0.184 | 0.178 | 0.3 | 0.112 | 0.146 | 0.443 |
| InternalIMT | dha | rs3780895 | 0.163 | -0.063 | 0.023 | 5.60E-03 | 0.045 | 0.049 | 0.364 | -0.046 | 0.036 | 0.203 |
| InternalIMT | dha | rs3780896 | 0.163 | -0.063 | 0.023 | 5.60E-03 | 0.045 | 0.049 | 0.365 | -0.046 | 0.036 | 0.203 |
| InternalIMT | dha | rs142747163 | 0.005 | . | . | . | . | . | . | . | . | . |
| InternalIMT | dha | rs189417584 | 0.007 | . | . | . | . | . | . | . | . | . |
| InternalIMT | dha | rs3780897 | 0.162 | -0.064 | 0.023 | 4.80E-03 | 0.045 | 0.049 | 0.368 | -0.043 | 0.036 | 0.23 |
| InternalIMT | dha | rs117509491 | 0.009 | . | . | . | . | . | . | . | . | . |
| InternalIMT | dha | rs117811514 | 0.009 | . | . | . | . | . | . | . | . | . |
| InternalIMT | dha | rs116500932 | 0.028 | -0.059 | 0.022 | 7.50E-03 | 0.112 | 0.134 | 0.403 | -0.063 | 0.094 | 0.501 |
| InternalIMT | dha | rs77584168 | 0.004 | . | . | . | . | . | . | . | . | . |
| InternalIMT | dha | rs2310776 | 0.163 | -0.063 | 0.023 | 5.50E-03 | 0.045 | 0.049 | 0.366 | -0.046 | 0.036 | 0.204 |
| InternalIMT | dha | rs17157736 | 0.163 | -0.063 | 0.023 | 5.50E-03 | 0.045 | 0.049 | 0.366 | -0.046 | 0.036 | 0.204 |
| InternalIMT | dha | rs72789735 | 0.065 | -0.065 | 0.02 | 1.50E-03 | 0.129 | 0.084 | 0.122 | -0.118 | 0.064 | 0.065 |
| InternalIMT | dha | rs183747187 | 0.002 | . | . | . | . | . | . | . | . | . |
| InternalIMT | dha | rs7077891 | 0.163 | -0.063 | 0.023 | 5.50E-03 | 0.045 | 0.049 | 0.366 | -0.046 | 0.036 | 0.204 |
| InternalIMT | dha | rs145063305 | 0.003 | . | . | . | . | . | . | . | . | . |
| InternalIMT | dha | rs3824613 | 0.164 | -0.063 | 0.023 | 5.10E-03 | 0.044 | 0.049 | 0.368 | -0.044 | 0.036 | 0.218 |
| InternalIMT | dha | rs145543199 | 0.009 | . | . | . | . | . | . | . | . | . |
| InternalIMT | dha | rs140361993 | 0.003 | . | . | . | . | . | . | . | . | . |
| InternalIMT | dha | rs11239496 | 0.065 | -0.064 | 0.02 | 1.50E-03 | 0.129 | 0.084 | 0.121 | -0.118 | 0.064 | 0.065 |
| InternalIMT | dha | rs11239497 | 0.158 | -0.059 | 0.023 | 9.00E-03 | 0.056 | 0.05 | 0.263 | -0.058 | 0.036 | 0.11 |
| InternalIMT | dha | rs10900212 | 0.184 | -0.063 | 0.023 | 5.70E-03 | 0.04 | 0.047 | 0.394 | -0.039 | 0.034 | 0.253 |
| InternalIMT | dha | rs186487136 | 0 | . | . | . | . | . | . | . | . | . |
| InternalIMT | dha | rs12774369 | 0.163 | -0.062 | 0.023 | 5.70E-03 | 0.045 | 0.049 | 0.362 | -0.046 | 0.036 | 0.2 |
| InternalIMT | dha | rs60842538 | 0.163 | -0.062 | 0.023 | 5.80E-03 | 0.046 | 0.05 | 0.356 | -0.047 | 0.036 | 0.194 |
| InternalIMT | dha | rs3780898 | 0.157 | -0.058 | 0.023 | 9.70E-03 | 0.057 | 0.05 | 0.253 | -0.06 | 0.037 | 0.101 |
| InternalIMT | dha | rs3780899 | 0.163 | -0.062 | 0.023 | 6.10E-03 | 0.047 | 0.05 | 0.348 | -0.048 | 0.036 | 0.186 |
| InternalIMT | dha | rs12252472 | 0.09 | -0.052 | 0.023 | 0.022 | 0.046 | 0.072 | 0.526 | -0.04 | 0.053 | 0.445 |
| InternalIMT | dha | rs185411011 | 0.002 | . | . | . | . | . | . | . | . | . |
| InternalIMT | dha | rs28395865 | 0.155 | -0.058 | 0.023 | 0.01 | 0.059 | 0.051 | 0.242 | -0.063 | 0.037 | 0.092 |
| InternalIMT | dha | rs191004074 | 0.03 | -0.055 | 0.023 | 0.015 | 0.075 | 0.162 | 0.641 | -0.068 | 0.12 | 0.568 |
| InternalIMT | dha | rs182401295 | 0.003 | . | . | . | . | . | . | . | . | . |
| InternalIMT | dha | rs2228064 | 0.093 | -0.078 | 0.021 | 1.70E-04 | -0.027 | 0.065 | 0.671 | 0.012 | 0.047 | 0.801 |
| InternalIMT | dha | rs188284159 | 0.001 | . | . | . | . | . | . | . | . | . |
| InternalIMT | dha | rs2303887 | 0.013 | -0.081 | 0.02 | 3.80E-05 | -0.292 | 0.11 | 8.20E-03 | 0.126 | 0.079 | 0.111 |
| InternalIMT | dha | rs2303886 | 0.016 | -0.083 | 0.02 | 2.60E-05 | -0.342 | 0.116 | 3.10E-03 | 0.187 | 0.084 | 0.027 |
| InternalIMT | dha | rs192621006 | 0.003 | . | . | . | . | . | . | . | . | . |
| InternalIMT | dha | rs2303885 | 0.252 | -0.057 | 0.024 | 0.015 | 0.037 | 0.041 | 0.36 | -0.039 | 0.03 | 0.192 |
| InternalIMT | dha | rs114466168 | 0.028 | -0.058 | 0.022 | 8.10E-03 | 0.14 | 0.134 | 0.297 | -0.08 | 0.094 | 0.393 |
| InternalIMT | dha | rs11239501 | 0.088 | -0.067 | 0.02 | 1.10E-03 | 0.091 | 0.067 | 0.178 | -0.061 | 0.052 | 0.24 |
| InternalIMT | dha | rs745986 | 0.18 | -0.059 | 0.023 | 9.60E-03 | 0.055 | 0.044 | 0.212 | -0.054 | 0.033 | 0.099 |
| InternalIMT | dha | rs75262268 | 0.002 | . | . | . | . | . | . | . | . | . |
| InternalIMT | dha | rs71494776 | 0.061 | -0.078 | 0.021 | 2.10E-04 | -0.073 | 0.061 | 0.231 | 0.012 | 0.044 | 0.782 |
| InternalIMT | dha | rs74386871 | 0.02 | -0.082 | 0.02 | 4.00E-05 | -0.199 | 0.106 | 0.061 | 0.08 | 0.076 | 0.291 |
| InternalIMT | dha | rs76502040 | 0.007 | . | . | . | . | . | . | . | . | . |
| InternalIMT | dha | rs142213734 | 0.001 | . | . | . | . | . | . | . | . | . |
| InternalIMT | dha | rs4948671 | 0.714 | -0.03 | 0.041 | 0.468 | 0.038 | 0.035 | 0.283 | -0.034 | 0.026 | 0.201 |
| InternalIMT | dha | rs146286085 | 0.001 | . | . | . | . | . | . | . | . | . |
| InternalIMT | dha | rs144080623 | 0 | . | . | . | . | . | . | . | . | . |
| InternalIMT | dha | rs115074014 | 0.044 | -0.053 | 0.022 | 0.017 | 0.062 | 0.112 | 0.577 | -0.068 | 0.08 | 0.394 |
| InternalIMT | dha | rs17157756 | 0.044 | -0.053 | 0.022 | 0.017 | 0.062 | 0.112 | 0.577 | -0.068 | 0.08 | 0.394 |
| InternalIMT | dha | rs190459780 | 0.001 | . | . | . | . | . | . | . | . | . |
| InternalIMT | dha | rs12358647 | 0.208 | -0.065 | 0.023 | 4.40E-03 | 0.031 | 0.043 | 0.462 | -0.031 | 0.031 | 0.329 |
| InternalIMT | dha | rs147080556 | 0.013 | -0.077 | 0.02 | 1.30E-04 | -0.011 | 0.152 | 0.942 | -0.11 | 0.111 | 0.322 |
| InternalIMT | dha | rs187543349 | 0.005 | . | . | . | . | . | . | . | . | . |
| InternalIMT | dha | rs114375595 | 0.01 | -0.084 | 0.02 | 1.90E-05 | -0.301 | 0.269 | 0.264 | 0.278 | 0.187 | 0.136 |
| InternalIMT | dha | rs117211200 | 0.006 | . | . | . | . | . | . | . | . | . |
| InternalIMT | dha | rs192963047 | 0.001 | . | . | . | . | . | . | . | . | . |
| InternalIMT | dha | rs11239505 | 0.209 | -0.064 | 0.023 | 5.30E-03 | 0.036 | 0.043 | 0.396 | -0.034 | 0.031 | 0.279 |
| InternalIMT | dha | rs184434116 | 0.002 | . | . | . | . | . | . | . | . | . |
| InternalIMT | dha | rs4948999 | 0.715 | -0.029 | 0.041 | 0.482 | 0.037 | 0.035 | 0.291 | -0.034 | 0.026 | 0.195 |
| InternalIMT | dha | rs11239506 | 0.102 | -0.065 | 0.021 | 1.90E-03 | 0.089 | 0.062 | 0.153 | -0.068 | 0.048 | 0.151 |
| InternalIMT | dha | rs190706620 | 0.004 | . | . | . | . | . | . | . | . | . |
| InternalIMT | dha | rs190770429 | 0.002 | . | . | . | . | . | . | . | . | . |
| InternalIMT | dha | rs181327133 | 0.005 | . | . | . | . | . | . | . | . | . |
| InternalIMT | dha | rs149251697 | 0.023 | -0.06 | 0.022 | 5.40E-03 | 0.119 | 0.174 | 0.495 | -0.046 | 0.119 | 0.696 |
| InternalIMT | dha | rs118081602 | 0.013 | -0.081 | 0.02 | 3.80E-05 | -0.292 | 0.111 | 8.30E-03 | 0.126 | 0.079 | 0.111 |
| InternalIMT | dha | rs188233691 | 0.002 | . | . | . | . | . | . | . | . | . |
| InternalIMT | dha | rs183778794 | 0.008 | . | . | . | . | . | . | . | . | . |
| InternalIMT | dha | rs188040078 | 0.006 | . | . | . | . | . | . | . | . | . |
| InternalIMT | dha | rs184675372 | 0.002 | . | . | . | . | . | . | . | . | . |
| InternalIMT | dha | rs150326891 | 0.069 | -0.068 | 0.02 | 8.10E-04 | 0.104 | 0.081 | 0.2 | -0.072 | 0.062 | 0.245 |

| InternalIMT | dha | rs185520021 | 0.006 | . | . | . | . | . | . | . | . | . |
| --- | --- | --- | --- | --- | --- | --- | --- | --- | --- | --- | --- | --- |
| InternalIMT | dha | rs7907779 | 0.102 | -0.065 | 0.021 | 1.90E-03 | 0.089 | 0.062 | 0.151 | -0.069 | 0.048 | 0.148 |
| InternalIMT | dha | rs184962860 | 0.001 | . | . | . | . | . | . | . | . | . |
| InternalIMT | dha | rs149762784 | 0.037 | -0.076 | 0.021 | 2.90E-04 | 0.013 | 0.107 | 0.902 | -0.03 | 0.085 | 0.722 |
| InternalIMT | dha | rs149203427 | 0.013 | -0.061 | 0.022 | 5.00E-03 | -9.30E-03 | 0.221 | 0.966 | 0.089 | 0.188 | 0.636 |
| InternalIMT | dha | rs60153083 | 0.016 | -0.059 | 0.022 | 6.60E-03 | 0.016 | 0.174 | 0.925 | -0.024 | 0.122 | 0.842 |
| InternalIMT | dha | rs112001069 | 0.076 | -0.075 | 0.021 | 4.20E-04 | -0.036 | 0.058 | 0.537 | -2.90E-03 | 0.041 | 0.945 |
| InternalIMT | dha | rs182355382 | 0.031 | -0.08 | 0.02 | 8.40E-05 | 6.00E-03 | 0.121 | 0.96 | 0.022 | 0.085 | 0.8 |
| InternalIMT | dha | rs186740974 | 0.125 | -0.087 | 0.024 | 3.00E-04 | -0.049 | 0.07 | 0.484 | 0.035 | 0.051 | 0.497 |
| InternalIMT | dha | rs191874916 | 0.036 | -0.077 | 0.02 | 9.60E-05 | 0.069 | 0.128 | 0.587 | -0.027 | 0.09 | 0.767 |
| InternalIMT | dha | rs182989412 | 0.146 | -0.093 | 0.023 | 7.20E-05 | -0.034 | 0.068 | 0.621 | 0.057 | 0.052 | 0.269 |
| InternalIMT | dha | rs188819476 | 0.002 | . | . | . | . | . | . | . | . | . |
| InternalIMT | dha | rs141217183 | 0.093 | -0.083 | 0.022 | 1.60E-04 | -0.088 | 0.056 | 0.117 | 0.034 | 0.04 | 0.398 |
| InternalIMT | dha | rs192922652 | 0.077 | -0.076 | 0.021 | 3.60E-04 | -0.052 | 0.06 | 0.379 | 7.10E-03 | 0.042 | 0.865 |
| InternalIMT | dha | rs185092343 | 0.02 | -0.086 | 0.02 | 1.40E-05 | -0.076 | 0.194 | 0.695 | 0.183 | 0.156 | 0.241 |
| InternalIMT | dha | rs145193311 | 0.08 | -0.075 | 0.021 | 4.60E-04 | -0.03 | 0.06 | 0.61 | -3.90E-03 | 0.042 | 0.927 |
| InternalIMT | dha | rs55789336 | 0.104 | -0.067 | 0.021 | 1.40E-03 | 0.077 | 0.062 | 0.216 | -0.056 | 0.047 | 0.238 |
| InternalIMT | dha | rs55837212 | 0.104 | -0.068 | 0.021 | 1.20E-03 | 0.071 | 0.056 | 0.21 | -0.053 | 0.044 | 0.226 |
| InternalIMT | dha | rs75408574 | 0.36 | -0.075 | 0.027 | 6.20E-03 | 8.60E-03 | 0.037 | 0.818 | -3.70E-03 | 0.027 | 0.892 |
| InternalIMT | dha | rs149306926 | 0.11 | -0.065 | 0.021 | 2.00E-03 | 0.081 | 0.055 | 0.139 | -0.06 | 0.042 | 0.161 |
| InternalIMT | dha | rs35990892 | 0.674 | -0.016 | 0.042 | 0.696 | 0.039 | 0.036 | 0.275 | -0.045 | 0.027 | 0.095 |
| InternalIMT | dha | rs192356207 | 0.05 | -0.081 | 0.02 | 6.80E-05 | 0.014 | 0.09 | 0.88 | 0.015 | 0.064 | 0.82 |
| InternalIMT | dha | rs183741163 | 0.102 | -0.069 | 0.021 | 1.20E-03 | 0.075 | 0.065 | 0.252 | -0.048 | 0.051 | 0.347 |
| InternalIMT | dha | rs34737297 | 0.555 | -0.067 | 0.037 | 0.068 | 0.019 | 0.039 | 0.63 | -0.011 | 0.029 | 0.708 |
| InternalIMT | dha | rs181346933 | 0.03 | -0.057 | 0.022 | 0.011 | 0.028 | 0.137 | 0.838 | -0.044 | 0.098 | 0.656 |
| InternalIMT | dha | rs36093253 | 0.361 | -0.067 | 0.029 | 0.019 | 0.015 | 0.039 | 0.69 | -0.015 | 0.028 | 0.605 |
| InternalIMT | dha | rs186307855 | 0.099 | -0.045 | 0.023 | 0.055 | 0.105 | 0.07 | 0.136 | -0.082 | 0.051 | 0.104 |
| InternalIMT | dha | rs189681865 | 0.034 | -0.055 | 0.022 | 0.012 | 0.084 | 0.153 | 0.582 | -0.068 | 0.101 | 0.503 |
| InternalIMT | dha | rs34942971 | 0.214 | -0.066 | 0.024 | 5.00E-03 | 0.028 | 0.041 | 0.495 | -0.027 | 0.03 | 0.367 |
| InternalIMT | dha | rs181816554 | 0.033 | -0.061 | 0.022 | 5.90E-03 | 0.045 | 0.151 | 0.765 | -2.70E-03 | 0.113 | 0.981 |
| InternalIMT | dha | rs186846881 | 0.094 | -0.052 | 0.023 | 0.024 | 0.072 | 0.074 | 0.329 | -0.054 | 0.053 | 0.309 |
| InternalIMT | dha | rs191178220 | 0.016 | -0.076 | 0.02 | 1.30E-04 | 0.129 | 0.197 | 0.511 | -0.048 | 0.145 | 0.741 |
| InternalIMT | dha | rs183305228 | 0.016 | -0.059 | 0.022 | 6.50E-03 | 0.014 | 0.172 | 0.935 | -0.022 | 0.121 | 0.858 |
| InternalIMT | dha | rs139912761 | 0.121 | -0.069 | 0.021 | 9.50E-04 | 0.061 | 0.054 | 0.257 | -0.037 | 0.04 | 0.361 |
| InternalIMT | dha | rs185499901 | 0.018 | -0.052 | 0.022 | 0.015 | 0.343 | 0.252 | 0.174 | -0.186 | 0.19 | 0.328 |
| InternalIMT | dha | rs189816197 | 0.087 | -0.076 | 0.021 | 4.40E-04 | -0.035 | 0.058 | 0.549 | -2.00E-03 | 0.042 | 0.962 |
| InternalIMT | dha | rs145656938 | 0.026 | -0.078 | 0.02 | 8.60E-05 | 0.011 | 0.185 | 0.951 | -5.30E-03 | 0.124 | 0.966 |
| InternalIMT | dha | rs35745197 | 0.076 | -0.076 | 0.021 | 3.80E-04 | -0.041 | 0.059 | 0.484 | 4.60E-04 | 0.042 | 0.991 |
| InternalIMT | dha | rs192856637 | 0.005 | . | . | . | . | . | . | . | . | . |
| InternalIMT | dha | rs2310775 | 0.415 | -0.073 | 0.029 | 0.012 | 0.011 | 0.034 | 0.741 | -5.40E-03 | 0.025 | 0.832 |
| InternalIMT | dha | rs11239507 | 0.506 | -0.069 | 0.032 | 0.031 | 0.011 | 0.034 | 0.741 | -9.10E-03 | 0.025 | 0.72 |
| InternalIMT | dha | rs74648467 | 0.062 | -0.076 | 0.021 | 2.80E-04 | -0.063 | 0.061 | 0.305 | 2.60E-03 | 0.045 | 0.954 |
| InternalIMT | dha | rs145815934 | 0.015 | -0.059 | 0.022 | 6.30E-03 | 0.044 | 0.212 | 0.837 | -1.80E-03 | 0.171 | 0.992 |
| InternalIMT | dha | rs145390687 | 0.02 | -0.067 | 0.022 | 2.30E-03 | -0.142 | 0.199 | 0.475 | 0.206 | 0.169 | 0.225 |
| InternalIMT | dha | rs184354913 | 0.034 | -0.054 | 0.022 | 0.013 | 0.108 | 0.139 | 0.438 | -0.067 | 0.108 | 0.535 |
| InternalIMT | dha | rs181244920 | 0.006 | . | . | . | . | . | . | . | . | . |
| InternalIMT | dha | rs11499087 | 0.055 | -0.051 | 0.022 | 0.022 | 0.031 | 0.096 | 0.746 | -0.054 | 0.068 | 0.43 |
| InternalIMT | dha | rs148263794 | 0.08 | -0.075 | 0.021 | 4.70E-04 | -0.036 | 0.058 | 0.534 | -2.90E-03 | 0.041 | 0.944 |
| InternalIMT | dha | rs150758903 | 0.036 | -0.057 | 0.022 | 9.40E-03 | 0.032 | 0.13 | 0.806 | -0.031 | 0.088 | 0.724 |
| InternalIMT | dha | rs147654382 | 0.179 | -0.074 | 0.023 | 1.10E-03 | 9.80E-03 | 0.043 | 0.821 | -0.011 | 0.032 | 0.733 |
| InternalIMT | dha | rs147495166 | 0.025 | -0.055 | 0.022 | 0.013 | 0.259 | 0.134 | 0.053 | -0.166 | 0.089 | 0.064 |
| InternalIMT | dha | rs35198778 | 0.106 | -0.076 | 0.022 | 5.20E-04 | -0.012 | 0.06 | 0.844 | -4.80E-03 | 0.043 | 0.911 |
| InternalIMT | dha | rs145207659 | 0.049 | -0.082 | 0.02 | 6.10E-05 | 8.60E-03 | 0.097 | 0.93 | 0.063 | 0.075 | 0.397 |
| InternalIMT | dha | rs186466464 | 0.01 | -0.055 | 0.021 | 0.011 | 0.417 | 0.484 | 0.388 | -0.277 | 0.334 | 0.408 |
| InternalIMT | dha | rs12778413 | 0.162 | -0.072 | 0.023 | 1.70E-03 | 0.028 | 0.047 | 0.555 | -0.02 | 0.035 | 0.568 |
| InternalIMT | dha | rs7895552 | 0.64 | -0.034 | 0.04 | 0.395 | 0.037 | 0.037 | 0.31 | -0.034 | 0.027 | 0.209 |
| InternalIMT | dha | rs12783895 | 0.078 | -0.075 | 0.021 | 4.30E-04 | -0.036 | 0.058 | 0.538 | -2.70E-03 | 0.042 | 0.948 |
| InternalIMT | dha | rs34929604 | 0.079 | -0.074 | 0.021 | 5.00E-04 | -0.025 | 0.058 | 0.661 | -8.60E-03 | 0.041 | 0.834 |
| InternalIMT | dha | rs12761961 | 0.075 | -0.076 | 0.021 | 3.70E-04 | -0.039 | 0.06 | 0.518 | 3.10E-05 | 0.043 | 0.999 |
| InternalIMT | dha | rs35536930 | 0.206 | -0.066 | 0.023 | 3.80E-03 | 0.03 | 0.04 | 0.454 | -0.029 | 0.03 | 0.336 |
| InternalIMT | dha | rs180951060 | 0.002 | . | . | . | . | . | . | . | . | . |
| InternalIMT | dha | rs148892128 | 0.015 | -0.059 | 0.022 | 6.30E-03 | 0.044 | 0.212 | 0.837 | -1.70E-03 | 0.171 | 0.992 |
| InternalIMT | dha | rs11239509 | 0.084 | -0.048 | 0.023 | 0.036 | 0.074 | 0.074 | 0.317 | -0.067 | 0.053 | 0.202 |
| InternalIMT | dha | rs41526545 | 0.093 | -0.066 | 0.021 | 1.40E-03 | 0.092 | 0.065 | 0.157 | -0.069 | 0.05 | 0.172 |
| InternalIMT | dha | rs2029253 | 0.421 | -0.078 | 0.029 | 7.20E-03 | 6.60E-03 | 0.034 | 0.847 | 5.90E-04 | 0.025 | 0.982 |
| InternalIMT | dha | rs17444064 | 0.092 | -0.067 | 0.021 | 1.20E-03 | 0.088 | 0.065 | 0.178 | -0.061 | 0.051 | 0.23 |
| InternalIMT | dha | rs71494779 | 0.08 | -0.074 | 0.021 | 4.80E-04 | -0.026 | 0.057 | 0.648 | -7.20E-03 | 0.041 | 0.86 |
| InternalIMT | dha | rs74991420 | 0.012 | -0.081 | 0.02 | 3.70E-05 | -0.297 | 0.113 | 8.60E-03 | 0.129 | 0.083 | 0.119 |
| InternalIMT | dha | rs182736551 | 0.012 | -0.081 | 0.02 | 3.70E-05 | -0.297 | 0.113 | 8.60E-03 | 0.129 | 0.083 | 0.119 |
| InternalIMT | dha | rs78702074 | 0.013 | -0.081 | 0.02 | 3.90E-05 | -0.303 | 0.115 | 8.20E-03 | 0.131 | 0.081 | 0.106 |
| InternalIMT | dha | rs187791466 | 0.004 | . | . | . | . | . | . | . | . | . |
| InternalIMT | dha | rs35300270 | 0.082 | -0.075 | 0.021 | 4.90E-04 | -0.031 | 0.056 | 0.587 | -4.50E-03 | 0.04 | 0.911 |
| InternalIMT | dha | rs6593484 | 0.102 | -0.065 | 0.021 | 1.90E-03 | 0.088 | 0.062 | 0.158 | -0.068 | 0.048 | 0.152 |
| InternalIMT | dha | rs12244601 | 0.023 | -0.057 | 0.022 | 9.10E-03 | 0.085 | 0.144 | 0.554 | -0.065 | 0.095 | 0.489 |
| InternalIMT | dha | rs184429574 | 0.005 | . | . | . | . | . | . | . | . | . |
| InternalIMT | dha | rs7080897 | 0.08 | -0.074 | 0.021 | 4.80E-04 | -0.026 | 0.057 | 0.649 | -7.20E-03 | 0.041 | 0.86 |
| InternalIMT | dha | rs7097429 | 0.083 | -0.075 | 0.021 | 4.90E-04 | -0.025 | 0.057 | 0.655 | -6.30E-03 | 0.041 | 0.877 |
| InternalIMT | dha | rs77979229 | 0.003 | . | . | . | . | . | . | . | . | . |
| InternalIMT | dha | rs11239512 | 0.103 | -0.064 | 0.021 | 2.00E-03 | 0.089 | 0.062 | 0.149 | -0.069 | 0.047 | 0.143 |
| InternalIMT | dha | rs60146827 | 0.08 | -0.074 | 0.021 | 4.90E-04 | -0.026 | 0.057 | 0.65 | -7.40E-03 | 0.041 | 0.855 |
| InternalIMT | dha | rs79032236 | 0.034 | -0.061 | 0.022 | 5.40E-03 | 0.107 | 0.121 | 0.374 | -0.042 | 0.087 | 0.631 |
| InternalIMT | dha | rs140854206 | 0.007 | . | . | . | . | . | . | . | . | . |
| InternalIMT | dha | rs184049550 | 0.004 | . | . | . | . | . | . | . | . | . |
| InternalIMT | dha | rs114433607 | 0.01 | -0.056 | 0.022 | 8.70E-03 | 0.249 | 0.257 | 0.332 | -0.196 | 0.172 | 0.255 |
| InternalIMT | dha | rs117653793 | 0.013 | -0.081 | 0.02 | 3.80E-05 | -0.294 | 0.112 | 8.40E-03 | 0.129 | 0.08 | 0.107 |
| InternalIMT | dha | rs117039533 | 0.013 | -0.081 | 0.02 | 3.80E-05 | -0.294 | 0.112 | 8.50E-03 | 0.128 | 0.08 | 0.108 |

| InternalIMT | dha | rs191660761 | 0.003 | . | . | . | . | . | . | . | . | . |
| --- | --- | --- | --- | --- | --- | --- | --- | --- | --- | --- | --- | --- |
| InternalIMT | dha | rs59514896 | 0.064 | -0.077 | 0.021 | 2.30E-04 | -0.07 | 0.06 | 0.246 | 0.011 | 0.044 | 0.796 |
| InternalIMT | dha | rs184661876 | 0.002 | . | . | . | . | . | . | . | . | . |
| InternalIMT | dha | rs61854073 | 0.027 | -0.062 | 0.022 | 4.70E-03 | -0.02 | 0.14 | 0.887 | 0.073 | 0.121 | 0.546 |
| InternalIMT | dha | rs2018518 | 0.299 | -0.075 | 0.026 | 4.70E-03 | 5.30E-03 | 0.037 | 0.887 | -5.80E-03 | 0.027 | 0.832 |
| InternalIMT | dha | rs934187 | 0.718 | -0.033 | 0.041 | 0.428 | 0.034 | 0.035 | 0.341 | -0.031 | 0.026 | 0.234 |
| InternalIMT | dha | rs7918542 | 0.214 | -0.06 | 0.023 | 9.10E-03 | 0.045 | 0.042 | 0.282 | -0.042 | 0.031 | 0.17 |
| InternalIMT | dha | rs144311920 | 0.002 | . | . | . | . | . | . | . | . | . |
| InternalIMT | dha | rs7099684 | 0.84 | -0.036 | 0.053 | 0.495 | 0.039 | 0.042 | 0.353 | -0.025 | 0.031 | 0.412 |
| InternalIMT | dha | rs150829171 | 0.002 | . | . | . | . | . | . | . | . | . |
| InternalIMT | dha | rs186060697 | 0.002 | . | . | . | . | . | . | . | . | . |
| InternalIMT | dha | rs11239513 | 0.097 | -0.052 | 0.023 | 0.024 | 0.074 | 0.071 | 0.303 | -0.053 | 0.052 | 0.304 |
| InternalIMT | dha | rs7917687 | 0.081 | -0.074 | 0.021 | 5.30E-04 | -0.025 | 0.057 | 0.665 | -9.40E-03 | 0.04 | 0.815 |
| InternalIMT | dha | rs61854076 | 0.01 | . | . | . | . | . | . | . | . | . |
| InternalIMT | dha | rs71494780 | 0.018 | -0.061 | 0.022 | 5.00E-03 | -0.058 | 0.097 | 0.554 | 0.032 | 0.083 | 0.704 |
| InternalIMT | dha | rs7919239 | 0.218 | -0.063 | 0.023 | 6.10E-03 | 0.035 | 0.04 | 0.39 | -0.035 | 0.029 | 0.237 |
| InternalIMT | dha | rs78334475 | 0.034 | -0.078 | 0.02 | 1.10E-04 | -0.159 | 0.096 | 0.096 | 0.038 | 0.064 | 0.553 |
| InternalIMT | dha | rs3780900 | 0.026 | -0.079 | 0.02 | 6.40E-05 | 5.80E-03 | 0.161 | 0.971 | 8.10E-03 | 0.112 | 0.943 |
| InternalIMT | dha | rs186729292 | 0.001 | . | . | . | . | . | . | . | . | . |
| InternalIMT | dha | rs12267438 | 0.026 | -0.057 | 0.022 | 8.00E-03 | 0.064 | 0.14 | 0.645 | -0.048 | 0.094 | 0.61 |
| InternalIMT | dha | rs1369214 | 0.568 | -0.075 | 0.036 | 0.036 | -9.10E-03 | 0.035 | 0.794 | -2.30E-03 | 0.026 | 0.93 |
| InternalIMT | dha | rs9282589 | 0.041 | -0.064 | 0.022 | 4.10E-03 | -0.041 | 0.069 | 0.55 | -0.015 | 0.062 | 0.809 |
| InternalIMT | dha | rs114522915 | 0.025 | -0.061 | 0.022 | 5.70E-03 | 0.116 | 0.139 | 0.404 | -0.052 | 0.097 | 0.592 |
| InternalIMT | dha | rs2115819 | 0.477 | -0.073 | 0.032 | 0.022 | -1.60E-03 | 0.034 | 0.962 | -5.40E-03 | 0.025 | 0.83 |
| InternalIMT | dha | rs117664603 | 0.013 | -0.081 | 0.02 | 3.90E-05 | -0.287 | 0.111 | 9.40E-03 | 0.124 | 0.079 | 0.118 |
| InternalIMT | dha | rs184979202 | 0.001 | . | . | . | . | . | . | . | . | . |
| InternalIMT | dha | rs190714671 | 0.002 | . | . | . | . | . | . | . | . | . |
| InternalIMT | dha | rs141538996 | 0 | . | . | . | . | . | . | . | . | . |
| InternalIMT | dha | rs150881741 | 0.002 | . | . | . | . | . | . | . | . | . |
| InternalIMT | dha | rs139076633 | 0.003 | . | . | . | . | . | . | . | . | . |
| InternalIMT | dha | rs12243649 | 0.059 | -0.083 | 0.02 | 4.50E-05 | -0.063 | 0.078 | 0.42 | 0.047 | 0.054 | 0.386 |
| InternalIMT | dha | rs140348708 | 0.001 | . | . | . | . | . | . | . | . | . |
| InternalIMT | dha | rs79023256 | 0.013 | -0.081 | 0.02 | 3.90E-05 | -0.287 | 0.111 | 9.60E-03 | 0.123 | 0.079 | 0.12 |
| InternalIMT | dha | rs11239515 | 0.094 | -0.062 | 0.021 | 2.60E-03 | 0.112 | 0.066 | 0.091 | -0.089 | 0.05 | 0.078 |
| InternalIMT | dha | rs55866086 | 0.157 | -0.065 | 0.021 | 2.60E-03 | 0.059 | 0.052 | 0.26 | -0.046 | 0.038 | 0.224 |
| InternalIMT | dha | rs181340150 | 0.003 | . | . | . | . | . | . | . | . | . |
| InternalIMT | dha | rs11239516 | 0.12 | -0.061 | 0.021 | 3.30E-03 | 0.097 | 0.06 | 0.108 | -0.073 | 0.044 | 0.099 |
| InternalIMT | dha | rs11239517 | 0.12 | -0.061 | 0.021 | 3.30E-03 | 0.097 | 0.06 | 0.107 | -0.073 | 0.044 | 0.099 |
| InternalIMT | dha | rs11239519 | 0.106 | -0.06 | 0.021 | 4.00E-03 | 0.112 | 0.063 | 0.079 | -0.09 | 0.048 | 0.059 |
| InternalIMT | dha | rs7909514 | 0.709 | -0.027 | 0.041 | 0.502 | 0.035 | 0.035 | 0.315 | -0.035 | 0.026 | 0.181 |
| InternalIMT | dha | rs7894352 | 0.835 | -0.034 | 0.052 | 0.509 | 0.039 | 0.042 | 0.355 | -0.026 | 0.031 | 0.395 |
| InternalIMT | dha | rs189384146 | 0.002 | . | . | . | . | . | . | . | . | . |
| InternalIMT | dha | rs11239520 | 0.105 | -0.062 | 0.021 | 2.60E-03 | 0.102 | 0.064 | 0.111 | -0.082 | 0.049 | 0.094 |
| InternalIMT | dha | rs12221233 | 0.406 | -0.072 | 0.029 | 0.014 | 0.012 | 0.035 | 0.738 | -6.70E-03 | 0.026 | 0.796 |
| InternalIMT | dha | rs74523293 | 0.032 | -0.083 | 0.02 | 3.80E-05 | -0.144 | 0.099 | 0.143 | 0.095 | 0.07 | 0.174 |
| InternalIMT | dha | rs79376040 | 0.032 | -0.083 | 0.02 | 3.80E-05 | -0.144 | 0.099 | 0.143 | 0.095 | 0.07 | 0.174 |
| InternalIMT | dha | rs4949000 | 0.406 | -0.072 | 0.029 | 0.014 | 0.012 | 0.035 | 0.738 | -6.80E-03 | 0.026 | 0.793 |
| InternalIMT | dha | rs7089406 | 0.025 | -0.058 | 0.022 | 7.90E-03 | -0.019 | 0.109 | 0.861 | -0.014 | 0.078 | 0.858 |
| InternalIMT | dha | rs138211056 | 0 | . | . | . | . | . | . | . | . | . |
| InternalIMT | dha | rs10900213 | 0.537 | -0.04 | 0.032 | 0.22 | 0.049 | 0.034 | 0.153 | -0.035 | 0.026 | 0.182 |
| InternalIMT | dha | rs145152827 | 0.003 | . | . | . | . | . | . | . | . | . |
| InternalIMT | dha | rs74443325 | 0.012 | -0.082 | 0.02 | 3.60E-05 | -0.314 | 0.1 | 1.60E-03 | 0.131 | 0.071 | 0.063 |
| InternalIMT | dha | rs188299446 | 0.011 | -0.055 | 0.022 | 0.011 | 0.282 | 0.263 | 0.284 | -0.134 | 0.195 | 0.492 |
| InternalIMT | dha | rs192699586 | 0.003 | . | . | . | . | . | . | . | . | . |
| InternalIMT | dha | rs72796464 | 0.086 | -0.065 | 0.02 | 1.50E-03 | 0.11 | 0.07 | 0.114 | -0.082 | 0.054 | 0.128 |
| InternalIMT | dha | rs4949001 | 0.266 | -0.077 | 0.026 | 3.20E-03 | -8.30E-03 | 0.039 | 0.832 | -5.40E-03 | 0.029 | 0.851 |
| InternalIMT | dha | rs147174641 | 0.008 | . | . | . | . | . | . | . | . | . |
| InternalIMT | dha | rs190835238 | 0.001 | . | . | . | . | . | . | . | . | . |
| InternalIMT | dha | rs76698944 | 0.022 | -0.054 | 0.022 | 0.013 | 0.177 | 0.158 | 0.261 | -0.134 | 0.121 | 0.27 |
| InternalIMT | dha | rs56071381 | 0.11 | -0.061 | 0.021 | 3.10E-03 | 0.101 | 0.062 | 0.107 | -0.082 | 0.047 | 0.082 |
| InternalIMT | dha | rs78130427 | 0.047 | -0.075 | 0.02 | 2.40E-04 | -0.112 | 0.081 | 0.163 | 0.027 | 0.055 | 0.626 |
| InternalIMT | dha | rs10900215 | 0.148 | -0.063 | 0.021 | 3.20E-03 | 0.078 | 0.055 | 0.158 | -0.058 | 0.041 | 0.157 |
| InternalIMT | dha | rs41491445 | 0.016 | -0.058 | 0.022 | 7.30E-03 | 0.042 | 0.17 | 0.803 | -0.048 | 0.12 | 0.686 |
| InternalIMT | dha | rs144995222 | 0.002 | . | . | . | . | . | . | . | . | . |
| InternalIMT | dha | rs149097803 | 0.003 | . | . | . | . | . | . | . | . | . |
| InternalIMT | dha | rs139429646 | 0.005 | . | . | . | . | . | . | . | . | . |
| InternalIMT | dha | rs7075620 | 0.681 | -0.021 | 0.04 | 0.593 | 0.044 | 0.035 | 0.216 | -0.041 | 0.027 | 0.12 |
| InternalIMT | dha | rs148299213 | 0.017 | -0.058 | 0.022 | 7.10E-03 | 0.041 | 0.17 | 0.809 | -0.043 | 0.12 | 0.722 |
| InternalIMT | dha | rs4949002 | 0.245 | -0.081 | 0.026 | 1.70E-03 | 9.10E-03 | 0.044 | 0.835 | 0.016 | 0.033 | 0.642 |
| InternalIMT | dha | rs150496007 | 0.021 | -0.055 | 0.022 | 0.014 | 0.097 | 0.125 | 0.439 | -0.111 | 0.089 | 0.213 |
| InternalIMT | dha | rs147303455 | 0.012 | -0.057 | 0.022 | 7.80E-03 | 0.191 | 0.207 | 0.355 | -0.125 | 0.129 | 0.335 |
| InternalIMT | dha | rs189518433 | 0.005 | . | . | . | . | . | . | . | . | . |
| InternalIMT | dha | rs186281355 | 0.005 | . | . | . | . | . | . | . | . | . |
| InternalIMT | dha | rs115297114 | 0.026 | -0.058 | 0.022 | 8.50E-03 | 0.181 | 0.135 | 0.178 | -0.101 | 0.092 | 0.273 |
| InternalIMT | dha | rs11239523 | 0.105 | -0.065 | 0.02 | 1.60E-03 | 0.089 | 0.064 | 0.169 | -0.069 | 0.049 | 0.161 |
| InternalIMT | dha | rs143704897 | 0.005 | . | . | . | . | . | . | . | . | . |
| InternalIMT | dha | rs188024096 | 0.003 | . | . | . | . | . | . | . | . | . |
| InternalIMT | dha | rs11239524 | 0.107 | -0.063 | 0.021 | 2.60E-03 | 0.091 | 0.066 | 0.166 | -0.077 | 0.05 | 0.122 |
| InternalIMT | dha | rs191656573 | 0.004 | . | . | . | . | . | . | . | . | . |
| InternalIMT | dha | rs193295875 | 0.002 | . | . | . | . | . | . | . | . | . |
| InternalIMT | dha | rs190458228 | 0.002 | . | . | . | . | . | . | . | . | . |
| InternalIMT | dha | rs139253993 | 0.002 | . | . | . | . | . | . | . | . | . |
| InternalIMT | dha | rs4948672 | 0.588 | -0.041 | 0.039 | 0.298 | 0.041 | 0.039 | 0.288 | -0.031 | 0.029 | 0.282 |
| InternalIMT | dha | rs180954921 | 0.002 | . | . | . | . | . | . | . | . | . |
| InternalIMT | dha | rs12264801 | 0.425 | -0.079 | 0.031 | 0.011 | 3.30E-03 | 0.041 | 0.936 | 1.40E-03 | 0.031 | 0.964 |
| InternalIMT | dha | rs73283184 | 0.02 | -0.056 | 0.022 | 9.60E-03 | 0.089 | 0.151 | 0.556 | -0.09 | 0.104 | 0.389 |

| InternalIMT | dha | rs142746187 | 0.009 | . | . | . | . | . | . | . | . | . |
| --- | --- | --- | --- | --- | --- | --- | --- | --- | --- | --- | --- | --- |
| InternalIMT | dha | rs58704739 | 0.019 | -0.056 | 0.022 | 9.80E-03 | 0.096 | 0.155 | 0.537 | -0.097 | 0.106 | 0.359 |
| InternalIMT | dha | rs61272335 | 0.014 | -0.057 | 0.022 | 7.90E-03 | 0.081 | 0.186 | 0.665 | -0.082 | 0.132 | 0.534 |
| InternalIMT | dha | rs60959489 | 0.035 | -0.049 | 0.022 | 0.026 | 0.161 | 0.108 | 0.137 | -0.143 | 0.072 | 0.046 |
| InternalIMT | dha | rs58502629 | 0.014 | -0.057 | 0.022 | 7.80E-03 | 0.081 | 0.186 | 0.663 | -0.082 | 0.132 | 0.533 |
| InternalIMT | dha | rs10900216 | 0.735 | -0.046 | 0.041 | 0.26 | 0.047 | 0.036 | 0.188 | -0.022 | 0.027 | 0.411 |
| InternalIMT | dha | rs181118141 | 0.002 | . | . | . | . | . | . | . | . | . |
| InternalIMT | dha | rs137882665 | 0.01 | . | . | . | . | . | . | . | . | . |
| InternalIMT | dha | rs149466736 | 0.003 | . | . | . | . | . | . | . | . | . |
| InternalIMT | dha | rs2099171 | 0.656 | -0.054 | 0.035 | 0.124 | 0.043 | 0.033 | 0.199 | -0.017 | 0.024 | 0.48 |
| InternalIMT | dha | rs141852280 | 0.006 | . | . | . | . | . | . | . | . | . |
| InternalIMT | dha | rs191539257 | 0.002 | . | . | . | . | . | . | . | . | . |
| InternalIMT | dha | rs17157771 | 0.034 | -0.079 | 0.02 | 7.00E-05 | -0.088 | 0.091 | 0.329 | 0.03 | 0.069 | 0.664 |
| InternalIMT | dha | rs111339241 | 0.032 | -0.079 | 0.02 | 6.50E-05 | -0.099 | 0.094 | 0.292 | 0.037 | 0.071 | 0.606 |
| InternalIMT | dha | rs116983255 | 0.008 | . | . | . | . | . | . | . | . | . |
| InternalIMT | dha | rs142324341 | 0.011 | -0.115 | 0.028 | 4.60E-05 | -0.532 | 0.374 | 0.156 | 0.316 | 0.242 | 0.192 |
| InternalIMT | dha | rs187191219 | 0.01 | . | . | . | . | . | . | . | . | . |
| InternalIMT | dha | rs151283973 | 0.002 | . | . | . | . | . | . | . | . | . |
| InternalIMT | dha | rs139316218 | 0.003 | . | . | . | . | . | . | . | . | . |
| InternalIMT | dha | rs6593485 | 0.732 | -0.064 | 0.04 | 0.11 | 0.022 | 0.035 | 0.538 | -9.80E-03 | 0.026 | 0.707 |
| InternalIMT | dha | rs6593486 | 0.732 | -0.066 | 0.04 | 0.098 | 0.019 | 0.035 | 0.584 | -8.20E-03 | 0.026 | 0.752 |
| InternalIMT | dha | rs145196618 | 0.014 | -0.06 | 0.021 | 5.10E-03 | 0.025 | 0.2 | 0.899 | -3.00E-03 | 0.136 | 0.982 |
| InternalIMT | dha | rs142598335 | 0.004 | . | . | . | . | . | . | . | . | . |
| InternalIMT | dha | rs892691 | 0.726 | -0.054 | 0.039 | 0.173 | 0.028 | 0.035 | 0.43 | -0.017 | 0.026 | 0.517 |
| InternalIMT | dha | rs7080713 | 0.671 | -0.044 | 0.036 | 0.222 | 0.051 | 0.034 | 0.13 | -0.025 | 0.025 | 0.314 |
| InternalIMT | dha | rs7077173 | 0.08 | -0.088 | 0.02 | 1.50E-05 | -0.118 | 0.057 | 0.038 | 0.076 | 0.043 | 0.075 |
| InternalIMT | dha | rs7080977 | 0.048 | -0.084 | 0.02 | 3.00E-05 | -0.139 | 0.082 | 0.09 | 0.084 | 0.061 | 0.169 |
| InternalIMT | dha | rs3780901 | 0.671 | -0.043 | 0.036 | 0.231 | 0.052 | 0.034 | 0.119 | -0.025 | 0.025 | 0.302 |
| InternalIMT | dha | rs61854091 | 0.051 | -0.067 | 0.022 | 3.10E-03 | -0.028 | 0.102 | 0.782 | 0.081 | 0.083 | 0.327 |
| InternalIMT | dha | rs77550209 | 0.005 | . | . | . | . | . | . | . | . | . |
| InternalIMT | dha | rs73283191 | 0.061 | -0.087 | 0.02 | 1.90E-05 | -0.178 | 0.07 | 0.011 | 0.1 | 0.051 | 0.051 |
| InternalIMT | dha | rs191866099 | 0.007 | . | . | . | . | . | . | . | . | . |
| InternalIMT | dha | rs147998395 | 0.002 | . | . | . | . | . | . | . | . | . |
| InternalIMT | dha | rs7090328 | 0.783 | -0.054 | 0.043 | 0.213 | 0.03 | 0.036 | 0.401 | -0.016 | 0.027 | 0.551 |
| InternalIMT | dha | rs79814535 | 0.012 | -0.081 | 0.02 | 4.40E-05 | -0.273 | 0.103 | 7.80E-03 | 0.102 | 0.069 | 0.14 |
| InternalIMT | dha | rs7073082 | 0.783 | -0.054 | 0.043 | 0.214 | 0.031 | 0.036 | 0.4 | -0.016 | 0.027 | 0.55 |
| InternalIMT | dha | rs11239526 | 0.036 | -0.079 | 0.02 | 7.70E-05 | 8.20E-03 | 0.088 | 0.925 | 0.03 | 0.065 | 0.65 |
| InternalIMT | dha | rs141839841 | 0.002 | . | . | . | . | . | . | . | . | . |
| InternalIMT | dha | rs75697371 | 0.01 | . | . | . | . | . | . | . | . | . |
| InternalIMT | dha | rs75972854 | 0.01 | . | . | . | . | . | . | . | . | . |
| InternalIMT | dha | rs139291975 | 0.001 | . | . | . | . | . | . | . | . | . |
| InternalIMT | dha | rs144064711 | 0.006 | . | . | . | . | . | . | . | . | . |
| InternalIMT | dha | rs3780902 | 0.062 | -0.086 | 0.02 | 2.30E-05 | -0.165 | 0.07 | 0.018 | 0.093 | 0.051 | 0.071 |
| InternalIMT | dha | rs190449801 | 0.002 | . | . | . | . | . | . | . | . | . |
| InternalIMT | dha | rs73283196 | 0.061 | -0.086 | 0.02 | 2.20E-05 | -0.173 | 0.07 | 0.014 | 0.097 | 0.052 | 0.061 |
| InternalIMT | dha | rs28395867 | 0.044 | -0.058 | 0.022 | 8.00E-03 | -0.023 | 0.104 | 0.821 | -1.30E-03 | 0.077 | 0.987 |
| InternalIMT | dha | rs28395866 | 0.102 | -0.083 | 0.021 | 6.30E-05 | -0.053 | 0.058 | 0.36 | 0.033 | 0.042 | 0.429 |
| InternalIMT | dha | rs55661611 | 0.047 | -0.084 | 0.02 | 3.10E-05 | -0.14 | 0.084 | 0.096 | 0.084 | 0.062 | 0.177 |
| InternalIMT | dha | rs55684518 | 0.02 | -0.067 | 0.022 | 2.10E-03 | -0.314 | 0.133 | 0.018 | 0.184 | 0.113 | 0.104 |
| InternalIMT | dha | rs78552493 | 0.047 | -0.051 | 0.022 | 0.021 | 0.064 | 0.081 | 0.425 | -0.09 | 0.062 | 0.144 |
| InternalIMT | dha | rs147665434 | 0.004 | . | . | . | . | . | . | . | . | . |
| InternalIMT | dha | rs34445001 | 0.01 | . | . | . | . | . | . | . | . | . |
| InternalIMT | dha | rs184019449 | 0.003 | . | . | . | . | . | . | . | . | . |
| InternalIMT | dha | rs3780903 | 0.714 | -0.013 | 0.039 | 0.747 | 0.073 | 0.035 | 0.036 | -0.045 | 0.026 | 0.081 |
| InternalIMT | dha | rs17157784 | 0.061 | -0.08 | 0.02 | 8.30E-05 | -0.064 | 0.075 | 0.392 | 0.03 | 0.053 | 0.574 |
| InternalIMT | dha | rs3780904 | 0.013 | -0.081 | 0.02 | 3.80E-05 | -0.273 | 0.107 | 0.011 | 0.137 | 0.075 | 0.068 |
| InternalIMT | dha | rs192064730 | 0 | . | . | . | . | . | . | . | . | . |
| InternalIMT | dha | rs12774534 | 0.01 | . | . | . | . | . | . | . | . | . |
| InternalIMT | dha | rs79179915 | 0.011 | -0.081 | 0.02 | 4.00E-05 | -0.298 | 0.102 | 3.60E-03 | 0.118 | 0.069 | 0.088 |
| InternalIMT | dha | rs117811571 | 0.011 | -0.055 | 0.022 | 0.011 | 0.289 | 0.266 | 0.277 | -0.125 | 0.197 | 0.525 |
| InternalIMT | dha | rs72796477 | 0.01 | -0.064 | 0.022 | 2.90E-03 | -0.188 | 0.175 | 0.283 | 0.205 | 0.146 | 0.159 |
| InternalIMT | dha | rs55729342 | 0.065 | -0.079 | 0.02 | 9.60E-05 | -0.052 | 0.073 | 0.475 | 0.025 | 0.051 | 0.625 |
| InternalIMT | dha | rs59317220 | 0.064 | -0.08 | 0.02 | 8.50E-05 | -0.054 | 0.074 | 0.463 | 0.028 | 0.052 | 0.592 |
| InternalIMT | dha | rs3780905 | 0.104 | -0.067 | 0.021 | 1.60E-03 | -0.106 | 0.055 | 0.053 | 0.018 | 0.036 | 0.625 |
| InternalIMT | dha | rs3780906 | 0.714 | -0.011 | 0.039 | 0.783 | 0.075 | 0.035 | 0.033 | -0.047 | 0.026 | 0.073 |
| InternalIMT | dha | rs149047975 | 0.036 | -0.063 | 0.022 | 4.40E-03 | -0.031 | 0.074 | 0.678 | -0.039 | 0.065 | 0.554 |
| InternalIMT | dha | rs3824614 | 0.011 | -0.081 | 0.02 | 3.90E-05 | -0.31 | 0.102 | 2.40E-03 | 0.123 | 0.07 | 0.078 |
| InternalIMT | dha | rs140254404 | 0.003 | . | . | . | . | . | . | . | . | . |
| InternalIMT | dha | rs145262684 | 0.014 | -0.053 | 0.022 | 0.015 | 0.356 | 0.249 | 0.153 | -0.164 | 0.189 | 0.386 |
| InternalIMT | dha | rs76735482 | 0.011 | -0.081 | 0.02 | 3.80E-05 | -0.315 | 0.102 | 2.00E-03 | 0.125 | 0.07 | 0.073 |
| InternalIMT | dha | rs73283198 | 0.034 | -0.08 | 0.02 | 6.30E-05 | -0.12 | 0.094 | 0.204 | 0.046 | 0.072 | 0.519 |
| InternalIMT | dha | rs74911239 | 0.035 | -0.068 | 0.023 | 2.80E-03 | -0.114 | 0.104 | 0.271 | 0.122 | 0.085 | 0.148 |
| InternalIMT | dha | rs150551647 | 0.006 | . | . | . | . | . | . | . | . | . |
| InternalIMT | dha | rs115200794 | 0.051 | -0.058 | 0.022 | 7.90E-03 | -0.017 | 0.093 | 0.856 | 7.90E-06 | 0.068 | 1 |
| InternalIMT | dha | rs74130823 | 0.168 | -0.074 | 0.022 | 6.20E-04 | 2.60E-03 | 0.053 | 0.96 | -0.011 | 0.039 | 0.779 |
| InternalIMT | dha | rs2279435 | 0.62 | -0.06 | 0.039 | 0.118 | 0.024 | 0.036 | 0.501 | -0.014 | 0.027 | 0.591 |
| InternalIMT | dha | rs147279673 | 0.014 | -0.075 | 0.02 | 1.90E-04 | 0.167 | 0.254 | 0.509 | -0.094 | 0.197 | 0.632 |
| InternalIMT | dha | rs183525146 | 0.004 | . | . | . | . | . | . | . | . | . |
| InternalIMT | dha | rs3740107 | 0.745 | -0.069 | 0.042 | 0.101 | 0.021 | 0.037 | 0.577 | -6.30E-03 | 0.027 | 0.816 |
| InternalIMT | dha | rs140697247 | 0.001 | . | . | . | . | . | . | . | . | . |
| InternalIMT | dha | rs1565096 | 0.81 | -0.052 | 0.046 | 0.257 | 0.025 | 0.038 | 0.51 | -0.016 | 0.028 | 0.562 |
| InternalIMT | dha | rs1565097 | 0.767 | -0.05 | 0.044 | 0.254 | 0.029 | 0.037 | 0.429 | -0.018 | 0.027 | 0.499 |
| InternalIMT | dha | rs144584548 | 0.02 | -0.067 | 0.022 | 2.20E-03 | -0.296 | 0.133 | 0.026 | 0.176 | 0.113 | 0.119 |
| InternalIMT | dha | rs75811053 | 0.011 | -0.081 | 0.02 | 3.60E-05 | -0.321 | 0.1 | 1.40E-03 | 0.12 | 0.071 | 0.09 |
| InternalIMT | dha | rs147462967 | 0.01 | . | . | . | . | . | . | . | . | . |
| InternalIMT | dha | rs74130824 | 0.049 | -0.08 | 0.02 | 7.10E-05 | -0.127 | 0.079 | 0.106 | 0.054 | 0.059 | 0.355 |

| InternalIMT | dha | rs116883184 | 0.018 | -0.055 | 0.022 | 0.013 | 0.22 | 0.219 | 0.314 | -0.087 | 0.17 | 0.609 |
| --- | --- | --- | --- | --- | --- | --- | --- | --- | --- | --- | --- | --- |
| InternalIMT | dha | rs117258049 | 0.018 | -0.055 | 0.022 | 0.013 | 0.22 | 0.219 | 0.314 | -0.087 | 0.17 | 0.609 |
| InternalIMT | dha | rs3780908 | 0.128 | -0.078 | 0.021 | 2.00E-04 | -5.70E-03 | 0.06 | 0.924 | -9.90E-04 | 0.045 | 0.982 |
| InternalIMT | dha | rs180938522 | 0.002 | . | . | . | . | . | . | . | . | . |
| InternalIMT | dha | rs76687372 | 0.008 | . | . | . | . | . | . | . | . | . |
| InternalIMT | dha | rs3780909 | 0.542 | -0.051 | 0.033 | 0.122 | 0.031 | 0.034 | 0.348 | -0.024 | 0.025 | 0.332 |
| InternalIMT | dha | rs78281156 | 0.011 | -0.081 | 0.02 | 3.60E-05 | -0.322 | 0.1 | 1.40E-03 | 0.12 | 0.071 | 0.089 |
| InternalIMT | dha | rs187840631 | 0.002 | . | . | . | . | . | . | . | . | . |
| InternalIMT | dha | rs76868242 | 0.011 | -0.081 | 0.02 | 3.60E-05 | -0.322 | 0.1 | 1.40E-03 | 0.12 | 0.071 | 0.088 |
| InternalIMT | dha | rs192794192 | 0.001 | . | . | . | . | . | . | . | . | . |
| InternalIMT | dha | rs12781248 | 0.01 | -0.058 | 0.022 | 7.10E-03 | 0.097 | 0.147 | 0.51 | -0.066 | 0.122 | 0.586 |
| InternalIMT | dha | rs12762375 | 0.01 | . | . | . | . | . | . | . | . | . |
| InternalIMT | dha | rs192256986 | 0.006 | . | . | . | . | . | . | . | . | . |
| InternalIMT | dha | rs184411030 | 0.012 | -0.064 | 0.021 | 2.80E-03 | -0.117 | 0.197 | 0.552 | 0.136 | 0.139 | 0.328 |
| InternalIMT | dha | rs189601483 | 0.011 | -0.082 | 0.02 | 3.30E-05 | -0.351 | 0.107 | 1.10E-03 | 0.144 | 0.079 | 0.069 |
| InternalIMT | dha | rs143254579 | 0.037 | -0.08 | 0.02 | 7.40E-05 | -3.30E-04 | 0.085 | 0.997 | 0.031 | 0.064 | 0.624 |
| InternalIMT | dha | rs12253903 | 0.028 | -0.053 | 0.022 | 0.016 | 0.231 | 0.129 | 0.073 | -0.175 | 0.091 | 0.053 |
| InternalIMT | dha | rs35489926 | 0.009 | . | . | . | . | . | . | . | . | . |
| InternalIMT | dha | rs71494783 | 0.048 | -0.08 | 0.02 | 7.40E-05 | -0.049 | 0.087 | 0.575 | 0.029 | 0.06 | 0.636 |
| InternalIMT | dha | rs180711602 | 0.003 | . | . | . | . | . | . | . | . | . |
| InternalIMT | dha | rs117312402 | 0.011 | -0.081 | 0.02 | 3.60E-05 | -0.323 | 0.1 | 1.30E-03 | 0.121 | 0.071 | 0.086 |
| InternalIMT | dha | rs74831694 | 0.008 | . | . | . | . | . | . | . | . | . |
| InternalIMT | dha | rs140301943 | 0.003 | . | . | . | . | . | . | . | . | . |
| InternalIMT | dha | rs78553679 | 0.016 | -0.06 | 0.022 | 5.80E-03 | 0.043 | 0.216 | 0.841 | 0.027 | 0.173 | 0.878 |
| InternalIMT | dha | rs78334250 | 0.011 | -0.081 | 0.02 | 3.60E-05 | -0.323 | 0.1 | 1.30E-03 | 0.121 | 0.071 | 0.086 |
| InternalIMT | dha | rs76000028 | 0.011 | -0.081 | 0.02 | 3.60E-05 | -0.323 | 0.1 | 1.30E-03 | 0.121 | 0.071 | 0.086 |
| InternalIMT | dha | rs145195254 | 0.002 | . | . | . | . | . | . | . | . | . |
| InternalIMT | dha | rs137888527 | 0.002 | . | . | . | . | . | . | . | . | . |
| InternalIMT | dha | rs149109592 | 0.005 | . | . | . | . | . | . | . | . | . |
| InternalIMT | dha | rs80280010 | 0.035 | -0.08 | 0.02 | 6.80E-05 | -7.30E-03 | 0.086 | 0.933 | 0.038 | 0.065 | 0.56 |
| InternalIMT | dha | rs189995374 | 0.002 | . | . | . | . | . | . | . | . | . |
| InternalIMT | dha | rs75467770 | 0.012 | -0.081 | 0.02 | 4.40E-05 | -0.292 | 0.099 | 3.30E-03 | 0.096 | 0.068 | 0.16 |
| InternalIMT | dha | rs1487562 | 0.167 | -0.077 | 0.021 | 3.30E-04 | -2.70E-03 | 0.052 | 0.958 | -3.40E-03 | 0.039 | 0.93 |
| InternalIMT | dha | rs12266963 | 0.048 | -0.08 | 0.02 | 7.40E-05 | -0.049 | 0.087 | 0.572 | 0.029 | 0.06 | 0.635 |
| InternalIMT | dha | rs7099874 | 0.764 | -0.061 | 0.042 | 0.152 | 0.014 | 0.036 | 0.702 | -0.012 | 0.027 | 0.668 |
| InternalIMT | dha | rs75687019 | 0.011 | -0.081 | 0.02 | 3.60E-05 | -0.323 | 0.1 | 1.30E-03 | 0.121 | 0.071 | 0.086 |
| InternalIMT | dha | rs148240462 | 0.038 | -0.07 | 0.023 | 2.20E-03 | -0.124 | 0.103 | 0.23 | 0.141 | 0.085 | 0.097 |
| InternalIMT | dha | rs141278174 | 0.002 | . | . | . | . | . | . | . | . | . |
| InternalIMT | dha | rs76404852 | 0.011 | -0.081 | 0.02 | 3.60E-05 | -0.323 | 0.1 | 1.30E-03 | 0.121 | 0.071 | 0.085 |
| InternalIMT | dha | rs139112240 | 0.008 | . | . | . | . | . | . | . | . | . |
| InternalIMT | dha | rs12783037 | 0.054 | -0.082 | 0.02 | 4.90E-05 | -0.05 | 0.084 | 0.557 | 0.041 | 0.058 | 0.483 |
| InternalIMT | dha | rs72796481 | 0.009 | . | . | . | . | . | . | . | . | . |
| InternalIMT | dha | rs76798749 | 0.017 | -0.083 | 0.02 | 2.80E-05 | -0.273 | 0.113 | 0.016 | 0.162 | 0.078 | 0.038 |
| InternalIMT | dha | rs187732548 | 0.001 | . | . | . | . | . | . | . | . | . |
| InternalIMT | dha | rs75675474 | 0.017 | -0.083 | 0.02 | 2.80E-05 | -0.273 | 0.113 | 0.016 | 0.162 | 0.078 | 0.038 |
| InternalIMT | dha | rs76552293 | 0.017 | -0.083 | 0.02 | 2.80E-05 | -0.273 | 0.113 | 0.016 | 0.162 | 0.078 | 0.038 |
| InternalIMT | dha | rs147445993 | 0.005 | . | . | . | . | . | . | . | . | . |
| InternalIMT | dha | rs115902283 | 0.017 | -0.083 | 0.02 | 2.80E-05 | -0.274 | 0.113 | 0.015 | 0.161 | 0.078 | 0.038 |
| InternalIMT | dha | rs4540799 | 0.762 | -0.059 | 0.042 | 0.167 | 0.016 | 0.036 | 0.653 | -0.013 | 0.027 | 0.63 |
| InternalIMT | dha | rs12765320 | 0.211 | -0.081 | 0.022 | 2.70E-04 | -0.017 | 0.046 | 0.717 | 8.90E-03 | 0.034 | 0.792 |
| InternalIMT | dha | rs12782256 | 0.009 | . | . | . | . | . | . | . | . | . |
| InternalIMT | dha | rs191727256 | 0.002 | . | . | . | . | . | . | . | . | . |
| InternalIMT | dha | rs2082111 | 0.123 | -0.078 | 0.021 | 1.80E-04 | -4.90E-04 | 0.06 | 0.993 | -8.90E-04 | 0.045 | 0.984 |
| InternalIMT | dha | rs2082110 | 0.175 | -0.084 | 0.022 | 1.10E-04 | -0.037 | 0.05 | 0.46 | 0.018 | 0.037 | 0.619 |
| InternalIMT | dha | rs2082109 | 0.175 | -0.084 | 0.022 | 1.10E-04 | -0.037 | 0.05 | 0.46 | 0.018 | 0.037 | 0.619 |
| InternalIMT | dha | rs117316662 | 0.011 | -0.081 | 0.02 | 3.60E-05 | -0.325 | 0.1 | 1.20E-03 | 0.122 | 0.071 | 0.083 |
| InternalIMT | dha | rs12247163 | 0.214 | -0.084 | 0.022 | 1.60E-04 | -0.023 | 0.045 | 0.606 | 0.016 | 0.034 | 0.638 |
| InternalIMT | dha | rs12355888 | 0.082 | -0.07 | 0.02 | 5.60E-04 | 0.069 | 0.073 | 0.344 | -0.049 | 0.056 | 0.381 |
| InternalIMT | dha | rs143772290 | 0.011 | -0.063 | 0.021 | 3.30E-03 | -0.112 | 0.272 | 0.68 | 0.151 | 0.18 | 0.4 |
| InternalIMT | dha | rs146849452 | 0.005 | . | . | . | . | . | . | . | . | . |
| InternalIMT | dha | rs140267121 | 0.004 | . | . | . | . | . | . | . | . | . |
| InternalIMT | dha | rs7080474 | 0.202 | -0.082 | 0.022 | 1.90E-04 | -3.80E-03 | 0.047 | 0.935 | 8.50E-03 | 0.035 | 0.807 |
| InternalIMT | dha | rs144066203 | 0.002 | . | . | . | . | . | . | . | . | . |
| InternalIMT | dha | rs12765209 | 0.012 | -0.082 | 0.02 | 3.10E-05 | -0.337 | 0.102 | 9.90E-04 | 0.163 | 0.075 | 0.031 |
| InternalIMT | dha | rs150930735 | 0.002 | . | . | . | . | . | . | . | . | . |
| InternalIMT | dha | rs7068039 | 0.216 | -0.085 | 0.022 | 1.30E-04 | -0.025 | 0.045 | 0.575 | 0.018 | 0.033 | 0.596 |
| InternalIMT | dha | rs117622652 | 0.011 | -0.081 | 0.02 | 3.50E-05 | -0.326 | 0.1 | 1.10E-03 | 0.123 | 0.071 | 0.082 |
| InternalIMT | dha | rs190191514 | 0.001 | . | . | . | . | . | . | . | . | . |
| InternalIMT | dha | rs74130835 | 0.04 | -0.057 | 0.022 | 9.40E-03 | -0.042 | 0.1 | 0.676 | 1.80E-03 | 0.075 | 0.981 |
| InternalIMT | dha | rs1811604 | 0.039 | -0.057 | 0.022 | 9.10E-03 | -0.051 | 0.101 | 0.615 | 5.90E-03 | 0.076 | 0.938 |
| InternalIMT | dha | rs146971256 | 0 | . | . | . | . | . | . | . | . | . |
| InternalIMT | dha | rs74336000 | 0.011 | -0.063 | 0.021 | 3.30E-03 | -0.115 | 0.272 | 0.672 | 0.153 | 0.18 | 0.395 |
| InternalIMT | dha | rs186046492 | 0.007 | . | . | . | . | . | . | . | . | . |
| InternalIMT | dha | rs34971823 | 0.011 | -0.06 | 0.022 | 5.80E-03 | 0.033 | 0.152 | 0.827 | -7.20E-03 | 0.126 | 0.954 |
| InternalIMT | dha | rs75227446 | 0.011 | -0.081 | 0.02 | 3.50E-05 | -0.329 | 0.1 | 1.00E-03 | 0.126 | 0.071 | 0.073 |
| InternalIMT | dha | rs190457599 | 0.001 | . | . | . | . | . | . | . | . | . |
| InternalIMT | dha | rs79591917 | 0.01 | -0.081 | 0.02 | 3.50E-05 | -0.327 | 0.1 | 1.10E-03 | 0.124 | 0.07 | 0.08 |
| InternalIMT | dha | rs702365 | 0.545 | -0.058 | 0.034 | 0.083 | 0.023 | 0.034 | 0.496 | -0.018 | 0.025 | 0.481 |
| InternalIMT | dha | rs79643836 | 0.011 | -0.081 | 0.02 | 4.10E-05 | -0.294 | 0.099 | 2.90E-03 | 0.098 | 0.07 | 0.162 |
| InternalIMT | dha | rs702366 | 0.546 | -0.057 | 0.034 | 0.092 | 0.026 | 0.034 | 0.447 | -0.019 | 0.025 | 0.445 |
| InternalIMT | dha | rs1059696 | 0.151 | -0.077 | 0.021 | 3.20E-04 | 0.011 | 0.057 | 0.84 | -4.80E-03 | 0.043 | 0.911 |
| InternalIMT | dha | rs901681 | 0.546 | -0.054 | 0.034 | 0.109 | 0.029 | 0.034 | 0.392 | -0.022 | 0.025 | 0.39 |
| InternalIMT | dha | rs61854092 | 0.15 | -0.077 | 0.021 | 3.20E-04 | 0.013 | 0.057 | 0.825 | -5.40E-03 | 0.043 | 0.898 |
| InternalIMT | dha | rs148080753 | 0.001 | . | . | . | . | . | . | . | . | . |
| InternalIMT | dha | rs61854093 | 0.186 | -0.077 | 0.022 | 4.10E-04 | 5.20E-03 | 0.05 | 0.917 | -4.30E-03 | 0.037 | 0.908 |
| InternalIMT | dha | rs141822774 | 0.001 | . | . | . | . | . | . | . | . | . |

| InternalIMT | dha | rs74130836 | 0.04 | -0.057 | 0.022 | 9.50E-03 | -0.041 | 0.1 | 0.685 | 7.00E-04 | 0.075 | 0.993 |
| --- | --- | --- | --- | --- | --- | --- | --- | --- | --- | --- | --- | --- |
| InternalIMT | dha | rs150620963 | 0.013 | -0.063 | 0.022 | 4.30E-03 | -0.11 | 0.148 | 0.457 | 0.096 | 0.131 | 0.462 |
| InternalIMT | dha | rs3780913 | 0.753 | -0.061 | 0.041 | 0.142 | 0.012 | 0.036 | 0.732 | -0.012 | 0.027 | 0.666 |
| InternalIMT | dha | rs142255723 | 0.002 | . | . | . | . | . | . | . | . | . |
| InternalIMT | dha | rs3780914 | 0.542 | -0.06 | 0.033 | 0.073 | 0.022 | 0.034 | 0.511 | -0.016 | 0.025 | 0.516 |
| InternalIMT | dha | rs182656507 | 0.002 | . | . | . | . | . | . | . | . | . |
| InternalIMT | dha | rs2291427 | 0.735 | -0.048 | 0.04 | 0.231 | 0.031 | 0.036 | 0.383 | -0.021 | 0.026 | 0.432 |
| InternalIMT | dha | rs10751382 | 0.733 | -0.044 | 0.04 | 0.272 | 0.032 | 0.036 | 0.363 | -0.024 | 0.026 | 0.37 |
| InternalIMT | dha | rs117610108 | 0.01 | -0.082 | 0.02 | 3.50E-05 | -0.334 | 0.1 | 8.20E-04 | 0.127 | 0.07 | 0.072 |
| InternalIMT | dha | rs144966941 | 0.023 | -0.08 | 0.02 | 7.20E-05 | -0.023 | 0.113 | 0.842 | 0.075 | 0.099 | 0.451 |
| InternalIMT | dha | rs41502051 | 0.03 | -0.075 | 0.02 | 2.30E-04 | 0.07 | 0.097 | 0.47 | -0.043 | 0.073 | 0.56 |
| InternalIMT | dha | rs41426046 | 0.011 | -0.081 | 0.02 | 3.70E-05 | -0.337 | 0.1 | 7.80E-04 | 0.125 | 0.071 | 0.077 |
| InternalIMT | dha | rs41519248 | 0.01 | -0.082 | 0.02 | 3.50E-05 | -0.336 | 0.1 | 7.40E-04 | 0.128 | 0.07 | 0.07 |
| InternalIMT | dha | rs189108245 | 0.004 | . | . | . | . | . | . | . | . | . |
| InternalIMT | dha | rs7896431 | 0.532 | -0.062 | 0.033 | 0.063 | 0.018 | 0.034 | 0.601 | -0.015 | 0.025 | 0.544 |
| InternalIMT | dha | rs183724547 | 0.003 | . | . | . | . | . | . | . | . | . |
| InternalIMT | dha | rs142044990 | 0.008 | . | . | . | . | . | . | . | . | . |
| InternalIMT | dha | rs188140929 | 0.007 | . | . | . | . | . | . | . | . | . |
| InternalIMT | dha | rs76819096 | 0.014 | -0.058 | 0.022 | 7.40E-03 | 0.274 | 0.198 | 0.167 | -0.164 | 0.142 | 0.247 |
| InternalIMT | dha | rs10751383 | 0.601 | -0.072 | 0.036 | 0.046 | 0.015 | 0.034 | 0.658 | -4.70E-03 | 0.025 | 0.854 |
| InternalIMT | dha | rs117819321 | 0.014 | -0.05 | 0.022 | 0.019 | 0.41 | 0.303 | 0.176 | -0.267 | 0.226 | 0.236 |
| InternalIMT | dha | rs2242332 | 0.702 | -0.046 | 0.039 | 0.237 | 0.024 | 0.035 | 0.488 | -0.023 | 0.026 | 0.376 |
| InternalIMT | dha | rs2242333 | 0.096 | -0.071 | 0.021 | 8.10E-04 | -0.113 | 0.051 | 0.028 | 0.024 | 0.034 | 0.478 |
| InternalIMT | dha | rs2242334 | 0.703 | -0.046 | 0.039 | 0.237 | 0.024 | 0.035 | 0.493 | -0.023 | 0.026 | 0.379 |
| InternalIMT | dha | rs189465041 | 0.001 | . | . | . | . | . | . | . | . | . |
| InternalIMT | dha | rs28395868 | 0.025 | -0.058 | 0.022 | 8.10E-03 | 0.062 | 0.118 | 0.601 | -0.045 | 0.099 | 0.646 |
| InternalIMT | dha | rs56067550 | 0.02 | -0.067 | 0.022 | 2.20E-03 | -0.295 | 0.134 | 0.028 | 0.176 | 0.114 | 0.124 |
| InternalIMT | dha | rs1051713 | 0.154 | -0.076 | 0.022 | 4.10E-04 | 7.70E-03 | 0.055 | 0.889 | -7.10E-03 | 0.041 | 0.861 |
| InternalIMT | dha | rs115074210 | 0.016 | -0.059 | 0.022 | 6.10E-03 | 0.162 | 0.189 | 0.39 | -0.087 | 0.137 | 0.527 |
| InternalIMT | dha | rs7900977 | 0.802 | -0.052 | 0.047 | 0.267 | 0.029 | 0.038 | 0.45 | -0.017 | 0.028 | 0.549 |
| InternalIMT | dha | rs147613891 | 0.003 | . | . | . | . | . | . | . | . | . |
| InternalIMT | dha | rs2229136 | 0.084 | -0.082 | 0.021 | 9.20E-05 | 0.015 | 0.063 | 0.818 | 0.015 | 0.047 | 0.751 |
| InternalIMT | dha | rs185482841 | 0.003 | . | . | . | . | . | . | . | . | . |
| InternalIMT | dha | rs2288619 | 0.083 | -0.08 | 0.021 | 1.20E-04 | 0.021 | 0.064 | 0.746 | 7.00E-03 | 0.047 | 0.88 |
| InternalIMT | dha | rs189891524 | 0.001 | . | . | . | . | . | . | . | . | . |
| InternalIMT | dha | rs56145077 | 0.143 | -0.072 | 0.021 | 6.90E-04 | 0.038 | 0.056 | 0.494 | -0.024 | 0.041 | 0.561 |
| InternalIMT | dha | rs143216970 | 0.005 | . | . | . | . | . | . | . | . | . |
| InternalIMT | dha | rs142246478 | 0.001 | . | . | . | . | . | . | . | . | . |
| InternalIMT | dha | rs13134 | 0.012 | -0.059 | 0.022 | 6.50E-03 | 0.058 | 0.151 | 0.701 | -0.038 | 0.117 | 0.746 |
| InternalIMT | dha | rs41314980 | 0.027 | -0.065 | 0.022 | 3.50E-03 | -0.101 | 0.111 | 0.363 | 0.118 | 0.095 | 0.214 |
| InternalIMT | dha | rs41304611 | 0.013 | -0.064 | 0.022 | 3.50E-03 | -0.189 | 0.175 | 0.281 | 0.161 | 0.147 | 0.273 |
| InternalIMT | dha | rs193299617 | 0.001 | . | . | . | . | . | . | . | . | . |
| InternalIMT | dha | rs12772724 | 0.012 | -0.059 | 0.022 | 6.50E-03 | 0.058 | 0.151 | 0.701 | -0.038 | 0.117 | 0.745 |
| InternalIMT | dha | rs139489537 | 0.033 | -0.082 | 0.02 | 5.00E-05 | -0.025 | 0.086 | 0.769 | 0.061 | 0.065 | 0.351 |
| InternalIMT | dha | rs12776062 | 0.009 | . | . | . | . | . | . | . | . | . |
| InternalIMT | dha | rs752059 | 0.144 | -0.073 | 0.021 | 6.10E-04 | 0.031 | 0.056 | 0.585 | -0.02 | 0.041 | 0.618 |
| InternalIMT | dha | rs147649982 | 0.03 | -0.088 | 0.02 | 1.30E-05 | -0.185 | 0.116 | 0.113 | 0.178 | 0.097 | 0.068 |
| InternalIMT | dha | rs17157798 | 0.07 | -0.079 | 0.021 | 1.40E-04 | 0.026 | 0.067 | 0.698 | 2.70E-03 | 0.05 | 0.956 |
| InternalIMT | dha | rs142298619 | 0.003 | . | . | . | . | . | . | . | . | . |
| InternalIMT | dha | rs4948673 | 0.793 | -0.063 | 0.047 | 0.179 | 0.017 | 0.038 | 0.663 | -9.80E-03 | 0.028 | 0.727 |
| InternalIMT | dha | rs74231484 | 0.102 | -0.067 | 0.021 | 1.50E-03 | -0.085 | 0.051 | 0.096 | 6.70E-03 | 0.033 | 0.842 |
| InternalIMT | dha | rs72796489 | 0.002 | . | . | . | . | . | . | . | . | . |
| InternalIMT | dha | rs113447578 | 0.016 | -0.08 | 0.02 | 5.90E-05 | -0.179 | 0.106 | 0.09 | 0.054 | 0.078 | 0.49 |
| InternalIMT | dha | rs34063374 | 0.013 | -0.058 | 0.022 | 6.90E-03 | 0.076 | 0.151 | 0.616 | -0.051 | 0.117 | 0.659 |
| InternalIMT | dha | rs11239527 | 0.013 | -0.057 | 0.022 | 8.40E-03 | 0.194 | 0.243 | 0.426 | -0.085 | 0.19 | 0.653 |
| InternalIMT | dha | rs34371883 | 0.011 | -0.058 | 0.022 | 6.80E-03 | 0.067 | 0.156 | 0.666 | -0.063 | 0.128 | 0.624 |
| InternalIMT | dha | rs118168828 | 0.007 | . | . | . | . | . | . | . | . | . |
| InternalIMT | dha | rs34349618 | 0.009 | . | . | . | . | . | . | . | . | . |
| InternalIMT | dha | rs80041679 | 0.033 | -0.082 | 0.02 | 5.00E-05 | -0.025 | 0.086 | 0.768 | 0.061 | 0.065 | 0.35 |
| InternalIMT | dha | rs149640607 | 0.003 | . | . | . | . | . | . | . | . | . |
| InternalIMT | dha | rs4427523 | 0.888 | -0.01 | 0.091 | 0.909 | 7.50E-03 | 0.067 | 0.911 | -0.037 | 0.05 | 0.46 |
| InternalIMT | dha | rs185880109 | 0.002 | . | . | . | . | . | . | . | . | . |
| InternalIMT | dha | rs191523220 | 0.003 | . | . | . | . | . | . | . | . | . |
| InternalIMT | dha | rs144315284 | 0.006 | . | . | . | . | . | . | . | . | . |
| InternalIMT | dha | rs4948674 | 0.341 | -0.082 | 0.025 | 1.10E-03 | 0.023 | 0.036 | 0.519 | 0.012 | 0.027 | 0.665 |
| InternalIMT | dha | rs182984298 | 0.006 | . | . | . | . | . | . | . | . | . |
| InternalIMT | dha | rs56749023 | 0.025 | -0.055 | 0.022 | 0.011 | 0.169 | 0.132 | 0.202 | -0.126 | 0.094 | 0.182 |
| InternalIMT | dha | rs112508957 | 0.015 | -0.063 | 0.022 | 3.40E-03 | -0.032 | 0.169 | 0.851 | 0.066 | 0.116 | 0.567 |
| InternalIMT | dha | rs7089063 | 0.229 | -0.072 | 0.023 | 1.50E-03 | 0.016 | 0.042 | 0.706 | -0.013 | 0.031 | 0.66 |
| InternalIMT | dha | rs145000779 | 0.001 | . | . | . | . | . | . | . | . | . |
| InternalIMT | dha | rs80201572 | 0.034 | -0.081 | 0.02 | 5.10E-05 | -0.024 | 0.086 | 0.78 | 0.058 | 0.065 | 0.365 |
| InternalIMT | dha | rs140568227 | 0.005 | . | . | . | . | . | . | . | . | . |
| InternalIMT | dha | rs149756228 | 0.01 | . | . | . | . | . | . | . | . | . |
| InternalIMT | dha | rs112380685 | 0.007 | . | . | . | . | . | . | . | . | . |
| InternalIMT | dha | rs60481109 | 0.023 | -0.055 | 0.022 | 0.011 | 0.189 | 0.135 | 0.162 | -0.141 | 0.097 | 0.146 |
| InternalIMT | dha | rs138221843 | 0.003 | . | . | . | . | . | . | . | . | . |
| InternalIMT | dha | rs150283194 | 0.003 | . | . | . | . | . | . | . | . | . |
| InternalIMT | dha | rs138879661 | 0.004 | . | . | . | . | . | . | . | . | . |
| InternalIMT | dha | rs34960345 | 0.056 | -0.082 | 0.02 | 6.10E-05 | -0.012 | 0.069 | 0.867 | 0.036 | 0.052 | 0.491 |
| InternalIMT | dha | rs147514068 | 0.004 | . | . | . | . | . | . | . | . | . |
| InternalIMT | dha | rs35410535 | 0.078 | -0.08 | 0.021 | 1.30E-04 | 0.024 | 0.064 | 0.711 | 3.60E-03 | 0.047 | 0.939 |
| InternalIMT | dha | rs191613635 | 0.003 | . | . | . | . | . | . | . | . | . |
| InternalIMT | dha | rs17157800 | 0.007 | . | . | . | . | . | . | . | . | . |
| InternalIMT | dha | rs17523413 | 0.045 | -0.081 | 0.02 | 7.10E-05 | -5.50E-03 | 0.075 | 0.941 | 0.039 | 0.056 | 0.492 |
| InternalIMT | dha | rs7897036 | 0.035 | -0.066 | 0.02 | 8.50E-04 | 0.192 | 0.138 | 0.163 | -0.172 | 0.099 | 0.082 |
| InternalIMT | dha | rs76334323 | 0.01 | -0.082 | 0.02 | 3.40E-05 | -0.351 | 0.1 | 4.30E-04 | 0.134 | 0.07 | 0.057 |

| InternalIMT | dha | rs143535991 | 0.001 | . | . | . | . | . | . | . | . | . |
| --- | --- | --- | --- | --- | --- | --- | --- | --- | --- | --- | --- | --- |
| InternalIMT | dha | rs140344979 | 0.005 | . | . | . | . | . | . | . | . | . |
| InternalIMT | dha | rs36047811 | 0.078 | -0.08 | 0.021 | 1.30E-04 | 0.024 | 0.064 | 0.713 | 3.70E-03 | 0.047 | 0.937 |
| InternalIMT | dha | rs76493570 | 0.054 | -0.081 | 0.02 | 7.00E-05 | -5.60E-03 | 0.07 | 0.936 | 0.032 | 0.052 | 0.54 |
| InternalIMT | dha | rs192998920 | 0.003 | . | . | . | . | . | . | . | . | . |
| InternalIMT | dha | rs116840852 | 0.016 | -0.061 | 0.022 | 5.40E-03 | 0.023 | 0.214 | 0.913 | 0.042 | 0.171 | 0.807 |
| InternalIMT | dha | rs184824101 | 0.001 | . | . | . | . | . | . | . | . | . |
| InternalIMT | dha | rs41301621 | 0.005 | . | . | . | . | . | . | . | . | . |
| InternalIMT | dha | rs3740109 | 0.078 | -0.08 | 0.021 | 1.30E-04 | 0.023 | 0.064 | 0.716 | 3.80E-03 | 0.047 | 0.935 |
| InternalIMT | dha | rs7899415 | 0.033 | -0.073 | 0.02 | 2.60E-04 | 0.129 | 0.13 | 0.324 | -0.081 | 0.085 | 0.341 |
| InternalIMT | dha | rs184395284 | 0.002 | . | . | . | . | . | . | . | . | . |
| InternalIMT | dha | rs139209589 | 0.004 | . | . | . | . | . | . | . | . | . |
| InternalIMT | dha | rs149924163 | 0.003 | . | . | . | . | . | . | . | . | . |
| InternalIMT | dha | rs3740110 | 0.01 | -0.082 | 0.02 | 3.40E-05 | -0.351 | 0.1 | 4.40E-04 | 0.134 | 0.07 | 0.058 |
| InternalIMT | dha | rs144335113 | 0.003 | . | . | . | . | . | . | . | . | . |
| InternalIMT | dha | rs74743385 | 0.015 | -0.063 | 0.022 | 3.40E-03 | -0.033 | 0.169 | 0.847 | 0.067 | 0.116 | 0.566 |
| InternalIMT | dha | rs146545829 | 0.003 | . | . | . | . | . | . | . | . | . |
| InternalIMT | dha | rs181842689 | 0 | . | . | . | . | . | . | . | . | . |
| InternalIMT | dha | rs3740111 | 0.056 | -0.082 | 0.02 | 6.10E-05 | -0.012 | 0.069 | 0.864 | 0.036 | 0.052 | 0.49 |
| InternalIMT | dha | rs186261599 | 0.003 | . | . | . | . | . | . | . | . | . |
| InternalIMT | dha | rs73285348 | 0.009 | . | . | . | . | . | . | . | . | . |
| InternalIMT | dha | rs186356901 | 0.003 | . | . | . | . | . | . | . | . | . |
| InternalIMT | dha | rs3824618 | 0.033 | -0.082 | 0.02 | 5.00E-05 | -0.027 | 0.086 | 0.755 | 0.061 | 0.065 | 0.345 |
| InternalIMT | dha | rs3802548 | 0.219 | -0.084 | 0.023 | 2.50E-04 | -0.015 | 0.04 | 0.7 | 0.013 | 0.031 | 0.68 |
| InternalIMT | dha | rs3802547 | 0.01 | . | . | . | . | . | . | . | . | . |
| InternalIMT | dha | rs77677521 | 0.016 | -0.061 | 0.022 | 5.40E-03 | 0.023 | 0.214 | 0.913 | 0.042 | 0.171 | 0.806 |
| InternalIMT | dha | rs3824616 | 0.078 | -0.08 | 0.021 | 1.30E-04 | 0.023 | 0.064 | 0.719 | 4.00E-03 | 0.047 | 0.933 |
| InternalIMT | dha | rs191073818 | 0.003 | . | . | . | . | . | . | . | . | . |
| InternalIMT | dha | rs147078004 | 0.002 | . | . | . | . | . | . | . | . | . |
| InternalIMT | dha | rs7908745 | 0.31 | -0.083 | 0.025 | 7.00E-04 | -9.80E-03 | 0.035 | 0.782 | 8.70E-03 | 0.027 | 0.744 |
| InternalIMT | dha | rs17157811 | 0.007 | . | . | . | . | . | . | . | . | . |
| InternalIMT | dha | rs138506087 | 0.006 | . | . | . | . | . | . | . | . | . |
| InternalIMT | dha | rs77241269 | 0.025 | -0.056 | 0.022 | 0.01 | 0.163 | 0.132 | 0.217 | -0.121 | 0.095 | 0.202 |
| InternalIMT | dha | rs138374938 | 0.001 | . | . | . | . | . | . | . | . | . |
| InternalIMT | dha | rs2279433 | 0.056 | -0.082 | 0.021 | 6.60E-05 | -0.011 | 0.069 | 0.878 | 0.032 | 0.051 | 0.527 |
| InternalIMT | dha | rs73285358 | 0.025 | -0.056 | 0.022 | 0.01 | 0.163 | 0.132 | 0.218 | -0.121 | 0.095 | 0.204 |
| InternalIMT | dha | rs11239528 | 0.218 | -0.084 | 0.023 | 2.40E-04 | -0.016 | 0.04 | 0.691 | 0.013 | 0.031 | 0.665 |
| InternalIMT | dha | rs77453386 | 0.01 | -0.082 | 0.02 | 3.40E-05 | -0.35 | 0.1 | 4.60E-04 | 0.134 | 0.07 | 0.058 |
| InternalIMT | dha | rs2279434 | 0.061 | -0.082 | 0.021 | 7.10E-05 | -0.013 | 0.067 | 0.842 | 0.033 | 0.049 | 0.508 |
| InternalIMT | dha | rs149236525 | 0.005 | . | . | . | . | . | . | . | . | . |
| InternalIMT | dha | rs12573609 | 0.05 | -0.07 | 0.021 | 8.20E-04 | 0.053 | 0.096 | 0.583 | -0.073 | 0.065 | 0.261 |
| InternalIMT | dha | rs191716655 | 0.002 | . | . | . | . | . | . | . | . | . |
| InternalIMT | dha | rs17523660 | 0.009 | . | . | . | . | . | . | . | . | . |
| InternalIMT | dha | rs142671360 | 0.003 | . | . | . | . | . | . | . | . | . |
| InternalIMT | dha | rs3764990 | 0.044 | -0.081 | 0.02 | 7.30E-05 | -3.80E-03 | 0.075 | 0.959 | 0.038 | 0.057 | 0.501 |
| InternalIMT | dha | rs113682861 | 0.004 | . | . | . | . | . | . | . | . | . |
| InternalIMT | dha | rs117658270 | 0.01 | -0.082 | 0.02 | 3.40E-05 | -0.35 | 0.1 | 4.60E-04 | 0.134 | 0.07 | 0.058 |
| InternalIMT | dha | rs187309063 | 0 | . | . | . | . | . | . | . | . | . |
| InternalIMT | dha | rs73285360 | 0.025 | -0.056 | 0.022 | 0.01 | 0.163 | 0.132 | 0.219 | -0.12 | 0.095 | 0.205 |
| InternalIMT | dha | rs80133289 | 0.015 | -0.075 | 0.02 | 1.70E-04 | -0.309 | 0.158 | 0.051 | 0.084 | 0.099 | 0.4 |
| InternalIMT | dha | rs186693257 | 0.001 | . | . | . | . | . | . | . | . | . |
| InternalIMT | dha | rs35735968 | 0.062 | -0.082 | 0.021 | 7.40E-05 | -7.90E-03 | 0.068 | 0.907 | 0.028 | 0.05 | 0.582 |
| InternalIMT | dha | rs17157825 | 0.034 | -0.081 | 0.02 | 5.10E-05 | -0.024 | 0.086 | 0.781 | 0.061 | 0.065 | 0.352 |
| InternalIMT | dha | rs2291428 | 0.213 | -0.084 | 0.023 | 2.50E-04 | -0.014 | 0.04 | 0.738 | 0.014 | 0.031 | 0.651 |
| InternalIMT | dha | rs2291429 | 0.222 | -0.084 | 0.023 | 2.60E-04 | -0.016 | 0.04 | 0.682 | 0.014 | 0.031 | 0.653 |
| InternalIMT | dha | rs145714792 | 0.005 | . | . | . | . | . | . | . | . | . |
| InternalIMT | dha | rs12769244 | 0.056 | -0.082 | 0.021 | 6.80E-05 | -8.70E-03 | 0.068 | 0.899 | 0.031 | 0.051 | 0.542 |
| InternalIMT | dha | rs12771275 | 0.056 | -0.082 | 0.021 | 6.80E-05 | -8.70E-03 | 0.068 | 0.899 | 0.031 | 0.051 | 0.542 |
| InternalIMT | dha | rs146266174 | 0.001 | . | . | . | . | . | . | . | . | . |
| InternalIMT | dha | rs41301623 | 0.003 | . | . | . | . | . | . | . | . | . |
| InternalIMT | dha | rs2306814 | 0.011 | -0.082 | 0.02 | 3.10E-05 | -0.353 | 0.096 | 2.40E-04 | 0.14 | 0.069 | 0.044 |
| InternalIMT | dha | rs12244834 | 0.027 | -0.078 | 0.02 | 8.10E-05 | 0.111 | 0.157 | 0.48 | -0.052 | 0.104 | 0.615 |
| InternalIMT | dha | rs184006011 | 0.002 | . | . | . | . | . | . | . | . | . |
| InternalIMT | dha | rs78829066 | 0.01 | -0.082 | 0.02 | 3.30E-05 | -0.352 | 0.101 | 4.70E-04 | 0.136 | 0.072 | 0.058 |
| InternalIMT | dha | rs34731408 | 0.056 | -0.082 | 0.021 | 6.80E-05 | -8.60E-03 | 0.069 | 0.9 | 0.031 | 0.051 | 0.543 |
| InternalIMT | dha | rs17451516 | 0.012 | -0.059 | 0.022 | 6.40E-03 | 0.052 | 0.151 | 0.73 | -0.032 | 0.117 | 0.783 |
| InternalIMT | dha | rs139910133 | 0.003 | . | . | . | . | . | . | . | . | . |
| InternalIMT | dha | rs10900218 | 0.233 | -0.077 | 0.023 | 7.20E-04 | 0.01 | 0.042 | 0.806 | -3.20E-03 | 0.031 | 0.917 |
| InternalIMT | dha | rs146023999 | 0.009 | . | . | . | . | . | . | . | . | . |
| InternalIMT | dha | rs187296958 | 0.003 | . | . | . | . | . | . | . | . | . |
| InternalIMT | dha | rs142342833 | 0.015 | -0.064 | 0.057 | 0.265 | -0.664 | 0.64 | 0.3 | 0.343 | 0.419 | 0.413 |
| InternalIMT | dha | rs143489255 | 0.006 | . | . | . | . | . | . | . | . | . |
| InternalIMT | dha | rs138532518 | 0.006 | . | . | . | . | . | . | . | . | . |
| InternalIMT | dha | rs183768079 | 0.005 | . | . | . | . | . | . | . | . | . |
| InternalIMT | dha | rs117892272 | 0.012 | -0.044 | 0.053 | 0.403 | 0.441 | 0.957 | 0.645 | -0.257 | 0.58 | 0.657 |
| InternalIMT | dha | rs192993943 | 0.003 | . | . | . | . | . | . | . | . | . |
| InternalIMT | dha | rs182989043 | 0.004 | . | . | . | . | . | . | . | . | . |
| InternalIMT | dha | rs189703435 | 0.007 | . | . | . | . | . | . | . | . | . |
| InternalIMT | dha | rs188794040 | 0.007 | . | . | . | . | . | . | . | . | . |
| InternalIMT | dha | rs143920953 | 0.003 | . | . | . | . | . | . | . | . | . |
| InternalIMT | dha | rs191029121 | 0.006 | . | . | . | . | . | . | . | . | . |
| InternalIMT | dha | rs116875448 | 0.006 | . | . | . | . | . | . | . | . | . |
| InternalIMT | dha | rs190167877 | 0.002 | . | . | . | . | . | . | . | . | . |
| InternalIMT | dha | rs184993418 | 0.003 | . | . | . | . | . | . | . | . | . |
| InternalIMT | dha | rs143241158 | 0.01 | . | . | . | . | . | . | . | . | . |
| InternalIMT | dha | rs11239500 | 0.038 | -0.053 | 0.025 | 0.034 | 0.057 | 0.148 | 0.698 | -3.40E-03 | 0.097 | 0.972 |

| InternalIMT | dha | rs140568994 | 0.001 | . | . | . | . | . | . | . | . | . |
| --- | --- | --- | --- | --- | --- | --- | --- | --- | --- | --- | --- | --- |
| InternalIMT | dha | rs187530317 | 0.003 | . | . | . | . | . | . | . | . | . |
| InternalIMT | dha | rs145189326 | 0.002 | . | . | . | . | . | . | . | . | . |
| InternalIMT | dha | rs186203332 | 0.009 | . | . | . | . | . | . | . | . | . |
| InternalIMT | dha | rs189921909 | 0.004 | . | . | . | . | . | . | . | . | . |
| InternalIMT | dha | rs187260989 | 0.01 | -0.046 | 0.057 | 0.422 | 0.694 | 0.885 | 0.433 | -0.424 | 0.577 | 0.462 |
| InternalIMT | dha | rs114376586 | 0.037 | -0.052 | 0.025 | 0.036 | 0.071 | 0.148 | 0.633 | -0.015 | 0.098 | 0.879 |
| InternalIMT | dha | rs151070808 | 0.019 | -0.053 | 0.06 | 0.383 | 0.178 | 0.476 | 0.709 | -0.129 | 0.294 | 0.661 |
| InternalIMT | dha | rs140927225 | 0.038 | -0.052 | 0.025 | 0.037 | 0.068 | 0.148 | 0.645 | -0.014 | 0.098 | 0.883 |
| InternalIMT | dha | rs191555615 | 0.012 | -0.038 | 0.053 | 0.476 | 0.623 | 0.827 | 0.451 | -0.367 | 0.51 | 0.472 |
| InternalIMT | dha | rs80278898 | 0.012 | -0.051 | 0.025 | 0.042 | -0.422 | 0.308 | 0.171 | 0.175 | 0.21 | 0.406 |
| InternalIMT | dha | rs190849503 | 0.004 | . | . | . | . | . | . | . | . | . |
| InternalIMT | dha | rs150080315 | 0.006 | . | . | . | . | . | . | . | . | . |
| InternalIMT | dha | rs143623885 | 0.011 | -0.061 | 0.058 | 0.29 | -0.598 | 0.707 | 0.398 | 0.303 | 0.454 | 0.504 |
| InternalIMT | dha | rs187760040 | 0.002 | . | . | . | . | . | . | . | . | . |
| InternalIMT | dha | rs143917143 | 0.01 | -0.037 | 0.031 | 0.227 | -0.08 | 0.281 | 0.776 | -0.023 | 0.201 | 0.909 |
| InternalIMT | dha | rs149851136 | 0.001 | . | . | . | . | . | . | . | . | . |
| InternalIMT | dha | rs181612054 | 0.001 | . | . | . | . | . | . | . | . | . |
| InternalIMT | dha | rs191006548 | 0.001 | . | . | . | . | . | . | . | . | . |
| InternalIMT | dha | rs117377631 | 0.012 | -0.048 | 0.025 | 0.051 | 0.032 | 0.236 | 0.89 | 0.106 | 0.196 | 0.587 |
| InternalIMT | dha | rs182107634 | 0.002 | . | . | . | . | . | . | . | . | . |
| InternalIMT | dha | rs190211867 | 0.002 | . | . | . | . | . | . | . | . | . |
| InternalIMT | dha | rs148293457 | 0.031 | -0.048 | 0.061 | 0.429 | 0.214 | 0.314 | 0.495 | -0.13 | 0.19 | 0.495 |
| InternalIMT | dha | rs191811733 | 0.006 | . | . | . | . | . | . | . | . | . |
| InternalIMT | dha | rs146109272 | 0.008 | . | . | . | . | . | . | . | . | . |
| InternalIMT | dha | rs148397915 | 0.006 | . | . | . | . | . | . | . | . | . |
| InternalIMT | dha | rs142233290 | 0.007 | . | . | . | . | . | . | . | . | . |
| InternalIMT | dha | rs80226874 | 0.022 | -0.074 | 0.058 | 0.203 | -0.526 | 0.729 | 0.471 | 0.404 | 0.442 | 0.361 |
| InternalIMT | dha | rs144496203 | 0.004 | . | . | . | . | . | . | . | . | . |
| InternalIMT | dha | rs191062130 | 0.001 | . | . | . | . | . | . | . | . | . |
| InternalIMT | dha | rs185807817 | 0.004 | . | . | . | . | . | . | . | . | . |
| InternalIMT | dha | rs117846213 | 0.012 | -0.106 | 0.038 | 5.10E-03 | -0.033 | 0.367 | 0.929 | -0.06 | 0.235 | 0.797 |
| InternalIMT | dha | rs190545473 | 0.002 | . | . | . | . | . | . | . | . | . |
| InternalIMT | dha | rs192364918 | 0.01 | -0.09 | 0.057 | 0.116 | -3.59 | 2.13 | 0.093 | 2.13 | 1.42 | 0.134 |
| InternalIMT | dha | rs182939241 | 0.003 | . | . | . | . | . | . | . | . | . |
| InternalIMT | dha | rs12571125 | 0.011 | -0.102 | 0.039 | 8.10E-03 | -0.127 | 0.388 | 0.744 | -0.02 | 0.228 | 0.929 |
| InternalIMT | dha | rs192418941 | 0.002 | . | . | . | . | . | . | . | . | . |
| InternalIMT | dha | rs141270243 | 0.015 | -0.05 | 0.06 | 0.403 | -0.017 | 0.445 | 0.969 | -0.112 | 0.248 | 0.651 |
| InternalIMT | dha | rs182615502 | 0.006 | . | . | . | . | . | . | . | . | . |
| InternalIMT | dha | rs146669856 | 0.01 | -0.053 | 0.024 | 0.029 | -0.101 | 0.361 | 0.78 | 0.152 | 0.272 | 0.575 |
| InternalIMT | dha | rs78290029 | 0.053 | -0.047 | 0.025 | 0.061 | 0.231 | 0.143 | 0.105 | -0.106 | 0.094 | 0.26 |
| InternalIMT | dha | rs139955565 | 0.004 | . | . | . | . | . | . | . | . | . |
| InternalIMT | dha | rs12267314 | 0.047 | -0.049 | 0.025 | 0.047 | 0.159 | 0.153 | 0.3 | -0.066 | 0.1 | 0.512 |
| InternalIMT | dha | rs74477197 | 0.004 | . | . | . | . | . | . | . | . | . |
| InternalIMT | dha | rs188147236 | 0.005 | . | . | . | . | . | . | . | . | . |
| InternalIMT | dha | rs2228065 | 0.046 | -0.048 | 0.025 | 0.051 | 0.189 | 0.157 | 0.229 | -0.085 | 0.103 | 0.409 |
| InternalIMT | dha | rs116961353 | 0.01 | . | . | . | . | . | . | . | . | . |
| InternalIMT | dha | rs190222272 | 0.007 | . | . | . | . | . | . | . | . | . |
| InternalIMT | dha | rs182719940 | 0.006 | . | . | . | . | . | . | . | . | . |
| InternalIMT | dha | rs184477413 | 0.004 | . | . | . | . | . | . | . | . | . |
| InternalIMT | dha | rs12265175 | 0.048 | -0.05 | 0.025 | 0.045 | 0.171 | 0.155 | 0.27 | -0.069 | 0.102 | 0.497 |
| InternalIMT | dha | rs182928276 | 0.003 | . | . | . | . | . | . | . | . | . |
| InternalIMT | dha | rs144409895 | 0.011 | -0.093 | 0.058 | 0.106 | -3.68 | 2.12 | 0.083 | 2.17 | 1.41 | 0.122 |
| InternalIMT | dha | rs58998447 | 0.031 | -0.045 | 0.025 | 0.074 | -0.159 | 0.188 | 0.397 | 0.043 | 0.111 | 0.699 |
| InternalIMT | dha | rs188668832 | 0.005 | . | . | . | . | . | . | . | . | . |
| InternalIMT | dha | rs148641686 | 0.002 | . | . | . | . | . | . | . | . | . |
| InternalIMT | dha | rs75468791 | 0.054 | -0.077 | 0.063 | 0.222 | -0.291 | 0.489 | 0.552 | 0.216 | 0.29 | 0.456 |
| InternalIMT | dha | rs147327397 | 0.003 | . | . | . | . | . | . | . | . | . |
| InternalIMT | dha | rs77354597 | 0.007 | . | . | . | . | . | . | . | . | . |
| InternalIMT | dha | rs41365544 | 0.045 | -0.048 | 0.025 | 0.055 | 0.176 | 0.157 | 0.261 | -0.082 | 0.103 | 0.425 |
| InternalIMT | dha | rs187564188 | 0.016 | -0.028 | 0.059 | 0.634 | 1.61 | 1.33 | 0.228 | -0.953 | 0.743 | 0.2 |
| InternalIMT | dha | rs183511984 | 0.002 | . | . | . | . | . | . | . | . | . |
| InternalIMT | dha | rs12262783 | 0.038 | -0.049 | 0.025 | 0.045 | 0.201 | 0.166 | 0.225 | -0.078 | 0.11 | 0.478 |
| InternalIMT | dha | rs188817687 | 0.002 | . | . | . | . | . | . | . | . | . |
| InternalIMT | dha | rs3815968 | 0.005 | . | . | . | . | . | . | . | . | . |
| InternalIMT | dha | rs187822300 | 0.001 | . | . | . | . | . | . | . | . | . |
| InternalIMT | dha | rs188902172 | 0.008 | . | . | . | . | . | . | . | . | . |
| InternalIMT | dha | rs181190448 | 0.006 | . | . | . | . | . | . | . | . | . |
| InternalIMT | dha | rs182699093 | 0.001 | . | . | . | . | . | . | . | . | . |
| InternalIMT | dha | rs182631303 | 0.005 | . | . | . | . | . | . | . | . | . |
| InternalIMT | dha | rs187014172 | 0.003 | . | . | . | . | . | . | . | . | . |
| InternalIMT | dha | rs142575406 | 0.009 | . | . | . | . | . | . | . | . | . |
| InternalIMT | dha | rs180711033 | 0.007 | . | . | . | . | . | . | . | . | . |
| InternalIMT | dha | rs137894823 | 0.002 | . | . | . | . | . | . | . | . | . |
| InternalIMT | dha | rs189053273 | 0.001 | . | . | . | . | . | . | . | . | . |
| InternalIMT | dha | rs143499388 | 0.002 | . | . | . | . | . | . | . | . | . |
| InternalIMT | dha | rs182936001 | 0.003 | . | . | . | . | . | . | . | . | . |
| InternalIMT | dha | rs148456406 | 0.003 | . | . | . | . | . | . | . | . | . |
| InternalIMT | dha | rs182499531 | 0.005 | . | . | . | . | . | . | . | . | . |
| InternalIMT | dha | rs74884915 | 0.016 | -0.013 | 0.029 | 0.642 | 0.036 | 0.175 | 0.838 | -0.061 | 0.116 | 0.598 |
| InternalIMT | dha | rs184046374 | 0.001 | . | . | . | . | . | . | . | . | . |
| InternalIMT | dha | rs74333993 | 0.019 | -0.016 | 0.028 | 0.574 | 0.156 | 0.233 | 0.503 | -0.076 | 0.159 | 0.63 |
| InternalIMT | dha | rs138889719 | 0.001 | . | . | . | . | . | . | . | . | . |
| InternalIMT | dha | rs75330330 | 0.013 | -0.017 | 0.029 | 0.543 | 0.135 | 0.322 | 0.675 | -0.042 | 0.209 | 0.842 |
| InternalIMT | dha | rs193051693 | 0 | . | . | . | . | . | . | . | . | . |
| InternalIMT | dha | rs145929633 | 0 | . | . | . | . | . | . | . | . | . |

| InternalIMT | dha | rs184076904 | 0.003 | . | . | . | . | . | . | . | . | . |
| --- | --- | --- | --- | --- | --- | --- | --- | --- | --- | --- | --- | --- |
| InternalIMT | dha | rs141802494 | 0.011 | -0.018 | 0.028 | 0.522 | 0.075 | 0.373 | 0.841 | 0.017 | 0.241 | 0.943 |
| InternalIMT | dha | rs149519759 | 0.001 | . | . | . | . | . | . | . | . | . |
| InternalIMT | dha | rs145467260 | 0.008 | . | . | . | . | . | . | . | . | . |
| InternalIMT | dha | rs79620914 | 0.017 | -8.30E-03 | 0.029 | 0.774 | 0.373 | 0.238 | 0.117 | -0.279 | 0.15 | 0.062 |
| InternalIMT | dha | rs140538633 | 0.011 | -0.014 | 0.029 | 0.619 | -0.035 | 0.177 | 0.844 | -0.036 | 0.113 | 0.746 |
| InternalIMT | dha | rs140862301 | 0.005 | . | . | . | . | . | . | . | . | . |
| InternalIMT | dha | rs114615847 | 0.006 | . | . | . | . | . | . | . | . | . |
| InternalIMT | dha | rs141800259 | 0.016 | -0.015 | 0.029 | 0.608 | 0.23 | 0.289 | 0.425 | -0.126 | 0.191 | 0.509 |
| InternalIMT | dha | rs149779059 | 0.011 | -0.02 | 0.029 | 0.485 | -8.90E-03 | 0.334 | 0.979 | 0.065 | 0.226 | 0.773 |
| InternalIMT | dha | rs116539253 | 0.004 | . | . | . | . | . | . | . | . | . |
| InternalIMT | dha | rs184979550 | 0.001 | . | . | . | . | . | . | . | . | . |
| InternalIMT | dha | rs190128940 | 0.003 | . | . | . | . | . | . | . | . | . |
| InternalIMT | dha | rs146594816 | 0.002 | . | . | . | . | . | . | . | . | . |
| InternalIMT | dha | rs141370766 | 0.013 | -0.017 | 0.029 | 0.543 | 0.136 | 0.323 | 0.673 | -0.041 | 0.209 | 0.845 |
| InternalIMT | dha | rs144670033 | 0.002 | . | . | . | . | . | . | . | . | . |
| InternalIMT | dha | rs187152174 | 0.005 | . | . | . | . | . | . | . | . | . |
| InternalIMT | dha | rs184180263 | 0.003 | . | . | . | . | . | . | . | . | . |
| InternalIMT | dha | rs140075861 | 0.007 | . | . | . | . | . | . | . | . | . |
| InternalIMT | dha | rs186483129 | 0 | . | . | . | . | . | . | . | . | . |
| InternalIMT | dha | rs186634282 | 0.008 | . | . | . | . | . | . | . | . | . |
| InternalIMT | dha | rs9988663 | 0.019 | -0.016 | 0.028 | 0.574 | 0.157 | 0.234 | 0.5 | -0.077 | 0.159 | 0.628 |
| InternalIMT | dha | rs138828529 | 0.013 | -0.019 | 0.028 | 0.506 | -0.062 | 0.4 | 0.878 | 0.088 | 0.32 | 0.783 |
| InternalIMT | dha | rs188777615 | 0.003 | . | . | . | . | . | . | . | . | . |
| InternalIMT | dha | rs192321882 | 0.005 | . | . | . | . | . | . | . | . | . |
| InternalIMT | dha | rs150812270 | 0.016 | -0.012 | 0.029 | 0.68 | 0.342 | 0.297 | 0.249 | -0.229 | 0.207 | 0.269 |
| InternalIMT | dha | rs185906603 | 0.001 | . | . | . | . | . | . | . | . | . |
| InternalIMT | dha | rs191070862 | 0.004 | . | . | . | . | . | . | . | . | . |
| InternalIMT | dha | rs182904978 | 0 | . | . | . | . | . | . | . | . | . |
| InternalIMT | dha | rs192679655 | 0.001 | . | . | . | . | . | . | . | . | . |
| InternalIMT | dha | rs189673086 | 0.005 | . | . | . | . | . | . | . | . | . |
| InternalIMT | dha | rs181141892 | 0.002 | . | . | . | . | . | . | . | . | . |
| InternalIMT | dha | rs186404123 | 0 | . | . | . | . | . | . | . | . | . |
| InternalIMT | dha | rs192117287 | 0.003 | . | . | . | . | . | . | . | . | . |
| InternalIMT | dha | rs182748835 | 0.001 | . | . | . | . | . | . | . | . | . |
| InternalIMT | dha | rs141854549 | 0.011 | -0.014 | 0.029 | 0.619 | -0.031 | 0.178 | 0.861 | -0.038 | 0.113 | 0.735 |
| InternalIMT | dha | rs140163397 | 0.003 | . | . | . | . | . | . | . | . | . |
| InternalIMT | dha | rs146347300 | 0.005 | . | . | . | . | . | . | . | . | . |
| InternalIMT | dha | rs139362377 | 0.011 | -0.014 | 0.029 | 0.62 | -0.031 | 0.178 | 0.863 | -0.038 | 0.113 | 0.734 |
| InternalIMT | dha | rs185087671 | 0.001 | . | . | . | . | . | . | . | . | . |
| InternalIMT | dha | rs190466574 | 0.003 | . | . | . | . | . | . | . | . | . |
| InternalIMT | dha | rs147012778 | 0.003 | . | . | . | . | . | . | . | . | . |
| InternalIMT | dha | rs138236960 | 0.006 | . | . | . | . | . | . | . | . | . |
| InternalIMT | dha | rs116310311 | 0.011 | -0.014 | 0.029 | 0.62 | -0.03 | 0.178 | 0.866 | -0.039 | 0.113 | 0.732 |
| InternalIMT | dha | rs142868356 | 0.008 | . | . | . | . | . | . | . | . | . |
| InternalIMT | dha | rs144956240 | 0.002 | . | . | . | . | . | . | . | . | . |
| InternalIMT | dha | rs188527776 | 0.002 | . | . | . | . | . | . | . | . | . |
| InternalIMT | dha | rs183971165 | 0.005 | . | . | . | . | . | . | . | . | . |
| InternalIMT | dha | rs76748216 | 0.006 | . | . | . | . | . | . | . | . | . |
| InternalIMT | dha | rs148554990 | 0.001 | . | . | . | . | . | . | . | . | . |
| InternalIMT | dha | rs114446477 | 0.017 | -8.50E-03 | 0.029 | 0.769 | 0.363 | 0.234 | 0.121 | -0.271 | 0.148 | 0.067 |
| InternalIMT | dha | rs185223933 | 0.004 | . | . | . | . | . | . | . | . | . |
| InternalIMT | dha | rs76869726 | 0.006 | . | . | . | . | . | . | . | . | . |
| InternalIMT | dha | rs151200830 | 0.011 | -0.014 | 0.029 | 0.624 | -9.50E-03 | 0.179 | 0.958 | -0.05 | 0.114 | 0.661 |
| InternalIMT | dha | rs17157731 | 0.011 | -0.014 | 0.029 | 0.625 | -7.50E-03 | 0.18 | 0.967 | -0.051 | 0.114 | 0.654 |
| InternalIMT | dha | rs181243469 | 0 | . | . | . | . | . | . | . | . | . |
| InternalIMT | dha | rs186890707 | 0 | . | . | . | . | . | . | . | . | . |
| InternalIMT | dha | rs184063360 | 0 | . | . | . | . | . | . | . | . | . |
| InternalIMT | dha | rs188550237 | 0.004 | . | . | . | . | . | . | . | . | . |
| InternalIMT | dha | rs140931068 | 0.01 | -0.021 | 0.028 | 0.462 | -0.052 | 0.408 | 0.899 | 0.155 | 0.316 | 0.624 |
| InternalIMT | dha | rs74867877 | 0.004 | . | . | . | . | . | . | . | . | . |
| InternalIMT | dha | rs11239495 | 0.036 | -0.017 | 0.029 | 0.564 | 0.123 | 0.171 | 0.471 | -0.045 | 0.117 | 0.697 |
| InternalIMT | dha | rs149745800 | 0 | . | . | . | . | . | . | . | . | . |
| InternalIMT | dha | rs145044458 | 0.01 | -0.014 | 0.029 | 0.614 | 4.50E-03 | 0.189 | 0.981 | -0.053 | 0.119 | 0.657 |
| InternalIMT | dha | rs188067427 | 0.001 | . | . | . | . | . | . | . | . | . |
| InternalIMT | dha | rs146827800 | 0.001 | . | . | . | . | . | . | . | . | . |
| InternalIMT | dha | rs140619817 | 0.01 | -0.014 | 0.029 | 0.614 | 6.40E-03 | 0.19 | 0.973 | -0.052 | 0.119 | 0.661 |
| InternalIMT | dha | rs892689 | 0.997 | . | . | . | . | . | . | . | . | . |
| InternalIMT | dha | rs143642005 | 0.004 | . | . | . | . | . | . | . | . | . |
| InternalIMT | dha | rs181929304 | 0.004 | . | . | . | . | . | . | . | . | . |
| InternalIMT | dha | rs191086173 | 0.001 | . | . | . | . | . | . | . | . | . |
| InternalIMT | dha | rs114613116 | 0.012 | -0.014 | 0.029 | 0.626 | -1.40E-03 | 0.182 | 0.994 | -0.051 | 0.116 | 0.657 |
| InternalIMT | dha | rs141076768 | 0.002 | . | . | . | . | . | . | . | . | . |
| InternalIMT | dha | rs187316531 | 0 | . | . | . | . | . | . | . | . | . |
| InternalIMT | dha | rs11239499 | 0.046 | -0.015 | 0.029 | 0.603 | 0.1 | 0.149 | 0.501 | -0.051 | 0.099 | 0.61 |
| InternalIMT | dha | rs192726371 | 0.002 | . | . | . | . | . | . | . | . | . |
| InternalIMT | dha | rs74128481 | 0.012 | -0.023 | 0.028 | 0.411 | -0.328 | 0.348 | 0.346 | 0.318 | 0.279 | 0.255 |
| InternalIMT | dha | rs74128482 | 0.008 | . | . | . | . | . | . | . | . | . |
| InternalIMT | dha | rs11239502 | 0.033 | -0.017 | 0.029 | 0.547 | 0.146 | 0.192 | 0.448 | -0.059 | 0.129 | 0.65 |
| InternalIMT | dha | rs116330285 | 0.002 | . | . | . | . | . | . | . | . | . |
| InternalIMT | dha | rs189926729 | 0.002 | . | . | . | . | . | . | . | . | . |
| InternalIMT | dha | rs188975779 | 0.008 | . | . | . | . | . | . | . | . | . |
| InternalIMT | dha | rs143846038 | 0.002 | . | . | . | . | . | . | . | . | . |
| InternalIMT | dha | rs74128483 | 0.012 | -0.023 | 0.028 | 0.41 | -0.33 | 0.348 | 0.343 | 0.319 | 0.279 | 0.253 |
| InternalIMT | dha | rs139292055 | 0.045 | -0.014 | 0.029 | 0.621 | 0.116 | 0.151 | 0.44 | -0.062 | 0.1 | 0.539 |
| InternalIMT | dha | rs11239503 | 0.045 | -0.014 | 0.029 | 0.62 | 0.116 | 0.151 | 0.44 | -0.061 | 0.1 | 0.539 |

| InternalIMT | dha | rs58838686 | 0.008 | . | . | . | . | . | . | . | . | . |
| --- | --- | --- | --- | --- | --- | --- | --- | --- | --- | --- | --- | --- |
| InternalIMT | dha | rs185825373 | 0.005 | . | . | . | . | . | . | . | . | . |
| InternalIMT | dha | rs11239504 | 0.045 | -0.014 | 0.029 | 0.62 | 0.116 | 0.151 | 0.44 | -0.062 | 0.1 | 0.539 |
| InternalIMT | dha | rs150012354 | 0.003 | . | . | . | . | . | . | . | . | . |
| InternalIMT | dha | rs187763096 | 0.005 | . | . | . | . | . | . | . | . | . |
| InternalIMT | dha | rs193043982 | 0.005 | . | . | . | . | . | . | . | . | . |
| InternalIMT | dha | rs79186884 | 0.014 | -0.023 | 0.029 | 0.431 | -0.112 | 0.307 | 0.714 | 0.138 | 0.212 | 0.516 |
| InternalIMT | dha | rs74128484 | 0.008 | . | . | . | . | . | . | . | . | . |
| InternalIMT | dha | rs58461537 | 0.045 | -0.014 | 0.029 | 0.62 | 0.116 | 0.15 | 0.44 | -0.061 | 0.1 | 0.54 |
| InternalIMT | dha | rs188527474 | 0.005 | . | . | . | . | . | . | . | . | . |
| InternalIMT | dha | rs141659976 | 0.005 | . | . | . | . | . | . | . | . | . |
| InternalIMT | dha | rs115846407 | 0.045 | -0.014 | 0.029 | 0.62 | 0.116 | 0.15 | 0.44 | -0.061 | 0.1 | 0.539 |
| InternalIMT | dha | rs138378816 | 0.045 | -0.014 | 0.029 | 0.62 | 0.116 | 0.15 | 0.44 | -0.062 | 0.1 | 0.539 |
| InternalIMT | dha | rs146188136 | 0.033 | -0.017 | 0.029 | 0.546 | 0.146 | 0.192 | 0.449 | -0.058 | 0.129 | 0.652 |
| InternalIMT | dha | rs139383871 | 0.008 | . | . | . | . | . | . | . | . | . |
| InternalIMT | dha | rs182953869 | 0.004 | . | . | . | . | . | . | . | . | . |
| InternalIMT | dha | rs192430299 | 0.012 | -0.023 | 0.028 | 0.419 | -0.118 | 0.237 | 0.62 | 0.207 | 0.197 | 0.293 |
| InternalIMT | dha | rs144152844 | 0.045 | -0.014 | 0.029 | 0.62 | 0.116 | 0.15 | 0.441 | -0.061 | 0.1 | 0.54 |
| InternalIMT | dha | rs145987786 | 0.033 | -5.90E-03 | 0.029 | 0.84 | 0.42 | 0.176 | 0.017 | -0.272 | 0.115 | 0.018 |
| InternalIMT | dha | rs181132926 | 0.002 | . | . | . | . | . | . | . | . | . |
| InternalIMT | dha | rs147882253 | 0.045 | -0.014 | 0.029 | 0.62 | 0.116 | 0.15 | 0.439 | -0.062 | 0.1 | 0.539 |
| InternalIMT | dha | rs141807621 | 0.015 | -0.017 | 0.028 | 0.533 | -4.10E-03 | 0.326 | 0.99 | 0.02 | 0.258 | 0.939 |
| InternalIMT | dha | rs147116052 | 0.045 | -0.014 | 0.029 | 0.62 | 0.116 | 0.15 | 0.439 | -0.062 | 0.1 | 0.539 |
| InternalIMT | dha | rs147716618 | 0.045 | -0.014 | 0.029 | 0.62 | 0.116 | 0.15 | 0.439 | -0.062 | 0.1 | 0.539 |
| InternalIMT | dha | rs187198855 | 0.029 | -0.011 | 0.029 | 0.699 | 0.365 | 0.197 | 0.064 | -0.209 | 0.131 | 0.112 |
| InternalIMT | dha | rs189409638 | 0.011 | -0.024 | 0.028 | 0.392 | -0.309 | 0.297 | 0.298 | 0.307 | 0.234 | 0.189 |
| InternalIMT | dha | rs182647339 | 0.001 | . | . | . | . | . | . | . | . | . |
| InternalIMT | dha | rs187995988 | 0.004 | . | . | . | . | . | . | . | . | . |
| InternalIMT | dha | rs141034184 | 0.048 | -0.017 | 0.029 | 0.565 | 0.027 | 0.146 | 0.855 | -0.012 | 0.098 | 0.904 |
| InternalIMT | dha | rs187310430 | 0.005 | . | . | . | . | . | . | . | . | . |
| InternalIMT | dha | rs192608488 | 0.045 | -0.015 | 0.029 | 0.618 | 0.116 | 0.151 | 0.442 | -0.061 | 0.1 | 0.544 |
| InternalIMT | dha | rs148840366 | 0.006 | . | . | . | . | . | . | . | . | . |
| InternalIMT | dha | rs186785022 | 0.012 | -0.023 | 0.028 | 0.409 | -0.334 | 0.346 | 0.334 | 0.323 | 0.278 | 0.245 |
| InternalIMT | dha | rs192348728 | 0.008 | . | . | . | . | . | . | . | . | . |
| InternalIMT | dha | rs189656631 | 0.045 | -0.015 | 0.029 | 0.618 | 0.116 | 0.151 | 0.442 | -0.061 | 0.1 | 0.544 |
| InternalIMT | dha | rs185289750 | 0.004 | . | . | . | . | . | . | . | . | . |
| InternalIMT | dha | rs142382395 | 0.045 | -0.015 | 0.029 | 0.618 | 0.116 | 0.151 | 0.442 | -0.061 | 0.1 | 0.544 |
| InternalIMT | dha | rs144866696 | 0.012 | -0.023 | 0.028 | 0.409 | -0.334 | 0.346 | 0.334 | 0.324 | 0.278 | 0.245 |
| InternalIMT | dha | rs141371289 | 0.045 | -0.015 | 0.029 | 0.618 | 0.116 | 0.151 | 0.442 | -0.061 | 0.1 | 0.544 |
| InternalIMT | dha | rs114750008 | 0.045 | -0.015 | 0.029 | 0.618 | 0.116 | 0.151 | 0.442 | -0.061 | 0.1 | 0.544 |
| InternalIMT | dha | rs189063597 | 0.001 | . | . | . | . | . | . | . | . | . |
| InternalIMT | dha | rs139151571 | 0.041 | -0.012 | 0.029 | 0.669 | 0.155 | 0.161 | 0.337 | -0.09 | 0.107 | 0.401 |
| InternalIMT | dha | rs139846368 | 0.046 | -0.013 | 0.029 | 0.648 | 0.127 | 0.154 | 0.411 | -0.07 | 0.102 | 0.49 |
| InternalIMT | dha | rs182057653 | 0.012 | -0.017 | 0.029 | 0.552 | -0.085 | 0.216 | 0.694 | 0.036 | 0.162 | 0.826 |
| InternalIMT | dha | rs148785420 | 0.045 | -0.015 | 0.029 | 0.618 | 0.116 | 0.151 | 0.442 | -0.061 | 0.1 | 0.543 |
| InternalIMT | dha | rs138383251 | 0.007 | . | . | . | . | . | . | . | . | . |
| InternalIMT | dha | rs116526705 | 0.012 | -0.014 | 0.029 | 0.625 | 8.20E-03 | 0.188 | 0.965 | -0.053 | 0.119 | 0.659 |
| InternalIMT | dha | rs185946436 | 0 | . | . | . | . | . | . | . | . | . |
| InternalIMT | dha | rs190455427 | 0.004 | . | . | . | . | . | . | . | . | . |
| InternalIMT | dha | rs9282584 | 0.045 | -0.015 | 0.029 | 0.618 | 0.116 | 0.151 | 0.442 | -0.061 | 0.1 | 0.544 |
| InternalIMT | dha | rs17153289 | 0.045 | -0.015 | 0.029 | 0.618 | 0.116 | 0.151 | 0.442 | -0.061 | 0.1 | 0.544 |
| InternalIMT | dha | rs138359103 | 0.005 | . | . | . | . | . | . | . | . | . |
| InternalIMT | dha | rs150605268 | 0.002 | . | . | . | . | . | . | . | . | . |
| InternalIMT | dha | rs79242910 | 0.046 | -0.015 | 0.029 | 0.606 | 0.092 | 0.14 | 0.513 | -0.046 | 0.094 | 0.625 |
| InternalIMT | dha | rs139716904 | 0.013 | -0.013 | 0.029 | 0.643 | 0.072 | 0.209 | 0.731 | -0.107 | 0.15 | 0.476 |
| InternalIMT | dha | rs76238498 | 0.034 | -6.60E-03 | 0.029 | 0.822 | 0.393 | 0.175 | 0.024 | -0.252 | 0.114 | 0.027 |
| InternalIMT | dha | rs184140639 | 0 | . | . | . | . | . | . | . | . | . |
| InternalIMT | dha | rs188517278 | 0.006 | . | . | . | . | . | . | . | . | . |
| InternalIMT | dha | rs139678913 | 0.002 | . | . | . | . | . | . | . | . | . |
| InternalIMT | dha | rs116697955 | 0.004 | . | . | . | . | . | . | . | . | . |
| InternalIMT | dha | rs79267994 | 0.007 | . | . | . | . | . | . | . | . | . |
| InternalIMT | dha | rs11239510 | 0.013 | -0.011 | 0.029 | 0.711 | 0.415 | 0.252 | 0.1 | -0.324 | 0.17 | 0.056 |
| InternalIMT | dha | rs75831645 | 0.012 | -0.014 | 0.029 | 0.625 | 7.60E-03 | 0.188 | 0.968 | -0.052 | 0.12 | 0.664 |
| InternalIMT | dha | rs78452276 | 0.031 | -9.90E-03 | 0.029 | 0.734 | 0.286 | 0.198 | 0.148 | -0.186 | 0.133 | 0.163 |
| InternalIMT | dha | rs144366924 | 0 | . | . | . | . | . | . | . | . | . |
| InternalIMT | dha | rs77519841 | 0.033 | -0.018 | 0.029 | 0.542 | 0.142 | 0.192 | 0.461 | -0.055 | 0.129 | 0.668 |
| InternalIMT | dha | rs76944454 | 0.045 | -0.015 | 0.029 | 0.615 | 0.113 | 0.151 | 0.452 | -0.059 | 0.1 | 0.556 |
| InternalIMT | dha | rs76443855 | 0.034 | -6.60E-03 | 0.029 | 0.822 | 0.393 | 0.175 | 0.025 | -0.252 | 0.114 | 0.027 |
| InternalIMT | dha | rs146613174 | 0.001 | . | . | . | . | . | . | . | . | . |
| InternalIMT | dha | rs181424761 | 0.001 | . | . | . | . | . | . | . | . | . |
| InternalIMT | dha | rs142119241 | 0.001 | . | . | . | . | . | . | . | . | . |
| InternalIMT | dha | rs140137954 | 0.012 | -0.014 | 0.029 | 0.626 | 9.70E-03 | 0.188 | 0.959 | -0.054 | 0.12 | 0.655 |
| InternalIMT | dha | rs187319549 | 0.002 | . | . | . | . | . | . | . | . | . |
| InternalIMT | dha | rs12241545 | 0.021 | -0.011 | 0.029 | 0.711 | 0.035 | 0.221 | 0.874 | -0.102 | 0.164 | 0.533 |
| InternalIMT | dha | rs10128228 | 0.045 | -0.015 | 0.029 | 0.615 | 0.114 | 0.151 | 0.451 | -0.059 | 0.1 | 0.554 |
| InternalIMT | dha | rs58580249 | 0.006 | . | . | . | . | . | . | . | . | . |
| InternalIMT | dha | rs193148863 | 0.001 | . | . | . | . | . | . | . | . | . |
| InternalIMT | dha | rs185638946 | 0.005 | . | . | . | . | . | . | . | . | . |
| InternalIMT | dha | rs117752921 | 0.001 | . | . | . | . | . | . | . | . | . |
| InternalIMT | dha | rs180841236 | 0.001 | . | . | . | . | . | . | . | . | . |
| InternalIMT | dha | rs73283155 | 0.006 | . | . | . | . | . | . | . | . | . |
| InternalIMT | dha | rs76560599 | 0.038 | -0.01 | 0.029 | 0.729 | 0.189 | 0.164 | 0.248 | -0.12 | 0.11 | 0.272 |
| InternalIMT | dha | rs76273620 | 0.038 | -0.01 | 0.029 | 0.729 | 0.189 | 0.164 | 0.248 | -0.12 | 0.11 | 0.272 |
| InternalIMT | dha | rs141432923 | 0.012 | -0.022 | 0.029 | 0.442 | -0.382 | 0.461 | 0.408 | 0.302 | 0.353 | 0.392 |
| InternalIMT | dha | rs113529322 | 0.012 | -0.024 | 0.028 | 0.409 | -0.336 | 0.345 | 0.331 | 0.325 | 0.278 | 0.243 |
| InternalIMT | dha | rs10128306 | 0.045 | -0.015 | 0.029 | 0.614 | 0.115 | 0.151 | 0.448 | -0.06 | 0.1 | 0.552 |

| InternalIMT | dha | rs149691688 | 0.001 | . | . | . | . | . | . | . | . | . |
| --- | --- | --- | --- | --- | --- | --- | --- | --- | --- | --- | --- | --- |
| InternalIMT | dha | rs75872495 | 0.045 | -0.015 | 0.029 | 0.614 | 0.115 | 0.151 | 0.446 | -0.06 | 0.1 | 0.552 |
| InternalIMT | dha | rs191873960 | 0.004 | . | . | . | . | . | . | . | . | . |
| InternalIMT | dha | rs80155622 | 0.045 | -0.015 | 0.029 | 0.614 | 0.116 | 0.151 | 0.444 | -0.06 | 0.1 | 0.55 |
| InternalIMT | dha | rs113914656 | 0.005 | . | . | . | . | . | . | . | . | . |
| InternalIMT | dha | rs12242840 | 0.045 | -0.015 | 0.029 | 0.614 | 0.116 | 0.151 | 0.443 | -0.06 | 0.1 | 0.549 |
| InternalIMT | dha | rs147824078 | 0.006 | . | . | . | . | . | . | . | . | . |
| InternalIMT | dha | rs59051493 | 0.045 | -0.015 | 0.029 | 0.613 | 0.115 | 0.151 | 0.446 | -0.059 | 0.1 | 0.554 |
| InternalIMT | dha | rs9282588 | 0.005 | . | . | . | . | . | . | . | . | . |
| InternalIMT | dha | rs150215207 | 0.006 | . | . | . | . | . | . | . | . | . |
| InternalIMT | dha | rs138772243 | 0.002 | . | . | . | . | . | . | . | . | . |
| InternalIMT | dha | rs149383244 | 0.014 | -0.012 | 0.029 | 0.667 | 0.175 | 0.231 | 0.45 | -0.13 | 0.156 | 0.402 |
| InternalIMT | dha | rs17522720 | 0.011 | -0.015 | 0.029 | 0.612 | 0.12 | 0.274 | 0.661 | -0.088 | 0.189 | 0.641 |
| InternalIMT | dha | rs79245105 | 0.045 | -0.015 | 0.029 | 0.613 | 0.115 | 0.151 | 0.447 | -0.059 | 0.1 | 0.556 |
| InternalIMT | dha | rs138222133 | 0.002 | . | . | . | . | . | . | . | . | . |
| InternalIMT | dha | rs12247314 | 0.05 | -0.015 | 0.029 | 0.62 | 0.112 | 0.145 | 0.44 | -0.059 | 0.098 | 0.547 |
| InternalIMT | dha | rs12263894 | 0.049 | -0.013 | 0.029 | 0.649 | 0.14 | 0.15 | 0.35 | -0.077 | 0.1 | 0.442 |
| InternalIMT | dha | rs149907880 | 0.005 | . | . | . | . | . | . | . | . | . |
| InternalIMT | dha | rs188153885 | 0.005 | . | . | . | . | . | . | . | . | . |
| InternalIMT | dha | rs73283159 | 0.003 | . | . | . | . | . | . | . | . | . |
| InternalIMT | dha | rs140476687 | 0.002 | . | . | . | . | . | . | . | . | . |
| InternalIMT | dha | rs186603148 | 0 | . | . | . | . | . | . | . | . | . |
| InternalIMT | dha | rs183296063 | 0.002 | . | . | . | . | . | . | . | . | . |
| InternalIMT | dha | rs145733694 | 0.006 | . | . | . | . | . | . | . | . | . |
| InternalIMT | dha | rs189807449 | 0 | . | . | . | . | . | . | . | . | . |
| InternalIMT | dha | rs12253223 | 0.038 | -0.018 | 0.029 | 0.542 | 0.131 | 0.183 | 0.475 | -0.052 | 0.124 | 0.678 |
| InternalIMT | dha | rs182113637 | 0 | . | . | . | . | . | . | . | . | . |
| InternalIMT | dha | rs142792099 | 0.01 | -0.019 | 0.028 | 0.515 | 0.05 | 0.374 | 0.893 | 0.04 | 0.24 | 0.869 |
| InternalIMT | dha | rs12249910 | 0.038 | -0.018 | 0.029 | 0.541 | 0.13 | 0.183 | 0.477 | -0.051 | 0.124 | 0.68 |
| InternalIMT | dha | rs149436339 | 0.001 | . | . | . | . | . | . | . | . | . |
| InternalIMT | dha | rs77632210 | 0 | . | . | . | . | . | . | . | . | . |
| InternalIMT | dha | rs74130818 | 0.008 | . | . | . | . | . | . | . | . | . |
| InternalIMT | dha | rs187583648 | 0 | . | . | . | . | . | . | . | . | . |
| InternalIMT | dha | rs191295987 | 0.004 | . | . | . | . | . | . | . | . | . |
| InternalIMT | dha | rs184200686 | 0.006 | . | . | . | . | . | . | . | . | . |
| InternalIMT | dha | rs76117263 | 0.033 | -6.30E-03 | 0.029 | 0.829 | 0.419 | 0.181 | 0.02 | -0.265 | 0.117 | 0.024 |
| InternalIMT | dha | rs185393149 | 0.001 | . | . | . | . | . | . | . | . | . |
| InternalIMT | dha | rs144760014 | 0.003 | . | . | . | . | . | . | . | . | . |
| InternalIMT | dha | rs7922292 | 0.005 | . | . | . | . | . | . | . | . | . |
| InternalIMT | dha | rs115372844 | 0.006 | . | . | . | . | . | . | . | . | . |
| InternalIMT | dha | rs111460364 | 0.033 | -6.30E-03 | 0.029 | 0.831 | 0.423 | 0.182 | 0.02 | -0.268 | 0.118 | 0.023 |
| InternalIMT | dha | rs182052255 | 0.001 | . | . | . | . | . | . | . | . | . |
| InternalIMT | dha | rs180983889 | 0.004 | . | . | . | . | . | . | . | . | . |
| InternalIMT | dha | rs139175327 | 0.006 | . | . | . | . | . | . | . | . | . |
| InternalIMT | dha | rs149959620 | 0.011 | -0.013 | 0.029 | 0.646 | 0.162 | 0.229 | 0.48 | -0.159 | 0.158 | 0.315 |
| InternalIMT | dha | rs114653117 | 0.005 | . | . | . | . | . | . | . | . | . |
| InternalIMT | dha | rs144270018 | 0.003 | . | . | . | . | . | . | . | . | . |
| InternalIMT | dha | rs182723204 | 0.005 | . | . | . | . | . | . | . | . | . |
| InternalIMT | dha | rs57204067 | 0.008 | . | . | . | . | . | . | . | . | . |
| InternalIMT | dha | rs144956226 | 0.038 | -0.018 | 0.029 | 0.539 | 0.12 | 0.185 | 0.518 | -0.046 | 0.125 | 0.714 |
| InternalIMT | dha | rs187842704 | 0.01 | -0.015 | 0.029 | 0.608 | 0.641 | 0.413 | 0.121 | -0.333 | 0.254 | 0.19 |
| InternalIMT | dha | rs184620511 | 0.008 | . | . | . | . | . | . | . | . | . |
| InternalIMT | dha | rs144031303 | 0.012 | -0.014 | 0.029 | 0.636 | 0.042 | 0.195 | 0.829 | -0.073 | 0.122 | 0.548 |
| InternalIMT | dha | rs187526473 | 0 | . | . | . | . | . | . | . | . | . |
| InternalIMT | dha | rs193002805 | 0.002 | . | . | . | . | . | . | . | . | . |
| InternalIMT | dha | rs138707426 | 0.014 | -0.012 | 0.029 | 0.67 | 0.199 | 0.229 | 0.386 | -0.142 | 0.153 | 0.353 |
| InternalIMT | dha | rs142754243 | 0.03 | -0.013 | 0.029 | 0.647 | 0.118 | 0.173 | 0.494 | -0.084 | 0.123 | 0.496 |
| InternalIMT | dha | rs181600331 | 0.005 | . | . | . | . | . | . | . | . | . |
| InternalIMT | dha | rs144421264 | 0.018 | -0.011 | 0.029 | 0.716 | 0.324 | 0.269 | 0.228 | -0.233 | 0.181 | 0.198 |
| InternalIMT | dha | rs184414519 | 0.004 | . | . | . | . | . | . | . | . | . |
| InternalIMT | dha | rs150596349 | 0.031 | -4.90E-03 | 0.029 | 0.868 | 0.492 | 0.192 | 0.011 | -0.313 | 0.124 | 0.012 |
| InternalIMT | dha | rs190753747 | 0.001 | . | . | . | . | . | . | . | . | . |
| InternalIMT | dha | rs139560940 | 0.004 | . | . | . | . | . | . | . | . | . |
| InternalIMT | dha | rs75640804 | 0.033 | -0.018 | 0.029 | 0.529 | 0.127 | 0.194 | 0.511 | -0.045 | 0.129 | 0.729 |
| InternalIMT | dha | rs73283175 | 0.052 | -7.50E-03 | 0.03 | 0.803 | 0.124 | 0.121 | 0.308 | -0.1 | 0.085 | 0.235 |
| InternalIMT | dha | rs143337229 | 0.002 | . | . | . | . | . | . | . | . | . |
| InternalIMT | dha | rs73283177 | 0.03 | -0.014 | 0.029 | 0.635 | 0.112 | 0.176 | 0.525 | -0.077 | 0.126 | 0.541 |
| InternalIMT | dha | rs76053611 | 0.029 | -4.20E-03 | 0.029 | 0.887 | 0.518 | 0.195 | 7.90E-03 | -0.333 | 0.126 | 8.10E-03 |
| InternalIMT | dha | rs116325983 | 0.004 | . | . | . | . | . | . | . | . | . |
| InternalIMT | dha | rs115451825 | 0.005 | . | . | . | . | . | . | . | . | . |
| InternalIMT | dha | rs183845246 | 0.001 | . | . | . | . | . | . | . | . | . |
| InternalIMT | dha | rs79756542 | 0.033 | -4.60E-03 | 0.03 | 0.876 | 0.474 | 0.188 | 0.012 | -0.303 | 0.121 | 0.012 |
| InternalIMT | dha | rs187242860 | 0.002 | . | . | . | . | . | . | . | . | . |
| InternalIMT | dha | rs57318980 | 0.03 | -0.015 | 0.029 | 0.61 | 0.106 | 0.183 | 0.563 | -0.064 | 0.132 | 0.627 |
| InternalIMT | dha | rs59969732 | 0.027 | -0.015 | 0.029 | 0.599 | 0.077 | 0.187 | 0.68 | -0.049 | 0.135 | 0.717 |
| InternalIMT | dha | rs143811488 | 0.006 | . | . | . | . | . | . | . | . | . |
| InternalIMT | dha | rs189067644 | 0.006 | . | . | . | . | . | . | . | . | . |
| InternalIMT | dha | rs60449178 | 0.025 | -0.014 | 0.029 | 0.629 | 0.091 | 0.204 | 0.657 | -0.074 | 0.148 | 0.618 |
| InternalIMT | dha | rs59243389 | 0.025 | -0.014 | 0.029 | 0.629 | 0.091 | 0.204 | 0.657 | -0.074 | 0.148 | 0.618 |
| InternalIMT | dha | rs184946540 | 0.027 | -7.60E-03 | 0.029 | 0.794 | 0.263 | 0.199 | 0.185 | -0.211 | 0.139 | 0.13 |
| InternalIMT | dha | rs188685881 | 0.001 | . | . | . | . | . | . | . | . | . |
| InternalIMT | dha | rs150335797 | 0.004 | . | . | . | . | . | . | . | . | . |
| InternalIMT | dha | rs143879260 | 0.004 | . | . | . | . | . | . | . | . | . |
| InternalIMT | dha | rs186044491 | 0.006 | . | . | . | . | . | . | . | . | . |
| InternalIMT | dha | rs11819084 | 0.018 | -7.10E-03 | 0.029 | 0.805 | 0.477 | 0.207 | 0.021 | -0.311 | 0.129 | 0.016 |
| InternalIMT | dha | rs146915670 | 0.006 | . | . | . | . | . | . | . | . | . |

| InternalIMT | dha | rs113995695 | 0.017 | -7.70E-03 | 0.029 | 0.789 | 0.463 | 0.208 | 0.026 | -0.298 | 0.13 | 0.022 |
| --- | --- | --- | --- | --- | --- | --- | --- | --- | --- | --- | --- | --- |
| InternalIMT | dha | rs143489451 | 0.003 | . | . | . | . | . | . | . | . | . |
| InternalIMT | dha | rs182653839 | 0 | . | . | . | . | . | . | . | . | . |
| InternalIMT | dha | rs141692564 | 0.004 | . | . | . | . | . | . | . | . | . |
| InternalIMT | dha | rs146281742 | 0.003 | . | . | . | . | . | . | . | . | . |
| InternalIMT | dha | rs192914353 | 0.003 | . | . | . | . | . | . | . | . | . |
| InternalIMT | dha | rs185182848 | 0.002 | . | . | . | . | . | . | . | . | . |
| InternalIMT | dha | rs28395858 | 0.017 | -8.10E-03 | 0.029 | 0.78 | 0.45 | 0.21 | 0.032 | -0.288 | 0.132 | 0.03 |
| InternalIMT | dha | rs41429645 | 0.005 | . | . | . | . | . | . | . | . | . |
| InternalIMT | dha | rs28395874 | 0.017 | -8.30E-03 | 0.029 | 0.775 | 0.442 | 0.211 | 0.036 | -0.282 | 0.134 | 0.035 |
| InternalIMT | dha | rs189663634 | 0.001 | . | . | . | . | . | . | . | . | . |
| InternalIMT | dha | rs77666700 | 0.006 | . | . | . | . | . | . | . | . | . |
| InternalIMT | dha | rs146711717 | 0.002 | . | . | . | . | . | . | . | . | . |
| InternalIMT | dha | rs144904251 | 0.001 | . | . | . | . | . | . | . | . | . |
| InternalIMT | dha | rs189893153 | 0.001 | . | . | . | . | . | . | . | . | . |
| InternalIMT | dha | rs150447740 | 0.006 | . | . | . | . | . | . | . | . | . |
| InternalIMT | dha | rs143099649 | 0.001 | . | . | . | . | . | . | . | . | . |
| InternalIMT | dha | rs191288436 | 0.007 | . | . | . | . | . | . | . | . | . |
| InternalIMT | dha | rs188577862 | 0.004 | . | . | . | . | . | . | . | . | . |
| InternalIMT | dha | rs192695334 | 0.004 | . | . | . | . | . | . | . | . | . |
| InternalIMT | dha | rs138612906 | 0.022 | -7.40E-03 | 0.029 | 0.797 | 0.328 | 0.179 | 0.067 | -0.224 | 0.115 | 0.051 |
| InternalIMT | dha | rs191290051 | 0.022 | -0.017 | 0.029 | 0.549 | 0.215 | 0.278 | 0.438 | -0.087 | 0.19 | 0.647 |
| InternalIMT | dha | rs189631459 | 0.001 | . | . | . | . | . | . | . | . | . |
| InternalIMT | dha | rs114620874 | 0.005 | . | . | . | . | . | . | . | . | . |
| InternalIMT | dha | rs78246652 | 0.013 | -0.019 | 0.029 | 0.497 | 0.05 | 0.318 | 0.876 | 0.06 | 0.212 | 0.779 |
| InternalIMT | dha | rs79432451 | 0.005 | . | . | . | . | . | . | . | . | . |
| InternalIMT | dha | rs186022585 | 0.001 | . | . | . | . | . | . | . | . | . |
| InternalIMT | dha | rs142978891 | 0.005 | . | . | . | . | . | . | . | . | . |
| InternalIMT | dha | rs151086840 | 0.007 | . | . | . | . | . | . | . | . | . |
| InternalIMT | dha | rs183270259 | 0.006 | . | . | . | . | . | . | . | . | . |
| InternalIMT | dha | rs188544289 | 0.006 | . | . | . | . | . | . | . | . | . |
| InternalIMT | dha | rs188500808 | 0.001 | . | . | . | . | . | . | . | . | . |
| InternalIMT | dha | rs189424589 | 0.002 | . | . | . | . | . | . | . | . | . |
| InternalIMT | dha | rs78622303 | 0.025 | -0.011 | 0.029 | 0.699 | 0.376 | 0.201 | 0.062 | -0.219 | 0.143 | 0.126 |
| InternalIMT | dha | rs143210835 | 0.008 | . | . | . | . | . | . | . | . | . |
| InternalIMT | dha | rs114761840 | 0.004 | . | . | . | . | . | . | . | . | . |
| InternalIMT | dha | rs185805402 | 0.003 | . | . | . | . | . | . | . | . | . |
| InternalIMT | dha | rs139273744 | 0.002 | . | . | . | . | . | . | . | . | . |
| InternalIMT | dha | rs184654150 | 0.004 | . | . | . | . | . | . | . | . | . |
| InternalIMT | dha | rs189069710 | 0 | . | . | . | . | . | . | . | . | . |
| InternalIMT | dha | rs114752115 | 0.018 | -0.018 | 0.029 | 0.531 | 0.098 | 0.258 | 0.703 | -8.90E-03 | 0.177 | 0.96 |
| InternalIMT | dha | rs150162515 | 0.001 | . | . | . | . | . | . | . | . | . |
| InternalIMT | dha | rs180982454 | 0.004 | . | . | . | . | . | . | . | . | . |
| InternalIMT | dha | rs184932088 | 0.001 | . | . | . | . | . | . | . | . | . |
| InternalIMT | dha | rs181109625 | 0 | . | . | . | . | . | . | . | . | . |
| InternalIMT | dha | rs138121134 | 0.001 | . | . | . | . | . | . | . | . | . |
| InternalIMT | dha | rs183253861 | 0.003 | . | . | . | . | . | . | . | . | . |
| InternalIMT | dha | rs147073946 | 0.001 | . | . | . | . | . | . | . | . | . |
| InternalIMT | dha | rs139938489 | 0.005 | . | . | . | . | . | . | . | . | . |
| InternalIMT | dha | rs146362560 | 0.008 | . | . | . | . | . | . | . | . | . |
| InternalIMT | dha | rs193155956 | 0.004 | . | . | . | . | . | . | . | . | . |
| InternalIMT | dha | rs77663092 | 0.004 | . | . | . | . | . | . | . | . | . |
| InternalIMT | dha | rs73285319 | 0.021 | -0.014 | 0.029 | 0.633 | 0.05 | 0.214 | 0.815 | -0.065 | 0.156 | 0.68 |
| InternalIMT | dha | rs184019276 | 0.007 | . | . | . | . | . | . | . | . | . |
| InternalIMT | dha | rs28395863 | 0.016 | -0.012 | 0.029 | 0.671 | 0.321 | 0.28 | 0.251 | -0.205 | 0.184 | 0.266 |
| InternalIMT | dha | rs182121645 | 0.011 | -0.013 | 0.029 | 0.655 | 0.299 | 0.299 | 0.317 | -0.224 | 0.221 | 0.312 |
| InternalIMT | dha | rs73285327 | 0.016 | -0.014 | 0.029 | 0.64 | 0.161 | 0.245 | 0.511 | -0.13 | 0.176 | 0.459 |
| InternalIMT | dha | rs149589584 | 0.001 | . | . | . | . | . | . | . | . | . |
| InternalIMT | dha | rs148310335 | 0.005 | . | . | . | . | . | . | . | . | . |
| InternalIMT | dha | rs187246093 | 0.007 | . | . | . | . | . | . | . | . | . |
| InternalIMT | dha | rs184668894 | 0 | . | . | . | . | . | . | . | . | . |
| InternalIMT | dha | rs114269961 | 0.001 | . | . | . | . | . | . | . | . | . |
| InternalIMT | dha | rs149694374 | 0.02 | -0.01 | 0.029 | 0.716 | 0.251 | 0.233 | 0.281 | -0.184 | 0.159 | 0.248 |
| InternalIMT | dha | rs145284227 | 0.004 | . | . | . | . | . | . | . | . | . |
| InternalIMT | dha | rs189983820 | 0.004 | . | . | . | . | . | . | . | . | . |
| InternalIMT | dha | rs181200444 | 0 | . | . | . | . | . | . | . | . | . |
| InternalIMT | dha | rs185486385 | 0 | . | . | . | . | . | . | . | . | . |
| InternalIMT | dha | rs151198241 | 0.006 | . | . | . | . | . | . | . | . | . |
| InternalIMT | dha | rs189959341 | 0.006 | . | . | . | . | . | . | . | . | . |
| InternalIMT | dha | rs140549257 | 0.005 | . | . | . | . | . | . | . | . | . |
| InternalIMT | dha | rs186453139 | 0.016 | -0.012 | 0.029 | 0.667 | 0.101 | 0.23 | 0.66 | -0.114 | 0.163 | 0.484 |
| InternalIMT | dha | rs180922711 | 0.027 | -7.10E-03 | 0.029 | 0.809 | 0.231 | 0.205 | 0.26 | -0.195 | 0.146 | 0.182 |
| InternalIMT | dha | rs56659903 | 0.004 | . | . | . | . | . | . | . | . | . |
| InternalIMT | dha | rs141202128 | 0.004 | . | . | . | . | . | . | . | . | . |
| InternalIMT | dha | rs59085314 | 0.033 | -0.021 | 0.029 | 0.478 | -0.053 | 0.177 | 0.763 | 0.051 | 0.127 | 0.687 |
| InternalIMT | dha | rs145564996 | 0.004 | . | . | . | . | . | . | . | . | . |
| InternalIMT | dha | rs114961009 | 0.006 | . | . | . | . | . | . | . | . | . |
| InternalIMT | dha | rs182294189 | 0.003 | . | . | . | . | . | . | . | . | . |
| InternalIMT | dha | rs187586636 | 0.005 | . | . | . | . | . | . | . | . | . |
| InternalIMT | dha | rs192254907 | 0.003 | . | . | . | . | . | . | . | . | . |
| InternalIMT | dha | rs149145663 | 0.001 | . | . | . | . | . | . | . | . | . |
| InternalIMT | dha | rs180713064 | 0.002 | . | . | . | . | . | . | . | . | . |
| InternalIMT | dha | rs183009023 | 0.004 | . | . | . | . | . | . | . | . | . |
| InternalIMT | dha | rs143385347 | 0.01 | -0.012 | 0.029 | 0.667 | 0.355 | 0.29 | 0.221 | -0.254 | 0.217 | 0.242 |
| InternalIMT | dha | rs185786329 | 0.004 | . | . | . | . | . | . | . | . | . |
| InternalIMT | dha | rs192341030 | 0.003 | . | . | . | . | . | . | . | . | . |

| InternalIMT | dha | rs74661429 | 0.009 | . | . | . | . | . | . | . | . | . |
| --- | --- | --- | --- | --- | --- | --- | --- | --- | --- | --- | --- | --- |
| InternalIMT | dha | rs188034853 | 0.001 | . | . | . | . | . | . | . | . | . |
| InternalIMT | dha | rs116808430 | 0.016 | -0.012 | 0.029 | 0.671 | 0.087 | 0.226 | 0.699 | -0.108 | 0.159 | 0.496 |
| InternalIMT | dha | rs148469010 | 0 | . | . | . | . | . | . | . | . | . |
| InternalIMT | dha | rs78621663 | 0.006 | . | . | . | . | . | . | . | . | . |
| InternalIMT | dha | rs192654886 | 0.001 | . | . | . | . | . | . | . | . | . |
| InternalIMT | dha | rs145996035 | 0.005 | . | . | . | . | . | . | . | . | . |
| InternalIMT | dha | rs139942226 | 0 | . | . | . | . | . | . | . | . | . |
| InternalIMT | dha | rs113645632 | 0.006 | . | . | . | . | . | . | . | . | . |
| InternalIMT | dha | rs74940661 | 0.016 | -0.014 | 0.029 | 0.636 | 0.185 | 0.248 | 0.455 | -0.131 | 0.173 | 0.449 |
| InternalIMT | dha | rs115355800 | 0.013 | -0.02 | 0.029 | 0.489 | -0.021 | 0.316 | 0.947 | 0.103 | 0.215 | 0.631 |
| InternalIMT | dha | rs150285898 | 0.005 | . | . | . | . | . | . | . | . | . |
| InternalIMT | dha | rs188117932 | 0.004 | . | . | . | . | . | . | . | . | . |
| InternalIMT | dha | rs139956566 | 0 | . | . | . | . | . | . | . | . | . |
| InternalIMT | dha | rs147673885 | 0 | . | . | . | . | . | . | . | . | . |
| InternalIMT | dha | rs189127928 | 0.005 | . | . | . | . | . | . | . | . | . |
| InternalIMT | dha | rs187558772 | 0.002 | . | . | . | . | . | . | . | . | . |
| InternalIMT | dha | rs181948587 | 0 | . | . | . | . | . | . | . | . | . |
| InternalIMT | dha | rs189983269 | 0.002 | . | . | . | . | . | . | . | . | . |
| InternalIMT | dha | rs187250069 | 0.002 | . | . | . | . | . | . | . | . | . |
| InternalIMT | dha | rs191485859 | 0.001 | . | . | . | . | . | . | . | . | . |
| InternalIMT | dha | rs185499555 | 0.002 | . | . | . | . | . | . | . | . | . |
| InternalIMT | dha | rs180862110 | 0.003 | . | . | . | . | . | . | . | . | . |
| InternalIMT | dha | rs190472603 | 0.005 | . | . | . | . | . | . | . | . | . |
| InternalIMT | dha | rs189226167 | 0.005 | . | . | . | . | . | . | . | . | . |
| InternalIMT | dha | rs181623256 | 0.002 | . | . | . | . | . | . | . | . | . |
| InternalIMT | dha | rs186079557 | 0.001 | . | . | . | . | . | . | . | . | . |
| InternalIMT | dha | rs147760994 | 0.002 | . | . | . | . | . | . | . | . | . |
| InternalIMT | dha | rs189176167 | 0 | . | . | . | . | . | . | . | . | . |
| InternalIMT | dha | rs117901000 | 0.001 | . | . | . | . | . | . | . | . | . |
| InternalIMT | dha | rs141984888 | 0 | . | . | . | . | . | . | . | . | . |
| InternalIMT | dha | rs192365061 | 0.007 | . | . | . | . | . | . | . | . | . |
| InternalIMT | dha | rs185976038 | 0 | . | . | . | . | . | . | . | . | . |
| InternalIMT | dha | rs184697303 | 0.002 | . | . | . | . | . | . | . | . | . |
| InternalIMT | dha | rs143383132 | 0.002 | . | . | . | . | . | . | . | . | . |
| InternalIMT | dha | rs141873881 | 0.004 | . | . | . | . | . | . | . | . | . |
| InternalIMT | dha | rs185507255 | 0 | . | . | . | . | . | . | . | . | . |
| InternalIMT | dha | rs191379425 | 0.001 | . | . | . | . | . | . | . | . | . |
| InternalIMT | dha | rs184199584 | 0.002 | . | . | . | . | . | . | . | . | . |
| InternalIMT | dha | rs183181360 | 0.002 | . | . | . | . | . | . | . | . | . |
| InternalIMT | dha | rs184495673 | 0.001 | . | . | . | . | . | . | . | . | . |
| InternalIMT | dha | rs182946297 | 0.001 | . | . | . | . | . | . | . | . | . |
| InternalIMT | dha | rs187743615 | 0 | . | . | . | . | . | . | . | . | . |
| InternalIMT | dha | rs183259862 | 0.001 | . | . | . | . | . | . | . | . | . |
| InternalIMT | dha | rs112433566 | 0 | . | . | . | . | . | . | . | . | . |
| InternalIMT | dha | rs182351085 | 0.001 | . | . | . | . | . | . | . | . | . |
| InternalIMT | dha | rs146248508 | 0.001 | . | . | . | . | . | . | . | . | . |
| InternalIMT | dha | rs188528439 | 0.001 | . | . | . | . | . | . | . | . | . |
| InternalIMT | dha | rs146167018 | 0.005 | . | . | . | . | . | . | . | . | . |
| InternalIMT | dha | rs192440980 | 0.004 | . | . | . | . | . | . | . | . | . |
| InternalIMT | dha | rs185601882 | 0.001 | . | . | . | . | . | . | . | . | . |
| InternalIMT | dha | rs117272563 | 0.002 | . | . | . | . | . | . | . | . | . |
| InternalIMT | dha | rs10430734 | 0.003 | . | . | . | . | . | . | . | . | . |
| InternalIMT | dha | rs181320952 | 0.001 | . | . | . | . | . | . | . | . | . |
| InternalIMT | dha | rs185480660 | 0.001 | . | . | . | . | . | . | . | . | . |
| InternalIMT | dha | rs188077917 | 0.001 | . | . | . | . | . | . | . | . | . |
| InternalIMT | dha | rs187562953 | 0.002 | . | . | . | . | . | . | . | . | . |
| InternalIMT | dha | rs182044631 | 0 | . | . | . | . | . | . | . | . | . |
| InternalIMT | dha | rs145455596 | 0.001 | . | . | . | . | . | . | . | . | . |
| InternalIMT | dha | rs181475877 | 0.004 | . | . | . | . | . | . | . | . | . |
| InternalIMT | dha | rs149413085 | 0.002 | . | . | . | . | . | . | . | . | . |
| InternalIMT | dha | rs12253656 | 0 | . | . | . | . | . | . | . | . | . |
| InternalIMT | dha | rs182718239 | 0 | . | . | . | . | . | . | . | . | . |
| InternalIMT | dha | rs192435695 | 0.002 | . | . | . | . | . | . | . | . | . |
| InternalIMT | dha | rs12248277 | 0 | . | . | . | . | . | . | . | . | . |
| InternalIMT | dha | rs143521710 | 0.001 | . | . | . | . | . | . | . | . | . |
| InternalIMT | dha | rs144454454 | 0 | . | . | . | . | . | . | . | . | . |
| InternalIMT | dha | rs190441610 | 0.004 | . | . | . | . | . | . | . | . | . |
| InternalIMT | dha | rs114690462 | 0 | . | . | . | . | . | . | . | . | . |
| InternalIMT | dha | rs184541357 | 0.001 | . | . | . | . | . | . | . | . | . |
| InternalIMT | dha | rs142657653 | 0 | . | . | . | . | . | . | . | . | . |
| InternalIMT | dha | rsurf1 | 2.145 | . | . | . | . | . | . | . | . | . |
| InternalIMT | dha | rsten1 | 2.303 | . | . | . | . | . | . | . | . | . |
| InternalIMT | dha | chr10:45852312:D | 0.086 | -0.072 | 0.021 | 8.20E-04 | 7.60E-03 | 0.055 | 0.89 | -0.025 | 0.039 | 0.524 |
| InternalIMT | dha | chr10:45853232:I | 0.1 | -0.077 | 0.022 | 3.80E-04 | -0.013 | 0.055 | 0.808 | -1.70E-03 | 0.04 | 0.966 |
| InternalIMT | dha | chr10:45853234:I | 0.168 | -0.064 | 0.023 | 4.30E-03 | 0.038 | 0.049 | 0.439 | -0.039 | 0.036 | 0.271 |
| InternalIMT | dha | chr10:45854796:D | 0.013 | -0.081 | 0.02 | 3.80E-05 | -0.293 | 0.111 | 8.40E-03 | 0.127 | 0.081 | 0.116 |
| InternalIMT | dha | chr10:45854994:I | 0.019 | -0.082 | 0.02 | 3.70E-05 | -0.222 | 0.114 | 0.052 | 0.122 | 0.078 | 0.119 |
| InternalIMT | dha | chr10:45858290:D | 0.045 | -0.074 | 0.021 | 3.90E-04 | 0.059 | 0.106 | 0.576 | -0.046 | 0.085 | 0.592 |
| InternalIMT | dha | chr10:45858820:I | 0.015 | -0.08 | 0.02 | 6.60E-05 | -0.052 | 0.201 | 0.794 | 0.06 | 0.136 | 0.66 |
| InternalIMT | dha | chr10:45860888:D | 0.081 | -0.059 | 0.022 | 6.80E-03 | 0.13 | 0.092 | 0.159 | -0.116 | 0.066 | 0.077 |
| InternalIMT | dha | chr10:45863913:I | 0.165 | -0.06 | 0.023 | 7.50E-03 | 0.055 | 0.049 | 0.26 | -0.052 | 0.035 | 0.14 |
| InternalIMT | dha | chr10:45866601:D | 0.036 | -0.06 | 0.022 | 6.70E-03 | 0.077 | 0.127 | 0.544 | -0.038 | 0.086 | 0.657 |
| InternalIMT | dha | chr10:45867197:I | 0.006 | . | . | . | . | . | . | . | . | . |
| InternalIMT | dha | chr10:45867538:D | 0.171 | -0.062 | 0.023 | 6.20E-03 | 0.044 | 0.048 | 0.359 | -0.045 | 0.035 | 0.2 |
| InternalIMT | dha | chr10:45868356:D | 0.161 | -0.062 | 0.023 | 5.60E-03 | 0.045 | 0.048 | 0.351 | -0.046 | 0.035 | 0.189 |

| InternalIMT | dha | chr10:45871158:I | 0.167 | -0.062 | 0.023 | 6.50E-03 | 0.049 | 0.049 | 0.315 | -0.048 | 0.035 | 0.172 |
| --- | --- | --- | --- | --- | --- | --- | --- | --- | --- | --- | --- | --- |
| InternalIMT | dha | chr10:45871637:D | 0.084 | -0.07 | 0.021 | 9.20E-04 | 0.015 | 0.082 | 0.853 | -0.037 | 0.058 | 0.52 |
| InternalIMT | dha | chr10:45871638:I | 0.132 | -0.066 | 0.022 | 2.80E-03 | 0.039 | 0.062 | 0.53 | -0.043 | 0.045 | 0.332 |
| InternalIMT | dha | chr10:45873629:I | 0.158 | -0.059 | 0.023 | 9.20E-03 | 0.056 | 0.05 | 0.26 | -0.059 | 0.036 | 0.108 |
| InternalIMT | dha | chr10:45873701:D | 0.019 | -0.057 | 0.021 | 7.10E-03 | 0.157 | 0.223 | 0.481 | -0.095 | 0.15 | 0.526 |
| InternalIMT | dha | chr10:45874997:I | 0.021 | -0.052 | 0.022 | 0.015 | 0.21 | 0.16 | 0.19 | -0.178 | 0.12 | 0.14 |
| InternalIMT | dha | chr10:45882030:I | 0.045 | -0.057 | 0.022 | 0.012 | 0.034 | 0.114 | 0.765 | -0.031 | 0.078 | 0.691 |
| InternalIMT | dha | chr10:45883774:D | 0.102 | -0.065 | 0.021 | 1.90E-03 | 0.089 | 0.062 | 0.152 | -0.068 | 0.048 | 0.15 |
| InternalIMT | dha | chr10:45885169:D | 0.067 | -0.073 | 0.021 | 5.40E-04 | 4.50E-03 | 0.067 | 0.947 | -0.024 | 0.046 | 0.599 |
| InternalIMT | dha | chr10:45886451:D | 0.67 | -0.02 | 0.042 | 0.629 | 0.065 | 0.039 | 0.094 | -0.044 | 0.029 | 0.133 |
| InternalIMT | dha | chr10:45891800:I | 0.685 | -0.032 | 0.04 | 0.419 | 0.038 | 0.036 | 0.29 | -0.033 | 0.027 | 0.213 |
| InternalIMT | dha | chr10:45904771:I | 0.685 | -0.029 | 0.039 | 0.455 | 0.04 | 0.035 | 0.252 | -0.036 | 0.026 | 0.172 |
| InternalIMT | dha | chr10:45904959:I | 0.113 | -0.072 | 0.022 | 9.50E-04 | 0.038 | 0.068 | 0.573 | -0.027 | 0.049 | 0.576 |
| InternalIMT | dha | chr10:45911028:I | 0.077 | -0.075 | 0.021 | 4.20E-04 | 0.042 | 0.095 | 0.657 | -0.028 | 0.069 | 0.686 |
| InternalIMT | dha | chr10:45911031:I | 0.355 | -0.067 | 0.029 | 0.023 | 0.03 | 0.041 | 0.472 | -0.015 | 0.031 | 0.631 |
| InternalIMT | dha | chr10:45912154:D | 0.103 | -0.063 | 0.021 | 2.30E-03 | 0.102 | 0.064 | 0.113 | -0.08 | 0.049 | 0.103 |
| InternalIMT | dha | chr10:45913885:I | 0.011 | -0.081 | 0.02 | 3.80E-05 | -0.299 | 0.101 | 3.20E-03 | 0.112 | 0.071 | 0.116 |
| InternalIMT | dha | chr10:45914753:D | 0.245 | -0.074 | 0.024 | 1.90E-03 | 0.047 | 0.047 | 0.325 | -0.015 | 0.035 | 0.668 |
| InternalIMT | dha | chr10:45919621 | 0.003 | . | . | . | . | . | . | . | . | . |
| InternalIMT | dha | chr10:45921739:I | 0.066 | -0.08 | 0.02 | 8.60E-05 | -0.057 | 0.072 | 0.433 | 0.028 | 0.051 | 0.577 |
| InternalIMT | dha | chr10:45923527:I | 0.057 | -0.073 | 0.021 | 5.30E-04 | 0.051 | 0.101 | 0.615 | -0.045 | 0.073 | 0.544 |
| InternalIMT | dha | chr10:45927001:I | 0.019 | -0.082 | 0.02 | 3.70E-05 | -0.09 | 0.192 | 0.641 | 0.11 | 0.135 | 0.414 |
| InternalIMT | dha | chr10:45930354:I | 0.017 | -0.083 | 0.02 | 2.80E-05 | -0.273 | 0.113 | 0.016 | 0.162 | 0.078 | 0.038 |
| InternalIMT | dha | chr10:45932864:I | 0.04 | -0.083 | 0.02 | 4.60E-05 | -0.086 | 0.107 | 0.424 | 0.068 | 0.081 | 0.404 |
| InternalIMT | dha | chr10:45936163:I | 0.029 | -0.081 | 0.02 | 5.00E-05 | -0.141 | 0.103 | 0.17 | 0.063 | 0.079 | 0.423 |
| InternalIMT | dha | chr10:45938095:D | 0.144 | -0.073 | 0.021 | 6.00E-04 | 0.031 | 0.056 | 0.577 | -0.021 | 0.041 | 0.616 |
| InternalIMT | dha | chr10:45938886 | 0 | . | . | . | . | . | . | . | . | . |
| InternalIMT | dha | chr10:45939320 | 0.001 | . | . | . | . | . | . | . | . | . |
| InternalIMT | dha | chr10:45939659 | 0.002 | . | . | . | . | . | . | . | . | . |
| InternalIMT | dha | chr10:45941150:D | 0.012 | -0.081 | 0.02 | 4.10E-05 | -0.294 | 0.099 | 3.00E-03 | 0.102 | 0.072 | 0.158 |
| InternalIMT | dha | chr10:45947128:D | 0.058 | -0.083 | 0.021 | 5.70E-05 | -0.015 | 0.069 | 0.831 | 0.04 | 0.052 | 0.439 |
| InternalIMT | dha | chr10:45950990:I | 0.047 | -0.087 | 0.021 | 2.30E-05 | -0.137 | 0.099 | 0.167 | 0.087 | 0.075 | 0.248 |
| InternalIMT | dha | chr10:45951854:I | 0.03 | -0.054 | 0.022 | 0.015 | 0.099 | 0.101 | 0.323 | -0.08 | 0.077 | 0.302 |
| InternalIMT | dha | chr10:45952670:I | 0.049 | -0.068 | 0.02 | 9.10E-04 | 0.099 | 0.076 | 0.189 | -0.109 | 0.055 | 0.046 |
| InternalIMT | dha | chr10:45956741 | 0.001 | . | . | . | . | . | . | . | . | . |
| InternalIMT | dha | chr10:45957247:I | 0.219 | -0.084 | 0.023 | 2.30E-04 | -0.017 | 0.04 | 0.668 | 0.014 | 0.031 | 0.643 |
| InternalIMT | dha | chr10:45958082:I | 0.216 | -0.082 | 0.023 | 3.60E-04 | -0.01 | 0.041 | 0.805 | 8.50E-03 | 0.031 | 0.786 |
| InternalIMT | dha | chr10:45905041:D | 0.053 | -0.055 | 0.026 | 0.031 | -0.067 | 0.101 | 0.509 | 0.058 | 0.072 | 0.424 |
| InternalIMT | dha | chr10:45938871 | 0.003 | . | . | . | . | . | . | . | . | . |
| InternalIMT | dha | chr10:45939160 | 0.004 | . | . | . | . | . | . | . | . | . |
| InternalIMT | dha | chr10:45869910 | 0.008 | . | . | . | . | . | . | . | . | . |
| InternalIMT | dha | chr10:45876214:D | 0.012 | -0.014 | 0.029 | 0.627 | -1.90E-03 | 0.18 | 0.992 | -0.054 | 0.114 | 0.635 |
| InternalIMT | dha | chr10:45880237:I | 0.056 | -7.40E-03 | 0.03 | 0.806 | 0.282 | 0.133 | 0.034 | -0.164 | 0.092 | 0.076 |
| InternalIMT | dha | chr10:45884371:I | 0.045 | -0.014 | 0.029 | 0.62 | 0.116 | 0.15 | 0.439 | -0.062 | 0.1 | 0.539 |
| InternalIMT | dha | chr10:45891535:D | 0.012 | -0.024 | 0.028 | 0.409 | -0.335 | 0.345 | 0.332 | 0.324 | 0.278 | 0.243 |
| InternalIMT | dha | chr10:45893516:D | 0.045 | -0.015 | 0.029 | 0.615 | 0.114 | 0.151 | 0.451 | -0.059 | 0.1 | 0.554 |
| InternalIMT | dha | chr10:45893782:D | 0.01 | -0.014 | 0.029 | 0.623 | 0.621 | 0.409 | 0.129 | -0.337 | 0.247 | 0.173 |
| InternalIMT | dha | chr10:45895254:I | 0.012 | -0.024 | 0.028 | 0.408 | -0.336 | 0.345 | 0.331 | 0.325 | 0.278 | 0.242 |
| InternalIMT | dha | chr10:45895848:D | 0.035 | -0.011 | 0.029 | 0.712 | 0.141 | 0.164 | 0.39 | -0.099 | 0.106 | 0.349 |
| InternalIMT | dha | chr10:45896316:D | 0.045 | -0.015 | 0.029 | 0.615 | 0.114 | 0.151 | 0.45 | -0.059 | 0.1 | 0.554 |
| InternalIMT | dha | chr10:45896317:D | 0.045 | -0.015 | 0.029 | 0.615 | 0.114 | 0.151 | 0.45 | -0.059 | 0.1 | 0.554 |
| InternalIMT | dha | chr10:45902683:I | 0.013 | -0.014 | 0.029 | 0.63 | 0.015 | 0.189 | 0.938 | -0.056 | 0.121 | 0.64 |
| InternalIMT | dha | chr10:45905003:D | 0.006 | . | . | . | . | . | . | . | . | . |
| InternalIMT | dha | chr10:45927476:D | 0.021 | -8.00E-03 | 0.029 | 0.78 | 0.321 | 0.207 | 0.12 | -0.229 | 0.14 | 0.103 |
| InternalIMT | dha | chr10:45935882 | 0.001 | . | . | . | . | . | . | . | . | . |
| InternalIMT | dha | chr10:45936100 | 0.001 | . | . | . | . | . | . | . | . | . |
| InternalIMT | dha | chr10:45939553 | 0.003 | . | . | . | . | . | . | . | . | . |
| InternalIMT | dha | chr10:45939631 | 0 | . | . | . | . | . | . | . | . | . |
| InternalIMT | dha | chr10:45944193:D | 0.008 | . | . | . | . | . | . | . | . | . |
| InternalIMT | dha | chr10:45954030 | 0 | . | . | . | . | . | . | . | . | . |

Supplemental table S22.

| phen | covar(fa) | rs_id | maf | beta_FA | se_FA | p_FA | beta_SNP | se_SNP | p_SNP | beta_inter | se_inter | p_inter |
| --- | --- | --- | --- | --- | --- | --- | --- | --- | --- | --- | --- | --- |
| InternalIMT | dha | rs76035182 | 0.001 | . | . | . | . | . | . | . | . | . |
| InternalIMT | dha | rs12359547 | 0.104 | -0.049 | 0.034 | 0.146 | 0.238 | 0.118 | 0.043 | -0.22 | 0.089 | 0.013 |
| InternalIMT | dha | rs74595484 | 0 | . | . | . | . | . | . | . | . | . |
| InternalIMT | dha | rs1864415 | 0.605 | -0.045 | 0.057 | 0.43 | 0.06 | 0.053 | 0.255 | -0.039 | 0.042 | 0.352 |
| InternalIMT | dha | rs72788101 | 0.104 | -0.049 | 0.034 | 0.146 | 0.238 | 0.118 | 0.043 | -0.22 | 0.089 | 0.013 |
| InternalIMT | dha | rs11239481 | 0.183 | -0.098 | 0.036 | 7.10E-03 | -6.60E-03 | 0.07 | 0.925 | 0.018 | 0.055 | 0.736 |
| InternalIMT | dha | rs72788102 | 0.104 | -0.049 | 0.034 | 0.146 | 0.238 | 0.118 | 0.043 | -0.22 | 0.089 | 0.013 |
| InternalIMT | dha | rs72789705 | 0.104 | -0.049 | 0.034 | 0.146 | 0.238 | 0.118 | 0.043 | -0.22 | 0.089 | 0.013 |
| InternalIMT | dha | rs141529858 | 0.008 | . | . | . | . | . | . | . | . | . |
| InternalIMT | dha | rs145376292 | 0 | . | . | . | . | . | . | . | . | . |
| InternalIMT | dha | rs190418301 | 0.001 | . | . | . | . | . | . | . | . | . |
| InternalIMT | dha | rs11239482 | 0.104 | -0.049 | 0.034 | 0.146 | 0.238 | 0.118 | 0.043 | -0.22 | 0.089 | 0.013 |
| InternalIMT | dha | rs61855872 | 0.014 | -0.109 | 0.032 | 6.70E-04 | -0.817 | 0.291 | 5.00E-03 | 0.718 | 0.246 | 3.50E-03 |
| InternalIMT | dha | rs148991144 | 0.011 | -0.091 | 0.032 | 5.10E-03 | 0.07 | 0.205 | 0.734 | -0.047 | 0.143 | 0.742 |
| InternalIMT | dha | rs191932221 | 0.007 | . | . | . | . | . | . | . | . | . |
| InternalIMT | dha | rs55978138 | 0.16 | -0.05 | 0.036 | 0.169 | 0.138 | 0.086 | 0.109 | -0.136 | 0.066 | 0.04 |
| InternalIMT | dha | rs73281167 | 0 | . | . | . | . | . | . | . | . | . |
| InternalIMT | dha | rs12768010 | 0.055 | -0.095 | 0.034 | 5.60E-03 | -0.045 | 0.094 | 0.631 | 0.028 | 0.078 | 0.724 |
| InternalIMT | dha | rs11239484 | 0.605 | -0.046 | 0.057 | 0.426 | 0.06 | 0.053 | 0.256 | -0.039 | 0.042 | 0.356 |
| InternalIMT | dha | rs35906360 | 0.16 | -0.05 | 0.036 | 0.169 | 0.138 | 0.086 | 0.109 | -0.136 | 0.066 | 0.041 |
| InternalIMT | dha | rs186250512 | 0.004 | . | . | . | . | . | . | . | . | . |
| InternalIMT | dha | rs150810743 | 0 | . | . | . | . | . | . | . | . | . |
| InternalIMT | dha | rs55965812 | 0.16 | -0.05 | 0.036 | 0.169 | 0.138 | 0.086 | 0.109 | -0.136 | 0.066 | 0.04 |
| InternalIMT | dha | rs56180358 | 0.16 | -0.05 | 0.036 | 0.169 | 0.138 | 0.086 | 0.109 | -0.136 | 0.066 | 0.04 |
| InternalIMT | dha | rs55864145 | 0.055 | -0.095 | 0.034 | 5.60E-03 | -0.045 | 0.094 | 0.631 | 0.027 | 0.078 | 0.724 |
| InternalIMT | dha | rs55986122 | 0.16 | -0.05 | 0.036 | 0.169 | 0.138 | 0.086 | 0.109 | -0.136 | 0.066 | 0.04 |
| InternalIMT | dha | rs7477350 | 0.605 | -0.046 | 0.057 | 0.424 | 0.06 | 0.053 | 0.257 | -0.039 | 0.042 | 0.358 |
| InternalIMT | dha | rs2099170 | 0.159 | -0.05 | 0.036 | 0.169 | 0.137 | 0.086 | 0.11 | -0.136 | 0.066 | 0.041 |
| InternalIMT | dha | rs2082108 | 0.16 | -0.05 | 0.036 | 0.169 | 0.138 | 0.086 | 0.109 | -0.136 | 0.066 | 0.041 |
| InternalIMT | dha | rs55902404 | 0 | . | . | . | . | . | . | . | . | . |
| InternalIMT | dha | rs58402662 | 0 | . | . | . | . | . | . | . | . | . |
| InternalIMT | dha | rs11511618 | 0.182 | -0.098 | 0.036 | 7.30E-03 | -7.50E-03 | 0.07 | 0.915 | 0.018 | 0.055 | 0.747 |
| InternalIMT | dha | rs1816492 | 0.605 | -0.046 | 0.057 | 0.423 | 0.06 | 0.053 | 0.258 | -0.038 | 0.042 | 0.359 |
| InternalIMT | dha | rs146611093 | 0.01 | -0.085 | 0.032 | 8.50E-03 | 0.226 | 0.173 | 0.191 | -0.294 | 0.142 | 0.039 |
| InternalIMT | dha | rs186897689 | 0 | . | . | . | . | . | . | . | . | . |
| InternalIMT | dha | rs10793616 | 0.605 | -0.046 | 0.057 | 0.422 | 0.06 | 0.053 | 0.258 | -0.038 | 0.042 | 0.36 |
| InternalIMT | dha | rs116700479 | 0.001 | . | . | . | . | . | . | . | . | . |
| InternalIMT | dha | rs192535936 | 0 | . | . | . | . | . | . | . | . | . |
| InternalIMT | dha | rs113363297 | 0.57 | -0.05 | 0.055 | 0.365 | 0.055 | 0.053 | 0.293 | -0.037 | 0.042 | 0.369 |
| InternalIMT | dha | rs12354994 | 0.152 | -0.054 | 0.036 | 0.137 | 0.13 | 0.077 | 0.093 | -0.128 | 0.06 | 0.033 |
| InternalIMT | dha | rs148068329 | 0.001 | . | . | . | . | . | . | . | . | . |
| InternalIMT | dha | rs12356109 | 0.16 | -0.05 | 0.036 | 0.169 | 0.138 | 0.086 | 0.108 | -0.136 | 0.066 | 0.04 |
| InternalIMT | dha | rs55908141 | 0 | . | . | . | . | . | . | . | . | . |
| InternalIMT | dha | rs12354712 | 0.159 | -0.05 | 0.036 | 0.171 | 0.139 | 0.086 | 0.105 | -0.137 | 0.066 | 0.039 |
| InternalIMT | dha | rs147282684 | 0.004 | . | . | . | . | . | . | . | . | . |
| InternalIMT | dha | rs12356140 | 0.16 | -0.05 | 0.036 | 0.17 | 0.139 | 0.086 | 0.107 | -0.136 | 0.066 | 0.04 |
| InternalIMT | dha | rs185476187 | 0 | . | . | . | . | . | . | . | . | . |
| InternalIMT | dha | rs145720057 | 0.039 | -0.092 | 0.033 | 5.10E-03 | 7.20E-04 | 0.158 | 0.996 | 7.60E-03 | 0.138 | 0.956 |
| InternalIMT | dha | rs12354742 | 0.159 | -0.05 | 0.036 | 0.17 | 0.139 | 0.086 | 0.106 | -0.137 | 0.066 | 0.04 |
| InternalIMT | dha | rs180913842 | 0 | . | . | . | . | . | . | . | . | . |
| InternalIMT | dha | rs142976914 | 0.006 | . | . | . | . | . | . | . | . | . |
| InternalIMT | dha | rs11511619 | 0.163 | -0.048 | 0.037 | 0.193 | 0.139 | 0.086 | 0.104 | -0.138 | 0.066 | 0.036 |
| InternalIMT | dha | rs146702870 | 0 | . | . | . | . | . | . | . | . | . |
| InternalIMT | dha | rs150287496 | 0.002 | . | . | . | . | . | . | . | . | . |
| InternalIMT | dha | rs190917973 | 0.006 | . | . | . | . | . | . | . | . | . |
| InternalIMT | dha | rs11517281 | 0.16 | -0.05 | 0.036 | 0.169 | 0.138 | 0.086 | 0.109 | -0.136 | 0.066 | 0.04 |
| InternalIMT | dha | rs12359299 | 0.16 | -0.05 | 0.036 | 0.17 | 0.138 | 0.086 | 0.108 | -0.136 | 0.066 | 0.04 |
| InternalIMT | dha | rs182307932 | 0 | . | . | . | . | . | . | . | . | . |
| InternalIMT | dha | rs61855873 | 0.035 | -0.114 | 0.033 | 6.40E-04 | -0.333 | 0.214 | 0.12 | 0.337 | 0.182 | 0.064 |
| InternalIMT | dha | rs12359302 | 0.156 | -0.056 | 0.036 | 0.125 | 0.117 | 0.075 | 0.118 | -0.12 | 0.059 | 0.041 |
| InternalIMT | dha | rs143208140 | 0.003 | . | . | . | . | . | . | . | . | . |
| InternalIMT | dha | rs9988764 | 0.594 | -0.049 | 0.057 | 0.39 | 0.056 | 0.054 | 0.294 | -0.037 | 0.043 | 0.39 |
| InternalIMT | dha | rs192875767 | 0.008 | . | . | . | . | . | . | . | . | . |
| InternalIMT | dha | rs147268846 | 0.015 | -0.11 | 0.033 | 7.50E-04 | -0.64 | 0.312 | 0.04 | 0.641 | 0.288 | 0.026 |
| InternalIMT | dha | rs11239485 | 0.111 | -0.046 | 0.035 | 0.197 | 0.225 | 0.123 | 0.069 | -0.227 | 0.096 | 0.018 |
| InternalIMT | dha | rs151317885 | 0.435 | -0.119 | 0.05 | 0.017 | -0.012 | 0.057 | 0.835 | 0.034 | 0.046 | 0.455 |
| InternalIMT | dha | rs11499085 | 0 | . | . | . | . | . | . | . | . | . |
| InternalIMT | dha | rs188130606 | 0 | . | . | . | . | . | . | . | . | . |
| InternalIMT | dha | rs11511620 | 0.16 | -0.05 | 0.036 | 0.169 | 0.138 | 0.086 | 0.108 | -0.136 | 0.066 | 0.04 |
| InternalIMT | dha | rs181498390 | 0 | . | . | . | . | . | . | . | . | . |
| InternalIMT | dha | rs186280701 | 0 | . | . | . | . | . | . | . | . | . |
| InternalIMT | dha | rs150059185 | 0.011 | -0.093 | 0.032 | 3.80E-03 | 0.074 | 0.326 | 0.82 | 0.056 | 0.295 | 0.85 |
| InternalIMT | dha | rs183338707 | 0.004 | . | . | . | . | . | . | . | . | . |
| InternalIMT | dha | rs11499038 | 0.002 | . | . | . | . | . | . | . | . | . |
| InternalIMT | dha | rs190699595 | 0.008 | . | . | . | . | . | . | . | . | . |
| InternalIMT | dha | rs11523408 | 0.16 | -0.05 | 0.036 | 0.169 | 0.138 | 0.086 | 0.108 | -0.136 | 0.066 | 0.04 |
| InternalIMT | dha | rs188049317 | 0 | . | . | . | . | . | . | . | . | . |
| InternalIMT | dha | rs144948634 | 0.427 | -0.105 | 0.05 | 0.038 | 5.80E-03 | 0.059 | 0.921 | 0.017 | 0.047 | 0.717 |
| InternalIMT | dha | rs143833576 | 0.001 | . | . | . | . | . | . | . | . | . |
| InternalIMT | dha | rs151239879 | 0.16 | -0.05 | 0.036 | 0.167 | 0.136 | 0.086 | 0.112 | -0.135 | 0.066 | 0.042 |
| InternalIMT | dha | rs7073743 | 0.438 | -0.121 | 0.05 | 0.017 | -0.015 | 0.057 | 0.795 | 0.036 | 0.046 | 0.437 |
| InternalIMT | dha | rs142563349 | 0 | . | . | . | . | . | . | . | . | . |
| InternalIMT | dha | rs187246403 | 0 | . | . | . | . | . | . | . | . | . |
| InternalIMT | dha | rs150423942 | 0.158 | -0.049 | 0.036 | 0.179 | 0.144 | 0.088 | 0.101 | -0.142 | 0.068 | 0.036 |

| InternalIMT | dha | rs184706102 | 0.002 | . | . | . | . | . | . | . | . | . |
| --- | --- | --- | --- | --- | --- | --- | --- | --- | --- | --- | --- | --- |
| InternalIMT | dha | rs189626288 | 0.03 | -0.091 | 0.032 | 4.40E-03 | 0.046 | 0.248 | 0.852 | 0.035 | 0.207 | 0.865 |
| InternalIMT | dha | rs140749834 | 0 | . | . | . | . | . | . | . | . | . |
| InternalIMT | dha | rs7078822 | 0.131 | -0.052 | 0.036 | 0.153 | 0.133 | 0.088 | 0.13 | -0.15 | 0.068 | 0.026 |
| InternalIMT | dha | rs186227617 | 0.159 | -0.05 | 0.036 | 0.169 | 0.137 | 0.086 | 0.11 | -0.136 | 0.066 | 0.041 |
| InternalIMT | dha | rs188984368 | 0.024 | -0.082 | 0.034 | 0.015 | 0.246 | 0.222 | 0.268 | -0.172 | 0.188 | 0.361 |
| InternalIMT | dha | rs182078109 | 0.027 | -0.081 | 0.034 | 0.017 | 0.266 | 0.224 | 0.235 | -0.172 | 0.189 | 0.365 |
| InternalIMT | dha | rs141456274 | 0.002 | . | . | . | . | . | . | . | . | . |
| InternalIMT | dha | rs146976512 | 0.16 | -0.05 | 0.036 | 0.169 | 0.138 | 0.086 | 0.108 | -0.136 | 0.066 | 0.04 |
| InternalIMT | dha | rs138036816 | 0 | . | . | . | . | . | . | . | . | . |
| InternalIMT | dha | rs114879777 | 0.001 | . | . | . | . | . | . | . | . | . |
| InternalIMT | dha | rs149366475 | 0 | . | . | . | . | . | . | . | . | . |
| InternalIMT | dha | rs182463043 | 0 | . | . | . | . | . | . | . | . | . |
| InternalIMT | dha | rs144754632 | 0.001 | . | . | . | . | . | . | . | . | . |
| InternalIMT | dha | rs55717607 | 0.16 | -0.05 | 0.036 | 0.169 | 0.138 | 0.086 | 0.108 | -0.136 | 0.066 | 0.04 |
| InternalIMT | dha | rs187537973 | 0 | . | . | . | . | . | . | . | . | . |
| InternalIMT | dha | rs148085971 | 0 | . | . | . | . | . | . | . | . | . |
| InternalIMT | dha | rs11239491 | 0.16 | -0.05 | 0.036 | 0.169 | 0.138 | 0.086 | 0.108 | -0.136 | 0.066 | 0.04 |
| InternalIMT | dha | rs184412184 | 0.008 | . | . | . | . | . | . | . | . | . |
| InternalIMT | dha | rs4948998 | 0.606 | -0.047 | 0.057 | 0.416 | 0.06 | 0.053 | 0.262 | -0.038 | 0.042 | 0.372 |
| InternalIMT | dha | rs7076696 | 0.607 | -0.047 | 0.057 | 0.417 | 0.06 | 0.053 | 0.262 | -0.038 | 0.042 | 0.371 |
| InternalIMT | dha | rs11239492 | 0.16 | -0.05 | 0.036 | 0.169 | 0.138 | 0.086 | 0.108 | -0.136 | 0.066 | 0.04 |
| InternalIMT | dha | rs79462470 | 0 | . | . | . | . | . | . | . | . | . |
| InternalIMT | dha | rs187804654 | 0.003 | . | . | . | . | . | . | . | . | . |
| InternalIMT | dha | rs7093687 | 0.16 | -0.05 | 0.036 | 0.169 | 0.138 | 0.086 | 0.108 | -0.136 | 0.066 | 0.04 |
| InternalIMT | dha | rs7081004 | 0.16 | -0.05 | 0.036 | 0.169 | 0.138 | 0.086 | 0.108 | -0.136 | 0.066 | 0.04 |
| InternalIMT | dha | rs7084793 | 0.16 | -0.05 | 0.036 | 0.169 | 0.138 | 0.086 | 0.108 | -0.136 | 0.066 | 0.04 |
| InternalIMT | dha | rs61556080 | 0 | . | . | . | . | . | . | . | . | . |
| InternalIMT | dha | rs115552064 | . | . | . | . | . | . | . | . | . | . |
| InternalIMT | dha | rs7921503 | 0.16 | -0.05 | 0.036 | 0.169 | 0.138 | 0.086 | 0.108 | -0.136 | 0.066 | 0.04 |
| InternalIMT | dha | rs7909740 | 0.16 | -0.05 | 0.036 | 0.169 | 0.138 | 0.086 | 0.108 | -0.136 | 0.066 | 0.04 |
| InternalIMT | dha | rs7909768 | 0.16 | -0.05 | 0.036 | 0.169 | 0.138 | 0.086 | 0.108 | -0.136 | 0.066 | 0.04 |
| InternalIMT | dha | rs144168004 | 0 | . | . | . | . | . | . | . | . | . |
| InternalIMT | dha | rs75850448 | 0 | . | . | . | . | . | . | . | . | . |
| InternalIMT | dha | rs147777574 | 0 | . | . | . | . | . | . | . | . | . |
| InternalIMT | dha | rs7913488 | 0.16 | -0.05 | 0.036 | 0.169 | 0.138 | 0.086 | 0.108 | -0.136 | 0.066 | 0.04 |
| InternalIMT | dha | rs74390874 | 0.071 | -0.107 | 0.034 | 2.00E-03 | -0.075 | 0.115 | 0.518 | 0.122 | 0.094 | 0.195 |
| InternalIMT | dha | rs141391142 | 0.009 | . | . | . | . | . | . | . | . | . |
| InternalIMT | dha | rs115294747 | 0.001 | . | . | . | . | . | . | . | . | . |
| InternalIMT | dha | rs148031781 | 0.003 | . | . | . | . | . | . | . | . | . |
| InternalIMT | dha | rs181340128 | 0.021 | -0.106 | 0.033 | 1.20E-03 | -0.399 | 0.218 | 0.068 | 0.323 | 0.201 | 0.108 |
| InternalIMT | dha | rs189930007 | 0.002 | . | . | . | . | . | . | . | . | . |
| InternalIMT | dha | rs12242128 | 0 | . | . | . | . | . | . | . | . | . |
| InternalIMT | dha | rs78421750 | 0.002 | . | . | . | . | . | . | . | . | . |
| InternalIMT | dha | rs114370747 | 0.002 | . | . | . | . | . | . | . | . | . |
| InternalIMT | dha | rs12264166 | 0 | . | . | . | . | . | . | . | . | . |
| InternalIMT | dha | rs182964822 | 0.001 | . | . | . | . | . | . | . | . | . |
| InternalIMT | dha | rs67491417 | 0.15 | -0.054 | 0.036 | 0.132 | 0.137 | 0.078 | 0.079 | -0.132 | 0.061 | 0.031 |
| InternalIMT | dha | rs72789720 | 0.023 | -0.095 | 0.033 | 4.20E-03 | -0.084 | 0.176 | 0.633 | 0.071 | 0.149 | 0.636 |
| InternalIMT | dha | rs6413416 | 0.16 | -0.05 | 0.036 | 0.167 | 0.136 | 0.086 | 0.114 | -0.135 | 0.066 | 0.042 |
| InternalIMT | dha | rs4986832 | 0.16 | -0.05 | 0.036 | 0.169 | 0.138 | 0.086 | 0.108 | -0.136 | 0.066 | 0.04 |
| InternalIMT | dha | rs144866316 | 0.005 | . | . | . | . | . | . | . | . | . |
| InternalIMT | dha | rs187769540 | 0.001 | . | . | . | . | . | . | . | . | . |
| InternalIMT | dha | rs6593482 | 0.16 | -0.049 | 0.037 | 0.18 | 0.135 | 0.085 | 0.114 | -0.136 | 0.066 | 0.039 |
| InternalIMT | dha | rs7913948 | 0.16 | -0.05 | 0.036 | 0.17 | 0.138 | 0.086 | 0.108 | -0.136 | 0.066 | 0.04 |
| InternalIMT | dha | rs77185151 | 0.001 | . | . | . | . | . | . | . | . | . |
| InternalIMT | dha | rs12762303 | 0.16 | -0.05 | 0.036 | 0.17 | 0.138 | 0.086 | 0.108 | -0.136 | 0.066 | 0.04 |
| InternalIMT | dha | rs73283136 | 0.003 | . | . | . | . | . | . | . | . | . |
| InternalIMT | dha | rs187180218 | 0 | . | . | . | . | . | . | . | . | . |
| InternalIMT | dha | rs78137087 | 0.159 | -0.05 | 0.036 | 0.173 | 0.139 | 0.086 | 0.105 | -0.138 | 0.066 | 0.038 |
| InternalIMT | dha | rs72561472 | 0.158 | -0.053 | 0.037 | 0.145 | 0.127 | 0.087 | 0.146 | -0.126 | 0.067 | 0.062 |
| InternalIMT | dha | rs4987106 | 0.152 | -0.054 | 0.036 | 0.142 | 0.128 | 0.09 | 0.156 | -0.13 | 0.07 | 0.062 |
| InternalIMT | dha | rs4987105 | 0.155 | -0.052 | 0.036 | 0.153 | 0.135 | 0.087 | 0.12 | -0.133 | 0.067 | 0.048 |
| InternalIMT | dha | rs111916157 | 0.188 | -0.052 | 0.037 | 0.167 | 0.119 | 0.081 | 0.143 | -0.112 | 0.063 | 0.076 |
| InternalIMT | dha | rs111528152 | 0.16 | -0.05 | 0.036 | 0.168 | 0.136 | 0.086 | 0.111 | -0.135 | 0.066 | 0.041 |
| InternalIMT | dha | rs12763738 | 0.16 | -0.05 | 0.036 | 0.169 | 0.138 | 0.086 | 0.108 | -0.136 | 0.066 | 0.04 |
| InternalIMT | dha | rs191675886 | 0.006 | . | . | . | . | . | . | . | . | . |
| InternalIMT | dha | rs66489870 | 0.16 | -0.05 | 0.036 | 0.17 | 0.138 | 0.086 | 0.108 | -0.136 | 0.066 | 0.04 |
| InternalIMT | dha | rs71494775 | 0.16 | -0.05 | 0.036 | 0.17 | 0.138 | 0.086 | 0.108 | -0.136 | 0.066 | 0.04 |
| InternalIMT | dha | rs61855875 | 0.033 | -0.112 | 0.033 | 7.50E-04 | -0.323 | 0.222 | 0.145 | 0.323 | 0.188 | 0.085 |
| InternalIMT | dha | rs116356837 | 0 | . | . | . | . | . | . | . | . | . |
| InternalIMT | dha | rs1864414 | 0.16 | -0.05 | 0.036 | 0.169 | 0.138 | 0.086 | 0.108 | -0.136 | 0.066 | 0.04 |
| InternalIMT | dha | rs1864413 | 0.16 | -0.05 | 0.036 | 0.173 | 0.139 | 0.086 | 0.105 | -0.137 | 0.066 | 0.039 |
| InternalIMT | dha | rs139497212 | 0 | . | . | . | . | . | . | . | . | . |
| InternalIMT | dha | rs892690 | 0.358 | -0.128 | 0.046 | 6.00E-03 | -0.034 | 0.061 | 0.575 | 0.052 | 0.049 | 0.293 |
| InternalIMT | dha | rs1864412 | 0.16 | -0.05 | 0.036 | 0.17 | 0.138 | 0.086 | 0.108 | -0.136 | 0.066 | 0.04 |
| InternalIMT | dha | rs77827771 | 0.004 | . | . | . | . | . | . | . | . | . |
| InternalIMT | dha | rs145294963 | 0.004 | . | . | . | . | . | . | . | . | . |
| InternalIMT | dha | rs149081327 | 0.012 | -0.093 | 0.033 | 4.30E-03 | -0.014 | 0.293 | 0.963 | 0.078 | 0.225 | 0.73 |
| InternalIMT | dha | rs67008983 | 0.16 | -0.05 | 0.036 | 0.17 | 0.138 | 0.086 | 0.107 | -0.136 | 0.066 | 0.04 |
| InternalIMT | dha | rs56150414 | 0.168 | -0.046 | 0.037 | 0.206 | 0.141 | 0.083 | 0.09 | -0.139 | 0.064 | 0.032 |
| InternalIMT | dha | rs55787481 | 0.16 | -0.05 | 0.036 | 0.174 | 0.139 | 0.086 | 0.105 | -0.137 | 0.066 | 0.038 |
| InternalIMT | dha | rs141270101 | 0.001 | . | . | . | . | . | . | . | . | . |
| InternalIMT | dha | rs114980075 | 0.002 | . | . | . | . | . | . | . | . | . |
| InternalIMT | dha | rs12783095 | 0.159 | -0.05 | 0.036 | 0.169 | 0.138 | 0.086 | 0.108 | -0.136 | 0.066 | 0.04 |
| InternalIMT | dha | rs182342688 | 0.005 | . | . | . | . | . | . | . | . | . |

| InternalIMT | dha | rs17157728 | 0.159 | -0.05 | 0.036 | 0.169 | 0.138 | 0.086 | 0.108 | -0.136 | 0.066 | 0.04 |
| --- | --- | --- | --- | --- | --- | --- | --- | --- | --- | --- | --- | --- |
| InternalIMT | dha | rs114835563 | 0 | . | . | . | . | . | . | . | . | . |
| InternalIMT | dha | rs3824612 | 0.358 | -0.129 | 0.046 | 5.40E-03 | -0.036 | 0.062 | 0.559 | 0.054 | 0.05 | 0.277 |
| InternalIMT | dha | rs17157733 | 0.158 | -0.05 | 0.036 | 0.168 | 0.138 | 0.086 | 0.109 | -0.136 | 0.066 | 0.04 |
| InternalIMT | dha | rs74128478 | 0 | . | . | . | . | . | . | . | . | . |
| InternalIMT | dha | rs184973589 | 0.001 | . | . | . | . | . | . | . | . | . |
| InternalIMT | dha | rs150479461 | 0 | . | . | . | . | . | . | . | . | . |
| InternalIMT | dha | rs138310407 | 0.004 | . | . | . | . | . | . | . | . | . |
| InternalIMT | dha | rs187426603 | 0.001 | . | . | . | . | . | . | . | . | . |
| InternalIMT | dha | rs190843143 | 0.003 | . | . | . | . | . | . | . | . | . |
| InternalIMT | dha | rs191883449 | 0.002 | . | . | . | . | . | . | . | . | . |
| InternalIMT | dha | rs58238740 | 0.158 | -0.05 | 0.036 | 0.168 | 0.138 | 0.086 | 0.107 | -0.136 | 0.066 | 0.04 |
| InternalIMT | dha | rs3780894 | 0.158 | -0.05 | 0.036 | 0.168 | 0.139 | 0.086 | 0.107 | -0.136 | 0.066 | 0.04 |
| InternalIMT | dha | rs149646626 | 0.027 | -0.077 | 0.032 | 0.017 | 0.387 | 0.287 | 0.177 | -0.254 | 0.22 | 0.25 |
| InternalIMT | dha | rs79615212 | 0.023 | -0.102 | 0.033 | 1.90E-03 | -0.352 | 0.228 | 0.123 | 0.222 | 0.183 | 0.226 |
| InternalIMT | dha | rs3780895 | 0.158 | -0.05 | 0.036 | 0.168 | 0.139 | 0.086 | 0.107 | -0.136 | 0.066 | 0.04 |
| InternalIMT | dha | rs3780896 | 0.158 | -0.05 | 0.036 | 0.168 | 0.139 | 0.086 | 0.107 | -0.136 | 0.066 | 0.04 |
| InternalIMT | dha | rs142747163 | 0.006 | . | . | . | . | . | . | . | . | . |
| InternalIMT | dha | rs189417584 | 0.005 | . | . | . | . | . | . | . | . | . |
| InternalIMT | dha | rs3780897 | 0.158 | -0.05 | 0.036 | 0.17 | 0.14 | 0.086 | 0.104 | -0.137 | 0.066 | 0.039 |
| InternalIMT | dha | rs117509491 | 0.004 | . | . | . | . | . | . | . | . | . |
| InternalIMT | dha | rs117811514 | 0.004 | . | . | . | . | . | . | . | . | . |
| InternalIMT | dha | rs116500932 | 0 | . | . | . | . | . | . | . | . | . |
| InternalIMT | dha | rs77584168 | 0.001 | . | . | . | . | . | . | . | . | . |
| InternalIMT | dha | rs2310776 | 0.158 | -0.05 | 0.036 | 0.168 | 0.139 | 0.086 | 0.107 | -0.136 | 0.066 | 0.04 |
| InternalIMT | dha | rs17157736 | 0.158 | -0.05 | 0.036 | 0.168 | 0.139 | 0.086 | 0.107 | -0.136 | 0.066 | 0.04 |
| InternalIMT | dha | rs72789735 | 0.105 | -0.049 | 0.034 | 0.146 | 0.237 | 0.118 | 0.044 | -0.219 | 0.089 | 0.014 |
| InternalIMT | dha | rs183747187 | 0.002 | . | . | . | . | . | . | . | . | . |
| InternalIMT | dha | rs7077891 | 0.158 | -0.05 | 0.036 | 0.168 | 0.139 | 0.086 | 0.107 | -0.136 | 0.066 | 0.04 |
| InternalIMT | dha | rs145063305 | 0.006 | . | . | . | . | . | . | . | . | . |
| InternalIMT | dha | rs3824613 | 0.159 | -0.05 | 0.036 | 0.167 | 0.138 | 0.086 | 0.108 | -0.136 | 0.066 | 0.041 |
| InternalIMT | dha | rs145543199 | 0.014 | -0.094 | 0.033 | 4.10E-03 | -0.119 | 0.268 | 0.657 | 0.088 | 0.228 | 0.699 |
| InternalIMT | dha | rs140361993 | 0.005 | . | . | . | . | . | . | . | . | . |
| InternalIMT | dha | rs11239496 | 0.105 | -0.049 | 0.034 | 0.146 | 0.237 | 0.118 | 0.044 | -0.219 | 0.089 | 0.014 |
| InternalIMT | dha | rs11239497 | 0.158 | -0.05 | 0.036 | 0.169 | 0.139 | 0.086 | 0.106 | -0.136 | 0.066 | 0.04 |
| InternalIMT | dha | rs10900212 | 0.158 | -0.05 | 0.036 | 0.169 | 0.139 | 0.086 | 0.106 | -0.136 | 0.066 | 0.04 |
| InternalIMT | dha | rs186487136 | 0 | . | . | . | . | . | . | . | . | . |
| InternalIMT | dha | rs12774369 | 0.158 | -0.05 | 0.036 | 0.173 | 0.14 | 0.086 | 0.103 | -0.138 | 0.066 | 0.038 |
| InternalIMT | dha | rs60842538 | 0.158 | -0.049 | 0.036 | 0.178 | 0.142 | 0.086 | 0.1 | -0.14 | 0.066 | 0.036 |
| InternalIMT | dha | rs3780898 | 0.158 | -0.048 | 0.036 | 0.185 | 0.144 | 0.086 | 0.095 | -0.142 | 0.067 | 0.033 |
| InternalIMT | dha | rs3780899 | 0.159 | -0.048 | 0.036 | 0.187 | 0.145 | 0.086 | 0.094 | -0.143 | 0.067 | 0.032 |
| InternalIMT | dha | rs12252472 | 0 | . | . | . | . | . | . | . | . | . |
| InternalIMT | dha | rs185411011 | 0.003 | . | . | . | . | . | . | . | . | . |
| InternalIMT | dha | rs28395865 | 0.156 | -0.046 | 0.036 | 0.202 | 0.154 | 0.088 | 0.08 | -0.151 | 0.068 | 0.026 |
| InternalIMT | dha | rs191004074 | 0.024 | -0.081 | 0.034 | 0.016 | 0.226 | 0.229 | 0.324 | -0.205 | 0.145 | 0.16 |
| InternalIMT | dha | rs182401295 | 0.004 | . | . | . | . | . | . | . | . | . |
| InternalIMT | dha | rs2228064 | 0.002 | . | . | . | . | . | . | . | . | . |
| InternalIMT | dha | rs188284159 | 0.001 | . | . | . | . | . | . | . | . | . |
| InternalIMT | dha | rs2303887 | 0.001 | . | . | . | . | . | . | . | . | . |
| InternalIMT | dha | rs2303886 | 0.001 | . | . | . | . | . | . | . | . | . |
| InternalIMT | dha | rs192621006 | 0.003 | . | . | . | . | . | . | . | . | . |
| InternalIMT | dha | rs2303885 | 0.154 | -0.045 | 0.036 | 0.208 | 0.157 | 0.088 | 0.074 | -0.159 | 0.067 | 0.017 |
| InternalIMT | dha | rs114466168 | 0 | . | . | . | . | . | . | . | . | . |
| InternalIMT | dha | rs11239501 | 0.149 | -0.043 | 0.035 | 0.212 | 0.204 | 0.091 | 0.025 | -0.18 | 0.07 | 0.011 |
| InternalIMT | dha | rs745986 | 0.204 | -0.04 | 0.037 | 0.283 | 0.141 | 0.071 | 0.047 | -0.138 | 0.056 | 0.013 |
| InternalIMT | dha | rs75262268 | 0 | . | . | . | . | . | . | . | . | . |
| InternalIMT | dha | rs71494776 | 0.05 | -0.09 | 0.034 | 7.70E-03 | -0.027 | 0.097 | 0.78 | -0.012 | 0.079 | 0.876 |
| InternalIMT | dha | rs74386871 | 0.011 | -0.09 | 0.032 | 5.30E-03 | 0.288 | 0.348 | 0.408 | -0.055 | 0.258 | 0.832 |
| InternalIMT | dha | rs76502040 | 0.01 | -0.097 | 0.033 | 3.00E-03 | -0.171 | 0.239 | 0.475 | 0.231 | 0.226 | 0.306 |
| InternalIMT | dha | rs142213734 | 0.001 | . | . | . | . | . | . | . | . | . |
| InternalIMT | dha | rs4948671 | 0.604 | -0.057 | 0.057 | 0.313 | 0.053 | 0.054 | 0.322 | -0.029 | 0.043 | 0.497 |
| InternalIMT | dha | rs146286085 | 0.002 | . | . | . | . | . | . | . | . | . |
| InternalIMT | dha | rs144080623 | 0 | . | . | . | . | . | . | . | . | . |
| InternalIMT | dha | rs115074014 | 0 | . | . | . | . | . | . | . | . | . |
| InternalIMT | dha | rs17157756 | 0 | . | . | . | . | . | . | . | . | . |
| InternalIMT | dha | rs190459780 | 0.001 | . | . | . | . | . | . | . | . | . |
| InternalIMT | dha | rs12358647 | 0.204 | -0.04 | 0.037 | 0.281 | 0.141 | 0.071 | 0.047 | -0.137 | 0.056 | 0.014 |
| InternalIMT | dha | rs147080556 | 0.011 | -0.095 | 0.033 | 3.70E-03 | -0.215 | 0.197 | 0.277 | 0.052 | 0.155 | 0.737 |
| InternalIMT | dha | rs187543349 | 0.004 | . | . | . | . | . | . | . | . | . |
| InternalIMT | dha | rs114375595 | 0.004 | . | . | . | . | . | . | . | . | . |
| InternalIMT | dha | rs117211200 | 0.01 | . | . | . | . | . | . | . | . | . |
| InternalIMT | dha | rs192963047 | 0.001 | . | . | . | . | . | . | . | . | . |
| InternalIMT | dha | rs11239505 | 0.204 | -0.04 | 0.037 | 0.281 | 0.142 | 0.071 | 0.046 | -0.138 | 0.056 | 0.014 |
| InternalIMT | dha | rs184434116 | 0.003 | . | . | . | . | . | . | . | . | . |
| InternalIMT | dha | rs4948999 | 0.604 | -0.057 | 0.057 | 0.311 | 0.053 | 0.054 | 0.32 | -0.029 | 0.043 | 0.501 |
| InternalIMT | dha | rs11239506 | 0.154 | -0.044 | 0.035 | 0.21 | 0.194 | 0.088 | 0.027 | -0.172 | 0.068 | 0.012 |
| InternalIMT | dha | rs190706620 | 0.006 | . | . | . | . | . | . | . | . | . |
| InternalIMT | dha | rs190770429 | 0.002 | . | . | . | . | . | . | . | . | . |
| InternalIMT | dha | rs181327133 | 0.006 | . | . | . | . | . | . | . | . | . |
| InternalIMT | dha | rs149251697 | 0.008 | . | . | . | . | . | . | . | . | . |
| InternalIMT | dha | rs118081602 | 0.001 | . | . | . | . | . | . | . | . | . |
| InternalIMT | dha | rs188233691 | 0.001 | . | . | . | . | . | . | . | . | . |
| InternalIMT | dha | rs183778794 | 0.005 | . | . | . | . | . | . | . | . | . |
| InternalIMT | dha | rs188040078 | 0.007 | . | . | . | . | . | . | . | . | . |
| InternalIMT | dha | rs184675372 | 0.003 | . | . | . | . | . | . | . | . | . |
| InternalIMT | dha | rs150326891 | 0.116 | -0.043 | 0.034 | 0.205 | 0.248 | 0.107 | 0.02 | -0.22 | 0.08 | 6.30E-03 |

| InternalIMT | dha | rs185520021 | 0.002 | . | . | . | . | . | . | . | . | . |
| --- | --- | --- | --- | --- | --- | --- | --- | --- | --- | --- | --- | --- |
| InternalIMT | dha | rs7907779 | 0.154 | -0.044 | 0.035 | 0.21 | 0.195 | 0.088 | 0.027 | -0.172 | 0.068 | 0.011 |
| InternalIMT | dha | rs184962860 | 0.001 | . | . | . | . | . | . | . | . | . |
| InternalIMT | dha | rs149762784 | 0.025 | -0.086 | 0.034 | 0.01 | 0.133 | 0.218 | 0.542 | -0.104 | 0.185 | 0.574 |
| InternalIMT | dha | rs149203427 | 0.021 | -0.093 | 0.033 | 4.60E-03 | -3.60E-04 | 0.245 | 0.999 | 0.067 | 0.211 | 0.752 |
| InternalIMT | dha | rs60153083 | 0 | . | . | . | . | . | . | . | . | . |
| InternalIMT | dha | rs112001069 | 0.05 | -0.09 | 0.034 | 7.70E-03 | -0.025 | 0.098 | 0.797 | -0.013 | 0.079 | 0.869 |
| InternalIMT | dha | rs182355382 | 0.021 | -0.097 | 0.033 | 3.10E-03 | -0.067 | 0.193 | 0.727 | 0.123 | 0.166 | 0.459 |
| InternalIMT | dha | rs186740974 | 0.117 | -0.122 | 0.039 | 1.90E-03 | -0.149 | 0.111 | 0.178 | 0.122 | 0.087 | 0.158 |
| InternalIMT | dha | rs191874916 | 0.014 | -0.091 | 0.032 | 4.30E-03 | 0.108 | 0.334 | 0.746 | 0.019 | 0.276 | 0.944 |
| InternalIMT | dha | rs182989412 | 0.152 | -0.101 | 0.038 | 8.00E-03 | -0.018 | 0.123 | 0.885 | 0.035 | 0.099 | 0.724 |
| InternalIMT | dha | rs188819476 | 0.002 | . | . | . | . | . | . | . | . | . |
| InternalIMT | dha | rs141217183 | 0.078 | -0.114 | 0.036 | 1.40E-03 | -0.225 | 0.096 | 0.02 | 0.142 | 0.079 | 0.074 |
| InternalIMT | dha | rs192922652 | 0.045 | -0.093 | 0.034 | 5.70E-03 | -0.085 | 0.106 | 0.424 | 0.022 | 0.087 | 0.803 |
| InternalIMT | dha | rs185092343 | 0.024 | -0.105 | 0.033 | 1.20E-03 | -0.198 | 0.277 | 0.474 | 0.297 | 0.25 | 0.234 |
| InternalIMT | dha | rs145193311 | 0.051 | -0.094 | 0.034 | 6.00E-03 | -0.079 | 0.105 | 0.455 | 0.026 | 0.086 | 0.764 |
| InternalIMT | dha | rs55789336 | 0.155 | -0.05 | 0.035 | 0.157 | 0.172 | 0.088 | 0.052 | -0.15 | 0.069 | 0.029 |
| InternalIMT | dha | rs55837212 | 0.152 | -0.052 | 0.035 | 0.138 | 0.166 | 0.078 | 0.034 | -0.147 | 0.062 | 0.017 |
| InternalIMT | dha | rs75408574 | 0.366 | -0.144 | 0.047 | 2.00E-03 | -0.052 | 0.061 | 0.393 | 0.073 | 0.049 | 0.133 |
| InternalIMT | dha | rs149306926 | 0.161 | -0.041 | 0.035 | 0.25 | 0.192 | 0.076 | 0.012 | -0.175 | 0.06 | 3.70E-03 |
| InternalIMT | dha | rs35990892 | 0.597 | -0.055 | 0.059 | 0.352 | 0.043 | 0.055 | 0.436 | -0.031 | 0.044 | 0.484 |
| InternalIMT | dha | rs192356207 | 0.019 | -0.094 | 0.033 | 5.00E-03 | -0.074 | 0.173 | 0.671 | 0.056 | 0.145 | 0.7 |
| InternalIMT | dha | rs183741163 | 0.129 | -0.051 | 0.035 | 0.146 | 0.214 | 0.095 | 0.024 | -0.175 | 0.073 | 0.016 |
| InternalIMT | dha | rs34737297 | 0.48 | -0.115 | 0.054 | 0.033 | -8.10E-03 | 0.059 | 0.89 | 0.025 | 0.046 | 0.593 |
| InternalIMT | dha | rs181346933 | 0.026 | -0.082 | 0.033 | 0.013 | 0.135 | 0.245 | 0.58 | -0.148 | 0.175 | 0.398 |
| InternalIMT | dha | rs36093253 | 0.343 | -0.124 | 0.047 | 8.40E-03 | -0.035 | 0.065 | 0.586 | 0.049 | 0.053 | 0.352 |
| InternalIMT | dha | rs186307855 | 0.02 | -0.086 | 0.033 | 8.60E-03 | 0.24 | 0.257 | 0.352 | -0.082 | 0.195 | 0.675 |
| InternalIMT | dha | rs189681865 | 0.003 | . | . | . | . | . | . | . | . | . |
| InternalIMT | dha | rs34942971 | 0.207 | -0.04 | 0.037 | 0.286 | 0.14 | 0.065 | 0.03 | -0.132 | 0.051 | 9.90E-03 |
| InternalIMT | dha | rs181816554 | 0.019 | -0.105 | 0.033 | 1.30E-03 | -0.346 | 0.285 | 0.225 | 0.371 | 0.268 | 0.166 |
| InternalIMT | dha | rs186846881 | 0.004 | . | . | . | . | . | . | . | . | . |
| InternalIMT | dha | rs191178220 | 0.024 | -0.089 | 0.033 | 6.10E-03 | 0.171 | 0.23 | 0.458 | -0.059 | 0.17 | 0.73 |
| InternalIMT | dha | rs183305228 | 0 | . | . | . | . | . | . | . | . | . |
| InternalIMT | dha | rs139912761 | 0.15 | -0.049 | 0.035 | 0.16 | 0.173 | 0.079 | 0.029 | -0.155 | 0.062 | 0.013 |
| InternalIMT | dha | rs185499901 | 0.019 | -0.074 | 0.032 | 0.021 | 0.56 | 0.365 | 0.125 | -0.37 | 0.286 | 0.196 |
| InternalIMT | dha | rs189816197 | 0.061 | -0.09 | 0.034 | 8.10E-03 | -0.018 | 0.101 | 0.856 | -0.014 | 0.081 | 0.866 |
| InternalIMT | dha | rs145656938 | 0.014 | -0.095 | 0.032 | 3.20E-03 | -0.141 | 0.565 | 0.803 | 0.123 | 0.453 | 0.786 |
| InternalIMT | dha | rs35745197 | 0.05 | -0.09 | 0.034 | 7.70E-03 | -0.028 | 0.097 | 0.772 | -0.012 | 0.079 | 0.878 |
| InternalIMT | dha | rs192856637 | 0.006 | . | . | . | . | . | . | . | . | . |
| InternalIMT | dha | rs2310775 | 0.401 | -0.138 | 0.048 | 4.00E-03 | -0.04 | 0.057 | 0.485 | 0.06 | 0.046 | 0.194 |
| InternalIMT | dha | rs11239507 | 0.403 | -0.139 | 0.048 | 3.90E-03 | -0.039 | 0.057 | 0.498 | 0.061 | 0.046 | 0.189 |
| InternalIMT | dha | rs74648467 | 0.049 | -0.09 | 0.034 | 7.60E-03 | -0.028 | 0.098 | 0.772 | -0.012 | 0.079 | 0.883 |
| InternalIMT | dha | rs145815934 | 0.022 | -0.091 | 0.033 | 5.50E-03 | 2.70E-03 | 0.238 | 0.991 | -0.011 | 0.187 | 0.953 |
| InternalIMT | dha | rs145390687 | 0.029 | -0.102 | 0.033 | 1.80E-03 | -0.139 | 0.238 | 0.559 | 0.23 | 0.204 | 0.26 |
| InternalIMT | dha | rs184354913 | 0.049 | -0.086 | 0.033 | 8.90E-03 | 0.099 | 0.181 | 0.587 | -0.046 | 0.139 | 0.74 |
| InternalIMT | dha | rs181244920 | 0.007 | . | . | . | . | . | . | . | . | . |
| InternalIMT | dha | rs11499087 | 0 | . | . | . | . | . | . | . | . | . |
| InternalIMT | dha | rs148263794 | 0.05 | -0.09 | 0.034 | 7.70E-03 | -0.027 | 0.097 | 0.781 | -0.013 | 0.079 | 0.867 |
| InternalIMT | dha | rs150758903 | 0.01 | -0.086 | 0.032 | 8.50E-03 | 0.312 | 0.425 | 0.464 | -0.301 | 0.291 | 0.3 |
| InternalIMT | dha | rs147654382 | 0.183 | -0.052 | 0.036 | 0.153 | 0.125 | 0.065 | 0.056 | -0.117 | 0.052 | 0.024 |
| InternalIMT | dha | rs147495166 | 0 | . | . | . | . | . | . | . | . | . |
| InternalIMT | dha | rs35198778 | 0.092 | -0.075 | 0.035 | 0.034 | 0.07 | 0.096 | 0.464 | -0.089 | 0.073 | 0.226 |
| InternalIMT | dha | rs145207659 | 0.071 | -0.112 | 0.034 | 1.10E-03 | -0.108 | 0.127 | 0.394 | 0.165 | 0.104 | 0.114 |
| InternalIMT | dha | rs186466464 | 0.003 | . | . | . | . | . | . | . | . | . |
| InternalIMT | dha | rs12778413 | 0.157 | -0.054 | 0.037 | 0.142 | 0.157 | 0.078 | 0.044 | -0.133 | 0.062 | 0.032 |
| InternalIMT | dha | rs7895552 | 0.531 | -0.08 | 0.056 | 0.15 | 0.042 | 0.056 | 0.449 | -0.011 | 0.045 | 0.81 |
| InternalIMT | dha | rs12783895 | 0.05 | -0.09 | 0.034 | 7.70E-03 | -0.028 | 0.097 | 0.773 | -0.013 | 0.079 | 0.874 |
| InternalIMT | dha | rs34929604 | 0.05 | -0.09 | 0.034 | 7.70E-03 | -0.028 | 0.097 | 0.775 | -0.013 | 0.079 | 0.872 |
| InternalIMT | dha | rs12761961 | 0.049 | -0.09 | 0.034 | 7.70E-03 | -0.026 | 0.098 | 0.79 | -0.012 | 0.079 | 0.876 |
| InternalIMT | dha | rs35536930 | 0.198 | -0.046 | 0.037 | 0.214 | 0.128 | 0.063 | 0.042 | -0.126 | 0.05 | 0.013 |
| InternalIMT | dha | rs180951060 | 0 | . | . | . | . | . | . | . | . | . |
| InternalIMT | dha | rs148892128 | 0.022 | -0.091 | 0.033 | 5.50E-03 | 2.80E-03 | 0.238 | 0.99 | -0.011 | 0.187 | 0.952 |
| InternalIMT | dha | rs11239509 | 0.001 | . | . | . | . | . | . | . | . | . |
| InternalIMT | dha | rs41526545 | 0.154 | -0.044 | 0.035 | 0.211 | 0.196 | 0.088 | 0.026 | -0.173 | 0.068 | 0.011 |
| InternalIMT | dha | rs2029253 | 0.401 | -0.139 | 0.048 | 3.80E-03 | -0.042 | 0.058 | 0.462 | 0.061 | 0.046 | 0.186 |
| InternalIMT | dha | rs17444064 | 0.154 | -0.043 | 0.035 | 0.213 | 0.198 | 0.088 | 0.024 | -0.174 | 0.068 | 0.011 |
| InternalIMT | dha | rs71494779 | 0.05 | -0.09 | 0.034 | 7.70E-03 | -0.027 | 0.097 | 0.781 | -0.013 | 0.079 | 0.87 |
| InternalIMT | dha | rs74991420 | 0.001 | . | . | . | . | . | . | . | . | . |
| InternalIMT | dha | rs182736551 | 0.001 | . | . | . | . | . | . | . | . | . |
| InternalIMT | dha | rs78702074 | 0.001 | . | . | . | . | . | . | . | . | . |
| InternalIMT | dha | rs187791466 | 0.005 | . | . | . | . | . | . | . | . | . |
| InternalIMT | dha | rs35300270 | 0.054 | -0.091 | 0.034 | 7.60E-03 | -0.04 | 0.095 | 0.673 | -8.50E-04 | 0.078 | 0.991 |
| InternalIMT | dha | rs6593484 | 0.155 | -0.044 | 0.035 | 0.211 | 0.197 | 0.088 | 0.025 | -0.173 | 0.068 | 0.011 |
| InternalIMT | dha | rs12244601 | 0.004 | . | . | . | . | . | . | . | . | . |
| InternalIMT | dha | rs184429574 | 0.005 | . | . | . | . | . | . | . | . | . |
| InternalIMT | dha | rs7080897 | 0.05 | -0.09 | 0.034 | 7.70E-03 | -0.027 | 0.097 | 0.781 | -0.013 | 0.079 | 0.87 |
| InternalIMT | dha | rs7097429 | 0.054 | -0.094 | 0.034 | 5.60E-03 | -0.057 | 0.097 | 0.557 | 0.024 | 0.079 | 0.76 |
| InternalIMT | dha | rs77979229 | 0.001 | . | . | . | . | . | . | . | . | . |
| InternalIMT | dha | rs11239512 | 0.155 | -0.044 | 0.035 | 0.212 | 0.197 | 0.088 | 0.025 | -0.173 | 0.068 | 0.011 |
| InternalIMT | dha | rs60146827 | 0.05 | -0.09 | 0.034 | 7.70E-03 | -0.027 | 0.097 | 0.783 | -0.013 | 0.079 | 0.869 |
| InternalIMT | dha | rs79032236 | 0 | . | . | . | . | . | . | . | . | . |
| InternalIMT | dha | rs140854206 | 0.009 | . | . | . | . | . | . | . | . | . |
| InternalIMT | dha | rs184049550 | 0 | . | . | . | . | . | . | . | . | . |
| InternalIMT | dha | rs114433607 | 0 | . | . | . | . | . | . | . | . | . |
| InternalIMT | dha | rs117653793 | 0.001 | . | . | . | . | . | . | . | . | . |
| InternalIMT | dha | rs117039533 | 0.001 | . | . | . | . | . | . | . | . | . |

| InternalIMT | dha | rs191660761 | 0.006 | . | . | . | . | . | . | . | . | . |
| --- | --- | --- | --- | --- | --- | --- | --- | --- | --- | --- | --- | --- |
| InternalIMT | dha | rs59514896 | 0.05 | -0.09 | 0.034 | 7.70E-03 | -0.027 | 0.097 | 0.783 | -0.013 | 0.079 | 0.873 |
| InternalIMT | dha | rs184661876 | 0.002 | . | . | . | . | . | . | . | . | . |
| InternalIMT | dha | rs61854073 | 0.037 | -0.114 | 0.033 | 6.30E-04 | -0.267 | 0.181 | 0.141 | 0.312 | 0.161 | 0.053 |
| InternalIMT | dha | rs2018518 | 0.315 | -0.139 | 0.045 | 1.90E-03 | -0.063 | 0.066 | 0.339 | 0.076 | 0.053 | 0.151 |
| InternalIMT | dha | rs934187 | 0.607 | -0.058 | 0.057 | 0.31 | 0.052 | 0.054 | 0.331 | -0.028 | 0.043 | 0.506 |
| InternalIMT | dha | rs7918542 | 0.205 | -0.039 | 0.037 | 0.285 | 0.145 | 0.071 | 0.042 | -0.139 | 0.056 | 0.013 |
| InternalIMT | dha | rs144311920 | 0 | . | . | . | . | . | . | . | . | . |
| InternalIMT | dha | rs7099684 | 0.79 | -0.041 | 0.074 | 0.585 | 0.069 | 0.061 | 0.26 | -0.032 | 0.047 | 0.499 |
| InternalIMT | dha | rs150829171 | 0.002 | . | . | . | . | . | . | . | . | . |
| InternalIMT | dha | rs186060697 | 0 | . | . | . | . | . | . | . | . | . |
| InternalIMT | dha | rs11239513 | 0.009 | . | . | . | . | . | . | . | . | . |
| InternalIMT | dha | rs7917687 | 0.05 | -0.09 | 0.034 | 7.70E-03 | -0.026 | 0.097 | 0.79 | -0.014 | 0.079 | 0.863 |
| InternalIMT | dha | rs61854076 | 0.014 | -0.11 | 0.032 | 6.40E-04 | -0.824 | 0.292 | 4.70E-03 | 0.731 | 0.247 | 3.10E-03 |
| InternalIMT | dha | rs71494780 | 0.019 | -0.09 | 0.033 | 5.80E-03 | 1.70E-03 | 0.14 | 0.99 | -0.045 | 0.109 | 0.677 |
| InternalIMT | dha | rs7919239 | 0.205 | -0.04 | 0.037 | 0.28 | 0.146 | 0.071 | 0.04 | -0.138 | 0.056 | 0.014 |
| InternalIMT | dha | rs78334475 | 0.003 | . | . | . | . | . | . | . | . | . |
| InternalIMT | dha | rs3780900 | 0.011 | -0.091 | 0.032 | 4.30E-03 | 0.186 | 0.418 | 0.656 | 0.019 | 0.329 | 0.954 |
| InternalIMT | dha | rs186729292 | 0.001 | . | . | . | . | . | . | . | . | . |
| InternalIMT | dha | rs12267438 | 0.001 | . | . | . | . | . | . | . | . | . |
| InternalIMT | dha | rs1369214 | 0.454 | -0.143 | 0.052 | 5.80E-03 | -0.044 | 0.056 | 0.433 | 0.057 | 0.045 | 0.201 |
| InternalIMT | dha | rs9282589 | 0.011 | -0.102 | 0.032 | 1.40E-03 | -0.685 | 0.288 | 0.017 | 0.577 | 0.287 | 0.044 |
| InternalIMT | dha | rs114522915 | 0 | . | . | . | . | . | . | . | . | . |
| InternalIMT | dha | rs2115819 | 0.452 | -0.143 | 0.052 | 5.80E-03 | -0.047 | 0.057 | 0.403 | 0.057 | 0.045 | 0.2 |
| InternalIMT | dha | rs117664603 | 0.001 | . | . | . | . | . | . | . | . | . |
| InternalIMT | dha | rs184979202 | 0.001 | . | . | . | . | . | . | . | . | . |
| InternalIMT | dha | rs190714671 | 0.001 | . | . | . | . | . | . | . | . | . |
| InternalIMT | dha | rs141538996 | 0 | . | . | . | . | . | . | . | . | . |
| InternalIMT | dha | rs150881741 | 0.003 | . | . | . | . | . | . | . | . | . |
| InternalIMT | dha | rs139076633 | 0 | . | . | . | . | . | . | . | . | . |
| InternalIMT | dha | rs12243649 | 0.002 | . | . | . | . | . | . | . | . | . |
| InternalIMT | dha | rs140348708 | 0.001 | . | . | . | . | . | . | . | . | . |
| InternalIMT | dha | rs79023256 | 0.001 | . | . | . | . | . | . | . | . | . |
| InternalIMT | dha | rs11239515 | 0.146 | -0.043 | 0.034 | 0.209 | 0.212 | 0.093 | 0.023 | -0.187 | 0.072 | 9.40E-03 |
| InternalIMT | dha | rs55866086 | 0.148 | -0.042 | 0.034 | 0.218 | 0.216 | 0.093 | 0.02 | -0.188 | 0.071 | 8.50E-03 |
| InternalIMT | dha | rs181340150 | 0.005 | . | . | . | . | . | . | . | . | . |
| InternalIMT | dha | rs11239516 | 0.146 | -0.043 | 0.034 | 0.209 | 0.212 | 0.093 | 0.023 | -0.187 | 0.072 | 9.40E-03 |
| InternalIMT | dha | rs11239517 | 0.146 | -0.043 | 0.034 | 0.209 | 0.212 | 0.093 | 0.023 | -0.187 | 0.072 | 9.40E-03 |
| InternalIMT | dha | rs11239519 | 0.135 | -0.054 | 0.034 | 0.118 | 0.191 | 0.098 | 0.052 | -0.164 | 0.077 | 0.034 |
| InternalIMT | dha | rs7909514 | 0.601 | -0.057 | 0.056 | 0.313 | 0.052 | 0.054 | 0.34 | -0.03 | 0.043 | 0.492 |
| InternalIMT | dha | rs7894352 | 0.782 | -0.042 | 0.073 | 0.569 | 0.069 | 0.062 | 0.264 | -0.032 | 0.048 | 0.503 |
| InternalIMT | dha | rs189384146 | 0.004 | . | . | . | . | . | . | . | . | . |
| InternalIMT | dha | rs11239520 | 0.146 | -0.043 | 0.034 | 0.209 | 0.212 | 0.093 | 0.023 | -0.187 | 0.072 | 9.40E-03 |
| InternalIMT | dha | rs12221233 | 0.404 | -0.14 | 0.049 | 3.90E-03 | -0.041 | 0.058 | 0.478 | 0.062 | 0.047 | 0.187 |
| InternalIMT | dha | rs74523293 | 0.001 | . | . | . | . | . | . | . | . | . |
| InternalIMT | dha | rs79376040 | 0.001 | . | . | . | . | . | . | . | . | . |
| InternalIMT | dha | rs4949000 | 0.404 | -0.14 | 0.049 | 3.90E-03 | -0.042 | 0.059 | 0.478 | 0.062 | 0.047 | 0.186 |
| InternalIMT | dha | rs7089406 | 0.018 | -0.095 | 0.033 | 3.60E-03 | -0.201 | 0.16 | 0.21 | 0.131 | 0.122 | 0.28 |
| InternalIMT | dha | rs138211056 | 0 | . | . | . | . | . | . | . | . | . |
| InternalIMT | dha | rs10900213 | 0.548 | -0.058 | 0.053 | 0.27 | 0.06 | 0.056 | 0.276 | -0.031 | 0.044 | 0.485 |
| InternalIMT | dha | rs145152827 | 0 | . | . | . | . | . | . | . | . | . |
| InternalIMT | dha | rs74443325 | 0.001 | . | . | . | . | . | . | . | . | . |
| InternalIMT | dha | rs188299446 | 0.016 | -0.081 | 0.033 | 0.013 | 0.443 | 0.338 | 0.19 | -0.289 | 0.243 | 0.234 |
| InternalIMT | dha | rs192699586 | 0.002 | . | . | . | . | . | . | . | . | . |
| InternalIMT | dha | rs72796464 | 0.145 | -0.043 | 0.034 | 0.208 | 0.213 | 0.094 | 0.023 | -0.188 | 0.072 | 9.40E-03 |
| InternalIMT | dha | rs4949001 | 0.268 | -0.117 | 0.044 | 8.20E-03 | -0.044 | 0.074 | 0.549 | 0.044 | 0.06 | 0.458 |
| InternalIMT | dha | rs147174641 | 0.013 | -0.082 | 0.032 | 0.01 | 0.579 | 0.366 | 0.114 | -0.457 | 0.279 | 0.102 |
| InternalIMT | dha | rs190835238 | 0.001 | . | . | . | . | . | . | . | . | . |
| InternalIMT | dha | rs76698944 | 0.03 | -0.086 | 0.033 | 8.70E-03 | 0.115 | 0.162 | 0.478 | -0.099 | 0.126 | 0.43 |
| InternalIMT | dha | rs56071381 | 0.142 | -0.044 | 0.034 | 0.2 | 0.213 | 0.095 | 0.025 | -0.188 | 0.073 | 0.01 |
| InternalIMT | dha | rs78130427 | 0.031 | -0.092 | 0.033 | 5.20E-03 | -0.053 | 0.137 | 0.701 | 0.015 | 0.11 | 0.893 |
| InternalIMT | dha | rs10900215 | 0.146 | -0.044 | 0.034 | 0.2 | 0.207 | 0.094 | 0.027 | -0.184 | 0.072 | 0.011 |
| InternalIMT | dha | rs41491445 | 0 | . | . | . | . | . | . | . | . | . |
| InternalIMT | dha | rs144995222 | . | . | . | . | . | . | . | . | . | . |
| InternalIMT | dha | rs149097803 | 0.003 | . | . | . | . | . | . | . | . | . |
| InternalIMT | dha | rs139429646 | 0.007 | . | . | . | . | . | . | . | . | . |
| InternalIMT | dha | rs7075620 | 0.576 | -0.062 | 0.055 | 0.262 | 0.054 | 0.056 | 0.338 | -0.027 | 0.044 | 0.547 |
| InternalIMT | dha | rs148299213 | 0 | . | . | . | . | . | . | . | . | . |
| InternalIMT | dha | rs4949002 | 0.31 | -0.14 | 0.045 | 2.00E-03 | -0.061 | 0.069 | 0.376 | 0.079 | 0.056 | 0.154 |
| InternalIMT | dha | rs150496007 | 0.011 | -0.09 | 0.033 | 6.00E-03 | 0.19 | 0.296 | 0.522 | -0.054 | 0.256 | 0.833 |
| InternalIMT | dha | rs147303455 | 0.004 | . | . | . | . | . | . | . | . | . |
| InternalIMT | dha | rs189518433 | 0.005 | . | . | . | . | . | . | . | . | . |
| InternalIMT | dha | rs186281355 | 0.005 | . | . | . | . | . | . | . | . | . |
| InternalIMT | dha | rs115297114 | 0 | . | . | . | . | . | . | . | . | . |
| InternalIMT | dha | rs11239523 | 0.146 | -0.049 | 0.034 | 0.143 | 0.183 | 0.095 | 0.055 | -0.161 | 0.073 | 0.028 |
| InternalIMT | dha | rs143704897 | 0.004 | . | . | . | . | . | . | . | . | . |
| InternalIMT | dha | rs188024096 | 0.004 | . | . | . | . | . | . | . | . | . |
| InternalIMT | dha | rs11239524 | 0.134 | -0.045 | 0.035 | 0.194 | 0.217 | 0.101 | 0.032 | -0.194 | 0.078 | 0.013 |
| InternalIMT | dha | rs191656573 | 0.005 | . | . | . | . | . | . | . | . | . |
| InternalIMT | dha | rs193295875 | 0 | . | . | . | . | . | . | . | . | . |
| InternalIMT | dha | rs190458228 | 0.002 | . | . | . | . | . | . | . | . | . |
| InternalIMT | dha | rs139253993 | 0.002 | . | . | . | . | . | . | . | . | . |
| InternalIMT | dha | rs4948672 | 0.565 | -0.152 | 0.06 | 0.012 | -0.016 | 0.063 | 0.794 | 0.056 | 0.05 | 0.261 |
| InternalIMT | dha | rs180954921 | 0.004 | . | . | . | . | . | . | . | . | . |
| InternalIMT | dha | rs12264801 | 0.431 | -0.036 | 0.048 | 0.458 | 0.081 | 0.066 | 0.221 | -0.068 | 0.051 | 0.189 |
| InternalIMT | dha | rs73283184 | 0 | . | . | . | . | . | . | . | . | . |

| InternalIMT | dha | rs142746187 | 0.002 | . | . | . | . | . | . | . | . | . |
| --- | --- | --- | --- | --- | --- | --- | --- | --- | --- | --- | --- | --- |
| InternalIMT | dha | rs58704739 | 0 | . | . | . | . | . | . | . | . | . |
| InternalIMT | dha | rs61272335 | 0 | . | . | . | . | . | . | . | . | . |
| InternalIMT | dha | rs60959489 | 0 | . | . | . | . | . | . | . | . | . |
| InternalIMT | dha | rs58502629 | 0 | . | . | . | . | . | . | . | . | . |
| InternalIMT | dha | rs10900216 | 0.695 | -0.072 | 0.056 | 0.203 | 0.063 | 0.054 | 0.243 | -0.013 | 0.043 | 0.761 |
| InternalIMT | dha | rs181118141 | 0 | . | . | . | . | . | . | . | . | . |
| InternalIMT | dha | rs137882665 | 0.004 | . | . | . | . | . | . | . | . | . |
| InternalIMT | dha | rs149466736 | 0.004 | . | . | . | . | . | . | . | . | . |
| InternalIMT | dha | rs2099171 | 0.662 | -0.072 | 0.053 | 0.176 | 0.074 | 0.053 | 0.167 | -0.012 | 0.042 | 0.767 |
| InternalIMT | dha | rs141852280 | 0.009 | . | . | . | . | . | . | . | . | . |
| InternalIMT | dha | rs191539257 | 0.002 | . | . | . | . | . | . | . | . | . |
| InternalIMT | dha | rs17157771 | 0.033 | -0.091 | 0.033 | 5.80E-03 | -0.111 | 0.124 | 0.369 | 0.011 | 0.096 | 0.911 |
| InternalIMT | dha | rs111339241 | 0.032 | -0.091 | 0.033 | 6.00E-03 | -0.104 | 0.131 | 0.43 | 7.50E-03 | 0.102 | 0.942 |
| InternalIMT | dha | rs116983255 | 0.013 | -0.088 | 0.032 | 6.40E-03 | 9.50E-03 | 0.198 | 0.962 | -0.1 | 0.146 | 0.492 |
| InternalIMT | dha | rs142324341 | 0.003 | . | . | . | . | . | . | . | . | . |
| InternalIMT | dha | rs187191219 | 0.009 | . | . | . | . | . | . | . | . | . |
| InternalIMT | dha | rs151283973 | . | . | . | . | . | . | . | . | . | . |
| InternalIMT | dha | rs139316218 | 0.005 | . | . | . | . | . | . | . | . | . |
| InternalIMT | dha | rs6593485 | 0.696 | -0.067 | 0.056 | 0.237 | 0.068 | 0.054 | 0.206 | -0.017 | 0.042 | 0.687 |
| InternalIMT | dha | rs6593486 | 0.696 | -0.067 | 0.056 | 0.235 | 0.067 | 0.054 | 0.209 | -0.017 | 0.042 | 0.692 |
| InternalIMT | dha | rs145196618 | 0.002 | . | . | . | . | . | . | . | . | . |
| InternalIMT | dha | rs142598335 | 0.006 | . | . | . | . | . | . | . | . | . |
| InternalIMT | dha | rs892691 | 0.704 | -0.078 | 0.057 | 0.167 | 0.062 | 0.053 | 0.243 | -8.80E-03 | 0.042 | 0.834 |
| InternalIMT | dha | rs7080713 | 0.663 | -0.071 | 0.054 | 0.188 | 0.074 | 0.053 | 0.168 | -0.014 | 0.042 | 0.742 |
| InternalIMT | dha | rs7077173 | 0.057 | -0.09 | 0.033 | 5.90E-03 | -0.027 | 0.098 | 0.783 | -5.50E-03 | 0.078 | 0.943 |
| InternalIMT | dha | rs7080977 | 0.032 | -0.091 | 0.033 | 5.70E-03 | -0.108 | 0.13 | 0.403 | 0.014 | 0.102 | 0.894 |
| InternalIMT | dha | rs3780901 | 0.663 | -0.068 | 0.053 | 0.203 | 0.076 | 0.054 | 0.155 | -0.016 | 0.042 | 0.705 |
| InternalIMT | dha | rs61854091 | 0.073 | -0.117 | 0.034 | 6.70E-04 | -0.13 | 0.124 | 0.295 | 0.168 | 0.101 | 0.095 |
| InternalIMT | dha | rs77550209 | 0.003 | . | . | . | . | . | . | . | . | . |
| InternalIMT | dha | rs73283191 | 0.033 | -0.091 | 0.033 | 6.20E-03 | -0.096 | 0.127 | 0.452 | 5.40E-03 | 0.099 | 0.957 |
| InternalIMT | dha | rs191866099 | 0.01 | -0.095 | 0.032 | 3.20E-03 | -0.208 | 0.457 | 0.649 | 0.181 | 0.402 | 0.652 |
| InternalIMT | dha | rs147998395 | . | . | . | . | . | . | . | . | . | . |
| InternalIMT | dha | rs7090328 | 0.699 | -0.065 | 0.056 | 0.247 | 0.069 | 0.054 | 0.198 | -0.018 | 0.042 | 0.667 |
| InternalIMT | dha | rs79814535 | 0.001 | . | . | . | . | . | . | . | . | . |
| InternalIMT | dha | rs7073082 | 0.699 | -0.065 | 0.056 | 0.248 | 0.069 | 0.054 | 0.198 | -0.018 | 0.042 | 0.666 |
| InternalIMT | dha | rs11239526 | 0.052 | -0.1 | 0.034 | 3.30E-03 | -0.054 | 0.102 | 0.596 | 0.081 | 0.081 | 0.32 |
| InternalIMT | dha | rs141839841 | . | . | . | . | . | . | . | . | . | . |
| InternalIMT | dha | rs75697371 | 0.014 | -0.09 | 0.032 | 5.50E-03 | 0.057 | 0.2 | 0.777 | -0.051 | 0.171 | 0.765 |
| InternalIMT | dha | rs75972854 | 0.016 | -0.099 | 0.032 | 2.00E-03 | -0.38 | 0.266 | 0.153 | 0.323 | 0.243 | 0.185 |
| InternalIMT | dha | rs139291975 | 0.001 | . | . | . | . | . | . | . | . | . |
| InternalIMT | dha | rs144064711 | 0.009 | . | . | . | . | . | . | . | . | . |
| InternalIMT | dha | rs3780902 | 0.033 | -0.09 | 0.033 | 6.70E-03 | -0.089 | 0.125 | 0.475 | -2.60E-03 | 0.096 | 0.978 |
| InternalIMT | dha | rs190449801 | 0 | . | . | . | . | . | . | . | . | . |
| InternalIMT | dha | rs73283196 | 0.033 | -0.09 | 0.033 | 6.60E-03 | -0.09 | 0.126 | 0.477 | -2.80E-03 | 0.098 | 0.977 |
| InternalIMT | dha | rs28395867 | 0.005 | . | . | . | . | . | . | . | . | . |
| InternalIMT | dha | rs28395866 | 0.036 | -0.092 | 0.033 | 5.60E-03 | -0.106 | 0.124 | 0.392 | 0.013 | 0.1 | 0.896 |
| InternalIMT | dha | rs55661611 | 0.032 | -0.09 | 0.033 | 6.20E-03 | -0.101 | 0.129 | 0.434 | 3.00E-03 | 0.1 | 0.976 |
| InternalIMT | dha | rs55684518 | 0.03 | -0.102 | 0.033 | 2.10E-03 | -0.386 | 0.159 | 0.015 | 0.193 | 0.132 | 0.144 |
| InternalIMT | dha | rs78552493 | 0.057 | -0.09 | 0.034 | 7.70E-03 | -0.05 | 0.096 | 0.601 | -4.90E-03 | 0.077 | 0.949 |
| InternalIMT | dha | rs147665434 | 0.007 | . | . | . | . | . | . | . | . | . |
| InternalIMT | dha | rs34445001 | 0.014 | -0.09 | 0.032 | 5.50E-03 | 0.059 | 0.201 | 0.77 | -0.053 | 0.172 | 0.757 |
| InternalIMT | dha | rs184019449 | 0.004 | . | . | . | . | . | . | . | . | . |
| InternalIMT | dha | rs3780903 | 0.719 | -0.062 | 0.06 | 0.303 | 0.072 | 0.055 | 0.193 | -0.02 | 0.043 | 0.642 |
| InternalIMT | dha | rs17157784 | 0.005 | . | . | . | . | . | . | . | . | . |
| InternalIMT | dha | rs3780904 | 0.001 | . | . | . | . | . | . | . | . | . |
| InternalIMT | dha | rs192064730 | 0 | . | . | . | . | . | . | . | . | . |
| InternalIMT | dha | rs12774534 | 0.014 | -0.09 | 0.032 | 5.50E-03 | 0.061 | 0.201 | 0.764 | -0.055 | 0.172 | 0.75 |
| InternalIMT | dha | rs79179915 | 0 | . | . | . | . | . | . | . | . | . |
| InternalIMT | dha | rs117811571 | 0.016 | -0.081 | 0.033 | 0.013 | 0.47 | 0.339 | 0.167 | -0.285 | 0.244 | 0.242 |
| InternalIMT | dha | rs72796477 | 0.015 | -0.098 | 0.032 | 2.50E-03 | -0.212 | 0.199 | 0.288 | 0.243 | 0.163 | 0.136 |
| InternalIMT | dha | rs55729342 | 0.005 | . | . | . | . | . | . | . | . | . |
| InternalIMT | dha | rs59317220 | 0.004 | . | . | . | . | . | . | . | . | . |
| InternalIMT | dha | rs3780905 | 0.042 | -0.085 | 0.034 | 0.012 | -3.00E-03 | 0.101 | 0.976 | -0.055 | 0.077 | 0.476 |
| InternalIMT | dha | rs3780906 | 0.718 | -0.061 | 0.06 | 0.307 | 0.072 | 0.055 | 0.195 | -0.021 | 0.043 | 0.635 |
| InternalIMT | dha | rs149047975 | 0.006 | . | . | . | . | . | . | . | . | . |
| InternalIMT | dha | rs3824614 | 0.001 | . | . | . | . | . | . | . | . | . |
| InternalIMT | dha | rs140254404 | 0.003 | . | . | . | . | . | . | . | . | . |
| InternalIMT | dha | rs145262684 | 0.02 | -0.077 | 0.033 | 0.019 | 0.537 | 0.302 | 0.076 | -0.313 | 0.227 | 0.167 |
| InternalIMT | dha | rs76735482 | 0.001 | . | . | . | . | . | . | . | . | . |
| InternalIMT | dha | rs73283198 | 0.032 | -0.09 | 0.033 | 6.30E-03 | -0.097 | 0.128 | 0.45 | -3.20E-04 | 0.099 | 0.997 |
| InternalIMT | dha | rs74911239 | 0.052 | -0.105 | 0.035 | 3.10E-03 | -0.194 | 0.118 | 0.099 | 0.121 | 0.096 | 0.209 |
| InternalIMT | dha | rs150551647 | 0.008 | . | . | . | . | . | . | . | . | . |
| InternalIMT | dha | rs115200794 | 0.004 | . | . | . | . | . | . | . | . | . |
| InternalIMT | dha | rs74130823 | 0.177 | -0.047 | 0.036 | 0.197 | 0.136 | 0.087 | 0.117 | -0.138 | 0.067 | 0.041 |
| InternalIMT | dha | rs2279435 | 0.563 | -0.175 | 0.057 | 2.30E-03 | -0.055 | 0.058 | 0.346 | 0.076 | 0.045 | 0.093 |
| InternalIMT | dha | rs147279673 | 0.02 | -0.083 | 0.033 | 0.013 | 0.288 | 0.344 | 0.402 | -0.228 | 0.279 | 0.414 |
| InternalIMT | dha | rs183525146 | 0.005 | . | . | . | . | . | . | . | . | . |
| InternalIMT | dha | rs3740107 | 0.727 | -0.078 | 0.063 | 0.215 | 0.034 | 0.056 | 0.547 | -9.60E-03 | 0.044 | 0.828 |
| InternalIMT | dha | rs140697247 | 0.002 | . | . | . | . | . | . | . | . | . |
| InternalIMT | dha | rs1565096 | 0.744 | -0.062 | 0.063 | 0.321 | 0.05 | 0.056 | 0.366 | -0.02 | 0.043 | 0.647 |
| InternalIMT | dha | rs1565097 | 0.741 | -0.058 | 0.063 | 0.36 | 0.055 | 0.056 | 0.321 | -0.023 | 0.044 | 0.597 |
| InternalIMT | dha | rs144584548 | 0.03 | -0.101 | 0.033 | 2.20E-03 | -0.365 | 0.159 | 0.022 | 0.185 | 0.132 | 0.162 |
| InternalIMT | dha | rs75811053 | 0.001 | . | . | . | . | . | . | . | . | . |
| InternalIMT | dha | rs147462967 | 0.01 | -0.088 | 0.032 | 6.40E-03 | 0.264 | 0.303 | 0.385 | -0.158 | 0.232 | 0.496 |
| InternalIMT | dha | rs74130824 | 0.004 | . | . | . | . | . | . | . | . | . |

| InternalIMT | dha | rs116883184 | 0.025 | -0.078 | 0.033 | 0.018 | 0.383 | 0.266 | 0.15 | -0.228 | 0.205 | 0.266 |
| --- | --- | --- | --- | --- | --- | --- | --- | --- | --- | --- | --- | --- |
| InternalIMT | dha | rs117258049 | 0.025 | -0.078 | 0.033 | 0.018 | 0.383 | 0.266 | 0.15 | -0.228 | 0.205 | 0.266 |
| InternalIMT | dha | rs3780908 | 0.171 | -0.045 | 0.036 | 0.207 | 0.15 | 0.087 | 0.088 | -0.147 | 0.067 | 0.028 |
| InternalIMT | dha | rs180938522 | 0.004 | . | . | . | . | . | . | . | . | . |
| InternalIMT | dha | rs76687372 | 0 | . | . | . | . | . | . | . | . | . |
| InternalIMT | dha | rs3780909 | 0.504 | -0.131 | 0.05 | 9.40E-03 | -0.017 | 0.055 | 0.757 | 0.041 | 0.044 | 0.356 |
| InternalIMT | dha | rs78281156 | 0.001 | . | . | . | . | . | . | . | . | . |
| InternalIMT | dha | rs187840631 | 0.002 | . | . | . | . | . | . | . | . | . |
| InternalIMT | dha | rs76868242 | 0.001 | . | . | . | . | . | . | . | . | . |
| InternalIMT | dha | rs192794192 | 0.001 | . | . | . | . | . | . | . | . | . |
| InternalIMT | dha | rs12781248 | 0.015 | -0.09 | 0.032 | 5.50E-03 | 0.06 | 0.186 | 0.749 | -0.052 | 0.164 | 0.749 |
| InternalIMT | dha | rs12762375 | 0.014 | -0.09 | 0.032 | 5.40E-03 | 0.057 | 0.2 | 0.777 | -0.049 | 0.171 | 0.776 |
| InternalIMT | dha | rs192256986 | 0.006 | . | . | . | . | . | . | . | . | . |
| InternalIMT | dha | rs184411030 | 0 | . | . | . | . | . | . | . | . | . |
| InternalIMT | dha | rs189601483 | 0.001 | . | . | . | . | . | . | . | . | . |
| InternalIMT | dha | rs143254579 | 0.05 | -0.102 | 0.034 | 2.60E-03 | -0.093 | 0.102 | 0.366 | 0.109 | 0.084 | 0.194 |
| InternalIMT | dha | rs12253903 | 0 | . | . | . | . | . | . | . | . | . |
| InternalIMT | dha | rs35489926 | 0.014 | -0.09 | 0.032 | 5.40E-03 | 0.06 | 0.2 | 0.764 | -0.052 | 0.171 | 0.762 |
| InternalIMT | dha | rs71494783 | 0.015 | -0.09 | 0.032 | 5.80E-03 | 0.079 | 0.191 | 0.678 | -0.06 | 0.161 | 0.708 |
| InternalIMT | dha | rs180711602 | 0.004 | . | . | . | . | . | . | . | . | . |
| InternalIMT | dha | rs117312402 | 0.001 | . | . | . | . | . | . | . | . | . |
| InternalIMT | dha | rs74831694 | 0.001 | . | . | . | . | . | . | . | . | . |
| InternalIMT | dha | rs140301943 | 0.004 | . | . | . | . | . | . | . | . | . |
| InternalIMT | dha | rs78553679 | 0.023 | -0.092 | 0.033 | 5.10E-03 | -8.50E-05 | 0.242 | 1 | 0.011 | 0.189 | 0.954 |
| InternalIMT | dha | rs78334250 | 0.001 | . | . | . | . | . | . | . | . | . |
| InternalIMT | dha | rs76000028 | 0.001 | . | . | . | . | . | . | . | . | . |
| InternalIMT | dha | rs145195254 | 0 | . | . | . | . | . | . | . | . | . |
| InternalIMT | dha | rs137888527 | 0 | . | . | . | . | . | . | . | . | . |
| InternalIMT | dha | rs149109592 | 0.005 | . | . | . | . | . | . | . | . | . |
| InternalIMT | dha | rs80280010 | 0.049 | -0.102 | 0.034 | 2.60E-03 | -0.092 | 0.102 | 0.37 | 0.109 | 0.084 | 0.195 |
| InternalIMT | dha | rs189995374 | 0.001 | . | . | . | . | . | . | . | . | . |
| InternalIMT | dha | rs75467770 | 0.001 | . | . | . | . | . | . | . | . | . |
| InternalIMT | dha | rs1487562 | 0.178 | -0.045 | 0.036 | 0.212 | 0.145 | 0.085 | 0.088 | -0.141 | 0.066 | 0.031 |
| InternalIMT | dha | rs12266963 | 0.015 | -0.089 | 0.032 | 5.80E-03 | 0.08 | 0.191 | 0.674 | -0.061 | 0.161 | 0.706 |
| InternalIMT | dha | rs7099874 | 0.692 | -0.04 | 0.057 | 0.48 | 0.064 | 0.054 | 0.234 | -0.038 | 0.042 | 0.371 |
| InternalIMT | dha | rs75687019 | 0.001 | . | . | . | . | . | . | . | . | . |
| InternalIMT | dha | rs148240462 | 0.056 | -0.109 | 0.036 | 2.20E-03 | -0.218 | 0.121 | 0.071 | 0.156 | 0.101 | 0.122 |
| InternalIMT | dha | rs141278174 | 0.003 | . | . | . | . | . | . | . | . | . |
| InternalIMT | dha | rs76404852 | 0.001 | . | . | . | . | . | . | . | . | . |
| InternalIMT | dha | rs139112240 | 0.012 | -0.095 | 0.033 | 3.40E-03 | -0.09 | 0.374 | 0.81 | 0.153 | 0.305 | 0.615 |
| InternalIMT | dha | rs12783037 | 0.015 | -0.089 | 0.032 | 5.80E-03 | 0.081 | 0.191 | 0.673 | -0.061 | 0.161 | 0.704 |
| InternalIMT | dha | rs72796481 | 0.013 | -0.101 | 0.032 | 1.80E-03 | -0.307 | 0.312 | 0.325 | 0.355 | 0.228 | 0.119 |
| InternalIMT | dha | rs76798749 | 0.001 | . | . | . | . | . | . | . | . | . |
| InternalIMT | dha | rs187732548 | 0.001 | . | . | . | . | . | . | . | . | . |
| InternalIMT | dha | rs75675474 | 0.001 | . | . | . | . | . | . | . | . | . |
| InternalIMT | dha | rs76552293 | 0.001 | . | . | . | . | . | . | . | . | . |
| InternalIMT | dha | rs147445993 | 0.005 | . | . | . | . | . | . | . | . | . |
| InternalIMT | dha | rs115902283 | 0.001 | . | . | . | . | . | . | . | . | . |
| InternalIMT | dha | rs4540799 | 0.692 | -0.038 | 0.057 | 0.499 | 0.065 | 0.054 | 0.228 | -0.039 | 0.042 | 0.354 |
| InternalIMT | dha | rs12765320 | 0.179 | -0.042 | 0.036 | 0.251 | 0.153 | 0.083 | 0.065 | -0.148 | 0.065 | 0.022 |
| InternalIMT | dha | rs12782256 | 0.014 | -0.09 | 0.032 | 5.50E-03 | 0.063 | 0.2 | 0.754 | -0.054 | 0.172 | 0.752 |
| InternalIMT | dha | rs191727256 | 0 | . | . | . | . | . | . | . | . | . |
| InternalIMT | dha | rs2082111 | 0.165 | -0.052 | 0.035 | 0.143 | 0.136 | 0.087 | 0.121 | -0.131 | 0.067 | 0.052 |
| InternalIMT | dha | rs2082110 | 0.166 | -0.051 | 0.035 | 0.146 | 0.136 | 0.087 | 0.117 | -0.131 | 0.067 | 0.051 |
| InternalIMT | dha | rs2082109 | 0.166 | -0.051 | 0.035 | 0.146 | 0.136 | 0.087 | 0.117 | -0.131 | 0.067 | 0.051 |
| InternalIMT | dha | rs117316662 | 0.001 | . | . | . | . | . | . | . | . | . |
| InternalIMT | dha | rs12247163 | 0.181 | -0.049 | 0.036 | 0.174 | 0.133 | 0.081 | 0.102 | -0.126 | 0.063 | 0.044 |
| InternalIMT | dha | rs12355888 | 0.139 | -0.05 | 0.034 | 0.147 | 0.193 | 0.097 | 0.047 | -0.167 | 0.075 | 0.026 |
| InternalIMT | dha | rs143772290 | 0 | . | . | . | . | . | . | . | . | . |
| InternalIMT | dha | rs146849452 | 0.005 | . | . | . | . | . | . | . | . | . |
| InternalIMT | dha | rs140267121 | 0.006 | . | . | . | . | . | . | . | . | . |
| InternalIMT | dha | rs7080474 | 0.18 | -0.049 | 0.036 | 0.169 | 0.132 | 0.081 | 0.104 | -0.126 | 0.063 | 0.046 |
| InternalIMT | dha | rs144066203 | 0 | . | . | . | . | . | . | . | . | . |
| InternalIMT | dha | rs12765209 | 0.001 | . | . | . | . | . | . | . | . | . |
| InternalIMT | dha | rs150930735 | 0 | . | . | . | . | . | . | . | . | . |
| InternalIMT | dha | rs7068039 | 0.181 | -0.049 | 0.036 | 0.176 | 0.134 | 0.081 | 0.1 | -0.127 | 0.063 | 0.043 |
| InternalIMT | dha | rs117622652 | 0.001 | . | . | . | . | . | . | . | . | . |
| InternalIMT | dha | rs190191514 | 0.002 | . | . | . | . | . | . | . | . | . |
| InternalIMT | dha | rs74130835 | 0 | . | . | . | . | . | . | . | . | . |
| InternalIMT | dha | rs1811604 | 0.001 | . | . | . | . | . | . | . | . | . |
| InternalIMT | dha | rs146971256 | 0 | . | . | . | . | . | . | . | . | . |
| InternalIMT | dha | rs74336000 | 0 | . | . | . | . | . | . | . | . | . |
| InternalIMT | dha | rs186046492 | 0.009 | . | . | . | . | . | . | . | . | . |
| InternalIMT | dha | rs34971823 | 0.014 | -0.09 | 0.032 | 5.50E-03 | 0.064 | 0.2 | 0.75 | -0.055 | 0.172 | 0.751 |
| InternalIMT | dha | rs75227446 | 0.001 | . | . | . | . | . | . | . | . | . |
| InternalIMT | dha | rs190457599 | 0.001 | . | . | . | . | . | . | . | . | . |
| InternalIMT | dha | rs79591917 | 0.001 | . | . | . | . | . | . | . | . | . |
| InternalIMT | dha | rs702365 | 0.51 | -0.131 | 0.052 | 0.011 | -0.023 | 0.056 | 0.688 | 0.04 | 0.044 | 0.373 |
| InternalIMT | dha | rs79643836 | 0.001 | . | . | . | . | . | . | . | . | . |
| InternalIMT | dha | rs702366 | 0.511 | -0.126 | 0.051 | 0.015 | -0.015 | 0.056 | 0.782 | 0.034 | 0.044 | 0.434 |
| InternalIMT | dha | rs1059696 | 0.166 | -0.051 | 0.035 | 0.149 | 0.138 | 0.087 | 0.114 | -0.132 | 0.067 | 0.048 |
| InternalIMT | dha | rs901681 | 0.515 | -0.123 | 0.051 | 0.017 | -0.011 | 0.056 | 0.844 | 0.032 | 0.044 | 0.463 |
| InternalIMT | dha | rs61854092 | 0.165 | -0.051 | 0.035 | 0.149 | 0.139 | 0.087 | 0.111 | -0.133 | 0.067 | 0.046 |
| InternalIMT | dha | rs148080753 | 0.001 | . | . | . | . | . | . | . | . | . |
| InternalIMT | dha | rs61854093 | 0.166 | -0.051 | 0.035 | 0.145 | 0.138 | 0.087 | 0.115 | -0.132 | 0.067 | 0.049 |
| InternalIMT | dha | rs141822774 | 0.001 | . | . | . | . | . | . | . | . | . |

| InternalIMT | dha | rs74130836 | 0 | . | . | . | . | . | . | . | . | . |
| --- | --- | --- | --- | --- | --- | --- | --- | --- | --- | --- | --- | --- |
| InternalIMT | dha | rs150620963 | 0.019 | -0.093 | 0.033 | 4.70E-03 | -0.041 | 0.169 | 0.809 | 0.04 | 0.156 | 0.797 |
| InternalIMT | dha | rs3780913 | 0.678 | -0.041 | 0.056 | 0.472 | 0.063 | 0.054 | 0.247 | -0.039 | 0.043 | 0.365 |
| InternalIMT | dha | rs142255723 | 0.002 | . | . | . | . | . | . | . | . | . |
| InternalIMT | dha | rs3780914 | 0.511 | -0.127 | 0.051 | 0.014 | -0.016 | 0.056 | 0.768 | 0.036 | 0.044 | 0.42 |
| InternalIMT | dha | rs182656507 | 0.002 | . | . | . | . | . | . | . | . | . |
| InternalIMT | dha | rs2291427 | 0.677 | -0.039 | 0.056 | 0.483 | 0.065 | 0.054 | 0.235 | -0.04 | 0.043 | 0.356 |
| InternalIMT | dha | rs10751382 | 0.677 | -0.04 | 0.056 | 0.473 | 0.063 | 0.054 | 0.249 | -0.039 | 0.043 | 0.364 |
| InternalIMT | dha | rs117610108 | 0.001 | . | . | . | . | . | . | . | . | . |
| InternalIMT | dha | rs144966941 | 0.031 | -0.092 | 0.033 | 5.70E-03 | 0.034 | 0.161 | 0.832 | 4.80E-03 | 0.135 | 0.972 |
| InternalIMT | dha | rs41502051 | 0.052 | -0.084 | 0.035 | 0.016 | 0.084 | 0.112 | 0.452 | -0.069 | 0.085 | 0.414 |
| InternalIMT | dha | rs41426046 | 0.001 | . | . | . | . | . | . | . | . | . |
| InternalIMT | dha | rs41519248 | 0.001 | . | . | . | . | . | . | . | . | . |
| InternalIMT | dha | rs189108245 | 0.008 | . | . | . | . | . | . | . | . | . |
| InternalIMT | dha | rs7896431 | 0.476 | -0.11 | 0.051 | 0.03 | -1.90E-03 | 0.058 | 0.973 | 0.02 | 0.046 | 0.663 |
| InternalIMT | dha | rs183724547 | 0.001 | . | . | . | . | . | . | . | . | . |
| InternalIMT | dha | rs142044990 | 0.012 | -0.096 | 0.033 | 3.10E-03 | -0.162 | 0.368 | 0.66 | 0.196 | 0.302 | 0.517 |
| InternalIMT | dha | rs188140929 | 0.002 | . | . | . | . | . | . | . | . | . |
| InternalIMT | dha | rs76819096 | 0 | . | . | . | . | . | . | . | . | . |
| InternalIMT | dha | rs10751383 | 0.56 | -0.152 | 0.056 | 7.00E-03 | -0.033 | 0.056 | 0.555 | 0.056 | 0.044 | 0.206 |
| InternalIMT | dha | rs117819321 | 0.019 | -0.077 | 0.032 | 0.017 | 0.545 | 0.408 | 0.182 | -0.289 | 0.305 | 0.345 |
| InternalIMT | dha | rs2242332 | 0.675 | -0.034 | 0.057 | 0.553 | 0.068 | 0.055 | 0.212 | -0.044 | 0.043 | 0.305 |
| InternalIMT | dha | rs2242333 | 0.046 | -0.085 | 0.034 | 0.012 | -0.022 | 0.098 | 0.825 | -0.047 | 0.074 | 0.529 |
| InternalIMT | dha | rs2242334 | 0.677 | -0.031 | 0.057 | 0.582 | 0.07 | 0.054 | 0.197 | -0.046 | 0.043 | 0.286 |
| InternalIMT | dha | rs189465041 | 0.001 | . | . | . | . | . | . | . | . | . |
| InternalIMT | dha | rs28395868 | 0.041 | -0.09 | 0.033 | 6.30E-03 | 0.048 | 0.149 | 0.748 | -0.025 | 0.127 | 0.84 |
| InternalIMT | dha | rs56067550 | 0.03 | -0.102 | 0.033 | 2.10E-03 | -0.368 | 0.16 | 0.021 | 0.189 | 0.133 | 0.154 |
| InternalIMT | dha | rs1051713 | 0.165 | -0.046 | 0.035 | 0.197 | 0.157 | 0.087 | 0.071 | -0.151 | 0.067 | 0.024 |
| InternalIMT | dha | rs115074210 | 0 | . | . | . | . | . | . | . | . | . |
| InternalIMT | dha | rs7900977 | 0.74 | -0.047 | 0.063 | 0.453 | 0.063 | 0.056 | 0.262 | -0.03 | 0.043 | 0.488 |
| InternalIMT | dha | rs147613891 | 0.004 | . | . | . | . | . | . | . | . | . |
| InternalIMT | dha | rs2229136 | 0.063 | -0.1 | 0.034 | 3.40E-03 | -0.055 | 0.093 | 0.557 | 0.072 | 0.076 | 0.347 |
| InternalIMT | dha | rs185482841 | 0 | . | . | . | . | . | . | . | . | . |
| InternalIMT | dha | rs2288619 | 0.063 | -0.1 | 0.034 | 3.40E-03 | -0.055 | 0.093 | 0.557 | 0.072 | 0.076 | 0.348 |
| InternalIMT | dha | rs189891524 | 0.001 | . | . | . | . | . | . | . | . | . |
| InternalIMT | dha | rs56145077 | 0.164 | -0.047 | 0.035 | 0.184 | 0.153 | 0.088 | 0.08 | -0.148 | 0.067 | 0.028 |
| InternalIMT | dha | rs143216970 | 0 | . | . | . | . | . | . | . | . | . |
| InternalIMT | dha | rs142246478 | 0.002 | . | . | . | . | . | . | . | . | . |
| InternalIMT | dha | rs13134 | 0.014 | -0.09 | 0.032 | 5.50E-03 | 0.071 | 0.201 | 0.726 | -0.058 | 0.172 | 0.735 |
| InternalIMT | dha | rs41314980 | 0.034 | -0.113 | 0.033 | 6.80E-04 | -0.355 | 0.122 | 3.60E-03 | 0.326 | 0.108 | 2.60E-03 |
| InternalIMT | dha | rs41304611 | 0.02 | -0.096 | 0.033 | 3.60E-03 | -0.231 | 0.185 | 0.21 | 0.108 | 0.153 | 0.482 |
| InternalIMT | dha | rs193299617 | 0 | . | . | . | . | . | . | . | . | . |
| InternalIMT | dha | rs12772724 | 0.014 | -0.09 | 0.032 | 5.50E-03 | 0.071 | 0.201 | 0.725 | -0.058 | 0.172 | 0.735 |
| InternalIMT | dha | rs139489537 | 0.048 | -0.104 | 0.034 | 2.00E-03 | -0.121 | 0.103 | 0.238 | 0.139 | 0.084 | 0.099 |
| InternalIMT | dha | rs12776062 | 0.014 | -0.09 | 0.032 | 5.50E-03 | 0.07 | 0.201 | 0.726 | -0.058 | 0.172 | 0.737 |
| InternalIMT | dha | rs752059 | 0.164 | -0.046 | 0.035 | 0.188 | 0.154 | 0.088 | 0.079 | -0.149 | 0.067 | 0.027 |
| InternalIMT | dha | rs147649982 | 0.035 | -0.102 | 0.033 | 2.10E-03 | -0.182 | 0.198 | 0.358 | 0.162 | 0.169 | 0.338 |
| InternalIMT | dha | rs17157798 | 0.062 | -0.101 | 0.034 | 3.10E-03 | -0.06 | 0.093 | 0.516 | 0.079 | 0.076 | 0.301 |
| InternalIMT | dha | rs142298619 | 0.001 | . | . | . | . | . | . | . | . | . |
| InternalIMT | dha | rs4948673 | 0.72 | -0.057 | 0.064 | 0.368 | 0.051 | 0.055 | 0.358 | -0.024 | 0.043 | 0.572 |
| InternalIMT | dha | rs74231484 | 0.041 | -0.08 | 0.034 | 0.018 | 0.048 | 0.098 | 0.628 | -0.101 | 0.074 | 0.171 |
| InternalIMT | dha | rs72796489 | 0.004 | . | . | . | . | . | . | . | . | . |
| InternalIMT | dha | rs113447578 | 0.021 | -0.092 | 0.033 | 4.90E-03 | -0.136 | 0.151 | 0.367 | 0.026 | 0.117 | 0.821 |
| InternalIMT | dha | rs34063374 | 0.014 | -0.089 | 0.032 | 6.00E-03 | 0.112 | 0.204 | 0.584 | -0.085 | 0.173 | 0.624 |
| InternalIMT | dha | rs11239527 | 0.019 | -0.091 | 0.033 | 5.40E-03 | 0.169 | 0.292 | 0.563 | -0.032 | 0.227 | 0.887 |
| InternalIMT | dha | rs34371883 | 0.014 | -0.09 | 0.032 | 5.50E-03 | 0.071 | 0.201 | 0.723 | -0.059 | 0.172 | 0.733 |
| InternalIMT | dha | rs118168828 | 0.012 | -0.091 | 0.032 | 5.20E-03 | 0.034 | 0.354 | 0.924 | -0.044 | 0.279 | 0.875 |
| InternalIMT | dha | rs34349618 | 0.014 | -0.09 | 0.032 | 5.50E-03 | 0.071 | 0.201 | 0.722 | -0.059 | 0.172 | 0.733 |
| InternalIMT | dha | rs80041679 | 0.048 | -0.104 | 0.034 | 2.00E-03 | -0.121 | 0.103 | 0.239 | 0.139 | 0.084 | 0.099 |
| InternalIMT | dha | rs149640607 | 0 | . | . | . | . | . | . | . | . | . |
| InternalIMT | dha | rs4427523 | 0.834 | -0.109 | 0.116 | 0.347 | -0.028 | 0.089 | 0.757 | 0.01 | 0.068 | 0.882 |
| InternalIMT | dha | rs185880109 | 0.002 | . | . | . | . | . | . | . | . | . |
| InternalIMT | dha | rs191523220 | 0.004 | . | . | . | . | . | . | . | . | . |
| InternalIMT | dha | rs144315284 | 0 | . | . | . | . | . | . | . | . | . |
| InternalIMT | dha | rs4948674 | 0.404 | -0.12 | 0.042 | 4.90E-03 | -2.20E-03 | 0.056 | 0.968 | 0.039 | 0.045 | 0.378 |
| InternalIMT | dha | rs182984298 | 0.01 | -0.089 | 0.032 | 6.20E-03 | 0.065 | 0.332 | 0.845 | -0.118 | 0.23 | 0.608 |
| InternalIMT | dha | rs56749023 | 0 | . | . | . | . | . | . | . | . | . |
| InternalIMT | dha | rs112508957 | 0 | . | . | . | . | . | . | . | . | . |
| InternalIMT | dha | rs7089063 | 0.224 | -0.051 | 0.038 | 0.176 | 0.101 | 0.07 | 0.149 | -0.095 | 0.055 | 0.083 |
| InternalIMT | dha | rs145000779 | 0.001 | . | . | . | . | . | . | . | . | . |
| InternalIMT | dha | rs80201572 | 0.048 | -0.104 | 0.034 | 2.00E-03 | -0.121 | 0.103 | 0.24 | 0.139 | 0.084 | 0.099 |
| InternalIMT | dha | rs140568227 | 0.009 | . | . | . | . | . | . | . | . | . |
| InternalIMT | dha | rs149756228 | 0.014 | -0.096 | 0.032 | 3.00E-03 | -0.126 | 0.318 | 0.692 | 0.131 | 0.257 | 0.611 |
| InternalIMT | dha | rs112380685 | 0.009 | . | . | . | . | . | . | . | . | . |
| InternalIMT | dha | rs60481109 | 0 | . | . | . | . | . | . | . | . | . |
| InternalIMT | dha | rs138221843 | 0.004 | . | . | . | . | . | . | . | . | . |
| InternalIMT | dha | rs150283194 | 0 | . | . | . | . | . | . | . | . | . |
| InternalIMT | dha | rs138879661 | 0.003 | . | . | . | . | . | . | . | . | . |
| InternalIMT | dha | rs34960345 | 0.062 | -0.101 | 0.034 | 3.10E-03 | -0.059 | 0.093 | 0.528 | 0.08 | 0.076 | 0.296 |
| InternalIMT | dha | rs147514068 | 0.004 | . | . | . | . | . | . | . | . | . |
| InternalIMT | dha | rs35410535 | 0.063 | -0.101 | 0.034 | 3.10E-03 | -0.06 | 0.093 | 0.518 | 0.079 | 0.076 | 0.3 |
| InternalIMT | dha | rs191613635 | 0.005 | . | . | . | . | . | . | . | . | . |
| InternalIMT | dha | rs17157800 | 0.009 | . | . | . | . | . | . | . | . | . |
| InternalIMT | dha | rs17523413 | 0.062 | -0.101 | 0.034 | 3.10E-03 | -0.059 | 0.093 | 0.524 | 0.08 | 0.076 | 0.293 |
| InternalIMT | dha | rs7897036 | 0.058 | -0.051 | 0.033 | 0.119 | 0.382 | 0.19 | 0.045 | -0.338 | 0.132 | 0.011 |
| InternalIMT | dha | rs76334323 | 0.001 | . | . | . | . | . | . | . | . | . |

| InternalIMT | dha | rs143535991 | 0.001 | . | . | . | . | . | . | . | . | . |
| --- | --- | --- | --- | --- | --- | --- | --- | --- | --- | --- | --- | --- |
| InternalIMT | dha | rs140344979 | 0.007 | . | . | . | . | . | . | . | . | . |
| InternalIMT | dha | rs36047811 | 0.063 | -0.101 | 0.034 | 3.10E-03 | -0.06 | 0.093 | 0.517 | 0.079 | 0.076 | 0.299 |
| InternalIMT | dha | rs76493570 | 0.062 | -0.101 | 0.034 | 3.10E-03 | -0.059 | 0.093 | 0.525 | 0.08 | 0.076 | 0.293 |
| InternalIMT | dha | rs192998920 | 0.003 | . | . | . | . | . | . | . | . | . |
| InternalIMT | dha | rs116840852 | 0.023 | -0.092 | 0.033 | 5.20E-03 | -4.70E-03 | 0.241 | 0.984 | 9.40E-03 | 0.188 | 0.96 |
| InternalIMT | dha | rs184824101 | 0.001 | . | . | . | . | . | . | . | . | . |
| InternalIMT | dha | rs41301621 | 0.008 | . | . | . | . | . | . | . | . | . |
| InternalIMT | dha | rs3740109 | 0.063 | -0.101 | 0.034 | 3.10E-03 | -0.061 | 0.093 | 0.515 | 0.079 | 0.076 | 0.297 |
| InternalIMT | dha | rs7899415 | 0.02 | -0.088 | 0.032 | 6.20E-03 | 0.278 | 0.264 | 0.293 | -0.032 | 0.218 | 0.882 |
| InternalIMT | dha | rs184395284 | 0.001 | . | . | . | . | . | . | . | . | . |
| InternalIMT | dha | rs139209589 | 0.006 | . | . | . | . | . | . | . | . | . |
| InternalIMT | dha | rs149924163 | 0 | . | . | . | . | . | . | . | . | . |
| InternalIMT | dha | rs3740110 | 0.001 | . | . | . | . | . | . | . | . | . |
| InternalIMT | dha | rs144335113 | 0 | . | . | . | . | . | . | . | . | . |
| InternalIMT | dha | rs74743385 | 0 | . | . | . | . | . | . | . | . | . |
| InternalIMT | dha | rs146545829 | 0 | . | . | . | . | . | . | . | . | . |
| InternalIMT | dha | rs181842689 | 0 | . | . | . | . | . | . | . | . | . |
| InternalIMT | dha | rs3740111 | 0.062 | -0.101 | 0.034 | 3.10E-03 | -0.06 | 0.093 | 0.523 | 0.081 | 0.076 | 0.292 |
| InternalIMT | dha | rs186261599 | 0 | . | . | . | . | . | . | . | . | . |
| InternalIMT | dha | rs73285348 | 0.012 | -0.091 | 0.032 | 4.80E-03 | -0.101 | 0.2 | 0.614 | -0.031 | 0.163 | 0.849 |
| InternalIMT | dha | rs186356901 | 0 | . | . | . | . | . | . | . | . | . |
| InternalIMT | dha | rs3824618 | 0.048 | -0.104 | 0.034 | 2.00E-03 | -0.121 | 0.103 | 0.239 | 0.139 | 0.084 | 0.099 |
| InternalIMT | dha | rs3802548 | 0.231 | -0.056 | 0.037 | 0.138 | 0.072 | 0.069 | 0.293 | -0.082 | 0.054 | 0.13 |
| InternalIMT | dha | rs3802547 | 0.001 | . | . | . | . | . | . | . | . | . |
| InternalIMT | dha | rs77677521 | 0.023 | -0.092 | 0.033 | 5.20E-03 | -4.70E-03 | 0.241 | 0.984 | 9.40E-03 | 0.188 | 0.96 |
| InternalIMT | dha | rs3824616 | 0.063 | -0.101 | 0.034 | 3.10E-03 | -0.061 | 0.093 | 0.511 | 0.08 | 0.076 | 0.294 |
| InternalIMT | dha | rs191073818 | 0 | . | . | . | . | . | . | . | . | . |
| InternalIMT | dha | rs147078004 | 0.002 | . | . | . | . | . | . | . | . | . |
| InternalIMT | dha | rs7908745 | 0.297 | -0.055 | 0.04 | 0.168 | 0.066 | 0.06 | 0.268 | -0.065 | 0.047 | 0.165 |
| InternalIMT | dha | rs17157811 | 0.009 | . | . | . | . | . | . | . | . | . |
| InternalIMT | dha | rs138506087 | 0.009 | . | . | . | . | . | . | . | . | . |
| InternalIMT | dha | rs77241269 | 0 | . | . | . | . | . | . | . | . | . |
| InternalIMT | dha | rs138374938 | 0.002 | . | . | . | . | . | . | . | . | . |
| InternalIMT | dha | rs2279433 | 0.062 | -0.101 | 0.034 | 3.20E-03 | -0.054 | 0.094 | 0.563 | 0.077 | 0.077 | 0.317 |
| InternalIMT | dha | rs73285358 | 0 | . | . | . | . | . | . | . | . | . |
| InternalIMT | dha | rs11239528 | 0.231 | -0.056 | 0.038 | 0.133 | 0.069 | 0.069 | 0.314 | -0.079 | 0.054 | 0.142 |
| InternalIMT | dha | rs77453386 | 0.001 | . | . | . | . | . | . | . | . | . |
| InternalIMT | dha | rs2279434 | 0.069 | -0.098 | 0.035 | 4.70E-03 | -0.027 | 0.092 | 0.769 | 0.045 | 0.075 | 0.55 |
| InternalIMT | dha | rs149236525 | 0.006 | . | . | . | . | . | . | . | . | . |
| InternalIMT | dha | rs12573609 | 0.052 | -0.092 | 0.035 | 7.80E-03 | -0.01 | 0.126 | 0.935 | 7.70E-03 | 0.1 | 0.939 |
| InternalIMT | dha | rs191716655 | 0 | . | . | . | . | . | . | . | . | . |
| InternalIMT | dha | rs17523660 | 0.014 | -0.09 | 0.032 | 5.40E-03 | 0.066 | 0.202 | 0.745 | -0.053 | 0.173 | 0.758 |
| InternalIMT | dha | rs142671360 | 0.004 | . | . | . | . | . | . | . | . | . |
| InternalIMT | dha | rs3764990 | 0.062 | -0.101 | 0.034 | 3.20E-03 | -0.054 | 0.094 | 0.57 | 0.077 | 0.077 | 0.319 |
| InternalIMT | dha | rs113682861 | 0.004 | . | . | . | . | . | . | . | . | . |
| InternalIMT | dha | rs117658270 | 0.001 | . | . | . | . | . | . | . | . | . |
| InternalIMT | dha | rs187309063 | 0 | . | . | . | . | . | . | . | . | . |
| InternalIMT | dha | rs73285360 | 0 | . | . | . | . | . | . | . | . | . |
| InternalIMT | dha | rs80133289 | 0.001 | . | . | . | . | . | . | . | . | . |
| InternalIMT | dha | rs186693257 | 0.001 | . | . | . | . | . | . | . | . | . |
| InternalIMT | dha | rs35735968 | 0.063 | -0.1 | 0.034 | 3.30E-03 | -0.051 | 0.094 | 0.586 | 0.076 | 0.077 | 0.329 |
| InternalIMT | dha | rs17157825 | 0.048 | -0.104 | 0.034 | 2.00E-03 | -0.115 | 0.104 | 0.269 | 0.138 | 0.086 | 0.107 |
| InternalIMT | dha | rs2291428 | 0.229 | -0.057 | 0.038 | 0.129 | 0.068 | 0.07 | 0.336 | -0.079 | 0.055 | 0.154 |
| InternalIMT | dha | rs2291429 | 0.231 | -0.057 | 0.038 | 0.132 | 0.068 | 0.069 | 0.324 | -0.079 | 0.054 | 0.147 |
| InternalIMT | dha | rs145714792 | 0.007 | . | . | . | . | . | . | . | . | . |
| InternalIMT | dha | rs12769244 | 0.062 | -0.101 | 0.034 | 3.30E-03 | -0.053 | 0.094 | 0.575 | 0.077 | 0.077 | 0.323 |
| InternalIMT | dha | rs12771275 | 0.062 | -0.101 | 0.034 | 3.30E-03 | -0.053 | 0.094 | 0.575 | 0.077 | 0.077 | 0.323 |
| InternalIMT | dha | rs146266174 | 0.002 | . | . | . | . | . | . | . | . | . |
| InternalIMT | dha | rs41301623 | 0.004 | . | . | . | . | . | . | . | . | . |
| InternalIMT | dha | rs2306814 | 0.001 | . | . | . | . | . | . | . | . | . |
| InternalIMT | dha | rs12244834 | 0 | . | . | . | . | . | . | . | . | . |
| InternalIMT | dha | rs184006011 | 0.004 | . | . | . | . | . | . | . | . | . |
| InternalIMT | dha | rs78829066 | 0.001 | . | . | . | . | . | . | . | . | . |
| InternalIMT | dha | rs34731408 | 0.062 | -0.101 | 0.034 | 3.30E-03 | -0.053 | 0.094 | 0.576 | 0.077 | 0.077 | 0.323 |
| InternalIMT | dha | rs17451516 | 0.014 | -0.09 | 0.032 | 5.40E-03 | 0.066 | 0.202 | 0.743 | -0.054 | 0.173 | 0.755 |
| InternalIMT | dha | rs139910133 | . | . | . | . | . | . | . | . | . | . |
| InternalIMT | dha | rs10900218 | 0.229 | -0.057 | 0.038 | 0.134 | 0.09 | 0.069 | 0.192 | -0.08 | 0.054 | 0.134 |
| InternalIMT | dha | rs146023999 | . | . | . | . | . | . | . | . | . | . |
| InternalIMT | dha | rs187296958 | . | . | . | . | . | . | . | . | . | . |
| InternalIMT | dha | rs142342833 | . | . | . | . | . | . | . | . | . | . |
| InternalIMT | dha | rs143489255 | . | . | . | . | . | . | . | . | . | . |
| InternalIMT | dha | rs138532518 | . | . | . | . | . | . | . | . | . | . |
| InternalIMT | dha | rs183768079 | . | . | . | . | . | . | . | . | . | . |
| InternalIMT | dha | rs117892272 | . | . | . | . | . | . | . | . | . | . |
| InternalIMT | dha | rs192993943 | . | . | . | . | . | . | . | . | . | . |
| InternalIMT | dha | rs182989043 | . | . | . | . | . | . | . | . | . | . |
| InternalIMT | dha | rs189703435 | . | . | . | . | . | . | . | . | . | . |
| InternalIMT | dha | rs188794040 | . | . | . | . | . | . | . | . | . | . |
| InternalIMT | dha | rs143920953 | . | . | . | . | . | . | . | . | . | . |
| InternalIMT | dha | rs191029121 | . | . | . | . | . | . | . | . | . | . |
| InternalIMT | dha | rs116875448 | . | . | . | . | . | . | . | . | . | . |
| InternalIMT | dha | rs190167877 | . | . | . | . | . | . | . | . | . | . |
| InternalIMT | dha | rs184993418 | . | . | . | . | . | . | . | . | . | . |
| InternalIMT | dha | rs143241158 | . | . | . | . | . | . | . | . | . | . |
| InternalIMT | dha | rs11239500 | . | . | . | . | . | . | . | . | . | . |

| InternalIMT | dha | rs140568994 | . | . | . | . | . | . | . | . | . | . |
| --- | --- | --- | --- | --- | --- | --- | --- | --- | --- | --- | --- | --- |
| InternalIMT | dha | rs187530317 | . | . | . | . | . | . | . | . | . | . |
| InternalIMT | dha | rs145189326 | . | . | . | . | . | . | . | . | . | . |
| InternalIMT | dha | rs186203332 | . | . | . | . | . | . | . | . | . | . |
| InternalIMT | dha | rs189921909 | . | . | . | . | . | . | . | . | . | . |
| InternalIMT | dha | rs187260989 | . | . | . | . | . | . | . | . | . | . |
| InternalIMT | dha | rs114376586 | . | . | . | . | . | . | . | . | . | . |
| InternalIMT | dha | rs151070808 | . | . | . | . | . | . | . | . | . | . |
| InternalIMT | dha | rs140927225 | . | . | . | . | . | . | . | . | . | . |
| InternalIMT | dha | rs191555615 | . | . | . | . | . | . | . | . | . | . |
| InternalIMT | dha | rs80278898 | . | . | . | . | . | . | . | . | . | . |
| InternalIMT | dha | rs190849503 | . | . | . | . | . | . | . | . | . | . |
| InternalIMT | dha | rs150080315 | . | . | . | . | . | . | . | . | . | . |
| InternalIMT | dha | rs143623885 | . | . | . | . | . | . | . | . | . | . |
| InternalIMT | dha | rs187760040 | . | . | . | . | . | . | . | . | . | . |
| InternalIMT | dha | rs143917143 | . | . | . | . | . | . | . | . | . | . |
| InternalIMT | dha | rs149851136 | . | . | . | . | . | . | . | . | . | . |
| InternalIMT | dha | rs181612054 | . | . | . | . | . | . | . | . | . | . |
| InternalIMT | dha | rs191006548 | . | . | . | . | . | . | . | . | . | . |
| InternalIMT | dha | rs117377631 | . | . | . | . | . | . | . | . | . | . |
| InternalIMT | dha | rs182107634 | . | . | . | . | . | . | . | . | . | . |
| InternalIMT | dha | rs190211867 | . | . | . | . | . | . | . | . | . | . |
| InternalIMT | dha | rs148293457 | . | . | . | . | . | . | . | . | . | . |
| InternalIMT | dha | rs191811733 | . | . | . | . | . | . | . | . | . | . |
| InternalIMT | dha | rs146109272 | . | . | . | . | . | . | . | . | . | . |
| InternalIMT | dha | rs148397915 | . | . | . | . | . | . | . | . | . | . |
| InternalIMT | dha | rs142233290 | . | . | . | . | . | . | . | . | . | . |
| InternalIMT | dha | rs80226874 | . | . | . | . | . | . | . | . | . | . |
| InternalIMT | dha | rs144496203 | . | . | . | . | . | . | . | . | . | . |
| InternalIMT | dha | rs191062130 | . | . | . | . | . | . | . | . | . | . |
| InternalIMT | dha | rs185807817 | . | . | . | . | . | . | . | . | . | . |
| InternalIMT | dha | rs117846213 | . | . | . | . | . | . | . | . | . | . |
| InternalIMT | dha | rs190545473 | . | . | . | . | . | . | . | . | . | . |
| InternalIMT | dha | rs192364918 | . | . | . | . | . | . | . | . | . | . |
| InternalIMT | dha | rs182939241 | . | . | . | . | . | . | . | . | . | . |
| InternalIMT | dha | rs12571125 | . | . | . | . | . | . | . | . | . | . |
| InternalIMT | dha | rs192418941 | . | . | . | . | . | . | . | . | . | . |
| InternalIMT | dha | rs141270243 | . | . | . | . | . | . | . | . | . | . |
| InternalIMT | dha | rs182615502 | . | . | . | . | . | . | . | . | . | . |
| InternalIMT | dha | rs146669856 | . | . | . | . | . | . | . | . | . | . |
| InternalIMT | dha | rs78290029 | . | . | . | . | . | . | . | . | . | . |
| InternalIMT | dha | rs139955565 | . | . | . | . | . | . | . | . | . | . |
| InternalIMT | dha | rs12267314 | . | . | . | . | . | . | . | . | . | . |
| InternalIMT | dha | rs74477197 | . | . | . | . | . | . | . | . | . | . |
| InternalIMT | dha | rs188147236 | . | . | . | . | . | . | . | . | . | . |
| InternalIMT | dha | rs2228065 | . | . | . | . | . | . | . | . | . | . |
| InternalIMT | dha | rs116961353 | . | . | . | . | . | . | . | . | . | . |
| InternalIMT | dha | rs190222272 | . | . | . | . | . | . | . | . | . | . |
| InternalIMT | dha | rs182719940 | . | . | . | . | . | . | . | . | . | . |
| InternalIMT | dha | rs184477413 | . | . | . | . | . | . | . | . | . | . |
| InternalIMT | dha | rs12265175 | . | . | . | . | . | . | . | . | . | . |
| InternalIMT | dha | rs182928276 | . | . | . | . | . | . | . | . | . | . |
| InternalIMT | dha | rs144409895 | . | . | . | . | . | . | . | . | . | . |
| InternalIMT | dha | rs58998447 | . | . | . | . | . | . | . | . | . | . |
| InternalIMT | dha | rs188668832 | . | . | . | . | . | . | . | . | . | . |
| InternalIMT | dha | rs148641686 | . | . | . | . | . | . | . | . | . | . |
| InternalIMT | dha | rs75468791 | . | . | . | . | . | . | . | . | . | . |
| InternalIMT | dha | rs147327397 | . | . | . | . | . | . | . | . | . | . |
| InternalIMT | dha | rs77354597 | . | . | . | . | . | . | . | . | . | . |
| InternalIMT | dha | rs41365544 | . | . | . | . | . | . | . | . | . | . |
| InternalIMT | dha | rs187564188 | . | . | . | . | . | . | . | . | . | . |
| InternalIMT | dha | rs183511984 | . | . | . | . | . | . | . | . | . | . |
| InternalIMT | dha | rs12262783 | . | . | . | . | . | . | . | . | . | . |
| InternalIMT | dha | rs188817687 | . | . | . | . | . | . | . | . | . | . |
| InternalIMT | dha | rs3815968 | . | . | . | . | . | . | . | . | . | . |
| InternalIMT | dha | rs187822300 | . | . | . | . | . | . | . | . | . | . |
| InternalIMT | dha | rs188902172 | . | . | . | . | . | . | . | . | . | . |
| InternalIMT | dha | rs181190448 | . | . | . | . | . | . | . | . | . | . |
| InternalIMT | dha | rs182699093 | . | . | . | . | . | . | . | . | . | . |
| InternalIMT | dha | rs182631303 | . | . | . | . | . | . | . | . | . | . |
| InternalIMT | dha | rs187014172 | . | . | . | . | . | . | . | . | . | . |
| InternalIMT | dha | rs142575406 | . | . | . | . | . | . | . | . | . | . |
| InternalIMT | dha | rs180711033 | . | . | . | . | . | . | . | . | . | . |
| InternalIMT | dha | rs137894823 | . | . | . | . | . | . | . | . | . | . |
| InternalIMT | dha | rs189053273 | . | . | . | . | . | . | . | . | . | . |
| InternalIMT | dha | rs143499388 | . | . | . | . | . | . | . | . | . | . |
| InternalIMT | dha | rs182936001 | . | . | . | . | . | . | . | . | . | . |
| InternalIMT | dha | rs148456406 | . | . | . | . | . | . | . | . | . | . |
| InternalIMT | dha | rs182499531 | . | . | . | . | . | . | . | . | . | . |
| InternalIMT | dha | rs74884915 | . | . | . | . | . | . | . | . | . | . |
| InternalIMT | dha | rs184046374 | . | . | . | . | . | . | . | . | . | . |
| InternalIMT | dha | rs74333993 | . | . | . | . | . | . | . | . | . | . |
| InternalIMT | dha | rs138889719 | . | . | . | . | . | . | . | . | . | . |
| InternalIMT | dha | rs75330330 | . | . | . | . | . | . | . | . | . | . |
| InternalIMT | dha | rs193051693 | . | . | . | . | . | . | . | . | . | . |
| InternalIMT | dha | rs145929633 | . | . | . | . | . | . | . | . | . | . |

| InternalIMT | dha | rs184076904 | . | . | . | . | . | . | . | . | . | . |
| --- | --- | --- | --- | --- | --- | --- | --- | --- | --- | --- | --- | --- |
| InternalIMT | dha | rs141802494 | . | . | . | . | . | . | . | . | . | . |
| InternalIMT | dha | rs149519759 | . | . | . | . | . | . | . | . | . | . |
| InternalIMT | dha | rs145467260 | . | . | . | . | . | . | . | . | . | . |
| InternalIMT | dha | rs79620914 | . | . | . | . | . | . | . | . | . | . |
| InternalIMT | dha | rs140538633 | . | . | . | . | . | . | . | . | . | . |
| InternalIMT | dha | rs140862301 | . | . | . | . | . | . | . | . | . | . |
| InternalIMT | dha | rs114615847 | . | . | . | . | . | . | . | . | . | . |
| InternalIMT | dha | rs141800259 | . | . | . | . | . | . | . | . | . | . |
| InternalIMT | dha | rs149779059 | . | . | . | . | . | . | . | . | . | . |
| InternalIMT | dha | rs116539253 | . | . | . | . | . | . | . | . | . | . |
| InternalIMT | dha | rs184979550 | . | . | . | . | . | . | . | . | . | . |
| InternalIMT | dha | rs190128940 | . | . | . | . | . | . | . | . | . | . |
| InternalIMT | dha | rs146594816 | . | . | . | . | . | . | . | . | . | . |
| InternalIMT | dha | rs141370766 | . | . | . | . | . | . | . | . | . | . |
| InternalIMT | dha | rs144670033 | . | . | . | . | . | . | . | . | . | . |
| InternalIMT | dha | rs187152174 | . | . | . | . | . | . | . | . | . | . |
| InternalIMT | dha | rs184180263 | . | . | . | . | . | . | . | . | . | . |
| InternalIMT | dha | rs140075861 | . | . | . | . | . | . | . | . | . | . |
| InternalIMT | dha | rs186483129 | . | . | . | . | . | . | . | . | . | . |
| InternalIMT | dha | rs186634282 | . | . | . | . | . | . | . | . | . | . |
| InternalIMT | dha | rs9988663 | . | . | . | . | . | . | . | . | . | . |
| InternalIMT | dha | rs138828529 | . | . | . | . | . | . | . | . | . | . |
| InternalIMT | dha | rs188777615 | . | . | . | . | . | . | . | . | . | . |
| InternalIMT | dha | rs192321882 | . | . | . | . | . | . | . | . | . | . |
| InternalIMT | dha | rs150812270 | . | . | . | . | . | . | . | . | . | . |
| InternalIMT | dha | rs185906603 | . | . | . | . | . | . | . | . | . | . |
| InternalIMT | dha | rs191070862 | . | . | . | . | . | . | . | . | . | . |
| InternalIMT | dha | rs182904978 | . | . | . | . | . | . | . | . | . | . |
| InternalIMT | dha | rs192679655 | . | . | . | . | . | . | . | . | . | . |
| InternalIMT | dha | rs189673086 | . | . | . | . | . | . | . | . | . | . |
| InternalIMT | dha | rs181141892 | . | . | . | . | . | . | . | . | . | . |
| InternalIMT | dha | rs186404123 | . | . | . | . | . | . | . | . | . | . |
| InternalIMT | dha | rs192117287 | . | . | . | . | . | . | . | . | . | . |
| InternalIMT | dha | rs182748835 | . | . | . | . | . | . | . | . | . | . |
| InternalIMT | dha | rs141854549 | . | . | . | . | . | . | . | . | . | . |
| InternalIMT | dha | rs140163397 | . | . | . | . | . | . | . | . | . | . |
| InternalIMT | dha | rs146347300 | . | . | . | . | . | . | . | . | . | . |
| InternalIMT | dha | rs139362377 | . | . | . | . | . | . | . | . | . | . |
| InternalIMT | dha | rs185087671 | . | . | . | . | . | . | . | . | . | . |
| InternalIMT | dha | rs190466574 | . | . | . | . | . | . | . | . | . | . |
| InternalIMT | dha | rs147012778 | . | . | . | . | . | . | . | . | . | . |
| InternalIMT | dha | rs138236960 | . | . | . | . | . | . | . | . | . | . |
| InternalIMT | dha | rs116310311 | . | . | . | . | . | . | . | . | . | . |
| InternalIMT | dha | rs142868356 | . | . | . | . | . | . | . | . | . | . |
| InternalIMT | dha | rs144956240 | . | . | . | . | . | . | . | . | . | . |
| InternalIMT | dha | rs188527776 | . | . | . | . | . | . | . | . | . | . |
| InternalIMT | dha | rs183971165 | . | . | . | . | . | . | . | . | . | . |
| InternalIMT | dha | rs76748216 | . | . | . | . | . | . | . | . | . | . |
| InternalIMT | dha | rs148554990 | . | . | . | . | . | . | . | . | . | . |
| InternalIMT | dha | rs114446477 | . | . | . | . | . | . | . | . | . | . |
| InternalIMT | dha | rs185223933 | . | . | . | . | . | . | . | . | . | . |
| InternalIMT | dha | rs76869726 | . | . | . | . | . | . | . | . | . | . |
| InternalIMT | dha | rs151200830 | . | . | . | . | . | . | . | . | . | . |
| InternalIMT | dha | rs17157731 | . | . | . | . | . | . | . | . | . | . |
| InternalIMT | dha | rs181243469 | . | . | . | . | . | . | . | . | . | . |
| InternalIMT | dha | rs186890707 | . | . | . | . | . | . | . | . | . | . |
| InternalIMT | dha | rs184063360 | . | . | . | . | . | . | . | . | . | . |
| InternalIMT | dha | rs188550237 | . | . | . | . | . | . | . | . | . | . |
| InternalIMT | dha | rs140931068 | . | . | . | . | . | . | . | . | . | . |
| InternalIMT | dha | rs74867877 | . | . | . | . | . | . | . | . | . | . |
| InternalIMT | dha | rs11239495 | . | . | . | . | . | . | . | . | . | . |
| InternalIMT | dha | rs149745800 | . | . | . | . | . | . | . | . | . | . |
| InternalIMT | dha | rs145044458 | . | . | . | . | . | . | . | . | . | . |
| InternalIMT | dha | rs188067427 | . | . | . | . | . | . | . | . | . | . |
| InternalIMT | dha | rs146827800 | . | . | . | . | . | . | . | . | . | . |
| InternalIMT | dha | rs140619817 | . | . | . | . | . | . | . | . | . | . |
| InternalIMT | dha | rs892689 | . | . | . | . | . | . | . | . | . | . |
| InternalIMT | dha | rs143642005 | . | . | . | . | . | . | . | . | . | . |
| InternalIMT | dha | rs181929304 | . | . | . | . | . | . | . | . | . | . |
| InternalIMT | dha | rs191086173 | . | . | . | . | . | . | . | . | . | . |
| InternalIMT | dha | rs114613116 | . | . | . | . | . | . | . | . | . | . |
| InternalIMT | dha | rs141076768 | . | . | . | . | . | . | . | . | . | . |
| InternalIMT | dha | rs187316531 | . | . | . | . | . | . | . | . | . | . |
| InternalIMT | dha | rs11239499 | . | . | . | . | . | . | . | . | . | . |
| InternalIMT | dha | rs192726371 | . | . | . | . | . | . | . | . | . | . |
| InternalIMT | dha | rs74128481 | . | . | . | . | . | . | . | . | . | . |
| InternalIMT | dha | rs74128482 | . | . | . | . | . | . | . | . | . | . |
| InternalIMT | dha | rs11239502 | . | . | . | . | . | . | . | . | . | . |
| InternalIMT | dha | rs116330285 | . | . | . | . | . | . | . | . | . | . |
| InternalIMT | dha | rs189926729 | . | . | . | . | . | . | . | . | . | . |
| InternalIMT | dha | rs188975779 | . | . | . | . | . | . | . | . | . | . |
| InternalIMT | dha | rs143846038 | . | . | . | . | . | . | . | . | . | . |
| InternalIMT | dha | rs74128483 | . | . | . | . | . | . | . | . | . | . |
| InternalIMT | dha | rs139292055 | . | . | . | . | . | . | . | . | . | . |
| InternalIMT | dha | rs11239503 | . | . | . | . | . | . | . | . | . | . |

| InternalIMT | dha | rs58838686 | . | . | . | . | . | . | . | . | . | . |
| --- | --- | --- | --- | --- | --- | --- | --- | --- | --- | --- | --- | --- |
| InternalIMT | dha | rs185825373 | . | . | . | . | . | . | . | . | . | . |
| InternalIMT | dha | rs11239504 | . | . | . | . | . | . | . | . | . | . |
| InternalIMT | dha | rs150012354 | . | . | . | . | . | . | . | . | . | . |
| InternalIMT | dha | rs187763096 | . | . | . | . | . | . | . | . | . | . |
| InternalIMT | dha | rs193043982 | . | . | . | . | . | . | . | . | . | . |
| InternalIMT | dha | rs79186884 | . | . | . | . | . | . | . | . | . | . |
| InternalIMT | dha | rs74128484 | . | . | . | . | . | . | . | . | . | . |
| InternalIMT | dha | rs58461537 | . | . | . | . | . | . | . | . | . | . |
| InternalIMT | dha | rs188527474 | . | . | . | . | . | . | . | . | . | . |
| InternalIMT | dha | rs141659976 | . | . | . | . | . | . | . | . | . | . |
| InternalIMT | dha | rs115846407 | . | . | . | . | . | . | . | . | . | . |
| InternalIMT | dha | rs138378816 | . | . | . | . | . | . | . | . | . | . |
| InternalIMT | dha | rs146188136 | . | . | . | . | . | . | . | . | . | . |
| InternalIMT | dha | rs139383871 | . | . | . | . | . | . | . | . | . | . |
| InternalIMT | dha | rs182953869 | . | . | . | . | . | . | . | . | . | . |
| InternalIMT | dha | rs192430299 | . | . | . | . | . | . | . | . | . | . |
| InternalIMT | dha | rs144152844 | . | . | . | . | . | . | . | . | . | . |
| InternalIMT | dha | rs145987786 | . | . | . | . | . | . | . | . | . | . |
| InternalIMT | dha | rs181132926 | . | . | . | . | . | . | . | . | . | . |
| InternalIMT | dha | rs147882253 | . | . | . | . | . | . | . | . | . | . |
| InternalIMT | dha | rs141807621 | . | . | . | . | . | . | . | . | . | . |
| InternalIMT | dha | rs147116052 | . | . | . | . | . | . | . | . | . | . |
| InternalIMT | dha | rs147716618 | . | . | . | . | . | . | . | . | . | . |
| InternalIMT | dha | rs187198855 | . | . | . | . | . | . | . | . | . | . |
| InternalIMT | dha | rs189409638 | . | . | . | . | . | . | . | . | . | . |
| InternalIMT | dha | rs182647339 | . | . | . | . | . | . | . | . | . | . |
| InternalIMT | dha | rs187995988 | . | . | . | . | . | . | . | . | . | . |
| InternalIMT | dha | rs141034184 | . | . | . | . | . | . | . | . | . | . |
| InternalIMT | dha | rs187310430 | . | . | . | . | . | . | . | . | . | . |
| InternalIMT | dha | rs192608488 | . | . | . | . | . | . | . | . | . | . |
| InternalIMT | dha | rs148840366 | . | . | . | . | . | . | . | . | . | . |
| InternalIMT | dha | rs186785022 | . | . | . | . | . | . | . | . | . | . |
| InternalIMT | dha | rs192348728 | . | . | . | . | . | . | . | . | . | . |
| InternalIMT | dha | rs189656631 | . | . | . | . | . | . | . | . | . | . |
| InternalIMT | dha | rs185289750 | . | . | . | . | . | . | . | . | . | . |
| InternalIMT | dha | rs142382395 | . | . | . | . | . | . | . | . | . | . |
| InternalIMT | dha | rs144866696 | . | . | . | . | . | . | . | . | . | . |
| InternalIMT | dha | rs141371289 | . | . | . | . | . | . | . | . | . | . |
| InternalIMT | dha | rs114750008 | . | . | . | . | . | . | . | . | . | . |
| InternalIMT | dha | rs189063597 | . | . | . | . | . | . | . | . | . | . |
| InternalIMT | dha | rs139151571 | . | . | . | . | . | . | . | . | . | . |
| InternalIMT | dha | rs139846368 | . | . | . | . | . | . | . | . | . | . |
| InternalIMT | dha | rs182057653 | . | . | . | . | . | . | . | . | . | . |
| InternalIMT | dha | rs148785420 | . | . | . | . | . | . | . | . | . | . |
| InternalIMT | dha | rs138383251 | . | . | . | . | . | . | . | . | . | . |
| InternalIMT | dha | rs116526705 | . | . | . | . | . | . | . | . | . | . |
| InternalIMT | dha | rs185946436 | . | . | . | . | . | . | . | . | . | . |
| InternalIMT | dha | rs190455427 | . | . | . | . | . | . | . | . | . | . |
| InternalIMT | dha | rs9282584 | . | . | . | . | . | . | . | . | . | . |
| InternalIMT | dha | rs17153289 | . | . | . | . | . | . | . | . | . | . |
| InternalIMT | dha | rs138359103 | . | . | . | . | . | . | . | . | . | . |
| InternalIMT | dha | rs150605268 | . | . | . | . | . | . | . | . | . | . |
| InternalIMT | dha | rs79242910 | . | . | . | . | . | . | . | . | . | . |
| InternalIMT | dha | rs139716904 | . | . | . | . | . | . | . | . | . | . |
| InternalIMT | dha | rs76238498 | . | . | . | . | . | . | . | . | . | . |
| InternalIMT | dha | rs184140639 | . | . | . | . | . | . | . | . | . | . |
| InternalIMT | dha | rs188517278 | . | . | . | . | . | . | . | . | . | . |
| InternalIMT | dha | rs139678913 | . | . | . | . | . | . | . | . | . | . |
| InternalIMT | dha | rs116697955 | . | . | . | . | . | . | . | . | . | . |
| InternalIMT | dha | rs79267994 | . | . | . | . | . | . | . | . | . | . |
| InternalIMT | dha | rs11239510 | . | . | . | . | . | . | . | . | . | . |
| InternalIMT | dha | rs75831645 | . | . | . | . | . | . | . | . | . | . |
| InternalIMT | dha | rs78452276 | . | . | . | . | . | . | . | . | . | . |
| InternalIMT | dha | rs144366924 | . | . | . | . | . | . | . | . | . | . |
| InternalIMT | dha | rs77519841 | . | . | . | . | . | . | . | . | . | . |
| InternalIMT | dha | rs76944454 | . | . | . | . | . | . | . | . | . | . |
| InternalIMT | dha | rs76443855 | . | . | . | . | . | . | . | . | . | . |
| InternalIMT | dha | rs146613174 | . | . | . | . | . | . | . | . | . | . |
| InternalIMT | dha | rs181424761 | . | . | . | . | . | . | . | . | . | . |
| InternalIMT | dha | rs142119241 | . | . | . | . | . | . | . | . | . | . |
| InternalIMT | dha | rs140137954 | . | . | . | . | . | . | . | . | . | . |
| InternalIMT | dha | rs187319549 | . | . | . | . | . | . | . | . | . | . |
| InternalIMT | dha | rs12241545 | . | . | . | . | . | . | . | . | . | . |
| InternalIMT | dha | rs10128228 | . | . | . | . | . | . | . | . | . | . |
| InternalIMT | dha | rs58580249 | . | . | . | . | . | . | . | . | . | . |
| InternalIMT | dha | rs193148863 | . | . | . | . | . | . | . | . | . | . |
| InternalIMT | dha | rs185638946 | . | . | . | . | . | . | . | . | . | . |
| InternalIMT | dha | rs117752921 | . | . | . | . | . | . | . | . | . | . |
| InternalIMT | dha | rs180841236 | . | . | . | . | . | . | . | . | . | . |
| InternalIMT | dha | rs73283155 | . | . | . | . | . | . | . | . | . | . |
| InternalIMT | dha | rs76560599 | . | . | . | . | . | . | . | . | . | . |
| InternalIMT | dha | rs76273620 | . | . | . | . | . | . | . | . | . | . |
| InternalIMT | dha | rs141432923 | . | . | . | . | . | . | . | . | . | . |
| InternalIMT | dha | rs113529322 | . | . | . | . | . | . | . | . | . | . |
| InternalIMT | dha | rs10128306 | . | . | . | . | . | . | . | . | . | . |

| InternalIMT | dha | rs149691688 | . | . | . | . | . | . | . | . | . | . |
| --- | --- | --- | --- | --- | --- | --- | --- | --- | --- | --- | --- | --- |
| InternalIMT | dha | rs75872495 | . | . | . | . | . | . | . | . | . | . |
| InternalIMT | dha | rs191873960 | . | . | . | . | . | . | . | . | . | . |
| InternalIMT | dha | rs80155622 | . | . | . | . | . | . | . | . | . | . |
| InternalIMT | dha | rs113914656 | . | . | . | . | . | . | . | . | . | . |
| InternalIMT | dha | rs12242840 | . | . | . | . | . | . | . | . | . | . |
| InternalIMT | dha | rs147824078 | . | . | . | . | . | . | . | . | . | . |
| InternalIMT | dha | rs59051493 | . | . | . | . | . | . | . | . | . | . |
| InternalIMT | dha | rs9282588 | . | . | . | . | . | . | . | . | . | . |
| InternalIMT | dha | rs150215207 | . | . | . | . | . | . | . | . | . | . |
| InternalIMT | dha | rs138772243 | . | . | . | . | . | . | . | . | . | . |
| InternalIMT | dha | rs149383244 | . | . | . | . | . | . | . | . | . | . |
| InternalIMT | dha | rs17522720 | . | . | . | . | . | . | . | . | . | . |
| InternalIMT | dha | rs79245105 | . | . | . | . | . | . | . | . | . | . |
| InternalIMT | dha | rs138222133 | . | . | . | . | . | . | . | . | . | . |
| InternalIMT | dha | rs12247314 | . | . | . | . | . | . | . | . | . | . |
| InternalIMT | dha | rs12263894 | . | . | . | . | . | . | . | . | . | . |
| InternalIMT | dha | rs149907880 | . | . | . | . | . | . | . | . | . | . |
| InternalIMT | dha | rs188153885 | . | . | . | . | . | . | . | . | . | . |
| InternalIMT | dha | rs73283159 | . | . | . | . | . | . | . | . | . | . |
| InternalIMT | dha | rs140476687 | . | . | . | . | . | . | . | . | . | . |
| InternalIMT | dha | rs186603148 | . | . | . | . | . | . | . | . | . | . |
| InternalIMT | dha | rs183296063 | . | . | . | . | . | . | . | . | . | . |
| InternalIMT | dha | rs145733694 | . | . | . | . | . | . | . | . | . | . |
| InternalIMT | dha | rs189807449 | . | . | . | . | . | . | . | . | . | . |
| InternalIMT | dha | rs12253223 | . | . | . | . | . | . | . | . | . | . |
| InternalIMT | dha | rs182113637 | . | . | . | . | . | . | . | . | . | . |
| InternalIMT | dha | rs142792099 | . | . | . | . | . | . | . | . | . | . |
| InternalIMT | dha | rs12249910 | . | . | . | . | . | . | . | . | . | . |
| InternalIMT | dha | rs149436339 | . | . | . | . | . | . | . | . | . | . |
| InternalIMT | dha | rs77632210 | . | . | . | . | . | . | . | . | . | . |
| InternalIMT | dha | rs74130818 | . | . | . | . | . | . | . | . | . | . |
| InternalIMT | dha | rs187583648 | . | . | . | . | . | . | . | . | . | . |
| InternalIMT | dha | rs191295987 | . | . | . | . | . | . | . | . | . | . |
| InternalIMT | dha | rs184200686 | . | . | . | . | . | . | . | . | . | . |
| InternalIMT | dha | rs76117263 | . | . | . | . | . | . | . | . | . | . |
| InternalIMT | dha | rs185393149 | . | . | . | . | . | . | . | . | . | . |
| InternalIMT | dha | rs144760014 | . | . | . | . | . | . | . | . | . | . |
| InternalIMT | dha | rs7922292 | . | . | . | . | . | . | . | . | . | . |
| InternalIMT | dha | rs115372844 | . | . | . | . | . | . | . | . | . | . |
| InternalIMT | dha | rs111460364 | . | . | . | . | . | . | . | . | . | . |
| InternalIMT | dha | rs182052255 | . | . | . | . | . | . | . | . | . | . |
| InternalIMT | dha | rs180983889 | . | . | . | . | . | . | . | . | . | . |
| InternalIMT | dha | rs139175327 | . | . | . | . | . | . | . | . | . | . |
| InternalIMT | dha | rs149959620 | . | . | . | . | . | . | . | . | . | . |
| InternalIMT | dha | rs114653117 | . | . | . | . | . | . | . | . | . | . |
| InternalIMT | dha | rs144270018 | . | . | . | . | . | . | . | . | . | . |
| InternalIMT | dha | rs182723204 | . | . | . | . | . | . | . | . | . | . |
| InternalIMT | dha | rs57204067 | . | . | . | . | . | . | . | . | . | . |
| InternalIMT | dha | rs144956226 | . | . | . | . | . | . | . | . | . | . |
| InternalIMT | dha | rs187842704 | . | . | . | . | . | . | . | . | . | . |
| InternalIMT | dha | rs184620511 | . | . | . | . | . | . | . | . | . | . |
| InternalIMT | dha | rs144031303 | . | . | . | . | . | . | . | . | . | . |
| InternalIMT | dha | rs187526473 | . | . | . | . | . | . | . | . | . | . |
| InternalIMT | dha | rs193002805 | . | . | . | . | . | . | . | . | . | . |
| InternalIMT | dha | rs138707426 | . | . | . | . | . | . | . | . | . | . |
| InternalIMT | dha | rs142754243 | . | . | . | . | . | . | . | . | . | . |
| InternalIMT | dha | rs181600331 | . | . | . | . | . | . | . | . | . | . |
| InternalIMT | dha | rs144421264 | . | . | . | . | . | . | . | . | . | . |
| InternalIMT | dha | rs184414519 | . | . | . | . | . | . | . | . | . | . |
| InternalIMT | dha | rs150596349 | . | . | . | . | . | . | . | . | . | . |
| InternalIMT | dha | rs190753747 | . | . | . | . | . | . | . | . | . | . |
| InternalIMT | dha | rs139560940 | . | . | . | . | . | . | . | . | . | . |
| InternalIMT | dha | rs75640804 | . | . | . | . | . | . | . | . | . | . |
| InternalIMT | dha | rs73283175 | . | . | . | . | . | . | . | . | . | . |
| InternalIMT | dha | rs143337229 | . | . | . | . | . | . | . | . | . | . |
| InternalIMT | dha | rs73283177 | . | . | . | . | . | . | . | . | . | . |
| InternalIMT | dha | rs76053611 | . | . | . | . | . | . | . | . | . | . |
| InternalIMT | dha | rs116325983 | . | . | . | . | . | . | . | . | . | . |
| InternalIMT | dha | rs115451825 | . | . | . | . | . | . | . | . | . | . |
| InternalIMT | dha | rs183845246 | . | . | . | . | . | . | . | . | . | . |
| InternalIMT | dha | rs79756542 | . | . | . | . | . | . | . | . | . | . |
| InternalIMT | dha | rs187242860 | . | . | . | . | . | . | . | . | . | . |
| InternalIMT | dha | rs57318980 | . | . | . | . | . | . | . | . | . | . |
| InternalIMT | dha | rs59969732 | . | . | . | . | . | . | . | . | . | . |
| InternalIMT | dha | rs143811488 | . | . | . | . | . | . | . | . | . | . |
| InternalIMT | dha | rs189067644 | . | . | . | . | . | . | . | . | . | . |
| InternalIMT | dha | rs60449178 | . | . | . | . | . | . | . | . | . | . |
| InternalIMT | dha | rs59243389 | . | . | . | . | . | . | . | . | . | . |
| InternalIMT | dha | rs184946540 | . | . | . | . | . | . | . | . | . | . |
| InternalIMT | dha | rs188685881 | . | . | . | . | . | . | . | . | . | . |
| InternalIMT | dha | rs150335797 | . | . | . | . | . | . | . | . | . | . |
| InternalIMT | dha | rs143879260 | . | . | . | . | . | . | . | . | . | . |
| InternalIMT | dha | rs186044491 | . | . | . | . | . | . | . | . | . | . |
| InternalIMT | dha | rs11819084 | . | . | . | . | . | . | . | . | . | . |
| InternalIMT | dha | rs146915670 | . | . | . | . | . | . | . | . | . | . |

| InternalIMT | dha | rs113995695 | . | . | . | . | . | . | . | . | . | . |
| --- | --- | --- | --- | --- | --- | --- | --- | --- | --- | --- | --- | --- |
| InternalIMT | dha | rs143489451 | . | . | . | . | . | . | . | . | . | . |
| InternalIMT | dha | rs182653839 | . | . | . | . | . | . | . | . | . | . |
| InternalIMT | dha | rs141692564 | . | . | . | . | . | . | . | . | . | . |
| InternalIMT | dha | rs146281742 | . | . | . | . | . | . | . | . | . | . |
| InternalIMT | dha | rs192914353 | . | . | . | . | . | . | . | . | . | . |
| InternalIMT | dha | rs185182848 | . | . | . | . | . | . | . | . | . | . |
| InternalIMT | dha | rs28395858 | . | . | . | . | . | . | . | . | . | . |
| InternalIMT | dha | rs41429645 | . | . | . | . | . | . | . | . | . | . |
| InternalIMT | dha | rs28395874 | . | . | . | . | . | . | . | . | . | . |
| InternalIMT | dha | rs189663634 | . | . | . | . | . | . | . | . | . | . |
| InternalIMT | dha | rs77666700 | . | . | . | . | . | . | . | . | . | . |
| InternalIMT | dha | rs146711717 | . | . | . | . | . | . | . | . | . | . |
| InternalIMT | dha | rs144904251 | . | . | . | . | . | . | . | . | . | . |
| InternalIMT | dha | rs189893153 | . | . | . | . | . | . | . | . | . | . |
| InternalIMT | dha | rs150447740 | . | . | . | . | . | . | . | . | . | . |
| InternalIMT | dha | rs143099649 | . | . | . | . | . | . | . | . | . | . |
| InternalIMT | dha | rs191288436 | . | . | . | . | . | . | . | . | . | . |
| InternalIMT | dha | rs188577862 | . | . | . | . | . | . | . | . | . | . |
| InternalIMT | dha | rs192695334 | . | . | . | . | . | . | . | . | . | . |
| InternalIMT | dha | rs138612906 | . | . | . | . | . | . | . | . | . | . |
| InternalIMT | dha | rs191290051 | . | . | . | . | . | . | . | . | . | . |
| InternalIMT | dha | rs189631459 | . | . | . | . | . | . | . | . | . | . |
| InternalIMT | dha | rs114620874 | . | . | . | . | . | . | . | . | . | . |
| InternalIMT | dha | rs78246652 | . | . | . | . | . | . | . | . | . | . |
| InternalIMT | dha | rs79432451 | . | . | . | . | . | . | . | . | . | . |
| InternalIMT | dha | rs186022585 | . | . | . | . | . | . | . | . | . | . |
| InternalIMT | dha | rs142978891 | . | . | . | . | . | . | . | . | . | . |
| InternalIMT | dha | rs151086840 | . | . | . | . | . | . | . | . | . | . |
| InternalIMT | dha | rs183270259 | . | . | . | . | . | . | . | . | . | . |
| InternalIMT | dha | rs188544289 | . | . | . | . | . | . | . | . | . | . |
| InternalIMT | dha | rs188500808 | . | . | . | . | . | . | . | . | . | . |
| InternalIMT | dha | rs189424589 | . | . | . | . | . | . | . | . | . | . |
| InternalIMT | dha | rs78622303 | . | . | . | . | . | . | . | . | . | . |
| InternalIMT | dha | rs143210835 | . | . | . | . | . | . | . | . | . | . |
| InternalIMT | dha | rs114761840 | . | . | . | . | . | . | . | . | . | . |
| InternalIMT | dha | rs185805402 | . | . | . | . | . | . | . | . | . | . |
| InternalIMT | dha | rs139273744 | . | . | . | . | . | . | . | . | . | . |
| InternalIMT | dha | rs184654150 | . | . | . | . | . | . | . | . | . | . |
| InternalIMT | dha | rs189069710 | . | . | . | . | . | . | . | . | . | . |
| InternalIMT | dha | rs114752115 | . | . | . | . | . | . | . | . | . | . |
| InternalIMT | dha | rs150162515 | . | . | . | . | . | . | . | . | . | . |
| InternalIMT | dha | rs180982454 | . | . | . | . | . | . | . | . | . | . |
| InternalIMT | dha | rs184932088 | . | . | . | . | . | . | . | . | . | . |
| InternalIMT | dha | rs181109625 | . | . | . | . | . | . | . | . | . | . |
| InternalIMT | dha | rs138121134 | . | . | . | . | . | . | . | . | . | . |
| InternalIMT | dha | rs183253861 | . | . | . | . | . | . | . | . | . | . |
| InternalIMT | dha | rs147073946 | . | . | . | . | . | . | . | . | . | . |
| InternalIMT | dha | rs139938489 | . | . | . | . | . | . | . | . | . | . |
| InternalIMT | dha | rs146362560 | . | . | . | . | . | . | . | . | . | . |
| InternalIMT | dha | rs193155956 | . | . | . | . | . | . | . | . | . | . |
| InternalIMT | dha | rs77663092 | . | . | . | . | . | . | . | . | . | . |
| InternalIMT | dha | rs73285319 | . | . | . | . | . | . | . | . | . | . |
| InternalIMT | dha | rs184019276 | . | . | . | . | . | . | . | . | . | . |
| InternalIMT | dha | rs28395863 | . | . | . | . | . | . | . | . | . | . |
| InternalIMT | dha | rs182121645 | . | . | . | . | . | . | . | . | . | . |
| InternalIMT | dha | rs73285327 | . | . | . | . | . | . | . | . | . | . |
| InternalIMT | dha | rs149589584 | . | . | . | . | . | . | . | . | . | . |
| InternalIMT | dha | rs148310335 | . | . | . | . | . | . | . | . | . | . |
| InternalIMT | dha | rs187246093 | . | . | . | . | . | . | . | . | . | . |
| InternalIMT | dha | rs184668894 | . | . | . | . | . | . | . | . | . | . |
| InternalIMT | dha | rs114269961 | . | . | . | . | . | . | . | . | . | . |
| InternalIMT | dha | rs149694374 | . | . | . | . | . | . | . | . | . | . |
| InternalIMT | dha | rs145284227 | . | . | . | . | . | . | . | . | . | . |
| InternalIMT | dha | rs189983820 | . | . | . | . | . | . | . | . | . | . |
| InternalIMT | dha | rs181200444 | . | . | . | . | . | . | . | . | . | . |
| InternalIMT | dha | rs185486385 | . | . | . | . | . | . | . | . | . | . |
| InternalIMT | dha | rs151198241 | . | . | . | . | . | . | . | . | . | . |
| InternalIMT | dha | rs189959341 | . | . | . | . | . | . | . | . | . | . |
| InternalIMT | dha | rs140549257 | . | . | . | . | . | . | . | . | . | . |
| InternalIMT | dha | rs186453139 | . | . | . | . | . | . | . | . | . | . |
| InternalIMT | dha | rs180922711 | . | . | . | . | . | . | . | . | . | . |
| InternalIMT | dha | rs56659903 | . | . | . | . | . | . | . | . | . | . |
| InternalIMT | dha | rs141202128 | . | . | . | . | . | . | . | . | . | . |
| InternalIMT | dha | rs59085314 | . | . | . | . | . | . | . | . | . | . |
| InternalIMT | dha | rs145564996 | . | . | . | . | . | . | . | . | . | . |
| InternalIMT | dha | rs114961009 | . | . | . | . | . | . | . | . | . | . |
| InternalIMT | dha | rs182294189 | . | . | . | . | . | . | . | . | . | . |
| InternalIMT | dha | rs187586636 | . | . | . | . | . | . | . | . | . | . |
| InternalIMT | dha | rs192254907 | . | . | . | . | . | . | . | . | . | . |
| InternalIMT | dha | rs149145663 | . | . | . | . | . | . | . | . | . | . |
| InternalIMT | dha | rs180713064 | . | . | . | . | . | . | . | . | . | . |
| InternalIMT | dha | rs183009023 | . | . | . | . | . | . | . | . | . | . |
| InternalIMT | dha | rs143385347 | . | . | . | . | . | . | . | . | . | . |
| InternalIMT | dha | rs185786329 | . | . | . | . | . | . | . | . | . | . |
| InternalIMT | dha | rs192341030 | . | . | . | . | . | . | . | . | . | . |

| InternalIMT | dha | rs74661429 | . | . | . | . | . | . | . | . | . | . |
| --- | --- | --- | --- | --- | --- | --- | --- | --- | --- | --- | --- | --- |
| InternalIMT | dha | rs188034853 | . | . | . | . | . | . | . | . | . | . |
| InternalIMT | dha | rs116808430 | . | . | . | . | . | . | . | . | . | . |
| InternalIMT | dha | rs148469010 | . | . | . | . | . | . | . | . | . | . |
| InternalIMT | dha | rs78621663 | . | . | . | . | . | . | . | . | . | . |
| InternalIMT | dha | rs192654886 | . | . | . | . | . | . | . | . | . | . |
| InternalIMT | dha | rs145996035 | . | . | . | . | . | . | . | . | . | . |
| InternalIMT | dha | rs139942226 | . | . | . | . | . | . | . | . | . | . |
| InternalIMT | dha | rs113645632 | . | . | . | . | . | . | . | . | . | . |
| InternalIMT | dha | rs74940661 | . | . | . | . | . | . | . | . | . | . |
| InternalIMT | dha | rs115355800 | . | . | . | . | . | . | . | . | . | . |
| InternalIMT | dha | rs150285898 | . | . | . | . | . | . | . | . | . | . |
| InternalIMT | dha | rs188117932 | . | . | . | . | . | . | . | . | . | . |
| InternalIMT | dha | rs139956566 | . | . | . | . | . | . | . | . | . | . |
| InternalIMT | dha | rs147673885 | . | . | . | . | . | . | . | . | . | . |
| InternalIMT | dha | rs189127928 | . | . | . | . | . | . | . | . | . | . |
| InternalIMT | dha | rs187558772 | . | . | . | . | . | . | . | . | . | . |
| InternalIMT | dha | rs181948587 | . | . | . | . | . | . | . | . | . | . |
| InternalIMT | dha | rs189983269 | . | . | . | . | . | . | . | . | . | . |
| InternalIMT | dha | rs187250069 | . | . | . | . | . | . | . | . | . | . |
| InternalIMT | dha | rs191485859 | . | . | . | . | . | . | . | . | . | . |
| InternalIMT | dha | rs185499555 | . | . | . | . | . | . | . | . | . | . |
| InternalIMT | dha | rs180862110 | . | . | . | . | . | . | . | . | . | . |
| InternalIMT | dha | rs190472603 | . | . | . | . | . | . | . | . | . | . |
| InternalIMT | dha | rs189226167 | . | . | . | . | . | . | . | . | . | . |
| InternalIMT | dha | rs181623256 | . | . | . | . | . | . | . | . | . | . |
| InternalIMT | dha | rs186079557 | . | . | . | . | . | . | . | . | . | . |
| InternalIMT | dha | rs147760994 | . | . | . | . | . | . | . | . | . | . |
| InternalIMT | dha | rs189176167 | . | . | . | . | . | . | . | . | . | . |
| InternalIMT | dha | rs117901000 | . | . | . | . | . | . | . | . | . | . |
| InternalIMT | dha | rs141984888 | . | . | . | . | . | . | . | . | . | . |
| InternalIMT | dha | rs192365061 | . | . | . | . | . | . | . | . | . | . |
| InternalIMT | dha | rs185976038 | . | . | . | . | . | . | . | . | . | . |
| InternalIMT | dha | rs184697303 | . | . | . | . | . | . | . | . | . | . |
| InternalIMT | dha | rs143383132 | . | . | . | . | . | . | . | . | . | . |
| InternalIMT | dha | rs141873881 | . | . | . | . | . | . | . | . | . | . |
| InternalIMT | dha | rs185507255 | . | . | . | . | . | . | . | . | . | . |
| InternalIMT | dha | rs191379425 | . | . | . | . | . | . | . | . | . | . |
| InternalIMT | dha | rs184199584 | . | . | . | . | . | . | . | . | . | . |
| InternalIMT | dha | rs183181360 | . | . | . | . | . | . | . | . | . | . |
| InternalIMT | dha | rs184495673 | . | . | . | . | . | . | . | . | . | . |
| InternalIMT | dha | rs182946297 | . | . | . | . | . | . | . | . | . | . |
| InternalIMT | dha | rs187743615 | . | . | . | . | . | . | . | . | . | . |
| InternalIMT | dha | rs183259862 | . | . | . | . | . | . | . | . | . | . |
| InternalIMT | dha | rs112433566 | . | . | . | . | . | . | . | . | . | . |
| InternalIMT | dha | rs182351085 | . | . | . | . | . | . | . | . | . | . |
| InternalIMT | dha | rs146248508 | . | . | . | . | . | . | . | . | . | . |
| InternalIMT | dha | rs188528439 | . | . | . | . | . | . | . | . | . | . |
| InternalIMT | dha | rs146167018 | . | . | . | . | . | . | . | . | . | . |
| InternalIMT | dha | rs192440980 | . | . | . | . | . | . | . | . | . | . |
| InternalIMT | dha | rs185601882 | . | . | . | . | . | . | . | . | . | . |
| InternalIMT | dha | rs117272563 | . | . | . | . | . | . | . | . | . | . |
| InternalIMT | dha | rs10430734 | . | . | . | . | . | . | . | . | . | . |
| InternalIMT | dha | rs181320952 | . | . | . | . | . | . | . | . | . | . |
| InternalIMT | dha | rs185480660 | . | . | . | . | . | . | . | . | . | . |
| InternalIMT | dha | rs188077917 | . | . | . | . | . | . | . | . | . | . |
| InternalIMT | dha | rs187562953 | . | . | . | . | . | . | . | . | . | . |
| InternalIMT | dha | rs182044631 | . | . | . | . | . | . | . | . | . | . |
| InternalIMT | dha | rs145455596 | . | . | . | . | . | . | . | . | . | . |
| InternalIMT | dha | rs181475877 | . | . | . | . | . | . | . | . | . | . |
| InternalIMT | dha | rs149413085 | . | . | . | . | . | . | . | . | . | . |
| InternalIMT | dha | rs12253656 | . | . | . | . | . | . | . | . | . | . |
| InternalIMT | dha | rs182718239 | . | . | . | . | . | . | . | . | . | . |
| InternalIMT | dha | rs192435695 | . | . | . | . | . | . | . | . | . | . |
| InternalIMT | dha | rs12248277 | . | . | . | . | . | . | . | . | . | . |
| InternalIMT | dha | rs143521710 | . | . | . | . | . | . | . | . | . | . |
| InternalIMT | dha | rs144454454 | . | . | . | . | . | . | . | . | . | . |
| InternalIMT | dha | rs190441610 | . | . | . | . | . | . | . | . | . | . |
| InternalIMT | dha | rs114690462 | . | . | . | . | . | . | . | . | . | . |
| InternalIMT | dha | rs184541357 | . | . | . | . | . | . | . | . | . | . |
| InternalIMT | dha | rs142657653 | . | . | . | . | . | . | . | . | . | . |
| InternalIMT | dha | rsurf1 | 2.019 | . | . | . | . | . | . | . | . | . |
| InternalIMT | dha | rsten1 | 2.195 | . | . | . | . | . | . | . | . | . |
| InternalIMT | dha | chr10:45852312:D | 0.055 | -0.095 | 0.034 | 5.70E-03 | -0.043 | 0.094 | 0.649 | 0.027 | 0.078 | 0.733 |
| InternalIMT | dha | chr10:45853232:I | 0.071 | -0.089 | 0.034 | 0.01 | 0.012 | 0.095 | 0.897 | -0.02 | 0.079 | 0.798 |
| InternalIMT | dha | chr10:45853234:I | 0.157 | -0.052 | 0.036 | 0.153 | 0.132 | 0.087 | 0.13 | -0.133 | 0.067 | 0.048 |
| InternalIMT | dha | chr10:45854796:D | 0.001 | . | . | . | . | . | . | . | . | . |
| InternalIMT | dha | chr10:45854994:I | 0.001 | . | . | . | . | . | . | . | . | . |
| InternalIMT | dha | chr10:45858290:D | 0.04 | -0.082 | 0.034 | 0.016 | 0.179 | 0.145 | 0.217 | -0.13 | 0.12 | 0.278 |
| InternalIMT | dha | chr10:45858820:I | 0.015 | -0.097 | 0.033 | 3.40E-03 | -0.122 | 0.383 | 0.749 | 0.165 | 0.285 | 0.563 |
| InternalIMT | dha | chr10:45860888:D | 0.067 | -0.052 | 0.035 | 0.138 | 0.346 | 0.188 | 0.066 | -0.308 | 0.144 | 0.033 |
| InternalIMT | dha | chr10:45863913:I | 0.159 | -0.05 | 0.036 | 0.17 | 0.138 | 0.086 | 0.107 | -0.136 | 0.066 | 0.04 |
| InternalIMT | dha | chr10:45866601:D | 0 | . | . | . | . | . | . | . | . | . |
| InternalIMT | dha | chr10:45867197:I | 0.008 | . | . | . | . | . | . | . | . | . |
| InternalIMT | dha | chr10:45867538:D | 0.167 | -0.052 | 0.037 | 0.158 | 0.124 | 0.084 | 0.141 | -0.123 | 0.065 | 0.061 |
| InternalIMT | dha | chr10:45868356:D | 0.151 | -0.057 | 0.036 | 0.116 | 0.117 | 0.086 | 0.176 | -0.119 | 0.067 | 0.077 |

| InternalIMT | dha | chr10:45871158:I | 0.16 | -0.05 | 0.036 | 0.17 | 0.138 | 0.086 | 0.108 | -0.136 | 0.066 | 0.04 |
| --- | --- | --- | --- | --- | --- | --- | --- | --- | --- | --- | --- | --- |
| InternalIMT | dha | chr10:45871637:D | 0.061 | -0.064 | 0.034 | 0.062 | 0.124 | 0.191 | 0.518 | -0.216 | 0.145 | 0.138 |
| InternalIMT | dha | chr10:45871638:I | 0.115 | -0.051 | 0.036 | 0.147 | 0.171 | 0.124 | 0.168 | -0.177 | 0.094 | 0.059 |
| InternalIMT | dha | chr10:45873629:I | 0.159 | -0.05 | 0.036 | 0.168 | 0.139 | 0.086 | 0.107 | -0.136 | 0.066 | 0.04 |
| InternalIMT | dha | chr10:45873701:D | 0.003 | . | . | . | . | . | . | . | . | . |
| InternalIMT | dha | chr10:45874997:I | 0.03 | -0.086 | 0.033 | 8.80E-03 | 0.125 | 0.164 | 0.447 | -0.098 | 0.126 | 0.435 |
| InternalIMT | dha | chr10:45882030:I | 0.021 | -0.087 | 0.034 | 9.50E-03 | 0.062 | 0.275 | 0.823 | -0.092 | 0.212 | 0.663 |
| InternalIMT | dha | chr10:45883774:D | 0.154 | -0.044 | 0.035 | 0.21 | 0.194 | 0.088 | 0.027 | -0.172 | 0.068 | 0.012 |
| InternalIMT | dha | chr10:45885169:D | 0.035 | -0.089 | 0.033 | 7.40E-03 | 0.048 | 0.132 | 0.714 | -0.039 | 0.104 | 0.709 |
| InternalIMT | dha | chr10:45886451:D | 0.588 | -0.057 | 0.061 | 0.352 | 0.075 | 0.061 | 0.218 | -0.03 | 0.048 | 0.538 |
| InternalIMT | dha | chr10:45891800:I | 0.592 | -0.056 | 0.056 | 0.313 | 0.055 | 0.054 | 0.314 | -0.031 | 0.043 | 0.472 |
| InternalIMT | dha | chr10:45904771:I | 0.573 | -0.069 | 0.055 | 0.21 | 0.046 | 0.053 | 0.394 | -0.021 | 0.043 | 0.628 |
| InternalIMT | dha | chr10:45904959:I | 0.103 | -0.085 | 0.035 | 0.015 | 0.059 | 0.115 | 0.607 | -0.035 | 0.091 | 0.698 |
| InternalIMT | dha | chr10:45911028:I | 0.034 | -0.08 | 0.034 | 0.018 | 0.141 | 0.205 | 0.492 | -0.191 | 0.161 | 0.235 |
| InternalIMT | dha | chr10:45911031:I | 0.363 | -0.117 | 0.048 | 0.014 | -0.016 | 0.065 | 0.811 | 0.037 | 0.051 | 0.474 |
| InternalIMT | dha | chr10:45912154:D | 0.145 | -0.044 | 0.034 | 0.206 | 0.212 | 0.094 | 0.024 | -0.188 | 0.072 | 9.60E-03 |
| InternalIMT | dha | chr10:45913885:I | 0.001 | . | . | . | . | . | . | . | . | . |
| InternalIMT | dha | chr10:45914753:D | 0.243 | -0.037 | 0.039 | 0.344 | 0.152 | 0.075 | 0.044 | -0.122 | 0.058 | 0.036 |
| InternalIMT | dha | chr10:45919621 | 0.003 | . | . | . | . | . | . | . | . | . |
| InternalIMT | dha | chr10:45921739:I | 0.004 | . | . | . | . | . | . | . | . | . |
| InternalIMT | dha | chr10:45923527:I | 0.052 | -0.104 | 0.034 | 2.60E-03 | -0.044 | 0.175 | 0.8 | 0.116 | 0.137 | 0.398 |
| InternalIMT | dha | chr10:45927001:I | 0.014 | -0.093 | 0.033 | 5.20E-03 | -0.166 | 0.318 | 0.601 | 0.023 | 0.259 | 0.929 |
| InternalIMT | dha | chr10:45930354:I | 0.001 | . | . | . | . | . | . | . | . | . |
| InternalIMT | dha | chr10:45932864:I | 0.027 | -0.086 | 0.034 | 0.01 | 0.244 | 0.233 | 0.295 | -0.134 | 0.163 | 0.409 |
| InternalIMT | dha | chr10:45936163:I | 0.026 | -0.093 | 0.033 | 4.40E-03 | -0.154 | 0.145 | 0.289 | 0.053 | 0.115 | 0.645 |
| InternalIMT | dha | chr10:45938095:D | 0.164 | -0.047 | 0.035 | 0.183 | 0.153 | 0.088 | 0.08 | -0.148 | 0.067 | 0.028 |
| InternalIMT | dha | chr10:45938886 | 0 | . | . | . | . | . | . | . | . | . |
| InternalIMT | dha | chr10:45939320 | 0.001 | . | . | . | . | . | . | . | . | . |
| InternalIMT | dha | chr10:45939659 | 0.001 | . | . | . | . | . | . | . | . | . |
| InternalIMT | dha | chr10:45941150:D | 0.001 | . | . | . | . | . | . | . | . | . |
| InternalIMT | dha | chr10:45947128:D | 0.064 | -0.104 | 0.034 | 2.50E-03 | -0.076 | 0.093 | 0.416 | 0.097 | 0.076 | 0.204 |
| InternalIMT | dha | chr10:45950990:I | 0.044 | -0.086 | 0.033 | 0.01 | 0.1 | 0.228 | 0.661 | -0.067 | 0.178 | 0.707 |
| InternalIMT | dha | chr10:45951854:I | 0.048 | -0.083 | 0.035 | 0.017 | 0.104 | 0.117 | 0.374 | -0.085 | 0.089 | 0.34 |
| InternalIMT | dha | chr10:45952670:I | 0.061 | -0.086 | 0.034 | 0.012 | -0.017 | 0.095 | 0.855 | -0.042 | 0.075 | 0.577 |
| InternalIMT | dha | chr10:45956741 | 0.001 | . | . | . | . | . | . | . | . | . |
| InternalIMT | dha | chr10:45957247:I | 0.233 | -0.057 | 0.038 | 0.127 | 0.066 | 0.068 | 0.334 | -0.077 | 0.054 | 0.154 |
| InternalIMT | dha | chr10:45958082:I | 0.232 | -0.053 | 0.037 | 0.16 | 0.081 | 0.07 | 0.245 | -0.088 | 0.054 | 0.106 |
| InternalIMT | dha | chr10:45905041:D | . | . | . | . | . | . | . | . | . | . |
| InternalIMT | dha | chr10:45938871 | . | . | . | . | . | . | . | . | . | . |
| InternalIMT | dha | chr10:45939160 | . | . | . | . | . | . | . | . | . | . |
| InternalIMT | dha | chr10:45869910 | . | . | . | . | . | . | . | . | . | . |
| InternalIMT | dha | chr10:45876214:D | . | . | . | . | . | . | . | . | . | . |
| InternalIMT | dha | chr10:45880237:I | . | . | . | . | . | . | . | . | . | . |
| InternalIMT | dha | chr10:45884371:I | . | . | . | . | . | . | . | . | . | . |
| InternalIMT | dha | chr10:45891535:D | . | . | . | . | . | . | . | . | . | . |
| InternalIMT | dha | chr10:45893516:D | . | . | . | . | . | . | . | . | . | . |
| InternalIMT | dha | chr10:45893782:D | . | . | . | . | . | . | . | . | . | . |
| InternalIMT | dha | chr10:45895254:I | . | . | . | . | . | . | . | . | . | . |
| InternalIMT | dha | chr10:45895848:D | . | . | . | . | . | . | . | . | . | . |
| InternalIMT | dha | chr10:45896316:D | . | . | . | . | . | . | . | . | . | . |
| InternalIMT | dha | chr10:45896317:D | . | . | . | . | . | . | . | . | . | . |
| InternalIMT | dha | chr10:45902683:I | . | . | . | . | . | . | . | . | . | . |
| InternalIMT | dha | chr10:45905003:D | . | . | . | . | . | . | . | . | . | . |
| InternalIMT | dha | chr10:45927476:D | . | . | . | . | . | . | . | . | . | . |
| InternalIMT | dha | chr10:45935882 | . | . | . | . | . | . | . | . | . | . |
| InternalIMT | dha | chr10:45936100 | . | . | . | . | . | . | . | . | . | . |
| InternalIMT | dha | chr10:45939553 | . | . | . | . | . | . | . | . | . | . |
| InternalIMT | dha | chr10:45939631 | . | . | . | . | . | . | . | . | . | . |
| InternalIMT | dha | chr10:45944193:D | . | . | . | . | . | . | . | . | . | . |
| InternalIMT | dha | chr10:45954030 | . | . | . | . | . | . | . | . | . | . |

Supplemental table S23.

| phen | covar(fa) | rs_id | maf | beta_FA | se_FA | p_FA | beta_SNP | se_SNP | p_SNP | beta_inter | se_inter | p_inter |
| --- | --- | --- | --- | --- | --- | --- | --- | --- | --- | --- | --- | --- |
| InternalIMT | dha | rs76035182 | 0.068 | -0.076 | 0.052 | 0.143 | 0.337 | 0.191 | 0.077 | -0.201 | 0.126 | 0.11 |
| InternalIMT | dha | rs12359547 | 0.052 | -0.083 | 0.049 | 0.091 | 0.405 | 0.223 | 0.069 | -0.256 | 0.155 | 0.098 |
| InternalIMT | dha | rs74595484 | 0.12 | -0.082 | 0.054 | 0.132 | 0.192 | 0.15 | 0.199 | -0.107 | 0.1 | 0.284 |
| InternalIMT | dha | rs1864415 | 0.749 | -4.80E-03 | 0.113 | 0.966 | 0.13 | 0.104 | 0.214 | -0.067 | 0.07 | 0.337 |
| InternalIMT | dha | rs72788101 | 0.064 | -0.099 | 0.049 | 0.044 | 0.131 | 0.215 | 0.544 | -0.036 | 0.158 | 0.82 |
| InternalIMT | dha | rs11239481 | 0.081 | -0.123 | 0.05 | 0.014 | -0.151 | 0.18 | 0.403 | 0.109 | 0.125 | 0.385 |
| InternalIMT | dha | rs72788102 | 0.064 | -0.099 | 0.049 | 0.044 | 0.13 | 0.215 | 0.544 | -0.036 | 0.158 | 0.82 |
| InternalIMT | dha | rs72789705 | 0.064 | -0.099 | 0.049 | 0.044 | 0.13 | 0.215 | 0.545 | -0.036 | 0.158 | 0.822 |
| InternalIMT | dha | rs141529858 | 0.002 | . | . | . | . | . | . | . | . | . |
| InternalIMT | dha | rs145376292 | 0 | . | . | . | . | . | . | . | . | . |
| InternalIMT | dha | rs190418301 | 0 | . | . | . | . | . | . | . | . | . |
| InternalIMT | dha | rs11239482 | 0.064 | -0.099 | 0.049 | 0.044 | 0.13 | 0.215 | 0.546 | -0.035 | 0.157 | 0.823 |
| InternalIMT | dha | rs61855872 | 0.004 | . | . | . | . | . | . | . | . | . |
| InternalIMT | dha | rs148991144 | 0.003 | . | . | . | . | . | . | . | . | . |
| InternalIMT | dha | rs191932221 | 0.002 | . | . | . | . | . | . | . | . | . |
| InternalIMT | dha | rs55978138 | 0.179 | -0.065 | 0.055 | 0.24 | 0.201 | 0.112 | 0.073 | -0.108 | 0.077 | 0.162 |
| InternalIMT | dha | rs73281167 | 0.045 | -0.109 | 0.05 | 0.03 | -0.054 | 0.207 | 0.793 | 0.018 | 0.14 | 0.897 |
| InternalIMT | dha | rs12768010 | 0.092 | -0.077 | 0.052 | 0.142 | 0.271 | 0.147 | 0.064 | -0.17 | 0.097 | 0.082 |
| InternalIMT | dha | rs11239484 | 0.749 | -5.80E-03 | 0.113 | 0.959 | 0.129 | 0.104 | 0.217 | -0.066 | 0.07 | 0.342 |
| InternalIMT | dha | rs35906360 | 0.18 | -0.068 | 0.055 | 0.218 | 0.194 | 0.113 | 0.085 | -0.102 | 0.077 | 0.188 |
| InternalIMT | dha | rs186250512 | 0.001 | . | . | . | . | . | . | . | . | . |
| InternalIMT | dha | rs150810743 | 0.007 | . | . | . | . | . | . | . | . | . |
| InternalIMT | dha | rs55965812 | 0.161 | -0.055 | 0.055 | 0.317 | 0.253 | 0.118 | 0.031 | -0.154 | 0.08 | 0.055 |
| InternalIMT | dha | rs56180358 | 0.181 | -0.068 | 0.055 | 0.218 | 0.193 | 0.112 | 0.086 | -0.101 | 0.077 | 0.191 |
| InternalIMT | dha | rs55864145 | 0.092 | -0.077 | 0.052 | 0.142 | 0.271 | 0.147 | 0.065 | -0.169 | 0.097 | 0.083 |
| InternalIMT | dha | rs55986122 | 0.181 | -0.068 | 0.055 | 0.217 | 0.193 | 0.112 | 0.086 | -0.101 | 0.077 | 0.191 |
| InternalIMT | dha | rs7477350 | 0.749 | -6.30E-03 | 0.113 | 0.956 | 0.128 | 0.104 | 0.218 | -0.066 | 0.07 | 0.344 |
| InternalIMT | dha | rs2099170 | 0.18 | -0.068 | 0.055 | 0.213 | 0.191 | 0.112 | 0.09 | -0.099 | 0.077 | 0.199 |
| InternalIMT | dha | rs2082108 | 0.181 | -0.068 | 0.055 | 0.216 | 0.192 | 0.112 | 0.087 | -0.101 | 0.077 | 0.193 |
| InternalIMT | dha | rs55902404 | 0.02 | -0.119 | 0.048 | 0.013 | -0.39 | 0.382 | 0.307 | 0.4 | 0.292 | 0.17 |
| InternalIMT | dha | rs58402662 | 0.02 | -0.119 | 0.048 | 0.013 | -0.391 | 0.382 | 0.306 | 0.401 | 0.292 | 0.169 |
| InternalIMT | dha | rs11511618 | 0.084 | -0.124 | 0.05 | 0.013 | -0.167 | 0.18 | 0.353 | 0.114 | 0.125 | 0.363 |
| InternalIMT | dha | rs1816492 | 0.747 | -7.60E-03 | 0.113 | 0.946 | 0.129 | 0.104 | 0.217 | -0.065 | 0.07 | 0.35 |
| InternalIMT | dha | rs146611093 | 0.002 | . | . | . | . | . | . | . | . | . |
| InternalIMT | dha | rs186897689 | 0.006 | . | . | . | . | . | . | . | . | . |
| InternalIMT | dha | rs10793616 | 0.749 | -6.70E-03 | 0.113 | 0.953 | 0.128 | 0.104 | 0.219 | -0.066 | 0.07 | 0.345 |
| InternalIMT | dha | rs116700479 | 0.052 | -0.073 | 0.051 | 0.151 | 0.544 | 0.218 | 0.013 | -0.333 | 0.141 | 0.018 |
| InternalIMT | dha | rs192535936 | 0.004 | . | . | . | . | . | . | . | . | . |
| InternalIMT | dha | rs113363297 | 0.723 | -0.011 | 0.113 | 0.921 | 0.125 | 0.105 | 0.234 | -0.065 | 0.071 | 0.361 |
| InternalIMT | dha | rs12354994 | 0.172 | -0.073 | 0.055 | 0.181 | 0.184 | 0.117 | 0.116 | -0.089 | 0.081 | 0.27 |
| InternalIMT | dha | rs148068329 | 0.025 | -0.106 | 0.048 | 0.027 | 0.049 | 0.311 | 0.876 | 0.022 | 0.205 | 0.916 |
| InternalIMT | dha | rs12356109 | 0.198 | -0.06 | 0.056 | 0.288 | 0.202 | 0.107 | 0.06 | -0.113 | 0.074 | 0.126 |
| InternalIMT | dha | rs55908141 | 0.02 | -0.119 | 0.048 | 0.013 | -0.392 | 0.381 | 0.303 | 0.401 | 0.291 | 0.168 |
| InternalIMT | dha | rs12354712 | 0.161 | -0.055 | 0.055 | 0.313 | 0.252 | 0.118 | 0.032 | -0.153 | 0.08 | 0.056 |
| InternalIMT | dha | rs147282684 | 0.002 | . | . | . | . | . | . | . | . | . |
| InternalIMT | dha | rs12356140 | 0.191 | -0.07 | 0.055 | 0.205 | 0.175 | 0.111 | 0.116 | -0.088 | 0.077 | 0.253 |
| InternalIMT | dha | rs185476187 | 0.004 | . | . | . | . | . | . | . | . | . |
| InternalIMT | dha | rs145720057 | 0.012 | -0.094 | 0.048 | 0.051 | 0.663 | 0.393 | 0.092 | -0.443 | 0.256 | 0.084 |
| InternalIMT | dha | rs12354742 | 0.181 | -0.068 | 0.055 | 0.214 | 0.191 | 0.112 | 0.089 | -0.1 | 0.077 | 0.197 |
| InternalIMT | dha | rs180913842 | 0.02 | -0.119 | 0.048 | 0.013 | -0.392 | 0.381 | 0.303 | 0.401 | 0.291 | 0.167 |
| InternalIMT | dha | rs142976914 | 0.002 | . | . | . | . | . | . | . | . | . |
| InternalIMT | dha | rs11511619 | 0.201 | -0.06 | 0.057 | 0.287 | 0.196 | 0.107 | 0.067 | -0.11 | 0.073 | 0.133 |
| InternalIMT | dha | rs146702870 | 0.003 | . | . | . | . | . | . | . | . | . |
| InternalIMT | dha | rs150287496 | 0.001 | . | . | . | . | . | . | . | . | . |
| InternalIMT | dha | rs190917973 | 0.002 | . | . | . | . | . | . | . | . | . |
| InternalIMT | dha | rs11517281 | 0.181 | -0.068 | 0.055 | 0.213 | 0.191 | 0.112 | 0.089 | -0.099 | 0.077 | 0.198 |
| InternalIMT | dha | rs12359299 | 0.181 | -0.068 | 0.055 | 0.213 | 0.191 | 0.112 | 0.089 | -0.099 | 0.077 | 0.198 |
| InternalIMT | dha | rs182307932 | 0.071 | -0.109 | 0.051 | 0.031 | -0.062 | 0.173 | 0.718 | 3.60E-03 | 0.126 | 0.978 |
| InternalIMT | dha | rs61855873 | 0.011 | -0.12 | 0.048 | 0.013 | -1.03 | 0.647 | 0.11 | 0.665 | 0.425 | 0.118 |
| InternalIMT | dha | rs12359302 | 0.18 | -0.074 | 0.055 | 0.175 | 0.178 | 0.113 | 0.116 | -0.083 | 0.078 | 0.283 |
| InternalIMT | dha | rs143208140 | 0.028 | -0.105 | 0.048 | 0.03 | 0.088 | 0.305 | 0.772 | -0.015 | 0.201 | 0.94 |
| InternalIMT | dha | rs9988764 | 0.712 | -0.049 | 0.106 | 0.64 | 0.09 | 0.101 | 0.376 | -0.039 | 0.069 | 0.568 |
| InternalIMT | dha | rs192875767 | 0.002 | . | . | . | . | . | . | . | . | . |
| InternalIMT | dha | rs147268846 | 0.005 | . | . | . | . | . | . | . | . | . |
| InternalIMT | dha | rs11239485 | 0.109 | -0.079 | 0.053 | 0.138 | 0.201 | 0.149 | 0.179 | -0.113 | 0.103 | 0.269 |
| InternalIMT | dha | rs151317885 | 0.24 | -0.158 | 0.057 | 6.00E-03 | -0.165 | 0.104 | 0.113 | 0.107 | 0.072 | 0.136 |
| InternalIMT | dha | rs11499085 | 0.322 | -0.057 | 0.066 | 0.389 | 0.119 | 0.103 | 0.249 | -0.078 | 0.072 | 0.275 |
| InternalIMT | dha | rs188130606 | 0.086 | -0.095 | 0.052 | 0.066 | 0.164 | 0.163 | 0.315 | -0.082 | 0.113 | 0.469 |
| InternalIMT | dha | rs11511620 | 0.161 | -0.055 | 0.055 | 0.311 | 0.25 | 0.117 | 0.033 | -0.152 | 0.08 | 0.058 |
| InternalIMT | dha | rs181498390 | 0.057 | -0.106 | 0.052 | 0.039 | -0.041 | 0.177 | 0.816 | -2.50E-03 | 0.12 | 0.983 |
| InternalIMT | dha | rs186280701 | 0.02 | -0.119 | 0.048 | 0.013 | -0.394 | 0.379 | 0.299 | 0.402 | 0.289 | 0.166 |
| InternalIMT | dha | rs150059185 | 0.034 | -0.103 | 0.049 | 0.035 | 0.083 | 0.307 | 0.786 | -0.074 | 0.216 | 0.732 |
| InternalIMT | dha | rs183338707 | 0.001 | . | . | . | . | . | . | . | . | . |
| InternalIMT | dha | rs11499038 | 0.306 | -0.067 | 0.064 | 0.295 | 0.105 | 0.107 | 0.325 | -0.066 | 0.074 | 0.375 |
| InternalIMT | dha | rs190699595 | 0.004 | . | . | . | . | . | . | . | . | . |
| InternalIMT | dha | rs11523408 | 0.175 | -0.069 | 0.055 | 0.204 | 0.192 | 0.114 | 0.094 | -0.1 | 0.079 | 0.205 |
| InternalIMT | dha | rs188049317 | 0.007 | . | . | . | . | . | . | . | . | . |
| InternalIMT | dha | rs144948634 | 0.214 | -0.164 | 0.056 | 3.80E-03 | -0.212 | 0.102 | 0.038 | 0.128 | 0.072 | 0.074 |
| InternalIMT | dha | rs143833576 | . | . | . | . | . | . | . | . | . | . |
| InternalIMT | dha | rs151239879 | 0.175 | -0.068 | 0.055 | 0.214 | 0.197 | 0.114 | 0.086 | -0.103 | 0.079 | 0.19 |
| InternalIMT | dha | rs7073743 | 0.212 | -0.157 | 0.056 | 5.20E-03 | -0.189 | 0.101 | 0.062 | 0.114 | 0.071 | 0.108 |
| InternalIMT | dha | rs142563349 | 0.092 | -0.1 | 0.052 | 0.055 | 0.098 | 0.16 | 0.54 | -0.044 | 0.112 | 0.692 |
| InternalIMT | dha | rs187246403 | 0.02 | -0.119 | 0.048 | 0.013 | -0.394 | 0.378 | 0.297 | 0.402 | 0.289 | 0.164 |
| InternalIMT | dha | rs150423942 | 0.178 | -0.068 | 0.055 | 0.214 | 0.197 | 0.113 | 0.082 | -0.102 | 0.078 | 0.189 |

| InternalIMT | dha | rs184706102 | . | . | . | . | . | . | . | . | . | . |
| --- | --- | --- | --- | --- | --- | --- | --- | --- | --- | --- | --- | --- |
| InternalIMT | dha | rs189626288 | 0.007 | . | . | . | . | . | . | . | . | . |
| InternalIMT | dha | rs140749834 | 0.142 | -0.09 | 0.055 | 0.098 | 0.057 | 0.147 | 0.7 | -0.059 | 0.102 | 0.558 |
| InternalIMT | dha | rs7078822 | 0.136 | -0.07 | 0.055 | 0.199 | 0.195 | 0.129 | 0.133 | -0.123 | 0.089 | 0.169 |
| InternalIMT | dha | rs186227617 | 0.177 | -0.07 | 0.055 | 0.204 | 0.186 | 0.115 | 0.107 | -0.099 | 0.08 | 0.217 |
| InternalIMT | dha | rs188984368 | 0.018 | -0.092 | 0.049 | 0.059 | 0.495 | 0.297 | 0.096 | -0.332 | 0.182 | 0.068 |
| InternalIMT | dha | rs182078109 | 0.04 | -0.092 | 0.051 | 0.072 | 0.358 | 0.353 | 0.31 | -0.199 | 0.233 | 0.394 |
| InternalIMT | dha | rs141456274 | 0.001 | . | . | . | . | . | . | . | . | . |
| InternalIMT | dha | rs146976512 | 0.181 | -0.068 | 0.055 | 0.212 | 0.191 | 0.113 | 0.093 | -0.099 | 0.078 | 0.204 |
| InternalIMT | dha | rs138036816 | 0.305 | -0.067 | 0.064 | 0.296 | 0.106 | 0.107 | 0.322 | -0.066 | 0.074 | 0.371 |
| InternalIMT | dha | rs114879777 | 0.051 | -0.072 | 0.051 | 0.157 | 0.557 | 0.221 | 0.012 | -0.346 | 0.144 | 0.016 |
| InternalIMT | dha | rs149366475 | 0.307 | -0.071 | 0.065 | 0.273 | 0.092 | 0.107 | 0.389 | -0.059 | 0.074 | 0.426 |
| InternalIMT | dha | rs182463043 | 0.007 | . | . | . | . | . | . | . | . | . |
| InternalIMT | dha | rs144754632 | 0.025 | -0.106 | 0.048 | 0.027 | 0.045 | 0.311 | 0.886 | 0.024 | 0.206 | 0.906 |
| InternalIMT | dha | rs55717607 | 0.181 | -0.069 | 0.055 | 0.207 | 0.188 | 0.112 | 0.094 | -0.097 | 0.077 | 0.208 |
| InternalIMT | dha | rs187537973 | 0.001 | . | . | . | . | . | . | . | . | . |
| InternalIMT | dha | rs148085971 | 0.007 | . | . | . | . | . | . | . | . | . |
| InternalIMT | dha | rs11239491 | 0.181 | -0.069 | 0.055 | 0.207 | 0.187 | 0.112 | 0.094 | -0.097 | 0.077 | 0.209 |
| InternalIMT | dha | rs184412184 | 0.002 | . | . | . | . | . | . | . | . | . |
| InternalIMT | dha | rs4948998 | 0.798 | 4.50E-03 | 0.126 | 0.971 | 0.131 | 0.107 | 0.222 | -0.069 | 0.073 | 0.347 |
| InternalIMT | dha | rs7076696 | 0.798 | 6.00E-03 | 0.126 | 0.962 | 0.133 | 0.107 | 0.216 | -0.069 | 0.073 | 0.341 |
| InternalIMT | dha | rs11239492 | 0.198 | -0.061 | 0.056 | 0.278 | 0.198 | 0.107 | 0.064 | -0.109 | 0.074 | 0.137 |
| InternalIMT | dha | rs79462470 | 0.02 | -0.119 | 0.048 | 0.013 | -0.396 | 0.375 | 0.291 | 0.402 | 0.287 | 0.161 |
| InternalIMT | dha | rs187804654 | . | . | . | . | . | . | . | . | . | . |
| InternalIMT | dha | rs7093687 | 0.161 | -0.058 | 0.055 | 0.288 | 0.239 | 0.117 | 0.041 | -0.144 | 0.08 | 0.072 |
| InternalIMT | dha | rs7081004 | 0.162 | -0.056 | 0.055 | 0.303 | 0.247 | 0.117 | 0.035 | -0.149 | 0.08 | 0.062 |
| InternalIMT | dha | rs7084793 | 0.181 | -0.07 | 0.055 | 0.204 | 0.185 | 0.112 | 0.098 | -0.095 | 0.077 | 0.216 |
| InternalIMT | dha | rs61556080 | 0.02 | -0.119 | 0.048 | 0.013 | -0.396 | 0.375 | 0.29 | 0.402 | 0.286 | 0.16 |
| InternalIMT | dha | rs115552064 | 0.019 | -0.118 | 0.048 | 0.014 | -0.22 | 0.308 | 0.475 | 0.282 | 0.24 | 0.241 |
| InternalIMT | dha | rs7921503 | 0.18 | -0.069 | 0.055 | 0.209 | 0.19 | 0.112 | 0.091 | -0.098 | 0.077 | 0.204 |
| InternalIMT | dha | rs7909740 | 0.181 | -0.07 | 0.055 | 0.201 | 0.183 | 0.112 | 0.101 | -0.094 | 0.077 | 0.222 |
| InternalIMT | dha | rs7909768 | 0.181 | -0.07 | 0.055 | 0.2 | 0.183 | 0.112 | 0.102 | -0.094 | 0.077 | 0.223 |
| InternalIMT | dha | rs144168004 | 0.001 | . | . | . | . | . | . | . | . | . |
| InternalIMT | dha | rs75850448 | 0.02 | -0.119 | 0.048 | 0.013 | -0.397 | 0.374 | 0.289 | 0.403 | 0.286 | 0.159 |
| InternalIMT | dha | rs147777574 | 0.016 | -0.105 | 0.048 | 0.028 | 0.114 | 0.424 | 0.788 | -0.05 | 0.282 | 0.859 |
| InternalIMT | dha | rs7913488 | 0.181 | -0.07 | 0.055 | 0.199 | 0.182 | 0.112 | 0.103 | -0.093 | 0.077 | 0.225 |
| InternalIMT | dha | rs74390874 | 0.019 | -0.121 | 0.049 | 0.014 | -0.619 | 0.378 | 0.101 | 0.39 | 0.291 | 0.18 |
| InternalIMT | dha | rs141391142 | 0.002 | . | . | . | . | . | . | . | . | . |
| InternalIMT | dha | rs115294747 | 0.017 | -0.1 | 0.048 | 0.038 | 0.207 | 0.256 | 0.417 | -0.171 | 0.155 | 0.272 |
| InternalIMT | dha | rs148031781 | 0.001 | . | . | . | . | . | . | . | . | . |
| InternalIMT | dha | rs181340128 | 0.007 | . | . | . | . | . | . | . | . | . |
| InternalIMT | dha | rs189930007 | 0 | . | . | . | . | . | . | . | . | . |
| InternalIMT | dha | rs12242128 | 0.304 | -0.068 | 0.064 | 0.29 | 0.103 | 0.107 | 0.335 | -0.065 | 0.075 | 0.38 |
| InternalIMT | dha | rs78421750 | 0.016 | -0.117 | 0.048 | 0.016 | -0.447 | 0.29 | 0.123 | 0.259 | 0.209 | 0.214 |
| InternalIMT | dha | rs114370747 | 0.007 | . | . | . | . | . | . | . | . | . |
| InternalIMT | dha | rs12264166 | 0.304 | -0.068 | 0.064 | 0.291 | 0.104 | 0.107 | 0.332 | -0.066 | 0.074 | 0.377 |
| InternalIMT | dha | rs182964822 | 0 | . | . | . | . | . | . | . | . | . |
| InternalIMT | dha | rs67491417 | 0.178 | -0.074 | 0.055 | 0.176 | 0.175 | 0.113 | 0.124 | -0.082 | 0.078 | 0.293 |
| InternalIMT | dha | rs72789720 | 0.006 | . | . | . | . | . | . | . | . | . |
| InternalIMT | dha | rs6413416 | 0.182 | -0.072 | 0.055 | 0.19 | 0.175 | 0.111 | 0.116 | -0.089 | 0.077 | 0.247 |
| InternalIMT | dha | rs4986832 | 0.182 | -0.072 | 0.055 | 0.191 | 0.176 | 0.111 | 0.114 | -0.089 | 0.077 | 0.245 |
| InternalIMT | dha | rs144866316 | . | . | . | . | . | . | . | . | . | . |
| InternalIMT | dha | rs187769540 | 0.001 | . | . | . | . | . | . | . | . | . |
| InternalIMT | dha | rs6593482 | 0.182 | -0.072 | 0.055 | 0.189 | 0.173 | 0.111 | 0.119 | -0.087 | 0.076 | 0.253 |
| InternalIMT | dha | rs7913948 | 0.183 | -0.071 | 0.055 | 0.194 | 0.176 | 0.111 | 0.113 | -0.09 | 0.076 | 0.237 |
| InternalIMT | dha | rs77185151 | 0.025 | -0.106 | 0.048 | 0.027 | 0.044 | 0.311 | 0.887 | 0.025 | 0.206 | 0.905 |
| InternalIMT | dha | rs12762303 | 0.182 | -0.072 | 0.055 | 0.19 | 0.175 | 0.111 | 0.115 | -0.089 | 0.077 | 0.246 |
| InternalIMT | dha | rs73283136 | 0.008 | . | . | . | . | . | . | . | . | . |
| InternalIMT | dha | rs187180218 | 0.007 | . | . | . | . | . | . | . | . | . |
| InternalIMT | dha | rs78137087 | 0.182 | -0.072 | 0.055 | 0.19 | 0.176 | 0.111 | 0.114 | -0.089 | 0.077 | 0.246 |
| InternalIMT | dha | rs72561472 | 0.181 | -0.069 | 0.056 | 0.216 | 0.181 | 0.113 | 0.112 | -0.097 | 0.077 | 0.209 |
| InternalIMT | dha | rs4987106 | 0.18 | -0.075 | 0.055 | 0.171 | 0.165 | 0.112 | 0.141 | -0.083 | 0.077 | 0.285 |
| InternalIMT | dha | rs4987105 | 0.159 | -0.058 | 0.055 | 0.284 | 0.239 | 0.118 | 0.042 | -0.144 | 0.08 | 0.074 |
| InternalIMT | dha | rs111916157 | 0.187 | -0.068 | 0.055 | 0.218 | 0.187 | 0.113 | 0.097 | -0.1 | 0.078 | 0.202 |
| InternalIMT | dha | rs111528152 | 0.184 | -0.074 | 0.055 | 0.18 | 0.159 | 0.118 | 0.177 | -0.081 | 0.082 | 0.322 |
| InternalIMT | dha | rs12763738 | 0.178 | -0.075 | 0.055 | 0.172 | 0.167 | 0.113 | 0.14 | -0.082 | 0.078 | 0.297 |
| InternalIMT | dha | rs191675886 | 0.011 | -0.103 | 0.048 | 0.032 | 0.43 | 0.716 | 0.548 | -0.249 | 0.494 | 0.615 |
| InternalIMT | dha | rs66489870 | 0.182 | -0.072 | 0.055 | 0.19 | 0.175 | 0.111 | 0.115 | -0.089 | 0.077 | 0.246 |
| InternalIMT | dha | rs71494775 | 0.182 | -0.072 | 0.055 | 0.19 | 0.175 | 0.111 | 0.115 | -0.089 | 0.077 | 0.246 |
| InternalIMT | dha | rs61855875 | 0.01 | -0.12 | 0.048 | 0.013 | -1.14 | 0.697 | 0.103 | 0.718 | 0.474 | 0.13 |
| InternalIMT | dha | rs116356837 | 0.019 | -0.118 | 0.048 | 0.014 | -0.236 | 0.305 | 0.441 | 0.29 | 0.238 | 0.223 |
| InternalIMT | dha | rs1864414 | 0.179 | -0.073 | 0.055 | 0.185 | 0.176 | 0.112 | 0.117 | -0.087 | 0.077 | 0.258 |
| InternalIMT | dha | rs1864413 | 0.182 | -0.072 | 0.055 | 0.19 | 0.175 | 0.111 | 0.115 | -0.089 | 0.077 | 0.248 |
| InternalIMT | dha | rs139497212 | 0 | . | . | . | . | . | . | . | . | . |
| InternalIMT | dha | rs892690 | 0.104 | -0.138 | 0.052 | 7.80E-03 | -0.23 | 0.139 | 0.099 | 0.147 | 0.097 | 0.128 |
| InternalIMT | dha | rs1864412 | 0.182 | -0.072 | 0.055 | 0.19 | 0.176 | 0.111 | 0.114 | -0.089 | 0.077 | 0.246 |
| InternalIMT | dha | rs77827771 | 0.087 | -0.094 | 0.052 | 0.071 | 0.177 | 0.162 | 0.275 | -0.091 | 0.113 | 0.419 |
| InternalIMT | dha | rs145294963 | . | . | . | . | . | . | . | . | . | . |
| InternalIMT | dha | rs149081327 | 0.003 | . | . | . | . | . | . | . | . | . |
| InternalIMT | dha | rs67008983 | 0.182 | -0.072 | 0.055 | 0.191 | 0.176 | 0.111 | 0.113 | -0.089 | 0.077 | 0.246 |
| InternalIMT | dha | rs56150414 | 0.182 | -0.073 | 0.055 | 0.187 | 0.169 | 0.111 | 0.128 | -0.084 | 0.076 | 0.269 |
| InternalIMT | dha | rs55787481 | 0.182 | -0.07 | 0.055 | 0.204 | 0.182 | 0.112 | 0.104 | -0.093 | 0.077 | 0.227 |
| InternalIMT | dha | rs141270101 | 0.001 | . | . | . | . | . | . | . | . | . |
| InternalIMT | dha | rs114980075 | 0.022 | -0.11 | 0.049 | 0.024 | -0.02 | 0.296 | 0.946 | 0.037 | 0.205 | 0.856 |
| InternalIMT | dha | rs12783095 | 0.181 | -0.072 | 0.055 | 0.191 | 0.177 | 0.111 | 0.111 | -0.089 | 0.077 | 0.247 |
| InternalIMT | dha | rs182342688 | 0.001 | . | . | . | . | . | . | . | . | . |

| InternalIMT | dha | rs17157728 | 0.181 | -0.072 | 0.055 | 0.191 | 0.177 | 0.111 | 0.111 | -0.089 | 0.077 | 0.247 |
| --- | --- | --- | --- | --- | --- | --- | --- | --- | --- | --- | --- | --- |
| InternalIMT | dha | rs114835563 | 0.086 | -0.094 | 0.052 | 0.07 | 0.181 | 0.162 | 0.263 | -0.093 | 0.113 | 0.41 |
| InternalIMT | dha | rs3824612 | 0.104 | -0.138 | 0.052 | 7.90E-03 | -0.231 | 0.14 | 0.099 | 0.147 | 0.097 | 0.129 |
| InternalIMT | dha | rs17157733 | 0.181 | -0.072 | 0.055 | 0.191 | 0.178 | 0.111 | 0.111 | -0.089 | 0.077 | 0.247 |
| InternalIMT | dha | rs74128478 | 0.019 | -0.12 | 0.048 | 0.012 | -0.416 | 0.377 | 0.271 | 0.417 | 0.29 | 0.15 |
| InternalIMT | dha | rs184973589 | 0.001 | . | . | . | . | . | . | . | . | . |
| InternalIMT | dha | rs150479461 | 0 | . | . | . | . | . | . | . | . | . |
| InternalIMT | dha | rs138310407 | . | . | . | . | . | . | . | . | . | . |
| InternalIMT | dha | rs187426603 | . | . | . | . | . | . | . | . | . | . |
| InternalIMT | dha | rs190843143 | 0.001 | . | . | . | . | . | . | . | . | . |
| InternalIMT | dha | rs191883449 | 0 | . | . | . | . | . | . | . | . | . |
| InternalIMT | dha | rs58238740 | 0.173 | -0.073 | 0.054 | 0.182 | 0.185 | 0.119 | 0.119 | -0.091 | 0.082 | 0.269 |
| InternalIMT | dha | rs3780894 | 0.173 | -0.073 | 0.054 | 0.182 | 0.185 | 0.119 | 0.119 | -0.091 | 0.082 | 0.269 |
| InternalIMT | dha | rs149646626 | 0.006 | . | . | . | . | . | . | . | . | . |
| InternalIMT | dha | rs79615212 | 0.006 | . | . | . | . | . | . | . | . | . |
| InternalIMT | dha | rs3780895 | 0.173 | -0.073 | 0.054 | 0.182 | 0.186 | 0.119 | 0.118 | -0.091 | 0.082 | 0.268 |
| InternalIMT | dha | rs3780896 | 0.173 | -0.073 | 0.054 | 0.182 | 0.185 | 0.119 | 0.119 | -0.091 | 0.082 | 0.269 |
| InternalIMT | dha | rs142747163 | 0.003 | . | . | . | . | . | . | . | . | . |
| InternalIMT | dha | rs189417584 | 0.01 | -0.098 | 0.048 | 0.041 | 0.733 | 0.493 | 0.138 | -0.523 | 0.315 | 0.097 |
| InternalIMT | dha | rs3780897 | 0.17 | -0.074 | 0.054 | 0.174 | 0.192 | 0.119 | 0.108 | -0.088 | 0.083 | 0.288 |
| InternalIMT | dha | rs117509491 | 0.002 | . | . | . | . | . | . | . | . | . |
| InternalIMT | dha | rs117811514 | 0.002 | . | . | . | . | . | . | . | . | . |
| InternalIMT | dha | rs116500932 | 0.086 | -0.094 | 0.052 | 0.07 | 0.181 | 0.162 | 0.264 | -0.093 | 0.113 | 0.411 |
| InternalIMT | dha | rs77584168 | . | . | . | . | . | . | . | . | . | . |
| InternalIMT | dha | rs2310776 | 0.173 | -0.073 | 0.054 | 0.182 | 0.185 | 0.119 | 0.119 | -0.091 | 0.082 | 0.269 |
| InternalIMT | dha | rs17157736 | 0.173 | -0.073 | 0.054 | 0.182 | 0.185 | 0.119 | 0.119 | -0.091 | 0.082 | 0.269 |
| InternalIMT | dha | rs72789735 | 0.036 | -0.086 | 0.049 | 0.078 | 0.482 | 0.274 | 0.079 | -0.345 | 0.191 | 0.072 |
| InternalIMT | dha | rs183747187 | 0.001 | . | . | . | . | . | . | . | . | . |
| InternalIMT | dha | rs7077891 | 0.173 | -0.073 | 0.054 | 0.182 | 0.185 | 0.119 | 0.119 | -0.091 | 0.082 | 0.269 |
| InternalIMT | dha | rs145063305 | 0.002 | . | . | . | . | . | . | . | . | . |
| InternalIMT | dha | rs3824613 | 0.174 | -0.073 | 0.054 | 0.176 | 0.185 | 0.117 | 0.114 | -0.088 | 0.081 | 0.277 |
| InternalIMT | dha | rs145543199 | 0.004 | . | . | . | . | . | . | . | . | . |
| InternalIMT | dha | rs140361993 | 0.001 | . | . | . | . | . | . | . | . | . |
| InternalIMT | dha | rs11239496 | 0.036 | -0.086 | 0.049 | 0.078 | 0.482 | 0.274 | 0.079 | -0.345 | 0.191 | 0.071 |
| InternalIMT | dha | rs11239497 | 0.154 | -0.058 | 0.054 | 0.289 | 0.258 | 0.126 | 0.041 | -0.152 | 0.086 | 0.078 |
| InternalIMT | dha | rs10900212 | 0.245 | -0.048 | 0.059 | 0.413 | 0.209 | 0.104 | 0.044 | -0.112 | 0.071 | 0.116 |
| InternalIMT | dha | rs186487136 | . | . | . | . | . | . | . | . | . | . |
| InternalIMT | dha | rs12774369 | 0.173 | -0.073 | 0.055 | 0.183 | 0.187 | 0.119 | 0.118 | -0.092 | 0.083 | 0.266 |
| InternalIMT | dha | rs60842538 | 0.173 | -0.073 | 0.055 | 0.183 | 0.187 | 0.12 | 0.118 | -0.093 | 0.083 | 0.264 |
| InternalIMT | dha | rs3780898 | 0.153 | -0.058 | 0.055 | 0.289 | 0.259 | 0.127 | 0.041 | -0.154 | 0.086 | 0.075 |
| InternalIMT | dha | rs3780899 | 0.172 | -0.073 | 0.055 | 0.183 | 0.188 | 0.12 | 0.118 | -0.094 | 0.083 | 0.261 |
| InternalIMT | dha | rs12252472 | 0.273 | -0.081 | 0.06 | 0.173 | 0.081 | 0.104 | 0.437 | -0.05 | 0.074 | 0.499 |
| InternalIMT | dha | rs185411011 | 0.001 | . | . | . | . | . | . | . | . | . |
| InternalIMT | dha | rs28395865 | 0.152 | -0.059 | 0.055 | 0.28 | 0.255 | 0.127 | 0.045 | -0.152 | 0.087 | 0.08 |
| InternalIMT | dha | rs191004074 | 0.034 | -0.107 | 0.049 | 0.03 | 0.129 | 0.422 | 0.76 | -0.044 | 0.29 | 0.879 |
| InternalIMT | dha | rs182401295 | . | . | . | . | . | . | . | . | . | . |
| InternalIMT | dha | rs2228064 | 0.277 | -0.075 | 0.06 | 0.207 | 0.1 | 0.104 | 0.336 | -0.06 | 0.073 | 0.41 |
| InternalIMT | dha | rs188284159 | . | . | . | . | . | . | . | . | . | . |
| InternalIMT | dha | rs2303887 | 0.003 | . | . | . | . | . | . | . | . | . |
| InternalIMT | dha | rs2303886 | 0.014 | -0.114 | 0.048 | 0.017 | -0.17 | 0.595 | 0.776 | 0.19 | 0.388 | 0.624 |
| InternalIMT | dha | rs192621006 | . | . | . | . | . | . | . | . | . | . |
| InternalIMT | dha | rs2303885 | 0.491 | -0.023 | 0.074 | 0.758 | 0.151 | 0.084 | 0.074 | -0.087 | 0.059 | 0.142 |
| InternalIMT | dha | rs114466168 | 0.086 | -0.089 | 0.052 | 0.084 | 0.215 | 0.162 | 0.184 | -0.114 | 0.113 | 0.313 |
| InternalIMT | dha | rs11239501 | 0.038 | -0.09 | 0.049 | 0.065 | 0.416 | 0.277 | 0.134 | -0.26 | 0.191 | 0.173 |
| InternalIMT | dha | rs745986 | 0.156 | -0.052 | 0.055 | 0.345 | 0.268 | 0.124 | 0.031 | -0.174 | 0.083 | 0.036 |
| InternalIMT | dha | rs75262268 | 0.004 | . | . | . | . | . | . | . | . | . |
| InternalIMT | dha | rs71494776 | 0.022 | -0.106 | 0.048 | 0.028 | -7.00E-03 | 0.352 | 0.984 | -0.013 | 0.241 | 0.957 |
| InternalIMT | dha | rs74386871 | 0.006 | . | . | . | . | . | . | . | . | . |
| InternalIMT | dha | rs76502040 | 0.004 | . | . | . | . | . | . | . | . | . |
| InternalIMT | dha | rs142213734 | 0.001 | . | . | . | . | . | . | . | . | . |
| InternalIMT | dha | rs4948671 | 0.796 | 6.70E-03 | 0.126 | 0.958 | 0.134 | 0.107 | 0.209 | -0.07 | 0.073 | 0.338 |
| InternalIMT | dha | rs146286085 | 0 | . | . | . | . | . | . | . | . | . |
| InternalIMT | dha | rs144080623 | 0 | . | . | . | . | . | . | . | . | . |
| InternalIMT | dha | rs115074014 | 0.133 | -0.09 | 0.053 | 0.091 | 0.075 | 0.147 | 0.611 | -0.065 | 0.103 | 0.528 |
| InternalIMT | dha | rs17157756 | 0.133 | -0.09 | 0.053 | 0.091 | 0.075 | 0.147 | 0.611 | -0.065 | 0.103 | 0.528 |
| InternalIMT | dha | rs190459780 | 0.001 | . | . | . | . | . | . | . | . | . |
| InternalIMT | dha | rs12358647 | 0.25 | -0.067 | 0.06 | 0.262 | 0.136 | 0.104 | 0.191 | -0.076 | 0.07 | 0.277 |
| InternalIMT | dha | rs147080556 | 0.009 | . | . | . | . | . | . | . | . | . |
| InternalIMT | dha | rs187543349 | 0.002 | . | . | . | . | . | . | . | . | . |
| InternalIMT | dha | rs114375595 | 0.024 | -0.112 | 0.048 | 0.02 | -0.113 | 0.31 | 0.715 | 0.141 | 0.204 | 0.492 |
| InternalIMT | dha | rs117211200 | 0.003 | . | . | . | . | . | . | . | . | . |
| InternalIMT | dha | rs192963047 | . | . | . | . | . | . | . | . | . | . |
| InternalIMT | dha | rs11239505 | 0.254 | -0.056 | 0.06 | 0.353 | 0.167 | 0.103 | 0.106 | -0.095 | 0.069 | 0.17 |
| InternalIMT | dha | rs184434116 | 0.001 | . | . | . | . | . | . | . | . | . |
| InternalIMT | dha | rs4948999 | 0.801 | -5.00E-03 | 0.127 | 0.969 | 0.117 | 0.108 | 0.278 | -0.062 | 0.073 | 0.395 |
| InternalIMT | dha | rs11239506 | 0.078 | -0.089 | 0.051 | 0.079 | 0.138 | 0.179 | 0.442 | -0.108 | 0.123 | 0.383 |
| InternalIMT | dha | rs190706620 | 0.001 | . | . | . | . | . | . | . | . | . |
| InternalIMT | dha | rs190770429 | 0.001 | . | . | . | . | . | . | . | . | . |
| InternalIMT | dha | rs181327133 | 0.003 | . | . | . | . | . | . | . | . | . |
| InternalIMT | dha | rs149251697 | 0.057 | -0.103 | 0.05 | 0.039 | 0.104 | 0.205 | 0.612 | -0.039 | 0.133 | 0.773 |
| InternalIMT | dha | rs118081602 | 0.003 | . | . | . | . | . | . | . | . | . |
| InternalIMT | dha | rs188233691 | . | . | . | . | . | . | . | . | . | . |
| InternalIMT | dha | rs183778794 | 0.006 | . | . | . | . | . | . | . | . | . |
| InternalIMT | dha | rs188040078 | 0.003 | . | . | . | . | . | . | . | . | . |
| InternalIMT | dha | rs184675372 | 0.001 | . | . | . | . | . | . | . | . | . |
| InternalIMT | dha | rs150326891 | 0.03 | -0.096 | 0.048 | 0.045 | 0.44 | 0.423 | 0.298 | -0.222 | 0.296 | 0.453 |

| InternalIMT | dha | rs185520021 | . | . | . | . | . | . | . | . | . | . |
| --- | --- | --- | --- | --- | --- | --- | --- | --- | --- | --- | --- | --- |
| InternalIMT | dha | rs7907779 | 0.078 | -0.089 | 0.051 | 0.079 | 0.136 | 0.179 | 0.446 | -0.107 | 0.123 | 0.385 |
| InternalIMT | dha | rs184962860 | . | . | . | . | . | . | . | . | . | . |
| InternalIMT | dha | rs149762784 | 0.034 | -0.084 | 0.05 | 0.092 | 0.519 | 0.292 | 0.076 | -0.309 | 0.187 | 0.097 |
| InternalIMT | dha | rs149203427 | 0.005 | . | . | . | . | . | . | . | . | . |
| InternalIMT | dha | rs60153083 | 0.045 | -0.111 | 0.05 | 0.028 | -0.084 | 0.207 | 0.686 | 0.039 | 0.14 | 0.779 |
| InternalIMT | dha | rs112001069 | 0.073 | -0.08 | 0.051 | 0.118 | 0.324 | 0.187 | 0.083 | -0.196 | 0.124 | 0.116 |
| InternalIMT | dha | rs182355382 | 0.057 | -0.103 | 0.051 | 0.044 | 0.072 | 0.213 | 0.733 | -0.036 | 0.14 | 0.796 |
| InternalIMT | dha | rs186740974 | 0.157 | -0.111 | 0.059 | 0.06 | -0.064 | 0.159 | 0.687 | 0.018 | 0.108 | 0.87 |
| InternalIMT | dha | rs191874916 | 0.06 | -0.106 | 0.049 | 0.032 | 0.055 | 0.231 | 0.811 | 0.01 | 0.166 | 0.95 |
| InternalIMT | dha | rs182989412 | 0.126 | -0.094 | 0.056 | 0.095 | 0.067 | 0.181 | 0.713 | -0.052 | 0.119 | 0.664 |
| InternalIMT | dha | rs188819476 | 0.003 | . | . | . | . | . | . | . | . | . |
| InternalIMT | dha | rs141217183 | 0.083 | -0.076 | 0.052 | 0.149 | 0.319 | 0.183 | 0.081 | -0.2 | 0.121 | 0.099 |
| InternalIMT | dha | rs192922652 | 0.081 | -0.074 | 0.052 | 0.151 | 0.369 | 0.173 | 0.034 | -0.226 | 0.114 | 0.048 |
| InternalIMT | dha | rs185092343 | 0.025 | -0.117 | 0.049 | 0.016 | -0.192 | 0.492 | 0.697 | 0.195 | 0.351 | 0.579 |
| InternalIMT | dha | rs145193311 | 0.082 | -0.076 | 0.052 | 0.141 | 0.336 | 0.17 | 0.048 | -0.205 | 0.112 | 0.067 |
| InternalIMT | dha | rs55789336 | 0.084 | -0.084 | 0.051 | 0.098 | 0.173 | 0.174 | 0.32 | -0.13 | 0.12 | 0.281 |
| InternalIMT | dha | rs55837212 | 0.083 | -0.095 | 0.051 | 0.066 | 0.09 | 0.173 | 0.603 | -0.073 | 0.121 | 0.549 |
| InternalIMT | dha | rs75408574 | 0.208 | -0.171 | 0.056 | 2.40E-03 | -0.243 | 0.108 | 0.024 | 0.145 | 0.076 | 0.056 |
| InternalIMT | dha | rs149306926 | 0.089 | -0.09 | 0.052 | 0.085 | 0.122 | 0.173 | 0.483 | -0.093 | 0.119 | 0.434 |
| InternalIMT | dha | rs35990892 | 0.683 | -0.035 | 0.108 | 0.744 | 0.063 | 0.099 | 0.522 | -0.054 | 0.069 | 0.435 |
| InternalIMT | dha | rs192356207 | 0.128 | -0.097 | 0.055 | 0.079 | 0.105 | 0.144 | 0.465 | -0.045 | 0.097 | 0.643 |
| InternalIMT | dha | rs183741163 | 0.097 | -0.09 | 0.052 | 0.085 | 0.122 | 0.169 | 0.471 | -0.081 | 0.12 | 0.501 |
| InternalIMT | dha | rs34737297 | 0.591 | -0.067 | 0.096 | 0.489 | 0.102 | 0.107 | 0.341 | -0.033 | 0.073 | 0.658 |
| InternalIMT | dha | rs181346933 | 0.042 | -0.094 | 0.05 | 0.059 | 0.221 | 0.256 | 0.39 | -0.153 | 0.178 | 0.389 |
| InternalIMT | dha | rs36093253 | 0.24 | -0.135 | 0.062 | 0.03 | -0.099 | 0.104 | 0.342 | 0.05 | 0.073 | 0.49 |
| InternalIMT | dha | rs186307855 | 0.262 | -0.055 | 0.059 | 0.351 | 0.15 | 0.103 | 0.145 | -0.101 | 0.072 | 0.161 |
| InternalIMT | dha | rs189681865 | 0.096 | -0.087 | 0.053 | 0.103 | 0.15 | 0.161 | 0.351 | -0.095 | 0.104 | 0.363 |
| InternalIMT | dha | rs34942971 | 0.259 | -0.058 | 0.063 | 0.362 | 0.15 | 0.107 | 0.162 | -0.089 | 0.073 | 0.218 |
| InternalIMT | dha | rs181816554 | 0.064 | -0.097 | 0.051 | 0.059 | 0.109 | 0.225 | 0.627 | -0.076 | 0.149 | 0.61 |
| InternalIMT | dha | rs186846881 | 0.278 | -0.058 | 0.061 | 0.347 | 0.147 | 0.109 | 0.177 | -0.091 | 0.075 | 0.226 |
| InternalIMT | dha | rs191178220 | 0.008 | . | . | . | . | . | . | . | . | . |
| InternalIMT | dha | rs183305228 | 0.046 | -0.111 | 0.05 | 0.028 | -0.085 | 0.205 | 0.679 | 0.041 | 0.139 | 0.765 |
| InternalIMT | dha | rs139912761 | 0.149 | -0.076 | 0.056 | 0.179 | 0.14 | 0.142 | 0.325 | -0.092 | 0.094 | 0.329 |
| InternalIMT | dha | rs185499901 | 0.018 | -0.1 | 0.049 | 0.04 | 0.317 | 0.525 | 0.545 | -0.176 | 0.335 | 0.599 |
| InternalIMT | dha | rs189816197 | 0.086 | -0.09 | 0.053 | 0.086 | 0.164 | 0.161 | 0.309 | -0.099 | 0.108 | 0.363 |
| InternalIMT | dha | rs145656938 | 0.058 | -0.093 | 0.051 | 0.067 | 0.212 | 0.231 | 0.36 | -0.121 | 0.149 | 0.415 |
| InternalIMT | dha | rs35745197 | 0.072 | -0.084 | 0.051 | 0.103 | 0.285 | 0.186 | 0.125 | -0.173 | 0.124 | 0.165 |
| InternalIMT | dha | rs192856637 | . | . | . | . | . | . | . | . | . | . |
| InternalIMT | dha | rs2310775 | 0.267 | -0.168 | 0.061 | 5.70E-03 | -0.179 | 0.094 | 0.057 | 0.112 | 0.067 | 0.096 |
| InternalIMT | dha | rs11239507 | 0.538 | -0.134 | 0.081 | 0.098 | -0.043 | 0.088 | 0.628 | 0.025 | 0.061 | 0.682 |
| InternalIMT | dha | rs74648467 | 0.027 | -0.096 | 0.049 | 0.051 | 0.244 | 0.353 | 0.49 | -0.21 | 0.247 | 0.395 |
| InternalIMT | dha | rs145815934 | 0.005 | . | . | . | . | . | . | . | . | . |
| InternalIMT | dha | rs145390687 | 0.012 | -0.117 | 0.048 | 0.016 | -0.925 | 0.736 | 0.209 | 0.605 | 0.655 | 0.356 |
| InternalIMT | dha | rs184354913 | 0.012 | -0.107 | 0.048 | 0.027 | -0.012 | 0.568 | 0.983 | -0.049 | 0.388 | 0.899 |
| InternalIMT | dha | rs181244920 | 0.003 | . | . | . | . | . | . | . | . | . |
| InternalIMT | dha | rs11499087 | 0.167 | -0.092 | 0.053 | 0.084 | 0.028 | 0.125 | 0.825 | -0.044 | 0.087 | 0.61 |
| InternalIMT | dha | rs148263794 | 0.079 | -0.081 | 0.052 | 0.117 | 0.296 | 0.174 | 0.089 | -0.18 | 0.115 | 0.119 |
| InternalIMT | dha | rs150758903 | 0.089 | -0.09 | 0.053 | 0.09 | 0.145 | 0.169 | 0.39 | -0.09 | 0.11 | 0.416 |
| InternalIMT | dha | rs147654382 | 0.192 | -0.11 | 0.057 | 0.054 | 0.04 | 0.129 | 0.759 | 0.01 | 0.088 | 0.905 |
| InternalIMT | dha | rs147495166 | 0.077 | -0.072 | 0.052 | 0.165 | 0.38 | 0.159 | 0.017 | -0.226 | 0.106 | 0.033 |
| InternalIMT | dha | rs35198778 | 0.14 | -0.101 | 0.054 | 0.061 | 0.108 | 0.159 | 0.496 | -0.023 | 0.107 | 0.832 |
| InternalIMT | dha | rs145207659 | 0.02 | -0.116 | 0.049 | 0.018 | -0.482 | 0.478 | 0.314 | 0.292 | 0.36 | 0.418 |
| InternalIMT | dha | rs186466464 | 0.024 | -0.079 | 0.049 | 0.108 | 0.91 | 0.558 | 0.104 | -0.574 | 0.352 | 0.103 |
| InternalIMT | dha | rs12778413 | 0.199 | -0.087 | 0.058 | 0.137 | 0.108 | 0.113 | 0.341 | -0.046 | 0.077 | 0.553 |
| InternalIMT | dha | rs7895552 | 0.724 | -0.066 | 0.12 | 0.583 | 0.06 | 0.106 | 0.571 | -0.027 | 0.073 | 0.713 |
| InternalIMT | dha | rs12783895 | 0.076 | -0.081 | 0.051 | 0.114 | 0.32 | 0.182 | 0.08 | -0.196 | 0.121 | 0.105 |
| InternalIMT | dha | rs34929604 | 0.081 | -0.075 | 0.051 | 0.146 | 0.369 | 0.175 | 0.035 | -0.228 | 0.116 | 0.049 |
| InternalIMT | dha | rs12761961 | 0.072 | -0.083 | 0.051 | 0.107 | 0.312 | 0.194 | 0.109 | -0.188 | 0.13 | 0.149 |
| InternalIMT | dha | rs35536930 | 0.252 | -0.058 | 0.06 | 0.337 | 0.164 | 0.107 | 0.124 | -0.093 | 0.072 | 0.193 |
| InternalIMT | dha | rs180951060 | 0.006 | . | . | . | . | . | . | . | . | . |
| InternalIMT | dha | rs148892128 | 0.005 | . | . | . | . | . | . | . | . | . |
| InternalIMT | dha | rs11239509 | 0.255 | -0.058 | 0.058 | 0.31 | 0.134 | 0.102 | 0.189 | -0.096 | 0.071 | 0.175 |
| InternalIMT | dha | rs41526545 | 0.045 | -0.087 | 0.049 | 0.076 | 0.378 | 0.26 | 0.146 | -0.273 | 0.177 | 0.123 |
| InternalIMT | dha | rs2029253 | 0.286 | -0.181 | 0.061 | 2.80E-03 | -0.191 | 0.09 | 0.034 | 0.132 | 0.064 | 0.04 |
| InternalIMT | dha | rs17444064 | 0.041 | -0.088 | 0.049 | 0.069 | 0.413 | 0.269 | 0.125 | -0.269 | 0.184 | 0.145 |
| InternalIMT | dha | rs71494779 | 0.084 | -0.075 | 0.052 | 0.148 | 0.343 | 0.167 | 0.041 | -0.21 | 0.11 | 0.057 |
| InternalIMT | dha | rs74991420 | 0.003 | . | . | . | . | . | . | . | . | . |
| InternalIMT | dha | rs182736551 | 0.003 | . | . | . | . | . | . | . | . | . |
| InternalIMT | dha | rs78702074 | 0.003 | . | . | . | . | . | . | . | . | . |
| InternalIMT | dha | rs187791466 | . | . | . | . | . | . | . | . | . | . |
| InternalIMT | dha | rs35300270 | 0.085 | -0.075 | 0.052 | 0.149 | 0.337 | 0.167 | 0.044 | -0.208 | 0.11 | 0.06 |
| InternalIMT | dha | rs6593484 | 0.078 | -0.091 | 0.051 | 0.075 | 0.122 | 0.179 | 0.497 | -0.1 | 0.124 | 0.419 |
| InternalIMT | dha | rs12244601 | 0.064 | -0.099 | 0.051 | 0.053 | 0.116 | 0.187 | 0.534 | -0.062 | 0.122 | 0.612 |
| InternalIMT | dha | rs184429574 | 0.006 | . | . | . | . | . | . | . | . | . |
| InternalIMT | dha | rs7080897 | 0.084 | -0.075 | 0.052 | 0.149 | 0.343 | 0.167 | 0.04 | -0.211 | 0.11 | 0.056 |
| InternalIMT | dha | rs7097429 | 0.085 | -0.078 | 0.052 | 0.135 | 0.303 | 0.163 | 0.063 | -0.184 | 0.108 | 0.088 |
| InternalIMT | dha | rs77979229 | . | . | . | . | . | . | . | . | . | . |
| InternalIMT | dha | rs11239512 | 0.079 | -0.09 | 0.051 | 0.079 | 0.13 | 0.176 | 0.459 | -0.106 | 0.122 | 0.382 |
| InternalIMT | dha | rs60146827 | 0.084 | -0.075 | 0.052 | 0.149 | 0.343 | 0.167 | 0.04 | -0.211 | 0.11 | 0.056 |
| InternalIMT | dha | rs79032236 | 0.105 | -0.098 | 0.053 | 0.067 | 0.171 | 0.158 | 0.281 | -0.063 | 0.113 | 0.575 |
| InternalIMT | dha | rs140854206 | . | . | . | . | . | . | . | . | . | . |
| InternalIMT | dha | rs184049550 | 0.012 | -0.107 | 0.048 | 0.026 | -0.15 | 0.242 | 0.535 | 0.046 | 0.172 | 0.787 |
| InternalIMT | dha | rs114433607 | 0.029 | -0.1 | 0.05 | 0.044 | 0.098 | 0.292 | 0.738 | -0.109 | 0.189 | 0.562 |
| InternalIMT | dha | rs117653793 | 0.003 | . | . | . | . | . | . | . | . | . |
| InternalIMT | dha | rs117039533 | 0.003 | . | . | . | . | . | . | . | . | . |

| InternalIMT | dha | rs191660761 | 0.002 | . | . | . | . | . | . | . | . | . |
| --- | --- | --- | --- | --- | --- | --- | --- | --- | --- | --- | --- | --- |
| InternalIMT | dha | rs59514896 | 0.03 | -0.107 | 0.049 | 0.029 | 0.014 | 0.308 | 0.964 | -1.80E-03 | 0.212 | 0.993 |
| InternalIMT | dha | rs184661876 | 0.001 | . | . | . | . | . | . | . | . | . |
| InternalIMT | dha | rs61854073 | 0.011 | -0.118 | 0.048 | 0.015 | -1.08 | 0.597 | 0.071 | 0.601 | 0.407 | 0.14 |
| InternalIMT | dha | rs2018518 | 0.09 | -0.139 | 0.052 | 7.90E-03 | -0.294 | 0.151 | 0.051 | 0.159 | 0.107 | 0.138 |
| InternalIMT | dha | rs934187 | 0.802 | -0.032 | 0.127 | 0.803 | 0.09 | 0.107 | 0.403 | -0.046 | 0.073 | 0.529 |
| InternalIMT | dha | rs7918542 | 0.269 | -0.034 | 0.061 | 0.578 | 0.208 | 0.1 | 0.038 | -0.128 | 0.067 | 0.056 |
| InternalIMT | dha | rs144311920 | 0.004 | . | . | . | . | . | . | . | . | . |
| InternalIMT | dha | rs7099684 | 0.883 | -0.057 | 0.177 | 0.746 | 0.083 | 0.143 | 0.559 | -0.027 | 0.098 | 0.781 |
| InternalIMT | dha | rs150829171 | 0.001 | . | . | . | . | . | . | . | . | . |
| InternalIMT | dha | rs186060697 | 0.006 | . | . | . | . | . | . | . | . | . |
| InternalIMT | dha | rs11239513 | 0.277 | -0.077 | 0.059 | 0.196 | 0.093 | 0.103 | 0.366 | -0.058 | 0.073 | 0.427 |
| InternalIMT | dha | rs7917687 | 0.084 | -0.075 | 0.052 | 0.148 | 0.34 | 0.167 | 0.042 | -0.209 | 0.11 | 0.058 |
| InternalIMT | dha | rs61854076 | 0.004 | . | . | . | . | . | . | . | . | . |
| InternalIMT | dha | rs71494780 | 0.007 | . | . | . | . | . | . | . | . | . |
| InternalIMT | dha | rs7919239 | 0.257 | -0.057 | 0.06 | 0.342 | 0.159 | 0.102 | 0.118 | -0.092 | 0.068 | 0.179 |
| InternalIMT | dha | rs78334475 | 0.004 | . | . | . | . | . | . | . | . | . |
| InternalIMT | dha | rs3780900 | 0.035 | -0.105 | 0.048 | 0.029 | 0.031 | 0.306 | 0.92 | -0.032 | 0.214 | 0.88 |
| InternalIMT | dha | rs186729292 | 0.001 | . | . | . | . | . | . | . | . | . |
| InternalIMT | dha | rs12267438 | 0.073 | -0.095 | 0.051 | 0.063 | 0.136 | 0.177 | 0.443 | -0.077 | 0.115 | 0.502 |
| InternalIMT | dha | rs1369214 | 0.564 | -0.187 | 0.085 | 0.029 | -0.108 | 0.093 | 0.247 | 0.071 | 0.065 | 0.27 |
| InternalIMT | dha | rs9282589 | 0.005 | . | . | . | . | . | . | . | . | . |
| InternalIMT | dha | rs114522915 | 0.078 | -0.098 | 0.051 | 0.056 | 0.188 | 0.172 | 0.275 | -0.08 | 0.119 | 0.5 |
| InternalIMT | dha | rs2115819 | 0.292 | -0.178 | 0.062 | 4.50E-03 | -0.191 | 0.094 | 0.042 | 0.119 | 0.068 | 0.079 |
| InternalIMT | dha | rs117664603 | 0.003 | . | . | . | . | . | . | . | . | . |
| InternalIMT | dha | rs184979202 | 0.001 | . | . | . | . | . | . | . | . | . |
| InternalIMT | dha | rs190714671 | . | . | . | . | . | . | . | . | . | . |
| InternalIMT | dha | rs141538996 | . | . | . | . | . | . | . | . | . | . |
| InternalIMT | dha | rs150881741 | 0.001 | . | . | . | . | . | . | . | . | . |
| InternalIMT | dha | rs139076633 | . | . | . | . | . | . | . | . | . | . |
| InternalIMT | dha | rs12243649 | 0.156 | -0.077 | 0.056 | 0.165 | 0.202 | 0.127 | 0.112 | -0.095 | 0.085 | 0.264 |
| InternalIMT | dha | rs140348708 | . | . | . | . | . | . | . | . | . | . |
| InternalIMT | dha | rs79023256 | 0.003 | . | . | . | . | . | . | . | . | . |
| InternalIMT | dha | rs11239515 | 0.071 | -0.081 | 0.05 | 0.108 | 0.236 | 0.191 | 0.218 | -0.173 | 0.129 | 0.18 |
| InternalIMT | dha | rs55866086 | 0.24 | -0.037 | 0.061 | 0.544 | 0.232 | 0.111 | 0.037 | -0.138 | 0.074 | 0.063 |
| InternalIMT | dha | rs181340150 | 0.002 | . | . | . | . | . | . | . | . | . |
| InternalIMT | dha | rs11239516 | 0.158 | -0.057 | 0.057 | 0.31 | 0.201 | 0.138 | 0.145 | -0.143 | 0.091 | 0.117 |
| InternalIMT | dha | rs11239517 | 0.158 | -0.057 | 0.057 | 0.311 | 0.202 | 0.138 | 0.144 | -0.144 | 0.091 | 0.117 |
| InternalIMT | dha | rs11239519 | 0.128 | -0.056 | 0.056 | 0.31 | 0.231 | 0.145 | 0.112 | -0.181 | 0.097 | 0.062 |
| InternalIMT | dha | rs7909514 | 0.788 | -0.024 | 0.12 | 0.842 | 0.093 | 0.104 | 0.374 | -0.052 | 0.071 | 0.464 |
| InternalIMT | dha | rs7894352 | 0.885 | -0.045 | 0.179 | 0.802 | 0.101 | 0.146 | 0.489 | -0.034 | 0.099 | 0.735 |
| InternalIMT | dha | rs189384146 | 0.001 | . | . | . | . | . | . | . | . | . |
| InternalIMT | dha | rs11239520 | 0.107 | -0.082 | 0.052 | 0.112 | 0.136 | 0.157 | 0.388 | -0.114 | 0.109 | 0.296 |
| InternalIMT | dha | rs12221233 | 0.234 | -0.162 | 0.06 | 7.10E-03 | -0.195 | 0.103 | 0.057 | 0.114 | 0.073 | 0.12 |
| InternalIMT | dha | rs74523293 | 0.066 | -0.09 | 0.051 | 0.076 | 0.295 | 0.21 | 0.162 | -0.127 | 0.144 | 0.377 |
| InternalIMT | dha | rs79376040 | 0.066 | -0.09 | 0.051 | 0.076 | 0.295 | 0.21 | 0.162 | -0.127 | 0.144 | 0.378 |
| InternalIMT | dha | rs4949000 | 0.234 | -0.162 | 0.06 | 7.10E-03 | -0.195 | 0.103 | 0.057 | 0.114 | 0.073 | 0.119 |
| InternalIMT | dha | rs7089406 | 0.039 | -0.093 | 0.049 | 0.057 | 0.235 | 0.245 | 0.338 | -0.203 | 0.163 | 0.215 |
| InternalIMT | dha | rs138211056 | 0 | . | . | . | . | . | . | . | . | . |
| InternalIMT | dha | rs10900213 | 0.434 | -0.124 | 0.077 | 0.107 | -0.049 | 0.094 | 0.604 | 0.016 | 0.066 | 0.813 |
| InternalIMT | dha | rs145152827 | . | . | . | . | . | . | . | . | . | . |
| InternalIMT | dha | rs74443325 | 0.003 | . | . | . | . | . | . | . | . | . |
| InternalIMT | dha | rs188299446 | 0.004 | . | . | . | . | . | . | . | . | . |
| InternalIMT | dha | rs192699586 | . | . | . | . | . | . | . | . | . | . |
| InternalIMT | dha | rs72796464 | 0.041 | -0.088 | 0.049 | 0.069 | 0.42 | 0.27 | 0.12 | -0.277 | 0.185 | 0.133 |
| InternalIMT | dha | rs4949001 | 0.077 | -0.137 | 0.052 | 8.40E-03 | -0.311 | 0.173 | 0.073 | 0.175 | 0.122 | 0.151 |
| InternalIMT | dha | rs147174641 | 0.003 | . | . | . | . | . | . | . | . | . |
| InternalIMT | dha | rs190835238 | 0.001 | . | . | . | . | . | . | . | . | . |
| InternalIMT | dha | rs76698944 | 0.013 | -0.105 | 0.048 | 0.028 | 0.225 | 0.593 | 0.704 | -0.105 | 0.4 | 0.793 |
| InternalIMT | dha | rs56071381 | 0.129 | -0.075 | 0.054 | 0.161 | 0.146 | 0.141 | 0.302 | -0.121 | 0.096 | 0.209 |
| InternalIMT | dha | rs78130427 | 0.019 | -0.105 | 0.049 | 0.032 | -0.045 | 0.356 | 0.9 | -0.052 | 0.238 | 0.826 |
| InternalIMT | dha | rs10900215 | 0.249 | -0.059 | 0.062 | 0.343 | 0.116 | 0.107 | 0.277 | -0.09 | 0.073 | 0.22 |
| InternalIMT | dha | rs41491445 | 0.046 | -0.11 | 0.05 | 0.029 | -0.075 | 0.196 | 0.704 | 0.028 | 0.134 | 0.837 |
| InternalIMT | dha | rs144995222 | 0.006 | . | . | . | . | . | . | . | . | . |
| InternalIMT | dha | rs149097803 | 0.002 | . | . | . | . | . | . | . | . | . |
| InternalIMT | dha | rs139429646 | 0.002 | . | . | . | . | . | . | . | . | . |
| InternalIMT | dha | rs7075620 | 0.764 | -6.80E-03 | 0.129 | 0.958 | 0.101 | 0.111 | 0.361 | -0.064 | 0.077 | 0.404 |
| InternalIMT | dha | rs148299213 | 0.049 | -0.109 | 0.051 | 0.031 | -0.058 | 0.197 | 0.768 | 0.02 | 0.135 | 0.885 |
| InternalIMT | dha | rs4949002 | 0.084 | -0.14 | 0.052 | 7.00E-03 | -0.337 | 0.173 | 0.052 | 0.191 | 0.123 | 0.12 |
| InternalIMT | dha | rs150496007 | 0.023 | -0.097 | 0.049 | 0.047 | 0.258 | 0.272 | 0.343 | -0.191 | 0.166 | 0.251 |
| InternalIMT | dha | rs147303455 | 0.028 | -0.099 | 0.049 | 0.045 | 0.169 | 0.212 | 0.426 | -0.121 | 0.138 | 0.379 |
| InternalIMT | dha | rs189518433 | . | . | . | . | . | . | . | . | . | . |
| InternalIMT | dha | rs186281355 | . | . | . | . | . | . | . | . | . | . |
| InternalIMT | dha | rs115297114 | 0.077 | -0.083 | 0.052 | 0.111 | 0.294 | 0.161 | 0.069 | -0.15 | 0.11 | 0.172 |
| InternalIMT | dha | rs11239523 | 0.106 | -0.085 | 0.052 | 0.1 | 0.126 | 0.156 | 0.42 | -0.105 | 0.108 | 0.334 |
| InternalIMT | dha | rs143704897 | 0.007 | . | . | . | . | . | . | . | . | . |
| InternalIMT | dha | rs188024096 | 0.001 | . | . | . | . | . | . | . | . | . |
| InternalIMT | dha | rs11239524 | 0.133 | -0.08 | 0.055 | 0.148 | 0.114 | 0.139 | 0.414 | -0.097 | 0.096 | 0.316 |
| InternalIMT | dha | rs191656573 | 0.002 | . | . | . | . | . | . | . | . | . |
| InternalIMT | dha | rs193295875 | 0.001 | . | . | . | . | . | . | . | . | . |
| InternalIMT | dha | rs190458228 | . | . | . | . | . | . | . | . | . | . |
| InternalIMT | dha | rs139253993 | 0.001 | . | . | . | . | . | . | . | . | . |
| InternalIMT | dha | rs4948672 | 0.497 | -0.02 | 0.083 | 0.806 | 0.115 | 0.1 | 0.252 | -0.091 | 0.069 | 0.185 |
| InternalIMT | dha | rs180954921 | 0.001 | . | . | . | . | . | . | . | . | . |
| InternalIMT | dha | rs12264801 | 0.383 | -0.098 | 0.074 | 0.185 | 0.068 | 0.111 | 0.54 | -0.01 | 0.077 | 0.894 |
| InternalIMT | dha | rs73283184 | 0.057 | -0.101 | 0.051 | 0.047 | 0.041 | 0.177 | 0.815 | -0.054 | 0.118 | 0.645 |

| InternalIMT | dha | rs142746187 | 0.022 | -0.145 | 0.048 | 2.40E-03 | -0.974 | 0.356 | 6.30E-03 | 0.761 | 0.271 | 5.00E-03 |
| --- | --- | --- | --- | --- | --- | --- | --- | --- | --- | --- | --- | --- |
| InternalIMT | dha | rs58704739 | 0.053 | -0.102 | 0.051 | 0.045 | 0.033 | 0.178 | 0.852 | -0.049 | 0.118 | 0.675 |
| InternalIMT | dha | rs61272335 | 0.04 | -0.107 | 0.05 | 0.031 | -0.048 | 0.209 | 0.82 | -1.10E-03 | 0.143 | 0.994 |
| InternalIMT | dha | rs60959489 | 0.103 | -0.079 | 0.054 | 0.148 | 0.149 | 0.138 | 0.279 | -0.119 | 0.089 | 0.182 |
| InternalIMT | dha | rs58502629 | 0.04 | -0.107 | 0.05 | 0.031 | -0.047 | 0.209 | 0.822 | -1.10E-03 | 0.143 | 0.994 |
| InternalIMT | dha | rs10900216 | 0.744 | -7.90E-03 | 0.13 | 0.952 | 0.113 | 0.116 | 0.332 | -0.066 | 0.08 | 0.415 |
| InternalIMT | dha | rs181118141 | 0.004 | . | . | . | . | . | . | . | . | . |
| InternalIMT | dha | rs137882665 | 0.019 | -0.099 | 0.049 | 0.041 | 0.342 | 0.278 | 0.219 | -0.216 | 0.174 | 0.215 |
| InternalIMT | dha | rs149466736 | 0.002 | . | . | . | . | . | . | . | . | . |
| InternalIMT | dha | rs2099171 | 0.553 | -0.081 | 0.08 | 0.306 | 0.045 | 0.092 | 0.622 | -0.024 | 0.061 | 0.696 |
| InternalIMT | dha | rs141852280 | 0.002 | . | . | . | . | . | . | . | . | . |
| InternalIMT | dha | rs191539257 | . | . | . | . | . | . | . | . | . | . |
| InternalIMT | dha | rs17157771 | 0.045 | -0.115 | 0.049 | 0.018 | -0.15 | 0.225 | 0.505 | 0.078 | 0.164 | 0.634 |
| InternalIMT | dha | rs111339241 | 0.041 | -0.115 | 0.048 | 0.018 | -0.155 | 0.23 | 0.501 | 0.079 | 0.165 | 0.63 |
| InternalIMT | dha | rs116983255 | 0.004 | . | . | . | . | . | . | . | . | . |
| InternalIMT | dha | rs142324341 | . | . | . | . | . | . | . | . | . | . |
| InternalIMT | dha | rs187191219 | 0.015 | -0.103 | 0.048 | 0.033 | 0.211 | 0.306 | 0.49 | -0.139 | 0.192 | 0.469 |
| InternalIMT | dha | rs151283973 | 0.005 | . | . | . | . | . | . | . | . | . |
| InternalIMT | dha | rs139316218 | 0.001 | . | . | . | . | . | . | . | . | . |
| InternalIMT | dha | rs6593485 | 0.697 | -0.104 | 0.103 | 0.313 | 0.015 | 0.1 | 0.88 | -2.20E-03 | 0.067 | 0.973 |
| InternalIMT | dha | rs6593486 | 0.697 | -0.104 | 0.103 | 0.313 | 0.015 | 0.1 | 0.881 | -2.30E-03 | 0.067 | 0.973 |
| InternalIMT | dha | rs145196618 | 0.036 | -0.104 | 0.049 | 0.033 | 0.102 | 0.251 | 0.683 | -0.033 | 0.165 | 0.842 |
| InternalIMT | dha | rs142598335 | 0.001 | . | . | . | . | . | . | . | . | . |
| InternalIMT | dha | rs892691 | 0.663 | -0.071 | 0.095 | 0.457 | 0.028 | 0.093 | 0.762 | -0.028 | 0.062 | 0.656 |
| InternalIMT | dha | rs7080713 | 0.597 | -0.058 | 0.087 | 0.508 | 0.058 | 0.091 | 0.525 | -0.042 | 0.06 | 0.489 |
| InternalIMT | dha | rs7077173 | 0.135 | -0.148 | 0.051 | 3.60E-03 | -0.201 | 0.11 | 0.067 | 0.146 | 0.079 | 0.066 |
| InternalIMT | dha | rs7080977 | 0.088 | -0.129 | 0.05 | 9.50E-03 | -0.163 | 0.16 | 0.31 | 0.126 | 0.114 | 0.269 |
| InternalIMT | dha | rs3780901 | 0.599 | -0.065 | 0.088 | 0.461 | 0.051 | 0.091 | 0.578 | -0.036 | 0.061 | 0.556 |
| InternalIMT | dha | rs61854091 | 0.021 | -0.12 | 0.049 | 0.014 | -0.408 | 0.453 | 0.368 | 0.31 | 0.31 | 0.317 |
| InternalIMT | dha | rs77550209 | 0.011 | -0.105 | 0.048 | 0.031 | -0.035 | 0.225 | 0.875 | -0.053 | 0.15 | 0.723 |
| InternalIMT | dha | rs73283191 | 0.097 | -0.129 | 0.05 | 0.01 | -0.132 | 0.158 | 0.402 | 0.121 | 0.112 | 0.282 |
| InternalIMT | dha | rs191866099 | 0.003 | . | . | . | . | . | . | . | . | . |
| InternalIMT | dha | rs147998395 | 0.005 | . | . | . | . | . | . | . | . | . |
| InternalIMT | dha | rs7090328 | 0.861 | -0.072 | 0.169 | 0.669 | 0.056 | 0.141 | 0.692 | -0.019 | 0.095 | 0.837 |
| InternalIMT | dha | rs79814535 | 0.003 | . | . | . | . | . | . | . | . | . |
| InternalIMT | dha | rs7073082 | 0.861 | -0.071 | 0.169 | 0.677 | 0.058 | 0.141 | 0.683 | -0.021 | 0.095 | 0.829 |
| InternalIMT | dha | rs11239526 | 0.012 | -0.108 | 0.048 | 0.024 | -0.131 | 0.376 | 0.726 | -0.068 | 0.271 | 0.803 |
| InternalIMT | dha | rs141839841 | 0.005 | . | . | . | . | . | . | . | . | . |
| InternalIMT | dha | rs75697371 | 0.005 | . | . | . | . | . | . | . | . | . |
| InternalIMT | dha | rs75972854 | 0.005 | . | . | . | . | . | . | . | . | . |
| InternalIMT | dha | rs139291975 | . | . | . | . | . | . | . | . | . | . |
| InternalIMT | dha | rs144064711 | 0.002 | . | . | . | . | . | . | . | . | . |
| InternalIMT | dha | rs3780902 | 0.098 | -0.126 | 0.05 | 0.012 | -0.105 | 0.16 | 0.511 | 0.106 | 0.113 | 0.349 |
| InternalIMT | dha | rs190449801 | 0.004 | . | . | . | . | . | . | . | . | . |
| InternalIMT | dha | rs73283196 | 0.096 | -0.127 | 0.05 | 0.012 | -0.116 | 0.161 | 0.471 | 0.111 | 0.114 | 0.331 |
| InternalIMT | dha | rs28395867 | 0.118 | -0.12 | 0.052 | 0.022 | -0.08 | 0.139 | 0.566 | 0.055 | 0.101 | 0.587 |
| InternalIMT | dha | rs28395866 | 0.264 | -0.133 | 0.058 | 0.021 | -0.047 | 0.097 | 0.626 | 0.05 | 0.067 | 0.455 |
| InternalIMT | dha | rs55661611 | 0.088 | -0.126 | 0.05 | 0.011 | -0.142 | 0.166 | 0.392 | 0.113 | 0.117 | 0.335 |
| InternalIMT | dha | rs55684518 | 0.008 | . | . | . | . | . | . | . | . | . |
| InternalIMT | dha | rs78552493 | 0.041 | -0.099 | 0.049 | 0.044 | 0.129 | 0.25 | 0.605 | -0.117 | 0.173 | 0.498 |
| InternalIMT | dha | rs147665434 | 0.001 | . | . | . | . | . | . | . | . | . |
| InternalIMT | dha | rs34445001 | 0.005 | . | . | . | . | . | . | . | . | . |
| InternalIMT | dha | rs184019449 | 0.001 | . | . | . | . | . | . | . | . | . |
| InternalIMT | dha | rs3780903 | 0.645 | 0.014 | 0.09 | 0.878 | 0.122 | 0.088 | 0.162 | -0.095 | 0.059 | 0.11 |
| InternalIMT | dha | rs17157784 | 0.159 | -0.108 | 0.054 | 0.043 | 0.013 | 0.123 | 0.918 | 4.90E-03 | 0.085 | 0.954 |
| InternalIMT | dha | rs3780904 | 0.01 | . | . | . | . | . | . | . | . | . |
| InternalIMT | dha | rs192064730 | 0 | . | . | . | . | . | . | . | . | . |
| InternalIMT | dha | rs12774534 | 0.005 | . | . | . | . | . | . | . | . | . |
| InternalIMT | dha | rs79179915 | 0.003 | . | . | . | . | . | . | . | . | . |
| InternalIMT | dha | rs117811571 | 0.004 | . | . | . | . | . | . | . | . | . |
| InternalIMT | dha | rs72796477 | 0.004 | . | . | . | . | . | . | . | . | . |
| InternalIMT | dha | rs55729342 | 0.172 | -0.11 | 0.055 | 0.047 | 0.017 | 0.118 | 0.885 | 9.70E-03 | 0.081 | 0.905 |
| InternalIMT | dha | rs59317220 | 0.172 | -0.11 | 0.055 | 0.047 | 0.017 | 0.118 | 0.882 | 9.50E-03 | 0.081 | 0.907 |
| InternalIMT | dha | rs3780905 | 0.018 | -0.106 | 0.048 | 0.028 | -0.239 | 0.392 | 0.542 | 0.047 | 0.276 | 0.865 |
| InternalIMT | dha | rs3780906 | 0.645 | 0.02 | 0.09 | 0.822 | 0.13 | 0.088 | 0.139 | -0.1 | 0.059 | 0.091 |
| InternalIMT | dha | rs149047975 | 0.003 | . | . | . | . | . | . | . | . | . |
| InternalIMT | dha | rs3824614 | 0.002 | . | . | . | . | . | . | . | . | . |
| InternalIMT | dha | rs140254404 | . | . | . | . | . | . | . | . | . | . |
| InternalIMT | dha | rs145262684 | 0.005 | . | . | . | . | . | . | . | . | . |
| InternalIMT | dha | rs76735482 | 0.002 | . | . | . | . | . | . | . | . | . |
| InternalIMT | dha | rs73283198 | 0.045 | -0.114 | 0.049 | 0.019 | -0.142 | 0.223 | 0.523 | 0.068 | 0.159 | 0.671 |
| InternalIMT | dha | rs74911239 | 0.013 | -0.117 | 0.048 | 0.016 | -0.677 | 0.481 | 0.159 | 0.408 | 0.348 | 0.24 |
| InternalIMT | dha | rs150551647 | 0.004 | . | . | . | . | . | . | . | . | . |
| InternalIMT | dha | rs115200794 | 0.143 | -0.122 | 0.054 | 0.023 | -0.067 | 0.121 | 0.58 | 0.051 | 0.087 | 0.553 |
| InternalIMT | dha | rs74130823 | 0.245 | -0.162 | 0.056 | 4.10E-03 | -0.165 | 0.108 | 0.127 | 0.116 | 0.076 | 0.125 |
| InternalIMT | dha | rs2279435 | 0.604 | 0.025 | 0.091 | 0.783 | 0.135 | 0.09 | 0.134 | -0.108 | 0.061 | 0.077 |
| InternalIMT | dha | rs147279673 | 0.007 | . | . | . | . | . | . | . | . | . |
| InternalIMT | dha | rs183525146 | 0.002 | . | . | . | . | . | . | . | . | . |
| InternalIMT | dha | rs3740107 | 0.769 | 0.088 | 0.115 | 0.445 | 0.19 | 0.1 | 0.059 | -0.127 | 0.07 | 0.068 |
| InternalIMT | dha | rs140697247 | 0 | . | . | . | . | . | . | . | . | . |
| InternalIMT | dha | rs1565096 | 0.903 | 0.267 | 0.182 | 0.143 | 0.311 | 0.141 | 0.027 | -0.207 | 0.099 | 0.038 |
| InternalIMT | dha | rs1565097 | 0.766 | 0.082 | 0.114 | 0.472 | 0.185 | 0.1 | 0.065 | -0.124 | 0.07 | 0.076 |
| InternalIMT | dha | rs144584548 | 0.008 | . | . | . | . | . | . | . | . | . |
| InternalIMT | dha | rs75811053 | 0.001 | . | . | . | . | . | . | . | . | . |
| InternalIMT | dha | rs147462967 | 0.009 | . | . | . | . | . | . | . | . | . |
| InternalIMT | dha | rs74130824 | 0.121 | -0.116 | 0.053 | 0.03 | -0.063 | 0.133 | 0.637 | 0.036 | 0.097 | 0.709 |

| InternalIMT | dha | rs116883184 | 0.006 | . | . | . | . | . | . | . | . | . |
| --- | --- | --- | --- | --- | --- | --- | --- | --- | --- | --- | --- | --- |
| InternalIMT | dha | rs117258049 | 0.006 | . | . | . | . | . | . | . | . | . |
| InternalIMT | dha | rs3780908 | 0.129 | -0.122 | 0.051 | 0.017 | -0.07 | 0.153 | 0.646 | 0.067 | 0.107 | 0.533 |
| InternalIMT | dha | rs180938522 | 0.001 | . | . | . | . | . | . | . | . | . |
| InternalIMT | dha | rs76687372 | 0.024 | -0.111 | 0.048 | 0.021 | -0.103 | 0.296 | 0.728 | 0.135 | 0.201 | 0.501 |
| InternalIMT | dha | rs3780909 | 0.483 | -0.027 | 0.077 | 0.724 | 0.096 | 0.087 | 0.272 | -0.085 | 0.059 | 0.153 |
| InternalIMT | dha | rs78281156 | 0.001 | . | . | . | . | . | . | . | . | . |
| InternalIMT | dha | rs187840631 | . | . | . | . | . | . | . | . | . | . |
| InternalIMT | dha | rs76868242 | 0.001 | . | . | . | . | . | . | . | . | . |
| InternalIMT | dha | rs192794192 | 0 | . | . | . | . | . | . | . | . | . |
| InternalIMT | dha | rs12781248 | 0.005 | . | . | . | . | . | . | . | . | . |
| InternalIMT | dha | rs12762375 | 0.005 | . | . | . | . | . | . | . | . | . |
| InternalIMT | dha | rs192256986 | . | . | . | . | . | . | . | . | . | . |
| InternalIMT | dha | rs184411030 | 0.033 | -0.114 | 0.048 | 0.018 | 0.024 | 0.247 | 0.921 | 0.094 | 0.173 | 0.586 |
| InternalIMT | dha | rs189601483 | 0.001 | . | . | . | . | . | . | . | . | . |
| InternalIMT | dha | rs143254579 | 0.015 | -0.106 | 0.048 | 0.029 | -0.039 | 0.354 | 0.913 | -0.046 | 0.236 | 0.846 |
| InternalIMT | dha | rs12253903 | 0.081 | -0.087 | 0.053 | 0.1 | 0.16 | 0.161 | 0.321 | -0.123 | 0.109 | 0.259 |
| InternalIMT | dha | rs35489926 | 0.005 | . | . | . | . | . | . | . | . | . |
| InternalIMT | dha | rs71494783 | 0.095 | -0.09 | 0.051 | 0.08 | 0.183 | 0.185 | 0.323 | -0.09 | 0.119 | 0.452 |
| InternalIMT | dha | rs180711602 | 0.001 | . | . | . | . | . | . | . | . | . |
| InternalIMT | dha | rs117312402 | 0.001 | . | . | . | . | . | . | . | . | . |
| InternalIMT | dha | rs74831694 | 0.001 | . | . | . | . | . | . | . | . | . |
| InternalIMT | dha | rs140301943 | . | . | . | . | . | . | . | . | . | . |
| InternalIMT | dha | rs78553679 | 0.006 | . | . | . | . | . | . | . | . | . |
| InternalIMT | dha | rs78334250 | 0.001 | . | . | . | . | . | . | . | . | . |
| InternalIMT | dha | rs76000028 | 0.001 | . | . | . | . | . | . | . | . | . |
| InternalIMT | dha | rs145195254 | 0.005 | . | . | . | . | . | . | . | . | . |
| InternalIMT | dha | rs137888527 | 0.005 | . | . | . | . | . | . | . | . | . |
| InternalIMT | dha | rs149109592 | . | . | . | . | . | . | . | . | . | . |
| InternalIMT | dha | rs80280010 | 0.013 | -0.106 | 0.048 | 0.027 | -0.024 | 0.382 | 0.95 | -0.129 | 0.266 | 0.629 |
| InternalIMT | dha | rs189995374 | . | . | . | . | . | . | . | . | . | . |
| InternalIMT | dha | rs75467770 | 0.002 | . | . | . | . | . | . | . | . | . |
| InternalIMT | dha | rs1487562 | 0.242 | -0.131 | 0.057 | 0.023 | -0.055 | 0.109 | 0.616 | 0.052 | 0.076 | 0.497 |
| InternalIMT | dha | rs12266963 | 0.095 | -0.09 | 0.051 | 0.08 | 0.183 | 0.185 | 0.323 | -0.09 | 0.12 | 0.451 |
| InternalIMT | dha | rs7099874 | 0.857 | 0.058 | 0.157 | 0.714 | 0.178 | 0.13 | 0.17 | -0.097 | 0.089 | 0.281 |
| InternalIMT | dha | rs75687019 | 0.001 | . | . | . | . | . | . | . | . | . |
| InternalIMT | dha | rs148240462 | 0.014 | -0.121 | 0.049 | 0.013 | -0.776 | 0.416 | 0.062 | 0.506 | 0.291 | 0.082 |
| InternalIMT | dha | rs141278174 | 0.001 | . | . | . | . | . | . | . | . | . |
| InternalIMT | dha | rs76404852 | 0.001 | . | . | . | . | . | . | . | . | . |
| InternalIMT | dha | rs139112240 | 0.004 | . | . | . | . | . | . | . | . | . |
| InternalIMT | dha | rs12783037 | 0.118 | -0.094 | 0.052 | 0.07 | 0.14 | 0.164 | 0.393 | -0.051 | 0.107 | 0.634 |
| InternalIMT | dha | rs72796481 | 0.003 | . | . | . | . | . | . | . | . | . |
| InternalIMT | dha | rs76798749 | 0.025 | -0.11 | 0.048 | 0.022 | -0.073 | 0.301 | 0.808 | 0.123 | 0.202 | 0.541 |
| InternalIMT | dha | rs187732548 | 0.001 | . | . | . | . | . | . | . | . | . |
| InternalIMT | dha | rs75675474 | 0.025 | -0.11 | 0.048 | 0.022 | -0.073 | 0.301 | 0.808 | 0.123 | 0.202 | 0.541 |
| InternalIMT | dha | rs76552293 | 0.025 | -0.11 | 0.048 | 0.022 | -0.073 | 0.301 | 0.807 | 0.124 | 0.202 | 0.541 |
| InternalIMT | dha | rs147445993 | 0.008 | . | . | . | . | . | . | . | . | . |
| InternalIMT | dha | rs115902283 | 0.025 | -0.11 | 0.048 | 0.022 | -0.076 | 0.301 | 0.8 | 0.125 | 0.202 | 0.535 |
| InternalIMT | dha | rs4540799 | 0.855 | 0.056 | 0.156 | 0.72 | 0.177 | 0.128 | 0.165 | -0.096 | 0.089 | 0.28 |
| InternalIMT | dha | rs12765320 | 0.35 | -0.108 | 0.063 | 0.088 | 0.033 | 0.099 | 0.738 | 4.70E-03 | 0.068 | 0.945 |
| InternalIMT | dha | rs12782256 | 0.005 | . | . | . | . | . | . | . | . | . |
| InternalIMT | dha | rs191727256 | 0.007 | . | . | . | . | . | . | . | . | . |
| InternalIMT | dha | rs2082111 | 0.113 | -0.108 | 0.05 | 0.032 | 0.017 | 0.157 | 0.916 | 0.016 | 0.109 | 0.886 |
| InternalIMT | dha | rs2082110 | 0.257 | -0.127 | 0.058 | 0.028 | -0.038 | 0.107 | 0.723 | 0.044 | 0.074 | 0.549 |
| InternalIMT | dha | rs2082109 | 0.257 | -0.127 | 0.058 | 0.029 | -0.038 | 0.107 | 0.723 | 0.044 | 0.074 | 0.549 |
| InternalIMT | dha | rs117316662 | 0.001 | . | . | . | . | . | . | . | . | . |
| InternalIMT | dha | rs12247163 | 0.357 | -0.111 | 0.063 | 0.078 | 0.028 | 0.098 | 0.775 | 9.90E-03 | 0.067 | 0.882 |
| InternalIMT | dha | rs12355888 | 0.037 | -0.105 | 0.048 | 0.029 | 0.088 | 0.29 | 0.761 | -0.037 | 0.2 | 0.853 |
| InternalIMT | dha | rs143772290 | 0.033 | -0.116 | 0.049 | 0.017 | -0.178 | 0.272 | 0.512 | 0.199 | 0.18 | 0.271 |
| InternalIMT | dha | rs146849452 | 0.009 | . | . | . | . | . | . | . | . | . |
| InternalIMT | dha | rs140267121 | 0.003 | . | . | . | . | . | . | . | . | . |
| InternalIMT | dha | rs7080474 | 0.352 | -0.116 | 0.063 | 0.063 | 0.015 | 0.098 | 0.875 | 0.017 | 0.067 | 0.801 |
| InternalIMT | dha | rs144066203 | 0.007 | . | . | . | . | . | . | . | . | . |
| InternalIMT | dha | rs12765209 | 0.005 | . | . | . | . | . | . | . | . | . |
| InternalIMT | dha | rs150930735 | 0.007 | . | . | . | . | . | . | . | . | . |
| InternalIMT | dha | rs7068039 | 0.365 | -0.119 | 0.063 | 0.061 | 0.017 | 0.097 | 0.863 | 0.02 | 0.067 | 0.768 |
| InternalIMT | dha | rs117622652 | 0.001 | . | . | . | . | . | . | . | . | . |
| InternalIMT | dha | rs190191514 | . | . | . | . | . | . | . | . | . | . |
| InternalIMT | dha | rs74130835 | 0.118 | -0.116 | 0.053 | 0.03 | -0.063 | 0.135 | 0.639 | 0.035 | 0.098 | 0.725 |
| InternalIMT | dha | rs1811604 | 0.114 | -0.118 | 0.053 | 0.026 | -0.081 | 0.136 | 0.552 | 0.045 | 0.1 | 0.657 |
| InternalIMT | dha | rs146971256 | . | . | . | . | . | . | . | . | . | . |
| InternalIMT | dha | rs74336000 | 0.033 | -0.116 | 0.049 | 0.017 | -0.18 | 0.272 | 0.507 | 0.2 | 0.181 | 0.269 |
| InternalIMT | dha | rs186046492 | 0.003 | . | . | . | . | . | . | . | . | . |
| InternalIMT | dha | rs34971823 | 0.009 | . | . | . | . | . | . | . | . | . |
| InternalIMT | dha | rs75227446 | 0.001 | . | . | . | . | . | . | . | . | . |
| InternalIMT | dha | rs190457599 | 0.001 | . | . | . | . | . | . | . | . | . |
| InternalIMT | dha | rs79591917 | 0.001 | . | . | . | . | . | . | . | . | . |
| InternalIMT | dha | rs702365 | 0.492 | -0.053 | 0.079 | 0.507 | 0.059 | 0.09 | 0.516 | -0.056 | 0.061 | 0.359 |
| InternalIMT | dha | rs79643836 | 0.004 | . | . | . | . | . | . | . | . | . |
| InternalIMT | dha | rs702366 | 0.493 | -0.051 | 0.079 | 0.521 | 0.06 | 0.09 | 0.506 | -0.058 | 0.061 | 0.345 |
| InternalIMT | dha | rs1059696 | 0.197 | -0.075 | 0.056 | 0.182 | 0.155 | 0.137 | 0.257 | -0.075 | 0.093 | 0.424 |
| InternalIMT | dha | rs901681 | 0.489 | -0.051 | 0.08 | 0.526 | 0.055 | 0.091 | 0.544 | -0.058 | 0.062 | 0.345 |
| InternalIMT | dha | rs61854092 | 0.196 | -0.075 | 0.056 | 0.183 | 0.157 | 0.137 | 0.253 | -0.075 | 0.093 | 0.419 |
| InternalIMT | dha | rs148080753 | . | . | . | . | . | . | . | . | . | . |
| InternalIMT | dha | rs61854093 | 0.315 | -0.084 | 0.062 | 0.173 | 0.078 | 0.103 | 0.45 | -0.034 | 0.071 | 0.628 |
| InternalIMT | dha | rs141822774 | 0 | . | . | . | . | . | . | . | . | . |

| InternalIMT | dha | rs74130836 | 0.118 | -0.115 | 0.053 | 0.031 | -0.06 | 0.135 | 0.655 | 0.033 | 0.099 | 0.742 |
| --- | --- | --- | --- | --- | --- | --- | --- | --- | --- | --- | --- | --- |
| InternalIMT | dha | rs150620963 | 0.006 | . | . | . | . | . | . | . | . | . |
| InternalIMT | dha | rs3780913 | 0.849 | 0.111 | 0.151 | 0.462 | 0.219 | 0.125 | 0.08 | -0.129 | 0.086 | 0.134 |
| InternalIMT | dha | rs142255723 | 0.001 | . | . | . | . | . | . | . | . | . |
| InternalIMT | dha | rs3780914 | 0.479 | -0.059 | 0.078 | 0.452 | 0.054 | 0.092 | 0.558 | -0.051 | 0.062 | 0.413 |
| InternalIMT | dha | rs182656507 | 0.001 | . | . | . | . | . | . | . | . | . |
| InternalIMT | dha | rs2291427 | 0.821 | 0.113 | 0.141 | 0.422 | 0.213 | 0.118 | 0.073 | -0.135 | 0.082 | 0.099 |
| InternalIMT | dha | rs10751382 | 0.816 | 0.122 | 0.133 | 0.36 | 0.211 | 0.113 | 0.062 | -0.141 | 0.078 | 0.069 |
| InternalIMT | dha | rs117610108 | 0.001 | . | . | . | . | . | . | . | . | . |
| InternalIMT | dha | rs144966941 | 0.009 | . | . | . | . | . | . | . | . | . |
| InternalIMT | dha | rs41502051 | 0.014 | -0.113 | 0.048 | 0.02 | -0.427 | 0.356 | 0.231 | 0.219 | 0.251 | 0.382 |
| InternalIMT | dha | rs41426046 | 0.001 | . | . | . | . | . | . | . | . | . |
| InternalIMT | dha | rs41519248 | 0.001 | . | . | . | . | . | . | . | . | . |
| InternalIMT | dha | rs189108245 | 0.001 | . | . | . | . | . | . | . | . | . |
| InternalIMT | dha | rs7896431 | 0.497 | -0.077 | 0.08 | 0.333 | 0.035 | 0.093 | 0.709 | -0.031 | 0.063 | 0.623 |
| InternalIMT | dha | rs183724547 | 0.009 | . | . | . | . | . | . | . | . | . |
| InternalIMT | dha | rs142044990 | 0.004 | . | . | . | . | . | . | . | . | . |
| InternalIMT | dha | rs188140929 | . | . | . | . | . | . | . | . | . | . |
| InternalIMT | dha | rs76819096 | 0.041 | -0.096 | 0.05 | 0.055 | 0.193 | 0.222 | 0.386 | -0.113 | 0.155 | 0.468 |
| InternalIMT | dha | rs10751383 | 0.58 | -0.085 | 0.086 | 0.324 | 7.20E-03 | 0.088 | 0.935 | -0.019 | 0.06 | 0.749 |
| InternalIMT | dha | rs117819321 | 0.005 | . | . | . | . | . | . | . | . | . |
| InternalIMT | dha | rs2242332 | 0.681 | 0.053 | 0.096 | 0.581 | 0.18 | 0.092 | 0.052 | -0.12 | 0.063 | 0.059 |
| InternalIMT | dha | rs2242333 | 0.02 | -0.104 | 0.048 | 0.031 | -0.097 | 0.382 | 0.799 | -0.038 | 0.268 | 0.887 |
| InternalIMT | dha | rs2242334 | 0.681 | 0.052 | 0.096 | 0.593 | 0.178 | 0.092 | 0.054 | -0.118 | 0.063 | 0.062 |
| InternalIMT | dha | rs189465041 | . | . | . | . | . | . | . | . | . | . |
| InternalIMT | dha | rs28395868 | 0.009 | . | . | . | . | . | . | . | . | . |
| InternalIMT | dha | rs56067550 | 0.008 | . | . | . | . | . | . | . | . | . |
| InternalIMT | dha | rs1051713 | 0.182 | -0.057 | 0.055 | 0.303 | 0.26 | 0.136 | 0.055 | -0.132 | 0.091 | 0.148 |
| InternalIMT | dha | rs115074210 | 0.048 | -0.103 | 0.05 | 0.041 | 0.083 | 0.212 | 0.697 | -0.04 | 0.151 | 0.79 |
| InternalIMT | dha | rs7900977 | 0.893 | 0.148 | 0.17 | 0.384 | 0.245 | 0.133 | 0.066 | -0.145 | 0.094 | 0.122 |
| InternalIMT | dha | rs147613891 | 0.001 | . | . | . | . | . | . | . | . | . |
| InternalIMT | dha | rs2229136 | 0.161 | -0.125 | 0.057 | 0.029 | -0.08 | 0.128 | 0.535 | 0.056 | 0.089 | 0.526 |
| InternalIMT | dha | rs185482841 | 0.009 | . | . | . | . | . | . | . | . | . |
| InternalIMT | dha | rs2288619 | 0.156 | -0.119 | 0.057 | 0.035 | -0.064 | 0.128 | 0.615 | 0.038 | 0.089 | 0.664 |
| InternalIMT | dha | rs189891524 | . | . | . | . | . | . | . | . | . | . |
| InternalIMT | dha | rs56145077 | 0.176 | -0.059 | 0.054 | 0.277 | 0.254 | 0.136 | 0.063 | -0.133 | 0.091 | 0.142 |
| InternalIMT | dha | rs143216970 | 0.013 | -0.102 | 0.048 | 0.035 | 0.294 | 0.303 | 0.332 | -0.206 | 0.189 | 0.276 |
| InternalIMT | dha | rs142246478 | 0 | . | . | . | . | . | . | . | . | . |
| InternalIMT | dha | rs13134 | 0.012 | -0.115 | 0.048 | 0.016 | -0.512 | 0.452 | 0.257 | 0.375 | 0.29 | 0.196 |
| InternalIMT | dha | rs41314980 | 0.012 | -0.107 | 0.048 | 0.026 | -7.10E-03 | 0.628 | 0.991 | -0.038 | 0.437 | 0.93 |
| InternalIMT | dha | rs41304611 | 0.005 | . | . | . | . | . | . | . | . | . |
| InternalIMT | dha | rs193299617 | . | . | . | . | . | . | . | . | . | . |
| InternalIMT | dha | rs12772724 | 0.012 | -0.115 | 0.048 | 0.016 | -0.511 | 0.452 | 0.258 | 0.375 | 0.29 | 0.197 |
| InternalIMT | dha | rs139489537 | 0.011 | -0.109 | 0.048 | 0.023 | -0.182 | 0.374 | 0.627 | 2.70E-03 | 0.266 | 0.992 |
| InternalIMT | dha | rs12776062 | 0.005 | . | . | . | . | . | . | . | . | . |
| InternalIMT | dha | rs752059 | 0.181 | -0.067 | 0.054 | 0.216 | 0.208 | 0.136 | 0.126 | -0.108 | 0.091 | 0.235 |
| InternalIMT | dha | rs147649982 | 0.013 | -0.109 | 0.049 | 0.025 | -0.098 | 0.788 | 0.901 | 0.084 | 0.561 | 0.881 |
| InternalIMT | dha | rs17157798 | 0.113 | -0.118 | 0.054 | 0.031 | -0.088 | 0.146 | 0.548 | 0.043 | 0.102 | 0.67 |
| InternalIMT | dha | rs142298619 | 0.009 | . | . | . | . | . | . | . | . | . |
| InternalIMT | dha | rs4948673 | 0.893 | 0.135 | 0.162 | 0.404 | 0.246 | 0.126 | 0.052 | -0.139 | 0.09 | 0.122 |
| InternalIMT | dha | rs74231484 | 0.045 | -0.092 | 0.05 | 0.065 | 0.168 | 0.222 | 0.447 | -0.161 | 0.153 | 0.295 |
| InternalIMT | dha | rs72796489 | 0.001 | . | . | . | . | . | . | . | . | . |
| InternalIMT | dha | rs113447578 | 0.006 | . | . | . | . | . | . | . | . | . |
| InternalIMT | dha | rs34063374 | 0.014 | -0.116 | 0.048 | 0.016 | -0.526 | 0.443 | 0.235 | 0.386 | 0.286 | 0.176 |
| InternalIMT | dha | rs11239527 | 0.006 | . | . | . | . | . | . | . | . | . |
| InternalIMT | dha | rs34371883 | 0.007 | . | . | . | . | . | . | . | . | . |
| InternalIMT | dha | rs118168828 | 0.004 | . | . | . | . | . | . | . | . | . |
| InternalIMT | dha | rs34349618 | 0.005 | . | . | . | . | . | . | . | . | . |
| InternalIMT | dha | rs80041679 | 0.011 | -0.109 | 0.048 | 0.023 | -0.185 | 0.372 | 0.62 | 5.30E-03 | 0.264 | 0.984 |
| InternalIMT | dha | rs149640607 | 0.01 | -0.115 | 0.048 | 0.017 | -0.569 | 0.624 | 0.362 | 0.413 | 0.435 | 0.342 |
| InternalIMT | dha | rs4427523 | 0.932 | 0.345 | 0.281 | 0.22 | 0.38 | 0.215 | 0.077 | -0.242 | 0.15 | 0.106 |
| InternalIMT | dha | rs185880109 | . | . | . | . | . | . | . | . | . | . |
| InternalIMT | dha | rs191523220 | 0.001 | . | . | . | . | . | . | . | . | . |
| InternalIMT | dha | rs144315284 | 0.02 | -0.111 | 0.049 | 0.023 | -0.214 | 0.289 | 0.458 | 0.102 | 0.203 | 0.614 |
| InternalIMT | dha | rs4948674 | 0.329 | -0.115 | 0.063 | 0.07 | -9.80E-03 | 0.1 | 0.922 | 0.012 | 0.068 | 0.863 |
| InternalIMT | dha | rs182984298 | 0.002 | . | . | . | . | . | . | . | . | . |
| InternalIMT | dha | rs56749023 | 0.071 | -0.099 | 0.052 | 0.056 | 0.07 | 0.156 | 0.653 | -0.058 | 0.107 | 0.59 |
| InternalIMT | dha | rs112508957 | 0.045 | -0.107 | 0.049 | 0.028 | 0.102 | 0.212 | 0.632 | 0.015 | 0.143 | 0.916 |
| InternalIMT | dha | rs7089063 | 0.313 | -0.061 | 0.064 | 0.343 | 0.119 | 0.103 | 0.248 | -0.073 | 0.069 | 0.29 |
| InternalIMT | dha | rs145000779 | . | . | . | . | . | . | . | . | . | . |
| InternalIMT | dha | rs80201572 | 0.013 | -0.108 | 0.048 | 0.025 | -0.081 | 0.374 | 0.828 | -0.059 | 0.261 | 0.822 |
| InternalIMT | dha | rs140568227 | 0.003 | . | . | . | . | . | . | . | . | . |
| InternalIMT | dha | rs149756228 | 0.004 | . | . | . | . | . | . | . | . | . |
| InternalIMT | dha | rs112380685 | 0.003 | . | . | . | . | . | . | . | . | . |
| InternalIMT | dha | rs60481109 | 0.067 | -0.096 | 0.052 | 0.063 | 0.096 | 0.159 | 0.548 | -0.077 | 0.111 | 0.485 |
| InternalIMT | dha | rs138221843 | . | . | . | . | . | . | . | . | . | . |
| InternalIMT | dha | rs150283194 | 0.009 | . | . | . | . | . | . | . | . | . |
| InternalIMT | dha | rs138879661 | 0.008 | . | . | . | . | . | . | . | . | . |
| InternalIMT | dha | rs34960345 | 0.067 | -0.124 | 0.05 | 0.014 | -0.205 | 0.181 | 0.258 | 0.135 | 0.124 | 0.277 |
| InternalIMT | dha | rs147514068 | . | . | . | . | . | . | . | . | . | . |
| InternalIMT | dha | rs35410535 | 0.138 | -0.116 | 0.056 | 0.038 | -0.059 | 0.132 | 0.655 | 0.031 | 0.092 | 0.733 |
| InternalIMT | dha | rs191613635 | 0.001 | . | . | . | . | . | . | . | . | . |
| InternalIMT | dha | rs17157800 | 0.004 | . | . | . | . | . | . | . | . | . |
| InternalIMT | dha | rs17523413 | 0.027 | -0.117 | 0.049 | 0.016 | -0.33 | 0.296 | 0.264 | 0.187 | 0.197 | 0.341 |
| InternalIMT | dha | rs7897036 | 0.013 | -0.102 | 0.047 | 0.032 | 0.819 | 0.664 | 0.217 | -0.44 | 0.476 | 0.356 |
| InternalIMT | dha | rs76334323 | 0.001 | . | . | . | . | . | . | . | . | . |

| InternalIMT | dha | rs143535991 | 0 | . | . | . | . | . | . | . | . | . |
| --- | --- | --- | --- | --- | --- | --- | --- | --- | --- | --- | --- | --- |
| InternalIMT | dha | rs140344979 | 0.002 | . | . | . | . | . | . | . | . | . |
| InternalIMT | dha | rs36047811 | 0.138 | -0.116 | 0.056 | 0.038 | -0.06 | 0.132 | 0.651 | 0.032 | 0.092 | 0.729 |
| InternalIMT | dha | rs76493570 | 0.057 | -0.118 | 0.05 | 0.018 | -0.177 | 0.192 | 0.356 | 0.108 | 0.131 | 0.409 |
| InternalIMT | dha | rs192998920 | . | . | . | . | . | . | . | . | . | . |
| InternalIMT | dha | rs116840852 | 0.007 | . | . | . | . | . | . | . | . | . |
| InternalIMT | dha | rs184824101 | 0 | . | . | . | . | . | . | . | . | . |
| InternalIMT | dha | rs41301621 | 0.002 | . | . | . | . | . | . | . | . | . |
| InternalIMT | dha | rs3740109 | 0.138 | -0.116 | 0.056 | 0.037 | -0.06 | 0.132 | 0.648 | 0.032 | 0.092 | 0.727 |
| InternalIMT | dha | rs7899415 | 0.032 | -0.119 | 0.049 | 0.015 | -0.335 | 0.332 | 0.314 | 0.218 | 0.235 | 0.353 |
| InternalIMT | dha | rs184395284 | 0.001 | . | . | . | . | . | . | . | . | . |
| InternalIMT | dha | rs139209589 | 0.002 | . | . | . | . | . | . | . | . | . |
| InternalIMT | dha | rs149924163 | 0.009 | . | . | . | . | . | . | . | . | . |
| InternalIMT | dha | rs3740110 | 0.001 | . | . | . | . | . | . | . | . | . |
| InternalIMT | dha | rs144335113 | 0.009 | . | . | . | . | . | . | . | . | . |
| InternalIMT | dha | rs74743385 | 0.045 | -0.107 | 0.049 | 0.028 | 0.101 | 0.212 | 0.634 | 0.015 | 0.144 | 0.914 |
| InternalIMT | dha | rs146545829 | 0.009 | . | . | . | . | . | . | . | . | . |
| InternalIMT | dha | rs181842689 | . | . | . | . | . | . | . | . | . | . |
| InternalIMT | dha | rs3740111 | 0.067 | -0.124 | 0.05 | 0.014 | -0.206 | 0.181 | 0.255 | 0.135 | 0.124 | 0.276 |
| InternalIMT | dha | rs186261599 | 0.009 | . | . | . | . | . | . | . | . | . |
| InternalIMT | dha | rs73285348 | 0.004 | . | . | . | . | . | . | . | . | . |
| InternalIMT | dha | rs186356901 | 0.009 | . | . | . | . | . | . | . | . | . |
| InternalIMT | dha | rs3824618 | 0.011 | -0.109 | 0.048 | 0.023 | -0.202 | 0.349 | 0.563 | 0.015 | 0.251 | 0.954 |
| InternalIMT | dha | rs3802548 | 0.201 | -0.061 | 0.055 | 0.268 | 0.197 | 0.128 | 0.124 | -0.111 | 0.086 | 0.198 |
| InternalIMT | dha | rs3802547 | 0.001 | . | . | . | . | . | . | . | . | . |
| InternalIMT | dha | rs77677521 | 0.007 | . | . | . | . | . | . | . | . | . |
| InternalIMT | dha | rs3824616 | 0.138 | -0.116 | 0.056 | 0.037 | -0.061 | 0.132 | 0.645 | 0.032 | 0.092 | 0.725 |
| InternalIMT | dha | rs191073818 | 0.009 | . | . | . | . | . | . | . | . | . |
| InternalIMT | dha | rs147078004 | . | . | . | . | . | . | . | . | . | . |
| InternalIMT | dha | rs7908745 | 0.342 | -0.066 | 0.067 | 0.321 | 0.102 | 0.101 | 0.313 | -0.057 | 0.069 | 0.408 |
| InternalIMT | dha | rs17157811 | 0.004 | . | . | . | . | . | . | . | . | . |
| InternalIMT | dha | rs138506087 | 0.002 | . | . | . | . | . | . | . | . | . |
| InternalIMT | dha | rs77241269 | 0.071 | -0.099 | 0.052 | 0.055 | 0.065 | 0.157 | 0.68 | -0.054 | 0.108 | 0.618 |
| InternalIMT | dha | rs138374938 | 0.001 | . | . | . | . | . | . | . | . | . |
| InternalIMT | dha | rs2279433 | 0.068 | -0.126 | 0.051 | 0.013 | -0.22 | 0.166 | 0.184 | 0.142 | 0.113 | 0.209 |
| InternalIMT | dha | rs73285358 | 0.071 | -0.099 | 0.052 | 0.055 | 0.064 | 0.157 | 0.682 | -0.054 | 0.109 | 0.62 |
| InternalIMT | dha | rs11239528 | 0.198 | -0.059 | 0.055 | 0.281 | 0.21 | 0.13 | 0.106 | -0.119 | 0.088 | 0.177 |
| InternalIMT | dha | rs77453386 | 0.001 | . | . | . | . | . | . | . | . | . |
| InternalIMT | dha | rs2279434 | 0.071 | -0.126 | 0.051 | 0.013 | -0.21 | 0.16 | 0.19 | 0.139 | 0.109 | 0.203 |
| InternalIMT | dha | rs149236525 | 0.003 | . | . | . | . | . | . | . | . | . |
| InternalIMT | dha | rs12573609 | 0.018 | -0.099 | 0.049 | 0.044 | 0.186 | 0.411 | 0.651 | -0.21 | 0.299 | 0.482 |
| InternalIMT | dha | rs191716655 | 0.006 | . | . | . | . | . | . | . | . | . |
| InternalIMT | dha | rs17523660 | 0.005 | . | . | . | . | . | . | . | . | . |
| InternalIMT | dha | rs142671360 | 0.001 | . | . | . | . | . | . | . | . | . |
| InternalIMT | dha | rs3764990 | 0.025 | -0.119 | 0.049 | 0.014 | -0.404 | 0.286 | 0.157 | 0.244 | 0.189 | 0.198 |
| InternalIMT | dha | rs113682861 | 0.001 | . | . | . | . | . | . | . | . | . |
| InternalIMT | dha | rs117658270 | 0.001 | . | . | . | . | . | . | . | . | . |
| InternalIMT | dha | rs187309063 | . | . | . | . | . | . | . | . | . | . |
| InternalIMT | dha | rs73285360 | 0.072 | -0.099 | 0.052 | 0.055 | 0.064 | 0.157 | 0.683 | -0.054 | 0.109 | 0.621 |
| InternalIMT | dha | rs80133289 | 0.002 | . | . | . | . | . | . | . | . | . |
| InternalIMT | dha | rs186693257 | . | . | . | . | . | . | . | . | . | . |
| InternalIMT | dha | rs35735968 | 0.081 | -0.124 | 0.052 | 0.017 | -0.183 | 0.16 | 0.253 | 0.111 | 0.109 | 0.308 |
| InternalIMT | dha | rs17157825 | 0.011 | -0.11 | 0.048 | 0.022 | -0.218 | 0.338 | 0.52 | 0.035 | 0.245 | 0.887 |
| InternalIMT | dha | rs2291428 | 0.183 | -0.05 | 0.055 | 0.358 | 0.275 | 0.134 | 0.041 | -0.151 | 0.09 | 0.095 |
| InternalIMT | dha | rs2291429 | 0.21 | -0.056 | 0.056 | 0.313 | 0.21 | 0.126 | 0.096 | -0.12 | 0.085 | 0.159 |
| InternalIMT | dha | rs145714792 | 0.002 | . | . | . | . | . | . | . | . | . |
| InternalIMT | dha | rs12769244 | 0.068 | -0.125 | 0.051 | 0.014 | -0.21 | 0.163 | 0.197 | 0.135 | 0.111 | 0.222 |
| InternalIMT | dha | rs12771275 | 0.068 | -0.125 | 0.051 | 0.014 | -0.21 | 0.163 | 0.197 | 0.135 | 0.111 | 0.222 |
| InternalIMT | dha | rs146266174 | 0.001 | . | . | . | . | . | . | . | . | . |
| InternalIMT | dha | rs41301623 | 0.001 | . | . | . | . | . | . | . | . | . |
| InternalIMT | dha | rs2306814 | 0.001 | . | . | . | . | . | . | . | . | . |
| InternalIMT | dha | rs12244834 | 0.08 | -0.08 | 0.051 | 0.115 | 0.269 | 0.197 | 0.172 | -0.151 | 0.128 | 0.237 |
| InternalIMT | dha | rs184006011 | 0.001 | . | . | . | . | . | . | . | . | . |
| InternalIMT | dha | rs78829066 | 0.001 | . | . | . | . | . | . | . | . | . |
| InternalIMT | dha | rs34731408 | 0.068 | -0.125 | 0.051 | 0.014 | -0.21 | 0.163 | 0.197 | 0.135 | 0.111 | 0.222 |
| InternalIMT | dha | rs17451516 | 0.012 | -0.115 | 0.048 | 0.016 | -0.506 | 0.45 | 0.261 | 0.374 | 0.289 | 0.196 |
| InternalIMT | dha | rs139910133 | 0.009 | . | . | . | . | . | . | . | . | . |
| InternalIMT | dha | rs10900218 | 0.319 | -0.076 | 0.065 | 0.24 | 0.093 | 0.105 | 0.377 | -0.047 | 0.072 | 0.515 |
| InternalIMT | dha | rs146023999 | . | . | . | . | . | . | . | . | . | . |
| InternalIMT | dha | rs187296958 | 0 | . | . | . | . | . | . | . | . | . |
| InternalIMT | dha | rs142342833 | . | . | . | . | . | . | . | . | . | . |
| InternalIMT | dha | rs143489255 | . | . | . | . | . | . | . | . | . | . |
| InternalIMT | dha | rs138532518 | . | . | . | . | . | . | . | . | . | . |
| InternalIMT | dha | rs183768079 | 0.007 | . | . | . | . | . | . | . | . | . |
| InternalIMT | dha | rs117892272 | . | . | . | . | . | . | . | . | . | . |
| InternalIMT | dha | rs192993943 | . | . | . | . | . | . | . | . | . | . |
| InternalIMT | dha | rs182989043 | 0.001 | . | . | . | . | . | . | . | . | . |
| InternalIMT | dha | rs189703435 | 0.001 | . | . | . | . | . | . | . | . | . |
| InternalIMT | dha | rs188794040 | 0 | . | . | . | . | . | . | . | . | . |
| InternalIMT | dha | rs143920953 | . | . | . | . | . | . | . | . | . | . |
| InternalIMT | dha | rs191029121 | . | . | . | . | . | . | . | . | . | . |
| InternalIMT | dha | rs116875448 | . | . | . | . | . | . | . | . | . | . |
| InternalIMT | dha | rs190167877 | 0.003 | . | . | . | . | . | . | . | . | . |
| InternalIMT | dha | rs184993418 | . | . | . | . | . | . | . | . | . | . |
| InternalIMT | dha | rs143241158 | . | . | . | . | . | . | . | . | . | . |
| InternalIMT | dha | rs11239500 | 0.074 | -0.094 | 0.051 | 0.065 | 0.138 | 0.177 | 0.435 | -0.08 | 0.115 | 0.488 |

| InternalIMT | dha | rs140568994 | . | . | . | . | . | . | . | . | . | . |
| --- | --- | --- | --- | --- | --- | --- | --- | --- | --- | --- | --- | --- |
| InternalIMT | dha | rs187530317 | 0.001 | . | . | . | . | . | . | . | . | . |
| InternalIMT | dha | rs145189326 | 0.001 | . | . | . | . | . | . | . | . | . |
| InternalIMT | dha | rs186203332 | . | . | . | . | . | . | . | . | . | . |
| InternalIMT | dha | rs189921909 | . | . | . | . | . | . | . | . | . | . |
| InternalIMT | dha | rs187260989 | . | . | . | . | . | . | . | . | . | . |
| InternalIMT | dha | rs114376586 | 0.073 | -0.094 | 0.051 | 0.065 | 0.139 | 0.177 | 0.434 | -0.08 | 0.115 | 0.486 |
| InternalIMT | dha | rs151070808 | . | . | . | . | . | . | . | . | . | . |
| InternalIMT | dha | rs140927225 | 0.074 | -0.096 | 0.051 | 0.06 | 0.129 | 0.177 | 0.466 | -0.071 | 0.114 | 0.535 |
| InternalIMT | dha | rs191555615 | . | . | . | . | . | . | . | . | . | . |
| InternalIMT | dha | rs80278898 | 0.001 | . | . | . | . | . | . | . | . | . |
| InternalIMT | dha | rs190849503 | . | . | . | . | . | . | . | . | . | . |
| InternalIMT | dha | rs150080315 | 0.007 | . | . | . | . | . | . | . | . | . |
| InternalIMT | dha | rs143623885 | . | . | . | . | . | . | . | . | . | . |
| InternalIMT | dha | rs187760040 | . | . | . | . | . | . | . | . | . | . |
| InternalIMT | dha | rs143917143 | . | . | . | . | . | . | . | . | . | . |
| InternalIMT | dha | rs149851136 | 0.002 | . | . | . | . | . | . | . | . | . |
| InternalIMT | dha | rs181612054 | . | . | . | . | . | . | . | . | . | . |
| InternalIMT | dha | rs191006548 | . | . | . | . | . | . | . | . | . | . |
| InternalIMT | dha | rs117377631 | 0.003 | . | . | . | . | . | . | . | . | . |
| InternalIMT | dha | rs182107634 | . | . | . | . | . | . | . | . | . | . |
| InternalIMT | dha | rs190211867 | . | . | . | . | . | . | . | . | . | . |
| InternalIMT | dha | rs148293457 | . | . | . | . | . | . | . | . | . | . |
| InternalIMT | dha | rs191811733 | . | . | . | . | . | . | . | . | . | . |
| InternalIMT | dha | rs146109272 | . | . | . | . | . | . | . | . | . | . |
| InternalIMT | dha | rs148397915 | . | . | . | . | . | . | . | . | . | . |
| InternalIMT | dha | rs142233290 | . | . | . | . | . | . | . | . | . | . |
| InternalIMT | dha | rs80226874 | . | . | . | . | . | . | . | . | . | . |
| InternalIMT | dha | rs144496203 | 0 | . | . | . | . | . | . | . | . | . |
| InternalIMT | dha | rs191062130 | . | . | . | . | . | . | . | . | . | . |
| InternalIMT | dha | rs185807817 | . | . | . | . | . | . | . | . | . | . |
| InternalIMT | dha | rs117846213 | 0.001 | . | . | . | . | . | . | . | . | . |
| InternalIMT | dha | rs190545473 | . | . | . | . | . | . | . | . | . | . |
| InternalIMT | dha | rs192364918 | . | . | . | . | . | . | . | . | . | . |
| InternalIMT | dha | rs182939241 | . | . | . | . | . | . | . | . | . | . |
| InternalIMT | dha | rs12571125 | 0.001 | . | . | . | . | . | . | . | . | . |
| InternalIMT | dha | rs192418941 | 0 | . | . | . | . | . | . | . | . | . |
| InternalIMT | dha | rs141270243 | . | . | . | . | . | . | . | . | . | . |
| InternalIMT | dha | rs182615502 | . | . | . | . | . | . | . | . | . | . |
| InternalIMT | dha | rs146669856 | 0.02 | -0.115 | 0.048 | 0.017 | -0.132 | 0.389 | 0.733 | 0.176 | 0.287 | 0.539 |
| InternalIMT | dha | rs78290029 | 0.098 | -0.069 | 0.052 | 0.187 | 0.339 | 0.18 | 0.06 | -0.181 | 0.116 | 0.12 |
| InternalIMT | dha | rs139955565 | . | . | . | . | . | . | . | . | . | . |
| InternalIMT | dha | rs12267314 | 0.087 | -0.078 | 0.051 | 0.125 | 0.292 | 0.194 | 0.133 | -0.161 | 0.125 | 0.198 |
| InternalIMT | dha | rs74477197 | . | . | . | . | . | . | . | . | . | . |
| InternalIMT | dha | rs188147236 | . | . | . | . | . | . | . | . | . | . |
| InternalIMT | dha | rs2228065 | 0.086 | -0.079 | 0.051 | 0.118 | 0.283 | 0.195 | 0.147 | -0.155 | 0.125 | 0.216 |
| InternalIMT | dha | rs116961353 | . | . | . | . | . | . | . | . | . | . |
| InternalIMT | dha | rs190222272 | . | . | . | . | . | . | . | . | . | . |
| InternalIMT | dha | rs182719940 | . | . | . | . | . | . | . | . | . | . |
| InternalIMT | dha | rs184477413 | . | . | . | . | . | . | . | . | . | . |
| InternalIMT | dha | rs12265175 | 0.089 | -0.085 | 0.051 | 0.096 | 0.241 | 0.192 | 0.208 | -0.122 | 0.124 | 0.326 |
| InternalIMT | dha | rs182928276 | . | . | . | . | . | . | . | . | . | . |
| InternalIMT | dha | rs144409895 | . | . | . | . | . | . | . | . | . | . |
| InternalIMT | dha | rs58998447 | 0.003 | . | . | . | . | . | . | . | . | . |
| InternalIMT | dha | rs188668832 | . | . | . | . | . | . | . | . | . | . |
| InternalIMT | dha | rs148641686 | . | . | . | . | . | . | . | . | . | . |
| InternalIMT | dha | rs75468791 | . | . | . | . | . | . | . | . | . | . |
| InternalIMT | dha | rs147327397 | . | . | . | . | . | . | . | . | . | . |
| InternalIMT | dha | rs77354597 | 0.002 | . | . | . | . | . | . | . | . | . |
| InternalIMT | dha | rs41365544 | 0.082 | -0.079 | 0.051 | 0.122 | 0.268 | 0.195 | 0.169 | -0.152 | 0.126 | 0.227 |
| InternalIMT | dha | rs187564188 | . | . | . | . | . | . | . | . | . | . |
| InternalIMT | dha | rs183511984 | 0 | . | . | . | . | . | . | . | . | . |
| InternalIMT | dha | rs12262783 | 0.08 | -0.077 | 0.051 | 0.129 | 0.293 | 0.196 | 0.136 | -0.163 | 0.127 | 0.199 |
| InternalIMT | dha | rs188817687 | 0.002 | . | . | . | . | . | . | . | . | . |
| InternalIMT | dha | rs3815968 | . | . | . | . | . | . | . | . | . | . |
| InternalIMT | dha | rs187822300 | . | . | . | . | . | . | . | . | . | . |
| InternalIMT | dha | rs188902172 | 0.002 | . | . | . | . | . | . | . | . | . |
| InternalIMT | dha | rs181190448 | . | . | . | . | . | . | . | . | . | . |
| InternalIMT | dha | rs182699093 | . | . | . | . | . | . | . | . | . | . |
| InternalIMT | dha | rs182631303 | 0.001 | . | . | . | . | . | . | . | . | . |
| InternalIMT | dha | rs187014172 | . | . | . | . | . | . | . | . | . | . |
| InternalIMT | dha | rs142575406 | . | . | . | . | . | . | . | . | . | . |
| InternalIMT | dha | rs180711033 | . | . | . | . | . | . | . | . | . | . |
| InternalIMT | dha | rs137894823 | 0.004 | . | . | . | . | . | . | . | . | . |
| InternalIMT | dha | rs189053273 | 0.001 | . | . | . | . | . | . | . | . | . |
| InternalIMT | dha | rs143499388 | 0.002 | . | . | . | . | . | . | . | . | . |
| InternalIMT | dha | rs182936001 | 0.006 | . | . | . | . | . | . | . | . | . |
| InternalIMT | dha | rs148456406 | 0.006 | . | . | . | . | . | . | . | . | . |
| InternalIMT | dha | rs182499531 | 0.008 | . | . | . | . | . | . | . | . | . |
| InternalIMT | dha | rs74884915 | 0.026 | -0.1 | 0.049 | 0.043 | 0.138 | 0.226 | 0.541 | -0.11 | 0.147 | 0.456 |
| InternalIMT | dha | rs184046374 | 0.001 | . | . | . | . | . | . | . | . | . |
| InternalIMT | dha | rs74333993 | 0.032 | -0.095 | 0.049 | 0.052 | 0.212 | 0.261 | 0.417 | -0.139 | 0.164 | 0.397 |
| InternalIMT | dha | rs138889719 | 0.001 | . | . | . | . | . | . | . | . | . |
| InternalIMT | dha | rs75330330 | 0.022 | -0.108 | 0.048 | 0.024 | 4.30E-03 | 0.318 | 0.989 | 0.051 | 0.209 | 0.807 |
| InternalIMT | dha | rs193051693 | 0 | . | . | . | . | . | . | . | . | . |
| InternalIMT | dha | rs145929633 | 0 | . | . | . | . | . | . | . | . | . |

| InternalIMT | dha | rs184076904 | 0.004 | . | . | . | . | . | . | . | . | . |
| --- | --- | --- | --- | --- | --- | --- | --- | --- | --- | --- | --- | --- |
| InternalIMT | dha | rs141802494 | 0.018 | -0.108 | 0.048 | 0.024 | -1.70E-03 | 0.373 | 0.996 | 0.061 | 0.243 | 0.801 |
| InternalIMT | dha | rs149519759 | 0.002 | . | . | . | . | . | . | . | . | . |
| InternalIMT | dha | rs145467260 | 0.013 | -0.094 | 0.048 | 0.05 | 0.558 | 0.429 | 0.193 | -0.401 | 0.267 | 0.133 |
| InternalIMT | dha | rs79620914 | 0.027 | -0.096 | 0.049 | 0.048 | 0.168 | 0.297 | 0.572 | -0.169 | 0.19 | 0.372 |
| InternalIMT | dha | rs140538633 | 0.017 | -0.101 | 0.048 | 0.037 | 0.185 | 0.254 | 0.467 | -0.158 | 0.154 | 0.306 |
| InternalIMT | dha | rs140862301 | 0.008 | . | . | . | . | . | . | . | . | . |
| InternalIMT | dha | rs114615847 | 0.009 | . | . | . | . | . | . | . | . | . |
| InternalIMT | dha | rs141800259 | 0.026 | -0.104 | 0.048 | 0.032 | 0.11 | 0.29 | 0.704 | -0.041 | 0.194 | 0.835 |
| InternalIMT | dha | rs149779059 | 0.019 | -0.111 | 0.048 | 0.02 | 0.012 | 0.391 | 0.975 | 0.075 | 0.259 | 0.773 |
| InternalIMT | dha | rs116539253 | 0.007 | . | . | . | . | . | . | . | . | . |
| InternalIMT | dha | rs184979550 | 0.001 | . | . | . | . | . | . | . | . | . |
| InternalIMT | dha | rs190128940 | 0.004 | . | . | . | . | . | . | . | . | . |
| InternalIMT | dha | rs146594816 | 0.002 | . | . | . | . | . | . | . | . | . |
| InternalIMT | dha | rs141370766 | 0.022 | -0.108 | 0.048 | 0.024 | 2.90E-03 | 0.319 | 0.993 | 0.053 | 0.209 | 0.798 |
| InternalIMT | dha | rs144670033 | 0.002 | . | . | . | . | . | . | . | . | . |
| InternalIMT | dha | rs187152174 | 0.007 | . | . | . | . | . | . | . | . | . |
| InternalIMT | dha | rs184180263 | 0.005 | . | . | . | . | . | . | . | . | . |
| InternalIMT | dha | rs140075861 | 0.01 | -0.102 | 0.048 | 0.033 | 0.295 | 0.331 | 0.373 | -0.206 | 0.201 | 0.307 |
| InternalIMT | dha | rs186483129 | 0 | . | . | . | . | . | . | . | . | . |
| InternalIMT | dha | rs186634282 | 0.012 | -0.122 | 0.047 | 9.80E-03 | -0.915 | 0.455 | 0.045 | 0.851 | 0.372 | 0.022 |
| InternalIMT | dha | rs9988663 | 0.032 | -0.095 | 0.049 | 0.052 | 0.213 | 0.262 | 0.417 | -0.139 | 0.164 | 0.398 |
| InternalIMT | dha | rs138828529 | 0.02 | -0.107 | 0.048 | 0.027 | 0.023 | 0.374 | 0.951 | -0.026 | 0.272 | 0.924 |
| InternalIMT | dha | rs188777615 | 0.004 | . | . | . | . | . | . | . | . | . |
| InternalIMT | dha | rs192321882 | 0.008 | . | . | . | . | . | . | . | . | . |
| InternalIMT | dha | rs150812270 | 0.027 | -0.1 | 0.049 | 0.04 | 0.188 | 0.328 | 0.567 | -0.127 | 0.226 | 0.574 |
| InternalIMT | dha | rs185906603 | 0.001 | . | . | . | . | . | . | . | . | . |
| InternalIMT | dha | rs191070862 | 0.004 | . | . | . | . | . | . | . | . | . |
| InternalIMT | dha | rs182904978 | 0 | . | . | . | . | . | . | . | . | . |
| InternalIMT | dha | rs192679655 | 0.001 | . | . | . | . | . | . | . | . | . |
| InternalIMT | dha | rs189673086 | 0.007 | . | . | . | . | . | . | . | . | . |
| InternalIMT | dha | rs181141892 | 0.002 | . | . | . | . | . | . | . | . | . |
| InternalIMT | dha | rs186404123 | 0 | . | . | . | . | . | . | . | . | . |
| InternalIMT | dha | rs192117287 | 0.005 | . | . | . | . | . | . | . | . | . |
| InternalIMT | dha | rs182748835 | 0.001 | . | . | . | . | . | . | . | . | . |
| InternalIMT | dha | rs141854549 | 0.017 | -0.101 | 0.048 | 0.037 | 0.181 | 0.254 | 0.477 | -0.154 | 0.154 | 0.317 |
| InternalIMT | dha | rs140163397 | 0.005 | . | . | . | . | . | . | . | . | . |
| InternalIMT | dha | rs146347300 | 0.008 | . | . | . | . | . | . | . | . | . |
| InternalIMT | dha | rs139362377 | 0.017 | -0.101 | 0.048 | 0.037 | 0.18 | 0.254 | 0.478 | -0.154 | 0.154 | 0.318 |
| InternalIMT | dha | rs185087671 | 0.001 | . | . | . | . | . | . | . | . | . |
| InternalIMT | dha | rs190466574 | 0.005 | . | . | . | . | . | . | . | . | . |
| InternalIMT | dha | rs147012778 | 0.005 | . | . | . | . | . | . | . | . | . |
| InternalIMT | dha | rs138236960 | 0.009 | . | . | . | . | . | . | . | . | . |
| InternalIMT | dha | rs116310311 | 0.017 | -0.101 | 0.048 | 0.037 | 0.18 | 0.254 | 0.479 | -0.153 | 0.154 | 0.319 |
| InternalIMT | dha | rs142868356 | 0.012 | -0.111 | 0.047 | 0.019 | -0.153 | 0.523 | 0.77 | 0.222 | 0.409 | 0.587 |
| InternalIMT | dha | rs144956240 | 0.003 | . | . | . | . | . | . | . | . | . |
| InternalIMT | dha | rs188527776 | 0.002 | . | . | . | . | . | . | . | . | . |
| InternalIMT | dha | rs183971165 | 0.007 | . | . | . | . | . | . | . | . | . |
| InternalIMT | dha | rs76748216 | 0.009 | . | . | . | . | . | . | . | . | . |
| InternalIMT | dha | rs148554990 | 0.001 | . | . | . | . | . | . | . | . | . |
| InternalIMT | dha | rs114446477 | 0.027 | -0.096 | 0.049 | 0.05 | 0.187 | 0.298 | 0.531 | -0.187 | 0.19 | 0.326 |
| InternalIMT | dha | rs185223933 | 0.006 | . | . | . | . | . | . | . | . | . |
| InternalIMT | dha | rs76869726 | 0.009 | . | . | . | . | . | . | . | . | . |
| InternalIMT | dha | rs151200830 | 0.017 | -0.101 | 0.048 | 0.038 | 0.197 | 0.255 | 0.439 | -0.163 | 0.154 | 0.292 |
| InternalIMT | dha | rs17157731 | 0.017 | -0.101 | 0.048 | 0.038 | 0.196 | 0.255 | 0.442 | -0.162 | 0.154 | 0.295 |
| InternalIMT | dha | rs181243469 | 0 | . | . | . | . | . | . | . | . | . |
| InternalIMT | dha | rs186890707 | 0 | . | . | . | . | . | . | . | . | . |
| InternalIMT | dha | rs184063360 | 0 | . | . | . | . | . | . | . | . | . |
| InternalIMT | dha | rs188550237 | 0.007 | . | . | . | . | . | . | . | . | . |
| InternalIMT | dha | rs140931068 | 0.017 | -0.111 | 0.048 | 0.02 | -0.018 | 0.498 | 0.971 | 0.185 | 0.375 | 0.622 |
| InternalIMT | dha | rs74867877 | 0.007 | . | . | . | . | . | . | . | . | . |
| InternalIMT | dha | rs11239495 | 0.055 | -0.098 | 0.05 | 0.049 | 0.152 | 0.208 | 0.465 | -0.077 | 0.134 | 0.563 |
| InternalIMT | dha | rs149745800 | 0 | . | . | . | . | . | . | . | . | . |
| InternalIMT | dha | rs145044458 | 0.015 | -0.101 | 0.048 | 0.037 | 0.221 | 0.267 | 0.408 | -0.17 | 0.16 | 0.289 |
| InternalIMT | dha | rs188067427 | 0.002 | . | . | . | . | . | . | . | . | . |
| InternalIMT | dha | rs146827800 | 0.001 | . | . | . | . | . | . | . | . | . |
| InternalIMT | dha | rs140619817 | 0.015 | -0.101 | 0.048 | 0.036 | 0.208 | 0.267 | 0.437 | -0.159 | 0.161 | 0.321 |
| InternalIMT | dha | rs892689 | 0.997 | . | . | . | . | . | . | . | . | . |
| InternalIMT | dha | rs143642005 | 0.008 | . | . | . | . | . | . | . | . | . |
| InternalIMT | dha | rs181929304 | 0.007 | . | . | . | . | . | . | . | . | . |
| InternalIMT | dha | rs191086173 | 0.002 | . | . | . | . | . | . | . | . | . |
| InternalIMT | dha | rs114613116 | 0.018 | -0.102 | 0.048 | 0.036 | 0.154 | 0.255 | 0.545 | -0.131 | 0.156 | 0.401 |
| InternalIMT | dha | rs141076768 | 0.002 | . | . | . | . | . | . | . | . | . |
| InternalIMT | dha | rs187316531 | 0 | . | . | . | . | . | . | . | . | . |
| InternalIMT | dha | rs11239499 | 0.075 | -0.094 | 0.051 | 0.065 | 0.142 | 0.177 | 0.422 | -0.079 | 0.114 | 0.49 |
| InternalIMT | dha | rs192726371 | 0.003 | . | . | . | . | . | . | . | . | . |
| InternalIMT | dha | rs74128481 | 0.019 | -0.122 | 0.048 | 0.011 | -0.437 | 0.364 | 0.231 | 0.417 | 0.289 | 0.148 |
| InternalIMT | dha | rs74128482 | 0.012 | -0.121 | 0.047 | 0.01 | -0.944 | 0.483 | 0.051 | 0.88 | 0.39 | 0.024 |
| InternalIMT | dha | rs11239502 | 0.055 | -0.099 | 0.05 | 0.045 | 0.134 | 0.206 | 0.518 | -0.064 | 0.133 | 0.629 |
| InternalIMT | dha | rs116330285 | 0.002 | . | . | . | . | . | . | . | . | . |
| InternalIMT | dha | rs189926729 | 0.003 | . | . | . | . | . | . | . | . | . |
| InternalIMT | dha | rs188975779 | 0.012 | -0.111 | 0.047 | 0.019 | -0.156 | 0.524 | 0.766 | 0.229 | 0.41 | 0.576 |
| InternalIMT | dha | rs143846038 | 0.002 | . | . | . | . | . | . | . | . | . |
| InternalIMT | dha | rs74128483 | 0.019 | -0.122 | 0.048 | 0.011 | -0.439 | 0.364 | 0.228 | 0.419 | 0.288 | 0.147 |
| InternalIMT | dha | rs139292055 | 0.073 | -0.094 | 0.051 | 0.065 | 0.14 | 0.178 | 0.431 | -0.081 | 0.115 | 0.481 |
| InternalIMT | dha | rs11239503 | 0.073 | -0.094 | 0.051 | 0.065 | 0.14 | 0.178 | 0.431 | -0.081 | 0.115 | 0.481 |

| InternalIMT | dha | rs58838686 | 0.012 | -0.121 | 0.047 | 0.01 | -0.944 | 0.48 | 0.049 | 0.881 | 0.388 | 0.023 |
| --- | --- | --- | --- | --- | --- | --- | --- | --- | --- | --- | --- | --- |
| InternalIMT | dha | rs185825373 | 0.008 | . | . | . | . | . | . | . | . | . |
| InternalIMT | dha | rs11239504 | 0.073 | -0.094 | 0.051 | 0.065 | 0.14 | 0.178 | 0.431 | -0.081 | 0.115 | 0.482 |
| InternalIMT | dha | rs150012354 | 0.006 | . | . | . | . | . | . | . | . | . |
| InternalIMT | dha | rs187763096 | 0.01 | . | . | . | . | . | . | . | . | . |
| InternalIMT | dha | rs193043982 | 0.008 | . | . | . | . | . | . | . | . | . |
| InternalIMT | dha | rs79186884 | 0.023 | -0.117 | 0.048 | 0.015 | -0.104 | 0.365 | 0.775 | 0.152 | 0.247 | 0.539 |
| InternalIMT | dha | rs74128484 | 0.012 | -0.121 | 0.047 | 0.01 | -0.944 | 0.479 | 0.049 | 0.881 | 0.387 | 0.023 |
| InternalIMT | dha | rs58461537 | 0.073 | -0.094 | 0.051 | 0.065 | 0.139 | 0.177 | 0.433 | -0.08 | 0.115 | 0.484 |
| InternalIMT | dha | rs188527474 | 0.008 | . | . | . | . | . | . | . | . | . |
| InternalIMT | dha | rs141659976 | 0.008 | . | . | . | . | . | . | . | . | . |
| InternalIMT | dha | rs115846407 | 0.073 | -0.094 | 0.051 | 0.065 | 0.139 | 0.177 | 0.433 | -0.08 | 0.115 | 0.484 |
| InternalIMT | dha | rs138378816 | 0.073 | -0.094 | 0.051 | 0.065 | 0.139 | 0.177 | 0.433 | -0.08 | 0.115 | 0.484 |
| InternalIMT | dha | rs146188136 | 0.055 | -0.1 | 0.05 | 0.045 | 0.133 | 0.206 | 0.521 | -0.064 | 0.133 | 0.633 |
| InternalIMT | dha | rs139383871 | 0.014 | -0.107 | 0.048 | 0.026 | 0.023 | 0.383 | 0.951 | -0.095 | 0.26 | 0.716 |
| InternalIMT | dha | rs182953869 | 0.004 | . | . | . | . | . | . | . | . | . |
| InternalIMT | dha | rs192430299 | 0.019 | -0.118 | 0.048 | 0.013 | -0.25 | 0.311 | 0.421 | 0.312 | 0.245 | 0.204 |
| InternalIMT | dha | rs144152844 | 0.073 | -0.094 | 0.051 | 0.064 | 0.138 | 0.177 | 0.436 | -0.08 | 0.115 | 0.487 |
| InternalIMT | dha | rs145987786 | 0.051 | -0.069 | 0.051 | 0.174 | 0.624 | 0.218 | 4.30E-03 | -0.388 | 0.141 | 5.90E-03 |
| InternalIMT | dha | rs181132926 | 0.003 | . | . | . | . | . | . | . | . | . |
| InternalIMT | dha | rs147882253 | 0.073 | -0.094 | 0.051 | 0.065 | 0.139 | 0.177 | 0.435 | -0.08 | 0.115 | 0.486 |
| InternalIMT | dha | rs141807621 | 0.023 | -0.108 | 0.048 | 0.025 | -0.023 | 0.328 | 0.945 | -7.60E-03 | 0.236 | 0.974 |
| InternalIMT | dha | rs147116052 | 0.073 | -0.094 | 0.051 | 0.065 | 0.139 | 0.177 | 0.435 | -0.08 | 0.115 | 0.486 |
| InternalIMT | dha | rs147716618 | 0.073 | -0.094 | 0.051 | 0.065 | 0.139 | 0.177 | 0.435 | -0.08 | 0.115 | 0.486 |
| InternalIMT | dha | rs187198855 | 0.046 | -0.081 | 0.051 | 0.111 | 0.516 | 0.248 | 0.038 | -0.294 | 0.163 | 0.072 |
| InternalIMT | dha | rs189409638 | 0.016 | -0.122 | 0.048 | 0.01 | -0.495 | 0.393 | 0.208 | 0.465 | 0.304 | 0.127 |
| InternalIMT | dha | rs182647339 | 0.001 | . | . | . | . | . | . | . | . | . |
| InternalIMT | dha | rs187995988 | 0.004 | . | . | . | . | . | . | . | . | . |
| InternalIMT | dha | rs141034184 | 0.079 | -0.103 | 0.051 | 0.044 | 0.042 | 0.175 | 0.812 | -0.026 | 0.114 | 0.823 |
| InternalIMT | dha | rs187310430 | 0.008 | . | . | . | . | . | . | . | . | . |
| InternalIMT | dha | rs192608488 | 0.073 | -0.095 | 0.051 | 0.064 | 0.137 | 0.178 | 0.441 | -0.079 | 0.115 | 0.495 |
| InternalIMT | dha | rs148840366 | 0.009 | . | . | . | . | . | . | . | . | . |
| InternalIMT | dha | rs186785022 | 0.019 | -0.122 | 0.048 | 0.011 | -0.444 | 0.362 | 0.219 | 0.423 | 0.287 | 0.141 |
| InternalIMT | dha | rs192348728 | 0.014 | -0.11 | 0.048 | 0.022 | -0.099 | 0.381 | 0.794 | 0.067 | 0.262 | 0.798 |
| InternalIMT | dha | rs189656631 | 0.073 | -0.095 | 0.051 | 0.064 | 0.137 | 0.178 | 0.441 | -0.078 | 0.115 | 0.495 |
| InternalIMT | dha | rs185289750 | 0.004 | . | . | . | . | . | . | . | . | . |
| InternalIMT | dha | rs142382395 | 0.073 | -0.095 | 0.051 | 0.064 | 0.137 | 0.178 | 0.442 | -0.078 | 0.115 | 0.496 |
| InternalIMT | dha | rs144866696 | 0.019 | -0.122 | 0.048 | 0.011 | -0.444 | 0.361 | 0.219 | 0.424 | 0.287 | 0.14 |
| InternalIMT | dha | rs141371289 | 0.073 | -0.095 | 0.051 | 0.064 | 0.137 | 0.178 | 0.442 | -0.078 | 0.115 | 0.496 |
| InternalIMT | dha | rs114750008 | 0.073 | -0.095 | 0.051 | 0.064 | 0.137 | 0.178 | 0.442 | -0.078 | 0.115 | 0.496 |
| InternalIMT | dha | rs189063597 | 0.001 | . | . | . | . | . | . | . | . | . |
| InternalIMT | dha | rs139151571 | 0.067 | -0.09 | 0.051 | 0.077 | 0.182 | 0.188 | 0.332 | -0.112 | 0.121 | 0.353 |
| InternalIMT | dha | rs139846368 | 0.075 | -0.092 | 0.051 | 0.073 | 0.159 | 0.184 | 0.388 | -0.094 | 0.118 | 0.426 |
| InternalIMT | dha | rs182057653 | 0.019 | -0.111 | 0.049 | 0.024 | -0.107 | 0.238 | 0.652 | 0.075 | 0.171 | 0.66 |
| InternalIMT | dha | rs148785420 | 0.073 | -0.095 | 0.051 | 0.064 | 0.137 | 0.178 | 0.442 | -0.078 | 0.115 | 0.496 |
| InternalIMT | dha | rs138383251 | 0.011 | -0.103 | 0.048 | 0.032 | 0.266 | 0.335 | 0.426 | -0.181 | 0.205 | 0.376 |
| InternalIMT | dha | rs116526705 | 0.018 | -0.103 | 0.048 | 0.033 | 0.105 | 0.26 | 0.687 | -0.093 | 0.161 | 0.561 |
| InternalIMT | dha | rs185946436 | 0 | . | . | . | . | . | . | . | . | . |
| InternalIMT | dha | rs190455427 | 0.004 | . | . | . | . | . | . | . | . | . |
| InternalIMT | dha | rs9282584 | 0.073 | -0.095 | 0.051 | 0.064 | 0.136 | 0.178 | 0.443 | -0.078 | 0.115 | 0.497 |
| InternalIMT | dha | rs17153289 | 0.073 | -0.095 | 0.051 | 0.064 | 0.137 | 0.178 | 0.443 | -0.078 | 0.115 | 0.497 |
| InternalIMT | dha | rs138359103 | 0.008 | . | . | . | . | . | . | . | . | . |
| InternalIMT | dha | rs150605268 | 0.003 | . | . | . | . | . | . | . | . | . |
| InternalIMT | dha | rs79242910 | 0.075 | -0.096 | 0.051 | 0.062 | 0.132 | 0.176 | 0.456 | -0.073 | 0.114 | 0.52 |
| InternalIMT | dha | rs139716904 | 0.021 | -0.109 | 0.048 | 0.025 | -0.145 | 0.299 | 0.627 | 0.028 | 0.223 | 0.9 |
| InternalIMT | dha | rs76238498 | 0.053 | -0.071 | 0.051 | 0.162 | 0.564 | 0.218 | 9.80E-03 | -0.347 | 0.14 | 0.013 |
| InternalIMT | dha | rs184140639 | 0 | . | . | . | . | . | . | . | . | . |
| InternalIMT | dha | rs188517278 | 0.009 | . | . | . | . | . | . | . | . | . |
| InternalIMT | dha | rs139678913 | 0.004 | . | . | . | . | . | . | . | . | . |
| InternalIMT | dha | rs116697955 | 0.004 | . | . | . | . | . | . | . | . | . |
| InternalIMT | dha | rs79267994 | 0.012 | -0.121 | 0.047 | 0.01 | -0.943 | 0.471 | 0.045 | 0.881 | 0.382 | 0.021 |
| InternalIMT | dha | rs11239510 | 0.022 | -0.098 | 0.049 | 0.045 | 0.31 | 0.348 | 0.373 | -0.254 | 0.226 | 0.26 |
| InternalIMT | dha | rs75831645 | 0.018 | -0.103 | 0.048 | 0.033 | 0.102 | 0.26 | 0.695 | -0.091 | 0.161 | 0.57 |
| InternalIMT | dha | rs78452276 | 0.052 | -0.093 | 0.049 | 0.061 | 0.231 | 0.227 | 0.309 | -0.149 | 0.151 | 0.322 |
| InternalIMT | dha | rs144366924 | 0 | . | . | . | . | . | . | . | . | . |
| InternalIMT | dha | rs77519841 | 0.056 | -0.1 | 0.05 | 0.045 | 0.132 | 0.206 | 0.522 | -0.063 | 0.133 | 0.636 |
| InternalIMT | dha | rs76944454 | 0.073 | -0.095 | 0.051 | 0.064 | 0.136 | 0.178 | 0.443 | -0.078 | 0.115 | 0.497 |
| InternalIMT | dha | rs76443855 | 0.053 | -0.071 | 0.051 | 0.162 | 0.565 | 0.218 | 9.70E-03 | -0.348 | 0.14 | 0.013 |
| InternalIMT | dha | rs146613174 | 0.002 | . | . | . | . | . | . | . | . | . |
| InternalIMT | dha | rs181424761 | 0.002 | . | . | . | . | . | . | . | . | . |
| InternalIMT | dha | rs142119241 | 0.001 | . | . | . | . | . | . | . | . | . |
| InternalIMT | dha | rs140137954 | 0.018 | -0.103 | 0.048 | 0.033 | 0.104 | 0.26 | 0.689 | -0.093 | 0.161 | 0.564 |
| InternalIMT | dha | rs187319549 | 0.002 | . | . | . | . | . | . | . | . | . |
| InternalIMT | dha | rs12241545 | 0.035 | -0.104 | 0.048 | 0.031 | -0.061 | 0.288 | 0.832 | -0.039 | 0.205 | 0.847 |
| InternalIMT | dha | rs10128228 | 0.073 | -0.095 | 0.051 | 0.064 | 0.137 | 0.178 | 0.443 | -0.078 | 0.115 | 0.497 |
| InternalIMT | dha | rs58580249 | 0.009 | . | . | . | . | . | . | . | . | . |
| InternalIMT | dha | rs193148863 | 0.001 | . | . | . | . | . | . | . | . | . |
| InternalIMT | dha | rs185638946 | 0.005 | . | . | . | . | . | . | . | . | . |
| InternalIMT | dha | rs117752921 | 0.001 | . | . | . | . | . | . | . | . | . |
| InternalIMT | dha | rs180841236 | 0.002 | . | . | . | . | . | . | . | . | . |
| InternalIMT | dha | rs73283155 | 0.009 | . | . | . | . | . | . | . | . | . |
| InternalIMT | dha | rs76560599 | 0.061 | -0.087 | 0.051 | 0.09 | 0.22 | 0.192 | 0.251 | -0.147 | 0.124 | 0.234 |
| InternalIMT | dha | rs76273620 | 0.061 | -0.087 | 0.051 | 0.09 | 0.22 | 0.192 | 0.251 | -0.147 | 0.124 | 0.234 |
| InternalIMT | dha | rs141432923 | 0.02 | -0.12 | 0.048 | 0.012 | -0.544 | 0.519 | 0.294 | 0.427 | 0.391 | 0.276 |
| InternalIMT | dha | rs113529322 | 0.019 | -0.122 | 0.048 | 0.011 | -0.447 | 0.361 | 0.215 | 0.426 | 0.287 | 0.138 |
| InternalIMT | dha | rs10128306 | 0.073 | -0.095 | 0.051 | 0.063 | 0.136 | 0.178 | 0.443 | -0.078 | 0.115 | 0.498 |

| InternalIMT | dha | rs149691688 | 0.002 | . | . | . | . | . | . | . | . | . |
| --- | --- | --- | --- | --- | --- | --- | --- | --- | --- | --- | --- | --- |
| InternalIMT | dha | rs75872495 | 0.073 | -0.095 | 0.051 | 0.063 | 0.137 | 0.178 | 0.442 | -0.078 | 0.115 | 0.498 |
| InternalIMT | dha | rs191873960 | 0.007 | . | . | . | . | . | . | . | . | . |
| InternalIMT | dha | rs80155622 | 0.073 | -0.095 | 0.051 | 0.063 | 0.136 | 0.178 | 0.444 | -0.078 | 0.115 | 0.5 |
| InternalIMT | dha | rs113914656 | 0.008 | . | . | . | . | . | . | . | . | . |
| InternalIMT | dha | rs12242840 | 0.073 | -0.095 | 0.051 | 0.063 | 0.136 | 0.178 | 0.446 | -0.078 | 0.115 | 0.501 |
| InternalIMT | dha | rs147824078 | 0.009 | . | . | . | . | . | . | . | . | . |
| InternalIMT | dha | rs59051493 | 0.073 | -0.095 | 0.051 | 0.063 | 0.137 | 0.179 | 0.445 | -0.078 | 0.116 | 0.5 |
| InternalIMT | dha | rs9282588 | 0.008 | . | . | . | . | . | . | . | . | . |
| InternalIMT | dha | rs150215207 | 0.009 | . | . | . | . | . | . | . | . | . |
| InternalIMT | dha | rs138772243 | 0.003 | . | . | . | . | . | . | . | . | . |
| InternalIMT | dha | rs149383244 | 0.025 | -0.104 | 0.049 | 0.035 | 0.053 | 0.277 | 0.849 | -0.043 | 0.184 | 0.815 |
| InternalIMT | dha | rs17522720 | 0.016 | -0.11 | 0.048 | 0.023 | -0.073 | 0.36 | 0.84 | 0.05 | 0.243 | 0.836 |
| InternalIMT | dha | rs79245105 | 0.073 | -0.095 | 0.051 | 0.064 | 0.137 | 0.179 | 0.445 | -0.078 | 0.116 | 0.5 |
| InternalIMT | dha | rs138222133 | 0.004 | . | . | . | . | . | . | . | . | . |
| InternalIMT | dha | rs12247314 | 0.082 | -0.096 | 0.052 | 0.063 | 0.128 | 0.173 | 0.46 | -0.067 | 0.113 | 0.552 |
| InternalIMT | dha | rs12263894 | 0.08 | -0.096 | 0.052 | 0.064 | 0.136 | 0.175 | 0.437 | -0.072 | 0.114 | 0.528 |
| InternalIMT | dha | rs149907880 | 0.008 | . | . | . | . | . | . | . | . | . |
| InternalIMT | dha | rs188153885 | 0.007 | . | . | . | . | . | . | . | . | . |
| InternalIMT | dha | rs73283159 | 0.005 | . | . | . | . | . | . | . | . | . |
| InternalIMT | dha | rs140476687 | 0.002 | . | . | . | . | . | . | . | . | . |
| InternalIMT | dha | rs186603148 | 0 | . | . | . | . | . | . | . | . | . |
| InternalIMT | dha | rs183296063 | 0.003 | . | . | . | . | . | . | . | . | . |
| InternalIMT | dha | rs145733694 | 0.009 | . | . | . | . | . | . | . | . | . |
| InternalIMT | dha | rs189807449 | 0 | . | . | . | . | . | . | . | . | . |
| InternalIMT | dha | rs12253223 | 0.064 | -0.101 | 0.05 | 0.044 | 0.114 | 0.198 | 0.565 | -0.047 | 0.129 | 0.716 |
| InternalIMT | dha | rs182113637 | 0 | . | . | . | . | . | . | . | . | . |
| InternalIMT | dha | rs142792099 | 0.018 | -0.109 | 0.048 | 0.022 | -0.035 | 0.371 | 0.925 | 0.088 | 0.24 | 0.715 |
| InternalIMT | dha | rs12249910 | 0.064 | -0.101 | 0.05 | 0.043 | 0.113 | 0.198 | 0.567 | -0.047 | 0.129 | 0.717 |
| InternalIMT | dha | rs149436339 | 0.001 | . | . | . | . | . | . | . | . | . |
| InternalIMT | dha | rs77632210 | 0 | . | . | . | . | . | . | . | . | . |
| InternalIMT | dha | rs74130818 | 0.012 | -0.122 | 0.047 | 9.70E-03 | -1 | 0.469 | 0.033 | 0.923 | 0.383 | 0.016 |
| InternalIMT | dha | rs187583648 | 0 | . | . | . | . | . | . | . | . | . |
| InternalIMT | dha | rs191295987 | 0.008 | . | . | . | . | . | . | . | . | . |
| InternalIMT | dha | rs184200686 | 0.01 | . | . | . | . | . | . | . | . | . |
| InternalIMT | dha | rs76117263 | 0.052 | -0.069 | 0.051 | 0.173 | 0.622 | 0.227 | 6.30E-03 | -0.379 | 0.145 | 9.00E-03 |
| InternalIMT | dha | rs185393149 | 0.001 | . | . | . | . | . | . | . | . | . |
| InternalIMT | dha | rs144760014 | 0.006 | . | . | . | . | . | . | . | . | . |
| InternalIMT | dha | rs7922292 | 0.008 | . | . | . | . | . | . | . | . | . |
| InternalIMT | dha | rs115372844 | 0.009 | . | . | . | . | . | . | . | . | . |
| InternalIMT | dha | rs111460364 | 0.052 | -0.069 | 0.051 | 0.174 | 0.625 | 0.229 | 6.30E-03 | -0.381 | 0.146 | 9.00E-03 |
| InternalIMT | dha | rs182052255 | 0.002 | . | . | . | . | . | . | . | . | . |
| InternalIMT | dha | rs180983889 | 0.006 | . | . | . | . | . | . | . | . | . |
| InternalIMT | dha | rs139175327 | 0.009 | . | . | . | . | . | . | . | . | . |
| InternalIMT | dha | rs149959620 | 0.018 | -0.107 | 0.048 | 0.027 | -0.048 | 0.34 | 0.888 | -0.026 | 0.248 | 0.916 |
| InternalIMT | dha | rs114653117 | 0.009 | . | . | . | . | . | . | . | . | . |
| InternalIMT | dha | rs144270018 | 0.005 | . | . | . | . | . | . | . | . | . |
| InternalIMT | dha | rs182723204 | 0.007 | . | . | . | . | . | . | . | . | . |
| InternalIMT | dha | rs57204067 | 0.012 | -0.123 | 0.047 | 9.20E-03 | -1.04 | 0.467 | 0.026 | 0.952 | 0.383 | 0.013 |
| InternalIMT | dha | rs144956226 | 0.064 | -0.101 | 0.05 | 0.044 | 0.107 | 0.2 | 0.592 | -0.046 | 0.13 | 0.723 |
| InternalIMT | dha | rs187842704 | 0.019 | -0.1 | 0.048 | 0.036 | 0.49 | 0.445 | 0.27 | -0.243 | 0.271 | 0.371 |
| InternalIMT | dha | rs184620511 | 0.013 | -0.106 | 0.049 | 0.031 | 0.159 | 0.428 | 0.71 | -0.062 | 0.283 | 0.826 |
| InternalIMT | dha | rs144031303 | 0.019 | -0.102 | 0.049 | 0.036 | 0.148 | 0.265 | 0.576 | -0.118 | 0.162 | 0.467 |
| InternalIMT | dha | rs187526473 | 0 | . | . | . | . | . | . | . | . | . |
| InternalIMT | dha | rs193002805 | 0.002 | . | . | . | . | . | . | . | . | . |
| InternalIMT | dha | rs138707426 | 0.024 | -0.103 | 0.049 | 0.036 | 0.075 | 0.275 | 0.786 | -0.055 | 0.182 | 0.764 |
| InternalIMT | dha | rs142754243 | 0.049 | -0.109 | 0.051 | 0.03 | -0.06 | 0.198 | 0.762 | 0.022 | 0.136 | 0.874 |
| InternalIMT | dha | rs181600331 | 0.008 | . | . | . | . | . | . | . | . | . |
| InternalIMT | dha | rs144421264 | 0.029 | -0.102 | 0.05 | 0.041 | 0.062 | 0.302 | 0.836 | -0.085 | 0.196 | 0.666 |
| InternalIMT | dha | rs184414519 | 0.006 | . | . | . | . | . | . | . | . | . |
| InternalIMT | dha | rs150596349 | 0.048 | -0.067 | 0.051 | 0.187 | 0.698 | 0.241 | 3.90E-03 | -0.425 | 0.153 | 5.40E-03 |
| InternalIMT | dha | rs190753747 | 0.001 | . | . | . | . | . | . | . | . | . |
| InternalIMT | dha | rs139560940 | 0.004 | . | . | . | . | . | . | . | . | . |
| InternalIMT | dha | rs75640804 | 0.057 | -0.099 | 0.05 | 0.046 | 0.127 | 0.209 | 0.542 | -0.063 | 0.134 | 0.639 |
| InternalIMT | dha | rs73283175 | 0.082 | -0.103 | 0.053 | 0.052 | 9.70E-03 | 0.148 | 0.948 | -0.021 | 0.1 | 0.831 |
| InternalIMT | dha | rs143337229 | 0.003 | . | . | . | . | . | . | . | . | . |
| InternalIMT | dha | rs73283177 | 0.048 | -0.11 | 0.051 | 0.029 | -0.067 | 0.201 | 0.739 | 0.029 | 0.139 | 0.836 |
| InternalIMT | dha | rs76053611 | 0.045 | -0.064 | 0.051 | 0.204 | 0.799 | 0.244 | 1.10E-03 | -0.494 | 0.156 | 1.50E-03 |
| InternalIMT | dha | rs116325983 | 0.004 | . | . | . | . | . | . | . | . | . |
| InternalIMT | dha | rs115451825 | 0.008 | . | . | . | . | . | . | . | . | . |
| InternalIMT | dha | rs183845246 | 0.002 | . | . | . | . | . | . | . | . | . |
| InternalIMT | dha | rs79756542 | 0.051 | -0.066 | 0.051 | 0.192 | 0.684 | 0.237 | 4.00E-03 | -0.419 | 0.15 | 5.40E-03 |
| InternalIMT | dha | rs187242860 | 0.002 | . | . | . | . | . | . | . | . | . |
| InternalIMT | dha | rs57318980 | 0.048 | -0.111 | 0.051 | 0.029 | -0.066 | 0.206 | 0.747 | 0.036 | 0.145 | 0.803 |
| InternalIMT | dha | rs59969732 | 0.043 | -0.113 | 0.05 | 0.025 | -0.107 | 0.209 | 0.61 | 0.062 | 0.147 | 0.674 |
| InternalIMT | dha | rs143811488 | 0.009 | . | . | . | . | . | . | . | . | . |
| InternalIMT | dha | rs189067644 | 0.01 | . | . | . | . | . | . | . | . | . |
| InternalIMT | dha | rs60449178 | 0.038 | -0.11 | 0.05 | 0.028 | -0.086 | 0.226 | 0.704 | 0.025 | 0.159 | 0.876 |
| InternalIMT | dha | rs59243389 | 0.038 | -0.11 | 0.05 | 0.028 | -0.086 | 0.226 | 0.704 | 0.025 | 0.159 | 0.876 |
| InternalIMT | dha | rs184946540 | 0.043 | -0.095 | 0.05 | 0.059 | 0.157 | 0.213 | 0.463 | -0.158 | 0.141 | 0.264 |
| InternalIMT | dha | rs188685881 | 0.001 | . | . | . | . | . | . | . | . | . |
| InternalIMT | dha | rs150335797 | 0.006 | . | . | . | . | . | . | . | . | . |
| InternalIMT | dha | rs143879260 | 0.006 | . | . | . | . | . | . | . | . | . |
| InternalIMT | dha | rs186044491 | 0.009 | . | . | . | . | . | . | . | . | . |
| InternalIMT | dha | rs11819084 | 0.03 | -0.09 | 0.049 | 0.07 | 0.373 | 0.25 | 0.136 | -0.226 | 0.153 | 0.14 |
| InternalIMT | dha | rs146915670 | 0.006 | . | . | . | . | . | . | . | . | . |

| InternalIMT | dha | rs113995695 | 0.029 | -0.091 | 0.049 | 0.064 | 0.352 | 0.252 | 0.162 | -0.21 | 0.155 | 0.176 |
| --- | --- | --- | --- | --- | --- | --- | --- | --- | --- | --- | --- | --- |
| InternalIMT | dha | rs143489451 | 0.005 | . | . | . | . | . | . | . | . | . |
| InternalIMT | dha | rs182653839 | 0 | . | . | . | . | . | . | . | . | . |
| InternalIMT | dha | rs141692564 | 0.007 | . | . | . | . | . | . | . | . | . |
| InternalIMT | dha | rs146281742 | 0.005 | . | . | . | . | . | . | . | . | . |
| InternalIMT | dha | rs192914353 | 0.005 | . | . | . | . | . | . | . | . | . |
| InternalIMT | dha | rs185182848 | 0.003 | . | . | . | . | . | . | . | . | . |
| InternalIMT | dha | rs28395858 | 0.029 | -0.092 | 0.049 | 0.061 | 0.34 | 0.255 | 0.183 | -0.2 | 0.158 | 0.207 |
| InternalIMT | dha | rs41429645 | 0.001 | . | . | . | . | . | . | . | . | . |
| InternalIMT | dha | rs28395874 | 0.029 | -0.093 | 0.049 | 0.06 | 0.331 | 0.256 | 0.197 | -0.193 | 0.16 | 0.228 |
| InternalIMT | dha | rs189663634 | 0.001 | . | . | . | . | . | . | . | . | . |
| InternalIMT | dha | rs77666700 | 0.01 | . | . | . | . | . | . | . | . | . |
| InternalIMT | dha | rs146711717 | 0.003 | . | . | . | . | . | . | . | . | . |
| InternalIMT | dha | rs144904251 | 0.001 | . | . | . | . | . | . | . | . | . |
| InternalIMT | dha | rs189893153 | 0.001 | . | . | . | . | . | . | . | . | . |
| InternalIMT | dha | rs150447740 | 0.009 | . | . | . | . | . | . | . | . | . |
| InternalIMT | dha | rs143099649 | 0.001 | . | . | . | . | . | . | . | . | . |
| InternalIMT | dha | rs191288436 | 0.01 | -0.108 | 0.048 | 0.025 | -0.047 | 0.309 | 0.88 | 0.034 | 0.231 | 0.885 |
| InternalIMT | dha | rs188577862 | 0.006 | . | . | . | . | . | . | . | . | . |
| InternalIMT | dha | rs192695334 | 0.006 | . | . | . | . | . | . | . | . | . |
| InternalIMT | dha | rs138612906 | 0.036 | -0.092 | 0.05 | 0.062 | 0.279 | 0.223 | 0.211 | -0.166 | 0.139 | 0.235 |
| InternalIMT | dha | rs191290051 | 0.037 | -0.107 | 0.049 | 0.028 | 0.017 | 0.269 | 0.95 | 0.025 | 0.185 | 0.893 |
| InternalIMT | dha | rs189631459 | 0.001 | . | . | . | . | . | . | . | . | . |
| InternalIMT | dha | rs114620874 | 0.007 | . | . | . | . | . | . | . | . | . |
| InternalIMT | dha | rs78246652 | 0.023 | -0.109 | 0.048 | 0.023 | -0.062 | 0.305 | 0.84 | 0.115 | 0.206 | 0.577 |
| InternalIMT | dha | rs79432451 | 0.007 | . | . | . | . | . | . | . | . | . |
| InternalIMT | dha | rs186022585 | 0.001 | . | . | . | . | . | . | . | . | . |
| InternalIMT | dha | rs142978891 | 0.009 | . | . | . | . | . | . | . | . | . |
| InternalIMT | dha | rs151086840 | 0.011 | -0.112 | 0.048 | 0.019 | -0.401 | 0.424 | 0.345 | 0.202 | 0.332 | 0.542 |
| InternalIMT | dha | rs183270259 | 0.01 | . | . | . | . | . | . | . | . | . |
| InternalIMT | dha | rs188544289 | 0.01 | . | . | . | . | . | . | . | . | . |
| InternalIMT | dha | rs188500808 | 0.001 | . | . | . | . | . | . | . | . | . |
| InternalIMT | dha | rs189424589 | 0.003 | . | . | . | . | . | . | . | . | . |
| InternalIMT | dha | rs78622303 | 0.04 | -0.096 | 0.051 | 0.058 | 0.208 | 0.227 | 0.361 | -0.119 | 0.157 | 0.448 |
| InternalIMT | dha | rs143210835 | 0.013 | -0.099 | 0.049 | 0.042 | 0.531 | 0.297 | 0.074 | -0.234 | 0.227 | 0.302 |
| InternalIMT | dha | rs114761840 | 0.007 | . | . | . | . | . | . | . | . | . |
| InternalIMT | dha | rs185805402 | 0.003 | . | . | . | . | . | . | . | . | . |
| InternalIMT | dha | rs139273744 | 0.002 | . | . | . | . | . | . | . | . | . |
| InternalIMT | dha | rs184654150 | 0.006 | . | . | . | . | . | . | . | . | . |
| InternalIMT | dha | rs189069710 | 0 | . | . | . | . | . | . | . | . | . |
| InternalIMT | dha | rs114752115 | 0.031 | -0.11 | 0.049 | 0.024 | -0.042 | 0.251 | 0.866 | 0.073 | 0.173 | 0.676 |
| InternalIMT | dha | rs150162515 | 0.002 | . | . | . | . | . | . | . | . | . |
| InternalIMT | dha | rs180982454 | 0.008 | . | . | . | . | . | . | . | . | . |
| InternalIMT | dha | rs184932088 | 0.001 | . | . | . | . | . | . | . | . | . |
| InternalIMT | dha | rs181109625 | 0 | . | . | . | . | . | . | . | . | . |
| InternalIMT | dha | rs138121134 | 0.001 | . | . | . | . | . | . | . | . | . |
| InternalIMT | dha | rs183253861 | 0.004 | . | . | . | . | . | . | . | . | . |
| InternalIMT | dha | rs147073946 | 0.001 | . | . | . | . | . | . | . | . | . |
| InternalIMT | dha | rs139938489 | 0.008 | . | . | . | . | . | . | . | . | . |
| InternalIMT | dha | rs146362560 | 0.013 | -0.111 | 0.048 | 0.021 | -0.04 | 0.487 | 0.934 | 0.128 | 0.333 | 0.701 |
| InternalIMT | dha | rs193155956 | 0.007 | . | . | . | . | . | . | . | . | . |
| InternalIMT | dha | rs77663092 | 0.007 | . | . | . | . | . | . | . | . | . |
| InternalIMT | dha | rs73285319 | 0.033 | -0.106 | 0.049 | 0.032 | -0.017 | 0.28 | 0.953 | -0.011 | 0.195 | 0.957 |
| InternalIMT | dha | rs184019276 | 0.007 | . | . | . | . | . | . | . | . | . |
| InternalIMT | dha | rs28395863 | 0.027 | -0.104 | 0.048 | 0.031 | 0.149 | 0.366 | 0.685 | -0.072 | 0.236 | 0.759 |
| InternalIMT | dha | rs182121645 | 0.017 | -0.098 | 0.048 | 0.041 | 0.349 | 0.302 | 0.249 | -0.249 | 0.22 | 0.259 |
| InternalIMT | dha | rs73285327 | 0.025 | -0.1 | 0.049 | 0.042 | 0.238 | 0.331 | 0.471 | -0.172 | 0.222 | 0.438 |
| InternalIMT | dha | rs149589584 | 0.001 | . | . | . | . | . | . | . | . | . |
| InternalIMT | dha | rs148310335 | 0.005 | . | . | . | . | . | . | . | . | . |
| InternalIMT | dha | rs187246093 | 0.01 | -0.101 | 0.048 | 0.036 | 0.425 | 0.368 | 0.249 | -0.31 | 0.222 | 0.163 |
| InternalIMT | dha | rs184668894 | 0 | . | . | . | . | . | . | . | . | . |
| InternalIMT | dha | rs114269961 | 0.001 | . | . | . | . | . | . | . | . | . |
| InternalIMT | dha | rs149694374 | 0.034 | -0.104 | 0.049 | 0.032 | 0.052 | 0.296 | 0.862 | -0.039 | 0.198 | 0.844 |
| InternalIMT | dha | rs145284227 | 0.004 | . | . | . | . | . | . | . | . | . |
| InternalIMT | dha | rs189983820 | 0.004 | . | . | . | . | . | . | . | . | . |
| InternalIMT | dha | rs181200444 | 0 | . | . | . | . | . | . | . | . | . |
| InternalIMT | dha | rs185486385 | 0 | . | . | . | . | . | . | . | . | . |
| InternalIMT | dha | rs151198241 | 0.01 | -0.11 | 0.048 | 0.022 | -0.182 | 0.408 | 0.656 | 0.022 | 0.304 | 0.942 |
| InternalIMT | dha | rs189959341 | 0.01 | -0.11 | 0.048 | 0.022 | -0.182 | 0.408 | 0.656 | 0.022 | 0.304 | 0.942 |
| InternalIMT | dha | rs140549257 | 0.007 | . | . | . | . | . | . | . | . | . |
| InternalIMT | dha | rs186453139 | 0.027 | -0.104 | 0.049 | 0.034 | 0.039 | 0.305 | 0.897 | -0.06 | 0.207 | 0.774 |
| InternalIMT | dha | rs180922711 | 0.044 | -0.094 | 0.051 | 0.066 | 0.184 | 0.255 | 0.47 | -0.159 | 0.174 | 0.361 |
| InternalIMT | dha | rs56659903 | 0.005 | . | . | . | . | . | . | . | . | . |
| InternalIMT | dha | rs141202128 | 0.006 | . | . | . | . | . | . | . | . | . |
| InternalIMT | dha | rs59085314 | 0.056 | -0.118 | 0.049 | 0.017 | -0.136 | 0.206 | 0.509 | 0.1 | 0.143 | 0.483 |
| InternalIMT | dha | rs145564996 | 0.004 | . | . | . | . | . | . | . | . | . |
| InternalIMT | dha | rs114961009 | 0.01 | -0.109 | 0.048 | 0.022 | -0.177 | 0.409 | 0.666 | 0.02 | 0.304 | 0.948 |
| InternalIMT | dha | rs182294189 | 0.004 | . | . | . | . | . | . | . | . | . |
| InternalIMT | dha | rs187586636 | 0.008 | . | . | . | . | . | . | . | . | . |
| InternalIMT | dha | rs192254907 | 0.002 | . | . | . | . | . | . | . | . | . |
| InternalIMT | dha | rs149145663 | 0.001 | . | . | . | . | . | . | . | . | . |
| InternalIMT | dha | rs180713064 | 0.001 | . | . | . | . | . | . | . | . | . |
| InternalIMT | dha | rs183009023 | 0.007 | . | . | . | . | . | . | . | . | . |
| InternalIMT | dha | rs143385347 | 0.017 | -0.097 | 0.048 | 0.044 | 0.402 | 0.285 | 0.158 | -0.276 | 0.212 | 0.194 |
| InternalIMT | dha | rs185786329 | 0.007 | . | . | . | . | . | . | . | . | . |
| InternalIMT | dha | rs192341030 | 0.004 | . | . | . | . | . | . | . | . | . |

| InternalIMT | dha | rs74661429 | 0.017 | -0.101 | 0.047 | 0.033 | 0.49 | 0.449 | 0.276 | -0.225 | 0.278 | 0.419 |
| --- | --- | --- | --- | --- | --- | --- | --- | --- | --- | --- | --- | --- |
| InternalIMT | dha | rs188034853 | 0.002 | . | . | . | . | . | . | . | . | . |
| InternalIMT | dha | rs116808430 | 0.027 | -0.104 | 0.049 | 0.035 | 0.029 | 0.299 | 0.922 | -0.057 | 0.202 | 0.777 |
| InternalIMT | dha | rs148469010 | 0 | . | . | . | . | . | . | . | . | . |
| InternalIMT | dha | rs78621663 | 0.01 | . | . | . | . | . | . | . | . | . |
| InternalIMT | dha | rs192654886 | 0.001 | . | . | . | . | . | . | . | . | . |
| InternalIMT | dha | rs145996035 | 0.008 | . | . | . | . | . | . | . | . | . |
| InternalIMT | dha | rs139942226 | 0.001 | . | . | . | . | . | . | . | . | . |
| InternalIMT | dha | rs113645632 | 0.01 | -0.104 | 0.048 | 0.03 | 0.183 | 0.57 | 0.749 | -0.022 | 0.421 | 0.959 |
| InternalIMT | dha | rs74940661 | 0.027 | -0.111 | 0.048 | 0.021 | -0.097 | 0.32 | 0.763 | 0.075 | 0.218 | 0.732 |
| InternalIMT | dha | rs115355800 | 0.022 | -0.111 | 0.048 | 0.02 | -0.13 | 0.305 | 0.67 | 0.159 | 0.209 | 0.448 |
| InternalIMT | dha | rs150285898 | 0.008 | . | . | . | . | . | . | . | . | . |
| InternalIMT | dha | rs188117932 | 0.004 | . | . | . | . | . | . | . | . | . |
| InternalIMT | dha | rs139956566 | . | . | . | . | . | . | . | . | . | . |
| InternalIMT | dha | rs147673885 | . | . | . | . | . | . | . | . | . | . |
| InternalIMT | dha | rs189127928 | . | . | . | . | . | . | . | . | . | . |
| InternalIMT | dha | rs187558772 | . | . | . | . | . | . | . | . | . | . |
| InternalIMT | dha | rs181948587 | . | . | . | . | . | . | . | . | . | . |
| InternalIMT | dha | rs189983269 | . | . | . | . | . | . | . | . | . | . |
| InternalIMT | dha | rs187250069 | . | . | . | . | . | . | . | . | . | . |
| InternalIMT | dha | rs191485859 | . | . | . | . | . | . | . | . | . | . |
| InternalIMT | dha | rs185499555 | . | . | . | . | . | . | . | . | . | . |
| InternalIMT | dha | rs180862110 | . | . | . | . | . | . | . | . | . | . |
| InternalIMT | dha | rs190472603 | . | . | . | . | . | . | . | . | . | . |
| InternalIMT | dha | rs189226167 | . | . | . | . | . | . | . | . | . | . |
| InternalIMT | dha | rs181623256 | . | . | . | . | . | . | . | . | . | . |
| InternalIMT | dha | rs186079557 | . | . | . | . | . | . | . | . | . | . |
| InternalIMT | dha | rs147760994 | . | . | . | . | . | . | . | . | . | . |
| InternalIMT | dha | rs189176167 | . | . | . | . | . | . | . | . | . | . |
| InternalIMT | dha | rs117901000 | . | . | . | . | . | . | . | . | . | . |
| InternalIMT | dha | rs141984888 | . | . | . | . | . | . | . | . | . | . |
| InternalIMT | dha | rs192365061 | . | . | . | . | . | . | . | . | . | . |
| InternalIMT | dha | rs185976038 | . | . | . | . | . | . | . | . | . | . |
| InternalIMT | dha | rs184697303 | . | . | . | . | . | . | . | . | . | . |
| InternalIMT | dha | rs143383132 | . | . | . | . | . | . | . | . | . | . |
| InternalIMT | dha | rs141873881 | . | . | . | . | . | . | . | . | . | . |
| InternalIMT | dha | rs185507255 | . | . | . | . | . | . | . | . | . | . |
| InternalIMT | dha | rs191379425 | . | . | . | . | . | . | . | . | . | . |
| InternalIMT | dha | rs184199584 | . | . | . | . | . | . | . | . | . | . |
| InternalIMT | dha | rs183181360 | . | . | . | . | . | . | . | . | . | . |
| InternalIMT | dha | rs184495673 | . | . | . | . | . | . | . | . | . | . |
| InternalIMT | dha | rs182946297 | . | . | . | . | . | . | . | . | . | . |
| InternalIMT | dha | rs187743615 | . | . | . | . | . | . | . | . | . | . |
| InternalIMT | dha | rs183259862 | . | . | . | . | . | . | . | . | . | . |
| InternalIMT | dha | rs112433566 | . | . | . | . | . | . | . | . | . | . |
| InternalIMT | dha | rs182351085 | . | . | . | . | . | . | . | . | . | . |
| InternalIMT | dha | rs146248508 | . | . | . | . | . | . | . | . | . | . |
| InternalIMT | dha | rs188528439 | . | . | . | . | . | . | . | . | . | . |
| InternalIMT | dha | rs146167018 | . | . | . | . | . | . | . | . | . | . |
| InternalIMT | dha | rs192440980 | . | . | . | . | . | . | . | . | . | . |
| InternalIMT | dha | rs185601882 | . | . | . | . | . | . | . | . | . | . |
| InternalIMT | dha | rs117272563 | . | . | . | . | . | . | . | . | . | . |
| InternalIMT | dha | rs10430734 | . | . | . | . | . | . | . | . | . | . |
| InternalIMT | dha | rs181320952 | . | . | . | . | . | . | . | . | . | . |
| InternalIMT | dha | rs185480660 | . | . | . | . | . | . | . | . | . | . |
| InternalIMT | dha | rs188077917 | . | . | . | . | . | . | . | . | . | . |
| InternalIMT | dha | rs187562953 | . | . | . | . | . | . | . | . | . | . |
| InternalIMT | dha | rs182044631 | . | . | . | . | . | . | . | . | . | . |
| InternalIMT | dha | rs145455596 | . | . | . | . | . | . | . | . | . | . |
| InternalIMT | dha | rs181475877 | . | . | . | . | . | . | . | . | . | . |
| InternalIMT | dha | rs149413085 | . | . | . | . | . | . | . | . | . | . |
| InternalIMT | dha | rs12253656 | . | . | . | . | . | . | . | . | . | . |
| InternalIMT | dha | rs182718239 | . | . | . | . | . | . | . | . | . | . |
| InternalIMT | dha | rs192435695 | . | . | . | . | . | . | . | . | . | . |
| InternalIMT | dha | rs12248277 | . | . | . | . | . | . | . | . | . | . |
| InternalIMT | dha | rs143521710 | . | . | . | . | . | . | . | . | . | . |
| InternalIMT | dha | rs144454454 | . | . | . | . | . | . | . | . | . | . |
| InternalIMT | dha | rs190441610 | . | . | . | . | . | . | . | . | . | . |
| InternalIMT | dha | rs114690462 | . | . | . | . | . | . | . | . | . | . |
| InternalIMT | dha | rs184541357 | . | . | . | . | . | . | . | . | . | . |
| InternalIMT | dha | rs142657653 | . | . | . | . | . | . | . | . | . | . |
| InternalIMT | dha | rsurf1 | 2.117 | . | . | . | . | . | . | . | . | . |
| InternalIMT | dha | rsten1 | 2.28 | . | . | . | . | . | . | . | . | . |
| InternalIMT | dha | chr10:45852312:D | 0.092 | -0.076 | 0.052 | 0.143 | 0.273 | 0.147 | 0.064 | -0.171 | 0.098 | 0.08 |
| InternalIMT | dha | chr10:45853232:I | 0.108 | -0.094 | 0.052 | 0.071 | 0.166 | 0.143 | 0.246 | -0.067 | 0.098 | 0.496 |
| InternalIMT | dha | chr10:45853234:I | 0.198 | -0.078 | 0.056 | 0.16 | 0.144 | 0.111 | 0.194 | -0.069 | 0.076 | 0.369 |
| InternalIMT | dha | chr10:45854796:D | 0.003 | . | . | . | . | . | . | . | . | . |
| InternalIMT | dha | chr10:45854994:I | 0.025 | -0.106 | 0.048 | 0.027 | 0.047 | 0.311 | 0.881 | 0.023 | 0.205 | 0.912 |
| InternalIMT | dha | chr10:45858290:D | 0.052 | -0.102 | 0.051 | 0.045 | 0.109 | 0.277 | 0.695 | -0.046 | 0.194 | 0.814 |
| InternalIMT | dha | chr10:45858820:I | 0.022 | -0.109 | 0.049 | 0.025 | -0.113 | 0.308 | 0.714 | 0.054 | 0.205 | 0.793 |
| InternalIMT | dha | chr10:45860888:D | 0.122 | -0.067 | 0.057 | 0.239 | 0.225 | 0.159 | 0.156 | -0.158 | 0.104 | 0.13 |
| InternalIMT | dha | chr10:45863913:I | 0.179 | -0.065 | 0.055 | 0.237 | 0.207 | 0.115 | 0.071 | -0.109 | 0.079 | 0.167 |
| InternalIMT | dha | chr10:45866601:D | 0.111 | -0.087 | 0.054 | 0.109 | 0.169 | 0.153 | 0.268 | -0.093 | 0.102 | 0.363 |
| InternalIMT | dha | chr10:45867197:I | 0.002 | . | . | . | . | . | . | . | . | . |
| InternalIMT | dha | chr10:45867538:D | 0.182 | -0.072 | 0.055 | 0.189 | 0.172 | 0.112 | 0.125 | -0.089 | 0.077 | 0.25 |
| InternalIMT | dha | chr10:45868356:D | 0.18 | -0.07 | 0.055 | 0.202 | 0.176 | 0.112 | 0.114 | -0.094 | 0.077 | 0.221 |

| InternalIMT | dha | chr10:45871158:I | 0.182 | -0.072 | 0.055 | 0.19 | 0.176 | 0.111 | 0.114 | -0.089 | 0.077 | 0.246 |
| --- | --- | --- | --- | --- | --- | --- | --- | --- | --- | --- | --- | --- |
| InternalIMT | dha | chr10:45871637:D | 0.127 | -0.078 | 0.053 | 0.145 | 0.213 | 0.136 | 0.119 | -0.104 | 0.092 | 0.261 |
| InternalIMT | dha | chr10:45871638:I | 0.164 | -0.079 | 0.054 | 0.145 | 0.184 | 0.118 | 0.121 | -0.078 | 0.081 | 0.336 |
| InternalIMT | dha | chr10:45873629:I | 0.154 | -0.058 | 0.054 | 0.29 | 0.258 | 0.126 | 0.041 | -0.152 | 0.086 | 0.078 |
| InternalIMT | dha | chr10:45873701:D | 0.055 | -0.096 | 0.05 | 0.054 | 0.165 | 0.207 | 0.425 | -0.092 | 0.134 | 0.493 |
| InternalIMT | dha | chr10:45874997:I | 0.011 | -0.108 | 0.048 | 0.024 | -0.353 | 0.483 | 0.465 | 0.112 | 0.354 | 0.752 |
| InternalIMT | dha | chr10:45882030:I | 0.096 | -0.087 | 0.053 | 0.099 | 0.153 | 0.17 | 0.368 | -0.099 | 0.11 | 0.368 |
| InternalIMT | dha | chr10:45883774:D | 0.078 | -0.089 | 0.051 | 0.079 | 0.137 | 0.179 | 0.443 | -0.108 | 0.123 | 0.383 |
| InternalIMT | dha | chr10:45885169:D | 0.075 | -0.075 | 0.052 | 0.148 | 0.372 | 0.177 | 0.036 | -0.229 | 0.116 | 0.048 |
| InternalIMT | dha | chr10:45886451:D | 0.736 | 0.061 | 0.121 | 0.612 | 0.173 | 0.109 | 0.111 | -0.112 | 0.076 | 0.141 |
| InternalIMT | dha | chr10:45891800:I | 0.768 | -0.022 | 0.126 | 0.862 | 0.097 | 0.108 | 0.37 | -0.054 | 0.075 | 0.471 |
| InternalIMT | dha | chr10:45904771:I | 0.772 | -0.063 | 0.122 | 0.606 | 0.064 | 0.107 | 0.552 | -0.027 | 0.073 | 0.712 |
| InternalIMT | dha | chr10:45904959:I | 0.156 | -0.051 | 0.058 | 0.374 | 0.231 | 0.152 | 0.129 | -0.166 | 0.1 | 0.099 |
| InternalIMT | dha | chr10:45911028:I | 0.161 | -0.097 | 0.058 | 0.095 | 0.078 | 0.155 | 0.614 | -0.034 | 0.105 | 0.749 |
| InternalIMT | dha | chr10:45911031:I | 0.259 | -0.16 | 0.066 | 0.015 | -0.185 | 0.127 | 0.146 | 0.104 | 0.09 | 0.249 |
| InternalIMT | dha | chr10:45912154:D | 0.104 | -0.084 | 0.052 | 0.101 | 0.129 | 0.157 | 0.41 | -0.107 | 0.109 | 0.325 |
| InternalIMT | dha | chr10:45913885:I | 0.003 | . | . | . | . | . | . | . | . | . |
| InternalIMT | dha | chr10:45914753:D | 0.351 | -0.098 | 0.07 | 0.162 | 0.076 | 0.113 | 0.504 | -0.015 | 0.079 | 0.848 |
| InternalIMT | dha | chr10:45919621 | . | . | . | . | . | . | . | . | . | . |
| InternalIMT | dha | chr10:45921739:I | 0.178 | -0.109 | 0.055 | 0.05 | 0.02 | 0.115 | 0.865 | 5.80E-03 | 0.079 | 0.941 |
| InternalIMT | dha | chr10:45923527:I | 0.066 | -0.117 | 0.052 | 0.024 | -0.177 | 0.168 | 0.291 | 0.063 | 0.117 | 0.592 |
| InternalIMT | dha | chr10:45927001:I | 0.04 | -0.117 | 0.048 | 0.015 | -0.151 | 0.265 | 0.57 | 0.132 | 0.18 | 0.462 |
| InternalIMT | dha | chr10:45930354:I | 0.025 | -0.11 | 0.048 | 0.022 | -0.073 | 0.301 | 0.808 | 0.123 | 0.202 | 0.541 |
| InternalIMT | dha | chr10:45932864:I | 0.043 | -0.089 | 0.05 | 0.078 | 0.474 | 0.432 | 0.273 | -0.207 | 0.304 | 0.497 |
| InternalIMT | dha | chr10:45936163:I | 0.038 | -0.097 | 0.049 | 0.048 | 0.161 | 0.271 | 0.553 | -0.133 | 0.188 | 0.48 |
| InternalIMT | dha | chr10:45938095:D | 0.179 | -0.066 | 0.054 | 0.218 | 0.212 | 0.136 | 0.12 | -0.109 | 0.091 | 0.23 |
| InternalIMT | dha | chr10:45938886 | . | . | . | . | . | . | . | . | . | . |
| InternalIMT | dha | chr10:45939320 | 0.001 | . | . | . | . | . | . | . | . | . |
| InternalIMT | dha | chr10:45939659 | 0.003 | . | . | . | . | . | . | . | . | . |
| InternalIMT | dha | chr10:45941150:D | 0.008 | . | . | . | . | . | . | . | . | . |
| InternalIMT | dha | chr10:45947128:D | 0.067 | -0.124 | 0.05 | 0.014 | -0.205 | 0.181 | 0.256 | 0.134 | 0.124 | 0.278 |
| InternalIMT | dha | chr10:45950990:I | 0.039 | -0.108 | 0.051 | 0.036 | 0.072 | 0.241 | 0.764 | 6.90E-03 | 0.172 | 0.968 |
| InternalIMT | dha | chr10:45951854:I | 0.012 | -0.111 | 0.048 | 0.022 | -0.393 | 0.426 | 0.356 | 0.196 | 0.288 | 0.495 |
| InternalIMT | dha | chr10:45952670:I | 0.061 | -0.079 | 0.05 | 0.116 | 0.264 | 0.201 | 0.188 | -0.221 | 0.131 | 0.092 |
| InternalIMT | dha | chr10:45956741 | . | . | . | . | . | . | . | . | . | . |
| InternalIMT | dha | chr10:45957247:I | 0.198 | -0.059 | 0.055 | 0.282 | 0.21 | 0.13 | 0.106 | -0.119 | 0.088 | 0.177 |
| InternalIMT | dha | chr10:45958082:I | 0.188 | -0.068 | 0.055 | 0.216 | 0.189 | 0.133 | 0.154 | -0.103 | 0.092 | 0.263 |
| InternalIMT | dha | chr10:45905041:D | 0.068 | -0.088 | 0.051 | 0.084 | 0.297 | 0.209 | 0.155 | -0.13 | 0.143 | 0.364 |
| InternalIMT | dha | chr10:45938871 | . | . | . | . | . | . | . | . | . | . |
| InternalIMT | dha | chr10:45939160 | . | . | . | . | . | . | . | . | . | . |
| InternalIMT | dha | chr10:45869910 | 0.015 | -0.115 | 0.048 | 0.017 | -0.316 | 0.336 | 0.346 | 0.209 | 0.258 | 0.418 |
| InternalIMT | dha | chr10:45876214:D | 0.017 | -0.101 | 0.048 | 0.037 | 0.191 | 0.254 | 0.452 | -0.159 | 0.154 | 0.304 |
| InternalIMT | dha | chr10:45880237:I | 0.094 | -0.081 | 0.052 | 0.12 | 0.283 | 0.157 | 0.072 | -0.154 | 0.108 | 0.154 |
| InternalIMT | dha | chr10:45884371:I | 0.073 | -0.094 | 0.051 | 0.065 | 0.139 | 0.177 | 0.434 | -0.08 | 0.115 | 0.485 |
| InternalIMT | dha | chr10:45891535:D | 0.019 | -0.122 | 0.048 | 0.011 | -0.446 | 0.361 | 0.217 | 0.425 | 0.287 | 0.139 |
| InternalIMT | dha | chr10:45893516:D | 0.073 | -0.095 | 0.051 | 0.064 | 0.136 | 0.178 | 0.443 | -0.078 | 0.115 | 0.497 |
| InternalIMT | dha | chr10:45893782:D | 0.018 | -0.099 | 0.048 | 0.038 | 0.481 | 0.437 | 0.271 | -0.254 | 0.262 | 0.332 |
| InternalIMT | dha | chr10:45895254:I | 0.019 | -0.122 | 0.048 | 0.011 | -0.447 | 0.36 | 0.215 | 0.426 | 0.287 | 0.138 |
| InternalIMT | dha | chr10:45895848:D | 0.057 | -0.098 | 0.051 | 0.053 | 0.112 | 0.193 | 0.562 | -0.07 | 0.126 | 0.577 |
| InternalIMT | dha | chr10:45896316:D | 0.073 | -0.095 | 0.051 | 0.063 | 0.137 | 0.178 | 0.443 | -0.078 | 0.115 | 0.498 |
| InternalIMT | dha | chr10:45896317:D | 0.073 | -0.095 | 0.051 | 0.063 | 0.137 | 0.178 | 0.443 | -0.078 | 0.115 | 0.498 |
| InternalIMT | dha | chr10:45902683:I | 0.019 | -0.103 | 0.048 | 0.034 | 0.103 | 0.258 | 0.689 | -0.093 | 0.161 | 0.564 |
| InternalIMT | dha | chr10:45905003:D | 0.01 | . | . | . | . | . | . | . | . | . |
| InternalIMT | dha | chr10:45927476:D | 0.036 | -0.097 | 0.049 | 0.047 | 0.158 | 0.253 | 0.532 | -0.112 | 0.168 | 0.508 |
| InternalIMT | dha | chr10:45935882 | 0.001 | . | . | . | . | . | . | . | . | . |
| InternalIMT | dha | chr10:45936100 | 0.001 | . | . | . | . | . | . | . | . | . |
| InternalIMT | dha | chr10:45939553 | 0.001 | . | . | . | . | . | . | . | . | . |
| InternalIMT | dha | chr10:45939631 | 0 | . | . | . | . | . | . | . | . | . |
| InternalIMT | dha | chr10:45944193:D | 0.012 | -0.102 | 0.048 | 0.035 | 0.352 | 0.315 | 0.264 | -0.224 | 0.198 | 0.257 |
| InternalIMT | dha | chr10:45954030 | 0 | . | . | . | . | . | . | . | . | . |

Supplemental table S24.

| phen | covar(fa) | rs_id | maf | beta_FA | se_FA | p_FA | beta_SNP | se_SNP | p_SNP | beta_inter | se_inter | p_inter |
| --- | --- | --- | --- | --- | --- | --- | --- | --- | --- | --- | --- | --- |
| InternalIMT | dha | rs76035182 | 0.016 | 0.034 | 0.043 | 0.429 | 0.053 | 0.187 | 0.777 | -0.054 | 0.139 | 0.701 |
| InternalIMT | dha | rs12359547 | 0.066 | 0.035 | 0.045 | 0.44 | -0.037 | 0.101 | 0.717 | -0.034 | 0.093 | 0.716 |
| InternalIMT | dha | rs74595484 | 0.017 | 0.022 | 0.041 | 0.602 | -0.271 | 0.285 | 0.343 | 0.219 | 0.258 | 0.397 |
| InternalIMT | dha | rs1864415 | 0.722 | 0.152 | 0.102 | 0.134 | 0.076 | 0.069 | 0.272 | -0.081 | 0.063 | 0.202 |
| InternalIMT | dha | rs72788101 | 0.069 | 0.035 | 0.045 | 0.436 | -0.044 | 0.1 | 0.657 | -0.031 | 0.089 | 0.728 |
| InternalIMT | dha | rs11239481 | 0.129 | 0.04 | 0.047 | 0.39 | 0.023 | 0.097 | 0.817 | -0.047 | 0.087 | 0.592 |
| InternalIMT | dha | rs72788102 | 0.069 | 0.035 | 0.045 | 0.436 | -0.044 | 0.1 | 0.657 | -0.031 | 0.089 | 0.728 |
| InternalIMT | dha | rs72789705 | 0.069 | 0.035 | 0.045 | 0.436 | -0.044 | 0.099 | 0.656 | -0.031 | 0.089 | 0.729 |
| InternalIMT | dha | rs141529858 | 0.005 | . | . | . | . | . | . | . | . | . |
| InternalIMT | dha | rs145376292 | 0 | . | . | . | . | . | . | . | . | . |
| InternalIMT | dha | rs190418301 | 0.001 | . | . | . | . | . | . | . | . | . |
| InternalIMT | dha | rs11239482 | 0.069 | 0.035 | 0.045 | 0.436 | -0.044 | 0.099 | 0.655 | -0.031 | 0.089 | 0.729 |
| InternalIMT | dha | rs61855872 | 0.011 | 0.034 | 0.043 | 0.43 | 0.447 | 0.48 | 0.352 | -0.134 | 0.453 | 0.768 |
| InternalIMT | dha | rs148991144 | 0.005 | . | . | . | . | . | . | . | . | . |
| InternalIMT | dha | rs191932221 | 0.005 | . | . | . | . | . | . | . | . | . |
| InternalIMT | dha | rs55978138 | 0.144 | 0.044 | 0.05 | 0.377 | -0.017 | 0.077 | 0.826 | -0.045 | 0.069 | 0.509 |
| InternalIMT | dha | rs73281167 | 0.01 | . | . | . | . | . | . | . | . | . |
| InternalIMT | dha | rs12768010 | 0.072 | 0.037 | 0.046 | 0.413 | 6.30E-03 | 0.097 | 0.948 | -0.038 | 0.086 | 0.66 |
| InternalIMT | dha | rs11239484 | 0.722 | 0.151 | 0.102 | 0.138 | 0.075 | 0.069 | 0.277 | -0.08 | 0.063 | 0.208 |
| InternalIMT | dha | rs35906360 | 0.144 | 0.045 | 0.05 | 0.373 | -0.013 | 0.076 | 0.869 | -0.045 | 0.067 | 0.504 |
| InternalIMT | dha | rs186250512 | 0.004 | . | . | . | . | . | . | . | . | . |
| InternalIMT | dha | rs150810743 | 0.001 | . | . | . | . | . | . | . | . | . |
| InternalIMT | dha | rs55965812 | 0.14 | 0.043 | 0.05 | 0.391 | -0.012 | 0.078 | 0.875 | -0.043 | 0.07 | 0.538 |
| InternalIMT | dha | rs56180358 | 0.144 | 0.045 | 0.05 | 0.373 | -0.013 | 0.076 | 0.868 | -0.045 | 0.067 | 0.504 |
| InternalIMT | dha | rs55864145 | 0.073 | 0.037 | 0.046 | 0.413 | 6.10E-03 | 0.097 | 0.95 | -0.037 | 0.086 | 0.662 |
| InternalIMT | dha | rs55986122 | 0.144 | 0.045 | 0.05 | 0.373 | -0.013 | 0.076 | 0.867 | -0.045 | 0.067 | 0.505 |
| InternalIMT | dha | rs7477350 | 0.722 | 0.151 | 0.102 | 0.138 | 0.075 | 0.069 | 0.277 | -0.08 | 0.063 | 0.208 |
| InternalIMT | dha | rs2099170 | 0.143 | 0.046 | 0.05 | 0.363 | -9.20E-03 | 0.076 | 0.905 | -0.047 | 0.068 | 0.483 |
| InternalIMT | dha | rs2082108 | 0.144 | 0.045 | 0.05 | 0.375 | -0.013 | 0.076 | 0.861 | -0.044 | 0.067 | 0.511 |
| InternalIMT | dha | rs55902404 | 0.004 | . | . | . | . | . | . | . | . | . |
| InternalIMT | dha | rs58402662 | 0.004 | . | . | . | . | . | . | . | . | . |
| InternalIMT | dha | rs11511618 | 0.129 | 0.039 | 0.047 | 0.41 | 0.014 | 0.099 | 0.885 | -0.038 | 0.089 | 0.67 |
| InternalIMT | dha | rs1816492 | 0.721 | 0.156 | 0.102 | 0.126 | 0.08 | 0.069 | 0.246 | -0.083 | 0.063 | 0.19 |
| InternalIMT | dha | rs146611093 | 0.006 | . | . | . | . | . | . | . | . | . |
| InternalIMT | dha | rs186897689 | 0.001 | . | . | . | . | . | . | . | . | . |
| InternalIMT | dha | rs10793616 | 0.722 | 0.151 | 0.102 | 0.138 | 0.075 | 0.069 | 0.276 | -0.08 | 0.063 | 0.207 |
| InternalIMT | dha | rs116700479 | 0.013 | 0.032 | 0.043 | 0.448 | 0.021 | 0.233 | 0.929 | -0.019 | 0.169 | 0.91 |
| InternalIMT | dha | rs192535936 | . | . | . | . | . | . | . | . | . | . |
| InternalIMT | dha | rs113363297 | 0.653 | 0.09 | 0.093 | 0.335 | 0.045 | 0.07 | 0.52 | -0.043 | 0.064 | 0.502 |
| InternalIMT | dha | rs12354994 | 0.137 | 0.045 | 0.05 | 0.365 | -4.80E-03 | 0.078 | 0.952 | -0.049 | 0.069 | 0.479 |
| InternalIMT | dha | rs148068329 | 0.025 | 0.015 | 0.043 | 0.731 | -0.5 | 0.206 | 0.015 | 0.442 | 0.244 | 0.07 |
| InternalIMT | dha | rs12356109 | 0.149 | 0.044 | 0.051 | 0.387 | -0.027 | 0.076 | 0.718 | -0.039 | 0.067 | 0.563 |
| InternalIMT | dha | rs55908141 | 0.004 | . | . | . | . | . | . | . | . | . |
| InternalIMT | dha | rs12354712 | 0.14 | 0.043 | 0.05 | 0.394 | -0.014 | 0.077 | 0.857 | -0.042 | 0.07 | 0.546 |
| InternalIMT | dha | rs147282684 | 0.003 | . | . | . | . | . | . | . | . | . |
| InternalIMT | dha | rs12356140 | 0.145 | 0.044 | 0.05 | 0.381 | -0.018 | 0.076 | 0.813 | -0.042 | 0.068 | 0.529 |
| InternalIMT | dha | rs185476187 | 0.001 | . | . | . | . | . | . | . | . | . |
| InternalIMT | dha | rs145720057 | 0.018 | 0.023 | 0.043 | 0.584 | -0.223 | 0.238 | 0.349 | 0.304 | 0.239 | 0.205 |
| InternalIMT | dha | rs12354742 | 0.144 | 0.044 | 0.05 | 0.376 | -0.014 | 0.076 | 0.849 | -0.044 | 0.067 | 0.512 |
| InternalIMT | dha | rs180913842 | 0.004 | . | . | . | . | . | . | . | . | . |
| InternalIMT | dha | rs142976914 | 0.002 | . | . | . | . | . | . | . | . | . |
| InternalIMT | dha | rs11511619 | 0.151 | 0.044 | 0.051 | 0.389 | -0.027 | 0.076 | 0.725 | -0.038 | 0.067 | 0.569 |
| InternalIMT | dha | rs146702870 | 0.002 | . | . | . | . | . | . | . | . | . |
| InternalIMT | dha | rs150287496 | . | . | . | . | . | . | . | . | . | . |
| InternalIMT | dha | rs190917973 | 0.004 | . | . | . | . | . | . | . | . | . |
| InternalIMT | dha | rs11517281 | 0.144 | 0.044 | 0.05 | 0.378 | -0.015 | 0.076 | 0.84 | -0.043 | 0.067 | 0.52 |
| InternalIMT | dha | rs12359299 | 0.144 | 0.044 | 0.05 | 0.378 | -0.015 | 0.076 | 0.841 | -0.043 | 0.067 | 0.519 |
| InternalIMT | dha | rs182307932 | 0.014 | 0.043 | 0.043 | 0.323 | 0.133 | 0.217 | 0.542 | -0.178 | 0.15 | 0.234 |
| InternalIMT | dha | rs61855873 | 0.026 | 0.035 | 0.043 | 0.425 | 0.222 | 0.28 | 0.427 | -0.057 | 0.246 | 0.817 |
| InternalIMT | dha | rs12359302 | 0.143 | 0.044 | 0.05 | 0.385 | -0.013 | 0.077 | 0.87 | -0.041 | 0.068 | 0.542 |
| InternalIMT | dha | rs143208140 | 0.027 | 0.014 | 0.043 | 0.737 | -0.488 | 0.202 | 0.016 | 0.449 | 0.236 | 0.057 |
| InternalIMT | dha | rs9988764 | 0.708 | 0.176 | 0.105 | 0.094 | 0.087 | 0.07 | 0.214 | -0.099 | 0.064 | 0.123 |
| InternalIMT | dha | rs192875767 | 0.003 | . | . | . | . | . | . | . | . | . |
| InternalIMT | dha | rs147268846 | 0.014 | 0.031 | 0.043 | 0.464 | 8.70E-03 | 0.478 | 0.986 | 0.089 | 0.468 | 0.85 |
| InternalIMT | dha | rs11239485 | 0.094 | 0.037 | 0.049 | 0.45 | -0.044 | 0.098 | 0.656 | -0.031 | 0.09 | 0.727 |
| InternalIMT | dha | rs151317885 | 0.476 | 0.046 | 0.062 | 0.452 | 0.056 | 0.065 | 0.389 | -0.011 | 0.057 | 0.844 |
| InternalIMT | dha | rs11499085 | 0.055 | 0.034 | 0.044 | 0.436 | -0.06 | 0.117 | 0.61 | 0.018 | 0.093 | 0.849 |
| InternalIMT | dha | rs188130606 | 0.012 | 0.036 | 0.043 | 0.399 | -2.00E-03 | 0.23 | 0.993 | -0.054 | 0.167 | 0.748 |
| InternalIMT | dha | rs11511620 | 0.141 | 0.042 | 0.05 | 0.398 | -0.015 | 0.077 | 0.844 | -0.041 | 0.07 | 0.557 |
| InternalIMT | dha | rs181498390 | 0.012 | 0.031 | 0.042 | 0.46 | 0.16 | 0.362 | 0.658 | -0.05 | 0.315 | 0.875 |
| InternalIMT | dha | rs186280701 | 0.004 | . | . | . | . | . | . | . | . | . |
| InternalIMT | dha | rs150059185 | 0.02 | 0.022 | 0.042 | 0.599 | -0.209 | 0.541 | 0.699 | 0.246 | 0.547 | 0.653 |
| InternalIMT | dha | rs183338707 | . | . | . | . | . | . | . | . | . | . |
| InternalIMT | dha | rs11499038 | 0.052 | 0.035 | 0.044 | 0.424 | -0.011 | 0.139 | 0.938 | -7.60E-03 | 0.116 | 0.947 |
| InternalIMT | dha | rs190699595 | 0.004 | . | . | . | . | . | . | . | . | . |
| InternalIMT | dha | rs11523408 | 0.143 | 0.042 | 0.05 | 0.402 | -0.02 | 0.076 | 0.791 | -0.036 | 0.067 | 0.59 |
| InternalIMT | dha | rs188049317 | 0.001 | . | . | . | . | . | . | . | . | . |
| InternalIMT | dha | rs144948634 | 0.435 | 0.088 | 0.06 | 0.144 | 0.118 | 0.065 | 0.07 | -0.059 | 0.056 | 0.291 |
| InternalIMT | dha | rs143833576 | 0.001 | . | . | . | . | . | . | . | . | . |
| InternalIMT | dha | rs151239879 | 0.144 | 0.043 | 0.05 | 0.396 | -0.019 | 0.076 | 0.801 | -0.038 | 0.067 | 0.573 |
| InternalIMT | dha | rs7073743 | 0.455 | 0.075 | 0.061 | 0.216 | 0.094 | 0.065 | 0.148 | -0.043 | 0.057 | 0.448 |
| InternalIMT | dha | rs142563349 | 0.013 | 0.038 | 0.043 | 0.383 | -0.018 | 0.229 | 0.937 | -0.055 | 0.166 | 0.742 |
| InternalIMT | dha | rs187246403 | 0.004 | . | . | . | . | . | . | . | . | . |
| InternalIMT | dha | rs150423942 | 0.134 | 0.045 | 0.05 | 0.36 | -0.015 | 0.081 | 0.855 | -0.046 | 0.071 | 0.515 |

| InternalIMT | dha | rs184706102 | . | . | . | . | . | . | . | . | . | . |
| --- | --- | --- | --- | --- | --- | --- | --- | --- | --- | --- | --- | --- |
| InternalIMT | dha | rs189626288 | 0.014 | 0.022 | 0.043 | 0.605 | -0.228 | 0.269 | 0.398 | 0.216 | 0.254 | 0.394 |
| InternalIMT | dha | rs140749834 | 0.027 | 0.044 | 0.044 | 0.314 | 0.281 | 0.179 | 0.116 | -0.21 | 0.126 | 0.095 |
| InternalIMT | dha | rs7078822 | 0.115 | 0.039 | 0.049 | 0.427 | -0.02 | 0.086 | 0.815 | -0.033 | 0.078 | 0.673 |
| InternalIMT | dha | rs186227617 | 0.143 | 0.044 | 0.05 | 0.377 | -0.015 | 0.076 | 0.847 | -0.043 | 0.067 | 0.519 |
| InternalIMT | dha | rs188984368 | 0.022 | 0.038 | 0.043 | 0.385 | 0.108 | 0.247 | 0.66 | -0.135 | 0.211 | 0.524 |
| InternalIMT | dha | rs182078109 | 0.027 | 0.032 | 0.042 | 0.455 | -0.022 | 0.292 | 0.94 | 8.00E-03 | 0.282 | 0.977 |
| InternalIMT | dha | rs141456274 | 0.006 | . | . | . | . | . | . | . | . | . |
| InternalIMT | dha | rs146976512 | 0.144 | 0.044 | 0.05 | 0.381 | -0.017 | 0.076 | 0.823 | -0.042 | 0.067 | 0.53 |
| InternalIMT | dha | rs138036816 | 0.05 | 0.035 | 0.044 | 0.424 | -6.60E-03 | 0.139 | 0.962 | -9.40E-03 | 0.116 | 0.935 |
| InternalIMT | dha | rs114879777 | 0.013 | 0.031 | 0.043 | 0.47 | -0.079 | 0.23 | 0.731 | 0.057 | 0.168 | 0.733 |
| InternalIMT | dha | rs149366475 | 0.052 | 0.036 | 0.044 | 0.41 | -3.20E-03 | 0.139 | 0.982 | -0.015 | 0.116 | 0.895 |
| InternalIMT | dha | rs182463043 | 0.001 | . | . | . | . | . | . | . | . | . |
| InternalIMT | dha | rs144754632 | 0.025 | 0.015 | 0.043 | 0.732 | -0.499 | 0.205 | 0.015 | 0.443 | 0.243 | 0.069 |
| InternalIMT | dha | rs55717607 | 0.145 | 0.044 | 0.05 | 0.38 | -0.016 | 0.076 | 0.829 | -0.042 | 0.067 | 0.528 |
| InternalIMT | dha | rs187537973 | . | . | . | . | . | . | . | . | . | . |
| InternalIMT | dha | rs148085971 | 0.001 | . | . | . | . | . | . | . | . | . |
| InternalIMT | dha | rs11239491 | 0.145 | 0.044 | 0.05 | 0.381 | -0.017 | 0.076 | 0.828 | -0.042 | 0.067 | 0.529 |
| InternalIMT | dha | rs184412184 | 0.002 | . | . | . | . | . | . | . | . | . |
| InternalIMT | dha | rs4948998 | 0.733 | 0.157 | 0.103 | 0.127 | 0.084 | 0.069 | 0.225 | -0.083 | 0.064 | 0.194 |
| InternalIMT | dha | rs7076696 | 0.733 | 0.157 | 0.103 | 0.127 | 0.084 | 0.069 | 0.225 | -0.083 | 0.064 | 0.194 |
| InternalIMT | dha | rs11239492 | 0.15 | 0.044 | 0.051 | 0.389 | -0.028 | 0.075 | 0.707 | -0.038 | 0.066 | 0.571 |
| InternalIMT | dha | rs79462470 | 0.004 | . | . | . | . | . | . | . | . | . |
| InternalIMT | dha | rs187804654 | 0.004 | . | . | . | . | . | . | . | . | . |
| InternalIMT | dha | rs7093687 | 0.141 | 0.042 | 0.05 | 0.403 | -0.017 | 0.077 | 0.827 | -0.039 | 0.069 | 0.574 |
| InternalIMT | dha | rs7081004 | 0.141 | 0.042 | 0.05 | 0.4 | -0.016 | 0.077 | 0.835 | -0.04 | 0.069 | 0.565 |
| InternalIMT | dha | rs7084793 | 0.145 | 0.044 | 0.05 | 0.38 | -0.017 | 0.076 | 0.827 | -0.042 | 0.067 | 0.528 |
| InternalIMT | dha | rs61556080 | 0.004 | . | . | . | . | . | . | . | . | . |
| InternalIMT | dha | rs115552064 | 0.004 | . | . | . | . | . | . | . | . | . |
| InternalIMT | dha | rs7921503 | 0.145 | 0.044 | 0.05 | 0.379 | -0.017 | 0.076 | 0.827 | -0.043 | 0.067 | 0.523 |
| InternalIMT | dha | rs7909740 | 0.145 | 0.044 | 0.05 | 0.379 | -0.017 | 0.076 | 0.827 | -0.042 | 0.067 | 0.526 |
| InternalIMT | dha | rs7909768 | 0.145 | 0.044 | 0.05 | 0.379 | -0.017 | 0.076 | 0.827 | -0.042 | 0.067 | 0.526 |
| InternalIMT | dha | rs144168004 | . | . | . | . | . | . | . | . | . | . |
| InternalIMT | dha | rs75850448 | 0.004 | . | . | . | . | . | . | . | . | . |
| InternalIMT | dha | rs147777574 | 0.003 | . | . | . | . | . | . | . | . | . |
| InternalIMT | dha | rs7913488 | 0.145 | 0.044 | 0.05 | 0.379 | -0.017 | 0.076 | 0.827 | -0.042 | 0.067 | 0.525 |
| InternalIMT | dha | rs74390874 | 0.042 | 0.042 | 0.044 | 0.343 | 0.259 | 0.217 | 0.232 | -0.121 | 0.187 | 0.518 |
| InternalIMT | dha | rs141391142 | 0.004 | . | . | . | . | . | . | . | . | . |
| InternalIMT | dha | rs115294747 | 0.006 | . | . | . | . | . | . | . | . | . |
| InternalIMT | dha | rs148031781 | 0.001 | . | . | . | . | . | . | . | . | . |
| InternalIMT | dha | rs181340128 | 0.025 | 0.038 | 0.043 | 0.374 | 0.185 | 0.285 | 0.517 | -0.11 | 0.261 | 0.672 |
| InternalIMT | dha | rs189930007 | 0.001 | . | . | . | . | . | . | . | . | . |
| InternalIMT | dha | rs12242128 | 0.049 | 0.035 | 0.044 | 0.431 | -5.50E-03 | 0.14 | 0.969 | -7.90E-03 | 0.117 | 0.946 |
| InternalIMT | dha | rs78421750 | 0.003 | . | . | . | . | . | . | . | . | . |
| InternalIMT | dha | rs114370747 | 0.002 | . | . | . | . | . | . | . | . | . |
| InternalIMT | dha | rs12264166 | 0.049 | 0.034 | 0.044 | 0.432 | -5.30E-03 | 0.14 | 0.97 | -7.70E-03 | 0.117 | 0.947 |
| InternalIMT | dha | rs182964822 | 0.001 | . | . | . | . | . | . | . | . | . |
| InternalIMT | dha | rs67491417 | 0.139 | 0.046 | 0.05 | 0.359 | -5.80E-04 | 0.077 | 0.994 | -0.052 | 0.068 | 0.445 |
| InternalIMT | dha | rs72789720 | 0.008 | . | . | . | . | . | . | . | . | . |
| InternalIMT | dha | rs6413416 | 0.145 | 0.044 | 0.05 | 0.378 | -0.016 | 0.076 | 0.828 | -0.043 | 0.067 | 0.52 |
| InternalIMT | dha | rs4986832 | 0.145 | 0.044 | 0.05 | 0.377 | -0.016 | 0.076 | 0.828 | -0.043 | 0.067 | 0.519 |
| InternalIMT | dha | rs144866316 | 0.004 | . | . | . | . | . | . | . | . | . |
| InternalIMT | dha | rs187769540 | 0.007 | . | . | . | . | . | . | . | . | . |
| InternalIMT | dha | rs6593482 | 0.146 | 0.048 | 0.05 | 0.338 | -3.00E-03 | 0.076 | 0.969 | -0.056 | 0.067 | 0.404 |
| InternalIMT | dha | rs7913948 | 0.146 | 0.048 | 0.05 | 0.337 | 4.60E-03 | 0.077 | 0.952 | -0.057 | 0.067 | 0.397 |
| InternalIMT | dha | rs77185151 | 0.025 | 0.015 | 0.043 | 0.73 | -0.497 | 0.205 | 0.016 | 0.44 | 0.243 | 0.07 |
| InternalIMT | dha | rs12762303 | 0.145 | 0.044 | 0.05 | 0.379 | -0.017 | 0.076 | 0.82 | -0.042 | 0.067 | 0.523 |
| InternalIMT | dha | rs73283136 | 0.003 | . | . | . | . | . | . | . | . | . |
| InternalIMT | dha | rs187180218 | 0.001 | . | . | . | . | . | . | . | . | . |
| InternalIMT | dha | rs78137087 | 0.145 | 0.044 | 0.05 | 0.377 | -0.017 | 0.076 | 0.817 | -0.043 | 0.067 | 0.52 |
| InternalIMT | dha | rs72561472 | 0.146 | 0.043 | 0.05 | 0.392 | -0.019 | 0.076 | 0.803 | -0.04 | 0.068 | 0.556 |
| InternalIMT | dha | rs4987106 | 0.142 | 0.044 | 0.05 | 0.376 | -0.017 | 0.077 | 0.823 | -0.044 | 0.068 | 0.513 |
| InternalIMT | dha | rs4987105 | 0.139 | 0.043 | 0.05 | 0.392 | -0.014 | 0.077 | 0.856 | -0.044 | 0.07 | 0.533 |
| InternalIMT | dha | rs111916157 | 0.158 | 0.037 | 0.051 | 0.466 | -0.039 | 0.074 | 0.597 | -0.016 | 0.065 | 0.807 |
| InternalIMT | dha | rs111528152 | 0.162 | 0.049 | 0.052 | 0.347 | -9.30E-03 | 0.076 | 0.903 | -0.055 | 0.067 | 0.417 |
| InternalIMT | dha | rs12763738 | 0.145 | 0.044 | 0.05 | 0.378 | -0.017 | 0.076 | 0.825 | -0.043 | 0.067 | 0.522 |
| InternalIMT | dha | rs191675886 | 0.004 | . | . | . | . | . | . | . | . | . |
| InternalIMT | dha | rs66489870 | 0.145 | 0.044 | 0.05 | 0.379 | -0.017 | 0.076 | 0.819 | -0.042 | 0.067 | 0.524 |
| InternalIMT | dha | rs71494775 | 0.145 | 0.044 | 0.05 | 0.379 | -0.017 | 0.076 | 0.819 | -0.042 | 0.067 | 0.524 |
| InternalIMT | dha | rs61855875 | 0.024 | 0.036 | 0.044 | 0.405 | 0.213 | 0.277 | 0.442 | -0.084 | 0.243 | 0.73 |
| InternalIMT | dha | rs116356837 | 0.004 | . | . | . | . | . | . | . | . | . |
| InternalIMT | dha | rs1864414 | 0.145 | 0.042 | 0.05 | 0.398 | -0.021 | 0.076 | 0.778 | -0.037 | 0.067 | 0.578 |
| InternalIMT | dha | rs1864413 | 0.145 | 0.044 | 0.05 | 0.378 | -0.018 | 0.076 | 0.811 | -0.043 | 0.067 | 0.522 |
| InternalIMT | dha | rs139497212 | 0 | . | . | . | . | . | . | . | . | . |
| InternalIMT | dha | rs892690 | 0.424 | 0.088 | 0.064 | 0.166 | 0.102 | 0.064 | 0.112 | -0.06 | 0.058 | 0.303 |
| InternalIMT | dha | rs1864412 | 0.145 | 0.044 | 0.05 | 0.379 | -0.018 | 0.076 | 0.816 | -0.042 | 0.066 | 0.525 |
| InternalIMT | dha | rs77827771 | 0.016 | 0.036 | 0.043 | 0.403 | 0.158 | 0.238 | 0.507 | -0.114 | 0.175 | 0.516 |
| InternalIMT | dha | rs145294963 | . | . | . | . | . | . | . | . | . | . |
| InternalIMT | dha | rs149081327 | 0.008 | . | . | . | . | . | . | . | . | . |
| InternalIMT | dha | rs67008983 | 0.145 | 0.044 | 0.05 | 0.381 | -0.019 | 0.076 | 0.802 | -0.042 | 0.066 | 0.531 |
| InternalIMT | dha | rs56150414 | 0.147 | 0.041 | 0.05 | 0.412 | -0.028 | 0.075 | 0.708 | -0.032 | 0.066 | 0.631 |
| InternalIMT | dha | rs55787481 | 0.145 | 0.043 | 0.05 | 0.391 | -0.022 | 0.076 | 0.77 | -0.038 | 0.067 | 0.568 |
| InternalIMT | dha | rs141270101 | 0.001 | . | . | . | . | . | . | . | . | . |
| InternalIMT | dha | rs114980075 | 0.003 | . | . | . | . | . | . | . | . | . |
| InternalIMT | dha | rs12783095 | 0.144 | 0.047 | 0.05 | 0.346 | -0.013 | 0.075 | 0.865 | -0.055 | 0.066 | 0.403 |
| InternalIMT | dha | rs182342688 | . | . | . | . | . | . | . | . | . | . |

| InternalIMT | dha | rs17157728 | 0.144 | 0.047 | 0.05 | 0.346 | -0.013 | 0.075 | 0.864 | -0.055 | 0.066 | 0.404 |
| --- | --- | --- | --- | --- | --- | --- | --- | --- | --- | --- | --- | --- |
| InternalIMT | dha | rs114835563 | 0.012 | 0.036 | 0.043 | 0.402 | -0.025 | 0.234 | 0.917 | -0.041 | 0.173 | 0.811 |
| InternalIMT | dha | rs3824612 | 0.424 | 0.088 | 0.064 | 0.168 | 0.101 | 0.064 | 0.113 | -0.059 | 0.058 | 0.309 |
| InternalIMT | dha | rs17157733 | 0.144 | 0.047 | 0.05 | 0.348 | -0.014 | 0.075 | 0.85 | -0.055 | 0.066 | 0.409 |
| InternalIMT | dha | rs74128478 | 0.004 | . | . | . | . | . | . | . | . | . |
| InternalIMT | dha | rs184973589 | 0.002 | . | . | . | . | . | . | . | . | . |
| InternalIMT | dha | rs150479461 | 0 | . | . | . | . | . | . | . | . | . |
| InternalIMT | dha | rs138310407 | 0.001 | . | . | . | . | . | . | . | . | . |
| InternalIMT | dha | rs187426603 | . | . | . | . | . | . | . | . | . | . |
| InternalIMT | dha | rs190843143 | 0.002 | . | . | . | . | . | . | . | . | . |
| InternalIMT | dha | rs191883449 | 0.001 | . | . | . | . | . | . | . | . | . |
| InternalIMT | dha | rs58238740 | 0.143 | 0.047 | 0.05 | 0.353 | -0.018 | 0.076 | 0.816 | -0.053 | 0.066 | 0.427 |
| InternalIMT | dha | rs3780894 | 0.143 | 0.047 | 0.05 | 0.353 | -0.018 | 0.076 | 0.814 | -0.053 | 0.066 | 0.428 |
| InternalIMT | dha | rs149646626 | 0.018 | 0.04 | 0.043 | 0.357 | 0.184 | 0.228 | 0.42 | -0.271 | 0.2 | 0.175 |
| InternalIMT | dha | rs79615212 | 0.015 | 0.033 | 0.043 | 0.443 | 0.048 | 0.307 | 0.877 | -0.036 | 0.319 | 0.91 |
| InternalIMT | dha | rs3780895 | 0.143 | 0.047 | 0.05 | 0.352 | -0.018 | 0.076 | 0.816 | -0.053 | 0.066 | 0.425 |
| InternalIMT | dha | rs3780896 | 0.143 | 0.047 | 0.05 | 0.353 | -0.018 | 0.076 | 0.814 | -0.052 | 0.066 | 0.428 |
| InternalIMT | dha | rs142747163 | 0.005 | . | . | . | . | . | . | . | . | . |
| InternalIMT | dha | rs189417584 | 0.005 | . | . | . | . | . | . | . | . | . |
| InternalIMT | dha | rs3780897 | 0.141 | 0.045 | 0.05 | 0.374 | -0.024 | 0.076 | 0.754 | -0.046 | 0.066 | 0.49 |
| InternalIMT | dha | rs117509491 | 0.025 | 0.03 | 0.043 | 0.497 | 0.089 | 0.252 | 0.724 | 0.092 | 0.231 | 0.689 |
| InternalIMT | dha | rs117811514 | 0.025 | 0.03 | 0.043 | 0.497 | 0.089 | 0.252 | 0.724 | 0.092 | 0.231 | 0.689 |
| InternalIMT | dha | rs116500932 | 0.012 | 0.036 | 0.043 | 0.402 | -0.025 | 0.234 | 0.914 | -0.04 | 0.173 | 0.815 |
| InternalIMT | dha | rs77584168 | . | . | . | . | . | . | . | . | . | . |
| InternalIMT | dha | rs2310776 | 0.143 | 0.047 | 0.05 | 0.353 | -0.018 | 0.076 | 0.812 | -0.052 | 0.066 | 0.429 |
| InternalIMT | dha | rs17157736 | 0.143 | 0.047 | 0.05 | 0.353 | -0.018 | 0.076 | 0.812 | -0.052 | 0.066 | 0.429 |
| InternalIMT | dha | rs72789735 | 0.063 | 0.033 | 0.045 | 0.464 | -0.052 | 0.105 | 0.621 | -0.024 | 0.098 | 0.809 |
| InternalIMT | dha | rs183747187 | 0.001 | . | . | . | . | . | . | . | . | . |
| InternalIMT | dha | rs7077891 | 0.143 | 0.047 | 0.05 | 0.353 | -0.018 | 0.076 | 0.81 | -0.052 | 0.066 | 0.43 |
| InternalIMT | dha | rs145063305 | 0.002 | . | . | . | . | . | . | . | . | . |
| InternalIMT | dha | rs3824613 | 0.143 | 0.047 | 0.05 | 0.345 | -0.016 | 0.075 | 0.833 | -0.055 | 0.066 | 0.409 |
| InternalIMT | dha | rs145543199 | 0.006 | . | . | . | . | . | . | . | . | . |
| InternalIMT | dha | rs140361993 | 0.003 | . | . | . | . | . | . | . | . | . |
| InternalIMT | dha | rs11239496 | 0.063 | 0.033 | 0.045 | 0.464 | -0.052 | 0.105 | 0.622 | -0.024 | 0.098 | 0.808 |
| InternalIMT | dha | rs11239497 | 0.139 | 0.045 | 0.05 | 0.368 | -0.015 | 0.077 | 0.842 | -0.053 | 0.069 | 0.444 |
| InternalIMT | dha | rs10900212 | 0.156 | 0.033 | 0.049 | 0.501 | -0.072 | 0.078 | 0.358 | 3.40E-03 | 0.069 | 0.961 |
| InternalIMT | dha | rs186487136 | . | . | . | . | . | . | . | . | . | . |
| InternalIMT | dha | rs12774369 | 0.143 | 0.047 | 0.05 | 0.351 | -0.017 | 0.076 | 0.82 | -0.053 | 0.066 | 0.424 |
| InternalIMT | dha | rs60842538 | 0.142 | 0.047 | 0.05 | 0.348 | -0.016 | 0.076 | 0.833 | -0.054 | 0.067 | 0.416 |
| InternalIMT | dha | rs3780898 | 0.138 | 0.046 | 0.05 | 0.359 | -0.012 | 0.078 | 0.88 | -0.056 | 0.07 | 0.421 |
| InternalIMT | dha | rs3780899 | 0.142 | 0.047 | 0.05 | 0.343 | -0.014 | 0.076 | 0.854 | -0.056 | 0.067 | 0.404 |
| InternalIMT | dha | rs12252472 | 0.045 | 0.048 | 0.045 | 0.292 | 0.086 | 0.127 | 0.5 | -0.104 | 0.095 | 0.273 |
| InternalIMT | dha | rs185411011 | . | . | . | . | . | . | . | . | . | . |
| InternalIMT | dha | rs28395865 | 0.137 | 0.047 | 0.05 | 0.346 | -8.20E-03 | 0.078 | 0.917 | -0.061 | 0.07 | 0.386 |
| InternalIMT | dha | rs191004074 | 0.038 | 0.05 | 0.045 | 0.272 | 0.233 | 0.296 | 0.432 | -0.255 | 0.268 | 0.342 |
| InternalIMT | dha | rs182401295 | 0.002 | . | . | . | . | . | . | . | . | . |
| InternalIMT | dha | rs2228064 | 0.068 | 0.03 | 0.046 | 0.523 | -0.102 | 0.1 | 0.304 | 0.037 | 0.083 | 0.658 |
| InternalIMT | dha | rs188284159 | . | . | . | . | . | . | . | . | . | . |
| InternalIMT | dha | rs2303887 | 0.022 | 0.015 | 0.043 | 0.726 | -0.569 | 0.229 | 0.013 | 0.512 | 0.271 | 0.059 |
| InternalIMT | dha | rs2303886 | 0.024 | 0.014 | 0.043 | 0.75 | -0.545 | 0.193 | 4.80E-03 | 0.476 | 0.223 | 0.033 |
| InternalIMT | dha | rs192621006 | . | . | . | . | . | . | . | . | . | . |
| InternalIMT | dha | rs2303885 | 0.191 | 0.058 | 0.053 | 0.271 | -3.80E-03 | 0.064 | 0.953 | -0.05 | 0.055 | 0.365 |
| InternalIMT | dha | rs114466168 | 0.012 | 0.036 | 0.043 | 0.4 | -0.026 | 0.233 | 0.911 | -0.042 | 0.171 | 0.805 |
| InternalIMT | dha | rs11239501 | 0.083 | 0.013 | 0.046 | 0.769 | -0.143 | 0.097 | 0.138 | 0.102 | 0.09 | 0.258 |
| InternalIMT | dha | rs745986 | 0.156 | 0.018 | 0.05 | 0.714 | -0.097 | 0.072 | 0.177 | 0.043 | 0.065 | 0.512 |
| InternalIMT | dha | rs75262268 | . | . | . | . | . | . | . | . | . | . |
| InternalIMT | dha | rs71494776 | 0.051 | 0.027 | 0.044 | 0.545 | -0.093 | 0.113 | 0.409 | 0.045 | 0.106 | 0.672 |
| InternalIMT | dha | rs74386871 | 0.03 | 0.017 | 0.043 | 0.697 | -0.375 | 0.184 | 0.042 | 0.266 | 0.214 | 0.214 |
| InternalIMT | dha | rs76502040 | 0.006 | . | . | . | . | . | . | . | . | . |
| InternalIMT | dha | rs142213734 | 0.001 | . | . | . | . | . | . | . | . | . |
| InternalIMT | dha | rs4948671 | 0.731 | 0.183 | 0.104 | 0.078 | 0.096 | 0.069 | 0.165 | -0.1 | 0.064 | 0.119 |
| InternalIMT | dha | rs146286085 | 0.001 | . | . | . | . | . | . | . | . | . |
| InternalIMT | dha | rs144080623 | . | . | . | . | . | . | . | . | . | . |
| InternalIMT | dha | rs115074014 | 0.023 | 0.045 | 0.044 | 0.309 | 0.155 | 0.19 | 0.413 | -0.168 | 0.136 | 0.215 |
| InternalIMT | dha | rs17157756 | 0.023 | 0.045 | 0.044 | 0.309 | 0.155 | 0.19 | 0.413 | -0.168 | 0.136 | 0.215 |
| InternalIMT | dha | rs190459780 | . | . | . | . | . | . | . | . | . | . |
| InternalIMT | dha | rs12358647 | 0.174 | 6.10E-03 | 0.049 | 0.901 | -0.133 | 0.073 | 0.07 | 0.078 | 0.067 | 0.243 |
| InternalIMT | dha | rs147080556 | 0.024 | 0.037 | 0.045 | 0.403 | 0.063 | 0.269 | 0.816 | -0.104 | 0.225 | 0.646 |
| InternalIMT | dha | rs187543349 | 0.01 | 0.031 | 0.043 | 0.466 | -3.20E-03 | 0.271 | 0.991 | -0.1 | 0.295 | 0.735 |
| InternalIMT | dha | rs114375595 | 0.006 | . | . | . | . | . | . | . | . | . |
| InternalIMT | dha | rs117211200 | 0.005 | . | . | . | . | . | . | . | . | . |
| InternalIMT | dha | rs192963047 | . | . | . | . | . | . | . | . | . | . |
| InternalIMT | dha | rs11239505 | 0.175 | 8.40E-03 | 0.049 | 0.864 | -0.129 | 0.073 | 0.077 | 0.072 | 0.066 | 0.277 |
| InternalIMT | dha | rs184434116 | 0 | . | . | . | . | . | . | . | . | . |
| InternalIMT | dha | rs4948999 | 0.733 | 0.184 | 0.105 | 0.079 | 0.096 | 0.07 | 0.166 | -0.1 | 0.064 | 0.119 |
| InternalIMT | dha | rs11239506 | 0.092 | 0.019 | 0.047 | 0.685 | -0.113 | 0.09 | 0.211 | 0.064 | 0.083 | 0.441 |
| InternalIMT | dha | rs190706620 | 0.003 | . | . | . | . | . | . | . | . | . |
| InternalIMT | dha | rs190770429 | . | . | . | . | . | . | . | . | . | . |
| InternalIMT | dha | rs181327133 | 0.005 | . | . | . | . | . | . | . | . | . |
| InternalIMT | dha | rs149251697 | 0.011 | 0.015 | 0.041 | 0.717 | -0.707 | 0.701 | 0.313 | 0.72 | 0.633 | 0.256 |
| InternalIMT | dha | rs118081602 | 0.022 | 0.015 | 0.043 | 0.726 | -0.567 | 0.229 | 0.013 | 0.51 | 0.271 | 0.061 |
| InternalIMT | dha | rs188233691 | 0.002 | . | . | . | . | . | . | . | . | . |
| InternalIMT | dha | rs183778794 | 0.003 | . | . | . | . | . | . | . | . | . |
| InternalIMT | dha | rs188040078 | 0.007 | . | . | . | . | . | . | . | . | . |
| InternalIMT | dha | rs184675372 | 0 | . | . | . | . | . | . | . | . | . |
| InternalIMT | dha | rs150326891 | 0.067 | 0.018 | 0.045 | 0.697 | -0.164 | 0.11 | 0.134 | 0.096 | 0.101 | 0.345 |

| InternalIMT | dha | rs185520021 | 0.014 | 0.025 | 0.041 | 0.543 | -0.334 | 0.469 | 0.477 | 0.24 | 0.465 | 0.606 |
| --- | --- | --- | --- | --- | --- | --- | --- | --- | --- | --- | --- | --- |
| InternalIMT | dha | rs7907779 | 0.092 | 0.019 | 0.047 | 0.681 | -0.112 | 0.09 | 0.214 | 0.063 | 0.083 | 0.45 |
| InternalIMT | dha | rs184962860 | 0.001 | . | . | . | . | . | . | . | . | . |
| InternalIMT | dha | rs149762784 | 0.066 | 0.058 | 0.049 | 0.233 | 0.156 | 0.163 | 0.34 | -0.228 | 0.152 | 0.133 |
| InternalIMT | dha | rs149203427 | 0.008 | . | . | . | . | . | . | . | . | . |
| InternalIMT | dha | rs60153083 | 0.01 | 0.031 | 0.042 | 0.467 | 0.162 | 0.399 | 0.685 | -0.019 | 0.356 | 0.957 |
| InternalIMT | dha | rs112001069 | 0.062 | 0.029 | 0.045 | 0.516 | -0.079 | 0.102 | 0.437 | 0.023 | 0.091 | 0.797 |
| InternalIMT | dha | rs182355382 | 0.032 | 0.039 | 0.044 | 0.377 | 0.168 | 0.27 | 0.534 | -0.12 | 0.237 | 0.611 |
| InternalIMT | dha | rs186740974 | 0.111 | 0.026 | 0.054 | 0.628 | -2.20E-03 | 0.129 | 0.986 | 0.025 | 0.123 | 0.838 |
| InternalIMT | dha | rs191874916 | 0.026 | 0.026 | 0.042 | 0.528 | -0.07 | 0.389 | 0.857 | 0.104 | 0.406 | 0.798 |
| InternalIMT | dha | rs182989412 | 0.168 | -0.016 | 0.053 | 0.76 | -0.091 | 0.103 | 0.378 | 0.164 | 0.102 | 0.107 |
| InternalIMT | dha | rs188819476 | 0.001 | . | . | . | . | . | . | . | . | . |
| InternalIMT | dha | rs141217183 | 0.08 | 0.028 | 0.047 | 0.555 | -0.088 | 0.095 | 0.352 | 0.025 | 0.086 | 0.769 |
| InternalIMT | dha | rs192922652 | 0.064 | 0.032 | 0.045 | 0.481 | -0.08 | 0.102 | 0.429 | 5.80E-03 | 0.088 | 0.947 |
| InternalIMT | dha | rs185092343 | 0.014 | 0.035 | 0.043 | 0.423 | 0.111 | 0.411 | 0.786 | -0.092 | 0.317 | 0.772 |
| InternalIMT | dha | rs145193311 | 0.065 | 0.035 | 0.045 | 0.433 | -0.025 | 0.103 | 0.809 | -0.026 | 0.087 | 0.766 |
| InternalIMT | dha | rs55789336 | 0.094 | 0.018 | 0.047 | 0.699 | -0.12 | 0.09 | 0.186 | 0.066 | 0.083 | 0.422 |
| InternalIMT | dha | rs55837212 | 0.094 | 0.017 | 0.047 | 0.712 | -0.116 | 0.092 | 0.204 | 0.074 | 0.083 | 0.374 |
| InternalIMT | dha | rs75408574 | 0.406 | 0.1 | 0.059 | 0.091 | 0.127 | 0.067 | 0.058 | -0.08 | 0.06 | 0.189 |
| InternalIMT | dha | rs149306926 | 0.099 | 0.012 | 0.047 | 0.797 | -0.124 | 0.091 | 0.173 | 0.092 | 0.084 | 0.274 |
| InternalIMT | dha | rs35990892 | 0.706 | 0.152 | 0.104 | 0.144 | 0.091 | 0.069 | 0.19 | -0.082 | 0.063 | 0.194 |
| InternalIMT | dha | rs192356207 | 0.036 | 0.017 | 0.042 | 0.684 | -0.14 | 0.198 | 0.481 | 0.171 | 0.184 | 0.353 |
| InternalIMT | dha | rs183741163 | 0.092 | 6.00E-03 | 0.045 | 0.894 | -0.179 | 0.118 | 0.128 | 0.133 | 0.114 | 0.242 |
| InternalIMT | dha | rs34737297 | 0.558 | 0.097 | 0.085 | 0.252 | 0.076 | 0.079 | 0.335 | -0.058 | 0.072 | 0.424 |
| InternalIMT | dha | rs181346933 | 0.022 | 0.027 | 0.044 | 0.539 | -0.243 | 0.205 | 0.235 | 0.123 | 0.171 | 0.474 |
| InternalIMT | dha | rs36093253 | 0.408 | 0.092 | 0.061 | 0.134 | 0.117 | 0.071 | 0.1 | -0.068 | 0.065 | 0.292 |
| InternalIMT | dha | rs186307855 | 0.059 | 0.047 | 0.046 | 0.31 | 0.02 | 0.109 | 0.851 | -0.075 | 0.082 | 0.363 |
| InternalIMT | dha | rs189681865 | 0.019 | 0.022 | 0.041 | 0.584 | -0.387 | 0.281 | 0.169 | 0.27 | 0.25 | 0.281 |
| InternalIMT | dha | rs34942971 | 0.176 | -6.40E-03 | 0.05 | 0.898 | -0.157 | 0.077 | 0.042 | 0.111 | 0.071 | 0.121 |
| InternalIMT | dha | rs181816554 | 0.025 | 0.031 | 0.042 | 0.461 | 0.191 | 0.449 | 0.67 | -0.055 | 0.394 | 0.888 |
| InternalIMT | dha | rs186846881 | 0.049 | 0.041 | 0.044 | 0.353 | 0.052 | 0.133 | 0.698 | -0.057 | 0.106 | 0.59 |
| InternalIMT | dha | rs191178220 | 0.016 | 0.046 | 0.043 | 0.282 | 0.537 | 0.45 | 0.233 | -0.502 | 0.374 | 0.179 |
| InternalIMT | dha | rs183305228 | 0.01 | 0.031 | 0.042 | 0.465 | 0.164 | 0.399 | 0.681 | -0.025 | 0.356 | 0.944 |
| InternalIMT | dha | rs139912761 | 0.102 | 2.60E-03 | 0.045 | 0.955 | -0.169 | 0.097 | 0.08 | 0.133 | 0.09 | 0.139 |
| InternalIMT | dha | rs185499901 | 0.016 | 0.024 | 0.042 | 0.566 | -0.219 | 0.526 | 0.677 | 0.252 | 0.525 | 0.631 |
| InternalIMT | dha | rs189816197 | 0.072 | 0.029 | 0.046 | 0.532 | -0.083 | 0.106 | 0.434 | 0.026 | 0.095 | 0.783 |
| InternalIMT | dha | rs145656938 | 0.022 | 0.034 | 0.043 | 0.437 | -0.087 | 0.224 | 0.698 | 8.50E-03 | 0.162 | 0.958 |
| InternalIMT | dha | rs35745197 | 0.062 | 0.029 | 0.045 | 0.521 | -0.082 | 0.104 | 0.43 | 0.026 | 0.093 | 0.781 |
| InternalIMT | dha | rs192856637 | 0.004 | . | . | . | . | . | . | . | . | . |
| InternalIMT | dha | rs2310775 | 0.492 | 0.138 | 0.063 | 0.028 | 0.147 | 0.061 | 0.015 | -0.103 | 0.054 | 0.057 |
| InternalIMT | dha | rs11239507 | 0.558 | 0.169 | 0.076 | 0.026 | 0.149 | 0.064 | 0.019 | -0.117 | 0.057 | 0.04 |
| InternalIMT | dha | rs74648467 | 0.053 | 0.028 | 0.044 | 0.527 | -0.08 | 0.114 | 0.482 | 0.027 | 0.105 | 0.794 |
| InternalIMT | dha | rs145815934 | 0.012 | 0.039 | 0.043 | 0.362 | 0.386 | 0.51 | 0.449 | -0.387 | 0.412 | 0.348 |
| InternalIMT | dha | rs145390687 | 0.015 | 0.019 | 0.043 | 0.666 | -0.268 | 0.284 | 0.345 | 0.327 | 0.246 | 0.185 |
| InternalIMT | dha | rs184354913 | 0.033 | 0.041 | 0.044 | 0.347 | 0.143 | 0.194 | 0.461 | -0.166 | 0.183 | 0.364 |
| InternalIMT | dha | rs181244920 | 0.007 | . | . | . | . | . | . | . | . | . |
| InternalIMT | dha | rs11499087 | 0.028 | 0.044 | 0.044 | 0.316 | 0.122 | 0.159 | 0.445 | -0.14 | 0.119 | 0.241 |
| InternalIMT | dha | rs148263794 | 0.064 | 0.031 | 0.045 | 0.486 | -0.065 | 0.102 | 0.526 | 5.20E-03 | 0.089 | 0.953 |
| InternalIMT | dha | rs150758903 | 0.021 | 0.019 | 0.041 | 0.637 | -0.412 | 0.337 | 0.221 | 0.329 | 0.297 | 0.268 |
| InternalIMT | dha | rs147654382 | 0.152 | 0.021 | 0.051 | 0.682 | -0.101 | 0.076 | 0.183 | 0.04 | 0.069 | 0.564 |
| InternalIMT | dha | rs147495166 | 0.011 | 0.038 | 0.043 | 0.373 | 0.024 | 0.224 | 0.915 | -0.098 | 0.152 | 0.52 |
| InternalIMT | dha | rs35198778 | 0.081 | 0.027 | 0.048 | 0.577 | -0.17 | 0.104 | 0.102 | 0.055 | 0.089 | 0.537 |
| InternalIMT | dha | rs145207659 | 0.043 | 0.04 | 0.045 | 0.366 | 0.228 | 0.215 | 0.291 | -0.097 | 0.185 | 0.601 |
| InternalIMT | dha | rs186466464 | 0.007 | . | . | . | . | . | . | . | . | . |
| InternalIMT | dha | rs12778413 | 0.146 | 0.032 | 0.052 | 0.53 | -0.081 | 0.08 | 0.308 | 2.90E-03 | 0.071 | 0.968 |
| InternalIMT | dha | rs7895552 | 0.632 | 0.171 | 0.094 | 0.07 | 0.099 | 0.072 | 0.169 | -0.105 | 0.064 | 0.1 |
| InternalIMT | dha | rs12783895 | 0.064 | 0.031 | 0.045 | 0.488 | -0.066 | 0.102 | 0.52 | 6.70E-03 | 0.089 | 0.94 |
| InternalIMT | dha | rs34929604 | 0.065 | 0.032 | 0.045 | 0.473 | -0.058 | 0.1 | 0.562 | -1.70E-03 | 0.087 | 0.985 |
| InternalIMT | dha | rs12761961 | 0.063 | 0.029 | 0.045 | 0.512 | -0.074 | 0.104 | 0.478 | 0.02 | 0.093 | 0.826 |
| InternalIMT | dha | rs35536930 | 0.173 | 9.10E-03 | 0.049 | 0.853 | -0.125 | 0.074 | 0.091 | 0.072 | 0.067 | 0.285 |
| InternalIMT | dha | rs180951060 | 0.001 | . | . | . | . | . | . | . | . | . |
| InternalIMT | dha | rs148892128 | 0.012 | 0.039 | 0.043 | 0.362 | 0.386 | 0.51 | 0.45 | -0.387 | 0.412 | 0.348 |
| InternalIMT | dha | rs11239509 | 0.043 | 0.049 | 0.045 | 0.277 | 0.071 | 0.13 | 0.582 | -0.106 | 0.097 | 0.274 |
| InternalIMT | dha | rs41526545 | 0.088 | 0.016 | 0.046 | 0.726 | -0.132 | 0.095 | 0.164 | 0.082 | 0.088 | 0.351 |
| InternalIMT | dha | rs2029253 | 0.496 | 0.141 | 0.063 | 0.026 | 0.151 | 0.061 | 0.014 | -0.105 | 0.054 | 0.054 |
| InternalIMT | dha | rs17444064 | 0.086 | 0.014 | 0.046 | 0.757 | -0.142 | 0.096 | 0.138 | 0.095 | 0.089 | 0.287 |
| InternalIMT | dha | rs71494779 | 0.066 | 0.033 | 0.045 | 0.471 | -0.056 | 0.099 | 0.569 | -3.20E-03 | 0.086 | 0.971 |
| InternalIMT | dha | rs74991420 | 0.022 | 0.015 | 0.043 | 0.721 | -0.566 | 0.233 | 0.015 | 0.502 | 0.277 | 0.07 |
| InternalIMT | dha | rs182736551 | 0.022 | 0.015 | 0.043 | 0.721 | -0.566 | 0.233 | 0.015 | 0.502 | 0.277 | 0.07 |
| InternalIMT | dha | rs78702074 | 0.021 | 0.015 | 0.042 | 0.73 | -0.609 | 0.238 | 0.011 | 0.543 | 0.28 | 0.053 |
| InternalIMT | dha | rs187791466 | 0.002 | . | . | . | . | . | . | . | . | . |
| InternalIMT | dha | rs35300270 | 0.068 | 0.034 | 0.045 | 0.459 | -0.049 | 0.097 | 0.615 | -0.013 | 0.085 | 0.874 |
| InternalIMT | dha | rs6593484 | 0.093 | 0.019 | 0.047 | 0.683 | -0.115 | 0.091 | 0.206 | 0.063 | 0.083 | 0.451 |
| InternalIMT | dha | rs12244601 | 0.013 | 0.032 | 0.042 | 0.442 | -0.125 | 0.297 | 0.673 | 0.052 | 0.199 | 0.793 |
| InternalIMT | dha | rs184429574 | 0.004 | . | . | . | . | . | . | . | . | . |
| InternalIMT | dha | rs7080897 | 0.066 | 0.033 | 0.045 | 0.471 | -0.056 | 0.099 | 0.568 | -3.10E-03 | 0.086 | 0.971 |
| InternalIMT | dha | rs7097429 | 0.07 | 0.034 | 0.045 | 0.447 | -0.037 | 0.101 | 0.718 | -0.022 | 0.089 | 0.803 |
| InternalIMT | dha | rs77979229 | 0.001 | . | . | . | . | . | . | . | . | . |
| InternalIMT | dha | rs11239512 | 0.093 | 0.02 | 0.047 | 0.674 | -0.112 | 0.09 | 0.214 | 0.06 | 0.083 | 0.468 |
| InternalIMT | dha | rs60146827 | 0.066 | 0.033 | 0.045 | 0.47 | -0.056 | 0.099 | 0.569 | -3.60E-03 | 0.086 | 0.967 |
| InternalIMT | dha | rs79032236 | 0.017 | 0.037 | 0.043 | 0.395 | 0.038 | 0.186 | 0.839 | -0.067 | 0.14 | 0.634 |
| InternalIMT | dha | rs140854206 | 0.005 | . | . | . | . | . | . | . | . | . |
| InternalIMT | dha | rs184049550 | 0.002 | . | . | . | . | . | . | . | . | . |
| InternalIMT | dha | rs114433607 | 0.007 | . | . | . | . | . | . | . | . | . |
| InternalIMT | dha | rs117653793 | 0.022 | 0.015 | 0.043 | 0.726 | -0.572 | 0.232 | 0.014 | 0.515 | 0.275 | 0.062 |
| InternalIMT | dha | rs117039533 | 0.022 | 0.015 | 0.043 | 0.725 | -0.572 | 0.232 | 0.014 | 0.514 | 0.275 | 0.062 |

| InternalIMT | dha | rs191660761 | 0.002 | . | . | . | . | . | . | . | . | . |
| --- | --- | --- | --- | --- | --- | --- | --- | --- | --- | --- | --- | --- |
| InternalIMT | dha | rs59514896 | 0.053 | 0.029 | 0.044 | 0.505 | -0.065 | 0.113 | 0.564 | 0.01 | 0.105 | 0.921 |
| InternalIMT | dha | rs184661876 | 0.001 | . | . | . | . | . | . | . | . | . |
| InternalIMT | dha | rs61854073 | 0.028 | 0.047 | 0.044 | 0.281 | 0.388 | 0.248 | 0.118 | -0.289 | 0.219 | 0.186 |
| InternalIMT | dha | rs2018518 | 0.391 | 0.108 | 0.061 | 0.075 | 0.129 | 0.064 | 0.043 | -0.09 | 0.058 | 0.123 |
| InternalIMT | dha | rs934187 | 0.734 | 0.172 | 0.104 | 0.099 | 0.089 | 0.069 | 0.198 | -0.092 | 0.064 | 0.152 |
| InternalIMT | dha | rs7918542 | 0.179 | 0.013 | 0.049 | 0.798 | -0.119 | 0.073 | 0.101 | 0.061 | 0.066 | 0.358 |
| InternalIMT | dha | rs144311920 | 0.002 | . | . | . | . | . | . | . | . | . |
| InternalIMT | dha | rs7099684 | 0.864 | 0.293 | 0.144 | 0.042 | 0.13 | 0.083 | 0.119 | -0.15 | 0.08 | 0.06 |
| InternalIMT | dha | rs150829171 | . | . | . | . | . | . | . | . | . | . |
| InternalIMT | dha | rs186060697 | 0.001 | . | . | . | . | . | . | . | . | . |
| InternalIMT | dha | rs11239513 | 0.054 | 0.046 | 0.046 | 0.317 | 0.098 | 0.123 | 0.425 | -0.095 | 0.093 | 0.308 |
| InternalIMT | dha | rs7917687 | 0.067 | 0.033 | 0.045 | 0.465 | -0.054 | 0.098 | 0.583 | -6.60E-03 | 0.085 | 0.938 |
| InternalIMT | dha | rs61854076 | 0.01 | 0.032 | 0.043 | 0.454 | 0.322 | 0.469 | 0.493 | -0.047 | 0.449 | 0.916 |
| InternalIMT | dha | rs71494780 | 0.029 | 0.032 | 0.043 | 0.465 | -9.90E-03 | 0.145 | 0.946 | 5.90E-03 | 0.147 | 0.968 |
| InternalIMT | dha | rs7919239 | 0.187 | 0.023 | 0.049 | 0.643 | -0.084 | 0.068 | 0.218 | 0.029 | 0.062 | 0.645 |
| InternalIMT | dha | rs78334475 | 0.047 | 0.04 | 0.043 | 0.352 | 0.025 | 0.139 | 0.858 | -0.084 | 0.132 | 0.522 |
| InternalIMT | dha | rs3780900 | 0.02 | 0.021 | 0.041 | 0.612 | -0.241 | 0.523 | 0.645 | 0.279 | 0.535 | 0.603 |
| InternalIMT | dha | rs186729292 | 0.001 | . | . | . | . | . | . | . | . | . |
| InternalIMT | dha | rs12267438 | 0.015 | 0.026 | 0.041 | 0.525 | -0.268 | 0.326 | 0.41 | 0.204 | 0.281 | 0.467 |
| InternalIMT | dha | rs1369214 | 0.61 | 0.16 | 0.083 | 0.055 | 0.127 | 0.066 | 0.056 | -0.102 | 0.059 | 0.087 |
| InternalIMT | dha | rs9282589 | 0.134 | 0.063 | 0.048 | 0.188 | 0.113 | 0.079 | 0.153 | -0.126 | 0.071 | 0.076 |
| InternalIMT | dha | rs114522915 | 0.011 | 0.036 | 0.043 | 0.4 | -0.048 | 0.224 | 0.83 | -0.033 | 0.165 | 0.843 |
| InternalIMT | dha | rs2115819 | 0.542 | 0.144 | 0.069 | 0.036 | 0.139 | 0.063 | 0.027 | -0.1 | 0.056 | 0.072 |
| InternalIMT | dha | rs117664603 | 0.022 | 0.015 | 0.043 | 0.725 | -0.563 | 0.229 | 0.014 | 0.506 | 0.272 | 0.063 |
| InternalIMT | dha | rs184979202 | . | . | . | . | . | . | . | . | . | . |
| InternalIMT | dha | rs190714671 | 0.002 | . | . | . | . | . | . | . | . | . |
| InternalIMT | dha | rs141538996 | . | . | . | . | . | . | . | . | . | . |
| InternalIMT | dha | rs150881741 | . | . | . | . | . | . | . | . | . | . |
| InternalIMT | dha | rs139076633 | . | . | . | . | . | . | . | . | . | . |
| InternalIMT | dha | rs12243649 | 0.054 | 0.011 | 0.042 | 0.791 | -0.328 | 0.138 | 0.018 | 0.231 | 0.132 | 0.08 |
| InternalIMT | dha | rs140348708 | 0.001 | . | . | . | . | . | . | . | . | . |
| InternalIMT | dha | rs79023256 | 0.022 | 0.015 | 0.043 | 0.725 | -0.562 | 0.229 | 0.014 | 0.505 | 0.272 | 0.064 |
| InternalIMT | dha | rs11239515 | 0.081 | 0.027 | 0.046 | 0.561 | -0.085 | 0.096 | 0.375 | 0.023 | 0.089 | 0.796 |
| InternalIMT | dha | rs55866086 | 0.137 | 6.80E-03 | 0.047 | 0.885 | -0.172 | 0.08 | 0.031 | 0.095 | 0.074 | 0.201 |
| InternalIMT | dha | rs181340150 | 0.001 | . | . | . | . | . | . | . | . | . |
| InternalIMT | dha | rs11239516 | 0.097 | 0.017 | 0.045 | 0.711 | -0.131 | 0.097 | 0.175 | 0.075 | 0.09 | 0.404 |
| InternalIMT | dha | rs11239517 | 0.096 | 0.017 | 0.045 | 0.712 | -0.131 | 0.097 | 0.175 | 0.075 | 0.09 | 0.402 |
| InternalIMT | dha | rs11239519 | 0.089 | 0.03 | 0.046 | 0.518 | -0.078 | 0.094 | 0.41 | 0.015 | 0.084 | 0.855 |
| InternalIMT | dha | rs7909514 | 0.721 | 0.197 | 0.106 | 0.064 | 0.103 | 0.071 | 0.144 | -0.109 | 0.066 | 0.1 |
| InternalIMT | dha | rs7894352 | 0.853 | 0.318 | 0.148 | 0.031 | 0.138 | 0.085 | 0.105 | -0.166 | 0.083 | 0.045 |
| InternalIMT | dha | rs189384146 | 0.002 | . | . | . | . | . | . | . | . | . |
| InternalIMT | dha | rs11239520 | 0.088 | 0.023 | 0.045 | 0.612 | -0.103 | 0.102 | 0.309 | 0.05 | 0.097 | 0.603 |
| InternalIMT | dha | rs12221233 | 0.482 | 0.146 | 0.069 | 0.034 | 0.156 | 0.064 | 0.015 | -0.113 | 0.059 | 0.055 |
| InternalIMT | dha | rs74523293 | 0.038 | 0.019 | 0.044 | 0.658 | -0.326 | 0.118 | 6.00E-03 | 0.203 | 0.115 | 0.078 |
| InternalIMT | dha | rs79376040 | 0.038 | 0.019 | 0.044 | 0.658 | -0.326 | 0.118 | 6.00E-03 | 0.203 | 0.115 | 0.079 |
| InternalIMT | dha | rs4949000 | 0.482 | 0.147 | 0.069 | 0.033 | 0.157 | 0.064 | 0.015 | -0.114 | 0.059 | 0.052 |
| InternalIMT | dha | rs7089406 | 0.023 | 0.03 | 0.042 | 0.476 | -0.044 | 0.195 | 0.823 | 0.046 | 0.188 | 0.806 |
| InternalIMT | dha | rs138211056 | 0.001 | . | . | . | . | . | . | . | . | . |
| InternalIMT | dha | rs10900213 | 0.586 | 0.146 | 0.073 | 0.048 | 0.12 | 0.061 | 0.051 | -0.092 | 0.057 | 0.108 |
| InternalIMT | dha | rs145152827 | . | . | . | . | . | . | . | . | . | . |
| InternalIMT | dha | rs74443325 | 0.022 | 0.016 | 0.043 | 0.711 | -0.558 | 0.237 | 0.019 | 0.492 | 0.282 | 0.081 |
| InternalIMT | dha | rs188299446 | 0.011 | 0.016 | 0.043 | 0.703 | -0.557 | 0.414 | 0.179 | 0.867 | 0.476 | 0.068 |
| InternalIMT | dha | rs192699586 | 0.005 | . | . | . | . | . | . | . | . | . |
| InternalIMT | dha | rs72796464 | 0.076 | 0.023 | 0.046 | 0.613 | -0.103 | 0.099 | 0.299 | 0.048 | 0.092 | 0.602 |
| InternalIMT | dha | rs4949001 | 0.366 | 0.092 | 0.06 | 0.126 | 0.102 | 0.065 | 0.118 | -0.077 | 0.06 | 0.195 |
| InternalIMT | dha | rs147174641 | 0.005 | . | . | . | . | . | . | . | . | . |
| InternalIMT | dha | rs190835238 | 0.001 | . | . | . | . | . | . | . | . | . |
| InternalIMT | dha | rs76698944 | 0.017 | 0.042 | 0.042 | 0.315 | 0.293 | 0.501 | 0.558 | -0.281 | 0.364 | 0.44 |
| InternalIMT | dha | rs56071381 | 0.092 | 0.025 | 0.045 | 0.582 | -0.095 | 0.099 | 0.336 | 0.04 | 0.093 | 0.667 |
| InternalIMT | dha | rs78130427 | 0.023 | 0.027 | 0.043 | 0.526 | -0.262 | 0.192 | 0.173 | 0.15 | 0.169 | 0.374 |
| InternalIMT | dha | rs10900215 | 0.118 | 0.013 | 0.046 | 0.781 | -0.103 | 0.108 | 0.343 | 0.077 | 0.105 | 0.464 |
| InternalIMT | dha | rs41491445 | 0.01 | 0.031 | 0.042 | 0.455 | 0.252 | 0.412 | 0.541 | -0.105 | 0.359 | 0.771 |
| InternalIMT | dha | rs144995222 | 0.002 | . | . | . | . | . | . | . | . | . |
| InternalIMT | dha | rs149097803 | 0.002 | . | . | . | . | . | . | . | . | . |
| InternalIMT | dha | rs139429646 | 0.005 | . | . | . | . | . | . | . | . | . |
| InternalIMT | dha | rs7075620 | 0.675 | 0.154 | 0.096 | 0.108 | 0.08 | 0.066 | 0.225 | -0.085 | 0.062 | 0.171 |
| InternalIMT | dha | rs148299213 | 0.01 | 0.031 | 0.042 | 0.455 | 0.254 | 0.416 | 0.542 | -0.106 | 0.363 | 0.771 |
| InternalIMT | dha | rs4949002 | 0.313 | 0.079 | 0.059 | 0.183 | 0.129 | 0.074 | 0.084 | -0.067 | 0.067 | 0.318 |
| InternalIMT | dha | rs150496007 | 0.036 | 0.05 | 0.045 | 0.263 | 0.204 | 0.177 | 0.248 | -0.249 | 0.144 | 0.083 |
| InternalIMT | dha | rs147303455 | 0.008 | . | . | . | . | . | . | . | . | . |
| InternalIMT | dha | rs189518433 | . | . | . | . | . | . | . | . | . | . |
| InternalIMT | dha | rs186281355 | . | . | . | . | . | . | . | . | . | . |
| InternalIMT | dha | rs115297114 | 0.014 | 0.036 | 0.043 | 0.399 | -0.035 | 0.214 | 0.868 | -0.038 | 0.149 | 0.798 |
| InternalIMT | dha | rs11239523 | 0.087 | 0.021 | 0.044 | 0.638 | -0.106 | 0.1 | 0.288 | 0.059 | 0.095 | 0.537 |
| InternalIMT | dha | rs143704897 | 0.005 | . | . | . | . | . | . | . | . | . |
| InternalIMT | dha | rs188024096 | 0.002 | . | . | . | . | . | . | . | . | . |
| InternalIMT | dha | rs11239524 | 0.087 | 0.024 | 0.045 | 0.596 | -0.12 | 0.103 | 0.246 | 0.05 | 0.098 | 0.613 |
| InternalIMT | dha | rs191656573 | 0.004 | . | . | . | . | . | . | . | . | . |
| InternalIMT | dha | rs193295875 | . | . | . | . | . | . | . | . | . | . |
| InternalIMT | dha | rs190458228 | . | . | . | . | . | . | . | . | . | . |
| InternalIMT | dha | rs139253993 | 0.002 | . | . | . | . | . | . | . | . | . |
| InternalIMT | dha | rs4948672 | 0.604 | 0.097 | 0.093 | 0.297 | 0.065 | 0.074 | 0.382 | -0.054 | 0.07 | 0.438 |
| InternalIMT | dha | rs180954921 | 0.002 | . | . | . | . | . | . | . | . | . |
| InternalIMT | dha | rs12264801 | 0.447 | -0.066 | 0.069 | 0.343 | -0.125 | 0.07 | 0.074 | 0.107 | 0.066 | 0.104 |
| InternalIMT | dha | rs73283184 | 0.014 | 0.032 | 0.042 | 0.441 | 0.22 | 0.43 | 0.609 | -0.136 | 0.346 | 0.696 |

| InternalIMT | dha | rs142746187 | 0.007 | . | . | . | . | . | . | . | . | . |
| --- | --- | --- | --- | --- | --- | --- | --- | --- | --- | --- | --- | --- |
| InternalIMT | dha | rs58704739 | 0.014 | 0.033 | 0.042 | 0.428 | 0.26 | 0.416 | 0.531 | -0.172 | 0.338 | 0.61 |
| InternalIMT | dha | rs61272335 | 0.011 | 0.031 | 0.042 | 0.459 | 0.315 | 0.429 | 0.463 | -0.15 | 0.363 | 0.679 |
| InternalIMT | dha | rs60959489 | 0.021 | 0.042 | 0.043 | 0.32 | 0.332 | 0.251 | 0.187 | -0.282 | 0.193 | 0.145 |
| InternalIMT | dha | rs58502629 | 0.011 | 0.031 | 0.042 | 0.459 | 0.315 | 0.428 | 0.462 | -0.15 | 0.362 | 0.678 |
| InternalIMT | dha | rs10900216 | 0.739 | 0.109 | 0.1 | 0.275 | 0.043 | 0.066 | 0.519 | -0.051 | 0.062 | 0.405 |
| InternalIMT | dha | rs181118141 | 0.001 | . | . | . | . | . | . | . | . | . |
| InternalIMT | dha | rs137882665 | 0.01 | 0.043 | 0.042 | 0.31 | 0.278 | 0.336 | 0.409 | -0.336 | 0.267 | 0.208 |
| InternalIMT | dha | rs149466736 | 0.004 | . | . | . | . | . | . | . | . | . |
| InternalIMT | dha | rs2099171 | 0.675 | 0.058 | 0.085 | 0.496 | 0.015 | 0.06 | 0.801 | -0.02 | 0.054 | 0.712 |
| InternalIMT | dha | rs141852280 | 0.005 | . | . | . | . | . | . | . | . | . |
| InternalIMT | dha | rs191539257 | . | . | . | . | . | . | . | . | . | . |
| InternalIMT | dha | rs17157771 | 0.027 | 0.031 | 0.041 | 0.45 | -0.017 | 0.242 | 0.945 | 0.014 | 0.221 | 0.948 |
| InternalIMT | dha | rs111339241 | 0.025 | 0.03 | 0.041 | 0.47 | -0.071 | 0.256 | 0.782 | 0.051 | 0.238 | 0.829 |
| InternalIMT | dha | rs116983255 | 0.006 | . | . | . | . | . | . | . | . | . |
| InternalIMT | dha | rs142324341 | . | . | . | . | . | . | . | . | . | . |
| InternalIMT | dha | rs187191219 | 0.007 | . | . | . | . | . | . | . | . | . |
| InternalIMT | dha | rs151283973 | 0 | . | . | . | . | . | . | . | . | . |
| InternalIMT | dha | rs139316218 | 0.003 | . | . | . | . | . | . | . | . | . |
| InternalIMT | dha | rs6593485 | 0.748 | 0.01 | 0.1 | 0.919 | -0.036 | 0.068 | 0.602 | 0.013 | 0.061 | 0.831 |
| InternalIMT | dha | rs6593486 | 0.751 | 3.10E-03 | 0.1 | 0.975 | -0.042 | 0.068 | 0.54 | 0.018 | 0.061 | 0.773 |
| InternalIMT | dha | rs145196618 | 0.011 | 0.036 | 0.042 | 0.386 | 0.246 | 0.381 | 0.518 | -0.221 | 0.28 | 0.429 |
| InternalIMT | dha | rs142598335 | . | . | . | . | . | . | . | . | . | . |
| InternalIMT | dha | rs892691 | 0.746 | 8.90E-03 | 0.094 | 0.925 | -0.03 | 0.066 | 0.648 | 0.014 | 0.059 | 0.808 |
| InternalIMT | dha | rs7080713 | 0.689 | 0.061 | 0.087 | 0.483 | 0.021 | 0.06 | 0.725 | -0.021 | 0.055 | 0.703 |
| InternalIMT | dha | rs7077173 | 0.068 | 0.036 | 0.043 | 0.409 | -0.085 | 0.107 | 0.428 | 0.018 | 0.088 | 0.84 |
| InternalIMT | dha | rs7080977 | 0.04 | 0.031 | 0.043 | 0.461 | -0.119 | 0.173 | 0.49 | 0.056 | 0.144 | 0.697 |
| InternalIMT | dha | rs3780901 | 0.689 | 0.066 | 0.087 | 0.447 | 0.028 | 0.06 | 0.649 | -0.024 | 0.055 | 0.656 |
| InternalIMT | dha | rs61854091 | 0.046 | 0.041 | 0.045 | 0.36 | 0.173 | 0.2 | 0.386 | -0.121 | 0.174 | 0.488 |
| InternalIMT | dha | rs77550209 | 0.003 | . | . | . | . | . | . | . | . | . |
| InternalIMT | dha | rs73283191 | 0.062 | 0.015 | 0.043 | 0.722 | -0.257 | 0.127 | 0.043 | 0.159 | 0.115 | 0.166 |
| InternalIMT | dha | rs191866099 | . | . | . | . | . | . | . | . | . | . |
| InternalIMT | dha | rs147998395 | 0 | . | . | . | . | . | . | . | . | . |
| InternalIMT | dha | rs7090328 | 0.789 | 0.123 | 0.12 | 0.306 | 0.028 | 0.076 | 0.715 | -0.056 | 0.07 | 0.423 |
| InternalIMT | dha | rs79814535 | 0.021 | 0.018 | 0.042 | 0.676 | -0.488 | 0.209 | 0.02 | 0.395 | 0.245 | 0.107 |
| InternalIMT | dha | rs7073082 | 0.789 | 0.123 | 0.12 | 0.307 | 0.028 | 0.076 | 0.716 | -0.056 | 0.07 | 0.424 |
| InternalIMT | dha | rs11239526 | 0.033 | 0.029 | 0.044 | 0.512 | 0.069 | 0.207 | 0.738 | 0.03 | 0.172 | 0.863 |
| InternalIMT | dha | rs141839841 | 0 | . | . | . | . | . | . | . | . | . |
| InternalIMT | dha | rs75697371 | 0.006 | . | . | . | . | . | . | . | . | . |
| InternalIMT | dha | rs75972854 | 0.005 | . | . | . | . | . | . | . | . | . |
| InternalIMT | dha | rs139291975 | . | . | . | . | . | . | . | . | . | . |
| InternalIMT | dha | rs144064711 | 0.004 | . | . | . | . | . | . | . | . | . |
| InternalIMT | dha | rs3780902 | 0.062 | 0.017 | 0.043 | 0.691 | -0.239 | 0.125 | 0.057 | 0.145 | 0.113 | 0.199 |
| InternalIMT | dha | rs190449801 | 0.001 | . | . | . | . | . | . | . | . | . |
| InternalIMT | dha | rs73283196 | 0.061 | 0.016 | 0.043 | 0.717 | -0.256 | 0.127 | 0.044 | 0.159 | 0.115 | 0.169 |
| InternalIMT | dha | rs28395867 | 0.031 | 0.047 | 0.043 | 0.275 | 0.176 | 0.207 | 0.396 | -0.18 | 0.15 | 0.229 |
| InternalIMT | dha | rs28395866 | 0.079 | 0.056 | 0.044 | 0.2 | 0.058 | 0.121 | 0.633 | -0.088 | 0.093 | 0.343 |
| InternalIMT | dha | rs55661611 | 0.04 | 0.031 | 0.043 | 0.468 | -0.136 | 0.176 | 0.438 | 0.067 | 0.146 | 0.647 |
| InternalIMT | dha | rs55684518 | 0.016 | 0.022 | 0.043 | 0.609 | -0.51 | 0.346 | 0.141 | 0.475 | 0.354 | 0.18 |
| InternalIMT | dha | rs78552493 | 0.038 | 0.056 | 0.043 | 0.196 | 0.292 | 0.186 | 0.117 | -0.301 | 0.139 | 0.031 |
| InternalIMT | dha | rs147665434 | 0.001 | . | . | . | . | . | . | . | . | . |
| InternalIMT | dha | rs34445001 | 0.006 | . | . | . | . | . | . | . | . | . |
| InternalIMT | dha | rs184019449 | 0.003 | . | . | . | . | . | . | . | . | . |
| InternalIMT | dha | rs3780903 | 0.731 | 0.14 | 0.099 | 0.158 | 0.081 | 0.066 | 0.221 | -0.074 | 0.062 | 0.232 |
| InternalIMT | dha | rs17157784 | 0.058 | 0.04 | 0.044 | 0.364 | -0.029 | 0.12 | 0.81 | -0.043 | 0.099 | 0.665 |
| InternalIMT | dha | rs3780904 | 0.021 | 0.017 | 0.042 | 0.685 | -0.539 | 0.213 | 0.011 | 0.444 | 0.247 | 0.072 |
| InternalIMT | dha | rs192064730 | . | . | . | . | . | . | . | . | . | . |
| InternalIMT | dha | rs12774534 | 0.006 | . | . | . | . | . | . | . | . | . |
| InternalIMT | dha | rs79179915 | 0.02 | 0.017 | 0.042 | 0.693 | -0.564 | 0.233 | 0.016 | 0.475 | 0.275 | 0.084 |
| InternalIMT | dha | rs117811571 | 0.011 | 0.017 | 0.043 | 0.7 | -0.558 | 0.405 | 0.169 | 0.848 | 0.47 | 0.072 |
| InternalIMT | dha | rs72796477 | 0.009 | . | . | . | . | . | . | . | . | . |
| InternalIMT | dha | rs55729342 | 0.058 | 0.041 | 0.044 | 0.353 | -0.013 | 0.121 | 0.913 | -0.051 | 0.1 | 0.612 |
| InternalIMT | dha | rs59317220 | 0.058 | 0.041 | 0.044 | 0.352 | -0.013 | 0.121 | 0.916 | -0.051 | 0.1 | 0.608 |
| InternalIMT | dha | rs3780905 | 0.057 | 0.028 | 0.043 | 0.508 | -0.101 | 0.132 | 0.446 | 0.024 | 0.126 | 0.848 |
| InternalIMT | dha | rs3780906 | 0.731 | 0.141 | 0.099 | 0.156 | 0.083 | 0.066 | 0.214 | -0.074 | 0.062 | 0.23 |
| InternalIMT | dha | rs149047975 | 0.125 | 0.057 | 0.047 | 0.225 | 0.103 | 0.082 | 0.212 | -0.115 | 0.073 | 0.118 |
| InternalIMT | dha | rs3824614 | 0.02 | 0.016 | 0.042 | 0.697 | -0.57 | 0.234 | 0.015 | 0.479 | 0.276 | 0.083 |
| InternalIMT | dha | rs140254404 | . | . | . | . | . | . | . | . | . | . |
| InternalIMT | dha | rs145262684 | 0.013 | 0.016 | 0.043 | 0.701 | -0.454 | 0.347 | 0.191 | 0.66 | 0.373 | 0.077 |
| InternalIMT | dha | rs76735482 | 0.02 | 0.016 | 0.042 | 0.7 | -0.572 | 0.234 | 0.015 | 0.48 | 0.277 | 0.083 |
| InternalIMT | dha | rs73283198 | 0.027 | 0.03 | 0.041 | 0.471 | -0.129 | 0.264 | 0.626 | 0.074 | 0.238 | 0.756 |
| InternalIMT | dha | rs74911239 | 0.029 | 0.011 | 0.044 | 0.803 | -0.066 | 0.254 | 0.795 | 0.332 | 0.221 | 0.134 |
| InternalIMT | dha | rs150551647 | 0.005 | . | . | . | . | . | . | . | . | . |
| InternalIMT | dha | rs115200794 | 0.034 | 0.049 | 0.043 | 0.258 | 0.196 | 0.204 | 0.339 | -0.192 | 0.147 | 0.191 |
| InternalIMT | dha | rs74130823 | 0.139 | 0.045 | 0.05 | 0.363 | -0.056 | 0.081 | 0.488 | -0.01 | 0.066 | 0.878 |
| InternalIMT | dha | rs2279435 | 0.662 | 0.227 | 0.092 | 0.013 | 0.181 | 0.068 | 7.70E-03 | -0.147 | 0.062 | 0.019 |
| InternalIMT | dha | rs147279673 | 0.015 | 8.50E-03 | 0.044 | 0.845 | -0.745 | 0.403 | 0.065 | 0.821 | 0.376 | 0.029 |
| InternalIMT | dha | rs183525146 | 0.004 | . | . | . | . | . | . | . | . | . |
| InternalIMT | dha | rs3740107 | 0.754 | 0.162 | 0.113 | 0.153 | 0.069 | 0.072 | 0.34 | -0.086 | 0.07 | 0.219 |
| InternalIMT | dha | rs140697247 | . | . | . | . | . | . | . | . | . | . |
| InternalIMT | dha | rs1565096 | 0.825 | 0.26 | 0.139 | 0.062 | 0.104 | 0.081 | 0.199 | -0.135 | 0.08 | 0.093 |
| InternalIMT | dha | rs1565097 | 0.793 | 0.127 | 0.119 | 0.285 | 0.037 | 0.075 | 0.616 | -0.06 | 0.071 | 0.401 |
| InternalIMT | dha | rs144584548 | 0.016 | 0.022 | 0.043 | 0.605 | -0.489 | 0.346 | 0.158 | 0.456 | 0.353 | 0.197 |
| InternalIMT | dha | rs75811053 | 0.02 | 0.016 | 0.042 | 0.7 | -0.563 | 0.233 | 0.016 | 0.468 | 0.277 | 0.091 |
| InternalIMT | dha | rs147462967 | 0.009 | . | . | . | . | . | . | . | . | . |
| InternalIMT | dha | rs74130824 | 0.051 | 0.033 | 0.044 | 0.454 | -0.109 | 0.127 | 0.39 | 0.013 | 0.109 | 0.902 |

| InternalIMT | dha | rs116883184 | 0.017 | 0.01 | 0.043 | 0.816 | -0.559 | 0.31 | 0.072 | 0.684 | 0.317 | 0.031 |
| --- | --- | --- | --- | --- | --- | --- | --- | --- | --- | --- | --- | --- |
| InternalIMT | dha | rs117258049 | 0.017 | 0.01 | 0.043 | 0.816 | -0.559 | 0.31 | 0.072 | 0.684 | 0.317 | 0.031 |
| InternalIMT | dha | rs3780908 | 0.107 | 9.20E-03 | 0.046 | 0.842 | -0.216 | 0.089 | 0.016 | 0.117 | 0.077 | 0.129 |
| InternalIMT | dha | rs180938522 | 0.002 | . | . | . | . | . | . | . | . | . |
| InternalIMT | dha | rs76687372 | 0.003 | . | . | . | . | . | . | . | . | . |
| InternalIMT | dha | rs3780909 | 0.606 | 0.159 | 0.082 | 0.052 | 0.126 | 0.063 | 0.048 | -0.107 | 0.059 | 0.069 |
| InternalIMT | dha | rs78281156 | 0.02 | 0.016 | 0.042 | 0.699 | -0.56 | 0.232 | 0.016 | 0.465 | 0.276 | 0.092 |
| InternalIMT | dha | rs187840631 | . | . | . | . | . | . | . | . | . | . |
| InternalIMT | dha | rs76868242 | 0.02 | 0.016 | 0.042 | 0.699 | -0.56 | 0.232 | 0.016 | 0.464 | 0.276 | 0.093 |
| InternalIMT | dha | rs192794192 | 0.001 | . | . | . | . | . | . | . | . | . |
| InternalIMT | dha | rs12781248 | 0.007 | . | . | . | . | . | . | . | . | . |
| InternalIMT | dha | rs12762375 | 0.006 | . | . | . | . | . | . | . | . | . |
| InternalIMT | dha | rs192256986 | 0.004 | . | . | . | . | . | . | . | . | . |
| InternalIMT | dha | rs184411030 | 0.01 | . | . | . | . | . | . | . | . | . |
| InternalIMT | dha | rs189601483 | 0.02 | 0.016 | 0.042 | 0.699 | -0.559 | 0.232 | 0.016 | 0.463 | 0.276 | 0.093 |
| InternalIMT | dha | rs143254579 | 0.033 | 0.028 | 0.043 | 0.52 | 0.062 | 0.202 | 0.76 | 0.031 | 0.166 | 0.851 |
| InternalIMT | dha | rs12253903 | 0.017 | 0.032 | 0.042 | 0.449 | 0.371 | 0.284 | 0.192 | -0.203 | 0.249 | 0.414 |
| InternalIMT | dha | rs35489926 | 0.006 | . | . | . | . | . | . | . | . | . |
| InternalIMT | dha | rs71494783 | 0.039 | 8.60E-03 | 0.042 | 0.838 | -0.354 | 0.181 | 0.05 | 0.296 | 0.195 | 0.13 |
| InternalIMT | dha | rs180711602 | 0.002 | . | . | . | . | . | . | . | . | . |
| InternalIMT | dha | rs117312402 | 0.02 | 0.016 | 0.042 | 0.699 | -0.56 | 0.232 | 0.016 | 0.464 | 0.276 | 0.093 |
| InternalIMT | dha | rs74831694 | 0.004 | . | . | . | . | . | . | . | . | . |
| InternalIMT | dha | rs140301943 | 0.002 | . | . | . | . | . | . | . | . | . |
| InternalIMT | dha | rs78553679 | 0.013 | 0.035 | 0.043 | 0.413 | 0.274 | 0.542 | 0.613 | -0.197 | 0.446 | 0.659 |
| InternalIMT | dha | rs78334250 | 0.02 | 0.016 | 0.042 | 0.699 | -0.56 | 0.232 | 0.016 | 0.464 | 0.276 | 0.093 |
| InternalIMT | dha | rs76000028 | 0.02 | 0.016 | 0.042 | 0.699 | -0.56 | 0.232 | 0.016 | 0.464 | 0.276 | 0.093 |
| InternalIMT | dha | rs145195254 | 0.001 | . | . | . | . | . | . | . | . | . |
| InternalIMT | dha | rs137888527 | 0.001 | . | . | . | . | . | . | . | . | . |
| InternalIMT | dha | rs149109592 | 0.005 | . | . | . | . | . | . | . | . | . |
| InternalIMT | dha | rs80280010 | 0.032 | 0.029 | 0.043 | 0.507 | 0.071 | 0.203 | 0.725 | 0.022 | 0.167 | 0.895 |
| InternalIMT | dha | rs189995374 | . | . | . | . | . | . | . | . | . | . |
| InternalIMT | dha | rs75467770 | 0.021 | 0.016 | 0.042 | 0.708 | -0.568 | 0.233 | 0.015 | 0.474 | 0.277 | 0.087 |
| InternalIMT | dha | rs1487562 | 0.139 | 0.032 | 0.047 | 0.5 | -0.131 | 0.08 | 0.103 | 0.036 | 0.068 | 0.597 |
| InternalIMT | dha | rs12266963 | 0.039 | 8.60E-03 | 0.042 | 0.838 | -0.356 | 0.181 | 0.049 | 0.296 | 0.195 | 0.129 |
| InternalIMT | dha | rs7099874 | 0.787 | 0.203 | 0.125 | 0.105 | 0.063 | 0.078 | 0.415 | -0.107 | 0.076 | 0.157 |
| InternalIMT | dha | rs75687019 | 0.02 | 0.016 | 0.042 | 0.699 | -0.56 | 0.232 | 0.016 | 0.463 | 0.276 | 0.093 |
| InternalIMT | dha | rs148240462 | 0.031 | 0.014 | 0.044 | 0.743 | -0.012 | 0.25 | 0.962 | 0.263 | 0.219 | 0.23 |
| InternalIMT | dha | rs141278174 | . | . | . | . | . | . | . | . | . | . |
| InternalIMT | dha | rs76404852 | 0.02 | 0.016 | 0.042 | 0.699 | -0.56 | 0.232 | 0.016 | 0.463 | 0.276 | 0.093 |
| InternalIMT | dha | rs139112240 | 0.006 | . | . | . | . | . | . | . | . | . |
| InternalIMT | dha | rs12783037 | 0.042 | 6.50E-03 | 0.042 | 0.877 | -0.362 | 0.18 | 0.044 | 0.325 | 0.193 | 0.093 |
| InternalIMT | dha | rs72796481 | 0.006 | . | . | . | . | . | . | . | . | . |
| InternalIMT | dha | rs76798749 | 0.023 | 0.015 | 0.042 | 0.728 | -0.626 | 0.244 | 0.01 | 0.576 | 0.293 | 0.049 |
| InternalIMT | dha | rs187732548 | . | . | . | . | . | . | . | . | . | . |
| InternalIMT | dha | rs75675474 | 0.023 | 0.015 | 0.042 | 0.728 | -0.626 | 0.244 | 0.01 | 0.576 | 0.293 | 0.049 |
| InternalIMT | dha | rs76552293 | 0.023 | 0.015 | 0.042 | 0.728 | -0.626 | 0.244 | 0.01 | 0.575 | 0.292 | 0.049 |
| InternalIMT | dha | rs147445993 | 0.002 | . | . | . | . | . | . | . | . | . |
| InternalIMT | dha | rs115902283 | 0.023 | 0.015 | 0.042 | 0.727 | -0.621 | 0.242 | 0.011 | 0.568 | 0.291 | 0.051 |
| InternalIMT | dha | rs4540799 | 0.786 | 0.202 | 0.125 | 0.106 | 0.063 | 0.078 | 0.416 | -0.107 | 0.075 | 0.158 |
| InternalIMT | dha | rs12765320 | 0.172 | -6.10E-04 | 0.049 | 0.99 | -0.198 | 0.074 | 7.70E-03 | 0.103 | 0.065 | 0.112 |
| InternalIMT | dha | rs12782256 | 0.006 | . | . | . | . | . | . | . | . | . |
| InternalIMT | dha | rs191727256 | 0 | . | . | . | . | . | . | . | . | . |
| InternalIMT | dha | rs2082111 | 0.111 | 0.012 | 0.045 | 0.796 | -0.196 | 0.091 | 0.031 | 0.101 | 0.079 | 0.201 |
| InternalIMT | dha | rs2082110 | 0.157 | -2.60E-04 | 0.048 | 0.996 | -0.217 | 0.076 | 4.30E-03 | 0.114 | 0.066 | 0.084 |
| InternalIMT | dha | rs2082109 | 0.157 | -2.70E-04 | 0.048 | 0.996 | -0.217 | 0.076 | 4.30E-03 | 0.114 | 0.066 | 0.084 |
| InternalIMT | dha | rs117316662 | 0.02 | 0.016 | 0.042 | 0.699 | -0.561 | 0.232 | 0.016 | 0.464 | 0.276 | 0.092 |
| InternalIMT | dha | rs12247163 | 0.177 | -4.10E-03 | 0.049 | 0.933 | -0.2 | 0.081 | 0.014 | 0.112 | 0.075 | 0.137 |
| InternalIMT | dha | rs12355888 | 0.075 | 0.023 | 0.046 | 0.62 | -0.134 | 0.107 | 0.209 | 0.057 | 0.095 | 0.549 |
| InternalIMT | dha | rs143772290 | 0.005 | . | . | . | . | . | . | . | . | . |
| InternalIMT | dha | rs146849452 | 0.003 | . | . | . | . | . | . | . | . | . |
| InternalIMT | dha | rs140267121 | 0.003 | . | . | . | . | . | . | . | . | . |
| InternalIMT | dha | rs7080474 | 0.155 | 0.016 | 0.048 | 0.734 | -0.141 | 0.085 | 0.098 | 0.069 | 0.078 | 0.378 |
| InternalIMT | dha | rs144066203 | 0.001 | . | . | . | . | . | . | . | . | . |
| InternalIMT | dha | rs12765209 | 0.021 | 0.017 | 0.042 | 0.697 | -0.55 | 0.231 | 0.017 | 0.452 | 0.274 | 0.1 |
| InternalIMT | dha | rs150930735 | 0.001 | . | . | . | . | . | . | . | . | . |
| InternalIMT | dha | rs7068039 | 0.177 | -2.90E-03 | 0.049 | 0.953 | -0.198 | 0.081 | 0.014 | 0.109 | 0.075 | 0.145 |
| InternalIMT | dha | rs117622652 | 0.02 | 0.016 | 0.042 | 0.7 | -0.561 | 0.232 | 0.016 | 0.465 | 0.276 | 0.092 |
| InternalIMT | dha | rs190191514 | 0.001 | . | . | . | . | . | . | . | . | . |
| InternalIMT | dha | rs74130835 | 0.023 | 0.042 | 0.043 | 0.329 | 0.018 | 0.152 | 0.908 | -0.102 | 0.117 | 0.382 |
| InternalIMT | dha | rs1811604 | 0.022 | 0.042 | 0.043 | 0.331 | -0.011 | 0.153 | 0.941 | -0.087 | 0.114 | 0.446 |
| InternalIMT | dha | rs146971256 | . | . | . | . | . | . | . | . | . | . |
| InternalIMT | dha | rs74336000 | 0.005 | . | . | . | . | . | . | . | . | . |
| InternalIMT | dha | rs186046492 | 0.005 | . | . | . | . | . | . | . | . | . |
| InternalIMT | dha | rs34971823 | 0.006 | . | . | . | . | . | . | . | . | . |
| InternalIMT | dha | rs75227446 | 0.02 | 0.016 | 0.042 | 0.704 | -0.57 | 0.233 | 0.015 | 0.476 | 0.277 | 0.086 |
| InternalIMT | dha | rs190457599 | 0.003 | . | . | . | . | . | . | . | . | . |
| InternalIMT | dha | rs79591917 | 0.02 | 0.016 | 0.042 | 0.7 | -0.562 | 0.232 | 0.016 | 0.466 | 0.276 | 0.091 |
| InternalIMT | dha | rs702365 | 0.609 | 0.182 | 0.084 | 0.03 | 0.144 | 0.064 | 0.024 | -0.125 | 0.06 | 0.037 |
| InternalIMT | dha | rs79643836 | 0.021 | 0.016 | 0.042 | 0.701 | -0.542 | 0.229 | 0.018 | 0.451 | 0.273 | 0.099 |
| InternalIMT | dha | rs702366 | 0.61 | 0.17 | 0.084 | 0.042 | 0.137 | 0.064 | 0.032 | -0.115 | 0.06 | 0.055 |
| InternalIMT | dha | rs1059696 | 0.124 | 3.70E-03 | 0.047 | 0.938 | -0.213 | 0.1 | 0.034 | 0.123 | 0.094 | 0.193 |
| InternalIMT | dha | rs901681 | 0.61 | 0.17 | 0.084 | 0.043 | 0.138 | 0.064 | 0.031 | -0.115 | 0.06 | 0.056 |
| InternalIMT | dha | rs61854092 | 0.122 | 3.30E-03 | 0.047 | 0.944 | -0.215 | 0.1 | 0.032 | 0.125 | 0.094 | 0.186 |
| InternalIMT | dha | rs148080753 | 0.001 | . | . | . | . | . | . | . | . | . |
| InternalIMT | dha | rs61854093 | 0.145 | 0.013 | 0.048 | 0.783 | -0.179 | 0.09 | 0.046 | 0.088 | 0.082 | 0.287 |
| InternalIMT | dha | rs141822774 | 0.002 | . | . | . | . | . | . | . | . | . |

| InternalIMT | dha | rs74130836 | 0.023 | 0.042 | 0.043 | 0.33 | 0.015 | 0.152 | 0.923 | -0.1 | 0.116 | 0.39 |
| --- | --- | --- | --- | --- | --- | --- | --- | --- | --- | --- | --- | --- |
| InternalIMT | dha | rs150620963 | 0.01 | . | . | . | . | . | . | . | . | . |
| InternalIMT | dha | rs3780913 | 0.781 | 0.178 | 0.119 | 0.134 | 0.046 | 0.075 | 0.545 | -0.093 | 0.073 | 0.202 |
| InternalIMT | dha | rs142255723 | 0.002 | . | . | . | . | . | . | . | . | . |
| InternalIMT | dha | rs3780914 | 0.607 | 0.167 | 0.082 | 0.041 | 0.133 | 0.062 | 0.033 | -0.114 | 0.058 | 0.052 |
| InternalIMT | dha | rs182656507 | 0.002 | . | . | . | . | . | . | . | . | . |
| InternalIMT | dha | rs2291427 | 0.756 | 0.252 | 0.114 | 0.028 | 0.108 | 0.073 | 0.137 | -0.142 | 0.071 | 0.046 |
| InternalIMT | dha | rs10751382 | 0.756 | 0.25 | 0.114 | 0.029 | 0.107 | 0.072 | 0.139 | -0.141 | 0.071 | 0.047 |
| InternalIMT | dha | rs117610108 | 0.02 | 0.016 | 0.042 | 0.701 | -0.566 | 0.233 | 0.015 | 0.471 | 0.277 | 0.089 |
| InternalIMT | dha | rs144966941 | 0.037 | 0.02 | 0.042 | 0.639 | -0.113 | 0.199 | 0.57 | 0.233 | 0.215 | 0.279 |
| InternalIMT | dha | rs41502051 | 0.022 | 0.035 | 0.043 | 0.419 | 0.037 | 0.271 | 0.891 | -0.083 | 0.234 | 0.722 |
| InternalIMT | dha | rs41426046 | 0.02 | 0.016 | 0.042 | 0.701 | -0.566 | 0.233 | 0.015 | 0.471 | 0.277 | 0.089 |
| InternalIMT | dha | rs41519248 | 0.02 | 0.016 | 0.042 | 0.702 | -0.568 | 0.233 | 0.015 | 0.474 | 0.277 | 0.088 |
| InternalIMT | dha | rs189108245 | 0.002 | . | . | . | . | . | . | . | . | . |
| InternalIMT | dha | rs7896431 | 0.591 | 0.167 | 0.081 | 0.04 | 0.132 | 0.065 | 0.042 | -0.117 | 0.06 | 0.053 |
| InternalIMT | dha | rs183724547 | 0.003 | . | . | . | . | . | . | . | . | . |
| InternalIMT | dha | rs142044990 | 0.006 | . | . | . | . | . | . | . | . | . |
| InternalIMT | dha | rs188140929 | . | . | . | . | . | . | . | . | . | . |
| InternalIMT | dha | rs76819096 | 0.008 | . | . | . | . | . | . | . | . | . |
| InternalIMT | dha | rs10751383 | 0.654 | 0.189 | 0.089 | 0.034 | 0.153 | 0.065 | 0.018 | -0.12 | 0.061 | 0.047 |
| InternalIMT | dha | rs117819321 | 0.016 | 0.045 | 0.043 | 0.298 | 0.313 | 0.278 | 0.261 | -0.436 | 0.206 | 0.035 |
| InternalIMT | dha | rs2242332 | 0.75 | 0.109 | 0.105 | 0.3 | 0.012 | 0.07 | 0.863 | -0.054 | 0.064 | 0.4 |
| InternalIMT | dha | rs2242333 | 0.06 | 0.03 | 0.043 | 0.487 | -0.075 | 0.126 | 0.55 | 0.011 | 0.121 | 0.928 |
| InternalIMT | dha | rs2242334 | 0.752 | 0.114 | 0.107 | 0.288 | 0.012 | 0.07 | 0.865 | -0.057 | 0.065 | 0.381 |
| InternalIMT | dha | rs189465041 | 0.002 | . | . | . | . | . | . | . | . | . |
| InternalIMT | dha | rs28395868 | 0.014 | 0.026 | 0.043 | 0.547 | -0.318 | 0.241 | 0.187 | 0.311 | 0.24 | 0.195 |
| InternalIMT | dha | rs56067550 | 0.016 | 0.022 | 0.043 | 0.602 | -0.497 | 0.357 | 0.164 | 0.459 | 0.361 | 0.204 |
| InternalIMT | dha | rs1051713 | 0.134 | -0.018 | 0.047 | 0.697 | -0.285 | 0.095 | 2.70E-03 | 0.183 | 0.09 | 0.043 |
| InternalIMT | dha | rs115074210 | 0.009 | . | . | . | . | . | . | . | . | . |
| InternalIMT | dha | rs7900977 | 0.822 | 0.294 | 0.15 | 0.05 | 0.122 | 0.086 | 0.156 | -0.157 | 0.084 | 0.062 |
| InternalIMT | dha | rs147613891 | 0.004 | . | . | . | . | . | . | . | . | . |
| InternalIMT | dha | rs2229136 | 0.064 | 0.036 | 0.044 | 0.42 | 0.183 | 0.148 | 0.216 | -0.076 | 0.119 | 0.526 |
| InternalIMT | dha | rs185482841 | 0.001 | . | . | . | . | . | . | . | . | . |
| InternalIMT | dha | rs2288619 | 0.063 | 0.036 | 0.044 | 0.421 | 0.182 | 0.148 | 0.219 | -0.075 | 0.119 | 0.532 |
| InternalIMT | dha | rs189891524 | . | . | . | . | . | . | . | . | . | . |
| InternalIMT | dha | rs56145077 | 0.115 | 0.011 | 0.046 | 0.806 | -0.193 | 0.087 | 0.027 | 0.102 | 0.076 | 0.178 |
| InternalIMT | dha | rs143216970 | 0.003 | . | . | . | . | . | . | . | . | . |
| InternalIMT | dha | rs142246478 | 0.001 | . | . | . | . | . | . | . | . | . |
| InternalIMT | dha | rs13134 | 0.008 | . | . | . | . | . | . | . | . | . |
| InternalIMT | dha | rs41314980 | 0.031 | 0.051 | 0.043 | 0.243 | 0.393 | 0.222 | 0.077 | -0.286 | 0.21 | 0.175 |
| InternalIMT | dha | rs41304611 | 0.009 | . | . | . | . | . | . | . | . | . |
| InternalIMT | dha | rs193299617 | . | . | . | . | . | . | . | . | . | . |
| InternalIMT | dha | rs12772724 | 0.008 | . | . | . | . | . | . | . | . | . |
| InternalIMT | dha | rs139489537 | 0.03 | 0.028 | 0.043 | 0.523 | 0.071 | 0.203 | 0.726 | 0.039 | 0.169 | 0.819 |
| InternalIMT | dha | rs12776062 | 0.006 | . | . | . | . | . | . | . | . | . |
| InternalIMT | dha | rs752059 | 0.116 | 9.70E-03 | 0.046 | 0.833 | -0.2 | 0.087 | 0.021 | 0.107 | 0.075 | 0.156 |
| InternalIMT | dha | rs147649982 | 0.049 | 0.028 | 0.045 | 0.529 | -0.032 | 0.155 | 0.836 | 0.036 | 0.138 | 0.792 |
| InternalIMT | dha | rs17157798 | 0.056 | 0.037 | 0.044 | 0.397 | 0.196 | 0.158 | 0.216 | -0.087 | 0.128 | 0.494 |
| InternalIMT | dha | rs142298619 | 0.001 | . | . | . | . | . | . | . | . | . |
| InternalIMT | dha | rs4948673 | 0.819 | 0.277 | 0.15 | 0.065 | 0.109 | 0.087 | 0.208 | -0.147 | 0.084 | 0.082 |
| InternalIMT | dha | rs74231484 | 0.066 | 0.03 | 0.043 | 0.485 | -0.085 | 0.115 | 0.457 | 0.015 | 0.107 | 0.889 |
| InternalIMT | dha | rs72796489 | 0.002 | . | . | . | . | . | . | . | . | . |
| InternalIMT | dha | rs113447578 | 0.014 | 0.026 | 0.043 | 0.551 | -0.432 | 0.215 | 0.045 | 0.254 | 0.19 | 0.182 |
| InternalIMT | dha | rs34063374 | 0.008 | . | . | . | . | . | . | . | . | . |
| InternalIMT | dha | rs11239527 | 0.011 | 0.035 | 0.042 | 0.403 | 0.235 | 0.709 | 0.741 | -0.251 | 0.651 | 0.7 |
| InternalIMT | dha | rs34371883 | 0.008 | . | . | . | . | . | . | . | . | . |
| InternalIMT | dha | rs118168828 | 0.006 | . | . | . | . | . | . | . | . | . |
| InternalIMT | dha | rs34349618 | 0.006 | . | . | . | . | . | . | . | . | . |
| InternalIMT | dha | rs80041679 | 0.03 | 0.028 | 0.043 | 0.523 | 0.07 | 0.202 | 0.73 | 0.039 | 0.169 | 0.817 |
| InternalIMT | dha | rs149640607 | 0.001 | . | . | . | . | . | . | . | . | . |
| InternalIMT | dha | rs4427523 | 0.896 | 0.166 | 0.22 | 0.451 | 0.064 | 0.135 | 0.634 | -0.074 | 0.12 | 0.536 |
| InternalIMT | dha | rs185880109 | . | . | . | . | . | . | . | . | . | . |
| InternalIMT | dha | rs191523220 | 0.001 | . | . | . | . | . | . | . | . | . |
| InternalIMT | dha | rs144315284 | 0.003 | . | . | . | . | . | . | . | . | . |
| InternalIMT | dha | rs4948674 | 0.345 | 0.095 | 0.06 | 0.113 | 0.109 | 0.065 | 0.096 | -0.094 | 0.058 | 0.109 |
| InternalIMT | dha | rs182984298 | 0.003 | . | . | . | . | . | . | . | . | . |
| InternalIMT | dha | rs56749023 | 0.016 | 0.03 | 0.042 | 0.471 | 0.336 | 0.331 | 0.31 | -0.162 | 0.281 | 0.564 |
| InternalIMT | dha | rs112508957 | 0.01 | 0.034 | 0.043 | 0.429 | -0.371 | 0.181 | 0.041 | 0.147 | 0.136 | 0.278 |
| InternalIMT | dha | rs7089063 | 0.195 | 1.20E-03 | 0.049 | 0.981 | -0.126 | 0.068 | 0.064 | 0.079 | 0.059 | 0.182 |
| InternalIMT | dha | rs145000779 | . | . | . | . | . | . | . | . | . | . |
| InternalIMT | dha | rs80201572 | 0.03 | 0.028 | 0.043 | 0.522 | 0.07 | 0.201 | 0.728 | 0.038 | 0.168 | 0.823 |
| InternalIMT | dha | rs140568227 | 0.002 | . | . | . | . | . | . | . | . | . |
| InternalIMT | dha | rs149756228 | 0.009 | . | . | . | . | . | . | . | . | . |
| InternalIMT | dha | rs112380685 | 0.008 | . | . | . | . | . | . | . | . | . |
| InternalIMT | dha | rs60481109 | 0.015 | 0.03 | 0.042 | 0.468 | 0.347 | 0.332 | 0.297 | -0.172 | 0.284 | 0.546 |
| InternalIMT | dha | rs138221843 | 0.002 | . | . | . | . | . | . | . | . | . |
| InternalIMT | dha | rs150283194 | 0.001 | . | . | . | . | . | . | . | . | . |
| InternalIMT | dha | rs138879661 | 0.004 | . | . | . | . | . | . | . | . | . |
| InternalIMT | dha | rs34960345 | 0.045 | 0.038 | 0.044 | 0.385 | 0.145 | 0.155 | 0.349 | -0.072 | 0.125 | 0.566 |
| InternalIMT | dha | rs147514068 | 0.004 | . | . | . | . | . | . | . | . | . |
| InternalIMT | dha | rs35410535 | 0.06 | 0.037 | 0.044 | 0.4 | 0.172 | 0.145 | 0.236 | -0.077 | 0.118 | 0.511 |
| InternalIMT | dha | rs191613635 | 0.001 | . | . | . | . | . | . | . | . | . |
| InternalIMT | dha | rs17157800 | 0.005 | . | . | . | . | . | . | . | . | . |
| InternalIMT | dha | rs17523413 | 0.039 | 0.034 | 0.044 | 0.442 | 0.131 | 0.172 | 0.444 | -0.031 | 0.141 | 0.825 |
| InternalIMT | dha | rs7897036 | 0.037 | 0.04 | 0.044 | 0.363 | -0.025 | 0.141 | 0.86 | -0.102 | 0.123 | 0.403 |
| InternalIMT | dha | rs76334323 | 0.02 | 0.016 | 0.042 | 0.703 | -0.571 | 0.234 | 0.015 | 0.475 | 0.279 | 0.089 |

| InternalIMT | dha | rs143535991 | 0.001 | . | . | . | . | . | . | . | . | . |
| --- | --- | --- | --- | --- | --- | --- | --- | --- | --- | --- | --- | --- |
| InternalIMT | dha | rs140344979 | 0.003 | . | . | . | . | . | . | . | . | . |
| InternalIMT | dha | rs36047811 | 0.06 | 0.037 | 0.044 | 0.4 | 0.172 | 0.145 | 0.236 | -0.077 | 0.118 | 0.511 |
| InternalIMT | dha | rs76493570 | 0.043 | 0.036 | 0.044 | 0.417 | 0.13 | 0.156 | 0.404 | -0.049 | 0.127 | 0.698 |
| InternalIMT | dha | rs192998920 | . | . | . | . | . | . | . | . | . | . |
| InternalIMT | dha | rs116840852 | 0.013 | 0.034 | 0.043 | 0.429 | 0.235 | 0.547 | 0.668 | -0.144 | 0.452 | 0.75 |
| InternalIMT | dha | rs184824101 | 0 | . | . | . | . | . | . | . | . | . |
| InternalIMT | dha | rs41301621 | 0.003 | . | . | . | . | . | . | . | . | . |
| InternalIMT | dha | rs3740109 | 0.06 | 0.037 | 0.044 | 0.4 | 0.172 | 0.145 | 0.236 | -0.077 | 0.118 | 0.511 |
| InternalIMT | dha | rs7899415 | 0.025 | 0.03 | 0.044 | 0.496 | 6.70E-03 | 0.26 | 0.98 | 0.059 | 0.237 | 0.804 |
| InternalIMT | dha | rs184395284 | 0.003 | . | . | . | . | . | . | . | . | . |
| InternalIMT | dha | rs139209589 | 0.004 | . | . | . | . | . | . | . | . | . |
| InternalIMT | dha | rs149924163 | 0.001 | . | . | . | . | . | . | . | . | . |
| InternalIMT | dha | rs3740110 | 0.02 | 0.016 | 0.042 | 0.702 | -0.571 | 0.234 | 0.015 | 0.476 | 0.279 | 0.089 |
| InternalIMT | dha | rs144335113 | 0.001 | . | . | . | . | . | . | . | . | . |
| InternalIMT | dha | rs74743385 | 0.01 | 0.034 | 0.043 | 0.429 | -0.372 | 0.181 | 0.04 | 0.148 | 0.136 | 0.277 |
| InternalIMT | dha | rs146545829 | 0.001 | . | . | . | . | . | . | . | . | . |
| InternalIMT | dha | rs181842689 | 0.001 | . | . | . | . | . | . | . | . | . |
| InternalIMT | dha | rs3740111 | 0.045 | 0.038 | 0.044 | 0.384 | 0.145 | 0.155 | 0.348 | -0.072 | 0.125 | 0.565 |
| InternalIMT | dha | rs186261599 | 0.001 | . | . | . | . | . | . | . | . | . |
| InternalIMT | dha | rs73285348 | 0.008 | . | . | . | . | . | . | . | . | . |
| InternalIMT | dha | rs186356901 | 0.001 | . | . | . | . | . | . | . | . | . |
| InternalIMT | dha | rs3824618 | 0.03 | 0.028 | 0.043 | 0.521 | 0.07 | 0.202 | 0.728 | 0.037 | 0.168 | 0.824 |
| InternalIMT | dha | rs3802548 | 0.304 | 0.02 | 0.054 | 0.713 | -0.041 | 0.061 | 0.504 | 0.017 | 0.055 | 0.76 |
| InternalIMT | dha | rs3802547 | 0.02 | 0.016 | 0.042 | 0.702 | -0.573 | 0.235 | 0.015 | 0.478 | 0.281 | 0.089 |
| InternalIMT | dha | rs77677521 | 0.013 | 0.034 | 0.043 | 0.429 | 0.234 | 0.547 | 0.669 | -0.143 | 0.452 | 0.751 |
| InternalIMT | dha | rs3824616 | 0.06 | 0.037 | 0.044 | 0.4 | 0.172 | 0.145 | 0.237 | -0.077 | 0.118 | 0.511 |
| InternalIMT | dha | rs191073818 | 0.001 | . | . | . | . | . | . | . | . | . |
| InternalIMT | dha | rs147078004 | . | . | . | . | . | . | . | . | . | . |
| InternalIMT | dha | rs7908745 | 0.388 | 8.50E-03 | 0.059 | 0.886 | -0.045 | 0.058 | 0.437 | 0.028 | 0.052 | 0.583 |
| InternalIMT | dha | rs17157811 | 0.005 | . | . | . | . | . | . | . | . | . |
| InternalIMT | dha | rs138506087 | 0.006 | . | . | . | . | . | . | . | . | . |
| InternalIMT | dha | rs77241269 | 0.016 | 0.03 | 0.042 | 0.479 | 0.319 | 0.317 | 0.315 | -0.144 | 0.268 | 0.589 |
| InternalIMT | dha | rs138374938 | 0.001 | . | . | . | . | . | . | . | . | . |
| InternalIMT | dha | rs2279433 | 0.045 | 0.04 | 0.044 | 0.36 | 0.156 | 0.154 | 0.313 | -0.087 | 0.123 | 0.479 |
| InternalIMT | dha | rs73285358 | 0.016 | 0.03 | 0.042 | 0.48 | 0.317 | 0.315 | 0.314 | -0.143 | 0.266 | 0.591 |
| InternalIMT | dha | rs11239528 | 0.303 | 0.018 | 0.054 | 0.732 | -0.043 | 0.061 | 0.479 | 0.019 | 0.054 | 0.729 |
| InternalIMT | dha | rs77453386 | 0.02 | 0.016 | 0.042 | 0.703 | -0.573 | 0.233 | 0.014 | 0.479 | 0.279 | 0.086 |
| InternalIMT | dha | rs2279434 | 0.05 | 0.039 | 0.045 | 0.385 | 0.129 | 0.147 | 0.38 | -0.064 | 0.12 | 0.592 |
| InternalIMT | dha | rs149236525 | 0.005 | . | . | . | . | . | . | . | . | . |
| InternalIMT | dha | rs12573609 | 0.037 | 0.026 | 0.045 | 0.568 | -0.149 | 0.146 | 0.309 | 0.084 | 0.127 | 0.508 |
| InternalIMT | dha | rs191716655 | . | . | . | . | . | . | . | . | . | . |
| InternalIMT | dha | rs17523660 | 0.006 | . | . | . | . | . | . | . | . | . |
| InternalIMT | dha | rs142671360 | 0.004 | . | . | . | . | . | . | . | . | . |
| InternalIMT | dha | rs3764990 | 0.038 | 0.035 | 0.044 | 0.418 | 0.146 | 0.173 | 0.398 | -0.048 | 0.141 | 0.732 |
| InternalIMT | dha | rs113682861 | 0.005 | . | . | . | . | . | . | . | . | . |
| InternalIMT | dha | rs117658270 | 0.02 | 0.016 | 0.042 | 0.703 | -0.573 | 0.233 | 0.014 | 0.479 | 0.279 | 0.086 |
| InternalIMT | dha | rs187309063 | . | . | . | . | . | . | . | . | . | . |
| InternalIMT | dha | rs73285360 | 0.016 | 0.03 | 0.042 | 0.48 | 0.316 | 0.313 | 0.314 | -0.142 | 0.264 | 0.592 |
| InternalIMT | dha | rs80133289 | 0.007 | . | . | . | . | . | . | . | . | . |
| InternalIMT | dha | rs186693257 | . | . | . | . | . | . | . | . | . | . |
| InternalIMT | dha | rs35735968 | 0.049 | 0.038 | 0.044 | 0.385 | 0.134 | 0.152 | 0.38 | -0.07 | 0.122 | 0.567 |
| InternalIMT | dha | rs17157825 | 0.031 | 0.029 | 0.043 | 0.501 | 0.083 | 0.204 | 0.684 | 0.018 | 0.169 | 0.917 |
| InternalIMT | dha | rs2291428 | 0.299 | 0.021 | 0.053 | 0.69 | -0.038 | 0.061 | 0.535 | 0.014 | 0.055 | 0.797 |
| InternalIMT | dha | rs2291429 | 0.304 | 0.015 | 0.054 | 0.777 | -0.046 | 0.061 | 0.448 | 0.024 | 0.054 | 0.663 |
| InternalIMT | dha | rs145714792 | 0.003 | . | . | . | . | . | . | . | . | . |
| InternalIMT | dha | rs12769244 | 0.044 | 0.041 | 0.044 | 0.357 | 0.157 | 0.154 | 0.308 | -0.089 | 0.123 | 0.467 |
| InternalIMT | dha | rs12771275 | 0.044 | 0.041 | 0.044 | 0.357 | 0.157 | 0.154 | 0.308 | -0.089 | 0.123 | 0.467 |
| InternalIMT | dha | rs146266174 | 0 | . | . | . | . | . | . | . | . | . |
| InternalIMT | dha | rs41301623 | 0.003 | . | . | . | . | . | . | . | . | . |
| InternalIMT | dha | rs2306814 | 0.021 | 0.014 | 0.042 | 0.733 | -0.62 | 0.225 | 5.90E-03 | 0.539 | 0.264 | 0.041 |
| InternalIMT | dha | rs12244834 | 0.012 | 0.021 | 0.041 | 0.609 | -0.485 | 0.441 | 0.271 | 0.416 | 0.415 | 0.317 |
| InternalIMT | dha | rs184006011 | 0.002 | . | . | . | . | . | . | . | . | . |
| InternalIMT | dha | rs78829066 | 0.02 | 0.016 | 0.042 | 0.706 | -0.575 | 0.234 | 0.014 | 0.48 | 0.279 | 0.085 |
| InternalIMT | dha | rs34731408 | 0.044 | 0.041 | 0.044 | 0.357 | 0.158 | 0.154 | 0.306 | -0.09 | 0.123 | 0.465 |
| InternalIMT | dha | rs17451516 | 0.008 | . | . | . | . | . | . | . | . | . |
| InternalIMT | dha | rs139910133 | 0.002 | . | . | . | . | . | . | . | . | . |
| InternalIMT | dha | rs10900218 | 0.197 | -1.20E-03 | 0.048 | 0.98 | -0.124 | 0.069 | 0.071 | 0.084 | 0.059 | 0.157 |
| InternalIMT | dha | rs146023999 | 0.001 | . | . | . | . | . | . | . | . | . |
| InternalIMT | dha | rs187296958 | . | . | . | . | . | . | . | . | . | . |
| InternalIMT | dha | rs142342833 | . | . | . | . | . | . | . | . | . | . |
| InternalIMT | dha | rs143489255 | . | . | . | . | . | . | . | . | . | . |
| InternalIMT | dha | rs138532518 | . | . | . | . | . | . | . | . | . | . |
| InternalIMT | dha | rs183768079 | 0.001 | . | . | . | . | . | . | . | . | . |
| InternalIMT | dha | rs117892272 | . | . | . | . | . | . | . | . | . | . |
| InternalIMT | dha | rs192993943 | . | . | . | . | . | . | . | . | . | . |
| InternalIMT | dha | rs182989043 | 0.001 | . | . | . | . | . | . | . | . | . |
| InternalIMT | dha | rs189703435 | . | . | . | . | . | . | . | . | . | . |
| InternalIMT | dha | rs188794040 | . | . | . | . | . | . | . | . | . | . |
| InternalIMT | dha | rs143920953 | . | . | . | . | . | . | . | . | . | . |
| InternalIMT | dha | rs191029121 | . | . | . | . | . | . | . | . | . | . |
| InternalIMT | dha | rs116875448 | . | . | . | . | . | . | . | . | . | . |
| InternalIMT | dha | rs190167877 | 0.001 | . | . | . | . | . | . | . | . | . |
| InternalIMT | dha | rs184993418 | . | . | . | . | . | . | . | . | . | . |
| InternalIMT | dha | rs143241158 | . | . | . | . | . | . | . | . | . | . |
| InternalIMT | dha | rs11239500 | 0.015 | 0.022 | 0.041 | 0.597 | -0.351 | 0.36 | 0.33 | 0.307 | 0.316 | 0.331 |

| InternalIMT | dha | rs140568994 | . | . | . | . | . | . | . | . | . | . |
| --- | --- | --- | --- | --- | --- | --- | --- | --- | --- | --- | --- | --- |
| InternalIMT | dha | rs187530317 | . | . | . | . | . | . | . | . | . | . |
| InternalIMT | dha | rs145189326 | . | . | . | . | . | . | . | . | . | . |
| InternalIMT | dha | rs186203332 | . | . | . | . | . | . | . | . | . | . |
| InternalIMT | dha | rs189921909 | . | . | . | . | . | . | . | . | . | . |
| InternalIMT | dha | rs187260989 | . | . | . | . | . | . | . | . | . | . |
| InternalIMT | dha | rs114376586 | 0.013 | 0.022 | 0.041 | 0.587 | -0.345 | 0.366 | 0.346 | 0.309 | 0.318 | 0.331 |
| InternalIMT | dha | rs151070808 | . | . | . | . | . | . | . | . | . | . |
| InternalIMT | dha | rs140927225 | 0.015 | 0.022 | 0.041 | 0.588 | -0.334 | 0.365 | 0.36 | 0.297 | 0.317 | 0.349 |
| InternalIMT | dha | rs191555615 | . | . | . | . | . | . | . | . | . | . |
| InternalIMT | dha | rs80278898 | 0.005 | . | . | . | . | . | . | . | . | . |
| InternalIMT | dha | rs190849503 | . | . | . | . | . | . | . | . | . | . |
| InternalIMT | dha | rs150080315 | 0.003 | . | . | . | . | . | . | . | . | . |
| InternalIMT | dha | rs143623885 | . | . | . | . | . | . | . | . | . | . |
| InternalIMT | dha | rs187760040 | . | . | . | . | . | . | . | . | . | . |
| InternalIMT | dha | rs143917143 | 0.005 | . | . | . | . | . | . | . | . | . |
| InternalIMT | dha | rs149851136 | 0.001 | . | . | . | . | . | . | . | . | . |
| InternalIMT | dha | rs181612054 | . | . | . | . | . | . | . | . | . | . |
| InternalIMT | dha | rs191006548 | . | . | . | . | . | . | . | . | . | . |
| InternalIMT | dha | rs117377631 | 0.026 | 0.03 | 0.044 | 0.491 | 0.09 | 0.252 | 0.721 | 0.09 | 0.232 | 0.698 |
| InternalIMT | dha | rs182107634 | . | . | . | . | . | . | . | . | . | . |
| InternalIMT | dha | rs190211867 | . | . | . | . | . | . | . | . | . | . |
| InternalIMT | dha | rs148293457 | . | . | . | . | . | . | . | . | . | . |
| InternalIMT | dha | rs191811733 | . | . | . | . | . | . | . | . | . | . |
| InternalIMT | dha | rs146109272 | . | . | . | . | . | . | . | . | . | . |
| InternalIMT | dha | rs148397915 | . | . | . | . | . | . | . | . | . | . |
| InternalIMT | dha | rs142233290 | . | . | . | . | . | . | . | . | . | . |
| InternalIMT | dha | rs80226874 | . | . | . | . | . | . | . | . | . | . |
| InternalIMT | dha | rs144496203 | . | . | . | . | . | . | . | . | . | . |
| InternalIMT | dha | rs191062130 | . | . | . | . | . | . | . | . | . | . |
| InternalIMT | dha | rs185807817 | . | . | . | . | . | . | . | . | . | . |
| InternalIMT | dha | rs117846213 | . | . | . | . | . | . | . | . | . | . |
| InternalIMT | dha | rs190545473 | . | . | . | . | . | . | . | . | . | . |
| InternalIMT | dha | rs192364918 | . | . | . | . | . | . | . | . | . | . |
| InternalIMT | dha | rs182939241 | . | . | . | . | . | . | . | . | . | . |
| InternalIMT | dha | rs12571125 | . | . | . | . | . | . | . | . | . | . |
| InternalIMT | dha | rs192418941 | . | . | . | . | . | . | . | . | . | . |
| InternalIMT | dha | rs141270243 | . | . | . | . | . | . | . | . | . | . |
| InternalIMT | dha | rs182615502 | . | . | . | . | . | . | . | . | . | . |
| InternalIMT | dha | rs146669856 | 0.004 | . | . | . | . | . | . | . | . | . |
| InternalIMT | dha | rs78290029 | 0.015 | 0.016 | 0.041 | 0.693 | -0.511 | 0.387 | 0.187 | 0.465 | 0.379 | 0.22 |
| InternalIMT | dha | rs139955565 | . | . | . | . | . | . | . | . | . | . |
| InternalIMT | dha | rs12267314 | 0.013 | 0.018 | 0.041 | 0.667 | -0.56 | 0.394 | 0.156 | 0.475 | 0.393 | 0.227 |
| InternalIMT | dha | rs74477197 | . | . | . | . | . | . | . | . | . | . |
| InternalIMT | dha | rs188147236 | . | . | . | . | . | . | . | . | . | . |
| InternalIMT | dha | rs2228065 | 0.012 | 0.02 | 0.041 | 0.635 | -0.469 | 0.446 | 0.293 | 0.418 | 0.43 | 0.331 |
| InternalIMT | dha | rs116961353 | 0.007 | . | . | . | . | . | . | . | . | . |
| InternalIMT | dha | rs190222272 | . | . | . | . | . | . | . | . | . | . |
| InternalIMT | dha | rs182719940 | . | . | . | . | . | . | . | . | . | . |
| InternalIMT | dha | rs184477413 | . | . | . | . | . | . | . | . | . | . |
| InternalIMT | dha | rs12265175 | 0.013 | 0.023 | 0.041 | 0.584 | -0.394 | 0.451 | 0.382 | 0.332 | 0.429 | 0.439 |
| InternalIMT | dha | rs182928276 | . | . | . | . | . | . | . | . | . | . |
| InternalIMT | dha | rs144409895 | . | . | . | . | . | . | . | . | . | . |
| InternalIMT | dha | rs58998447 | 0.003 | . | . | . | . | . | . | . | . | . |
| InternalIMT | dha | rs188668832 | . | . | . | . | . | . | . | . | . | . |
| InternalIMT | dha | rs148641686 | . | . | . | . | . | . | . | . | . | . |
| InternalIMT | dha | rs75468791 | . | . | . | . | . | . | . | . | . | . |
| InternalIMT | dha | rs147327397 | . | . | . | . | . | . | . | . | . | . |
| InternalIMT | dha | rs77354597 | 0.001 | . | . | . | . | . | . | . | . | . |
| InternalIMT | dha | rs41365544 | 0.012 | 0.021 | 0.041 | 0.601 | -0.465 | 0.447 | 0.299 | 0.395 | 0.42 | 0.347 |
| InternalIMT | dha | rs187564188 | . | . | . | . | . | . | . | . | . | . |
| InternalIMT | dha | rs183511984 | . | . | . | . | . | . | . | . | . | . |
| InternalIMT | dha | rs12262783 | 0.011 | 0.021 | 0.041 | 0.603 | -0.486 | 0.471 | 0.302 | 0.416 | 0.444 | 0.349 |
| InternalIMT | dha | rs188817687 | 0.001 | . | . | . | . | . | . | . | . | . |
| InternalIMT | dha | rs3815968 | . | . | . | . | . | . | . | . | . | . |
| InternalIMT | dha | rs187822300 | . | . | . | . | . | . | . | . | . | . |
| InternalIMT | dha | rs188902172 | 0.001 | . | . | . | . | . | . | . | . | . |
| InternalIMT | dha | rs181190448 | . | . | . | . | . | . | . | . | . | . |
| InternalIMT | dha | rs182699093 | . | . | . | . | . | . | . | . | . | . |
| InternalIMT | dha | rs182631303 | . | . | . | . | . | . | . | . | . | . |
| InternalIMT | dha | rs187014172 | 0.001 | . | . | . | . | . | . | . | . | . |
| InternalIMT | dha | rs142575406 | . | . | . | . | . | . | . | . | . | . |
| InternalIMT | dha | rs180711033 | . | . | . | . | . | . | . | . | . | . |
| InternalIMT | dha | rs137894823 | 0.001 | . | . | . | . | . | . | . | . | . |
| InternalIMT | dha | rs189053273 | 0 | . | . | . | . | . | . | . | . | . |
| InternalIMT | dha | rs143499388 | . | . | . | . | . | . | . | . | . | . |
| InternalIMT | dha | rs182936001 | 0.001 | . | . | . | . | . | . | . | . | . |
| InternalIMT | dha | rs148456406 | 0 | . | . | . | . | . | . | . | . | . |
| InternalIMT | dha | rs182499531 | 0.001 | . | . | . | . | . | . | . | . | . |
| InternalIMT | dha | rs74884915 | 0.005 | . | . | . | . | . | . | . | . | . |
| InternalIMT | dha | rs184046374 | . | . | . | . | . | . | . | . | . | . |
| InternalIMT | dha | rs74333993 | 0.004 | . | . | . | . | . | . | . | . | . |
| InternalIMT | dha | rs138889719 | . | . | . | . | . | . | . | . | . | . |
| InternalIMT | dha | rs75330330 | 0.003 | . | . | . | . | . | . | . | . | . |
| InternalIMT | dha | rs193051693 | . | . | . | . | . | . | . | . | . | . |
| InternalIMT | dha | rs145929633 | . | . | . | . | . | . | . | . | . | . |

| InternalIMT | dha | rs184076904 | 0.001 | . | . | . | . | . | . | . | . | . |
| --- | --- | --- | --- | --- | --- | --- | --- | --- | --- | --- | --- | --- |
| InternalIMT | dha | rs141802494 | 0.002 | . | . | . | . | . | . | . | . | . |
| InternalIMT | dha | rs149519759 | 0 | . | . | . | . | . | . | . | . | . |
| InternalIMT | dha | rs145467260 | 0.002 | . | . | . | . | . | . | . | . | . |
| InternalIMT | dha | rs79620914 | 0.006 | . | . | . | . | . | . | . | . | . |
| InternalIMT | dha | rs140538633 | 0.005 | . | . | . | . | . | . | . | . | . |
| InternalIMT | dha | rs140862301 | 0.001 | . | . | . | . | . | . | . | . | . |
| InternalIMT | dha | rs114615847 | 0.003 | . | . | . | . | . | . | . | . | . |
| InternalIMT | dha | rs141800259 | 0.004 | . | . | . | . | . | . | . | . | . |
| InternalIMT | dha | rs149779059 | 0.003 | . | . | . | . | . | . | . | . | . |
| InternalIMT | dha | rs116539253 | 0.001 | . | . | . | . | . | . | . | . | . |
| InternalIMT | dha | rs184979550 | 0 | . | . | . | . | . | . | . | . | . |
| InternalIMT | dha | rs190128940 | 0.001 | . | . | . | . | . | . | . | . | . |
| InternalIMT | dha | rs146594816 | . | . | . | . | . | . | . | . | . | . |
| InternalIMT | dha | rs141370766 | 0.003 | . | . | . | . | . | . | . | . | . |
| InternalIMT | dha | rs144670033 | . | . | . | . | . | . | . | . | . | . |
| InternalIMT | dha | rs187152174 | 0.003 | . | . | . | . | . | . | . | . | . |
| InternalIMT | dha | rs184180263 | 0.002 | . | . | . | . | . | . | . | . | . |
| InternalIMT | dha | rs140075861 | 0.002 | . | . | . | . | . | . | . | . | . |
| InternalIMT | dha | rs186483129 | . | . | . | . | . | . | . | . | . | . |
| InternalIMT | dha | rs186634282 | 0.003 | . | . | . | . | . | . | . | . | . |
| InternalIMT | dha | rs9988663 | 0.004 | . | . | . | . | . | . | . | . | . |
| InternalIMT | dha | rs138828529 | 0.004 | . | . | . | . | . | . | . | . | . |
| InternalIMT | dha | rs188777615 | 0.001 | . | . | . | . | . | . | . | . | . |
| InternalIMT | dha | rs192321882 | 0.001 | . | . | . | . | . | . | . | . | . |
| InternalIMT | dha | rs150812270 | 0.003 | . | . | . | . | . | . | . | . | . |
| InternalIMT | dha | rs185906603 | . | . | . | . | . | . | . | . | . | . |
| InternalIMT | dha | rs191070862 | . | . | . | . | . | . | . | . | . | . |
| InternalIMT | dha | rs182904978 | . | . | . | . | . | . | . | . | . | . |
| InternalIMT | dha | rs192679655 | . | . | . | . | . | . | . | . | . | . |
| InternalIMT | dha | rs189673086 | 0.003 | . | . | . | . | . | . | . | . | . |
| InternalIMT | dha | rs181141892 | 0.001 | . | . | . | . | . | . | . | . | . |
| InternalIMT | dha | rs186404123 | . | . | . | . | . | . | . | . | . | . |
| InternalIMT | dha | rs192117287 | 0.001 | . | . | . | . | . | . | . | . | . |
| InternalIMT | dha | rs182748835 | 0 | . | . | . | . | . | . | . | . | . |
| InternalIMT | dha | rs141854549 | 0.005 | . | . | . | . | . | . | . | . | . |
| InternalIMT | dha | rs140163397 | 0.001 | . | . | . | . | . | . | . | . | . |
| InternalIMT | dha | rs146347300 | 0.001 | . | . | . | . | . | . | . | . | . |
| InternalIMT | dha | rs139362377 | 0.005 | . | . | . | . | . | . | . | . | . |
| InternalIMT | dha | rs185087671 | 0 | . | . | . | . | . | . | . | . | . |
| InternalIMT | dha | rs190466574 | 0 | . | . | . | . | . | . | . | . | . |
| InternalIMT | dha | rs147012778 | 0.001 | . | . | . | . | . | . | . | . | . |
| InternalIMT | dha | rs138236960 | 0.003 | . | . | . | . | . | . | . | . | . |
| InternalIMT | dha | rs116310311 | 0.005 | . | . | . | . | . | . | . | . | . |
| InternalIMT | dha | rs142868356 | 0.003 | . | . | . | . | . | . | . | . | . |
| InternalIMT | dha | rs144956240 | 0.001 | . | . | . | . | . | . | . | . | . |
| InternalIMT | dha | rs188527776 | . | . | . | . | . | . | . | . | . | . |
| InternalIMT | dha | rs183971165 | 0.003 | . | . | . | . | . | . | . | . | . |
| InternalIMT | dha | rs76748216 | 0.003 | . | . | . | . | . | . | . | . | . |
| InternalIMT | dha | rs148554990 | . | . | . | . | . | . | . | . | . | . |
| InternalIMT | dha | rs114446477 | 0.007 | . | . | . | . | . | . | . | . | . |
| InternalIMT | dha | rs185223933 | 0.002 | . | . | . | . | . | . | . | . | . |
| InternalIMT | dha | rs76869726 | 0.003 | . | . | . | . | . | . | . | . | . |
| InternalIMT | dha | rs151200830 | 0.005 | . | . | . | . | . | . | . | . | . |
| InternalIMT | dha | rs17157731 | 0.005 | . | . | . | . | . | . | . | . | . |
| InternalIMT | dha | rs181243469 | . | . | . | . | . | . | . | . | . | . |
| InternalIMT | dha | rs186890707 | . | . | . | . | . | . | . | . | . | . |
| InternalIMT | dha | rs184063360 | . | . | . | . | . | . | . | . | . | . |
| InternalIMT | dha | rs188550237 | 0.001 | . | . | . | . | . | . | . | . | . |
| InternalIMT | dha | rs140931068 | 0.003 | . | . | . | . | . | . | . | . | . |
| InternalIMT | dha | rs74867877 | 0.001 | . | . | . | . | . | . | . | . | . |
| InternalIMT | dha | rs11239495 | 0.015 | 0.018 | 0.041 | 0.652 | -0.355 | 0.449 | 0.43 | 0.433 | 0.444 | 0.33 |
| InternalIMT | dha | rs149745800 | . | . | . | . | . | . | . | . | . | . |
| InternalIMT | dha | rs145044458 | 0.005 | . | . | . | . | . | . | . | . | . |
| InternalIMT | dha | rs188067427 | 0 | . | . | . | . | . | . | . | . | . |
| InternalIMT | dha | rs146827800 | . | . | . | . | . | . | . | . | . | . |
| InternalIMT | dha | rs140619817 | 0.005 | . | . | . | . | . | . | . | . | . |
| InternalIMT | dha | rs892689 | 0.998 | . | . | . | . | . | . | . | . | . |
| InternalIMT | dha | rs143642005 | 0.001 | . | . | . | . | . | . | . | . | . |
| InternalIMT | dha | rs181929304 | 0.001 | . | . | . | . | . | . | . | . | . |
| InternalIMT | dha | rs191086173 | 0.001 | . | . | . | . | . | . | . | . | . |
| InternalIMT | dha | rs114613116 | 0.005 | . | . | . | . | . | . | . | . | . |
| InternalIMT | dha | rs141076768 | . | . | . | . | . | . | . | . | . | . |
| InternalIMT | dha | rs187316531 | . | . | . | . | . | . | . | . | . | . |
| InternalIMT | dha | rs11239499 | 0.014 | 0.022 | 0.041 | 0.589 | -0.416 | 0.366 | 0.256 | 0.344 | 0.321 | 0.284 |
| InternalIMT | dha | rs192726371 | 0.001 | . | . | . | . | . | . | . | . | . |
| InternalIMT | dha | rs74128481 | 0.004 | . | . | . | . | . | . | . | . | . |
| InternalIMT | dha | rs74128482 | 0.003 | . | . | . | . | . | . | . | . | . |
| InternalIMT | dha | rs11239502 | 0.008 | . | . | . | . | . | . | . | . | . |
| InternalIMT | dha | rs116330285 | . | . | . | . | . | . | . | . | . | . |
| InternalIMT | dha | rs189926729 | 0.001 | . | . | . | . | . | . | . | . | . |
| InternalIMT | dha | rs188975779 | 0.003 | . | . | . | . | . | . | . | . | . |
| InternalIMT | dha | rs143846038 | . | . | . | . | . | . | . | . | . | . |
| InternalIMT | dha | rs74128483 | 0.004 | . | . | . | . | . | . | . | . | . |
| InternalIMT | dha | rs139292055 | 0.013 | 0.022 | 0.041 | 0.589 | -0.352 | 0.367 | 0.338 | 0.315 | 0.32 | 0.326 |
| InternalIMT | dha | rs11239503 | 0.013 | 0.022 | 0.041 | 0.589 | -0.351 | 0.367 | 0.339 | 0.314 | 0.32 | 0.326 |

| InternalIMT | dha | rs58838686 | 0.003 | . | . | . | . | . | . | . | . | . |
| --- | --- | --- | --- | --- | --- | --- | --- | --- | --- | --- | --- | --- |
| InternalIMT | dha | rs185825373 | 0.001 | . | . | . | . | . | . | . | . | . |
| InternalIMT | dha | rs11239504 | 0.013 | 0.022 | 0.041 | 0.589 | -0.35 | 0.366 | 0.34 | 0.313 | 0.319 | 0.327 |
| InternalIMT | dha | rs150012354 | 0 | . | . | . | . | . | . | . | . | . |
| InternalIMT | dha | rs187763096 | 0.001 | . | . | . | . | . | . | . | . | . |
| InternalIMT | dha | rs193043982 | 0.002 | . | . | . | . | . | . | . | . | . |
| InternalIMT | dha | rs79186884 | 0.004 | . | . | . | . | . | . | . | . | . |
| InternalIMT | dha | rs74128484 | 0.003 | . | . | . | . | . | . | . | . | . |
| InternalIMT | dha | rs58461537 | 0.013 | 0.022 | 0.041 | 0.588 | -0.348 | 0.366 | 0.343 | 0.311 | 0.319 | 0.329 |
| InternalIMT | dha | rs188527474 | 0.002 | . | . | . | . | . | . | . | . | . |
| InternalIMT | dha | rs141659976 | 0.002 | . | . | . | . | . | . | . | . | . |
| InternalIMT | dha | rs115846407 | 0.013 | 0.022 | 0.041 | 0.588 | -0.346 | 0.366 | 0.345 | 0.31 | 0.319 | 0.33 |
| InternalIMT | dha | rs138378816 | 0.013 | 0.022 | 0.041 | 0.588 | -0.346 | 0.366 | 0.345 | 0.31 | 0.319 | 0.331 |
| InternalIMT | dha | rs146188136 | 0.008 | . | . | . | . | . | . | . | . | . |
| InternalIMT | dha | rs139383871 | 0.002 | . | . | . | . | . | . | . | . | . |
| InternalIMT | dha | rs182953869 | . | . | . | . | . | . | . | . | . | . |
| InternalIMT | dha | rs192430299 | 0.004 | . | . | . | . | . | . | . | . | . |
| InternalIMT | dha | rs144152844 | 0.013 | 0.022 | 0.041 | 0.588 | -0.344 | 0.366 | 0.347 | 0.309 | 0.318 | 0.332 |
| InternalIMT | dha | rs145987786 | 0.013 | 0.036 | 0.043 | 0.404 | -0.09 | 0.208 | 0.664 | -0.016 | 0.153 | 0.915 |
| InternalIMT | dha | rs181132926 | 0.001 | . | . | . | . | . | . | . | . | . |
| InternalIMT | dha | rs147882253 | 0.013 | 0.022 | 0.041 | 0.587 | -0.343 | 0.366 | 0.349 | 0.307 | 0.318 | 0.334 |
| InternalIMT | dha | rs141807621 | 0.005 | . | . | . | . | . | . | . | . | . |
| InternalIMT | dha | rs147116052 | 0.013 | 0.022 | 0.041 | 0.587 | -0.342 | 0.366 | 0.349 | 0.307 | 0.318 | 0.334 |
| InternalIMT | dha | rs147716618 | 0.013 | 0.022 | 0.041 | 0.587 | -0.342 | 0.366 | 0.349 | 0.307 | 0.318 | 0.334 |
| InternalIMT | dha | rs187198855 | 0.01 | 0.032 | 0.043 | 0.456 | -0.225 | 0.233 | 0.334 | 0.114 | 0.178 | 0.523 |
| InternalIMT | dha | rs189409638 | 0.005 | . | . | . | . | . | . | . | . | . |
| InternalIMT | dha | rs182647339 | . | . | . | . | . | . | . | . | . | . |
| InternalIMT | dha | rs187995988 | . | . | . | . | . | . | . | . | . | . |
| InternalIMT | dha | rs141034184 | 0.015 | 0.021 | 0.041 | 0.611 | -0.4 | 0.341 | 0.241 | 0.34 | 0.303 | 0.263 |
| InternalIMT | dha | rs187310430 | 0.002 | . | . | . | . | . | . | . | . | . |
| InternalIMT | dha | rs192608488 | 0.013 | 0.022 | 0.041 | 0.586 | -0.335 | 0.365 | 0.359 | 0.301 | 0.316 | 0.34 |
| InternalIMT | dha | rs148840366 | 0.003 | . | . | . | . | . | . | . | . | . |
| InternalIMT | dha | rs186785022 | 0.004 | . | . | . | . | . | . | . | . | . |
| InternalIMT | dha | rs192348728 | 0.002 | . | . | . | . | . | . | . | . | . |
| InternalIMT | dha | rs189656631 | 0.013 | 0.022 | 0.041 | 0.585 | -0.334 | 0.365 | 0.359 | 0.301 | 0.316 | 0.341 |
| InternalIMT | dha | rs185289750 | . | . | . | . | . | . | . | . | . | . |
| InternalIMT | dha | rs142382395 | 0.013 | 0.022 | 0.041 | 0.585 | -0.334 | 0.365 | 0.36 | 0.3 | 0.315 | 0.341 |
| InternalIMT | dha | rs144866696 | 0.004 | . | . | . | . | . | . | . | . | . |
| InternalIMT | dha | rs141371289 | 0.013 | 0.022 | 0.041 | 0.585 | -0.334 | 0.365 | 0.36 | 0.3 | 0.315 | 0.341 |
| InternalIMT | dha | rs114750008 | 0.013 | 0.022 | 0.041 | 0.585 | -0.333 | 0.365 | 0.361 | 0.3 | 0.315 | 0.342 |
| InternalIMT | dha | rs189063597 | 0.002 | . | . | . | . | . | . | . | . | . |
| InternalIMT | dha | rs139151571 | 0.013 | 0.022 | 0.041 | 0.582 | -0.327 | 0.388 | 0.4 | 0.298 | 0.325 | 0.36 |
| InternalIMT | dha | rs139846368 | 0.014 | 0.022 | 0.041 | 0.595 | -0.337 | 0.354 | 0.341 | 0.296 | 0.309 | 0.338 |
| InternalIMT | dha | rs182057653 | 0.004 | . | . | . | . | . | . | . | . | . |
| InternalIMT | dha | rs148785420 | 0.013 | 0.022 | 0.041 | 0.585 | -0.332 | 0.364 | 0.362 | 0.299 | 0.315 | 0.343 |
| InternalIMT | dha | rs138383251 | 0.002 | . | . | . | . | . | . | . | . | . |
| InternalIMT | dha | rs116526705 | 0.006 | . | . | . | . | . | . | . | . | . |
| InternalIMT | dha | rs185946436 | . | . | . | . | . | . | . | . | . | . |
| InternalIMT | dha | rs190455427 | . | . | . | . | . | . | . | . | . | . |
| InternalIMT | dha | rs9282584 | 0.013 | 0.022 | 0.041 | 0.584 | -0.331 | 0.364 | 0.364 | 0.298 | 0.315 | 0.344 |
| InternalIMT | dha | rs17153289 | 0.013 | 0.022 | 0.041 | 0.584 | -0.33 | 0.364 | 0.364 | 0.298 | 0.315 | 0.344 |
| InternalIMT | dha | rs138359103 | 0.001 | . | . | . | . | . | . | . | . | . |
| InternalIMT | dha | rs150605268 | 0.001 | . | . | . | . | . | . | . | . | . |
| InternalIMT | dha | rs79242910 | 0.015 | 0.023 | 0.041 | 0.574 | -0.262 | 0.299 | 0.382 | 0.233 | 0.275 | 0.397 |
| InternalIMT | dha | rs139716904 | 0.004 | . | . | . | . | . | . | . | . | . |
| InternalIMT | dha | rs76238498 | 0.013 | 0.036 | 0.043 | 0.404 | -0.071 | 0.211 | 0.736 | -0.025 | 0.154 | 0.874 |
| InternalIMT | dha | rs184140639 | . | . | . | . | . | . | . | . | . | . |
| InternalIMT | dha | rs188517278 | 0.002 | . | . | . | . | . | . | . | . | . |
| InternalIMT | dha | rs139678913 | 0.001 | . | . | . | . | . | . | . | . | . |
| InternalIMT | dha | rs116697955 | . | . | . | . | . | . | . | . | . | . |
| InternalIMT | dha | rs79267994 | 0.003 | . | . | . | . | . | . | . | . | . |
| InternalIMT | dha | rs11239510 | 0.003 | . | . | . | . | . | . | . | . | . |
| InternalIMT | dha | rs75831645 | 0.006 | . | . | . | . | . | . | . | . | . |
| InternalIMT | dha | rs78452276 | 0.008 | . | . | . | . | . | . | . | . | . |
| InternalIMT | dha | rs144366924 | . | . | . | . | . | . | . | . | . | . |
| InternalIMT | dha | rs77519841 | 0.008 | . | . | . | . | . | . | . | . | . |
| InternalIMT | dha | rs76944454 | 0.013 | 0.022 | 0.041 | 0.587 | -0.343 | 0.365 | 0.347 | 0.307 | 0.314 | 0.329 |
| InternalIMT | dha | rs76443855 | 0.013 | 0.036 | 0.043 | 0.405 | -0.076 | 0.211 | 0.719 | -0.021 | 0.154 | 0.892 |
| InternalIMT | dha | rs146613174 | 0.001 | . | . | . | . | . | . | . | . | . |
| InternalIMT | dha | rs181424761 | 0 | . | . | . | . | . | . | . | . | . |
| InternalIMT | dha | rs142119241 | 0 | . | . | . | . | . | . | . | . | . |
| InternalIMT | dha | rs140137954 | 0.006 | . | . | . | . | . | . | . | . | . |
| InternalIMT | dha | rs187319549 | . | . | . | . | . | . | . | . | . | . |
| InternalIMT | dha | rs12241545 | 0.006 | . | . | . | . | . | . | . | . | . |
| InternalIMT | dha | rs10128228 | 0.013 | 0.022 | 0.041 | 0.587 | -0.341 | 0.364 | 0.35 | 0.305 | 0.314 | 0.331 |
| InternalIMT | dha | rs58580249 | 0.003 | . | . | . | . | . | . | . | . | . |
| InternalIMT | dha | rs193148863 | . | . | . | . | . | . | . | . | . | . |
| InternalIMT | dha | rs185638946 | . | . | . | . | . | . | . | . | . | . |
| InternalIMT | dha | rs117752921 | 0 | . | . | . | . | . | . | . | . | . |
| InternalIMT | dha | rs180841236 | 0 | . | . | . | . | . | . | . | . | . |
| InternalIMT | dha | rs73283155 | 0.003 | . | . | . | . | . | . | . | . | . |
| InternalIMT | dha | rs76560599 | 0.012 | 0.023 | 0.041 | 0.573 | -0.307 | 0.392 | 0.434 | 0.284 | 0.333 | 0.394 |
| InternalIMT | dha | rs76273620 | 0.012 | 0.023 | 0.041 | 0.573 | -0.307 | 0.392 | 0.434 | 0.284 | 0.333 | 0.394 |
| InternalIMT | dha | rs141432923 | 0.003 | . | . | . | . | . | . | . | . | . |
| InternalIMT | dha | rs113529322 | 0.004 | . | . | . | . | . | . | . | . | . |
| InternalIMT | dha | rs10128306 | 0.013 | 0.022 | 0.041 | 0.587 | -0.336 | 0.364 | 0.356 | 0.303 | 0.313 | 0.334 |

| InternalIMT | dha | rs149691688 | 0.001 | . | . | . | . | . | . | . | . | . |
| --- | --- | --- | --- | --- | --- | --- | --- | --- | --- | --- | --- | --- |
| InternalIMT | dha | rs75872495 | 0.013 | 0.022 | 0.041 | 0.587 | -0.334 | 0.363 | 0.358 | 0.301 | 0.312 | 0.335 |
| InternalIMT | dha | rs191873960 | 0.001 | . | . | . | . | . | . | . | . | . |
| InternalIMT | dha | rs80155622 | 0.013 | 0.022 | 0.041 | 0.587 | -0.331 | 0.364 | 0.364 | 0.3 | 0.313 | 0.338 |
| InternalIMT | dha | rs113914656 | 0.001 | . | . | . | . | . | . | . | . | . |
| InternalIMT | dha | rs12242840 | 0.013 | 0.022 | 0.041 | 0.587 | -0.325 | 0.364 | 0.372 | 0.298 | 0.314 | 0.342 |
| InternalIMT | dha | rs147824078 | 0.002 | . | . | . | . | . | . | . | . | . |
| InternalIMT | dha | rs59051493 | 0.013 | 0.022 | 0.041 | 0.589 | -0.329 | 0.365 | 0.367 | 0.302 | 0.315 | 0.338 |
| InternalIMT | dha | rs9282588 | 0.001 | . | . | . | . | . | . | . | . | . |
| InternalIMT | dha | rs150215207 | 0.003 | . | . | . | . | . | . | . | . | . |
| InternalIMT | dha | rs138772243 | 0.001 | . | . | . | . | . | . | . | . | . |
| InternalIMT | dha | rs149383244 | 0.002 | . | . | . | . | . | . | . | . | . |
| InternalIMT | dha | rs17522720 | 0.006 | . | . | . | . | . | . | . | . | . |
| InternalIMT | dha | rs79245105 | 0.013 | 0.022 | 0.041 | 0.591 | -0.332 | 0.366 | 0.364 | 0.305 | 0.316 | 0.334 |
| InternalIMT | dha | rs138222133 | 0.001 | . | . | . | . | . | . | . | . | . |
| InternalIMT | dha | rs12247314 | 0.015 | 0.024 | 0.041 | 0.551 | -0.273 | 0.352 | 0.438 | 0.231 | 0.304 | 0.447 |
| InternalIMT | dha | rs12263894 | 0.014 | 0.026 | 0.041 | 0.528 | -0.186 | 0.378 | 0.622 | 0.174 | 0.32 | 0.586 |
| InternalIMT | dha | rs149907880 | 0.002 | . | . | . | . | . | . | . | . | . |
| InternalIMT | dha | rs188153885 | 0.002 | . | . | . | . | . | . | . | . | . |
| InternalIMT | dha | rs73283159 | 0.002 | . | . | . | . | . | . | . | . | . |
| InternalIMT | dha | rs140476687 | . | . | . | . | . | . | . | . | . | . |
| InternalIMT | dha | rs186603148 | . | . | . | . | . | . | . | . | . | . |
| InternalIMT | dha | rs183296063 | 0.001 | . | . | . | . | . | . | . | . | . |
| InternalIMT | dha | rs145733694 | 0.003 | . | . | . | . | . | . | . | . | . |
| InternalIMT | dha | rs189807449 | . | . | . | . | . | . | . | . | . | . |
| InternalIMT | dha | rs12253223 | 0.009 | . | . | . | . | . | . | . | . | . |
| InternalIMT | dha | rs182113637 | . | . | . | . | . | . | . | . | . | . |
| InternalIMT | dha | rs142792099 | 0.002 | . | . | . | . | . | . | . | . | . |
| InternalIMT | dha | rs12249910 | 0.009 | . | . | . | . | . | . | . | . | . |
| InternalIMT | dha | rs149436339 | 0 | . | . | . | . | . | . | . | . | . |
| InternalIMT | dha | rs77632210 | . | . | . | . | . | . | . | . | . | . |
| InternalIMT | dha | rs74130818 | 0.003 | . | . | . | . | . | . | . | . | . |
| InternalIMT | dha | rs187583648 | . | . | . | . | . | . | . | . | . | . |
| InternalIMT | dha | rs191295987 | 0.001 | . | . | . | . | . | . | . | . | . |
| InternalIMT | dha | rs184200686 | 0.002 | . | . | . | . | . | . | . | . | . |
| InternalIMT | dha | rs76117263 | 0.013 | 0.036 | 0.043 | 0.402 | -0.079 | 0.202 | 0.695 | -0.026 | 0.148 | 0.862 |
| InternalIMT | dha | rs185393149 | 0.001 | . | . | . | . | . | . | . | . | . |
| InternalIMT | dha | rs144760014 | 0 | . | . | . | . | . | . | . | . | . |
| InternalIMT | dha | rs7922292 | 0.002 | . | . | . | . | . | . | . | . | . |
| InternalIMT | dha | rs115372844 | 0.003 | . | . | . | . | . | . | . | . | . |
| InternalIMT | dha | rs111460364 | 0.013 | 0.036 | 0.043 | 0.401 | -0.076 | 0.202 | 0.705 | -0.028 | 0.147 | 0.847 |
| InternalIMT | dha | rs182052255 | 0 | . | . | . | . | . | . | . | . | . |
| InternalIMT | dha | rs180983889 | 0.002 | . | . | . | . | . | . | . | . | . |
| InternalIMT | dha | rs139175327 | 0.002 | . | . | . | . | . | . | . | . | . |
| InternalIMT | dha | rs149959620 | 0.004 | . | . | . | . | . | . | . | . | . |
| InternalIMT | dha | rs114653117 | 0.002 | . | . | . | . | . | . | . | . | . |
| InternalIMT | dha | rs144270018 | 0 | . | . | . | . | . | . | . | . | . |
| InternalIMT | dha | rs182723204 | 0.002 | . | . | . | . | . | . | . | . | . |
| InternalIMT | dha | rs57204067 | 0.003 | . | . | . | . | . | . | . | . | . |
| InternalIMT | dha | rs144956226 | 0.009 | . | . | . | . | . | . | . | . | . |
| InternalIMT | dha | rs187842704 | 0.001 | . | . | . | . | . | . | . | . | . |
| InternalIMT | dha | rs184620511 | 0.003 | . | . | . | . | . | . | . | . | . |
| InternalIMT | dha | rs144031303 | 0.005 | . | . | . | . | . | . | . | . | . |
| InternalIMT | dha | rs187526473 | . | . | . | . | . | . | . | . | . | . |
| InternalIMT | dha | rs193002805 | . | . | . | . | . | . | . | . | . | . |
| InternalIMT | dha | rs138707426 | 0.002 | . | . | . | . | . | . | . | . | . |
| InternalIMT | dha | rs142754243 | 0.01 | 0.031 | 0.042 | 0.456 | 0.259 | 0.421 | 0.539 | -0.108 | 0.366 | 0.767 |
| InternalIMT | dha | rs181600331 | 0.002 | . | . | . | . | . | . | . | . | . |
| InternalIMT | dha | rs144421264 | 0.006 | . | . | . | . | . | . | . | . | . |
| InternalIMT | dha | rs184414519 | 0.002 | . | . | . | . | . | . | . | . | . |
| InternalIMT | dha | rs150596349 | 0.012 | 0.038 | 0.043 | 0.38 | -0.028 | 0.203 | 0.891 | -0.095 | 0.155 | 0.541 |
| InternalIMT | dha | rs190753747 | . | . | . | . | . | . | . | . | . | . |
| InternalIMT | dha | rs139560940 | . | . | . | . | . | . | . | . | . | . |
| InternalIMT | dha | rs75640804 | 0.008 | . | . | . | . | . | . | . | . | . |
| InternalIMT | dha | rs73283175 | 0.018 | 0.036 | 0.043 | 0.403 | 0.131 | 0.265 | 0.621 | -0.112 | 0.207 | 0.589 |
| InternalIMT | dha | rs143337229 | 0.001 | . | . | . | . | . | . | . | . | . |
| InternalIMT | dha | rs73283177 | 0.01 | 0.031 | 0.042 | 0.456 | 0.269 | 0.433 | 0.534 | -0.115 | 0.375 | 0.76 |
| InternalIMT | dha | rs76053611 | 0.011 | 0.037 | 0.043 | 0.392 | -0.095 | 0.166 | 0.569 | -0.046 | 0.13 | 0.723 |
| InternalIMT | dha | rs116325983 | . | . | . | . | . | . | . | . | . | . |
| InternalIMT | dha | rs115451825 | 0.001 | . | . | . | . | . | . | . | . | . |
| InternalIMT | dha | rs183845246 | 0 | . | . | . | . | . | . | . | . | . |
| InternalIMT | dha | rs79756542 | 0.013 | 0.037 | 0.043 | 0.386 | -0.06 | 0.172 | 0.726 | -0.059 | 0.129 | 0.646 |
| InternalIMT | dha | rs187242860 | . | . | . | . | . | . | . | . | . | . |
| InternalIMT | dha | rs57318980 | 0.01 | 0.031 | 0.042 | 0.457 | 0.298 | 0.463 | 0.52 | -0.134 | 0.397 | 0.736 |
| InternalIMT | dha | rs59969732 | 0.01 | 0.031 | 0.042 | 0.456 | 0.265 | 0.475 | 0.578 | -0.118 | 0.406 | 0.772 |
| InternalIMT | dha | rs143811488 | 0.003 | . | . | . | . | . | . | . | . | . |
| InternalIMT | dha | rs189067644 | 0.002 | . | . | . | . | . | . | . | . | . |
| InternalIMT | dha | rs60449178 | 0.01 | . | . | . | . | . | . | . | . | . |
| InternalIMT | dha | rs59243389 | 0.01 | . | . | . | . | . | . | . | . | . |
| InternalIMT | dha | rs184946540 | 0.009 | . | . | . | . | . | . | . | . | . |
| InternalIMT | dha | rs188685881 | 0 | . | . | . | . | . | . | . | . | . |
| InternalIMT | dha | rs150335797 | 0.002 | . | . | . | . | . | . | . | . | . |
| InternalIMT | dha | rs143879260 | 0.002 | . | . | . | . | . | . | . | . | . |
| InternalIMT | dha | rs186044491 | 0.003 | . | . | . | . | . | . | . | . | . |
| InternalIMT | dha | rs11819084 | 0.004 | . | . | . | . | . | . | . | . | . |
| InternalIMT | dha | rs146915670 | . | . | . | . | . | . | . | . | . | . |

| InternalIMT | dha | rs113995695 | 0.004 | . | . | . | . | . | . | . | . | . |
| --- | --- | --- | --- | --- | --- | --- | --- | --- | --- | --- | --- | --- |
| InternalIMT | dha | rs143489451 | 0.001 | . | . | . | . | . | . | . | . | . |
| InternalIMT | dha | rs182653839 | . | . | . | . | . | . | . | . | . | . |
| InternalIMT | dha | rs141692564 | 0.002 | . | . | . | . | . | . | . | . | . |
| InternalIMT | dha | rs146281742 | 0.001 | . | . | . | . | . | . | . | . | . |
| InternalIMT | dha | rs192914353 | 0.001 | . | . | . | . | . | . | . | . | . |
| InternalIMT | dha | rs185182848 | 0 | . | . | . | . | . | . | . | . | . |
| InternalIMT | dha | rs28395858 | 0.004 | . | . | . | . | . | . | . | . | . |
| InternalIMT | dha | rs41429645 | 0.01 | . | . | . | . | . | . | . | . | . |
| InternalIMT | dha | rs28395874 | 0.004 | . | . | . | . | . | . | . | . | . |
| InternalIMT | dha | rs189663634 | . | . | . | . | . | . | . | . | . | . |
| InternalIMT | dha | rs77666700 | 0.003 | . | . | . | . | . | . | . | . | . |
| InternalIMT | dha | rs146711717 | 0.001 | . | . | . | . | . | . | . | . | . |
| InternalIMT | dha | rs144904251 | . | . | . | . | . | . | . | . | . | . |
| InternalIMT | dha | rs189893153 | . | . | . | . | . | . | . | . | . | . |
| InternalIMT | dha | rs150447740 | 0.002 | . | . | . | . | . | . | . | . | . |
| InternalIMT | dha | rs143099649 | . | . | . | . | . | . | . | . | . | . |
| InternalIMT | dha | rs191288436 | 0.003 | . | . | . | . | . | . | . | . | . |
| InternalIMT | dha | rs188577862 | 0.001 | . | . | . | . | . | . | . | . | . |
| InternalIMT | dha | rs192695334 | 0.001 | . | . | . | . | . | . | . | . | . |
| InternalIMT | dha | rs138612906 | 0.007 | . | . | . | . | . | . | . | . | . |
| InternalIMT | dha | rs191290051 | 0.006 | . | . | . | . | . | . | . | . | . |
| InternalIMT | dha | rs189631459 | 0 | . | . | . | . | . | . | . | . | . |
| InternalIMT | dha | rs114620874 | 0.002 | . | . | . | . | . | . | . | . | . |
| InternalIMT | dha | rs78246652 | 0.003 | . | . | . | . | . | . | . | . | . |
| InternalIMT | dha | rs79432451 | 0.002 | . | . | . | . | . | . | . | . | . |
| InternalIMT | dha | rs186022585 | . | . | . | . | . | . | . | . | . | . |
| InternalIMT | dha | rs142978891 | 0.002 | . | . | . | . | . | . | . | . | . |
| InternalIMT | dha | rs151086840 | 0.003 | . | . | . | . | . | . | . | . | . |
| InternalIMT | dha | rs183270259 | 0.002 | . | . | . | . | . | . | . | . | . |
| InternalIMT | dha | rs188544289 | 0.002 | . | . | . | . | . | . | . | . | . |
| InternalIMT | dha | rs188500808 | 0 | . | . | . | . | . | . | . | . | . |
| InternalIMT | dha | rs189424589 | 0 | . | . | . | . | . | . | . | . | . |
| InternalIMT | dha | rs78622303 | 0.008 | . | . | . | . | . | . | . | . | . |
| InternalIMT | dha | rs143210835 | 0.002 | . | . | . | . | . | . | . | . | . |
| InternalIMT | dha | rs114761840 | 0.001 | . | . | . | . | . | . | . | . | . |
| InternalIMT | dha | rs185805402 | . | . | . | . | . | . | . | . | . | . |
| InternalIMT | dha | rs139273744 | 0.001 | . | . | . | . | . | . | . | . | . |
| InternalIMT | dha | rs184654150 | 0.001 | . | . | . | . | . | . | . | . | . |
| InternalIMT | dha | rs189069710 | . | . | . | . | . | . | . | . | . | . |
| InternalIMT | dha | rs114752115 | 0.004 | . | . | . | . | . | . | . | . | . |
| InternalIMT | dha | rs150162515 | 0 | . | . | . | . | . | . | . | . | . |
| InternalIMT | dha | rs180982454 | 0.001 | . | . | . | . | . | . | . | . | . |
| InternalIMT | dha | rs184932088 | . | . | . | . | . | . | . | . | . | . |
| InternalIMT | dha | rs181109625 | . | . | . | . | . | . | . | . | . | . |
| InternalIMT | dha | rs138121134 | . | . | . | . | . | . | . | . | . | . |
| InternalIMT | dha | rs183253861 | 0 | . | . | . | . | . | . | . | . | . |
| InternalIMT | dha | rs147073946 | . | . | . | . | . | . | . | . | . | . |
| InternalIMT | dha | rs139938489 | 0.002 | . | . | . | . | . | . | . | . | . |
| InternalIMT | dha | rs146362560 | 0.003 | . | . | . | . | . | . | . | . | . |
| InternalIMT | dha | rs193155956 | 0.001 | . | . | . | . | . | . | . | . | . |
| InternalIMT | dha | rs77663092 | 0.001 | . | . | . | . | . | . | . | . | . |
| InternalIMT | dha | rs73285319 | 0.007 | . | . | . | . | . | . | . | . | . |
| InternalIMT | dha | rs184019276 | . | . | . | . | . | . | . | . | . | . |
| InternalIMT | dha | rs28395863 | 0.004 | . | . | . | . | . | . | . | . | . |
| InternalIMT | dha | rs182121645 | 0.004 | . | . | . | . | . | . | . | . | . |
| InternalIMT | dha | rs73285327 | 0.006 | . | . | . | . | . | . | . | . | . |
| InternalIMT | dha | rs149589584 | . | . | . | . | . | . | . | . | . | . |
| InternalIMT | dha | rs148310335 | . | . | . | . | . | . | . | . | . | . |
| InternalIMT | dha | rs187246093 | 0.003 | . | . | . | . | . | . | . | . | . |
| InternalIMT | dha | rs184668894 | . | . | . | . | . | . | . | . | . | . |
| InternalIMT | dha | rs114269961 | . | . | . | . | . | . | . | . | . | . |
| InternalIMT | dha | rs149694374 | 0.005 | . | . | . | . | . | . | . | . | . |
| InternalIMT | dha | rs145284227 | . | . | . | . | . | . | . | . | . | . |
| InternalIMT | dha | rs189983820 | . | . | . | . | . | . | . | . | . | . |
| InternalIMT | dha | rs181200444 | . | . | . | . | . | . | . | . | . | . |
| InternalIMT | dha | rs185486385 | . | . | . | . | . | . | . | . | . | . |
| InternalIMT | dha | rs151198241 | 0.002 | . | . | . | . | . | . | . | . | . |
| InternalIMT | dha | rs189959341 | 0.002 | . | . | . | . | . | . | . | . | . |
| InternalIMT | dha | rs140549257 | 0.002 | . | . | . | . | . | . | . | . | . |
| InternalIMT | dha | rs186453139 | 0.005 | . | . | . | . | . | . | . | . | . |
| InternalIMT | dha | rs180922711 | 0.009 | . | . | . | . | . | . | . | . | . |
| InternalIMT | dha | rs56659903 | 0.002 | . | . | . | . | . | . | . | . | . |
| InternalIMT | dha | rs141202128 | 0.002 | . | . | . | . | . | . | . | . | . |
| InternalIMT | dha | rs59085314 | 0.008 | . | . | . | . | . | . | . | . | . |
| InternalIMT | dha | rs145564996 | . | . | . | . | . | . | . | . | . | . |
| InternalIMT | dha | rs114961009 | 0.002 | . | . | . | . | . | . | . | . | . |
| InternalIMT | dha | rs182294189 | 0.001 | . | . | . | . | . | . | . | . | . |
| InternalIMT | dha | rs187586636 | 0.002 | . | . | . | . | . | . | . | . | . |
| InternalIMT | dha | rs192254907 | 0.005 | . | . | . | . | . | . | . | . | . |
| InternalIMT | dha | rs149145663 | . | . | . | . | . | . | . | . | . | . |
| InternalIMT | dha | rs180713064 | 0.002 | . | . | . | . | . | . | . | . | . |
| InternalIMT | dha | rs183009023 | 0.001 | . | . | . | . | . | . | . | . | . |
| InternalIMT | dha | rs143385347 | 0.004 | . | . | . | . | . | . | . | . | . |
| InternalIMT | dha | rs185786329 | 0.001 | . | . | . | . | . | . | . | . | . |
| InternalIMT | dha | rs192341030 | 0.001 | . | . | . | . | . | . | . | . | . |

| InternalIMT | dha | rs74661429 | 0.002 | . | . | . | . | . | . | . | . | . |
| --- | --- | --- | --- | --- | --- | --- | --- | --- | --- | --- | --- | --- |
| InternalIMT | dha | rs188034853 | 0 | . | . | . | . | . | . | . | . | . |
| InternalIMT | dha | rs116808430 | 0.005 | . | . | . | . | . | . | . | . | . |
| InternalIMT | dha | rs148469010 | . | . | . | . | . | . | . | . | . | . |
| InternalIMT | dha | rs78621663 | 0.002 | . | . | . | . | . | . | . | . | . |
| InternalIMT | dha | rs192654886 | . | . | . | . | . | . | . | . | . | . |
| InternalIMT | dha | rs145996035 | 0.002 | . | . | . | . | . | . | . | . | . |
| InternalIMT | dha | rs139942226 | 0 | . | . | . | . | . | . | . | . | . |
| InternalIMT | dha | rs113645632 | 0.002 | . | . | . | . | . | . | . | . | . |
| InternalIMT | dha | rs74940661 | 0.005 | . | . | . | . | . | . | . | . | . |
| InternalIMT | dha | rs115355800 | 0.003 | . | . | . | . | . | . | . | . | . |
| InternalIMT | dha | rs150285898 | 0.001 | . | . | . | . | . | . | . | . | . |
| InternalIMT | dha | rs188117932 | . | . | . | . | . | . | . | . | . | . |
| InternalIMT | dha | rs139956566 | 0 | . | . | . | . | . | . | . | . | . |
| InternalIMT | dha | rs147673885 | 0 | . | . | . | . | . | . | . | . | . |
| InternalIMT | dha | rs189127928 | 0.005 | . | . | . | . | . | . | . | . | . |
| InternalIMT | dha | rs187558772 | 0.002 | . | . | . | . | . | . | . | . | . |
| InternalIMT | dha | rs181948587 | 0 | . | . | . | . | . | . | . | . | . |
| InternalIMT | dha | rs189983269 | 0.002 | . | . | . | . | . | . | . | . | . |
| InternalIMT | dha | rs187250069 | 0.002 | . | . | . | . | . | . | . | . | . |
| InternalIMT | dha | rs191485859 | 0.001 | . | . | . | . | . | . | . | . | . |
| InternalIMT | dha | rs185499555 | 0.002 | . | . | . | . | . | . | . | . | . |
| InternalIMT | dha | rs180862110 | 0.003 | . | . | . | . | . | . | . | . | . |
| InternalIMT | dha | rs190472603 | 0.005 | . | . | . | . | . | . | . | . | . |
| InternalIMT | dha | rs189226167 | 0.005 | . | . | . | . | . | . | . | . | . |
| InternalIMT | dha | rs181623256 | 0.002 | . | . | . | . | . | . | . | . | . |
| InternalIMT | dha | rs186079557 | 0.001 | . | . | . | . | . | . | . | . | . |
| InternalIMT | dha | rs147760994 | 0.002 | . | . | . | . | . | . | . | . | . |
| InternalIMT | dha | rs189176167 | 0 | . | . | . | . | . | . | . | . | . |
| InternalIMT | dha | rs117901000 | 0.001 | . | . | . | . | . | . | . | . | . |
| InternalIMT | dha | rs141984888 | 0 | . | . | . | . | . | . | . | . | . |
| InternalIMT | dha | rs192365061 | 0.007 | . | . | . | . | . | . | . | . | . |
| InternalIMT | dha | rs185976038 | 0 | . | . | . | . | . | . | . | . | . |
| InternalIMT | dha | rs184697303 | 0.002 | . | . | . | . | . | . | . | . | . |
| InternalIMT | dha | rs143383132 | 0.002 | . | . | . | . | . | . | . | . | . |
| InternalIMT | dha | rs141873881 | 0.004 | . | . | . | . | . | . | . | . | . |
| InternalIMT | dha | rs185507255 | 0 | . | . | . | . | . | . | . | . | . |
| InternalIMT | dha | rs191379425 | 0.001 | . | . | . | . | . | . | . | . | . |
| InternalIMT | dha | rs184199584 | 0.002 | . | . | . | . | . | . | . | . | . |
| InternalIMT | dha | rs183181360 | 0.002 | . | . | . | . | . | . | . | . | . |
| InternalIMT | dha | rs184495673 | 0.001 | . | . | . | . | . | . | . | . | . |
| InternalIMT | dha | rs182946297 | 0.001 | . | . | . | . | . | . | . | . | . |
| InternalIMT | dha | rs187743615 | 0 | . | . | . | . | . | . | . | . | . |
| InternalIMT | dha | rs183259862 | 0.001 | . | . | . | . | . | . | . | . | . |
| InternalIMT | dha | rs112433566 | 0 | . | . | . | . | . | . | . | . | . |
| InternalIMT | dha | rs182351085 | 0.001 | . | . | . | . | . | . | . | . | . |
| InternalIMT | dha | rs146248508 | 0.001 | . | . | . | . | . | . | . | . | . |
| InternalIMT | dha | rs188528439 | 0.001 | . | . | . | . | . | . | . | . | . |
| InternalIMT | dha | rs146167018 | 0.005 | . | . | . | . | . | . | . | . | . |
| InternalIMT | dha | rs192440980 | 0.004 | . | . | . | . | . | . | . | . | . |
| InternalIMT | dha | rs185601882 | 0.001 | . | . | . | . | . | . | . | . | . |
| InternalIMT | dha | rs117272563 | 0.002 | . | . | . | . | . | . | . | . | . |
| InternalIMT | dha | rs10430734 | 0.003 | . | . | . | . | . | . | . | . | . |
| InternalIMT | dha | rs181320952 | 0.001 | . | . | . | . | . | . | . | . | . |
| InternalIMT | dha | rs185480660 | 0.001 | . | . | . | . | . | . | . | . | . |
| InternalIMT | dha | rs188077917 | 0.001 | . | . | . | . | . | . | . | . | . |
| InternalIMT | dha | rs187562953 | 0.002 | . | . | . | . | . | . | . | . | . |
| InternalIMT | dha | rs182044631 | 0 | . | . | . | . | . | . | . | . | . |
| InternalIMT | dha | rs145455596 | 0.001 | . | . | . | . | . | . | . | . | . |
| InternalIMT | dha | rs181475877 | 0.004 | . | . | . | . | . | . | . | . | . |
| InternalIMT | dha | rs149413085 | 0.002 | . | . | . | . | . | . | . | . | . |
| InternalIMT | dha | rs12253656 | 0 | . | . | . | . | . | . | . | . | . |
| InternalIMT | dha | rs182718239 | 0 | . | . | . | . | . | . | . | . | . |
| InternalIMT | dha | rs192435695 | 0.002 | . | . | . | . | . | . | . | . | . |
| InternalIMT | dha | rs12248277 | 0 | . | . | . | . | . | . | . | . | . |
| InternalIMT | dha | rs143521710 | 0.001 | . | . | . | . | . | . | . | . | . |
| InternalIMT | dha | rs144454454 | 0 | . | . | . | . | . | . | . | . | . |
| InternalIMT | dha | rs190441610 | 0.004 | . | . | . | . | . | . | . | . | . |
| InternalIMT | dha | rs114690462 | 0 | . | . | . | . | . | . | . | . | . |
| InternalIMT | dha | rs184541357 | 0.001 | . | . | . | . | . | . | . | . | . |
| InternalIMT | dha | rs142657653 | 0 | . | . | . | . | . | . | . | . | . |
| InternalIMT | dha | rsurf1 | 2.224 | . | . | . | . | . | . | . | . | . |
| InternalIMT | dha | rsten1 | 2.372 | . | . | . | . | . | . | . | . | . |
| InternalIMT | dha | chr10:45852312:D | 0.072 | 0.037 | 0.046 | 0.41 | 8.00E-03 | 0.097 | 0.934 | -0.039 | 0.086 | 0.649 |
| InternalIMT | dha | chr10:45853232:I | 0.086 | 0.034 | 0.046 | 0.47 | -0.065 | 0.098 | 0.507 | -3.20E-03 | 0.087 | 0.971 |
| InternalIMT | dha | chr10:45853234:I | 0.141 | 0.037 | 0.05 | 0.457 | -0.042 | 0.077 | 0.584 | -0.018 | 0.068 | 0.792 |
| InternalIMT | dha | chr10:45854796:D | 0.022 | 0.015 | 0.043 | 0.73 | -0.58 | 0.228 | 0.011 | 0.521 | 0.27 | 0.054 |
| InternalIMT | dha | chr10:45854994:I | 0.025 | 0.015 | 0.043 | 0.731 | -0.5 | 0.206 | 0.015 | 0.443 | 0.244 | 0.069 |
| InternalIMT | dha | chr10:45858290:D | 0.045 | 0.031 | 0.043 | 0.477 | -0.056 | 0.256 | 0.827 | 0.011 | 0.252 | 0.964 |
| InternalIMT | dha | chr10:45858820:I | 0.012 | 0.034 | 0.043 | 0.437 | 0.055 | 0.361 | 0.879 | -0.082 | 0.382 | 0.831 |
| InternalIMT | dha | chr10:45860888:D | 0.072 | 0.036 | 0.047 | 0.447 | -0.06 | 0.146 | 0.682 | -0.019 | 0.131 | 0.887 |
| InternalIMT | dha | chr10:45863913:I | 0.144 | 0.042 | 0.05 | 0.401 | -0.022 | 0.076 | 0.771 | -0.036 | 0.067 | 0.587 |
| InternalIMT | dha | chr10:45866601:D | 0.016 | 0.021 | 0.041 | 0.618 | -0.285 | 0.314 | 0.364 | 0.243 | 0.282 | 0.389 |
| InternalIMT | dha | chr10:45867197:I | 0.005 | . | . | . | . | . | . | . | . | . |
| InternalIMT | dha | chr10:45867538:D | 0.15 | 0.046 | 0.05 | 0.363 | -0.013 | 0.076 | 0.866 | -0.048 | 0.067 | 0.471 |
| InternalIMT | dha | chr10:45868356:D | 0.14 | 0.045 | 0.05 | 0.366 | -6.80E-03 | 0.077 | 0.93 | -0.048 | 0.067 | 0.475 |

| InternalIMT | dha | chr10:45871158:I | 0.145 | 0.044 | 0.05 | 0.38 | -0.019 | 0.076 | 0.806 | -0.042 | 0.066 | 0.529 |
| --- | --- | --- | --- | --- | --- | --- | --- | --- | --- | --- | --- | --- |
| InternalIMT | dha | chr10:45871637:D | 0.066 | 0.046 | 0.047 | 0.33 | -0.039 | 0.125 | 0.756 | -0.06 | 0.103 | 0.559 |
| InternalIMT | dha | chr10:45871638:I | 0.111 | 0.047 | 0.049 | 0.339 | -0.035 | 0.093 | 0.708 | -0.058 | 0.078 | 0.456 |
| InternalIMT | dha | chr10:45873629:I | 0.139 | 0.045 | 0.05 | 0.367 | -0.014 | 0.077 | 0.851 | -0.053 | 0.069 | 0.441 |
| InternalIMT | dha | chr10:45873701:D | 0.009 | . | . | . | . | . | . | . | . | . |
| InternalIMT | dha | chr10:45874997:I | 0.016 | 0.042 | 0.042 | 0.319 | 0.366 | 0.562 | 0.515 | -0.323 | 0.417 | 0.439 |
| InternalIMT | dha | chr10:45882030:I | 0.028 | 0.015 | 0.042 | 0.725 | -0.303 | 0.228 | 0.184 | 0.265 | 0.228 | 0.246 |
| InternalIMT | dha | chr10:45883774:D | 0.092 | 0.019 | 0.047 | 0.685 | -0.113 | 0.09 | 0.211 | 0.064 | 0.083 | 0.442 |
| InternalIMT | dha | chr10:45885169:D | 0.054 | 0.034 | 0.045 | 0.443 | -0.039 | 0.112 | 0.724 | -0.026 | 0.098 | 0.791 |
| InternalIMT | dha | chr10:45886451:D | 0.701 | 0.189 | 0.105 | 0.071 | 0.128 | 0.074 | 0.084 | -0.109 | 0.069 | 0.114 |
| InternalIMT | dha | chr10:45891800:I | 0.669 | 0.091 | 0.094 | 0.334 | 0.044 | 0.069 | 0.523 | -0.042 | 0.063 | 0.502 |
| InternalIMT | dha | chr10:45904771:I | 0.683 | 0.186 | 0.097 | 0.055 | 0.11 | 0.068 | 0.105 | -0.107 | 0.063 | 0.091 |
| InternalIMT | dha | chr10:45904959:I | 0.083 | -5.20E-03 | 0.045 | 0.909 | -0.262 | 0.127 | 0.04 | 0.21 | 0.119 | 0.077 |
| InternalIMT | dha | chr10:45911028:I | 0.045 | 0.029 | 0.045 | 0.513 | 0.1 | 0.255 | 0.694 | -0.024 | 0.229 | 0.916 |
| InternalIMT | dha | chr10:45911031:I | 0.411 | 0.138 | 0.072 | 0.057 | 0.168 | 0.072 | 0.02 | -0.126 | 0.066 | 0.056 |
| InternalIMT | dha | chr10:45912154:D | 0.086 | 0.021 | 0.044 | 0.642 | -0.107 | 0.101 | 0.292 | 0.06 | 0.097 | 0.533 |
| InternalIMT | dha | chr10:45913885:I | 0.021 | 0.017 | 0.043 | 0.688 | -0.497 | 0.226 | 0.028 | 0.415 | 0.27 | 0.124 |
| InternalIMT | dha | chr10:45914753:D | 0.181 | -7.30E-04 | 0.05 | 0.989 | -0.115 | 0.092 | 0.211 | 0.088 | 0.085 | 0.298 |
| InternalIMT | dha | chr10:45919621 | . | . | . | . | . | . | . | . | . | . |
| InternalIMT | dha | chr10:45921739:I | 0.06 | 0.04 | 0.044 | 0.366 | -0.032 | 0.119 | 0.788 | -0.037 | 0.098 | 0.703 |
| InternalIMT | dha | chr10:45923527:I | 0.054 | 0.048 | 0.045 | 0.292 | 0.196 | 0.199 | 0.326 | -0.157 | 0.166 | 0.346 |
| InternalIMT | dha | chr10:45927001:I | 0.011 | 0.018 | 0.043 | 0.679 | -0.085 | 0.686 | 0.902 | 0.478 | 0.688 | 0.488 |
| InternalIMT | dha | chr10:45930354:I | 0.023 | 0.015 | 0.042 | 0.728 | -0.626 | 0.244 | 0.01 | 0.576 | 0.293 | 0.049 |
| InternalIMT | dha | chr10:45932864:I | 0.046 | -0.025 | 0.043 | 0.564 | -0.697 | 0.247 | 4.80E-03 | 0.696 | 0.281 | 0.013 |
| InternalIMT | dha | chr10:45936163:I | 0.025 | 0.023 | 0.041 | 0.574 | -0.342 | 0.287 | 0.235 | 0.22 | 0.276 | 0.424 |
| InternalIMT | dha | chr10:45938095:D | 0.116 | 0.011 | 0.046 | 0.817 | -0.197 | 0.087 | 0.023 | 0.104 | 0.075 | 0.168 |
| InternalIMT | dha | chr10:45938886 | . | . | . | . | . | . | . | . | . | . |
| InternalIMT | dha | chr10:45939320 | . | . | . | . | . | . | . | . | . | . |
| InternalIMT | dha | chr10:45939659 | 0.002 | . | . | . | . | . | . | . | . | . |
| InternalIMT | dha | chr10:45941150:D | 0.021 | 0.016 | 0.042 | 0.701 | -0.557 | 0.231 | 0.016 | 0.468 | 0.275 | 0.089 |
| InternalIMT | dha | chr10:45947128:D | 0.046 | 0.039 | 0.044 | 0.381 | 0.152 | 0.155 | 0.327 | -0.074 | 0.125 | 0.553 |
| InternalIMT | dha | chr10:45950990:I | 0.06 | -9.40E-03 | 0.044 | 0.832 | -0.404 | 0.132 | 2.30E-03 | 0.316 | 0.144 | 0.028 |
| InternalIMT | dha | chr10:45951854:I | 0.02 | 0.033 | 0.043 | 0.433 | 8.70E-03 | 0.267 | 0.974 | -0.07 | 0.24 | 0.771 |
| InternalIMT | dha | chr10:45952670:I | 0.042 | 0.058 | 0.043 | 0.179 | 0.319 | 0.183 | 0.082 | -0.305 | 0.139 | 0.028 |
| InternalIMT | dha | chr10:45956741 | . | . | . | . | . | . | . | . | . | . |
| InternalIMT | dha | chr10:45957247:I | 0.304 | 0.019 | 0.054 | 0.724 | -0.042 | 0.061 | 0.496 | 0.017 | 0.055 | 0.75 |
| InternalIMT | dha | chr10:45958082:I | 0.302 | 0.026 | 0.055 | 0.645 | -0.033 | 0.061 | 0.592 | 8.10E-03 | 0.054 | 0.882 |
| InternalIMT | dha | chr10:45905041:D | 0.039 | 0.02 | 0.044 | 0.655 | -0.317 | 0.118 | 7.30E-03 | 0.195 | 0.115 | 0.09 |
| InternalIMT | dha | chr10:45938871 | . | . | . | . | . | . | . | . | . | . |
| InternalIMT | dha | chr10:45939160 | . | . | . | . | . | . | . | . | . | . |
| InternalIMT | dha | chr10:45869910 | 0.001 | . | . | . | . | . | . | . | . | . |
| InternalIMT | dha | chr10:45876214:D | 0.005 | . | . | . | . | . | . | . | . | . |
| InternalIMT | dha | chr10:45880237:I | 0.014 | 0.038 | 0.043 | 0.381 | -0.04 | 0.23 | 0.862 | -0.051 | 0.169 | 0.764 |
| InternalIMT | dha | chr10:45884371:I | 0.013 | 0.022 | 0.041 | 0.587 | -0.345 | 0.366 | 0.346 | 0.309 | 0.318 | 0.332 |
| InternalIMT | dha | chr10:45891535:D | 0.004 | . | . | . | . | . | . | . | . | . |
| InternalIMT | dha | chr10:45893516:D | 0.013 | 0.022 | 0.041 | 0.587 | -0.342 | 0.364 | 0.348 | 0.306 | 0.314 | 0.331 |
| InternalIMT | dha | chr10:45893782:D | 0.001 | . | . | . | . | . | . | . | . | . |
| InternalIMT | dha | chr10:45895254:I | 0.004 | . | . | . | . | . | . | . | . | . |
| InternalIMT | dha | chr10:45895848:D | 0.011 | 0.032 | 0.042 | 0.442 | -0.106 | 0.311 | 0.734 | 0.046 | 0.2 | 0.817 |
| InternalIMT | dha | chr10:45896316:D | 0.013 | 0.022 | 0.041 | 0.587 | -0.34 | 0.364 | 0.351 | 0.304 | 0.314 | 0.332 |
| InternalIMT | dha | chr10:45896317:D | 0.013 | 0.022 | 0.041 | 0.587 | -0.34 | 0.364 | 0.351 | 0.304 | 0.314 | 0.332 |
| InternalIMT | dha | chr10:45902683:I | 0.006 | . | . | . | . | . | . | . | . | . |
| InternalIMT | dha | chr10:45905003:D | 0.002 | . | . | . | . | . | . | . | . | . |
| InternalIMT | dha | chr10:45927476:D | 0.005 | . | . | . | . | . | . | . | . | . |
| InternalIMT | dha | chr10:45935882 | . | . | . | . | . | . | . | . | . | . |
| InternalIMT | dha | chr10:45936100 | . | . | . | . | . | . | . | . | . | . |
| InternalIMT | dha | chr10:45939553 | 0.004 | . | . | . | . | . | . | . | . | . |
| InternalIMT | dha | chr10:45939631 | . | . | . | . | . | . | . | . | . | . |
| InternalIMT | dha | chr10:45944193:D | 0.003 | . | . | . | . | . | . | . | . | . |
| InternalIMT | dha | chr10:45954030 | . | . | . | . | . | . | . | . | . | . |

Supplemental table S25.

| phen | covar(fa) | rs_id | maf | beta_FA | se_FA | p_FA | beta_SNP | se_SNP | p_SNP | beta_inter | se_inter | p_inter |
| --- | --- | --- | --- | --- | --- | --- | --- | --- | --- | --- | --- | --- |
| InternalIMT | dha | rs76035182 | . | . | . | . | . | . | . | . | . | . |
| InternalIMT | dha | rs12359547 | 0.001 | . | . | . | . | . | . | . | . | . |
| InternalIMT | dha | rs74595484 | . | . | . | . | . | . | . | . | . | . |
| InternalIMT | dha | rs1864415 | 0.873 | -0.228 | 0.181 | 0.207 | -0.14 | 0.182 | 0.442 | 0.097 | 0.108 | 0.366 |
| InternalIMT | dha | rs72788101 | 0.001 | . | . | . | . | . | . | . | . | . |
| InternalIMT | dha | rs11239481 | . | . | . | . | . | . | . | . | . | . |
| InternalIMT | dha | rs72788102 | 0.001 | . | . | . | . | . | . | . | . | . |
| InternalIMT | dha | rs72789705 | 0.001 | . | . | . | . | . | . | . | . | . |
| InternalIMT | dha | rs141529858 | . | . | . | . | . | . | . | . | . | . |
| InternalIMT | dha | rs145376292 | . | . | . | . | . | . | . | . | . | . |
| InternalIMT | dha | rs190418301 | . | . | . | . | . | . | . | . | . | . |
| InternalIMT | dha | rs11239482 | 0.001 | . | . | . | . | . | . | . | . | . |
| InternalIMT | dha | rs61855872 | . | . | . | . | . | . | . | . | . | . |
| InternalIMT | dha | rs148991144 | . | . | . | . | . | . | . | . | . | . |
| InternalIMT | dha | rs191932221 | . | . | . | . | . | . | . | . | . | . |
| InternalIMT | dha | rs55978138 | 0.201 | -0.037 | 0.074 | 0.619 | 0.08 | 0.179 | 0.656 | -0.045 | 0.111 | 0.685 |
| InternalIMT | dha | rs73281167 | . | . | . | . | . | . | . | . | . | . |
| InternalIMT | dha | rs12768010 | 0.2 | -0.033 | 0.073 | 0.656 | 0.099 | 0.178 | 0.58 | -0.056 | 0.111 | 0.615 |
| InternalIMT | dha | rs11239484 | 0.873 | -0.228 | 0.181 | 0.208 | -0.139 | 0.182 | 0.443 | 0.097 | 0.108 | 0.368 |
| InternalIMT | dha | rs35906360 | 0.201 | -0.037 | 0.074 | 0.619 | 0.08 | 0.179 | 0.656 | -0.045 | 0.111 | 0.685 |
| InternalIMT | dha | rs186250512 | . | . | . | . | . | . | . | . | . | . |
| InternalIMT | dha | rs150810743 | . | . | . | . | . | . | . | . | . | . |
| InternalIMT | dha | rs55965812 | 0.201 | -0.037 | 0.074 | 0.619 | 0.08 | 0.179 | 0.656 | -0.045 | 0.111 | 0.685 |
| InternalIMT | dha | rs56180358 | 0.203 | -0.036 | 0.074 | 0.625 | 0.081 | 0.179 | 0.65 | -0.046 | 0.112 | 0.681 |
| InternalIMT | dha | rs55864145 | 0.2 | -0.033 | 0.073 | 0.656 | 0.099 | 0.178 | 0.58 | -0.056 | 0.111 | 0.615 |
| InternalIMT | dha | rs55986122 | 0.201 | -0.037 | 0.074 | 0.619 | 0.08 | 0.179 | 0.656 | -0.045 | 0.111 | 0.685 |
| InternalIMT | dha | rs7477350 | 0.873 | -0.227 | 0.181 | 0.209 | -0.139 | 0.182 | 0.444 | 0.097 | 0.108 | 0.368 |
| InternalIMT | dha | rs2099170 | 0.201 | -0.037 | 0.074 | 0.619 | 0.08 | 0.179 | 0.656 | -0.045 | 0.111 | 0.685 |
| InternalIMT | dha | rs2082108 | 0.201 | -0.037 | 0.074 | 0.619 | 0.08 | 0.179 | 0.656 | -0.045 | 0.111 | 0.685 |
| InternalIMT | dha | rs55902404 | . | . | . | . | . | . | . | . | . | . |
| InternalIMT | dha | rs58402662 | . | . | . | . | . | . | . | . | . | . |
| InternalIMT | dha | rs11511618 | . | . | . | . | . | . | . | . | . | . |
| InternalIMT | dha | rs1816492 | 0.873 | -0.227 | 0.181 | 0.209 | -0.139 | 0.181 | 0.444 | 0.097 | 0.108 | 0.369 |
| InternalIMT | dha | rs146611093 | . | . | . | . | . | . | . | . | . | . |
| InternalIMT | dha | rs186897689 | . | . | . | . | . | . | . | . | . | . |
| InternalIMT | dha | rs10793616 | 0.873 | -0.227 | 0.181 | 0.209 | -0.139 | 0.181 | 0.444 | 0.097 | 0.108 | 0.369 |
| InternalIMT | dha | rs116700479 | . | . | . | . | . | . | . | . | . | . |
| InternalIMT | dha | rs192535936 | . | . | . | . | . | . | . | . | . | . |
| InternalIMT | dha | rs113363297 | 0.852 | -0.281 | 0.179 | 0.117 | -0.195 | 0.187 | 0.297 | 0.131 | 0.111 | 0.238 |
| InternalIMT | dha | rs12354994 | 0.186 | -0.047 | 0.072 | 0.512 | 0.04 | 0.177 | 0.821 | -0.024 | 0.108 | 0.824 |
| InternalIMT | dha | rs148068329 | 0.059 | -0.08 | 0.063 | 0.204 | -0.471 | 0.242 | 0.052 | 0.244 | 0.158 | 0.123 |
| InternalIMT | dha | rs12356109 | 0.198 | -0.041 | 0.074 | 0.582 | 0.063 | 0.18 | 0.725 | -0.037 | 0.112 | 0.739 |
| InternalIMT | dha | rs55908141 | . | . | . | . | . | . | . | . | . | . |
| InternalIMT | dha | rs12354712 | 0.196 | -0.05 | 0.073 | 0.496 | 0.029 | 0.182 | 0.871 | -0.017 | 0.113 | 0.88 |
| InternalIMT | dha | rs147282684 | . | . | . | . | . | . | . | . | . | . |
| InternalIMT | dha | rs12356140 | 0.194 | -0.051 | 0.074 | 0.491 | 0.023 | 0.186 | 0.9 | -0.015 | 0.116 | 0.896 |
| InternalIMT | dha | rs185476187 | . | . | . | . | . | . | . | . | . | . |
| InternalIMT | dha | rs145720057 | . | . | . | . | . | . | . | . | . | . |
| InternalIMT | dha | rs12354742 | 0.196 | -0.05 | 0.073 | 0.496 | 0.029 | 0.182 | 0.871 | -0.017 | 0.113 | 0.88 |
| InternalIMT | dha | rs180913842 | . | . | . | . | . | . | . | . | . | . |
| InternalIMT | dha | rs142976914 | 0.001 | . | . | . | . | . | . | . | . | . |
| InternalIMT | dha | rs11511619 | 0.198 | -0.041 | 0.074 | 0.582 | 0.064 | 0.18 | 0.721 | -0.037 | 0.112 | 0.739 |
| InternalIMT | dha | rs146702870 | . | . | . | . | . | . | . | . | . | . |
| InternalIMT | dha | rs150287496 | 0.024 | -0.052 | 0.061 | 0.393 | 0.117 | 0.481 | 0.808 | -0.094 | 0.28 | 0.738 |
| InternalIMT | dha | rs190917973 | . | . | . | . | . | . | . | . | . | . |
| InternalIMT | dha | rs11517281 | 0.197 | -0.041 | 0.074 | 0.582 | 0.063 | 0.18 | 0.725 | -0.037 | 0.112 | 0.739 |
| InternalIMT | dha | rs12359299 | 0.208 | -0.04 | 0.075 | 0.597 | 0.061 | 0.179 | 0.733 | -0.038 | 0.112 | 0.737 |
| InternalIMT | dha | rs182307932 | . | . | . | . | . | . | . | . | . | . |
| InternalIMT | dha | rs61855873 | . | . | . | . | . | . | . | . | . | . |
| InternalIMT | dha | rs12359302 | 0.193 | -0.041 | 0.074 | 0.575 | 0.063 | 0.182 | 0.728 | -0.036 | 0.113 | 0.749 |
| InternalIMT | dha | rs143208140 | 0.054 | -0.078 | 0.062 | 0.209 | -0.454 | 0.241 | 0.061 | 0.241 | 0.158 | 0.127 |
| InternalIMT | dha | rs9988764 | 0.858 | -0.217 | 0.177 | 0.221 | -0.133 | 0.181 | 0.461 | 0.092 | 0.107 | 0.391 |
| InternalIMT | dha | rs192875767 | 0.015 | -0.057 | 0.059 | 0.338 | 0.037 | 0.528 | 0.944 | -7.80E-03 | 0.322 | 0.981 |
| InternalIMT | dha | rs147268846 | . | . | . | . | . | . | . | . | . | . |
| InternalIMT | dha | rs11239485 | 0.1 | -0.06 | 0.067 | 0.377 | -0.02 | 0.293 | 0.945 | 9.70E-03 | 0.194 | 0.96 |
| InternalIMT | dha | rs151317885 | 0.66 | -0.148 | 0.124 | 0.234 | -0.11 | 0.149 | 0.463 | 0.071 | 0.09 | 0.431 |
| InternalIMT | dha | rs11499085 | . | . | . | . | . | . | . | . | . | . |
| InternalIMT | dha | rs188130606 | . | . | . | . | . | . | . | . | . | . |
| InternalIMT | dha | rs11511620 | 0.196 | -0.047 | 0.074 | 0.528 | 0.042 | 0.18 | 0.817 | -0.024 | 0.112 | 0.829 |
| InternalIMT | dha | rs181498390 | 0.002 | . | . | . | . | . | . | . | . | . |
| InternalIMT | dha | rs186280701 | . | . | . | . | . | . | . | . | . | . |
| InternalIMT | dha | rs150059185 | 0.082 | -0.041 | 0.058 | 0.481 | 0.169 | 0.276 | 0.542 | -0.092 | 0.166 | 0.582 |
| InternalIMT | dha | rs183338707 | . | . | . | . | . | . | . | . | . | . |
| InternalIMT | dha | rs11499038 | 0.006 | . | . | . | . | . | . | . | . | . |
| InternalIMT | dha | rs190699595 | . | . | . | . | . | . | . | . | . | . |
| InternalIMT | dha | rs11523408 | 0.197 | -0.041 | 0.074 | 0.582 | 0.063 | 0.18 | 0.725 | -0.037 | 0.112 | 0.739 |
| InternalIMT | dha | rs188049317 | . | . | . | . | . | . | . | . | . | . |
| InternalIMT | dha | rs144948634 | 0.584 | -0.08 | 0.111 | 0.471 | -0.014 | 0.15 | 0.926 | 0.021 | 0.092 | 0.819 |
| InternalIMT | dha | rs143833576 | 0.016 | -0.051 | 0.059 | 0.383 | 0.264 | 0.423 | 0.532 | -0.132 | 0.237 | 0.578 |
| InternalIMT | dha | rs151239879 | 0.2 | -0.041 | 0.074 | 0.582 | 0.06 | 0.181 | 0.739 | -0.036 | 0.112 | 0.747 |
| InternalIMT | dha | rs7073743 | 0.61 | -0.086 | 0.112 | 0.444 | -0.017 | 0.144 | 0.907 | 0.025 | 0.088 | 0.779 |
| InternalIMT | dha | rs142563349 | . | . | . | . | . | . | . | . | . | . |
| InternalIMT | dha | rs187246403 | . | . | . | . | . | . | . | . | . | . |
| InternalIMT | dha | rs150423942 | 0.197 | -0.041 | 0.074 | 0.583 | 0.064 | 0.18 | 0.723 | -0.037 | 0.112 | 0.739 |

| InternalIMT | dha | rs184706102 | . | . | . | . | . | . | . | . | . | . |
| --- | --- | --- | --- | --- | --- | --- | --- | --- | --- | --- | --- | --- |
| InternalIMT | dha | rs189626288 | . | . | . | . | . | . | . | . | . | . |
| InternalIMT | dha | rs140749834 | . | . | . | . | . | . | . | . | . | . |
| InternalIMT | dha | rs7078822 | 0.163 | -0.04 | 0.072 | 0.574 | 0.09 | 0.21 | 0.668 | -0.047 | 0.131 | 0.719 |
| InternalIMT | dha | rs186227617 | 0.188 | -0.039 | 0.074 | 0.6 | 0.066 | 0.183 | 0.717 | -0.043 | 0.114 | 0.709 |
| InternalIMT | dha | rs188984368 | 0.024 | -0.038 | 0.055 | 0.485 | 0.48 | 0.924 | 0.604 | -0.255 | 0.549 | 0.643 |
| InternalIMT | dha | rs182078109 | . | . | . | . | . | . | . | . | . | . |
| InternalIMT | dha | rs141456274 | 0.023 | -0.067 | 0.06 | 0.271 | -0.494 | 0.305 | 0.105 | 0.25 | 0.191 | 0.19 |
| InternalIMT | dha | rs146976512 | 0.198 | -0.041 | 0.074 | 0.582 | 0.063 | 0.18 | 0.725 | -0.037 | 0.112 | 0.739 |
| InternalIMT | dha | rs138036816 | . | . | . | . | . | . | . | . | . | . |
| InternalIMT | dha | rs114879777 | . | . | . | . | . | . | . | . | . | . |
| InternalIMT | dha | rs149366475 | . | . | . | . | . | . | . | . | . | . |
| InternalIMT | dha | rs182463043 | . | . | . | . | . | . | . | . | . | . |
| InternalIMT | dha | rs144754632 | 0.055 | -0.08 | 0.062 | 0.197 | -0.479 | 0.235 | 0.042 | 0.255 | 0.152 | 0.095 |
| InternalIMT | dha | rs55717607 | 0.198 | -0.041 | 0.074 | 0.582 | 0.063 | 0.18 | 0.725 | -0.037 | 0.112 | 0.739 |
| InternalIMT | dha | rs187537973 | . | . | . | . | . | . | . | . | . | . |
| InternalIMT | dha | rs148085971 | . | . | . | . | . | . | . | . | . | . |
| InternalIMT | dha | rs11239491 | 0.198 | -0.041 | 0.074 | 0.582 | 0.063 | 0.18 | 0.725 | -0.037 | 0.112 | 0.739 |
| InternalIMT | dha | rs184412184 | . | . | . | . | . | . | . | . | . | . |
| InternalIMT | dha | rs4948998 | 0.868 | -0.232 | 0.179 | 0.195 | -0.142 | 0.181 | 0.435 | 0.1 | 0.107 | 0.352 |
| InternalIMT | dha | rs7076696 | 0.873 | -0.229 | 0.18 | 0.203 | -0.141 | 0.181 | 0.438 | 0.098 | 0.107 | 0.362 |
| InternalIMT | dha | rs11239492 | 0.197 | -0.041 | 0.074 | 0.583 | 0.064 | 0.18 | 0.723 | -0.037 | 0.112 | 0.738 |
| InternalIMT | dha | rs79462470 | . | . | . | . | . | . | . | . | . | . |
| InternalIMT | dha | rs187804654 | . | . | . | . | . | . | . | . | . | . |
| InternalIMT | dha | rs7093687 | 0.198 | -0.041 | 0.074 | 0.582 | 0.063 | 0.18 | 0.725 | -0.037 | 0.112 | 0.739 |
| InternalIMT | dha | rs7081004 | 0.198 | -0.041 | 0.074 | 0.582 | 0.063 | 0.18 | 0.725 | -0.037 | 0.112 | 0.739 |
| InternalIMT | dha | rs7084793 | 0.198 | -0.041 | 0.074 | 0.582 | 0.063 | 0.18 | 0.725 | -0.037 | 0.112 | 0.739 |
| InternalIMT | dha | rs61556080 | . | . | . | . | . | . | . | . | . | . |
| InternalIMT | dha | rs115552064 | . | . | . | . | . | . | . | . | . | . |
| InternalIMT | dha | rs7921503 | 0.198 | -0.041 | 0.074 | 0.582 | 0.063 | 0.18 | 0.725 | -0.037 | 0.112 | 0.739 |
| InternalIMT | dha | rs7909740 | 0.198 | -0.041 | 0.074 | 0.582 | 0.063 | 0.18 | 0.725 | -0.037 | 0.112 | 0.739 |
| InternalIMT | dha | rs7909768 | 0.198 | -0.041 | 0.074 | 0.582 | 0.063 | 0.18 | 0.725 | -0.037 | 0.112 | 0.739 |
| InternalIMT | dha | rs144168004 | . | . | . | . | . | . | . | . | . | . |
| InternalIMT | dha | rs75850448 | . | . | . | . | . | . | . | . | . | . |
| InternalIMT | dha | rs147777574 | . | . | . | . | . | . | . | . | . | . |
| InternalIMT | dha | rs7913488 | 0.198 | -0.041 | 0.074 | 0.582 | 0.063 | 0.18 | 0.725 | -0.037 | 0.112 | 0.739 |
| InternalIMT | dha | rs74390874 | 0.048 | -0.072 | 0.061 | 0.234 | -0.21 | 0.357 | 0.557 | 0.157 | 0.22 | 0.477 |
| InternalIMT | dha | rs141391142 | . | . | . | . | . | . | . | . | . | . |
| InternalIMT | dha | rs115294747 | . | . | . | . | . | . | . | . | . | . |
| InternalIMT | dha | rs148031781 | . | . | . | . | . | . | . | . | . | . |
| InternalIMT | dha | rs181340128 | . | . | . | . | . | . | . | . | . | . |
| InternalIMT | dha | rs189930007 | . | . | . | . | . | . | . | . | . | . |
| InternalIMT | dha | rs12242128 | . | . | . | . | . | . | . | . | . | . |
| InternalIMT | dha | rs78421750 | . | . | . | . | . | . | . | . | . | . |
| InternalIMT | dha | rs114370747 | . | . | . | . | . | . | . | . | . | . |
| InternalIMT | dha | rs12264166 | . | . | . | . | . | . | . | . | . | . |
| InternalIMT | dha | rs182964822 | . | . | . | . | . | . | . | . | . | . |
| InternalIMT | dha | rs67491417 | 0.188 | -0.05 | 0.074 | 0.498 | 0.031 | 0.184 | 0.865 | -0.018 | 0.114 | 0.878 |
| InternalIMT | dha | rs72789720 | . | . | . | . | . | . | . | . | . | . |
| InternalIMT | dha | rs6413416 | 0.198 | -0.041 | 0.074 | 0.582 | 0.063 | 0.18 | 0.725 | -0.037 | 0.112 | 0.739 |
| InternalIMT | dha | rs4986832 | 0.198 | -0.041 | 0.074 | 0.582 | 0.063 | 0.18 | 0.725 | -0.037 | 0.112 | 0.739 |
| InternalIMT | dha | rs144866316 | . | . | . | . | . | . | . | . | . | . |
| InternalIMT | dha | rs187769540 | . | . | . | . | . | . | . | . | . | . |
| InternalIMT | dha | rs6593482 | 0.198 | -0.04 | 0.075 | 0.592 | 0.065 | 0.18 | 0.719 | -0.038 | 0.112 | 0.734 |
| InternalIMT | dha | rs7913948 | 0.199 | -0.042 | 0.074 | 0.572 | 0.057 | 0.18 | 0.75 | -0.034 | 0.112 | 0.758 |
| InternalIMT | dha | rs77185151 | 0.055 | -0.08 | 0.062 | 0.197 | -0.479 | 0.235 | 0.042 | 0.255 | 0.152 | 0.095 |
| InternalIMT | dha | rs12762303 | 0.198 | -0.041 | 0.074 | 0.582 | 0.063 | 0.18 | 0.725 | -0.037 | 0.112 | 0.739 |
| InternalIMT | dha | rs73283136 | . | . | . | . | . | . | . | . | . | . |
| InternalIMT | dha | rs187180218 | . | . | . | . | . | . | . | . | . | . |
| InternalIMT | dha | rs78137087 | 0.198 | -0.041 | 0.074 | 0.582 | 0.063 | 0.18 | 0.725 | -0.037 | 0.112 | 0.739 |
| InternalIMT | dha | rs72561472 | 0.193 | -0.041 | 0.074 | 0.58 | 0.063 | 0.184 | 0.733 | -0.037 | 0.114 | 0.745 |
| InternalIMT | dha | rs4987106 | 0.193 | -0.033 | 0.073 | 0.656 | 0.092 | 0.176 | 0.603 | -0.056 | 0.108 | 0.606 |
| InternalIMT | dha | rs4987105 | 0.198 | -0.041 | 0.074 | 0.582 | 0.063 | 0.18 | 0.725 | -0.037 | 0.112 | 0.739 |
| InternalIMT | dha | rs111916157 | 0.199 | -0.04 | 0.074 | 0.591 | 0.065 | 0.181 | 0.719 | -0.039 | 0.112 | 0.73 |
| InternalIMT | dha | rs111528152 | 0.198 | -0.041 | 0.074 | 0.582 | 0.063 | 0.18 | 0.725 | -0.037 | 0.112 | 0.739 |
| InternalIMT | dha | rs12763738 | 0.197 | -0.041 | 0.074 | 0.582 | 0.063 | 0.18 | 0.725 | -0.037 | 0.112 | 0.74 |
| InternalIMT | dha | rs191675886 | . | . | . | . | . | . | . | . | . | . |
| InternalIMT | dha | rs66489870 | 0.198 | -0.041 | 0.074 | 0.582 | 0.063 | 0.18 | 0.725 | -0.037 | 0.112 | 0.739 |
| InternalIMT | dha | rs71494775 | 0.198 | -0.041 | 0.074 | 0.582 | 0.063 | 0.18 | 0.725 | -0.037 | 0.112 | 0.739 |
| InternalIMT | dha | rs61855875 | . | . | . | . | . | . | . | . | . | . |
| InternalIMT | dha | rs116356837 | . | . | . | . | . | . | . | . | . | . |
| InternalIMT | dha | rs1864414 | 0.198 | -0.041 | 0.074 | 0.582 | 0.063 | 0.18 | 0.725 | -0.037 | 0.112 | 0.739 |
| InternalIMT | dha | rs1864413 | 0.198 | -0.041 | 0.074 | 0.583 | 0.063 | 0.18 | 0.724 | -0.037 | 0.112 | 0.738 |
| InternalIMT | dha | rs139497212 | . | . | . | . | . | . | . | . | . | . |
| InternalIMT | dha | rs892690 | 0.504 | -0.108 | 0.096 | 0.258 | -0.061 | 0.133 | 0.648 | 0.05 | 0.081 | 0.534 |
| InternalIMT | dha | rs1864412 | 0.198 | -0.041 | 0.074 | 0.582 | 0.063 | 0.18 | 0.725 | -0.037 | 0.112 | 0.739 |
| InternalIMT | dha | rs77827771 | . | . | . | . | . | . | . | . | . | . |
| InternalIMT | dha | rs145294963 | . | . | . | . | . | . | . | . | . | . |
| InternalIMT | dha | rs149081327 | . | . | . | . | . | . | . | . | . | . |
| InternalIMT | dha | rs67008983 | 0.198 | -0.041 | 0.074 | 0.582 | 0.063 | 0.18 | 0.725 | -0.037 | 0.112 | 0.74 |
| InternalIMT | dha | rs56150414 | 0.198 | -0.04 | 0.074 | 0.587 | 0.065 | 0.18 | 0.721 | -0.038 | 0.112 | 0.735 |
| InternalIMT | dha | rs55787481 | 0.199 | -0.041 | 0.074 | 0.584 | 0.063 | 0.18 | 0.725 | -0.037 | 0.112 | 0.738 |
| InternalIMT | dha | rs141270101 | . | . | . | . | . | . | . | . | . | . |
| InternalIMT | dha | rs114980075 | . | . | . | . | . | . | . | . | . | . |
| InternalIMT | dha | rs12783095 | 0.198 | -0.041 | 0.074 | 0.582 | 0.063 | 0.18 | 0.726 | -0.037 | 0.112 | 0.74 |
| InternalIMT | dha | rs182342688 | . | . | . | . | . | . | . | . | . | . |

| InternalIMT | dha | rs17157728 | 0.198 | -0.041 | 0.074 | 0.582 | 0.063 | 0.18 | 0.726 | -0.037 | 0.112 | 0.74 |
| --- | --- | --- | --- | --- | --- | --- | --- | --- | --- | --- | --- | --- |
| InternalIMT | dha | rs114835563 | . | . | . | . | . | . | . | . | . | . |
| InternalIMT | dha | rs3824612 | 0.504 | -0.106 | 0.096 | 0.268 | -0.058 | 0.133 | 0.665 | 0.048 | 0.081 | 0.552 |
| InternalIMT | dha | rs17157733 | 0.196 | -0.041 | 0.074 | 0.582 | 0.064 | 0.181 | 0.726 | -0.037 | 0.112 | 0.739 |
| InternalIMT | dha | rs74128478 | . | . | . | . | . | . | . | . | . | . |
| InternalIMT | dha | rs184973589 | . | . | . | . | . | . | . | . | . | . |
| InternalIMT | dha | rs150479461 | . | . | . | . | . | . | . | . | . | . |
| InternalIMT | dha | rs138310407 | . | . | . | . | . | . | . | . | . | . |
| InternalIMT | dha | rs187426603 | . | . | . | . | . | . | . | . | . | . |
| InternalIMT | dha | rs190843143 | . | . | . | . | . | . | . | . | . | . |
| InternalIMT | dha | rs191883449 | . | . | . | . | . | . | . | . | . | . |
| InternalIMT | dha | rs58238740 | 0.198 | -0.041 | 0.074 | 0.582 | 0.063 | 0.18 | 0.726 | -0.037 | 0.112 | 0.74 |
| InternalIMT | dha | rs3780894 | 0.198 | -0.041 | 0.074 | 0.582 | 0.063 | 0.18 | 0.726 | -0.037 | 0.112 | 0.74 |
| InternalIMT | dha | rs149646626 | . | . | . | . | . | . | . | . | . | . |
| InternalIMT | dha | rs79615212 | . | . | . | . | . | . | . | . | . | . |
| InternalIMT | dha | rs3780895 | 0.197 | -0.041 | 0.074 | 0.583 | 0.064 | 0.18 | 0.723 | -0.037 | 0.112 | 0.739 |
| InternalIMT | dha | rs3780896 | 0.198 | -0.041 | 0.074 | 0.582 | 0.063 | 0.18 | 0.726 | -0.037 | 0.112 | 0.74 |
| InternalIMT | dha | rs142747163 | 0.003 | . | . | . | . | . | . | . | . | . |
| InternalIMT | dha | rs189417584 | . | . | . | . | . | . | . | . | . | . |
| InternalIMT | dha | rs3780897 | 0.197 | -0.041 | 0.074 | 0.582 | 0.063 | 0.18 | 0.725 | -0.037 | 0.112 | 0.74 |
| InternalIMT | dha | rs117509491 | . | . | . | . | . | . | . | . | . | . |
| InternalIMT | dha | rs117811514 | . | . | . | . | . | . | . | . | . | . |
| InternalIMT | dha | rs116500932 | . | . | . | . | . | . | . | . | . | . |
| InternalIMT | dha | rs77584168 | 0.014 | -0.045 | 0.06 | 0.45 | 0.29 | 0.529 | 0.583 | -0.234 | 0.292 | 0.423 |
| InternalIMT | dha | rs2310776 | 0.198 | -0.041 | 0.074 | 0.582 | 0.063 | 0.18 | 0.726 | -0.037 | 0.112 | 0.74 |
| InternalIMT | dha | rs17157736 | 0.198 | -0.041 | 0.074 | 0.582 | 0.063 | 0.18 | 0.726 | -0.037 | 0.112 | 0.74 |
| InternalIMT | dha | rs72789735 | 0.001 | . | . | . | . | . | . | . | . | . |
| InternalIMT | dha | rs183747187 | . | . | . | . | . | . | . | . | . | . |
| InternalIMT | dha | rs7077891 | 0.198 | -0.041 | 0.074 | 0.582 | 0.063 | 0.18 | 0.726 | -0.037 | 0.112 | 0.74 |
| InternalIMT | dha | rs145063305 | 0.001 | . | . | . | . | . | . | . | . | . |
| InternalIMT | dha | rs3824613 | 0.198 | -0.042 | 0.074 | 0.575 | 0.06 | 0.18 | 0.739 | -0.035 | 0.112 | 0.756 |
| InternalIMT | dha | rs145543199 | . | . | . | . | . | . | . | . | . | . |
| InternalIMT | dha | rs140361993 | . | . | . | . | . | . | . | . | . | . |
| InternalIMT | dha | rs11239496 | 0.001 | . | . | . | . | . | . | . | . | . |
| InternalIMT | dha | rs11239497 | 0.197 | -0.041 | 0.074 | 0.582 | 0.063 | 0.18 | 0.726 | -0.037 | 0.112 | 0.74 |
| InternalIMT | dha | rs10900212 | 0.198 | -0.041 | 0.074 | 0.582 | 0.063 | 0.18 | 0.725 | -0.037 | 0.112 | 0.74 |
| InternalIMT | dha | rs186487136 | . | . | . | . | . | . | . | . | . | . |
| InternalIMT | dha | rs12774369 | 0.197 | -0.041 | 0.074 | 0.582 | 0.063 | 0.18 | 0.725 | -0.037 | 0.112 | 0.74 |
| InternalIMT | dha | rs60842538 | 0.197 | -0.041 | 0.074 | 0.582 | 0.064 | 0.181 | 0.725 | -0.037 | 0.112 | 0.739 |
| InternalIMT | dha | rs3780898 | 0.197 | -0.041 | 0.074 | 0.582 | 0.064 | 0.181 | 0.724 | -0.037 | 0.112 | 0.739 |
| InternalIMT | dha | rs3780899 | 0.197 | -0.041 | 0.074 | 0.582 | 0.064 | 0.181 | 0.724 | -0.037 | 0.112 | 0.739 |
| InternalIMT | dha | rs12252472 | . | . | . | . | . | . | . | . | . | . |
| InternalIMT | dha | rs185411011 | . | . | . | . | . | . | . | . | . | . |
| InternalIMT | dha | rs28395865 | 0.193 | -0.042 | 0.074 | 0.57 | 0.061 | 0.184 | 0.741 | -0.035 | 0.114 | 0.756 |
| InternalIMT | dha | rs191004074 | . | . | . | . | . | . | . | . | . | . |
| InternalIMT | dha | rs182401295 | . | . | . | . | . | . | . | . | . | . |
| InternalIMT | dha | rs2228064 | 0.057 | -0.081 | 0.062 | 0.195 | -0.484 | 0.236 | 0.04 | 0.256 | 0.153 | 0.094 |
| InternalIMT | dha | rs188284159 | . | . | . | . | . | . | . | . | . | . |
| InternalIMT | dha | rs2303887 | 0.055 | -0.08 | 0.062 | 0.197 | -0.479 | 0.236 | 0.042 | 0.255 | 0.153 | 0.096 |
| InternalIMT | dha | rs2303886 | 0.055 | -0.08 | 0.062 | 0.197 | -0.479 | 0.236 | 0.042 | 0.255 | 0.153 | 0.096 |
| InternalIMT | dha | rs192621006 | . | . | . | . | . | . | . | . | . | . |
| InternalIMT | dha | rs2303885 | 0.195 | -0.047 | 0.074 | 0.528 | 0.042 | 0.182 | 0.817 | -0.025 | 0.113 | 0.828 |
| InternalIMT | dha | rs114466168 | . | . | . | . | . | . | . | . | . | . |
| InternalIMT | dha | rs11239501 | 0.003 | . | . | . | . | . | . | . | . | . |
| InternalIMT | dha | rs745986 | 0.196 | -0.042 | 0.074 | 0.573 | 0.069 | 0.184 | 0.706 | -0.036 | 0.113 | 0.749 |
| InternalIMT | dha | rs75262268 | . | . | . | . | . | . | . | . | . | . |
| InternalIMT | dha | rs71494776 | 0.192 | -0.042 | 0.073 | 0.56 | 0.061 | 0.183 | 0.736 | -0.035 | 0.113 | 0.757 |
| InternalIMT | dha | rs74386871 | 0.055 | -0.08 | 0.062 | 0.197 | -0.479 | 0.236 | 0.042 | 0.255 | 0.153 | 0.096 |
| InternalIMT | dha | rs76502040 | . | . | . | . | . | . | . | . | . | . |
| InternalIMT | dha | rs142213734 | . | . | . | . | . | . | . | . | . | . |
| InternalIMT | dha | rs4948671 | 0.869 | -0.232 | 0.172 | 0.178 | -0.149 | 0.174 | 0.393 | 0.1 | 0.103 | 0.332 |
| InternalIMT | dha | rs146286085 | . | . | . | . | . | . | . | . | . | . |
| InternalIMT | dha | rs144080623 | . | . | . | . | . | . | . | . | . | . |
| InternalIMT | dha | rs115074014 | . | . | . | . | . | . | . | . | . | . |
| InternalIMT | dha | rs17157756 | . | . | . | . | . | . | . | . | . | . |
| InternalIMT | dha | rs190459780 | . | . | . | . | . | . | . | . | . | . |
| InternalIMT | dha | rs12358647 | 0.195 | -0.042 | 0.074 | 0.573 | 0.07 | 0.185 | 0.704 | -0.036 | 0.113 | 0.748 |
| InternalIMT | dha | rs147080556 | 0.009 | . | . | . | . | . | . | . | . | . |
| InternalIMT | dha | rs187543349 | . | . | . | . | . | . | . | . | . | . |
| InternalIMT | dha | rs114375595 | 0.01 | . | . | . | . | . | . | . | . | . |
| InternalIMT | dha | rs117211200 | . | . | . | . | . | . | . | . | . | . |
| InternalIMT | dha | rs192963047 | . | . | . | . | . | . | . | . | . | . |
| InternalIMT | dha | rs11239505 | 0.196 | -0.042 | 0.074 | 0.572 | 0.07 | 0.184 | 0.705 | -0.036 | 0.113 | 0.749 |
| InternalIMT | dha | rs184434116 | . | . | . | . | . | . | . | . | . | . |
| InternalIMT | dha | rs4948999 | 0.869 | -0.231 | 0.172 | 0.179 | -0.148 | 0.174 | 0.395 | 0.099 | 0.103 | 0.334 |
| InternalIMT | dha | rs11239506 | 0.003 | . | . | . | . | . | . | . | . | . |
| InternalIMT | dha | rs190706620 | . | . | . | . | . | . | . | . | . | . |
| InternalIMT | dha | rs190770429 | . | . | . | . | . | . | . | . | . | . |
| InternalIMT | dha | rs181327133 | . | . | . | . | . | . | . | . | . | . |
| InternalIMT | dha | rs149251697 | . | . | . | . | . | . | . | . | . | . |
| InternalIMT | dha | rs118081602 | 0.055 | -0.08 | 0.062 | 0.197 | -0.48 | 0.236 | 0.042 | 0.255 | 0.153 | 0.096 |
| InternalIMT | dha | rs188233691 | . | . | . | . | . | . | . | . | . | . |
| InternalIMT | dha | rs183778794 | 0.032 | -0.036 | 0.058 | 0.538 | 0.693 | 0.411 | 0.092 | -0.459 | 0.257 | 0.074 |
| InternalIMT | dha | rs188040078 | . | . | . | . | . | . | . | . | . | . |
| InternalIMT | dha | rs184675372 | . | . | . | . | . | . | . | . | . | . |
| InternalIMT | dha | rs150326891 | 0.004 | . | . | . | . | . | . | . | . | . |

| InternalIMT | dha | rs185520021 | . | . | . | . | . | . | . | . | . | . |
| --- | --- | --- | --- | --- | --- | --- | --- | --- | --- | --- | --- | --- |
| InternalIMT | dha | rs7907779 | 0.003 | . | . | . | . | . | . | . | . | . |
| InternalIMT | dha | rs184962860 | . | . | . | . | . | . | . | . | . | . |
| InternalIMT | dha | rs149762784 | 0.027 | -0.04 | 0.055 | 0.466 | 0.357 | 0.754 | 0.635 | -0.229 | 0.448 | 0.609 |
| InternalIMT | dha | rs149203427 | . | . | . | . | . | . | . | . | . | . |
| InternalIMT | dha | rs60153083 | . | . | . | . | . | . | . | . | . | . |
| InternalIMT | dha | rs112001069 | 0.194 | -0.051 | 0.072 | 0.476 | 0.024 | 0.182 | 0.895 | -0.015 | 0.112 | 0.891 |
| InternalIMT | dha | rs182355382 | 0.012 | -0.069 | 0.056 | 0.212 | -0.823 | 1.19 | 0.488 | 0.611 | 0.811 | 0.451 |
| InternalIMT | dha | rs186740974 | 0.109 | 6.90E-04 | 0.071 | 0.992 | 0.515 | 0.31 | 0.097 | -0.289 | 0.188 | 0.124 |
| InternalIMT | dha | rs191874916 | 0.081 | -0.043 | 0.058 | 0.465 | 0.156 | 0.277 | 0.575 | -0.084 | 0.167 | 0.615 |
| InternalIMT | dha | rs182989412 | 0.122 | -0.099 | 0.072 | 0.171 | -0.223 | 0.335 | 0.505 | 0.19 | 0.201 | 0.344 |
| InternalIMT | dha | rs188819476 | . | . | . | . | . | . | . | . | . | . |
| InternalIMT | dha | rs141217183 | 0.186 | -0.029 | 0.074 | 0.691 | 0.116 | 0.191 | 0.543 | -0.067 | 0.115 | 0.564 |
| InternalIMT | dha | rs192922652 | 0.199 | -0.043 | 0.073 | 0.559 | 0.057 | 0.182 | 0.755 | -0.033 | 0.113 | 0.77 |
| InternalIMT | dha | rs185092343 | 0.012 | -0.039 | 0.059 | 0.507 | 2.65 | 1.84 | 0.15 | -1.45 | 1.03 | 0.16 |
| InternalIMT | dha | rs145193311 | 0.194 | -0.043 | 0.073 | 0.557 | 0.059 | 0.183 | 0.745 | -0.033 | 0.113 | 0.767 |
| InternalIMT | dha | rs55789336 | 0.004 | . | . | . | . | . | . | . | . | . |
| InternalIMT | dha | rs55837212 | 0.009 | . | . | . | . | . | . | . | . | . |
| InternalIMT | dha | rs75408574 | 0.565 | -0.117 | 0.107 | 0.277 | -0.081 | 0.134 | 0.547 | 0.054 | 0.083 | 0.512 |
| InternalIMT | dha | rs149306926 | 0.01 | -0.046 | 0.059 | 0.433 | 0.526 | 0.634 | 0.407 | -0.204 | 0.448 | 0.649 |
| InternalIMT | dha | rs35990892 | 0.843 | -0.171 | 0.193 | 0.377 | -0.103 | 0.18 | 0.569 | 0.068 | 0.108 | 0.533 |
| InternalIMT | dha | rs192356207 | 0.015 | -0.03 | 0.053 | 0.571 | 0.821 | 0.83 | 0.323 | -0.525 | 0.508 | 0.302 |
| InternalIMT | dha | rs183741163 | 0.049 | -0.061 | 0.065 | 0.345 | 7.10E-04 | 0.325 | 0.998 | 0.03 | 0.182 | 0.87 |
| InternalIMT | dha | rs34737297 | 0.719 | -0.234 | 0.185 | 0.205 | -0.218 | 0.205 | 0.288 | 0.125 | 0.121 | 0.301 |
| InternalIMT | dha | rs181346933 | . | . | . | . | . | . | . | . | . | . |
| InternalIMT | dha | rs36093253 | 0.576 | -0.106 | 0.112 | 0.345 | -0.072 | 0.14 | 0.607 | 0.043 | 0.085 | 0.616 |
| InternalIMT | dha | rs186307855 | . | . | . | . | . | . | . | . | . | . |
| InternalIMT | dha | rs189681865 | . | . | . | . | . | . | . | . | . | . |
| InternalIMT | dha | rs34942971 | 0.214 | -0.052 | 0.076 | 0.494 | 0.03 | 0.188 | 0.875 | -0.013 | 0.115 | 0.912 |
| InternalIMT | dha | rs181816554 | . | . | . | . | . | . | . | . | . | . |
| InternalIMT | dha | rs186846881 | . | . | . | . | . | . | . | . | . | . |
| InternalIMT | dha | rs191178220 | 0.003 | . | . | . | . | . | . | . | . | . |
| InternalIMT | dha | rs183305228 | . | . | . | . | . | . | . | . | . | . |
| InternalIMT | dha | rs139912761 | 0.004 | . | . | . | . | . | . | . | . | . |
| InternalIMT | dha | rs185499901 | . | . | . | . | . | . | . | . | . | . |
| InternalIMT | dha | rs189816197 | 0.194 | -0.045 | 0.074 | 0.544 | 0.052 | 0.188 | 0.78 | -0.029 | 0.116 | 0.804 |
| InternalIMT | dha | rs145656938 | 0.009 | . | . | . | . | . | . | . | . | . |
| InternalIMT | dha | rs35745197 | 0.193 | -0.042 | 0.073 | 0.566 | 0.063 | 0.183 | 0.729 | -0.036 | 0.113 | 0.748 |
| InternalIMT | dha | rs192856637 | 0.006 | . | . | . | . | . | . | . | . | . |
| InternalIMT | dha | rs2310775 | 0.617 | -0.088 | 0.112 | 0.433 | -0.028 | 0.143 | 0.843 | 0.025 | 0.086 | 0.768 |
| InternalIMT | dha | rs11239507 | 0.673 | -0.149 | 0.124 | 0.231 | -0.116 | 0.148 | 0.433 | 0.07 | 0.088 | 0.428 |
| InternalIMT | dha | rs74648467 | 0.192 | -0.042 | 0.073 | 0.565 | 0.063 | 0.184 | 0.73 | -0.036 | 0.113 | 0.749 |
| InternalIMT | dha | rs145815934 | . | . | . | . | . | . | . | . | . | . |
| InternalIMT | dha | rs145390687 | . | . | . | . | . | . | . | . | . | . |
| InternalIMT | dha | rs184354913 | . | . | . | . | . | . | . | . | . | . |
| InternalIMT | dha | rs181244920 | . | . | . | . | . | . | . | . | . | . |
| InternalIMT | dha | rs11499087 | . | . | . | . | . | . | . | . | . | . |
| InternalIMT | dha | rs148263794 | 0.207 | -0.041 | 0.075 | 0.587 | 0.065 | 0.177 | 0.714 | -0.036 | 0.11 | 0.745 |
| InternalIMT | dha | rs150758903 | . | . | . | . | . | . | . | . | . | . |
| InternalIMT | dha | rs147654382 | 0.187 | -0.064 | 0.073 | 0.381 | -0.02 | 0.19 | 0.918 | 0.016 | 0.117 | 0.893 |
| InternalIMT | dha | rs147495166 | . | . | . | . | . | . | . | . | . | . |
| InternalIMT | dha | rs35198778 | 0.131 | -0.025 | 0.07 | 0.72 | 0.153 | 0.203 | 0.452 | -0.102 | 0.12 | 0.395 |
| InternalIMT | dha | rs145207659 | 0.048 | -0.073 | 0.061 | 0.233 | -0.209 | 0.358 | 0.56 | 0.157 | 0.221 | 0.477 |
| InternalIMT | dha | rs186466464 | . | . | . | . | . | . | . | . | . | . |
| InternalIMT | dha | rs12778413 | 0.131 | -0.038 | 0.07 | 0.585 | 0.146 | 0.237 | 0.537 | -0.07 | 0.138 | 0.612 |
| InternalIMT | dha | rs7895552 | 0.833 | -0.21 | 0.167 | 0.21 | -0.131 | 0.176 | 0.456 | 0.091 | 0.105 | 0.384 |
| InternalIMT | dha | rs12783895 | 0.193 | -0.042 | 0.073 | 0.566 | 0.063 | 0.182 | 0.729 | -0.036 | 0.113 | 0.748 |
| InternalIMT | dha | rs34929604 | 0.193 | -0.042 | 0.073 | 0.565 | 0.063 | 0.182 | 0.729 | -0.036 | 0.113 | 0.748 |
| InternalIMT | dha | rs12761961 | 0.185 | -0.05 | 0.072 | 0.494 | 0.04 | 0.189 | 0.832 | -0.02 | 0.117 | 0.866 |
| InternalIMT | dha | rs35536930 | 0.197 | -0.042 | 0.074 | 0.572 | 0.07 | 0.184 | 0.704 | -0.036 | 0.113 | 0.749 |
| InternalIMT | dha | rs180951060 | . | . | . | . | . | . | . | . | . | . |
| InternalIMT | dha | rs148892128 | . | . | . | . | . | . | . | . | . | . |
| InternalIMT | dha | rs11239509 | . | . | . | . | . | . | . | . | . | . |
| InternalIMT | dha | rs41526545 | 0.003 | . | . | . | . | . | . | . | . | . |
| InternalIMT | dha | rs2029253 | 0.617 | -0.088 | 0.112 | 0.433 | -0.028 | 0.143 | 0.843 | 0.025 | 0.086 | 0.769 |
| InternalIMT | dha | rs17444064 | 0.003 | . | . | . | . | . | . | . | . | . |
| InternalIMT | dha | rs71494779 | 0.194 | -0.042 | 0.073 | 0.561 | 0.062 | 0.182 | 0.732 | -0.035 | 0.112 | 0.754 |
| InternalIMT | dha | rs74991420 | 0.05 | -0.08 | 0.062 | 0.2 | -0.496 | 0.25 | 0.047 | 0.264 | 0.163 | 0.107 |
| InternalIMT | dha | rs182736551 | 0.05 | -0.08 | 0.062 | 0.2 | -0.496 | 0.25 | 0.047 | 0.264 | 0.163 | 0.107 |
| InternalIMT | dha | rs78702074 | 0.055 | -0.08 | 0.062 | 0.195 | -0.486 | 0.236 | 0.04 | 0.26 | 0.154 | 0.091 |
| InternalIMT | dha | rs187791466 | . | . | . | . | . | . | . | . | . | . |
| InternalIMT | dha | rs35300270 | 0.194 | -0.042 | 0.073 | 0.561 | 0.062 | 0.182 | 0.732 | -0.035 | 0.112 | 0.754 |
| InternalIMT | dha | rs6593484 | 0.003 | . | . | . | . | . | . | . | . | . |
| InternalIMT | dha | rs12244601 | . | . | . | . | . | . | . | . | . | . |
| InternalIMT | dha | rs184429574 | . | . | . | . | . | . | . | . | . | . |
| InternalIMT | dha | rs7080897 | 0.194 | -0.042 | 0.073 | 0.561 | 0.062 | 0.182 | 0.732 | -0.035 | 0.112 | 0.754 |
| InternalIMT | dha | rs7097429 | 0.196 | -0.043 | 0.074 | 0.56 | 0.056 | 0.184 | 0.762 | -0.033 | 0.115 | 0.771 |
| InternalIMT | dha | rs77979229 | 0.016 | -0.05 | 0.059 | 0.39 | 0.338 | 0.491 | 0.492 | -0.177 | 0.275 | 0.52 |
| InternalIMT | dha | rs11239512 | 0.003 | . | . | . | . | . | . | . | . | . |
| InternalIMT | dha | rs60146827 | 0.195 | -0.042 | 0.073 | 0.565 | 0.063 | 0.181 | 0.729 | -0.036 | 0.112 | 0.749 |
| InternalIMT | dha | rs79032236 | . | . | . | . | . | . | . | . | . | . |
| InternalIMT | dha | rs140854206 | . | . | . | . | . | . | . | . | . | . |
| InternalIMT | dha | rs184049550 | . | . | . | . | . | . | . | . | . | . |
| InternalIMT | dha | rs114433607 | . | . | . | . | . | . | . | . | . | . |
| InternalIMT | dha | rs117653793 | 0.055 | -0.08 | 0.062 | 0.195 | -0.487 | 0.237 | 0.04 | 0.26 | 0.154 | 0.091 |
| InternalIMT | dha | rs117039533 | 0.055 | -0.08 | 0.062 | 0.195 | -0.487 | 0.237 | 0.04 | 0.26 | 0.154 | 0.091 |

| InternalIMT | dha | rs191660761 | 0.001 | . | . | . | . | . | . | . | . | . |
| --- | --- | --- | --- | --- | --- | --- | --- | --- | --- | --- | --- | --- |
| InternalIMT | dha | rs59514896 | 0.197 | -0.041 | 0.073 | 0.574 | 0.064 | 0.181 | 0.722 | -0.038 | 0.112 | 0.735 |
| InternalIMT | dha | rs184661876 | . | . | . | . | . | . | . | . | . | . |
| InternalIMT | dha | rs61854073 | . | . | . | . | . | . | . | . | . | . |
| InternalIMT | dha | rs2018518 | 0.5 | -0.091 | 0.098 | 0.35 | -0.047 | 0.142 | 0.74 | 0.033 | 0.086 | 0.696 |
| InternalIMT | dha | rs934187 | 0.872 | -0.228 | 0.17 | 0.181 | -0.146 | 0.172 | 0.397 | 0.097 | 0.102 | 0.338 |
| InternalIMT | dha | rs7918542 | 0.202 | -0.04 | 0.074 | 0.587 | 0.072 | 0.182 | 0.69 | -0.039 | 0.111 | 0.725 |
| InternalIMT | dha | rs144311920 | . | . | . | . | . | . | . | . | . | . |
| InternalIMT | dha | rs7099684 | 0.871 | -0.227 | 0.17 | 0.182 | -0.146 | 0.172 | 0.396 | 0.097 | 0.102 | 0.34 |
| InternalIMT | dha | rs150829171 | . | . | . | . | . | . | . | . | . | . |
| InternalIMT | dha | rs186060697 | . | . | . | . | . | . | . | . | . | . |
| InternalIMT | dha | rs11239513 | . | . | . | . | . | . | . | . | . | . |
| InternalIMT | dha | rs7917687 | 0.2 | -0.039 | 0.073 | 0.59 | 0.067 | 0.178 | 0.704 | -0.041 | 0.11 | 0.71 |
| InternalIMT | dha | rs61854076 | . | . | . | . | . | . | . | . | . | . |
| InternalIMT | dha | rs71494780 | . | . | . | . | . | . | . | . | . | . |
| InternalIMT | dha | rs7919239 | 0.235 | -0.048 | 0.073 | 0.508 | 0.037 | 0.142 | 0.796 | -0.017 | 0.088 | 0.848 |
| InternalIMT | dha | rs78334475 | 0.166 | -0.101 | 0.084 | 0.227 | -0.196 | 0.202 | 0.333 | 0.122 | 0.122 | 0.317 |
| InternalIMT | dha | rs3780900 | 0.068 | -0.061 | 0.06 | 0.305 | -0.029 | 0.302 | 0.922 | 0.051 | 0.186 | 0.784 |
| InternalIMT | dha | rs186729292 | . | . | . | . | . | . | . | . | . | . |
| InternalIMT | dha | rs12267438 | . | . | . | . | . | . | . | . | . | . |
| InternalIMT | dha | rs1369214 | 0.868 | -0.219 | 0.169 | 0.195 | -0.148 | 0.17 | 0.386 | 0.093 | 0.101 | 0.357 |
| InternalIMT | dha | rs9282589 | . | . | . | . | . | . | . | . | . | . |
| InternalIMT | dha | rs114522915 | . | . | . | . | . | . | . | . | . | . |
| InternalIMT | dha | rs2115819 | 0.812 | -0.079 | 0.135 | 0.56 | 4.40E-03 | 0.145 | 0.976 | 0.013 | 0.088 | 0.885 |
| InternalIMT | dha | rs117664603 | 0.055 | -0.08 | 0.062 | 0.198 | -0.476 | 0.234 | 0.042 | 0.253 | 0.152 | 0.096 |
| InternalIMT | dha | rs184979202 | . | . | . | . | . | . | . | . | . | . |
| InternalIMT | dha | rs190714671 | . | . | . | . | . | . | . | . | . | . |
| InternalIMT | dha | rs141538996 | . | . | . | . | . | . | . | . | . | . |
| InternalIMT | dha | rs150881741 | . | . | . | . | . | . | . | . | . | . |
| InternalIMT | dha | rs139076633 | 0.011 | -0.057 | 0.059 | 0.328 | -0.088 | 0.723 | 0.903 | 6.50E-03 | 0.408 | 0.987 |
| InternalIMT | dha | rs12243649 | 0.056 | -0.08 | 0.062 | 0.202 | -0.457 | 0.234 | 0.052 | 0.242 | 0.152 | 0.111 |
| InternalIMT | dha | rs140348708 | . | . | . | . | . | . | . | . | . | . |
| InternalIMT | dha | rs79023256 | 0.055 | -0.08 | 0.062 | 0.199 | -0.475 | 0.234 | 0.043 | 0.252 | 0.152 | 0.097 |
| InternalIMT | dha | rs11239515 | 0.003 | . | . | . | . | . | . | . | . | . |
| InternalIMT | dha | rs55866086 | 0.059 | -0.08 | 0.063 | 0.2 | -0.403 | 0.228 | 0.078 | 0.224 | 0.145 | 0.124 |
| InternalIMT | dha | rs181340150 | . | . | . | . | . | . | . | . | . | . |
| InternalIMT | dha | rs11239516 | 0.003 | . | . | . | . | . | . | . | . | . |
| InternalIMT | dha | rs11239517 | 0.003 | . | . | . | . | . | . | . | . | . |
| InternalIMT | dha | rs11239519 | 0.003 | . | . | . | . | . | . | . | . | . |
| InternalIMT | dha | rs7909514 | 0.872 | -0.23 | 0.175 | 0.188 | -0.149 | 0.177 | 0.4 | 0.099 | 0.104 | 0.344 |
| InternalIMT | dha | rs7894352 | 0.872 | -0.23 | 0.175 | 0.189 | -0.148 | 0.177 | 0.402 | 0.098 | 0.104 | 0.346 |
| InternalIMT | dha | rs189384146 | 0 | . | . | . | . | . | . | . | . | . |
| InternalIMT | dha | rs11239520 | 0.004 | . | . | . | . | . | . | . | . | . |
| InternalIMT | dha | rs12221233 | 0.617 | -0.109 | 0.112 | 0.334 | -0.052 | 0.144 | 0.719 | 0.043 | 0.087 | 0.621 |
| InternalIMT | dha | rs74523293 | 0.049 | -0.072 | 0.062 | 0.247 | -0.375 | 0.222 | 0.091 | 0.179 | 0.14 | 0.201 |
| InternalIMT | dha | rs79376040 | 0.049 | -0.072 | 0.062 | 0.247 | -0.375 | 0.222 | 0.091 | 0.179 | 0.14 | 0.201 |
| InternalIMT | dha | rs4949000 | 0.617 | -0.109 | 0.112 | 0.334 | -0.052 | 0.144 | 0.72 | 0.043 | 0.087 | 0.622 |
| InternalIMT | dha | rs7089406 | . | . | . | . | . | . | . | . | . | . |
| InternalIMT | dha | rs138211056 | . | . | . | . | . | . | . | . | . | . |
| InternalIMT | dha | rs10900213 | 0.621 | -0.109 | 0.115 | 0.343 | -0.048 | 0.146 | 0.744 | 0.043 | 0.088 | 0.625 |
| InternalIMT | dha | rs145152827 | 0.011 | -0.057 | 0.059 | 0.328 | -0.092 | 0.727 | 0.899 | 8.30E-03 | 0.41 | 0.984 |
| InternalIMT | dha | rs74443325 | 0.048 | -0.071 | 0.062 | 0.247 | -0.38 | 0.221 | 0.086 | 0.181 | 0.139 | 0.194 |
| InternalIMT | dha | rs188299446 | . | . | . | . | . | . | . | . | . | . |
| InternalIMT | dha | rs192699586 | . | . | . | . | . | . | . | . | . | . |
| InternalIMT | dha | rs72796464 | 0.003 | . | . | . | . | . | . | . | . | . |
| InternalIMT | dha | rs4949001 | 0.455 | -0.079 | 0.091 | 0.388 | -0.035 | 0.142 | 0.806 | 0.023 | 0.085 | 0.783 |
| InternalIMT | dha | rs147174641 | . | . | . | . | . | . | . | . | . | . |
| InternalIMT | dha | rs190835238 | . | . | . | . | . | . | . | . | . | . |
| InternalIMT | dha | rs76698944 | . | . | . | . | . | . | . | . | . | . |
| InternalIMT | dha | rs56071381 | 0.003 | . | . | . | . | . | . | . | . | . |
| InternalIMT | dha | rs78130427 | 0.2 | -0.039 | 0.073 | 0.59 | 0.066 | 0.181 | 0.715 | -0.041 | 0.112 | 0.716 |
| InternalIMT | dha | rs10900215 | 0.007 | . | . | . | . | . | . | . | . | . |
| InternalIMT | dha | rs41491445 | . | . | . | . | . | . | . | . | . | . |
| InternalIMT | dha | rs144995222 | . | . | . | . | . | . | . | . | . | . |
| InternalIMT | dha | rs149097803 | . | . | . | . | . | . | . | . | . | . |
| InternalIMT | dha | rs139429646 | . | . | . | . | . | . | . | . | . | . |
| InternalIMT | dha | rs7075620 | 0.861 | -0.258 | 0.179 | 0.151 | -0.171 | 0.183 | 0.353 | 0.116 | 0.108 | 0.283 |
| InternalIMT | dha | rs148299213 | . | . | . | . | . | . | . | . | . | . |
| InternalIMT | dha | rs4949002 | 0.238 | -0.018 | 0.07 | 0.797 | 0.179 | 0.178 | 0.317 | -0.098 | 0.107 | 0.358 |
| InternalIMT | dha | rs150496007 | . | . | . | . | . | . | . | . | . | . |
| InternalIMT | dha | rs147303455 | . | . | . | . | . | . | . | . | . | . |
| InternalIMT | dha | rs189518433 | . | . | . | . | . | . | . | . | . | . |
| InternalIMT | dha | rs186281355 | . | . | . | . | . | . | . | . | . | . |
| InternalIMT | dha | rs115297114 | . | . | . | . | . | . | . | . | . | . |
| InternalIMT | dha | rs11239523 | 0.003 | . | . | . | . | . | . | . | . | . |
| InternalIMT | dha | rs143704897 | . | . | . | . | . | . | . | . | . | . |
| InternalIMT | dha | rs188024096 | . | . | . | . | . | . | . | . | . | . |
| InternalIMT | dha | rs11239524 | 0.003 | . | . | . | . | . | . | . | . | . |
| InternalIMT | dha | rs191656573 | . | . | . | . | . | . | . | . | . | . |
| InternalIMT | dha | rs193295875 | 0.011 | -0.06 | 0.058 | 0.301 | -0.517 | 0.528 | 0.328 | 0.252 | 0.353 | 0.476 |
| InternalIMT | dha | rs190458228 | . | . | . | . | . | . | . | . | . | . |
| InternalIMT | dha | rs139253993 | 0.002 | . | . | . | . | . | . | . | . | . |
| InternalIMT | dha | rs4948672 | 0.812 | -0.33 | 0.166 | 0.047 | -0.254 | 0.174 | 0.146 | 0.167 | 0.104 | 0.108 |
| InternalIMT | dha | rs180954921 | . | . | . | . | . | . | . | . | . | . |
| InternalIMT | dha | rs12264801 | 0.447 | 0.038 | 0.095 | 0.686 | 0.158 | 0.157 | 0.314 | -0.109 | 0.101 | 0.28 |
| InternalIMT | dha | rs73283184 | . | . | . | . | . | . | . | . | . | . |

| InternalIMT | dha | rs142746187 | . | . | . | . | . | . | . | . | . | . |
| --- | --- | --- | --- | --- | --- | --- | --- | --- | --- | --- | --- | --- |
| InternalIMT | dha | rs58704739 | . | . | . | . | . | . | . | . | . | . |
| InternalIMT | dha | rs61272335 | . | . | . | . | . | . | . | . | . | . |
| InternalIMT | dha | rs60959489 | . | . | . | . | . | . | . | . | . | . |
| InternalIMT | dha | rs58502629 | . | . | . | . | . | . | . | . | . | . |
| InternalIMT | dha | rs10900216 | 0.838 | -0.135 | 0.153 | 0.378 | -2.40E-03 | 0.158 | 0.988 | 0.045 | 0.097 | 0.641 |
| InternalIMT | dha | rs181118141 | . | . | . | . | . | . | . | . | . | . |
| InternalIMT | dha | rs137882665 | . | . | . | . | . | . | . | . | . | . |
| InternalIMT | dha | rs149466736 | . | . | . | . | . | . | . | . | . | . |
| InternalIMT | dha | rs2099171 | 0.806 | -0.209 | 0.147 | 0.157 | -0.096 | 0.155 | 0.537 | 0.092 | 0.095 | 0.334 |
| InternalIMT | dha | rs141852280 | . | . | . | . | . | . | . | . | . | . |
| InternalIMT | dha | rs191539257 | 0.001 | . | . | . | . | . | . | . | . | . |
| InternalIMT | dha | rs17157771 | 0.029 | -0.039 | 0.059 | 0.506 | 0.483 | 0.386 | 0.211 | -0.284 | 0.23 | 0.217 |
| InternalIMT | dha | rs111339241 | 0.029 | -0.039 | 0.059 | 0.503 | 0.487 | 0.393 | 0.215 | -0.281 | 0.233 | 0.228 |
| InternalIMT | dha | rs116983255 | . | . | . | . | . | . | . | . | . | . |
| InternalIMT | dha | rs142324341 | 0.037 | -0.059 | 0.061 | 0.333 | 0.012 | 0.51 | 0.982 | 0.032 | 0.317 | 0.92 |
| InternalIMT | dha | rs187191219 | . | . | . | . | . | . | . | . | . | . |
| InternalIMT | dha | rs151283973 | . | . | . | . | . | . | . | . | . | . |
| InternalIMT | dha | rs139316218 | . | . | . | . | . | . | . | . | . | . |
| InternalIMT | dha | rs6593485 | 0.886 | -0.21 | 0.193 | 0.279 | -0.081 | 0.19 | 0.669 | 0.086 | 0.114 | 0.449 |
| InternalIMT | dha | rs6593486 | 0.886 | -0.208 | 0.193 | 0.281 | -0.08 | 0.189 | 0.674 | 0.085 | 0.114 | 0.453 |
| InternalIMT | dha | rs145196618 | . | . | . | . | . | . | . | . | . | . |
| InternalIMT | dha | rs142598335 | . | . | . | . | . | . | . | . | . | . |
| InternalIMT | dha | rs892691 | 0.885 | -0.213 | 0.191 | 0.264 | -0.083 | 0.187 | 0.658 | 0.088 | 0.112 | 0.435 |
| InternalIMT | dha | rs7080713 | 0.809 | -0.194 | 0.147 | 0.186 | -0.079 | 0.155 | 0.611 | 0.083 | 0.095 | 0.383 |
| InternalIMT | dha | rs7077173 | 0.065 | -0.046 | 0.06 | 0.447 | 0.199 | 0.273 | 0.465 | -0.091 | 0.163 | 0.575 |
| InternalIMT | dha | rs7080977 | 0.03 | -0.037 | 0.059 | 0.526 | 0.521 | 0.388 | 0.179 | -0.305 | 0.23 | 0.185 |
| InternalIMT | dha | rs3780901 | 0.809 | -0.198 | 0.147 | 0.178 | -0.083 | 0.155 | 0.592 | 0.085 | 0.095 | 0.369 |
| InternalIMT | dha | rs61854091 | . | . | . | . | . | . | . | . | . | . |
| InternalIMT | dha | rs77550209 | . | . | . | . | . | . | . | . | . | . |
| InternalIMT | dha | rs73283191 | 0.077 | -0.052 | 0.063 | 0.408 | 0.041 | 0.226 | 0.856 | -0.049 | 0.136 | 0.718 |
| InternalIMT | dha | rs191866099 | . | . | . | . | . | . | . | . | . | . |
| InternalIMT | dha | rs147998395 | . | . | . | . | . | . | . | . | . | . |
| InternalIMT | dha | rs7090328 | 0.885 | -0.215 | 0.193 | 0.266 | -0.087 | 0.19 | 0.648 | 0.089 | 0.113 | 0.432 |
| InternalIMT | dha | rs79814535 | 0.048 | -0.069 | 0.061 | 0.261 | -0.322 | 0.208 | 0.123 | 0.146 | 0.13 | 0.264 |
| InternalIMT | dha | rs7073082 | 0.885 | -0.215 | 0.193 | 0.265 | -0.087 | 0.19 | 0.646 | 0.089 | 0.113 | 0.431 |
| InternalIMT | dha | rs11239526 | 0.039 | -0.08 | 0.059 | 0.176 | -0.429 | 0.391 | 0.274 | 0.318 | 0.248 | 0.2 |
| InternalIMT | dha | rs141839841 | . | . | . | . | . | . | . | . | . | . |
| InternalIMT | dha | rs75697371 | . | . | . | . | . | . | . | . | . | . |
| InternalIMT | dha | rs75972854 | . | . | . | . | . | . | . | . | . | . |
| InternalIMT | dha | rs139291975 | . | . | . | . | . | . | . | . | . | . |
| InternalIMT | dha | rs144064711 | . | . | . | . | . | . | . | . | . | . |
| InternalIMT | dha | rs3780902 | 0.078 | -0.052 | 0.063 | 0.406 | 0.038 | 0.226 | 0.867 | -0.048 | 0.136 | 0.726 |
| InternalIMT | dha | rs190449801 | . | . | . | . | . | . | . | . | . | . |
| InternalIMT | dha | rs73283196 | 0.077 | -0.052 | 0.063 | 0.405 | 0.037 | 0.226 | 0.869 | -0.047 | 0.136 | 0.731 |
| InternalIMT | dha | rs28395867 | . | . | . | . | . | . | . | . | . | . |
| InternalIMT | dha | rs28395866 | 0.029 | -0.04 | 0.059 | 0.501 | 0.481 | 0.392 | 0.221 | -0.278 | 0.234 | 0.236 |
| InternalIMT | dha | rs55661611 | 0.028 | -0.04 | 0.059 | 0.491 | 0.463 | 0.391 | 0.237 | -0.267 | 0.234 | 0.254 |
| InternalIMT | dha | rs55684518 | . | . | . | . | . | . | . | . | . | . |
| InternalIMT | dha | rs78552493 | . | . | . | . | . | . | . | . | . | . |
| InternalIMT | dha | rs147665434 | . | . | . | . | . | . | . | . | . | . |
| InternalIMT | dha | rs34445001 | . | . | . | . | . | . | . | . | . | . |
| InternalIMT | dha | rs184019449 | . | . | . | . | . | . | . | . | . | . |
| InternalIMT | dha | rs3780903 | 0.808 | -0.196 | 0.147 | 0.183 | -0.081 | 0.156 | 0.604 | 0.084 | 0.095 | 0.379 |
| InternalIMT | dha | rs17157784 | 0.048 | -0.07 | 0.061 | 0.257 | -0.331 | 0.208 | 0.112 | 0.154 | 0.13 | 0.236 |
| InternalIMT | dha | rs3780904 | 0.048 | -0.07 | 0.061 | 0.257 | -0.331 | 0.208 | 0.112 | 0.154 | 0.13 | 0.236 |
| InternalIMT | dha | rs192064730 | . | . | . | . | . | . | . | . | . | . |
| InternalIMT | dha | rs12774534 | . | . | . | . | . | . | . | . | . | . |
| InternalIMT | dha | rs79179915 | 0.047 | -0.069 | 0.061 | 0.258 | -0.329 | 0.21 | 0.116 | 0.153 | 0.131 | 0.244 |
| InternalIMT | dha | rs117811571 | . | . | . | . | . | . | . | . | . | . |
| InternalIMT | dha | rs72796477 | . | . | . | . | . | . | . | . | . | . |
| InternalIMT | dha | rs55729342 | 0.057 | -0.072 | 0.062 | 0.25 | -0.332 | 0.207 | 0.11 | 0.152 | 0.127 | 0.232 |
| InternalIMT | dha | rs59317220 | 0.047 | -0.069 | 0.061 | 0.258 | -0.327 | 0.21 | 0.12 | 0.151 | 0.131 | 0.249 |
| InternalIMT | dha | rs3780905 | 0.562 | -0.062 | 0.09 | 0.494 | -4.80E-03 | 0.128 | 0.97 | 3.70E-03 | 0.079 | 0.962 |
| InternalIMT | dha | rs3780906 | 0.807 | -0.19 | 0.147 | 0.197 | -0.074 | 0.156 | 0.636 | 0.08 | 0.095 | 0.401 |
| InternalIMT | dha | rs149047975 | . | . | . | . | . | . | . | . | . | . |
| InternalIMT | dha | rs3824614 | 0.047 | -0.069 | 0.061 | 0.258 | -0.327 | 0.21 | 0.119 | 0.152 | 0.132 | 0.249 |
| InternalIMT | dha | rs140254404 | . | . | . | . | . | . | . | . | . | . |
| InternalIMT | dha | rs145262684 | . | . | . | . | . | . | . | . | . | . |
| InternalIMT | dha | rs76735482 | 0.047 | -0.069 | 0.061 | 0.258 | -0.327 | 0.21 | 0.12 | 0.151 | 0.132 | 0.251 |
| InternalIMT | dha | rs73283198 | 0.028 | -0.046 | 0.059 | 0.438 | 0.336 | 0.386 | 0.385 | -0.192 | 0.238 | 0.419 |
| InternalIMT | dha | rs74911239 | . | . | . | . | . | . | . | . | . | . |
| InternalIMT | dha | rs150551647 | . | . | . | . | . | . | . | . | . | . |
| InternalIMT | dha | rs115200794 | . | . | . | . | . | . | . | . | . | . |
| InternalIMT | dha | rs74130823 | 0.038 | -0.044 | 0.061 | 0.47 | 0.271 | 0.35 | 0.439 | -0.16 | 0.211 | 0.448 |
| InternalIMT | dha | rs2279435 | 0.754 | -0.377 | 0.205 | 0.066 | -0.316 | 0.213 | 0.139 | 0.214 | 0.126 | 0.088 |
| InternalIMT | dha | rs147279673 | 0.01 | . | . | . | . | . | . | . | . | . |
| InternalIMT | dha | rs183525146 | 0.006 | . | . | . | . | . | . | . | . | . |
| InternalIMT | dha | rs3740107 | 0.737 | -0.374 | 0.19 | 0.05 | -0.293 | 0.2 | 0.144 | 0.218 | 0.118 | 0.066 |
| InternalIMT | dha | rs140697247 | . | . | . | . | . | . | . | . | . | . |
| InternalIMT | dha | rs1565096 | 0.806 | -0.379 | 0.238 | 0.112 | -0.292 | 0.237 | 0.218 | 0.202 | 0.138 | 0.144 |
| InternalIMT | dha | rs1565097 | 0.806 | -0.375 | 0.239 | 0.117 | -0.288 | 0.237 | 0.225 | 0.199 | 0.138 | 0.149 |
| InternalIMT | dha | rs144584548 | . | . | . | . | . | . | . | . | . | . |
| InternalIMT | dha | rs75811053 | 0.044 | -0.068 | 0.061 | 0.266 | -0.285 | 0.212 | 0.179 | 0.122 | 0.134 | 0.364 |
| InternalIMT | dha | rs147462967 | . | . | . | . | . | . | . | . | . | . |
| InternalIMT | dha | rs74130824 | 0.044 | -0.068 | 0.061 | 0.266 | -0.283 | 0.212 | 0.182 | 0.121 | 0.134 | 0.368 |

| InternalIMT | dha | rs116883184 | . | . | . | . | . | . | . | . | . | . |
| --- | --- | --- | --- | --- | --- | --- | --- | --- | --- | --- | --- | --- |
| InternalIMT | dha | rs117258049 | . | . | . | . | . | . | . | . | . | . |
| InternalIMT | dha | rs3780908 | 0.029 | -0.05 | 0.059 | 0.399 | 0.176 | 0.366 | 0.631 | -0.072 | 0.227 | 0.752 |
| InternalIMT | dha | rs180938522 | . | . | . | . | . | . | . | . | . | . |
| InternalIMT | dha | rs76687372 | . | . | . | . | . | . | . | . | . | . |
| InternalIMT | dha | rs3780909 | 0.666 | -0.2 | 0.164 | 0.222 | -0.153 | 0.178 | 0.39 | 0.104 | 0.106 | 0.327 |
| InternalIMT | dha | rs78281156 | 0.044 | -0.068 | 0.061 | 0.266 | -0.284 | 0.212 | 0.18 | 0.122 | 0.134 | 0.365 |
| InternalIMT | dha | rs187840631 | . | . | . | . | . | . | . | . | . | . |
| InternalIMT | dha | rs76868242 | 0.044 | -0.068 | 0.061 | 0.266 | -0.284 | 0.212 | 0.181 | 0.122 | 0.134 | 0.366 |
| InternalIMT | dha | rs192794192 | . | . | . | . | . | . | . | . | . | . |
| InternalIMT | dha | rs12781248 | . | . | . | . | . | . | . | . | . | . |
| InternalIMT | dha | rs12762375 | . | . | . | . | . | . | . | . | . | . |
| InternalIMT | dha | rs192256986 | . | . | . | . | . | . | . | . | . | . |
| InternalIMT | dha | rs184411030 | . | . | . | . | . | . | . | . | . | . |
| InternalIMT | dha | rs189601483 | 0.049 | -0.078 | 0.06 | 0.191 | -0.477 | 0.251 | 0.058 | 0.25 | 0.166 | 0.133 |
| InternalIMT | dha | rs143254579 | 0.045 | -0.081 | 0.06 | 0.175 | -0.433 | 0.375 | 0.249 | 0.303 | 0.238 | 0.203 |
| InternalIMT | dha | rs12253903 | . | . | . | . | . | . | . | . | . | . |
| InternalIMT | dha | rs35489926 | . | . | . | . | . | . | . | . | . | . |
| InternalIMT | dha | rs71494783 | 0.073 | -0.07 | 0.063 | 0.265 | -0.17 | 0.185 | 0.358 | 0.069 | 0.117 | 0.555 |
| InternalIMT | dha | rs180711602 | . | . | . | . | . | . | . | . | . | . |
| InternalIMT | dha | rs117312402 | 0.044 | -0.068 | 0.061 | 0.267 | -0.283 | 0.212 | 0.183 | 0.121 | 0.134 | 0.368 |
| InternalIMT | dha | rs74831694 | 0.053 | -0.098 | 0.065 | 0.132 | -0.464 | 0.343 | 0.176 | 0.332 | 0.224 | 0.139 |
| InternalIMT | dha | rs140301943 | . | . | . | . | . | . | . | . | . | . |
| InternalIMT | dha | rs78553679 | . | . | . | . | . | . | . | . | . | . |
| InternalIMT | dha | rs78334250 | 0.044 | -0.068 | 0.061 | 0.267 | -0.282 | 0.212 | 0.183 | 0.121 | 0.134 | 0.368 |
| InternalIMT | dha | rs76000028 | 0.044 | -0.068 | 0.061 | 0.267 | -0.282 | 0.212 | 0.183 | 0.121 | 0.134 | 0.368 |
| InternalIMT | dha | rs145195254 | . | . | . | . | . | . | . | . | . | . |
| InternalIMT | dha | rs137888527 | . | . | . | . | . | . | . | . | . | . |
| InternalIMT | dha | rs149109592 | . | . | . | . | . | . | . | . | . | . |
| InternalIMT | dha | rs80280010 | 0.038 | -0.078 | 0.059 | 0.186 | -0.395 | 0.388 | 0.309 | 0.295 | 0.245 | 0.229 |
| InternalIMT | dha | rs189995374 | 0.005 | . | . | . | . | . | . | . | . | . |
| InternalIMT | dha | rs75467770 | 0.05 | -0.065 | 0.062 | 0.294 | -0.237 | 0.206 | 0.25 | 0.088 | 0.129 | 0.495 |
| InternalIMT | dha | rs1487562 | 0.029 | -0.05 | 0.059 | 0.396 | 0.17 | 0.367 | 0.643 | -0.067 | 0.228 | 0.769 |
| InternalIMT | dha | rs12266963 | 0.073 | -0.07 | 0.063 | 0.265 | -0.17 | 0.185 | 0.358 | 0.069 | 0.117 | 0.554 |
| InternalIMT | dha | rs7099874 | 0.769 | -0.254 | 0.222 | 0.253 | -0.196 | 0.223 | 0.38 | 0.129 | 0.131 | 0.327 |
| InternalIMT | dha | rs75687019 | 0.044 | -0.068 | 0.061 | 0.267 | -0.282 | 0.212 | 0.183 | 0.121 | 0.134 | 0.369 |
| InternalIMT | dha | rs148240462 | . | . | . | . | . | . | . | . | . | . |
| InternalIMT | dha | rs141278174 | . | . | . | . | . | . | . | . | . | . |
| InternalIMT | dha | rs76404852 | 0.044 | -0.068 | 0.061 | 0.267 | -0.282 | 0.212 | 0.184 | 0.121 | 0.134 | 0.369 |
| InternalIMT | dha | rs139112240 | . | . | . | . | . | . | . | . | . | . |
| InternalIMT | dha | rs12783037 | 0.073 | -0.07 | 0.063 | 0.264 | -0.171 | 0.185 | 0.357 | 0.07 | 0.117 | 0.553 |
| InternalIMT | dha | rs72796481 | . | . | . | . | . | . | . | . | . | . |
| InternalIMT | dha | rs76798749 | 0.044 | -0.068 | 0.061 | 0.267 | -0.281 | 0.212 | 0.185 | 0.12 | 0.134 | 0.37 |
| InternalIMT | dha | rs187732548 | . | . | . | . | . | . | . | . | . | . |
| InternalIMT | dha | rs75675474 | 0.044 | -0.068 | 0.061 | 0.267 | -0.281 | 0.212 | 0.185 | 0.12 | 0.134 | 0.37 |
| InternalIMT | dha | rs76552293 | 0.044 | -0.068 | 0.061 | 0.267 | -0.281 | 0.212 | 0.185 | 0.12 | 0.134 | 0.37 |
| InternalIMT | dha | rs147445993 | 0.001 | . | . | . | . | . | . | . | . | . |
| InternalIMT | dha | rs115902283 | 0.044 | -0.068 | 0.061 | 0.265 | -0.285 | 0.212 | 0.179 | 0.122 | 0.135 | 0.364 |
| InternalIMT | dha | rs4540799 | 0.759 | -0.182 | 0.228 | 0.425 | -0.122 | 0.227 | 0.59 | 0.082 | 0.134 | 0.538 |
| InternalIMT | dha | rs12765320 | 0.102 | -0.057 | 0.064 | 0.373 | 6.20E-03 | 0.17 | 0.971 | -0.02 | 0.107 | 0.854 |
| InternalIMT | dha | rs12782256 | . | . | . | . | . | . | . | . | . | . |
| InternalIMT | dha | rs191727256 | . | . | . | . | . | . | . | . | . | . |
| InternalIMT | dha | rs2082111 | 0.028 | -0.051 | 0.059 | 0.395 | 0.172 | 0.367 | 0.639 | -0.066 | 0.228 | 0.773 |
| InternalIMT | dha | rs2082110 | 0.072 | -0.061 | 0.063 | 0.33 | -0.056 | 0.213 | 0.795 | 0.019 | 0.131 | 0.885 |
| InternalIMT | dha | rs2082109 | 0.072 | -0.061 | 0.063 | 0.33 | -0.056 | 0.213 | 0.795 | 0.019 | 0.131 | 0.885 |
| InternalIMT | dha | rs117316662 | 0.044 | -0.068 | 0.061 | 0.266 | -0.284 | 0.211 | 0.178 | 0.122 | 0.134 | 0.361 |
| InternalIMT | dha | rs12247163 | 0.102 | -0.062 | 0.064 | 0.333 | -0.041 | 0.17 | 0.81 | 0.012 | 0.106 | 0.913 |
| InternalIMT | dha | rs12355888 | 0.002 | . | . | . | . | . | . | . | . | . |
| InternalIMT | dha | rs143772290 | . | . | . | . | . | . | . | . | . | . |
| InternalIMT | dha | rs146849452 | . | . | . | . | . | . | . | . | . | . |
| InternalIMT | dha | rs140267121 | . | . | . | . | . | . | . | . | . | . |
| InternalIMT | dha | rs7080474 | 0.058 | -0.051 | 0.06 | 0.4 | 0.083 | 0.228 | 0.714 | -0.034 | 0.144 | 0.816 |
| InternalIMT | dha | rs144066203 | . | . | . | . | . | . | . | . | . | . |
| InternalIMT | dha | rs12765209 | 0.044 | -0.068 | 0.061 | 0.265 | -0.287 | 0.211 | 0.173 | 0.124 | 0.134 | 0.353 |
| InternalIMT | dha | rs150930735 | . | . | . | . | . | . | . | . | . | . |
| InternalIMT | dha | rs7068039 | 0.102 | -0.062 | 0.064 | 0.332 | -0.042 | 0.17 | 0.805 | 0.012 | 0.106 | 0.908 |
| InternalIMT | dha | rs117622652 | 0.043 | -0.068 | 0.061 | 0.265 | -0.289 | 0.21 | 0.17 | 0.125 | 0.134 | 0.35 |
| InternalIMT | dha | rs190191514 | . | . | . | . | . | . | . | . | . | . |
| InternalIMT | dha | rs74130835 | . | . | . | . | . | . | . | . | . | . |
| InternalIMT | dha | rs1811604 | . | . | . | . | . | . | . | . | . | . |
| InternalIMT | dha | rs146971256 | 0.001 | . | . | . | . | . | . | . | . | . |
| InternalIMT | dha | rs74336000 | . | . | . | . | . | . | . | . | . | . |
| InternalIMT | dha | rs186046492 | . | . | . | . | . | . | . | . | . | . |
| InternalIMT | dha | rs34971823 | . | . | . | . | . | . | . | . | . | . |
| InternalIMT | dha | rs75227446 | 0.043 | -0.068 | 0.061 | 0.263 | -0.297 | 0.209 | 0.156 | 0.13 | 0.133 | 0.33 |
| InternalIMT | dha | rs190457599 | . | . | . | . | . | . | . | . | . | . |
| InternalIMT | dha | rs79591917 | 0.043 | -0.068 | 0.061 | 0.263 | -0.297 | 0.209 | 0.156 | 0.13 | 0.133 | 0.33 |
| InternalIMT | dha | rs702365 | 0.647 | -0.174 | 0.162 | 0.283 | -0.12 | 0.174 | 0.491 | 0.086 | 0.104 | 0.407 |
| InternalIMT | dha | rs79643836 | 0.043 | -0.068 | 0.061 | 0.263 | -0.297 | 0.209 | 0.156 | 0.13 | 0.133 | 0.329 |
| InternalIMT | dha | rs702366 | 0.649 | -0.174 | 0.162 | 0.283 | -0.12 | 0.174 | 0.492 | 0.087 | 0.104 | 0.408 |
| InternalIMT | dha | rs1059696 | 0.059 | -0.051 | 0.06 | 0.398 | 0.08 | 0.227 | 0.726 | -0.032 | 0.144 | 0.825 |
| InternalIMT | dha | rs901681 | 0.648 | -0.175 | 0.162 | 0.279 | -0.122 | 0.174 | 0.485 | 0.088 | 0.104 | 0.402 |
| InternalIMT | dha | rs61854092 | 0.058 | -0.051 | 0.06 | 0.4 | 0.083 | 0.228 | 0.715 | -0.034 | 0.144 | 0.815 |
| InternalIMT | dha | rs148080753 | . | . | . | . | . | . | . | . | . | . |
| InternalIMT | dha | rs61854093 | 0.064 | -0.048 | 0.061 | 0.425 | 0.114 | 0.231 | 0.621 | -0.052 | 0.146 | 0.723 |
| InternalIMT | dha | rs141822774 | . | . | . | . | . | . | . | . | . | . |

| InternalIMT | dha | rs74130836 | . | . | . | . | . | . | . | . | . | . |
| --- | --- | --- | --- | --- | --- | --- | --- | --- | --- | --- | --- | --- |
| InternalIMT | dha | rs150620963 | . | . | . | . | . | . | . | . | . | . |
| InternalIMT | dha | rs3780913 | 0.749 | -0.223 | 0.213 | 0.296 | -0.169 | 0.213 | 0.427 | 0.111 | 0.126 | 0.378 |
| InternalIMT | dha | rs142255723 | . | . | . | . | . | . | . | . | . | . |
| InternalIMT | dha | rs3780914 | 0.647 | -0.174 | 0.162 | 0.281 | -0.122 | 0.174 | 0.484 | 0.087 | 0.104 | 0.404 |
| InternalIMT | dha | rs182656507 | . | . | . | . | . | . | . | . | . | . |
| InternalIMT | dha | rs2291427 | 0.703 | -0.185 | 0.19 | 0.331 | -0.117 | 0.195 | 0.551 | 0.09 | 0.117 | 0.443 |
| InternalIMT | dha | rs10751382 | 0.703 | -0.185 | 0.19 | 0.331 | -0.117 | 0.195 | 0.55 | 0.09 | 0.117 | 0.444 |
| InternalIMT | dha | rs117610108 | 0.043 | -0.069 | 0.061 | 0.258 | -0.325 | 0.205 | 0.114 | 0.146 | 0.131 | 0.263 |
| InternalIMT | dha | rs144966941 | 0.002 | . | . | . | . | . | . | . | . | . |
| InternalIMT | dha | rs41502051 | 0.007 | . | . | . | . | . | . | . | . | . |
| InternalIMT | dha | rs41426046 | 0.047 | -0.072 | 0.06 | 0.236 | -0.387 | 0.203 | 0.057 | 0.183 | 0.129 | 0.159 |
| InternalIMT | dha | rs41519248 | 0.043 | -0.069 | 0.061 | 0.255 | -0.335 | 0.204 | 0.101 | 0.152 | 0.13 | 0.242 |
| InternalIMT | dha | rs189108245 | . | . | . | . | . | . | . | . | . | . |
| InternalIMT | dha | rs7896431 | 0.668 | -0.182 | 0.173 | 0.291 | -0.131 | 0.184 | 0.477 | 0.092 | 0.11 | 0.402 |
| InternalIMT | dha | rs183724547 | . | . | . | . | . | . | . | . | . | . |
| InternalIMT | dha | rs142044990 | . | . | . | . | . | . | . | . | . | . |
| InternalIMT | dha | rs188140929 | 0.021 | -0.074 | 0.058 | 0.206 | -0.639 | 0.499 | 0.201 | 0.479 | 0.32 | 0.134 |
| InternalIMT | dha | rs76819096 | . | . | . | . | . | . | . | . | . | . |
| InternalIMT | dha | rs10751383 | 0.681 | -0.235 | 0.166 | 0.158 | -0.173 | 0.176 | 0.326 | 0.126 | 0.105 | 0.229 |
| InternalIMT | dha | rs117819321 | . | . | . | . | . | . | . | . | . | . |
| InternalIMT | dha | rs2242332 | 0.744 | -0.223 | 0.211 | 0.291 | -0.173 | 0.211 | 0.413 | 0.111 | 0.125 | 0.372 |
| InternalIMT | dha | rs2242333 | 0.476 | -0.108 | 0.106 | 0.31 | -0.086 | 0.139 | 0.534 | 0.053 | 0.082 | 0.524 |
| InternalIMT | dha | rs2242334 | 0.744 | -0.223 | 0.21 | 0.29 | -0.173 | 0.211 | 0.411 | 0.112 | 0.125 | 0.371 |
| InternalIMT | dha | rs189465041 | . | . | . | . | . | . | . | . | . | . |
| InternalIMT | dha | rs28395868 | . | . | . | . | . | . | . | . | . | . |
| InternalIMT | dha | rs56067550 | . | . | . | . | . | . | . | . | . | . |
| InternalIMT | dha | rs1051713 | 0.1 | -0.062 | 0.064 | 0.336 | -0.037 | 0.173 | 0.832 | 7.10E-03 | 0.108 | 0.948 |
| InternalIMT | dha | rs115074210 | . | . | . | . | . | . | . | . | . | . |
| InternalIMT | dha | rs7900977 | 0.782 | -0.316 | 0.224 | 0.159 | -0.244 | 0.221 | 0.27 | 0.167 | 0.13 | 0.199 |
| InternalIMT | dha | rs147613891 | . | . | . | . | . | . | . | . | . | . |
| InternalIMT | dha | rs2229136 | 0.038 | -0.079 | 0.059 | 0.18 | -0.42 | 0.392 | 0.284 | 0.31 | 0.248 | 0.211 |
| InternalIMT | dha | rs185482841 | . | . | . | . | . | . | . | . | . | . |
| InternalIMT | dha | rs2288619 | 0.038 | -0.079 | 0.059 | 0.18 | -0.42 | 0.392 | 0.284 | 0.31 | 0.248 | 0.211 |
| InternalIMT | dha | rs189891524 | 0.003 | . | . | . | . | . | . | . | . | . |
| InternalIMT | dha | rs56145077 | 0.058 | -0.051 | 0.06 | 0.398 | 0.08 | 0.227 | 0.725 | -0.031 | 0.144 | 0.828 |
| InternalIMT | dha | rs143216970 | . | . | . | . | . | . | . | . | . | . |
| InternalIMT | dha | rs142246478 | . | . | . | . | . | . | . | . | . | . |
| InternalIMT | dha | rs13134 | . | . | . | . | . | . | . | . | . | . |
| InternalIMT | dha | rs41314980 | . | . | . | . | . | . | . | . | . | . |
| InternalIMT | dha | rs41304611 | . | . | . | . | . | . | . | . | . | . |
| InternalIMT | dha | rs193299617 | 0.004 | . | . | . | . | . | . | . | . | . |
| InternalIMT | dha | rs12772724 | . | . | . | . | . | . | . | . | . | . |
| InternalIMT | dha | rs139489537 | 0.038 | -0.078 | 0.059 | 0.182 | -0.414 | 0.393 | 0.292 | 0.306 | 0.248 | 0.217 |
| InternalIMT | dha | rs12776062 | . | . | . | . | . | . | . | . | . | . |
| InternalIMT | dha | rs752059 | 0.059 | -0.051 | 0.06 | 0.398 | 0.081 | 0.227 | 0.723 | -0.032 | 0.144 | 0.822 |
| InternalIMT | dha | rs147649982 | 0.015 | -0.081 | 0.06 | 0.178 | -1.03 | 0.632 | 0.104 | 0.67 | 0.35 | 0.056 |
| InternalIMT | dha | rs17157798 | 0.038 | -0.078 | 0.059 | 0.182 | -0.413 | 0.392 | 0.292 | 0.306 | 0.247 | 0.217 |
| InternalIMT | dha | rs142298619 | . | . | . | . | . | . | . | . | . | . |
| InternalIMT | dha | rs4948673 | 0.777 | -0.313 | 0.224 | 0.164 | -0.245 | 0.223 | 0.271 | 0.165 | 0.13 | 0.205 |
| InternalIMT | dha | rs74231484 | 0.476 | -0.109 | 0.106 | 0.305 | -0.088 | 0.138 | 0.524 | 0.054 | 0.082 | 0.515 |
| InternalIMT | dha | rs72796489 | . | . | . | . | . | . | . | . | . | . |
| InternalIMT | dha | rs113447578 | 0.026 | -0.05 | 0.059 | 0.393 | 0.211 | 0.398 | 0.596 | -0.112 | 0.248 | 0.651 |
| InternalIMT | dha | rs34063374 | . | . | . | . | . | . | . | . | . | . |
| InternalIMT | dha | rs11239527 | . | . | . | . | . | . | . | . | . | . |
| InternalIMT | dha | rs34371883 | . | . | . | . | . | . | . | . | . | . |
| InternalIMT | dha | rs118168828 | 0.002 | . | . | . | . | . | . | . | . | . |
| InternalIMT | dha | rs34349618 | . | . | . | . | . | . | . | . | . | . |
| InternalIMT | dha | rs80041679 | 0.038 | -0.078 | 0.059 | 0.182 | -0.414 | 0.392 | 0.292 | 0.306 | 0.248 | 0.217 |
| InternalIMT | dha | rs149640607 | . | . | . | . | . | . | . | . | . | . |
| InternalIMT | dha | rs4427523 | 0.957 | 0.168 | 0.704 | 0.812 | 0.085 | 0.612 | 0.889 | -0.118 | 0.367 | 0.749 |
| InternalIMT | dha | rs185880109 | . | . | . | . | . | . | . | . | . | . |
| InternalIMT | dha | rs191523220 | . | . | . | . | . | . | . | . | . | . |
| InternalIMT | dha | rs144315284 | . | . | . | . | . | . | . | . | . | . |
| InternalIMT | dha | rs4948674 | 0.156 | -0.075 | 0.067 | 0.264 | -0.059 | 0.134 | 0.659 | 0.053 | 0.081 | 0.518 |
| InternalIMT | dha | rs182984298 | . | . | . | . | . | . | . | . | . | . |
| InternalIMT | dha | rs56749023 | . | . | . | . | . | . | . | . | . | . |
| InternalIMT | dha | rs112508957 | . | . | . | . | . | . | . | . | . | . |
| InternalIMT | dha | rs7089063 | 0.138 | -0.086 | 0.066 | 0.191 | -0.19 | 0.166 | 0.253 | 0.124 | 0.105 | 0.237 |
| InternalIMT | dha | rs145000779 | . | . | . | . | . | . | . | . | . | . |
| InternalIMT | dha | rs80201572 | 0.038 | -0.078 | 0.059 | 0.182 | -0.414 | 0.391 | 0.29 | 0.306 | 0.247 | 0.216 |
| InternalIMT | dha | rs140568227 | . | . | . | . | . | . | . | . | . | . |
| InternalIMT | dha | rs149756228 | . | . | . | . | . | . | . | . | . | . |
| InternalIMT | dha | rs112380685 | . | . | . | . | . | . | . | . | . | . |
| InternalIMT | dha | rs60481109 | . | . | . | . | . | . | . | . | . | . |
| InternalIMT | dha | rs138221843 | . | . | . | . | . | . | . | . | . | . |
| InternalIMT | dha | rs150283194 | . | . | . | . | . | . | . | . | . | . |
| InternalIMT | dha | rs138879661 | . | . | . | . | . | . | . | . | . | . |
| InternalIMT | dha | rs34960345 | 0.038 | -0.078 | 0.059 | 0.182 | -0.415 | 0.39 | 0.288 | 0.306 | 0.247 | 0.214 |
| InternalIMT | dha | rs147514068 | . | . | . | . | . | . | . | . | . | . |
| InternalIMT | dha | rs35410535 | 0.038 | -0.078 | 0.059 | 0.182 | -0.415 | 0.39 | 0.288 | 0.306 | 0.246 | 0.214 |
| InternalIMT | dha | rs191613635 | . | . | . | . | . | . | . | . | . | . |
| InternalIMT | dha | rs17157800 | . | . | . | . | . | . | . | . | . | . |
| InternalIMT | dha | rs17523413 | 0.038 | -0.078 | 0.059 | 0.182 | -0.414 | 0.391 | 0.29 | 0.306 | 0.247 | 0.216 |
| InternalIMT | dha | rs7897036 | 0.001 | . | . | . | . | . | . | . | . | . |
| InternalIMT | dha | rs76334323 | 0.042 | -0.07 | 0.061 | 0.249 | -0.372 | 0.203 | 0.067 | 0.173 | 0.129 | 0.18 |

| InternalIMT | dha | rs143535991 | . | . | . | . | . | . | . | . | . | . |
| --- | --- | --- | --- | --- | --- | --- | --- | --- | --- | --- | --- | --- |
| InternalIMT | dha | rs140344979 | . | . | . | . | . | . | . | . | . | . |
| InternalIMT | dha | rs36047811 | 0.038 | -0.078 | 0.059 | 0.182 | -0.415 | 0.39 | 0.288 | 0.306 | 0.246 | 0.214 |
| InternalIMT | dha | rs76493570 | 0.038 | -0.078 | 0.059 | 0.182 | -0.415 | 0.39 | 0.288 | 0.306 | 0.247 | 0.214 |
| InternalIMT | dha | rs192998920 | . | . | . | . | . | . | . | . | . | . |
| InternalIMT | dha | rs116840852 | . | . | . | . | . | . | . | . | . | . |
| InternalIMT | dha | rs184824101 | . | . | . | . | . | . | . | . | . | . |
| InternalIMT | dha | rs41301621 | . | . | . | . | . | . | . | . | . | . |
| InternalIMT | dha | rs3740109 | 0.038 | -0.078 | 0.059 | 0.182 | -0.415 | 0.39 | 0.288 | 0.306 | 0.246 | 0.214 |
| InternalIMT | dha | rs7899415 | 0.089 | -0.053 | 0.065 | 0.413 | 0.048 | 0.228 | 0.833 | -0.023 | 0.14 | 0.868 |
| InternalIMT | dha | rs184395284 | 0.004 | . | . | . | . | . | . | . | . | . |
| InternalIMT | dha | rs139209589 | . | . | . | . | . | . | . | . | . | . |
| InternalIMT | dha | rs149924163 | . | . | . | . | . | . | . | . | . | . |
| InternalIMT | dha | rs3740110 | 0.042 | -0.07 | 0.061 | 0.249 | -0.372 | 0.203 | 0.067 | 0.174 | 0.129 | 0.18 |
| InternalIMT | dha | rs144335113 | . | . | . | . | . | . | . | . | . | . |
| InternalIMT | dha | rs74743385 | . | . | . | . | . | . | . | . | . | . |
| InternalIMT | dha | rs146545829 | . | . | . | . | . | . | . | . | . | . |
| InternalIMT | dha | rs181842689 | . | . | . | . | . | . | . | . | . | . |
| InternalIMT | dha | rs3740111 | 0.038 | -0.078 | 0.059 | 0.182 | -0.415 | 0.39 | 0.288 | 0.306 | 0.247 | 0.214 |
| InternalIMT | dha | rs186261599 | . | . | . | . | . | . | . | . | . | . |
| InternalIMT | dha | rs73285348 | 0.013 | -0.047 | 0.058 | 0.415 | 0.704 | 0.686 | 0.305 | -0.463 | 0.425 | 0.276 |
| InternalIMT | dha | rs186356901 | . | . | . | . | . | . | . | . | . | . |
| InternalIMT | dha | rs3824618 | 0.038 | -0.078 | 0.059 | 0.182 | -0.414 | 0.391 | 0.29 | 0.306 | 0.247 | 0.216 |
| InternalIMT | dha | rs3802548 | 0.065 | -0.041 | 0.061 | 0.497 | 0.221 | 0.228 | 0.334 | -0.128 | 0.139 | 0.358 |
| InternalIMT | dha | rs3802547 | 0.04 | -0.068 | 0.061 | 0.265 | -0.341 | 0.207 | 0.101 | 0.152 | 0.132 | 0.25 |
| InternalIMT | dha | rs77677521 | . | . | . | . | . | . | . | . | . | . |
| InternalIMT | dha | rs3824616 | 0.038 | -0.078 | 0.059 | 0.182 | -0.415 | 0.39 | 0.288 | 0.306 | 0.246 | 0.214 |
| InternalIMT | dha | rs191073818 | . | . | . | . | . | . | . | . | . | . |
| InternalIMT | dha | rs147078004 | . | . | . | . | . | . | . | . | . | . |
| InternalIMT | dha | rs7908745 | 0.145 | -0.074 | 0.066 | 0.259 | -0.108 | 0.163 | 0.507 | 0.069 | 0.101 | 0.498 |
| InternalIMT | dha | rs17157811 | . | . | . | . | . | . | . | . | . | . |
| InternalIMT | dha | rs138506087 | . | . | . | . | . | . | . | . | . | . |
| InternalIMT | dha | rs77241269 | . | . | . | . | . | . | . | . | . | . |
| InternalIMT | dha | rs138374938 | . | . | . | . | . | . | . | . | . | . |
| InternalIMT | dha | rs2279433 | 0.038 | -0.078 | 0.059 | 0.182 | -0.416 | 0.39 | 0.287 | 0.307 | 0.247 | 0.213 |
| InternalIMT | dha | rs73285358 | . | . | . | . | . | . | . | . | . | . |
| InternalIMT | dha | rs11239528 | 0.065 | -0.041 | 0.061 | 0.497 | 0.22 | 0.228 | 0.334 | -0.128 | 0.139 | 0.356 |
| InternalIMT | dha | rs77453386 | 0.042 | -0.07 | 0.061 | 0.249 | -0.374 | 0.203 | 0.066 | 0.175 | 0.129 | 0.178 |
| InternalIMT | dha | rs2279434 | 0.04 | -0.079 | 0.059 | 0.182 | -0.399 | 0.39 | 0.306 | 0.297 | 0.246 | 0.229 |
| InternalIMT | dha | rs149236525 | . | . | . | . | . | . | . | . | . | . |
| InternalIMT | dha | rs12573609 | 0.13 | -6.60E-04 | 0.065 | 0.992 | 0.328 | 0.369 | 0.374 | -0.216 | 0.219 | 0.324 |
| InternalIMT | dha | rs191716655 | . | . | . | . | . | . | . | . | . | . |
| InternalIMT | dha | rs17523660 | . | . | . | . | . | . | . | . | . | . |
| InternalIMT | dha | rs142671360 | . | . | . | . | . | . | . | . | . | . |
| InternalIMT | dha | rs3764990 | 0.037 | -0.078 | 0.059 | 0.182 | -0.415 | 0.391 | 0.289 | 0.307 | 0.247 | 0.214 |
| InternalIMT | dha | rs113682861 | . | . | . | . | . | . | . | . | . | . |
| InternalIMT | dha | rs117658270 | 0.042 | -0.07 | 0.061 | 0.249 | -0.374 | 0.203 | 0.066 | 0.175 | 0.129 | 0.178 |
| InternalIMT | dha | rs187309063 | . | . | . | . | . | . | . | . | . | . |
| InternalIMT | dha | rs73285360 | . | . | . | . | . | . | . | . | . | . |
| InternalIMT | dha | rs80133289 | 0.1 | -0.076 | 0.075 | 0.314 | -0.142 | 0.253 | 0.575 | 0.081 | 0.152 | 0.592 |
| InternalIMT | dha | rs186693257 | 0.003 | . | . | . | . | . | . | . | . | . |
| InternalIMT | dha | rs35735968 | 0.05 | -0.094 | 0.059 | 0.113 | -0.484 | 0.383 | 0.207 | 0.354 | 0.238 | 0.138 |
| InternalIMT | dha | rs17157825 | 0.038 | -0.078 | 0.059 | 0.183 | -0.406 | 0.391 | 0.298 | 0.304 | 0.247 | 0.219 |
| InternalIMT | dha | rs2291428 | 0.065 | -0.041 | 0.061 | 0.497 | 0.22 | 0.228 | 0.334 | -0.129 | 0.139 | 0.356 |
| InternalIMT | dha | rs2291429 | 0.065 | -0.041 | 0.061 | 0.498 | 0.222 | 0.228 | 0.331 | -0.13 | 0.139 | 0.351 |
| InternalIMT | dha | rs145714792 | . | . | . | . | . | . | . | . | . | . |
| InternalIMT | dha | rs12769244 | 0.038 | -0.078 | 0.059 | 0.182 | -0.416 | 0.39 | 0.287 | 0.307 | 0.247 | 0.213 |
| InternalIMT | dha | rs12771275 | 0.038 | -0.078 | 0.059 | 0.182 | -0.416 | 0.39 | 0.287 | 0.307 | 0.247 | 0.213 |
| InternalIMT | dha | rs146266174 | . | . | . | . | . | . | . | . | . | . |
| InternalIMT | dha | rs41301623 | . | . | . | . | . | . | . | . | . | . |
| InternalIMT | dha | rs2306814 | 0.043 | -0.071 | 0.061 | 0.242 | -0.387 | 0.201 | 0.054 | 0.182 | 0.128 | 0.155 |
| InternalIMT | dha | rs12244834 | 0.03 | -0.058 | 0.059 | 0.325 | -6.60E-03 | 0.295 | 0.982 | -2.80E-03 | 0.189 | 0.988 |
| InternalIMT | dha | rs184006011 | 0 | . | . | . | . | . | . | . | . | . |
| InternalIMT | dha | rs78829066 | 0.042 | -0.071 | 0.061 | 0.243 | -0.388 | 0.207 | 0.062 | 0.184 | 0.133 | 0.167 |
| InternalIMT | dha | rs34731408 | 0.038 | -0.078 | 0.059 | 0.182 | -0.417 | 0.391 | 0.287 | 0.308 | 0.247 | 0.213 |
| InternalIMT | dha | rs17451516 | . | . | . | . | . | . | . | . | . | . |
| InternalIMT | dha | rs139910133 | . | . | . | . | . | . | . | . | . | . |
| InternalIMT | dha | rs10900218 | 0.138 | -0.087 | 0.066 | 0.188 | -0.203 | 0.183 | 0.267 | 0.132 | 0.114 | 0.247 |
| InternalIMT | dha | rs146023999 | 0.025 | -0.052 | 0.06 | 0.379 | 0.327 | 0.572 | 0.568 | -0.138 | 0.349 | 0.691 |
| InternalIMT | dha | rs187296958 | 0.007 | . | . | . | . | . | . | . | . | . |
| InternalIMT | dha | rs142342833 | 0.015 | -0.064 | 0.057 | 0.265 | -0.664 | 0.64 | 0.3 | 0.343 | 0.419 | 0.413 |
| InternalIMT | dha | rs143489255 | 0.006 | . | . | . | . | . | . | . | . | . |
| InternalIMT | dha | rs138532518 | 0.006 | . | . | . | . | . | . | . | . | . |
| InternalIMT | dha | rs183768079 | 0.007 | . | . | . | . | . | . | . | . | . |
| InternalIMT | dha | rs117892272 | 0.012 | -0.044 | 0.053 | 0.403 | 0.441 | 0.957 | 0.645 | -0.257 | 0.58 | 0.657 |
| InternalIMT | dha | rs192993943 | 0.003 | . | . | . | . | . | . | . | . | . |
| InternalIMT | dha | rs182989043 | 0.015 | -0.067 | 0.061 | 0.269 | -0.334 | 0.529 | 0.529 | 0.249 | 0.315 | 0.43 |
| InternalIMT | dha | rs189703435 | 0.021 | -0.06 | 0.058 | 0.305 | -0.152 | 0.475 | 0.75 | 0.086 | 0.303 | 0.776 |
| InternalIMT | dha | rs188794040 | 0.02 | -0.06 | 0.059 | 0.311 | -0.084 | 0.419 | 0.841 | 0.026 | 0.275 | 0.924 |
| InternalIMT | dha | rs143920953 | 0.003 | . | . | . | . | . | . | . | . | . |
| InternalIMT | dha | rs191029121 | 0.006 | . | . | . | . | . | . | . | . | . |
| InternalIMT | dha | rs116875448 | 0.006 | . | . | . | . | . | . | . | . | . |
| InternalIMT | dha | rs190167877 | 0.002 | . | . | . | . | . | . | . | . | . |
| InternalIMT | dha | rs184993418 | 0.003 | . | . | . | . | . | . | . | . | . |
| InternalIMT | dha | rs143241158 | 0.01 | . | . | . | . | . | . | . | . | . |
| InternalIMT | dha | rs11239500 | 0.007 | . | . | . | . | . | . | . | . | . |

| InternalIMT | dha | rs140568994 | 0.001 | . | . | . | . | . | . | . | . | . |
| --- | --- | --- | --- | --- | --- | --- | --- | --- | --- | --- | --- | --- |
| InternalIMT | dha | rs187530317 | 0.008 | . | . | . | . | . | . | . | . | . |
| InternalIMT | dha | rs145189326 | 0.003 | . | . | . | . | . | . | . | . | . |
| InternalIMT | dha | rs186203332 | 0.009 | . | . | . | . | . | . | . | . | . |
| InternalIMT | dha | rs189921909 | 0.004 | . | . | . | . | . | . | . | . | . |
| InternalIMT | dha | rs187260989 | 0.01 | -0.046 | 0.057 | 0.422 | 0.694 | 0.885 | 0.433 | -0.424 | 0.577 | 0.462 |
| InternalIMT | dha | rs114376586 | 0.006 | . | . | . | . | . | . | . | . | . |
| InternalIMT | dha | rs151070808 | 0.019 | -0.053 | 0.06 | 0.383 | 0.178 | 0.476 | 0.709 | -0.129 | 0.294 | 0.661 |
| InternalIMT | dha | rs140927225 | 0.006 | . | . | . | . | . | . | . | . | . |
| InternalIMT | dha | rs191555615 | 0.012 | -0.038 | 0.053 | 0.476 | 0.623 | 0.827 | 0.451 | -0.367 | 0.51 | 0.472 |
| InternalIMT | dha | rs80278898 | 0.047 | -0.095 | 0.059 | 0.108 | -0.394 | 0.362 | 0.276 | 0.319 | 0.238 | 0.182 |
| InternalIMT | dha | rs190849503 | 0.004 | . | . | . | . | . | . | . | . | . |
| InternalIMT | dha | rs150080315 | 0.009 | . | . | . | . | . | . | . | . | . |
| InternalIMT | dha | rs143623885 | 0.011 | -0.061 | 0.058 | 0.29 | -0.598 | 0.707 | 0.398 | 0.303 | 0.454 | 0.504 |
| InternalIMT | dha | rs187760040 | 0.002 | . | . | . | . | . | . | . | . | . |
| InternalIMT | dha | rs143917143 | 0.02 | -0.053 | 0.06 | 0.382 | 0.173 | 0.475 | 0.715 | -0.126 | 0.293 | 0.667 |
| InternalIMT | dha | rs149851136 | 0.002 | . | . | . | . | . | . | . | . | . |
| InternalIMT | dha | rs181612054 | 0.001 | . | . | . | . | . | . | . | . | . |
| InternalIMT | dha | rs191006548 | 0.001 | . | . | . | . | . | . | . | . | . |
| InternalIMT | dha | rs117377631 | 0.006 | . | . | . | . | . | . | . | . | . |
| InternalIMT | dha | rs182107634 | 0.002 | . | . | . | . | . | . | . | . | . |
| InternalIMT | dha | rs190211867 | 0.002 | . | . | . | . | . | . | . | . | . |
| InternalIMT | dha | rs148293457 | 0.031 | -0.048 | 0.061 | 0.429 | 0.214 | 0.314 | 0.495 | -0.13 | 0.19 | 0.495 |
| InternalIMT | dha | rs191811733 | 0.006 | . | . | . | . | . | . | . | . | . |
| InternalIMT | dha | rs146109272 | 0.008 | . | . | . | . | . | . | . | . | . |
| InternalIMT | dha | rs148397915 | 0.006 | . | . | . | . | . | . | . | . | . |
| InternalIMT | dha | rs142233290 | 0.007 | . | . | . | . | . | . | . | . | . |
| InternalIMT | dha | rs80226874 | 0.022 | -0.074 | 0.058 | 0.203 | -0.526 | 0.729 | 0.471 | 0.404 | 0.442 | 0.361 |
| InternalIMT | dha | rs144496203 | 0.012 | -0.051 | 0.058 | 0.381 | 0.422 | 0.698 | 0.546 | -0.315 | 0.424 | 0.458 |
| InternalIMT | dha | rs191062130 | 0.001 | . | . | . | . | . | . | . | . | . |
| InternalIMT | dha | rs185807817 | 0.004 | . | . | . | . | . | . | . | . | . |
| InternalIMT | dha | rs117846213 | 0.032 | -0.051 | 0.058 | 0.384 | 0.266 | 0.341 | 0.436 | -0.199 | 0.213 | 0.35 |
| InternalIMT | dha | rs190545473 | 0.002 | . | . | . | . | . | . | . | . | . |
| InternalIMT | dha | rs192364918 | 0.01 | -0.09 | 0.057 | 0.116 | -3.59 | 2.13 | 0.093 | 2.13 | 1.42 | 0.134 |
| InternalIMT | dha | rs182939241 | 0.003 | . | . | . | . | . | . | . | . | . |
| InternalIMT | dha | rs12571125 | 0.031 | -0.038 | 0.062 | 0.537 | 0.469 | 0.39 | 0.229 | -0.306 | 0.229 | 0.181 |
| InternalIMT | dha | rs192418941 | 0.005 | . | . | . | . | . | . | . | . | . |
| InternalIMT | dha | rs141270243 | 0.015 | -0.05 | 0.06 | 0.403 | -0.017 | 0.445 | 0.969 | -0.112 | 0.248 | 0.651 |
| InternalIMT | dha | rs182615502 | 0.006 | . | . | . | . | . | . | . | . | . |
| InternalIMT | dha | rs146669856 | 0.001 | . | . | . | . | . | . | . | . | . |
| InternalIMT | dha | rs78290029 | 0.034 | -0.051 | 0.059 | 0.385 | 0.246 | 0.325 | 0.449 | -0.179 | 0.203 | 0.377 |
| InternalIMT | dha | rs139955565 | 0.004 | . | . | . | . | . | . | . | . | . |
| InternalIMT | dha | rs12267314 | 0.03 | -0.057 | 0.059 | 0.328 | 0.017 | 0.304 | 0.956 | -0.022 | 0.194 | 0.911 |
| InternalIMT | dha | rs74477197 | 0.004 | . | . | . | . | . | . | . | . | . |
| InternalIMT | dha | rs188147236 | 0.005 | . | . | . | . | . | . | . | . | . |
| InternalIMT | dha | rs2228065 | 0.03 | -0.058 | 0.059 | 0.328 | 0.012 | 0.299 | 0.968 | -0.017 | 0.191 | 0.929 |
| InternalIMT | dha | rs116961353 | 0.015 | -0.083 | 0.059 | 0.157 | -1.18 | 1.02 | 0.244 | 0.842 | 0.618 | 0.173 |
| InternalIMT | dha | rs190222272 | 0.007 | . | . | . | . | . | . | . | . | . |
| InternalIMT | dha | rs182719940 | 0.006 | . | . | . | . | . | . | . | . | . |
| InternalIMT | dha | rs184477413 | 0.004 | . | . | . | . | . | . | . | . | . |
| InternalIMT | dha | rs12265175 | 0.03 | -0.058 | 0.059 | 0.327 | 2.30E-03 | 0.296 | 0.994 | -8.90E-03 | 0.189 | 0.962 |
| InternalIMT | dha | rs182928276 | 0.003 | . | . | . | . | . | . | . | . | . |
| InternalIMT | dha | rs144409895 | 0.011 | -0.093 | 0.058 | 0.106 | -3.68 | 2.12 | 0.083 | 2.17 | 1.41 | 0.122 |
| InternalIMT | dha | rs58998447 | 0.136 | -0.04 | 0.068 | 0.555 | 0.081 | 0.21 | 0.701 | -0.055 | 0.124 | 0.66 |
| InternalIMT | dha | rs188668832 | 0.005 | . | . | . | . | . | . | . | . | . |
| InternalIMT | dha | rs148641686 | 0.002 | . | . | . | . | . | . | . | . | . |
| InternalIMT | dha | rs75468791 | 0.054 | -0.077 | 0.063 | 0.222 | -0.291 | 0.489 | 0.552 | 0.216 | 0.29 | 0.456 |
| InternalIMT | dha | rs147327397 | 0.003 | . | . | . | . | . | . | . | . | . |
| InternalIMT | dha | rs77354597 | 0.029 | -0.058 | 0.059 | 0.327 | 3.70E-03 | 0.305 | 0.99 | -0.011 | 0.195 | 0.957 |
| InternalIMT | dha | rs41365544 | 0.03 | -0.058 | 0.059 | 0.325 | -7.20E-03 | 0.295 | 0.981 | -2.70E-03 | 0.189 | 0.989 |
| InternalIMT | dha | rs187564188 | 0.016 | -0.028 | 0.059 | 0.634 | 1.61 | 1.33 | 0.228 | -0.953 | 0.743 | 0.2 |
| InternalIMT | dha | rs183511984 | 0.007 | . | . | . | . | . | . | . | . | . |
| InternalIMT | dha | rs12262783 | 0.003 | . | . | . | . | . | . | . | . | . |
| InternalIMT | dha | rs188817687 | 0.003 | . | . | . | . | . | . | . | . | . |
| InternalIMT | dha | rs3815968 | 0.005 | . | . | . | . | . | . | . | . | . |
| InternalIMT | dha | rs187822300 | 0.001 | . | . | . | . | . | . | . | . | . |
| InternalIMT | dha | rs188902172 | 0.032 | -0.058 | 0.059 | 0.328 | 8.90E-03 | 0.299 | 0.976 | -0.02 | 0.19 | 0.916 |
| InternalIMT | dha | rs181190448 | 0.006 | . | . | . | . | . | . | . | . | . |
| InternalIMT | dha | rs182699093 | 0.001 | . | . | . | . | . | . | . | . | . |
| InternalIMT | dha | rs182631303 | 0.014 | -0.058 | 0.059 | 0.328 | -0.075 | 0.416 | 0.856 | 0.082 | 0.26 | 0.752 |
| InternalIMT | dha | rs187014172 | 0.006 | . | . | . | . | . | . | . | . | . |
| InternalIMT | dha | rs142575406 | 0.009 | . | . | . | . | . | . | . | . | . |
| InternalIMT | dha | rs180711033 | 0.007 | . | . | . | . | . | . | . | . | . |
| InternalIMT | dha | rs137894823 | . | . | . | . | . | . | . | . | . | . |
| InternalIMT | dha | rs189053273 | . | . | . | . | . | . | . | . | . | . |
| InternalIMT | dha | rs143499388 | . | . | . | . | . | . | . | . | . | . |
| InternalIMT | dha | rs182936001 | . | . | . | . | . | . | . | . | . | . |
| InternalIMT | dha | rs148456406 | . | . | . | . | . | . | . | . | . | . |
| InternalIMT | dha | rs182499531 | . | . | . | . | . | . | . | . | . | . |
| InternalIMT | dha | rs74884915 | . | . | . | . | . | . | . | . | . | . |
| InternalIMT | dha | rs184046374 | . | . | . | . | . | . | . | . | . | . |
| InternalIMT | dha | rs74333993 | . | . | . | . | . | . | . | . | . | . |
| InternalIMT | dha | rs138889719 | . | . | . | . | . | . | . | . | . | . |
| InternalIMT | dha | rs75330330 | . | . | . | . | . | . | . | . | . | . |
| InternalIMT | dha | rs193051693 | . | . | . | . | . | . | . | . | . | . |
| InternalIMT | dha | rs145929633 | . | . | . | . | . | . | . | . | . | . |

| InternalIMT | dha | rs184076904 | . | . | . | . | . | . | . | . | . | . |
| --- | --- | --- | --- | --- | --- | --- | --- | --- | --- | --- | --- | --- |
| InternalIMT | dha | rs141802494 | . | . | . | . | . | . | . | . | . | . |
| InternalIMT | dha | rs149519759 | . | . | . | . | . | . | . | . | . | . |
| InternalIMT | dha | rs145467260 | . | . | . | . | . | . | . | . | . | . |
| InternalIMT | dha | rs79620914 | . | . | . | . | . | . | . | . | . | . |
| InternalIMT | dha | rs140538633 | . | . | . | . | . | . | . | . | . | . |
| InternalIMT | dha | rs140862301 | . | . | . | . | . | . | . | . | . | . |
| InternalIMT | dha | rs114615847 | . | . | . | . | . | . | . | . | . | . |
| InternalIMT | dha | rs141800259 | . | . | . | . | . | . | . | . | . | . |
| InternalIMT | dha | rs149779059 | . | . | . | . | . | . | . | . | . | . |
| InternalIMT | dha | rs116539253 | . | . | . | . | . | . | . | . | . | . |
| InternalIMT | dha | rs184979550 | . | . | . | . | . | . | . | . | . | . |
| InternalIMT | dha | rs190128940 | . | . | . | . | . | . | . | . | . | . |
| InternalIMT | dha | rs146594816 | . | . | . | . | . | . | . | . | . | . |
| InternalIMT | dha | rs141370766 | . | . | . | . | . | . | . | . | . | . |
| InternalIMT | dha | rs144670033 | . | . | . | . | . | . | . | . | . | . |
| InternalIMT | dha | rs187152174 | . | . | . | . | . | . | . | . | . | . |
| InternalIMT | dha | rs184180263 | . | . | . | . | . | . | . | . | . | . |
| InternalIMT | dha | rs140075861 | . | . | . | . | . | . | . | . | . | . |
| InternalIMT | dha | rs186483129 | . | . | . | . | . | . | . | . | . | . |
| InternalIMT | dha | rs186634282 | . | . | . | . | . | . | . | . | . | . |
| InternalIMT | dha | rs9988663 | . | . | . | . | . | . | . | . | . | . |
| InternalIMT | dha | rs138828529 | . | . | . | . | . | . | . | . | . | . |
| InternalIMT | dha | rs188777615 | . | . | . | . | . | . | . | . | . | . |
| InternalIMT | dha | rs192321882 | . | . | . | . | . | . | . | . | . | . |
| InternalIMT | dha | rs150812270 | . | . | . | . | . | . | . | . | . | . |
| InternalIMT | dha | rs185906603 | . | . | . | . | . | . | . | . | . | . |
| InternalIMT | dha | rs191070862 | . | . | . | . | . | . | . | . | . | . |
| InternalIMT | dha | rs182904978 | . | . | . | . | . | . | . | . | . | . |
| InternalIMT | dha | rs192679655 | . | . | . | . | . | . | . | . | . | . |
| InternalIMT | dha | rs189673086 | . | . | . | . | . | . | . | . | . | . |
| InternalIMT | dha | rs181141892 | . | . | . | . | . | . | . | . | . | . |
| InternalIMT | dha | rs186404123 | . | . | . | . | . | . | . | . | . | . |
| InternalIMT | dha | rs192117287 | . | . | . | . | . | . | . | . | . | . |
| InternalIMT | dha | rs182748835 | . | . | . | . | . | . | . | . | . | . |
| InternalIMT | dha | rs141854549 | . | . | . | . | . | . | . | . | . | . |
| InternalIMT | dha | rs140163397 | . | . | . | . | . | . | . | . | . | . |
| InternalIMT | dha | rs146347300 | . | . | . | . | . | . | . | . | . | . |
| InternalIMT | dha | rs139362377 | . | . | . | . | . | . | . | . | . | . |
| InternalIMT | dha | rs185087671 | . | . | . | . | . | . | . | . | . | . |
| InternalIMT | dha | rs190466574 | . | . | . | . | . | . | . | . | . | . |
| InternalIMT | dha | rs147012778 | . | . | . | . | . | . | . | . | . | . |
| InternalIMT | dha | rs138236960 | . | . | . | . | . | . | . | . | . | . |
| InternalIMT | dha | rs116310311 | . | . | . | . | . | . | . | . | . | . |
| InternalIMT | dha | rs142868356 | . | . | . | . | . | . | . | . | . | . |
| InternalIMT | dha | rs144956240 | . | . | . | . | . | . | . | . | . | . |
| InternalIMT | dha | rs188527776 | . | . | . | . | . | . | . | . | . | . |
| InternalIMT | dha | rs183971165 | . | . | . | . | . | . | . | . | . | . |
| InternalIMT | dha | rs76748216 | . | . | . | . | . | . | . | . | . | . |
| InternalIMT | dha | rs148554990 | . | . | . | . | . | . | . | . | . | . |
| InternalIMT | dha | rs114446477 | . | . | . | . | . | . | . | . | . | . |
| InternalIMT | dha | rs185223933 | . | . | . | . | . | . | . | . | . | . |
| InternalIMT | dha | rs76869726 | . | . | . | . | . | . | . | . | . | . |
| InternalIMT | dha | rs151200830 | . | . | . | . | . | . | . | . | . | . |
| InternalIMT | dha | rs17157731 | . | . | . | . | . | . | . | . | . | . |
| InternalIMT | dha | rs181243469 | . | . | . | . | . | . | . | . | . | . |
| InternalIMT | dha | rs186890707 | . | . | . | . | . | . | . | . | . | . |
| InternalIMT | dha | rs184063360 | . | . | . | . | . | . | . | . | . | . |
| InternalIMT | dha | rs188550237 | . | . | . | . | . | . | . | . | . | . |
| InternalIMT | dha | rs140931068 | . | . | . | . | . | . | . | . | . | . |
| InternalIMT | dha | rs74867877 | . | . | . | . | . | . | . | . | . | . |
| InternalIMT | dha | rs11239495 | . | . | . | . | . | . | . | . | . | . |
| InternalIMT | dha | rs149745800 | . | . | . | . | . | . | . | . | . | . |
| InternalIMT | dha | rs145044458 | . | . | . | . | . | . | . | . | . | . |
| InternalIMT | dha | rs188067427 | . | . | . | . | . | . | . | . | . | . |
| InternalIMT | dha | rs146827800 | . | . | . | . | . | . | . | . | . | . |
| InternalIMT | dha | rs140619817 | . | . | . | . | . | . | . | . | . | . |
| InternalIMT | dha | rs892689 | . | . | . | . | . | . | . | . | . | . |
| InternalIMT | dha | rs143642005 | . | . | . | . | . | . | . | . | . | . |
| InternalIMT | dha | rs181929304 | . | . | . | . | . | . | . | . | . | . |
| InternalIMT | dha | rs191086173 | . | . | . | . | . | . | . | . | . | . |
| InternalIMT | dha | rs114613116 | . | . | . | . | . | . | . | . | . | . |
| InternalIMT | dha | rs141076768 | . | . | . | . | . | . | . | . | . | . |
| InternalIMT | dha | rs187316531 | . | . | . | . | . | . | . | . | . | . |
| InternalIMT | dha | rs11239499 | . | . | . | . | . | . | . | . | . | . |
| InternalIMT | dha | rs192726371 | . | . | . | . | . | . | . | . | . | . |
| InternalIMT | dha | rs74128481 | . | . | . | . | . | . | . | . | . | . |
| InternalIMT | dha | rs74128482 | . | . | . | . | . | . | . | . | . | . |
| InternalIMT | dha | rs11239502 | . | . | . | . | . | . | . | . | . | . |
| InternalIMT | dha | rs116330285 | . | . | . | . | . | . | . | . | . | . |
| InternalIMT | dha | rs189926729 | . | . | . | . | . | . | . | . | . | . |
| InternalIMT | dha | rs188975779 | . | . | . | . | . | . | . | . | . | . |
| InternalIMT | dha | rs143846038 | . | . | . | . | . | . | . | . | . | . |
| InternalIMT | dha | rs74128483 | . | . | . | . | . | . | . | . | . | . |
| InternalIMT | dha | rs139292055 | . | . | . | . | . | . | . | . | . | . |
| InternalIMT | dha | rs11239503 | . | . | . | . | . | . | . | . | . | . |

| InternalIMT | dha | rs58838686 | . | . | . | . | . | . | . | . | . | . |
| --- | --- | --- | --- | --- | --- | --- | --- | --- | --- | --- | --- | --- |
| InternalIMT | dha | rs185825373 | . | . | . | . | . | . | . | . | . | . |
| InternalIMT | dha | rs11239504 | . | . | . | . | . | . | . | . | . | . |
| InternalIMT | dha | rs150012354 | . | . | . | . | . | . | . | . | . | . |
| InternalIMT | dha | rs187763096 | . | . | . | . | . | . | . | . | . | . |
| InternalIMT | dha | rs193043982 | . | . | . | . | . | . | . | . | . | . |
| InternalIMT | dha | rs79186884 | . | . | . | . | . | . | . | . | . | . |
| InternalIMT | dha | rs74128484 | . | . | . | . | . | . | . | . | . | . |
| InternalIMT | dha | rs58461537 | . | . | . | . | . | . | . | . | . | . |
| InternalIMT | dha | rs188527474 | . | . | . | . | . | . | . | . | . | . |
| InternalIMT | dha | rs141659976 | . | . | . | . | . | . | . | . | . | . |
| InternalIMT | dha | rs115846407 | . | . | . | . | . | . | . | . | . | . |
| InternalIMT | dha | rs138378816 | . | . | . | . | . | . | . | . | . | . |
| InternalIMT | dha | rs146188136 | . | . | . | . | . | . | . | . | . | . |
| InternalIMT | dha | rs139383871 | . | . | . | . | . | . | . | . | . | . |
| InternalIMT | dha | rs182953869 | . | . | . | . | . | . | . | . | . | . |
| InternalIMT | dha | rs192430299 | . | . | . | . | . | . | . | . | . | . |
| InternalIMT | dha | rs144152844 | . | . | . | . | . | . | . | . | . | . |
| InternalIMT | dha | rs145987786 | . | . | . | . | . | . | . | . | . | . |
| InternalIMT | dha | rs181132926 | . | . | . | . | . | . | . | . | . | . |
| InternalIMT | dha | rs147882253 | . | . | . | . | . | . | . | . | . | . |
| InternalIMT | dha | rs141807621 | . | . | . | . | . | . | . | . | . | . |
| InternalIMT | dha | rs147116052 | . | . | . | . | . | . | . | . | . | . |
| InternalIMT | dha | rs147716618 | . | . | . | . | . | . | . | . | . | . |
| InternalIMT | dha | rs187198855 | . | . | . | . | . | . | . | . | . | . |
| InternalIMT | dha | rs189409638 | . | . | . | . | . | . | . | . | . | . |
| InternalIMT | dha | rs182647339 | . | . | . | . | . | . | . | . | . | . |
| InternalIMT | dha | rs187995988 | . | . | . | . | . | . | . | . | . | . |
| InternalIMT | dha | rs141034184 | . | . | . | . | . | . | . | . | . | . |
| InternalIMT | dha | rs187310430 | . | . | . | . | . | . | . | . | . | . |
| InternalIMT | dha | rs192608488 | . | . | . | . | . | . | . | . | . | . |
| InternalIMT | dha | rs148840366 | . | . | . | . | . | . | . | . | . | . |
| InternalIMT | dha | rs186785022 | . | . | . | . | . | . | . | . | . | . |
| InternalIMT | dha | rs192348728 | . | . | . | . | . | . | . | . | . | . |
| InternalIMT | dha | rs189656631 | . | . | . | . | . | . | . | . | . | . |
| InternalIMT | dha | rs185289750 | . | . | . | . | . | . | . | . | . | . |
| InternalIMT | dha | rs142382395 | . | . | . | . | . | . | . | . | . | . |
| InternalIMT | dha | rs144866696 | . | . | . | . | . | . | . | . | . | . |
| InternalIMT | dha | rs141371289 | . | . | . | . | . | . | . | . | . | . |
| InternalIMT | dha | rs114750008 | . | . | . | . | . | . | . | . | . | . |
| InternalIMT | dha | rs189063597 | . | . | . | . | . | . | . | . | . | . |
| InternalIMT | dha | rs139151571 | . | . | . | . | . | . | . | . | . | . |
| InternalIMT | dha | rs139846368 | . | . | . | . | . | . | . | . | . | . |
| InternalIMT | dha | rs182057653 | . | . | . | . | . | . | . | . | . | . |
| InternalIMT | dha | rs148785420 | . | . | . | . | . | . | . | . | . | . |
| InternalIMT | dha | rs138383251 | . | . | . | . | . | . | . | . | . | . |
| InternalIMT | dha | rs116526705 | . | . | . | . | . | . | . | . | . | . |
| InternalIMT | dha | rs185946436 | . | . | . | . | . | . | . | . | . | . |
| InternalIMT | dha | rs190455427 | . | . | . | . | . | . | . | . | . | . |
| InternalIMT | dha | rs9282584 | . | . | . | . | . | . | . | . | . | . |
| InternalIMT | dha | rs17153289 | . | . | . | . | . | . | . | . | . | . |
| InternalIMT | dha | rs138359103 | . | . | . | . | . | . | . | . | . | . |
| InternalIMT | dha | rs150605268 | . | . | . | . | . | . | . | . | . | . |
| InternalIMT | dha | rs79242910 | . | . | . | . | . | . | . | . | . | . |
| InternalIMT | dha | rs139716904 | . | . | . | . | . | . | . | . | . | . |
| InternalIMT | dha | rs76238498 | . | . | . | . | . | . | . | . | . | . |
| InternalIMT | dha | rs184140639 | . | . | . | . | . | . | . | . | . | . |
| InternalIMT | dha | rs188517278 | . | . | . | . | . | . | . | . | . | . |
| InternalIMT | dha | rs139678913 | . | . | . | . | . | . | . | . | . | . |
| InternalIMT | dha | rs116697955 | . | . | . | . | . | . | . | . | . | . |
| InternalIMT | dha | rs79267994 | . | . | . | . | . | . | . | . | . | . |
| InternalIMT | dha | rs11239510 | . | . | . | . | . | . | . | . | . | . |
| InternalIMT | dha | rs75831645 | . | . | . | . | . | . | . | . | . | . |
| InternalIMT | dha | rs78452276 | . | . | . | . | . | . | . | . | . | . |
| InternalIMT | dha | rs144366924 | . | . | . | . | . | . | . | . | . | . |
| InternalIMT | dha | rs77519841 | . | . | . | . | . | . | . | . | . | . |
| InternalIMT | dha | rs76944454 | . | . | . | . | . | . | . | . | . | . |
| InternalIMT | dha | rs76443855 | . | . | . | . | . | . | . | . | . | . |
| InternalIMT | dha | rs146613174 | . | . | . | . | . | . | . | . | . | . |
| InternalIMT | dha | rs181424761 | . | . | . | . | . | . | . | . | . | . |
| InternalIMT | dha | rs142119241 | . | . | . | . | . | . | . | . | . | . |
| InternalIMT | dha | rs140137954 | . | . | . | . | . | . | . | . | . | . |
| InternalIMT | dha | rs187319549 | . | . | . | . | . | . | . | . | . | . |
| InternalIMT | dha | rs12241545 | . | . | . | . | . | . | . | . | . | . |
| InternalIMT | dha | rs10128228 | . | . | . | . | . | . | . | . | . | . |
| InternalIMT | dha | rs58580249 | . | . | . | . | . | . | . | . | . | . |
| InternalIMT | dha | rs193148863 | . | . | . | . | . | . | . | . | . | . |
| InternalIMT | dha | rs185638946 | . | . | . | . | . | . | . | . | . | . |
| InternalIMT | dha | rs117752921 | . | . | . | . | . | . | . | . | . | . |
| InternalIMT | dha | rs180841236 | . | . | . | . | . | . | . | . | . | . |
| InternalIMT | dha | rs73283155 | . | . | . | . | . | . | . | . | . | . |
| InternalIMT | dha | rs76560599 | . | . | . | . | . | . | . | . | . | . |
| InternalIMT | dha | rs76273620 | . | . | . | . | . | . | . | . | . | . |
| InternalIMT | dha | rs141432923 | . | . | . | . | . | . | . | . | . | . |
| InternalIMT | dha | rs113529322 | . | . | . | . | . | . | . | . | . | . |
| InternalIMT | dha | rs10128306 | . | . | . | . | . | . | . | . | . | . |

| InternalIMT | dha | rs149691688 | . | . | . | . | . | . | . | . | . | . |
| --- | --- | --- | --- | --- | --- | --- | --- | --- | --- | --- | --- | --- |
| InternalIMT | dha | rs75872495 | . | . | . | . | . | . | . | . | . | . |
| InternalIMT | dha | rs191873960 | . | . | . | . | . | . | . | . | . | . |
| InternalIMT | dha | rs80155622 | . | . | . | . | . | . | . | . | . | . |
| InternalIMT | dha | rs113914656 | . | . | . | . | . | . | . | . | . | . |
| InternalIMT | dha | rs12242840 | . | . | . | . | . | . | . | . | . | . |
| InternalIMT | dha | rs147824078 | . | . | . | . | . | . | . | . | . | . |
| InternalIMT | dha | rs59051493 | . | . | . | . | . | . | . | . | . | . |
| InternalIMT | dha | rs9282588 | . | . | . | . | . | . | . | . | . | . |
| InternalIMT | dha | rs150215207 | . | . | . | . | . | . | . | . | . | . |
| InternalIMT | dha | rs138772243 | . | . | . | . | . | . | . | . | . | . |
| InternalIMT | dha | rs149383244 | . | . | . | . | . | . | . | . | . | . |
| InternalIMT | dha | rs17522720 | . | . | . | . | . | . | . | . | . | . |
| InternalIMT | dha | rs79245105 | . | . | . | . | . | . | . | . | . | . |
| InternalIMT | dha | rs138222133 | . | . | . | . | . | . | . | . | . | . |
| InternalIMT | dha | rs12247314 | . | . | . | . | . | . | . | . | . | . |
| InternalIMT | dha | rs12263894 | . | . | . | . | . | . | . | . | . | . |
| InternalIMT | dha | rs149907880 | . | . | . | . | . | . | . | . | . | . |
| InternalIMT | dha | rs188153885 | . | . | . | . | . | . | . | . | . | . |
| InternalIMT | dha | rs73283159 | . | . | . | . | . | . | . | . | . | . |
| InternalIMT | dha | rs140476687 | . | . | . | . | . | . | . | . | . | . |
| InternalIMT | dha | rs186603148 | . | . | . | . | . | . | . | . | . | . |
| InternalIMT | dha | rs183296063 | . | . | . | . | . | . | . | . | . | . |
| InternalIMT | dha | rs145733694 | . | . | . | . | . | . | . | . | . | . |
| InternalIMT | dha | rs189807449 | . | . | . | . | . | . | . | . | . | . |
| InternalIMT | dha | rs12253223 | . | . | . | . | . | . | . | . | . | . |
| InternalIMT | dha | rs182113637 | . | . | . | . | . | . | . | . | . | . |
| InternalIMT | dha | rs142792099 | . | . | . | . | . | . | . | . | . | . |
| InternalIMT | dha | rs12249910 | . | . | . | . | . | . | . | . | . | . |
| InternalIMT | dha | rs149436339 | . | . | . | . | . | . | . | . | . | . |
| InternalIMT | dha | rs77632210 | . | . | . | . | . | . | . | . | . | . |
| InternalIMT | dha | rs74130818 | . | . | . | . | . | . | . | . | . | . |
| InternalIMT | dha | rs187583648 | . | . | . | . | . | . | . | . | . | . |
| InternalIMT | dha | rs191295987 | . | . | . | . | . | . | . | . | . | . |
| InternalIMT | dha | rs184200686 | . | . | . | . | . | . | . | . | . | . |
| InternalIMT | dha | rs76117263 | . | . | . | . | . | . | . | . | . | . |
| InternalIMT | dha | rs185393149 | . | . | . | . | . | . | . | . | . | . |
| InternalIMT | dha | rs144760014 | . | . | . | . | . | . | . | . | . | . |
| InternalIMT | dha | rs7922292 | . | . | . | . | . | . | . | . | . | . |
| InternalIMT | dha | rs115372844 | . | . | . | . | . | . | . | . | . | . |
| InternalIMT | dha | rs111460364 | . | . | . | . | . | . | . | . | . | . |
| InternalIMT | dha | rs182052255 | . | . | . | . | . | . | . | . | . | . |
| InternalIMT | dha | rs180983889 | . | . | . | . | . | . | . | . | . | . |
| InternalIMT | dha | rs139175327 | . | . | . | . | . | . | . | . | . | . |
| InternalIMT | dha | rs149959620 | . | . | . | . | . | . | . | . | . | . |
| InternalIMT | dha | rs114653117 | . | . | . | . | . | . | . | . | . | . |
| InternalIMT | dha | rs144270018 | . | . | . | . | . | . | . | . | . | . |
| InternalIMT | dha | rs182723204 | . | . | . | . | . | . | . | . | . | . |
| InternalIMT | dha | rs57204067 | . | . | . | . | . | . | . | . | . | . |
| InternalIMT | dha | rs144956226 | . | . | . | . | . | . | . | . | . | . |
| InternalIMT | dha | rs187842704 | . | . | . | . | . | . | . | . | . | . |
| InternalIMT | dha | rs184620511 | . | . | . | . | . | . | . | . | . | . |
| InternalIMT | dha | rs144031303 | . | . | . | . | . | . | . | . | . | . |
| InternalIMT | dha | rs187526473 | . | . | . | . | . | . | . | . | . | . |
| InternalIMT | dha | rs193002805 | . | . | . | . | . | . | . | . | . | . |
| InternalIMT | dha | rs138707426 | . | . | . | . | . | . | . | . | . | . |
| InternalIMT | dha | rs142754243 | . | . | . | . | . | . | . | . | . | . |
| InternalIMT | dha | rs181600331 | . | . | . | . | . | . | . | . | . | . |
| InternalIMT | dha | rs144421264 | . | . | . | . | . | . | . | . | . | . |
| InternalIMT | dha | rs184414519 | . | . | . | . | . | . | . | . | . | . |
| InternalIMT | dha | rs150596349 | . | . | . | . | . | . | . | . | . | . |
| InternalIMT | dha | rs190753747 | . | . | . | . | . | . | . | . | . | . |
| InternalIMT | dha | rs139560940 | . | . | . | . | . | . | . | . | . | . |
| InternalIMT | dha | rs75640804 | . | . | . | . | . | . | . | . | . | . |
| InternalIMT | dha | rs73283175 | . | . | . | . | . | . | . | . | . | . |
| InternalIMT | dha | rs143337229 | . | . | . | . | . | . | . | . | . | . |
| InternalIMT | dha | rs73283177 | . | . | . | . | . | . | . | . | . | . |
| InternalIMT | dha | rs76053611 | . | . | . | . | . | . | . | . | . | . |
| InternalIMT | dha | rs116325983 | . | . | . | . | . | . | . | . | . | . |
| InternalIMT | dha | rs115451825 | . | . | . | . | . | . | . | . | . | . |
| InternalIMT | dha | rs183845246 | . | . | . | . | . | . | . | . | . | . |
| InternalIMT | dha | rs79756542 | . | . | . | . | . | . | . | . | . | . |
| InternalIMT | dha | rs187242860 | . | . | . | . | . | . | . | . | . | . |
| InternalIMT | dha | rs57318980 | . | . | . | . | . | . | . | . | . | . |
| InternalIMT | dha | rs59969732 | . | . | . | . | . | . | . | . | . | . |
| InternalIMT | dha | rs143811488 | . | . | . | . | . | . | . | . | . | . |
| InternalIMT | dha | rs189067644 | . | . | . | . | . | . | . | . | . | . |
| InternalIMT | dha | rs60449178 | . | . | . | . | . | . | . | . | . | . |
| InternalIMT | dha | rs59243389 | . | . | . | . | . | . | . | . | . | . |
| InternalIMT | dha | rs184946540 | . | . | . | . | . | . | . | . | . | . |
| InternalIMT | dha | rs188685881 | . | . | . | . | . | . | . | . | . | . |
| InternalIMT | dha | rs150335797 | . | . | . | . | . | . | . | . | . | . |
| InternalIMT | dha | rs143879260 | . | . | . | . | . | . | . | . | . | . |
| InternalIMT | dha | rs186044491 | . | . | . | . | . | . | . | . | . | . |
| InternalIMT | dha | rs11819084 | . | . | . | . | . | . | . | . | . | . |
| InternalIMT | dha | rs146915670 | . | . | . | . | . | . | . | . | . | . |

| InternalIMT | dha | rs113995695 | . | . | . | . | . | . | . | . | . | . |
| --- | --- | --- | --- | --- | --- | --- | --- | --- | --- | --- | --- | --- |
| InternalIMT | dha | rs143489451 | . | . | . | . | . | . | . | . | . | . |
| InternalIMT | dha | rs182653839 | . | . | . | . | . | . | . | . | . | . |
| InternalIMT | dha | rs141692564 | . | . | . | . | . | . | . | . | . | . |
| InternalIMT | dha | rs146281742 | . | . | . | . | . | . | . | . | . | . |
| InternalIMT | dha | rs192914353 | . | . | . | . | . | . | . | . | . | . |
| InternalIMT | dha | rs185182848 | . | . | . | . | . | . | . | . | . | . |
| InternalIMT | dha | rs28395858 | . | . | . | . | . | . | . | . | . | . |
| InternalIMT | dha | rs41429645 | . | . | . | . | . | . | . | . | . | . |
| InternalIMT | dha | rs28395874 | . | . | . | . | . | . | . | . | . | . |
| InternalIMT | dha | rs189663634 | . | . | . | . | . | . | . | . | . | . |
| InternalIMT | dha | rs77666700 | . | . | . | . | . | . | . | . | . | . |
| InternalIMT | dha | rs146711717 | . | . | . | . | . | . | . | . | . | . |
| InternalIMT | dha | rs144904251 | . | . | . | . | . | . | . | . | . | . |
| InternalIMT | dha | rs189893153 | . | . | . | . | . | . | . | . | . | . |
| InternalIMT | dha | rs150447740 | . | . | . | . | . | . | . | . | . | . |
| InternalIMT | dha | rs143099649 | . | . | . | . | . | . | . | . | . | . |
| InternalIMT | dha | rs191288436 | . | . | . | . | . | . | . | . | . | . |
| InternalIMT | dha | rs188577862 | . | . | . | . | . | . | . | . | . | . |
| InternalIMT | dha | rs192695334 | . | . | . | . | . | . | . | . | . | . |
| InternalIMT | dha | rs138612906 | . | . | . | . | . | . | . | . | . | . |
| InternalIMT | dha | rs191290051 | . | . | . | . | . | . | . | . | . | . |
| InternalIMT | dha | rs189631459 | . | . | . | . | . | . | . | . | . | . |
| InternalIMT | dha | rs114620874 | . | . | . | . | . | . | . | . | . | . |
| InternalIMT | dha | rs78246652 | . | . | . | . | . | . | . | . | . | . |
| InternalIMT | dha | rs79432451 | . | . | . | . | . | . | . | . | . | . |
| InternalIMT | dha | rs186022585 | . | . | . | . | . | . | . | . | . | . |
| InternalIMT | dha | rs142978891 | . | . | . | . | . | . | . | . | . | . |
| InternalIMT | dha | rs151086840 | . | . | . | . | . | . | . | . | . | . |
| InternalIMT | dha | rs183270259 | . | . | . | . | . | . | . | . | . | . |
| InternalIMT | dha | rs188544289 | . | . | . | . | . | . | . | . | . | . |
| InternalIMT | dha | rs188500808 | . | . | . | . | . | . | . | . | . | . |
| InternalIMT | dha | rs189424589 | . | . | . | . | . | . | . | . | . | . |
| InternalIMT | dha | rs78622303 | . | . | . | . | . | . | . | . | . | . |
| InternalIMT | dha | rs143210835 | . | . | . | . | . | . | . | . | . | . |
| InternalIMT | dha | rs114761840 | . | . | . | . | . | . | . | . | . | . |
| InternalIMT | dha | rs185805402 | . | . | . | . | . | . | . | . | . | . |
| InternalIMT | dha | rs139273744 | . | . | . | . | . | . | . | . | . | . |
| InternalIMT | dha | rs184654150 | . | . | . | . | . | . | . | . | . | . |
| InternalIMT | dha | rs189069710 | . | . | . | . | . | . | . | . | . | . |
| InternalIMT | dha | rs114752115 | . | . | . | . | . | . | . | . | . | . |
| InternalIMT | dha | rs150162515 | . | . | . | . | . | . | . | . | . | . |
| InternalIMT | dha | rs180982454 | . | . | . | . | . | . | . | . | . | . |
| InternalIMT | dha | rs184932088 | . | . | . | . | . | . | . | . | . | . |
| InternalIMT | dha | rs181109625 | . | . | . | . | . | . | . | . | . | . |
| InternalIMT | dha | rs138121134 | . | . | . | . | . | . | . | . | . | . |
| InternalIMT | dha | rs183253861 | . | . | . | . | . | . | . | . | . | . |
| InternalIMT | dha | rs147073946 | . | . | . | . | . | . | . | . | . | . |
| InternalIMT | dha | rs139938489 | . | . | . | . | . | . | . | . | . | . |
| InternalIMT | dha | rs146362560 | . | . | . | . | . | . | . | . | . | . |
| InternalIMT | dha | rs193155956 | . | . | . | . | . | . | . | . | . | . |
| InternalIMT | dha | rs77663092 | . | . | . | . | . | . | . | . | . | . |
| InternalIMT | dha | rs73285319 | . | . | . | . | . | . | . | . | . | . |
| InternalIMT | dha | rs184019276 | . | . | . | . | . | . | . | . | . | . |
| InternalIMT | dha | rs28395863 | . | . | . | . | . | . | . | . | . | . |
| InternalIMT | dha | rs182121645 | . | . | . | . | . | . | . | . | . | . |
| InternalIMT | dha | rs73285327 | . | . | . | . | . | . | . | . | . | . |
| InternalIMT | dha | rs149589584 | . | . | . | . | . | . | . | . | . | . |
| InternalIMT | dha | rs148310335 | . | . | . | . | . | . | . | . | . | . |
| InternalIMT | dha | rs187246093 | . | . | . | . | . | . | . | . | . | . |
| InternalIMT | dha | rs184668894 | . | . | . | . | . | . | . | . | . | . |
| InternalIMT | dha | rs114269961 | . | . | . | . | . | . | . | . | . | . |
| InternalIMT | dha | rs149694374 | . | . | . | . | . | . | . | . | . | . |
| InternalIMT | dha | rs145284227 | . | . | . | . | . | . | . | . | . | . |
| InternalIMT | dha | rs189983820 | . | . | . | . | . | . | . | . | . | . |
| InternalIMT | dha | rs181200444 | . | . | . | . | . | . | . | . | . | . |
| InternalIMT | dha | rs185486385 | . | . | . | . | . | . | . | . | . | . |
| InternalIMT | dha | rs151198241 | . | . | . | . | . | . | . | . | . | . |
| InternalIMT | dha | rs189959341 | . | . | . | . | . | . | . | . | . | . |
| InternalIMT | dha | rs140549257 | . | . | . | . | . | . | . | . | . | . |
| InternalIMT | dha | rs186453139 | . | . | . | . | . | . | . | . | . | . |
| InternalIMT | dha | rs180922711 | . | . | . | . | . | . | . | . | . | . |
| InternalIMT | dha | rs56659903 | . | . | . | . | . | . | . | . | . | . |
| InternalIMT | dha | rs141202128 | . | . | . | . | . | . | . | . | . | . |
| InternalIMT | dha | rs59085314 | . | . | . | . | . | . | . | . | . | . |
| InternalIMT | dha | rs145564996 | . | . | . | . | . | . | . | . | . | . |
| InternalIMT | dha | rs114961009 | . | . | . | . | . | . | . | . | . | . |
| InternalIMT | dha | rs182294189 | . | . | . | . | . | . | . | . | . | . |
| InternalIMT | dha | rs187586636 | . | . | . | . | . | . | . | . | . | . |
| InternalIMT | dha | rs192254907 | . | . | . | . | . | . | . | . | . | . |
| InternalIMT | dha | rs149145663 | . | . | . | . | . | . | . | . | . | . |
| InternalIMT | dha | rs180713064 | . | . | . | . | . | . | . | . | . | . |
| InternalIMT | dha | rs183009023 | . | . | . | . | . | . | . | . | . | . |
| InternalIMT | dha | rs143385347 | . | . | . | . | . | . | . | . | . | . |
| InternalIMT | dha | rs185786329 | . | . | . | . | . | . | . | . | . | . |
| InternalIMT | dha | rs192341030 | . | . | . | . | . | . | . | . | . | . |

| InternalIMT | dha | rs74661429 | . | . | . | . | . | . | . | . | . | . |
| --- | --- | --- | --- | --- | --- | --- | --- | --- | --- | --- | --- | --- |
| InternalIMT | dha | rs188034853 | . | . | . | . | . | . | . | . | . | . |
| InternalIMT | dha | rs116808430 | . | . | . | . | . | . | . | . | . | . |
| InternalIMT | dha | rs148469010 | . | . | . | . | . | . | . | . | . | . |
| InternalIMT | dha | rs78621663 | . | . | . | . | . | . | . | . | . | . |
| InternalIMT | dha | rs192654886 | . | . | . | . | . | . | . | . | . | . |
| InternalIMT | dha | rs145996035 | . | . | . | . | . | . | . | . | . | . |
| InternalIMT | dha | rs139942226 | . | . | . | . | . | . | . | . | . | . |
| InternalIMT | dha | rs113645632 | . | . | . | . | . | . | . | . | . | . |
| InternalIMT | dha | rs74940661 | . | . | . | . | . | . | . | . | . | . |
| InternalIMT | dha | rs115355800 | . | . | . | . | . | . | . | . | . | . |
| InternalIMT | dha | rs150285898 | . | . | . | . | . | . | . | . | . | . |
| InternalIMT | dha | rs188117932 | . | . | . | . | . | . | . | . | . | . |
| InternalIMT | dha | rs139956566 | . | . | . | . | . | . | . | . | . | . |
| InternalIMT | dha | rs147673885 | . | . | . | . | . | . | . | . | . | . |
| InternalIMT | dha | rs189127928 | . | . | . | . | . | . | . | . | . | . |
| InternalIMT | dha | rs187558772 | . | . | . | . | . | . | . | . | . | . |
| InternalIMT | dha | rs181948587 | . | . | . | . | . | . | . | . | . | . |
| InternalIMT | dha | rs189983269 | . | . | . | . | . | . | . | . | . | . |
| InternalIMT | dha | rs187250069 | . | . | . | . | . | . | . | . | . | . |
| InternalIMT | dha | rs191485859 | . | . | . | . | . | . | . | . | . | . |
| InternalIMT | dha | rs185499555 | . | . | . | . | . | . | . | . | . | . |
| InternalIMT | dha | rs180862110 | . | . | . | . | . | . | . | . | . | . |
| InternalIMT | dha | rs190472603 | . | . | . | . | . | . | . | . | . | . |
| InternalIMT | dha | rs189226167 | . | . | . | . | . | . | . | . | . | . |
| InternalIMT | dha | rs181623256 | . | . | . | . | . | . | . | . | . | . |
| InternalIMT | dha | rs186079557 | . | . | . | . | . | . | . | . | . | . |
| InternalIMT | dha | rs147760994 | . | . | . | . | . | . | . | . | . | . |
| InternalIMT | dha | rs189176167 | . | . | . | . | . | . | . | . | . | . |
| InternalIMT | dha | rs117901000 | . | . | . | . | . | . | . | . | . | . |
| InternalIMT | dha | rs141984888 | . | . | . | . | . | . | . | . | . | . |
| InternalIMT | dha | rs192365061 | . | . | . | . | . | . | . | . | . | . |
| InternalIMT | dha | rs185976038 | . | . | . | . | . | . | . | . | . | . |
| InternalIMT | dha | rs184697303 | . | . | . | . | . | . | . | . | . | . |
| InternalIMT | dha | rs143383132 | . | . | . | . | . | . | . | . | . | . |
| InternalIMT | dha | rs141873881 | . | . | . | . | . | . | . | . | . | . |
| InternalIMT | dha | rs185507255 | . | . | . | . | . | . | . | . | . | . |
| InternalIMT | dha | rs191379425 | . | . | . | . | . | . | . | . | . | . |
| InternalIMT | dha | rs184199584 | . | . | . | . | . | . | . | . | . | . |
| InternalIMT | dha | rs183181360 | . | . | . | . | . | . | . | . | . | . |
| InternalIMT | dha | rs184495673 | . | . | . | . | . | . | . | . | . | . |
| InternalIMT | dha | rs182946297 | . | . | . | . | . | . | . | . | . | . |
| InternalIMT | dha | rs187743615 | . | . | . | . | . | . | . | . | . | . |
| InternalIMT | dha | rs183259862 | . | . | . | . | . | . | . | . | . | . |
| InternalIMT | dha | rs112433566 | . | . | . | . | . | . | . | . | . | . |
| InternalIMT | dha | rs182351085 | . | . | . | . | . | . | . | . | . | . |
| InternalIMT | dha | rs146248508 | . | . | . | . | . | . | . | . | . | . |
| InternalIMT | dha | rs188528439 | . | . | . | . | . | . | . | . | . | . |
| InternalIMT | dha | rs146167018 | . | . | . | . | . | . | . | . | . | . |
| InternalIMT | dha | rs192440980 | . | . | . | . | . | . | . | . | . | . |
| InternalIMT | dha | rs185601882 | . | . | . | . | . | . | . | . | . | . |
| InternalIMT | dha | rs117272563 | . | . | . | . | . | . | . | . | . | . |
| InternalIMT | dha | rs10430734 | . | . | . | . | . | . | . | . | . | . |
| InternalIMT | dha | rs181320952 | . | . | . | . | . | . | . | . | . | . |
| InternalIMT | dha | rs185480660 | . | . | . | . | . | . | . | . | . | . |
| InternalIMT | dha | rs188077917 | . | . | . | . | . | . | . | . | . | . |
| InternalIMT | dha | rs187562953 | . | . | . | . | . | . | . | . | . | . |
| InternalIMT | dha | rs182044631 | . | . | . | . | . | . | . | . | . | . |
| InternalIMT | dha | rs145455596 | . | . | . | . | . | . | . | . | . | . |
| InternalIMT | dha | rs181475877 | . | . | . | . | . | . | . | . | . | . |
| InternalIMT | dha | rs149413085 | . | . | . | . | . | . | . | . | . | . |
| InternalIMT | dha | rs12253656 | . | . | . | . | . | . | . | . | . | . |
| InternalIMT | dha | rs182718239 | . | . | . | . | . | . | . | . | . | . |
| InternalIMT | dha | rs192435695 | . | . | . | . | . | . | . | . | . | . |
| InternalIMT | dha | rs12248277 | . | . | . | . | . | . | . | . | . | . |
| InternalIMT | dha | rs143521710 | . | . | . | . | . | . | . | . | . | . |
| InternalIMT | dha | rs144454454 | . | . | . | . | . | . | . | . | . | . |
| InternalIMT | dha | rs190441610 | . | . | . | . | . | . | . | . | . | . |
| InternalIMT | dha | rs114690462 | . | . | . | . | . | . | . | . | . | . |
| InternalIMT | dha | rs184541357 | . | . | . | . | . | . | . | . | . | . |
| InternalIMT | dha | rs142657653 | . | . | . | . | . | . | . | . | . | . |
| InternalIMT | dha | rsurf1 | 2.462 | . | . | . | . | . | . | . | . | . |
| InternalIMT | dha | rsten1 | 2.569 | . | . | . | . | . | . | . | . | . |
| InternalIMT | dha | chr10:45852312:D | 0.198 | -0.042 | 0.073 | 0.565 | 0.066 | 0.18 | 0.717 | -0.036 | 0.112 | 0.749 |
| InternalIMT | dha | chr10:45853232:I | 0.2 | -0.033 | 0.074 | 0.652 | 0.097 | 0.179 | 0.589 | -0.055 | 0.111 | 0.624 |
| InternalIMT | dha | chr10:45853234:I | 0.194 | -0.029 | 0.073 | 0.687 | 0.112 | 0.18 | 0.535 | -0.065 | 0.112 | 0.563 |
| InternalIMT | dha | chr10:45854796:D | 0.053 | -0.078 | 0.062 | 0.209 | -0.453 | 0.241 | 0.061 | 0.24 | 0.158 | 0.128 |
| InternalIMT | dha | chr10:45854994:I | 0.054 | -0.078 | 0.062 | 0.209 | -0.454 | 0.241 | 0.061 | 0.241 | 0.158 | 0.127 |
| InternalIMT | dha | chr10:45858290:D | 0.048 | -0.06 | 0.061 | 0.325 | -0.044 | 0.513 | 0.931 | 0.023 | 0.321 | 0.943 |
| InternalIMT | dha | chr10:45858820:I | 0.005 | . | . | . | . | . | . | . | . | . |
| InternalIMT | dha | chr10:45860888:D | 0.063 | -0.047 | 0.061 | 0.444 | 0.097 | 0.295 | 0.741 | -0.074 | 0.174 | 0.672 |
| InternalIMT | dha | chr10:45863913:I | 0.195 | -0.036 | 0.073 | 0.627 | 0.081 | 0.175 | 0.643 | -0.049 | 0.107 | 0.648 |
| InternalIMT | dha | chr10:45866601:D | . | . | . | . | . | . | . | . | . | . |
| InternalIMT | dha | chr10:45867197:I | . | . | . | . | . | . | . | . | . | . |
| InternalIMT | dha | chr10:45867538:D | 0.198 | -0.041 | 0.074 | 0.58 | 0.062 | 0.18 | 0.73 | -0.037 | 0.112 | 0.743 |
| InternalIMT | dha | chr10:45868356:D | 0.197 | -0.04 | 0.074 | 0.584 | 0.064 | 0.18 | 0.722 | -0.038 | 0.112 | 0.736 |

| InternalIMT | dha | chr10:45871158:I | 0.198 | -0.041 | 0.074 | 0.582 | 0.063 | 0.18 | 0.725 | -0.037 | 0.112 | 0.74 |
| --- | --- | --- | --- | --- | --- | --- | --- | --- | --- | --- | --- | --- |
| InternalIMT | dha | chr10:45871637:D | 0.1 | -0.061 | 0.066 | 0.359 | -0.031 | 0.277 | 0.912 | 0.016 | 0.174 | 0.927 |
| InternalIMT | dha | chr10:45871638:I | 0.161 | -0.059 | 0.071 | 0.407 | -2.60E-03 | 0.202 | 0.99 | 5.40E-03 | 0.126 | 0.966 |
| InternalIMT | dha | chr10:45873629:I | 0.198 | -0.041 | 0.074 | 0.582 | 0.063 | 0.18 | 0.726 | -0.037 | 0.112 | 0.74 |
| InternalIMT | dha | chr10:45873701:D | . | . | . | . | . | . | . | . | . | . |
| InternalIMT | dha | chr10:45874997:I | . | . | . | . | . | . | . | . | . | . |
| InternalIMT | dha | chr10:45882030:I | . | . | . | . | . | . | . | . | . | . |
| InternalIMT | dha | chr10:45883774:D | 0.003 | . | . | . | . | . | . | . | . | . |
| InternalIMT | dha | chr10:45885169:D | 0.176 | -0.045 | 0.071 | 0.53 | 0.051 | 0.186 | 0.785 | -0.031 | 0.114 | 0.784 |
| InternalIMT | dha | chr10:45886451:D | 0.744 | -0.22 | 0.146 | 0.13 | -0.164 | 0.145 | 0.259 | 0.109 | 0.09 | 0.222 |
| InternalIMT | dha | chr10:45891800:I | 0.85 | -0.262 | 0.17 | 0.124 | -0.183 | 0.176 | 0.299 | 0.12 | 0.104 | 0.251 |
| InternalIMT | dha | chr10:45904771:I | 0.87 | -0.246 | 0.175 | 0.161 | -0.164 | 0.177 | 0.354 | 0.108 | 0.105 | 0.303 |
| InternalIMT | dha | chr10:45904959:I | 0.11 | -0.073 | 0.071 | 0.306 | -0.094 | 0.275 | 0.733 | 0.064 | 0.169 | 0.703 |
| InternalIMT | dha | chr10:45911028:I | 0.105 | -0.092 | 0.067 | 0.173 | -0.212 | 0.313 | 0.497 | 0.171 | 0.198 | 0.388 |
| InternalIMT | dha | chr10:45911031:I | 0.419 | -0.045 | 0.101 | 0.655 | 0.064 | 0.215 | 0.768 | -0.018 | 0.128 | 0.887 |
| InternalIMT | dha | chr10:45912154:D | 0.003 | . | . | . | . | . | . | . | . | . |
| InternalIMT | dha | chr10:45913885:I | 0.043 | -0.072 | 0.061 | 0.239 | -0.434 | 0.225 | 0.054 | 0.205 | 0.142 | 0.148 |
| InternalIMT | dha | chr10:45914753:D | 0.159 | -0.058 | 0.068 | 0.398 | 0.036 | 0.196 | 0.852 | 0.011 | 0.121 | 0.927 |
| InternalIMT | dha | chr10:45919621 | . | . | . | . | . | . | . | . | . | . |
| InternalIMT | dha | chr10:45921739:I | 0.048 | -0.069 | 0.061 | 0.261 | -0.316 | 0.21 | 0.132 | 0.145 | 0.131 | 0.271 |
| InternalIMT | dha | chr10:45923527:I | 0.062 | -0.078 | 0.067 | 0.246 | -0.334 | 0.314 | 0.288 | 0.178 | 0.194 | 0.357 |
| InternalIMT | dha | chr10:45927001:I | 0.005 | . | . | . | . | . | . | . | . | . |
| InternalIMT | dha | chr10:45930354:I | 0.044 | -0.068 | 0.061 | 0.267 | -0.281 | 0.212 | 0.185 | 0.12 | 0.134 | 0.37 |
| InternalIMT | dha | chr10:45932864:I | 0.065 | -0.061 | 0.064 | 0.344 | -0.132 | 0.223 | 0.556 | 0.018 | 0.14 | 0.896 |
| InternalIMT | dha | chr10:45936163:I | 0.027 | -0.05 | 0.059 | 0.396 | 0.213 | 0.395 | 0.589 | -0.115 | 0.247 | 0.641 |
| InternalIMT | dha | chr10:45938095:D | 0.058 | -0.051 | 0.06 | 0.397 | 0.079 | 0.227 | 0.727 | -0.031 | 0.144 | 0.83 |
| InternalIMT | dha | chr10:45938886 | . | . | . | . | . | . | . | . | . | . |
| InternalIMT | dha | chr10:45939320 | . | . | . | . | . | . | . | . | . | . |
| InternalIMT | dha | chr10:45939659 | . | . | . | . | . | . | . | . | . | . |
| InternalIMT | dha | chr10:45941150:D | 0.042 | -0.07 | 0.061 | 0.25 | -0.367 | 0.203 | 0.071 | 0.171 | 0.129 | 0.188 |
| InternalIMT | dha | chr10:45947128:D | 0.038 | -0.078 | 0.059 | 0.183 | -0.41 | 0.39 | 0.293 | 0.303 | 0.246 | 0.219 |
| InternalIMT | dha | chr10:45950990:I | 0.052 | -0.074 | 0.062 | 0.232 | -0.41 | 0.214 | 0.055 | 0.207 | 0.133 | 0.121 |
| InternalIMT | dha | chr10:45951854:I | . | . | . | . | . | . | . | . | . | . |
| InternalIMT | dha | chr10:45952670:I | 0.001 | . | . | . | . | . | . | . | . | . |
| InternalIMT | dha | chr10:45956741 | . | . | . | . | . | . | . | . | . | . |
| InternalIMT | dha | chr10:45957247:I | 0.065 | -0.041 | 0.061 | 0.499 | 0.223 | 0.228 | 0.33 | -0.13 | 0.139 | 0.35 |
| InternalIMT | dha | chr10:45958082:I | 0.065 | -0.041 | 0.061 | 0.499 | 0.222 | 0.229 | 0.333 | -0.129 | 0.14 | 0.356 |
| InternalIMT | dha | chr10:45905041:D | 0.05 | -0.072 | 0.062 | 0.247 | -0.374 | 0.222 | 0.092 | 0.177 | 0.14 | 0.205 |
| InternalIMT | dha | chr10:45938871 | 0.003 | . | . | . | . | . | . | . | . | . |
| InternalIMT | dha | chr10:45939160 | 0.004 | . | . | . | . | . | . | . | . | . |
| InternalIMT | dha | chr10:45869910 | . | . | . | . | . | . | . | . | . | . |
| InternalIMT | dha | chr10:45876214:D | . | . | . | . | . | . | . | . | . | . |
| InternalIMT | dha | chr10:45880237:I | . | . | . | . | . | . | . | . | . | . |
| InternalIMT | dha | chr10:45884371:I | . | . | . | . | . | . | . | . | . | . |
| InternalIMT | dha | chr10:45891535:D | . | . | . | . | . | . | . | . | . | . |
| InternalIMT | dha | chr10:45893516:D | . | . | . | . | . | . | . | . | . | . |
| InternalIMT | dha | chr10:45893782:D | . | . | . | . | . | . | . | . | . | . |
| InternalIMT | dha | chr10:45895254:I | . | . | . | . | . | . | . | . | . | . |
| InternalIMT | dha | chr10:45895848:D | . | . | . | . | . | . | . | . | . | . |
| InternalIMT | dha | chr10:45896316:D | . | . | . | . | . | . | . | . | . | . |
| InternalIMT | dha | chr10:45896317:D | . | . | . | . | . | . | . | . | . | . |
| InternalIMT | dha | chr10:45902683:I | . | . | . | . | . | . | . | . | . | . |
| InternalIMT | dha | chr10:45905003:D | . | . | . | . | . | . | . | . | . | . |
| InternalIMT | dha | chr10:45927476:D | . | . | . | . | . | . | . | . | . | . |
| InternalIMT | dha | chr10:45935882 | . | . | . | . | . | . | . | . | . | . |
| InternalIMT | dha | chr10:45936100 | . | . | . | . | . | . | . | . | . | . |
| InternalIMT | dha | chr10:45939553 | . | . | . | . | . | . | . | . | . | . |
| InternalIMT | dha | chr10:45939631 | . | . | . | . | . | . | . | . | . | . |
| InternalIMT | dha | chr10:45944193:D | . | . | . | . | . | . | . | . | . | . |
| InternalIMT | dha | chr10:45954030 | . | . | . | . | . | . | . | . | . | . |

Supplemental table S26.

| phen | covar(fa) | rs_id | maf | beta_FA | se_FA | p_FA | beta_SNP | se_SNP | p_SNP | beta_inter | se_inter | p_inter |
| --- | --- | --- | --- | --- | --- | --- | --- | --- | --- | --- | --- | --- |
| InternalIMT | aa | rs76035182 | 0.024 | 0.051 | 0.037 | 0.173 | 0.179 | 0.461 | 0.699 | -0.067 | 0.181 | 0.71 |
| InternalIMT | aa | rs12359547 | 0.069 | 0.07 | 0.035 | 0.044 | -0.457 | 0.24 | 0.057 | 0.186 | 0.099 | 0.061 |
| InternalIMT | aa | rs74595484 | 0.039 | 0.06 | 0.038 | 0.117 | 0.431 | 0.369 | 0.243 | -0.167 | 0.145 | 0.25 |
| InternalIMT | aa | rs1864415 | 0.701 | -0.034 | 0.08 | 0.667 | -0.222 | 0.124 | 0.074 | 0.088 | 0.051 | 0.083 |
| InternalIMT | aa | rs72788101 | 0.073 | 0.064 | 0.035 | 0.065 | -0.528 | 0.235 | 0.025 | 0.217 | 0.097 | 0.025 |
| InternalIMT | aa | rs11239481 | 0.14 | 0.057 | 0.042 | 0.167 | 0.079 | 0.172 | 0.644 | -0.028 | 0.072 | 0.701 |
| InternalIMT | aa | rs72788102 | 0.073 | 0.064 | 0.035 | 0.065 | -0.528 | 0.235 | 0.025 | 0.217 | 0.097 | 0.025 |
| InternalIMT | aa | rs72789705 | 0.073 | 0.064 | 0.035 | 0.065 | -0.528 | 0.235 | 0.025 | 0.217 | 0.097 | 0.025 |
| InternalIMT | aa | rs141529858 | 0.005 | . | . | . | . | . | . | . | . | . |
| InternalIMT | aa | rs145376292 | 0 | . | . | . | . | . | . | . | . | . |
| InternalIMT | aa | rs190418301 | 0.001 | . | . | . | . | . | . | . | . | . |
| InternalIMT | aa | rs11239482 | 0.073 | 0.064 | 0.035 | 0.065 | -0.527 | 0.235 | 0.025 | 0.217 | 0.097 | 0.025 |
| InternalIMT | aa | rs61855872 | 0.01 | 0.055 | 0.037 | 0.143 | 0.803 | 0.973 | 0.41 | -0.281 | 0.403 | 0.486 |
| InternalIMT | aa | rs148991144 | 0.007 | . | . | . | . | . | . | . | . | . |
| InternalIMT | aa | rs191932221 | 0.005 | . | . | . | . | . | . | . | . | . |
| InternalIMT | aa | rs55978138 | 0.166 | 0.04 | 0.038 | 0.3 | -0.408 | 0.151 | 6.80E-03 | 0.161 | 0.062 | 9.90E-03 |
| InternalIMT | aa | rs73281167 | 0.015 | 0.053 | 0.037 | 0.155 | 0.321 | 0.528 | 0.543 | -0.135 | 0.204 | 0.507 |
| InternalIMT | aa | rs12768010 | 0.086 | 0.067 | 0.036 | 0.059 | -0.397 | 0.182 | 0.029 | 0.152 | 0.076 | 0.046 |
| InternalIMT | aa | rs11239484 | 0.701 | -0.034 | 0.08 | 0.671 | -0.222 | 0.124 | 0.074 | 0.088 | 0.051 | 0.084 |
| InternalIMT | aa | rs35906360 | 0.166 | 0.039 | 0.038 | 0.314 | -0.413 | 0.15 | 5.90E-03 | 0.164 | 0.062 | 8.30E-03 |
| InternalIMT | aa | rs186250512 | 0.003 | . | . | . | . | . | . | . | . | . |
| InternalIMT | aa | rs150810743 | 0.002 | . | . | . | . | . | . | . | . | . |
| InternalIMT | aa | rs55965812 | 0.161 | 0.045 | 0.038 | 0.235 | -0.377 | 0.152 | 0.013 | 0.147 | 0.063 | 0.02 |
| InternalIMT | aa | rs56180358 | 0.167 | 0.038 | 0.038 | 0.317 | -0.415 | 0.15 | 5.70E-03 | 0.164 | 0.062 | 8.10E-03 |
| InternalIMT | aa | rs55864145 | 0.086 | 0.067 | 0.036 | 0.059 | -0.397 | 0.182 | 0.029 | 0.152 | 0.076 | 0.046 |
| InternalIMT | aa | rs55986122 | 0.166 | 0.039 | 0.038 | 0.313 | -0.413 | 0.15 | 5.90E-03 | 0.163 | 0.062 | 8.30E-03 |
| InternalIMT | aa | rs7477350 | 0.701 | -0.034 | 0.08 | 0.671 | -0.222 | 0.124 | 0.074 | 0.088 | 0.051 | 0.084 |
| InternalIMT | aa | rs2099170 | 0.166 | 0.039 | 0.038 | 0.314 | -0.413 | 0.15 | 5.80E-03 | 0.164 | 0.062 | 8.20E-03 |
| InternalIMT | aa | rs2082108 | 0.167 | 0.039 | 0.038 | 0.314 | -0.413 | 0.15 | 5.90E-03 | 0.163 | 0.062 | 8.30E-03 |
| InternalIMT | aa | rs55902404 | 0.007 | . | . | . | . | . | . | . | . | . |
| InternalIMT | aa | rs58402662 | 0.007 | . | . | . | . | . | . | . | . | . |
| InternalIMT | aa | rs11511618 | 0.14 | 0.06 | 0.042 | 0.154 | 0.1 | 0.171 | 0.56 | -0.037 | 0.071 | 0.604 |
| InternalIMT | aa | rs1816492 | 0.7 | -0.033 | 0.08 | 0.679 | -0.22 | 0.124 | 0.077 | 0.088 | 0.051 | 0.086 |
| InternalIMT | aa | rs146611093 | 0.007 | . | . | . | . | . | . | . | . | . |
| InternalIMT | aa | rs186897689 | 0.002 | . | . | . | . | . | . | . | . | . |
| InternalIMT | aa | rs10793616 | 0.701 | -0.034 | 0.08 | 0.672 | -0.221 | 0.124 | 0.075 | 0.088 | 0.051 | 0.084 |
| InternalIMT | aa | rs116700479 | 0.019 | 0.045 | 0.037 | 0.229 | -0.232 | 0.653 | 0.722 | 0.098 | 0.254 | 0.701 |
| InternalIMT | aa | rs192535936 | 0.002 | . | . | . | . | . | . | . | . | . |
| InternalIMT | aa | rs113363297 | 0.662 | -0.02 | 0.078 | 0.795 | -0.212 | 0.127 | 0.094 | 0.084 | 0.052 | 0.108 |
| InternalIMT | aa | rs12354994 | 0.158 | 0.044 | 0.038 | 0.254 | -0.393 | 0.156 | 0.012 | 0.156 | 0.064 | 0.015 |
| InternalIMT | aa | rs148068329 | 0.02 | 0.074 | 0.034 | 0.027 | -1.19 | 0.385 | 2.00E-03 | 0.466 | 0.166 | 4.90E-03 |
| InternalIMT | aa | rs12356109 | 0.171 | 0.041 | 0.039 | 0.293 | -0.393 | 0.146 | 7.20E-03 | 0.153 | 0.06 | 0.011 |
| InternalIMT | aa | rs55908141 | 0.007 | . | . | . | . | . | . | . | . | . |
| InternalIMT | aa | rs12354712 | 0.16 | 0.046 | 0.038 | 0.23 | -0.375 | 0.153 | 0.014 | 0.146 | 0.063 | 0.021 |
| InternalIMT | aa | rs147282684 | 0.003 | . | . | . | . | . | . | . | . | . |
| InternalIMT | aa | rs12356140 | 0.168 | 0.037 | 0.039 | 0.337 | -0.423 | 0.151 | 5.10E-03 | 0.167 | 0.062 | 7.30E-03 |
| InternalIMT | aa | rs185476187 | 0.002 | . | . | . | . | . | . | . | . | . |
| InternalIMT | aa | rs145720057 | 0.026 | 0.046 | 0.037 | 0.216 | -0.062 | 0.495 | 0.901 | 0.046 | 0.204 | 0.824 |
| InternalIMT | aa | rs12354742 | 0.166 | 0.039 | 0.038 | 0.308 | -0.411 | 0.15 | 6.10E-03 | 0.163 | 0.062 | 8.70E-03 |
| InternalIMT | aa | rs180913842 | 0.007 | . | . | . | . | . | . | . | . | . |
| InternalIMT | aa | rs142976914 | 0.003 | . | . | . | . | . | . | . | . | . |
| InternalIMT | aa | rs11511619 | 0.174 | 0.04 | 0.039 | 0.3 | -0.391 | 0.146 | 7.30E-03 | 0.152 | 0.06 | 0.011 |
| InternalIMT | aa | rs146702870 | 0.001 | . | . | . | . | . | . | . | . | . |
| InternalIMT | aa | rs150287496 | 0.005 | . | . | . | . | . | . | . | . | . |
| InternalIMT | aa | rs190917973 | 0.005 | . | . | . | . | . | . | . | . | . |
| InternalIMT | aa | rs11517281 | 0.166 | 0.039 | 0.038 | 0.305 | -0.408 | 0.149 | 6.30E-03 | 0.161 | 0.062 | 9.00E-03 |
| InternalIMT | aa | rs12359299 | 0.168 | 0.039 | 0.039 | 0.311 | -0.405 | 0.149 | 6.50E-03 | 0.16 | 0.062 | 9.50E-03 |
| InternalIMT | aa | rs182307932 | 0.024 | 0.045 | 0.038 | 0.23 | -0.83 | 0.417 | 0.047 | 0.292 | 0.166 | 0.078 |
| InternalIMT | aa | rs61855873 | 0.025 | 0.054 | 0.038 | 0.156 | 0.255 | 0.659 | 0.698 | -0.066 | 0.273 | 0.808 |
| InternalIMT | aa | rs12359302 | 0.164 | 0.043 | 0.038 | 0.262 | -0.391 | 0.15 | 9.30E-03 | 0.155 | 0.062 | 0.012 |
| InternalIMT | aa | rs143208140 | 0.021 | 0.074 | 0.034 | 0.03 | -0.986 | 0.383 | 1.00E-02 | 0.388 | 0.164 | 0.018 |
| InternalIMT | aa | rs9988764 | 0.682 | -0.038 | 0.078 | 0.629 | -0.237 | 0.125 | 0.057 | 0.094 | 0.051 | 0.067 |
| InternalIMT | aa | rs192875767 | 0.006 | . | . | . | . | . | . | . | . | . |
| InternalIMT | aa | rs147268846 | 0.012 | 0.038 | 0.038 | 0.312 | -1.23 | 0.987 | 0.213 | 0.551 | 0.413 | 0.182 |
| InternalIMT | aa | rs11239485 | 0.105 | 0.056 | 0.037 | 0.133 | -0.458 | 0.227 | 0.043 | 0.178 | 0.094 | 0.058 |
| InternalIMT | aa | rs151317885 | 0.424 | 0.07 | 0.052 | 0.172 | -0.066 | 0.112 | 0.559 | 0.032 | 0.046 | 0.488 |
| InternalIMT | aa | rs11499085 | 0.106 | 0.071 | 0.041 | 0.085 | 0.136 | 0.201 | 0.497 | -0.063 | 0.079 | 0.42 |
| InternalIMT | aa | rs188130606 | 0.028 | 0.062 | 0.038 | 0.1 | 0.658 | 0.355 | 0.064 | -0.255 | 0.138 | 0.064 |
| InternalIMT | aa | rs11511620 | 0.16 | 0.046 | 0.038 | 0.224 | -0.369 | 0.152 | 0.015 | 0.144 | 0.063 | 0.022 |
| InternalIMT | aa | rs181498390 | 0.017 | 0.103 | 0.034 | 2.40E-03 | 0.638 | 0.469 | 0.173 | -0.266 | 0.183 | 0.145 |
| InternalIMT | aa | rs186280701 | 0.007 | . | . | . | . | . | . | . | . | . |
| InternalIMT | aa | rs150059185 | 0.028 | 0.094 | 0.035 | 6.50E-03 | 0.047 | 0.414 | 0.909 | -0.026 | 0.17 | 0.88 |
| InternalIMT | aa | rs183338707 | 0.003 | . | . | . | . | . | . | . | . | . |
| InternalIMT | aa | rs11499038 | 0.09 | 0.114 | 0.036 | 1.80E-03 | 0.237 | 0.209 | 0.256 | -0.098 | 0.082 | 0.229 |
| InternalIMT | aa | rs190699595 | 0.006 | . | . | . | . | . | . | . | . | . |
| InternalIMT | aa | rs11523408 | 0.164 | 0.039 | 0.038 | 0.304 | -0.411 | 0.151 | 6.50E-03 | 0.163 | 0.062 | 9.20E-03 |
| InternalIMT | aa | rs188049317 | 0.002 | . | . | . | . | . | . | . | . | . |
| InternalIMT | aa | rs144948634 | 0.395 | 0.096 | 0.05 | 0.057 | 7.80E-03 | 0.114 | 0.945 | 3.80E-03 | 0.047 | 0.935 |
| InternalIMT | aa | rs143833576 | 0.003 | . | . | . | . | . | . | . | . | . |
| InternalIMT | aa | rs151239879 | 0.165 | 0.039 | 0.038 | 0.315 | -0.416 | 0.151 | 5.90E-03 | 0.165 | 0.063 | 8.50E-03 |
| InternalIMT | aa | rs7073743 | 0.407 | 0.091 | 0.05 | 0.071 | -7.80E-03 | 0.111 | 0.944 | 9.90E-03 | 0.045 | 0.827 |
| InternalIMT | aa | rs142563349 | 0.03 | 0.064 | 0.038 | 0.093 | 0.628 | 0.352 | 0.075 | -0.247 | 0.137 | 0.07 |
| InternalIMT | aa | rs187246403 | 0.007 | . | . | . | . | . | . | . | . | . |
| InternalIMT | aa | rs150423942 | 0.162 | 0.041 | 0.038 | 0.289 | -0.411 | 0.154 | 7.60E-03 | 0.162 | 0.063 | 0.011 |

| InternalIMT | aa | rs184706102 | 0.002 | . | . | . | . | . | . | . | . | . |
| --- | --- | --- | --- | --- | --- | --- | --- | --- | --- | --- | --- | --- |
| InternalIMT | aa | rs189626288 | 0.02 | 0.042 | 0.038 | 0.263 | -0.418 | 0.559 | 0.455 | 0.201 | 0.232 | 0.386 |
| InternalIMT | aa | rs140749834 | 0.048 | 0.061 | 0.039 | 0.121 | 0.107 | 0.257 | 0.676 | -0.059 | 0.101 | 0.558 |
| InternalIMT | aa | rs7078822 | 0.133 | 0.058 | 0.038 | 0.125 | -0.337 | 0.171 | 0.049 | 0.126 | 0.071 | 0.075 |
| InternalIMT | aa | rs186227617 | 0.164 | 0.043 | 0.038 | 0.257 | -0.388 | 0.151 | 0.01 | 0.152 | 0.062 | 0.015 |
| InternalIMT | aa | rs188984368 | 0.022 | 0.09 | 0.035 | 9.10E-03 | -0.084 | 0.57 | 0.882 | 0.054 | 0.238 | 0.819 |
| InternalIMT | aa | rs182078109 | 0.031 | 0.049 | 0.039 | 0.202 | 0.095 | 0.525 | 0.857 | -0.018 | 0.214 | 0.933 |
| InternalIMT | aa | rs141456274 | 0.005 | . | . | . | . | . | . | . | . | . |
| InternalIMT | aa | rs146976512 | 0.166 | 0.04 | 0.038 | 0.3 | -0.405 | 0.149 | 6.70E-03 | 0.16 | 0.062 | 9.60E-03 |
| InternalIMT | aa | rs138036816 | 0.1 | 0.071 | 0.041 | 0.086 | 0.177 | 0.212 | 0.402 | -0.077 | 0.083 | 0.351 |
| InternalIMT | aa | rs114879777 | 0.019 | 0.044 | 0.037 | 0.239 | -0.38 | 0.647 | 0.557 | 0.154 | 0.252 | 0.542 |
| InternalIMT | aa | rs149366475 | 0.101 | 0.072 | 0.041 | 0.079 | 0.181 | 0.213 | 0.396 | -0.08 | 0.083 | 0.338 |
| InternalIMT | aa | rs182463043 | 0.002 | . | . | . | . | . | . | . | . | . |
| InternalIMT | aa | rs144754632 | 0.019 | 0.075 | 0.034 | 0.026 | -1.15 | 0.376 | 2.30E-03 | 0.45 | 0.163 | 5.70E-03 |
| InternalIMT | aa | rs55717607 | 0.166 | 0.04 | 0.038 | 0.303 | -0.406 | 0.149 | 6.60E-03 | 0.16 | 0.062 | 9.30E-03 |
| InternalIMT | aa | rs187537973 | 0 | . | . | . | . | . | . | . | . | . |
| InternalIMT | aa | rs148085971 | 0.002 | . | . | . | . | . | . | . | . | . |
| InternalIMT | aa | rs11239491 | 0.166 | 0.04 | 0.038 | 0.303 | -0.405 | 0.149 | 6.60E-03 | 0.16 | 0.062 | 9.40E-03 |
| InternalIMT | aa | rs184412184 | 0.005 | . | . | . | . | . | . | . | . | . |
| InternalIMT | aa | rs4948998 | 0.716 | -0.021 | 0.082 | 0.801 | -0.195 | 0.124 | 0.115 | 0.077 | 0.051 | 0.128 |
| InternalIMT | aa | rs7076696 | 0.716 | -0.022 | 0.082 | 0.786 | -0.198 | 0.124 | 0.11 | 0.078 | 0.051 | 0.123 |
| InternalIMT | aa | rs11239492 | 0.172 | 0.041 | 0.039 | 0.293 | -0.393 | 0.146 | 7.20E-03 | 0.153 | 0.06 | 0.011 |
| InternalIMT | aa | rs79462470 | 0.007 | . | . | . | . | . | . | . | . | . |
| InternalIMT | aa | rs187804654 | 0.003 | . | . | . | . | . | . | . | . | . |
| InternalIMT | aa | rs7093687 | 0.161 | 0.047 | 0.038 | 0.221 | -0.365 | 0.151 | 0.016 | 0.142 | 0.063 | 0.023 |
| InternalIMT | aa | rs7081004 | 0.161 | 0.046 | 0.038 | 0.224 | -0.367 | 0.151 | 0.015 | 0.143 | 0.063 | 0.022 |
| InternalIMT | aa | rs7084793 | 0.166 | 0.04 | 0.038 | 0.302 | -0.405 | 0.149 | 6.60E-03 | 0.16 | 0.062 | 9.40E-03 |
| InternalIMT | aa | rs61556080 | 0.007 | . | . | . | . | . | . | . | . | . |
| InternalIMT | aa | rs115552064 | 0.007 | . | . | . | . | . | . | . | . | . |
| InternalIMT | aa | rs7921503 | 0.166 | 0.038 | 0.038 | 0.317 | -0.414 | 0.149 | 5.60E-03 | 0.164 | 0.062 | 8.00E-03 |
| InternalIMT | aa | rs7909740 | 0.166 | 0.04 | 0.038 | 0.302 | -0.405 | 0.149 | 6.60E-03 | 0.16 | 0.062 | 9.40E-03 |
| InternalIMT | aa | rs7909768 | 0.166 | 0.04 | 0.038 | 0.302 | -0.405 | 0.149 | 6.60E-03 | 0.16 | 0.062 | 9.40E-03 |
| InternalIMT | aa | rs144168004 | 0 | . | . | . | . | . | . | . | . | . |
| InternalIMT | aa | rs75850448 | 0.007 | . | . | . | . | . | . | . | . | . |
| InternalIMT | aa | rs147777574 | 0.006 | . | . | . | . | . | . | . | . | . |
| InternalIMT | aa | rs7913488 | 0.166 | 0.04 | 0.038 | 0.302 | -0.405 | 0.149 | 6.60E-03 | 0.16 | 0.062 | 9.40E-03 |
| InternalIMT | aa | rs74390874 | 0.049 | 0.093 | 0.035 | 7.30E-03 | 0.06 | 0.32 | 0.851 | 9.10E-03 | 0.13 | 0.944 |
| InternalIMT | aa | rs141391142 | 0.006 | . | . | . | . | . | . | . | . | . |
| InternalIMT | aa | rs115294747 | 0.007 | . | . | . | . | . | . | . | . | . |
| InternalIMT | aa | rs148031781 | 0.002 | . | . | . | . | . | . | . | . | . |
| InternalIMT | aa | rs181340128 | 0.018 | 0.038 | 0.038 | 0.315 | -0.794 | 0.679 | 0.243 | 0.347 | 0.289 | 0.231 |
| InternalIMT | aa | rs189930007 | 0.001 | . | . | . | . | . | . | . | . | . |
| InternalIMT | aa | rs12242128 | 0.1 | 0.071 | 0.041 | 0.085 | 0.181 | 0.213 | 0.395 | -0.079 | 0.083 | 0.343 |
| InternalIMT | aa | rs78421750 | 0.006 | . | . | . | . | . | . | . | . | . |
| InternalIMT | aa | rs114370747 | 0.003 | . | . | . | . | . | . | . | . | . |
| InternalIMT | aa | rs12264166 | 0.1 | 0.071 | 0.041 | 0.085 | 0.181 | 0.213 | 0.395 | -0.079 | 0.083 | 0.343 |
| InternalIMT | aa | rs182964822 | 0.001 | . | . | . | . | . | . | . | . | . |
| InternalIMT | aa | rs67491417 | 0.159 | 0.043 | 0.038 | 0.265 | -0.393 | 0.153 | 0.01 | 0.157 | 0.063 | 0.013 |
| InternalIMT | aa | rs72789720 | 0.015 | 0.042 | 0.038 | 0.27 | -0.665 | 0.538 | 0.216 | 0.273 | 0.222 | 0.22 |
| InternalIMT | aa | rs6413416 | 0.167 | 0.041 | 0.038 | 0.291 | -0.399 | 0.149 | 7.40E-03 | 0.157 | 0.061 | 0.011 |
| InternalIMT | aa | rs4986832 | 0.167 | 0.04 | 0.038 | 0.3 | -0.404 | 0.149 | 6.60E-03 | 0.16 | 0.061 | 9.50E-03 |
| InternalIMT | aa | rs144866316 | 0.005 | . | . | . | . | . | . | . | . | . |
| InternalIMT | aa | rs187769540 | 0.002 | . | . | . | . | . | . | . | . | . |
| InternalIMT | aa | rs6593482 | 0.167 | 0.041 | 0.038 | 0.287 | -0.395 | 0.148 | 7.80E-03 | 0.155 | 0.061 | 0.011 |
| InternalIMT | aa | rs7913948 | 0.167 | 0.04 | 0.038 | 0.3 | -0.4 | 0.148 | 7.00E-03 | 0.158 | 0.061 | 1.00E-02 |
| InternalIMT | aa | rs77185151 | 0.019 | 0.075 | 0.034 | 0.026 | -1.14 | 0.377 | 2.40E-03 | 0.449 | 0.163 | 5.90E-03 |
| InternalIMT | aa | rs12762303 | 0.167 | 0.04 | 0.038 | 0.301 | -0.404 | 0.149 | 6.60E-03 | 0.16 | 0.061 | 9.40E-03 |
| InternalIMT | aa | rs73283136 | 0.005 | . | . | . | . | . | . | . | . | . |
| InternalIMT | aa | rs187180218 | 0.002 | . | . | . | . | . | . | . | . | . |
| InternalIMT | aa | rs78137087 | 0.166 | 0.039 | 0.038 | 0.304 | -0.408 | 0.149 | 6.20E-03 | 0.161 | 0.061 | 8.90E-03 |
| InternalIMT | aa | rs72561472 | 0.165 | 0.034 | 0.039 | 0.377 | -0.45 | 0.153 | 3.20E-03 | 0.178 | 0.063 | 5.00E-03 |
| InternalIMT | aa | rs4987106 | 0.162 | 0.041 | 0.038 | 0.288 | -0.409 | 0.152 | 7.00E-03 | 0.16 | 0.063 | 0.01 |
| InternalIMT | aa | rs4987105 | 0.158 | 0.045 | 0.038 | 0.234 | -0.379 | 0.153 | 0.013 | 0.148 | 0.063 | 0.02 |
| InternalIMT | aa | rs111916157 | 0.182 | 0.036 | 0.039 | 0.36 | -0.388 | 0.149 | 9.10E-03 | 0.156 | 0.061 | 0.011 |
| InternalIMT | aa | rs111528152 | 0.171 | 0.043 | 0.039 | 0.263 | -0.372 | 0.149 | 0.013 | 0.145 | 0.061 | 0.019 |
| InternalIMT | aa | rs12763738 | 0.166 | 0.039 | 0.038 | 0.312 | -0.412 | 0.149 | 5.90E-03 | 0.163 | 0.062 | 8.40E-03 |
| InternalIMT | aa | rs191675886 | 0.007 | . | . | . | . | . | . | . | . | . |
| InternalIMT | aa | rs66489870 | 0.167 | 0.04 | 0.038 | 0.301 | -0.404 | 0.149 | 6.60E-03 | 0.16 | 0.061 | 9.40E-03 |
| InternalIMT | aa | rs71494775 | 0.167 | 0.04 | 0.038 | 0.301 | -0.404 | 0.149 | 6.60E-03 | 0.16 | 0.061 | 9.40E-03 |
| InternalIMT | aa | rs61855875 | 0.024 | 0.051 | 0.038 | 0.182 | 0.105 | 0.68 | 0.877 | -0.012 | 0.281 | 0.966 |
| InternalIMT | aa | rs116356837 | 0.007 | . | . | . | . | . | . | . | . | . |
| InternalIMT | aa | rs1864414 | 0.166 | 0.038 | 0.038 | 0.316 | -0.421 | 0.15 | 5.00E-03 | 0.167 | 0.062 | 7.10E-03 |
| InternalIMT | aa | rs1864413 | 0.167 | 0.039 | 0.038 | 0.305 | -0.406 | 0.149 | 6.30E-03 | 0.16 | 0.061 | 9.10E-03 |
| InternalIMT | aa | rs139497212 | 0 | . | . | . | . | . | . | . | . | . |
| InternalIMT | aa | rs892690 | 0.328 | 0.118 | 0.047 | 0.013 | 0.079 | 0.121 | 0.512 | -0.025 | 0.05 | 0.608 |
| InternalIMT | aa | rs1864412 | 0.167 | 0.04 | 0.038 | 0.302 | -0.405 | 0.149 | 6.50E-03 | 0.16 | 0.061 | 9.40E-03 |
| InternalIMT | aa | rs77827771 | 0.031 | 0.068 | 0.038 | 0.074 | 0.889 | 0.344 | 9.80E-03 | -0.34 | 0.134 | 0.011 |
| InternalIMT | aa | rs145294963 | 0.004 | . | . | . | . | . | . | . | . | . |
| InternalIMT | aa | rs149081327 | 0.008 | . | . | . | . | . | . | . | . | . |
| InternalIMT | aa | rs67008983 | 0.167 | 0.04 | 0.038 | 0.304 | -0.406 | 0.149 | 6.40E-03 | 0.16 | 0.062 | 9.20E-03 |
| InternalIMT | aa | rs56150414 | 0.17 | 0.043 | 0.039 | 0.263 | -0.371 | 0.148 | 0.012 | 0.147 | 0.061 | 0.016 |
| InternalIMT | aa | rs55787481 | 0.167 | 0.039 | 0.038 | 0.309 | -0.409 | 0.149 | 6.20E-03 | 0.161 | 0.062 | 8.90E-03 |
| InternalIMT | aa | rs141270101 | 0.001 | . | . | . | . | . | . | . | . | . |
| InternalIMT | aa | rs114980075 | 0.008 | . | . | . | . | . | . | . | . | . |
| InternalIMT | aa | rs12783095 | 0.166 | 0.04 | 0.038 | 0.301 | -0.407 | 0.149 | 6.30E-03 | 0.16 | 0.062 | 9.30E-03 |
| InternalIMT | aa | rs182342688 | 0.003 | . | . | . | . | . | . | . | . | . |

| InternalIMT | aa | rs17157728 | 0.166 | 0.04 | 0.038 | 0.301 | -0.407 | 0.149 | 6.30E-03 | 0.16 | 0.062 | 9.30E-03 |
| --- | --- | --- | --- | --- | --- | --- | --- | --- | --- | --- | --- | --- |
| InternalIMT | aa | rs114835563 | 0.028 | 0.062 | 0.038 | 0.101 | 0.655 | 0.362 | 0.07 | -0.254 | 0.141 | 0.072 |
| InternalIMT | aa | rs3824612 | 0.328 | 0.118 | 0.047 | 0.012 | 0.081 | 0.121 | 0.502 | -0.026 | 0.05 | 0.6 |
| InternalIMT | aa | rs17157733 | 0.165 | 0.04 | 0.038 | 0.298 | -0.407 | 0.149 | 6.50E-03 | 0.16 | 0.062 | 9.60E-03 |
| InternalIMT | aa | rs74128478 | 0.007 | . | . | . | . | . | . | . | . | . |
| InternalIMT | aa | rs184973589 | 0.001 | . | . | . | . | . | . | . | . | . |
| InternalIMT | aa | rs150479461 | 0 | . | . | . | . | . | . | . | . | . |
| InternalIMT | aa | rs138310407 | 0.003 | . | . | . | . | . | . | . | . | . |
| InternalIMT | aa | rs187426603 | 0.001 | . | . | . | . | . | . | . | . | . |
| InternalIMT | aa | rs190843143 | 0.002 | . | . | . | . | . | . | . | . | . |
| InternalIMT | aa | rs191883449 | 0.001 | . | . | . | . | . | . | . | . | . |
| InternalIMT | aa | rs58238740 | 0.163 | 0.04 | 0.038 | 0.294 | -0.407 | 0.15 | 6.80E-03 | 0.16 | 0.062 | 9.80E-03 |
| InternalIMT | aa | rs3780894 | 0.163 | 0.04 | 0.038 | 0.294 | -0.407 | 0.15 | 6.80E-03 | 0.16 | 0.062 | 9.80E-03 |
| InternalIMT | aa | rs149646626 | 0.018 | 0.07 | 0.038 | 0.061 | 1.52 | 0.757 | 0.045 | -0.603 | 0.307 | 0.05 |
| InternalIMT | aa | rs79615212 | 0.016 | 0.055 | 0.038 | 0.145 | 0.557 | 0.647 | 0.389 | -0.25 | 0.274 | 0.363 |
| InternalIMT | aa | rs3780895 | 0.163 | 0.04 | 0.038 | 0.294 | -0.407 | 0.15 | 6.80E-03 | 0.16 | 0.062 | 9.90E-03 |
| InternalIMT | aa | rs3780896 | 0.163 | 0.04 | 0.038 | 0.294 | -0.407 | 0.15 | 6.80E-03 | 0.16 | 0.062 | 9.80E-03 |
| InternalIMT | aa | rs142747163 | 0.005 | . | . | . | . | . | . | . | . | . |
| InternalIMT | aa | rs189417584 | 0.007 | . | . | . | . | . | . | . | . | . |
| InternalIMT | aa | rs3780897 | 0.162 | 0.038 | 0.038 | 0.323 | -0.422 | 0.151 | 5.20E-03 | 0.168 | 0.062 | 7.20E-03 |
| InternalIMT | aa | rs117509491 | 0.009 | . | . | . | . | . | . | . | . | . |
| InternalIMT | aa | rs117811514 | 0.009 | . | . | . | . | . | . | . | . | . |
| InternalIMT | aa | rs116500932 | 0.028 | 0.062 | 0.038 | 0.101 | 0.654 | 0.362 | 0.071 | -0.253 | 0.141 | 0.073 |
| InternalIMT | aa | rs77584168 | 0.004 | . | . | . | . | . | . | . | . | . |
| InternalIMT | aa | rs2310776 | 0.163 | 0.04 | 0.038 | 0.294 | -0.407 | 0.15 | 6.70E-03 | 0.161 | 0.062 | 9.80E-03 |
| InternalIMT | aa | rs17157736 | 0.163 | 0.04 | 0.038 | 0.294 | -0.407 | 0.15 | 6.70E-03 | 0.161 | 0.062 | 9.80E-03 |
| InternalIMT | aa | rs72789735 | 0.065 | 0.071 | 0.035 | 0.041 | -0.463 | 0.254 | 0.068 | 0.186 | 0.106 | 0.078 |
| InternalIMT | aa | rs183747187 | 0.002 | . | . | . | . | . | . | . | . | . |
| InternalIMT | aa | rs7077891 | 0.163 | 0.04 | 0.038 | 0.294 | -0.407 | 0.15 | 6.70E-03 | 0.161 | 0.062 | 9.70E-03 |
| InternalIMT | aa | rs145063305 | 0.003 | . | . | . | . | . | . | . | . | . |
| InternalIMT | aa | rs3824613 | 0.164 | 0.038 | 0.038 | 0.32 | -0.42 | 0.149 | 4.90E-03 | 0.166 | 0.062 | 7.00E-03 |
| InternalIMT | aa | rs145543199 | 0.009 | . | . | . | . | . | . | . | . | . |
| InternalIMT | aa | rs140361993 | 0.003 | . | . | . | . | . | . | . | . | . |
| InternalIMT | aa | rs11239496 | 0.065 | 0.071 | 0.035 | 0.041 | -0.464 | 0.254 | 0.068 | 0.186 | 0.106 | 0.078 |
| InternalIMT | aa | rs11239497 | 0.158 | 0.047 | 0.038 | 0.219 | -0.37 | 0.153 | 0.016 | 0.144 | 0.063 | 0.023 |
| InternalIMT | aa | rs10900212 | 0.184 | 0.033 | 0.039 | 0.399 | -0.422 | 0.143 | 3.20E-03 | 0.167 | 0.059 | 4.60E-03 |
| InternalIMT | aa | rs186487136 | 0 | . | . | . | . | . | . | . | . | . |
| InternalIMT | aa | rs12774369 | 0.163 | 0.04 | 0.038 | 0.292 | -0.406 | 0.151 | 7.00E-03 | 0.16 | 0.062 | 0.01 |
| InternalIMT | aa | rs60842538 | 0.163 | 0.041 | 0.038 | 0.289 | -0.405 | 0.151 | 7.20E-03 | 0.159 | 0.062 | 0.011 |
| InternalIMT | aa | rs3780898 | 0.157 | 0.048 | 0.038 | 0.209 | -0.364 | 0.154 | 0.018 | 0.141 | 0.064 | 0.027 |
| InternalIMT | aa | rs3780899 | 0.163 | 0.041 | 0.038 | 0.283 | -0.402 | 0.151 | 7.80E-03 | 0.158 | 0.062 | 0.011 |
| InternalIMT | aa | rs12252472 | 0.09 | 0.072 | 0.041 | 0.079 | 0.201 | 0.213 | 0.345 | -0.088 | 0.083 | 0.292 |
| InternalIMT | aa | rs185411011 | 0.002 | . | . | . | . | . | . | . | . | . |
| InternalIMT | aa | rs28395865 | 0.155 | 0.049 | 0.038 | 0.202 | -0.362 | 0.156 | 0.02 | 0.14 | 0.065 | 0.031 |
| InternalIMT | aa | rs191004074 | 0.03 | 0.044 | 0.039 | 0.255 | -0.161 | 0.507 | 0.751 | 0.06 | 0.206 | 0.769 |
| InternalIMT | aa | rs182401295 | 0.003 | . | . | . | . | . | . | . | . | . |
| InternalIMT | aa | rs2228064 | 0.093 | 0.101 | 0.037 | 5.80E-03 | -0.012 | 0.19 | 0.951 | -6.30E-03 | 0.076 | 0.934 |
| InternalIMT | aa | rs188284159 | 0.001 | . | . | . | . | . | . | . | . | . |
| InternalIMT | aa | rs2303887 | 0.013 | 0.08 | 0.034 | 0.018 | -0.954 | 0.394 | 0.015 | 0.358 | 0.175 | 0.041 |
| InternalIMT | aa | rs2303886 | 0.016 | 0.079 | 0.034 | 0.019 | -0.876 | 0.321 | 6.40E-03 | 0.334 | 0.14 | 0.017 |
| InternalIMT | aa | rs192621006 | 0.003 | . | . | . | . | . | . | . | . | . |
| InternalIMT | aa | rs2303885 | 0.252 | 0.076 | 0.042 | 0.07 | -0.149 | 0.125 | 0.235 | 0.051 | 0.051 | 0.319 |
| InternalIMT | aa | rs114466168 | 0.028 | 0.061 | 0.038 | 0.108 | 0.631 | 0.369 | 0.087 | -0.243 | 0.144 | 0.092 |
| InternalIMT | aa | rs11239501 | 0.088 | 0.066 | 0.035 | 0.058 | -0.42 | 0.216 | 0.052 | 0.182 | 0.09 | 0.043 |
| InternalIMT | aa | rs745986 | 0.18 | 0.044 | 0.038 | 0.251 | -0.355 | 0.141 | 0.012 | 0.14 | 0.059 | 0.017 |
| InternalIMT | aa | rs75262268 | 0.002 | . | . | . | . | . | . | . | . | . |
| InternalIMT | aa | rs71494776 | 0.061 | 0.066 | 0.035 | 0.062 | -0.512 | 0.217 | 0.018 | 0.192 | 0.093 | 0.039 |
| InternalIMT | aa | rs74386871 | 0.02 | 0.081 | 0.034 | 0.018 | -0.627 | 0.363 | 0.084 | 0.23 | 0.16 | 0.151 |
| InternalIMT | aa | rs76502040 | 0.007 | . | . | . | . | . | . | . | . | . |
| InternalIMT | aa | rs142213734 | 0.001 | . | . | . | . | . | . | . | . | . |
| InternalIMT | aa | rs4948671 | 0.714 | -0.027 | 0.082 | 0.741 | -0.205 | 0.124 | 0.098 | 0.082 | 0.051 | 0.108 |
| InternalIMT | aa | rs146286085 | 0.001 | . | . | . | . | . | . | . | . | . |
| InternalIMT | aa | rs144080623 | 0 | . | . | . | . | . | . | . | . | . |
| InternalIMT | aa | rs115074014 | 0.044 | 0.054 | 0.039 | 0.165 | -0.061 | 0.273 | 0.822 | 6.50E-03 | 0.108 | 0.952 |
| InternalIMT | aa | rs17157756 | 0.044 | 0.054 | 0.039 | 0.165 | -0.061 | 0.273 | 0.822 | 6.50E-03 | 0.108 | 0.952 |
| InternalIMT | aa | rs190459780 | 0.001 | . | . | . | . | . | . | . | . | . |
| InternalIMT | aa | rs12358647 | 0.208 | 0.029 | 0.039 | 0.46 | -0.404 | 0.136 | 3.00E-03 | 0.161 | 0.057 | 4.40E-03 |
| InternalIMT | aa | rs147080556 | 0.013 | 0.092 | 0.034 | 6.80E-03 | 7.50E-03 | 0.471 | 0.987 | -0.058 | 0.197 | 0.767 |
| InternalIMT | aa | rs187543349 | 0.005 | . | . | . | . | . | . | . | . | . |
| InternalIMT | aa | rs114375595 | 0.01 | 0.084 | 0.034 | 0.012 | -0.935 | 0.879 | 0.287 | 0.394 | 0.352 | 0.262 |
| InternalIMT | aa | rs117211200 | 0.006 | . | . | . | . | . | . | . | . | . |
| InternalIMT | aa | rs192963047 | 0.001 | . | . | . | . | . | . | . | . | . |
| InternalIMT | aa | rs11239505 | 0.209 | 0.029 | 0.039 | 0.469 | -0.405 | 0.136 | 2.90E-03 | 0.162 | 0.056 | 4.10E-03 |
| InternalIMT | aa | rs184434116 | 0.002 | . | . | . | . | . | . | . | . | . |
| InternalIMT | aa | rs4948999 | 0.715 | -0.02 | 0.082 | 0.811 | -0.194 | 0.125 | 0.119 | 0.077 | 0.051 | 0.133 |
| InternalIMT | aa | rs11239506 | 0.102 | 0.08 | 0.036 | 0.026 | -0.143 | 0.19 | 0.452 | 0.062 | 0.079 | 0.431 |
| InternalIMT | aa | rs190706620 | 0.004 | . | . | . | . | . | . | . | . | . |
| InternalIMT | aa | rs190770429 | 0.002 | . | . | . | . | . | . | . | . | . |
| InternalIMT | aa | rs181327133 | 0.005 | . | . | . | . | . | . | . | . | . |
| InternalIMT | aa | rs149251697 | 0.023 | 0.046 | 0.037 | 0.222 | 9.40E-03 | 0.652 | 0.989 | 0.013 | 0.258 | 0.959 |
| InternalIMT | aa | rs118081602 | 0.013 | 0.08 | 0.034 | 0.018 | -0.955 | 0.395 | 0.015 | 0.359 | 0.175 | 0.041 |
| InternalIMT | aa | rs188233691 | 0.002 | . | . | . | . | . | . | . | . | . |
| InternalIMT | aa | rs183778794 | 0.008 | . | . | . | . | . | . | . | . | . |
| InternalIMT | aa | rs188040078 | 0.006 | . | . | . | . | . | . | . | . | . |
| InternalIMT | aa | rs184675372 | 0.002 | . | . | . | . | . | . | . | . | . |
| InternalIMT | aa | rs150326891 | 0.069 | 0.058 | 0.035 | 0.095 | -0.707 | 0.241 | 3.40E-03 | 0.301 | 0.101 | 3.00E-03 |

| InternalIMT | aa | rs185520021 | 0.006 | . | . | . | . | . | . | . | . | . |
| --- | --- | --- | --- | --- | --- | --- | --- | --- | --- | --- | --- | --- |
| InternalIMT | aa | rs7907779 | 0.102 | 0.08 | 0.036 | 0.026 | -0.142 | 0.19 | 0.454 | 0.062 | 0.079 | 0.432 |
| InternalIMT | aa | rs184962860 | 0.001 | . | . | . | . | . | . | . | . | . |
| InternalIMT | aa | rs149762784 | 0.037 | 0.082 | 0.036 | 0.021 | -0.318 | 0.416 | 0.444 | 0.128 | 0.175 | 0.466 |
| InternalIMT | aa | rs149203427 | 0.013 | 0.045 | 0.037 | 0.226 | -0.374 | 0.833 | 0.653 | 0.194 | 0.34 | 0.568 |
| InternalIMT | aa | rs60153083 | 0.016 | 0.053 | 0.037 | 0.154 | 0.288 | 0.513 | 0.575 | -0.125 | 0.199 | 0.532 |
| InternalIMT | aa | rs112001069 | 0.076 | 0.062 | 0.036 | 0.081 | -0.523 | 0.193 | 6.80E-03 | 0.199 | 0.081 | 0.014 |
| InternalIMT | aa | rs182355382 | 0.031 | 0.103 | 0.034 | 2.50E-03 | 0.562 | 0.569 | 0.324 | -0.216 | 0.231 | 0.35 |
| InternalIMT | aa | rs186740974 | 0.125 | 0.112 | 0.042 | 7.80E-03 | 0.171 | 0.244 | 0.482 | -0.073 | 0.1 | 0.462 |
| InternalIMT | aa | rs191874916 | 0.036 | 0.089 | 0.035 | 0.011 | -0.078 | 0.365 | 0.831 | 0.041 | 0.148 | 0.78 |
| InternalIMT | aa | rs182989412 | 0.146 | 0.087 | 0.041 | 0.037 | -0.04 | 0.229 | 0.861 | 0.035 | 0.096 | 0.714 |
| InternalIMT | aa | rs188819476 | 0.002 | . | . | . | . | . | . | . | . | . |
| InternalIMT | aa | rs141217183 | 0.093 | 0.063 | 0.037 | 0.084 | -0.41 | 0.197 | 0.037 | 0.151 | 0.083 | 0.068 |
| InternalIMT | aa | rs192922652 | 0.077 | 0.064 | 0.036 | 0.071 | -0.501 | 0.192 | 9.20E-03 | 0.187 | 0.081 | 0.02 |
| InternalIMT | aa | rs185092343 | 0.02 | 0.102 | 0.034 | 3.00E-03 | 1.09 | 0.67 | 0.102 | -0.387 | 0.27 | 0.152 |
| InternalIMT | aa | rs145193311 | 0.08 | 0.065 | 0.036 | 0.07 | -0.471 | 0.191 | 0.014 | 0.178 | 0.08 | 0.027 |
| InternalIMT | aa | rs55789336 | 0.104 | 0.075 | 0.036 | 0.036 | -0.186 | 0.191 | 0.331 | 0.08 | 0.079 | 0.308 |
| InternalIMT | aa | rs55837212 | 0.104 | 0.08 | 0.036 | 0.025 | -0.134 | 0.189 | 0.478 | 0.058 | 0.078 | 0.455 |
| InternalIMT | aa | rs75408574 | 0.36 | 0.122 | 0.049 | 0.014 | 0.097 | 0.118 | 0.413 | -0.036 | 0.048 | 0.456 |
| InternalIMT | aa | rs149306926 | 0.11 | 0.088 | 0.036 | 0.016 | -0.041 | 0.182 | 0.822 | 0.021 | 0.075 | 0.778 |
| InternalIMT | aa | rs35990892 | 0.674 | 0.038 | 0.081 | 0.638 | -0.111 | 0.127 | 0.38 | 0.039 | 0.052 | 0.457 |
| InternalIMT | aa | rs192356207 | 0.05 | 0.087 | 0.035 | 0.014 | -0.072 | 0.349 | 0.836 | 0.036 | 0.138 | 0.793 |
| InternalIMT | aa | rs183741163 | 0.102 | 0.089 | 0.037 | 0.016 | -0.018 | 0.23 | 0.938 | 0.014 | 0.095 | 0.887 |
| InternalIMT | aa | rs34737297 | 0.555 | -0.068 | 0.07 | 0.328 | -0.347 | 0.134 | 9.60E-03 | 0.144 | 0.055 | 8.80E-03 |
| InternalIMT | aa | rs181346933 | 0.03 | 0.037 | 0.039 | 0.346 | -0.543 | 0.379 | 0.152 | 0.204 | 0.152 | 0.179 |
| InternalIMT | aa | rs36093253 | 0.361 | 0.11 | 0.051 | 0.03 | 0.058 | 0.121 | 0.634 | -0.023 | 0.049 | 0.641 |
| InternalIMT | aa | rs186307855 | 0.099 | 0.072 | 0.042 | 0.084 | 0.215 | 0.204 | 0.292 | -0.092 | 0.081 | 0.254 |
| InternalIMT | aa | rs189681865 | 0.034 | 0.041 | 0.038 | 0.283 | -0.477 | 0.431 | 0.268 | 0.18 | 0.172 | 0.295 |
| InternalIMT | aa | rs34942971 | 0.214 | 0.034 | 0.04 | 0.404 | -0.361 | 0.141 | 0.01 | 0.143 | 0.058 | 0.014 |
| InternalIMT | aa | rs181816554 | 0.033 | 0.055 | 0.038 | 0.149 | 0.42 | 0.605 | 0.488 | -0.154 | 0.239 | 0.52 |
| InternalIMT | aa | rs186846881 | 0.094 | 0.07 | 0.041 | 0.088 | 0.216 | 0.215 | 0.316 | -0.091 | 0.085 | 0.283 |
| InternalIMT | aa | rs191178220 | 0.016 | 0.1 | 0.034 | 3.10E-03 | 0.669 | 0.558 | 0.231 | -0.244 | 0.23 | 0.289 |
| InternalIMT | aa | rs183305228 | 0.016 | 0.053 | 0.037 | 0.155 | 0.286 | 0.51 | 0.575 | -0.123 | 0.198 | 0.533 |
| InternalIMT | aa | rs139912761 | 0.121 | 0.077 | 0.037 | 0.036 | -0.138 | 0.189 | 0.465 | 0.062 | 0.078 | 0.428 |
| InternalIMT | aa | rs185499901 | 0.018 | 0.062 | 0.038 | 0.105 | 1.02 | 0.797 | 0.2 | -0.369 | 0.32 | 0.248 |
| InternalIMT | aa | rs189816197 | 0.087 | 0.065 | 0.037 | 0.078 | -0.395 | 0.188 | 0.036 | 0.147 | 0.079 | 0.063 |
| InternalIMT | aa | rs145656938 | 0.026 | 0.08 | 0.034 | 0.02 | -0.73 | 0.471 | 0.121 | 0.287 | 0.192 | 0.136 |
| InternalIMT | aa | rs35745197 | 0.076 | 0.061 | 0.036 | 0.084 | -0.542 | 0.193 | 5.00E-03 | 0.207 | 0.081 | 0.011 |
| InternalIMT | aa | rs192856637 | 0.005 | . | . | . | . | . | . | . | . | . |
| InternalIMT | aa | rs2310775 | 0.415 | 0.114 | 0.051 | 0.026 | 0.061 | 0.108 | 0.573 | -0.021 | 0.044 | 0.634 |
| InternalIMT | aa | rs11239507 | 0.506 | 0.128 | 0.06 | 0.033 | 0.082 | 0.113 | 0.467 | -0.034 | 0.046 | 0.462 |
| InternalIMT | aa | rs74648467 | 0.062 | 0.07 | 0.035 | 0.048 | -0.362 | 0.222 | 0.103 | 0.127 | 0.095 | 0.18 |
| InternalIMT | aa | rs145815934 | 0.015 | 0.047 | 0.038 | 0.212 | -0.118 | 0.606 | 0.845 | 0.07 | 0.248 | 0.778 |
| InternalIMT | aa | rs145390687 | 0.02 | 0.05 | 0.038 | 0.186 | 0.138 | 0.599 | 0.817 | -0.017 | 0.243 | 0.943 |
| InternalIMT | aa | rs184354913 | 0.034 | 0.076 | 0.039 | 0.049 | 0.984 | 0.492 | 0.046 | -0.391 | 0.204 | 0.055 |
| InternalIMT | aa | rs181244920 | 0.006 | . | . | . | . | . | . | . | . | . |
| InternalIMT | aa | rs11499087 | 0.055 | 0.062 | 0.04 | 0.119 | 7.20E-03 | 0.243 | 0.976 | -0.025 | 0.096 | 0.794 |
| InternalIMT | aa | rs148263794 | 0.08 | 0.061 | 0.036 | 0.089 | -0.527 | 0.187 | 4.70E-03 | 0.2 | 0.079 | 0.011 |
| InternalIMT | aa | rs150758903 | 0.036 | 0.044 | 0.038 | 0.25 | -0.317 | 0.468 | 0.499 | 0.116 | 0.186 | 0.534 |
| InternalIMT | aa | rs147654382 | 0.179 | 0.031 | 0.039 | 0.428 | -0.435 | 0.15 | 3.80E-03 | 0.176 | 0.063 | 4.90E-03 |
| InternalIMT | aa | rs147495166 | 0.025 | 0.058 | 0.038 | 0.127 | 0.501 | 0.377 | 0.185 | -0.192 | 0.148 | 0.194 |
| InternalIMT | aa | rs35198778 | 0.106 | 0.045 | 0.038 | 0.23 | -0.62 | 0.202 | 2.20E-03 | 0.244 | 0.085 | 4.00E-03 |
| InternalIMT | aa | rs145207659 | 0.049 | 0.094 | 0.035 | 7.10E-03 | 0.077 | 0.326 | 0.813 | 5.20E-03 | 0.133 | 0.969 |
| InternalIMT | aa | rs186466464 | 0.01 | 0.044 | 0.038 | 0.245 | -0.482 | 1.12 | 0.666 | 0.206 | 0.44 | 0.64 |
| InternalIMT | aa | rs12778413 | 0.162 | 0.041 | 0.039 | 0.301 | -0.398 | 0.176 | 0.023 | 0.162 | 0.072 | 0.025 |
| InternalIMT | aa | rs7895552 | 0.64 | -0.018 | 0.077 | 0.815 | -0.213 | 0.127 | 0.092 | 0.084 | 0.052 | 0.106 |
| InternalIMT | aa | rs12783895 | 0.078 | 0.063 | 0.036 | 0.077 | -0.514 | 0.19 | 6.80E-03 | 0.195 | 0.08 | 0.015 |
| InternalIMT | aa | rs34929604 | 0.079 | 0.064 | 0.036 | 0.073 | -0.493 | 0.188 | 8.60E-03 | 0.187 | 0.079 | 0.018 |
| InternalIMT | aa | rs12761961 | 0.075 | 0.061 | 0.036 | 0.084 | -0.549 | 0.197 | 5.30E-03 | 0.21 | 0.083 | 0.011 |
| InternalIMT | aa | rs35536930 | 0.206 | 0.034 | 0.039 | 0.393 | -0.38 | 0.136 | 5.30E-03 | 0.152 | 0.056 | 7.20E-03 |
| InternalIMT | aa | rs180951060 | 0.002 | . | . | . | . | . | . | . | . | . |
| InternalIMT | aa | rs148892128 | 0.015 | 0.047 | 0.038 | 0.212 | -0.118 | 0.606 | 0.846 | 0.07 | 0.248 | 0.778 |
| InternalIMT | aa | rs11239509 | 0.084 | 0.074 | 0.041 | 0.067 | 0.213 | 0.209 | 0.308 | -0.096 | 0.082 | 0.243 |
| InternalIMT | aa | rs41526545 | 0.093 | 0.069 | 0.035 | 0.048 | -0.349 | 0.206 | 0.091 | 0.149 | 0.086 | 0.083 |
| InternalIMT | aa | rs2029253 | 0.421 | 0.106 | 0.052 | 0.039 | 0.041 | 0.109 | 0.708 | -0.012 | 0.044 | 0.792 |
| InternalIMT | aa | rs17444064 | 0.092 | 0.068 | 0.035 | 0.053 | -0.381 | 0.209 | 0.068 | 0.165 | 0.087 | 0.058 |
| InternalIMT | aa | rs71494779 | 0.08 | 0.063 | 0.036 | 0.078 | -0.504 | 0.186 | 6.80E-03 | 0.192 | 0.078 | 0.014 |
| InternalIMT | aa | rs74991420 | 0.012 | 0.08 | 0.034 | 0.018 | -1.04 | 0.408 | 0.011 | 0.393 | 0.181 | 0.03 |
| InternalIMT | aa | rs182736551 | 0.012 | 0.08 | 0.034 | 0.018 | -1.04 | 0.408 | 0.011 | 0.393 | 0.181 | 0.03 |
| InternalIMT | aa | rs78702074 | 0.013 | 0.08 | 0.034 | 0.017 | -0.944 | 0.413 | 0.022 | 0.353 | 0.183 | 0.054 |
| InternalIMT | aa | rs187791466 | 0.004 | . | . | . | . | . | . | . | . | . |
| InternalIMT | aa | rs35300270 | 0.082 | 0.061 | 0.036 | 0.086 | -0.517 | 0.185 | 5.20E-03 | 0.197 | 0.078 | 0.011 |
| InternalIMT | aa | rs6593484 | 0.102 | 0.08 | 0.036 | 0.026 | -0.144 | 0.189 | 0.445 | 0.062 | 0.078 | 0.427 |
| InternalIMT | aa | rs12244601 | 0.023 | 0.04 | 0.037 | 0.288 | -0.717 | 0.476 | 0.132 | 0.276 | 0.192 | 0.151 |
| InternalIMT | aa | rs184429574 | 0.005 | . | . | . | . | . | . | . | . | . |
| InternalIMT | aa | rs7080897 | 0.08 | 0.063 | 0.036 | 0.078 | -0.505 | 0.186 | 6.80E-03 | 0.192 | 0.078 | 0.014 |
| InternalIMT | aa | rs7097429 | 0.083 | 0.06 | 0.036 | 0.094 | -0.528 | 0.183 | 3.90E-03 | 0.203 | 0.077 | 8.40E-03 |
| InternalIMT | aa | rs77979229 | 0.003 | . | . | . | . | . | . | . | . | . |
| InternalIMT | aa | rs11239512 | 0.103 | 0.08 | 0.036 | 0.026 | -0.142 | 0.188 | 0.45 | 0.061 | 0.078 | 0.43 |
| InternalIMT | aa | rs60146827 | 0.08 | 0.063 | 0.036 | 0.077 | -0.503 | 0.186 | 6.90E-03 | 0.191 | 0.078 | 0.014 |
| InternalIMT | aa | rs79032236 | 0.034 | 0.057 | 0.038 | 0.131 | 0.572 | 0.358 | 0.11 | -0.211 | 0.139 | 0.13 |
| InternalIMT | aa | rs140854206 | 0.007 | . | . | . | . | . | . | . | . | . |
| InternalIMT | aa | rs184049550 | 0.004 | . | . | . | . | . | . | . | . | . |
| InternalIMT | aa | rs114433607 | 0.01 | 0.054 | 0.037 | 0.147 | 0.629 | 0.661 | 0.341 | -0.261 | 0.255 | 0.307 |
| InternalIMT | aa | rs117653793 | 0.013 | 0.08 | 0.034 | 0.018 | -0.963 | 0.397 | 0.015 | 0.363 | 0.176 | 0.04 |
| InternalIMT | aa | rs117039533 | 0.013 | 0.08 | 0.034 | 0.018 | -0.964 | 0.397 | 0.015 | 0.363 | 0.176 | 0.039 |

| InternalIMT | aa | rs191660761 | 0.003 | . | . | . | . | . | . | . | . | . |
| --- | --- | --- | --- | --- | --- | --- | --- | --- | --- | --- | --- | --- |
| InternalIMT | aa | rs59514896 | 0.064 | 0.066 | 0.035 | 0.059 | -0.481 | 0.21 | 0.022 | 0.179 | 0.09 | 0.046 |
| InternalIMT | aa | rs184661876 | 0.002 | . | . | . | . | . | . | . | . | . |
| InternalIMT | aa | rs61854073 | 0.027 | 0.062 | 0.038 | 0.107 | 0.615 | 0.612 | 0.315 | -0.225 | 0.251 | 0.369 |
| InternalIMT | aa | rs2018518 | 0.299 | 0.126 | 0.046 | 6.70E-03 | 0.127 | 0.122 | 0.297 | -0.05 | 0.05 | 0.317 |
| InternalIMT | aa | rs934187 | 0.718 | -0.019 | 0.082 | 0.82 | -0.192 | 0.124 | 0.122 | 0.076 | 0.051 | 0.137 |
| InternalIMT | aa | rs7918542 | 0.214 | 0.028 | 0.04 | 0.481 | -0.404 | 0.134 | 2.50E-03 | 0.161 | 0.055 | 3.70E-03 |
| InternalIMT | aa | rs144311920 | 0.002 | . | . | . | . | . | . | . | . | . |
| InternalIMT | aa | rs7099684 | 0.84 | -6.00E-03 | 0.116 | 0.959 | -0.134 | 0.159 | 0.4 | 0.058 | 0.065 | 0.373 |
| InternalIMT | aa | rs150829171 | 0.002 | . | . | . | . | . | . | . | . | . |
| InternalIMT | aa | rs186060697 | 0.002 | . | . | . | . | . | . | . | . | . |
| InternalIMT | aa | rs11239513 | 0.097 | 0.079 | 0.041 | 0.054 | 0.341 | 0.208 | 0.101 | -0.139 | 0.082 | 0.088 |
| InternalIMT | aa | rs7917687 | 0.081 | 0.063 | 0.036 | 0.075 | -0.49 | 0.184 | 7.80E-03 | 0.185 | 0.077 | 0.017 |
| InternalIMT | aa | rs61854076 | 0.01 | . | . | . | . | . | . | . | . | . |
| InternalIMT | aa | rs71494780 | 0.018 | 0.038 | 0.038 | 0.305 | -0.724 | 0.417 | 0.083 | 0.297 | 0.175 | 0.089 |
| InternalIMT | aa | rs7919239 | 0.218 | 0.032 | 0.039 | 0.42 | -0.37 | 0.126 | 3.20E-03 | 0.147 | 0.052 | 4.90E-03 |
| InternalIMT | aa | rs78334475 | 0.034 | 0.091 | 0.035 | 9.60E-03 | 0.249 | 0.267 | 0.351 | -0.151 | 0.113 | 0.182 |
| InternalIMT | aa | rs3780900 | 0.026 | 0.096 | 0.034 | 5.00E-03 | 0.185 | 0.439 | 0.674 | -0.07 | 0.179 | 0.694 |
| InternalIMT | aa | rs186729292 | 0.001 | . | . | . | . | . | . | . | . | . |
| InternalIMT | aa | rs12267438 | 0.026 | 0.041 | 0.037 | 0.272 | -0.507 | 0.485 | 0.296 | 0.195 | 0.192 | 0.31 |
| InternalIMT | aa | rs1369214 | 0.568 | 0.065 | 0.066 | 0.322 | -0.064 | 0.114 | 0.575 | 0.021 | 0.047 | 0.646 |
| InternalIMT | aa | rs9282589 | 0.041 | 0.036 | 0.038 | 0.344 | -0.211 | 0.309 | 0.494 | 0.072 | 0.13 | 0.579 |
| InternalIMT | aa | rs114522915 | 0.025 | 0.055 | 0.038 | 0.144 | 0.63 | 0.501 | 0.209 | -0.236 | 0.196 | 0.229 |
| InternalIMT | aa | rs2115819 | 0.477 | 0.06 | 0.055 | 0.272 | -0.082 | 0.106 | 0.44 | 0.033 | 0.043 | 0.454 |
| InternalIMT | aa | rs117664603 | 0.013 | 0.079 | 0.034 | 0.018 | -0.964 | 0.398 | 0.015 | 0.363 | 0.177 | 0.04 |
| InternalIMT | aa | rs184979202 | 0.001 | . | . | . | . | . | . | . | . | . |
| InternalIMT | aa | rs190714671 | 0.002 | . | . | . | . | . | . | . | . | . |
| InternalIMT | aa | rs141538996 | 0 | . | . | . | . | . | . | . | . | . |
| InternalIMT | aa | rs150881741 | 0.002 | . | . | . | . | . | . | . | . | . |
| InternalIMT | aa | rs139076633 | 0.003 | . | . | . | . | . | . | . | . | . |
| InternalIMT | aa | rs12243649 | 0.059 | 0.065 | 0.035 | 0.062 | -0.72 | 0.249 | 3.80E-03 | 0.283 | 0.101 | 4.90E-03 |
| InternalIMT | aa | rs140348708 | 0.001 | . | . | . | . | . | . | . | . | . |
| InternalIMT | aa | rs79023256 | 0.013 | 0.079 | 0.034 | 0.018 | -0.962 | 0.398 | 0.016 | 0.362 | 0.177 | 0.041 |
| InternalIMT | aa | rs11239515 | 0.094 | 0.086 | 0.035 | 0.016 | -0.079 | 0.199 | 0.691 | 0.035 | 0.082 | 0.673 |
| InternalIMT | aa | rs55866086 | 0.157 | 0.059 | 0.038 | 0.122 | -0.281 | 0.16 | 0.078 | 0.113 | 0.065 | 0.086 |
| InternalIMT | aa | rs181340150 | 0.003 | . | . | . | . | . | . | . | . | . |
| InternalIMT | aa | rs11239516 | 0.12 | 0.084 | 0.037 | 0.022 | -0.084 | 0.181 | 0.642 | 0.035 | 0.075 | 0.636 |
| InternalIMT | aa | rs11239517 | 0.12 | 0.084 | 0.037 | 0.022 | -0.083 | 0.181 | 0.646 | 0.035 | 0.074 | 0.639 |
| InternalIMT | aa | rs11239519 | 0.106 | 0.09 | 0.036 | 0.013 | -0.033 | 0.189 | 0.863 | 0.013 | 0.078 | 0.867 |
| InternalIMT | aa | rs7909514 | 0.709 | 3.30E-03 | 0.082 | 0.967 | -0.161 | 0.125 | 0.198 | 0.062 | 0.051 | 0.23 |
| InternalIMT | aa | rs7894352 | 0.835 | 8.90E-03 | 0.118 | 0.94 | -0.114 | 0.162 | 0.48 | 0.049 | 0.066 | 0.458 |
| InternalIMT | aa | rs189384146 | 0.002 | . | . | . | . | . | . | . | . | . |
| InternalIMT | aa | rs11239520 | 0.105 | 0.094 | 0.036 | 8.90E-03 | 0.022 | 0.188 | 0.906 | -8.60E-03 | 0.078 | 0.912 |
| InternalIMT | aa | rs12221233 | 0.406 | 0.102 | 0.052 | 0.048 | 0.027 | 0.112 | 0.81 | -7.30E-03 | 0.046 | 0.874 |
| InternalIMT | aa | rs74523293 | 0.032 | 0.069 | 0.034 | 0.043 | -1.05 | 0.301 | 5.10E-04 | 0.412 | 0.125 | 9.60E-04 |
| InternalIMT | aa | rs79376040 | 0.032 | 0.069 | 0.034 | 0.043 | -1.05 | 0.301 | 5.10E-04 | 0.412 | 0.125 | 9.60E-04 |
| InternalIMT | aa | rs4949000 | 0.406 | 0.101 | 0.052 | 0.049 | 0.025 | 0.112 | 0.823 | -6.50E-03 | 0.046 | 0.887 |
| InternalIMT | aa | rs7089406 | 0.025 | 0.049 | 0.038 | 0.19 | -0.063 | 0.335 | 0.852 | 7.80E-03 | 0.137 | 0.954 |
| InternalIMT | aa | rs138211056 | 0 | . | . | . | . | . | . | . | . | . |
| InternalIMT | aa | rs10900213 | 0.537 | 0.097 | 0.061 | 0.111 | 0.015 | 0.115 | 0.894 | -2.30E-03 | 0.047 | 0.96 |
| InternalIMT | aa | rs145152827 | 0.003 | . | . | . | . | . | . | . | . | . |
| InternalIMT | aa | rs74443325 | 0.012 | 0.08 | 0.034 | 0.018 | -0.94 | 0.408 | 0.021 | 0.347 | 0.182 | 0.057 |
| InternalIMT | aa | rs188299446 | 0.011 | 0.054 | 0.038 | 0.154 | 0.519 | 0.923 | 0.574 | -0.162 | 0.384 | 0.674 |
| InternalIMT | aa | rs192699586 | 0.003 | . | . | . | . | . | . | . | . | . |
| InternalIMT | aa | rs72796464 | 0.086 | 0.077 | 0.035 | 0.029 | -0.244 | 0.21 | 0.246 | 0.107 | 0.087 | 0.223 |
| InternalIMT | aa | rs4949001 | 0.266 | 0.113 | 0.045 | 0.013 | 0.099 | 0.129 | 0.442 | -0.044 | 0.053 | 0.409 |
| InternalIMT | aa | rs147174641 | 0.008 | . | . | . | . | . | . | . | . | . |
| InternalIMT | aa | rs190835238 | 0.001 | . | . | . | . | . | . | . | . | . |
| InternalIMT | aa | rs76698944 | 0.022 | 0.03 | 0.038 | 0.433 | -1.02 | 0.478 | 0.033 | 0.424 | 0.205 | 0.038 |
| InternalIMT | aa | rs56071381 | 0.11 | 0.094 | 0.036 | 9.50E-03 | 0.014 | 0.183 | 0.941 | -6.50E-03 | 0.075 | 0.932 |
| InternalIMT | aa | rs78130427 | 0.047 | 0.073 | 0.035 | 0.035 | -0.429 | 0.244 | 0.079 | 0.148 | 0.106 | 0.162 |
| InternalIMT | aa | rs10900215 | 0.148 | 0.093 | 0.038 | 0.013 | 1.30E-03 | 0.166 | 0.994 | -1.20E-03 | 0.068 | 0.986 |
| InternalIMT | aa | rs41491445 | 0.016 | 0.054 | 0.037 | 0.148 | 0.345 | 0.523 | 0.509 | -0.149 | 0.204 | 0.463 |
| InternalIMT | aa | rs144995222 | 0.002 | . | . | . | . | . | . | . | . | . |
| InternalIMT | aa | rs149097803 | 0.003 | . | . | . | . | . | . | . | . | . |
| InternalIMT | aa | rs139429646 | 0.005 | . | . | . | . | . | . | . | . | . |
| InternalIMT | aa | rs7075620 | 0.681 | 0.011 | 0.079 | 0.892 | -0.154 | 0.125 | 0.217 | 0.059 | 0.051 | 0.251 |
| InternalIMT | aa | rs148299213 | 0.017 | 0.054 | 0.038 | 0.149 | 0.333 | 0.522 | 0.524 | -0.142 | 0.203 | 0.483 |
| InternalIMT | aa | rs4949002 | 0.245 | 0.138 | 0.044 | 1.80E-03 | 0.21 | 0.143 | 0.142 | -0.071 | 0.058 | 0.22 |
| InternalIMT | aa | rs150496007 | 0.021 | 0.039 | 0.038 | 0.305 | -0.533 | 0.542 | 0.326 | 0.201 | 0.223 | 0.368 |
| InternalIMT | aa | rs147303455 | 0.012 | 0.044 | 0.037 | 0.234 | -0.441 | 0.723 | 0.542 | 0.178 | 0.284 | 0.531 |
| InternalIMT | aa | rs189518433 | 0.005 | . | . | . | . | . | . | . | . | . |
| InternalIMT | aa | rs186281355 | 0.005 | . | . | . | . | . | . | . | . | . |
| InternalIMT | aa | rs115297114 | 0.026 | 0.052 | 0.038 | 0.167 | 0.319 | 0.414 | 0.44 | -0.115 | 0.163 | 0.478 |
| InternalIMT | aa | rs11239523 | 0.105 | 0.094 | 0.036 | 9.00E-03 | 0.024 | 0.185 | 0.899 | -8.10E-03 | 0.076 | 0.915 |
| InternalIMT | aa | rs143704897 | 0.005 | . | . | . | . | . | . | . | . | . |
| InternalIMT | aa | rs188024096 | 0.003 | . | . | . | . | . | . | . | . | . |
| InternalIMT | aa | rs11239524 | 0.107 | 0.097 | 0.037 | 8.10E-03 | 0.035 | 0.195 | 0.857 | -0.017 | 0.08 | 0.833 |
| InternalIMT | aa | rs191656573 | 0.004 | . | . | . | . | . | . | . | . | . |
| InternalIMT | aa | rs193295875 | 0.002 | . | . | . | . | . | . | . | . | . |
| InternalIMT | aa | rs190458228 | 0.002 | . | . | . | . | . | . | . | . | . |
| InternalIMT | aa | rs139253993 | 0.002 | . | . | . | . | . | . | . | . | . |
| InternalIMT | aa | rs4948672 | 0.588 | 0.098 | 0.076 | 0.197 | 0.012 | 0.132 | 0.926 | -2.60E-03 | 0.054 | 0.962 |
| InternalIMT | aa | rs180954921 | 0.002 | . | . | . | . | . | . | . | . | . |
| InternalIMT | aa | rs12264801 | 0.425 | 0.057 | 0.056 | 0.308 | -0.094 | 0.133 | 0.48 | 0.042 | 0.055 | 0.447 |
| InternalIMT | aa | rs73283184 | 0.02 | 0.053 | 0.038 | 0.156 | 0.011 | 0.483 | 0.982 | -0.023 | 0.189 | 0.903 |

| InternalIMT | aa | rs142746187 | 0.009 | . | . | . | . | . | . | . | . | . |
| --- | --- | --- | --- | --- | --- | --- | --- | --- | --- | --- | --- | --- |
| InternalIMT | aa | rs58704739 | 0.019 | 0.054 | 0.038 | 0.148 | 0.123 | 0.507 | 0.809 | -0.068 | 0.199 | 0.732 |
| InternalIMT | aa | rs61272335 | 0.014 | 0.056 | 0.037 | 0.137 | 0.525 | 0.601 | 0.382 | -0.222 | 0.235 | 0.344 |
| InternalIMT | aa | rs60959489 | 0.035 | 0.069 | 0.038 | 0.073 | 0.423 | 0.277 | 0.126 | -0.187 | 0.109 | 0.086 |
| InternalIMT | aa | rs58502629 | 0.014 | 0.055 | 0.037 | 0.138 | 0.521 | 0.601 | 0.386 | -0.22 | 0.235 | 0.348 |
| InternalIMT | aa | rs10900216 | 0.735 | 0.162 | 0.084 | 0.053 | 0.131 | 0.125 | 0.296 | -0.046 | 0.051 | 0.37 |
| InternalIMT | aa | rs181118141 | 0.002 | . | . | . | . | . | . | . | . | . |
| InternalIMT | aa | rs137882665 | 0.01 | . | . | . | . | . | . | . | . | . |
| InternalIMT | aa | rs149466736 | 0.003 | . | . | . | . | . | . | . | . | . |
| InternalIMT | aa | rs2099171 | 0.656 | 0.107 | 0.069 | 0.12 | 0.04 | 0.11 | 0.72 | -6.20E-03 | 0.045 | 0.891 |
| InternalIMT | aa | rs141852280 | 0.006 | . | . | . | . | . | . | . | . | . |
| InternalIMT | aa | rs191539257 | 0.002 | . | . | . | . | . | . | . | . | . |
| InternalIMT | aa | rs17157771 | 0.034 | 0.11 | 0.034 | 1.40E-03 | 0.512 | 0.311 | 0.1 | -0.231 | 0.127 | 0.07 |
| InternalIMT | aa | rs111339241 | 0.032 | 0.109 | 0.034 | 1.40E-03 | 0.54 | 0.339 | 0.111 | -0.243 | 0.139 | 0.08 |
| InternalIMT | aa | rs116983255 | 0.008 | . | . | . | . | . | . | . | . | . |
| InternalIMT | aa | rs142324341 | 0.011 | 0.16 | 0.05 | 1.40E-03 | -0.476 | 0.835 | 0.569 | 0.181 | 0.356 | 0.612 |
| InternalIMT | aa | rs187191219 | 0.01 | . | . | . | . | . | . | . | . | . |
| InternalIMT | aa | rs151283973 | 0.002 | . | . | . | . | . | . | . | . | . |
| InternalIMT | aa | rs139316218 | 0.003 | . | . | . | . | . | . | . | . | . |
| InternalIMT | aa | rs6593485 | 0.732 | 0.135 | 0.083 | 0.104 | 0.078 | 0.123 | 0.527 | -0.028 | 0.051 | 0.582 |
| InternalIMT | aa | rs6593486 | 0.732 | 0.137 | 0.083 | 0.1 | 0.08 | 0.123 | 0.515 | -0.029 | 0.05 | 0.567 |
| InternalIMT | aa | rs145196618 | 0.014 | 0.04 | 0.037 | 0.284 | -1.11 | 0.626 | 0.076 | 0.441 | 0.251 | 0.079 |
| InternalIMT | aa | rs142598335 | 0.004 | . | . | . | . | . | . | . | . | . |
| InternalIMT | aa | rs892691 | 0.726 | 0.152 | 0.081 | 0.059 | 0.104 | 0.12 | 0.385 | -0.039 | 0.049 | 0.423 |
| InternalIMT | aa | rs7080713 | 0.671 | 0.126 | 0.072 | 0.078 | 0.075 | 0.112 | 0.505 | -0.022 | 0.046 | 0.638 |
| InternalIMT | aa | rs7077173 | 0.08 | 0.11 | 0.035 | 1.90E-03 | 0.195 | 0.167 | 0.242 | -0.089 | 0.068 | 0.191 |
| InternalIMT | aa | rs7080977 | 0.048 | 0.107 | 0.035 | 2.10E-03 | 0.252 | 0.269 | 0.349 | -0.117 | 0.109 | 0.284 |
| InternalIMT | aa | rs3780901 | 0.671 | 0.139 | 0.072 | 0.052 | 0.098 | 0.112 | 0.383 | -0.031 | 0.046 | 0.503 |
| InternalIMT | aa | rs61854091 | 0.051 | 0.051 | 0.039 | 0.191 | 0.052 | 0.366 | 0.888 | 7.60E-03 | 0.149 | 0.959 |
| InternalIMT | aa | rs77550209 | 0.005 | . | . | . | . | . | . | . | . | . |
| InternalIMT | aa | rs73283191 | 0.061 | 0.092 | 0.035 | 8.80E-03 | -0.13 | 0.22 | 0.555 | 0.032 | 0.09 | 0.723 |
| InternalIMT | aa | rs191866099 | 0.007 | . | . | . | . | . | . | . | . | . |
| InternalIMT | aa | rs147998395 | 0.002 | . | . | . | . | . | . | . | . | . |
| InternalIMT | aa | rs7090328 | 0.783 | 0.119 | 0.096 | 0.214 | 0.048 | 0.135 | 0.725 | -0.017 | 0.056 | 0.763 |
| InternalIMT | aa | rs79814535 | 0.012 | 0.08 | 0.034 | 0.017 | -0.878 | 0.41 | 0.032 | 0.322 | 0.183 | 0.078 |
| InternalIMT | aa | rs7073082 | 0.783 | 0.119 | 0.096 | 0.214 | 0.048 | 0.135 | 0.725 | -0.017 | 0.056 | 0.763 |
| InternalIMT | aa | rs11239526 | 0.036 | 0.098 | 0.035 | 4.80E-03 | 0.193 | 0.301 | 0.521 | -0.06 | 0.123 | 0.628 |
| InternalIMT | aa | rs141839841 | 0.002 | . | . | . | . | . | . | . | . | . |
| InternalIMT | aa | rs75697371 | 0.01 | . | . | . | . | . | . | . | . | . |
| InternalIMT | aa | rs75972854 | 0.01 | . | . | . | . | . | . | . | . | . |
| InternalIMT | aa | rs139291975 | 0.001 | . | . | . | . | . | . | . | . | . |
| InternalIMT | aa | rs144064711 | 0.006 | . | . | . | . | . | . | . | . | . |
| InternalIMT | aa | rs3780902 | 0.062 | 0.093 | 0.035 | 8.60E-03 | -0.121 | 0.219 | 0.58 | 0.029 | 0.09 | 0.743 |
| InternalIMT | aa | rs190449801 | 0.002 | . | . | . | . | . | . | . | . | . |
| InternalIMT | aa | rs73283196 | 0.061 | 0.093 | 0.035 | 8.60E-03 | -0.129 | 0.221 | 0.56 | 0.032 | 0.091 | 0.728 |
| InternalIMT | aa | rs28395867 | 0.044 | 0.036 | 0.038 | 0.341 | -0.689 | 0.309 | 0.026 | 0.256 | 0.122 | 0.035 |
| InternalIMT | aa | rs28395866 | 0.102 | 0.096 | 0.036 | 8.20E-03 | -0.08 | 0.188 | 0.67 | 0.023 | 0.075 | 0.762 |
| InternalIMT | aa | rs55661611 | 0.047 | 0.107 | 0.035 | 2.20E-03 | 0.244 | 0.276 | 0.376 | -0.114 | 0.111 | 0.308 |
| InternalIMT | aa | rs55684518 | 0.02 | 0.048 | 0.038 | 0.207 | -0.152 | 0.444 | 0.732 | 0.024 | 0.181 | 0.894 |
| InternalIMT | aa | rs78552493 | 0.047 | 0.035 | 0.039 | 0.364 | -0.394 | 0.264 | 0.136 | 0.143 | 0.111 | 0.197 |
| InternalIMT | aa | rs147665434 | 0.004 | . | . | . | . | . | . | . | . | . |
| InternalIMT | aa | rs34445001 | 0.01 | . | . | . | . | . | . | . | . | . |
| InternalIMT | aa | rs184019449 | 0.003 | . | . | . | . | . | . | . | . | . |
| InternalIMT | aa | rs3780903 | 0.714 | 0.137 | 0.08 | 0.089 | 0.088 | 0.121 | 0.469 | -0.028 | 0.05 | 0.569 |
| InternalIMT | aa | rs17157784 | 0.061 | 0.072 | 0.035 | 0.039 | -0.529 | 0.238 | 0.026 | 0.199 | 0.096 | 0.039 |
| InternalIMT | aa | rs3780904 | 0.013 | 0.078 | 0.034 | 0.02 | -1.18 | 0.36 | 1.00E-03 | 0.467 | 0.159 | 3.30E-03 |
| InternalIMT | aa | rs192064730 | 0 | . | . | . | . | . | . | . | . | . |
| InternalIMT | aa | rs12774534 | 0.01 | . | . | . | . | . | . | . | . | . |
| InternalIMT | aa | rs79179915 | 0.011 | 0.08 | 0.034 | 0.017 | -0.937 | 0.424 | 0.027 | 0.347 | 0.19 | 0.067 |
| InternalIMT | aa | rs117811571 | 0.011 | 0.053 | 0.037 | 0.154 | 0.517 | 0.95 | 0.586 | -0.153 | 0.395 | 0.699 |
| InternalIMT | aa | rs72796477 | 0.01 | 0.05 | 0.037 | 0.176 | 0.17 | 0.648 | 0.793 | -0.052 | 0.266 | 0.844 |
| InternalIMT | aa | rs55729342 | 0.065 | 0.068 | 0.035 | 0.054 | -0.58 | 0.239 | 0.015 | 0.221 | 0.097 | 0.023 |
| InternalIMT | aa | rs59317220 | 0.064 | 0.069 | 0.035 | 0.051 | -0.583 | 0.243 | 0.017 | 0.222 | 0.098 | 0.024 |
| InternalIMT | aa | rs3780905 | 0.104 | 0.062 | 0.037 | 0.092 | -0.151 | 0.137 | 0.267 | 0.029 | 0.058 | 0.623 |
| InternalIMT | aa | rs3780906 | 0.714 | 0.134 | 0.081 | 0.096 | 0.084 | 0.122 | 0.492 | -0.027 | 0.05 | 0.593 |
| InternalIMT | aa | rs149047975 | 0.036 | 0.039 | 0.038 | 0.3 | -0.109 | 0.328 | 0.74 | 0.023 | 0.137 | 0.868 |
| InternalIMT | aa | rs3824614 | 0.011 | 0.08 | 0.034 | 0.017 | -0.902 | 0.427 | 0.035 | 0.33 | 0.191 | 0.084 |
| InternalIMT | aa | rs140254404 | 0.003 | . | . | . | . | . | . | . | . | . |
| InternalIMT | aa | rs145262684 | 0.014 | 0.053 | 0.038 | 0.158 | 0.371 | 0.81 | 0.647 | -0.083 | 0.336 | 0.806 |
| InternalIMT | aa | rs76735482 | 0.011 | 0.08 | 0.034 | 0.017 | -0.883 | 0.428 | 0.039 | 0.32 | 0.191 | 0.094 |
| InternalIMT | aa | rs73283198 | 0.034 | 0.109 | 0.034 | 1.40E-03 | 0.5 | 0.338 | 0.14 | -0.23 | 0.138 | 0.096 |
| InternalIMT | aa | rs74911239 | 0.035 | 0.049 | 0.038 | 0.197 | -5.20E-03 | 0.378 | 0.989 | 0.017 | 0.158 | 0.916 |
| InternalIMT | aa | rs150551647 | 0.006 | . | . | . | . | . | . | . | . | . |
| InternalIMT | aa | rs115200794 | 0.051 | 0.028 | 0.038 | 0.464 | -0.853 | 0.276 | 2.00E-03 | 0.324 | 0.109 | 3.10E-03 |
| InternalIMT | aa | rs74130823 | 0.168 | 0.083 | 0.039 | 0.033 | -0.133 | 0.155 | 0.392 | 0.047 | 0.064 | 0.459 |
| InternalIMT | aa | rs2279435 | 0.62 | 0.171 | 0.075 | 0.023 | 0.152 | 0.123 | 0.217 | -0.059 | 0.051 | 0.241 |
| InternalIMT | aa | rs147279673 | 0.014 | 0.082 | 0.034 | 0.016 | -0.886 | 0.605 | 0.143 | 0.391 | 0.247 | 0.114 |
| InternalIMT | aa | rs183525146 | 0.004 | . | . | . | . | . | . | . | . | . |
| InternalIMT | aa | rs3740107 | 0.745 | 0.232 | 0.088 | 8.40E-03 | 0.234 | 0.13 | 0.071 | -0.092 | 0.053 | 0.085 |
| InternalIMT | aa | rs140697247 | 0.001 | . | . | . | . | . | . | . | . | . |
| InternalIMT | aa | rs1565096 | 0.81 | 0.105 | 0.106 | 0.324 | 0.019 | 0.148 | 0.899 | -7.40E-03 | 0.061 | 0.903 |
| InternalIMT | aa | rs1565097 | 0.767 | 0.168 | 0.093 | 0.069 | 0.124 | 0.135 | 0.358 | -0.049 | 0.055 | 0.379 |
| InternalIMT | aa | rs144584548 | 0.02 | 0.047 | 0.038 | 0.209 | -0.157 | 0.446 | 0.725 | 0.03 | 0.182 | 0.871 |
| InternalIMT | aa | rs75811053 | 0.011 | 0.081 | 0.034 | 0.016 | -0.79 | 0.435 | 0.069 | 0.275 | 0.194 | 0.157 |
| InternalIMT | aa | rs147462967 | 0.01 | . | . | . | . | . | . | . | . | . |
| InternalIMT | aa | rs74130824 | 0.049 | 0.067 | 0.035 | 0.054 | -0.928 | 0.217 | 2.00E-05 | 0.347 | 0.089 | 1.10E-04 |

| InternalIMT | aa | rs116883184 | 0.018 | 0.05 | 0.038 | 0.189 | 0.05 | 0.653 | 0.939 | 0.033 | 0.269 | 0.903 |
| --- | --- | --- | --- | --- | --- | --- | --- | --- | --- | --- | --- | --- |
| InternalIMT | aa | rs117258049 | 0.018 | 0.05 | 0.038 | 0.189 | 0.05 | 0.652 | 0.939 | 0.033 | 0.269 | 0.903 |
| InternalIMT | aa | rs3780908 | 0.128 | 0.093 | 0.037 | 0.011 | -0.012 | 0.183 | 0.947 | 1.50E-03 | 0.076 | 0.985 |
| InternalIMT | aa | rs180938522 | 0.002 | . | . | . | . | . | . | . | . | . |
| InternalIMT | aa | rs76687372 | 0.008 | . | . | . | . | . | . | . | . | . |
| InternalIMT | aa | rs3780909 | 0.542 | 0.136 | 0.063 | 0.03 | 0.097 | 0.115 | 0.398 | -0.039 | 0.047 | 0.414 |
| InternalIMT | aa | rs78281156 | 0.011 | 0.081 | 0.034 | 0.016 | -0.793 | 0.434 | 0.068 | 0.276 | 0.194 | 0.154 |
| InternalIMT | aa | rs187840631 | 0.002 | . | . | . | . | . | . | . | . | . |
| InternalIMT | aa | rs76868242 | 0.011 | 0.081 | 0.034 | 0.016 | -0.793 | 0.434 | 0.068 | 0.276 | 0.194 | 0.155 |
| InternalIMT | aa | rs192794192 | 0.001 | . | . | . | . | . | . | . | . | . |
| InternalIMT | aa | rs12781248 | 0.01 | 0.047 | 0.037 | 0.203 | -0.132 | 0.451 | 0.771 | 0.064 | 0.183 | 0.727 |
| InternalIMT | aa | rs12762375 | 0.01 | . | . | . | . | . | . | . | . | . |
| InternalIMT | aa | rs192256986 | 0.006 | . | . | . | . | . | . | . | . | . |
| InternalIMT | aa | rs184411030 | 0.012 | 0.041 | 0.037 | 0.273 | -0.67 | 0.619 | 0.279 | 0.283 | 0.244 | 0.245 |
| InternalIMT | aa | rs189601483 | 0.011 | 0.078 | 0.034 | 0.02 | -1.06 | 0.45 | 0.019 | 0.39 | 0.201 | 0.052 |
| InternalIMT | aa | rs143254579 | 0.037 | 0.099 | 0.035 | 4.40E-03 | 0.228 | 0.29 | 0.432 | -0.077 | 0.118 | 0.515 |
| InternalIMT | aa | rs12253903 | 0.028 | 0.055 | 0.038 | 0.146 | 0.272 | 0.386 | 0.482 | -0.114 | 0.151 | 0.45 |
| InternalIMT | aa | rs35489926 | 0.009 | . | . | . | . | . | . | . | . | . |
| InternalIMT | aa | rs71494783 | 0.048 | 0.075 | 0.035 | 0.034 | -0.501 | 0.255 | 0.05 | 0.197 | 0.106 | 0.064 |
| InternalIMT | aa | rs180711602 | 0.003 | . | . | . | . | . | . | . | . | . |
| InternalIMT | aa | rs117312402 | 0.011 | 0.081 | 0.034 | 0.016 | -0.791 | 0.433 | 0.068 | 0.275 | 0.194 | 0.155 |
| InternalIMT | aa | rs74831694 | 0.008 | . | . | . | . | . | . | . | . | . |
| InternalIMT | aa | rs140301943 | 0.003 | . | . | . | . | . | . | . | . | . |
| InternalIMT | aa | rs78553679 | 0.016 | 0.045 | 0.038 | 0.236 | -0.247 | 0.611 | 0.685 | 0.136 | 0.25 | 0.585 |
| InternalIMT | aa | rs78334250 | 0.011 | 0.081 | 0.034 | 0.016 | -0.791 | 0.433 | 0.068 | 0.275 | 0.194 | 0.155 |
| InternalIMT | aa | rs76000028 | 0.011 | 0.081 | 0.034 | 0.016 | -0.791 | 0.433 | 0.068 | 0.275 | 0.194 | 0.155 |
| InternalIMT | aa | rs145195254 | 0.002 | . | . | . | . | . | . | . | . | . |
| InternalIMT | aa | rs137888527 | 0.002 | . | . | . | . | . | . | . | . | . |
| InternalIMT | aa | rs149109592 | 0.005 | . | . | . | . | . | . | . | . | . |
| InternalIMT | aa | rs80280010 | 0.035 | 0.102 | 0.035 | 3.30E-03 | 0.326 | 0.293 | 0.266 | -0.117 | 0.12 | 0.327 |
| InternalIMT | aa | rs189995374 | 0.002 | . | . | . | . | . | . | . | . | . |
| InternalIMT | aa | rs75467770 | 0.012 | 0.079 | 0.034 | 0.019 | -0.908 | 0.416 | 0.029 | 0.325 | 0.186 | 0.08 |
| InternalIMT | aa | rs1487562 | 0.167 | 0.079 | 0.038 | 0.037 | -0.149 | 0.156 | 0.34 | 0.055 | 0.064 | 0.386 |
| InternalIMT | aa | rs12266963 | 0.048 | 0.075 | 0.035 | 0.034 | -0.499 | 0.255 | 0.051 | 0.196 | 0.106 | 0.065 |
| InternalIMT | aa | rs7099874 | 0.764 | 0.024 | 0.093 | 0.795 | -0.113 | 0.138 | 0.413 | 0.045 | 0.057 | 0.425 |
| InternalIMT | aa | rs75687019 | 0.011 | 0.081 | 0.034 | 0.016 | -0.79 | 0.433 | 0.068 | 0.275 | 0.194 | 0.156 |
| InternalIMT | aa | rs148240462 | 0.038 | 0.044 | 0.038 | 0.252 | -0.188 | 0.392 | 0.631 | 0.098 | 0.164 | 0.553 |
| InternalIMT | aa | rs141278174 | 0.002 | . | . | . | . | . | . | . | . | . |
| InternalIMT | aa | rs76404852 | 0.011 | 0.081 | 0.034 | 0.016 | -0.79 | 0.433 | 0.068 | 0.275 | 0.193 | 0.156 |
| InternalIMT | aa | rs139112240 | 0.008 | . | . | . | . | . | . | . | . | . |
| InternalIMT | aa | rs12783037 | 0.054 | 0.07 | 0.035 | 0.045 | -0.533 | 0.247 | 0.031 | 0.215 | 0.102 | 0.035 |
| InternalIMT | aa | rs72796481 | 0.009 | . | . | . | . | . | . | . | . | . |
| InternalIMT | aa | rs76798749 | 0.017 | 0.077 | 0.034 | 0.021 | -1.14 | 0.386 | 3.20E-03 | 0.449 | 0.168 | 7.60E-03 |
| InternalIMT | aa | rs187732548 | 0.001 | . | . | . | . | . | . | . | . | . |
| InternalIMT | aa | rs75675474 | 0.017 | 0.077 | 0.034 | 0.021 | -1.14 | 0.386 | 3.20E-03 | 0.449 | 0.168 | 7.60E-03 |
| InternalIMT | aa | rs76552293 | 0.017 | 0.077 | 0.034 | 0.021 | -1.14 | 0.386 | 3.20E-03 | 0.449 | 0.168 | 7.60E-03 |
| InternalIMT | aa | rs147445993 | 0.005 | . | . | . | . | . | . | . | . | . |
| InternalIMT | aa | rs115902283 | 0.017 | 0.077 | 0.034 | 0.022 | -1.16 | 0.386 | 2.70E-03 | 0.456 | 0.168 | 6.80E-03 |
| InternalIMT | aa | rs4540799 | 0.762 | 0.044 | 0.091 | 0.628 | -0.08 | 0.136 | 0.555 | 0.032 | 0.056 | 0.568 |
| InternalIMT | aa | rs12765320 | 0.211 | 0.047 | 0.04 | 0.238 | -0.327 | 0.141 | 0.02 | 0.128 | 0.058 | 0.026 |
| InternalIMT | aa | rs12782256 | 0.009 | . | . | . | . | . | . | . | . | . |
| InternalIMT | aa | rs191727256 | 0.002 | . | . | . | . | . | . | . | . | . |
| InternalIMT | aa | rs2082111 | 0.123 | 0.087 | 0.036 | 0.017 | -0.07 | 0.184 | 0.706 | 0.028 | 0.076 | 0.718 |
| InternalIMT | aa | rs2082110 | 0.175 | 0.059 | 0.038 | 0.124 | -0.314 | 0.153 | 0.039 | 0.12 | 0.063 | 0.055 |
| InternalIMT | aa | rs2082109 | 0.175 | 0.059 | 0.038 | 0.124 | -0.314 | 0.153 | 0.039 | 0.12 | 0.063 | 0.055 |
| InternalIMT | aa | rs117316662 | 0.011 | 0.081 | 0.034 | 0.016 | -0.789 | 0.433 | 0.069 | 0.274 | 0.193 | 0.157 |
| InternalIMT | aa | rs12247163 | 0.214 | 0.055 | 0.04 | 0.17 | -0.267 | 0.141 | 0.058 | 0.104 | 0.058 | 0.07 |
| InternalIMT | aa | rs12355888 | 0.082 | 0.079 | 0.035 | 0.022 | -0.217 | 0.222 | 0.328 | 0.095 | 0.092 | 0.303 |
| InternalIMT | aa | rs143772290 | 0.011 | 0.048 | 0.037 | 0.194 | 0.968 | 1.16 | 0.403 | -0.345 | 0.449 | 0.443 |
| InternalIMT | aa | rs146849452 | 0.005 | . | . | . | . | . | . | . | . | . |
| InternalIMT | aa | rs140267121 | 0.004 | . | . | . | . | . | . | . | . | . |
| InternalIMT | aa | rs7080474 | 0.202 | 0.07 | 0.039 | 0.075 | -0.152 | 0.145 | 0.295 | 0.062 | 0.059 | 0.299 |
| InternalIMT | aa | rs144066203 | 0.002 | . | . | . | . | . | . | . | . | . |
| InternalIMT | aa | rs12765209 | 0.012 | 0.08 | 0.034 | 0.017 | -1.11 | 0.486 | 0.022 | 0.426 | 0.218 | 0.051 |
| InternalIMT | aa | rs150930735 | 0.002 | . | . | . | . | . | . | . | . | . |
| InternalIMT | aa | rs7068039 | 0.216 | 0.054 | 0.04 | 0.174 | -0.268 | 0.14 | 0.056 | 0.105 | 0.057 | 0.067 |
| InternalIMT | aa | rs117622652 | 0.011 | 0.081 | 0.034 | 0.016 | -0.787 | 0.433 | 0.069 | 0.273 | 0.193 | 0.158 |
| InternalIMT | aa | rs190191514 | 0.001 | . | . | . | . | . | . | . | . | . |
| InternalIMT | aa | rs74130835 | 0.04 | 0.036 | 0.038 | 0.351 | -0.958 | 0.316 | 2.40E-03 | 0.353 | 0.125 | 4.70E-03 |
| InternalIMT | aa | rs1811604 | 0.039 | 0.035 | 0.038 | 0.358 | -0.973 | 0.314 | 1.90E-03 | 0.358 | 0.124 | 3.90E-03 |
| InternalIMT | aa | rs146971256 | 0 | . | . | . | . | . | . | . | . | . |
| InternalIMT | aa | rs74336000 | 0.011 | 0.048 | 0.037 | 0.193 | 0.981 | 1.16 | 0.397 | -0.35 | 0.45 | 0.436 |
| InternalIMT | aa | rs186046492 | 0.007 | . | . | . | . | . | . | . | . | . |
| InternalIMT | aa | rs34971823 | 0.011 | 0.047 | 0.037 | 0.21 | -0.196 | 0.492 | 0.69 | 0.091 | 0.205 | 0.655 |
| InternalIMT | aa | rs75227446 | 0.011 | 0.081 | 0.034 | 0.016 | -0.8 | 0.432 | 0.064 | 0.279 | 0.193 | 0.148 |
| InternalIMT | aa | rs190457599 | 0.001 | . | . | . | . | . | . | . | . | . |
| InternalIMT | aa | rs79591917 | 0.01 | 0.081 | 0.034 | 0.016 | -0.784 | 0.433 | 0.07 | 0.272 | 0.193 | 0.16 |
| InternalIMT | aa | rs702365 | 0.545 | 0.144 | 0.064 | 0.023 | 0.115 | 0.116 | 0.323 | -0.046 | 0.048 | 0.336 |
| InternalIMT | aa | rs79643836 | 0.011 | 0.081 | 0.034 | 0.016 | -0.756 | 0.417 | 0.07 | 0.258 | 0.186 | 0.164 |
| InternalIMT | aa | rs702366 | 0.546 | 0.141 | 0.064 | 0.027 | 0.108 | 0.116 | 0.354 | -0.043 | 0.048 | 0.371 |
| InternalIMT | aa | rs1059696 | 0.151 | 0.082 | 0.038 | 0.03 | -0.095 | 0.173 | 0.585 | 0.039 | 0.071 | 0.582 |
| InternalIMT | aa | rs901681 | 0.546 | 0.146 | 0.063 | 0.021 | 0.119 | 0.116 | 0.304 | -0.047 | 0.048 | 0.321 |
| InternalIMT | aa | rs61854092 | 0.15 | 0.081 | 0.038 | 0.032 | -0.101 | 0.173 | 0.561 | 0.042 | 0.071 | 0.557 |
| InternalIMT | aa | rs148080753 | 0.001 | . | . | . | . | . | . | . | . | . |
| InternalIMT | aa | rs61854093 | 0.186 | 0.069 | 0.039 | 0.076 | -0.198 | 0.151 | 0.19 | 0.077 | 0.062 | 0.211 |
| InternalIMT | aa | rs141822774 | 0.001 | . | . | . | . | . | . | . | . | . |

| InternalIMT | aa | rs74130836 | 0.04 | 0.036 | 0.038 | 0.35 | -0.959 | 0.316 | 2.50E-03 | 0.354 | 0.125 | 4.70E-03 |
| --- | --- | --- | --- | --- | --- | --- | --- | --- | --- | --- | --- | --- |
| InternalIMT | aa | rs150620963 | 0.013 | 0.045 | 0.037 | 0.229 | -0.273 | 0.482 | 0.571 | 0.119 | 0.204 | 0.561 |
| InternalIMT | aa | rs3780913 | 0.753 | 0.045 | 0.089 | 0.613 | -0.083 | 0.134 | 0.536 | 0.032 | 0.055 | 0.56 |
| InternalIMT | aa | rs142255723 | 0.002 | . | . | . | . | . | . | . | . | . |
| InternalIMT | aa | rs3780914 | 0.542 | 0.132 | 0.063 | 0.036 | 0.089 | 0.116 | 0.442 | -0.035 | 0.048 | 0.465 |
| InternalIMT | aa | rs182656507 | 0.002 | . | . | . | . | . | . | . | . | . |
| InternalIMT | aa | rs2291427 | 0.735 | 0.086 | 0.086 | 0.315 | -8.40E-03 | 0.131 | 0.948 | 4.30E-03 | 0.054 | 0.937 |
| InternalIMT | aa | rs10751382 | 0.733 | 0.091 | 0.085 | 0.285 | -2.70E-03 | 0.13 | 0.983 | 9.70E-04 | 0.054 | 0.986 |
| InternalIMT | aa | rs117610108 | 0.01 | 0.081 | 0.034 | 0.016 | -0.774 | 0.432 | 0.074 | 0.266 | 0.193 | 0.168 |
| InternalIMT | aa | rs144966941 | 0.023 | 0.094 | 0.034 | 6.10E-03 | 0.053 | 0.421 | 0.9 | 9.30E-03 | 0.177 | 0.958 |
| InternalIMT | aa | rs41502051 | 0.03 | 0.109 | 0.034 | 1.40E-03 | 0.778 | 0.486 | 0.109 | -0.311 | 0.196 | 0.113 |
| InternalIMT | aa | rs41426046 | 0.011 | 0.079 | 0.034 | 0.019 | -0.925 | 0.431 | 0.032 | 0.329 | 0.192 | 0.087 |
| InternalIMT | aa | rs41519248 | 0.01 | 0.081 | 0.034 | 0.016 | -0.77 | 0.432 | 0.075 | 0.264 | 0.193 | 0.171 |
| InternalIMT | aa | rs189108245 | 0.004 | . | . | . | . | . | . | . | . | . |
| InternalIMT | aa | rs7896431 | 0.532 | 0.117 | 0.063 | 0.064 | 0.056 | 0.118 | 0.637 | -0.023 | 0.049 | 0.638 |
| InternalIMT | aa | rs183724547 | 0.003 | . | . | . | . | . | . | . | . | . |
| InternalIMT | aa | rs142044990 | 0.008 | . | . | . | . | . | . | . | . | . |
| InternalIMT | aa | rs188140929 | 0.007 | . | . | . | . | . | . | . | . | . |
| InternalIMT | aa | rs76819096 | 0.014 | 0.052 | 0.037 | 0.163 | 0.687 | 0.547 | 0.209 | -0.256 | 0.216 | 0.238 |
| InternalIMT | aa | rs10751383 | 0.601 | 0.167 | 0.069 | 0.015 | 0.156 | 0.117 | 0.184 | -0.06 | 0.048 | 0.216 |
| InternalIMT | aa | rs117819321 | 0.014 | 0.058 | 0.038 | 0.123 | 0.731 | 0.897 | 0.415 | -0.258 | 0.37 | 0.485 |
| InternalIMT | aa | rs2242332 | 0.702 | 0.047 | 0.079 | 0.556 | -0.082 | 0.124 | 0.507 | 0.033 | 0.051 | 0.52 |
| InternalIMT | aa | rs2242333 | 0.096 | 0.071 | 0.036 | 0.051 | -0.095 | 0.144 | 0.508 | 6.00E-03 | 0.061 | 0.921 |
| InternalIMT | aa | rs2242334 | 0.703 | 0.046 | 0.079 | 0.565 | -0.084 | 0.124 | 0.496 | 0.033 | 0.051 | 0.51 |
| InternalIMT | aa | rs189465041 | 0.001 | . | . | . | . | . | . | . | . | . |
| InternalIMT | aa | rs28395868 | 0.025 | 0.056 | 0.037 | 0.132 | 0.477 | 0.471 | 0.311 | -0.191 | 0.193 | 0.323 |
| InternalIMT | aa | rs56067550 | 0.02 | 0.048 | 0.038 | 0.199 | -0.08 | 0.45 | 0.858 | -1.20E-03 | 0.183 | 0.995 |
| InternalIMT | aa | rs1051713 | 0.154 | 0.053 | 0.038 | 0.165 | -0.36 | 0.167 | 0.031 | 0.146 | 0.069 | 0.034 |
| InternalIMT | aa | rs115074210 | 0.016 | 0.056 | 0.037 | 0.134 | 0.955 | 0.552 | 0.083 | -0.366 | 0.218 | 0.093 |
| InternalIMT | aa | rs7900977 | 0.802 | 0.047 | 0.102 | 0.648 | -0.064 | 0.145 | 0.66 | 0.028 | 0.06 | 0.637 |
| InternalIMT | aa | rs147613891 | 0.003 | . | . | . | . | . | . | . | . | . |
| InternalIMT | aa | rs2229136 | 0.084 | 0.106 | 0.036 | 3.40E-03 | 0.307 | 0.201 | 0.127 | -0.113 | 0.081 | 0.16 |
| InternalIMT | aa | rs185482841 | 0.003 | . | . | . | . | . | . | . | . | . |
| InternalIMT | aa | rs2288619 | 0.083 | 0.108 | 0.036 | 3.00E-03 | 0.315 | 0.201 | 0.118 | -0.118 | 0.081 | 0.144 |
| InternalIMT | aa | rs189891524 | 0.001 | . | . | . | . | . | . | . | . | . |
| InternalIMT | aa | rs56145077 | 0.143 | 0.064 | 0.037 | 0.081 | -0.261 | 0.16 | 0.104 | 0.108 | 0.066 | 0.102 |
| InternalIMT | aa | rs143216970 | 0.005 | . | . | . | . | . | . | . | . | . |
| InternalIMT | aa | rs142246478 | 0.001 | . | . | . | . | . | . | . | . | . |
| InternalIMT | aa | rs13134 | 0.012 | 0.05 | 0.037 | 0.181 | 0.168 | 0.464 | 0.717 | -0.064 | 0.186 | 0.732 |
| InternalIMT | aa | rs41314980 | 0.027 | 0.039 | 0.038 | 0.305 | -0.47 | 0.411 | 0.253 | 0.213 | 0.173 | 0.218 |
| InternalIMT | aa | rs41304611 | 0.013 | 0.051 | 0.037 | 0.172 | 0.239 | 0.522 | 0.647 | -0.097 | 0.214 | 0.65 |
| InternalIMT | aa | rs193299617 | 0.001 | . | . | . | . | . | . | . | . | . |
| InternalIMT | aa | rs12772724 | 0.012 | 0.05 | 0.037 | 0.181 | 0.168 | 0.464 | 0.717 | -0.064 | 0.186 | 0.732 |
| InternalIMT | aa | rs139489537 | 0.033 | 0.103 | 0.034 | 2.80E-03 | 0.372 | 0.307 | 0.225 | -0.132 | 0.125 | 0.291 |
| InternalIMT | aa | rs12776062 | 0.009 | . | . | . | . | . | . | . | . | . |
| InternalIMT | aa | rs752059 | 0.144 | 0.07 | 0.037 | 0.06 | -0.218 | 0.159 | 0.172 | 0.089 | 0.066 | 0.175 |
| InternalIMT | aa | rs147649982 | 0.03 | 0.092 | 0.035 | 8.30E-03 | -0.046 | 0.427 | 0.914 | 0.033 | 0.182 | 0.854 |
| InternalIMT | aa | rs17157798 | 0.07 | 0.111 | 0.036 | 2.00E-03 | 0.398 | 0.212 | 0.061 | -0.151 | 0.085 | 0.076 |
| InternalIMT | aa | rs142298619 | 0.003 | . | . | . | . | . | . | . | . | . |
| InternalIMT | aa | rs4948673 | 0.793 | -8.70E-03 | 0.1 | 0.931 | -0.154 | 0.142 | 0.278 | 0.064 | 0.058 | 0.273 |
| InternalIMT | aa | rs74231484 | 0.102 | 0.079 | 0.037 | 0.031 | -0.034 | 0.143 | 0.815 | -0.019 | 0.061 | 0.756 |
| InternalIMT | aa | rs72796489 | 0.002 | . | . | . | . | . | . | . | . | . |
| InternalIMT | aa | rs113447578 | 0.016 | 0.097 | 0.034 | 4.00E-03 | 0.406 | 0.366 | 0.267 | -0.214 | 0.153 | 0.161 |
| InternalIMT | aa | rs34063374 | 0.013 | 0.05 | 0.037 | 0.185 | 0.123 | 0.458 | 0.788 | -0.045 | 0.184 | 0.807 |
| InternalIMT | aa | rs11239527 | 0.013 | 0.052 | 0.037 | 0.161 | 0.317 | 0.67 | 0.636 | -0.091 | 0.281 | 0.746 |
| InternalIMT | aa | rs34371883 | 0.011 | 0.046 | 0.037 | 0.213 | -0.23 | 0.44 | 0.601 | 0.093 | 0.178 | 0.601 |
| InternalIMT | aa | rs118168828 | 0.007 | . | . | . | . | . | . | . | . | . |
| InternalIMT | aa | rs34349618 | 0.009 | . | . | . | . | . | . | . | . | . |
| InternalIMT | aa | rs80041679 | 0.033 | 0.103 | 0.034 | 2.80E-03 | 0.372 | 0.306 | 0.225 | -0.132 | 0.125 | 0.291 |
| InternalIMT | aa | rs149640607 | 0.003 | . | . | . | . | . | . | . | . | . |
| InternalIMT | aa | rs4427523 | 0.888 | -0.109 | 0.188 | 0.562 | -0.315 | 0.251 | 0.209 | 0.112 | 0.102 | 0.271 |
| InternalIMT | aa | rs185880109 | 0.002 | . | . | . | . | . | . | . | . | . |
| InternalIMT | aa | rs191523220 | 0.003 | . | . | . | . | . | . | . | . | . |
| InternalIMT | aa | rs144315284 | 0.006 | . | . | . | . | . | . | . | . | . |
| InternalIMT | aa | rs4948674 | 0.341 | 0.123 | 0.047 | 8.00E-03 | 0.155 | 0.119 | 0.194 | -0.047 | 0.049 | 0.334 |
| InternalIMT | aa | rs182984298 | 0.006 | . | . | . | . | . | . | . | . | . |
| InternalIMT | aa | rs56749023 | 0.025 | 0.054 | 0.038 | 0.151 | 0.293 | 0.389 | 0.451 | -0.12 | 0.152 | 0.431 |
| InternalIMT | aa | rs112508957 | 0.015 | 0.04 | 0.037 | 0.281 | -0.6 | 0.56 | 0.284 | 0.251 | 0.219 | 0.253 |
| InternalIMT | aa | rs7089063 | 0.229 | 0.077 | 0.041 | 0.061 | -0.103 | 0.126 | 0.411 | 0.04 | 0.052 | 0.44 |
| InternalIMT | aa | rs145000779 | 0.001 | . | . | . | . | . | . | . | . | . |
| InternalIMT | aa | rs80201572 | 0.034 | 0.103 | 0.034 | 2.90E-03 | 0.37 | 0.303 | 0.222 | -0.132 | 0.124 | 0.286 |
| InternalIMT | aa | rs140568227 | 0.005 | . | . | . | . | . | . | . | . | . |
| InternalIMT | aa | rs149756228 | 0.01 | . | . | . | . | . | . | . | . | . |
| InternalIMT | aa | rs112380685 | 0.007 | . | . | . | . | . | . | . | . | . |
| InternalIMT | aa | rs60481109 | 0.023 | 0.054 | 0.038 | 0.15 | 0.318 | 0.399 | 0.426 | -0.13 | 0.157 | 0.408 |
| InternalIMT | aa | rs138221843 | 0.003 | . | . | . | . | . | . | . | . | . |
| InternalIMT | aa | rs150283194 | 0.003 | . | . | . | . | . | . | . | . | . |
| InternalIMT | aa | rs138879661 | 0.004 | . | . | . | . | . | . | . | . | . |
| InternalIMT | aa | rs34960345 | 0.056 | 0.103 | 0.035 | 3.40E-03 | 0.286 | 0.229 | 0.211 | -0.104 | 0.092 | 0.259 |
| InternalIMT | aa | rs147514068 | 0.004 | . | . | . | . | . | . | . | . | . |
| InternalIMT | aa | rs35410535 | 0.078 | 0.108 | 0.036 | 2.70E-03 | 0.327 | 0.201 | 0.104 | -0.123 | 0.081 | 0.126 |
| InternalIMT | aa | rs191613635 | 0.003 | . | . | . | . | . | . | . | . | . |
| InternalIMT | aa | rs17157800 | 0.007 | . | . | . | . | . | . | . | . | . |
| InternalIMT | aa | rs17523413 | 0.045 | 0.104 | 0.035 | 2.70E-03 | 0.346 | 0.252 | 0.171 | -0.124 | 0.103 | 0.226 |
| InternalIMT | aa | rs7897036 | 0.035 | 0.067 | 0.034 | 0.051 | -1.03 | 0.365 | 4.70E-03 | 0.417 | 0.154 | 6.80E-03 |
| InternalIMT | aa | rs76334323 | 0.01 | 0.081 | 0.034 | 0.016 | -0.744 | 0.433 | 0.085 | 0.25 | 0.193 | 0.196 |

| InternalIMT | aa | rs143535991 | 0.001 | . | . | . | . | . | . | . | . | . |
| --- | --- | --- | --- | --- | --- | --- | --- | --- | --- | --- | --- | --- |
| InternalIMT | aa | rs140344979 | 0.005 | . | . | . | . | . | . | . | . | . |
| InternalIMT | aa | rs36047811 | 0.078 | 0.108 | 0.036 | 2.70E-03 | 0.327 | 0.201 | 0.104 | -0.123 | 0.081 | 0.126 |
| InternalIMT | aa | rs76493570 | 0.054 | 0.102 | 0.035 | 3.60E-03 | 0.279 | 0.232 | 0.228 | -0.1 | 0.093 | 0.283 |
| InternalIMT | aa | rs192998920 | 0.003 | . | . | . | . | . | . | . | . | . |
| InternalIMT | aa | rs116840852 | 0.016 | 0.045 | 0.038 | 0.236 | -0.249 | 0.611 | 0.684 | 0.136 | 0.25 | 0.586 |
| InternalIMT | aa | rs184824101 | 0.001 | . | . | . | . | . | . | . | . | . |
| InternalIMT | aa | rs41301621 | 0.005 | . | . | . | . | . | . | . | . | . |
| InternalIMT | aa | rs3740109 | 0.078 | 0.108 | 0.036 | 2.70E-03 | 0.328 | 0.201 | 0.103 | -0.124 | 0.081 | 0.125 |
| InternalIMT | aa | rs7899415 | 0.033 | 0.076 | 0.035 | 0.03 | -0.589 | 0.349 | 0.092 | 0.249 | 0.148 | 0.093 |
| InternalIMT | aa | rs184395284 | 0.002 | . | . | . | . | . | . | . | . | . |
| InternalIMT | aa | rs139209589 | 0.004 | . | . | . | . | . | . | . | . | . |
| InternalIMT | aa | rs149924163 | 0.003 | . | . | . | . | . | . | . | . | . |
| InternalIMT | aa | rs3740110 | 0.01 | 0.081 | 0.034 | 0.016 | -0.743 | 0.433 | 0.086 | 0.249 | 0.193 | 0.197 |
| InternalIMT | aa | rs144335113 | 0.003 | . | . | . | . | . | . | . | . | . |
| InternalIMT | aa | rs74743385 | 0.015 | 0.04 | 0.037 | 0.281 | -0.604 | 0.561 | 0.281 | 0.252 | 0.219 | 0.25 |
| InternalIMT | aa | rs146545829 | 0.003 | . | . | . | . | . | . | . | . | . |
| InternalIMT | aa | rs181842689 | 0 | . | . | . | . | . | . | . | . | . |
| InternalIMT | aa | rs3740111 | 0.056 | 0.103 | 0.035 | 3.40E-03 | 0.289 | 0.229 | 0.207 | -0.105 | 0.092 | 0.253 |
| InternalIMT | aa | rs186261599 | 0.003 | . | . | . | . | . | . | . | . | . |
| InternalIMT | aa | rs73285348 | 0.009 | . | . | . | . | . | . | . | . | . |
| InternalIMT | aa | rs186356901 | 0.003 | . | . | . | . | . | . | . | . | . |
| InternalIMT | aa | rs3824618 | 0.033 | 0.103 | 0.034 | 2.80E-03 | 0.371 | 0.305 | 0.225 | -0.132 | 0.125 | 0.29 |
| InternalIMT | aa | rs3802548 | 0.219 | 0.07 | 0.039 | 0.074 | -0.127 | 0.127 | 0.316 | 0.054 | 0.053 | 0.308 |
| InternalIMT | aa | rs3802547 | 0.01 | . | . | . | . | . | . | . | . | . |
| InternalIMT | aa | rs77677521 | 0.016 | 0.045 | 0.038 | 0.236 | -0.25 | 0.611 | 0.683 | 0.136 | 0.25 | 0.585 |
| InternalIMT | aa | rs3824616 | 0.078 | 0.108 | 0.036 | 2.70E-03 | 0.33 | 0.201 | 0.101 | -0.124 | 0.081 | 0.123 |
| InternalIMT | aa | rs191073818 | 0.003 | . | . | . | . | . | . | . | . | . |
| InternalIMT | aa | rs147078004 | 0.002 | . | . | . | . | . | . | . | . | . |
| InternalIMT | aa | rs7908745 | 0.31 | 0.073 | 0.044 | 0.096 | -0.077 | 0.113 | 0.496 | 0.033 | 0.047 | 0.485 |
| InternalIMT | aa | rs17157811 | 0.007 | . | . | . | . | . | . | . | . | . |
| InternalIMT | aa | rs138506087 | 0.006 | . | . | . | . | . | . | . | . | . |
| InternalIMT | aa | rs77241269 | 0.025 | 0.054 | 0.038 | 0.149 | 0.312 | 0.387 | 0.419 | -0.127 | 0.152 | 0.401 |
| InternalIMT | aa | rs138374938 | 0.001 | . | . | . | . | . | . | . | . | . |
| InternalIMT | aa | rs2279433 | 0.056 | 0.104 | 0.035 | 3.10E-03 | 0.306 | 0.227 | 0.178 | -0.113 | 0.091 | 0.216 |
| InternalIMT | aa | rs73285358 | 0.025 | 0.054 | 0.038 | 0.149 | 0.315 | 0.386 | 0.415 | -0.128 | 0.152 | 0.397 |
| InternalIMT | aa | rs11239528 | 0.218 | 0.069 | 0.039 | 0.077 | -0.132 | 0.127 | 0.299 | 0.056 | 0.053 | 0.29 |
| InternalIMT | aa | rs77453386 | 0.01 | 0.081 | 0.034 | 0.016 | -0.744 | 0.433 | 0.086 | 0.25 | 0.193 | 0.196 |
| InternalIMT | aa | rs2279434 | 0.061 | 0.106 | 0.035 | 2.80E-03 | 0.312 | 0.223 | 0.163 | -0.117 | 0.09 | 0.195 |
| InternalIMT | aa | rs149236525 | 0.005 | . | . | . | . | . | . | . | . | . |
| InternalIMT | aa | rs12573609 | 0.05 | 0.078 | 0.036 | 0.032 | -0.26 | 0.258 | 0.313 | 0.092 | 0.107 | 0.393 |
| InternalIMT | aa | rs191716655 | 0.002 | . | . | . | . | . | . | . | . | . |
| InternalIMT | aa | rs17523660 | 0.009 | . | . | . | . | . | . | . | . | . |
| InternalIMT | aa | rs142671360 | 0.003 | . | . | . | . | . | . | . | . | . |
| InternalIMT | aa | rs3764990 | 0.044 | 0.104 | 0.035 | 2.70E-03 | 0.346 | 0.253 | 0.172 | -0.124 | 0.103 | 0.229 |
| InternalIMT | aa | rs113682861 | 0.004 | . | . | . | . | . | . | . | . | . |
| InternalIMT | aa | rs117658270 | 0.01 | 0.081 | 0.034 | 0.016 | -0.744 | 0.433 | 0.086 | 0.25 | 0.193 | 0.196 |
| InternalIMT | aa | rs187309063 | 0 | . | . | . | . | . | . | . | . | . |
| InternalIMT | aa | rs73285360 | 0.025 | 0.054 | 0.038 | 0.149 | 0.316 | 0.386 | 0.413 | -0.129 | 0.151 | 0.395 |
| InternalIMT | aa | rs80133289 | 0.015 | 0.093 | 0.034 | 6.60E-03 | 0.6 | 0.465 | 0.197 | -0.336 | 0.198 | 0.09 |
| InternalIMT | aa | rs186693257 | 0.001 | . | . | . | . | . | . | . | . | . |
| InternalIMT | aa | rs35735968 | 0.062 | 0.106 | 0.036 | 3.00E-03 | 0.307 | 0.222 | 0.166 | -0.115 | 0.089 | 0.195 |
| InternalIMT | aa | rs17157825 | 0.034 | 0.103 | 0.034 | 2.80E-03 | 0.38 | 0.305 | 0.212 | -0.135 | 0.125 | 0.278 |
| InternalIMT | aa | rs2291428 | 0.213 | 0.059 | 0.039 | 0.129 | -0.193 | 0.131 | 0.141 | 0.083 | 0.054 | 0.129 |
| InternalIMT | aa | rs2291429 | 0.222 | 0.067 | 0.039 | 0.088 | -0.141 | 0.126 | 0.265 | 0.059 | 0.052 | 0.258 |
| InternalIMT | aa | rs145714792 | 0.005 | . | . | . | . | . | . | . | . | . |
| InternalIMT | aa | rs12769244 | 0.056 | 0.104 | 0.035 | 3.10E-03 | 0.302 | 0.227 | 0.183 | -0.112 | 0.091 | 0.221 |
| InternalIMT | aa | rs12771275 | 0.056 | 0.104 | 0.035 | 3.10E-03 | 0.302 | 0.227 | 0.182 | -0.112 | 0.091 | 0.221 |
| InternalIMT | aa | rs146266174 | 0.001 | . | . | . | . | . | . | . | . | . |
| InternalIMT | aa | rs41301623 | 0.003 | . | . | . | . | . | . | . | . | . |
| InternalIMT | aa | rs2306814 | 0.011 | 0.081 | 0.034 | 0.016 | -0.77 | 0.429 | 0.073 | 0.263 | 0.191 | 0.169 |
| InternalIMT | aa | rs12244834 | 0.027 | 0.087 | 0.034 | 0.011 | -0.196 | 0.397 | 0.621 | 0.087 | 0.16 | 0.588 |
| InternalIMT | aa | rs184006011 | 0.002 | . | . | . | . | . | . | . | . | . |
| InternalIMT | aa | rs78829066 | 0.01 | 0.081 | 0.034 | 0.015 | -0.752 | 0.437 | 0.085 | 0.253 | 0.195 | 0.193 |
| InternalIMT | aa | rs34731408 | 0.056 | 0.104 | 0.035 | 3.10E-03 | 0.303 | 0.227 | 0.181 | -0.112 | 0.091 | 0.22 |
| InternalIMT | aa | rs17451516 | 0.012 | 0.05 | 0.037 | 0.18 | 0.178 | 0.462 | 0.7 | -0.067 | 0.185 | 0.716 |
| InternalIMT | aa | rs139910133 | 0.003 | . | . | . | . | . | . | . | . | . |
| InternalIMT | aa | rs10900218 | 0.233 | 0.074 | 0.041 | 0.067 | -0.098 | 0.125 | 0.434 | 0.041 | 0.051 | 0.428 |
| InternalIMT | aa | rs146023999 | 0.009 | . | . | . | . | . | . | . | . | . |
| InternalIMT | aa | rs187296958 | 0.003 | . | . | . | . | . | . | . | . | . |
| InternalIMT | aa | rs142342833 | 0.015 | 0.082 | 0.083 | 0.32 | 1.46 | 1.51 | 0.333 | -0.663 | 0.611 | 0.279 |
| InternalIMT | aa | rs143489255 | 0.006 | . | . | . | . | . | . | . | . | . |
| InternalIMT | aa | rs138532518 | 0.006 | . | . | . | . | . | . | . | . | . |
| InternalIMT | aa | rs183768079 | 0.005 | . | . | . | . | . | . | . | . | . |
| InternalIMT | aa | rs117892272 | 0.012 | 0.075 | 0.081 | 0.354 | 1.74 | 2.04 | 0.396 | -0.717 | 0.868 | 0.409 |
| InternalIMT | aa | rs192993943 | 0.003 | . | . | . | . | . | . | . | . | . |
| InternalIMT | aa | rs182989043 | 0.004 | . | . | . | . | . | . | . | . | . |
| InternalIMT | aa | rs189703435 | 0.007 | . | . | . | . | . | . | . | . | . |
| InternalIMT | aa | rs188794040 | 0.007 | . | . | . | . | . | . | . | . | . |
| InternalIMT | aa | rs143920953 | 0.003 | . | . | . | . | . | . | . | . | . |
| InternalIMT | aa | rs191029121 | 0.006 | . | . | . | . | . | . | . | . | . |
| InternalIMT | aa | rs116875448 | 0.006 | . | . | . | . | . | . | . | . | . |
| InternalIMT | aa | rs190167877 | 0.002 | . | . | . | . | . | . | . | . | . |
| InternalIMT | aa | rs184993418 | 0.003 | . | . | . | . | . | . | . | . | . |
| InternalIMT | aa | rs143241158 | 0.01 | . | . | . | . | . | . | . | . | . |
| InternalIMT | aa | rs11239500 | 0.038 | 0.079 | 0.041 | 0.054 | -0.251 | 0.514 | 0.626 | 0.116 | 0.204 | 0.57 |

| InternalIMT | aa | rs140568994 | 0.001 | . | . | . | . | . | . | . | . | . |
| --- | --- | --- | --- | --- | --- | --- | --- | --- | --- | --- | --- | --- |
| InternalIMT | aa | rs187530317 | 0.003 | . | . | . | . | . | . | . | . | . |
| InternalIMT | aa | rs145189326 | 0.002 | . | . | . | . | . | . | . | . | . |
| InternalIMT | aa | rs186203332 | 0.009 | . | . | . | . | . | . | . | . | . |
| InternalIMT | aa | rs189921909 | 0.004 | . | . | . | . | . | . | . | . | . |
| InternalIMT | aa | rs187260989 | 0.01 | 0.065 | 0.081 | 0.426 | 0.578 | 1.29 | 0.655 | -0.197 | 0.507 | 0.698 |
| InternalIMT | aa | rs114376586 | 0.037 | 0.078 | 0.041 | 0.057 | -0.323 | 0.522 | 0.536 | 0.143 | 0.207 | 0.489 |
| InternalIMT | aa | rs151070808 | 0.019 | 0.044 | 0.083 | 0.597 | -1.29 | 1 | 0.197 | 0.548 | 0.433 | 0.206 |
| InternalIMT | aa | rs140927225 | 0.038 | 0.075 | 0.041 | 0.065 | -0.405 | 0.516 | 0.432 | 0.175 | 0.204 | 0.393 |
| InternalIMT | aa | rs191555615 | 0.012 | 0.082 | 0.082 | 0.315 | 2.51 | 2.18 | 0.25 | -1.05 | 0.959 | 0.274 |
| InternalIMT | aa | rs80278898 | 0.012 | 0.07 | 0.041 | 0.09 | -1.66 | 1.1 | 0.132 | 0.641 | 0.478 | 0.18 |
| InternalIMT | aa | rs190849503 | 0.004 | . | . | . | . | . | . | . | . | . |
| InternalIMT | aa | rs150080315 | 0.006 | . | . | . | . | . | . | . | . | . |
| InternalIMT | aa | rs143623885 | 0.011 | 0.079 | 0.083 | 0.341 | 1.85 | 2.34 | 0.43 | -0.814 | 0.951 | 0.392 |
| InternalIMT | aa | rs187760040 | 0.002 | . | . | . | . | . | . | . | . | . |
| InternalIMT | aa | rs143917143 | 0.01 | 0.07 | 0.052 | 0.178 | -1.34 | 0.934 | 0.151 | 0.525 | 0.397 | 0.186 |
| InternalIMT | aa | rs149851136 | 0.001 | . | . | . | . | . | . | . | . | . |
| InternalIMT | aa | rs181612054 | 0.001 | . | . | . | . | . | . | . | . | . |
| InternalIMT | aa | rs191006548 | 0.001 | . | . | . | . | . | . | . | . | . |
| InternalIMT | aa | rs117377631 | 0.012 | 0.095 | 0.041 | 0.02 | 0.212 | 0.718 | 0.768 | -0.016 | 0.304 | 0.958 |
| InternalIMT | aa | rs182107634 | 0.002 | . | . | . | . | . | . | . | . | . |
| InternalIMT | aa | rs190211867 | 0.002 | . | . | . | . | . | . | . | . | . |
| InternalIMT | aa | rs148293457 | 0.031 | 0.058 | 0.086 | 0.498 | -0.071 | 0.695 | 0.919 | 0.038 | 0.31 | 0.901 |
| InternalIMT | aa | rs191811733 | 0.006 | . | . | . | . | . | . | . | . | . |
| InternalIMT | aa | rs146109272 | 0.008 | . | . | . | . | . | . | . | . | . |
| InternalIMT | aa | rs148397915 | 0.006 | . | . | . | . | . | . | . | . | . |
| InternalIMT | aa | rs142233290 | 0.007 | . | . | . | . | . | . | . | . | . |
| InternalIMT | aa | rs80226874 | 0.022 | 0.033 | 0.081 | 0.686 | -1.07 | 1.41 | 0.449 | 0.485 | 0.593 | 0.414 |
| InternalIMT | aa | rs144496203 | 0.004 | . | . | . | . | . | . | . | . | . |
| InternalIMT | aa | rs191062130 | 0.001 | . | . | . | . | . | . | . | . | . |
| InternalIMT | aa | rs185807817 | 0.004 | . | . | . | . | . | . | . | . | . |
| InternalIMT | aa | rs117846213 | 0.012 | 0.152 | 0.053 | 4.50E-03 | 0.11 | 0.706 | 0.876 | -0.092 | 0.302 | 0.76 |
| InternalIMT | aa | rs190545473 | 0.002 | . | . | . | . | . | . | . | . | . |
| InternalIMT | aa | rs192364918 | 0.01 | 0.019 | 0.079 | 0.808 | -3.73 | 1.7 | 0.028 | 1.52 | 0.796 | 0.057 |
| InternalIMT | aa | rs182939241 | 0.003 | . | . | . | . | . | . | . | . | . |
| InternalIMT | aa | rs12571125 | 0.011 | 0.16 | 0.055 | 3.50E-03 | 1.51 | 0.938 | 0.108 | -0.716 | 0.403 | 0.075 |
| InternalIMT | aa | rs192418941 | 0.002 | . | . | . | . | . | . | . | . | . |
| InternalIMT | aa | rs141270243 | 0.015 | 0.055 | 0.083 | 0.51 | -0.509 | 0.977 | 0.602 | 0.132 | 0.427 | 0.758 |
| InternalIMT | aa | rs182615502 | 0.006 | . | . | . | . | . | . | . | . | . |
| InternalIMT | aa | rs146669856 | 0.01 | 0.08 | 0.04 | 0.048 | -1.01 | 1.08 | 0.349 | 0.433 | 0.431 | 0.315 |
| InternalIMT | aa | rs78290029 | 0.053 | 0.074 | 0.042 | 0.077 | -0.13 | 0.398 | 0.744 | 0.08 | 0.16 | 0.616 |
| InternalIMT | aa | rs139955565 | 0.004 | . | . | . | . | . | . | . | . | . |
| InternalIMT | aa | rs12267314 | 0.047 | 0.078 | 0.042 | 0.061 | -0.112 | 0.413 | 0.786 | 0.067 | 0.166 | 0.687 |
| InternalIMT | aa | rs74477197 | 0.004 | . | . | . | . | . | . | . | . | . |
| InternalIMT | aa | rs188147236 | 0.005 | . | . | . | . | . | . | . | . | . |
| InternalIMT | aa | rs2228065 | 0.046 | 0.08 | 0.042 | 0.056 | -0.052 | 0.418 | 0.901 | 0.044 | 0.168 | 0.793 |
| InternalIMT | aa | rs116961353 | 0.01 | . | . | . | . | . | . | . | . | . |
| InternalIMT | aa | rs190222272 | 0.007 | . | . | . | . | . | . | . | . | . |
| InternalIMT | aa | rs182719940 | 0.006 | . | . | . | . | . | . | . | . | . |
| InternalIMT | aa | rs184477413 | 0.004 | . | . | . | . | . | . | . | . | . |
| InternalIMT | aa | rs12265175 | 0.048 | 0.079 | 0.042 | 0.061 | -0.062 | 0.406 | 0.878 | 0.049 | 0.163 | 0.761 |
| InternalIMT | aa | rs182928276 | 0.003 | . | . | . | . | . | . | . | . | . |
| InternalIMT | aa | rs144409895 | 0.011 | 0.019 | 0.079 | 0.807 | -3.5 | 1.62 | 0.031 | 1.41 | 0.759 | 0.063 |
| InternalIMT | aa | rs58998447 | 0.031 | 0.069 | 0.042 | 0.095 | -0.547 | 0.386 | 0.156 | 0.196 | 0.171 | 0.252 |
| InternalIMT | aa | rs188668832 | 0.005 | . | . | . | . | . | . | . | . | . |
| InternalIMT | aa | rs148641686 | 0.002 | . | . | . | . | . | . | . | . | . |
| InternalIMT | aa | rs75468791 | 0.054 | 0.039 | 0.085 | 0.642 | -0.347 | 0.752 | 0.645 | 0.174 | 0.335 | 0.603 |
| InternalIMT | aa | rs147327397 | 0.003 | . | . | . | . | . | . | . | . | . |
| InternalIMT | aa | rs77354597 | 0.007 | . | . | . | . | . | . | . | . | . |
| InternalIMT | aa | rs41365544 | 0.045 | 0.078 | 0.042 | 0.06 | -0.145 | 0.401 | 0.717 | 0.078 | 0.162 | 0.629 |
| InternalIMT | aa | rs187564188 | 0.016 | -1.60E-03 | 0.082 | 0.985 | -3.54 | 2.02 | 0.081 | 1.57 | 0.97 | 0.106 |
| InternalIMT | aa | rs183511984 | 0.002 | . | . | . | . | . | . | . | . | . |
| InternalIMT | aa | rs12262783 | 0.038 | 0.082 | 0.042 | 0.048 | 0.149 | 0.51 | 0.77 | -0.027 | 0.201 | 0.893 |
| InternalIMT | aa | rs188817687 | 0.002 | . | . | . | . | . | . | . | . | . |
| InternalIMT | aa | rs3815968 | 0.005 | . | . | . | . | . | . | . | . | . |
| InternalIMT | aa | rs187822300 | 0.001 | . | . | . | . | . | . | . | . | . |
| InternalIMT | aa | rs188902172 | 0.008 | . | . | . | . | . | . | . | . | . |
| InternalIMT | aa | rs181190448 | 0.006 | . | . | . | . | . | . | . | . | . |
| InternalIMT | aa | rs182699093 | 0.001 | . | . | . | . | . | . | . | . | . |
| InternalIMT | aa | rs182631303 | 0.005 | . | . | . | . | . | . | . | . | . |
| InternalIMT | aa | rs187014172 | 0.003 | . | . | . | . | . | . | . | . | . |
| InternalIMT | aa | rs142575406 | 0.009 | . | . | . | . | . | . | . | . | . |
| InternalIMT | aa | rs180711033 | 0.007 | . | . | . | . | . | . | . | . | . |
| InternalIMT | aa | rs137894823 | 0.002 | . | . | . | . | . | . | . | . | . |
| InternalIMT | aa | rs189053273 | 0.001 | . | . | . | . | . | . | . | . | . |
| InternalIMT | aa | rs143499388 | 0.002 | . | . | . | . | . | . | . | . | . |
| InternalIMT | aa | rs182936001 | 0.003 | . | . | . | . | . | . | . | . | . |
| InternalIMT | aa | rs148456406 | 0.003 | . | . | . | . | . | . | . | . | . |
| InternalIMT | aa | rs182499531 | 0.005 | . | . | . | . | . | . | . | . | . |
| InternalIMT | aa | rs74884915 | 0.016 | 0.021 | 0.05 | 0.671 | -0.175 | 0.418 | 0.675 | 0.048 | 0.168 | 0.775 |
| InternalIMT | aa | rs184046374 | 0.001 | . | . | . | . | . | . | . | . | . |
| InternalIMT | aa | rs74333993 | 0.019 | 0.019 | 0.048 | 0.699 | -0.293 | 0.93 | 0.753 | 0.135 | 0.367 | 0.714 |
| InternalIMT | aa | rs138889719 | 0.001 | . | . | . | . | . | . | . | . | . |
| InternalIMT | aa | rs75330330 | 0.013 | 0.015 | 0.047 | 0.753 | -0.936 | 1.04 | 0.37 | 0.395 | 0.416 | 0.343 |
| InternalIMT | aa | rs193051693 | 0 | . | . | . | . | . | . | . | . | . |
| InternalIMT | aa | rs145929633 | 0 | . | . | . | . | . | . | . | . | . |

| InternalIMT | aa | rs184076904 | 0.003 | . | . | . | . | . | . | . | . | . |
| --- | --- | --- | --- | --- | --- | --- | --- | --- | --- | --- | --- | --- |
| InternalIMT | aa | rs141802494 | 0.011 | 0.016 | 0.047 | 0.741 | -1.26 | 1.48 | 0.392 | 0.531 | 0.587 | 0.366 |
| InternalIMT | aa | rs149519759 | 0.001 | . | . | . | . | . | . | . | . | . |
| InternalIMT | aa | rs145467260 | 0.008 | . | . | . | . | . | . | . | . | . |
| InternalIMT | aa | rs79620914 | 0.017 | 0.025 | 0.048 | 0.606 | 0.036 | 0.547 | 0.948 | -0.024 | 0.217 | 0.912 |
| InternalIMT | aa | rs140538633 | 0.011 | 0.022 | 0.048 | 0.651 | -0.494 | 0.626 | 0.43 | 0.159 | 0.254 | 0.53 |
| InternalIMT | aa | rs140862301 | 0.005 | . | . | . | . | . | . | . | . | . |
| InternalIMT | aa | rs114615847 | 0.006 | . | . | . | . | . | . | . | . | . |
| InternalIMT | aa | rs141800259 | 0.016 | 0.023 | 0.048 | 0.628 | 0.15 | 0.801 | 0.852 | -0.036 | 0.32 | 0.909 |
| InternalIMT | aa | rs149779059 | 0.011 | 0.02 | 0.048 | 0.68 | -0.283 | 0.801 | 0.724 | 0.143 | 0.315 | 0.649 |
| InternalIMT | aa | rs116539253 | 0.004 | . | . | . | . | . | . | . | . | . |
| InternalIMT | aa | rs184979550 | 0.001 | . | . | . | . | . | . | . | . | . |
| InternalIMT | aa | rs190128940 | 0.003 | . | . | . | . | . | . | . | . | . |
| InternalIMT | aa | rs146594816 | 0.002 | . | . | . | . | . | . | . | . | . |
| InternalIMT | aa | rs141370766 | 0.013 | 0.015 | 0.047 | 0.753 | -0.932 | 1.05 | 0.374 | 0.394 | 0.418 | 0.346 |
| InternalIMT | aa | rs144670033 | 0.002 | . | . | . | . | . | . | . | . | . |
| InternalIMT | aa | rs187152174 | 0.005 | . | . | . | . | . | . | . | . | . |
| InternalIMT | aa | rs184180263 | 0.003 | . | . | . | . | . | . | . | . | . |
| InternalIMT | aa | rs140075861 | 0.007 | . | . | . | . | . | . | . | . | . |
| InternalIMT | aa | rs186483129 | 0 | . | . | . | . | . | . | . | . | . |
| InternalIMT | aa | rs186634282 | 0.008 | . | . | . | . | . | . | . | . | . |
| InternalIMT | aa | rs9988663 | 0.019 | 0.019 | 0.048 | 0.699 | -0.294 | 0.929 | 0.752 | 0.135 | 0.367 | 0.712 |
| InternalIMT | aa | rs138828529 | 0.013 | 0.029 | 0.047 | 0.544 | 0.942 | 1.53 | 0.538 | -0.347 | 0.589 | 0.556 |
| InternalIMT | aa | rs188777615 | 0.003 | . | . | . | . | . | . | . | . | . |
| InternalIMT | aa | rs192321882 | 0.005 | . | . | . | . | . | . | . | . | . |
| InternalIMT | aa | rs150812270 | 0.016 | 0.041 | 0.048 | 0.392 | 1.84 | 0.734 | 0.012 | -0.718 | 0.281 | 0.011 |
| InternalIMT | aa | rs185906603 | 0.001 | . | . | . | . | . | . | . | . | . |
| InternalIMT | aa | rs191070862 | 0.004 | . | . | . | . | . | . | . | . | . |
| InternalIMT | aa | rs182904978 | 0 | . | . | . | . | . | . | . | . | . |
| InternalIMT | aa | rs192679655 | 0.001 | . | . | . | . | . | . | . | . | . |
| InternalIMT | aa | rs189673086 | 0.005 | . | . | . | . | . | . | . | . | . |
| InternalIMT | aa | rs181141892 | 0.002 | . | . | . | . | . | . | . | . | . |
| InternalIMT | aa | rs186404123 | 0 | . | . | . | . | . | . | . | . | . |
| InternalIMT | aa | rs192117287 | 0.003 | . | . | . | . | . | . | . | . | . |
| InternalIMT | aa | rs182748835 | 0.001 | . | . | . | . | . | . | . | . | . |
| InternalIMT | aa | rs141854549 | 0.011 | 0.022 | 0.048 | 0.647 | -0.528 | 0.628 | 0.4 | 0.173 | 0.254 | 0.497 |
| InternalIMT | aa | rs140163397 | 0.003 | . | . | . | . | . | . | . | . | . |
| InternalIMT | aa | rs146347300 | 0.005 | . | . | . | . | . | . | . | . | . |
| InternalIMT | aa | rs139362377 | 0.011 | 0.022 | 0.048 | 0.647 | -0.53 | 0.627 | 0.398 | 0.174 | 0.254 | 0.494 |
| InternalIMT | aa | rs185087671 | 0.001 | . | . | . | . | . | . | . | . | . |
| InternalIMT | aa | rs190466574 | 0.003 | . | . | . | . | . | . | . | . | . |
| InternalIMT | aa | rs147012778 | 0.003 | . | . | . | . | . | . | . | . | . |
| InternalIMT | aa | rs138236960 | 0.006 | . | . | . | . | . | . | . | . | . |
| InternalIMT | aa | rs116310311 | 0.011 | 0.022 | 0.048 | 0.647 | -0.53 | 0.627 | 0.398 | 0.174 | 0.254 | 0.494 |
| InternalIMT | aa | rs142868356 | 0.008 | . | . | . | . | . | . | . | . | . |
| InternalIMT | aa | rs144956240 | 0.002 | . | . | . | . | . | . | . | . | . |
| InternalIMT | aa | rs188527776 | 0.002 | . | . | . | . | . | . | . | . | . |
| InternalIMT | aa | rs183971165 | 0.005 | . | . | . | . | . | . | . | . | . |
| InternalIMT | aa | rs76748216 | 0.006 | . | . | . | . | . | . | . | . | . |
| InternalIMT | aa | rs148554990 | 0.001 | . | . | . | . | . | . | . | . | . |
| InternalIMT | aa | rs114446477 | 0.017 | 0.023 | 0.048 | 0.634 | -0.107 | 0.546 | 0.844 | 0.034 | 0.218 | 0.877 |
| InternalIMT | aa | rs185223933 | 0.004 | . | . | . | . | . | . | . | . | . |
| InternalIMT | aa | rs76869726 | 0.006 | . | . | . | . | . | . | . | . | . |
| InternalIMT | aa | rs151200830 | 0.011 | 0.022 | 0.048 | 0.652 | -0.558 | 0.63 | 0.376 | 0.187 | 0.256 | 0.465 |
| InternalIMT | aa | rs17157731 | 0.011 | 0.022 | 0.048 | 0.652 | -0.558 | 0.63 | 0.376 | 0.187 | 0.256 | 0.465 |
| InternalIMT | aa | rs181243469 | 0 | . | . | . | . | . | . | . | . | . |
| InternalIMT | aa | rs186890707 | 0 | . | . | . | . | . | . | . | . | . |
| InternalIMT | aa | rs184063360 | 0 | . | . | . | . | . | . | . | . | . |
| InternalIMT | aa | rs188550237 | 0.004 | . | . | . | . | . | . | . | . | . |
| InternalIMT | aa | rs140931068 | 0.01 | 0.02 | 0.048 | 0.677 | 0.103 | 1.19 | 0.931 | 0.018 | 0.467 | 0.969 |
| InternalIMT | aa | rs74867877 | 0.004 | . | . | . | . | . | . | . | . | . |
| InternalIMT | aa | rs11239495 | 0.036 | 0.011 | 0.048 | 0.824 | -0.428 | 0.63 | 0.497 | 0.194 | 0.251 | 0.441 |
| InternalIMT | aa | rs149745800 | 0 | . | . | . | . | . | . | . | . | . |
| InternalIMT | aa | rs145044458 | 0.01 | 0.021 | 0.048 | 0.656 | -0.581 | 0.694 | 0.402 | 0.2 | 0.282 | 0.478 |
| InternalIMT | aa | rs188067427 | 0.001 | . | . | . | . | . | . | . | . | . |
| InternalIMT | aa | rs146827800 | 0.001 | . | . | . | . | . | . | . | . | . |
| InternalIMT | aa | rs140619817 | 0.01 | 0.021 | 0.048 | 0.656 | -0.575 | 0.694 | 0.408 | 0.198 | 0.282 | 0.482 |
| InternalIMT | aa | rs892689 | 0.997 | . | . | . | . | . | . | . | . | . |
| InternalIMT | aa | rs143642005 | 0.004 | . | . | . | . | . | . | . | . | . |
| InternalIMT | aa | rs181929304 | 0.004 | . | . | . | . | . | . | . | . | . |
| InternalIMT | aa | rs191086173 | 0.001 | . | . | . | . | . | . | . | . | . |
| InternalIMT | aa | rs114613116 | 0.012 | 0.022 | 0.048 | 0.651 | -0.539 | 0.628 | 0.391 | 0.182 | 0.254 | 0.475 |
| InternalIMT | aa | rs141076768 | 0.002 | . | . | . | . | . | . | . | . | . |
| InternalIMT | aa | rs187316531 | 0 | . | . | . | . | . | . | . | . | . |
| InternalIMT | aa | rs11239499 | 0.046 | 8.20E-03 | 0.048 | 0.866 | -0.46 | 0.527 | 0.383 | 0.192 | 0.209 | 0.357 |
| InternalIMT | aa | rs192726371 | 0.002 | . | . | . | . | . | . | . | . | . |
| InternalIMT | aa | rs74128481 | 0.012 | 0.01 | 0.048 | 0.827 | -1.55 | 1.02 | 0.126 | 0.647 | 0.405 | 0.11 |
| InternalIMT | aa | rs74128482 | 0.008 | . | . | . | . | . | . | . | . | . |
| InternalIMT | aa | rs11239502 | 0.033 | 0.01 | 0.048 | 0.833 | -0.489 | 0.712 | 0.493 | 0.217 | 0.281 | 0.439 |
| InternalIMT | aa | rs116330285 | 0.002 | . | . | . | . | . | . | . | . | . |
| InternalIMT | aa | rs189926729 | 0.002 | . | . | . | . | . | . | . | . | . |
| InternalIMT | aa | rs188975779 | 0.008 | . | . | . | . | . | . | . | . | . |
| InternalIMT | aa | rs143846038 | 0.002 | . | . | . | . | . | . | . | . | . |
| InternalIMT | aa | rs74128483 | 0.012 | 0.01 | 0.048 | 0.828 | -1.56 | 1.02 | 0.125 | 0.648 | 0.405 | 0.109 |
| InternalIMT | aa | rs139292055 | 0.045 | 8.40E-03 | 0.048 | 0.862 | -0.465 | 0.528 | 0.379 | 0.194 | 0.209 | 0.353 |
| InternalIMT | aa | rs11239503 | 0.045 | 8.40E-03 | 0.048 | 0.862 | -0.465 | 0.528 | 0.379 | 0.194 | 0.209 | 0.353 |

| InternalIMT | aa | rs58838686 | 0.008 | . | . | . | . | . | . | . | . | . |
| --- | --- | --- | --- | --- | --- | --- | --- | --- | --- | --- | --- | --- |
| InternalIMT | aa | rs185825373 | 0.005 | . | . | . | . | . | . | . | . | . |
| InternalIMT | aa | rs11239504 | 0.045 | 8.40E-03 | 0.048 | 0.862 | -0.465 | 0.528 | 0.379 | 0.194 | 0.209 | 0.353 |
| InternalIMT | aa | rs150012354 | 0.003 | . | . | . | . | . | . | . | . | . |
| InternalIMT | aa | rs187763096 | 0.005 | . | . | . | . | . | . | . | . | . |
| InternalIMT | aa | rs193043982 | 0.005 | . | . | . | . | . | . | . | . | . |
| InternalIMT | aa | rs79186884 | 0.014 | 0.021 | 0.048 | 0.655 | -0.016 | 0.664 | 0.981 | 0.037 | 0.259 | 0.887 |
| InternalIMT | aa | rs74128484 | 0.008 | . | . | . | . | . | . | . | . | . |
| InternalIMT | aa | rs58461537 | 0.045 | 8.40E-03 | 0.048 | 0.863 | -0.465 | 0.528 | 0.378 | 0.194 | 0.209 | 0.352 |
| InternalIMT | aa | rs188527474 | 0.005 | . | . | . | . | . | . | . | . | . |
| InternalIMT | aa | rs141659976 | 0.005 | . | . | . | . | . | . | . | . | . |
| InternalIMT | aa | rs115846407 | 0.045 | 8.40E-03 | 0.048 | 0.863 | -0.465 | 0.528 | 0.378 | 0.194 | 0.209 | 0.352 |
| InternalIMT | aa | rs138378816 | 0.045 | 8.40E-03 | 0.048 | 0.863 | -0.465 | 0.528 | 0.378 | 0.194 | 0.209 | 0.352 |
| InternalIMT | aa | rs146188136 | 0.033 | 0.01 | 0.048 | 0.833 | -0.489 | 0.711 | 0.491 | 0.218 | 0.28 | 0.438 |
| InternalIMT | aa | rs139383871 | 0.008 | . | . | . | . | . | . | . | . | . |
| InternalIMT | aa | rs182953869 | 0.004 | . | . | . | . | . | . | . | . | . |
| InternalIMT | aa | rs192430299 | 0.012 | 0.019 | 0.047 | 0.686 | 0.519 | 1.18 | 0.66 | -0.14 | 0.45 | 0.756 |
| InternalIMT | aa | rs144152844 | 0.045 | 8.40E-03 | 0.048 | 0.863 | -0.465 | 0.528 | 0.378 | 0.194 | 0.209 | 0.352 |
| InternalIMT | aa | rs145987786 | 0.033 | 9.70E-03 | 0.048 | 0.84 | -0.647 | 0.623 | 0.299 | 0.266 | 0.245 | 0.277 |
| InternalIMT | aa | rs181132926 | 0.002 | . | . | . | . | . | . | . | . | . |
| InternalIMT | aa | rs147882253 | 0.045 | 8.40E-03 | 0.048 | 0.863 | -0.465 | 0.528 | 0.379 | 0.194 | 0.209 | 0.352 |
| InternalIMT | aa | rs141807621 | 0.015 | 0.03 | 0.047 | 0.529 | 0.795 | 1.33 | 0.55 | -0.302 | 0.511 | 0.554 |
| InternalIMT | aa | rs147116052 | 0.045 | 8.40E-03 | 0.048 | 0.863 | -0.465 | 0.528 | 0.378 | 0.194 | 0.209 | 0.352 |
| InternalIMT | aa | rs147716618 | 0.045 | 8.40E-03 | 0.048 | 0.863 | -0.465 | 0.528 | 0.378 | 0.194 | 0.209 | 0.352 |
| InternalIMT | aa | rs187198855 | 0.029 | 6.70E-03 | 0.048 | 0.889 | -0.802 | 0.758 | 0.29 | 0.339 | 0.296 | 0.252 |
| InternalIMT | aa | rs189409638 | 0.011 | 0.016 | 0.048 | 0.74 | -0.617 | 1.09 | 0.571 | 0.283 | 0.435 | 0.514 |
| InternalIMT | aa | rs182647339 | 0.001 | . | . | . | . | . | . | . | . | . |
| InternalIMT | aa | rs187995988 | 0.004 | . | . | . | . | . | . | . | . | . |
| InternalIMT | aa | rs141034184 | 0.048 | 0.015 | 0.049 | 0.751 | -0.245 | 0.503 | 0.626 | 0.1 | 0.199 | 0.617 |
| InternalIMT | aa | rs187310430 | 0.005 | . | . | . | . | . | . | . | . | . |
| InternalIMT | aa | rs192608488 | 0.045 | 8.40E-03 | 0.048 | 0.863 | -0.463 | 0.527 | 0.38 | 0.194 | 0.208 | 0.353 |
| InternalIMT | aa | rs148840366 | 0.006 | . | . | . | . | . | . | . | . | . |
| InternalIMT | aa | rs186785022 | 0.012 | 0.01 | 0.048 | 0.829 | -1.57 | 1.02 | 0.122 | 0.653 | 0.404 | 0.106 |
| InternalIMT | aa | rs192348728 | 0.008 | . | . | . | . | . | . | . | . | . |
| InternalIMT | aa | rs189656631 | 0.045 | 8.40E-03 | 0.048 | 0.863 | -0.462 | 0.527 | 0.38 | 0.194 | 0.208 | 0.353 |
| InternalIMT | aa | rs185289750 | 0.004 | . | . | . | . | . | . | . | . | . |
| InternalIMT | aa | rs142382395 | 0.045 | 8.40E-03 | 0.048 | 0.863 | -0.462 | 0.526 | 0.38 | 0.194 | 0.208 | 0.353 |
| InternalIMT | aa | rs144866696 | 0.012 | 0.01 | 0.048 | 0.829 | -1.57 | 1.02 | 0.122 | 0.654 | 0.404 | 0.106 |
| InternalIMT | aa | rs141371289 | 0.045 | 8.40E-03 | 0.048 | 0.863 | -0.462 | 0.526 | 0.38 | 0.194 | 0.208 | 0.353 |
| InternalIMT | aa | rs114750008 | 0.045 | 8.40E-03 | 0.048 | 0.863 | -0.462 | 0.526 | 0.38 | 0.194 | 0.208 | 0.353 |
| InternalIMT | aa | rs189063597 | 0.001 | . | . | . | . | . | . | . | . | . |
| InternalIMT | aa | rs139151571 | 0.041 | 0.011 | 0.048 | 0.819 | -0.412 | 0.586 | 0.482 | 0.173 | 0.231 | 0.455 |
| InternalIMT | aa | rs139846368 | 0.046 | 7.80E-03 | 0.048 | 0.872 | -0.469 | 0.525 | 0.371 | 0.196 | 0.208 | 0.347 |
| InternalIMT | aa | rs182057653 | 0.012 | 0.034 | 0.05 | 0.492 | 0.612 | 0.53 | 0.248 | -0.259 | 0.207 | 0.212 |
| InternalIMT | aa | rs148785420 | 0.045 | 8.40E-03 | 0.048 | 0.863 | -0.462 | 0.526 | 0.38 | 0.193 | 0.208 | 0.353 |
| InternalIMT | aa | rs138383251 | 0.007 | . | . | . | . | . | . | . | . | . |
| InternalIMT | aa | rs116526705 | 0.012 | 0.022 | 0.048 | 0.65 | -0.502 | 0.626 | 0.422 | 0.17 | 0.253 | 0.502 |
| InternalIMT | aa | rs185946436 | 0 | . | . | . | . | . | . | . | . | . |
| InternalIMT | aa | rs190455427 | 0.004 | . | . | . | . | . | . | . | . | . |
| InternalIMT | aa | rs9282584 | 0.045 | 8.40E-03 | 0.048 | 0.863 | -0.462 | 0.526 | 0.38 | 0.194 | 0.208 | 0.353 |
| InternalIMT | aa | rs17153289 | 0.045 | 8.40E-03 | 0.048 | 0.863 | -0.462 | 0.526 | 0.38 | 0.193 | 0.208 | 0.353 |
| InternalIMT | aa | rs138359103 | 0.005 | . | . | . | . | . | . | . | . | . |
| InternalIMT | aa | rs150605268 | 0.002 | . | . | . | . | . | . | . | . | . |
| InternalIMT | aa | rs79242910 | 0.046 | 8.10E-03 | 0.049 | 0.868 | -0.465 | 0.508 | 0.36 | 0.194 | 0.201 | 0.336 |
| InternalIMT | aa | rs139716904 | 0.013 | 0.022 | 0.048 | 0.645 | -0.487 | 0.579 | 0.4 | 0.159 | 0.235 | 0.498 |
| InternalIMT | aa | rs76238498 | 0.034 | 0.013 | 0.048 | 0.791 | -0.405 | 0.646 | 0.53 | 0.173 | 0.252 | 0.494 |
| InternalIMT | aa | rs184140639 | 0 | . | . | . | . | . | . | . | . | . |
| InternalIMT | aa | rs188517278 | 0.006 | . | . | . | . | . | . | . | . | . |
| InternalIMT | aa | rs139678913 | 0.002 | . | . | . | . | . | . | . | . | . |
| InternalIMT | aa | rs116697955 | 0.004 | . | . | . | . | . | . | . | . | . |
| InternalIMT | aa | rs79267994 | 0.007 | . | . | . | . | . | . | . | . | . |
| InternalIMT | aa | rs11239510 | 0.013 | 0.023 | 0.048 | 0.629 | -0.178 | 0.661 | 0.788 | 0.056 | 0.253 | 0.825 |
| InternalIMT | aa | rs75831645 | 0.012 | 0.022 | 0.048 | 0.65 | -0.499 | 0.625 | 0.424 | 0.169 | 0.253 | 0.504 |
| InternalIMT | aa | rs78452276 | 0.031 | 0.044 | 0.05 | 0.383 | 0.624 | 0.325 | 0.055 | -0.24 | 0.126 | 0.057 |
| InternalIMT | aa | rs144366924 | 0 | . | . | . | . | . | . | . | . | . |
| InternalIMT | aa | rs77519841 | 0.033 | 9.90E-03 | 0.048 | 0.837 | -0.501 | 0.71 | 0.48 | 0.222 | 0.28 | 0.428 |
| InternalIMT | aa | rs76944454 | 0.045 | 8.20E-03 | 0.048 | 0.866 | -0.471 | 0.526 | 0.371 | 0.197 | 0.208 | 0.345 |
| InternalIMT | aa | rs76443855 | 0.034 | 0.013 | 0.048 | 0.79 | -0.405 | 0.646 | 0.531 | 0.172 | 0.252 | 0.495 |
| InternalIMT | aa | rs146613174 | 0.001 | . | . | . | . | . | . | . | . | . |
| InternalIMT | aa | rs181424761 | 0.001 | . | . | . | . | . | . | . | . | . |
| InternalIMT | aa | rs142119241 | 0.001 | . | . | . | . | . | . | . | . | . |
| InternalIMT | aa | rs140137954 | 0.012 | 0.022 | 0.048 | 0.65 | -0.501 | 0.625 | 0.423 | 0.169 | 0.253 | 0.503 |
| InternalIMT | aa | rs187319549 | 0.002 | . | . | . | . | . | . | . | . | . |
| InternalIMT | aa | rs12241545 | 0.021 | 0.034 | 0.048 | 0.486 | 0.395 | 0.574 | 0.491 | -0.197 | 0.222 | 0.375 |
| InternalIMT | aa | rs10128228 | 0.045 | 8.20E-03 | 0.048 | 0.865 | -0.47 | 0.526 | 0.372 | 0.196 | 0.208 | 0.346 |
| InternalIMT | aa | rs58580249 | 0.006 | . | . | . | . | . | . | . | . | . |
| InternalIMT | aa | rs193148863 | 0.001 | . | . | . | . | . | . | . | . | . |
| InternalIMT | aa | rs185638946 | 0.005 | . | . | . | . | . | . | . | . | . |
| InternalIMT | aa | rs117752921 | 0.001 | . | . | . | . | . | . | . | . | . |
| InternalIMT | aa | rs180841236 | 0.001 | . | . | . | . | . | . | . | . | . |
| InternalIMT | aa | rs73283155 | 0.006 | . | . | . | . | . | . | . | . | . |
| InternalIMT | aa | rs76560599 | 0.038 | 0.015 | 0.048 | 0.763 | -0.326 | 0.596 | 0.584 | 0.135 | 0.235 | 0.565 |
| InternalIMT | aa | rs76273620 | 0.038 | 0.015 | 0.048 | 0.763 | -0.326 | 0.596 | 0.584 | 0.135 | 0.235 | 0.565 |
| InternalIMT | aa | rs141432923 | 0.012 | 0.013 | 0.048 | 0.78 | -1.69 | 1.35 | 0.213 | 0.665 | 0.521 | 0.202 |
| InternalIMT | aa | rs113529322 | 0.012 | 0.01 | 0.048 | 0.831 | -1.59 | 1.02 | 0.119 | 0.661 | 0.406 | 0.104 |
| InternalIMT | aa | rs10128306 | 0.045 | 8.30E-03 | 0.048 | 0.865 | -0.467 | 0.528 | 0.376 | 0.196 | 0.209 | 0.349 |

| InternalIMT | aa | rs149691688 | 0.001 | . | . | . | . | . | . | . | . | . |
| --- | --- | --- | --- | --- | --- | --- | --- | --- | --- | --- | --- | --- |
| InternalIMT | aa | rs75872495 | 0.045 | 8.20E-03 | 0.048 | 0.865 | -0.468 | 0.529 | 0.376 | 0.196 | 0.209 | 0.349 |
| InternalIMT | aa | rs191873960 | 0.004 | . | . | . | . | . | . | . | . | . |
| InternalIMT | aa | rs80155622 | 0.045 | 8.40E-03 | 0.048 | 0.862 | -0.462 | 0.53 | 0.383 | 0.193 | 0.209 | 0.356 |
| InternalIMT | aa | rs113914656 | 0.005 | . | . | . | . | . | . | . | . | . |
| InternalIMT | aa | rs12242840 | 0.045 | 8.60E-03 | 0.048 | 0.859 | -0.453 | 0.529 | 0.391 | 0.19 | 0.209 | 0.363 |
| InternalIMT | aa | rs147824078 | 0.006 | . | . | . | . | . | . | . | . | . |
| InternalIMT | aa | rs59051493 | 0.045 | 8.60E-03 | 0.048 | 0.86 | -0.449 | 0.529 | 0.396 | 0.189 | 0.209 | 0.367 |
| InternalIMT | aa | rs9282588 | 0.005 | . | . | . | . | . | . | . | . | . |
| InternalIMT | aa | rs150215207 | 0.006 | . | . | . | . | . | . | . | . | . |
| InternalIMT | aa | rs138772243 | 0.002 | . | . | . | . | . | . | . | . | . |
| InternalIMT | aa | rs149383244 | 0.014 | 0.041 | 0.049 | 0.402 | 0.606 | 0.358 | 0.091 | -0.249 | 0.145 | 0.086 |
| InternalIMT | aa | rs17522720 | 0.011 | 0.031 | 0.048 | 0.518 | 0.372 | 0.484 | 0.443 | -0.154 | 0.204 | 0.452 |
| InternalIMT | aa | rs79245105 | 0.045 | 8.60E-03 | 0.048 | 0.86 | -0.447 | 0.529 | 0.398 | 0.188 | 0.209 | 0.369 |
| InternalIMT | aa | rs138222133 | 0.002 | . | . | . | . | . | . | . | . | . |
| InternalIMT | aa | rs12247314 | 0.05 | 1.00E-02 | 0.049 | 0.838 | -0.339 | 0.493 | 0.492 | 0.144 | 0.194 | 0.458 |
| InternalIMT | aa | rs12263894 | 0.049 | 0.013 | 0.049 | 0.797 | -0.249 | 0.509 | 0.625 | 0.109 | 0.2 | 0.585 |
| InternalIMT | aa | rs149907880 | 0.005 | . | . | . | . | . | . | . | . | . |
| InternalIMT | aa | rs188153885 | 0.005 | . | . | . | . | . | . | . | . | . |
| InternalIMT | aa | rs73283159 | 0.003 | . | . | . | . | . | . | . | . | . |
| InternalIMT | aa | rs140476687 | 0.002 | . | . | . | . | . | . | . | . | . |
| InternalIMT | aa | rs186603148 | 0 | . | . | . | . | . | . | . | . | . |
| InternalIMT | aa | rs183296063 | 0.002 | . | . | . | . | . | . | . | . | . |
| InternalIMT | aa | rs145733694 | 0.006 | . | . | . | . | . | . | . | . | . |
| InternalIMT | aa | rs189807449 | 0 | . | . | . | . | . | . | . | . | . |
| InternalIMT | aa | rs12253223 | 0.038 | 0.011 | 0.048 | 0.815 | -0.321 | 0.658 | 0.626 | 0.149 | 0.259 | 0.566 |
| InternalIMT | aa | rs182113637 | 0 | . | . | . | . | . | . | . | . | . |
| InternalIMT | aa | rs142792099 | 0.01 | 0.016 | 0.047 | 0.734 | -1.16 | 1.52 | 0.444 | 0.493 | 0.603 | 0.413 |
| InternalIMT | aa | rs12249910 | 0.038 | 0.011 | 0.048 | 0.814 | -0.32 | 0.658 | 0.627 | 0.148 | 0.259 | 0.567 |
| InternalIMT | aa | rs149436339 | 0.001 | . | . | . | . | . | . | . | . | . |
| InternalIMT | aa | rs77632210 | 0 | . | . | . | . | . | . | . | . | . |
| InternalIMT | aa | rs74130818 | 0.008 | . | . | . | . | . | . | . | . | . |
| InternalIMT | aa | rs187583648 | 0 | . | . | . | . | . | . | . | . | . |
| InternalIMT | aa | rs191295987 | 0.004 | . | . | . | . | . | . | . | . | . |
| InternalIMT | aa | rs184200686 | 0.006 | . | . | . | . | . | . | . | . | . |
| InternalIMT | aa | rs76117263 | 0.033 | 0.012 | 0.048 | 0.81 | -0.441 | 0.664 | 0.507 | 0.189 | 0.26 | 0.467 |
| InternalIMT | aa | rs185393149 | 0.001 | . | . | . | . | . | . | . | . | . |
| InternalIMT | aa | rs144760014 | 0.003 | . | . | . | . | . | . | . | . | . |
| InternalIMT | aa | rs7922292 | 0.005 | . | . | . | . | . | . | . | . | . |
| InternalIMT | aa | rs115372844 | 0.006 | . | . | . | . | . | . | . | . | . |
| InternalIMT | aa | rs111460364 | 0.033 | 0.011 | 0.048 | 0.812 | -0.447 | 0.668 | 0.503 | 0.192 | 0.261 | 0.463 |
| InternalIMT | aa | rs182052255 | 0.001 | . | . | . | . | . | . | . | . | . |
| InternalIMT | aa | rs180983889 | 0.004 | . | . | . | . | . | . | . | . | . |
| InternalIMT | aa | rs139175327 | 0.006 | . | . | . | . | . | . | . | . | . |
| InternalIMT | aa | rs149959620 | 0.011 | 0.023 | 0.048 | 0.638 | -0.345 | 0.602 | 0.566 | 0.109 | 0.244 | 0.654 |
| InternalIMT | aa | rs114653117 | 0.005 | . | . | . | . | . | . | . | . | . |
| InternalIMT | aa | rs144270018 | 0.003 | . | . | . | . | . | . | . | . | . |
| InternalIMT | aa | rs182723204 | 0.005 | . | . | . | . | . | . | . | . | . |
| InternalIMT | aa | rs57204067 | 0.008 | . | . | . | . | . | . | . | . | . |
| InternalIMT | aa | rs144956226 | 0.038 | 0.012 | 0.048 | 0.797 | -0.281 | 0.672 | 0.675 | 0.132 | 0.264 | 0.618 |
| InternalIMT | aa | rs187842704 | 0.01 | 0.022 | 0.048 | 0.649 | 1.03 | 1.37 | 0.454 | -0.336 | 0.535 | 0.53 |
| InternalIMT | aa | rs184620511 | 0.008 | . | . | . | . | . | . | . | . | . |
| InternalIMT | aa | rs144031303 | 0.012 | 0.022 | 0.048 | 0.648 | -0.455 | 0.648 | 0.483 | 0.153 | 0.26 | 0.557 |
| InternalIMT | aa | rs187526473 | 0 | . | . | . | . | . | . | . | . | . |
| InternalIMT | aa | rs193002805 | 0.002 | . | . | . | . | . | . | . | . | . |
| InternalIMT | aa | rs138707426 | 0.014 | 0.04 | 0.049 | 0.418 | 0.59 | 0.374 | 0.115 | -0.239 | 0.151 | 0.113 |
| InternalIMT | aa | rs142754243 | 0.03 | 0.029 | 0.049 | 0.546 | 0.409 | 0.528 | 0.438 | -0.159 | 0.205 | 0.438 |
| InternalIMT | aa | rs181600331 | 0.005 | . | . | . | . | . | . | . | . | . |
| InternalIMT | aa | rs144421264 | 0.018 | 0.03 | 0.048 | 0.539 | 0.648 | 0.709 | 0.361 | -0.254 | 0.275 | 0.357 |
| InternalIMT | aa | rs184414519 | 0.004 | . | . | . | . | . | . | . | . | . |
| InternalIMT | aa | rs150596349 | 0.031 | 9.90E-03 | 0.048 | 0.837 | -0.559 | 0.707 | 0.429 | 0.237 | 0.277 | 0.393 |
| InternalIMT | aa | rs190753747 | 0.001 | . | . | . | . | . | . | . | . | . |
| InternalIMT | aa | rs139560940 | 0.004 | . | . | . | . | . | . | . | . | . |
| InternalIMT | aa | rs75640804 | 0.033 | 0.012 | 0.048 | 0.8 | -0.358 | 0.744 | 0.63 | 0.166 | 0.293 | 0.572 |
| InternalIMT | aa | rs73283175 | 0.052 | 0.029 | 0.05 | 0.561 | 0.091 | 0.382 | 0.811 | -0.043 | 0.149 | 0.775 |
| InternalIMT | aa | rs143337229 | 0.002 | . | . | . | . | . | . | . | . | . |
| InternalIMT | aa | rs73283177 | 0.03 | 0.03 | 0.049 | 0.543 | 0.453 | 0.544 | 0.405 | -0.175 | 0.211 | 0.408 |
| InternalIMT | aa | rs76053611 | 0.029 | 4.50E-03 | 0.048 | 0.925 | -1.03 | 0.664 | 0.12 | 0.419 | 0.262 | 0.11 |
| InternalIMT | aa | rs116325983 | 0.004 | . | . | . | . | . | . | . | . | . |
| InternalIMT | aa | rs115451825 | 0.005 | . | . | . | . | . | . | . | . | . |
| InternalIMT | aa | rs183845246 | 0.001 | . | . | . | . | . | . | . | . | . |
| InternalIMT | aa | rs79756542 | 0.033 | 8.30E-03 | 0.048 | 0.863 | -0.657 | 0.681 | 0.335 | 0.273 | 0.267 | 0.306 |
| InternalIMT | aa | rs187242860 | 0.002 | . | . | . | . | . | . | . | . | . |
| InternalIMT | aa | rs57318980 | 0.03 | 0.028 | 0.049 | 0.566 | 0.431 | 0.582 | 0.459 | -0.162 | 0.227 | 0.475 |
| InternalIMT | aa | rs59969732 | 0.027 | 0.03 | 0.049 | 0.533 | 0.573 | 0.606 | 0.345 | -0.221 | 0.236 | 0.35 |
| InternalIMT | aa | rs143811488 | 0.006 | . | . | . | . | . | . | . | . | . |
| InternalIMT | aa | rs189067644 | 0.006 | . | . | . | . | . | . | . | . | . |
| InternalIMT | aa | rs60449178 | 0.025 | 0.033 | 0.049 | 0.498 | 0.674 | 0.647 | 0.297 | -0.268 | 0.251 | 0.286 |
| InternalIMT | aa | rs59243389 | 0.025 | 0.033 | 0.049 | 0.498 | 0.674 | 0.647 | 0.297 | -0.268 | 0.251 | 0.286 |
| InternalIMT | aa | rs184946540 | 0.027 | 0.031 | 0.049 | 0.521 | 0.296 | 0.568 | 0.602 | -0.129 | 0.219 | 0.557 |
| InternalIMT | aa | rs188685881 | 0.001 | . | . | . | . | . | . | . | . | . |
| InternalIMT | aa | rs150335797 | 0.004 | . | . | . | . | . | . | . | . | . |
| InternalIMT | aa | rs143879260 | 0.004 | . | . | . | . | . | . | . | . | . |
| InternalIMT | aa | rs186044491 | 0.006 | . | . | . | . | . | . | . | . | . |
| InternalIMT | aa | rs11819084 | 0.018 | 0.033 | 0.048 | 0.489 | 0.482 | 0.405 | 0.235 | -0.177 | 0.161 | 0.273 |
| InternalIMT | aa | rs146915670 | 0.006 | . | . | . | . | . | . | . | . | . |

| InternalIMT | aa | rs113995695 | 0.017 | 0.033 | 0.048 | 0.491 | 0.48 | 0.407 | 0.238 | -0.175 | 0.162 | 0.279 |
| --- | --- | --- | --- | --- | --- | --- | --- | --- | --- | --- | --- | --- |
| InternalIMT | aa | rs143489451 | 0.003 | . | . | . | . | . | . | . | . | . |
| InternalIMT | aa | rs182653839 | 0 | . | . | . | . | . | . | . | . | . |
| InternalIMT | aa | rs141692564 | 0.004 | . | . | . | . | . | . | . | . | . |
| InternalIMT | aa | rs146281742 | 0.003 | . | . | . | . | . | . | . | . | . |
| InternalIMT | aa | rs192914353 | 0.003 | . | . | . | . | . | . | . | . | . |
| InternalIMT | aa | rs185182848 | 0.002 | . | . | . | . | . | . | . | . | . |
| InternalIMT | aa | rs28395858 | 0.017 | 0.033 | 0.048 | 0.488 | 0.489 | 0.405 | 0.227 | -0.179 | 0.161 | 0.267 |
| InternalIMT | aa | rs41429645 | 0.005 | . | . | . | . | . | . | . | . | . |
| InternalIMT | aa | rs28395874 | 0.017 | 0.033 | 0.048 | 0.486 | 0.497 | 0.404 | 0.218 | -0.182 | 0.16 | 0.258 |
| InternalIMT | aa | rs189663634 | 0.001 | . | . | . | . | . | . | . | . | . |
| InternalIMT | aa | rs77666700 | 0.006 | . | . | . | . | . | . | . | . | . |
| InternalIMT | aa | rs146711717 | 0.002 | . | . | . | . | . | . | . | . | . |
| InternalIMT | aa | rs144904251 | 0.001 | . | . | . | . | . | . | . | . | . |
| InternalIMT | aa | rs189893153 | 0.001 | . | . | . | . | . | . | . | . | . |
| InternalIMT | aa | rs150447740 | 0.006 | . | . | . | . | . | . | . | . | . |
| InternalIMT | aa | rs143099649 | 0.001 | . | . | . | . | . | . | . | . | . |
| InternalIMT | aa | rs191288436 | 0.007 | . | . | . | . | . | . | . | . | . |
| InternalIMT | aa | rs188577862 | 0.004 | . | . | . | . | . | . | . | . | . |
| InternalIMT | aa | rs192695334 | 0.004 | . | . | . | . | . | . | . | . | . |
| InternalIMT | aa | rs138612906 | 0.022 | 0.03 | 0.048 | 0.534 | 0.293 | 0.45 | 0.514 | -0.111 | 0.177 | 0.529 |
| InternalIMT | aa | rs191290051 | 0.022 | 0.021 | 0.048 | 0.665 | 0.571 | 0.811 | 0.482 | -0.186 | 0.317 | 0.558 |
| InternalIMT | aa | rs189631459 | 0.001 | . | . | . | . | . | . | . | . | . |
| InternalIMT | aa | rs114620874 | 0.005 | . | . | . | . | . | . | . | . | . |
| InternalIMT | aa | rs78246652 | 0.013 | 0.016 | 0.047 | 0.73 | -0.136 | 1.18 | 0.908 | 0.102 | 0.46 | 0.824 |
| InternalIMT | aa | rs79432451 | 0.005 | . | . | . | . | . | . | . | . | . |
| InternalIMT | aa | rs186022585 | 0.001 | . | . | . | . | . | . | . | . | . |
| InternalIMT | aa | rs142978891 | 0.005 | . | . | . | . | . | . | . | . | . |
| InternalIMT | aa | rs151086840 | 0.007 | . | . | . | . | . | . | . | . | . |
| InternalIMT | aa | rs183270259 | 0.006 | . | . | . | . | . | . | . | . | . |
| InternalIMT | aa | rs188544289 | 0.006 | . | . | . | . | . | . | . | . | . |
| InternalIMT | aa | rs188500808 | 0.001 | . | . | . | . | . | . | . | . | . |
| InternalIMT | aa | rs189424589 | 0.002 | . | . | . | . | . | . | . | . | . |
| InternalIMT | aa | rs78622303 | 0.025 | 0.027 | 0.048 | 0.581 | 0.645 | 0.561 | 0.25 | -0.224 | 0.222 | 0.313 |
| InternalIMT | aa | rs143210835 | 0.008 | . | . | . | . | . | . | . | . | . |
| InternalIMT | aa | rs114761840 | 0.004 | . | . | . | . | . | . | . | . | . |
| InternalIMT | aa | rs185805402 | 0.003 | . | . | . | . | . | . | . | . | . |
| InternalIMT | aa | rs139273744 | 0.002 | . | . | . | . | . | . | . | . | . |
| InternalIMT | aa | rs184654150 | 0.004 | . | . | . | . | . | . | . | . | . |
| InternalIMT | aa | rs189069710 | 0 | . | . | . | . | . | . | . | . | . |
| InternalIMT | aa | rs114752115 | 0.018 | 0.018 | 0.048 | 0.713 | -0.167 | 0.905 | 0.853 | 0.098 | 0.355 | 0.783 |
| InternalIMT | aa | rs150162515 | 0.001 | . | . | . | . | . | . | . | . | . |
| InternalIMT | aa | rs180982454 | 0.004 | . | . | . | . | . | . | . | . | . |
| InternalIMT | aa | rs184932088 | 0.001 | . | . | . | . | . | . | . | . | . |
| InternalIMT | aa | rs181109625 | 0 | . | . | . | . | . | . | . | . | . |
| InternalIMT | aa | rs138121134 | 0.001 | . | . | . | . | . | . | . | . | . |
| InternalIMT | aa | rs183253861 | 0.003 | . | . | . | . | . | . | . | . | . |
| InternalIMT | aa | rs147073946 | 0.001 | . | . | . | . | . | . | . | . | . |
| InternalIMT | aa | rs139938489 | 0.005 | . | . | . | . | . | . | . | . | . |
| InternalIMT | aa | rs146362560 | 0.008 | . | . | . | . | . | . | . | . | . |
| InternalIMT | aa | rs193155956 | 0.004 | . | . | . | . | . | . | . | . | . |
| InternalIMT | aa | rs77663092 | 0.004 | . | . | . | . | . | . | . | . | . |
| InternalIMT | aa | rs73285319 | 0.021 | 0.016 | 0.048 | 0.748 | -0.859 | 0.566 | 0.129 | 0.322 | 0.218 | 0.141 |
| InternalIMT | aa | rs184019276 | 0.007 | . | . | . | . | . | . | . | . | . |
| InternalIMT | aa | rs28395863 | 0.016 | 0.026 | 0.048 | 0.592 | 0.274 | 0.652 | 0.674 | -0.099 | 0.258 | 0.702 |
| InternalIMT | aa | rs182121645 | 0.011 | 0.038 | 0.048 | 0.431 | 0.793 | 0.463 | 0.087 | -0.32 | 0.177 | 0.071 |
| InternalIMT | aa | rs73285327 | 0.016 | 0.017 | 0.048 | 0.722 | -0.726 | 0.66 | 0.271 | 0.28 | 0.255 | 0.272 |
| InternalIMT | aa | rs149589584 | 0.001 | . | . | . | . | . | . | . | . | . |
| InternalIMT | aa | rs148310335 | 0.005 | . | . | . | . | . | . | . | . | . |
| InternalIMT | aa | rs187246093 | 0.007 | . | . | . | . | . | . | . | . | . |
| InternalIMT | aa | rs184668894 | 0 | . | . | . | . | . | . | . | . | . |
| InternalIMT | aa | rs114269961 | 0.001 | . | . | . | . | . | . | . | . | . |
| InternalIMT | aa | rs149694374 | 0.02 | 0.028 | 0.048 | 0.555 | 0.361 | 0.548 | 0.511 | -0.147 | 0.216 | 0.496 |
| InternalIMT | aa | rs145284227 | 0.004 | . | . | . | . | . | . | . | . | . |
| InternalIMT | aa | rs189983820 | 0.004 | . | . | . | . | . | . | . | . | . |
| InternalIMT | aa | rs181200444 | 0 | . | . | . | . | . | . | . | . | . |
| InternalIMT | aa | rs185486385 | 0 | . | . | . | . | . | . | . | . | . |
| InternalIMT | aa | rs151198241 | 0.006 | . | . | . | . | . | . | . | . | . |
| InternalIMT | aa | rs189959341 | 0.006 | . | . | . | . | . | . | . | . | . |
| InternalIMT | aa | rs140549257 | 0.005 | . | . | . | . | . | . | . | . | . |
| InternalIMT | aa | rs186453139 | 0.016 | 0.018 | 0.048 | 0.702 | -0.792 | 0.62 | 0.201 | 0.289 | 0.239 | 0.228 |
| InternalIMT | aa | rs180922711 | 0.027 | 0.023 | 0.049 | 0.641 | -0.278 | 0.541 | 0.608 | 0.092 | 0.209 | 0.66 |
| InternalIMT | aa | rs56659903 | 0.004 | . | . | . | . | . | . | . | . | . |
| InternalIMT | aa | rs141202128 | 0.004 | . | . | . | . | . | . | . | . | . |
| InternalIMT | aa | rs59085314 | 0.033 | 0.048 | 0.05 | 0.345 | 0.823 | 0.405 | 0.042 | -0.318 | 0.155 | 0.041 |
| InternalIMT | aa | rs145564996 | 0.004 | . | . | . | . | . | . | . | . | . |
| InternalIMT | aa | rs114961009 | 0.006 | . | . | . | . | . | . | . | . | . |
| InternalIMT | aa | rs182294189 | 0.003 | . | . | . | . | . | . | . | . | . |
| InternalIMT | aa | rs187586636 | 0.005 | . | . | . | . | . | . | . | . | . |
| InternalIMT | aa | rs192254907 | 0.003 | . | . | . | . | . | . | . | . | . |
| InternalIMT | aa | rs149145663 | 0.001 | . | . | . | . | . | . | . | . | . |
| InternalIMT | aa | rs180713064 | 0.002 | . | . | . | . | . | . | . | . | . |
| InternalIMT | aa | rs183009023 | 0.004 | . | . | . | . | . | . | . | . | . |
| InternalIMT | aa | rs143385347 | 0.01 | 0.038 | 0.048 | 0.421 | 0.858 | 0.449 | 0.056 | -0.34 | 0.172 | 0.048 |
| InternalIMT | aa | rs185786329 | 0.004 | . | . | . | . | . | . | . | . | . |
| InternalIMT | aa | rs192341030 | 0.003 | . | . | . | . | . | . | . | . | . |

| InternalIMT | aa | rs74661429 | 0.009 | . | . | . | . | . | . | . | . | . |
| --- | --- | --- | --- | --- | --- | --- | --- | --- | --- | --- | --- | --- |
| InternalIMT | aa | rs188034853 | 0.001 | . | . | . | . | . | . | . | . | . |
| InternalIMT | aa | rs116808430 | 0.016 | 0.019 | 0.048 | 0.692 | -0.78 | 0.613 | 0.203 | 0.281 | 0.237 | 0.235 |
| InternalIMT | aa | rs148469010 | 0 | . | . | . | . | . | . | . | . | . |
| InternalIMT | aa | rs78621663 | 0.006 | . | . | . | . | . | . | . | . | . |
| InternalIMT | aa | rs192654886 | 0.001 | . | . | . | . | . | . | . | . | . |
| InternalIMT | aa | rs145996035 | 0.005 | . | . | . | . | . | . | . | . | . |
| InternalIMT | aa | rs139942226 | 0 | . | . | . | . | . | . | . | . | . |
| InternalIMT | aa | rs113645632 | 0.006 | . | . | . | . | . | . | . | . | . |
| InternalIMT | aa | rs74940661 | 0.016 | 0.022 | 0.048 | 0.638 | -0.068 | 0.641 | 0.915 | 0.029 | 0.256 | 0.909 |
| InternalIMT | aa | rs115355800 | 0.013 | 0.018 | 0.047 | 0.697 | 0.134 | 1.21 | 0.912 | -7.10E-03 | 0.473 | 0.988 |
| InternalIMT | aa | rs150285898 | 0.005 | . | . | . | . | . | . | . | . | . |
| InternalIMT | aa | rs188117932 | 0.004 | . | . | . | . | . | . | . | . | . |
| InternalIMT | aa | rs139956566 | 0 | . | . | . | . | . | . | . | . | . |
| InternalIMT | aa | rs147673885 | 0 | . | . | . | . | . | . | . | . | . |
| InternalIMT | aa | rs189127928 | 0.005 | . | . | . | . | . | . | . | . | . |
| InternalIMT | aa | rs187558772 | 0.002 | . | . | . | . | . | . | . | . | . |
| InternalIMT | aa | rs181948587 | 0 | . | . | . | . | . | . | . | . | . |
| InternalIMT | aa | rs189983269 | 0.002 | . | . | . | . | . | . | . | . | . |
| InternalIMT | aa | rs187250069 | 0.002 | . | . | . | . | . | . | . | . | . |
| InternalIMT | aa | rs191485859 | 0.001 | . | . | . | . | . | . | . | . | . |
| InternalIMT | aa | rs185499555 | 0.002 | . | . | . | . | . | . | . | . | . |
| InternalIMT | aa | rs180862110 | 0.003 | . | . | . | . | . | . | . | . | . |
| InternalIMT | aa | rs190472603 | 0.005 | . | . | . | . | . | . | . | . | . |
| InternalIMT | aa | rs189226167 | 0.005 | . | . | . | . | . | . | . | . | . |
| InternalIMT | aa | rs181623256 | 0.002 | . | . | . | . | . | . | . | . | . |
| InternalIMT | aa | rs186079557 | 0.001 | . | . | . | . | . | . | . | . | . |
| InternalIMT | aa | rs147760994 | 0.002 | . | . | . | . | . | . | . | . | . |
| InternalIMT | aa | rs189176167 | 0 | . | . | . | . | . | . | . | . | . |
| InternalIMT | aa | rs117901000 | 0.001 | . | . | . | . | . | . | . | . | . |
| InternalIMT | aa | rs141984888 | 0 | . | . | . | . | . | . | . | . | . |
| InternalIMT | aa | rs192365061 | 0.007 | . | . | . | . | . | . | . | . | . |
| InternalIMT | aa | rs185976038 | 0 | . | . | . | . | . | . | . | . | . |
| InternalIMT | aa | rs184697303 | 0.002 | . | . | . | . | . | . | . | . | . |
| InternalIMT | aa | rs143383132 | 0.002 | . | . | . | . | . | . | . | . | . |
| InternalIMT | aa | rs141873881 | 0.004 | . | . | . | . | . | . | . | . | . |
| InternalIMT | aa | rs185507255 | 0 | . | . | . | . | . | . | . | . | . |
| InternalIMT | aa | rs191379425 | 0.001 | . | . | . | . | . | . | . | . | . |
| InternalIMT | aa | rs184199584 | 0.002 | . | . | . | . | . | . | . | . | . |
| InternalIMT | aa | rs183181360 | 0.002 | . | . | . | . | . | . | . | . | . |
| InternalIMT | aa | rs184495673 | 0.001 | . | . | . | . | . | . | . | . | . |
| InternalIMT | aa | rs182946297 | 0.001 | . | . | . | . | . | . | . | . | . |
| InternalIMT | aa | rs187743615 | 0 | . | . | . | . | . | . | . | . | . |
| InternalIMT | aa | rs183259862 | 0.001 | . | . | . | . | . | . | . | . | . |
| InternalIMT | aa | rs112433566 | 0 | . | . | . | . | . | . | . | . | . |
| InternalIMT | aa | rs182351085 | 0.001 | . | . | . | . | . | . | . | . | . |
| InternalIMT | aa | rs146248508 | 0.001 | . | . | . | . | . | . | . | . | . |
| InternalIMT | aa | rs188528439 | 0.001 | . | . | . | . | . | . | . | . | . |
| InternalIMT | aa | rs146167018 | 0.005 | . | . | . | . | . | . | . | . | . |
| InternalIMT | aa | rs192440980 | 0.004 | . | . | . | . | . | . | . | . | . |
| InternalIMT | aa | rs185601882 | 0.001 | . | . | . | . | . | . | . | . | . |
| InternalIMT | aa | rs117272563 | 0.002 | . | . | . | . | . | . | . | . | . |
| InternalIMT | aa | rs10430734 | 0.003 | . | . | . | . | . | . | . | . | . |
| InternalIMT | aa | rs181320952 | 0.001 | . | . | . | . | . | . | . | . | . |
| InternalIMT | aa | rs185480660 | 0.001 | . | . | . | . | . | . | . | . | . |
| InternalIMT | aa | rs188077917 | 0.001 | . | . | . | . | . | . | . | . | . |
| InternalIMT | aa | rs187562953 | 0.002 | . | . | . | . | . | . | . | . | . |
| InternalIMT | aa | rs182044631 | 0 | . | . | . | . | . | . | . | . | . |
| InternalIMT | aa | rs145455596 | 0.001 | . | . | . | . | . | . | . | . | . |
| InternalIMT | aa | rs181475877 | 0.004 | . | . | . | . | . | . | . | . | . |
| InternalIMT | aa | rs149413085 | 0.002 | . | . | . | . | . | . | . | . | . |
| InternalIMT | aa | rs12253656 | 0 | . | . | . | . | . | . | . | . | . |
| InternalIMT | aa | rs182718239 | 0 | . | . | . | . | . | . | . | . | . |
| InternalIMT | aa | rs192435695 | 0.002 | . | . | . | . | . | . | . | . | . |
| InternalIMT | aa | rs12248277 | 0 | . | . | . | . | . | . | . | . | . |
| InternalIMT | aa | rs143521710 | 0.001 | . | . | . | . | . | . | . | . | . |
| InternalIMT | aa | rs144454454 | 0 | . | . | . | . | . | . | . | . | . |
| InternalIMT | aa | rs190441610 | 0.004 | . | . | . | . | . | . | . | . | . |
| InternalIMT | aa | rs114690462 | 0 | . | . | . | . | . | . | . | . | . |
| InternalIMT | aa | rs184541357 | 0.001 | . | . | . | . | . | . | . | . | . |
| InternalIMT | aa | rs142657653 | 0 | . | . | . | . | . | . | . | . | . |
| InternalIMT | aa | rsurf1 | 2.145 | . | . | . | . | . | . | . | . | . |
| InternalIMT | aa | rsten1 | 2.303 | . | . | . | . | . | . | . | . | . |
| InternalIMT | aa | chr10:45852312:D | 0.086 | 0.067 | 0.036 | 0.06 | -0.403 | 0.183 | 0.028 | 0.154 | 0.076 | 0.044 |
| InternalIMT | aa | chr10:45853232:I | 0.1 | 0.048 | 0.036 | 0.184 | -0.607 | 0.183 | 9.40E-04 | 0.242 | 0.076 | 1.60E-03 |
| InternalIMT | aa | chr10:45853234:I | 0.168 | 0.04 | 0.039 | 0.303 | -0.408 | 0.152 | 7.10E-03 | 0.161 | 0.063 | 0.01 |
| InternalIMT | aa | chr10:45854796:D | 0.013 | 0.08 | 0.034 | 0.018 | -0.958 | 0.401 | 0.017 | 0.359 | 0.178 | 0.044 |
| InternalIMT | aa | chr10:45854994:I | 0.019 | 0.075 | 0.034 | 0.025 | -1.15 | 0.385 | 2.70E-03 | 0.453 | 0.166 | 6.30E-03 |
| InternalIMT | aa | chr10:45858290:D | 0.045 | 0.077 | 0.036 | 0.031 | -0.463 | 0.475 | 0.329 | 0.189 | 0.194 | 0.331 |
| InternalIMT | aa | chr10:45858820:I | 0.015 | 0.096 | 0.034 | 4.60E-03 | 0.355 | 0.965 | 0.713 | -0.136 | 0.38 | 0.722 |
| InternalIMT | aa | chr10:45860888:D | 0.081 | 0.083 | 0.037 | 0.027 | -0.251 | 0.286 | 0.38 | 0.09 | 0.116 | 0.439 |
| InternalIMT | aa | chr10:45863913:I | 0.165 | 0.039 | 0.038 | 0.314 | -0.411 | 0.152 | 6.60E-03 | 0.163 | 0.063 | 9.20E-03 |
| InternalIMT | aa | chr10:45866601:D | 0.036 | 0.059 | 0.038 | 0.125 | 0.406 | 0.389 | 0.296 | -0.157 | 0.153 | 0.306 |
| InternalIMT | aa | chr10:45867197:I | 0.006 | . | . | . | . | . | . | . | . | . |
| InternalIMT | aa | chr10:45867538:D | 0.171 | 0.04 | 0.039 | 0.301 | -0.397 | 0.148 | 7.50E-03 | 0.156 | 0.061 | 0.011 |
| InternalIMT | aa | chr10:45868356:D | 0.161 | 0.044 | 0.038 | 0.249 | -0.382 | 0.15 | 0.011 | 0.15 | 0.062 | 0.016 |

| InternalIMT | aa | chr10:45871158:I | 0.167 | 0.04 | 0.038 | 0.303 | -0.406 | 0.149 | 6.40E-03 | 0.16 | 0.061 | 9.20E-03 |
| --- | --- | --- | --- | --- | --- | --- | --- | --- | --- | --- | --- | --- |
| InternalIMT | aa | chr10:45871637:D | 0.084 | 0.06 | 0.037 | 0.101 | -0.544 | 0.259 | 0.036 | 0.203 | 0.106 | 0.055 |
| InternalIMT | aa | chr10:45871638:I | 0.132 | 0.031 | 0.038 | 0.41 | -0.596 | 0.182 | 1.10E-03 | 0.234 | 0.075 | 1.80E-03 |
| InternalIMT | aa | chr10:45873629:I | 0.158 | 0.047 | 0.038 | 0.219 | -0.369 | 0.153 | 0.016 | 0.143 | 0.063 | 0.024 |
| InternalIMT | aa | chr10:45873701:D | 0.019 | 0.037 | 0.037 | 0.318 | -0.816 | 0.633 | 0.197 | 0.331 | 0.252 | 0.19 |
| InternalIMT | aa | chr10:45874997:I | 0.021 | 0.036 | 0.038 | 0.347 | -0.72 | 0.457 | 0.115 | 0.295 | 0.194 | 0.13 |
| InternalIMT | aa | chr10:45882030:I | 0.045 | 0.035 | 0.038 | 0.36 | -0.504 | 0.402 | 0.209 | 0.193 | 0.16 | 0.227 |
| InternalIMT | aa | chr10:45883774:D | 0.102 | 0.08 | 0.036 | 0.026 | -0.143 | 0.19 | 0.453 | 0.062 | 0.079 | 0.431 |
| InternalIMT | aa | chr10:45885169:D | 0.067 | 0.067 | 0.035 | 0.059 | -0.516 | 0.212 | 0.015 | 0.199 | 0.089 | 0.025 |
| InternalIMT | aa | chr10:45886451:D | 0.67 | 0.064 | 0.086 | 0.456 | -0.042 | 0.139 | 0.764 | 0.021 | 0.057 | 0.718 |
| InternalIMT | aa | chr10:45891800:I | 0.685 | -0.031 | 0.08 | 0.697 | -0.222 | 0.126 | 0.078 | 0.089 | 0.052 | 0.087 |
| InternalIMT | aa | chr10:45904771:I | 0.685 | 0.028 | 0.079 | 0.721 | -0.12 | 0.124 | 0.333 | 0.046 | 0.051 | 0.366 |
| InternalIMT | aa | chr10:45904959:I | 0.113 | 0.082 | 0.038 | 0.031 | -0.125 | 0.231 | 0.588 | 0.049 | 0.094 | 0.603 |
| InternalIMT | aa | chr10:45911028:I | 0.077 | 0.108 | 0.038 | 5.00E-03 | 0.213 | 0.312 | 0.495 | -0.091 | 0.124 | 0.467 |
| InternalIMT | aa | chr10:45911031:I | 0.355 | 0.165 | 0.054 | 2.00E-03 | 0.25 | 0.139 | 0.073 | -0.096 | 0.057 | 0.089 |
| InternalIMT | aa | chr10:45912154:D | 0.103 | 0.095 | 0.036 | 8.10E-03 | 0.037 | 0.188 | 0.845 | -0.014 | 0.077 | 0.861 |
| InternalIMT | aa | chr10:45913885:I | 0.011 | 0.08 | 0.034 | 0.018 | -0.936 | 0.436 | 0.032 | 0.341 | 0.195 | 0.08 |
| InternalIMT | aa | chr10:45914753:D | 0.245 | 0.103 | 0.044 | 0.019 | 0.106 | 0.148 | 0.474 | -0.035 | 0.061 | 0.564 |
| InternalIMT | aa | chr10:45919621 | 0.003 | . | . | . | . | . | . | . | . | . |
| InternalIMT | aa | chr10:45921739:I | 0.066 | 0.068 | 0.035 | 0.053 | -0.59 | 0.238 | 0.013 | 0.224 | 0.096 | 0.019 |
| InternalIMT | aa | chr10:45923527:I | 0.057 | 0.087 | 0.037 | 0.019 | -0.122 | 0.321 | 0.704 | 0.046 | 0.132 | 0.725 |
| InternalIMT | aa | chr10:45927001:I | 0.019 | 0.098 | 0.034 | 4.30E-03 | 0.611 | 0.59 | 0.301 | -0.225 | 0.236 | 0.341 |
| InternalIMT | aa | chr10:45930354:I | 0.017 | 0.077 | 0.034 | 0.021 | -1.14 | 0.386 | 3.20E-03 | 0.449 | 0.168 | 7.60E-03 |
| InternalIMT | aa | chr10:45932864:I | 0.04 | 0.067 | 0.035 | 0.056 | -0.819 | 0.406 | 0.044 | 0.346 | 0.173 | 0.046 |
| InternalIMT | aa | chr10:45936163:I | 0.029 | 0.106 | 0.034 | 2.00E-03 | 0.439 | 0.38 | 0.248 | -0.206 | 0.156 | 0.188 |
| InternalIMT | aa | chr10:45938095:D | 0.144 | 0.069 | 0.037 | 0.061 | -0.219 | 0.16 | 0.171 | 0.09 | 0.066 | 0.173 |
| InternalIMT | aa | chr10:45938886 | 0 | . | . | . | . | . | . | . | . | . |
| InternalIMT | aa | chr10:45939320 | 0.001 | . | . | . | . | . | . | . | . | . |
| InternalIMT | aa | chr10:45939659 | 0.002 | . | . | . | . | . | . | . | . | . |
| InternalIMT | aa | chr10:45941150:D | 0.012 | 0.081 | 0.034 | 0.016 | -0.756 | 0.368 | 0.04 | 0.257 | 0.162 | 0.112 |
| InternalIMT | aa | chr10:45947128:D | 0.058 | 0.103 | 0.035 | 3.50E-03 | 0.285 | 0.228 | 0.212 | -0.103 | 0.092 | 0.265 |
| InternalIMT | aa | chr10:45950990:I | 0.047 | 0.082 | 0.035 | 0.019 | -0.249 | 0.321 | 0.438 | 0.094 | 0.134 | 0.484 |
| InternalIMT | aa | chr10:45951854:I | 0.03 | 0.064 | 0.038 | 0.09 | 0.692 | 0.499 | 0.166 | -0.282 | 0.202 | 0.163 |
| InternalIMT | aa | chr10:45952670:I | 0.049 | 0.095 | 0.035 | 6.50E-03 | -0.01 | 0.24 | 0.966 | -0.013 | 0.1 | 0.899 |
| InternalIMT | aa | chr10:45956741 | 0.001 | . | . | . | . | . | . | . | . | . |
| InternalIMT | aa | chr10:45957247:I | 0.219 | 0.069 | 0.039 | 0.076 | -0.129 | 0.127 | 0.309 | 0.055 | 0.053 | 0.299 |
| InternalIMT | aa | chr10:45958082:I | 0.216 | 0.069 | 0.039 | 0.077 | -0.134 | 0.127 | 0.291 | 0.057 | 0.053 | 0.28 |
| InternalIMT | aa | chr10:45905041:D | 0.053 | 0.05 | 0.042 | 0.227 | -1.05 | 0.31 | 7.10E-04 | 0.427 | 0.128 | 8.90E-04 |
| InternalIMT | aa | chr10:45938871 | 0.003 | . | . | . | . | . | . | . | . | . |
| InternalIMT | aa | chr10:45939160 | 0.004 | . | . | . | . | . | . | . | . | . |
| InternalIMT | aa | chr10:45869910 | 0.008 | . | . | . | . | . | . | . | . | . |
| InternalIMT | aa | chr10:45876214:D | 0.012 | 0.022 | 0.048 | 0.652 | -0.554 | 0.629 | 0.379 | 0.186 | 0.255 | 0.467 |
| InternalIMT | aa | chr10:45880237:I | 0.056 | 0.04 | 0.05 | 0.421 | 0.623 | 0.373 | 0.095 | -0.225 | 0.146 | 0.122 |
| InternalIMT | aa | chr10:45884371:I | 0.045 | 8.40E-03 | 0.048 | 0.863 | -0.465 | 0.528 | 0.379 | 0.194 | 0.209 | 0.352 |
| InternalIMT | aa | chr10:45891535:D | 0.012 | 0.01 | 0.048 | 0.83 | -1.57 | 1.02 | 0.121 | 0.655 | 0.404 | 0.106 |
| InternalIMT | aa | chr10:45893516:D | 0.045 | 8.20E-03 | 0.048 | 0.866 | -0.47 | 0.526 | 0.372 | 0.197 | 0.208 | 0.345 |
| InternalIMT | aa | chr10:45893782:D | 0.01 | 0.023 | 0.048 | 0.632 | 1.12 | 1.36 | 0.411 | -0.38 | 0.53 | 0.474 |
| InternalIMT | aa | chr10:45895254:I | 0.012 | 0.01 | 0.048 | 0.83 | -1.58 | 1.02 | 0.12 | 0.659 | 0.405 | 0.104 |
| InternalIMT | aa | chr10:45895848:D | 0.035 | 7.20E-03 | 0.048 | 0.881 | -0.833 | 0.524 | 0.112 | 0.327 | 0.211 | 0.121 |
| InternalIMT | aa | chr10:45896316:D | 0.045 | 8.20E-03 | 0.048 | 0.865 | -0.469 | 0.526 | 0.373 | 0.196 | 0.208 | 0.346 |
| InternalIMT | aa | chr10:45896317:D | 0.045 | 8.20E-03 | 0.048 | 0.865 | -0.469 | 0.526 | 0.373 | 0.196 | 0.208 | 0.346 |
| InternalIMT | aa | chr10:45902683:I | 0.013 | 0.022 | 0.048 | 0.643 | -0.455 | 0.614 | 0.459 | 0.151 | 0.247 | 0.54 |
| InternalIMT | aa | chr10:45905003:D | 0.006 | . | . | . | . | . | . | . | . | . |
| InternalIMT | aa | chr10:45927476:D | 0.021 | 0.037 | 0.048 | 0.442 | 0.597 | 0.404 | 0.139 | -0.239 | 0.16 | 0.135 |
| InternalIMT | aa | chr10:45935882 | 0.001 | . | . | . | . | . | . | . | . | . |
| InternalIMT | aa | chr10:45936100 | 0.001 | . | . | . | . | . | . | . | . | . |
| InternalIMT | aa | chr10:45939553 | 0.003 | . | . | . | . | . | . | . | . | . |
| InternalIMT | aa | chr10:45939631 | 0 | . | . | . | . | . | . | . | . | . |
| InternalIMT | aa | chr10:45944193:D | 0.008 | . | . | . | . | . | . | . | . | . |
| InternalIMT | aa | chr10:45954030 | 0 | . | . | . | . | . | . | . | . | . |

Supplemental table S27.

| phen | covar(fa) | rs_id | maf | beta_FA | se_FA | p_FA | beta_SNP | se_SNP | p_SNP | beta_inter | se_inter | p_inter |
| --- | --- | --- | --- | --- | --- | --- | --- | --- | --- | --- | --- | --- |
| InternalIMT | aa | rs76035182 | 0.001 | . | . | . | . | . | . | . | . | . |
| InternalIMT | aa | rs12359547 | 0.104 | 0.114 | 0.066 | 0.082 | -0.119 | 0.357 | 0.739 | 0.036 | 0.148 | 0.81 |
| InternalIMT | aa | rs74595484 | 0 | . | . | . | . | . | . | . | . | . |
| InternalIMT | aa | rs1864415 | 0.605 | 0.081 | 0.123 | 0.511 | -0.055 | 0.204 | 0.787 | 0.029 | 0.085 | 0.734 |
| InternalIMT | aa | rs72788101 | 0.104 | 0.114 | 0.066 | 0.082 | -0.119 | 0.357 | 0.738 | 0.036 | 0.148 | 0.809 |
| InternalIMT | aa | rs11239481 | 0.183 | 0.098 | 0.071 | 0.164 | -0.125 | 0.264 | 0.635 | 0.06 | 0.112 | 0.593 |
| InternalIMT | aa | rs72788102 | 0.104 | 0.114 | 0.066 | 0.082 | -0.119 | 0.357 | 0.738 | 0.036 | 0.148 | 0.809 |
| InternalIMT | aa | rs72789705 | 0.104 | 0.114 | 0.066 | 0.082 | -0.119 | 0.357 | 0.738 | 0.036 | 0.148 | 0.809 |
| InternalIMT | aa | rs141529858 | 0.008 | . | . | . | . | . | . | . | . | . |
| InternalIMT | aa | rs145376292 | 0 | . | . | . | . | . | . | . | . | . |
| InternalIMT | aa | rs190418301 | 0.001 | . | . | . | . | . | . | . | . | . |
| InternalIMT | aa | rs11239482 | 0.104 | 0.114 | 0.066 | 0.082 | -0.119 | 0.357 | 0.738 | 0.036 | 0.148 | 0.809 |
| InternalIMT | aa | rs61855872 | 0.014 | 0.141 | 0.062 | 0.023 | 2.08 | 1.39 | 0.135 | -0.857 | 0.58 | 0.139 |
| InternalIMT | aa | rs148991144 | 0.011 | 0.121 | 0.061 | 0.049 | 0.619 | 0.954 | 0.516 | -0.252 | 0.396 | 0.525 |
| InternalIMT | aa | rs191932221 | 0.007 | . | . | . | . | . | . | . | . | . |
| InternalIMT | aa | rs55978138 | 0.16 | 0.109 | 0.068 | 0.11 | -0.127 | 0.29 | 0.661 | 0.041 | 0.12 | 0.731 |
| InternalIMT | aa | rs73281167 | 0 | . | . | . | . | . | . | . | . | . |
| InternalIMT | aa | rs12768010 | 0.055 | 0.112 | 0.063 | 0.078 | -0.17 | 0.488 | 0.728 | 0.065 | 0.202 | 0.749 |
| InternalIMT | aa | rs11239484 | 0.605 | 0.082 | 0.123 | 0.508 | -0.054 | 0.204 | 0.792 | 0.028 | 0.085 | 0.738 |
| InternalIMT | aa | rs35906360 | 0.16 | 0.109 | 0.068 | 0.11 | -0.128 | 0.29 | 0.66 | 0.041 | 0.12 | 0.73 |
| InternalIMT | aa | rs186250512 | 0.004 | . | . | . | . | . | . | . | . | . |
| InternalIMT | aa | rs150810743 | 0 | . | . | . | . | . | . | . | . | . |
| InternalIMT | aa | rs55965812 | 0.16 | 0.109 | 0.068 | 0.11 | -0.127 | 0.29 | 0.661 | 0.041 | 0.12 | 0.731 |
| InternalIMT | aa | rs56180358 | 0.16 | 0.109 | 0.068 | 0.111 | -0.129 | 0.29 | 0.658 | 0.042 | 0.12 | 0.728 |
| InternalIMT | aa | rs55864145 | 0.055 | 0.112 | 0.063 | 0.078 | -0.17 | 0.488 | 0.728 | 0.065 | 0.203 | 0.749 |
| InternalIMT | aa | rs55986122 | 0.16 | 0.109 | 0.068 | 0.11 | -0.128 | 0.29 | 0.66 | 0.041 | 0.12 | 0.73 |
| InternalIMT | aa | rs7477350 | 0.605 | 0.081 | 0.123 | 0.508 | -0.054 | 0.204 | 0.792 | 0.028 | 0.085 | 0.738 |
| InternalIMT | aa | rs2099170 | 0.159 | 0.108 | 0.068 | 0.112 | -0.133 | 0.29 | 0.647 | 0.043 | 0.12 | 0.718 |
| InternalIMT | aa | rs2082108 | 0.16 | 0.109 | 0.068 | 0.11 | -0.127 | 0.29 | 0.66 | 0.041 | 0.12 | 0.73 |
| InternalIMT | aa | rs55902404 | 0 | . | . | . | . | . | . | . | . | . |
| InternalIMT | aa | rs58402662 | 0 | . | . | . | . | . | . | . | . | . |
| InternalIMT | aa | rs11511618 | 0.182 | 0.103 | 0.071 | 0.145 | -0.094 | 0.262 | 0.721 | 0.046 | 0.111 | 0.681 |
| InternalIMT | aa | rs1816492 | 0.605 | 0.082 | 0.123 | 0.508 | -0.054 | 0.204 | 0.793 | 0.028 | 0.085 | 0.738 |
| InternalIMT | aa | rs146611093 | 0.01 | 0.111 | 0.062 | 0.073 | -0.345 | 0.793 | 0.663 | 0.107 | 0.344 | 0.756 |
| InternalIMT | aa | rs186897689 | 0 | . | . | . | . | . | . | . | . | . |
| InternalIMT | aa | rs10793616 | 0.605 | 0.082 | 0.123 | 0.508 | -0.054 | 0.204 | 0.793 | 0.028 | 0.085 | 0.739 |
| InternalIMT | aa | rs116700479 | 0.001 | . | . | . | . | . | . | . | . | . |
| InternalIMT | aa | rs192535936 | 0 | . | . | . | . | . | . | . | . | . |
| InternalIMT | aa | rs113363297 | 0.57 | 0.119 | 0.12 | 0.321 | 0.016 | 0.208 | 0.939 | -2.60E-03 | 0.086 | 0.976 |
| InternalIMT | aa | rs12354994 | 0.152 | 0.12 | 0.068 | 0.077 | -0.037 | 0.301 | 0.903 | 3.50E-03 | 0.123 | 0.978 |
| InternalIMT | aa | rs148068329 | 0.001 | . | . | . | . | . | . | . | . | . |
| InternalIMT | aa | rs12356109 | 0.16 | 0.109 | 0.068 | 0.11 | -0.127 | 0.29 | 0.661 | 0.041 | 0.12 | 0.731 |
| InternalIMT | aa | rs55908141 | 0 | . | . | . | . | . | . | . | . | . |
| InternalIMT | aa | rs12354712 | 0.159 | 0.109 | 0.068 | 0.111 | -0.127 | 0.29 | 0.662 | 0.041 | 0.12 | 0.73 |
| InternalIMT | aa | rs147282684 | 0.004 | . | . | . | . | . | . | . | . | . |
| InternalIMT | aa | rs12356140 | 0.16 | 0.109 | 0.068 | 0.11 | -0.125 | 0.291 | 0.667 | 0.041 | 0.12 | 0.735 |
| InternalIMT | aa | rs185476187 | 0 | . | . | . | . | . | . | . | . | . |
| InternalIMT | aa | rs145720057 | 0.039 | 0.138 | 0.062 | 0.027 | 0.689 | 0.613 | 0.261 | -0.28 | 0.251 | 0.264 |
| InternalIMT | aa | rs12354742 | 0.159 | 0.109 | 0.068 | 0.111 | -0.127 | 0.29 | 0.661 | 0.041 | 0.12 | 0.73 |
| InternalIMT | aa | rs180913842 | 0 | . | . | . | . | . | . | . | . | . |
| InternalIMT | aa | rs142976914 | 0.006 | . | . | . | . | . | . | . | . | . |
| InternalIMT | aa | rs11511619 | 0.163 | 0.106 | 0.069 | 0.123 | -0.153 | 0.289 | 0.597 | 0.051 | 0.12 | 0.669 |
| InternalIMT | aa | rs146702870 | 0 | . | . | . | . | . | . | . | . | . |
| InternalIMT | aa | rs150287496 | 0.002 | . | . | . | . | . | . | . | . | . |
| InternalIMT | aa | rs190917973 | 0.006 | . | . | . | . | . | . | . | . | . |
| InternalIMT | aa | rs11517281 | 0.16 | 0.109 | 0.068 | 0.11 | -0.127 | 0.29 | 0.661 | 0.041 | 0.12 | 0.731 |
| InternalIMT | aa | rs12359299 | 0.16 | 0.109 | 0.068 | 0.111 | -0.127 | 0.29 | 0.661 | 0.041 | 0.12 | 0.73 |
| InternalIMT | aa | rs182307932 | 0 | . | . | . | . | . | . | . | . | . |
| InternalIMT | aa | rs61855873 | 0.035 | 0.13 | 0.064 | 0.042 | 0.504 | 0.95 | 0.596 | -0.189 | 0.395 | 0.631 |
| InternalIMT | aa | rs12359302 | 0.156 | 0.117 | 0.068 | 0.083 | -0.061 | 0.284 | 0.831 | 0.013 | 0.117 | 0.914 |
| InternalIMT | aa | rs143208140 | 0.003 | . | . | . | . | . | . | . | . | . |
| InternalIMT | aa | rs9988764 | 0.594 | 0.095 | 0.122 | 0.434 | -0.029 | 0.205 | 0.887 | 0.017 | 0.085 | 0.837 |
| InternalIMT | aa | rs192875767 | 0.008 | . | . | . | . | . | . | . | . | . |
| InternalIMT | aa | rs147268846 | 0.015 | 0.111 | 0.063 | 0.077 | -0.527 | 1.4 | 0.706 | 0.243 | 0.584 | 0.677 |
| InternalIMT | aa | rs11239485 | 0.111 | 0.114 | 0.067 | 0.087 | -0.134 | 0.404 | 0.741 | 0.035 | 0.167 | 0.836 |
| InternalIMT | aa | rs151317885 | 0.435 | 0.105 | 0.105 | 0.314 | -4.10E-04 | 0.213 | 0.998 | 0.012 | 0.088 | 0.889 |
| InternalIMT | aa | rs11499085 | 0 | . | . | . | . | . | . | . | . | . |
| InternalIMT | aa | rs188130606 | 0 | . | . | . | . | . | . | . | . | . |
| InternalIMT | aa | rs11511620 | 0.16 | 0.109 | 0.068 | 0.11 | -0.127 | 0.29 | 0.662 | 0.041 | 0.12 | 0.732 |
| InternalIMT | aa | rs181498390 | 0 | . | . | . | . | . | . | . | . | . |
| InternalIMT | aa | rs186280701 | 0 | . | . | . | . | . | . | . | . | . |
| InternalIMT | aa | rs150059185 | 0.011 | 0.116 | 0.062 | 0.061 | -0.018 | 1.2 | 0.988 | 0.063 | 0.476 | 0.894 |
| InternalIMT | aa | rs183338707 | 0.004 | . | . | . | . | . | . | . | . | . |
| InternalIMT | aa | rs11499038 | 0.002 | . | . | . | . | . | . | . | . | . |
| InternalIMT | aa | rs190699595 | 0.008 | . | . | . | . | . | . | . | . | . |
| InternalIMT | aa | rs11523408 | 0.16 | 0.109 | 0.068 | 0.11 | -0.127 | 0.29 | 0.661 | 0.041 | 0.12 | 0.731 |
| InternalIMT | aa | rs188049317 | 0 | . | . | . | . | . | . | . | . | . |
| InternalIMT | aa | rs144948634 | 0.427 | 0.118 | 0.105 | 0.263 | 0.031 | 0.221 | 0.888 | -1.80E-03 | 0.091 | 0.984 |
| InternalIMT | aa | rs143833576 | 0.001 | . | . | . | . | . | . | . | . | . |
| InternalIMT | aa | rs151239879 | 0.16 | 0.108 | 0.068 | 0.113 | -0.133 | 0.29 | 0.648 | 0.044 | 0.12 | 0.717 |
| InternalIMT | aa | rs7073743 | 0.438 | 0.11 | 0.105 | 0.297 | 0.011 | 0.213 | 0.958 | 7.00E-03 | 0.088 | 0.937 |
| InternalIMT | aa | rs142563349 | 0 | . | . | . | . | . | . | . | . | . |
| InternalIMT | aa | rs187246403 | 0 | . | . | . | . | . | . | . | . | . |
| InternalIMT | aa | rs150423942 | 0.158 | 0.111 | 0.068 | 0.104 | -0.114 | 0.298 | 0.701 | 0.035 | 0.123 | 0.773 |

| InternalIMT | aa | rs184706102 | 0.002 | . | . | . | . | . | . | . | . | . |
| --- | --- | --- | --- | --- | --- | --- | --- | --- | --- | --- | --- | --- |
| InternalIMT | aa | rs189626288 | 0.03 | 0.116 | 0.063 | 0.068 | 0.031 | 0.737 | 0.967 | 0.025 | 0.308 | 0.935 |
| InternalIMT | aa | rs140749834 | 0 | . | . | . | . | . | . | . | . | . |
| InternalIMT | aa | rs7078822 | 0.131 | 0.126 | 0.068 | 0.064 | -7.40E-03 | 0.322 | 0.982 | -0.019 | 0.132 | 0.886 |
| InternalIMT | aa | rs186227617 | 0.159 | 0.11 | 0.068 | 0.106 | -0.116 | 0.291 | 0.69 | 0.036 | 0.12 | 0.763 |
| InternalIMT | aa | rs188984368 | 0.024 | 0.097 | 0.063 | 0.126 | -0.829 | 1.01 | 0.414 | 0.373 | 0.429 | 0.384 |
| InternalIMT | aa | rs182078109 | 0.027 | 0.095 | 0.064 | 0.138 | -0.881 | 0.987 | 0.372 | 0.404 | 0.418 | 0.334 |
| InternalIMT | aa | rs141456274 | 0.002 | . | . | . | . | . | . | . | . | . |
| InternalIMT | aa | rs146976512 | 0.16 | 0.109 | 0.068 | 0.11 | -0.127 | 0.29 | 0.662 | 0.041 | 0.12 | 0.732 |
| InternalIMT | aa | rs138036816 | 0 | . | . | . | . | . | . | . | . | . |
| InternalIMT | aa | rs114879777 | 0.001 | . | . | . | . | . | . | . | . | . |
| InternalIMT | aa | rs149366475 | 0 | . | . | . | . | . | . | . | . | . |
| InternalIMT | aa | rs182463043 | 0 | . | . | . | . | . | . | . | . | . |
| InternalIMT | aa | rs144754632 | 0.001 | . | . | . | . | . | . | . | . | . |
| InternalIMT | aa | rs55717607 | 0.16 | 0.109 | 0.068 | 0.11 | -0.127 | 0.29 | 0.662 | 0.041 | 0.12 | 0.732 |
| InternalIMT | aa | rs187537973 | 0 | . | . | . | . | . | . | . | . | . |
| InternalIMT | aa | rs148085971 | 0 | . | . | . | . | . | . | . | . | . |
| InternalIMT | aa | rs11239491 | 0.16 | 0.109 | 0.068 | 0.11 | -0.127 | 0.29 | 0.662 | 0.041 | 0.12 | 0.732 |
| InternalIMT | aa | rs184412184 | 0.008 | . | . | . | . | . | . | . | . | . |
| InternalIMT | aa | rs4948998 | 0.606 | 0.09 | 0.121 | 0.459 | -0.037 | 0.2 | 0.854 | 0.021 | 0.083 | 0.796 |
| InternalIMT | aa | rs7076696 | 0.607 | 0.089 | 0.121 | 0.46 | -0.037 | 0.2 | 0.852 | 0.022 | 0.083 | 0.794 |
| InternalIMT | aa | rs11239492 | 0.16 | 0.109 | 0.068 | 0.11 | -0.127 | 0.29 | 0.662 | 0.041 | 0.12 | 0.732 |
| InternalIMT | aa | rs79462470 | 0 | . | . | . | . | . | . | . | . | . |
| InternalIMT | aa | rs187804654 | 0.003 | . | . | . | . | . | . | . | . | . |
| InternalIMT | aa | rs7093687 | 0.16 | 0.109 | 0.068 | 0.11 | -0.126 | 0.29 | 0.664 | 0.041 | 0.12 | 0.734 |
| InternalIMT | aa | rs7081004 | 0.16 | 0.109 | 0.068 | 0.11 | -0.126 | 0.29 | 0.663 | 0.041 | 0.12 | 0.733 |
| InternalIMT | aa | rs7084793 | 0.16 | 0.109 | 0.068 | 0.11 | -0.127 | 0.29 | 0.662 | 0.041 | 0.12 | 0.733 |
| InternalIMT | aa | rs61556080 | 0 | . | . | . | . | . | . | . | . | . |
| InternalIMT | aa | rs115552064 | . | . | . | . | . | . | . | . | . | . |
| InternalIMT | aa | rs7921503 | 0.16 | 0.109 | 0.068 | 0.11 | -0.127 | 0.29 | 0.662 | 0.041 | 0.12 | 0.732 |
| InternalIMT | aa | rs7909740 | 0.16 | 0.109 | 0.068 | 0.11 | -0.126 | 0.29 | 0.663 | 0.041 | 0.12 | 0.733 |
| InternalIMT | aa | rs7909768 | 0.16 | 0.109 | 0.068 | 0.11 | -0.126 | 0.29 | 0.663 | 0.041 | 0.12 | 0.733 |
| InternalIMT | aa | rs144168004 | 0 | . | . | . | . | . | . | . | . | . |
| InternalIMT | aa | rs75850448 | 0 | . | . | . | . | . | . | . | . | . |
| InternalIMT | aa | rs147777574 | 0 | . | . | . | . | . | . | . | . | . |
| InternalIMT | aa | rs7913488 | 0.16 | 0.109 | 0.068 | 0.11 | -0.126 | 0.29 | 0.663 | 0.041 | 0.12 | 0.733 |
| InternalIMT | aa | rs74390874 | 0.071 | 0.11 | 0.067 | 0.1 | -0.022 | 0.419 | 0.958 | 0.036 | 0.171 | 0.835 |
| InternalIMT | aa | rs141391142 | 0.009 | . | . | . | . | . | . | . | . | . |
| InternalIMT | aa | rs115294747 | 0.001 | . | . | . | . | . | . | . | . | . |
| InternalIMT | aa | rs148031781 | 0.003 | . | . | . | . | . | . | . | . | . |
| InternalIMT | aa | rs181340128 | 0.021 | 0.106 | 0.063 | 0.093 | -0.709 | 1.05 | 0.501 | 0.281 | 0.44 | 0.524 |
| InternalIMT | aa | rs189930007 | 0.002 | . | . | . | . | . | . | . | . | . |
| InternalIMT | aa | rs12242128 | 0 | . | . | . | . | . | . | . | . | . |
| InternalIMT | aa | rs78421750 | 0.002 | . | . | . | . | . | . | . | . | . |
| InternalIMT | aa | rs114370747 | 0.002 | . | . | . | . | . | . | . | . | . |
| InternalIMT | aa | rs12264166 | 0 | . | . | . | . | . | . | . | . | . |
| InternalIMT | aa | rs182964822 | 0.001 | . | . | . | . | . | . | . | . | . |
| InternalIMT | aa | rs67491417 | 0.15 | 0.119 | 0.067 | 0.076 | -0.038 | 0.298 | 0.898 | 5.30E-03 | 0.123 | 0.966 |
| InternalIMT | aa | rs72789720 | 0.023 | 0.111 | 0.063 | 0.08 | -0.413 | 0.746 | 0.58 | 0.167 | 0.307 | 0.586 |
| InternalIMT | aa | rs6413416 | 0.16 | 0.111 | 0.068 | 0.105 | -0.113 | 0.29 | 0.698 | 0.035 | 0.12 | 0.771 |
| InternalIMT | aa | rs4986832 | 0.16 | 0.109 | 0.068 | 0.11 | -0.126 | 0.29 | 0.663 | 0.041 | 0.12 | 0.734 |
| InternalIMT | aa | rs144866316 | 0.005 | . | . | . | . | . | . | . | . | . |
| InternalIMT | aa | rs187769540 | 0.001 | . | . | . | . | . | . | . | . | . |
| InternalIMT | aa | rs6593482 | 0.16 | 0.107 | 0.068 | 0.118 | -0.151 | 0.29 | 0.603 | 0.05 | 0.12 | 0.679 |
| InternalIMT | aa | rs7913948 | 0.16 | 0.109 | 0.068 | 0.109 | -0.124 | 0.289 | 0.668 | 0.04 | 0.12 | 0.739 |
| InternalIMT | aa | rs77185151 | 0.001 | . | . | . | . | . | . | . | . | . |
| InternalIMT | aa | rs12762303 | 0.16 | 0.109 | 0.068 | 0.11 | -0.127 | 0.29 | 0.662 | 0.041 | 0.12 | 0.732 |
| InternalIMT | aa | rs73283136 | 0.003 | . | . | . | . | . | . | . | . | . |
| InternalIMT | aa | rs187180218 | 0 | . | . | . | . | . | . | . | . | . |
| InternalIMT | aa | rs78137087 | 0.159 | 0.107 | 0.068 | 0.116 | -0.142 | 0.291 | 0.625 | 0.047 | 0.12 | 0.695 |
| InternalIMT | aa | rs72561472 | 0.158 | 0.101 | 0.069 | 0.143 | -0.196 | 0.297 | 0.51 | 0.07 | 0.123 | 0.57 |
| InternalIMT | aa | rs4987106 | 0.152 | 0.11 | 0.068 | 0.106 | -0.126 | 0.298 | 0.673 | 0.039 | 0.123 | 0.75 |
| InternalIMT | aa | rs4987105 | 0.155 | 0.106 | 0.068 | 0.12 | -0.152 | 0.296 | 0.608 | 0.052 | 0.122 | 0.671 |
| InternalIMT | aa | rs111916157 | 0.188 | 0.115 | 0.071 | 0.106 | -0.045 | 0.281 | 0.872 | 0.013 | 0.116 | 0.914 |
| InternalIMT | aa | rs111528152 | 0.16 | 0.109 | 0.068 | 0.11 | -0.124 | 0.29 | 0.667 | 0.04 | 0.12 | 0.738 |
| InternalIMT | aa | rs12763738 | 0.16 | 0.109 | 0.068 | 0.11 | -0.126 | 0.29 | 0.663 | 0.041 | 0.12 | 0.733 |
| InternalIMT | aa | rs191675886 | 0.006 | . | . | . | . | . | . | . | . | . |
| InternalIMT | aa | rs66489870 | 0.16 | 0.109 | 0.068 | 0.11 | -0.126 | 0.29 | 0.663 | 0.041 | 0.12 | 0.733 |
| InternalIMT | aa | rs71494775 | 0.16 | 0.109 | 0.068 | 0.11 | -0.126 | 0.29 | 0.663 | 0.041 | 0.12 | 0.733 |
| InternalIMT | aa | rs61855875 | 0.033 | 0.131 | 0.064 | 0.04 | 0.575 | 0.986 | 0.56 | -0.222 | 0.409 | 0.587 |
| InternalIMT | aa | rs116356837 | 0 | . | . | . | . | . | . | . | . | . |
| InternalIMT | aa | rs1864414 | 0.16 | 0.109 | 0.068 | 0.11 | -0.126 | 0.29 | 0.663 | 0.041 | 0.12 | 0.733 |
| InternalIMT | aa | rs1864413 | 0.16 | 0.109 | 0.068 | 0.112 | -0.13 | 0.29 | 0.655 | 0.042 | 0.12 | 0.725 |
| InternalIMT | aa | rs139497212 | 0 | . | . | . | . | . | . | . | . | . |
| InternalIMT | aa | rs892690 | 0.358 | 0.166 | 0.096 | 0.083 | 0.187 | 0.224 | 0.404 | -0.067 | 0.092 | 0.469 |
| InternalIMT | aa | rs1864412 | 0.16 | 0.109 | 0.068 | 0.11 | -0.126 | 0.29 | 0.665 | 0.041 | 0.12 | 0.735 |
| InternalIMT | aa | rs77827771 | 0.004 | . | . | . | . | . | . | . | . | . |
| InternalIMT | aa | rs145294963 | 0.004 | . | . | . | . | . | . | . | . | . |
| InternalIMT | aa | rs149081327 | 0.012 | 0.099 | 0.062 | 0.11 | -1.85 | 1.05 | 0.078 | 0.786 | 0.427 | 0.066 |
| InternalIMT | aa | rs67008983 | 0.16 | 0.11 | 0.068 | 0.108 | -0.122 | 0.29 | 0.675 | 0.039 | 0.12 | 0.745 |
| InternalIMT | aa | rs56150414 | 0.168 | 0.119 | 0.069 | 0.084 | -0.043 | 0.284 | 0.88 | 6.70E-03 | 0.118 | 0.955 |
| InternalIMT | aa | rs55787481 | 0.16 | 0.11 | 0.068 | 0.108 | -0.122 | 0.29 | 0.676 | 0.039 | 0.12 | 0.746 |
| InternalIMT | aa | rs141270101 | 0.001 | . | . | . | . | . | . | . | . | . |
| InternalIMT | aa | rs114980075 | 0.002 | . | . | . | . | . | . | . | . | . |
| InternalIMT | aa | rs12783095 | 0.159 | 0.112 | 0.068 | 0.101 | -0.103 | 0.291 | 0.723 | 0.031 | 0.12 | 0.794 |
| InternalIMT | aa | rs182342688 | 0.005 | . | . | . | . | . | . | . | . | . |

| InternalIMT | aa | rs17157728 | 0.159 | 0.112 | 0.068 | 0.101 | -0.103 | 0.291 | 0.724 | 0.031 | 0.12 | 0.795 |
| --- | --- | --- | --- | --- | --- | --- | --- | --- | --- | --- | --- | --- |
| InternalIMT | aa | rs114835563 | 0 | . | . | . | . | . | . | . | . | . |
| InternalIMT | aa | rs3824612 | 0.358 | 0.166 | 0.096 | 0.083 | 0.188 | 0.224 | 0.4 | -0.067 | 0.092 | 0.468 |
| InternalIMT | aa | rs17157733 | 0.158 | 0.113 | 0.068 | 0.097 | -0.091 | 0.291 | 0.754 | 0.026 | 0.12 | 0.828 |
| InternalIMT | aa | rs74128478 | 0 | . | . | . | . | . | . | . | . | . |
| InternalIMT | aa | rs184973589 | 0.001 | . | . | . | . | . | . | . | . | . |
| InternalIMT | aa | rs150479461 | 0 | . | . | . | . | . | . | . | . | . |
| InternalIMT | aa | rs138310407 | 0.004 | . | . | . | . | . | . | . | . | . |
| InternalIMT | aa | rs187426603 | 0.001 | . | . | . | . | . | . | . | . | . |
| InternalIMT | aa | rs190843143 | 0.003 | . | . | . | . | . | . | . | . | . |
| InternalIMT | aa | rs191883449 | 0.002 | . | . | . | . | . | . | . | . | . |
| InternalIMT | aa | rs58238740 | 0.158 | 0.113 | 0.068 | 0.098 | -0.096 | 0.291 | 0.74 | 0.029 | 0.12 | 0.812 |
| InternalIMT | aa | rs3780894 | 0.158 | 0.113 | 0.068 | 0.098 | -0.096 | 0.291 | 0.742 | 0.028 | 0.12 | 0.814 |
| InternalIMT | aa | rs149646626 | 0.027 | 0.149 | 0.062 | 0.016 | 1.5 | 1.08 | 0.163 | -0.584 | 0.443 | 0.188 |
| InternalIMT | aa | rs79615212 | 0.023 | 0.112 | 0.063 | 0.075 | -0.309 | 0.881 | 0.726 | 0.094 | 0.379 | 0.805 |
| InternalIMT | aa | rs3780895 | 0.158 | 0.113 | 0.068 | 0.098 | -0.096 | 0.291 | 0.742 | 0.028 | 0.12 | 0.814 |
| InternalIMT | aa | rs3780896 | 0.158 | 0.113 | 0.068 | 0.098 | -0.095 | 0.29 | 0.742 | 0.028 | 0.12 | 0.814 |
| InternalIMT | aa | rs142747163 | 0.006 | . | . | . | . | . | . | . | . | . |
| InternalIMT | aa | rs189417584 | 0.005 | . | . | . | . | . | . | . | . | . |
| InternalIMT | aa | rs3780897 | 0.158 | 0.113 | 0.068 | 0.097 | -0.093 | 0.291 | 0.749 | 0.027 | 0.12 | 0.82 |
| InternalIMT | aa | rs117509491 | 0.004 | . | . | . | . | . | . | . | . | . |
| InternalIMT | aa | rs117811514 | 0.004 | . | . | . | . | . | . | . | . | . |
| InternalIMT | aa | rs116500932 | 0 | . | . | . | . | . | . | . | . | . |
| InternalIMT | aa | rs77584168 | 0.001 | . | . | . | . | . | . | . | . | . |
| InternalIMT | aa | rs2310776 | 0.158 | 0.113 | 0.068 | 0.098 | -0.095 | 0.29 | 0.744 | 0.028 | 0.12 | 0.816 |
| InternalIMT | aa | rs17157736 | 0.158 | 0.113 | 0.068 | 0.098 | -0.095 | 0.29 | 0.744 | 0.028 | 0.12 | 0.816 |
| InternalIMT | aa | rs72789735 | 0.105 | 0.114 | 0.066 | 0.081 | -0.112 | 0.358 | 0.754 | 0.033 | 0.148 | 0.825 |
| InternalIMT | aa | rs183747187 | 0.002 | . | . | . | . | . | . | . | . | . |
| InternalIMT | aa | rs7077891 | 0.158 | 0.113 | 0.068 | 0.098 | -0.094 | 0.29 | 0.745 | 0.028 | 0.12 | 0.817 |
| InternalIMT | aa | rs145063305 | 0.006 | . | . | . | . | . | . | . | . | . |
| InternalIMT | aa | rs3824613 | 0.159 | 0.113 | 0.068 | 0.097 | -0.092 | 0.29 | 0.751 | 0.027 | 0.12 | 0.823 |
| InternalIMT | aa | rs145543199 | 0.014 | 0.105 | 0.062 | 0.091 | -1.1 | 1.01 | 0.275 | 0.45 | 0.422 | 0.287 |
| InternalIMT | aa | rs140361993 | 0.005 | . | . | . | . | . | . | . | . | . |
| InternalIMT | aa | rs11239496 | 0.105 | 0.114 | 0.066 | 0.081 | -0.112 | 0.358 | 0.754 | 0.033 | 0.148 | 0.825 |
| InternalIMT | aa | rs11239497 | 0.158 | 0.113 | 0.068 | 0.098 | -0.094 | 0.29 | 0.745 | 0.028 | 0.12 | 0.817 |
| InternalIMT | aa | rs10900212 | 0.158 | 0.113 | 0.068 | 0.098 | -0.094 | 0.29 | 0.745 | 0.028 | 0.12 | 0.817 |
| InternalIMT | aa | rs186487136 | 0 | . | . | . | . | . | . | . | . | . |
| InternalIMT | aa | rs12774369 | 0.158 | 0.113 | 0.068 | 0.098 | -0.096 | 0.291 | 0.741 | 0.028 | 0.12 | 0.815 |
| InternalIMT | aa | rs60842538 | 0.158 | 0.113 | 0.068 | 0.098 | -0.098 | 0.291 | 0.737 | 0.029 | 0.12 | 0.811 |
| InternalIMT | aa | rs3780898 | 0.158 | 0.113 | 0.068 | 0.098 | -0.1 | 0.291 | 0.731 | 0.029 | 0.12 | 0.807 |
| InternalIMT | aa | rs3780899 | 0.159 | 0.113 | 0.068 | 0.099 | -0.101 | 0.291 | 0.728 | 0.03 | 0.12 | 0.805 |
| InternalIMT | aa | rs12252472 | 0 | . | . | . | . | . | . | . | . | . |
| InternalIMT | aa | rs185411011 | 0.003 | . | . | . | . | . | . | . | . | . |
| InternalIMT | aa | rs28395865 | 0.156 | 0.11 | 0.068 | 0.106 | -0.123 | 0.296 | 0.679 | 0.039 | 0.123 | 0.754 |
| InternalIMT | aa | rs191004074 | 0.024 | 0.106 | 0.064 | 0.097 | -0.478 | 0.76 | 0.53 | 0.194 | 0.328 | 0.553 |
| InternalIMT | aa | rs182401295 | 0.004 | . | . | . | . | . | . | . | . | . |
| InternalIMT | aa | rs2228064 | 0.002 | . | . | . | . | . | . | . | . | . |
| InternalIMT | aa | rs188284159 | 0.001 | . | . | . | . | . | . | . | . | . |
| InternalIMT | aa | rs2303887 | 0.001 | . | . | . | . | . | . | . | . | . |
| InternalIMT | aa | rs2303886 | 0.001 | . | . | . | . | . | . | . | . | . |
| InternalIMT | aa | rs192621006 | 0.003 | . | . | . | . | . | . | . | . | . |
| InternalIMT | aa | rs2303885 | 0.154 | 0.108 | 0.068 | 0.113 | -0.169 | 0.293 | 0.563 | 0.054 | 0.122 | 0.656 |
| InternalIMT | aa | rs114466168 | 0 | . | . | . | . | . | . | . | . | . |
| InternalIMT | aa | rs11239501 | 0.149 | 0.106 | 0.067 | 0.113 | -0.132 | 0.281 | 0.639 | 0.049 | 0.117 | 0.675 |
| InternalIMT | aa | rs745986 | 0.204 | 0.103 | 0.069 | 0.137 | -0.151 | 0.236 | 0.523 | 0.052 | 0.098 | 0.594 |
| InternalIMT | aa | rs75262268 | 0 | . | . | . | . | . | . | . | . | . |
| InternalIMT | aa | rs71494776 | 0.05 | 0.11 | 0.063 | 0.08 | -0.299 | 0.513 | 0.56 | 0.105 | 0.213 | 0.622 |
| InternalIMT | aa | rs74386871 | 0.011 | 0.114 | 0.062 | 0.064 | -0.1 | 1.46 | 0.945 | 0.135 | 0.617 | 0.827 |
| InternalIMT | aa | rs76502040 | 0.01 | 0.135 | 0.061 | 0.027 | 1.87 | 1.48 | 0.207 | -0.754 | 0.617 | 0.221 |
| InternalIMT | aa | rs142213734 | 0.001 | . | . | . | . | . | . | . | . | . |
| InternalIMT | aa | rs4948671 | 0.604 | 0.085 | 0.123 | 0.489 | -0.042 | 0.203 | 0.838 | 0.025 | 0.084 | 0.767 |
| InternalIMT | aa | rs146286085 | 0.002 | . | . | . | . | . | . | . | . | . |
| InternalIMT | aa | rs144080623 | 0 | . | . | . | . | . | . | . | . | . |
| InternalIMT | aa | rs115074014 | 0 | . | . | . | . | . | . | . | . | . |
| InternalIMT | aa | rs17157756 | 0 | . | . | . | . | . | . | . | . | . |
| InternalIMT | aa | rs190459780 | 0.001 | . | . | . | . | . | . | . | . | . |
| InternalIMT | aa | rs12358647 | 0.204 | 0.104 | 0.069 | 0.135 | -0.144 | 0.235 | 0.54 | 0.05 | 0.098 | 0.611 |
| InternalIMT | aa | rs147080556 | 0.011 | 0.114 | 0.062 | 0.065 | -0.438 | 0.743 | 0.555 | 0.121 | 0.315 | 0.7 |
| InternalIMT | aa | rs187543349 | 0.004 | . | . | . | . | . | . | . | . | . |
| InternalIMT | aa | rs114375595 | 0.004 | . | . | . | . | . | . | . | . | . |
| InternalIMT | aa | rs117211200 | 0.01 | . | . | . | . | . | . | . | . | . |
| InternalIMT | aa | rs192963047 | 0.001 | . | . | . | . | . | . | . | . | . |
| InternalIMT | aa | rs11239505 | 0.204 | 0.103 | 0.069 | 0.138 | -0.15 | 0.235 | 0.523 | 0.053 | 0.098 | 0.591 |
| InternalIMT | aa | rs184434116 | 0.003 | . | . | . | . | . | . | . | . | . |
| InternalIMT | aa | rs4948999 | 0.604 | 0.084 | 0.122 | 0.492 | -0.042 | 0.203 | 0.837 | 0.025 | 0.084 | 0.764 |
| InternalIMT | aa | rs11239506 | 0.154 | 0.109 | 0.067 | 0.106 | -0.106 | 0.272 | 0.697 | 0.038 | 0.113 | 0.735 |
| InternalIMT | aa | rs190706620 | 0.006 | . | . | . | . | . | . | . | . | . |
| InternalIMT | aa | rs190770429 | 0.002 | . | . | . | . | . | . | . | . | . |
| InternalIMT | aa | rs181327133 | 0.006 | . | . | . | . | . | . | . | . | . |
| InternalIMT | aa | rs149251697 | 0.008 | . | . | . | . | . | . | . | . | . |
| InternalIMT | aa | rs118081602 | 0.001 | . | . | . | . | . | . | . | . | . |
| InternalIMT | aa | rs188233691 | 0.001 | . | . | . | . | . | . | . | . | . |
| InternalIMT | aa | rs183778794 | 0.005 | . | . | . | . | . | . | . | . | . |
| InternalIMT | aa | rs188040078 | 0.007 | . | . | . | . | . | . | . | . | . |
| InternalIMT | aa | rs184675372 | 0.003 | . | . | . | . | . | . | . | . | . |
| InternalIMT | aa | rs150326891 | 0.116 | 0.086 | 0.067 | 0.197 | -0.416 | 0.312 | 0.182 | 0.165 | 0.131 | 0.207 |

| InternalIMT | aa | rs185520021 | 0.002 | . | . | . | . | . | . | . | . | . |
| --- | --- | --- | --- | --- | --- | --- | --- | --- | --- | --- | --- | --- |
| InternalIMT | aa | rs7907779 | 0.154 | 0.109 | 0.067 | 0.106 | -0.106 | 0.271 | 0.696 | 0.038 | 0.113 | 0.733 |
| InternalIMT | aa | rs184962860 | 0.001 | . | . | . | . | . | . | . | . | . |
| InternalIMT | aa | rs149762784 | 0.025 | 0.103 | 0.065 | 0.112 | -0.557 | 0.919 | 0.544 | 0.241 | 0.389 | 0.536 |
| InternalIMT | aa | rs149203427 | 0.021 | 0.116 | 0.063 | 0.063 | 0.053 | 0.956 | 0.956 | 0.011 | 0.395 | 0.978 |
| InternalIMT | aa | rs60153083 | 0 | . | . | . | . | . | . | . | . | . |
| InternalIMT | aa | rs112001069 | 0.05 | 0.111 | 0.063 | 0.079 | -0.278 | 0.509 | 0.585 | 0.097 | 0.212 | 0.647 |
| InternalIMT | aa | rs182355382 | 0.021 | 0.116 | 0.061 | 0.059 | 0.018 | 1.25 | 0.989 | 0.024 | 0.524 | 0.964 |
| InternalIMT | aa | rs186740974 | 0.117 | 0.121 | 0.073 | 0.099 | 0.035 | 0.488 | 0.943 | -0.015 | 0.204 | 0.941 |
| InternalIMT | aa | rs191874916 | 0.014 | 0.106 | 0.062 | 0.088 | -0.944 | 1.15 | 0.412 | 0.441 | 0.457 | 0.335 |
| InternalIMT | aa | rs182989412 | 0.152 | 0.104 | 0.079 | 0.188 | -0.102 | 0.414 | 0.805 | 0.056 | 0.175 | 0.751 |
| InternalIMT | aa | rs188819476 | 0.002 | . | . | . | . | . | . | . | . | . |
| InternalIMT | aa | rs141217183 | 0.078 | 0.11 | 0.067 | 0.099 | -0.202 | 0.459 | 0.659 | 0.06 | 0.193 | 0.756 |
| InternalIMT | aa | rs192922652 | 0.045 | 0.117 | 0.063 | 0.063 | -0.136 | 0.565 | 0.809 | 0.031 | 0.235 | 0.895 |
| InternalIMT | aa | rs185092343 | 0.024 | 0.116 | 0.064 | 0.068 | 0.181 | 1.05 | 0.863 | -0.018 | 0.439 | 0.968 |
| InternalIMT | aa | rs145193311 | 0.051 | 0.12 | 0.064 | 0.06 | -0.039 | 0.532 | 0.942 | -5.10E-03 | 0.221 | 0.982 |
| InternalIMT | aa | rs55789336 | 0.155 | 0.1 | 0.068 | 0.141 | -0.18 | 0.277 | 0.516 | 0.071 | 0.115 | 0.539 |
| InternalIMT | aa | rs55837212 | 0.152 | 0.113 | 0.067 | 0.093 | -0.072 | 0.266 | 0.788 | 0.024 | 0.11 | 0.825 |
| InternalIMT | aa | rs75408574 | 0.366 | 0.146 | 0.097 | 0.134 | 0.125 | 0.222 | 0.573 | -0.037 | 0.091 | 0.681 |
| InternalIMT | aa | rs149306926 | 0.161 | 0.112 | 0.068 | 0.101 | -0.08 | 0.258 | 0.755 | 0.025 | 0.107 | 0.812 |
| InternalIMT | aa | rs35990892 | 0.597 | 0.134 | 0.13 | 0.303 | 0.041 | 0.22 | 0.853 | -0.014 | 0.09 | 0.873 |
| InternalIMT | aa | rs192356207 | 0.019 | 0.126 | 0.062 | 0.043 | 0.495 | 1.06 | 0.642 | -0.209 | 0.436 | 0.632 |
| InternalIMT | aa | rs183741163 | 0.129 | 0.106 | 0.067 | 0.112 | -0.123 | 0.324 | 0.705 | 0.05 | 0.134 | 0.71 |
| InternalIMT | aa | rs34737297 | 0.48 | 0.101 | 0.113 | 0.37 | -0.018 | 0.22 | 0.934 | 0.016 | 0.091 | 0.859 |
| InternalIMT | aa | rs181346933 | 0.026 | 0.082 | 0.064 | 0.198 | -1.59 | 0.654 | 0.015 | 0.633 | 0.277 | 0.022 |
| InternalIMT | aa | rs36093253 | 0.343 | 0.128 | 0.095 | 0.178 | 0.06 | 0.228 | 0.794 | -0.015 | 0.093 | 0.871 |
| InternalIMT | aa | rs186307855 | 0.02 | 0.117 | 0.063 | 0.062 | 0.29 | 0.944 | 0.759 | -0.058 | 0.401 | 0.886 |
| InternalIMT | aa | rs189681865 | 0.003 | . | . | . | . | . | . | . | . | . |
| InternalIMT | aa | rs34942971 | 0.207 | 0.111 | 0.07 | 0.113 | -0.084 | 0.241 | 0.729 | 0.026 | 0.099 | 0.791 |
| InternalIMT | aa | rs181816554 | 0.019 | 0.134 | 0.062 | 0.03 | 1.13 | 1.21 | 0.35 | -0.439 | 0.505 | 0.385 |
| InternalIMT | aa | rs186846881 | 0.004 | . | . | . | . | . | . | . | . | . |
| InternalIMT | aa | rs191178220 | 0.024 | 0.123 | 0.063 | 0.05 | 0.324 | 0.835 | 0.698 | -0.095 | 0.353 | 0.788 |
| InternalIMT | aa | rs183305228 | 0 | . | . | . | . | . | . | . | . | . |
| InternalIMT | aa | rs139912761 | 0.15 | 0.118 | 0.067 | 0.079 | -0.023 | 0.278 | 0.934 | 3.50E-03 | 0.115 | 0.976 |
| InternalIMT | aa | rs185499901 | 0.019 | 0.128 | 0.062 | 0.04 | 0.658 | 1.29 | 0.61 | -0.206 | 0.544 | 0.704 |
| InternalIMT | aa | rs189816197 | 0.061 | 0.112 | 0.064 | 0.08 | -0.185 | 0.452 | 0.682 | 0.062 | 0.189 | 0.742 |
| InternalIMT | aa | rs145656938 | 0.014 | 0.096 | 0.063 | 0.128 | -1.75 | 1.74 | 0.315 | 0.733 | 0.751 | 0.329 |
| InternalIMT | aa | rs35745197 | 0.05 | 0.111 | 0.063 | 0.078 | -0.274 | 0.509 | 0.591 | 0.094 | 0.212 | 0.656 |
| InternalIMT | aa | rs192856637 | 0.006 | . | . | . | . | . | . | . | . | . |
| InternalIMT | aa | rs2310775 | 0.401 | 0.128 | 0.096 | 0.184 | 0.062 | 0.201 | 0.757 | -0.013 | 0.082 | 0.878 |
| InternalIMT | aa | rs11239507 | 0.403 | 0.121 | 0.099 | 0.222 | 0.045 | 0.207 | 0.829 | -4.60E-03 | 0.085 | 0.957 |
| InternalIMT | aa | rs74648467 | 0.049 | 0.11 | 0.063 | 0.08 | -0.295 | 0.516 | 0.568 | 0.103 | 0.214 | 0.63 |
| InternalIMT | aa | rs145815934 | 0.022 | 0.133 | 0.064 | 0.036 | 0.578 | 0.686 | 0.4 | -0.243 | 0.28 | 0.385 |
| InternalIMT | aa | rs145390687 | 0.029 | 0.129 | 0.063 | 0.043 | 0.636 | 0.692 | 0.358 | -0.216 | 0.284 | 0.448 |
| InternalIMT | aa | rs184354913 | 0.049 | 0.141 | 0.064 | 0.026 | 0.637 | 0.739 | 0.389 | -0.244 | 0.309 | 0.429 |
| InternalIMT | aa | rs181244920 | 0.007 | . | . | . | . | . | . | . | . | . |
| InternalIMT | aa | rs11499087 | 0 | . | . | . | . | . | . | . | . | . |
| InternalIMT | aa | rs148263794 | 0.05 | 0.111 | 0.063 | 0.078 | -0.275 | 0.508 | 0.588 | 0.095 | 0.211 | 0.653 |
| InternalIMT | aa | rs150758903 | 0.01 | 0.115 | 0.062 | 0.065 | -0.482 | 1.28 | 0.707 | 0.158 | 0.53 | 0.765 |
| InternalIMT | aa | rs147654382 | 0.183 | 0.107 | 0.068 | 0.116 | -0.114 | 0.24 | 0.636 | 0.04 | 0.099 | 0.689 |
| InternalIMT | aa | rs147495166 | 0 | . | . | . | . | . | . | . | . | . |
| InternalIMT | aa | rs35198778 | 0.092 | 0.114 | 0.067 | 0.091 | -0.127 | 0.341 | 0.71 | 0.036 | 0.142 | 0.8 |
| InternalIMT | aa | rs145207659 | 0.071 | 0.112 | 0.067 | 0.094 | 0.024 | 0.428 | 0.955 | 0.023 | 0.175 | 0.895 |
| InternalIMT | aa | rs186466464 | 0.003 | . | . | . | . | . | . | . | . | . |
| InternalIMT | aa | rs12778413 | 0.157 | 0.096 | 0.069 | 0.164 | -0.197 | 0.302 | 0.514 | 0.079 | 0.125 | 0.527 |
| InternalIMT | aa | rs7895552 | 0.531 | 0.047 | 0.119 | 0.692 | -0.123 | 0.217 | 0.572 | 0.063 | 0.09 | 0.483 |
| InternalIMT | aa | rs12783895 | 0.05 | 0.111 | 0.063 | 0.078 | -0.273 | 0.507 | 0.591 | 0.094 | 0.211 | 0.656 |
| InternalIMT | aa | rs34929604 | 0.05 | 0.111 | 0.063 | 0.078 | -0.274 | 0.507 | 0.589 | 0.094 | 0.211 | 0.654 |
| InternalIMT | aa | rs12761961 | 0.049 | 0.111 | 0.063 | 0.079 | -0.282 | 0.513 | 0.583 | 0.098 | 0.214 | 0.645 |
| InternalIMT | aa | rs35536930 | 0.198 | 0.109 | 0.069 | 0.113 | -0.107 | 0.231 | 0.641 | 0.035 | 0.096 | 0.716 |
| InternalIMT | aa | rs180951060 | 0 | . | . | . | . | . | . | . | . | . |
| InternalIMT | aa | rs148892128 | 0.022 | 0.133 | 0.064 | 0.036 | 0.578 | 0.686 | 0.399 | -0.243 | 0.28 | 0.385 |
| InternalIMT | aa | rs11239509 | 0.001 | . | . | . | . | . | . | . | . | . |
| InternalIMT | aa | rs41526545 | 0.154 | 0.108 | 0.067 | 0.107 | -0.108 | 0.27 | 0.688 | 0.04 | 0.112 | 0.722 |
| InternalIMT | aa | rs2029253 | 0.401 | 0.12 | 0.099 | 0.225 | 0.038 | 0.206 | 0.855 | -3.00E-03 | 0.084 | 0.972 |
| InternalIMT | aa | rs17444064 | 0.154 | 0.109 | 0.067 | 0.107 | -0.106 | 0.27 | 0.694 | 0.039 | 0.112 | 0.727 |
| InternalIMT | aa | rs71494779 | 0.05 | 0.111 | 0.063 | 0.078 | -0.276 | 0.504 | 0.584 | 0.096 | 0.209 | 0.648 |
| InternalIMT | aa | rs74991420 | 0.001 | . | . | . | . | . | . | . | . | . |
| InternalIMT | aa | rs182736551 | 0.001 | . | . | . | . | . | . | . | . | . |
| InternalIMT | aa | rs78702074 | 0.001 | . | . | . | . | . | . | . | . | . |
| InternalIMT | aa | rs187791466 | 0.005 | . | . | . | . | . | . | . | . | . |
| InternalIMT | aa | rs35300270 | 0.054 | 0.106 | 0.064 | 0.095 | -0.374 | 0.486 | 0.442 | 0.136 | 0.202 | 0.501 |
| InternalIMT | aa | rs6593484 | 0.155 | 0.108 | 0.067 | 0.107 | -0.108 | 0.269 | 0.688 | 0.04 | 0.112 | 0.722 |
| InternalIMT | aa | rs12244601 | 0.004 | . | . | . | . | . | . | . | . | . |
| InternalIMT | aa | rs184429574 | 0.005 | . | . | . | . | . | . | . | . | . |
| InternalIMT | aa | rs7080897 | 0.05 | 0.111 | 0.063 | 0.078 | -0.276 | 0.504 | 0.583 | 0.096 | 0.209 | 0.648 |
| InternalIMT | aa | rs7097429 | 0.054 | 0.107 | 0.063 | 0.091 | -0.347 | 0.48 | 0.469 | 0.131 | 0.199 | 0.512 |
| InternalIMT | aa | rs77979229 | 0.001 | . | . | . | . | . | . | . | . | . |
| InternalIMT | aa | rs11239512 | 0.155 | 0.108 | 0.067 | 0.107 | -0.108 | 0.269 | 0.687 | 0.04 | 0.112 | 0.72 |
| InternalIMT | aa | rs60146827 | 0.05 | 0.111 | 0.063 | 0.078 | -0.277 | 0.504 | 0.582 | 0.096 | 0.209 | 0.647 |
| InternalIMT | aa | rs79032236 | 0 | . | . | . | . | . | . | . | . | . |
| InternalIMT | aa | rs140854206 | 0.009 | . | . | . | . | . | . | . | . | . |
| InternalIMT | aa | rs184049550 | 0 | . | . | . | . | . | . | . | . | . |
| InternalIMT | aa | rs114433607 | 0 | . | . | . | . | . | . | . | . | . |
| InternalIMT | aa | rs117653793 | 0.001 | . | . | . | . | . | . | . | . | . |
| InternalIMT | aa | rs117039533 | 0.001 | . | . | . | . | . | . | . | . | . |

| InternalIMT | aa | rs191660761 | 0.006 | . | . | . | . | . | . | . | . | . |
| --- | --- | --- | --- | --- | --- | --- | --- | --- | --- | --- | --- | --- |
| InternalIMT | aa | rs59514896 | 0.05 | 0.11 | 0.063 | 0.081 | -0.301 | 0.511 | 0.556 | 0.106 | 0.212 | 0.618 |
| InternalIMT | aa | rs184661876 | 0.002 | . | . | . | . | . | . | . | . | . |
| InternalIMT | aa | rs61854073 | 0.037 | 0.136 | 0.064 | 0.034 | 0.658 | 0.89 | 0.46 | -0.24 | 0.367 | 0.513 |
| InternalIMT | aa | rs2018518 | 0.315 | 0.182 | 0.09 | 0.044 | 0.263 | 0.225 | 0.243 | -0.098 | 0.092 | 0.286 |
| InternalIMT | aa | rs934187 | 0.607 | 0.082 | 0.123 | 0.505 | -0.047 | 0.203 | 0.816 | 0.027 | 0.084 | 0.746 |
| InternalIMT | aa | rs7918542 | 0.205 | 0.102 | 0.069 | 0.141 | -0.152 | 0.232 | 0.513 | 0.054 | 0.096 | 0.577 |
| InternalIMT | aa | rs144311920 | 0 | . | . | . | . | . | . | . | . | . |
| InternalIMT | aa | rs7099684 | 0.79 | 0.015 | 0.179 | 0.934 | -0.124 | 0.255 | 0.626 | 0.065 | 0.105 | 0.536 |
| InternalIMT | aa | rs150829171 | 0.002 | . | . | . | . | . | . | . | . | . |
| InternalIMT | aa | rs186060697 | 0 | . | . | . | . | . | . | . | . | . |
| InternalIMT | aa | rs11239513 | 0.009 | . | . | . | . | . | . | . | . | . |
| InternalIMT | aa | rs7917687 | 0.05 | 0.111 | 0.063 | 0.079 | -0.28 | 0.505 | 0.579 | 0.097 | 0.21 | 0.643 |
| InternalIMT | aa | rs61854076 | 0.014 | 0.14 | 0.062 | 0.024 | 2 | 1.4 | 0.153 | -0.819 | 0.583 | 0.16 |
| InternalIMT | aa | rs71494780 | 0.019 | 0.107 | 0.062 | 0.084 | -0.82 | 0.534 | 0.125 | 0.319 | 0.227 | 0.159 |
| InternalIMT | aa | rs7919239 | 0.205 | 0.101 | 0.069 | 0.146 | -0.156 | 0.231 | 0.5 | 0.057 | 0.096 | 0.556 |
| InternalIMT | aa | rs78334475 | 0.003 | . | . | . | . | . | . | . | . | . |
| InternalIMT | aa | rs3780900 | 0.011 | 0.115 | 0.062 | 0.061 | 0.214 | 1.32 | 0.871 | -2.60E-03 | 0.517 | 0.996 |
| InternalIMT | aa | rs186729292 | 0.001 | . | . | . | . | . | . | . | . | . |
| InternalIMT | aa | rs12267438 | 0.001 | . | . | . | . | . | . | . | . | . |
| InternalIMT | aa | rs1369214 | 0.454 | 0.11 | 0.104 | 0.29 | 7.40E-03 | 0.205 | 0.971 | 6.90E-03 | 0.084 | 0.934 |
| InternalIMT | aa | rs9282589 | 0.011 | 0.117 | 0.062 | 0.057 | -0.08 | 1.02 | 0.938 | 0.021 | 0.429 | 0.96 |
| InternalIMT | aa | rs114522915 | 0 | . | . | . | . | . | . | . | . | . |
| InternalIMT | aa | rs2115819 | 0.452 | 0.108 | 0.103 | 0.296 | -3.30E-04 | 0.204 | 0.999 | 8.90E-03 | 0.084 | 0.915 |
| InternalIMT | aa | rs117664603 | 0.001 | . | . | . | . | . | . | . | . | . |
| InternalIMT | aa | rs184979202 | 0.001 | . | . | . | . | . | . | . | . | . |
| InternalIMT | aa | rs190714671 | 0.001 | . | . | . | . | . | . | . | . | . |
| InternalIMT | aa | rs141538996 | 0 | . | . | . | . | . | . | . | . | . |
| InternalIMT | aa | rs150881741 | 0.003 | . | . | . | . | . | . | . | . | . |
| InternalIMT | aa | rs139076633 | 0 | . | . | . | . | . | . | . | . | . |
| InternalIMT | aa | rs12243649 | 0.002 | . | . | . | . | . | . | . | . | . |
| InternalIMT | aa | rs140348708 | 0.001 | . | . | . | . | . | . | . | . | . |
| InternalIMT | aa | rs79023256 | 0.001 | . | . | . | . | . | . | . | . | . |
| InternalIMT | aa | rs11239515 | 0.146 | 0.119 | 0.067 | 0.075 | -0.015 | 0.273 | 0.957 | -4.50E-06 | 0.113 | 1 |
| InternalIMT | aa | rs55866086 | 0.148 | 0.12 | 0.067 | 0.074 | 7.50E-04 | 0.272 | 0.998 | -5.10E-03 | 0.113 | 0.964 |
| InternalIMT | aa | rs181340150 | 0.005 | . | . | . | . | . | . | . | . | . |
| InternalIMT | aa | rs11239516 | 0.146 | 0.119 | 0.067 | 0.075 | -0.015 | 0.273 | 0.957 | -2.50E-05 | 0.113 | 1 |
| InternalIMT | aa | rs11239517 | 0.146 | 0.119 | 0.067 | 0.075 | -0.015 | 0.272 | 0.957 | -2.90E-06 | 0.113 | 1 |
| InternalIMT | aa | rs11239519 | 0.135 | 0.119 | 0.067 | 0.074 | 5.80E-03 | 0.291 | 0.984 | -4.00E-03 | 0.121 | 0.973 |
| InternalIMT | aa | rs7909514 | 0.601 | 0.104 | 0.123 | 0.396 | -4.90E-03 | 0.205 | 0.981 | 8.70E-03 | 0.085 | 0.918 |
| InternalIMT | aa | rs7894352 | 0.782 | 0.037 | 0.18 | 0.837 | -0.09 | 0.259 | 0.727 | 0.051 | 0.107 | 0.634 |
| InternalIMT | aa | rs189384146 | 0.004 | . | . | . | . | . | . | . | . | . |
| InternalIMT | aa | rs11239520 | 0.146 | 0.119 | 0.067 | 0.075 | -0.015 | 0.272 | 0.956 | 9.40E-05 | 0.113 | 0.999 |
| InternalIMT | aa | rs12221233 | 0.404 | 0.118 | 0.099 | 0.234 | 0.035 | 0.208 | 0.867 | -1.00E-03 | 0.085 | 0.99 |
| InternalIMT | aa | rs74523293 | 0.001 | . | . | . | . | . | . | . | . | . |
| InternalIMT | aa | rs79376040 | 0.001 | . | . | . | . | . | . | . | . | . |
| InternalIMT | aa | rs4949000 | 0.404 | 0.118 | 0.099 | 0.235 | 0.035 | 0.209 | 0.867 | -9.60E-04 | 0.085 | 0.991 |
| InternalIMT | aa | rs7089406 | 0.018 | 0.103 | 0.062 | 0.097 | -0.965 | 0.555 | 0.082 | 0.387 | 0.243 | 0.111 |
| InternalIMT | aa | rs138211056 | 0 | . | . | . | . | . | . | . | . | . |
| InternalIMT | aa | rs10900213 | 0.548 | 0.116 | 0.118 | 0.325 | 0.026 | 0.209 | 0.902 | -8.70E-04 | 0.086 | 0.992 |
| InternalIMT | aa | rs145152827 | 0 | . | . | . | . | . | . | . | . | . |
| InternalIMT | aa | rs74443325 | 0.001 | . | . | . | . | . | . | . | . | . |
| InternalIMT | aa | rs188299446 | 0.016 | 0.121 | 0.062 | 0.051 | 0.371 | 1.18 | 0.754 | -0.118 | 0.497 | 0.812 |
| InternalIMT | aa | rs192699586 | 0.002 | . | . | . | . | . | . | . | . | . |
| InternalIMT | aa | rs72796464 | 0.145 | 0.119 | 0.067 | 0.075 | -0.013 | 0.272 | 0.961 | -4.90E-04 | 0.113 | 0.997 |
| InternalIMT | aa | rs4949001 | 0.268 | 0.177 | 0.088 | 0.043 | 0.256 | 0.252 | 0.309 | -0.104 | 0.104 | 0.317 |
| InternalIMT | aa | rs147174641 | 0.013 | 0.111 | 0.062 | 0.074 | -0.643 | 1.12 | 0.568 | 0.275 | 0.465 | 0.554 |
| InternalIMT | aa | rs190835238 | 0.001 | . | . | . | . | . | . | . | . | . |
| InternalIMT | aa | rs76698944 | 0.03 | 0.087 | 0.063 | 0.166 | -1.17 | 0.634 | 0.065 | 0.489 | 0.276 | 0.076 |
| InternalIMT | aa | rs56071381 | 0.142 | 0.115 | 0.067 | 0.087 | -0.059 | 0.277 | 0.83 | 0.018 | 0.115 | 0.875 |
| InternalIMT | aa | rs78130427 | 0.031 | 0.121 | 0.062 | 0.051 | 0.047 | 0.754 | 0.95 | -0.036 | 0.312 | 0.909 |
| InternalIMT | aa | rs10900215 | 0.146 | 0.119 | 0.067 | 0.076 | -0.021 | 0.271 | 0.937 | 2.00E-03 | 0.112 | 0.986 |
| InternalIMT | aa | rs41491445 | 0 | . | . | . | . | . | . | . | . | . |
| InternalIMT | aa | rs144995222 | . | . | . | . | . | . | . | . | . | . |
| InternalIMT | aa | rs149097803 | 0.003 | . | . | . | . | . | . | . | . | . |
| InternalIMT | aa | rs139429646 | 0.007 | . | . | . | . | . | . | . | . | . |
| InternalIMT | aa | rs7075620 | 0.576 | 0.108 | 0.119 | 0.365 | 6.80E-03 | 0.207 | 0.974 | 5.90E-03 | 0.086 | 0.945 |
| InternalIMT | aa | rs148299213 | 0 | . | . | . | . | . | . | . | . | . |
| InternalIMT | aa | rs4949002 | 0.31 | 0.189 | 0.09 | 0.035 | 0.305 | 0.232 | 0.19 | -0.114 | 0.095 | 0.233 |
| InternalIMT | aa | rs150496007 | 0.011 | 0.113 | 0.062 | 0.068 | -0.406 | 1.47 | 0.782 | 0.228 | 0.624 | 0.715 |
| InternalIMT | aa | rs147303455 | 0.004 | . | . | . | . | . | . | . | . | . |
| InternalIMT | aa | rs189518433 | 0.005 | . | . | . | . | . | . | . | . | . |
| InternalIMT | aa | rs186281355 | 0.005 | . | . | . | . | . | . | . | . | . |
| InternalIMT | aa | rs115297114 | 0 | . | . | . | . | . | . | . | . | . |
| InternalIMT | aa | rs11239523 | 0.146 | 0.115 | 0.067 | 0.087 | -0.05 | 0.268 | 0.851 | 0.015 | 0.111 | 0.893 |
| InternalIMT | aa | rs143704897 | 0.004 | . | . | . | . | . | . | . | . | . |
| InternalIMT | aa | rs188024096 | 0.004 | . | . | . | . | . | . | . | . | . |
| InternalIMT | aa | rs11239524 | 0.134 | 0.115 | 0.067 | 0.087 | -0.067 | 0.314 | 0.831 | 0.02 | 0.13 | 0.877 |
| InternalIMT | aa | rs191656573 | 0.005 | . | . | . | . | . | . | . | . | . |
| InternalIMT | aa | rs193295875 | 0 | . | . | . | . | . | . | . | . | . |
| InternalIMT | aa | rs190458228 | 0.002 | . | . | . | . | . | . | . | . | . |
| InternalIMT | aa | rs139253993 | 0.002 | . | . | . | . | . | . | . | . | . |
| InternalIMT | aa | rs4948672 | 0.565 | 0.122 | 0.124 | 0.326 | 0.044 | 0.227 | 0.846 | 3.40E-03 | 0.094 | 0.971 |
| InternalIMT | aa | rs180954921 | 0.004 | . | . | . | . | . | . | . | . | . |
| InternalIMT | aa | rs12264801 | 0.431 | 0.203 | 0.101 | 0.044 | 0.24 | 0.232 | 0.3 | -0.1 | 0.096 | 0.295 |
| InternalIMT | aa | rs73283184 | 0 | . | . | . | . | . | . | . | . | . |

| InternalIMT | aa | rs142746187 | 0.002 | . | . | . | . | . | . | . | . | . |
| --- | --- | --- | --- | --- | --- | --- | --- | --- | --- | --- | --- | --- |
| InternalIMT | aa | rs58704739 | 0 | . | . | . | . | . | . | . | . | . |
| InternalIMT | aa | rs61272335 | 0 | . | . | . | . | . | . | . | . | . |
| InternalIMT | aa | rs60959489 | 0 | . | . | . | . | . | . | . | . | . |
| InternalIMT | aa | rs58502629 | 0 | . | . | . | . | . | . | . | . | . |
| InternalIMT | aa | rs10900216 | 0.695 | 0.138 | 0.133 | 0.3 | 0.097 | 0.212 | 0.646 | -0.021 | 0.088 | 0.814 |
| InternalIMT | aa | rs181118141 | 0 | . | . | . | . | . | . | . | . | . |
| InternalIMT | aa | rs137882665 | 0.004 | . | . | . | . | . | . | . | . | . |
| InternalIMT | aa | rs149466736 | 0.004 | . | . | . | . | . | . | . | . | . |
| InternalIMT | aa | rs2099171 | 0.662 | 0.105 | 0.123 | 0.393 | 0.052 | 0.205 | 0.799 | 2.90E-03 | 0.085 | 0.973 |
| InternalIMT | aa | rs141852280 | 0.009 | . | . | . | . | . | . | . | . | . |
| InternalIMT | aa | rs191539257 | 0.002 | . | . | . | . | . | . | . | . | . |
| InternalIMT | aa | rs17157771 | 0.033 | 0.13 | 0.063 | 0.038 | 0.367 | 0.429 | 0.393 | -0.193 | 0.179 | 0.279 |
| InternalIMT | aa | rs111339241 | 0.032 | 0.13 | 0.063 | 0.038 | 0.373 | 0.439 | 0.396 | -0.195 | 0.182 | 0.286 |
| InternalIMT | aa | rs116983255 | 0.013 | 0.128 | 0.061 | 0.038 | 1.24 | 0.672 | 0.065 | -0.557 | 0.268 | 0.038 |
| InternalIMT | aa | rs142324341 | 0.003 | . | . | . | . | . | . | . | . | . |
| InternalIMT | aa | rs187191219 | 0.009 | . | . | . | . | . | . | . | . | . |
| InternalIMT | aa | rs151283973 | . | . | . | . | . | . | . | . | . | . |
| InternalIMT | aa | rs139316218 | 0.005 | . | . | . | . | . | . | . | . | . |
| InternalIMT | aa | rs6593485 | 0.696 | 0.14 | 0.131 | 0.287 | 0.101 | 0.209 | 0.63 | -0.022 | 0.087 | 0.796 |
| InternalIMT | aa | rs6593486 | 0.696 | 0.14 | 0.131 | 0.287 | 0.101 | 0.209 | 0.629 | -0.022 | 0.087 | 0.796 |
| InternalIMT | aa | rs145196618 | 0.002 | . | . | . | . | . | . | . | . | . |
| InternalIMT | aa | rs142598335 | 0.006 | . | . | . | . | . | . | . | . | . |
| InternalIMT | aa | rs892691 | 0.704 | 0.113 | 0.133 | 0.393 | 0.058 | 0.211 | 0.783 | -3.00E-03 | 0.088 | 0.973 |
| InternalIMT | aa | rs7080713 | 0.663 | 0.106 | 0.124 | 0.392 | 0.052 | 0.206 | 0.801 | 2.30E-03 | 0.085 | 0.978 |
| InternalIMT | aa | rs7077173 | 0.057 | 0.124 | 0.063 | 0.051 | 0.057 | 0.273 | 0.834 | -0.039 | 0.113 | 0.729 |
| InternalIMT | aa | rs7080977 | 0.032 | 0.13 | 0.063 | 0.038 | 0.374 | 0.423 | 0.376 | -0.194 | 0.176 | 0.27 |
| InternalIMT | aa | rs3780901 | 0.663 | 0.109 | 0.124 | 0.378 | 0.057 | 0.206 | 0.781 | 3.00E-05 | 0.085 | 1 |
| InternalIMT | aa | rs61854091 | 0.073 | 0.132 | 0.066 | 0.046 | 0.265 | 0.45 | 0.556 | -0.084 | 0.185 | 0.65 |
| InternalIMT | aa | rs77550209 | 0.003 | . | . | . | . | . | . | . | . | . |
| InternalIMT | aa | rs73283191 | 0.033 | 0.131 | 0.063 | 0.038 | 0.37 | 0.422 | 0.381 | -0.191 | 0.176 | 0.276 |
| InternalIMT | aa | rs191866099 | 0.01 | 0.112 | 0.061 | 0.068 | -0.816 | 1.78 | 0.647 | 0.34 | 0.748 | 0.649 |
| InternalIMT | aa | rs147998395 | . | . | . | . | . | . | . | . | . | . |
| InternalIMT | aa | rs7090328 | 0.699 | 0.145 | 0.133 | 0.275 | 0.11 | 0.211 | 0.602 | -0.026 | 0.088 | 0.765 |
| InternalIMT | aa | rs79814535 | 0.001 | . | . | . | . | . | . | . | . | . |
| InternalIMT | aa | rs7073082 | 0.699 | 0.145 | 0.133 | 0.275 | 0.11 | 0.211 | 0.602 | -0.026 | 0.088 | 0.764 |
| InternalIMT | aa | rs11239526 | 0.052 | 0.12 | 0.065 | 0.065 | 0.111 | 0.405 | 0.784 | -0.029 | 0.166 | 0.86 |
| InternalIMT | aa | rs141839841 | . | . | . | . | . | . | . | . | . | . |
| InternalIMT | aa | rs75697371 | 0.014 | 0.108 | 0.062 | 0.082 | -0.739 | 0.574 | 0.198 | 0.307 | 0.237 | 0.195 |
| InternalIMT | aa | rs75972854 | 0.016 | 0.096 | 0.062 | 0.124 | -1.7 | 1.1 | 0.123 | 0.706 | 0.477 | 0.139 |
| InternalIMT | aa | rs139291975 | 0.001 | . | . | . | . | . | . | . | . | . |
| InternalIMT | aa | rs144064711 | 0.009 | . | . | . | . | . | . | . | . | . |
| InternalIMT | aa | rs3780902 | 0.033 | 0.13 | 0.063 | 0.038 | 0.356 | 0.427 | 0.405 | -0.187 | 0.178 | 0.293 |
| InternalIMT | aa | rs190449801 | 0 | . | . | . | . | . | . | . | . | . |
| InternalIMT | aa | rs73283196 | 0.033 | 0.13 | 0.063 | 0.039 | 0.331 | 0.423 | 0.435 | -0.177 | 0.176 | 0.315 |
| InternalIMT | aa | rs28395867 | 0.005 | . | . | . | . | . | . | . | . | . |
| InternalIMT | aa | rs28395866 | 0.036 | 0.13 | 0.063 | 0.039 | 0.306 | 0.405 | 0.45 | -0.165 | 0.168 | 0.327 |
| InternalIMT | aa | rs55661611 | 0.032 | 0.129 | 0.063 | 0.039 | 0.325 | 0.427 | 0.447 | -0.176 | 0.178 | 0.322 |
| InternalIMT | aa | rs55684518 | 0.03 | 0.138 | 0.064 | 0.031 | 0.465 | 0.55 | 0.398 | -0.257 | 0.224 | 0.25 |
| InternalIMT | aa | rs78552493 | 0.057 | 0.082 | 0.065 | 0.21 | -0.648 | 0.379 | 0.087 | 0.252 | 0.165 | 0.127 |
| InternalIMT | aa | rs147665434 | 0.007 | . | . | . | . | . | . | . | . | . |
| InternalIMT | aa | rs34445001 | 0.014 | 0.108 | 0.062 | 0.081 | -0.724 | 0.576 | 0.209 | 0.301 | 0.238 | 0.205 |
| InternalIMT | aa | rs184019449 | 0.004 | . | . | . | . | . | . | . | . | . |
| InternalIMT | aa | rs3780903 | 0.719 | 0.012 | 0.147 | 0.935 | -0.125 | 0.227 | 0.583 | 0.072 | 0.094 | 0.441 |
| InternalIMT | aa | rs17157784 | 0.005 | . | . | . | . | . | . | . | . | . |
| InternalIMT | aa | rs3780904 | 0.001 | . | . | . | . | . | . | . | . | . |
| InternalIMT | aa | rs192064730 | 0 | . | . | . | . | . | . | . | . | . |
| InternalIMT | aa | rs12774534 | 0.014 | 0.108 | 0.062 | 0.081 | -0.714 | 0.578 | 0.217 | 0.297 | 0.238 | 0.213 |
| InternalIMT | aa | rs79179915 | 0 | . | . | . | . | . | . | . | . | . |
| InternalIMT | aa | rs117811571 | 0.016 | 0.119 | 0.062 | 0.053 | 0.317 | 1.23 | 0.796 | -0.082 | 0.516 | 0.874 |
| InternalIMT | aa | rs72796477 | 0.015 | 0.136 | 0.062 | 0.029 | 1.24 | 0.666 | 0.063 | -0.492 | 0.277 | 0.076 |
| InternalIMT | aa | rs55729342 | 0.005 | . | . | . | . | . | . | . | . | . |
| InternalIMT | aa | rs59317220 | 0.004 | . | . | . | . | . | . | . | . | . |
| InternalIMT | aa | rs3780905 | 0.042 | 0.096 | 0.064 | 0.134 | -0.623 | 0.39 | 0.11 | 0.23 | 0.164 | 0.16 |
| InternalIMT | aa | rs3780906 | 0.718 | 0.011 | 0.147 | 0.941 | -0.127 | 0.227 | 0.574 | 0.073 | 0.094 | 0.436 |
| InternalIMT | aa | rs149047975 | 0.006 | . | . | . | . | . | . | . | . | . |
| InternalIMT | aa | rs3824614 | 0.001 | . | . | . | . | . | . | . | . | . |
| InternalIMT | aa | rs140254404 | 0.003 | . | . | . | . | . | . | . | . | . |
| InternalIMT | aa | rs145262684 | 0.02 | 0.117 | 0.062 | 0.059 | 0.238 | 1.04 | 0.818 | -0.03 | 0.432 | 0.944 |
| InternalIMT | aa | rs76735482 | 0.001 | . | . | . | . | . | . | . | . | . |
| InternalIMT | aa | rs73283198 | 0.032 | 0.129 | 0.063 | 0.039 | 0.326 | 0.434 | 0.453 | -0.176 | 0.181 | 0.329 |
| InternalIMT | aa | rs74911239 | 0.052 | 0.109 | 0.066 | 0.096 | -0.192 | 0.393 | 0.625 | 0.059 | 0.164 | 0.72 |
| InternalIMT | aa | rs150551647 | 0.008 | . | . | . | . | . | . | . | . | . |
| InternalIMT | aa | rs115200794 | 0.004 | . | . | . | . | . | . | . | . | . |
| InternalIMT | aa | rs74130823 | 0.177 | 0.148 | 0.071 | 0.037 | 0.183 | 0.268 | 0.496 | -0.088 | 0.111 | 0.431 |
| InternalIMT | aa | rs2279435 | 0.563 | 0.028 | 0.12 | 0.818 | -0.156 | 0.211 | 0.461 | 0.081 | 0.088 | 0.359 |
| InternalIMT | aa | rs147279673 | 0.02 | 0.115 | 0.063 | 0.068 | -0.151 | 0.736 | 0.838 | 0.073 | 0.301 | 0.808 |
| InternalIMT | aa | rs183525146 | 0.005 | . | . | . | . | . | . | . | . | . |
| InternalIMT | aa | rs3740107 | 0.727 | 0.091 | 0.152 | 0.547 | -0.02 | 0.231 | 0.929 | 0.018 | 0.096 | 0.852 |
| InternalIMT | aa | rs140697247 | 0.002 | . | . | . | . | . | . | . | . | . |
| InternalIMT | aa | rs1565096 | 0.744 | 0.074 | 0.154 | 0.63 | -0.041 | 0.232 | 0.858 | 0.028 | 0.096 | 0.766 |
| InternalIMT | aa | rs1565097 | 0.741 | 0.064 | 0.153 | 0.675 | -0.057 | 0.23 | 0.803 | 0.036 | 0.095 | 0.708 |
| InternalIMT | aa | rs144584548 | 0.03 | 0.136 | 0.064 | 0.032 | 0.445 | 0.552 | 0.42 | -0.244 | 0.224 | 0.276 |
| InternalIMT | aa | rs75811053 | 0.001 | . | . | . | . | . | . | . | . | . |
| InternalIMT | aa | rs147462967 | 0.01 | 0.127 | 0.061 | 0.038 | 1.54 | 1.57 | 0.328 | -0.601 | 0.652 | 0.356 |
| InternalIMT | aa | rs74130824 | 0.004 | . | . | . | . | . | . | . | . | . |

| InternalIMT | aa | rs116883184 | 0.025 | 0.118 | 0.063 | 0.06 | 0.19 | 0.815 | 0.815 | -0.029 | 0.338 | 0.932 |
| --- | --- | --- | --- | --- | --- | --- | --- | --- | --- | --- | --- | --- |
| InternalIMT | aa | rs117258049 | 0.025 | 0.118 | 0.063 | 0.06 | 0.19 | 0.815 | 0.816 | -0.029 | 0.338 | 0.932 |
| InternalIMT | aa | rs3780908 | 0.171 | 0.147 | 0.069 | 0.034 | 0.179 | 0.264 | 0.498 | -0.087 | 0.11 | 0.431 |
| InternalIMT | aa | rs180938522 | 0.004 | . | . | . | . | . | . | . | . | . |
| InternalIMT | aa | rs76687372 | 0 | . | . | . | . | . | . | . | . | . |
| InternalIMT | aa | rs3780909 | 0.504 | 0.05 | 0.105 | 0.632 | -0.133 | 0.206 | 0.521 | 0.069 | 0.086 | 0.423 |
| InternalIMT | aa | rs78281156 | 0.001 | . | . | . | . | . | . | . | . | . |
| InternalIMT | aa | rs187840631 | 0.002 | . | . | . | . | . | . | . | . | . |
| InternalIMT | aa | rs76868242 | 0.001 | . | . | . | . | . | . | . | . | . |
| InternalIMT | aa | rs192794192 | 0.001 | . | . | . | . | . | . | . | . | . |
| InternalIMT | aa | rs12781248 | 0.015 | 0.108 | 0.062 | 0.081 | -0.717 | 0.553 | 0.194 | 0.299 | 0.225 | 0.184 |
| InternalIMT | aa | rs12762375 | 0.014 | 0.109 | 0.062 | 0.078 | -0.669 | 0.588 | 0.256 | 0.28 | 0.242 | 0.248 |
| InternalIMT | aa | rs192256986 | 0.006 | . | . | . | . | . | . | . | . | . |
| InternalIMT | aa | rs184411030 | 0 | . | . | . | . | . | . | . | . | . |
| InternalIMT | aa | rs189601483 | 0.001 | . | . | . | . | . | . | . | . | . |
| InternalIMT | aa | rs143254579 | 0.05 | 0.127 | 0.065 | 0.05 | 0.264 | 0.399 | 0.508 | -0.095 | 0.163 | 0.559 |
| InternalIMT | aa | rs12253903 | 0 | . | . | . | . | . | . | . | . | . |
| InternalIMT | aa | rs35489926 | 0.014 | 0.109 | 0.062 | 0.078 | -0.651 | 0.589 | 0.269 | 0.272 | 0.242 | 0.261 |
| InternalIMT | aa | rs71494783 | 0.015 | 0.11 | 0.062 | 0.076 | -0.566 | 0.592 | 0.34 | 0.24 | 0.243 | 0.325 |
| InternalIMT | aa | rs180711602 | 0.004 | . | . | . | . | . | . | . | . | . |
| InternalIMT | aa | rs117312402 | 0.001 | . | . | . | . | . | . | . | . | . |
| InternalIMT | aa | rs74831694 | 0.001 | . | . | . | . | . | . | . | . | . |
| InternalIMT | aa | rs140301943 | 0.004 | . | . | . | . | . | . | . | . | . |
| InternalIMT | aa | rs78553679 | 0.023 | 0.128 | 0.064 | 0.044 | 0.403 | 0.684 | 0.556 | -0.161 | 0.28 | 0.566 |
| InternalIMT | aa | rs78334250 | 0.001 | . | . | . | . | . | . | . | . | . |
| InternalIMT | aa | rs76000028 | 0.001 | . | . | . | . | . | . | . | . | . |
| InternalIMT | aa | rs145195254 | 0 | . | . | . | . | . | . | . | . | . |
| InternalIMT | aa | rs137888527 | 0 | . | . | . | . | . | . | . | . | . |
| InternalIMT | aa | rs149109592 | 0.005 | . | . | . | . | . | . | . | . | . |
| InternalIMT | aa | rs80280010 | 0.049 | 0.127 | 0.065 | 0.05 | 0.268 | 0.399 | 0.502 | -0.097 | 0.163 | 0.554 |
| InternalIMT | aa | rs189995374 | 0.001 | . | . | . | . | . | . | . | . | . |
| InternalIMT | aa | rs75467770 | 0.001 | . | . | . | . | . | . | . | . | . |
| InternalIMT | aa | rs1487562 | 0.178 | 0.149 | 0.07 | 0.033 | 0.191 | 0.26 | 0.463 | -0.09 | 0.108 | 0.404 |
| InternalIMT | aa | rs12266963 | 0.015 | 0.11 | 0.062 | 0.075 | -0.56 | 0.593 | 0.345 | 0.238 | 0.243 | 0.329 |
| InternalIMT | aa | rs7099874 | 0.692 | 0.067 | 0.139 | 0.628 | -0.068 | 0.225 | 0.761 | 0.037 | 0.093 | 0.694 |
| InternalIMT | aa | rs75687019 | 0.001 | . | . | . | . | . | . | . | . | . |
| InternalIMT | aa | rs148240462 | 0.056 | 0.097 | 0.066 | 0.139 | -0.4 | 0.439 | 0.363 | 0.152 | 0.185 | 0.413 |
| InternalIMT | aa | rs141278174 | 0.003 | . | . | . | . | . | . | . | . | . |
| InternalIMT | aa | rs76404852 | 0.001 | . | . | . | . | . | . | . | . | . |
| InternalIMT | aa | rs139112240 | 0.012 | 0.121 | 0.063 | 0.054 | 0.787 | 2.22 | 0.723 | -0.29 | 0.937 | 0.757 |
| InternalIMT | aa | rs12783037 | 0.015 | 0.11 | 0.062 | 0.075 | -0.552 | 0.593 | 0.352 | 0.234 | 0.243 | 0.336 |
| InternalIMT | aa | rs72796481 | 0.013 | 0.119 | 0.062 | 0.055 | 0.262 | 1.34 | 0.845 | -0.061 | 0.544 | 0.911 |
| InternalIMT | aa | rs76798749 | 0.001 | . | . | . | . | . | . | . | . | . |
| InternalIMT | aa | rs187732548 | 0.001 | . | . | . | . | . | . | . | . | . |
| InternalIMT | aa | rs75675474 | 0.001 | . | . | . | . | . | . | . | . | . |
| InternalIMT | aa | rs76552293 | 0.001 | . | . | . | . | . | . | . | . | . |
| InternalIMT | aa | rs147445993 | 0.005 | . | . | . | . | . | . | . | . | . |
| InternalIMT | aa | rs115902283 | 0.001 | . | . | . | . | . | . | . | . | . |
| InternalIMT | aa | rs4540799 | 0.692 | 0.067 | 0.139 | 0.63 | -0.07 | 0.225 | 0.755 | 0.037 | 0.093 | 0.691 |
| InternalIMT | aa | rs12765320 | 0.179 | 0.133 | 0.071 | 0.06 | 0.072 | 0.26 | 0.781 | -0.04 | 0.108 | 0.714 |
| InternalIMT | aa | rs12782256 | 0.014 | 0.11 | 0.062 | 0.077 | -0.63 | 0.589 | 0.285 | 0.264 | 0.242 | 0.277 |
| InternalIMT | aa | rs191727256 | 0 | . | . | . | . | . | . | . | . | . |
| InternalIMT | aa | rs2082111 | 0.165 | 0.145 | 0.069 | 0.035 | 0.178 | 0.264 | 0.502 | -0.083 | 0.11 | 0.449 |
| InternalIMT | aa | rs2082110 | 0.166 | 0.145 | 0.069 | 0.034 | 0.183 | 0.264 | 0.487 | -0.085 | 0.11 | 0.437 |
| InternalIMT | aa | rs2082109 | 0.166 | 0.145 | 0.069 | 0.034 | 0.183 | 0.264 | 0.488 | -0.085 | 0.11 | 0.438 |
| InternalIMT | aa | rs117316662 | 0.001 | . | . | . | . | . | . | . | . | . |
| InternalIMT | aa | rs12247163 | 0.181 | 0.139 | 0.07 | 0.049 | 0.117 | 0.252 | 0.643 | -0.057 | 0.105 | 0.588 |
| InternalIMT | aa | rs12355888 | 0.139 | 0.136 | 0.067 | 0.042 | 0.163 | 0.293 | 0.578 | -0.071 | 0.122 | 0.56 |
| InternalIMT | aa | rs143772290 | 0 | . | . | . | . | . | . | . | . | . |
| InternalIMT | aa | rs146849452 | 0.005 | . | . | . | . | . | . | . | . | . |
| InternalIMT | aa | rs140267121 | 0.006 | . | . | . | . | . | . | . | . | . |
| InternalIMT | aa | rs7080474 | 0.18 | 0.139 | 0.07 | 0.048 | 0.12 | 0.252 | 0.633 | -0.058 | 0.105 | 0.58 |
| InternalIMT | aa | rs144066203 | 0 | . | . | . | . | . | . | . | . | . |
| InternalIMT | aa | rs12765209 | 0.001 | . | . | . | . | . | . | . | . | . |
| InternalIMT | aa | rs150930735 | 0 | . | . | . | . | . | . | . | . | . |
| InternalIMT | aa | rs7068039 | 0.181 | 0.138 | 0.07 | 0.05 | 0.113 | 0.252 | 0.653 | -0.055 | 0.105 | 0.597 |
| InternalIMT | aa | rs117622652 | 0.001 | . | . | . | . | . | . | . | . | . |
| InternalIMT | aa | rs190191514 | 0.002 | . | . | . | . | . | . | . | . | . |
| InternalIMT | aa | rs74130835 | 0 | . | . | . | . | . | . | . | . | . |
| InternalIMT | aa | rs1811604 | 0.001 | . | . | . | . | . | . | . | . | . |
| InternalIMT | aa | rs146971256 | 0 | . | . | . | . | . | . | . | . | . |
| InternalIMT | aa | rs74336000 | 0 | . | . | . | . | . | . | . | . | . |
| InternalIMT | aa | rs186046492 | 0.009 | . | . | . | . | . | . | . | . | . |
| InternalIMT | aa | rs34971823 | 0.014 | 0.11 | 0.062 | 0.077 | -0.633 | 0.584 | 0.279 | 0.265 | 0.24 | 0.27 |
| InternalIMT | aa | rs75227446 | 0.001 | . | . | . | . | . | . | . | . | . |
| InternalIMT | aa | rs190457599 | 0.001 | . | . | . | . | . | . | . | . | . |
| InternalIMT | aa | rs79591917 | 0.001 | . | . | . | . | . | . | . | . | . |
| InternalIMT | aa | rs702365 | 0.51 | 0.047 | 0.106 | 0.656 | -0.143 | 0.209 | 0.492 | 0.071 | 0.087 | 0.417 |
| InternalIMT | aa | rs79643836 | 0.001 | . | . | . | . | . | . | . | . | . |
| InternalIMT | aa | rs702366 | 0.511 | 0.05 | 0.106 | 0.639 | -0.136 | 0.208 | 0.513 | 0.068 | 0.086 | 0.432 |
| InternalIMT | aa | rs1059696 | 0.166 | 0.144 | 0.069 | 0.037 | 0.167 | 0.262 | 0.524 | -0.079 | 0.109 | 0.47 |
| InternalIMT | aa | rs901681 | 0.515 | 0.062 | 0.106 | 0.557 | -0.104 | 0.206 | 0.613 | 0.056 | 0.086 | 0.518 |
| InternalIMT | aa | rs61854092 | 0.165 | 0.143 | 0.069 | 0.038 | 0.162 | 0.262 | 0.538 | -0.077 | 0.109 | 0.483 |
| InternalIMT | aa | rs148080753 | 0.001 | . | . | . | . | . | . | . | . | . |
| InternalIMT | aa | rs61854093 | 0.166 | 0.143 | 0.069 | 0.038 | 0.163 | 0.262 | 0.533 | -0.077 | 0.109 | 0.48 |
| InternalIMT | aa | rs141822774 | 0.001 | . | . | . | . | . | . | . | . | . |

| InternalIMT | aa | rs74130836 | 0 | . | . | . | . | . | . | . | . | . |
| --- | --- | --- | --- | --- | --- | --- | --- | --- | --- | --- | --- | --- |
| InternalIMT | aa | rs150620963 | 0.019 | 0.104 | 0.062 | 0.098 | -0.705 | 0.623 | 0.258 | 0.307 | 0.268 | 0.252 |
| InternalIMT | aa | rs3780913 | 0.678 | 0.079 | 0.135 | 0.556 | -0.051 | 0.223 | 0.818 | 0.028 | 0.092 | 0.757 |
| InternalIMT | aa | rs142255723 | 0.002 | . | . | . | . | . | . | . | . | . |
| InternalIMT | aa | rs3780914 | 0.511 | 0.05 | 0.106 | 0.637 | -0.135 | 0.208 | 0.515 | 0.068 | 0.086 | 0.433 |
| InternalIMT | aa | rs182656507 | 0.002 | . | . | . | . | . | . | . | . | . |
| InternalIMT | aa | rs2291427 | 0.677 | 0.077 | 0.135 | 0.567 | -0.054 | 0.223 | 0.807 | 0.03 | 0.092 | 0.744 |
| InternalIMT | aa | rs10751382 | 0.677 | 0.073 | 0.135 | 0.585 | -0.062 | 0.222 | 0.78 | 0.033 | 0.092 | 0.721 |
| InternalIMT | aa | rs117610108 | 0.001 | . | . | . | . | . | . | . | . | . |
| InternalIMT | aa | rs144966941 | 0.031 | 0.104 | 0.063 | 0.102 | -0.393 | 0.606 | 0.517 | 0.179 | 0.255 | 0.484 |
| InternalIMT | aa | rs41502051 | 0.052 | 0.156 | 0.063 | 0.013 | 0.897 | 0.586 | 0.126 | -0.372 | 0.239 | 0.12 |
| InternalIMT | aa | rs41426046 | 0.001 | . | . | . | . | . | . | . | . | . |
| InternalIMT | aa | rs41519248 | 0.001 | . | . | . | . | . | . | . | . | . |
| InternalIMT | aa | rs189108245 | 0.008 | . | . | . | . | . | . | . | . | . |
| InternalIMT | aa | rs7896431 | 0.476 | 0.05 | 0.105 | 0.633 | -0.15 | 0.216 | 0.486 | 0.072 | 0.09 | 0.422 |
| InternalIMT | aa | rs183724547 | 0.001 | . | . | . | . | . | . | . | . | . |
| InternalIMT | aa | rs142044990 | 0.012 | 0.121 | 0.063 | 0.054 | 0.701 | 2.2 | 0.749 | -0.263 | 0.929 | 0.777 |
| InternalIMT | aa | rs188140929 | 0.002 | . | . | . | . | . | . | . | . | . |
| InternalIMT | aa | rs76819096 | 0 | . | . | . | . | . | . | . | . | . |
| InternalIMT | aa | rs10751383 | 0.56 | 0.084 | 0.115 | 0.468 | -0.042 | 0.207 | 0.838 | 0.032 | 0.086 | 0.707 |
| InternalIMT | aa | rs117819321 | 0.019 | 0.126 | 0.062 | 0.043 | 0.358 | 1.39 | 0.796 | -0.053 | 0.587 | 0.928 |
| InternalIMT | aa | rs2242332 | 0.675 | 0.061 | 0.135 | 0.651 | -0.085 | 0.224 | 0.702 | 0.042 | 0.092 | 0.647 |
| InternalIMT | aa | rs2242333 | 0.046 | 0.098 | 0.064 | 0.128 | -0.498 | 0.378 | 0.188 | 0.175 | 0.159 | 0.273 |
| InternalIMT | aa | rs2242334 | 0.677 | 0.062 | 0.135 | 0.648 | -0.084 | 0.223 | 0.705 | 0.042 | 0.092 | 0.649 |
| InternalIMT | aa | rs189465041 | 0.001 | . | . | . | . | . | . | . | . | . |
| InternalIMT | aa | rs28395868 | 0.041 | 0.138 | 0.062 | 0.028 | 0.729 | 0.575 | 0.205 | -0.293 | 0.235 | 0.214 |
| InternalIMT | aa | rs56067550 | 0.03 | 0.137 | 0.064 | 0.031 | 0.475 | 0.555 | 0.393 | -0.256 | 0.225 | 0.256 |
| InternalIMT | aa | rs1051713 | 0.165 | 0.134 | 0.069 | 0.053 | 0.084 | 0.265 | 0.751 | -0.046 | 0.11 | 0.678 |
| InternalIMT | aa | rs115074210 | 0 | . | . | . | . | . | . | . | . | . |
| InternalIMT | aa | rs7900977 | 0.74 | 0.085 | 0.154 | 0.583 | -0.024 | 0.233 | 0.917 | 0.022 | 0.096 | 0.822 |
| InternalIMT | aa | rs147613891 | 0.004 | . | . | . | . | . | . | . | . | . |
| InternalIMT | aa | rs2229136 | 0.063 | 0.129 | 0.065 | 0.047 | 0.239 | 0.347 | 0.492 | -0.086 | 0.142 | 0.543 |
| InternalIMT | aa | rs185482841 | 0 | . | . | . | . | . | . | . | . | . |
| InternalIMT | aa | rs2288619 | 0.063 | 0.129 | 0.065 | 0.047 | 0.239 | 0.347 | 0.491 | -0.086 | 0.142 | 0.542 |
| InternalIMT | aa | rs189891524 | 0.001 | . | . | . | . | . | . | . | . | . |
| InternalIMT | aa | rs56145077 | 0.164 | 0.134 | 0.069 | 0.054 | 0.08 | 0.265 | 0.763 | -0.044 | 0.11 | 0.69 |
| InternalIMT | aa | rs143216970 | 0 | . | . | . | . | . | . | . | . | . |
| InternalIMT | aa | rs142246478 | 0.002 | . | . | . | . | . | . | . | . | . |
| InternalIMT | aa | rs13134 | 0.014 | 0.11 | 0.062 | 0.076 | -0.622 | 0.558 | 0.265 | 0.26 | 0.227 | 0.252 |
| InternalIMT | aa | rs41314980 | 0.034 | 0.111 | 0.064 | 0.08 | -0.224 | 0.552 | 0.685 | 0.098 | 0.233 | 0.673 |
| InternalIMT | aa | rs41304611 | 0.02 | 0.116 | 0.063 | 0.067 | -0.121 | 0.522 | 0.816 | 7.50E-03 | 0.217 | 0.972 |
| InternalIMT | aa | rs193299617 | 0 | . | . | . | . | . | . | . | . | . |
| InternalIMT | aa | rs12772724 | 0.014 | 0.11 | 0.062 | 0.076 | -0.622 | 0.558 | 0.266 | 0.26 | 0.227 | 0.252 |
| InternalIMT | aa | rs139489537 | 0.048 | 0.138 | 0.064 | 0.032 | 0.5 | 0.422 | 0.236 | -0.19 | 0.173 | 0.271 |
| InternalIMT | aa | rs12776062 | 0.014 | 0.11 | 0.062 | 0.076 | -0.627 | 0.558 | 0.261 | 0.263 | 0.227 | 0.247 |
| InternalIMT | aa | rs752059 | 0.164 | 0.134 | 0.069 | 0.053 | 0.08 | 0.265 | 0.763 | -0.044 | 0.11 | 0.689 |
| InternalIMT | aa | rs147649982 | 0.035 | 0.095 | 0.064 | 0.139 | -0.86 | 0.898 | 0.338 | 0.363 | 0.384 | 0.346 |
| InternalIMT | aa | rs17157798 | 0.062 | 0.129 | 0.065 | 0.047 | 0.241 | 0.347 | 0.488 | -0.086 | 0.142 | 0.544 |
| InternalIMT | aa | rs142298619 | 0.001 | . | . | . | . | . | . | . | . | . |
| InternalIMT | aa | rs4948673 | 0.72 | 6.90E-03 | 0.151 | 0.964 | -0.163 | 0.229 | 0.477 | 0.077 | 0.095 | 0.416 |
| InternalIMT | aa | rs74231484 | 0.041 | 0.098 | 0.064 | 0.125 | -0.539 | 0.38 | 0.157 | 0.193 | 0.16 | 0.228 |
| InternalIMT | aa | rs72796489 | 0.004 | . | . | . | . | . | . | . | . | . |
| InternalIMT | aa | rs113447578 | 0.021 | 0.124 | 0.062 | 0.046 | 0.212 | 0.487 | 0.664 | -0.132 | 0.205 | 0.518 |
| InternalIMT | aa | rs34063374 | 0.014 | 0.11 | 0.062 | 0.077 | -0.624 | 0.561 | 0.266 | 0.266 | 0.228 | 0.245 |
| InternalIMT | aa | rs11239527 | 0.019 | 0.12 | 0.062 | 0.056 | 0.18 | 0.921 | 0.845 | -0.021 | 0.395 | 0.958 |
| InternalIMT | aa | rs34371883 | 0.014 | 0.11 | 0.062 | 0.076 | -0.617 | 0.559 | 0.269 | 0.258 | 0.227 | 0.255 |
| InternalIMT | aa | rs118168828 | 0.012 | 0.122 | 0.063 | 0.052 | 0.348 | 0.866 | 0.688 | -0.158 | 0.37 | 0.67 |
| InternalIMT | aa | rs34349618 | 0.014 | 0.11 | 0.062 | 0.076 | -0.617 | 0.559 | 0.269 | 0.258 | 0.227 | 0.256 |
| InternalIMT | aa | rs80041679 | 0.048 | 0.138 | 0.064 | 0.032 | 0.503 | 0.422 | 0.233 | -0.191 | 0.173 | 0.268 |
| InternalIMT | aa | rs149640607 | 0 | . | . | . | . | . | . | . | . | . |
| InternalIMT | aa | rs4427523 | 0.834 | -8.20E-03 | 0.257 | 0.974 | -0.193 | 0.36 | 0.592 | 0.075 | 0.148 | 0.614 |
| InternalIMT | aa | rs185880109 | 0.002 | . | . | . | . | . | . | . | . | . |
| InternalIMT | aa | rs191523220 | 0.004 | . | . | . | . | . | . | . | . | . |
| InternalIMT | aa | rs144315284 | 0 | . | . | . | . | . | . | . | . | . |
| InternalIMT | aa | rs4948674 | 0.404 | 0.111 | 0.089 | 0.214 | 0.013 | 0.212 | 0.949 | 0.014 | 0.089 | 0.877 |
| InternalIMT | aa | rs182984298 | 0.01 | 0.118 | 0.061 | 0.055 | 0.038 | 0.959 | 0.968 | -0.049 | 0.415 | 0.907 |
| InternalIMT | aa | rs56749023 | 0 | . | . | . | . | . | . | . | . | . |
| InternalIMT | aa | rs112508957 | 0 | . | . | . | . | . | . | . | . | . |
| InternalIMT | aa | rs7089063 | 0.224 | 0.143 | 0.074 | 0.054 | 0.125 | 0.228 | 0.584 | -0.057 | 0.094 | 0.547 |
| InternalIMT | aa | rs145000779 | 0.001 | . | . | . | . | . | . | . | . | . |
| InternalIMT | aa | rs80201572 | 0.048 | 0.138 | 0.064 | 0.032 | 0.502 | 0.422 | 0.234 | -0.191 | 0.173 | 0.269 |
| InternalIMT | aa | rs140568227 | 0.009 | . | . | . | . | . | . | . | . | . |
| InternalIMT | aa | rs149756228 | 0.014 | 0.127 | 0.063 | 0.043 | 0.519 | 0.912 | 0.57 | -0.208 | 0.372 | 0.575 |
| InternalIMT | aa | rs112380685 | 0.009 | . | . | . | . | . | . | . | . | . |
| InternalIMT | aa | rs60481109 | 0 | . | . | . | . | . | . | . | . | . |
| InternalIMT | aa | rs138221843 | 0.004 | . | . | . | . | . | . | . | . | . |
| InternalIMT | aa | rs150283194 | 0 | . | . | . | . | . | . | . | . | . |
| InternalIMT | aa | rs138879661 | 0.003 | . | . | . | . | . | . | . | . | . |
| InternalIMT | aa | rs34960345 | 0.062 | 0.129 | 0.065 | 0.048 | 0.241 | 0.347 | 0.489 | -0.085 | 0.142 | 0.55 |
| InternalIMT | aa | rs147514068 | 0.004 | . | . | . | . | . | . | . | . | . |
| InternalIMT | aa | rs35410535 | 0.063 | 0.129 | 0.065 | 0.047 | 0.243 | 0.347 | 0.484 | -0.087 | 0.142 | 0.541 |
| InternalIMT | aa | rs191613635 | 0.005 | . | . | . | . | . | . | . | . | . |
| InternalIMT | aa | rs17157800 | 0.009 | . | . | . | . | . | . | . | . | . |
| InternalIMT | aa | rs17523413 | 0.062 | 0.129 | 0.065 | 0.048 | 0.239 | 0.348 | 0.492 | -0.084 | 0.142 | 0.553 |
| InternalIMT | aa | rs7897036 | 0.058 | 0.099 | 0.065 | 0.127 | -0.557 | 0.52 | 0.285 | 0.207 | 0.218 | 0.343 |
| InternalIMT | aa | rs76334323 | 0.001 | . | . | . | . | . | . | . | . | . |

| InternalIMT | aa | rs143535991 | 0.001 | . | . | . | . | . | . | . | . | . |
| --- | --- | --- | --- | --- | --- | --- | --- | --- | --- | --- | --- | --- |
| InternalIMT | aa | rs140344979 | 0.007 | . | . | . | . | . | . | . | . | . |
| InternalIMT | aa | rs36047811 | 0.063 | 0.129 | 0.065 | 0.047 | 0.243 | 0.347 | 0.484 | -0.087 | 0.142 | 0.54 |
| InternalIMT | aa | rs76493570 | 0.062 | 0.129 | 0.065 | 0.048 | 0.24 | 0.347 | 0.491 | -0.084 | 0.142 | 0.552 |
| InternalIMT | aa | rs192998920 | 0.003 | . | . | . | . | . | . | . | . | . |
| InternalIMT | aa | rs116840852 | 0.023 | 0.128 | 0.064 | 0.044 | 0.401 | 0.685 | 0.558 | -0.162 | 0.28 | 0.562 |
| InternalIMT | aa | rs184824101 | 0.001 | . | . | . | . | . | . | . | . | . |
| InternalIMT | aa | rs41301621 | 0.008 | . | . | . | . | . | . | . | . | . |
| InternalIMT | aa | rs3740109 | 0.063 | 0.129 | 0.065 | 0.047 | 0.243 | 0.347 | 0.483 | -0.087 | 0.142 | 0.54 |
| InternalIMT | aa | rs7899415 | 0.02 | 0.097 | 0.062 | 0.12 | -0.944 | 1.26 | 0.453 | 0.48 | 0.508 | 0.345 |
| InternalIMT | aa | rs184395284 | 0.001 | . | . | . | . | . | . | . | . | . |
| InternalIMT | aa | rs139209589 | 0.006 | . | . | . | . | . | . | . | . | . |
| InternalIMT | aa | rs149924163 | 0 | . | . | . | . | . | . | . | . | . |
| InternalIMT | aa | rs3740110 | 0.001 | . | . | . | . | . | . | . | . | . |
| InternalIMT | aa | rs144335113 | 0 | . | . | . | . | . | . | . | . | . |
| InternalIMT | aa | rs74743385 | 0 | . | . | . | . | . | . | . | . | . |
| InternalIMT | aa | rs146545829 | 0 | . | . | . | . | . | . | . | . | . |
| InternalIMT | aa | rs181842689 | 0 | . | . | . | . | . | . | . | . | . |
| InternalIMT | aa | rs3740111 | 0.062 | 0.129 | 0.065 | 0.048 | 0.241 | 0.347 | 0.487 | -0.085 | 0.142 | 0.549 |
| InternalIMT | aa | rs186261599 | 0 | . | . | . | . | . | . | . | . | . |
| InternalIMT | aa | rs73285348 | 0.012 | 0.117 | 0.062 | 0.059 | -0.188 | 0.612 | 0.758 | 0.023 | 0.26 | 0.93 |
| InternalIMT | aa | rs186356901 | 0 | . | . | . | . | . | . | . | . | . |
| InternalIMT | aa | rs3824618 | 0.048 | 0.138 | 0.064 | 0.032 | 0.501 | 0.422 | 0.235 | -0.19 | 0.173 | 0.27 |
| InternalIMT | aa | rs3802548 | 0.231 | 0.127 | 0.075 | 0.089 | -7.70E-04 | 0.221 | 0.997 | -0.011 | 0.092 | 0.904 |
| InternalIMT | aa | rs3802547 | 0.001 | . | . | . | . | . | . | . | . | . |
| InternalIMT | aa | rs77677521 | 0.023 | 0.128 | 0.064 | 0.044 | 0.401 | 0.685 | 0.558 | -0.162 | 0.28 | 0.562 |
| InternalIMT | aa | rs3824616 | 0.063 | 0.129 | 0.065 | 0.047 | 0.244 | 0.347 | 0.482 | -0.087 | 0.142 | 0.539 |
| InternalIMT | aa | rs191073818 | 0 | . | . | . | . | . | . | . | . | . |
| InternalIMT | aa | rs147078004 | 0.002 | . | . | . | . | . | . | . | . | . |
| InternalIMT | aa | rs7908745 | 0.297 | 0.137 | 0.081 | 0.091 | 0.061 | 0.203 | 0.766 | -0.029 | 0.084 | 0.725 |
| InternalIMT | aa | rs17157811 | 0.009 | . | . | . | . | . | . | . | . | . |
| InternalIMT | aa | rs138506087 | 0.009 | . | . | . | . | . | . | . | . | . |
| InternalIMT | aa | rs77241269 | 0 | . | . | . | . | . | . | . | . | . |
| InternalIMT | aa | rs138374938 | 0.002 | . | . | . | . | . | . | . | . | . |
| InternalIMT | aa | rs2279433 | 0.062 | 0.129 | 0.065 | 0.047 | 0.251 | 0.347 | 0.47 | -0.088 | 0.142 | 0.533 |
| InternalIMT | aa | rs73285358 | 0 | . | . | . | . | . | . | . | . | . |
| InternalIMT | aa | rs11239528 | 0.231 | 0.127 | 0.075 | 0.09 | -2.60E-03 | 0.221 | 0.99 | -0.01 | 0.092 | 0.911 |
| InternalIMT | aa | rs77453386 | 0.001 | . | . | . | . | . | . | . | . | . |
| InternalIMT | aa | rs2279434 | 0.069 | 0.128 | 0.066 | 0.052 | 0.193 | 0.341 | 0.573 | -0.069 | 0.14 | 0.622 |
| InternalIMT | aa | rs149236525 | 0.006 | . | . | . | . | . | . | . | . | . |
| InternalIMT | aa | rs12573609 | 0.052 | 0.08 | 0.066 | 0.225 | -0.842 | 0.427 | 0.049 | 0.352 | 0.179 | 0.049 |
| InternalIMT | aa | rs191716655 | 0 | . | . | . | . | . | . | . | . | . |
| InternalIMT | aa | rs17523660 | 0.014 | 0.11 | 0.062 | 0.075 | -0.583 | 0.559 | 0.297 | 0.245 | 0.227 | 0.282 |
| InternalIMT | aa | rs142671360 | 0.004 | . | . | . | . | . | . | . | . | . |
| InternalIMT | aa | rs3764990 | 0.062 | 0.129 | 0.065 | 0.047 | 0.248 | 0.347 | 0.476 | -0.087 | 0.142 | 0.541 |
| InternalIMT | aa | rs113682861 | 0.004 | . | . | . | . | . | . | . | . | . |
| InternalIMT | aa | rs117658270 | 0.001 | . | . | . | . | . | . | . | . | . |
| InternalIMT | aa | rs187309063 | 0 | . | . | . | . | . | . | . | . | . |
| InternalIMT | aa | rs73285360 | 0 | . | . | . | . | . | . | . | . | . |
| InternalIMT | aa | rs80133289 | 0.001 | . | . | . | . | . | . | . | . | . |
| InternalIMT | aa | rs186693257 | 0.001 | . | . | . | . | . | . | . | . | . |
| InternalIMT | aa | rs35735968 | 0.063 | 0.129 | 0.065 | 0.048 | 0.244 | 0.347 | 0.483 | -0.085 | 0.141 | 0.548 |
| InternalIMT | aa | rs17157825 | 0.048 | 0.138 | 0.064 | 0.032 | 0.512 | 0.419 | 0.222 | -0.193 | 0.172 | 0.261 |
| InternalIMT | aa | rs2291428 | 0.229 | 0.124 | 0.075 | 0.099 | -0.021 | 0.228 | 0.925 | -3.00E-03 | 0.095 | 0.975 |
| InternalIMT | aa | rs2291429 | 0.231 | 0.128 | 0.075 | 0.088 | 1.80E-03 | 0.221 | 0.993 | -0.012 | 0.092 | 0.893 |
| InternalIMT | aa | rs145714792 | 0.007 | . | . | . | . | . | . | . | . | . |
| InternalIMT | aa | rs12769244 | 0.062 | 0.129 | 0.065 | 0.047 | 0.25 | 0.347 | 0.471 | -0.088 | 0.142 | 0.535 |
| InternalIMT | aa | rs12771275 | 0.062 | 0.129 | 0.065 | 0.047 | 0.25 | 0.347 | 0.471 | -0.088 | 0.142 | 0.535 |
| InternalIMT | aa | rs146266174 | 0.002 | . | . | . | . | . | . | . | . | . |
| InternalIMT | aa | rs41301623 | 0.004 | . | . | . | . | . | . | . | . | . |
| InternalIMT | aa | rs2306814 | 0.001 | . | . | . | . | . | . | . | . | . |
| InternalIMT | aa | rs12244834 | 0 | . | . | . | . | . | . | . | . | . |
| InternalIMT | aa | rs184006011 | 0.004 | . | . | . | . | . | . | . | . | . |
| InternalIMT | aa | rs78829066 | 0.001 | . | . | . | . | . | . | . | . | . |
| InternalIMT | aa | rs34731408 | 0.062 | 0.129 | 0.065 | 0.047 | 0.25 | 0.347 | 0.472 | -0.088 | 0.142 | 0.537 |
| InternalIMT | aa | rs17451516 | 0.014 | 0.11 | 0.062 | 0.075 | -0.576 | 0.559 | 0.303 | 0.241 | 0.227 | 0.289 |
| InternalIMT | aa | rs139910133 | . | . | . | . | . | . | . | . | . | . |
| InternalIMT | aa | rs10900218 | 0.229 | 0.145 | 0.075 | 0.052 | 0.143 | 0.222 | 0.519 | -0.062 | 0.092 | 0.502 |
| InternalIMT | aa | rs146023999 | . | . | . | . | . | . | . | . | . | . |
| InternalIMT | aa | rs187296958 | . | . | . | . | . | . | . | . | . | . |
| InternalIMT | aa | rs142342833 | . | . | . | . | . | . | . | . | . | . |
| InternalIMT | aa | rs143489255 | . | . | . | . | . | . | . | . | . | . |
| InternalIMT | aa | rs138532518 | . | . | . | . | . | . | . | . | . | . |
| InternalIMT | aa | rs183768079 | . | . | . | . | . | . | . | . | . | . |
| InternalIMT | aa | rs117892272 | . | . | . | . | . | . | . | . | . | . |
| InternalIMT | aa | rs192993943 | . | . | . | . | . | . | . | . | . | . |
| InternalIMT | aa | rs182989043 | . | . | . | . | . | . | . | . | . | . |
| InternalIMT | aa | rs189703435 | . | . | . | . | . | . | . | . | . | . |
| InternalIMT | aa | rs188794040 | . | . | . | . | . | . | . | . | . | . |
| InternalIMT | aa | rs143920953 | . | . | . | . | . | . | . | . | . | . |
| InternalIMT | aa | rs191029121 | . | . | . | . | . | . | . | . | . | . |
| InternalIMT | aa | rs116875448 | . | . | . | . | . | . | . | . | . | . |
| InternalIMT | aa | rs190167877 | . | . | . | . | . | . | . | . | . | . |
| InternalIMT | aa | rs184993418 | . | . | . | . | . | . | . | . | . | . |
| InternalIMT | aa | rs143241158 | . | . | . | . | . | . | . | . | . | . |
| InternalIMT | aa | rs11239500 | . | . | . | . | . | . | . | . | . | . |

| InternalIMT | aa | rs140568994 | . | . | . | . | . | . | . | . | . | . |
| --- | --- | --- | --- | --- | --- | --- | --- | --- | --- | --- | --- | --- |
| InternalIMT | aa | rs187530317 | . | . | . | . | . | . | . | . | . | . |
| InternalIMT | aa | rs145189326 | . | . | . | . | . | . | . | . | . | . |
| InternalIMT | aa | rs186203332 | . | . | . | . | . | . | . | . | . | . |
| InternalIMT | aa | rs189921909 | . | . | . | . | . | . | . | . | . | . |
| InternalIMT | aa | rs187260989 | . | . | . | . | . | . | . | . | . | . |
| InternalIMT | aa | rs114376586 | . | . | . | . | . | . | . | . | . | . |
| InternalIMT | aa | rs151070808 | . | . | . | . | . | . | . | . | . | . |
| InternalIMT | aa | rs140927225 | . | . | . | . | . | . | . | . | . | . |
| InternalIMT | aa | rs191555615 | . | . | . | . | . | . | . | . | . | . |
| InternalIMT | aa | rs80278898 | . | . | . | . | . | . | . | . | . | . |
| InternalIMT | aa | rs190849503 | . | . | . | . | . | . | . | . | . | . |
| InternalIMT | aa | rs150080315 | . | . | . | . | . | . | . | . | . | . |
| InternalIMT | aa | rs143623885 | . | . | . | . | . | . | . | . | . | . |
| InternalIMT | aa | rs187760040 | . | . | . | . | . | . | . | . | . | . |
| InternalIMT | aa | rs143917143 | . | . | . | . | . | . | . | . | . | . |
| InternalIMT | aa | rs149851136 | . | . | . | . | . | . | . | . | . | . |
| InternalIMT | aa | rs181612054 | . | . | . | . | . | . | . | . | . | . |
| InternalIMT | aa | rs191006548 | . | . | . | . | . | . | . | . | . | . |
| InternalIMT | aa | rs117377631 | . | . | . | . | . | . | . | . | . | . |
| InternalIMT | aa | rs182107634 | . | . | . | . | . | . | . | . | . | . |
| InternalIMT | aa | rs190211867 | . | . | . | . | . | . | . | . | . | . |
| InternalIMT | aa | rs148293457 | . | . | . | . | . | . | . | . | . | . |
| InternalIMT | aa | rs191811733 | . | . | . | . | . | . | . | . | . | . |
| InternalIMT | aa | rs146109272 | . | . | . | . | . | . | . | . | . | . |
| InternalIMT | aa | rs148397915 | . | . | . | . | . | . | . | . | . | . |
| InternalIMT | aa | rs142233290 | . | . | . | . | . | . | . | . | . | . |
| InternalIMT | aa | rs80226874 | . | . | . | . | . | . | . | . | . | . |
| InternalIMT | aa | rs144496203 | . | . | . | . | . | . | . | . | . | . |
| InternalIMT | aa | rs191062130 | . | . | . | . | . | . | . | . | . | . |
| InternalIMT | aa | rs185807817 | . | . | . | . | . | . | . | . | . | . |
| InternalIMT | aa | rs117846213 | . | . | . | . | . | . | . | . | . | . |
| InternalIMT | aa | rs190545473 | . | . | . | . | . | . | . | . | . | . |
| InternalIMT | aa | rs192364918 | . | . | . | . | . | . | . | . | . | . |
| InternalIMT | aa | rs182939241 | . | . | . | . | . | . | . | . | . | . |
| InternalIMT | aa | rs12571125 | . | . | . | . | . | . | . | . | . | . |
| InternalIMT | aa | rs192418941 | . | . | . | . | . | . | . | . | . | . |
| InternalIMT | aa | rs141270243 | . | . | . | . | . | . | . | . | . | . |
| InternalIMT | aa | rs182615502 | . | . | . | . | . | . | . | . | . | . |
| InternalIMT | aa | rs146669856 | . | . | . | . | . | . | . | . | . | . |
| InternalIMT | aa | rs78290029 | . | . | . | . | . | . | . | . | . | . |
| InternalIMT | aa | rs139955565 | . | . | . | . | . | . | . | . | . | . |
| InternalIMT | aa | rs12267314 | . | . | . | . | . | . | . | . | . | . |
| InternalIMT | aa | rs74477197 | . | . | . | . | . | . | . | . | . | . |
| InternalIMT | aa | rs188147236 | . | . | . | . | . | . | . | . | . | . |
| InternalIMT | aa | rs2228065 | . | . | . | . | . | . | . | . | . | . |
| InternalIMT | aa | rs116961353 | . | . | . | . | . | . | . | . | . | . |
| InternalIMT | aa | rs190222272 | . | . | . | . | . | . | . | . | . | . |
| InternalIMT | aa | rs182719940 | . | . | . | . | . | . | . | . | . | . |
| InternalIMT | aa | rs184477413 | . | . | . | . | . | . | . | . | . | . |
| InternalIMT | aa | rs12265175 | . | . | . | . | . | . | . | . | . | . |
| InternalIMT | aa | rs182928276 | . | . | . | . | . | . | . | . | . | . |
| InternalIMT | aa | rs144409895 | . | . | . | . | . | . | . | . | . | . |
| InternalIMT | aa | rs58998447 | . | . | . | . | . | . | . | . | . | . |
| InternalIMT | aa | rs188668832 | . | . | . | . | . | . | . | . | . | . |
| InternalIMT | aa | rs148641686 | . | . | . | . | . | . | . | . | . | . |
| InternalIMT | aa | rs75468791 | . | . | . | . | . | . | . | . | . | . |
| InternalIMT | aa | rs147327397 | . | . | . | . | . | . | . | . | . | . |
| InternalIMT | aa | rs77354597 | . | . | . | . | . | . | . | . | . | . |
| InternalIMT | aa | rs41365544 | . | . | . | . | . | . | . | . | . | . |
| InternalIMT | aa | rs187564188 | . | . | . | . | . | . | . | . | . | . |
| InternalIMT | aa | rs183511984 | . | . | . | . | . | . | . | . | . | . |
| InternalIMT | aa | rs12262783 | . | . | . | . | . | . | . | . | . | . |
| InternalIMT | aa | rs188817687 | . | . | . | . | . | . | . | . | . | . |
| InternalIMT | aa | rs3815968 | . | . | . | . | . | . | . | . | . | . |
| InternalIMT | aa | rs187822300 | . | . | . | . | . | . | . | . | . | . |
| InternalIMT | aa | rs188902172 | . | . | . | . | . | . | . | . | . | . |
| InternalIMT | aa | rs181190448 | . | . | . | . | . | . | . | . | . | . |
| InternalIMT | aa | rs182699093 | . | . | . | . | . | . | . | . | . | . |
| InternalIMT | aa | rs182631303 | . | . | . | . | . | . | . | . | . | . |
| InternalIMT | aa | rs187014172 | . | . | . | . | . | . | . | . | . | . |
| InternalIMT | aa | rs142575406 | . | . | . | . | . | . | . | . | . | . |
| InternalIMT | aa | rs180711033 | . | . | . | . | . | . | . | . | . | . |
| InternalIMT | aa | rs137894823 | . | . | . | . | . | . | . | . | . | . |
| InternalIMT | aa | rs189053273 | . | . | . | . | . | . | . | . | . | . |
| InternalIMT | aa | rs143499388 | . | . | . | . | . | . | . | . | . | . |
| InternalIMT | aa | rs182936001 | . | . | . | . | . | . | . | . | . | . |
| InternalIMT | aa | rs148456406 | . | . | . | . | . | . | . | . | . | . |
| InternalIMT | aa | rs182499531 | . | . | . | . | . | . | . | . | . | . |
| InternalIMT | aa | rs74884915 | . | . | . | . | . | . | . | . | . | . |
| InternalIMT | aa | rs184046374 | . | . | . | . | . | . | . | . | . | . |
| InternalIMT | aa | rs74333993 | . | . | . | . | . | . | . | . | . | . |
| InternalIMT | aa | rs138889719 | . | . | . | . | . | . | . | . | . | . |
| InternalIMT | aa | rs75330330 | . | . | . | . | . | . | . | . | . | . |
| InternalIMT | aa | rs193051693 | . | . | . | . | . | . | . | . | . | . |
| InternalIMT | aa | rs145929633 | . | . | . | . | . | . | . | . | . | . |

| InternalIMT | aa | rs184076904 | . | . | . | . | . | . | . | . | . | . |
| --- | --- | --- | --- | --- | --- | --- | --- | --- | --- | --- | --- | --- |
| InternalIMT | aa | rs141802494 | . | . | . | . | . | . | . | . | . | . |
| InternalIMT | aa | rs149519759 | . | . | . | . | . | . | . | . | . | . |
| InternalIMT | aa | rs145467260 | . | . | . | . | . | . | . | . | . | . |
| InternalIMT | aa | rs79620914 | . | . | . | . | . | . | . | . | . | . |
| InternalIMT | aa | rs140538633 | . | . | . | . | . | . | . | . | . | . |
| InternalIMT | aa | rs140862301 | . | . | . | . | . | . | . | . | . | . |
| InternalIMT | aa | rs114615847 | . | . | . | . | . | . | . | . | . | . |
| InternalIMT | aa | rs141800259 | . | . | . | . | . | . | . | . | . | . |
| InternalIMT | aa | rs149779059 | . | . | . | . | . | . | . | . | . | . |
| InternalIMT | aa | rs116539253 | . | . | . | . | . | . | . | . | . | . |
| InternalIMT | aa | rs184979550 | . | . | . | . | . | . | . | . | . | . |
| InternalIMT | aa | rs190128940 | . | . | . | . | . | . | . | . | . | . |
| InternalIMT | aa | rs146594816 | . | . | . | . | . | . | . | . | . | . |
| InternalIMT | aa | rs141370766 | . | . | . | . | . | . | . | . | . | . |
| InternalIMT | aa | rs144670033 | . | . | . | . | . | . | . | . | . | . |
| InternalIMT | aa | rs187152174 | . | . | . | . | . | . | . | . | . | . |
| InternalIMT | aa | rs184180263 | . | . | . | . | . | . | . | . | . | . |
| InternalIMT | aa | rs140075861 | . | . | . | . | . | . | . | . | . | . |
| InternalIMT | aa | rs186483129 | . | . | . | . | . | . | . | . | . | . |
| InternalIMT | aa | rs186634282 | . | . | . | . | . | . | . | . | . | . |
| InternalIMT | aa | rs9988663 | . | . | . | . | . | . | . | . | . | . |
| InternalIMT | aa | rs138828529 | . | . | . | . | . | . | . | . | . | . |
| InternalIMT | aa | rs188777615 | . | . | . | . | . | . | . | . | . | . |
| InternalIMT | aa | rs192321882 | . | . | . | . | . | . | . | . | . | . |
| InternalIMT | aa | rs150812270 | . | . | . | . | . | . | . | . | . | . |
| InternalIMT | aa | rs185906603 | . | . | . | . | . | . | . | . | . | . |
| InternalIMT | aa | rs191070862 | . | . | . | . | . | . | . | . | . | . |
| InternalIMT | aa | rs182904978 | . | . | . | . | . | . | . | . | . | . |
| InternalIMT | aa | rs192679655 | . | . | . | . | . | . | . | . | . | . |
| InternalIMT | aa | rs189673086 | . | . | . | . | . | . | . | . | . | . |
| InternalIMT | aa | rs181141892 | . | . | . | . | . | . | . | . | . | . |
| InternalIMT | aa | rs186404123 | . | . | . | . | . | . | . | . | . | . |
| InternalIMT | aa | rs192117287 | . | . | . | . | . | . | . | . | . | . |
| InternalIMT | aa | rs182748835 | . | . | . | . | . | . | . | . | . | . |
| InternalIMT | aa | rs141854549 | . | . | . | . | . | . | . | . | . | . |
| InternalIMT | aa | rs140163397 | . | . | . | . | . | . | . | . | . | . |
| InternalIMT | aa | rs146347300 | . | . | . | . | . | . | . | . | . | . |
| InternalIMT | aa | rs139362377 | . | . | . | . | . | . | . | . | . | . |
| InternalIMT | aa | rs185087671 | . | . | . | . | . | . | . | . | . | . |
| InternalIMT | aa | rs190466574 | . | . | . | . | . | . | . | . | . | . |
| InternalIMT | aa | rs147012778 | . | . | . | . | . | . | . | . | . | . |
| InternalIMT | aa | rs138236960 | . | . | . | . | . | . | . | . | . | . |
| InternalIMT | aa | rs116310311 | . | . | . | . | . | . | . | . | . | . |
| InternalIMT | aa | rs142868356 | . | . | . | . | . | . | . | . | . | . |
| InternalIMT | aa | rs144956240 | . | . | . | . | . | . | . | . | . | . |
| InternalIMT | aa | rs188527776 | . | . | . | . | . | . | . | . | . | . |
| InternalIMT | aa | rs183971165 | . | . | . | . | . | . | . | . | . | . |
| InternalIMT | aa | rs76748216 | . | . | . | . | . | . | . | . | . | . |
| InternalIMT | aa | rs148554990 | . | . | . | . | . | . | . | . | . | . |
| InternalIMT | aa | rs114446477 | . | . | . | . | . | . | . | . | . | . |
| InternalIMT | aa | rs185223933 | . | . | . | . | . | . | . | . | . | . |
| InternalIMT | aa | rs76869726 | . | . | . | . | . | . | . | . | . | . |
| InternalIMT | aa | rs151200830 | . | . | . | . | . | . | . | . | . | . |
| InternalIMT | aa | rs17157731 | . | . | . | . | . | . | . | . | . | . |
| InternalIMT | aa | rs181243469 | . | . | . | . | . | . | . | . | . | . |
| InternalIMT | aa | rs186890707 | . | . | . | . | . | . | . | . | . | . |
| InternalIMT | aa | rs184063360 | . | . | . | . | . | . | . | . | . | . |
| InternalIMT | aa | rs188550237 | . | . | . | . | . | . | . | . | . | . |
| InternalIMT | aa | rs140931068 | . | . | . | . | . | . | . | . | . | . |
| InternalIMT | aa | rs74867877 | . | . | . | . | . | . | . | . | . | . |
| InternalIMT | aa | rs11239495 | . | . | . | . | . | . | . | . | . | . |
| InternalIMT | aa | rs149745800 | . | . | . | . | . | . | . | . | . | . |
| InternalIMT | aa | rs145044458 | . | . | . | . | . | . | . | . | . | . |
| InternalIMT | aa | rs188067427 | . | . | . | . | . | . | . | . | . | . |
| InternalIMT | aa | rs146827800 | . | . | . | . | . | . | . | . | . | . |
| InternalIMT | aa | rs140619817 | . | . | . | . | . | . | . | . | . | . |
| InternalIMT | aa | rs892689 | . | . | . | . | . | . | . | . | . | . |
| InternalIMT | aa | rs143642005 | . | . | . | . | . | . | . | . | . | . |
| InternalIMT | aa | rs181929304 | . | . | . | . | . | . | . | . | . | . |
| InternalIMT | aa | rs191086173 | . | . | . | . | . | . | . | . | . | . |
| InternalIMT | aa | rs114613116 | . | . | . | . | . | . | . | . | . | . |
| InternalIMT | aa | rs141076768 | . | . | . | . | . | . | . | . | . | . |
| InternalIMT | aa | rs187316531 | . | . | . | . | . | . | . | . | . | . |
| InternalIMT | aa | rs11239499 | . | . | . | . | . | . | . | . | . | . |
| InternalIMT | aa | rs192726371 | . | . | . | . | . | . | . | . | . | . |
| InternalIMT | aa | rs74128481 | . | . | . | . | . | . | . | . | . | . |
| InternalIMT | aa | rs74128482 | . | . | . | . | . | . | . | . | . | . |
| InternalIMT | aa | rs11239502 | . | . | . | . | . | . | . | . | . | . |
| InternalIMT | aa | rs116330285 | . | . | . | . | . | . | . | . | . | . |
| InternalIMT | aa | rs189926729 | . | . | . | . | . | . | . | . | . | . |
| InternalIMT | aa | rs188975779 | . | . | . | . | . | . | . | . | . | . |
| InternalIMT | aa | rs143846038 | . | . | . | . | . | . | . | . | . | . |
| InternalIMT | aa | rs74128483 | . | . | . | . | . | . | . | . | . | . |
| InternalIMT | aa | rs139292055 | . | . | . | . | . | . | . | . | . | . |
| InternalIMT | aa | rs11239503 | . | . | . | . | . | . | . | . | . | . |

| InternalIMT | aa | rs58838686 | . | . | . | . | . | . | . | . | . | . |
| --- | --- | --- | --- | --- | --- | --- | --- | --- | --- | --- | --- | --- |
| InternalIMT | aa | rs185825373 | . | . | . | . | . | . | . | . | . | . |
| InternalIMT | aa | rs11239504 | . | . | . | . | . | . | . | . | . | . |
| InternalIMT | aa | rs150012354 | . | . | . | . | . | . | . | . | . | . |
| InternalIMT | aa | rs187763096 | . | . | . | . | . | . | . | . | . | . |
| InternalIMT | aa | rs193043982 | . | . | . | . | . | . | . | . | . | . |
| InternalIMT | aa | rs79186884 | . | . | . | . | . | . | . | . | . | . |
| InternalIMT | aa | rs74128484 | . | . | . | . | . | . | . | . | . | . |
| InternalIMT | aa | rs58461537 | . | . | . | . | . | . | . | . | . | . |
| InternalIMT | aa | rs188527474 | . | . | . | . | . | . | . | . | . | . |
| InternalIMT | aa | rs141659976 | . | . | . | . | . | . | . | . | . | . |
| InternalIMT | aa | rs115846407 | . | . | . | . | . | . | . | . | . | . |
| InternalIMT | aa | rs138378816 | . | . | . | . | . | . | . | . | . | . |
| InternalIMT | aa | rs146188136 | . | . | . | . | . | . | . | . | . | . |
| InternalIMT | aa | rs139383871 | . | . | . | . | . | . | . | . | . | . |
| InternalIMT | aa | rs182953869 | . | . | . | . | . | . | . | . | . | . |
| InternalIMT | aa | rs192430299 | . | . | . | . | . | . | . | . | . | . |
| InternalIMT | aa | rs144152844 | . | . | . | . | . | . | . | . | . | . |
| InternalIMT | aa | rs145987786 | . | . | . | . | . | . | . | . | . | . |
| InternalIMT | aa | rs181132926 | . | . | . | . | . | . | . | . | . | . |
| InternalIMT | aa | rs147882253 | . | . | . | . | . | . | . | . | . | . |
| InternalIMT | aa | rs141807621 | . | . | . | . | . | . | . | . | . | . |
| InternalIMT | aa | rs147116052 | . | . | . | . | . | . | . | . | . | . |
| InternalIMT | aa | rs147716618 | . | . | . | . | . | . | . | . | . | . |
| InternalIMT | aa | rs187198855 | . | . | . | . | . | . | . | . | . | . |
| InternalIMT | aa | rs189409638 | . | . | . | . | . | . | . | . | . | . |
| InternalIMT | aa | rs182647339 | . | . | . | . | . | . | . | . | . | . |
| InternalIMT | aa | rs187995988 | . | . | . | . | . | . | . | . | . | . |
| InternalIMT | aa | rs141034184 | . | . | . | . | . | . | . | . | . | . |
| InternalIMT | aa | rs187310430 | . | . | . | . | . | . | . | . | . | . |
| InternalIMT | aa | rs192608488 | . | . | . | . | . | . | . | . | . | . |
| InternalIMT | aa | rs148840366 | . | . | . | . | . | . | . | . | . | . |
| InternalIMT | aa | rs186785022 | . | . | . | . | . | . | . | . | . | . |
| InternalIMT | aa | rs192348728 | . | . | . | . | . | . | . | . | . | . |
| InternalIMT | aa | rs189656631 | . | . | . | . | . | . | . | . | . | . |
| InternalIMT | aa | rs185289750 | . | . | . | . | . | . | . | . | . | . |
| InternalIMT | aa | rs142382395 | . | . | . | . | . | . | . | . | . | . |
| InternalIMT | aa | rs144866696 | . | . | . | . | . | . | . | . | . | . |
| InternalIMT | aa | rs141371289 | . | . | . | . | . | . | . | . | . | . |
| InternalIMT | aa | rs114750008 | . | . | . | . | . | . | . | . | . | . |
| InternalIMT | aa | rs189063597 | . | . | . | . | . | . | . | . | . | . |
| InternalIMT | aa | rs139151571 | . | . | . | . | . | . | . | . | . | . |
| InternalIMT | aa | rs139846368 | . | . | . | . | . | . | . | . | . | . |
| InternalIMT | aa | rs182057653 | . | . | . | . | . | . | . | . | . | . |
| InternalIMT | aa | rs148785420 | . | . | . | . | . | . | . | . | . | . |
| InternalIMT | aa | rs138383251 | . | . | . | . | . | . | . | . | . | . |
| InternalIMT | aa | rs116526705 | . | . | . | . | . | . | . | . | . | . |
| InternalIMT | aa | rs185946436 | . | . | . | . | . | . | . | . | . | . |
| InternalIMT | aa | rs190455427 | . | . | . | . | . | . | . | . | . | . |
| InternalIMT | aa | rs9282584 | . | . | . | . | . | . | . | . | . | . |
| InternalIMT | aa | rs17153289 | . | . | . | . | . | . | . | . | . | . |
| InternalIMT | aa | rs138359103 | . | . | . | . | . | . | . | . | . | . |
| InternalIMT | aa | rs150605268 | . | . | . | . | . | . | . | . | . | . |
| InternalIMT | aa | rs79242910 | . | . | . | . | . | . | . | . | . | . |
| InternalIMT | aa | rs139716904 | . | . | . | . | . | . | . | . | . | . |
| InternalIMT | aa | rs76238498 | . | . | . | . | . | . | . | . | . | . |
| InternalIMT | aa | rs184140639 | . | . | . | . | . | . | . | . | . | . |
| InternalIMT | aa | rs188517278 | . | . | . | . | . | . | . | . | . | . |
| InternalIMT | aa | rs139678913 | . | . | . | . | . | . | . | . | . | . |
| InternalIMT | aa | rs116697955 | . | . | . | . | . | . | . | . | . | . |
| InternalIMT | aa | rs79267994 | . | . | . | . | . | . | . | . | . | . |
| InternalIMT | aa | rs11239510 | . | . | . | . | . | . | . | . | . | . |
| InternalIMT | aa | rs75831645 | . | . | . | . | . | . | . | . | . | . |
| InternalIMT | aa | rs78452276 | . | . | . | . | . | . | . | . | . | . |
| InternalIMT | aa | rs144366924 | . | . | . | . | . | . | . | . | . | . |
| InternalIMT | aa | rs77519841 | . | . | . | . | . | . | . | . | . | . |
| InternalIMT | aa | rs76944454 | . | . | . | . | . | . | . | . | . | . |
| InternalIMT | aa | rs76443855 | . | . | . | . | . | . | . | . | . | . |
| InternalIMT | aa | rs146613174 | . | . | . | . | . | . | . | . | . | . |
| InternalIMT | aa | rs181424761 | . | . | . | . | . | . | . | . | . | . |
| InternalIMT | aa | rs142119241 | . | . | . | . | . | . | . | . | . | . |
| InternalIMT | aa | rs140137954 | . | . | . | . | . | . | . | . | . | . |
| InternalIMT | aa | rs187319549 | . | . | . | . | . | . | . | . | . | . |
| InternalIMT | aa | rs12241545 | . | . | . | . | . | . | . | . | . | . |
| InternalIMT | aa | rs10128228 | . | . | . | . | . | . | . | . | . | . |
| InternalIMT | aa | rs58580249 | . | . | . | . | . | . | . | . | . | . |
| InternalIMT | aa | rs193148863 | . | . | . | . | . | . | . | . | . | . |
| InternalIMT | aa | rs185638946 | . | . | . | . | . | . | . | . | . | . |
| InternalIMT | aa | rs117752921 | . | . | . | . | . | . | . | . | . | . |
| InternalIMT | aa | rs180841236 | . | . | . | . | . | . | . | . | . | . |
| InternalIMT | aa | rs73283155 | . | . | . | . | . | . | . | . | . | . |
| InternalIMT | aa | rs76560599 | . | . | . | . | . | . | . | . | . | . |
| InternalIMT | aa | rs76273620 | . | . | . | . | . | . | . | . | . | . |
| InternalIMT | aa | rs141432923 | . | . | . | . | . | . | . | . | . | . |
| InternalIMT | aa | rs113529322 | . | . | . | . | . | . | . | . | . | . |
| InternalIMT | aa | rs10128306 | . | . | . | . | . | . | . | . | . | . |

| InternalIMT | aa | rs149691688 | . | . | . | . | . | . | . | . | . | . |
| --- | --- | --- | --- | --- | --- | --- | --- | --- | --- | --- | --- | --- |
| InternalIMT | aa | rs75872495 | . | . | . | . | . | . | . | . | . | . |
| InternalIMT | aa | rs191873960 | . | . | . | . | . | . | . | . | . | . |
| InternalIMT | aa | rs80155622 | . | . | . | . | . | . | . | . | . | . |
| InternalIMT | aa | rs113914656 | . | . | . | . | . | . | . | . | . | . |
| InternalIMT | aa | rs12242840 | . | . | . | . | . | . | . | . | . | . |
| InternalIMT | aa | rs147824078 | . | . | . | . | . | . | . | . | . | . |
| InternalIMT | aa | rs59051493 | . | . | . | . | . | . | . | . | . | . |
| InternalIMT | aa | rs9282588 | . | . | . | . | . | . | . | . | . | . |
| InternalIMT | aa | rs150215207 | . | . | . | . | . | . | . | . | . | . |
| InternalIMT | aa | rs138772243 | . | . | . | . | . | . | . | . | . | . |
| InternalIMT | aa | rs149383244 | . | . | . | . | . | . | . | . | . | . |
| InternalIMT | aa | rs17522720 | . | . | . | . | . | . | . | . | . | . |
| InternalIMT | aa | rs79245105 | . | . | . | . | . | . | . | . | . | . |
| InternalIMT | aa | rs138222133 | . | . | . | . | . | . | . | . | . | . |
| InternalIMT | aa | rs12247314 | . | . | . | . | . | . | . | . | . | . |
| InternalIMT | aa | rs12263894 | . | . | . | . | . | . | . | . | . | . |
| InternalIMT | aa | rs149907880 | . | . | . | . | . | . | . | . | . | . |
| InternalIMT | aa | rs188153885 | . | . | . | . | . | . | . | . | . | . |
| InternalIMT | aa | rs73283159 | . | . | . | . | . | . | . | . | . | . |
| InternalIMT | aa | rs140476687 | . | . | . | . | . | . | . | . | . | . |
| InternalIMT | aa | rs186603148 | . | . | . | . | . | . | . | . | . | . |
| InternalIMT | aa | rs183296063 | . | . | . | . | . | . | . | . | . | . |
| InternalIMT | aa | rs145733694 | . | . | . | . | . | . | . | . | . | . |
| InternalIMT | aa | rs189807449 | . | . | . | . | . | . | . | . | . | . |
| InternalIMT | aa | rs12253223 | . | . | . | . | . | . | . | . | . | . |
| InternalIMT | aa | rs182113637 | . | . | . | . | . | . | . | . | . | . |
| InternalIMT | aa | rs142792099 | . | . | . | . | . | . | . | . | . | . |
| InternalIMT | aa | rs12249910 | . | . | . | . | . | . | . | . | . | . |
| InternalIMT | aa | rs149436339 | . | . | . | . | . | . | . | . | . | . |
| InternalIMT | aa | rs77632210 | . | . | . | . | . | . | . | . | . | . |
| InternalIMT | aa | rs74130818 | . | . | . | . | . | . | . | . | . | . |
| InternalIMT | aa | rs187583648 | . | . | . | . | . | . | . | . | . | . |
| InternalIMT | aa | rs191295987 | . | . | . | . | . | . | . | . | . | . |
| InternalIMT | aa | rs184200686 | . | . | . | . | . | . | . | . | . | . |
| InternalIMT | aa | rs76117263 | . | . | . | . | . | . | . | . | . | . |
| InternalIMT | aa | rs185393149 | . | . | . | . | . | . | . | . | . | . |
| InternalIMT | aa | rs144760014 | . | . | . | . | . | . | . | . | . | . |
| InternalIMT | aa | rs7922292 | . | . | . | . | . | . | . | . | . | . |
| InternalIMT | aa | rs115372844 | . | . | . | . | . | . | . | . | . | . |
| InternalIMT | aa | rs111460364 | . | . | . | . | . | . | . | . | . | . |
| InternalIMT | aa | rs182052255 | . | . | . | . | . | . | . | . | . | . |
| InternalIMT | aa | rs180983889 | . | . | . | . | . | . | . | . | . | . |
| InternalIMT | aa | rs139175327 | . | . | . | . | . | . | . | . | . | . |
| InternalIMT | aa | rs149959620 | . | . | . | . | . | . | . | . | . | . |
| InternalIMT | aa | rs114653117 | . | . | . | . | . | . | . | . | . | . |
| InternalIMT | aa | rs144270018 | . | . | . | . | . | . | . | . | . | . |
| InternalIMT | aa | rs182723204 | . | . | . | . | . | . | . | . | . | . |
| InternalIMT | aa | rs57204067 | . | . | . | . | . | . | . | . | . | . |
| InternalIMT | aa | rs144956226 | . | . | . | . | . | . | . | . | . | . |
| InternalIMT | aa | rs187842704 | . | . | . | . | . | . | . | . | . | . |
| InternalIMT | aa | rs184620511 | . | . | . | . | . | . | . | . | . | . |
| InternalIMT | aa | rs144031303 | . | . | . | . | . | . | . | . | . | . |
| InternalIMT | aa | rs187526473 | . | . | . | . | . | . | . | . | . | . |
| InternalIMT | aa | rs193002805 | . | . | . | . | . | . | . | . | . | . |
| InternalIMT | aa | rs138707426 | . | . | . | . | . | . | . | . | . | . |
| InternalIMT | aa | rs142754243 | . | . | . | . | . | . | . | . | . | . |
| InternalIMT | aa | rs181600331 | . | . | . | . | . | . | . | . | . | . |
| InternalIMT | aa | rs144421264 | . | . | . | . | . | . | . | . | . | . |
| InternalIMT | aa | rs184414519 | . | . | . | . | . | . | . | . | . | . |
| InternalIMT | aa | rs150596349 | . | . | . | . | . | . | . | . | . | . |
| InternalIMT | aa | rs190753747 | . | . | . | . | . | . | . | . | . | . |
| InternalIMT | aa | rs139560940 | . | . | . | . | . | . | . | . | . | . |
| InternalIMT | aa | rs75640804 | . | . | . | . | . | . | . | . | . | . |
| InternalIMT | aa | rs73283175 | . | . | . | . | . | . | . | . | . | . |
| InternalIMT | aa | rs143337229 | . | . | . | . | . | . | . | . | . | . |
| InternalIMT | aa | rs73283177 | . | . | . | . | . | . | . | . | . | . |
| InternalIMT | aa | rs76053611 | . | . | . | . | . | . | . | . | . | . |
| InternalIMT | aa | rs116325983 | . | . | . | . | . | . | . | . | . | . |
| InternalIMT | aa | rs115451825 | . | . | . | . | . | . | . | . | . | . |
| InternalIMT | aa | rs183845246 | . | . | . | . | . | . | . | . | . | . |
| InternalIMT | aa | rs79756542 | . | . | . | . | . | . | . | . | . | . |
| InternalIMT | aa | rs187242860 | . | . | . | . | . | . | . | . | . | . |
| InternalIMT | aa | rs57318980 | . | . | . | . | . | . | . | . | . | . |
| InternalIMT | aa | rs59969732 | . | . | . | . | . | . | . | . | . | . |
| InternalIMT | aa | rs143811488 | . | . | . | . | . | . | . | . | . | . |
| InternalIMT | aa | rs189067644 | . | . | . | . | . | . | . | . | . | . |
| InternalIMT | aa | rs60449178 | . | . | . | . | . | . | . | . | . | . |
| InternalIMT | aa | rs59243389 | . | . | . | . | . | . | . | . | . | . |
| InternalIMT | aa | rs184946540 | . | . | . | . | . | . | . | . | . | . |
| InternalIMT | aa | rs188685881 | . | . | . | . | . | . | . | . | . | . |
| InternalIMT | aa | rs150335797 | . | . | . | . | . | . | . | . | . | . |
| InternalIMT | aa | rs143879260 | . | . | . | . | . | . | . | . | . | . |
| InternalIMT | aa | rs186044491 | . | . | . | . | . | . | . | . | . | . |
| InternalIMT | aa | rs11819084 | . | . | . | . | . | . | . | . | . | . |
| InternalIMT | aa | rs146915670 | . | . | . | . | . | . | . | . | . | . |

| InternalIMT | aa | rs113995695 | . | . | . | . | . | . | . | . | . | . |
| --- | --- | --- | --- | --- | --- | --- | --- | --- | --- | --- | --- | --- |
| InternalIMT | aa | rs143489451 | . | . | . | . | . | . | . | . | . | . |
| InternalIMT | aa | rs182653839 | . | . | . | . | . | . | . | . | . | . |
| InternalIMT | aa | rs141692564 | . | . | . | . | . | . | . | . | . | . |
| InternalIMT | aa | rs146281742 | . | . | . | . | . | . | . | . | . | . |
| InternalIMT | aa | rs192914353 | . | . | . | . | . | . | . | . | . | . |
| InternalIMT | aa | rs185182848 | . | . | . | . | . | . | . | . | . | . |
| InternalIMT | aa | rs28395858 | . | . | . | . | . | . | . | . | . | . |
| InternalIMT | aa | rs41429645 | . | . | . | . | . | . | . | . | . | . |
| InternalIMT | aa | rs28395874 | . | . | . | . | . | . | . | . | . | . |
| InternalIMT | aa | rs189663634 | . | . | . | . | . | . | . | . | . | . |
| InternalIMT | aa | rs77666700 | . | . | . | . | . | . | . | . | . | . |
| InternalIMT | aa | rs146711717 | . | . | . | . | . | . | . | . | . | . |
| InternalIMT | aa | rs144904251 | . | . | . | . | . | . | . | . | . | . |
| InternalIMT | aa | rs189893153 | . | . | . | . | . | . | . | . | . | . |
| InternalIMT | aa | rs150447740 | . | . | . | . | . | . | . | . | . | . |
| InternalIMT | aa | rs143099649 | . | . | . | . | . | . | . | . | . | . |
| InternalIMT | aa | rs191288436 | . | . | . | . | . | . | . | . | . | . |
| InternalIMT | aa | rs188577862 | . | . | . | . | . | . | . | . | . | . |
| InternalIMT | aa | rs192695334 | . | . | . | . | . | . | . | . | . | . |
| InternalIMT | aa | rs138612906 | . | . | . | . | . | . | . | . | . | . |
| InternalIMT | aa | rs191290051 | . | . | . | . | . | . | . | . | . | . |
| InternalIMT | aa | rs189631459 | . | . | . | . | . | . | . | . | . | . |
| InternalIMT | aa | rs114620874 | . | . | . | . | . | . | . | . | . | . |
| InternalIMT | aa | rs78246652 | . | . | . | . | . | . | . | . | . | . |
| InternalIMT | aa | rs79432451 | . | . | . | . | . | . | . | . | . | . |
| InternalIMT | aa | rs186022585 | . | . | . | . | . | . | . | . | . | . |
| InternalIMT | aa | rs142978891 | . | . | . | . | . | . | . | . | . | . |
| InternalIMT | aa | rs151086840 | . | . | . | . | . | . | . | . | . | . |
| InternalIMT | aa | rs183270259 | . | . | . | . | . | . | . | . | . | . |
| InternalIMT | aa | rs188544289 | . | . | . | . | . | . | . | . | . | . |
| InternalIMT | aa | rs188500808 | . | . | . | . | . | . | . | . | . | . |
| InternalIMT | aa | rs189424589 | . | . | . | . | . | . | . | . | . | . |
| InternalIMT | aa | rs78622303 | . | . | . | . | . | . | . | . | . | . |
| InternalIMT | aa | rs143210835 | . | . | . | . | . | . | . | . | . | . |
| InternalIMT | aa | rs114761840 | . | . | . | . | . | . | . | . | . | . |
| InternalIMT | aa | rs185805402 | . | . | . | . | . | . | . | . | . | . |
| InternalIMT | aa | rs139273744 | . | . | . | . | . | . | . | . | . | . |
| InternalIMT | aa | rs184654150 | . | . | . | . | . | . | . | . | . | . |
| InternalIMT | aa | rs189069710 | . | . | . | . | . | . | . | . | . | . |
| InternalIMT | aa | rs114752115 | . | . | . | . | . | . | . | . | . | . |
| InternalIMT | aa | rs150162515 | . | . | . | . | . | . | . | . | . | . |
| InternalIMT | aa | rs180982454 | . | . | . | . | . | . | . | . | . | . |
| InternalIMT | aa | rs184932088 | . | . | . | . | . | . | . | . | . | . |
| InternalIMT | aa | rs181109625 | . | . | . | . | . | . | . | . | . | . |
| InternalIMT | aa | rs138121134 | . | . | . | . | . | . | . | . | . | . |
| InternalIMT | aa | rs183253861 | . | . | . | . | . | . | . | . | . | . |
| InternalIMT | aa | rs147073946 | . | . | . | . | . | . | . | . | . | . |
| InternalIMT | aa | rs139938489 | . | . | . | . | . | . | . | . | . | . |
| InternalIMT | aa | rs146362560 | . | . | . | . | . | . | . | . | . | . |
| InternalIMT | aa | rs193155956 | . | . | . | . | . | . | . | . | . | . |
| InternalIMT | aa | rs77663092 | . | . | . | . | . | . | . | . | . | . |
| InternalIMT | aa | rs73285319 | . | . | . | . | . | . | . | . | . | . |
| InternalIMT | aa | rs184019276 | . | . | . | . | . | . | . | . | . | . |
| InternalIMT | aa | rs28395863 | . | . | . | . | . | . | . | . | . | . |
| InternalIMT | aa | rs182121645 | . | . | . | . | . | . | . | . | . | . |
| InternalIMT | aa | rs73285327 | . | . | . | . | . | . | . | . | . | . |
| InternalIMT | aa | rs149589584 | . | . | . | . | . | . | . | . | . | . |
| InternalIMT | aa | rs148310335 | . | . | . | . | . | . | . | . | . | . |
| InternalIMT | aa | rs187246093 | . | . | . | . | . | . | . | . | . | . |
| InternalIMT | aa | rs184668894 | . | . | . | . | . | . | . | . | . | . |
| InternalIMT | aa | rs114269961 | . | . | . | . | . | . | . | . | . | . |
| InternalIMT | aa | rs149694374 | . | . | . | . | . | . | . | . | . | . |
| InternalIMT | aa | rs145284227 | . | . | . | . | . | . | . | . | . | . |
| InternalIMT | aa | rs189983820 | . | . | . | . | . | . | . | . | . | . |
| InternalIMT | aa | rs181200444 | . | . | . | . | . | . | . | . | . | . |
| InternalIMT | aa | rs185486385 | . | . | . | . | . | . | . | . | . | . |
| InternalIMT | aa | rs151198241 | . | . | . | . | . | . | . | . | . | . |
| InternalIMT | aa | rs189959341 | . | . | . | . | . | . | . | . | . | . |
| InternalIMT | aa | rs140549257 | . | . | . | . | . | . | . | . | . | . |
| InternalIMT | aa | rs186453139 | . | . | . | . | . | . | . | . | . | . |
| InternalIMT | aa | rs180922711 | . | . | . | . | . | . | . | . | . | . |
| InternalIMT | aa | rs56659903 | . | . | . | . | . | . | . | . | . | . |
| InternalIMT | aa | rs141202128 | . | . | . | . | . | . | . | . | . | . |
| InternalIMT | aa | rs59085314 | . | . | . | . | . | . | . | . | . | . |
| InternalIMT | aa | rs145564996 | . | . | . | . | . | . | . | . | . | . |
| InternalIMT | aa | rs114961009 | . | . | . | . | . | . | . | . | . | . |
| InternalIMT | aa | rs182294189 | . | . | . | . | . | . | . | . | . | . |
| InternalIMT | aa | rs187586636 | . | . | . | . | . | . | . | . | . | . |
| InternalIMT | aa | rs192254907 | . | . | . | . | . | . | . | . | . | . |
| InternalIMT | aa | rs149145663 | . | . | . | . | . | . | . | . | . | . |
| InternalIMT | aa | rs180713064 | . | . | . | . | . | . | . | . | . | . |
| InternalIMT | aa | rs183009023 | . | . | . | . | . | . | . | . | . | . |
| InternalIMT | aa | rs143385347 | . | . | . | . | . | . | . | . | . | . |
| InternalIMT | aa | rs185786329 | . | . | . | . | . | . | . | . | . | . |
| InternalIMT | aa | rs192341030 | . | . | . | . | . | . | . | . | . | . |

| InternalIMT | aa | rs74661429 | . | . | . | . | . | . | . | . | . | . |
| --- | --- | --- | --- | --- | --- | --- | --- | --- | --- | --- | --- | --- |
| InternalIMT | aa | rs188034853 | . | . | . | . | . | . | . | . | . | . |
| InternalIMT | aa | rs116808430 | . | . | . | . | . | . | . | . | . | . |
| InternalIMT | aa | rs148469010 | . | . | . | . | . | . | . | . | . | . |
| InternalIMT | aa | rs78621663 | . | . | . | . | . | . | . | . | . | . |
| InternalIMT | aa | rs192654886 | . | . | . | . | . | . | . | . | . | . |
| InternalIMT | aa | rs145996035 | . | . | . | . | . | . | . | . | . | . |
| InternalIMT | aa | rs139942226 | . | . | . | . | . | . | . | . | . | . |
| InternalIMT | aa | rs113645632 | . | . | . | . | . | . | . | . | . | . |
| InternalIMT | aa | rs74940661 | . | . | . | . | . | . | . | . | . | . |
| InternalIMT | aa | rs115355800 | . | . | . | . | . | . | . | . | . | . |
| InternalIMT | aa | rs150285898 | . | . | . | . | . | . | . | . | . | . |
| InternalIMT | aa | rs188117932 | . | . | . | . | . | . | . | . | . | . |
| InternalIMT | aa | rs139956566 | . | . | . | . | . | . | . | . | . | . |
| InternalIMT | aa | rs147673885 | . | . | . | . | . | . | . | . | . | . |
| InternalIMT | aa | rs189127928 | . | . | . | . | . | . | . | . | . | . |
| InternalIMT | aa | rs187558772 | . | . | . | . | . | . | . | . | . | . |
| InternalIMT | aa | rs181948587 | . | . | . | . | . | . | . | . | . | . |
| InternalIMT | aa | rs189983269 | . | . | . | . | . | . | . | . | . | . |
| InternalIMT | aa | rs187250069 | . | . | . | . | . | . | . | . | . | . |
| InternalIMT | aa | rs191485859 | . | . | . | . | . | . | . | . | . | . |
| InternalIMT | aa | rs185499555 | . | . | . | . | . | . | . | . | . | . |
| InternalIMT | aa | rs180862110 | . | . | . | . | . | . | . | . | . | . |
| InternalIMT | aa | rs190472603 | . | . | . | . | . | . | . | . | . | . |
| InternalIMT | aa | rs189226167 | . | . | . | . | . | . | . | . | . | . |
| InternalIMT | aa | rs181623256 | . | . | . | . | . | . | . | . | . | . |
| InternalIMT | aa | rs186079557 | . | . | . | . | . | . | . | . | . | . |
| InternalIMT | aa | rs147760994 | . | . | . | . | . | . | . | . | . | . |
| InternalIMT | aa | rs189176167 | . | . | . | . | . | . | . | . | . | . |
| InternalIMT | aa | rs117901000 | . | . | . | . | . | . | . | . | . | . |
| InternalIMT | aa | rs141984888 | . | . | . | . | . | . | . | . | . | . |
| InternalIMT | aa | rs192365061 | . | . | . | . | . | . | . | . | . | . |
| InternalIMT | aa | rs185976038 | . | . | . | . | . | . | . | . | . | . |
| InternalIMT | aa | rs184697303 | . | . | . | . | . | . | . | . | . | . |
| InternalIMT | aa | rs143383132 | . | . | . | . | . | . | . | . | . | . |
| InternalIMT | aa | rs141873881 | . | . | . | . | . | . | . | . | . | . |
| InternalIMT | aa | rs185507255 | . | . | . | . | . | . | . | . | . | . |
| InternalIMT | aa | rs191379425 | . | . | . | . | . | . | . | . | . | . |
| InternalIMT | aa | rs184199584 | . | . | . | . | . | . | . | . | . | . |
| InternalIMT | aa | rs183181360 | . | . | . | . | . | . | . | . | . | . |
| InternalIMT | aa | rs184495673 | . | . | . | . | . | . | . | . | . | . |
| InternalIMT | aa | rs182946297 | . | . | . | . | . | . | . | . | . | . |
| InternalIMT | aa | rs187743615 | . | . | . | . | . | . | . | . | . | . |
| InternalIMT | aa | rs183259862 | . | . | . | . | . | . | . | . | . | . |
| InternalIMT | aa | rs112433566 | . | . | . | . | . | . | . | . | . | . |
| InternalIMT | aa | rs182351085 | . | . | . | . | . | . | . | . | . | . |
| InternalIMT | aa | rs146248508 | . | . | . | . | . | . | . | . | . | . |
| InternalIMT | aa | rs188528439 | . | . | . | . | . | . | . | . | . | . |
| InternalIMT | aa | rs146167018 | . | . | . | . | . | . | . | . | . | . |
| InternalIMT | aa | rs192440980 | . | . | . | . | . | . | . | . | . | . |
| InternalIMT | aa | rs185601882 | . | . | . | . | . | . | . | . | . | . |
| InternalIMT | aa | rs117272563 | . | . | . | . | . | . | . | . | . | . |
| InternalIMT | aa | rs10430734 | . | . | . | . | . | . | . | . | . | . |
| InternalIMT | aa | rs181320952 | . | . | . | . | . | . | . | . | . | . |
| InternalIMT | aa | rs185480660 | . | . | . | . | . | . | . | . | . | . |
| InternalIMT | aa | rs188077917 | . | . | . | . | . | . | . | . | . | . |
| InternalIMT | aa | rs187562953 | . | . | . | . | . | . | . | . | . | . |
| InternalIMT | aa | rs182044631 | . | . | . | . | . | . | . | . | . | . |
| InternalIMT | aa | rs145455596 | . | . | . | . | . | . | . | . | . | . |
| InternalIMT | aa | rs181475877 | . | . | . | . | . | . | . | . | . | . |
| InternalIMT | aa | rs149413085 | . | . | . | . | . | . | . | . | . | . |
| InternalIMT | aa | rs12253656 | . | . | . | . | . | . | . | . | . | . |
| InternalIMT | aa | rs182718239 | . | . | . | . | . | . | . | . | . | . |
| InternalIMT | aa | rs192435695 | . | . | . | . | . | . | . | . | . | . |
| InternalIMT | aa | rs12248277 | . | . | . | . | . | . | . | . | . | . |
| InternalIMT | aa | rs143521710 | . | . | . | . | . | . | . | . | . | . |
| InternalIMT | aa | rs144454454 | . | . | . | . | . | . | . | . | . | . |
| InternalIMT | aa | rs190441610 | . | . | . | . | . | . | . | . | . | . |
| InternalIMT | aa | rs114690462 | . | . | . | . | . | . | . | . | . | . |
| InternalIMT | aa | rs184541357 | . | . | . | . | . | . | . | . | . | . |
| InternalIMT | aa | rs142657653 | . | . | . | . | . | . | . | . | . | . |
| InternalIMT | aa | rsurf1 | 2.019 | . | . | . | . | . | . | . | . | . |
| InternalIMT | aa | rsten1 | 2.195 | . | . | . | . | . | . | . | . | . |
| InternalIMT | aa | chr10:45852312:D | 0.055 | 0.112 | 0.063 | 0.078 | -0.171 | 0.489 | 0.727 | 0.066 | 0.203 | 0.746 |
| InternalIMT | aa | chr10:45853232:I | 0.071 | 0.108 | 0.065 | 0.096 | -0.216 | 0.471 | 0.647 | 0.083 | 0.194 | 0.67 |
| InternalIMT | aa | chr10:45853234:I | 0.157 | 0.109 | 0.068 | 0.11 | -0.139 | 0.294 | 0.636 | 0.045 | 0.122 | 0.714 |
| InternalIMT | aa | chr10:45854796:D | 0.001 | . | . | . | . | . | . | . | . | . |
| InternalIMT | aa | chr10:45854994:I | 0.001 | . | . | . | . | . | . | . | . | . |
| InternalIMT | aa | chr10:45858290:D | 0.04 | 0.112 | 0.064 | 0.081 | -0.142 | 0.973 | 0.884 | 0.068 | 0.4 | 0.864 |
| InternalIMT | aa | chr10:45858820:I | 0.015 | 0.147 | 0.062 | 0.017 | 2.5 | 1.57 | 0.111 | -1.02 | 0.64 | 0.111 |
| InternalIMT | aa | chr10:45860888:D | 0.067 | 0.131 | 0.067 | 0.049 | 0.255 | 0.747 | 0.732 | -0.114 | 0.309 | 0.711 |
| InternalIMT | aa | chr10:45863913:I | 0.159 | 0.109 | 0.068 | 0.11 | -0.127 | 0.29 | 0.662 | 0.041 | 0.12 | 0.731 |
| InternalIMT | aa | chr10:45866601:D | 0 | . | . | . | . | . | . | . | . | . |
| InternalIMT | aa | chr10:45867197:I | 0.008 | . | . | . | . | . | . | . | . | . |
| InternalIMT | aa | chr10:45867538:D | 0.167 | 0.102 | 0.069 | 0.137 | -0.175 | 0.284 | 0.538 | 0.062 | 0.117 | 0.599 |
| InternalIMT | aa | chr10:45868356:D | 0.151 | 0.108 | 0.068 | 0.112 | -0.138 | 0.3 | 0.644 | 0.046 | 0.124 | 0.71 |

| InternalIMT | aa | chr10:45871158:I | 0.16 | 0.109 | 0.068 | 0.109 | -0.123 | 0.29 | 0.672 | 0.039 | 0.12 | 0.742 |
| --- | --- | --- | --- | --- | --- | --- | --- | --- | --- | --- | --- | --- |
| InternalIMT | aa | chr10:45871637:D | 0.061 | 0.108 | 0.066 | 0.103 | -0.464 | 0.548 | 0.397 | 0.134 | 0.23 | 0.562 |
| InternalIMT | aa | chr10:45871638:I | 0.115 | 0.101 | 0.067 | 0.134 | -0.298 | 0.379 | 0.432 | 0.102 | 0.157 | 0.517 |
| InternalIMT | aa | chr10:45873629:I | 0.159 | 0.113 | 0.068 | 0.099 | -0.097 | 0.291 | 0.738 | 0.029 | 0.12 | 0.809 |
| InternalIMT | aa | chr10:45873701:D | 0.003 | . | . | . | . | . | . | . | . | . |
| InternalIMT | aa | chr10:45874997:I | 0.03 | 0.088 | 0.063 | 0.164 | -1.11 | 0.614 | 0.071 | 0.469 | 0.267 | 0.079 |
| InternalIMT | aa | chr10:45882030:I | 0.021 | 0.12 | 0.063 | 0.058 | 0.088 | 0.967 | 0.927 | -0.054 | 0.4 | 0.893 |
| InternalIMT | aa | chr10:45883774:D | 0.154 | 0.109 | 0.067 | 0.106 | -0.106 | 0.272 | 0.697 | 0.038 | 0.113 | 0.735 |
| InternalIMT | aa | chr10:45885169:D | 0.035 | 0.113 | 0.062 | 0.069 | -0.196 | 0.811 | 0.809 | 0.079 | 0.333 | 0.814 |
| InternalIMT | aa | chr10:45886451:D | 0.588 | 0.059 | 0.136 | 0.662 | -0.075 | 0.24 | 0.755 | 0.047 | 0.099 | 0.631 |
| InternalIMT | aa | chr10:45891800:I | 0.592 | 0.076 | 0.122 | 0.535 | -0.064 | 0.206 | 0.757 | 0.034 | 0.085 | 0.693 |
| InternalIMT | aa | chr10:45904771:I | 0.573 | 0.148 | 0.118 | 0.21 | 0.091 | 0.205 | 0.658 | -0.03 | 0.085 | 0.728 |
| InternalIMT | aa | chr10:45904959:I | 0.103 | 0.118 | 0.067 | 0.077 | 0.035 | 0.422 | 0.934 | -8.10E-03 | 0.174 | 0.963 |
| InternalIMT | aa | chr10:45911028:I | 0.034 | 0.094 | 0.065 | 0.15 | -0.924 | 0.789 | 0.242 | 0.356 | 0.334 | 0.287 |
| InternalIMT | aa | chr10:45911031:I | 0.363 | 0.202 | 0.096 | 0.036 | 0.307 | 0.231 | 0.184 | -0.116 | 0.095 | 0.22 |
| InternalIMT | aa | chr10:45912154:D | 0.145 | 0.12 | 0.067 | 0.074 | -0.012 | 0.272 | 0.964 | -1.20E-03 | 0.113 | 0.991 |
| InternalIMT | aa | chr10:45913885:I | 0.001 | . | . | . | . | . | . | . | . | . |
| InternalIMT | aa | chr10:45914753:D | 0.243 | 0.139 | 0.076 | 0.069 | 0.123 | 0.235 | 0.601 | -0.049 | 0.098 | 0.613 |
| InternalIMT | aa | chr10:45919621 | 0.003 | . | . | . | . | . | . | . | . | . |
| InternalIMT | aa | chr10:45921739:I | 0.004 | . | . | . | . | . | . | . | . | . |
| InternalIMT | aa | chr10:45923527:I | 0.052 | 0.109 | 0.067 | 0.104 | -0.04 | 0.605 | 0.948 | 0.053 | 0.251 | 0.833 |
| InternalIMT | aa | chr10:45927001:I | 0.014 | 0.12 | 0.064 | 0.062 | 0.127 | 0.769 | 0.868 | -0.108 | 0.327 | 0.74 |
| InternalIMT | aa | chr10:45930354:I | 0.001 | . | . | . | . | . | . | . | . | . |
| InternalIMT | aa | chr10:45932864:I | 0.027 | 0.106 | 0.063 | 0.095 | -0.599 | 1.04 | 0.563 | 0.286 | 0.448 | 0.524 |
| InternalIMT | aa | chr10:45936163:I | 0.026 | 0.125 | 0.062 | 0.045 | 0.165 | 0.485 | 0.734 | -0.107 | 0.203 | 0.598 |
| InternalIMT | aa | chr10:45938095:D | 0.164 | 0.134 | 0.069 | 0.053 | 0.082 | 0.265 | 0.758 | -0.045 | 0.11 | 0.686 |
| InternalIMT | aa | chr10:45938886 | 0 | . | . | . | . | . | . | . | . | . |
| InternalIMT | aa | chr10:45939320 | 0.001 | . | . | . | . | . | . | . | . | . |
| InternalIMT | aa | chr10:45939659 | 0.001 | . | . | . | . | . | . | . | . | . |
| InternalIMT | aa | chr10:45941150:D | 0.001 | . | . | . | . | . | . | . | . | . |
| InternalIMT | aa | chr10:45947128:D | 0.064 | 0.128 | 0.065 | 0.05 | 0.222 | 0.346 | 0.522 | -0.076 | 0.141 | 0.592 |
| InternalIMT | aa | chr10:45950990:I | 0.044 | 0.165 | 0.063 | 8.30E-03 | 1.32 | 0.7 | 0.06 | -0.539 | 0.281 | 0.055 |
| InternalIMT | aa | chr10:45951854:I | 0.048 | 0.152 | 0.062 | 0.015 | 0.867 | 0.59 | 0.142 | -0.359 | 0.242 | 0.137 |
| InternalIMT | aa | chr10:45952670:I | 0.061 | 0.088 | 0.066 | 0.182 | -0.524 | 0.368 | 0.155 | 0.193 | 0.159 | 0.224 |
| InternalIMT | aa | chr10:45956741 | 0.001 | . | . | . | . | . | . | . | . | . |
| InternalIMT | aa | chr10:45957247:I | 0.233 | 0.127 | 0.075 | 0.089 | 1.60E-03 | 0.22 | 0.994 | -0.012 | 0.092 | 0.896 |
| InternalIMT | aa | chr10:45958082:I | 0.232 | 0.131 | 0.075 | 0.082 | 0.024 | 0.224 | 0.916 | -0.02 | 0.093 | 0.826 |
| InternalIMT | aa | chr10:45905041:D | . | . | . | . | . | . | . | . | . | . |
| InternalIMT | aa | chr10:45938871 | . | . | . | . | . | . | . | . | . | . |
| InternalIMT | aa | chr10:45939160 | . | . | . | . | . | . | . | . | . | . |
| InternalIMT | aa | chr10:45869910 | . | . | . | . | . | . | . | . | . | . |
| InternalIMT | aa | chr10:45876214:D | . | . | . | . | . | . | . | . | . | . |
| InternalIMT | aa | chr10:45880237:I | . | . | . | . | . | . | . | . | . | . |
| InternalIMT | aa | chr10:45884371:I | . | . | . | . | . | . | . | . | . | . |
| InternalIMT | aa | chr10:45891535:D | . | . | . | . | . | . | . | . | . | . |
| InternalIMT | aa | chr10:45893516:D | . | . | . | . | . | . | . | . | . | . |
| InternalIMT | aa | chr10:45893782:D | . | . | . | . | . | . | . | . | . | . |
| InternalIMT | aa | chr10:45895254:I | . | . | . | . | . | . | . | . | . | . |
| InternalIMT | aa | chr10:45895848:D | . | . | . | . | . | . | . | . | . | . |
| InternalIMT | aa | chr10:45896316:D | . | . | . | . | . | . | . | . | . | . |
| InternalIMT | aa | chr10:45896317:D | . | . | . | . | . | . | . | . | . | . |
| InternalIMT | aa | chr10:45902683:I | . | . | . | . | . | . | . | . | . | . |
| InternalIMT | aa | chr10:45905003:D | . | . | . | . | . | . | . | . | . | . |
| InternalIMT | aa | chr10:45927476:D | . | . | . | . | . | . | . | . | . | . |
| InternalIMT | aa | chr10:45935882 | . | . | . | . | . | . | . | . | . | . |
| InternalIMT | aa | chr10:45936100 | . | . | . | . | . | . | . | . | . | . |
| InternalIMT | aa | chr10:45939553 | . | . | . | . | . | . | . | . | . | . |
| InternalIMT | aa | chr10:45939631 | . | . | . | . | . | . | . | . | . | . |
| InternalIMT | aa | chr10:45944193:D | . | . | . | . | . | . | . | . | . | . |
| InternalIMT | aa | chr10:45954030 | . | . | . | . | . | . | . | . | . | . |

Supplemental table S28.

| phen | covar(fa) | rs_id | maf | beta_FA | se_FA | p_FA | beta_SNP | se_SNP | p_SNP | beta_inter | se_inter | p_inter |
| --- | --- | --- | --- | --- | --- | --- | --- | --- | --- | --- | --- | --- |
| InternalIMT | aa | rs76035182 | 0.068 | 0.02 | 0.077 | 0.795 | 0.067 | 0.544 | 0.902 | -6.80E-03 | 0.213 | 0.975 |
| InternalIMT | aa | rs12359547 | 0.052 | -0.029 | 0.075 | 0.694 | -1.4 | 0.852 | 0.102 | 0.562 | 0.331 | 0.09 |
| InternalIMT | aa | rs74595484 | 0.12 | 0.082 | 0.084 | 0.333 | 0.712 | 0.402 | 0.077 | -0.264 | 0.157 | 0.093 |
| InternalIMT | aa | rs1864415 | 0.749 | -0.164 | 0.176 | 0.351 | -0.26 | 0.289 | 0.367 | 0.116 | 0.112 | 0.302 |
| InternalIMT | aa | rs72788101 | 0.064 | -0.048 | 0.075 | 0.522 | -1.53 | 0.764 | 0.045 | 0.627 | 0.297 | 0.035 |
| InternalIMT | aa | rs11239481 | 0.081 | 0.037 | 0.081 | 0.647 | 0.352 | 0.381 | 0.356 | -0.138 | 0.149 | 0.354 |
| InternalIMT | aa | rs72788102 | 0.064 | -0.048 | 0.075 | 0.521 | -1.53 | 0.764 | 0.045 | 0.627 | 0.297 | 0.035 |
| InternalIMT | aa | rs72789705 | 0.064 | -0.048 | 0.075 | 0.52 | -1.53 | 0.764 | 0.046 | 0.627 | 0.297 | 0.035 |
| InternalIMT | aa | rs141529858 | 0.002 | . | . | . | . | . | . | . | . | . |
| InternalIMT | aa | rs145376292 | 0 | . | . | . | . | . | . | . | . | . |
| InternalIMT | aa | rs190418301 | 0 | . | . | . | . | . | . | . | . | . |
| InternalIMT | aa | rs11239482 | 0.064 | -0.048 | 0.075 | 0.519 | -1.53 | 0.764 | 0.046 | 0.626 | 0.297 | 0.035 |
| InternalIMT | aa | rs61855872 | 0.004 | . | . | . | . | . | . | . | . | . |
| InternalIMT | aa | rs148991144 | 0.003 | . | . | . | . | . | . | . | . | . |
| InternalIMT | aa | rs191932221 | 0.002 | . | . | . | . | . | . | . | . | . |
| InternalIMT | aa | rs55978138 | 0.179 | -0.069 | 0.09 | 0.444 | -0.47 | 0.368 | 0.202 | 0.204 | 0.144 | 0.156 |
| InternalIMT | aa | rs73281167 | 0.045 | 0.027 | 0.077 | 0.728 | 0.327 | 0.64 | 0.609 | -0.138 | 0.246 | 0.574 |
| InternalIMT | aa | rs12768010 | 0.092 | -5.60E-03 | 0.076 | 0.941 | -0.396 | 0.517 | 0.444 | 0.166 | 0.202 | 0.412 |
| InternalIMT | aa | rs11239484 | 0.749 | -0.163 | 0.176 | 0.353 | -0.259 | 0.289 | 0.369 | 0.115 | 0.112 | 0.304 |
| InternalIMT | aa | rs35906360 | 0.18 | -0.067 | 0.09 | 0.456 | -0.457 | 0.367 | 0.213 | 0.199 | 0.143 | 0.165 |
| InternalIMT | aa | rs186250512 | 0.001 | . | . | . | . | . | . | . | . | . |
| InternalIMT | aa | rs150810743 | 0.007 | . | . | . | . | . | . | . | . | . |
| InternalIMT | aa | rs55965812 | 0.161 | -0.044 | 0.089 | 0.62 | -0.356 | 0.379 | 0.348 | 0.154 | 0.148 | 0.3 |
| InternalIMT | aa | rs56180358 | 0.181 | -0.067 | 0.09 | 0.457 | -0.455 | 0.366 | 0.214 | 0.199 | 0.143 | 0.165 |
| InternalIMT | aa | rs55864145 | 0.092 | -5.70E-03 | 0.076 | 0.941 | -0.397 | 0.517 | 0.443 | 0.166 | 0.202 | 0.411 |
| InternalIMT | aa | rs55986122 | 0.181 | -0.067 | 0.09 | 0.457 | -0.455 | 0.366 | 0.214 | 0.198 | 0.143 | 0.165 |
| InternalIMT | aa | rs7477350 | 0.749 | -0.163 | 0.176 | 0.354 | -0.259 | 0.289 | 0.369 | 0.115 | 0.112 | 0.304 |
| InternalIMT | aa | rs2099170 | 0.18 | -0.066 | 0.09 | 0.465 | -0.448 | 0.366 | 0.221 | 0.196 | 0.143 | 0.17 |
| InternalIMT | aa | rs2082108 | 0.181 | -0.067 | 0.09 | 0.457 | -0.454 | 0.366 | 0.214 | 0.198 | 0.143 | 0.165 |
| InternalIMT | aa | rs55902404 | 0.02 | 2.40E-03 | 0.074 | 0.975 | -1.18 | 1.3 | 0.366 | 0.517 | 0.505 | 0.306 |
| InternalIMT | aa | rs58402662 | 0.02 | 2.40E-03 | 0.074 | 0.974 | -1.18 | 1.3 | 0.365 | 0.518 | 0.505 | 0.306 |
| InternalIMT | aa | rs11511618 | 0.084 | 0.038 | 0.081 | 0.636 | 0.361 | 0.376 | 0.337 | -0.145 | 0.146 | 0.323 |
| InternalIMT | aa | rs1816492 | 0.747 | -0.166 | 0.176 | 0.345 | -0.263 | 0.29 | 0.363 | 0.118 | 0.113 | 0.297 |
| InternalIMT | aa | rs146611093 | 0.002 | . | . | . | . | . | . | . | . | . |
| InternalIMT | aa | rs186897689 | 0.006 | . | . | . | . | . | . | . | . | . |
| InternalIMT | aa | rs10793616 | 0.749 | -0.163 | 0.176 | 0.354 | -0.259 | 0.289 | 0.37 | 0.115 | 0.112 | 0.305 |
| InternalIMT | aa | rs116700479 | 0.052 | -4.80E-03 | 0.074 | 0.949 | -0.652 | 0.785 | 0.406 | 0.277 | 0.306 | 0.366 |
| InternalIMT | aa | rs192535936 | 0.004 | . | . | . | . | . | . | . | . | . |
| InternalIMT | aa | rs113363297 | 0.723 | -0.266 | 0.18 | 0.139 | -0.44 | 0.302 | 0.145 | 0.185 | 0.117 | 0.114 |
| InternalIMT | aa | rs12354994 | 0.172 | -0.071 | 0.09 | 0.428 | -0.486 | 0.377 | 0.198 | 0.214 | 0.147 | 0.147 |
| InternalIMT | aa | rs148068329 | 0.025 | 3.70E-03 | 0.074 | 0.96 | -0.8 | 1.03 | 0.437 | 0.343 | 0.41 | 0.402 |
| InternalIMT | aa | rs12356109 | 0.198 | -0.071 | 0.092 | 0.436 | -0.44 | 0.337 | 0.192 | 0.19 | 0.131 | 0.149 |
| InternalIMT | aa | rs55908141 | 0.02 | 2.30E-03 | 0.074 | 0.975 | -1.19 | 1.3 | 0.36 | 0.522 | 0.504 | 0.301 |
| InternalIMT | aa | rs12354712 | 0.161 | -0.043 | 0.089 | 0.627 | -0.349 | 0.377 | 0.354 | 0.151 | 0.147 | 0.305 |
| InternalIMT | aa | rs147282684 | 0.002 | . | . | . | . | . | . | . | . | . |
| InternalIMT | aa | rs12356140 | 0.191 | -0.075 | 0.092 | 0.415 | -0.481 | 0.362 | 0.184 | 0.209 | 0.141 | 0.139 |
| InternalIMT | aa | rs185476187 | 0.004 | . | . | . | . | . | . | . | . | . |
| InternalIMT | aa | rs145720057 | 0.012 | 8.00E-03 | 0.074 | 0.914 | -0.938 | 1.51 | 0.536 | 0.389 | 0.599 | 0.517 |
| InternalIMT | aa | rs12354742 | 0.181 | -0.067 | 0.09 | 0.46 | -0.45 | 0.364 | 0.217 | 0.197 | 0.142 | 0.167 |
| InternalIMT | aa | rs180913842 | 0.02 | 2.30E-03 | 0.074 | 0.975 | -1.19 | 1.3 | 0.359 | 0.523 | 0.504 | 0.3 |
| InternalIMT | aa | rs142976914 | 0.002 | . | . | . | . | . | . | . | . | . |
| InternalIMT | aa | rs11511619 | 0.201 | -0.074 | 0.093 | 0.427 | -0.446 | 0.336 | 0.185 | 0.191 | 0.131 | 0.146 |
| InternalIMT | aa | rs146702870 | 0.003 | . | . | . | . | . | . | . | . | . |
| InternalIMT | aa | rs150287496 | 0.001 | . | . | . | . | . | . | . | . | . |
| InternalIMT | aa | rs190917973 | 0.002 | . | . | . | . | . | . | . | . | . |
| InternalIMT | aa | rs11517281 | 0.181 | -0.067 | 0.09 | 0.461 | -0.449 | 0.364 | 0.217 | 0.196 | 0.142 | 0.167 |
| InternalIMT | aa | rs12359299 | 0.181 | -0.067 | 0.09 | 0.461 | -0.449 | 0.364 | 0.217 | 0.196 | 0.142 | 0.167 |
| InternalIMT | aa | rs182307932 | 0.071 | -0.021 | 0.076 | 0.782 | -0.997 | 0.516 | 0.054 | 0.366 | 0.204 | 0.072 |
| InternalIMT | aa | rs61855873 | 0.011 | 0.018 | 0.075 | 0.811 | 0.206 | 1.82 | 0.91 | -0.096 | 0.708 | 0.892 |
| InternalIMT | aa | rs12359302 | 0.18 | -0.053 | 0.088 | 0.55 | -0.386 | 0.383 | 0.314 | 0.175 | 0.15 | 0.243 |
| InternalIMT | aa | rs143208140 | 0.028 | -5.40E-03 | 0.079 | 0.946 | -0.767 | 0.903 | 0.396 | 0.327 | 0.36 | 0.364 |
| InternalIMT | aa | rs9988764 | 0.712 | -0.153 | 0.165 | 0.353 | -0.264 | 0.302 | 0.382 | 0.117 | 0.117 | 0.319 |
| InternalIMT | aa | rs192875767 | 0.002 | . | . | . | . | . | . | . | . | . |
| InternalIMT | aa | rs147268846 | 0.005 | . | . | . | . | . | . | . | . | . |
| InternalIMT | aa | rs11239485 | 0.109 | -0.032 | 0.088 | 0.717 | -0.377 | 0.489 | 0.442 | 0.165 | 0.192 | 0.392 |
| InternalIMT | aa | rs151317885 | 0.24 | -0.067 | 0.082 | 0.412 | -0.498 | 0.305 | 0.102 | 0.19 | 0.12 | 0.113 |
| InternalIMT | aa | rs11499085 | 0.322 | 0.167 | 0.11 | 0.129 | 0.572 | 0.312 | 0.067 | -0.22 | 0.121 | 0.069 |
| InternalIMT | aa | rs188130606 | 0.086 | 0.099 | 0.081 | 0.224 | 1.17 | 0.413 | 4.50E-03 | -0.439 | 0.159 | 5.60E-03 |
| InternalIMT | aa | rs11511620 | 0.161 | -0.043 | 0.089 | 0.631 | -0.345 | 0.375 | 0.358 | 0.149 | 0.146 | 0.308 |
| InternalIMT | aa | rs181498390 | 0.057 | 0.044 | 0.079 | 0.577 | 0.691 | 0.566 | 0.222 | -0.287 | 0.219 | 0.191 |
| InternalIMT | aa | rs186280701 | 0.02 | 2.20E-03 | 0.074 | 0.977 | -1.21 | 1.29 | 0.352 | 0.528 | 0.503 | 0.294 |
| InternalIMT | aa | rs150059185 | 0.034 | 1.70E-03 | 0.075 | 0.982 | -0.606 | 0.999 | 0.544 | 0.234 | 0.392 | 0.55 |
| InternalIMT | aa | rs183338707 | 0.001 | . | . | . | . | . | . | . | . | . |
| InternalIMT | aa | rs11499038 | 0.306 | 0.173 | 0.107 | 0.107 | 0.634 | 0.321 | 0.048 | -0.243 | 0.124 | 0.051 |
| InternalIMT | aa | rs190699595 | 0.004 | . | . | . | . | . | . | . | . | . |
| InternalIMT | aa | rs11523408 | 0.175 | -0.064 | 0.09 | 0.477 | -0.448 | 0.373 | 0.229 | 0.196 | 0.145 | 0.178 |
| InternalIMT | aa | rs188049317 | 0.007 | . | . | . | . | . | . | . | . | . |
| InternalIMT | aa | rs144948634 | 0.214 | -0.07 | 0.08 | 0.384 | -0.574 | 0.291 | 0.048 | 0.213 | 0.114 | 0.062 |
| InternalIMT | aa | rs143833576 | . | . | . | . | . | . | . | . | . | . |
| InternalIMT | aa | rs151239879 | 0.175 | -0.065 | 0.09 | 0.471 | -0.452 | 0.372 | 0.225 | 0.198 | 0.145 | 0.174 |
| InternalIMT | aa | rs7073743 | 0.212 | -0.071 | 0.08 | 0.375 | -0.578 | 0.286 | 0.043 | 0.216 | 0.112 | 0.054 |
| InternalIMT | aa | rs142563349 | 0.092 | 0.103 | 0.082 | 0.21 | 1.16 | 0.414 | 5.20E-03 | -0.438 | 0.159 | 5.80E-03 |
| InternalIMT | aa | rs187246403 | 0.02 | 2.10E-03 | 0.074 | 0.978 | -1.22 | 1.29 | 0.347 | 0.532 | 0.502 | 0.29 |
| InternalIMT | aa | rs150423942 | 0.178 | -0.062 | 0.09 | 0.487 | -0.43 | 0.365 | 0.239 | 0.19 | 0.143 | 0.184 |

| InternalIMT | aa | rs184706102 | . | . | . | . | . | . | . | . | . | . |
| --- | --- | --- | --- | --- | --- | --- | --- | --- | --- | --- | --- | --- |
| InternalIMT | aa | rs189626288 | 0.007 | . | . | . | . | . | . | . | . | . |
| InternalIMT | aa | rs140749834 | 0.142 | 0.039 | 0.092 | 0.672 | 0.137 | 0.338 | 0.686 | -0.065 | 0.132 | 0.623 |
| InternalIMT | aa | rs7078822 | 0.136 | -0.019 | 0.089 | 0.831 | -0.233 | 0.395 | 0.556 | 0.099 | 0.154 | 0.517 |
| InternalIMT | aa | rs186227617 | 0.177 | -0.053 | 0.09 | 0.553 | -0.384 | 0.369 | 0.298 | 0.169 | 0.144 | 0.239 |
| InternalIMT | aa | rs188984368 | 0.018 | 0.029 | 0.077 | 0.71 | 0.845 | 1.06 | 0.425 | -0.322 | 0.424 | 0.447 |
| InternalIMT | aa | rs182078109 | 0.04 | 0.048 | 0.079 | 0.547 | 1.19 | 0.968 | 0.217 | -0.438 | 0.376 | 0.245 |
| InternalIMT | aa | rs141456274 | 0.001 | . | . | . | . | . | . | . | . | . |
| InternalIMT | aa | rs146976512 | 0.181 | -0.065 | 0.09 | 0.472 | -0.436 | 0.363 | 0.229 | 0.191 | 0.141 | 0.176 |
| InternalIMT | aa | rs138036816 | 0.305 | 0.173 | 0.107 | 0.107 | 0.635 | 0.321 | 0.048 | -0.243 | 0.125 | 0.052 |
| InternalIMT | aa | rs114879777 | 0.051 | -6.80E-03 | 0.074 | 0.927 | -0.755 | 0.798 | 0.344 | 0.315 | 0.311 | 0.311 |
| InternalIMT | aa | rs149366475 | 0.307 | 0.18 | 0.108 | 0.096 | 0.654 | 0.324 | 0.044 | -0.252 | 0.126 | 0.045 |
| InternalIMT | aa | rs182463043 | 0.007 | . | . | . | . | . | . | . | . | . |
| InternalIMT | aa | rs144754632 | 0.025 | 3.90E-03 | 0.074 | 0.958 | -0.784 | 1.03 | 0.446 | 0.337 | 0.41 | 0.41 |
| InternalIMT | aa | rs55717607 | 0.181 | -0.066 | 0.09 | 0.468 | -0.439 | 0.36 | 0.223 | 0.192 | 0.14 | 0.171 |
| InternalIMT | aa | rs187537973 | 0.001 | . | . | . | . | . | . | . | . | . |
| InternalIMT | aa | rs148085971 | 0.007 | . | . | . | . | . | . | . | . | . |
| InternalIMT | aa | rs11239491 | 0.181 | -0.066 | 0.09 | 0.468 | -0.438 | 0.359 | 0.223 | 0.192 | 0.14 | 0.171 |
| InternalIMT | aa | rs184412184 | 0.002 | . | . | . | . | . | . | . | . | . |
| InternalIMT | aa | rs4948998 | 0.798 | -0.142 | 0.191 | 0.458 | -0.209 | 0.295 | 0.478 | 0.095 | 0.115 | 0.406 |
| InternalIMT | aa | rs7076696 | 0.798 | -0.145 | 0.191 | 0.447 | -0.214 | 0.295 | 0.468 | 0.098 | 0.115 | 0.396 |
| InternalIMT | aa | rs11239492 | 0.198 | -0.072 | 0.092 | 0.434 | -0.441 | 0.337 | 0.19 | 0.19 | 0.131 | 0.148 |
| InternalIMT | aa | rs79462470 | 0.02 | 1.90E-03 | 0.074 | 0.98 | -1.24 | 1.29 | 0.335 | 0.541 | 0.5 | 0.279 |
| InternalIMT | aa | rs187804654 | . | . | . | . | . | . | . | . | . | . |
| InternalIMT | aa | rs7093687 | 0.161 | -0.04 | 0.089 | 0.654 | -0.324 | 0.37 | 0.382 | 0.141 | 0.144 | 0.329 |
| InternalIMT | aa | rs7081004 | 0.162 | -0.042 | 0.09 | 0.642 | -0.333 | 0.37 | 0.368 | 0.145 | 0.144 | 0.316 |
| InternalIMT | aa | rs7084793 | 0.181 | -0.066 | 0.09 | 0.467 | -0.439 | 0.359 | 0.222 | 0.192 | 0.14 | 0.17 |
| InternalIMT | aa | rs61556080 | 0.02 | 1.80E-03 | 0.074 | 0.98 | -1.24 | 1.29 | 0.334 | 0.542 | 0.5 | 0.278 |
| InternalIMT | aa | rs115552064 | 0.019 | 0.022 | 0.074 | 0.771 | 1.44 | 1.55 | 0.351 | -0.49 | 0.585 | 0.403 |
| InternalIMT | aa | rs7921503 | 0.18 | -0.072 | 0.09 | 0.425 | -0.477 | 0.36 | 0.186 | 0.207 | 0.141 | 0.141 |
| InternalIMT | aa | rs7909740 | 0.181 | -0.066 | 0.09 | 0.465 | -0.44 | 0.358 | 0.22 | 0.193 | 0.14 | 0.168 |
| InternalIMT | aa | rs7909768 | 0.181 | -0.066 | 0.09 | 0.465 | -0.44 | 0.358 | 0.22 | 0.193 | 0.14 | 0.168 |
| InternalIMT | aa | rs144168004 | 0.001 | . | . | . | . | . | . | . | . | . |
| InternalIMT | aa | rs75850448 | 0.02 | 1.80E-03 | 0.074 | 0.98 | -1.24 | 1.29 | 0.333 | 0.542 | 0.5 | 0.278 |
| InternalIMT | aa | rs147777574 | 0.016 | 0.014 | 0.074 | 0.851 | -0.214 | 1.31 | 0.87 | 0.101 | 0.498 | 0.839 |
| InternalIMT | aa | rs7913488 | 0.181 | -0.066 | 0.09 | 0.464 | -0.441 | 0.358 | 0.219 | 0.193 | 0.14 | 0.168 |
| InternalIMT | aa | rs74390874 | 0.019 | -6.70E-03 | 0.074 | 0.928 | -2.27 | 1.37 | 0.099 | 0.864 | 0.532 | 0.105 |
| InternalIMT | aa | rs141391142 | 0.002 | . | . | . | . | . | . | . | . | . |
| InternalIMT | aa | rs115294747 | 0.017 | 0.012 | 0.075 | 0.87 | -0.381 | 0.902 | 0.673 | 0.13 | 0.36 | 0.718 |
| InternalIMT | aa | rs148031781 | 0.001 | . | . | . | . | . | . | . | . | . |
| InternalIMT | aa | rs181340128 | 0.007 | . | . | . | . | . | . | . | . | . |
| InternalIMT | aa | rs189930007 | 0 | . | . | . | . | . | . | . | . | . |
| InternalIMT | aa | rs12242128 | 0.304 | 0.175 | 0.108 | 0.104 | 0.642 | 0.323 | 0.047 | -0.246 | 0.125 | 0.05 |
| InternalIMT | aa | rs78421750 | 0.016 | 0.013 | 0.075 | 0.858 | -0.587 | 1.13 | 0.605 | 0.193 | 0.444 | 0.664 |
| InternalIMT | aa | rs114370747 | 0.007 | . | . | . | . | . | . | . | . | . |
| InternalIMT | aa | rs12264166 | 0.304 | 0.176 | 0.108 | 0.103 | 0.643 | 0.323 | 0.047 | -0.247 | 0.125 | 0.049 |
| InternalIMT | aa | rs182964822 | 0 | . | . | . | . | . | . | . | . | . |
| InternalIMT | aa | rs67491417 | 0.178 | -0.062 | 0.09 | 0.49 | -0.416 | 0.361 | 0.249 | 0.187 | 0.141 | 0.186 |
| InternalIMT | aa | rs72789720 | 0.006 | . | . | . | . | . | . | . | . | . |
| InternalIMT | aa | rs6413416 | 0.182 | -0.066 | 0.09 | 0.462 | -0.443 | 0.357 | 0.215 | 0.193 | 0.139 | 0.165 |
| InternalIMT | aa | rs4986832 | 0.182 | -0.067 | 0.09 | 0.459 | -0.445 | 0.357 | 0.213 | 0.194 | 0.139 | 0.163 |
| InternalIMT | aa | rs144866316 | . | . | . | . | . | . | . | . | . | . |
| InternalIMT | aa | rs187769540 | 0.001 | . | . | . | . | . | . | . | . | . |
| InternalIMT | aa | rs6593482 | 0.182 | -0.066 | 0.09 | 0.462 | -0.443 | 0.357 | 0.215 | 0.193 | 0.139 | 0.165 |
| InternalIMT | aa | rs7913948 | 0.183 | -0.075 | 0.091 | 0.414 | -0.485 | 0.357 | 0.175 | 0.209 | 0.14 | 0.134 |
| InternalIMT | aa | rs77185151 | 0.025 | 4.00E-03 | 0.074 | 0.957 | -0.782 | 1.03 | 0.448 | 0.336 | 0.41 | 0.412 |
| InternalIMT | aa | rs12762303 | 0.182 | -0.067 | 0.09 | 0.459 | -0.445 | 0.357 | 0.213 | 0.194 | 0.139 | 0.163 |
| InternalIMT | aa | rs73283136 | 0.008 | . | . | . | . | . | . | . | . | . |
| InternalIMT | aa | rs187180218 | 0.007 | . | . | . | . | . | . | . | . | . |
| InternalIMT | aa | rs78137087 | 0.182 | -0.066 | 0.09 | 0.467 | -0.439 | 0.357 | 0.219 | 0.192 | 0.139 | 0.168 |
| InternalIMT | aa | rs72561472 | 0.181 | -0.103 | 0.092 | 0.263 | -0.646 | 0.371 | 0.081 | 0.27 | 0.145 | 0.063 |
| InternalIMT | aa | rs4987106 | 0.18 | -0.077 | 0.09 | 0.393 | -0.507 | 0.36 | 0.159 | 0.218 | 0.14 | 0.121 |
| InternalIMT | aa | rs4987105 | 0.159 | -0.05 | 0.089 | 0.575 | -0.388 | 0.373 | 0.299 | 0.166 | 0.146 | 0.255 |
| InternalIMT | aa | rs111916157 | 0.187 | -0.073 | 0.091 | 0.422 | -0.475 | 0.362 | 0.19 | 0.205 | 0.141 | 0.147 |
| InternalIMT | aa | rs111528152 | 0.184 | -0.049 | 0.091 | 0.594 | -0.337 | 0.361 | 0.351 | 0.15 | 0.141 | 0.288 |
| InternalIMT | aa | rs12763738 | 0.178 | -0.07 | 0.09 | 0.435 | -0.468 | 0.362 | 0.196 | 0.205 | 0.141 | 0.148 |
| InternalIMT | aa | rs191675886 | 0.011 | 0.048 | 0.075 | 0.521 | 4.01 | 1.79 | 0.025 | -1.56 | 0.689 | 0.024 |
| InternalIMT | aa | rs66489870 | 0.182 | -0.067 | 0.09 | 0.459 | -0.445 | 0.357 | 0.213 | 0.194 | 0.139 | 0.163 |
| InternalIMT | aa | rs71494775 | 0.182 | -0.067 | 0.09 | 0.459 | -0.445 | 0.357 | 0.213 | 0.194 | 0.139 | 0.163 |
| InternalIMT | aa | rs61855875 | 0.01 | 0.013 | 0.075 | 0.864 | -0.848 | 2.42 | 0.726 | 0.293 | 0.931 | 0.753 |
| InternalIMT | aa | rs116356837 | 0.019 | 0.022 | 0.074 | 0.768 | 1.48 | 1.56 | 0.343 | -0.505 | 0.59 | 0.392 |
| InternalIMT | aa | rs1864414 | 0.179 | -0.067 | 0.089 | 0.454 | -0.479 | 0.382 | 0.21 | 0.209 | 0.149 | 0.162 |
| InternalIMT | aa | rs1864413 | 0.182 | -0.066 | 0.09 | 0.461 | -0.443 | 0.357 | 0.215 | 0.194 | 0.139 | 0.165 |
| InternalIMT | aa | rs139497212 | 0 | . | . | . | . | . | . | . | . | . |
| InternalIMT | aa | rs892690 | 0.104 | -0.013 | 0.079 | 0.866 | -0.377 | 0.428 | 0.378 | 0.139 | 0.168 | 0.407 |
| InternalIMT | aa | rs1864412 | 0.182 | -0.067 | 0.09 | 0.459 | -0.445 | 0.357 | 0.214 | 0.194 | 0.139 | 0.164 |
| InternalIMT | aa | rs77827771 | 0.087 | 0.102 | 0.081 | 0.212 | 1.21 | 0.424 | 4.40E-03 | -0.453 | 0.163 | 5.40E-03 |
| InternalIMT | aa | rs145294963 | . | . | . | . | . | . | . | . | . | . |
| InternalIMT | aa | rs149081327 | 0.003 | . | . | . | . | . | . | . | . | . |
| InternalIMT | aa | rs67008983 | 0.182 | -0.067 | 0.09 | 0.46 | -0.444 | 0.357 | 0.215 | 0.194 | 0.139 | 0.164 |
| InternalIMT | aa | rs56150414 | 0.182 | -0.07 | 0.091 | 0.44 | -0.462 | 0.358 | 0.197 | 0.201 | 0.14 | 0.151 |
| InternalIMT | aa | rs55787481 | 0.182 | -0.068 | 0.09 | 0.448 | -0.451 | 0.358 | 0.208 | 0.197 | 0.14 | 0.159 |
| InternalIMT | aa | rs141270101 | 0.001 | . | . | . | . | . | . | . | . | . |
| InternalIMT | aa | rs114980075 | 0.022 | 0.058 | 0.082 | 0.481 | 0.591 | 0.442 | 0.181 | -0.225 | 0.179 | 0.208 |
| InternalIMT | aa | rs12783095 | 0.181 | -0.066 | 0.09 | 0.464 | -0.44 | 0.357 | 0.219 | 0.193 | 0.139 | 0.166 |
| InternalIMT | aa | rs182342688 | 0.001 | . | . | . | . | . | . | . | . | . |

| InternalIMT | aa | rs17157728 | 0.181 | -0.066 | 0.09 | 0.464 | -0.44 | 0.357 | 0.219 | 0.193 | 0.139 | 0.166 |
| --- | --- | --- | --- | --- | --- | --- | --- | --- | --- | --- | --- | --- |
| InternalIMT | aa | rs114835563 | 0.086 | 0.1 | 0.081 | 0.219 | 1.2 | 0.425 | 4.90E-03 | -0.448 | 0.163 | 6.10E-03 |
| InternalIMT | aa | rs3824612 | 0.104 | -0.014 | 0.079 | 0.861 | -0.384 | 0.43 | 0.372 | 0.142 | 0.169 | 0.402 |
| InternalIMT | aa | rs17157733 | 0.181 | -0.066 | 0.09 | 0.466 | -0.438 | 0.357 | 0.22 | 0.193 | 0.139 | 0.167 |
| InternalIMT | aa | rs74128478 | 0.019 | 1.80E-03 | 0.074 | 0.981 | -1.27 | 1.3 | 0.328 | 0.552 | 0.504 | 0.274 |
| InternalIMT | aa | rs184973589 | 0.001 | . | . | . | . | . | . | . | . | . |
| InternalIMT | aa | rs150479461 | 0 | . | . | . | . | . | . | . | . | . |
| InternalIMT | aa | rs138310407 | . | . | . | . | . | . | . | . | . | . |
| InternalIMT | aa | rs187426603 | . | . | . | . | . | . | . | . | . | . |
| InternalIMT | aa | rs190843143 | 0.001 | . | . | . | . | . | . | . | . | . |
| InternalIMT | aa | rs191883449 | 0 | . | . | . | . | . | . | . | . | . |
| InternalIMT | aa | rs58238740 | 0.173 | -0.056 | 0.089 | 0.532 | -0.387 | 0.369 | 0.295 | 0.174 | 0.144 | 0.226 |
| InternalIMT | aa | rs3780894 | 0.173 | -0.056 | 0.089 | 0.532 | -0.386 | 0.369 | 0.295 | 0.174 | 0.144 | 0.226 |
| InternalIMT | aa | rs149646626 | 0.006 | . | . | . | . | . | . | . | . | . |
| InternalIMT | aa | rs79615212 | 0.006 | . | . | . | . | . | . | . | . | . |
| InternalIMT | aa | rs3780895 | 0.173 | -0.056 | 0.089 | 0.532 | -0.387 | 0.369 | 0.295 | 0.174 | 0.144 | 0.226 |
| InternalIMT | aa | rs3780896 | 0.173 | -0.056 | 0.089 | 0.532 | -0.386 | 0.369 | 0.295 | 0.174 | 0.144 | 0.226 |
| InternalIMT | aa | rs142747163 | 0.003 | . | . | . | . | . | . | . | . | . |
| InternalIMT | aa | rs189417584 | 0.01 | 0.021 | 0.075 | 0.774 | 0.883 | 1.34 | 0.509 | -0.351 | 0.503 | 0.486 |
| InternalIMT | aa | rs3780897 | 0.17 | -0.055 | 0.089 | 0.537 | -0.378 | 0.37 | 0.307 | 0.175 | 0.144 | 0.225 |
| InternalIMT | aa | rs117509491 | 0.002 | . | . | . | . | . | . | . | . | . |
| InternalIMT | aa | rs117811514 | 0.002 | . | . | . | . | . | . | . | . | . |
| InternalIMT | aa | rs116500932 | 0.086 | 0.1 | 0.081 | 0.22 | 1.2 | 0.426 | 5.00E-03 | -0.448 | 0.164 | 6.20E-03 |
| InternalIMT | aa | rs77584168 | . | . | . | . | . | . | . | . | . | . |
| InternalIMT | aa | rs2310776 | 0.173 | -0.056 | 0.089 | 0.533 | -0.386 | 0.369 | 0.295 | 0.174 | 0.144 | 0.226 |
| InternalIMT | aa | rs17157736 | 0.173 | -0.056 | 0.089 | 0.533 | -0.386 | 0.369 | 0.295 | 0.174 | 0.144 | 0.226 |
| InternalIMT | aa | rs72789735 | 0.036 | -0.049 | 0.079 | 0.536 | -2.4 | 1.1 | 0.03 | 0.935 | 0.434 | 0.031 |
| InternalIMT | aa | rs183747187 | 0.001 | . | . | . | . | . | . | . | . | . |
| InternalIMT | aa | rs7077891 | 0.173 | -0.056 | 0.089 | 0.533 | -0.386 | 0.369 | 0.296 | 0.174 | 0.144 | 0.226 |
| InternalIMT | aa | rs145063305 | 0.002 | . | . | . | . | . | . | . | . | . |
| InternalIMT | aa | rs3824613 | 0.174 | -0.058 | 0.089 | 0.512 | -0.392 | 0.363 | 0.281 | 0.177 | 0.141 | 0.209 |
| InternalIMT | aa | rs145543199 | 0.004 | . | . | . | . | . | . | . | . | . |
| InternalIMT | aa | rs140361993 | 0.001 | . | . | . | . | . | . | . | . | . |
| InternalIMT | aa | rs11239496 | 0.036 | -0.049 | 0.079 | 0.536 | -2.4 | 1.1 | 0.03 | 0.935 | 0.434 | 0.031 |
| InternalIMT | aa | rs11239497 | 0.154 | -0.032 | 0.089 | 0.715 | -0.279 | 0.38 | 0.463 | 0.126 | 0.148 | 0.394 |
| InternalIMT | aa | rs10900212 | 0.245 | -0.125 | 0.096 | 0.192 | -0.615 | 0.329 | 0.061 | 0.261 | 0.128 | 0.042 |
| InternalIMT | aa | rs186487136 | . | . | . | . | . | . | . | . | . | . |
| InternalIMT | aa | rs12774369 | 0.173 | -0.055 | 0.089 | 0.538 | -0.382 | 0.369 | 0.3 | 0.172 | 0.144 | 0.23 |
| InternalIMT | aa | rs60842538 | 0.173 | -0.054 | 0.09 | 0.543 | -0.378 | 0.369 | 0.305 | 0.171 | 0.144 | 0.235 |
| InternalIMT | aa | rs3780898 | 0.153 | -0.03 | 0.089 | 0.732 | -0.267 | 0.379 | 0.481 | 0.121 | 0.148 | 0.414 |
| InternalIMT | aa | rs3780899 | 0.172 | -0.053 | 0.09 | 0.553 | -0.371 | 0.369 | 0.315 | 0.167 | 0.144 | 0.243 |
| InternalIMT | aa | rs12252472 | 0.273 | 0.191 | 0.102 | 0.061 | 0.766 | 0.319 | 0.016 | -0.295 | 0.124 | 0.017 |
| InternalIMT | aa | rs185411011 | 0.001 | . | . | . | . | . | . | . | . | . |
| InternalIMT | aa | rs28395865 | 0.152 | -0.029 | 0.089 | 0.747 | -0.259 | 0.381 | 0.497 | 0.117 | 0.148 | 0.431 |
| InternalIMT | aa | rs191004074 | 0.034 | 0.011 | 0.075 | 0.881 | -0.102 | 1.36 | 0.94 | 0.06 | 0.527 | 0.909 |
| InternalIMT | aa | rs182401295 | . | . | . | . | . | . | . | . | . | . |
| InternalIMT | aa | rs2228064 | 0.277 | 0.191 | 0.102 | 0.062 | 0.766 | 0.319 | 0.016 | -0.293 | 0.124 | 0.018 |
| InternalIMT | aa | rs188284159 | . | . | . | . | . | . | . | . | . | . |
| InternalIMT | aa | rs2303887 | 0.003 | . | . | . | . | . | . | . | . | . |
| InternalIMT | aa | rs2303886 | 0.014 | 0.021 | 0.076 | 0.783 | 0.151 | 0.849 | 0.859 | -0.016 | 0.316 | 0.959 |
| InternalIMT | aa | rs192621006 | . | . | . | . | . | . | . | . | . | . |
| InternalIMT | aa | rs2303885 | 0.491 | 0.12 | 0.117 | 0.307 | 0.273 | 0.212 | 0.198 | -0.096 | 0.083 | 0.252 |
| InternalIMT | aa | rs114466168 | 0.086 | 0.098 | 0.081 | 0.229 | 1.18 | 0.432 | 6.40E-03 | -0.439 | 0.166 | 8.30E-03 |
| InternalIMT | aa | rs11239501 | 0.038 | -0.025 | 0.077 | 0.743 | -1.59 | 1.35 | 0.238 | 0.643 | 0.531 | 0.226 |
| InternalIMT | aa | rs745986 | 0.156 | -0.014 | 0.089 | 0.873 | -0.183 | 0.372 | 0.622 | 0.08 | 0.146 | 0.582 |
| InternalIMT | aa | rs75262268 | 0.004 | . | . | . | . | . | . | . | . | . |
| InternalIMT | aa | rs71494776 | 0.022 | 5.90E-03 | 0.074 | 0.936 | -0.835 | 1.15 | 0.469 | 0.32 | 0.45 | 0.478 |
| InternalIMT | aa | rs74386871 | 0.006 | . | . | . | . | . | . | . | . | . |
| InternalIMT | aa | rs76502040 | 0.004 | . | . | . | . | . | . | . | . | . |
| InternalIMT | aa | rs142213734 | 0.001 | . | . | . | . | . | . | . | . | . |
| InternalIMT | aa | rs4948671 | 0.796 | -0.162 | 0.19 | 0.395 | -0.24 | 0.294 | 0.414 | 0.108 | 0.115 | 0.345 |
| InternalIMT | aa | rs146286085 | 0 | . | . | . | . | . | . | . | . | . |
| InternalIMT | aa | rs144080623 | 0 | . | . | . | . | . | . | . | . | . |
| InternalIMT | aa | rs115074014 | 0.133 | 0.027 | 0.09 | 0.762 | 0.068 | 0.34 | 0.84 | -0.034 | 0.133 | 0.801 |
| InternalIMT | aa | rs17157756 | 0.133 | 0.027 | 0.09 | 0.762 | 0.068 | 0.34 | 0.84 | -0.034 | 0.133 | 0.801 |
| InternalIMT | aa | rs190459780 | 0.001 | . | . | . | . | . | . | . | . | . |
| InternalIMT | aa | rs12358647 | 0.25 | -0.116 | 0.099 | 0.243 | -0.578 | 0.335 | 0.084 | 0.238 | 0.132 | 0.071 |
| InternalIMT | aa | rs147080556 | 0.009 | . | . | . | . | . | . | . | . | . |
| InternalIMT | aa | rs187543349 | 0.002 | . | . | . | . | . | . | . | . | . |
| InternalIMT | aa | rs114375595 | 0.024 | 1.10E-03 | 0.074 | 0.989 | -0.97 | 1.07 | 0.365 | 0.409 | 0.429 | 0.34 |
| InternalIMT | aa | rs117211200 | 0.003 | . | . | . | . | . | . | . | . | . |
| InternalIMT | aa | rs192963047 | . | . | . | . | . | . | . | . | . | . |
| InternalIMT | aa | rs11239505 | 0.254 | -0.115 | 0.1 | 0.25 | -0.564 | 0.328 | 0.086 | 0.233 | 0.129 | 0.071 |
| InternalIMT | aa | rs184434116 | 0.001 | . | . | . | . | . | . | . | . | . |
| InternalIMT | aa | rs4948999 | 0.801 | -0.142 | 0.194 | 0.465 | -0.213 | 0.297 | 0.474 | 0.095 | 0.116 | 0.414 |
| InternalIMT | aa | rs11239506 | 0.078 | 0.028 | 0.087 | 0.751 | 0.09 | 0.413 | 0.828 | -0.039 | 0.163 | 0.81 |
| InternalIMT | aa | rs190706620 | 0.001 | . | . | . | . | . | . | . | . | . |
| InternalIMT | aa | rs190770429 | 0.001 | . | . | . | . | . | . | . | . | . |
| InternalIMT | aa | rs181327133 | 0.003 | . | . | . | . | . | . | . | . | . |
| InternalIMT | aa | rs149251697 | 0.057 | -0.018 | 0.075 | 0.809 | -0.945 | 0.629 | 0.133 | 0.389 | 0.253 | 0.124 |
| InternalIMT | aa | rs118081602 | 0.003 | . | . | . | . | . | . | . | . | . |
| InternalIMT | aa | rs188233691 | . | . | . | . | . | . | . | . | . | . |
| InternalIMT | aa | rs183778794 | 0.006 | . | . | . | . | . | . | . | . | . |
| InternalIMT | aa | rs188040078 | 0.003 | . | . | . | . | . | . | . | . | . |
| InternalIMT | aa | rs184675372 | 0.001 | . | . | . | . | . | . | . | . | . |
| InternalIMT | aa | rs150326891 | 0.03 | -0.039 | 0.078 | 0.617 | -2.63 | 1.72 | 0.127 | 1.08 | 0.676 | 0.112 |

| InternalIMT | aa | rs185520021 | . | . | . | . | . | . | . | . | . | . |
| --- | --- | --- | --- | --- | --- | --- | --- | --- | --- | --- | --- | --- |
| InternalIMT | aa | rs7907779 | 0.078 | 0.028 | 0.087 | 0.747 | 0.093 | 0.412 | 0.821 | -0.041 | 0.163 | 0.802 |
| InternalIMT | aa | rs184962860 | . | . | . | . | . | . | . | . | . | . |
| InternalIMT | aa | rs149762784 | 0.034 | 0.012 | 0.076 | 0.875 | -0.121 | 0.902 | 0.894 | 0.077 | 0.36 | 0.83 |
| InternalIMT | aa | rs149203427 | 0.005 | . | . | . | . | . | . | . | . | . |
| InternalIMT | aa | rs60153083 | 0.045 | 0.025 | 0.077 | 0.745 | 0.257 | 0.625 | 0.681 | -0.111 | 0.241 | 0.644 |
| InternalIMT | aa | rs112001069 | 0.073 | -8.30E-03 | 0.075 | 0.912 | -0.543 | 0.642 | 0.398 | 0.229 | 0.25 | 0.358 |
| InternalIMT | aa | rs182355382 | 0.057 | 0.037 | 0.078 | 0.632 | 0.534 | 0.78 | 0.494 | -0.202 | 0.31 | 0.515 |
| InternalIMT | aa | rs186740974 | 0.157 | 0.022 | 0.101 | 0.83 | -3.10E-03 | 0.427 | 0.994 | -0.015 | 0.166 | 0.928 |
| InternalIMT | aa | rs191874916 | 0.06 | 9.70E-03 | 0.076 | 0.899 | -2.70E-03 | 0.77 | 0.997 | 0.03 | 0.299 | 0.921 |
| InternalIMT | aa | rs182989412 | 0.126 | 0.014 | 0.087 | 0.868 | -0.032 | 0.497 | 0.949 | 9.00E-03 | 0.196 | 0.964 |
| InternalIMT | aa | rs188819476 | 0.003 | . | . | . | . | . | . | . | . | . |
| InternalIMT | aa | rs141217183 | 0.083 | -2.90E-03 | 0.076 | 0.969 | -0.382 | 0.658 | 0.562 | 0.162 | 0.257 | 0.527 |
| InternalIMT | aa | rs192922652 | 0.081 | -7.90E-03 | 0.075 | 0.917 | -0.491 | 0.608 | 0.419 | 0.209 | 0.237 | 0.379 |
| InternalIMT | aa | rs185092343 | 0.025 | 0.075 | 0.077 | 0.328 | 4.16 | 1.44 | 4.00E-03 | -1.6 | 0.555 | 3.90E-03 |
| InternalIMT | aa | rs145193311 | 0.082 | -0.012 | 0.075 | 0.876 | -0.574 | 0.594 | 0.335 | 0.24 | 0.232 | 0.302 |
| InternalIMT | aa | rs55789336 | 0.084 | 0.03 | 0.087 | 0.734 | 0.104 | 0.405 | 0.797 | -0.043 | 0.16 | 0.787 |
| InternalIMT | aa | rs55837212 | 0.083 | 0.032 | 0.088 | 0.713 | 0.126 | 0.408 | 0.757 | -0.053 | 0.16 | 0.742 |
| InternalIMT | aa | rs75408574 | 0.208 | -0.056 | 0.082 | 0.495 | -0.502 | 0.298 | 0.092 | 0.181 | 0.116 | 0.119 |
| InternalIMT | aa | rs149306926 | 0.089 | 0.07 | 0.086 | 0.416 | 0.428 | 0.378 | 0.258 | -0.17 | 0.149 | 0.253 |
| InternalIMT | aa | rs35990892 | 0.683 | 0.099 | 0.167 | 0.555 | 0.131 | 0.268 | 0.624 | -0.056 | 0.105 | 0.597 |
| InternalIMT | aa | rs192356207 | 0.128 | -0.033 | 0.085 | 0.7 | -0.509 | 0.448 | 0.256 | 0.214 | 0.176 | 0.224 |
| InternalIMT | aa | rs183741163 | 0.097 | 0.066 | 0.084 | 0.435 | 0.369 | 0.354 | 0.298 | -0.14 | 0.145 | 0.335 |
| InternalIMT | aa | rs34737297 | 0.591 | -0.348 | 0.152 | 0.022 | -0.748 | 0.318 | 0.019 | 0.314 | 0.124 | 0.011 |
| InternalIMT | aa | rs181346933 | 0.042 | 0.051 | 0.083 | 0.538 | 0.931 | 0.688 | 0.176 | -0.355 | 0.262 | 0.175 |
| InternalIMT | aa | rs36093253 | 0.24 | -0.056 | 0.083 | 0.499 | -0.469 | 0.295 | 0.112 | 0.174 | 0.115 | 0.132 |
| InternalIMT | aa | rs186307855 | 0.262 | 0.177 | 0.103 | 0.085 | 0.717 | 0.318 | 0.024 | -0.277 | 0.124 | 0.025 |
| InternalIMT | aa | rs189681865 | 0.096 | -0.023 | 0.079 | 0.77 | -0.607 | 0.465 | 0.192 | 0.242 | 0.186 | 0.193 |
| InternalIMT | aa | rs34942971 | 0.259 | -0.098 | 0.105 | 0.352 | -0.452 | 0.327 | 0.167 | 0.186 | 0.129 | 0.149 |
| InternalIMT | aa | rs181816554 | 0.064 | -0.02 | 0.076 | 0.795 | -0.956 | 0.851 | 0.261 | 0.371 | 0.332 | 0.265 |
| InternalIMT | aa | rs186846881 | 0.278 | 0.169 | 0.107 | 0.114 | 0.665 | 0.329 | 0.043 | -0.252 | 0.128 | 0.048 |
| InternalIMT | aa | rs191178220 | 0.008 | . | . | . | . | . | . | . | . | . |
| InternalIMT | aa | rs183305228 | 0.046 | 0.025 | 0.077 | 0.744 | 0.262 | 0.62 | 0.673 | -0.112 | 0.24 | 0.639 |
| InternalIMT | aa | rs139912761 | 0.149 | -0.031 | 0.097 | 0.748 | -0.283 | 0.407 | 0.486 | 0.115 | 0.161 | 0.475 |
| InternalIMT | aa | rs185499901 | 0.018 | 0.014 | 0.076 | 0.856 | -0.185 | 1.2 | 0.878 | 0.095 | 0.467 | 0.839 |
| InternalIMT | aa | rs189816197 | 0.086 | -7.30E-03 | 0.085 | 0.931 | -0.299 | 0.495 | 0.546 | 0.126 | 0.194 | 0.515 |
| InternalIMT | aa | rs145656938 | 0.058 | -0.023 | 0.075 | 0.758 | -1.16 | 0.754 | 0.123 | 0.47 | 0.301 | 0.119 |
| InternalIMT | aa | rs35745197 | 0.072 | -0.014 | 0.075 | 0.857 | -0.684 | 0.639 | 0.285 | 0.282 | 0.249 | 0.257 |
| InternalIMT | aa | rs192856637 | . | . | . | . | . | . | . | . | . | . |
| InternalIMT | aa | rs2310775 | 0.267 | -0.046 | 0.084 | 0.589 | -0.354 | 0.267 | 0.185 | 0.13 | 0.104 | 0.212 |
| InternalIMT | aa | rs11239507 | 0.538 | 0.103 | 0.136 | 0.449 | 0.197 | 0.277 | 0.476 | -0.08 | 0.108 | 0.461 |
| InternalIMT | aa | rs74648467 | 0.027 | 0.052 | 0.08 | 0.516 | 0.834 | 0.721 | 0.248 | -0.35 | 0.28 | 0.21 |
| InternalIMT | aa | rs145815934 | 0.005 | . | . | . | . | . | . | . | . | . |
| InternalIMT | aa | rs145390687 | 0.012 | 0.012 | 0.075 | 0.876 | -1.81 | 2.62 | 0.489 | 0.661 | 0.957 | 0.49 |
| InternalIMT | aa | rs184354913 | 0.012 | 0.038 | 0.076 | 0.614 | 3.4 | 1.62 | 0.036 | -1.33 | 0.62 | 0.032 |
| InternalIMT | aa | rs181244920 | 0.003 | . | . | . | . | . | . | . | . | . |
| InternalIMT | aa | rs11499087 | 0.167 | 0.049 | 0.093 | 0.601 | 0.178 | 0.322 | 0.58 | -0.083 | 0.126 | 0.506 |
| InternalIMT | aa | rs148263794 | 0.079 | -0.018 | 0.075 | 0.808 | -0.729 | 0.59 | 0.217 | 0.299 | 0.23 | 0.194 |
| InternalIMT | aa | rs150758903 | 0.089 | -0.032 | 0.079 | 0.682 | -0.819 | 0.51 | 0.108 | 0.326 | 0.204 | 0.11 |
| InternalIMT | aa | rs147654382 | 0.192 | -0.032 | 0.094 | 0.732 | -0.224 | 0.384 | 0.56 | 0.109 | 0.152 | 0.472 |
| InternalIMT | aa | rs147495166 | 0.077 | 0.078 | 0.08 | 0.332 | 0.982 | 0.423 | 0.02 | -0.36 | 0.163 | 0.027 |
| InternalIMT | aa | rs35198778 | 0.14 | -0.041 | 0.078 | 0.605 | -0.533 | 0.49 | 0.277 | 0.238 | 0.195 | 0.222 |
| InternalIMT | aa | rs145207659 | 0.02 | -5.80E-03 | 0.074 | 0.938 | -2.25 | 1.54 | 0.143 | 0.858 | 0.594 | 0.149 |
| InternalIMT | aa | rs186466464 | 0.024 | -7.80E-03 | 0.075 | 0.917 | -1.64 | 1.16 | 0.158 | 0.67 | 0.455 | 0.142 |
| InternalIMT | aa | rs12778413 | 0.199 | -0.025 | 0.097 | 0.799 | -0.188 | 0.355 | 0.596 | 0.092 | 0.141 | 0.515 |
| InternalIMT | aa | rs7895552 | 0.724 | -0.166 | 0.178 | 0.351 | -0.279 | 0.291 | 0.338 | 0.119 | 0.114 | 0.297 |
| InternalIMT | aa | rs12783895 | 0.076 | -0.014 | 0.075 | 0.856 | -0.671 | 0.619 | 0.278 | 0.277 | 0.241 | 0.251 |
| InternalIMT | aa | rs34929604 | 0.081 | -6.00E-03 | 0.075 | 0.936 | -0.458 | 0.597 | 0.443 | 0.195 | 0.232 | 0.402 |
| InternalIMT | aa | rs12761961 | 0.072 | -0.013 | 0.075 | 0.86 | -0.689 | 0.673 | 0.306 | 0.285 | 0.261 | 0.274 |
| InternalIMT | aa | rs35536930 | 0.252 | -0.102 | 0.1 | 0.307 | -0.514 | 0.336 | 0.127 | 0.214 | 0.132 | 0.106 |
| InternalIMT | aa | rs180951060 | 0.006 | . | . | . | . | . | . | . | . | . |
| InternalIMT | aa | rs148892128 | 0.005 | . | . | . | . | . | . | . | . | . |
| InternalIMT | aa | rs11239509 | 0.255 | 0.176 | 0.099 | 0.074 | 0.715 | 0.306 | 0.02 | -0.279 | 0.119 | 0.019 |
| InternalIMT | aa | rs41526545 | 0.045 | -0.025 | 0.078 | 0.752 | -1.37 | 1.18 | 0.246 | 0.534 | 0.465 | 0.251 |
| InternalIMT | aa | rs2029253 | 0.286 | -0.037 | 0.084 | 0.659 | -0.281 | 0.254 | 0.27 | 0.107 | 0.099 | 0.28 |
| InternalIMT | aa | rs17444064 | 0.041 | -0.02 | 0.077 | 0.793 | -1.33 | 1.28 | 0.3 | 0.534 | 0.506 | 0.291 |
| InternalIMT | aa | rs71494779 | 0.084 | -0.011 | 0.075 | 0.886 | -0.531 | 0.571 | 0.352 | 0.223 | 0.223 | 0.317 |
| InternalIMT | aa | rs74991420 | 0.003 | . | . | . | . | . | . | . | . | . |
| InternalIMT | aa | rs182736551 | 0.003 | . | . | . | . | . | . | . | . | . |
| InternalIMT | aa | rs78702074 | 0.003 | . | . | . | . | . | . | . | . | . |
| InternalIMT | aa | rs187791466 | . | . | . | . | . | . | . | . | . | . |
| InternalIMT | aa | rs35300270 | 0.085 | -0.01 | 0.075 | 0.89 | -0.52 | 0.571 | 0.362 | 0.218 | 0.223 | 0.328 |
| InternalIMT | aa | rs6593484 | 0.078 | 0.027 | 0.087 | 0.758 | 0.08 | 0.414 | 0.847 | -0.037 | 0.164 | 0.819 |
| InternalIMT | aa | rs12244601 | 0.064 | -0.019 | 0.076 | 0.805 | -0.846 | 0.596 | 0.156 | 0.341 | 0.239 | 0.154 |
| InternalIMT | aa | rs184429574 | 0.006 | . | . | . | . | . | . | . | . | . |
| InternalIMT | aa | rs7080897 | 0.084 | -0.011 | 0.075 | 0.886 | -0.532 | 0.571 | 0.352 | 0.223 | 0.223 | 0.316 |
| InternalIMT | aa | rs7097429 | 0.085 | -0.015 | 0.075 | 0.84 | -0.609 | 0.554 | 0.271 | 0.254 | 0.217 | 0.241 |
| InternalIMT | aa | rs77979229 | . | . | . | . | . | . | . | . | . | . |
| InternalIMT | aa | rs11239512 | 0.079 | 0.028 | 0.087 | 0.743 | 0.094 | 0.409 | 0.819 | -0.043 | 0.161 | 0.792 |
| InternalIMT | aa | rs60146827 | 0.084 | -0.011 | 0.075 | 0.886 | -0.531 | 0.571 | 0.352 | 0.223 | 0.223 | 0.317 |
| InternalIMT | aa | rs79032236 | 0.105 | 0.104 | 0.083 | 0.209 | 1.21 | 0.432 | 5.20E-03 | -0.44 | 0.166 | 8.10E-03 |
| InternalIMT | aa | rs140854206 | . | . | . | . | . | . | . | . | . | . |
| InternalIMT | aa | rs184049550 | 0.012 | 0.04 | 0.074 | 0.587 | 2.56 | 1.26 | 0.043 | -1.04 | 0.494 | 0.035 |
| InternalIMT | aa | rs114433607 | 0.029 | 0.022 | 0.076 | 0.774 | 0.176 | 0.693 | 0.8 | -0.091 | 0.268 | 0.736 |
| InternalIMT | aa | rs117653793 | 0.003 | . | . | . | . | . | . | . | . | . |
| InternalIMT | aa | rs117039533 | 0.003 | . | . | . | . | . | . | . | . | . |

| InternalIMT | aa | rs191660761 | 0.002 | . | . | . | . | . | . | . | . | . |
| --- | --- | --- | --- | --- | --- | --- | --- | --- | --- | --- | --- | --- |
| InternalIMT | aa | rs59514896 | 0.03 | 8.70E-03 | 0.075 | 0.908 | -0.427 | 0.941 | 0.65 | 0.172 | 0.365 | 0.638 |
| InternalIMT | aa | rs184661876 | 0.001 | . | . | . | . | . | . | . | . | . |
| InternalIMT | aa | rs61854073 | 0.011 | 0.019 | 0.075 | 0.796 | -0.13 | 1.94 | 0.947 | -0.031 | 0.732 | 0.967 |
| InternalIMT | aa | rs2018518 | 0.09 | -0.031 | 0.079 | 0.696 | -0.648 | 0.362 | 0.074 | 0.228 | 0.142 | 0.109 |
| InternalIMT | aa | rs934187 | 0.802 | -0.137 | 0.194 | 0.48 | -0.209 | 0.296 | 0.481 | 0.092 | 0.116 | 0.427 |
| InternalIMT | aa | rs7918542 | 0.269 | -0.136 | 0.101 | 0.177 | -0.632 | 0.319 | 0.048 | 0.258 | 0.126 | 0.04 |
| InternalIMT | aa | rs144311920 | 0.004 | . | . | . | . | . | . | . | . | . |
| InternalIMT | aa | rs7099684 | 0.883 | 0.013 | 0.269 | 0.962 | 0.038 | 0.379 | 0.919 | 3.20E-03 | 0.147 | 0.982 |
| InternalIMT | aa | rs150829171 | 0.001 | . | . | . | . | . | . | . | . | . |
| InternalIMT | aa | rs186060697 | 0.006 | . | . | . | . | . | . | . | . | . |
| InternalIMT | aa | rs11239513 | 0.277 | 0.208 | 0.103 | 0.044 | 0.835 | 0.321 | 9.40E-03 | -0.321 | 0.125 | 0.01 |
| InternalIMT | aa | rs7917687 | 0.084 | -0.011 | 0.075 | 0.889 | -0.525 | 0.571 | 0.358 | 0.221 | 0.223 | 0.322 |
| InternalIMT | aa | rs61854076 | 0.004 | . | . | . | . | . | . | . | . | . |
| InternalIMT | aa | rs71494780 | 0.007 | . | . | . | . | . | . | . | . | . |
| InternalIMT | aa | rs7919239 | 0.257 | -0.11 | 0.099 | 0.271 | -0.538 | 0.324 | 0.097 | 0.222 | 0.127 | 0.081 |
| InternalIMT | aa | rs78334475 | 0.004 | . | . | . | . | . | . | . | . | . |
| InternalIMT | aa | rs3780900 | 0.035 | 4.80E-03 | 0.075 | 0.949 | -0.458 | 0.956 | 0.632 | 0.177 | 0.375 | 0.638 |
| InternalIMT | aa | rs186729292 | 0.001 | . | . | . | . | . | . | . | . | . |
| InternalIMT | aa | rs12267438 | 0.073 | -0.022 | 0.076 | 0.775 | -0.792 | 0.513 | 0.123 | 0.319 | 0.206 | 0.12 |
| InternalIMT | aa | rs1369214 | 0.564 | 0.112 | 0.139 | 0.422 | 0.213 | 0.287 | 0.457 | -0.086 | 0.112 | 0.443 |
| InternalIMT | aa | rs9282589 | 0.005 | . | . | . | . | . | . | . | . | . |
| InternalIMT | aa | rs114522915 | 0.078 | 0.071 | 0.078 | 0.367 | 1.22 | 0.63 | 0.054 | -0.445 | 0.244 | 0.068 |
| InternalIMT | aa | rs2115819 | 0.292 | -0.062 | 0.085 | 0.464 | -0.411 | 0.272 | 0.131 | 0.151 | 0.106 | 0.154 |
| InternalIMT | aa | rs117664603 | 0.003 | . | . | . | . | . | . | . | . | . |
| InternalIMT | aa | rs184979202 | 0.001 | . | . | . | . | . | . | . | . | . |
| InternalIMT | aa | rs190714671 | . | . | . | . | . | . | . | . | . | . |
| InternalIMT | aa | rs141538996 | . | . | . | . | . | . | . | . | . | . |
| InternalIMT | aa | rs150881741 | 0.001 | . | . | . | . | . | . | . | . | . |
| InternalIMT | aa | rs139076633 | . | . | . | . | . | . | . | . | . | . |
| InternalIMT | aa | rs12243649 | 0.156 | -0.072 | 0.078 | 0.351 | -0.841 | 0.381 | 0.027 | 0.355 | 0.151 | 0.018 |
| InternalIMT | aa | rs140348708 | . | . | . | . | . | . | . | . | . | . |
| InternalIMT | aa | rs79023256 | 0.003 | . | . | . | . | . | . | . | . | . |
| InternalIMT | aa | rs11239515 | 0.071 | 0.033 | 0.086 | 0.702 | 0.158 | 0.492 | 0.748 | -0.064 | 0.196 | 0.743 |
| InternalIMT | aa | rs55866086 | 0.24 | -0.087 | 0.101 | 0.388 | -0.441 | 0.337 | 0.19 | 0.188 | 0.133 | 0.157 |
| InternalIMT | aa | rs181340150 | 0.002 | . | . | . | . | . | . | . | . | . |
| InternalIMT | aa | rs11239516 | 0.158 | -0.015 | 0.098 | 0.877 | -0.186 | 0.389 | 0.633 | 0.073 | 0.154 | 0.637 |
| InternalIMT | aa | rs11239517 | 0.158 | -0.015 | 0.098 | 0.878 | -0.185 | 0.39 | 0.635 | 0.072 | 0.154 | 0.639 |
| InternalIMT | aa | rs11239519 | 0.128 | 8.70E-03 | 0.094 | 0.927 | -0.065 | 0.396 | 0.869 | 0.017 | 0.157 | 0.916 |
| InternalIMT | aa | rs7909514 | 0.788 | -0.08 | 0.191 | 0.675 | -0.129 | 0.295 | 0.662 | 0.058 | 0.115 | 0.612 |
| InternalIMT | aa | rs7894352 | 0.885 | 0.03 | 0.273 | 0.912 | 0.073 | 0.384 | 0.849 | -6.70E-03 | 0.149 | 0.964 |
| InternalIMT | aa | rs189384146 | 0.001 | . | . | . | . | . | . | . | . | . |
| InternalIMT | aa | rs11239520 | 0.107 | 0.028 | 0.09 | 0.755 | 0.069 | 0.404 | 0.865 | -0.034 | 0.16 | 0.832 |
| InternalIMT | aa | rs12221233 | 0.234 | -0.039 | 0.087 | 0.652 | -0.362 | 0.299 | 0.227 | 0.128 | 0.117 | 0.275 |
| InternalIMT | aa | rs74523293 | 0.066 | -0.02 | 0.074 | 0.785 | -0.805 | 0.705 | 0.254 | 0.357 | 0.275 | 0.194 |
| InternalIMT | aa | rs79376040 | 0.066 | -0.02 | 0.074 | 0.784 | -0.804 | 0.705 | 0.254 | 0.357 | 0.275 | 0.194 |
| InternalIMT | aa | rs4949000 | 0.234 | -0.039 | 0.087 | 0.652 | -0.361 | 0.3 | 0.228 | 0.128 | 0.117 | 0.276 |
| InternalIMT | aa | rs7089406 | 0.039 | 0.019 | 0.076 | 0.802 | 0.021 | 0.546 | 0.969 | -0.029 | 0.214 | 0.89 |
| InternalIMT | aa | rs138211056 | 0 | . | . | . | . | . | . | . | . | . |
| InternalIMT | aa | rs10900213 | 0.434 | -0.163 | 0.134 | 0.224 | -0.505 | 0.299 | 0.091 | 0.187 | 0.117 | 0.11 |
| InternalIMT | aa | rs145152827 | . | . | . | . | . | . | . | . | . | . |
| InternalIMT | aa | rs74443325 | 0.003 | . | . | . | . | . | . | . | . | . |
| InternalIMT | aa | rs188299446 | 0.004 | . | . | . | . | . | . | . | . | . |
| InternalIMT | aa | rs192699586 | . | . | . | . | . | . | . | . | . | . |
| InternalIMT | aa | rs72796464 | 0.041 | -0.016 | 0.078 | 0.843 | -1.03 | 1.24 | 0.403 | 0.418 | 0.487 | 0.391 |
| InternalIMT | aa | rs4949001 | 0.077 | -0.023 | 0.08 | 0.77 | -0.583 | 0.394 | 0.139 | 0.205 | 0.155 | 0.187 |
| InternalIMT | aa | rs147174641 | 0.003 | . | . | . | . | . | . | . | . | . |
| InternalIMT | aa | rs190835238 | 0.001 | . | . | . | . | . | . | . | . | . |
| InternalIMT | aa | rs76698944 | 0.013 | -3.40E-03 | 0.074 | 0.964 | -2.39 | 1.46 | 0.102 | 0.944 | 0.564 | 0.095 |
| InternalIMT | aa | rs56071381 | 0.129 | 0.022 | 0.093 | 0.816 | 0.013 | 0.381 | 0.973 | -0.013 | 0.15 | 0.93 |
| InternalIMT | aa | rs78130427 | 0.019 | -9.20E-04 | 0.075 | 0.99 | -1.69 | 1.44 | 0.241 | 0.621 | 0.559 | 0.267 |
| InternalIMT | aa | rs10900215 | 0.249 | -0.029 | 0.105 | 0.782 | -0.204 | 0.322 | 0.526 | 0.077 | 0.127 | 0.547 |
| InternalIMT | aa | rs41491445 | 0.046 | 0.026 | 0.077 | 0.738 | 0.277 | 0.635 | 0.663 | -0.122 | 0.246 | 0.621 |
| InternalIMT | aa | rs144995222 | 0.006 | . | . | . | . | . | . | . | . | . |
| InternalIMT | aa | rs149097803 | 0.002 | . | . | . | . | . | . | . | . | . |
| InternalIMT | aa | rs139429646 | 0.002 | . | . | . | . | . | . | . | . | . |
| InternalIMT | aa | rs7075620 | 0.764 | -0.227 | 0.204 | 0.266 | -0.374 | 0.317 | 0.238 | 0.152 | 0.124 | 0.22 |
| InternalIMT | aa | rs148299213 | 0.049 | 0.028 | 0.078 | 0.721 | 0.325 | 0.626 | 0.604 | -0.138 | 0.242 | 0.569 |
| InternalIMT | aa | rs4949002 | 0.084 | -0.026 | 0.08 | 0.74 | -0.607 | 0.419 | 0.148 | 0.212 | 0.165 | 0.198 |
| InternalIMT | aa | rs150496007 | 0.023 | 0.011 | 0.076 | 0.882 | -0.326 | 0.891 | 0.714 | 0.119 | 0.355 | 0.738 |
| InternalIMT | aa | rs147303455 | 0.028 | 3.40E-03 | 0.076 | 0.965 | -0.594 | 0.772 | 0.442 | 0.231 | 0.307 | 0.453 |
| InternalIMT | aa | rs189518433 | . | . | . | . | . | . | . | . | . | . |
| InternalIMT | aa | rs186281355 | . | . | . | . | . | . | . | . | . | . |
| InternalIMT | aa | rs115297114 | 0.077 | 0.063 | 0.08 | 0.435 | 0.785 | 0.446 | 0.079 | -0.275 | 0.174 | 0.114 |
| InternalIMT | aa | rs11239523 | 0.106 | 0.034 | 0.09 | 0.703 | 0.114 | 0.394 | 0.773 | -0.05 | 0.156 | 0.747 |
| InternalIMT | aa | rs143704897 | 0.007 | . | . | . | . | . | . | . | . | . |
| InternalIMT | aa | rs188024096 | 0.001 | . | . | . | . | . | . | . | . | . |
| InternalIMT | aa | rs11239524 | 0.133 | 0.037 | 0.095 | 0.7 | 0.113 | 0.382 | 0.767 | -0.052 | 0.151 | 0.733 |
| InternalIMT | aa | rs191656573 | 0.002 | . | . | . | . | . | . | . | . | . |
| InternalIMT | aa | rs193295875 | 0.001 | . | . | . | . | . | . | . | . | . |
| InternalIMT | aa | rs190458228 | . | . | . | . | . | . | . | . | . | . |
| InternalIMT | aa | rs139253993 | 0.001 | . | . | . | . | . | . | . | . | . |
| InternalIMT | aa | rs4948672 | 0.497 | 0.186 | 0.166 | 0.262 | 0.383 | 0.332 | 0.249 | -0.155 | 0.129 | 0.232 |
| InternalIMT | aa | rs180954921 | 0.001 | . | . | . | . | . | . | . | . | . |
| InternalIMT | aa | rs12264801 | 0.383 | 0.071 | 0.121 | 0.56 | 0.209 | 0.322 | 0.517 | -0.061 | 0.127 | 0.634 |
| InternalIMT | aa | rs73283184 | 0.057 | 0.012 | 0.078 | 0.879 | -0.22 | 0.574 | 0.701 | 0.073 | 0.225 | 0.747 |

| InternalIMT | aa | rs142746187 | 0.022 | 0.018 | 0.074 | 0.813 | 0.503 | 1.58 | 0.75 | -0.158 | 0.61 | 0.795 |
| --- | --- | --- | --- | --- | --- | --- | --- | --- | --- | --- | --- | --- |
| InternalIMT | aa | rs58704739 | 0.053 | 0.011 | 0.078 | 0.883 | -0.216 | 0.578 | 0.709 | 0.07 | 0.228 | 0.758 |
| InternalIMT | aa | rs61272335 | 0.04 | 0.022 | 0.077 | 0.771 | 0.144 | 0.666 | 0.829 | -0.075 | 0.261 | 0.775 |
| InternalIMT | aa | rs60959489 | 0.103 | 0.072 | 0.085 | 0.393 | 0.514 | 0.32 | 0.108 | -0.211 | 0.125 | 0.093 |
| InternalIMT | aa | rs58502629 | 0.04 | 0.022 | 0.077 | 0.773 | 0.136 | 0.666 | 0.838 | -0.072 | 0.261 | 0.784 |
| InternalIMT | aa | rs10900216 | 0.744 | 0.193 | 0.213 | 0.367 | 0.301 | 0.327 | 0.358 | -0.109 | 0.127 | 0.391 |
| InternalIMT | aa | rs181118141 | 0.004 | . | . | . | . | . | . | . | . | . |
| InternalIMT | aa | rs137882665 | 0.019 | -5.20E-03 | 0.074 | 0.944 | -2.01 | 0.956 | 0.036 | 0.793 | 0.378 | 0.036 |
| InternalIMT | aa | rs149466736 | 0.002 | . | . | . | . | . | . | . | . | . |
| InternalIMT | aa | rs2099171 | 0.553 | 0.128 | 0.132 | 0.332 | 0.249 | 0.239 | 0.297 | -0.093 | 0.093 | 0.319 |
| InternalIMT | aa | rs141852280 | 0.002 | . | . | . | . | . | . | . | . | . |
| InternalIMT | aa | rs191539257 | . | . | . | . | . | . | . | . | . | . |
| InternalIMT | aa | rs17157771 | 0.045 | 0.012 | 0.075 | 0.87 | -0.201 | 0.692 | 0.771 | 0.063 | 0.271 | 0.817 |
| InternalIMT | aa | rs111339241 | 0.041 | 6.80E-03 | 0.074 | 0.927 | -0.411 | 0.768 | 0.592 | 0.144 | 0.302 | 0.635 |
| InternalIMT | aa | rs116983255 | 0.004 | . | . | . | . | . | . | . | . | . |
| InternalIMT | aa | rs142324341 | . | . | . | . | . | . | . | . | . | . |
| InternalIMT | aa | rs187191219 | 0.015 | 2.10E-03 | 0.074 | 0.978 | -1.22 | 0.763 | 0.11 | 0.485 | 0.305 | 0.112 |
| InternalIMT | aa | rs151283973 | 0.005 | . | . | . | . | . | . | . | . | . |
| InternalIMT | aa | rs139316218 | 0.001 | . | . | . | . | . | . | . | . | . |
| InternalIMT | aa | rs6593485 | 0.697 | 0.199 | 0.173 | 0.251 | 0.339 | 0.278 | 0.222 | -0.128 | 0.108 | 0.237 |
| InternalIMT | aa | rs6593486 | 0.697 | 0.199 | 0.173 | 0.251 | 0.339 | 0.278 | 0.222 | -0.128 | 0.108 | 0.237 |
| InternalIMT | aa | rs145196618 | 0.036 | -7.80E-03 | 0.074 | 0.916 | -1.17 | 0.801 | 0.143 | 0.48 | 0.32 | 0.133 |
| InternalIMT | aa | rs142598335 | 0.001 | . | . | . | . | . | . | . | . | . |
| InternalIMT | aa | rs892691 | 0.663 | 0.214 | 0.158 | 0.176 | 0.356 | 0.254 | 0.161 | -0.144 | 0.099 | 0.147 |
| InternalIMT | aa | rs7080713 | 0.597 | 0.154 | 0.145 | 0.288 | 0.277 | 0.244 | 0.257 | -0.109 | 0.095 | 0.252 |
| InternalIMT | aa | rs7077173 | 0.135 | 9.20E-03 | 0.079 | 0.907 | -0.081 | 0.321 | 0.801 | 0.032 | 0.126 | 0.802 |
| InternalIMT | aa | rs7080977 | 0.088 | 9.10E-03 | 0.078 | 0.906 | -0.092 | 0.515 | 0.858 | 0.041 | 0.201 | 0.84 |
| InternalIMT | aa | rs3780901 | 0.599 | 0.188 | 0.144 | 0.191 | 0.347 | 0.243 | 0.153 | -0.136 | 0.094 | 0.151 |
| InternalIMT | aa | rs61854091 | 0.021 | 4.70E-03 | 0.074 | 0.949 | -0.898 | 1.49 | 0.546 | 0.358 | 0.585 | 0.541 |
| InternalIMT | aa | rs77550209 | 0.011 | 0.036 | 0.074 | 0.627 | 2.33 | 1.24 | 0.061 | -0.969 | 0.489 | 0.048 |
| InternalIMT | aa | rs73283191 | 0.097 | 0.012 | 0.078 | 0.88 | 0.022 | 0.515 | 0.966 | 5.50E-03 | 0.2 | 0.978 |
| InternalIMT | aa | rs191866099 | 0.003 | . | . | . | . | . | . | . | . | . |
| InternalIMT | aa | rs147998395 | 0.005 | . | . | . | . | . | . | . | . | . |
| InternalIMT | aa | rs7090328 | 0.861 | 0.152 | 0.245 | 0.534 | 0.228 | 0.351 | 0.516 | -0.078 | 0.137 | 0.571 |
| InternalIMT | aa | rs79814535 | 0.003 | . | . | . | . | . | . | . | . | . |
| InternalIMT | aa | rs7073082 | 0.861 | 0.154 | 0.245 | 0.529 | 0.23 | 0.351 | 0.512 | -0.079 | 0.137 | 0.566 |
| InternalIMT | aa | rs11239526 | 0.012 | 0.016 | 0.075 | 0.834 | -0.422 | 0.999 | 0.673 | 0.079 | 0.395 | 0.841 |
| InternalIMT | aa | rs141839841 | 0.005 | . | . | . | . | . | . | . | . | . |
| InternalIMT | aa | rs75697371 | 0.005 | . | . | . | . | . | . | . | . | . |
| InternalIMT | aa | rs75972854 | 0.005 | . | . | . | . | . | . | . | . | . |
| InternalIMT | aa | rs139291975 | . | . | . | . | . | . | . | . | . | . |
| InternalIMT | aa | rs144064711 | 0.002 | . | . | . | . | . | . | . | . | . |
| InternalIMT | aa | rs3780902 | 0.098 | 0.016 | 0.078 | 0.836 | 0.12 | 0.517 | 0.816 | -0.03 | 0.2 | 0.881 |
| InternalIMT | aa | rs190449801 | 0.004 | . | . | . | . | . | . | . | . | . |
| InternalIMT | aa | rs73283196 | 0.096 | 0.014 | 0.078 | 0.857 | 0.069 | 0.52 | 0.895 | -0.012 | 0.202 | 0.954 |
| InternalIMT | aa | rs28395867 | 0.118 | -0.029 | 0.078 | 0.713 | -0.661 | 0.433 | 0.128 | 0.255 | 0.169 | 0.131 |
| InternalIMT | aa | rs28395866 | 0.264 | -0.058 | 0.087 | 0.501 | -0.335 | 0.3 | 0.264 | 0.14 | 0.116 | 0.23 |
| InternalIMT | aa | rs55661611 | 0.088 | 0.01 | 0.078 | 0.892 | -0.059 | 0.527 | 0.91 | 0.029 | 0.206 | 0.887 |
| InternalIMT | aa | rs55684518 | 0.008 | . | . | . | . | . | . | . | . | . |
| InternalIMT | aa | rs78552493 | 0.041 | -5.90E-03 | 0.076 | 0.938 | -0.857 | 0.673 | 0.203 | 0.325 | 0.267 | 0.225 |
| InternalIMT | aa | rs147665434 | 0.001 | . | . | . | . | . | . | . | . | . |
| InternalIMT | aa | rs34445001 | 0.005 | . | . | . | . | . | . | . | . | . |
| InternalIMT | aa | rs184019449 | 0.001 | . | . | . | . | . | . | . | . | . |
| InternalIMT | aa | rs3780903 | 0.645 | 0.19 | 0.156 | 0.223 | 0.321 | 0.247 | 0.195 | -0.13 | 0.096 | 0.179 |
| InternalIMT | aa | rs17157784 | 0.159 | -3.00E-03 | 0.084 | 0.971 | -0.12 | 0.373 | 0.748 | 0.055 | 0.145 | 0.705 |
| InternalIMT | aa | rs3780904 | 0.01 | . | . | . | . | . | . | . | . | . |
| InternalIMT | aa | rs192064730 | 0 | . | . | . | . | . | . | . | . | . |
| InternalIMT | aa | rs12774534 | 0.005 | . | . | . | . | . | . | . | . | . |
| InternalIMT | aa | rs79179915 | 0.003 | . | . | . | . | . | . | . | . | . |
| InternalIMT | aa | rs117811571 | 0.004 | . | . | . | . | . | . | . | . | . |
| InternalIMT | aa | rs72796477 | 0.004 | . | . | . | . | . | . | . | . | . |
| InternalIMT | aa | rs55729342 | 0.172 | -0.033 | 0.084 | 0.698 | -0.309 | 0.381 | 0.417 | 0.133 | 0.148 | 0.37 |
| InternalIMT | aa | rs59317220 | 0.172 | -0.033 | 0.084 | 0.698 | -0.309 | 0.381 | 0.417 | 0.133 | 0.148 | 0.37 |
| InternalIMT | aa | rs3780905 | 0.018 | 5.20E-03 | 0.075 | 0.945 | -0.757 | 0.566 | 0.182 | 0.228 | 0.221 | 0.303 |
| InternalIMT | aa | rs3780906 | 0.645 | 0.184 | 0.159 | 0.247 | 0.308 | 0.252 | 0.221 | -0.125 | 0.098 | 0.203 |
| InternalIMT | aa | rs149047975 | 0.003 | . | . | . | . | . | . | . | . | . |
| InternalIMT | aa | rs3824614 | 0.002 | . | . | . | . | . | . | . | . | . |
| InternalIMT | aa | rs140254404 | . | . | . | . | . | . | . | . | . | . |
| InternalIMT | aa | rs145262684 | 0.005 | . | . | . | . | . | . | . | . | . |
| InternalIMT | aa | rs76735482 | 0.002 | . | . | . | . | . | . | . | . | . |
| InternalIMT | aa | rs73283198 | 0.045 | 8.00E-03 | 0.075 | 0.915 | -0.36 | 0.764 | 0.637 | 0.123 | 0.301 | 0.684 |
| InternalIMT | aa | rs74911239 | 0.013 | 0.021 | 0.075 | 0.782 | 0.701 | 1.49 | 0.638 | -0.324 | 0.591 | 0.583 |
| InternalIMT | aa | rs150551647 | 0.004 | . | . | . | . | . | . | . | . | . |
| InternalIMT | aa | rs115200794 | 0.143 | -0.071 | 0.077 | 0.359 | -0.997 | 0.361 | 5.90E-03 | 0.389 | 0.142 | 6.00E-03 |
| InternalIMT | aa | rs74130823 | 0.245 | -0.083 | 0.085 | 0.326 | -0.641 | 0.337 | 0.058 | 0.249 | 0.131 | 0.058 |
| InternalIMT | aa | rs2279435 | 0.604 | 0.367 | 0.168 | 0.028 | 0.657 | 0.272 | 0.016 | -0.263 | 0.107 | 0.014 |
| InternalIMT | aa | rs147279673 | 0.007 | . | . | . | . | . | . | . | . | . |
| InternalIMT | aa | rs183525146 | 0.002 | . | . | . | . | . | . | . | . | . |
| InternalIMT | aa | rs3740107 | 0.769 | 0.488 | 0.191 | 0.011 | 0.752 | 0.284 | 8.10E-03 | -0.289 | 0.111 | 9.20E-03 |
| InternalIMT | aa | rs140697247 | 0 | . | . | . | . | . | . | . | . | . |
| InternalIMT | aa | rs1565096 | 0.903 | 0.176 | 0.292 | 0.545 | 0.243 | 0.409 | 0.552 | -0.088 | 0.159 | 0.582 |
| InternalIMT | aa | rs1565097 | 0.766 | 0.518 | 0.192 | 7.00E-03 | 0.81 | 0.292 | 5.60E-03 | -0.311 | 0.114 | 6.50E-03 |
| InternalIMT | aa | rs144584548 | 0.008 | . | . | . | . | . | . | . | . | . |
| InternalIMT | aa | rs75811053 | 0.001 | . | . | . | . | . | . | . | . | . |
| InternalIMT | aa | rs147462967 | 0.009 | . | . | . | . | . | . | . | . | . |
| InternalIMT | aa | rs74130824 | 0.121 | -0.061 | 0.077 | 0.427 | -1.06 | 0.391 | 6.80E-03 | 0.407 | 0.153 | 8.00E-03 |

| InternalIMT | aa | rs116883184 | 0.006 | . | . | . | . | . | . | . | . | . |
| --- | --- | --- | --- | --- | --- | --- | --- | --- | --- | --- | --- | --- |
| InternalIMT | aa | rs117258049 | 0.006 | . | . | . | . | . | . | . | . | . |
| InternalIMT | aa | rs3780908 | 0.129 | -0.02 | 0.082 | 0.808 | -0.373 | 0.552 | 0.499 | 0.155 | 0.217 | 0.475 |
| InternalIMT | aa | rs180938522 | 0.001 | . | . | . | . | . | . | . | . | . |
| InternalIMT | aa | rs76687372 | 0.024 | 7.70E-03 | 0.074 | 0.917 | -0.472 | 1.14 | 0.68 | 0.217 | 0.45 | 0.63 |
| InternalIMT | aa | rs3780909 | 0.483 | 0.205 | 0.13 | 0.115 | 0.465 | 0.273 | 0.088 | -0.191 | 0.107 | 0.073 |
| InternalIMT | aa | rs78281156 | 0.001 | . | . | . | . | . | . | . | . | . |
| InternalIMT | aa | rs187840631 | . | . | . | . | . | . | . | . | . | . |
| InternalIMT | aa | rs76868242 | 0.001 | . | . | . | . | . | . | . | . | . |
| InternalIMT | aa | rs192794192 | 0 | . | . | . | . | . | . | . | . | . |
| InternalIMT | aa | rs12781248 | 0.005 | . | . | . | . | . | . | . | . | . |
| InternalIMT | aa | rs12762375 | 0.005 | . | . | . | . | . | . | . | . | . |
| InternalIMT | aa | rs192256986 | . | . | . | . | . | . | . | . | . | . |
| InternalIMT | aa | rs184411030 | 0.033 | 4.40E-03 | 0.075 | 0.953 | -0.22 | 0.872 | 0.8 | 0.146 | 0.34 | 0.668 |
| InternalIMT | aa | rs189601483 | 0.001 | . | . | . | . | . | . | . | . | . |
| InternalIMT | aa | rs143254579 | 0.015 | 0.012 | 0.075 | 0.877 | -0.984 | 1.06 | 0.352 | 0.34 | 0.42 | 0.419 |
| InternalIMT | aa | rs12253903 | 0.081 | 0.024 | 0.079 | 0.764 | 0.13 | 0.464 | 0.78 | -0.055 | 0.181 | 0.762 |
| InternalIMT | aa | rs35489926 | 0.005 | . | . | . | . | . | . | . | . | . |
| InternalIMT | aa | rs71494783 | 0.095 | 0.01 | 0.08 | 0.899 | -0.029 | 0.497 | 0.953 | 0.032 | 0.196 | 0.871 |
| InternalIMT | aa | rs180711602 | 0.001 | . | . | . | . | . | . | . | . | . |
| InternalIMT | aa | rs117312402 | 0.001 | . | . | . | . | . | . | . | . | . |
| InternalIMT | aa | rs74831694 | 0.001 | . | . | . | . | . | . | . | . | . |
| InternalIMT | aa | rs140301943 | . | . | . | . | . | . | . | . | . | . |
| InternalIMT | aa | rs78553679 | 0.006 | . | . | . | . | . | . | . | . | . |
| InternalIMT | aa | rs78334250 | 0.001 | . | . | . | . | . | . | . | . | . |
| InternalIMT | aa | rs76000028 | 0.001 | . | . | . | . | . | . | . | . | . |
| InternalIMT | aa | rs145195254 | 0.005 | . | . | . | . | . | . | . | . | . |
| InternalIMT | aa | rs137888527 | 0.005 | . | . | . | . | . | . | . | . | . |
| InternalIMT | aa | rs149109592 | . | . | . | . | . | . | . | . | . | . |
| InternalIMT | aa | rs80280010 | 0.013 | 0.014 | 0.079 | 0.856 | -0.273 | 0.872 | 0.755 | 0.029 | 0.341 | 0.932 |
| InternalIMT | aa | rs189995374 | . | . | . | . | . | . | . | . | . | . |
| InternalIMT | aa | rs75467770 | 0.002 | . | . | . | . | . | . | . | . | . |
| InternalIMT | aa | rs1487562 | 0.242 | -0.104 | 0.08 | 0.193 | -0.748 | 0.338 | 0.027 | 0.299 | 0.132 | 0.024 |
| InternalIMT | aa | rs12266963 | 0.095 | 0.011 | 0.08 | 0.894 | -0.022 | 0.498 | 0.965 | 0.029 | 0.196 | 0.883 |
| InternalIMT | aa | rs7099874 | 0.857 | -0.164 | 0.187 | 0.381 | -0.235 | 0.306 | 0.444 | 0.109 | 0.12 | 0.362 |
| InternalIMT | aa | rs75687019 | 0.001 | . | . | . | . | . | . | . | . | . |
| InternalIMT | aa | rs148240462 | 0.014 | 0.019 | 0.075 | 0.802 | 0.29 | 1.36 | 0.831 | -0.141 | 0.535 | 0.792 |
| InternalIMT | aa | rs141278174 | 0.001 | . | . | . | . | . | . | . | . | . |
| InternalIMT | aa | rs76404852 | 0.001 | . | . | . | . | . | . | . | . | . |
| InternalIMT | aa | rs139112240 | 0.004 | . | . | . | . | . | . | . | . | . |
| InternalIMT | aa | rs12783037 | 0.118 | -8.30E-04 | 0.08 | 0.992 | -0.128 | 0.469 | 0.784 | 0.076 | 0.185 | 0.679 |
| InternalIMT | aa | rs72796481 | 0.003 | . | . | . | . | . | . | . | . | . |
| InternalIMT | aa | rs76798749 | 0.025 | 0.011 | 0.074 | 0.885 | -0.141 | 1.18 | 0.905 | 0.094 | 0.464 | 0.839 |
| InternalIMT | aa | rs187732548 | 0.001 | . | . | . | . | . | . | . | . | . |
| InternalIMT | aa | rs75675474 | 0.025 | 0.011 | 0.074 | 0.885 | -0.141 | 1.18 | 0.905 | 0.094 | 0.464 | 0.839 |
| InternalIMT | aa | rs76552293 | 0.025 | 0.011 | 0.074 | 0.885 | -0.142 | 1.18 | 0.904 | 0.094 | 0.464 | 0.839 |
| InternalIMT | aa | rs147445993 | 0.008 | . | . | . | . | . | . | . | . | . |
| InternalIMT | aa | rs115902283 | 0.025 | 0.011 | 0.074 | 0.885 | -0.134 | 1.18 | 0.91 | 0.091 | 0.465 | 0.845 |
| InternalIMT | aa | rs4540799 | 0.855 | -0.16 | 0.186 | 0.388 | -0.23 | 0.306 | 0.453 | 0.107 | 0.119 | 0.369 |
| InternalIMT | aa | rs12765320 | 0.35 | -0.137 | 0.085 | 0.106 | -0.622 | 0.286 | 0.03 | 0.259 | 0.112 | 0.021 |
| InternalIMT | aa | rs12782256 | 0.005 | . | . | . | . | . | . | . | . | . |
| InternalIMT | aa | rs191727256 | 0.007 | . | . | . | . | . | . | . | . | . |
| InternalIMT | aa | rs2082111 | 0.113 | -0.021 | 0.077 | 0.781 | -0.452 | 0.551 | 0.412 | 0.192 | 0.217 | 0.377 |
| InternalIMT | aa | rs2082110 | 0.257 | -0.119 | 0.08 | 0.139 | -0.796 | 0.328 | 0.015 | 0.32 | 0.129 | 0.013 |
| InternalIMT | aa | rs2082109 | 0.257 | -0.119 | 0.08 | 0.139 | -0.796 | 0.328 | 0.015 | 0.32 | 0.129 | 0.013 |
| InternalIMT | aa | rs117316662 | 0.001 | . | . | . | . | . | . | . | . | . |
| InternalIMT | aa | rs12247163 | 0.357 | -0.134 | 0.085 | 0.116 | -0.59 | 0.286 | 0.039 | 0.247 | 0.112 | 0.027 |
| InternalIMT | aa | rs12355888 | 0.037 | -0.015 | 0.073 | 0.832 | -1.46 | 1.21 | 0.228 | 0.585 | 0.481 | 0.224 |
| InternalIMT | aa | rs143772290 | 0.033 | 0.019 | 0.075 | 0.805 | 0.515 | 1.24 | 0.677 | -0.161 | 0.482 | 0.739 |
| InternalIMT | aa | rs146849452 | 0.009 | . | . | . | . | . | . | . | . | . |
| InternalIMT | aa | rs140267121 | 0.003 | . | . | . | . | . | . | . | . | . |
| InternalIMT | aa | rs7080474 | 0.352 | -0.13 | 0.085 | 0.126 | -0.586 | 0.287 | 0.041 | 0.245 | 0.112 | 0.03 |
| InternalIMT | aa | rs144066203 | 0.007 | . | . | . | . | . | . | . | . | . |
| InternalIMT | aa | rs12765209 | 0.005 | . | . | . | . | . | . | . | . | . |
| InternalIMT | aa | rs150930735 | 0.007 | . | . | . | . | . | . | . | . | . |
| InternalIMT | aa | rs7068039 | 0.365 | -0.134 | 0.085 | 0.115 | -0.577 | 0.283 | 0.042 | 0.243 | 0.111 | 0.029 |
| InternalIMT | aa | rs117622652 | 0.001 | . | . | . | . | . | . | . | . | . |
| InternalIMT | aa | rs190191514 | . | . | . | . | . | . | . | . | . | . |
| InternalIMT | aa | rs74130835 | 0.118 | -0.058 | 0.077 | 0.456 | -1.05 | 0.397 | 8.30E-03 | 0.401 | 0.156 | 1.00E-02 |
| InternalIMT | aa | rs1811604 | 0.114 | -0.053 | 0.077 | 0.497 | -1 | 0.4 | 0.012 | 0.383 | 0.157 | 0.015 |
| InternalIMT | aa | rs146971256 | . | . | . | . | . | . | . | . | . | . |
| InternalIMT | aa | rs74336000 | 0.033 | 0.018 | 0.075 | 0.808 | 0.494 | 1.24 | 0.691 | -0.153 | 0.484 | 0.752 |
| InternalIMT | aa | rs186046492 | 0.003 | . | . | . | . | . | . | . | . | . |
| InternalIMT | aa | rs34971823 | 0.009 | . | . | . | . | . | . | . | . | . |
| InternalIMT | aa | rs75227446 | 0.001 | . | . | . | . | . | . | . | . | . |
| InternalIMT | aa | rs190457599 | 0.001 | . | . | . | . | . | . | . | . | . |
| InternalIMT | aa | rs79591917 | 0.001 | . | . | . | . | . | . | . | . | . |
| InternalIMT | aa | rs702365 | 0.492 | 0.202 | 0.132 | 0.126 | 0.449 | 0.275 | 0.102 | -0.184 | 0.108 | 0.087 |
| InternalIMT | aa | rs79643836 | 0.004 | . | . | . | . | . | . | . | . | . |
| InternalIMT | aa | rs702366 | 0.493 | 0.2 | 0.132 | 0.129 | 0.444 | 0.274 | 0.106 | -0.183 | 0.108 | 0.09 |
| InternalIMT | aa | rs1059696 | 0.197 | -0.039 | 0.083 | 0.636 | -0.354 | 0.41 | 0.388 | 0.159 | 0.161 | 0.326 |
| InternalIMT | aa | rs901681 | 0.489 | 0.189 | 0.132 | 0.153 | 0.417 | 0.275 | 0.13 | -0.174 | 0.108 | 0.107 |
| InternalIMT | aa | rs61854092 | 0.196 | -0.039 | 0.083 | 0.641 | -0.348 | 0.41 | 0.396 | 0.156 | 0.161 | 0.333 |
| InternalIMT | aa | rs148080753 | . | . | . | . | . | . | . | . | . | . |
| InternalIMT | aa | rs61854093 | 0.315 | -0.133 | 0.084 | 0.114 | -0.681 | 0.3 | 0.023 | 0.277 | 0.117 | 0.018 |
| InternalIMT | aa | rs141822774 | 0 | . | . | . | . | . | . | . | . | . |

| InternalIMT | aa | rs74130836 | 0.118 | -0.057 | 0.077 | 0.458 | -1.05 | 0.398 | 8.70E-03 | 0.401 | 0.156 | 0.01 |
| --- | --- | --- | --- | --- | --- | --- | --- | --- | --- | --- | --- | --- |
| InternalIMT | aa | rs150620963 | 0.006 | . | . | . | . | . | . | . | . | . |
| InternalIMT | aa | rs3780913 | 0.849 | -0.163 | 0.186 | 0.381 | -0.242 | 0.307 | 0.432 | 0.11 | 0.12 | 0.362 |
| InternalIMT | aa | rs142255723 | 0.001 | . | . | . | . | . | . | . | . | . |
| InternalIMT | aa | rs3780914 | 0.479 | 0.174 | 0.13 | 0.181 | 0.396 | 0.282 | 0.161 | -0.162 | 0.11 | 0.143 |
| InternalIMT | aa | rs182656507 | 0.001 | . | . | . | . | . | . | . | . | . |
| InternalIMT | aa | rs2291427 | 0.821 | -0.151 | 0.188 | 0.424 | -0.247 | 0.312 | 0.428 | 0.106 | 0.122 | 0.386 |
| InternalIMT | aa | rs10751382 | 0.816 | -0.148 | 0.187 | 0.429 | -0.257 | 0.311 | 0.408 | 0.106 | 0.122 | 0.385 |
| InternalIMT | aa | rs117610108 | 0.001 | . | . | . | . | . | . | . | . | . |
| InternalIMT | aa | rs144966941 | 0.009 | . | . | . | . | . | . | . | . | . |
| InternalIMT | aa | rs41502051 | 0.014 | -6.10E-04 | 0.075 | 0.994 | -1.69 | 1.1 | 0.126 | 0.623 | 0.436 | 0.154 |
| InternalIMT | aa | rs41426046 | 0.001 | . | . | . | . | . | . | . | . | . |
| InternalIMT | aa | rs41519248 | 0.001 | . | . | . | . | . | . | . | . | . |
| InternalIMT | aa | rs189108245 | 0.001 | . | . | . | . | . | . | . | . | . |
| InternalIMT | aa | rs7896431 | 0.497 | 0.141 | 0.133 | 0.286 | 0.308 | 0.281 | 0.272 | -0.124 | 0.11 | 0.26 |
| InternalIMT | aa | rs183724547 | 0.009 | . | . | . | . | . | . | . | . | . |
| InternalIMT | aa | rs142044990 | 0.004 | . | . | . | . | . | . | . | . | . |
| InternalIMT | aa | rs188140929 | . | . | . | . | . | . | . | . | . | . |
| InternalIMT | aa | rs76819096 | 0.041 | 0.024 | 0.077 | 0.757 | 0.279 | 0.533 | 0.6 | -0.093 | 0.212 | 0.66 |
| InternalIMT | aa | rs10751383 | 0.58 | 0.201 | 0.147 | 0.173 | 0.393 | 0.277 | 0.157 | -0.162 | 0.109 | 0.137 |
| InternalIMT | aa | rs117819321 | 0.005 | . | . | . | . | . | . | . | . | . |
| InternalIMT | aa | rs2242332 | 0.681 | 0.087 | 0.155 | 0.576 | 0.148 | 0.277 | 0.594 | -0.053 | 0.108 | 0.623 |
| InternalIMT | aa | rs2242333 | 0.02 | 8.90E-03 | 0.075 | 0.906 | -0.498 | 0.599 | 0.406 | 0.134 | 0.235 | 0.568 |
| InternalIMT | aa | rs2242334 | 0.681 | 0.093 | 0.156 | 0.552 | 0.16 | 0.278 | 0.566 | -0.057 | 0.108 | 0.596 |
| InternalIMT | aa | rs189465041 | . | . | . | . | . | . | . | . | . | . |
| InternalIMT | aa | rs28395868 | 0.009 | . | . | . | . | . | . | . | . | . |
| InternalIMT | aa | rs56067550 | 0.008 | . | . | . | . | . | . | . | . | . |
| InternalIMT | aa | rs1051713 | 0.182 | -0.057 | 0.08 | 0.472 | -0.557 | 0.435 | 0.2 | 0.247 | 0.171 | 0.148 |
| InternalIMT | aa | rs115074210 | 0.048 | 0.04 | 0.078 | 0.61 | 0.681 | 0.549 | 0.215 | -0.257 | 0.218 | 0.238 |
| InternalIMT | aa | rs7900977 | 0.893 | -0.117 | 0.225 | 0.603 | -0.162 | 0.347 | 0.64 | 0.079 | 0.136 | 0.561 |
| InternalIMT | aa | rs147613891 | 0.001 | . | . | . | . | . | . | . | . | . |
| InternalIMT | aa | rs2229136 | 0.161 | 0.019 | 0.084 | 0.819 | 0.033 | 0.367 | 0.929 | -0.012 | 0.144 | 0.932 |
| InternalIMT | aa | rs185482841 | 0.009 | . | . | . | . | . | . | . | . | . |
| InternalIMT | aa | rs2288619 | 0.156 | 0.018 | 0.084 | 0.828 | 2.90E-03 | 0.366 | 0.994 | -4.50E-03 | 0.143 | 0.975 |
| InternalIMT | aa | rs189891524 | . | . | . | . | . | . | . | . | . | . |
| InternalIMT | aa | rs56145077 | 0.176 | -0.058 | 0.078 | 0.454 | -0.612 | 0.399 | 0.125 | 0.264 | 0.156 | 0.091 |
| InternalIMT | aa | rs143216970 | 0.013 | 8.60E-03 | 0.074 | 0.908 | -1.11 | 1.05 | 0.287 | 0.432 | 0.406 | 0.287 |
| InternalIMT | aa | rs142246478 | 0 | . | . | . | . | . | . | . | . | . |
| InternalIMT | aa | rs13134 | 0.012 | 0.027 | 0.075 | 0.72 | 1.52 | 1.59 | 0.338 | -0.596 | 0.601 | 0.322 |
| InternalIMT | aa | rs41314980 | 0.012 | 7.00E-03 | 0.074 | 0.925 | -0.876 | 0.868 | 0.313 | 0.326 | 0.338 | 0.335 |
| InternalIMT | aa | rs41304611 | 0.005 | . | . | . | . | . | . | . | . | . |
| InternalIMT | aa | rs193299617 | . | . | . | . | . | . | . | . | . | . |
| InternalIMT | aa | rs12772724 | 0.012 | 0.027 | 0.075 | 0.72 | 1.52 | 1.59 | 0.339 | -0.595 | 0.601 | 0.322 |
| InternalIMT | aa | rs139489537 | 0.011 | 0.017 | 0.074 | 0.82 | -0.353 | 0.861 | 0.682 | 0.07 | 0.342 | 0.839 |
| InternalIMT | aa | rs12776062 | 0.005 | . | . | . | . | . | . | . | . | . |
| InternalIMT | aa | rs752059 | 0.181 | -0.041 | 0.079 | 0.599 | -0.444 | 0.393 | 0.259 | 0.195 | 0.154 | 0.206 |
| InternalIMT | aa | rs147649982 | 0.013 | 0.04 | 0.076 | 0.598 | 2.35 | 2.08 | 0.258 | -0.941 | 0.809 | 0.245 |
| InternalIMT | aa | rs17157798 | 0.113 | 0.031 | 0.083 | 0.705 | 0.172 | 0.406 | 0.673 | -0.077 | 0.158 | 0.627 |
| InternalIMT | aa | rs142298619 | 0.009 | . | . | . | . | . | . | . | . | . |
| InternalIMT | aa | rs4948673 | 0.893 | -0.083 | 0.197 | 0.672 | -0.095 | 0.304 | 0.753 | 0.057 | 0.12 | 0.632 |
| InternalIMT | aa | rs74231484 | 0.045 | 0.053 | 0.077 | 0.496 | 0.613 | 0.457 | 0.18 | -0.266 | 0.18 | 0.139 |
| InternalIMT | aa | rs72796489 | 0.001 | . | . | . | . | . | . | . | . | . |
| InternalIMT | aa | rs113447578 | 0.006 | . | . | . | . | . | . | . | . | . |
| InternalIMT | aa | rs34063374 | 0.014 | 0.026 | 0.075 | 0.731 | 1.28 | 1.52 | 0.399 | -0.503 | 0.579 | 0.386 |
| InternalIMT | aa | rs11239527 | 0.006 | . | . | . | . | . | . | . | . | . |
| InternalIMT | aa | rs34371883 | 0.007 | . | . | . | . | . | . | . | . | . |
| InternalIMT | aa | rs118168828 | 0.004 | . | . | . | . | . | . | . | . | . |
| InternalIMT | aa | rs34349618 | 0.005 | . | . | . | . | . | . | . | . | . |
| InternalIMT | aa | rs80041679 | 0.011 | 0.017 | 0.074 | 0.82 | -0.362 | 0.855 | 0.672 | 0.074 | 0.34 | 0.828 |
| InternalIMT | aa | rs149640607 | 0.01 | 0.021 | 0.074 | 0.776 | 1.31 | 2.73 | 0.633 | -0.5 | 1.04 | 0.632 |
| InternalIMT | aa | rs4427523 | 0.932 | -0.202 | 0.409 | 0.621 | -0.268 | 0.58 | 0.644 | 0.119 | 0.226 | 0.598 |
| InternalIMT | aa | rs185880109 | . | . | . | . | . | . | . | . | . | . |
| InternalIMT | aa | rs191523220 | 0.001 | . | . | . | . | . | . | . | . | . |
| InternalIMT | aa | rs144315284 | 0.02 | 0.035 | 0.075 | 0.635 | 1.63 | 1.24 | 0.189 | -0.667 | 0.485 | 0.169 |
| InternalIMT | aa | rs4948674 | 0.329 | 0.048 | 0.11 | 0.659 | 0.124 | 0.298 | 0.677 | -0.047 | 0.116 | 0.689 |
| InternalIMT | aa | rs182984298 | 0.002 | . | . | . | . | . | . | . | . | . |
| InternalIMT | aa | rs56749023 | 0.071 | 0.021 | 0.079 | 0.789 | 0.085 | 0.439 | 0.846 | -0.037 | 0.172 | 0.831 |
| InternalIMT | aa | rs112508957 | 0.045 | 1.10E-04 | 0.075 | 0.999 | -0.344 | 0.724 | 0.635 | 0.181 | 0.282 | 0.52 |
| InternalIMT | aa | rs7089063 | 0.313 | -0.016 | 0.091 | 0.864 | -0.142 | 0.287 | 0.62 | 0.062 | 0.112 | 0.58 |
| InternalIMT | aa | rs145000779 | . | . | . | . | . | . | . | . | . | . |
| InternalIMT | aa | rs80201572 | 0.013 | 0.017 | 0.074 | 0.823 | -0.386 | 0.861 | 0.654 | 0.09 | 0.336 | 0.79 |
| InternalIMT | aa | rs140568227 | 0.003 | . | . | . | . | . | . | . | . | . |
| InternalIMT | aa | rs149756228 | 0.004 | . | . | . | . | . | . | . | . | . |
| InternalIMT | aa | rs112380685 | 0.003 | . | . | . | . | . | . | . | . | . |
| InternalIMT | aa | rs60481109 | 0.067 | 0.021 | 0.079 | 0.789 | 0.089 | 0.45 | 0.843 | -0.038 | 0.177 | 0.828 |
| InternalIMT | aa | rs138221843 | . | . | . | . | . | . | . | . | . | . |
| InternalIMT | aa | rs150283194 | 0.009 | . | . | . | . | . | . | . | . | . |
| InternalIMT | aa | rs138879661 | 0.008 | . | . | . | . | . | . | . | . | . |
| InternalIMT | aa | rs34960345 | 0.067 | 0.025 | 0.077 | 0.748 | 0.209 | 0.592 | 0.724 | -0.088 | 0.227 | 0.698 |
| InternalIMT | aa | rs147514068 | . | . | . | . | . | . | . | . | . | . |
| InternalIMT | aa | rs35410535 | 0.138 | 0.03 | 0.083 | 0.72 | 0.14 | 0.371 | 0.706 | -0.06 | 0.144 | 0.676 |
| InternalIMT | aa | rs191613635 | 0.001 | . | . | . | . | . | . | . | . | . |
| InternalIMT | aa | rs17157800 | 0.004 | . | . | . | . | . | . | . | . | . |
| InternalIMT | aa | rs17523413 | 0.027 | 0.026 | 0.076 | 0.735 | 0.508 | 0.939 | 0.589 | -0.226 | 0.358 | 0.527 |
| InternalIMT | aa | rs7897036 | 0.013 | -0.031 | 0.072 | 0.667 | -6.86 | 2.08 | 9.90E-04 | 2.74 | 0.831 | 1.00E-03 |
| InternalIMT | aa | rs76334323 | 0.001 | . | . | . | . | . | . | . | . | . |

| InternalIMT | aa | rs143535991 | 0 | . | . | . | . | . | . | . | . | . |
| --- | --- | --- | --- | --- | --- | --- | --- | --- | --- | --- | --- | --- |
| InternalIMT | aa | rs140344979 | 0.002 | . | . | . | . | . | . | . | . | . |
| InternalIMT | aa | rs36047811 | 0.138 | 0.03 | 0.083 | 0.719 | 0.14 | 0.371 | 0.705 | -0.06 | 0.144 | 0.675 |
| InternalIMT | aa | rs76493570 | 0.057 | 0.023 | 0.077 | 0.763 | 0.157 | 0.597 | 0.792 | -0.072 | 0.23 | 0.754 |
| InternalIMT | aa | rs192998920 | . | . | . | . | . | . | . | . | . | . |
| InternalIMT | aa | rs116840852 | 0.007 | . | . | . | . | . | . | . | . | . |
| InternalIMT | aa | rs184824101 | 0 | . | . | . | . | . | . | . | . | . |
| InternalIMT | aa | rs41301621 | 0.002 | . | . | . | . | . | . | . | . | . |
| InternalIMT | aa | rs3740109 | 0.138 | 0.03 | 0.083 | 0.718 | 0.142 | 0.371 | 0.703 | -0.061 | 0.144 | 0.672 |
| InternalIMT | aa | rs7899415 | 0.032 | 2.40E-03 | 0.081 | 0.977 | -0.536 | 1.17 | 0.647 | 0.199 | 0.457 | 0.664 |
| InternalIMT | aa | rs184395284 | 0.001 | . | . | . | . | . | . | . | . | . |
| InternalIMT | aa | rs139209589 | 0.002 | . | . | . | . | . | . | . | . | . |
| InternalIMT | aa | rs149924163 | 0.009 | . | . | . | . | . | . | . | . | . |
| InternalIMT | aa | rs3740110 | 0.001 | . | . | . | . | . | . | . | . | . |
| InternalIMT | aa | rs144335113 | 0.009 | . | . | . | . | . | . | . | . | . |
| InternalIMT | aa | rs74743385 | 0.045 | 1.00E-04 | 0.075 | 0.999 | -0.344 | 0.725 | 0.636 | 0.181 | 0.282 | 0.521 |
| InternalIMT | aa | rs146545829 | 0.009 | . | . | . | . | . | . | . | . | . |
| InternalIMT | aa | rs181842689 | . | . | . | . | . | . | . | . | . | . |
| InternalIMT | aa | rs3740111 | 0.067 | 0.025 | 0.077 | 0.745 | 0.216 | 0.592 | 0.716 | -0.091 | 0.227 | 0.689 |
| InternalIMT | aa | rs186261599 | 0.009 | . | . | . | . | . | . | . | . | . |
| InternalIMT | aa | rs73285348 | 0.004 | . | . | . | . | . | . | . | . | . |
| InternalIMT | aa | rs186356901 | 0.009 | . | . | . | . | . | . | . | . | . |
| InternalIMT | aa | rs3824618 | 0.011 | 0.017 | 0.074 | 0.817 | -0.313 | 0.804 | 0.697 | 0.053 | 0.32 | 0.868 |
| InternalIMT | aa | rs3802548 | 0.201 | -0.028 | 0.081 | 0.729 | -0.308 | 0.371 | 0.408 | 0.136 | 0.146 | 0.35 |
| InternalIMT | aa | rs3802547 | 0.001 | . | . | . | . | . | . | . | . | . |
| InternalIMT | aa | rs77677521 | 0.007 | . | . | . | . | . | . | . | . | . |
| InternalIMT | aa | rs3824616 | 0.138 | 0.03 | 0.083 | 0.716 | 0.144 | 0.371 | 0.698 | -0.062 | 0.144 | 0.667 |
| InternalIMT | aa | rs191073818 | 0.009 | . | . | . | . | . | . | . | . | . |
| InternalIMT | aa | rs147078004 | . | . | . | . | . | . | . | . | . | . |
| InternalIMT | aa | rs7908745 | 0.342 | -0.017 | 0.092 | 0.856 | -0.128 | 0.28 | 0.648 | 0.058 | 0.109 | 0.593 |
| InternalIMT | aa | rs17157811 | 0.004 | . | . | . | . | . | . | . | . | . |
| InternalIMT | aa | rs138506087 | 0.002 | . | . | . | . | . | . | . | . | . |
| InternalIMT | aa | rs77241269 | 0.071 | 0.021 | 0.079 | 0.786 | 0.091 | 0.438 | 0.835 | -0.039 | 0.172 | 0.819 |
| InternalIMT | aa | rs138374938 | 0.001 | . | . | . | . | . | . | . | . | . |
| InternalIMT | aa | rs2279433 | 0.068 | 0.026 | 0.077 | 0.741 | 0.214 | 0.563 | 0.703 | -0.093 | 0.217 | 0.669 |
| InternalIMT | aa | rs73285358 | 0.071 | 0.022 | 0.079 | 0.786 | 0.092 | 0.438 | 0.833 | -0.04 | 0.172 | 0.817 |
| InternalIMT | aa | rs11239528 | 0.198 | -0.029 | 0.08 | 0.719 | -0.316 | 0.374 | 0.399 | 0.14 | 0.146 | 0.338 |
| InternalIMT | aa | rs77453386 | 0.001 | . | . | . | . | . | . | . | . | . |
| InternalIMT | aa | rs2279434 | 0.071 | 0.03 | 0.078 | 0.696 | 0.358 | 0.567 | 0.528 | -0.146 | 0.218 | 0.503 |
| InternalIMT | aa | rs149236525 | 0.003 | . | . | . | . | . | . | . | . | . |
| InternalIMT | aa | rs12573609 | 0.018 | 0.056 | 0.084 | 0.505 | 0.671 | 0.495 | 0.176 | -0.306 | 0.187 | 0.102 |
| InternalIMT | aa | rs191716655 | 0.006 | . | . | . | . | . | . | . | . | . |
| InternalIMT | aa | rs17523660 | 0.005 | . | . | . | . | . | . | . | . | . |
| InternalIMT | aa | rs142671360 | 0.001 | . | . | . | . | . | . | . | . | . |
| InternalIMT | aa | rs3764990 | 0.025 | 0.026 | 0.076 | 0.735 | 0.561 | 0.958 | 0.559 | -0.246 | 0.366 | 0.502 |
| InternalIMT | aa | rs113682861 | 0.001 | . | . | . | . | . | . | . | . | . |
| InternalIMT | aa | rs117658270 | 0.001 | . | . | . | . | . | . | . | . | . |
| InternalIMT | aa | rs187309063 | . | . | . | . | . | . | . | . | . | . |
| InternalIMT | aa | rs73285360 | 0.072 | 0.022 | 0.079 | 0.785 | 0.093 | 0.438 | 0.832 | -0.04 | 0.172 | 0.817 |
| InternalIMT | aa | rs80133289 | 0.002 | . | . | . | . | . | . | . | . | . |
| InternalIMT | aa | rs186693257 | . | . | . | . | . | . | . | . | . | . |
| InternalIMT | aa | rs35735968 | 0.081 | 0.036 | 0.079 | 0.652 | 0.386 | 0.518 | 0.457 | -0.161 | 0.199 | 0.419 |
| InternalIMT | aa | rs17157825 | 0.011 | 0.018 | 0.074 | 0.81 | -0.173 | 0.826 | 0.834 | 3.40E-03 | 0.327 | 0.992 |
| InternalIMT | aa | rs2291428 | 0.183 | -0.059 | 0.079 | 0.453 | -0.617 | 0.406 | 0.129 | 0.266 | 0.16 | 0.097 |
| InternalIMT | aa | rs2291429 | 0.21 | -0.038 | 0.081 | 0.637 | -0.375 | 0.369 | 0.31 | 0.163 | 0.144 | 0.259 |
| InternalIMT | aa | rs145714792 | 0.002 | . | . | . | . | . | . | . | . | . |
| InternalIMT | aa | rs12769244 | 0.068 | 0.025 | 0.077 | 0.744 | 0.209 | 0.555 | 0.707 | -0.09 | 0.214 | 0.673 |
| InternalIMT | aa | rs12771275 | 0.068 | 0.025 | 0.077 | 0.744 | 0.209 | 0.555 | 0.707 | -0.09 | 0.214 | 0.673 |
| InternalIMT | aa | rs146266174 | 0.001 | . | . | . | . | . | . | . | . | . |
| InternalIMT | aa | rs41301623 | 0.001 | . | . | . | . | . | . | . | . | . |
| InternalIMT | aa | rs2306814 | 0.001 | . | . | . | . | . | . | . | . | . |
| InternalIMT | aa | rs12244834 | 0.08 | -2.30E-03 | 0.078 | 0.977 | -0.316 | 0.539 | 0.558 | 0.144 | 0.213 | 0.498 |
| InternalIMT | aa | rs184006011 | 0.001 | . | . | . | . | . | . | . | . | . |
| InternalIMT | aa | rs78829066 | 0.001 | . | . | . | . | . | . | . | . | . |
| InternalIMT | aa | rs34731408 | 0.068 | 0.025 | 0.077 | 0.744 | 0.209 | 0.555 | 0.707 | -0.09 | 0.214 | 0.673 |
| InternalIMT | aa | rs17451516 | 0.012 | 0.026 | 0.075 | 0.727 | 1.49 | 1.58 | 0.347 | -0.583 | 0.6 | 0.332 |
| InternalIMT | aa | rs139910133 | 0.009 | . | . | . | . | . | . | . | . | . |
| InternalIMT | aa | rs10900218 | 0.319 | -0.029 | 0.089 | 0.741 | -0.197 | 0.286 | 0.49 | 0.088 | 0.112 | 0.432 |
| InternalIMT | aa | rs146023999 | . | . | . | . | . | . | . | . | . | . |
| InternalIMT | aa | rs187296958 | 0 | . | . | . | . | . | . | . | . | . |
| InternalIMT | aa | rs142342833 | . | . | . | . | . | . | . | . | . | . |
| InternalIMT | aa | rs143489255 | . | . | . | . | . | . | . | . | . | . |
| InternalIMT | aa | rs138532518 | . | . | . | . | . | . | . | . | . | . |
| InternalIMT | aa | rs183768079 | 0.007 | . | . | . | . | . | . | . | . | . |
| InternalIMT | aa | rs117892272 | . | . | . | . | . | . | . | . | . | . |
| InternalIMT | aa | rs192993943 | . | . | . | . | . | . | . | . | . | . |
| InternalIMT | aa | rs182989043 | 0.001 | . | . | . | . | . | . | . | . | . |
| InternalIMT | aa | rs189703435 | 0.001 | . | . | . | . | . | . | . | . | . |
| InternalIMT | aa | rs188794040 | 0 | . | . | . | . | . | . | . | . | . |
| InternalIMT | aa | rs143920953 | . | . | . | . | . | . | . | . | . | . |
| InternalIMT | aa | rs191029121 | . | . | . | . | . | . | . | . | . | . |
| InternalIMT | aa | rs116875448 | . | . | . | . | . | . | . | . | . | . |
| InternalIMT | aa | rs190167877 | 0.003 | . | . | . | . | . | . | . | . | . |
| InternalIMT | aa | rs184993418 | . | . | . | . | . | . | . | . | . | . |
| InternalIMT | aa | rs143241158 | . | . | . | . | . | . | . | . | . | . |
| InternalIMT | aa | rs11239500 | 0.074 | -0.022 | 0.076 | 0.772 | -0.795 | 0.517 | 0.125 | 0.32 | 0.207 | 0.123 |

| InternalIMT | aa | rs140568994 | . | . | . | . | . | . | . | . | . | . |
| --- | --- | --- | --- | --- | --- | --- | --- | --- | --- | --- | --- | --- |
| InternalIMT | aa | rs187530317 | 0.001 | . | . | . | . | . | . | . | . | . |
| InternalIMT | aa | rs145189326 | 0.001 | . | . | . | . | . | . | . | . | . |
| InternalIMT | aa | rs186203332 | . | . | . | . | . | . | . | . | . | . |
| InternalIMT | aa | rs189921909 | . | . | . | . | . | . | . | . | . | . |
| InternalIMT | aa | rs187260989 | . | . | . | . | . | . | . | . | . | . |
| InternalIMT | aa | rs114376586 | 0.073 | -0.022 | 0.076 | 0.778 | -0.789 | 0.516 | 0.127 | 0.318 | 0.207 | 0.125 |
| InternalIMT | aa | rs151070808 | . | . | . | . | . | . | . | . | . | . |
| InternalIMT | aa | rs140927225 | 0.074 | -0.024 | 0.076 | 0.756 | -0.815 | 0.515 | 0.114 | 0.329 | 0.206 | 0.111 |
| InternalIMT | aa | rs191555615 | . | . | . | . | . | . | . | . | . | . |
| InternalIMT | aa | rs80278898 | 0.001 | . | . | . | . | . | . | . | . | . |
| InternalIMT | aa | rs190849503 | . | . | . | . | . | . | . | . | . | . |
| InternalIMT | aa | rs150080315 | 0.007 | . | . | . | . | . | . | . | . | . |
| InternalIMT | aa | rs143623885 | . | . | . | . | . | . | . | . | . | . |
| InternalIMT | aa | rs187760040 | . | . | . | . | . | . | . | . | . | . |
| InternalIMT | aa | rs143917143 | . | . | . | . | . | . | . | . | . | . |
| InternalIMT | aa | rs149851136 | 0.002 | . | . | . | . | . | . | . | . | . |
| InternalIMT | aa | rs181612054 | . | . | . | . | . | . | . | . | . | . |
| InternalIMT | aa | rs191006548 | . | . | . | . | . | . | . | . | . | . |
| InternalIMT | aa | rs117377631 | 0.003 | . | . | . | . | . | . | . | . | . |
| InternalIMT | aa | rs182107634 | . | . | . | . | . | . | . | . | . | . |
| InternalIMT | aa | rs190211867 | . | . | . | . | . | . | . | . | . | . |
| InternalIMT | aa | rs148293457 | . | . | . | . | . | . | . | . | . | . |
| InternalIMT | aa | rs191811733 | . | . | . | . | . | . | . | . | . | . |
| InternalIMT | aa | rs146109272 | . | . | . | . | . | . | . | . | . | . |
| InternalIMT | aa | rs148397915 | . | . | . | . | . | . | . | . | . | . |
| InternalIMT | aa | rs142233290 | . | . | . | . | . | . | . | . | . | . |
| InternalIMT | aa | rs80226874 | . | . | . | . | . | . | . | . | . | . |
| InternalIMT | aa | rs144496203 | 0 | . | . | . | . | . | . | . | . | . |
| InternalIMT | aa | rs191062130 | . | . | . | . | . | . | . | . | . | . |
| InternalIMT | aa | rs185807817 | . | . | . | . | . | . | . | . | . | . |
| InternalIMT | aa | rs117846213 | 0.001 | . | . | . | . | . | . | . | . | . |
| InternalIMT | aa | rs190545473 | . | . | . | . | . | . | . | . | . | . |
| InternalIMT | aa | rs192364918 | . | . | . | . | . | . | . | . | . | . |
| InternalIMT | aa | rs182939241 | . | . | . | . | . | . | . | . | . | . |
| InternalIMT | aa | rs12571125 | 0.001 | . | . | . | . | . | . | . | . | . |
| InternalIMT | aa | rs192418941 | 0 | . | . | . | . | . | . | . | . | . |
| InternalIMT | aa | rs141270243 | . | . | . | . | . | . | . | . | . | . |
| InternalIMT | aa | rs182615502 | . | . | . | . | . | . | . | . | . | . |
| InternalIMT | aa | rs146669856 | 0.02 | 2.50E-03 | 0.074 | 0.974 | -0.876 | 1.17 | 0.455 | 0.386 | 0.464 | 0.405 |
| InternalIMT | aa | rs78290029 | 0.098 | 0.011 | 0.081 | 0.893 | -5.20E-03 | 0.51 | 0.992 | 0.033 | 0.201 | 0.871 |
| InternalIMT | aa | rs139955565 | . | . | . | . | . | . | . | . | . | . |
| InternalIMT | aa | rs12267314 | 0.087 | 0.011 | 0.079 | 0.892 | -0.022 | 0.539 | 0.967 | 0.032 | 0.212 | 0.879 |
| InternalIMT | aa | rs74477197 | . | . | . | . | . | . | . | . | . | . |
| InternalIMT | aa | rs188147236 | . | . | . | . | . | . | . | . | . | . |
| InternalIMT | aa | rs2228065 | 0.086 | 9.10E-03 | 0.079 | 0.908 | -0.056 | 0.538 | 0.917 | 0.045 | 0.212 | 0.832 |
| InternalIMT | aa | rs116961353 | . | . | . | . | . | . | . | . | . | . |
| InternalIMT | aa | rs190222272 | . | . | . | . | . | . | . | . | . | . |
| InternalIMT | aa | rs182719940 | . | . | . | . | . | . | . | . | . | . |
| InternalIMT | aa | rs184477413 | . | . | . | . | . | . | . | . | . | . |
| InternalIMT | aa | rs12265175 | 0.089 | 9.30E-03 | 0.079 | 0.907 | -0.033 | 0.534 | 0.95 | 0.038 | 0.21 | 0.855 |
| InternalIMT | aa | rs182928276 | . | . | . | . | . | . | . | . | . | . |
| InternalIMT | aa | rs144409895 | . | . | . | . | . | . | . | . | . | . |
| InternalIMT | aa | rs58998447 | 0.003 | . | . | . | . | . | . | . | . | . |
| InternalIMT | aa | rs188668832 | . | . | . | . | . | . | . | . | . | . |
| InternalIMT | aa | rs148641686 | . | . | . | . | . | . | . | . | . | . |
| InternalIMT | aa | rs75468791 | . | . | . | . | . | . | . | . | . | . |
| InternalIMT | aa | rs147327397 | . | . | . | . | . | . | . | . | . | . |
| InternalIMT | aa | rs77354597 | 0.002 | . | . | . | . | . | . | . | . | . |
| InternalIMT | aa | rs41365544 | 0.082 | 2.70E-04 | 0.078 | 0.997 | -0.258 | 0.537 | 0.631 | 0.121 | 0.212 | 0.568 |
| InternalIMT | aa | rs187564188 | . | . | . | . | . | . | . | . | . | . |
| InternalIMT | aa | rs183511984 | 0 | . | . | . | . | . | . | . | . | . |
| InternalIMT | aa | rs12262783 | 0.08 | 4.20E-03 | 0.078 | 0.958 | -0.174 | 0.541 | 0.749 | 0.091 | 0.214 | 0.669 |
| InternalIMT | aa | rs188817687 | 0.002 | . | . | . | . | . | . | . | . | . |
| InternalIMT | aa | rs3815968 | . | . | . | . | . | . | . | . | . | . |
| InternalIMT | aa | rs187822300 | . | . | . | . | . | . | . | . | . | . |
| InternalIMT | aa | rs188902172 | 0.002 | . | . | . | . | . | . | . | . | . |
| InternalIMT | aa | rs181190448 | . | . | . | . | . | . | . | . | . | . |
| InternalIMT | aa | rs182699093 | . | . | . | . | . | . | . | . | . | . |
| InternalIMT | aa | rs182631303 | 0.001 | . | . | . | . | . | . | . | . | . |
| InternalIMT | aa | rs187014172 | . | . | . | . | . | . | . | . | . | . |
| InternalIMT | aa | rs142575406 | . | . | . | . | . | . | . | . | . | . |
| InternalIMT | aa | rs180711033 | . | . | . | . | . | . | . | . | . | . |
| InternalIMT | aa | rs137894823 | 0.004 | . | . | . | . | . | . | . | . | . |
| InternalIMT | aa | rs189053273 | 0.001 | . | . | . | . | . | . | . | . | . |
| InternalIMT | aa | rs143499388 | 0.002 | . | . | . | . | . | . | . | . | . |
| InternalIMT | aa | rs182936001 | 0.006 | . | . | . | . | . | . | . | . | . |
| InternalIMT | aa | rs148456406 | 0.006 | . | . | . | . | . | . | . | . | . |
| InternalIMT | aa | rs182499531 | 0.008 | . | . | . | . | . | . | . | . | . |
| InternalIMT | aa | rs74884915 | 0.026 | 0.014 | 0.084 | 0.87 | -0.06 | 0.456 | 0.896 | 0.014 | 0.182 | 0.938 |
| InternalIMT | aa | rs184046374 | 0.001 | . | . | . | . | . | . | . | . | . |
| InternalIMT | aa | rs74333993 | 0.032 | -4.10E-03 | 0.075 | 0.956 | -0.944 | 0.734 | 0.198 | 0.376 | 0.296 | 0.204 |
| InternalIMT | aa | rs138889719 | 0.001 | . | . | . | . | . | . | . | . | . |
| InternalIMT | aa | rs75330330 | 0.022 | 4.40E-03 | 0.074 | 0.953 | -0.835 | 1.07 | 0.434 | 0.355 | 0.428 | 0.407 |
| InternalIMT | aa | rs193051693 | 0 | . | . | . | . | . | . | . | . | . |
| InternalIMT | aa | rs145929633 | 0 | . | . | . | . | . | . | . | . | . |

| InternalIMT | aa | rs184076904 | 0.004 | . | . | . | . | . | . | . | . | . |
| --- | --- | --- | --- | --- | --- | --- | --- | --- | --- | --- | --- | --- |
| InternalIMT | aa | rs141802494 | 0.018 | 3.40E-03 | 0.073 | 0.963 | -1.5 | 1.54 | 0.33 | 0.615 | 0.613 | 0.315 |
| InternalIMT | aa | rs149519759 | 0.002 | . | . | . | . | . | . | . | . | . |
| InternalIMT | aa | rs145467260 | 0.013 | 2.70E-03 | 0.074 | 0.97 | -1.55 | 1.32 | 0.241 | 0.594 | 0.526 | 0.259 |
| InternalIMT | aa | rs79620914 | 0.027 | 6.70E-03 | 0.075 | 0.929 | -0.52 | 0.546 | 0.342 | 0.171 | 0.22 | 0.436 |
| InternalIMT | aa | rs140538633 | 0.017 | 0.012 | 0.076 | 0.872 | -0.34 | 0.891 | 0.703 | 0.113 | 0.356 | 0.752 |
| InternalIMT | aa | rs140862301 | 0.008 | . | . | . | . | . | . | . | . | . |
| InternalIMT | aa | rs114615847 | 0.009 | . | . | . | . | . | . | . | . | . |
| InternalIMT | aa | rs141800259 | 0.026 | 0.029 | 0.075 | 0.7 | 0.449 | 0.767 | 0.558 | -0.152 | 0.309 | 0.623 |
| InternalIMT | aa | rs149779059 | 0.019 | 0.017 | 0.075 | 0.824 | -0.154 | 0.945 | 0.871 | 0.106 | 0.371 | 0.774 |
| InternalIMT | aa | rs116539253 | 0.007 | . | . | . | . | . | . | . | . | . |
| InternalIMT | aa | rs184979550 | 0.001 | . | . | . | . | . | . | . | . | . |
| InternalIMT | aa | rs190128940 | 0.004 | . | . | . | . | . | . | . | . | . |
| InternalIMT | aa | rs146594816 | 0.002 | . | . | . | . | . | . | . | . | . |
| InternalIMT | aa | rs141370766 | 0.022 | 4.40E-03 | 0.074 | 0.953 | -0.843 | 1.07 | 0.432 | 0.359 | 0.43 | 0.404 |
| InternalIMT | aa | rs144670033 | 0.002 | . | . | . | . | . | . | . | . | . |
| InternalIMT | aa | rs187152174 | 0.007 | . | . | . | . | . | . | . | . | . |
| InternalIMT | aa | rs184180263 | 0.005 | . | . | . | . | . | . | . | . | . |
| InternalIMT | aa | rs140075861 | 0.01 | 8.60E-03 | 0.074 | 0.907 | -1.17 | 1.12 | 0.294 | 0.459 | 0.441 | 0.298 |
| InternalIMT | aa | rs186483129 | 0 | . | . | . | . | . | . | . | . | . |
| InternalIMT | aa | rs186634282 | 0.012 | 1.60E-03 | 0.073 | 0.982 | -2.09 | 1.82 | 0.252 | 0.898 | 0.716 | 0.21 |
| InternalIMT | aa | rs9988663 | 0.032 | -4.10E-03 | 0.075 | 0.956 | -0.945 | 0.734 | 0.198 | 0.376 | 0.296 | 0.203 |
| InternalIMT | aa | rs138828529 | 0.02 | 3.10E-03 | 0.074 | 0.966 | -0.849 | 1.32 | 0.52 | 0.33 | 0.519 | 0.525 |
| InternalIMT | aa | rs188777615 | 0.004 | . | . | . | . | . | . | . | . | . |
| InternalIMT | aa | rs192321882 | 0.008 | . | . | . | . | . | . | . | . | . |
| InternalIMT | aa | rs150812270 | 0.027 | 0.059 | 0.076 | 0.434 | 2.01 | 0.802 | 0.012 | -0.787 | 0.307 | 0.011 |
| InternalIMT | aa | rs185906603 | 0.001 | . | . | . | . | . | . | . | . | . |
| InternalIMT | aa | rs191070862 | 0.004 | . | . | . | . | . | . | . | . | . |
| InternalIMT | aa | rs182904978 | 0 | . | . | . | . | . | . | . | . | . |
| InternalIMT | aa | rs192679655 | 0.001 | . | . | . | . | . | . | . | . | . |
| InternalIMT | aa | rs189673086 | 0.007 | . | . | . | . | . | . | . | . | . |
| InternalIMT | aa | rs181141892 | 0.002 | . | . | . | . | . | . | . | . | . |
| InternalIMT | aa | rs186404123 | 0 | . | . | . | . | . | . | . | . | . |
| InternalIMT | aa | rs192117287 | 0.005 | . | . | . | . | . | . | . | . | . |
| InternalIMT | aa | rs182748835 | 0.001 | . | . | . | . | . | . | . | . | . |
| InternalIMT | aa | rs141854549 | 0.017 | 0.012 | 0.075 | 0.869 | -0.393 | 0.891 | 0.659 | 0.134 | 0.356 | 0.708 |
| InternalIMT | aa | rs140163397 | 0.005 | . | . | . | . | . | . | . | . | . |
| InternalIMT | aa | rs146347300 | 0.008 | . | . | . | . | . | . | . | . | . |
| InternalIMT | aa | rs139362377 | 0.017 | 0.012 | 0.075 | 0.869 | -0.396 | 0.89 | 0.656 | 0.135 | 0.356 | 0.704 |
| InternalIMT | aa | rs185087671 | 0.001 | . | . | . | . | . | . | . | . | . |
| InternalIMT | aa | rs190466574 | 0.005 | . | . | . | . | . | . | . | . | . |
| InternalIMT | aa | rs147012778 | 0.005 | . | . | . | . | . | . | . | . | . |
| InternalIMT | aa | rs138236960 | 0.009 | . | . | . | . | . | . | . | . | . |
| InternalIMT | aa | rs116310311 | 0.017 | 0.012 | 0.075 | 0.869 | -0.396 | 0.89 | 0.656 | 0.135 | 0.356 | 0.704 |
| InternalIMT | aa | rs142868356 | 0.012 | 0.019 | 0.074 | 0.8 | 1.51 | 2 | 0.452 | -0.52 | 0.749 | 0.488 |
| InternalIMT | aa | rs144956240 | 0.003 | . | . | . | . | . | . | . | . | . |
| InternalIMT | aa | rs188527776 | 0.002 | . | . | . | . | . | . | . | . | . |
| InternalIMT | aa | rs183971165 | 0.007 | . | . | . | . | . | . | . | . | . |
| InternalIMT | aa | rs76748216 | 0.009 | . | . | . | . | . | . | . | . | . |
| InternalIMT | aa | rs148554990 | 0.001 | . | . | . | . | . | . | . | . | . |
| InternalIMT | aa | rs114446477 | 0.027 | 6.10E-03 | 0.075 | 0.935 | -0.547 | 0.549 | 0.319 | 0.179 | 0.221 | 0.418 |
| InternalIMT | aa | rs185223933 | 0.006 | . | . | . | . | . | . | . | . | . |
| InternalIMT | aa | rs76869726 | 0.009 | . | . | . | . | . | . | . | . | . |
| InternalIMT | aa | rs151200830 | 0.017 | 0.012 | 0.075 | 0.875 | -0.44 | 0.895 | 0.623 | 0.154 | 0.358 | 0.668 |
| InternalIMT | aa | rs17157731 | 0.017 | 0.012 | 0.075 | 0.875 | -0.441 | 0.895 | 0.623 | 0.154 | 0.358 | 0.667 |
| InternalIMT | aa | rs181243469 | 0 | . | . | . | . | . | . | . | . | . |
| InternalIMT | aa | rs186890707 | 0 | . | . | . | . | . | . | . | . | . |
| InternalIMT | aa | rs184063360 | 0 | . | . | . | . | . | . | . | . | . |
| InternalIMT | aa | rs188550237 | 0.007 | . | . | . | . | . | . | . | . | . |
| InternalIMT | aa | rs140931068 | 0.017 | 0.023 | 0.075 | 0.762 | 0.553 | 1.44 | 0.702 | -0.126 | 0.564 | 0.823 |
| InternalIMT | aa | rs74867877 | 0.007 | . | . | . | . | . | . | . | . | . |
| InternalIMT | aa | rs11239495 | 0.055 | -0.017 | 0.075 | 0.817 | -0.969 | 0.627 | 0.122 | 0.396 | 0.252 | 0.116 |
| InternalIMT | aa | rs149745800 | 0 | . | . | . | . | . | . | . | . | . |
| InternalIMT | aa | rs145044458 | 0.015 | 0.011 | 0.075 | 0.882 | -0.5 | 0.976 | 0.608 | 0.183 | 0.392 | 0.641 |
| InternalIMT | aa | rs188067427 | 0.002 | . | . | . | . | . | . | . | . | . |
| InternalIMT | aa | rs146827800 | 0.001 | . | . | . | . | . | . | . | . | . |
| InternalIMT | aa | rs140619817 | 0.015 | 0.011 | 0.075 | 0.881 | -0.494 | 0.976 | 0.613 | 0.181 | 0.392 | 0.644 |
| InternalIMT | aa | rs892689 | 0.997 | . | . | . | . | . | . | . | . | . |
| InternalIMT | aa | rs143642005 | 0.008 | . | . | . | . | . | . | . | . | . |
| InternalIMT | aa | rs181929304 | 0.007 | . | . | . | . | . | . | . | . | . |
| InternalIMT | aa | rs191086173 | 0.002 | . | . | . | . | . | . | . | . | . |
| InternalIMT | aa | rs114613116 | 0.018 | 0.012 | 0.075 | 0.874 | -0.425 | 0.892 | 0.634 | 0.149 | 0.356 | 0.676 |
| InternalIMT | aa | rs141076768 | 0.002 | . | . | . | . | . | . | . | . | . |
| InternalIMT | aa | rs187316531 | 0 | . | . | . | . | . | . | . | . | . |
| InternalIMT | aa | rs11239499 | 0.075 | -0.022 | 0.076 | 0.772 | -0.783 | 0.518 | 0.131 | 0.317 | 0.208 | 0.127 |
| InternalIMT | aa | rs192726371 | 0.003 | . | . | . | . | . | . | . | . | . |
| InternalIMT | aa | rs74128481 | 0.019 | -6.00E-03 | 0.074 | 0.935 | -1.92 | 1.1 | 0.081 | 0.806 | 0.439 | 0.067 |
| InternalIMT | aa | rs74128482 | 0.012 | 1.80E-03 | 0.073 | 0.981 | -2.01 | 1.88 | 0.284 | 0.873 | 0.735 | 0.236 |
| InternalIMT | aa | rs11239502 | 0.055 | -0.017 | 0.075 | 0.823 | -0.945 | 0.621 | 0.128 | 0.386 | 0.249 | 0.121 |
| InternalIMT | aa | rs116330285 | 0.002 | . | . | . | . | . | . | . | . | . |
| InternalIMT | aa | rs189926729 | 0.003 | . | . | . | . | . | . | . | . | . |
| InternalIMT | aa | rs188975779 | 0.012 | 0.019 | 0.074 | 0.798 | 1.6 | 2.02 | 0.429 | -0.553 | 0.757 | 0.466 |
| InternalIMT | aa | rs143846038 | 0.002 | . | . | . | . | . | . | . | . | . |
| InternalIMT | aa | rs74128483 | 0.019 | -6.10E-03 | 0.074 | 0.934 | -1.92 | 1.1 | 0.081 | 0.808 | 0.439 | 0.066 |
| InternalIMT | aa | rs139292055 | 0.073 | -0.022 | 0.076 | 0.778 | -0.788 | 0.517 | 0.127 | 0.317 | 0.207 | 0.126 |
| InternalIMT | aa | rs11239503 | 0.073 | -0.022 | 0.076 | 0.778 | -0.788 | 0.517 | 0.127 | 0.317 | 0.207 | 0.126 |

| InternalIMT | aa | rs58838686 | 0.012 | 1.70E-03 | 0.073 | 0.982 | -2.02 | 1.87 | 0.28 | 0.876 | 0.734 | 0.232 |
| --- | --- | --- | --- | --- | --- | --- | --- | --- | --- | --- | --- | --- |
| InternalIMT | aa | rs185825373 | 0.008 | . | . | . | . | . | . | . | . | . |
| InternalIMT | aa | rs11239504 | 0.073 | -0.022 | 0.076 | 0.778 | -0.788 | 0.517 | 0.127 | 0.317 | 0.207 | 0.126 |
| InternalIMT | aa | rs150012354 | 0.006 | . | . | . | . | . | . | . | . | . |
| InternalIMT | aa | rs187763096 | 0.01 | . | . | . | . | . | . | . | . | . |
| InternalIMT | aa | rs193043982 | 0.008 | . | . | . | . | . | . | . | . | . |
| InternalIMT | aa | rs79186884 | 0.023 | 0.022 | 0.076 | 0.769 | 0.131 | 0.771 | 0.865 | -9.00E-03 | 0.3 | 0.976 |
| InternalIMT | aa | rs74128484 | 0.012 | 1.60E-03 | 0.073 | 0.982 | -2.02 | 1.87 | 0.279 | 0.878 | 0.733 | 0.231 |
| InternalIMT | aa | rs58461537 | 0.073 | -0.022 | 0.076 | 0.778 | -0.788 | 0.516 | 0.127 | 0.317 | 0.207 | 0.125 |
| InternalIMT | aa | rs188527474 | 0.008 | . | . | . | . | . | . | . | . | . |
| InternalIMT | aa | rs141659976 | 0.008 | . | . | . | . | . | . | . | . | . |
| InternalIMT | aa | rs115846407 | 0.073 | -0.022 | 0.076 | 0.778 | -0.788 | 0.516 | 0.127 | 0.317 | 0.207 | 0.125 |
| InternalIMT | aa | rs138378816 | 0.073 | -0.022 | 0.076 | 0.778 | -0.788 | 0.516 | 0.127 | 0.317 | 0.207 | 0.125 |
| InternalIMT | aa | rs146188136 | 0.055 | -0.017 | 0.075 | 0.822 | -0.945 | 0.62 | 0.128 | 0.386 | 0.249 | 0.121 |
| InternalIMT | aa | rs139383871 | 0.014 | 6.90E-03 | 0.074 | 0.926 | -1.18 | 0.975 | 0.227 | 0.417 | 0.384 | 0.277 |
| InternalIMT | aa | rs182953869 | 0.004 | . | . | . | . | . | . | . | . | . |
| InternalIMT | aa | rs192430299 | 0.019 | 0.023 | 0.074 | 0.761 | 1.72 | 1.66 | 0.299 | -0.593 | 0.627 | 0.345 |
| InternalIMT | aa | rs144152844 | 0.073 | -0.022 | 0.076 | 0.778 | -0.788 | 0.516 | 0.127 | 0.317 | 0.207 | 0.125 |
| InternalIMT | aa | rs145987786 | 0.051 | -0.011 | 0.074 | 0.878 | -0.887 | 0.748 | 0.236 | 0.369 | 0.293 | 0.208 |
| InternalIMT | aa | rs181132926 | 0.003 | . | . | . | . | . | . | . | . | . |
| InternalIMT | aa | rs147882253 | 0.073 | -0.022 | 0.076 | 0.778 | -0.788 | 0.516 | 0.127 | 0.317 | 0.207 | 0.125 |
| InternalIMT | aa | rs141807621 | 0.023 | 5.40E-03 | 0.074 | 0.941 | -0.676 | 1.14 | 0.554 | 0.253 | 0.447 | 0.572 |
| InternalIMT | aa | rs147116052 | 0.073 | -0.022 | 0.076 | 0.778 | -0.788 | 0.516 | 0.127 | 0.317 | 0.207 | 0.125 |
| InternalIMT | aa | rs147716618 | 0.073 | -0.022 | 0.076 | 0.778 | -0.788 | 0.516 | 0.127 | 0.317 | 0.207 | 0.125 |
| InternalIMT | aa | rs187198855 | 0.046 | -0.01 | 0.074 | 0.893 | -0.894 | 0.884 | 0.312 | 0.382 | 0.343 | 0.266 |
| InternalIMT | aa | rs189409638 | 0.016 | 5.70E-03 | 0.074 | 0.938 | -0.806 | 1.34 | 0.546 | 0.374 | 0.529 | 0.479 |
| InternalIMT | aa | rs182647339 | 0.001 | . | . | . | . | . | . | . | . | . |
| InternalIMT | aa | rs187995988 | 0.004 | . | . | . | . | . | . | . | . | . |
| InternalIMT | aa | rs141034184 | 0.079 | -6.90E-03 | 0.077 | 0.929 | -0.458 | 0.509 | 0.368 | 0.18 | 0.203 | 0.375 |
| InternalIMT | aa | rs187310430 | 0.008 | . | . | . | . | . | . | . | . | . |
| InternalIMT | aa | rs192608488 | 0.073 | -0.021 | 0.076 | 0.779 | -0.785 | 0.516 | 0.128 | 0.316 | 0.207 | 0.126 |
| InternalIMT | aa | rs148840366 | 0.009 | . | . | . | . | . | . | . | . | . |
| InternalIMT | aa | rs186785022 | 0.019 | -6.30E-03 | 0.074 | 0.932 | -1.94 | 1.1 | 0.078 | 0.814 | 0.439 | 0.064 |
| InternalIMT | aa | rs192348728 | 0.014 | 0.039 | 0.075 | 0.605 | 0.587 | 0.483 | 0.225 | -0.243 | 0.198 | 0.221 |
| InternalIMT | aa | rs189656631 | 0.073 | -0.021 | 0.076 | 0.779 | -0.785 | 0.515 | 0.128 | 0.316 | 0.207 | 0.126 |
| InternalIMT | aa | rs185289750 | 0.004 | . | . | . | . | . | . | . | . | . |
| InternalIMT | aa | rs142382395 | 0.073 | -0.021 | 0.076 | 0.779 | -0.785 | 0.515 | 0.128 | 0.316 | 0.207 | 0.126 |
| InternalIMT | aa | rs144866696 | 0.019 | -6.30E-03 | 0.074 | 0.932 | -1.94 | 1.1 | 0.078 | 0.814 | 0.439 | 0.064 |
| InternalIMT | aa | rs141371289 | 0.073 | -0.021 | 0.076 | 0.779 | -0.785 | 0.515 | 0.128 | 0.316 | 0.207 | 0.126 |
| InternalIMT | aa | rs114750008 | 0.073 | -0.021 | 0.076 | 0.779 | -0.785 | 0.515 | 0.128 | 0.316 | 0.207 | 0.126 |
| InternalIMT | aa | rs189063597 | 0.001 | . | . | . | . | . | . | . | . | . |
| InternalIMT | aa | rs139151571 | 0.067 | -0.019 | 0.076 | 0.799 | -0.823 | 0.563 | 0.145 | 0.329 | 0.226 | 0.145 |
| InternalIMT | aa | rs139846368 | 0.075 | -0.022 | 0.076 | 0.772 | -0.772 | 0.517 | 0.135 | 0.311 | 0.207 | 0.133 |
| InternalIMT | aa | rs182057653 | 0.019 | 0.044 | 0.083 | 0.596 | 0.717 | 0.605 | 0.236 | -0.286 | 0.237 | 0.229 |
| InternalIMT | aa | rs148785420 | 0.073 | -0.021 | 0.076 | 0.779 | -0.785 | 0.515 | 0.128 | 0.316 | 0.207 | 0.126 |
| InternalIMT | aa | rs138383251 | 0.011 | 8.80E-03 | 0.074 | 0.905 | -1.16 | 1.1 | 0.293 | 0.457 | 0.435 | 0.294 |
| InternalIMT | aa | rs116526705 | 0.018 | 0.012 | 0.075 | 0.872 | -0.399 | 0.885 | 0.652 | 0.141 | 0.354 | 0.69 |
| InternalIMT | aa | rs185946436 | 0 | . | . | . | . | . | . | . | . | . |
| InternalIMT | aa | rs190455427 | 0.004 | . | . | . | . | . | . | . | . | . |
| InternalIMT | aa | rs9282584 | 0.073 | -0.021 | 0.076 | 0.779 | -0.785 | 0.515 | 0.128 | 0.316 | 0.207 | 0.126 |
| InternalIMT | aa | rs17153289 | 0.073 | -0.021 | 0.076 | 0.779 | -0.785 | 0.515 | 0.128 | 0.316 | 0.207 | 0.126 |
| InternalIMT | aa | rs138359103 | 0.008 | . | . | . | . | . | . | . | . | . |
| InternalIMT | aa | rs150605268 | 0.003 | . | . | . | . | . | . | . | . | . |
| InternalIMT | aa | rs79242910 | 0.075 | -0.019 | 0.077 | 0.8 | -0.726 | 0.513 | 0.157 | 0.294 | 0.206 | 0.153 |
| InternalIMT | aa | rs139716904 | 0.021 | 0.011 | 0.076 | 0.88 | -0.536 | 0.643 | 0.405 | 0.168 | 0.258 | 0.516 |
| InternalIMT | aa | rs76238498 | 0.053 | -4.20E-03 | 0.075 | 0.955 | -0.593 | 0.786 | 0.451 | 0.255 | 0.306 | 0.404 |
| InternalIMT | aa | rs184140639 | 0 | . | . | . | . | . | . | . | . | . |
| InternalIMT | aa | rs188517278 | 0.009 | . | . | . | . | . | . | . | . | . |
| InternalIMT | aa | rs139678913 | 0.004 | . | . | . | . | . | . | . | . | . |
| InternalIMT | aa | rs116697955 | 0.004 | . | . | . | . | . | . | . | . | . |
| InternalIMT | aa | rs79267994 | 0.012 | 1.40E-03 | 0.073 | 0.984 | -2.05 | 1.86 | 0.27 | 0.888 | 0.728 | 0.223 |
| InternalIMT | aa | rs11239510 | 0.022 | 0.017 | 0.075 | 0.815 | -0.025 | 0.965 | 0.979 | -0.011 | 0.367 | 0.975 |
| InternalIMT | aa | rs75831645 | 0.018 | 0.012 | 0.075 | 0.871 | -0.395 | 0.884 | 0.655 | 0.139 | 0.353 | 0.693 |
| InternalIMT | aa | rs78452276 | 0.052 | 0.078 | 0.085 | 0.36 | 0.786 | 0.378 | 0.038 | -0.306 | 0.147 | 0.037 |
| InternalIMT | aa | rs144366924 | 0 | . | . | . | . | . | . | . | . | . |
| InternalIMT | aa | rs77519841 | 0.056 | -0.017 | 0.075 | 0.825 | -0.936 | 0.619 | 0.131 | 0.383 | 0.248 | 0.123 |
| InternalIMT | aa | rs76944454 | 0.073 | -0.021 | 0.076 | 0.782 | -0.779 | 0.515 | 0.13 | 0.314 | 0.206 | 0.128 |
| InternalIMT | aa | rs76443855 | 0.053 | -4.10E-03 | 0.075 | 0.956 | -0.591 | 0.786 | 0.452 | 0.255 | 0.306 | 0.405 |
| InternalIMT | aa | rs146613174 | 0.002 | . | . | . | . | . | . | . | . | . |
| InternalIMT | aa | rs181424761 | 0.002 | . | . | . | . | . | . | . | . | . |
| InternalIMT | aa | rs142119241 | 0.001 | . | . | . | . | . | . | . | . | . |
| InternalIMT | aa | rs140137954 | 0.018 | 0.012 | 0.075 | 0.872 | -0.398 | 0.884 | 0.653 | 0.14 | 0.353 | 0.691 |
| InternalIMT | aa | rs187319549 | 0.002 | . | . | . | . | . | . | . | . | . |
| InternalIMT | aa | rs12241545 | 0.035 | 0.034 | 0.076 | 0.652 | 0.695 | 0.836 | 0.405 | -0.317 | 0.32 | 0.322 |
| InternalIMT | aa | rs10128228 | 0.073 | -0.021 | 0.076 | 0.782 | -0.778 | 0.515 | 0.131 | 0.313 | 0.206 | 0.129 |
| InternalIMT | aa | rs58580249 | 0.009 | . | . | . | . | . | . | . | . | . |
| InternalIMT | aa | rs193148863 | 0.001 | . | . | . | . | . | . | . | . | . |
| InternalIMT | aa | rs185638946 | 0.005 | . | . | . | . | . | . | . | . | . |
| InternalIMT | aa | rs117752921 | 0.001 | . | . | . | . | . | . | . | . | . |
| InternalIMT | aa | rs180841236 | 0.002 | . | . | . | . | . | . | . | . | . |
| InternalIMT | aa | rs73283155 | 0.009 | . | . | . | . | . | . | . | . | . |
| InternalIMT | aa | rs76560599 | 0.061 | -0.013 | 0.077 | 0.862 | -0.725 | 0.55 | 0.188 | 0.286 | 0.221 | 0.196 |
| InternalIMT | aa | rs76273620 | 0.061 | -0.013 | 0.077 | 0.862 | -0.725 | 0.55 | 0.188 | 0.286 | 0.221 | 0.196 |
| InternalIMT | aa | rs141432923 | 0.02 | -2.10E-03 | 0.073 | 0.977 | -1.9 | 1.62 | 0.24 | 0.752 | 0.621 | 0.226 |
| InternalIMT | aa | rs113529322 | 0.019 | -6.40E-03 | 0.074 | 0.931 | -1.95 | 1.1 | 0.077 | 0.818 | 0.44 | 0.063 |
| InternalIMT | aa | rs10128306 | 0.073 | -0.021 | 0.076 | 0.783 | -0.778 | 0.515 | 0.131 | 0.314 | 0.206 | 0.129 |

| InternalIMT | aa | rs149691688 | 0.002 | . | . | . | . | . | . | . | . | . |
| --- | --- | --- | --- | --- | --- | --- | --- | --- | --- | --- | --- | --- |
| InternalIMT | aa | rs75872495 | 0.073 | -0.021 | 0.076 | 0.781 | -0.781 | 0.515 | 0.13 | 0.315 | 0.206 | 0.128 |
| InternalIMT | aa | rs191873960 | 0.007 | . | . | . | . | . | . | . | . | . |
| InternalIMT | aa | rs80155622 | 0.073 | -0.021 | 0.076 | 0.783 | -0.779 | 0.515 | 0.131 | 0.314 | 0.206 | 0.129 |
| InternalIMT | aa | rs113914656 | 0.008 | . | . | . | . | . | . | . | . | . |
| InternalIMT | aa | rs12242840 | 0.073 | -0.021 | 0.076 | 0.783 | -0.779 | 0.515 | 0.131 | 0.314 | 0.206 | 0.129 |
| InternalIMT | aa | rs147824078 | 0.009 | . | . | . | . | . | . | . | . | . |
| InternalIMT | aa | rs59051493 | 0.073 | -0.021 | 0.076 | 0.779 | -0.782 | 0.516 | 0.13 | 0.315 | 0.207 | 0.128 |
| InternalIMT | aa | rs9282588 | 0.008 | . | . | . | . | . | . | . | . | . |
| InternalIMT | aa | rs150215207 | 0.009 | . | . | . | . | . | . | . | . | . |
| InternalIMT | aa | rs138772243 | 0.003 | . | . | . | . | . | . | . | . | . |
| InternalIMT | aa | rs149383244 | 0.025 | 0.067 | 0.083 | 0.417 | 0.725 | 0.395 | 0.067 | -0.296 | 0.158 | 0.061 |
| InternalIMT | aa | rs17522720 | 0.016 | 0.034 | 0.076 | 0.656 | 0.402 | 0.516 | 0.436 | -0.166 | 0.214 | 0.439 |
| InternalIMT | aa | rs79245105 | 0.073 | -0.022 | 0.077 | 0.779 | -0.781 | 0.517 | 0.131 | 0.315 | 0.207 | 0.129 |
| InternalIMT | aa | rs138222133 | 0.004 | . | . | . | . | . | . | . | . | . |
| InternalIMT | aa | rs12247314 | 0.082 | -0.019 | 0.077 | 0.804 | -0.63 | 0.492 | 0.2 | 0.258 | 0.196 | 0.189 |
| InternalIMT | aa | rs12263894 | 0.08 | -0.017 | 0.077 | 0.829 | -0.587 | 0.494 | 0.235 | 0.241 | 0.197 | 0.22 |
| InternalIMT | aa | rs149907880 | 0.008 | . | . | . | . | . | . | . | . | . |
| InternalIMT | aa | rs188153885 | 0.007 | . | . | . | . | . | . | . | . | . |
| InternalIMT | aa | rs73283159 | 0.005 | . | . | . | . | . | . | . | . | . |
| InternalIMT | aa | rs140476687 | 0.002 | . | . | . | . | . | . | . | . | . |
| InternalIMT | aa | rs186603148 | 0 | . | . | . | . | . | . | . | . | . |
| InternalIMT | aa | rs183296063 | 0.003 | . | . | . | . | . | . | . | . | . |
| InternalIMT | aa | rs145733694 | 0.009 | . | . | . | . | . | . | . | . | . |
| InternalIMT | aa | rs189807449 | 0 | . | . | . | . | . | . | . | . | . |
| InternalIMT | aa | rs12253223 | 0.064 | -0.016 | 0.076 | 0.83 | -0.76 | 0.588 | 0.196 | 0.316 | 0.235 | 0.179 |
| InternalIMT | aa | rs182113637 | 0 | . | . | . | . | . | . | . | . | . |
| InternalIMT | aa | rs142792099 | 0.018 | 4.10E-03 | 0.073 | 0.956 | -1.43 | 1.54 | 0.352 | 0.591 | 0.613 | 0.335 |
| InternalIMT | aa | rs12249910 | 0.064 | -0.016 | 0.076 | 0.83 | -0.76 | 0.588 | 0.196 | 0.315 | 0.235 | 0.179 |
| InternalIMT | aa | rs149436339 | 0.001 | . | . | . | . | . | . | . | . | . |
| InternalIMT | aa | rs77632210 | 0 | . | . | . | . | . | . | . | . | . |
| InternalIMT | aa | rs74130818 | 0.012 | 1.50E-03 | 0.073 | 0.983 | -2.06 | 1.91 | 0.281 | 0.891 | 0.749 | 0.234 |
| InternalIMT | aa | rs187583648 | 0 | . | . | . | . | . | . | . | . | . |
| InternalIMT | aa | rs191295987 | 0.008 | . | . | . | . | . | . | . | . | . |
| InternalIMT | aa | rs184200686 | 0.01 | . | . | . | . | . | . | . | . | . |
| InternalIMT | aa | rs76117263 | 0.052 | -4.10E-03 | 0.075 | 0.957 | -0.57 | 0.811 | 0.483 | 0.25 | 0.316 | 0.429 |
| InternalIMT | aa | rs185393149 | 0.001 | . | . | . | . | . | . | . | . | . |
| InternalIMT | aa | rs144760014 | 0.006 | . | . | . | . | . | . | . | . | . |
| InternalIMT | aa | rs7922292 | 0.008 | . | . | . | . | . | . | . | . | . |
| InternalIMT | aa | rs115372844 | 0.009 | . | . | . | . | . | . | . | . | . |
| InternalIMT | aa | rs111460364 | 0.052 | -4.20E-03 | 0.075 | 0.955 | -0.573 | 0.814 | 0.482 | 0.252 | 0.317 | 0.427 |
| InternalIMT | aa | rs182052255 | 0.002 | . | . | . | . | . | . | . | . | . |
| InternalIMT | aa | rs180983889 | 0.006 | . | . | . | . | . | . | . | . | . |
| InternalIMT | aa | rs139175327 | 0.009 | . | . | . | . | . | . | . | . | . |
| InternalIMT | aa | rs149959620 | 0.018 | 0.014 | 0.076 | 0.852 | -0.347 | 0.658 | 0.599 | 0.102 | 0.265 | 0.701 |
| InternalIMT | aa | rs114653117 | 0.009 | . | . | . | . | . | . | . | . | . |
| InternalIMT | aa | rs144270018 | 0.005 | . | . | . | . | . | . | . | . | . |
| InternalIMT | aa | rs182723204 | 0.007 | . | . | . | . | . | . | . | . | . |
| InternalIMT | aa | rs57204067 | 0.012 | 1.60E-03 | 0.073 | 0.983 | -2.06 | 1.94 | 0.288 | 0.892 | 0.762 | 0.242 |
| InternalIMT | aa | rs144956226 | 0.064 | -0.015 | 0.076 | 0.842 | -0.743 | 0.598 | 0.214 | 0.307 | 0.239 | 0.199 |
| InternalIMT | aa | rs187842704 | 0.019 | 0.02 | 0.075 | 0.791 | 1.19 | 1.71 | 0.484 | -0.408 | 0.657 | 0.535 |
| InternalIMT | aa | rs184620511 | 0.013 | 0.024 | 0.083 | 0.775 | 0.271 | 0.827 | 0.743 | -0.085 | 0.324 | 0.793 |
| InternalIMT | aa | rs144031303 | 0.019 | 0.014 | 0.076 | 0.858 | -0.26 | 0.895 | 0.772 | 0.089 | 0.356 | 0.801 |
| InternalIMT | aa | rs187526473 | 0 | . | . | . | . | . | . | . | . | . |
| InternalIMT | aa | rs193002805 | 0.002 | . | . | . | . | . | . | . | . | . |
| InternalIMT | aa | rs138707426 | 0.024 | 0.064 | 0.082 | 0.435 | 0.713 | 0.407 | 0.08 | -0.289 | 0.162 | 0.075 |
| InternalIMT | aa | rs142754243 | 0.049 | 0.028 | 0.078 | 0.72 | 0.332 | 0.629 | 0.598 | -0.14 | 0.244 | 0.565 |
| InternalIMT | aa | rs181600331 | 0.008 | . | . | . | . | . | . | . | . | . |
| InternalIMT | aa | rs144421264 | 0.029 | 0.021 | 0.076 | 0.783 | 0.145 | 0.745 | 0.846 | -0.079 | 0.29 | 0.786 |
| InternalIMT | aa | rs184414519 | 0.006 | . | . | . | . | . | . | . | . | . |
| InternalIMT | aa | rs150596349 | 0.048 | -3.60E-03 | 0.075 | 0.962 | -0.556 | 0.855 | 0.516 | 0.248 | 0.334 | 0.456 |
| InternalIMT | aa | rs190753747 | 0.001 | . | . | . | . | . | . | . | . | . |
| InternalIMT | aa | rs139560940 | 0.004 | . | . | . | . | . | . | . | . | . |
| InternalIMT | aa | rs75640804 | 0.057 | -0.014 | 0.076 | 0.851 | -0.856 | 0.643 | 0.184 | 0.35 | 0.258 | 0.175 |
| InternalIMT | aa | rs73283175 | 0.082 | 0.023 | 0.081 | 0.776 | 0.081 | 0.473 | 0.863 | -0.04 | 0.183 | 0.827 |
| InternalIMT | aa | rs143337229 | 0.003 | . | . | . | . | . | . | . | . | . |
| InternalIMT | aa | rs73283177 | 0.048 | 0.028 | 0.078 | 0.719 | 0.35 | 0.638 | 0.584 | -0.146 | 0.247 | 0.554 |
| InternalIMT | aa | rs76053611 | 0.045 | -0.014 | 0.074 | 0.852 | -1 | 0.794 | 0.207 | 0.419 | 0.312 | 0.179 |
| InternalIMT | aa | rs116325983 | 0.004 | . | . | . | . | . | . | . | . | . |
| InternalIMT | aa | rs115451825 | 0.008 | . | . | . | . | . | . | . | . | . |
| InternalIMT | aa | rs183845246 | 0.002 | . | . | . | . | . | . | . | . | . |
| InternalIMT | aa | rs79756542 | 0.051 | -7.10E-03 | 0.075 | 0.925 | -0.659 | 0.825 | 0.425 | 0.286 | 0.321 | 0.374 |
| InternalIMT | aa | rs187242860 | 0.002 | . | . | . | . | . | . | . | . | . |
| InternalIMT | aa | rs57318980 | 0.048 | 0.024 | 0.078 | 0.758 | 0.231 | 0.664 | 0.727 | -0.096 | 0.258 | 0.71 |
| InternalIMT | aa | rs59969732 | 0.043 | 0.026 | 0.077 | 0.735 | 0.345 | 0.686 | 0.615 | -0.142 | 0.266 | 0.593 |
| InternalIMT | aa | rs143811488 | 0.009 | . | . | . | . | . | . | . | . | . |
| InternalIMT | aa | rs189067644 | 0.01 | . | . | . | . | . | . | . | . | . |
| InternalIMT | aa | rs60449178 | 0.038 | 0.026 | 0.077 | 0.739 | 0.279 | 0.702 | 0.691 | -0.128 | 0.273 | 0.639 |
| InternalIMT | aa | rs59243389 | 0.038 | 0.026 | 0.077 | 0.739 | 0.279 | 0.702 | 0.691 | -0.128 | 0.273 | 0.639 |
| InternalIMT | aa | rs184946540 | 0.043 | 0.029 | 0.078 | 0.712 | 0.249 | 0.659 | 0.706 | -0.121 | 0.255 | 0.634 |
| InternalIMT | aa | rs188685881 | 0.001 | . | . | . | . | . | . | . | . | . |
| InternalIMT | aa | rs150335797 | 0.006 | . | . | . | . | . | . | . | . | . |
| InternalIMT | aa | rs143879260 | 0.006 | . | . | . | . | . | . | . | . | . |
| InternalIMT | aa | rs186044491 | 0.009 | . | . | . | . | . | . | . | . | . |
| InternalIMT | aa | rs11819084 | 0.03 | 0.05 | 0.078 | 0.522 | 0.605 | 0.409 | 0.139 | -0.223 | 0.161 | 0.168 |
| InternalIMT | aa | rs146915670 | 0.006 | . | . | . | . | . | . | . | . | . |

| InternalIMT | aa | rs113995695 | 0.029 | 0.05 | 0.078 | 0.524 | 0.601 | 0.411 | 0.143 | -0.22 | 0.162 | 0.175 |
| --- | --- | --- | --- | --- | --- | --- | --- | --- | --- | --- | --- | --- |
| InternalIMT | aa | rs143489451 | 0.005 | . | . | . | . | . | . | . | . | . |
| InternalIMT | aa | rs182653839 | 0 | . | . | . | . | . | . | . | . | . |
| InternalIMT | aa | rs141692564 | 0.007 | . | . | . | . | . | . | . | . | . |
| InternalIMT | aa | rs146281742 | 0.005 | . | . | . | . | . | . | . | . | . |
| InternalIMT | aa | rs192914353 | 0.005 | . | . | . | . | . | . | . | . | . |
| InternalIMT | aa | rs185182848 | 0.003 | . | . | . | . | . | . | . | . | . |
| InternalIMT | aa | rs28395858 | 0.029 | 0.051 | 0.078 | 0.516 | 0.617 | 0.409 | 0.131 | -0.226 | 0.161 | 0.162 |
| InternalIMT | aa | rs41429645 | 0.001 | . | . | . | . | . | . | . | . | . |
| InternalIMT | aa | rs28395874 | 0.029 | 0.051 | 0.078 | 0.512 | 0.626 | 0.408 | 0.125 | -0.229 | 0.161 | 0.155 |
| InternalIMT | aa | rs189663634 | 0.001 | . | . | . | . | . | . | . | . | . |
| InternalIMT | aa | rs77666700 | 0.01 | . | . | . | . | . | . | . | . | . |
| InternalIMT | aa | rs146711717 | 0.003 | . | . | . | . | . | . | . | . | . |
| InternalIMT | aa | rs144904251 | 0.001 | . | . | . | . | . | . | . | . | . |
| InternalIMT | aa | rs189893153 | 0.001 | . | . | . | . | . | . | . | . | . |
| InternalIMT | aa | rs150447740 | 0.009 | . | . | . | . | . | . | . | . | . |
| InternalIMT | aa | rs143099649 | 0.001 | . | . | . | . | . | . | . | . | . |
| InternalIMT | aa | rs191288436 | 0.01 | -2.00E-03 | 0.073 | 0.979 | -2.83 | 1.12 | 0.011 | 1.1 | 0.461 | 0.017 |
| InternalIMT | aa | rs188577862 | 0.006 | . | . | . | . | . | . | . | . | . |
| InternalIMT | aa | rs192695334 | 0.006 | . | . | . | . | . | . | . | . | . |
| InternalIMT | aa | rs138612906 | 0.036 | 0.035 | 0.077 | 0.652 | 0.312 | 0.48 | 0.516 | -0.109 | 0.189 | 0.565 |
| InternalIMT | aa | rs191290051 | 0.037 | 0.016 | 0.076 | 0.829 | 0.175 | 0.799 | 0.826 | -0.047 | 0.316 | 0.883 |
| InternalIMT | aa | rs189631459 | 0.001 | . | . | . | . | . | . | . | . | . |
| InternalIMT | aa | rs114620874 | 0.007 | . | . | . | . | . | . | . | . | . |
| InternalIMT | aa | rs78246652 | 0.023 | 7.30E-03 | 0.074 | 0.921 | -0.477 | 1.15 | 0.678 | 0.224 | 0.453 | 0.621 |
| InternalIMT | aa | rs79432451 | 0.007 | . | . | . | . | . | . | . | . | . |
| InternalIMT | aa | rs186022585 | 0.001 | . | . | . | . | . | . | . | . | . |
| InternalIMT | aa | rs142978891 | 0.009 | . | . | . | . | . | . | . | . | . |
| InternalIMT | aa | rs151086840 | 0.011 | 0.029 | 0.075 | 0.694 | 1.39 | 1.63 | 0.394 | -0.579 | 0.613 | 0.345 |
| InternalIMT | aa | rs183270259 | 0.01 | . | . | . | . | . | . | . | . | . |
| InternalIMT | aa | rs188544289 | 0.01 | . | . | . | . | . | . | . | . | . |
| InternalIMT | aa | rs188500808 | 0.001 | . | . | . | . | . | . | . | . | . |
| InternalIMT | aa | rs189424589 | 0.003 | . | . | . | . | . | . | . | . | . |
| InternalIMT | aa | rs78622303 | 0.04 | 0.023 | 0.077 | 0.763 | 0.265 | 0.547 | 0.628 | -0.086 | 0.218 | 0.693 |
| InternalIMT | aa | rs143210835 | 0.013 | 0.02 | 0.075 | 0.787 | 0.284 | 1.28 | 0.824 | -0.027 | 0.515 | 0.958 |
| InternalIMT | aa | rs114761840 | 0.007 | . | . | . | . | . | . | . | . | . |
| InternalIMT | aa | rs185805402 | 0.003 | . | . | . | . | . | . | . | . | . |
| InternalIMT | aa | rs139273744 | 0.002 | . | . | . | . | . | . | . | . | . |
| InternalIMT | aa | rs184654150 | 0.006 | . | . | . | . | . | . | . | . | . |
| InternalIMT | aa | rs189069710 | 0 | . | . | . | . | . | . | . | . | . |
| InternalIMT | aa | rs114752115 | 0.031 | 5.10E-03 | 0.075 | 0.945 | -0.582 | 0.888 | 0.513 | 0.251 | 0.352 | 0.477 |
| InternalIMT | aa | rs150162515 | 0.002 | . | . | . | . | . | . | . | . | . |
| InternalIMT | aa | rs180982454 | 0.008 | . | . | . | . | . | . | . | . | . |
| InternalIMT | aa | rs184932088 | 0.001 | . | . | . | . | . | . | . | . | . |
| InternalIMT | aa | rs181109625 | 0 | . | . | . | . | . | . | . | . | . |
| InternalIMT | aa | rs138121134 | 0.001 | . | . | . | . | . | . | . | . | . |
| InternalIMT | aa | rs183253861 | 0.004 | . | . | . | . | . | . | . | . | . |
| InternalIMT | aa | rs147073946 | 0.001 | . | . | . | . | . | . | . | . | . |
| InternalIMT | aa | rs139938489 | 0.008 | . | . | . | . | . | . | . | . | . |
| InternalIMT | aa | rs146362560 | 0.013 | 0.016 | 0.074 | 0.831 | 0.582 | 1.82 | 0.75 | -0.171 | 0.69 | 0.804 |
| InternalIMT | aa | rs193155956 | 0.007 | . | . | . | . | . | . | . | . | . |
| InternalIMT | aa | rs77663092 | 0.007 | . | . | . | . | . | . | . | . | . |
| InternalIMT | aa | rs73285319 | 0.033 | 0.012 | 0.076 | 0.877 | -0.37 | 0.832 | 0.656 | 0.131 | 0.316 | 0.678 |
| InternalIMT | aa | rs184019276 | 0.007 | . | . | . | . | . | . | . | . | . |
| InternalIMT | aa | rs28395863 | 0.027 | 0.027 | 0.077 | 0.721 | 0.374 | 0.713 | 0.6 | -0.135 | 0.281 | 0.633 |
| InternalIMT | aa | rs182121645 | 0.017 | 0.061 | 0.075 | 0.416 | 1.34 | 0.489 | 6.40E-03 | -0.529 | 0.183 | 3.90E-03 |
| InternalIMT | aa | rs73285327 | 0.025 | 0.014 | 0.075 | 0.855 | -0.211 | 0.994 | 0.832 | 0.081 | 0.379 | 0.831 |
| InternalIMT | aa | rs149589584 | 0.001 | . | . | . | . | . | . | . | . | . |
| InternalIMT | aa | rs148310335 | 0.005 | . | . | . | . | . | . | . | . | . |
| InternalIMT | aa | rs187246093 | 0.01 | 0.042 | 0.074 | 0.575 | 2.29 | 1.42 | 0.107 | -0.893 | 0.541 | 0.099 |
| InternalIMT | aa | rs184668894 | 0 | . | . | . | . | . | . | . | . | . |
| InternalIMT | aa | rs114269961 | 0.001 | . | . | . | . | . | . | . | . | . |
| InternalIMT | aa | rs149694374 | 0.034 | 0.028 | 0.077 | 0.71 | 0.416 | 0.593 | 0.483 | -0.169 | 0.234 | 0.47 |
| InternalIMT | aa | rs145284227 | 0.004 | . | . | . | . | . | . | . | . | . |
| InternalIMT | aa | rs189983820 | 0.004 | . | . | . | . | . | . | . | . | . |
| InternalIMT | aa | rs181200444 | 0 | . | . | . | . | . | . | . | . | . |
| InternalIMT | aa | rs185486385 | 0 | . | . | . | . | . | . | . | . | . |
| InternalIMT | aa | rs151198241 | 0.01 | 0.031 | 0.075 | 0.675 | 1.56 | 1.41 | 0.269 | -0.653 | 0.531 | 0.219 |
| InternalIMT | aa | rs189959341 | 0.01 | 0.031 | 0.075 | 0.675 | 1.56 | 1.41 | 0.269 | -0.653 | 0.531 | 0.219 |
| InternalIMT | aa | rs140549257 | 0.007 | . | . | . | . | . | . | . | . | . |
| InternalIMT | aa | rs186453139 | 0.027 | 0.013 | 0.075 | 0.86 | -0.325 | 0.854 | 0.704 | 0.108 | 0.326 | 0.74 |
| InternalIMT | aa | rs180922711 | 0.044 | 0.019 | 0.077 | 0.807 | -9.90E-03 | 0.699 | 0.989 | -0.013 | 0.267 | 0.962 |
| InternalIMT | aa | rs56659903 | 0.005 | . | . | . | . | . | . | . | . | . |
| InternalIMT | aa | rs141202128 | 0.006 | . | . | . | . | . | . | . | . | . |
| InternalIMT | aa | rs59085314 | 0.056 | 0.087 | 0.084 | 0.303 | 1.06 | 0.474 | 0.026 | -0.413 | 0.181 | 0.023 |
| InternalIMT | aa | rs145564996 | 0.004 | . | . | . | . | . | . | . | . | . |
| InternalIMT | aa | rs114961009 | 0.01 | 0.032 | 0.075 | 0.673 | 1.59 | 1.4 | 0.254 | -0.666 | 0.525 | 0.205 |
| InternalIMT | aa | rs182294189 | 0.004 | . | . | . | . | . | . | . | . | . |
| InternalIMT | aa | rs187586636 | 0.008 | . | . | . | . | . | . | . | . | . |
| InternalIMT | aa | rs192254907 | 0.002 | . | . | . | . | . | . | . | . | . |
| InternalIMT | aa | rs149145663 | 0.001 | . | . | . | . | . | . | . | . | . |
| InternalIMT | aa | rs180713064 | 0.001 | . | . | . | . | . | . | . | . | . |
| InternalIMT | aa | rs183009023 | 0.007 | . | . | . | . | . | . | . | . | . |
| InternalIMT | aa | rs143385347 | 0.017 | 0.064 | 0.076 | 0.394 | 1.42 | 0.492 | 4.10E-03 | -0.555 | 0.186 | 2.90E-03 |
| InternalIMT | aa | rs185786329 | 0.007 | . | . | . | . | . | . | . | . | . |
| InternalIMT | aa | rs192341030 | 0.004 | . | . | . | . | . | . | . | . | . |

| InternalIMT | aa | rs74661429 | 0.017 | 0.016 | 0.075 | 0.831 | 0.686 | 1.44 | 0.635 | -0.203 | 0.562 | 0.718 |
| --- | --- | --- | --- | --- | --- | --- | --- | --- | --- | --- | --- | --- |
| InternalIMT | aa | rs188034853 | 0.002 | . | . | . | . | . | . | . | . | . |
| InternalIMT | aa | rs116808430 | 0.027 | 0.013 | 0.075 | 0.859 | -0.322 | 0.842 | 0.702 | 0.104 | 0.322 | 0.746 |
| InternalIMT | aa | rs148469010 | 0 | . | . | . | . | . | . | . | . | . |
| InternalIMT | aa | rs78621663 | 0.01 | . | . | . | . | . | . | . | . | . |
| InternalIMT | aa | rs192654886 | 0.001 | . | . | . | . | . | . | . | . | . |
| InternalIMT | aa | rs145996035 | 0.008 | . | . | . | . | . | . | . | . | . |
| InternalIMT | aa | rs139942226 | 0.001 | . | . | . | . | . | . | . | . | . |
| InternalIMT | aa | rs113645632 | 0.01 | 0.031 | 0.074 | 0.673 | 0.927 | 1.84 | 0.614 | -0.302 | 0.748 | 0.687 |
| InternalIMT | aa | rs74940661 | 0.027 | 0.015 | 0.075 | 0.845 | -0.1 | 0.693 | 0.885 | 0.043 | 0.276 | 0.877 |
| InternalIMT | aa | rs115355800 | 0.022 | 4.00E-03 | 0.074 | 0.957 | -0.895 | 1.17 | 0.446 | 0.383 | 0.467 | 0.413 |
| InternalIMT | aa | rs150285898 | 0.008 | . | . | . | . | . | . | . | . | . |
| InternalIMT | aa | rs188117932 | 0.004 | . | . | . | . | . | . | . | . | . |
| InternalIMT | aa | rs139956566 | . | . | . | . | . | . | . | . | . | . |
| InternalIMT | aa | rs147673885 | . | . | . | . | . | . | . | . | . | . |
| InternalIMT | aa | rs189127928 | . | . | . | . | . | . | . | . | . | . |
| InternalIMT | aa | rs187558772 | . | . | . | . | . | . | . | . | . | . |
| InternalIMT | aa | rs181948587 | . | . | . | . | . | . | . | . | . | . |
| InternalIMT | aa | rs189983269 | . | . | . | . | . | . | . | . | . | . |
| InternalIMT | aa | rs187250069 | . | . | . | . | . | . | . | . | . | . |
| InternalIMT | aa | rs191485859 | . | . | . | . | . | . | . | . | . | . |
| InternalIMT | aa | rs185499555 | . | . | . | . | . | . | . | . | . | . |
| InternalIMT | aa | rs180862110 | . | . | . | . | . | . | . | . | . | . |
| InternalIMT | aa | rs190472603 | . | . | . | . | . | . | . | . | . | . |
| InternalIMT | aa | rs189226167 | . | . | . | . | . | . | . | . | . | . |
| InternalIMT | aa | rs181623256 | . | . | . | . | . | . | . | . | . | . |
| InternalIMT | aa | rs186079557 | . | . | . | . | . | . | . | . | . | . |
| InternalIMT | aa | rs147760994 | . | . | . | . | . | . | . | . | . | . |
| InternalIMT | aa | rs189176167 | . | . | . | . | . | . | . | . | . | . |
| InternalIMT | aa | rs117901000 | . | . | . | . | . | . | . | . | . | . |
| InternalIMT | aa | rs141984888 | . | . | . | . | . | . | . | . | . | . |
| InternalIMT | aa | rs192365061 | . | . | . | . | . | . | . | . | . | . |
| InternalIMT | aa | rs185976038 | . | . | . | . | . | . | . | . | . | . |
| InternalIMT | aa | rs184697303 | . | . | . | . | . | . | . | . | . | . |
| InternalIMT | aa | rs143383132 | . | . | . | . | . | . | . | . | . | . |
| InternalIMT | aa | rs141873881 | . | . | . | . | . | . | . | . | . | . |
| InternalIMT | aa | rs185507255 | . | . | . | . | . | . | . | . | . | . |
| InternalIMT | aa | rs191379425 | . | . | . | . | . | . | . | . | . | . |
| InternalIMT | aa | rs184199584 | . | . | . | . | . | . | . | . | . | . |
| InternalIMT | aa | rs183181360 | . | . | . | . | . | . | . | . | . | . |
| InternalIMT | aa | rs184495673 | . | . | . | . | . | . | . | . | . | . |
| InternalIMT | aa | rs182946297 | . | . | . | . | . | . | . | . | . | . |
| InternalIMT | aa | rs187743615 | . | . | . | . | . | . | . | . | . | . |
| InternalIMT | aa | rs183259862 | . | . | . | . | . | . | . | . | . | . |
| InternalIMT | aa | rs112433566 | . | . | . | . | . | . | . | . | . | . |
| InternalIMT | aa | rs182351085 | . | . | . | . | . | . | . | . | . | . |
| InternalIMT | aa | rs146248508 | . | . | . | . | . | . | . | . | . | . |
| InternalIMT | aa | rs188528439 | . | . | . | . | . | . | . | . | . | . |
| InternalIMT | aa | rs146167018 | . | . | . | . | . | . | . | . | . | . |
| InternalIMT | aa | rs192440980 | . | . | . | . | . | . | . | . | . | . |
| InternalIMT | aa | rs185601882 | . | . | . | . | . | . | . | . | . | . |
| InternalIMT | aa | rs117272563 | . | . | . | . | . | . | . | . | . | . |
| InternalIMT | aa | rs10430734 | . | . | . | . | . | . | . | . | . | . |
| InternalIMT | aa | rs181320952 | . | . | . | . | . | . | . | . | . | . |
| InternalIMT | aa | rs185480660 | . | . | . | . | . | . | . | . | . | . |
| InternalIMT | aa | rs188077917 | . | . | . | . | . | . | . | . | . | . |
| InternalIMT | aa | rs187562953 | . | . | . | . | . | . | . | . | . | . |
| InternalIMT | aa | rs182044631 | . | . | . | . | . | . | . | . | . | . |
| InternalIMT | aa | rs145455596 | . | . | . | . | . | . | . | . | . | . |
| InternalIMT | aa | rs181475877 | . | . | . | . | . | . | . | . | . | . |
| InternalIMT | aa | rs149413085 | . | . | . | . | . | . | . | . | . | . |
| InternalIMT | aa | rs12253656 | . | . | . | . | . | . | . | . | . | . |
| InternalIMT | aa | rs182718239 | . | . | . | . | . | . | . | . | . | . |
| InternalIMT | aa | rs192435695 | . | . | . | . | . | . | . | . | . | . |
| InternalIMT | aa | rs12248277 | . | . | . | . | . | . | . | . | . | . |
| InternalIMT | aa | rs143521710 | . | . | . | . | . | . | . | . | . | . |
| InternalIMT | aa | rs144454454 | . | . | . | . | . | . | . | . | . | . |
| InternalIMT | aa | rs190441610 | . | . | . | . | . | . | . | . | . | . |
| InternalIMT | aa | rs114690462 | . | . | . | . | . | . | . | . | . | . |
| InternalIMT | aa | rs184541357 | . | . | . | . | . | . | . | . | . | . |
| InternalIMT | aa | rs142657653 | . | . | . | . | . | . | . | . | . | . |
| InternalIMT | aa | rsurf1 | 2.117 | . | . | . | . | . | . | . | . | . |
| InternalIMT | aa | rsten1 | 2.28 | . | . | . | . | . | . | . | . | . |
| InternalIMT | aa | chr10:45852312:D | 0.092 | -5.60E-03 | 0.076 | 0.941 | -0.396 | 0.517 | 0.444 | 0.166 | 0.202 | 0.412 |
| InternalIMT | aa | chr10:45853232:I | 0.108 | -0.05 | 0.074 | 0.499 | -1.03 | 0.518 | 0.048 | 0.43 | 0.204 | 0.035 |
| InternalIMT | aa | chr10:45853234:I | 0.198 | -0.061 | 0.091 | 0.502 | -0.396 | 0.361 | 0.273 | 0.174 | 0.141 | 0.217 |
| InternalIMT | aa | chr10:45854796:D | 0.003 | . | . | . | . | . | . | . | . | . |
| InternalIMT | aa | chr10:45854994:I | 0.025 | 3.70E-03 | 0.074 | 0.96 | -0.802 | 1.03 | 0.435 | 0.344 | 0.41 | 0.401 |
| InternalIMT | aa | chr10:45858290:D | 0.052 | -2.70E-03 | 0.078 | 0.972 | -0.531 | 1.04 | 0.609 | 0.225 | 0.407 | 0.58 |
| InternalIMT | aa | chr10:45858820:I | 0.022 | -3.40E-03 | 0.074 | 0.964 | -2.26 | 0.888 | 0.011 | 0.858 | 0.35 | 0.014 |
| InternalIMT | aa | chr10:45860888:D | 0.122 | 0.013 | 0.085 | 0.882 | -0.056 | 0.522 | 0.915 | 0.02 | 0.202 | 0.923 |
| InternalIMT | aa | chr10:45863913:I | 0.179 | -0.073 | 0.091 | 0.417 | -0.48 | 0.368 | 0.192 | 0.21 | 0.144 | 0.145 |
| InternalIMT | aa | chr10:45866601:D | 0.111 | 0.071 | 0.083 | 0.393 | 0.631 | 0.412 | 0.126 | -0.233 | 0.162 | 0.151 |
| InternalIMT | aa | chr10:45867197:I | 0.002 | . | . | . | . | . | . | . | . | . |
| InternalIMT | aa | chr10:45867538:D | 0.182 | -0.064 | 0.091 | 0.482 | -0.427 | 0.358 | 0.233 | 0.186 | 0.14 | 0.183 |
| InternalIMT | aa | chr10:45868356:D | 0.18 | -0.045 | 0.091 | 0.618 | -0.331 | 0.348 | 0.342 | 0.147 | 0.136 | 0.278 |

| InternalIMT | aa | chr10:45871158:I | 0.182 | -0.067 | 0.09 | 0.46 | -0.444 | 0.357 | 0.214 | 0.194 | 0.139 | 0.164 |
| --- | --- | --- | --- | --- | --- | --- | --- | --- | --- | --- | --- | --- |
| InternalIMT | aa | chr10:45871637:D | 0.127 | -0.048 | 0.087 | 0.576 | -0.469 | 0.454 | 0.301 | 0.21 | 0.178 | 0.238 |
| InternalIMT | aa | chr10:45871638:I | 0.164 | -0.079 | 0.088 | 0.369 | -0.579 | 0.401 | 0.149 | 0.256 | 0.157 | 0.103 |
| InternalIMT | aa | chr10:45873629:I | 0.154 | -0.033 | 0.089 | 0.713 | -0.281 | 0.38 | 0.461 | 0.127 | 0.148 | 0.393 |
| InternalIMT | aa | chr10:45873701:D | 0.055 | -0.02 | 0.075 | 0.79 | -1.04 | 0.621 | 0.095 | 0.421 | 0.25 | 0.092 |
| InternalIMT | aa | chr10:45874997:I | 0.011 | 0.012 | 0.074 | 0.868 | -2.21 | 1.32 | 0.095 | 0.768 | 0.517 | 0.138 |
| InternalIMT | aa | chr10:45882030:I | 0.096 | -0.033 | 0.078 | 0.672 | -0.834 | 0.505 | 0.099 | 0.329 | 0.2 | 0.1 |
| InternalIMT | aa | chr10:45883774:D | 0.078 | 0.028 | 0.087 | 0.75 | 0.09 | 0.412 | 0.827 | -0.039 | 0.163 | 0.809 |
| InternalIMT | aa | chr10:45885169:D | 0.075 | -8.80E-03 | 0.075 | 0.906 | -0.553 | 0.617 | 0.37 | 0.232 | 0.241 | 0.335 |
| InternalIMT | aa | chr10:45886451:D | 0.736 | -6.80E-03 | 0.183 | 0.97 | -0.021 | 0.288 | 0.943 | 0.015 | 0.114 | 0.896 |
| InternalIMT | aa | chr10:45891800:I | 0.768 | -0.292 | 0.201 | 0.147 | -0.465 | 0.313 | 0.138 | 0.191 | 0.122 | 0.118 |
| InternalIMT | aa | chr10:45904771:I | 0.772 | -0.071 | 0.193 | 0.712 | -0.109 | 0.302 | 0.719 | 0.054 | 0.118 | 0.649 |
| InternalIMT | aa | chr10:45904959:I | 0.156 | -0.052 | 0.096 | 0.584 | -0.471 | 0.497 | 0.343 | 0.182 | 0.194 | 0.35 |
| InternalIMT | aa | chr10:45911028:I | 0.161 | 0.115 | 0.093 | 0.216 | 0.739 | 0.47 | 0.116 | -0.277 | 0.183 | 0.13 |
| InternalIMT | aa | chr10:45911031:I | 0.259 | 0.089 | 0.112 | 0.425 | 0.333 | 0.401 | 0.407 | -0.145 | 0.156 | 0.352 |
| InternalIMT | aa | chr10:45912154:D | 0.104 | 0.032 | 0.09 | 0.722 | 0.099 | 0.402 | 0.805 | -0.045 | 0.159 | 0.779 |
| InternalIMT | aa | chr10:45913885:I | 0.003 | . | . | . | . | . | . | . | . | . |
| InternalIMT | aa | chr10:45914753:D | 0.351 | 0.021 | 0.117 | 0.857 | 0.074 | 0.349 | 0.833 | -7.80E-03 | 0.137 | 0.955 |
| InternalIMT | aa | chr10:45919621 | . | . | . | . | . | . | . | . | . | . |
| InternalIMT | aa | chr10:45921739:I | 0.178 | -0.037 | 0.084 | 0.655 | -0.341 | 0.374 | 0.362 | 0.144 | 0.146 | 0.322 |
| InternalIMT | aa | chr10:45923527:I | 0.066 | -0.045 | 0.083 | 0.591 | -0.979 | 0.483 | 0.043 | 0.353 | 0.196 | 0.072 |
| InternalIMT | aa | chr10:45927001:I | 0.04 | 0.032 | 0.076 | 0.67 | 0.859 | 0.92 | 0.351 | -0.319 | 0.36 | 0.374 |
| InternalIMT | aa | chr10:45930354:I | 0.025 | 0.011 | 0.074 | 0.885 | -0.141 | 1.18 | 0.905 | 0.094 | 0.464 | 0.839 |
| InternalIMT | aa | chr10:45932864:I | 0.043 | 0.017 | 0.077 | 0.828 | 0.081 | 1.11 | 0.941 | 0.045 | 0.438 | 0.917 |
| InternalIMT | aa | chr10:45936163:I | 0.038 | 3.80E-03 | 0.076 | 0.96 | -0.41 | 0.831 | 0.622 | 0.155 | 0.331 | 0.64 |
| InternalIMT | aa | chr10:45938095:D | 0.179 | -0.043 | 0.079 | 0.586 | -0.453 | 0.394 | 0.25 | 0.199 | 0.155 | 0.197 |
| InternalIMT | aa | chr10:45938886 | . | . | . | . | . | . | . | . | . | . |
| InternalIMT | aa | chr10:45939320 | 0.001 | . | . | . | . | . | . | . | . | . |
| InternalIMT | aa | chr10:45939659 | 0.003 | . | . | . | . | . | . | . | . | . |
| InternalIMT | aa | chr10:45941150:D | 0.008 | . | . | . | . | . | . | . | . | . |
| InternalIMT | aa | chr10:45947128:D | 0.067 | 0.024 | 0.077 | 0.754 | 0.185 | 0.59 | 0.754 | -0.079 | 0.227 | 0.726 |
| InternalIMT | aa | chr10:45950990:I | 0.039 | -0.018 | 0.078 | 0.814 | -1.2 | 0.886 | 0.174 | 0.503 | 0.355 | 0.157 |
| InternalIMT | aa | chr10:45951854:I | 0.012 | -1.00E-03 | 0.077 | 0.99 | -1.75 | 1.52 | 0.252 | 0.646 | 0.599 | 0.281 |
| InternalIMT | aa | chr10:45952670:I | 0.061 | 0.032 | 0.078 | 0.685 | 0.371 | 0.592 | 0.531 | -0.166 | 0.231 | 0.474 |
| InternalIMT | aa | chr10:45956741 | . | . | . | . | . | . | . | . | . | . |
| InternalIMT | aa | chr10:45957247:I | 0.198 | -0.029 | 0.08 | 0.72 | -0.314 | 0.374 | 0.4 | 0.14 | 0.146 | 0.34 |
| InternalIMT | aa | chr10:45958082:I | 0.188 | -0.039 | 0.079 | 0.623 | -0.425 | 0.399 | 0.287 | 0.184 | 0.156 | 0.239 |
| InternalIMT | aa | chr10:45905041:D | 0.068 | -0.02 | 0.075 | 0.791 | -0.768 | 0.701 | 0.274 | 0.343 | 0.273 | 0.21 |
| InternalIMT | aa | chr10:45938871 | . | . | . | . | . | . | . | . | . | . |
| InternalIMT | aa | chr10:45939160 | . | . | . | . | . | . | . | . | . | . |
| InternalIMT | aa | chr10:45869910 | 0.015 | 0.025 | 0.075 | 0.738 | 0.835 | 1.2 | 0.487 | -0.341 | 0.472 | 0.47 |
| InternalIMT | aa | chr10:45876214:D | 0.017 | 0.012 | 0.075 | 0.875 | -0.439 | 0.894 | 0.623 | 0.153 | 0.357 | 0.668 |
| InternalIMT | aa | chr10:45880237:I | 0.094 | 0.095 | 0.082 | 0.247 | 1.1 | 0.434 | 0.011 | -0.406 | 0.167 | 0.015 |
| InternalIMT | aa | chr10:45884371:I | 0.073 | -0.022 | 0.076 | 0.778 | -0.788 | 0.516 | 0.127 | 0.317 | 0.207 | 0.125 |
| InternalIMT | aa | chr10:45891535:D | 0.019 | -6.30E-03 | 0.074 | 0.932 | -1.94 | 1.1 | 0.078 | 0.815 | 0.439 | 0.064 |
| InternalIMT | aa | chr10:45893516:D | 0.073 | -0.021 | 0.076 | 0.782 | -0.779 | 0.515 | 0.13 | 0.314 | 0.206 | 0.128 |
| InternalIMT | aa | chr10:45893782:D | 0.018 | 0.022 | 0.075 | 0.773 | 1.31 | 1.68 | 0.437 | -0.46 | 0.647 | 0.477 |
| InternalIMT | aa | chr10:45895254:I | 0.019 | -6.40E-03 | 0.074 | 0.931 | -1.94 | 1.1 | 0.077 | 0.817 | 0.44 | 0.063 |
| InternalIMT | aa | chr10:45895848:D | 0.057 | -0.017 | 0.076 | 0.826 | -0.913 | 0.631 | 0.148 | 0.361 | 0.253 | 0.154 |
| InternalIMT | aa | chr10:45896316:D | 0.073 | -0.021 | 0.076 | 0.783 | -0.778 | 0.515 | 0.131 | 0.313 | 0.206 | 0.129 |
| InternalIMT | aa | chr10:45896317:D | 0.073 | -0.021 | 0.076 | 0.783 | -0.778 | 0.515 | 0.131 | 0.313 | 0.206 | 0.129 |
| InternalIMT | aa | chr10:45902683:I | 0.019 | 0.013 | 0.075 | 0.858 | -0.296 | 0.858 | 0.73 | 0.1 | 0.341 | 0.769 |
| InternalIMT | aa | chr10:45905003:D | 0.01 | . | . | . | . | . | . | . | . | . |
| InternalIMT | aa | chr10:45927476:D | 0.036 | 0.054 | 0.078 | 0.492 | 0.706 | 0.423 | 0.095 | -0.282 | 0.166 | 0.09 |
| InternalIMT | aa | chr10:45935882 | 0.001 | . | . | . | . | . | . | . | . | . |
| InternalIMT | aa | chr10:45936100 | 0.001 | . | . | . | . | . | . | . | . | . |
| InternalIMT | aa | chr10:45939553 | 0.001 | . | . | . | . | . | . | . | . | . |
| InternalIMT | aa | chr10:45939631 | 0 | . | . | . | . | . | . | . | . | . |
| InternalIMT | aa | chr10:45944193:D | 0.012 | 6.00E-03 | 0.074 | 0.935 | -1.38 | 1.12 | 0.216 | 0.548 | 0.439 | 0.212 |
| InternalIMT | aa | chr10:45954030 | 0 | . | . | . | . | . | . | . | . | . |

Supplemental table S29.

| phen | covar(fa) | rs_id | maf | beta_FA | se_FA | p_FA | beta_SNP | se_SNP | p_SNP | beta_inter | se_inter | p_inter |
| --- | --- | --- | --- | --- | --- | --- | --- | --- | --- | --- | --- | --- |
| InternalIMT | aa | rs76035182 | 0.016 | 0.051 | 0.071 | 0.474 | 2.02 | 0.952 | 0.034 | -0.801 | 0.358 | 0.026 |
| InternalIMT | aa | rs12359547 | 0.066 | -0.011 | 0.076 | 0.889 | -0.776 | 0.35 | 0.027 | 0.296 | 0.148 | 0.046 |
| InternalIMT | aa | rs74595484 | 0.017 | 0.027 | 0.072 | 0.703 | -0.08 | 1.12 | 0.943 | 0.04 | 0.438 | 0.927 |
| InternalIMT | aa | rs1864415 | 0.722 | -0.11 | 0.163 | 0.5 | -0.237 | 0.25 | 0.342 | 0.095 | 0.103 | 0.36 |
| InternalIMT | aa | rs72788101 | 0.069 | -4.20E-03 | 0.076 | 0.957 | -0.673 | 0.336 | 0.045 | 0.249 | 0.141 | 0.079 |
| InternalIMT | aa | rs11239481 | 0.129 | 0.092 | 0.08 | 0.251 | 0.515 | 0.324 | 0.113 | -0.228 | 0.137 | 0.096 |
| InternalIMT | aa | rs72788102 | 0.069 | -4.20E-03 | 0.076 | 0.957 | -0.673 | 0.336 | 0.045 | 0.249 | 0.141 | 0.079 |
| InternalIMT | aa | rs72789705 | 0.069 | -4.10E-03 | 0.076 | 0.957 | -0.672 | 0.336 | 0.046 | 0.249 | 0.141 | 0.079 |
| InternalIMT | aa | rs141529858 | 0.005 | . | . | . | . | . | . | . | . | . |
| InternalIMT | aa | rs145376292 | 0 | . | . | . | . | . | . | . | . | . |
| InternalIMT | aa | rs190418301 | 0.001 | . | . | . | . | . | . | . | . | . |
| InternalIMT | aa | rs11239482 | 0.069 | -4.10E-03 | 0.076 | 0.957 | -0.672 | 0.336 | 0.046 | 0.249 | 0.141 | 0.079 |
| InternalIMT | aa | rs61855872 | 0.011 | 0.012 | 0.07 | 0.861 | -1.45 | 1.58 | 0.359 | 0.736 | 0.682 | 0.281 |
| InternalIMT | aa | rs148991144 | 0.005 | . | . | . | . | . | . | . | . | . |
| InternalIMT | aa | rs191932221 | 0.005 | . | . | . | . | . | . | . | . | . |
| InternalIMT | aa | rs55978138 | 0.144 | 0.03 | 0.083 | 0.723 | -0.118 | 0.272 | 0.664 | 0.022 | 0.113 | 0.849 |
| InternalIMT | aa | rs73281167 | 0.01 | . | . | . | . | . | . | . | . | . |
| InternalIMT | aa | rs12768010 | 0.072 | 0.051 | 0.075 | 0.496 | 0.289 | 0.365 | 0.429 | -0.135 | 0.15 | 0.369 |
| InternalIMT | aa | rs11239484 | 0.722 | -0.11 | 0.163 | 0.499 | -0.237 | 0.249 | 0.342 | 0.095 | 0.103 | 0.359 |
| InternalIMT | aa | rs35906360 | 0.144 | 0.024 | 0.082 | 0.775 | -0.155 | 0.267 | 0.561 | 0.039 | 0.111 | 0.724 |
| InternalIMT | aa | rs186250512 | 0.004 | . | . | . | . | . | . | . | . | . |
| InternalIMT | aa | rs150810743 | 0.001 | . | . | . | . | . | . | . | . | . |
| InternalIMT | aa | rs55965812 | 0.14 | 0.017 | 0.082 | 0.833 | -0.19 | 0.276 | 0.491 | 0.055 | 0.115 | 0.633 |
| InternalIMT | aa | rs56180358 | 0.144 | 0.024 | 0.082 | 0.774 | -0.155 | 0.267 | 0.562 | 0.039 | 0.111 | 0.726 |
| InternalIMT | aa | rs55864145 | 0.073 | 0.051 | 0.075 | 0.496 | 0.289 | 0.365 | 0.428 | -0.135 | 0.15 | 0.369 |
| InternalIMT | aa | rs55986122 | 0.144 | 0.024 | 0.082 | 0.774 | -0.155 | 0.267 | 0.562 | 0.039 | 0.111 | 0.726 |
| InternalIMT | aa | rs7477350 | 0.722 | -0.11 | 0.163 | 0.499 | -0.237 | 0.249 | 0.342 | 0.095 | 0.103 | 0.358 |
| InternalIMT | aa | rs2099170 | 0.143 | 0.024 | 0.082 | 0.772 | -0.153 | 0.268 | 0.568 | 0.038 | 0.111 | 0.73 |
| InternalIMT | aa | rs2082108 | 0.144 | 0.024 | 0.082 | 0.775 | -0.155 | 0.267 | 0.562 | 0.039 | 0.111 | 0.725 |
| InternalIMT | aa | rs55902404 | 0.004 | . | . | . | . | . | . | . | . | . |
| InternalIMT | aa | rs58402662 | 0.004 | . | . | . | . | . | . | . | . | . |
| InternalIMT | aa | rs11511618 | 0.129 | 0.088 | 0.08 | 0.272 | 0.479 | 0.324 | 0.14 | -0.212 | 0.137 | 0.121 |
| InternalIMT | aa | rs1816492 | 0.721 | -0.098 | 0.163 | 0.546 | -0.217 | 0.25 | 0.385 | 0.087 | 0.103 | 0.4 |
| InternalIMT | aa | rs146611093 | 0.006 | . | . | . | . | . | . | . | . | . |
| InternalIMT | aa | rs186897689 | 0.001 | . | . | . | . | . | . | . | . | . |
| InternalIMT | aa | rs10793616 | 0.722 | -0.11 | 0.163 | 0.499 | -0.237 | 0.249 | 0.342 | 0.095 | 0.103 | 0.359 |
| InternalIMT | aa | rs116700479 | 0.013 | 0.05 | 0.071 | 0.486 | 2.33 | 1.21 | 0.054 | -0.914 | 0.451 | 0.043 |
| InternalIMT | aa | rs192535936 | . | . | . | . | . | . | . | . | . | . |
| InternalIMT | aa | rs113363297 | 0.653 | -0.073 | 0.156 | 0.638 | -0.187 | 0.261 | 0.474 | 0.078 | 0.108 | 0.47 |
| InternalIMT | aa | rs12354994 | 0.137 | 0.029 | 0.082 | 0.727 | -0.114 | 0.276 | 0.681 | 0.023 | 0.115 | 0.841 |
| InternalIMT | aa | rs148068329 | 0.025 | 0.014 | 0.071 | 0.843 | -1 | 0.801 | 0.21 | 0.397 | 0.346 | 0.251 |
| InternalIMT | aa | rs12356109 | 0.149 | 0.026 | 0.084 | 0.751 | -0.146 | 0.262 | 0.578 | 0.032 | 0.109 | 0.77 |
| InternalIMT | aa | rs55908141 | 0.004 | . | . | . | . | . | . | . | . | . |
| InternalIMT | aa | rs12354712 | 0.14 | 0.017 | 0.082 | 0.836 | -0.191 | 0.274 | 0.486 | 0.055 | 0.114 | 0.631 |
| InternalIMT | aa | rs147282684 | 0.003 | . | . | . | . | . | . | . | . | . |
| InternalIMT | aa | rs12356140 | 0.145 | 0.021 | 0.083 | 0.796 | -0.175 | 0.265 | 0.51 | 0.046 | 0.11 | 0.676 |
| InternalIMT | aa | rs185476187 | 0.001 | . | . | . | . | . | . | . | . | . |
| InternalIMT | aa | rs145720057 | 0.018 | 4.50E-03 | 0.071 | 0.949 | -1.58 | 0.914 | 0.083 | 0.705 | 0.391 | 0.072 |
| InternalIMT | aa | rs12354742 | 0.144 | 0.024 | 0.082 | 0.775 | -0.155 | 0.265 | 0.558 | 0.039 | 0.11 | 0.726 |
| InternalIMT | aa | rs180913842 | 0.004 | . | . | . | . | . | . | . | . | . |
| InternalIMT | aa | rs142976914 | 0.002 | . | . | . | . | . | . | . | . | . |
| InternalIMT | aa | rs11511619 | 0.151 | 0.027 | 0.084 | 0.745 | -0.135 | 0.262 | 0.607 | 0.028 | 0.109 | 0.799 |
| InternalIMT | aa | rs146702870 | 0.002 | . | . | . | . | . | . | . | . | . |
| InternalIMT | aa | rs150287496 | . | . | . | . | . | . | . | . | . | . |
| InternalIMT | aa | rs190917973 | 0.004 | . | . | . | . | . | . | . | . | . |
| InternalIMT | aa | rs11517281 | 0.144 | 0.024 | 0.082 | 0.774 | -0.154 | 0.264 | 0.56 | 0.038 | 0.11 | 0.729 |
| InternalIMT | aa | rs12359299 | 0.144 | 0.024 | 0.082 | 0.774 | -0.154 | 0.264 | 0.56 | 0.038 | 0.11 | 0.728 |
| InternalIMT | aa | rs182307932 | 0.014 | 0.023 | 0.071 | 0.743 | -0.789 | 0.738 | 0.285 | 0.282 | 0.304 | 0.353 |
| InternalIMT | aa | rs61855873 | 0.026 | 0.024 | 0.073 | 0.741 | -0.209 | 1.18 | 0.86 | 0.158 | 0.499 | 0.752 |
| InternalIMT | aa | rs12359302 | 0.143 | 0.024 | 0.082 | 0.766 | -0.145 | 0.266 | 0.587 | 0.036 | 0.111 | 0.743 |
| InternalIMT | aa | rs143208140 | 0.027 | 0.02 | 0.071 | 0.781 | -0.586 | 0.843 | 0.487 | 0.23 | 0.361 | 0.524 |
| InternalIMT | aa | rs9988764 | 0.708 | -0.143 | 0.163 | 0.379 | -0.303 | 0.25 | 0.225 | 0.119 | 0.103 | 0.25 |
| InternalIMT | aa | rs192875767 | 0.003 | . | . | . | . | . | . | . | . | . |
| InternalIMT | aa | rs147268846 | 0.014 | 3.50E-03 | 0.073 | 0.961 | -2.25 | 1.86 | 0.227 | 0.979 | 0.8 | 0.221 |
| InternalIMT | aa | rs11239485 | 0.094 | 1.20E-03 | 0.08 | 0.988 | -0.458 | 0.371 | 0.217 | 0.16 | 0.157 | 0.307 |
| InternalIMT | aa | rs151317885 | 0.476 | 5.50E-03 | 0.102 | 0.957 | -0.045 | 0.234 | 0.848 | 0.037 | 0.096 | 0.698 |
| InternalIMT | aa | rs11499085 | 0.055 | 0.015 | 0.075 | 0.845 | -0.381 | 0.452 | 0.399 | 0.142 | 0.179 | 0.428 |
| InternalIMT | aa | rs188130606 | 0.012 | 0.018 | 0.072 | 0.801 | -1.01 | 0.602 | 0.093 | 0.387 | 0.25 | 0.121 |
| InternalIMT | aa | rs11511620 | 0.141 | 0.017 | 0.082 | 0.834 | -0.189 | 0.273 | 0.489 | 0.054 | 0.114 | 0.634 |
| InternalIMT | aa | rs181498390 | 0.012 | 0.028 | 0.071 | 0.697 | 0.279 | 0.923 | 0.762 | -0.071 | 0.365 | 0.845 |
| InternalIMT | aa | rs186280701 | 0.004 | . | . | . | . | . | . | . | . | . |
| InternalIMT | aa | rs150059185 | 0.02 | 0.08 | 0.074 | 0.28 | 2.93 | 1.65 | 0.076 | -1.17 | 0.642 | 0.068 |
| InternalIMT | aa | rs183338707 | . | . | . | . | . | . | . | . | . | . |
| InternalIMT | aa | rs11499038 | 0.052 | 0.013 | 0.075 | 0.864 | -0.393 | 0.513 | 0.444 | 0.154 | 0.205 | 0.452 |
| InternalIMT | aa | rs190699595 | 0.004 | . | . | . | . | . | . | . | . | . |
| InternalIMT | aa | rs11523408 | 0.143 | 0.021 | 0.082 | 0.799 | -0.168 | 0.266 | 0.527 | 0.045 | 0.111 | 0.682 |
| InternalIMT | aa | rs188049317 | 0.001 | . | . | . | . | . | . | . | . | . |
| InternalIMT | aa | rs144948634 | 0.435 | 0.015 | 0.098 | 0.874 | -0.015 | 0.232 | 0.949 | 0.029 | 0.095 | 0.762 |
| InternalIMT | aa | rs143833576 | 0.001 | . | . | . | . | . | . | . | . | . |
| InternalIMT | aa | rs151239879 | 0.144 | 0.021 | 0.082 | 0.798 | -0.168 | 0.266 | 0.528 | 0.045 | 0.111 | 0.685 |
| InternalIMT | aa | rs7073743 | 0.455 | 0.02 | 0.1 | 0.84 | -7.20E-03 | 0.233 | 0.975 | 0.023 | 0.095 | 0.811 |
| InternalIMT | aa | rs142563349 | 0.013 | 0.018 | 0.072 | 0.806 | -1.07 | 0.591 | 0.07 | 0.405 | 0.246 | 0.1 |
| InternalIMT | aa | rs187246403 | 0.004 | . | . | . | . | . | . | . | . | . |
| InternalIMT | aa | rs150423942 | 0.134 | 0.027 | 0.082 | 0.739 | -0.153 | 0.276 | 0.58 | 0.036 | 0.114 | 0.75 |

| InternalIMT | aa | rs184706102 | . | . | . | . | . | . | . | . | . | . |
| --- | --- | --- | --- | --- | --- | --- | --- | --- | --- | --- | --- | --- |
| InternalIMT | aa | rs189626288 | 0.014 | 3.40E-03 | 0.07 | 0.962 | -1.36 | 0.75 | 0.071 | 0.576 | 0.319 | 0.071 |
| InternalIMT | aa | rs140749834 | 0.027 | 0.026 | 0.072 | 0.717 | -0.07 | 0.61 | 0.908 | 0.038 | 0.245 | 0.876 |
| InternalIMT | aa | rs7078822 | 0.115 | 0.015 | 0.081 | 0.857 | -0.24 | 0.304 | 0.429 | 0.078 | 0.128 | 0.544 |
| InternalIMT | aa | rs186227617 | 0.143 | 0.023 | 0.082 | 0.775 | -0.154 | 0.266 | 0.564 | 0.038 | 0.111 | 0.73 |
| InternalIMT | aa | rs188984368 | 0.022 | 0.029 | 0.072 | 0.684 | 6.60E-03 | 1 | 0.995 | -0.018 | 0.418 | 0.966 |
| InternalIMT | aa | rs182078109 | 0.027 | 0.048 | 0.073 | 0.506 | 0.791 | 1.05 | 0.451 | -0.341 | 0.433 | 0.431 |
| InternalIMT | aa | rs141456274 | 0.006 | . | . | . | . | . | . | . | . | . |
| InternalIMT | aa | rs146976512 | 0.144 | 0.024 | 0.082 | 0.767 | -0.147 | 0.264 | 0.578 | 0.035 | 0.11 | 0.75 |
| InternalIMT | aa | rs138036816 | 0.05 | 0.012 | 0.075 | 0.87 | -0.411 | 0.514 | 0.424 | 0.163 | 0.206 | 0.429 |
| InternalIMT | aa | rs114879777 | 0.013 | 0.047 | 0.071 | 0.512 | 1.81 | 1.08 | 0.094 | -0.717 | 0.405 | 0.077 |
| InternalIMT | aa | rs149366475 | 0.052 | 0.011 | 0.075 | 0.88 | -0.435 | 0.526 | 0.409 | 0.171 | 0.211 | 0.417 |
| InternalIMT | aa | rs182463043 | 0.001 | . | . | . | . | . | . | . | . | . |
| InternalIMT | aa | rs144754632 | 0.025 | 0.014 | 0.071 | 0.842 | -0.997 | 0.802 | 0.214 | 0.395 | 0.347 | 0.255 |
| InternalIMT | aa | rs55717607 | 0.145 | 0.024 | 0.082 | 0.77 | -0.151 | 0.263 | 0.566 | 0.037 | 0.109 | 0.736 |
| InternalIMT | aa | rs187537973 | . | . | . | . | . | . | . | . | . | . |
| InternalIMT | aa | rs148085971 | 0.001 | . | . | . | . | . | . | . | . | . |
| InternalIMT | aa | rs11239491 | 0.145 | 0.024 | 0.082 | 0.77 | -0.151 | 0.263 | 0.567 | 0.037 | 0.109 | 0.737 |
| InternalIMT | aa | rs184412184 | 0.002 | . | . | . | . | . | . | . | . | . |
| InternalIMT | aa | rs4948998 | 0.733 | -0.121 | 0.167 | 0.467 | -0.246 | 0.249 | 0.323 | 0.101 | 0.103 | 0.326 |
| InternalIMT | aa | rs7076696 | 0.733 | -0.121 | 0.167 | 0.47 | -0.245 | 0.249 | 0.325 | 0.101 | 0.103 | 0.329 |
| InternalIMT | aa | rs11239492 | 0.15 | 0.027 | 0.084 | 0.749 | -0.144 | 0.26 | 0.581 | 0.031 | 0.108 | 0.774 |
| InternalIMT | aa | rs79462470 | 0.004 | . | . | . | . | . | . | . | . | . |
| InternalIMT | aa | rs187804654 | 0.004 | . | . | . | . | . | . | . | . | . |
| InternalIMT | aa | rs7093687 | 0.141 | 0.017 | 0.082 | 0.832 | -0.187 | 0.271 | 0.491 | 0.054 | 0.113 | 0.636 |
| InternalIMT | aa | rs7081004 | 0.141 | 0.017 | 0.082 | 0.832 | -0.187 | 0.272 | 0.492 | 0.053 | 0.113 | 0.638 |
| InternalIMT | aa | rs7084793 | 0.145 | 0.024 | 0.082 | 0.769 | -0.15 | 0.263 | 0.569 | 0.036 | 0.109 | 0.74 |
| InternalIMT | aa | rs61556080 | 0.004 | . | . | . | . | . | . | . | . | . |
| InternalIMT | aa | rs115552064 | 0.004 | . | . | . | . | . | . | . | . | . |
| InternalIMT | aa | rs7921503 | 0.145 | 0.024 | 0.082 | 0.769 | -0.15 | 0.264 | 0.57 | 0.036 | 0.11 | 0.741 |
| InternalIMT | aa | rs7909740 | 0.145 | 0.024 | 0.082 | 0.767 | -0.149 | 0.263 | 0.571 | 0.036 | 0.109 | 0.742 |
| InternalIMT | aa | rs7909768 | 0.145 | 0.024 | 0.082 | 0.767 | -0.149 | 0.263 | 0.571 | 0.036 | 0.109 | 0.742 |
| InternalIMT | aa | rs144168004 | . | . | . | . | . | . | . | . | . | . |
| InternalIMT | aa | rs75850448 | 0.004 | . | . | . | . | . | . | . | . | . |
| InternalIMT | aa | rs147777574 | 0.003 | . | . | . | . | . | . | . | . | . |
| InternalIMT | aa | rs7913488 | 0.145 | 0.024 | 0.082 | 0.767 | -0.149 | 0.263 | 0.571 | 0.036 | 0.109 | 0.743 |
| InternalIMT | aa | rs74390874 | 0.042 | 0.031 | 0.07 | 0.655 | 0.216 | 0.802 | 0.788 | -0.036 | 0.323 | 0.912 |
| InternalIMT | aa | rs141391142 | 0.004 | . | . | . | . | . | . | . | . | . |
| InternalIMT | aa | rs115294747 | 0.006 | . | . | . | . | . | . | . | . | . |
| InternalIMT | aa | rs148031781 | 0.001 | . | . | . | . | . | . | . | . | . |
| InternalIMT | aa | rs181340128 | 0.025 | 0.015 | 0.073 | 0.839 | -0.727 | 1.16 | 0.532 | 0.341 | 0.513 | 0.507 |
| InternalIMT | aa | rs189930007 | 0.001 | . | . | . | . | . | . | . | . | . |
| InternalIMT | aa | rs12242128 | 0.049 | 0.011 | 0.075 | 0.887 | -0.446 | 0.526 | 0.397 | 0.179 | 0.211 | 0.397 |
| InternalIMT | aa | rs78421750 | 0.003 | . | . | . | . | . | . | . | . | . |
| InternalIMT | aa | rs114370747 | 0.002 | . | . | . | . | . | . | . | . | . |
| InternalIMT | aa | rs12264166 | 0.049 | 0.01 | 0.075 | 0.889 | -0.449 | 0.526 | 0.393 | 0.18 | 0.211 | 0.393 |
| InternalIMT | aa | rs182964822 | 0.001 | . | . | . | . | . | . | . | . | . |
| InternalIMT | aa | rs67491417 | 0.139 | 0.028 | 0.082 | 0.735 | -0.117 | 0.271 | 0.666 | 0.025 | 0.113 | 0.825 |
| InternalIMT | aa | rs72789720 | 0.008 | . | . | . | . | . | . | . | . | . |
| InternalIMT | aa | rs6413416 | 0.145 | 0.025 | 0.082 | 0.759 | -0.143 | 0.262 | 0.585 | 0.033 | 0.109 | 0.76 |
| InternalIMT | aa | rs4986832 | 0.145 | 0.025 | 0.082 | 0.762 | -0.146 | 0.262 | 0.577 | 0.034 | 0.109 | 0.751 |
| InternalIMT | aa | rs144866316 | 0.004 | . | . | . | . | . | . | . | . | . |
| InternalIMT | aa | rs187769540 | 0.007 | . | . | . | . | . | . | . | . | . |
| InternalIMT | aa | rs6593482 | 0.146 | 0.032 | 0.082 | 0.702 | -0.091 | 0.262 | 0.728 | 0.011 | 0.109 | 0.917 |
| InternalIMT | aa | rs7913948 | 0.146 | 0.026 | 0.082 | 0.754 | -0.128 | 0.262 | 0.625 | 0.029 | 0.109 | 0.787 |
| InternalIMT | aa | rs77185151 | 0.025 | 0.015 | 0.071 | 0.836 | -0.974 | 0.805 | 0.226 | 0.385 | 0.348 | 0.268 |
| InternalIMT | aa | rs12762303 | 0.145 | 0.025 | 0.082 | 0.762 | -0.146 | 0.262 | 0.576 | 0.035 | 0.109 | 0.751 |
| InternalIMT | aa | rs73283136 | 0.003 | . | . | . | . | . | . | . | . | . |
| InternalIMT | aa | rs187180218 | 0.001 | . | . | . | . | . | . | . | . | . |
| InternalIMT | aa | rs78137087 | 0.145 | 0.026 | 0.082 | 0.755 | -0.141 | 0.261 | 0.59 | 0.032 | 0.109 | 0.769 |
| InternalIMT | aa | rs72561472 | 0.146 | 0.024 | 0.083 | 0.773 | -0.158 | 0.268 | 0.555 | 0.04 | 0.112 | 0.721 |
| InternalIMT | aa | rs4987106 | 0.142 | 0.029 | 0.082 | 0.723 | -0.113 | 0.267 | 0.672 | 0.02 | 0.111 | 0.858 |
| InternalIMT | aa | rs4987105 | 0.139 | 0.023 | 0.082 | 0.782 | -0.145 | 0.272 | 0.596 | 0.035 | 0.114 | 0.759 |
| InternalIMT | aa | rs111916157 | 0.158 | 3.80E-03 | 0.084 | 0.964 | -0.272 | 0.279 | 0.331 | 0.09 | 0.116 | 0.437 |
| InternalIMT | aa | rs111528152 | 0.162 | 0.021 | 0.084 | 0.801 | -0.155 | 0.264 | 0.556 | 0.036 | 0.11 | 0.743 |
| InternalIMT | aa | rs12763738 | 0.145 | 0.024 | 0.082 | 0.767 | -0.15 | 0.263 | 0.569 | 0.036 | 0.109 | 0.743 |
| InternalIMT | aa | rs191675886 | 0.004 | . | . | . | . | . | . | . | . | . |
| InternalIMT | aa | rs66489870 | 0.145 | 0.025 | 0.082 | 0.763 | -0.147 | 0.262 | 0.575 | 0.035 | 0.109 | 0.75 |
| InternalIMT | aa | rs71494775 | 0.145 | 0.025 | 0.082 | 0.763 | -0.147 | 0.262 | 0.574 | 0.035 | 0.109 | 0.749 |
| InternalIMT | aa | rs61855875 | 0.024 | 0.013 | 0.072 | 0.858 | -0.812 | 1.17 | 0.488 | 0.396 | 0.495 | 0.424 |
| InternalIMT | aa | rs116356837 | 0.004 | . | . | . | . | . | . | . | . | . |
| InternalIMT | aa | rs1864414 | 0.145 | 0.022 | 0.082 | 0.794 | -0.168 | 0.262 | 0.523 | 0.044 | 0.109 | 0.686 |
| InternalIMT | aa | rs1864413 | 0.145 | 0.025 | 0.082 | 0.764 | -0.149 | 0.262 | 0.568 | 0.035 | 0.109 | 0.745 |
| InternalIMT | aa | rs139497212 | 0 | . | . | . | . | . | . | . | . | . |
| InternalIMT | aa | rs892690 | 0.424 | 0.035 | 0.101 | 0.728 | 0.014 | 0.246 | 0.956 | 9.70E-03 | 0.1 | 0.923 |
| InternalIMT | aa | rs1864412 | 0.145 | 0.025 | 0.082 | 0.764 | -0.149 | 0.262 | 0.57 | 0.035 | 0.109 | 0.745 |
| InternalIMT | aa | rs77827771 | 0.016 | 0.028 | 0.072 | 0.703 | -0.077 | 0.852 | 0.928 | 0.039 | 0.342 | 0.909 |
| InternalIMT | aa | rs145294963 | . | . | . | . | . | . | . | . | . | . |
| InternalIMT | aa | rs149081327 | 0.008 | . | . | . | . | . | . | . | . | . |
| InternalIMT | aa | rs67008983 | 0.145 | 0.024 | 0.082 | 0.769 | -0.154 | 0.262 | 0.555 | 0.037 | 0.109 | 0.73 |
| InternalIMT | aa | rs56150414 | 0.147 | 0.028 | 0.083 | 0.735 | -0.12 | 0.262 | 0.648 | 0.024 | 0.109 | 0.829 |
| InternalIMT | aa | rs55787481 | 0.145 | 0.022 | 0.083 | 0.788 | -0.17 | 0.263 | 0.519 | 0.044 | 0.11 | 0.686 |
| InternalIMT | aa | rs141270101 | 0.001 | . | . | . | . | . | . | . | . | . |
| InternalIMT | aa | rs114980075 | 0.003 | . | . | . | . | . | . | . | . | . |
| InternalIMT | aa | rs12783095 | 0.144 | 0.026 | 0.082 | 0.753 | -0.152 | 0.261 | 0.559 | 0.033 | 0.108 | 0.761 |
| InternalIMT | aa | rs182342688 | . | . | . | . | . | . | . | . | . | . |

| InternalIMT | aa | rs17157728 | 0.144 | 0.026 | 0.082 | 0.753 | -0.153 | 0.261 | 0.558 | 0.033 | 0.108 | 0.759 |
| --- | --- | --- | --- | --- | --- | --- | --- | --- | --- | --- | --- | --- |
| InternalIMT | aa | rs114835563 | 0.012 | 0.018 | 0.071 | 0.803 | -1.04 | 0.609 | 0.089 | 0.396 | 0.254 | 0.12 |
| InternalIMT | aa | rs3824612 | 0.424 | 0.036 | 0.101 | 0.724 | 0.015 | 0.246 | 0.952 | 9.40E-03 | 0.1 | 0.926 |
| InternalIMT | aa | rs17157733 | 0.144 | 0.025 | 0.082 | 0.758 | -0.158 | 0.261 | 0.544 | 0.035 | 0.109 | 0.746 |
| InternalIMT | aa | rs74128478 | 0.004 | . | . | . | . | . | . | . | . | . |
| InternalIMT | aa | rs184973589 | 0.002 | . | . | . | . | . | . | . | . | . |
| InternalIMT | aa | rs150479461 | 0 | . | . | . | . | . | . | . | . | . |
| InternalIMT | aa | rs138310407 | 0.001 | . | . | . | . | . | . | . | . | . |
| InternalIMT | aa | rs187426603 | . | . | . | . | . | . | . | . | . | . |
| InternalIMT | aa | rs190843143 | 0.002 | . | . | . | . | . | . | . | . | . |
| InternalIMT | aa | rs191883449 | 0.001 | . | . | . | . | . | . | . | . | . |
| InternalIMT | aa | rs58238740 | 0.143 | 0.025 | 0.082 | 0.761 | -0.161 | 0.262 | 0.539 | 0.036 | 0.109 | 0.742 |
| InternalIMT | aa | rs3780894 | 0.143 | 0.025 | 0.082 | 0.761 | -0.162 | 0.262 | 0.537 | 0.036 | 0.109 | 0.74 |
| InternalIMT | aa | rs149646626 | 0.018 | 0.056 | 0.072 | 0.435 | 1.41 | 0.889 | 0.112 | -0.624 | 0.363 | 0.086 |
| InternalIMT | aa | rs79615212 | 0.015 | 0.068 | 0.072 | 0.351 | 2.2 | 0.958 | 0.022 | -0.937 | 0.399 | 0.019 |
| InternalIMT | aa | rs3780895 | 0.143 | 0.025 | 0.082 | 0.761 | -0.162 | 0.262 | 0.536 | 0.036 | 0.109 | 0.74 |
| InternalIMT | aa | rs3780896 | 0.143 | 0.025 | 0.082 | 0.761 | -0.162 | 0.262 | 0.536 | 0.036 | 0.109 | 0.739 |
| InternalIMT | aa | rs142747163 | 0.005 | . | . | . | . | . | . | . | . | . |
| InternalIMT | aa | rs189417584 | 0.005 | . | . | . | . | . | . | . | . | . |
| InternalIMT | aa | rs3780897 | 0.141 | 0.023 | 0.082 | 0.775 | -0.169 | 0.262 | 0.518 | 0.04 | 0.109 | 0.714 |
| InternalIMT | aa | rs117509491 | 0.025 | 0.019 | 0.072 | 0.796 | -0.519 | 0.766 | 0.499 | 0.303 | 0.328 | 0.355 |
| InternalIMT | aa | rs117811514 | 0.025 | 0.019 | 0.072 | 0.796 | -0.519 | 0.766 | 0.499 | 0.303 | 0.328 | 0.355 |
| InternalIMT | aa | rs116500932 | 0.012 | 0.018 | 0.071 | 0.803 | -1.04 | 0.609 | 0.089 | 0.395 | 0.254 | 0.12 |
| InternalIMT | aa | rs77584168 | . | . | . | . | . | . | . | . | . | . |
| InternalIMT | aa | rs2310776 | 0.143 | 0.025 | 0.082 | 0.762 | -0.163 | 0.262 | 0.534 | 0.036 | 0.109 | 0.737 |
| InternalIMT | aa | rs17157736 | 0.143 | 0.025 | 0.082 | 0.762 | -0.163 | 0.262 | 0.534 | 0.037 | 0.109 | 0.737 |
| InternalIMT | aa | rs72789735 | 0.063 | -0.014 | 0.076 | 0.857 | -0.845 | 0.362 | 0.02 | 0.324 | 0.154 | 0.036 |
| InternalIMT | aa | rs183747187 | 0.001 | . | . | . | . | . | . | . | . | . |
| InternalIMT | aa | rs7077891 | 0.143 | 0.025 | 0.082 | 0.762 | -0.163 | 0.262 | 0.533 | 0.037 | 0.109 | 0.736 |
| InternalIMT | aa | rs145063305 | 0.002 | . | . | . | . | . | . | . | . | . |
| InternalIMT | aa | rs3824613 | 0.143 | 0.024 | 0.082 | 0.77 | -0.169 | 0.261 | 0.519 | 0.039 | 0.109 | 0.721 |
| InternalIMT | aa | rs145543199 | 0.006 | . | . | . | . | . | . | . | . | . |
| InternalIMT | aa | rs140361993 | 0.003 | . | . | . | . | . | . | . | . | . |
| InternalIMT | aa | rs11239496 | 0.063 | -0.014 | 0.076 | 0.857 | -0.845 | 0.362 | 0.02 | 0.324 | 0.154 | 0.036 |
| InternalIMT | aa | rs11239497 | 0.139 | 0.018 | 0.082 | 0.825 | -0.198 | 0.271 | 0.466 | 0.053 | 0.113 | 0.643 |
| InternalIMT | aa | rs10900212 | 0.156 | 0.032 | 0.083 | 0.703 | -0.098 | 0.259 | 0.704 | 0.013 | 0.107 | 0.905 |
| InternalIMT | aa | rs186487136 | . | . | . | . | . | . | . | . | . | . |
| InternalIMT | aa | rs12774369 | 0.143 | 0.025 | 0.082 | 0.762 | -0.163 | 0.263 | 0.534 | 0.037 | 0.109 | 0.736 |
| InternalIMT | aa | rs60842538 | 0.142 | 0.025 | 0.082 | 0.762 | -0.163 | 0.264 | 0.535 | 0.037 | 0.11 | 0.737 |
| InternalIMT | aa | rs3780898 | 0.138 | 0.019 | 0.082 | 0.82 | -0.195 | 0.274 | 0.475 | 0.052 | 0.114 | 0.652 |
| InternalIMT | aa | rs3780899 | 0.142 | 0.025 | 0.082 | 0.76 | -0.163 | 0.265 | 0.538 | 0.037 | 0.11 | 0.739 |
| InternalIMT | aa | rs12252472 | 0.045 | 2.20E-03 | 0.074 | 0.976 | -0.788 | 0.421 | 0.062 | 0.306 | 0.175 | 0.08 |
| InternalIMT | aa | rs185411011 | . | . | . | . | . | . | . | . | . | . |
| InternalIMT | aa | rs28395865 | 0.137 | 0.02 | 0.082 | 0.805 | -0.186 | 0.275 | 0.499 | 0.047 | 0.115 | 0.682 |
| InternalIMT | aa | rs191004074 | 0.038 | 0.041 | 0.075 | 0.583 | 0.337 | 0.932 | 0.717 | -0.155 | 0.379 | 0.683 |
| InternalIMT | aa | rs182401295 | 0.002 | . | . | . | . | . | . | . | . | . |
| InternalIMT | aa | rs2228064 | 0.068 | -0.01 | 0.076 | 0.89 | -0.816 | 0.378 | 0.031 | 0.314 | 0.157 | 0.046 |
| InternalIMT | aa | rs188284159 | . | . | . | . | . | . | . | . | . | . |
| InternalIMT | aa | rs2303887 | 0.022 | 0.017 | 0.071 | 0.812 | -0.856 | 0.832 | 0.303 | 0.328 | 0.357 | 0.358 |
| InternalIMT | aa | rs2303886 | 0.024 | 0.02 | 0.071 | 0.777 | -0.571 | 0.768 | 0.458 | 0.207 | 0.327 | 0.527 |
| InternalIMT | aa | rs192621006 | . | . | . | . | . | . | . | . | . | . |
| InternalIMT | aa | rs2303885 | 0.191 | -9.60E-03 | 0.087 | 0.912 | -0.355 | 0.217 | 0.102 | 0.123 | 0.09 | 0.171 |
| InternalIMT | aa | rs114466168 | 0.012 | 0.018 | 0.071 | 0.801 | -1.03 | 0.601 | 0.087 | 0.391 | 0.251 | 0.119 |
| InternalIMT | aa | rs11239501 | 0.083 | -0.029 | 0.077 | 0.702 | -0.919 | 0.347 | 8.10E-03 | 0.374 | 0.148 | 0.012 |
| InternalIMT | aa | rs745986 | 0.156 | -9.50E-03 | 0.082 | 0.907 | -0.381 | 0.26 | 0.142 | 0.138 | 0.108 | 0.202 |
| InternalIMT | aa | rs75262268 | . | . | . | . | . | . | . | . | . | . |
| InternalIMT | aa | rs71494776 | 0.051 | 0.022 | 0.073 | 0.757 | -0.176 | 0.473 | 0.711 | 0.054 | 0.2 | 0.787 |
| InternalIMT | aa | rs74386871 | 0.03 | 0.025 | 0.073 | 0.732 | -0.214 | 0.705 | 0.762 | 0.04 | 0.302 | 0.895 |
| InternalIMT | aa | rs76502040 | 0.006 | . | . | . | . | . | . | . | . | . |
| InternalIMT | aa | rs142213734 | 0.001 | . | . | . | . | . | . | . | . | . |
| InternalIMT | aa | rs4948671 | 0.731 | -0.117 | 0.163 | 0.472 | -0.245 | 0.243 | 0.315 | 0.098 | 0.101 | 0.33 |
| InternalIMT | aa | rs146286085 | 0.001 | . | . | . | . | . | . | . | . | . |
| InternalIMT | aa | rs144080623 | . | . | . | . | . | . | . | . | . | . |
| InternalIMT | aa | rs115074014 | 0.023 | 0.022 | 0.072 | 0.764 | -0.71 | 0.619 | 0.251 | 0.265 | 0.249 | 0.287 |
| InternalIMT | aa | rs17157756 | 0.023 | 0.022 | 0.072 | 0.764 | -0.71 | 0.619 | 0.251 | 0.265 | 0.249 | 0.287 |
| InternalIMT | aa | rs190459780 | . | . | . | . | . | . | . | . | . | . |
| InternalIMT | aa | rs12358647 | 0.174 | -4.80E-03 | 0.083 | 0.954 | -0.312 | 0.247 | 0.206 | 0.111 | 0.103 | 0.28 |
| InternalIMT | aa | rs147080556 | 0.024 | 5.90E-03 | 0.072 | 0.935 | -1.34 | 1.07 | 0.21 | 0.545 | 0.457 | 0.232 |
| InternalIMT | aa | rs187543349 | 0.01 | 0.018 | 0.071 | 0.801 | -1.24 | 1.51 | 0.411 | 0.501 | 0.657 | 0.446 |
| InternalIMT | aa | rs114375595 | 0.006 | . | . | . | . | . | . | . | . | . |
| InternalIMT | aa | rs117211200 | 0.005 | . | . | . | . | . | . | . | . | . |
| InternalIMT | aa | rs192963047 | . | . | . | . | . | . | . | . | . | . |
| InternalIMT | aa | rs11239505 | 0.175 | -1.90E-03 | 0.083 | 0.982 | -0.302 | 0.253 | 0.232 | 0.105 | 0.105 | 0.314 |
| InternalIMT | aa | rs184434116 | 0 | . | . | . | . | . | . | . | . | . |
| InternalIMT | aa | rs4948999 | 0.733 | -0.134 | 0.164 | 0.412 | -0.272 | 0.244 | 0.266 | 0.11 | 0.101 | 0.279 |
| InternalIMT | aa | rs11239506 | 0.092 | -0.029 | 0.077 | 0.703 | -0.867 | 0.328 | 8.40E-03 | 0.346 | 0.139 | 0.013 |
| InternalIMT | aa | rs190706620 | 0.003 | . | . | . | . | . | . | . | . | . |
| InternalIMT | aa | rs190770429 | . | . | . | . | . | . | . | . | . | . |
| InternalIMT | aa | rs181327133 | 0.005 | . | . | . | . | . | . | . | . | . |
| InternalIMT | aa | rs149251697 | 0.011 | 0.04 | 0.07 | 0.573 | 1.58 | 2.51 | 0.528 | -0.585 | 1.02 | 0.565 |
| InternalIMT | aa | rs118081602 | 0.022 | 0.017 | 0.071 | 0.812 | -0.857 | 0.835 | 0.305 | 0.329 | 0.359 | 0.36 |
| InternalIMT | aa | rs188233691 | 0.002 | . | . | . | . | . | . | . | . | . |
| InternalIMT | aa | rs183778794 | 0.003 | . | . | . | . | . | . | . | . | . |
| InternalIMT | aa | rs188040078 | 0.007 | . | . | . | . | . | . | . | . | . |
| InternalIMT | aa | rs184675372 | 0 | . | . | . | . | . | . | . | . | . |
| InternalIMT | aa | rs150326891 | 0.067 | -0.017 | 0.076 | 0.827 | -0.892 | 0.376 | 0.018 | 0.352 | 0.161 | 0.029 |

| InternalIMT | aa | rs185520021 | 0.014 | 0.031 | 0.07 | 0.658 | 0.364 | 1.01 | 0.72 | -0.191 | 0.414 | 0.645 |
| --- | --- | --- | --- | --- | --- | --- | --- | --- | --- | --- | --- | --- |
| InternalIMT | aa | rs7907779 | 0.092 | -0.03 | 0.077 | 0.698 | -0.871 | 0.328 | 8.00E-03 | 0.348 | 0.139 | 0.012 |
| InternalIMT | aa | rs184962860 | 0.001 | . | . | . | . | . | . | . | . | . |
| InternalIMT | aa | rs149762784 | 0.066 | 0.029 | 0.077 | 0.709 | 0.075 | 0.621 | 0.904 | -0.069 | 0.263 | 0.793 |
| InternalIMT | aa | rs149203427 | 0.008 | . | . | . | . | . | . | . | . | . |
| InternalIMT | aa | rs60153083 | 0.01 | 0.027 | 0.071 | 0.697 | 0.326 | 0.942 | 0.73 | -0.075 | 0.375 | 0.84 |
| InternalIMT | aa | rs112001069 | 0.062 | 0.034 | 0.074 | 0.65 | 9.90E-03 | 0.398 | 0.98 | -0.027 | 0.166 | 0.87 |
| InternalIMT | aa | rs182355382 | 0.032 | 0.049 | 0.075 | 0.519 | 0.823 | 1.14 | 0.47 | -0.325 | 0.463 | 0.482 |
| InternalIMT | aa | rs186740974 | 0.111 | 0.019 | 0.096 | 0.843 | -0.077 | 0.624 | 0.902 | 0.043 | 0.258 | 0.868 |
| InternalIMT | aa | rs191874916 | 0.026 | 0.071 | 0.074 | 0.335 | 1.96 | 1.44 | 0.175 | -0.783 | 0.571 | 0.17 |
| InternalIMT | aa | rs182989412 | 0.168 | 0.047 | 0.084 | 0.575 | 0.151 | 0.429 | 0.726 | -0.031 | 0.181 | 0.865 |
| InternalIMT | aa | rs188819476 | 0.001 | . | . | . | . | . | . | . | . | . |
| InternalIMT | aa | rs141217183 | 0.08 | 0.053 | 0.077 | 0.486 | 0.276 | 0.367 | 0.452 | -0.141 | 0.153 | 0.355 |
| InternalIMT | aa | rs192922652 | 0.064 | 0.04 | 0.075 | 0.595 | 0.059 | 0.377 | 0.876 | -0.056 | 0.156 | 0.721 |
| InternalIMT | aa | rs185092343 | 0.014 | 0.018 | 0.072 | 0.799 | -1.01 | 1.2 | 0.4 | 0.415 | 0.477 | 0.384 |
| InternalIMT | aa | rs145193311 | 0.065 | 0.041 | 0.075 | 0.583 | 0.106 | 0.38 | 0.781 | -0.066 | 0.157 | 0.672 |
| InternalIMT | aa | rs55789336 | 0.094 | -0.037 | 0.078 | 0.632 | -0.948 | 0.326 | 3.80E-03 | 0.379 | 0.138 | 6.30E-03 |
| InternalIMT | aa | rs55837212 | 0.094 | -0.034 | 0.077 | 0.659 | -0.948 | 0.332 | 4.40E-03 | 0.383 | 0.141 | 6.50E-03 |
| InternalIMT | aa | rs75408574 | 0.406 | 0.085 | 0.096 | 0.378 | 0.177 | 0.25 | 0.48 | -0.056 | 0.102 | 0.583 |
| InternalIMT | aa | rs149306926 | 0.099 | -0.032 | 0.078 | 0.683 | -0.847 | 0.341 | 0.013 | 0.346 | 0.144 | 0.017 |
| InternalIMT | aa | rs35990892 | 0.706 | -0.21 | 0.158 | 0.185 | -0.401 | 0.246 | 0.104 | 0.17 | 0.102 | 0.097 |
| InternalIMT | aa | rs192356207 | 0.036 | 0.025 | 0.073 | 0.727 | 0.053 | 0.819 | 0.949 | 5.30E-03 | 0.325 | 0.987 |
| InternalIMT | aa | rs183741163 | 0.092 | -1.50E-03 | 0.078 | 0.985 | -0.496 | 0.432 | 0.251 | 0.194 | 0.179 | 0.28 |
| InternalIMT | aa | rs34737297 | 0.558 | -0.029 | 0.142 | 0.836 | -0.113 | 0.293 | 0.699 | 0.053 | 0.121 | 0.659 |
| InternalIMT | aa | rs181346933 | 0.022 | 0.017 | 0.071 | 0.815 | -0.975 | 0.917 | 0.288 | 0.363 | 0.381 | 0.341 |
| InternalIMT | aa | rs36093253 | 0.408 | 0.094 | 0.102 | 0.357 | 0.201 | 0.263 | 0.446 | -0.066 | 0.108 | 0.541 |
| InternalIMT | aa | rs186307855 | 0.059 | -2.40E-03 | 0.077 | 0.975 | -0.71 | 0.403 | 0.079 | 0.265 | 0.167 | 0.112 |
| InternalIMT | aa | rs189681865 | 0.019 | 0.04 | 0.072 | 0.576 | 0.467 | 1.09 | 0.669 | -0.213 | 0.429 | 0.62 |
| InternalIMT | aa | rs34942971 | 0.176 | -0.017 | 0.084 | 0.841 | -0.388 | 0.274 | 0.156 | 0.147 | 0.114 | 0.196 |
| InternalIMT | aa | rs181816554 | 0.025 | 0.059 | 0.073 | 0.42 | 1.69 | 1.3 | 0.192 | -0.646 | 0.506 | 0.202 |
| InternalIMT | aa | rs186846881 | 0.049 | 0.017 | 0.074 | 0.819 | -0.314 | 0.484 | 0.517 | 0.122 | 0.195 | 0.531 |
| InternalIMT | aa | rs191178220 | 0.016 | 0.062 | 0.072 | 0.39 | 2.48 | 1.48 | 0.094 | -1.03 | 0.594 | 0.084 |
| InternalIMT | aa | rs183305228 | 0.01 | 0.028 | 0.071 | 0.697 | 0.323 | 0.938 | 0.731 | -0.076 | 0.373 | 0.838 |
| InternalIMT | aa | rs139912761 | 0.102 | -0.021 | 0.078 | 0.79 | -0.67 | 0.356 | 0.06 | 0.272 | 0.148 | 0.067 |
| InternalIMT | aa | rs185499901 | 0.016 | 0.067 | 0.073 | 0.359 | 2.61 | 1.76 | 0.139 | -1.04 | 0.688 | 0.129 |
| InternalIMT | aa | rs189816197 | 0.072 | 0.047 | 0.075 | 0.532 | 0.22 | 0.404 | 0.587 | -0.115 | 0.167 | 0.492 |
| InternalIMT | aa | rs145656938 | 0.022 | 0.036 | 0.074 | 0.629 | 0.107 | 0.635 | 0.867 | -0.073 | 0.252 | 0.771 |
| InternalIMT | aa | rs35745197 | 0.062 | 0.034 | 0.074 | 0.651 | 8.10E-03 | 0.4 | 0.984 | -0.026 | 0.166 | 0.874 |
| InternalIMT | aa | rs192856637 | 0.004 | . | . | . | . | . | . | . | . | . |
| InternalIMT | aa | rs2310775 | 0.492 | 0.073 | 0.102 | 0.473 | 0.115 | 0.215 | 0.592 | -0.033 | 0.088 | 0.708 |
| InternalIMT | aa | rs11239507 | 0.558 | 6.30E-03 | 0.116 | 0.957 | -0.037 | 0.22 | 0.866 | 0.025 | 0.09 | 0.781 |
| InternalIMT | aa | rs74648467 | 0.053 | 0.019 | 0.073 | 0.794 | -0.243 | 0.468 | 0.603 | 0.081 | 0.198 | 0.682 |
| InternalIMT | aa | rs145815934 | 0.012 | 0.02 | 0.071 | 0.775 | -1.02 | 1.63 | 0.535 | 0.399 | 0.659 | 0.545 |
| InternalIMT | aa | rs145390687 | 0.015 | 2.10E-03 | 0.07 | 0.976 | -1.66 | 0.822 | 0.044 | 0.732 | 0.328 | 0.026 |
| InternalIMT | aa | rs184354913 | 0.033 | 0.085 | 0.074 | 0.252 | 1.62 | 0.642 | 0.012 | -0.692 | 0.265 | 9.10E-03 |
| InternalIMT | aa | rs181244920 | 0.007 | . | . | . | . | . | . | . | . | . |
| InternalIMT | aa | rs11499087 | 0.028 | 0.021 | 0.072 | 0.772 | -0.512 | 0.493 | 0.299 | 0.191 | 0.201 | 0.344 |
| InternalIMT | aa | rs148263794 | 0.064 | 0.04 | 0.074 | 0.594 | 0.097 | 0.379 | 0.798 | -0.065 | 0.157 | 0.677 |
| InternalIMT | aa | rs150758903 | 0.021 | 0.055 | 0.072 | 0.446 | 1.47 | 1.25 | 0.24 | -0.603 | 0.487 | 0.216 |
| InternalIMT | aa | rs147654382 | 0.152 | -0.023 | 0.082 | 0.777 | -0.52 | 0.279 | 0.062 | 0.194 | 0.117 | 0.097 |
| InternalIMT | aa | rs147495166 | 0.011 | 0.021 | 0.071 | 0.771 | -0.874 | 0.598 | 0.144 | 0.319 | 0.247 | 0.197 |
| InternalIMT | aa | rs35198778 | 0.081 | 0.019 | 0.079 | 0.815 | -0.415 | 0.349 | 0.235 | 0.127 | 0.144 | 0.377 |
| InternalIMT | aa | rs145207659 | 0.043 | 0.03 | 0.07 | 0.671 | 0.175 | 0.802 | 0.827 | -0.021 | 0.323 | 0.948 |
| InternalIMT | aa | rs186466464 | 0.007 | . | . | . | . | . | . | . | . | . |
| InternalIMT | aa | rs12778413 | 0.146 | -9.80E-03 | 0.085 | 0.908 | -0.444 | 0.298 | 0.137 | 0.153 | 0.123 | 0.214 |
| InternalIMT | aa | rs7895552 | 0.632 | -0.083 | 0.149 | 0.576 | -0.224 | 0.253 | 0.376 | 0.088 | 0.104 | 0.4 |
| InternalIMT | aa | rs12783895 | 0.064 | 0.039 | 0.074 | 0.596 | 0.094 | 0.379 | 0.804 | -0.064 | 0.157 | 0.684 |
| InternalIMT | aa | rs34929604 | 0.065 | 0.04 | 0.074 | 0.586 | 0.103 | 0.371 | 0.782 | -0.068 | 0.153 | 0.657 |
| InternalIMT | aa | rs12761961 | 0.063 | 0.034 | 0.074 | 0.648 | 0.013 | 0.404 | 0.974 | -0.028 | 0.168 | 0.869 |
| InternalIMT | aa | rs35536930 | 0.173 | -1.50E-03 | 0.083 | 0.986 | -0.299 | 0.256 | 0.242 | 0.106 | 0.106 | 0.318 |
| InternalIMT | aa | rs180951060 | 0.001 | . | . | . | . | . | . | . | . | . |
| InternalIMT | aa | rs148892128 | 0.012 | 0.02 | 0.071 | 0.775 | -1.01 | 1.64 | 0.535 | 0.398 | 0.659 | 0.546 |
| InternalIMT | aa | rs11239509 | 0.043 | 0.014 | 0.074 | 0.851 | -0.536 | 0.372 | 0.149 | 0.197 | 0.152 | 0.197 |
| InternalIMT | aa | rs41526545 | 0.088 | -0.033 | 0.077 | 0.671 | -0.929 | 0.338 | 6.10E-03 | 0.373 | 0.144 | 9.50E-03 |
| InternalIMT | aa | rs2029253 | 0.496 | 0.06 | 0.102 | 0.556 | 0.085 | 0.216 | 0.692 | -0.02 | 0.088 | 0.821 |
| InternalIMT | aa | rs17444064 | 0.086 | -0.035 | 0.077 | 0.652 | -0.966 | 0.341 | 4.70E-03 | 0.391 | 0.145 | 7.20E-03 |
| InternalIMT | aa | rs71494779 | 0.066 | 0.041 | 0.074 | 0.58 | 0.114 | 0.37 | 0.758 | -0.073 | 0.153 | 0.634 |
| InternalIMT | aa | rs74991420 | 0.022 | 0.015 | 0.071 | 0.827 | -0.97 | 0.833 | 0.244 | 0.373 | 0.358 | 0.297 |
| InternalIMT | aa | rs182736551 | 0.022 | 0.015 | 0.071 | 0.827 | -0.97 | 0.833 | 0.244 | 0.373 | 0.358 | 0.297 |
| InternalIMT | aa | rs78702074 | 0.021 | 0.017 | 0.071 | 0.805 | -0.812 | 0.878 | 0.355 | 0.307 | 0.378 | 0.416 |
| InternalIMT | aa | rs187791466 | 0.002 | . | . | . | . | . | . | . | . | . |
| InternalIMT | aa | rs35300270 | 0.068 | 0.042 | 0.074 | 0.572 | 0.117 | 0.368 | 0.749 | -0.076 | 0.152 | 0.619 |
| InternalIMT | aa | rs6593484 | 0.093 | -0.032 | 0.077 | 0.675 | -0.893 | 0.325 | 6.20E-03 | 0.356 | 0.138 | 1.00E-02 |
| InternalIMT | aa | rs12244601 | 0.013 | 0.029 | 0.07 | 0.684 | -0.238 | 0.777 | 0.759 | 0.075 | 0.309 | 0.809 |
| InternalIMT | aa | rs184429574 | 0.004 | . | . | . | . | . | . | . | . | . |
| InternalIMT | aa | rs7080897 | 0.066 | 0.041 | 0.074 | 0.58 | 0.114 | 0.37 | 0.757 | -0.073 | 0.153 | 0.634 |
| InternalIMT | aa | rs7097429 | 0.07 | 0.041 | 0.075 | 0.582 | 0.108 | 0.367 | 0.768 | -0.071 | 0.153 | 0.643 |
| InternalIMT | aa | rs77979229 | 0.001 | . | . | . | . | . | . | . | . | . |
| InternalIMT | aa | rs11239512 | 0.093 | -0.033 | 0.077 | 0.672 | -0.895 | 0.324 | 5.90E-03 | 0.356 | 0.137 | 9.60E-03 |
| InternalIMT | aa | rs60146827 | 0.066 | 0.041 | 0.074 | 0.582 | 0.111 | 0.369 | 0.764 | -0.071 | 0.152 | 0.639 |
| InternalIMT | aa | rs79032236 | 0.017 | 0.01 | 0.071 | 0.888 | -1.17 | 0.616 | 0.057 | 0.458 | 0.261 | 0.079 |
| InternalIMT | aa | rs140854206 | 0.005 | . | . | . | . | . | . | . | . | . |
| InternalIMT | aa | rs184049550 | 0.002 | . | . | . | . | . | . | . | . | . |
| InternalIMT | aa | rs114433607 | 0.007 | . | . | . | . | . | . | . | . | . |
| InternalIMT | aa | rs117653793 | 0.022 | 0.017 | 0.071 | 0.815 | -0.874 | 0.84 | 0.298 | 0.336 | 0.36 | 0.352 |
| InternalIMT | aa | rs117039533 | 0.022 | 0.017 | 0.071 | 0.814 | -0.872 | 0.84 | 0.3 | 0.335 | 0.36 | 0.353 |

| InternalIMT | aa | rs191660761 | 0.002 | . | . | . | . | . | . | . | . | . |
| --- | --- | --- | --- | --- | --- | --- | --- | --- | --- | --- | --- | --- |
| InternalIMT | aa | rs59514896 | 0.053 | 0.025 | 0.073 | 0.73 | -0.118 | 0.46 | 0.799 | 0.026 | 0.194 | 0.892 |
| InternalIMT | aa | rs184661876 | 0.001 | . | . | . | . | . | . | . | . | . |
| InternalIMT | aa | rs61854073 | 0.028 | 0.027 | 0.072 | 0.712 | -0.077 | 1.06 | 0.942 | 0.067 | 0.445 | 0.88 |
| InternalIMT | aa | rs2018518 | 0.391 | 0.07 | 0.097 | 0.469 | 0.12 | 0.243 | 0.621 | -0.037 | 0.099 | 0.709 |
| InternalIMT | aa | rs934187 | 0.734 | -0.146 | 0.164 | 0.371 | -0.289 | 0.244 | 0.237 | 0.118 | 0.101 | 0.245 |
| InternalIMT | aa | rs7918542 | 0.179 | -6.40E-03 | 0.084 | 0.939 | -0.329 | 0.251 | 0.189 | 0.116 | 0.104 | 0.265 |
| InternalIMT | aa | rs144311920 | 0.002 | . | . | . | . | . | . | . | . | . |
| InternalIMT | aa | rs7099684 | 0.864 | 0.07 | 0.258 | 0.788 | 0.025 | 0.335 | 0.94 | -0.025 | 0.14 | 0.857 |
| InternalIMT | aa | rs150829171 | . | . | . | . | . | . | . | . | . | . |
| InternalIMT | aa | rs186060697 | 0.001 | . | . | . | . | . | . | . | . | . |
| InternalIMT | aa | rs11239513 | 0.054 | 2.10E-03 | 0.076 | 0.978 | -0.594 | 0.443 | 0.181 | 0.237 | 0.18 | 0.19 |
| InternalIMT | aa | rs7917687 | 0.067 | 0.04 | 0.074 | 0.593 | 0.09 | 0.365 | 0.805 | -0.063 | 0.151 | 0.676 |
| InternalIMT | aa | rs61854076 | 0.01 | 0.014 | 0.07 | 0.844 | -1.37 | 1.57 | 0.381 | 0.688 | 0.676 | 0.309 |
| InternalIMT | aa | rs71494780 | 0.029 | 0.014 | 0.071 | 0.841 | -0.737 | 0.726 | 0.31 | 0.314 | 0.308 | 0.307 |
| InternalIMT | aa | rs7919239 | 0.187 | -0.014 | 0.083 | 0.865 | -0.358 | 0.228 | 0.117 | 0.128 | 0.095 | 0.179 |
| InternalIMT | aa | rs78334475 | 0.047 | 0.026 | 0.072 | 0.721 | -0.015 | 0.524 | 0.977 | -0.02 | 0.217 | 0.925 |
| InternalIMT | aa | rs3780900 | 0.02 | 0.076 | 0.074 | 0.302 | 2.73 | 1.62 | 0.092 | -1.09 | 0.63 | 0.083 |
| InternalIMT | aa | rs186729292 | 0.001 | . | . | . | . | . | . | . | . | . |
| InternalIMT | aa | rs12267438 | 0.015 | 0.028 | 0.07 | 0.691 | -0.105 | 1.13 | 0.926 | 0.041 | 0.449 | 0.928 |
| InternalIMT | aa | rs1369214 | 0.61 | -0.035 | 0.129 | 0.789 | -0.115 | 0.232 | 0.62 | 0.055 | 0.095 | 0.564 |
| InternalIMT | aa | rs9282589 | 0.134 | 2.60E-03 | 0.076 | 0.973 | -0.237 | 0.351 | 0.499 | 0.094 | 0.147 | 0.523 |
| InternalIMT | aa | rs114522915 | 0.011 | 0.024 | 0.071 | 0.738 | -0.799 | 0.693 | 0.249 | 0.288 | 0.28 | 0.305 |
| InternalIMT | aa | rs2115819 | 0.542 | 0.061 | 0.112 | 0.584 | 0.078 | 0.223 | 0.725 | -0.02 | 0.091 | 0.823 |
| InternalIMT | aa | rs117664603 | 0.022 | 0.017 | 0.071 | 0.815 | -0.868 | 0.845 | 0.304 | 0.334 | 0.363 | 0.358 |
| InternalIMT | aa | rs184979202 | . | . | . | . | . | . | . | . | . | . |
| InternalIMT | aa | rs190714671 | 0.002 | . | . | . | . | . | . | . | . | . |
| InternalIMT | aa | rs141538996 | . | . | . | . | . | . | . | . | . | . |
| InternalIMT | aa | rs150881741 | . | . | . | . | . | . | . | . | . | . |
| InternalIMT | aa | rs139076633 | . | . | . | . | . | . | . | . | . | . |
| InternalIMT | aa | rs12243649 | 0.054 | 0.049 | 0.074 | 0.51 | 0.265 | 0.541 | 0.624 | -0.135 | 0.213 | 0.528 |
| InternalIMT | aa | rs140348708 | 0.001 | . | . | . | . | . | . | . | . | . |
| InternalIMT | aa | rs79023256 | 0.022 | 0.017 | 0.071 | 0.815 | -0.87 | 0.846 | 0.304 | 0.334 | 0.363 | 0.358 |
| InternalIMT | aa | rs11239515 | 0.081 | -0.015 | 0.077 | 0.85 | -0.705 | 0.33 | 0.033 | 0.271 | 0.139 | 0.051 |
| InternalIMT | aa | rs55866086 | 0.137 | 5.00E-03 | 0.082 | 0.951 | -0.338 | 0.285 | 0.236 | 0.113 | 0.118 | 0.337 |
| InternalIMT | aa | rs181340150 | 0.001 | . | . | . | . | . | . | . | . | . |
| InternalIMT | aa | rs11239516 | 0.097 | -9.30E-03 | 0.078 | 0.905 | -0.536 | 0.336 | 0.111 | 0.205 | 0.139 | 0.141 |
| InternalIMT | aa | rs11239517 | 0.096 | -9.10E-03 | 0.078 | 0.907 | -0.534 | 0.336 | 0.113 | 0.204 | 0.139 | 0.144 |
| InternalIMT | aa | rs11239519 | 0.089 | -0.012 | 0.078 | 0.878 | -0.651 | 0.321 | 0.042 | 0.247 | 0.134 | 0.066 |
| InternalIMT | aa | rs7909514 | 0.721 | -0.126 | 0.164 | 0.443 | -0.261 | 0.246 | 0.289 | 0.104 | 0.102 | 0.308 |
| InternalIMT | aa | rs7894352 | 0.853 | 0.13 | 0.265 | 0.624 | 0.1 | 0.344 | 0.771 | -0.06 | 0.144 | 0.677 |
| InternalIMT | aa | rs189384146 | 0.002 | . | . | . | . | . | . | . | . | . |
| InternalIMT | aa | rs11239520 | 0.088 | 1.50E-03 | 0.077 | 0.984 | -0.433 | 0.356 | 0.224 | 0.162 | 0.148 | 0.274 |
| InternalIMT | aa | rs12221233 | 0.482 | 0.039 | 0.104 | 0.71 | 0.033 | 0.222 | 0.883 | 8.90E-04 | 0.091 | 0.992 |
| InternalIMT | aa | rs74523293 | 0.038 | 0.037 | 0.073 | 0.609 | -0.046 | 0.537 | 0.931 | -0.025 | 0.218 | 0.909 |
| InternalIMT | aa | rs79376040 | 0.038 | 0.037 | 0.073 | 0.609 | -0.047 | 0.537 | 0.931 | -0.025 | 0.219 | 0.909 |
| InternalIMT | aa | rs4949000 | 0.482 | 0.036 | 0.104 | 0.729 | 0.026 | 0.222 | 0.906 | 3.30E-03 | 0.091 | 0.971 |
| InternalIMT | aa | rs7089406 | 0.023 | 0.033 | 0.07 | 0.637 | 0.305 | 0.905 | 0.736 | -0.123 | 0.371 | 0.74 |
| InternalIMT | aa | rs138211056 | 0.001 | . | . | . | . | . | . | . | . | . |
| InternalIMT | aa | rs10900213 | 0.586 | -4.50E-03 | 0.123 | 0.971 | -0.057 | 0.223 | 0.798 | 0.033 | 0.092 | 0.718 |
| InternalIMT | aa | rs145152827 | . | . | . | . | . | . | . | . | . | . |
| InternalIMT | aa | rs74443325 | 0.022 | 0.016 | 0.071 | 0.816 | -0.874 | 0.852 | 0.305 | 0.332 | 0.366 | 0.365 |
| InternalIMT | aa | rs188299446 | 0.011 | 0.021 | 0.072 | 0.77 | -0.899 | 1.72 | 0.6 | 0.518 | 0.731 | 0.478 |
| InternalIMT | aa | rs192699586 | 0.005 | . | . | . | . | . | . | . | . | . |
| InternalIMT | aa | rs72796464 | 0.076 | -0.015 | 0.077 | 0.848 | -0.716 | 0.339 | 0.035 | 0.28 | 0.143 | 0.05 |
| InternalIMT | aa | rs4949001 | 0.366 | 0.043 | 0.098 | 0.665 | 0.037 | 0.246 | 0.879 | -8.30E-03 | 0.101 | 0.935 |
| InternalIMT | aa | rs147174641 | 0.005 | . | . | . | . | . | . | . | . | . |
| InternalIMT | aa | rs190835238 | 0.001 | . | . | . | . | . | . | . | . | . |
| InternalIMT | aa | rs76698944 | 0.017 | 0.021 | 0.071 | 0.761 | -0.703 | 1.12 | 0.53 | 0.263 | 0.448 | 0.558 |
| InternalIMT | aa | rs56071381 | 0.092 | 7.50E-03 | 0.078 | 0.923 | -0.346 | 0.351 | 0.324 | 0.124 | 0.145 | 0.393 |
| InternalIMT | aa | rs78130427 | 0.023 | 0.035 | 0.071 | 0.622 | 0.12 | 0.514 | 0.816 | -0.09 | 0.221 | 0.683 |
| InternalIMT | aa | rs10900215 | 0.118 | 0.016 | 0.079 | 0.838 | -0.159 | 0.368 | 0.667 | 0.06 | 0.149 | 0.688 |
| InternalIMT | aa | rs41491445 | 0.01 | 0.028 | 0.071 | 0.687 | 0.49 | 0.958 | 0.609 | -0.146 | 0.379 | 0.7 |
| InternalIMT | aa | rs144995222 | 0.002 | . | . | . | . | . | . | . | . | . |
| InternalIMT | aa | rs149097803 | 0.002 | . | . | . | . | . | . | . | . | . |
| InternalIMT | aa | rs139429646 | 0.005 | . | . | . | . | . | . | . | . | . |
| InternalIMT | aa | rs7075620 | 0.675 | -0.032 | 0.153 | 0.835 | -0.115 | 0.239 | 0.63 | 0.044 | 0.099 | 0.659 |
| InternalIMT | aa | rs148299213 | 0.01 | 0.029 | 0.071 | 0.68 | 0.615 | 1.02 | 0.546 | -0.196 | 0.402 | 0.626 |
| InternalIMT | aa | rs4949002 | 0.313 | 0.077 | 0.092 | 0.401 | 0.189 | 0.273 | 0.488 | -0.055 | 0.111 | 0.617 |
| InternalIMT | aa | rs150496007 | 0.036 | 2.50E-03 | 0.072 | 0.972 | -0.979 | 0.641 | 0.127 | 0.379 | 0.266 | 0.154 |
| InternalIMT | aa | rs147303455 | 0.008 | . | . | . | . | . | . | . | . | . |
| InternalIMT | aa | rs189518433 | . | . | . | . | . | . | . | . | . | . |
| InternalIMT | aa | rs186281355 | . | . | . | . | . | . | . | . | . | . |
| InternalIMT | aa | rs115297114 | 0.014 | 0.018 | 0.072 | 0.8 | -0.991 | 0.551 | 0.073 | 0.369 | 0.228 | 0.106 |
| InternalIMT | aa | rs11239523 | 0.087 | 4.10E-03 | 0.077 | 0.957 | -0.389 | 0.35 | 0.268 | 0.146 | 0.146 | 0.316 |
| InternalIMT | aa | rs143704897 | 0.005 | . | . | . | . | . | . | . | . | . |
| InternalIMT | aa | rs188024096 | 0.002 | . | . | . | . | . | . | . | . | . |
| InternalIMT | aa | rs11239524 | 0.087 | 9.30E-03 | 0.078 | 0.905 | -0.356 | 0.356 | 0.318 | 0.122 | 0.147 | 0.408 |
| InternalIMT | aa | rs191656573 | 0.004 | . | . | . | . | . | . | . | . | . |
| InternalIMT | aa | rs193295875 | . | . | . | . | . | . | . | . | . | . |
| InternalIMT | aa | rs190458228 | . | . | . | . | . | . | . | . | . | . |
| InternalIMT | aa | rs139253993 | 0.002 | . | . | . | . | . | . | . | . | . |
| InternalIMT | aa | rs4948672 | 0.604 | 8.00E-03 | 0.158 | 0.96 | -0.037 | 0.265 | 0.888 | 0.018 | 0.109 | 0.871 |
| InternalIMT | aa | rs180954921 | 0.002 | . | . | . | . | . | . | . | . | . |
| InternalIMT | aa | rs12264801 | 0.447 | -0.027 | 0.118 | 0.817 | -0.148 | 0.243 | 0.542 | 0.058 | 0.101 | 0.564 |
| InternalIMT | aa | rs73283184 | 0.014 | 0.038 | 0.071 | 0.591 | 1.33 | 1 | 0.185 | -0.503 | 0.381 | 0.186 |

| InternalIMT | aa | rs142746187 | 0.007 | . | . | . | . | . | . | . | . | . |
| --- | --- | --- | --- | --- | --- | --- | --- | --- | --- | --- | --- | --- |
| InternalIMT | aa | rs58704739 | 0.014 | 0.039 | 0.071 | 0.579 | 1.73 | 1.09 | 0.115 | -0.658 | 0.415 | 0.113 |
| InternalIMT | aa | rs61272335 | 0.011 | 0.034 | 0.07 | 0.627 | 2.03 | 1.33 | 0.126 | -0.751 | 0.512 | 0.142 |
| InternalIMT | aa | rs60959489 | 0.021 | 0.041 | 0.072 | 0.572 | 0.673 | 0.834 | 0.42 | -0.273 | 0.321 | 0.395 |
| InternalIMT | aa | rs58502629 | 0.011 | 0.034 | 0.07 | 0.627 | 2.05 | 1.33 | 0.124 | -0.756 | 0.511 | 0.14 |
| InternalIMT | aa | rs10900216 | 0.739 | 0.18 | 0.186 | 0.335 | 0.219 | 0.251 | 0.383 | -0.097 | 0.104 | 0.351 |
| InternalIMT | aa | rs181118141 | 0.001 | . | . | . | . | . | . | . | . | . |
| InternalIMT | aa | rs137882665 | 0.01 | 0.03 | 0.07 | 0.669 | -0.642 | 0.948 | 0.499 | 0.197 | 0.386 | 0.61 |
| InternalIMT | aa | rs149466736 | 0.004 | . | . | . | . | . | . | . | . | . |
| InternalIMT | aa | rs2099171 | 0.675 | 1.90E-03 | 0.154 | 0.99 | -0.05 | 0.218 | 0.819 | 0.018 | 0.091 | 0.845 |
| InternalIMT | aa | rs141852280 | 0.005 | . | . | . | . | . | . | . | . | . |
| InternalIMT | aa | rs191539257 | . | . | . | . | . | . | . | . | . | . |
| InternalIMT | aa | rs17157771 | 0.027 | 0.061 | 0.071 | 0.391 | 1.09 | 0.623 | 0.079 | -0.46 | 0.259 | 0.076 |
| InternalIMT | aa | rs111339241 | 0.025 | 0.063 | 0.07 | 0.367 | 1.37 | 0.688 | 0.047 | -0.577 | 0.282 | 0.041 |
| InternalIMT | aa | rs116983255 | 0.006 | . | . | . | . | . | . | . | . | . |
| InternalIMT | aa | rs142324341 | . | . | . | . | . | . | . | . | . | . |
| InternalIMT | aa | rs187191219 | 0.007 | . | . | . | . | . | . | . | . | . |
| InternalIMT | aa | rs151283973 | 0 | . | . | . | . | . | . | . | . | . |
| InternalIMT | aa | rs139316218 | 0.003 | . | . | . | . | . | . | . | . | . |
| InternalIMT | aa | rs6593485 | 0.748 | 0.115 | 0.194 | 0.553 | 0.115 | 0.26 | 0.659 | -0.057 | 0.108 | 0.598 |
| InternalIMT | aa | rs6593486 | 0.751 | 0.125 | 0.194 | 0.518 | 0.129 | 0.259 | 0.618 | -0.063 | 0.108 | 0.555 |
| InternalIMT | aa | rs145196618 | 0.011 | 0.029 | 0.07 | 0.675 | -0.101 | 1.11 | 0.927 | 0.031 | 0.438 | 0.943 |
| InternalIMT | aa | rs142598335 | . | . | . | . | . | . | . | . | . | . |
| InternalIMT | aa | rs892691 | 0.746 | 0.102 | 0.181 | 0.575 | 0.102 | 0.244 | 0.675 | -0.049 | 0.101 | 0.628 |
| InternalIMT | aa | rs7080713 | 0.689 | 0.034 | 0.163 | 0.834 | 6.30E-03 | 0.226 | 0.978 | -3.60E-03 | 0.095 | 0.969 |
| InternalIMT | aa | rs7077173 | 0.068 | 0.075 | 0.073 | 0.304 | 0.556 | 0.314 | 0.077 | -0.255 | 0.127 | 0.046 |
| InternalIMT | aa | rs7080977 | 0.04 | 0.065 | 0.072 | 0.368 | 0.731 | 0.518 | 0.159 | -0.318 | 0.208 | 0.126 |
| InternalIMT | aa | rs3780901 | 0.689 | 0.052 | 0.162 | 0.75 | 0.037 | 0.225 | 0.87 | -0.015 | 0.094 | 0.87 |
| InternalIMT | aa | rs61854091 | 0.046 | 0.025 | 0.075 | 0.738 | -0.087 | 0.764 | 0.909 | 0.054 | 0.314 | 0.863 |
| InternalIMT | aa | rs77550209 | 0.003 | . | . | . | . | . | . | . | . | . |
| InternalIMT | aa | rs73283191 | 0.062 | 0.048 | 0.073 | 0.51 | 0.222 | 0.422 | 0.6 | -0.121 | 0.173 | 0.484 |
| InternalIMT | aa | rs191866099 | . | . | . | . | . | . | . | . | . | . |
| InternalIMT | aa | rs147998395 | 0 | . | . | . | . | . | . | . | . | . |
| InternalIMT | aa | rs7090328 | 0.789 | 0.079 | 0.227 | 0.727 | 0.044 | 0.296 | 0.882 | -0.033 | 0.122 | 0.79 |
| InternalIMT | aa | rs79814535 | 0.021 | 0.015 | 0.071 | 0.83 | -0.847 | 0.843 | 0.315 | 0.316 | 0.364 | 0.385 |
| InternalIMT | aa | rs7073082 | 0.789 | 0.078 | 0.227 | 0.729 | 0.043 | 0.296 | 0.885 | -0.032 | 0.122 | 0.793 |
| InternalIMT | aa | rs11239526 | 0.033 | 0.016 | 0.071 | 0.819 | -0.281 | 0.746 | 0.707 | 0.16 | 0.301 | 0.595 |
| InternalIMT | aa | rs141839841 | 0 | . | . | . | . | . | . | . | . | . |
| InternalIMT | aa | rs75697371 | 0.006 | . | . | . | . | . | . | . | . | . |
| InternalIMT | aa | rs75972854 | 0.005 | . | . | . | . | . | . | . | . | . |
| InternalIMT | aa | rs139291975 | . | . | . | . | . | . | . | . | . | . |
| InternalIMT | aa | rs144064711 | 0.004 | . | . | . | . | . | . | . | . | . |
| InternalIMT | aa | rs3780902 | 0.062 | 0.049 | 0.073 | 0.503 | 0.233 | 0.412 | 0.571 | -0.125 | 0.169 | 0.457 |
| InternalIMT | aa | rs190449801 | 0.001 | . | . | . | . | . | . | . | . | . |
| InternalIMT | aa | rs73283196 | 0.061 | 0.049 | 0.073 | 0.501 | 0.24 | 0.426 | 0.573 | -0.129 | 0.174 | 0.461 |
| InternalIMT | aa | rs28395867 | 0.031 | 9.30E-03 | 0.071 | 0.896 | -0.921 | 0.581 | 0.113 | 0.365 | 0.243 | 0.133 |
| InternalIMT | aa | rs28395866 | 0.079 | 0.046 | 0.074 | 0.537 | 0.081 | 0.36 | 0.822 | -0.053 | 0.146 | 0.718 |
| InternalIMT | aa | rs55661611 | 0.04 | 0.064 | 0.072 | 0.375 | 0.729 | 0.535 | 0.173 | -0.319 | 0.214 | 0.137 |
| InternalIMT | aa | rs55684518 | 0.016 | 0.016 | 0.07 | 0.824 | -1.15 | 0.875 | 0.19 | 0.48 | 0.376 | 0.201 |
| InternalIMT | aa | rs78552493 | 0.038 | 0.049 | 0.072 | 0.494 | 0.53 | 0.697 | 0.448 | -0.239 | 0.278 | 0.391 |
| InternalIMT | aa | rs147665434 | 0.001 | . | . | . | . | . | . | . | . | . |
| InternalIMT | aa | rs34445001 | 0.006 | . | . | . | . | . | . | . | . | . |
| InternalIMT | aa | rs184019449 | 0.003 | . | . | . | . | . | . | . | . | . |
| InternalIMT | aa | rs3780903 | 0.731 | 0.108 | 0.177 | 0.543 | 0.123 | 0.242 | 0.612 | -0.052 | 0.101 | 0.61 |
| InternalIMT | aa | rs17157784 | 0.058 | -4.90E-03 | 0.072 | 0.946 | -0.963 | 0.44 | 0.029 | 0.371 | 0.187 | 0.047 |
| InternalIMT | aa | rs3780904 | 0.021 | 0.013 | 0.07 | 0.849 | -0.982 | 0.774 | 0.205 | 0.374 | 0.335 | 0.265 |
| InternalIMT | aa | rs192064730 | . | . | . | . | . | . | . | . | . | . |
| InternalIMT | aa | rs12774534 | 0.006 | . | . | . | . | . | . | . | . | . |
| InternalIMT | aa | rs79179915 | 0.02 | 0.014 | 0.07 | 0.839 | -1.01 | 0.871 | 0.246 | 0.385 | 0.377 | 0.308 |
| InternalIMT | aa | rs117811571 | 0.011 | 0.023 | 0.072 | 0.747 | -0.726 | 1.71 | 0.671 | 0.434 | 0.729 | 0.551 |
| InternalIMT | aa | rs72796477 | 0.009 | . | . | . | . | . | . | . | . | . |
| InternalIMT | aa | rs55729342 | 0.058 | -2.70E-03 | 0.073 | 0.97 | -0.9 | 0.446 | 0.044 | 0.348 | 0.19 | 0.067 |
| InternalIMT | aa | rs59317220 | 0.058 | -2.60E-03 | 0.073 | 0.972 | -0.899 | 0.446 | 0.044 | 0.347 | 0.189 | 0.067 |
| InternalIMT | aa | rs3780905 | 0.057 | 0.034 | 0.072 | 0.643 | 0.094 | 0.457 | 0.837 | -0.071 | 0.19 | 0.709 |
| InternalIMT | aa | rs3780906 | 0.731 | 0.101 | 0.178 | 0.571 | 0.113 | 0.243 | 0.643 | -0.047 | 0.102 | 0.644 |
| InternalIMT | aa | rs149047975 | 0.125 | 2.10E-03 | 0.075 | 0.978 | -0.27 | 0.363 | 0.457 | 0.108 | 0.152 | 0.478 |
| InternalIMT | aa | rs3824614 | 0.02 | 0.014 | 0.07 | 0.841 | -1.02 | 0.872 | 0.24 | 0.389 | 0.378 | 0.303 |
| InternalIMT | aa | rs140254404 | . | . | . | . | . | . | . | . | . | . |
| InternalIMT | aa | rs145262684 | 0.013 | 0.024 | 0.072 | 0.745 | -0.557 | 1.48 | 0.707 | 0.337 | 0.634 | 0.595 |
| InternalIMT | aa | rs76735482 | 0.02 | 0.014 | 0.07 | 0.842 | -1.03 | 0.873 | 0.238 | 0.391 | 0.378 | 0.301 |
| InternalIMT | aa | rs73283198 | 0.027 | 0.067 | 0.071 | 0.341 | 1.43 | 0.708 | 0.044 | -0.611 | 0.288 | 0.034 |
| InternalIMT | aa | rs74911239 | 0.029 | 0.028 | 0.068 | 0.686 | -0.226 | 1.37 | 0.869 | 0.226 | 0.588 | 0.701 |
| InternalIMT | aa | rs150551647 | 0.005 | . | . | . | . | . | . | . | . | . |
| InternalIMT | aa | rs115200794 | 0.034 | 9.30E-03 | 0.071 | 0.897 | -0.866 | 0.542 | 0.11 | 0.342 | 0.225 | 0.128 |
| InternalIMT | aa | rs74130823 | 0.139 | -0.024 | 0.083 | 0.775 | -0.538 | 0.277 | 0.053 | 0.197 | 0.116 | 0.089 |
| InternalIMT | aa | rs2279435 | 0.662 | 0.231 | 0.157 | 0.141 | 0.368 | 0.243 | 0.131 | -0.147 | 0.101 | 0.146 |
| InternalIMT | aa | rs147279673 | 0.015 | -2.50E-05 | 0.073 | 1 | -2.11 | 1.41 | 0.136 | 0.959 | 0.574 | 0.095 |
| InternalIMT | aa | rs183525146 | 0.004 | . | . | . | . | . | . | . | . | . |
| InternalIMT | aa | rs3740107 | 0.754 | 0.281 | 0.201 | 0.163 | 0.352 | 0.267 | 0.189 | -0.158 | 0.112 | 0.159 |
| InternalIMT | aa | rs140697247 | . | . | . | . | . | . | . | . | . | . |
| InternalIMT | aa | rs1565096 | 0.825 | 0.137 | 0.251 | 0.585 | 0.11 | 0.321 | 0.732 | -0.063 | 0.135 | 0.641 |
| InternalIMT | aa | rs1565097 | 0.793 | 0.191 | 0.214 | 0.372 | 0.208 | 0.28 | 0.459 | -0.099 | 0.117 | 0.398 |
| InternalIMT | aa | rs144584548 | 0.016 | 0.016 | 0.07 | 0.821 | -1.11 | 0.875 | 0.205 | 0.465 | 0.376 | 0.216 |
| InternalIMT | aa | rs75811053 | 0.02 | 0.014 | 0.07 | 0.843 | -1.06 | 0.87 | 0.223 | 0.402 | 0.377 | 0.287 |
| InternalIMT | aa | rs147462967 | 0.009 | . | . | . | . | . | . | . | . | . |
| InternalIMT | aa | rs74130824 | 0.051 | -6.40E-03 | 0.072 | 0.929 | -1.06 | 0.462 | 0.022 | 0.402 | 0.195 | 0.04 |

| InternalIMT | aa | rs116883184 | 0.017 | 0.014 | 0.073 | 0.853 | -0.996 | 1.26 | 0.429 | 0.494 | 0.536 | 0.356 |
| --- | --- | --- | --- | --- | --- | --- | --- | --- | --- | --- | --- | --- |
| InternalIMT | aa | rs117258049 | 0.017 | 0.014 | 0.073 | 0.853 | -0.996 | 1.26 | 0.429 | 0.494 | 0.536 | 0.356 |
| InternalIMT | aa | rs3780908 | 0.107 | 0.022 | 0.08 | 0.779 | -0.193 | 0.312 | 0.536 | 0.048 | 0.129 | 0.712 |
| InternalIMT | aa | rs180938522 | 0.002 | . | . | . | . | . | . | . | . | . |
| InternalIMT | aa | rs76687372 | 0.003 | . | . | . | . | . | . | . | . | . |
| InternalIMT | aa | rs3780909 | 0.606 | 0.121 | 0.141 | 0.391 | 0.181 | 0.228 | 0.428 | -0.073 | 0.095 | 0.443 |
| InternalIMT | aa | rs78281156 | 0.02 | 0.014 | 0.07 | 0.844 | -1.07 | 0.866 | 0.217 | 0.406 | 0.375 | 0.28 |
| InternalIMT | aa | rs187840631 | . | . | . | . | . | . | . | . | . | . |
| InternalIMT | aa | rs76868242 | 0.02 | 0.014 | 0.07 | 0.844 | -1.07 | 0.866 | 0.217 | 0.406 | 0.375 | 0.28 |
| InternalIMT | aa | rs192794192 | 0.001 | . | . | . | . | . | . | . | . | . |
| InternalIMT | aa | rs12781248 | 0.007 | . | . | . | . | . | . | . | . | . |
| InternalIMT | aa | rs12762375 | 0.006 | . | . | . | . | . | . | . | . | . |
| InternalIMT | aa | rs192256986 | 0.004 | . | . | . | . | . | . | . | . | . |
| InternalIMT | aa | rs184411030 | 0.01 | . | . | . | . | . | . | . | . | . |
| InternalIMT | aa | rs189601483 | 0.02 | 0.014 | 0.07 | 0.844 | -1.07 | 0.866 | 0.218 | 0.405 | 0.376 | 0.282 |
| InternalIMT | aa | rs143254579 | 0.033 | 0.019 | 0.071 | 0.784 | -0.165 | 0.737 | 0.822 | 0.109 | 0.297 | 0.714 |
| InternalIMT | aa | rs12253903 | 0.017 | 0.034 | 0.071 | 0.628 | 1.06 | 0.749 | 0.156 | -0.375 | 0.295 | 0.204 |
| InternalIMT | aa | rs35489926 | 0.006 | . | . | . | . | . | . | . | . | . |
| InternalIMT | aa | rs71494783 | 0.039 | 0.03 | 0.072 | 0.677 | 0.056 | 0.612 | 0.926 | -0.041 | 0.253 | 0.87 |
| InternalIMT | aa | rs180711602 | 0.002 | . | . | . | . | . | . | . | . | . |
| InternalIMT | aa | rs117312402 | 0.02 | 0.014 | 0.07 | 0.844 | -1.07 | 0.865 | 0.216 | 0.406 | 0.375 | 0.28 |
| InternalIMT | aa | rs74831694 | 0.004 | . | . | . | . | . | . | . | . | . |
| InternalIMT | aa | rs140301943 | 0.002 | . | . | . | . | . | . | . | . | . |
| InternalIMT | aa | rs78553679 | 0.013 | 0.015 | 0.072 | 0.833 | -1.32 | 1.79 | 0.461 | 0.567 | 0.72 | 0.431 |
| InternalIMT | aa | rs78334250 | 0.02 | 0.014 | 0.07 | 0.844 | -1.07 | 0.865 | 0.216 | 0.405 | 0.375 | 0.28 |
| InternalIMT | aa | rs76000028 | 0.02 | 0.014 | 0.07 | 0.844 | -1.07 | 0.865 | 0.216 | 0.405 | 0.375 | 0.28 |
| InternalIMT | aa | rs145195254 | 0.001 | . | . | . | . | . | . | . | . | . |
| InternalIMT | aa | rs137888527 | 0.001 | . | . | . | . | . | . | . | . | . |
| InternalIMT | aa | rs149109592 | 0.005 | . | . | . | . | . | . | . | . | . |
| InternalIMT | aa | rs80280010 | 0.032 | 0.021 | 0.071 | 0.765 | -0.113 | 0.752 | 0.881 | 0.087 | 0.303 | 0.774 |
| InternalIMT | aa | rs189995374 | . | . | . | . | . | . | . | . | . | . |
| InternalIMT | aa | rs75467770 | 0.021 | 0.014 | 0.07 | 0.847 | -1.07 | 0.865 | 0.217 | 0.406 | 0.375 | 0.28 |
| InternalIMT | aa | rs1487562 | 0.139 | 9.20E-03 | 0.082 | 0.91 | -0.314 | 0.272 | 0.249 | 0.094 | 0.112 | 0.401 |
| InternalIMT | aa | rs12266963 | 0.039 | 0.03 | 0.072 | 0.678 | 0.054 | 0.611 | 0.93 | -0.041 | 0.253 | 0.872 |
| InternalIMT | aa | rs7099874 | 0.787 | 0.143 | 0.239 | 0.551 | 0.119 | 0.319 | 0.711 | -0.071 | 0.133 | 0.594 |
| InternalIMT | aa | rs75687019 | 0.02 | 0.014 | 0.07 | 0.844 | -1.07 | 0.865 | 0.216 | 0.405 | 0.375 | 0.28 |
| InternalIMT | aa | rs148240462 | 0.031 | 0.029 | 0.068 | 0.676 | -0.054 | 1.26 | 0.966 | 0.142 | 0.539 | 0.792 |
| InternalIMT | aa | rs141278174 | . | . | . | . | . | . | . | . | . | . |
| InternalIMT | aa | rs76404852 | 0.02 | 0.014 | 0.07 | 0.844 | -1.07 | 0.865 | 0.216 | 0.405 | 0.375 | 0.28 |
| InternalIMT | aa | rs139112240 | 0.006 | . | . | . | . | . | . | . | . | . |
| InternalIMT | aa | rs12783037 | 0.042 | 0.032 | 0.072 | 0.654 | 0.102 | 0.612 | 0.868 | -0.05 | 0.255 | 0.844 |
| InternalIMT | aa | rs72796481 | 0.006 | . | . | . | . | . | . | . | . | . |
| InternalIMT | aa | rs76798749 | 0.023 | 0.016 | 0.07 | 0.825 | -0.95 | 0.876 | 0.279 | 0.375 | 0.384 | 0.329 |
| InternalIMT | aa | rs187732548 | . | . | . | . | . | . | . | . | . | . |
| InternalIMT | aa | rs75675474 | 0.023 | 0.016 | 0.07 | 0.825 | -0.949 | 0.876 | 0.279 | 0.375 | 0.384 | 0.329 |
| InternalIMT | aa | rs76552293 | 0.023 | 0.016 | 0.07 | 0.824 | -0.947 | 0.876 | 0.28 | 0.374 | 0.384 | 0.33 |
| InternalIMT | aa | rs147445993 | 0.002 | . | . | . | . | . | . | . | . | . |
| InternalIMT | aa | rs115902283 | 0.023 | 0.015 | 0.07 | 0.827 | -0.956 | 0.876 | 0.275 | 0.377 | 0.384 | 0.327 |
| InternalIMT | aa | rs4540799 | 0.786 | 0.149 | 0.238 | 0.532 | 0.128 | 0.319 | 0.688 | -0.075 | 0.133 | 0.574 |
| InternalIMT | aa | rs12765320 | 0.172 | 4.50E-03 | 0.084 | 0.958 | -0.286 | 0.266 | 0.284 | 0.086 | 0.111 | 0.439 |
| InternalIMT | aa | rs12782256 | 0.006 | . | . | . | . | . | . | . | . | . |
| InternalIMT | aa | rs191727256 | 0 | . | . | . | . | . | . | . | . | . |
| InternalIMT | aa | rs2082111 | 0.111 | 0.019 | 0.079 | 0.808 | -0.222 | 0.312 | 0.477 | 0.059 | 0.129 | 0.646 |
| InternalIMT | aa | rs2082110 | 0.157 | -9.20E-03 | 0.082 | 0.911 | -0.427 | 0.271 | 0.115 | 0.142 | 0.112 | 0.206 |
| InternalIMT | aa | rs2082109 | 0.157 | -9.20E-03 | 0.082 | 0.911 | -0.427 | 0.271 | 0.115 | 0.142 | 0.112 | 0.206 |
| InternalIMT | aa | rs117316662 | 0.02 | 0.014 | 0.07 | 0.844 | -1.07 | 0.865 | 0.216 | 0.405 | 0.375 | 0.28 |
| InternalIMT | aa | rs12247163 | 0.177 | 0.018 | 0.084 | 0.834 | -0.18 | 0.275 | 0.512 | 0.046 | 0.112 | 0.682 |
| InternalIMT | aa | rs12355888 | 0.075 | -0.019 | 0.076 | 0.804 | -0.823 | 0.333 | 0.013 | 0.316 | 0.14 | 0.025 |
| InternalIMT | aa | rs143772290 | 0.005 | . | . | . | . | . | . | . | . | . |
| InternalIMT | aa | rs146849452 | 0.003 | . | . | . | . | . | . | . | . | . |
| InternalIMT | aa | rs140267121 | 0.003 | . | . | . | . | . | . | . | . | . |
| InternalIMT | aa | rs7080474 | 0.155 | 0.034 | 0.083 | 0.681 | -0.07 | 0.284 | 0.804 | 4.60E-03 | 0.115 | 0.968 |
| InternalIMT | aa | rs144066203 | 0.001 | . | . | . | . | . | . | . | . | . |
| InternalIMT | aa | rs12765209 | 0.021 | 0.014 | 0.07 | 0.845 | -1.07 | 0.865 | 0.217 | 0.404 | 0.375 | 0.281 |
| InternalIMT | aa | rs150930735 | 0.001 | . | . | . | . | . | . | . | . | . |
| InternalIMT | aa | rs7068039 | 0.177 | 0.018 | 0.084 | 0.835 | -0.182 | 0.275 | 0.507 | 0.046 | 0.112 | 0.679 |
| InternalIMT | aa | rs117622652 | 0.02 | 0.014 | 0.07 | 0.844 | -1.07 | 0.865 | 0.217 | 0.405 | 0.375 | 0.28 |
| InternalIMT | aa | rs190191514 | 0.001 | . | . | . | . | . | . | . | . | . |
| InternalIMT | aa | rs74130835 | 0.023 | 0.018 | 0.071 | 0.806 | -1.04 | 0.595 | 0.081 | 0.378 | 0.245 | 0.123 |
| InternalIMT | aa | rs1811604 | 0.022 | 0.015 | 0.072 | 0.837 | -1.08 | 0.58 | 0.062 | 0.394 | 0.241 | 0.102 |
| InternalIMT | aa | rs146971256 | . | . | . | . | . | . | . | . | . | . |
| InternalIMT | aa | rs74336000 | 0.005 | . | . | . | . | . | . | . | . | . |
| InternalIMT | aa | rs186046492 | 0.005 | . | . | . | . | . | . | . | . | . |
| InternalIMT | aa | rs34971823 | 0.006 | . | . | . | . | . | . | . | . | . |
| InternalIMT | aa | rs75227446 | 0.02 | 0.014 | 0.07 | 0.845 | -1.07 | 0.864 | 0.215 | 0.407 | 0.375 | 0.278 |
| InternalIMT | aa | rs190457599 | 0.003 | . | . | . | . | . | . | . | . | . |
| InternalIMT | aa | rs79591917 | 0.02 | 0.014 | 0.07 | 0.843 | -1.07 | 0.865 | 0.217 | 0.404 | 0.375 | 0.281 |
| InternalIMT | aa | rs702365 | 0.609 | 0.141 | 0.144 | 0.328 | 0.216 | 0.23 | 0.349 | -0.088 | 0.095 | 0.359 |
| InternalIMT | aa | rs79643836 | 0.021 | 0.014 | 0.07 | 0.847 | -1.02 | 0.856 | 0.233 | 0.387 | 0.372 | 0.298 |
| InternalIMT | aa | rs702366 | 0.61 | 0.111 | 0.143 | 0.438 | 0.162 | 0.231 | 0.483 | -0.064 | 0.096 | 0.506 |
| InternalIMT | aa | rs1059696 | 0.124 | 0.037 | 0.081 | 0.647 | -0.031 | 0.336 | 0.925 | -0.016 | 0.136 | 0.907 |
| InternalIMT | aa | rs901681 | 0.61 | 0.102 | 0.143 | 0.478 | 0.146 | 0.231 | 0.528 | -0.056 | 0.096 | 0.559 |
| InternalIMT | aa | rs61854092 | 0.122 | 0.036 | 0.081 | 0.66 | -0.044 | 0.336 | 0.897 | -0.011 | 0.136 | 0.937 |
| InternalIMT | aa | rs148080753 | 0.001 | . | . | . | . | . | . | . | . | . |
| InternalIMT | aa | rs61854093 | 0.145 | 0.024 | 0.083 | 0.77 | -0.176 | 0.303 | 0.563 | 0.042 | 0.123 | 0.732 |
| InternalIMT | aa | rs141822774 | 0.002 | . | . | . | . | . | . | . | . | . |

| InternalIMT | aa | rs74130836 | 0.023 | 0.017 | 0.071 | 0.808 | -1.06 | 0.596 | 0.077 | 0.384 | 0.245 | 0.118 |
| --- | --- | --- | --- | --- | --- | --- | --- | --- | --- | --- | --- | --- |
| InternalIMT | aa | rs150620963 | 0.01 | . | . | . | . | . | . | . | . | . |
| InternalIMT | aa | rs3780913 | 0.781 | 0.135 | 0.236 | 0.568 | 0.104 | 0.317 | 0.743 | -0.066 | 0.132 | 0.617 |
| InternalIMT | aa | rs142255723 | 0.002 | . | . | . | . | . | . | . | . | . |
| InternalIMT | aa | rs3780914 | 0.607 | 0.111 | 0.143 | 0.435 | 0.162 | 0.231 | 0.484 | -0.064 | 0.096 | 0.502 |
| InternalIMT | aa | rs182656507 | 0.002 | . | . | . | . | . | . | . | . | . |
| InternalIMT | aa | rs2291427 | 0.756 | 0.164 | 0.219 | 0.455 | 0.161 | 0.296 | 0.588 | -0.085 | 0.124 | 0.492 |
| InternalIMT | aa | rs10751382 | 0.756 | 0.159 | 0.219 | 0.468 | 0.155 | 0.297 | 0.602 | -0.082 | 0.124 | 0.508 |
| InternalIMT | aa | rs117610108 | 0.02 | 0.014 | 0.07 | 0.842 | -1.06 | 0.865 | 0.221 | 0.401 | 0.375 | 0.285 |
| InternalIMT | aa | rs144966941 | 0.037 | 0.022 | 0.073 | 0.764 | -0.237 | 0.656 | 0.718 | 0.154 | 0.277 | 0.578 |
| InternalIMT | aa | rs41502051 | 0.022 | 0.049 | 0.071 | 0.492 | 1.04 | 1.1 | 0.344 | -0.446 | 0.44 | 0.31 |
| InternalIMT | aa | rs41426046 | 0.02 | 0.014 | 0.07 | 0.841 | -1.06 | 0.865 | 0.222 | 0.4 | 0.375 | 0.287 |
| InternalIMT | aa | rs41519248 | 0.02 | 0.014 | 0.07 | 0.841 | -1.06 | 0.865 | 0.222 | 0.4 | 0.375 | 0.287 |
| InternalIMT | aa | rs189108245 | 0.002 | . | . | . | . | . | . | . | . | . |
| InternalIMT | aa | rs7896431 | 0.591 | 0.141 | 0.145 | 0.332 | 0.218 | 0.24 | 0.365 | -0.09 | 0.099 | 0.366 |
| InternalIMT | aa | rs183724547 | 0.003 | . | . | . | . | . | . | . | . | . |
| InternalIMT | aa | rs142044990 | 0.006 | . | . | . | . | . | . | . | . | . |
| InternalIMT | aa | rs188140929 | . | . | . | . | . | . | . | . | . | . |
| InternalIMT | aa | rs76819096 | 0.008 | . | . | . | . | . | . | . | . | . |
| InternalIMT | aa | rs10751383 | 0.654 | 0.123 | 0.149 | 0.41 | 0.18 | 0.231 | 0.436 | -0.067 | 0.095 | 0.482 |
| InternalIMT | aa | rs117819321 | 0.016 | 0.036 | 0.073 | 0.62 | 0.303 | 1.02 | 0.767 | -0.199 | 0.432 | 0.645 |
| InternalIMT | aa | rs2242332 | 0.75 | 0.035 | 0.211 | 0.868 | -0.029 | 0.287 | 0.918 | -7.30E-03 | 0.119 | 0.951 |
| InternalIMT | aa | rs2242333 | 0.06 | 0.034 | 0.072 | 0.635 | 0.108 | 0.436 | 0.805 | -0.072 | 0.182 | 0.691 |
| InternalIMT | aa | rs2242334 | 0.752 | 0.029 | 0.213 | 0.893 | -0.042 | 0.29 | 0.884 | -3.30E-03 | 0.12 | 0.978 |
| InternalIMT | aa | rs189465041 | 0.002 | . | . | . | . | . | . | . | . | . |
| InternalIMT | aa | rs28395868 | 0.014 | 0.017 | 0.071 | 0.809 | -0.941 | 0.978 | 0.336 | 0.394 | 0.418 | 0.346 |
| InternalIMT | aa | rs56067550 | 0.016 | 0.016 | 0.07 | 0.816 | -1.09 | 0.89 | 0.221 | 0.456 | 0.381 | 0.232 |
| InternalIMT | aa | rs1051713 | 0.134 | 0.014 | 0.082 | 0.862 | -0.228 | 0.321 | 0.478 | 0.063 | 0.13 | 0.627 |
| InternalIMT | aa | rs115074210 | 0.009 | . | . | . | . | . | . | . | . | . |
| InternalIMT | aa | rs7900977 | 0.822 | 0.041 | 0.267 | 0.878 | -0.029 | 0.343 | 0.933 | -7.40E-03 | 0.143 | 0.959 |
| InternalIMT | aa | rs147613891 | 0.004 | . | . | . | . | . | . | . | . | . |
| InternalIMT | aa | rs2229136 | 0.064 | 0.034 | 0.073 | 0.639 | 0.318 | 0.472 | 0.501 | -0.091 | 0.188 | 0.63 |
| InternalIMT | aa | rs185482841 | 0.001 | . | . | . | . | . | . | . | . | . |
| InternalIMT | aa | rs2288619 | 0.063 | 0.034 | 0.073 | 0.639 | 0.318 | 0.472 | 0.5 | -0.091 | 0.188 | 0.629 |
| InternalIMT | aa | rs189891524 | . | . | . | . | . | . | . | . | . | . |
| InternalIMT | aa | rs56145077 | 0.115 | 0.015 | 0.08 | 0.853 | -0.258 | 0.271 | 0.341 | 0.077 | 0.112 | 0.495 |
| InternalIMT | aa | rs143216970 | 0.003 | . | . | . | . | . | . | . | . | . |
| InternalIMT | aa | rs142246478 | 0.001 | . | . | . | . | . | . | . | . | . |
| InternalIMT | aa | rs13134 | 0.008 | . | . | . | . | . | . | . | . | . |
| InternalIMT | aa | rs41314980 | 0.031 | 5.50E-03 | 0.074 | 0.94 | -0.863 | 0.925 | 0.351 | 0.411 | 0.398 | 0.302 |
| InternalIMT | aa | rs41304611 | 0.009 | . | . | . | . | . | . | . | . | . |
| InternalIMT | aa | rs193299617 | . | . | . | . | . | . | . | . | . | . |
| InternalIMT | aa | rs12772724 | 0.008 | . | . | . | . | . | . | . | . | . |
| InternalIMT | aa | rs139489537 | 0.03 | 0.018 | 0.071 | 0.797 | -0.243 | 0.74 | 0.742 | 0.149 | 0.299 | 0.618 |
| InternalIMT | aa | rs12776062 | 0.006 | . | . | . | . | . | . | . | . | . |
| InternalIMT | aa | rs752059 | 0.116 | 0.015 | 0.08 | 0.855 | -0.257 | 0.269 | 0.339 | 0.075 | 0.111 | 0.499 |
| InternalIMT | aa | rs147649982 | 0.049 | 0.029 | 0.076 | 0.704 | -0.012 | 0.52 | 0.981 | 7.50E-03 | 0.22 | 0.973 |
| InternalIMT | aa | rs17157798 | 0.056 | 0.031 | 0.072 | 0.665 | 0.265 | 0.524 | 0.612 | -0.069 | 0.209 | 0.74 |
| InternalIMT | aa | rs142298619 | 0.001 | . | . | . | . | . | . | . | . | . |
| InternalIMT | aa | rs4948673 | 0.819 | -0.026 | 0.267 | 0.924 | -0.122 | 0.345 | 0.723 | 0.031 | 0.144 | 0.828 |
| InternalIMT | aa | rs74231484 | 0.066 | 0.03 | 0.073 | 0.686 | -0.021 | 0.394 | 0.958 | -0.02 | 0.165 | 0.902 |
| InternalIMT | aa | rs72796489 | 0.002 | . | . | . | . | . | . | . | . | . |
| InternalIMT | aa | rs113447578 | 0.014 | 0.037 | 0.07 | 0.599 | 0.632 | 0.849 | 0.457 | -0.331 | 0.357 | 0.353 |
| InternalIMT | aa | rs34063374 | 0.008 | . | . | . | . | . | . | . | . | . |
| InternalIMT | aa | rs11239527 | 0.011 | 0.042 | 0.071 | 0.552 | 1.31 | 1.5 | 0.381 | -0.56 | 0.611 | 0.359 |
| InternalIMT | aa | rs34371883 | 0.008 | . | . | . | . | . | . | . | . | . |
| InternalIMT | aa | rs118168828 | 0.006 | . | . | . | . | . | . | . | . | . |
| InternalIMT | aa | rs34349618 | 0.006 | . | . | . | . | . | . | . | . | . |
| InternalIMT | aa | rs80041679 | 0.03 | 0.018 | 0.071 | 0.798 | -0.249 | 0.735 | 0.735 | 0.151 | 0.297 | 0.611 |
| InternalIMT | aa | rs149640607 | 0.001 | . | . | . | . | . | . | . | . | . |
| InternalIMT | aa | rs4427523 | 0.896 | -0.221 | 0.392 | 0.573 | -0.349 | 0.513 | 0.496 | 0.137 | 0.21 | 0.516 |
| InternalIMT | aa | rs185880109 | . | . | . | . | . | . | . | . | . | . |
| InternalIMT | aa | rs191523220 | 0.001 | . | . | . | . | . | . | . | . | . |
| InternalIMT | aa | rs144315284 | 0.003 | . | . | . | . | . | . | . | . | . |
| InternalIMT | aa | rs4948674 | 0.345 | 0.09 | 0.106 | 0.392 | 0.208 | 0.237 | 0.38 | -0.084 | 0.098 | 0.391 |
| InternalIMT | aa | rs182984298 | 0.003 | . | . | . | . | . | . | . | . | . |
| InternalIMT | aa | rs56749023 | 0.016 | 0.035 | 0.071 | 0.62 | 1.49 | 0.963 | 0.122 | -0.539 | 0.375 | 0.151 |
| InternalIMT | aa | rs112508957 | 0.01 | 0.033 | 0.07 | 0.636 | -1.08 | 0.677 | 0.112 | 0.353 | 0.277 | 0.202 |
| InternalIMT | aa | rs7089063 | 0.195 | -8.70E-03 | 0.084 | 0.918 | -0.273 | 0.224 | 0.224 | 0.099 | 0.093 | 0.287 |
| InternalIMT | aa | rs145000779 | . | . | . | . | . | . | . | . | . | . |
| InternalIMT | aa | rs80201572 | 0.03 | 0.018 | 0.071 | 0.799 | -0.254 | 0.731 | 0.728 | 0.153 | 0.295 | 0.605 |
| InternalIMT | aa | rs140568227 | 0.002 | . | . | . | . | . | . | . | . | . |
| InternalIMT | aa | rs149756228 | 0.009 | . | . | . | . | . | . | . | . | . |
| InternalIMT | aa | rs112380685 | 0.008 | . | . | . | . | . | . | . | . | . |
| InternalIMT | aa | rs60481109 | 0.015 | 0.035 | 0.071 | 0.616 | 1.54 | 0.97 | 0.112 | -0.559 | 0.377 | 0.138 |
| InternalIMT | aa | rs138221843 | 0.002 | . | . | . | . | . | . | . | . | . |
| InternalIMT | aa | rs150283194 | 0.001 | . | . | . | . | . | . | . | . | . |
| InternalIMT | aa | rs138879661 | 0.004 | . | . | . | . | . | . | . | . | . |
| InternalIMT | aa | rs34960345 | 0.045 | 0.021 | 0.072 | 0.771 | -0.125 | 0.512 | 0.807 | 0.079 | 0.206 | 0.701 |
| InternalIMT | aa | rs147514068 | 0.004 | . | . | . | . | . | . | . | . | . |
| InternalIMT | aa | rs35410535 | 0.06 | 0.026 | 0.073 | 0.716 | 0.135 | 0.46 | 0.769 | -0.021 | 0.183 | 0.909 |
| InternalIMT | aa | rs191613635 | 0.001 | . | . | . | . | . | . | . | . | . |
| InternalIMT | aa | rs17157800 | 0.005 | . | . | . | . | . | . | . | . | . |
| InternalIMT | aa | rs17523413 | 0.039 | 0.025 | 0.071 | 0.731 | -0.033 | 0.614 | 0.957 | 0.055 | 0.25 | 0.827 |
| InternalIMT | aa | rs7897036 | 0.037 | -9.00E-05 | 0.075 | 0.999 | -0.905 | 0.408 | 0.027 | 0.326 | 0.174 | 0.061 |
| InternalIMT | aa | rs76334323 | 0.02 | 0.014 | 0.07 | 0.838 | -1.07 | 0.868 | 0.22 | 0.403 | 0.376 | 0.284 |

| InternalIMT | aa | rs143535991 | 0.001 | . | . | . | . | . | . | . | . | . |
| --- | --- | --- | --- | --- | --- | --- | --- | --- | --- | --- | --- | --- |
| InternalIMT | aa | rs140344979 | 0.003 | . | . | . | . | . | . | . | . | . |
| InternalIMT | aa | rs36047811 | 0.06 | 0.026 | 0.073 | 0.716 | 0.136 | 0.46 | 0.768 | -0.021 | 0.183 | 0.909 |
| InternalIMT | aa | rs76493570 | 0.043 | 0.02 | 0.072 | 0.775 | -0.128 | 0.519 | 0.806 | 0.085 | 0.209 | 0.684 |
| InternalIMT | aa | rs192998920 | . | . | . | . | . | . | . | . | . | . |
| InternalIMT | aa | rs116840852 | 0.013 | 0.016 | 0.072 | 0.822 | -1.17 | 1.8 | 0.516 | 0.514 | 0.725 | 0.478 |
| InternalIMT | aa | rs184824101 | 0 | . | . | . | . | . | . | . | . | . |
| InternalIMT | aa | rs41301621 | 0.003 | . | . | . | . | . | . | . | . | . |
| InternalIMT | aa | rs3740109 | 0.06 | 0.026 | 0.073 | 0.716 | 0.136 | 0.46 | 0.768 | -0.021 | 0.183 | 0.908 |
| InternalIMT | aa | rs7899415 | 0.025 | 0.018 | 0.075 | 0.807 | -0.452 | 1.14 | 0.692 | 0.218 | 0.483 | 0.652 |
| InternalIMT | aa | rs184395284 | 0.003 | . | . | . | . | . | . | . | . | . |
| InternalIMT | aa | rs139209589 | 0.004 | . | . | . | . | . | . | . | . | . |
| InternalIMT | aa | rs149924163 | 0.001 | . | . | . | . | . | . | . | . | . |
| InternalIMT | aa | rs3740110 | 0.02 | 0.014 | 0.07 | 0.837 | -1.06 | 0.869 | 0.223 | 0.401 | 0.377 | 0.287 |
| InternalIMT | aa | rs144335113 | 0.001 | . | . | . | . | . | . | . | . | . |
| InternalIMT | aa | rs74743385 | 0.01 | 0.033 | 0.07 | 0.636 | -1.08 | 0.676 | 0.11 | 0.355 | 0.276 | 0.199 |
| InternalIMT | aa | rs146545829 | 0.001 | . | . | . | . | . | . | . | . | . |
| InternalIMT | aa | rs181842689 | 0.001 | . | . | . | . | . | . | . | . | . |
| InternalIMT | aa | rs3740111 | 0.045 | 0.021 | 0.072 | 0.771 | -0.125 | 0.512 | 0.807 | 0.079 | 0.206 | 0.7 |
| InternalIMT | aa | rs186261599 | 0.001 | . | . | . | . | . | . | . | . | . |
| InternalIMT | aa | rs73285348 | 0.008 | . | . | . | . | . | . | . | . | . |
| InternalIMT | aa | rs186356901 | 0.001 | . | . | . | . | . | . | . | . | . |
| InternalIMT | aa | rs3824618 | 0.03 | 0.018 | 0.071 | 0.799 | -0.254 | 0.731 | 0.728 | 0.153 | 0.295 | 0.605 |
| InternalIMT | aa | rs3802548 | 0.304 | -8.90E-03 | 0.091 | 0.922 | -0.153 | 0.214 | 0.476 | 0.055 | 0.09 | 0.543 |
| InternalIMT | aa | rs3802547 | 0.02 | 0.015 | 0.07 | 0.836 | -1.07 | 0.871 | 0.221 | 0.403 | 0.377 | 0.286 |
| InternalIMT | aa | rs77677521 | 0.013 | 0.016 | 0.072 | 0.822 | -1.17 | 1.8 | 0.515 | 0.514 | 0.725 | 0.478 |
| InternalIMT | aa | rs3824616 | 0.06 | 0.026 | 0.073 | 0.716 | 0.136 | 0.46 | 0.768 | -0.021 | 0.183 | 0.908 |
| InternalIMT | aa | rs191073818 | 0.001 | . | . | . | . | . | . | . | . | . |
| InternalIMT | aa | rs147078004 | . | . | . | . | . | . | . | . | . | . |
| InternalIMT | aa | rs7908745 | 0.388 | -0.031 | 0.098 | 0.75 | -0.189 | 0.202 | 0.35 | 0.073 | 0.085 | 0.386 |
| InternalIMT | aa | rs17157811 | 0.005 | . | . | . | . | . | . | . | . | . |
| InternalIMT | aa | rs138506087 | 0.006 | . | . | . | . | . | . | . | . | . |
| InternalIMT | aa | rs77241269 | 0.016 | 0.035 | 0.071 | 0.616 | 1.53 | 0.932 | 0.101 | -0.554 | 0.364 | 0.128 |
| InternalIMT | aa | rs138374938 | 0.001 | . | . | . | . | . | . | . | . | . |
| InternalIMT | aa | rs2279433 | 0.045 | 0.021 | 0.072 | 0.768 | -0.123 | 0.508 | 0.809 | 0.075 | 0.205 | 0.713 |
| InternalIMT | aa | rs73285358 | 0.016 | 0.036 | 0.071 | 0.615 | 1.53 | 0.927 | 0.099 | -0.555 | 0.362 | 0.125 |
| InternalIMT | aa | rs11239528 | 0.303 | -6.90E-03 | 0.091 | 0.94 | -0.145 | 0.214 | 0.499 | 0.051 | 0.09 | 0.568 |
| InternalIMT | aa | rs77453386 | 0.02 | 0.015 | 0.07 | 0.834 | -1.04 | 0.872 | 0.233 | 0.393 | 0.378 | 0.298 |
| InternalIMT | aa | rs2279434 | 0.05 | 0.024 | 0.072 | 0.743 | -0.059 | 0.504 | 0.906 | 0.049 | 0.203 | 0.81 |
| InternalIMT | aa | rs149236525 | 0.005 | . | . | . | . | . | . | . | . | . |
| InternalIMT | aa | rs12573609 | 0.037 | 0.016 | 0.072 | 0.826 | -0.724 | 0.767 | 0.345 | 0.277 | 0.32 | 0.387 |
| InternalIMT | aa | rs191716655 | . | . | . | . | . | . | . | . | . | . |
| InternalIMT | aa | rs17523660 | 0.006 | . | . | . | . | . | . | . | . | . |
| InternalIMT | aa | rs142671360 | 0.004 | . | . | . | . | . | . | . | . | . |
| InternalIMT | aa | rs3764990 | 0.038 | 0.024 | 0.071 | 0.731 | -0.04 | 0.612 | 0.949 | 0.056 | 0.249 | 0.823 |
| InternalIMT | aa | rs113682861 | 0.005 | . | . | . | . | . | . | . | . | . |
| InternalIMT | aa | rs117658270 | 0.02 | 0.015 | 0.07 | 0.834 | -1.04 | 0.872 | 0.233 | 0.393 | 0.378 | 0.298 |
| InternalIMT | aa | rs187309063 | . | . | . | . | . | . | . | . | . | . |
| InternalIMT | aa | rs73285360 | 0.016 | 0.036 | 0.071 | 0.615 | 1.53 | 0.925 | 0.097 | -0.556 | 0.361 | 0.124 |
| InternalIMT | aa | rs80133289 | 0.007 | . | . | . | . | . | . | . | . | . |
| InternalIMT | aa | rs186693257 | . | . | . | . | . | . | . | . | . | . |
| InternalIMT | aa | rs35735968 | 0.049 | 0.02 | 0.072 | 0.778 | -0.118 | 0.491 | 0.811 | 0.072 | 0.197 | 0.716 |
| InternalIMT | aa | rs17157825 | 0.031 | 0.019 | 0.071 | 0.791 | -0.227 | 0.735 | 0.758 | 0.138 | 0.297 | 0.643 |
| InternalIMT | aa | rs2291428 | 0.299 | 1.70E-03 | 0.091 | 0.985 | -0.113 | 0.216 | 0.601 | 0.038 | 0.09 | 0.676 |
| InternalIMT | aa | rs2291429 | 0.304 | -0.012 | 0.092 | 0.898 | -0.161 | 0.213 | 0.45 | 0.059 | 0.09 | 0.509 |
| InternalIMT | aa | rs145714792 | 0.003 | . | . | . | . | . | . | . | . | . |
| InternalIMT | aa | rs12769244 | 0.044 | 0.021 | 0.072 | 0.772 | -0.135 | 0.507 | 0.791 | 0.08 | 0.204 | 0.697 |
| InternalIMT | aa | rs12771275 | 0.044 | 0.021 | 0.072 | 0.772 | -0.135 | 0.507 | 0.791 | 0.08 | 0.204 | 0.697 |
| InternalIMT | aa | rs146266174 | 0 | . | . | . | . | . | . | . | . | . |
| InternalIMT | aa | rs41301623 | 0.003 | . | . | . | . | . | . | . | . | . |
| InternalIMT | aa | rs2306814 | 0.021 | 0.014 | 0.07 | 0.841 | -1.09 | 0.867 | 0.208 | 0.422 | 0.375 | 0.261 |
| InternalIMT | aa | rs12244834 | 0.012 | 0.044 | 0.071 | 0.534 | 0.995 | 1.1 | 0.365 | -0.401 | 0.424 | 0.344 |
| InternalIMT | aa | rs184006011 | 0.002 | . | . | . | . | . | . | . | . | . |
| InternalIMT | aa | rs78829066 | 0.02 | 0.014 | 0.07 | 0.838 | -1.06 | 0.871 | 0.225 | 0.401 | 0.377 | 0.289 |
| InternalIMT | aa | rs34731408 | 0.044 | 0.021 | 0.072 | 0.769 | -0.129 | 0.507 | 0.799 | 0.077 | 0.204 | 0.705 |
| InternalIMT | aa | rs17451516 | 0.008 | . | . | . | . | . | . | . | . | . |
| InternalIMT | aa | rs139910133 | 0.002 | . | . | . | . | . | . | . | . | . |
| InternalIMT | aa | rs10900218 | 0.197 | -9.00E-04 | 0.084 | 0.991 | -0.214 | 0.225 | 0.34 | 0.078 | 0.093 | 0.402 |
| InternalIMT | aa | rs146023999 | 0.001 | . | . | . | . | . | . | . | . | . |
| InternalIMT | aa | rs187296958 | . | . | . | . | . | . | . | . | . | . |
| InternalIMT | aa | rs142342833 | . | . | . | . | . | . | . | . | . | . |
| InternalIMT | aa | rs143489255 | . | . | . | . | . | . | . | . | . | . |
| InternalIMT | aa | rs138532518 | . | . | . | . | . | . | . | . | . | . |
| InternalIMT | aa | rs183768079 | 0.001 | . | . | . | . | . | . | . | . | . |
| InternalIMT | aa | rs117892272 | . | . | . | . | . | . | . | . | . | . |
| InternalIMT | aa | rs192993943 | . | . | . | . | . | . | . | . | . | . |
| InternalIMT | aa | rs182989043 | 0.001 | . | . | . | . | . | . | . | . | . |
| InternalIMT | aa | rs189703435 | . | . | . | . | . | . | . | . | . | . |
| InternalIMT | aa | rs188794040 | . | . | . | . | . | . | . | . | . | . |
| InternalIMT | aa | rs143920953 | . | . | . | . | . | . | . | . | . | . |
| InternalIMT | aa | rs191029121 | . | . | . | . | . | . | . | . | . | . |
| InternalIMT | aa | rs116875448 | . | . | . | . | . | . | . | . | . | . |
| InternalIMT | aa | rs190167877 | 0.001 | . | . | . | . | . | . | . | . | . |
| InternalIMT | aa | rs184993418 | . | . | . | . | . | . | . | . | . | . |
| InternalIMT | aa | rs143241158 | . | . | . | . | . | . | . | . | . | . |
| InternalIMT | aa | rs11239500 | 0.015 | 0.038 | 0.07 | 0.588 | 0.838 | 1.41 | 0.554 | -0.331 | 0.554 | 0.551 |

| InternalIMT | aa | rs140568994 | . | . | . | . | . | . | . | . | . | . |
| --- | --- | --- | --- | --- | --- | --- | --- | --- | --- | --- | --- | --- |
| InternalIMT | aa | rs187530317 | . | . | . | . | . | . | . | . | . | . |
| InternalIMT | aa | rs145189326 | . | . | . | . | . | . | . | . | . | . |
| InternalIMT | aa | rs186203332 | . | . | . | . | . | . | . | . | . | . |
| InternalIMT | aa | rs189921909 | . | . | . | . | . | . | . | . | . | . |
| InternalIMT | aa | rs187260989 | . | . | . | . | . | . | . | . | . | . |
| InternalIMT | aa | rs114376586 | 0.013 | 0.037 | 0.07 | 0.601 | 0.783 | 1.42 | 0.58 | -0.304 | 0.554 | 0.583 |
| InternalIMT | aa | rs151070808 | . | . | . | . | . | . | . | . | . | . |
| InternalIMT | aa | rs140927225 | 0.015 | 0.038 | 0.071 | 0.594 | 0.795 | 1.41 | 0.572 | -0.311 | 0.55 | 0.572 |
| InternalIMT | aa | rs191555615 | . | . | . | . | . | . | . | . | . | . |
| InternalIMT | aa | rs80278898 | 0.005 | . | . | . | . | . | . | . | . | . |
| InternalIMT | aa | rs190849503 | . | . | . | . | . | . | . | . | . | . |
| InternalIMT | aa | rs150080315 | 0.003 | . | . | . | . | . | . | . | . | . |
| InternalIMT | aa | rs143623885 | . | . | . | . | . | . | . | . | . | . |
| InternalIMT | aa | rs187760040 | . | . | . | . | . | . | . | . | . | . |
| InternalIMT | aa | rs143917143 | 0.005 | . | . | . | . | . | . | . | . | . |
| InternalIMT | aa | rs149851136 | 0.001 | . | . | . | . | . | . | . | . | . |
| InternalIMT | aa | rs181612054 | . | . | . | . | . | . | . | . | . | . |
| InternalIMT | aa | rs191006548 | . | . | . | . | . | . | . | . | . | . |
| InternalIMT | aa | rs117377631 | 0.026 | 0.019 | 0.072 | 0.791 | -0.5 | 0.765 | 0.513 | 0.294 | 0.327 | 0.368 |
| InternalIMT | aa | rs182107634 | . | . | . | . | . | . | . | . | . | . |
| InternalIMT | aa | rs190211867 | . | . | . | . | . | . | . | . | . | . |
| InternalIMT | aa | rs148293457 | . | . | . | . | . | . | . | . | . | . |
| InternalIMT | aa | rs191811733 | . | . | . | . | . | . | . | . | . | . |
| InternalIMT | aa | rs146109272 | . | . | . | . | . | . | . | . | . | . |
| InternalIMT | aa | rs148397915 | . | . | . | . | . | . | . | . | . | . |
| InternalIMT | aa | rs142233290 | . | . | . | . | . | . | . | . | . | . |
| InternalIMT | aa | rs80226874 | . | . | . | . | . | . | . | . | . | . |
| InternalIMT | aa | rs144496203 | . | . | . | . | . | . | . | . | . | . |
| InternalIMT | aa | rs191062130 | . | . | . | . | . | . | . | . | . | . |
| InternalIMT | aa | rs185807817 | . | . | . | . | . | . | . | . | . | . |
| InternalIMT | aa | rs117846213 | . | . | . | . | . | . | . | . | . | . |
| InternalIMT | aa | rs190545473 | . | . | . | . | . | . | . | . | . | . |
| InternalIMT | aa | rs192364918 | . | . | . | . | . | . | . | . | . | . |
| InternalIMT | aa | rs182939241 | . | . | . | . | . | . | . | . | . | . |
| InternalIMT | aa | rs12571125 | . | . | . | . | . | . | . | . | . | . |
| InternalIMT | aa | rs192418941 | . | . | . | . | . | . | . | . | . | . |
| InternalIMT | aa | rs141270243 | . | . | . | . | . | . | . | . | . | . |
| InternalIMT | aa | rs182615502 | . | . | . | . | . | . | . | . | . | . |
| InternalIMT | aa | rs146669856 | 0.004 | . | . | . | . | . | . | . | . | . |
| InternalIMT | aa | rs78290029 | 0.015 | 0.038 | 0.071 | 0.592 | 0.632 | 1.15 | 0.584 | -0.246 | 0.453 | 0.587 |
| InternalIMT | aa | rs139955565 | . | . | . | . | . | . | . | . | . | . |
| InternalIMT | aa | rs12267314 | 0.013 | 0.038 | 0.071 | 0.59 | 0.628 | 1.12 | 0.574 | -0.262 | 0.436 | 0.547 |
| InternalIMT | aa | rs74477197 | . | . | . | . | . | . | . | . | . | . |
| InternalIMT | aa | rs188147236 | . | . | . | . | . | . | . | . | . | . |
| InternalIMT | aa | rs2228065 | 0.012 | 0.043 | 0.071 | 0.544 | 1.06 | 1.25 | 0.395 | -0.427 | 0.488 | 0.381 |
| InternalIMT | aa | rs116961353 | 0.007 | . | . | . | . | . | . | . | . | . |
| InternalIMT | aa | rs190222272 | . | . | . | . | . | . | . | . | . | . |
| InternalIMT | aa | rs182719940 | . | . | . | . | . | . | . | . | . | . |
| InternalIMT | aa | rs184477413 | . | . | . | . | . | . | . | . | . | . |
| InternalIMT | aa | rs12265175 | 0.013 | 0.045 | 0.071 | 0.526 | 1.12 | 1.22 | 0.358 | -0.461 | 0.475 | 0.332 |
| InternalIMT | aa | rs182928276 | . | . | . | . | . | . | . | . | . | . |
| InternalIMT | aa | rs144409895 | . | . | . | . | . | . | . | . | . | . |
| InternalIMT | aa | rs58998447 | 0.003 | . | . | . | . | . | . | . | . | . |
| InternalIMT | aa | rs188668832 | . | . | . | . | . | . | . | . | . | . |
| InternalIMT | aa | rs148641686 | . | . | . | . | . | . | . | . | . | . |
| InternalIMT | aa | rs75468791 | . | . | . | . | . | . | . | . | . | . |
| InternalIMT | aa | rs147327397 | . | . | . | . | . | . | . | . | . | . |
| InternalIMT | aa | rs77354597 | 0.001 | . | . | . | . | . | . | . | . | . |
| InternalIMT | aa | rs41365544 | 0.012 | 0.045 | 0.071 | 0.526 | 1.05 | 1.11 | 0.344 | -0.428 | 0.43 | 0.32 |
| InternalIMT | aa | rs187564188 | . | . | . | . | . | . | . | . | . | . |
| InternalIMT | aa | rs183511984 | . | . | . | . | . | . | . | . | . | . |
| InternalIMT | aa | rs12262783 | 0.011 | 0.047 | 0.071 | 0.507 | 1.28 | 1.22 | 0.296 | -0.517 | 0.471 | 0.272 |
| InternalIMT | aa | rs188817687 | 0.001 | . | . | . | . | . | . | . | . | . |
| InternalIMT | aa | rs3815968 | . | . | . | . | . | . | . | . | . | . |
| InternalIMT | aa | rs187822300 | . | . | . | . | . | . | . | . | . | . |
| InternalIMT | aa | rs188902172 | 0.001 | . | . | . | . | . | . | . | . | . |
| InternalIMT | aa | rs181190448 | . | . | . | . | . | . | . | . | . | . |
| InternalIMT | aa | rs182699093 | . | . | . | . | . | . | . | . | . | . |
| InternalIMT | aa | rs182631303 | . | . | . | . | . | . | . | . | . | . |
| InternalIMT | aa | rs187014172 | 0.001 | . | . | . | . | . | . | . | . | . |
| InternalIMT | aa | rs142575406 | . | . | . | . | . | . | . | . | . | . |
| InternalIMT | aa | rs180711033 | . | . | . | . | . | . | . | . | . | . |
| InternalIMT | aa | rs137894823 | 0.001 | . | . | . | . | . | . | . | . | . |
| InternalIMT | aa | rs189053273 | 0 | . | . | . | . | . | . | . | . | . |
| InternalIMT | aa | rs143499388 | . | . | . | . | . | . | . | . | . | . |
| InternalIMT | aa | rs182936001 | 0.001 | . | . | . | . | . | . | . | . | . |
| InternalIMT | aa | rs148456406 | 0 | . | . | . | . | . | . | . | . | . |
| InternalIMT | aa | rs182499531 | 0.001 | . | . | . | . | . | . | . | . | . |
| InternalIMT | aa | rs74884915 | 0.005 | . | . | . | . | . | . | . | . | . |
| InternalIMT | aa | rs184046374 | . | . | . | . | . | . | . | . | . | . |
| InternalIMT | aa | rs74333993 | 0.004 | . | . | . | . | . | . | . | . | . |
| InternalIMT | aa | rs138889719 | . | . | . | . | . | . | . | . | . | . |
| InternalIMT | aa | rs75330330 | 0.003 | . | . | . | . | . | . | . | . | . |
| InternalIMT | aa | rs193051693 | . | . | . | . | . | . | . | . | . | . |
| InternalIMT | aa | rs145929633 | . | . | . | . | . | . | . | . | . | . |

| InternalIMT | aa | rs184076904 | 0.001 | . | . | . | . | . | . | . | . | . |
| --- | --- | --- | --- | --- | --- | --- | --- | --- | --- | --- | --- | --- |
| InternalIMT | aa | rs141802494 | 0.002 | . | . | . | . | . | . | . | . | . |
| InternalIMT | aa | rs149519759 | 0 | . | . | . | . | . | . | . | . | . |
| InternalIMT | aa | rs145467260 | 0.002 | . | . | . | . | . | . | . | . | . |
| InternalIMT | aa | rs79620914 | 0.006 | . | . | . | . | . | . | . | . | . |
| InternalIMT | aa | rs140538633 | 0.005 | . | . | . | . | . | . | . | . | . |
| InternalIMT | aa | rs140862301 | 0.001 | . | . | . | . | . | . | . | . | . |
| InternalIMT | aa | rs114615847 | 0.003 | . | . | . | . | . | . | . | . | . |
| InternalIMT | aa | rs141800259 | 0.004 | . | . | . | . | . | . | . | . | . |
| InternalIMT | aa | rs149779059 | 0.003 | . | . | . | . | . | . | . | . | . |
| InternalIMT | aa | rs116539253 | 0.001 | . | . | . | . | . | . | . | . | . |
| InternalIMT | aa | rs184979550 | 0 | . | . | . | . | . | . | . | . | . |
| InternalIMT | aa | rs190128940 | 0.001 | . | . | . | . | . | . | . | . | . |
| InternalIMT | aa | rs146594816 | . | . | . | . | . | . | . | . | . | . |
| InternalIMT | aa | rs141370766 | 0.003 | . | . | . | . | . | . | . | . | . |
| InternalIMT | aa | rs144670033 | . | . | . | . | . | . | . | . | . | . |
| InternalIMT | aa | rs187152174 | 0.003 | . | . | . | . | . | . | . | . | . |
| InternalIMT | aa | rs184180263 | 0.002 | . | . | . | . | . | . | . | . | . |
| InternalIMT | aa | rs140075861 | 0.002 | . | . | . | . | . | . | . | . | . |
| InternalIMT | aa | rs186483129 | . | . | . | . | . | . | . | . | . | . |
| InternalIMT | aa | rs186634282 | 0.003 | . | . | . | . | . | . | . | . | . |
| InternalIMT | aa | rs9988663 | 0.004 | . | . | . | . | . | . | . | . | . |
| InternalIMT | aa | rs138828529 | 0.004 | . | . | . | . | . | . | . | . | . |
| InternalIMT | aa | rs188777615 | 0.001 | . | . | . | . | . | . | . | . | . |
| InternalIMT | aa | rs192321882 | 0.001 | . | . | . | . | . | . | . | . | . |
| InternalIMT | aa | rs150812270 | 0.003 | . | . | . | . | . | . | . | . | . |
| InternalIMT | aa | rs185906603 | . | . | . | . | . | . | . | . | . | . |
| InternalIMT | aa | rs191070862 | . | . | . | . | . | . | . | . | . | . |
| InternalIMT | aa | rs182904978 | . | . | . | . | . | . | . | . | . | . |
| InternalIMT | aa | rs192679655 | . | . | . | . | . | . | . | . | . | . |
| InternalIMT | aa | rs189673086 | 0.003 | . | . | . | . | . | . | . | . | . |
| InternalIMT | aa | rs181141892 | 0.001 | . | . | . | . | . | . | . | . | . |
| InternalIMT | aa | rs186404123 | . | . | . | . | . | . | . | . | . | . |
| InternalIMT | aa | rs192117287 | 0.001 | . | . | . | . | . | . | . | . | . |
| InternalIMT | aa | rs182748835 | 0 | . | . | . | . | . | . | . | . | . |
| InternalIMT | aa | rs141854549 | 0.005 | . | . | . | . | . | . | . | . | . |
| InternalIMT | aa | rs140163397 | 0.001 | . | . | . | . | . | . | . | . | . |
| InternalIMT | aa | rs146347300 | 0.001 | . | . | . | . | . | . | . | . | . |
| InternalIMT | aa | rs139362377 | 0.005 | . | . | . | . | . | . | . | . | . |
| InternalIMT | aa | rs185087671 | 0 | . | . | . | . | . | . | . | . | . |
| InternalIMT | aa | rs190466574 | 0 | . | . | . | . | . | . | . | . | . |
| InternalIMT | aa | rs147012778 | 0.001 | . | . | . | . | . | . | . | . | . |
| InternalIMT | aa | rs138236960 | 0.003 | . | . | . | . | . | . | . | . | . |
| InternalIMT | aa | rs116310311 | 0.005 | . | . | . | . | . | . | . | . | . |
| InternalIMT | aa | rs142868356 | 0.003 | . | . | . | . | . | . | . | . | . |
| InternalIMT | aa | rs144956240 | 0.001 | . | . | . | . | . | . | . | . | . |
| InternalIMT | aa | rs188527776 | . | . | . | . | . | . | . | . | . | . |
| InternalIMT | aa | rs183971165 | 0.003 | . | . | . | . | . | . | . | . | . |
| InternalIMT | aa | rs76748216 | 0.003 | . | . | . | . | . | . | . | . | . |
| InternalIMT | aa | rs148554990 | . | . | . | . | . | . | . | . | . | . |
| InternalIMT | aa | rs114446477 | 0.007 | . | . | . | . | . | . | . | . | . |
| InternalIMT | aa | rs185223933 | 0.002 | . | . | . | . | . | . | . | . | . |
| InternalIMT | aa | rs76869726 | 0.003 | . | . | . | . | . | . | . | . | . |
| InternalIMT | aa | rs151200830 | 0.005 | . | . | . | . | . | . | . | . | . |
| InternalIMT | aa | rs17157731 | 0.005 | . | . | . | . | . | . | . | . | . |
| InternalIMT | aa | rs181243469 | . | . | . | . | . | . | . | . | . | . |
| InternalIMT | aa | rs186890707 | . | . | . | . | . | . | . | . | . | . |
| InternalIMT | aa | rs184063360 | . | . | . | . | . | . | . | . | . | . |
| InternalIMT | aa | rs188550237 | 0.001 | . | . | . | . | . | . | . | . | . |
| InternalIMT | aa | rs140931068 | 0.003 | . | . | . | . | . | . | . | . | . |
| InternalIMT | aa | rs74867877 | 0.001 | . | . | . | . | . | . | . | . | . |
| InternalIMT | aa | rs11239495 | 0.015 | 0.038 | 0.07 | 0.586 | 0.71 | 1.66 | 0.669 | -0.241 | 0.697 | 0.729 |
| InternalIMT | aa | rs149745800 | . | . | . | . | . | . | . | . | . | . |
| InternalIMT | aa | rs145044458 | 0.005 | . | . | . | . | . | . | . | . | . |
| InternalIMT | aa | rs188067427 | 0 | . | . | . | . | . | . | . | . | . |
| InternalIMT | aa | rs146827800 | . | . | . | . | . | . | . | . | . | . |
| InternalIMT | aa | rs140619817 | 0.005 | . | . | . | . | . | . | . | . | . |
| InternalIMT | aa | rs892689 | 0.998 | . | . | . | . | . | . | . | . | . |
| InternalIMT | aa | rs143642005 | 0.001 | . | . | . | . | . | . | . | . | . |
| InternalIMT | aa | rs181929304 | 0.001 | . | . | . | . | . | . | . | . | . |
| InternalIMT | aa | rs191086173 | 0.001 | . | . | . | . | . | . | . | . | . |
| InternalIMT | aa | rs114613116 | 0.005 | . | . | . | . | . | . | . | . | . |
| InternalIMT | aa | rs141076768 | . | . | . | . | . | . | . | . | . | . |
| InternalIMT | aa | rs187316531 | . | . | . | . | . | . | . | . | . | . |
| InternalIMT | aa | rs11239499 | 0.014 | 0.04 | 0.07 | 0.571 | 0.945 | 1.39 | 0.498 | -0.383 | 0.546 | 0.484 |
| InternalIMT | aa | rs192726371 | 0.001 | . | . | . | . | . | . | . | . | . |
| InternalIMT | aa | rs74128481 | 0.004 | . | . | . | . | . | . | . | . | . |
| InternalIMT | aa | rs74128482 | 0.003 | . | . | . | . | . | . | . | . | . |
| InternalIMT | aa | rs11239502 | 0.008 | . | . | . | . | . | . | . | . | . |
| InternalIMT | aa | rs116330285 | . | . | . | . | . | . | . | . | . | . |
| InternalIMT | aa | rs189926729 | 0.001 | . | . | . | . | . | . | . | . | . |
| InternalIMT | aa | rs188975779 | 0.003 | . | . | . | . | . | . | . | . | . |
| InternalIMT | aa | rs143846038 | . | . | . | . | . | . | . | . | . | . |
| InternalIMT | aa | rs74128483 | 0.004 | . | . | . | . | . | . | . | . | . |
| InternalIMT | aa | rs139292055 | 0.013 | 0.037 | 0.07 | 0.603 | 0.769 | 1.42 | 0.588 | -0.298 | 0.556 | 0.591 |
| InternalIMT | aa | rs11239503 | 0.013 | 0.037 | 0.07 | 0.603 | 0.769 | 1.42 | 0.588 | -0.298 | 0.555 | 0.592 |

| InternalIMT | aa | rs58838686 | 0.003 | . | . | . | . | . | . | . | . | . |
| --- | --- | --- | --- | --- | --- | --- | --- | --- | --- | --- | --- | --- |
| InternalIMT | aa | rs185825373 | 0.001 | . | . | . | . | . | . | . | . | . |
| InternalIMT | aa | rs11239504 | 0.013 | 0.037 | 0.07 | 0.603 | 0.769 | 1.42 | 0.588 | -0.298 | 0.555 | 0.592 |
| InternalIMT | aa | rs150012354 | 0 | . | . | . | . | . | . | . | . | . |
| InternalIMT | aa | rs187763096 | 0.001 | . | . | . | . | . | . | . | . | . |
| InternalIMT | aa | rs193043982 | 0.002 | . | . | . | . | . | . | . | . | . |
| InternalIMT | aa | rs79186884 | 0.004 | . | . | . | . | . | . | . | . | . |
| InternalIMT | aa | rs74128484 | 0.003 | . | . | . | . | . | . | . | . | . |
| InternalIMT | aa | rs58461537 | 0.013 | 0.037 | 0.07 | 0.603 | 0.768 | 1.42 | 0.588 | -0.298 | 0.555 | 0.592 |
| InternalIMT | aa | rs188527474 | 0.002 | . | . | . | . | . | . | . | . | . |
| InternalIMT | aa | rs141659976 | 0.002 | . | . | . | . | . | . | . | . | . |
| InternalIMT | aa | rs115846407 | 0.013 | 0.037 | 0.07 | 0.603 | 0.767 | 1.42 | 0.588 | -0.297 | 0.555 | 0.592 |
| InternalIMT | aa | rs138378816 | 0.013 | 0.037 | 0.07 | 0.603 | 0.767 | 1.42 | 0.588 | -0.297 | 0.555 | 0.592 |
| InternalIMT | aa | rs146188136 | 0.008 | . | . | . | . | . | . | . | . | . |
| InternalIMT | aa | rs139383871 | 0.002 | . | . | . | . | . | . | . | . | . |
| InternalIMT | aa | rs182953869 | . | . | . | . | . | . | . | . | . | . |
| InternalIMT | aa | rs192430299 | 0.004 | . | . | . | . | . | . | . | . | . |
| InternalIMT | aa | rs144152844 | 0.013 | 0.037 | 0.07 | 0.603 | 0.765 | 1.42 | 0.589 | -0.296 | 0.554 | 0.593 |
| InternalIMT | aa | rs145987786 | 0.013 | 0.047 | 0.071 | 0.513 | 1.06 | 0.877 | 0.226 | -0.462 | 0.335 | 0.168 |
| InternalIMT | aa | rs181132926 | 0.001 | . | . | . | . | . | . | . | . | . |
| InternalIMT | aa | rs147882253 | 0.013 | 0.037 | 0.07 | 0.603 | 0.767 | 1.42 | 0.588 | -0.297 | 0.554 | 0.592 |
| InternalIMT | aa | rs141807621 | 0.005 | . | . | . | . | . | . | . | . | . |
| InternalIMT | aa | rs147116052 | 0.013 | 0.037 | 0.07 | 0.603 | 0.766 | 1.42 | 0.588 | -0.297 | 0.554 | 0.592 |
| InternalIMT | aa | rs147716618 | 0.013 | 0.037 | 0.07 | 0.603 | 0.766 | 1.42 | 0.588 | -0.297 | 0.554 | 0.592 |
| InternalIMT | aa | rs187198855 | 0.01 | 0.041 | 0.071 | 0.566 | 1.09 | 1.23 | 0.374 | -0.463 | 0.473 | 0.328 |
| InternalIMT | aa | rs189409638 | 0.005 | . | . | . | . | . | . | . | . | . |
| InternalIMT | aa | rs182647339 | . | . | . | . | . | . | . | . | . | . |
| InternalIMT | aa | rs187995988 | . | . | . | . | . | . | . | . | . | . |
| InternalIMT | aa | rs141034184 | 0.015 | 0.037 | 0.071 | 0.601 | 0.656 | 1.26 | 0.603 | -0.262 | 0.498 | 0.598 |
| InternalIMT | aa | rs187310430 | 0.002 | . | . | . | . | . | . | . | . | . |
| InternalIMT | aa | rs192608488 | 0.013 | 0.037 | 0.07 | 0.603 | 0.763 | 1.41 | 0.588 | -0.295 | 0.552 | 0.593 |
| InternalIMT | aa | rs148840366 | 0.003 | . | . | . | . | . | . | . | . | . |
| InternalIMT | aa | rs186785022 | 0.004 | . | . | . | . | . | . | . | . | . |
| InternalIMT | aa | rs192348728 | 0.002 | . | . | . | . | . | . | . | . | . |
| InternalIMT | aa | rs189656631 | 0.013 | 0.037 | 0.07 | 0.603 | 0.763 | 1.41 | 0.588 | -0.295 | 0.552 | 0.593 |
| InternalIMT | aa | rs185289750 | . | . | . | . | . | . | . | . | . | . |
| InternalIMT | aa | rs142382395 | 0.013 | 0.037 | 0.07 | 0.603 | 0.762 | 1.41 | 0.588 | -0.295 | 0.552 | 0.593 |
| InternalIMT | aa | rs144866696 | 0.004 | . | . | . | . | . | . | . | . | . |
| InternalIMT | aa | rs141371289 | 0.013 | 0.037 | 0.07 | 0.603 | 0.762 | 1.41 | 0.589 | -0.295 | 0.552 | 0.593 |
| InternalIMT | aa | rs114750008 | 0.013 | 0.037 | 0.07 | 0.603 | 0.762 | 1.41 | 0.588 | -0.295 | 0.552 | 0.593 |
| InternalIMT | aa | rs189063597 | 0.002 | . | . | . | . | . | . | . | . | . |
| InternalIMT | aa | rs139151571 | 0.013 | 0.038 | 0.07 | 0.587 | 1.01 | 1.55 | 0.512 | -0.392 | 0.602 | 0.515 |
| InternalIMT | aa | rs139846368 | 0.014 | 0.037 | 0.07 | 0.596 | 0.783 | 1.4 | 0.576 | -0.308 | 0.549 | 0.575 |
| InternalIMT | aa | rs182057653 | 0.004 | . | . | . | . | . | . | . | . | . |
| InternalIMT | aa | rs148785420 | 0.013 | 0.037 | 0.07 | 0.603 | 0.762 | 1.41 | 0.588 | -0.295 | 0.551 | 0.593 |
| InternalIMT | aa | rs138383251 | 0.002 | . | . | . | . | . | . | . | . | . |
| InternalIMT | aa | rs116526705 | 0.006 | . | . | . | . | . | . | . | . | . |
| InternalIMT | aa | rs185946436 | . | . | . | . | . | . | . | . | . | . |
| InternalIMT | aa | rs190455427 | . | . | . | . | . | . | . | . | . | . |
| InternalIMT | aa | rs9282584 | 0.013 | 0.037 | 0.07 | 0.603 | 0.762 | 1.41 | 0.588 | -0.295 | 0.551 | 0.593 |
| InternalIMT | aa | rs17153289 | 0.013 | 0.037 | 0.07 | 0.603 | 0.762 | 1.41 | 0.588 | -0.295 | 0.551 | 0.593 |
| InternalIMT | aa | rs138359103 | 0.001 | . | . | . | . | . | . | . | . | . |
| InternalIMT | aa | rs150605268 | 0.001 | . | . | . | . | . | . | . | . | . |
| InternalIMT | aa | rs79242910 | 0.015 | 0.035 | 0.07 | 0.615 | 0.547 | 1.24 | 0.66 | -0.218 | 0.491 | 0.657 |
| InternalIMT | aa | rs139716904 | 0.004 | . | . | . | . | . | . | . | . | . |
| InternalIMT | aa | rs76238498 | 0.013 | 0.047 | 0.071 | 0.508 | 1.12 | 0.864 | 0.194 | -0.483 | 0.33 | 0.144 |
| InternalIMT | aa | rs184140639 | . | . | . | . | . | . | . | . | . | . |
| InternalIMT | aa | rs188517278 | 0.002 | . | . | . | . | . | . | . | . | . |
| InternalIMT | aa | rs139678913 | 0.001 | . | . | . | . | . | . | . | . | . |
| InternalIMT | aa | rs116697955 | . | . | . | . | . | . | . | . | . | . |
| InternalIMT | aa | rs79267994 | 0.003 | . | . | . | . | . | . | . | . | . |
| InternalIMT | aa | rs11239510 | 0.003 | . | . | . | . | . | . | . | . | . |
| InternalIMT | aa | rs75831645 | 0.006 | . | . | . | . | . | . | . | . | . |
| InternalIMT | aa | rs78452276 | 0.008 | . | . | . | . | . | . | . | . | . |
| InternalIMT | aa | rs144366924 | . | . | . | . | . | . | . | . | . | . |
| InternalIMT | aa | rs77519841 | 0.008 | . | . | . | . | . | . | . | . | . |
| InternalIMT | aa | rs76944454 | 0.013 | 0.036 | 0.07 | 0.61 | 0.706 | 1.41 | 0.616 | -0.272 | 0.552 | 0.622 |
| InternalIMT | aa | rs76443855 | 0.013 | 0.047 | 0.071 | 0.509 | 1.12 | 0.864 | 0.195 | -0.481 | 0.33 | 0.145 |
| InternalIMT | aa | rs146613174 | 0.001 | . | . | . | . | . | . | . | . | . |
| InternalIMT | aa | rs181424761 | 0 | . | . | . | . | . | . | . | . | . |
| InternalIMT | aa | rs142119241 | 0 | . | . | . | . | . | . | . | . | . |
| InternalIMT | aa | rs140137954 | 0.006 | . | . | . | . | . | . | . | . | . |
| InternalIMT | aa | rs187319549 | . | . | . | . | . | . | . | . | . | . |
| InternalIMT | aa | rs12241545 | 0.006 | . | . | . | . | . | . | . | . | . |
| InternalIMT | aa | rs10128228 | 0.013 | 0.036 | 0.07 | 0.61 | 0.706 | 1.41 | 0.616 | -0.272 | 0.551 | 0.622 |
| InternalIMT | aa | rs58580249 | 0.003 | . | . | . | . | . | . | . | . | . |
| InternalIMT | aa | rs193148863 | . | . | . | . | . | . | . | . | . | . |
| InternalIMT | aa | rs185638946 | . | . | . | . | . | . | . | . | . | . |
| InternalIMT | aa | rs117752921 | 0 | . | . | . | . | . | . | . | . | . |
| InternalIMT | aa | rs180841236 | 0 | . | . | . | . | . | . | . | . | . |
| InternalIMT | aa | rs73283155 | 0.003 | . | . | . | . | . | . | . | . | . |
| InternalIMT | aa | rs76560599 | 0.012 | 0.037 | 0.07 | 0.6 | 0.935 | 1.63 | 0.567 | -0.357 | 0.63 | 0.571 |
| InternalIMT | aa | rs76273620 | 0.012 | 0.037 | 0.07 | 0.6 | 0.935 | 1.63 | 0.567 | -0.357 | 0.63 | 0.571 |
| InternalIMT | aa | rs141432923 | 0.003 | . | . | . | . | . | . | . | . | . |
| InternalIMT | aa | rs113529322 | 0.004 | . | . | . | . | . | . | . | . | . |
| InternalIMT | aa | rs10128306 | 0.013 | 0.036 | 0.07 | 0.61 | 0.722 | 1.43 | 0.613 | -0.278 | 0.558 | 0.619 |

| InternalIMT | aa | rs149691688 | 0.001 | . | . | . | . | . | . | . | . | . |
| --- | --- | --- | --- | --- | --- | --- | --- | --- | --- | --- | --- | --- |
| InternalIMT | aa | rs75872495 | 0.013 | 0.036 | 0.07 | 0.609 | 0.729 | 1.43 | 0.611 | -0.281 | 0.559 | 0.616 |
| InternalIMT | aa | rs191873960 | 0.001 | . | . | . | . | . | . | . | . | . |
| InternalIMT | aa | rs80155622 | 0.013 | 0.036 | 0.07 | 0.608 | 0.744 | 1.43 | 0.604 | -0.286 | 0.56 | 0.61 |
| InternalIMT | aa | rs113914656 | 0.001 | . | . | . | . | . | . | . | . | . |
| InternalIMT | aa | rs12242840 | 0.013 | 0.036 | 0.07 | 0.606 | 0.76 | 1.41 | 0.59 | -0.292 | 0.551 | 0.596 |
| InternalIMT | aa | rs147824078 | 0.002 | . | . | . | . | . | . | . | . | . |
| InternalIMT | aa | rs59051493 | 0.013 | 0.036 | 0.07 | 0.604 | 0.775 | 1.4 | 0.579 | -0.298 | 0.546 | 0.585 |
| InternalIMT | aa | rs9282588 | 0.001 | . | . | . | . | . | . | . | . | . |
| InternalIMT | aa | rs150215207 | 0.003 | . | . | . | . | . | . | . | . | . |
| InternalIMT | aa | rs138772243 | 0.001 | . | . | . | . | . | . | . | . | . |
| InternalIMT | aa | rs149383244 | 0.002 | . | . | . | . | . | . | . | . | . |
| InternalIMT | aa | rs17522720 | 0.006 | . | . | . | . | . | . | . | . | . |
| InternalIMT | aa | rs79245105 | 0.013 | 0.036 | 0.07 | 0.604 | 0.783 | 1.4 | 0.576 | -0.301 | 0.547 | 0.582 |
| InternalIMT | aa | rs138222133 | 0.001 | . | . | . | . | . | . | . | . | . |
| InternalIMT | aa | rs12247314 | 0.015 | 0.041 | 0.071 | 0.563 | 1.01 | 1.35 | 0.455 | -0.405 | 0.524 | 0.439 |
| InternalIMT | aa | rs12263894 | 0.014 | 0.045 | 0.07 | 0.526 | 1.53 | 1.47 | 0.299 | -0.61 | 0.571 | 0.286 |
| InternalIMT | aa | rs149907880 | 0.002 | . | . | . | . | . | . | . | . | . |
| InternalIMT | aa | rs188153885 | 0.002 | . | . | . | . | . | . | . | . | . |
| InternalIMT | aa | rs73283159 | 0.002 | . | . | . | . | . | . | . | . | . |
| InternalIMT | aa | rs140476687 | . | . | . | . | . | . | . | . | . | . |
| InternalIMT | aa | rs186603148 | . | . | . | . | . | . | . | . | . | . |
| InternalIMT | aa | rs183296063 | 0.001 | . | . | . | . | . | . | . | . | . |
| InternalIMT | aa | rs145733694 | 0.003 | . | . | . | . | . | . | . | . | . |
| InternalIMT | aa | rs189807449 | . | . | . | . | . | . | . | . | . | . |
| InternalIMT | aa | rs12253223 | 0.009 | . | . | . | . | . | . | . | . | . |
| InternalIMT | aa | rs182113637 | . | . | . | . | . | . | . | . | . | . |
| InternalIMT | aa | rs142792099 | 0.002 | . | . | . | . | . | . | . | . | . |
| InternalIMT | aa | rs12249910 | 0.009 | . | . | . | . | . | . | . | . | . |
| InternalIMT | aa | rs149436339 | 0 | . | . | . | . | . | . | . | . | . |
| InternalIMT | aa | rs77632210 | . | . | . | . | . | . | . | . | . | . |
| InternalIMT | aa | rs74130818 | 0.003 | . | . | . | . | . | . | . | . | . |
| InternalIMT | aa | rs187583648 | . | . | . | . | . | . | . | . | . | . |
| InternalIMT | aa | rs191295987 | 0.001 | . | . | . | . | . | . | . | . | . |
| InternalIMT | aa | rs184200686 | 0.002 | . | . | . | . | . | . | . | . | . |
| InternalIMT | aa | rs76117263 | 0.013 | 0.046 | 0.071 | 0.517 | 0.985 | 0.848 | 0.246 | -0.432 | 0.324 | 0.183 |
| InternalIMT | aa | rs185393149 | 0.001 | . | . | . | . | . | . | . | . | . |
| InternalIMT | aa | rs144760014 | 0 | . | . | . | . | . | . | . | . | . |
| InternalIMT | aa | rs7922292 | 0.002 | . | . | . | . | . | . | . | . | . |
| InternalIMT | aa | rs115372844 | 0.003 | . | . | . | . | . | . | . | . | . |
| InternalIMT | aa | rs111460364 | 0.013 | 0.046 | 0.071 | 0.518 | 0.981 | 0.849 | 0.248 | -0.431 | 0.325 | 0.185 |
| InternalIMT | aa | rs182052255 | 0 | . | . | . | . | . | . | . | . | . |
| InternalIMT | aa | rs180983889 | 0.002 | . | . | . | . | . | . | . | . | . |
| InternalIMT | aa | rs139175327 | 0.002 | . | . | . | . | . | . | . | . | . |
| InternalIMT | aa | rs149959620 | 0.004 | . | . | . | . | . | . | . | . | . |
| InternalIMT | aa | rs114653117 | 0.002 | . | . | . | . | . | . | . | . | . |
| InternalIMT | aa | rs144270018 | 0 | . | . | . | . | . | . | . | . | . |
| InternalIMT | aa | rs182723204 | 0.002 | . | . | . | . | . | . | . | . | . |
| InternalIMT | aa | rs57204067 | 0.003 | . | . | . | . | . | . | . | . | . |
| InternalIMT | aa | rs144956226 | 0.009 | . | . | . | . | . | . | . | . | . |
| InternalIMT | aa | rs187842704 | 0.001 | . | . | . | . | . | . | . | . | . |
| InternalIMT | aa | rs184620511 | 0.003 | . | . | . | . | . | . | . | . | . |
| InternalIMT | aa | rs144031303 | 0.005 | . | . | . | . | . | . | . | . | . |
| InternalIMT | aa | rs187526473 | . | . | . | . | . | . | . | . | . | . |
| InternalIMT | aa | rs193002805 | . | . | . | . | . | . | . | . | . | . |
| InternalIMT | aa | rs138707426 | 0.002 | . | . | . | . | . | . | . | . | . |
| InternalIMT | aa | rs142754243 | 0.01 | 0.029 | 0.071 | 0.677 | 0.666 | 1.04 | 0.523 | -0.215 | 0.41 | 0.6 |
| InternalIMT | aa | rs181600331 | 0.002 | . | . | . | . | . | . | . | . | . |
| InternalIMT | aa | rs144421264 | 0.006 | . | . | . | . | . | . | . | . | . |
| InternalIMT | aa | rs184414519 | 0.002 | . | . | . | . | . | . | . | . | . |
| InternalIMT | aa | rs150596349 | 0.012 | 0.043 | 0.071 | 0.545 | 0.586 | 0.752 | 0.436 | -0.287 | 0.292 | 0.326 |
| InternalIMT | aa | rs190753747 | . | . | . | . | . | . | . | . | . | . |
| InternalIMT | aa | rs139560940 | . | . | . | . | . | . | . | . | . | . |
| InternalIMT | aa | rs75640804 | 0.008 | . | . | . | . | . | . | . | . | . |
| InternalIMT | aa | rs73283175 | 0.018 | 0.035 | 0.072 | 0.623 | 0.381 | 0.726 | 0.6 | -0.154 | 0.282 | 0.586 |
| InternalIMT | aa | rs143337229 | 0.001 | . | . | . | . | . | . | . | . | . |
| InternalIMT | aa | rs73283177 | 0.01 | 0.03 | 0.071 | 0.668 | 0.825 | 1.11 | 0.457 | -0.278 | 0.436 | 0.524 |
| InternalIMT | aa | rs76053611 | 0.011 | 0.039 | 0.071 | 0.587 | 0.13 | 0.763 | 0.865 | -0.11 | 0.3 | 0.713 |
| InternalIMT | aa | rs116325983 | . | . | . | . | . | . | . | . | . | . |
| InternalIMT | aa | rs115451825 | 0.001 | . | . | . | . | . | . | . | . | . |
| InternalIMT | aa | rs183845246 | 0 | . | . | . | . | . | . | . | . | . |
| InternalIMT | aa | rs79756542 | 0.013 | 0.042 | 0.071 | 0.556 | 0.442 | 0.72 | 0.54 | -0.226 | 0.281 | 0.422 |
| InternalIMT | aa | rs187242860 | . | . | . | . | . | . | . | . | . | . |
| InternalIMT | aa | rs57318980 | 0.01 | 0.033 | 0.071 | 0.645 | 1.28 | 1.27 | 0.316 | -0.455 | 0.497 | 0.36 |
| InternalIMT | aa | rs59969732 | 0.01 | 0.033 | 0.071 | 0.642 | 1.25 | 1.28 | 0.329 | -0.451 | 0.501 | 0.368 |
| InternalIMT | aa | rs143811488 | 0.003 | . | . | . | . | . | . | . | . | . |
| InternalIMT | aa | rs189067644 | 0.002 | . | . | . | . | . | . | . | . | . |
| InternalIMT | aa | rs60449178 | 0.01 | . | . | . | . | . | . | . | . | . |
| InternalIMT | aa | rs59243389 | 0.01 | . | . | . | . | . | . | . | . | . |
| InternalIMT | aa | rs184946540 | 0.009 | . | . | . | . | . | . | . | . | . |
| InternalIMT | aa | rs188685881 | 0 | . | . | . | . | . | . | . | . | . |
| InternalIMT | aa | rs150335797 | 0.002 | . | . | . | . | . | . | . | . | . |
| InternalIMT | aa | rs143879260 | 0.002 | . | . | . | . | . | . | . | . | . |
| InternalIMT | aa | rs186044491 | 0.003 | . | . | . | . | . | . | . | . | . |
| InternalIMT | aa | rs11819084 | 0.004 | . | . | . | . | . | . | . | . | . |
| InternalIMT | aa | rs146915670 | . | . | . | . | . | . | . | . | . | . |

| InternalIMT | aa | rs113995695 | 0.004 | . | . | . | . | . | . | . | . | . |
| --- | --- | --- | --- | --- | --- | --- | --- | --- | --- | --- | --- | --- |
| InternalIMT | aa | rs143489451 | 0.001 | . | . | . | . | . | . | . | . | . |
| InternalIMT | aa | rs182653839 | . | . | . | . | . | . | . | . | . | . |
| InternalIMT | aa | rs141692564 | 0.002 | . | . | . | . | . | . | . | . | . |
| InternalIMT | aa | rs146281742 | 0.001 | . | . | . | . | . | . | . | . | . |
| InternalIMT | aa | rs192914353 | 0.001 | . | . | . | . | . | . | . | . | . |
| InternalIMT | aa | rs185182848 | 0 | . | . | . | . | . | . | . | . | . |
| InternalIMT | aa | rs28395858 | 0.004 | . | . | . | . | . | . | . | . | . |
| InternalIMT | aa | rs41429645 | 0.01 | . | . | . | . | . | . | . | . | . |
| InternalIMT | aa | rs28395874 | 0.004 | . | . | . | . | . | . | . | . | . |
| InternalIMT | aa | rs189663634 | . | . | . | . | . | . | . | . | . | . |
| InternalIMT | aa | rs77666700 | 0.003 | . | . | . | . | . | . | . | . | . |
| InternalIMT | aa | rs146711717 | 0.001 | . | . | . | . | . | . | . | . | . |
| InternalIMT | aa | rs144904251 | . | . | . | . | . | . | . | . | . | . |
| InternalIMT | aa | rs189893153 | . | . | . | . | . | . | . | . | . | . |
| InternalIMT | aa | rs150447740 | 0.002 | . | . | . | . | . | . | . | . | . |
| InternalIMT | aa | rs143099649 | . | . | . | . | . | . | . | . | . | . |
| InternalIMT | aa | rs191288436 | 0.003 | . | . | . | . | . | . | . | . | . |
| InternalIMT | aa | rs188577862 | 0.001 | . | . | . | . | . | . | . | . | . |
| InternalIMT | aa | rs192695334 | 0.001 | . | . | . | . | . | . | . | . | . |
| InternalIMT | aa | rs138612906 | 0.007 | . | . | . | . | . | . | . | . | . |
| InternalIMT | aa | rs191290051 | 0.006 | . | . | . | . | . | . | . | . | . |
| InternalIMT | aa | rs189631459 | 0 | . | . | . | . | . | . | . | . | . |
| InternalIMT | aa | rs114620874 | 0.002 | . | . | . | . | . | . | . | . | . |
| InternalIMT | aa | rs78246652 | 0.003 | . | . | . | . | . | . | . | . | . |
| InternalIMT | aa | rs79432451 | 0.002 | . | . | . | . | . | . | . | . | . |
| InternalIMT | aa | rs186022585 | . | . | . | . | . | . | . | . | . | . |
| InternalIMT | aa | rs142978891 | 0.002 | . | . | . | . | . | . | . | . | . |
| InternalIMT | aa | rs151086840 | 0.003 | . | . | . | . | . | . | . | . | . |
| InternalIMT | aa | rs183270259 | 0.002 | . | . | . | . | . | . | . | . | . |
| InternalIMT | aa | rs188544289 | 0.002 | . | . | . | . | . | . | . | . | . |
| InternalIMT | aa | rs188500808 | 0 | . | . | . | . | . | . | . | . | . |
| InternalIMT | aa | rs189424589 | 0 | . | . | . | . | . | . | . | . | . |
| InternalIMT | aa | rs78622303 | 0.008 | . | . | . | . | . | . | . | . | . |
| InternalIMT | aa | rs143210835 | 0.002 | . | . | . | . | . | . | . | . | . |
| InternalIMT | aa | rs114761840 | 0.001 | . | . | . | . | . | . | . | . | . |
| InternalIMT | aa | rs185805402 | . | . | . | . | . | . | . | . | . | . |
| InternalIMT | aa | rs139273744 | 0.001 | . | . | . | . | . | . | . | . | . |
| InternalIMT | aa | rs184654150 | 0.001 | . | . | . | . | . | . | . | . | . |
| InternalIMT | aa | rs189069710 | . | . | . | . | . | . | . | . | . | . |
| InternalIMT | aa | rs114752115 | 0.004 | . | . | . | . | . | . | . | . | . |
| InternalIMT | aa | rs150162515 | 0 | . | . | . | . | . | . | . | . | . |
| InternalIMT | aa | rs180982454 | 0.001 | . | . | . | . | . | . | . | . | . |
| InternalIMT | aa | rs184932088 | . | . | . | . | . | . | . | . | . | . |
| InternalIMT | aa | rs181109625 | . | . | . | . | . | . | . | . | . | . |
| InternalIMT | aa | rs138121134 | . | . | . | . | . | . | . | . | . | . |
| InternalIMT | aa | rs183253861 | 0 | . | . | . | . | . | . | . | . | . |
| InternalIMT | aa | rs147073946 | . | . | . | . | . | . | . | . | . | . |
| InternalIMT | aa | rs139938489 | 0.002 | . | . | . | . | . | . | . | . | . |
| InternalIMT | aa | rs146362560 | 0.003 | . | . | . | . | . | . | . | . | . |
| InternalIMT | aa | rs193155956 | 0.001 | . | . | . | . | . | . | . | . | . |
| InternalIMT | aa | rs77663092 | 0.001 | . | . | . | . | . | . | . | . | . |
| InternalIMT | aa | rs73285319 | 0.007 | . | . | . | . | . | . | . | . | . |
| InternalIMT | aa | rs184019276 | . | . | . | . | . | . | . | . | . | . |
| InternalIMT | aa | rs28395863 | 0.004 | . | . | . | . | . | . | . | . | . |
| InternalIMT | aa | rs182121645 | 0.004 | . | . | . | . | . | . | . | . | . |
| InternalIMT | aa | rs73285327 | 0.006 | . | . | . | . | . | . | . | . | . |
| InternalIMT | aa | rs149589584 | . | . | . | . | . | . | . | . | . | . |
| InternalIMT | aa | rs148310335 | . | . | . | . | . | . | . | . | . | . |
| InternalIMT | aa | rs187246093 | 0.003 | . | . | . | . | . | . | . | . | . |
| InternalIMT | aa | rs184668894 | . | . | . | . | . | . | . | . | . | . |
| InternalIMT | aa | rs114269961 | . | . | . | . | . | . | . | . | . | . |
| InternalIMT | aa | rs149694374 | 0.005 | . | . | . | . | . | . | . | . | . |
| InternalIMT | aa | rs145284227 | . | . | . | . | . | . | . | . | . | . |
| InternalIMT | aa | rs189983820 | . | . | . | . | . | . | . | . | . | . |
| InternalIMT | aa | rs181200444 | . | . | . | . | . | . | . | . | . | . |
| InternalIMT | aa | rs185486385 | . | . | . | . | . | . | . | . | . | . |
| InternalIMT | aa | rs151198241 | 0.002 | . | . | . | . | . | . | . | . | . |
| InternalIMT | aa | rs189959341 | 0.002 | . | . | . | . | . | . | . | . | . |
| InternalIMT | aa | rs140549257 | 0.002 | . | . | . | . | . | . | . | . | . |
| InternalIMT | aa | rs186453139 | 0.005 | . | . | . | . | . | . | . | . | . |
| InternalIMT | aa | rs180922711 | 0.009 | . | . | . | . | . | . | . | . | . |
| InternalIMT | aa | rs56659903 | 0.002 | . | . | . | . | . | . | . | . | . |
| InternalIMT | aa | rs141202128 | 0.002 | . | . | . | . | . | . | . | . | . |
| InternalIMT | aa | rs59085314 | 0.008 | . | . | . | . | . | . | . | . | . |
| InternalIMT | aa | rs145564996 | . | . | . | . | . | . | . | . | . | . |
| InternalIMT | aa | rs114961009 | 0.002 | . | . | . | . | . | . | . | . | . |
| InternalIMT | aa | rs182294189 | 0.001 | . | . | . | . | . | . | . | . | . |
| InternalIMT | aa | rs187586636 | 0.002 | . | . | . | . | . | . | . | . | . |
| InternalIMT | aa | rs192254907 | 0.005 | . | . | . | . | . | . | . | . | . |
| InternalIMT | aa | rs149145663 | . | . | . | . | . | . | . | . | . | . |
| InternalIMT | aa | rs180713064 | 0.002 | . | . | . | . | . | . | . | . | . |
| InternalIMT | aa | rs183009023 | 0.001 | . | . | . | . | . | . | . | . | . |
| InternalIMT | aa | rs143385347 | 0.004 | . | . | . | . | . | . | . | . | . |
| InternalIMT | aa | rs185786329 | 0.001 | . | . | . | . | . | . | . | . | . |
| InternalIMT | aa | rs192341030 | 0.001 | . | . | . | . | . | . | . | . | . |

| InternalIMT | aa | rs74661429 | 0.002 | . | . | . | . | . | . | . | . | . |
| --- | --- | --- | --- | --- | --- | --- | --- | --- | --- | --- | --- | --- |
| InternalIMT | aa | rs188034853 | 0 | . | . | . | . | . | . | . | . | . |
| InternalIMT | aa | rs116808430 | 0.005 | . | . | . | . | . | . | . | . | . |
| InternalIMT | aa | rs148469010 | . | . | . | . | . | . | . | . | . | . |
| InternalIMT | aa | rs78621663 | 0.002 | . | . | . | . | . | . | . | . | . |
| InternalIMT | aa | rs192654886 | . | . | . | . | . | . | . | . | . | . |
| InternalIMT | aa | rs145996035 | 0.002 | . | . | . | . | . | . | . | . | . |
| InternalIMT | aa | rs139942226 | 0 | . | . | . | . | . | . | . | . | . |
| InternalIMT | aa | rs113645632 | 0.002 | . | . | . | . | . | . | . | . | . |
| InternalIMT | aa | rs74940661 | 0.005 | . | . | . | . | . | . | . | . | . |
| InternalIMT | aa | rs115355800 | 0.003 | . | . | . | . | . | . | . | . | . |
| InternalIMT | aa | rs150285898 | 0.001 | . | . | . | . | . | . | . | . | . |
| InternalIMT | aa | rs188117932 | . | . | . | . | . | . | . | . | . | . |
| InternalIMT | aa | rs139956566 | 0 | . | . | . | . | . | . | . | . | . |
| InternalIMT | aa | rs147673885 | 0 | . | . | . | . | . | . | . | . | . |
| InternalIMT | aa | rs189127928 | 0.005 | . | . | . | . | . | . | . | . | . |
| InternalIMT | aa | rs187558772 | 0.002 | . | . | . | . | . | . | . | . | . |
| InternalIMT | aa | rs181948587 | 0 | . | . | . | . | . | . | . | . | . |
| InternalIMT | aa | rs189983269 | 0.002 | . | . | . | . | . | . | . | . | . |
| InternalIMT | aa | rs187250069 | 0.002 | . | . | . | . | . | . | . | . | . |
| InternalIMT | aa | rs191485859 | 0.001 | . | . | . | . | . | . | . | . | . |
| InternalIMT | aa | rs185499555 | 0.002 | . | . | . | . | . | . | . | . | . |
| InternalIMT | aa | rs180862110 | 0.003 | . | . | . | . | . | . | . | . | . |
| InternalIMT | aa | rs190472603 | 0.005 | . | . | . | . | . | . | . | . | . |
| InternalIMT | aa | rs189226167 | 0.005 | . | . | . | . | . | . | . | . | . |
| InternalIMT | aa | rs181623256 | 0.002 | . | . | . | . | . | . | . | . | . |
| InternalIMT | aa | rs186079557 | 0.001 | . | . | . | . | . | . | . | . | . |
| InternalIMT | aa | rs147760994 | 0.002 | . | . | . | . | . | . | . | . | . |
| InternalIMT | aa | rs189176167 | 0 | . | . | . | . | . | . | . | . | . |
| InternalIMT | aa | rs117901000 | 0.001 | . | . | . | . | . | . | . | . | . |
| InternalIMT | aa | rs141984888 | 0 | . | . | . | . | . | . | . | . | . |
| InternalIMT | aa | rs192365061 | 0.007 | . | . | . | . | . | . | . | . | . |
| InternalIMT | aa | rs185976038 | 0 | . | . | . | . | . | . | . | . | . |
| InternalIMT | aa | rs184697303 | 0.002 | . | . | . | . | . | . | . | . | . |
| InternalIMT | aa | rs143383132 | 0.002 | . | . | . | . | . | . | . | . | . |
| InternalIMT | aa | rs141873881 | 0.004 | . | . | . | . | . | . | . | . | . |
| InternalIMT | aa | rs185507255 | 0 | . | . | . | . | . | . | . | . | . |
| InternalIMT | aa | rs191379425 | 0.001 | . | . | . | . | . | . | . | . | . |
| InternalIMT | aa | rs184199584 | 0.002 | . | . | . | . | . | . | . | . | . |
| InternalIMT | aa | rs183181360 | 0.002 | . | . | . | . | . | . | . | . | . |
| InternalIMT | aa | rs184495673 | 0.001 | . | . | . | . | . | . | . | . | . |
| InternalIMT | aa | rs182946297 | 0.001 | . | . | . | . | . | . | . | . | . |
| InternalIMT | aa | rs187743615 | 0 | . | . | . | . | . | . | . | . | . |
| InternalIMT | aa | rs183259862 | 0.001 | . | . | . | . | . | . | . | . | . |
| InternalIMT | aa | rs112433566 | 0 | . | . | . | . | . | . | . | . | . |
| InternalIMT | aa | rs182351085 | 0.001 | . | . | . | . | . | . | . | . | . |
| InternalIMT | aa | rs146248508 | 0.001 | . | . | . | . | . | . | . | . | . |
| InternalIMT | aa | rs188528439 | 0.001 | . | . | . | . | . | . | . | . | . |
| InternalIMT | aa | rs146167018 | 0.005 | . | . | . | . | . | . | . | . | . |
| InternalIMT | aa | rs192440980 | 0.004 | . | . | . | . | . | . | . | . | . |
| InternalIMT | aa | rs185601882 | 0.001 | . | . | . | . | . | . | . | . | . |
| InternalIMT | aa | rs117272563 | 0.002 | . | . | . | . | . | . | . | . | . |
| InternalIMT | aa | rs10430734 | 0.003 | . | . | . | . | . | . | . | . | . |
| InternalIMT | aa | rs181320952 | 0.001 | . | . | . | . | . | . | . | . | . |
| InternalIMT | aa | rs185480660 | 0.001 | . | . | . | . | . | . | . | . | . |
| InternalIMT | aa | rs188077917 | 0.001 | . | . | . | . | . | . | . | . | . |
| InternalIMT | aa | rs187562953 | 0.002 | . | . | . | . | . | . | . | . | . |
| InternalIMT | aa | rs182044631 | 0 | . | . | . | . | . | . | . | . | . |
| InternalIMT | aa | rs145455596 | 0.001 | . | . | . | . | . | . | . | . | . |
| InternalIMT | aa | rs181475877 | 0.004 | . | . | . | . | . | . | . | . | . |
| InternalIMT | aa | rs149413085 | 0.002 | . | . | . | . | . | . | . | . | . |
| InternalIMT | aa | rs12253656 | 0 | . | . | . | . | . | . | . | . | . |
| InternalIMT | aa | rs182718239 | 0 | . | . | . | . | . | . | . | . | . |
| InternalIMT | aa | rs192435695 | 0.002 | . | . | . | . | . | . | . | . | . |
| InternalIMT | aa | rs12248277 | 0 | . | . | . | . | . | . | . | . | . |
| InternalIMT | aa | rs143521710 | 0.001 | . | . | . | . | . | . | . | . | . |
| InternalIMT | aa | rs144454454 | 0 | . | . | . | . | . | . | . | . | . |
| InternalIMT | aa | rs190441610 | 0.004 | . | . | . | . | . | . | . | . | . |
| InternalIMT | aa | rs114690462 | 0 | . | . | . | . | . | . | . | . | . |
| InternalIMT | aa | rs184541357 | 0.001 | . | . | . | . | . | . | . | . | . |
| InternalIMT | aa | rs142657653 | 0 | . | . | . | . | . | . | . | . | . |
| InternalIMT | aa | rsurf1 | 2.224 | . | . | . | . | . | . | . | . | . |
| InternalIMT | aa | rsten1 | 2.372 | . | . | . | . | . | . | . | . | . |
| InternalIMT | aa | chr10:45852312:D | 0.072 | 0.051 | 0.075 | 0.497 | 0.288 | 0.366 | 0.43 | -0.135 | 0.151 | 0.371 |
| InternalIMT | aa | chr10:45853232:I | 0.086 | 0.055 | 0.077 | 0.48 | 0.194 | 0.351 | 0.58 | -0.11 | 0.144 | 0.447 |
| InternalIMT | aa | chr10:45853234:I | 0.141 | 0.015 | 0.083 | 0.858 | -0.231 | 0.269 | 0.39 | 0.071 | 0.112 | 0.527 |
| InternalIMT | aa | chr10:45854796:D | 0.022 | 0.016 | 0.071 | 0.817 | -0.892 | 0.813 | 0.272 | 0.342 | 0.348 | 0.326 |
| InternalIMT | aa | chr10:45854994:I | 0.025 | 0.014 | 0.071 | 0.843 | -1.01 | 0.8 | 0.207 | 0.4 | 0.346 | 0.248 |
| InternalIMT | aa | chr10:45858290:D | 0.045 | 0.039 | 0.076 | 0.609 | 0.224 | 0.745 | 0.764 | -0.113 | 0.303 | 0.71 |
| InternalIMT | aa | chr10:45858820:I | 0.012 | 3.10E-03 | 0.071 | 0.965 | -2.8 | 1.48 | 0.058 | 1.16 | 0.627 | 0.065 |
| InternalIMT | aa | chr10:45860888:D | 0.072 | 0.03 | 0.081 | 0.711 | -0.138 | 0.461 | 0.764 | 0.024 | 0.191 | 0.899 |
| InternalIMT | aa | chr10:45863913:I | 0.144 | 0.022 | 0.082 | 0.787 | -0.161 | 0.265 | 0.542 | 0.042 | 0.11 | 0.705 |
| InternalIMT | aa | chr10:45866601:D | 0.016 | 0.027 | 0.072 | 0.708 | -0.1 | 1.2 | 0.934 | 0.056 | 0.474 | 0.906 |
| InternalIMT | aa | chr10:45867197:I | 0.005 | . | . | . | . | . | . | . | . | . |
| InternalIMT | aa | chr10:45867538:D | 0.15 | 0.031 | 0.083 | 0.71 | -0.101 | 0.263 | 0.7 | 0.015 | 0.109 | 0.893 |
| InternalIMT | aa | chr10:45868356:D | 0.14 | 0.025 | 0.082 | 0.762 | -0.142 | 0.271 | 0.6 | 0.035 | 0.113 | 0.759 |

| InternalIMT | aa | chr10:45871158:I | 0.145 | 0.024 | 0.082 | 0.768 | -0.153 | 0.262 | 0.559 | 0.037 | 0.109 | 0.735 |
| --- | --- | --- | --- | --- | --- | --- | --- | --- | --- | --- | --- | --- |
| InternalIMT | aa | chr10:45871637:D | 0.066 | 0.048 | 0.078 | 0.536 | 2.30E-03 | 0.433 | 0.996 | -0.046 | 0.177 | 0.797 |
| InternalIMT | aa | chr10:45871638:I | 0.111 | 0.029 | 0.081 | 0.723 | -0.223 | 0.305 | 0.464 | 0.051 | 0.125 | 0.684 |
| InternalIMT | aa | chr10:45873629:I | 0.139 | 0.018 | 0.082 | 0.822 | -0.194 | 0.271 | 0.475 | 0.051 | 0.113 | 0.652 |
| InternalIMT | aa | chr10:45873701:D | 0.009 | . | . | . | . | . | . | . | . | . |
| InternalIMT | aa | chr10:45874997:I | 0.016 | 0.018 | 0.071 | 0.796 | -0.77 | 1.09 | 0.481 | 0.303 | 0.443 | 0.494 |
| InternalIMT | aa | chr10:45882030:I | 0.028 | 0.032 | 0.072 | 0.652 | 0.14 | 0.93 | 0.88 | -0.055 | 0.369 | 0.881 |
| InternalIMT | aa | chr10:45883774:D | 0.092 | -0.03 | 0.077 | 0.702 | -0.867 | 0.328 | 8.30E-03 | 0.346 | 0.139 | 0.013 |
| InternalIMT | aa | chr10:45885169:D | 0.054 | 0.056 | 0.074 | 0.453 | 0.439 | 0.428 | 0.305 | -0.211 | 0.174 | 0.225 |
| InternalIMT | aa | chr10:45886451:D | 0.701 | -0.063 | 0.183 | 0.73 | -0.149 | 0.286 | 0.603 | 0.067 | 0.118 | 0.573 |
| InternalIMT | aa | chr10:45891800:I | 0.669 | -0.114 | 0.155 | 0.462 | -0.255 | 0.252 | 0.313 | 0.106 | 0.104 | 0.308 |
| InternalIMT | aa | chr10:45904771:I | 0.683 | -0.063 | 0.153 | 0.679 | -0.163 | 0.241 | 0.5 | 0.067 | 0.1 | 0.505 |
| InternalIMT | aa | chr10:45904959:I | 0.083 | 0.031 | 0.078 | 0.69 | -0.025 | 0.479 | 0.958 | 1.10E-03 | 0.192 | 0.996 |
| InternalIMT | aa | chr10:45911028:I | 0.045 | 0.031 | 0.078 | 0.691 | 0.209 | 1.14 | 0.855 | -0.056 | 0.451 | 0.901 |
| InternalIMT | aa | chr10:45911031:I | 0.411 | 0.048 | 0.11 | 0.664 | 0.066 | 0.264 | 0.804 | -0.013 | 0.108 | 0.902 |
| InternalIMT | aa | chr10:45912154:D | 0.086 | 3.30E-03 | 0.077 | 0.966 | -0.402 | 0.357 | 0.261 | 0.152 | 0.149 | 0.307 |
| InternalIMT | aa | chr10:45913885:I | 0.021 | 0.014 | 0.071 | 0.841 | -0.99 | 0.863 | 0.251 | 0.377 | 0.371 | 0.309 |
| InternalIMT | aa | chr10:45914753:D | 0.181 | 0.057 | 0.088 | 0.514 | 0.14 | 0.304 | 0.644 | -0.064 | 0.123 | 0.601 |
| InternalIMT | aa | chr10:45919621 | . | . | . | . | . | . | . | . | . | . |
| InternalIMT | aa | chr10:45921739:I | 0.06 | -3.70E-03 | 0.073 | 0.96 | -0.9 | 0.43 | 0.037 | 0.346 | 0.182 | 0.058 |
| InternalIMT | aa | chr10:45923527:I | 0.054 | 0.068 | 0.076 | 0.374 | 0.877 | 0.707 | 0.215 | -0.358 | 0.288 | 0.213 |
| InternalIMT | aa | chr10:45927001:I | 0.011 | 0.035 | 0.07 | 0.617 | 3.03 | 2.92 | 0.3 | -1.08 | 1.23 | 0.382 |
| InternalIMT | aa | chr10:45930354:I | 0.023 | 0.016 | 0.07 | 0.825 | -0.951 | 0.876 | 0.278 | 0.375 | 0.384 | 0.329 |
| InternalIMT | aa | chr10:45932864:I | 0.046 | 0.013 | 0.074 | 0.861 | -0.513 | 0.879 | 0.559 | 0.213 | 0.364 | 0.559 |
| InternalIMT | aa | chr10:45936163:I | 0.025 | 0.072 | 0.071 | 0.31 | 1.73 | 0.737 | 0.019 | -0.761 | 0.293 | 9.50E-03 |
| InternalIMT | aa | chr10:45938095:D | 0.116 | 0.015 | 0.08 | 0.853 | -0.253 | 0.268 | 0.346 | 0.074 | 0.111 | 0.507 |
| InternalIMT | aa | chr10:45938886 | . | . | . | . | . | . | . | . | . | . |
| InternalIMT | aa | chr10:45939320 | . | . | . | . | . | . | . | . | . | . |
| InternalIMT | aa | chr10:45939659 | 0.002 | . | . | . | . | . | . | . | . | . |
| InternalIMT | aa | chr10:45941150:D | 0.021 | 0.014 | 0.07 | 0.842 | -1.06 | 0.805 | 0.188 | 0.403 | 0.347 | 0.246 |
| InternalIMT | aa | chr10:45947128:D | 0.046 | 0.021 | 0.072 | 0.766 | -0.101 | 0.513 | 0.844 | 0.071 | 0.207 | 0.732 |
| InternalIMT | aa | chr10:45950990:I | 0.06 | -9.80E-03 | 0.075 | 0.896 | -0.631 | 0.538 | 0.241 | 0.233 | 0.23 | 0.309 |
| InternalIMT | aa | chr10:45951854:I | 0.02 | 0.044 | 0.071 | 0.541 | 0.691 | 1.15 | 0.548 | -0.309 | 0.461 | 0.503 |
| InternalIMT | aa | chr10:45952670:I | 0.042 | 0.04 | 0.072 | 0.575 | 0.2 | 0.655 | 0.76 | -0.1 | 0.265 | 0.705 |
| InternalIMT | aa | chr10:45956741 | . | . | . | . | . | . | . | . | . | . |
| InternalIMT | aa | chr10:45957247:I | 0.304 | -7.50E-03 | 0.091 | 0.935 | -0.147 | 0.215 | 0.493 | 0.052 | 0.09 | 0.562 |
| InternalIMT | aa | chr10:45958082:I | 0.302 | -0.011 | 0.092 | 0.903 | -0.165 | 0.217 | 0.449 | 0.059 | 0.091 | 0.517 |
| InternalIMT | aa | chr10:45905041:D | 0.039 | 0.037 | 0.073 | 0.609 | -0.048 | 0.537 | 0.929 | -0.025 | 0.219 | 0.91 |
| InternalIMT | aa | chr10:45938871 | . | . | . | . | . | . | . | . | . | . |
| InternalIMT | aa | chr10:45939160 | . | . | . | . | . | . | . | . | . | . |
| InternalIMT | aa | chr10:45869910 | 0.001 | . | . | . | . | . | . | . | . | . |
| InternalIMT | aa | chr10:45876214:D | 0.005 | . | . | . | . | . | . | . | . | . |
| InternalIMT | aa | chr10:45880237:I | 0.014 | 0.022 | 0.072 | 0.757 | -0.704 | 0.587 | 0.231 | 0.247 | 0.242 | 0.309 |
| InternalIMT | aa | chr10:45884371:I | 0.013 | 0.037 | 0.07 | 0.603 | 0.767 | 1.42 | 0.588 | -0.297 | 0.554 | 0.592 |
| InternalIMT | aa | chr10:45891535:D | 0.004 | . | . | . | . | . | . | . | . | . |
| InternalIMT | aa | chr10:45893516:D | 0.013 | 0.036 | 0.07 | 0.609 | 0.709 | 1.41 | 0.615 | -0.273 | 0.551 | 0.62 |
| InternalIMT | aa | chr10:45893782:D | 0.001 | . | . | . | . | . | . | . | . | . |
| InternalIMT | aa | chr10:45895254:I | 0.004 | . | . | . | . | . | . | . | . | . |
| InternalIMT | aa | chr10:45895848:D | 0.011 | 0.029 | 0.07 | 0.678 | -0.187 | 0.814 | 0.819 | 0.058 | 0.316 | 0.854 |
| InternalIMT | aa | chr10:45896316:D | 0.013 | 0.036 | 0.07 | 0.61 | 0.707 | 1.41 | 0.616 | -0.273 | 0.552 | 0.621 |
| InternalIMT | aa | chr10:45896317:D | 0.013 | 0.036 | 0.07 | 0.61 | 0.707 | 1.41 | 0.616 | -0.273 | 0.552 | 0.621 |
| InternalIMT | aa | chr10:45902683:I | 0.006 | . | . | . | . | . | . | . | . | . |
| InternalIMT | aa | chr10:45905003:D | 0.002 | . | . | . | . | . | . | . | . | . |
| InternalIMT | aa | chr10:45927476:D | 0.005 | . | . | . | . | . | . | . | . | . |
| InternalIMT | aa | chr10:45935882 | . | . | . | . | . | . | . | . | . | . |
| InternalIMT | aa | chr10:45936100 | . | . | . | . | . | . | . | . | . | . |
| InternalIMT | aa | chr10:45939553 | 0.004 | . | . | . | . | . | . | . | . | . |
| InternalIMT | aa | chr10:45939631 | . | . | . | . | . | . | . | . | . | . |
| InternalIMT | aa | chr10:45944193:D | 0.003 | . | . | . | . | . | . | . | . | . |
| InternalIMT | aa | chr10:45954030 | . | . | . | . | . | . | . | . | . | . |

Supplemental table S30.

| phen | covar(fa) | rs_id | maf | beta_FA | se_FA | p_FA | beta_SNP | se_SNP | p_SNP | beta_inter | se_inter | p_inter |
| --- | --- | --- | --- | --- | --- | --- | --- | --- | --- | --- | --- | --- |
| InternalIMT | aa | rs76035182 | . | . | . | . | . | . | . | . | . | . |
| InternalIMT | aa | rs12359547 | 0.001 | . | . | . | . | . | . | . | . | . |
| InternalIMT | aa | rs74595484 | . | . | . | . | . | . | . | . | . | . |
| InternalIMT | aa | rs1864415 | 0.873 | -0.142 | 0.265 | 0.593 | -0.254 | 0.359 | 0.479 | 0.116 | 0.154 | 0.451 |
| InternalIMT | aa | rs72788101 | 0.001 | . | . | . | . | . | . | . | . | . |
| InternalIMT | aa | rs11239481 | . | . | . | . | . | . | . | . | . | . |
| InternalIMT | aa | rs72788102 | 0.001 | . | . | . | . | . | . | . | . | . |
| InternalIMT | aa | rs72789705 | 0.001 | . | . | . | . | . | . | . | . | . |
| InternalIMT | aa | rs141529858 | . | . | . | . | . | . | . | . | . | . |
| InternalIMT | aa | rs145376292 | . | . | . | . | . | . | . | . | . | . |
| InternalIMT | aa | rs190418301 | . | . | . | . | . | . | . | . | . | . |
| InternalIMT | aa | rs11239482 | 0.001 | . | . | . | . | . | . | . | . | . |
| InternalIMT | aa | rs61855872 | . | . | . | . | . | . | . | . | . | . |
| InternalIMT | aa | rs148991144 | . | . | . | . | . | . | . | . | . | . |
| InternalIMT | aa | rs191932221 | . | . | . | . | . | . | . | . | . | . |
| InternalIMT | aa | rs55978138 | 0.201 | -0.056 | 0.096 | 0.562 | -0.651 | 0.357 | 0.069 | 0.283 | 0.161 | 0.078 |
| InternalIMT | aa | rs73281167 | . | . | . | . | . | . | . | . | . | . |
| InternalIMT | aa | rs12768010 | 0.2 | -0.054 | 0.095 | 0.574 | -0.645 | 0.36 | 0.073 | 0.281 | 0.162 | 0.082 |
| InternalIMT | aa | rs11239484 | 0.873 | -0.142 | 0.265 | 0.592 | -0.254 | 0.358 | 0.478 | 0.116 | 0.154 | 0.451 |
| InternalIMT | aa | rs35906360 | 0.201 | -0.056 | 0.096 | 0.562 | -0.651 | 0.357 | 0.069 | 0.283 | 0.161 | 0.078 |
| InternalIMT | aa | rs186250512 | . | . | . | . | . | . | . | . | . | . |
| InternalIMT | aa | rs150810743 | . | . | . | . | . | . | . | . | . | . |
| InternalIMT | aa | rs55965812 | 0.201 | -0.056 | 0.096 | 0.562 | -0.651 | 0.357 | 0.069 | 0.283 | 0.161 | 0.078 |
| InternalIMT | aa | rs56180358 | 0.203 | -0.057 | 0.096 | 0.554 | -0.654 | 0.356 | 0.067 | 0.285 | 0.16 | 0.076 |
| InternalIMT | aa | rs55864145 | 0.2 | -0.054 | 0.095 | 0.574 | -0.645 | 0.36 | 0.074 | 0.281 | 0.162 | 0.082 |
| InternalIMT | aa | rs55986122 | 0.201 | -0.056 | 0.096 | 0.562 | -0.651 | 0.357 | 0.069 | 0.283 | 0.161 | 0.078 |
| InternalIMT | aa | rs7477350 | 0.873 | -0.142 | 0.265 | 0.591 | -0.255 | 0.358 | 0.477 | 0.116 | 0.154 | 0.45 |
| InternalIMT | aa | rs2099170 | 0.201 | -0.056 | 0.096 | 0.562 | -0.651 | 0.357 | 0.069 | 0.283 | 0.161 | 0.078 |
| InternalIMT | aa | rs2082108 | 0.201 | -0.056 | 0.096 | 0.562 | -0.651 | 0.357 | 0.069 | 0.283 | 0.161 | 0.078 |
| InternalIMT | aa | rs55902404 | . | . | . | . | . | . | . | . | . | . |
| InternalIMT | aa | rs58402662 | . | . | . | . | . | . | . | . | . | . |
| InternalIMT | aa | rs11511618 | . | . | . | . | . | . | . | . | . | . |
| InternalIMT | aa | rs1816492 | 0.873 | -0.143 | 0.265 | 0.59 | -0.255 | 0.358 | 0.476 | 0.117 | 0.154 | 0.449 |
| InternalIMT | aa | rs146611093 | . | . | . | . | . | . | . | . | . | . |
| InternalIMT | aa | rs186897689 | . | . | . | . | . | . | . | . | . | . |
| InternalIMT | aa | rs10793616 | 0.873 | -0.143 | 0.265 | 0.59 | -0.255 | 0.358 | 0.476 | 0.117 | 0.154 | 0.449 |
| InternalIMT | aa | rs116700479 | . | . | . | . | . | . | . | . | . | . |
| InternalIMT | aa | rs192535936 | . | . | . | . | . | . | . | . | . | . |
| InternalIMT | aa | rs113363297 | 0.852 | -0.073 | 0.264 | 0.783 | -0.167 | 0.365 | 0.646 | 0.078 | 0.157 | 0.619 |
| InternalIMT | aa | rs12354994 | 0.186 | -0.041 | 0.094 | 0.66 | -0.619 | 0.36 | 0.086 | 0.267 | 0.161 | 0.097 |
| InternalIMT | aa | rs148068329 | 0.059 | 0.022 | 0.086 | 0.796 | -0.982 | 0.579 | 0.09 | 0.389 | 0.263 | 0.14 |
| InternalIMT | aa | rs12356109 | 0.198 | -0.048 | 0.095 | 0.61 | -0.626 | 0.362 | 0.084 | 0.271 | 0.163 | 0.096 |
| InternalIMT | aa | rs55908141 | . | . | . | . | . | . | . | . | . | . |
| InternalIMT | aa | rs12354712 | 0.196 | -0.052 | 0.095 | 0.588 | -0.652 | 0.368 | 0.077 | 0.282 | 0.165 | 0.089 |
| InternalIMT | aa | rs147282684 | . | . | . | . | . | . | . | . | . | . |
| InternalIMT | aa | rs12356140 | 0.194 | -0.048 | 0.096 | 0.616 | -0.64 | 0.378 | 0.091 | 0.275 | 0.17 | 0.106 |
| InternalIMT | aa | rs185476187 | . | . | . | . | . | . | . | . | . | . |
| InternalIMT | aa | rs145720057 | . | . | . | . | . | . | . | . | . | . |
| InternalIMT | aa | rs12354742 | 0.196 | -0.052 | 0.095 | 0.588 | -0.652 | 0.368 | 0.077 | 0.282 | 0.165 | 0.089 |
| InternalIMT | aa | rs180913842 | . | . | . | . | . | . | . | . | . | . |
| InternalIMT | aa | rs142976914 | 0.001 | . | . | . | . | . | . | . | . | . |
| InternalIMT | aa | rs11511619 | 0.198 | -0.049 | 0.095 | 0.604 | -0.628 | 0.361 | 0.083 | 0.272 | 0.162 | 0.094 |
| InternalIMT | aa | rs146702870 | . | . | . | . | . | . | . | . | . | . |
| InternalIMT | aa | rs150287496 | 0.024 | 0.078 | 0.087 | 0.367 | 0.823 | 1.15 | 0.474 | -0.377 | 0.492 | 0.444 |
| InternalIMT | aa | rs190917973 | . | . | . | . | . | . | . | . | . | . |
| InternalIMT | aa | rs11517281 | 0.197 | -0.048 | 0.095 | 0.61 | -0.626 | 0.362 | 0.084 | 0.271 | 0.163 | 0.096 |
| InternalIMT | aa | rs12359299 | 0.208 | -0.05 | 0.098 | 0.614 | -0.591 | 0.358 | 0.099 | 0.255 | 0.161 | 0.113 |
| InternalIMT | aa | rs182307932 | . | . | . | . | . | . | . | . | . | . |
| InternalIMT | aa | rs61855873 | . | . | . | . | . | . | . | . | . | . |
| InternalIMT | aa | rs12359302 | 0.193 | -0.049 | 0.095 | 0.605 | -0.637 | 0.367 | 0.083 | 0.277 | 0.165 | 0.094 |
| InternalIMT | aa | rs143208140 | 0.054 | 0.024 | 0.085 | 0.777 | -0.98 | 0.58 | 0.092 | 0.395 | 0.265 | 0.136 |
| InternalIMT | aa | rs9988764 | 0.858 | -0.061 | 0.26 | 0.814 | -0.151 | 0.355 | 0.67 | 0.071 | 0.152 | 0.641 |
| InternalIMT | aa | rs192875767 | 0.015 | 0.049 | 0.083 | 0.551 | -1.2 | 1.3 | 0.357 | 0.506 | 0.536 | 0.345 |
| InternalIMT | aa | rs147268846 | . | . | . | . | . | . | . | . | . | . |
| InternalIMT | aa | rs11239485 | 0.1 | -0.015 | 0.09 | 0.867 | -0.942 | 0.565 | 0.096 | 0.4 | 0.254 | 0.115 |
| InternalIMT | aa | rs151317885 | 0.66 | 0.287 | 0.192 | 0.135 | 0.404 | 0.288 | 0.16 | -0.172 | 0.128 | 0.18 |
| InternalIMT | aa | rs11499085 | . | . | . | . | . | . | . | . | . | . |
| InternalIMT | aa | rs188130606 | . | . | . | . | . | . | . | . | . | . |
| InternalIMT | aa | rs11511620 | 0.196 | -0.044 | 0.095 | 0.642 | -0.604 | 0.363 | 0.097 | 0.261 | 0.163 | 0.11 |
| InternalIMT | aa | rs181498390 | 0.002 | . | . | . | . | . | . | . | . | . |
| InternalIMT | aa | rs186280701 | . | . | . | . | . | . | . | . | . | . |
| InternalIMT | aa | rs150059185 | 0.082 | 0.043 | 0.089 | 0.624 | -0.196 | 0.445 | 0.66 | 0.095 | 0.198 | 0.632 |
| InternalIMT | aa | rs183338707 | . | . | . | . | . | . | . | . | . | . |
| InternalIMT | aa | rs11499038 | 0.006 | . | . | . | . | . | . | . | . | . |
| InternalIMT | aa | rs190699595 | . | . | . | . | . | . | . | . | . | . |
| InternalIMT | aa | rs11523408 | 0.197 | -0.048 | 0.095 | 0.61 | -0.626 | 0.362 | 0.084 | 0.271 | 0.163 | 0.096 |
| InternalIMT | aa | rs188049317 | . | . | . | . | . | . | . | . | . | . |
| InternalIMT | aa | rs144948634 | 0.584 | 0.351 | 0.17 | 0.04 | 0.588 | 0.273 | 0.032 | -0.245 | 0.121 | 0.043 |
| InternalIMT | aa | rs143833576 | 0.016 | 0.051 | 0.082 | 0.532 | -0.656 | 0.986 | 0.506 | 0.308 | 0.431 | 0.475 |
| InternalIMT | aa | rs151239879 | 0.2 | -0.049 | 0.096 | 0.607 | -0.627 | 0.364 | 0.086 | 0.271 | 0.164 | 0.099 |
| InternalIMT | aa | rs7073743 | 0.61 | 0.327 | 0.173 | 0.059 | 0.522 | 0.276 | 0.059 | -0.215 | 0.123 | 0.08 |
| InternalIMT | aa | rs142563349 | . | . | . | . | . | . | . | . | . | . |
| InternalIMT | aa | rs187246403 | . | . | . | . | . | . | . | . | . | . |
| InternalIMT | aa | rs150423942 | 0.197 | -0.048 | 0.095 | 0.61 | -0.627 | 0.363 | 0.085 | 0.271 | 0.163 | 0.097 |

| InternalIMT | aa | rs184706102 | . | . | . | . | . | . | . | . | . | . |
| --- | --- | --- | --- | --- | --- | --- | --- | --- | --- | --- | --- | --- |
| InternalIMT | aa | rs189626288 | . | . | . | . | . | . | . | . | . | . |
| InternalIMT | aa | rs140749834 | . | . | . | . | . | . | . | . | . | . |
| InternalIMT | aa | rs7078822 | 0.163 | -0.063 | 0.091 | 0.487 | -0.837 | 0.443 | 0.059 | 0.368 | 0.2 | 0.066 |
| InternalIMT | aa | rs186227617 | 0.188 | -0.049 | 0.094 | 0.601 | -0.663 | 0.371 | 0.074 | 0.285 | 0.167 | 0.089 |
| InternalIMT | aa | rs188984368 | 0.024 | 0.057 | 0.085 | 0.503 | -0.19 | 1.88 | 0.92 | 0.129 | 0.82 | 0.875 |
| InternalIMT | aa | rs182078109 | . | . | . | . | . | . | . | . | . | . |
| InternalIMT | aa | rs141456274 | 0.023 | 0.047 | 0.083 | 0.574 | -0.863 | 0.785 | 0.272 | 0.336 | 0.35 | 0.338 |
| InternalIMT | aa | rs146976512 | 0.198 | -0.048 | 0.095 | 0.611 | -0.625 | 0.362 | 0.084 | 0.271 | 0.163 | 0.096 |
| InternalIMT | aa | rs138036816 | . | . | . | . | . | . | . | . | . | . |
| InternalIMT | aa | rs114879777 | . | . | . | . | . | . | . | . | . | . |
| InternalIMT | aa | rs149366475 | . | . | . | . | . | . | . | . | . | . |
| InternalIMT | aa | rs182463043 | . | . | . | . | . | . | . | . | . | . |
| InternalIMT | aa | rs144754632 | 0.055 | 0.023 | 0.085 | 0.785 | -0.967 | 0.554 | 0.081 | 0.388 | 0.253 | 0.125 |
| InternalIMT | aa | rs55717607 | 0.198 | -0.048 | 0.095 | 0.611 | -0.625 | 0.362 | 0.084 | 0.271 | 0.163 | 0.096 |
| InternalIMT | aa | rs187537973 | . | . | . | . | . | . | . | . | . | . |
| InternalIMT | aa | rs148085971 | . | . | . | . | . | . | . | . | . | . |
| InternalIMT | aa | rs11239491 | 0.198 | -0.048 | 0.095 | 0.611 | -0.625 | 0.362 | 0.084 | 0.271 | 0.163 | 0.096 |
| InternalIMT | aa | rs184412184 | . | . | . | . | . | . | . | . | . | . |
| InternalIMT | aa | rs4948998 | 0.868 | -0.147 | 0.26 | 0.572 | -0.259 | 0.354 | 0.464 | 0.12 | 0.152 | 0.432 |
| InternalIMT | aa | rs7076696 | 0.873 | -0.147 | 0.263 | 0.577 | -0.26 | 0.356 | 0.465 | 0.119 | 0.153 | 0.437 |
| InternalIMT | aa | rs11239492 | 0.197 | -0.048 | 0.095 | 0.611 | -0.626 | 0.363 | 0.085 | 0.271 | 0.163 | 0.097 |
| InternalIMT | aa | rs79462470 | . | . | . | . | . | . | . | . | . | . |
| InternalIMT | aa | rs187804654 | . | . | . | . | . | . | . | . | . | . |
| InternalIMT | aa | rs7093687 | 0.198 | -0.048 | 0.095 | 0.611 | -0.625 | 0.362 | 0.084 | 0.271 | 0.163 | 0.096 |
| InternalIMT | aa | rs7081004 | 0.198 | -0.048 | 0.095 | 0.611 | -0.625 | 0.362 | 0.084 | 0.271 | 0.163 | 0.096 |
| InternalIMT | aa | rs7084793 | 0.198 | -0.048 | 0.095 | 0.611 | -0.625 | 0.362 | 0.084 | 0.271 | 0.163 | 0.096 |
| InternalIMT | aa | rs61556080 | . | . | . | . | . | . | . | . | . | . |
| InternalIMT | aa | rs115552064 | . | . | . | . | . | . | . | . | . | . |
| InternalIMT | aa | rs7921503 | 0.198 | -0.048 | 0.095 | 0.611 | -0.625 | 0.362 | 0.084 | 0.271 | 0.163 | 0.096 |
| InternalIMT | aa | rs7909740 | 0.198 | -0.048 | 0.095 | 0.611 | -0.625 | 0.362 | 0.084 | 0.271 | 0.163 | 0.096 |
| InternalIMT | aa | rs7909768 | 0.198 | -0.048 | 0.095 | 0.611 | -0.625 | 0.362 | 0.084 | 0.271 | 0.163 | 0.096 |
| InternalIMT | aa | rs144168004 | . | . | . | . | . | . | . | . | . | . |
| InternalIMT | aa | rs75850448 | . | . | . | . | . | . | . | . | . | . |
| InternalIMT | aa | rs147777574 | . | . | . | . | . | . | . | . | . | . |
| InternalIMT | aa | rs7913488 | 0.198 | -0.048 | 0.095 | 0.611 | -0.625 | 0.362 | 0.084 | 0.271 | 0.163 | 0.096 |
| InternalIMT | aa | rs74390874 | 0.048 | 0.083 | 0.084 | 0.323 | 0.626 | 0.755 | 0.407 | -0.251 | 0.326 | 0.442 |
| InternalIMT | aa | rs141391142 | . | . | . | . | . | . | . | . | . | . |
| InternalIMT | aa | rs115294747 | . | . | . | . | . | . | . | . | . | . |
| InternalIMT | aa | rs148031781 | . | . | . | . | . | . | . | . | . | . |
| InternalIMT | aa | rs181340128 | . | . | . | . | . | . | . | . | . | . |
| InternalIMT | aa | rs189930007 | . | . | . | . | . | . | . | . | . | . |
| InternalIMT | aa | rs12242128 | . | . | . | . | . | . | . | . | . | . |
| InternalIMT | aa | rs78421750 | . | . | . | . | . | . | . | . | . | . |
| InternalIMT | aa | rs114370747 | . | . | . | . | . | . | . | . | . | . |
| InternalIMT | aa | rs12264166 | . | . | . | . | . | . | . | . | . | . |
| InternalIMT | aa | rs182964822 | . | . | . | . | . | . | . | . | . | . |
| InternalIMT | aa | rs67491417 | 0.188 | -0.062 | 0.094 | 0.512 | -0.736 | 0.368 | 0.046 | 0.318 | 0.166 | 0.056 |
| InternalIMT | aa | rs72789720 | . | . | . | . | . | . | . | . | . | . |
| InternalIMT | aa | rs6413416 | 0.198 | -0.048 | 0.095 | 0.611 | -0.625 | 0.362 | 0.084 | 0.271 | 0.163 | 0.096 |
| InternalIMT | aa | rs4986832 | 0.198 | -0.048 | 0.095 | 0.611 | -0.625 | 0.362 | 0.084 | 0.271 | 0.163 | 0.096 |
| InternalIMT | aa | rs144866316 | . | . | . | . | . | . | . | . | . | . |
| InternalIMT | aa | rs187769540 | . | . | . | . | . | . | . | . | . | . |
| InternalIMT | aa | rs6593482 | 0.198 | -0.048 | 0.096 | 0.614 | -0.618 | 0.363 | 0.089 | 0.268 | 0.163 | 0.101 |
| InternalIMT | aa | rs7913948 | 0.199 | -0.047 | 0.095 | 0.621 | -0.614 | 0.36 | 0.089 | 0.265 | 0.162 | 0.102 |
| InternalIMT | aa | rs77185151 | 0.055 | 0.023 | 0.085 | 0.785 | -0.968 | 0.554 | 0.081 | 0.389 | 0.253 | 0.125 |
| InternalIMT | aa | rs12762303 | 0.198 | -0.048 | 0.095 | 0.611 | -0.625 | 0.362 | 0.084 | 0.271 | 0.163 | 0.096 |
| InternalIMT | aa | rs73283136 | . | . | . | . | . | . | . | . | . | . |
| InternalIMT | aa | rs187180218 | . | . | . | . | . | . | . | . | . | . |
| InternalIMT | aa | rs78137087 | 0.198 | -0.048 | 0.095 | 0.611 | -0.625 | 0.362 | 0.084 | 0.271 | 0.163 | 0.096 |
| InternalIMT | aa | rs72561472 | 0.193 | -0.048 | 0.095 | 0.614 | -0.636 | 0.37 | 0.086 | 0.275 | 0.166 | 0.098 |
| InternalIMT | aa | rs4987106 | 0.193 | -0.041 | 0.094 | 0.664 | -0.597 | 0.36 | 0.098 | 0.258 | 0.161 | 0.11 |
| InternalIMT | aa | rs4987105 | 0.198 | -0.048 | 0.095 | 0.611 | -0.625 | 0.362 | 0.084 | 0.271 | 0.163 | 0.096 |
| InternalIMT | aa | rs111916157 | 0.199 | -0.051 | 0.095 | 0.593 | -0.634 | 0.363 | 0.081 | 0.274 | 0.163 | 0.093 |
| InternalIMT | aa | rs111528152 | 0.198 | -0.048 | 0.095 | 0.611 | -0.625 | 0.362 | 0.084 | 0.271 | 0.163 | 0.096 |
| InternalIMT | aa | rs12763738 | 0.197 | -0.048 | 0.095 | 0.611 | -0.625 | 0.362 | 0.084 | 0.271 | 0.163 | 0.096 |
| InternalIMT | aa | rs191675886 | . | . | . | . | . | . | . | . | . | . |
| InternalIMT | aa | rs66489870 | 0.198 | -0.048 | 0.095 | 0.611 | -0.625 | 0.362 | 0.084 | 0.271 | 0.163 | 0.096 |
| InternalIMT | aa | rs71494775 | 0.198 | -0.048 | 0.095 | 0.611 | -0.625 | 0.362 | 0.084 | 0.271 | 0.163 | 0.096 |
| InternalIMT | aa | rs61855875 | . | . | . | . | . | . | . | . | . | . |
| InternalIMT | aa | rs116356837 | . | . | . | . | . | . | . | . | . | . |
| InternalIMT | aa | rs1864414 | 0.198 | -0.048 | 0.095 | 0.611 | -0.625 | 0.362 | 0.084 | 0.271 | 0.163 | 0.096 |
| InternalIMT | aa | rs1864413 | 0.198 | -0.048 | 0.095 | 0.611 | -0.625 | 0.362 | 0.084 | 0.271 | 0.163 | 0.097 |
| InternalIMT | aa | rs139497212 | . | . | . | . | . | . | . | . | . | . |
| InternalIMT | aa | rs892690 | 0.504 | 0.33 | 0.146 | 0.024 | 0.629 | 0.272 | 0.021 | -0.263 | 0.12 | 0.029 |
| InternalIMT | aa | rs1864412 | 0.198 | -0.048 | 0.095 | 0.611 | -0.625 | 0.362 | 0.084 | 0.271 | 0.163 | 0.096 |
| InternalIMT | aa | rs77827771 | . | . | . | . | . | . | . | . | . | . |
| InternalIMT | aa | rs145294963 | . | . | . | . | . | . | . | . | . | . |
| InternalIMT | aa | rs149081327 | . | . | . | . | . | . | . | . | . | . |
| InternalIMT | aa | rs67008983 | 0.198 | -0.048 | 0.095 | 0.611 | -0.625 | 0.362 | 0.084 | 0.271 | 0.163 | 0.097 |
| InternalIMT | aa | rs56150414 | 0.198 | -0.048 | 0.095 | 0.611 | -0.625 | 0.362 | 0.084 | 0.271 | 0.163 | 0.096 |
| InternalIMT | aa | rs55787481 | 0.199 | -0.048 | 0.095 | 0.614 | -0.621 | 0.363 | 0.087 | 0.269 | 0.163 | 0.1 |
| InternalIMT | aa | rs141270101 | . | . | . | . | . | . | . | . | . | . |
| InternalIMT | aa | rs114980075 | . | . | . | . | . | . | . | . | . | . |
| InternalIMT | aa | rs12783095 | 0.198 | -0.048 | 0.095 | 0.61 | -0.626 | 0.363 | 0.085 | 0.271 | 0.163 | 0.097 |
| InternalIMT | aa | rs182342688 | . | . | . | . | . | . | . | . | . | . |

| InternalIMT | aa | rs17157728 | 0.198 | -0.048 | 0.095 | 0.61 | -0.626 | 0.363 | 0.085 | 0.271 | 0.163 | 0.097 |
| --- | --- | --- | --- | --- | --- | --- | --- | --- | --- | --- | --- | --- |
| InternalIMT | aa | rs114835563 | . | . | . | . | . | . | . | . | . | . |
| InternalIMT | aa | rs3824612 | 0.504 | 0.332 | 0.146 | 0.024 | 0.632 | 0.273 | 0.021 | -0.264 | 0.12 | 0.029 |
| InternalIMT | aa | rs17157733 | 0.196 | -0.05 | 0.095 | 0.597 | -0.641 | 0.366 | 0.08 | 0.278 | 0.165 | 0.092 |
| InternalIMT | aa | rs74128478 | . | . | . | . | . | . | . | . | . | . |
| InternalIMT | aa | rs184973589 | . | . | . | . | . | . | . | . | . | . |
| InternalIMT | aa | rs150479461 | . | . | . | . | . | . | . | . | . | . |
| InternalIMT | aa | rs138310407 | . | . | . | . | . | . | . | . | . | . |
| InternalIMT | aa | rs187426603 | . | . | . | . | . | . | . | . | . | . |
| InternalIMT | aa | rs190843143 | . | . | . | . | . | . | . | . | . | . |
| InternalIMT | aa | rs191883449 | . | . | . | . | . | . | . | . | . | . |
| InternalIMT | aa | rs58238740 | 0.198 | -0.048 | 0.095 | 0.61 | -0.626 | 0.363 | 0.085 | 0.271 | 0.163 | 0.097 |
| InternalIMT | aa | rs3780894 | 0.198 | -0.048 | 0.095 | 0.61 | -0.626 | 0.363 | 0.085 | 0.271 | 0.163 | 0.097 |
| InternalIMT | aa | rs149646626 | . | . | . | . | . | . | . | . | . | . |
| InternalIMT | aa | rs79615212 | . | . | . | . | . | . | . | . | . | . |
| InternalIMT | aa | rs3780895 | 0.197 | -0.049 | 0.095 | 0.61 | -0.627 | 0.364 | 0.085 | 0.272 | 0.163 | 0.097 |
| InternalIMT | aa | rs3780896 | 0.198 | -0.048 | 0.095 | 0.61 | -0.626 | 0.363 | 0.085 | 0.271 | 0.163 | 0.097 |
| InternalIMT | aa | rs142747163 | 0.003 | . | . | . | . | . | . | . | . | . |
| InternalIMT | aa | rs189417584 | . | . | . | . | . | . | . | . | . | . |
| InternalIMT | aa | rs3780897 | 0.197 | -0.049 | 0.095 | 0.61 | -0.626 | 0.363 | 0.085 | 0.271 | 0.163 | 0.097 |
| InternalIMT | aa | rs117509491 | . | . | . | . | . | . | . | . | . | . |
| InternalIMT | aa | rs117811514 | . | . | . | . | . | . | . | . | . | . |
| InternalIMT | aa | rs116500932 | . | . | . | . | . | . | . | . | . | . |
| InternalIMT | aa | rs77584168 | 0.014 | 0.049 | 0.082 | 0.556 | -1.42 | 1.53 | 0.355 | 0.561 | 0.625 | 0.37 |
| InternalIMT | aa | rs2310776 | 0.198 | -0.048 | 0.095 | 0.61 | -0.626 | 0.363 | 0.085 | 0.271 | 0.163 | 0.097 |
| InternalIMT | aa | rs17157736 | 0.198 | -0.048 | 0.095 | 0.61 | -0.626 | 0.363 | 0.085 | 0.271 | 0.163 | 0.097 |
| InternalIMT | aa | rs72789735 | 0.001 | . | . | . | . | . | . | . | . | . |
| InternalIMT | aa | rs183747187 | . | . | . | . | . | . | . | . | . | . |
| InternalIMT | aa | rs7077891 | 0.198 | -0.048 | 0.095 | 0.61 | -0.626 | 0.363 | 0.085 | 0.271 | 0.163 | 0.097 |
| InternalIMT | aa | rs145063305 | 0.001 | . | . | . | . | . | . | . | . | . |
| InternalIMT | aa | rs3824613 | 0.198 | -0.048 | 0.095 | 0.611 | -0.624 | 0.362 | 0.085 | 0.271 | 0.163 | 0.097 |
| InternalIMT | aa | rs145543199 | . | . | . | . | . | . | . | . | . | . |
| InternalIMT | aa | rs140361993 | . | . | . | . | . | . | . | . | . | . |
| InternalIMT | aa | rs11239496 | 0.001 | . | . | . | . | . | . | . | . | . |
| InternalIMT | aa | rs11239497 | 0.197 | -0.049 | 0.095 | 0.61 | -0.626 | 0.363 | 0.085 | 0.271 | 0.163 | 0.097 |
| InternalIMT | aa | rs10900212 | 0.198 | -0.048 | 0.095 | 0.611 | -0.626 | 0.363 | 0.085 | 0.271 | 0.163 | 0.097 |
| InternalIMT | aa | rs186487136 | . | . | . | . | . | . | . | . | . | . |
| InternalIMT | aa | rs12774369 | 0.197 | -0.049 | 0.095 | 0.609 | -0.628 | 0.365 | 0.086 | 0.272 | 0.164 | 0.098 |
| InternalIMT | aa | rs60842538 | 0.197 | -0.049 | 0.095 | 0.609 | -0.63 | 0.368 | 0.088 | 0.273 | 0.166 | 0.1 |
| InternalIMT | aa | rs3780898 | 0.197 | -0.049 | 0.096 | 0.608 | -0.633 | 0.372 | 0.089 | 0.274 | 0.167 | 0.102 |
| InternalIMT | aa | rs3780899 | 0.197 | -0.049 | 0.096 | 0.608 | -0.633 | 0.373 | 0.09 | 0.275 | 0.168 | 0.102 |
| InternalIMT | aa | rs12252472 | . | . | . | . | . | . | . | . | . | . |
| InternalIMT | aa | rs185411011 | . | . | . | . | . | . | . | . | . | . |
| InternalIMT | aa | rs28395865 | 0.193 | -0.046 | 0.095 | 0.627 | -0.63 | 0.38 | 0.098 | 0.273 | 0.171 | 0.11 |
| InternalIMT | aa | rs191004074 | . | . | . | . | . | . | . | . | . | . |
| InternalIMT | aa | rs182401295 | . | . | . | . | . | . | . | . | . | . |
| InternalIMT | aa | rs2228064 | 0.057 | 0.022 | 0.086 | 0.793 | -0.967 | 0.561 | 0.085 | 0.387 | 0.256 | 0.131 |
| InternalIMT | aa | rs188284159 | . | . | . | . | . | . | . | . | . | . |
| InternalIMT | aa | rs2303887 | 0.055 | 0.023 | 0.085 | 0.79 | -0.979 | 0.56 | 0.081 | 0.394 | 0.256 | 0.124 |
| InternalIMT | aa | rs2303886 | 0.055 | 0.023 | 0.085 | 0.791 | -0.979 | 0.56 | 0.081 | 0.394 | 0.256 | 0.124 |
| InternalIMT | aa | rs192621006 | . | . | . | . | . | . | . | . | . | . |
| InternalIMT | aa | rs2303885 | 0.195 | -0.044 | 0.096 | 0.648 | -0.608 | 0.381 | 0.112 | 0.263 | 0.172 | 0.126 |
| InternalIMT | aa | rs114466168 | . | . | . | . | . | . | . | . | . | . |
| InternalIMT | aa | rs11239501 | 0.003 | . | . | . | . | . | . | . | . | . |
| InternalIMT | aa | rs745986 | 0.196 | -0.065 | 0.097 | 0.5 | -0.726 | 0.404 | 0.073 | 0.318 | 0.183 | 0.082 |
| InternalIMT | aa | rs75262268 | . | . | . | . | . | . | . | . | . | . |
| InternalIMT | aa | rs71494776 | 0.192 | -0.043 | 0.096 | 0.653 | -0.612 | 0.388 | 0.116 | 0.266 | 0.175 | 0.129 |
| InternalIMT | aa | rs74386871 | 0.055 | 0.023 | 0.085 | 0.791 | -0.98 | 0.56 | 0.081 | 0.394 | 0.256 | 0.124 |
| InternalIMT | aa | rs76502040 | . | . | . | . | . | . | . | . | . | . |
| InternalIMT | aa | rs142213734 | . | . | . | . | . | . | . | . | . | . |
| InternalIMT | aa | rs4948671 | 0.869 | -0.123 | 0.258 | 0.635 | -0.233 | 0.349 | 0.504 | 0.105 | 0.15 | 0.482 |
| InternalIMT | aa | rs146286085 | . | . | . | . | . | . | . | . | . | . |
| InternalIMT | aa | rs144080623 | . | . | . | . | . | . | . | . | . | . |
| InternalIMT | aa | rs115074014 | . | . | . | . | . | . | . | . | . | . |
| InternalIMT | aa | rs17157756 | . | . | . | . | . | . | . | . | . | . |
| InternalIMT | aa | rs190459780 | . | . | . | . | . | . | . | . | . | . |
| InternalIMT | aa | rs12358647 | 0.195 | -0.067 | 0.097 | 0.488 | -0.742 | 0.407 | 0.069 | 0.325 | 0.184 | 0.078 |
| InternalIMT | aa | rs147080556 | 0.009 | . | . | . | . | . | . | . | . | . |
| InternalIMT | aa | rs187543349 | . | . | . | . | . | . | . | . | . | . |
| InternalIMT | aa | rs114375595 | 0.01 | . | . | . | . | . | . | . | . | . |
| InternalIMT | aa | rs117211200 | . | . | . | . | . | . | . | . | . | . |
| InternalIMT | aa | rs192963047 | . | . | . | . | . | . | . | . | . | . |
| InternalIMT | aa | rs11239505 | 0.196 | -0.066 | 0.097 | 0.5 | -0.725 | 0.404 | 0.073 | 0.318 | 0.183 | 0.082 |
| InternalIMT | aa | rs184434116 | . | . | . | . | . | . | . | . | . | . |
| InternalIMT | aa | rs4948999 | 0.869 | -0.123 | 0.258 | 0.632 | -0.234 | 0.349 | 0.502 | 0.106 | 0.149 | 0.48 |
| InternalIMT | aa | rs11239506 | 0.003 | . | . | . | . | . | . | . | . | . |
| InternalIMT | aa | rs190706620 | . | . | . | . | . | . | . | . | . | . |
| InternalIMT | aa | rs190770429 | . | . | . | . | . | . | . | . | . | . |
| InternalIMT | aa | rs181327133 | . | . | . | . | . | . | . | . | . | . |
| InternalIMT | aa | rs149251697 | . | . | . | . | . | . | . | . | . | . |
| InternalIMT | aa | rs118081602 | 0.055 | 0.023 | 0.085 | 0.792 | -0.981 | 0.561 | 0.081 | 0.395 | 0.256 | 0.124 |
| InternalIMT | aa | rs188233691 | . | . | . | . | . | . | . | . | . | . |
| InternalIMT | aa | rs183778794 | 0.032 | 0.051 | 0.086 | 0.552 | -0.446 | 0.93 | 0.631 | 0.187 | 0.403 | 0.643 |
| InternalIMT | aa | rs188040078 | . | . | . | . | . | . | . | . | . | . |
| InternalIMT | aa | rs184675372 | . | . | . | . | . | . | . | . | . | . |
| InternalIMT | aa | rs150326891 | 0.004 | . | . | . | . | . | . | . | . | . |

| InternalIMT | aa | rs185520021 | . | . | . | . | . | . | . | . | . | . |
| --- | --- | --- | --- | --- | --- | --- | --- | --- | --- | --- | --- | --- |
| InternalIMT | aa | rs7907779 | 0.003 | . | . | . | . | . | . | . | . | . |
| InternalIMT | aa | rs184962860 | . | . | . | . | . | . | . | . | . | . |
| InternalIMT | aa | rs149762784 | 0.027 | 0.051 | 0.086 | 0.555 | -0.515 | 1.87 | 0.783 | 0.233 | 0.829 | 0.778 |
| InternalIMT | aa | rs149203427 | . | . | . | . | . | . | . | . | . | . |
| InternalIMT | aa | rs60153083 | . | . | . | . | . | . | . | . | . | . |
| InternalIMT | aa | rs112001069 | 0.194 | -0.037 | 0.096 | 0.696 | -0.572 | 0.389 | 0.142 | 0.247 | 0.175 | 0.159 |
| InternalIMT | aa | rs182355382 | 0.012 | 0.03 | 0.085 | 0.725 | -3.76 | 3.5 | 0.283 | 1.62 | 1.49 | 0.277 |
| InternalIMT | aa | rs186740974 | 0.109 | 0.081 | 0.102 | 0.428 | 0.307 | 0.618 | 0.619 | -0.109 | 0.265 | 0.68 |
| InternalIMT | aa | rs191874916 | 0.081 | 0.042 | 0.089 | 0.637 | -0.223 | 0.451 | 0.621 | 0.106 | 0.201 | 0.598 |
| InternalIMT | aa | rs182989412 | 0.122 | -0.183 | 0.095 | 0.053 | -2.1 | 0.641 | 1.10E-03 | 0.94 | 0.289 | 1.20E-03 |
| InternalIMT | aa | rs188819476 | . | . | . | . | . | . | . | . | . | . |
| InternalIMT | aa | rs141217183 | 0.186 | -0.047 | 0.097 | 0.629 | -0.649 | 0.414 | 0.117 | 0.284 | 0.186 | 0.128 |
| InternalIMT | aa | rs192922652 | 0.199 | -0.044 | 0.097 | 0.648 | -0.607 | 0.387 | 0.117 | 0.263 | 0.174 | 0.131 |
| InternalIMT | aa | rs185092343 | 0.012 | 0.034 | 0.083 | 0.677 | -2.57 | 2.88 | 0.372 | 1.24 | 1.27 | 0.331 |
| InternalIMT | aa | rs145193311 | 0.194 | -0.044 | 0.096 | 0.644 | -0.616 | 0.389 | 0.113 | 0.268 | 0.175 | 0.126 |
| InternalIMT | aa | rs55789336 | 0.004 | . | . | . | . | . | . | . | . | . |
| InternalIMT | aa | rs55837212 | 0.009 | . | . | . | . | . | . | . | . | . |
| InternalIMT | aa | rs75408574 | 0.565 | 0.347 | 0.182 | 0.057 | 0.577 | 0.298 | 0.053 | -0.246 | 0.134 | 0.065 |
| InternalIMT | aa | rs149306926 | 0.01 | 0.016 | 0.083 | 0.847 | -5.4 | 3.25 | 0.097 | 2.45 | 1.48 | 0.098 |
| InternalIMT | aa | rs35990892 | 0.843 | -0.214 | 0.251 | 0.395 | -0.37 | 0.344 | 0.282 | 0.161 | 0.149 | 0.278 |
| InternalIMT | aa | rs192356207 | 0.015 | 0.094 | 0.082 | 0.252 | 3.01 | 1.64 | 0.067 | -1.28 | 0.702 | 0.068 |
| InternalIMT | aa | rs183741163 | 0.049 | -7.90E-03 | 0.098 | 0.936 | -1.01 | 0.839 | 0.229 | 0.46 | 0.397 | 0.247 |
| InternalIMT | aa | rs34737297 | 0.719 | -0.315 | 0.203 | 0.122 | -0.64 | 0.333 | 0.055 | 0.267 | 0.145 | 0.066 |
| InternalIMT | aa | rs181346933 | . | . | . | . | . | . | . | . | . | . |
| InternalIMT | aa | rs36093253 | 0.576 | 0.459 | 0.203 | 0.024 | 0.776 | 0.317 | 0.015 | -0.335 | 0.141 | 0.017 |
| InternalIMT | aa | rs186307855 | . | . | . | . | . | . | . | . | . | . |
| InternalIMT | aa | rs189681865 | . | . | . | . | . | . | . | . | . | . |
| InternalIMT | aa | rs34942971 | 0.214 | -0.077 | 0.099 | 0.434 | -0.726 | 0.395 | 0.066 | 0.317 | 0.178 | 0.075 |
| InternalIMT | aa | rs181816554 | . | . | . | . | . | . | . | . | . | . |
| InternalIMT | aa | rs186846881 | . | . | . | . | . | . | . | . | . | . |
| InternalIMT | aa | rs191178220 | 0.003 | . | . | . | . | . | . | . | . | . |
| InternalIMT | aa | rs183305228 | . | . | . | . | . | . | . | . | . | . |
| InternalIMT | aa | rs139912761 | 0.004 | . | . | . | . | . | . | . | . | . |
| InternalIMT | aa | rs185499901 | . | . | . | . | . | . | . | . | . | . |
| InternalIMT | aa | rs189816197 | 0.194 | -0.055 | 0.098 | 0.578 | -0.657 | 0.403 | 0.103 | 0.287 | 0.182 | 0.115 |
| InternalIMT | aa | rs145656938 | 0.009 | . | . | . | . | . | . | . | . | . |
| InternalIMT | aa | rs35745197 | 0.193 | -0.043 | 0.096 | 0.65 | -0.616 | 0.39 | 0.115 | 0.268 | 0.176 | 0.128 |
| InternalIMT | aa | rs192856637 | 0.006 | . | . | . | . | . | . | . | . | . |
| InternalIMT | aa | rs2310775 | 0.617 | 0.356 | 0.186 | 0.055 | 0.557 | 0.287 | 0.053 | -0.235 | 0.128 | 0.068 |
| InternalIMT | aa | rs11239507 | 0.673 | 0.297 | 0.207 | 0.153 | 0.404 | 0.302 | 0.182 | -0.175 | 0.135 | 0.197 |
| InternalIMT | aa | rs74648467 | 0.192 | -0.044 | 0.096 | 0.648 | -0.618 | 0.391 | 0.114 | 0.269 | 0.176 | 0.127 |
| InternalIMT | aa | rs145815934 | . | . | . | . | . | . | . | . | . | . |
| InternalIMT | aa | rs145390687 | . | . | . | . | . | . | . | . | . | . |
| InternalIMT | aa | rs184354913 | . | . | . | . | . | . | . | . | . | . |
| InternalIMT | aa | rs181244920 | . | . | . | . | . | . | . | . | . | . |
| InternalIMT | aa | rs11499087 | . | . | . | . | . | . | . | . | . | . |
| InternalIMT | aa | rs148263794 | 0.207 | -0.045 | 0.098 | 0.642 | -0.588 | 0.381 | 0.124 | 0.257 | 0.172 | 0.135 |
| InternalIMT | aa | rs150758903 | . | . | . | . | . | . | . | . | . | . |
| InternalIMT | aa | rs147654382 | 0.187 | -0.078 | 0.097 | 0.425 | -0.838 | 0.42 | 0.047 | 0.364 | 0.191 | 0.057 |
| InternalIMT | aa | rs147495166 | . | . | . | . | . | . | . | . | . | . |
| InternalIMT | aa | rs35198778 | 0.131 | -0.029 | 0.095 | 0.756 | -0.765 | 0.552 | 0.166 | 0.33 | 0.251 | 0.189 |
| InternalIMT | aa | rs145207659 | 0.048 | 0.083 | 0.084 | 0.326 | 0.622 | 0.768 | 0.419 | -0.248 | 0.331 | 0.454 |
| InternalIMT | aa | rs186466464 | . | . | . | . | . | . | . | . | . | . |
| InternalIMT | aa | rs12778413 | 0.131 | -0.095 | 0.092 | 0.304 | -1.41 | 0.581 | 0.015 | 0.622 | 0.261 | 0.018 |
| InternalIMT | aa | rs7895552 | 0.833 | -0.035 | 0.237 | 0.883 | -0.118 | 0.333 | 0.722 | 0.057 | 0.144 | 0.69 |
| InternalIMT | aa | rs12783895 | 0.193 | -0.043 | 0.096 | 0.653 | -0.612 | 0.388 | 0.115 | 0.266 | 0.175 | 0.129 |
| InternalIMT | aa | rs34929604 | 0.193 | -0.043 | 0.096 | 0.654 | -0.611 | 0.388 | 0.116 | 0.266 | 0.175 | 0.129 |
| InternalIMT | aa | rs12761961 | 0.185 | -0.049 | 0.096 | 0.608 | -0.666 | 0.409 | 0.104 | 0.291 | 0.185 | 0.115 |
| InternalIMT | aa | rs35536930 | 0.197 | -0.065 | 0.097 | 0.501 | -0.723 | 0.403 | 0.074 | 0.317 | 0.182 | 0.083 |
| InternalIMT | aa | rs180951060 | . | . | . | . | . | . | . | . | . | . |
| InternalIMT | aa | rs148892128 | . | . | . | . | . | . | . | . | . | . |
| InternalIMT | aa | rs11239509 | . | . | . | . | . | . | . | . | . | . |
| InternalIMT | aa | rs41526545 | 0.003 | . | . | . | . | . | . | . | . | . |
| InternalIMT | aa | rs2029253 | 0.617 | 0.356 | 0.185 | 0.055 | 0.556 | 0.287 | 0.053 | -0.235 | 0.128 | 0.068 |
| InternalIMT | aa | rs17444064 | 0.003 | . | . | . | . | . | . | . | . | . |
| InternalIMT | aa | rs71494779 | 0.194 | -0.043 | 0.096 | 0.655 | -0.608 | 0.387 | 0.117 | 0.265 | 0.175 | 0.129 |
| InternalIMT | aa | rs74991420 | 0.05 | 0.023 | 0.085 | 0.789 | -1.08 | 0.577 | 0.061 | 0.436 | 0.263 | 0.097 |
| InternalIMT | aa | rs182736551 | 0.05 | 0.023 | 0.085 | 0.789 | -1.08 | 0.577 | 0.061 | 0.436 | 0.263 | 0.097 |
| InternalIMT | aa | rs78702074 | 0.055 | 0.022 | 0.085 | 0.794 | -0.988 | 0.563 | 0.08 | 0.398 | 0.257 | 0.122 |
| InternalIMT | aa | rs187791466 | . | . | . | . | . | . | . | . | . | . |
| InternalIMT | aa | rs35300270 | 0.194 | -0.043 | 0.096 | 0.655 | -0.608 | 0.387 | 0.117 | 0.265 | 0.175 | 0.129 |
| InternalIMT | aa | rs6593484 | 0.003 | . | . | . | . | . | . | . | . | . |
| InternalIMT | aa | rs12244601 | . | . | . | . | . | . | . | . | . | . |
| InternalIMT | aa | rs184429574 | . | . | . | . | . | . | . | . | . | . |
| InternalIMT | aa | rs7080897 | 0.194 | -0.043 | 0.096 | 0.655 | -0.608 | 0.387 | 0.116 | 0.265 | 0.174 | 0.129 |
| InternalIMT | aa | rs7097429 | 0.196 | -0.048 | 0.096 | 0.618 | -0.627 | 0.38 | 0.099 | 0.272 | 0.172 | 0.114 |
| InternalIMT | aa | rs77979229 | 0.016 | 0.052 | 0.082 | 0.529 | -0.772 | 1.35 | 0.567 | 0.363 | 0.598 | 0.544 |
| InternalIMT | aa | rs11239512 | 0.003 | . | . | . | . | . | . | . | . | . |
| InternalIMT | aa | rs60146827 | 0.195 | -0.041 | 0.096 | 0.665 | -0.598 | 0.386 | 0.121 | 0.26 | 0.174 | 0.135 |
| InternalIMT | aa | rs79032236 | . | . | . | . | . | . | . | . | . | . |
| InternalIMT | aa | rs140854206 | . | . | . | . | . | . | . | . | . | . |
| InternalIMT | aa | rs184049550 | . | . | . | . | . | . | . | . | . | . |
| InternalIMT | aa | rs114433607 | . | . | . | . | . | . | . | . | . | . |
| InternalIMT | aa | rs117653793 | 0.055 | 0.022 | 0.085 | 0.795 | -0.99 | 0.563 | 0.079 | 0.399 | 0.257 | 0.121 |
| InternalIMT | aa | rs117039533 | 0.055 | 0.022 | 0.085 | 0.795 | -0.99 | 0.563 | 0.079 | 0.399 | 0.257 | 0.121 |

| InternalIMT | aa | rs191660761 | 0.001 | . | . | . | . | . | . | . | . | . |
| --- | --- | --- | --- | --- | --- | --- | --- | --- | --- | --- | --- | --- |
| InternalIMT | aa | rs59514896 | 0.197 | -0.037 | 0.096 | 0.697 | -0.57 | 0.382 | 0.136 | 0.247 | 0.172 | 0.151 |
| InternalIMT | aa | rs184661876 | . | . | . | . | . | . | . | . | . | . |
| InternalIMT | aa | rs61854073 | . | . | . | . | . | . | . | . | . | . |
| InternalIMT | aa | rs2018518 | 0.5 | 0.373 | 0.155 | 0.017 | 0.714 | 0.283 | 0.012 | -0.305 | 0.125 | 0.015 |
| InternalIMT | aa | rs934187 | 0.872 | -0.118 | 0.255 | 0.643 | -0.228 | 0.345 | 0.508 | 0.103 | 0.148 | 0.488 |
| InternalIMT | aa | rs7918542 | 0.202 | -0.058 | 0.097 | 0.548 | -0.672 | 0.395 | 0.089 | 0.294 | 0.179 | 0.101 |
| InternalIMT | aa | rs144311920 | . | . | . | . | . | . | . | . | . | . |
| InternalIMT | aa | rs7099684 | 0.871 | -0.123 | 0.255 | 0.631 | -0.235 | 0.345 | 0.496 | 0.105 | 0.148 | 0.477 |
| InternalIMT | aa | rs150829171 | . | . | . | . | . | . | . | . | . | . |
| InternalIMT | aa | rs186060697 | . | . | . | . | . | . | . | . | . | . |
| InternalIMT | aa | rs11239513 | . | . | . | . | . | . | . | . | . | . |
| InternalIMT | aa | rs7917687 | 0.2 | -0.031 | 0.096 | 0.748 | -0.525 | 0.375 | 0.161 | 0.227 | 0.169 | 0.178 |
| InternalIMT | aa | rs61854076 | . | . | . | . | . | . | . | . | . | . |
| InternalIMT | aa | rs71494780 | . | . | . | . | . | . | . | . | . | . |
| InternalIMT | aa | rs7919239 | 0.235 | -2.50E-03 | 0.094 | 0.978 | -0.294 | 0.301 | 0.33 | 0.131 | 0.134 | 0.33 |
| InternalIMT | aa | rs78334475 | 0.166 | 0.245 | 0.113 | 0.03 | 1.27 | 0.415 | 2.30E-03 | -0.547 | 0.181 | 2.60E-03 |
| InternalIMT | aa | rs3780900 | 0.068 | 0.039 | 0.086 | 0.654 | -0.276 | 0.489 | 0.573 | 0.14 | 0.217 | 0.519 |
| InternalIMT | aa | rs186729292 | . | . | . | . | . | . | . | . | . | . |
| InternalIMT | aa | rs12267438 | . | . | . | . | . | . | . | . | . | . |
| InternalIMT | aa | rs1369214 | 0.868 | 0.048 | 0.279 | 0.864 | -0.016 | 0.364 | 0.965 | 7.30E-03 | 0.158 | 0.963 |
| InternalIMT | aa | rs9282589 | . | . | . | . | . | . | . | . | . | . |
| InternalIMT | aa | rs114522915 | . | . | . | . | . | . | . | . | . | . |
| InternalIMT | aa | rs2115819 | 0.812 | 0.256 | 0.232 | 0.27 | 0.298 | 0.313 | 0.341 | -0.118 | 0.139 | 0.394 |
| InternalIMT | aa | rs117664603 | 0.055 | 0.022 | 0.085 | 0.794 | -0.987 | 0.565 | 0.081 | 0.397 | 0.258 | 0.124 |
| InternalIMT | aa | rs184979202 | . | . | . | . | . | . | . | . | . | . |
| InternalIMT | aa | rs190714671 | . | . | . | . | . | . | . | . | . | . |
| InternalIMT | aa | rs141538996 | . | . | . | . | . | . | . | . | . | . |
| InternalIMT | aa | rs150881741 | . | . | . | . | . | . | . | . | . | . |
| InternalIMT | aa | rs139076633 | 0.011 | 0.068 | 0.083 | 0.408 | 0.831 | 0.847 | 0.327 | -0.413 | 0.422 | 0.328 |
| InternalIMT | aa | rs12243649 | 0.056 | 0.023 | 0.086 | 0.793 | -0.965 | 0.566 | 0.089 | 0.389 | 0.259 | 0.133 |
| InternalIMT | aa | rs140348708 | . | . | . | . | . | . | . | . | . | . |
| InternalIMT | aa | rs79023256 | 0.055 | 0.022 | 0.085 | 0.794 | -0.986 | 0.567 | 0.082 | 0.397 | 0.259 | 0.126 |
| InternalIMT | aa | rs11239515 | 0.003 | . | . | . | . | . | . | . | . | . |
| InternalIMT | aa | rs55866086 | 0.059 | -2.70E-04 | 0.084 | 0.997 | -1.44 | 0.645 | 0.026 | 0.605 | 0.294 | 0.04 |
| InternalIMT | aa | rs181340150 | . | . | . | . | . | . | . | . | . | . |
| InternalIMT | aa | rs11239516 | 0.003 | . | . | . | . | . | . | . | . | . |
| InternalIMT | aa | rs11239517 | 0.003 | . | . | . | . | . | . | . | . | . |
| InternalIMT | aa | rs11239519 | 0.003 | . | . | . | . | . | . | . | . | . |
| InternalIMT | aa | rs7909514 | 0.872 | -0.088 | 0.264 | 0.738 | -0.189 | 0.357 | 0.597 | 0.085 | 0.153 | 0.576 |
| InternalIMT | aa | rs7894352 | 0.872 | -0.09 | 0.264 | 0.733 | -0.191 | 0.357 | 0.593 | 0.086 | 0.153 | 0.572 |
| InternalIMT | aa | rs189384146 | 0 | . | . | . | . | . | . | . | . | . |
| InternalIMT | aa | rs11239520 | 0.004 | . | . | . | . | . | . | . | . | . |
| InternalIMT | aa | rs12221233 | 0.617 | 0.319 | 0.189 | 0.092 | 0.496 | 0.296 | 0.095 | -0.207 | 0.133 | 0.119 |
| InternalIMT | aa | rs74523293 | 0.049 | 0.031 | 0.085 | 0.717 | -0.795 | 0.504 | 0.115 | 0.31 | 0.23 | 0.178 |
| InternalIMT | aa | rs79376040 | 0.049 | 0.031 | 0.085 | 0.717 | -0.795 | 0.504 | 0.115 | 0.31 | 0.23 | 0.178 |
| InternalIMT | aa | rs4949000 | 0.617 | 0.32 | 0.189 | 0.092 | 0.497 | 0.296 | 0.094 | -0.207 | 0.133 | 0.119 |
| InternalIMT | aa | rs7089406 | . | . | . | . | . | . | . | . | . | . |
| InternalIMT | aa | rs138211056 | . | . | . | . | . | . | . | . | . | . |
| InternalIMT | aa | rs10900213 | 0.621 | 0.274 | 0.181 | 0.132 | 0.414 | 0.286 | 0.148 | -0.169 | 0.127 | 0.184 |
| InternalIMT | aa | rs145152827 | 0.011 | 0.068 | 0.083 | 0.409 | 0.83 | 0.857 | 0.333 | -0.413 | 0.426 | 0.333 |
| InternalIMT | aa | rs74443325 | 0.048 | 0.031 | 0.085 | 0.716 | -0.801 | 0.506 | 0.114 | 0.312 | 0.231 | 0.176 |
| InternalIMT | aa | rs188299446 | . | . | . | . | . | . | . | . | . | . |
| InternalIMT | aa | rs192699586 | . | . | . | . | . | . | . | . | . | . |
| InternalIMT | aa | rs72796464 | 0.003 | . | . | . | . | . | . | . | . | . |
| InternalIMT | aa | rs4949001 | 0.455 | 0.323 | 0.147 | 0.028 | 0.664 | 0.283 | 0.019 | -0.285 | 0.125 | 0.023 |
| InternalIMT | aa | rs147174641 | . | . | . | . | . | . | . | . | . | . |
| InternalIMT | aa | rs190835238 | . | . | . | . | . | . | . | . | . | . |
| InternalIMT | aa | rs76698944 | . | . | . | . | . | . | . | . | . | . |
| InternalIMT | aa | rs56071381 | 0.003 | . | . | . | . | . | . | . | . | . |
| InternalIMT | aa | rs78130427 | 0.2 | -0.026 | 0.096 | 0.785 | -0.497 | 0.378 | 0.189 | 0.214 | 0.17 | 0.208 |
| InternalIMT | aa | rs10900215 | 0.007 | . | . | . | . | . | . | . | . | . |
| InternalIMT | aa | rs41491445 | . | . | . | . | . | . | . | . | . | . |
| InternalIMT | aa | rs144995222 | . | . | . | . | . | . | . | . | . | . |
| InternalIMT | aa | rs149097803 | . | . | . | . | . | . | . | . | . | . |
| InternalIMT | aa | rs139429646 | . | . | . | . | . | . | . | . | . | . |
| InternalIMT | aa | rs7075620 | 0.861 | -0.125 | 0.271 | 0.645 | -0.236 | 0.375 | 0.529 | 0.108 | 0.161 | 0.502 |
| InternalIMT | aa | rs148299213 | . | . | . | . | . | . | . | . | . | . |
| InternalIMT | aa | rs4949002 | 0.238 | 0.114 | 0.114 | 0.317 | 0.265 | 0.378 | 0.483 | -0.105 | 0.165 | 0.523 |
| InternalIMT | aa | rs150496007 | . | . | . | . | . | . | . | . | . | . |
| InternalIMT | aa | rs147303455 | . | . | . | . | . | . | . | . | . | . |
| InternalIMT | aa | rs189518433 | . | . | . | . | . | . | . | . | . | . |
| InternalIMT | aa | rs186281355 | . | . | . | . | . | . | . | . | . | . |
| InternalIMT | aa | rs115297114 | . | . | . | . | . | . | . | . | . | . |
| InternalIMT | aa | rs11239523 | 0.003 | . | . | . | . | . | . | . | . | . |
| InternalIMT | aa | rs143704897 | . | . | . | . | . | . | . | . | . | . |
| InternalIMT | aa | rs188024096 | . | . | . | . | . | . | . | . | . | . |
| InternalIMT | aa | rs11239524 | 0.003 | . | . | . | . | . | . | . | . | . |
| InternalIMT | aa | rs191656573 | . | . | . | . | . | . | . | . | . | . |
| InternalIMT | aa | rs193295875 | 0.011 | 0.051 | 0.082 | 0.536 | -0.919 | 1.03 | 0.371 | 0.353 | 0.478 | 0.46 |
| InternalIMT | aa | rs190458228 | . | . | . | . | . | . | . | . | . | . |
| InternalIMT | aa | rs139253993 | 0.002 | . | . | . | . | . | . | . | . | . |
| InternalIMT | aa | rs4948672 | 0.812 | -0.018 | 0.266 | 0.947 | -0.106 | 0.381 | 0.781 | 0.048 | 0.163 | 0.767 |
| InternalIMT | aa | rs180954921 | . | . | . | . | . | . | . | . | . | . |
| InternalIMT | aa | rs12264801 | 0.447 | -0.213 | 0.154 | 0.168 | -0.749 | 0.361 | 0.038 | 0.315 | 0.159 | 0.048 |
| InternalIMT | aa | rs73283184 | . | . | . | . | . | . | . | . | . | . |

| InternalIMT | aa | rs142746187 | . | . | . | . | . | . | . | . | . | . |
| --- | --- | --- | --- | --- | --- | --- | --- | --- | --- | --- | --- | --- |
| InternalIMT | aa | rs58704739 | . | . | . | . | . | . | . | . | . | . |
| InternalIMT | aa | rs61272335 | . | . | . | . | . | . | . | . | . | . |
| InternalIMT | aa | rs60959489 | . | . | . | . | . | . | . | . | . | . |
| InternalIMT | aa | rs58502629 | . | . | . | . | . | . | . | . | . | . |
| InternalIMT | aa | rs10900216 | 0.838 | 0.235 | 0.214 | 0.272 | 0.312 | 0.295 | 0.29 | -0.106 | 0.13 | 0.415 |
| InternalIMT | aa | rs181118141 | . | . | . | . | . | . | . | . | . | . |
| InternalIMT | aa | rs137882665 | . | . | . | . | . | . | . | . | . | . |
| InternalIMT | aa | rs149466736 | . | . | . | . | . | . | . | . | . | . |
| InternalIMT | aa | rs2099171 | 0.806 | 0.109 | 0.201 | 0.586 | 0.116 | 0.284 | 0.684 | -0.029 | 0.125 | 0.815 |
| InternalIMT | aa | rs141852280 | . | . | . | . | . | . | . | . | . | . |
| InternalIMT | aa | rs191539257 | 0.001 | . | . | . | . | . | . | . | . | . |
| InternalIMT | aa | rs17157771 | 0.029 | 0.086 | 0.082 | 0.3 | 1.55 | 0.952 | 0.103 | -0.642 | 0.392 | 0.102 |
| InternalIMT | aa | rs111339241 | 0.029 | 0.085 | 0.082 | 0.302 | 1.59 | 0.969 | 0.1 | -0.656 | 0.398 | 0.1 |
| InternalIMT | aa | rs116983255 | . | . | . | . | . | . | . | . | . | . |
| InternalIMT | aa | rs142324341 | 0.037 | 0.058 | 0.085 | 0.499 | 0.013 | 0.852 | 0.988 | 0.021 | 0.369 | 0.955 |
| InternalIMT | aa | rs187191219 | . | . | . | . | . | . | . | . | . | . |
| InternalIMT | aa | rs151283973 | . | . | . | . | . | . | . | . | . | . |
| InternalIMT | aa | rs139316218 | . | . | . | . | . | . | . | . | . | . |
| InternalIMT | aa | rs6593485 | 0.886 | 0.098 | 0.271 | 0.717 | 0.103 | 0.359 | 0.775 | -0.021 | 0.155 | 0.894 |
| InternalIMT | aa | rs6593486 | 0.886 | 0.097 | 0.271 | 0.721 | 0.101 | 0.359 | 0.779 | -0.02 | 0.155 | 0.898 |
| InternalIMT | aa | rs145196618 | . | . | . | . | . | . | . | . | . | . |
| InternalIMT | aa | rs142598335 | . | . | . | . | . | . | . | . | . | . |
| InternalIMT | aa | rs892691 | 0.885 | 0.084 | 0.269 | 0.755 | 0.085 | 0.356 | 0.812 | -0.013 | 0.153 | 0.934 |
| InternalIMT | aa | rs7080713 | 0.809 | 0.091 | 0.208 | 0.662 | 0.093 | 0.293 | 0.752 | -0.018 | 0.128 | 0.887 |
| InternalIMT | aa | rs7077173 | 0.065 | 0.097 | 0.082 | 0.24 | 0.844 | 0.522 | 0.106 | -0.338 | 0.217 | 0.12 |
| InternalIMT | aa | rs7080977 | 0.03 | 0.084 | 0.083 | 0.31 | 1.35 | 0.938 | 0.149 | -0.557 | 0.386 | 0.15 |
| InternalIMT | aa | rs3780901 | 0.809 | 0.094 | 0.208 | 0.65 | 0.097 | 0.293 | 0.74 | -0.02 | 0.128 | 0.874 |
| InternalIMT | aa | rs61854091 | . | . | . | . | . | . | . | . | . | . |
| InternalIMT | aa | rs77550209 | . | . | . | . | . | . | . | . | . | . |
| InternalIMT | aa | rs73283191 | 0.077 | 0.054 | 0.087 | 0.534 | -0.15 | 0.436 | 0.731 | 0.049 | 0.189 | 0.794 |
| InternalIMT | aa | rs191866099 | . | . | . | . | . | . | . | . | . | . |
| InternalIMT | aa | rs147998395 | . | . | . | . | . | . | . | . | . | . |
| InternalIMT | aa | rs7090328 | 0.885 | 0.1 | 0.272 | 0.713 | 0.105 | 0.361 | 0.771 | -0.022 | 0.156 | 0.889 |
| InternalIMT | aa | rs79814535 | 0.048 | 0.029 | 0.085 | 0.729 | -0.796 | 0.495 | 0.108 | 0.312 | 0.225 | 0.166 |
| InternalIMT | aa | rs7073082 | 0.885 | 0.101 | 0.273 | 0.713 | 0.105 | 0.362 | 0.771 | -0.022 | 0.156 | 0.888 |
| InternalIMT | aa | rs11239526 | 0.039 | 0.08 | 0.086 | 0.351 | 0.568 | 0.59 | 0.336 | -0.21 | 0.253 | 0.407 |
| InternalIMT | aa | rs141839841 | . | . | . | . | . | . | . | . | . | . |
| InternalIMT | aa | rs75697371 | . | . | . | . | . | . | . | . | . | . |
| InternalIMT | aa | rs75972854 | . | . | . | . | . | . | . | . | . | . |
| InternalIMT | aa | rs139291975 | . | . | . | . | . | . | . | . | . | . |
| InternalIMT | aa | rs144064711 | . | . | . | . | . | . | . | . | . | . |
| InternalIMT | aa | rs3780902 | 0.078 | 0.054 | 0.087 | 0.534 | -0.151 | 0.432 | 0.727 | 0.05 | 0.187 | 0.791 |
| InternalIMT | aa | rs190449801 | . | . | . | . | . | . | . | . | . | . |
| InternalIMT | aa | rs73283196 | 0.077 | 0.053 | 0.086 | 0.539 | -0.161 | 0.433 | 0.711 | 0.054 | 0.188 | 0.774 |
| InternalIMT | aa | rs28395867 | . | . | . | . | . | . | . | . | . | . |
| InternalIMT | aa | rs28395866 | 0.029 | 0.084 | 0.083 | 0.31 | 1.51 | 0.935 | 0.106 | -0.621 | 0.384 | 0.106 |
| InternalIMT | aa | rs55661611 | 0.028 | 0.084 | 0.083 | 0.311 | 1.51 | 0.944 | 0.11 | -0.619 | 0.388 | 0.111 |
| InternalIMT | aa | rs55684518 | . | . | . | . | . | . | . | . | . | . |
| InternalIMT | aa | rs78552493 | . | . | . | . | . | . | . | . | . | . |
| InternalIMT | aa | rs147665434 | . | . | . | . | . | . | . | . | . | . |
| InternalIMT | aa | rs34445001 | . | . | . | . | . | . | . | . | . | . |
| InternalIMT | aa | rs184019449 | . | . | . | . | . | . | . | . | . | . |
| InternalIMT | aa | rs3780903 | 0.808 | 0.114 | 0.208 | 0.584 | 0.125 | 0.295 | 0.672 | -0.032 | 0.13 | 0.805 |
| InternalIMT | aa | rs17157784 | 0.048 | 0.029 | 0.085 | 0.735 | -0.821 | 0.491 | 0.095 | 0.325 | 0.223 | 0.147 |
| InternalIMT | aa | rs3780904 | 0.048 | 0.029 | 0.085 | 0.735 | -0.821 | 0.491 | 0.095 | 0.325 | 0.223 | 0.147 |
| InternalIMT | aa | rs192064730 | . | . | . | . | . | . | . | . | . | . |
| InternalIMT | aa | rs12774534 | . | . | . | . | . | . | . | . | . | . |
| InternalIMT | aa | rs79179915 | 0.047 | 0.028 | 0.085 | 0.737 | -0.835 | 0.495 | 0.092 | 0.331 | 0.225 | 0.143 |
| InternalIMT | aa | rs117811571 | . | . | . | . | . | . | . | . | . | . |
| InternalIMT | aa | rs72796477 | . | . | . | . | . | . | . | . | . | . |
| InternalIMT | aa | rs55729342 | 0.057 | 0.027 | 0.087 | 0.753 | -0.648 | 0.48 | 0.177 | 0.247 | 0.218 | 0.258 |
| InternalIMT | aa | rs59317220 | 0.047 | 0.029 | 0.085 | 0.736 | -0.835 | 0.496 | 0.093 | 0.33 | 0.226 | 0.144 |
| InternalIMT | aa | rs3780905 | 0.562 | 0.211 | 0.138 | 0.128 | 0.309 | 0.251 | 0.22 | -0.133 | 0.112 | 0.235 |
| InternalIMT | aa | rs3780906 | 0.807 | 0.135 | 0.206 | 0.512 | 0.156 | 0.294 | 0.597 | -0.045 | 0.13 | 0.73 |
| InternalIMT | aa | rs149047975 | . | . | . | . | . | . | . | . | . | . |
| InternalIMT | aa | rs3824614 | 0.047 | 0.029 | 0.085 | 0.735 | -0.836 | 0.497 | 0.093 | 0.331 | 0.226 | 0.145 |
| InternalIMT | aa | rs140254404 | . | . | . | . | . | . | . | . | . | . |
| InternalIMT | aa | rs145262684 | . | . | . | . | . | . | . | . | . | . |
| InternalIMT | aa | rs76735482 | 0.047 | 0.029 | 0.085 | 0.734 | -0.835 | 0.499 | 0.094 | 0.331 | 0.227 | 0.146 |
| InternalIMT | aa | rs73283198 | 0.028 | 0.077 | 0.083 | 0.352 | 1.18 | 0.83 | 0.157 | -0.48 | 0.341 | 0.16 |
| InternalIMT | aa | rs74911239 | . | . | . | . | . | . | . | . | . | . |
| InternalIMT | aa | rs150551647 | . | . | . | . | . | . | . | . | . | . |
| InternalIMT | aa | rs115200794 | . | . | . | . | . | . | . | . | . | . |
| InternalIMT | aa | rs74130823 | 0.038 | 0.052 | 0.084 | 0.536 | -0.214 | 0.855 | 0.802 | 0.1 | 0.37 | 0.788 |
| InternalIMT | aa | rs2279435 | 0.754 | -0.021 | 0.212 | 0.921 | -0.103 | 0.313 | 0.741 | 0.055 | 0.133 | 0.681 |
| InternalIMT | aa | rs147279673 | 0.01 | . | . | . | . | . | . | . | . | . |
| InternalIMT | aa | rs183525146 | 0.006 | . | . | . | . | . | . | . | . | . |
| InternalIMT | aa | rs3740107 | 0.737 | 0.018 | 0.185 | 0.921 | -0.01 | 0.289 | 0.971 | 0.026 | 0.126 | 0.835 |
| InternalIMT | aa | rs140697247 | . | . | . | . | . | . | . | . | . | . |
| InternalIMT | aa | rs1565096 | 0.806 | -0.161 | 0.231 | 0.487 | -0.285 | 0.334 | 0.393 | 0.136 | 0.142 | 0.339 |
| InternalIMT | aa | rs1565097 | 0.806 | -0.16 | 0.232 | 0.491 | -0.284 | 0.335 | 0.397 | 0.135 | 0.142 | 0.343 |
| InternalIMT | aa | rs144584548 | . | . | . | . | . | . | . | . | . | . |
| InternalIMT | aa | rs75811053 | 0.044 | 0.034 | 0.084 | 0.686 | -0.741 | 0.515 | 0.151 | 0.287 | 0.235 | 0.223 |
| InternalIMT | aa | rs147462967 | . | . | . | . | . | . | . | . | . | . |
| InternalIMT | aa | rs74130824 | 0.044 | 0.034 | 0.084 | 0.688 | -0.746 | 0.516 | 0.149 | 0.289 | 0.236 | 0.22 |

| InternalIMT | aa | rs116883184 | . | . | . | . | . | . | . | . | . | . |
| --- | --- | --- | --- | --- | --- | --- | --- | --- | --- | --- | --- | --- |
| InternalIMT | aa | rs117258049 | . | . | . | . | . | . | . | . | . | . |
| InternalIMT | aa | rs3780908 | 0.029 | 0.048 | 0.082 | 0.555 | -0.281 | 0.996 | 0.778 | 0.147 | 0.43 | 0.733 |
| InternalIMT | aa | rs180938522 | . | . | . | . | . | . | . | . | . | . |
| InternalIMT | aa | rs76687372 | . | . | . | . | . | . | . | . | . | . |
| InternalIMT | aa | rs3780909 | 0.666 | 0.058 | 0.176 | 0.741 | 6.70E-03 | 0.296 | 0.982 | 2.00E-03 | 0.128 | 0.988 |
| InternalIMT | aa | rs78281156 | 0.044 | 0.034 | 0.084 | 0.686 | -0.743 | 0.516 | 0.15 | 0.288 | 0.236 | 0.222 |
| InternalIMT | aa | rs187840631 | . | . | . | . | . | . | . | . | . | . |
| InternalIMT | aa | rs76868242 | 0.044 | 0.034 | 0.084 | 0.685 | -0.742 | 0.516 | 0.151 | 0.288 | 0.236 | 0.223 |
| InternalIMT | aa | rs192794192 | . | . | . | . | . | . | . | . | . | . |
| InternalIMT | aa | rs12781248 | . | . | . | . | . | . | . | . | . | . |
| InternalIMT | aa | rs12762375 | . | . | . | . | . | . | . | . | . | . |
| InternalIMT | aa | rs192256986 | . | . | . | . | . | . | . | . | . | . |
| InternalIMT | aa | rs184411030 | . | . | . | . | . | . | . | . | . | . |
| InternalIMT | aa | rs189601483 | 0.049 | 8.80E-03 | 0.082 | 0.914 | -1.32 | 0.622 | 0.034 | 0.544 | 0.284 | 0.056 |
| InternalIMT | aa | rs143254579 | 0.045 | 0.084 | 0.086 | 0.331 | 0.524 | 0.548 | 0.339 | -0.204 | 0.235 | 0.387 |
| InternalIMT | aa | rs12253903 | . | . | . | . | . | . | . | . | . | . |
| InternalIMT | aa | rs35489926 | . | . | . | . | . | . | . | . | . | . |
| InternalIMT | aa | rs71494783 | 0.073 | 0.03 | 0.088 | 0.731 | -0.531 | 0.418 | 0.205 | 0.206 | 0.187 | 0.271 |
| InternalIMT | aa | rs180711602 | . | . | . | . | . | . | . | . | . | . |
| InternalIMT | aa | rs117312402 | 0.044 | 0.034 | 0.084 | 0.683 | -0.738 | 0.516 | 0.153 | 0.286 | 0.236 | 0.225 |
| InternalIMT | aa | rs74831694 | 0.053 | 0.054 | 0.086 | 0.533 | -0.062 | 1.14 | 0.956 | 0.049 | 0.494 | 0.921 |
| InternalIMT | aa | rs140301943 | . | . | . | . | . | . | . | . | . | . |
| InternalIMT | aa | rs78553679 | . | . | . | . | . | . | . | . | . | . |
| InternalIMT | aa | rs78334250 | 0.044 | 0.034 | 0.084 | 0.683 | -0.738 | 0.516 | 0.153 | 0.286 | 0.236 | 0.226 |
| InternalIMT | aa | rs76000028 | 0.044 | 0.034 | 0.084 | 0.683 | -0.738 | 0.516 | 0.153 | 0.286 | 0.236 | 0.226 |
| InternalIMT | aa | rs145195254 | . | . | . | . | . | . | . | . | . | . |
| InternalIMT | aa | rs137888527 | . | . | . | . | . | . | . | . | . | . |
| InternalIMT | aa | rs149109592 | . | . | . | . | . | . | . | . | . | . |
| InternalIMT | aa | rs80280010 | 0.038 | 0.079 | 0.086 | 0.356 | 0.53 | 0.573 | 0.355 | -0.195 | 0.245 | 0.426 |
| InternalIMT | aa | rs189995374 | 0.005 | . | . | . | . | . | . | . | . | . |
| InternalIMT | aa | rs75467770 | 0.05 | 0.029 | 0.085 | 0.733 | -0.759 | 0.483 | 0.117 | 0.292 | 0.221 | 0.186 |
| InternalIMT | aa | rs1487562 | 0.029 | 0.048 | 0.082 | 0.563 | -0.321 | 1.01 | 0.75 | 0.164 | 0.435 | 0.705 |
| InternalIMT | aa | rs12266963 | 0.073 | 0.03 | 0.088 | 0.732 | -0.53 | 0.416 | 0.204 | 0.206 | 0.187 | 0.271 |
| InternalIMT | aa | rs7099874 | 0.769 | -0.195 | 0.206 | 0.344 | -0.381 | 0.315 | 0.227 | 0.168 | 0.135 | 0.213 |
| InternalIMT | aa | rs75687019 | 0.044 | 0.034 | 0.084 | 0.683 | -0.736 | 0.516 | 0.154 | 0.285 | 0.236 | 0.227 |
| InternalIMT | aa | rs148240462 | . | . | . | . | . | . | . | . | . | . |
| InternalIMT | aa | rs141278174 | . | . | . | . | . | . | . | . | . | . |
| InternalIMT | aa | rs76404852 | 0.044 | 0.034 | 0.084 | 0.682 | -0.736 | 0.516 | 0.154 | 0.285 | 0.236 | 0.227 |
| InternalIMT | aa | rs139112240 | . | . | . | . | . | . | . | . | . | . |
| InternalIMT | aa | rs12783037 | 0.073 | 0.03 | 0.088 | 0.732 | -0.529 | 0.415 | 0.203 | 0.205 | 0.186 | 0.27 |
| InternalIMT | aa | rs72796481 | . | . | . | . | . | . | . | . | . | . |
| InternalIMT | aa | rs76798749 | 0.044 | 0.034 | 0.084 | 0.681 | -0.733 | 0.516 | 0.156 | 0.284 | 0.236 | 0.229 |
| InternalIMT | aa | rs187732548 | . | . | . | . | . | . | . | . | . | . |
| InternalIMT | aa | rs75675474 | 0.044 | 0.034 | 0.084 | 0.681 | -0.733 | 0.516 | 0.156 | 0.284 | 0.236 | 0.229 |
| InternalIMT | aa | rs76552293 | 0.044 | 0.034 | 0.084 | 0.681 | -0.733 | 0.516 | 0.156 | 0.284 | 0.236 | 0.229 |
| InternalIMT | aa | rs147445993 | 0.001 | . | . | . | . | . | . | . | . | . |
| InternalIMT | aa | rs115902283 | 0.044 | 0.033 | 0.084 | 0.695 | -0.758 | 0.518 | 0.143 | 0.295 | 0.236 | 0.213 |
| InternalIMT | aa | rs4540799 | 0.759 | -0.032 | 0.199 | 0.872 | -0.135 | 0.294 | 0.646 | 0.062 | 0.126 | 0.621 |
| InternalIMT | aa | rs12765320 | 0.102 | 0.015 | 0.089 | 0.867 | -0.622 | 0.403 | 0.123 | 0.26 | 0.179 | 0.147 |
| InternalIMT | aa | rs12782256 | . | . | . | . | . | . | . | . | . | . |
| InternalIMT | aa | rs191727256 | . | . | . | . | . | . | . | . | . | . |
| InternalIMT | aa | rs2082111 | 0.028 | 0.047 | 0.082 | 0.57 | -0.356 | 1.01 | 0.726 | 0.181 | 0.439 | 0.68 |
| InternalIMT | aa | rs2082110 | 0.072 | 0.02 | 0.085 | 0.818 | -0.809 | 0.475 | 0.089 | 0.339 | 0.212 | 0.111 |
| InternalIMT | aa | rs2082109 | 0.072 | 0.02 | 0.085 | 0.818 | -0.809 | 0.475 | 0.089 | 0.339 | 0.212 | 0.111 |
| InternalIMT | aa | rs117316662 | 0.044 | 0.034 | 0.084 | 0.681 | -0.732 | 0.516 | 0.157 | 0.283 | 0.236 | 0.23 |
| InternalIMT | aa | rs12247163 | 0.102 | 0.017 | 0.089 | 0.851 | -0.596 | 0.387 | 0.124 | 0.248 | 0.172 | 0.148 |
| InternalIMT | aa | rs12355888 | 0.002 | . | . | . | . | . | . | . | . | . |
| InternalIMT | aa | rs143772290 | . | . | . | . | . | . | . | . | . | . |
| InternalIMT | aa | rs146849452 | . | . | . | . | . | . | . | . | . | . |
| InternalIMT | aa | rs140267121 | . | . | . | . | . | . | . | . | . | . |
| InternalIMT | aa | rs7080474 | 0.058 | 0.047 | 0.085 | 0.579 | -0.208 | 0.549 | 0.706 | 0.103 | 0.239 | 0.666 |
| InternalIMT | aa | rs144066203 | . | . | . | . | . | . | . | . | . | . |
| InternalIMT | aa | rs12765209 | 0.044 | 0.034 | 0.084 | 0.681 | -0.731 | 0.516 | 0.157 | 0.283 | 0.236 | 0.231 |
| InternalIMT | aa | rs150930735 | . | . | . | . | . | . | . | . | . | . |
| InternalIMT | aa | rs7068039 | 0.102 | 0.017 | 0.089 | 0.851 | -0.596 | 0.387 | 0.124 | 0.248 | 0.172 | 0.148 |
| InternalIMT | aa | rs117622652 | 0.043 | 0.034 | 0.084 | 0.681 | -0.731 | 0.516 | 0.158 | 0.283 | 0.236 | 0.231 |
| InternalIMT | aa | rs190191514 | . | . | . | . | . | . | . | . | . | . |
| InternalIMT | aa | rs74130835 | . | . | . | . | . | . | . | . | . | . |
| InternalIMT | aa | rs1811604 | . | . | . | . | . | . | . | . | . | . |
| InternalIMT | aa | rs146971256 | 0.001 | . | . | . | . | . | . | . | . | . |
| InternalIMT | aa | rs74336000 | . | . | . | . | . | . | . | . | . | . |
| InternalIMT | aa | rs186046492 | . | . | . | . | . | . | . | . | . | . |
| InternalIMT | aa | rs34971823 | . | . | . | . | . | . | . | . | . | . |
| InternalIMT | aa | rs75227446 | 0.043 | 0.035 | 0.084 | 0.681 | -0.728 | 0.517 | 0.159 | 0.282 | 0.236 | 0.233 |
| InternalIMT | aa | rs190457599 | . | . | . | . | . | . | . | . | . | . |
| InternalIMT | aa | rs79591917 | 0.043 | 0.035 | 0.084 | 0.681 | -0.729 | 0.517 | 0.159 | 0.282 | 0.236 | 0.233 |
| InternalIMT | aa | rs702365 | 0.647 | 0.094 | 0.163 | 0.566 | 0.076 | 0.275 | 0.782 | -0.026 | 0.119 | 0.826 |
| InternalIMT | aa | rs79643836 | 0.043 | 0.035 | 0.084 | 0.681 | -0.729 | 0.517 | 0.159 | 0.282 | 0.236 | 0.233 |
| InternalIMT | aa | rs702366 | 0.649 | 0.099 | 0.164 | 0.545 | 0.086 | 0.275 | 0.756 | -0.03 | 0.119 | 0.8 |
| InternalIMT | aa | rs1059696 | 0.059 | 0.048 | 0.085 | 0.575 | -0.197 | 0.55 | 0.72 | 0.098 | 0.24 | 0.682 |
| InternalIMT | aa | rs901681 | 0.648 | 0.098 | 0.163 | 0.547 | 0.085 | 0.275 | 0.759 | -0.03 | 0.119 | 0.802 |
| InternalIMT | aa | rs61854092 | 0.058 | 0.047 | 0.085 | 0.581 | -0.215 | 0.551 | 0.696 | 0.106 | 0.24 | 0.658 |
| InternalIMT | aa | rs148080753 | . | . | . | . | . | . | . | . | . | . |
| InternalIMT | aa | rs61854093 | 0.064 | 0.037 | 0.085 | 0.666 | -0.391 | 0.553 | 0.48 | 0.182 | 0.241 | 0.449 |
| InternalIMT | aa | rs141822774 | . | . | . | . | . | . | . | . | . | . |

| InternalIMT | aa | rs74130836 | . | . | . | . | . | . | . | . | . | . |
| --- | --- | --- | --- | --- | --- | --- | --- | --- | --- | --- | --- | --- |
| InternalIMT | aa | rs150620963 | . | . | . | . | . | . | . | . | . | . |
| InternalIMT | aa | rs3780913 | 0.749 | -0.056 | 0.189 | 0.766 | -0.178 | 0.281 | 0.528 | 0.08 | 0.121 | 0.508 |
| InternalIMT | aa | rs142255723 | . | . | . | . | . | . | . | . | . | . |
| InternalIMT | aa | rs3780914 | 0.647 | 0.097 | 0.161 | 0.547 | 0.082 | 0.271 | 0.763 | -0.029 | 0.117 | 0.806 |
| InternalIMT | aa | rs182656507 | . | . | . | . | . | . | . | . | . | . |
| InternalIMT | aa | rs2291427 | 0.703 | 0.039 | 0.174 | 0.825 | -7.60E-03 | 0.269 | 0.977 | 0.014 | 0.117 | 0.906 |
| InternalIMT | aa | rs10751382 | 0.703 | 0.038 | 0.173 | 0.825 | -8.10E-03 | 0.268 | 0.976 | 0.014 | 0.117 | 0.904 |
| InternalIMT | aa | rs117610108 | 0.043 | 0.035 | 0.084 | 0.68 | -0.722 | 0.518 | 0.163 | 0.278 | 0.237 | 0.24 |
| InternalIMT | aa | rs144966941 | 0.002 | . | . | . | . | . | . | . | . | . |
| InternalIMT | aa | rs41502051 | 0.007 | . | . | . | . | . | . | . | . | . |
| InternalIMT | aa | rs41426046 | 0.047 | 0.019 | 0.084 | 0.823 | -1.01 | 0.531 | 0.058 | 0.402 | 0.241 | 0.096 |
| InternalIMT | aa | rs41519248 | 0.043 | 0.035 | 0.084 | 0.68 | -0.72 | 0.518 | 0.165 | 0.277 | 0.237 | 0.243 |
| InternalIMT | aa | rs189108245 | . | . | . | . | . | . | . | . | . | . |
| InternalIMT | aa | rs7896431 | 0.668 | 0.1 | 0.161 | 0.533 | 0.086 | 0.257 | 0.738 | -0.031 | 0.111 | 0.782 |
| InternalIMT | aa | rs183724547 | . | . | . | . | . | . | . | . | . | . |
| InternalIMT | aa | rs142044990 | . | . | . | . | . | . | . | . | . | . |
| InternalIMT | aa | rs188140929 | 0.021 | 0.084 | 0.085 | 0.322 | 1.07 | 0.799 | 0.182 | -0.405 | 0.342 | 0.237 |
| InternalIMT | aa | rs76819096 | . | . | . | . | . | . | . | . | . | . |
| InternalIMT | aa | rs10751383 | 0.681 | 0.149 | 0.179 | 0.406 | 0.179 | 0.285 | 0.529 | -0.066 | 0.122 | 0.588 |
| InternalIMT | aa | rs117819321 | . | . | . | . | . | . | . | . | . | . |
| InternalIMT | aa | rs2242332 | 0.744 | -0.041 | 0.178 | 0.818 | -0.158 | 0.263 | 0.548 | 0.07 | 0.113 | 0.535 |
| InternalIMT | aa | rs2242333 | 0.476 | 0.2 | 0.12 | 0.095 | 0.338 | 0.233 | 0.147 | -0.148 | 0.102 | 0.148 |
| InternalIMT | aa | rs2242334 | 0.744 | -0.042 | 0.178 | 0.814 | -0.16 | 0.263 | 0.544 | 0.071 | 0.113 | 0.531 |
| InternalIMT | aa | rs189465041 | . | . | . | . | . | . | . | . | . | . |
| InternalIMT | aa | rs28395868 | . | . | . | . | . | . | . | . | . | . |
| InternalIMT | aa | rs56067550 | . | . | . | . | . | . | . | . | . | . |
| InternalIMT | aa | rs1051713 | 0.1 | 0.015 | 0.089 | 0.865 | -0.621 | 0.389 | 0.111 | 0.257 | 0.172 | 0.135 |
| InternalIMT | aa | rs115074210 | . | . | . | . | . | . | . | . | . | . |
| InternalIMT | aa | rs7900977 | 0.782 | -0.014 | 0.224 | 0.95 | -0.088 | 0.32 | 0.782 | 0.047 | 0.137 | 0.73 |
| InternalIMT | aa | rs147613891 | . | . | . | . | . | . | . | . | . | . |
| InternalIMT | aa | rs2229136 | 0.038 | 0.076 | 0.086 | 0.373 | 0.487 | 0.575 | 0.398 | -0.178 | 0.246 | 0.471 |
| InternalIMT | aa | rs185482841 | . | . | . | . | . | . | . | . | . | . |
| InternalIMT | aa | rs2288619 | 0.038 | 0.076 | 0.086 | 0.373 | 0.487 | 0.575 | 0.398 | -0.178 | 0.246 | 0.471 |
| InternalIMT | aa | rs189891524 | 0.003 | . | . | . | . | . | . | . | . | . |
| InternalIMT | aa | rs56145077 | 0.058 | 0.047 | 0.085 | 0.58 | -0.21 | 0.549 | 0.702 | 0.104 | 0.239 | 0.663 |
| InternalIMT | aa | rs143216970 | . | . | . | . | . | . | . | . | . | . |
| InternalIMT | aa | rs142246478 | . | . | . | . | . | . | . | . | . | . |
| InternalIMT | aa | rs13134 | . | . | . | . | . | . | . | . | . | . |
| InternalIMT | aa | rs41314980 | . | . | . | . | . | . | . | . | . | . |
| InternalIMT | aa | rs41304611 | . | . | . | . | . | . | . | . | . | . |
| InternalIMT | aa | rs193299617 | 0.004 | . | . | . | . | . | . | . | . | . |
| InternalIMT | aa | rs12772724 | . | . | . | . | . | . | . | . | . | . |
| InternalIMT | aa | rs139489537 | 0.038 | 0.077 | 0.086 | 0.372 | 0.492 | 0.577 | 0.394 | -0.18 | 0.247 | 0.467 |
| InternalIMT | aa | rs12776062 | . | . | . | . | . | . | . | . | . | . |
| InternalIMT | aa | rs752059 | 0.059 | 0.047 | 0.086 | 0.587 | -0.23 | 0.549 | 0.675 | 0.112 | 0.239 | 0.638 |
| InternalIMT | aa | rs147649982 | 0.015 | 0.052 | 0.083 | 0.528 | -0.592 | 1.36 | 0.663 | 0.275 | 0.583 | 0.638 |
| InternalIMT | aa | rs17157798 | 0.038 | 0.077 | 0.086 | 0.372 | 0.493 | 0.577 | 0.393 | -0.18 | 0.247 | 0.466 |
| InternalIMT | aa | rs142298619 | . | . | . | . | . | . | . | . | . | . |
| InternalIMT | aa | rs4948673 | 0.777 | -0.038 | 0.224 | 0.865 | -0.127 | 0.32 | 0.691 | 0.063 | 0.137 | 0.646 |
| InternalIMT | aa | rs74231484 | 0.476 | 0.199 | 0.12 | 0.096 | 0.336 | 0.232 | 0.148 | -0.147 | 0.102 | 0.149 |
| InternalIMT | aa | rs72796489 | . | . | . | . | . | . | . | . | . | . |
| InternalIMT | aa | rs113447578 | 0.026 | 0.07 | 0.083 | 0.404 | 0.785 | 0.813 | 0.335 | -0.315 | 0.336 | 0.348 |
| InternalIMT | aa | rs34063374 | . | . | . | . | . | . | . | . | . | . |
| InternalIMT | aa | rs11239527 | . | . | . | . | . | . | . | . | . | . |
| InternalIMT | aa | rs34371883 | . | . | . | . | . | . | . | . | . | . |
| InternalIMT | aa | rs118168828 | 0.002 | . | . | . | . | . | . | . | . | . |
| InternalIMT | aa | rs34349618 | . | . | . | . | . | . | . | . | . | . |
| InternalIMT | aa | rs80041679 | 0.038 | 0.077 | 0.086 | 0.372 | 0.492 | 0.577 | 0.394 | -0.18 | 0.247 | 0.467 |
| InternalIMT | aa | rs149640607 | . | . | . | . | . | . | . | . | . | . |
| InternalIMT | aa | rs4427523 | 0.957 | 2.06 | 1.1 | 0.062 | 2.34 | 1.3 | 0.072 | -1.04 | 0.565 | 0.065 |
| InternalIMT | aa | rs185880109 | . | . | . | . | . | . | . | . | . | . |
| InternalIMT | aa | rs191523220 | . | . | . | . | . | . | . | . | . | . |
| InternalIMT | aa | rs144315284 | . | . | . | . | . | . | . | . | . | . |
| InternalIMT | aa | rs4948674 | 0.156 | 9.30E-03 | 0.089 | 0.917 | -0.364 | 0.302 | 0.228 | 0.167 | 0.135 | 0.216 |
| InternalIMT | aa | rs182984298 | . | . | . | . | . | . | . | . | . | . |
| InternalIMT | aa | rs56749023 | . | . | . | . | . | . | . | . | . | . |
| InternalIMT | aa | rs112508957 | . | . | . | . | . | . | . | . | . | . |
| InternalIMT | aa | rs7089063 | 0.138 | 0.029 | 0.096 | 0.763 | -0.254 | 0.35 | 0.468 | 0.111 | 0.153 | 0.468 |
| InternalIMT | aa | rs145000779 | . | . | . | . | . | . | . | . | . | . |
| InternalIMT | aa | rs80201572 | 0.038 | 0.076 | 0.086 | 0.373 | 0.487 | 0.575 | 0.398 | -0.178 | 0.246 | 0.471 |
| InternalIMT | aa | rs140568227 | . | . | . | . | . | . | . | . | . | . |
| InternalIMT | aa | rs149756228 | . | . | . | . | . | . | . | . | . | . |
| InternalIMT | aa | rs112380685 | . | . | . | . | . | . | . | . | . | . |
| InternalIMT | aa | rs60481109 | . | . | . | . | . | . | . | . | . | . |
| InternalIMT | aa | rs138221843 | . | . | . | . | . | . | . | . | . | . |
| InternalIMT | aa | rs150283194 | . | . | . | . | . | . | . | . | . | . |
| InternalIMT | aa | rs138879661 | . | . | . | . | . | . | . | . | . | . |
| InternalIMT | aa | rs34960345 | 0.038 | 0.076 | 0.086 | 0.374 | 0.48 | 0.573 | 0.402 | -0.175 | 0.245 | 0.476 |
| InternalIMT | aa | rs147514068 | . | . | . | . | . | . | . | . | . | . |
| InternalIMT | aa | rs35410535 | 0.038 | 0.076 | 0.086 | 0.374 | 0.48 | 0.573 | 0.402 | -0.175 | 0.245 | 0.476 |
| InternalIMT | aa | rs191613635 | . | . | . | . | . | . | . | . | . | . |
| InternalIMT | aa | rs17157800 | . | . | . | . | . | . | . | . | . | . |
| InternalIMT | aa | rs17523413 | 0.038 | 0.076 | 0.086 | 0.373 | 0.487 | 0.575 | 0.398 | -0.178 | 0.246 | 0.471 |
| InternalIMT | aa | rs7897036 | 0.001 | . | . | . | . | . | . | . | . | . |
| InternalIMT | aa | rs76334323 | 0.042 | 0.035 | 0.084 | 0.678 | -0.705 | 0.518 | 0.174 | 0.269 | 0.237 | 0.256 |

| InternalIMT | aa | rs143535991 | . | . | . | . | . | . | . | . | . | . |
| --- | --- | --- | --- | --- | --- | --- | --- | --- | --- | --- | --- | --- |
| InternalIMT | aa | rs140344979 | . | . | . | . | . | . | . | . | . | . |
| InternalIMT | aa | rs36047811 | 0.038 | 0.076 | 0.086 | 0.374 | 0.48 | 0.573 | 0.402 | -0.175 | 0.245 | 0.476 |
| InternalIMT | aa | rs76493570 | 0.038 | 0.076 | 0.086 | 0.374 | 0.48 | 0.573 | 0.402 | -0.175 | 0.245 | 0.476 |
| InternalIMT | aa | rs192998920 | . | . | . | . | . | . | . | . | . | . |
| InternalIMT | aa | rs116840852 | . | . | . | . | . | . | . | . | . | . |
| InternalIMT | aa | rs184824101 | . | . | . | . | . | . | . | . | . | . |
| InternalIMT | aa | rs41301621 | . | . | . | . | . | . | . | . | . | . |
| InternalIMT | aa | rs3740109 | 0.038 | 0.076 | 0.086 | 0.374 | 0.48 | 0.573 | 0.402 | -0.175 | 0.245 | 0.476 |
| InternalIMT | aa | rs7899415 | 0.089 | 0.028 | 0.088 | 0.751 | -0.432 | 0.447 | 0.334 | 0.19 | 0.197 | 0.334 |
| InternalIMT | aa | rs184395284 | 0.004 | . | . | . | . | . | . | . | . | . |
| InternalIMT | aa | rs139209589 | . | . | . | . | . | . | . | . | . | . |
| InternalIMT | aa | rs149924163 | . | . | . | . | . | . | . | . | . | . |
| InternalIMT | aa | rs3740110 | 0.042 | 0.035 | 0.084 | 0.678 | -0.705 | 0.518 | 0.174 | 0.269 | 0.237 | 0.256 |
| InternalIMT | aa | rs144335113 | . | . | . | . | . | . | . | . | . | . |
| InternalIMT | aa | rs74743385 | . | . | . | . | . | . | . | . | . | . |
| InternalIMT | aa | rs146545829 | . | . | . | . | . | . | . | . | . | . |
| InternalIMT | aa | rs181842689 | . | . | . | . | . | . | . | . | . | . |
| InternalIMT | aa | rs3740111 | 0.038 | 0.076 | 0.086 | 0.374 | 0.48 | 0.573 | 0.402 | -0.175 | 0.245 | 0.476 |
| InternalIMT | aa | rs186261599 | . | . | . | . | . | . | . | . | . | . |
| InternalIMT | aa | rs73285348 | 0.013 | 0.073 | 0.082 | 0.372 | 1.46 | 1.03 | 0.157 | -0.618 | 0.417 | 0.139 |
| InternalIMT | aa | rs186356901 | . | . | . | . | . | . | . | . | . | . |
| InternalIMT | aa | rs3824618 | 0.038 | 0.076 | 0.086 | 0.373 | 0.487 | 0.575 | 0.398 | -0.178 | 0.246 | 0.471 |
| InternalIMT | aa | rs3802548 | 0.065 | 0.034 | 0.086 | 0.692 | -0.493 | 0.541 | 0.363 | 0.221 | 0.238 | 0.352 |
| InternalIMT | aa | rs3802547 | 0.04 | 0.036 | 0.084 | 0.668 | -0.701 | 0.553 | 0.205 | 0.266 | 0.253 | 0.292 |
| InternalIMT | aa | rs77677521 | . | . | . | . | . | . | . | . | . | . |
| InternalIMT | aa | rs3824616 | 0.038 | 0.076 | 0.086 | 0.374 | 0.48 | 0.573 | 0.402 | -0.175 | 0.245 | 0.476 |
| InternalIMT | aa | rs191073818 | . | . | . | . | . | . | . | . | . | . |
| InternalIMT | aa | rs147078004 | . | . | . | . | . | . | . | . | . | . |
| InternalIMT | aa | rs7908745 | 0.145 | 0.015 | 0.097 | 0.875 | -0.362 | 0.338 | 0.284 | 0.156 | 0.149 | 0.295 |
| InternalIMT | aa | rs17157811 | . | . | . | . | . | . | . | . | . | . |
| InternalIMT | aa | rs138506087 | . | . | . | . | . | . | . | . | . | . |
| InternalIMT | aa | rs77241269 | . | . | . | . | . | . | . | . | . | . |
| InternalIMT | aa | rs138374938 | . | . | . | . | . | . | . | . | . | . |
| InternalIMT | aa | rs2279433 | 0.038 | 0.076 | 0.086 | 0.375 | 0.475 | 0.573 | 0.408 | -0.173 | 0.246 | 0.482 |
| InternalIMT | aa | rs73285358 | . | . | . | . | . | . | . | . | . | . |
| InternalIMT | aa | rs11239528 | 0.065 | 0.035 | 0.086 | 0.689 | -0.487 | 0.539 | 0.367 | 0.218 | 0.237 | 0.357 |
| InternalIMT | aa | rs77453386 | 0.042 | 0.035 | 0.084 | 0.678 | -0.705 | 0.518 | 0.174 | 0.269 | 0.237 | 0.256 |
| InternalIMT | aa | rs2279434 | 0.04 | 0.077 | 0.086 | 0.37 | 0.47 | 0.568 | 0.408 | -0.171 | 0.244 | 0.484 |
| InternalIMT | aa | rs149236525 | . | . | . | . | . | . | . | . | . | . |
| InternalIMT | aa | rs12573609 | 0.13 | 0.084 | 0.096 | 0.38 | 0.199 | 0.472 | 0.673 | -0.091 | 0.2 | 0.649 |
| InternalIMT | aa | rs191716655 | . | . | . | . | . | . | . | . | . | . |
| InternalIMT | aa | rs17523660 | . | . | . | . | . | . | . | . | . | . |
| InternalIMT | aa | rs142671360 | . | . | . | . | . | . | . | . | . | . |
| InternalIMT | aa | rs3764990 | 0.037 | 0.076 | 0.086 | 0.374 | 0.481 | 0.576 | 0.403 | -0.175 | 0.247 | 0.478 |
| InternalIMT | aa | rs113682861 | . | . | . | . | . | . | . | . | . | . |
| InternalIMT | aa | rs117658270 | 0.042 | 0.035 | 0.084 | 0.678 | -0.705 | 0.518 | 0.174 | 0.269 | 0.237 | 0.256 |
| InternalIMT | aa | rs187309063 | . | . | . | . | . | . | . | . | . | . |
| InternalIMT | aa | rs73285360 | . | . | . | . | . | . | . | . | . | . |
| InternalIMT | aa | rs80133289 | 0.1 | 0.175 | 0.096 | 0.069 | 1.33 | 0.593 | 0.025 | -0.577 | 0.259 | 0.026 |
| InternalIMT | aa | rs186693257 | 0.003 | . | . | . | . | . | . | . | . | . |
| InternalIMT | aa | rs35735968 | 0.05 | 0.065 | 0.086 | 0.45 | 0.239 | 0.557 | 0.668 | -0.07 | 0.24 | 0.769 |
| InternalIMT | aa | rs17157825 | 0.038 | 0.074 | 0.085 | 0.384 | 0.445 | 0.573 | 0.438 | -0.158 | 0.246 | 0.52 |
| InternalIMT | aa | rs2291428 | 0.065 | 0.034 | 0.086 | 0.69 | -0.491 | 0.54 | 0.364 | 0.22 | 0.237 | 0.355 |
| InternalIMT | aa | rs2291429 | 0.065 | 0.034 | 0.086 | 0.689 | -0.486 | 0.539 | 0.368 | 0.218 | 0.237 | 0.358 |
| InternalIMT | aa | rs145714792 | . | . | . | . | . | . | . | . | . | . |
| InternalIMT | aa | rs12769244 | 0.038 | 0.076 | 0.086 | 0.376 | 0.474 | 0.573 | 0.409 | -0.172 | 0.246 | 0.483 |
| InternalIMT | aa | rs12771275 | 0.038 | 0.076 | 0.086 | 0.376 | 0.474 | 0.573 | 0.409 | -0.172 | 0.246 | 0.483 |
| InternalIMT | aa | rs146266174 | . | . | . | . | . | . | . | . | . | . |
| InternalIMT | aa | rs41301623 | . | . | . | . | . | . | . | . | . | . |
| InternalIMT | aa | rs2306814 | 0.043 | 0.035 | 0.084 | 0.677 | -0.7 | 0.514 | 0.174 | 0.265 | 0.235 | 0.259 |
| InternalIMT | aa | rs12244834 | 0.03 | 0.06 | 0.084 | 0.474 | -0.011 | 0.606 | 0.986 | 2.20E-03 | 0.264 | 0.993 |
| InternalIMT | aa | rs184006011 | 0 | . | . | . | . | . | . | . | . | . |
| InternalIMT | aa | rs78829066 | 0.042 | 0.036 | 0.084 | 0.671 | -0.703 | 0.522 | 0.178 | 0.268 | 0.238 | 0.262 |
| InternalIMT | aa | rs34731408 | 0.038 | 0.076 | 0.086 | 0.376 | 0.472 | 0.573 | 0.41 | -0.172 | 0.246 | 0.485 |
| InternalIMT | aa | rs17451516 | . | . | . | . | . | . | . | . | . | . |
| InternalIMT | aa | rs139910133 | . | . | . | . | . | . | . | . | . | . |
| InternalIMT | aa | rs10900218 | 0.138 | 0.029 | 0.097 | 0.767 | -0.259 | 0.359 | 0.471 | 0.114 | 0.158 | 0.47 |
| InternalIMT | aa | rs146023999 | 0.025 | 0.047 | 0.084 | 0.577 | -0.353 | 0.916 | 0.7 | 0.193 | 0.394 | 0.624 |
| InternalIMT | aa | rs187296958 | 0.007 | . | . | . | . | . | . | . | . | . |
| InternalIMT | aa | rs142342833 | 0.015 | 0.082 | 0.083 | 0.32 | 1.46 | 1.51 | 0.333 | -0.663 | 0.611 | 0.279 |
| InternalIMT | aa | rs143489255 | 0.006 | . | . | . | . | . | . | . | . | . |
| InternalIMT | aa | rs138532518 | 0.006 | . | . | . | . | . | . | . | . | . |
| InternalIMT | aa | rs183768079 | 0.007 | . | . | . | . | . | . | . | . | . |
| InternalIMT | aa | rs117892272 | 0.012 | 0.075 | 0.081 | 0.354 | 1.74 | 2.04 | 0.396 | -0.717 | 0.868 | 0.409 |
| InternalIMT | aa | rs192993943 | 0.003 | . | . | . | . | . | . | . | . | . |
| InternalIMT | aa | rs182989043 | 0.015 | 0.078 | 0.085 | 0.362 | 0.805 | 0.808 | 0.32 | -0.325 | 0.373 | 0.384 |
| InternalIMT | aa | rs189703435 | 0.021 | 0.058 | 0.084 | 0.486 | -0.188 | 1 | 0.851 | 0.073 | 0.438 | 0.867 |
| InternalIMT | aa | rs188794040 | 0.02 | 0.06 | 0.084 | 0.473 | -0.095 | 0.99 | 0.923 | 0.024 | 0.433 | 0.956 |
| InternalIMT | aa | rs143920953 | 0.003 | . | . | . | . | . | . | . | . | . |
| InternalIMT | aa | rs191029121 | 0.006 | . | . | . | . | . | . | . | . | . |
| InternalIMT | aa | rs116875448 | 0.006 | . | . | . | . | . | . | . | . | . |
| InternalIMT | aa | rs190167877 | 0.002 | . | . | . | . | . | . | . | . | . |
| InternalIMT | aa | rs184993418 | 0.003 | . | . | . | . | . | . | . | . | . |
| InternalIMT | aa | rs143241158 | 0.01 | . | . | . | . | . | . | . | . | . |
| InternalIMT | aa | rs11239500 | 0.007 | . | . | . | . | . | . | . | . | . |

| InternalIMT | aa | rs140568994 | 0.001 | . | . | . | . | . | . | . | . | . |
| --- | --- | --- | --- | --- | --- | --- | --- | --- | --- | --- | --- | --- |
| InternalIMT | aa | rs187530317 | 0.008 | . | . | . | . | . | . | . | . | . |
| InternalIMT | aa | rs145189326 | 0.003 | . | . | . | . | . | . | . | . | . |
| InternalIMT | aa | rs186203332 | 0.009 | . | . | . | . | . | . | . | . | . |
| InternalIMT | aa | rs189921909 | 0.004 | . | . | . | . | . | . | . | . | . |
| InternalIMT | aa | rs187260989 | 0.01 | 0.065 | 0.081 | 0.426 | 0.578 | 1.29 | 0.655 | -0.197 | 0.507 | 0.698 |
| InternalIMT | aa | rs114376586 | 0.006 | . | . | . | . | . | . | . | . | . |
| InternalIMT | aa | rs151070808 | 0.019 | 0.044 | 0.083 | 0.597 | -1.29 | 1 | 0.197 | 0.548 | 0.433 | 0.206 |
| InternalIMT | aa | rs140927225 | 0.006 | . | . | . | . | . | . | . | . | . |
| InternalIMT | aa | rs191555615 | 0.012 | 0.082 | 0.082 | 0.315 | 2.51 | 2.18 | 0.25 | -1.05 | 0.959 | 0.274 |
| InternalIMT | aa | rs80278898 | 0.047 | -3.30E-03 | 0.085 | 0.969 | -1.43 | 1.27 | 0.262 | 0.649 | 0.554 | 0.242 |
| InternalIMT | aa | rs190849503 | 0.004 | . | . | . | . | . | . | . | . | . |
| InternalIMT | aa | rs150080315 | 0.009 | . | . | . | . | . | . | . | . | . |
| InternalIMT | aa | rs143623885 | 0.011 | 0.079 | 0.083 | 0.341 | 1.85 | 2.34 | 0.43 | -0.814 | 0.951 | 0.392 |
| InternalIMT | aa | rs187760040 | 0.002 | . | . | . | . | . | . | . | . | . |
| InternalIMT | aa | rs143917143 | 0.02 | 0.044 | 0.083 | 0.596 | -1.28 | 1 | 0.2 | 0.543 | 0.432 | 0.209 |
| InternalIMT | aa | rs149851136 | 0.002 | . | . | . | . | . | . | . | . | . |
| InternalIMT | aa | rs181612054 | 0.001 | . | . | . | . | . | . | . | . | . |
| InternalIMT | aa | rs191006548 | 0.001 | . | . | . | . | . | . | . | . | . |
| InternalIMT | aa | rs117377631 | 0.006 | . | . | . | . | . | . | . | . | . |
| InternalIMT | aa | rs182107634 | 0.002 | . | . | . | . | . | . | . | . | . |
| InternalIMT | aa | rs190211867 | 0.002 | . | . | . | . | . | . | . | . | . |
| InternalIMT | aa | rs148293457 | 0.031 | 0.058 | 0.086 | 0.498 | -0.071 | 0.695 | 0.919 | 0.038 | 0.31 | 0.901 |
| InternalIMT | aa | rs191811733 | 0.006 | . | . | . | . | . | . | . | . | . |
| InternalIMT | aa | rs146109272 | 0.008 | . | . | . | . | . | . | . | . | . |
| InternalIMT | aa | rs148397915 | 0.006 | . | . | . | . | . | . | . | . | . |
| InternalIMT | aa | rs142233290 | 0.007 | . | . | . | . | . | . | . | . | . |
| InternalIMT | aa | rs80226874 | 0.022 | 0.033 | 0.081 | 0.686 | -1.07 | 1.41 | 0.449 | 0.485 | 0.593 | 0.414 |
| InternalIMT | aa | rs144496203 | 0.012 | 0.077 | 0.082 | 0.345 | 1.65 | 1.03 | 0.109 | -0.739 | 0.429 | 0.085 |
| InternalIMT | aa | rs191062130 | 0.001 | . | . | . | . | . | . | . | . | . |
| InternalIMT | aa | rs185807817 | 0.004 | . | . | . | . | . | . | . | . | . |
| InternalIMT | aa | rs117846213 | 0.032 | 0.056 | 0.085 | 0.51 | -0.245 | 0.727 | 0.736 | 0.09 | 0.314 | 0.775 |
| InternalIMT | aa | rs190545473 | 0.002 | . | . | . | . | . | . | . | . | . |
| InternalIMT | aa | rs192364918 | 0.01 | 0.019 | 0.079 | 0.808 | -3.73 | 1.7 | 0.028 | 1.52 | 0.796 | 0.057 |
| InternalIMT | aa | rs182939241 | 0.003 | . | . | . | . | . | . | . | . | . |
| InternalIMT | aa | rs12571125 | 0.031 | 0.088 | 0.087 | 0.314 | 1.05 | 1.02 | 0.305 | -0.472 | 0.436 | 0.28 |
| InternalIMT | aa | rs192418941 | 0.005 | . | . | . | . | . | . | . | . | . |
| InternalIMT | aa | rs141270243 | 0.015 | 0.055 | 0.083 | 0.51 | -0.509 | 0.977 | 0.602 | 0.132 | 0.427 | 0.758 |
| InternalIMT | aa | rs182615502 | 0.006 | . | . | . | . | . | . | . | . | . |
| InternalIMT | aa | rs146669856 | 0.001 | . | . | . | . | . | . | . | . | . |
| InternalIMT | aa | rs78290029 | 0.034 | 0.058 | 0.085 | 0.494 | -0.142 | 0.686 | 0.836 | 0.05 | 0.297 | 0.865 |
| InternalIMT | aa | rs139955565 | 0.004 | . | . | . | . | . | . | . | . | . |
| InternalIMT | aa | rs12267314 | 0.03 | 0.063 | 0.084 | 0.458 | 0.073 | 0.674 | 0.914 | -0.036 | 0.292 | 0.902 |
| InternalIMT | aa | rs74477197 | 0.004 | . | . | . | . | . | . | . | . | . |
| InternalIMT | aa | rs188147236 | 0.005 | . | . | . | . | . | . | . | . | . |
| InternalIMT | aa | rs2228065 | 0.03 | 0.063 | 0.084 | 0.457 | 0.084 | 0.662 | 0.899 | -0.04 | 0.287 | 0.889 |
| InternalIMT | aa | rs116961353 | 0.015 | 0.046 | 0.081 | 0.57 | -0.422 | 1.98 | 0.831 | 0.252 | 0.83 | 0.762 |
| InternalIMT | aa | rs190222272 | 0.007 | . | . | . | . | . | . | . | . | . |
| InternalIMT | aa | rs182719940 | 0.006 | . | . | . | . | . | . | . | . | . |
| InternalIMT | aa | rs184477413 | 0.004 | . | . | . | . | . | . | . | . | . |
| InternalIMT | aa | rs12265175 | 0.03 | 0.061 | 0.084 | 0.47 | 9.70E-03 | 0.623 | 0.988 | -6.80E-03 | 0.271 | 0.98 |
| InternalIMT | aa | rs182928276 | 0.003 | . | . | . | . | . | . | . | . | . |
| InternalIMT | aa | rs144409895 | 0.011 | 0.019 | 0.079 | 0.807 | -3.5 | 1.62 | 0.031 | 1.41 | 0.759 | 0.063 |
| InternalIMT | aa | rs58998447 | 0.136 | 3.60E-03 | 0.091 | 0.969 | -0.479 | 0.447 | 0.284 | 0.203 | 0.199 | 0.308 |
| InternalIMT | aa | rs188668832 | 0.005 | . | . | . | . | . | . | . | . | . |
| InternalIMT | aa | rs148641686 | 0.002 | . | . | . | . | . | . | . | . | . |
| InternalIMT | aa | rs75468791 | 0.054 | 0.039 | 0.085 | 0.642 | -0.347 | 0.752 | 0.645 | 0.174 | 0.335 | 0.603 |
| InternalIMT | aa | rs147327397 | 0.003 | . | . | . | . | . | . | . | . | . |
| InternalIMT | aa | rs77354597 | 0.029 | 0.06 | 0.084 | 0.476 | -0.026 | 0.632 | 0.968 | 8.10E-03 | 0.276 | 0.977 |
| InternalIMT | aa | rs41365544 | 0.03 | 0.06 | 0.084 | 0.475 | -0.017 | 0.605 | 0.978 | 4.80E-03 | 0.264 | 0.986 |
| InternalIMT | aa | rs187564188 | 0.016 | -1.60E-03 | 0.082 | 0.985 | -3.54 | 2.02 | 0.081 | 1.57 | 0.97 | 0.106 |
| InternalIMT | aa | rs183511984 | 0.007 | . | . | . | . | . | . | . | . | . |
| InternalIMT | aa | rs12262783 | 0.003 | . | . | . | . | . | . | . | . | . |
| InternalIMT | aa | rs188817687 | 0.003 | . | . | . | . | . | . | . | . | . |
| InternalIMT | aa | rs3815968 | 0.005 | . | . | . | . | . | . | . | . | . |
| InternalIMT | aa | rs187822300 | 0.001 | . | . | . | . | . | . | . | . | . |
| InternalIMT | aa | rs188902172 | 0.032 | 0.064 | 0.085 | 0.452 | 0.114 | 0.62 | 0.855 | -0.056 | 0.27 | 0.835 |
| InternalIMT | aa | rs181190448 | 0.006 | . | . | . | . | . | . | . | . | . |
| InternalIMT | aa | rs182699093 | 0.001 | . | . | . | . | . | . | . | . | . |
| InternalIMT | aa | rs182631303 | 0.014 | 0.062 | 0.082 | 0.452 | 0.093 | 1.18 | 0.937 | -0.015 | 0.529 | 0.978 |
| InternalIMT | aa | rs187014172 | 0.006 | . | . | . | . | . | . | . | . | . |
| InternalIMT | aa | rs142575406 | 0.009 | . | . | . | . | . | . | . | . | . |
| InternalIMT | aa | rs180711033 | 0.007 | . | . | . | . | . | . | . | . | . |
| InternalIMT | aa | rs137894823 | . | . | . | . | . | . | . | . | . | . |
| InternalIMT | aa | rs189053273 | . | . | . | . | . | . | . | . | . | . |
| InternalIMT | aa | rs143499388 | . | . | . | . | . | . | . | . | . | . |
| InternalIMT | aa | rs182936001 | . | . | . | . | . | . | . | . | . | . |
| InternalIMT | aa | rs148456406 | . | . | . | . | . | . | . | . | . | . |
| InternalIMT | aa | rs182499531 | . | . | . | . | . | . | . | . | . | . |
| InternalIMT | aa | rs74884915 | . | . | . | . | . | . | . | . | . | . |
| InternalIMT | aa | rs184046374 | . | . | . | . | . | . | . | . | . | . |
| InternalIMT | aa | rs74333993 | . | . | . | . | . | . | . | . | . | . |
| InternalIMT | aa | rs138889719 | . | . | . | . | . | . | . | . | . | . |
| InternalIMT | aa | rs75330330 | . | . | . | . | . | . | . | . | . | . |
| InternalIMT | aa | rs193051693 | . | . | . | . | . | . | . | . | . | . |
| InternalIMT | aa | rs145929633 | . | . | . | . | . | . | . | . | . | . |

| InternalIMT | aa | rs184076904 | . | . | . | . | . | . | . | . | . | . |
| --- | --- | --- | --- | --- | --- | --- | --- | --- | --- | --- | --- | --- |
| InternalIMT | aa | rs141802494 | . | . | . | . | . | . | . | . | . | . |
| InternalIMT | aa | rs149519759 | . | . | . | . | . | . | . | . | . | . |
| InternalIMT | aa | rs145467260 | . | . | . | . | . | . | . | . | . | . |
| InternalIMT | aa | rs79620914 | . | . | . | . | . | . | . | . | . | . |
| InternalIMT | aa | rs140538633 | . | . | . | . | . | . | . | . | . | . |
| InternalIMT | aa | rs140862301 | . | . | . | . | . | . | . | . | . | . |
| InternalIMT | aa | rs114615847 | . | . | . | . | . | . | . | . | . | . |
| InternalIMT | aa | rs141800259 | . | . | . | . | . | . | . | . | . | . |
| InternalIMT | aa | rs149779059 | . | . | . | . | . | . | . | . | . | . |
| InternalIMT | aa | rs116539253 | . | . | . | . | . | . | . | . | . | . |
| InternalIMT | aa | rs184979550 | . | . | . | . | . | . | . | . | . | . |
| InternalIMT | aa | rs190128940 | . | . | . | . | . | . | . | . | . | . |
| InternalIMT | aa | rs146594816 | . | . | . | . | . | . | . | . | . | . |
| InternalIMT | aa | rs141370766 | . | . | . | . | . | . | . | . | . | . |
| InternalIMT | aa | rs144670033 | . | . | . | . | . | . | . | . | . | . |
| InternalIMT | aa | rs187152174 | . | . | . | . | . | . | . | . | . | . |
| InternalIMT | aa | rs184180263 | . | . | . | . | . | . | . | . | . | . |
| InternalIMT | aa | rs140075861 | . | . | . | . | . | . | . | . | . | . |
| InternalIMT | aa | rs186483129 | . | . | . | . | . | . | . | . | . | . |
| InternalIMT | aa | rs186634282 | . | . | . | . | . | . | . | . | . | . |
| InternalIMT | aa | rs9988663 | . | . | . | . | . | . | . | . | . | . |
| InternalIMT | aa | rs138828529 | . | . | . | . | . | . | . | . | . | . |
| InternalIMT | aa | rs188777615 | . | . | . | . | . | . | . | . | . | . |
| InternalIMT | aa | rs192321882 | . | . | . | . | . | . | . | . | . | . |
| InternalIMT | aa | rs150812270 | . | . | . | . | . | . | . | . | . | . |
| InternalIMT | aa | rs185906603 | . | . | . | . | . | . | . | . | . | . |
| InternalIMT | aa | rs191070862 | . | . | . | . | . | . | . | . | . | . |
| InternalIMT | aa | rs182904978 | . | . | . | . | . | . | . | . | . | . |
| InternalIMT | aa | rs192679655 | . | . | . | . | . | . | . | . | . | . |
| InternalIMT | aa | rs189673086 | . | . | . | . | . | . | . | . | . | . |
| InternalIMT | aa | rs181141892 | . | . | . | . | . | . | . | . | . | . |
| InternalIMT | aa | rs186404123 | . | . | . | . | . | . | . | . | . | . |
| InternalIMT | aa | rs192117287 | . | . | . | . | . | . | . | . | . | . |
| InternalIMT | aa | rs182748835 | . | . | . | . | . | . | . | . | . | . |
| InternalIMT | aa | rs141854549 | . | . | . | . | . | . | . | . | . | . |
| InternalIMT | aa | rs140163397 | . | . | . | . | . | . | . | . | . | . |
| InternalIMT | aa | rs146347300 | . | . | . | . | . | . | . | . | . | . |
| InternalIMT | aa | rs139362377 | . | . | . | . | . | . | . | . | . | . |
| InternalIMT | aa | rs185087671 | . | . | . | . | . | . | . | . | . | . |
| InternalIMT | aa | rs190466574 | . | . | . | . | . | . | . | . | . | . |
| InternalIMT | aa | rs147012778 | . | . | . | . | . | . | . | . | . | . |
| InternalIMT | aa | rs138236960 | . | . | . | . | . | . | . | . | . | . |
| InternalIMT | aa | rs116310311 | . | . | . | . | . | . | . | . | . | . |
| InternalIMT | aa | rs142868356 | . | . | . | . | . | . | . | . | . | . |
| InternalIMT | aa | rs144956240 | . | . | . | . | . | . | . | . | . | . |
| InternalIMT | aa | rs188527776 | . | . | . | . | . | . | . | . | . | . |
| InternalIMT | aa | rs183971165 | . | . | . | . | . | . | . | . | . | . |
| InternalIMT | aa | rs76748216 | . | . | . | . | . | . | . | . | . | . |
| InternalIMT | aa | rs148554990 | . | . | . | . | . | . | . | . | . | . |
| InternalIMT | aa | rs114446477 | . | . | . | . | . | . | . | . | . | . |
| InternalIMT | aa | rs185223933 | . | . | . | . | . | . | . | . | . | . |
| InternalIMT | aa | rs76869726 | . | . | . | . | . | . | . | . | . | . |
| InternalIMT | aa | rs151200830 | . | . | . | . | . | . | . | . | . | . |
| InternalIMT | aa | rs17157731 | . | . | . | . | . | . | . | . | . | . |
| InternalIMT | aa | rs181243469 | . | . | . | . | . | . | . | . | . | . |
| InternalIMT | aa | rs186890707 | . | . | . | . | . | . | . | . | . | . |
| InternalIMT | aa | rs184063360 | . | . | . | . | . | . | . | . | . | . |
| InternalIMT | aa | rs188550237 | . | . | . | . | . | . | . | . | . | . |
| InternalIMT | aa | rs140931068 | . | . | . | . | . | . | . | . | . | . |
| InternalIMT | aa | rs74867877 | . | . | . | . | . | . | . | . | . | . |
| InternalIMT | aa | rs11239495 | . | . | . | . | . | . | . | . | . | . |
| InternalIMT | aa | rs149745800 | . | . | . | . | . | . | . | . | . | . |
| InternalIMT | aa | rs145044458 | . | . | . | . | . | . | . | . | . | . |
| InternalIMT | aa | rs188067427 | . | . | . | . | . | . | . | . | . | . |
| InternalIMT | aa | rs146827800 | . | . | . | . | . | . | . | . | . | . |
| InternalIMT | aa | rs140619817 | . | . | . | . | . | . | . | . | . | . |
| InternalIMT | aa | rs892689 | . | . | . | . | . | . | . | . | . | . |
| InternalIMT | aa | rs143642005 | . | . | . | . | . | . | . | . | . | . |
| InternalIMT | aa | rs181929304 | . | . | . | . | . | . | . | . | . | . |
| InternalIMT | aa | rs191086173 | . | . | . | . | . | . | . | . | . | . |
| InternalIMT | aa | rs114613116 | . | . | . | . | . | . | . | . | . | . |
| InternalIMT | aa | rs141076768 | . | . | . | . | . | . | . | . | . | . |
| InternalIMT | aa | rs187316531 | . | . | . | . | . | . | . | . | . | . |
| InternalIMT | aa | rs11239499 | . | . | . | . | . | . | . | . | . | . |
| InternalIMT | aa | rs192726371 | . | . | . | . | . | . | . | . | . | . |
| InternalIMT | aa | rs74128481 | . | . | . | . | . | . | . | . | . | . |
| InternalIMT | aa | rs74128482 | . | . | . | . | . | . | . | . | . | . |
| InternalIMT | aa | rs11239502 | . | . | . | . | . | . | . | . | . | . |
| InternalIMT | aa | rs116330285 | . | . | . | . | . | . | . | . | . | . |
| InternalIMT | aa | rs189926729 | . | . | . | . | . | . | . | . | . | . |
| InternalIMT | aa | rs188975779 | . | . | . | . | . | . | . | . | . | . |
| InternalIMT | aa | rs143846038 | . | . | . | . | . | . | . | . | . | . |
| InternalIMT | aa | rs74128483 | . | . | . | . | . | . | . | . | . | . |
| InternalIMT | aa | rs139292055 | . | . | . | . | . | . | . | . | . | . |
| InternalIMT | aa | rs11239503 | . | . | . | . | . | . | . | . | . | . |

| InternalIMT | aa | rs58838686 | . | . | . | . | . | . | . | . | . | . |
| --- | --- | --- | --- | --- | --- | --- | --- | --- | --- | --- | --- | --- |
| InternalIMT | aa | rs185825373 | . | . | . | . | . | . | . | . | . | . |
| InternalIMT | aa | rs11239504 | . | . | . | . | . | . | . | . | . | . |
| InternalIMT | aa | rs150012354 | . | . | . | . | . | . | . | . | . | . |
| InternalIMT | aa | rs187763096 | . | . | . | . | . | . | . | . | . | . |
| InternalIMT | aa | rs193043982 | . | . | . | . | . | . | . | . | . | . |
| InternalIMT | aa | rs79186884 | . | . | . | . | . | . | . | . | . | . |
| InternalIMT | aa | rs74128484 | . | . | . | . | . | . | . | . | . | . |
| InternalIMT | aa | rs58461537 | . | . | . | . | . | . | . | . | . | . |
| InternalIMT | aa | rs188527474 | . | . | . | . | . | . | . | . | . | . |
| InternalIMT | aa | rs141659976 | . | . | . | . | . | . | . | . | . | . |
| InternalIMT | aa | rs115846407 | . | . | . | . | . | . | . | . | . | . |
| InternalIMT | aa | rs138378816 | . | . | . | . | . | . | . | . | . | . |
| InternalIMT | aa | rs146188136 | . | . | . | . | . | . | . | . | . | . |
| InternalIMT | aa | rs139383871 | . | . | . | . | . | . | . | . | . | . |
| InternalIMT | aa | rs182953869 | . | . | . | . | . | . | . | . | . | . |
| InternalIMT | aa | rs192430299 | . | . | . | . | . | . | . | . | . | . |
| InternalIMT | aa | rs144152844 | . | . | . | . | . | . | . | . | . | . |
| InternalIMT | aa | rs145987786 | . | . | . | . | . | . | . | . | . | . |
| InternalIMT | aa | rs181132926 | . | . | . | . | . | . | . | . | . | . |
| InternalIMT | aa | rs147882253 | . | . | . | . | . | . | . | . | . | . |
| InternalIMT | aa | rs141807621 | . | . | . | . | . | . | . | . | . | . |
| InternalIMT | aa | rs147116052 | . | . | . | . | . | . | . | . | . | . |
| InternalIMT | aa | rs147716618 | . | . | . | . | . | . | . | . | . | . |
| InternalIMT | aa | rs187198855 | . | . | . | . | . | . | . | . | . | . |
| InternalIMT | aa | rs189409638 | . | . | . | . | . | . | . | . | . | . |
| InternalIMT | aa | rs182647339 | . | . | . | . | . | . | . | . | . | . |
| InternalIMT | aa | rs187995988 | . | . | . | . | . | . | . | . | . | . |
| InternalIMT | aa | rs141034184 | . | . | . | . | . | . | . | . | . | . |
| InternalIMT | aa | rs187310430 | . | . | . | . | . | . | . | . | . | . |
| InternalIMT | aa | rs192608488 | . | . | . | . | . | . | . | . | . | . |
| InternalIMT | aa | rs148840366 | . | . | . | . | . | . | . | . | . | . |
| InternalIMT | aa | rs186785022 | . | . | . | . | . | . | . | . | . | . |
| InternalIMT | aa | rs192348728 | . | . | . | . | . | . | . | . | . | . |
| InternalIMT | aa | rs189656631 | . | . | . | . | . | . | . | . | . | . |
| InternalIMT | aa | rs185289750 | . | . | . | . | . | . | . | . | . | . |
| InternalIMT | aa | rs142382395 | . | . | . | . | . | . | . | . | . | . |
| InternalIMT | aa | rs144866696 | . | . | . | . | . | . | . | . | . | . |
| InternalIMT | aa | rs141371289 | . | . | . | . | . | . | . | . | . | . |
| InternalIMT | aa | rs114750008 | . | . | . | . | . | . | . | . | . | . |
| InternalIMT | aa | rs189063597 | . | . | . | . | . | . | . | . | . | . |
| InternalIMT | aa | rs139151571 | . | . | . | . | . | . | . | . | . | . |
| InternalIMT | aa | rs139846368 | . | . | . | . | . | . | . | . | . | . |
| InternalIMT | aa | rs182057653 | . | . | . | . | . | . | . | . | . | . |
| InternalIMT | aa | rs148785420 | . | . | . | . | . | . | . | . | . | . |
| InternalIMT | aa | rs138383251 | . | . | . | . | . | . | . | . | . | . |
| InternalIMT | aa | rs116526705 | . | . | . | . | . | . | . | . | . | . |
| InternalIMT | aa | rs185946436 | . | . | . | . | . | . | . | . | . | . |
| InternalIMT | aa | rs190455427 | . | . | . | . | . | . | . | . | . | . |
| InternalIMT | aa | rs9282584 | . | . | . | . | . | . | . | . | . | . |
| InternalIMT | aa | rs17153289 | . | . | . | . | . | . | . | . | . | . |
| InternalIMT | aa | rs138359103 | . | . | . | . | . | . | . | . | . | . |
| InternalIMT | aa | rs150605268 | . | . | . | . | . | . | . | . | . | . |
| InternalIMT | aa | rs79242910 | . | . | . | . | . | . | . | . | . | . |
| InternalIMT | aa | rs139716904 | . | . | . | . | . | . | . | . | . | . |
| InternalIMT | aa | rs76238498 | . | . | . | . | . | . | . | . | . | . |
| InternalIMT | aa | rs184140639 | . | . | . | . | . | . | . | . | . | . |
| InternalIMT | aa | rs188517278 | . | . | . | . | . | . | . | . | . | . |
| InternalIMT | aa | rs139678913 | . | . | . | . | . | . | . | . | . | . |
| InternalIMT | aa | rs116697955 | . | . | . | . | . | . | . | . | . | . |
| InternalIMT | aa | rs79267994 | . | . | . | . | . | . | . | . | . | . |
| InternalIMT | aa | rs11239510 | . | . | . | . | . | . | . | . | . | . |
| InternalIMT | aa | rs75831645 | . | . | . | . | . | . | . | . | . | . |
| InternalIMT | aa | rs78452276 | . | . | . | . | . | . | . | . | . | . |
| InternalIMT | aa | rs144366924 | . | . | . | . | . | . | . | . | . | . |
| InternalIMT | aa | rs77519841 | . | . | . | . | . | . | . | . | . | . |
| InternalIMT | aa | rs76944454 | . | . | . | . | . | . | . | . | . | . |
| InternalIMT | aa | rs76443855 | . | . | . | . | . | . | . | . | . | . |
| InternalIMT | aa | rs146613174 | . | . | . | . | . | . | . | . | . | . |
| InternalIMT | aa | rs181424761 | . | . | . | . | . | . | . | . | . | . |
| InternalIMT | aa | rs142119241 | . | . | . | . | . | . | . | . | . | . |
| InternalIMT | aa | rs140137954 | . | . | . | . | . | . | . | . | . | . |
| InternalIMT | aa | rs187319549 | . | . | . | . | . | . | . | . | . | . |
| InternalIMT | aa | rs12241545 | . | . | . | . | . | . | . | . | . | . |
| InternalIMT | aa | rs10128228 | . | . | . | . | . | . | . | . | . | . |
| InternalIMT | aa | rs58580249 | . | . | . | . | . | . | . | . | . | . |
| InternalIMT | aa | rs193148863 | . | . | . | . | . | . | . | . | . | . |
| InternalIMT | aa | rs185638946 | . | . | . | . | . | . | . | . | . | . |
| InternalIMT | aa | rs117752921 | . | . | . | . | . | . | . | . | . | . |
| InternalIMT | aa | rs180841236 | . | . | . | . | . | . | . | . | . | . |
| InternalIMT | aa | rs73283155 | . | . | . | . | . | . | . | . | . | . |
| InternalIMT | aa | rs76560599 | . | . | . | . | . | . | . | . | . | . |
| InternalIMT | aa | rs76273620 | . | . | . | . | . | . | . | . | . | . |
| InternalIMT | aa | rs141432923 | . | . | . | . | . | . | . | . | . | . |
| InternalIMT | aa | rs113529322 | . | . | . | . | . | . | . | . | . | . |
| InternalIMT | aa | rs10128306 | . | . | . | . | . | . | . | . | . | . |

| InternalIMT | aa | rs149691688 | . | . | . | . | . | . | . | . | . | . |
| --- | --- | --- | --- | --- | --- | --- | --- | --- | --- | --- | --- | --- |
| InternalIMT | aa | rs75872495 | . | . | . | . | . | . | . | . | . | . |
| InternalIMT | aa | rs191873960 | . | . | . | . | . | . | . | . | . | . |
| InternalIMT | aa | rs80155622 | . | . | . | . | . | . | . | . | . | . |
| InternalIMT | aa | rs113914656 | . | . | . | . | . | . | . | . | . | . |
| InternalIMT | aa | rs12242840 | . | . | . | . | . | . | . | . | . | . |
| InternalIMT | aa | rs147824078 | . | . | . | . | . | . | . | . | . | . |
| InternalIMT | aa | rs59051493 | . | . | . | . | . | . | . | . | . | . |
| InternalIMT | aa | rs9282588 | . | . | . | . | . | . | . | . | . | . |
| InternalIMT | aa | rs150215207 | . | . | . | . | . | . | . | . | . | . |
| InternalIMT | aa | rs138772243 | . | . | . | . | . | . | . | . | . | . |
| InternalIMT | aa | rs149383244 | . | . | . | . | . | . | . | . | . | . |
| InternalIMT | aa | rs17522720 | . | . | . | . | . | . | . | . | . | . |
| InternalIMT | aa | rs79245105 | . | . | . | . | . | . | . | . | . | . |
| InternalIMT | aa | rs138222133 | . | . | . | . | . | . | . | . | . | . |
| InternalIMT | aa | rs12247314 | . | . | . | . | . | . | . | . | . | . |
| InternalIMT | aa | rs12263894 | . | . | . | . | . | . | . | . | . | . |
| InternalIMT | aa | rs149907880 | . | . | . | . | . | . | . | . | . | . |
| InternalIMT | aa | rs188153885 | . | . | . | . | . | . | . | . | . | . |
| InternalIMT | aa | rs73283159 | . | . | . | . | . | . | . | . | . | . |
| InternalIMT | aa | rs140476687 | . | . | . | . | . | . | . | . | . | . |
| InternalIMT | aa | rs186603148 | . | . | . | . | . | . | . | . | . | . |
| InternalIMT | aa | rs183296063 | . | . | . | . | . | . | . | . | . | . |
| InternalIMT | aa | rs145733694 | . | . | . | . | . | . | . | . | . | . |
| InternalIMT | aa | rs189807449 | . | . | . | . | . | . | . | . | . | . |
| InternalIMT | aa | rs12253223 | . | . | . | . | . | . | . | . | . | . |
| InternalIMT | aa | rs182113637 | . | . | . | . | . | . | . | . | . | . |
| InternalIMT | aa | rs142792099 | . | . | . | . | . | . | . | . | . | . |
| InternalIMT | aa | rs12249910 | . | . | . | . | . | . | . | . | . | . |
| InternalIMT | aa | rs149436339 | . | . | . | . | . | . | . | . | . | . |
| InternalIMT | aa | rs77632210 | . | . | . | . | . | . | . | . | . | . |
| InternalIMT | aa | rs74130818 | . | . | . | . | . | . | . | . | . | . |
| InternalIMT | aa | rs187583648 | . | . | . | . | . | . | . | . | . | . |
| InternalIMT | aa | rs191295987 | . | . | . | . | . | . | . | . | . | . |
| InternalIMT | aa | rs184200686 | . | . | . | . | . | . | . | . | . | . |
| InternalIMT | aa | rs76117263 | . | . | . | . | . | . | . | . | . | . |
| InternalIMT | aa | rs185393149 | . | . | . | . | . | . | . | . | . | . |
| InternalIMT | aa | rs144760014 | . | . | . | . | . | . | . | . | . | . |
| InternalIMT | aa | rs7922292 | . | . | . | . | . | . | . | . | . | . |
| InternalIMT | aa | rs115372844 | . | . | . | . | . | . | . | . | . | . |
| InternalIMT | aa | rs111460364 | . | . | . | . | . | . | . | . | . | . |
| InternalIMT | aa | rs182052255 | . | . | . | . | . | . | . | . | . | . |
| InternalIMT | aa | rs180983889 | . | . | . | . | . | . | . | . | . | . |
| InternalIMT | aa | rs139175327 | . | . | . | . | . | . | . | . | . | . |
| InternalIMT | aa | rs149959620 | . | . | . | . | . | . | . | . | . | . |
| InternalIMT | aa | rs114653117 | . | . | . | . | . | . | . | . | . | . |
| InternalIMT | aa | rs144270018 | . | . | . | . | . | . | . | . | . | . |
| InternalIMT | aa | rs182723204 | . | . | . | . | . | . | . | . | . | . |
| InternalIMT | aa | rs57204067 | . | . | . | . | . | . | . | . | . | . |
| InternalIMT | aa | rs144956226 | . | . | . | . | . | . | . | . | . | . |
| InternalIMT | aa | rs187842704 | . | . | . | . | . | . | . | . | . | . |
| InternalIMT | aa | rs184620511 | . | . | . | . | . | . | . | . | . | . |
| InternalIMT | aa | rs144031303 | . | . | . | . | . | . | . | . | . | . |
| InternalIMT | aa | rs187526473 | . | . | . | . | . | . | . | . | . | . |
| InternalIMT | aa | rs193002805 | . | . | . | . | . | . | . | . | . | . |
| InternalIMT | aa | rs138707426 | . | . | . | . | . | . | . | . | . | . |
| InternalIMT | aa | rs142754243 | . | . | . | . | . | . | . | . | . | . |
| InternalIMT | aa | rs181600331 | . | . | . | . | . | . | . | . | . | . |
| InternalIMT | aa | rs144421264 | . | . | . | . | . | . | . | . | . | . |
| InternalIMT | aa | rs184414519 | . | . | . | . | . | . | . | . | . | . |
| InternalIMT | aa | rs150596349 | . | . | . | . | . | . | . | . | . | . |
| InternalIMT | aa | rs190753747 | . | . | . | . | . | . | . | . | . | . |
| InternalIMT | aa | rs139560940 | . | . | . | . | . | . | . | . | . | . |
| InternalIMT | aa | rs75640804 | . | . | . | . | . | . | . | . | . | . |
| InternalIMT | aa | rs73283175 | . | . | . | . | . | . | . | . | . | . |
| InternalIMT | aa | rs143337229 | . | . | . | . | . | . | . | . | . | . |
| InternalIMT | aa | rs73283177 | . | . | . | . | . | . | . | . | . | . |
| InternalIMT | aa | rs76053611 | . | . | . | . | . | . | . | . | . | . |
| InternalIMT | aa | rs116325983 | . | . | . | . | . | . | . | . | . | . |
| InternalIMT | aa | rs115451825 | . | . | . | . | . | . | . | . | . | . |
| InternalIMT | aa | rs183845246 | . | . | . | . | . | . | . | . | . | . |
| InternalIMT | aa | rs79756542 | . | . | . | . | . | . | . | . | . | . |
| InternalIMT | aa | rs187242860 | . | . | . | . | . | . | . | . | . | . |
| InternalIMT | aa | rs57318980 | . | . | . | . | . | . | . | . | . | . |
| InternalIMT | aa | rs59969732 | . | . | . | . | . | . | . | . | . | . |
| InternalIMT | aa | rs143811488 | . | . | . | . | . | . | . | . | . | . |
| InternalIMT | aa | rs189067644 | . | . | . | . | . | . | . | . | . | . |
| InternalIMT | aa | rs60449178 | . | . | . | . | . | . | . | . | . | . |
| InternalIMT | aa | rs59243389 | . | . | . | . | . | . | . | . | . | . |
| InternalIMT | aa | rs184946540 | . | . | . | . | . | . | . | . | . | . |
| InternalIMT | aa | rs188685881 | . | . | . | . | . | . | . | . | . | . |
| InternalIMT | aa | rs150335797 | . | . | . | . | . | . | . | . | . | . |
| InternalIMT | aa | rs143879260 | . | . | . | . | . | . | . | . | . | . |
| InternalIMT | aa | rs186044491 | . | . | . | . | . | . | . | . | . | . |
| InternalIMT | aa | rs11819084 | . | . | . | . | . | . | . | . | . | . |
| InternalIMT | aa | rs146915670 | . | . | . | . | . | . | . | . | . | . |

| InternalIMT | aa | rs113995695 | . | . | . | . | . | . | . | . | . | . |
| --- | --- | --- | --- | --- | --- | --- | --- | --- | --- | --- | --- | --- |
| InternalIMT | aa | rs143489451 | . | . | . | . | . | . | . | . | . | . |
| InternalIMT | aa | rs182653839 | . | . | . | . | . | . | . | . | . | . |
| InternalIMT | aa | rs141692564 | . | . | . | . | . | . | . | . | . | . |
| InternalIMT | aa | rs146281742 | . | . | . | . | . | . | . | . | . | . |
| InternalIMT | aa | rs192914353 | . | . | . | . | . | . | . | . | . | . |
| InternalIMT | aa | rs185182848 | . | . | . | . | . | . | . | . | . | . |
| InternalIMT | aa | rs28395858 | . | . | . | . | . | . | . | . | . | . |
| InternalIMT | aa | rs41429645 | . | . | . | . | . | . | . | . | . | . |
| InternalIMT | aa | rs28395874 | . | . | . | . | . | . | . | . | . | . |
| InternalIMT | aa | rs189663634 | . | . | . | . | . | . | . | . | . | . |
| InternalIMT | aa | rs77666700 | . | . | . | . | . | . | . | . | . | . |
| InternalIMT | aa | rs146711717 | . | . | . | . | . | . | . | . | . | . |
| InternalIMT | aa | rs144904251 | . | . | . | . | . | . | . | . | . | . |
| InternalIMT | aa | rs189893153 | . | . | . | . | . | . | . | . | . | . |
| InternalIMT | aa | rs150447740 | . | . | . | . | . | . | . | . | . | . |
| InternalIMT | aa | rs143099649 | . | . | . | . | . | . | . | . | . | . |
| InternalIMT | aa | rs191288436 | . | . | . | . | . | . | . | . | . | . |
| InternalIMT | aa | rs188577862 | . | . | . | . | . | . | . | . | . | . |
| InternalIMT | aa | rs192695334 | . | . | . | . | . | . | . | . | . | . |
| InternalIMT | aa | rs138612906 | . | . | . | . | . | . | . | . | . | . |
| InternalIMT | aa | rs191290051 | . | . | . | . | . | . | . | . | . | . |
| InternalIMT | aa | rs189631459 | . | . | . | . | . | . | . | . | . | . |
| InternalIMT | aa | rs114620874 | . | . | . | . | . | . | . | . | . | . |
| InternalIMT | aa | rs78246652 | . | . | . | . | . | . | . | . | . | . |
| InternalIMT | aa | rs79432451 | . | . | . | . | . | . | . | . | . | . |
| InternalIMT | aa | rs186022585 | . | . | . | . | . | . | . | . | . | . |
| InternalIMT | aa | rs142978891 | . | . | . | . | . | . | . | . | . | . |
| InternalIMT | aa | rs151086840 | . | . | . | . | . | . | . | . | . | . |
| InternalIMT | aa | rs183270259 | . | . | . | . | . | . | . | . | . | . |
| InternalIMT | aa | rs188544289 | . | . | . | . | . | . | . | . | . | . |
| InternalIMT | aa | rs188500808 | . | . | . | . | . | . | . | . | . | . |
| InternalIMT | aa | rs189424589 | . | . | . | . | . | . | . | . | . | . |
| InternalIMT | aa | rs78622303 | . | . | . | . | . | . | . | . | . | . |
| InternalIMT | aa | rs143210835 | . | . | . | . | . | . | . | . | . | . |
| InternalIMT | aa | rs114761840 | . | . | . | . | . | . | . | . | . | . |
| InternalIMT | aa | rs185805402 | . | . | . | . | . | . | . | . | . | . |
| InternalIMT | aa | rs139273744 | . | . | . | . | . | . | . | . | . | . |
| InternalIMT | aa | rs184654150 | . | . | . | . | . | . | . | . | . | . |
| InternalIMT | aa | rs189069710 | . | . | . | . | . | . | . | . | . | . |
| InternalIMT | aa | rs114752115 | . | . | . | . | . | . | . | . | . | . |
| InternalIMT | aa | rs150162515 | . | . | . | . | . | . | . | . | . | . |
| InternalIMT | aa | rs180982454 | . | . | . | . | . | . | . | . | . | . |
| InternalIMT | aa | rs184932088 | . | . | . | . | . | . | . | . | . | . |
| InternalIMT | aa | rs181109625 | . | . | . | . | . | . | . | . | . | . |
| InternalIMT | aa | rs138121134 | . | . | . | . | . | . | . | . | . | . |
| InternalIMT | aa | rs183253861 | . | . | . | . | . | . | . | . | . | . |
| InternalIMT | aa | rs147073946 | . | . | . | . | . | . | . | . | . | . |
| InternalIMT | aa | rs139938489 | . | . | . | . | . | . | . | . | . | . |
| InternalIMT | aa | rs146362560 | . | . | . | . | . | . | . | . | . | . |
| InternalIMT | aa | rs193155956 | . | . | . | . | . | . | . | . | . | . |
| InternalIMT | aa | rs77663092 | . | . | . | . | . | . | . | . | . | . |
| InternalIMT | aa | rs73285319 | . | . | . | . | . | . | . | . | . | . |
| InternalIMT | aa | rs184019276 | . | . | . | . | . | . | . | . | . | . |
| InternalIMT | aa | rs28395863 | . | . | . | . | . | . | . | . | . | . |
| InternalIMT | aa | rs182121645 | . | . | . | . | . | . | . | . | . | . |
| InternalIMT | aa | rs73285327 | . | . | . | . | . | . | . | . | . | . |
| InternalIMT | aa | rs149589584 | . | . | . | . | . | . | . | . | . | . |
| InternalIMT | aa | rs148310335 | . | . | . | . | . | . | . | . | . | . |
| InternalIMT | aa | rs187246093 | . | . | . | . | . | . | . | . | . | . |
| InternalIMT | aa | rs184668894 | . | . | . | . | . | . | . | . | . | . |
| InternalIMT | aa | rs114269961 | . | . | . | . | . | . | . | . | . | . |
| InternalIMT | aa | rs149694374 | . | . | . | . | . | . | . | . | . | . |
| InternalIMT | aa | rs145284227 | . | . | . | . | . | . | . | . | . | . |
| InternalIMT | aa | rs189983820 | . | . | . | . | . | . | . | . | . | . |
| InternalIMT | aa | rs181200444 | . | . | . | . | . | . | . | . | . | . |
| InternalIMT | aa | rs185486385 | . | . | . | . | . | . | . | . | . | . |
| InternalIMT | aa | rs151198241 | . | . | . | . | . | . | . | . | . | . |
| InternalIMT | aa | rs189959341 | . | . | . | . | . | . | . | . | . | . |
| InternalIMT | aa | rs140549257 | . | . | . | . | . | . | . | . | . | . |
| InternalIMT | aa | rs186453139 | . | . | . | . | . | . | . | . | . | . |
| InternalIMT | aa | rs180922711 | . | . | . | . | . | . | . | . | . | . |
| InternalIMT | aa | rs56659903 | . | . | . | . | . | . | . | . | . | . |
| InternalIMT | aa | rs141202128 | . | . | . | . | . | . | . | . | . | . |
| InternalIMT | aa | rs59085314 | . | . | . | . | . | . | . | . | . | . |
| InternalIMT | aa | rs145564996 | . | . | . | . | . | . | . | . | . | . |
| InternalIMT | aa | rs114961009 | . | . | . | . | . | . | . | . | . | . |
| InternalIMT | aa | rs182294189 | . | . | . | . | . | . | . | . | . | . |
| InternalIMT | aa | rs187586636 | . | . | . | . | . | . | . | . | . | . |
| InternalIMT | aa | rs192254907 | . | . | . | . | . | . | . | . | . | . |
| InternalIMT | aa | rs149145663 | . | . | . | . | . | . | . | . | . | . |
| InternalIMT | aa | rs180713064 | . | . | . | . | . | . | . | . | . | . |
| InternalIMT | aa | rs183009023 | . | . | . | . | . | . | . | . | . | . |
| InternalIMT | aa | rs143385347 | . | . | . | . | . | . | . | . | . | . |
| InternalIMT | aa | rs185786329 | . | . | . | . | . | . | . | . | . | . |
| InternalIMT | aa | rs192341030 | . | . | . | . | . | . | . | . | . | . |

| InternalIMT | aa | rs74661429 | . | . | . | . | . | . | . | . | . | . |
| --- | --- | --- | --- | --- | --- | --- | --- | --- | --- | --- | --- | --- |
| InternalIMT | aa | rs188034853 | . | . | . | . | . | . | . | . | . | . |
| InternalIMT | aa | rs116808430 | . | . | . | . | . | . | . | . | . | . |
| InternalIMT | aa | rs148469010 | . | . | . | . | . | . | . | . | . | . |
| InternalIMT | aa | rs78621663 | . | . | . | . | . | . | . | . | . | . |
| InternalIMT | aa | rs192654886 | . | . | . | . | . | . | . | . | . | . |
| InternalIMT | aa | rs145996035 | . | . | . | . | . | . | . | . | . | . |
| InternalIMT | aa | rs139942226 | . | . | . | . | . | . | . | . | . | . |
| InternalIMT | aa | rs113645632 | . | . | . | . | . | . | . | . | . | . |
| InternalIMT | aa | rs74940661 | . | . | . | . | . | . | . | . | . | . |
| InternalIMT | aa | rs115355800 | . | . | . | . | . | . | . | . | . | . |
| InternalIMT | aa | rs150285898 | . | . | . | . | . | . | . | . | . | . |
| InternalIMT | aa | rs188117932 | . | . | . | . | . | . | . | . | . | . |
| InternalIMT | aa | rs139956566 | . | . | . | . | . | . | . | . | . | . |
| InternalIMT | aa | rs147673885 | . | . | . | . | . | . | . | . | . | . |
| InternalIMT | aa | rs189127928 | . | . | . | . | . | . | . | . | . | . |
| InternalIMT | aa | rs187558772 | . | . | . | . | . | . | . | . | . | . |
| InternalIMT | aa | rs181948587 | . | . | . | . | . | . | . | . | . | . |
| InternalIMT | aa | rs189983269 | . | . | . | . | . | . | . | . | . | . |
| InternalIMT | aa | rs187250069 | . | . | . | . | . | . | . | . | . | . |
| InternalIMT | aa | rs191485859 | . | . | . | . | . | . | . | . | . | . |
| InternalIMT | aa | rs185499555 | . | . | . | . | . | . | . | . | . | . |
| InternalIMT | aa | rs180862110 | . | . | . | . | . | . | . | . | . | . |
| InternalIMT | aa | rs190472603 | . | . | . | . | . | . | . | . | . | . |
| InternalIMT | aa | rs189226167 | . | . | . | . | . | . | . | . | . | . |
| InternalIMT | aa | rs181623256 | . | . | . | . | . | . | . | . | . | . |
| InternalIMT | aa | rs186079557 | . | . | . | . | . | . | . | . | . | . |
| InternalIMT | aa | rs147760994 | . | . | . | . | . | . | . | . | . | . |
| InternalIMT | aa | rs189176167 | . | . | . | . | . | . | . | . | . | . |
| InternalIMT | aa | rs117901000 | . | . | . | . | . | . | . | . | . | . |
| InternalIMT | aa | rs141984888 | . | . | . | . | . | . | . | . | . | . |
| InternalIMT | aa | rs192365061 | . | . | . | . | . | . | . | . | . | . |
| InternalIMT | aa | rs185976038 | . | . | . | . | . | . | . | . | . | . |
| InternalIMT | aa | rs184697303 | . | . | . | . | . | . | . | . | . | . |
| InternalIMT | aa | rs143383132 | . | . | . | . | . | . | . | . | . | . |
| InternalIMT | aa | rs141873881 | . | . | . | . | . | . | . | . | . | . |
| InternalIMT | aa | rs185507255 | . | . | . | . | . | . | . | . | . | . |
| InternalIMT | aa | rs191379425 | . | . | . | . | . | . | . | . | . | . |
| InternalIMT | aa | rs184199584 | . | . | . | . | . | . | . | . | . | . |
| InternalIMT | aa | rs183181360 | . | . | . | . | . | . | . | . | . | . |
| InternalIMT | aa | rs184495673 | . | . | . | . | . | . | . | . | . | . |
| InternalIMT | aa | rs182946297 | . | . | . | . | . | . | . | . | . | . |
| InternalIMT | aa | rs187743615 | . | . | . | . | . | . | . | . | . | . |
| InternalIMT | aa | rs183259862 | . | . | . | . | . | . | . | . | . | . |
| InternalIMT | aa | rs112433566 | . | . | . | . | . | . | . | . | . | . |
| InternalIMT | aa | rs182351085 | . | . | . | . | . | . | . | . | . | . |
| InternalIMT | aa | rs146248508 | . | . | . | . | . | . | . | . | . | . |
| InternalIMT | aa | rs188528439 | . | . | . | . | . | . | . | . | . | . |
| InternalIMT | aa | rs146167018 | . | . | . | . | . | . | . | . | . | . |
| InternalIMT | aa | rs192440980 | . | . | . | . | . | . | . | . | . | . |
| InternalIMT | aa | rs185601882 | . | . | . | . | . | . | . | . | . | . |
| InternalIMT | aa | rs117272563 | . | . | . | . | . | . | . | . | . | . |
| InternalIMT | aa | rs10430734 | . | . | . | . | . | . | . | . | . | . |
| InternalIMT | aa | rs181320952 | . | . | . | . | . | . | . | . | . | . |
| InternalIMT | aa | rs185480660 | . | . | . | . | . | . | . | . | . | . |
| InternalIMT | aa | rs188077917 | . | . | . | . | . | . | . | . | . | . |
| InternalIMT | aa | rs187562953 | . | . | . | . | . | . | . | . | . | . |
| InternalIMT | aa | rs182044631 | . | . | . | . | . | . | . | . | . | . |
| InternalIMT | aa | rs145455596 | . | . | . | . | . | . | . | . | . | . |
| InternalIMT | aa | rs181475877 | . | . | . | . | . | . | . | . | . | . |
| InternalIMT | aa | rs149413085 | . | . | . | . | . | . | . | . | . | . |
| InternalIMT | aa | rs12253656 | . | . | . | . | . | . | . | . | . | . |
| InternalIMT | aa | rs182718239 | . | . | . | . | . | . | . | . | . | . |
| InternalIMT | aa | rs192435695 | . | . | . | . | . | . | . | . | . | . |
| InternalIMT | aa | rs12248277 | . | . | . | . | . | . | . | . | . | . |
| InternalIMT | aa | rs143521710 | . | . | . | . | . | . | . | . | . | . |
| InternalIMT | aa | rs144454454 | . | . | . | . | . | . | . | . | . | . |
| InternalIMT | aa | rs190441610 | . | . | . | . | . | . | . | . | . | . |
| InternalIMT | aa | rs114690462 | . | . | . | . | . | . | . | . | . | . |
| InternalIMT | aa | rs184541357 | . | . | . | . | . | . | . | . | . | . |
| InternalIMT | aa | rs142657653 | . | . | . | . | . | . | . | . | . | . |
| InternalIMT | aa | rsurf1 | 2.462 | . | . | . | . | . | . | . | . | . |
| InternalIMT | aa | rsten1 | 2.569 | . | . | . | . | . | . | . | . | . |
| InternalIMT | aa | chr10:45852312:D | 0.198 | -0.057 | 0.095 | 0.552 | -0.672 | 0.366 | 0.066 | 0.292 | 0.164 | 0.075 |
| InternalIMT | aa | chr10:45853232:I | 0.2 | -0.054 | 0.096 | 0.57 | -0.648 | 0.36 | 0.073 | 0.283 | 0.162 | 0.081 |
| InternalIMT | aa | chr10:45853234:I | 0.194 | -0.048 | 0.094 | 0.608 | -0.629 | 0.355 | 0.077 | 0.275 | 0.159 | 0.085 |
| InternalIMT | aa | chr10:45854796:D | 0.053 | 0.024 | 0.085 | 0.777 | -0.981 | 0.58 | 0.091 | 0.395 | 0.264 | 0.136 |
| InternalIMT | aa | chr10:45854994:I | 0.054 | 0.024 | 0.085 | 0.777 | -0.98 | 0.58 | 0.091 | 0.394 | 0.264 | 0.136 |
| InternalIMT | aa | chr10:45858290:D | 0.048 | -0.069 | 0.09 | 0.444 | -3.34 | 1.5 | 0.027 | 1.43 | 0.679 | 0.036 |
| InternalIMT | aa | chr10:45858820:I | 0.005 | . | . | . | . | . | . | . | . | . |
| InternalIMT | aa | chr10:45860888:D | 0.063 | -0.056 | 0.087 | 0.522 | -2.17 | 0.905 | 0.017 | 0.924 | 0.406 | 0.023 |
| InternalIMT | aa | chr10:45863913:I | 0.195 | -0.039 | 0.094 | 0.682 | -0.58 | 0.357 | 0.104 | 0.251 | 0.16 | 0.117 |
| InternalIMT | aa | chr10:45866601:D | . | . | . | . | . | . | . | . | . | . |
| InternalIMT | aa | chr10:45867197:I | . | . | . | . | . | . | . | . | . | . |
| InternalIMT | aa | chr10:45867538:D | 0.198 | -0.049 | 0.095 | 0.604 | -0.63 | 0.362 | 0.083 | 0.273 | 0.163 | 0.095 |
| InternalIMT | aa | chr10:45868356:D | 0.197 | -0.048 | 0.095 | 0.611 | -0.625 | 0.362 | 0.085 | 0.271 | 0.163 | 0.097 |

| InternalIMT | aa | chr10:45871158:I | 0.198 | -0.048 | 0.095 | 0.611 | -0.625 | 0.362 | 0.084 | 0.271 | 0.163 | 0.097 |
| --- | --- | --- | --- | --- | --- | --- | --- | --- | --- | --- | --- | --- |
| InternalIMT | aa | chr10:45871637:D | 0.1 | 0.048 | 0.091 | 0.598 | -0.157 | 0.548 | 0.775 | 0.065 | 0.239 | 0.787 |
| InternalIMT | aa | chr10:45871638:I | 0.161 | -0.039 | 0.094 | 0.677 | -0.695 | 0.437 | 0.112 | 0.302 | 0.196 | 0.123 |
| InternalIMT | aa | chr10:45873629:I | 0.198 | -0.048 | 0.095 | 0.61 | -0.626 | 0.363 | 0.085 | 0.271 | 0.163 | 0.097 |
| InternalIMT | aa | chr10:45873701:D | . | . | . | . | . | . | . | . | . | . |
| InternalIMT | aa | chr10:45874997:I | . | . | . | . | . | . | . | . | . | . |
| InternalIMT | aa | chr10:45882030:I | . | . | . | . | . | . | . | . | . | . |
| InternalIMT | aa | chr10:45883774:D | 0.003 | . | . | . | . | . | . | . | . | . |
| InternalIMT | aa | chr10:45885169:D | 0.176 | -0.05 | 0.094 | 0.591 | -0.711 | 0.396 | 0.073 | 0.308 | 0.18 | 0.086 |
| InternalIMT | aa | chr10:45886451:D | 0.744 | 0.197 | 0.211 | 0.351 | 0.222 | 0.296 | 0.454 | -0.092 | 0.13 | 0.479 |
| InternalIMT | aa | chr10:45891800:I | 0.85 | -0.098 | 0.254 | 0.699 | -0.207 | 0.347 | 0.552 | 0.093 | 0.15 | 0.536 |
| InternalIMT | aa | chr10:45904771:I | 0.87 | -0.104 | 0.264 | 0.695 | -0.211 | 0.358 | 0.555 | 0.095 | 0.153 | 0.538 |
| InternalIMT | aa | chr10:45904959:I | 0.11 | 0.029 | 0.091 | 0.751 | -0.307 | 0.576 | 0.594 | 0.136 | 0.258 | 0.598 |
| InternalIMT | aa | chr10:45911028:I | 0.105 | 0.14 | 0.097 | 0.147 | 1.1 | 0.678 | 0.106 | -0.443 | 0.29 | 0.128 |
| InternalIMT | aa | chr10:45911031:I | 0.419 | 0.192 | 0.129 | 0.135 | 0.396 | 0.308 | 0.199 | -0.156 | 0.134 | 0.243 |
| InternalIMT | aa | chr10:45912154:D | 0.003 | . | . | . | . | . | . | . | . | . |
| InternalIMT | aa | chr10:45913885:I | 0.043 | 0.034 | 0.084 | 0.69 | -0.743 | 0.563 | 0.187 | 0.28 | 0.257 | 0.275 |
| InternalIMT | aa | chr10:45914753:D | 0.159 | 0.079 | 0.095 | 0.407 | 0.27 | 0.447 | 0.546 | -0.092 | 0.191 | 0.63 |
| InternalIMT | aa | chr10:45919621 | . | . | . | . | . | . | . | . | . | . |
| InternalIMT | aa | chr10:45921739:I | 0.048 | 0.029 | 0.085 | 0.733 | -0.823 | 0.496 | 0.097 | 0.325 | 0.226 | 0.15 |
| InternalIMT | aa | chr10:45923527:I | 0.062 | 0.034 | 0.094 | 0.72 | -0.542 | 0.805 | 0.501 | 0.211 | 0.359 | 0.557 |
| InternalIMT | aa | chr10:45927001:I | 0.005 | . | . | . | . | . | . | . | . | . |
| InternalIMT | aa | chr10:45930354:I | 0.044 | 0.034 | 0.084 | 0.682 | -0.733 | 0.516 | 0.156 | 0.284 | 0.236 | 0.228 |
| InternalIMT | aa | chr10:45932864:I | 0.065 | 0.031 | 0.085 | 0.72 | -0.545 | 0.564 | 0.334 | 0.196 | 0.257 | 0.446 |
| InternalIMT | aa | chr10:45936163:I | 0.027 | 0.07 | 0.083 | 0.405 | 0.755 | 0.79 | 0.339 | -0.303 | 0.325 | 0.351 |
| InternalIMT | aa | chr10:45938095:D | 0.058 | 0.047 | 0.085 | 0.58 | -0.212 | 0.549 | 0.699 | 0.105 | 0.239 | 0.66 |
| InternalIMT | aa | chr10:45938886 | . | . | . | . | . | . | . | . | . | . |
| InternalIMT | aa | chr10:45939320 | . | . | . | . | . | . | . | . | . | . |
| InternalIMT | aa | chr10:45939659 | . | . | . | . | . | . | . | . | . | . |
| InternalIMT | aa | chr10:45941150:D | 0.042 | 0.035 | 0.084 | 0.679 | -0.708 | 0.518 | 0.172 | 0.271 | 0.237 | 0.254 |
| InternalIMT | aa | chr10:45947128:D | 0.038 | 0.076 | 0.086 | 0.375 | 0.471 | 0.572 | 0.41 | -0.172 | 0.245 | 0.484 |
| InternalIMT | aa | chr10:45950990:I | 0.052 | 0.03 | 0.085 | 0.728 | -0.762 | 0.545 | 0.162 | 0.299 | 0.249 | 0.23 |
| InternalIMT | aa | chr10:45951854:I | . | . | . | . | . | . | . | . | . | . |
| InternalIMT | aa | chr10:45952670:I | 0.001 | . | . | . | . | . | . | . | . | . |
| InternalIMT | aa | chr10:45956741 | . | . | . | . | . | . | . | . | . | . |
| InternalIMT | aa | chr10:45957247:I | 0.065 | 0.035 | 0.086 | 0.688 | -0.482 | 0.538 | 0.371 | 0.216 | 0.236 | 0.361 |
| InternalIMT | aa | chr10:45958082:I | 0.065 | 0.034 | 0.086 | 0.696 | -0.497 | 0.54 | 0.357 | 0.223 | 0.237 | 0.347 |
| InternalIMT | aa | chr10:45905041:D | 0.05 | 0.031 | 0.085 | 0.716 | -0.783 | 0.505 | 0.121 | 0.304 | 0.23 | 0.187 |
| InternalIMT | aa | chr10:45938871 | 0.003 | . | . | . | . | . | . | . | . | . |
| InternalIMT | aa | chr10:45939160 | 0.004 | . | . | . | . | . | . | . | . | . |
| InternalIMT | aa | chr10:45869910 | . | . | . | . | . | . | . | . | . | . |
| InternalIMT | aa | chr10:45876214:D | . | . | . | . | . | . | . | . | . | . |
| InternalIMT | aa | chr10:45880237:I | . | . | . | . | . | . | . | . | . | . |
| InternalIMT | aa | chr10:45884371:I | . | . | . | . | . | . | . | . | . | . |
| InternalIMT | aa | chr10:45891535:D | . | . | . | . | . | . | . | . | . | . |
| InternalIMT | aa | chr10:45893516:D | . | . | . | . | . | . | . | . | . | . |
| InternalIMT | aa | chr10:45893782:D | . | . | . | . | . | . | . | . | . | . |
| InternalIMT | aa | chr10:45895254:I | . | . | . | . | . | . | . | . | . | . |
| InternalIMT | aa | chr10:45895848:D | . | . | . | . | . | . | . | . | . | . |
| InternalIMT | aa | chr10:45896316:D | . | . | . | . | . | . | . | . | . | . |
| InternalIMT | aa | chr10:45896317:D | . | . | . | . | . | . | . | . | . | . |
| InternalIMT | aa | chr10:45902683:I | . | . | . | . | . | . | . | . | . | . |
| InternalIMT | aa | chr10:45905003:D | . | . | . | . | . | . | . | . | . | . |
| InternalIMT | aa | chr10:45927476:D | . | . | . | . | . | . | . | . | . | . |
| InternalIMT | aa | chr10:45935882 | . | . | . | . | . | . | . | . | . | . |
| InternalIMT | aa | chr10:45936100 | . | . | . | . | . | . | . | . | . | . |
| InternalIMT | aa | chr10:45939553 | . | . | . | . | . | . | . | . | . | . |
| InternalIMT | aa | chr10:45939631 | . | . | . | . | . | . | . | . | . | . |
| InternalIMT | aa | chr10:45944193:D | . | . | . | . | . | . | . | . | . | . |
| InternalIMT | aa | chr10:45954030 | . | . | . | . | . | . | . | . | . | . |
