# Supplementary material for: 5‐Lipoxygenase Gene Variants Are Not Associated With Atherosclerosis or Incident Coronary Heart Disease in the Multi‐Ethnic Study of Atherosclerosis Cohort
Source: J Am Heart Assoc. 2016 Mar 29;5(3):e002814. doi: 10.1161/JAHA.115.002814 (PMC4943258; doi:10.1161/JAHA.115.002814)
Supplement: Supplementary file 3 — Table S7A. Main and Interaction Effects of ALOX5 SNPs and Levels of EPA on CHD Risk in All Race/Ethnicity Groups. SNPs with minor allele frequency of <1% were excluded. HR indicates hazard ratio; SE, standard error Table S7B. Main and Interaction Effects of ALOX5 SNPs and Levels of EPA on CHD Risk in Caucasians Table S7C. Main and Interaction Effects of ALOX5 SNPs and Levels of EPA on CHD Risk in Blacks Table S7D. Main and Interaction Effects of ALOX5 SNPs and Levels of EPA on CHD Risk in Hispanics Table S7E. Main and Interaction Effects of ALOX5 SNPs and Levels of EPA on CHD Risk in Chinese Table S8A. Main and Interaction Effects of ALOX5 SNPs and Levels of DHA on CHD Risk in All Race/Ethnicity Groups. SNPs with minor allele frequency of <1% were excluded. HR indicates hazard ratio; SE, standard error Table S8B. Main and Interaction Effects of ALOX5 SNPs and Levels of DHA on CHD Risk in Caucasians Table S8C. Main and Interaction Effects of ALOX5 SNPs and Levels of DHA on CHD Risk in Blacks Table S8D. Main and Interaction Effects of ALOX5 SNPs and Levels of DHA on CHD Risk in Hispanics Table S8E. Main and Interaction Effects of ALOX5 SNPs and Levels of DHA on CHD Risk in Chinese [file JAH3-5-e002814-s003.docx]

Supplemental table S31.

| fa | rs_id | freq | HR_logepa | se_logepa | p_logepa | HR_snp | se_snp | p_snp | HR_inter | se_inter | p_inter | RERI |
| --- | --- | --- | --- | --- | --- | --- | --- | --- | --- | --- | --- | --- |
| logepa | rs76035182 | 0.024 | 0.767 | 0.084 | 0.015 | 1.24 | 0.418 | 0.525 | 1.07 | 0.534 | 0.896 | 8.40E-03 |
| logepa | rs12359547 | 0.069 | 0.711 | 0.078 | 1.90E-03 | 0.855 | 0.172 | 0.437 | 1.26 | 0.403 | 0.462 | 0.203 |
| logepa | rs74595484 | 0.039 | 0.763 | 0.085 | 0.015 | 0.728 | 0.233 | 0.322 | 1.15 | 0.501 | 0.747 | 0.148 |
| logepa | rs1864415 | 0.701 | 1.04 | 0.213 | 0.846 | 0.887 | 0.091 | 0.244 | 0.771 | 0.102 | 0.05 | -0.216 |
| logepa | rs72788101 | 0.073 | 0.708 | 0.078 | 1.80E-03 | 0.836 | 0.167 | 0.371 | 1.29 | 0.406 | 0.416 | 0.22 |
| logepa | rs11239481 | 0.14 | 0.795 | 0.098 | 0.062 | 0.932 | 0.141 | 0.643 | 0.884 | 0.176 | 0.537 | -0.072 |
| logepa | rs72788102 | 0.073 | 0.708 | 0.078 | 1.80E-03 | 0.836 | 0.167 | 0.371 | 1.29 | 0.406 | 0.416 | 0.22 |
| logepa | rs72789705 | 0.073 | 0.708 | 0.078 | 1.80E-03 | 0.836 | 0.167 | 0.371 | 1.29 | 0.406 | 0.416 | 0.22 |
| logepa | rs141529858 | 0.005 | . | . | . | . | . | . | . | . | . | . |
| logepa | rs145376292 | 0 | . | . | . | . | . | . | . | . | . | . |
| logepa | rs190418301 | 0.001 | . | . | . | . | . | . | . | . | . | . |
| logepa | rs11239482 | 0.073 | 0.708 | 0.078 | 1.80E-03 | 0.836 | 0.167 | 0.371 | 1.29 | 0.406 | 0.416 | 0.22 |
| logepa | rs61855872 | 0.01 | 0.78 | 0.086 | 0.024 | 0.527 | 0.364 | 0.354 | 0.508 | 0.277 | 0.214 | -0.098 |
| logepa | rs148991144 | 0.007 | . | . | . | . | . | . | . | . | . | . |
| logepa | rs191932221 | 0.005 | . | . | . | . | . | . | . | . | . | . |
| logepa | rs55978138 | 0.166 | 0.756 | 0.087 | 0.015 | 0.886 | 0.127 | 0.4 | 0.899 | 0.17 | 0.573 | -0.04 |
| logepa | rs73281167 | 0.015 | 0.743 | 0.081 | 6.10E-03 | 0.958 | 0.403 | 0.918 | 3.06 | 2.12 | 0.106 | 1.47 |
| logepa | rs12768010 | 0.086 | 0.771 | 0.083 | 0.015 | 0.921 | 0.192 | 0.695 | 0.72 | 0.18 | 0.189 | -0.181 |
| logepa | rs11239484 | 0.701 | 1.04 | 0.213 | 0.845 | 0.887 | 0.091 | 0.243 | 0.771 | 0.102 | 0.05 | -0.216 |
| logepa | rs35906360 | 0.166 | 0.755 | 0.087 | 0.014 | 0.889 | 0.127 | 0.412 | 0.901 | 0.172 | 0.584 | -0.039 |
| logepa | rs186250512 | 0.003 | . | . | . | . | . | . | . | . | . | . |
| logepa | rs150810743 | 0.002 | . | . | . | . | . | . | . | . | . | . |
| logepa | rs55965812 | 0.161 | 0.758 | 0.087 | 0.016 | 0.911 | 0.13 | 0.511 | 0.889 | 0.17 | 0.54 | -0.055 |
| logepa | rs56180358 | 0.167 | 0.755 | 0.087 | 0.014 | 0.888 | 0.127 | 0.407 | 0.901 | 0.172 | 0.584 | -0.039 |
| logepa | rs55864145 | 0.086 | 0.771 | 0.083 | 0.015 | 0.921 | 0.192 | 0.694 | 0.72 | 0.18 | 0.188 | -0.181 |
| logepa | rs55986122 | 0.166 | 0.755 | 0.087 | 0.014 | 0.889 | 0.127 | 0.41 | 0.901 | 0.172 | 0.584 | -0.039 |
| logepa | rs7477350 | 0.701 | 1.04 | 0.213 | 0.845 | 0.887 | 0.091 | 0.244 | 0.771 | 0.102 | 0.05 | -0.216 |
| logepa | rs2099170 | 0.166 | 0.755 | 0.087 | 0.014 | 0.891 | 0.127 | 0.421 | 0.901 | 0.172 | 0.584 | -0.04 |
| logepa | rs2082108 | 0.167 | 0.755 | 0.087 | 0.014 | 0.889 | 0.127 | 0.409 | 0.901 | 0.172 | 0.583 | -0.039 |
| logepa | rs55902404 | 0.007 | . | . | . | . | . | . | . | . | . | . |
| logepa | rs58402662 | 0.006 | . | . | . | . | . | . | . | . | . | . |
| logepa | rs11511618 | 0.14 | 0.797 | 0.098 | 0.065 | 0.916 | 0.14 | 0.568 | 0.876 | 0.177 | 0.514 | -0.073 |
| logepa | rs1816492 | 0.7 | 1.04 | 0.214 | 0.84 | 0.889 | 0.091 | 0.251 | 0.771 | 0.102 | 0.049 | -0.217 |
| logepa | rs146611093 | 0.007 | . | . | . | . | . | . | . | . | . | . |
| logepa | rs186897689 | 0.002 | . | . | . | . | . | . | . | . | . | . |
| logepa | rs10793616 | 0.701 | 1.04 | 0.213 | 0.845 | 0.887 | 0.091 | 0.243 | 0.771 | 0.102 | 0.05 | -0.216 |
| logepa | rs116700479 | 0.019 | 0.764 | 0.083 | 0.013 | 0.881 | 0.354 | 0.752 | 1.25 | 0.799 | 0.722 | 0.199 |
| logepa | rs192535936 | 0.002 | . | . | . | . | . | . | . | . | . | . |
| logepa | rs113363297 | 0.663 | 1.03 | 0.203 | 0.882 | 0.911 | 0.093 | 0.359 | 0.769 | 0.1 | 0.043 | -0.22 |
| logepa | rs12354994 | 0.158 | 0.752 | 0.086 | 0.013 | 0.893 | 0.132 | 0.443 | 0.907 | 0.175 | 0.614 | -0.036 |
| logepa | rs148068329 | 0.02 | 0.727 | 0.075 | 1.90E-03 | 1.14 | 0.488 | 0.768 | 1.15 | 0.84 | 0.849 | 0.086 |
| logepa | rs12356109 | 0.171 | 0.752 | 0.087 | 0.014 | 0.876 | 0.126 | 0.356 | 0.915 | 0.173 | 0.638 | -0.025 |
| logepa | rs55908141 | 0.006 | . | . | . | . | . | . | . | . | . | . |
| logepa | rs12354712 | 0.16 | 0.756 | 0.087 | 0.015 | 0.916 | 0.13 | 0.535 | 0.896 | 0.171 | 0.566 | -0.051 |
| logepa | rs147282684 | 0.003 | . | . | . | . | . | . | . | . | . | . |
| logepa | rs12356140 | 0.168 | 0.757 | 0.087 | 0.016 | 0.891 | 0.127 | 0.419 | 0.894 | 0.17 | 0.554 | -0.045 |
| logepa | rs185476187 | 0.002 | . | . | . | . | . | . | . | . | . | . |
| logepa | rs145720057 | 0.026 | 0.769 | 0.084 | 0.016 | 0.873 | 0.34 | 0.728 | 1.01 | 0.611 | 0.99 | 0.035 |
| logepa | rs12354742 | 0.166 | 0.753 | 0.087 | 0.014 | 0.894 | 0.127 | 0.431 | 0.908 | 0.172 | 0.612 | -0.036 |
| logepa | rs180913842 | 0.006 | . | . | . | . | . | . | . | . | . | . |
| logepa | rs142976914 | 0.003 | . | . | . | . | . | . | . | . | . | . |
| logepa | rs11511619 | 0.174 | 0.754 | 0.088 | 0.016 | 0.871 | 0.125 | 0.337 | 0.909 | 0.171 | 0.611 | -0.028 |
| logepa | rs146702870 | 0.001 | . | . | . | . | . | . | . | . | . | . |
| logepa | rs150287496 | 0.005 | . | . | . | . | . | . | . | . | . | . |
| logepa | rs190917973 | 0.005 | . | . | . | . | . | . | . | . | . | . |
| logepa | rs11517281 | 0.166 | 0.753 | 0.087 | 0.014 | 0.893 | 0.127 | 0.424 | 0.907 | 0.172 | 0.608 | -0.036 |
| logepa | rs12359299 | 0.168 | 0.755 | 0.087 | 0.015 | 0.89 | 0.127 | 0.414 | 0.899 | 0.171 | 0.576 | -0.041 |
| logepa | rs182307932 | 0.024 | 0.752 | 0.082 | 9.30E-03 | 0.958 | 0.309 | 0.894 | 2.12 | 1.15 | 0.169 | 0.814 |
| logepa | rs61855873 | 0.025 | 0.772 | 0.087 | 0.022 | 0.941 | 0.419 | 0.892 | 0.942 | 0.529 | 0.915 | -0.029 |
| logepa | rs12359302 | 0.164 | 0.756 | 0.087 | 0.015 | 0.889 | 0.13 | 0.421 | 0.894 | 0.174 | 0.563 | -0.045 |
| logepa | rs143208140 | 0.021 | 0.726 | 0.075 | 2.00E-03 | 1.11 | 0.475 | 0.806 | 1.14 | 0.811 | 0.85 | 0.086 |
| logepa | rs9988764 | 0.683 | 1.04 | 0.212 | 0.849 | 0.887 | 0.093 | 0.251 | 0.767 | 0.103 | 0.049 | -0.22 |
| logepa | rs192875767 | 0.006 | . | . | . | . | . | . | . | . | . | . |
| logepa | rs147268846 | 0.012 | 0.779 | 0.086 | 0.024 | 0.217 | 0.249 | 0.183 | 0.337 | 0.375 | 0.328 | 0.061 |
| logepa | rs11239485 | 0.105 | 0.741 | 0.085 | 8.60E-03 | 0.991 | 0.183 | 0.96 | 0.934 | 0.238 | 0.787 | -0.046 |
| logepa | rs151317885 | 0.424 | 0.956 | 0.145 | 0.766 | 0.992 | 0.101 | 0.936 | 0.739 | 0.092 | 0.016 | -0.247 |
| logepa | rs11499085 | 0.107 | 0.727 | 0.084 | 5.80E-03 | 0.828 | 0.154 | 0.311 | 1.45 | 0.282 | 0.056 | 0.319 |
| logepa | rs188130606 | 0.028 | 0.778 | 0.086 | 0.023 | 0.389 | 0.193 | 0.057 | 0.584 | 0.35 | 0.369 | 9.90E-03 |
| logepa | rs11511620 | 0.16 | 0.756 | 0.087 | 0.015 | 0.915 | 0.129 | 0.528 | 0.895 | 0.17 | 0.562 | -0.052 |
| logepa | rs181498390 | 0.017 | 0.708 | 0.074 | 9.60E-04 | 1.05 | 0.402 | 0.891 | 2.34 | 1.43 | 0.166 | 0.981 |
| logepa | rs186280701 | 0.007 | . | . | . | . | . | . | . | . | . | . |
| logepa | rs150059185 | 0.028 | 0.779 | 0.082 | 0.017 | 0.831 | 0.427 | 0.719 | 0.288 | 0.194 | 0.065 | -0.424 |
| logepa | rs183338707 | 0.003 | . | . | . | . | . | . | . | . | . | . |
| logepa | rs11499038 | 0.09 | 0.69 | 0.076 | 7.80E-04 | 0.877 | 0.174 | 0.509 | 1.53 | 0.33 | 0.05 | 0.357 |
| logepa | rs190699595 | 0.006 | . | . | . | . | . | . | . | . | . | . |
| logepa | rs11523408 | 0.164 | 0.752 | 0.087 | 0.013 | 0.904 | 0.129 | 0.479 | 0.913 | 0.173 | 0.63 | -0.035 |
| logepa | rs188049317 | 0.002 | . | . | . | . | . | . | . | . | . | . |
| logepa | rs144948634 | 0.395 | 0.932 | 0.143 | 0.646 | 0.99 | 0.105 | 0.921 | 0.748 | 0.096 | 0.023 | -0.232 |
| logepa | rs143833576 | 0.003 | . | . | . | . | . | . | . | . | . | . |
| logepa | rs151239879 | 0.165 | 0.755 | 0.087 | 0.015 | 0.9 | 0.129 | 0.462 | 0.899 | 0.17 | 0.572 | -0.044 |
| logepa | rs7073743 | 0.407 | 0.933 | 0.145 | 0.655 | 0.996 | 0.105 | 0.968 | 0.753 | 0.096 | 0.026 | -0.229 |
| logepa | rs142563349 | 0.03 | 0.781 | 0.086 | 0.025 | 0.445 | 0.199 | 0.07 | 0.572 | 0.321 | 0.32 | -0.027 |
| logepa | rs187246403 | 0.007 | . | . | . | . | . | . | . | . | . | . |
| logepa | rs150423942 | 0.162 | 0.749 | 0.086 | 0.012 | 0.901 | 0.128 | 0.464 | 0.923 | 0.173 | 0.671 | -0.027 |

| logepa | rs184706102 | 0.002 | . | . | . | . | . | . | . | . | . | . |
| --- | --- | --- | --- | --- | --- | --- | --- | --- | --- | --- | --- | --- |
| logepa | rs189626288 | 0.02 | 0.769 | 0.088 | 0.021 | 1.8 | 0.681 | 0.118 | 1.15 | 0.477 | 0.729 | 0.028 |
| logepa | rs140749834 | 0.048 | 0.715 | 0.081 | 2.90E-03 | 1.16 | 0.261 | 0.521 | 2.51 | 0.639 | 3.20E-04 | 1.2 |
| logepa | rs7078822 | 0.133 | 0.735 | 0.085 | 8.00E-03 | 0.958 | 0.149 | 0.783 | 0.979 | 0.199 | 0.916 | -3.80E-03 |
| logepa | rs186227617 | 0.164 | 0.75 | 0.087 | 0.013 | 0.905 | 0.129 | 0.485 | 0.92 | 0.176 | 0.663 | -0.03 |
| logepa | rs188984368 | 0.022 | 0.712 | 0.078 | 1.90E-03 | 1.2 | 0.595 | 0.711 | 1.78 | 1.2 | 0.39 | 0.611 |
| logepa | rs182078109 | 0.031 | 0.757 | 0.088 | 0.016 | 1.03 | 0.487 | 0.948 | 1.32 | 0.873 | 0.68 | 0.238 |
| logepa | rs141456274 | 0.005 | . | . | . | . | . | . | . | . | . | . |
| logepa | rs146976512 | 0.166 | 0.753 | 0.087 | 0.014 | 0.893 | 0.126 | 0.425 | 0.907 | 0.172 | 0.607 | -0.036 |
| logepa | rs138036816 | 0.101 | 0.728 | 0.084 | 6.10E-03 | 0.878 | 0.175 | 0.511 | 1.45 | 0.315 | 0.085 | 0.323 |
| logepa | rs114879777 | 0.019 | 0.763 | 0.083 | 0.013 | 0.897 | 0.367 | 0.79 | 1.31 | 0.851 | 0.677 | 0.237 |
| logepa | rs149366475 | 0.101 | 0.73 | 0.084 | 6.40E-03 | 0.864 | 0.171 | 0.462 | 1.44 | 0.313 | 0.095 | 0.314 |
| logepa | rs182463043 | 0.002 | . | . | . | . | . | . | . | . | . | . |
| logepa | rs144754632 | 0.02 | 0.726 | 0.075 | 1.90E-03 | 1.15 | 0.479 | 0.742 | 1.15 | 0.818 | 0.843 | 0.085 |
| logepa | rs55717607 | 0.166 | 0.753 | 0.087 | 0.014 | 0.892 | 0.126 | 0.419 | 0.907 | 0.171 | 0.603 | -0.036 |
| logepa | rs187537973 | 0 | . | . | . | . | . | . | . | . | . | . |
| logepa | rs148085971 | 0.002 | . | . | . | . | . | . | . | . | . | . |
| logepa | rs11239491 | 0.166 | 0.753 | 0.087 | 0.014 | 0.892 | 0.126 | 0.418 | 0.906 | 0.171 | 0.602 | -0.036 |
| logepa | rs184412184 | 0.005 | . | . | . | . | . | . | . | . | . | . |
| logepa | rs4948998 | 0.716 | 0.985 | 0.203 | 0.943 | 0.898 | 0.094 | 0.303 | 0.807 | 0.107 | 0.106 | -0.169 |
| logepa | rs7076696 | 0.717 | 0.986 | 0.204 | 0.947 | 0.897 | 0.094 | 0.298 | 0.807 | 0.107 | 0.105 | -0.17 |
| logepa | rs11239492 | 0.172 | 0.752 | 0.087 | 0.014 | 0.874 | 0.125 | 0.348 | 0.913 | 0.171 | 0.627 | -0.026 |
| logepa | rs79462470 | 0.007 | . | . | . | . | . | . | . | . | . | . |
| logepa | rs187804654 | 0.003 | . | . | . | . | . | . | . | . | . | . |
| logepa | rs7093687 | 0.161 | 0.757 | 0.087 | 0.015 | 0.913 | 0.128 | 0.519 | 0.894 | 0.169 | 0.554 | -0.052 |
| logepa | rs7081004 | 0.161 | 0.756 | 0.087 | 0.015 | 0.913 | 0.129 | 0.519 | 0.895 | 0.169 | 0.557 | -0.052 |
| logepa | rs7084793 | 0.166 | 0.753 | 0.087 | 0.014 | 0.892 | 0.126 | 0.417 | 0.907 | 0.171 | 0.603 | -0.036 |
| logepa | rs61556080 | 0.007 | . | . | . | . | . | . | . | . | . | . |
| logepa | rs115552064 | 0.007 | . | . | . | . | . | . | . | . | . | . |
| logepa | rs7921503 | 0.166 | 0.756 | 0.087 | 0.015 | 0.886 | 0.125 | 0.392 | 0.898 | 0.169 | 0.565 | -0.041 |
| logepa | rs7909740 | 0.166 | 0.753 | 0.087 | 0.014 | 0.892 | 0.126 | 0.417 | 0.907 | 0.171 | 0.604 | -0.036 |
| logepa | rs7909768 | 0.166 | 0.753 | 0.087 | 0.014 | 0.892 | 0.126 | 0.417 | 0.907 | 0.171 | 0.604 | -0.036 |
| logepa | rs144168004 | 0 | . | . | . | . | . | . | . | . | . | . |
| logepa | rs75850448 | 0.007 | . | . | . | . | . | . | . | . | . | . |
| logepa | rs147777574 | 0.006 | . | . | . | . | . | . | . | . | . | . |
| logepa | rs7913488 | 0.166 | 0.753 | 0.087 | 0.014 | 0.892 | 0.126 | 0.417 | 0.907 | 0.171 | 0.604 | -0.036 |
| logepa | rs74390874 | 0.049 | 0.745 | 0.085 | 9.90E-03 | 1.23 | 0.319 | 0.415 | 0.851 | 0.286 | 0.631 | -0.197 |
| logepa | rs141391142 | 0.006 | . | . | . | . | . | . | . | . | . | . |
| logepa | rs115294747 | 0.007 | . | . | . | . | . | . | . | . | . | . |
| logepa | rs148031781 | 0.002 | . | . | . | . | . | . | . | . | . | . |
| logepa | rs181340128 | 0.018 | 0.777 | 0.087 | 0.024 | 0.642 | 0.506 | 0.574 | 0.691 | 0.636 | 0.688 | -0.074 |
| logepa | rs189930007 | 0.001 | . | . | . | . | . | . | . | . | . | . |
| logepa | rs12242128 | 0.1 | 0.729 | 0.084 | 6.20E-03 | 0.876 | 0.175 | 0.507 | 1.45 | 0.316 | 0.086 | 0.323 |
| logepa | rs78421750 | 0.006 | . | . | . | . | . | . | . | . | . | . |
| logepa | rs114370747 | 0.003 | . | . | . | . | . | . | . | . | . | . |
| logepa | rs12264166 | 0.1 | 0.729 | 0.084 | 6.20E-03 | 0.877 | 0.175 | 0.51 | 1.45 | 0.316 | 0.086 | 0.322 |
| logepa | rs182964822 | 0.001 | . | . | . | . | . | . | . | . | . | . |
| logepa | rs67491417 | 0.159 | 0.75 | 0.085 | 0.012 | 0.87 | 0.129 | 0.349 | 0.914 | 0.182 | 0.651 | -0.024 |
| logepa | rs72789720 | 0.015 | 0.739 | 0.083 | 6.80E-03 | 1.52 | 0.645 | 0.325 | 3.46 | 2.39 | 0.073 | 2.62 |
| logepa | rs6413416 | 0.167 | 0.755 | 0.087 | 0.015 | 0.899 | 0.126 | 0.448 | 0.9 | 0.168 | 0.573 | -0.043 |
| logepa | rs4986832 | 0.167 | 0.753 | 0.087 | 0.014 | 0.891 | 0.125 | 0.414 | 0.908 | 0.171 | 0.607 | -0.035 |
| logepa | rs144866316 | 0.005 | . | . | . | . | . | . | . | . | . | . |
| logepa | rs187769540 | 0.002 | . | . | . | . | . | . | . | . | . | . |
| logepa | rs6593482 | 0.167 | 0.758 | 0.088 | 0.017 | 0.878 | 0.124 | 0.358 | 0.893 | 0.167 | 0.545 | -0.042 |
| logepa | rs7913948 | 0.167 | 0.753 | 0.087 | 0.014 | 0.888 | 0.125 | 0.4 | 0.907 | 0.171 | 0.604 | -0.035 |
| logepa | rs77185151 | 0.019 | 0.726 | 0.075 | 1.90E-03 | 1.14 | 0.479 | 0.749 | 1.15 | 0.823 | 0.842 | 0.087 |
| logepa | rs12762303 | 0.167 | 0.753 | 0.087 | 0.014 | 0.891 | 0.125 | 0.413 | 0.908 | 0.171 | 0.606 | -0.035 |
| logepa | rs73283136 | 0.005 | . | . | . | . | . | . | . | . | . | . |
| logepa | rs187180218 | 0.002 | . | . | . | . | . | . | . | . | . | . |
| logepa | rs78137087 | 0.166 | 0.753 | 0.087 | 0.014 | 0.89 | 0.126 | 0.411 | 0.909 | 0.171 | 0.614 | -0.034 |
| logepa | rs72561472 | 0.165 | 0.758 | 0.088 | 0.017 | 0.861 | 0.126 | 0.304 | 0.887 | 0.172 | 0.538 | -0.04 |
| logepa | rs4987106 | 0.162 | 0.763 | 0.088 | 0.019 | 0.841 | 0.124 | 0.24 | 0.861 | 0.169 | 0.446 | -0.052 |
| logepa | rs4987105 | 0.158 | 0.768 | 0.088 | 0.021 | 0.869 | 0.127 | 0.337 | 0.847 | 0.164 | 0.392 | -0.072 |
| logepa | rs111916157 | 0.182 | 0.767 | 0.092 | 0.028 | 0.922 | 0.125 | 0.55 | 0.873 | 0.154 | 0.44 | -0.072 |
| logepa | rs111528152 | 0.171 | 0.763 | 0.088 | 0.02 | 0.855 | 0.122 | 0.273 | 0.869 | 0.163 | 0.453 | -0.051 |
| logepa | rs12763738 | 0.166 | 0.754 | 0.087 | 0.014 | 0.891 | 0.126 | 0.413 | 0.903 | 0.17 | 0.586 | -0.039 |
| logepa | rs191675886 | 0.007 | . | . | . | . | . | . | . | . | . | . |
| logepa | rs66489870 | 0.167 | 0.753 | 0.087 | 0.014 | 0.891 | 0.125 | 0.413 | 0.908 | 0.171 | 0.606 | -0.035 |
| logepa | rs71494775 | 0.167 | 0.753 | 0.087 | 0.014 | 0.891 | 0.125 | 0.413 | 0.908 | 0.171 | 0.606 | -0.035 |
| logepa | rs61855875 | 0.024 | 0.769 | 0.087 | 0.02 | 0.962 | 0.432 | 0.931 | 1.02 | 0.592 | 0.974 | 0.023 |
| logepa | rs116356837 | 0.007 | . | . | . | . | . | . | . | . | . | . |
| logepa | rs1864414 | 0.166 | 0.754 | 0.087 | 0.014 | 0.895 | 0.127 | 0.436 | 0.904 | 0.172 | 0.597 | -0.039 |
| logepa | rs1864413 | 0.167 | 0.754 | 0.087 | 0.014 | 0.894 | 0.126 | 0.425 | 0.904 | 0.169 | 0.59 | -0.039 |
| logepa | rs139497212 | 0 | . | . | . | . | . | . | . | . | . | . |
| logepa | rs892690 | 0.328 | 0.856 | 0.126 | 0.29 | 1.01 | 0.115 | 0.951 | 0.801 | 0.106 | 0.094 | -0.173 |
| logepa | rs1864412 | 0.167 | 0.753 | 0.087 | 0.014 | 0.892 | 0.125 | 0.415 | 0.908 | 0.171 | 0.608 | -0.035 |
| logepa | rs77827771 | 0.031 | 0.775 | 0.086 | 0.022 | 0.523 | 0.2 | 0.09 | 0.743 | 0.365 | 0.544 | 3.00E-03 |
| logepa | rs145294963 | 0.004 | . | . | . | . | . | . | . | . | . | . |
| logepa | rs149081327 | 0.008 | . | . | . | . | . | . | . | . | . | . |
| logepa | rs67008983 | 0.166 | 0.753 | 0.087 | 0.014 | 0.893 | 0.126 | 0.421 | 0.91 | 0.171 | 0.614 | -0.034 |
| logepa | rs56150414 | 0.17 | 0.752 | 0.088 | 0.014 | 0.889 | 0.124 | 0.397 | 0.915 | 0.171 | 0.632 | -0.03 |
| logepa | rs55787481 | 0.167 | 0.752 | 0.087 | 0.013 | 0.892 | 0.126 | 0.419 | 0.911 | 0.172 | 0.623 | -0.033 |
| logepa | rs141270101 | 0.001 | . | . | . | . | . | . | . | . | . | . |
| logepa | rs114980075 | 0.008 | . | . | . | . | . | . | . | . | . | . |
| logepa | rs12783095 | 0.166 | 0.749 | 0.087 | 0.013 | 0.897 | 0.126 | 0.441 | 0.921 | 0.173 | 0.662 | -0.027 |
| logepa | rs182342688 | 0.003 | . | . | . | . | . | . | . | . | . | . |

| logepa | rs17157728 | 0.166 | 0.749 | 0.087 | 0.013 | 0.897 | 0.126 | 0.441 | 0.921 | 0.173 | 0.663 | -0.027 |
| --- | --- | --- | --- | --- | --- | --- | --- | --- | --- | --- | --- | --- |
| logepa | rs114835563 | 0.028 | 0.782 | 0.086 | 0.026 | 0.396 | 0.192 | 0.056 | 0.507 | 0.303 | 0.256 | -0.021 |
| logepa | rs3824612 | 0.328 | 0.856 | 0.126 | 0.29 | 1.01 | 0.115 | 0.95 | 0.801 | 0.106 | 0.095 | -0.173 |
| logepa | rs17157733 | 0.165 | 0.745 | 0.086 | 0.011 | 0.901 | 0.126 | 0.456 | 0.938 | 0.175 | 0.731 | -0.016 |
| logepa | rs74128478 | 0.006 | . | . | . | . | . | . | . | . | . | . |
| logepa | rs184973589 | 0.001 | . | . | . | . | . | . | . | . | . | . |
| logepa | rs150479461 | 0 | . | . | . | . | . | . | . | . | . | . |
| logepa | rs138310407 | 0.003 | . | . | . | . | . | . | . | . | . | . |
| logepa | rs187426603 | 0.001 | . | . | . | . | . | . | . | . | . | . |
| logepa | rs190843143 | 0.002 | . | . | . | . | . | . | . | . | . | . |
| logepa | rs191883449 | 0.001 | . | . | . | . | . | . | . | . | . | . |
| logepa | rs58238740 | 0.163 | 0.748 | 0.086 | 0.012 | 0.913 | 0.129 | 0.52 | 0.926 | 0.175 | 0.685 | -0.029 |
| logepa | rs3780894 | 0.163 | 0.748 | 0.086 | 0.012 | 0.913 | 0.129 | 0.521 | 0.926 | 0.175 | 0.685 | -0.029 |
| logepa | rs149646626 | 0.018 | 0.799 | 0.088 | 0.042 | 0.27 | 0.168 | 0.035 | 0.195 | 0.146 | 0.029 | -0.027 |
| logepa | rs79615212 | 0.016 | 0.82 | 0.09 | 0.072 | 0.145 | 0.122 | 0.022 | 0.067 | 0.067 | 6.90E-03 | 0.043 |
| logepa | rs3780895 | 0.163 | 0.748 | 0.086 | 0.012 | 0.913 | 0.129 | 0.518 | 0.925 | 0.175 | 0.68 | -0.029 |
| logepa | rs3780896 | 0.163 | 0.748 | 0.086 | 0.012 | 0.913 | 0.129 | 0.521 | 0.926 | 0.175 | 0.686 | -0.029 |
| logepa | rs142747163 | 0.005 | . | . | . | . | . | . | . | . | . | . |
| logepa | rs189417584 | 0.007 | . | . | . | . | . | . | . | . | . | . |
| logepa | rs3780897 | 0.162 | 0.742 | 0.086 | 9.90E-03 | 0.921 | 0.13 | 0.56 | 0.948 | 0.179 | 0.779 | -0.015 |
| logepa | rs117509491 | 0.009 | . | . | . | . | . | . | . | . | . | . |
| logepa | rs117811514 | 0.009 | . | . | . | . | . | . | . | . | . | . |
| logepa | rs116500932 | 0.028 | 0.782 | 0.086 | 0.025 | 0.402 | 0.192 | 0.056 | 0.516 | 0.306 | 0.265 | -0.021 |
| logepa | rs77584168 | 0.004 | . | . | . | . | . | . | . | . | . | . |
| logepa | rs2310776 | 0.163 | 0.748 | 0.086 | 0.012 | 0.914 | 0.129 | 0.522 | 0.926 | 0.175 | 0.687 | -0.028 |
| logepa | rs17157736 | 0.163 | 0.748 | 0.086 | 0.012 | 0.914 | 0.129 | 0.522 | 0.926 | 0.175 | 0.687 | -0.028 |
| logepa | rs72789735 | 0.065 | 0.712 | 0.078 | 1.90E-03 | 0.871 | 0.179 | 0.503 | 1.27 | 0.417 | 0.473 | 0.202 |
| logepa | rs183747187 | 0.002 | . | . | . | . | . | . | . | . | . | . |
| logepa | rs7077891 | 0.163 | 0.748 | 0.086 | 0.012 | 0.914 | 0.129 | 0.522 | 0.927 | 0.175 | 0.687 | -0.028 |
| logepa | rs145063305 | 0.003 | . | . | . | . | . | . | . | . | . | . |
| logepa | rs3824613 | 0.164 | 0.751 | 0.087 | 0.013 | 0.916 | 0.129 | 0.535 | 0.915 | 0.173 | 0.64 | -0.037 |
| logepa | rs145543199 | 0.009 | . | . | . | . | . | . | . | . | . | . |
| logepa | rs140361993 | 0.003 | . | . | . | . | . | . | . | . | . | . |
| logepa | rs11239496 | 0.065 | 0.712 | 0.078 | 1.90E-03 | 0.872 | 0.179 | 0.503 | 1.27 | 0.417 | 0.472 | 0.203 |
| logepa | rs11239497 | 0.158 | 0.751 | 0.087 | 0.013 | 0.936 | 0.132 | 0.639 | 0.914 | 0.174 | 0.639 | -0.044 |
| logepa | rs10900212 | 0.184 | 0.731 | 0.085 | 7.30E-03 | 0.922 | 0.121 | 0.533 | 1 | 0.176 | 0.998 | 0.021 |
| logepa | rs186487136 | 0 | . | . | . | . | . | . | . | . | . | . |
| logepa | rs12774369 | 0.163 | 0.748 | 0.086 | 0.012 | 0.915 | 0.13 | 0.531 | 0.927 | 0.177 | 0.69 | -0.029 |
| logepa | rs60842538 | 0.163 | 0.748 | 0.086 | 0.012 | 0.917 | 0.131 | 0.541 | 0.927 | 0.178 | 0.694 | -0.029 |
| logepa | rs3780898 | 0.157 | 0.751 | 0.087 | 0.013 | 0.94 | 0.135 | 0.667 | 0.916 | 0.178 | 0.651 | -0.045 |
| logepa | rs3780899 | 0.163 | 0.747 | 0.086 | 0.012 | 0.919 | 0.132 | 0.559 | 0.928 | 0.18 | 0.7 | -0.029 |
| logepa | rs12252472 | 0.09 | 0.745 | 0.086 | 0.011 | 0.836 | 0.172 | 0.384 | 1.3 | 0.318 | 0.279 | 0.23 |
| logepa | rs185411011 | 0.002 | . | . | . | . | . | . | . | . | . | . |
| logepa | rs28395865 | 0.155 | 0.747 | 0.087 | 0.012 | 0.947 | 0.138 | 0.71 | 0.929 | 0.184 | 0.708 | -0.037 |
| logepa | rs191004074 | 0.03 | 0.756 | 0.086 | 0.014 | 0.956 | 0.429 | 0.92 | 1.32 | 0.791 | 0.645 | 0.241 |
| logepa | rs182401295 | 0.003 | . | . | . | . | . | . | . | . | . | . |
| logepa | rs2228064 | 0.093 | 0.7 | 0.076 | 1.00E-03 | 0.922 | 0.179 | 0.675 | 1.34 | 0.342 | 0.256 | 0.241 |
| logepa | rs188284159 | 0.001 | . | . | . | . | . | . | . | . | . | . |
| logepa | rs2303887 | 0.013 | 0.726 | 0.075 | 1.90E-03 | 1.54 | 0.698 | 0.339 | 1.26 | 0.749 | 0.703 | 0.138 |
| logepa | rs2303886 | 0.016 | 0.726 | 0.075 | 1.90E-03 | 1.4 | 0.567 | 0.405 | 1.2 | 0.682 | 0.749 | 0.093 |
| logepa | rs192621006 | 0.003 | . | . | . | . | . | . | . | . | . | . |
| logepa | rs2303885 | 0.252 | 0.69 | 0.083 | 2.00E-03 | 0.887 | 0.108 | 0.324 | 1.14 | 0.174 | 0.393 | 0.12 |
| logepa | rs114466168 | 0.028 | 0.782 | 0.086 | 0.026 | 0.394 | 0.193 | 0.057 | 0.503 | 0.304 | 0.255 | -0.021 |
| logepa | rs11239501 | 0.088 | 0.715 | 0.081 | 3.20E-03 | 0.962 | 0.154 | 0.809 | 1.13 | 0.253 | 0.583 | 0.101 |
| logepa | rs745986 | 0.18 | 0.757 | 0.091 | 0.02 | 0.966 | 0.124 | 0.79 | 0.905 | 0.161 | 0.575 | -0.061 |
| logepa | rs75262268 | 0.002 | . | . | . | . | . | . | . | . | . | . |
| logepa | rs71494776 | 0.061 | 0.766 | 0.081 | 0.012 | 0.934 | 0.224 | 0.775 | 0.675 | 0.176 | 0.132 | -0.217 |
| logepa | rs74386871 | 0.02 | 0.724 | 0.075 | 1.90E-03 | 1.24 | 0.565 | 0.638 | 1.29 | 0.833 | 0.69 | 0.196 |
| logepa | rs76502040 | 0.007 | . | . | . | . | . | . | . | . | . | . |
| logepa | rs142213734 | 0.001 | . | . | . | . | . | . | . | . | . | . |
| logepa | rs4948671 | 0.714 | 0.966 | 0.199 | 0.867 | 0.908 | 0.094 | 0.351 | 0.819 | 0.108 | 0.13 | -0.156 |
| logepa | rs146286085 | 0.001 | . | . | . | . | . | . | . | . | . | . |
| logepa | rs144080623 | 0 | . | . | . | . | . | . | . | . | . | . |
| logepa | rs115074014 | 0.044 | 0.725 | 0.081 | 4.20E-03 | 1.18 | 0.282 | 0.491 | 2.28 | 0.618 | 2.40E-03 | 1.04 |
| logepa | rs17157756 | 0.044 | 0.725 | 0.081 | 4.20E-03 | 1.18 | 0.282 | 0.491 | 2.28 | 0.618 | 2.40E-03 | 1.04 |
| logepa | rs190459780 | 0.001 | . | . | . | . | . | . | . | . | . | . |
| logepa | rs12358647 | 0.208 | 0.729 | 0.089 | 9.90E-03 | 0.956 | 0.114 | 0.708 | 1.01 | 0.165 | 0.96 | 0.018 |
| logepa | rs147080556 | 0.013 | 0.732 | 0.078 | 3.40E-03 | 1.27 | 0.572 | 0.593 | 0.916 | 0.386 | 0.835 | -0.151 |
| logepa | rs187543349 | 0.005 | . | . | . | . | . | . | . | . | . | . |
| logepa | rs114375595 | 0.01 | 0.729 | 0.077 | 2.60E-03 | 0.945 | 0.526 | 0.918 | 1.14 | 1.31 | 0.907 | 0.114 |
| logepa | rs117211200 | 0.006 | . | . | . | . | . | . | . | . | . | . |
| logepa | rs192963047 | 0.001 | . | . | . | . | . | . | . | . | . | . |
| logepa | rs11239505 | 0.209 | 0.736 | 0.09 | 0.013 | 0.941 | 0.113 | 0.614 | 0.985 | 0.161 | 0.929 | 5.40E-03 |
| logepa | rs184434116 | 0.002 | . | . | . | . | . | . | . | . | . | . |
| logepa | rs4948999 | 0.716 | 0.966 | 0.2 | 0.868 | 0.902 | 0.094 | 0.321 | 0.819 | 0.108 | 0.129 | -0.154 |
| logepa | rs11239506 | 0.102 | 0.719 | 0.084 | 4.70E-03 | 0.973 | 0.15 | 0.86 | 1.08 | 0.234 | 0.737 | 0.061 |
| logepa | rs190706620 | 0.004 | . | . | . | . | . | . | . | . | . | . |
| logepa | rs190770429 | 0.002 | . | . | . | . | . | . | . | . | . | . |
| logepa | rs181327133 | 0.005 | . | . | . | . | . | . | . | . | . | . |
| logepa | rs149251697 | 0.023 | 0.756 | 0.083 | 0.011 | 1.05 | 0.372 | 0.889 | 1.51 | 0.87 | 0.477 | 0.391 |
| logepa | rs118081602 | 0.013 | 0.726 | 0.075 | 1.90E-03 | 1.54 | 0.696 | 0.34 | 1.26 | 0.75 | 0.701 | 0.14 |
| logepa | rs188233691 | 0.002 | . | . | . | . | . | . | . | . | . | . |
| logepa | rs183778794 | 0.008 | . | . | . | . | . | . | . | . | . | . |
| logepa | rs188040078 | 0.006 | . | . | . | . | . | . | . | . | . | . |
| logepa | rs184675372 | 0.002 | . | . | . | . | . | . | . | . | . | . |
| logepa | rs150326891 | 0.069 | 0.727 | 0.082 | 4.60E-03 | 0.938 | 0.17 | 0.726 | 1.04 | 0.255 | 0.875 | 0.044 |

| logepa | rs185520021 | 0.006 | . | . | . | . | . | . | . | . | . | . |
| --- | --- | --- | --- | --- | --- | --- | --- | --- | --- | --- | --- | --- |
| logepa | rs7907779 | 0.102 | 0.719 | 0.084 | 4.60E-03 | 0.974 | 0.15 | 0.865 | 1.08 | 0.234 | 0.731 | 0.062 |
| logepa | rs184962860 | 0.001 | . | . | . | . | . | . | . | . | . | . |
| logepa | rs149762784 | 0.037 | 0.739 | 0.081 | 5.70E-03 | 0.569 | 0.249 | 0.197 | 0.774 | 0.456 | 0.663 | 0.017 |
| logepa | rs149203427 | 0.013 | 0.775 | 0.087 | 0.023 | 2.17 | 1 | 0.093 | 1.11 | 0.62 | 0.846 | -0.071 |
| logepa | rs60153083 | 0.016 | 0.743 | 0.08 | 6.00E-03 | 0.894 | 0.385 | 0.795 | 3.22 | 2.23 | 0.091 | 1.5 |
| logepa | rs112001069 | 0.076 | 0.764 | 0.081 | 0.011 | 0.918 | 0.201 | 0.696 | 0.725 | 0.189 | 0.216 | -0.174 |
| logepa | rs182355382 | 0.031 | 0.73 | 0.079 | 3.40E-03 | 0.863 | 0.294 | 0.665 | 1.03 | 0.613 | 0.958 | 0.057 |
| logepa | rs186740974 | 0.125 | 0.817 | 0.107 | 0.122 | 0.87 | 0.189 | 0.522 | 0.642 | 0.187 | 0.128 | -0.23 |
| logepa | rs191874916 | 0.036 | 0.788 | 0.083 | 0.024 | 0.669 | 0.301 | 0.372 | 0.289 | 0.181 | 0.047 | -0.305 |
| logepa | rs182989412 | 0.145 | 0.703 | 0.093 | 7.80E-03 | 1.06 | 0.198 | 0.759 | 1.13 | 0.265 | 0.599 | 0.08 |
| logepa | rs188819476 | 0.002 | . | . | . | . | . | . | . | . | . | . |
| logepa | rs141217183 | 0.093 | 0.781 | 0.086 | 0.024 | 0.852 | 0.19 | 0.472 | 0.675 | 0.185 | 0.151 | -0.184 |
| logepa | rs192922652 | 0.077 | 0.77 | 0.082 | 0.014 | 0.898 | 0.2 | 0.629 | 0.682 | 0.18 | 0.146 | -0.197 |
| logepa | rs185092343 | 0.02 | 0.72 | 0.079 | 2.70E-03 | 0.847 | 0.465 | 0.762 | 1.55 | 1.14 | 0.547 | 0.381 |
| logepa | rs145193311 | 0.08 | 0.763 | 0.082 | 0.012 | 0.945 | 0.208 | 0.796 | 0.738 | 0.202 | 0.268 | -0.176 |
| logepa | rs55789336 | 0.104 | 0.714 | 0.084 | 4.10E-03 | 0.97 | 0.152 | 0.846 | 1.11 | 0.245 | 0.632 | 0.086 |
| logepa | rs55837212 | 0.104 | 0.72 | 0.084 | 4.70E-03 | 0.971 | 0.15 | 0.848 | 1.07 | 0.235 | 0.748 | 0.059 |
| logepa | rs75408574 | 0.36 | 0.943 | 0.135 | 0.682 | 0.906 | 0.1 | 0.373 | 0.705 | 0.092 | 7.60E-03 | -0.247 |
| logepa | rs149306926 | 0.11 | 0.702 | 0.083 | 2.70E-03 | 0.991 | 0.151 | 0.952 | 1.19 | 0.253 | 0.422 | 0.132 |
| logepa | rs35990892 | 0.674 | 0.999 | 0.208 | 0.997 | 0.97 | 0.104 | 0.78 | 0.794 | 0.108 | 0.089 | -0.2 |
| logepa | rs192356207 | 0.05 | 0.714 | 0.078 | 2.00E-03 | 0.786 | 0.21 | 0.367 | 1.4 | 0.392 | 0.225 | 0.288 |
| logepa | rs183741163 | 0.102 | 0.739 | 0.088 | 0.011 | 0.957 | 0.176 | 0.809 | 0.949 | 0.245 | 0.839 | -0.025 |
| logepa | rs34737297 | 0.556 | 0.872 | 0.164 | 0.467 | 0.937 | 0.108 | 0.575 | 0.851 | 0.129 | 0.287 | -0.114 |
| logepa | rs181346933 | 0.03 | 0.721 | 0.082 | 4.00E-03 | 0.989 | 0.335 | 0.973 | 3.62 | 1.92 | 0.015 | 1.87 |
| logepa | rs36093253 | 0.361 | 0.828 | 0.122 | 0.2 | 1.03 | 0.12 | 0.815 | 0.851 | 0.119 | 0.247 | -0.132 |
| logepa | rs186307855 | 0.099 | 0.745 | 0.088 | 0.013 | 0.872 | 0.174 | 0.493 | 1.24 | 0.3 | 0.384 | 0.185 |
| logepa | rs189681865 | 0.034 | 0.76 | 0.085 | 0.014 | 0.877 | 0.273 | 0.674 | 1.28 | 0.494 | 0.521 | 0.217 |
| logepa | rs34942971 | 0.214 | 0.751 | 0.095 | 0.023 | 0.958 | 0.118 | 0.729 | 0.94 | 0.157 | 0.713 | -0.032 |
| logepa | rs181816554 | 0.033 | 0.794 | 0.091 | 0.043 | 0.989 | 0.406 | 0.979 | 0.6 | 0.377 | 0.417 | -0.312 |
| logepa | rs186846881 | 0.094 | 0.741 | 0.087 | 0.01 | 0.9 | 0.185 | 0.607 | 1.32 | 0.313 | 0.239 | 0.24 |
| logepa | rs191178220 | 0.016 | 0.738 | 0.079 | 4.40E-03 | 1.05 | 0.647 | 0.936 | 0.684 | 0.555 | 0.639 | -0.259 |
| logepa | rs183305228 | 0.016 | 0.743 | 0.081 | 6.10E-03 | 0.879 | 0.38 | 0.765 | 3.17 | 2.2 | 0.097 | 1.45 |
| logepa | rs139912761 | 0.121 | 0.703 | 0.084 | 3.20E-03 | 0.97 | 0.141 | 0.833 | 1.17 | 0.239 | 0.452 | 0.122 |
| logepa | rs185499901 | 0.018 | 0.786 | 0.088 | 0.032 | 0.426 | 0.296 | 0.22 | 0.405 | 0.348 | 0.293 | -0.077 |
| logepa | rs189816197 | 0.087 | 0.761 | 0.083 | 0.012 | 0.934 | 0.192 | 0.741 | 0.777 | 0.191 | 0.305 | -0.143 |
| logepa | rs145656938 | 0.026 | 0.72 | 0.077 | 2.00E-03 | 0.671 | 0.261 | 0.305 | 1.56 | 0.908 | 0.444 | 0.363 |
| logepa | rs35745197 | 0.076 | 0.763 | 0.081 | 0.011 | 0.93 | 0.203 | 0.741 | 0.734 | 0.191 | 0.234 | -0.172 |
| logepa | rs192856637 | 0.005 | . | . | . | . | . | . | . | . | . | . |
| logepa | rs2310775 | 0.416 | 0.883 | 0.131 | 0.401 | 0.982 | 0.1 | 0.861 | 0.803 | 0.1 | 0.078 | -0.169 |
| logepa | rs11239507 | 0.506 | 0.839 | 0.135 | 0.275 | 0.99 | 0.096 | 0.915 | 0.872 | 0.108 | 0.267 | -0.105 |
| logepa | rs74648467 | 0.062 | 0.774 | 0.082 | 0.016 | 0.817 | 0.198 | 0.404 | 0.601 | 0.149 | 0.041 | -0.211 |
| logepa | rs145815934 | 0.015 | 0.766 | 0.085 | 0.016 | 0.861 | 0.488 | 0.792 | 1.12 | 0.633 | 0.846 | 0.109 |
| logepa | rs145390687 | 0.02 | 0.767 | 0.087 | 0.019 | 1.77 | 0.685 | 0.14 | 1.25 | 0.56 | 0.623 | 0.156 |
| logepa | rs184354913 | 0.034 | 0.834 | 0.093 | 0.104 | 0.201 | 0.108 | 2.90E-03 | 0.135 | 0.089 | 2.30E-03 | -0.012 |
| logepa | rs181244920 | 0.006 | . | . | . | . | . | . | . | . | . | . |
| logepa | rs11499087 | 0.055 | 0.728 | 0.082 | 4.80E-03 | 1.06 | 0.231 | 0.785 | 1.94 | 0.471 | 6.70E-03 | 0.706 |
| logepa | rs148263794 | 0.08 | 0.766 | 0.082 | 0.013 | 0.944 | 0.204 | 0.79 | 0.725 | 0.188 | 0.216 | -0.186 |
| logepa | rs150758903 | 0.036 | 0.754 | 0.086 | 0.013 | 0.692 | 0.224 | 0.255 | 1.48 | 0.626 | 0.357 | 0.325 |
| logepa | rs147654382 | 0.179 | 0.765 | 0.093 | 0.027 | 0.93 | 0.125 | 0.587 | 0.881 | 0.161 | 0.487 | -0.068 |
| logepa | rs147495166 | 0.025 | 0.783 | 0.086 | 0.026 | 0.444 | 0.233 | 0.122 | 0.498 | 0.318 | 0.276 | -0.053 |
| logepa | rs35198778 | 0.106 | 0.758 | 0.086 | 0.015 | 0.762 | 0.15 | 0.167 | 0.851 | 0.226 | 0.543 | -0.028 |
| logepa | rs145207659 | 0.049 | 0.74 | 0.084 | 8.00E-03 | 1.23 | 0.314 | 0.428 | 0.901 | 0.301 | 0.756 | -0.148 |
| logepa | rs186466464 | 0.01 | 0.764 | 0.084 | 0.014 | 1.18 | 0.675 | 0.773 | 1.44 | 0.763 | 0.49 | 0.355 |
| logepa | rs12778413 | 0.162 | 0.746 | 0.09 | 0.015 | 0.896 | 0.14 | 0.482 | 0.943 | 0.197 | 0.778 | -0.012 |
| logepa | rs7895552 | 0.64 | 0.949 | 0.189 | 0.792 | 0.935 | 0.103 | 0.543 | 0.814 | 0.113 | 0.137 | -0.162 |
| logepa | rs12783895 | 0.077 | 0.763 | 0.081 | 0.011 | 0.949 | 0.205 | 0.808 | 0.743 | 0.194 | 0.256 | -0.174 |
| logepa | rs34929604 | 0.079 | 0.76 | 0.081 | 0.01 | 0.951 | 0.205 | 0.815 | 0.763 | 0.203 | 0.308 | -0.16 |
| logepa | rs12761961 | 0.075 | 0.763 | 0.081 | 0.011 | 0.926 | 0.206 | 0.729 | 0.736 | 0.195 | 0.246 | -0.169 |
| logepa | rs35536930 | 0.206 | 0.739 | 0.091 | 0.014 | 0.944 | 0.114 | 0.634 | 0.975 | 0.161 | 0.878 | -2.90E-03 |
| logepa | rs180951060 | 0.002 | . | . | . | . | . | . | . | . | . | . |
| logepa | rs148892128 | 0.015 | 0.766 | 0.085 | 0.016 | 0.86 | 0.488 | 0.791 | 1.11 | 0.633 | 0.848 | 0.108 |
| logepa | rs11239509 | 0.084 | 0.737 | 0.085 | 7.80E-03 | 0.881 | 0.177 | 0.527 | 1.43 | 0.334 | 0.126 | 0.31 |
| logepa | rs41526545 | 0.093 | 0.716 | 0.082 | 3.60E-03 | 0.975 | 0.152 | 0.871 | 1.11 | 0.247 | 0.644 | 0.083 |
| logepa | rs2029253 | 0.421 | 0.895 | 0.134 | 0.46 | 0.975 | 0.099 | 0.802 | 0.791 | 0.098 | 0.06 | -0.18 |
| logepa | rs17444064 | 0.092 | 0.711 | 0.082 | 3.00E-03 | 0.993 | 0.155 | 0.965 | 1.15 | 0.253 | 0.534 | 0.106 |
| logepa | rs71494779 | 0.08 | 0.761 | 0.081 | 0.01 | 0.944 | 0.202 | 0.787 | 0.759 | 0.202 | 0.3 | -0.159 |
| logepa | rs74991420 | 0.012 | 0.725 | 0.075 | 1.80E-03 | 1.81 | 1.01 | 0.291 | 1.42 | 1.11 | 0.656 | 0.326 |
| logepa | rs182736551 | 0.012 | 0.725 | 0.075 | 1.80E-03 | 1.81 | 1.01 | 0.291 | 1.42 | 1.11 | 0.656 | 0.326 |
| logepa | rs78702074 | 0.013 | 0.727 | 0.075 | 1.90E-03 | 1.54 | 0.721 | 0.359 | 1.22 | 0.75 | 0.747 | 0.099 |
| logepa | rs187791466 | 0.004 | . | . | . | . | . | . | . | . | . | . |
| logepa | rs35300270 | 0.082 | 0.761 | 0.081 | 0.011 | 0.932 | 0.199 | 0.74 | 0.758 | 0.202 | 0.297 | -0.155 |
| logepa | rs6593484 | 0.103 | 0.719 | 0.084 | 4.60E-03 | 0.978 | 0.15 | 0.885 | 1.08 | 0.232 | 0.722 | 0.062 |
| logepa | rs12244601 | 0.023 | 0.754 | 0.084 | 0.011 | 0.655 | 0.259 | 0.285 | 2.03 | 1.21 | 0.237 | 0.593 |
| logepa | rs184429574 | 0.005 | . | . | . | . | . | . | . | . | . | . |
| logepa | rs7080897 | 0.08 | 0.761 | 0.081 | 0.01 | 0.944 | 0.202 | 0.787 | 0.759 | 0.202 | 0.299 | -0.159 |
| logepa | rs7097429 | 0.083 | 0.759 | 0.081 | 9.80E-03 | 1.04 | 0.209 | 0.848 | 0.796 | 0.198 | 0.359 | -0.171 |
| logepa | rs77979229 | 0.003 | . | . | . | . | . | . | . | . | . | . |
| logepa | rs11239512 | 0.103 | 0.718 | 0.084 | 4.60E-03 | 0.977 | 0.149 | 0.879 | 1.08 | 0.232 | 0.717 | 0.063 |
| logepa | rs60146827 | 0.08 | 0.761 | 0.081 | 0.01 | 0.943 | 0.202 | 0.783 | 0.758 | 0.201 | 0.297 | -0.16 |
| logepa | rs79032236 | 0.035 | 0.79 | 0.088 | 0.033 | 0.46 | 0.2 | 0.074 | 0.498 | 0.275 | 0.206 | -0.069 |
| logepa | rs140854206 | 0.007 | . | . | . | . | . | . | . | . | . | . |
| logepa | rs184049550 | 0.004 | . | . | . | . | . | . | . | . | . | . |
| logepa | rs114433607 | 0.01 | 0.745 | 0.08 | 6.20E-03 | 0.98 | 0.478 | 0.967 | 5.32 | 5.1 | 0.081 | 3.16 |
| logepa | rs117653793 | 0.013 | 0.727 | 0.075 | 1.90E-03 | 1.55 | 0.708 | 0.336 | 1.24 | 0.746 | 0.716 | 0.124 |
| logepa | rs117039533 | 0.013 | 0.727 | 0.075 | 1.90E-03 | 1.55 | 0.708 | 0.334 | 1.24 | 0.744 | 0.715 | 0.124 |

| logepa | rs191660761 | 0.003 | . | . | . | . | . | . | . | . | . | . |
| --- | --- | --- | --- | --- | --- | --- | --- | --- | --- | --- | --- | --- |
| logepa | rs59514896 | 0.064 | 0.772 | 0.082 | 0.015 | 0.892 | 0.213 | 0.631 | 0.635 | 0.167 | 0.084 | -0.226 |
| logepa | rs184661876 | 0.002 | . | . | . | . | . | . | . | . | . | . |
| logepa | rs61854073 | 0.027 | 0.775 | 0.088 | 0.025 | 1.1 | 0.427 | 0.802 | 0.896 | 0.43 | 0.819 | -0.112 |
| logepa | rs2018518 | 0.299 | 0.827 | 0.116 | 0.178 | 1.04 | 0.122 | 0.725 | 0.832 | 0.109 | 0.16 | -0.152 |
| logepa | rs934187 | 0.718 | 0.953 | 0.2 | 0.819 | 0.928 | 0.097 | 0.476 | 0.829 | 0.109 | 0.154 | -0.148 |
| logepa | rs7918542 | 0.214 | 0.73 | 0.09 | 0.011 | 0.95 | 0.111 | 0.662 | 1 | 0.161 | 0.977 | 0.017 |
| logepa | rs144311920 | 0.002 | . | . | . | . | . | . | . | . | . | . |
| logepa | rs7099684 | 0.841 | 1.24 | 0.369 | 0.475 | 0.861 | 0.105 | 0.218 | 0.726 | 0.123 | 0.06 | -0.325 |
| logepa | rs150829171 | 0.002 | . | . | . | . | . | . | . | . | . | . |
| logepa | rs186060697 | 0.002 | . | . | . | . | . | . | . | . | . | . |
| logepa | rs11239513 | 0.098 | 0.73 | 0.086 | 7.50E-03 | 0.897 | 0.177 | 0.581 | 1.43 | 0.326 | 0.117 | 0.309 |
| logepa | rs7917687 | 0.081 | 0.762 | 0.081 | 0.011 | 0.935 | 0.2 | 0.753 | 0.753 | 0.199 | 0.284 | -0.161 |
| logepa | rs61854076 | 0.01 | . | . | . | . | . | . | . | . | . | . |
| logepa | rs71494780 | 0.018 | 0.788 | 0.086 | 0.029 | 0.798 | 0.39 | 0.644 | 0.493 | 0.263 | 0.185 | -0.276 |
| logepa | rs7919239 | 0.218 | 0.75 | 0.092 | 0.018 | 0.93 | 0.107 | 0.531 | 0.944 | 0.149 | 0.716 | -0.022 |
| logepa | rs78334475 | 0.034 | 0.764 | 0.081 | 0.011 | 0.342 | 0.194 | 0.058 | 0.356 | 0.195 | 0.059 | -0.013 |
| logepa | rs3780900 | 0.026 | 0.769 | 0.081 | 0.012 | 0.865 | 0.438 | 0.774 | 0.329 | 0.232 | 0.115 | -0.415 |
| logepa | rs186729292 | 0.001 | . | . | . | . | . | . | . | . | . | . |
| logepa | rs12267438 | 0.026 | 0.76 | 0.084 | 0.013 | 0.833 | 0.266 | 0.569 | 1.41 | 0.641 | 0.446 | 0.301 |
| logepa | rs1369214 | 0.569 | 0.958 | 0.159 | 0.795 | 0.965 | 0.097 | 0.72 | 0.784 | 0.097 | 0.049 | -0.198 |
| logepa | rs9282589 | 0.041 | 0.781 | 0.086 | 0.025 | 1.02 | 0.405 | 0.963 | 0.86 | 0.362 | 0.719 | -0.116 |
| logepa | rs114522915 | 0.025 | 0.782 | 0.086 | 0.026 | 0.45 | 0.244 | 0.141 | 0.52 | 0.366 | 0.353 | -0.049 |
| logepa | rs2115819 | 0.478 | 0.996 | 0.152 | 0.979 | 0.976 | 0.103 | 0.818 | 0.728 | 0.089 | 9.70E-03 | -0.264 |
| logepa | rs117664603 | 0.013 | 0.726 | 0.075 | 1.90E-03 | 1.55 | 0.689 | 0.325 | 1.28 | 0.748 | 0.677 | 0.16 |
| logepa | rs184979202 | 0.001 | . | . | . | . | . | . | . | . | . | . |
| logepa | rs190714671 | 0.002 | . | . | . | . | . | . | . | . | . | . |
| logepa | rs141538996 | 0 | . | . | . | . | . | . | . | . | . | . |
| logepa | rs150881741 | 0.002 | . | . | . | . | . | . | . | . | . | . |
| logepa | rs139076633 | 0.003 | . | . | . | . | . | . | . | . | . | . |
| logepa | rs12243649 | 0.059 | 0.701 | 0.073 | 7.00E-04 | 0.956 | 0.202 | 0.83 | 1.43 | 0.42 | 0.22 | 0.303 |
| logepa | rs140348708 | 0.001 | . | . | . | . | . | . | . | . | . | . |
| logepa | rs79023256 | 0.013 | 0.726 | 0.075 | 1.80E-03 | 1.55 | 0.686 | 0.319 | 1.28 | 0.748 | 0.667 | 0.169 |
| logepa | rs11239515 | 0.094 | 0.72 | 0.083 | 4.60E-03 | 1 | 0.158 | 0.994 | 1.07 | 0.238 | 0.769 | 0.048 |
| logepa | rs55866086 | 0.158 | 0.686 | 0.08 | 1.30E-03 | 0.979 | 0.127 | 0.868 | 1.21 | 0.219 | 0.281 | 0.151 |
| logepa | rs181340150 | 0.003 | . | . | . | . | . | . | . | . | . | . |
| logepa | rs11239516 | 0.12 | 0.705 | 0.084 | 3.20E-03 | 0.963 | 0.14 | 0.795 | 1.16 | 0.236 | 0.47 | 0.119 |
| logepa | rs11239517 | 0.12 | 0.705 | 0.084 | 3.20E-03 | 0.963 | 0.14 | 0.796 | 1.16 | 0.236 | 0.473 | 0.118 |
| logepa | rs11239519 | 0.106 | 0.706 | 0.083 | 3.00E-03 | 0.959 | 0.153 | 0.793 | 1.17 | 0.26 | 0.471 | 0.129 |
| logepa | rs7909514 | 0.709 | 0.972 | 0.2 | 0.89 | 0.935 | 0.099 | 0.528 | 0.815 | 0.108 | 0.122 | -0.166 |
| logepa | rs7894352 | 0.835 | 1.18 | 0.343 | 0.564 | 0.866 | 0.108 | 0.247 | 0.745 | 0.124 | 0.077 | -0.286 |
| logepa | rs189384146 | 0.002 | . | . | . | . | . | . | . | . | . | . |
| logepa | rs11239520 | 0.105 | 0.73 | 0.085 | 6.50E-03 | 1 | 0.153 | 0.983 | 1 | 0.22 | 0.989 | 1.20E-03 |
| logepa | rs12221233 | 0.406 | 0.862 | 0.127 | 0.316 | 0.996 | 0.102 | 0.972 | 0.823 | 0.101 | 0.113 | -0.151 |
| logepa | rs74523293 | 0.032 | 0.718 | 0.074 | 1.20E-03 | 0.96 | 0.326 | 0.905 | 1.34 | 0.758 | 0.61 | 0.243 |
| logepa | rs79376040 | 0.032 | 0.718 | 0.074 | 1.20E-03 | 0.96 | 0.325 | 0.904 | 1.34 | 0.758 | 0.609 | 0.243 |
| logepa | rs4949000 | 0.406 | 0.863 | 0.127 | 0.318 | 0.996 | 0.102 | 0.973 | 0.822 | 0.101 | 0.112 | -0.152 |
| logepa | rs7089406 | 0.025 | 0.745 | 0.083 | 8.20E-03 | 1.19 | 0.309 | 0.514 | 1.93 | 0.67 | 0.059 | 0.772 |
| logepa | rs138211056 | 0 | . | . | . | . | . | . | . | . | . | . |
| logepa | rs10900213 | 0.537 | 0.9 | 0.163 | 0.559 | 0.982 | 0.099 | 0.858 | 0.832 | 0.108 | 0.155 | -0.147 |
| logepa | rs145152827 | 0.003 | . | . | . | . | . | . | . | . | . | . |
| logepa | rs74443325 | 0.012 | 0.733 | 0.075 | 2.40E-03 | 0.982 | 0.88 | 0.984 | 0.866 | 1.05 | 0.905 | -0.092 |
| logepa | rs188299446 | 0.011 | 0.774 | 0.086 | 0.022 | 1.48 | 0.877 | 0.508 | 0.803 | 0.659 | 0.789 | -0.334 |
| logepa | rs192699586 | 0.003 | . | . | . | . | . | . | . | . | . | . |
| logepa | rs72796464 | 0.086 | 0.712 | 0.081 | 2.90E-03 | 0.989 | 0.158 | 0.946 | 1.15 | 0.262 | 0.546 | 0.107 |
| logepa | rs4949001 | 0.266 | 0.821 | 0.111 | 0.143 | 0.988 | 0.124 | 0.925 | 0.82 | 0.115 | 0.155 | -0.144 |
| logepa | rs147174641 | 0.008 | . | . | . | . | . | . | . | . | . | . |
| logepa | rs190835238 | 0.001 | . | . | . | . | . | . | . | . | . | . |
| logepa | rs76698944 | 0.022 | 0.779 | 0.086 | 0.024 | 0.574 | 0.244 | 0.191 | 0.705 | 0.372 | 0.508 | -0.038 |
| logepa | rs56071381 | 0.11 | 0.728 | 0.086 | 6.90E-03 | 0.98 | 0.153 | 0.896 | 1.02 | 0.226 | 0.931 | 0.019 |
| logepa | rs78130427 | 0.047 | 0.75 | 0.079 | 6.40E-03 | 0.902 | 0.248 | 0.707 | 0.721 | 0.199 | 0.237 | -0.164 |
| logepa | rs10900215 | 0.148 | 0.702 | 0.084 | 3.20E-03 | 1 | 0.132 | 0.987 | 1.14 | 0.222 | 0.515 | 0.095 |
| logepa | rs41491445 | 0.016 | 0.747 | 0.081 | 6.90E-03 | 0.908 | 0.367 | 0.811 | 2.77 | 1.96 | 0.15 | 1.22 |
| logepa | rs144995222 | 0.002 | . | . | . | . | . | . | . | . | . | . |
| logepa | rs149097803 | 0.003 | . | . | . | . | . | . | . | . | . | . |
| logepa | rs139429646 | 0.005 | . | . | . | . | . | . | . | . | . | . |
| logepa | rs7075620 | 0.681 | 0.897 | 0.186 | 0.601 | 0.924 | 0.099 | 0.458 | 0.859 | 0.116 | 0.258 | -0.109 |
| logepa | rs148299213 | 0.017 | 0.749 | 0.081 | 7.70E-03 | 1.02 | 0.396 | 0.966 | 2.19 | 1.53 | 0.26 | 0.905 |
| logepa | rs4949002 | 0.246 | 0.781 | 0.111 | 0.083 | 1.2 | 0.149 | 0.136 | 0.903 | 0.131 | 0.481 | -0.136 |
| logepa | rs150496007 | 0.021 | 0.771 | 0.087 | 0.021 | 0.489 | 0.279 | 0.21 | 1.02 | 0.584 | 0.973 | 0.124 |
| logepa | rs147303455 | 0.012 | 0.768 | 0.085 | 0.018 | 0.649 | 0.378 | 0.458 | 1.23 | 0.56 | 0.648 | 0.196 |
| logepa | rs189518433 | 0.005 | . | . | . | . | . | . | . | . | . | . |
| logepa | rs186281355 | 0.005 | . | . | . | . | . | . | . | . | . | . |
| logepa | rs115297114 | 0.026 | 0.774 | 0.085 | 0.02 | 0.54 | 0.244 | 0.172 | 0.754 | 0.457 | 0.642 | 1.50E-03 |
| logepa | rs11239523 | 0.105 | 0.727 | 0.084 | 5.60E-03 | 0.996 | 0.151 | 0.979 | 1.02 | 0.224 | 0.919 | 0.017 |
| logepa | rs143704897 | 0.005 | . | . | . | . | . | . | . | . | . | . |
| logepa | rs188024096 | 0.003 | . | . | . | . | . | . | . | . | . | . |
| logepa | rs11239524 | 0.107 | 0.727 | 0.086 | 7.20E-03 | 0.977 | 0.161 | 0.887 | 1.02 | 0.235 | 0.925 | 0.022 |
| logepa | rs191656573 | 0.004 | . | . | . | . | . | . | . | . | . | . |
| logepa | rs193295875 | 0.002 | . | . | . | . | . | . | . | . | . | . |
| logepa | rs190458228 | 0.002 | . | . | . | . | . | . | . | . | . | . |
| logepa | rs139253993 | 0.002 | . | . | . | . | . | . | . | . | . | . |
| logepa | rs4948672 | 0.588 | 0.947 | 0.185 | 0.78 | 0.927 | 0.099 | 0.479 | 0.803 | 0.116 | 0.128 | -0.169 |
| logepa | rs180954921 | 0.002 | . | . | . | . | . | . | . | . | . | . |
| logepa | rs12264801 | 0.425 | 0.689 | 0.116 | 0.027 | 1.03 | 0.112 | 0.763 | 1.07 | 0.156 | 0.659 | 0.037 |
| logepa | rs73283184 | 0.02 | 0.744 | 0.08 | 5.80E-03 | 0.731 | 0.289 | 0.428 | 3.61 | 2.99 | 0.122 | 1.49 |

| logepa | rs142746187 | 0.009 | . | . | . | . | . | . | . | . | . | . |
| --- | --- | --- | --- | --- | --- | --- | --- | --- | --- | --- | --- | --- |
| logepa | rs58704739 | 0.019 | 0.741 | 0.079 | 5.20E-03 | 0.58 | 0.267 | 0.236 | 5.52 | 4.76 | 0.048 | 2.05 |
| logepa | rs61272335 | 0.014 | 0.74 | 0.079 | 4.80E-03 | 0.759 | 0.375 | 0.577 | 7.44 | 7.96 | 0.061 | 3.68 |
| logepa | rs60959489 | 0.035 | 0.741 | 0.081 | 6.00E-03 | 1.15 | 0.293 | 0.592 | 1.59 | 0.626 | 0.243 | 0.46 |
| logepa | rs58502629 | 0.014 | 0.74 | 0.079 | 4.80E-03 | 0.76 | 0.375 | 0.577 | 7.45 | 7.97 | 0.06 | 3.69 |
| logepa | rs10900216 | 0.736 | 0.85 | 0.2 | 0.489 | 0.969 | 0.099 | 0.759 | 0.903 | 0.126 | 0.467 | -0.075 |
| logepa | rs181118141 | 0.002 | . | . | . | . | . | . | . | . | . | . |
| logepa | rs137882665 | 0.01 | . | . | . | . | . | . | . | . | . | . |
| logepa | rs149466736 | 0.003 | . | . | . | . | . | . | . | . | . | . |
| logepa | rs2099171 | 0.656 | 0.899 | 0.183 | 0.6 | 0.961 | 0.088 | 0.66 | 0.857 | 0.104 | 0.205 | -0.119 |
| logepa | rs141852280 | 0.006 | . | . | . | . | . | . | . | . | . | . |
| logepa | rs191539257 | 0.002 | . | . | . | . | . | . | . | . | . | . |
| logepa | rs17157771 | 0.034 | 0.747 | 0.079 | 5.70E-03 | 0.93 | 0.288 | 0.814 | 0.647 | 0.305 | 0.357 | -0.227 |
| logepa | rs111339241 | 0.032 | 0.748 | 0.079 | 5.90E-03 | 0.958 | 0.301 | 0.891 | 0.617 | 0.299 | 0.32 | -0.263 |
| logepa | rs116983255 | 0.008 | . | . | . | . | . | . | . | . | . | . |
| logepa | rs142324341 | 0.011 | 0.695 | 0.087 | 3.50E-03 | 0.235 | 0.25 | 0.174 | 0.851 | 1.42 | 0.923 | 0.209 |
| logepa | rs187191219 | 0.01 | . | . | . | . | . | . | . | . | . | . |
| logepa | rs151283973 | 0.002 | . | . | . | . | . | . | . | . | . | . |
| logepa | rs139316218 | 0.003 | . | . | . | . | . | . | . | . | . | . |
| logepa | rs6593485 | 0.732 | 0.858 | 0.197 | 0.504 | 0.958 | 0.097 | 0.675 | 0.896 | 0.126 | 0.433 | -0.079 |
| logepa | rs6593486 | 0.733 | 0.859 | 0.197 | 0.507 | 0.957 | 0.097 | 0.668 | 0.895 | 0.126 | 0.43 | -0.08 |
| logepa | rs145196618 | 0.014 | 0.774 | 0.084 | 0.018 | 0.412 | 0.288 | 0.204 | 0.762 | 0.837 | 0.805 | 0.057 |
| logepa | rs142598335 | 0.004 | . | . | . | . | . | . | . | . | . | . |
| logepa | rs892691 | 0.726 | 0.881 | 0.204 | 0.585 | 0.976 | 0.1 | 0.812 | 0.88 | 0.124 | 0.367 | -0.1 |
| logepa | rs7080713 | 0.671 | 0.825 | 0.178 | 0.373 | 0.956 | 0.095 | 0.649 | 0.914 | 0.122 | 0.499 | -0.06 |
| logepa | rs7077173 | 0.08 | 0.755 | 0.082 | 9.30E-03 | 0.869 | 0.163 | 0.455 | 0.771 | 0.228 | 0.38 | -0.118 |
| logepa | rs7080977 | 0.048 | 0.745 | 0.079 | 5.50E-03 | 0.869 | 0.228 | 0.594 | 0.769 | 0.322 | 0.53 | -0.116 |
| logepa | rs3780901 | 0.671 | 0.824 | 0.175 | 0.362 | 0.974 | 0.097 | 0.788 | 0.916 | 0.12 | 0.501 | -0.063 |
| logepa | rs61854091 | 0.051 | 0.76 | 0.089 | 0.018 | 1.2 | 0.312 | 0.482 | 1.11 | 0.352 | 0.731 | 0.056 |
| logepa | rs77550209 | 0.005 | . | . | . | . | . | . | . | . | . | . |
| logepa | rs73283191 | 0.061 | 0.742 | 0.078 | 4.30E-03 | 0.889 | 0.218 | 0.631 | 0.844 | 0.337 | 0.671 | -0.074 |
| logepa | rs191866099 | 0.007 | . | . | . | . | . | . | . | . | . | . |
| logepa | rs147998395 | 0.002 | . | . | . | . | . | . | . | . | . | . |
| logepa | rs7090328 | 0.784 | 0.768 | 0.193 | 0.292 | 0.988 | 0.11 | 0.916 | 0.969 | 0.144 | 0.83 | -0.021 |
| logepa | rs79814535 | 0.012 | 0.728 | 0.075 | 2.00E-03 | 1.11 | 0.648 | 0.863 | 1.17 | 1.04 | 0.859 | 0.108 |
| logepa | rs7073082 | 0.784 | 0.768 | 0.193 | 0.292 | 0.989 | 0.11 | 0.917 | 0.968 | 0.144 | 0.829 | -0.021 |
| logepa | rs11239526 | 0.036 | 0.715 | 0.078 | 2.10E-03 | 1.5 | 0.362 | 0.094 | 1.33 | 0.493 | 0.445 | 0.209 |
| logepa | rs141839841 | 0.002 | . | . | . | . | . | . | . | . | . | . |
| logepa | rs75697371 | 0.01 | . | . | . | . | . | . | . | . | . | . |
| logepa | rs75972854 | 0.01 | . | . | . | . | . | . | . | . | . | . |
| logepa | rs139291975 | 0.001 | . | . | . | . | . | . | . | . | . | . |
| logepa | rs144064711 | 0.006 | . | . | . | . | . | . | . | . | . | . |
| logepa | rs3780902 | 0.062 | 0.741 | 0.077 | 4.10E-03 | 0.886 | 0.216 | 0.62 | 0.858 | 0.34 | 0.7 | -0.064 |
| logepa | rs190449801 | 0.002 | . | . | . | . | . | . | . | . | . | . |
| logepa | rs73283196 | 0.061 | 0.742 | 0.077 | 4.30E-03 | 0.885 | 0.219 | 0.622 | 0.846 | 0.34 | 0.678 | -0.072 |
| logepa | rs28395867 | 0.044 | 0.756 | 0.083 | 0.011 | 0.904 | 0.219 | 0.676 | 1.38 | 0.591 | 0.457 | 0.281 |
| logepa | rs28395866 | 0.102 | 0.715 | 0.078 | 2.10E-03 | 0.984 | 0.158 | 0.92 | 1.14 | 0.281 | 0.596 | 0.103 |
| logepa | rs55661611 | 0.047 | 0.745 | 0.079 | 5.40E-03 | 0.865 | 0.231 | 0.587 | 0.771 | 0.332 | 0.545 | -0.113 |
| logepa | rs55684518 | 0.02 | 0.78 | 0.086 | 0.024 | 0.89 | 0.427 | 0.807 | 0.706 | 0.454 | 0.588 | -0.18 |
| logepa | rs78552493 | 0.048 | 0.748 | 0.085 | 0.01 | 1.1 | 0.243 | 0.658 | 1.34 | 0.43 | 0.362 | 0.254 |
| logepa | rs147665434 | 0.004 | . | . | . | . | . | . | . | . | . | . |
| logepa | rs34445001 | 0.01 | . | . | . | . | . | . | . | . | . | . |
| logepa | rs184019449 | 0.003 | . | . | . | . | . | . | . | . | . | . |
| logepa | rs3780903 | 0.715 | 0.714 | 0.18 | 0.181 | 1.02 | 0.111 | 0.853 | 1.02 | 0.153 | 0.915 | 6.00E-03 |
| logepa | rs17157784 | 0.061 | 0.704 | 0.074 | 8.10E-04 | 1.12 | 0.207 | 0.536 | 1.37 | 0.41 | 0.295 | 0.255 |
| logepa | rs3780904 | 0.013 | 0.728 | 0.075 | 2.00E-03 | 0.936 | 0.585 | 0.916 | 1.11 | 1.11 | 0.916 | 0.093 |
| logepa | rs192064730 | 0 | . | . | . | . | . | . | . | . | . | . |
| logepa | rs12774534 | 0.01 | . | . | . | . | . | . | . | . | . | . |
| logepa | rs79179915 | 0.011 | 0.728 | 0.075 | 2.00E-03 | 1.17 | 0.656 | 0.776 | 1.18 | 0.97 | 0.841 | 0.107 |
| logepa | rs117811571 | 0.011 | 0.773 | 0.086 | 0.021 | 1.58 | 0.902 | 0.422 | 0.845 | 0.666 | 0.831 | -0.321 |
| logepa | rs72796477 | 0.01 | 0.766 | 0.085 | 0.016 | 1.23 | 0.691 | 0.711 | 1.27 | 0.704 | 0.665 | 0.201 |
| logepa | rs55729342 | 0.065 | 0.697 | 0.074 | 6.40E-04 | 1.11 | 0.209 | 0.586 | 1.45 | 0.419 | 0.201 | 0.313 |
| logepa | rs59317220 | 0.064 | 0.696 | 0.073 | 5.90E-04 | 1.11 | 0.209 | 0.578 | 1.48 | 0.425 | 0.175 | 0.335 |
| logepa | rs3780905 | 0.104 | 0.793 | 0.086 | 0.032 | 0.557 | 0.134 | 0.015 | 0.593 | 0.13 | 0.017 | -0.089 |
| logepa | rs3780906 | 0.714 | 0.73 | 0.184 | 0.211 | 1.02 | 0.111 | 0.886 | 1 | 0.152 | 0.994 | -3.40E-03 |
| logepa | rs149047975 | 0.036 | 0.77 | 0.085 | 0.018 | 1.17 | 0.509 | 0.717 | 1.04 | 0.487 | 0.938 | -5.80E-03 |
| logepa | rs3824614 | 0.011 | 0.728 | 0.075 | 2.00E-03 | 1.2 | 0.656 | 0.743 | 1.21 | 0.955 | 0.814 | 0.125 |
| logepa | rs140254404 | 0.003 | . | . | . | . | . | . | . | . | . | . |
| logepa | rs145262684 | 0.014 | 0.778 | 0.087 | 0.026 | 1.49 | 0.75 | 0.428 | 0.722 | 0.441 | 0.594 | -0.431 |
| logepa | rs76735482 | 0.011 | 0.727 | 0.075 | 1.90E-03 | 1.21 | 0.653 | 0.726 | 1.22 | 0.945 | 0.799 | 0.135 |
| logepa | rs73283198 | 0.034 | 0.748 | 0.079 | 6.00E-03 | 0.911 | 0.293 | 0.772 | 0.615 | 0.309 | 0.334 | -0.24 |
| logepa | rs74911239 | 0.035 | 0.768 | 0.087 | 0.02 | 0.567 | 0.221 | 0.146 | 1.1 | 0.483 | 0.836 | 0.142 |
| logepa | rs150551647 | 0.006 | . | . | . | . | . | . | . | . | . | . |
| logepa | rs115200794 | 0.052 | 0.745 | 0.083 | 8.20E-03 | 0.941 | 0.208 | 0.782 | 1.52 | 0.534 | 0.23 | 0.382 |
| logepa | rs74130823 | 0.168 | 0.686 | 0.085 | 2.40E-03 | 1.01 | 0.132 | 0.917 | 1.21 | 0.233 | 0.313 | 0.144 |
| logepa | rs2279435 | 0.62 | 0.73 | 0.142 | 0.106 | 1.01 | 0.107 | 0.948 | 1 | 0.143 | 1 | -1.80E-03 |
| logepa | rs147279673 | 0.014 | 0.76 | 0.081 | 0.01 | 0.971 | 0.561 | 0.96 | 0.289 | 0.212 | 0.09 | -0.518 |
| logepa | rs183525146 | 0.004 | . | . | . | . | . | . | . | . | . | . |
| logepa | rs3740107 | 0.745 | 0.766 | 0.19 | 0.281 | 0.954 | 0.102 | 0.657 | 0.968 | 0.147 | 0.83 | -0.012 |
| logepa | rs140697247 | 0.001 | . | . | . | . | . | . | . | . | . | . |
| logepa | rs1565096 | 0.81 | 0.581 | 0.16 | 0.049 | 0.978 | 0.118 | 0.856 | 1.15 | 0.19 | 0.391 | 0.095 |
| logepa | rs1565097 | 0.768 | 0.693 | 0.174 | 0.144 | 0.979 | 0.109 | 0.851 | 1.03 | 0.159 | 0.826 | 0.03 |
| logepa | rs144584548 | 0.02 | 0.78 | 0.086 | 0.025 | 0.913 | 0.432 | 0.848 | 0.704 | 0.443 | 0.576 | -0.192 |
| logepa | rs75811053 | 0.011 | 0.727 | 0.075 | 2.00E-03 | 1.31 | 0.668 | 0.602 | 1.23 | 0.888 | 0.776 | 0.134 |
| logepa | rs147462967 | 0.01 | . | . | . | . | . | . | . | . | . | . |
| logepa | rs74130824 | 0.049 | 0.701 | 0.074 | 7.60E-04 | 1.06 | 0.222 | 0.795 | 1.61 | 0.566 | 0.176 | 0.435 |

| logepa | rs116883184 | 0.018 | 0.782 | 0.089 | 0.031 | 1.61 | 0.73 | 0.291 | 0.733 | 0.391 | 0.56 | -0.47 |
| --- | --- | --- | --- | --- | --- | --- | --- | --- | --- | --- | --- | --- |
| logepa | rs117258049 | 0.018 | 0.782 | 0.089 | 0.031 | 1.61 | 0.73 | 0.291 | 0.733 | 0.391 | 0.56 | -0.47 |
| logepa | rs3780908 | 0.128 | 0.717 | 0.086 | 5.60E-03 | 0.992 | 0.135 | 0.954 | 1.08 | 0.212 | 0.703 | 0.058 |
| logepa | rs180938522 | 0.002 | . | . | . | . | . | . | . | . | . | . |
| logepa | rs76687372 | 0.008 | . | . | . | . | . | . | . | . | . | . |
| logepa | rs3780909 | 0.542 | 0.848 | 0.143 | 0.327 | 0.872 | 0.085 | 0.163 | 0.863 | 0.115 | 0.27 | -0.082 |
| logepa | rs78281156 | 0.011 | 0.727 | 0.075 | 2.00E-03 | 1.31 | 0.668 | 0.601 | 1.23 | 0.888 | 0.776 | 0.134 |
| logepa | rs187840631 | 0.002 | . | . | . | . | . | . | . | . | . | . |
| logepa | rs76868242 | 0.011 | 0.727 | 0.075 | 2.00E-03 | 1.31 | 0.667 | 0.6 | 1.23 | 0.888 | 0.774 | 0.135 |
| logepa | rs192794192 | 0.001 | . | . | . | . | . | . | . | . | . | . |
| logepa | rs12781248 | 0.01 | 0.768 | 0.084 | 0.016 | 0.807 | 0.415 | 0.677 | 1.11 | 0.686 | 0.86 | 0.116 |
| logepa | rs12762375 | 0.01 | . | . | . | . | . | . | . | . | . | . |
| logepa | rs192256986 | 0.006 | . | . | . | . | . | . | . | . | . | . |
| logepa | rs184411030 | 0.012 | 0.761 | 0.082 | 0.011 | 0.779 | 0.38 | 0.609 | 2.06 | 1.71 | 0.384 | 0.682 |
| logepa | rs189601483 | 0.011 | 0.728 | 0.075 | 2.00E-03 | 1.33 | 0.687 | 0.577 | 1.19 | 0.857 | 0.809 | 0.094 |
| logepa | rs143254579 | 0.037 | 0.712 | 0.078 | 1.80E-03 | 1.46 | 0.343 | 0.107 | 1.4 | 0.497 | 0.346 | 0.281 |
| logepa | rs12253903 | 0.028 | 0.736 | 0.08 | 4.60E-03 | 1.07 | 0.311 | 0.819 | 2.65 | 1.53 | 0.092 | 1.28 |
| logepa | rs35489926 | 0.009 | . | . | . | . | . | . | . | . | . | . |
| logepa | rs71494783 | 0.048 | 0.707 | 0.074 | 9.60E-04 | 1.11 | 0.265 | 0.654 | 1.4 | 0.487 | 0.338 | 0.279 |
| logepa | rs180711602 | 0.003 | . | . | . | . | . | . | . | . | . | . |
| logepa | rs117312402 | 0.011 | 0.727 | 0.075 | 2.00E-03 | 1.31 | 0.665 | 0.598 | 1.23 | 0.888 | 0.771 | 0.138 |
| logepa | rs74831694 | 0.008 | . | . | . | . | . | . | . | . | . | . |
| logepa | rs140301943 | 0.003 | . | . | . | . | . | . | . | . | . | . |
| logepa | rs78553679 | 0.016 | 0.763 | 0.084 | 0.014 | 1.03 | 0.541 | 0.954 | 1.28 | 0.738 | 0.673 | 0.21 |
| logepa | rs78334250 | 0.011 | 0.727 | 0.075 | 2.00E-03 | 1.31 | 0.665 | 0.598 | 1.23 | 0.888 | 0.771 | 0.138 |
| logepa | rs76000028 | 0.011 | 0.727 | 0.075 | 2.00E-03 | 1.31 | 0.665 | 0.598 | 1.23 | 0.888 | 0.771 | 0.138 |
| logepa | rs145195254 | 0.002 | . | . | . | . | . | . | . | . | . | . |
| logepa | rs137888527 | 0.002 | . | . | . | . | . | . | . | . | . | . |
| logepa | rs149109592 | 0.005 | . | . | . | . | . | . | . | . | . | . |
| logepa | rs80280010 | 0.035 | 0.715 | 0.078 | 2.10E-03 | 1.5 | 0.357 | 0.091 | 1.34 | 0.496 | 0.424 | 0.227 |
| logepa | rs189995374 | 0.002 | . | . | . | . | . | . | . | . | . | . |
| logepa | rs75467770 | 0.012 | 0.728 | 0.075 | 2.00E-03 | 1.16 | 0.682 | 0.802 | 1.18 | 0.977 | 0.845 | 0.105 |
| logepa | rs1487562 | 0.167 | 0.695 | 0.088 | 4.10E-03 | 0.992 | 0.128 | 0.948 | 1.18 | 0.224 | 0.397 | 0.123 |
| logepa | rs12266963 | 0.048 | 0.707 | 0.074 | 9.50E-04 | 1.11 | 0.265 | 0.667 | 1.4 | 0.489 | 0.336 | 0.281 |
| logepa | rs7099874 | 0.765 | 0.72 | 0.179 | 0.187 | 0.858 | 0.093 | 0.157 | 1.01 | 0.159 | 0.96 | 0.045 |
| logepa | rs75687019 | 0.011 | 0.727 | 0.075 | 2.00E-03 | 1.31 | 0.664 | 0.597 | 1.24 | 0.888 | 0.769 | 0.14 |
| logepa | rs148240462 | 0.038 | 0.782 | 0.089 | 0.031 | 0.549 | 0.208 | 0.113 | 0.835 | 0.366 | 0.68 | 0.027 |
| logepa | rs141278174 | 0.002 | . | . | . | . | . | . | . | . | . | . |
| logepa | rs76404852 | 0.011 | 0.727 | 0.075 | 2.00E-03 | 1.31 | 0.663 | 0.597 | 1.24 | 0.888 | 0.768 | 0.141 |
| logepa | rs139112240 | 0.008 | . | . | . | . | . | . | . | . | . | . |
| logepa | rs12783037 | 0.054 | 0.712 | 0.074 | 1.10E-03 | 1.04 | 0.244 | 0.875 | 1.28 | 0.466 | 0.49 | 0.199 |
| logepa | rs72796481 | 0.009 | . | . | . | . | . | . | . | . | . | . |
| logepa | rs76798749 | 0.017 | 0.731 | 0.075 | 2.20E-03 | 0.969 | 0.525 | 0.954 | 0.945 | 0.814 | 0.948 | -0.031 |
| logepa | rs187732548 | 0.001 | . | . | . | . | . | . | . | . | . | . |
| logepa | rs75675474 | 0.017 | 0.731 | 0.075 | 2.20E-03 | 0.969 | 0.525 | 0.954 | 0.945 | 0.814 | 0.948 | -0.031 |
| logepa | rs76552293 | 0.017 | 0.731 | 0.075 | 2.20E-03 | 0.969 | 0.526 | 0.954 | 0.944 | 0.813 | 0.947 | -0.032 |
| logepa | rs147445993 | 0.005 | . | . | . | . | . | . | . | . | . | . |
| logepa | rs115902283 | 0.017 | 0.731 | 0.075 | 2.20E-03 | 0.97 | 0.524 | 0.955 | 0.948 | 0.815 | 0.951 | -0.029 |
| logepa | rs4540799 | 0.763 | 0.717 | 0.179 | 0.183 | 0.857 | 0.092 | 0.151 | 1.01 | 0.16 | 0.949 | 0.047 |
| logepa | rs12765320 | 0.211 | 0.671 | 0.084 | 1.50E-03 | 1.04 | 0.124 | 0.747 | 1.23 | 0.211 | 0.239 | 0.144 |
| logepa | rs12782256 | 0.009 | . | . | . | . | . | . | . | . | . | . |
| logepa | rs191727256 | 0.002 | . | . | . | . | . | . | . | . | . | . |
| logepa | rs2082111 | 0.123 | 0.729 | 0.088 | 8.70E-03 | 1 | 0.139 | 0.976 | 1.01 | 0.202 | 0.966 | 5.10E-03 |
| logepa | rs2082110 | 0.175 | 0.697 | 0.086 | 3.50E-03 | 1.02 | 0.129 | 0.867 | 1.14 | 0.217 | 0.477 | 0.096 |
| logepa | rs2082109 | 0.175 | 0.697 | 0.086 | 3.50E-03 | 1.02 | 0.129 | 0.867 | 1.14 | 0.217 | 0.477 | 0.096 |
| logepa | rs117316662 | 0.011 | 0.727 | 0.075 | 2.00E-03 | 1.31 | 0.661 | 0.593 | 1.24 | 0.886 | 0.764 | 0.144 |
| logepa | rs12247163 | 0.214 | 0.664 | 0.082 | 9.30E-04 | 1.06 | 0.12 | 0.582 | 1.25 | 0.203 | 0.169 | 0.155 |
| logepa | rs12355888 | 0.082 | 0.71 | 0.083 | 3.60E-03 | 1.06 | 0.164 | 0.705 | 1.15 | 0.236 | 0.497 | 0.095 |
| logepa | rs143772290 | 0.011 | 0.765 | 0.083 | 0.013 | 1.39 | 0.686 | 0.509 | 1.35 | 1.41 | 0.772 | 0.282 |
| logepa | rs146849452 | 0.005 | . | . | . | . | . | . | . | . | . | . |
| logepa | rs140267121 | 0.004 | . | . | . | . | . | . | . | . | . | . |
| logepa | rs7080474 | 0.202 | 0.674 | 0.085 | 1.80E-03 | 1.06 | 0.122 | 0.641 | 1.22 | 0.203 | 0.232 | 0.138 |
| logepa | rs144066203 | 0.002 | . | . | . | . | . | . | . | . | . | . |
| logepa | rs12765209 | 0.012 | 0.728 | 0.075 | 2.00E-03 | 1.11 | 0.595 | 0.845 | 1.14 | 0.883 | 0.866 | 0.083 |
| logepa | rs150930735 | 0.002 | . | . | . | . | . | . | . | . | . | . |
| logepa | rs7068039 | 0.216 | 0.667 | 0.083 | 1.10E-03 | 1.06 | 0.12 | 0.618 | 1.24 | 0.2 | 0.193 | 0.147 |
| logepa | rs117622652 | 0.011 | 0.727 | 0.075 | 2.00E-03 | 1.31 | 0.661 | 0.592 | 1.24 | 0.886 | 0.764 | 0.144 |
| logepa | rs190191514 | 0.001 | . | . | . | . | . | . | . | . | . | . |
| logepa | rs74130835 | 0.04 | 0.737 | 0.082 | 6.30E-03 | 1.06 | 0.252 | 0.805 | 1.96 | 0.743 | 0.075 | 0.736 |
| logepa | rs1811604 | 0.039 | 0.747 | 0.083 | 8.60E-03 | 1.14 | 0.269 | 0.584 | 1.63 | 0.64 | 0.217 | 0.497 |
| logepa | rs146971256 | 0 | . | . | . | . | . | . | . | . | . | . |
| logepa | rs74336000 | 0.011 | 0.766 | 0.083 | 0.013 | 1.38 | 0.692 | 0.523 | 1.29 | 1.37 | 0.807 | 0.223 |
| logepa | rs186046492 | 0.007 | . | . | . | . | . | . | . | . | . | . |
| logepa | rs34971823 | 0.011 | 0.767 | 0.084 | 0.015 | 0.729 | 0.347 | 0.506 | 1.2 | 0.565 | 0.695 | 0.176 |
| logepa | rs75227446 | 0.011 | 0.727 | 0.075 | 1.90E-03 | 1.3 | 0.663 | 0.601 | 1.24 | 0.891 | 0.767 | 0.143 |
| logepa | rs190457599 | 0.001 | . | . | . | . | . | . | . | . | . | . |
| logepa | rs79591917 | 0.011 | 0.727 | 0.075 | 1.90E-03 | 1.31 | 0.661 | 0.591 | 1.24 | 0.886 | 0.762 | 0.145 |
| logepa | rs702365 | 0.545 | 0.846 | 0.145 | 0.329 | 0.847 | 0.084 | 0.095 | 0.863 | 0.117 | 0.277 | -0.074 |
| logepa | rs79643836 | 0.011 | 0.727 | 0.075 | 1.90E-03 | 1.23 | 0.652 | 0.702 | 1.24 | 0.938 | 0.776 | 0.152 |
| logepa | rs702366 | 0.546 | 0.845 | 0.144 | 0.324 | 0.843 | 0.084 | 0.084 | 0.864 | 0.117 | 0.28 | -0.072 |
| logepa | rs1059696 | 0.151 | 0.709 | 0.086 | 4.60E-03 | 1.03 | 0.133 | 0.801 | 1.1 | 0.205 | 0.606 | 0.064 |
| logepa | rs901681 | 0.547 | 0.842 | 0.143 | 0.312 | 0.836 | 0.084 | 0.073 | 0.867 | 0.118 | 0.291 | -0.068 |
| logepa | rs61854092 | 0.15 | 0.709 | 0.086 | 4.60E-03 | 1.03 | 0.133 | 0.804 | 1.1 | 0.205 | 0.61 | 0.064 |
| logepa | rs148080753 | 0.001 | . | . | . | . | . | . | . | . | . | . |
| logepa | rs61854093 | 0.186 | 0.677 | 0.085 | 1.90E-03 | 1.05 | 0.126 | 0.709 | 1.23 | 0.216 | 0.237 | 0.148 |
| logepa | rs141822774 | 0.001 | . | . | . | . | . | . | . | . | . | . |

| logepa | rs74130836 | 0.04 | 0.737 | 0.082 | 6.30E-03 | 1.06 | 0.252 | 0.81 | 1.97 | 0.746 | 0.074 | 0.742 |
| --- | --- | --- | --- | --- | --- | --- | --- | --- | --- | --- | --- | --- |
| logepa | rs150620963 | 0.013 | 0.767 | 0.085 | 0.017 | 1.16 | 0.677 | 0.797 | 1.11 | 0.994 | 0.908 | 0.059 |
| logepa | rs3780913 | 0.753 | 0.752 | 0.182 | 0.239 | 0.858 | 0.092 | 0.153 | 0.978 | 0.153 | 0.887 | 0.021 |
| logepa | rs142255723 | 0.002 | . | . | . | . | . | . | . | . | . | . |
| logepa | rs3780914 | 0.542 | 0.854 | 0.147 | 0.359 | 0.847 | 0.084 | 0.096 | 0.854 | 0.116 | 0.246 | -0.083 |
| logepa | rs182656507 | 0.002 | . | . | . | . | . | . | . | . | . | . |
| logepa | rs2291427 | 0.735 | 0.787 | 0.201 | 0.349 | 0.828 | 0.091 | 0.085 | 0.947 | 0.156 | 0.741 | 2.30E-03 |
| logepa | rs10751382 | 0.733 | 0.78 | 0.196 | 0.322 | 0.839 | 0.091 | 0.104 | 0.953 | 0.154 | 0.765 | 4.70E-03 |
| logepa | rs117610108 | 0.01 | 0.727 | 0.075 | 1.90E-03 | 1.31 | 0.66 | 0.585 | 1.24 | 0.883 | 0.759 | 0.147 |
| logepa | rs144966941 | 0.023 | 0.757 | 0.08 | 8.60E-03 | 0.889 | 0.349 | 0.765 | 0.468 | 0.238 | 0.135 | -0.331 |
| logepa | rs41502051 | 0.03 | 0.709 | 0.077 | 1.60E-03 | 1.6 | 0.374 | 0.046 | 1.36 | 0.453 | 0.354 | 0.235 |
| logepa | rs41426046 | 0.011 | 0.727 | 0.075 | 1.90E-03 | 1.34 | 0.653 | 0.546 | 1.24 | 0.858 | 0.752 | 0.144 |
| logepa | rs41519248 | 0.01 | 0.727 | 0.075 | 1.90E-03 | 1.32 | 0.659 | 0.583 | 1.24 | 0.882 | 0.758 | 0.148 |
| logepa | rs189108245 | 0.004 | . | . | . | . | . | . | . | . | . | . |
| logepa | rs7896431 | 0.532 | 0.872 | 0.145 | 0.412 | 0.814 | 0.082 | 0.04 | 0.829 | 0.112 | 0.166 | -0.097 |
| logepa | rs183724547 | 0.003 | . | . | . | . | . | . | . | . | . | . |
| logepa | rs142044990 | 0.008 | . | . | . | . | . | . | . | . | . | . |
| logepa | rs188140929 | 0.007 | . | . | . | . | . | . | . | . | . | . |
| logepa | rs76819096 | 0.014 | 0.755 | 0.08 | 8.20E-03 | 0.593 | 0.324 | 0.338 | 3.57 | 4.45 | 0.307 | 1.25 |
| logepa | rs10751383 | 0.602 | 0.838 | 0.153 | 0.332 | 0.903 | 0.089 | 0.301 | 0.887 | 0.118 | 0.37 | -0.07 |
| logepa | rs117819321 | 0.014 | 0.787 | 0.088 | 0.032 | 0.391 | 0.295 | 0.213 | 0.322 | 0.3 | 0.225 | -0.08 |
| logepa | rs2242332 | 0.702 | 0.825 | 0.188 | 0.399 | 0.856 | 0.088 | 0.13 | 0.912 | 0.135 | 0.535 | -0.037 |
| logepa | rs2242333 | 0.096 | 0.803 | 0.086 | 0.041 | 0.523 | 0.133 | 0.011 | 0.524 | 0.126 | 7.20E-03 | -0.106 |
| logepa | rs2242334 | 0.704 | 0.829 | 0.189 | 0.41 | 0.852 | 0.087 | 0.118 | 0.909 | 0.135 | 0.522 | -0.039 |
| logepa | rs189465041 | 0.001 | . | . | . | . | . | . | . | . | . | . |
| logepa | rs28395868 | 0.025 | 0.766 | 0.086 | 0.018 | 1.2 | 0.379 | 0.559 | 1.11 | 0.419 | 0.779 | 0.055 |
| logepa | rs56067550 | 0.02 | 0.779 | 0.086 | 0.024 | 0.93 | 0.435 | 0.877 | 0.719 | 0.448 | 0.596 | -0.189 |
| logepa | rs1051713 | 0.154 | 0.698 | 0.084 | 2.90E-03 | 1.06 | 0.133 | 0.662 | 1.15 | 0.204 | 0.437 | 0.092 |
| logepa | rs115074210 | 0.016 | 0.754 | 0.08 | 7.90E-03 | 0.482 | 0.264 | 0.182 | 3.66 | 4.68 | 0.311 | 1.09 |
| logepa | rs7900977 | 0.802 | 0.61 | 0.173 | 0.081 | 0.928 | 0.111 | 0.531 | 1.12 | 0.189 | 0.508 | 0.095 |
| logepa | rs147613891 | 0.003 | . | . | . | . | . | . | . | . | . | . |
| logepa | rs2229136 | 0.084 | 0.68 | 0.074 | 4.00E-04 | 1.22 | 0.191 | 0.206 | 1.48 | 0.356 | 0.1 | 0.331 |
| logepa | rs185482841 | 0.003 | . | . | . | . | . | . | . | . | . | . |
| logepa | rs2288619 | 0.083 | 0.68 | 0.074 | 3.80E-04 | 1.24 | 0.194 | 0.16 | 1.5 | 0.363 | 0.092 | 0.347 |
| logepa | rs189891524 | 0.001 | . | . | . | . | . | . | . | . | . | . |
| logepa | rs56145077 | 0.143 | 0.702 | 0.086 | 3.70E-03 | 1.03 | 0.135 | 0.806 | 1.14 | 0.205 | 0.453 | 0.095 |
| logepa | rs143216970 | 0.005 | . | . | . | . | . | . | . | . | . | . |
| logepa | rs142246478 | 0.001 | . | . | . | . | . | . | . | . | . | . |
| logepa | rs13134 | 0.012 | 0.753 | 0.083 | 9.70E-03 | 1.42 | 0.54 | 0.353 | 2.59 | 1.54 | 0.109 | 1.6 |
| logepa | rs41314980 | 0.027 | 0.77 | 0.087 | 0.022 | 1.21 | 0.471 | 0.616 | 1.02 | 0.444 | 0.956 | -0.027 |
| logepa | rs41304611 | 0.013 | 0.76 | 0.084 | 0.013 | 0.451 | 0.377 | 0.34 | 1.65 | 1.99 | 0.675 | 0.356 |
| logepa | rs193299617 | 0.001 | . | . | . | . | . | . | . | . | . | . |
| logepa | rs12772724 | 0.012 | 0.753 | 0.083 | 9.70E-03 | 1.42 | 0.539 | 0.354 | 2.59 | 1.54 | 0.109 | 1.6 |
| logepa | rs139489537 | 0.033 | 0.715 | 0.077 | 2.00E-03 | 1.5 | 0.353 | 0.088 | 1.36 | 0.498 | 0.399 | 0.245 |
| logepa | rs12776062 | 0.009 | . | . | . | . | . | . | . | . | . | . |
| logepa | rs752059 | 0.144 | 0.701 | 0.086 | 3.70E-03 | 1.02 | 0.134 | 0.859 | 1.15 | 0.206 | 0.439 | 0.1 |
| logepa | rs147649982 | 0.03 | 0.751 | 0.08 | 7.40E-03 | 0.368 | 0.191 | 0.054 | 0.478 | 0.237 | 0.136 | 0.013 |
| logepa | rs17157798 | 0.07 | 0.669 | 0.073 | 2.30E-04 | 1.32 | 0.224 | 0.099 | 1.79 | 0.51 | 0.042 | 0.59 |
| logepa | rs142298619 | 0.003 | . | . | . | . | . | . | . | . | . | . |
| logepa | rs4948673 | 0.793 | 0.62 | 0.172 | 0.085 | 0.933 | 0.111 | 0.559 | 1.11 | 0.185 | 0.54 | 0.088 |
| logepa | rs74231484 | 0.102 | 0.805 | 0.087 | 0.044 | 0.572 | 0.133 | 0.016 | 0.542 | 0.123 | 7.00E-03 | -0.127 |
| logepa | rs72796489 | 0.002 | . | . | . | . | . | . | . | . | . | . |
| logepa | rs113447578 | 0.016 | 0.74 | 0.078 | 4.20E-03 | 0.588 | 0.255 | 0.22 | 0.578 | 0.234 | 0.176 | -0.077 |
| logepa | rs34063374 | 0.013 | 0.751 | 0.083 | 9.30E-03 | 1.41 | 0.525 | 0.36 | 2.7 | 1.56 | 0.085 | 1.69 |
| logepa | rs11239527 | 0.013 | 0.769 | 0.086 | 0.019 | 2.08 | 1.03 | 0.142 | 1.05 | 0.583 | 0.933 | -0.172 |
| logepa | rs34371883 | 0.011 | 0.764 | 0.084 | 0.014 | 0.761 | 0.399 | 0.603 | 1.53 | 0.903 | 0.471 | 0.365 |
| logepa | rs118168828 | 0.007 | . | . | . | . | . | . | . | . | . | . |
| logepa | rs34349618 | 0.009 | . | . | . | . | . | . | . | . | . | . |
| logepa | rs80041679 | 0.033 | 0.715 | 0.077 | 1.90E-03 | 1.49 | 0.352 | 0.089 | 1.36 | 0.496 | 0.397 | 0.245 |
| logepa | rs149640607 | 0.003 | . | . | . | . | . | . | . | . | . | . |
| logepa | rs4427523 | 0.888 | 0.555 | 0.222 | 0.141 | 0.68 | 0.117 | 0.024 | 1.17 | 0.27 | 0.506 | 0.205 |
| logepa | rs185880109 | 0.002 | . | . | . | . | . | . | . | . | . | . |
| logepa | rs191523220 | 0.003 | . | . | . | . | . | . | . | . | . | . |
| logepa | rs144315284 | 0.006 | . | . | . | . | . | . | . | . | . | . |
| logepa | rs4948674 | 0.341 | 0.713 | 0.107 | 0.025 | 0.923 | 0.098 | 0.45 | 1.03 | 0.152 | 0.829 | 0.043 |
| logepa | rs182984298 | 0.006 | . | . | . | . | . | . | . | . | . | . |
| logepa | rs56749023 | 0.025 | 0.734 | 0.078 | 3.90E-03 | 0.685 | 0.255 | 0.309 | 4.08 | 2.78 | 0.039 | 1.63 |
| logepa | rs112508957 | 0.015 | 0.766 | 0.083 | 0.014 | 0.81 | 0.382 | 0.656 | 1.28 | 0.997 | 0.75 | 0.219 |
| logepa | rs7089063 | 0.229 | 0.631 | 0.084 | 5.10E-04 | 1.13 | 0.133 | 0.301 | 1.35 | 0.232 | 0.076 | 0.205 |
| logepa | rs145000779 | 0.001 | . | . | . | . | . | . | . | . | . | . |
| logepa | rs80201572 | 0.034 | 0.714 | 0.077 | 1.90E-03 | 1.48 | 0.349 | 0.094 | 1.37 | 0.497 | 0.386 | 0.254 |
| logepa | rs140568227 | 0.005 | . | . | . | . | . | . | . | . | . | . |
| logepa | rs149756228 | 0.01 | . | . | . | . | . | . | . | . | . | . |
| logepa | rs112380685 | 0.007 | . | . | . | . | . | . | . | . | . | . |
| logepa | rs60481109 | 0.023 | 0.734 | 0.078 | 3.80E-03 | 0.71 | 0.268 | 0.364 | 4.14 | 2.8 | 0.036 | 1.71 |
| logepa | rs138221843 | 0.003 | . | . | . | . | . | . | . | . | . | . |
| logepa | rs150283194 | 0.003 | . | . | . | . | . | . | . | . | . | . |
| logepa | rs138879661 | 0.004 | . | . | . | . | . | . | . | . | . | . |
| logepa | rs34960345 | 0.056 | 0.698 | 0.078 | 1.30E-03 | 1.43 | 0.252 | 0.045 | 1.41 | 0.343 | 0.163 | 0.275 |
| logepa | rs147514068 | 0.004 | . | . | . | . | . | . | . | . | . | . |
| logepa | rs35410535 | 0.078 | 0.671 | 0.074 | 2.70E-04 | 1.27 | 0.197 | 0.128 | 1.63 | 0.37 | 0.03 | 0.45 |
| logepa | rs191613635 | 0.003 | . | . | . | . | . | . | . | . | . | . |
| logepa | rs17157800 | 0.007 | . | . | . | . | . | . | . | . | . | . |
| logepa | rs17523413 | 0.045 | 0.699 | 0.077 | 1.10E-03 | 1.49 | 0.296 | 0.047 | 1.56 | 0.477 | 0.143 | 0.438 |
| logepa | rs7897036 | 0.035 | 0.71 | 0.077 | 1.70E-03 | 0.941 | 0.22 | 0.795 | 1.51 | 0.426 | 0.144 | 0.358 |
| logepa | rs76334323 | 0.01 | 0.727 | 0.075 | 1.90E-03 | 1.32 | 0.655 | 0.578 | 1.25 | 0.883 | 0.751 | 0.154 |

| logepa | rs143535991 | 0.001 | . | . | . | . | . | . | . | . | . | . |
| --- | --- | --- | --- | --- | --- | --- | --- | --- | --- | --- | --- | --- |
| logepa | rs140344979 | 0.005 | . | . | . | . | . | . | . | . | . | . |
| logepa | rs36047811 | 0.078 | 0.671 | 0.074 | 2.70E-04 | 1.27 | 0.197 | 0.128 | 1.63 | 0.37 | 0.031 | 0.45 |
| logepa | rs76493570 | 0.054 | 0.694 | 0.078 | 1.10E-03 | 1.47 | 0.266 | 0.034 | 1.48 | 0.376 | 0.12 | 0.349 |
| logepa | rs192998920 | 0.003 | . | . | . | . | . | . | . | . | . | . |
| logepa | rs116840852 | 0.016 | 0.765 | 0.085 | 0.016 | 0.94 | 0.515 | 0.91 | 1.15 | 0.672 | 0.812 | 0.121 |
| logepa | rs184824101 | 0.001 | . | . | . | . | . | . | . | . | . | . |
| logepa | rs41301621 | 0.005 | . | . | . | . | . | . | . | . | . | . |
| logepa | rs3740109 | 0.078 | 0.671 | 0.074 | 2.80E-04 | 1.27 | 0.197 | 0.13 | 1.63 | 0.369 | 0.031 | 0.448 |
| logepa | rs7899415 | 0.033 | 0.749 | 0.08 | 6.70E-03 | 0.59 | 0.304 | 0.306 | 0.584 | 0.465 | 0.499 | -0.081 |
| logepa | rs184395284 | 0.002 | . | . | . | . | . | . | . | . | . | . |
| logepa | rs139209589 | 0.004 | . | . | . | . | . | . | . | . | . | . |
| logepa | rs149924163 | 0.003 | . | . | . | . | . | . | . | . | . | . |
| logepa | rs3740110 | 0.01 | 0.727 | 0.075 | 1.90E-03 | 1.32 | 0.655 | 0.578 | 1.25 | 0.883 | 0.751 | 0.154 |
| logepa | rs144335113 | 0.003 | . | . | . | . | . | . | . | . | . | . |
| logepa | rs74743385 | 0.015 | 0.766 | 0.083 | 0.014 | 0.811 | 0.383 | 0.656 | 1.28 | 0.999 | 0.749 | 0.22 |
| logepa | rs146545829 | 0.003 | . | . | . | . | . | . | . | . | . | . |
| logepa | rs181842689 | 0 | . | . | . | . | . | . | . | . | . | . |
| logepa | rs3740111 | 0.056 | 0.698 | 0.078 | 1.40E-03 | 1.42 | 0.252 | 0.047 | 1.4 | 0.343 | 0.165 | 0.274 |
| logepa | rs186261599 | 0.003 | . | . | . | . | . | . | . | . | . | . |
| logepa | rs73285348 | 0.009 | . | . | . | . | . | . | . | . | . | . |
| logepa | rs186356901 | 0.003 | . | . | . | . | . | . | . | . | . | . |
| logepa | rs3824618 | 0.033 | 0.715 | 0.077 | 1.90E-03 | 1.49 | 0.351 | 0.089 | 1.36 | 0.495 | 0.395 | 0.246 |
| logepa | rs3802548 | 0.219 | 0.731 | 0.094 | 0.014 | 1.07 | 0.123 | 0.555 | 0.998 | 0.153 | 0.989 | -0.02 |
| logepa | rs3802547 | 0.01 | . | . | . | . | . | . | . | . | . | . |
| logepa | rs77677521 | 0.016 | 0.765 | 0.085 | 0.016 | 0.941 | 0.515 | 0.911 | 1.15 | 0.672 | 0.812 | 0.121 |
| logepa | rs3824616 | 0.078 | 0.671 | 0.074 | 2.80E-04 | 1.26 | 0.197 | 0.133 | 1.63 | 0.369 | 0.032 | 0.446 |
| logepa | rs191073818 | 0.003 | . | . | . | . | . | . | . | . | . | . |
| logepa | rs147078004 | 0.002 | . | . | . | . | . | . | . | . | . | . |
| logepa | rs7908745 | 0.31 | 0.648 | 0.091 | 2.10E-03 | 1.17 | 0.127 | 0.155 | 1.2 | 0.182 | 0.235 | 0.091 |
| logepa | rs17157811 | 0.007 | . | . | . | . | . | . | . | . | . | . |
| logepa | rs138506087 | 0.006 | . | . | . | . | . | . | . | . | . | . |
| logepa | rs77241269 | 0.025 | 0.735 | 0.079 | 4.00E-03 | 0.695 | 0.251 | 0.314 | 3.9 | 2.64 | 0.045 | 1.56 |
| logepa | rs138374938 | 0.001 | . | . | . | . | . | . | . | . | . | . |
| logepa | rs2279433 | 0.056 | 0.7 | 0.078 | 1.40E-03 | 1.39 | 0.242 | 0.055 | 1.37 | 0.324 | 0.184 | 0.243 |
| logepa | rs73285358 | 0.025 | 0.736 | 0.079 | 4.00E-03 | 0.695 | 0.251 | 0.313 | 3.89 | 2.63 | 0.045 | 1.56 |
| logepa | rs11239528 | 0.219 | 0.731 | 0.094 | 0.014 | 1.07 | 0.123 | 0.53 | 0.998 | 0.153 | 0.992 | -0.021 |
| logepa | rs77453386 | 0.01 | 0.727 | 0.075 | 1.90E-03 | 1.31 | 0.65 | 0.587 | 1.26 | 0.891 | 0.746 | 0.161 |
| logepa | rs2279434 | 0.061 | 0.706 | 0.08 | 2.00E-03 | 1.34 | 0.234 | 0.099 | 1.25 | 0.296 | 0.337 | 0.142 |
| logepa | rs149236525 | 0.005 | . | . | . | . | . | . | . | . | . | . |
| logepa | rs12573609 | 0.05 | 0.741 | 0.083 | 7.40E-03 | 1.55 | 0.372 | 0.065 | 0.905 | 0.265 | 0.734 | -0.253 |
| logepa | rs191716655 | 0.002 | . | . | . | . | . | . | . | . | . | . |
| logepa | rs17523660 | 0.009 | . | . | . | . | . | . | . | . | . | . |
| logepa | rs142671360 | 0.003 | . | . | . | . | . | . | . | . | . | . |
| logepa | rs3764990 | 0.044 | 0.699 | 0.077 | 1.10E-03 | 1.46 | 0.296 | 0.059 | 1.56 | 0.483 | 0.152 | 0.432 |
| logepa | rs113682861 | 0.004 | . | . | . | . | . | . | . | . | . | . |
| logepa | rs117658270 | 0.01 | 0.727 | 0.075 | 1.90E-03 | 1.31 | 0.65 | 0.587 | 1.26 | 0.891 | 0.746 | 0.161 |
| logepa | rs187309063 | 0 | . | . | . | . | . | . | . | . | . | . |
| logepa | rs73285360 | 0.025 | 0.736 | 0.079 | 4.00E-03 | 0.695 | 0.25 | 0.313 | 3.88 | 2.63 | 0.045 | 1.55 |
| logepa | rs80133289 | 0.015 | 0.753 | 0.078 | 6.50E-03 | 0.201 | 0.217 | 0.137 | 0.184 | 0.15 | 0.038 | 0.074 |
| logepa | rs186693257 | 0.001 | . | . | . | . | . | . | . | . | . | . |
| logepa | rs35735968 | 0.062 | 0.697 | 0.078 | 1.20E-03 | 1.34 | 0.229 | 0.09 | 1.39 | 0.329 | 0.169 | 0.257 |
| logepa | rs17157825 | 0.034 | 0.714 | 0.077 | 1.90E-03 | 1.45 | 0.351 | 0.121 | 1.37 | 0.509 | 0.39 | 0.259 |
| logepa | rs2291428 | 0.213 | 0.723 | 0.093 | 0.011 | 1.11 | 0.128 | 0.374 | 1.02 | 0.159 | 0.886 | -0.012 |
| logepa | rs2291429 | 0.222 | 0.725 | 0.094 | 0.013 | 1.09 | 0.125 | 0.453 | 1.02 | 0.155 | 0.921 | -0.013 |
| logepa | rs145714792 | 0.005 | . | . | . | . | . | . | . | . | . | . |
| logepa | rs12769244 | 0.056 | 0.701 | 0.078 | 1.40E-03 | 1.39 | 0.239 | 0.057 | 1.37 | 0.321 | 0.183 | 0.24 |
| logepa | rs12771275 | 0.056 | 0.701 | 0.078 | 1.40E-03 | 1.39 | 0.239 | 0.057 | 1.37 | 0.321 | 0.183 | 0.24 |
| logepa | rs146266174 | 0.001 | . | . | . | . | . | . | . | . | . | . |
| logepa | rs41301623 | 0.003 | . | . | . | . | . | . | . | . | . | . |
| logepa | rs2306814 | 0.011 | 0.728 | 0.075 | 2.00E-03 | 1.22 | 0.646 | 0.705 | 1.19 | 0.906 | 0.822 | 0.106 |
| logepa | rs12244834 | 0.027 | 0.718 | 0.075 | 1.50E-03 | 1.24 | 0.372 | 0.473 | 1.4 | 0.639 | 0.46 | 0.289 |
| logepa | rs184006011 | 0.002 | . | . | . | . | . | . | . | . | . | . |
| logepa | rs78829066 | 0.01 | 0.727 | 0.075 | 1.90E-03 | 1.31 | 0.652 | 0.587 | 1.26 | 0.895 | 0.744 | 0.164 |
| logepa | rs34731408 | 0.056 | 0.701 | 0.078 | 1.40E-03 | 1.39 | 0.24 | 0.057 | 1.37 | 0.321 | 0.183 | 0.241 |
| logepa | rs17451516 | 0.012 | 0.753 | 0.083 | 0.01 | 1.39 | 0.538 | 0.398 | 2.52 | 1.53 | 0.128 | 1.49 |
| logepa | rs139910133 | 0.003 | . | . | . | . | . | . | . | . | . | . |
| logepa | rs10900218 | 0.233 | 0.628 | 0.084 | 4.80E-04 | 1.16 | 0.133 | 0.203 | 1.35 | 0.227 | 0.069 | 0.199 |
| logepa | rs146023999 | 0.009 | . | . | . | . | . | . | . | . | . | . |
| logepa | rs187296958 | 0.003 | . | . | . | . | . | . | . | . | . | . |
| logepa | rs142342833 | 0.015 | 0.544 | 0.203 | 0.103 | 5.00E-04 | 2.10E-03 | 0.074 | 0.01 | 0.045 | 0.301 | 0.455 |
| logepa | rs143489255 | 0.006 | . | . | . | . | . | . | . | . | . | . |
| logepa | rs138532518 | 0.006 | . | . | . | . | . | . | . | . | . | . |
| logepa | rs183768079 | 0.005 | . | . | . | . | . | . | . | . | . | . |
| logepa | rs117892272 | 0.012 | 0.54 | 0.224 | 0.138 | 10.7 | 10.3 | 0.013 | 2.22 | 2.48 | 0.475 | 2.58 |
| logepa | rs192993943 | 0.003 | . | . | . | . | . | . | . | . | . | . |
| logepa | rs182989043 | 0.004 | . | . | . | . | . | . | . | . | . | . |
| logepa | rs189703435 | 0.007 | . | . | . | . | . | . | . | . | . | . |
| logepa | rs188794040 | 0.007 | . | . | . | . | . | . | . | . | . | . |
| logepa | rs143920953 | 0.003 | . | . | . | . | . | . | . | . | . | . |
| logepa | rs191029121 | 0.006 | . | . | . | . | . | . | . | . | . | . |
| logepa | rs116875448 | 0.006 | . | . | . | . | . | . | . | . | . | . |
| logepa | rs190167877 | 0.002 | . | . | . | . | . | . | . | . | . | . |
| logepa | rs184993418 | 0.003 | . | . | . | . | . | . | . | . | . | . |
| logepa | rs143241158 | 0.01 | . | . | . | . | . | . | . | . | . | . |
| logepa | rs11239500 | 0.038 | 0.682 | 0.113 | 0.021 | 0.89 | 0.297 | 0.727 | 1.8 | 0.784 | 0.18 | 0.518 |

| logepa | rs140568994 | 0.001 | . | . | . | . | . | . | . | . | . | . |
| --- | --- | --- | --- | --- | --- | --- | --- | --- | --- | --- | --- | --- |
| logepa | rs187530317 | 0.003 | . | . | . | . | . | . | . | . | . | . |
| logepa | rs145189326 | 0.002 | . | . | . | . | . | . | . | . | . | . |
| logepa | rs186203332 | 0.009 | . | . | . | . | . | . | . | . | . | . |
| logepa | rs189921909 | 0.004 | . | . | . | . | . | . | . | . | . | . |
| logepa | rs187260989 | 0.01 | 0.498 | 0.192 | 0.07 | 4.38 | 8.02 | 0.419 | 17.2 | 41 | 0.231 | 33.7 |
| logepa | rs114376586 | 0.037 | 0.686 | 0.114 | 0.023 | 0.859 | 0.294 | 0.657 | 1.74 | 0.78 | 0.216 | 0.481 |
| logepa | rs151070808 | 0.019 | 0.537 | 0.202 | 0.099 | 1.29 | 1.08 | 0.765 | 0.316 | 0.536 | 0.497 | -0.605 |
| logepa | rs140927225 | 0.038 | 0.682 | 0.113 | 0.02 | 0.878 | 0.295 | 0.7 | 1.83 | 0.803 | 0.171 | 0.533 |
| logepa | rs191555615 | 0.012 | 0.531 | 0.211 | 0.112 | 6.52 | 6.07 | 0.044 | 2.26 | 2.76 | 0.504 | 1.78 |
| logepa | rs80278898 | 0.012 | 0.721 | 0.118 | 0.045 | 0.255 | 0.528 | 0.509 | 0.461 | 0.804 | 0.657 | 0.108 |
| logepa | rs190849503 | 0.004 | . | . | . | . | . | . | . | . | . | . |
| logepa | rs150080315 | 0.006 | . | . | . | . | . | . | . | . | . | . |
| logepa | rs143623885 | 0.011 | 0.561 | 0.205 | 0.114 | 1.60E-12 | 2.80E-11 | 0.12 | 7.10E-12 | 1.10E-10 | 0.108 | 0.439 |
| logepa | rs187760040 | 0.002 | . | . | . | . | . | . | . | . | . | . |
| logepa | rs143917143 | 0.01 | 0.551 | 0.118 | 5.40E-03 | 0.911 | 0.918 | 0.926 | 2.27 | 4.43 | 0.675 | 0.678 |
| logepa | rs149851136 | 0.001 | . | . | . | . | . | . | . | . | . | . |
| logepa | rs181612054 | 0.001 | . | . | . | . | . | . | . | . | . | . |
| logepa | rs191006548 | 0.001 | . | . | . | . | . | . | . | . | . | . |
| logepa | rs117377631 | 0.012 | 0.72 | 0.114 | 0.038 | 0.052 | 0.083 | 0.063 | 0.17 | 0.21 | 0.151 | 0.234 |
| logepa | rs182107634 | 0.002 | . | . | . | . | . | . | . | . | . | . |
| logepa | rs190211867 | 0.002 | . | . | . | . | . | . | . | . | . | . |
| logepa | rs148293457 | 0.031 | 0.561 | 0.224 | 0.148 | 0.788 | 0.769 | 0.807 | 0.335 | 0.424 | 0.388 | -0.201 |
| logepa | rs191811733 | 0.006 | . | . | . | . | . | . | . | . | . | . |
| logepa | rs146109272 | 0.008 | . | . | . | . | . | . | . | . | . | . |
| logepa | rs148397915 | 0.006 | . | . | . | . | . | . | . | . | . | . |
| logepa | rs142233290 | 0.007 | . | . | . | . | . | . | . | . | . | . |
| logepa | rs80226874 | 0.022 | 0.526 | 0.195 | 0.083 | 0.026 | 0.076 | 0.212 | 0.967 | 3.12 | 0.992 | 0.461 |
| logepa | rs144496203 | 0.004 | . | . | . | . | . | . | . | . | . | . |
| logepa | rs191062130 | 0.001 | . | . | . | . | . | . | . | . | . | . |
| logepa | rs185807817 | 0.004 | . | . | . | . | . | . | . | . | . | . |
| logepa | rs117846213 | 0.012 | 0.874 | 0.168 | 0.481 | 0.153 | 0.296 | 0.331 | 0.025 | 0.046 | 0.043 | -0.023 |
| logepa | rs190545473 | 0.002 | . | . | . | . | . | . | . | . | . | . |
| logepa | rs192364918 | 0.01 | 0.503 | 0.196 | 0.078 | 7.99 | 11 | 0.133 | 3.53 | 11.8 | 0.706 | 6.71 |
| logepa | rs182939241 | 0.003 | . | . | . | . | . | . | . | . | . | . |
| logepa | rs12571125 | 0.011 | 0.818 | 0.16 | 0.303 | 0.423 | 0.792 | 0.646 | 1.11 | 2.18 | 0.957 | 0.143 |
| logepa | rs192418941 | 0.002 | . | . | . | . | . | . | . | . | . | . |
| logepa | rs141270243 | 0.015 | 0.545 | 0.214 | 0.122 | 2.87 | 2.72 | 0.266 | 0.286 | 0.884 | 0.686 | -1.97 |
| logepa | rs182615502 | 0.006 | . | . | . | . | . | . | . | . | . | . |
| logepa | rs146669856 | 0.01 | 0.704 | 0.113 | 0.028 | 0.489 | 0.311 | 0.261 | 2.09 | 1.83 | 0.4 | 0.527 |
| logepa | rs78290029 | 0.053 | 0.672 | 0.11 | 0.015 | 1.2 | 0.384 | 0.56 | 1.71 | 0.727 | 0.206 | 0.508 |
| logepa | rs139955565 | 0.004 | . | . | . | . | . | . | . | . | . | . |
| logepa | rs12267314 | 0.047 | 0.679 | 0.111 | 0.018 | 1.32 | 0.419 | 0.384 | 1.6 | 0.673 | 0.264 | 0.435 |
| logepa | rs74477197 | 0.004 | . | . | . | . | . | . | . | . | . | . |
| logepa | rs188147236 | 0.005 | . | . | . | . | . | . | . | . | . | . |
| logepa | rs2228065 | 0.046 | 0.681 | 0.111 | 0.019 | 1.31 | 0.419 | 0.392 | 1.57 | 0.675 | 0.294 | 0.41 |
| logepa | rs116961353 | 0.01 | . | . | . | . | . | . | . | . | . | . |
| logepa | rs190222272 | 0.007 | . | . | . | . | . | . | . | . | . | . |
| logepa | rs182719940 | 0.006 | . | . | . | . | . | . | . | . | . | . |
| logepa | rs184477413 | 0.004 | . | . | . | . | . | . | . | . | . | . |
| logepa | rs12265175 | 0.048 | 0.682 | 0.112 | 0.019 | 1.19 | 0.383 | 0.598 | 1.52 | 0.65 | 0.331 | 0.358 |
| logepa | rs182928276 | 0.003 | . | . | . | . | . | . | . | . | . | . |
| logepa | rs144409895 | 0.011 | 0.533 | 0.207 | 0.106 | 3.69 | 5.42 | 0.374 | 0.387 | 1.22 | 0.764 | -2.46 |
| logepa | rs58998447 | 0.031 | 0.726 | 0.116 | 0.046 | 0.578 | 0.343 | 0.355 | 0.635 | 0.32 | 0.367 | -0.038 |
| logepa | rs188668832 | 0.005 | . | . | . | . | . | . | . | . | . | . |
| logepa | rs148641686 | 0.002 | . | . | . | . | . | . | . | . | . | . |
| logepa | rs75468791 | 0.054 | 0.514 | 0.202 | 0.091 | 0.503 | 0.539 | 0.521 | 2.17 | 1.59 | 0.292 | 0.544 |
| logepa | rs147327397 | 0.003 | . | . | . | . | . | . | . | . | . | . |
| logepa | rs77354597 | 0.007 | . | . | . | . | . | . | . | . | . | . |
| logepa | rs41365544 | 0.045 | 0.684 | 0.112 | 0.02 | 1.31 | 0.411 | 0.382 | 1.52 | 0.705 | 0.367 | 0.368 |
| logepa | rs187564188 | 0.016 | 0.51 | 0.214 | 0.109 | 1.72 | 3.17 | 0.77 | 1.52 | 2.49 | 0.8 | 0.101 |
| logepa | rs183511984 | 0.002 | . | . | . | . | . | . | . | . | . | . |
| logepa | rs12262783 | 0.038 | 0.668 | 0.109 | 0.013 | 1.34 | 0.434 | 0.368 | 2.14 | 1.02 | 0.111 | 0.904 |
| logepa | rs188817687 | 0.002 | . | . | . | . | . | . | . | . | . | . |
| logepa | rs3815968 | 0.005 | . | . | . | . | . | . | . | . | . | . |
| logepa | rs187822300 | 0.001 | . | . | . | . | . | . | . | . | . | . |
| logepa | rs188902172 | 0.008 | . | . | . | . | . | . | . | . | . | . |
| logepa | rs181190448 | 0.006 | . | . | . | . | . | . | . | . | . | . |
| logepa | rs182699093 | 0.001 | . | . | . | . | . | . | . | . | . | . |
| logepa | rs182631303 | 0.005 | . | . | . | . | . | . | . | . | . | . |
| logepa | rs187014172 | 0.003 | . | . | . | . | . | . | . | . | . | . |
| logepa | rs142575406 | 0.009 | . | . | . | . | . | . | . | . | . | . |
| logepa | rs180711033 | 0.007 | . | . | . | . | . | . | . | . | . | . |
| logepa | rs137894823 | 0.002 | . | . | . | . | . | . | . | . | . | . |
| logepa | rs189053273 | 0.001 | . | . | . | . | . | . | . | . | . | . |
| logepa | rs143499388 | 0.002 | . | . | . | . | . | . | . | . | . | . |
| logepa | rs182936001 | 0.003 | . | . | . | . | . | . | . | . | . | . |
| logepa | rs148456406 | 0.003 | . | . | . | . | . | . | . | . | . | . |
| logepa | rs182499531 | 0.005 | . | . | . | . | . | . | . | . | . | . |
| logepa | rs74884915 | 0.016 | 0.759 | 0.138 | 0.129 | 0.191 | 0.24 | 0.187 | 2.86 | 1.03 | 3.60E-03 | 0.464 |
| logepa | rs184046374 | 0.001 | . | . | . | . | . | . | . | . | . | . |
| logepa | rs74333993 | 0.019 | 0.73 | 0.132 | 0.083 | 1.43 | 0.654 | 0.434 | 2.19 | 1.18 | 0.148 | 1.12 |
| logepa | rs138889719 | 0.001 | . | . | . | . | . | . | . | . | . | . |
| logepa | rs75330330 | 0.013 | 0.766 | 0.137 | 0.138 | 0.724 | 0.505 | 0.643 | 1.03 | 1.4 | 0.981 | 0.083 |
| logepa | rs193051693 | 0 | . | . | . | . | . | . | . | . | . | . |
| logepa | rs145929633 | 0 | . | . | . | . | . | . | . | . | . | . |

| logepa | rs184076904 | 0.003 | . | . | . | . | . | . | . | . | . | . |
| --- | --- | --- | --- | --- | --- | --- | --- | --- | --- | --- | --- | --- |
| logepa | rs141802494 | 0.011 | 0.768 | 0.136 | 0.136 | 0.367 | 0.525 | 0.484 | 0.781 | 2.22 | 0.931 | 0.086 |
| logepa | rs149519759 | 0.001 | . | . | . | . | . | . | . | . | . | . |
| logepa | rs145467260 | 0.008 | . | . | . | . | . | . | . | . | . | . |
| logepa | rs79620914 | 0.017 | 0.733 | 0.134 | 0.088 | 1.36 | 0.579 | 0.466 | 2.73 | 0.984 | 5.20E-03 | 1.63 |
| logepa | rs140538633 | 0.011 | 0.762 | 0.137 | 0.131 | 0.287 | 0.35 | 0.306 | 2.6 | 1.04 | 0.017 | 0.52 |
| logepa | rs140862301 | 0.005 | . | . | . | . | . | . | . | . | . | . |
| logepa | rs114615847 | 0.006 | . | . | . | . | . | . | . | . | . | . |
| logepa | rs141800259 | 0.016 | 0.766 | 0.138 | 0.14 | 0.629 | 0.477 | 0.54 | 1.1 | 1.57 | 0.949 | 0.133 |
| logepa | rs149779059 | 0.011 | 0.751 | 0.134 | 0.108 | 2.06 | 1.23 | 0.225 | 1.71 | 2.08 | 0.661 | 0.828 |
| logepa | rs116539253 | 0.004 | . | . | . | . | . | . | . | . | . | . |
| logepa | rs184979550 | 0.001 | . | . | . | . | . | . | . | . | . | . |
| logepa | rs190128940 | 0.003 | . | . | . | . | . | . | . | . | . | . |
| logepa | rs146594816 | 0.002 | . | . | . | . | . | . | . | . | . | . |
| logepa | rs141370766 | 0.013 | 0.766 | 0.137 | 0.137 | 0.739 | 0.52 | 0.667 | 1.04 | 1.42 | 0.979 | 0.082 |
| logepa | rs144670033 | 0.002 | . | . | . | . | . | . | . | . | . | . |
| logepa | rs187152174 | 0.005 | . | . | . | . | . | . | . | . | . | . |
| logepa | rs184180263 | 0.003 | . | . | . | . | . | . | . | . | . | . |
| logepa | rs140075861 | 0.007 | . | . | . | . | . | . | . | . | . | . |
| logepa | rs186483129 | 0 | . | . | . | . | . | . | . | . | . | . |
| logepa | rs186634282 | 0.008 | . | . | . | . | . | . | . | . | . | . |
| logepa | rs9988663 | 0.019 | 0.73 | 0.132 | 0.083 | 1.43 | 0.653 | 0.438 | 2.2 | 1.19 | 0.146 | 1.13 |
| logepa | rs138828529 | 0.013 | 0.786 | 0.143 | 0.185 | 2.01 | 1.33 | 0.29 | 0.391 | 0.39 | 0.347 | -1.18 |
| logepa | rs188777615 | 0.003 | . | . | . | . | . | . | . | . | . | . |
| logepa | rs192321882 | 0.005 | . | . | . | . | . | . | . | . | . | . |
| logepa | rs150812270 | 0.016 | 0.757 | 0.139 | 0.128 | 1.23 | 0.607 | 0.681 | 1.27 | 0.6 | 0.614 | 0.195 |
| logepa | rs185906603 | 0.001 | . | . | . | . | . | . | . | . | . | . |
| logepa | rs191070862 | 0.004 | . | . | . | . | . | . | . | . | . | . |
| logepa | rs182904978 | 0 | . | . | . | . | . | . | . | . | . | . |
| logepa | rs192679655 | 0.001 | . | . | . | . | . | . | . | . | . | . |
| logepa | rs189673086 | 0.005 | . | . | . | . | . | . | . | . | . | . |
| logepa | rs181141892 | 0.002 | . | . | . | . | . | . | . | . | . | . |
| logepa | rs186404123 | 0 | . | . | . | . | . | . | . | . | . | . |
| logepa | rs192117287 | 0.003 | . | . | . | . | . | . | . | . | . | . |
| logepa | rs182748835 | 0.001 | . | . | . | . | . | . | . | . | . | . |
| logepa | rs141854549 | 0.011 | 0.762 | 0.137 | 0.132 | 0.291 | 0.35 | 0.305 | 2.56 | 1 | 0.016 | 0.514 |
| logepa | rs140163397 | 0.003 | . | . | . | . | . | . | . | . | . | . |
| logepa | rs146347300 | 0.005 | . | . | . | . | . | . | . | . | . | . |
| logepa | rs139362377 | 0.011 | 0.762 | 0.137 | 0.132 | 0.291 | 0.35 | 0.305 | 2.55 | 0.999 | 0.016 | 0.513 |
| logepa | rs185087671 | 0.001 | . | . | . | . | . | . | . | . | . | . |
| logepa | rs190466574 | 0.003 | . | . | . | . | . | . | . | . | . | . |
| logepa | rs147012778 | 0.003 | . | . | . | . | . | . | . | . | . | . |
| logepa | rs138236960 | 0.006 | . | . | . | . | . | . | . | . | . | . |
| logepa | rs116310311 | 0.011 | 0.762 | 0.137 | 0.132 | 0.29 | 0.35 | 0.305 | 2.55 | 0.999 | 0.016 | 0.513 |
| logepa | rs142868356 | 0.008 | . | . | . | . | . | . | . | . | . | . |
| logepa | rs144956240 | 0.002 | . | . | . | . | . | . | . | . | . | . |
| logepa | rs188527776 | 0.002 | . | . | . | . | . | . | . | . | . | . |
| logepa | rs183971165 | 0.005 | . | . | . | . | . | . | . | . | . | . |
| logepa | rs76748216 | 0.006 | . | . | . | . | . | . | . | . | . | . |
| logepa | rs148554990 | 0.001 | . | . | . | . | . | . | . | . | . | . |
| logepa | rs114446477 | 0.017 | 0.734 | 0.134 | 0.089 | 1.31 | 0.561 | 0.526 | 2.72 | 0.996 | 6.30E-03 | 1.57 |
| logepa | rs185223933 | 0.004 | . | . | . | . | . | . | . | . | . | . |
| logepa | rs76869726 | 0.006 | . | . | . | . | . | . | . | . | . | . |
| logepa | rs151200830 | 0.011 | 0.764 | 0.138 | 0.135 | 0.264 | 0.342 | 0.303 | 2.55 | 1.01 | 0.018 | 0.486 |
| logepa | rs17157731 | 0.011 | 0.764 | 0.138 | 0.135 | 0.264 | 0.342 | 0.303 | 2.54 | 1 | 0.018 | 0.485 |
| logepa | rs181243469 | 0 | . | . | . | . | . | . | . | . | . | . |
| logepa | rs186890707 | 0 | . | . | . | . | . | . | . | . | . | . |
| logepa | rs184063360 | 0 | . | . | . | . | . | . | . | . | . | . |
| logepa | rs188550237 | 0.004 | . | . | . | . | . | . | . | . | . | . |
| logepa | rs140931068 | 0.01 | 0.746 | 0.134 | 0.103 | 3.34 | 2.14 | 0.059 | 2.54 | 3.44 | 0.492 | 3.24 |
| logepa | rs74867877 | 0.004 | . | . | . | . | . | . | . | . | . | . |
| logepa | rs11239495 | 0.036 | 0.712 | 0.132 | 0.068 | 1.31 | 0.457 | 0.434 | 2.16 | 1.07 | 0.122 | 0.992 |
| logepa | rs149745800 | 0 | . | . | . | . | . | . | . | . | . | . |
| logepa | rs145044458 | 0.01 | 0.764 | 0.138 | 0.136 | 0.279 | 0.362 | 0.325 | 2.43 | 0.943 | 0.022 | 0.475 |
| logepa | rs188067427 | 0.001 | . | . | . | . | . | . | . | . | . | . |
| logepa | rs146827800 | 0.001 | . | . | . | . | . | . | . | . | . | . |
| logepa | rs140619817 | 0.01 | 0.766 | 0.139 | 0.141 | 0.264 | 0.358 | 0.326 | 2.22 | 0.852 | 0.039 | 0.418 |
| logepa | rs892689 | 0.997 | . | . | . | . | . | . | . | . | . | . |
| logepa | rs143642005 | 0.004 | . | . | . | . | . | . | . | . | . | . |
| logepa | rs181929304 | 0.004 | . | . | . | . | . | . | . | . | . | . |
| logepa | rs191086173 | 0.001 | . | . | . | . | . | . | . | . | . | . |
| logepa | rs114613116 | 0.012 | 0.769 | 0.14 | 0.149 | 0.234 | 0.325 | 0.295 | 2.02 | 0.867 | 0.101 | 0.36 |
| logepa | rs141076768 | 0.002 | . | . | . | . | . | . | . | . | . | . |
| logepa | rs187316531 | 0 | . | . | . | . | . | . | . | . | . | . |
| logepa | rs11239499 | 0.046 | 0.741 | 0.14 | 0.112 | 0.872 | 0.3 | 0.691 | 1.46 | 0.689 | 0.427 | 0.328 |
| logepa | rs192726371 | 0.002 | . | . | . | . | . | . | . | . | . | . |
| logepa | rs74128481 | 0.012 | 0.755 | 0.137 | 0.121 | 0.45 | 0.352 | 0.307 | 1.75 | 0.735 | 0.185 | 0.389 |
| logepa | rs74128482 | 0.008 | . | . | . | . | . | . | . | . | . | . |
| logepa | rs11239502 | 0.033 | 0.73 | 0.135 | 0.088 | 1.06 | 0.413 | 0.876 | 1.88 | 1.11 | 0.285 | 0.666 |
| logepa | rs116330285 | 0.002 | . | . | . | . | . | . | . | . | . | . |
| logepa | rs189926729 | 0.002 | . | . | . | . | . | . | . | . | . | . |
| logepa | rs188975779 | 0.008 | . | . | . | . | . | . | . | . | . | . |
| logepa | rs143846038 | 0.002 | . | . | . | . | . | . | . | . | . | . |
| logepa | rs74128483 | 0.012 | 0.754 | 0.137 | 0.121 | 0.457 | 0.357 | 0.316 | 1.76 | 0.739 | 0.181 | 0.394 |
| logepa | rs139292055 | 0.045 | 0.738 | 0.138 | 0.105 | 0.83 | 0.288 | 0.591 | 1.6 | 0.735 | 0.307 | 0.412 |
| logepa | rs11239503 | 0.045 | 0.738 | 0.138 | 0.105 | 0.83 | 0.288 | 0.591 | 1.6 | 0.735 | 0.306 | 0.412 |

| logepa | rs58838686 | 0.008 | . | . | . | . | . | . | . | . | . | . |
| --- | --- | --- | --- | --- | --- | --- | --- | --- | --- | --- | --- | --- |
| logepa | rs185825373 | 0.005 | . | . | . | . | . | . | . | . | . | . |
| logepa | rs11239504 | 0.045 | 0.738 | 0.138 | 0.105 | 0.83 | 0.288 | 0.591 | 1.6 | 0.735 | 0.307 | 0.412 |
| logepa | rs150012354 | 0.003 | . | . | . | . | . | . | . | . | . | . |
| logepa | rs187763096 | 0.005 | . | . | . | . | . | . | . | . | . | . |
| logepa | rs193043982 | 0.005 | . | . | . | . | . | . | . | . | . | . |
| logepa | rs79186884 | 0.014 | 0.753 | 0.134 | 0.112 | 1.68 | 0.854 | 0.31 | 1.46 | 1.42 | 0.699 | 0.411 |
| logepa | rs74128484 | 0.008 | . | . | . | . | . | . | . | . | . | . |
| logepa | rs58461537 | 0.045 | 0.738 | 0.138 | 0.105 | 0.829 | 0.288 | 0.59 | 1.6 | 0.735 | 0.306 | 0.412 |
| logepa | rs188527474 | 0.005 | . | . | . | . | . | . | . | . | . | . |
| logepa | rs141659976 | 0.005 | . | . | . | . | . | . | . | . | . | . |
| logepa | rs115846407 | 0.045 | 0.738 | 0.138 | 0.105 | 0.829 | 0.288 | 0.589 | 1.6 | 0.734 | 0.306 | 0.411 |
| logepa | rs138378816 | 0.045 | 0.738 | 0.138 | 0.105 | 0.829 | 0.288 | 0.589 | 1.6 | 0.734 | 0.306 | 0.411 |
| logepa | rs146188136 | 0.033 | 0.73 | 0.135 | 0.088 | 1.06 | 0.412 | 0.879 | 1.88 | 1.11 | 0.283 | 0.669 |
| logepa | rs139383871 | 0.008 | . | . | . | . | . | . | . | . | . | . |
| logepa | rs182953869 | 0.004 | . | . | . | . | . | . | . | . | . | . |
| logepa | rs192430299 | 0.012 | 0.784 | 0.141 | 0.177 | 0.614 | 0.541 | 0.58 | 0.335 | 0.418 | 0.381 | -0.237 |
| logepa | rs144152844 | 0.045 | 0.738 | 0.138 | 0.105 | 0.829 | 0.288 | 0.589 | 1.6 | 0.734 | 0.307 | 0.411 |
| logepa | rs145987786 | 0.033 | 0.745 | 0.134 | 0.1 | 0.992 | 0.42 | 0.985 | 1.57 | 1.14 | 0.53 | 0.427 |
| logepa | rs181132926 | 0.002 | . | . | . | . | . | . | . | . | . | . |
| logepa | rs147882253 | 0.045 | 0.738 | 0.138 | 0.105 | 0.829 | 0.287 | 0.588 | 1.6 | 0.734 | 0.306 | 0.411 |
| logepa | rs141807621 | 0.015 | 0.788 | 0.143 | 0.189 | 1.41 | 0.943 | 0.612 | 0.387 | 0.397 | 0.355 | -0.765 |
| logepa | rs147116052 | 0.045 | 0.738 | 0.138 | 0.105 | 0.829 | 0.287 | 0.588 | 1.6 | 0.734 | 0.306 | 0.411 |
| logepa | rs147716618 | 0.045 | 0.738 | 0.138 | 0.105 | 0.829 | 0.287 | 0.588 | 1.6 | 0.734 | 0.306 | 0.411 |
| logepa | rs187198855 | 0.029 | 0.748 | 0.135 | 0.107 | 0.873 | 0.39 | 0.761 | 1.52 | 1.02 | 0.536 | 0.37 |
| logepa | rs189409638 | 0.011 | 0.758 | 0.138 | 0.128 | 0.625 | 0.556 | 0.597 | 1.75 | 1.1 | 0.376 | 0.444 |
| logepa | rs182647339 | 0.001 | . | . | . | . | . | . | . | . | . | . |
| logepa | rs187995988 | 0.004 | . | . | . | . | . | . | . | . | . | . |
| logepa | rs141034184 | 0.048 | 0.735 | 0.138 | 0.102 | 0.771 | 0.269 | 0.456 | 1.69 | 0.782 | 0.255 | 0.453 |
| logepa | rs187310430 | 0.005 | . | . | . | . | . | . | . | . | . | . |
| logepa | rs192608488 | 0.045 | 0.738 | 0.139 | 0.106 | 0.826 | 0.286 | 0.581 | 1.59 | 0.727 | 0.31 | 0.406 |
| logepa | rs148840366 | 0.006 | . | . | . | . | . | . | . | . | . | . |
| logepa | rs186785022 | 0.012 | 0.754 | 0.137 | 0.12 | 0.487 | 0.376 | 0.351 | 1.79 | 0.753 | 0.169 | 0.415 |
| logepa | rs192348728 | 0.008 | . | . | . | . | . | . | . | . | . | . |
| logepa | rs189656631 | 0.045 | 0.739 | 0.139 | 0.106 | 0.826 | 0.286 | 0.581 | 1.59 | 0.727 | 0.31 | 0.406 |
| logepa | rs185289750 | 0.004 | . | . | . | . | . | . | . | . | . | . |
| logepa | rs142382395 | 0.045 | 0.739 | 0.139 | 0.106 | 0.826 | 0.285 | 0.58 | 1.59 | 0.727 | 0.31 | 0.406 |
| logepa | rs144866696 | 0.012 | 0.754 | 0.137 | 0.12 | 0.488 | 0.377 | 0.353 | 1.79 | 0.753 | 0.168 | 0.416 |
| logepa | rs141371289 | 0.045 | 0.739 | 0.139 | 0.106 | 0.826 | 0.285 | 0.58 | 1.59 | 0.727 | 0.31 | 0.406 |
| logepa | rs114750008 | 0.045 | 0.739 | 0.139 | 0.106 | 0.826 | 0.285 | 0.58 | 1.59 | 0.727 | 0.31 | 0.406 |
| logepa | rs189063597 | 0.001 | . | . | . | . | . | . | . | . | . | . |
| logepa | rs139151571 | 0.041 | 0.735 | 0.138 | 0.101 | 0.813 | 0.298 | 0.573 | 1.71 | 0.843 | 0.274 | 0.476 |
| logepa | rs139846368 | 0.046 | 0.736 | 0.138 | 0.103 | 0.797 | 0.278 | 0.516 | 1.66 | 0.769 | 0.276 | 0.44 |
| logepa | rs182057653 | 0.012 | 0.762 | 0.138 | 0.135 | 0.399 | 0.331 | 0.269 | 1.37 | 0.909 | 0.632 | 0.256 |
| logepa | rs148785420 | 0.045 | 0.739 | 0.139 | 0.106 | 0.826 | 0.285 | 0.58 | 1.59 | 0.727 | 0.31 | 0.406 |
| logepa | rs138383251 | 0.007 | . | . | . | . | . | . | . | . | . | . |
| logepa | rs116526705 | 0.012 | 0.774 | 0.141 | 0.16 | 0.218 | 0.295 | 0.26 | 1.74 | 0.785 | 0.218 | 0.302 |
| logepa | rs185946436 | 0 | . | . | . | . | . | . | . | . | . | . |
| logepa | rs190455427 | 0.004 | . | . | . | . | . | . | . | . | . | . |
| logepa | rs9282584 | 0.045 | 0.739 | 0.139 | 0.106 | 0.826 | 0.285 | 0.58 | 1.59 | 0.727 | 0.31 | 0.406 |
| logepa | rs17153289 | 0.045 | 0.739 | 0.139 | 0.106 | 0.826 | 0.285 | 0.58 | 1.59 | 0.727 | 0.31 | 0.406 |
| logepa | rs138359103 | 0.005 | . | . | . | . | . | . | . | . | . | . |
| logepa | rs150605268 | 0.002 | . | . | . | . | . | . | . | . | . | . |
| logepa | rs79242910 | 0.046 | 0.735 | 0.138 | 0.102 | 0.801 | 0.279 | 0.524 | 1.66 | 0.755 | 0.263 | 0.443 |
| logepa | rs139716904 | 0.013 | 0.777 | 0.137 | 0.152 | 0.145 | 0.115 | 0.014 | 0.166 | 0.175 | 0.089 | 0.096 |
| logepa | rs76238498 | 0.034 | 0.735 | 0.132 | 0.087 | 1.09 | 0.443 | 0.838 | 1.86 | 1.31 | 0.38 | 0.661 |
| logepa | rs184140639 | 0 | . | . | . | . | . | . | . | . | . | . |
| logepa | rs188517278 | 0.006 | . | . | . | . | . | . | . | . | . | . |
| logepa | rs139678913 | 0.002 | . | . | . | . | . | . | . | . | . | . |
| logepa | rs116697955 | 0.004 | . | . | . | . | . | . | . | . | . | . |
| logepa | rs79267994 | 0.007 | . | . | . | . | . | . | . | . | . | . |
| logepa | rs11239510 | 0.013 | 0.777 | 0.141 | 0.165 | 0.803 | 0.603 | 0.77 | 0.465 | 0.362 | 0.326 | -0.29 |
| logepa | rs75831645 | 0.012 | 0.774 | 0.141 | 0.16 | 0.217 | 0.294 | 0.259 | 1.75 | 0.787 | 0.214 | 0.303 |
| logepa | rs78452276 | 0.031 | 0.718 | 0.134 | 0.076 | 1.43 | 0.504 | 0.304 | 2.3 | 0.793 | 0.016 | 1.22 |
| logepa | rs144366924 | 0 | . | . | . | . | . | . | . | . | . | . |
| logepa | rs77519841 | 0.033 | 0.73 | 0.135 | 0.088 | 1.06 | 0.41 | 0.887 | 1.89 | 1.12 | 0.28 | 0.673 |
| logepa | rs76944454 | 0.045 | 0.739 | 0.139 | 0.106 | 0.824 | 0.284 | 0.575 | 1.59 | 0.725 | 0.31 | 0.405 |
| logepa | rs76443855 | 0.034 | 0.735 | 0.132 | 0.086 | 1.09 | 0.444 | 0.829 | 1.87 | 1.32 | 0.374 | 0.674 |
| logepa | rs146613174 | 0.001 | . | . | . | . | . | . | . | . | . | . |
| logepa | rs181424761 | 0.001 | . | . | . | . | . | . | . | . | . | . |
| logepa | rs142119241 | 0.001 | . | . | . | . | . | . | . | . | . | . |
| logepa | rs140137954 | 0.012 | 0.774 | 0.141 | 0.161 | 0.219 | 0.295 | 0.259 | 1.73 | 0.779 | 0.225 | 0.3 |
| logepa | rs187319549 | 0.002 | . | . | . | . | . | . | . | . | . | . |
| logepa | rs12241545 | 0.021 | 0.772 | 0.14 | 0.155 | 0.483 | 0.332 | 0.29 | 0.72 | 0.532 | 0.656 | 0.013 |
| logepa | rs10128228 | 0.045 | 0.739 | 0.139 | 0.106 | 0.824 | 0.284 | 0.575 | 1.59 | 0.724 | 0.31 | 0.404 |
| logepa | rs58580249 | 0.006 | . | . | . | . | . | . | . | . | . | . |
| logepa | rs193148863 | 0.001 | . | . | . | . | . | . | . | . | . | . |
| logepa | rs185638946 | 0.005 | . | . | . | . | . | . | . | . | . | . |
| logepa | rs117752921 | 0.001 | . | . | . | . | . | . | . | . | . | . |
| logepa | rs180841236 | 0.001 | . | . | . | . | . | . | . | . | . | . |
| logepa | rs73283155 | 0.006 | . | . | . | . | . | . | . | . | . | . |
| logepa | rs76560599 | 0.038 | 0.732 | 0.138 | 0.098 | 0.776 | 0.311 | 0.527 | 1.92 | 0.858 | 0.147 | 0.58 |
| logepa | rs76273620 | 0.038 | 0.732 | 0.138 | 0.098 | 0.776 | 0.311 | 0.527 | 1.92 | 0.858 | 0.147 | 0.58 |
| logepa | rs141432923 | 0.012 | 0.745 | 0.132 | 0.096 | 0.328 | 0.322 | 0.256 | 47.5 | 40.1 | 4.80E-06 | 11.6 |
| logepa | rs113529322 | 0.012 | 0.754 | 0.137 | 0.12 | 0.511 | 0.393 | 0.382 | 1.8 | 0.76 | 0.161 | 0.43 |
| logepa | rs10128306 | 0.045 | 0.739 | 0.139 | 0.107 | 0.825 | 0.284 | 0.576 | 1.59 | 0.723 | 0.311 | 0.403 |

| logepa | rs149691688 | 0.001 | . | . | . | . | . | . | . | . | . | . |
| --- | --- | --- | --- | --- | --- | --- | --- | --- | --- | --- | --- | --- |
| logepa | rs75872495 | 0.045 | 0.739 | 0.139 | 0.107 | 0.826 | 0.285 | 0.579 | 1.58 | 0.721 | 0.313 | 0.401 |
| logepa | rs191873960 | 0.004 | . | . | . | . | . | . | . | . | . | . |
| logepa | rs80155622 | 0.045 | 0.739 | 0.139 | 0.106 | 0.828 | 0.286 | 0.585 | 1.59 | 0.721 | 0.31 | 0.404 |
| logepa | rs113914656 | 0.005 | . | . | . | . | . | . | . | . | . | . |
| logepa | rs12242840 | 0.045 | 0.738 | 0.138 | 0.105 | 0.837 | 0.289 | 0.607 | 1.59 | 0.72 | 0.304 | 0.408 |
| logepa | rs147824078 | 0.006 | . | . | . | . | . | . | . | . | . | . |
| logepa | rs59051493 | 0.045 | 0.737 | 0.138 | 0.104 | 0.847 | 0.291 | 0.629 | 1.59 | 0.715 | 0.305 | 0.407 |
| logepa | rs9282588 | 0.005 | . | . | . | . | . | . | . | . | . | . |
| logepa | rs150215207 | 0.006 | . | . | . | . | . | . | . | . | . | . |
| logepa | rs138772243 | 0.002 | . | . | . | . | . | . | . | . | . | . |
| logepa | rs149383244 | 0.014 | 0.776 | 0.143 | 0.168 | 1.55 | 0.827 | 0.409 | 0.756 | 0.459 | 0.644 | -0.418 |
| logepa | rs17522720 | 0.011 | 0.774 | 0.142 | 0.162 | 2.09 | 0.899 | 0.087 | 0.717 | 0.376 | 0.526 | -0.704 |
| logepa | rs79245105 | 0.045 | 0.737 | 0.138 | 0.104 | 0.849 | 0.292 | 0.634 | 1.58 | 0.714 | 0.307 | 0.405 |
| logepa | rs138222133 | 0.002 | . | . | . | . | . | . | . | . | . | . |
| logepa | rs12247314 | 0.05 | 0.751 | 0.141 | 0.127 | 0.85 | 0.284 | 0.626 | 1.27 | 0.605 | 0.609 | 0.213 |
| logepa | rs12263894 | 0.049 | 0.751 | 0.141 | 0.128 | 0.866 | 0.29 | 0.667 | 1.26 | 0.596 | 0.625 | 0.203 |
| logepa | rs149907880 | 0.005 | . | . | . | . | . | . | . | . | . | . |
| logepa | rs188153885 | 0.005 | . | . | . | . | . | . | . | . | . | . |
| logepa | rs73283159 | 0.003 | . | . | . | . | . | . | . | . | . | . |
| logepa | rs140476687 | 0.002 | . | . | . | . | . | . | . | . | . | . |
| logepa | rs186603148 | 0 | . | . | . | . | . | . | . | . | . | . |
| logepa | rs183296063 | 0.002 | . | . | . | . | . | . | . | . | . | . |
| logepa | rs145733694 | 0.006 | . | . | . | . | . | . | . | . | . | . |
| logepa | rs189807449 | 0 | . | . | . | . | . | . | . | . | . | . |
| logepa | rs12253223 | 0.038 | 0.742 | 0.137 | 0.107 | 1.07 | 0.413 | 0.868 | 1.45 | 0.907 | 0.558 | 0.335 |
| logepa | rs182113637 | 0 | . | . | . | . | . | . | . | . | . | . |
| logepa | rs142792099 | 0.01 | 0.766 | 0.136 | 0.134 | 0.425 | 0.618 | 0.556 | 0.851 | 2.43 | 0.955 | 0.086 |
| logepa | rs12249910 | 0.038 | 0.742 | 0.137 | 0.107 | 1.07 | 0.413 | 0.866 | 1.45 | 0.908 | 0.557 | 0.336 |
| logepa | rs149436339 | 0.001 | . | . | . | . | . | . | . | . | . | . |
| logepa | rs77632210 | 0 | . | . | . | . | . | . | . | . | . | . |
| logepa | rs74130818 | 0.008 | . | . | . | . | . | . | . | . | . | . |
| logepa | rs187583648 | 0 | . | . | . | . | . | . | . | . | . | . |
| logepa | rs191295987 | 0.004 | . | . | . | . | . | . | . | . | . | . |
| logepa | rs184200686 | 0.006 | . | . | . | . | . | . | . | . | . | . |
| logepa | rs76117263 | 0.033 | 0.733 | 0.132 | 0.084 | 1.09 | 0.451 | 0.834 | 1.96 | 1.38 | 0.341 | 0.742 |
| logepa | rs185393149 | 0.001 | . | . | . | . | . | . | . | . | . | . |
| logepa | rs144760014 | 0.003 | . | . | . | . | . | . | . | . | . | . |
| logepa | rs7922292 | 0.005 | . | . | . | . | . | . | . | . | . | . |
| logepa | rs115372844 | 0.006 | . | . | . | . | . | . | . | . | . | . |
| logepa | rs111460364 | 0.033 | 0.733 | 0.132 | 0.083 | 1.09 | 0.453 | 0.827 | 1.97 | 1.39 | 0.337 | 0.753 |
| logepa | rs182052255 | 0.001 | . | . | . | . | . | . | . | . | . | . |
| logepa | rs180983889 | 0.004 | . | . | . | . | . | . | . | . | . | . |
| logepa | rs139175327 | 0.006 | . | . | . | . | . | . | . | . | . | . |
| logepa | rs149959620 | 0.011 | 0.777 | 0.137 | 0.152 | 0.131 | 0.112 | 0.018 | 0.134 | 0.147 | 0.066 | 0.106 |
| logepa | rs114653117 | 0.005 | . | . | . | . | . | . | . | . | . | . |
| logepa | rs144270018 | 0.003 | . | . | . | . | . | . | . | . | . | . |
| logepa | rs182723204 | 0.005 | . | . | . | . | . | . | . | . | . | . |
| logepa | rs57204067 | 0.008 | . | . | . | . | . | . | . | . | . | . |
| logepa | rs144956226 | 0.038 | 0.74 | 0.137 | 0.104 | 1.1 | 0.425 | 0.809 | 1.48 | 0.934 | 0.532 | 0.367 |
| logepa | rs187842704 | 0.01 | 0.753 | 0.135 | 0.114 | 0.033 | 0.041 | 5.80E-03 | 1.37 | 1.75 | 0.807 | 0.248 |
| logepa | rs184620511 | 0.008 | . | . | . | . | . | . | . | . | . | . |
| logepa | rs144031303 | 0.012 | 0.773 | 0.141 | 0.159 | 0.308 | 0.324 | 0.263 | 1.29 | 0.798 | 0.684 | 0.225 |
| logepa | rs187526473 | 0 | . | . | . | . | . | . | . | . | . | . |
| logepa | rs193002805 | 0.002 | . | . | . | . | . | . | . | . | . | . |
| logepa | rs138707426 | 0.014 | 0.777 | 0.143 | 0.169 | 1.6 | 0.832 | 0.363 | 0.758 | 0.449 | 0.64 | -0.436 |
| logepa | rs142754243 | 0.03 | 0.725 | 0.131 | 0.075 | 1.02 | 0.403 | 0.967 | 2.17 | 1.58 | 0.29 | 0.854 |
| logepa | rs181600331 | 0.005 | . | . | . | . | . | . | . | . | . | . |
| logepa | rs144421264 | 0.018 | 0.719 | 0.128 | 0.063 | 0.895 | 0.45 | 0.825 | 5.12 | 5.37 | 0.12 | 2.68 |
| logepa | rs184414519 | 0.004 | . | . | . | . | . | . | . | . | . | . |
| logepa | rs150596349 | 0.031 | 0.722 | 0.129 | 0.068 | 1.2 | 0.491 | 0.66 | 2.44 | 1.61 | 0.175 | 1.19 |
| logepa | rs190753747 | 0.001 | . | . | . | . | . | . | . | . | . | . |
| logepa | rs139560940 | 0.004 | . | . | . | . | . | . | . | . | . | . |
| logepa | rs75640804 | 0.033 | 0.725 | 0.134 | 0.082 | 1.16 | 0.445 | 0.7 | 1.99 | 1.14 | 0.233 | 0.784 |
| logepa | rs73283175 | 0.052 | 0.739 | 0.14 | 0.109 | 0.765 | 0.253 | 0.418 | 1.44 | 0.767 | 0.497 | 0.309 |
| logepa | rs143337229 | 0.002 | . | . | . | . | . | . | . | . | . | . |
| logepa | rs73283177 | 0.03 | 0.727 | 0.131 | 0.078 | 0.987 | 0.395 | 0.973 | 2.13 | 1.58 | 0.306 | 0.815 |
| logepa | rs76053611 | 0.029 | 0.738 | 0.131 | 0.087 | 0.855 | 0.38 | 0.724 | 2.03 | 1.46 | 0.328 | 0.686 |
| logepa | rs116325983 | 0.004 | . | . | . | . | . | . | . | . | . | . |
| logepa | rs115451825 | 0.005 | . | . | . | . | . | . | . | . | . | . |
| logepa | rs183845246 | 0.001 | . | . | . | . | . | . | . | . | . | . |
| logepa | rs79756542 | 0.033 | 0.72 | 0.128 | 0.066 | 1.19 | 0.473 | 0.653 | 2.4 | 1.52 | 0.167 | 1.15 |
| logepa | rs187242860 | 0.002 | . | . | . | . | . | . | . | . | . | . |
| logepa | rs57318980 | 0.03 | 0.731 | 0.132 | 0.082 | 0.896 | 0.368 | 0.789 | 2.1 | 1.59 | 0.325 | 0.75 |
| logepa | rs59969732 | 0.028 | 0.729 | 0.131 | 0.079 | 0.739 | 0.321 | 0.486 | 2.72 | 2.15 | 0.205 | 0.999 |
| logepa | rs143811488 | 0.006 | . | . | . | . | . | . | . | . | . | . |
| logepa | rs189067644 | 0.006 | . | . | . | . | . | . | . | . | . | . |
| logepa | rs60449178 | 0.025 | 0.728 | 0.13 | 0.075 | 0.676 | 0.329 | 0.421 | 4.25 | 4.76 | 0.196 | 1.69 |
| logepa | rs59243389 | 0.025 | 0.728 | 0.13 | 0.075 | 0.676 | 0.329 | 0.421 | 4.25 | 4.75 | 0.197 | 1.69 |
| logepa | rs184946540 | 0.027 | 0.769 | 0.136 | 0.139 | 0.48 | 0.25 | 0.16 | 1.14 | 1.07 | 0.891 | 0.171 |
| logepa | rs188685881 | 0.001 | . | . | . | . | . | . | . | . | . | . |
| logepa | rs150335797 | 0.004 | . | . | . | . | . | . | . | . | . | . |
| logepa | rs143879260 | 0.004 | . | . | . | . | . | . | . | . | . | . |
| logepa | rs186044491 | 0.006 | . | . | . | . | . | . | . | . | . | . |
| logepa | rs11819084 | 0.018 | 0.732 | 0.134 | 0.089 | 2.23 | 0.821 | 0.029 | 1.59 | 0.982 | 0.45 | 0.637 |
| logepa | rs146915670 | 0.006 | . | . | . | . | . | . | . | . | . | . |

| logepa | rs113995695 | 0.017 | 0.729 | 0.134 | 0.085 | 2.25 | 0.824 | 0.028 | 1.66 | 1.04 | 0.42 | 0.74 |
| --- | --- | --- | --- | --- | --- | --- | --- | --- | --- | --- | --- | --- |
| logepa | rs143489451 | 0.003 | . | . | . | . | . | . | . | . | . | . |
| logepa | rs182653839 | 0 | . | . | . | . | . | . | . | . | . | . |
| logepa | rs141692564 | 0.004 | . | . | . | . | . | . | . | . | . | . |
| logepa | rs146281742 | 0.003 | . | . | . | . | . | . | . | . | . | . |
| logepa | rs192914353 | 0.003 | . | . | . | . | . | . | . | . | . | . |
| logepa | rs185182848 | 0.002 | . | . | . | . | . | . | . | . | . | . |
| logepa | rs28395858 | 0.017 | 0.727 | 0.134 | 0.083 | 2.26 | 0.826 | 0.026 | 1.68 | 1.06 | 0.411 | 0.776 |
| logepa | rs41429645 | 0.005 | . | . | . | . | . | . | . | . | . | . |
| logepa | rs28395874 | 0.018 | 0.727 | 0.134 | 0.083 | 2.26 | 0.827 | 0.026 | 1.69 | 1.07 | 0.41 | 0.783 |
| logepa | rs189663634 | 0.001 | . | . | . | . | . | . | . | . | . | . |
| logepa | rs77666700 | 0.006 | . | . | . | . | . | . | . | . | . | . |
| logepa | rs146711717 | 0.002 | . | . | . | . | . | . | . | . | . | . |
| logepa | rs144904251 | 0.001 | . | . | . | . | . | . | . | . | . | . |
| logepa | rs189893153 | 0.001 | . | . | . | . | . | . | . | . | . | . |
| logepa | rs150447740 | 0.006 | . | . | . | . | . | . | . | . | . | . |
| logepa | rs143099649 | 0.001 | . | . | . | . | . | . | . | . | . | . |
| logepa | rs191288436 | 0.007 | . | . | . | . | . | . | . | . | . | . |
| logepa | rs188577862 | 0.004 | . | . | . | . | . | . | . | . | . | . |
| logepa | rs192695334 | 0.004 | . | . | . | . | . | . | . | . | . | . |
| logepa | rs138612906 | 0.022 | 0.728 | 0.134 | 0.085 | 1.67 | 0.581 | 0.14 | 1.6 | 0.844 | 0.37 | 0.551 |
| logepa | rs191290051 | 0.022 | 0.76 | 0.133 | 0.117 | 0.73 | 0.42 | 0.585 | 1.31 | 1.76 | 0.841 | 0.236 |
| logepa | rs189631459 | 0.001 | . | . | . | . | . | . | . | . | . | . |
| logepa | rs114620874 | 0.005 | . | . | . | . | . | . | . | . | . | . |
| logepa | rs78246652 | 0.013 | 0.768 | 0.136 | 0.135 | 0.711 | 0.61 | 0.691 | 0.771 | 1.12 | 0.858 | -0.058 |
| logepa | rs79432451 | 0.005 | . | . | . | . | . | . | . | . | . | . |
| logepa | rs186022585 | 0.001 | . | . | . | . | . | . | . | . | . | . |
| logepa | rs142978891 | 0.005 | . | . | . | . | . | . | . | . | . | . |
| logepa | rs151086840 | 0.007 | . | . | . | . | . | . | . | . | . | . |
| logepa | rs183270259 | 0.006 | . | . | . | . | . | . | . | . | . | . |
| logepa | rs188544289 | 0.006 | . | . | . | . | . | . | . | . | . | . |
| logepa | rs188500808 | 0.001 | . | . | . | . | . | . | . | . | . | . |
| logepa | rs189424589 | 0.002 | . | . | . | . | . | . | . | . | . | . |
| logepa | rs78622303 | 0.025 | 0.732 | 0.129 | 0.077 | 0.632 | 0.339 | 0.393 | 3.65 | 3.81 | 0.214 | 1.33 |
| logepa | rs143210835 | 0.008 | . | . | . | . | . | . | . | . | . | . |
| logepa | rs114761840 | 0.004 | . | . | . | . | . | . | . | . | . | . |
| logepa | rs185805402 | 0.003 | . | . | . | . | . | . | . | . | . | . |
| logepa | rs139273744 | 0.002 | . | . | . | . | . | . | . | . | . | . |
| logepa | rs184654150 | 0.004 | . | . | . | . | . | . | . | . | . | . |
| logepa | rs189069710 | 0 | . | . | . | . | . | . | . | . | . | . |
| logepa | rs114752115 | 0.018 | 0.77 | 0.137 | 0.142 | 0.698 | 0.478 | 0.6 | 0.767 | 0.748 | 0.786 | -0.056 |
| logepa | rs150162515 | 0.001 | . | . | . | . | . | . | . | . | . | . |
| logepa | rs180982454 | 0.004 | . | . | . | . | . | . | . | . | . | . |
| logepa | rs184932088 | 0.001 | . | . | . | . | . | . | . | . | . | . |
| logepa | rs181109625 | 0 | . | . | . | . | . | . | . | . | . | . |
| logepa | rs138121134 | 0.001 | . | . | . | . | . | . | . | . | . | . |
| logepa | rs183253861 | 0.003 | . | . | . | . | . | . | . | . | . | . |
| logepa | rs147073946 | 0.001 | . | . | . | . | . | . | . | . | . | . |
| logepa | rs139938489 | 0.005 | . | . | . | . | . | . | . | . | . | . |
| logepa | rs146362560 | 0.008 | . | . | . | . | . | . | . | . | . | . |
| logepa | rs193155956 | 0.004 | . | . | . | . | . | . | . | . | . | . |
| logepa | rs77663092 | 0.004 | . | . | . | . | . | . | . | . | . | . |
| logepa | rs73285319 | 0.021 | 0.758 | 0.142 | 0.14 | 1.52 | 0.754 | 0.401 | 0.993 | 0.413 | 0.987 | -0.133 |
| logepa | rs184019276 | 0.007 | . | . | . | . | . | . | . | . | . | . |
| logepa | rs28395863 | 0.017 | 0.723 | 0.133 | 0.079 | 2.39 | 0.854 | 0.014 | 1.74 | 1.04 | 0.356 | 0.891 |
| logepa | rs182121645 | 0.011 | 0.771 | 0.139 | 0.149 | 1.18 | 0.698 | 0.786 | 0.75 | 0.498 | 0.664 | -0.267 |
| logepa | rs73285327 | 0.016 | 0.763 | 0.141 | 0.143 | 1.1 | 0.673 | 0.872 | 1.04 | 0.437 | 0.919 | 0.012 |
| logepa | rs149589584 | 0.001 | . | . | . | . | . | . | . | . | . | . |
| logepa | rs148310335 | 0.005 | . | . | . | . | . | . | . | . | . | . |
| logepa | rs187246093 | 0.007 | . | . | . | . | . | . | . | . | . | . |
| logepa | rs184668894 | 0 | . | . | . | . | . | . | . | . | . | . |
| logepa | rs114269961 | 0.001 | . | . | . | . | . | . | . | . | . | . |
| logepa | rs149694374 | 0.02 | 0.723 | 0.133 | 0.079 | 1.99 | 0.752 | 0.067 | 1.96 | 1.25 | 0.294 | 1.1 |
| logepa | rs145284227 | 0.004 | . | . | . | . | . | . | . | . | . | . |
| logepa | rs189983820 | 0.004 | . | . | . | . | . | . | . | . | . | . |
| logepa | rs181200444 | 0 | . | . | . | . | . | . | . | . | . | . |
| logepa | rs185486385 | 0 | . | . | . | . | . | . | . | . | . | . |
| logepa | rs151198241 | 0.006 | . | . | . | . | . | . | . | . | . | . |
| logepa | rs189959341 | 0.006 | . | . | . | . | . | . | . | . | . | . |
| logepa | rs140549257 | 0.005 | . | . | . | . | . | . | . | . | . | . |
| logepa | rs186453139 | 0.016 | 0.755 | 0.142 | 0.136 | 1.93 | 0.897 | 0.156 | 0.965 | 0.391 | 0.931 | -0.279 |
| logepa | rs180922711 | 0.027 | 0.749 | 0.139 | 0.121 | 1.41 | 0.552 | 0.383 | 1.18 | 0.543 | 0.713 | 0.092 |
| logepa | rs56659903 | 0.004 | . | . | . | . | . | . | . | . | . | . |
| logepa | rs141202128 | 0.004 | . | . | . | . | . | . | . | . | . | . |
| logepa | rs59085314 | 0.033 | 0.731 | 0.133 | 0.085 | 0.804 | 0.322 | 0.586 | 2.47 | 0.81 | 5.90E-03 | 0.916 |
| logepa | rs145564996 | 0.004 | . | . | . | . | . | . | . | . | . | . |
| logepa | rs114961009 | 0.006 | . | . | . | . | . | . | . | . | . | . |
| logepa | rs182294189 | 0.003 | . | . | . | . | . | . | . | . | . | . |
| logepa | rs187586636 | 0.005 | . | . | . | . | . | . | . | . | . | . |
| logepa | rs192254907 | 0.003 | . | . | . | . | . | . | . | . | . | . |
| logepa | rs149145663 | 0.001 | . | . | . | . | . | . | . | . | . | . |
| logepa | rs180713064 | 0.002 | . | . | . | . | . | . | . | . | . | . |
| logepa | rs183009023 | 0.004 | . | . | . | . | . | . | . | . | . | . |
| logepa | rs143385347 | 0.01 | 0.771 | 0.139 | 0.15 | 1.2 | 0.702 | 0.753 | 0.735 | 0.464 | 0.625 | -0.292 |
| logepa | rs185786329 | 0.004 | . | . | . | . | . | . | . | . | . | . |
| logepa | rs192341030 | 0.003 | . | . | . | . | . | . | . | . | . | . |

| logepa | rs74661429 | 0.009 | . | . | . | . | . | . | . | . | . | . |
| --- | --- | --- | --- | --- | --- | --- | --- | --- | --- | --- | --- | --- |
| logepa | rs188034853 | 0.001 | . | . | . | . | . | . | . | . | . | . |
| logepa | rs116808430 | 0.016 | 0.756 | 0.143 | 0.138 | 2.01 | 0.918 | 0.127 | 0.96 | 0.392 | 0.92 | -0.307 |
| logepa | rs148469010 | 0 | . | . | . | . | . | . | . | . | . | . |
| logepa | rs78621663 | 0.006 | . | . | . | . | . | . | . | . | . | . |
| logepa | rs192654886 | 0.001 | . | . | . | . | . | . | . | . | . | . |
| logepa | rs145996035 | 0.005 | . | . | . | . | . | . | . | . | . | . |
| logepa | rs139942226 | 0 | . | . | . | . | . | . | . | . | . | . |
| logepa | rs113645632 | 0.006 | . | . | . | . | . | . | . | . | . | . |
| logepa | rs74940661 | 0.016 | 0.731 | 0.133 | 0.086 | 2.24 | 0.87 | 0.037 | 1.87 | 1.37 | 0.391 | 1.1 |
| logepa | rs115355800 | 0.013 | 0.783 | 0.137 | 0.164 | 0.469 | 0.527 | 0.5 | 0.306 | 0.475 | 0.445 | -0.14 |
| logepa | rs150285898 | 0.005 | . | . | . | . | . | . | . | . | . | . |
| logepa | rs188117932 | 0.004 | . | . | . | . | . | . | . | . | . | . |
| logepa | rs139956566 | 0 | . | . | . | . | . | . | . | . | . | . |
| logepa | rs147673885 | 0 | . | . | . | . | . | . | . | . | . | . |
| logepa | rs189127928 | 0.005 | . | . | . | . | . | . | . | . | . | . |
| logepa | rs187558772 | 0.002 | . | . | . | . | . | . | . | . | . | . |
| logepa | rs181948587 | 0 | . | . | . | . | . | . | . | . | . | . |
| logepa | rs189983269 | 0.002 | . | . | . | . | . | . | . | . | . | . |
| logepa | rs187250069 | 0.002 | . | . | . | . | . | . | . | . | . | . |
| logepa | rs191485859 | 0.001 | . | . | . | . | . | . | . | . | . | . |
| logepa | rs185499555 | 0.002 | . | . | . | . | . | . | . | . | . | . |
| logepa | rs180862110 | 0.003 | . | . | . | . | . | . | . | . | . | . |
| logepa | rs190472603 | 0.005 | . | . | . | . | . | . | . | . | . | . |
| logepa | rs189226167 | 0.005 | . | . | . | . | . | . | . | . | . | . |
| logepa | rs181623256 | 0.002 | . | . | . | . | . | . | . | . | . | . |
| logepa | rs186079557 | 0.001 | . | . | . | . | . | . | . | . | . | . |
| logepa | rs147760994 | 0.002 | . | . | . | . | . | . | . | . | . | . |
| logepa | rs189176167 | 0 | . | . | . | . | . | . | . | . | . | . |
| logepa | rs117901000 | 0.001 | . | . | . | . | . | . | . | . | . | . |
| logepa | rs141984888 | 0 | . | . | . | . | . | . | . | . | . | . |
| logepa | rs192365061 | 0.007 | . | . | . | . | . | . | . | . | . | . |
| logepa | rs185976038 | 0 | . | . | . | . | . | . | . | . | . | . |
| logepa | rs184697303 | 0.002 | . | . | . | . | . | . | . | . | . | . |
| logepa | rs143383132 | 0.002 | . | . | . | . | . | . | . | . | . | . |
| logepa | rs141873881 | 0.004 | . | . | . | . | . | . | . | . | . | . |
| logepa | rs185507255 | 0 | . | . | . | . | . | . | . | . | . | . |
| logepa | rs191379425 | 0.001 | . | . | . | . | . | . | . | . | . | . |
| logepa | rs184199584 | 0.002 | . | . | . | . | . | . | . | . | . | . |
| logepa | rs183181360 | 0.002 | . | . | . | . | . | . | . | . | . | . |
| logepa | rs184495673 | 0.001 | . | . | . | . | . | . | . | . | . | . |
| logepa | rs182946297 | 0.001 | . | . | . | . | . | . | . | . | . | . |
| logepa | rs187743615 | 0 | . | . | . | . | . | . | . | . | . | . |
| logepa | rs183259862 | 0.001 | . | . | . | . | . | . | . | . | . | . |
| logepa | rs112433566 | 0 | . | . | . | . | . | . | . | . | . | . |
| logepa | rs182351085 | 0.001 | . | . | . | . | . | . | . | . | . | . |
| logepa | rs146248508 | 0.001 | . | . | . | . | . | . | . | . | . | . |
| logepa | rs188528439 | 0.001 | . | . | . | . | . | . | . | . | . | . |
| logepa | rs146167018 | 0.005 | . | . | . | . | . | . | . | . | . | . |
| logepa | rs192440980 | 0.004 | . | . | . | . | . | . | . | . | . | . |
| logepa | rs185601882 | 0.001 | . | . | . | . | . | . | . | . | . | . |
| logepa | rs117272563 | 0.002 | . | . | . | . | . | . | . | . | . | . |
| logepa | rs10430734 | 0.003 | . | . | . | . | . | . | . | . | . | . |
| logepa | rs181320952 | 0.001 | . | . | . | . | . | . | . | . | . | . |
| logepa | rs185480660 | 0.001 | . | . | . | . | . | . | . | . | . | . |
| logepa | rs188077917 | 0.001 | . | . | . | . | . | . | . | . | . | . |
| logepa | rs187562953 | 0.002 | . | . | . | . | . | . | . | . | . | . |
| logepa | rs182044631 | 0 | . | . | . | . | . | . | . | . | . | . |
| logepa | rs145455596 | 0.001 | . | . | . | . | . | . | . | . | . | . |
| logepa | rs181475877 | 0.004 | . | . | . | . | . | . | . | . | . | . |
| logepa | rs149413085 | 0.002 | . | . | . | . | . | . | . | . | . | . |
| logepa | rs12253656 | 0 | . | . | . | . | . | . | . | . | . | . |
| logepa | rs182718239 | 0 | . | . | . | . | . | . | . | . | . | . |
| logepa | rs192435695 | 0.002 | . | . | . | . | . | . | . | . | . | . |
| logepa | rs12248277 | 0 | . | . | . | . | . | . | . | . | . | . |
| logepa | rs143521710 | 0.001 | . | . | . | . | . | . | . | . | . | . |
| logepa | rs144454454 | 0 | . | . | . | . | . | . | . | . | . | . |
| logepa | rs190441610 | 0.004 | . | . | . | . | . | . | . | . | . | . |
| logepa | rs114690462 | 0 | . | . | . | . | . | . | . | . | . | . |
| logepa | rs184541357 | 0.001 | . | . | . | . | . | . | . | . | . | . |
| logepa | rs142657653 | 0 | . | . | . | . | . | . | . | . | . | . |
| logepa | rsurf1 | 2.146 | . | . | . | . | . | . | . | . | . | . |
| logepa | rsten1 | 2.304 | . | . | . | . | . | . | . | . | . | . |
| logepa | chr10:45852312:D | 0.086 | 0.77 | 0.083 | 0.015 | 0.925 | 0.193 | 0.708 | 0.721 | 0.18 | 0.191 | -0.181 |
| logepa | chr10:45853232:I | 0.1 | 0.768 | 0.084 | 0.015 | 0.882 | 0.18 | 0.539 | 0.764 | 0.187 | 0.27 | -0.133 |
| logepa | chr10:45853234:I | 0.168 | 0.751 | 0.087 | 0.013 | 0.869 | 0.127 | 0.338 | 0.913 | 0.178 | 0.641 | -0.024 |
| logepa | chr10:45854796:D | 0.013 | 0.727 | 0.075 | 1.90E-03 | 1.6 | 0.722 | 0.297 | 1.26 | 0.734 | 0.688 | 0.142 |
| logepa | chr10:45854994:I | 0.019 | 0.726 | 0.075 | 1.90E-03 | 1.16 | 0.488 | 0.721 | 1.16 | 0.825 | 0.836 | 0.09 |
| logepa | chr10:45858290:D | 0.045 | 0.756 | 0.081 | 9.30E-03 | 0.925 | 0.296 | 0.808 | 0.695 | 0.302 | 0.402 | -0.195 |
| logepa | chr10:45858820:I | 0.015 | 0.764 | 0.081 | 0.011 | 0.36 | 0.289 | 0.203 | 0.161 | 0.124 | 0.017 | -0.08 |
| logepa | chr10:45860888:D | 0.081 | 0.705 | 0.076 | 1.30E-03 | 0.955 | 0.201 | 0.829 | 1.25 | 0.404 | 0.498 | 0.179 |
| logepa | chr10:45863913:I | 0.165 | 0.751 | 0.087 | 0.013 | 0.891 | 0.127 | 0.419 | 0.916 | 0.174 | 0.646 | -0.029 |
| logepa | chr10:45866601:D | 0.036 | 0.762 | 0.085 | 0.015 | 0.77 | 0.256 | 0.433 | 1.18 | 0.523 | 0.711 | 0.16 |
| logepa | chr10:45867197:I | 0.006 | . | . | . | . | . | . | . | . | . | . |
| logepa | chr10:45867538:D | 0.171 | 0.741 | 0.086 | 0.01 | 0.924 | 0.125 | 0.561 | 0.957 | 0.175 | 0.808 | -0.01 |
| logepa | chr10:45868356:D | 0.161 | 0.759 | 0.087 | 0.016 | 0.874 | 0.128 | 0.357 | 0.878 | 0.172 | 0.507 | -0.051 |

| logepa | chr10:45871158:I | 0.167 | 0.753 | 0.087 | 0.014 | 0.893 | 0.126 | 0.42 | 0.909 | 0.171 | 0.613 | -0.034 |
| --- | --- | --- | --- | --- | --- | --- | --- | --- | --- | --- | --- | --- |
| logepa | chr10:45871637:D | 0.084 | 0.726 | 0.082 | 4.30E-03 | 0.978 | 0.196 | 0.913 | 1.04 | 0.271 | 0.881 | 0.034 |
| logepa | chr10:45871638:I | 0.132 | 0.737 | 0.085 | 8.10E-03 | 0.973 | 0.153 | 0.86 | 0.967 | 0.206 | 0.874 | -0.017 |
| logepa | chr10:45873629:I | 0.158 | 0.751 | 0.087 | 0.013 | 0.935 | 0.132 | 0.634 | 0.913 | 0.174 | 0.634 | -0.045 |
| logepa | chr10:45873701:D | 0.019 | 0.751 | 0.082 | 9.00E-03 | 1.07 | 0.396 | 0.853 | 1.95 | 1.05 | 0.216 | 0.744 |
| logepa | chr10:45874997:I | 0.021 | 0.781 | 0.086 | 0.025 | 0.594 | 0.257 | 0.228 | 0.66 | 0.36 | 0.447 | -0.068 |
| logepa | chr10:45882030:I | 0.045 | 0.747 | 0.085 | 0.01 | 0.946 | 0.276 | 0.849 | 1.45 | 0.531 | 0.309 | 0.332 |
| logepa | chr10:45883774:D | 0.102 | 0.719 | 0.084 | 4.70E-03 | 0.973 | 0.15 | 0.861 | 1.08 | 0.234 | 0.736 | 0.061 |
| logepa | chr10:45885169:D | 0.067 | 0.751 | 0.079 | 6.70E-03 | 0.89 | 0.221 | 0.637 | 0.783 | 0.245 | 0.434 | -0.118 |
| logepa | chr10:45886451:D | 0.671 | 1.01 | 0.243 | 0.976 | 0.821 | 0.101 | 0.11 | 0.78 | 0.133 | 0.143 | -0.184 |
| logepa | chr10:45891800:I | 0.686 | 0.967 | 0.204 | 0.873 | 0.94 | 0.1 | 0.559 | 0.814 | 0.11 | 0.129 | -0.167 |
| logepa | chr10:45904771:I | 0.685 | 0.932 | 0.19 | 0.729 | 0.925 | 0.097 | 0.46 | 0.836 | 0.11 | 0.173 | -0.136 |
| logepa | chr10:45904959:I | 0.113 | 0.726 | 0.092 | 0.011 | 1.43 | 0.247 | 0.039 | 1 | 0.226 | 0.982 | -0.112 |
| logepa | chr10:45911028:I | 0.077 | 0.723 | 0.083 | 4.60E-03 | 0.871 | 0.206 | 0.56 | 1.1 | 0.384 | 0.788 | 0.098 |
| logepa | chr10:45911031:I | 0.355 | 0.827 | 0.129 | 0.223 | 1.07 | 0.126 | 0.567 | 0.852 | 0.12 | 0.253 | -0.143 |
| logepa | chr10:45912154:D | 0.104 | 0.728 | 0.084 | 5.80E-03 | 1 | 0.153 | 0.976 | 1.02 | 0.223 | 0.945 | 1.00E-02 |
| logepa | chr10:45913885:I | 0.011 | 0.729 | 0.075 | 2.00E-03 | 1.18 | 0.754 | 0.799 | 1.13 | 1.05 | 0.892 | 0.067 |
| logepa | chr10:45914753:D | 0.246 | 0.667 | 0.09 | 2.80E-03 | 1.24 | 0.133 | 0.049 | 1.17 | 0.174 | 0.29 | 0.062 |
| logepa | chr10:45919621 | 0.003 | . | . | . | . | . | . | . | . | . | . |
| logepa | chr10:45921739:I | 0.066 | 0.695 | 0.073 | 5.70E-04 | 1.09 | 0.201 | 0.647 | 1.49 | 0.424 | 0.165 | 0.341 |
| logepa | chr10:45923527:I | 0.057 | 0.706 | 0.083 | 3.00E-03 | 1.46 | 0.398 | 0.17 | 1.33 | 0.523 | 0.475 | 0.201 |
| logepa | chr10:45927001:I | 0.019 | 0.732 | 0.077 | 3.10E-03 | 0.537 | 0.332 | 0.315 | 0.821 | 0.821 | 0.844 | 0.054 |
| logepa | chr10:45930354:I | 0.017 | 0.731 | 0.075 | 2.20E-03 | 0.969 | 0.525 | 0.954 | 0.945 | 0.814 | 0.948 | -0.03 |
| logepa | chr10:45932864:I | 0.04 | 0.732 | 0.078 | 3.50E-03 | 1.67 | 0.49 | 0.083 | 0.976 | 0.396 | 0.951 | -0.208 |
| logepa | chr10:45936163:I | 0.029 | 0.751 | 0.08 | 6.90E-03 | 0.865 | 0.309 | 0.684 | 0.541 | 0.266 | 0.212 | -0.265 |
| logepa | chr10:45938095:D | 0.144 | 0.7 | 0.085 | 3.50E-03 | 1.02 | 0.133 | 0.856 | 1.16 | 0.207 | 0.421 | 0.104 |
| logepa | chr10:45938886 | 0 | . | . | . | . | . | . | . | . | . | . |
| logepa | chr10:45939320 | 0.001 | . | . | . | . | . | . | . | . | . | . |
| logepa | chr10:45939659 | 0.002 | . | . | . | . | . | . | . | . | . | . |
| logepa | chr10:45941150:D | 0.012 | 0.725 | 0.075 | 1.80E-03 | 1.18 | 0.528 | 0.711 | 1.3 | 0.853 | 0.69 | 0.206 |
| logepa | chr10:45947128:D | 0.058 | 0.705 | 0.079 | 1.70E-03 | 1.41 | 0.254 | 0.058 | 1.31 | 0.33 | 0.277 | 0.19 |
| logepa | chr10:45950990:I | 0.047 | 0.705 | 0.076 | 1.10E-03 | 1.14 | 0.283 | 0.594 | 1.44 | 0.562 | 0.348 | 0.314 |
| logepa | chr10:45951854:I | 0.03 | 0.749 | 0.085 | 0.011 | 1.58 | 0.389 | 0.063 | 1.32 | 0.463 | 0.431 | 0.231 |
| logepa | chr10:45952670:I | 0.049 | 0.704 | 0.077 | 1.30E-03 | 1.02 | 0.21 | 0.914 | 1.47 | 0.409 | 0.167 | 0.331 |
| logepa | chr10:45956741 | 0.001 | . | . | . | . | . | . | . | . | . | . |
| logepa | chr10:45957247:I | 0.22 | 0.73 | 0.094 | 0.014 | 1.07 | 0.123 | 0.537 | 1 | 0.153 | 0.994 | -0.019 |
| logepa | chr10:45958082:I | 0.216 | 0.726 | 0.094 | 0.013 | 1.08 | 0.127 | 0.539 | 1.01 | 0.159 | 0.942 | -0.012 |
| logepa | chr10:45905041:D | 0.053 | 0.686 | 0.106 | 0.015 | 0.986 | 0.36 | 0.97 | 1.46 | 0.945 | 0.561 | 0.314 |
| logepa | chr10:45938871 | 0.003 | . | . | . | . | . | . | . | . | . | . |
| logepa | chr10:45939160 | 0.004 | . | . | . | . | . | . | . | . | . | . |
| logepa | chr10:45869910 | 0.008 | . | . | . | . | . | . | . | . | . | . |
| logepa | chr10:45876214:D | 0.012 | 0.764 | 0.138 | 0.136 | 0.263 | 0.341 | 0.303 | 2.52 | 0.987 | 0.019 | 0.479 |
| logepa | chr10:45880237:I | 0.056 | 0.788 | 0.147 | 0.201 | 0.448 | 0.197 | 0.068 | 0.574 | 0.324 | 0.325 | -0.033 |
| logepa | chr10:45884371:I | 0.045 | 0.738 | 0.138 | 0.105 | 0.829 | 0.288 | 0.589 | 1.6 | 0.734 | 0.306 | 0.412 |
| logepa | chr10:45891535:D | 0.012 | 0.754 | 0.137 | 0.12 | 0.495 | 0.381 | 0.361 | 1.79 | 0.756 | 0.166 | 0.42 |
| logepa | chr10:45893516:D | 0.045 | 0.739 | 0.139 | 0.106 | 0.824 | 0.284 | 0.575 | 1.59 | 0.724 | 0.311 | 0.404 |
| logepa | chr10:45893782:D | 0.01 | 0.759 | 0.137 | 0.126 | 5.00E-03 | 0.015 | 0.079 | 0.124 | 0.396 | 0.513 | 0.237 |
| logepa | chr10:45895254:I | 0.012 | 0.754 | 0.137 | 0.12 | 0.506 | 0.389 | 0.376 | 1.8 | 0.76 | 0.162 | 0.428 |
| logepa | chr10:45895848:D | 0.035 | 0.734 | 0.136 | 0.095 | 0.713 | 0.297 | 0.417 | 2.06 | 1.29 | 0.25 | 0.631 |
| logepa | chr10:45896316:D | 0.045 | 0.739 | 0.139 | 0.106 | 0.824 | 0.284 | 0.575 | 1.59 | 0.724 | 0.311 | 0.404 |
| logepa | chr10:45896317:D | 0.045 | 0.739 | 0.139 | 0.106 | 0.824 | 0.284 | 0.575 | 1.59 | 0.724 | 0.311 | 0.404 |
| logepa | chr10:45902683:I | 0.013 | 0.774 | 0.141 | 0.161 | 0.239 | 0.277 | 0.218 | 1.5 | 0.783 | 0.442 | 0.264 |
| logepa | chr10:45905003:D | 0.006 | . | . | . | . | . | . | . | . | . | . |
| logepa | chr10:45927476:D | 0.021 | 0.725 | 0.134 | 0.081 | 1.93 | 0.733 | 0.084 | 1.91 | 1.27 | 0.333 | 1.01 |
| logepa | chr10:45935882 | 0.001 | . | . | . | . | . | . | . | . | . | . |
| logepa | chr10:45936100 | 0.001 | . | . | . | . | . | . | . | . | . | . |
| logepa | chr10:45939553 | 0.003 | . | . | . | . | . | . | . | . | . | . |
| logepa | chr10:45939631 | 0 | . | . | . | . | . | . | . | . | . | . |
| logepa | chr10:45944193:D | 0.008 | . | . | . | . | . | . | . | . | . | . |
| logepa | chr10:45954030 | 0 | . | . | . | . | . | . | . | . | . | . |

Supplemental table S32.

| fa | rs_id | freq | HR_logepa | se_logepa | p_logepa | HR_snp | se_snp | p_snp | HR_inter | se_inter | p_inter | RERI |
| --- | --- | --- | --- | --- | --- | --- | --- | --- | --- | --- | --- | --- |
| logepa | rs76035182 | 0.001 | . | . | . | . | . | . | . | . | . | . |
| logepa | rs12359547 | 0.105 | 0.746 | 0.104 | 0.035 | 0.981 | 0.222 | 0.932 | 1.23 | 0.459 | 0.571 | 0.177 |
| logepa | rs74595484 | 0 | . | . | . | . | . | . | . | . | . | . |
| logepa | rs1864415 | 0.605 | 0.896 | 0.246 | 0.69 | 1.08 | 0.15 | 0.592 | 0.892 | 0.163 | 0.533 | -0.112 |
| logepa | rs72788101 | 0.105 | 0.746 | 0.104 | 0.036 | 0.982 | 0.222 | 0.935 | 1.23 | 0.458 | 0.573 | 0.176 |
| logepa | rs11239481 | 0.183 | 0.827 | 0.122 | 0.2 | 0.79 | 0.158 | 0.239 | 0.796 | 0.203 | 0.372 | -0.097 |
| logepa | rs72788102 | 0.105 | 0.747 | 0.104 | 0.036 | 0.982 | 0.222 | 0.936 | 1.23 | 0.458 | 0.573 | 0.175 |
| logepa | rs72789705 | 0.105 | 0.747 | 0.104 | 0.036 | 0.982 | 0.222 | 0.936 | 1.23 | 0.458 | 0.573 | 0.175 |
| logepa | rs141529858 | 0.008 | . | . | . | . | . | . | . | . | . | . |
| logepa | rs145376292 | 0 | . | . | . | . | . | . | . | . | . | . |
| logepa | rs190418301 | 0.001 | . | . | . | . | . | . | . | . | . | . |
| logepa | rs11239482 | 0.105 | 0.747 | 0.104 | 0.036 | 0.982 | 0.223 | 0.936 | 1.23 | 0.458 | 0.573 | 0.175 |
| logepa | rs61855872 | 0.014 | 0.804 | 0.108 | 0.106 | 0.462 | 0.406 | 0.38 | 0.256 | 0.168 | 0.038 | -0.171 |
| logepa | rs148991144 | 0.011 | 0.785 | 0.104 | 0.066 | 0.022 | 0.022 | 1.50E-04 | 0.275 | 0.289 | 0.22 | 0.198 |
| logepa | rs191932221 | 0.007 | . | . | . | . | . | . | . | . | . | . |
| logepa | rs55978138 | 0.159 | 0.769 | 0.112 | 0.071 | 0.969 | 0.193 | 0.875 | 1.04 | 0.306 | 0.882 | 0.04 |
| logepa | rs73281167 | 0 | . | . | . | . | . | . | . | . | . | . |
| logepa | rs12768010 | 0.055 | 0.8 | 0.111 | 0.107 | 0.906 | 0.321 | 0.78 | 0.707 | 0.287 | 0.393 | -0.193 |
| logepa | rs11239484 | 0.605 | 0.895 | 0.246 | 0.685 | 1.08 | 0.15 | 0.59 | 0.894 | 0.163 | 0.538 | -0.111 |
| logepa | rs35906360 | 0.16 | 0.769 | 0.112 | 0.071 | 0.969 | 0.193 | 0.875 | 1.04 | 0.306 | 0.883 | 0.04 |
| logepa | rs186250512 | 0.004 | . | . | . | . | . | . | . | . | . | . |
| logepa | rs150810743 | 0 | . | . | . | . | . | . | . | . | . | . |
| logepa | rs55965812 | 0.16 | 0.769 | 0.112 | 0.071 | 0.969 | 0.193 | 0.875 | 1.04 | 0.306 | 0.883 | 0.04 |
| logepa | rs56180358 | 0.16 | 0.769 | 0.112 | 0.071 | 0.969 | 0.193 | 0.874 | 1.04 | 0.306 | 0.883 | 0.04 |
| logepa | rs55864145 | 0.055 | 0.8 | 0.111 | 0.107 | 0.906 | 0.321 | 0.78 | 0.707 | 0.287 | 0.393 | -0.194 |
| logepa | rs55986122 | 0.16 | 0.769 | 0.112 | 0.071 | 0.969 | 0.193 | 0.875 | 1.04 | 0.306 | 0.883 | 0.04 |
| logepa | rs7477350 | 0.605 | 0.894 | 0.245 | 0.683 | 1.08 | 0.15 | 0.588 | 0.894 | 0.164 | 0.541 | -0.11 |
| logepa | rs2099170 | 0.159 | 0.769 | 0.112 | 0.072 | 0.972 | 0.194 | 0.887 | 1.04 | 0.306 | 0.886 | 0.039 |
| logepa | rs2082108 | 0.16 | 0.769 | 0.112 | 0.071 | 0.969 | 0.193 | 0.876 | 1.04 | 0.306 | 0.883 | 0.04 |
| logepa | rs55902404 | 0 | . | . | . | . | . | . | . | . | . | . |
| logepa | rs58402662 | 0 | . | . | . | . | . | . | . | . | . | . |
| logepa | rs11511618 | 0.182 | 0.829 | 0.122 | 0.205 | 0.774 | 0.158 | 0.209 | 0.785 | 0.203 | 0.35 | -0.1 |
| logepa | rs1816492 | 0.605 | 0.893 | 0.245 | 0.682 | 1.08 | 0.15 | 0.587 | 0.894 | 0.164 | 0.542 | -0.11 |
| logepa | rs146611093 | 0.01 | 0.759 | 0.1 | 0.037 | 1.2 | 1 | 0.83 | 4.92 | 8.45 | 0.353 | 3.51 |
| logepa | rs186897689 | 0 | . | . | . | . | . | . | . | . | . | . |
| logepa | rs10793616 | 0.605 | 0.893 | 0.245 | 0.681 | 1.08 | 0.15 | 0.587 | 0.895 | 0.164 | 0.543 | -0.11 |
| logepa | rs116700479 | 0.001 | . | . | . | . | . | . | . | . | . | . |
| logepa | rs192535936 | 0 | . | . | . | . | . | . | . | . | . | . |
| logepa | rs113363297 | 0.571 | 0.863 | 0.232 | 0.583 | 1.11 | 0.157 | 0.466 | 0.915 | 0.171 | 0.633 | -0.096 |
| logepa | rs12354994 | 0.152 | 0.769 | 0.111 | 0.07 | 0.968 | 0.198 | 0.873 | 1.05 | 0.314 | 0.883 | 0.041 |
| logepa | rs148068329 | 0.001 | . | . | . | . | . | . | . | . | . | . |
| logepa | rs12356109 | 0.16 | 0.769 | 0.112 | 0.071 | 0.969 | 0.193 | 0.875 | 1.04 | 0.306 | 0.884 | 0.04 |
| logepa | rs55908141 | 0 | . | . | . | . | . | . | . | . | . | . |
| logepa | rs12354712 | 0.159 | 0.769 | 0.112 | 0.071 | 0.97 | 0.193 | 0.878 | 1.04 | 0.306 | 0.884 | 0.04 |
| logepa | rs147282684 | 0.004 | . | . | . | . | . | . | . | . | . | . |
| logepa | rs12356140 | 0.16 | 0.77 | 0.112 | 0.072 | 0.97 | 0.194 | 0.878 | 1.04 | 0.305 | 0.891 | 0.038 |
| logepa | rs185476187 | 0 | . | . | . | . | . | . | . | . | . | . |
| logepa | rs145720057 | 0.039 | 0.791 | 0.103 | 0.071 | 0.929 | 0.398 | 0.863 | 0.794 | 0.569 | 0.748 | -0.136 |
| logepa | rs12354742 | 0.159 | 0.769 | 0.112 | 0.071 | 0.97 | 0.193 | 0.878 | 1.04 | 0.306 | 0.884 | 0.04 |
| logepa | rs180913842 | 0 | . | . | . | . | . | . | . | . | . | . |
| logepa | rs142976914 | 0.006 | . | . | . | . | . | . | . | . | . | . |
| logepa | rs11511619 | 0.163 | 0.773 | 0.114 | 0.08 | 0.961 | 0.191 | 0.84 | 1.03 | 0.3 | 0.932 | 0.028 |
| logepa | rs146702870 | 0 | . | . | . | . | . | . | . | . | . | . |
| logepa | rs150287496 | 0.002 | . | . | . | . | . | . | . | . | . | . |
| logepa | rs190917973 | 0.006 | . | . | . | . | . | . | . | . | . | . |
| logepa | rs11517281 | 0.16 | 0.769 | 0.112 | 0.071 | 0.969 | 0.193 | 0.876 | 1.04 | 0.306 | 0.885 | 0.039 |
| logepa | rs12359299 | 0.16 | 0.769 | 0.112 | 0.072 | 0.968 | 0.193 | 0.872 | 1.04 | 0.306 | 0.886 | 0.039 |
| logepa | rs182307932 | 0 | . | . | . | . | . | . | . | . | . | . |
| logepa | rs61855873 | 0.035 | 0.828 | 0.114 | 0.172 | 0.524 | 0.323 | 0.295 | 0.331 | 0.232 | 0.114 | -0.209 |
| logepa | rs12359302 | 0.156 | 0.77 | 0.112 | 0.072 | 0.984 | 0.197 | 0.934 | 1.04 | 0.307 | 0.892 | 0.035 |
| logepa | rs143208140 | 0.003 | . | . | . | . | . | . | . | . | . | . |
| logepa | rs9988764 | 0.595 | 0.88 | 0.242 | 0.641 | 1.09 | 0.154 | 0.56 | 0.905 | 0.169 | 0.591 | -0.102 |
| logepa | rs192875767 | 0.008 | . | . | . | . | . | . | . | . | . | . |
| logepa | rs147268846 | 0.015 | 0.805 | 0.107 | 0.103 | 0.032 | 0.047 | 0.02 | 0.033 | 0.038 | 2.70E-03 | 0.164 |
| logepa | rs11239485 | 0.111 | 0.733 | 0.104 | 0.029 | 1.06 | 0.249 | 0.791 | 1.32 | 0.473 | 0.437 | 0.233 |
| logepa | rs151317885 | 0.436 | 0.88 | 0.211 | 0.594 | 1.1 | 0.153 | 0.48 | 0.878 | 0.163 | 0.483 | -0.131 |
| logepa | rs11499085 | 0 | . | . | . | . | . | . | . | . | . | . |
| logepa | rs188130606 | 0 | . | . | . | . | . | . | . | . | . | . |
| logepa | rs11511620 | 0.16 | 0.769 | 0.112 | 0.071 | 0.97 | 0.193 | 0.877 | 1.04 | 0.306 | 0.885 | 0.039 |
| logepa | rs181498390 | 0 | . | . | . | . | . | . | . | . | . | . |
| logepa | rs186280701 | 0 | . | . | . | . | . | . | . | . | . | . |
| logepa | rs150059185 | 0.011 | 0.792 | 0.105 | 0.077 | 0.907 | 0.944 | 0.925 | 0.476 | 0.741 | 0.634 | -0.357 |
| logepa | rs183338707 | 0.004 | . | . | . | . | . | . | . | . | . | . |
| logepa | rs11499038 | 0.002 | . | . | . | . | . | . | . | . | . | . |
| logepa | rs190699595 | 0.008 | . | . | . | . | . | . | . | . | . | . |
| logepa | rs11523408 | 0.16 | 0.769 | 0.112 | 0.071 | 0.97 | 0.193 | 0.877 | 1.04 | 0.306 | 0.885 | 0.039 |
| logepa | rs188049317 | 0 | . | . | . | . | . | . | . | . | . | . |
| logepa | rs144948634 | 0.428 | 0.923 | 0.218 | 0.735 | 1.04 | 0.147 | 0.758 | 0.83 | 0.158 | 0.327 | -0.167 |
| logepa | rs143833576 | 0.001 | . | . | . | . | . | . | . | . | . | . |
| logepa | rs151239879 | 0.16 | 0.774 | 0.112 | 0.077 | 0.97 | 0.194 | 0.88 | 1.02 | 0.299 | 0.942 | 0.023 |
| logepa | rs7073743 | 0.438 | 0.915 | 0.22 | 0.712 | 1.04 | 0.147 | 0.756 | 0.841 | 0.161 | 0.366 | -0.156 |
| logepa | rs142563349 | 0 | . | . | . | . | . | . | . | . | . | . |
| logepa | rs187246403 | 0 | . | . | . | . | . | . | . | . | . | . |
| logepa | rs150423942 | 0.158 | 0.769 | 0.112 | 0.071 | 0.959 | 0.192 | 0.833 | 1.05 | 0.312 | 0.881 | 0.043 |

| logepa | rs184706102 | 0.002 | . | . | . | . | . | . | . | . | . | . |
| --- | --- | --- | --- | --- | --- | --- | --- | --- | --- | --- | --- | --- |
| logepa | rs189626288 | 0.031 | 0.781 | 0.109 | 0.076 | 1.69 | 0.817 | 0.281 | 1.16 | 0.655 | 0.786 | 0.066 |
| logepa | rs140749834 | 0 | . | . | . | . | . | . | . | . | . | . |
| logepa | rs7078822 | 0.131 | 0.754 | 0.108 | 0.047 | 0.99 | 0.214 | 0.965 | 1.15 | 0.354 | 0.654 | 0.113 |
| logepa | rs186227617 | 0.159 | 0.769 | 0.112 | 0.072 | 0.972 | 0.194 | 0.887 | 1.04 | 0.306 | 0.886 | 0.039 |
| logepa | rs188984368 | 0.025 | 0.778 | 0.107 | 0.068 | 0.42 | 0.259 | 0.16 | 1.02 | 0.729 | 0.976 | 0.136 |
| logepa | rs182078109 | 0.027 | 0.783 | 0.109 | 0.078 | 0.307 | 0.223 | 0.104 | 0.859 | 0.71 | 0.854 | 0.116 |
| logepa | rs141456274 | 0.002 | . | . | . | . | . | . | . | . | . | . |
| logepa | rs146976512 | 0.16 | 0.769 | 0.112 | 0.072 | 0.97 | 0.193 | 0.878 | 1.04 | 0.306 | 0.886 | 0.039 |
| logepa | rs138036816 | 0 | . | . | . | . | . | . | . | . | . | . |
| logepa | rs114879777 | 0.001 | . | . | . | . | . | . | . | . | . | . |
| logepa | rs149366475 | 0 | . | . | . | . | . | . | . | . | . | . |
| logepa | rs182463043 | 0 | . | . | . | . | . | . | . | . | . | . |
| logepa | rs144754632 | 0.001 | . | . | . | . | . | . | . | . | . | . |
| logepa | rs55717607 | 0.16 | 0.769 | 0.112 | 0.072 | 0.97 | 0.193 | 0.878 | 1.04 | 0.306 | 0.887 | 0.039 |
| logepa | rs187537973 | 0 | . | . | . | . | . | . | . | . | . | . |
| logepa | rs148085971 | 0 | . | . | . | . | . | . | . | . | . | . |
| logepa | rs11239491 | 0.16 | 0.769 | 0.112 | 0.072 | 0.97 | 0.193 | 0.879 | 1.04 | 0.306 | 0.887 | 0.039 |
| logepa | rs184412184 | 0.008 | . | . | . | . | . | . | . | . | . | . |
| logepa | rs4948998 | 0.607 | 0.896 | 0.247 | 0.692 | 1.08 | 0.152 | 0.572 | 0.892 | 0.164 | 0.535 | -0.113 |
| logepa | rs7076696 | 0.607 | 0.896 | 0.247 | 0.69 | 1.08 | 0.152 | 0.575 | 0.893 | 0.164 | 0.537 | -0.112 |
| logepa | rs11239492 | 0.16 | 0.769 | 0.112 | 0.071 | 0.97 | 0.193 | 0.879 | 1.04 | 0.306 | 0.884 | 0.039 |
| logepa | rs79462470 | 0 | . | . | . | . | . | . | . | . | . | . |
| logepa | rs187804654 | 0.003 | . | . | . | . | . | . | . | . | . | . |
| logepa | rs7093687 | 0.16 | 0.769 | 0.112 | 0.072 | 0.97 | 0.193 | 0.88 | 1.04 | 0.305 | 0.887 | 0.038 |
| logepa | rs7081004 | 0.16 | 0.769 | 0.112 | 0.072 | 0.97 | 0.193 | 0.88 | 1.04 | 0.305 | 0.887 | 0.039 |
| logepa | rs7084793 | 0.16 | 0.769 | 0.112 | 0.072 | 0.97 | 0.193 | 0.879 | 1.04 | 0.306 | 0.886 | 0.039 |
| logepa | rs61556080 | 0 | . | . | . | . | . | . | . | . | . | . |
| logepa | rs115552064 | . | . | . | . | . | . | . | . | . | . | . |
| logepa | rs7921503 | 0.16 | 0.769 | 0.112 | 0.071 | 0.97 | 0.193 | 0.88 | 1.04 | 0.306 | 0.885 | 0.039 |
| logepa | rs7909740 | 0.16 | 0.769 | 0.112 | 0.071 | 0.97 | 0.193 | 0.88 | 1.04 | 0.306 | 0.885 | 0.039 |
| logepa | rs7909768 | 0.16 | 0.769 | 0.112 | 0.071 | 0.97 | 0.193 | 0.88 | 1.04 | 0.306 | 0.884 | 0.039 |
| logepa | rs144168004 | 0 | . | . | . | . | . | . | . | . | . | . |
| logepa | rs75850448 | 0 | . | . | . | . | . | . | . | . | . | . |
| logepa | rs147777574 | 0 | . | . | . | . | . | . | . | . | . | . |
| logepa | rs7913488 | 0.16 | 0.769 | 0.112 | 0.071 | 0.97 | 0.193 | 0.88 | 1.04 | 0.306 | 0.884 | 0.039 |
| logepa | rs74390874 | 0.071 | 0.786 | 0.116 | 0.102 | 1.28 | 0.397 | 0.434 | 0.97 | 0.4 | 0.942 | -0.089 |
| logepa | rs141391142 | 0.009 | . | . | . | . | . | . | . | . | . | . |
| logepa | rs115294747 | 0.001 | . | . | . | . | . | . | . | . | . | . |
| logepa | rs148031781 | 0.003 | . | . | . | . | . | . | . | . | . | . |
| logepa | rs181340128 | 0.021 | 0.846 | 0.112 | 0.206 | 0.03 | 0.04 | 7.60E-03 | 0.015 | 0.018 | 6.90E-04 | 0.125 |
| logepa | rs189930007 | 0.002 | . | . | . | . | . | . | . | . | . | . |
| logepa | rs12242128 | 0 | . | . | . | . | . | . | . | . | . | . |
| logepa | rs78421750 | 0.002 | . | . | . | . | . | . | . | . | . | . |
| logepa | rs114370747 | 0.002 | . | . | . | . | . | . | . | . | . | . |
| logepa | rs12264166 | 0 | . | . | . | . | . | . | . | . | . | . |
| logepa | rs182964822 | 0.001 | . | . | . | . | . | . | . | . | . | . |
| logepa | rs67491417 | 0.15 | 0.775 | 0.113 | 0.079 | 1.01 | 0.206 | 0.944 | 1.01 | 0.305 | 0.965 | 7.10E-03 |
| logepa | rs72789720 | 0.024 | 0.726 | 0.101 | 0.021 | 1.55 | 0.757 | 0.369 | 3.97 | 3.32 | 0.099 | 3.2 |
| logepa | rs6413416 | 0.16 | 0.776 | 0.114 | 0.083 | 0.987 | 0.197 | 0.948 | 1.01 | 0.297 | 0.968 | 0.012 |
| logepa | rs4986832 | 0.16 | 0.769 | 0.112 | 0.071 | 0.971 | 0.193 | 0.881 | 1.05 | 0.306 | 0.88 | 0.04 |
| logepa | rs144866316 | 0.005 | . | . | . | . | . | . | . | . | . | . |
| logepa | rs187769540 | 0.001 | . | . | . | . | . | . | . | . | . | . |
| logepa | rs6593482 | 0.16 | 0.779 | 0.115 | 0.089 | 0.948 | 0.19 | 0.79 | 1 | 0.286 | 0.997 | 0.012 |
| logepa | rs7913948 | 0.16 | 0.769 | 0.112 | 0.072 | 0.97 | 0.193 | 0.877 | 1.04 | 0.306 | 0.887 | 0.039 |
| logepa | rs77185151 | 0.001 | . | . | . | . | . | . | . | . | . | . |
| logepa | rs12762303 | 0.16 | 0.769 | 0.112 | 0.071 | 0.97 | 0.193 | 0.879 | 1.05 | 0.306 | 0.878 | 0.041 |
| logepa | rs73283136 | 0.003 | . | . | . | . | . | . | . | . | . | . |
| logepa | rs187180218 | 0 | . | . | . | . | . | . | . | . | . | . |
| logepa | rs78137087 | 0.159 | 0.767 | 0.112 | 0.069 | 0.972 | 0.194 | 0.888 | 1.05 | 0.309 | 0.861 | 0.046 |
| logepa | rs72561472 | 0.158 | 0.783 | 0.117 | 0.1 | 0.918 | 0.194 | 0.686 | 0.982 | 0.299 | 0.952 | 4.80E-03 |
| logepa | rs4987106 | 0.152 | 0.79 | 0.115 | 0.105 | 0.91 | 0.195 | 0.659 | 0.951 | 0.296 | 0.871 | -0.016 |
| logepa | rs4987105 | 0.155 | 0.787 | 0.115 | 0.101 | 0.898 | 0.191 | 0.613 | 0.962 | 0.295 | 0.9 | -5.10E-03 |
| logepa | rs111916157 | 0.188 | 0.771 | 0.118 | 0.09 | 1.07 | 0.202 | 0.738 | 1.03 | 0.269 | 0.92 | 6.90E-03 |
| logepa | rs111528152 | 0.16 | 0.768 | 0.112 | 0.071 | 0.969 | 0.193 | 0.876 | 1.05 | 0.307 | 0.875 | 0.042 |
| logepa | rs12763738 | 0.16 | 0.769 | 0.112 | 0.071 | 0.97 | 0.193 | 0.879 | 1.05 | 0.306 | 0.878 | 0.041 |
| logepa | rs191675886 | 0.006 | . | . | . | . | . | . | . | . | . | . |
| logepa | rs66489870 | 0.16 | 0.769 | 0.112 | 0.071 | 0.97 | 0.193 | 0.879 | 1.05 | 0.306 | 0.877 | 0.041 |
| logepa | rs71494775 | 0.16 | 0.769 | 0.112 | 0.071 | 0.97 | 0.193 | 0.879 | 1.05 | 0.306 | 0.877 | 0.041 |
| logepa | rs61855875 | 0.033 | 0.821 | 0.113 | 0.152 | 0.543 | 0.336 | 0.323 | 0.367 | 0.264 | 0.163 | -0.2 |
| logepa | rs116356837 | 0 | . | . | . | . | . | . | . | . | . | . |
| logepa | rs1864414 | 0.16 | 0.769 | 0.112 | 0.071 | 0.97 | 0.193 | 0.88 | 1.05 | 0.306 | 0.877 | 0.041 |
| logepa | rs1864413 | 0.16 | 0.768 | 0.112 | 0.07 | 0.973 | 0.193 | 0.89 | 1.05 | 0.306 | 0.867 | 0.044 |
| logepa | rs139497212 | 0 | . | . | . | . | . | . | . | . | . | . |
| logepa | rs892690 | 0.359 | 0.888 | 0.194 | 0.586 | 1.09 | 0.159 | 0.537 | 0.847 | 0.157 | 0.37 | -0.16 |
| logepa | rs1864412 | 0.16 | 0.768 | 0.112 | 0.07 | 0.97 | 0.193 | 0.88 | 1.05 | 0.306 | 0.875 | 0.042 |
| logepa | rs77827771 | 0.004 | . | . | . | . | . | . | . | . | . | . |
| logepa | rs145294963 | 0.004 | . | . | . | . | . | . | . | . | . | . |
| logepa | rs149081327 | 0.012 | 0.783 | 0.106 | 0.072 | 1.76 | 0.864 | 0.25 | 0.576 | 0.492 | 0.519 | -0.749 |
| logepa | rs67008983 | 0.16 | 0.768 | 0.112 | 0.069 | 0.971 | 0.194 | 0.881 | 1.05 | 0.307 | 0.864 | 0.045 |
| logepa | rs56150414 | 0.168 | 0.765 | 0.113 | 0.071 | 0.973 | 0.188 | 0.89 | 1.06 | 0.3 | 0.837 | 0.051 |
| logepa | rs55787481 | 0.16 | 0.768 | 0.112 | 0.07 | 0.969 | 0.194 | 0.877 | 1.05 | 0.308 | 0.866 | 0.045 |
| logepa | rs141270101 | 0.001 | . | . | . | . | . | . | . | . | . | . |
| logepa | rs114980075 | 0.002 | . | . | . | . | . | . | . | . | . | . |
| logepa | rs12783095 | 0.159 | 0.758 | 0.111 | 0.059 | 0.97 | 0.193 | 0.88 | 1.1 | 0.32 | 0.744 | 0.081 |
| logepa | rs182342688 | 0.005 | . | . | . | . | . | . | . | . | . | . |

| logepa | rs17157728 | 0.159 | 0.758 | 0.111 | 0.059 | 0.97 | 0.193 | 0.88 | 1.1 | 0.32 | 0.742 | 0.081 |
| --- | --- | --- | --- | --- | --- | --- | --- | --- | --- | --- | --- | --- |
| logepa | rs114835563 | 0 | . | . | . | . | . | . | . | . | . | . |
| logepa | rs3824612 | 0.359 | 0.884 | 0.193 | 0.574 | 1.09 | 0.159 | 0.533 | 0.851 | 0.158 | 0.383 | -0.156 |
| logepa | rs17157733 | 0.158 | 0.757 | 0.111 | 0.058 | 0.972 | 0.193 | 0.886 | 1.1 | 0.321 | 0.732 | 0.084 |
| logepa | rs74128478 | 0 | . | . | . | . | . | . | . | . | . | . |
| logepa | rs184973589 | 0.001 | . | . | . | . | . | . | . | . | . | . |
| logepa | rs150479461 | 0 | . | . | . | . | . | . | . | . | . | . |
| logepa | rs138310407 | 0.004 | . | . | . | . | . | . | . | . | . | . |
| logepa | rs187426603 | 0.001 | . | . | . | . | . | . | . | . | . | . |
| logepa | rs190843143 | 0.003 | . | . | . | . | . | . | . | . | . | . |
| logepa | rs191883449 | 0.003 | . | . | . | . | . | . | . | . | . | . |
| logepa | rs58238740 | 0.158 | 0.757 | 0.111 | 0.057 | 0.971 | 0.193 | 0.882 | 1.11 | 0.322 | 0.726 | 0.086 |
| logepa | rs3780894 | 0.158 | 0.757 | 0.111 | 0.057 | 0.971 | 0.193 | 0.883 | 1.11 | 0.322 | 0.724 | 0.086 |
| logepa | rs149646626 | 0.027 | 0.822 | 0.11 | 0.141 | 0.148 | 0.12 | 0.018 | 0.119 | 0.09 | 4.80E-03 | 0.045 |
| logepa | rs79615212 | 0.023 | 0.83 | 0.111 | 0.165 | 0.147 | 0.16 | 0.079 | 0.103 | 0.142 | 0.098 | 0.036 |
| logepa | rs3780895 | 0.158 | 0.757 | 0.111 | 0.057 | 0.971 | 0.193 | 0.883 | 1.11 | 0.322 | 0.724 | 0.087 |
| logepa | rs3780896 | 0.158 | 0.757 | 0.111 | 0.057 | 0.971 | 0.193 | 0.883 | 1.11 | 0.322 | 0.724 | 0.087 |
| logepa | rs142747163 | 0.006 | . | . | . | . | . | . | . | . | . | . |
| logepa | rs189417584 | 0.005 | . | . | . | . | . | . | . | . | . | . |
| logepa | rs3780897 | 0.158 | 0.757 | 0.111 | 0.057 | 0.972 | 0.193 | 0.886 | 1.11 | 0.322 | 0.724 | 0.086 |
| logepa | rs117509491 | 0.004 | . | . | . | . | . | . | . | . | . | . |
| logepa | rs117811514 | 0.004 | . | . | . | . | . | . | . | . | . | . |
| logepa | rs116500932 | 0 | . | . | . | . | . | . | . | . | . | . |
| logepa | rs77584168 | 0.001 | . | . | . | . | . | . | . | . | . | . |
| logepa | rs2310776 | 0.158 | 0.756 | 0.111 | 0.057 | 0.971 | 0.193 | 0.883 | 1.11 | 0.322 | 0.722 | 0.087 |
| logepa | rs17157736 | 0.158 | 0.756 | 0.111 | 0.057 | 0.971 | 0.193 | 0.883 | 1.11 | 0.322 | 0.722 | 0.087 |
| logepa | rs72789735 | 0.105 | 0.747 | 0.104 | 0.036 | 0.988 | 0.223 | 0.959 | 1.23 | 0.455 | 0.577 | 0.172 |
| logepa | rs183747187 | 0.002 | . | . | . | . | . | . | . | . | . | . |
| logepa | rs7077891 | 0.158 | 0.756 | 0.111 | 0.057 | 0.971 | 0.193 | 0.883 | 1.11 | 0.323 | 0.721 | 0.087 |
| logepa | rs145063305 | 0.006 | . | . | . | . | . | . | . | . | . | . |
| logepa | rs3824613 | 0.159 | 0.757 | 0.111 | 0.057 | 0.971 | 0.193 | 0.882 | 1.11 | 0.322 | 0.724 | 0.087 |
| logepa | rs145543199 | 0.014 | 0.758 | 0.104 | 0.044 | 1.31 | 0.894 | 0.691 | 2.16 | 2.83 | 0.554 | 1.08 |
| logepa | rs140361993 | 0.005 | . | . | . | . | . | . | . | . | . | . |
| logepa | rs11239496 | 0.105 | 0.747 | 0.104 | 0.036 | 0.988 | 0.223 | 0.959 | 1.23 | 0.455 | 0.576 | 0.173 |
| logepa | rs11239497 | 0.158 | 0.756 | 0.111 | 0.057 | 0.971 | 0.193 | 0.882 | 1.11 | 0.323 | 0.72 | 0.088 |
| logepa | rs10900212 | 0.158 | 0.756 | 0.111 | 0.057 | 0.971 | 0.193 | 0.882 | 1.11 | 0.323 | 0.72 | 0.088 |
| logepa | rs186487136 | 0 | . | . | . | . | . | . | . | . | . | . |
| logepa | rs12774369 | 0.158 | 0.756 | 0.111 | 0.057 | 0.97 | 0.193 | 0.878 | 1.11 | 0.324 | 0.717 | 0.089 |
| logepa | rs60842538 | 0.158 | 0.756 | 0.111 | 0.057 | 0.969 | 0.193 | 0.874 | 1.11 | 0.326 | 0.712 | 0.091 |
| logepa | rs3780898 | 0.158 | 0.755 | 0.111 | 0.057 | 0.967 | 0.194 | 0.868 | 1.12 | 0.327 | 0.707 | 0.093 |
| logepa | rs3780899 | 0.158 | 0.755 | 0.111 | 0.057 | 0.966 | 0.194 | 0.864 | 1.12 | 0.328 | 0.705 | 0.094 |
| logepa | rs12252472 | 0 | . | . | . | . | . | . | . | . | . | . |
| logepa | rs185411011 | 0.003 | . | . | . | . | . | . | . | . | . | . |
| logepa | rs28395865 | 0.156 | 0.748 | 0.11 | 0.049 | 0.971 | 0.196 | 0.885 | 1.16 | 0.338 | 0.603 | 0.126 |
| logepa | rs191004074 | 0.024 | 0.767 | 0.104 | 0.052 | 0.861 | 0.586 | 0.826 | 1.39 | 1.73 | 0.79 | 0.292 |
| logepa | rs182401295 | 0.004 | . | . | . | . | . | . | . | . | . | . |
| logepa | rs2228064 | 0.002 | . | . | . | . | . | . | . | . | . | . |
| logepa | rs188284159 | 0.001 | . | . | . | . | . | . | . | . | . | . |
| logepa | rs2303887 | 0.001 | . | . | . | . | . | . | . | . | . | . |
| logepa | rs2303886 | 0.001 | . | . | . | . | . | . | . | . | . | . |
| logepa | rs192621006 | 0.003 | . | . | . | . | . | . | . | . | . | . |
| logepa | rs2303885 | 0.154 | 0.751 | 0.11 | 0.05 | 0.933 | 0.191 | 0.735 | 1.16 | 0.371 | 0.64 | 0.13 |
| logepa | rs114466168 | 0 | . | . | . | . | . | . | . | . | . | . |
| logepa | rs11239501 | 0.149 | 0.776 | 0.113 | 0.081 | 0.896 | 0.171 | 0.567 | 1.03 | 0.282 | 0.917 | 0.043 |
| logepa | rs745986 | 0.204 | 0.779 | 0.12 | 0.104 | 0.891 | 0.157 | 0.512 | 1.02 | 0.257 | 0.945 | 0.036 |
| logepa | rs75262268 | 0 | . | . | . | . | . | . | . | . | . | . |
| logepa | rs71494776 | 0.049 | 0.787 | 0.107 | 0.078 | 0.757 | 0.26 | 0.418 | 0.843 | 0.325 | 0.657 | -0.042 |
| logepa | rs74386871 | 0.011 | 0.735 | 0.101 | 0.025 | 1.99 | 1.71 | 0.426 | 6.55 | 4.38 | 5.00E-03 | 7.84 |
| logepa | rs76502040 | 0.01 | 0.748 | 0.1 | 0.029 | 2.56 | 1.62 | 0.139 | 5.4 | 5.67 | 0.108 | 8.03 |
| logepa | rs142213734 | 0.001 | . | . | . | . | . | . | . | . | . | . |
| logepa | rs4948671 | 0.604 | 0.845 | 0.234 | 0.544 | 1.08 | 0.152 | 0.573 | 0.934 | 0.172 | 0.709 | -0.073 |
| logepa | rs146286085 | 0.002 | . | . | . | . | . | . | . | . | . | . |
| logepa | rs144080623 | 0 | . | . | . | . | . | . | . | . | . | . |
| logepa | rs115074014 | 0 | . | . | . | . | . | . | . | . | . | . |
| logepa | rs17157756 | 0 | . | . | . | . | . | . | . | . | . | . |
| logepa | rs190459780 | 0.001 | . | . | . | . | . | . | . | . | . | . |
| logepa | rs12358647 | 0.204 | 0.778 | 0.12 | 0.104 | 0.895 | 0.157 | 0.529 | 1.02 | 0.256 | 0.941 | 0.036 |
| logepa | rs147080556 | 0.011 | 0.779 | 0.104 | 0.061 | 1.08 | 0.735 | 0.913 | 0.989 | 0.76 | 0.988 | -0.027 |
| logepa | rs187543349 | 0.004 | . | . | . | . | . | . | . | . | . | . |
| logepa | rs114375595 | 0.004 | . | . | . | . | . | . | . | . | . | . |
| logepa | rs117211200 | 0.01 | . | . | . | . | . | . | . | . | . | . |
| logepa | rs192963047 | 0.001 | . | . | . | . | . | . | . | . | . | . |
| logepa | rs11239505 | 0.204 | 0.778 | 0.12 | 0.104 | 0.895 | 0.157 | 0.529 | 1.02 | 0.256 | 0.94 | 0.037 |
| logepa | rs184434116 | 0.003 | . | . | . | . | . | . | . | . | . | . |
| logepa | rs4948999 | 0.604 | 0.843 | 0.234 | 0.539 | 1.09 | 0.152 | 0.558 | 0.936 | 0.172 | 0.717 | -0.072 |
| logepa | rs11239506 | 0.155 | 0.768 | 0.113 | 0.072 | 0.943 | 0.176 | 0.752 | 1.06 | 0.284 | 0.841 | 0.053 |
| logepa | rs190706620 | 0.006 | . | . | . | . | . | . | . | . | . | . |
| logepa | rs190770429 | 0.002 | . | . | . | . | . | . | . | . | . | . |
| logepa | rs181327133 | 0.006 | . | . | . | . | . | . | . | . | . | . |
| logepa | rs149251697 | 0.008 | . | . | . | . | . | . | . | . | . | . |
| logepa | rs118081602 | 0.001 | . | . | . | . | . | . | . | . | . | . |
| logepa | rs188233691 | 0.001 | . | . | . | . | . | . | . | . | . | . |
| logepa | rs183778794 | 0.005 | . | . | . | . | . | . | . | . | . | . |
| logepa | rs188040078 | 0.007 | . | . | . | . | . | . | . | . | . | . |
| logepa | rs184675372 | 0.003 | . | . | . | . | . | . | . | . | . | . |
| logepa | rs150326891 | 0.116 | 0.798 | 0.114 | 0.116 | 0.85 | 0.183 | 0.45 | 0.927 | 0.276 | 0.798 | -0.019 |

| logepa | rs185520021 | 0.002 | . | . | . | . | . | . | . | . | . | . |
| --- | --- | --- | --- | --- | --- | --- | --- | --- | --- | --- | --- | --- |
| logepa | rs7907779 | 0.155 | 0.767 | 0.113 | 0.072 | 0.945 | 0.176 | 0.76 | 1.06 | 0.283 | 0.84 | 0.053 |
| logepa | rs184962860 | 0.001 | . | . | . | . | . | . | . | . | . | . |
| logepa | rs149762784 | 0.025 | 0.789 | 0.109 | 0.087 | 0.276 | 0.216 | 0.1 | 0.665 | 0.546 | 0.619 | 0.08 |
| logepa | rs149203427 | 0.021 | 0.786 | 0.108 | 0.081 | 2.08 | 1.2 | 0.204 | 1.19 | 0.834 | 0.806 | 0.076 |
| logepa | rs60153083 | 0 | . | . | . | . | . | . | . | . | . | . |
| logepa | rs112001069 | 0.05 | 0.787 | 0.107 | 0.079 | 0.758 | 0.261 | 0.421 | 0.841 | 0.323 | 0.652 | -0.043 |
| logepa | rs182355382 | 0.021 | 0.791 | 0.108 | 0.084 | 0.631 | 0.465 | 0.532 | 0.59 | 0.789 | 0.693 | -0.127 |
| logepa | rs186740974 | 0.117 | 0.897 | 0.151 | 0.518 | 0.942 | 0.314 | 0.859 | 0.576 | 0.219 | 0.147 | -0.352 |
| logepa | rs191874916 | 0.014 | 0.799 | 0.105 | 0.09 | 0.585 | 0.583 | 0.591 | 0.339 | 0.544 | 0.5 | -0.226 |
| logepa | rs182989412 | 0.152 | 0.768 | 0.135 | 0.132 | 1.01 | 0.282 | 0.96 | 1.04 | 0.351 | 0.909 | 0.027 |
| logepa | rs188819476 | 0.002 | . | . | . | . | . | . | . | . | . | . |
| logepa | rs141217183 | 0.078 | 0.808 | 0.117 | 0.141 | 0.59 | 0.216 | 0.15 | 0.706 | 0.313 | 0.433 | -0.061 |
| logepa | rs192922652 | 0.045 | 0.782 | 0.107 | 0.072 | 0.802 | 0.289 | 0.54 | 0.918 | 0.356 | 0.825 | -8.60E-03 |
| logepa | rs185092343 | 0.024 | 0.764 | 0.108 | 0.056 | 1.33 | 0.88 | 0.671 | 1.49 | 1.4 | 0.672 | 0.417 |
| logepa | rs145193311 | 0.051 | 0.783 | 0.107 | 0.074 | 0.774 | 0.279 | 0.478 | 0.927 | 0.353 | 0.842 | 4.70E-03 |
| logepa | rs55789336 | 0.155 | 0.764 | 0.114 | 0.071 | 0.96 | 0.183 | 0.83 | 1.07 | 0.292 | 0.812 | 0.059 |
| logepa | rs55837212 | 0.152 | 0.769 | 0.113 | 0.074 | 0.963 | 0.179 | 0.842 | 1.05 | 0.28 | 0.866 | 0.043 |
| logepa | rs75408574 | 0.366 | 0.88 | 0.188 | 0.549 | 1.13 | 0.163 | 0.381 | 0.864 | 0.161 | 0.433 | -0.152 |
| logepa | rs149306926 | 0.162 | 0.754 | 0.111 | 0.055 | 0.96 | 0.177 | 0.824 | 1.11 | 0.297 | 0.702 | 0.088 |
| logepa | rs35990892 | 0.598 | 0.769 | 0.219 | 0.357 | 1.2 | 0.171 | 0.196 | 1.01 | 0.185 | 0.973 | -0.041 |
| logepa | rs192356207 | 0.019 | 0.774 | 0.104 | 0.055 | 0.951 | 0.515 | 0.926 | 1.29 | 0.738 | 0.654 | 0.226 |
| logepa | rs183741163 | 0.129 | 0.752 | 0.112 | 0.056 | 0.943 | 0.207 | 0.789 | 1.15 | 0.348 | 0.64 | 0.122 |
| logepa | rs34737297 | 0.48 | 0.718 | 0.186 | 0.202 | 1.12 | 0.176 | 0.481 | 1.08 | 0.216 | 0.69 | 0.033 |
| logepa | rs181346933 | 0.026 | 0.702 | 0.096 | 9.90E-03 | 1.28 | 0.644 | 0.629 | 4.82 | 2.56 | 3.10E-03 | 3.33 |
| logepa | rs36093253 | 0.343 | 0.744 | 0.168 | 0.191 | 1.23 | 0.198 | 0.205 | 1.07 | 0.217 | 0.744 | 4.50E-03 |
| logepa | rs186307855 | 0.02 | 0.801 | 0.11 | 0.106 | 0.653 | 0.416 | 0.503 | 0.454 | 0.318 | 0.259 | -0.216 |
| logepa | rs189681865 | 0.003 | . | . | . | . | . | . | . | . | . | . |
| logepa | rs34942971 | 0.207 | 0.786 | 0.126 | 0.135 | 0.928 | 0.17 | 0.686 | 0.987 | 0.247 | 0.958 | 5.70E-03 |
| logepa | rs181816554 | 0.019 | 0.811 | 0.111 | 0.128 | 0.771 | 0.502 | 0.689 | 0.376 | 0.212 | 0.082 | -0.347 |
| logepa | rs186846881 | 0.004 | . | . | . | . | . | . | . | . | . | . |
| logepa | rs191178220 | 0.024 | 0.83 | 0.113 | 0.17 | 0.324 | 0.416 | 0.38 | 0.114 | 0.195 | 0.203 | -0.123 |
| logepa | rs183305228 | 0 | . | . | . | . | . | . | . | . | . | . |
| logepa | rs139912761 | 0.15 | 0.765 | 0.114 | 0.072 | 0.982 | 0.186 | 0.925 | 1.06 | 0.286 | 0.829 | 0.049 |
| logepa | rs185499901 | 0.019 | 0.809 | 0.108 | 0.112 | 0.053 | 0.072 | 0.031 | 0.068 | 0.063 | 3.80E-03 | 0.141 |
| logepa | rs189816197 | 0.061 | 0.78 | 0.109 | 0.076 | 0.859 | 0.26 | 0.617 | 0.96 | 0.331 | 0.906 | 4.20E-03 |
| logepa | rs145656938 | 0.014 | 0.781 | 0.105 | 0.065 | 0.469 | 0.399 | 0.374 | 0.807 | 0.515 | 0.736 | 0.045 |
| logepa | rs35745197 | 0.05 | 0.787 | 0.107 | 0.078 | 0.758 | 0.261 | 0.422 | 0.844 | 0.324 | 0.658 | -0.042 |
| logepa | rs192856637 | 0.006 | . | . | . | . | . | . | . | . | . | . |
| logepa | rs2310775 | 0.402 | 0.852 | 0.189 | 0.472 | 1.15 | 0.159 | 0.31 | 0.907 | 0.163 | 0.588 | -0.113 |
| logepa | rs11239507 | 0.404 | 0.818 | 0.184 | 0.372 | 1.21 | 0.163 | 0.161 | 0.953 | 0.168 | 0.783 | -0.085 |
| logepa | rs74648467 | 0.049 | 0.787 | 0.107 | 0.079 | 0.757 | 0.262 | 0.421 | 0.841 | 0.324 | 0.653 | -0.043 |
| logepa | rs145815934 | 0.022 | 0.791 | 0.108 | 0.085 | 0.311 | 0.247 | 0.141 | 0.491 | 0.274 | 0.203 | 0.019 |
| logepa | rs145390687 | 0.029 | 0.77 | 0.108 | 0.061 | 2.08 | 0.969 | 0.115 | 1.51 | 0.849 | 0.459 | 0.576 |
| logepa | rs184354913 | 0.049 | 0.86 | 0.117 | 0.266 | 0.127 | 0.08 | 1.00E-03 | 0.102 | 0.07 | 8.40E-04 | 0.024 |
| logepa | rs181244920 | 0.007 | . | . | . | . | . | . | . | . | . | . |
| logepa | rs11499087 | 0 | . | . | . | . | . | . | . | . | . | . |
| logepa | rs148263794 | 0.05 | 0.787 | 0.107 | 0.079 | 0.756 | 0.261 | 0.417 | 0.84 | 0.323 | 0.65 | -0.043 |
| logepa | rs150758903 | 0.01 | 0.786 | 0.105 | 0.072 | 0.439 | 0.411 | 0.379 | 0.758 | 0.732 | 0.774 | 0.036 |
| logepa | rs147654382 | 0.183 | 0.8 | 0.122 | 0.143 | 0.928 | 0.166 | 0.675 | 0.93 | 0.24 | 0.779 | -0.037 |
| logepa | rs147495166 | 0 | . | . | . | . | . | . | . | . | . | . |
| logepa | rs35198778 | 0.092 | 0.797 | 0.117 | 0.124 | 0.771 | 0.233 | 0.39 | 0.913 | 0.348 | 0.811 | -7.10E-03 |
| logepa | rs145207659 | 0.071 | 0.768 | 0.113 | 0.073 | 1.28 | 0.391 | 0.417 | 1.14 | 0.454 | 0.741 | 0.073 |
| logepa | rs186466464 | 0.003 | . | . | . | . | . | . | . | . | . | . |
| logepa | rs12778413 | 0.157 | 0.757 | 0.115 | 0.067 | 0.916 | 0.205 | 0.694 | 1.13 | 0.343 | 0.689 | 0.11 |
| logepa | rs7895552 | 0.532 | 0.771 | 0.212 | 0.343 | 1.19 | 0.177 | 0.236 | 1.01 | 0.199 | 0.98 | -0.039 |
| logepa | rs12783895 | 0.05 | 0.787 | 0.107 | 0.078 | 0.758 | 0.261 | 0.421 | 0.843 | 0.323 | 0.656 | -0.042 |
| logepa | rs34929604 | 0.05 | 0.787 | 0.107 | 0.078 | 0.758 | 0.26 | 0.42 | 0.843 | 0.323 | 0.657 | -0.042 |
| logepa | rs12761961 | 0.049 | 0.787 | 0.107 | 0.079 | 0.758 | 0.262 | 0.423 | 0.838 | 0.323 | 0.646 | -0.045 |
| logepa | rs35536930 | 0.198 | 0.78 | 0.121 | 0.109 | 0.916 | 0.162 | 0.617 | 1.01 | 0.257 | 0.978 | 0.024 |
| logepa | rs180951060 | 0 | . | . | . | . | . | . | . | . | . | . |
| logepa | rs148892128 | 0.022 | 0.791 | 0.108 | 0.085 | 0.311 | 0.247 | 0.141 | 0.491 | 0.274 | 0.203 | 0.019 |
| logepa | rs11239509 | 0.001 | . | . | . | . | . | . | . | . | . | . |
| logepa | rs41526545 | 0.155 | 0.767 | 0.113 | 0.072 | 0.952 | 0.176 | 0.792 | 1.06 | 0.282 | 0.836 | 0.052 |
| logepa | rs2029253 | 0.402 | 0.852 | 0.189 | 0.47 | 1.15 | 0.159 | 0.307 | 0.908 | 0.163 | 0.591 | -0.113 |
| logepa | rs17444064 | 0.155 | 0.767 | 0.113 | 0.072 | 0.954 | 0.177 | 0.797 | 1.06 | 0.282 | 0.838 | 0.052 |
| logepa | rs71494779 | 0.05 | 0.787 | 0.107 | 0.078 | 0.756 | 0.259 | 0.415 | 0.844 | 0.323 | 0.657 | -0.041 |
| logepa | rs74991420 | 0.001 | . | . | . | . | . | . | . | . | . | . |
| logepa | rs182736551 | 0.001 | . | . | . | . | . | . | . | . | . | . |
| logepa | rs78702074 | 0.001 | . | . | . | . | . | . | . | . | . | . |
| logepa | rs187791466 | 0.005 | . | . | . | . | . | . | . | . | . | . |
| logepa | rs35300270 | 0.054 | 0.788 | 0.108 | 0.081 | 0.746 | 0.247 | 0.376 | 0.853 | 0.317 | 0.669 | -0.032 |
| logepa | rs6593484 | 0.155 | 0.767 | 0.113 | 0.071 | 0.954 | 0.177 | 0.799 | 1.06 | 0.281 | 0.835 | 0.052 |
| logepa | rs12244601 | 0.004 | . | . | . | . | . | . | . | . | . | . |
| logepa | rs184429574 | 0.005 | . | . | . | . | . | . | . | . | . | . |
| logepa | rs7080897 | 0.05 | 0.787 | 0.107 | 0.078 | 0.757 | 0.259 | 0.416 | 0.844 | 0.323 | 0.657 | -0.041 |
| logepa | rs7097429 | 0.054 | 0.771 | 0.105 | 0.057 | 1.1 | 0.33 | 0.751 | 1.1 | 0.452 | 0.818 | 0.061 |
| logepa | rs77979229 | 0.001 | . | . | . | . | . | . | . | . | . | . |
| logepa | rs11239512 | 0.155 | 0.767 | 0.113 | 0.071 | 0.955 | 0.177 | 0.803 | 1.06 | 0.281 | 0.835 | 0.052 |
| logepa | rs60146827 | 0.05 | 0.787 | 0.107 | 0.078 | 0.757 | 0.259 | 0.416 | 0.843 | 0.323 | 0.656 | -0.042 |
| logepa | rs79032236 | 0 | . | . | . | . | . | . | . | . | . | . |
| logepa | rs140854206 | 0.009 | . | . | . | . | . | . | . | . | . | . |
| logepa | rs184049550 | 0 | . | . | . | . | . | . | . | . | . | . |
| logepa | rs114433607 | 0 | . | . | . | . | . | . | . | . | . | . |
| logepa | rs117653793 | 0.001 | . | . | . | . | . | . | . | . | . | . |
| logepa | rs117039533 | 0.001 | . | . | . | . | . | . | . | . | . | . |

| logepa | rs191660761 | 0.006 | . | . | . | . | . | . | . | . | . | . |
| --- | --- | --- | --- | --- | --- | --- | --- | --- | --- | --- | --- | --- |
| logepa | rs59514896 | 0.049 | 0.787 | 0.107 | 0.079 | 0.757 | 0.26 | 0.418 | 0.84 | 0.323 | 0.65 | -0.044 |
| logepa | rs184661876 | 0.002 | . | . | . | . | . | . | . | . | . | . |
| logepa | rs61854073 | 0.037 | 0.821 | 0.115 | 0.161 | 0.846 | 0.417 | 0.734 | 0.495 | 0.276 | 0.207 | -0.323 |
| logepa | rs2018518 | 0.315 | 0.823 | 0.172 | 0.351 | 1.3 | 0.198 | 0.086 | 0.937 | 0.172 | 0.722 | -0.121 |
| logepa | rs934187 | 0.608 | 0.808 | 0.231 | 0.457 | 1.14 | 0.161 | 0.364 | 0.968 | 0.179 | 0.859 | -0.056 |
| logepa | rs7918542 | 0.205 | 0.777 | 0.121 | 0.104 | 0.908 | 0.158 | 0.579 | 1.02 | 0.254 | 0.937 | 0.035 |
| logepa | rs144311920 | 0 | . | . | . | . | . | . | . | . | . | . |
| logepa | rs7099684 | 0.791 | 1.15 | 0.482 | 0.738 | 0.978 | 0.169 | 0.895 | 0.782 | 0.194 | 0.322 | -0.249 |
| logepa | rs150829171 | 0.002 | . | . | . | . | . | . | . | . | . | . |
| logepa | rs186060697 | 0 | . | . | . | . | . | . | . | . | . | . |
| logepa | rs11239513 | 0.009 | . | . | . | . | . | . | . | . | . | . |
| logepa | rs7917687 | 0.05 | 0.787 | 0.107 | 0.078 | 0.759 | 0.26 | 0.421 | 0.843 | 0.323 | 0.656 | -0.042 |
| logepa | rs61854076 | 0.014 | 0.805 | 0.108 | 0.107 | 0.463 | 0.407 | 0.381 | 0.246 | 0.165 | 0.037 | -0.176 |
| logepa | rs71494780 | 0.019 | 0.792 | 0.105 | 0.08 | 1.09 | 0.519 | 0.858 | 0.506 | 0.316 | 0.275 | -0.445 |
| logepa | rs7919239 | 0.205 | 0.776 | 0.121 | 0.103 | 0.91 | 0.158 | 0.587 | 1.02 | 0.254 | 0.933 | 0.035 |
| logepa | rs78334475 | 0.003 | . | . | . | . | . | . | . | . | . | . |
| logepa | rs3780900 | 0.011 | 0.776 | 0.103 | 0.056 | 1 | 0.959 | 0.997 | 1.17 | 2 | 0.928 | 0.129 |
| logepa | rs186729292 | 0.001 | . | . | . | . | . | . | . | . | . | . |
| logepa | rs12267438 | 0.001 | . | . | . | . | . | . | . | . | . | . |
| logepa | rs1369214 | 0.454 | 0.851 | 0.197 | 0.485 | 1.15 | 0.153 | 0.285 | 0.914 | 0.158 | 0.602 | -0.107 |
| logepa | rs9282589 | 0.011 | 0.772 | 0.104 | 0.054 | 1.25 | 1.03 | 0.79 | 1.41 | 1.01 | 0.625 | 0.343 |
| logepa | rs114522915 | 0 | . | . | . | . | . | . | . | . | . | . |
| logepa | rs2115819 | 0.452 | 0.886 | 0.202 | 0.597 | 1.1 | 0.151 | 0.48 | 0.873 | 0.153 | 0.437 | -0.136 |
| logepa | rs117664603 | 0.001 | . | . | . | . | . | . | . | . | . | . |
| logepa | rs184979202 | 0.001 | . | . | . | . | . | . | . | . | . | . |
| logepa | rs190714671 | 0.001 | . | . | . | . | . | . | . | . | . | . |
| logepa | rs141538996 | 0 | . | . | . | . | . | . | . | . | . | . |
| logepa | rs150881741 | 0.003 | . | . | . | . | . | . | . | . | . | . |
| logepa | rs139076633 | 0 | . | . | . | . | . | . | . | . | . | . |
| logepa | rs12243649 | 0.002 | . | . | . | . | . | . | . | . | . | . |
| logepa | rs140348708 | 0.001 | . | . | . | . | . | . | . | . | . | . |
| logepa | rs79023256 | 0.001 | . | . | . | . | . | . | . | . | . | . |
| logepa | rs11239515 | 0.146 | 0.768 | 0.113 | 0.072 | 0.96 | 0.182 | 0.83 | 1.05 | 0.285 | 0.849 | 0.048 |
| logepa | rs55866086 | 0.148 | 0.743 | 0.111 | 0.046 | 1.03 | 0.192 | 0.859 | 1.15 | 0.297 | 0.587 | 0.107 |
| logepa | rs181340150 | 0.005 | . | . | . | . | . | . | . | . | . | . |
| logepa | rs11239516 | 0.146 | 0.767 | 0.113 | 0.072 | 0.959 | 0.182 | 0.827 | 1.05 | 0.285 | 0.847 | 0.049 |
| logepa | rs11239517 | 0.146 | 0.767 | 0.113 | 0.071 | 0.959 | 0.182 | 0.826 | 1.05 | 0.285 | 0.845 | 0.049 |
| logepa | rs11239519 | 0.135 | 0.77 | 0.112 | 0.073 | 0.948 | 0.191 | 0.793 | 1.05 | 0.296 | 0.87 | 0.046 |
| logepa | rs7909514 | 0.601 | 0.854 | 0.242 | 0.577 | 1.14 | 0.166 | 0.359 | 0.927 | 0.172 | 0.684 | -0.092 |
| logepa | rs7894352 | 0.782 | 1.12 | 0.435 | 0.762 | 0.999 | 0.173 | 0.995 | 0.791 | 0.182 | 0.309 | -0.235 |
| logepa | rs189384146 | 0.004 | . | . | . | . | . | . | . | . | . | . |
| logepa | rs11239520 | 0.146 | 0.767 | 0.113 | 0.071 | 0.959 | 0.182 | 0.826 | 1.05 | 0.285 | 0.846 | 0.049 |
| logepa | rs12221233 | 0.405 | 0.846 | 0.189 | 0.452 | 1.21 | 0.164 | 0.163 | 0.918 | 0.162 | 0.629 | -0.116 |
| logepa | rs74523293 | 0.001 | . | . | . | . | . | . | . | . | . | . |
| logepa | rs79376040 | 0.001 | . | . | . | . | . | . | . | . | . | . |
| logepa | rs4949000 | 0.405 | 0.846 | 0.189 | 0.452 | 1.21 | 0.164 | 0.162 | 0.918 | 0.162 | 0.63 | -0.116 |
| logepa | rs7089406 | 0.018 | 0.753 | 0.103 | 0.037 | 1.5 | 0.621 | 0.33 | 2 | 0.767 | 0.071 | 1 |
| logepa | rs138211056 | 0 | . | . | . | . | . | . | . | . | . | . |
| logepa | rs10900213 | 0.549 | 0.815 | 0.216 | 0.44 | 1.19 | 0.17 | 0.229 | 0.961 | 0.174 | 0.824 | -0.073 |
| logepa | rs145152827 | 0 | . | . | . | . | . | . | . | . | . | . |
| logepa | rs74443325 | 0.001 | . | . | . | . | . | . | . | . | . | . |
| logepa | rs188299446 | 0.016 | 0.791 | 0.107 | 0.083 | 1.47 | 0.968 | 0.558 | 0.586 | 0.646 | 0.628 | -0.58 |
| logepa | rs192699586 | 0.002 | . | . | . | . | . | . | . | . | . | . |
| logepa | rs72796464 | 0.145 | 0.766 | 0.112 | 0.07 | 0.956 | 0.181 | 0.813 | 1.06 | 0.287 | 0.832 | 0.054 |
| logepa | rs4949001 | 0.268 | 0.842 | 0.164 | 0.378 | 1.23 | 0.199 | 0.196 | 0.881 | 0.174 | 0.521 | -0.16 |
| logepa | rs147174641 | 0.013 | 0.764 | 0.1 | 0.039 | 0.484 | 0.455 | 0.44 | 3.82 | 3.1 | 0.098 | 1.17 |
| logepa | rs190835238 | 0.001 | . | . | . | . | . | . | . | . | . | . |
| logepa | rs76698944 | 0.03 | 0.804 | 0.108 | 0.104 | 0.668 | 0.331 | 0.415 | 0.505 | 0.344 | 0.315 | -0.201 |
| logepa | rs56071381 | 0.142 | 0.757 | 0.111 | 0.057 | 0.952 | 0.184 | 0.8 | 1.11 | 0.306 | 0.701 | 0.092 |
| logepa | rs78130427 | 0.03 | 0.771 | 0.104 | 0.053 | 0.539 | 0.281 | 0.236 | 1.54 | 0.432 | 0.127 | 0.328 |
| logepa | rs10900215 | 0.146 | 0.765 | 0.112 | 0.067 | 0.946 | 0.179 | 0.771 | 1.07 | 0.29 | 0.804 | 0.063 |
| logepa | rs41491445 | 0 | . | . | . | . | . | . | . | . | . | . |
| logepa | rs144995222 | . | . | . | . | . | . | . | . | . | . | . |
| logepa | rs149097803 | 0.003 | . | . | . | . | . | . | . | . | . | . |
| logepa | rs139429646 | 0.007 | . | . | . | . | . | . | . | . | . | . |
| logepa | rs7075620 | 0.577 | 0.829 | 0.227 | 0.492 | 1.11 | 0.161 | 0.48 | 0.948 | 0.175 | 0.771 | -0.067 |
| logepa | rs148299213 | 0 | . | . | . | . | . | . | . | . | . | . |
| logepa | rs4949002 | 0.311 | 0.808 | 0.171 | 0.314 | 1.35 | 0.208 | 0.049 | 0.962 | 0.181 | 0.838 | -0.109 |
| logepa | rs150496007 | 0.011 | 0.792 | 0.107 | 0.084 | 0.093 | 0.111 | 0.046 | 0.381 | 0.39 | 0.346 | 0.143 |
| logepa | rs147303455 | 0.004 | . | . | . | . | . | . | . | . | . | . |
| logepa | rs189518433 | 0.005 | . | . | . | . | . | . | . | . | . | . |
| logepa | rs186281355 | 0.005 | . | . | . | . | . | . | . | . | . | . |
| logepa | rs115297114 | 0 | . | . | . | . | . | . | . | . | . | . |
| logepa | rs11239523 | 0.147 | 0.764 | 0.112 | 0.066 | 0.945 | 0.179 | 0.767 | 1.07 | 0.29 | 0.796 | 0.065 |
| logepa | rs143704897 | 0.004 | . | . | . | . | . | . | . | . | . | . |
| logepa | rs188024096 | 0.004 | . | . | . | . | . | . | . | . | . | . |
| logepa | rs11239524 | 0.134 | 0.765 | 0.112 | 0.069 | 0.97 | 0.2 | 0.884 | 1.07 | 0.321 | 0.828 | 0.057 |
| logepa | rs191656573 | 0.005 | . | . | . | . | . | . | . | . | . | . |
| logepa | rs193295875 | 0 | . | . | . | . | . | . | . | . | . | . |
| logepa | rs190458228 | 0.002 | . | . | . | . | . | . | . | . | . | . |
| logepa | rs139253993 | 0.002 | . | . | . | . | . | . | . | . | . | . |
| logepa | rs4948672 | 0.565 | 0.973 | 0.256 | 0.918 | 1 | 0.141 | 0.987 | 0.828 | 0.162 | 0.334 | -0.168 |
| logepa | rs180954921 | 0.004 | . | . | . | . | . | . | . | . | . | . |
| logepa | rs12264801 | 0.432 | 0.72 | 0.175 | 0.176 | 1.02 | 0.148 | 0.91 | 1.09 | 0.215 | 0.669 | 0.06 |
| logepa | rs73283184 | 0 | . | . | . | . | . | . | . | . | . | . |

| logepa | rs142746187 | 0.002 | . | . | . | . | . | . | . | . | . | . |
| --- | --- | --- | --- | --- | --- | --- | --- | --- | --- | --- | --- | --- |
| logepa | rs58704739 | 0 | . | . | . | . | . | . | . | . | . | . |
| logepa | rs61272335 | 0 | . | . | . | . | . | . | . | . | . | . |
| logepa | rs60959489 | 0 | . | . | . | . | . | . | . | . | . | . |
| logepa | rs58502629 | 0 | . | . | . | . | . | . | . | . | . | . |
| logepa | rs10900216 | 0.696 | 0.937 | 0.294 | 0.837 | 0.966 | 0.132 | 0.803 | 0.876 | 0.172 | 0.501 | -0.11 |
| logepa | rs181118141 | 0 | . | . | . | . | . | . | . | . | . | . |
| logepa | rs137882665 | 0.004 | . | . | . | . | . | . | . | . | . | . |
| logepa | rs149466736 | 0.004 | . | . | . | . | . | . | . | . | . | . |
| logepa | rs2099171 | 0.663 | 0.923 | 0.271 | 0.785 | 1 | 0.132 | 0.985 | 0.884 | 0.167 | 0.514 | -0.108 |
| logepa | rs141852280 | 0.009 | . | . | . | . | . | . | . | . | . | . |
| logepa | rs191539257 | 0.002 | . | . | . | . | . | . | . | . | . | . |
| logepa | rs17157771 | 0.033 | 0.774 | 0.103 | 0.055 | 0.675 | 0.277 | 0.339 | 1.23 | 0.382 | 0.5 | 0.195 |
| logepa | rs111339241 | 0.031 | 0.775 | 0.103 | 0.056 | 0.685 | 0.286 | 0.364 | 1.22 | 0.388 | 0.537 | 0.186 |
| logepa | rs116983255 | 0.013 | 0.79 | 0.104 | 0.072 | 0.102 | 0.141 | 0.097 | 0.176 | 0.092 | 9.20E-04 | 0.122 |
| logepa | rs142324341 | 0.003 | . | . | . | . | . | . | . | . | . | . |
| logepa | rs187191219 | 0.009 | . | . | . | . | . | . | . | . | . | . |
| logepa | rs151283973 | . | . | . | . | . | . | . | . | . | . | . |
| logepa | rs139316218 | 0.005 | . | . | . | . | . | . | . | . | . | . |
| logepa | rs6593485 | 0.696 | 0.923 | 0.289 | 0.798 | 0.971 | 0.131 | 0.827 | 0.886 | 0.173 | 0.536 | -0.1 |
| logepa | rs6593486 | 0.696 | 0.922 | 0.289 | 0.795 | 0.972 | 0.132 | 0.833 | 0.887 | 0.173 | 0.539 | -0.099 |
| logepa | rs145196618 | 0.002 | . | . | . | . | . | . | . | . | . | . |
| logepa | rs142598335 | 0.006 | . | . | . | . | . | . | . | . | . | . |
| logepa | rs892691 | 0.704 | 0.98 | 0.308 | 0.95 | 0.957 | 0.128 | 0.741 | 0.85 | 0.165 | 0.403 | -0.14 |
| logepa | rs7080713 | 0.664 | 0.928 | 0.275 | 0.802 | 1.01 | 0.134 | 0.928 | 0.881 | 0.167 | 0.503 | -0.113 |
| logepa | rs7077173 | 0.057 | 0.796 | 0.107 | 0.089 | 0.798 | 0.212 | 0.395 | 0.703 | 0.193 | 0.2 | -0.147 |
| logepa | rs7080977 | 0.032 | 0.774 | 0.104 | 0.056 | 0.706 | 0.291 | 0.398 | 1.22 | 0.377 | 0.519 | 0.187 |
| logepa | rs3780901 | 0.663 | 0.929 | 0.275 | 0.805 | 1.01 | 0.135 | 0.928 | 0.88 | 0.167 | 0.5 | -0.114 |
| logepa | rs61854091 | 0.073 | 0.787 | 0.112 | 0.094 | 0.899 | 0.279 | 0.732 | 0.926 | 0.348 | 0.837 | -0.031 |
| logepa | rs77550209 | 0.003 | . | . | . | . | . | . | . | . | . | . |
| logepa | rs73283191 | 0.033 | 0.775 | 0.104 | 0.056 | 0.694 | 0.285 | 0.374 | 1.2 | 0.367 | 0.542 | 0.179 |
| logepa | rs191866099 | 0.01 | 0.802 | 0.108 | 0.101 | 3.10E-03 | 6.90E-03 | 8.60E-03 | 8.80E-03 | 0.02 | 0.037 | 0.195 |
| logepa | rs147998395 | . | . | . | . | . | . | . | . | . | . | . |
| logepa | rs7090328 | 0.699 | 0.933 | 0.29 | 0.825 | 0.962 | 0.131 | 0.777 | 0.879 | 0.171 | 0.507 | -0.106 |
| logepa | rs79814535 | 0.001 | . | . | . | . | . | . | . | . | . | . |
| logepa | rs7073082 | 0.699 | 0.932 | 0.29 | 0.822 | 0.962 | 0.131 | 0.778 | 0.88 | 0.171 | 0.509 | -0.105 |
| logepa | rs11239526 | 0.052 | 0.754 | 0.105 | 0.043 | 1.2 | 0.346 | 0.521 | 1.44 | 0.552 | 0.336 | 0.353 |
| logepa | rs141839841 | . | . | . | . | . | . | . | . | . | . | . |
| logepa | rs75697371 | 0.014 | 0.774 | 0.103 | 0.055 | 0.939 | 0.537 | 0.912 | 1.22 | 0.776 | 0.757 | 0.172 |
| logepa | rs75972854 | 0.016 | 0.77 | 0.102 | 0.048 | 1.04 | 0.822 | 0.965 | 1.53 | 1.67 | 0.698 | 0.414 |
| logepa | rs139291975 | 0.001 | . | . | . | . | . | . | . | . | . | . |
| logepa | rs144064711 | 0.009 | . | . | . | . | . | . | . | . | . | . |
| logepa | rs3780902 | 0.033 | 0.775 | 0.103 | 0.056 | 0.67 | 0.275 | 0.329 | 1.21 | 0.37 | 0.542 | 0.181 |
| logepa | rs190449801 | 0 | . | . | . | . | . | . | . | . | . | . |
| logepa | rs73283196 | 0.033 | 0.775 | 0.104 | 0.056 | 0.679 | 0.281 | 0.349 | 1.2 | 0.371 | 0.546 | 0.18 |
| logepa | rs28395867 | 0.005 | . | . | . | . | . | . | . | . | . | . |
| logepa | rs28395866 | 0.036 | 0.777 | 0.104 | 0.058 | 0.668 | 0.264 | 0.307 | 1.07 | 0.371 | 0.841 | 0.112 |
| logepa | rs55661611 | 0.032 | 0.775 | 0.103 | 0.056 | 0.684 | 0.284 | 0.362 | 1.22 | 0.383 | 0.524 | 0.188 |
| logepa | rs55684518 | 0.03 | 0.78 | 0.104 | 0.063 | 0.779 | 0.379 | 0.608 | 1.02 | 0.575 | 0.973 | 0.06 |
| logepa | rs78552493 | 0.057 | 0.743 | 0.103 | 0.033 | 1.28 | 0.346 | 0.355 | 1.41 | 0.532 | 0.361 | 0.319 |
| logepa | rs147665434 | 0.007 | . | . | . | . | . | . | . | . | . | . |
| logepa | rs34445001 | 0.014 | 0.774 | 0.103 | 0.055 | 0.943 | 0.537 | 0.918 | 1.22 | 0.776 | 0.749 | 0.177 |
| logepa | rs184019449 | 0.004 | . | . | . | . | . | . | . | . | . | . |
| logepa | rs3780903 | 0.72 | 0.807 | 0.292 | 0.553 | 1.09 | 0.167 | 0.573 | 0.978 | 0.218 | 0.922 | -0.036 |
| logepa | rs17157784 | 0.005 | . | . | . | . | . | . | . | . | . | . |
| logepa | rs3780904 | 0.001 | . | . | . | . | . | . | . | . | . | . |
| logepa | rs192064730 | 0 | . | . | . | . | . | . | . | . | . | . |
| logepa | rs12774534 | 0.014 | 0.774 | 0.103 | 0.055 | 0.947 | 0.537 | 0.923 | 1.23 | 0.777 | 0.741 | 0.182 |
| logepa | rs79179915 | 0 | . | . | . | . | . | . | . | . | . | . |
| logepa | rs117811571 | 0.016 | 0.788 | 0.107 | 0.079 | 1.59 | 0.995 | 0.46 | 0.629 | 0.657 | 0.657 | -0.59 |
| logepa | rs72796477 | 0.015 | 0.773 | 0.104 | 0.055 | 1.18 | 0.816 | 0.813 | 1.32 | 0.993 | 0.712 | 0.252 |
| logepa | rs55729342 | 0.005 | . | . | . | . | . | . | . | . | . | . |
| logepa | rs59317220 | 0.004 | . | . | . | . | . | . | . | . | . | . |
| logepa | rs3780905 | 0.042 | 0.814 | 0.112 | 0.133 | 0.579 | 0.211 | 0.133 | 0.604 | 0.252 | 0.228 | -0.108 |
| logepa | rs3780906 | 0.719 | 0.802 | 0.291 | 0.543 | 1.09 | 0.168 | 0.562 | 0.982 | 0.219 | 0.936 | -0.034 |
| logepa | rs149047975 | 0.006 | . | . | . | . | . | . | . | . | . | . |
| logepa | rs3824614 | 0.001 | . | . | . | . | . | . | . | . | . | . |
| logepa | rs140254404 | 0.003 | . | . | . | . | . | . | . | . | . | . |
| logepa | rs145262684 | 0.02 | 0.802 | 0.109 | 0.104 | 1.52 | 0.853 | 0.453 | 0.511 | 0.384 | 0.371 | -0.701 |
| logepa | rs76735482 | 0.001 | . | . | . | . | . | . | . | . | . | . |
| logepa | rs73283198 | 0.032 | 0.775 | 0.103 | 0.056 | 0.675 | 0.285 | 0.351 | 1.22 | 0.388 | 0.527 | 0.189 |
| logepa | rs74911239 | 0.052 | 0.774 | 0.107 | 0.064 | 0.475 | 0.207 | 0.088 | 0.931 | 0.488 | 0.892 | 0.093 |
| logepa | rs150551647 | 0.008 | . | . | . | . | . | . | . | . | . | . |
| logepa | rs115200794 | 0.004 | . | . | . | . | . | . | . | . | . | . |
| logepa | rs74130823 | 0.177 | 0.785 | 0.124 | 0.127 | 0.909 | 0.172 | 0.614 | 0.978 | 0.245 | 0.929 | 3.80E-03 |
| logepa | rs2279435 | 0.564 | 0.802 | 0.207 | 0.391 | 1 | 0.142 | 0.993 | 0.975 | 0.194 | 0.897 | -0.021 |
| logepa | rs147279673 | 0.02 | 0.833 | 0.112 | 0.175 | 1.05 | 0.716 | 0.942 | 0.239 | 0.205 | 0.096 | -0.675 |
| logepa | rs183525146 | 0.005 | . | . | . | . | . | . | . | . | . | . |
| logepa | rs3740107 | 0.728 | 0.854 | 0.306 | 0.66 | 0.948 | 0.141 | 0.721 | 0.939 | 0.208 | 0.774 | -0.042 |
| logepa | rs140697247 | 0.002 | . | . | . | . | . | . | . | . | . | . |
| logepa | rs1565096 | 0.745 | 0.731 | 0.266 | 0.388 | 0.957 | 0.145 | 0.771 | 1.04 | 0.233 | 0.856 | 0.041 |
| logepa | rs1565097 | 0.742 | 0.726 | 0.265 | 0.38 | 0.966 | 0.147 | 0.819 | 1.05 | 0.235 | 0.84 | 0.042 |
| logepa | rs144584548 | 0.03 | 0.781 | 0.105 | 0.064 | 0.81 | 0.388 | 0.659 | 0.992 | 0.55 | 0.989 | 0.037 |
| logepa | rs75811053 | 0.001 | . | . | . | . | . | . | . | . | . | . |
| logepa | rs147462967 | 0.01 | 0.749 | 0.099 | 0.029 | 0.025 | 0.046 | 0.044 | 33 | 70.8 | 0.103 | 0.84 |
| logepa | rs74130824 | 0.004 | . | . | . | . | . | . | . | . | . | . |

| logepa | rs116883184 | 0.025 | 0.809 | 0.111 | 0.122 | 1.69 | 0.855 | 0.296 | 0.593 | 0.37 | 0.403 | -0.69 |
| --- | --- | --- | --- | --- | --- | --- | --- | --- | --- | --- | --- | --- |
| logepa | rs117258049 | 0.025 | 0.809 | 0.111 | 0.122 | 1.69 | 0.855 | 0.296 | 0.593 | 0.37 | 0.403 | -0.69 |
| logepa | rs3780908 | 0.171 | 0.777 | 0.124 | 0.114 | 0.927 | 0.168 | 0.677 | 1.01 | 0.244 | 0.958 | 0.025 |
| logepa | rs180938522 | 0.004 | . | . | . | . | . | . | . | . | . | . |
| logepa | rs76687372 | 0 | . | . | . | . | . | . | . | . | . | . |
| logepa | rs3780909 | 0.504 | 0.804 | 0.181 | 0.333 | 1.01 | 0.136 | 0.953 | 0.969 | 0.182 | 0.868 | -0.026 |
| logepa | rs78281156 | 0.001 | . | . | . | . | . | . | . | . | . | . |
| logepa | rs187840631 | 0.002 | . | . | . | . | . | . | . | . | . | . |
| logepa | rs76868242 | 0.001 | . | . | . | . | . | . | . | . | . | . |
| logepa | rs192794192 | 0.001 | . | . | . | . | . | . | . | . | . | . |
| logepa | rs12781248 | 0.015 | 0.78 | 0.104 | 0.061 | 0.969 | 0.531 | 0.955 | 0.918 | 0.631 | 0.901 | -0.055 |
| logepa | rs12762375 | 0.014 | 0.773 | 0.103 | 0.054 | 0.976 | 0.537 | 0.965 | 1.31 | 0.809 | 0.66 | 0.24 |
| logepa | rs192256986 | 0.006 | . | . | . | . | . | . | . | . | . | . |
| logepa | rs184411030 | 0 | . | . | . | . | . | . | . | . | . | . |
| logepa | rs189601483 | 0.001 | . | . | . | . | . | . | . | . | . | . |
| logepa | rs143254579 | 0.05 | 0.752 | 0.105 | 0.041 | 1.23 | 0.356 | 0.479 | 1.52 | 0.609 | 0.295 | 0.425 |
| logepa | rs12253903 | 0 | . | . | . | . | . | . | . | . | . | . |
| logepa | rs35489926 | 0.014 | 0.773 | 0.103 | 0.054 | 0.981 | 0.538 | 0.971 | 1.31 | 0.806 | 0.659 | 0.24 |
| logepa | rs71494783 | 0.015 | 0.772 | 0.103 | 0.053 | 1.01 | 0.527 | 0.982 | 1.33 | 0.726 | 0.596 | 0.258 |
| logepa | rs180711602 | 0.004 | . | . | . | . | . | . | . | . | . | . |
| logepa | rs117312402 | 0.001 | . | . | . | . | . | . | . | . | . | . |
| logepa | rs74831694 | 0.001 | . | . | . | . | . | . | . | . | . | . |
| logepa | rs140301943 | 0.004 | . | . | . | . | . | . | . | . | . | . |
| logepa | rs78553679 | 0.023 | 0.777 | 0.104 | 0.059 | 0.515 | 0.348 | 0.326 | 0.863 | 0.588 | 0.828 | 0.053 |
| logepa | rs78334250 | 0.001 | . | . | . | . | . | . | . | . | . | . |
| logepa | rs76000028 | 0.001 | . | . | . | . | . | . | . | . | . | . |
| logepa | rs145195254 | 0 | . | . | . | . | . | . | . | . | . | . |
| logepa | rs137888527 | 0 | . | . | . | . | . | . | . | . | . | . |
| logepa | rs149109592 | 0.005 | . | . | . | . | . | . | . | . | . | . |
| logepa | rs80280010 | 0.049 | 0.752 | 0.105 | 0.041 | 1.23 | 0.357 | 0.475 | 1.53 | 0.61 | 0.291 | 0.428 |
| logepa | rs189995374 | 0.001 | . | . | . | . | . | . | . | . | . | . |
| logepa | rs75467770 | 0.001 | . | . | . | . | . | . | . | . | . | . |
| logepa | rs1487562 | 0.178 | 0.772 | 0.124 | 0.106 | 0.937 | 0.166 | 0.713 | 1.03 | 0.246 | 0.888 | 0.039 |
| logepa | rs12266963 | 0.015 | 0.772 | 0.103 | 0.053 | 1.02 | 0.527 | 0.975 | 1.34 | 0.724 | 0.592 | 0.26 |
| logepa | rs7099874 | 0.693 | 0.825 | 0.274 | 0.563 | 0.927 | 0.138 | 0.614 | 0.959 | 0.21 | 0.847 | -0.019 |
| logepa | rs75687019 | 0.001 | . | . | . | . | . | . | . | . | . | . |
| logepa | rs148240462 | 0.056 | 0.787 | 0.108 | 0.082 | 0.465 | 0.201 | 0.076 | 0.786 | 0.41 | 0.644 | 0.035 |
| logepa | rs141278174 | 0.003 | . | . | . | . | . | . | . | . | . | . |
| logepa | rs76404852 | 0.001 | . | . | . | . | . | . | . | . | . | . |
| logepa | rs139112240 | 0.012 | 0.799 | 0.109 | 0.1 | 0.827 | 0.85 | 0.853 | 0.375 | 0.4 | 0.358 | -0.379 |
| logepa | rs12783037 | 0.015 | 0.772 | 0.103 | 0.053 | 1.02 | 0.527 | 0.969 | 1.34 | 0.724 | 0.587 | 0.264 |
| logepa | rs72796481 | 0.013 | 0.771 | 0.104 | 0.054 | 1 | 0.734 | 0.995 | 1.3 | 0.968 | 0.726 | 0.23 |
| logepa | rs76798749 | 0.001 | . | . | . | . | . | . | . | . | . | . |
| logepa | rs187732548 | 0.001 | . | . | . | . | . | . | . | . | . | . |
| logepa | rs75675474 | 0.001 | . | . | . | . | . | . | . | . | . | . |
| logepa | rs76552293 | 0.001 | . | . | . | . | . | . | . | . | . | . |
| logepa | rs147445993 | 0.005 | . | . | . | . | . | . | . | . | . | . |
| logepa | rs115902283 | 0.001 | . | . | . | . | . | . | . | . | . | . |
| logepa | rs4540799 | 0.692 | 0.826 | 0.274 | 0.565 | 0.929 | 0.139 | 0.623 | 0.958 | 0.209 | 0.845 | -0.02 |
| logepa | rs12765320 | 0.179 | 0.751 | 0.124 | 0.082 | 0.968 | 0.176 | 0.856 | 1.11 | 0.272 | 0.671 | 0.088 |
| logepa | rs12782256 | 0.014 | 0.773 | 0.103 | 0.053 | 0.994 | 0.538 | 0.992 | 1.32 | 0.803 | 0.65 | 0.246 |
| logepa | rs191727256 | 0 | . | . | . | . | . | . | . | . | . | . |
| logepa | rs2082111 | 0.165 | 0.773 | 0.122 | 0.104 | 0.955 | 0.172 | 0.797 | 1.03 | 0.252 | 0.916 | 0.03 |
| logepa | rs2082110 | 0.166 | 0.774 | 0.123 | 0.106 | 0.955 | 0.172 | 0.798 | 1.02 | 0.251 | 0.923 | 0.028 |
| logepa | rs2082109 | 0.166 | 0.774 | 0.123 | 0.106 | 0.955 | 0.172 | 0.798 | 1.02 | 0.251 | 0.923 | 0.028 |
| logepa | rs117316662 | 0.001 | . | . | . | . | . | . | . | . | . | . |
| logepa | rs12247163 | 0.181 | 0.766 | 0.124 | 0.098 | 0.962 | 0.169 | 0.824 | 1.05 | 0.242 | 0.828 | 0.047 |
| logepa | rs12355888 | 0.14 | 0.782 | 0.121 | 0.111 | 0.993 | 0.19 | 0.971 | 0.985 | 0.257 | 0.954 | -0.01 |
| logepa | rs143772290 | 0 | . | . | . | . | . | . | . | . | . | . |
| logepa | rs146849452 | 0.004 | . | . | . | . | . | . | . | . | . | . |
| logepa | rs140267121 | 0.006 | . | . | . | . | . | . | . | . | . | . |
| logepa | rs7080474 | 0.18 | 0.765 | 0.123 | 0.095 | 0.962 | 0.169 | 0.827 | 1.06 | 0.243 | 0.813 | 0.05 |
| logepa | rs144066203 | 0 | . | . | . | . | . | . | . | . | . | . |
| logepa | rs12765209 | 0.001 | . | . | . | . | . | . | . | . | . | . |
| logepa | rs150930735 | 0 | . | . | . | . | . | . | . | . | . | . |
| logepa | rs7068039 | 0.181 | 0.766 | 0.123 | 0.098 | 0.961 | 0.169 | 0.821 | 1.05 | 0.242 | 0.828 | 0.047 |
| logepa | rs117622652 | 0.001 | . | . | . | . | . | . | . | . | . | . |
| logepa | rs190191514 | 0.002 | . | . | . | . | . | . | . | . | . | . |
| logepa | rs74130835 | 0 | . | . | . | . | . | . | . | . | . | . |
| logepa | rs1811604 | 0.001 | . | . | . | . | . | . | . | . | . | . |
| logepa | rs146971256 | 0 | . | . | . | . | . | . | . | . | . | . |
| logepa | rs74336000 | 0 | . | . | . | . | . | . | . | . | . | . |
| logepa | rs186046492 | 0.009 | . | . | . | . | . | . | . | . | . | . |
| logepa | rs34971823 | 0.014 | 0.773 | 0.103 | 0.053 | 0.993 | 0.537 | 0.99 | 1.32 | 0.801 | 0.643 | 0.25 |
| logepa | rs75227446 | 0.001 | . | . | . | . | . | . | . | . | . | . |
| logepa | rs190457599 | 0.001 | . | . | . | . | . | . | . | . | . | . |
| logepa | rs79591917 | 0.001 | . | . | . | . | . | . | . | . | . | . |
| logepa | rs702365 | 0.51 | 0.833 | 0.193 | 0.43 | 0.967 | 0.134 | 0.811 | 0.936 | 0.183 | 0.735 | -0.046 |
| logepa | rs79643836 | 0.001 | . | . | . | . | . | . | . | . | . | . |
| logepa | rs702366 | 0.512 | 0.827 | 0.192 | 0.413 | 0.965 | 0.133 | 0.796 | 0.942 | 0.183 | 0.76 | -0.04 |
| logepa | rs1059696 | 0.166 | 0.773 | 0.123 | 0.105 | 0.954 | 0.173 | 0.796 | 1.03 | 0.252 | 0.911 | 0.031 |
| logepa | rs901681 | 0.515 | 0.822 | 0.191 | 0.4 | 0.959 | 0.132 | 0.762 | 0.947 | 0.184 | 0.781 | -0.034 |
| logepa | rs61854092 | 0.166 | 0.772 | 0.122 | 0.102 | 0.953 | 0.173 | 0.793 | 1.03 | 0.253 | 0.896 | 0.035 |
| logepa | rs148080753 | 0.001 | . | . | . | . | . | . | . | . | . | . |
| logepa | rs61854093 | 0.166 | 0.772 | 0.122 | 0.103 | 0.957 | 0.173 | 0.808 | 1.03 | 0.252 | 0.898 | 0.033 |
| logepa | rs141822774 | 0.001 | . | . | . | . | . | . | . | . | . | . |

| logepa | rs74130836 | 0 | . | . | . | . | . | . | . | . | . | . |
| --- | --- | --- | --- | --- | --- | --- | --- | --- | --- | --- | --- | --- |
| logepa | rs150620963 | 0.019 | 0.767 | 0.103 | 0.048 | 1.23 | 0.837 | 0.765 | 1.58 | 1.99 | 0.718 | 0.489 |
| logepa | rs3780913 | 0.678 | 0.848 | 0.276 | 0.612 | 0.932 | 0.138 | 0.635 | 0.94 | 0.205 | 0.776 | -0.037 |
| logepa | rs142255723 | 0.002 | . | . | . | . | . | . | . | . | . | . |
| logepa | rs3780914 | 0.511 | 0.827 | 0.192 | 0.414 | 0.97 | 0.133 | 0.823 | 0.942 | 0.183 | 0.757 | -0.041 |
| logepa | rs182656507 | 0.002 | . | . | . | . | . | . | . | . | . | . |
| logepa | rs2291427 | 0.678 | 0.847 | 0.275 | 0.609 | 0.931 | 0.139 | 0.633 | 0.94 | 0.206 | 0.778 | -0.037 |
| logepa | rs10751382 | 0.678 | 0.85 | 0.276 | 0.616 | 0.935 | 0.139 | 0.652 | 0.938 | 0.205 | 0.77 | -0.039 |
| logepa | rs117610108 | 0.001 | . | . | . | . | . | . | . | . | . | . |
| logepa | rs144966941 | 0.031 | 0.808 | 0.109 | 0.113 | 0.952 | 0.391 | 0.904 | 0.54 | 0.31 | 0.284 | -0.344 |
| logepa | rs41502051 | 0.051 | 0.75 | 0.105 | 0.041 | 1.38 | 0.415 | 0.28 | 1.37 | 0.608 | 0.485 | 0.283 |
| logepa | rs41426046 | 0.001 | . | . | . | . | . | . | . | . | . | . |
| logepa | rs41519248 | 0.001 | . | . | . | . | . | . | . | . | . | . |
| logepa | rs189108245 | 0.008 | . | . | . | . | . | . | . | . | . | . |
| logepa | rs7896431 | 0.477 | 0.812 | 0.181 | 0.349 | 0.963 | 0.135 | 0.789 | 0.956 | 0.184 | 0.813 | -0.028 |
| logepa | rs183724547 | 0.001 | . | . | . | . | . | . | . | . | . | . |
| logepa | rs142044990 | 0.012 | 0.801 | 0.109 | 0.103 | 0.791 | 0.836 | 0.824 | 0.335 | 0.373 | 0.326 | -0.38 |
| logepa | rs188140929 | 0.002 | . | . | . | . | . | . | . | . | . | . |
| logepa | rs76819096 | 0 | . | . | . | . | . | . | . | . | . | . |
| logepa | rs10751383 | 0.561 | 0.785 | 0.194 | 0.327 | 0.997 | 0.134 | 0.983 | 0.992 | 0.188 | 0.968 | -5.30E-03 |
| logepa | rs117819321 | 0.019 | 0.807 | 0.108 | 0.109 | 0.124 | 0.148 | 0.081 | 0.154 | 0.153 | 0.06 | 0.085 |
| logepa | rs2242332 | 0.676 | 0.866 | 0.281 | 0.657 | 0.944 | 0.141 | 0.699 | 0.926 | 0.202 | 0.723 | -0.053 |
| logepa | rs2242333 | 0.046 | 0.834 | 0.115 | 0.188 | 0.594 | 0.214 | 0.148 | 0.504 | 0.208 | 0.098 | -0.178 |
| logepa | rs2242334 | 0.678 | 0.867 | 0.282 | 0.661 | 0.94 | 0.14 | 0.677 | 0.925 | 0.202 | 0.72 | -0.053 |
| logepa | rs189465041 | 0.001 | . | . | . | . | . | . | . | . | . | . |
| logepa | rs28395868 | 0.041 | 0.787 | 0.109 | 0.085 | 0.975 | 0.353 | 0.944 | 0.855 | 0.421 | 0.75 | -0.106 |
| logepa | rs56067550 | 0.03 | 0.781 | 0.105 | 0.064 | 0.802 | 0.389 | 0.65 | 0.995 | 0.558 | 0.993 | 0.04 |
| logepa | rs1051713 | 0.165 | 0.773 | 0.123 | 0.105 | 0.942 | 0.173 | 0.747 | 1.03 | 0.254 | 0.912 | 0.033 |
| logepa | rs115074210 | 0 | . | . | . | . | . | . | . | . | . | . |
| logepa | rs7900977 | 0.74 | 0.748 | 0.269 | 0.421 | 0.976 | 0.149 | 0.872 | 1.03 | 0.228 | 0.91 | 0.025 |
| logepa | rs147613891 | 0.004 | . | . | . | . | . | . | . | . | . | . |
| logepa | rs2229136 | 0.063 | 0.746 | 0.105 | 0.038 | 1.16 | 0.288 | 0.556 | 1.49 | 0.498 | 0.231 | 0.384 |
| logepa | rs185482841 | 0 | . | . | . | . | . | . | . | . | . | . |
| logepa | rs2288619 | 0.063 | 0.746 | 0.105 | 0.038 | 1.16 | 0.288 | 0.557 | 1.49 | 0.498 | 0.231 | 0.384 |
| logepa | rs189891524 | 0.001 | . | . | . | . | . | . | . | . | . | . |
| logepa | rs56145077 | 0.164 | 0.773 | 0.123 | 0.105 | 0.946 | 0.173 | 0.762 | 1.03 | 0.254 | 0.911 | 0.033 |
| logepa | rs143216970 | 0 | . | . | . | . | . | . | . | . | . | . |
| logepa | rs142246478 | 0.002 | . | . | . | . | . | . | . | . | . | . |
| logepa | rs13134 | 0.014 | 0.772 | 0.103 | 0.053 | 0.978 | 0.53 | 0.968 | 1.34 | 0.791 | 0.617 | 0.264 |
| logepa | rs41314980 | 0.034 | 0.799 | 0.109 | 0.099 | 0.658 | 0.387 | 0.477 | 0.582 | 0.396 | 0.426 | -0.151 |
| logepa | rs41304611 | 0.02 | 0.773 | 0.103 | 0.053 | 0.148 | 0.145 | 0.051 | 0.517 | 0.659 | 0.605 | 0.138 |
| logepa | rs193299617 | 0 | . | . | . | . | . | . | . | . | . | . |
| logepa | rs12772724 | 0.014 | 0.772 | 0.103 | 0.053 | 0.977 | 0.529 | 0.966 | 1.34 | 0.792 | 0.617 | 0.264 |
| logepa | rs139489537 | 0.048 | 0.75 | 0.104 | 0.038 | 1.24 | 0.348 | 0.445 | 1.59 | 0.621 | 0.232 | 0.492 |
| logepa | rs12776062 | 0.014 | 0.772 | 0.103 | 0.053 | 0.977 | 0.529 | 0.966 | 1.34 | 0.791 | 0.619 | 0.262 |
| logepa | rs752059 | 0.164 | 0.774 | 0.123 | 0.106 | 0.946 | 0.173 | 0.763 | 1.03 | 0.254 | 0.917 | 0.031 |
| logepa | rs147649982 | 0.035 | 0.806 | 0.111 | 0.116 | 0.712 | 0.393 | 0.538 | 0.547 | 0.342 | 0.335 | -0.204 |
| logepa | rs17157798 | 0.063 | 0.744 | 0.105 | 0.036 | 1.17 | 0.289 | 0.528 | 1.53 | 0.512 | 0.203 | 0.418 |
| logepa | rs142298619 | 0.001 | . | . | . | . | . | . | . | . | . | . |
| logepa | rs4948673 | 0.721 | 0.748 | 0.257 | 0.398 | 0.968 | 0.146 | 0.83 | 1.03 | 0.222 | 0.904 | 0.027 |
| logepa | rs74231484 | 0.041 | 0.825 | 0.112 | 0.157 | 0.559 | 0.214 | 0.129 | 0.522 | 0.231 | 0.141 | -0.143 |
| logepa | rs72796489 | 0.004 | . | . | . | . | . | . | . | . | . | . |
| logepa | rs113447578 | 0.021 | 0.78 | 0.103 | 0.062 | 0.459 | 0.282 | 0.205 | 1.2 | 0.392 | 0.568 | 0.192 |
| logepa | rs34063374 | 0.014 | 0.772 | 0.103 | 0.053 | 0.967 | 0.527 | 0.95 | 1.36 | 0.806 | 0.609 | 0.273 |
| logepa | rs11239527 | 0.019 | 0.79 | 0.107 | 0.081 | 1.17 | 0.759 | 0.806 | 0.666 | 0.516 | 0.6 | -0.346 |
| logepa | rs34371883 | 0.014 | 0.772 | 0.103 | 0.053 | 0.974 | 0.529 | 0.961 | 1.34 | 0.795 | 0.618 | 0.264 |
| logepa | rs118168828 | 0.012 | 0.739 | 0.1 | 0.025 | 1.45 | 1.17 | 0.647 | 7.59 | 7.99 | 0.054 | 6.94 |
| logepa | rs34349618 | 0.014 | 0.772 | 0.103 | 0.053 | 0.974 | 0.529 | 0.961 | 1.34 | 0.795 | 0.618 | 0.264 |
| logepa | rs80041679 | 0.048 | 0.75 | 0.104 | 0.038 | 1.23 | 0.346 | 0.452 | 1.59 | 0.62 | 0.234 | 0.488 |
| logepa | rs149640607 | 0 | . | . | . | . | . | . | . | . | . | . |
| logepa | rs4427523 | 0.834 | 0.462 | 0.236 | 0.131 | 0.805 | 0.175 | 0.32 | 1.36 | 0.415 | 0.308 | 0.24 |
| logepa | rs185880109 | 0.002 | . | . | . | . | . | . | . | . | . | . |
| logepa | rs191523220 | 0.004 | . | . | . | . | . | . | . | . | . | . |
| logepa | rs144315284 | 0 | . | . | . | . | . | . | . | . | . | . |
| logepa | rs4948674 | 0.405 | 0.797 | 0.152 | 0.234 | 0.947 | 0.133 | 0.697 | 0.966 | 0.187 | 0.858 | -0.015 |
| logepa | rs182984298 | 0.01 | 0.78 | 0.104 | 0.061 | 0.537 | 0.411 | 0.416 | 0.925 | 0.514 | 0.888 | 0.07 |
| logepa | rs56749023 | 0 | . | . | . | . | . | . | . | . | . | . |
| logepa | rs112508957 | 0 | . | . | . | . | . | . | . | . | . | . |
| logepa | rs7089063 | 0.224 | 0.734 | 0.124 | 0.067 | 0.993 | 0.155 | 0.963 | 1.14 | 0.239 | 0.527 | 0.105 |
| logepa | rs145000779 | 0.001 | . | . | . | . | . | . | . | . | . | . |
| logepa | rs80201572 | 0.048 | 0.751 | 0.104 | 0.039 | 1.24 | 0.346 | 0.448 | 1.57 | 0.615 | 0.245 | 0.475 |
| logepa | rs140568227 | 0.009 | . | . | . | . | . | . | . | . | . | . |
| logepa | rs149756228 | 0.014 | 0.786 | 0.107 | 0.077 | 0.723 | 0.549 | 0.669 | 0.705 | 0.411 | 0.549 | -0.109 |
| logepa | rs112380685 | 0.009 | . | . | . | . | . | . | . | . | . | . |
| logepa | rs60481109 | 0 | . | . | . | . | . | . | . | . | . | . |
| logepa | rs138221843 | 0.004 | . | . | . | . | . | . | . | . | . | . |
| logepa | rs150283194 | 0 | . | . | . | . | . | . | . | . | . | . |
| logepa | rs138879661 | 0.003 | . | . | . | . | . | . | . | . | . | . |
| logepa | rs34960345 | 0.062 | 0.746 | 0.105 | 0.037 | 1.17 | 0.29 | 0.519 | 1.5 | 0.503 | 0.223 | 0.396 |
| logepa | rs147514068 | 0.004 | . | . | . | . | . | . | . | . | . | . |
| logepa | rs35410535 | 0.063 | 0.746 | 0.105 | 0.037 | 1.17 | 0.289 | 0.531 | 1.5 | 0.504 | 0.225 | 0.395 |
| logepa | rs191613635 | 0.005 | . | . | . | . | . | . | . | . | . | . |
| logepa | rs17157800 | 0.009 | . | . | . | . | . | . | . | . | . | . |
| logepa | rs17523413 | 0.062 | 0.745 | 0.105 | 0.037 | 1.17 | 0.29 | 0.526 | 1.51 | 0.505 | 0.218 | 0.401 |
| logepa | rs7897036 | 0.058 | 0.761 | 0.104 | 0.046 | 1.05 | 0.276 | 0.852 | 1.18 | 0.4 | 0.622 | 0.133 |
| logepa | rs76334323 | 0.001 | . | . | . | . | . | . | . | . | . | . |

| logepa | rs143535991 | 0.001 | . | . | . | . | . | . | . | . | . | . |
| --- | --- | --- | --- | --- | --- | --- | --- | --- | --- | --- | --- | --- |
| logepa | rs140344979 | 0.007 | . | . | . | . | . | . | . | . | . | . |
| logepa | rs36047811 | 0.063 | 0.746 | 0.105 | 0.037 | 1.17 | 0.289 | 0.533 | 1.5 | 0.503 | 0.227 | 0.393 |
| logepa | rs76493570 | 0.062 | 0.746 | 0.105 | 0.037 | 1.17 | 0.29 | 0.526 | 1.5 | 0.503 | 0.227 | 0.392 |
| logepa | rs192998920 | 0.003 | . | . | . | . | . | . | . | . | . | . |
| logepa | rs116840852 | 0.024 | 0.778 | 0.104 | 0.061 | 0.511 | 0.345 | 0.32 | 0.84 | 0.575 | 0.799 | 0.045 |
| logepa | rs184824101 | 0.001 | . | . | . | . | . | . | . | . | . | . |
| logepa | rs41301621 | 0.008 | . | . | . | . | . | . | . | . | . | . |
| logepa | rs3740109 | 0.063 | 0.746 | 0.105 | 0.038 | 1.16 | 0.289 | 0.541 | 1.49 | 0.502 | 0.234 | 0.386 |
| logepa | rs7899415 | 0.02 | 0.767 | 0.103 | 0.048 | 0.776 | 0.62 | 0.751 | 1.57 | 2.5 | 0.778 | 0.391 |
| logepa | rs184395284 | 0.001 | . | . | . | . | . | . | . | . | . | . |
| logepa | rs139209589 | 0.006 | . | . | . | . | . | . | . | . | . | . |
| logepa | rs149924163 | 0 | . | . | . | . | . | . | . | . | . | . |
| logepa | rs3740110 | 0.001 | . | . | . | . | . | . | . | . | . | . |
| logepa | rs144335113 | 0 | . | . | . | . | . | . | . | . | . | . |
| logepa | rs74743385 | 0 | . | . | . | . | . | . | . | . | . | . |
| logepa | rs146545829 | 0 | . | . | . | . | . | . | . | . | . | . |
| logepa | rs181842689 | 0 | . | . | . | . | . | . | . | . | . | . |
| logepa | rs3740111 | 0.062 | 0.746 | 0.105 | 0.038 | 1.17 | 0.29 | 0.536 | 1.49 | 0.502 | 0.232 | 0.388 |
| logepa | rs186261599 | 0 | . | . | . | . | . | . | . | . | . | . |
| logepa | rs73285348 | 0.012 | 0.77 | 0.103 | 0.05 | 0.839 | 0.624 | 0.814 | 1.93 | 0.835 | 0.126 | 0.641 |
| logepa | rs186356901 | 0 | . | . | . | . | . | . | . | . | . | . |
| logepa | rs3824618 | 0.048 | 0.751 | 0.104 | 0.039 | 1.24 | 0.346 | 0.446 | 1.58 | 0.615 | 0.244 | 0.476 |
| logepa | rs3802548 | 0.231 | 0.748 | 0.129 | 0.091 | 1.09 | 0.169 | 0.565 | 1.07 | 0.211 | 0.726 | 0.035 |
| logepa | rs3802547 | 0.001 | . | . | . | . | . | . | . | . | . | . |
| logepa | rs77677521 | 0.024 | 0.778 | 0.104 | 0.061 | 0.511 | 0.345 | 0.321 | 0.841 | 0.575 | 0.8 | 0.045 |
| logepa | rs3824616 | 0.063 | 0.747 | 0.105 | 0.038 | 1.16 | 0.289 | 0.552 | 1.48 | 0.499 | 0.246 | 0.375 |
| logepa | rs191073818 | 0 | . | . | . | . | . | . | . | . | . | . |
| logepa | rs147078004 | 0.002 | . | . | . | . | . | . | . | . | . | . |
| logepa | rs7908745 | 0.297 | 0.719 | 0.131 | 0.071 | 1.11 | 0.156 | 0.462 | 1.14 | 0.207 | 0.486 | 0.077 |
| logepa | rs17157811 | 0.009 | . | . | . | . | . | . | . | . | . | . |
| logepa | rs138506087 | 0.009 | . | . | . | . | . | . | . | . | . | . |
| logepa | rs77241269 | 0 | . | . | . | . | . | . | . | . | . | . |
| logepa | rs138374938 | 0.002 | . | . | . | . | . | . | . | . | . | . |
| logepa | rs2279433 | 0.062 | 0.745 | 0.105 | 0.036 | 1.13 | 0.285 | 0.641 | 1.51 | 0.523 | 0.229 | 0.4 |
| logepa | rs73285358 | 0 | . | . | . | . | . | . | . | . | . | . |
| logepa | rs11239528 | 0.232 | 0.747 | 0.128 | 0.09 | 1.11 | 0.17 | 0.504 | 1.07 | 0.21 | 0.722 | 0.033 |
| logepa | rs77453386 | 0.001 | . | . | . | . | . | . | . | . | . | . |
| logepa | rs2279434 | 0.069 | 0.757 | 0.108 | 0.051 | 1.01 | 0.261 | 0.97 | 1.25 | 0.422 | 0.511 | 0.188 |
| logepa | rs149236525 | 0.006 | . | . | . | . | . | . | . | . | . | . |
| logepa | rs12573609 | 0.052 | 0.776 | 0.111 | 0.075 | 2.03 | 0.66 | 0.029 | 0.988 | 0.435 | 0.977 | -0.251 |
| logepa | rs191716655 | 0 | . | . | . | . | . | . | . | . | . | . |
| logepa | rs17523660 | 0.014 | 0.774 | 0.103 | 0.054 | 0.904 | 0.524 | 0.861 | 1.25 | 0.778 | 0.718 | 0.198 |
| logepa | rs142671360 | 0.004 | . | . | . | . | . | . | . | . | . | . |
| logepa | rs3764990 | 0.062 | 0.745 | 0.104 | 0.036 | 1.12 | 0.284 | 0.658 | 1.53 | 0.529 | 0.219 | 0.411 |
| logepa | rs113682861 | 0.004 | . | . | . | . | . | . | . | . | . | . |
| logepa | rs117658270 | 0.001 | . | . | . | . | . | . | . | . | . | . |
| logepa | rs187309063 | 0 | . | . | . | . | . | . | . | . | . | . |
| logepa | rs73285360 | 0 | . | . | . | . | . | . | . | . | . | . |
| logepa | rs80133289 | 0.001 | . | . | . | . | . | . | . | . | . | . |
| logepa | rs186693257 | 0.001 | . | . | . | . | . | . | . | . | . | . |
| logepa | rs35735968 | 0.063 | 0.746 | 0.105 | 0.037 | 1.12 | 0.284 | 0.656 | 1.5 | 0.519 | 0.238 | 0.39 |
| logepa | rs17157825 | 0.048 | 0.749 | 0.104 | 0.037 | 1.18 | 0.341 | 0.573 | 1.59 | 0.643 | 0.247 | 0.48 |
| logepa | rs2291428 | 0.229 | 0.741 | 0.128 | 0.081 | 1.13 | 0.176 | 0.43 | 1.09 | 0.218 | 0.66 | 0.043 |
| logepa | rs2291429 | 0.231 | 0.751 | 0.129 | 0.095 | 1.11 | 0.17 | 0.505 | 1.06 | 0.208 | 0.76 | 0.025 |
| logepa | rs145714792 | 0.007 | . | . | . | . | . | . | . | . | . | . |
| logepa | rs12769244 | 0.062 | 0.745 | 0.105 | 0.036 | 1.12 | 0.285 | 0.655 | 1.52 | 0.527 | 0.226 | 0.404 |
| logepa | rs12771275 | 0.062 | 0.745 | 0.105 | 0.036 | 1.12 | 0.285 | 0.655 | 1.52 | 0.527 | 0.226 | 0.404 |
| logepa | rs146266174 | 0.002 | . | . | . | . | . | . | . | . | . | . |
| logepa | rs41301623 | 0.004 | . | . | . | . | . | . | . | . | . | . |
| logepa | rs2306814 | 0.001 | . | . | . | . | . | . | . | . | . | . |
| logepa | rs12244834 | 0 | . | . | . | . | . | . | . | . | . | . |
| logepa | rs184006011 | 0.004 | . | . | . | . | . | . | . | . | . | . |
| logepa | rs78829066 | 0.001 | . | . | . | . | . | . | . | . | . | . |
| logepa | rs34731408 | 0.062 | 0.745 | 0.105 | 0.036 | 1.12 | 0.285 | 0.655 | 1.52 | 0.527 | 0.226 | 0.404 |
| logepa | rs17451516 | 0.014 | 0.774 | 0.103 | 0.054 | 0.903 | 0.524 | 0.861 | 1.25 | 0.78 | 0.717 | 0.199 |
| logepa | rs139910133 | . | . | . | . | . | . | . | . | . | . | . |
| logepa | rs10900218 | 0.229 | 0.738 | 0.124 | 0.07 | 1.01 | 0.154 | 0.971 | 1.13 | 0.233 | 0.559 | 0.094 |
| logepa | rs146023999 | . | . | . | . | . | . | . | . | . | . | . |
| logepa | rs187296958 | . | . | . | . | . | . | . | . | . | . | . |
| logepa | rs142342833 | . | . | . | . | . | . | . | . | . | . | . |
| logepa | rs143489255 | . | . | . | . | . | . | . | . | . | . | . |
| logepa | rs138532518 | . | . | . | . | . | . | . | . | . | . | . |
| logepa | rs183768079 | . | . | . | . | . | . | . | . | . | . | . |
| logepa | rs117892272 | . | . | . | . | . | . | . | . | . | . | . |
| logepa | rs192993943 | . | . | . | . | . | . | . | . | . | . | . |
| logepa | rs182989043 | . | . | . | . | . | . | . | . | . | . | . |
| logepa | rs189703435 | . | . | . | . | . | . | . | . | . | . | . |
| logepa | rs188794040 | . | . | . | . | . | . | . | . | . | . | . |
| logepa | rs143920953 | . | . | . | . | . | . | . | . | . | . | . |
| logepa | rs191029121 | . | . | . | . | . | . | . | . | . | . | . |
| logepa | rs116875448 | . | . | . | . | . | . | . | . | . | . | . |
| logepa | rs190167877 | . | . | . | . | . | . | . | . | . | . | . |
| logepa | rs184993418 | . | . | . | . | . | . | . | . | . | . | . |
| logepa | rs143241158 | . | . | . | . | . | . | . | . | . | . | . |
| logepa | rs11239500 | . | . | . | . | . | . | . | . | . | . | . |

| logepa | rs140568994 | . | . | . | . | . | . | . | . | . | . | . |
| --- | --- | --- | --- | --- | --- | --- | --- | --- | --- | --- | --- | --- |
| logepa | rs187530317 | . | . | . | . | . | . | . | . | . | . | . |
| logepa | rs145189326 | . | . | . | . | . | . | . | . | . | . | . |
| logepa | rs186203332 | . | . | . | . | . | . | . | . | . | . | . |
| logepa | rs189921909 | . | . | . | . | . | . | . | . | . | . | . |
| logepa | rs187260989 | . | . | . | . | . | . | . | . | . | . | . |
| logepa | rs114376586 | . | . | . | . | . | . | . | . | . | . | . |
| logepa | rs151070808 | . | . | . | . | . | . | . | . | . | . | . |
| logepa | rs140927225 | . | . | . | . | . | . | . | . | . | . | . |
| logepa | rs191555615 | . | . | . | . | . | . | . | . | . | . | . |
| logepa | rs80278898 | . | . | . | . | . | . | . | . | . | . | . |
| logepa | rs190849503 | . | . | . | . | . | . | . | . | . | . | . |
| logepa | rs150080315 | . | . | . | . | . | . | . | . | . | . | . |
| logepa | rs143623885 | . | . | . | . | . | . | . | . | . | . | . |
| logepa | rs187760040 | . | . | . | . | . | . | . | . | . | . | . |
| logepa | rs143917143 | . | . | . | . | . | . | . | . | . | . | . |
| logepa | rs149851136 | . | . | . | . | . | . | . | . | . | . | . |
| logepa | rs181612054 | . | . | . | . | . | . | . | . | . | . | . |
| logepa | rs191006548 | . | . | . | . | . | . | . | . | . | . | . |
| logepa | rs117377631 | . | . | . | . | . | . | . | . | . | . | . |
| logepa | rs182107634 | . | . | . | . | . | . | . | . | . | . | . |
| logepa | rs190211867 | . | . | . | . | . | . | . | . | . | . | . |
| logepa | rs148293457 | . | . | . | . | . | . | . | . | . | . | . |
| logepa | rs191811733 | . | . | . | . | . | . | . | . | . | . | . |
| logepa | rs146109272 | . | . | . | . | . | . | . | . | . | . | . |
| logepa | rs148397915 | . | . | . | . | . | . | . | . | . | . | . |
| logepa | rs142233290 | . | . | . | . | . | . | . | . | . | . | . |
| logepa | rs80226874 | . | . | . | . | . | . | . | . | . | . | . |
| logepa | rs144496203 | . | . | . | . | . | . | . | . | . | . | . |
| logepa | rs191062130 | . | . | . | . | . | . | . | . | . | . | . |
| logepa | rs185807817 | . | . | . | . | . | . | . | . | . | . | . |
| logepa | rs117846213 | . | . | . | . | . | . | . | . | . | . | . |
| logepa | rs190545473 | . | . | . | . | . | . | . | . | . | . | . |
| logepa | rs192364918 | . | . | . | . | . | . | . | . | . | . | . |
| logepa | rs182939241 | . | . | . | . | . | . | . | . | . | . | . |
| logepa | rs12571125 | . | . | . | . | . | . | . | . | . | . | . |
| logepa | rs192418941 | . | . | . | . | . | . | . | . | . | . | . |
| logepa | rs141270243 | . | . | . | . | . | . | . | . | . | . | . |
| logepa | rs182615502 | . | . | . | . | . | . | . | . | . | . | . |
| logepa | rs146669856 | . | . | . | . | . | . | . | . | . | . | . |
| logepa | rs78290029 | . | . | . | . | . | . | . | . | . | . | . |
| logepa | rs139955565 | . | . | . | . | . | . | . | . | . | . | . |
| logepa | rs12267314 | . | . | . | . | . | . | . | . | . | . | . |
| logepa | rs74477197 | . | . | . | . | . | . | . | . | . | . | . |
| logepa | rs188147236 | . | . | . | . | . | . | . | . | . | . | . |
| logepa | rs2228065 | . | . | . | . | . | . | . | . | . | . | . |
| logepa | rs116961353 | . | . | . | . | . | . | . | . | . | . | . |
| logepa | rs190222272 | . | . | . | . | . | . | . | . | . | . | . |
| logepa | rs182719940 | . | . | . | . | . | . | . | . | . | . | . |
| logepa | rs184477413 | . | . | . | . | . | . | . | . | . | . | . |
| logepa | rs12265175 | . | . | . | . | . | . | . | . | . | . | . |
| logepa | rs182928276 | . | . | . | . | . | . | . | . | . | . | . |
| logepa | rs144409895 | . | . | . | . | . | . | . | . | . | . | . |
| logepa | rs58998447 | . | . | . | . | . | . | . | . | . | . | . |
| logepa | rs188668832 | . | . | . | . | . | . | . | . | . | . | . |
| logepa | rs148641686 | . | . | . | . | . | . | . | . | . | . | . |
| logepa | rs75468791 | . | . | . | . | . | . | . | . | . | . | . |
| logepa | rs147327397 | . | . | . | . | . | . | . | . | . | . | . |
| logepa | rs77354597 | . | . | . | . | . | . | . | . | . | . | . |
| logepa | rs41365544 | . | . | . | . | . | . | . | . | . | . | . |
| logepa | rs187564188 | . | . | . | . | . | . | . | . | . | . | . |
| logepa | rs183511984 | . | . | . | . | . | . | . | . | . | . | . |
| logepa | rs12262783 | . | . | . | . | . | . | . | . | . | . | . |
| logepa | rs188817687 | . | . | . | . | . | . | . | . | . | . | . |
| logepa | rs3815968 | . | . | . | . | . | . | . | . | . | . | . |
| logepa | rs187822300 | . | . | . | . | . | . | . | . | . | . | . |
| logepa | rs188902172 | . | . | . | . | . | . | . | . | . | . | . |
| logepa | rs181190448 | . | . | . | . | . | . | . | . | . | . | . |
| logepa | rs182699093 | . | . | . | . | . | . | . | . | . | . | . |
| logepa | rs182631303 | . | . | . | . | . | . | . | . | . | . | . |
| logepa | rs187014172 | . | . | . | . | . | . | . | . | . | . | . |
| logepa | rs142575406 | . | . | . | . | . | . | . | . | . | . | . |
| logepa | rs180711033 | . | . | . | . | . | . | . | . | . | . | . |
| logepa | rs137894823 | . | . | . | . | . | . | . | . | . | . | . |
| logepa | rs189053273 | . | . | . | . | . | . | . | . | . | . | . |
| logepa | rs143499388 | . | . | . | . | . | . | . | . | . | . | . |
| logepa | rs182936001 | . | . | . | . | . | . | . | . | . | . | . |
| logepa | rs148456406 | . | . | . | . | . | . | . | . | . | . | . |
| logepa | rs182499531 | . | . | . | . | . | . | . | . | . | . | . |
| logepa | rs74884915 | . | . | . | . | . | . | . | . | . | . | . |
| logepa | rs184046374 | . | . | . | . | . | . | . | . | . | . | . |
| logepa | rs74333993 | . | . | . | . | . | . | . | . | . | . | . |
| logepa | rs138889719 | . | . | . | . | . | . | . | . | . | . | . |
| logepa | rs75330330 | . | . | . | . | . | . | . | . | . | . | . |
| logepa | rs193051693 | . | . | . | . | . | . | . | . | . | . | . |
| logepa | rs145929633 | . | . | . | . | . | . | . | . | . | . | . |

| logepa | rs184076904 | . | . | . | . | . | . | . | . | . | . | . |
| --- | --- | --- | --- | --- | --- | --- | --- | --- | --- | --- | --- | --- |
| logepa | rs141802494 | . | . | . | . | . | . | . | . | . | . | . |
| logepa | rs149519759 | . | . | . | . | . | . | . | . | . | . | . |
| logepa | rs145467260 | . | . | . | . | . | . | . | . | . | . | . |
| logepa | rs79620914 | . | . | . | . | . | . | . | . | . | . | . |
| logepa | rs140538633 | . | . | . | . | . | . | . | . | . | . | . |
| logepa | rs140862301 | . | . | . | . | . | . | . | . | . | . | . |
| logepa | rs114615847 | . | . | . | . | . | . | . | . | . | . | . |
| logepa | rs141800259 | . | . | . | . | . | . | . | . | . | . | . |
| logepa | rs149779059 | . | . | . | . | . | . | . | . | . | . | . |
| logepa | rs116539253 | . | . | . | . | . | . | . | . | . | . | . |
| logepa | rs184979550 | . | . | . | . | . | . | . | . | . | . | . |
| logepa | rs190128940 | . | . | . | . | . | . | . | . | . | . | . |
| logepa | rs146594816 | . | . | . | . | . | . | . | . | . | . | . |
| logepa | rs141370766 | . | . | . | . | . | . | . | . | . | . | . |
| logepa | rs144670033 | . | . | . | . | . | . | . | . | . | . | . |
| logepa | rs187152174 | . | . | . | . | . | . | . | . | . | . | . |
| logepa | rs184180263 | . | . | . | . | . | . | . | . | . | . | . |
| logepa | rs140075861 | . | . | . | . | . | . | . | . | . | . | . |
| logepa | rs186483129 | . | . | . | . | . | . | . | . | . | . | . |
| logepa | rs186634282 | . | . | . | . | . | . | . | . | . | . | . |
| logepa | rs9988663 | . | . | . | . | . | . | . | . | . | . | . |
| logepa | rs138828529 | . | . | . | . | . | . | . | . | . | . | . |
| logepa | rs188777615 | . | . | . | . | . | . | . | . | . | . | . |
| logepa | rs192321882 | . | . | . | . | . | . | . | . | . | . | . |
| logepa | rs150812270 | . | . | . | . | . | . | . | . | . | . | . |
| logepa | rs185906603 | . | . | . | . | . | . | . | . | . | . | . |
| logepa | rs191070862 | . | . | . | . | . | . | . | . | . | . | . |
| logepa | rs182904978 | . | . | . | . | . | . | . | . | . | . | . |
| logepa | rs192679655 | . | . | . | . | . | . | . | . | . | . | . |
| logepa | rs189673086 | . | . | . | . | . | . | . | . | . | . | . |
| logepa | rs181141892 | . | . | . | . | . | . | . | . | . | . | . |
| logepa | rs186404123 | . | . | . | . | . | . | . | . | . | . | . |
| logepa | rs192117287 | . | . | . | . | . | . | . | . | . | . | . |
| logepa | rs182748835 | . | . | . | . | . | . | . | . | . | . | . |
| logepa | rs141854549 | . | . | . | . | . | . | . | . | . | . | . |
| logepa | rs140163397 | . | . | . | . | . | . | . | . | . | . | . |
| logepa | rs146347300 | . | . | . | . | . | . | . | . | . | . | . |
| logepa | rs139362377 | . | . | . | . | . | . | . | . | . | . | . |
| logepa | rs185087671 | . | . | . | . | . | . | . | . | . | . | . |
| logepa | rs190466574 | . | . | . | . | . | . | . | . | . | . | . |
| logepa | rs147012778 | . | . | . | . | . | . | . | . | . | . | . |
| logepa | rs138236960 | . | . | . | . | . | . | . | . | . | . | . |
| logepa | rs116310311 | . | . | . | . | . | . | . | . | . | . | . |
| logepa | rs142868356 | . | . | . | . | . | . | . | . | . | . | . |
| logepa | rs144956240 | . | . | . | . | . | . | . | . | . | . | . |
| logepa | rs188527776 | . | . | . | . | . | . | . | . | . | . | . |
| logepa | rs183971165 | . | . | . | . | . | . | . | . | . | . | . |
| logepa | rs76748216 | . | . | . | . | . | . | . | . | . | . | . |
| logepa | rs148554990 | . | . | . | . | . | . | . | . | . | . | . |
| logepa | rs114446477 | . | . | . | . | . | . | . | . | . | . | . |
| logepa | rs185223933 | . | . | . | . | . | . | . | . | . | . | . |
| logepa | rs76869726 | . | . | . | . | . | . | . | . | . | . | . |
| logepa | rs151200830 | . | . | . | . | . | . | . | . | . | . | . |
| logepa | rs17157731 | . | . | . | . | . | . | . | . | . | . | . |
| logepa | rs181243469 | . | . | . | . | . | . | . | . | . | . | . |
| logepa | rs186890707 | . | . | . | . | . | . | . | . | . | . | . |
| logepa | rs184063360 | . | . | . | . | . | . | . | . | . | . | . |
| logepa | rs188550237 | . | . | . | . | . | . | . | . | . | . | . |
| logepa | rs140931068 | . | . | . | . | . | . | . | . | . | . | . |
| logepa | rs74867877 | . | . | . | . | . | . | . | . | . | . | . |
| logepa | rs11239495 | . | . | . | . | . | . | . | . | . | . | . |
| logepa | rs149745800 | . | . | . | . | . | . | . | . | . | . | . |
| logepa | rs145044458 | . | . | . | . | . | . | . | . | . | . | . |
| logepa | rs188067427 | . | . | . | . | . | . | . | . | . | . | . |
| logepa | rs146827800 | . | . | . | . | . | . | . | . | . | . | . |
| logepa | rs140619817 | . | . | . | . | . | . | . | . | . | . | . |
| logepa | rs892689 | . | . | . | . | . | . | . | . | . | . | . |
| logepa | rs143642005 | . | . | . | . | . | . | . | . | . | . | . |
| logepa | rs181929304 | . | . | . | . | . | . | . | . | . | . | . |
| logepa | rs191086173 | . | . | . | . | . | . | . | . | . | . | . |
| logepa | rs114613116 | . | . | . | . | . | . | . | . | . | . | . |
| logepa | rs141076768 | . | . | . | . | . | . | . | . | . | . | . |
| logepa | rs187316531 | . | . | . | . | . | . | . | . | . | . | . |
| logepa | rs11239499 | . | . | . | . | . | . | . | . | . | . | . |
| logepa | rs192726371 | . | . | . | . | . | . | . | . | . | . | . |
| logepa | rs74128481 | . | . | . | . | . | . | . | . | . | . | . |
| logepa | rs74128482 | . | . | . | . | . | . | . | . | . | . | . |
| logepa | rs11239502 | . | . | . | . | . | . | . | . | . | . | . |
| logepa | rs116330285 | . | . | . | . | . | . | . | . | . | . | . |
| logepa | rs189926729 | . | . | . | . | . | . | . | . | . | . | . |
| logepa | rs188975779 | . | . | . | . | . | . | . | . | . | . | . |
| logepa | rs143846038 | . | . | . | . | . | . | . | . | . | . | . |
| logepa | rs74128483 | . | . | . | . | . | . | . | . | . | . | . |
| logepa | rs139292055 | . | . | . | . | . | . | . | . | . | . | . |
| logepa | rs11239503 | . | . | . | . | . | . | . | . | . | . | . |

| logepa | rs58838686 | . | . | . | . | . | . | . | . | . | . | . |
| --- | --- | --- | --- | --- | --- | --- | --- | --- | --- | --- | --- | --- |
| logepa | rs185825373 | . | . | . | . | . | . | . | . | . | . | . |
| logepa | rs11239504 | . | . | . | . | . | . | . | . | . | . | . |
| logepa | rs150012354 | . | . | . | . | . | . | . | . | . | . | . |
| logepa | rs187763096 | . | . | . | . | . | . | . | . | . | . | . |
| logepa | rs193043982 | . | . | . | . | . | . | . | . | . | . | . |
| logepa | rs79186884 | . | . | . | . | . | . | . | . | . | . | . |
| logepa | rs74128484 | . | . | . | . | . | . | . | . | . | . | . |
| logepa | rs58461537 | . | . | . | . | . | . | . | . | . | . | . |
| logepa | rs188527474 | . | . | . | . | . | . | . | . | . | . | . |
| logepa | rs141659976 | . | . | . | . | . | . | . | . | . | . | . |
| logepa | rs115846407 | . | . | . | . | . | . | . | . | . | . | . |
| logepa | rs138378816 | . | . | . | . | . | . | . | . | . | . | . |
| logepa | rs146188136 | . | . | . | . | . | . | . | . | . | . | . |
| logepa | rs139383871 | . | . | . | . | . | . | . | . | . | . | . |
| logepa | rs182953869 | . | . | . | . | . | . | . | . | . | . | . |
| logepa | rs192430299 | . | . | . | . | . | . | . | . | . | . | . |
| logepa | rs144152844 | . | . | . | . | . | . | . | . | . | . | . |
| logepa | rs145987786 | . | . | . | . | . | . | . | . | . | . | . |
| logepa | rs181132926 | . | . | . | . | . | . | . | . | . | . | . |
| logepa | rs147882253 | . | . | . | . | . | . | . | . | . | . | . |
| logepa | rs141807621 | . | . | . | . | . | . | . | . | . | . | . |
| logepa | rs147116052 | . | . | . | . | . | . | . | . | . | . | . |
| logepa | rs147716618 | . | . | . | . | . | . | . | . | . | . | . |
| logepa | rs187198855 | . | . | . | . | . | . | . | . | . | . | . |
| logepa | rs189409638 | . | . | . | . | . | . | . | . | . | . | . |
| logepa | rs182647339 | . | . | . | . | . | . | . | . | . | . | . |
| logepa | rs187995988 | . | . | . | . | . | . | . | . | . | . | . |
| logepa | rs141034184 | . | . | . | . | . | . | . | . | . | . | . |
| logepa | rs187310430 | . | . | . | . | . | . | . | . | . | . | . |
| logepa | rs192608488 | . | . | . | . | . | . | . | . | . | . | . |
| logepa | rs148840366 | . | . | . | . | . | . | . | . | . | . | . |
| logepa | rs186785022 | . | . | . | . | . | . | . | . | . | . | . |
| logepa | rs192348728 | . | . | . | . | . | . | . | . | . | . | . |
| logepa | rs189656631 | . | . | . | . | . | . | . | . | . | . | . |
| logepa | rs185289750 | . | . | . | . | . | . | . | . | . | . | . |
| logepa | rs142382395 | . | . | . | . | . | . | . | . | . | . | . |
| logepa | rs144866696 | . | . | . | . | . | . | . | . | . | . | . |
| logepa | rs141371289 | . | . | . | . | . | . | . | . | . | . | . |
| logepa | rs114750008 | . | . | . | . | . | . | . | . | . | . | . |
| logepa | rs189063597 | . | . | . | . | . | . | . | . | . | . | . |
| logepa | rs139151571 | . | . | . | . | . | . | . | . | . | . | . |
| logepa | rs139846368 | . | . | . | . | . | . | . | . | . | . | . |
| logepa | rs182057653 | . | . | . | . | . | . | . | . | . | . | . |
| logepa | rs148785420 | . | . | . | . | . | . | . | . | . | . | . |
| logepa | rs138383251 | . | . | . | . | . | . | . | . | . | . | . |
| logepa | rs116526705 | . | . | . | . | . | . | . | . | . | . | . |
| logepa | rs185946436 | . | . | . | . | . | . | . | . | . | . | . |
| logepa | rs190455427 | . | . | . | . | . | . | . | . | . | . | . |
| logepa | rs9282584 | . | . | . | . | . | . | . | . | . | . | . |
| logepa | rs17153289 | . | . | . | . | . | . | . | . | . | . | . |
| logepa | rs138359103 | . | . | . | . | . | . | . | . | . | . | . |
| logepa | rs150605268 | . | . | . | . | . | . | . | . | . | . | . |
| logepa | rs79242910 | . | . | . | . | . | . | . | . | . | . | . |
| logepa | rs139716904 | . | . | . | . | . | . | . | . | . | . | . |
| logepa | rs76238498 | . | . | . | . | . | . | . | . | . | . | . |
| logepa | rs184140639 | . | . | . | . | . | . | . | . | . | . | . |
| logepa | rs188517278 | . | . | . | . | . | . | . | . | . | . | . |
| logepa | rs139678913 | . | . | . | . | . | . | . | . | . | . | . |
| logepa | rs116697955 | . | . | . | . | . | . | . | . | . | . | . |
| logepa | rs79267994 | . | . | . | . | . | . | . | . | . | . | . |
| logepa | rs11239510 | . | . | . | . | . | . | . | . | . | . | . |
| logepa | rs75831645 | . | . | . | . | . | . | . | . | . | . | . |
| logepa | rs78452276 | . | . | . | . | . | . | . | . | . | . | . |
| logepa | rs144366924 | . | . | . | . | . | . | . | . | . | . | . |
| logepa | rs77519841 | . | . | . | . | . | . | . | . | . | . | . |
| logepa | rs76944454 | . | . | . | . | . | . | . | . | . | . | . |
| logepa | rs76443855 | . | . | . | . | . | . | . | . | . | . | . |
| logepa | rs146613174 | . | . | . | . | . | . | . | . | . | . | . |
| logepa | rs181424761 | . | . | . | . | . | . | . | . | . | . | . |
| logepa | rs142119241 | . | . | . | . | . | . | . | . | . | . | . |
| logepa | rs140137954 | . | . | . | . | . | . | . | . | . | . | . |
| logepa | rs187319549 | . | . | . | . | . | . | . | . | . | . | . |
| logepa | rs12241545 | . | . | . | . | . | . | . | . | . | . | . |
| logepa | rs10128228 | . | . | . | . | . | . | . | . | . | . | . |
| logepa | rs58580249 | . | . | . | . | . | . | . | . | . | . | . |
| logepa | rs193148863 | . | . | . | . | . | . | . | . | . | . | . |
| logepa | rs185638946 | . | . | . | . | . | . | . | . | . | . | . |
| logepa | rs117752921 | . | . | . | . | . | . | . | . | . | . | . |
| logepa | rs180841236 | . | . | . | . | . | . | . | . | . | . | . |
| logepa | rs73283155 | . | . | . | . | . | . | . | . | . | . | . |
| logepa | rs76560599 | . | . | . | . | . | . | . | . | . | . | . |
| logepa | rs76273620 | . | . | . | . | . | . | . | . | . | . | . |
| logepa | rs141432923 | . | . | . | . | . | . | . | . | . | . | . |
| logepa | rs113529322 | . | . | . | . | . | . | . | . | . | . | . |
| logepa | rs10128306 | . | . | . | . | . | . | . | . | . | . | . |

| logepa | rs149691688 | . | . | . | . | . | . | . | . | . | . | . |
| --- | --- | --- | --- | --- | --- | --- | --- | --- | --- | --- | --- | --- |
| logepa | rs75872495 | . | . | . | . | . | . | . | . | . | . | . |
| logepa | rs191873960 | . | . | . | . | . | . | . | . | . | . | . |
| logepa | rs80155622 | . | . | . | . | . | . | . | . | . | . | . |
| logepa | rs113914656 | . | . | . | . | . | . | . | . | . | . | . |
| logepa | rs12242840 | . | . | . | . | . | . | . | . | . | . | . |
| logepa | rs147824078 | . | . | . | . | . | . | . | . | . | . | . |
| logepa | rs59051493 | . | . | . | . | . | . | . | . | . | . | . |
| logepa | rs9282588 | . | . | . | . | . | . | . | . | . | . | . |
| logepa | rs150215207 | . | . | . | . | . | . | . | . | . | . | . |
| logepa | rs138772243 | . | . | . | . | . | . | . | . | . | . | . |
| logepa | rs149383244 | . | . | . | . | . | . | . | . | . | . | . |
| logepa | rs17522720 | . | . | . | . | . | . | . | . | . | . | . |
| logepa | rs79245105 | . | . | . | . | . | . | . | . | . | . | . |
| logepa | rs138222133 | . | . | . | . | . | . | . | . | . | . | . |
| logepa | rs12247314 | . | . | . | . | . | . | . | . | . | . | . |
| logepa | rs12263894 | . | . | . | . | . | . | . | . | . | . | . |
| logepa | rs149907880 | . | . | . | . | . | . | . | . | . | . | . |
| logepa | rs188153885 | . | . | . | . | . | . | . | . | . | . | . |
| logepa | rs73283159 | . | . | . | . | . | . | . | . | . | . | . |
| logepa | rs140476687 | . | . | . | . | . | . | . | . | . | . | . |
| logepa | rs186603148 | . | . | . | . | . | . | . | . | . | . | . |
| logepa | rs183296063 | . | . | . | . | . | . | . | . | . | . | . |
| logepa | rs145733694 | . | . | . | . | . | . | . | . | . | . | . |
| logepa | rs189807449 | . | . | . | . | . | . | . | . | . | . | . |
| logepa | rs12253223 | . | . | . | . | . | . | . | . | . | . | . |
| logepa | rs182113637 | . | . | . | . | . | . | . | . | . | . | . |
| logepa | rs142792099 | . | . | . | . | . | . | . | . | . | . | . |
| logepa | rs12249910 | . | . | . | . | . | . | . | . | . | . | . |
| logepa | rs149436339 | . | . | . | . | . | . | . | . | . | . | . |
| logepa | rs77632210 | . | . | . | . | . | . | . | . | . | . | . |
| logepa | rs74130818 | . | . | . | . | . | . | . | . | . | . | . |
| logepa | rs187583648 | . | . | . | . | . | . | . | . | . | . | . |
| logepa | rs191295987 | . | . | . | . | . | . | . | . | . | . | . |
| logepa | rs184200686 | . | . | . | . | . | . | . | . | . | . | . |
| logepa | rs76117263 | . | . | . | . | . | . | . | . | . | . | . |
| logepa | rs185393149 | . | . | . | . | . | . | . | . | . | . | . |
| logepa | rs144760014 | . | . | . | . | . | . | . | . | . | . | . |
| logepa | rs7922292 | . | . | . | . | . | . | . | . | . | . | . |
| logepa | rs115372844 | . | . | . | . | . | . | . | . | . | . | . |
| logepa | rs111460364 | . | . | . | . | . | . | . | . | . | . | . |
| logepa | rs182052255 | . | . | . | . | . | . | . | . | . | . | . |
| logepa | rs180983889 | . | . | . | . | . | . | . | . | . | . | . |
| logepa | rs139175327 | . | . | . | . | . | . | . | . | . | . | . |
| logepa | rs149959620 | . | . | . | . | . | . | . | . | . | . | . |
| logepa | rs114653117 | . | . | . | . | . | . | . | . | . | . | . |
| logepa | rs144270018 | . | . | . | . | . | . | . | . | . | . | . |
| logepa | rs182723204 | . | . | . | . | . | . | . | . | . | . | . |
| logepa | rs57204067 | . | . | . | . | . | . | . | . | . | . | . |
| logepa | rs144956226 | . | . | . | . | . | . | . | . | . | . | . |
| logepa | rs187842704 | . | . | . | . | . | . | . | . | . | . | . |
| logepa | rs184620511 | . | . | . | . | . | . | . | . | . | . | . |
| logepa | rs144031303 | . | . | . | . | . | . | . | . | . | . | . |
| logepa | rs187526473 | . | . | . | . | . | . | . | . | . | . | . |
| logepa | rs193002805 | . | . | . | . | . | . | . | . | . | . | . |
| logepa | rs138707426 | . | . | . | . | . | . | . | . | . | . | . |
| logepa | rs142754243 | . | . | . | . | . | . | . | . | . | . | . |
| logepa | rs181600331 | . | . | . | . | . | . | . | . | . | . | . |
| logepa | rs144421264 | . | . | . | . | . | . | . | . | . | . | . |
| logepa | rs184414519 | . | . | . | . | . | . | . | . | . | . | . |
| logepa | rs150596349 | . | . | . | . | . | . | . | . | . | . | . |
| logepa | rs190753747 | . | . | . | . | . | . | . | . | . | . | . |
| logepa | rs139560940 | . | . | . | . | . | . | . | . | . | . | . |
| logepa | rs75640804 | . | . | . | . | . | . | . | . | . | . | . |
| logepa | rs73283175 | . | . | . | . | . | . | . | . | . | . | . |
| logepa | rs143337229 | . | . | . | . | . | . | . | . | . | . | . |
| logepa | rs73283177 | . | . | . | . | . | . | . | . | . | . | . |
| logepa | rs76053611 | . | . | . | . | . | . | . | . | . | . | . |
| logepa | rs116325983 | . | . | . | . | . | . | . | . | . | . | . |
| logepa | rs115451825 | . | . | . | . | . | . | . | . | . | . | . |
| logepa | rs183845246 | . | . | . | . | . | . | . | . | . | . | . |
| logepa | rs79756542 | . | . | . | . | . | . | . | . | . | . | . |
| logepa | rs187242860 | . | . | . | . | . | . | . | . | . | . | . |
| logepa | rs57318980 | . | . | . | . | . | . | . | . | . | . | . |
| logepa | rs59969732 | . | . | . | . | . | . | . | . | . | . | . |
| logepa | rs143811488 | . | . | . | . | . | . | . | . | . | . | . |
| logepa | rs189067644 | . | . | . | . | . | . | . | . | . | . | . |
| logepa | rs60449178 | . | . | . | . | . | . | . | . | . | . | . |
| logepa | rs59243389 | . | . | . | . | . | . | . | . | . | . | . |
| logepa | rs184946540 | . | . | . | . | . | . | . | . | . | . | . |
| logepa | rs188685881 | . | . | . | . | . | . | . | . | . | . | . |
| logepa | rs150335797 | . | . | . | . | . | . | . | . | . | . | . |
| logepa | rs143879260 | . | . | . | . | . | . | . | . | . | . | . |
| logepa | rs186044491 | . | . | . | . | . | . | . | . | . | . | . |
| logepa | rs11819084 | . | . | . | . | . | . | . | . | . | . | . |
| logepa | rs146915670 | . | . | . | . | . | . | . | . | . | . | . |

| logepa | rs113995695 | . | . | . | . | . | . | . | . | . | . | . |
| --- | --- | --- | --- | --- | --- | --- | --- | --- | --- | --- | --- | --- |
| logepa | rs143489451 | . | . | . | . | . | . | . | . | . | . | . |
| logepa | rs182653839 | . | . | . | . | . | . | . | . | . | . | . |
| logepa | rs141692564 | . | . | . | . | . | . | . | . | . | . | . |
| logepa | rs146281742 | . | . | . | . | . | . | . | . | . | . | . |
| logepa | rs192914353 | . | . | . | . | . | . | . | . | . | . | . |
| logepa | rs185182848 | . | . | . | . | . | . | . | . | . | . | . |
| logepa | rs28395858 | . | . | . | . | . | . | . | . | . | . | . |
| logepa | rs41429645 | . | . | . | . | . | . | . | . | . | . | . |
| logepa | rs28395874 | . | . | . | . | . | . | . | . | . | . | . |
| logepa | rs189663634 | . | . | . | . | . | . | . | . | . | . | . |
| logepa | rs77666700 | . | . | . | . | . | . | . | . | . | . | . |
| logepa | rs146711717 | . | . | . | . | . | . | . | . | . | . | . |
| logepa | rs144904251 | . | . | . | . | . | . | . | . | . | . | . |
| logepa | rs189893153 | . | . | . | . | . | . | . | . | . | . | . |
| logepa | rs150447740 | . | . | . | . | . | . | . | . | . | . | . |
| logepa | rs143099649 | . | . | . | . | . | . | . | . | . | . | . |
| logepa | rs191288436 | . | . | . | . | . | . | . | . | . | . | . |
| logepa | rs188577862 | . | . | . | . | . | . | . | . | . | . | . |
| logepa | rs192695334 | . | . | . | . | . | . | . | . | . | . | . |
| logepa | rs138612906 | . | . | . | . | . | . | . | . | . | . | . |
| logepa | rs191290051 | . | . | . | . | . | . | . | . | . | . | . |
| logepa | rs189631459 | . | . | . | . | . | . | . | . | . | . | . |
| logepa | rs114620874 | . | . | . | . | . | . | . | . | . | . | . |
| logepa | rs78246652 | . | . | . | . | . | . | . | . | . | . | . |
| logepa | rs79432451 | . | . | . | . | . | . | . | . | . | . | . |
| logepa | rs186022585 | . | . | . | . | . | . | . | . | . | . | . |
| logepa | rs142978891 | . | . | . | . | . | . | . | . | . | . | . |
| logepa | rs151086840 | . | . | . | . | . | . | . | . | . | . | . |
| logepa | rs183270259 | . | . | . | . | . | . | . | . | . | . | . |
| logepa | rs188544289 | . | . | . | . | . | . | . | . | . | . | . |
| logepa | rs188500808 | . | . | . | . | . | . | . | . | . | . | . |
| logepa | rs189424589 | . | . | . | . | . | . | . | . | . | . | . |
| logepa | rs78622303 | . | . | . | . | . | . | . | . | . | . | . |
| logepa | rs143210835 | . | . | . | . | . | . | . | . | . | . | . |
| logepa | rs114761840 | . | . | . | . | . | . | . | . | . | . | . |
| logepa | rs185805402 | . | . | . | . | . | . | . | . | . | . | . |
| logepa | rs139273744 | . | . | . | . | . | . | . | . | . | . | . |
| logepa | rs184654150 | . | . | . | . | . | . | . | . | . | . | . |
| logepa | rs189069710 | . | . | . | . | . | . | . | . | . | . | . |
| logepa | rs114752115 | . | . | . | . | . | . | . | . | . | . | . |
| logepa | rs150162515 | . | . | . | . | . | . | . | . | . | . | . |
| logepa | rs180982454 | . | . | . | . | . | . | . | . | . | . | . |
| logepa | rs184932088 | . | . | . | . | . | . | . | . | . | . | . |
| logepa | rs181109625 | . | . | . | . | . | . | . | . | . | . | . |
| logepa | rs138121134 | . | . | . | . | . | . | . | . | . | . | . |
| logepa | rs183253861 | . | . | . | . | . | . | . | . | . | . | . |
| logepa | rs147073946 | . | . | . | . | . | . | . | . | . | . | . |
| logepa | rs139938489 | . | . | . | . | . | . | . | . | . | . | . |
| logepa | rs146362560 | . | . | . | . | . | . | . | . | . | . | . |
| logepa | rs193155956 | . | . | . | . | . | . | . | . | . | . | . |
| logepa | rs77663092 | . | . | . | . | . | . | . | . | . | . | . |
| logepa | rs73285319 | . | . | . | . | . | . | . | . | . | . | . |
| logepa | rs184019276 | . | . | . | . | . | . | . | . | . | . | . |
| logepa | rs28395863 | . | . | . | . | . | . | . | . | . | . | . |
| logepa | rs182121645 | . | . | . | . | . | . | . | . | . | . | . |
| logepa | rs73285327 | . | . | . | . | . | . | . | . | . | . | . |
| logepa | rs149589584 | . | . | . | . | . | . | . | . | . | . | . |
| logepa | rs148310335 | . | . | . | . | . | . | . | . | . | . | . |
| logepa | rs187246093 | . | . | . | . | . | . | . | . | . | . | . |
| logepa | rs184668894 | . | . | . | . | . | . | . | . | . | . | . |
| logepa | rs114269961 | . | . | . | . | . | . | . | . | . | . | . |
| logepa | rs149694374 | . | . | . | . | . | . | . | . | . | . | . |
| logepa | rs145284227 | . | . | . | . | . | . | . | . | . | . | . |
| logepa | rs189983820 | . | . | . | . | . | . | . | . | . | . | . |
| logepa | rs181200444 | . | . | . | . | . | . | . | . | . | . | . |
| logepa | rs185486385 | . | . | . | . | . | . | . | . | . | . | . |
| logepa | rs151198241 | . | . | . | . | . | . | . | . | . | . | . |
| logepa | rs189959341 | . | . | . | . | . | . | . | . | . | . | . |
| logepa | rs140549257 | . | . | . | . | . | . | . | . | . | . | . |
| logepa | rs186453139 | . | . | . | . | . | . | . | . | . | . | . |
| logepa | rs180922711 | . | . | . | . | . | . | . | . | . | . | . |
| logepa | rs56659903 | . | . | . | . | . | . | . | . | . | . | . |
| logepa | rs141202128 | . | . | . | . | . | . | . | . | . | . | . |
| logepa | rs59085314 | . | . | . | . | . | . | . | . | . | . | . |
| logepa | rs145564996 | . | . | . | . | . | . | . | . | . | . | . |
| logepa | rs114961009 | . | . | . | . | . | . | . | . | . | . | . |
| logepa | rs182294189 | . | . | . | . | . | . | . | . | . | . | . |
| logepa | rs187586636 | . | . | . | . | . | . | . | . | . | . | . |
| logepa | rs192254907 | . | . | . | . | . | . | . | . | . | . | . |
| logepa | rs149145663 | . | . | . | . | . | . | . | . | . | . | . |
| logepa | rs180713064 | . | . | . | . | . | . | . | . | . | . | . |
| logepa | rs183009023 | . | . | . | . | . | . | . | . | . | . | . |
| logepa | rs143385347 | . | . | . | . | . | . | . | . | . | . | . |
| logepa | rs185786329 | . | . | . | . | . | . | . | . | . | . | . |
| logepa | rs192341030 | . | . | . | . | . | . | . | . | . | . | . |

| logepa | rs74661429 | . | . | . | . | . | . | . | . | . | . | . |
| --- | --- | --- | --- | --- | --- | --- | --- | --- | --- | --- | --- | --- |
| logepa | rs188034853 | . | . | . | . | . | . | . | . | . | . | . |
| logepa | rs116808430 | . | . | . | . | . | . | . | . | . | . | . |
| logepa | rs148469010 | . | . | . | . | . | . | . | . | . | . | . |
| logepa | rs78621663 | . | . | . | . | . | . | . | . | . | . | . |
| logepa | rs192654886 | . | . | . | . | . | . | . | . | . | . | . |
| logepa | rs145996035 | . | . | . | . | . | . | . | . | . | . | . |
| logepa | rs139942226 | . | . | . | . | . | . | . | . | . | . | . |
| logepa | rs113645632 | . | . | . | . | . | . | . | . | . | . | . |
| logepa | rs74940661 | . | . | . | . | . | . | . | . | . | . | . |
| logepa | rs115355800 | . | . | . | . | . | . | . | . | . | . | . |
| logepa | rs150285898 | . | . | . | . | . | . | . | . | . | . | . |
| logepa | rs188117932 | . | . | . | . | . | . | . | . | . | . | . |
| logepa | rs139956566 | . | . | . | . | . | . | . | . | . | . | . |
| logepa | rs147673885 | . | . | . | . | . | . | . | . | . | . | . |
| logepa | rs189127928 | . | . | . | . | . | . | . | . | . | . | . |
| logepa | rs187558772 | . | . | . | . | . | . | . | . | . | . | . |
| logepa | rs181948587 | . | . | . | . | . | . | . | . | . | . | . |
| logepa | rs189983269 | . | . | . | . | . | . | . | . | . | . | . |
| logepa | rs187250069 | . | . | . | . | . | . | . | . | . | . | . |
| logepa | rs191485859 | . | . | . | . | . | . | . | . | . | . | . |
| logepa | rs185499555 | . | . | . | . | . | . | . | . | . | . | . |
| logepa | rs180862110 | . | . | . | . | . | . | . | . | . | . | . |
| logepa | rs190472603 | . | . | . | . | . | . | . | . | . | . | . |
| logepa | rs189226167 | . | . | . | . | . | . | . | . | . | . | . |
| logepa | rs181623256 | . | . | . | . | . | . | . | . | . | . | . |
| logepa | rs186079557 | . | . | . | . | . | . | . | . | . | . | . |
| logepa | rs147760994 | . | . | . | . | . | . | . | . | . | . | . |
| logepa | rs189176167 | . | . | . | . | . | . | . | . | . | . | . |
| logepa | rs117901000 | . | . | . | . | . | . | . | . | . | . | . |
| logepa | rs141984888 | . | . | . | . | . | . | . | . | . | . | . |
| logepa | rs192365061 | . | . | . | . | . | . | . | . | . | . | . |
| logepa | rs185976038 | . | . | . | . | . | . | . | . | . | . | . |
| logepa | rs184697303 | . | . | . | . | . | . | . | . | . | . | . |
| logepa | rs143383132 | . | . | . | . | . | . | . | . | . | . | . |
| logepa | rs141873881 | . | . | . | . | . | . | . | . | . | . | . |
| logepa | rs185507255 | . | . | . | . | . | . | . | . | . | . | . |
| logepa | rs191379425 | . | . | . | . | . | . | . | . | . | . | . |
| logepa | rs184199584 | . | . | . | . | . | . | . | . | . | . | . |
| logepa | rs183181360 | . | . | . | . | . | . | . | . | . | . | . |
| logepa | rs184495673 | . | . | . | . | . | . | . | . | . | . | . |
| logepa | rs182946297 | . | . | . | . | . | . | . | . | . | . | . |
| logepa | rs187743615 | . | . | . | . | . | . | . | . | . | . | . |
| logepa | rs183259862 | . | . | . | . | . | . | . | . | . | . | . |
| logepa | rs112433566 | . | . | . | . | . | . | . | . | . | . | . |
| logepa | rs182351085 | . | . | . | . | . | . | . | . | . | . | . |
| logepa | rs146248508 | . | . | . | . | . | . | . | . | . | . | . |
| logepa | rs188528439 | . | . | . | . | . | . | . | . | . | . | . |
| logepa | rs146167018 | . | . | . | . | . | . | . | . | . | . | . |
| logepa | rs192440980 | . | . | . | . | . | . | . | . | . | . | . |
| logepa | rs185601882 | . | . | . | . | . | . | . | . | . | . | . |
| logepa | rs117272563 | . | . | . | . | . | . | . | . | . | . | . |
| logepa | rs10430734 | . | . | . | . | . | . | . | . | . | . | . |
| logepa | rs181320952 | . | . | . | . | . | . | . | . | . | . | . |
| logepa | rs185480660 | . | . | . | . | . | . | . | . | . | . | . |
| logepa | rs188077917 | . | . | . | . | . | . | . | . | . | . | . |
| logepa | rs187562953 | . | . | . | . | . | . | . | . | . | . | . |
| logepa | rs182044631 | . | . | . | . | . | . | . | . | . | . | . |
| logepa | rs145455596 | . | . | . | . | . | . | . | . | . | . | . |
| logepa | rs181475877 | . | . | . | . | . | . | . | . | . | . | . |
| logepa | rs149413085 | . | . | . | . | . | . | . | . | . | . | . |
| logepa | rs12253656 | . | . | . | . | . | . | . | . | . | . | . |
| logepa | rs182718239 | . | . | . | . | . | . | . | . | . | . | . |
| logepa | rs192435695 | . | . | . | . | . | . | . | . | . | . | . |
| logepa | rs12248277 | . | . | . | . | . | . | . | . | . | . | . |
| logepa | rs143521710 | . | . | . | . | . | . | . | . | . | . | . |
| logepa | rs144454454 | . | . | . | . | . | . | . | . | . | . | . |
| logepa | rs190441610 | . | . | . | . | . | . | . | . | . | . | . |
| logepa | rs114690462 | . | . | . | . | . | . | . | . | . | . | . |
| logepa | rs184541357 | . | . | . | . | . | . | . | . | . | . | . |
| logepa | rs142657653 | . | . | . | . | . | . | . | . | . | . | . |
| logepa | rsurf1 | 2.02 | . | . | . | . | . | . | . | . | . | . |
| logepa | rsten1 | 2.196 | . | . | . | . | . | . | . | . | . | . |
| logepa | chr10:45852312:D | 0.055 | 0.8 | 0.111 | 0.107 | 0.908 | 0.322 | 0.785 | 0.708 | 0.287 | 0.393 | -0.194 |
| logepa | chr10:45853232:I | 0.071 | 0.785 | 0.11 | 0.085 | 1.04 | 0.318 | 0.904 | 0.925 | 0.318 | 0.822 | -0.069 |
| logepa | chr10:45853234:I | 0.157 | 0.761 | 0.111 | 0.061 | 0.961 | 0.195 | 0.846 | 1.09 | 0.335 | 0.779 | 0.075 |
| logepa | chr10:45854796:D | 0.001 | . | . | . | . | . | . | . | . | . | . |
| logepa | chr10:45854994:I | 0.001 | . | . | . | . | . | . | . | . | . | . |
| logepa | chr10:45858290:D | 0.04 | 0.768 | 0.102 | 0.047 | 1.7 | 0.63 | 0.152 | 1.16 | 0.675 | 0.797 | 0.048 |
| logepa | chr10:45858820:I | 0.015 | 0.817 | 0.111 | 0.137 | 0.201 | 0.243 | 0.184 | 0.094 | 0.111 | 0.044 | -2.70E-03 |
| logepa | chr10:45860888:D | 0.067 | 0.748 | 0.101 | 0.032 | 0.976 | 0.285 | 0.933 | 1.38 | 0.585 | 0.448 | 0.283 |
| logepa | chr10:45863913:I | 0.159 | 0.77 | 0.112 | 0.072 | 0.969 | 0.193 | 0.876 | 1.04 | 0.306 | 0.888 | 0.038 |
| logepa | chr10:45866601:D | 0 | . | . | . | . | . | . | . | . | . | . |
| logepa | chr10:45867197:I | 0.008 | . | . | . | . | . | . | . | . | . | . |
| logepa | chr10:45867538:D | 0.167 | 0.751 | 0.111 | 0.053 | 1.03 | 0.192 | 0.891 | 1.12 | 0.309 | 0.674 | 0.088 |
| logepa | chr10:45868356:D | 0.151 | 0.776 | 0.111 | 0.076 | 0.923 | 0.199 | 0.71 | 1.02 | 0.323 | 0.956 | 0.03 |

| logepa | chr10:45871158:I | 0.16 | 0.768 | 0.112 | 0.07 | 0.97 | 0.194 | 0.881 | 1.05 | 0.307 | 0.867 | 0.044 |
| --- | --- | --- | --- | --- | --- | --- | --- | --- | --- | --- | --- | --- |
| logepa | chr10:45871637:D | 0.061 | 0.749 | 0.102 | 0.034 | 1.27 | 0.394 | 0.448 | 1.28 | 0.546 | 0.56 | 0.201 |
| logepa | chr10:45871638:I | 0.115 | 0.73 | 0.105 | 0.028 | 1.15 | 0.252 | 0.525 | 1.31 | 0.398 | 0.379 | 0.217 |
| logepa | chr10:45873629:I | 0.159 | 0.757 | 0.111 | 0.058 | 0.971 | 0.193 | 0.882 | 1.11 | 0.321 | 0.729 | 0.085 |
| logepa | chr10:45873701:D | 0.003 | . | . | . | . | . | . | . | . | . | . |
| logepa | chr10:45874997:I | 0.03 | 0.805 | 0.109 | 0.108 | 0.656 | 0.338 | 0.413 | 0.491 | 0.35 | 0.319 | -0.202 |
| logepa | chr10:45882030:I | 0.021 | 0.768 | 0.106 | 0.056 | 0.991 | 0.878 | 0.992 | 1.38 | 1.58 | 0.78 | 0.29 |
| logepa | chr10:45883774:D | 0.155 | 0.768 | 0.113 | 0.072 | 0.943 | 0.176 | 0.753 | 1.06 | 0.284 | 0.841 | 0.053 |
| logepa | chr10:45885169:D | 0.034 | 0.784 | 0.106 | 0.072 | 0.747 | 0.303 | 0.473 | 0.887 | 0.34 | 0.754 | -0.012 |
| logepa | chr10:45886451:D | 0.589 | 0.989 | 0.314 | 0.972 | 0.973 | 0.159 | 0.868 | 0.818 | 0.191 | 0.389 | -0.175 |
| logepa | chr10:45891800:I | 0.592 | 0.771 | 0.225 | 0.373 | 1.18 | 0.171 | 0.258 | 1 | 0.191 | 0.987 | -0.038 |
| logepa | chr10:45904771:I | 0.573 | 0.814 | 0.221 | 0.449 | 1.14 | 0.161 | 0.362 | 0.962 | 0.176 | 0.83 | -0.061 |
| logepa | chr10:45904959:I | 0.103 | 0.809 | 0.13 | 0.186 | 1.66 | 0.418 | 0.046 | 0.82 | 0.278 | 0.559 | -0.367 |
| logepa | chr10:45911028:I | 0.034 | 0.767 | 0.107 | 0.058 | 0.936 | 0.479 | 0.897 | 1.22 | 0.841 | 0.775 | 0.172 |
| logepa | chr10:45911031:I | 0.364 | 0.783 | 0.174 | 0.271 | 1.32 | 0.197 | 0.064 | 1 | 0.181 | 0.994 | -0.068 |
| logepa | chr10:45912154:D | 0.145 | 0.766 | 0.112 | 0.069 | 0.952 | 0.18 | 0.796 | 1.06 | 0.287 | 0.829 | 0.055 |
| logepa | chr10:45913885:I | 0.001 | . | . | . | . | . | . | . | . | . | . |
| logepa | chr10:45914753:D | 0.243 | 0.749 | 0.131 | 0.099 | 1.05 | 0.161 | 0.769 | 1.08 | 0.225 | 0.728 | 0.048 |
| logepa | chr10:45919621 | 0.003 | . | . | . | . | . | . | . | . | . | . |
| logepa | chr10:45921739:I | 0.004 | . | . | . | . | . | . | . | . | . | . |
| logepa | chr10:45923527:I | 0.052 | 0.753 | 0.11 | 0.053 | 1.66 | 0.623 | 0.173 | 1.25 | 0.488 | 0.56 | 0.155 |
| logepa | chr10:45927001:I | 0.014 | 0.768 | 0.104 | 0.051 | 0.219 | 0.264 | 0.209 | 1.33 | 1.36 | 0.783 | 0.236 |
| logepa | chr10:45930354:I | 0.001 | . | . | . | . | . | . | . | . | . | . |
| logepa | chr10:45932864:I | 0.027 | 0.781 | 0.106 | 0.068 | 2.01 | 0.86 | 0.101 | 0.86 | 0.505 | 0.798 | -0.442 |
| logepa | chr10:45936163:I | 0.025 | 0.773 | 0.103 | 0.054 | 0.699 | 0.339 | 0.46 | 1.46 | 0.413 | 0.184 | 0.315 |
| logepa | chr10:45938095:D | 0.164 | 0.773 | 0.122 | 0.103 | 0.945 | 0.173 | 0.756 | 1.03 | 0.254 | 0.905 | 0.034 |
| logepa | chr10:45938886 | 0 | . | . | . | . | . | . | . | . | . | . |
| logepa | chr10:45939320 | 0.001 | . | . | . | . | . | . | . | . | . | . |
| logepa | chr10:45939659 | 0.001 | . | . | . | . | . | . | . | . | . | . |
| logepa | chr10:45941150:D | 0.001 | . | . | . | . | . | . | . | . | . | . |
| logepa | chr10:45947128:D | 0.065 | 0.742 | 0.105 | 0.035 | 1.17 | 0.289 | 0.515 | 1.54 | 0.513 | 0.191 | 0.428 |
| logepa | chr10:45950990:I | 0.044 | 0.756 | 0.105 | 0.045 | 1.31 | 0.405 | 0.378 | 1.36 | 0.622 | 0.501 | 0.281 |
| logepa | chr10:45951854:I | 0.047 | 0.742 | 0.105 | 0.034 | 1.41 | 0.438 | 0.273 | 1.51 | 0.691 | 0.364 | 0.431 |
| logepa | chr10:45952670:I | 0.061 | 0.749 | 0.104 | 0.038 | 1.17 | 0.318 | 0.554 | 1.32 | 0.504 | 0.467 | 0.238 |
| logepa | chr10:45956741 | 0.001 | . | . | . | . | . | . | . | . | . | . |
| logepa | chr10:45957247:I | 0.233 | 0.746 | 0.128 | 0.088 | 1.11 | 0.169 | 0.512 | 1.08 | 0.211 | 0.702 | 0.037 |
| logepa | chr10:45958082:I | 0.233 | 0.749 | 0.129 | 0.094 | 1.11 | 0.173 | 0.508 | 1.07 | 0.213 | 0.742 | 0.029 |
| logepa | chr10:45905041:D | . | . | . | . | . | . | . | . | . | . | . |
| logepa | chr10:45938871 | . | . | . | . | . | . | . | . | . | . | . |
| logepa | chr10:45939160 | . | . | . | . | . | . | . | . | . | . | . |
| logepa | chr10:45869910 | . | . | . | . | . | . | . | . | . | . | . |
| logepa | chr10:45876214:D | . | . | . | . | . | . | . | . | . | . | . |
| logepa | chr10:45880237:I | . | . | . | . | . | . | . | . | . | . | . |
| logepa | chr10:45884371:I | . | . | . | . | . | . | . | . | . | . | . |
| logepa | chr10:45891535:D | . | . | . | . | . | . | . | . | . | . | . |
| logepa | chr10:45893516:D | . | . | . | . | . | . | . | . | . | . | . |
| logepa | chr10:45893782:D | . | . | . | . | . | . | . | . | . | . | . |
| logepa | chr10:45895254:I | . | . | . | . | . | . | . | . | . | . | . |
| logepa | chr10:45895848:D | . | . | . | . | . | . | . | . | . | . | . |
| logepa | chr10:45896316:D | . | . | . | . | . | . | . | . | . | . | . |
| logepa | chr10:45896317:D | . | . | . | . | . | . | . | . | . | . | . |
| logepa | chr10:45902683:I | . | . | . | . | . | . | . | . | . | . | . |
| logepa | chr10:45905003:D | . | . | . | . | . | . | . | . | . | . | . |
| logepa | chr10:45927476:D | . | . | . | . | . | . | . | . | . | . | . |
| logepa | chr10:45935882 | . | . | . | . | . | . | . | . | . | . | . |
| logepa | chr10:45936100 | . | . | . | . | . | . | . | . | . | . | . |
| logepa | chr10:45939553 | . | . | . | . | . | . | . | . | . | . | . |
| logepa | chr10:45939631 | . | . | . | . | . | . | . | . | . | . | . |
| logepa | chr10:45944193:D | . | . | . | . | . | . | . | . | . | . | . |
| logepa | chr10:45954030 | . | . | . | . | . | . | . | . | . | . | . |

Supplemental table S33.

| fa | rs_id | freq | HR_logepa | se_logepa | p_logepa | HR_snp | se_snp | p_snp | HR_inter | se_inter | p_inter | RERI |
| --- | --- | --- | --- | --- | --- | --- | --- | --- | --- | --- | --- | --- |
| logepa | rs76035182 | 0.068 | 0.963 | 0.238 | 0.879 | 1.27 | 0.44 | 0.484 | 1.2 | 0.574 | 0.698 | 0.24 |
| logepa | rs12359547 | 0.052 | 1.04 | 0.237 | 0.865 | 0.368 | 0.214 | 0.085 | 0.449 | 0.309 | 0.245 | -0.236 |
| logepa | rs74595484 | 0.12 | 1.01 | 0.251 | 0.964 | 0.68 | 0.231 | 0.256 | 0.919 | 0.475 | 0.87 | -0.059 |
| logepa | rs1864415 | 0.749 | 1.36 | 0.589 | 0.483 | 0.802 | 0.155 | 0.252 | 0.796 | 0.235 | 0.439 | -0.293 |
| logepa | rs72788101 | 0.064 | 1.03 | 0.238 | 0.913 | 0.384 | 0.198 | 0.063 | 0.562 | 0.333 | 0.331 | -0.188 |
| logepa | rs11239481 | 0.081 | 1.08 | 0.264 | 0.739 | 1.37 | 0.396 | 0.284 | 0.676 | 0.329 | 0.421 | -0.449 |
| logepa | rs72788102 | 0.064 | 1.03 | 0.238 | 0.913 | 0.384 | 0.198 | 0.063 | 0.562 | 0.333 | 0.331 | -0.188 |
| logepa | rs72789705 | 0.064 | 1.03 | 0.238 | 0.913 | 0.384 | 0.198 | 0.063 | 0.562 | 0.333 | 0.331 | -0.188 |
| logepa | rs141529858 | 0.002 | . | . | . | . | . | . | . | . | . | . |
| logepa | rs145376292 | 0 | . | . | . | . | . | . | . | . | . | . |
| logepa | rs190418301 | 0 | . | . | . | . | . | . | . | . | . | . |
| logepa | rs11239482 | 0.064 | 1.03 | 0.238 | 0.913 | 0.384 | 0.198 | 0.063 | 0.562 | 0.333 | 0.331 | -0.188 |
| logepa | rs61855872 | 0.004 | . | . | . | . | . | . | . | . | . | . |
| logepa | rs148991144 | 0.003 | . | . | . | . | . | . | . | . | . | . |
| logepa | rs191932221 | 0.002 | . | . | . | . | . | . | . | . | . | . |
| logepa | rs55978138 | 0.179 | 1.04 | 0.26 | 0.862 | 0.873 | 0.21 | 0.572 | 0.902 | 0.297 | 0.755 | -0.095 |
| logepa | rs73281167 | 0.045 | 0.878 | 0.208 | 0.583 | 0.912 | 0.415 | 0.84 | 3.34 | 2.58 | 0.117 | 1.89 |
| logepa | rs12768010 | 0.092 | 1 | 0.222 | 0.996 | 1.08 | 0.299 | 0.791 | 1 | 0.435 | 1 | -8.60E-05 |
| logepa | rs11239484 | 0.749 | 1.36 | 0.591 | 0.474 | 0.801 | 0.154 | 0.249 | 0.793 | 0.233 | 0.43 | -0.298 |
| logepa | rs35906360 | 0.18 | 1.03 | 0.251 | 0.919 | 0.889 | 0.21 | 0.618 | 0.939 | 0.311 | 0.85 | -0.058 |
| logepa | rs186250512 | 0.001 | . | . | . | . | . | . | . | . | . | . |
| logepa | rs150810743 | 0.007 | . | . | . | . | . | . | . | . | . | . |
| logepa | rs55965812 | 0.161 | 1.05 | 0.251 | 0.844 | 0.945 | 0.222 | 0.81 | 0.885 | 0.305 | 0.723 | -0.116 |
| logepa | rs56180358 | 0.181 | 1.03 | 0.251 | 0.919 | 0.888 | 0.21 | 0.616 | 0.939 | 0.31 | 0.85 | -0.058 |
| logepa | rs55864145 | 0.092 | 1 | 0.222 | 0.994 | 1.08 | 0.298 | 0.794 | 0.998 | 0.433 | 0.996 | -2.30E-03 |
| logepa | rs55986122 | 0.181 | 1.03 | 0.251 | 0.919 | 0.888 | 0.21 | 0.615 | 0.939 | 0.31 | 0.849 | -0.058 |
| logepa | rs7477350 | 0.749 | 1.37 | 0.592 | 0.471 | 0.801 | 0.154 | 0.249 | 0.792 | 0.233 | 0.427 | -0.301 |
| logepa | rs2099170 | 0.18 | 1.02 | 0.251 | 0.921 | 0.89 | 0.21 | 0.622 | 0.941 | 0.31 | 0.854 | -0.056 |
| logepa | rs2082108 | 0.181 | 1.03 | 0.251 | 0.918 | 0.888 | 0.21 | 0.614 | 0.938 | 0.309 | 0.847 | -0.059 |
| logepa | rs55902404 | 0.02 | 0.981 | 0.226 | 0.934 | 0.345 | 0.39 | 0.346 | 1.3 | 0.548 | 0.533 | 0.114 |
| logepa | rs58402662 | 0.02 | 0.981 | 0.226 | 0.934 | 0.347 | 0.39 | 0.347 | 1.3 | 0.545 | 0.539 | 0.113 |
| logepa | rs11511618 | 0.084 | 1.08 | 0.263 | 0.751 | 1.34 | 0.398 | 0.324 | 0.683 | 0.346 | 0.453 | -0.431 |
| logepa | rs1816492 | 0.747 | 1.37 | 0.596 | 0.466 | 0.805 | 0.155 | 0.26 | 0.789 | 0.233 | 0.422 | -0.305 |
| logepa | rs146611093 | 0.002 | . | . | . | . | . | . | . | . | . | . |
| logepa | rs186897689 | 0.006 | . | . | . | . | . | . | . | . | . | . |
| logepa | rs10793616 | 0.749 | 1.37 | 0.592 | 0.469 | 0.801 | 0.154 | 0.248 | 0.791 | 0.232 | 0.424 | -0.302 |
| logepa | rs116700479 | 0.052 | 0.97 | 0.223 | 0.896 | 0.847 | 0.352 | 0.689 | 1.33 | 0.874 | 0.666 | 0.274 |
| logepa | rs192535936 | 0.004 | . | . | . | . | . | . | . | . | . | . |
| logepa | rs113363297 | 0.723 | 1.27 | 0.511 | 0.548 | 0.795 | 0.143 | 0.202 | 0.828 | 0.219 | 0.476 | -0.23 |
| logepa | rs12354994 | 0.172 | 1.03 | 0.251 | 0.907 | 0.88 | 0.222 | 0.614 | 0.926 | 0.322 | 0.826 | -0.07 |
| logepa | rs148068329 | 0.025 | 0.983 | 0.225 | 0.941 | 1.2 | 0.776 | 0.782 | 1.83 | 2.96 | 0.71 | 0.969 |
| logepa | rs12356109 | 0.198 | 1.03 | 0.261 | 0.911 | 0.83 | 0.204 | 0.447 | 0.942 | 0.306 | 0.853 | -0.055 |
| logepa | rs55908141 | 0.02 | 0.981 | 0.226 | 0.934 | 0.351 | 0.391 | 0.347 | 1.3 | 0.545 | 0.531 | 0.116 |
| logepa | rs12354712 | 0.161 | 1.05 | 0.251 | 0.84 | 0.943 | 0.22 | 0.8 | 0.883 | 0.301 | 0.715 | -0.119 |
| logepa | rs147282684 | 0.002 | . | . | . | . | . | . | . | . | . | . |
| logepa | rs12356140 | 0.191 | 1.06 | 0.259 | 0.799 | 0.887 | 0.209 | 0.61 | 0.858 | 0.278 | 0.636 | -0.141 |
| logepa | rs185476187 | 0.004 | . | . | . | . | . | . | . | . | . | . |
| logepa | rs145720057 | 0.012 | 1.01 | 0.233 | 0.96 | 0.746 | 1 | 0.828 | 0.537 | 0.506 | 0.509 | -0.353 |
| logepa | rs12354742 | 0.181 | 1.03 | 0.251 | 0.915 | 0.886 | 0.208 | 0.608 | 0.937 | 0.307 | 0.842 | -0.061 |
| logepa | rs180913842 | 0.02 | 0.981 | 0.226 | 0.934 | 0.351 | 0.391 | 0.347 | 1.3 | 0.545 | 0.529 | 0.116 |
| logepa | rs142976914 | 0.002 | . | . | . | . | . | . | . | . | . | . |
| logepa | rs11511619 | 0.201 | 1.03 | 0.262 | 0.895 | 0.819 | 0.201 | 0.416 | 0.932 | 0.305 | 0.829 | -0.064 |
| logepa | rs146702870 | 0.003 | . | . | . | . | . | . | . | . | . | . |
| logepa | rs150287496 | 0.001 | . | . | . | . | . | . | . | . | . | . |
| logepa | rs190917973 | 0.002 | . | . | . | . | . | . | . | . | . | . |
| logepa | rs11517281 | 0.181 | 1.03 | 0.251 | 0.915 | 0.886 | 0.208 | 0.607 | 0.937 | 0.306 | 0.841 | -0.061 |
| logepa | rs12359299 | 0.181 | 1.03 | 0.251 | 0.915 | 0.886 | 0.208 | 0.606 | 0.937 | 0.307 | 0.842 | -0.06 |
| logepa | rs182307932 | 0.071 | 0.955 | 0.229 | 0.847 | 0.953 | 0.335 | 0.891 | 1.56 | 1.12 | 0.537 | 0.509 |
| logepa | rs61855873 | 0.011 | 0.998 | 0.229 | 0.992 | 6.94 | 5.44 | 0.013 | 0.668 | 0.804 | 0.737 | -2.32 |
| logepa | rs12359302 | 0.18 | 1.04 | 0.252 | 0.887 | 0.857 | 0.214 | 0.537 | 0.913 | 0.319 | 0.795 | -0.082 |
| logepa | rs143208140 | 0.028 | 0.982 | 0.227 | 0.939 | 1.11 | 0.701 | 0.867 | 1.62 | 2.23 | 0.726 | 0.675 |
| logepa | rs9988764 | 0.712 | 1.39 | 0.589 | 0.44 | 0.796 | 0.158 | 0.253 | 0.771 | 0.235 | 0.392 | -0.333 |
| logepa | rs192875767 | 0.002 | . | . | . | . | . | . | . | . | . | . |
| logepa | rs147268846 | 0.005 | . | . | . | . | . | . | . | . | . | . |
| logepa | rs11239485 | 0.109 | 1.06 | 0.261 | 0.808 | 0.863 | 0.282 | 0.651 | 0.78 | 0.341 | 0.569 | -0.21 |
| logepa | rs151317885 | 0.24 | 1.39 | 0.334 | 0.175 | 1.02 | 0.234 | 0.946 | 0.467 | 0.178 | 0.046 | -0.745 |
| logepa | rs11499085 | 0.322 | 0.866 | 0.272 | 0.648 | 0.776 | 0.156 | 0.206 | 1.31 | 0.388 | 0.362 | 0.239 |
| logepa | rs188130606 | 0.086 | 1.06 | 0.253 | 0.793 | 0.389 | 0.197 | 0.063 | 0.434 | 0.294 | 0.217 | -0.274 |
| logepa | rs11511620 | 0.161 | 1.05 | 0.251 | 0.839 | 0.942 | 0.219 | 0.796 | 0.882 | 0.299 | 0.711 | -0.12 |
| logepa | rs181498390 | 0.057 | 0.901 | 0.219 | 0.67 | 1.04 | 0.423 | 0.915 | 2.05 | 1.37 | 0.285 | 0.983 |
| logepa | rs186280701 | 0.02 | 0.981 | 0.226 | 0.934 | 0.357 | 0.392 | 0.348 | 1.31 | 0.544 | 0.517 | 0.12 |
| logepa | rs150059185 | 0.034 | 1.11 | 0.25 | 0.646 | 1.29 | 0.801 | 0.678 | 0.342 | 0.318 | 0.248 | -0.912 |
| logepa | rs183338707 | 0.001 | . | . | . | . | . | . | . | . | . | . |
| logepa | rs11499038 | 0.306 | 0.873 | 0.272 | 0.663 | 0.815 | 0.177 | 0.347 | 1.31 | 0.42 | 0.406 | 0.241 |
| logepa | rs190699595 | 0.004 | . | . | . | . | . | . | . | . | . | . |
| logepa | rs11523408 | 0.175 | 1.02 | 0.252 | 0.935 | 0.906 | 0.216 | 0.678 | 0.953 | 0.312 | 0.883 | -0.045 |
| logepa | rs188049317 | 0.007 | . | . | . | . | . | . | . | . | . | . |
| logepa | rs144948634 | 0.214 | 1.21 | 0.287 | 0.415 | 1.11 | 0.269 | 0.667 | 0.615 | 0.242 | 0.217 | -0.494 |
| logepa | rs143833576 | . | . | . | . | . | . | . | . | . | . | . |
| logepa | rs151239879 | 0.175 | 1.03 | 0.253 | 0.899 | 0.903 | 0.216 | 0.671 | 0.926 | 0.301 | 0.814 | -0.072 |
| logepa | rs7073743 | 0.212 | 1.28 | 0.309 | 0.3 | 1.13 | 0.278 | 0.627 | 0.538 | 0.21 | 0.112 | -0.632 |
| logepa | rs142563349 | 0.092 | 1.06 | 0.254 | 0.802 | 0.439 | 0.204 | 0.076 | 0.487 | 0.306 | 0.252 | -0.274 |
| logepa | rs187246403 | 0.02 | 0.981 | 0.226 | 0.935 | 0.36 | 0.393 | 0.349 | 1.31 | 0.544 | 0.512 | 0.122 |
| logepa | rs150423942 | 0.178 | 1.03 | 0.253 | 0.904 | 0.893 | 0.213 | 0.634 | 0.929 | 0.308 | 0.824 | -0.069 |
| logepa | rs184706102 | . | . | . | . | . | . | . | . | . | . | . |
| logepa | rs189626288 | 0.007 | . | . | . | . | . | . | . | . | . | . |
| logepa | rs140749834 | 0.142 | 0.836 | 0.226 | 0.508 | 1.09 | 0.268 | 0.719 | 2.09 | 0.789 | 0.051 | 0.982 |

| logepa | rs7078822 | 0.136 | 1.03 | 0.253 | 0.915 | 0.938 | 0.249 | 0.808 | 0.928 | 0.344 | 0.841 | -0.071 |
| --- | --- | --- | --- | --- | --- | --- | --- | --- | --- | --- | --- | --- |
| logepa | rs186227617 | 0.177 | 1.03 | 0.253 | 0.895 | 0.898 | 0.213 | 0.65 | 0.923 | 0.302 | 0.806 | -0.075 |
| logepa | rs188984368 | 0.018 | 0.907 | 0.214 | 0.68 | 2.06 | 1.52 | 0.325 | 14.4 | 13.5 | 4.40E-03 | 25 |
| logepa | rs182078109 | 0.04 | 0.968 | 0.243 | 0.897 | 2.21 | 1.3 | 0.177 | 1.31 | 1.15 | 0.755 | 0.635 |
| logepa | rs141456274 | 0.001 | . | . | . | . | . | . | . | . | . | . |
| logepa | rs146976512 | 0.181 | 1.03 | 0.251 | 0.913 | 0.886 | 0.208 | 0.605 | 0.935 | 0.306 | 0.838 | -0.062 |
| logepa | rs138036816 | 0.305 | 0.874 | 0.272 | 0.665 | 0.815 | 0.177 | 0.347 | 1.3 | 0.419 | 0.408 | 0.24 |
| logepa | rs114879777 | 0.051 | 0.967 | 0.223 | 0.882 | 0.857 | 0.363 | 0.715 | 1.38 | 0.943 | 0.641 | 0.317 |
| logepa | rs149366475 | 0.307 | 0.877 | 0.272 | 0.671 | 0.802 | 0.174 | 0.308 | 1.3 | 0.419 | 0.419 | 0.234 |
| logepa | rs182463043 | 0.007 | . | . | . | . | . | . | . | . | . | . |
| logepa | rs144754632 | 0.025 | 0.983 | 0.225 | 0.94 | 1.2 | 0.779 | 0.778 | 1.84 | 2.97 | 0.707 | 0.983 |
| logepa | rs55717607 | 0.181 | 1.03 | 0.251 | 0.909 | 0.884 | 0.205 | 0.594 | 0.933 | 0.301 | 0.83 | -0.064 |
| logepa | rs187537973 | 0.001 | . | . | . | . | . | . | . | . | . | . |
| logepa | rs148085971 | 0.007 | . | . | . | . | . | . | . | . | . | . |
| logepa | rs11239491 | 0.181 | 1.03 | 0.251 | 0.908 | 0.883 | 0.205 | 0.593 | 0.933 | 0.3 | 0.829 | -0.064 |
| logepa | rs184412184 | 0.002 | . | . | . | . | . | . | . | . | . | . |
| logepa | rs4948998 | 0.798 | 1.09 | 0.469 | 0.836 | 0.799 | 0.15 | 0.231 | 0.939 | 0.257 | 0.819 | -0.072 |
| logepa | rs7076696 | 0.798 | 1.09 | 0.469 | 0.841 | 0.8 | 0.15 | 0.234 | 0.941 | 0.258 | 0.824 | -0.07 |
| logepa | rs11239492 | 0.198 | 1.03 | 0.262 | 0.897 | 0.825 | 0.2 | 0.428 | 0.933 | 0.297 | 0.827 | -0.063 |
| logepa | rs79462470 | 0.02 | 0.981 | 0.226 | 0.934 | 0.371 | 0.394 | 0.351 | 1.33 | 0.545 | 0.492 | 0.13 |
| logepa | rs187804654 | . | . | . | . | . | . | . | . | . | . | . |
| logepa | rs7093687 | 0.161 | 1.05 | 0.251 | 0.834 | 0.938 | 0.215 | 0.781 | 0.88 | 0.294 | 0.701 | -0.122 |
| logepa | rs7081004 | 0.162 | 1.05 | 0.251 | 0.833 | 0.938 | 0.215 | 0.78 | 0.879 | 0.293 | 0.699 | -0.123 |
| logepa | rs7084793 | 0.181 | 1.03 | 0.251 | 0.908 | 0.883 | 0.205 | 0.591 | 0.933 | 0.3 | 0.829 | -0.064 |
| logepa | rs61556080 | 0.02 | 0.981 | 0.226 | 0.935 | 0.371 | 0.395 | 0.352 | 1.32 | 0.544 | 0.494 | 0.13 |
| logepa | rs115552064 | 0.019 | 1.01 | 0.234 | 0.95 | 0.835 | 0.562 | 0.789 | 0.715 | 0.492 | 0.626 | -0.243 |
| logepa | rs7921503 | 0.18 | 1.04 | 0.254 | 0.872 | 0.868 | 0.202 | 0.543 | 0.904 | 0.29 | 0.753 | -0.092 |
| logepa | rs7909740 | 0.181 | 1.03 | 0.251 | 0.909 | 0.883 | 0.205 | 0.591 | 0.933 | 0.299 | 0.829 | -0.064 |
| logepa | rs7909768 | 0.181 | 1.03 | 0.251 | 0.909 | 0.883 | 0.205 | 0.591 | 0.933 | 0.299 | 0.83 | -0.064 |
| logepa | rs144168004 | 0.001 | . | . | . | . | . | . | . | . | . | . |
| logepa | rs75850448 | 0.02 | 0.981 | 0.226 | 0.935 | 0.371 | 0.395 | 0.351 | 1.32 | 0.542 | 0.501 | 0.128 |
| logepa | rs147777574 | 0.016 | 0.974 | 0.226 | 0.909 | 0.721 | 0.523 | 0.652 | 1.91 | 1.22 | 0.312 | 0.646 |
| logepa | rs7913488 | 0.181 | 1.03 | 0.251 | 0.909 | 0.883 | 0.205 | 0.59 | 0.933 | 0.299 | 0.83 | -0.064 |
| logepa | rs74390874 | 0.019 | 1.02 | 0.235 | 0.944 | 0.759 | 0.745 | 0.779 | 0.6 | 0.911 | 0.736 | -0.313 |
| logepa | rs141391142 | 0.002 | . | . | . | . | . | . | . | . | . | . |
| logepa | rs115294747 | 0.017 | 1.01 | 0.231 | 0.982 | 0.283 | 0.35 | 0.307 | 1.89 | 0.902 | 0.18 | 0.251 |
| logepa | rs148031781 | 0.001 | . | . | . | . | . | . | . | . | . | . |
| logepa | rs181340128 | 0.007 | . | . | . | . | . | . | . | . | . | . |
| logepa | rs189930007 | 0 | . | . | . | . | . | . | . | . | . | . |
| logepa | rs12242128 | 0.304 | 0.878 | 0.273 | 0.676 | 0.812 | 0.178 | 0.341 | 1.29 | 0.417 | 0.424 | 0.233 |
| logepa | rs78421750 | 0.016 | 1.04 | 0.236 | 0.874 | 0.094 | 0.214 | 0.3 | 0.092 | 0.119 | 0.066 | -0.122 |
| logepa | rs114370747 | 0.007 | . | . | . | . | . | . | . | . | . | . |
| logepa | rs12264166 | 0.304 | 0.878 | 0.273 | 0.677 | 0.813 | 0.178 | 0.342 | 1.29 | 0.417 | 0.425 | 0.232 |
| logepa | rs182964822 | 0 | . | . | . | . | . | . | . | . | . | . |
| logepa | rs67491417 | 0.178 | 1 | 0.246 | 0.988 | 0.849 | 0.207 | 0.502 | 0.991 | 0.324 | 0.979 | -7.80E-03 |
| logepa | rs72789720 | 0.006 | . | . | . | . | . | . | . | . | . | . |
| logepa | rs6413416 | 0.182 | 1.03 | 0.251 | 0.911 | 0.882 | 0.204 | 0.587 | 0.935 | 0.299 | 0.833 | -0.063 |
| logepa | rs4986832 | 0.182 | 1.03 | 0.251 | 0.911 | 0.882 | 0.204 | 0.587 | 0.935 | 0.299 | 0.833 | -0.062 |
| logepa | rs144866316 | . | . | . | . | . | . | . | . | . | . | . |
| logepa | rs187769540 | 0.001 | . | . | . | . | . | . | . | . | . | . |
| logepa | rs6593482 | 0.182 | 1.03 | 0.251 | 0.913 | 0.882 | 0.204 | 0.587 | 0.936 | 0.299 | 0.837 | -0.061 |
| logepa | rs7913948 | 0.183 | 1.03 | 0.251 | 0.912 | 0.878 | 0.204 | 0.576 | 0.935 | 0.3 | 0.834 | -0.062 |
| logepa | rs77185151 | 0.025 | 0.983 | 0.225 | 0.94 | 1.2 | 0.779 | 0.778 | 1.84 | 2.98 | 0.707 | 0.988 |
| logepa | rs12762303 | 0.182 | 1.03 | 0.251 | 0.911 | 0.882 | 0.204 | 0.587 | 0.935 | 0.299 | 0.833 | -0.062 |
| logepa | rs73283136 | 0.008 | . | . | . | . | . | . | . | . | . | . |
| logepa | rs187180218 | 0.007 | . | . | . | . | . | . | . | . | . | . |
| logepa | rs78137087 | 0.182 | 1.03 | 0.251 | 0.915 | 0.88 | 0.204 | 0.58 | 0.937 | 0.301 | 0.84 | -0.06 |
| logepa | rs72561472 | 0.181 | 1.03 | 0.247 | 0.912 | 0.858 | 0.201 | 0.514 | 0.937 | 0.306 | 0.841 | -0.06 |
| logepa | rs4987106 | 0.18 | 1.01 | 0.249 | 0.974 | 0.812 | 0.189 | 0.371 | 0.979 | 0.315 | 0.948 | -0.019 |
| logepa | rs4987105 | 0.159 | 1.06 | 0.252 | 0.8 | 0.9 | 0.211 | 0.653 | 0.852 | 0.29 | 0.638 | -0.148 |
| logepa | rs111916157 | 0.187 | 1.11 | 0.275 | 0.681 | 0.816 | 0.199 | 0.405 | 0.761 | 0.245 | 0.396 | -0.236 |
| logepa | rs111528152 | 0.184 | 1.08 | 0.266 | 0.759 | 0.785 | 0.192 | 0.322 | 0.805 | 0.259 | 0.501 | -0.182 |
| logepa | rs12763738 | 0.178 | 1.03 | 0.253 | 0.889 | 0.882 | 0.208 | 0.594 | 0.918 | 0.296 | 0.79 | -0.079 |
| logepa | rs191675886 | 0.011 | 1.09 | 0.248 | 0.701 | 0.22 | 0.308 | 0.279 | 0.015 | 0.03 | 0.037 | -0.307 |
| logepa | rs66489870 | 0.182 | 1.03 | 0.251 | 0.911 | 0.882 | 0.204 | 0.588 | 0.935 | 0.299 | 0.833 | -0.062 |
| logepa | rs71494775 | 0.182 | 1.03 | 0.251 | 0.911 | 0.882 | 0.204 | 0.588 | 0.935 | 0.299 | 0.833 | -0.062 |
| logepa | rs61855875 | 0.01 | 0.988 | 0.227 | 0.958 | 6.75 | 5.77 | 0.026 | 0.9 | 1.17 | 0.936 | -0.737 |
| logepa | rs116356837 | 0.019 | 1.01 | 0.234 | 0.951 | 0.801 | 0.55 | 0.746 | 0.718 | 0.503 | 0.636 | -0.232 |
| logepa | rs1864414 | 0.179 | 1.03 | 0.252 | 0.905 | 0.896 | 0.213 | 0.643 | 0.93 | 0.308 | 0.826 | -0.068 |
| logepa | rs1864413 | 0.182 | 1.03 | 0.251 | 0.911 | 0.882 | 0.205 | 0.589 | 0.935 | 0.299 | 0.833 | -0.063 |
| logepa | rs139497212 | 0 | . | . | . | . | . | . | . | . | . | . |
| logepa | rs892690 | 0.104 | 1.1 | 0.272 | 0.691 | 1.34 | 0.451 | 0.382 | 0.618 | 0.355 | 0.402 | -0.53 |
| logepa | rs1864412 | 0.182 | 1.03 | 0.251 | 0.911 | 0.883 | 0.205 | 0.591 | 0.935 | 0.299 | 0.833 | -0.062 |
| logepa | rs77827771 | 0.087 | 1.07 | 0.253 | 0.79 | 0.382 | 0.195 | 0.059 | 0.428 | 0.292 | 0.214 | -0.273 |
| logepa | rs145294963 | . | . | . | . | . | . | . | . | . | . | . |
| logepa | rs149081327 | 0.003 | . | . | . | . | . | . | . | . | . | . |
| logepa | rs67008983 | 0.182 | 1.03 | 0.251 | 0.911 | 0.886 | 0.205 | 0.602 | 0.935 | 0.299 | 0.833 | -0.063 |
| logepa | rs56150414 | 0.182 | 1.02 | 0.251 | 0.92 | 0.889 | 0.206 | 0.611 | 0.942 | 0.3 | 0.85 | -0.056 |
| logepa | rs55787481 | 0.182 | 1.03 | 0.251 | 0.92 | 0.881 | 0.205 | 0.585 | 0.942 | 0.303 | 0.852 | -0.056 |
| logepa | rs141270101 | 0.001 | . | . | . | . | . | . | . | . | . | . |
| logepa | rs114980075 | 0.022 | 1.02 | 0.247 | 0.949 | 1.77 | 0.89 | 0.258 | 0.791 | 0.426 | 0.663 | -0.363 |
| logepa | rs12783095 | 0.181 | 1.03 | 0.252 | 0.912 | 0.895 | 0.208 | 0.632 | 0.936 | 0.299 | 0.835 | -0.062 |
| logepa | rs182342688 | 0.001 | . | . | . | . | . | . | . | . | . | . |
| logepa | rs17157728 | 0.181 | 1.03 | 0.252 | 0.912 | 0.895 | 0.208 | 0.634 | 0.936 | 0.299 | 0.835 | -0.062 |
| logepa | rs114835563 | 0.086 | 1.07 | 0.253 | 0.783 | 0.387 | 0.196 | 0.061 | 0.424 | 0.289 | 0.208 | -0.28 |
| logepa | rs3824612 | 0.104 | 1.11 | 0.273 | 0.679 | 1.34 | 0.451 | 0.39 | 0.608 | 0.35 | 0.387 | -0.544 |
| logepa | rs17157733 | 0.181 | 1.03 | 0.252 | 0.912 | 0.899 | 0.209 | 0.645 | 0.936 | 0.298 | 0.835 | -0.062 |
| logepa | rs74128478 | 0.019 | 0.982 | 0.226 | 0.936 | 0.365 | 0.399 | 0.356 | 1.28 | 0.54 | 0.552 | 0.113 |
| logepa | rs184973589 | 0.001 | . | . | . | . | . | . | . | . | . | . |

| logepa | rs150479461 | 0 | . | . | . | . | . | . | . | . | . | . |
| --- | --- | --- | --- | --- | --- | --- | --- | --- | --- | --- | --- | --- |
| logepa | rs138310407 | . | . | . | . | . | . | . | . | . | . | . |
| logepa | rs187426603 | . | . | . | . | . | . | . | . | . | . | . |
| logepa | rs190843143 | 0.001 | . | . | . | . | . | . | . | . | . | . |
| logepa | rs191883449 | 0 | . | . | . | . | . | . | . | . | . | . |
| logepa | rs58238740 | 0.173 | 1.03 | 0.246 | 0.901 | 0.941 | 0.221 | 0.795 | 0.927 | 0.306 | 0.818 | -0.073 |
| logepa | rs3780894 | 0.173 | 1.03 | 0.246 | 0.901 | 0.941 | 0.221 | 0.796 | 0.927 | 0.306 | 0.818 | -0.073 |
| logepa | rs149646626 | 0.006 | . | . | . | . | . | . | . | . | . | . |
| logepa | rs79615212 | 0.006 | . | . | . | . | . | . | . | . | . | . |
| logepa | rs3780895 | 0.173 | 1.03 | 0.246 | 0.894 | 0.939 | 0.221 | 0.788 | 0.921 | 0.303 | 0.803 | -0.078 |
| logepa | rs3780896 | 0.173 | 1.03 | 0.246 | 0.901 | 0.941 | 0.221 | 0.796 | 0.927 | 0.306 | 0.818 | -0.073 |
| logepa | rs142747163 | 0.003 | . | . | . | . | . | . | . | . | . | . |
| logepa | rs189417584 | 0.01 | 1 | 0.225 | 0.995 | 9.40E-04 | 5.00E-03 | 0.193 | 0.032 | 0.157 | 0.482 | -2.30E-03 |
| logepa | rs3780897 | 0.17 | 0.998 | 0.241 | 0.992 | 0.942 | 0.22 | 0.797 | 1.01 | 0.332 | 0.974 | 0.01 |
| logepa | rs117509491 | 0.002 | . | . | . | . | . | . | . | . | . | . |
| logepa | rs117811514 | 0.002 | . | . | . | . | . | . | . | . | . | . |
| logepa | rs116500932 | 0.086 | 1.07 | 0.253 | 0.787 | 0.394 | 0.195 | 0.06 | 0.435 | 0.292 | 0.215 | -0.278 |
| logepa | rs77584168 | . | . | . | . | . | . | . | . | . | . | . |
| logepa | rs2310776 | 0.173 | 1.03 | 0.246 | 0.901 | 0.941 | 0.221 | 0.797 | 0.927 | 0.306 | 0.818 | -0.073 |
| logepa | rs17157736 | 0.173 | 1.03 | 0.246 | 0.901 | 0.942 | 0.221 | 0.797 | 0.927 | 0.306 | 0.818 | -0.073 |
| logepa | rs72789735 | 0.036 | 1.06 | 0.238 | 0.78 | 0.283 | 0.223 | 0.11 | 0.182 | 0.17 | 0.068 | -0.293 |
| logepa | rs183747187 | 0.001 | . | . | . | . | . | . | . | . | . | . |
| logepa | rs7077891 | 0.173 | 1.03 | 0.246 | 0.901 | 0.942 | 0.221 | 0.798 | 0.927 | 0.306 | 0.818 | -0.073 |
| logepa | rs145063305 | 0.002 | . | . | . | . | . | . | . | . | . | . |
| logepa | rs3824613 | 0.174 | 1.06 | 0.251 | 0.819 | 0.958 | 0.226 | 0.857 | 0.87 | 0.298 | 0.685 | -0.134 |
| logepa | rs145543199 | 0.004 | . | . | . | . | . | . | . | . | . | . |
| logepa | rs140361993 | 0.001 | . | . | . | . | . | . | . | . | . | . |
| logepa | rs11239496 | 0.036 | 1.06 | 0.238 | 0.78 | 0.284 | 0.223 | 0.11 | 0.182 | 0.17 | 0.068 | -0.293 |
| logepa | rs11239497 | 0.154 | 1.05 | 0.246 | 0.826 | 1 | 0.234 | 0.99 | 0.868 | 0.301 | 0.684 | -0.139 |
| logepa | rs10900212 | 0.245 | 0.966 | 0.241 | 0.89 | 0.934 | 0.194 | 0.745 | 1.07 | 0.301 | 0.797 | 0.07 |
| logepa | rs186487136 | . | . | . | . | . | . | . | . | . | . | . |
| logepa | rs12774369 | 0.173 | 1.03 | 0.246 | 0.902 | 0.946 | 0.225 | 0.814 | 0.927 | 0.312 | 0.822 | -0.073 |
| logepa | rs60842538 | 0.173 | 1.03 | 0.246 | 0.903 | 0.95 | 0.228 | 0.832 | 0.928 | 0.319 | 0.827 | -0.072 |
| logepa | rs3780898 | 0.153 | 1.05 | 0.246 | 0.833 | 1.02 | 0.247 | 0.946 | 0.871 | 0.322 | 0.708 | -0.137 |
| logepa | rs3780899 | 0.172 | 1.03 | 0.246 | 0.906 | 0.959 | 0.235 | 0.864 | 0.93 | 0.33 | 0.837 | -0.071 |
| logepa | rs12252472 | 0.273 | 0.947 | 0.283 | 0.857 | 0.758 | 0.174 | 0.227 | 1.12 | 0.385 | 0.74 | 0.099 |
| logepa | rs185411011 | 0.001 | . | . | . | . | . | . | . | . | . | . |
| logepa | rs28395865 | 0.152 | 1.05 | 0.246 | 0.841 | 1.03 | 0.257 | 0.898 | 0.875 | 0.338 | 0.73 | -0.133 |
| logepa | rs191004074 | 0.034 | 1.03 | 0.243 | 0.91 | 1.02 | 0.655 | 0.979 | 0.752 | 0.497 | 0.666 | -0.259 |
| logepa | rs182401295 | . | . | . | . | . | . | . | . | . | . | . |
| logepa | rs2228064 | 0.277 | 0.934 | 0.283 | 0.822 | 0.787 | 0.177 | 0.289 | 1.16 | 0.391 | 0.67 | 0.128 |
| logepa | rs188284159 | . | . | . | . | . | . | . | . | . | . | . |
| logepa | rs2303887 | 0.003 | . | . | . | . | . | . | . | . | . | . |
| logepa | rs2303886 | 0.014 | 1.01 | 0.232 | 0.971 | 1.41 | 0.992 | 0.622 | 0.773 | 0.432 | 0.645 | -0.32 |
| logepa | rs192621006 | . | . | . | . | . | . | . | . | . | . | . |
| logepa | rs2303885 | 0.491 | 0.688 | 0.227 | 0.256 | 0.811 | 0.142 | 0.231 | 1.48 | 0.326 | 0.078 | 0.325 |
| logepa | rs114466168 | 0.086 | 1.07 | 0.255 | 0.772 | 0.384 | 0.198 | 0.063 | 0.411 | 0.287 | 0.202 | -0.286 |
| logepa | rs11239501 | 0.038 | 1.03 | 0.236 | 0.911 | 1.01 | 0.539 | 0.988 | 0.655 | 0.613 | 0.651 | -0.357 |
| logepa | rs745986 | 0.156 | 1.03 | 0.237 | 0.896 | 1.18 | 0.273 | 0.478 | 0.923 | 0.353 | 0.833 | -0.089 |
| logepa | rs75262268 | 0.004 | . | . | . | . | . | . | . | . | . | . |
| logepa | rs71494776 | 0.022 | 0.973 | 0.209 | 0.898 | 2.24 | 1.24 | 0.147 | 1.21 | 1.29 | 0.858 | 0.424 |
| logepa | rs74386871 | 0.006 | . | . | . | . | . | . | . | . | . | . |
| logepa | rs76502040 | 0.004 | . | . | . | . | . | . | . | . | . | . |
| logepa | rs142213734 | 0.001 | . | . | . | . | . | . | . | . | . | . |
| logepa | rs4948671 | 0.796 | 1.13 | 0.491 | 0.779 | 0.841 | 0.158 | 0.358 | 0.92 | 0.254 | 0.764 | -0.096 |
| logepa | rs146286085 | 0 | . | . | . | . | . | . | . | . | . | . |
| logepa | rs144080623 | 0 | . | . | . | . | . | . | . | . | . | . |
| logepa | rs115074014 | 0.133 | 0.864 | 0.23 | 0.584 | 1.08 | 0.286 | 0.761 | 1.92 | 0.757 | 0.1 | 0.846 |
| logepa | rs17157756 | 0.133 | 0.864 | 0.23 | 0.584 | 1.08 | 0.286 | 0.761 | 1.92 | 0.757 | 0.1 | 0.846 |
| logepa | rs190459780 | 0.001 | . | . | . | . | . | . | . | . | . | . |
| logepa | rs12358647 | 0.25 | 0.926 | 0.23 | 0.757 | 1.06 | 0.207 | 0.748 | 1.15 | 0.303 | 0.591 | 0.145 |
| logepa | rs147080556 | 0.009 | . | . | . | . | . | . | . | . | . | . |
| logepa | rs187543349 | 0.002 | . | . | . | . | . | . | . | . | . | . |
| logepa | rs114375595 | 0.024 | 1 | 0.229 | 0.993 | 0.986 | 0.744 | 0.985 | 0.985 | 1.49 | 0.992 | -0.015 |
| logepa | rs117211200 | 0.003 | . | . | . | . | . | . | . | . | . | . |
| logepa | rs192963047 | . | . | . | . | . | . | . | . | . | . | . |
| logepa | rs11239505 | 0.254 | 0.945 | 0.24 | 0.825 | 1.02 | 0.2 | 0.923 | 1.11 | 0.295 | 0.697 | 0.104 |
| logepa | rs184434116 | 0.001 | . | . | . | . | . | . | . | . | . | . |
| logepa | rs4948999 | 0.801 | 1.1 | 0.476 | 0.821 | 0.824 | 0.155 | 0.302 | 0.935 | 0.256 | 0.805 | -0.078 |
| logepa | rs11239506 | 0.078 | 1.06 | 0.258 | 0.816 | 1.01 | 0.387 | 0.983 | 0.694 | 0.372 | 0.495 | -0.326 |
| logepa | rs190706620 | 0.001 | . | . | . | . | . | . | . | . | . | . |
| logepa | rs190770429 | 0.001 | . | . | . | . | . | . | . | . | . | . |
| logepa | rs181327133 | 0.003 | . | . | . | . | . | . | . | . | . | . |
| logepa | rs149251697 | 0.057 | 0.942 | 0.223 | 0.799 | 1.14 | 0.458 | 0.74 | 1.67 | 1.05 | 0.418 | 0.708 |
| logepa | rs118081602 | 0.003 | . | . | . | . | . | . | . | . | . | . |
| logepa | rs188233691 | . | . | . | . | . | . | . | . | . | . | . |
| logepa | rs183778794 | 0.006 | . | . | . | . | . | . | . | . | . | . |
| logepa | rs188040078 | 0.003 | . | . | . | . | . | . | . | . | . | . |
| logepa | rs184675372 | 0.001 | . | . | . | . | . | . | . | . | . | . |
| logepa | rs150326891 | 0.03 | 1.02 | 0.231 | 0.942 | 0.915 | 0.635 | 0.899 | 0.695 | 0.962 | 0.793 | -0.285 |
| logepa | rs185520021 | . | . | . | . | . | . | . | . | . | . | . |
| logepa | rs7907779 | 0.078 | 1.06 | 0.258 | 0.819 | 1.01 | 0.386 | 0.985 | 0.699 | 0.373 | 0.502 | -0.321 |
| logepa | rs184962860 | . | . | . | . | . | . | . | . | . | . | . |
| logepa | rs149762784 | 0.034 | 1.03 | 0.245 | 0.885 | 0.565 | 0.419 | 0.441 | 0.652 | 0.57 | 0.625 | -0.218 |
| logepa | rs149203427 | 0.005 | . | . | . | . | . | . | . | . | . | . |
| logepa | rs60153083 | 0.045 | 0.873 | 0.206 | 0.566 | 0.878 | 0.405 | 0.778 | 3.61 | 2.74 | 0.089 | 2.02 |
| logepa | rs112001069 | 0.073 | 0.979 | 0.212 | 0.92 | 1.22 | 0.406 | 0.545 | 1.14 | 0.664 | 0.818 | 0.167 |
| logepa | rs182355382 | 0.057 | 1.06 | 0.25 | 0.816 | 0.849 | 0.423 | 0.742 | 0.625 | 0.515 | 0.569 | -0.345 |
| logepa | rs186740974 | 0.157 | 1.11 | 0.304 | 0.709 | 0.862 | 0.285 | 0.653 | 0.721 | 0.418 | 0.573 | -0.281 |

| logepa | rs191874916 | 0.06 | 1.11 | 0.259 | 0.645 | 0.994 | 0.475 | 0.99 | 0.448 | 0.337 | 0.286 | -0.611 |
| --- | --- | --- | --- | --- | --- | --- | --- | --- | --- | --- | --- | --- |
| logepa | rs182989412 | 0.126 | 0.994 | 0.268 | 0.983 | 1.44 | 0.435 | 0.224 | 1.03 | 0.396 | 0.949 | 0.033 |
| logepa | rs188819476 | 0.003 | . | . | . | . | . | . | . | . | . | . |
| logepa | rs141217183 | 0.083 | 0.999 | 0.216 | 0.997 | 1.24 | 0.4 | 0.496 | 1.01 | 0.585 | 0.983 | 0.015 |
| logepa | rs192922652 | 0.081 | 1.01 | 0.222 | 0.949 | 1.12 | 0.369 | 0.728 | 0.94 | 0.532 | 0.913 | -0.066 |
| logepa | rs185092343 | 0.025 | 1.05 | 0.241 | 0.836 | 0.118 | 0.126 | 0.045 | 0.193 | 0.285 | 0.266 | -0.143 |
| logepa | rs145193311 | 0.082 | 0.972 | 0.212 | 0.894 | 1.22 | 0.378 | 0.524 | 1.16 | 0.645 | 0.785 | 0.187 |
| logepa | rs55789336 | 0.084 | 1.04 | 0.256 | 0.877 | 0.974 | 0.37 | 0.945 | 0.792 | 0.417 | 0.658 | -0.212 |
| logepa | rs55837212 | 0.083 | 1.06 | 0.259 | 0.803 | 0.927 | 0.371 | 0.85 | 0.675 | 0.376 | 0.481 | -0.325 |
| logepa | rs75408574 | 0.208 | 1.42 | 0.323 | 0.122 | 0.713 | 0.194 | 0.215 | 0.349 | 0.136 | 6.80E-03 | -0.781 |
| logepa | rs149306926 | 0.089 | 1.01 | 0.257 | 0.98 | 1.1 | 0.398 | 0.791 | 0.973 | 0.479 | 0.956 | -0.029 |
| logepa | rs35990892 | 0.683 | 1.24 | 0.546 | 0.623 | 0.944 | 0.204 | 0.789 | 0.848 | 0.265 | 0.597 | -0.192 |
| logepa | rs192356207 | 0.128 | 0.96 | 0.261 | 0.88 | 0.725 | 0.246 | 0.344 | 1.27 | 0.479 | 0.534 | 0.196 |
| logepa | rs183741163 | 0.097 | 1.15 | 0.282 | 0.568 | 0.864 | 0.363 | 0.728 | 0.46 | 0.275 | 0.194 | -0.557 |
| logepa | rs34737297 | 0.591 | 0.836 | 0.321 | 0.641 | 0.915 | 0.188 | 0.667 | 1.17 | 0.409 | 0.645 | 0.147 |
| logepa | rs181346933 | 0.042 | 1 | 0.231 | 0.993 | 0.402 | 0.329 | 0.266 | 0.674 | 0.913 | 0.771 | -0.133 |
| logepa | rs36093253 | 0.24 | 1.09 | 0.282 | 0.754 | 0.964 | 0.231 | 0.877 | 0.836 | 0.343 | 0.663 | -0.174 |
| logepa | rs186307855 | 0.262 | 0.941 | 0.286 | 0.841 | 0.842 | 0.193 | 0.452 | 1.14 | 0.394 | 0.702 | 0.121 |
| logepa | rs189681865 | 0.096 | 0.992 | 0.251 | 0.974 | 0.898 | 0.317 | 0.76 | 1.07 | 0.488 | 0.877 | 0.066 |
| logepa | rs34942971 | 0.259 | 0.968 | 0.251 | 0.901 | 0.981 | 0.196 | 0.923 | 1.07 | 0.303 | 0.816 | 0.065 |
| logepa | rs181816554 | 0.064 | 1.03 | 0.254 | 0.901 | 1.19 | 0.524 | 0.692 | 0.819 | 0.641 | 0.799 | -0.216 |
| logepa | rs186846881 | 0.278 | 0.955 | 0.301 | 0.885 | 0.808 | 0.189 | 0.362 | 1.1 | 0.388 | 0.78 | 0.088 |
| logepa | rs191178220 | 0.008 | . | . | . | . | . | . | . | . | . | . |
| logepa | rs183305228 | 0.046 | 0.873 | 0.206 | 0.566 | 0.867 | 0.401 | 0.758 | 3.56 | 2.71 | 0.094 | 1.96 |
| logepa | rs139912761 | 0.149 | 1 | 0.267 | 0.989 | 0.911 | 0.26 | 0.744 | 1 | 0.428 | 0.999 | -3.50E-05 |
| logepa | rs185499901 | 0.018 | 1 | 0.243 | 0.988 | 1.23 | 1.16 | 0.827 | 0.917 | 0.646 | 0.902 | -0.101 |
| logepa | rs189816197 | 0.086 | 0.978 | 0.217 | 0.919 | 1.23 | 0.389 | 0.521 | 1.12 | 0.581 | 0.824 | 0.141 |
| logepa | rs145656938 | 0.058 | 0.985 | 0.226 | 0.947 | 0.662 | 0.307 | 0.374 | 1.28 | 0.958 | 0.738 | 0.19 |
| logepa | rs35745197 | 0.072 | 0.977 | 0.212 | 0.916 | 1.25 | 0.419 | 0.513 | 1.15 | 0.684 | 0.813 | 0.178 |
| logepa | rs192856637 | . | . | . | . | . | . | . | . | . | . | . |
| logepa | rs2310775 | 0.267 | 1.2 | 0.28 | 0.442 | 0.96 | 0.193 | 0.84 | 0.7 | 0.238 | 0.295 | -0.353 |
| logepa | rs11239507 | 0.538 | 1.12 | 0.367 | 0.74 | 0.902 | 0.156 | 0.552 | 0.89 | 0.225 | 0.647 | -0.122 |
| logepa | rs74648467 | 0.027 | 1.04 | 0.226 | 0.849 | 1.17 | 0.8 | 0.815 | 0.513 | 0.588 | 0.561 | -0.588 |
| logepa | rs145815934 | 0.005 | . | . | . | . | . | . | . | . | . | . |
| logepa | rs145390687 | 0.012 | 1.02 | 0.234 | 0.919 | 0.951 | 0.852 | 0.955 | 0.312 | 0.363 | 0.317 | -0.671 |
| logepa | rs184354913 | 0.012 | 1.01 | 0.235 | 0.962 | 0.98 | 1.21 | 0.987 | 0.699 | 1.52 | 0.87 | -0.298 |
| logepa | rs181244920 | 0.003 | . | . | . | . | . | . | . | . | . | . |
| logepa | rs11499087 | 0.167 | 0.881 | 0.241 | 0.644 | 0.967 | 0.234 | 0.891 | 1.59 | 0.584 | 0.204 | 0.51 |
| logepa | rs148263794 | 0.079 | 0.982 | 0.214 | 0.933 | 1.25 | 0.401 | 0.482 | 1.11 | 0.623 | 0.857 | 0.127 |
| logepa | rs150758903 | 0.089 | 0.975 | 0.243 | 0.919 | 0.749 | 0.277 | 0.435 | 1.21 | 0.676 | 0.732 | 0.16 |
| logepa | rs147654382 | 0.192 | 0.972 | 0.244 | 0.909 | 1.07 | 0.258 | 0.785 | 1.08 | 0.385 | 0.83 | 0.081 |
| logepa | rs147495166 | 0.077 | 1.07 | 0.256 | 0.771 | 0.437 | 0.243 | 0.137 | 0.416 | 0.307 | 0.235 | -0.314 |
| logepa | rs35198778 | 0.14 | 0.981 | 0.223 | 0.933 | 0.738 | 0.206 | 0.277 | 1.09 | 0.486 | 0.852 | 0.068 |
| logepa | rs145207659 | 0.02 | 1.02 | 0.236 | 0.929 | 0.79 | 0.693 | 0.788 | 0.54 | 0.748 | 0.656 | -0.375 |
| logepa | rs186466464 | 0.024 | 1.02 | 0.245 | 0.923 | 1.51 | 1.1 | 0.575 | 0.627 | 0.48 | 0.542 | -0.563 |
| logepa | rs12778413 | 0.199 | 0.986 | 0.253 | 0.955 | 1.03 | 0.249 | 0.912 | 1.04 | 0.32 | 0.906 | 0.037 |
| logepa | rs7895552 | 0.724 | 0.933 | 0.402 | 0.872 | 0.831 | 0.168 | 0.359 | 1.05 | 0.313 | 0.877 | 0.048 |
| logepa | rs12783895 | 0.076 | 0.983 | 0.214 | 0.935 | 1.29 | 0.419 | 0.432 | 1.1 | 0.623 | 0.861 | 0.126 |
| logepa | rs34929604 | 0.081 | 0.97 | 0.211 | 0.888 | 1.26 | 0.392 | 0.459 | 1.17 | 0.643 | 0.772 | 0.202 |
| logepa | rs12761961 | 0.072 | 0.969 | 0.212 | 0.885 | 1.2 | 0.422 | 0.595 | 1.21 | 0.737 | 0.756 | 0.237 |
| logepa | rs35536930 | 0.252 | 0.94 | 0.237 | 0.807 | 1 | 0.198 | 0.998 | 1.12 | 0.307 | 0.672 | 0.115 |
| logepa | rs180951060 | 0.006 | . | . | . | . | . | . | . | . | . | . |
| logepa | rs148892128 | 0.005 | . | . | . | . | . | . | . | . | . | . |
| logepa | rs11239509 | 0.255 | 0.925 | 0.269 | 0.787 | 0.799 | 0.184 | 0.328 | 1.2 | 0.411 | 0.592 | 0.164 |
| logepa | rs41526545 | 0.045 | 1.05 | 0.239 | 0.816 | 0.848 | 0.464 | 0.763 | 0.473 | 0.433 | 0.414 | -0.48 |
| logepa | rs2029253 | 0.286 | 1.21 | 0.287 | 0.427 | 0.949 | 0.183 | 0.788 | 0.704 | 0.214 | 0.249 | -0.349 |
| logepa | rs17444064 | 0.041 | 1.02 | 0.236 | 0.918 | 0.973 | 0.507 | 0.959 | 0.692 | 0.631 | 0.687 | -0.307 |
| logepa | rs71494779 | 0.084 | 0.969 | 0.211 | 0.884 | 1.23 | 0.376 | 0.496 | 1.18 | 0.636 | 0.763 | 0.204 |
| logepa | rs74991420 | 0.003 | . | . | . | . | . | . | . | . | . | . |
| logepa | rs182736551 | 0.003 | . | . | . | . | . | . | . | . | . | . |
| logepa | rs78702074 | 0.003 | . | . | . | . | . | . | . | . | . | . |
| logepa | rs187791466 | . | . | . | . | . | . | . | . | . | . | . |
| logepa | rs35300270 | 0.085 | 0.969 | 0.212 | 0.886 | 1.22 | 0.376 | 0.519 | 1.17 | 0.638 | 0.771 | 0.196 |
| logepa | rs6593484 | 0.078 | 1.05 | 0.257 | 0.829 | 1.01 | 0.386 | 0.979 | 0.715 | 0.379 | 0.526 | -0.303 |
| logepa | rs12244601 | 0.064 | 0.945 | 0.228 | 0.815 | 0.746 | 0.32 | 0.494 | 1.7 | 1.18 | 0.445 | 0.506 |
| logepa | rs184429574 | 0.006 | . | . | . | . | . | . | . | . | . | . |
| logepa | rs7080897 | 0.084 | 0.969 | 0.211 | 0.884 | 1.23 | 0.376 | 0.496 | 1.18 | 0.635 | 0.763 | 0.203 |
| logepa | rs7097429 | 0.085 | 0.968 | 0.211 | 0.882 | 1.22 | 0.373 | 0.517 | 1.18 | 0.638 | 0.76 | 0.205 |
| logepa | rs77979229 | . | . | . | . | . | . | . | . | . | . | . |
| logepa | rs11239512 | 0.079 | 1.05 | 0.257 | 0.834 | 1 | 0.379 | 0.999 | 0.725 | 0.378 | 0.538 | -0.289 |
| logepa | rs60146827 | 0.084 | 0.969 | 0.212 | 0.884 | 1.23 | 0.376 | 0.498 | 1.18 | 0.635 | 0.764 | 0.203 |
| logepa | rs79032236 | 0.105 | 1.08 | 0.263 | 0.749 | 0.499 | 0.198 | 0.079 | 0.524 | 0.277 | 0.222 | -0.297 |
| logepa | rs140854206 | . | . | . | . | . | . | . | . | . | . | . |
| logepa | rs184049550 | 0.012 | 1.03 | 0.238 | 0.907 | 0.999 | 0.836 | 0.999 | 0.482 | 0.44 | 0.424 | -0.531 |
| logepa | rs114433607 | 0.029 | 0.872 | 0.199 | 0.55 | 0.81 | 0.431 | 0.692 | 7.53 | 7.36 | 0.039 | 4.64 |
| logepa | rs117653793 | 0.003 | . | . | . | . | . | . | . | . | . | . |
| logepa | rs117039533 | 0.003 | . | . | . | . | . | . | . | . | . | . |
| logepa | rs191660761 | 0.002 | . | . | . | . | . | . | . | . | . | . |
| logepa | rs59514896 | 0.03 | 1.01 | 0.217 | 0.951 | 1.68 | 0.975 | 0.37 | 0.839 | 0.933 | 0.874 | -0.266 |
| logepa | rs184661876 | 0.001 | . | . | . | . | . | . | . | . | . | . |
| logepa | rs61854073 | 0.011 | 1.01 | 0.228 | 0.978 | 6.23 | 4.55 | 0.012 | 0.933 | 1.06 | 0.951 | -0.389 |
| logepa | rs2018518 | 0.09 | 1.13 | 0.268 | 0.595 | 1.05 | 0.373 | 0.89 | 0.476 | 0.281 | 0.209 | -0.617 |
| logepa | rs934187 | 0.802 | 1.1 | 0.477 | 0.827 | 0.827 | 0.156 | 0.313 | 0.937 | 0.257 | 0.811 | -0.075 |
| logepa | rs7918542 | 0.269 | 0.921 | 0.241 | 0.753 | 1.03 | 0.195 | 0.894 | 1.15 | 0.294 | 0.582 | 0.141 |
| logepa | rs144311920 | 0.004 | . | . | . | . | . | . | . | . | . | . |
| logepa | rs7099684 | 0.883 | 1.85 | 1.05 | 0.274 | 1 | 0.241 | 0.989 | 0.7 | 0.232 | 0.281 | -0.555 |
| logepa | rs150829171 | 0.001 | . | . | . | . | . | . | . | . | . | . |
| logepa | rs186060697 | 0.006 | . | . | . | . | . | . | . | . | . | . |
| logepa | rs11239513 | 0.277 | 0.936 | 0.285 | 0.828 | 0.792 | 0.183 | 0.311 | 1.15 | 0.393 | 0.679 | 0.126 |

| logepa | rs7917687 | 0.084 | 0.97 | 0.212 | 0.887 | 1.22 | 0.377 | 0.513 | 1.17 | 0.635 | 0.772 | 0.195 |
| --- | --- | --- | --- | --- | --- | --- | --- | --- | --- | --- | --- | --- |
| logepa | rs61854076 | 0.004 | . | . | . | . | . | . | . | . | . | . |
| logepa | rs71494780 | 0.007 | . | . | . | . | . | . | . | . | . | . |
| logepa | rs7919239 | 0.257 | 0.947 | 0.239 | 0.829 | 1 | 0.194 | 0.982 | 1.11 | 0.285 | 0.698 | 0.1 |
| logepa | rs78334475 | 0.004 | . | . | . | . | . | . | . | . | . | . |
| logepa | rs3780900 | 0.035 | 1.1 | 0.249 | 0.66 | 1.15 | 0.747 | 0.833 | 0.331 | 0.327 | 0.263 | -0.832 |
| logepa | rs186729292 | 0.001 | . | . | . | . | . | . | . | . | . | . |
| logepa | rs12267438 | 0.073 | 0.959 | 0.233 | 0.864 | 0.86 | 0.31 | 0.675 | 1.38 | 0.696 | 0.52 | 0.321 |
| logepa | rs1369214 | 0.564 | 1.12 | 0.363 | 0.722 | 0.899 | 0.166 | 0.566 | 0.892 | 0.251 | 0.686 | -0.121 |
| logepa | rs9282589 | 0.005 | . | . | . | . | . | . | . | . | . | . |
| logepa | rs114522915 | 0.078 | 1.07 | 0.257 | 0.773 | 0.433 | 0.245 | 0.139 | 0.42 | 0.325 | 0.262 | -0.31 |
| logepa | rs2115819 | 0.292 | 1.19 | 0.292 | 0.481 | 1.04 | 0.233 | 0.851 | 0.738 | 0.306 | 0.463 | -0.317 |
| logepa | rs117664603 | 0.003 | . | . | . | . | . | . | . | . | . | . |
| logepa | rs184979202 | 0.001 | . | . | . | . | . | . | . | . | . | . |
| logepa | rs190714671 | . | . | . | . | . | . | . | . | . | . | . |
| logepa | rs141538996 | . | . | . | . | . | . | . | . | . | . | . |
| logepa | rs150881741 | 0.001 | . | . | . | . | . | . | . | . | . | . |
| logepa | rs139076633 | . | . | . | . | . | . | . | . | . | . | . |
| logepa | rs12243649 | 0.156 | 0.908 | 0.232 | 0.707 | 0.901 | 0.223 | 0.675 | 1.33 | 0.434 | 0.382 | 0.279 |
| logepa | rs140348708 | . | . | . | . | . | . | . | . | . | . | . |
| logepa | rs79023256 | 0.003 | . | . | . | . | . | . | . | . | . | . |
| logepa | rs11239515 | 0.071 | 1.08 | 0.263 | 0.744 | 1.11 | 0.439 | 0.789 | 0.595 | 0.34 | 0.363 | -0.479 |
| logepa | rs55866086 | 0.24 | 0.973 | 0.272 | 0.923 | 0.953 | 0.208 | 0.825 | 1.06 | 0.319 | 0.855 | 0.054 |
| logepa | rs181340150 | 0.002 | . | . | . | . | . | . | . | . | . | . |
| logepa | rs11239516 | 0.158 | 1.03 | 0.275 | 0.904 | 0.953 | 0.275 | 0.866 | 0.91 | 0.372 | 0.817 | -0.09 |
| logepa | rs11239517 | 0.158 | 1.03 | 0.275 | 0.898 | 0.953 | 0.276 | 0.869 | 0.904 | 0.37 | 0.805 | -0.096 |
| logepa | rs11239519 | 0.128 | 1 | 0.268 | 0.998 | 0.976 | 0.335 | 0.943 | 1.01 | 0.495 | 0.992 | 5.00E-03 |
| logepa | rs7909514 | 0.788 | 1.24 | 0.492 | 0.584 | 0.878 | 0.165 | 0.49 | 0.86 | 0.237 | 0.584 | -0.182 |
| logepa | rs7894352 | 0.885 | 1.66 | 0.906 | 0.357 | 0.976 | 0.239 | 0.921 | 0.745 | 0.243 | 0.368 | -0.427 |
| logepa | rs189384146 | 0.001 | . | . | . | . | . | . | . | . | . | . |
| logepa | rs11239520 | 0.107 | 1.1 | 0.272 | 0.693 | 1.14 | 0.355 | 0.666 | 0.662 | 0.284 | 0.336 | -0.411 |
| logepa | rs12221233 | 0.234 | 1.16 | 0.269 | 0.514 | 0.894 | 0.203 | 0.623 | 0.697 | 0.279 | 0.368 | -0.333 |
| logepa | rs74523293 | 0.066 | 0.916 | 0.217 | 0.711 | 1.06 | 0.388 | 0.865 | 1.86 | 1.04 | 0.27 | 0.828 |
| logepa | rs79376040 | 0.066 | 0.916 | 0.217 | 0.711 | 1.06 | 0.388 | 0.866 | 1.86 | 1.04 | 0.27 | 0.829 |
| logepa | rs4949000 | 0.234 | 1.16 | 0.269 | 0.513 | 0.894 | 0.203 | 0.621 | 0.696 | 0.279 | 0.367 | -0.333 |
| logepa | rs7089406 | 0.039 | 0.947 | 0.222 | 0.817 | 0.824 | 0.345 | 0.644 | 2.3 | 0.86 | 0.026 | 1.03 |
| logepa | rs138211056 | 0 | . | . | . | . | . | . | . | . | . | . |
| logepa | rs10900213 | 0.434 | 1.48 | 0.462 | 0.211 | 0.942 | 0.183 | 0.758 | 0.633 | 0.208 | 0.163 | -0.539 |
| logepa | rs145152827 | . | . | . | . | . | . | . | . | . | . | . |
| logepa | rs74443325 | 0.003 | . | . | . | . | . | . | . | . | . | . |
| logepa | rs188299446 | 0.004 | . | . | . | . | . | . | . | . | . | . |
| logepa | rs192699586 | . | . | . | . | . | . | . | . | . | . | . |
| logepa | rs72796464 | 0.041 | 1.04 | 0.237 | 0.871 | 0.966 | 0.516 | 0.948 | 0.567 | 0.497 | 0.518 | -0.435 |
| logepa | rs4949001 | 0.077 | 1.15 | 0.273 | 0.557 | 1.21 | 0.471 | 0.616 | 0.43 | 0.28 | 0.195 | -0.763 |
| logepa | rs147174641 | 0.003 | . | . | . | . | . | . | . | . | . | . |
| logepa | rs190835238 | 0.001 | . | . | . | . | . | . | . | . | . | . |
| logepa | rs76698944 | 0.013 | 1.02 | 0.233 | 0.928 | 5.90E-06 | 3.70E-05 | 0.057 | 0.029 | 0.253 | 0.685 | -0.021 |
| logepa | rs56071381 | 0.129 | 1.16 | 0.301 | 0.56 | 1.04 | 0.326 | 0.899 | 0.595 | 0.247 | 0.211 | -0.484 |
| logepa | rs78130427 | 0.019 | 0.967 | 0.201 | 0.873 | 2.29 | 1.65 | 0.248 | 1.71 | 3.66 | 0.803 | 1.53 |
| logepa | rs10900215 | 0.249 | 1.02 | 0.306 | 0.953 | 1.09 | 0.244 | 0.704 | 0.971 | 0.361 | 0.936 | -0.031 |
| logepa | rs41491445 | 0.046 | 0.885 | 0.208 | 0.604 | 0.9 | 0.383 | 0.804 | 3.21 | 2.42 | 0.123 | 1.77 |
| logepa | rs144995222 | 0.006 | . | . | . | . | . | . | . | . | . | . |
| logepa | rs149097803 | 0.002 | . | . | . | . | . | . | . | . | . | . |
| logepa | rs139429646 | 0.002 | . | . | . | . | . | . | . | . | . | . |
| logepa | rs7075620 | 0.764 | 1.16 | 0.526 | 0.747 | 0.81 | 0.157 | 0.277 | 0.895 | 0.28 | 0.722 | -0.129 |
| logepa | rs148299213 | 0.049 | 0.903 | 0.21 | 0.663 | 1.07 | 0.432 | 0.875 | 2.38 | 1.77 | 0.244 | 1.32 |
| logepa | rs4949002 | 0.084 | 1.17 | 0.274 | 0.499 | 1 | 0.421 | 0.998 | 0.358 | 0.25 | 0.142 | -0.753 |
| logepa | rs150496007 | 0.023 | 1.01 | 0.239 | 0.964 | 0.579 | 0.457 | 0.489 | 1.12 | 0.571 | 0.828 | 0.064 |
| logepa | rs147303455 | 0.028 | 1.02 | 0.246 | 0.945 | 0.421 | 0.323 | 0.259 | 1.09 | 0.509 | 0.859 | 0.027 |
| logepa | rs189518433 | . | . | . | . | . | . | . | . | . | . | . |
| logepa | rs186281355 | . | . | . | . | . | . | . | . | . | . | . |
| logepa | rs115297114 | 0.077 | 1.06 | 0.255 | 0.794 | 0.477 | 0.254 | 0.164 | 0.459 | 0.348 | 0.304 | -0.308 |
| logepa | rs11239523 | 0.106 | 1.09 | 0.265 | 0.718 | 1.14 | 0.352 | 0.665 | 0.683 | 0.295 | 0.377 | -0.382 |
| logepa | rs143704897 | 0.007 | . | . | . | . | . | . | . | . | . | . |
| logepa | rs188024096 | 0.001 | . | . | . | . | . | . | . | . | . | . |
| logepa | rs11239524 | 0.133 | 1.14 | 0.307 | 0.617 | 0.974 | 0.313 | 0.935 | 0.644 | 0.268 | 0.291 | -0.4 |
| logepa | rs191656573 | 0.002 | . | . | . | . | . | . | . | . | . | . |
| logepa | rs193295875 | 0.001 | . | . | . | . | . | . | . | . | . | . |
| logepa | rs190458228 | . | . | . | . | . | . | . | . | . | . | . |
| logepa | rs139253993 | 0.001 | . | . | . | . | . | . | . | . | . | . |
| logepa | rs4948672 | 0.497 | 1.32 | 0.525 | 0.481 | 1.18 | 0.219 | 0.36 | 0.769 | 0.239 | 0.397 | -0.303 |
| logepa | rs180954921 | 0.001 | . | . | . | . | . | . | . | . | . | . |
| logepa | rs12264801 | 0.383 | 0.907 | 0.261 | 0.733 | 1.28 | 0.268 | 0.246 | 1.15 | 0.379 | 0.663 | 0.152 |
| logepa | rs73283184 | 0.057 | 0.873 | 0.196 | 0.545 | 0.706 | 0.301 | 0.414 | 3.88 | 3.12 | 0.092 | 1.81 |
| logepa | rs142746187 | 0.022 | 0.985 | 0.23 | 0.949 | 0.128 | 0.125 | 0.036 | 0.647 | 0.518 | 0.587 | -0.031 |
| logepa | rs58704739 | 0.053 | 0.857 | 0.194 | 0.494 | 0.494 | 0.25 | 0.163 | 6.58 | 5.45 | 0.023 | 2.44 |
| logepa | rs61272335 | 0.04 | 0.847 | 0.187 | 0.451 | 0.633 | 0.383 | 0.45 | 13.1 | 14 | 0.017 | 6.52 |
| logepa | rs60959489 | 0.103 | 0.867 | 0.207 | 0.55 | 1.15 | 0.323 | 0.625 | 1.67 | 0.652 | 0.193 | 0.642 |
| logepa | rs58502629 | 0.04 | 0.846 | 0.187 | 0.45 | 0.634 | 0.384 | 0.451 | 13.1 | 14.1 | 0.017 | 6.54 |
| logepa | rs10900216 | 0.744 | 0.863 | 0.385 | 0.742 | 1.1 | 0.206 | 0.618 | 1.11 | 0.322 | 0.728 | 0.088 |
| logepa | rs181118141 | 0.004 | . | . | . | . | . | . | . | . | . | . |
| logepa | rs137882665 | 0.019 | 0.998 | 0.231 | 0.992 | 0.051 | 0.045 | 8.10E-04 | 2.12 | 1.4 | 0.255 | 0.059 |
| logepa | rs149466736 | 0.002 | . | . | . | . | . | . | . | . | . | . |
| logepa | rs2099171 | 0.553 | 1.51 | 0.521 | 0.237 | 1.02 | 0.157 | 0.883 | 0.7 | 0.159 | 0.115 | -0.451 |
| logepa | rs141852280 | 0.002 | . | . | . | . | . | . | . | . | . | . |
| logepa | rs191539257 | . | . | . | . | . | . | . | . | . | . | . |
| logepa | rs17157771 | 0.045 | 0.984 | 0.225 | 0.945 | 1.02 | 0.449 | 0.966 | 1.28 | 1.27 | 0.803 | 0.28 |
| logepa | rs111339241 | 0.041 | 0.988 | 0.226 | 0.958 | 1.1 | 0.511 | 0.83 | 1.18 | 1.26 | 0.876 | 0.197 |
| logepa | rs116983255 | 0.004 | . | . | . | . | . | . | . | . | . | . |

| logepa | rs142324341 | . | . | . | . | . | . | . | . | . | . | . |
| --- | --- | --- | --- | --- | --- | --- | --- | --- | --- | --- | --- | --- |
| logepa | rs187191219 | 0.015 | 1.03 | 0.234 | 0.904 | 0.033 | 0.051 | 0.027 | 0.606 | 0.945 | 0.748 | -0.041 |
| logepa | rs151283973 | 0.005 | . | . | . | . | . | . | . | . | . | . |
| logepa | rs139316218 | 0.001 | . | . | . | . | . | . | . | . | . | . |
| logepa | rs6593485 | 0.697 | 0.988 | 0.399 | 0.975 | 1.01 | 0.188 | 0.958 | 1.01 | 0.281 | 0.969 | 0.011 |
| logepa | rs6593486 | 0.697 | 0.987 | 0.399 | 0.975 | 1.01 | 0.188 | 0.959 | 1.01 | 0.281 | 0.969 | 0.011 |
| logepa | rs145196618 | 0.036 | 1.02 | 0.23 | 0.934 | 0.506 | 0.353 | 0.329 | 0.677 | 0.708 | 0.709 | -0.176 |
| logepa | rs142598335 | 0.001 | . | . | . | . | . | . | . | . | . | . |
| logepa | rs892691 | 0.663 | 0.996 | 0.385 | 0.992 | 1.05 | 0.194 | 0.8 | 1.01 | 0.27 | 0.982 | 6.10E-03 |
| logepa | rs7080713 | 0.597 | 1.07 | 0.43 | 0.867 | 0.982 | 0.187 | 0.925 | 0.946 | 0.283 | 0.854 | -0.058 |
| logepa | rs7077173 | 0.135 | 1.01 | 0.248 | 0.961 | 0.841 | 0.225 | 0.517 | 0.975 | 0.486 | 0.959 | -0.023 |
| logepa | rs7080977 | 0.088 | 1.02 | 0.234 | 0.943 | 0.775 | 0.322 | 0.538 | 0.872 | 0.756 | 0.875 | -0.104 |
| logepa | rs3780901 | 0.599 | 1.06 | 0.405 | 0.883 | 1.05 | 0.201 | 0.802 | 0.959 | 0.269 | 0.881 | -0.043 |
| logepa | rs61854091 | 0.021 | 0.986 | 0.231 | 0.952 | 3.94 | 2.46 | 0.028 | 1.35 | 1.53 | 0.789 | 1.34 |
| logepa | rs77550209 | 0.011 | 1.03 | 0.237 | 0.884 | 0.55 | 0.481 | 0.494 | 0.242 | 0.17 | 0.043 | -0.446 |
| logepa | rs73283191 | 0.097 | 1.01 | 0.236 | 0.961 | 0.794 | 0.298 | 0.538 | 0.923 | 0.733 | 0.919 | -0.064 |
| logepa | rs191866099 | 0.003 | . | . | . | . | . | . | . | . | . | . |
| logepa | rs147998395 | 0.005 | . | . | . | . | . | . | . | . | . | . |
| logepa | rs7090328 | 0.861 | 0.457 | 0.284 | 0.207 | 1.29 | 0.351 | 0.351 | 1.59 | 0.569 | 0.197 | 0.189 |
| logepa | rs79814535 | 0.003 | . | . | . | . | . | . | . | . | . | . |
| logepa | rs7073082 | 0.861 | 0.456 | 0.284 | 0.207 | 1.29 | 0.352 | 0.349 | 1.59 | 0.57 | 0.197 | 0.189 |
| logepa | rs11239526 | 0.012 | 0.991 | 0.228 | 0.967 | 1.49 | 1.15 | 0.609 | 1.58 | 1.63 | 0.66 | 0.843 |
| logepa | rs141839841 | 0.005 | . | . | . | . | . | . | . | . | . | . |
| logepa | rs75697371 | 0.005 | . | . | . | . | . | . | . | . | . | . |
| logepa | rs75972854 | 0.005 | . | . | . | . | . | . | . | . | . | . |
| logepa | rs139291975 | . | . | . | . | . | . | . | . | . | . | . |
| logepa | rs144064711 | 0.002 | . | . | . | . | . | . | . | . | . | . |
| logepa | rs3780902 | 0.098 | 1.01 | 0.236 | 0.973 | 0.788 | 0.29 | 0.517 | 0.948 | 0.729 | 0.944 | -0.043 |
| logepa | rs190449801 | 0.004 | . | . | . | . | . | . | . | . | . | . |
| logepa | rs73283196 | 0.096 | 1.01 | 0.236 | 0.967 | 0.792 | 0.297 | 0.534 | 0.934 | 0.743 | 0.931 | -0.055 |
| logepa | rs28395867 | 0.118 | 0.937 | 0.23 | 0.793 | 0.993 | 0.252 | 0.978 | 1.4 | 0.653 | 0.468 | 0.375 |
| logepa | rs28395866 | 0.264 | 0.84 | 0.242 | 0.545 | 1.03 | 0.216 | 0.905 | 1.44 | 0.522 | 0.319 | 0.372 |
| logepa | rs55661611 | 0.088 | 1.02 | 0.234 | 0.948 | 0.773 | 0.325 | 0.541 | 0.879 | 0.781 | 0.885 | -0.099 |
| logepa | rs55684518 | 0.008 | . | . | . | . | . | . | . | . | . | . |
| logepa | rs78552493 | 0.041 | 1.07 | 0.242 | 0.778 | 0.901 | 0.435 | 0.83 | 0.408 | 0.366 | 0.318 | -0.575 |
| logepa | rs147665434 | 0.001 | . | . | . | . | . | . | . | . | . | . |
| logepa | rs34445001 | 0.005 | . | . | . | . | . | . | . | . | . | . |
| logepa | rs184019449 | 0.001 | . | . | . | . | . | . | . | . | . | . |
| logepa | rs3780903 | 0.645 | 1.05 | 0.448 | 0.917 | 1.12 | 0.211 | 0.535 | 0.973 | 0.289 | 0.927 | -0.026 |
| logepa | rs17157784 | 0.159 | 0.835 | 0.221 | 0.496 | 1.22 | 0.27 | 0.381 | 1.72 | 0.61 | 0.128 | 0.693 |
| logepa | rs3780904 | 0.01 | . | . | . | . | . | . | . | . | . | . |
| logepa | rs192064730 | 0 | . | . | . | . | . | . | . | . | . | . |
| logepa | rs12774534 | 0.005 | . | . | . | . | . | . | . | . | . | . |
| logepa | rs79179915 | 0.003 | . | . | . | . | . | . | . | . | . | . |
| logepa | rs117811571 | 0.004 | . | . | . | . | . | . | . | . | . | . |
| logepa | rs72796477 | 0.004 | . | . | . | . | . | . | . | . | . | . |
| logepa | rs55729342 | 0.172 | 0.811 | 0.23 | 0.461 | 1.23 | 0.286 | 0.374 | 1.73 | 0.627 | 0.134 | 0.68 |
| logepa | rs59317220 | 0.172 | 0.812 | 0.23 | 0.462 | 1.23 | 0.286 | 0.372 | 1.72 | 0.627 | 0.134 | 0.68 |
| logepa | rs3780905 | 0.018 | 0.966 | 0.215 | 0.878 | 1.31 | 0.88 | 0.684 | 2.37 | 4.12 | 0.62 | 1.73 |
| logepa | rs3780906 | 0.645 | 1.08 | 0.461 | 0.856 | 1.11 | 0.211 | 0.579 | 0.948 | 0.284 | 0.859 | -0.053 |
| logepa | rs149047975 | 0.003 | . | . | . | . | . | . | . | . | . | . |
| logepa | rs3824614 | 0.002 | . | . | . | . | . | . | . | . | . | . |
| logepa | rs140254404 | . | . | . | . | . | . | . | . | . | . | . |
| logepa | rs145262684 | 0.005 | . | . | . | . | . | . | . | . | . | . |
| logepa | rs76735482 | 0.002 | . | . | . | . | . | . | . | . | . | . |
| logepa | rs73283198 | 0.045 | 0.986 | 0.224 | 0.951 | 1.03 | 0.491 | 0.945 | 1.26 | 1.42 | 0.84 | 0.261 |
| logepa | rs74911239 | 0.013 | 1.11 | 0.26 | 0.654 | 3.90E-05 | 1.90E-04 | 0.042 | 5.60E-05 | 2.70E-04 | 0.039 | -0.111 |
| logepa | rs150551647 | 0.004 | . | . | . | . | . | . | . | . | . | . |
| logepa | rs115200794 | 0.143 | 0.901 | 0.236 | 0.69 | 1.03 | 0.25 | 0.907 | 1.48 | 0.601 | 0.331 | 0.445 |
| logepa | rs74130823 | 0.245 | 0.875 | 0.242 | 0.629 | 1.04 | 0.228 | 0.871 | 1.43 | 0.64 | 0.426 | 0.384 |
| logepa | rs2279435 | 0.604 | 0.853 | 0.395 | 0.732 | 1.29 | 0.26 | 0.215 | 1.14 | 0.377 | 0.699 | 0.108 |
| logepa | rs147279673 | 0.007 | . | . | . | . | . | . | . | . | . | . |
| logepa | rs183525146 | 0.002 | . | . | . | . | . | . | . | . | . | . |
| logepa | rs3740107 | 0.769 | 0.864 | 0.406 | 0.757 | 1.05 | 0.208 | 0.791 | 1.1 | 0.348 | 0.754 | 0.087 |
| logepa | rs140697247 | 0 | . | . | . | . | . | . | . | . | . | . |
| logepa | rs1565096 | 0.903 | 0.272 | 0.198 | 0.074 | 1.67 | 0.544 | 0.115 | 2.07 | 0.866 | 0.083 | -2.90E-03 |
| logepa | rs1565097 | 0.766 | 0.882 | 0.419 | 0.791 | 1.19 | 0.252 | 0.402 | 1.09 | 0.338 | 0.777 | 0.073 |
| logepa | rs144584548 | 0.008 | . | . | . | . | . | . | . | . | . | . |
| logepa | rs75811053 | 0.001 | . | . | . | . | . | . | . | . | . | . |
| logepa | rs147462967 | 0.009 | . | . | . | . | . | . | . | . | . | . |
| logepa | rs74130824 | 0.121 | 0.873 | 0.237 | 0.616 | 1.16 | 0.286 | 0.555 | 1.78 | 0.781 | 0.19 | 0.766 |
| logepa | rs116883184 | 0.006 | . | . | . | . | . | . | . | . | . | . |
| logepa | rs117258049 | 0.006 | . | . | . | . | . | . | . | . | . | . |
| logepa | rs3780908 | 0.129 | 1.02 | 0.241 | 0.93 | 0.876 | 0.27 | 0.668 | 0.889 | 0.583 | 0.857 | -0.102 |
| logepa | rs180938522 | 0.001 | . | . | . | . | . | . | . | . | . | . |
| logepa | rs76687372 | 0.024 | 1.01 | 0.226 | 0.976 | 0.776 | 0.639 | 0.758 | 0.785 | 1.12 | 0.866 | -0.169 |
| logepa | rs3780909 | 0.483 | 1.31 | 0.399 | 0.374 | 0.927 | 0.163 | 0.668 | 0.753 | 0.202 | 0.291 | -0.323 |
| logepa | rs78281156 | 0.001 | . | . | . | . | . | . | . | . | . | . |
| logepa | rs187840631 | . | . | . | . | . | . | . | . | . | . | . |
| logepa | rs76868242 | 0.001 | . | . | . | . | . | . | . | . | . | . |
| logepa | rs192794192 | 0 | . | . | . | . | . | . | . | . | . | . |
| logepa | rs12781248 | 0.005 | . | . | . | . | . | . | . | . | . | . |
| logepa | rs12762375 | 0.005 | . | . | . | . | . | . | . | . | . | . |
| logepa | rs192256986 | . | . | . | . | . | . | . | . | . | . | . |
| logepa | rs184411030 | 0.033 | 1.01 | 0.229 | 0.978 | 0.529 | 0.442 | 0.446 | 0.668 | 0.914 | 0.768 | -0.18 |
| logepa | rs189601483 | 0.001 | . | . | . | . | . | . | . | . | . | . |
| logepa | rs143254579 | 0.015 | 0.963 | 0.226 | 0.871 | 1.29 | 0.863 | 0.707 | 2.52 | 1.5 | 0.122 | 1.87 |
| logepa | rs12253903 | 0.081 | 0.83 | 0.196 | 0.43 | 1.18 | 0.37 | 0.588 | 3.11 | 1.89 | 0.062 | 2.05 |
| logepa | rs35489926 | 0.005 | . | . | . | . | . | . | . | . | . | . |

| logepa | rs71494783 | 0.095 | 0.916 | 0.226 | 0.722 | 1.16 | 0.379 | 0.659 | 1.52 | 0.652 | 0.327 | 0.539 |
| --- | --- | --- | --- | --- | --- | --- | --- | --- | --- | --- | --- | --- |
| logepa | rs180711602 | 0.001 | . | . | . | . | . | . | . | . | . | . |
| logepa | rs117312402 | 0.001 | . | . | . | . | . | . | . | . | . | . |
| logepa | rs74831694 | 0.001 | . | . | . | . | . | . | . | . | . | . |
| logepa | rs140301943 | . | . | . | . | . | . | . | . | . | . | . |
| logepa | rs78553679 | 0.006 | . | . | . | . | . | . | . | . | . | . |
| logepa | rs78334250 | 0.001 | . | . | . | . | . | . | . | . | . | . |
| logepa | rs76000028 | 0.001 | . | . | . | . | . | . | . | . | . | . |
| logepa | rs145195254 | 0.005 | . | . | . | . | . | . | . | . | . | . |
| logepa | rs137888527 | 0.005 | . | . | . | . | . | . | . | . | . | . |
| logepa | rs149109592 | . | . | . | . | . | . | . | . | . | . | . |
| logepa | rs80280010 | 0.013 | 0.988 | 0.228 | 0.958 | 1.48 | 1.05 | 0.586 | 1.63 | 1.29 | 0.533 | 0.918 |
| logepa | rs189995374 | . | . | . | . | . | . | . | . | . | . | . |
| logepa | rs75467770 | 0.002 | . | . | . | . | . | . | . | . | . | . |
| logepa | rs1487562 | 0.242 | 0.944 | 0.278 | 0.844 | 0.961 | 0.223 | 0.865 | 1.16 | 0.531 | 0.744 | 0.148 |
| logepa | rs12266963 | 0.095 | 0.916 | 0.225 | 0.721 | 1.15 | 0.378 | 0.681 | 1.53 | 0.657 | 0.326 | 0.54 |
| logepa | rs7099874 | 0.857 | 0.833 | 0.469 | 0.745 | 1.06 | 0.257 | 0.809 | 1.12 | 0.389 | 0.744 | 0.096 |
| logepa | rs75687019 | 0.001 | . | . | . | . | . | . | . | . | . | . |
| logepa | rs148240462 | 0.014 | 1.13 | 0.264 | 0.603 | 2.40E-05 | 1.20E-04 | 0.039 | 3.30E-05 | 1.60E-04 | 0.032 | -0.13 |
| logepa | rs141278174 | 0.001 | . | . | . | . | . | . | . | . | . | . |
| logepa | rs76404852 | 0.001 | . | . | . | . | . | . | . | . | . | . |
| logepa | rs139112240 | 0.004 | . | . | . | . | . | . | . | . | . | . |
| logepa | rs12783037 | 0.118 | 0.922 | 0.226 | 0.739 | 1.08 | 0.32 | 0.804 | 1.41 | 0.638 | 0.452 | 0.397 |
| logepa | rs72796481 | 0.003 | . | . | . | . | . | . | . | . | . | . |
| logepa | rs76798749 | 0.025 | 1.01 | 0.229 | 0.967 | 1.06 | 0.706 | 0.931 | 0.895 | 1.04 | 0.924 | -0.112 |
| logepa | rs187732548 | 0.001 | . | . | . | . | . | . | . | . | . | . |
| logepa | rs75675474 | 0.025 | 1.01 | 0.229 | 0.967 | 1.06 | 0.706 | 0.931 | 0.895 | 1.04 | 0.924 | -0.112 |
| logepa | rs76552293 | 0.025 | 1.01 | 0.229 | 0.967 | 1.06 | 0.706 | 0.931 | 0.895 | 1.04 | 0.924 | -0.112 |
| logepa | rs147445993 | 0.008 | . | . | . | . | . | . | . | . | . | . |
| logepa | rs115902283 | 0.025 | 1.01 | 0.229 | 0.967 | 1.05 | 0.706 | 0.937 | 0.889 | 1.04 | 0.92 | -0.117 |
| logepa | rs4540799 | 0.855 | 0.817 | 0.463 | 0.722 | 1.07 | 0.256 | 0.781 | 1.13 | 0.394 | 0.72 | 0.103 |
| logepa | rs12765320 | 0.35 | 0.837 | 0.264 | 0.573 | 1.05 | 0.199 | 0.782 | 1.33 | 0.454 | 0.408 | 0.28 |
| logepa | rs12782256 | 0.005 | . | . | . | . | . | . | . | . | . | . |
| logepa | rs191727256 | 0.007 | . | . | . | . | . | . | . | . | . | . |
| logepa | rs2082111 | 0.113 | 1.06 | 0.252 | 0.799 | 0.881 | 0.297 | 0.707 | 0.69 | 0.434 | 0.555 | -0.298 |
| logepa | rs2082110 | 0.257 | 0.944 | 0.278 | 0.844 | 1.03 | 0.228 | 0.887 | 1.14 | 0.476 | 0.745 | 0.139 |
| logepa | rs2082109 | 0.257 | 0.944 | 0.278 | 0.844 | 1.03 | 0.228 | 0.887 | 1.14 | 0.476 | 0.745 | 0.139 |
| logepa | rs117316662 | 0.001 | . | . | . | . | . | . | . | . | . | . |
| logepa | rs12247163 | 0.357 | 0.781 | 0.251 | 0.442 | 1.15 | 0.209 | 0.441 | 1.46 | 0.478 | 0.252 | 0.378 |
| logepa | rs12355888 | 0.037 | 1.02 | 0.236 | 0.929 | 1.13 | 0.559 | 0.802 | 0.705 | 0.639 | 0.7 | -0.338 |
| logepa | rs143772290 | 0.033 | 0.966 | 0.224 | 0.882 | 1.67 | 0.774 | 0.271 | 1.84 | 1.82 | 0.538 | 1.33 |
| logepa | rs146849452 | 0.009 | . | . | . | . | . | . | . | . | . | . |
| logepa | rs140267121 | 0.003 | . | . | . | . | . | . | . | . | . | . |
| logepa | rs7080474 | 0.352 | 0.823 | 0.252 | 0.524 | 1.13 | 0.21 | 0.517 | 1.37 | 0.447 | 0.34 | 0.318 |
| logepa | rs144066203 | 0.007 | . | . | . | . | . | . | . | . | . | . |
| logepa | rs12765209 | 0.005 | . | . | . | . | . | . | . | . | . | . |
| logepa | rs150930735 | 0.007 | . | . | . | . | . | . | . | . | . | . |
| logepa | rs7068039 | 0.365 | 0.805 | 0.261 | 0.502 | 1.14 | 0.21 | 0.487 | 1.39 | 0.461 | 0.326 | 0.327 |
| logepa | rs117622652 | 0.001 | . | . | . | . | . | . | . | . | . | . |
| logepa | rs190191514 | . | . | . | . | . | . | . | . | . | . | . |
| logepa | rs74130835 | 0.118 | 0.877 | 0.235 | 0.626 | 1.09 | 0.278 | 0.731 | 1.79 | 0.808 | 0.198 | 0.743 |
| logepa | rs1811604 | 0.114 | 0.906 | 0.237 | 0.705 | 1.13 | 0.284 | 0.637 | 1.62 | 0.75 | 0.302 | 0.616 |
| logepa | rs146971256 | . | . | . | . | . | . | . | . | . | . | . |
| logepa | rs74336000 | 0.033 | 0.966 | 0.224 | 0.881 | 1.67 | 0.773 | 0.271 | 1.85 | 1.83 | 0.534 | 1.35 |
| logepa | rs186046492 | 0.003 | . | . | . | . | . | . | . | . | . | . |
| logepa | rs34971823 | 0.009 | . | . | . | . | . | . | . | . | . | . |
| logepa | rs75227446 | 0.001 | . | . | . | . | . | . | . | . | . | . |
| logepa | rs190457599 | 0.001 | . | . | . | . | . | . | . | . | . | . |
| logepa | rs79591917 | 0.001 | . | . | . | . | . | . | . | . | . | . |
| logepa | rs702365 | 0.492 | 1.2 | 0.364 | 0.541 | 0.932 | 0.162 | 0.687 | 0.827 | 0.217 | 0.467 | -0.208 |
| logepa | rs79643836 | 0.004 | . | . | . | . | . | . | . | . | . | . |
| logepa | rs702366 | 0.493 | 1.2 | 0.362 | 0.549 | 0.934 | 0.161 | 0.691 | 0.83 | 0.217 | 0.477 | -0.203 |
| logepa | rs1059696 | 0.197 | 0.972 | 0.248 | 0.91 | 1.05 | 0.248 | 0.845 | 1.1 | 0.479 | 0.831 | 0.098 |
| logepa | rs901681 | 0.489 | 1.21 | 0.359 | 0.523 | 0.924 | 0.163 | 0.654 | 0.82 | 0.216 | 0.452 | -0.217 |
| logepa | rs61854092 | 0.196 | 0.972 | 0.248 | 0.911 | 1.04 | 0.247 | 0.857 | 1.1 | 0.481 | 0.833 | 0.097 |
| logepa | rs148080753 | . | . | . | . | . | . | . | . | . | . | . |
| logepa | rs61854093 | 0.315 | 0.826 | 0.258 | 0.541 | 1.08 | 0.211 | 0.684 | 1.4 | 0.511 | 0.352 | 0.346 |
| logepa | rs141822774 | 0 | . | . | . | . | . | . | . | . | . | . |
| logepa | rs74130836 | 0.118 | 0.877 | 0.235 | 0.625 | 1.09 | 0.278 | 0.737 | 1.79 | 0.811 | 0.197 | 0.746 |
| logepa | rs150620963 | 0.006 | . | . | . | . | . | . | . | . | . | . |
| logepa | rs3780913 | 0.849 | 0.8 | 0.442 | 0.686 | 1.13 | 0.271 | 0.61 | 1.15 | 0.389 | 0.678 | 0.11 |
| logepa | rs142255723 | 0.001 | . | . | . | . | . | . | . | . | . | . |
| logepa | rs3780914 | 0.479 | 1.27 | 0.399 | 0.442 | 0.956 | 0.169 | 0.797 | 0.78 | 0.21 | 0.355 | -0.28 |
| logepa | rs182656507 | 0.001 | . | . | . | . | . | . | . | . | . | . |
| logepa | rs2291427 | 0.821 | 0.975 | 0.501 | 0.96 | 0.943 | 0.201 | 0.783 | 1.01 | 0.331 | 0.964 | 0.015 |
| logepa | rs10751382 | 0.816 | 0.934 | 0.451 | 0.887 | 0.966 | 0.201 | 0.867 | 1.04 | 0.319 | 0.888 | 0.042 |
| logepa | rs117610108 | 0.001 | . | . | . | . | . | . | . | . | . | . |
| logepa | rs144966941 | 0.009 | . | . | . | . | . | . | . | . | . | . |
| logepa | rs41502051 | 0.014 | 1 | 0.234 | 0.999 | 1.56 | 0.891 | 0.432 | 0.867 | 0.538 | 0.818 | -0.208 |
| logepa | rs41426046 | 0.001 | . | . | . | . | . | . | . | . | . | . |
| logepa | rs41519248 | 0.001 | . | . | . | . | . | . | . | . | . | . |
| logepa | rs189108245 | 0.001 | . | . | . | . | . | . | . | . | . | . |
| logepa | rs7896431 | 0.497 | 1.11 | 0.344 | 0.726 | 0.867 | 0.152 | 0.415 | 0.886 | 0.228 | 0.64 | -0.125 |
| logepa | rs183724547 | 0.009 | . | . | . | . | . | . | . | . | . | . |
| logepa | rs142044990 | 0.004 | . | . | . | . | . | . | . | . | . | . |
| logepa | rs188140929 | . | . | . | . | . | . | . | . | . | . | . |
| logepa | rs76819096 | 0.041 | 0.913 | 0.199 | 0.676 | 0.614 | 0.393 | 0.446 | 5.23 | 6.68 | 0.195 | 2.41 |
| logepa | rs10751383 | 0.58 | 0.958 | 0.389 | 0.916 | 1.08 | 0.201 | 0.691 | 1.04 | 0.314 | 0.897 | 0.038 |
| logepa | rs117819321 | 0.005 | . | . | . | . | . | . | . | . | . | . |

| logepa | rs2242332 | 0.681 | 1.23 | 0.485 | 0.607 | 1 | 0.18 | 0.993 | 0.86 | 0.244 | 0.594 | -0.172 |
| --- | --- | --- | --- | --- | --- | --- | --- | --- | --- | --- | --- | --- |
| logepa | rs2242333 | 0.02 | 0.973 | 0.217 | 0.901 | 1.38 | 0.864 | 0.605 | 1.89 | 2.85 | 0.674 | 1.18 |
| logepa | rs2242334 | 0.681 | 1.23 | 0.485 | 0.607 | 1 | 0.18 | 0.997 | 0.859 | 0.244 | 0.594 | -0.172 |
| logepa | rs189465041 | . | . | . | . | . | . | . | . | . | . | . |
| logepa | rs28395868 | 0.009 | . | . | . | . | . | . | . | . | . | . |
| logepa | rs56067550 | 0.008 | . | . | . | . | . | . | . | . | . | . |
| logepa | rs1051713 | 0.182 | 1.03 | 0.263 | 0.903 | 1.1 | 0.263 | 0.705 | 0.928 | 0.381 | 0.855 | -0.079 |
| logepa | rs115074210 | 0.048 | 0.912 | 0.197 | 0.671 | 0.474 | 0.306 | 0.248 | 4.84 | 6.66 | 0.251 | 1.71 |
| logepa | rs7900977 | 0.893 | 0.302 | 0.237 | 0.128 | 1.32 | 0.381 | 0.333 | 1.97 | 0.863 | 0.12 | 0.163 |
| logepa | rs147613891 | 0.001 | . | . | . | . | . | . | . | . | . | . |
| logepa | rs2229136 | 0.161 | 0.788 | 0.194 | 0.335 | 1.15 | 0.253 | 0.536 | 2 | 0.614 | 0.025 | 0.868 |
| logepa | rs185482841 | 0.009 | . | . | . | . | . | . | . | . | . | . |
| logepa | rs2288619 | 0.156 | 0.789 | 0.193 | 0.334 | 1.18 | 0.256 | 0.454 | 2.03 | 0.628 | 0.022 | 0.921 |
| logepa | rs189891524 | . | . | . | . | . | . | . | . | . | . | . |
| logepa | rs56145077 | 0.176 | 1.02 | 0.254 | 0.926 | 1.01 | 0.254 | 0.969 | 0.935 | 0.402 | 0.875 | -0.067 |
| logepa | rs143216970 | 0.013 | 0.987 | 0.228 | 0.955 | 0.24 | 0.304 | 0.259 | 3.81 | 2.54 | 0.045 | 0.676 |
| logepa | rs142246478 | 0 | . | . | . | . | . | . | . | . | . | . |
| logepa | rs13134 | 0.012 | 0.956 | 0.22 | 0.845 | 2.08 | 1.18 | 0.197 | 8.17 | 10 | 0.087 | 14.2 |
| logepa | rs41314980 | 0.012 | 1.02 | 0.235 | 0.929 | 2.01 | 1.21 | 0.246 | 0.73 | 0.529 | 0.664 | -0.534 |
| logepa | rs41304611 | 0.005 | . | . | . | . | . | . | . | . | . | . |
| logepa | rs193299617 | . | . | . | . | . | . | . | . | . | . | . |
| logepa | rs12772724 | 0.012 | 0.956 | 0.22 | 0.845 | 2.08 | 1.18 | 0.197 | 8.18 | 10 | 0.086 | 14.2 |
| logepa | rs139489537 | 0.011 | 0.99 | 0.229 | 0.966 | 1.77 | 1.25 | 0.416 | 1.33 | 0.987 | 0.701 | 0.572 |
| logepa | rs12776062 | 0.005 | . | . | . | . | . | . | . | . | . | . |
| logepa | rs752059 | 0.181 | 1.02 | 0.255 | 0.937 | 0.978 | 0.244 | 0.93 | 0.941 | 0.4 | 0.887 | -0.059 |
| logepa | rs147649982 | 0.013 | 1.06 | 0.246 | 0.791 | 0.148 | 0.256 | 0.27 | 0.032 | 0.071 | 0.122 | -0.206 |
| logepa | rs17157798 | 0.113 | 0.754 | 0.181 | 0.24 | 1.24 | 0.334 | 0.42 | 3.44 | 1.77 | 0.016 | 2.23 |
| logepa | rs142298619 | 0.009 | . | . | . | . | . | . | . | . | . | . |
| logepa | rs4948673 | 0.893 | 0.293 | 0.236 | 0.128 | 1.33 | 0.387 | 0.33 | 2 | 0.896 | 0.121 | 0.159 |
| logepa | rs74231484 | 0.045 | 1.02 | 0.229 | 0.939 | 1.14 | 0.535 | 0.772 | 0.867 | 0.783 | 0.875 | -0.152 |
| logepa | rs72796489 | 0.001 | . | . | . | . | . | . | . | . | . | . |
| logepa | rs113447578 | 0.006 | . | . | . | . | . | . | . | . | . | . |
| logepa | rs34063374 | 0.014 | 0.951 | 0.22 | 0.828 | 2.24 | 1.16 | 0.12 | 7.62 | 8.46 | 0.067 | 14 |
| logepa | rs11239527 | 0.006 | . | . | . | . | . | . | . | . | . | . |
| logepa | rs34371883 | 0.007 | . | . | . | . | . | . | . | . | . | . |
| logepa | rs118168828 | 0.004 | . | . | . | . | . | . | . | . | . | . |
| logepa | rs34349618 | 0.005 | . | . | . | . | . | . | . | . | . | . |
| logepa | rs80041679 | 0.011 | 0.99 | 0.23 | 0.967 | 1.76 | 1.23 | 0.417 | 1.31 | 0.957 | 0.707 | 0.543 |
| logepa | rs149640607 | 0.01 | 1.03 | 0.231 | 0.911 | 0.337 | 0.272 | 0.178 | 0.115 | 0.153 | 0.104 | -0.322 |
| logepa | rs4427523 | 0.932 | 0.129 | 0.193 | 0.17 | 1.37 | 0.751 | 0.572 | 2.98 | 2.41 | 0.175 | 0.031 |
| logepa | rs185880109 | . | . | . | . | . | . | . | . | . | . | . |
| logepa | rs191523220 | 0.001 | . | . | . | . | . | . | . | . | . | . |
| logepa | rs144315284 | 0.02 | 1.06 | 0.242 | 0.805 | 0.484 | 0.281 | 0.211 | 0.182 | 0.127 | 0.015 | -0.449 |
| logepa | rs4948674 | 0.329 | 1.25 | 0.372 | 0.458 | 0.826 | 0.17 | 0.353 | 0.695 | 0.227 | 0.265 | -0.357 |
| logepa | rs182984298 | 0.002 | . | . | . | . | . | . | . | . | . | . |
| logepa | rs56749023 | 0.071 | 0.809 | 0.182 | 0.346 | 0.679 | 0.295 | 0.372 | 5.69 | 4.15 | 0.017 | 2.64 |
| logepa | rs112508957 | 0.045 | 1.04 | 0.238 | 0.869 | 0.519 | 0.314 | 0.279 | 0.41 | 0.328 | 0.264 | -0.337 |
| logepa | rs7089063 | 0.313 | 0.761 | 0.236 | 0.378 | 1.18 | 0.269 | 0.474 | 1.58 | 0.557 | 0.194 | 0.478 |
| logepa | rs145000779 | . | . | . | . | . | . | . | . | . | . | . |
| logepa | rs80201572 | 0.013 | 0.987 | 0.229 | 0.954 | 1.65 | 1.14 | 0.469 | 1.52 | 1.01 | 0.528 | 0.84 |
| logepa | rs140568227 | 0.003 | . | . | . | . | . | . | . | . | . | . |
| logepa | rs149756228 | 0.004 | . | . | . | . | . | . | . | . | . | . |
| logepa | rs112380685 | 0.003 | . | . | . | . | . | . | . | . | . | . |
| logepa | rs60481109 | 0.067 | 0.809 | 0.182 | 0.346 | 0.708 | 0.314 | 0.437 | 5.74 | 4.17 | 0.016 | 2.77 |
| logepa | rs138221843 | . | . | . | . | . | . | . | . | . | . | . |
| logepa | rs150283194 | 0.009 | . | . | . | . | . | . | . | . | . | . |
| logepa | rs138879661 | 0.008 | . | . | . | . | . | . | . | . | . | . |
| logepa | rs34960345 | 0.067 | 0.97 | 0.252 | 0.906 | 1.65 | 0.541 | 0.126 | 1.13 | 0.487 | 0.768 | 0.196 |
| logepa | rs147514068 | . | . | . | . | . | . | . | . | . | . | . |
| logepa | rs35410535 | 0.138 | 0.795 | 0.197 | 0.355 | 1.17 | 0.277 | 0.515 | 2.09 | 0.656 | 0.019 | 0.974 |
| logepa | rs191613635 | 0.001 | . | . | . | . | . | . | . | . | . | . |
| logepa | rs17157800 | 0.004 | . | . | . | . | . | . | . | . | . | . |
| logepa | rs17523413 | 0.027 | 0.937 | 0.223 | 0.785 | 1.93 | 0.882 | 0.15 | 2.88 | 2.22 | 0.171 | 3.34 |
| logepa | rs7897036 | 0.013 | 1.03 | 0.228 | 0.91 | 0.246 | 0.281 | 0.219 | 0.229 | 0.172 | 0.05 | -0.214 |
| logepa | rs76334323 | 0.001 | . | . | . | . | . | . | . | . | . | . |
| logepa | rs143535991 | 0 | . | . | . | . | . | . | . | . | . | . |
| logepa | rs140344979 | 0.002 | . | . | . | . | . | . | . | . | . | . |
| logepa | rs36047811 | 0.138 | 0.795 | 0.197 | 0.354 | 1.17 | 0.277 | 0.515 | 2.09 | 0.656 | 0.019 | 0.974 |
| logepa | rs76493570 | 0.057 | 0.942 | 0.245 | 0.82 | 1.79 | 0.605 | 0.087 | 1.36 | 0.571 | 0.464 | 0.561 |
| logepa | rs192998920 | . | . | . | . | . | . | . | . | . | . | . |
| logepa | rs116840852 | 0.007 | . | . | . | . | . | . | . | . | . | . |
| logepa | rs184824101 | 0 | . | . | . | . | . | . | . | . | . | . |
| logepa | rs41301621 | 0.002 | . | . | . | . | . | . | . | . | . | . |
| logepa | rs3740109 | 0.138 | 0.795 | 0.197 | 0.354 | 1.17 | 0.276 | 0.517 | 2.09 | 0.656 | 0.019 | 0.974 |
| logepa | rs7899415 | 0.032 | 1 | 0.232 | 0.993 | 0.498 | 0.436 | 0.426 | 1.23 | 2.41 | 0.917 | 0.112 |
| logepa | rs184395284 | 0.001 | . | . | . | . | . | . | . | . | . | . |
| logepa | rs139209589 | 0.002 | . | . | . | . | . | . | . | . | . | . |
| logepa | rs149924163 | 0.009 | . | . | . | . | . | . | . | . | . | . |
| logepa | rs3740110 | 0.001 | . | . | . | . | . | . | . | . | . | . |
| logepa | rs144335113 | 0.009 | . | . | . | . | . | . | . | . | . | . |
| logepa | rs74743385 | 0.045 | 1.04 | 0.238 | 0.869 | 0.52 | 0.314 | 0.279 | 0.41 | 0.328 | 0.265 | -0.337 |
| logepa | rs146545829 | 0.009 | . | . | . | . | . | . | . | . | . | . |
| logepa | rs181842689 | . | . | . | . | . | . | . | . | . | . | . |
| logepa | rs3740111 | 0.067 | 0.969 | 0.252 | 0.904 | 1.65 | 0.54 | 0.127 | 1.14 | 0.487 | 0.756 | 0.207 |
| logepa | rs186261599 | 0.009 | . | . | . | . | . | . | . | . | . | . |
| logepa | rs73285348 | 0.004 | . | . | . | . | . | . | . | . | . | . |
| logepa | rs186356901 | 0.009 | . | . | . | . | . | . | . | . | . | . |
| logepa | rs3824618 | 0.011 | 0.989 | 0.229 | 0.962 | 1.75 | 1.22 | 0.423 | 1.39 | 0.993 | 0.649 | 0.659 |
| logepa | rs3802548 | 0.201 | 1.1 | 0.277 | 0.715 | 1.07 | 0.242 | 0.776 | 0.792 | 0.304 | 0.544 | -0.237 |

| logepa | rs3802547 | 0.001 | . | . | . | . | . | . | . | . | . | . |
| --- | --- | --- | --- | --- | --- | --- | --- | --- | --- | --- | --- | --- |
| logepa | rs77677521 | 0.007 | . | . | . | . | . | . | . | . | . | . |
| logepa | rs3824616 | 0.138 | 0.795 | 0.197 | 0.353 | 1.17 | 0.276 | 0.518 | 2.09 | 0.655 | 0.019 | 0.973 |
| logepa | rs191073818 | 0.009 | . | . | . | . | . | . | . | . | . | . |
| logepa | rs147078004 | . | . | . | . | . | . | . | . | . | . | . |
| logepa | rs7908745 | 0.342 | 0.776 | 0.254 | 0.44 | 1.3 | 0.284 | 0.235 | 1.46 | 0.494 | 0.263 | 0.397 |
| logepa | rs17157811 | 0.004 | . | . | . | . | . | . | . | . | . | . |
| logepa | rs138506087 | 0.002 | . | . | . | . | . | . | . | . | . | . |
| logepa | rs77241269 | 0.071 | 0.811 | 0.182 | 0.351 | 0.675 | 0.291 | 0.362 | 5.65 | 4.12 | 0.018 | 2.6 |
| logepa | rs138374938 | 0.001 | . | . | . | . | . | . | . | . | . | . |
| logepa | rs2279433 | 0.068 | 0.977 | 0.252 | 0.927 | 1.64 | 0.519 | 0.118 | 1.09 | 0.456 | 0.829 | 0.136 |
| logepa | rs73285358 | 0.071 | 0.811 | 0.182 | 0.351 | 0.674 | 0.291 | 0.361 | 5.64 | 4.12 | 0.018 | 2.6 |
| logepa | rs11239528 | 0.198 | 1.09 | 0.275 | 0.743 | 1.07 | 0.244 | 0.77 | 0.809 | 0.317 | 0.589 | -0.216 |
| logepa | rs77453386 | 0.001 | . | . | . | . | . | . | . | . | . | . |
| logepa | rs2279434 | 0.071 | 0.976 | 0.252 | 0.924 | 1.6 | 0.51 | 0.141 | 1.11 | 0.454 | 0.802 | 0.154 |
| logepa | rs149236525 | 0.003 | . | . | . | . | . | . | . | . | . | . |
| logepa | rs12573609 | 0.018 | 1.08 | 0.259 | 0.756 | 0.361 | 0.532 | 0.489 | 0.245 | 0.279 | 0.217 | -0.344 |
| logepa | rs191716655 | 0.006 | . | . | . | . | . | . | . | . | . | . |
| logepa | rs17523660 | 0.005 | . | . | . | . | . | . | . | . | . | . |
| logepa | rs142671360 | 0.001 | . | . | . | . | . | . | . | . | . | . |
| logepa | rs3764990 | 0.025 | 0.939 | 0.224 | 0.791 | 2.04 | 0.93 | 0.118 | 2.77 | 2.19 | 0.197 | 3.33 |
| logepa | rs113682861 | 0.001 | . | . | . | . | . | . | . | . | . | . |
| logepa | rs117658270 | 0.001 | . | . | . | . | . | . | . | . | . | . |
| logepa | rs187309063 | . | . | . | . | . | . | . | . | . | . | . |
| logepa | rs73285360 | 0.072 | 0.811 | 0.182 | 0.352 | 0.674 | 0.291 | 0.361 | 5.64 | 4.12 | 0.018 | 2.6 |
| logepa | rs80133289 | 0.002 | . | . | . | . | . | . | . | . | . | . |
| logepa | rs186693257 | . | . | . | . | . | . | . | . | . | . | . |
| logepa | rs35735968 | 0.081 | 0.946 | 0.241 | 0.829 | 1.5 | 0.447 | 0.178 | 1.31 | 0.537 | 0.516 | 0.407 |
| logepa | rs17157825 | 0.011 | 0.987 | 0.229 | 0.956 | 1.78 | 1.25 | 0.411 | 1.51 | 1.07 | 0.563 | 0.879 |
| logepa | rs2291428 | 0.183 | 1.06 | 0.272 | 0.813 | 1.16 | 0.27 | 0.533 | 0.863 | 0.346 | 0.714 | -0.158 |
| logepa | rs2291429 | 0.21 | 1.08 | 0.292 | 0.781 | 1.13 | 0.255 | 0.591 | 0.842 | 0.312 | 0.643 | -0.182 |
| logepa | rs145714792 | 0.002 | . | . | . | . | . | . | . | . | . | . |
| logepa | rs12769244 | 0.068 | 0.98 | 0.253 | 0.938 | 1.63 | 0.514 | 0.12 | 1.07 | 0.446 | 0.865 | 0.104 |
| logepa | rs12771275 | 0.068 | 0.98 | 0.253 | 0.938 | 1.63 | 0.514 | 0.121 | 1.07 | 0.446 | 0.865 | 0.104 |
| logepa | rs146266174 | 0.001 | . | . | . | . | . | . | . | . | . | . |
| logepa | rs41301623 | 0.001 | . | . | . | . | . | . | . | . | . | . |
| logepa | rs2306814 | 0.001 | . | . | . | . | . | . | . | . | . | . |
| logepa | rs12244834 | 0.08 | 0.935 | 0.229 | 0.784 | 1.34 | 0.441 | 0.379 | 1.44 | 0.733 | 0.472 | 0.53 |
| logepa | rs184006011 | 0.001 | . | . | . | . | . | . | . | . | . | . |
| logepa | rs78829066 | 0.001 | . | . | . | . | . | . | . | . | . | . |
| logepa | rs34731408 | 0.068 | 0.98 | 0.253 | 0.937 | 1.63 | 0.514 | 0.12 | 1.07 | 0.446 | 0.865 | 0.104 |
| logepa | rs17451516 | 0.012 | 0.955 | 0.22 | 0.843 | 2.12 | 1.2 | 0.182 | 8.14 | 9.83 | 0.083 | 14.4 |
| logepa | rs139910133 | 0.009 | . | . | . | . | . | . | . | . | . | . |
| logepa | rs10900218 | 0.319 | 0.76 | 0.239 | 0.384 | 1.2 | 0.269 | 0.423 | 1.56 | 0.543 | 0.205 | 0.459 |
| logepa | rs146023999 | . | . | . | . | . | . | . | . | . | . | . |
| logepa | rs187296958 | 0 | . | . | . | . | . | . | . | . | . | . |
| logepa | rs142342833 | . | . | . | . | . | . | . | . | . | . | . |
| logepa | rs143489255 | . | . | . | . | . | . | . | . | . | . | . |
| logepa | rs138532518 | . | . | . | . | . | . | . | . | . | . | . |
| logepa | rs183768079 | 0.007 | . | . | . | . | . | . | . | . | . | . |
| logepa | rs117892272 | . | . | . | . | . | . | . | . | . | . | . |
| logepa | rs192993943 | . | . | . | . | . | . | . | . | . | . | . |
| logepa | rs182989043 | 0.001 | . | . | . | . | . | . | . | . | . | . |
| logepa | rs189703435 | 0.001 | . | . | . | . | . | . | . | . | . | . |
| logepa | rs188794040 | 0 | . | . | . | . | . | . | . | . | . | . |
| logepa | rs143920953 | . | . | . | . | . | . | . | . | . | . | . |
| logepa | rs191029121 | . | . | . | . | . | . | . | . | . | . | . |
| logepa | rs116875448 | . | . | . | . | . | . | . | . | . | . | . |
| logepa | rs190167877 | 0.003 | . | . | . | . | . | . | . | . | . | . |
| logepa | rs184993418 | . | . | . | . | . | . | . | . | . | . | . |
| logepa | rs143241158 | . | . | . | . | . | . | . | . | . | . | . |
| logepa | rs11239500 | 0.074 | 0.96 | 0.233 | 0.867 | 0.842 | 0.308 | 0.637 | 1.39 | 0.72 | 0.526 | 0.321 |
| logepa | rs140568994 | . | . | . | . | . | . | . | . | . | . | . |
| logepa | rs187530317 | 0.001 | . | . | . | . | . | . | . | . | . | . |
| logepa | rs145189326 | 0.001 | . | . | . | . | . | . | . | . | . | . |
| logepa | rs186203332 | . | . | . | . | . | . | . | . | . | . | . |
| logepa | rs189921909 | . | . | . | . | . | . | . | . | . | . | . |
| logepa | rs187260989 | . | . | . | . | . | . | . | . | . | . | . |
| logepa | rs114376586 | 0.073 | 0.962 | 0.233 | 0.873 | 0.839 | 0.306 | 0.63 | 1.38 | 0.713 | 0.534 | 0.312 |
| logepa | rs151070808 | . | . | . | . | . | . | . | . | . | . | . |
| logepa | rs140927225 | 0.074 | 0.96 | 0.233 | 0.867 | 0.827 | 0.304 | 0.605 | 1.4 | 0.731 | 0.516 | 0.326 |
| logepa | rs191555615 | . | . | . | . | . | . | . | . | . | . | . |
| logepa | rs80278898 | 0.001 | . | . | . | . | . | . | . | . | . | . |
| logepa | rs190849503 | . | . | . | . | . | . | . | . | . | . | . |
| logepa | rs150080315 | 0.007 | . | . | . | . | . | . | . | . | . | . |
| logepa | rs143623885 | . | . | . | . | . | . | . | . | . | . | . |
| logepa | rs187760040 | . | . | . | . | . | . | . | . | . | . | . |
| logepa | rs143917143 | . | . | . | . | . | . | . | . | . | . | . |
| logepa | rs149851136 | 0.002 | . | . | . | . | . | . | . | . | . | . |
| logepa | rs181612054 | . | . | . | . | . | . | . | . | . | . | . |
| logepa | rs191006548 | . | . | . | . | . | . | . | . | . | . | . |
| logepa | rs117377631 | 0.003 | . | . | . | . | . | . | . | . | . | . |
| logepa | rs182107634 | . | . | . | . | . | . | . | . | . | . | . |
| logepa | rs190211867 | . | . | . | . | . | . | . | . | . | . | . |
| logepa | rs148293457 | . | . | . | . | . | . | . | . | . | . | . |
| logepa | rs191811733 | . | . | . | . | . | . | . | . | . | . | . |
| logepa | rs146109272 | . | . | . | . | . | . | . | . | . | . | . |
| logepa | rs148397915 | . | . | . | . | . | . | . | . | . | . | . |
| logepa | rs142233290 | . | . | . | . | . | . | . | . | . | . | . |

| logepa | rs80226874 | . | . | . | . | . | . | . | . | . | . | . |
| --- | --- | --- | --- | --- | --- | --- | --- | --- | --- | --- | --- | --- |
| logepa | rs144496203 | 0 | . | . | . | . | . | . | . | . | . | . |
| logepa | rs191062130 | . | . | . | . | . | . | . | . | . | . | . |
| logepa | rs185807817 | . | . | . | . | . | . | . | . | . | . | . |
| logepa | rs117846213 | 0.001 | . | . | . | . | . | . | . | . | . | . |
| logepa | rs190545473 | . | . | . | . | . | . | . | . | . | . | . |
| logepa | rs192364918 | . | . | . | . | . | . | . | . | . | . | . |
| logepa | rs182939241 | . | . | . | . | . | . | . | . | . | . | . |
| logepa | rs12571125 | 0.001 | . | . | . | . | . | . | . | . | . | . |
| logepa | rs192418941 | 0 | . | . | . | . | . | . | . | . | . | . |
| logepa | rs141270243 | . | . | . | . | . | . | . | . | . | . | . |
| logepa | rs182615502 | . | . | . | . | . | . | . | . | . | . | . |
| logepa | rs146669856 | 0.02 | 0.997 | 0.228 | 0.989 | 0.446 | 0.305 | 0.238 | 0.906 | 0.671 | 0.894 | -0.04 |
| logepa | rs78290029 | 0.098 | 0.882 | 0.217 | 0.61 | 1.22 | 0.41 | 0.563 | 1.81 | 0.732 | 0.145 | 0.839 |
| logepa | rs139955565 | . | . | . | . | . | . | . | . | . | . | . |
| logepa | rs12267314 | 0.087 | 0.906 | 0.222 | 0.688 | 1.28 | 0.43 | 0.464 | 1.64 | 0.688 | 0.241 | 0.712 |
| logepa | rs74477197 | . | . | . | . | . | . | . | . | . | . | . |
| logepa | rs188147236 | . | . | . | . | . | . | . | . | . | . | . |
| logepa | rs2228065 | 0.086 | 0.91 | 0.223 | 0.702 | 1.27 | 0.43 | 0.475 | 1.61 | 0.691 | 0.266 | 0.685 |
| logepa | rs116961353 | . | . | . | . | . | . | . | . | . | . | . |
| logepa | rs190222272 | . | . | . | . | . | . | . | . | . | . | . |
| logepa | rs182719940 | . | . | . | . | . | . | . | . | . | . | . |
| logepa | rs184477413 | . | . | . | . | . | . | . | . | . | . | . |
| logepa | rs12265175 | 0.089 | 0.919 | 0.226 | 0.73 | 1.17 | 0.397 | 0.643 | 1.51 | 0.66 | 0.342 | 0.538 |
| logepa | rs182928276 | . | . | . | . | . | . | . | . | . | . | . |
| logepa | rs144409895 | . | . | . | . | . | . | . | . | . | . | . |
| logepa | rs58998447 | 0.003 | . | . | . | . | . | . | . | . | . | . |
| logepa | rs188668832 | . | . | . | . | . | . | . | . | . | . | . |
| logepa | rs148641686 | . | . | . | . | . | . | . | . | . | . | . |
| logepa | rs75468791 | . | . | . | . | . | . | . | . | . | . | . |
| logepa | rs147327397 | . | . | . | . | . | . | . | . | . | . | . |
| logepa | rs77354597 | 0.002 | . | . | . | . | . | . | . | . | . | . |
| logepa | rs41365544 | 0.082 | 0.931 | 0.229 | 0.772 | 1.33 | 0.445 | 0.398 | 1.46 | 0.732 | 0.447 | 0.55 |
| logepa | rs187564188 | . | . | . | . | . | . | . | . | . | . | . |
| logepa | rs183511984 | 0 | . | . | . | . | . | . | . | . | . | . |
| logepa | rs12262783 | 0.08 | 0.931 | 0.227 | 0.769 | 1.33 | 0.454 | 0.402 | 1.49 | 0.781 | 0.442 | 0.59 |
| logepa | rs188817687 | 0.002 | . | . | . | . | . | . | . | . | . | . |
| logepa | rs3815968 | . | . | . | . | . | . | . | . | . | . | . |
| logepa | rs187822300 | . | . | . | . | . | . | . | . | . | . | . |
| logepa | rs188902172 | 0.002 | . | . | . | . | . | . | . | . | . | . |
| logepa | rs181190448 | . | . | . | . | . | . | . | . | . | . | . |
| logepa | rs182699093 | . | . | . | . | . | . | . | . | . | . | . |
| logepa | rs182631303 | 0.001 | . | . | . | . | . | . | . | . | . | . |
| logepa | rs187014172 | . | . | . | . | . | . | . | . | . | . | . |
| logepa | rs142575406 | . | . | . | . | . | . | . | . | . | . | . |
| logepa | rs180711033 | . | . | . | . | . | . | . | . | . | . | . |
| logepa | rs137894823 | 0.004 | . | . | . | . | . | . | . | . | . | . |
| logepa | rs189053273 | 0.001 | . | . | . | . | . | . | . | . | . | . |
| logepa | rs143499388 | 0.002 | . | . | . | . | . | . | . | . | . | . |
| logepa | rs182936001 | 0.006 | . | . | . | . | . | . | . | . | . | . |
| logepa | rs148456406 | 0.006 | . | . | . | . | . | . | . | . | . | . |
| logepa | rs182499531 | 0.008 | . | . | . | . | . | . | . | . | . | . |
| logepa | rs74884915 | 0.026 | 0.999 | 0.233 | 0.996 | 0.205 | 0.242 | 0.18 | 2.07 | 0.845 | 0.074 | 0.221 |
| logepa | rs184046374 | 0.001 | . | . | . | . | . | . | . | . | . | . |
| logepa | rs74333993 | 0.032 | 0.956 | 0.223 | 0.846 | 1.28 | 0.625 | 0.617 | 1.66 | 1.02 | 0.406 | 0.798 |
| logepa | rs138889719 | 0.001 | . | . | . | . | . | . | . | . | . | . |
| logepa | rs75330330 | 0.022 | 0.99 | 0.224 | 0.963 | 0.966 | 0.732 | 0.963 | 1.5 | 2.82 | 0.831 | 0.474 |
| logepa | rs193051693 | 0 | . | . | . | . | . | . | . | . | . | . |
| logepa | rs145929633 | 0 | . | . | . | . | . | . | . | . | . | . |
| logepa | rs184076904 | 0.004 | . | . | . | . | . | . | . | . | . | . |
| logepa | rs141802494 | 0.018 | 0.994 | 0.221 | 0.979 | 0.472 | 0.658 | 0.591 | 0.952 | 3.01 | 0.988 | -0.02 |
| logepa | rs149519759 | 0.002 | . | . | . | . | . | . | . | . | . | . |
| logepa | rs145467260 | 0.013 | 0.988 | 0.228 | 0.957 | 1.14 | 0.803 | 0.856 | 1.46 | 0.84 | 0.51 | 0.515 |
| logepa | rs79620914 | 0.027 | 0.961 | 0.228 | 0.869 | 1.4 | 0.604 | 0.437 | 1.78 | 0.75 | 0.17 | 1.04 |
| logepa | rs140538633 | 0.017 | 1 | 0.231 | 0.994 | 0.314 | 0.365 | 0.319 | 1.88 | 0.88 | 0.179 | 0.275 |
| logepa | rs140862301 | 0.008 | . | . | . | . | . | . | . | . | . | . |
| logepa | rs114615847 | 0.009 | . | . | . | . | . | . | . | . | . | . |
| logepa | rs141800259 | 0.026 | 0.993 | 0.227 | 0.974 | 0.794 | 0.664 | 0.782 | 1.24 | 2.3 | 0.906 | 0.193 |
| logepa | rs149779059 | 0.019 | 0.988 | 0.225 | 0.957 | 1.95 | 1.15 | 0.259 | 1.16 | 1.48 | 0.908 | 0.293 |
| logepa | rs116539253 | 0.007 | . | . | . | . | . | . | . | . | . | . |
| logepa | rs184979550 | 0.001 | . | . | . | . | . | . | . | . | . | . |
| logepa | rs190128940 | 0.004 | . | . | . | . | . | . | . | . | . | . |
| logepa | rs146594816 | 0.002 | . | . | . | . | . | . | . | . | . | . |
| logepa | rs141370766 | 0.022 | 0.99 | 0.224 | 0.965 | 0.973 | 0.737 | 0.972 | 1.48 | 2.77 | 0.834 | 0.463 |
| logepa | rs144670033 | 0.002 | . | . | . | . | . | . | . | . | . | . |
| logepa | rs187152174 | 0.007 | . | . | . | . | . | . | . | . | . | . |
| logepa | rs184180263 | 0.005 | . | . | . | . | . | . | . | . | . | . |
| logepa | rs140075861 | 0.01 | 1.01 | 0.23 | 0.969 | 3.20E-13 | 6.80E-12 | 0.175 | 4.00E+07 | 6.00E+08 | 0.25 | -9.00E-03 |
| logepa | rs186483129 | 0 | . | . | . | . | . | . | . | . | . | . |
| logepa | rs186634282 | 0.012 | 0.987 | 0.226 | 0.956 | 0.516 | 0.563 | 0.544 | 1.37 | 0.658 | 0.509 | 0.196 |
| logepa | rs9988663 | 0.032 | 0.955 | 0.223 | 0.845 | 1.27 | 0.624 | 0.622 | 1.67 | 1.02 | 0.401 | 0.806 |
| logepa | rs138828529 | 0.02 | 1.02 | 0.24 | 0.928 | 2.36 | 1.39 | 0.145 | 0.644 | 0.663 | 0.669 | -0.831 |
| logepa | rs188777615 | 0.004 | . | . | . | . | . | . | . | . | . | . |
| logepa | rs192321882 | 0.008 | . | . | . | . | . | . | . | . | . | . |
| logepa | rs150812270 | 0.027 | 1.02 | 0.239 | 0.949 | 0.891 | 0.501 | 0.838 | 0.787 | 0.438 | 0.667 | -0.194 |
| logepa | rs185906603 | 0.001 | . | . | . | . | . | . | . | . | . | . |
| logepa | rs191070862 | 0.004 | . | . | . | . | . | . | . | . | . | . |
| logepa | rs182904978 | 0 | . | . | . | . | . | . | . | . | . | . |
| logepa | rs192679655 | 0.001 | . | . | . | . | . | . | . | . | . | . |

| logepa | rs189673086 | 0.007 | . | . | . | . | . | . | . | . | . | . |
| --- | --- | --- | --- | --- | --- | --- | --- | --- | --- | --- | --- | --- |
| logepa | rs181141892 | 0.002 | . | . | . | . | . | . | . | . | . | . |
| logepa | rs186404123 | 0 | . | . | . | . | . | . | . | . | . | . |
| logepa | rs192117287 | 0.005 | . | . | . | . | . | . | . | . | . | . |
| logepa | rs182748835 | 0.001 | . | . | . | . | . | . | . | . | . | . |
| logepa | rs141854549 | 0.017 | 1 | 0.231 | 0.993 | 0.319 | 0.365 | 0.318 | 1.85 | 0.856 | 0.182 | 0.271 |
| logepa | rs140163397 | 0.005 | . | . | . | . | . | . | . | . | . | . |
| logepa | rs146347300 | 0.008 | . | . | . | . | . | . | . | . | . | . |
| logepa | rs139362377 | 0.017 | 1 | 0.231 | 0.993 | 0.319 | 0.365 | 0.318 | 1.85 | 0.855 | 0.183 | 0.271 |
| logepa | rs185087671 | 0.001 | . | . | . | . | . | . | . | . | . | . |
| logepa | rs190466574 | 0.005 | . | . | . | . | . | . | . | . | . | . |
| logepa | rs147012778 | 0.005 | . | . | . | . | . | . | . | . | . | . |
| logepa | rs138236960 | 0.009 | . | . | . | . | . | . | . | . | . | . |
| logepa | rs116310311 | 0.017 | 1 | 0.231 | 0.993 | 0.319 | 0.365 | 0.318 | 1.85 | 0.855 | 0.183 | 0.271 |
| logepa | rs142868356 | 0.012 | 1 | 0.233 | 0.989 | 1.29 | 1 | 0.74 | 0.949 | 0.763 | 0.949 | -0.065 |
| logepa | rs144956240 | 0.003 | . | . | . | . | . | . | . | . | . | . |
| logepa | rs188527776 | 0.002 | . | . | . | . | . | . | . | . | . | . |
| logepa | rs183971165 | 0.007 | . | . | . | . | . | . | . | . | . | . |
| logepa | rs76748216 | 0.009 | . | . | . | . | . | . | . | . | . | . |
| logepa | rs148554990 | 0.001 | . | . | . | . | . | . | . | . | . | . |
| logepa | rs114446477 | 0.027 | 0.96 | 0.228 | 0.864 | 1.37 | 0.596 | 0.472 | 1.84 | 0.765 | 0.143 | 1.09 |
| logepa | rs185223933 | 0.006 | . | . | . | . | . | . | . | . | . | . |
| logepa | rs76869726 | 0.009 | . | . | . | . | . | . | . | . | . | . |
| logepa | rs151200830 | 0.017 | 1 | 0.231 | 0.989 | 0.299 | 0.358 | 0.313 | 1.89 | 0.894 | 0.177 | 0.265 |
| logepa | rs17157731 | 0.017 | 1 | 0.231 | 0.989 | 0.3 | 0.358 | 0.313 | 1.89 | 0.891 | 0.178 | 0.265 |
| logepa | rs181243469 | 0 | . | . | . | . | . | . | . | . | . | . |
| logepa | rs186890707 | 0 | . | . | . | . | . | . | . | . | . | . |
| logepa | rs184063360 | 0 | . | . | . | . | . | . | . | . | . | . |
| logepa | rs188550237 | 0.007 | . | . | . | . | . | . | . | . | . | . |
| logepa | rs140931068 | 0.017 | 0.959 | 0.223 | 0.856 | 2.78 | 1.68 | 0.093 | 2.23 | 2.87 | 0.533 | 3.2 |
| logepa | rs74867877 | 0.007 | . | . | . | . | . | . | . | . | . | . |
| logepa | rs11239495 | 0.055 | 0.935 | 0.221 | 0.777 | 1.19 | 0.471 | 0.659 | 1.82 | 1.12 | 0.332 | 0.897 |
| logepa | rs149745800 | 0 | . | . | . | . | . | . | . | . | . | . |
| logepa | rs145044458 | 0.015 | 1 | 0.231 | 0.988 | 0.313 | 0.379 | 0.338 | 1.83 | 0.86 | 0.201 | 0.258 |
| logepa | rs188067427 | 0.002 | . | . | . | . | . | . | . | . | . | . |
| logepa | rs146827800 | 0.001 | . | . | . | . | . | . | . | . | . | . |
| logepa | rs140619817 | 0.015 | 1.01 | 0.233 | 0.973 | 0.299 | 0.375 | 0.336 | 1.67 | 0.771 | 0.265 | 0.197 |
| logepa | rs892689 | 0.997 | . | . | . | . | . | . | . | . | . | . |
| logepa | rs143642005 | 0.008 | . | . | . | . | . | . | . | . | . | . |
| logepa | rs181929304 | 0.007 | . | . | . | . | . | . | . | . | . | . |
| logepa | rs191086173 | 0.002 | . | . | . | . | . | . | . | . | . | . |
| logepa | rs114613116 | 0.018 | 1.01 | 0.235 | 0.951 | 0.271 | 0.343 | 0.303 | 1.53 | 0.739 | 0.373 | 0.136 |
| logepa | rs141076768 | 0.002 | . | . | . | . | . | . | . | . | . | . |
| logepa | rs187316531 | 0 | . | . | . | . | . | . | . | . | . | . |
| logepa | rs11239499 | 0.075 | 0.955 | 0.233 | 0.849 | 0.841 | 0.31 | 0.639 | 1.43 | 0.755 | 0.493 | 0.356 |
| logepa | rs192726371 | 0.003 | . | . | . | . | . | . | . | . | . | . |
| logepa | rs74128481 | 0.019 | 0.981 | 0.233 | 0.935 | 0.518 | 0.401 | 0.396 | 1.39 | 0.625 | 0.467 | 0.206 |
| logepa | rs74128482 | 0.012 | 0.988 | 0.226 | 0.958 | 0.55 | 0.574 | 0.567 | 1.39 | 0.67 | 0.498 | 0.216 |
| logepa | rs11239502 | 0.055 | 0.942 | 0.224 | 0.802 | 1.09 | 0.454 | 0.827 | 1.7 | 1.1 | 0.41 | 0.721 |
| logepa | rs116330285 | 0.002 | . | . | . | . | . | . | . | . | . | . |
| logepa | rs189926729 | 0.003 | . | . | . | . | . | . | . | . | . | . |
| logepa | rs188975779 | 0.012 | 1 | 0.233 | 0.988 | 1.3 | 1 | 0.738 | 0.946 | 0.762 | 0.945 | -0.069 |
| logepa | rs143846038 | 0.002 | . | . | . | . | . | . | . | . | . | . |
| logepa | rs74128483 | 0.019 | 0.981 | 0.233 | 0.934 | 0.527 | 0.406 | 0.406 | 1.39 | 0.628 | 0.461 | 0.213 |
| logepa | rs139292055 | 0.073 | 0.962 | 0.233 | 0.873 | 0.84 | 0.306 | 0.632 | 1.38 | 0.713 | 0.536 | 0.311 |
| logepa | rs11239503 | 0.073 | 0.962 | 0.233 | 0.873 | 0.84 | 0.306 | 0.632 | 1.38 | 0.713 | 0.535 | 0.312 |
| logepa | rs58838686 | 0.012 | 0.988 | 0.226 | 0.958 | 0.552 | 0.575 | 0.568 | 1.39 | 0.669 | 0.498 | 0.216 |
| logepa | rs185825373 | 0.008 | . | . | . | . | . | . | . | . | . | . |
| logepa | rs11239504 | 0.073 | 0.962 | 0.233 | 0.873 | 0.84 | 0.306 | 0.632 | 1.38 | 0.713 | 0.535 | 0.312 |
| logepa | rs150012354 | 0.006 | . | . | . | . | . | . | . | . | . | . |
| logepa | rs187763096 | 0.01 | . | . | . | . | . | . | . | . | . | . |
| logepa | rs193043982 | 0.008 | . | . | . | . | . | . | . | . | . | . |
| logepa | rs79186884 | 0.023 | 0.992 | 0.226 | 0.973 | 1.65 | 0.846 | 0.328 | 1.08 | 1.12 | 0.941 | 0.125 |
| logepa | rs74128484 | 0.012 | 0.988 | 0.226 | 0.958 | 0.553 | 0.575 | 0.569 | 1.38 | 0.667 | 0.499 | 0.216 |
| logepa | rs58461537 | 0.073 | 0.962 | 0.233 | 0.873 | 0.839 | 0.306 | 0.631 | 1.38 | 0.713 | 0.534 | 0.312 |
| logepa | rs188527474 | 0.008 | . | . | . | . | . | . | . | . | . | . |
| logepa | rs141659976 | 0.008 | . | . | . | . | . | . | . | . | . | . |
| logepa | rs115846407 | 0.073 | 0.962 | 0.233 | 0.873 | 0.839 | 0.306 | 0.631 | 1.38 | 0.713 | 0.534 | 0.312 |
| logepa | rs138378816 | 0.073 | 0.962 | 0.233 | 0.873 | 0.839 | 0.306 | 0.631 | 1.38 | 0.713 | 0.534 | 0.312 |
| logepa | rs146188136 | 0.055 | 0.942 | 0.224 | 0.801 | 1.09 | 0.453 | 0.83 | 1.71 | 1.11 | 0.407 | 0.725 |
| logepa | rs139383871 | 0.014 | 0.999 | 0.231 | 0.998 | 2.35 | 1.46 | 0.172 | 0.935 | 1.17 | 0.957 | -0.153 |
| logepa | rs182953869 | 0.004 | . | . | . | . | . | . | . | . | . | . |
| logepa | rs192430299 | 0.019 | 1.01 | 0.234 | 0.955 | 0.821 | 0.566 | 0.775 | 0.73 | 0.52 | 0.659 | -0.227 |
| logepa | rs144152844 | 0.073 | 0.962 | 0.233 | 0.872 | 0.839 | 0.306 | 0.631 | 1.38 | 0.714 | 0.534 | 0.313 |
| logepa | rs145987786 | 0.051 | 0.96 | 0.22 | 0.857 | 0.971 | 0.406 | 0.944 | 1.47 | 1.04 | 0.582 | 0.442 |
| logepa | rs181132926 | 0.003 | . | . | . | . | . | . | . | . | . | . |
| logepa | rs147882253 | 0.073 | 0.962 | 0.233 | 0.872 | 0.839 | 0.306 | 0.63 | 1.38 | 0.713 | 0.534 | 0.312 |
| logepa | rs141807621 | 0.023 | 1.02 | 0.238 | 0.924 | 1.71 | 1.04 | 0.374 | 0.649 | 0.746 | 0.706 | -0.599 |
| logepa | rs147116052 | 0.073 | 0.962 | 0.233 | 0.872 | 0.839 | 0.306 | 0.63 | 1.38 | 0.713 | 0.534 | 0.312 |
| logepa | rs147716618 | 0.073 | 0.962 | 0.233 | 0.872 | 0.839 | 0.306 | 0.63 | 1.38 | 0.713 | 0.534 | 0.312 |
| logepa | rs187198855 | 0.046 | 0.966 | 0.223 | 0.882 | 0.873 | 0.395 | 0.764 | 1.43 | 0.992 | 0.603 | 0.37 |
| logepa | rs189409638 | 0.016 | 0.987 | 0.231 | 0.955 | 0.772 | 0.674 | 0.767 | 1.44 | 0.968 | 0.586 | 0.34 |
| logepa | rs182647339 | 0.001 | . | . | . | . | . | . | . | . | . | . |
| logepa | rs187995988 | 0.004 | . | . | . | . | . | . | . | . | . | . |
| logepa | rs141034184 | 0.079 | 0.955 | 0.233 | 0.85 | 0.778 | 0.285 | 0.493 | 1.45 | 0.755 | 0.473 | 0.346 |
| logepa | rs187310430 | 0.008 | . | . | . | . | . | . | . | . | . | . |
| logepa | rs192608488 | 0.073 | 0.963 | 0.233 | 0.875 | 0.837 | 0.304 | 0.626 | 1.37 | 0.707 | 0.538 | 0.307 |
| logepa | rs148840366 | 0.009 | . | . | . | . | . | . | . | . | . | . |
| logepa | rs186785022 | 0.019 | 0.98 | 0.233 | 0.932 | 0.559 | 0.427 | 0.447 | 1.42 | 0.639 | 0.44 | 0.237 |

| logepa | rs192348728 | 0.014 | 1.01 | 0.242 | 0.962 | 2.37 | 1.03 | 0.047 | 0.73 | 0.404 | 0.57 | -0.63 |
| --- | --- | --- | --- | --- | --- | --- | --- | --- | --- | --- | --- | --- |
| logepa | rs189656631 | 0.073 | 0.963 | 0.233 | 0.875 | 0.837 | 0.304 | 0.626 | 1.37 | 0.707 | 0.538 | 0.307 |
| logepa | rs185289750 | 0.004 | . | . | . | . | . | . | . | . | . | . |
| logepa | rs142382395 | 0.073 | 0.963 | 0.233 | 0.875 | 0.837 | 0.304 | 0.625 | 1.37 | 0.707 | 0.538 | 0.307 |
| logepa | rs144866696 | 0.019 | 0.98 | 0.233 | 0.932 | 0.561 | 0.429 | 0.449 | 1.42 | 0.64 | 0.44 | 0.238 |
| logepa | rs141371289 | 0.073 | 0.963 | 0.233 | 0.875 | 0.837 | 0.304 | 0.625 | 1.37 | 0.707 | 0.538 | 0.307 |
| logepa | rs114750008 | 0.073 | 0.963 | 0.233 | 0.875 | 0.837 | 0.304 | 0.625 | 1.37 | 0.707 | 0.538 | 0.307 |
| logepa | rs189063597 | 0.001 | . | . | . | . | . | . | . | . | . | . |
| logepa | rs139151571 | 0.067 | 0.954 | 0.232 | 0.847 | 0.828 | 0.322 | 0.626 | 1.48 | 0.817 | 0.478 | 0.387 |
| logepa | rs139846368 | 0.075 | 0.96 | 0.234 | 0.866 | 0.809 | 0.295 | 0.561 | 1.41 | 0.734 | 0.514 | 0.323 |
| logepa | rs182057653 | 0.019 | 1.01 | 0.237 | 0.96 | 0.371 | 0.33 | 0.265 | 0.865 | 0.689 | 0.856 | -0.058 |
| logepa | rs148785420 | 0.073 | 0.963 | 0.233 | 0.875 | 0.837 | 0.304 | 0.625 | 1.37 | 0.707 | 0.538 | 0.307 |
| logepa | rs138383251 | 0.011 | 1.03 | 0.238 | 0.882 | 6.10E-11 | 9.60E-10 | 0.135 | 0.068 | 0.788 | 0.817 | -0.035 |
| logepa | rs116526705 | 0.018 | 1.02 | 0.237 | 0.922 | 0.255 | 0.318 | 0.273 | 1.36 | 0.662 | 0.523 | 0.078 |
| logepa | rs185946436 | 0 | . | . | . | . | . | . | . | . | . | . |
| logepa | rs190455427 | 0.004 | . | . | . | . | . | . | . | . | . | . |
| logepa | rs9282584 | 0.073 | 0.963 | 0.233 | 0.875 | 0.837 | 0.304 | 0.625 | 1.37 | 0.707 | 0.538 | 0.307 |
| logepa | rs17153289 | 0.073 | 0.963 | 0.233 | 0.875 | 0.837 | 0.304 | 0.625 | 1.37 | 0.707 | 0.538 | 0.307 |
| logepa | rs138359103 | 0.008 | . | . | . | . | . | . | . | . | . | . |
| logepa | rs150605268 | 0.003 | . | . | . | . | . | . | . | . | . | . |
| logepa | rs79242910 | 0.075 | 0.959 | 0.233 | 0.863 | 0.821 | 0.3 | 0.589 | 1.41 | 0.718 | 0.505 | 0.327 |
| logepa | rs139716904 | 0.021 | 1.02 | 0.226 | 0.925 | 0.18 | 0.132 | 0.019 | 0.162 | 0.168 | 0.079 | -0.171 |
| logepa | rs76238498 | 0.053 | 0.939 | 0.216 | 0.784 | 1.06 | 0.43 | 0.889 | 1.73 | 1.2 | 0.427 | 0.723 |
| logepa | rs184140639 | 0 | . | . | . | . | . | . | . | . | . | . |
| logepa | rs188517278 | 0.009 | . | . | . | . | . | . | . | . | . | . |
| logepa | rs139678913 | 0.004 | . | . | . | . | . | . | . | . | . | . |
| logepa | rs116697955 | 0.004 | . | . | . | . | . | . | . | . | . | . |
| logepa | rs79267994 | 0.012 | 0.988 | 0.226 | 0.959 | 0.557 | 0.576 | 0.571 | 1.38 | 0.662 | 0.501 | 0.215 |
| logepa | rs11239510 | 0.022 | 1.04 | 0.242 | 0.86 | 0.728 | 0.566 | 0.683 | 0.315 | 0.297 | 0.22 | -0.531 |
| logepa | rs75831645 | 0.018 | 1.02 | 0.237 | 0.922 | 0.254 | 0.317 | 0.272 | 1.37 | 0.666 | 0.519 | 0.079 |
| logepa | rs78452276 | 0.052 | 0.95 | 0.232 | 0.835 | 1.04 | 0.396 | 0.914 | 1.68 | 0.71 | 0.222 | 0.667 |
| logepa | rs144366924 | 0 | . | . | . | . | . | . | . | . | . | . |
| logepa | rs77519841 | 0.056 | 0.942 | 0.224 | 0.8 | 1.09 | 0.451 | 0.837 | 1.71 | 1.11 | 0.405 | 0.725 |
| logepa | rs76944454 | 0.073 | 0.963 | 0.233 | 0.876 | 0.836 | 0.303 | 0.621 | 1.37 | 0.704 | 0.539 | 0.304 |
| logepa | rs76443855 | 0.053 | 0.938 | 0.216 | 0.782 | 1.06 | 0.431 | 0.881 | 1.74 | 1.2 | 0.421 | 0.736 |
| logepa | rs146613174 | 0.002 | . | . | . | . | . | . | . | . | . | . |
| logepa | rs181424761 | 0.002 | . | . | . | . | . | . | . | . | . | . |
| logepa | rs142119241 | 0.001 | . | . | . | . | . | . | . | . | . | . |
| logepa | rs140137954 | 0.018 | 1.02 | 0.237 | 0.921 | 0.256 | 0.318 | 0.273 | 1.36 | 0.658 | 0.529 | 0.076 |
| logepa | rs187319549 | 0.002 | . | . | . | . | . | . | . | . | . | . |
| logepa | rs12241545 | 0.035 | 1.03 | 0.242 | 0.896 | 0.451 | 0.313 | 0.251 | 0.468 | 0.386 | 0.357 | -0.264 |
| logepa | rs10128228 | 0.073 | 0.963 | 0.233 | 0.877 | 0.836 | 0.303 | 0.622 | 1.37 | 0.704 | 0.54 | 0.304 |
| logepa | rs58580249 | 0.009 | . | . | . | . | . | . | . | . | . | . |
| logepa | rs193148863 | 0.001 | . | . | . | . | . | . | . | . | . | . |
| logepa | rs185638946 | 0.005 | . | . | . | . | . | . | . | . | . | . |
| logepa | rs117752921 | 0.001 | . | . | . | . | . | . | . | . | . | . |
| logepa | rs180841236 | 0.002 | . | . | . | . | . | . | . | . | . | . |
| logepa | rs73283155 | 0.009 | . | . | . | . | . | . | . | . | . | . |
| logepa | rs76560599 | 0.061 | 0.948 | 0.232 | 0.826 | 0.787 | 0.332 | 0.57 | 1.64 | 0.831 | 0.325 | 0.492 |
| logepa | rs76273620 | 0.061 | 0.948 | 0.232 | 0.826 | 0.787 | 0.332 | 0.57 | 1.64 | 0.831 | 0.325 | 0.492 |
| logepa | rs141432923 | 0.02 | 0.963 | 0.218 | 0.867 | 0.354 | 0.325 | 0.258 | 20.1 | 17.6 | 6.10E-04 | 6.53 |
| logepa | rs113529322 | 0.019 | 0.98 | 0.233 | 0.931 | 0.585 | 0.444 | 0.48 | 1.43 | 0.647 | 0.427 | 0.256 |
| logepa | rs10128306 | 0.073 | 0.963 | 0.233 | 0.877 | 0.837 | 0.304 | 0.623 | 1.37 | 0.702 | 0.541 | 0.303 |
| logepa | rs149691688 | 0.002 | . | . | . | . | . | . | . | . | . | . |
| logepa | rs75872495 | 0.073 | 0.963 | 0.233 | 0.878 | 0.838 | 0.305 | 0.627 | 1.36 | 0.7 | 0.544 | 0.301 |
| logepa | rs191873960 | 0.007 | . | . | . | . | . | . | . | . | . | . |
| logepa | rs80155622 | 0.073 | 0.963 | 0.233 | 0.875 | 0.841 | 0.306 | 0.635 | 1.37 | 0.7 | 0.539 | 0.305 |
| logepa | rs113914656 | 0.008 | . | . | . | . | . | . | . | . | . | . |
| logepa | rs12242840 | 0.073 | 0.961 | 0.233 | 0.87 | 0.853 | 0.31 | 0.662 | 1.37 | 0.698 | 0.531 | 0.312 |
| logepa | rs147824078 | 0.009 | . | . | . | . | . | . | . | . | . | . |
| logepa | rs59051493 | 0.073 | 0.961 | 0.233 | 0.869 | 0.864 | 0.312 | 0.685 | 1.37 | 0.689 | 0.536 | 0.309 |
| logepa | rs9282588 | 0.008 | . | . | . | . | . | . | . | . | . | . |
| logepa | rs150215207 | 0.009 | . | . | . | . | . | . | . | . | . | . |
| logepa | rs138772243 | 0.003 | . | . | . | . | . | . | . | . | . | . |
| logepa | rs149383244 | 0.025 | 1.01 | 0.245 | 0.96 | 1.57 | 0.854 | 0.403 | 0.828 | 0.472 | 0.74 | -0.268 |
| logepa | rs17522720 | 0.016 | 1.01 | 0.242 | 0.962 | 2.23 | 1.04 | 0.084 | 0.741 | 0.428 | 0.603 | -0.571 |
| logepa | rs79245105 | 0.073 | 0.961 | 0.233 | 0.87 | 0.866 | 0.312 | 0.689 | 1.36 | 0.685 | 0.541 | 0.305 |
| logepa | rs138222133 | 0.004 | . | . | . | . | . | . | . | . | . | . |
| logepa | rs12247314 | 0.082 | 0.997 | 0.241 | 0.991 | 0.866 | 0.306 | 0.685 | 1.05 | 0.554 | 0.931 | 0.041 |
| logepa | rs12263894 | 0.08 | 0.998 | 0.242 | 0.993 | 0.884 | 0.313 | 0.728 | 1.04 | 0.547 | 0.941 | 0.035 |
| logepa | rs149907880 | 0.008 | . | . | . | . | . | . | . | . | . | . |
| logepa | rs188153885 | 0.007 | . | . | . | . | . | . | . | . | . | . |
| logepa | rs73283159 | 0.005 | . | . | . | . | . | . | . | . | . | . |
| logepa | rs140476687 | 0.002 | . | . | . | . | . | . | . | . | . | . |
| logepa | rs186603148 | 0 | . | . | . | . | . | . | . | . | . | . |
| logepa | rs183296063 | 0.003 | . | . | . | . | . | . | . | . | . | . |
| logepa | rs145733694 | 0.009 | . | . | . | . | . | . | . | . | . | . |
| logepa | rs189807449 | 0 | . | . | . | . | . | . | . | . | . | . |
| logepa | rs12253223 | 0.064 | 0.976 | 0.232 | 0.92 | 1.1 | 0.453 | 0.819 | 1.22 | 0.844 | 0.771 | 0.236 |
| logepa | rs182113637 | 0 | . | . | . | . | . | . | . | . | . | . |
| logepa | rs142792099 | 0.018 | 0.994 | 0.222 | 0.979 | 0.534 | 0.729 | 0.646 | 0.989 | 2.95 | 0.997 | -3.30E-03 |
| logepa | rs12249910 | 0.064 | 0.976 | 0.232 | 0.92 | 1.1 | 0.453 | 0.818 | 1.22 | 0.843 | 0.772 | 0.235 |
| logepa | rs149436339 | 0.001 | . | . | . | . | . | . | . | . | . | . |
| logepa | rs77632210 | 0 | . | . | . | . | . | . | . | . | . | . |
| logepa | rs74130818 | 0.012 | 0.987 | 0.226 | 0.956 | 0.468 | 0.511 | 0.487 | 1.36 | 0.666 | 0.527 | 0.174 |
| logepa | rs187583648 | 0 | . | . | . | . | . | . | . | . | . | . |
| logepa | rs191295987 | 0.008 | . | . | . | . | . | . | . | . | . | . |
| logepa | rs184200686 | 0.01 | . | . | . | . | . | . | . | . | . | . |
| logepa | rs76117263 | 0.052 | 0.935 | 0.216 | 0.772 | 1.07 | 0.443 | 0.867 | 1.79 | 1.24 | 0.399 | 0.789 |

| logepa | rs185393149 | 0.001 | . | . | . | . | . | . | . | . | . | . |
| --- | --- | --- | --- | --- | --- | --- | --- | --- | --- | --- | --- | --- |
| logepa | rs144760014 | 0.006 | . | . | . | . | . | . | . | . | . | . |
| logepa | rs7922292 | 0.008 | . | . | . | . | . | . | . | . | . | . |
| logepa | rs115372844 | 0.009 | . | . | . | . | . | . | . | . | . | . |
| logepa | rs111460364 | 0.052 | 0.935 | 0.216 | 0.77 | 1.08 | 0.445 | 0.858 | 1.8 | 1.24 | 0.395 | 0.8 |
| logepa | rs182052255 | 0.002 | . | . | . | . | . | . | . | . | . | . |
| logepa | rs180983889 | 0.006 | . | . | . | . | . | . | . | . | . | . |
| logepa | rs139175327 | 0.009 | . | . | . | . | . | . | . | . | . | . |
| logepa | rs149959620 | 0.018 | 1.02 | 0.227 | 0.915 | 0.165 | 0.132 | 0.024 | 0.13 | 0.139 | 0.057 | -0.167 |
| logepa | rs114653117 | 0.009 | . | . | . | . | . | . | . | . | . | . |
| logepa | rs144270018 | 0.005 | . | . | . | . | . | . | . | . | . | . |
| logepa | rs182723204 | 0.007 | . | . | . | . | . | . | . | . | . | . |
| logepa | rs57204067 | 0.012 | 0.987 | 0.226 | 0.954 | 0.408 | 0.458 | 0.425 | 1.35 | 0.668 | 0.546 | 0.148 |
| logepa | rs144956226 | 0.064 | 0.976 | 0.232 | 0.917 | 1.13 | 0.461 | 0.759 | 1.23 | 0.832 | 0.762 | 0.248 |
| logepa | rs187842704 | 0.019 | 0.976 | 0.223 | 0.915 | 0.02 | 0.023 | 5.60E-04 | 2.51 | 1.94 | 0.235 | 0.053 |
| logepa | rs184620511 | 0.013 | 0.966 | 0.221 | 0.879 | 0.143 | 0.225 | 0.216 | 6.23 | 6.82 | 0.095 | 0.751 |
| logepa | rs144031303 | 0.019 | 1.02 | 0.238 | 0.916 | 0.354 | 0.352 | 0.296 | 0.957 | 0.6 | 0.944 | -0.032 |
| logepa | rs187526473 | 0 | . | . | . | . | . | . | . | . | . | . |
| logepa | rs193002805 | 0.002 | . | . | . | . | . | . | . | . | . | . |
| logepa | rs138707426 | 0.024 | 1.01 | 0.245 | 0.954 | 1.62 | 0.863 | 0.361 | 0.825 | 0.467 | 0.735 | -0.279 |
| logepa | rs142754243 | 0.049 | 0.905 | 0.21 | 0.666 | 1.06 | 0.43 | 0.889 | 2.39 | 1.79 | 0.246 | 1.32 |
| logepa | rs181600331 | 0.008 | . | . | . | . | . | . | . | . | . | . |
| logepa | rs144421264 | 0.029 | 0.883 | 0.199 | 0.582 | 0.781 | 0.421 | 0.647 | 7.57 | 7.53 | 0.042 | 4.55 |
| logepa | rs184414519 | 0.006 | . | . | . | . | . | . | . | . | . | . |
| logepa | rs150596349 | 0.048 | 0.917 | 0.21 | 0.706 | 1.2 | 0.491 | 0.659 | 2.08 | 1.36 | 0.259 | 1.18 |
| logepa | rs190753747 | 0.001 | . | . | . | . | . | . | . | . | . | . |
| logepa | rs139560940 | 0.004 | . | . | . | . | . | . | . | . | . | . |
| logepa | rs75640804 | 0.057 | 0.937 | 0.225 | 0.788 | 1.2 | 0.478 | 0.641 | 1.68 | 0.992 | 0.378 | 0.757 |
| logepa | rs73283175 | 0.082 | 0.949 | 0.237 | 0.833 | 0.803 | 0.273 | 0.519 | 1.34 | 0.751 | 0.602 | 0.269 |
| logepa | rs143337229 | 0.003 | . | . | . | . | . | . | . | . | . | . |
| logepa | rs73283177 | 0.048 | 0.908 | 0.21 | 0.678 | 1.03 | 0.423 | 0.935 | 2.39 | 1.81 | 0.25 | 1.3 |
| logepa | rs76053611 | 0.045 | 0.95 | 0.212 | 0.818 | 0.858 | 0.379 | 0.729 | 1.74 | 1.2 | 0.426 | 0.608 |
| logepa | rs116325983 | 0.004 | . | . | . | . | . | . | . | . | . | . |
| logepa | rs115451825 | 0.008 | . | . | . | . | . | . | . | . | . | . |
| logepa | rs183845246 | 0.002 | . | . | . | . | . | . | . | . | . | . |
| logepa | rs79756542 | 0.051 | 0.915 | 0.208 | 0.697 | 1.21 | 0.48 | 0.627 | 2.04 | 1.27 | 0.252 | 1.14 |
| logepa | rs187242860 | 0.002 | . | . | . | . | . | . | . | . | . | . |
| logepa | rs57318980 | 0.048 | 0.914 | 0.209 | 0.693 | 0.953 | 0.398 | 0.908 | 2.45 | 1.88 | 0.243 | 1.27 |
| logepa | rs59969732 | 0.043 | 0.903 | 0.207 | 0.658 | 0.738 | 0.328 | 0.495 | 3.54 | 2.79 | 0.109 | 1.72 |
| logepa | rs143811488 | 0.009 | . | . | . | . | . | . | . | . | . | . |
| logepa | rs189067644 | 0.01 | . | . | . | . | . | . | . | . | . | . |
| logepa | rs60449178 | 0.038 | 0.897 | 0.202 | 0.631 | 0.641 | 0.339 | 0.401 | 7.11 | 7.57 | 0.066 | 3.55 |
| logepa | rs59243389 | 0.038 | 0.898 | 0.202 | 0.631 | 0.641 | 0.339 | 0.4 | 7.1 | 7.57 | 0.066 | 3.55 |
| logepa | rs184946540 | 0.043 | 0.997 | 0.22 | 0.989 | 0.458 | 0.249 | 0.152 | 1.22 | 1.3 | 0.853 | 0.102 |
| logepa | rs188685881 | 0.001 | . | . | . | . | . | . | . | . | . | . |
| logepa | rs150335797 | 0.006 | . | . | . | . | . | . | . | . | . | . |
| logepa | rs143879260 | 0.006 | . | . | . | . | . | . | . | . | . | . |
| logepa | rs186044491 | 0.009 | . | . | . | . | . | . | . | . | . | . |
| logepa | rs11819084 | 0.03 | 0.922 | 0.223 | 0.736 | 1.99 | 0.854 | 0.109 | 1.78 | 1.06 | 0.337 | 1.34 |
| logepa | rs146915670 | 0.006 | . | . | . | . | . | . | . | . | . | . |
| logepa | rs113995695 | 0.029 | 0.915 | 0.222 | 0.713 | 2 | 0.859 | 0.107 | 1.86 | 1.13 | 0.307 | 1.49 |
| logepa | rs143489451 | 0.005 | . | . | . | . | . | . | . | . | . | . |
| logepa | rs182653839 | 0 | . | . | . | . | . | . | . | . | . | . |
| logepa | rs141692564 | 0.007 | . | . | . | . | . | . | . | . | . | . |
| logepa | rs146281742 | 0.005 | . | . | . | . | . | . | . | . | . | . |
| logepa | rs192914353 | 0.005 | . | . | . | . | . | . | . | . | . | . |
| logepa | rs185182848 | 0.003 | . | . | . | . | . | . | . | . | . | . |
| logepa | rs28395858 | 0.029 | 0.911 | 0.221 | 0.702 | 2.02 | 0.868 | 0.1 | 1.88 | 1.16 | 0.302 | 1.54 |
| logepa | rs41429645 | 0.001 | . | . | . | . | . | . | . | . | . | . |
| logepa | rs28395874 | 0.029 | 0.911 | 0.221 | 0.7 | 2.03 | 0.869 | 0.099 | 1.88 | 1.16 | 0.303 | 1.54 |
| logepa | rs189663634 | 0.001 | . | . | . | . | . | . | . | . | . | . |
| logepa | rs77666700 | 0.01 | . | . | . | . | . | . | . | . | . | . |
| logepa | rs146711717 | 0.003 | . | . | . | . | . | . | . | . | . | . |
| logepa | rs144904251 | 0.001 | . | . | . | . | . | . | . | . | . | . |
| logepa | rs189893153 | 0.001 | . | . | . | . | . | . | . | . | . | . |
| logepa | rs150447740 | 0.009 | . | . | . | . | . | . | . | . | . | . |
| logepa | rs143099649 | 0.001 | . | . | . | . | . | . | . | . | . | . |
| logepa | rs191288436 | 0.01 | 1.03 | 0.237 | 0.906 | 1.47 | 0.809 | 0.481 | 0.333 | 0.289 | 0.205 | -0.996 |
| logepa | rs188577862 | 0.006 | . | . | . | . | . | . | . | . | . | . |
| logepa | rs192695334 | 0.006 | . | . | . | . | . | . | . | . | . | . |
| logepa | rs138612906 | 0.036 | 0.922 | 0.226 | 0.739 | 1.47 | 0.612 | 0.353 | 1.62 | 0.865 | 0.362 | 0.81 |
| logepa | rs191290051 | 0.037 | 0.97 | 0.211 | 0.89 | 0.826 | 0.457 | 0.73 | 1.76 | 2.56 | 0.698 | 0.614 |
| logepa | rs189631459 | 0.001 | . | . | . | . | . | . | . | . | . | . |
| logepa | rs114620874 | 0.007 | . | . | . | . | . | . | . | . | . | . |
| logepa | rs78246652 | 0.023 | 1.01 | 0.226 | 0.964 | 0.776 | 0.644 | 0.76 | 0.73 | 1.06 | 0.828 | -0.214 |
| logepa | rs79432451 | 0.007 | . | . | . | . | . | . | . | . | . | . |
| logepa | rs186022585 | 0.001 | . | . | . | . | . | . | . | . | . | . |
| logepa | rs142978891 | 0.009 | . | . | . | . | . | . | . | . | . | . |
| logepa | rs151086840 | 0.011 | 1.03 | 0.23 | 0.903 | 5.10E-12 | 6.90E-11 | 0.053 | 6.40E-12 | 7.40E-11 | 0.026 | -0.028 |
| logepa | rs183270259 | 0.01 | . | . | . | . | . | . | . | . | . | . |
| logepa | rs188544289 | 0.01 | . | . | . | . | . | . | . | . | . | . |
| logepa | rs188500808 | 0.001 | . | . | . | . | . | . | . | . | . | . |
| logepa | rs189424589 | 0.003 | . | . | . | . | . | . | . | . | . | . |
| logepa | rs78622303 | 0.04 | 0.904 | 0.2 | 0.647 | 0.678 | 0.407 | 0.517 | 5.85 | 5.88 | 0.079 | 3 |
| logepa | rs143210835 | 0.013 | 0.978 | 0.215 | 0.92 | 0.809 | 0.996 | 0.863 | 5.53 | 20.7 | 0.648 | 3.59 |
| logepa | rs114761840 | 0.007 | . | . | . | . | . | . | . | . | . | . |
| logepa | rs185805402 | 0.003 | . | . | . | . | . | . | . | . | . | . |
| logepa | rs139273744 | 0.002 | . | . | . | . | . | . | . | . | . | . |
| logepa | rs184654150 | 0.006 | . | . | . | . | . | . | . | . | . | . |

| logepa | rs189069710 | 0 | . | . | . | . | . | . | . | . | . | . |
| --- | --- | --- | --- | --- | --- | --- | --- | --- | --- | --- | --- | --- |
| logepa | rs114752115 | 0.031 | 0.999 | 0.23 | 0.998 | 0.789 | 0.482 | 0.698 | 0.947 | 0.889 | 0.954 | -0.042 |
| logepa | rs150162515 | 0.002 | . | . | . | . | . | . | . | . | . | . |
| logepa | rs180982454 | 0.008 | . | . | . | . | . | . | . | . | . | . |
| logepa | rs184932088 | 0.001 | . | . | . | . | . | . | . | . | . | . |
| logepa | rs181109625 | 0 | . | . | . | . | . | . | . | . | . | . |
| logepa | rs138121134 | 0.001 | . | . | . | . | . | . | . | . | . | . |
| logepa | rs183253861 | 0.004 | . | . | . | . | . | . | . | . | . | . |
| logepa | rs147073946 | 0.001 | . | . | . | . | . | . | . | . | . | . |
| logepa | rs139938489 | 0.008 | . | . | . | . | . | . | . | . | . | . |
| logepa | rs146362560 | 0.013 | 1 | 0.232 | 0.994 | 0.915 | 0.945 | 0.932 | 0.978 | 0.68 | 0.975 | -0.02 |
| logepa | rs193155956 | 0.007 | . | . | . | . | . | . | . | . | . | . |
| logepa | rs77663092 | 0.007 | . | . | . | . | . | . | . | . | . | . |
| logepa | rs73285319 | 0.033 | 1 | 0.245 | 0.993 | 1.8 | 0.836 | 0.206 | 0.886 | 0.444 | 0.809 | -0.203 |
| logepa | rs184019276 | 0.007 | . | . | . | . | . | . | . | . | . | . |
| logepa | rs28395863 | 0.027 | 0.905 | 0.222 | 0.684 | 2.11 | 0.9 | 0.079 | 1.86 | 1.06 | 0.278 | 1.54 |
| logepa | rs182121645 | 0.017 | 1.04 | 0.238 | 0.874 | 1.07 | 0.666 | 0.912 | 0.381 | 0.363 | 0.311 | -0.685 |
| logepa | rs73285327 | 0.025 | 1 | 0.24 | 0.99 | 1.34 | 0.751 | 0.598 | 0.92 | 0.461 | 0.868 | -0.107 |
| logepa | rs149589584 | 0.001 | . | . | . | . | . | . | . | . | . | . |
| logepa | rs148310335 | 0.005 | . | . | . | . | . | . | . | . | . | . |
| logepa | rs187246093 | 0.01 | 0.997 | 0.225 | 0.989 | 0.235 | 0.243 | 0.161 | 1.59 | 1.3 | 0.567 | 0.142 |
| logepa | rs184668894 | 0 | . | . | . | . | . | . | . | . | . | . |
| logepa | rs114269961 | 0.001 | . | . | . | . | . | . | . | . | . | . |
| logepa | rs149694374 | 0.034 | 0.903 | 0.22 | 0.675 | 1.68 | 0.75 | 0.248 | 2.16 | 1.28 | 0.194 | 1.69 |
| logepa | rs145284227 | 0.004 | . | . | . | . | . | . | . | . | . | . |
| logepa | rs189983820 | 0.004 | . | . | . | . | . | . | . | . | . | . |
| logepa | rs181200444 | 0 | . | . | . | . | . | . | . | . | . | . |
| logepa | rs185486385 | 0 | . | . | . | . | . | . | . | . | . | . |
| logepa | rs151198241 | 0.01 | 1.02 | 0.227 | 0.937 | 2.00E-06 | 7.10E-06 | 2.30E-04 | 5.60E-07 | 2.00E-06 | 9.10E-05 | -0.018 |
| logepa | rs189959341 | 0.01 | 1.02 | 0.227 | 0.937 | 2.00E-06 | 7.10E-06 | 2.30E-04 | 5.50E-07 | 2.00E-06 | 9.10E-05 | -0.018 |
| logepa | rs140549257 | 0.007 | . | . | . | . | . | . | . | . | . | . |
| logepa | rs186453139 | 0.027 | 0.999 | 0.246 | 0.996 | 2.16 | 0.965 | 0.084 | 0.902 | 0.446 | 0.835 | -0.212 |
| logepa | rs180922711 | 0.044 | 0.963 | 0.233 | 0.877 | 1.61 | 0.607 | 0.208 | 1.36 | 0.634 | 0.513 | 0.531 |
| logepa | rs56659903 | 0.005 | . | . | . | . | . | . | . | . | . | . |
| logepa | rs141202128 | 0.006 | . | . | . | . | . | . | . | . | . | . |
| logepa | rs59085314 | 0.056 | 0.958 | 0.225 | 0.856 | 0.57 | 0.261 | 0.22 | 1.88 | 0.824 | 0.149 | 0.499 |
| logepa | rs145564996 | 0.004 | . | . | . | . | . | . | . | . | . | . |
| logepa | rs114961009 | 0.01 | 1.02 | 0.228 | 0.934 | 2.30E-06 | 8.20E-06 | 2.80E-04 | 6.40E-07 | 2.30E-06 | 9.50E-05 | -0.019 |
| logepa | rs182294189 | 0.004 | . | . | . | . | . | . | . | . | . | . |
| logepa | rs187586636 | 0.008 | . | . | . | . | . | . | . | . | . | . |
| logepa | rs192254907 | 0.002 | . | . | . | . | . | . | . | . | . | . |
| logepa | rs149145663 | 0.001 | . | . | . | . | . | . | . | . | . | . |
| logepa | rs180713064 | 0.001 | . | . | . | . | . | . | . | . | . | . |
| logepa | rs183009023 | 0.007 | . | . | . | . | . | . | . | . | . | . |
| logepa | rs143385347 | 0.017 | 1.04 | 0.239 | 0.862 | 1.03 | 0.651 | 0.965 | 0.338 | 0.32 | 0.252 | -0.707 |
| logepa | rs185786329 | 0.007 | . | . | . | . | . | . | . | . | . | . |
| logepa | rs192341030 | 0.004 | . | . | . | . | . | . | . | . | . | . |
| logepa | rs74661429 | 0.017 | 0.979 | 0.225 | 0.925 | 0.097 | 0.1 | 0.024 | 1.58 | 1.05 | 0.493 | 0.074 |
| logepa | rs188034853 | 0.002 | . | . | . | . | . | . | . | . | . | . |
| logepa | rs116808430 | 0.027 | 1.01 | 0.249 | 0.98 | 2.26 | 0.995 | 0.065 | 0.841 | 0.415 | 0.725 | -0.353 |
| logepa | rs148469010 | 0 | . | . | . | . | . | . | . | . | . | . |
| logepa | rs78621663 | 0.01 | . | . | . | . | . | . | . | . | . | . |
| logepa | rs192654886 | 0.001 | . | . | . | . | . | . | . | . | . | . |
| logepa | rs145996035 | 0.008 | . | . | . | . | . | . | . | . | . | . |
| logepa | rs139942226 | 0.001 | . | . | . | . | . | . | . | . | . | . |
| logepa | rs113645632 | 0.01 | 1.01 | 0.23 | 0.967 | 0.955 | 0.931 | 0.962 | 0.587 | 0.591 | 0.597 | -0.398 |
| logepa | rs74940661 | 0.027 | 0.917 | 0.219 | 0.718 | 1.96 | 0.887 | 0.139 | 2.2 | 1.52 | 0.257 | 2.07 |
| logepa | rs115355800 | 0.022 | 1 | 0.224 | 0.996 | 0.7 | 0.617 | 0.686 | 0.83 | 1.31 | 0.906 | -0.119 |
| logepa | rs150285898 | 0.008 | . | . | . | . | . | . | . | . | . | . |
| logepa | rs188117932 | 0.004 | . | . | . | . | . | . | . | . | . | . |
| logepa | rs139956566 | . | . | . | . | . | . | . | . | . | . | . |
| logepa | rs147673885 | . | . | . | . | . | . | . | . | . | . | . |
| logepa | rs189127928 | . | . | . | . | . | . | . | . | . | . | . |
| logepa | rs187558772 | . | . | . | . | . | . | . | . | . | . | . |
| logepa | rs181948587 | . | . | . | . | . | . | . | . | . | . | . |
| logepa | rs189983269 | . | . | . | . | . | . | . | . | . | . | . |
| logepa | rs187250069 | . | . | . | . | . | . | . | . | . | . | . |
| logepa | rs191485859 | . | . | . | . | . | . | . | . | . | . | . |
| logepa | rs185499555 | . | . | . | . | . | . | . | . | . | . | . |
| logepa | rs180862110 | . | . | . | . | . | . | . | . | . | . | . |
| logepa | rs190472603 | . | . | . | . | . | . | . | . | . | . | . |
| logepa | rs189226167 | . | . | . | . | . | . | . | . | . | . | . |
| logepa | rs181623256 | . | . | . | . | . | . | . | . | . | . | . |
| logepa | rs186079557 | . | . | . | . | . | . | . | . | . | . | . |
| logepa | rs147760994 | . | . | . | . | . | . | . | . | . | . | . |
| logepa | rs189176167 | . | . | . | . | . | . | . | . | . | . | . |
| logepa | rs117901000 | . | . | . | . | . | . | . | . | . | . | . |
| logepa | rs141984888 | . | . | . | . | . | . | . | . | . | . | . |
| logepa | rs192365061 | . | . | . | . | . | . | . | . | . | . | . |
| logepa | rs185976038 | . | . | . | . | . | . | . | . | . | . | . |
| logepa | rs184697303 | . | . | . | . | . | . | . | . | . | . | . |
| logepa | rs143383132 | . | . | . | . | . | . | . | . | . | . | . |
| logepa | rs141873881 | . | . | . | . | . | . | . | . | . | . | . |
| logepa | rs185507255 | . | . | . | . | . | . | . | . | . | . | . |
| logepa | rs191379425 | . | . | . | . | . | . | . | . | . | . | . |
| logepa | rs184199584 | . | . | . | . | . | . | . | . | . | . | . |
| logepa | rs183181360 | . | . | . | . | . | . | . | . | . | . | . |
| logepa | rs184495673 | . | . | . | . | . | . | . | . | . | . | . |
| logepa | rs182946297 | . | . | . | . | . | . | . | . | . | . | . |

| logepa | rs187743615 | . | . | . | . | . | . | . | . | . | . | . |
| --- | --- | --- | --- | --- | --- | --- | --- | --- | --- | --- | --- | --- |
| logepa | rs183259862 | . | . | . | . | . | . | . | . | . | . | . |
| logepa | rs112433566 | . | . | . | . | . | . | . | . | . | . | . |
| logepa | rs182351085 | . | . | . | . | . | . | . | . | . | . | . |
| logepa | rs146248508 | . | . | . | . | . | . | . | . | . | . | . |
| logepa | rs188528439 | . | . | . | . | . | . | . | . | . | . | . |
| logepa | rs146167018 | . | . | . | . | . | . | . | . | . | . | . |
| logepa | rs192440980 | . | . | . | . | . | . | . | . | . | . | . |
| logepa | rs185601882 | . | . | . | . | . | . | . | . | . | . | . |
| logepa | rs117272563 | . | . | . | . | . | . | . | . | . | . | . |
| logepa | rs10430734 | . | . | . | . | . | . | . | . | . | . | . |
| logepa | rs181320952 | . | . | . | . | . | . | . | . | . | . | . |
| logepa | rs185480660 | . | . | . | . | . | . | . | . | . | . | . |
| logepa | rs188077917 | . | . | . | . | . | . | . | . | . | . | . |
| logepa | rs187562953 | . | . | . | . | . | . | . | . | . | . | . |
| logepa | rs182044631 | . | . | . | . | . | . | . | . | . | . | . |
| logepa | rs145455596 | . | . | . | . | . | . | . | . | . | . | . |
| logepa | rs181475877 | . | . | . | . | . | . | . | . | . | . | . |
| logepa | rs149413085 | . | . | . | . | . | . | . | . | . | . | . |
| logepa | rs12253656 | . | . | . | . | . | . | . | . | . | . | . |
| logepa | rs182718239 | . | . | . | . | . | . | . | . | . | . | . |
| logepa | rs192435695 | . | . | . | . | . | . | . | . | . | . | . |
| logepa | rs12248277 | . | . | . | . | . | . | . | . | . | . | . |
| logepa | rs143521710 | . | . | . | . | . | . | . | . | . | . | . |
| logepa | rs144454454 | . | . | . | . | . | . | . | . | . | . | . |
| logepa | rs190441610 | . | . | . | . | . | . | . | . | . | . | . |
| logepa | rs114690462 | . | . | . | . | . | . | . | . | . | . | . |
| logepa | rs184541357 | . | . | . | . | . | . | . | . | . | . | . |
| logepa | rs142657653 | . | . | . | . | . | . | . | . | . | . | . |
| logepa | rsurf1 | 2.117 | . | . | . | . | . | . | . | . | . | . |
| logepa | rsten1 | 2.28 | . | . | . | . | . | . | . | . | . | . |
| logepa | chr10:45852312:D | 0.092 | 1 | 0.222 | 0.997 | 1.08 | 0.3 | 0.789 | 1 | 0.438 | 0.998 | 1.50E-03 |
| logepa | chr10:45853232:I | 0.108 | 0.978 | 0.22 | 0.922 | 0.939 | 0.276 | 0.829 | 1.1 | 0.465 | 0.826 | 0.091 |
| logepa | chr10:45853234:I | 0.198 | 1.03 | 0.256 | 0.904 | 0.808 | 0.198 | 0.385 | 0.923 | 0.311 | 0.811 | -0.07 |
| logepa | chr10:45854796:D | 0.003 | . | . | . | . | . | . | . | . | . | . |
| logepa | chr10:45854994:I | 0.025 | 0.983 | 0.225 | 0.941 | 1.2 | 0.776 | 0.78 | 1.83 | 2.95 | 0.71 | 0.969 |
| logepa | chr10:45858290:D | 0.052 | 1.12 | 0.268 | 0.651 | 0.946 | 0.461 | 0.909 | 0.436 | 0.295 | 0.22 | -0.6 |
| logepa | chr10:45858820:I | 0.022 | 1.05 | 0.24 | 0.832 | 0.693 | 0.558 | 0.649 | 0.298 | 0.243 | 0.138 | -0.526 |
| logepa | chr10:45860888:D | 0.122 | 1 | 0.226 | 0.988 | 0.971 | 0.306 | 0.925 | 0.994 | 0.546 | 0.991 | -5.90E-03 |
| logepa | chr10:45863913:I | 0.179 | 1.01 | 0.25 | 0.968 | 0.882 | 0.21 | 0.597 | 0.977 | 0.317 | 0.942 | -0.022 |
| logepa | chr10:45866601:D | 0.111 | 1.01 | 0.251 | 0.971 | 0.715 | 0.252 | 0.34 | 0.938 | 0.494 | 0.903 | -0.047 |
| logepa | chr10:45867197:I | 0.002 | . | . | . | . | . | . | . | . | . | . |
| logepa | chr10:45867538:D | 0.182 | 1.03 | 0.251 | 0.904 | 0.87 | 0.205 | 0.555 | 0.929 | 0.302 | 0.821 | -0.068 |
| logepa | chr10:45868356:D | 0.18 | 1.03 | 0.251 | 0.917 | 0.899 | 0.209 | 0.647 | 0.939 | 0.3 | 0.845 | -0.058 |
| logepa | chr10:45871158:I | 0.182 | 1.03 | 0.251 | 0.911 | 0.886 | 0.205 | 0.6 | 0.935 | 0.299 | 0.833 | -0.062 |
| logepa | chr10:45871637:D | 0.127 | 0.975 | 0.254 | 0.923 | 0.858 | 0.257 | 0.609 | 1.09 | 0.412 | 0.819 | 0.079 |
| logepa | chr10:45871638:I | 0.164 | 0.979 | 0.249 | 0.934 | 0.872 | 0.22 | 0.586 | 1.06 | 0.377 | 0.864 | 0.056 |
| logepa | chr10:45873629:I | 0.154 | 1.05 | 0.246 | 0.827 | 1 | 0.233 | 0.997 | 0.869 | 0.301 | 0.684 | -0.138 |
| logepa | chr10:45873701:D | 0.055 | 0.941 | 0.223 | 0.798 | 1.23 | 0.514 | 0.627 | 1.7 | 1.04 | 0.387 | 0.794 |
| logepa | chr10:45874997:I | 0.011 | 1.03 | 0.232 | 0.911 | 2.50E-06 | 2.20E-05 | 0.151 | 7.30E-04 | 4.70E-03 | 0.266 | -0.026 |
| logepa | chr10:45882030:I | 0.096 | 1.01 | 0.254 | 0.98 | 0.971 | 0.323 | 0.929 | 0.983 | 0.45 | 0.971 | -0.016 |
| logepa | chr10:45883774:D | 0.078 | 1.06 | 0.258 | 0.817 | 1.01 | 0.386 | 0.984 | 0.694 | 0.372 | 0.495 | -0.326 |
| logepa | chr10:45885169:D | 0.075 | 0.952 | 0.207 | 0.82 | 1.21 | 0.387 | 0.545 | 1.31 | 0.722 | 0.627 | 0.345 |
| logepa | chr10:45886451:D | 0.736 | 0.758 | 0.414 | 0.612 | 0.968 | 0.223 | 0.888 | 1.21 | 0.421 | 0.578 | 0.164 |
| logepa | chr10:45891800:I | 0.768 | 1.23 | 0.533 | 0.629 | 0.806 | 0.153 | 0.254 | 0.86 | 0.242 | 0.592 | -0.184 |
| logepa | chr10:45904771:I | 0.772 | 1.33 | 0.554 | 0.49 | 0.785 | 0.151 | 0.207 | 0.812 | 0.228 | 0.459 | -0.268 |
| logepa | chr10:45904959:I | 0.156 | 1.11 | 0.29 | 0.681 | 1.28 | 0.35 | 0.36 | 0.738 | 0.292 | 0.443 | -0.342 |
| logepa | chr10:45911028:I | 0.161 | 0.998 | 0.276 | 0.993 | 0.852 | 0.231 | 0.556 | 1.01 | 0.481 | 0.978 | 0.011 |
| logepa | chr10:45911031:I | 0.259 | 1.32 | 0.304 | 0.233 | 0.815 | 0.213 | 0.434 | 0.509 | 0.191 | 0.072 | -0.586 |
| logepa | chr10:45912154:D | 0.104 | 1.09 | 0.265 | 0.716 | 1.16 | 0.357 | 0.634 | 0.683 | 0.292 | 0.373 | -0.386 |
| logepa | chr10:45913885:I | 0.003 | . | . | . | . | . | . | . | . | . | . |
| logepa | chr10:45914753:D | 0.351 | 0.917 | 0.291 | 0.784 | 1.2 | 0.244 | 0.36 | 1.14 | 0.381 | 0.687 | 0.142 |
| logepa | chr10:45919621 | . | . | . | . | . | . | . | . | . | . | . |
| logepa | chr10:45921739:I | 0.178 | 0.808 | 0.229 | 0.453 | 1.2 | 0.273 | 0.423 | 1.74 | 0.621 | 0.124 | 0.674 |
| logepa | chr10:45923527:I | 0.066 | 1.09 | 0.265 | 0.711 | 0.596 | 0.291 | 0.289 | 0.407 | 0.274 | 0.181 | -0.424 |
| logepa | chr10:45927001:I | 0.04 | 0.984 | 0.223 | 0.942 | 0.656 | 0.44 | 0.53 | 1.18 | 1.6 | 0.903 | 0.122 |
| logepa | chr10:45930354:I | 0.025 | 1.01 | 0.229 | 0.967 | 1.06 | 0.706 | 0.931 | 0.895 | 1.04 | 0.924 | -0.112 |
| logepa | chr10:45932864:I | 0.043 | 1.14 | 0.276 | 0.599 | 2.36 | 1.26 | 0.107 | 0.431 | 0.31 | 0.242 | -1.34 |
| logepa | chr10:45936163:I | 0.038 | 1.03 | 0.235 | 0.911 | 1.09 | 0.622 | 0.876 | 0.722 | 0.722 | 0.745 | -0.31 |
| logepa | chr10:45938095:D | 0.179 | 1.01 | 0.253 | 0.957 | 0.984 | 0.245 | 0.947 | 0.961 | 0.409 | 0.925 | -0.039 |
| logepa | chr10:45938886 | . | . | . | . | . | . | . | . | . | . | . |
| logepa | chr10:45939320 | 0.001 | . | . | . | . | . | . | . | . | . | . |
| logepa | chr10:45939659 | 0.003 | . | . | . | . | . | . | . | . | . | . |
| logepa | chr10:45941150:D | 0.008 | . | . | . | . | . | . | . | . | . | . |
| logepa | chr10:45947128:D | 0.067 | 0.974 | 0.253 | 0.919 | 1.66 | 0.542 | 0.12 | 1.11 | 0.474 | 0.815 | 0.154 |
| logepa | chr10:45950990:I | 0.039 | 0.963 | 0.23 | 0.876 | 1.62 | 0.89 | 0.376 | 1.57 | 1.09 | 0.515 | 0.871 |
| logepa | chr10:45951854:I | 0.012 | 1.01 | 0.234 | 0.974 | 1.74 | 1.06 | 0.366 | 0.637 | 0.457 | 0.53 | -0.631 |
| logepa | chr10:45952670:I | 0.061 | 1.03 | 0.242 | 0.896 | 0.742 | 0.32 | 0.488 | 0.721 | 0.455 | 0.604 | -0.222 |
| logepa | chr10:45956741 | . | . | . | . | . | . | . | . | . | . | . |
| logepa | chr10:45957247:I | 0.198 | 1.09 | 0.276 | 0.743 | 1.07 | 0.244 | 0.758 | 0.81 | 0.317 | 0.59 | -0.215 |
| logepa | chr10:45958082:I | 0.188 | 1.08 | 0.269 | 0.754 | 1.07 | 0.265 | 0.8 | 0.814 | 0.352 | 0.634 | -0.209 |
| logepa | chr10:45905041:D | 0.068 | 0.91 | 0.216 | 0.693 | 1.04 | 0.385 | 0.914 | 1.92 | 1.1 | 0.256 | 0.866 |
| logepa | chr10:45938871 | . | . | . | . | . | . | . | . | . | . | . |
| logepa | chr10:45939160 | . | . | . | . | . | . | . | . | . | . | . |
| logepa | chr10:45869910 | 0.015 | 1 | 0.23 | 0.999 | 3.00E-04 | 1.90E-03 | 0.213 | 16.6 | 56.7 | 0.41 | 4.90E-03 |
| logepa | chr10:45876214:D | 0.017 | 1 | 0.231 | 0.988 | 0.3 | 0.358 | 0.313 | 1.88 | 0.881 | 0.18 | 0.261 |
| logepa | chr10:45880237:I | 0.094 | 1.06 | 0.253 | 0.793 | 0.426 | 0.19 | 0.056 | 0.496 | 0.295 | 0.239 | -0.266 |
| logepa | chr10:45884371:I | 0.073 | 0.962 | 0.233 | 0.872 | 0.839 | 0.306 | 0.631 | 1.38 | 0.713 | 0.534 | 0.312 |
| logepa | chr10:45891535:D | 0.019 | 0.98 | 0.233 | 0.932 | 0.568 | 0.433 | 0.459 | 1.42 | 0.642 | 0.435 | 0.244 |

| logepa | chr10:45893516:D | 0.073 | 0.963 | 0.233 | 0.876 | 0.836 | 0.303 | 0.621 | 1.37 | 0.704 | 0.54 | 0.304 |
| --- | --- | --- | --- | --- | --- | --- | --- | --- | --- | --- | --- | --- |
| logepa | chr10:45893782:D | 0.018 | 0.979 | 0.224 | 0.927 | 9.30E-03 | 0.019 | 0.023 | 1.11 | 1.73 | 0.949 | 0.022 |
| logepa | chr10:45895254:I | 0.019 | 0.98 | 0.233 | 0.931 | 0.58 | 0.441 | 0.474 | 1.43 | 0.646 | 0.428 | 0.253 |
| logepa | chr10:45895848:D | 0.057 | 0.945 | 0.225 | 0.812 | 0.714 | 0.318 | 0.449 | 1.86 | 1.37 | 0.402 | 0.592 |
| logepa | chr10:45896316:D | 0.073 | 0.963 | 0.233 | 0.877 | 0.836 | 0.303 | 0.622 | 1.37 | 0.703 | 0.541 | 0.303 |
| logepa | chr10:45896317:D | 0.073 | 0.963 | 0.233 | 0.877 | 0.836 | 0.303 | 0.622 | 1.37 | 0.703 | 0.541 | 0.303 |
| logepa | chr10:45902683:I | 0.019 | 1.02 | 0.237 | 0.927 | 0.283 | 0.304 | 0.24 | 1.2 | 0.622 | 0.732 | 0.041 |
| logepa | chr10:45905003:D | 0.01 | . | . | . | . | . | . | . | . | . | . |
| logepa | chr10:45927476:D | 0.036 | 0.905 | 0.221 | 0.681 | 1.63 | 0.73 | 0.274 | 2.17 | 1.34 | 0.211 | 1.67 |
| logepa | chr10:45935882 | 0.001 | . | . | . | . | . | . | . | . | . | . |
| logepa | chr10:45936100 | 0.001 | . | . | . | . | . | . | . | . | . | . |
| logepa | chr10:45939553 | 0.001 | . | . | . | . | . | . | . | . | . | . |
| logepa | chr10:45939631 | 0 | . | . | . | . | . | . | . | . | . | . |
| logepa | chr10:45944193:D | 0.012 | 0.989 | 0.228 | 0.962 | 0.291 | 0.345 | 0.297 | 3.43 | 2.23 | 0.058 | 0.708 |
| logepa | chr10:45954030 | 0 | . | . | . | . | . | . | . | . | . | . |

Supplemental table S34.

| fa | rs_id | freq | HR_logepa | se_logepa | p_logepa | HR_snp | se_snp | p_snp | HR_inter | se_inter | p_inter | RERI |
| --- | --- | --- | --- | --- | --- | --- | --- | --- | --- | --- | --- | --- |
| logepa | rs76035182 | 0.016 | 0.606 | 0.166 | 0.068 | 3.20E-03 | 0.01 | 0.069 | 5.20E-03 | 0.011 | 0.015 | 0.391 |
| logepa | rs12359547 | 0.065 | 0.585 | 0.174 | 0.072 | 0.616 | 0.388 | 0.441 | 1.19 | 0.9 | 0.821 | 0.227 |
| logepa | rs74595484 | 0.017 | 0.573 | 0.165 | 0.053 | 1.19 | 1.12 | 0.856 | 1.27 | 1.03 | 0.771 | 0.102 |
| logepa | rs1864415 | 0.722 | 1.09 | 0.582 | 0.865 | 0.636 | 0.187 | 0.123 | 0.641 | 0.214 | 0.184 | -0.284 |
| logepa | rs72788101 | 0.068 | 0.592 | 0.177 | 0.079 | 0.565 | 0.361 | 0.371 | 1.14 | 0.886 | 0.862 | 0.226 |
| logepa | rs11239481 | 0.128 | 0.514 | 0.161 | 0.034 | 1.55 | 0.56 | 0.224 | 1.72 | 0.735 | 0.202 | 0.308 |
| logepa | rs72788102 | 0.068 | 0.592 | 0.177 | 0.079 | 0.565 | 0.361 | 0.371 | 1.14 | 0.886 | 0.862 | 0.226 |
| logepa | rs72789705 | 0.068 | 0.592 | 0.177 | 0.079 | 0.565 | 0.361 | 0.371 | 1.14 | 0.885 | 0.862 | 0.226 |
| logepa | rs141529858 | 0.005 | . | . | . | . | . | . | . | . | . | . |
| logepa | rs145376292 | 0 | . | . | . | . | . | . | . | . | . | . |
| logepa | rs190418301 | 0.001 | . | . | . | . | . | . | . | . | . | . |
| logepa | rs11239482 | 0.068 | 0.592 | 0.177 | 0.08 | 0.564 | 0.36 | 0.371 | 1.14 | 0.885 | 0.862 | 0.226 |
| logepa | rs61855872 | 0.011 | 0.588 | 0.164 | 0.057 | 0.357 | 0.584 | 0.529 | 0.821 | 0.89 | 0.855 | 0.227 |
| logepa | rs148991144 | 0.005 | . | . | . | . | . | . | . | . | . | . |
| logepa | rs191932221 | 0.005 | . | . | . | . | . | . | . | . | . | . |
| logepa | rs55978138 | 0.144 | 0.684 | 0.21 | 0.215 | 0.508 | 0.269 | 0.201 | 0.553 | 0.283 | 0.246 | 7.60E-04 |
| logepa | rs73281167 | 0.01 | . | . | . | . | . | . | . | . | . | . |
| logepa | rs12768010 | 0.073 | 0.64 | 0.18 | 0.112 | 0.491 | 0.429 | 0.416 | 0.392 | 0.315 | 0.244 | -7.90E-03 |
| logepa | rs11239484 | 0.722 | 1.09 | 0.583 | 0.865 | 0.637 | 0.187 | 0.123 | 0.641 | 0.214 | 0.184 | -0.284 |
| logepa | rs35906360 | 0.144 | 0.684 | 0.209 | 0.213 | 0.514 | 0.265 | 0.197 | 0.552 | 0.275 | 0.233 | -3.80E-03 |
| logepa | rs186250512 | 0.004 | . | . | . | . | . | . | . | . | . | . |
| logepa | rs150810743 | 0.001 | . | . | . | . | . | . | . | . | . | . |
| logepa | rs55965812 | 0.14 | 0.674 | 0.205 | 0.195 | 0.549 | 0.288 | 0.253 | 0.577 | 0.29 | 0.273 | -9.10E-03 |
| logepa | rs56180358 | 0.144 | 0.684 | 0.209 | 0.213 | 0.514 | 0.265 | 0.196 | 0.552 | 0.275 | 0.233 | -3.80E-03 |
| logepa | rs55864145 | 0.073 | 0.64 | 0.18 | 0.112 | 0.491 | 0.429 | 0.415 | 0.392 | 0.315 | 0.244 | -7.80E-03 |
| logepa | rs55986122 | 0.144 | 0.684 | 0.209 | 0.213 | 0.514 | 0.265 | 0.196 | 0.552 | 0.275 | 0.232 | -3.80E-03 |
| logepa | rs7477350 | 0.722 | 1.09 | 0.583 | 0.865 | 0.637 | 0.187 | 0.123 | 0.641 | 0.214 | 0.184 | -0.284 |
| logepa | rs2099170 | 0.143 | 0.684 | 0.209 | 0.213 | 0.515 | 0.266 | 0.2 | 0.553 | 0.275 | 0.234 | -4.50E-03 |
| logepa | rs2082108 | 0.144 | 0.685 | 0.209 | 0.215 | 0.512 | 0.264 | 0.195 | 0.551 | 0.274 | 0.231 | -3.60E-03 |
| logepa | rs55902404 | 0.004 | . | . | . | . | . | . | . | . | . | . |
| logepa | rs58402662 | 0.003 | . | . | . | . | . | . | . | . | . | . |
| logepa | rs11511618 | 0.129 | 0.513 | 0.163 | 0.035 | 1.53 | 0.559 | 0.24 | 1.7 | 0.725 | 0.214 | 0.291 |
| logepa | rs1816492 | 0.721 | 1.1 | 0.586 | 0.86 | 0.637 | 0.187 | 0.124 | 0.64 | 0.214 | 0.182 | -0.288 |
| logepa | rs146611093 | 0.006 | . | . | . | . | . | . | . | . | . | . |
| logepa | rs186897689 | 0.001 | . | . | . | . | . | . | . | . | . | . |
| logepa | rs10793616 | 0.722 | 1.09 | 0.583 | 0.865 | 0.637 | 0.187 | 0.123 | 0.642 | 0.214 | 0.184 | -0.284 |
| logepa | rs116700479 | 0.013 | 0.593 | 0.162 | 0.056 | 1.20E-03 | 4.00E-03 | 0.037 | 1.50E-03 | 4.00E-03 | 0.014 | 0.406 |
| logepa | rs192535936 | . | . | . | . | . | . | . | . | . | . | . |
| logepa | rs113363297 | 0.654 | 1.15 | 0.601 | 0.788 | 0.671 | 0.201 | 0.183 | 0.603 | 0.206 | 0.138 | -0.356 |
| logepa | rs12354994 | 0.136 | 0.663 | 0.202 | 0.178 | 0.555 | 0.297 | 0.271 | 0.591 | 0.316 | 0.325 | -1.00E-03 |
| logepa | rs148068329 | 0.025 | 0.68 | 0.192 | 0.172 | 8.90E-06 | 2.50E-05 | 3.20E-05 | 1.40E-04 | 2.40E-04 | 4.10E-07 | 0.32 |
| logepa | rs12356109 | 0.149 | 0.686 | 0.211 | 0.22 | 0.498 | 0.257 | 0.176 | 0.553 | 0.279 | 0.241 | 5.20E-03 |
| logepa | rs55908141 | 0.003 | . | . | . | . | . | . | . | . | . | . |
| logepa | rs12354712 | 0.14 | 0.671 | 0.205 | 0.191 | 0.552 | 0.29 | 0.259 | 0.586 | 0.297 | 0.291 | -6.00E-03 |
| logepa | rs147282684 | 0.003 | . | . | . | . | . | . | . | . | . | . |
| logepa | rs12356140 | 0.145 | 0.685 | 0.21 | 0.216 | 0.507 | 0.262 | 0.189 | 0.554 | 0.279 | 0.241 | 7.00E-04 |
| logepa | rs185476187 | 0.001 | . | . | . | . | . | . | . | . | . | . |
| logepa | rs145720057 | 0.018 | 0.574 | 0.161 | 0.048 | 0.599 | 0.99 | 0.757 | 8.84 | 10.1 | 0.056 | 2.87 |
| logepa | rs12354742 | 0.144 | 0.681 | 0.208 | 0.209 | 0.517 | 0.267 | 0.201 | 0.561 | 0.281 | 0.249 | -7.60E-04 |
| logepa | rs180913842 | 0.003 | . | . | . | . | . | . | . | . | . | . |
| logepa | rs142976914 | 0.002 | . | . | . | . | . | . | . | . | . | . |
| logepa | rs11511619 | 0.15 | 0.687 | 0.211 | 0.222 | 0.5 | 0.256 | 0.176 | 0.555 | 0.277 | 0.239 | 4.00E-03 |
| logepa | rs146702870 | 0.002 | . | . | . | . | . | . | . | . | . | . |
| logepa | rs150287496 | . | . | . | . | . | . | . | . | . | . | . |
| logepa | rs190917973 | 0.004 | . | . | . | . | . | . | . | . | . | . |
| logepa | rs11517281 | 0.144 | 0.682 | 0.208 | 0.209 | 0.516 | 0.266 | 0.199 | 0.56 | 0.28 | 0.247 | -6.20E-04 |
| logepa | rs12359299 | 0.144 | 0.682 | 0.208 | 0.209 | 0.516 | 0.266 | 0.198 | 0.56 | 0.28 | 0.247 | -5.70E-04 |
| logepa | rs182307932 | 0.014 | 0.588 | 0.159 | 0.05 | 4.90E-04 | 4.40E-03 | 0.401 | 0.092 | 0.929 | 0.813 | 0.411 |
| logepa | rs61855873 | 0.026 | 0.573 | 0.161 | 0.048 | 0.557 | 0.573 | 0.569 | 1.84 | 1.84 | 0.543 | 0.456 |
| logepa | rs12359302 | 0.143 | 0.674 | 0.206 | 0.196 | 0.532 | 0.28 | 0.231 | 0.567 | 0.291 | 0.27 | -3.00E-03 |
| logepa | rs143208140 | 0.027 | 0.689 | 0.194 | 0.185 | 9.20E-06 | 2.40E-05 | 9.60E-06 | 1.40E-04 | 2.30E-04 | 9.40E-08 | 0.311 |
| logepa | rs9988764 | 0.708 | 1.08 | 0.564 | 0.889 | 0.628 | 0.187 | 0.118 | 0.642 | 0.215 | 0.185 | -0.271 |
| logepa | rs192875767 | 0.003 | . | . | . | . | . | . | . | . | . | . |
| logepa | rs147268846 | 0.014 | 0.562 | 0.161 | 0.044 | 2.73 | 3.92 | 0.484 | 6.03 | 7.44 | 0.145 | 6.97 |
| logepa | rs11239485 | 0.094 | 0.623 | 0.191 | 0.123 | 0.739 | 0.481 | 0.642 | 0.684 | 0.437 | 0.553 | -0.047 |
| logepa | rs151317885 | 0.476 | 0.76 | 0.338 | 0.536 | 0.853 | 0.271 | 0.617 | 0.773 | 0.274 | 0.468 | -0.112 |
| logepa | rs11499085 | 0.055 | 0.557 | 0.163 | 0.046 | 1.14 | 0.724 | 0.836 | 1.51 | 0.936 | 0.507 | 0.261 |
| logepa | rs188130606 | 0.012 | 0.591 | 0.166 | 0.061 | 0.045 | 0.111 | 0.205 | 0.262 | 0.287 | 0.222 | 0.37 |
| logepa | rs11511620 | 0.141 | 0.671 | 0.205 | 0.192 | 0.552 | 0.289 | 0.257 | 0.585 | 0.296 | 0.289 | -6.00E-03 |
| logepa | rs181498390 | 0.012 | 0.595 | 0.166 | 0.063 | 0.319 | 0.42 | 0.385 | 0.194 | 0.169 | 0.059 | 0.124 |
| logepa | rs186280701 | 0.003 | . | . | . | . | . | . | . | . | . | . |
| logepa | rs150059185 | 0.02 | 0.684 | 0.192 | 0.176 | 2.80E-03 | 7.50E-03 | 0.03 | 2.90E-03 | 6.80E-03 | 0.011 | 0.314 |
| logepa | rs183338707 | . | . | . | . | . | . | . | . | . | . | . |
| logepa | rs11499038 | 0.052 | 0.554 | 0.164 | 0.047 | 1.26 | 0.872 | 0.742 | 1.55 | 1.24 | 0.584 | 0.269 |
| logepa | rs190699595 | 0.004 | . | . | . | . | . | . | . | . | . | . |
| logepa | rs11523408 | 0.143 | 0.679 | 0.207 | 0.205 | 0.522 | 0.271 | 0.21 | 0.564 | 0.285 | 0.257 | -7.20E-04 |
| logepa | rs188049317 | 0.001 | . | . | . | . | . | . | . | . | . | . |
| logepa | rs144948634 | 0.435 | 0.615 | 0.261 | 0.251 | 1.03 | 0.321 | 0.921 | 0.954 | 0.316 | 0.887 | -0.041 |
| logepa | rs143833576 | 0.001 | . | . | . | . | . | . | . | . | . | . |
| logepa | rs151239879 | 0.143 | 0.683 | 0.208 | 0.211 | 0.512 | 0.269 | 0.202 | 0.555 | 0.282 | 0.246 | -4.70E-04 |
| logepa | rs7073743 | 0.456 | 0.609 | 0.259 | 0.244 | 1.01 | 0.312 | 0.971 | 0.96 | 0.319 | 0.901 | -0.029 |
| logepa | rs142563349 | 0.013 | 0.598 | 0.17 | 0.07 | 0.361 | 0.481 | 0.444 | 0.365 | 0.263 | 0.162 | 0.12 |
| logepa | rs187246403 | 0.003 | . | . | . | . | . | . | . | . | . | . |
| logepa | rs150423942 | 0.134 | 0.662 | 0.204 | 0.18 | 0.561 | 0.305 | 0.288 | 0.614 | 0.333 | 0.368 | 5.20E-03 |

| logepa | rs184706102 | . | . | . | . | . | . | . | . | . | . | . |
| --- | --- | --- | --- | --- | --- | --- | --- | --- | --- | --- | --- | --- |
| logepa | rs189626288 | 0.014 | 0.574 | 0.167 | 0.057 | 1.16 | 1.18 | 0.886 | 1.18 | 0.947 | 0.835 | 0.054 |
| logepa | rs140749834 | 0.027 | 0.53 | 0.148 | 0.023 | 1.71 | 1.28 | 0.473 | 7.27 | 3.73 | 1.10E-04 | 5.34 |
| logepa | rs7078822 | 0.115 | 0.643 | 0.196 | 0.147 | 0.611 | 0.377 | 0.424 | 0.631 | 0.369 | 0.431 | -6.20E-03 |
| logepa | rs186227617 | 0.143 | 0.679 | 0.207 | 0.204 | 0.521 | 0.271 | 0.21 | 0.564 | 0.285 | 0.257 | -2.90E-04 |
| logepa | rs188984368 | 0.022 | 0.613 | 0.174 | 0.085 | 0.106 | 0.282 | 0.399 | 0.131 | 0.344 | 0.438 | 0.29 |
| logepa | rs182078109 | 0.027 | 0.602 | 0.17 | 0.072 | 0.327 | 0.483 | 0.449 | 0.408 | 0.666 | 0.583 | 0.152 |
| logepa | rs141456274 | 0.006 | . | . | . | . | . | . | . | . | . | . |
| logepa | rs146976512 | 0.144 | 0.681 | 0.208 | 0.208 | 0.517 | 0.266 | 0.199 | 0.561 | 0.281 | 0.248 | -5.40E-04 |
| logepa | rs138036816 | 0.05 | 0.554 | 0.164 | 0.046 | 1.26 | 0.872 | 0.733 | 1.56 | 1.24 | 0.578 | 0.273 |
| logepa | rs114879777 | 0.013 | 0.591 | 0.162 | 0.055 | 5.40E-03 | 0.016 | 0.072 | 9.00E-03 | 0.019 | 0.026 | 0.403 |
| logepa | rs149366475 | 0.052 | 0.559 | 0.164 | 0.048 | 1.23 | 0.793 | 0.744 | 1.38 | 0.999 | 0.653 | 0.161 |
| logepa | rs182463043 | 0.001 | . | . | . | . | . | . | . | . | . | . |
| logepa | rs144754632 | 0.025 | 0.68 | 0.192 | 0.172 | 7.80E-06 | 2.20E-05 | 3.30E-05 | 1.30E-04 | 2.30E-04 | 4.50E-07 | 0.32 |
| logepa | rs55717607 | 0.144 | 0.682 | 0.208 | 0.21 | 0.516 | 0.265 | 0.197 | 0.56 | 0.28 | 0.246 | -6.50E-04 |
| logepa | rs187537973 | . | . | . | . | . | . | . | . | . | . | . |
| logepa | rs148085971 | 0.001 | . | . | . | . | . | . | . | . | . | . |
| logepa | rs11239491 | 0.144 | 0.682 | 0.208 | 0.21 | 0.516 | 0.265 | 0.197 | 0.56 | 0.28 | 0.245 | -6.30E-04 |
| logepa | rs184412184 | 0.002 | . | . | . | . | . | . | . | . | . | . |
| logepa | rs4948998 | 0.733 | 1.3 | 0.799 | 0.665 | 0.585 | 0.193 | 0.104 | 0.577 | 0.221 | 0.151 | -0.449 |
| logepa | rs7076696 | 0.733 | 1.3 | 0.797 | 0.666 | 0.585 | 0.192 | 0.103 | 0.577 | 0.221 | 0.151 | -0.448 |
| logepa | rs11239492 | 0.149 | 0.686 | 0.211 | 0.22 | 0.498 | 0.256 | 0.175 | 0.554 | 0.279 | 0.241 | 5.30E-03 |
| logepa | rs79462470 | 0.003 | . | . | . | . | . | . | . | . | . | . |
| logepa | rs187804654 | 0.004 | . | . | . | . | . | . | . | . | . | . |
| logepa | rs7093687 | 0.141 | 0.671 | 0.205 | 0.193 | 0.553 | 0.288 | 0.255 | 0.585 | 0.294 | 0.286 | -7.00E-03 |
| logepa | rs7081004 | 0.141 | 0.671 | 0.205 | 0.192 | 0.551 | 0.288 | 0.254 | 0.585 | 0.295 | 0.287 | -6.00E-03 |
| logepa | rs7084793 | 0.145 | 0.682 | 0.208 | 0.21 | 0.516 | 0.264 | 0.196 | 0.56 | 0.279 | 0.245 | -6.80E-04 |
| logepa | rs61556080 | 0.003 | . | . | . | . | . | . | . | . | . | . |
| logepa | rs115552064 | 0.004 | . | . | . | . | . | . | . | . | . | . |
| logepa | rs7921503 | 0.144 | 0.682 | 0.208 | 0.21 | 0.516 | 0.265 | 0.197 | 0.56 | 0.279 | 0.245 | -1.00E-03 |
| logepa | rs7909740 | 0.145 | 0.682 | 0.208 | 0.21 | 0.516 | 0.264 | 0.196 | 0.56 | 0.279 | 0.245 | -7.30E-04 |
| logepa | rs7909768 | 0.145 | 0.682 | 0.208 | 0.21 | 0.516 | 0.264 | 0.196 | 0.56 | 0.279 | 0.245 | -7.50E-04 |
| logepa | rs144168004 | . | . | . | . | . | . | . | . | . | . | . |
| logepa | rs75850448 | 0.003 | . | . | . | . | . | . | . | . | . | . |
| logepa | rs147777574 | 0.003 | . | . | . | . | . | . | . | . | . | . |
| logepa | rs7913488 | 0.145 | 0.682 | 0.208 | 0.21 | 0.516 | 0.264 | 0.196 | 0.56 | 0.279 | 0.245 | -7.20E-04 |
| logepa | rs74390874 | 0.042 | 0.602 | 0.179 | 0.087 | 1.28 | 0.801 | 0.688 | 0.8 | 0.569 | 0.754 | -0.268 |
| logepa | rs141391142 | 0.004 | . | . | . | . | . | . | . | . | . | . |
| logepa | rs115294747 | 0.006 | . | . | . | . | . | . | . | . | . | . |
| logepa | rs148031781 | 0.001 | . | . | . | . | . | . | . | . | . | . |
| logepa | rs181340128 | 0.025 | 0.514 | 0.151 | 0.024 | 6.74 | 5.38 | 0.017 | 13.5 | 14.1 | 0.013 | 40.5 |
| logepa | rs189930007 | 0.001 | . | . | . | . | . | . | . | . | . | . |
| logepa | rs12242128 | 0.049 | 0.554 | 0.164 | 0.046 | 1.28 | 0.876 | 0.723 | 1.55 | 1.23 | 0.581 | 0.266 |
| logepa | rs78421750 | 0.003 | . | . | . | . | . | . | . | . | . | . |
| logepa | rs114370747 | 0.002 | . | . | . | . | . | . | . | . | . | . |
| logepa | rs12264166 | 0.049 | 0.554 | 0.164 | 0.046 | 1.28 | 0.876 | 0.721 | 1.55 | 1.23 | 0.581 | 0.266 |
| logepa | rs182964822 | 0.001 | . | . | . | . | . | . | . | . | . | . |
| logepa | rs67491417 | 0.139 | 0.707 | 0.209 | 0.241 | 0.354 | 0.192 | 0.055 | 0.424 | 0.221 | 0.099 | 0.045 |
| logepa | rs72789720 | 0.008 | . | . | . | . | . | . | . | . | . | . |
| logepa | rs6413416 | 0.145 | 0.682 | 0.208 | 0.21 | 0.516 | 0.263 | 0.194 | 0.56 | 0.278 | 0.243 | -1.20E-03 |
| logepa | rs4986832 | 0.145 | 0.682 | 0.208 | 0.21 | 0.516 | 0.263 | 0.195 | 0.56 | 0.279 | 0.244 | -9.30E-04 |
| logepa | rs144866316 | 0.004 | . | . | . | . | . | . | . | . | . | . |
| logepa | rs187769540 | 0.007 | . | . | . | . | . | . | . | . | . | . |
| logepa | rs6593482 | 0.146 | 0.688 | 0.21 | 0.22 | 0.5 | 0.253 | 0.171 | 0.549 | 0.271 | 0.224 | 9.70E-04 |
| logepa | rs7913948 | 0.146 | 0.683 | 0.208 | 0.211 | 0.508 | 0.26 | 0.185 | 0.555 | 0.277 | 0.239 | 1.90E-03 |
| logepa | rs77185151 | 0.025 | 0.679 | 0.192 | 0.171 | 7.90E-06 | 2.30E-05 | 3.80E-05 | 1.30E-04 | 2.30E-04 | 5.30E-07 | 0.321 |
| logepa | rs12762303 | 0.145 | 0.682 | 0.208 | 0.21 | 0.517 | 0.262 | 0.193 | 0.56 | 0.277 | 0.242 | -1.40E-03 |
| logepa | rs73283136 | 0.003 | . | . | . | . | . | . | . | . | . | . |
| logepa | rs187180218 | 0.001 | . | . | . | . | . | . | . | . | . | . |
| logepa | rs78137087 | 0.145 | 0.684 | 0.208 | 0.212 | 0.509 | 0.259 | 0.185 | 0.554 | 0.274 | 0.233 | -2.70E-04 |
| logepa | rs72561472 | 0.146 | 0.682 | 0.213 | 0.22 | 0.512 | 0.263 | 0.193 | 0.574 | 0.295 | 0.28 | 6.30E-03 |
| logepa | rs4987106 | 0.141 | 0.684 | 0.209 | 0.215 | 0.521 | 0.268 | 0.204 | 0.554 | 0.275 | 0.234 | -7.60E-03 |
| logepa | rs4987105 | 0.139 | 0.672 | 0.206 | 0.193 | 0.557 | 0.291 | 0.263 | 0.584 | 0.293 | 0.283 | -0.01 |
| logepa | rs111916157 | 0.158 | 0.681 | 0.223 | 0.242 | 0.582 | 0.275 | 0.252 | 0.624 | 0.306 | 0.336 | -0.016 |
| logepa | rs111528152 | 0.162 | 0.685 | 0.215 | 0.227 | 0.536 | 0.273 | 0.221 | 0.587 | 0.301 | 0.298 | -5.30E-03 |
| logepa | rs12763738 | 0.144 | 0.682 | 0.208 | 0.21 | 0.517 | 0.263 | 0.196 | 0.56 | 0.278 | 0.242 | -2.00E-03 |
| logepa | rs191675886 | 0.004 | . | . | . | . | . | . | . | . | . | . |
| logepa | rs66489870 | 0.145 | 0.682 | 0.208 | 0.21 | 0.517 | 0.262 | 0.194 | 0.56 | 0.277 | 0.242 | -1.50E-03 |
| logepa | rs71494775 | 0.145 | 0.682 | 0.208 | 0.21 | 0.517 | 0.262 | 0.194 | 0.56 | 0.277 | 0.242 | -1.40E-03 |
| logepa | rs61855875 | 0.024 | 0.569 | 0.161 | 0.046 | 0.629 | 0.64 | 0.649 | 2 | 2.02 | 0.495 | 0.517 |
| logepa | rs116356837 | 0.004 | . | . | . | . | . | . | . | . | . | . |
| logepa | rs1864414 | 0.144 | 0.682 | 0.208 | 0.21 | 0.518 | 0.263 | 0.196 | 0.561 | 0.277 | 0.242 | -2.30E-03 |
| logepa | rs1864413 | 0.145 | 0.684 | 0.209 | 0.215 | 0.527 | 0.264 | 0.201 | 0.558 | 0.271 | 0.229 | -9.80E-03 |
| logepa | rs139497212 | 0 | . | . | . | . | . | . | . | . | . | . |
| logepa | rs892690 | 0.425 | 0.691 | 0.271 | 0.347 | 0.835 | 0.274 | 0.583 | 0.814 | 0.288 | 0.561 | -0.056 |
| logepa | rs1864412 | 0.145 | 0.682 | 0.208 | 0.21 | 0.517 | 0.263 | 0.194 | 0.56 | 0.278 | 0.242 | -1.50E-03 |
| logepa | rs77827771 | 0.016 | 0.595 | 0.17 | 0.069 | 0.386 | 0.383 | 0.338 | 0.511 | 0.393 | 0.382 | 0.136 |
| logepa | rs145294963 | . | . | . | . | . | . | . | . | . | . | . |
| logepa | rs149081327 | 0.008 | . | . | . | . | . | . | . | . | . | . |
| logepa | rs67008983 | 0.145 | 0.682 | 0.208 | 0.21 | 0.517 | 0.263 | 0.195 | 0.561 | 0.278 | 0.243 | -1.70E-03 |
| logepa | rs56150414 | 0.147 | 0.684 | 0.21 | 0.215 | 0.509 | 0.261 | 0.189 | 0.558 | 0.279 | 0.243 | 9.90E-04 |
| logepa | rs55787481 | 0.145 | 0.682 | 0.208 | 0.211 | 0.518 | 0.264 | 0.196 | 0.56 | 0.278 | 0.243 | -2.00E-03 |
| logepa | rs141270101 | 0.001 | . | . | . | . | . | . | . | . | . | . |
| logepa | rs114980075 | 0.003 | . | . | . | . | . | . | . | . | . | . |
| logepa | rs12783095 | 0.144 | 0.681 | 0.21 | 0.213 | 0.541 | 0.27 | 0.218 | 0.567 | 0.275 | 0.243 | -0.013 |
| logepa | rs182342688 | . | . | . | . | . | . | . | . | . | . | . |

| logepa | rs17157728 | 0.144 | 0.681 | 0.21 | 0.213 | 0.541 | 0.27 | 0.218 | 0.568 | 0.276 | 0.243 | -0.013 |
| --- | --- | --- | --- | --- | --- | --- | --- | --- | --- | --- | --- | --- |
| logepa | rs114835563 | 0.012 | 0.597 | 0.169 | 0.068 | 0.4 | 0.518 | 0.479 | 0.388 | 0.279 | 0.188 | 0.096 |
| logepa | rs3824612 | 0.425 | 0.691 | 0.272 | 0.348 | 0.837 | 0.275 | 0.588 | 0.814 | 0.288 | 0.561 | -0.057 |
| logepa | rs17157733 | 0.143 | 0.681 | 0.21 | 0.213 | 0.542 | 0.271 | 0.22 | 0.568 | 0.276 | 0.244 | -0.014 |
| logepa | rs74128478 | 0.003 | . | . | . | . | . | . | . | . | . | . |
| logepa | rs184973589 | 0.002 | . | . | . | . | . | . | . | . | . | . |
| logepa | rs150479461 | 0 | . | . | . | . | . | . | . | . | . | . |
| logepa | rs138310407 | 0.001 | . | . | . | . | . | . | . | . | . | . |
| logepa | rs187426603 | . | . | . | . | . | . | . | . | . | . | . |
| logepa | rs190843143 | 0.002 | . | . | . | . | . | . | . | . | . | . |
| logepa | rs191883449 | 0.001 | . | . | . | . | . | . | . | . | . | . |
| logepa | rs58238740 | 0.143 | 0.68 | 0.21 | 0.211 | 0.544 | 0.272 | 0.223 | 0.57 | 0.277 | 0.247 | -0.014 |
| logepa | rs3780894 | 0.143 | 0.68 | 0.21 | 0.211 | 0.544 | 0.272 | 0.224 | 0.57 | 0.277 | 0.247 | -0.014 |
| logepa | rs149646626 | 0.018 | 0.593 | 0.169 | 0.066 | 1.03 | 0.941 | 0.974 | 0.429 | 0.47 | 0.439 | -0.361 |
| logepa | rs79615212 | 0.015 | 0.6 | 0.17 | 0.071 | 0.252 | 0.502 | 0.489 | 0.208 | 0.361 | 0.365 | 0.179 |
| logepa | rs3780895 | 0.143 | 0.68 | 0.209 | 0.211 | 0.545 | 0.273 | 0.225 | 0.571 | 0.278 | 0.249 | -0.014 |
| logepa | rs3780896 | 0.143 | 0.68 | 0.21 | 0.211 | 0.544 | 0.272 | 0.224 | 0.57 | 0.277 | 0.247 | -0.014 |
| logepa | rs142747163 | 0.005 | . | . | . | . | . | . | . | . | . | . |
| logepa | rs189417584 | 0.005 | . | . | . | . | . | . | . | . | . | . |
| logepa | rs3780897 | 0.141 | 0.673 | 0.207 | 0.198 | 0.564 | 0.287 | 0.26 | 0.585 | 0.289 | 0.278 | -0.015 |
| logepa | rs117509491 | 0.025 | 0.587 | 0.159 | 0.049 | 0.068 | 0.109 | 0.093 | 0.213 | 0.279 | 0.237 | 0.353 |
| logepa | rs117811514 | 0.025 | 0.587 | 0.159 | 0.049 | 0.068 | 0.109 | 0.093 | 0.213 | 0.279 | 0.237 | 0.353 |
| logepa | rs116500932 | 0.012 | 0.597 | 0.169 | 0.069 | 0.391 | 0.514 | 0.475 | 0.378 | 0.274 | 0.18 | 0.099 |
| logepa | rs77584168 | . | . | . | . | . | . | . | . | . | . | . |
| logepa | rs2310776 | 0.143 | 0.68 | 0.21 | 0.211 | 0.545 | 0.272 | 0.224 | 0.57 | 0.277 | 0.247 | -0.014 |
| logepa | rs17157736 | 0.143 | 0.68 | 0.21 | 0.211 | 0.545 | 0.272 | 0.224 | 0.57 | 0.277 | 0.247 | -0.014 |
| logepa | rs72789735 | 0.063 | 0.577 | 0.172 | 0.066 | 0.581 | 0.391 | 0.42 | 1.41 | 1.2 | 0.689 | 0.314 |
| logepa | rs183747187 | 0.001 | . | . | . | . | . | . | . | . | . | . |
| logepa | rs7077891 | 0.143 | 0.68 | 0.21 | 0.211 | 0.545 | 0.272 | 0.224 | 0.57 | 0.277 | 0.248 | -0.014 |
| logepa | rs145063305 | 0.002 | . | . | . | . | . | . | . | . | . | . |
| logepa | rs3824613 | 0.143 | 0.679 | 0.209 | 0.209 | 0.544 | 0.272 | 0.224 | 0.575 | 0.281 | 0.258 | -0.01 |
| logepa | rs145543199 | 0.006 | . | . | . | . | . | . | . | . | . | . |
| logepa | rs140361993 | 0.003 | . | . | . | . | . | . | . | . | . | . |
| logepa | rs11239496 | 0.063 | 0.577 | 0.172 | 0.066 | 0.582 | 0.392 | 0.421 | 1.41 | 1.21 | 0.689 | 0.314 |
| logepa | rs11239497 | 0.139 | 0.67 | 0.207 | 0.194 | 0.583 | 0.298 | 0.291 | 0.595 | 0.293 | 0.291 | -0.02 |
| logepa | rs10900212 | 0.155 | 0.673 | 0.214 | 0.213 | 0.6 | 0.268 | 0.252 | 0.646 | 0.296 | 0.341 | -0.012 |
| logepa | rs186487136 | . | . | . | . | . | . | . | . | . | . | . |
| logepa | rs12774369 | 0.142 | 0.68 | 0.21 | 0.211 | 0.546 | 0.274 | 0.228 | 0.571 | 0.278 | 0.25 | -0.014 |
| logepa | rs60842538 | 0.142 | 0.68 | 0.21 | 0.21 | 0.548 | 0.276 | 0.232 | 0.572 | 0.279 | 0.253 | -0.015 |
| logepa | rs3780898 | 0.138 | 0.669 | 0.206 | 0.192 | 0.589 | 0.304 | 0.305 | 0.599 | 0.296 | 0.3 | -0.022 |
| logepa | rs3780899 | 0.142 | 0.679 | 0.209 | 0.209 | 0.551 | 0.279 | 0.24 | 0.574 | 0.281 | 0.257 | -0.015 |
| logepa | rs12252472 | 0.045 | 0.572 | 0.164 | 0.052 | 1.1 | 0.831 | 0.902 | 1.24 | 1.17 | 0.82 | 0.109 |
| logepa | rs185411011 | . | . | . | . | . | . | . | . | . | . | . |
| logepa | rs28395865 | 0.137 | 0.669 | 0.206 | 0.192 | 0.59 | 0.307 | 0.31 | 0.597 | 0.297 | 0.299 | -0.023 |
| logepa | rs191004074 | 0.038 | 0.525 | 0.156 | 0.03 | 1.67 | 1.69 | 0.613 | 4.08 | 5.76 | 0.319 | 2.39 |
| logepa | rs182401295 | 0.002 | . | . | . | . | . | . | . | . | . | . |
| logepa | rs2228064 | 0.068 | 0.629 | 0.182 | 0.109 | 0.578 | 0.694 | 0.648 | 0.494 | 0.594 | 0.557 | -0.027 |
| logepa | rs188284159 | . | . | . | . | . | . | . | . | . | . | . |
| logepa | rs2303887 | 0.022 | 0.658 | 0.183 | 0.132 | 7.20E-06 | 2.00E-05 | 2.50E-05 | 1.20E-04 | 2.10E-04 | 3.10E-07 | 0.342 |
| logepa | rs2303886 | 0.024 | 0.657 | 0.184 | 0.134 | 5.40E-06 | 1.50E-05 | 1.20E-05 | 1.00E-04 | 1.70E-04 | 1.10E-07 | 0.343 |
| logepa | rs192621006 | . | . | . | . | . | . | . | . | . | . | . |
| logepa | rs2303885 | 0.191 | 0.674 | 0.223 | 0.233 | 0.682 | 0.306 | 0.393 | 0.684 | 0.31 | 0.402 | -0.042 |
| logepa | rs114466168 | 0.012 | 0.597 | 0.169 | 0.068 | 0.406 | 0.518 | 0.48 | 0.395 | 0.281 | 0.192 | 0.093 |
| logepa | rs11239501 | 0.083 | 0.543 | 0.169 | 0.05 | 1.05 | 0.504 | 0.927 | 1.52 | 0.854 | 0.457 | 0.274 |
| logepa | rs745986 | 0.156 | 0.633 | 0.205 | 0.158 | 0.81 | 0.37 | 0.644 | 0.768 | 0.35 | 0.563 | -0.049 |
| logepa | rs75262268 | . | . | . | . | . | . | . | . | . | . | . |
| logepa | rs71494776 | 0.051 | 0.617 | 0.175 | 0.088 | 0.842 | 0.737 | 0.844 | 0.592 | 0.486 | 0.522 | -0.152 |
| logepa | rs74386871 | 0.03 | 0.661 | 0.183 | 0.135 | 0.021 | 0.059 | 0.163 | 0.028 | 0.06 | 0.096 | 0.318 |
| logepa | rs76502040 | 0.006 | . | . | . | . | . | . | . | . | . | . |
| logepa | rs142213734 | 0.001 | . | . | . | . | . | . | . | . | . | . |
| logepa | rs4948671 | 0.731 | 1.33 | 0.823 | 0.644 | 0.611 | 0.199 | 0.13 | 0.574 | 0.218 | 0.143 | -0.475 |
| logepa | rs146286085 | 0.001 | . | . | . | . | . | . | . | . | . | . |
| logepa | rs144080623 | . | . | . | . | . | . | . | . | . | . | . |
| logepa | rs115074014 | 0.023 | 0.538 | 0.15 | 0.026 | 1.92 | 1.33 | 0.348 | 6.21 | 3.28 | 5.60E-04 | 4.94 |
| logepa | rs17157756 | 0.023 | 0.538 | 0.15 | 0.026 | 1.92 | 1.33 | 0.348 | 6.21 | 3.28 | 5.60E-04 | 4.95 |
| logepa | rs190459780 | . | . | . | . | . | . | . | . | . | . | . |
| logepa | rs12358647 | 0.174 | 0.641 | 0.218 | 0.192 | 0.781 | 0.327 | 0.555 | 0.778 | 0.333 | 0.558 | -0.033 |
| logepa | rs147080556 | 0.024 | 0.526 | 0.152 | 0.026 | 5.46 | 3.82 | 0.015 | 4.24 | 2.85 | 0.032 | 7.18 |
| logepa | rs187543349 | 0.01 | 0.624 | 0.169 | 0.081 | 1.10E-04 | 7.30E-04 | 0.159 | 6.00E-04 | 2.60E-03 | 0.086 | 0.376 |
| logepa | rs114375595 | 0.006 | . | . | . | . | . | . | . | . | . | . |
| logepa | rs117211200 | 0.005 | . | . | . | . | . | . | . | . | . | . |
| logepa | rs192963047 | . | . | . | . | . | . | . | . | . | . | . |
| logepa | rs11239505 | 0.175 | 0.64 | 0.219 | 0.192 | 0.778 | 0.321 | 0.543 | 0.783 | 0.333 | 0.565 | -0.028 |
| logepa | rs184434116 | 0 | . | . | . | . | . | . | . | . | . | . |
| logepa | rs4948999 | 0.733 | 1.37 | 0.857 | 0.612 | 0.603 | 0.194 | 0.117 | 0.565 | 0.214 | 0.131 | -0.508 |
| logepa | rs11239506 | 0.092 | 0.582 | 0.184 | 0.086 | 0.82 | 0.403 | 0.687 | 1.03 | 0.556 | 0.956 | 0.09 |
| logepa | rs190706620 | 0.003 | . | . | . | . | . | . | . | . | . | . |
| logepa | rs190770429 | . | . | . | . | . | . | . | . | . | . | . |
| logepa | rs181327133 | 0.005 | . | . | . | . | . | . | . | . | . | . |
| logepa | rs149251697 | 0.011 | 0.573 | 0.163 | 0.05 | 1.16 | 1.3 | 0.892 | 1.33 | 0.866 | 0.659 | 0.152 |
| logepa | rs118081602 | 0.022 | 0.657 | 0.183 | 0.132 | 6.80E-06 | 1.90E-05 | 2.10E-05 | 1.20E-04 | 2.00E-04 | 2.30E-07 | 0.343 |
| logepa | rs188233691 | 0.002 | . | . | . | . | . | . | . | . | . | . |
| logepa | rs183778794 | 0.003 | . | . | . | . | . | . | . | . | . | . |
| logepa | rs188040078 | 0.007 | . | . | . | . | . | . | . | . | . | . |
| logepa | rs184675372 | 0 | . | . | . | . | . | . | . | . | . | . |
| logepa | rs150326891 | 0.067 | 0.54 | 0.168 | 0.048 | 1.25 | 0.633 | 0.655 | 1.55 | 0.904 | 0.455 | 0.253 |

| logepa | rs185520021 | 0.014 | 0.58 | 0.16 | 0.048 | 1.2 | 2.71 | 0.937 | 1.16 | 2.95 | 0.953 | 0.031 |
| --- | --- | --- | --- | --- | --- | --- | --- | --- | --- | --- | --- | --- |
| logepa | rs7907779 | 0.092 | 0.582 | 0.184 | 0.086 | 0.819 | 0.403 | 0.685 | 1.03 | 0.556 | 0.956 | 0.09 |
| logepa | rs184962860 | 0.001 | . | . | . | . | . | . | . | . | . | . |
| logepa | rs149762784 | 0.066 | 0.525 | 0.152 | 0.026 | 1.1 | 1.08 | 0.92 | 2.42 | 3.69 | 0.562 | 0.772 |
| logepa | rs149203427 | 0.008 | . | . | . | . | . | . | . | . | . | . |
| logepa | rs60153083 | 0.01 | 0.593 | 0.166 | 0.062 | 0.336 | 0.462 | 0.427 | 0.15 | 0.134 | 0.034 | 0.1 |
| logepa | rs112001069 | 0.062 | 0.626 | 0.178 | 0.099 | 0.726 | 0.609 | 0.702 | 0.527 | 0.412 | 0.412 | -0.112 |
| logepa | rs182355382 | 0.032 | 0.541 | 0.159 | 0.036 | 0.783 | 0.751 | 0.799 | 3.95 | 5.19 | 0.295 | 1.35 |
| logepa | rs186740974 | 0.111 | 0.606 | 0.22 | 0.167 | 1.06 | 0.649 | 0.92 | 0.811 | 0.56 | 0.761 | -0.147 |
| logepa | rs191874916 | 0.026 | 0.718 | 0.201 | 0.236 | 5.70E-04 | 1.70E-03 | 0.01 | 5.70E-04 | 1.30E-03 | 1.00E-03 | 0.281 |
| logepa | rs182989412 | 0.168 | 0.666 | 0.235 | 0.25 | 0.442 | 0.258 | 0.162 | 0.607 | 0.35 | 0.386 | 0.071 |
| logepa | rs188819476 | 0.001 | . | . | . | . | . | . | . | . | . | . |
| logepa | rs141217183 | 0.08 | 0.627 | 0.182 | 0.108 | 0.895 | 0.703 | 0.888 | 0.588 | 0.445 | 0.482 | -0.192 |
| logepa | rs192922652 | 0.064 | 0.634 | 0.179 | 0.106 | 0.631 | 0.581 | 0.617 | 0.431 | 0.373 | 0.33 | -0.092 |
| logepa | rs185092343 | 0.014 | 0.588 | 0.163 | 0.056 | 0.206 | 0.25 | 0.192 | 1.06 | 1.84 | 0.975 | 0.334 |
| logepa | rs145193311 | 0.065 | 0.633 | 0.179 | 0.107 | 0.649 | 0.582 | 0.63 | 0.437 | 0.371 | 0.329 | -0.103 |
| logepa | rs55789336 | 0.093 | 0.586 | 0.185 | 0.09 | 0.791 | 0.393 | 0.637 | 1 | 0.548 | 0.997 | 0.087 |
| logepa | rs55837212 | 0.093 | 0.582 | 0.182 | 0.083 | 0.829 | 0.417 | 0.709 | 1.02 | 0.576 | 0.967 | 0.083 |
| logepa | rs75408574 | 0.406 | 0.647 | 0.244 | 0.248 | 0.839 | 0.264 | 0.575 | 0.873 | 0.285 | 0.678 | -0.012 |
| logepa | rs149306926 | 0.098 | 0.578 | 0.184 | 0.085 | 0.785 | 0.41 | 0.643 | 1.07 | 0.619 | 0.911 | 0.121 |
| logepa | rs35990892 | 0.706 | 1.5 | 0.867 | 0.482 | 0.624 | 0.194 | 0.129 | 0.518 | 0.187 | 0.069 | -0.64 |
| logepa | rs192356207 | 0.036 | 0.597 | 0.169 | 0.069 | 0.454 | 0.507 | 0.479 | 0.913 | 1.05 | 0.937 | 0.196 |
| logepa | rs183741163 | 0.092 | 0.654 | 0.203 | 0.171 | 0.667 | 0.435 | 0.535 | 0.565 | 0.403 | 0.424 | -0.075 |
| logepa | rs34737297 | 0.559 | 1.15 | 0.645 | 0.804 | 0.692 | 0.257 | 0.322 | 0.571 | 0.243 | 0.188 | -0.387 |
| logepa | rs181346933 | 0.022 | 0.549 | 0.156 | 0.035 | 1.28 | 1.08 | 0.768 | 3.92 | 4.62 | 0.247 | 1.93 |
| logepa | rs36093253 | 0.409 | 0.824 | 0.329 | 0.627 | 0.794 | 0.265 | 0.488 | 0.688 | 0.232 | 0.267 | -0.168 |
| logepa | rs186307855 | 0.059 | 0.575 | 0.165 | 0.054 | 0.903 | 0.847 | 0.913 | 1.18 | 1.23 | 0.874 | 0.135 |
| logepa | rs189681865 | 0.019 | 0.591 | 0.172 | 0.071 | 0.699 | 0.622 | 0.687 | 0.914 | 0.626 | 0.896 | 0.088 |
| logepa | rs34942971 | 0.176 | 0.624 | 0.216 | 0.173 | 0.922 | 0.39 | 0.848 | 0.831 | 0.363 | 0.672 | -0.068 |
| logepa | rs181816554 | 0.025 | 0.641 | 0.184 | 0.122 | 0.093 | 0.274 | 0.418 | 0.043 | 0.106 | 0.205 | 0.268 |
| logepa | rs186846881 | 0.049 | 0.56 | 0.163 | 0.046 | 1.25 | 0.847 | 0.743 | 1.48 | 1.27 | 0.646 | 0.227 |
| logepa | rs191178220 | 0.016 | 0.53 | 0.147 | 0.022 | 13.8 | 20 | 0.069 | 641 | 1.40E+03 | 4.20E-03 | 4.70E+03 |
| logepa | rs183305228 | 0.01 | 0.593 | 0.166 | 0.062 | 0.339 | 0.454 | 0.419 | 0.157 | 0.139 | 0.037 | 0.099 |
| logepa | rs139912761 | 0.102 | 0.573 | 0.183 | 0.081 | 0.867 | 0.355 | 0.727 | 1.11 | 0.506 | 0.812 | 0.113 |
| logepa | rs185499901 | 0.016 | 0.584 | 0.165 | 0.058 | 1.26 | 0.963 | 0.759 | 0.7 | 0.77 | 0.746 | -0.331 |
| logepa | rs189816197 | 0.072 | 0.646 | 0.189 | 0.135 | 0.566 | 0.487 | 0.508 | 0.436 | 0.345 | 0.294 | -0.053 |
| logepa | rs145656938 | 0.022 | 0.583 | 0.168 | 0.061 | 0.457 | 0.445 | 0.422 | 1.64 | 1.19 | 0.495 | 0.398 |
| logepa | rs35745197 | 0.062 | 0.626 | 0.178 | 0.1 | 0.722 | 0.608 | 0.699 | 0.523 | 0.411 | 0.409 | -0.112 |
| logepa | rs192856637 | 0.004 | . | . | . | . | . | . | . | . | . | . |
| logepa | rs2310775 | 0.492 | 0.674 | 0.28 | 0.343 | 0.863 | 0.272 | 0.64 | 0.861 | 0.283 | 0.649 | -0.036 |
| logepa | rs11239507 | 0.559 | 0.822 | 0.377 | 0.67 | 0.762 | 0.242 | 0.392 | 0.735 | 0.256 | 0.377 | -0.124 |
| logepa | rs74648467 | 0.053 | 0.617 | 0.175 | 0.089 | 0.828 | 0.733 | 0.831 | 0.596 | 0.493 | 0.531 | -0.141 |
| logepa | rs145815934 | 0.012 | 0.551 | 0.154 | 0.033 | 4.49 | 5.52 | 0.221 | 6.57 | 9.55 | 0.195 | 12.2 |
| logepa | rs145390687 | 0.015 | 0.568 | 0.164 | 0.05 | 1.8 | 2.03 | 0.602 | 1.49 | 1.44 | 0.679 | 0.155 |
| logepa | rs184354913 | 0.033 | 0.601 | 0.175 | 0.08 | 0.864 | 0.733 | 0.863 | 0.475 | 0.422 | 0.402 | -0.219 |
| logepa | rs181244920 | 0.007 | . | . | . | . | . | . | . | . | . | . |
| logepa | rs11499087 | 0.028 | 0.538 | 0.151 | 0.027 | 1.65 | 1.22 | 0.501 | 5.79 | 2.95 | 5.70E-04 | 3.95 |
| logepa | rs148263794 | 0.064 | 0.629 | 0.179 | 0.103 | 0.698 | 0.574 | 0.663 | 0.507 | 0.388 | 0.375 | -0.104 |
| logepa | rs150758903 | 0.021 | 0.569 | 0.162 | 0.047 | 0.434 | 0.568 | 0.524 | 4.19 | 2.23 | 7.20E-03 | 1.03 |
| logepa | rs147654382 | 0.152 | 0.698 | 0.228 | 0.27 | 0.534 | 0.273 | 0.219 | 0.547 | 0.262 | 0.207 | -0.028 |
| logepa | rs147495166 | 0.011 | 0.596 | 0.169 | 0.068 | 0.41 | 0.531 | 0.491 | 0.391 | 0.288 | 0.202 | 0.089 |
| logepa | rs35198778 | 0.081 | 0.636 | 0.199 | 0.147 | 0.617 | 0.566 | 0.598 | 0.602 | 0.467 | 0.513 | -0.017 |
| logepa | rs145207659 | 0.043 | 0.608 | 0.179 | 0.092 | 1.24 | 0.781 | 0.729 | 0.711 | 0.498 | 0.626 | -0.314 |
| logepa | rs186466464 | 0.007 | . | . | . | . | . | . | . | . | . | . |
| logepa | rs12778413 | 0.146 | 0.77 | 0.247 | 0.416 | 0.304 | 0.161 | 0.025 | 0.337 | 0.161 | 0.023 | 4.30E-03 |
| logepa | rs7895552 | 0.632 | 1.92 | 1.07 | 0.244 | 0.532 | 0.167 | 0.044 | 0.411 | 0.152 | 0.016 | -1.03 |
| logepa | rs12783895 | 0.064 | 0.628 | 0.179 | 0.102 | 0.703 | 0.576 | 0.667 | 0.509 | 0.389 | 0.377 | -0.106 |
| logepa | rs34929604 | 0.065 | 0.628 | 0.179 | 0.102 | 0.693 | 0.562 | 0.651 | 0.507 | 0.384 | 0.371 | -0.1 |
| logepa | rs12761961 | 0.063 | 0.628 | 0.178 | 0.102 | 0.699 | 0.588 | 0.67 | 0.506 | 0.396 | 0.384 | -0.105 |
| logepa | rs35536930 | 0.172 | 0.639 | 0.216 | 0.184 | 0.797 | 0.326 | 0.579 | 0.78 | 0.33 | 0.557 | -0.038 |
| logepa | rs180951060 | 0 | . | . | . | . | . | . | . | . | . | . |
| logepa | rs148892128 | 0.012 | 0.551 | 0.154 | 0.033 | 4.49 | 5.52 | 0.222 | 6.55 | 9.53 | 0.196 | 12.2 |
| logepa | rs11239509 | 0.043 | 0.551 | 0.157 | 0.036 | 1.18 | 0.744 | 0.796 | 2.37 | 1.42 | 0.151 | 0.808 |
| logepa | rs41526545 | 0.088 | 0.554 | 0.173 | 0.059 | 0.93 | 0.444 | 0.88 | 1.36 | 0.733 | 0.567 | 0.217 |
| logepa | rs2029253 | 0.496 | 0.725 | 0.303 | 0.442 | 0.826 | 0.26 | 0.545 | 0.805 | 0.267 | 0.512 | -0.069 |
| logepa | rs17444064 | 0.086 | 0.546 | 0.17 | 0.052 | 1.01 | 0.49 | 0.976 | 1.47 | 0.821 | 0.489 | 0.255 |
| logepa | rs71494779 | 0.066 | 0.629 | 0.179 | 0.102 | 0.69 | 0.558 | 0.646 | 0.506 | 0.382 | 0.366 | -0.099 |
| logepa | rs74991420 | 0.022 | 0.655 | 0.181 | 0.126 | 7.30E-06 | 2.00E-05 | 1.10E-05 | 1.20E-04 | 2.00E-04 | 9.80E-08 | 0.345 |
| logepa | rs182736551 | 0.022 | 0.655 | 0.181 | 0.126 | 7.30E-06 | 2.00E-05 | 1.10E-05 | 1.20E-04 | 2.00E-04 | 9.80E-08 | 0.345 |
| logepa | rs78702074 | 0.021 | 0.658 | 0.183 | 0.133 | 6.60E-06 | 1.80E-05 | 1.10E-05 | 1.10E-04 | 1.90E-04 | 1.20E-07 | 0.342 |
| logepa | rs187791466 | 0.002 | . | . | . | . | . | . | . | . | . | . |
| logepa | rs35300270 | 0.068 | 0.633 | 0.18 | 0.107 | 0.641 | 0.528 | 0.589 | 0.475 | 0.363 | 0.33 | -0.081 |
| logepa | rs6593484 | 0.093 | 0.584 | 0.184 | 0.088 | 0.806 | 0.391 | 0.656 | 1.01 | 0.537 | 0.981 | 0.087 |
| logepa | rs12244601 | 0.013 | 0.578 | 0.165 | 0.054 | 0.475 | 0.638 | 0.579 | 2.15 | 1.09 | 0.13 | 0.538 |
| logepa | rs184429574 | 0.004 | . | . | . | . | . | . | . | . | . | . |
| logepa | rs7080897 | 0.066 | 0.629 | 0.179 | 0.102 | 0.69 | 0.557 | 0.646 | 0.506 | 0.382 | 0.366 | -0.099 |
| logepa | rs7097429 | 0.07 | 0.649 | 0.184 | 0.127 | 0.604 | 0.491 | 0.536 | 0.419 | 0.309 | 0.239 | -0.089 |
| logepa | rs77979229 | 0.001 | . | . | . | . | . | . | . | . | . | . |
| logepa | rs11239512 | 0.093 | 0.584 | 0.184 | 0.088 | 0.805 | 0.391 | 0.655 | 1.01 | 0.537 | 0.981 | 0.087 |
| logepa | rs60146827 | 0.066 | 0.629 | 0.179 | 0.102 | 0.689 | 0.556 | 0.644 | 0.504 | 0.38 | 0.364 | -0.099 |
| logepa | rs79032236 | 0.017 | 0.626 | 0.178 | 0.1 | 0.184 | 0.232 | 0.18 | 0.139 | 0.136 | 0.043 | 0.206 |
| logepa | rs140854206 | 0.005 | . | . | . | . | . | . | . | . | . | . |
| logepa | rs184049550 | 0.002 | . | . | . | . | . | . | . | . | . | . |
| logepa | rs114433607 | 0.007 | . | . | . | . | . | . | . | . | . | . |
| logepa | rs117653793 | 0.022 | 0.659 | 0.184 | 0.136 | 6.60E-06 | 1.80E-05 | 9.60E-06 | 1.10E-04 | 1.90E-04 | 9.00E-08 | 0.341 |
| logepa | rs117039533 | 0.022 | 0.658 | 0.183 | 0.133 | 6.90E-06 | 1.80E-05 | 9.80E-06 | 1.20E-04 | 2.00E-04 | 9.20E-08 | 0.342 |

| logepa | rs191660761 | 0.002 | . | . | . | . | . | . | . | . | . | . |
| --- | --- | --- | --- | --- | --- | --- | --- | --- | --- | --- | --- | --- |
| logepa | rs59514896 | 0.053 | 0.62 | 0.175 | 0.091 | 0.807 | 0.701 | 0.806 | 0.569 | 0.462 | 0.488 | -0.142 |
| logepa | rs184661876 | 0.001 | . | . | . | . | . | . | . | . | . | . |
| logepa | rs61854073 | 0.028 | 0.578 | 0.163 | 0.051 | 0.463 | 0.451 | 0.429 | 1.78 | 1.88 | 0.582 | 0.436 |
| logepa | rs2018518 | 0.391 | 0.709 | 0.254 | 0.337 | 0.738 | 0.24 | 0.35 | 0.764 | 0.254 | 0.418 | -0.047 |
| logepa | rs934187 | 0.735 | 1.42 | 0.889 | 0.578 | 0.61 | 0.194 | 0.119 | 0.556 | 0.209 | 0.119 | -0.547 |
| logepa | rs7918542 | 0.179 | 0.646 | 0.22 | 0.199 | 0.756 | 0.309 | 0.494 | 0.768 | 0.324 | 0.531 | -0.027 |
| logepa | rs144311920 | 0.002 | . | . | . | . | . | . | . | . | . | . |
| logepa | rs7099684 | 0.864 | 1.2 | 1.18 | 0.852 | 0.642 | 0.27 | 0.292 | 0.655 | 0.346 | 0.423 | -0.338 |
| logepa | rs150829171 | . | . | . | . | . | . | . | . | . | . | . |
| logepa | rs186060697 | 0 | . | . | . | . | . | . | . | . | . | . |
| logepa | rs11239513 | 0.054 | 0.564 | 0.164 | 0.048 | 1.09 | 0.781 | 0.909 | 1.55 | 1.38 | 0.619 | 0.302 |
| logepa | rs7917687 | 0.067 | 0.63 | 0.179 | 0.105 | 0.681 | 0.546 | 0.631 | 0.496 | 0.373 | 0.351 | -0.098 |
| logepa | rs61854076 | 0.01 | 0.588 | 0.164 | 0.057 | 0.365 | 0.592 | 0.535 | 0.824 | 0.915 | 0.862 | 0.224 |
| logepa | rs71494780 | 0.029 | 0.638 | 0.174 | 0.099 | 0.061 | 0.107 | 0.11 | 0.073 | 0.097 | 0.048 | 0.304 |
| logepa | rs7919239 | 0.186 | 0.665 | 0.227 | 0.231 | 0.756 | 0.29 | 0.466 | 0.715 | 0.287 | 0.404 | -0.061 |
| logepa | rs78334475 | 0.047 | 0.636 | 0.181 | 0.111 | 0.195 | 0.227 | 0.161 | 0.299 | 0.318 | 0.256 | 0.206 |
| logepa | rs3780900 | 0.02 | 0.657 | 0.184 | 0.134 | 3.60E-03 | 0.01 | 0.055 | 4.50E-03 | 0.012 | 0.042 | 0.339 |
| logepa | rs186729292 | 0.001 | . | . | . | . | . | . | . | . | . | . |
| logepa | rs12267438 | 0.015 | 0.585 | 0.167 | 0.061 | 1.02 | 0.832 | 0.977 | 0.851 | 0.577 | 0.812 | -0.099 |
| logepa | rs1369214 | 0.61 | 1.02 | 0.483 | 0.964 | 0.755 | 0.238 | 0.372 | 0.64 | 0.222 | 0.198 | -0.283 |
| logepa | rs9282589 | 0.134 | 0.56 | 0.168 | 0.054 | 1.27 | 0.623 | 0.622 | 1.19 | 0.642 | 0.743 | 0.017 |
| logepa | rs114522915 | 0.011 | 0.598 | 0.169 | 0.069 | 0.389 | 0.437 | 0.401 | 0.3 | 0.281 | 0.199 | 0.083 |
| logepa | rs2115819 | 0.542 | 0.822 | 0.365 | 0.66 | 0.848 | 0.268 | 0.603 | 0.743 | 0.25 | 0.377 | -0.152 |
| logepa | rs117664603 | 0.022 | 0.655 | 0.182 | 0.128 | 6.00E-06 | 1.60E-05 | 9.50E-06 | 1.10E-04 | 1.90E-04 | 9.90E-08 | 0.345 |
| logepa | rs184979202 | . | . | . | . | . | . | . | . | . | . | . |
| logepa | rs190714671 | 0.002 | . | . | . | . | . | . | . | . | . | . |
| logepa | rs141538996 | . | . | . | . | . | . | . | . | . | . | . |
| logepa | rs150881741 | . | . | . | . | . | . | . | . | . | . | . |
| logepa | rs139076633 | . | . | . | . | . | . | . | . | . | . | . |
| logepa | rs12243649 | 0.054 | 0.652 | 0.187 | 0.135 | 0.145 | 0.302 | 0.353 | 0.181 | 0.328 | 0.346 | 0.22 |
| logepa | rs140348708 | 0.001 | . | . | . | . | . | . | . | . | . | . |
| logepa | rs79023256 | 0.022 | 0.655 | 0.182 | 0.127 | 5.70E-06 | 1.60E-05 | 9.30E-06 | 1.00E-04 | 1.80E-04 | 9.70E-08 | 0.345 |
| logepa | rs11239515 | 0.081 | 0.587 | 0.18 | 0.083 | 0.755 | 0.41 | 0.605 | 1.02 | 0.612 | 0.98 | 0.108 |
| logepa | rs55866086 | 0.137 | 0.667 | 0.213 | 0.205 | 0.508 | 0.255 | 0.177 | 0.633 | 0.341 | 0.397 | 0.039 |
| logepa | rs181340150 | 0.001 | . | . | . | . | . | . | . | . | . | . |
| logepa | rs11239516 | 0.096 | 0.585 | 0.186 | 0.091 | 0.751 | 0.323 | 0.505 | 1.09 | 0.533 | 0.856 | 0.144 |
| logepa | rs11239517 | 0.096 | 0.584 | 0.186 | 0.091 | 0.751 | 0.324 | 0.506 | 1.09 | 0.534 | 0.854 | 0.145 |
| logepa | rs11239519 | 0.088 | 0.591 | 0.186 | 0.095 | 0.724 | 0.327 | 0.475 | 1.03 | 0.533 | 0.96 | 0.124 |
| logepa | rs7909514 | 0.722 | 1.29 | 0.788 | 0.68 | 0.599 | 0.193 | 0.111 | 0.585 | 0.22 | 0.153 | -0.435 |
| logepa | rs7894352 | 0.853 | 1.14 | 1.14 | 0.893 | 0.647 | 0.282 | 0.318 | 0.672 | 0.362 | 0.461 | -0.293 |
| logepa | rs189384146 | 0.002 | . | . | . | . | . | . | . | . | . | . |
| logepa | rs11239520 | 0.088 | 0.633 | 0.196 | 0.14 | 0.62 | 0.337 | 0.379 | 0.709 | 0.414 | 0.556 | 0.026 |
| logepa | rs12221233 | 0.483 | 0.609 | 0.246 | 0.219 | 0.902 | 0.283 | 0.742 | 0.946 | 0.303 | 0.863 | 8.80E-03 |
| logepa | rs74523293 | 0.038 | 0.67 | 0.186 | 0.148 | 1.60E-04 | 5.60E-04 | 0.01 | 9.40E-04 | 2.10E-03 | 2.10E-03 | 0.33 |
| logepa | rs79376040 | 0.038 | 0.67 | 0.186 | 0.148 | 1.60E-04 | 5.50E-04 | 0.01 | 9.30E-04 | 2.10E-03 | 2.10E-03 | 0.33 |
| logepa | rs4949000 | 0.482 | 0.611 | 0.246 | 0.222 | 0.902 | 0.283 | 0.742 | 0.944 | 0.302 | 0.856 | 7.10E-03 |
| logepa | rs7089406 | 0.023 | 0.585 | 0.164 | 0.055 | 0.904 | 0.785 | 0.907 | 0.908 | 0.993 | 0.93 | -8.60E-03 |
| logepa | rs138211056 | 0.001 | . | . | . | . | . | . | . | . | . | . |
| logepa | rs10900213 | 0.586 | 0.722 | 0.375 | 0.53 | 0.767 | 0.232 | 0.38 | 0.839 | 0.28 | 0.599 | -0.024 |
| logepa | rs145152827 | . | . | . | . | . | . | . | . | . | . | . |
| logepa | rs74443325 | 0.022 | 0.651 | 0.18 | 0.121 | 2.60E-06 | 7.60E-06 | 1.20E-05 | 6.30E-05 | 1.20E-04 | 1.60E-07 | 0.349 |
| logepa | rs188299446 | 0.011 | 0.561 | 0.158 | 0.04 | 13.8 | 35.3 | 0.306 | 12.7 | 45.6 | 0.48 | 84.6 |
| logepa | rs192699586 | 0.005 | . | . | . | . | . | . | . | . | . | . |
| logepa | rs72796464 | 0.076 | 0.554 | 0.17 | 0.054 | 0.904 | 0.48 | 0.85 | 1.51 | 0.976 | 0.525 | 0.297 |
| logepa | rs4949001 | 0.366 | 0.663 | 0.225 | 0.225 | 0.716 | 0.233 | 0.305 | 0.801 | 0.262 | 0.497 | 1.10E-03 |
| logepa | rs147174641 | 0.005 | . | . | . | . | . | . | . | . | . | . |
| logepa | rs190835238 | 0.001 | . | . | . | . | . | . | . | . | . | . |
| logepa | rs76698944 | 0.017 | 0.549 | 0.153 | 0.032 | 1.28 | 1.16 | 0.783 | 11.3 | 9.53 | 4.20E-03 | 7.11 |
| logepa | rs56071381 | 0.091 | 0.635 | 0.199 | 0.148 | 0.592 | 0.322 | 0.334 | 0.714 | 0.425 | 0.572 | 0.042 |
| logepa | rs78130427 | 0.023 | 0.568 | 0.162 | 0.047 | 2.7 | 1.74 | 0.124 | 1.29 | 0.846 | 0.694 | -0.283 |
| logepa | rs10900215 | 0.118 | 0.625 | 0.203 | 0.148 | 0.733 | 0.283 | 0.42 | 0.831 | 0.369 | 0.677 | 0.023 |
| logepa | rs41491445 | 0.01 | 0.597 | 0.168 | 0.067 | 0.306 | 0.447 | 0.418 | 0.104 | 0.105 | 0.025 | 0.116 |
| logepa | rs144995222 | 0.002 | . | . | . | . | . | . | . | . | . | . |
| logepa | rs149097803 | 0.002 | . | . | . | . | . | . | . | . | . | . |
| logepa | rs139429646 | 0.005 | . | . | . | . | . | . | . | . | . | . |
| logepa | rs7075620 | 0.675 | 0.807 | 0.483 | 0.72 | 0.761 | 0.24 | 0.388 | 0.792 | 0.288 | 0.521 | -0.082 |
| logepa | rs148299213 | 0.01 | 0.599 | 0.169 | 0.069 | 0.27 | 0.413 | 0.392 | 0.087 | 0.091 | 0.02 | 0.145 |
| logepa | rs4949002 | 0.313 | 0.623 | 0.233 | 0.206 | 0.945 | 0.371 | 0.886 | 0.905 | 0.375 | 0.809 | -0.036 |
| logepa | rs150496007 | 0.036 | 0.553 | 0.163 | 0.044 | 0.775 | 0.665 | 0.766 | 1.94 | 2.17 | 0.555 | 0.502 |
| logepa | rs147303455 | 0.008 | . | . | . | . | . | . | . | . | . | . |
| logepa | rs189518433 | . | . | . | . | . | . | . | . | . | . | . |
| logepa | rs186281355 | . | . | . | . | . | . | . | . | . | . | . |
| logepa | rs115297114 | 0.014 | 0.574 | 0.158 | 0.044 | 0.657 | 0.639 | 0.666 | 2.03 | 2.73 | 0.599 | 0.535 |
| logepa | rs11239523 | 0.087 | 0.626 | 0.193 | 0.129 | 0.638 | 0.348 | 0.41 | 0.739 | 0.437 | 0.609 | 0.031 |
| logepa | rs143704897 | 0.005 | . | . | . | . | . | . | . | . | . | . |
| logepa | rs188024096 | 0.002 | . | . | . | . | . | . | . | . | . | . |
| logepa | rs11239524 | 0.087 | 0.63 | 0.198 | 0.142 | 0.62 | 0.349 | 0.395 | 0.732 | 0.442 | 0.605 | 0.036 |
| logepa | rs191656573 | 0.004 | . | . | . | . | . | . | . | . | . | . |
| logepa | rs193295875 | . | . | . | . | . | . | . | . | . | . | . |
| logepa | rs190458228 | . | . | . | . | . | . | . | . | . | . | . |
| logepa | rs139253993 | 0.002 | . | . | . | . | . | . | . | . | . | . |
| logepa | rs4948672 | 0.604 | 0.757 | 0.404 | 0.602 | 0.711 | 0.268 | 0.366 | 0.783 | 0.323 | 0.554 | -0.046 |
| logepa | rs180954921 | 0.002 | . | . | . | . | . | . | . | . | . | . |
| logepa | rs12264801 | 0.447 | 0.522 | 0.239 | 0.156 | 0.994 | 0.352 | 0.986 | 1.11 | 0.424 | 0.793 | 0.058 |
| logepa | rs73283184 | 0.014 | 0.606 | 0.172 | 0.077 | 0.253 | 0.377 | 0.356 | 0.25 | 0.219 | 0.113 | 0.179 |

| logepa | rs142746187 | 0.007 | . | . | . | . | . | . | . | . | . | . |
| --- | --- | --- | --- | --- | --- | --- | --- | --- | --- | --- | --- | --- |
| logepa | rs58704739 | 0.014 | 0.606 | 0.171 | 0.077 | 0.259 | 0.385 | 0.364 | 0.256 | 0.223 | 0.118 | 0.176 |
| logepa | rs61272335 | 0.011 | 0.604 | 0.169 | 0.072 | 0.206 | 0.314 | 0.3 | 0.074 | 0.074 | 9.70E-03 | 0.2 |
| logepa | rs60959489 | 0.021 | 0.61 | 0.175 | 0.085 | 0.66 | 0.671 | 0.682 | 0.418 | 0.403 | 0.366 | -0.101 |
| logepa | rs58502629 | 0.011 | 0.604 | 0.169 | 0.072 | 0.206 | 0.313 | 0.298 | 0.074 | 0.074 | 9.70E-03 | 0.2 |
| logepa | rs10900216 | 0.739 | 0.489 | 0.404 | 0.387 | 1.17 | 0.491 | 0.705 | 1.12 | 0.506 | 0.803 | -0.02 |
| logepa | rs181118141 | 0.001 | . | . | . | . | . | . | . | . | . | . |
| logepa | rs137882665 | 0.01 | 0.578 | 0.16 | 0.048 | 0.209 | 0.294 | 0.265 | 2.6 | 2.07 | 0.23 | 0.528 |
| logepa | rs149466736 | 0.004 | . | . | . | . | . | . | . | . | . | . |
| logepa | rs2099171 | 0.675 | 0.435 | 0.284 | 0.202 | 1.11 | 0.416 | 0.784 | 1.23 | 0.473 | 0.596 | 0.048 |
| logepa | rs141852280 | 0.005 | . | . | . | . | . | . | . | . | . | . |
| logepa | rs191539257 | . | . | . | . | . | . | . | . | . | . | . |
| logepa | rs17157771 | 0.027 | 0.617 | 0.18 | 0.099 | 1.23 | 0.846 | 0.76 | 0.338 | 0.237 | 0.121 | -0.593 |
| logepa | rs111339241 | 0.025 | 0.62 | 0.181 | 0.101 | 1.13 | 0.733 | 0.845 | 0.294 | 0.202 | 0.075 | -0.548 |
| logepa | rs116983255 | 0.006 | . | . | . | . | . | . | . | . | . | . |
| logepa | rs142324341 | . | . | . | . | . | . | . | . | . | . | . |
| logepa | rs187191219 | 0.007 | . | . | . | . | . | . | . | . | . | . |
| logepa | rs151283973 | 0 | . | . | . | . | . | . | . | . | . | . |
| logepa | rs139316218 | 0.003 | . | . | . | . | . | . | . | . | . | . |
| logepa | rs6593485 | 0.748 | 0.592 | 0.451 | 0.491 | 1.04 | 0.378 | 0.912 | 0.992 | 0.416 | 0.985 | -0.022 |
| logepa | rs6593486 | 0.751 | 0.602 | 0.458 | 0.505 | 1.03 | 0.372 | 0.931 | 0.981 | 0.412 | 0.964 | -0.024 |
| logepa | rs145196618 | 0.011 | 0.589 | 0.165 | 0.059 | 0.126 | 0.261 | 0.316 | 0.719 | 0.678 | 0.726 | 0.338 |
| logepa | rs142598335 | . | . | . | . | . | . | . | . | . | . | . |
| logepa | rs892691 | 0.745 | 0.749 | 0.625 | 0.729 | 0.958 | 0.372 | 0.912 | 0.855 | 0.403 | 0.739 | -0.094 |
| logepa | rs7080713 | 0.689 | 0.455 | 0.321 | 0.265 | 1.07 | 0.424 | 0.859 | 1.18 | 0.488 | 0.684 | 0.05 |
| logepa | rs7077173 | 0.068 | 0.619 | 0.178 | 0.095 | 0.941 | 0.479 | 0.905 | 0.626 | 0.453 | 0.517 | -0.196 |
| logepa | rs7080977 | 0.04 | 0.6 | 0.172 | 0.075 | 1.33 | 0.741 | 0.61 | 0.579 | 0.422 | 0.454 | -0.467 |
| logepa | rs3780901 | 0.688 | 0.467 | 0.33 | 0.282 | 1.07 | 0.426 | 0.857 | 1.16 | 0.48 | 0.714 | 0.042 |
| logepa | rs61854091 | 0.046 | 0.55 | 0.16 | 0.04 | 1.6 | 0.979 | 0.447 | 1.65 | 1.07 | 0.44 | 0.304 |
| logepa | rs77550209 | 0.003 | . | . | . | . | . | . | . | . | . | . |
| logepa | rs73283191 | 0.062 | 0.648 | 0.183 | 0.124 | 0.845 | 0.574 | 0.804 | 0.485 | 0.353 | 0.32 | -0.228 |
| logepa | rs191866099 | . | . | . | . | . | . | . | . | . | . | . |
| logepa | rs147998395 | 0 | . | . | . | . | . | . | . | . | . | . |
| logepa | rs7090328 | 0.789 | 0.833 | 0.676 | 0.822 | 0.875 | 0.326 | 0.721 | 0.803 | 0.354 | 0.619 | -0.122 |
| logepa | rs79814535 | 0.021 | 0.621 | 0.169 | 0.08 | 3.90E-07 | 1.40E-06 | 4.70E-05 | 2.20E-05 | 4.90E-05 | 1.50E-06 | 0.379 |
| logepa | rs7073082 | 0.789 | 0.838 | 0.679 | 0.827 | 0.874 | 0.325 | 0.717 | 0.801 | 0.352 | 0.613 | -0.126 |
| logepa | rs11239526 | 0.033 | 0.595 | 0.173 | 0.074 | 1.24 | 0.75 | 0.72 | 0.889 | 0.556 | 0.85 | -0.18 |
| logepa | rs141839841 | 0 | . | . | . | . | . | . | . | . | . | . |
| logepa | rs75697371 | 0.006 | . | . | . | . | . | . | . | . | . | . |
| logepa | rs75972854 | 0.005 | . | . | . | . | . | . | . | . | . | . |
| logepa | rs139291975 | . | . | . | . | . | . | . | . | . | . | . |
| logepa | rs144064711 | 0.004 | . | . | . | . | . | . | . | . | . | . |
| logepa | rs3780902 | 0.062 | 0.643 | 0.181 | 0.117 | 0.901 | 0.606 | 0.877 | 0.52 | 0.381 | 0.372 | -0.243 |
| logepa | rs190449801 | 0.001 | . | . | . | . | . | . | . | . | . | . |
| logepa | rs73283196 | 0.061 | 0.647 | 0.182 | 0.122 | 0.86 | 0.59 | 0.826 | 0.493 | 0.363 | 0.337 | -0.232 |
| logepa | rs28395867 | 0.031 | 0.617 | 0.173 | 0.085 | 0.147 | 0.229 | 0.217 | 0.169 | 0.283 | 0.288 | 0.251 |
| logepa | rs28395866 | 0.079 | 0.637 | 0.187 | 0.124 | 0.837 | 0.461 | 0.746 | 0.53 | 0.342 | 0.325 | -0.192 |
| logepa | rs55661611 | 0.04 | 0.598 | 0.172 | 0.073 | 1.35 | 0.773 | 0.598 | 0.584 | 0.445 | 0.48 | -0.478 |
| logepa | rs55684518 | 0.016 | 0.582 | 0.164 | 0.055 | 1.54 | 3.1 | 0.829 | 0.891 | 2.04 | 0.96 | -0.325 |
| logepa | rs78552493 | 0.038 | 0.54 | 0.15 | 0.026 | 0.835 | 0.54 | 0.78 | 11.4 | 7.44 | 1.90E-04 | 4.77 |
| logepa | rs147665434 | 0.001 | . | . | . | . | . | . | . | . | . | . |
| logepa | rs34445001 | 0.006 | . | . | . | . | . | . | . | . | . | . |
| logepa | rs184019449 | 0.003 | . | . | . | . | . | . | . | . | . | . |
| logepa | rs3780903 | 0.731 | 0.344 | 0.263 | 0.163 | 1.07 | 0.471 | 0.873 | 1.41 | 0.632 | 0.439 | 0.105 |
| logepa | rs17157784 | 0.058 | 0.67 | 0.187 | 0.151 | 0.123 | 0.217 | 0.236 | 0.122 | 0.191 | 0.178 | 0.218 |
| logepa | rs3780904 | 0.021 | 0.623 | 0.17 | 0.083 | 8.20E-07 | 2.80E-06 | 4.20E-05 | 3.50E-05 | 7.40E-05 | 1.60E-06 | 0.377 |
| logepa | rs192064730 | . | . | . | . | . | . | . | . | . | . | . |
| logepa | rs12774534 | 0.006 | . | . | . | . | . | . | . | . | . | . |
| logepa | rs79179915 | 0.02 | 0.62 | 0.17 | 0.081 | 6.50E-07 | 2.30E-06 | 4.20E-05 | 2.90E-05 | 6.40E-05 | 1.50E-06 | 0.38 |
| logepa | rs117811571 | 0.011 | 0.561 | 0.157 | 0.039 | 16 | 42.4 | 0.296 | 15.3 | 57.5 | 0.468 | 122 |
| logepa | rs72796477 | 0.009 | . | . | . | . | . | . | . | . | . | . |
| logepa | rs55729342 | 0.058 | 0.663 | 0.185 | 0.142 | 0.105 | 0.208 | 0.254 | 0.117 | 0.201 | 0.212 | 0.24 |
| logepa | rs59317220 | 0.058 | 0.663 | 0.185 | 0.141 | 0.106 | 0.209 | 0.253 | 0.118 | 0.202 | 0.212 | 0.239 |
| logepa | rs3780905 | 0.057 | 0.686 | 0.187 | 0.168 | 0.205 | 0.171 | 0.057 | 0.178 | 0.139 | 0.027 | 0.134 |
| logepa | rs3780906 | 0.731 | 0.361 | 0.277 | 0.184 | 1.06 | 0.466 | 0.897 | 1.37 | 0.619 | 0.487 | 0.104 |
| logepa | rs149047975 | 0.125 | 0.537 | 0.16 | 0.037 | 1.36 | 0.709 | 0.556 | 1.39 | 0.8 | 0.572 | 0.115 |
| logepa | rs3824614 | 0.02 | 0.62 | 0.169 | 0.08 | 4.40E-07 | 1.60E-06 | 4.70E-05 | 2.20E-05 | 5.00E-05 | 1.80E-06 | 0.38 |
| logepa | rs140254404 | . | . | . | . | . | . | . | . | . | . | . |
| logepa | rs145262684 | 0.013 | 0.563 | 0.159 | 0.042 | 8.11 | 19.8 | 0.391 | 6.03 | 19.4 | 0.577 | 19.9 |
| logepa | rs76735482 | 0.02 | 0.62 | 0.169 | 0.08 | 3.50E-07 | 1.30E-06 | 5.10E-05 | 1.90E-05 | 4.30E-05 | 2.10E-06 | 0.38 |
| logepa | rs73283198 | 0.027 | 0.629 | 0.184 | 0.113 | 0.969 | 0.619 | 0.96 | 0.257 | 0.176 | 0.047 | -0.441 |
| logepa | rs74911239 | 0.029 | 0.512 | 0.147 | 0.02 | 3.54 | 2.27 | 0.049 | 16.8 | 23.2 | 0.041 | 27.4 |
| logepa | rs150551647 | 0.005 | . | . | . | . | . | . | . | . | . | . |
| logepa | rs115200794 | 0.034 | 0.616 | 0.172 | 0.082 | 0.073 | 0.159 | 0.23 | 0.116 | 0.245 | 0.309 | 0.317 |
| logepa | rs74130823 | 0.139 | 0.549 | 0.176 | 0.062 | 1.11 | 0.419 | 0.786 | 1.21 | 0.542 | 0.67 | 0.079 |
| logepa | rs2279435 | 0.662 | 0.684 | 0.4 | 0.517 | 0.789 | 0.288 | 0.516 | 0.871 | 0.348 | 0.729 | -3.00E-03 |
| logepa | rs147279673 | 0.015 | 0.589 | 0.167 | 0.062 | 1.19 | 2.22 | 0.923 | 0.496 | 1.13 | 0.759 | -0.435 |
| logepa | rs183525146 | 0.004 | . | . | . | . | . | . | . | . | . | . |
| logepa | rs3740107 | 0.754 | 0.425 | 0.361 | 0.314 | 1.18 | 0.505 | 0.693 | 1.22 | 0.583 | 0.679 | 4.60E-03 |
| logepa | rs140697247 | . | . | . | . | . | . | . | . | . | . | . |
| logepa | rs1565096 | 0.825 | 0.874 | 0.711 | 0.868 | 0.691 | 0.259 | 0.324 | 0.777 | 0.35 | 0.576 | -0.096 |
| logepa | rs1565097 | 0.793 | 0.671 | 0.557 | 0.631 | 0.885 | 0.344 | 0.753 | 0.914 | 0.423 | 0.846 | -0.013 |
| logepa | rs144584548 | 0.016 | 0.582 | 0.164 | 0.055 | 1.56 | 3.15 | 0.825 | 0.924 | 2.15 | 0.973 | -0.303 |
| logepa | rs75811053 | 0.02 | 0.627 | 0.173 | 0.091 | 1.30E-06 | 4.30E-06 | 4.20E-05 | 4.70E-05 | 9.70E-05 | 1.50E-06 | 0.373 |
| logepa | rs147462967 | 0.009 | . | . | . | . | . | . | . | . | . | . |
| logepa | rs74130824 | 0.051 | 0.658 | 0.182 | 0.13 | 7.50E-03 | 0.027 | 0.17 | 0.017 | 0.046 | 0.138 | 0.334 |

| logepa | rs116883184 | 0.017 | 0.561 | 0.161 | 0.044 | 4.14 | 5.8 | 0.309 | 2.68 | 4.64 | 0.57 | 2.52 |
| --- | --- | --- | --- | --- | --- | --- | --- | --- | --- | --- | --- | --- |
| logepa | rs117258049 | 0.017 | 0.561 | 0.161 | 0.044 | 4.14 | 5.8 | 0.309 | 2.68 | 4.64 | 0.57 | 2.52 |
| logepa | rs3780908 | 0.107 | 0.519 | 0.164 | 0.037 | 1.45 | 0.504 | 0.282 | 1.39 | 0.551 | 0.409 | 0.074 |
| logepa | rs180938522 | 0.002 | . | . | . | . | . | . | . | . | . | . |
| logepa | rs76687372 | 0.003 | . | . | . | . | . | . | . | . | . | . |
| logepa | rs3780909 | 0.606 | 0.61 | 0.294 | 0.305 | 0.84 | 0.279 | 0.6 | 0.949 | 0.343 | 0.885 | 0.036 |
| logepa | rs78281156 | 0.02 | 0.627 | 0.173 | 0.092 | 1.30E-06 | 4.20E-06 | 4.30E-05 | 4.60E-05 | 9.60E-05 | 1.50E-06 | 0.373 |
| logepa | rs187840631 | . | . | . | . | . | . | . | . | . | . | . |
| logepa | rs76868242 | 0.02 | 0.627 | 0.173 | 0.092 | 1.30E-06 | 4.20E-06 | 4.50E-05 | 4.60E-05 | 9.50E-05 | 1.60E-06 | 0.373 |
| logepa | rs192794192 | 0.001 | . | . | . | . | . | . | . | . | . | . |
| logepa | rs12781248 | 0.007 | . | . | . | . | . | . | . | . | . | . |
| logepa | rs12762375 | 0.006 | . | . | . | . | . | . | . | . | . | . |
| logepa | rs192256986 | 0.004 | . | . | . | . | . | . | . | . | . | . |
| logepa | rs184411030 | 0.01 | . | . | . | . | . | . | . | . | . | . |
| logepa | rs189601483 | 0.02 | 0.627 | 0.173 | 0.091 | 1.10E-06 | 3.90E-06 | 5.00E-05 | 4.30E-05 | 9.10E-05 | 1.80E-06 | 0.373 |
| logepa | rs143254579 | 0.033 | 0.596 | 0.173 | 0.075 | 1.09 | 0.678 | 0.896 | 0.849 | 0.528 | 0.792 | -0.132 |
| logepa | rs12253903 | 0.017 | 0.615 | 0.174 | 0.085 | 0.195 | 0.262 | 0.225 | 0.1 | 0.075 | 2.20E-03 | 0.203 |
| logepa | rs35489926 | 0.006 | . | . | . | . | . | . | . | . | . | . |
| logepa | rs71494783 | 0.039 | 0.63 | 0.178 | 0.103 | 0.071 | 0.158 | 0.236 | 0.129 | 0.238 | 0.265 | 0.305 |
| logepa | rs180711602 | 0.002 | . | . | . | . | . | . | . | . | . | . |
| logepa | rs117312402 | 0.02 | 0.627 | 0.173 | 0.091 | 1.20E-06 | 4.00E-06 | 4.90E-05 | 4.40E-05 | 9.30E-05 | 1.70E-06 | 0.373 |
| logepa | rs74831694 | 0.004 | . | . | . | . | . | . | . | . | . | . |
| logepa | rs140301943 | 0.002 | . | . | . | . | . | . | . | . | . | . |
| logepa | rs78553679 | 0.013 | 0.558 | 0.157 | 0.038 | 3.72 | 4.72 | 0.301 | 3.35 | 4.76 | 0.395 | 3.68 |
| logepa | rs78334250 | 0.02 | 0.627 | 0.173 | 0.091 | 1.20E-06 | 4.00E-06 | 4.90E-05 | 4.40E-05 | 9.30E-05 | 1.80E-06 | 0.373 |
| logepa | rs76000028 | 0.02 | 0.627 | 0.173 | 0.091 | 1.20E-06 | 4.00E-06 | 4.90E-05 | 4.40E-05 | 9.30E-05 | 1.80E-06 | 0.373 |
| logepa | rs145195254 | 0.001 | . | . | . | . | . | . | . | . | . | . |
| logepa | rs137888527 | 0.001 | . | . | . | . | . | . | . | . | . | . |
| logepa | rs149109592 | 0.005 | . | . | . | . | . | . | . | . | . | . |
| logepa | rs80280010 | 0.032 | 0.598 | 0.174 | 0.078 | 1.15 | 0.715 | 0.824 | 0.84 | 0.523 | 0.78 | -0.169 |
| logepa | rs189995374 | . | . | . | . | . | . | . | . | . | . | . |
| logepa | rs75467770 | 0.021 | 0.631 | 0.175 | 0.097 | 8.10E-07 | 2.80E-06 | 5.60E-05 | 3.50E-05 | 7.50E-05 | 2.10E-06 | 0.369 |
| logepa | rs1487562 | 0.139 | 0.569 | 0.184 | 0.08 | 1.01 | 0.389 | 0.976 | 1.09 | 0.472 | 0.851 | 0.044 |
| logepa | rs12266963 | 0.039 | 0.63 | 0.178 | 0.103 | 0.071 | 0.159 | 0.237 | 0.13 | 0.239 | 0.268 | 0.305 |
| logepa | rs7099874 | 0.787 | 0.753 | 0.54 | 0.693 | 0.667 | 0.226 | 0.233 | 0.843 | 0.358 | 0.687 | 3.10E-03 |
| logepa | rs75687019 | 0.02 | 0.627 | 0.173 | 0.091 | 1.20E-06 | 4.00E-06 | 5.10E-05 | 4.40E-05 | 9.30E-05 | 1.80E-06 | 0.373 |
| logepa | rs148240462 | 0.031 | 0.511 | 0.149 | 0.021 | 3.76 | 2.38 | 0.036 | 7.39 | 9.49 | 0.119 | 10.9 |
| logepa | rs141278174 | . | . | . | . | . | . | . | . | . | . | . |
| logepa | rs76404852 | 0.02 | 0.627 | 0.173 | 0.091 | 1.20E-06 | 3.90E-06 | 5.20E-05 | 4.40E-05 | 9.20E-05 | 1.90E-06 | 0.373 |
| logepa | rs139112240 | 0.006 | . | . | . | . | . | . | . | . | . | . |
| logepa | rs12783037 | 0.042 | 0.643 | 0.18 | 0.115 | 0.06 | 0.133 | 0.203 | 0.104 | 0.183 | 0.199 | 0.301 |
| logepa | rs72796481 | 0.006 | . | . | . | . | . | . | . | . | . | . |
| logepa | rs76798749 | 0.023 | 0.645 | 0.177 | 0.109 | 1.30E-05 | 3.30E-05 | 6.90E-06 | 1.80E-04 | 3.00E-04 | 1.30E-07 | 0.355 |
| logepa | rs187732548 | . | . | . | . | . | . | . | . | . | . | . |
| logepa | rs75675474 | 0.023 | 0.645 | 0.177 | 0.109 | 1.30E-05 | 3.30E-05 | 6.80E-06 | 1.80E-04 | 3.00E-04 | 1.30E-07 | 0.355 |
| logepa | rs76552293 | 0.023 | 0.645 | 0.177 | 0.109 | 1.30E-05 | 3.30E-05 | 7.00E-06 | 1.90E-04 | 3.00E-04 | 1.40E-07 | 0.355 |
| logepa | rs147445993 | 0.002 | . | . | . | . | . | . | . | . | . | . |
| logepa | rs115902283 | 0.023 | 0.646 | 0.177 | 0.11 | 1.50E-05 | 3.70E-05 | 6.50E-06 | 2.00E-04 | 3.20E-04 | 1.20E-07 | 0.354 |
| logepa | rs4540799 | 0.786 | 0.773 | 0.554 | 0.719 | 0.659 | 0.22 | 0.212 | 0.829 | 0.349 | 0.655 | -9.90E-03 |
| logepa | rs12765320 | 0.172 | 0.625 | 0.204 | 0.15 | 0.836 | 0.359 | 0.677 | 0.83 | 0.363 | 0.67 | -0.027 |
| logepa | rs12782256 | 0.006 | . | . | . | . | . | . | . | . | . | . |
| logepa | rs191727256 | 0 | . | . | . | . | . | . | . | . | . | . |
| logepa | rs2082111 | 0.111 | 0.53 | 0.169 | 0.046 | 1.42 | 0.519 | 0.338 | 1.28 | 0.511 | 0.536 | 0.014 |
| logepa | rs2082110 | 0.157 | 0.618 | 0.198 | 0.133 | 0.887 | 0.396 | 0.788 | 0.835 | 0.417 | 0.718 | -0.047 |
| logepa | rs2082109 | 0.157 | 0.618 | 0.198 | 0.133 | 0.887 | 0.396 | 0.788 | 0.835 | 0.417 | 0.718 | -0.047 |
| logepa | rs117316662 | 0.02 | 0.626 | 0.173 | 0.09 | 1.10E-06 | 3.80E-06 | 5.50E-05 | 4.30E-05 | 9.10E-05 | 2.00E-06 | 0.374 |
| logepa | rs12247163 | 0.177 | 0.615 | 0.198 | 0.132 | 0.837 | 0.333 | 0.655 | 0.868 | 0.364 | 0.736 | -5.40E-03 |
| logepa | rs12355888 | 0.075 | 0.529 | 0.161 | 0.037 | 1.29 | 0.479 | 0.492 | 1.73 | 0.645 | 0.139 | 0.364 |
| logepa | rs143772290 | 0.005 | . | . | . | . | . | . | . | . | . | . |
| logepa | rs146849452 | 0.003 | . | . | . | . | . | . | . | . | . | . |
| logepa | rs140267121 | 0.003 | . | . | . | . | . | . | . | . | . | . |
| logepa | rs7080474 | 0.155 | 0.574 | 0.188 | 0.09 | 1.01 | 0.372 | 0.984 | 1.04 | 0.416 | 0.921 | 0.02 |
| logepa | rs144066203 | 0.001 | . | . | . | . | . | . | . | . | . | . |
| logepa | rs12765209 | 0.021 | 0.63 | 0.176 | 0.098 | 1.00E-06 | 3.50E-06 | 5.00E-05 | 4.00E-05 | 8.60E-05 | 1.70E-06 | 0.37 |
| logepa | rs150930735 | 0.001 | . | . | . | . | . | . | . | . | . | . |
| logepa | rs7068039 | 0.177 | 0.615 | 0.199 | 0.133 | 0.836 | 0.331 | 0.651 | 0.868 | 0.361 | 0.734 | -4.90E-03 |
| logepa | rs117622652 | 0.02 | 0.626 | 0.173 | 0.09 | 1.10E-06 | 3.70E-06 | 5.70E-05 | 4.20E-05 | 8.90E-05 | 2.10E-06 | 0.374 |
| logepa | rs190191514 | 0.001 | . | . | . | . | . | . | . | . | . | . |
| logepa | rs74130835 | 0.023 | 0.617 | 0.17 | 0.08 | 4.40E-09 | 3.80E-08 | 0.027 | 7.00E-07 | 4.10E-06 | 0.014 | 0.383 |
| logepa | rs1811604 | 0.022 | 0.62 | 0.172 | 0.085 | 1.40E-08 | 1.20E-07 | 0.05 | 1.50E-06 | 9.10E-06 | 0.028 | 0.38 |
| logepa | rs146971256 | . | . | . | . | . | . | . | . | . | . | . |
| logepa | rs74336000 | 0.005 | . | . | . | . | . | . | . | . | . | . |
| logepa | rs186046492 | 0.005 | . | . | . | . | . | . | . | . | . | . |
| logepa | rs34971823 | 0.006 | . | . | . | . | . | . | . | . | . | . |
| logepa | rs75227446 | 0.02 | 0.626 | 0.173 | 0.09 | 8.00E-07 | 2.80E-06 | 7.90E-05 | 3.50E-05 | 7.60E-05 | 3.10E-06 | 0.374 |
| logepa | rs190457599 | 0.003 | . | . | . | . | . | . | . | . | . | . |
| logepa | rs79591917 | 0.02 | 0.626 | 0.173 | 0.089 | 1.10E-06 | 3.70E-06 | 6.10E-05 | 4.20E-05 | 8.90E-05 | 2.30E-06 | 0.374 |
| logepa | rs702365 | 0.609 | 0.609 | 0.303 | 0.319 | 0.83 | 0.272 | 0.571 | 0.952 | 0.338 | 0.89 | 0.042 |
| logepa | rs79643836 | 0.021 | 0.627 | 0.173 | 0.091 | 8.90E-07 | 3.10E-06 | 5.60E-05 | 3.70E-05 | 8.00E-05 | 2.00E-06 | 0.373 |
| logepa | rs702366 | 0.61 | 0.629 | 0.306 | 0.341 | 0.802 | 0.257 | 0.491 | 0.925 | 0.326 | 0.825 | 0.036 |
| logepa | rs1059696 | 0.123 | 0.531 | 0.172 | 0.051 | 1.29 | 0.466 | 0.483 | 1.29 | 0.503 | 0.518 | 0.061 |
| logepa | rs901681 | 0.61 | 0.617 | 0.299 | 0.319 | 0.795 | 0.256 | 0.475 | 0.94 | 0.331 | 0.861 | 0.049 |
| logepa | rs61854092 | 0.122 | 0.531 | 0.172 | 0.051 | 1.3 | 0.469 | 0.465 | 1.29 | 0.499 | 0.516 | 0.057 |
| logepa | rs148080753 | 0.001 | . | . | . | . | . | . | . | . | . | . |
| logepa | rs61854093 | 0.145 | 0.557 | 0.182 | 0.073 | 1.05 | 0.404 | 0.895 | 1.15 | 0.483 | 0.736 | 0.066 |
| logepa | rs141822774 | 0.002 | . | . | . | . | . | . | . | . | . | . |

| logepa | rs74130836 | 0.023 | 0.62 | 0.171 | 0.083 | 2.40E-09 | 2.00E-08 | 0.02 | 4.60E-07 | 2.60E-06 | 0.01 | 0.38 |
| --- | --- | --- | --- | --- | --- | --- | --- | --- | --- | --- | --- | --- |
| logepa | rs150620963 | 0.01 | . | . | . | . | . | . | . | . | . | . |
| logepa | rs3780913 | 0.781 | 0.841 | 0.6 | 0.809 | 0.644 | 0.214 | 0.186 | 0.78 | 0.329 | 0.555 | -0.063 |
| logepa | rs142255723 | 0.002 | . | . | . | . | . | . | . | . | . | . |
| logepa | rs3780914 | 0.608 | 0.617 | 0.292 | 0.308 | 0.806 | 0.256 | 0.497 | 0.937 | 0.323 | 0.85 | 0.043 |
| logepa | rs182656507 | 0.002 | . | . | . | . | . | . | . | . | . | . |
| logepa | rs2291427 | 0.756 | 0.61 | 0.428 | 0.481 | 0.766 | 0.266 | 0.442 | 0.973 | 0.394 | 0.946 | 0.079 |
| logepa | rs10751382 | 0.755 | 0.607 | 0.427 | 0.478 | 0.77 | 0.268 | 0.454 | 0.976 | 0.396 | 0.952 | 0.079 |
| logepa | rs117610108 | 0.02 | 0.624 | 0.171 | 0.086 | 9.60E-07 | 3.40E-06 | 7.70E-05 | 3.90E-05 | 8.50E-05 | 3.10E-06 | 0.376 |
| logepa | rs144966941 | 0.037 | 0.663 | 0.187 | 0.144 | 0.139 | 0.188 | 0.144 | 0.101 | 0.129 | 0.073 | 0.207 |
| logepa | rs41502051 | 0.022 | 0.531 | 0.144 | 0.02 | 4.52 | 3.41 | 0.046 | 3.31 | 3.68 | 0.281 | 3.9 |
| logepa | rs41426046 | 0.02 | 0.623 | 0.171 | 0.085 | 1.90E-06 | 6.30E-06 | 5.50E-05 | 6.10E-05 | 1.20E-04 | 1.80E-06 | 0.377 |
| logepa | rs41519248 | 0.02 | 0.622 | 0.171 | 0.084 | 9.70E-07 | 3.40E-06 | 7.90E-05 | 3.90E-05 | 8.50E-05 | 3.20E-06 | 0.378 |
| logepa | rs189108245 | 0.002 | . | . | . | . | . | . | . | . | . | . |
| logepa | rs7896431 | 0.591 | 0.661 | 0.328 | 0.404 | 0.796 | 0.251 | 0.47 | 0.883 | 0.314 | 0.726 | 7.20E-03 |
| logepa | rs183724547 | 0.003 | . | . | . | . | . | . | . | . | . | . |
| logepa | rs142044990 | 0.006 | . | . | . | . | . | . | . | . | . | . |
| logepa | rs188140929 | . | . | . | . | . | . | . | . | . | . | . |
| logepa | rs76819096 | 0.008 | . | . | . | . | . | . | . | . | . | . |
| logepa | rs10751383 | 0.654 | 0.936 | 0.474 | 0.897 | 0.691 | 0.205 | 0.214 | 0.682 | 0.219 | 0.234 | -0.186 |
| logepa | rs117819321 | 0.015 | 0.594 | 0.169 | 0.068 | 1.13 | 1.09 | 0.898 | 0.365 | 0.406 | 0.365 | -0.48 |
| logepa | rs2242332 | 0.75 | 0.521 | 0.354 | 0.337 | 0.828 | 0.278 | 0.575 | 1.07 | 0.417 | 0.857 | 0.114 |
| logepa | rs2242333 | 0.06 | 0.709 | 0.194 | 0.209 | 0.225 | 0.165 | 0.042 | 0.164 | 0.116 | 0.01 | 0.092 |
| logepa | rs2242334 | 0.752 | 0.532 | 0.363 | 0.355 | 0.814 | 0.274 | 0.542 | 1.06 | 0.412 | 0.887 | 0.112 |
| logepa | rs189465041 | 0.002 | . | . | . | . | . | . | . | . | . | . |
| logepa | rs28395868 | 0.014 | 0.523 | 0.148 | 0.022 | 38.8 | 41.4 | 6.10E-04 | 226 | 344 | 3.70E-04 | 4.60E+03 |
| logepa | rs56067550 | 0.016 | 0.577 | 0.162 | 0.05 | 2 | 3.61 | 0.702 | 1.23 | 2.77 | 0.926 | -0.153 |
| logepa | rs1051713 | 0.134 | 0.511 | 0.162 | 0.035 | 1.26 | 0.46 | 0.522 | 1.5 | 0.557 | 0.273 | 0.195 |
| logepa | rs115074210 | 0.009 | . | . | . | . | . | . | . | . | . | . |
| logepa | rs7900977 | 0.822 | 0.855 | 0.686 | 0.845 | 0.642 | 0.221 | 0.198 | 0.779 | 0.334 | 0.561 | -0.069 |
| logepa | rs147613891 | 0.004 | . | . | . | . | . | . | . | . | . | . |
| logepa | rs2229136 | 0.063 | 0.679 | 0.208 | 0.206 | 0.45 | 0.293 | 0.221 | 0.393 | 0.221 | 0.096 | -9.60E-03 |
| logepa | rs185482841 | 0.001 | . | . | . | . | . | . | . | . | . | . |
| logepa | rs2288619 | 0.063 | 0.679 | 0.208 | 0.206 | 0.45 | 0.293 | 0.22 | 0.393 | 0.221 | 0.096 | -8.90E-03 |
| logepa | rs189891524 | . | . | . | . | . | . | . | . | . | . | . |
| logepa | rs56145077 | 0.115 | 0.481 | 0.153 | 0.021 | 1.49 | 0.534 | 0.268 | 1.86 | 0.62 | 0.063 | 0.362 |
| logepa | rs143216970 | 0.003 | . | . | . | . | . | . | . | . | . | . |
| logepa | rs142246478 | 0.001 | . | . | . | . | . | . | . | . | . | . |
| logepa | rs13134 | 0.008 | . | . | . | . | . | . | . | . | . | . |
| logepa | rs41314980 | 0.031 | 0.518 | 0.155 | 0.028 | 5.23 | 4.03 | 0.032 | 6.51 | 5.6 | 0.029 | 12.9 |
| logepa | rs41304611 | 0.009 | . | . | . | . | . | . | . | . | . | . |
| logepa | rs193299617 | . | . | . | . | . | . | . | . | . | . | . |
| logepa | rs12772724 | 0.008 | . | . | . | . | . | . | . | . | . | . |
| logepa | rs139489537 | 0.03 | 0.605 | 0.176 | 0.083 | 0.788 | 0.558 | 0.736 | 0.663 | 0.431 | 0.528 | -0.076 |
| logepa | rs12776062 | 0.006 | . | . | . | . | . | . | . | . | . | . |
| logepa | rs752059 | 0.116 | 0.481 | 0.153 | 0.021 | 1.48 | 0.533 | 0.275 | 1.86 | 0.622 | 0.062 | 0.366 |
| logepa | rs147649982 | 0.049 | 0.601 | 0.17 | 0.072 | 0.064 | 0.081 | 0.032 | 0.197 | 0.194 | 0.099 | 0.343 |
| logepa | rs17157798 | 0.055 | 0.64 | 0.195 | 0.143 | 0.523 | 0.358 | 0.344 | 0.525 | 0.288 | 0.24 | 0.013 |
| logepa | rs142298619 | 0.001 | . | . | . | . | . | . | . | . | . | . |
| logepa | rs4948673 | 0.819 | 0.874 | 0.708 | 0.868 | 0.647 | 0.225 | 0.21 | 0.768 | 0.332 | 0.542 | -0.086 |
| logepa | rs74231484 | 0.066 | 0.706 | 0.195 | 0.207 | 0.286 | 0.194 | 0.065 | 0.21 | 0.132 | 0.013 | 0.05 |
| logepa | rs72796489 | 0.002 | . | . | . | . | . | . | . | . | . | . |
| logepa | rs113447578 | 0.014 | 0.571 | 0.16 | 0.046 | 2.95 | 2.71 | 0.238 | 1.96 | 1.51 | 0.381 | 0.786 |
| logepa | rs34063374 | 0.008 | . | . | . | . | . | . | . | . | . | . |
| logepa | rs11239527 | 0.011 | 0.541 | 0.151 | 0.028 | 8.19 | 14.6 | 0.24 | 71.8 | 107 | 4.00E-03 | 310 |
| logepa | rs34371883 | 0.007 | . | . | . | . | . | . | . | . | . | . |
| logepa | rs118168828 | 0.006 | . | . | . | . | . | . | . | . | . | . |
| logepa | rs34349618 | 0.006 | . | . | . | . | . | . | . | . | . | . |
| logepa | rs80041679 | 0.03 | 0.604 | 0.176 | 0.083 | 0.782 | 0.556 | 0.73 | 0.666 | 0.433 | 0.531 | -0.072 |
| logepa | rs149640607 | 0.001 | . | . | . | . | . | . | . | . | . | . |
| logepa | rs4427523 | 0.896 | 3.84 | 3.54 | 0.145 | 0.2 | 0.076 | 2.30E-05 | 0.329 | 0.174 | 0.036 | -2.79 |
| logepa | rs185880109 | . | . | . | . | . | . | . | . | . | . | . |
| logepa | rs191523220 | 0.001 | . | . | . | . | . | . | . | . | . | . |
| logepa | rs144315284 | 0.002 | . | . | . | . | . | . | . | . | . | . |
| logepa | rs4948674 | 0.345 | 0.397 | 0.164 | 0.026 | 1.3 | 0.43 | 0.429 | 1.9 | 0.762 | 0.111 | 0.281 |
| logepa | rs182984298 | 0.003 | . | . | . | . | . | . | . | . | . | . |
| logepa | rs56749023 | 0.016 | 0.615 | 0.173 | 0.084 | 0.165 | 0.245 | 0.225 | 0.059 | 0.06 | 5.60E-03 | 0.227 |
| logepa | rs112508957 | 0.01 | 0.542 | 0.144 | 0.021 | 0.419 | 0.409 | 0.373 | 19.2 | 12.1 | 3.00E-06 | 4.39 |
| logepa | rs7089063 | 0.195 | 0.581 | 0.202 | 0.118 | 0.892 | 0.291 | 0.726 | 1.01 | 0.349 | 0.967 | 0.053 |
| logepa | rs145000779 | . | . | . | . | . | . | . | . | . | . | . |
| logepa | rs80201572 | 0.03 | 0.604 | 0.176 | 0.083 | 0.776 | 0.551 | 0.721 | 0.663 | 0.431 | 0.527 | -0.069 |
| logepa | rs140568227 | 0.002 | . | . | . | . | . | . | . | . | . | . |
| logepa | rs149756228 | 0.009 | . | . | . | . | . | . | . | . | . | . |
| logepa | rs112380685 | 0.008 | . | . | . | . | . | . | . | . | . | . |
| logepa | rs60481109 | 0.015 | 0.612 | 0.171 | 0.08 | 0.177 | 0.249 | 0.219 | 0.064 | 0.061 | 4.20E-03 | 0.218 |
| logepa | rs138221843 | 0.002 | . | . | . | . | . | . | . | . | . | . |
| logepa | rs150283194 | 0.001 | . | . | . | . | . | . | . | . | . | . |
| logepa | rs138879661 | 0.004 | . | . | . | . | . | . | . | . | . | . |
| logepa | rs34960345 | 0.044 | 0.615 | 0.184 | 0.105 | 0.572 | 0.358 | 0.372 | 0.617 | 0.343 | 0.385 | 0.03 |
| logepa | rs147514068 | 0.004 | . | . | . | . | . | . | . | . | . | . |
| logepa | rs35410535 | 0.06 | 0.649 | 0.2 | 0.159 | 0.477 | 0.308 | 0.252 | 0.504 | 0.264 | 0.191 | 0.03 |
| logepa | rs191613635 | 0.001 | . | . | . | . | . | . | . | . | . | . |
| logepa | rs17157800 | 0.005 | . | . | . | . | . | . | . | . | . | . |
| logepa | rs17523413 | 0.039 | 0.603 | 0.178 | 0.086 | 0.683 | 0.493 | 0.597 | 0.692 | 0.437 | 0.56 | -9.70E-04 |
| logepa | rs7897036 | 0.037 | 0.544 | 0.159 | 0.038 | 0.924 | 0.512 | 0.886 | 2.8 | 1.33 | 0.031 | 0.94 |
| logepa | rs76334323 | 0.02 | 0.619 | 0.169 | 0.079 | 9.00E-07 | 3.20E-06 | 9.50E-05 | 3.80E-05 | 8.30E-05 | 3.80E-06 | 0.381 |

| logepa | rs143535991 | 0.001 | . | . | . | . | . | . | . | . | . | . |
| --- | --- | --- | --- | --- | --- | --- | --- | --- | --- | --- | --- | --- |
| logepa | rs140344979 | 0.003 | . | . | . | . | . | . | . | . | . | . |
| logepa | rs36047811 | 0.06 | 0.649 | 0.2 | 0.159 | 0.477 | 0.308 | 0.252 | 0.504 | 0.264 | 0.191 | 0.03 |
| logepa | rs76493570 | 0.043 | 0.615 | 0.184 | 0.104 | 0.573 | 0.365 | 0.382 | 0.615 | 0.348 | 0.39 | 0.028 |
| logepa | rs192998920 | . | . | . | . | . | . | . | . | . | . | . |
| logepa | rs116840852 | 0.013 | 0.565 | 0.162 | 0.046 | 2.8 | 4.24 | 0.497 | 2.4 | 3.59 | 0.559 | 1.43 |
| logepa | rs184824101 | 0 | . | . | . | . | . | . | . | . | . | . |
| logepa | rs41301621 | 0.003 | . | . | . | . | . | . | . | . | . | . |
| logepa | rs3740109 | 0.06 | 0.649 | 0.2 | 0.159 | 0.477 | 0.308 | 0.252 | 0.504 | 0.264 | 0.191 | 0.03 |
| logepa | rs7899415 | 0.025 | 0.57 | 0.168 | 0.057 | 1.43 | 2.7 | 0.852 | 1.68 | 3.24 | 0.787 | 0.372 |
| logepa | rs184395284 | 0.003 | . | . | . | . | . | . | . | . | . | . |
| logepa | rs139209589 | 0.004 | . | . | . | . | . | . | . | . | . | . |
| logepa | rs149924163 | 0.001 | . | . | . | . | . | . | . | . | . | . |
| logepa | rs3740110 | 0.02 | 0.619 | 0.169 | 0.079 | 9.00E-07 | 3.20E-06 | 9.50E-05 | 3.80E-05 | 8.30E-05 | 3.80E-06 | 0.381 |
| logepa | rs144335113 | 0.001 | . | . | . | . | . | . | . | . | . | . |
| logepa | rs74743385 | 0.01 | 0.542 | 0.144 | 0.021 | 0.419 | 0.409 | 0.373 | 19.2 | 12.1 | 2.90E-06 | 4.4 |
| logepa | rs146545829 | 0.001 | . | . | . | . | . | . | . | . | . | . |
| logepa | rs181842689 | 0.001 | . | . | . | . | . | . | . | . | . | . |
| logepa | rs3740111 | 0.044 | 0.615 | 0.184 | 0.105 | 0.572 | 0.358 | 0.372 | 0.617 | 0.343 | 0.385 | 0.03 |
| logepa | rs186261599 | 0.001 | . | . | . | . | . | . | . | . | . | . |
| logepa | rs73285348 | 0.008 | . | . | . | . | . | . | . | . | . | . |
| logepa | rs186356901 | 0.001 | . | . | . | . | . | . | . | . | . | . |
| logepa | rs3824618 | 0.03 | 0.604 | 0.176 | 0.083 | 0.776 | 0.551 | 0.721 | 0.662 | 0.431 | 0.526 | -0.069 |
| logepa | rs3802548 | 0.304 | 0.523 | 0.191 | 0.075 | 1.15 | 0.36 | 0.666 | 1.18 | 0.416 | 0.647 | 0.036 |
| logepa | rs3802547 | 0.02 | 0.619 | 0.169 | 0.079 | 9.00E-07 | 3.20E-06 | 9.40E-05 | 3.80E-05 | 8.30E-05 | 3.70E-06 | 0.381 |
| logepa | rs77677521 | 0.013 | 0.565 | 0.162 | 0.046 | 2.8 | 4.24 | 0.497 | 2.39 | 3.58 | 0.56 | 1.42 |
| logepa | rs3824616 | 0.06 | 0.649 | 0.2 | 0.159 | 0.477 | 0.308 | 0.252 | 0.504 | 0.264 | 0.191 | 0.03 |
| logepa | rs191073818 | 0.001 | . | . | . | . | . | . | . | . | . | . |
| logepa | rs147078004 | . | . | . | . | . | . | . | . | . | . | . |
| logepa | rs7908745 | 0.388 | 0.674 | 0.271 | 0.326 | 0.824 | 0.224 | 0.476 | 0.834 | 0.273 | 0.579 | -0.035 |
| logepa | rs17157811 | 0.005 | . | . | . | . | . | . | . | . | . | . |
| logepa | rs138506087 | 0.006 | . | . | . | . | . | . | . | . | . | . |
| logepa | rs77241269 | 0.016 | 0.613 | 0.173 | 0.082 | 0.172 | 0.248 | 0.223 | 0.064 | 0.062 | 4.70E-03 | 0.221 |
| logepa | rs138374938 | 0.001 | . | . | . | . | . | . | . | . | . | . |
| logepa | rs2279433 | 0.044 | 0.616 | 0.184 | 0.105 | 0.569 | 0.349 | 0.359 | 0.612 | 0.334 | 0.368 | 0.029 |
| logepa | rs73285358 | 0.016 | 0.613 | 0.172 | 0.082 | 0.173 | 0.249 | 0.223 | 0.064 | 0.062 | 4.60E-03 | 0.22 |
| logepa | rs11239528 | 0.303 | 0.523 | 0.19 | 0.074 | 1.15 | 0.36 | 0.655 | 1.18 | 0.414 | 0.643 | 0.035 |
| logepa | rs77453386 | 0.02 | 0.618 | 0.169 | 0.078 | 5.50E-07 | 2.10E-06 | 1.80E-04 | 2.80E-05 | 6.50E-05 | 8.40E-06 | 0.382 |
| logepa | rs2279434 | 0.049 | 0.621 | 0.187 | 0.114 | 0.662 | 0.362 | 0.45 | 0.628 | 0.322 | 0.364 | -0.025 |
| logepa | rs149236525 | 0.005 | . | . | . | . | . | . | . | . | . | . |
| logepa | rs12573609 | 0.037 | 0.551 | 0.155 | 0.034 | 1.24 | 0.995 | 0.788 | 2 | 2.45 | 0.57 | 0.579 |
| logepa | rs191716655 | . | . | . | . | . | . | . | . | . | . | . |
| logepa | rs17523660 | 0.006 | . | . | . | . | . | . | . | . | . | . |
| logepa | rs142671360 | 0.004 | . | . | . | . | . | . | . | . | . | . |
| logepa | rs3764990 | 0.038 | 0.603 | 0.177 | 0.086 | 0.679 | 0.475 | 0.58 | 0.692 | 0.425 | 0.548 | 1.10E-03 |
| logepa | rs113682861 | 0.005 | . | . | . | . | . | . | . | . | . | . |
| logepa | rs117658270 | 0.02 | 0.618 | 0.169 | 0.078 | 5.50E-07 | 2.10E-06 | 1.80E-04 | 2.80E-05 | 6.50E-05 | 8.40E-06 | 0.382 |
| logepa | rs187309063 | . | . | . | . | . | . | . | . | . | . | . |
| logepa | rs73285360 | 0.016 | 0.613 | 0.172 | 0.082 | 0.174 | 0.249 | 0.222 | 0.065 | 0.062 | 4.60E-03 | 0.22 |
| logepa | rs80133289 | 0.007 | . | . | . | . | . | . | . | . | . | . |
| logepa | rs186693257 | . | . | . | . | . | . | . | . | . | . | . |
| logepa | rs35735968 | 0.048 | 0.624 | 0.19 | 0.121 | 0.533 | 0.352 | 0.34 | 0.597 | 0.326 | 0.345 | 0.042 |
| logepa | rs17157825 | 0.031 | 0.606 | 0.176 | 0.085 | 0.73 | 0.535 | 0.667 | 0.636 | 0.421 | 0.495 | -0.054 |
| logepa | rs2291428 | 0.299 | 0.521 | 0.186 | 0.068 | 1.16 | 0.36 | 0.626 | 1.19 | 0.415 | 0.623 | 0.036 |
| logepa | rs2291429 | 0.305 | 0.52 | 0.188 | 0.071 | 1.13 | 0.351 | 0.686 | 1.18 | 0.413 | 0.627 | 0.045 |
| logepa | rs145714792 | 0.003 | . | . | . | . | . | . | . | . | . | . |
| logepa | rs12769244 | 0.044 | 0.615 | 0.183 | 0.103 | 0.574 | 0.348 | 0.36 | 0.623 | 0.338 | 0.384 | 0.031 |
| logepa | rs12771275 | 0.044 | 0.615 | 0.183 | 0.103 | 0.574 | 0.348 | 0.36 | 0.623 | 0.338 | 0.384 | 0.031 |
| logepa | rs146266174 | 0 | . | . | . | . | . | . | . | . | . | . |
| logepa | rs41301623 | 0.002 | . | . | . | . | . | . | . | . | . | . |
| logepa | rs2306814 | 0.021 | 0.621 | 0.17 | 0.082 | 9.40E-07 | 3.20E-06 | 4.80E-05 | 3.80E-05 | 8.10E-05 | 1.70E-06 | 0.379 |
| logepa | rs12244834 | 0.012 | 0.583 | 0.162 | 0.052 | 5.30E-03 | 0.025 | 0.256 | 7.55 | 97.9 | 0.876 | 0.436 |
| logepa | rs184006011 | 0.002 | . | . | . | . | . | . | . | . | . | . |
| logepa | rs78829066 | 0.02 | 0.621 | 0.17 | 0.082 | 4.20E-07 | 1.70E-06 | 2.30E-04 | 2.30E-05 | 5.70E-05 | 1.20E-05 | 0.379 |
| logepa | rs34731408 | 0.044 | 0.615 | 0.183 | 0.103 | 0.574 | 0.349 | 0.362 | 0.624 | 0.339 | 0.385 | 0.031 |
| logepa | rs17451516 | 0.008 | . | . | . | . | . | . | . | . | . | . |
| logepa | rs139910133 | 0.002 | . | . | . | . | . | . | . | . | . | . |
| logepa | rs10900218 | 0.197 | 0.578 | 0.201 | 0.115 | 0.931 | 0.297 | 0.824 | 1.03 | 0.345 | 0.938 | 0.043 |
| logepa | rs146023999 | 0.001 | . | . | . | . | . | . | . | . | . | . |
| logepa | rs187296958 | . | . | . | . | . | . | . | . | . | . | . |
| logepa | rs142342833 | . | . | . | . | . | . | . | . | . | . | . |
| logepa | rs143489255 | . | . | . | . | . | . | . | . | . | . | . |
| logepa | rs138532518 | . | . | . | . | . | . | . | . | . | . | . |
| logepa | rs183768079 | 0.001 | . | . | . | . | . | . | . | . | . | . |
| logepa | rs117892272 | . | . | . | . | . | . | . | . | . | . | . |
| logepa | rs192993943 | . | . | . | . | . | . | . | . | . | . | . |
| logepa | rs182989043 | 0.001 | . | . | . | . | . | . | . | . | . | . |
| logepa | rs189703435 | . | . | . | . | . | . | . | . | . | . | . |
| logepa | rs188794040 | . | . | . | . | . | . | . | . | . | . | . |
| logepa | rs143920953 | . | . | . | . | . | . | . | . | . | . | . |
| logepa | rs191029121 | . | . | . | . | . | . | . | . | . | . | . |
| logepa | rs116875448 | . | . | . | . | . | . | . | . | . | . | . |
| logepa | rs190167877 | 0.001 | . | . | . | . | . | . | . | . | . | . |
| logepa | rs184993418 | . | . | . | . | . | . | . | . | . | . | . |
| logepa | rs143241158 | . | . | . | . | . | . | . | . | . | . | . |
| logepa | rs11239500 | 0.015 | 0.573 | 0.162 | 0.049 | 0.774 | 0.801 | 0.804 | 1.91 | 0.818 | 0.132 | 0.499 |

| logepa | rs140568994 | . | . | . | . | . | . | . | . | . | . | . |
| --- | --- | --- | --- | --- | --- | --- | --- | --- | --- | --- | --- | --- |
| logepa | rs187530317 | . | . | . | . | . | . | . | . | . | . | . |
| logepa | rs145189326 | . | . | . | . | . | . | . | . | . | . | . |
| logepa | rs186203332 | . | . | . | . | . | . | . | . | . | . | . |
| logepa | rs189921909 | . | . | . | . | . | . | . | . | . | . | . |
| logepa | rs187260989 | . | . | . | . | . | . | . | . | . | . | . |
| logepa | rs114376586 | 0.013 | 0.576 | 0.164 | 0.052 | 0.692 | 0.788 | 0.747 | 1.88 | 0.789 | 0.135 | 0.479 |
| logepa | rs151070808 | . | . | . | . | . | . | . | . | . | . | . |
| logepa | rs140927225 | 0.015 | 0.577 | 0.164 | 0.053 | 0.68 | 0.783 | 0.738 | 1.79 | 0.776 | 0.18 | 0.445 |
| logepa | rs191555615 | . | . | . | . | . | . | . | . | . | . | . |
| logepa | rs80278898 | 0.005 | . | . | . | . | . | . | . | . | . | . |
| logepa | rs190849503 | . | . | . | . | . | . | . | . | . | . | . |
| logepa | rs150080315 | 0.003 | . | . | . | . | . | . | . | . | . | . |
| logepa | rs143623885 | . | . | . | . | . | . | . | . | . | . | . |
| logepa | rs187760040 | . | . | . | . | . | . | . | . | . | . | . |
| logepa | rs143917143 | 0.005 | . | . | . | . | . | . | . | . | . | . |
| logepa | rs149851136 | 0.001 | . | . | . | . | . | . | . | . | . | . |
| logepa | rs181612054 | . | . | . | . | . | . | . | . | . | . | . |
| logepa | rs191006548 | . | . | . | . | . | . | . | . | . | . | . |
| logepa | rs117377631 | 0.026 | 0.587 | 0.159 | 0.049 | 0.065 | 0.106 | 0.093 | 0.209 | 0.278 | 0.239 | 0.356 |
| logepa | rs182107634 | . | . | . | . | . | . | . | . | . | . | . |
| logepa | rs190211867 | . | . | . | . | . | . | . | . | . | . | . |
| logepa | rs148293457 | . | . | . | . | . | . | . | . | . | . | . |
| logepa | rs191811733 | . | . | . | . | . | . | . | . | . | . | . |
| logepa | rs146109272 | . | . | . | . | . | . | . | . | . | . | . |
| logepa | rs148397915 | . | . | . | . | . | . | . | . | . | . | . |
| logepa | rs142233290 | . | . | . | . | . | . | . | . | . | . | . |
| logepa | rs80226874 | . | . | . | . | . | . | . | . | . | . | . |
| logepa | rs144496203 | . | . | . | . | . | . | . | . | . | . | . |
| logepa | rs191062130 | . | . | . | . | . | . | . | . | . | . | . |
| logepa | rs185807817 | . | . | . | . | . | . | . | . | . | . | . |
| logepa | rs117846213 | . | . | . | . | . | . | . | . | . | . | . |
| logepa | rs190545473 | . | . | . | . | . | . | . | . | . | . | . |
| logepa | rs192364918 | . | . | . | . | . | . | . | . | . | . | . |
| logepa | rs182939241 | . | . | . | . | . | . | . | . | . | . | . |
| logepa | rs12571125 | . | . | . | . | . | . | . | . | . | . | . |
| logepa | rs192418941 | . | . | . | . | . | . | . | . | . | . | . |
| logepa | rs141270243 | . | . | . | . | . | . | . | . | . | . | . |
| logepa | rs182615502 | . | . | . | . | . | . | . | . | . | . | . |
| logepa | rs146669856 | 0.004 | . | . | . | . | . | . | . | . | . | . |
| logepa | rs78290029 | 0.014 | 0.601 | 0.171 | 0.074 | 0.044 | 0.087 | 0.115 | 0.292 | 0.417 | 0.389 | 0.362 |
| logepa | rs139955565 | . | . | . | . | . | . | . | . | . | . | . |
| logepa | rs12267314 | 0.013 | 0.594 | 0.167 | 0.064 | 0.032 | 0.066 | 0.096 | 0.222 | 0.265 | 0.207 | 0.379 |
| logepa | rs74477197 | . | . | . | . | . | . | . | . | . | . | . |
| logepa | rs188147236 | . | . | . | . | . | . | . | . | . | . | . |
| logepa | rs2228065 | 0.012 | 0.592 | 0.166 | 0.062 | 0.036 | 0.064 | 0.059 | 0.289 | 0.286 | 0.209 | 0.378 |
| logepa | rs116961353 | 0.007 | . | . | . | . | . | . | . | . | . | . |
| logepa | rs190222272 | . | . | . | . | . | . | . | . | . | . | . |
| logepa | rs182719940 | . | . | . | . | . | . | . | . | . | . | . |
| logepa | rs184477413 | . | . | . | . | . | . | . | . | . | . | . |
| logepa | rs12265175 | 0.012 | 0.595 | 0.168 | 0.066 | 0.026 | 0.057 | 0.092 | 0.236 | 0.301 | 0.257 | 0.383 |
| logepa | rs182928276 | . | . | . | . | . | . | . | . | . | . | . |
| logepa | rs144409895 | . | . | . | . | . | . | . | . | . | . | . |
| logepa | rs58998447 | 0.003 | . | . | . | . | . | . | . | . | . | . |
| logepa | rs188668832 | . | . | . | . | . | . | . | . | . | . | . |
| logepa | rs148641686 | . | . | . | . | . | . | . | . | . | . | . |
| logepa | rs75468791 | . | . | . | . | . | . | . | . | . | . | . |
| logepa | rs147327397 | . | . | . | . | . | . | . | . | . | . | . |
| logepa | rs77354597 | 0.001 | . | . | . | . | . | . | . | . | . | . |
| logepa | rs41365544 | 0.012 | 0.58 | 0.163 | 0.053 | 1.40E-03 | 9.50E-03 | 0.325 | 28.2 | 253 | 0.711 | 0.442 |
| logepa | rs187564188 | . | . | . | . | . | . | . | . | . | . | . |
| logepa | rs183511984 | . | . | . | . | . | . | . | . | . | . | . |
| logepa | rs12262783 | 0.011 | 0.589 | 0.167 | 0.062 | 1.20E-11 | 3.10E-10 | 0.327 | 0.911 | 30.3 | 0.998 | 0.411 |
| logepa | rs188817687 | 0.001 | . | . | . | . | . | . | . | . | . | . |
| logepa | rs3815968 | . | . | . | . | . | . | . | . | . | . | . |
| logepa | rs187822300 | . | . | . | . | . | . | . | . | . | . | . |
| logepa | rs188902172 | 0.001 | . | . | . | . | . | . | . | . | . | . |
| logepa | rs181190448 | . | . | . | . | . | . | . | . | . | . | . |
| logepa | rs182699093 | . | . | . | . | . | . | . | . | . | . | . |
| logepa | rs182631303 | . | . | . | . | . | . | . | . | . | . | . |
| logepa | rs187014172 | 0.001 | . | . | . | . | . | . | . | . | . | . |
| logepa | rs142575406 | . | . | . | . | . | . | . | . | . | . | . |
| logepa | rs180711033 | . | . | . | . | . | . | . | . | . | . | . |
| logepa | rs137894823 | 0.001 | . | . | . | . | . | . | . | . | . | . |
| logepa | rs189053273 | 0 | . | . | . | . | . | . | . | . | . | . |
| logepa | rs143499388 | . | . | . | . | . | . | . | . | . | . | . |
| logepa | rs182936001 | 0.001 | . | . | . | . | . | . | . | . | . | . |
| logepa | rs148456406 | 0 | . | . | . | . | . | . | . | . | . | . |
| logepa | rs182499531 | 0.001 | . | . | . | . | . | . | . | . | . | . |
| logepa | rs74884915 | 0.005 | . | . | . | . | . | . | . | . | . | . |
| logepa | rs184046374 | . | . | . | . | . | . | . | . | . | . | . |
| logepa | rs74333993 | 0.004 | . | . | . | . | . | . | . | . | . | . |
| logepa | rs138889719 | . | . | . | . | . | . | . | . | . | . | . |
| logepa | rs75330330 | 0.003 | . | . | . | . | . | . | . | . | . | . |
| logepa | rs193051693 | . | . | . | . | . | . | . | . | . | . | . |
| logepa | rs145929633 | . | . | . | . | . | . | . | . | . | . | . |

| logepa | rs184076904 | 0.001 | . | . | . | . | . | . | . | . | . | . |
| --- | --- | --- | --- | --- | --- | --- | --- | --- | --- | --- | --- | --- |
| logepa | rs141802494 | 0.002 | . | . | . | . | . | . | . | . | . | . |
| logepa | rs149519759 | 0 | . | . | . | . | . | . | . | . | . | . |
| logepa | rs145467260 | 0.002 | . | . | . | . | . | . | . | . | . | . |
| logepa | rs79620914 | 0.006 | . | . | . | . | . | . | . | . | . | . |
| logepa | rs140538633 | 0.005 | . | . | . | . | . | . | . | . | . | . |
| logepa | rs140862301 | 0.001 | . | . | . | . | . | . | . | . | . | . |
| logepa | rs114615847 | 0.003 | . | . | . | . | . | . | . | . | . | . |
| logepa | rs141800259 | 0.004 | . | . | . | . | . | . | . | . | . | . |
| logepa | rs149779059 | 0.003 | . | . | . | . | . | . | . | . | . | . |
| logepa | rs116539253 | 0.001 | . | . | . | . | . | . | . | . | . | . |
| logepa | rs184979550 | 0 | . | . | . | . | . | . | . | . | . | . |
| logepa | rs190128940 | 0.001 | . | . | . | . | . | . | . | . | . | . |
| logepa | rs146594816 | . | . | . | . | . | . | . | . | . | . | . |
| logepa | rs141370766 | 0.003 | . | . | . | . | . | . | . | . | . | . |
| logepa | rs144670033 | . | . | . | . | . | . | . | . | . | . | . |
| logepa | rs187152174 | 0.003 | . | . | . | . | . | . | . | . | . | . |
| logepa | rs184180263 | 0.002 | . | . | . | . | . | . | . | . | . | . |
| logepa | rs140075861 | 0.002 | . | . | . | . | . | . | . | . | . | . |
| logepa | rs186483129 | . | . | . | . | . | . | . | . | . | . | . |
| logepa | rs186634282 | 0.003 | . | . | . | . | . | . | . | . | . | . |
| logepa | rs9988663 | 0.004 | . | . | . | . | . | . | . | . | . | . |
| logepa | rs138828529 | 0.004 | . | . | . | . | . | . | . | . | . | . |
| logepa | rs188777615 | 0.001 | . | . | . | . | . | . | . | . | . | . |
| logepa | rs192321882 | 0.001 | . | . | . | . | . | . | . | . | . | . |
| logepa | rs150812270 | 0.003 | . | . | . | . | . | . | . | . | . | . |
| logepa | rs185906603 | . | . | . | . | . | . | . | . | . | . | . |
| logepa | rs191070862 | . | . | . | . | . | . | . | . | . | . | . |
| logepa | rs182904978 | . | . | . | . | . | . | . | . | . | . | . |
| logepa | rs192679655 | . | . | . | . | . | . | . | . | . | . | . |
| logepa | rs189673086 | 0.003 | . | . | . | . | . | . | . | . | . | . |
| logepa | rs181141892 | 0.001 | . | . | . | . | . | . | . | . | . | . |
| logepa | rs186404123 | . | . | . | . | . | . | . | . | . | . | . |
| logepa | rs192117287 | 0.001 | . | . | . | . | . | . | . | . | . | . |
| logepa | rs182748835 | 0 | . | . | . | . | . | . | . | . | . | . |
| logepa | rs141854549 | 0.005 | . | . | . | . | . | . | . | . | . | . |
| logepa | rs140163397 | 0.001 | . | . | . | . | . | . | . | . | . | . |
| logepa | rs146347300 | 0.001 | . | . | . | . | . | . | . | . | . | . |
| logepa | rs139362377 | 0.005 | . | . | . | . | . | . | . | . | . | . |
| logepa | rs185087671 | 0 | . | . | . | . | . | . | . | . | . | . |
| logepa | rs190466574 | 0 | . | . | . | . | . | . | . | . | . | . |
| logepa | rs147012778 | 0.001 | . | . | . | . | . | . | . | . | . | . |
| logepa | rs138236960 | 0.003 | . | . | . | . | . | . | . | . | . | . |
| logepa | rs116310311 | 0.005 | . | . | . | . | . | . | . | . | . | . |
| logepa | rs142868356 | 0.003 | . | . | . | . | . | . | . | . | . | . |
| logepa | rs144956240 | 0.001 | . | . | . | . | . | . | . | . | . | . |
| logepa | rs188527776 | . | . | . | . | . | . | . | . | . | . | . |
| logepa | rs183971165 | 0.003 | . | . | . | . | . | . | . | . | . | . |
| logepa | rs76748216 | 0.003 | . | . | . | . | . | . | . | . | . | . |
| logepa | rs148554990 | . | . | . | . | . | . | . | . | . | . | . |
| logepa | rs114446477 | 0.007 | . | . | . | . | . | . | . | . | . | . |
| logepa | rs185223933 | 0.002 | . | . | . | . | . | . | . | . | . | . |
| logepa | rs76869726 | 0.003 | . | . | . | . | . | . | . | . | . | . |
| logepa | rs151200830 | 0.005 | . | . | . | . | . | . | . | . | . | . |
| logepa | rs17157731 | 0.005 | . | . | . | . | . | . | . | . | . | . |
| logepa | rs181243469 | . | . | . | . | . | . | . | . | . | . | . |
| logepa | rs186890707 | . | . | . | . | . | . | . | . | . | . | . |
| logepa | rs184063360 | . | . | . | . | . | . | . | . | . | . | . |
| logepa | rs188550237 | 0.001 | . | . | . | . | . | . | . | . | . | . |
| logepa | rs140931068 | 0.003 | . | . | . | . | . | . | . | . | . | . |
| logepa | rs74867877 | 0.001 | . | . | . | . | . | . | . | . | . | . |
| logepa | rs11239495 | 0.015 | 0.532 | 0.155 | 0.03 | 2.34 | 1.75 | 0.255 | 2.46 | 1.45 | 0.126 | 1.19 |
| logepa | rs149745800 | . | . | . | . | . | . | . | . | . | . | . |
| logepa | rs145044458 | 0.005 | . | . | . | . | . | . | . | . | . | . |
| logepa | rs188067427 | 0 | . | . | . | . | . | . | . | . | . | . |
| logepa | rs146827800 | . | . | . | . | . | . | . | . | . | . | . |
| logepa | rs140619817 | 0.005 | . | . | . | . | . | . | . | . | . | . |
| logepa | rs892689 | 0.998 | . | . | . | . | . | . | . | . | . | . |
| logepa | rs143642005 | 0.001 | . | . | . | . | . | . | . | . | . | . |
| logepa | rs181929304 | 0.001 | . | . | . | . | . | . | . | . | . | . |
| logepa | rs191086173 | 0.001 | . | . | . | . | . | . | . | . | . | . |
| logepa | rs114613116 | 0.005 | . | . | . | . | . | . | . | . | . | . |
| logepa | rs141076768 | . | . | . | . | . | . | . | . | . | . | . |
| logepa | rs187316531 | . | . | . | . | . | . | . | . | . | . | . |
| logepa | rs11239499 | 0.014 | 0.581 | 0.166 | 0.057 | 1.07 | 0.953 | 0.938 | 0.982 | 0.676 | 0.979 | -0.041 |
| logepa | rs192726371 | 0.001 | . | . | . | . | . | . | . | . | . | . |
| logepa | rs74128481 | 0.004 | . | . | . | . | . | . | . | . | . | . |
| logepa | rs74128482 | 0.003 | . | . | . | . | . | . | . | . | . | . |
| logepa | rs11239502 | 0.008 | . | . | . | . | . | . | . | . | . | . |
| logepa | rs116330285 | . | . | . | . | . | . | . | . | . | . | . |
| logepa | rs189926729 | 0.001 | . | . | . | . | . | . | . | . | . | . |
| logepa | rs188975779 | 0.003 | . | . | . | . | . | . | . | . | . | . |
| logepa | rs143846038 | . | . | . | . | . | . | . | . | . | . | . |
| logepa | rs74128483 | 0.004 | . | . | . | . | . | . | . | . | . | . |
| logepa | rs139292055 | 0.013 | 0.576 | 0.164 | 0.052 | 0.697 | 0.795 | 0.752 | 1.88 | 0.792 | 0.135 | 0.481 |
| logepa | rs11239503 | 0.013 | 0.576 | 0.164 | 0.052 | 0.697 | 0.795 | 0.752 | 1.88 | 0.792 | 0.135 | 0.481 |

| logepa | rs58838686 | 0.003 | . | . | . | . | . | . | . | . | . | . |
| --- | --- | --- | --- | --- | --- | --- | --- | --- | --- | --- | --- | --- |
| logepa | rs185825373 | 0.001 | . | . | . | . | . | . | . | . | . | . |
| logepa | rs11239504 | 0.013 | 0.576 | 0.164 | 0.052 | 0.698 | 0.794 | 0.752 | 1.88 | 0.79 | 0.136 | 0.48 |
| logepa | rs150012354 | 0 | . | . | . | . | . | . | . | . | . | . |
| logepa | rs187763096 | 0.001 | . | . | . | . | . | . | . | . | . | . |
| logepa | rs193043982 | 0.002 | . | . | . | . | . | . | . | . | . | . |
| logepa | rs79186884 | 0.004 | . | . | . | . | . | . | . | . | . | . |
| logepa | rs74128484 | 0.003 | . | . | . | . | . | . | . | . | . | . |
| logepa | rs58461537 | 0.013 | 0.576 | 0.164 | 0.052 | 0.697 | 0.793 | 0.751 | 1.88 | 0.79 | 0.136 | 0.48 |
| logepa | rs188527474 | 0.002 | . | . | . | . | . | . | . | . | . | . |
| logepa | rs141659976 | 0.002 | . | . | . | . | . | . | . | . | . | . |
| logepa | rs115846407 | 0.013 | 0.576 | 0.164 | 0.052 | 0.696 | 0.791 | 0.75 | 1.87 | 0.79 | 0.136 | 0.48 |
| logepa | rs138378816 | 0.013 | 0.576 | 0.164 | 0.052 | 0.696 | 0.791 | 0.75 | 1.87 | 0.79 | 0.136 | 0.48 |
| logepa | rs146188136 | 0.008 | . | . | . | . | . | . | . | . | . | . |
| logepa | rs139383871 | 0.002 | . | . | . | . | . | . | . | . | . | . |
| logepa | rs182953869 | . | . | . | . | . | . | . | . | . | . | . |
| logepa | rs192430299 | 0.004 | . | . | . | . | . | . | . | . | . | . |
| logepa | rs144152844 | 0.013 | 0.576 | 0.164 | 0.052 | 0.699 | 0.791 | 0.752 | 1.86 | 0.785 | 0.142 | 0.474 |
| logepa | rs145987786 | 0.013 | 0.591 | 0.163 | 0.056 | 0.036 | 0.092 | 0.19 | 0.04 | 0.063 | 0.042 | 0.374 |
| logepa | rs181132926 | 0.001 | . | . | . | . | . | . | . | . | . | . |
| logepa | rs147882253 | 0.013 | 0.576 | 0.164 | 0.052 | 0.695 | 0.789 | 0.749 | 1.87 | 0.788 | 0.136 | 0.479 |
| logepa | rs141807621 | 0.005 | . | . | . | . | . | . | . | . | . | . |
| logepa | rs147116052 | 0.013 | 0.576 | 0.164 | 0.052 | 0.695 | 0.789 | 0.749 | 1.87 | 0.788 | 0.136 | 0.479 |
| logepa | rs147716618 | 0.013 | 0.576 | 0.164 | 0.052 | 0.695 | 0.788 | 0.749 | 1.87 | 0.788 | 0.136 | 0.479 |
| logepa | rs187198855 | 0.01 | 0.592 | 0.164 | 0.058 | 0.018 | 0.066 | 0.264 | 0.022 | 0.05 | 0.096 | 0.39 |
| logepa | rs189409638 | 0.005 | . | . | . | . | . | . | . | . | . | . |
| logepa | rs182647339 | . | . | . | . | . | . | . | . | . | . | . |
| logepa | rs187995988 | . | . | . | . | . | . | . | . | . | . | . |
| logepa | rs141034184 | 0.015 | 0.577 | 0.164 | 0.053 | 0.642 | 0.742 | 0.702 | 1.96 | 0.83 | 0.11 | 0.508 |
| logepa | rs187310430 | 0.002 | . | . | . | . | . | . | . | . | . | . |
| logepa | rs192608488 | 0.013 | 0.576 | 0.164 | 0.053 | 0.692 | 0.781 | 0.744 | 1.87 | 0.784 | 0.136 | 0.477 |
| logepa | rs148840366 | 0.003 | . | . | . | . | . | . | . | . | . | . |
| logepa | rs186785022 | 0.004 | . | . | . | . | . | . | . | . | . | . |
| logepa | rs192348728 | 0.002 | . | . | . | . | . | . | . | . | . | . |
| logepa | rs189656631 | 0.013 | 0.576 | 0.164 | 0.053 | 0.691 | 0.781 | 0.744 | 1.87 | 0.784 | 0.136 | 0.477 |
| logepa | rs185289750 | . | . | . | . | . | . | . | . | . | . | . |
| logepa | rs142382395 | 0.013 | 0.576 | 0.164 | 0.053 | 0.691 | 0.78 | 0.744 | 1.87 | 0.784 | 0.136 | 0.477 |
| logepa | rs144866696 | 0.004 | . | . | . | . | . | . | . | . | . | . |
| logepa | rs141371289 | 0.013 | 0.576 | 0.164 | 0.053 | 0.691 | 0.78 | 0.743 | 1.87 | 0.784 | 0.136 | 0.477 |
| logepa | rs114750008 | 0.013 | 0.576 | 0.164 | 0.053 | 0.691 | 0.78 | 0.743 | 1.87 | 0.783 | 0.136 | 0.477 |
| logepa | rs189063597 | 0.002 | . | . | . | . | . | . | . | . | . | . |
| logepa | rs139151571 | 0.013 | 0.577 | 0.164 | 0.053 | 0.658 | 0.76 | 0.717 | 1.89 | 0.814 | 0.139 | 0.483 |
| logepa | rs139846368 | 0.014 | 0.575 | 0.163 | 0.052 | 0.624 | 0.763 | 0.7 | 2.16 | 0.969 | 0.085 | 0.578 |
| logepa | rs182057653 | 0.004 | . | . | . | . | . | . | . | . | . | . |
| logepa | rs148785420 | 0.013 | 0.576 | 0.164 | 0.053 | 0.691 | 0.779 | 0.743 | 1.87 | 0.783 | 0.136 | 0.477 |
| logepa | rs138383251 | 0.002 | . | . | . | . | . | . | . | . | . | . |
| logepa | rs116526705 | 0.006 | . | . | . | . | . | . | . | . | . | . |
| logepa | rs185946436 | . | . | . | . | . | . | . | . | . | . | . |
| logepa | rs190455427 | . | . | . | . | . | . | . | . | . | . | . |
| logepa | rs9282584 | 0.013 | 0.576 | 0.164 | 0.053 | 0.69 | 0.778 | 0.742 | 1.87 | 0.783 | 0.136 | 0.476 |
| logepa | rs17153289 | 0.013 | 0.576 | 0.164 | 0.053 | 0.69 | 0.778 | 0.742 | 1.87 | 0.783 | 0.136 | 0.476 |
| logepa | rs138359103 | 0.001 | . | . | . | . | . | . | . | . | . | . |
| logepa | rs150605268 | 0.001 | . | . | . | . | . | . | . | . | . | . |
| logepa | rs79242910 | 0.015 | 0.575 | 0.164 | 0.052 | 0.624 | 0.72 | 0.683 | 2.12 | 0.885 | 0.073 | 0.56 |
| logepa | rs139716904 | 0.004 | . | . | . | . | . | . | . | . | . | . |
| logepa | rs76238498 | 0.013 | 0.591 | 0.163 | 0.057 | 0.032 | 0.08 | 0.17 | 0.036 | 0.057 | 0.037 | 0.377 |
| logepa | rs184140639 | . | . | . | . | . | . | . | . | . | . | . |
| logepa | rs188517278 | 0.002 | . | . | . | . | . | . | . | . | . | . |
| logepa | rs139678913 | 0.001 | . | . | . | . | . | . | . | . | . | . |
| logepa | rs116697955 | . | . | . | . | . | . | . | . | . | . | . |
| logepa | rs79267994 | 0.003 | . | . | . | . | . | . | . | . | . | . |
| logepa | rs11239510 | 0.003 | . | . | . | . | . | . | . | . | . | . |
| logepa | rs75831645 | 0.006 | . | . | . | . | . | . | . | . | . | . |
| logepa | rs78452276 | 0.008 | . | . | . | . | . | . | . | . | . | . |
| logepa | rs144366924 | . | . | . | . | . | . | . | . | . | . | . |
| logepa | rs77519841 | 0.008 | . | . | . | . | . | . | . | . | . | . |
| logepa | rs76944454 | 0.013 | 0.576 | 0.164 | 0.053 | 0.685 | 0.776 | 0.738 | 1.89 | 0.795 | 0.128 | 0.486 |
| logepa | rs76443855 | 0.013 | 0.591 | 0.163 | 0.056 | 0.034 | 0.083 | 0.164 | 0.039 | 0.059 | 0.033 | 0.376 |
| logepa | rs146613174 | 0.001 | . | . | . | . | . | . | . | . | . | . |
| logepa | rs181424761 | 0 | . | . | . | . | . | . | . | . | . | . |
| logepa | rs142119241 | 0 | . | . | . | . | . | . | . | . | . | . |
| logepa | rs140137954 | 0.006 | . | . | . | . | . | . | . | . | . | . |
| logepa | rs187319549 | . | . | . | . | . | . | . | . | . | . | . |
| logepa | rs12241545 | 0.006 | . | . | . | . | . | . | . | . | . | . |
| logepa | rs10128228 | 0.013 | 0.576 | 0.164 | 0.053 | 0.684 | 0.774 | 0.737 | 1.89 | 0.794 | 0.128 | 0.486 |
| logepa | rs58580249 | 0.003 | . | . | . | . | . | . | . | . | . | . |
| logepa | rs193148863 | . | . | . | . | . | . | . | . | . | . | . |
| logepa | rs185638946 | . | . | . | . | . | . | . | . | . | . | . |
| logepa | rs117752921 | 0 | . | . | . | . | . | . | . | . | . | . |
| logepa | rs180841236 | 0 | . | . | . | . | . | . | . | . | . | . |
| logepa | rs73283155 | 0.003 | . | . | . | . | . | . | . | . | . | . |
| logepa | rs76560599 | 0.012 | 0.576 | 0.164 | 0.052 | 0.691 | 0.836 | 0.76 | 1.98 | 0.884 | 0.125 | 0.522 |
| logepa | rs76273620 | 0.012 | 0.576 | 0.164 | 0.052 | 0.691 | 0.836 | 0.76 | 1.98 | 0.884 | 0.125 | 0.522 |
| logepa | rs141432923 | 0.003 | . | . | . | . | . | . | . | . | . | . |
| logepa | rs113529322 | 0.004 | . | . | . | . | . | . | . | . | . | . |
| logepa | rs10128306 | 0.013 | 0.576 | 0.164 | 0.053 | 0.684 | 0.772 | 0.736 | 1.89 | 0.795 | 0.129 | 0.486 |

| logepa | rs149691688 | 0.001 | . | . | . | . | . | . | . | . | . | . |
| --- | --- | --- | --- | --- | --- | --- | --- | --- | --- | --- | --- | --- |
| logepa | rs75872495 | 0.013 | 0.577 | 0.164 | 0.053 | 0.683 | 0.768 | 0.735 | 1.89 | 0.796 | 0.129 | 0.486 |
| logepa | rs191873960 | 0.001 | . | . | . | . | . | . | . | . | . | . |
| logepa | rs80155622 | 0.013 | 0.577 | 0.164 | 0.053 | 0.685 | 0.771 | 0.736 | 1.89 | 0.795 | 0.13 | 0.485 |
| logepa | rs113914656 | 0.001 | . | . | . | . | . | . | . | . | . | . |
| logepa | rs12242840 | 0.013 | 0.577 | 0.164 | 0.053 | 0.687 | 0.772 | 0.738 | 1.89 | 0.799 | 0.13 | 0.487 |
| logepa | rs147824078 | 0.002 | . | . | . | . | . | . | . | . | . | . |
| logepa | rs59051493 | 0.013 | 0.576 | 0.164 | 0.053 | 0.692 | 0.781 | 0.744 | 1.9 | 0.812 | 0.131 | 0.491 |
| logepa | rs9282588 | 0.001 | . | . | . | . | . | . | . | . | . | . |
| logepa | rs150215207 | 0.003 | . | . | . | . | . | . | . | . | . | . |
| logepa | rs138772243 | 0.001 | . | . | . | . | . | . | . | . | . | . |
| logepa | rs149383244 | 0.002 | . | . | . | . | . | . | . | . | . | . |
| logepa | rs17522720 | 0.006 | . | . | . | . | . | . | . | . | . | . |
| logepa | rs79245105 | 0.013 | 0.576 | 0.164 | 0.052 | 0.694 | 0.786 | 0.747 | 1.91 | 0.82 | 0.13 | 0.495 |
| logepa | rs138222133 | 0.001 | . | . | . | . | . | . | . | . | . | . |
| logepa | rs12247314 | 0.015 | 0.577 | 0.164 | 0.053 | 0.649 | 0.744 | 0.706 | 1.97 | 0.84 | 0.113 | 0.511 |
| logepa | rs12263894 | 0.014 | 0.577 | 0.164 | 0.054 | 0.657 | 0.758 | 0.716 | 1.92 | 0.829 | 0.13 | 0.495 |
| logepa | rs149907880 | 0.002 | . | . | . | . | . | . | . | . | . | . |
| logepa | rs188153885 | 0.002 | . | . | . | . | . | . | . | . | . | . |
| logepa | rs73283159 | 0.002 | . | . | . | . | . | . | . | . | . | . |
| logepa | rs140476687 | . | . | . | . | . | . | . | . | . | . | . |
| logepa | rs186603148 | . | . | . | . | . | . | . | . | . | . | . |
| logepa | rs183296063 | 0.001 | . | . | . | . | . | . | . | . | . | . |
| logepa | rs145733694 | 0.003 | . | . | . | . | . | . | . | . | . | . |
| logepa | rs189807449 | . | . | . | . | . | . | . | . | . | . | . |
| logepa | rs12253223 | 0.009 | . | . | . | . | . | . | . | . | . | . |
| logepa | rs182113637 | . | . | . | . | . | . | . | . | . | . | . |
| logepa | rs142792099 | 0.002 | . | . | . | . | . | . | . | . | . | . |
| logepa | rs12249910 | 0.009 | . | . | . | . | . | . | . | . | . | . |
| logepa | rs149436339 | 0 | . | . | . | . | . | . | . | . | . | . |
| logepa | rs77632210 | . | . | . | . | . | . | . | . | . | . | . |
| logepa | rs74130818 | 0.003 | . | . | . | . | . | . | . | . | . | . |
| logepa | rs187583648 | . | . | . | . | . | . | . | . | . | . | . |
| logepa | rs191295987 | 0.001 | . | . | . | . | . | . | . | . | . | . |
| logepa | rs184200686 | 0.002 | . | . | . | . | . | . | . | . | . | . |
| logepa | rs76117263 | 0.013 | 0.592 | 0.163 | 0.057 | 0.016 | 0.051 | 0.192 | 0.022 | 0.044 | 0.057 | 0.392 |
| logepa | rs185393149 | 0.001 | . | . | . | . | . | . | . | . | . | . |
| logepa | rs144760014 | 0 | . | . | . | . | . | . | . | . | . | . |
| logepa | rs7922292 | 0.002 | . | . | . | . | . | . | . | . | . | . |
| logepa | rs115372844 | 0.003 | . | . | . | . | . | . | . | . | . | . |
| logepa | rs111460364 | 0.013 | 0.592 | 0.163 | 0.057 | 0.015 | 0.047 | 0.191 | 0.02 | 0.041 | 0.058 | 0.394 |
| logepa | rs182052255 | 0 | . | . | . | . | . | . | . | . | . | . |
| logepa | rs180983889 | 0.002 | . | . | . | . | . | . | . | . | . | . |
| logepa | rs139175327 | 0.002 | . | . | . | . | . | . | . | . | . | . |
| logepa | rs149959620 | 0.004 | . | . | . | . | . | . | . | . | . | . |
| logepa | rs114653117 | 0.002 | . | . | . | . | . | . | . | . | . | . |
| logepa | rs144270018 | 0 | . | . | . | . | . | . | . | . | . | . |
| logepa | rs182723204 | 0.002 | . | . | . | . | . | . | . | . | . | . |
| logepa | rs57204067 | 0.003 | . | . | . | . | . | . | . | . | . | . |
| logepa | rs144956226 | 0.009 | . | . | . | . | . | . | . | . | . | . |
| logepa | rs187842704 | 0.001 | . | . | . | . | . | . | . | . | . | . |
| logepa | rs184620511 | 0.003 | . | . | . | . | . | . | . | . | . | . |
| logepa | rs144031303 | 0.005 | . | . | . | . | . | . | . | . | . | . |
| logepa | rs187526473 | . | . | . | . | . | . | . | . | . | . | . |
| logepa | rs193002805 | . | . | . | . | . | . | . | . | . | . | . |
| logepa | rs138707426 | 0.002 | . | . | . | . | . | . | . | . | . | . |
| logepa | rs142754243 | 0.01 | 0.6 | 0.169 | 0.07 | 0.259 | 0.401 | 0.383 | 0.082 | 0.086 | 0.018 | 0.154 |
| logepa | rs181600331 | 0.002 | . | . | . | . | . | . | . | . | . | . |
| logepa | rs144421264 | 0.006 | . | . | . | . | . | . | . | . | . | . |
| logepa | rs184414519 | 0.002 | . | . | . | . | . | . | . | . | . | . |
| logepa | rs150596349 | 0.012 | 0.59 | 0.163 | 0.056 | 2.80E-03 | 0.014 | 0.236 | 0.012 | 0.042 | 0.202 | 0.407 |
| logepa | rs190753747 | . | . | . | . | . | . | . | . | . | . | . |
| logepa | rs139560940 | . | . | . | . | . | . | . | . | . | . | . |
| logepa | rs75640804 | 0.008 | . | . | . | . | . | . | . | . | . | . |
| logepa | rs73283175 | 0.018 | 0.606 | 0.173 | 0.079 | 0.251 | 0.373 | 0.353 | 0.299 | 0.239 | 0.132 | 0.189 |
| logepa | rs143337229 | 0.001 | . | . | . | . | . | . | . | . | . | . |
| logepa | rs73283177 | 0.01 | 0.601 | 0.17 | 0.071 | 0.231 | 0.366 | 0.355 | 0.069 | 0.073 | 0.012 | 0.177 |
| logepa | rs76053611 | 0.011 | 0.591 | 0.163 | 0.057 | 2.60E-03 | 0.013 | 0.238 | 9.60E-03 | 0.037 | 0.224 | 0.406 |
| logepa | rs116325983 | . | . | . | . | . | . | . | . | . | . | . |
| logepa | rs115451825 | 0.001 | . | . | . | . | . | . | . | . | . | . |
| logepa | rs183845246 | 0 | . | . | . | . | . | . | . | . | . | . |
| logepa | rs79756542 | 0.013 | 0.591 | 0.163 | 0.057 | 5.50E-03 | 0.025 | 0.248 | 0.022 | 0.075 | 0.272 | 0.404 |
| logepa | rs187242860 | . | . | . | . | . | . | . | . | . | . | . |
| logepa | rs57318980 | 0.01 | 0.604 | 0.171 | 0.074 | 0.192 | 0.3 | 0.291 | 0.05 | 0.053 | 4.80E-03 | 0.21 |
| logepa | rs59969732 | 0.01 | 0.604 | 0.171 | 0.075 | 0.181 | 0.288 | 0.283 | 0.045 | 0.049 | 4.20E-03 | 0.219 |
| logepa | rs143811488 | 0.003 | . | . | . | . | . | . | . | . | . | . |
| logepa | rs189067644 | 0.002 | . | . | . | . | . | . | . | . | . | . |
| logepa | rs60449178 | 0.01 | . | . | . | . | . | . | . | . | . | . |
| logepa | rs59243389 | 0.01 | . | . | . | . | . | . | . | . | . | . |
| logepa | rs184946540 | 0.009 | . | . | . | . | . | . | . | . | . | . |
| logepa | rs188685881 | 0 | . | . | . | . | . | . | . | . | . | . |
| logepa | rs150335797 | 0.002 | . | . | . | . | . | . | . | . | . | . |
| logepa | rs143879260 | 0.002 | . | . | . | . | . | . | . | . | . | . |
| logepa | rs186044491 | 0.003 | . | . | . | . | . | . | . | . | . | . |
| logepa | rs11819084 | 0.004 | . | . | . | . | . | . | . | . | . | . |
| logepa | rs146915670 | . | . | . | . | . | . | . | . | . | . | . |

| logepa | rs113995695 | 0.004 | . | . | . | . | . | . | . | . | . | . |
| --- | --- | --- | --- | --- | --- | --- | --- | --- | --- | --- | --- | --- |
| logepa | rs143489451 | 0.001 | . | . | . | . | . | . | . | . | . | . |
| logepa | rs182653839 | . | . | . | . | . | . | . | . | . | . | . |
| logepa | rs141692564 | 0.002 | . | . | . | . | . | . | . | . | . | . |
| logepa | rs146281742 | 0.001 | . | . | . | . | . | . | . | . | . | . |
| logepa | rs192914353 | 0.001 | . | . | . | . | . | . | . | . | . | . |
| logepa | rs185182848 | 0 | . | . | . | . | . | . | . | . | . | . |
| logepa | rs28395858 | 0.004 | . | . | . | . | . | . | . | . | . | . |
| logepa | rs41429645 | 0.01 | . | . | . | . | . | . | . | . | . | . |
| logepa | rs28395874 | 0.004 | . | . | . | . | . | . | . | . | . | . |
| logepa | rs189663634 | . | . | . | . | . | . | . | . | . | . | . |
| logepa | rs77666700 | 0.003 | . | . | . | . | . | . | . | . | . | . |
| logepa | rs146711717 | 0.001 | . | . | . | . | . | . | . | . | . | . |
| logepa | rs144904251 | . | . | . | . | . | . | . | . | . | . | . |
| logepa | rs189893153 | . | . | . | . | . | . | . | . | . | . | . |
| logepa | rs150447740 | 0.002 | . | . | . | . | . | . | . | . | . | . |
| logepa | rs143099649 | . | . | . | . | . | . | . | . | . | . | . |
| logepa | rs191288436 | 0.003 | . | . | . | . | . | . | . | . | . | . |
| logepa | rs188577862 | 0.001 | . | . | . | . | . | . | . | . | . | . |
| logepa | rs192695334 | 0.001 | . | . | . | . | . | . | . | . | . | . |
| logepa | rs138612906 | 0.007 | . | . | . | . | . | . | . | . | . | . |
| logepa | rs191290051 | 0.006 | . | . | . | . | . | . | . | . | . | . |
| logepa | rs189631459 | 0 | . | . | . | . | . | . | . | . | . | . |
| logepa | rs114620874 | 0.002 | . | . | . | . | . | . | . | . | . | . |
| logepa | rs78246652 | 0.003 | . | . | . | . | . | . | . | . | . | . |
| logepa | rs79432451 | 0.002 | . | . | . | . | . | . | . | . | . | . |
| logepa | rs186022585 | . | . | . | . | . | . | . | . | . | . | . |
| logepa | rs142978891 | 0.002 | . | . | . | . | . | . | . | . | . | . |
| logepa | rs151086840 | 0.003 | . | . | . | . | . | . | . | . | . | . |
| logepa | rs183270259 | 0.002 | . | . | . | . | . | . | . | . | . | . |
| logepa | rs188544289 | 0.002 | . | . | . | . | . | . | . | . | . | . |
| logepa | rs188500808 | 0 | . | . | . | . | . | . | . | . | . | . |
| logepa | rs189424589 | 0 | . | . | . | . | . | . | . | . | . | . |
| logepa | rs78622303 | 0.008 | . | . | . | . | . | . | . | . | . | . |
| logepa | rs143210835 | 0.002 | . | . | . | . | . | . | . | . | . | . |
| logepa | rs114761840 | 0.001 | . | . | . | . | . | . | . | . | . | . |
| logepa | rs185805402 | . | . | . | . | . | . | . | . | . | . | . |
| logepa | rs139273744 | 0.001 | . | . | . | . | . | . | . | . | . | . |
| logepa | rs184654150 | 0.001 | . | . | . | . | . | . | . | . | . | . |
| logepa | rs189069710 | . | . | . | . | . | . | . | . | . | . | . |
| logepa | rs114752115 | 0.004 | . | . | . | . | . | . | . | . | . | . |
| logepa | rs150162515 | 0 | . | . | . | . | . | . | . | . | . | . |
| logepa | rs180982454 | 0.001 | . | . | . | . | . | . | . | . | . | . |
| logepa | rs184932088 | . | . | . | . | . | . | . | . | . | . | . |
| logepa | rs181109625 | . | . | . | . | . | . | . | . | . | . | . |
| logepa | rs138121134 | . | . | . | . | . | . | . | . | . | . | . |
| logepa | rs183253861 | 0 | . | . | . | . | . | . | . | . | . | . |
| logepa | rs147073946 | . | . | . | . | . | . | . | . | . | . | . |
| logepa | rs139938489 | 0.002 | . | . | . | . | . | . | . | . | . | . |
| logepa | rs146362560 | 0.003 | . | . | . | . | . | . | . | . | . | . |
| logepa | rs193155956 | 0.001 | . | . | . | . | . | . | . | . | . | . |
| logepa | rs77663092 | 0.001 | . | . | . | . | . | . | . | . | . | . |
| logepa | rs73285319 | 0.007 | . | . | . | . | . | . | . | . | . | . |
| logepa | rs184019276 | . | . | . | . | . | . | . | . | . | . | . |
| logepa | rs28395863 | 0.004 | . | . | . | . | . | . | . | . | . | . |
| logepa | rs182121645 | 0.004 | . | . | . | . | . | . | . | . | . | . |
| logepa | rs73285327 | 0.006 | . | . | . | . | . | . | . | . | . | . |
| logepa | rs149589584 | . | . | . | . | . | . | . | . | . | . | . |
| logepa | rs148310335 | . | . | . | . | . | . | . | . | . | . | . |
| logepa | rs187246093 | 0.003 | . | . | . | . | . | . | . | . | . | . |
| logepa | rs184668894 | . | . | . | . | . | . | . | . | . | . | . |
| logepa | rs114269961 | . | . | . | . | . | . | . | . | . | . | . |
| logepa | rs149694374 | 0.005 | . | . | . | . | . | . | . | . | . | . |
| logepa | rs145284227 | . | . | . | . | . | . | . | . | . | . | . |
| logepa | rs189983820 | . | . | . | . | . | . | . | . | . | . | . |
| logepa | rs181200444 | . | . | . | . | . | . | . | . | . | . | . |
| logepa | rs185486385 | . | . | . | . | . | . | . | . | . | . | . |
| logepa | rs151198241 | 0.002 | . | . | . | . | . | . | . | . | . | . |
| logepa | rs189959341 | 0.002 | . | . | . | . | . | . | . | . | . | . |
| logepa | rs140549257 | 0.002 | . | . | . | . | . | . | . | . | . | . |
| logepa | rs186453139 | 0.005 | . | . | . | . | . | . | . | . | . | . |
| logepa | rs180922711 | 0.009 | . | . | . | . | . | . | . | . | . | . |
| logepa | rs56659903 | 0.002 | . | . | . | . | . | . | . | . | . | . |
| logepa | rs141202128 | 0.002 | . | . | . | . | . | . | . | . | . | . |
| logepa | rs59085314 | 0.008 | . | . | . | . | . | . | . | . | . | . |
| logepa | rs145564996 | . | . | . | . | . | . | . | . | . | . | . |
| logepa | rs114961009 | 0.002 | . | . | . | . | . | . | . | . | . | . |
| logepa | rs182294189 | 0.001 | . | . | . | . | . | . | . | . | . | . |
| logepa | rs187586636 | 0.002 | . | . | . | . | . | . | . | . | . | . |
| logepa | rs192254907 | 0.005 | . | . | . | . | . | . | . | . | . | . |
| logepa | rs149145663 | . | . | . | . | . | . | . | . | . | . | . |
| logepa | rs180713064 | 0.002 | . | . | . | . | . | . | . | . | . | . |
| logepa | rs183009023 | 0.001 | . | . | . | . | . | . | . | . | . | . |
| logepa | rs143385347 | 0.004 | . | . | . | . | . | . | . | . | . | . |
| logepa | rs185786329 | 0.001 | . | . | . | . | . | . | . | . | . | . |
| logepa | rs192341030 | 0.001 | . | . | . | . | . | . | . | . | . | . |

| logepa | rs74661429 | 0.002 | . | . | . | . | . | . | . | . | . | . |
| --- | --- | --- | --- | --- | --- | --- | --- | --- | --- | --- | --- | --- |
| logepa | rs188034853 | 0 | . | . | . | . | . | . | . | . | . | . |
| logepa | rs116808430 | 0.005 | . | . | . | . | . | . | . | . | . | . |
| logepa | rs148469010 | . | . | . | . | . | . | . | . | . | . | . |
| logepa | rs78621663 | 0.002 | . | . | . | . | . | . | . | . | . | . |
| logepa | rs192654886 | . | . | . | . | . | . | . | . | . | . | . |
| logepa | rs145996035 | 0.002 | . | . | . | . | . | . | . | . | . | . |
| logepa | rs139942226 | 0 | . | . | . | . | . | . | . | . | . | . |
| logepa | rs113645632 | 0.002 | . | . | . | . | . | . | . | . | . | . |
| logepa | rs74940661 | 0.005 | . | . | . | . | . | . | . | . | . | . |
| logepa | rs115355800 | 0.003 | . | . | . | . | . | . | . | . | . | . |
| logepa | rs150285898 | 0.001 | . | . | . | . | . | . | . | . | . | . |
| logepa | rs188117932 | . | . | . | . | . | . | . | . | . | . | . |
| logepa | rs139956566 | 0 | . | . | . | . | . | . | . | . | . | . |
| logepa | rs147673885 | 0 | . | . | . | . | . | . | . | . | . | . |
| logepa | rs189127928 | 0.005 | . | . | . | . | . | . | . | . | . | . |
| logepa | rs187558772 | 0.002 | . | . | . | . | . | . | . | . | . | . |
| logepa | rs181948587 | 0 | . | . | . | . | . | . | . | . | . | . |
| logepa | rs189983269 | 0.002 | . | . | . | . | . | . | . | . | . | . |
| logepa | rs187250069 | 0.002 | . | . | . | . | . | . | . | . | . | . |
| logepa | rs191485859 | 0.001 | . | . | . | . | . | . | . | . | . | . |
| logepa | rs185499555 | 0.002 | . | . | . | . | . | . | . | . | . | . |
| logepa | rs180862110 | 0.003 | . | . | . | . | . | . | . | . | . | . |
| logepa | rs190472603 | 0.005 | . | . | . | . | . | . | . | . | . | . |
| logepa | rs189226167 | 0.005 | . | . | . | . | . | . | . | . | . | . |
| logepa | rs181623256 | 0.002 | . | . | . | . | . | . | . | . | . | . |
| logepa | rs186079557 | 0.001 | . | . | . | . | . | . | . | . | . | . |
| logepa | rs147760994 | 0.002 | . | . | . | . | . | . | . | . | . | . |
| logepa | rs189176167 | 0 | . | . | . | . | . | . | . | . | . | . |
| logepa | rs117901000 | 0.001 | . | . | . | . | . | . | . | . | . | . |
| logepa | rs141984888 | 0 | . | . | . | . | . | . | . | . | . | . |
| logepa | rs192365061 | 0.007 | . | . | . | . | . | . | . | . | . | . |
| logepa | rs185976038 | 0 | . | . | . | . | . | . | . | . | . | . |
| logepa | rs184697303 | 0.002 | . | . | . | . | . | . | . | . | . | . |
| logepa | rs143383132 | 0.002 | . | . | . | . | . | . | . | . | . | . |
| logepa | rs141873881 | 0.004 | . | . | . | . | . | . | . | . | . | . |
| logepa | rs185507255 | 0 | . | . | . | . | . | . | . | . | . | . |
| logepa | rs191379425 | 0.001 | . | . | . | . | . | . | . | . | . | . |
| logepa | rs184199584 | 0.002 | . | . | . | . | . | . | . | . | . | . |
| logepa | rs183181360 | 0.002 | . | . | . | . | . | . | . | . | . | . |
| logepa | rs184495673 | 0.001 | . | . | . | . | . | . | . | . | . | . |
| logepa | rs182946297 | 0.001 | . | . | . | . | . | . | . | . | . | . |
| logepa | rs187743615 | 0 | . | . | . | . | . | . | . | . | . | . |
| logepa | rs183259862 | 0.001 | . | . | . | . | . | . | . | . | . | . |
| logepa | rs112433566 | 0 | . | . | . | . | . | . | . | . | . | . |
| logepa | rs182351085 | 0.001 | . | . | . | . | . | . | . | . | . | . |
| logepa | rs146248508 | 0.001 | . | . | . | . | . | . | . | . | . | . |
| logepa | rs188528439 | 0.001 | . | . | . | . | . | . | . | . | . | . |
| logepa | rs146167018 | 0.005 | . | . | . | . | . | . | . | . | . | . |
| logepa | rs192440980 | 0.004 | . | . | . | . | . | . | . | . | . | . |
| logepa | rs185601882 | 0.001 | . | . | . | . | . | . | . | . | . | . |
| logepa | rs117272563 | 0.002 | . | . | . | . | . | . | . | . | . | . |
| logepa | rs10430734 | 0.003 | . | . | . | . | . | . | . | . | . | . |
| logepa | rs181320952 | 0.001 | . | . | . | . | . | . | . | . | . | . |
| logepa | rs185480660 | 0.001 | . | . | . | . | . | . | . | . | . | . |
| logepa | rs188077917 | 0.001 | . | . | . | . | . | . | . | . | . | . |
| logepa | rs187562953 | 0.002 | . | . | . | . | . | . | . | . | . | . |
| logepa | rs182044631 | 0 | . | . | . | . | . | . | . | . | . | . |
| logepa | rs145455596 | 0.001 | . | . | . | . | . | . | . | . | . | . |
| logepa | rs181475877 | 0.004 | . | . | . | . | . | . | . | . | . | . |
| logepa | rs149413085 | 0.002 | . | . | . | . | . | . | . | . | . | . |
| logepa | rs12253656 | 0 | . | . | . | . | . | . | . | . | . | . |
| logepa | rs182718239 | 0 | . | . | . | . | . | . | . | . | . | . |
| logepa | rs192435695 | 0.002 | . | . | . | . | . | . | . | . | . | . |
| logepa | rs12248277 | 0 | . | . | . | . | . | . | . | . | . | . |
| logepa | rs143521710 | 0.001 | . | . | . | . | . | . | . | . | . | . |
| logepa | rs144454454 | 0 | . | . | . | . | . | . | . | . | . | . |
| logepa | rs190441610 | 0.004 | . | . | . | . | . | . | . | . | . | . |
| logepa | rs114690462 | 0 | . | . | . | . | . | . | . | . | . | . |
| logepa | rs184541357 | 0.001 | . | . | . | . | . | . | . | . | . | . |
| logepa | rs142657653 | 0 | . | . | . | . | . | . | . | . | . | . |
| logepa | rsurf1 | 2.225 | . | . | . | . | . | . | . | . | . | . |
| logepa | rsten1 | 2.374 | . | . | . | . | . | . | . | . | . | . |
| logepa | chr10:45852312:D | 0.072 | 0.639 | 0.18 | 0.112 | 0.494 | 0.432 | 0.42 | 0.394 | 0.317 | 0.247 | -9.10E-03 |
| logepa | chr10:45853232:I | 0.086 | 0.677 | 0.192 | 0.17 | 0.362 | 0.287 | 0.2 | 0.34 | 0.222 | 0.099 | 0.044 |
| logepa | chr10:45853234:I | 0.141 | 0.671 | 0.208 | 0.197 | 0.563 | 0.289 | 0.263 | 0.595 | 0.303 | 0.308 | -9.10E-03 |
| logepa | chr10:45854796:D | 0.022 | 0.66 | 0.184 | 0.136 | 1.30E-05 | 3.50E-05 | 3.30E-05 | 1.80E-04 | 3.10E-04 | 4.80E-07 | 0.34 |
| logepa | chr10:45854994:I | 0.025 | 0.68 | 0.192 | 0.172 | 9.70E-06 | 2.70E-05 | 3.20E-05 | 1.50E-04 | 2.60E-04 | 4.20E-07 | 0.32 |
| logepa | chr10:45858290:D | 0.045 | 0.704 | 0.194 | 0.203 | 0.031 | 0.046 | 0.019 | 0.079 | 0.076 | 8.40E-03 | 0.267 |
| logepa | chr10:45858820:I | 0.012 | 0.626 | 0.171 | 0.086 | 0.139 | 0.252 | 0.278 | 0.058 | 0.085 | 0.052 | 0.241 |
| logepa | chr10:45860888:D | 0.072 | 0.644 | 0.195 | 0.146 | 0.317 | 0.294 | 0.215 | 0.469 | 0.439 | 0.419 | 0.134 |
| logepa | chr10:45863913:I | 0.144 | 0.682 | 0.209 | 0.211 | 0.517 | 0.266 | 0.2 | 0.56 | 0.28 | 0.246 | -1.60E-03 |
| logepa | chr10:45866601:D | 0.016 | 0.572 | 0.165 | 0.052 | 1.28 | 1.24 | 0.796 | 1.27 | 1.07 | 0.772 | 0.08 |
| logepa | chr10:45867197:I | 0.005 | . | . | . | . | . | . | . | . | . | . |
| logepa | chr10:45867538:D | 0.149 | 0.655 | 0.204 | 0.174 | 0.614 | 0.303 | 0.323 | 0.674 | 0.349 | 0.446 | 1.60E-03 |
| logepa | chr10:45868356:D | 0.14 | 0.686 | 0.21 | 0.218 | 0.513 | 0.266 | 0.198 | 0.541 | 0.27 | 0.219 | -8.60E-03 |

| logepa | chr10:45871158:I | 0.145 | 0.682 | 0.208 | 0.21 | 0.517 | 0.263 | 0.195 | 0.561 | 0.278 | 0.243 | -1.60E-03 |
| --- | --- | --- | --- | --- | --- | --- | --- | --- | --- | --- | --- | --- |
| logepa | chr10:45871637:D | 0.066 | 0.605 | 0.187 | 0.104 | 0.872 | 0.657 | 0.856 | 0.732 | 0.587 | 0.697 | -0.091 |
| logepa | chr10:45871638:I | 0.111 | 0.686 | 0.213 | 0.225 | 0.564 | 0.318 | 0.31 | 0.508 | 0.283 | 0.224 | -0.053 |
| logepa | chr10:45873629:I | 0.139 | 0.67 | 0.207 | 0.194 | 0.582 | 0.297 | 0.289 | 0.595 | 0.292 | 0.29 | -0.02 |
| logepa | chr10:45873701:D | 0.009 | . | . | . | . | . | . | . | . | . | . |
| logepa | chr10:45874997:I | 0.016 | 0.557 | 0.156 | 0.036 | 1.21 | 1.1 | 0.835 | 7.07 | 6.51 | 0.034 | 3.99 |
| logepa | chr10:45882030:I | 0.028 | 0.557 | 0.16 | 0.042 | 0.418 | 0.552 | 0.509 | 4.9 | 3.52 | 0.027 | 1.17 |
| logepa | chr10:45883774:D | 0.092 | 0.582 | 0.184 | 0.086 | 0.82 | 0.403 | 0.686 | 1.03 | 0.556 | 0.956 | 0.09 |
| logepa | chr10:45885169:D | 0.054 | 0.651 | 0.18 | 0.121 | 0.202 | 0.179 | 0.071 | 0.19 | 0.146 | 0.03 | 0.173 |
| logepa | chr10:45886451:D | 0.701 | 1.42 | 0.861 | 0.561 | 0.494 | 0.189 | 0.065 | 0.517 | 0.215 | 0.113 | -0.553 |
| logepa | chr10:45891800:I | 0.669 | 1.54 | 0.901 | 0.464 | 0.59 | 0.178 | 0.08 | 0.502 | 0.184 | 0.06 | -0.671 |
| logepa | chr10:45904771:I | 0.683 | 0.864 | 0.511 | 0.805 | 0.754 | 0.241 | 0.378 | 0.756 | 0.277 | 0.446 | -0.125 |
| logepa | chr10:45904959:I | 0.083 | 0.507 | 0.171 | 0.043 | 1.93 | 1.01 | 0.211 | 1.8 | 1.05 | 0.311 | 0.329 |
| logepa | chr10:45911028:I | 0.045 | 0.59 | 0.176 | 0.078 | 0.839 | 1.1 | 0.893 | 0.853 | 1.6 | 0.932 | -6.80E-03 |
| logepa | chr10:45911031:I | 0.411 | 0.687 | 0.306 | 0.399 | 0.8 | 0.325 | 0.582 | 0.811 | 0.353 | 0.631 | -0.041 |
| logepa | chr10:45912154:D | 0.086 | 0.626 | 0.193 | 0.13 | 0.636 | 0.345 | 0.405 | 0.737 | 0.437 | 0.606 | 0.031 |
| logepa | chr10:45913885:I | 0.021 | 0.635 | 0.174 | 0.097 | 1.00E-05 | 3.00E-05 | 1.10E-04 | 1.60E-04 | 3.20E-04 | 6.80E-06 | 0.365 |
| logepa | chr10:45914753:D | 0.181 | 0.475 | 0.172 | 0.04 | 1.91 | 0.591 | 0.036 | 1.38 | 0.486 | 0.359 | -0.133 |
| logepa | chr10:45919621 | . | . | . | . | . | . | . | . | . | . | . |
| logepa | chr10:45921739:I | 0.06 | 0.662 | 0.184 | 0.138 | 0.105 | 0.204 | 0.245 | 0.12 | 0.203 | 0.211 | 0.241 |
| logepa | chr10:45923527:I | 0.054 | 0.542 | 0.167 | 0.047 | 2.16 | 0.987 | 0.092 | 1.66 | 1.19 | 0.48 | 0.24 |
| logepa | chr10:45927001:I | 0.011 | 0.605 | 0.167 | 0.069 | 0.177 | 0.5 | 0.54 | 0.018 | 0.073 | 0.31 | 0.22 |
| logepa | chr10:45930354:I | 0.023 | 0.645 | 0.177 | 0.109 | 1.30E-05 | 3.30E-05 | 6.90E-06 | 1.80E-04 | 3.00E-04 | 1.30E-07 | 0.355 |
| logepa | chr10:45932864:I | 0.046 | 0.672 | 0.187 | 0.154 | 6.20E-03 | 0.016 | 0.056 | 0.018 | 0.04 | 0.071 | 0.322 |
| logepa | chr10:45936163:I | 0.025 | 0.624 | 0.181 | 0.104 | 0.859 | 0.839 | 0.877 | 0.3 | 0.261 | 0.167 | -0.323 |
| logepa | chr10:45938095:D | 0.116 | 0.482 | 0.153 | 0.021 | 1.47 | 0.523 | 0.277 | 1.86 | 0.622 | 0.064 | 0.364 |
| logepa | chr10:45938886 | . | . | . | . | . | . | . | . | . | . | . |
| logepa | chr10:45939320 | . | . | . | . | . | . | . | . | . | . | . |
| logepa | chr10:45939659 | 0.002 | . | . | . | . | . | . | . | . | . | . |
| logepa | chr10:45941150:D | 0.021 | 0.62 | 0.17 | 0.081 | 9.00E-07 | 3.20E-06 | 9.20E-05 | 3.80E-05 | 8.30E-05 | 3.60E-06 | 0.38 |
| logepa | chr10:45947128:D | 0.046 | 0.642 | 0.191 | 0.136 | 0.484 | 0.336 | 0.295 | 0.468 | 0.284 | 0.212 | 0.019 |
| logepa | chr10:45950990:I | 0.06 | 0.687 | 0.197 | 0.191 | 0.034 | 0.065 | 0.076 | 0.067 | 0.104 | 0.084 | 0.281 |
| logepa | chr10:45951854:I | 0.02 | 0.529 | 0.144 | 0.019 | 5.61 | 4.09 | 0.018 | 3.78 | 4.09 | 0.22 | 6.06 |
| logepa | chr10:45952670:I | 0.042 | 0.519 | 0.147 | 0.02 | 1.12 | 0.623 | 0.835 | 13.2 | 8.43 | 5.40E-05 | 7.05 |
| logepa | chr10:45956741 | . | . | . | . | . | . | . | . | . | . | . |
| logepa | chr10:45957247:I | 0.304 | 0.523 | 0.19 | 0.074 | 1.14 | 0.358 | 0.666 | 1.18 | 0.415 | 0.644 | 0.037 |
| logepa | chr10:45958082:I | 0.302 | 0.505 | 0.186 | 0.063 | 1.16 | 0.378 | 0.655 | 1.24 | 0.452 | 0.555 | 0.063 |
| logepa | chr10:45905041:D | 0.039 | 0.675 | 0.187 | 0.155 | 9.60E-05 | 3.50E-04 | 9.80E-03 | 6.60E-04 | 1.60E-03 | 2.00E-03 | 0.324 |
| logepa | chr10:45938871 | . | . | . | . | . | . | . | . | . | . | . |
| logepa | chr10:45939160 | . | . | . | . | . | . | . | . | . | . | . |
| logepa | chr10:45869910 | 0.001 | . | . | . | . | . | . | . | . | . | . |
| logepa | chr10:45876214:D | 0.005 | . | . | . | . | . | . | . | . | . | . |
| logepa | chr10:45880237:I | 0.015 | 0.6 | 0.171 | 0.073 | 0.354 | 0.472 | 0.436 | 0.355 | 0.263 | 0.162 | 0.121 |
| logepa | chr10:45884371:I | 0.013 | 0.576 | 0.164 | 0.052 | 0.696 | 0.79 | 0.75 | 1.87 | 0.789 | 0.136 | 0.479 |
| logepa | chr10:45891535:D | 0.004 | . | . | . | . | . | . | . | . | . | . |
| logepa | chr10:45893516:D | 0.013 | 0.576 | 0.164 | 0.053 | 0.686 | 0.776 | 0.739 | 1.88 | 0.79 | 0.132 | 0.482 |
| logepa | chr10:45893782:D | 0.001 | . | . | . | . | . | . | . | . | . | . |
| logepa | chr10:45895254:I | 0.004 | . | . | . | . | . | . | . | . | . | . |
| logepa | chr10:45895848:D | 0.011 | 0.575 | 0.163 | 0.051 | 0.736 | 0.9 | 0.802 | 1.88 | 0.862 | 0.166 | 0.486 |
| logepa | chr10:45896316:D | 0.013 | 0.576 | 0.164 | 0.053 | 0.684 | 0.773 | 0.737 | 1.89 | 0.795 | 0.128 | 0.486 |
| logepa | chr10:45896317:D | 0.013 | 0.576 | 0.164 | 0.053 | 0.684 | 0.773 | 0.737 | 1.89 | 0.795 | 0.128 | 0.486 |
| logepa | chr10:45902683:I | 0.006 | . | . | . | . | . | . | . | . | . | . |
| logepa | chr10:45905003:D | 0.002 | . | . | . | . | . | . | . | . | . | . |
| logepa | chr10:45927476:D | 0.005 | . | . | . | . | . | . | . | . | . | . |
| logepa | chr10:45935882 | . | . | . | . | . | . | . | . | . | . | . |
| logepa | chr10:45936100 | . | . | . | . | . | . | . | . | . | . | . |
| logepa | chr10:45939553 | 0.004 | . | . | . | . | . | . | . | . | . | . |
| logepa | chr10:45939631 | . | . | . | . | . | . | . | . | . | . | . |
| logepa | chr10:45944193:D | 0.003 | . | . | . | . | . | . | . | . | . | . |
| logepa | chr10:45954030 | . | . | . | . | . | . | . | . | . | . | . |

Supplemental table S35.

| fa | rs_id | freq | HR_logepa | se_logepa | p_logepa | HR_snp | se_snp | p_snp | HR_inter | se_inter | p_inter | RERI |
| --- | --- | --- | --- | --- | --- | --- | --- | --- | --- | --- | --- | --- |
| logepa | rs76035182 | . | . | . | . | . | . | . | . | . | . | . |
| logepa | rs12359547 | 0.001 | . | . | . | . | . | . | . | . | . | . |
| logepa | rs74595484 | . | . | . | . | . | . | . | . | . | . | . |
| logepa | rs1864415 | 0.873 | 2.21 | 2.71 | 0.516 | 0.417 | 0.149 | 0.014 | 0.416 | 0.257 | 0.156 | -1.25 |
| logepa | rs72788101 | 0.001 | . | . | . | . | . | . | . | . | . | . |
| logepa | rs11239481 | . | . | . | . | . | . | . | . | . | . | . |
| logepa | rs72788102 | 0.001 | . | . | . | . | . | . | . | . | . | . |
| logepa | rs72789705 | 0.001 | . | . | . | . | . | . | . | . | . | . |
| logepa | rs141529858 | . | . | . | . | . | . | . | . | . | . | . |
| logepa | rs145376292 | . | . | . | . | . | . | . | . | . | . | . |
| logepa | rs190418301 | . | . | . | . | . | . | . | . | . | . | . |
| logepa | rs11239482 | 0.001 | . | . | . | . | . | . | . | . | . | . |
| logepa | rs61855872 | . | . | . | . | . | . | . | . | . | . | . |
| logepa | rs148991144 | . | . | . | . | . | . | . | . | . | . | . |
| logepa | rs191932221 | . | . | . | . | . | . | . | . | . | . | . |
| logepa | rs55978138 | 0.201 | 0.68 | 0.295 | 0.374 | 0.737 | 0.326 | 0.49 | 0.518 | 0.232 | 0.141 | -0.158 |
| logepa | rs73281167 | . | . | . | . | . | . | . | . | . | . | . |
| logepa | rs12768010 | 0.2 | 0.68 | 0.295 | 0.374 | 0.738 | 0.326 | 0.492 | 0.516 | 0.231 | 0.14 | -0.159 |
| logepa | rs11239484 | 0.873 | 2.21 | 2.71 | 0.517 | 0.418 | 0.149 | 0.014 | 0.417 | 0.257 | 0.157 | -1.25 |
| logepa | rs35906360 | 0.201 | 0.68 | 0.295 | 0.374 | 0.737 | 0.326 | 0.49 | 0.518 | 0.232 | 0.141 | -0.158 |
| logepa | rs186250512 | . | . | . | . | . | . | . | . | . | . | . |
| logepa | rs150810743 | . | . | . | . | . | . | . | . | . | . | . |
| logepa | rs55965812 | 0.201 | 0.68 | 0.295 | 0.374 | 0.737 | 0.326 | 0.49 | 0.518 | 0.232 | 0.141 | -0.158 |
| logepa | rs56180358 | 0.203 | 0.681 | 0.297 | 0.378 | 0.73 | 0.325 | 0.48 | 0.519 | 0.235 | 0.147 | -0.154 |
| logepa | rs55864145 | 0.2 | 0.68 | 0.295 | 0.374 | 0.738 | 0.326 | 0.492 | 0.516 | 0.231 | 0.14 | -0.159 |
| logepa | rs55986122 | 0.201 | 0.68 | 0.295 | 0.374 | 0.737 | 0.326 | 0.49 | 0.518 | 0.232 | 0.141 | -0.158 |
| logepa | rs7477350 | 0.873 | 2.21 | 2.71 | 0.516 | 0.418 | 0.149 | 0.014 | 0.416 | 0.257 | 0.156 | -1.25 |
| logepa | rs2099170 | 0.201 | 0.68 | 0.295 | 0.374 | 0.737 | 0.326 | 0.49 | 0.518 | 0.232 | 0.141 | -0.158 |
| logepa | rs2082108 | 0.201 | 0.68 | 0.295 | 0.374 | 0.737 | 0.326 | 0.49 | 0.518 | 0.232 | 0.141 | -0.158 |
| logepa | rs55902404 | . | . | . | . | . | . | . | . | . | . | . |
| logepa | rs58402662 | . | . | . | . | . | . | . | . | . | . | . |
| logepa | rs11511618 | . | . | . | . | . | . | . | . | . | . | . |
| logepa | rs1816492 | 0.873 | 2.22 | 2.72 | 0.516 | 0.418 | 0.149 | 0.014 | 0.416 | 0.257 | 0.156 | -1.25 |
| logepa | rs146611093 | . | . | . | . | . | . | . | . | . | . | . |
| logepa | rs186897689 | . | . | . | . | . | . | . | . | . | . | . |
| logepa | rs10793616 | 0.873 | 2.21 | 2.71 | 0.517 | 0.418 | 0.149 | 0.014 | 0.416 | 0.257 | 0.156 | -1.25 |
| logepa | rs116700479 | . | . | . | . | . | . | . | . | . | . | . |
| logepa | rs192535936 | . | . | . | . | . | . | . | . | . | . | . |
| logepa | rs113363297 | 0.852 | 2.34 | 2.93 | 0.498 | 0.44 | 0.165 | 0.029 | 0.398 | 0.255 | 0.151 | -1.37 |
| logepa | rs12354994 | 0.186 | 0.683 | 0.301 | 0.388 | 0.757 | 0.34 | 0.536 | 0.498 | 0.241 | 0.149 | -0.183 |
| logepa | rs148068329 | 0.059 | 0.473 | 0.144 | 0.014 | 0.911 | 0.785 | 0.914 | 2.81 | 3.35 | 0.386 | 0.825 |
| logepa | rs12356109 | 0.198 | 0.673 | 0.296 | 0.367 | 0.779 | 0.341 | 0.568 | 0.539 | 0.24 | 0.165 | -0.169 |
| logepa | rs55908141 | . | . | . | . | . | . | . | . | . | . | . |
| logepa | rs12354712 | 0.196 | 0.672 | 0.297 | 0.369 | 0.792 | 0.345 | 0.592 | 0.542 | 0.242 | 0.169 | -0.176 |
| logepa | rs147282684 | . | . | . | . | . | . | . | . | . | . | . |
| logepa | rs12356140 | 0.194 | 0.68 | 0.299 | 0.38 | 0.77 | 0.352 | 0.568 | 0.518 | 0.24 | 0.156 | -0.179 |
| logepa | rs185476187 | . | . | . | . | . | . | . | . | . | . | . |
| logepa | rs145720057 | . | . | . | . | . | . | . | . | . | . | . |
| logepa | rs12354742 | 0.196 | 0.672 | 0.297 | 0.369 | 0.792 | 0.345 | 0.592 | 0.542 | 0.242 | 0.169 | -0.176 |
| logepa | rs180913842 | . | . | . | . | . | . | . | . | . | . | . |
| logepa | rs142976914 | 0.001 | . | . | . | . | . | . | . | . | . | . |
| logepa | rs11511619 | 0.198 | 0.673 | 0.296 | 0.368 | 0.779 | 0.341 | 0.568 | 0.54 | 0.24 | 0.166 | -0.169 |
| logepa | rs146702870 | . | . | . | . | . | . | . | . | . | . | . |
| logepa | rs150287496 | 0.024 | 0.556 | 0.204 | 0.11 | 3.30E-05 | 2.10E-04 | 0.113 | 7.70E-03 | 0.065 | 0.561 | 0.444 |
| logepa | rs190917973 | . | . | . | . | . | . | . | . | . | . | . |
| logepa | rs11517281 | 0.197 | 0.673 | 0.296 | 0.367 | 0.779 | 0.341 | 0.568 | 0.539 | 0.24 | 0.165 | -0.169 |
| logepa | rs12359299 | 0.208 | 0.695 | 0.306 | 0.408 | 0.762 | 0.338 | 0.541 | 0.508 | 0.229 | 0.134 | -0.188 |
| logepa | rs182307932 | . | . | . | . | . | . | . | . | . | . | . |
| logepa | rs61855873 | . | . | . | . | . | . | . | . | . | . | . |
| logepa | rs12359302 | 0.193 | 0.666 | 0.293 | 0.356 | 0.789 | 0.349 | 0.593 | 0.55 | 0.245 | 0.18 | -0.166 |
| logepa | rs143208140 | 0.054 | 0.474 | 0.145 | 0.015 | 1.01 | 0.795 | 0.986 | 2.63 | 2.97 | 0.392 | 0.775 |
| logepa | rs9988764 | 0.858 | 2.23 | 2.76 | 0.515 | 0.434 | 0.162 | 0.026 | 0.41 | 0.259 | 0.157 | -1.27 |
| logepa | rs192875767 | 0.015 | 0.574 | 0.219 | 0.146 | 2.00E-116 | 3.00E-114 | 0.052 | 1.00E-68 | 1.00E-66 | 0.104 | 0.426 |
| logepa | rs147268846 | . | . | . | . | . | . | . | . | . | . | . |
| logepa | rs11239485 | 0.1 | 0.84 | 0.339 | 0.666 | 0.629 | 0.454 | 0.521 | 0.083 | 0.076 | 6.90E-03 | -0.426 |
| logepa | rs151317885 | 0.66 | 0.551 | 0.264 | 0.214 | 0.674 | 0.166 | 0.11 | 0.946 | 0.247 | 0.831 | 0.126 |
| logepa | rs11499085 | . | . | . | . | . | . | . | . | . | . | . |
| logepa | rs188130606 | . | . | . | . | . | . | . | . | . | . | . |
| logepa | rs11511620 | 0.196 | 0.672 | 0.295 | 0.365 | 0.783 | 0.342 | 0.575 | 0.54 | 0.241 | 0.167 | -0.17 |
| logepa | rs181498390 | 0.002 | . | . | . | . | . | . | . | . | . | . |
| logepa | rs186280701 | . | . | . | . | . | . | . | . | . | . | . |
| logepa | rs150059185 | 0.082 | 0.62 | 0.256 | 0.247 | 0.653 | 0.402 | 0.489 | 0.294 | 0.264 | 0.172 | -0.153 |
| logepa | rs183338707 | . | . | . | . | . | . | . | . | . | . | . |
| logepa | rs11499038 | 0.006 | . | . | . | . | . | . | . | . | . | . |
| logepa | rs190699595 | . | . | . | . | . | . | . | . | . | . | . |
| logepa | rs11523408 | 0.197 | 0.673 | 0.296 | 0.367 | 0.779 | 0.341 | 0.568 | 0.539 | 0.24 | 0.166 | -0.169 |
| logepa | rs188049317 | . | . | . | . | . | . | . | . | . | . | . |
| logepa | rs144948634 | 0.584 | 0.71 | 0.432 | 0.574 | 0.577 | 0.181 | 0.08 | 0.717 | 0.282 | 0.398 | 6.30E-03 |
| logepa | rs143833576 | 0.016 | 0.573 | 0.21 | 0.128 | 0.482 | 0.921 | 0.703 | 0.113 | 0.253 | 0.331 | -0.024 |
| logepa | rs151239879 | 0.2 | 0.675 | 0.298 | 0.374 | 0.768 | 0.341 | 0.552 | 0.535 | 0.241 | 0.165 | -0.166 |
| logepa | rs7073743 | 0.61 | 0.645 | 0.43 | 0.511 | 0.653 | 0.21 | 0.185 | 0.809 | 0.328 | 0.602 | 0.043 |
| logepa | rs142563349 | . | . | . | . | . | . | . | . | . | . | . |
| logepa | rs187246403 | . | . | . | . | . | . | . | . | . | . | . |
| logepa | rs150423942 | 0.197 | 0.673 | 0.296 | 0.368 | 0.781 | 0.342 | 0.573 | 0.539 | 0.24 | 0.166 | -0.171 |

| logepa | rs184706102 | . | . | . | . | . | . | . | . | . | . | . |
| --- | --- | --- | --- | --- | --- | --- | --- | --- | --- | --- | --- | --- |
| logepa | rs189626288 | . | . | . | . | . | . | . | . | . | . | . |
| logepa | rs140749834 | . | . | . | . | . | . | . | . | . | . | . |
| logepa | rs7078822 | 0.163 | 0.605 | 0.292 | 0.299 | 0.953 | 0.475 | 0.923 | 0.693 | 0.326 | 0.436 | -0.158 |
| logepa | rs186227617 | 0.188 | 0.647 | 0.291 | 0.333 | 0.791 | 0.357 | 0.603 | 0.574 | 0.26 | 0.22 | -0.144 |
| logepa | rs188984368 | 0.024 | 0.522 | 0.227 | 0.134 | 8.63 | 8.82 | 0.035 | 2.17 | 2.52 | 0.506 | 1.61 |
| logepa | rs182078109 | . | . | . | . | . | . | . | . | . | . | . |
| logepa | rs141456274 | 0.023 | 0.399 | 0.128 | 4.20E-03 | 0.716 | 1.72 | 0.89 | 60.8 | 163 | 0.124 | 17.3 |
| logepa | rs146976512 | 0.198 | 0.673 | 0.296 | 0.367 | 0.779 | 0.341 | 0.567 | 0.539 | 0.24 | 0.165 | -0.169 |
| logepa | rs138036816 | . | . | . | . | . | . | . | . | . | . | . |
| logepa | rs114879777 | . | . | . | . | . | . | . | . | . | . | . |
| logepa | rs149366475 | . | . | . | . | . | . | . | . | . | . | . |
| logepa | rs182463043 | . | . | . | . | . | . | . | . | . | . | . |
| logepa | rs144754632 | 0.055 | 0.474 | 0.145 | 0.015 | 0.988 | 0.763 | 0.988 | 2.61 | 3.01 | 0.404 | 0.762 |
| logepa | rs55717607 | 0.198 | 0.673 | 0.296 | 0.367 | 0.779 | 0.341 | 0.567 | 0.539 | 0.24 | 0.165 | -0.169 |
| logepa | rs187537973 | . | . | . | . | . | . | . | . | . | . | . |
| logepa | rs148085971 | . | . | . | . | . | . | . | . | . | . | . |
| logepa | rs11239491 | 0.198 | 0.673 | 0.296 | 0.367 | 0.779 | 0.341 | 0.567 | 0.539 | 0.24 | 0.165 | -0.169 |
| logepa | rs184412184 | . | . | . | . | . | . | . | . | . | . | . |
| logepa | rs4948998 | 0.868 | 2.23 | 2.75 | 0.516 | 0.436 | 0.157 | 0.021 | 0.416 | 0.259 | 0.159 | -1.26 |
| logepa | rs7076696 | 0.873 | 2.34 | 2.93 | 0.497 | 0.434 | 0.155 | 0.019 | 0.405 | 0.256 | 0.152 | -1.36 |
| logepa | rs11239492 | 0.197 | 0.673 | 0.296 | 0.368 | 0.781 | 0.342 | 0.573 | 0.539 | 0.24 | 0.166 | -0.171 |
| logepa | rs79462470 | . | . | . | . | . | . | . | . | . | . | . |
| logepa | rs187804654 | . | . | . | . | . | . | . | . | . | . | . |
| logepa | rs7093687 | 0.198 | 0.673 | 0.296 | 0.367 | 0.779 | 0.341 | 0.567 | 0.539 | 0.24 | 0.165 | -0.169 |
| logepa | rs7081004 | 0.198 | 0.673 | 0.296 | 0.367 | 0.779 | 0.341 | 0.567 | 0.539 | 0.24 | 0.165 | -0.169 |
| logepa | rs7084793 | 0.198 | 0.673 | 0.296 | 0.367 | 0.779 | 0.341 | 0.567 | 0.539 | 0.24 | 0.165 | -0.169 |
| logepa | rs61556080 | . | . | . | . | . | . | . | . | . | . | . |
| logepa | rs115552064 | . | . | . | . | . | . | . | . | . | . | . |
| logepa | rs7921503 | 0.198 | 0.673 | 0.296 | 0.367 | 0.779 | 0.341 | 0.567 | 0.539 | 0.24 | 0.165 | -0.169 |
| logepa | rs7909740 | 0.198 | 0.673 | 0.296 | 0.367 | 0.779 | 0.341 | 0.567 | 0.539 | 0.24 | 0.165 | -0.169 |
| logepa | rs7909768 | 0.198 | 0.673 | 0.296 | 0.367 | 0.779 | 0.341 | 0.567 | 0.539 | 0.24 | 0.165 | -0.169 |
| logepa | rs144168004 | . | . | . | . | . | . | . | . | . | . | . |
| logepa | rs75850448 | . | . | . | . | . | . | . | . | . | . | . |
| logepa | rs147777574 | . | . | . | . | . | . | . | . | . | . | . |
| logepa | rs7913488 | 0.198 | 0.673 | 0.296 | 0.367 | 0.779 | 0.341 | 0.567 | 0.539 | 0.24 | 0.165 | -0.169 |
| logepa | rs74390874 | 0.048 | 0.458 | 0.191 | 0.061 | 1.11 | 0.87 | 0.89 | 3.23 | 2.4 | 0.115 | 1.08 |
| logepa | rs141391142 | . | . | . | . | . | . | . | . | . | . | . |
| logepa | rs115294747 | . | . | . | . | . | . | . | . | . | . | . |
| logepa | rs148031781 | . | . | . | . | . | . | . | . | . | . | . |
| logepa | rs181340128 | . | . | . | . | . | . | . | . | . | . | . |
| logepa | rs189930007 | . | . | . | . | . | . | . | . | . | . | . |
| logepa | rs12242128 | . | . | . | . | . | . | . | . | . | . | . |
| logepa | rs78421750 | . | . | . | . | . | . | . | . | . | . | . |
| logepa | rs114370747 | . | . | . | . | . | . | . | . | . | . | . |
| logepa | rs12264166 | . | . | . | . | . | . | . | . | . | . | . |
| logepa | rs182964822 | . | . | . | . | . | . | . | . | . | . | . |
| logepa | rs67491417 | 0.188 | 0.656 | 0.281 | 0.324 | 0.68 | 0.33 | 0.427 | 0.534 | 0.247 | 0.176 | -0.098 |
| logepa | rs72789720 | . | . | . | . | . | . | . | . | . | . | . |
| logepa | rs6413416 | 0.198 | 0.673 | 0.296 | 0.367 | 0.779 | 0.341 | 0.567 | 0.539 | 0.24 | 0.165 | -0.169 |
| logepa | rs4986832 | 0.198 | 0.673 | 0.296 | 0.367 | 0.779 | 0.341 | 0.567 | 0.539 | 0.24 | 0.165 | -0.169 |
| logepa | rs144866316 | . | . | . | . | . | . | . | . | . | . | . |
| logepa | rs187769540 | . | . | . | . | . | . | . | . | . | . | . |
| logepa | rs6593482 | 0.198 | 0.671 | 0.296 | 0.366 | 0.778 | 0.341 | 0.567 | 0.543 | 0.242 | 0.171 | -0.166 |
| logepa | rs7913948 | 0.199 | 0.673 | 0.295 | 0.367 | 0.776 | 0.34 | 0.562 | 0.538 | 0.24 | 0.165 | -0.167 |
| logepa | rs77185151 | 0.055 | 0.474 | 0.145 | 0.015 | 0.989 | 0.763 | 0.988 | 2.61 | 3.01 | 0.404 | 0.763 |
| logepa | rs12762303 | 0.198 | 0.673 | 0.296 | 0.367 | 0.779 | 0.341 | 0.567 | 0.539 | 0.24 | 0.165 | -0.169 |
| logepa | rs73283136 | . | . | . | . | . | . | . | . | . | . | . |
| logepa | rs187180218 | . | . | . | . | . | . | . | . | . | . | . |
| logepa | rs78137087 | 0.198 | 0.673 | 0.296 | 0.367 | 0.779 | 0.341 | 0.567 | 0.539 | 0.24 | 0.165 | -0.169 |
| logepa | rs72561472 | 0.193 | 0.677 | 0.298 | 0.376 | 0.781 | 0.35 | 0.581 | 0.525 | 0.242 | 0.162 | -0.181 |
| logepa | rs4987106 | 0.193 | 0.67 | 0.292 | 0.358 | 0.749 | 0.327 | 0.509 | 0.532 | 0.239 | 0.161 | -0.152 |
| logepa | rs4987105 | 0.198 | 0.673 | 0.296 | 0.367 | 0.779 | 0.341 | 0.568 | 0.539 | 0.24 | 0.165 | -0.169 |
| logepa | rs111916157 | 0.199 | 0.674 | 0.299 | 0.374 | 0.789 | 0.339 | 0.582 | 0.541 | 0.241 | 0.168 | -0.176 |
| logepa | rs111528152 | 0.198 | 0.673 | 0.296 | 0.367 | 0.779 | 0.341 | 0.567 | 0.539 | 0.24 | 0.165 | -0.169 |
| logepa | rs12763738 | 0.197 | 0.673 | 0.296 | 0.367 | 0.779 | 0.341 | 0.568 | 0.539 | 0.24 | 0.165 | -0.169 |
| logepa | rs191675886 | . | . | . | . | . | . | . | . | . | . | . |
| logepa | rs66489870 | 0.198 | 0.673 | 0.296 | 0.367 | 0.779 | 0.341 | 0.567 | 0.539 | 0.24 | 0.165 | -0.169 |
| logepa | rs71494775 | 0.198 | 0.673 | 0.296 | 0.367 | 0.779 | 0.341 | 0.567 | 0.539 | 0.24 | 0.165 | -0.169 |
| logepa | rs61855875 | . | . | . | . | . | . | . | . | . | . | . |
| logepa | rs116356837 | . | . | . | . | . | . | . | . | . | . | . |
| logepa | rs1864414 | 0.198 | 0.673 | 0.296 | 0.367 | 0.779 | 0.341 | 0.567 | 0.539 | 0.24 | 0.165 | -0.169 |
| logepa | rs1864413 | 0.198 | 0.673 | 0.296 | 0.367 | 0.778 | 0.341 | 0.567 | 0.539 | 0.24 | 0.165 | -0.169 |
| logepa | rs139497212 | . | . | . | . | . | . | . | . | . | . | . |
| logepa | rs892690 | 0.504 | 0.465 | 0.331 | 0.283 | 0.668 | 0.262 | 0.304 | 1.12 | 0.565 | 0.829 | 0.214 |
| logepa | rs1864412 | 0.198 | 0.673 | 0.296 | 0.367 | 0.779 | 0.341 | 0.567 | 0.539 | 0.24 | 0.165 | -0.169 |
| logepa | rs77827771 | . | . | . | . | . | . | . | . | . | . | . |
| logepa | rs145294963 | . | . | . | . | . | . | . | . | . | . | . |
| logepa | rs149081327 | . | . | . | . | . | . | . | . | . | . | . |
| logepa | rs67008983 | 0.198 | 0.673 | 0.296 | 0.367 | 0.779 | 0.341 | 0.567 | 0.539 | 0.24 | 0.165 | -0.169 |
| logepa | rs56150414 | 0.198 | 0.673 | 0.296 | 0.367 | 0.779 | 0.341 | 0.568 | 0.539 | 0.24 | 0.166 | -0.169 |
| logepa | rs55787481 | 0.199 | 0.672 | 0.295 | 0.364 | 0.782 | 0.339 | 0.572 | 0.543 | 0.239 | 0.166 | -0.169 |
| logepa | rs141270101 | . | . | . | . | . | . | . | . | . | . | . |
| logepa | rs114980075 | . | . | . | . | . | . | . | . | . | . | . |
| logepa | rs12783095 | 0.198 | 0.673 | 0.296 | 0.367 | 0.779 | 0.341 | 0.567 | 0.539 | 0.24 | 0.165 | -0.169 |
| logepa | rs182342688 | . | . | . | . | . | . | . | . | . | . | . |

| logepa | rs17157728 | 0.198 | 0.673 | 0.296 | 0.367 | 0.779 | 0.341 | 0.567 | 0.539 | 0.24 | 0.165 | -0.169 |
| --- | --- | --- | --- | --- | --- | --- | --- | --- | --- | --- | --- | --- |
| logepa | rs114835563 | . | . | . | . | . | . | . | . | . | . | . |
| logepa | rs3824612 | 0.504 | 0.465 | 0.332 | 0.283 | 0.669 | 0.262 | 0.305 | 1.11 | 0.566 | 0.83 | 0.213 |
| logepa | rs17157733 | 0.196 | 0.642 | 0.29 | 0.325 | 0.787 | 0.342 | 0.581 | 0.597 | 0.261 | 0.238 | -0.127 |
| logepa | rs74128478 | . | . | . | . | . | . | . | . | . | . | . |
| logepa | rs184973589 | . | . | . | . | . | . | . | . | . | . | . |
| logepa | rs150479461 | . | . | . | . | . | . | . | . | . | . | . |
| logepa | rs138310407 | . | . | . | . | . | . | . | . | . | . | . |
| logepa | rs187426603 | . | . | . | . | . | . | . | . | . | . | . |
| logepa | rs190843143 | . | . | . | . | . | . | . | . | . | . | . |
| logepa | rs191883449 | . | . | . | . | . | . | . | . | . | . | . |
| logepa | rs58238740 | 0.198 | 0.673 | 0.296 | 0.367 | 0.779 | 0.341 | 0.567 | 0.539 | 0.24 | 0.165 | -0.169 |
| logepa | rs3780894 | 0.198 | 0.673 | 0.296 | 0.367 | 0.779 | 0.341 | 0.567 | 0.539 | 0.24 | 0.165 | -0.169 |
| logepa | rs149646626 | . | . | . | . | . | . | . | . | . | . | . |
| logepa | rs79615212 | . | . | . | . | . | . | . | . | . | . | . |
| logepa | rs3780895 | 0.197 | 0.673 | 0.296 | 0.368 | 0.781 | 0.342 | 0.573 | 0.539 | 0.24 | 0.166 | -0.171 |
| logepa | rs3780896 | 0.198 | 0.673 | 0.296 | 0.367 | 0.779 | 0.341 | 0.567 | 0.539 | 0.24 | 0.165 | -0.169 |
| logepa | rs142747163 | 0.003 | . | . | . | . | . | . | . | . | . | . |
| logepa | rs189417584 | . | . | . | . | . | . | . | . | . | . | . |
| logepa | rs3780897 | 0.197 | 0.673 | 0.296 | 0.367 | 0.779 | 0.341 | 0.567 | 0.539 | 0.24 | 0.165 | -0.169 |
| logepa | rs117509491 | . | . | . | . | . | . | . | . | . | . | . |
| logepa | rs117811514 | . | . | . | . | . | . | . | . | . | . | . |
| logepa | rs116500932 | . | . | . | . | . | . | . | . | . | . | . |
| logepa | rs77584168 | 0.014 | 0.661 | 0.24 | 0.254 | 1.10E-03 | 4.20E-03 | 0.081 | 1.20E-04 | 5.30E-04 | 0.034 | 0.338 |
| logepa | rs2310776 | 0.198 | 0.673 | 0.296 | 0.367 | 0.779 | 0.341 | 0.567 | 0.539 | 0.24 | 0.165 | -0.169 |
| logepa | rs17157736 | 0.198 | 0.673 | 0.296 | 0.367 | 0.779 | 0.341 | 0.567 | 0.539 | 0.24 | 0.165 | -0.169 |
| logepa | rs72789735 | 0.001 | . | . | . | . | . | . | . | . | . | . |
| logepa | rs183747187 | . | . | . | . | . | . | . | . | . | . | . |
| logepa | rs7077891 | 0.198 | 0.673 | 0.296 | 0.367 | 0.779 | 0.341 | 0.567 | 0.539 | 0.24 | 0.165 | -0.169 |
| logepa | rs145063305 | 0.001 | . | . | . | . | . | . | . | . | . | . |
| logepa | rs3824613 | 0.198 | 0.677 | 0.298 | 0.375 | 0.771 | 0.336 | 0.551 | 0.534 | 0.238 | 0.159 | -0.169 |
| logepa | rs145543199 | . | . | . | . | . | . | . | . | . | . | . |
| logepa | rs140361993 | . | . | . | . | . | . | . | . | . | . | . |
| logepa | rs11239496 | 0.001 | . | . | . | . | . | . | . | . | . | . |
| logepa | rs11239497 | 0.197 | 0.673 | 0.296 | 0.367 | 0.779 | 0.341 | 0.568 | 0.539 | 0.24 | 0.166 | -0.169 |
| logepa | rs10900212 | 0.198 | 0.673 | 0.296 | 0.367 | 0.778 | 0.341 | 0.567 | 0.539 | 0.24 | 0.165 | -0.169 |
| logepa | rs186487136 | . | . | . | . | . | . | . | . | . | . | . |
| logepa | rs12774369 | 0.197 | 0.673 | 0.296 | 0.367 | 0.779 | 0.341 | 0.569 | 0.54 | 0.24 | 0.166 | -0.169 |
| logepa | rs60842538 | 0.197 | 0.672 | 0.296 | 0.367 | 0.781 | 0.342 | 0.572 | 0.54 | 0.241 | 0.167 | -0.169 |
| logepa | rs3780898 | 0.197 | 0.672 | 0.296 | 0.366 | 0.782 | 0.343 | 0.575 | 0.541 | 0.241 | 0.168 | -0.17 |
| logepa | rs3780899 | 0.197 | 0.672 | 0.296 | 0.366 | 0.783 | 0.343 | 0.576 | 0.541 | 0.241 | 0.168 | -0.17 |
| logepa | rs12252472 | . | . | . | . | . | . | . | . | . | . | . |
| logepa | rs185411011 | . | . | . | . | . | . | . | . | . | . | . |
| logepa | rs28395865 | 0.193 | 0.675 | 0.296 | 0.37 | 0.765 | 0.343 | 0.55 | 0.524 | 0.24 | 0.158 | -0.17 |
| logepa | rs191004074 | . | . | . | . | . | . | . | . | . | . | . |
| logepa | rs182401295 | . | . | . | . | . | . | . | . | . | . | . |
| logepa | rs2228064 | 0.057 | 0.474 | 0.145 | 0.014 | 0.984 | 0.767 | 0.984 | 2.62 | 3.03 | 0.403 | 0.766 |
| logepa | rs188284159 | . | . | . | . | . | . | . | . | . | . | . |
| logepa | rs2303887 | 0.055 | 0.474 | 0.145 | 0.015 | 0.994 | 0.77 | 0.994 | 2.61 | 2.99 | 0.402 | 0.764 |
| logepa | rs2303886 | 0.055 | 0.474 | 0.145 | 0.015 | 0.994 | 0.77 | 0.994 | 2.61 | 2.99 | 0.402 | 0.764 |
| logepa | rs192621006 | . | . | . | . | . | . | . | . | . | . | . |
| logepa | rs2303885 | 0.195 | 0.67 | 0.295 | 0.363 | 0.792 | 0.345 | 0.592 | 0.545 | 0.242 | 0.172 | -0.173 |
| logepa | rs114466168 | . | . | . | . | . | . | . | . | . | . | . |
| logepa | rs11239501 | 0.003 | . | . | . | . | . | . | . | . | . | . |
| logepa | rs745986 | 0.196 | 0.672 | 0.294 | 0.364 | 0.775 | 0.33 | 0.549 | 0.538 | 0.238 | 0.161 | -0.167 |
| logepa | rs75262268 | . | . | . | . | . | . | . | . | . | . | . |
| logepa | rs71494776 | 0.192 | 0.667 | 0.294 | 0.358 | 0.796 | 0.347 | 0.601 | 0.548 | 0.243 | 0.174 | -0.172 |
| logepa | rs74386871 | 0.055 | 0.474 | 0.145 | 0.015 | 0.994 | 0.771 | 0.994 | 2.62 | 2.99 | 0.401 | 0.765 |
| logepa | rs76502040 | . | . | . | . | . | . | . | . | . | . | . |
| logepa | rs142213734 | . | . | . | . | . | . | . | . | . | . | . |
| logepa | rs4948671 | 0.869 | 2.11 | 2.57 | 0.539 | 0.44 | 0.151 | 0.016 | 0.427 | 0.262 | 0.165 | -1.15 |
| logepa | rs146286085 | . | . | . | . | . | . | . | . | . | . | . |
| logepa | rs144080623 | . | . | . | . | . | . | . | . | . | . | . |
| logepa | rs115074014 | . | . | . | . | . | . | . | . | . | . | . |
| logepa | rs17157756 | . | . | . | . | . | . | . | . | . | . | . |
| logepa | rs190459780 | . | . | . | . | . | . | . | . | . | . | . |
| logepa | rs12358647 | 0.195 | 0.641 | 0.288 | 0.323 | 0.782 | 0.331 | 0.561 | 0.595 | 0.259 | 0.232 | -0.125 |
| logepa | rs147080556 | 0.009 | . | . | . | . | . | . | . | . | . | . |
| logepa | rs187543349 | . | . | . | . | . | . | . | . | . | . | . |
| logepa | rs114375595 | 0.01 | . | . | . | . | . | . | . | . | . | . |
| logepa | rs117211200 | . | . | . | . | . | . | . | . | . | . | . |
| logepa | rs192963047 | . | . | . | . | . | . | . | . | . | . | . |
| logepa | rs11239505 | 0.196 | 0.672 | 0.294 | 0.364 | 0.773 | 0.329 | 0.546 | 0.537 | 0.238 | 0.16 | -0.167 |
| logepa | rs184434116 | . | . | . | . | . | . | . | . | . | . | . |
| logepa | rs4948999 | 0.869 | 2.11 | 2.57 | 0.539 | 0.44 | 0.15 | 0.016 | 0.427 | 0.262 | 0.165 | -1.15 |
| logepa | rs11239506 | 0.003 | . | . | . | . | . | . | . | . | . | . |
| logepa | rs190706620 | . | . | . | . | . | . | . | . | . | . | . |
| logepa | rs190770429 | . | . | . | . | . | . | . | . | . | . | . |
| logepa | rs181327133 | . | . | . | . | . | . | . | . | . | . | . |
| logepa | rs149251697 | . | . | . | . | . | . | . | . | . | . | . |
| logepa | rs118081602 | 0.055 | 0.474 | 0.145 | 0.015 | 0.996 | 0.771 | 0.995 | 2.61 | 2.99 | 0.401 | 0.764 |
| logepa | rs188233691 | . | . | . | . | . | . | . | . | . | . | . |
| logepa | rs183778794 | 0.032 | 0.684 | 0.25 | 0.3 | 0.116 | 0.245 | 0.308 | 0.012 | 0.022 | 0.014 | 0.201 |
| logepa | rs188040078 | . | . | . | . | . | . | . | . | . | . | . |
| logepa | rs184675372 | . | . | . | . | . | . | . | . | . | . | . |
| logepa | rs150326891 | 0.004 | . | . | . | . | . | . | . | . | . | . |

| logepa | rs185520021 | . | . | . | . | . | . | . | . | . | . | . |
| --- | --- | --- | --- | --- | --- | --- | --- | --- | --- | --- | --- | --- |
| logepa | rs7907779 | 0.003 | . | . | . | . | . | . | . | . | . | . |
| logepa | rs184962860 | . | . | . | . | . | . | . | . | . | . | . |
| logepa | rs149762784 | 0.027 | 0.541 | 0.222 | 0.135 | 1.69 | 2.01 | 0.658 | 0.686 | 0.859 | 0.764 | -0.604 |
| logepa | rs149203427 | . | . | . | . | . | . | . | . | . | . | . |
| logepa | rs60153083 | . | . | . | . | . | . | . | . | . | . | . |
| logepa | rs112001069 | 0.194 | 0.678 | 0.293 | 0.369 | 0.745 | 0.333 | 0.51 | 0.517 | 0.232 | 0.141 | -0.162 |
| logepa | rs182355382 | 0.012 | 0.498 | 0.188 | 0.064 | 13.7 | 19.5 | 0.066 | 6.71 | 22.3 | 0.567 | 32.6 |
| logepa | rs186740974 | 0.109 | 0.742 | 0.29 | 0.445 | 0.127 | 0.129 | 0.043 | 0.117 | 0.127 | 0.048 | 0.142 |
| logepa | rs191874916 | 0.081 | 0.622 | 0.255 | 0.247 | 0.632 | 0.392 | 0.459 | 0.274 | 0.242 | 0.142 | -0.147 |
| logepa | rs182989412 | 0.122 | 0.387 | 0.175 | 0.035 | 0.993 | 0.555 | 0.99 | 3.45 | 2.64 | 0.106 | 0.944 |
| logepa | rs188819476 | . | . | . | . | . | . | . | . | . | . | . |
| logepa | rs141217183 | 0.186 | 0.651 | 0.292 | 0.338 | 0.761 | 0.344 | 0.545 | 0.563 | 0.258 | 0.211 | -0.133 |
| logepa | rs192922652 | 0.199 | 0.675 | 0.295 | 0.368 | 0.766 | 0.335 | 0.542 | 0.532 | 0.237 | 0.157 | -0.165 |
| logepa | rs185092343 | 0.012 | 0.486 | 0.196 | 0.074 | 5.01 | 7.75 | 0.297 | 6.51 | 7.43 | 0.101 | 11.4 |
| logepa | rs145193311 | 0.194 | 0.67 | 0.295 | 0.363 | 0.796 | 0.347 | 0.601 | 0.546 | 0.243 | 0.174 | -0.175 |
| logepa | rs55789336 | 0.004 | . | . | . | . | . | . | . | . | . | . |
| logepa | rs55837212 | 0.009 | . | . | . | . | . | . | . | . | . | . |
| logepa | rs75408574 | 0.565 | 0.708 | 0.425 | 0.565 | 0.53 | 0.169 | 0.046 | 0.703 | 0.281 | 0.379 | 0.026 |
| logepa | rs149306926 | 0.01 | 0.564 | 0.217 | 0.136 | 2.50E-08 | 4.90E-07 | 0.366 | 2.80E-07 | 4.20E-06 | 0.316 | 0.436 |
| logepa | rs35990892 | 0.843 | 1.76 | 1.97 | 0.613 | 0.398 | 0.135 | 6.50E-03 | 0.466 | 0.274 | 0.194 | -0.831 |
| logepa | rs192356207 | 0.015 | 0.537 | 0.215 | 0.12 | 3.42 | 3.5 | 0.229 | 1.04 | 1.18 | 0.972 | -1.05 |
| logepa | rs183741163 | 0.049 | 0.48 | 0.225 | 0.118 | 1.45 | 1.38 | 0.693 | 2.03 | 1.89 | 0.447 | 0.481 |
| logepa | rs34737297 | 0.719 | 1.82 | 1.64 | 0.506 | 0.488 | 0.176 | 0.046 | 0.396 | 0.228 | 0.108 | -0.957 |
| logepa | rs181346933 | . | . | . | . | . | . | . | . | . | . | . |
| logepa | rs36093253 | 0.576 | 0.416 | 0.241 | 0.13 | 0.807 | 0.203 | 0.392 | 1.21 | 0.369 | 0.536 | 0.183 |
| logepa | rs186307855 | . | . | . | . | . | . | . | . | . | . | . |
| logepa | rs189681865 | . | . | . | . | . | . | . | . | . | . | . |
| logepa | rs34942971 | 0.214 | 0.695 | 0.297 | 0.395 | 0.706 | 0.311 | 0.429 | 0.498 | 0.219 | 0.114 | -0.156 |
| logepa | rs181816554 | . | . | . | . | . | . | . | . | . | . | . |
| logepa | rs186846881 | . | . | . | . | . | . | . | . | . | . | . |
| logepa | rs191178220 | 0.003 | . | . | . | . | . | . | . | . | . | . |
| logepa | rs183305228 | . | . | . | . | . | . | . | . | . | . | . |
| logepa | rs139912761 | 0.004 | . | . | . | . | . | . | . | . | . | . |
| logepa | rs185499901 | . | . | . | . | . | . | . | . | . | . | . |
| logepa | rs189816197 | 0.194 | 0.667 | 0.299 | 0.366 | 0.764 | 0.354 | 0.562 | 0.549 | 0.255 | 0.197 | -0.151 |
| logepa | rs145656938 | 0.009 | . | . | . | . | . | . | . | . | . | . |
| logepa | rs35745197 | 0.193 | 0.664 | 0.293 | 0.354 | 0.793 | 0.348 | 0.597 | 0.552 | 0.245 | 0.181 | -0.166 |
| logepa | rs192856637 | 0.006 | . | . | . | . | . | . | . | . | . | . |
| logepa | rs2310775 | 0.617 | 0.633 | 0.431 | 0.502 | 0.66 | 0.208 | 0.188 | 0.826 | 0.336 | 0.638 | 0.052 |
| logepa | rs11239507 | 0.673 | 0.544 | 0.266 | 0.212 | 0.68 | 0.158 | 0.097 | 0.958 | 0.244 | 0.867 | 0.13 |
| logepa | rs74648467 | 0.192 | 0.662 | 0.292 | 0.351 | 0.795 | 0.348 | 0.6 | 0.556 | 0.246 | 0.185 | -0.165 |
| logepa | rs145815934 | . | . | . | . | . | . | . | . | . | . | . |
| logepa | rs145390687 | . | . | . | . | . | . | . | . | . | . | . |
| logepa | rs184354913 | . | . | . | . | . | . | . | . | . | . | . |
| logepa | rs181244920 | . | . | . | . | . | . | . | . | . | . | . |
| logepa | rs11499087 | . | . | . | . | . | . | . | . | . | . | . |
| logepa | rs148263794 | 0.207 | 0.694 | 0.314 | 0.419 | 0.826 | 0.353 | 0.656 | 0.531 | 0.238 | 0.158 | -0.216 |
| logepa | rs150758903 | . | . | . | . | . | . | . | . | . | . | . |
| logepa | rs147654382 | 0.187 | 0.703 | 0.31 | 0.424 | 0.768 | 0.335 | 0.545 | 0.458 | 0.233 | 0.124 | -0.224 |
| logepa | rs147495166 | . | . | . | . | . | . | . | . | . | . | . |
| logepa | rs35198778 | 0.131 | 0.62 | 0.272 | 0.276 | 0.823 | 0.402 | 0.689 | 0.579 | 0.293 | 0.28 | -0.148 |
| logepa | rs145207659 | 0.048 | 0.459 | 0.191 | 0.062 | 1.13 | 0.884 | 0.872 | 3.17 | 2.38 | 0.124 | 1.06 |
| logepa | rs186466464 | . | . | . | . | . | . | . | . | . | . | . |
| logepa | rs12778413 | 0.131 | 0.623 | 0.271 | 0.276 | 0.724 | 0.432 | 0.589 | 0.52 | 0.303 | 0.262 | -0.112 |
| logepa | rs7895552 | 0.833 | 1.65 | 1.92 | 0.667 | 0.461 | 0.165 | 0.03 | 0.485 | 0.285 | 0.218 | -0.742 |
| logepa | rs12783895 | 0.193 | 0.664 | 0.293 | 0.353 | 0.791 | 0.346 | 0.592 | 0.552 | 0.245 | 0.18 | -0.165 |
| logepa | rs34929604 | 0.193 | 0.664 | 0.293 | 0.353 | 0.791 | 0.346 | 0.591 | 0.552 | 0.245 | 0.181 | -0.165 |
| logepa | rs12761961 | 0.185 | 0.663 | 0.296 | 0.357 | 0.831 | 0.374 | 0.682 | 0.56 | 0.252 | 0.198 | -0.186 |
| logepa | rs35536930 | 0.197 | 0.673 | 0.294 | 0.365 | 0.77 | 0.327 | 0.538 | 0.536 | 0.237 | 0.158 | -0.165 |
| logepa | rs180951060 | . | . | . | . | . | . | . | . | . | . | . |
| logepa | rs148892128 | . | . | . | . | . | . | . | . | . | . | . |
| logepa | rs11239509 | . | . | . | . | . | . | . | . | . | . | . |
| logepa | rs41526545 | 0.003 | . | . | . | . | . | . | . | . | . | . |
| logepa | rs2029253 | 0.617 | 0.634 | 0.432 | 0.503 | 0.66 | 0.208 | 0.188 | 0.825 | 0.336 | 0.637 | 0.051 |
| logepa | rs17444064 | 0.003 | . | . | . | . | . | . | . | . | . | . |
| logepa | rs71494779 | 0.194 | 0.67 | 0.294 | 0.362 | 0.79 | 0.344 | 0.589 | 0.543 | 0.241 | 0.169 | -0.173 |
| logepa | rs74991420 | 0.05 | 0.459 | 0.134 | 7.50E-03 | 1.2 | 1.01 | 0.824 | 3.68 | 6.74 | 0.476 | 1.37 |
| logepa | rs182736551 | 0.05 | 0.459 | 0.134 | 7.50E-03 | 1.2 | 1.01 | 0.824 | 3.68 | 6.74 | 0.476 | 1.37 |
| logepa | rs78702074 | 0.055 | 0.474 | 0.145 | 0.015 | 0.999 | 0.784 | 0.999 | 2.61 | 3.01 | 0.406 | 0.764 |
| logepa | rs187791466 | . | . | . | . | . | . | . | . | . | . | . |
| logepa | rs35300270 | 0.194 | 0.67 | 0.294 | 0.362 | 0.79 | 0.344 | 0.589 | 0.543 | 0.241 | 0.169 | -0.173 |
| logepa | rs6593484 | 0.003 | . | . | . | . | . | . | . | . | . | . |
| logepa | rs12244601 | . | . | . | . | . | . | . | . | . | . | . |
| logepa | rs184429574 | . | . | . | . | . | . | . | . | . | . | . |
| logepa | rs7080897 | 0.194 | 0.67 | 0.294 | 0.361 | 0.79 | 0.344 | 0.588 | 0.543 | 0.241 | 0.169 | -0.173 |
| logepa | rs7097429 | 0.196 | 0.676 | 0.299 | 0.376 | 0.779 | 0.336 | 0.562 | 0.534 | 0.237 | 0.157 | -0.174 |
| logepa | rs77979229 | 0.016 | 0.603 | 0.222 | 0.169 | 0.389 | 0.727 | 0.613 | 0.077 | 0.126 | 0.117 | 0.026 |
| logepa | rs11239512 | 0.003 | . | . | . | . | . | . | . | . | . | . |
| logepa | rs60146827 | 0.195 | 0.67 | 0.294 | 0.361 | 0.787 | 0.342 | 0.581 | 0.542 | 0.241 | 0.168 | -0.171 |
| logepa | rs79032236 | . | . | . | . | . | . | . | . | . | . | . |
| logepa | rs140854206 | . | . | . | . | . | . | . | . | . | . | . |
| logepa | rs184049550 | . | . | . | . | . | . | . | . | . | . | . |
| logepa | rs114433607 | . | . | . | . | . | . | . | . | . | . | . |
| logepa | rs117653793 | 0.055 | 0.474 | 0.145 | 0.015 | 1 | 0.784 | 1 | 2.61 | 3.01 | 0.406 | 0.763 |
| logepa | rs117039533 | 0.055 | 0.474 | 0.145 | 0.015 | 1 | 0.784 | 1 | 2.61 | 3.01 | 0.406 | 0.763 |

| logepa | rs191660761 | 0.001 | . | . | . | . | . | . | . | . | . | . |
| --- | --- | --- | --- | --- | --- | --- | --- | --- | --- | --- | --- | --- |
| logepa | rs59514896 | 0.197 | 0.667 | 0.293 | 0.357 | 0.78 | 0.336 | 0.564 | 0.547 | 0.24 | 0.169 | -0.163 |
| logepa | rs184661876 | . | . | . | . | . | . | . | . | . | . | . |
| logepa | rs61854073 | . | . | . | . | . | . | . | . | . | . | . |
| logepa | rs2018518 | 0.5 | 0.428 | 0.312 | 0.244 | 0.705 | 0.27 | 0.361 | 1.22 | 0.615 | 0.699 | 0.234 |
| logepa | rs934187 | 0.872 | 2 | 2.44 | 0.569 | 0.448 | 0.151 | 0.017 | 0.443 | 0.271 | 0.182 | -1.05 |
| logepa | rs7918542 | 0.202 | 0.672 | 0.293 | 0.363 | 0.758 | 0.316 | 0.507 | 0.536 | 0.235 | 0.155 | -0.157 |
| logepa | rs144311920 | . | . | . | . | . | . | . | . | . | . | . |
| logepa | rs7099684 | 0.871 | 2.01 | 2.45 | 0.569 | 0.448 | 0.151 | 0.018 | 0.442 | 0.27 | 0.182 | -1.06 |
| logepa | rs150829171 | . | . | . | . | . | . | . | . | . | . | . |
| logepa | rs186060697 | . | . | . | . | . | . | . | . | . | . | . |
| logepa | rs11239513 | . | . | . | . | . | . | . | . | . | . | . |
| logepa | rs7917687 | 0.2 | 0.669 | 0.293 | 0.358 | 0.769 | 0.326 | 0.535 | 0.545 | 0.238 | 0.164 | -0.157 |
| logepa | rs61854076 | . | . | . | . | . | . | . | . | . | . | . |
| logepa | rs71494780 | . | . | . | . | . | . | . | . | . | . | . |
| logepa | rs7919239 | 0.235 | 0.668 | 0.289 | 0.351 | 0.733 | 0.243 | 0.35 | 0.595 | 0.199 | 0.121 | -0.11 |
| logepa | rs78334475 | 0.166 | 0.646 | 0.351 | 0.421 | 0.349 | 0.287 | 0.2 | 0.395 | 0.48 | 0.444 | 0.094 |
| logepa | rs3780900 | 0.068 | 0.62 | 0.247 | 0.231 | 0.752 | 0.444 | 0.629 | 0.243 | 0.214 | 0.109 | -0.259 |
| logepa | rs186729292 | . | . | . | . | . | . | . | . | . | . | . |
| logepa | rs12267438 | . | . | . | . | . | . | . | . | . | . | . |
| logepa | rs1369214 | 0.868 | 1.88 | 2.33 | 0.608 | 0.448 | 0.157 | 0.022 | 0.458 | 0.283 | 0.206 | -0.946 |
| logepa | rs9282589 | . | . | . | . | . | . | . | . | . | . | . |
| logepa | rs114522915 | . | . | . | . | . | . | . | . | . | . | . |
| logepa | rs2115819 | 0.812 | 1.97 | 2.08 | 0.522 | 0.488 | 0.182 | 0.054 | 0.418 | 0.238 | 0.126 | -1.05 |
| logepa | rs117664603 | 0.055 | 0.473 | 0.144 | 0.014 | 1.04 | 0.782 | 0.962 | 2.61 | 2.88 | 0.383 | 0.77 |
| logepa | rs184979202 | . | . | . | . | . | . | . | . | . | . | . |
| logepa | rs190714671 | . | . | . | . | . | . | . | . | . | . | . |
| logepa | rs141538996 | . | . | . | . | . | . | . | . | . | . | . |
| logepa | rs150881741 | . | . | . | . | . | . | . | . | . | . | . |
| logepa | rs139076633 | 0.011 | 0.576 | 0.218 | 0.145 | 9.70E-07 | 5.40E-06 | 0.013 | 5.10E-11 | 4.90E-10 | 0.013 | 0.424 |
| logepa | rs12243649 | 0.056 | 0.473 | 0.144 | 0.014 | 1.06 | 0.773 | 0.936 | 2.54 | 2.79 | 0.397 | 0.739 |
| logepa | rs140348708 | . | . | . | . | . | . | . | . | . | . | . |
| logepa | rs79023256 | 0.055 | 0.472 | 0.144 | 0.014 | 1.05 | 0.785 | 0.949 | 2.61 | 2.84 | 0.378 | 0.771 |
| logepa | rs11239515 | 0.003 | . | . | . | . | . | . | . | . | . | . |
| logepa | rs55866086 | 0.059 | 0.473 | 0.144 | 0.014 | 0.989 | 0.731 | 0.988 | 2.62 | 2.98 | 0.398 | 0.763 |
| logepa | rs181340150 | . | . | . | . | . | . | . | . | . | . | . |
| logepa | rs11239516 | 0.003 | . | . | . | . | . | . | . | . | . | . |
| logepa | rs11239517 | 0.003 | . | . | . | . | . | . | . | . | . | . |
| logepa | rs11239519 | 0.003 | . | . | . | . | . | . | . | . | . | . |
| logepa | rs7909514 | 0.872 | 1.82 | 2.2 | 0.62 | 0.44 | 0.151 | 0.017 | 0.468 | 0.283 | 0.209 | -0.886 |
| logepa | rs7894352 | 0.872 | 1.82 | 2.21 | 0.619 | 0.439 | 0.151 | 0.017 | 0.468 | 0.283 | 0.209 | -0.888 |
| logepa | rs189384146 | 0 | . | . | . | . | . | . | . | . | . | . |
| logepa | rs11239520 | 0.004 | . | . | . | . | . | . | . | . | . | . |
| logepa | rs12221233 | 0.617 | 0.634 | 0.43 | 0.502 | 0.664 | 0.208 | 0.19 | 0.825 | 0.332 | 0.632 | 0.049 |
| logepa | rs74523293 | 0.049 | 0.471 | 0.145 | 0.014 | 1.12 | 0.838 | 0.878 | 2.57 | 2.68 | 0.364 | 0.767 |
| logepa | rs79376040 | 0.049 | 0.471 | 0.145 | 0.014 | 1.12 | 0.838 | 0.876 | 2.57 | 2.67 | 0.364 | 0.767 |
| logepa | rs4949000 | 0.617 | 0.634 | 0.429 | 0.501 | 0.664 | 0.208 | 0.19 | 0.825 | 0.332 | 0.633 | 0.05 |
| logepa | rs7089406 | . | . | . | . | . | . | . | . | . | . | . |
| logepa | rs138211056 | . | . | . | . | . | . | . | . | . | . | . |
| logepa | rs10900213 | 0.621 | 0.637 | 0.428 | 0.502 | 0.651 | 0.205 | 0.173 | 0.821 | 0.329 | 0.622 | 0.053 |
| logepa | rs145152827 | 0.011 | 0.576 | 0.218 | 0.145 | 9.90E-07 | 5.50E-06 | 0.013 | 5.20E-11 | 5.00E-10 | 0.013 | 0.424 |
| logepa | rs74443325 | 0.048 | 0.47 | 0.145 | 0.015 | 1.12 | 0.853 | 0.882 | 2.63 | 2.71 | 0.347 | 0.796 |
| logepa | rs188299446 | . | . | . | . | . | . | . | . | . | . | . |
| logepa | rs192699586 | . | . | . | . | . | . | . | . | . | . | . |
| logepa | rs72796464 | 0.003 | . | . | . | . | . | . | . | . | . | . |
| logepa | rs4949001 | 0.455 | 0.485 | 0.336 | 0.296 | 0.655 | 0.264 | 0.294 | 1.08 | 0.611 | 0.894 | 0.203 |
| logepa | rs147174641 | . | . | . | . | . | . | . | . | . | . | . |
| logepa | rs190835238 | . | . | . | . | . | . | . | . | . | . | . |
| logepa | rs76698944 | . | . | . | . | . | . | . | . | . | . | . |
| logepa | rs56071381 | 0.003 | . | . | . | . | . | . | . | . | . | . |
| logepa | rs78130427 | 0.2 | 0.657 | 0.289 | 0.34 | 0.736 | 0.316 | 0.475 | 0.557 | 0.24 | 0.174 | -0.124 |
| logepa | rs10900215 | 0.007 | . | . | . | . | . | . | . | . | . | . |
| logepa | rs41491445 | . | . | . | . | . | . | . | . | . | . | . |
| logepa | rs144995222 | . | . | . | . | . | . | . | . | . | . | . |
| logepa | rs149097803 | . | . | . | . | . | . | . | . | . | . | . |
| logepa | rs139429646 | . | . | . | . | . | . | . | . | . | . | . |
| logepa | rs7075620 | 0.861 | 1.64 | 2.03 | 0.69 | 0.455 | 0.162 | 0.027 | 0.499 | 0.309 | 0.261 | -0.723 |
| logepa | rs148299213 | . | . | . | . | . | . | . | . | . | . | . |
| logepa | rs4949002 | 0.238 | 0.355 | 0.171 | 0.032 | 0.945 | 0.397 | 0.893 | 2.18 | 0.985 | 0.084 | 0.432 |
| logepa | rs150496007 | . | . | . | . | . | . | . | . | . | . | . |
| logepa | rs147303455 | . | . | . | . | . | . | . | . | . | . | . |
| logepa | rs189518433 | . | . | . | . | . | . | . | . | . | . | . |
| logepa | rs186281355 | . | . | . | . | . | . | . | . | . | . | . |
| logepa | rs115297114 | . | . | . | . | . | . | . | . | . | . | . |
| logepa | rs11239523 | 0.003 | . | . | . | . | . | . | . | . | . | . |
| logepa | rs143704897 | . | . | . | . | . | . | . | . | . | . | . |
| logepa | rs188024096 | . | . | . | . | . | . | . | . | . | . | . |
| logepa | rs11239524 | 0.003 | . | . | . | . | . | . | . | . | . | . |
| logepa | rs191656573 | . | . | . | . | . | . | . | . | . | . | . |
| logepa | rs193295875 | 0.011 | 0.521 | 0.195 | 0.081 | 0.04 | 0.129 | 0.315 | 3.88 | 4.95 | 0.288 | 0.52 |
| logepa | rs190458228 | . | . | . | . | . | . | . | . | . | . | . |
| logepa | rs139253993 | 0.002 | . | . | . | . | . | . | . | . | . | . |
| logepa | rs4948672 | 0.812 | 0.59 | 0.513 | 0.544 | 0.433 | 0.2 | 0.07 | 0.906 | 0.561 | 0.873 | 0.209 |
| logepa | rs180954921 | . | . | . | . | . | . | . | . | . | . | . |
| logepa | rs12264801 | 0.447 | 0.729 | 0.458 | 0.615 | 0.678 | 0.272 | 0.332 | 0.689 | 0.414 | 0.536 | -0.066 |
| logepa | rs73283184 | . | . | . | . | . | . | . | . | . | . | . |

| logepa | rs142746187 | . | . | . | . | . | . | . | . | . | . | . |
| --- | --- | --- | --- | --- | --- | --- | --- | --- | --- | --- | --- | --- |
| logepa | rs58704739 | . | . | . | . | . | . | . | . | . | . | . |
| logepa | rs61272335 | . | . | . | . | . | . | . | . | . | . | . |
| logepa | rs60959489 | . | . | . | . | . | . | . | . | . | . | . |
| logepa | rs58502629 | . | . | . | . | . | . | . | . | . | . | . |
| logepa | rs10900216 | 0.838 | 1.7 | 1.81 | 0.617 | 0.627 | 0.211 | 0.166 | 0.49 | 0.248 | 0.159 | -0.808 |
| logepa | rs181118141 | . | . | . | . | . | . | . | . | . | . | . |
| logepa | rs137882665 | . | . | . | . | . | . | . | . | . | . | . |
| logepa | rs149466736 | . | . | . | . | . | . | . | . | . | . | . |
| logepa | rs2099171 | 0.806 | 1.06 | 0.913 | 0.946 | 0.684 | 0.2 | 0.195 | 0.638 | 0.262 | 0.273 | -0.281 |
| logepa | rs141852280 | . | . | . | . | . | . | . | . | . | . | . |
| logepa | rs191539257 | 0.001 | . | . | . | . | . | . | . | . | . | . |
| logepa | rs17157771 | 0.029 | 0.537 | 0.209 | 0.11 | 0.771 | 0.502 | 0.689 | 0.351 | 0.372 | 0.324 | -0.162 |
| logepa | rs111339241 | 0.029 | 0.536 | 0.209 | 0.11 | 0.793 | 0.529 | 0.728 | 0.363 | 0.391 | 0.346 | -0.175 |
| logepa | rs116983255 | . | . | . | . | . | . | . | . | . | . | . |
| logepa | rs142324341 | 0.037 | 0.516 | 0.183 | 0.062 | 1.00E-03 | 3.30E-03 | 0.035 | 442 | 1.30E+03 | 0.037 | 0.713 |
| logepa | rs187191219 | . | . | . | . | . | . | . | . | . | . | . |
| logepa | rs151283973 | . | . | . | . | . | . | . | . | . | . | . |
| logepa | rs139316218 | . | . | . | . | . | . | . | . | . | . | . |
| logepa | rs6593485 | 0.886 | 0.922 | 1.26 | 0.952 | 0.803 | 0.39 | 0.651 | 0.729 | 0.539 | 0.669 | -0.185 |
| logepa | rs6593486 | 0.886 | 0.921 | 1.26 | 0.952 | 0.804 | 0.39 | 0.653 | 0.73 | 0.539 | 0.67 | -0.185 |
| logepa | rs145196618 | . | . | . | . | . | . | . | . | . | . | . |
| logepa | rs142598335 | . | . | . | . | . | . | . | . | . | . | . |
| logepa | rs892691 | 0.885 | 0.146 | 0.129 | 0.029 | 1.55 | 0.795 | 0.396 | 1.99 | 1.06 | 0.195 | -0.243 |
| logepa | rs7080713 | 0.809 | 1.06 | 0.906 | 0.95 | 0.683 | 0.201 | 0.194 | 0.64 | 0.262 | 0.275 | -0.277 |
| logepa | rs7077173 | 0.065 | 0.545 | 0.21 | 0.115 | 0.61 | 0.32 | 0.345 | 0.506 | 0.327 | 0.291 | 0.013 |
| logepa | rs7080977 | 0.03 | 0.536 | 0.209 | 0.11 | 0.814 | 0.509 | 0.742 | 0.389 | 0.4 | 0.359 | -0.18 |
| logepa | rs3780901 | 0.809 | 1.05 | 0.907 | 0.952 | 0.683 | 0.201 | 0.195 | 0.64 | 0.263 | 0.278 | -0.276 |
| logepa | rs61854091 | . | . | . | . | . | . | . | . | . | . | . |
| logepa | rs77550209 | . | . | . | . | . | . | . | . | . | . | . |
| logepa | rs73283191 | 0.077 | 0.474 | 0.158 | 0.025 | 1.56 | 0.657 | 0.288 | 1.5 | 0.805 | 0.454 | 0.071 |
| logepa | rs191866099 | . | . | . | . | . | . | . | . | . | . | . |
| logepa | rs147998395 | . | . | . | . | . | . | . | . | . | . | . |
| logepa | rs7090328 | 0.885 | 0.902 | 1.24 | 0.94 | 0.806 | 0.392 | 0.658 | 0.738 | 0.548 | 0.683 | -0.171 |
| logepa | rs79814535 | 0.048 | 0.462 | 0.146 | 0.014 | 1.62 | 0.99 | 0.431 | 2.32 | 1.77 | 0.267 | 0.655 |
| logepa | rs7073082 | 0.885 | 0.898 | 1.23 | 0.938 | 0.807 | 0.393 | 0.659 | 0.74 | 0.55 | 0.685 | -0.169 |
| logepa | rs11239526 | 0.039 | 0.435 | 0.145 | 0.013 | 3.6 | 2.04 | 0.023 | 3.06 | 3.3 | 0.299 | 1.77 |
| logepa | rs141839841 | . | . | . | . | . | . | . | . | . | . | . |
| logepa | rs75697371 | . | . | . | . | . | . | . | . | . | . | . |
| logepa | rs75972854 | . | . | . | . | . | . | . | . | . | . | . |
| logepa | rs139291975 | . | . | . | . | . | . | . | . | . | . | . |
| logepa | rs144064711 | . | . | . | . | . | . | . | . | . | . | . |
| logepa | rs3780902 | 0.078 | 0.474 | 0.158 | 0.025 | 1.55 | 0.652 | 0.295 | 1.5 | 0.81 | 0.457 | 0.075 |
| logepa | rs190449801 | . | . | . | . | . | . | . | . | . | . | . |
| logepa | rs73283196 | 0.077 | 0.474 | 0.158 | 0.025 | 1.57 | 0.659 | 0.286 | 1.5 | 0.814 | 0.454 | 0.074 |
| logepa | rs28395867 | . | . | . | . | . | . | . | . | . | . | . |
| logepa | rs28395866 | 0.029 | 0.538 | 0.21 | 0.112 | 0.779 | 0.517 | 0.707 | 0.331 | 0.356 | 0.304 | -0.178 |
| logepa | rs55661611 | 0.028 | 0.538 | 0.21 | 0.112 | 0.779 | 0.525 | 0.711 | 0.326 | 0.354 | 0.302 | -0.181 |
| logepa | rs55684518 | . | . | . | . | . | . | . | . | . | . | . |
| logepa | rs78552493 | . | . | . | . | . | . | . | . | . | . | . |
| logepa | rs147665434 | . | . | . | . | . | . | . | . | . | . | . |
| logepa | rs34445001 | . | . | . | . | . | . | . | . | . | . | . |
| logepa | rs184019449 | . | . | . | . | . | . | . | . | . | . | . |
| logepa | rs3780903 | 0.808 | 1.04 | 0.981 | 0.968 | 0.667 | 0.201 | 0.178 | 0.645 | 0.294 | 0.336 | -0.259 |
| logepa | rs17157784 | 0.048 | 0.462 | 0.146 | 0.015 | 1.63 | 0.987 | 0.422 | 2.32 | 1.75 | 0.263 | 0.657 |
| logepa | rs3780904 | 0.048 | 0.462 | 0.146 | 0.015 | 1.63 | 0.987 | 0.422 | 2.32 | 1.75 | 0.263 | 0.657 |
| logepa | rs192064730 | . | . | . | . | . | . | . | . | . | . | . |
| logepa | rs12774534 | . | . | . | . | . | . | . | . | . | . | . |
| logepa | rs79179915 | 0.047 | 0.462 | 0.146 | 0.015 | 1.64 | 0.999 | 0.418 | 2.33 | 1.74 | 0.26 | 0.659 |
| logepa | rs117811571 | . | . | . | . | . | . | . | . | . | . | . |
| logepa | rs72796477 | . | . | . | . | . | . | . | . | . | . | . |
| logepa | rs55729342 | 0.057 | 0.468 | 0.148 | 0.016 | 1.72 | 0.952 | 0.325 | 1.97 | 1.51 | 0.377 | 0.397 |
| logepa | rs59317220 | 0.047 | 0.462 | 0.146 | 0.015 | 1.64 | 1 | 0.418 | 2.33 | 1.74 | 0.258 | 0.663 |
| logepa | rs3780905 | 0.562 | 0.773 | 0.585 | 0.734 | 0.555 | 0.219 | 0.135 | 0.655 | 0.357 | 0.437 | -0.047 |
| logepa | rs3780906 | 0.807 | 1.03 | 1.03 | 0.976 | 0.659 | 0.201 | 0.171 | 0.649 | 0.313 | 0.369 | -0.249 |
| logepa | rs149047975 | . | . | . | . | . | . | . | . | . | . | . |
| logepa | rs3824614 | 0.047 | 0.461 | 0.146 | 0.015 | 1.64 | 1.01 | 0.418 | 2.33 | 1.74 | 0.256 | 0.665 |
| logepa | rs140254404 | . | . | . | . | . | . | . | . | . | . | . |
| logepa | rs145262684 | . | . | . | . | . | . | . | . | . | . | . |
| logepa | rs76735482 | 0.047 | 0.461 | 0.146 | 0.015 | 1.65 | 1.01 | 0.418 | 2.34 | 1.74 | 0.254 | 0.668 |
| logepa | rs73283198 | 0.028 | 0.545 | 0.213 | 0.12 | 0.684 | 0.494 | 0.599 | 0.189 | 0.21 | 0.134 | -0.158 |
| logepa | rs74911239 | . | . | . | . | . | . | . | . | . | . | . |
| logepa | rs150551647 | . | . | . | . | . | . | . | . | . | . | . |
| logepa | rs115200794 | . | . | . | . | . | . | . | . | . | . | . |
| logepa | rs74130823 | 0.038 | 0.559 | 0.22 | 0.14 | 0.831 | 0.493 | 0.755 | 0.213 | 0.198 | 0.096 | -0.291 |
| logepa | rs2279435 | 0.754 | 0.319 | 0.224 | 0.104 | 0.809 | 0.316 | 0.587 | 1.39 | 0.696 | 0.51 | 0.231 |
| logepa | rs147279673 | 0.01 | . | . | . | . | . | . | . | . | . | . |
| logepa | rs183525146 | 0.006 | . | . | . | . | . | . | . | . | . | . |
| logepa | rs3740107 | 0.737 | 0.876 | 0.567 | 0.838 | 0.622 | 0.187 | 0.115 | 0.681 | 0.238 | 0.273 | -0.127 |
| logepa | rs140697247 | . | . | . | . | . | . | . | . | . | . | . |
| logepa | rs1565096 | 0.806 | 0.468 | 0.357 | 0.319 | 0.773 | 0.295 | 0.5 | 1.08 | 0.549 | 0.885 | 0.149 |
| logepa | rs1565097 | 0.806 | 0.468 | 0.357 | 0.32 | 0.772 | 0.295 | 0.499 | 1.08 | 0.548 | 0.886 | 0.149 |
| logepa | rs144584548 | . | . | . | . | . | . | . | . | . | . | . |
| logepa | rs75811053 | 0.044 | 0.462 | 0.146 | 0.015 | 1.72 | 1.03 | 0.363 | 2.31 | 1.68 | 0.251 | 0.653 |
| logepa | rs147462967 | . | . | . | . | . | . | . | . | . | . | . |
| logepa | rs74130824 | 0.044 | 0.462 | 0.146 | 0.015 | 1.72 | 1.03 | 0.362 | 2.31 | 1.68 | 0.251 | 0.652 |

| logepa | rs116883184 | . | . | . | . | . | . | . | . | . | . | . |
| --- | --- | --- | --- | --- | --- | --- | --- | --- | --- | --- | --- | --- |
| logepa | rs117258049 | . | . | . | . | . | . | . | . | . | . | . |
| logepa | rs3780908 | 0.029 | 0.553 | 0.214 | 0.125 | 0.597 | 0.435 | 0.479 | 0.152 | 0.164 | 0.08 | -0.1 |
| logepa | rs180938522 | . | . | . | . | . | . | . | . | . | . | . |
| logepa | rs76687372 | . | . | . | . | . | . | . | . | . | . | . |
| logepa | rs3780909 | 0.666 | 0.608 | 0.484 | 0.532 | 0.448 | 0.161 | 0.026 | 0.858 | 0.432 | 0.761 | 0.178 |
| logepa | rs78281156 | 0.044 | 0.462 | 0.146 | 0.015 | 1.72 | 1.03 | 0.363 | 2.31 | 1.68 | 0.251 | 0.653 |
| logepa | rs187840631 | . | . | . | . | . | . | . | . | . | . | . |
| logepa | rs76868242 | 0.044 | 0.462 | 0.146 | 0.015 | 1.73 | 1.03 | 0.362 | 2.31 | 1.68 | 0.251 | 0.653 |
| logepa | rs192794192 | . | . | . | . | . | . | . | . | . | . | . |
| logepa | rs12781248 | . | . | . | . | . | . | . | . | . | . | . |
| logepa | rs12762375 | . | . | . | . | . | . | . | . | . | . | . |
| logepa | rs192256986 | . | . | . | . | . | . | . | . | . | . | . |
| logepa | rs184411030 | . | . | . | . | . | . | . | . | . | . | . |
| logepa | rs189601483 | 0.049 | 0.463 | 0.147 | 0.015 | 1.95 | 1.06 | 0.216 | 2.05 | 1.48 | 0.319 | 0.438 |
| logepa | rs143254579 | 0.045 | 0.444 | 0.15 | 0.016 | 3.28 | 1.95 | 0.046 | 2.53 | 2.96 | 0.428 | 0.961 |
| logepa | rs12253903 | . | . | . | . | . | . | . | . | . | . | . |
| logepa | rs35489926 | . | . | . | . | . | . | . | . | . | . | . |
| logepa | rs71494783 | 0.073 | 0.499 | 0.153 | 0.024 | 1.82 | 0.88 | 0.216 | 1.27 | 1.08 | 0.774 | -0.161 |
| logepa | rs180711602 | . | . | . | . | . | . | . | . | . | . | . |
| logepa | rs117312402 | 0.044 | 0.462 | 0.146 | 0.015 | 1.73 | 1.03 | 0.36 | 2.31 | 1.68 | 0.251 | 0.651 |
| logepa | rs74831694 | 0.053 | 0.525 | 0.226 | 0.134 | 0.674 | 0.959 | 0.782 | 1 | 1.43 | 0.999 | 0.155 |
| logepa | rs140301943 | . | . | . | . | . | . | . | . | . | . | . |
| logepa | rs78553679 | . | . | . | . | . | . | . | . | . | . | . |
| logepa | rs78334250 | 0.044 | 0.462 | 0.146 | 0.015 | 1.73 | 1.03 | 0.36 | 2.31 | 1.68 | 0.251 | 0.651 |
| logepa | rs76000028 | 0.044 | 0.462 | 0.146 | 0.015 | 1.73 | 1.03 | 0.36 | 2.31 | 1.68 | 0.251 | 0.651 |
| logepa | rs145195254 | . | . | . | . | . | . | . | . | . | . | . |
| logepa | rs137888527 | . | . | . | . | . | . | . | . | . | . | . |
| logepa | rs149109592 | . | . | . | . | . | . | . | . | . | . | . |
| logepa | rs80280010 | 0.038 | 0.443 | 0.148 | 0.014 | 3.58 | 2.05 | 0.026 | 2.67 | 2.98 | 0.38 | 1.2 |
| logepa | rs189995374 | 0.005 | . | . | . | . | . | . | . | . | . | . |
| logepa | rs75467770 | 0.05 | 0.455 | 0.143 | 0.013 | 1.55 | 1.02 | 0.502 | 2.43 | 1.9 | 0.257 | 0.707 |
| logepa | rs1487562 | 0.029 | 0.554 | 0.214 | 0.126 | 0.592 | 0.435 | 0.476 | 0.149 | 0.161 | 0.078 | -0.097 |
| logepa | rs12266963 | 0.073 | 0.499 | 0.153 | 0.024 | 1.82 | 0.879 | 0.216 | 1.28 | 1.08 | 0.773 | -0.16 |
| logepa | rs7099874 | 0.769 | 0.64 | 0.446 | 0.522 | 0.513 | 0.153 | 0.026 | 0.854 | 0.334 | 0.687 | 0.127 |
| logepa | rs75687019 | 0.044 | 0.462 | 0.146 | 0.015 | 1.73 | 1.03 | 0.36 | 2.31 | 1.68 | 0.251 | 0.651 |
| logepa | rs148240462 | . | . | . | . | . | . | . | . | . | . | . |
| logepa | rs141278174 | . | . | . | . | . | . | . | . | . | . | . |
| logepa | rs76404852 | 0.044 | 0.462 | 0.146 | 0.015 | 1.73 | 1.03 | 0.359 | 2.31 | 1.68 | 0.251 | 0.651 |
| logepa | rs139112240 | . | . | . | . | . | . | . | . | . | . | . |
| logepa | rs12783037 | 0.073 | 0.499 | 0.153 | 0.024 | 1.82 | 0.878 | 0.217 | 1.28 | 1.08 | 0.773 | -0.159 |
| logepa | rs72796481 | . | . | . | . | . | . | . | . | . | . | . |
| logepa | rs76798749 | 0.044 | 0.462 | 0.146 | 0.015 | 1.73 | 1.03 | 0.359 | 2.31 | 1.68 | 0.251 | 0.651 |
| logepa | rs187732548 | . | . | . | . | . | . | . | . | . | . | . |
| logepa | rs75675474 | 0.044 | 0.462 | 0.146 | 0.015 | 1.73 | 1.03 | 0.359 | 2.31 | 1.68 | 0.251 | 0.651 |
| logepa | rs76552293 | 0.044 | 0.462 | 0.146 | 0.015 | 1.73 | 1.03 | 0.359 | 2.31 | 1.68 | 0.251 | 0.651 |
| logepa | rs147445993 | 0.001 | . | . | . | . | . | . | . | . | . | . |
| logepa | rs115902283 | 0.044 | 0.461 | 0.146 | 0.015 | 1.7 | 1.03 | 0.382 | 2.34 | 1.72 | 0.246 | 0.676 |
| logepa | rs4540799 | 0.759 | 0.602 | 0.399 | 0.444 | 0.483 | 0.142 | 0.014 | 0.885 | 0.342 | 0.752 | 0.172 |
| logepa | rs12765320 | 0.102 | 0.538 | 0.174 | 0.056 | 1.68 | 0.853 | 0.303 | 0.845 | 0.852 | 0.868 | -0.457 |
| logepa | rs12782256 | . | . | . | . | . | . | . | . | . | . | . |
| logepa | rs191727256 | . | . | . | . | . | . | . | . | . | . | . |
| logepa | rs2082111 | 0.028 | 0.554 | 0.214 | 0.126 | 0.595 | 0.437 | 0.48 | 0.15 | 0.162 | 0.078 | -0.1 |
| logepa | rs2082110 | 0.072 | 0.474 | 0.153 | 0.021 | 1.76 | 0.718 | 0.166 | 1.58 | 1.26 | 0.566 | 0.086 |
| logepa | rs2082109 | 0.072 | 0.474 | 0.153 | 0.021 | 1.76 | 0.718 | 0.166 | 1.58 | 1.26 | 0.566 | 0.086 |
| logepa | rs117316662 | 0.044 | 0.462 | 0.146 | 0.015 | 1.73 | 1.03 | 0.358 | 2.3 | 1.68 | 0.251 | 0.65 |
| logepa | rs12247163 | 0.102 | 0.524 | 0.17 | 0.047 | 1.62 | 0.752 | 0.301 | 0.961 | 0.859 | 0.964 | -0.327 |
| logepa | rs12355888 | 0.002 | . | . | . | . | . | . | . | . | . | . |
| logepa | rs143772290 | . | . | . | . | . | . | . | . | . | . | . |
| logepa | rs146849452 | . | . | . | . | . | . | . | . | . | . | . |
| logepa | rs140267121 | . | . | . | . | . | . | . | . | . | . | . |
| logepa | rs7080474 | 0.058 | 0.625 | 0.249 | 0.239 | 0.497 | 0.331 | 0.293 | 0.118 | 0.102 | 0.014 | -0.086 |
| logepa | rs144066203 | . | . | . | . | . | . | . | . | . | . | . |
| logepa | rs12765209 | 0.044 | 0.462 | 0.146 | 0.015 | 1.73 | 1.03 | 0.358 | 2.3 | 1.68 | 0.251 | 0.65 |
| logepa | rs150930735 | . | . | . | . | . | . | . | . | . | . | . |
| logepa | rs7068039 | 0.102 | 0.525 | 0.17 | 0.047 | 1.62 | 0.752 | 0.301 | 0.96 | 0.858 | 0.964 | -0.328 |
| logepa | rs117622652 | 0.043 | 0.462 | 0.146 | 0.015 | 1.73 | 1.03 | 0.358 | 2.3 | 1.68 | 0.252 | 0.65 |
| logepa | rs190191514 | . | . | . | . | . | . | . | . | . | . | . |
| logepa | rs74130835 | . | . | . | . | . | . | . | . | . | . | . |
| logepa | rs1811604 | . | . | . | . | . | . | . | . | . | . | . |
| logepa | rs146971256 | 0.001 | . | . | . | . | . | . | . | . | . | . |
| logepa | rs74336000 | . | . | . | . | . | . | . | . | . | . | . |
| logepa | rs186046492 | . | . | . | . | . | . | . | . | . | . | . |
| logepa | rs34971823 | . | . | . | . | . | . | . | . | . | . | . |
| logepa | rs75227446 | 0.043 | 0.462 | 0.146 | 0.015 | 1.73 | 1.03 | 0.359 | 2.31 | 1.68 | 0.251 | 0.651 |
| logepa | rs190457599 | . | . | . | . | . | . | . | . | . | . | . |
| logepa | rs79591917 | 0.043 | 0.462 | 0.146 | 0.015 | 1.73 | 1.03 | 0.359 | 2.31 | 1.68 | 0.251 | 0.651 |
| logepa | rs702365 | 0.647 | 0.668 | 0.477 | 0.572 | 0.369 | 0.137 | 7.10E-03 | 0.754 | 0.356 | 0.55 | 0.149 |
| logepa | rs79643836 | 0.043 | 0.462 | 0.146 | 0.015 | 1.73 | 1.03 | 0.359 | 2.31 | 1.68 | 0.251 | 0.651 |
| logepa | rs702366 | 0.649 | 0.671 | 0.479 | 0.576 | 0.366 | 0.136 | 6.70E-03 | 0.752 | 0.355 | 0.546 | 0.147 |
| logepa | rs1059696 | 0.059 | 0.623 | 0.248 | 0.235 | 0.549 | 0.338 | 0.33 | 0.134 | 0.109 | 0.013 | -0.126 |
| logepa | rs901681 | 0.648 | 0.672 | 0.479 | 0.577 | 0.366 | 0.136 | 6.70E-03 | 0.751 | 0.355 | 0.545 | 0.147 |
| logepa | rs61854092 | 0.058 | 0.625 | 0.25 | 0.239 | 0.523 | 0.335 | 0.312 | 0.125 | 0.105 | 0.014 | -0.107 |
| logepa | rs148080753 | . | . | . | . | . | . | . | . | . | . | . |
| logepa | rs61854093 | 0.064 | 0.651 | 0.273 | 0.305 | 0.499 | 0.332 | 0.296 | 0.113 | 0.104 | 0.018 | -0.113 |
| logepa | rs141822774 | . | . | . | . | . | . | . | . | . | . | . |

| logepa | rs74130836 | . | . | . | . | . | . | . | . | . | . | . |
| --- | --- | --- | --- | --- | --- | --- | --- | --- | --- | --- | --- | --- |
| logepa | rs150620963 | . | . | . | . | . | . | . | . | . | . | . |
| logepa | rs3780913 | 0.749 | 0.694 | 0.463 | 0.584 | 0.445 | 0.143 | 0.012 | 0.78 | 0.325 | 0.551 | 0.102 |
| logepa | rs142255723 | . | . | . | . | . | . | . | . | . | . | . |
| logepa | rs3780914 | 0.647 | 0.682 | 0.483 | 0.589 | 0.359 | 0.135 | 6.30E-03 | 0.734 | 0.347 | 0.513 | 0.138 |
| logepa | rs182656507 | . | . | . | . | . | . | . | . | . | . | . |
| logepa | rs2291427 | 0.703 | 0.94 | 0.656 | 0.93 | 0.38 | 0.133 | 5.50E-03 | 0.574 | 0.242 | 0.188 | -0.115 |
| logepa | rs10751382 | 0.703 | 0.942 | 0.656 | 0.932 | 0.379 | 0.132 | 5.50E-03 | 0.572 | 0.241 | 0.186 | -0.117 |
| logepa | rs117610108 | 0.043 | 0.462 | 0.146 | 0.015 | 1.73 | 1.03 | 0.357 | 2.3 | 1.68 | 0.252 | 0.649 |
| logepa | rs144966941 | 0.002 | . | . | . | . | . | . | . | . | . | . |
| logepa | rs41502051 | 0.007 | . | . | . | . | . | . | . | . | . | . |
| logepa | rs41426046 | 0.047 | 0.46 | 0.145 | 0.014 | 1.85 | 1.03 | 0.273 | 2.22 | 1.56 | 0.257 | 0.576 |
| logepa | rs41519248 | 0.043 | 0.462 | 0.146 | 0.015 | 1.73 | 1.04 | 0.357 | 2.3 | 1.68 | 0.252 | 0.649 |
| logepa | rs189108245 | . | . | . | . | . | . | . | . | . | . | . |
| logepa | rs7896431 | 0.668 | 0.856 | 0.595 | 0.823 | 0.352 | 0.129 | 4.20E-03 | 0.586 | 0.266 | 0.238 | -0.032 |
| logepa | rs183724547 | . | . | . | . | . | . | . | . | . | . | . |
| logepa | rs142044990 | . | . | . | . | . | . | . | . | . | . | . |
| logepa | rs188140929 | 0.021 | 0.507 | 0.169 | 0.042 | 3.55 | 3.22 | 0.161 | 1.17 | 1.85 | 0.923 | -0.958 |
| logepa | rs76819096 | . | . | . | . | . | . | . | . | . | . | . |
| logepa | rs10751383 | 0.681 | 0.615 | 0.407 | 0.463 | 0.505 | 0.168 | 0.04 | 0.856 | 0.372 | 0.72 | 0.146 |
| logepa | rs117819321 | . | . | . | . | . | . | . | . | . | . | . |
| logepa | rs2242332 | 0.744 | 0.727 | 0.486 | 0.633 | 0.435 | 0.144 | 0.012 | 0.741 | 0.324 | 0.492 | 0.072 |
| logepa | rs2242333 | 0.476 | 0.692 | 0.426 | 0.55 | 0.44 | 0.187 | 0.054 | 0.653 | 0.36 | 0.439 | 0.067 |
| logepa | rs2242334 | 0.744 | 0.734 | 0.491 | 0.643 | 0.434 | 0.144 | 0.012 | 0.735 | 0.32 | 0.48 | 0.066 |
| logepa | rs189465041 | . | . | . | . | . | . | . | . | . | . | . |
| logepa | rs28395868 | . | . | . | . | . | . | . | . | . | . | . |
| logepa | rs56067550 | . | . | . | . | . | . | . | . | . | . | . |
| logepa | rs1051713 | 0.1 | 0.527 | 0.172 | 0.049 | 1.62 | 0.776 | 0.315 | 0.941 | 0.863 | 0.947 | -0.343 |
| logepa | rs115074210 | . | . | . | . | . | . | . | . | . | . | . |
| logepa | rs7900977 | 0.782 | 0.667 | 0.539 | 0.617 | 0.59 | 0.229 | 0.174 | 0.838 | 0.49 | 0.762 | 0.073 |
| logepa | rs147613891 | . | . | . | . | . | . | . | . | . | . | . |
| logepa | rs2229136 | 0.038 | 0.441 | 0.147 | 0.014 | 3.57 | 2.02 | 0.024 | 2.73 | 3.02 | 0.363 | 1.29 |
| logepa | rs185482841 | . | . | . | . | . | . | . | . | . | . | . |
| logepa | rs2288619 | 0.038 | 0.441 | 0.147 | 0.014 | 3.57 | 2.02 | 0.024 | 2.73 | 3.02 | 0.363 | 1.29 |
| logepa | rs189891524 | 0.003 | . | . | . | . | . | . | . | . | . | . |
| logepa | rs56145077 | 0.058 | 0.627 | 0.25 | 0.242 | 0.503 | 0.33 | 0.295 | 0.118 | 0.1 | 0.011 | -0.093 |
| logepa | rs143216970 | . | . | . | . | . | . | . | . | . | . | . |
| logepa | rs142246478 | . | . | . | . | . | . | . | . | . | . | . |
| logepa | rs13134 | . | . | . | . | . | . | . | . | . | . | . |
| logepa | rs41314980 | . | . | . | . | . | . | . | . | . | . | . |
| logepa | rs41304611 | . | . | . | . | . | . | . | . | . | . | . |
| logepa | rs193299617 | 0.004 | . | . | . | . | . | . | . | . | . | . |
| logepa | rs12772724 | . | . | . | . | . | . | . | . | . | . | . |
| logepa | rs139489537 | 0.038 | 0.441 | 0.147 | 0.014 | 3.57 | 2.02 | 0.024 | 2.74 | 3.04 | 0.362 | 1.3 |
| logepa | rs12776062 | . | . | . | . | . | . | . | . | . | . | . |
| logepa | rs752059 | 0.059 | 0.628 | 0.251 | 0.244 | 0.51 | 0.331 | 0.299 | 0.12 | 0.1 | 0.011 | -0.099 |
| logepa | rs147649982 | 0.015 | 0.512 | 0.185 | 0.064 | 6.70E-13 | 8.00E-12 | 0.02 | 6.20E-06 | 4.30E-05 | 0.087 | 0.488 |
| logepa | rs17157798 | 0.038 | 0.441 | 0.147 | 0.014 | 3.56 | 2.01 | 0.024 | 2.74 | 3.04 | 0.362 | 1.31 |
| logepa | rs142298619 | . | . | . | . | . | . | . | . | . | . | . |
| logepa | rs4948673 | 0.777 | 0.672 | 0.562 | 0.634 | 0.615 | 0.242 | 0.218 | 0.835 | 0.496 | 0.762 | 0.058 |
| logepa | rs74231484 | 0.476 | 0.693 | 0.428 | 0.552 | 0.453 | 0.189 | 0.058 | 0.657 | 0.358 | 0.441 | 0.06 |
| logepa | rs72796489 | . | . | . | . | . | . | . | . | . | . | . |
| logepa | rs113447578 | 0.026 | 0.549 | 0.213 | 0.123 | 0.614 | 0.482 | 0.534 | 0.128 | 0.153 | 0.086 | -0.12 |
| logepa | rs34063374 | . | . | . | . | . | . | . | . | . | . | . |
| logepa | rs11239527 | . | . | . | . | . | . | . | . | . | . | . |
| logepa | rs34371883 | . | . | . | . | . | . | . | . | . | . | . |
| logepa | rs118168828 | 0.002 | . | . | . | . | . | . | . | . | . | . |
| logepa | rs34349618 | . | . | . | . | . | . | . | . | . | . | . |
| logepa | rs80041679 | 0.038 | 0.441 | 0.147 | 0.014 | 3.57 | 2.02 | 0.024 | 2.74 | 3.03 | 0.362 | 1.3 |
| logepa | rs149640607 | . | . | . | . | . | . | . | . | . | . | . |
| logepa | rs4427523 | 0.957 | 0.699 | 1.74 | 0.886 | 0.208 | 0.203 | 0.107 | 0.86 | 1.17 | 0.912 | 0.219 |
| logepa | rs185880109 | . | . | . | . | . | . | . | . | . | . | . |
| logepa | rs191523220 | . | . | . | . | . | . | . | . | . | . | . |
| logepa | rs144315284 | . | . | . | . | . | . | . | . | . | . | . |
| logepa | rs4948674 | 0.156 | 0.542 | 0.211 | 0.116 | 0.648 | 0.259 | 0.278 | 0.839 | 0.361 | 0.683 | 0.104 |
| logepa | rs182984298 | . | . | . | . | . | . | . | . | . | . | . |
| logepa | rs56749023 | . | . | . | . | . | . | . | . | . | . | . |
| logepa | rs112508957 | . | . | . | . | . | . | . | . | . | . | . |
| logepa | rs7089063 | 0.138 | 0.443 | 0.168 | 0.032 | 2.43 | 1.15 | 0.061 | 1.39 | 1.15 | 0.687 | -0.375 |
| logepa | rs145000779 | . | . | . | . | . | . | . | . | . | . | . |
| logepa | rs80201572 | 0.038 | 0.441 | 0.147 | 0.014 | 3.57 | 2.01 | 0.024 | 2.75 | 3.03 | 0.36 | 1.31 |
| logepa | rs140568227 | . | . | . | . | . | . | . | . | . | . | . |
| logepa | rs149756228 | . | . | . | . | . | . | . | . | . | . | . |
| logepa | rs112380685 | . | . | . | . | . | . | . | . | . | . | . |
| logepa | rs60481109 | . | . | . | . | . | . | . | . | . | . | . |
| logepa | rs138221843 | . | . | . | . | . | . | . | . | . | . | . |
| logepa | rs150283194 | . | . | . | . | . | . | . | . | . | . | . |
| logepa | rs138879661 | . | . | . | . | . | . | . | . | . | . | . |
| logepa | rs34960345 | 0.038 | 0.441 | 0.147 | 0.014 | 3.56 | 2.01 | 0.024 | 2.75 | 3.03 | 0.358 | 1.31 |
| logepa | rs147514068 | . | . | . | . | . | . | . | . | . | . | . |
| logepa | rs35410535 | 0.038 | 0.441 | 0.147 | 0.014 | 3.56 | 2.01 | 0.024 | 2.75 | 3.03 | 0.358 | 1.31 |
| logepa | rs191613635 | . | . | . | . | . | . | . | . | . | . | . |
| logepa | rs17157800 | . | . | . | . | . | . | . | . | . | . | . |
| logepa | rs17523413 | 0.038 | 0.441 | 0.147 | 0.014 | 3.56 | 2.01 | 0.024 | 2.75 | 3.03 | 0.36 | 1.31 |
| logepa | rs7897036 | 0.001 | . | . | . | . | . | . | . | . | . | . |
| logepa | rs76334323 | 0.042 | 0.462 | 0.146 | 0.015 | 1.73 | 1.03 | 0.356 | 2.3 | 1.68 | 0.252 | 0.649 |

| logepa | rs143535991 | . | . | . | . | . | . | . | . | . | . | . |
| --- | --- | --- | --- | --- | --- | --- | --- | --- | --- | --- | --- | --- |
| logepa | rs140344979 | . | . | . | . | . | . | . | . | . | . | . |
| logepa | rs36047811 | 0.038 | 0.441 | 0.147 | 0.014 | 3.56 | 2.01 | 0.024 | 2.75 | 3.03 | 0.358 | 1.31 |
| logepa | rs76493570 | 0.038 | 0.441 | 0.147 | 0.014 | 3.56 | 2.01 | 0.024 | 2.75 | 3.03 | 0.358 | 1.31 |
| logepa | rs192998920 | . | . | . | . | . | . | . | . | . | . | . |
| logepa | rs116840852 | . | . | . | . | . | . | . | . | . | . | . |
| logepa | rs184824101 | . | . | . | . | . | . | . | . | . | . | . |
| logepa | rs41301621 | . | . | . | . | . | . | . | . | . | . | . |
| logepa | rs3740109 | 0.038 | 0.441 | 0.147 | 0.014 | 3.56 | 2.01 | 0.024 | 2.75 | 3.03 | 0.358 | 1.31 |
| logepa | rs7899415 | 0.089 | 0.665 | 0.26 | 0.296 | 0.487 | 0.325 | 0.281 | 0.217 | 0.184 | 0.072 | -0.081 |
| logepa | rs184395284 | 0.004 | . | . | . | . | . | . | . | . | . | . |
| logepa | rs139209589 | . | . | . | . | . | . | . | . | . | . | . |
| logepa | rs149924163 | . | . | . | . | . | . | . | . | . | . | . |
| logepa | rs3740110 | 0.042 | 0.462 | 0.146 | 0.015 | 1.73 | 1.03 | 0.356 | 2.3 | 1.68 | 0.252 | 0.649 |
| logepa | rs144335113 | . | . | . | . | . | . | . | . | . | . | . |
| logepa | rs74743385 | . | . | . | . | . | . | . | . | . | . | . |
| logepa | rs146545829 | . | . | . | . | . | . | . | . | . | . | . |
| logepa | rs181842689 | . | . | . | . | . | . | . | . | . | . | . |
| logepa | rs3740111 | 0.038 | 0.441 | 0.147 | 0.014 | 3.56 | 2.01 | 0.024 | 2.75 | 3.03 | 0.358 | 1.31 |
| logepa | rs186261599 | . | . | . | . | . | . | . | . | . | . | . |
| logepa | rs73285348 | 0.013 | 0.552 | 0.215 | 0.127 | 0.588 | 0.702 | 0.657 | 0.021 | 0.038 | 0.031 | -0.133 |
| logepa | rs186356901 | . | . | . | . | . | . | . | . | . | . | . |
| logepa | rs3824618 | 0.038 | 0.441 | 0.147 | 0.014 | 3.57 | 2.01 | 0.024 | 2.75 | 3.03 | 0.36 | 1.31 |
| logepa | rs3802548 | 0.065 | 0.63 | 0.249 | 0.243 | 0.434 | 0.295 | 0.219 | 0.105 | 0.09 | 8.60E-03 | -0.035 |
| logepa | rs3802547 | 0.04 | 0.461 | 0.145 | 0.014 | 1.82 | 1.13 | 0.334 | 2.36 | 1.75 | 0.246 | 0.7 |
| logepa | rs77677521 | . | . | . | . | . | . | . | . | . | . | . |
| logepa | rs3824616 | 0.038 | 0.441 | 0.147 | 0.014 | 3.56 | 2.01 | 0.024 | 2.75 | 3.03 | 0.358 | 1.31 |
| logepa | rs191073818 | . | . | . | . | . | . | . | . | . | . | . |
| logepa | rs147078004 | . | . | . | . | . | . | . | . | . | . | . |
| logepa | rs7908745 | 0.145 | 0.45 | 0.166 | 0.031 | 2.29 | 1.06 | 0.071 | 1.33 | 1.05 | 0.717 | -0.37 |
| logepa | rs17157811 | . | . | . | . | . | . | . | . | . | . | . |
| logepa | rs138506087 | . | . | . | . | . | . | . | . | . | . | . |
| logepa | rs77241269 | . | . | . | . | . | . | . | . | . | . | . |
| logepa | rs138374938 | . | . | . | . | . | . | . | . | . | . | . |
| logepa | rs2279433 | 0.038 | 0.44 | 0.147 | 0.014 | 3.58 | 2.02 | 0.024 | 2.76 | 3.03 | 0.354 | 1.34 |
| logepa | rs73285358 | . | . | . | . | . | . | . | . | . | . | . |
| logepa | rs11239528 | 0.065 | 0.631 | 0.249 | 0.244 | 0.431 | 0.295 | 0.218 | 0.104 | 0.09 | 8.60E-03 | -0.033 |
| logepa | rs77453386 | 0.042 | 0.462 | 0.146 | 0.015 | 1.73 | 1.03 | 0.356 | 2.3 | 1.68 | 0.252 | 0.65 |
| logepa | rs2279434 | 0.04 | 0.435 | 0.144 | 0.012 | 3.62 | 2.01 | 0.021 | 2.89 | 3.11 | 0.324 | 1.5 |
| logepa | rs149236525 | . | . | . | . | . | . | . | . | . | . | . |
| logepa | rs12573609 | 0.13 | 0.544 | 0.269 | 0.219 | 1.61 | 0.836 | 0.357 | 0.913 | 0.527 | 0.875 | -0.355 |
| logepa | rs191716655 | . | . | . | . | . | . | . | . | . | . | . |
| logepa | rs17523660 | . | . | . | . | . | . | . | . | . | . | . |
| logepa | rs142671360 | . | . | . | . | . | . | . | . | . | . | . |
| logepa | rs3764990 | 0.037 | 0.44 | 0.147 | 0.014 | 3.59 | 2.03 | 0.024 | 2.76 | 3.04 | 0.354 | 1.34 |
| logepa | rs113682861 | . | . | . | . | . | . | . | . | . | . | . |
| logepa | rs117658270 | 0.042 | 0.462 | 0.146 | 0.015 | 1.73 | 1.03 | 0.356 | 2.3 | 1.68 | 0.252 | 0.65 |
| logepa | rs187309063 | . | . | . | . | . | . | . | . | . | . | . |
| logepa | rs73285360 | . | . | . | . | . | . | . | . | . | . | . |
| logepa | rs80133289 | 0.1 | 0.556 | 0.252 | 0.195 | 0.397 | 0.436 | 0.4 | 0.589 | 0.638 | 0.625 | 0.177 |
| logepa | rs186693257 | 0.003 | . | . | . | . | . | . | . | . | . | . |
| logepa | rs35735968 | 0.05 | 0.431 | 0.149 | 0.015 | 3.35 | 1.95 | 0.038 | 2.55 | 2.81 | 0.394 | 0.901 |
| logepa | rs17157825 | 0.038 | 0.44 | 0.147 | 0.014 | 3.58 | 2.03 | 0.024 | 2.76 | 3.04 | 0.358 | 1.33 |
| logepa | rs2291428 | 0.065 | 0.63 | 0.249 | 0.244 | 0.431 | 0.295 | 0.219 | 0.104 | 0.09 | 8.70E-03 | -0.033 |
| logepa | rs2291429 | 0.065 | 0.631 | 0.25 | 0.244 | 0.429 | 0.295 | 0.218 | 0.103 | 0.089 | 8.70E-03 | -0.032 |
| logepa | rs145714792 | . | . | . | . | . | . | . | . | . | . | . |
| logepa | rs12769244 | 0.038 | 0.44 | 0.147 | 0.014 | 3.58 | 2.02 | 0.024 | 2.77 | 3.03 | 0.353 | 1.34 |
| logepa | rs12771275 | 0.038 | 0.44 | 0.147 | 0.014 | 3.58 | 2.02 | 0.024 | 2.77 | 3.03 | 0.353 | 1.34 |
| logepa | rs146266174 | . | . | . | . | . | . | . | . | . | . | . |
| logepa | rs41301623 | . | . | . | . | . | . | . | . | . | . | . |
| logepa | rs2306814 | 0.043 | 0.458 | 0.146 | 0.014 | 1.56 | 0.929 | 0.453 | 2.54 | 1.78 | 0.183 | 0.797 |
| logepa | rs12244834 | 0.03 | 0.585 | 0.226 | 0.166 | 0.248 | 0.499 | 0.488 | 0.065 | 0.122 | 0.144 | 0.176 |
| logepa | rs184006011 | 0 | . | . | . | . | . | . | . | . | . | . |
| logepa | rs78829066 | 0.042 | 0.462 | 0.147 | 0.015 | 1.75 | 1.04 | 0.349 | 2.32 | 1.69 | 0.245 | 0.669 |
| logepa | rs34731408 | 0.038 | 0.44 | 0.147 | 0.014 | 3.59 | 2.02 | 0.024 | 2.77 | 3.04 | 0.352 | 1.35 |
| logepa | rs17451516 | . | . | . | . | . | . | . | . | . | . | . |
| logepa | rs139910133 | . | . | . | . | . | . | . | . | . | . | . |
| logepa | rs10900218 | 0.138 | 0.408 | 0.156 | 0.019 | 2.86 | 1.16 | 9.40E-03 | 1.58 | 1.08 | 0.502 | -0.421 |
| logepa | rs146023999 | 0.025 | 0.518 | 0.185 | 0.066 | 2.30E-04 | 8.30E-04 | 0.021 | 1.80E+03 | 5.10E+03 | 6.90E-03 | 0.698 |
| logepa | rs187296958 | 0.007 | . | . | . | . | . | . | . | . | . | . |
| logepa | rs142342833 | 0.015 | 0.544 | 0.203 | 0.103 | 5.00E-04 | 2.10E-03 | 0.074 | 0.01 | 0.045 | 0.301 | 0.455 |
| logepa | rs143489255 | 0.006 | . | . | . | . | . | . | . | . | . | . |
| logepa | rs138532518 | 0.006 | . | . | . | . | . | . | . | . | . | . |
| logepa | rs183768079 | 0.007 | . | . | . | . | . | . | . | . | . | . |
| logepa | rs117892272 | 0.012 | 0.54 | 0.224 | 0.138 | 10.7 | 10.3 | 0.013 | 2.22 | 2.48 | 0.475 | 2.58 |
| logepa | rs192993943 | 0.003 | . | . | . | . | . | . | . | . | . | . |
| logepa | rs182989043 | 0.015 | 0.494 | 0.203 | 0.086 | 2.76 | 4.25 | 0.51 | 4.11 | 3.44 | 0.092 | 3.34 |
| logepa | rs189703435 | 0.021 | 0.593 | 0.229 | 0.175 | 0.233 | 0.632 | 0.591 | 0.044 | 0.106 | 0.191 | 0.181 |
| logepa | rs188794040 | 0.02 | 0.596 | 0.23 | 0.179 | 0.2 | 0.54 | 0.551 | 0.032 | 0.074 | 0.137 | 0.208 |
| logepa | rs143920953 | 0.003 | . | . | . | . | . | . | . | . | . | . |
| logepa | rs191029121 | 0.006 | . | . | . | . | . | . | . | . | . | . |
| logepa | rs116875448 | 0.006 | . | . | . | . | . | . | . | . | . | . |
| logepa | rs190167877 | 0.002 | . | . | . | . | . | . | . | . | . | . |
| logepa | rs184993418 | 0.003 | . | . | . | . | . | . | . | . | . | . |
| logepa | rs143241158 | 0.01 | . | . | . | . | . | . | . | . | . | . |
| logepa | rs11239500 | 0.007 | . | . | . | . | . | . | . | . | . | . |

| logepa | rs140568994 | 0.001 | . | . | . | . | . | . | . | . | . | . |
| --- | --- | --- | --- | --- | --- | --- | --- | --- | --- | --- | --- | --- |
| logepa | rs187530317 | 0.008 | . | . | . | . | . | . | . | . | . | . |
| logepa | rs145189326 | 0.003 | . | . | . | . | . | . | . | . | . | . |
| logepa | rs186203332 | 0.009 | . | . | . | . | . | . | . | . | . | . |
| logepa | rs189921909 | 0.004 | . | . | . | . | . | . | . | . | . | . |
| logepa | rs187260989 | 0.01 | 0.498 | 0.192 | 0.07 | 4.38 | 8.02 | 0.419 | 17.2 | 41 | 0.231 | 33.7 |
| logepa | rs114376586 | 0.006 | . | . | . | . | . | . | . | . | . | . |
| logepa | rs151070808 | 0.019 | 0.537 | 0.202 | 0.099 | 1.29 | 1.08 | 0.765 | 0.316 | 0.536 | 0.497 | -0.605 |
| logepa | rs140927225 | 0.006 | . | . | . | . | . | . | . | . | . | . |
| logepa | rs191555615 | 0.012 | 0.531 | 0.211 | 0.112 | 6.52 | 6.07 | 0.044 | 2.26 | 2.76 | 0.504 | 1.78 |
| logepa | rs80278898 | 0.047 | 0.512 | 0.23 | 0.136 | 0.497 | 0.936 | 0.71 | 1.63 | 2.48 | 0.749 | 0.405 |
| logepa | rs190849503 | 0.004 | . | . | . | . | . | . | . | . | . | . |
| logepa | rs150080315 | 0.009 | . | . | . | . | . | . | . | . | . | . |
| logepa | rs143623885 | 0.011 | 0.561 | 0.205 | 0.114 | 1.60E-12 | 2.80E-11 | 0.12 | 7.10E-12 | 1.10E-10 | 0.108 | 0.439 |
| logepa | rs187760040 | 0.002 | . | . | . | . | . | . | . | . | . | . |
| logepa | rs143917143 | 0.02 | 0.537 | 0.202 | 0.099 | 1.27 | 1.07 | 0.772 | 0.324 | 0.548 | 0.505 | -0.59 |
| logepa | rs149851136 | 0.002 | . | . | . | . | . | . | . | . | . | . |
| logepa | rs181612054 | 0.001 | . | . | . | . | . | . | . | . | . | . |
| logepa | rs191006548 | 0.001 | . | . | . | . | . | . | . | . | . | . |
| logepa | rs117377631 | 0.006 | . | . | . | . | . | . | . | . | . | . |
| logepa | rs182107634 | 0.002 | . | . | . | . | . | . | . | . | . | . |
| logepa | rs190211867 | 0.002 | . | . | . | . | . | . | . | . | . | . |
| logepa | rs148293457 | 0.031 | 0.561 | 0.224 | 0.148 | 0.788 | 0.769 | 0.807 | 0.335 | 0.424 | 0.388 | -0.201 |
| logepa | rs191811733 | 0.006 | . | . | . | . | . | . | . | . | . | . |
| logepa | rs146109272 | 0.008 | . | . | . | . | . | . | . | . | . | . |
| logepa | rs148397915 | 0.006 | . | . | . | . | . | . | . | . | . | . |
| logepa | rs142233290 | 0.007 | . | . | . | . | . | . | . | . | . | . |
| logepa | rs80226874 | 0.022 | 0.526 | 0.195 | 0.083 | 0.026 | 0.076 | 0.212 | 0.967 | 3.12 | 0.992 | 0.461 |
| logepa | rs144496203 | 0.012 | 0.545 | 0.2 | 0.098 | 6.80E-33 | 3.50E-31 | 0.156 | 2.70E-19 | 8.40E-18 | 0.165 | 0.455 |
| logepa | rs191062130 | 0.001 | . | . | . | . | . | . | . | . | . | . |
| logepa | rs185807817 | 0.004 | . | . | . | . | . | . | . | . | . | . |
| logepa | rs117846213 | 0.032 | 0.633 | 0.233 | 0.214 | 0.088 | 0.23 | 0.352 | 0.015 | 0.035 | 0.074 | 0.28 |
| logepa | rs190545473 | 0.002 | . | . | . | . | . | . | . | . | . | . |
| logepa | rs192364918 | 0.01 | 0.503 | 0.196 | 0.078 | 7.99 | 11 | 0.133 | 3.53 | 11.8 | 0.706 | 6.71 |
| logepa | rs182939241 | 0.003 | . | . | . | . | . | . | . | . | . | . |
| logepa | rs12571125 | 0.031 | 0.487 | 0.19 | 0.066 | 0.953 | 1.5 | 0.975 | 3.79 | 5.76 | 0.38 | 1.32 |
| logepa | rs192418941 | 0.005 | . | . | . | . | . | . | . | . | . | . |
| logepa | rs141270243 | 0.015 | 0.545 | 0.214 | 0.122 | 2.87 | 2.72 | 0.266 | 0.286 | 0.884 | 0.686 | -1.97 |
| logepa | rs182615502 | 0.006 | . | . | . | . | . | . | . | . | . | . |
| logepa | rs146669856 | 0.001 | . | . | . | . | . | . | . | . | . | . |
| logepa | rs78290029 | 0.034 | 0.602 | 0.229 | 0.183 | 0.177 | 0.313 | 0.327 | 0.04 | 0.068 | 0.056 | 0.225 |
| logepa | rs139955565 | 0.004 | . | . | . | . | . | . | . | . | . | . |
| logepa | rs12267314 | 0.03 | 0.595 | 0.23 | 0.179 | 0.433 | 0.581 | 0.533 | 0.094 | 0.121 | 0.067 | -3.10E-03 |
| logepa | rs74477197 | 0.004 | . | . | . | . | . | . | . | . | . | . |
| logepa | rs188147236 | 0.005 | . | . | . | . | . | . | . | . | . | . |
| logepa | rs2228065 | 0.03 | 0.593 | 0.229 | 0.177 | 0.42 | 0.572 | 0.524 | 0.093 | 0.122 | 0.071 | 9.90E-03 |
| logepa | rs116961353 | 0.015 | 0.607 | 0.226 | 0.18 | 9.30E-03 | 0.049 | 0.374 | 2.20E-03 | 0.013 | 0.304 | 0.383 |
| logepa | rs190222272 | 0.007 | . | . | . | . | . | . | . | . | . | . |
| logepa | rs182719940 | 0.006 | . | . | . | . | . | . | . | . | . | . |
| logepa | rs184477413 | 0.004 | . | . | . | . | . | . | . | . | . | . |
| logepa | rs12265175 | 0.03 | 0.584 | 0.226 | 0.164 | 0.251 | 0.502 | 0.489 | 0.066 | 0.123 | 0.147 | 0.175 |
| logepa | rs182928276 | 0.003 | . | . | . | . | . | . | . | . | . | . |
| logepa | rs144409895 | 0.011 | 0.533 | 0.207 | 0.106 | 3.69 | 5.42 | 0.374 | 0.387 | 1.22 | 0.764 | -2.46 |
| logepa | rs58998447 | 0.136 | 0.584 | 0.246 | 0.201 | 0.496 | 0.304 | 0.253 | 0.6 | 0.339 | 0.366 | 0.094 |
| logepa | rs188668832 | 0.005 | . | . | . | . | . | . | . | . | . | . |
| logepa | rs148641686 | 0.002 | . | . | . | . | . | . | . | . | . | . |
| logepa | rs75468791 | 0.054 | 0.514 | 0.202 | 0.091 | 0.503 | 0.539 | 0.521 | 2.17 | 1.59 | 0.292 | 0.544 |
| logepa | rs147327397 | 0.003 | . | . | . | . | . | . | . | . | . | . |
| logepa | rs77354597 | 0.029 | 0.585 | 0.227 | 0.166 | 0.254 | 0.51 | 0.495 | 0.065 | 0.121 | 0.141 | 0.17 |
| logepa | rs41365544 | 0.03 | 0.585 | 0.226 | 0.166 | 0.253 | 0.502 | 0.488 | 0.066 | 0.122 | 0.142 | 0.172 |
| logepa | rs187564188 | 0.016 | 0.51 | 0.214 | 0.109 | 1.72 | 3.17 | 0.77 | 1.52 | 2.49 | 0.8 | 0.101 |
| logepa | rs183511984 | 0.007 | . | . | . | . | . | . | . | . | . | . |
| logepa | rs12262783 | 0.003 | . | . | . | . | . | . | . | . | . | . |
| logepa | rs188817687 | 0.003 | . | . | . | . | . | . | . | . | . | . |
| logepa | rs3815968 | 0.005 | . | . | . | . | . | . | . | . | . | . |
| logepa | rs187822300 | 0.001 | . | . | . | . | . | . | . | . | . | . |
| logepa | rs188902172 | 0.032 | 0.586 | 0.232 | 0.177 | 0.957 | 0.99 | 0.966 | 0.236 | 0.244 | 0.163 | -0.411 |
| logepa | rs181190448 | 0.006 | . | . | . | . | . | . | . | . | . | . |
| logepa | rs182699093 | 0.001 | . | . | . | . | . | . | . | . | . | . |
| logepa | rs182631303 | 0.014 | 0.551 | 0.212 | 0.122 | 1.39 | 1.39 | 0.746 | 0.382 | 0.545 | 0.5 | -0.645 |
| logepa | rs187014172 | 0.006 | . | . | . | . | . | . | . | . | . | . |
| logepa | rs142575406 | 0.009 | . | . | . | . | . | . | . | . | . | . |
| logepa | rs180711033 | 0.007 | . | . | . | . | . | . | . | . | . | . |
| logepa | rs137894823 | . | . | . | . | . | . | . | . | . | . | . |
| logepa | rs189053273 | . | . | . | . | . | . | . | . | . | . | . |
| logepa | rs143499388 | . | . | . | . | . | . | . | . | . | . | . |
| logepa | rs182936001 | . | . | . | . | . | . | . | . | . | . | . |
| logepa | rs148456406 | . | . | . | . | . | . | . | . | . | . | . |
| logepa | rs182499531 | . | . | . | . | . | . | . | . | . | . | . |
| logepa | rs74884915 | . | . | . | . | . | . | . | . | . | . | . |
| logepa | rs184046374 | . | . | . | . | . | . | . | . | . | . | . |
| logepa | rs74333993 | . | . | . | . | . | . | . | . | . | . | . |
| logepa | rs138889719 | . | . | . | . | . | . | . | . | . | . | . |
| logepa | rs75330330 | . | . | . | . | . | . | . | . | . | . | . |
| logepa | rs193051693 | . | . | . | . | . | . | . | . | . | . | . |
| logepa | rs145929633 | . | . | . | . | . | . | . | . | . | . | . |

| logepa | rs184076904 | . | . | . | . | . | . | . | . | . | . | . |
| --- | --- | --- | --- | --- | --- | --- | --- | --- | --- | --- | --- | --- |
| logepa | rs141802494 | . | . | . | . | . | . | . | . | . | . | . |
| logepa | rs149519759 | . | . | . | . | . | . | . | . | . | . | . |
| logepa | rs145467260 | . | . | . | . | . | . | . | . | . | . | . |
| logepa | rs79620914 | . | . | . | . | . | . | . | . | . | . | . |
| logepa | rs140538633 | . | . | . | . | . | . | . | . | . | . | . |
| logepa | rs140862301 | . | . | . | . | . | . | . | . | . | . | . |
| logepa | rs114615847 | . | . | . | . | . | . | . | . | . | . | . |
| logepa | rs141800259 | . | . | . | . | . | . | . | . | . | . | . |
| logepa | rs149779059 | . | . | . | . | . | . | . | . | . | . | . |
| logepa | rs116539253 | . | . | . | . | . | . | . | . | . | . | . |
| logepa | rs184979550 | . | . | . | . | . | . | . | . | . | . | . |
| logepa | rs190128940 | . | . | . | . | . | . | . | . | . | . | . |
| logepa | rs146594816 | . | . | . | . | . | . | . | . | . | . | . |
| logepa | rs141370766 | . | . | . | . | . | . | . | . | . | . | . |
| logepa | rs144670033 | . | . | . | . | . | . | . | . | . | . | . |
| logepa | rs187152174 | . | . | . | . | . | . | . | . | . | . | . |
| logepa | rs184180263 | . | . | . | . | . | . | . | . | . | . | . |
| logepa | rs140075861 | . | . | . | . | . | . | . | . | . | . | . |
| logepa | rs186483129 | . | . | . | . | . | . | . | . | . | . | . |
| logepa | rs186634282 | . | . | . | . | . | . | . | . | . | . | . |
| logepa | rs9988663 | . | . | . | . | . | . | . | . | . | . | . |
| logepa | rs138828529 | . | . | . | . | . | . | . | . | . | . | . |
| logepa | rs188777615 | . | . | . | . | . | . | . | . | . | . | . |
| logepa | rs192321882 | . | . | . | . | . | . | . | . | . | . | . |
| logepa | rs150812270 | . | . | . | . | . | . | . | . | . | . | . |
| logepa | rs185906603 | . | . | . | . | . | . | . | . | . | . | . |
| logepa | rs191070862 | . | . | . | . | . | . | . | . | . | . | . |
| logepa | rs182904978 | . | . | . | . | . | . | . | . | . | . | . |
| logepa | rs192679655 | . | . | . | . | . | . | . | . | . | . | . |
| logepa | rs189673086 | . | . | . | . | . | . | . | . | . | . | . |
| logepa | rs181141892 | . | . | . | . | . | . | . | . | . | . | . |
| logepa | rs186404123 | . | . | . | . | . | . | . | . | . | . | . |
| logepa | rs192117287 | . | . | . | . | . | . | . | . | . | . | . |
| logepa | rs182748835 | . | . | . | . | . | . | . | . | . | . | . |
| logepa | rs141854549 | . | . | . | . | . | . | . | . | . | . | . |
| logepa | rs140163397 | . | . | . | . | . | . | . | . | . | . | . |
| logepa | rs146347300 | . | . | . | . | . | . | . | . | . | . | . |
| logepa | rs139362377 | . | . | . | . | . | . | . | . | . | . | . |
| logepa | rs185087671 | . | . | . | . | . | . | . | . | . | . | . |
| logepa | rs190466574 | . | . | . | . | . | . | . | . | . | . | . |
| logepa | rs147012778 | . | . | . | . | . | . | . | . | . | . | . |
| logepa | rs138236960 | . | . | . | . | . | . | . | . | . | . | . |
| logepa | rs116310311 | . | . | . | . | . | . | . | . | . | . | . |
| logepa | rs142868356 | . | . | . | . | . | . | . | . | . | . | . |
| logepa | rs144956240 | . | . | . | . | . | . | . | . | . | . | . |
| logepa | rs188527776 | . | . | . | . | . | . | . | . | . | . | . |
| logepa | rs183971165 | . | . | . | . | . | . | . | . | . | . | . |
| logepa | rs76748216 | . | . | . | . | . | . | . | . | . | . | . |
| logepa | rs148554990 | . | . | . | . | . | . | . | . | . | . | . |
| logepa | rs114446477 | . | . | . | . | . | . | . | . | . | . | . |
| logepa | rs185223933 | . | . | . | . | . | . | . | . | . | . | . |
| logepa | rs76869726 | . | . | . | . | . | . | . | . | . | . | . |
| logepa | rs151200830 | . | . | . | . | . | . | . | . | . | . | . |
| logepa | rs17157731 | . | . | . | . | . | . | . | . | . | . | . |
| logepa | rs181243469 | . | . | . | . | . | . | . | . | . | . | . |
| logepa | rs186890707 | . | . | . | . | . | . | . | . | . | . | . |
| logepa | rs184063360 | . | . | . | . | . | . | . | . | . | . | . |
| logepa | rs188550237 | . | . | . | . | . | . | . | . | . | . | . |
| logepa | rs140931068 | . | . | . | . | . | . | . | . | . | . | . |
| logepa | rs74867877 | . | . | . | . | . | . | . | . | . | . | . |
| logepa | rs11239495 | . | . | . | . | . | . | . | . | . | . | . |
| logepa | rs149745800 | . | . | . | . | . | . | . | . | . | . | . |
| logepa | rs145044458 | . | . | . | . | . | . | . | . | . | . | . |
| logepa | rs188067427 | . | . | . | . | . | . | . | . | . | . | . |
| logepa | rs146827800 | . | . | . | . | . | . | . | . | . | . | . |
| logepa | rs140619817 | . | . | . | . | . | . | . | . | . | . | . |
| logepa | rs892689 | . | . | . | . | . | . | . | . | . | . | . |
| logepa | rs143642005 | . | . | . | . | . | . | . | . | . | . | . |
| logepa | rs181929304 | . | . | . | . | . | . | . | . | . | . | . |
| logepa | rs191086173 | . | . | . | . | . | . | . | . | . | . | . |
| logepa | rs114613116 | . | . | . | . | . | . | . | . | . | . | . |
| logepa | rs141076768 | . | . | . | . | . | . | . | . | . | . | . |
| logepa | rs187316531 | . | . | . | . | . | . | . | . | . | . | . |
| logepa | rs11239499 | . | . | . | . | . | . | . | . | . | . | . |
| logepa | rs192726371 | . | . | . | . | . | . | . | . | . | . | . |
| logepa | rs74128481 | . | . | . | . | . | . | . | . | . | . | . |
| logepa | rs74128482 | . | . | . | . | . | . | . | . | . | . | . |
| logepa | rs11239502 | . | . | . | . | . | . | . | . | . | . | . |
| logepa | rs116330285 | . | . | . | . | . | . | . | . | . | . | . |
| logepa | rs189926729 | . | . | . | . | . | . | . | . | . | . | . |
| logepa | rs188975779 | . | . | . | . | . | . | . | . | . | . | . |
| logepa | rs143846038 | . | . | . | . | . | . | . | . | . | . | . |
| logepa | rs74128483 | . | . | . | . | . | . | . | . | . | . | . |
| logepa | rs139292055 | . | . | . | . | . | . | . | . | . | . | . |
| logepa | rs11239503 | . | . | . | . | . | . | . | . | . | . | . |

| logepa | rs58838686 | . | . | . | . | . | . | . | . | . | . | . |
| --- | --- | --- | --- | --- | --- | --- | --- | --- | --- | --- | --- | --- |
| logepa | rs185825373 | . | . | . | . | . | . | . | . | . | . | . |
| logepa | rs11239504 | . | . | . | . | . | . | . | . | . | . | . |
| logepa | rs150012354 | . | . | . | . | . | . | . | . | . | . | . |
| logepa | rs187763096 | . | . | . | . | . | . | . | . | . | . | . |
| logepa | rs193043982 | . | . | . | . | . | . | . | . | . | . | . |
| logepa | rs79186884 | . | . | . | . | . | . | . | . | . | . | . |
| logepa | rs74128484 | . | . | . | . | . | . | . | . | . | . | . |
| logepa | rs58461537 | . | . | . | . | . | . | . | . | . | . | . |
| logepa | rs188527474 | . | . | . | . | . | . | . | . | . | . | . |
| logepa | rs141659976 | . | . | . | . | . | . | . | . | . | . | . |
| logepa | rs115846407 | . | . | . | . | . | . | . | . | . | . | . |
| logepa | rs138378816 | . | . | . | . | . | . | . | . | . | . | . |
| logepa | rs146188136 | . | . | . | . | . | . | . | . | . | . | . |
| logepa | rs139383871 | . | . | . | . | . | . | . | . | . | . | . |
| logepa | rs182953869 | . | . | . | . | . | . | . | . | . | . | . |
| logepa | rs192430299 | . | . | . | . | . | . | . | . | . | . | . |
| logepa | rs144152844 | . | . | . | . | . | . | . | . | . | . | . |
| logepa | rs145987786 | . | . | . | . | . | . | . | . | . | . | . |
| logepa | rs181132926 | . | . | . | . | . | . | . | . | . | . | . |
| logepa | rs147882253 | . | . | . | . | . | . | . | . | . | . | . |
| logepa | rs141807621 | . | . | . | . | . | . | . | . | . | . | . |
| logepa | rs147116052 | . | . | . | . | . | . | . | . | . | . | . |
| logepa | rs147716618 | . | . | . | . | . | . | . | . | . | . | . |
| logepa | rs187198855 | . | . | . | . | . | . | . | . | . | . | . |
| logepa | rs189409638 | . | . | . | . | . | . | . | . | . | . | . |
| logepa | rs182647339 | . | . | . | . | . | . | . | . | . | . | . |
| logepa | rs187995988 | . | . | . | . | . | . | . | . | . | . | . |
| logepa | rs141034184 | . | . | . | . | . | . | . | . | . | . | . |
| logepa | rs187310430 | . | . | . | . | . | . | . | . | . | . | . |
| logepa | rs192608488 | . | . | . | . | . | . | . | . | . | . | . |
| logepa | rs148840366 | . | . | . | . | . | . | . | . | . | . | . |
| logepa | rs186785022 | . | . | . | . | . | . | . | . | . | . | . |
| logepa | rs192348728 | . | . | . | . | . | . | . | . | . | . | . |
| logepa | rs189656631 | . | . | . | . | . | . | . | . | . | . | . |
| logepa | rs185289750 | . | . | . | . | . | . | . | . | . | . | . |
| logepa | rs142382395 | . | . | . | . | . | . | . | . | . | . | . |
| logepa | rs144866696 | . | . | . | . | . | . | . | . | . | . | . |
| logepa | rs141371289 | . | . | . | . | . | . | . | . | . | . | . |
| logepa | rs114750008 | . | . | . | . | . | . | . | . | . | . | . |
| logepa | rs189063597 | . | . | . | . | . | . | . | . | . | . | . |
| logepa | rs139151571 | . | . | . | . | . | . | . | . | . | . | . |
| logepa | rs139846368 | . | . | . | . | . | . | . | . | . | . | . |
| logepa | rs182057653 | . | . | . | . | . | . | . | . | . | . | . |
| logepa | rs148785420 | . | . | . | . | . | . | . | . | . | . | . |
| logepa | rs138383251 | . | . | . | . | . | . | . | . | . | . | . |
| logepa | rs116526705 | . | . | . | . | . | . | . | . | . | . | . |
| logepa | rs185946436 | . | . | . | . | . | . | . | . | . | . | . |
| logepa | rs190455427 | . | . | . | . | . | . | . | . | . | . | . |
| logepa | rs9282584 | . | . | . | . | . | . | . | . | . | . | . |
| logepa | rs17153289 | . | . | . | . | . | . | . | . | . | . | . |
| logepa | rs138359103 | . | . | . | . | . | . | . | . | . | . | . |
| logepa | rs150605268 | . | . | . | . | . | . | . | . | . | . | . |
| logepa | rs79242910 | . | . | . | . | . | . | . | . | . | . | . |
| logepa | rs139716904 | . | . | . | . | . | . | . | . | . | . | . |
| logepa | rs76238498 | . | . | . | . | . | . | . | . | . | . | . |
| logepa | rs184140639 | . | . | . | . | . | . | . | . | . | . | . |
| logepa | rs188517278 | . | . | . | . | . | . | . | . | . | . | . |
| logepa | rs139678913 | . | . | . | . | . | . | . | . | . | . | . |
| logepa | rs116697955 | . | . | . | . | . | . | . | . | . | . | . |
| logepa | rs79267994 | . | . | . | . | . | . | . | . | . | . | . |
| logepa | rs11239510 | . | . | . | . | . | . | . | . | . | . | . |
| logepa | rs75831645 | . | . | . | . | . | . | . | . | . | . | . |
| logepa | rs78452276 | . | . | . | . | . | . | . | . | . | . | . |
| logepa | rs144366924 | . | . | . | . | . | . | . | . | . | . | . |
| logepa | rs77519841 | . | . | . | . | . | . | . | . | . | . | . |
| logepa | rs76944454 | . | . | . | . | . | . | . | . | . | . | . |
| logepa | rs76443855 | . | . | . | . | . | . | . | . | . | . | . |
| logepa | rs146613174 | . | . | . | . | . | . | . | . | . | . | . |
| logepa | rs181424761 | . | . | . | . | . | . | . | . | . | . | . |
| logepa | rs142119241 | . | . | . | . | . | . | . | . | . | . | . |
| logepa | rs140137954 | . | . | . | . | . | . | . | . | . | . | . |
| logepa | rs187319549 | . | . | . | . | . | . | . | . | . | . | . |
| logepa | rs12241545 | . | . | . | . | . | . | . | . | . | . | . |
| logepa | rs10128228 | . | . | . | . | . | . | . | . | . | . | . |
| logepa | rs58580249 | . | . | . | . | . | . | . | . | . | . | . |
| logepa | rs193148863 | . | . | . | . | . | . | . | . | . | . | . |
| logepa | rs185638946 | . | . | . | . | . | . | . | . | . | . | . |
| logepa | rs117752921 | . | . | . | . | . | . | . | . | . | . | . |
| logepa | rs180841236 | . | . | . | . | . | . | . | . | . | . | . |
| logepa | rs73283155 | . | . | . | . | . | . | . | . | . | . | . |
| logepa | rs76560599 | . | . | . | . | . | . | . | . | . | . | . |
| logepa | rs76273620 | . | . | . | . | . | . | . | . | . | . | . |
| logepa | rs141432923 | . | . | . | . | . | . | . | . | . | . | . |
| logepa | rs113529322 | . | . | . | . | . | . | . | . | . | . | . |
| logepa | rs10128306 | . | . | . | . | . | . | . | . | . | . | . |

| logepa | rs149691688 | . | . | . | . | . | . | . | . | . | . | . |
| --- | --- | --- | --- | --- | --- | --- | --- | --- | --- | --- | --- | --- |
| logepa | rs75872495 | . | . | . | . | . | . | . | . | . | . | . |
| logepa | rs191873960 | . | . | . | . | . | . | . | . | . | . | . |
| logepa | rs80155622 | . | . | . | . | . | . | . | . | . | . | . |
| logepa | rs113914656 | . | . | . | . | . | . | . | . | . | . | . |
| logepa | rs12242840 | . | . | . | . | . | . | . | . | . | . | . |
| logepa | rs147824078 | . | . | . | . | . | . | . | . | . | . | . |
| logepa | rs59051493 | . | . | . | . | . | . | . | . | . | . | . |
| logepa | rs9282588 | . | . | . | . | . | . | . | . | . | . | . |
| logepa | rs150215207 | . | . | . | . | . | . | . | . | . | . | . |
| logepa | rs138772243 | . | . | . | . | . | . | . | . | . | . | . |
| logepa | rs149383244 | . | . | . | . | . | . | . | . | . | . | . |
| logepa | rs17522720 | . | . | . | . | . | . | . | . | . | . | . |
| logepa | rs79245105 | . | . | . | . | . | . | . | . | . | . | . |
| logepa | rs138222133 | . | . | . | . | . | . | . | . | . | . | . |
| logepa | rs12247314 | . | . | . | . | . | . | . | . | . | . | . |
| logepa | rs12263894 | . | . | . | . | . | . | . | . | . | . | . |
| logepa | rs149907880 | . | . | . | . | . | . | . | . | . | . | . |
| logepa | rs188153885 | . | . | . | . | . | . | . | . | . | . | . |
| logepa | rs73283159 | . | . | . | . | . | . | . | . | . | . | . |
| logepa | rs140476687 | . | . | . | . | . | . | . | . | . | . | . |
| logepa | rs186603148 | . | . | . | . | . | . | . | . | . | . | . |
| logepa | rs183296063 | . | . | . | . | . | . | . | . | . | . | . |
| logepa | rs145733694 | . | . | . | . | . | . | . | . | . | . | . |
| logepa | rs189807449 | . | . | . | . | . | . | . | . | . | . | . |
| logepa | rs12253223 | . | . | . | . | . | . | . | . | . | . | . |
| logepa | rs182113637 | . | . | . | . | . | . | . | . | . | . | . |
| logepa | rs142792099 | . | . | . | . | . | . | . | . | . | . | . |
| logepa | rs12249910 | . | . | . | . | . | . | . | . | . | . | . |
| logepa | rs149436339 | . | . | . | . | . | . | . | . | . | . | . |
| logepa | rs77632210 | . | . | . | . | . | . | . | . | . | . | . |
| logepa | rs74130818 | . | . | . | . | . | . | . | . | . | . | . |
| logepa | rs187583648 | . | . | . | . | . | . | . | . | . | . | . |
| logepa | rs191295987 | . | . | . | . | . | . | . | . | . | . | . |
| logepa | rs184200686 | . | . | . | . | . | . | . | . | . | . | . |
| logepa | rs76117263 | . | . | . | . | . | . | . | . | . | . | . |
| logepa | rs185393149 | . | . | . | . | . | . | . | . | . | . | . |
| logepa | rs144760014 | . | . | . | . | . | . | . | . | . | . | . |
| logepa | rs7922292 | . | . | . | . | . | . | . | . | . | . | . |
| logepa | rs115372844 | . | . | . | . | . | . | . | . | . | . | . |
| logepa | rs111460364 | . | . | . | . | . | . | . | . | . | . | . |
| logepa | rs182052255 | . | . | . | . | . | . | . | . | . | . | . |
| logepa | rs180983889 | . | . | . | . | . | . | . | . | . | . | . |
| logepa | rs139175327 | . | . | . | . | . | . | . | . | . | . | . |
| logepa | rs149959620 | . | . | . | . | . | . | . | . | . | . | . |
| logepa | rs114653117 | . | . | . | . | . | . | . | . | . | . | . |
| logepa | rs144270018 | . | . | . | . | . | . | . | . | . | . | . |
| logepa | rs182723204 | . | . | . | . | . | . | . | . | . | . | . |
| logepa | rs57204067 | . | . | . | . | . | . | . | . | . | . | . |
| logepa | rs144956226 | . | . | . | . | . | . | . | . | . | . | . |
| logepa | rs187842704 | . | . | . | . | . | . | . | . | . | . | . |
| logepa | rs184620511 | . | . | . | . | . | . | . | . | . | . | . |
| logepa | rs144031303 | . | . | . | . | . | . | . | . | . | . | . |
| logepa | rs187526473 | . | . | . | . | . | . | . | . | . | . | . |
| logepa | rs193002805 | . | . | . | . | . | . | . | . | . | . | . |
| logepa | rs138707426 | . | . | . | . | . | . | . | . | . | . | . |
| logepa | rs142754243 | . | . | . | . | . | . | . | . | . | . | . |
| logepa | rs181600331 | . | . | . | . | . | . | . | . | . | . | . |
| logepa | rs144421264 | . | . | . | . | . | . | . | . | . | . | . |
| logepa | rs184414519 | . | . | . | . | . | . | . | . | . | . | . |
| logepa | rs150596349 | . | . | . | . | . | . | . | . | . | . | . |
| logepa | rs190753747 | . | . | . | . | . | . | . | . | . | . | . |
| logepa | rs139560940 | . | . | . | . | . | . | . | . | . | . | . |
| logepa | rs75640804 | . | . | . | . | . | . | . | . | . | . | . |
| logepa | rs73283175 | . | . | . | . | . | . | . | . | . | . | . |
| logepa | rs143337229 | . | . | . | . | . | . | . | . | . | . | . |
| logepa | rs73283177 | . | . | . | . | . | . | . | . | . | . | . |
| logepa | rs76053611 | . | . | . | . | . | . | . | . | . | . | . |
| logepa | rs116325983 | . | . | . | . | . | . | . | . | . | . | . |
| logepa | rs115451825 | . | . | . | . | . | . | . | . | . | . | . |
| logepa | rs183845246 | . | . | . | . | . | . | . | . | . | . | . |
| logepa | rs79756542 | . | . | . | . | . | . | . | . | . | . | . |
| logepa | rs187242860 | . | . | . | . | . | . | . | . | . | . | . |
| logepa | rs57318980 | . | . | . | . | . | . | . | . | . | . | . |
| logepa | rs59969732 | . | . | . | . | . | . | . | . | . | . | . |
| logepa | rs143811488 | . | . | . | . | . | . | . | . | . | . | . |
| logepa | rs189067644 | . | . | . | . | . | . | . | . | . | . | . |
| logepa | rs60449178 | . | . | . | . | . | . | . | . | . | . | . |
| logepa | rs59243389 | . | . | . | . | . | . | . | . | . | . | . |
| logepa | rs184946540 | . | . | . | . | . | . | . | . | . | . | . |
| logepa | rs188685881 | . | . | . | . | . | . | . | . | . | . | . |
| logepa | rs150335797 | . | . | . | . | . | . | . | . | . | . | . |
| logepa | rs143879260 | . | . | . | . | . | . | . | . | . | . | . |
| logepa | rs186044491 | . | . | . | . | . | . | . | . | . | . | . |
| logepa | rs11819084 | . | . | . | . | . | . | . | . | . | . | . |
| logepa | rs146915670 | . | . | . | . | . | . | . | . | . | . | . |

| logepa | rs113995695 | . | . | . | . | . | . | . | . | . | . | . |
| --- | --- | --- | --- | --- | --- | --- | --- | --- | --- | --- | --- | --- |
| logepa | rs143489451 | . | . | . | . | . | . | . | . | . | . | . |
| logepa | rs182653839 | . | . | . | . | . | . | . | . | . | . | . |
| logepa | rs141692564 | . | . | . | . | . | . | . | . | . | . | . |
| logepa | rs146281742 | . | . | . | . | . | . | . | . | . | . | . |
| logepa | rs192914353 | . | . | . | . | . | . | . | . | . | . | . |
| logepa | rs185182848 | . | . | . | . | . | . | . | . | . | . | . |
| logepa | rs28395858 | . | . | . | . | . | . | . | . | . | . | . |
| logepa | rs41429645 | . | . | . | . | . | . | . | . | . | . | . |
| logepa | rs28395874 | . | . | . | . | . | . | . | . | . | . | . |
| logepa | rs189663634 | . | . | . | . | . | . | . | . | . | . | . |
| logepa | rs77666700 | . | . | . | . | . | . | . | . | . | . | . |
| logepa | rs146711717 | . | . | . | . | . | . | . | . | . | . | . |
| logepa | rs144904251 | . | . | . | . | . | . | . | . | . | . | . |
| logepa | rs189893153 | . | . | . | . | . | . | . | . | . | . | . |
| logepa | rs150447740 | . | . | . | . | . | . | . | . | . | . | . |
| logepa | rs143099649 | . | . | . | . | . | . | . | . | . | . | . |
| logepa | rs191288436 | . | . | . | . | . | . | . | . | . | . | . |
| logepa | rs188577862 | . | . | . | . | . | . | . | . | . | . | . |
| logepa | rs192695334 | . | . | . | . | . | . | . | . | . | . | . |
| logepa | rs138612906 | . | . | . | . | . | . | . | . | . | . | . |
| logepa | rs191290051 | . | . | . | . | . | . | . | . | . | . | . |
| logepa | rs189631459 | . | . | . | . | . | . | . | . | . | . | . |
| logepa | rs114620874 | . | . | . | . | . | . | . | . | . | . | . |
| logepa | rs78246652 | . | . | . | . | . | . | . | . | . | . | . |
| logepa | rs79432451 | . | . | . | . | . | . | . | . | . | . | . |
| logepa | rs186022585 | . | . | . | . | . | . | . | . | . | . | . |
| logepa | rs142978891 | . | . | . | . | . | . | . | . | . | . | . |
| logepa | rs151086840 | . | . | . | . | . | . | . | . | . | . | . |
| logepa | rs183270259 | . | . | . | . | . | . | . | . | . | . | . |
| logepa | rs188544289 | . | . | . | . | . | . | . | . | . | . | . |
| logepa | rs188500808 | . | . | . | . | . | . | . | . | . | . | . |
| logepa | rs189424589 | . | . | . | . | . | . | . | . | . | . | . |
| logepa | rs78622303 | . | . | . | . | . | . | . | . | . | . | . |
| logepa | rs143210835 | . | . | . | . | . | . | . | . | . | . | . |
| logepa | rs114761840 | . | . | . | . | . | . | . | . | . | . | . |
| logepa | rs185805402 | . | . | . | . | . | . | . | . | . | . | . |
| logepa | rs139273744 | . | . | . | . | . | . | . | . | . | . | . |
| logepa | rs184654150 | . | . | . | . | . | . | . | . | . | . | . |
| logepa | rs189069710 | . | . | . | . | . | . | . | . | . | . | . |
| logepa | rs114752115 | . | . | . | . | . | . | . | . | . | . | . |
| logepa | rs150162515 | . | . | . | . | . | . | . | . | . | . | . |
| logepa | rs180982454 | . | . | . | . | . | . | . | . | . | . | . |
| logepa | rs184932088 | . | . | . | . | . | . | . | . | . | . | . |
| logepa | rs181109625 | . | . | . | . | . | . | . | . | . | . | . |
| logepa | rs138121134 | . | . | . | . | . | . | . | . | . | . | . |
| logepa | rs183253861 | . | . | . | . | . | . | . | . | . | . | . |
| logepa | rs147073946 | . | . | . | . | . | . | . | . | . | . | . |
| logepa | rs139938489 | . | . | . | . | . | . | . | . | . | . | . |
| logepa | rs146362560 | . | . | . | . | . | . | . | . | . | . | . |
| logepa | rs193155956 | . | . | . | . | . | . | . | . | . | . | . |
| logepa | rs77663092 | . | . | . | . | . | . | . | . | . | . | . |
| logepa | rs73285319 | . | . | . | . | . | . | . | . | . | . | . |
| logepa | rs184019276 | . | . | . | . | . | . | . | . | . | . | . |
| logepa | rs28395863 | . | . | . | . | . | . | . | . | . | . | . |
| logepa | rs182121645 | . | . | . | . | . | . | . | . | . | . | . |
| logepa | rs73285327 | . | . | . | . | . | . | . | . | . | . | . |
| logepa | rs149589584 | . | . | . | . | . | . | . | . | . | . | . |
| logepa | rs148310335 | . | . | . | . | . | . | . | . | . | . | . |
| logepa | rs187246093 | . | . | . | . | . | . | . | . | . | . | . |
| logepa | rs184668894 | . | . | . | . | . | . | . | . | . | . | . |
| logepa | rs114269961 | . | . | . | . | . | . | . | . | . | . | . |
| logepa | rs149694374 | . | . | . | . | . | . | . | . | . | . | . |
| logepa | rs145284227 | . | . | . | . | . | . | . | . | . | . | . |
| logepa | rs189983820 | . | . | . | . | . | . | . | . | . | . | . |
| logepa | rs181200444 | . | . | . | . | . | . | . | . | . | . | . |
| logepa | rs185486385 | . | . | . | . | . | . | . | . | . | . | . |
| logepa | rs151198241 | . | . | . | . | . | . | . | . | . | . | . |
| logepa | rs189959341 | . | . | . | . | . | . | . | . | . | . | . |
| logepa | rs140549257 | . | . | . | . | . | . | . | . | . | . | . |
| logepa | rs186453139 | . | . | . | . | . | . | . | . | . | . | . |
| logepa | rs180922711 | . | . | . | . | . | . | . | . | . | . | . |
| logepa | rs56659903 | . | . | . | . | . | . | . | . | . | . | . |
| logepa | rs141202128 | . | . | . | . | . | . | . | . | . | . | . |
| logepa | rs59085314 | . | . | . | . | . | . | . | . | . | . | . |
| logepa | rs145564996 | . | . | . | . | . | . | . | . | . | . | . |
| logepa | rs114961009 | . | . | . | . | . | . | . | . | . | . | . |
| logepa | rs182294189 | . | . | . | . | . | . | . | . | . | . | . |
| logepa | rs187586636 | . | . | . | . | . | . | . | . | . | . | . |
| logepa | rs192254907 | . | . | . | . | . | . | . | . | . | . | . |
| logepa | rs149145663 | . | . | . | . | . | . | . | . | . | . | . |
| logepa | rs180713064 | . | . | . | . | . | . | . | . | . | . | . |
| logepa | rs183009023 | . | . | . | . | . | . | . | . | . | . | . |
| logepa | rs143385347 | . | . | . | . | . | . | . | . | . | . | . |
| logepa | rs185786329 | . | . | . | . | . | . | . | . | . | . | . |
| logepa | rs192341030 | . | . | . | . | . | . | . | . | . | . | . |

| logepa | rs74661429 | . | . | . | . | . | . | . | . | . | . | . |
| --- | --- | --- | --- | --- | --- | --- | --- | --- | --- | --- | --- | --- |
| logepa | rs188034853 | . | . | . | . | . | . | . | . | . | . | . |
| logepa | rs116808430 | . | . | . | . | . | . | . | . | . | . | . |
| logepa | rs148469010 | . | . | . | . | . | . | . | . | . | . | . |
| logepa | rs78621663 | . | . | . | . | . | . | . | . | . | . | . |
| logepa | rs192654886 | . | . | . | . | . | . | . | . | . | . | . |
| logepa | rs145996035 | . | . | . | . | . | . | . | . | . | . | . |
| logepa | rs139942226 | . | . | . | . | . | . | . | . | . | . | . |
| logepa | rs113645632 | . | . | . | . | . | . | . | . | . | . | . |
| logepa | rs74940661 | . | . | . | . | . | . | . | . | . | . | . |
| logepa | rs115355800 | . | . | . | . | . | . | . | . | . | . | . |
| logepa | rs150285898 | . | . | . | . | . | . | . | . | . | . | . |
| logepa | rs188117932 | . | . | . | . | . | . | . | . | . | . | . |
| logepa | rs139956566 | . | . | . | . | . | . | . | . | . | . | . |
| logepa | rs147673885 | . | . | . | . | . | . | . | . | . | . | . |
| logepa | rs189127928 | . | . | . | . | . | . | . | . | . | . | . |
| logepa | rs187558772 | . | . | . | . | . | . | . | . | . | . | . |
| logepa | rs181948587 | . | . | . | . | . | . | . | . | . | . | . |
| logepa | rs189983269 | . | . | . | . | . | . | . | . | . | . | . |
| logepa | rs187250069 | . | . | . | . | . | . | . | . | . | . | . |
| logepa | rs191485859 | . | . | . | . | . | . | . | . | . | . | . |
| logepa | rs185499555 | . | . | . | . | . | . | . | . | . | . | . |
| logepa | rs180862110 | . | . | . | . | . | . | . | . | . | . | . |
| logepa | rs190472603 | . | . | . | . | . | . | . | . | . | . | . |
| logepa | rs189226167 | . | . | . | . | . | . | . | . | . | . | . |
| logepa | rs181623256 | . | . | . | . | . | . | . | . | . | . | . |
| logepa | rs186079557 | . | . | . | . | . | . | . | . | . | . | . |
| logepa | rs147760994 | . | . | . | . | . | . | . | . | . | . | . |
| logepa | rs189176167 | . | . | . | . | . | . | . | . | . | . | . |
| logepa | rs117901000 | . | . | . | . | . | . | . | . | . | . | . |
| logepa | rs141984888 | . | . | . | . | . | . | . | . | . | . | . |
| logepa | rs192365061 | . | . | . | . | . | . | . | . | . | . | . |
| logepa | rs185976038 | . | . | . | . | . | . | . | . | . | . | . |
| logepa | rs184697303 | . | . | . | . | . | . | . | . | . | . | . |
| logepa | rs143383132 | . | . | . | . | . | . | . | . | . | . | . |
| logepa | rs141873881 | . | . | . | . | . | . | . | . | . | . | . |
| logepa | rs185507255 | . | . | . | . | . | . | . | . | . | . | . |
| logepa | rs191379425 | . | . | . | . | . | . | . | . | . | . | . |
| logepa | rs184199584 | . | . | . | . | . | . | . | . | . | . | . |
| logepa | rs183181360 | . | . | . | . | . | . | . | . | . | . | . |
| logepa | rs184495673 | . | . | . | . | . | . | . | . | . | . | . |
| logepa | rs182946297 | . | . | . | . | . | . | . | . | . | . | . |
| logepa | rs187743615 | . | . | . | . | . | . | . | . | . | . | . |
| logepa | rs183259862 | . | . | . | . | . | . | . | . | . | . | . |
| logepa | rs112433566 | . | . | . | . | . | . | . | . | . | . | . |
| logepa | rs182351085 | . | . | . | . | . | . | . | . | . | . | . |
| logepa | rs146248508 | . | . | . | . | . | . | . | . | . | . | . |
| logepa | rs188528439 | . | . | . | . | . | . | . | . | . | . | . |
| logepa | rs146167018 | . | . | . | . | . | . | . | . | . | . | . |
| logepa | rs192440980 | . | . | . | . | . | . | . | . | . | . | . |
| logepa | rs185601882 | . | . | . | . | . | . | . | . | . | . | . |
| logepa | rs117272563 | . | . | . | . | . | . | . | . | . | . | . |
| logepa | rs10430734 | . | . | . | . | . | . | . | . | . | . | . |
| logepa | rs181320952 | . | . | . | . | . | . | . | . | . | . | . |
| logepa | rs185480660 | . | . | . | . | . | . | . | . | . | . | . |
| logepa | rs188077917 | . | . | . | . | . | . | . | . | . | . | . |
| logepa | rs187562953 | . | . | . | . | . | . | . | . | . | . | . |
| logepa | rs182044631 | . | . | . | . | . | . | . | . | . | . | . |
| logepa | rs145455596 | . | . | . | . | . | . | . | . | . | . | . |
| logepa | rs181475877 | . | . | . | . | . | . | . | . | . | . | . |
| logepa | rs149413085 | . | . | . | . | . | . | . | . | . | . | . |
| logepa | rs12253656 | . | . | . | . | . | . | . | . | . | . | . |
| logepa | rs182718239 | . | . | . | . | . | . | . | . | . | . | . |
| logepa | rs192435695 | . | . | . | . | . | . | . | . | . | . | . |
| logepa | rs12248277 | . | . | . | . | . | . | . | . | . | . | . |
| logepa | rs143521710 | . | . | . | . | . | . | . | . | . | . | . |
| logepa | rs144454454 | . | . | . | . | . | . | . | . | . | . | . |
| logepa | rs190441610 | . | . | . | . | . | . | . | . | . | . | . |
| logepa | rs114690462 | . | . | . | . | . | . | . | . | . | . | . |
| logepa | rs184541357 | . | . | . | . | . | . | . | . | . | . | . |
| logepa | rs142657653 | . | . | . | . | . | . | . | . | . | . | . |
| logepa | rsurf1 | 2.462 | . | . | . | . | . | . | . | . | . | . |
| logepa | rsten1 | 2.569 | . | . | . | . | . | . | . | . | . | . |
| logepa | chr10:45852312:D | 0.198 | 0.68 | 0.296 | 0.376 | 0.75 | 0.331 | 0.514 | 0.519 | 0.233 | 0.143 | -0.165 |
| logepa | chr10:45853232:I | 0.2 | 0.68 | 0.295 | 0.374 | 0.738 | 0.326 | 0.492 | 0.516 | 0.231 | 0.14 | -0.159 |
| logepa | chr10:45853234:I | 0.194 | 0.686 | 0.297 | 0.384 | 0.74 | 0.342 | 0.514 | 0.499 | 0.234 | 0.138 | -0.172 |
| logepa | chr10:45854796:D | 0.053 | 0.474 | 0.145 | 0.015 | 1.02 | 0.794 | 0.985 | 2.63 | 2.96 | 0.392 | 0.773 |
| logepa | chr10:45854994:I | 0.054 | 0.474 | 0.145 | 0.015 | 1.01 | 0.794 | 0.985 | 2.63 | 2.96 | 0.392 | 0.774 |
| logepa | chr10:45858290:D | 0.048 | 0.702 | 0.286 | 0.385 | 0.082 | 0.143 | 0.152 | 0.022 | 0.051 | 0.104 | 0.217 |
| logepa | chr10:45858820:I | 0.005 | . | . | . | . | . | . | . | . | . | . |
| logepa | chr10:45860888:D | 0.063 | 0.636 | 0.24 | 0.23 | 0.381 | 0.354 | 0.298 | 0.15 | 0.167 | 0.088 | 0.019 |
| logepa | chr10:45863913:I | 0.195 | 0.671 | 0.292 | 0.359 | 0.746 | 0.325 | 0.501 | 0.53 | 0.238 | 0.158 | -0.152 |
| logepa | chr10:45866601:D | . | . | . | . | . | . | . | . | . | . | . |
| logepa | chr10:45867197:I | . | . | . | . | . | . | . | . | . | . | . |
| logepa | chr10:45867538:D | 0.198 | 0.672 | 0.297 | 0.369 | 0.782 | 0.341 | 0.572 | 0.545 | 0.244 | 0.175 | -0.167 |
| logepa | chr10:45868356:D | 0.197 | 0.673 | 0.296 | 0.367 | 0.779 | 0.341 | 0.568 | 0.539 | 0.24 | 0.165 | -0.169 |

| logepa | chr10:45871158:I | 0.198 | 0.673 | 0.296 | 0.367 | 0.779 | 0.341 | 0.567 | 0.539 | 0.24 | 0.165 | -0.169 |
| --- | --- | --- | --- | --- | --- | --- | --- | --- | --- | --- | --- | --- |
| logepa | chr10:45871637:D | 0.1 | 0.684 | 0.27 | 0.336 | 0.441 | 0.253 | 0.153 | 0.23 | 0.172 | 0.05 | -0.055 |
| logepa | chr10:45871638:I | 0.161 | 0.701 | 0.302 | 0.41 | 0.706 | 0.336 | 0.465 | 0.412 | 0.23 | 0.112 | -0.203 |
| logepa | chr10:45873629:I | 0.198 | 0.673 | 0.296 | 0.367 | 0.779 | 0.341 | 0.567 | 0.539 | 0.24 | 0.165 | -0.169 |
| logepa | chr10:45873701:D | . | . | . | . | . | . | . | . | . | . | . |
| logepa | chr10:45874997:I | . | . | . | . | . | . | . | . | . | . | . |
| logepa | chr10:45882030:I | . | . | . | . | . | . | . | . | . | . | . |
| logepa | chr10:45883774:D | 0.003 | . | . | . | . | . | . | . | . | . | . |
| logepa | chr10:45885169:D | 0.176 | 0.657 | 0.28 | 0.325 | 0.686 | 0.346 | 0.455 | 0.521 | 0.249 | 0.173 | -0.109 |
| logepa | chr10:45886451:D | 0.744 | 2.29 | 1.98 | 0.335 | 0.246 | 0.089 | 1.00E-04 | 0.3 | 0.168 | 0.032 | -1.37 |
| logepa | chr10:45891800:I | 0.85 | 2.16 | 2.63 | 0.526 | 0.478 | 0.171 | 0.039 | 0.419 | 0.258 | 0.158 | -1.21 |
| logepa | chr10:45904771:I | 0.87 | 1.8 | 2.19 | 0.627 | 0.444 | 0.154 | 0.019 | 0.471 | 0.285 | 0.214 | -0.87 |
| logepa | chr10:45904959:I | 0.11 | 0.594 | 0.281 | 0.272 | 0.497 | 0.485 | 0.474 | 0.558 | 0.49 | 0.507 | 0.073 |
| logepa | chr10:45911028:I | 0.105 | 0.613 | 0.301 | 0.318 | 0.437 | 0.53 | 0.495 | 0.386 | 0.491 | 0.454 | 0.054 |
| logepa | chr10:45911031:I | 0.419 | 0.417 | 0.296 | 0.218 | 0.793 | 0.361 | 0.611 | 1.31 | 0.704 | 0.62 | 0.222 |
| logepa | chr10:45912154:D | 0.003 | . | . | . | . | . | . | . | . | . | . |
| logepa | chr10:45913885:I | 0.043 | 0.467 | 0.146 | 0.015 | 1.59 | 1.08 | 0.494 | 2.34 | 1.91 | 0.3 | 0.678 |
| logepa | chr10:45914753:D | 0.159 | 0.503 | 0.215 | 0.107 | 1.82 | 0.784 | 0.164 | 1.04 | 0.777 | 0.956 | -0.369 |
| logepa | chr10:45919621 | . | . | . | . | . | . | . | . | . | . | . |
| logepa | chr10:45921739:I | 0.048 | 0.461 | 0.146 | 0.015 | 1.64 | 1 | 0.422 | 2.33 | 1.75 | 0.259 | 0.664 |
| logepa | chr10:45923527:I | 0.062 | 0.33 | 0.119 | 2.20E-03 | 1.68 | 2.03 | 0.669 | 35.6 | 70.9 | 0.073 | 18.7 |
| logepa | chr10:45927001:I | 0.005 | . | . | . | . | . | . | . | . | . | . |
| logepa | chr10:45930354:I | 0.044 | 0.462 | 0.146 | 0.015 | 1.73 | 1.03 | 0.359 | 2.31 | 1.68 | 0.251 | 0.651 |
| logepa | chr10:45932864:I | 0.065 | 0.457 | 0.143 | 0.013 | 1.48 | 0.953 | 0.546 | 2.32 | 1.97 | 0.322 | 0.63 |
| logepa | chr10:45936163:I | 0.027 | 0.55 | 0.213 | 0.123 | 0.602 | 0.471 | 0.517 | 0.129 | 0.152 | 0.083 | -0.109 |
| logepa | chr10:45938095:D | 0.058 | 0.627 | 0.25 | 0.242 | 0.503 | 0.33 | 0.295 | 0.118 | 0.1 | 0.011 | -0.093 |
| logepa | chr10:45938886 | . | . | . | . | . | . | . | . | . | . | . |
| logepa | chr10:45939320 | . | . | . | . | . | . | . | . | . | . | . |
| logepa | chr10:45939659 | . | . | . | . | . | . | . | . | . | . | . |
| logepa | chr10:45941150:D | 0.042 | 0.462 | 0.146 | 0.015 | 1.73 | 1.03 | 0.356 | 2.3 | 1.68 | 0.252 | 0.649 |
| logepa | chr10:45947128:D | 0.038 | 0.441 | 0.147 | 0.014 | 3.55 | 2 | 0.024 | 2.73 | 2.98 | 0.358 | 1.28 |
| logepa | chr10:45950990:I | 0.052 | 0.451 | 0.144 | 0.012 | 1.41 | 1.03 | 0.64 | 2.69 | 2.13 | 0.211 | 0.849 |
| logepa | chr10:45951854:I | . | . | . | . | . | . | . | . | . | . | . |
| logepa | chr10:45952670:I | 0.001 | . | . | . | . | . | . | . | . | . | . |
| logepa | chr10:45956741 | . | . | . | . | . | . | . | . | . | . | . |
| logepa | chr10:45957247:I | 0.065 | 0.631 | 0.25 | 0.244 | 0.428 | 0.294 | 0.217 | 0.103 | 0.089 | 8.50E-03 | -0.031 |
| logepa | chr10:45958082:I | 0.065 | 0.632 | 0.25 | 0.247 | 0.428 | 0.295 | 0.217 | 0.102 | 0.088 | 7.90E-03 | -0.033 |
| logepa | chr10:45905041:D | 0.05 | 0.465 | 0.142 | 0.012 | 1.16 | 0.861 | 0.844 | 2.7 | 2.6 | 0.302 | 0.832 |
| logepa | chr10:45938871 | 0.003 | . | . | . | . | . | . | . | . | . | . |
| logepa | chr10:45939160 | 0.004 | . | . | . | . | . | . | . | . | . | . |
| logepa | chr10:45869910 | . | . | . | . | . | . | . | . | . | . | . |
| logepa | chr10:45876214:D | . | . | . | . | . | . | . | . | . | . | . |
| logepa | chr10:45880237:I | . | . | . | . | . | . | . | . | . | . | . |
| logepa | chr10:45884371:I | . | . | . | . | . | . | . | . | . | . | . |
| logepa | chr10:45891535:D | . | . | . | . | . | . | . | . | . | . | . |
| logepa | chr10:45893516:D | . | . | . | . | . | . | . | . | . | . | . |
| logepa | chr10:45893782:D | . | . | . | . | . | . | . | . | . | . | . |
| logepa | chr10:45895254:I | . | . | . | . | . | . | . | . | . | . | . |
| logepa | chr10:45895848:D | . | . | . | . | . | . | . | . | . | . | . |
| logepa | chr10:45896316:D | . | . | . | . | . | . | . | . | . | . | . |
| logepa | chr10:45896317:D | . | . | . | . | . | . | . | . | . | . | . |
| logepa | chr10:45902683:I | . | . | . | . | . | . | . | . | . | . | . |
| logepa | chr10:45905003:D | . | . | . | . | . | . | . | . | . | . | . |
| logepa | chr10:45927476:D | . | . | . | . | . | . | . | . | . | . | . |
| logepa | chr10:45935882 | . | . | . | . | . | . | . | . | . | . | . |
| logepa | chr10:45936100 | . | . | . | . | . | . | . | . | . | . | . |
| logepa | chr10:45939553 | . | . | . | . | . | . | . | . | . | . | . |
| logepa | chr10:45939631 | . | . | . | . | . | . | . | . | . | . | . |
| logepa | chr10:45944193:D | . | . | . | . | . | . | . | . | . | . | . |
| logepa | chr10:45954030 | . | . | . | . | . | . | . | . | . | . | . |

Supplemental table S36.

| fa | rs_id | freq | HR_logdha | se_logdha | p_logdha | HR_snp | se_snp | p_snp | HR_inter | se_inter | p_inter | RERI |
| --- | --- | --- | --- | --- | --- | --- | --- | --- | --- | --- | --- | --- |
| logdha | rs76035182 | 0.024 | 0.769 | 0.122 | 0.098 | 2.53 | 2.5 | 0.347 | 0.585 | 0.414 | 0.449 | -1.16 |
| logdha | rs12359547 | 0.069 | 0.712 | 0.112 | 0.031 | 0.755 | 0.408 | 0.603 | 1.03 | 0.443 | 0.95 | 0.086 |
| logdha | rs74595484 | 0.039 | 0.78 | 0.125 | 0.121 | 1.54 | 1.55 | 0.668 | 0.561 | 0.407 | 0.426 | -0.647 |
| logdha | rs1864415 | 0.701 | 1.05 | 0.328 | 0.873 | 1.39 | 0.348 | 0.192 | 0.751 | 0.145 | 0.139 | -0.343 |
| logdha | rs72788101 | 0.073 | 0.719 | 0.113 | 0.035 | 0.808 | 0.431 | 0.69 | 0.947 | 0.406 | 0.898 | 0.023 |
| logdha | rs11239481 | 0.14 | 0.808 | 0.143 | 0.228 | 1.3 | 0.42 | 0.417 | 0.787 | 0.212 | 0.373 | -0.281 |
| logdha | rs72788102 | 0.073 | 0.719 | 0.113 | 0.035 | 0.808 | 0.431 | 0.69 | 0.947 | 0.406 | 0.898 | 0.023 |
| logdha | rs72789705 | 0.073 | 0.719 | 0.113 | 0.035 | 0.808 | 0.431 | 0.69 | 0.947 | 0.405 | 0.898 | 0.023 |
| logdha | rs141529858 | 0.005 | . | . | . | . | . | . | . | . | . | . |
| logdha | rs145376292 | 0 | . | . | . | . | . | . | . | . | . | . |
| logdha | rs190418301 | 0.001 | . | . | . | . | . | . | . | . | . | . |
| logdha | rs11239482 | 0.073 | 0.719 | 0.113 | 0.035 | 0.808 | 0.432 | 0.69 | 0.946 | 0.405 | 0.898 | 0.023 |
| logdha | rs61855872 | 0.01 | 0.798 | 0.127 | 0.156 | 20.4 | 37.6 | 0.102 | 0.039 | 0.071 | 0.075 | -19.6 |
| logdha | rs148991144 | 0.007 | . | . | . | . | . | . | . | . | . | . |
| logdha | rs191932221 | 0.005 | . | . | . | . | . | . | . | . | . | . |
| logdha | rs55978138 | 0.166 | 0.743 | 0.123 | 0.073 | 1.1 | 0.351 | 0.761 | 0.871 | 0.212 | 0.57 | -0.132 |
| logdha | rs73281167 | 0.015 | 0.726 | 0.114 | 0.041 | 0.041 | 0.086 | 0.124 | 7.25 | 9.69 | 0.138 | 0.45 |
| logdha | rs12768010 | 0.086 | 0.743 | 0.117 | 0.058 | 1.55 | 0.652 | 0.297 | 0.755 | 0.236 | 0.368 | -0.424 |
| logdha | rs11239484 | 0.701 | 1.05 | 0.328 | 0.874 | 1.39 | 0.348 | 0.193 | 0.752 | 0.145 | 0.14 | -0.342 |
| logdha | rs35906360 | 0.166 | 0.743 | 0.123 | 0.072 | 1.11 | 0.353 | 0.751 | 0.87 | 0.212 | 0.567 | -0.134 |
| logdha | rs186250512 | 0.003 | . | . | . | . | . | . | . | . | . | . |
| logdha | rs150810743 | 0.002 | . | . | . | . | . | . | . | . | . | . |
| logdha | rs55965812 | 0.161 | 0.735 | 0.122 | 0.063 | 1.08 | 0.342 | 0.8 | 0.905 | 0.218 | 0.678 | -0.098 |
| logdha | rs56180358 | 0.167 | 0.743 | 0.123 | 0.073 | 1.11 | 0.353 | 0.75 | 0.869 | 0.212 | 0.563 | -0.136 |
| logdha | rs55864145 | 0.086 | 0.743 | 0.117 | 0.058 | 1.55 | 0.652 | 0.296 | 0.754 | 0.235 | 0.366 | -0.425 |
| logdha | rs55986122 | 0.166 | 0.743 | 0.123 | 0.072 | 1.11 | 0.352 | 0.752 | 0.87 | 0.212 | 0.567 | -0.134 |
| logdha | rs7477350 | 0.701 | 1.05 | 0.328 | 0.875 | 1.39 | 0.348 | 0.193 | 0.752 | 0.145 | 0.14 | -0.342 |
| logdha | rs2099170 | 0.166 | 0.744 | 0.123 | 0.073 | 1.11 | 0.355 | 0.737 | 0.868 | 0.211 | 0.559 | -0.139 |
| logdha | rs2082108 | 0.167 | 0.743 | 0.123 | 0.073 | 1.11 | 0.352 | 0.752 | 0.87 | 0.211 | 0.566 | -0.134 |
| logdha | rs55902404 | 0.007 | . | . | . | . | . | . | . | . | . | . |
| logdha | rs58402662 | 0.006 | . | . | . | . | . | . | . | . | . | . |
| logdha | rs11511618 | 0.14 | 0.812 | 0.144 | 0.24 | 1.32 | 0.428 | 0.399 | 0.769 | 0.209 | 0.335 | -0.306 |
| logdha | rs1816492 | 0.7 | 1.05 | 0.328 | 0.877 | 1.39 | 0.349 | 0.192 | 0.752 | 0.146 | 0.142 | -0.341 |
| logdha | rs146611093 | 0.007 | . | . | . | . | . | . | . | . | . | . |
| logdha | rs186897689 | 0.002 | . | . | . | . | . | . | . | . | . | . |
| logdha | rs10793616 | 0.701 | 1.05 | 0.328 | 0.876 | 1.39 | 0.348 | 0.194 | 0.752 | 0.145 | 0.14 | -0.341 |
| logdha | rs116700479 | 0.019 | 0.764 | 0.12 | 0.087 | 1.59 | 2.45 | 0.764 | 0.625 | 0.68 | 0.665 | -0.595 |
| logdha | rs192535936 | 0.002 | . | . | . | . | . | . | . | . | . | . |
| logdha | rs113363297 | 0.663 | 1.01 | 0.312 | 0.968 | 1.4 | 0.36 | 0.188 | 0.762 | 0.15 | 0.169 | -0.332 |
| logdha | rs12354994 | 0.158 | 0.748 | 0.124 | 0.078 | 1.15 | 0.394 | 0.684 | 0.845 | 0.219 | 0.517 | -0.171 |
| logdha | rs148068329 | 0.02 | 0.717 | 0.108 | 0.027 | 1.32 | 1.5 | 0.807 | 0.85 | 0.734 | 0.851 | -0.232 |
| logdha | rs12356109 | 0.171 | 0.753 | 0.125 | 0.088 | 1.14 | 0.365 | 0.682 | 0.834 | 0.203 | 0.455 | -0.177 |
| logdha | rs55908141 | 0.006 | . | . | . | . | . | . | . | . | . | . |
| logdha | rs12354712 | 0.16 | 0.734 | 0.122 | 0.061 | 1.08 | 0.34 | 0.809 | 0.91 | 0.219 | 0.693 | -0.093 |
| logdha | rs147282684 | 0.003 | . | . | . | . | . | . | . | . | . | . |
| logdha | rs12356140 | 0.168 | 0.748 | 0.124 | 0.079 | 1.14 | 0.362 | 0.674 | 0.851 | 0.207 | 0.507 | -0.163 |
| logdha | rs185476187 | 0.002 | . | . | . | . | . | . | . | . | . | . |
| logdha | rs145720057 | 0.026 | 0.776 | 0.124 | 0.114 | 2.02 | 1.79 | 0.429 | 0.471 | 0.367 | 0.334 | -1.06 |
| logdha | rs12354742 | 0.166 | 0.742 | 0.123 | 0.071 | 1.1 | 0.351 | 0.761 | 0.875 | 0.212 | 0.581 | -0.129 |
| logdha | rs180913842 | 0.006 | . | . | . | . | . | . | . | . | . | . |
| logdha | rs142976914 | 0.003 | . | . | . | . | . | . | . | . | . | . |
| logdha | rs11511619 | 0.174 | 0.754 | 0.125 | 0.089 | 1.13 | 0.362 | 0.697 | 0.837 | 0.203 | 0.463 | -0.172 |
| logdha | rs146702870 | 0.001 | . | . | . | . | . | . | . | . | . | . |
| logdha | rs150287496 | 0.005 | . | . | . | . | . | . | . | . | . | . |
| logdha | rs190917973 | 0.005 | . | . | . | . | . | . | . | . | . | . |
| logdha | rs11517281 | 0.166 | 0.742 | 0.123 | 0.071 | 1.1 | 0.35 | 0.765 | 0.875 | 0.212 | 0.582 | -0.128 |
| logdha | rs12359299 | 0.168 | 0.742 | 0.123 | 0.071 | 1.1 | 0.349 | 0.767 | 0.876 | 0.212 | 0.586 | -0.126 |
| logdha | rs182307932 | 0.024 | 0.786 | 0.124 | 0.127 | 4.84 | 7.33 | 0.297 | 0.239 | 0.287 | 0.234 | -3.72 |
| logdha | rs61855873 | 0.025 | 0.745 | 0.124 | 0.078 | 0.765 | 0.97 | 0.833 | 1.22 | 1.3 | 0.849 | 0.188 |
| logdha | rs12359302 | 0.164 | 0.75 | 0.124 | 0.083 | 1.16 | 0.387 | 0.649 | 0.839 | 0.213 | 0.489 | -0.182 |
| logdha | rs143208140 | 0.021 | 0.716 | 0.108 | 0.027 | 1.2 | 1.3 | 0.866 | 0.899 | 0.741 | 0.897 | -0.144 |
| logdha | rs9988764 | 0.683 | 1.08 | 0.34 | 0.816 | 1.43 | 0.369 | 0.162 | 0.733 | 0.147 | 0.122 | -0.379 |
| logdha | rs192875767 | 0.006 | . | . | . | . | . | . | . | . | . | . |
| logdha | rs147268846 | 0.012 | 0.769 | 0.123 | 0.1 | 1.66 | 3.35 | 0.802 | 0.239 | 0.479 | 0.475 | -1.12 |
| logdha | rs11239485 | 0.105 | 0.718 | 0.118 | 0.044 | 1.06 | 0.445 | 0.897 | 0.97 | 0.311 | 0.925 | -0.038 |
| logdha | rs151317885 | 0.424 | 0.755 | 0.182 | 0.244 | 1.19 | 0.282 | 0.462 | 0.953 | 0.174 | 0.791 | -0.089 |
| logdha | rs11499085 | 0.107 | 0.804 | 0.131 | 0.183 | 1.34 | 0.863 | 0.652 | 0.648 | 0.295 | 0.341 | -0.445 |
| logdha | rs188130606 | 0.028 | 0.791 | 0.126 | 0.141 | 3.57 | 3.23 | 0.16 | 0.231 | 0.157 | 0.031 | -2.71 |
| logdha | rs11511620 | 0.16 | 0.733 | 0.121 | 0.061 | 1.08 | 0.339 | 0.813 | 0.91 | 0.218 | 0.695 | -0.091 |
| logdha | rs181498390 | 0.017 | 0.703 | 0.107 | 0.02 | 0.349 | 0.666 | 0.581 | 1.86 | 2.47 | 0.64 | 0.404 |
| logdha | rs186280701 | 0.007 | . | . | . | . | . | . | . | . | . | . |
| logdha | rs150059185 | 0.028 | 0.797 | 0.123 | 0.142 | 15.2 | 16 | 9.40E-03 | 0.154 | 0.132 | 0.029 | -13.2 |
| logdha | rs183338707 | 0.003 | . | . | . | . | . | . | . | . | . | . |
| logdha | rs11499038 | 0.09 | 0.748 | 0.118 | 0.064 | 1.2 | 0.801 | 0.781 | 0.716 | 0.337 | 0.477 | -0.307 |
| logdha | rs190699595 | 0.006 | . | . | . | . | . | . | . | . | . | . |
| logdha | rs11523408 | 0.164 | 0.738 | 0.122 | 0.067 | 1.09 | 0.347 | 0.797 | 0.89 | 0.218 | 0.634 | -0.11 |
| logdha | rs188049317 | 0.002 | . | . | . | . | . | . | . | . | . | . |
| logdha | rs144948634 | 0.395 | 0.735 | 0.173 | 0.19 | 1.15 | 0.27 | 0.555 | 0.976 | 0.18 | 0.897 | -0.059 |
| logdha | rs143833576 | 0.003 | . | . | . | . | . | . | . | . | . | . |
| logdha | rs151239879 | 0.165 | 0.744 | 0.123 | 0.074 | 1.12 | 0.357 | 0.711 | 0.867 | 0.212 | 0.558 | -0.143 |
| logdha | rs7073743 | 0.407 | 0.72 | 0.172 | 0.169 | 1.12 | 0.259 | 0.631 | 1 | 0.183 | 0.987 | -0.031 |
| logdha | rs142563349 | 0.03 | 0.797 | 0.127 | 0.155 | 4.29 | 3.46 | 0.07 | 0.222 | 0.131 | 0.011 | -3.33 |
| logdha | rs187246403 | 0.007 | . | . | . | . | . | . | . | . | . | . |
| logdha | rs150423942 | 0.162 | 0.743 | 0.123 | 0.072 | 1.11 | 0.362 | 0.749 | 0.869 | 0.216 | 0.573 | -0.136 |

| logdha | rs184706102 | 0.002 | . | . | . | . | . | . | . | . | . | . |
| --- | --- | --- | --- | --- | --- | --- | --- | --- | --- | --- | --- | --- |
| logdha | rs189626288 | 0.02 | 0.742 | 0.122 | 0.07 | 1.05 | 0.699 | 0.941 | 1.54 | 0.848 | 0.437 | 0.405 |
| logdha | rs140749834 | 0.048 | 0.757 | 0.121 | 0.082 | 0.988 | 0.917 | 0.989 | 0.946 | 0.632 | 0.933 | -0.038 |
| logdha | rs7078822 | 0.133 | 0.719 | 0.118 | 0.045 | 0.996 | 0.371 | 0.992 | 0.973 | 0.274 | 0.922 | -0.018 |
| logdha | rs186227617 | 0.164 | 0.741 | 0.123 | 0.07 | 1.1 | 0.353 | 0.766 | 0.879 | 0.216 | 0.602 | -0.124 |
| logdha | rs188984368 | 0.022 | 0.668 | 0.105 | 0.01 | 0.142 | 0.193 | 0.151 | 4.52 | 4.58 | 0.136 | 0.619 |
| logdha | rs182078109 | 0.031 | 0.715 | 0.118 | 0.043 | 0.281 | 0.366 | 0.33 | 2.58 | 2.6 | 0.345 | 0.523 |
| logdha | rs141456274 | 0.005 | . | . | . | . | . | . | . | . | . | . |
| logdha | rs146976512 | 0.166 | 0.742 | 0.123 | 0.072 | 1.1 | 0.35 | 0.76 | 0.874 | 0.211 | 0.577 | -0.129 |
| logdha | rs138036816 | 0.101 | 0.799 | 0.131 | 0.17 | 1.35 | 0.892 | 0.65 | 0.666 | 0.312 | 0.386 | -0.43 |
| logdha | rs114879777 | 0.019 | 0.763 | 0.12 | 0.086 | 1.53 | 2.38 | 0.786 | 0.643 | 0.707 | 0.688 | -0.54 |
| logdha | rs149366475 | 0.101 | 0.799 | 0.131 | 0.171 | 1.34 | 0.897 | 0.666 | 0.667 | 0.315 | 0.392 | -0.423 |
| logdha | rs182463043 | 0.002 | . | . | . | . | . | . | . | . | . | . |
| logdha | rs144754632 | 0.02 | 0.716 | 0.108 | 0.027 | 1.29 | 1.45 | 0.821 | 0.872 | 0.74 | 0.872 | -0.2 |
| logdha | rs55717607 | 0.166 | 0.743 | 0.123 | 0.072 | 1.1 | 0.349 | 0.76 | 0.874 | 0.21 | 0.575 | -0.13 |
| logdha | rs187537973 | 0 | . | . | . | . | . | . | . | . | . | . |
| logdha | rs148085971 | 0.002 | . | . | . | . | . | . | . | . | . | . |
| logdha | rs11239491 | 0.166 | 0.743 | 0.123 | 0.072 | 1.1 | 0.349 | 0.76 | 0.873 | 0.21 | 0.574 | -0.13 |
| logdha | rs184412184 | 0.005 | . | . | . | . | . | . | . | . | . | . |
| logdha | rs4948998 | 0.716 | 0.98 | 0.308 | 0.95 | 1.29 | 0.323 | 0.313 | 0.794 | 0.155 | 0.238 | -0.266 |
| logdha | rs7076696 | 0.717 | 0.981 | 0.308 | 0.952 | 1.29 | 0.323 | 0.313 | 0.794 | 0.155 | 0.236 | -0.266 |
| logdha | rs11239492 | 0.172 | 0.754 | 0.125 | 0.089 | 1.14 | 0.364 | 0.673 | 0.831 | 0.201 | 0.445 | -0.181 |
| logdha | rs79462470 | 0.007 | . | . | . | . | . | . | . | . | . | . |
| logdha | rs187804654 | 0.003 | . | . | . | . | . | . | . | . | . | . |
| logdha | rs7093687 | 0.161 | 0.734 | 0.122 | 0.062 | 1.08 | 0.339 | 0.802 | 0.906 | 0.216 | 0.68 | -0.096 |
| logdha | rs7081004 | 0.161 | 0.734 | 0.122 | 0.062 | 1.08 | 0.338 | 0.806 | 0.907 | 0.216 | 0.683 | -0.095 |
| logdha | rs7084793 | 0.166 | 0.743 | 0.123 | 0.072 | 1.1 | 0.349 | 0.76 | 0.873 | 0.21 | 0.574 | -0.13 |
| logdha | rs61556080 | 0.007 | . | . | . | . | . | . | . | . | . | . |
| logdha | rs115552064 | 0.007 | . | . | . | . | . | . | . | . | . | . |
| logdha | rs7921503 | 0.166 | 0.745 | 0.123 | 0.076 | 1.12 | 0.352 | 0.725 | 0.862 | 0.207 | 0.535 | -0.145 |
| logdha | rs7909740 | 0.166 | 0.743 | 0.123 | 0.072 | 1.1 | 0.348 | 0.761 | 0.874 | 0.21 | 0.574 | -0.129 |
| logdha | rs7909768 | 0.166 | 0.743 | 0.123 | 0.072 | 1.1 | 0.348 | 0.761 | 0.874 | 0.21 | 0.574 | -0.129 |
| logdha | rs144168004 | 0 | . | . | . | . | . | . | . | . | . | . |
| logdha | rs75850448 | 0.007 | . | . | . | . | . | . | . | . | . | . |
| logdha | rs147777574 | 0.006 | . | . | . | . | . | . | . | . | . | . |
| logdha | rs7913488 | 0.166 | 0.743 | 0.123 | 0.072 | 1.1 | 0.348 | 0.762 | 0.874 | 0.21 | 0.574 | -0.129 |
| logdha | rs74390874 | 0.049 | 0.704 | 0.116 | 0.034 | 1.12 | 0.546 | 0.812 | 1.16 | 0.472 | 0.712 | 0.092 |
| logdha | rs141391142 | 0.006 | . | . | . | . | . | . | . | . | . | . |
| logdha | rs115294747 | 0.007 | . | . | . | . | . | . | . | . | . | . |
| logdha | rs148031781 | 0.002 | . | . | . | . | . | . | . | . | . | . |
| logdha | rs181340128 | 0.018 | 0.762 | 0.124 | 0.094 | 1.15 | 1.6 | 0.918 | 0.697 | 0.902 | 0.78 | -0.303 |
| logdha | rs189930007 | 0.001 | . | . | . | . | . | . | . | . | . | . |
| logdha | rs12242128 | 0.1 | 0.799 | 0.131 | 0.17 | 1.36 | 0.912 | 0.651 | 0.664 | 0.315 | 0.388 | -0.436 |
| logdha | rs78421750 | 0.006 | . | . | . | . | . | . | . | . | . | . |
| logdha | rs114370747 | 0.003 | . | . | . | . | . | . | . | . | . | . |
| logdha | rs12264166 | 0.1 | 0.799 | 0.131 | 0.17 | 1.36 | 0.913 | 0.647 | 0.663 | 0.315 | 0.386 | -0.438 |
| logdha | rs182964822 | 0.001 | . | . | . | . | . | . | . | . | . | . |
| logdha | rs67491417 | 0.159 | 0.749 | 0.122 | 0.077 | 1.13 | 0.386 | 0.727 | 0.839 | 0.22 | 0.504 | -0.168 |
| logdha | rs72789720 | 0.015 | 0.724 | 0.117 | 0.046 | 0.195 | 0.286 | 0.264 | 4.33 | 4.67 | 0.174 | 0.693 |
| logdha | rs6413416 | 0.167 | 0.745 | 0.123 | 0.076 | 1.13 | 0.356 | 0.701 | 0.864 | 0.206 | 0.54 | -0.147 |
| logdha | rs4986832 | 0.167 | 0.742 | 0.123 | 0.071 | 1.1 | 0.347 | 0.765 | 0.874 | 0.21 | 0.575 | -0.128 |
| logdha | rs144866316 | 0.005 | . | . | . | . | . | . | . | . | . | . |
| logdha | rs187769540 | 0.002 | . | . | . | . | . | . | . | . | . | . |
| logdha | rs6593482 | 0.167 | 0.749 | 0.125 | 0.082 | 1.12 | 0.352 | 0.719 | 0.855 | 0.205 | 0.513 | -0.152 |
| logdha | rs7913948 | 0.167 | 0.741 | 0.123 | 0.07 | 1.09 | 0.344 | 0.788 | 0.878 | 0.211 | 0.589 | -0.121 |
| logdha | rs77185151 | 0.019 | 0.716 | 0.108 | 0.027 | 1.25 | 1.42 | 0.842 | 0.889 | 0.758 | 0.89 | -0.172 |
| logdha | rs12762303 | 0.167 | 0.742 | 0.123 | 0.071 | 1.1 | 0.347 | 0.766 | 0.874 | 0.21 | 0.575 | -0.128 |
| logdha | rs73283136 | 0.005 | . | . | . | . | . | . | . | . | . | . |
| logdha | rs187180218 | 0.002 | . | . | . | . | . | . | . | . | . | . |
| logdha | rs78137087 | 0.166 | 0.741 | 0.122 | 0.07 | 1.09 | 0.345 | 0.783 | 0.878 | 0.211 | 0.589 | -0.122 |
| logdha | rs72561472 | 0.165 | 0.742 | 0.122 | 0.071 | 1.07 | 0.344 | 0.823 | 0.873 | 0.213 | 0.578 | -0.12 |
| logdha | rs4987106 | 0.162 | 0.749 | 0.123 | 0.079 | 1.11 | 0.361 | 0.741 | 0.841 | 0.208 | 0.483 | -0.161 |
| logdha | rs4987105 | 0.158 | 0.738 | 0.122 | 0.066 | 1.08 | 0.341 | 0.803 | 0.888 | 0.214 | 0.622 | -0.111 |
| logdha | rs111916157 | 0.182 | 0.76 | 0.131 | 0.111 | 1.23 | 0.36 | 0.484 | 0.835 | 0.188 | 0.422 | -0.209 |
| logdha | rs111528152 | 0.171 | 0.748 | 0.125 | 0.082 | 1.11 | 0.351 | 0.743 | 0.852 | 0.205 | 0.506 | -0.15 |
| logdha | rs12763738 | 0.166 | 0.743 | 0.123 | 0.072 | 1.1 | 0.349 | 0.754 | 0.871 | 0.21 | 0.568 | -0.132 |
| logdha | rs191675886 | 0.007 | . | . | . | . | . | . | . | . | . | . |
| logdha | rs66489870 | 0.167 | 0.742 | 0.123 | 0.071 | 1.1 | 0.347 | 0.766 | 0.874 | 0.21 | 0.575 | -0.128 |
| logdha | rs71494775 | 0.167 | 0.742 | 0.123 | 0.071 | 1.1 | 0.347 | 0.766 | 0.874 | 0.21 | 0.575 | -0.128 |
| logdha | rs61855875 | 0.024 | 0.747 | 0.124 | 0.078 | 0.765 | 1.02 | 0.841 | 1.21 | 1.35 | 0.866 | 0.178 |
| logdha | rs116356837 | 0.007 | . | . | . | . | . | . | . | . | . | . |
| logdha | rs1864414 | 0.166 | 0.742 | 0.123 | 0.071 | 1.1 | 0.349 | 0.762 | 0.877 | 0.212 | 0.587 | -0.127 |
| logdha | rs1864413 | 0.167 | 0.743 | 0.123 | 0.072 | 1.1 | 0.348 | 0.752 | 0.874 | 0.209 | 0.574 | -0.13 |
| logdha | rs139497212 | 0 | . | . | . | . | . | . | . | . | . | . |
| logdha | rs892690 | 0.328 | 0.666 | 0.15 | 0.071 | 0.981 | 0.241 | 0.939 | 1.11 | 0.216 | 0.586 | 0.079 |
| logdha | rs1864412 | 0.167 | 0.742 | 0.123 | 0.071 | 1.1 | 0.347 | 0.766 | 0.874 | 0.21 | 0.576 | -0.128 |
| logdha | rs77827771 | 0.031 | 0.804 | 0.129 | 0.175 | 4.85 | 3.88 | 0.048 | 0.202 | 0.122 | 8.00E-03 | -3.87 |
| logdha | rs145294963 | 0.004 | . | . | . | . | . | . | . | . | . | . |
| logdha | rs149081327 | 0.008 | . | . | . | . | . | . | . | . | . | . |
| logdha | rs67008983 | 0.166 | 0.742 | 0.123 | 0.071 | 1.1 | 0.347 | 0.767 | 0.875 | 0.21 | 0.579 | -0.127 |
| logdha | rs56150414 | 0.17 | 0.744 | 0.124 | 0.076 | 1.09 | 0.337 | 0.776 | 0.874 | 0.206 | 0.567 | -0.126 |
| logdha | rs55787481 | 0.167 | 0.74 | 0.122 | 0.069 | 1.08 | 0.343 | 0.797 | 0.883 | 0.212 | 0.604 | -0.116 |
| logdha | rs141270101 | 0.001 | . | . | . | . | . | . | . | . | . | . |
| logdha | rs114980075 | 0.008 | . | . | . | . | . | . | . | . | . | . |
| logdha | rs12783095 | 0.166 | 0.738 | 0.122 | 0.066 | 1.07 | 0.34 | 0.823 | 0.891 | 0.214 | 0.63 | -0.106 |
| logdha | rs182342688 | 0.003 | . | . | . | . | . | . | . | . | . | . |

| logdha | rs17157728 | 0.166 | 0.738 | 0.122 | 0.066 | 1.07 | 0.34 | 0.823 | 0.891 | 0.214 | 0.631 | -0.106 |
| --- | --- | --- | --- | --- | --- | --- | --- | --- | --- | --- | --- | --- |
| logdha | rs114835563 | 0.028 | 0.794 | 0.127 | 0.149 | 4.17 | 3.53 | 0.091 | 0.22 | 0.138 | 0.016 | -3.24 |
| logdha | rs3824612 | 0.328 | 0.664 | 0.149 | 0.068 | 0.975 | 0.239 | 0.916 | 1.12 | 0.217 | 0.565 | 0.085 |
| logdha | rs17157733 | 0.165 | 0.739 | 0.122 | 0.068 | 1.08 | 0.342 | 0.817 | 0.886 | 0.214 | 0.616 | -0.111 |
| logdha | rs74128478 | 0.006 | . | . | . | . | . | . | . | . | . | . |
| logdha | rs184973589 | 0.001 | . | . | . | . | . | . | . | . | . | . |
| logdha | rs150479461 | 0 | . | . | . | . | . | . | . | . | . | . |
| logdha | rs138310407 | 0.003 | . | . | . | . | . | . | . | . | . | . |
| logdha | rs187426603 | 0.001 | . | . | . | . | . | . | . | . | . | . |
| logdha | rs190843143 | 0.002 | . | . | . | . | . | . | . | . | . | . |
| logdha | rs191883449 | 0.001 | . | . | . | . | . | . | . | . | . | . |
| logdha | rs58238740 | 0.163 | 0.734 | 0.121 | 0.062 | 1.06 | 0.338 | 0.845 | 0.909 | 0.219 | 0.691 | -0.088 |
| logdha | rs3780894 | 0.163 | 0.734 | 0.121 | 0.062 | 1.06 | 0.338 | 0.845 | 0.909 | 0.219 | 0.691 | -0.088 |
| logdha | rs149646626 | 0.018 | 0.791 | 0.129 | 0.149 | 3.33 | 3.46 | 0.247 | 0.21 | 0.19 | 0.085 | -2.57 |
| logdha | rs79615212 | 0.016 | 0.832 | 0.135 | 0.256 | 33.7 | 53.5 | 0.026 | 0.024 | 0.038 | 0.017 | -32.9 |
| logdha | rs3780895 | 0.163 | 0.734 | 0.122 | 0.062 | 1.07 | 0.338 | 0.842 | 0.908 | 0.218 | 0.688 | -0.089 |
| logdha | rs3780896 | 0.163 | 0.734 | 0.121 | 0.062 | 1.06 | 0.338 | 0.845 | 0.909 | 0.219 | 0.692 | -0.088 |
| logdha | rs142747163 | 0.005 | . | . | . | . | . | . | . | . | . | . |
| logdha | rs189417584 | 0.007 | . | . | . | . | . | . | . | . | . | . |
| logdha | rs3780897 | 0.162 | 0.729 | 0.121 | 0.057 | 1.04 | 0.331 | 0.913 | 0.928 | 0.224 | 0.757 | -0.064 |
| logdha | rs117509491 | 0.009 | . | . | . | . | . | . | . | . | . | . |
| logdha | rs117811514 | 0.009 | . | . | . | . | . | . | . | . | . | . |
| logdha | rs116500932 | 0.028 | 0.794 | 0.127 | 0.149 | 4.14 | 3.52 | 0.095 | 0.222 | 0.14 | 0.017 | -3.2 |
| logdha | rs77584168 | 0.004 | . | . | . | . | . | . | . | . | . | . |
| logdha | rs2310776 | 0.163 | 0.734 | 0.121 | 0.061 | 1.06 | 0.338 | 0.846 | 0.909 | 0.219 | 0.693 | -0.088 |
| logdha | rs17157736 | 0.163 | 0.734 | 0.121 | 0.062 | 1.06 | 0.338 | 0.846 | 0.909 | 0.219 | 0.693 | -0.088 |
| logdha | rs72789735 | 0.065 | 0.709 | 0.112 | 0.029 | 0.742 | 0.412 | 0.591 | 1.06 | 0.471 | 0.899 | 0.106 |
| logdha | rs183747187 | 0.002 | . | . | . | . | . | . | . | . | . | . |
| logdha | rs7077891 | 0.163 | 0.734 | 0.121 | 0.061 | 1.06 | 0.338 | 0.847 | 0.909 | 0.219 | 0.693 | -0.088 |
| logdha | rs145063305 | 0.003 | . | . | . | . | . | . | . | . | . | . |
| logdha | rs3824613 | 0.164 | 0.73 | 0.121 | 0.058 | 1.05 | 0.331 | 0.882 | 0.927 | 0.222 | 0.751 | -0.069 |
| logdha | rs145543199 | 0.009 | . | . | . | . | . | . | . | . | . | . |
| logdha | rs140361993 | 0.003 | . | . | . | . | . | . | . | . | . | . |
| logdha | rs11239496 | 0.065 | 0.709 | 0.112 | 0.029 | 0.742 | 0.412 | 0.591 | 1.06 | 0.471 | 0.899 | 0.106 |
| logdha | rs11239497 | 0.158 | 0.725 | 0.12 | 0.053 | 1.04 | 0.328 | 0.895 | 0.945 | 0.225 | 0.814 | -0.053 |
| logdha | rs10900212 | 0.184 | 0.731 | 0.121 | 0.058 | 1 | 0.316 | 0.989 | 0.933 | 0.218 | 0.766 | -0.05 |
| logdha | rs186487136 | 0 | . | . | . | . | . | . | . | . | . | . |
| logdha | rs12774369 | 0.163 | 0.734 | 0.122 | 0.062 | 1.07 | 0.34 | 0.842 | 0.909 | 0.22 | 0.693 | -0.089 |
| logdha | rs60842538 | 0.163 | 0.734 | 0.122 | 0.062 | 1.07 | 0.343 | 0.837 | 0.908 | 0.221 | 0.693 | -0.09 |
| logdha | rs3780898 | 0.157 | 0.725 | 0.12 | 0.053 | 1.05 | 0.335 | 0.888 | 0.946 | 0.23 | 0.819 | -0.054 |
| logdha | rs3780899 | 0.163 | 0.734 | 0.122 | 0.062 | 1.07 | 0.347 | 0.83 | 0.908 | 0.223 | 0.694 | -0.092 |
| logdha | rs12252472 | 0.09 | 0.812 | 0.132 | 0.202 | 1.8 | 1.23 | 0.39 | 0.528 | 0.265 | 0.203 | -0.841 |
| logdha | rs185411011 | 0.002 | . | . | . | . | . | . | . | . | . | . |
| logdha | rs28395865 | 0.155 | 0.722 | 0.12 | 0.05 | 1.03 | 0.334 | 0.932 | 0.96 | 0.238 | 0.87 | -0.037 |
| logdha | rs191004074 | 0.03 | 0.726 | 0.119 | 0.05 | 0.377 | 0.47 | 0.434 | 1.94 | 1.89 | 0.494 | 0.429 |
| logdha | rs182401295 | 0.003 | . | . | . | . | . | . | . | . | . | . |
| logdha | rs2228064 | 0.093 | 0.764 | 0.119 | 0.084 | 1.72 | 1 | 0.353 | 0.576 | 0.25 | 0.204 | -0.726 |
| logdha | rs188284159 | 0.001 | . | . | . | . | . | . | . | . | . | . |
| logdha | rs2303887 | 0.013 | 0.717 | 0.108 | 0.028 | 1.63 | 1.77 | 0.652 | 0.885 | 0.765 | 0.887 | -0.313 |
| logdha | rs2303886 | 0.016 | 0.722 | 0.109 | 0.031 | 2.36 | 2.19 | 0.353 | 0.628 | 0.457 | 0.522 | -1.01 |
| logdha | rs192621006 | 0.003 | . | . | . | . | . | . | . | . | . | . |
| logdha | rs2303885 | 0.252 | 0.775 | 0.131 | 0.13 | 1.08 | 0.323 | 0.807 | 0.824 | 0.185 | 0.388 | -0.164 |
| logdha | rs114466168 | 0.028 | 0.795 | 0.127 | 0.151 | 4.2 | 3.59 | 0.093 | 0.219 | 0.139 | 0.017 | -3.27 |
| logdha | rs11239501 | 0.088 | 0.706 | 0.116 | 0.035 | 0.846 | 0.386 | 0.714 | 1.06 | 0.381 | 0.862 | 0.084 |
| logdha | rs745986 | 0.18 | 0.718 | 0.125 | 0.057 | 1.03 | 0.319 | 0.933 | 0.982 | 0.231 | 0.937 | -0.021 |
| logdha | rs75262268 | 0.002 | . | . | . | . | . | . | . | . | . | . |
| logdha | rs71494776 | 0.061 | 0.721 | 0.113 | 0.037 | 1.3 | 0.573 | 0.546 | 0.904 | 0.293 | 0.756 | -0.175 |
| logdha | rs74386871 | 0.02 | 0.714 | 0.108 | 0.026 | 1.06 | 1.13 | 0.959 | 1.05 | 0.891 | 0.953 | 0.022 |
| logdha | rs76502040 | 0.007 | . | . | . | . | . | . | . | . | . | . |
| logdha | rs142213734 | 0.001 | . | . | . | . | . | . | . | . | . | . |
| logdha | rs4948671 | 0.714 | 0.926 | 0.289 | 0.806 | 1.23 | 0.304 | 0.4 | 0.828 | 0.16 | 0.328 | -0.213 |
| logdha | rs146286085 | 0.001 | . | . | . | . | . | . | . | . | . | . |
| logdha | rs144080623 | 0 | . | . | . | . | . | . | . | . | . | . |
| logdha | rs115074014 | 0.044 | 0.766 | 0.123 | 0.097 | 1.34 | 1.31 | 0.763 | 0.78 | 0.553 | 0.726 | -0.306 |
| logdha | rs17157756 | 0.044 | 0.766 | 0.123 | 0.097 | 1.34 | 1.31 | 0.763 | 0.78 | 0.553 | 0.726 | -0.306 |
| logdha | rs190459780 | 0.001 | . | . | . | . | . | . | . | . | . | . |
| logdha | rs12358647 | 0.208 | 0.73 | 0.127 | 0.07 | 1.02 | 0.311 | 0.957 | 0.945 | 0.214 | 0.804 | -0.045 |
| logdha | rs147080556 | 0.013 | 0.714 | 0.111 | 0.029 | 1.22 | 1.14 | 0.834 | 1.07 | 0.701 | 0.921 | -3.40E-03 |
| logdha | rs187543349 | 0.005 | . | . | . | . | . | . | . | . | . | . |
| logdha | rs114375595 | 0.01 | 0.705 | 0.108 | 0.022 | 0.321 | 0.649 | 0.574 | 2.04 | 2.75 | 0.596 | 0.436 |
| logdha | rs117211200 | 0.006 | . | . | . | . | . | . | . | . | . | . |
| logdha | rs192963047 | 0.001 | . | . | . | . | . | . | . | . | . | . |
| logdha | rs11239505 | 0.209 | 0.734 | 0.128 | 0.076 | 1.03 | 0.313 | 0.931 | 0.933 | 0.21 | 0.759 | -0.057 |
| logdha | rs184434116 | 0.002 | . | . | . | . | . | . | . | . | . | . |
| logdha | rs4948999 | 0.716 | 0.932 | 0.291 | 0.822 | 1.23 | 0.303 | 0.4 | 0.824 | 0.159 | 0.314 | -0.218 |
| logdha | rs11239506 | 0.102 | 0.711 | 0.12 | 0.044 | 0.918 | 0.386 | 0.838 | 1.02 | 0.338 | 0.958 | 0.035 |
| logdha | rs190706620 | 0.004 | . | . | . | . | . | . | . | . | . | . |
| logdha | rs190770429 | 0.002 | . | . | . | . | . | . | . | . | . | . |
| logdha | rs181327133 | 0.005 | . | . | . | . | . | . | . | . | . | . |
| logdha | rs149251697 | 0.023 | 0.735 | 0.117 | 0.054 | 0.348 | 0.488 | 0.452 | 2.01 | 1.92 | 0.465 | 0.431 |
| logdha | rs118081602 | 0.013 | 0.717 | 0.108 | 0.028 | 1.61 | 1.75 | 0.661 | 0.892 | 0.772 | 0.895 | -0.297 |
| logdha | rs188233691 | 0.002 | . | . | . | . | . | . | . | . | . | . |
| logdha | rs183778794 | 0.008 | . | . | . | . | . | . | . | . | . | . |
| logdha | rs188040078 | 0.006 | . | . | . | . | . | . | . | . | . | . |
| logdha | rs184675372 | 0.002 | . | . | . | . | . | . | . | . | . | . |
| logdha | rs150326891 | 0.069 | 0.7 | 0.115 | 0.029 | 0.785 | 0.402 | 0.636 | 1.14 | 0.45 | 0.748 | 0.139 |

| logdha | rs185520021 | 0.006 | . | . | . | . | . | . | . | . | . | . |
| --- | --- | --- | --- | --- | --- | --- | --- | --- | --- | --- | --- | --- |
| logdha | rs7907779 | 0.102 | 0.711 | 0.12 | 0.043 | 0.917 | 0.385 | 0.837 | 1.02 | 0.338 | 0.955 | 0.036 |
| logdha | rs184962860 | 0.001 | . | . | . | . | . | . | . | . | . | . |
| logdha | rs149762784 | 0.037 | 0.67 | 0.107 | 0.012 | 0.217 | 0.22 | 0.131 | 2.47 | 1.85 | 0.229 | 0.472 |
| logdha | rs149203427 | 0.013 | 0.753 | 0.122 | 0.08 | 1.34 | 1.05 | 0.708 | 1.5 | 0.993 | 0.536 | 0.425 |
| logdha | rs60153083 | 0.016 | 0.722 | 0.113 | 0.038 | 0.023 | 0.049 | 0.078 | 10 | 13.4 | 0.085 | 0.422 |
| logdha | rs112001069 | 0.076 | 0.728 | 0.114 | 0.044 | 1.35 | 0.594 | 0.498 | 0.843 | 0.271 | 0.595 | -0.249 |
| logdha | rs182355382 | 0.031 | 0.731 | 0.114 | 0.044 | 1.48 | 1.59 | 0.715 | 0.653 | 0.515 | 0.589 | -0.505 |
| logdha | rs186740974 | 0.125 | 0.817 | 0.15 | 0.272 | 2.03 | 0.999 | 0.15 | 0.591 | 0.226 | 0.169 | -0.867 |
| logdha | rs191874916 | 0.036 | 0.792 | 0.122 | 0.129 | 8.59 | 8.55 | 0.031 | 0.206 | 0.165 | 0.048 | -6.98 |
| logdha | rs182989412 | 0.145 | 0.73 | 0.134 | 0.087 | 1.1 | 0.49 | 0.835 | 0.927 | 0.323 | 0.828 | -0.085 |
| logdha | rs188819476 | 0.002 | . | . | . | . | . | . | . | . | . | . |
| logdha | rs141217183 | 0.093 | 0.748 | 0.121 | 0.073 | 1.49 | 0.691 | 0.39 | 0.749 | 0.258 | 0.402 | -0.403 |
| logdha | rs192922652 | 0.077 | 0.731 | 0.115 | 0.047 | 1.43 | 0.636 | 0.427 | 0.815 | 0.264 | 0.527 | -0.308 |
| logdha | rs185092343 | 0.02 | 0.694 | 0.109 | 0.02 | 0.25 | 0.332 | 0.296 | 2.27 | 2.17 | 0.391 | 0.45 |
| logdha | rs145193311 | 0.08 | 0.723 | 0.114 | 0.04 | 1.27 | 0.582 | 0.601 | 0.896 | 0.296 | 0.74 | -0.17 |
| logdha | rs55789336 | 0.104 | 0.72 | 0.122 | 0.052 | 0.963 | 0.41 | 0.929 | 0.966 | 0.328 | 0.92 | -0.013 |
| logdha | rs55837212 | 0.104 | 0.721 | 0.122 | 0.053 | 0.985 | 0.425 | 0.971 | 0.96 | 0.327 | 0.904 | -0.024 |
| logdha | rs75408574 | 0.36 | 0.779 | 0.173 | 0.262 | 1.2 | 0.284 | 0.444 | 0.9 | 0.166 | 0.566 | -0.138 |
| logdha | rs149306926 | 0.11 | 0.688 | 0.116 | 0.027 | 0.761 | 0.341 | 0.542 | 1.18 | 0.409 | 0.639 | 0.167 |
| logdha | rs35990892 | 0.674 | 0.858 | 0.274 | 0.632 | 1.25 | 0.323 | 0.388 | 0.876 | 0.177 | 0.512 | -0.169 |
| logdha | rs192356207 | 0.05 | 0.714 | 0.11 | 0.028 | 0.6 | 0.58 | 0.597 | 1.13 | 0.78 | 0.864 | 0.168 |
| logdha | rs183741163 | 0.102 | 0.733 | 0.125 | 0.068 | 1.12 | 0.513 | 0.805 | 0.885 | 0.316 | 0.733 | -0.126 |
| logdha | rs34737297 | 0.556 | 0.871 | 0.244 | 0.622 | 1.25 | 0.348 | 0.42 | 0.833 | 0.18 | 0.397 | -0.214 |
| logdha | rs181346933 | 0.03 | 0.706 | 0.113 | 0.029 | 0.066 | 0.102 | 0.08 | 5.97 | 6.1 | 0.081 | 0.506 |
| logdha | rs36093253 | 0.361 | 0.705 | 0.153 | 0.107 | 1.06 | 0.257 | 0.801 | 1.03 | 0.191 | 0.877 | 3.30E-03 |
| logdha | rs186307855 | 0.099 | 0.812 | 0.136 | 0.215 | 1.57 | 0.953 | 0.455 | 0.6 | 0.272 | 0.26 | -0.618 |
| logdha | rs189681865 | 0.034 | 0.774 | 0.124 | 0.11 | 1.39 | 1.31 | 0.724 | 0.673 | 0.453 | 0.556 | -0.441 |
| logdha | rs34942971 | 0.214 | 0.762 | 0.136 | 0.128 | 1.19 | 0.374 | 0.582 | 0.852 | 0.197 | 0.489 | -0.179 |
| logdha | rs181816554 | 0.033 | 0.857 | 0.138 | 0.335 | 15.8 | 15.1 | 4.00E-03 | 0.114 | 0.098 | 0.012 | -14.1 |
| logdha | rs186846881 | 0.094 | 0.812 | 0.134 | 0.206 | 1.76 | 1.14 | 0.385 | 0.563 | 0.268 | 0.228 | -0.768 |
| logdha | rs191178220 | 0.016 | 0.714 | 0.111 | 0.031 | 1.25 | 1.64 | 0.866 | 0.988 | 1.04 | 0.991 | -0.082 |
| logdha | rs183305228 | 0.016 | 0.722 | 0.113 | 0.038 | 0.023 | 0.048 | 0.076 | 10 | 13.4 | 0.084 | 0.42 |
| logdha | rs139912761 | 0.121 | 0.726 | 0.123 | 0.059 | 0.972 | 0.402 | 0.945 | 0.947 | 0.301 | 0.863 | -0.03 |
| logdha | rs185499901 | 0.018 | 0.761 | 0.125 | 0.096 | 1.07 | 1.76 | 0.965 | 0.627 | 0.905 | 0.746 | -0.323 |
| logdha | rs189816197 | 0.087 | 0.727 | 0.116 | 0.046 | 1.25 | 0.538 | 0.599 | 0.877 | 0.277 | 0.679 | -0.181 |
| logdha | rs145656938 | 0.026 | 0.742 | 0.114 | 0.051 | 1.72 | 2.23 | 0.673 | 0.425 | 0.421 | 0.388 | -0.923 |
| logdha | rs35745197 | 0.076 | 0.726 | 0.114 | 0.042 | 1.31 | 0.579 | 0.537 | 0.865 | 0.278 | 0.651 | -0.215 |
| logdha | rs192856637 | 0.005 | . | . | . | . | . | . | . | . | . | . |
| logdha | rs2310775 | 0.416 | 0.691 | 0.153 | 0.096 | 1.02 | 0.222 | 0.924 | 1.05 | 0.177 | 0.785 | 0.027 |
| logdha | rs11239507 | 0.506 | 0.782 | 0.183 | 0.295 | 1.16 | 0.263 | 0.5 | 0.918 | 0.156 | 0.614 | -0.111 |
| logdha | rs74648467 | 0.062 | 0.73 | 0.115 | 0.046 | 1.41 | 0.622 | 0.431 | 0.804 | 0.262 | 0.503 | -0.314 |
| logdha | rs145815934 | 0.015 | 0.71 | 0.116 | 0.036 | 0.074 | 0.116 | 0.098 | 7.75 | 8.52 | 0.062 | 0.622 |
| logdha | rs145390687 | 0.02 | 0.744 | 0.122 | 0.07 | 1.07 | 0.685 | 0.916 | 1.44 | 0.777 | 0.494 | 0.336 |
| logdha | rs184354913 | 0.034 | 0.85 | 0.14 | 0.324 | 7.41 | 6.19 | 0.017 | 0.09 | 0.069 | 1.70E-03 | -6.7 |
| logdha | rs181244920 | 0.006 | . | . | . | . | . | . | . | . | . | . |
| logdha | rs11499087 | 0.055 | 0.766 | 0.123 | 0.097 | 1.17 | 0.918 | 0.842 | 0.813 | 0.464 | 0.717 | -0.207 |
| logdha | rs148263794 | 0.08 | 0.728 | 0.115 | 0.044 | 1.37 | 0.601 | 0.472 | 0.85 | 0.271 | 0.609 | -0.251 |
| logdha | rs150758903 | 0.036 | 0.78 | 0.126 | 0.125 | 1.07 | 1.37 | 0.955 | 0.673 | 0.616 | 0.666 | -0.29 |
| logdha | rs147654382 | 0.179 | 0.752 | 0.131 | 0.101 | 1.18 | 0.397 | 0.623 | 0.857 | 0.216 | 0.541 | -0.171 |
| logdha | rs147495166 | 0.025 | 0.792 | 0.127 | 0.146 | 4.49 | 3.87 | 0.081 | 0.226 | 0.147 | 0.022 | -3.48 |
| logdha | rs35198778 | 0.106 | 0.746 | 0.122 | 0.074 | 1.06 | 0.524 | 0.9 | 0.803 | 0.291 | 0.544 | -0.173 |
| logdha | rs145207659 | 0.049 | 0.697 | 0.115 | 0.028 | 0.994 | 0.469 | 0.99 | 1.26 | 0.497 | 0.566 | 0.179 |
| logdha | rs186466464 | 0.01 | 0.736 | 0.118 | 0.055 | 0.201 | 0.257 | 0.209 | 3.36 | 3.04 | 0.18 | 0.561 |
| logdha | rs12778413 | 0.162 | 0.748 | 0.128 | 0.089 | 1.11 | 0.413 | 0.781 | 0.856 | 0.241 | 0.579 | -0.147 |
| logdha | rs7895552 | 0.64 | 0.962 | 0.286 | 0.896 | 1.36 | 0.348 | 0.235 | 0.787 | 0.16 | 0.239 | -0.291 |
| logdha | rs12783895 | 0.077 | 0.725 | 0.114 | 0.041 | 1.31 | 0.578 | 0.541 | 0.876 | 0.282 | 0.68 | -0.203 |
| logdha | rs34929604 | 0.079 | 0.724 | 0.114 | 0.04 | 1.28 | 0.568 | 0.58 | 0.885 | 0.287 | 0.706 | -0.184 |
| logdha | rs12761961 | 0.075 | 0.724 | 0.114 | 0.04 | 1.28 | 0.573 | 0.585 | 0.881 | 0.289 | 0.699 | -0.187 |
| logdha | rs35536930 | 0.206 | 0.743 | 0.129 | 0.088 | 1.08 | 0.341 | 0.812 | 0.903 | 0.21 | 0.661 | -0.098 |
| logdha | rs180951060 | 0.002 | . | . | . | . | . | . | . | . | . | . |
| logdha | rs148892128 | 0.015 | 0.71 | 0.116 | 0.036 | 0.074 | 0.116 | 0.098 | 7.76 | 8.52 | 0.062 | 0.621 |
| logdha | rs11239509 | 0.084 | 0.8 | 0.13 | 0.17 | 1.61 | 0.909 | 0.399 | 0.583 | 0.248 | 0.204 | -0.66 |
| logdha | rs41526545 | 0.093 | 0.704 | 0.117 | 0.035 | 0.856 | 0.375 | 0.723 | 1.07 | 0.371 | 0.841 | 0.086 |
| logdha | rs2029253 | 0.421 | 0.706 | 0.155 | 0.112 | 1.05 | 0.226 | 0.821 | 1.02 | 0.171 | 0.899 | 1.30E-03 |
| logdha | rs17444064 | 0.092 | 0.697 | 0.116 | 0.03 | 0.81 | 0.36 | 0.635 | 1.13 | 0.397 | 0.727 | 0.131 |
| logdha | rs71494779 | 0.08 | 0.724 | 0.114 | 0.04 | 1.27 | 0.56 | 0.586 | 0.885 | 0.284 | 0.705 | -0.18 |
| logdha | rs74991420 | 0.012 | 0.716 | 0.108 | 0.027 | 1.53 | 1.69 | 0.698 | 0.998 | 0.894 | 0.999 | -0.153 |
| logdha | rs182736551 | 0.012 | 0.716 | 0.108 | 0.027 | 1.53 | 1.69 | 0.698 | 0.998 | 0.894 | 0.999 | -0.153 |
| logdha | rs78702074 | 0.013 | 0.717 | 0.108 | 0.028 | 1.74 | 1.92 | 0.618 | 0.847 | 0.751 | 0.851 | -0.399 |
| logdha | rs187791466 | 0.004 | . | . | . | . | . | . | . | . | . | . |
| logdha | rs35300270 | 0.082 | 0.72 | 0.113 | 0.037 | 1.18 | 0.518 | 0.706 | 0.927 | 0.295 | 0.812 | -0.112 |
| logdha | rs6593484 | 0.103 | 0.71 | 0.12 | 0.043 | 0.916 | 0.383 | 0.834 | 1.02 | 0.338 | 0.946 | 0.039 |
| logdha | rs12244601 | 0.023 | 0.752 | 0.119 | 0.073 | 0.349 | 0.599 | 0.539 | 1.41 | 1.66 | 0.772 | 0.268 |
| logdha | rs184429574 | 0.005 | . | . | . | . | . | . | . | . | . | . |
| logdha | rs7080897 | 0.08 | 0.724 | 0.114 | 0.04 | 1.27 | 0.56 | 0.586 | 0.885 | 0.284 | 0.704 | -0.18 |
| logdha | rs7097429 | 0.083 | 0.714 | 0.113 | 0.033 | 1.19 | 0.487 | 0.665 | 0.984 | 0.292 | 0.956 | -0.069 |
| logdha | rs77979229 | 0.003 | . | . | . | . | . | . | . | . | . | . |
| logdha | rs11239512 | 0.103 | 0.711 | 0.12 | 0.043 | 0.916 | 0.383 | 0.834 | 1.02 | 0.337 | 0.949 | 0.038 |
| logdha | rs60146827 | 0.08 | 0.724 | 0.114 | 0.04 | 1.27 | 0.561 | 0.583 | 0.884 | 0.284 | 0.7 | -0.183 |
| logdha | rs79032236 | 0.035 | 0.807 | 0.128 | 0.177 | 4.83 | 4.65 | 0.102 | 0.221 | 0.163 | 0.041 | -3.78 |
| logdha | rs140854206 | 0.007 | . | . | . | . | . | . | . | . | . | . |
| logdha | rs184049550 | 0.004 | . | . | . | . | . | . | . | . | . | . |
| logdha | rs114433607 | 0.01 | 0.723 | 0.113 | 0.038 | 4.80E-03 | 0.013 | 0.054 | 28.8 | 48.7 | 0.047 | 0.372 |
| logdha | rs117653793 | 0.013 | 0.717 | 0.108 | 0.028 | 1.65 | 1.81 | 0.646 | 0.882 | 0.769 | 0.885 | -0.325 |
| logdha | rs117039533 | 0.013 | 0.717 | 0.108 | 0.028 | 1.65 | 1.8 | 0.648 | 0.885 | 0.772 | 0.889 | -0.319 |

| logdha | rs191660761 | 0.003 | . | . | . | . | . | . | . | . | . | . |
| --- | --- | --- | --- | --- | --- | --- | --- | --- | --- | --- | --- | --- |
| logdha | rs59514896 | 0.064 | 0.725 | 0.114 | 0.041 | 1.37 | 0.598 | 0.467 | 0.862 | 0.277 | 0.644 | -0.24 |
| logdha | rs184661876 | 0.002 | . | . | . | . | . | . | . | . | . | . |
| logdha | rs61854073 | 0.027 | 0.756 | 0.126 | 0.093 | 1.16 | 1.34 | 0.9 | 0.99 | 0.985 | 0.992 | -0.047 |
| logdha | rs2018518 | 0.299 | 0.674 | 0.141 | 0.06 | 1 | 0.241 | 0.987 | 1.11 | 0.206 | 0.574 | 0.073 |
| logdha | rs934187 | 0.718 | 0.933 | 0.295 | 0.826 | 1.26 | 0.313 | 0.358 | 0.826 | 0.16 | 0.324 | -0.221 |
| logdha | rs7918542 | 0.214 | 0.727 | 0.126 | 0.067 | 0.996 | 0.301 | 0.989 | 0.958 | 0.213 | 0.846 | -0.029 |
| logdha | rs144311920 | 0.002 | . | . | . | . | . | . | . | . | . | . |
| logdha | rs7099684 | 0.841 | 1.44 | 0.635 | 0.404 | 1.64 | 0.527 | 0.123 | 0.654 | 0.161 | 0.084 | -0.536 |
| logdha | rs150829171 | 0.002 | . | . | . | . | . | . | . | . | . | . |
| logdha | rs186060697 | 0.002 | . | . | . | . | . | . | . | . | . | . |
| logdha | rs11239513 | 0.098 | 0.822 | 0.136 | 0.235 | 1.89 | 1.27 | 0.342 | 0.52 | 0.259 | 0.19 | -0.907 |
| logdha | rs7917687 | 0.081 | 0.725 | 0.114 | 0.041 | 1.28 | 0.564 | 0.573 | 0.876 | 0.28 | 0.679 | -0.193 |
| logdha | rs61854076 | 0.01 | . | . | . | . | . | . | . | . | . | . |
| logdha | rs71494780 | 0.018 | 0.76 | 0.121 | 0.086 | 1.49 | 1.09 | 0.588 | 0.859 | 0.532 | 0.807 | -0.276 |
| logdha | rs7919239 | 0.218 | 0.734 | 0.127 | 0.074 | 1.03 | 0.296 | 0.92 | 0.936 | 0.198 | 0.754 | -0.056 |
| logdha | rs78334475 | 0.034 | 0.717 | 0.112 | 0.033 | 0.909 | 0.598 | 0.884 | 0.828 | 0.397 | 0.694 | -0.086 |
| logdha | rs3780900 | 0.026 | 0.776 | 0.12 | 0.1 | 8.88 | 9.33 | 0.038 | 0.23 | 0.192 | 0.078 | -7.07 |
| logdha | rs186729292 | 0.001 | . | . | . | . | . | . | . | . | . | . |
| logdha | rs12267438 | 0.026 | 0.752 | 0.119 | 0.072 | 0.527 | 0.61 | 0.58 | 1.29 | 1.02 | 0.746 | 0.233 |
| logdha | rs1369214 | 0.569 | 0.84 | 0.218 | 0.502 | 1.27 | 0.299 | 0.319 | 0.869 | 0.156 | 0.434 | -0.182 |
| logdha | rs9282589 | 0.041 | 0.718 | 0.118 | 0.043 | 0.57 | 0.306 | 0.295 | 1.94 | 0.824 | 0.119 | 0.505 |
| logdha | rs114522915 | 0.025 | 0.797 | 0.127 | 0.156 | 6.92 | 7.4 | 0.07 | 0.165 | 0.133 | 0.026 | -5.81 |
| logdha | rs2115819 | 0.478 | 0.709 | 0.174 | 0.162 | 1.09 | 0.248 | 0.705 | 1.02 | 0.183 | 0.914 | -0.011 |
| logdha | rs117664603 | 0.013 | 0.716 | 0.108 | 0.027 | 1.52 | 1.65 | 0.702 | 0.937 | 0.809 | 0.94 | -0.215 |
| logdha | rs184979202 | 0.001 | . | . | . | . | . | . | . | . | . | . |
| logdha | rs190714671 | 0.002 | . | . | . | . | . | . | . | . | . | . |
| logdha | rs141538996 | 0 | . | . | . | . | . | . | . | . | . | . |
| logdha | rs150881741 | 0.002 | . | . | . | . | . | . | . | . | . | . |
| logdha | rs139076633 | 0.003 | . | . | . | . | . | . | . | . | . | . |
| logdha | rs12243649 | 0.059 | 0.724 | 0.11 | 0.033 | 1.04 | 0.746 | 0.961 | 0.866 | 0.437 | 0.775 | -0.11 |
| logdha | rs140348708 | 0.001 | . | . | . | . | . | . | . | . | . | . |
| logdha | rs79023256 | 0.013 | 0.716 | 0.108 | 0.027 | 1.49 | 1.63 | 0.716 | 0.952 | 0.821 | 0.954 | -0.19 |
| logdha | rs11239515 | 0.094 | 0.7 | 0.117 | 0.033 | 0.868 | 0.364 | 0.736 | 1.09 | 0.361 | 0.788 | 0.096 |
| logdha | rs55866086 | 0.158 | 0.723 | 0.12 | 0.051 | 0.948 | 0.339 | 0.881 | 0.964 | 0.257 | 0.892 | -9.90E-03 |
| logdha | rs181340150 | 0.003 | . | . | . | . | . | . | . | . | . | . |
| logdha | rs11239516 | 0.12 | 0.709 | 0.119 | 0.04 | 0.86 | 0.345 | 0.708 | 1.04 | 0.32 | 0.897 | 0.066 |
| logdha | rs11239517 | 0.12 | 0.708 | 0.119 | 0.04 | 0.859 | 0.344 | 0.704 | 1.04 | 0.32 | 0.892 | 0.067 |
| logdha | rs11239519 | 0.106 | 0.716 | 0.12 | 0.046 | 0.901 | 0.385 | 0.807 | 0.993 | 0.33 | 0.984 | 0.024 |
| logdha | rs7909514 | 0.709 | 0.911 | 0.286 | 0.768 | 1.25 | 0.311 | 0.366 | 0.839 | 0.163 | 0.367 | -0.206 |
| logdha | rs7894352 | 0.835 | 1.43 | 0.622 | 0.414 | 1.63 | 0.52 | 0.126 | 0.656 | 0.161 | 0.086 | -0.532 |
| logdha | rs189384146 | 0.002 | . | . | . | . | . | . | . | . | . | . |
| logdha | rs11239520 | 0.105 | 0.733 | 0.122 | 0.062 | 1.14 | 0.452 | 0.748 | 0.892 | 0.281 | 0.716 | -0.127 |
| logdha | rs12221233 | 0.406 | 0.663 | 0.149 | 0.067 | 0.968 | 0.214 | 0.881 | 1.1 | 0.189 | 0.58 | 0.075 |
| logdha | rs74523293 | 0.032 | 0.721 | 0.109 | 0.03 | 1.24 | 1.26 | 0.83 | 0.758 | 0.567 | 0.711 | -0.285 |
| logdha | rs79376040 | 0.032 | 0.721 | 0.109 | 0.03 | 1.24 | 1.26 | 0.832 | 0.759 | 0.568 | 0.713 | -0.283 |
| logdha | rs4949000 | 0.406 | 0.663 | 0.149 | 0.067 | 0.969 | 0.214 | 0.887 | 1.1 | 0.188 | 0.584 | 0.074 |
| logdha | rs7089406 | 0.025 | 0.71 | 0.112 | 0.03 | 0.086 | 0.099 | 0.034 | 5.9 | 4.51 | 0.02 | 0.564 |
| logdha | rs138211056 | 0 | . | . | . | . | . | . | . | . | . | . |
| logdha | rs10900213 | 0.537 | 0.697 | 0.184 | 0.172 | 1.03 | 0.231 | 0.91 | 1.02 | 0.183 | 0.893 | 9.60E-03 |
| logdha | rs145152827 | 0.003 | . | . | . | . | . | . | . | . | . | . |
| logdha | rs74443325 | 0.012 | 0.717 | 0.108 | 0.028 | 1.58 | 1.82 | 0.693 | 0.733 | 0.693 | 0.742 | -0.466 |
| logdha | rs188299446 | 0.011 | 0.792 | 0.128 | 0.149 | 6.16 | 5.39 | 0.038 | 0.29 | 0.215 | 0.096 | -4.54 |
| logdha | rs192699586 | 0.003 | . | . | . | . | . | . | . | . | . | . |
| logdha | rs72796464 | 0.086 | 0.689 | 0.113 | 0.023 | 0.745 | 0.33 | 0.507 | 1.21 | 0.419 | 0.59 | 0.185 |
| logdha | rs4949001 | 0.266 | 0.68 | 0.135 | 0.052 | 0.974 | 0.248 | 0.919 | 1.1 | 0.216 | 0.637 | 0.073 |
| logdha | rs147174641 | 0.008 | . | . | . | . | . | . | . | . | . | . |
| logdha | rs190835238 | 0.001 | . | . | . | . | . | . | . | . | . | . |
| logdha | rs76698944 | 0.022 | 0.77 | 0.123 | 0.102 | 1.3 | 1.6 | 0.829 | 0.55 | 0.578 | 0.569 | -0.522 |
| logdha | rs56071381 | 0.11 | 0.738 | 0.124 | 0.07 | 1.14 | 0.448 | 0.745 | 0.87 | 0.271 | 0.654 | -0.145 |
| logdha | rs78130427 | 0.047 | 0.712 | 0.11 | 0.028 | 1 | 0.584 | 1 | 1.03 | 0.396 | 0.941 | 0.021 |
| logdha | rs10900215 | 0.148 | 0.705 | 0.118 | 0.037 | 0.891 | 0.334 | 0.759 | 1.05 | 0.301 | 0.868 | 0.063 |
| logdha | rs41491445 | 0.016 | 0.723 | 0.113 | 0.038 | 0.028 | 0.056 | 0.073 | 9.18 | 11.7 | 0.081 | 0.435 |
| logdha | rs144995222 | 0.002 | . | . | . | . | . | . | . | . | . | . |
| logdha | rs149097803 | 0.003 | . | . | . | . | . | . | . | . | . | . |
| logdha | rs139429646 | 0.005 | . | . | . | . | . | . | . | . | . | . |
| logdha | rs7075620 | 0.681 | 0.88 | 0.269 | 0.674 | 1.19 | 0.292 | 0.489 | 0.853 | 0.164 | 0.409 | -0.176 |
| logdha | rs148299213 | 0.017 | 0.721 | 0.113 | 0.037 | 0.045 | 0.077 | 0.069 | 7.4 | 8.18 | 0.07 | 0.474 |
| logdha | rs4949002 | 0.246 | 0.664 | 0.141 | 0.053 | 1.06 | 0.288 | 0.843 | 1.16 | 0.249 | 0.48 | 0.096 |
| logdha | rs150496007 | 0.021 | 0.768 | 0.125 | 0.105 | 0.931 | 0.942 | 0.944 | 0.566 | 0.364 | 0.375 | -0.295 |
| logdha | rs147303455 | 0.012 | 0.779 | 0.124 | 0.118 | 7.64 | 9.67 | 0.108 | 0.138 | 0.108 | 0.012 | -6.6 |
| logdha | rs189518433 | 0.005 | . | . | . | . | . | . | . | . | . | . |
| logdha | rs186281355 | 0.005 | . | . | . | . | . | . | . | . | . | . |
| logdha | rs115297114 | 0.026 | 0.787 | 0.126 | 0.134 | 3.29 | 3.07 | 0.201 | 0.28 | 0.198 | 0.072 | -2.36 |
| logdha | rs11239523 | 0.105 | 0.73 | 0.121 | 0.058 | 1.1 | 0.434 | 0.806 | 0.906 | 0.283 | 0.751 | -0.103 |
| logdha | rs143704897 | 0.005 | . | . | . | . | . | . | . | . | . | . |
| logdha | rs188024096 | 0.003 | . | . | . | . | . | . | . | . | . | . |
| logdha | rs11239524 | 0.107 | 0.753 | 0.126 | 0.09 | 1.26 | 0.509 | 0.563 | 0.794 | 0.254 | 0.471 | -0.261 |
| logdha | rs191656573 | 0.004 | . | . | . | . | . | . | . | . | . | . |
| logdha | rs193295875 | 0.002 | . | . | . | . | . | . | . | . | . | . |
| logdha | rs190458228 | 0.002 | . | . | . | . | . | . | . | . | . | . |
| logdha | rs139253993 | 0.002 | . | . | . | . | . | . | . | . | . | . |
| logdha | rs4948672 | 0.588 | 1.06 | 0.316 | 0.856 | 1.52 | 0.399 | 0.107 | 0.716 | 0.143 | 0.096 | -0.427 |
| logdha | rs180954921 | 0.002 | . | . | . | . | . | . | . | . | . | . |
| logdha | rs12264801 | 0.425 | 0.531 | 0.13 | 9.50E-03 | 0.657 | 0.195 | 0.156 | 1.41 | 0.322 | 0.129 | 0.305 |
| logdha | rs73283184 | 0.02 | 0.721 | 0.113 | 0.036 | 9.80E-03 | 0.019 | 0.014 | 15.4 | 17.6 | 0.017 | 0.378 |

| logdha | rs142746187 | 0.009 | . | . | . | . | . | . | . | . | . | . |
| --- | --- | --- | --- | --- | --- | --- | --- | --- | --- | --- | --- | --- |
| logdha | rs58704739 | 0.019 | 0.722 | 0.112 | 0.036 | 3.20E-03 | 6.90E-03 | 6.70E-03 | 27.2 | 34 | 8.20E-03 | 0.339 |
| logdha | rs61272335 | 0.014 | 0.718 | 0.112 | 0.033 | 1.50E-03 | 3.90E-03 | 0.014 | 54.3 | 86.4 | 0.012 | 0.339 |
| logdha | rs60959489 | 0.035 | 0.736 | 0.116 | 0.053 | 0.508 | 0.555 | 0.535 | 1.63 | 1.16 | 0.495 | 0.364 |
| logdha | rs58502629 | 0.014 | 0.718 | 0.112 | 0.033 | 1.50E-03 | 3.90E-03 | 0.014 | 54.7 | 86.9 | 0.012 | 0.338 |
| logdha | rs10900216 | 0.736 | 0.741 | 0.269 | 0.408 | 1.04 | 0.286 | 0.885 | 0.975 | 0.208 | 0.906 | -0.03 |
| logdha | rs181118141 | 0.002 | . | . | . | . | . | . | . | . | . | . |
| logdha | rs137882665 | 0.01 | . | . | . | . | . | . | . | . | . | . |
| logdha | rs149466736 | 0.003 | . | . | . | . | . | . | . | . | . | . |
| logdha | rs2099171 | 0.656 | 0.8 | 0.254 | 0.481 | 1.14 | 0.279 | 0.605 | 0.921 | 0.172 | 0.661 | -0.099 |
| logdha | rs141852280 | 0.006 | . | . | . | . | . | . | . | . | . | . |
| logdha | rs191539257 | 0.002 | . | . | . | . | . | . | . | . | . | . |
| logdha | rs17157771 | 0.034 | 0.708 | 0.108 | 0.024 | 0.955 | 0.668 | 0.947 | 1.11 | 0.57 | 0.844 | 0.085 |
| logdha | rs111339241 | 0.032 | 0.711 | 0.109 | 0.026 | 1.09 | 0.808 | 0.907 | 1.03 | 0.559 | 0.955 | -2.40E-03 |
| logdha | rs116983255 | 0.008 | . | . | . | . | . | . | . | . | . | . |
| logdha | rs142324341 | 0.011 | 0.741 | 0.147 | 0.13 | 176 | 905 | 0.313 | 4.00E-03 | 0.018 | 0.218 | -176 |
| logdha | rs187191219 | 0.01 | . | . | . | . | . | . | . | . | . | . |
| logdha | rs151283973 | 0.002 | . | . | . | . | . | . | . | . | . | . |
| logdha | rs139316218 | 0.003 | . | . | . | . | . | . | . | . | . | . |
| logdha | rs6593485 | 0.732 | 0.733 | 0.263 | 0.387 | 1.03 | 0.282 | 0.928 | 0.981 | 0.208 | 0.93 | -0.021 |
| logdha | rs6593486 | 0.733 | 0.733 | 0.262 | 0.385 | 1.02 | 0.281 | 0.931 | 0.982 | 0.208 | 0.931 | -0.02 |
| logdha | rs145196618 | 0.014 | 0.761 | 0.12 | 0.082 | 0.764 | 1.49 | 0.89 | 0.688 | 0.983 | 0.793 | -0.125 |
| logdha | rs142598335 | 0.004 | . | . | . | . | . | . | . | . | . | . |
| logdha | rs892691 | 0.726 | 0.722 | 0.257 | 0.36 | 1.03 | 0.28 | 0.899 | 0.994 | 0.208 | 0.977 | -0.014 |
| logdha | rs7080713 | 0.671 | 0.75 | 0.244 | 0.377 | 1.04 | 0.264 | 0.871 | 0.963 | 0.19 | 0.85 | -0.039 |
| logdha | rs7077173 | 0.08 | 0.712 | 0.111 | 0.029 | 0.933 | 0.489 | 0.895 | 1.02 | 0.393 | 0.962 | 0.032 |
| logdha | rs7080977 | 0.048 | 0.723 | 0.111 | 0.034 | 1.15 | 0.784 | 0.837 | 0.863 | 0.432 | 0.769 | -0.156 |
| logdha | rs3780901 | 0.671 | 0.727 | 0.235 | 0.324 | 1.03 | 0.258 | 0.914 | 0.988 | 0.193 | 0.949 | -0.017 |
| logdha | rs61854091 | 0.051 | 0.704 | 0.121 | 0.041 | 0.588 | 0.412 | 0.449 | 1.77 | 0.983 | 0.3 | 0.443 |
| logdha | rs77550209 | 0.005 | . | . | . | . | . | . | . | . | . | . |
| logdha | rs73283191 | 0.061 | 0.721 | 0.111 | 0.033 | 1.07 | 0.652 | 0.907 | 0.905 | 0.404 | 0.823 | -0.094 |
| logdha | rs191866099 | 0.007 | . | . | . | . | . | . | . | . | . | . |
| logdha | rs147998395 | 0.002 | . | . | . | . | . | . | . | . | . | . |
| logdha | rs7090328 | 0.784 | 0.885 | 0.328 | 0.74 | 1.19 | 0.33 | 0.535 | 0.87 | 0.189 | 0.521 | -0.159 |
| logdha | rs79814535 | 0.012 | 0.71 | 0.107 | 0.024 | 0.648 | 0.869 | 0.746 | 1.45 | 1.43 | 0.707 | 0.309 |
| logdha | rs7073082 | 0.784 | 0.885 | 0.328 | 0.742 | 1.19 | 0.33 | 0.534 | 0.869 | 0.189 | 0.52 | -0.159 |
| logdha | rs11239526 | 0.036 | 0.674 | 0.107 | 0.013 | 0.599 | 0.341 | 0.368 | 1.94 | 0.852 | 0.131 | 0.511 |
| logdha | rs141839841 | 0.002 | . | . | . | . | . | . | . | . | . | . |
| logdha | rs75697371 | 0.01 | . | . | . | . | . | . | . | . | . | . |
| logdha | rs75972854 | 0.01 | . | . | . | . | . | . | . | . | . | . |
| logdha | rs139291975 | 0.001 | . | . | . | . | . | . | . | . | . | . |
| logdha | rs144064711 | 0.006 | . | . | . | . | . | . | . | . | . | . |
| logdha | rs3780902 | 0.062 | 0.721 | 0.111 | 0.033 | 1.07 | 0.646 | 0.912 | 0.902 | 0.399 | 0.815 | -0.095 |
| logdha | rs190449801 | 0.002 | . | . | . | . | . | . | . | . | . | . |
| logdha | rs73283196 | 0.061 | 0.722 | 0.111 | 0.034 | 1.09 | 0.664 | 0.886 | 0.89 | 0.397 | 0.794 | -0.112 |
| logdha | rs28395867 | 0.044 | 0.772 | 0.121 | 0.099 | 1.41 | 1.41 | 0.734 | 0.664 | 0.497 | 0.585 | -0.457 |
| logdha | rs28395866 | 0.102 | 0.753 | 0.117 | 0.069 | 1.43 | 0.761 | 0.506 | 0.722 | 0.29 | 0.418 | -0.404 |
| logdha | rs55661611 | 0.047 | 0.724 | 0.111 | 0.036 | 1.19 | 0.812 | 0.804 | 0.839 | 0.422 | 0.727 | -0.189 |
| logdha | rs55684518 | 0.02 | 0.764 | 0.123 | 0.094 | 1.45 | 1.17 | 0.645 | 0.731 | 0.496 | 0.644 | -0.403 |
| logdha | rs78552493 | 0.048 | 0.763 | 0.123 | 0.094 | 1.14 | 0.754 | 0.838 | 0.889 | 0.472 | 0.825 | -0.131 |
| logdha | rs147665434 | 0.004 | . | . | . | . | . | . | . | . | . | . |
| logdha | rs34445001 | 0.01 | . | . | . | . | . | . | . | . | . | . |
| logdha | rs184019449 | 0.003 | . | . | . | . | . | . | . | . | . | . |
| logdha | rs3780903 | 0.715 | 0.637 | 0.23 | 0.211 | 0.923 | 0.248 | 0.766 | 1.08 | 0.231 | 0.712 | 0.076 |
| logdha | rs17157784 | 0.061 | 0.727 | 0.11 | 0.035 | 1.33 | 0.861 | 0.657 | 0.812 | 0.385 | 0.66 | -0.273 |
| logdha | rs3780904 | 0.013 | 0.712 | 0.108 | 0.025 | 0.772 | 1.03 | 0.846 | 1.13 | 1.12 | 0.902 | 0.137 |
| logdha | rs192064730 | 0 | . | . | . | . | . | . | . | . | . | . |
| logdha | rs12774534 | 0.01 | . | . | . | . | . | . | . | . | . | . |
| logdha | rs79179915 | 0.011 | 0.711 | 0.107 | 0.024 | 0.704 | 0.897 | 0.783 | 1.42 | 1.35 | 0.713 | 0.295 |
| logdha | rs117811571 | 0.011 | 0.791 | 0.128 | 0.146 | 5.85 | 5.05 | 0.041 | 0.315 | 0.231 | 0.116 | -4.18 |
| logdha | rs72796477 | 0.01 | 0.743 | 0.119 | 0.064 | 0.604 | 0.534 | 0.568 | 1.73 | 1.2 | 0.433 | 0.428 |
| logdha | rs55729342 | 0.065 | 0.734 | 0.112 | 0.043 | 1.49 | 1.1 | 0.59 | 0.733 | 0.398 | 0.567 | -0.423 |
| logdha | rs59317220 | 0.064 | 0.734 | 0.112 | 0.043 | 1.49 | 1.11 | 0.59 | 0.729 | 0.398 | 0.563 | -0.428 |
| logdha | rs3780905 | 0.104 | 0.773 | 0.123 | 0.105 | 1.66 | 0.692 | 0.223 | 0.532 | 0.171 | 0.05 | -0.751 |
| logdha | rs3780906 | 0.714 | 0.65 | 0.235 | 0.234 | 0.94 | 0.254 | 0.819 | 1.07 | 0.229 | 0.762 | 0.062 |
| logdha | rs149047975 | 0.036 | 0.718 | 0.117 | 0.043 | 0.528 | 0.305 | 0.268 | 2.09 | 0.942 | 0.102 | 0.546 |
| logdha | rs3824614 | 0.011 | 0.711 | 0.107 | 0.024 | 0.675 | 0.855 | 0.756 | 1.48 | 1.4 | 0.678 | 0.325 |
| logdha | rs140254404 | 0.003 | . | . | . | . | . | . | . | . | . | . |
| logdha | rs145262684 | 0.014 | 0.788 | 0.129 | 0.145 | 4.58 | 3.83 | 0.069 | 0.404 | 0.28 | 0.191 | -2.91 |
| logdha | rs76735482 | 0.011 | 0.711 | 0.107 | 0.024 | 0.659 | 0.831 | 0.741 | 1.52 | 1.43 | 0.658 | 0.34 |
| logdha | rs73283198 | 0.034 | 0.711 | 0.109 | 0.026 | 1.04 | 0.774 | 0.963 | 1.04 | 0.568 | 0.949 | 0.016 |
| logdha | rs74911239 | 0.035 | 0.72 | 0.118 | 0.045 | 0.171 | 0.167 | 0.07 | 2.57 | 1.94 | 0.212 | 0.425 |
| logdha | rs150551647 | 0.006 | . | . | . | . | . | . | . | . | . | . |
| logdha | rs115200794 | 0.052 | 0.786 | 0.124 | 0.127 | 1.99 | 2.09 | 0.512 | 0.514 | 0.409 | 0.402 | -0.974 |
| logdha | rs74130823 | 0.168 | 0.724 | 0.124 | 0.059 | 0.992 | 0.408 | 0.985 | 0.955 | 0.302 | 0.885 | -0.03 |
| logdha | rs2279435 | 0.62 | 0.689 | 0.209 | 0.219 | 0.976 | 0.255 | 0.927 | 1.03 | 0.208 | 0.886 | 0.027 |
| logdha | rs147279673 | 0.014 | 0.758 | 0.118 | 0.076 | 13.5 | 14.9 | 0.018 | 0.178 | 0.17 | 0.07 | -11.4 |
| logdha | rs183525146 | 0.004 | . | . | . | . | . | . | . | . | . | . |
| logdha | rs3740107 | 0.745 | 0.834 | 0.308 | 0.622 | 1.1 | 0.311 | 0.736 | 0.9 | 0.196 | 0.629 | -0.108 |
| logdha | rs140697247 | 0.001 | . | . | . | . | . | . | . | . | . | . |
| logdha | rs1565096 | 0.81 | 0.846 | 0.325 | 0.664 | 1.06 | 0.301 | 0.847 | 0.895 | 0.201 | 0.62 | -0.103 |
| logdha | rs1565097 | 0.768 | 0.691 | 0.266 | 0.336 | 0.942 | 0.271 | 0.836 | 1.02 | 0.229 | 0.932 | 0.031 |
| logdha | rs144584548 | 0.02 | 0.764 | 0.123 | 0.094 | 1.49 | 1.19 | 0.617 | 0.731 | 0.49 | 0.64 | -0.422 |
| logdha | rs75811053 | 0.011 | 0.71 | 0.107 | 0.024 | 0.655 | 0.803 | 0.73 | 1.62 | 1.47 | 0.595 | 0.388 |
| logdha | rs147462967 | 0.01 | . | . | . | . | . | . | . | . | . | . |
| logdha | rs74130824 | 0.049 | 0.735 | 0.111 | 0.042 | 1.65 | 1.54 | 0.59 | 0.629 | 0.439 | 0.507 | -0.623 |

| logdha | rs116883184 | 0.018 | 0.791 | 0.131 | 0.155 | 4.41 | 3.4 | 0.054 | 0.463 | 0.304 | 0.24 | -2.59 |
| --- | --- | --- | --- | --- | --- | --- | --- | --- | --- | --- | --- | --- |
| logdha | rs117258049 | 0.018 | 0.791 | 0.131 | 0.155 | 4.41 | 3.4 | 0.054 | 0.463 | 0.304 | 0.24 | -2.59 |
| logdha | rs3780908 | 0.128 | 0.694 | 0.116 | 0.029 | 0.842 | 0.341 | 0.671 | 1.11 | 0.336 | 0.737 | 0.111 |
| logdha | rs180938522 | 0.002 | . | . | . | . | . | . | . | . | . | . |
| logdha | rs76687372 | 0.008 | . | . | . | . | . | . | . | . | . | . |
| logdha | rs3780909 | 0.542 | 0.961 | 0.246 | 0.876 | 1.32 | 0.309 | 0.237 | 0.751 | 0.136 | 0.114 | -0.328 |
| logdha | rs78281156 | 0.011 | 0.71 | 0.107 | 0.024 | 0.655 | 0.804 | 0.73 | 1.62 | 1.47 | 0.595 | 0.388 |
| logdha | rs187840631 | 0.002 | . | . | . | . | . | . | . | . | . | . |
| logdha | rs76868242 | 0.011 | 0.71 | 0.107 | 0.024 | 0.654 | 0.802 | 0.729 | 1.62 | 1.47 | 0.594 | 0.389 |
| logdha | rs192794192 | 0.001 | . | . | . | . | . | . | . | . | . | . |
| logdha | rs12781248 | 0.01 | 0.74 | 0.117 | 0.056 | 0.207 | 0.281 | 0.246 | 2.92 | 3.04 | 0.303 | 0.5 |
| logdha | rs12762375 | 0.01 | . | . | . | . | . | . | . | . | . | . |
| logdha | rs192256986 | 0.006 | . | . | . | . | . | . | . | . | . | . |
| logdha | rs184411030 | 0.012 | 0.765 | 0.12 | 0.087 | 3 | 4.67 | 0.481 | 0.289 | 0.378 | 0.342 | -2.1 |
| logdha | rs189601483 | 0.011 | 0.709 | 0.107 | 0.023 | 0.612 | 0.74 | 0.685 | 1.75 | 1.54 | 0.524 | 0.44 |
| logdha | rs143254579 | 0.037 | 0.68 | 0.108 | 0.015 | 0.634 | 0.348 | 0.406 | 1.78 | 0.761 | 0.177 | 0.454 |
| logdha | rs12253903 | 0.028 | 0.713 | 0.112 | 0.031 | 0.062 | 0.093 | 0.063 | 6.15 | 5.99 | 0.062 | 0.498 |
| logdha | rs35489926 | 0.009 | . | . | . | . | . | . | . | . | . | . |
| logdha | rs71494783 | 0.048 | 0.672 | 0.103 | 9.30E-03 | 0.331 | 0.25 | 0.143 | 2.25 | 1.21 | 0.13 | 0.498 |
| logdha | rs180711602 | 0.003 | . | . | . | . | . | . | . | . | . | . |
| logdha | rs117312402 | 0.011 | 0.71 | 0.107 | 0.024 | 0.65 | 0.799 | 0.726 | 1.63 | 1.48 | 0.591 | 0.391 |
| logdha | rs74831694 | 0.008 | . | . | . | . | . | . | . | . | . | . |
| logdha | rs140301943 | 0.003 | . | . | . | . | . | . | . | . | . | . |
| logdha | rs78553679 | 0.016 | 0.706 | 0.116 | 0.033 | 0.086 | 0.132 | 0.11 | 7.43 | 7.95 | 0.061 | 0.66 |
| logdha | rs78334250 | 0.011 | 0.71 | 0.107 | 0.024 | 0.65 | 0.798 | 0.726 | 1.63 | 1.48 | 0.591 | 0.392 |
| logdha | rs76000028 | 0.011 | 0.71 | 0.107 | 0.024 | 0.65 | 0.798 | 0.726 | 1.63 | 1.48 | 0.591 | 0.392 |
| logdha | rs145195254 | 0.002 | . | . | . | . | . | . | . | . | . | . |
| logdha | rs137888527 | 0.002 | . | . | . | . | . | . | . | . | . | . |
| logdha | rs149109592 | 0.005 | . | . | . | . | . | . | . | . | . | . |
| logdha | rs80280010 | 0.035 | 0.677 | 0.108 | 0.014 | 0.621 | 0.339 | 0.383 | 1.88 | 0.799 | 0.139 | 0.492 |
| logdha | rs189995374 | 0.002 | . | . | . | . | . | . | . | . | . | . |
| logdha | rs75467770 | 0.012 | 0.71 | 0.107 | 0.023 | 0.644 | 0.818 | 0.729 | 1.52 | 1.43 | 0.66 | 0.339 |
| logdha | rs1487562 | 0.167 | 0.744 | 0.128 | 0.086 | 1.08 | 0.427 | 0.847 | 0.884 | 0.273 | 0.691 | -0.113 |
| logdha | rs12266963 | 0.048 | 0.672 | 0.103 | 9.20E-03 | 0.325 | 0.247 | 0.139 | 2.27 | 1.22 | 0.127 | 0.5 |
| logdha | rs7099874 | 0.765 | 1.09 | 0.372 | 0.808 | 1.22 | 0.336 | 0.467 | 0.746 | 0.161 | 0.174 | -0.318 |
| logdha | rs75687019 | 0.011 | 0.71 | 0.107 | 0.024 | 0.648 | 0.796 | 0.724 | 1.63 | 1.48 | 0.59 | 0.393 |
| logdha | rs148240462 | 0.038 | 0.738 | 0.122 | 0.066 | 0.314 | 0.286 | 0.203 | 1.67 | 1.2 | 0.473 | 0.336 |
| logdha | rs141278174 | 0.002 | . | . | . | . | . | . | . | . | . | . |
| logdha | rs76404852 | 0.011 | 0.71 | 0.107 | 0.024 | 0.647 | 0.795 | 0.723 | 1.63 | 1.48 | 0.589 | 0.393 |
| logdha | rs139112240 | 0.008 | . | . | . | . | . | . | . | . | . | . |
| logdha | rs12783037 | 0.054 | 0.671 | 0.103 | 9.10E-03 | 0.342 | 0.244 | 0.132 | 2.13 | 1.08 | 0.134 | 0.476 |
| logdha | rs72796481 | 0.009 | . | . | . | . | . | . | . | . | . | . |
| logdha | rs76798749 | 0.017 | 0.71 | 0.107 | 0.023 | 0.714 | 0.75 | 0.749 | 1.3 | 0.982 | 0.73 | 0.234 |
| logdha | rs187732548 | 0.001 | . | . | . | . | . | . | . | . | . | . |
| logdha | rs75675474 | 0.017 | 0.71 | 0.107 | 0.023 | 0.714 | 0.75 | 0.749 | 1.3 | 0.982 | 0.73 | 0.234 |
| logdha | rs76552293 | 0.017 | 0.71 | 0.107 | 0.024 | 0.715 | 0.751 | 0.75 | 1.3 | 0.981 | 0.731 | 0.233 |
| logdha | rs147445993 | 0.005 | . | . | . | . | . | . | . | . | . | . |
| logdha | rs115902283 | 0.017 | 0.71 | 0.107 | 0.023 | 0.706 | 0.743 | 0.741 | 1.31 | 0.99 | 0.723 | 0.24 |
| logdha | rs4540799 | 0.763 | 1.1 | 0.375 | 0.786 | 1.23 | 0.338 | 0.452 | 0.74 | 0.159 | 0.162 | -0.328 |
| logdha | rs12765320 | 0.211 | 0.69 | 0.117 | 0.029 | 0.861 | 0.293 | 0.66 | 1.09 | 0.285 | 0.738 | 0.097 |
| logdha | rs12782256 | 0.009 | . | . | . | . | . | . | . | . | . | . |
| logdha | rs191727256 | 0.002 | . | . | . | . | . | . | . | . | . | . |
| logdha | rs2082111 | 0.123 | 0.725 | 0.122 | 0.056 | 1.06 | 0.411 | 0.874 | 0.942 | 0.281 | 0.842 | -0.062 |
| logdha | rs2082110 | 0.175 | 0.746 | 0.127 | 0.084 | 1.14 | 0.423 | 0.728 | 0.875 | 0.254 | 0.646 | -0.141 |
| logdha | rs2082109 | 0.175 | 0.746 | 0.127 | 0.084 | 1.14 | 0.423 | 0.728 | 0.875 | 0.254 | 0.646 | -0.141 |
| logdha | rs117316662 | 0.011 | 0.71 | 0.107 | 0.024 | 0.644 | 0.792 | 0.72 | 1.64 | 1.49 | 0.586 | 0.396 |
| logdha | rs12247163 | 0.214 | 0.694 | 0.118 | 0.031 | 0.896 | 0.307 | 0.748 | 1.07 | 0.28 | 0.79 | 0.077 |
| logdha | rs12355888 | 0.082 | 0.694 | 0.115 | 0.027 | 0.844 | 0.378 | 0.705 | 1.15 | 0.404 | 0.686 | 0.137 |
| logdha | rs143772290 | 0.011 | 0.75 | 0.118 | 0.068 | 0.685 | 1.18 | 0.826 | 1.54 | 1.86 | 0.72 | 0.356 |
| logdha | rs146849452 | 0.005 | . | . | . | . | . | . | . | . | . | . |
| logdha | rs140267121 | 0.004 | . | . | . | . | . | . | . | . | . | . |
| logdha | rs7080474 | 0.202 | 0.701 | 0.119 | 0.037 | 0.92 | 0.322 | 0.812 | 1.05 | 0.281 | 0.86 | 0.055 |
| logdha | rs144066203 | 0.002 | . | . | . | . | . | . | . | . | . | . |
| logdha | rs12765209 | 0.012 | 0.711 | 0.108 | 0.024 | 0.738 | 0.825 | 0.786 | 1.32 | 1.09 | 0.735 | 0.245 |
| logdha | rs150930735 | 0.002 | . | . | . | . | . | . | . | . | . | . |
| logdha | rs7068039 | 0.216 | 0.698 | 0.119 | 0.035 | 0.911 | 0.311 | 0.785 | 1.06 | 0.275 | 0.834 | 0.063 |
| logdha | rs117622652 | 0.011 | 0.71 | 0.107 | 0.024 | 0.644 | 0.792 | 0.72 | 1.64 | 1.49 | 0.585 | 0.396 |
| logdha | rs190191514 | 0.001 | . | . | . | . | . | . | . | . | . | . |
| logdha | rs74130835 | 0.04 | 0.778 | 0.123 | 0.111 | 2.05 | 2.79 | 0.598 | 0.531 | 0.532 | 0.527 | -0.981 |
| logdha | rs1811604 | 0.039 | 0.789 | 0.125 | 0.133 | 3.06 | 3.6 | 0.342 | 0.426 | 0.375 | 0.332 | -1.82 |
| logdha | rs146971256 | 0 | . | . | . | . | . | . | . | . | . | . |
| logdha | rs74336000 | 0.011 | 0.75 | 0.118 | 0.068 | 0.688 | 1.18 | 0.827 | 1.54 | 1.85 | 0.718 | 0.358 |
| logdha | rs186046492 | 0.007 | . | . | . | . | . | . | . | . | . | . |
| logdha | rs34971823 | 0.011 | 0.74 | 0.117 | 0.056 | 0.19 | 0.217 | 0.147 | 2.71 | 2.26 | 0.231 | 0.451 |
| logdha | rs75227446 | 0.011 | 0.71 | 0.107 | 0.024 | 0.647 | 0.797 | 0.723 | 1.63 | 1.48 | 0.592 | 0.392 |
| logdha | rs190457599 | 0.001 | . | . | . | . | . | . | . | . | . | . |
| logdha | rs79591917 | 0.011 | 0.71 | 0.107 | 0.024 | 0.643 | 0.791 | 0.72 | 1.64 | 1.49 | 0.585 | 0.397 |
| logdha | rs702365 | 0.545 | 0.938 | 0.241 | 0.804 | 1.25 | 0.296 | 0.35 | 0.766 | 0.14 | 0.145 | -0.289 |
| logdha | rs79643836 | 0.011 | 0.71 | 0.107 | 0.024 | 0.66 | 0.821 | 0.739 | 1.52 | 1.42 | 0.651 | 0.345 |
| logdha | rs702366 | 0.546 | 0.938 | 0.239 | 0.802 | 1.24 | 0.292 | 0.36 | 0.766 | 0.139 | 0.143 | -0.287 |
| logdha | rs1059696 | 0.151 | 0.702 | 0.118 | 0.036 | 0.93 | 0.363 | 0.852 | 1.05 | 0.309 | 0.862 | 0.055 |
| logdha | rs901681 | 0.547 | 0.94 | 0.239 | 0.806 | 1.23 | 0.291 | 0.372 | 0.764 | 0.139 | 0.138 | -0.288 |
| logdha | rs61854092 | 0.15 | 0.703 | 0.118 | 0.037 | 0.934 | 0.365 | 0.861 | 1.05 | 0.308 | 0.872 | 0.051 |
| logdha | rs148080753 | 0.001 | . | . | . | . | . | . | . | . | . | . |
| logdha | rs61854093 | 0.186 | 0.732 | 0.124 | 0.067 | 1.05 | 0.402 | 0.889 | 0.931 | 0.273 | 0.808 | -0.068 |
| logdha | rs141822774 | 0.001 | . | . | . | . | . | . | . | . | . | . |

| logdha | rs74130836 | 0.04 | 0.777 | 0.123 | 0.111 | 2.03 | 2.77 | 0.603 | 0.534 | 0.536 | 0.532 | -0.966 |
| --- | --- | --- | --- | --- | --- | --- | --- | --- | --- | --- | --- | --- |
| logdha | rs150620963 | 0.013 | 0.699 | 0.112 | 0.025 | 0.05 | 0.079 | 0.057 | 10.6 | 10.9 | 0.022 | 0.624 |
| logdha | rs3780913 | 0.753 | 1.2 | 0.399 | 0.59 | 1.35 | 0.367 | 0.268 | 0.695 | 0.148 | 0.087 | -0.425 |
| logdha | rs142255723 | 0.002 | . | . | . | . | . | . | . | . | . | . |
| logdha | rs3780914 | 0.542 | 0.942 | 0.241 | 0.815 | 1.26 | 0.298 | 0.327 | 0.763 | 0.139 | 0.137 | -0.296 |
| logdha | rs182656507 | 0.002 | . | . | . | . | . | . | . | . | . | . |
| logdha | rs2291427 | 0.735 | 1.29 | 0.428 | 0.446 | 1.42 | 0.381 | 0.197 | 0.656 | 0.139 | 0.047 | -0.508 |
| logdha | rs10751382 | 0.733 | 1.26 | 0.41 | 0.47 | 1.41 | 0.373 | 0.193 | 0.664 | 0.138 | 0.049 | -0.491 |
| logdha | rs117610108 | 0.01 | 0.71 | 0.107 | 0.024 | 0.642 | 0.79 | 0.719 | 1.65 | 1.49 | 0.582 | 0.399 |
| logdha | rs144966941 | 0.023 | 0.726 | 0.113 | 0.04 | 1.72 | 1.14 | 0.413 | 0.784 | 0.432 | 0.659 | -0.466 |
| logdha | rs41502051 | 0.03 | 0.688 | 0.107 | 0.016 | 0.879 | 0.613 | 0.854 | 1.5 | 0.783 | 0.441 | 0.338 |
| logdha | rs41426046 | 0.011 | 0.71 | 0.107 | 0.023 | 0.625 | 0.761 | 0.699 | 1.71 | 1.52 | 0.547 | 0.423 |
| logdha | rs41519248 | 0.01 | 0.71 | 0.107 | 0.024 | 0.642 | 0.79 | 0.719 | 1.65 | 1.49 | 0.582 | 0.399 |
| logdha | rs189108245 | 0.004 | . | . | . | . | . | . | . | . | . | . |
| logdha | rs7896431 | 0.532 | 0.93 | 0.234 | 0.773 | 1.23 | 0.293 | 0.382 | 0.762 | 0.14 | 0.138 | -0.288 |
| logdha | rs183724547 | 0.003 | . | . | . | . | . | . | . | . | . | . |
| logdha | rs142044990 | 0.008 | . | . | . | . | . | . | . | . | . | . |
| logdha | rs188140929 | 0.007 | . | . | . | . | . | . | . | . | . | . |
| logdha | rs76819096 | 0.014 | 0.734 | 0.115 | 0.049 | 0.016 | 0.039 | 0.097 | 10.1 | 15.4 | 0.132 | 0.366 |
| logdha | rs10751383 | 0.602 | 0.852 | 0.246 | 0.578 | 1.15 | 0.285 | 0.585 | 0.86 | 0.163 | 0.426 | -0.159 |
| logdha | rs117819321 | 0.014 | 0.786 | 0.128 | 0.138 | 4.82 | 6.6 | 0.25 | 0.14 | 0.17 | 0.106 | -4.08 |
| logdha | rs2242332 | 0.702 | 1.01 | 0.323 | 0.977 | 1.22 | 0.313 | 0.432 | 0.773 | 0.156 | 0.2 | -0.278 |
| logdha | rs2242333 | 0.096 | 0.798 | 0.126 | 0.153 | 2.37 | 0.997 | 0.04 | 0.392 | 0.132 | 5.40E-03 | -1.43 |
| logdha | rs2242334 | 0.704 | 1.01 | 0.323 | 0.978 | 1.22 | 0.312 | 0.443 | 0.773 | 0.156 | 0.201 | -0.277 |
| logdha | rs189465041 | 0.001 | . | . | . | . | . | . | . | . | . | . |
| logdha | rs28395868 | 0.025 | 0.763 | 0.124 | 0.095 | 1.42 | 1.21 | 0.68 | 0.829 | 0.597 | 0.794 | -0.285 |
| logdha | rs56067550 | 0.02 | 0.762 | 0.122 | 0.091 | 1.39 | 1.13 | 0.682 | 0.781 | 0.523 | 0.712 | -0.327 |
| logdha | rs1051713 | 0.154 | 0.717 | 0.121 | 0.048 | 1.02 | 0.383 | 0.964 | 0.985 | 0.279 | 0.958 | -0.016 |
| logdha | rs115074210 | 0.016 | 0.735 | 0.115 | 0.05 | 0.014 | 0.037 | 0.112 | 9.5 | 15.7 | 0.172 | 0.346 |
| logdha | rs7900977 | 0.802 | 0.879 | 0.333 | 0.733 | 1.05 | 0.296 | 0.851 | 0.871 | 0.192 | 0.531 | -0.126 |
| logdha | rs147613891 | 0.003 | . | . | . | . | . | . | . | . | . | . |
| logdha | rs2229136 | 0.084 | 0.634 | 0.101 | 4.30E-03 | 0.415 | 0.187 | 0.051 | 2.05 | 0.669 | 0.027 | 0.492 |
| logdha | rs185482841 | 0.003 | . | . | . | . | . | . | . | . | . | . |
| logdha | rs2288619 | 0.083 | 0.633 | 0.101 | 4.10E-03 | 0.407 | 0.185 | 0.048 | 2.11 | 0.693 | 0.023 | 0.504 |
| logdha | rs189891524 | 0.001 | . | . | . | . | . | . | . | . | . | . |
| logdha | rs56145077 | 0.143 | 0.709 | 0.119 | 0.041 | 0.95 | 0.363 | 0.894 | 1.02 | 0.297 | 0.939 | 0.03 |
| logdha | rs143216970 | 0.005 | . | . | . | . | . | . | . | . | . | . |
| logdha | rs142246478 | 0.001 | . | . | . | . | . | . | . | . | . | . |
| logdha | rs13134 | 0.012 | 0.724 | 0.114 | 0.041 | 0.106 | 0.152 | 0.117 | 5.95 | 6.1 | 0.082 | 0.628 |
| logdha | rs41314980 | 0.027 | 0.739 | 0.121 | 0.064 | 0.742 | 0.582 | 0.704 | 1.55 | 1.01 | 0.503 | 0.368 |
| logdha | rs41304611 | 0.013 | 0.729 | 0.116 | 0.048 | 0.036 | 0.117 | 0.308 | 6.67 | 16 | 0.43 | 0.409 |
| logdha | rs193299617 | 0.001 | . | . | . | . | . | . | . | . | . | . |
| logdha | rs12772724 | 0.012 | 0.724 | 0.114 | 0.041 | 0.106 | 0.152 | 0.117 | 5.96 | 6.11 | 0.082 | 0.627 |
| logdha | rs139489537 | 0.033 | 0.672 | 0.107 | 0.012 | 0.54 | 0.301 | 0.269 | 2.1 | 0.907 | 0.087 | 0.548 |
| logdha | rs12776062 | 0.009 | . | . | . | . | . | . | . | . | . | . |
| logdha | rs752059 | 0.144 | 0.711 | 0.119 | 0.042 | 0.948 | 0.362 | 0.889 | 1.02 | 0.295 | 0.956 | 0.026 |
| logdha | rs147649982 | 0.03 | 0.711 | 0.114 | 0.033 | 0.73 | 0.46 | 0.617 | 0.889 | 0.464 | 0.822 | 0.021 |
| logdha | rs17157798 | 0.07 | 0.634 | 0.1 | 4.00E-03 | 0.356 | 0.188 | 0.05 | 2.36 | 0.908 | 0.026 | 0.542 |
| logdha | rs142298619 | 0.003 | . | . | . | . | . | . | . | . | . | . |
| logdha | rs4948673 | 0.793 | 0.849 | 0.313 | 0.656 | 1.04 | 0.286 | 0.895 | 0.89 | 0.193 | 0.591 | -0.102 |
| logdha | rs74231484 | 0.102 | 0.794 | 0.127 | 0.15 | 2.16 | 0.838 | 0.047 | 0.452 | 0.136 | 8.20E-03 | -1.18 |
| logdha | rs72796489 | 0.002 | . | . | . | . | . | . | . | . | . | . |
| logdha | rs113447578 | 0.016 | 0.717 | 0.11 | 0.03 | 0.874 | 0.714 | 0.869 | 0.856 | 0.506 | 0.793 | -0.055 |
| logdha | rs34063374 | 0.013 | 0.723 | 0.114 | 0.04 | 0.094 | 0.136 | 0.104 | 6.37 | 6.61 | 0.075 | 0.615 |
| logdha | rs11239527 | 0.013 | 0.755 | 0.121 | 0.08 | 2.22 | 2.78 | 0.523 | 0.941 | 0.917 | 0.95 | -0.4 |
| logdha | rs34371883 | 0.011 | 0.737 | 0.116 | 0.053 | 0.087 | 0.143 | 0.139 | 4.86 | 5.83 | 0.187 | 0.487 |
| logdha | rs118168828 | 0.007 | . | . | . | . | . | . | . | . | . | . |
| logdha | rs34349618 | 0.009 | . | . | . | . | . | . | . | . | . | . |
| logdha | rs80041679 | 0.033 | 0.672 | 0.106 | 0.012 | 0.538 | 0.3 | 0.266 | 2.1 | 0.907 | 0.087 | 0.548 |
| logdha | rs149640607 | 0.003 | . | . | . | . | . | . | . | . | . | . |
| logdha | rs4427523 | 0.888 | 0.861 | 0.438 | 0.769 | 0.739 | 0.284 | 0.432 | 0.891 | 0.265 | 0.699 | -0.033 |
| logdha | rs185880109 | 0.002 | . | . | . | . | . | . | . | . | . | . |
| logdha | rs191523220 | 0.003 | . | . | . | . | . | . | . | . | . | . |
| logdha | rs144315284 | 0.006 | . | . | . | . | . | . | . | . | . | . |
| logdha | rs4948674 | 0.341 | 0.757 | 0.163 | 0.195 | 1.02 | 0.251 | 0.95 | 0.916 | 0.181 | 0.656 | -0.069 |
| logdha | rs182984298 | 0.006 | . | . | . | . | . | . | . | . | . | . |
| logdha | rs56749023 | 0.025 | 0.726 | 0.113 | 0.04 | 0.022 | 0.055 | 0.122 | 8.98 | 14 | 0.158 | 0.397 |
| logdha | rs112508957 | 0.015 | 0.768 | 0.12 | 0.092 | 3.42 | 4.76 | 0.376 | 0.301 | 0.33 | 0.273 | -2.4 |
| logdha | rs7089063 | 0.229 | 0.615 | 0.111 | 6.90E-03 | 0.666 | 0.205 | 0.186 | 1.39 | 0.331 | 0.167 | 0.288 |
| logdha | rs145000779 | 0.001 | . | . | . | . | . | . | . | . | . | . |
| logdha | rs80201572 | 0.034 | 0.672 | 0.107 | 0.012 | 0.533 | 0.298 | 0.261 | 2.1 | 0.909 | 0.088 | 0.546 |
| logdha | rs140568227 | 0.005 | . | . | . | . | . | . | . | . | . | . |
| logdha | rs149756228 | 0.01 | . | . | . | . | . | . | . | . | . | . |
| logdha | rs112380685 | 0.007 | . | . | . | . | . | . | . | . | . | . |
| logdha | rs60481109 | 0.023 | 0.724 | 0.113 | 0.038 | 0.021 | 0.051 | 0.116 | 9.68 | 15.1 | 0.146 | 0.401 |
| logdha | rs138221843 | 0.003 | . | . | . | . | . | . | . | . | . | . |
| logdha | rs150283194 | 0.003 | . | . | . | . | . | . | . | . | . | . |
| logdha | rs138879661 | 0.004 | . | . | . | . | . | . | . | . | . | . |
| logdha | rs34960345 | 0.056 | 0.645 | 0.104 | 6.60E-03 | 0.478 | 0.222 | 0.112 | 2.14 | 0.752 | 0.03 | 0.538 |
| logdha | rs147514068 | 0.004 | . | . | . | . | . | . | . | . | . | . |
| logdha | rs35410535 | 0.078 | 0.634 | 0.101 | 4.20E-03 | 0.397 | 0.184 | 0.046 | 2.14 | 0.714 | 0.023 | 0.508 |
| logdha | rs191613635 | 0.003 | . | . | . | . | . | . | . | . | . | . |
| logdha | rs17157800 | 0.007 | . | . | . | . | . | . | . | . | . | . |
| logdha | rs17523413 | 0.045 | 0.647 | 0.103 | 6.20E-03 | 0.393 | 0.213 | 0.084 | 2.54 | 1.04 | 0.024 | 0.605 |
| logdha | rs7897036 | 0.035 | 0.694 | 0.107 | 0.018 | 0.498 | 0.38 | 0.361 | 1.52 | 0.879 | 0.473 | 0.332 |
| logdha | rs76334323 | 0.01 | 0.71 | 0.107 | 0.024 | 0.635 | 0.781 | 0.712 | 1.66 | 1.51 | 0.575 | 0.404 |

| logdha | rs143535991 | 0.001 | . | . | . | . | . | . | . | . | . | . |
| --- | --- | --- | --- | --- | --- | --- | --- | --- | --- | --- | --- | --- |
| logdha | rs140344979 | 0.005 | . | . | . | . | . | . | . | . | . | . |
| logdha | rs36047811 | 0.078 | 0.634 | 0.101 | 4.20E-03 | 0.398 | 0.184 | 0.046 | 2.14 | 0.713 | 0.023 | 0.507 |
| logdha | rs76493570 | 0.054 | 0.642 | 0.103 | 6.00E-03 | 0.451 | 0.215 | 0.095 | 2.26 | 0.815 | 0.024 | 0.562 |
| logdha | rs192998920 | 0.003 | . | . | . | . | . | . | . | . | . | . |
| logdha | rs116840852 | 0.016 | 0.707 | 0.116 | 0.035 | 0.09 | 0.138 | 0.117 | 6.95 | 7.46 | 0.071 | 0.644 |
| logdha | rs184824101 | 0.001 | . | . | . | . | . | . | . | . | . | . |
| logdha | rs41301621 | 0.005 | . | . | . | . | . | . | . | . | . | . |
| logdha | rs3740109 | 0.078 | 0.635 | 0.101 | 4.30E-03 | 0.4 | 0.185 | 0.048 | 2.13 | 0.709 | 0.024 | 0.505 |
| logdha | rs7899415 | 0.033 | 0.709 | 0.113 | 0.03 | 0.631 | 0.861 | 0.736 | 1.11 | 0.978 | 0.904 | 0.158 |
| logdha | rs184395284 | 0.002 | . | . | . | . | . | . | . | . | . | . |
| logdha | rs139209589 | 0.004 | . | . | . | . | . | . | . | . | . | . |
| logdha | rs149924163 | 0.003 | . | . | . | . | . | . | . | . | . | . |
| logdha | rs3740110 | 0.01 | 0.71 | 0.107 | 0.024 | 0.636 | 0.783 | 0.713 | 1.66 | 1.51 | 0.577 | 0.403 |
| logdha | rs144335113 | 0.003 | . | . | . | . | . | . | . | . | . | . |
| logdha | rs74743385 | 0.015 | 0.768 | 0.12 | 0.092 | 3.42 | 4.76 | 0.376 | 0.301 | 0.33 | 0.273 | -2.4 |
| logdha | rs146545829 | 0.003 | . | . | . | . | . | . | . | . | . | . |
| logdha | rs181842689 | 0 | . | . | . | . | . | . | . | . | . | . |
| logdha | rs3740111 | 0.056 | 0.645 | 0.104 | 6.70E-03 | 0.481 | 0.224 | 0.115 | 2.13 | 0.747 | 0.031 | 0.535 |
| logdha | rs186261599 | 0.003 | . | . | . | . | . | . | . | . | . | . |
| logdha | rs73285348 | 0.009 | . | . | . | . | . | . | . | . | . | . |
| logdha | rs186356901 | 0.003 | . | . | . | . | . | . | . | . | . | . |
| logdha | rs3824618 | 0.033 | 0.672 | 0.107 | 0.012 | 0.539 | 0.3 | 0.267 | 2.09 | 0.903 | 0.087 | 0.547 |
| logdha | rs3802548 | 0.219 | 0.701 | 0.128 | 0.052 | 1.01 | 0.283 | 0.959 | 1.04 | 0.23 | 0.851 | 0.026 |
| logdha | rs3802547 | 0.01 | . | . | . | . | . | . | . | . | . | . |
| logdha | rs77677521 | 0.016 | 0.707 | 0.116 | 0.035 | 0.09 | 0.138 | 0.117 | 6.94 | 7.45 | 0.071 | 0.644 |
| logdha | rs3824616 | 0.078 | 0.636 | 0.101 | 4.40E-03 | 0.404 | 0.187 | 0.05 | 2.11 | 0.704 | 0.025 | 0.502 |
| logdha | rs191073818 | 0.003 | . | . | . | . | . | . | . | . | . | . |
| logdha | rs147078004 | 0.002 | . | . | . | . | . | . | . | . | . | . |
| logdha | rs7908745 | 0.31 | 0.599 | 0.119 | 9.80E-03 | 0.776 | 0.199 | 0.324 | 1.31 | 0.267 | 0.183 | 0.234 |
| logdha | rs17157811 | 0.007 | . | . | . | . | . | . | . | . | . | . |
| logdha | rs138506087 | 0.006 | . | . | . | . | . | . | . | . | . | . |
| logdha | rs77241269 | 0.025 | 0.728 | 0.113 | 0.041 | 0.031 | 0.073 | 0.143 | 7.34 | 11.2 | 0.191 | 0.405 |
| logdha | rs138374938 | 0.001 | . | . | . | . | . | . | . | . | . | . |
| logdha | rs2279433 | 0.056 | 0.644 | 0.104 | 6.50E-03 | 0.473 | 0.217 | 0.103 | 2.14 | 0.737 | 0.028 | 0.534 |
| logdha | rs73285358 | 0.025 | 0.728 | 0.113 | 0.042 | 0.031 | 0.074 | 0.144 | 7.27 | 11.1 | 0.193 | 0.406 |
| logdha | rs11239528 | 0.219 | 0.706 | 0.129 | 0.057 | 1.04 | 0.288 | 0.901 | 1.03 | 0.227 | 0.899 | 0.01 |
| logdha | rs77453386 | 0.01 | 0.71 | 0.107 | 0.023 | 0.615 | 0.766 | 0.696 | 1.69 | 1.55 | 0.566 | 0.413 |
| logdha | rs2279434 | 0.061 | 0.649 | 0.106 | 8.00E-03 | 0.549 | 0.241 | 0.171 | 1.89 | 0.63 | 0.056 | 0.475 |
| logdha | rs149236525 | 0.005 | . | . | . | . | . | . | . | . | . | . |
| logdha | rs12573609 | 0.05 | 0.663 | 0.105 | 9.60E-03 | 0.746 | 0.445 | 0.622 | 1.85 | 0.779 | 0.144 | 0.507 |
| logdha | rs191716655 | 0.002 | . | . | . | . | . | . | . | . | . | . |
| logdha | rs17523660 | 0.009 | . | . | . | . | . | . | . | . | . | . |
| logdha | rs142671360 | 0.003 | . | . | . | . | . | . | . | . | . | . |
| logdha | rs3764990 | 0.044 | 0.644 | 0.102 | 5.70E-03 | 0.362 | 0.199 | 0.065 | 2.67 | 1.11 | 0.018 | 0.617 |
| logdha | rs113682861 | 0.004 | . | . | . | . | . | . | . | . | . | . |
| logdha | rs117658270 | 0.01 | 0.71 | 0.107 | 0.023 | 0.615 | 0.766 | 0.696 | 1.69 | 1.55 | 0.566 | 0.413 |
| logdha | rs187309063 | 0 | . | . | . | . | . | . | . | . | . | . |
| logdha | rs73285360 | 0.025 | 0.728 | 0.113 | 0.042 | 0.031 | 0.075 | 0.145 | 7.23 | 11 | 0.194 | 0.406 |
| logdha | rs80133289 | 0.015 | 0.73 | 0.112 | 0.04 | 4.42 | 4.93 | 0.183 | 0.231 | 0.22 | 0.124 | -3.4 |
| logdha | rs186693257 | 0.001 | . | . | . | . | . | . | . | . | . | . |
| logdha | rs35735968 | 0.062 | 0.642 | 0.104 | 6.00E-03 | 0.455 | 0.21 | 0.089 | 2.12 | 0.722 | 0.028 | 0.521 |
| logdha | rs17157825 | 0.034 | 0.668 | 0.106 | 0.011 | 0.478 | 0.271 | 0.194 | 2.25 | 0.991 | 0.065 | 0.573 |
| logdha | rs2291428 | 0.213 | 0.698 | 0.127 | 0.049 | 1.03 | 0.289 | 0.921 | 1.05 | 0.235 | 0.818 | 0.029 |
| logdha | rs2291429 | 0.222 | 0.703 | 0.13 | 0.056 | 1.03 | 0.288 | 0.905 | 1.04 | 0.23 | 0.876 | 0.016 |
| logdha | rs145714792 | 0.005 | . | . | . | . | . | . | . | . | . | . |
| logdha | rs12769244 | 0.056 | 0.645 | 0.104 | 6.50E-03 | 0.472 | 0.217 | 0.102 | 2.13 | 0.733 | 0.027 | 0.533 |
| logdha | rs12771275 | 0.056 | 0.645 | 0.104 | 6.50E-03 | 0.472 | 0.217 | 0.102 | 2.13 | 0.733 | 0.027 | 0.533 |
| logdha | rs146266174 | 0.001 | . | . | . | . | . | . | . | . | . | . |
| logdha | rs41301623 | 0.003 | . | . | . | . | . | . | . | . | . | . |
| logdha | rs2306814 | 0.011 | 0.71 | 0.107 | 0.023 | 0.613 | 0.761 | 0.694 | 1.64 | 1.51 | 0.592 | 0.389 |
| logdha | rs12244834 | 0.027 | 0.714 | 0.107 | 0.025 | 1.14 | 1.45 | 0.92 | 0.988 | 0.899 | 0.99 | -0.049 |
| logdha | rs184006011 | 0.002 | . | . | . | . | . | . | . | . | . | . |
| logdha | rs78829066 | 0.01 | 0.71 | 0.107 | 0.023 | 0.606 | 0.761 | 0.69 | 1.71 | 1.58 | 0.561 | 0.419 |
| logdha | rs34731408 | 0.056 | 0.645 | 0.104 | 6.50E-03 | 0.472 | 0.217 | 0.102 | 2.14 | 0.734 | 0.027 | 0.533 |
| logdha | rs17451516 | 0.012 | 0.725 | 0.115 | 0.042 | 0.113 | 0.161 | 0.127 | 5.62 | 5.8 | 0.094 | 0.622 |
| logdha | rs139910133 | 0.003 | . | . | . | . | . | . | . | . | . | . |
| logdha | rs10900218 | 0.233 | 0.62 | 0.111 | 7.50E-03 | 0.705 | 0.21 | 0.242 | 1.35 | 0.311 | 0.186 | 0.267 |
| logdha | rs146023999 | 0.009 | . | . | . | . | . | . | . | . | . | . |
| logdha | rs187296958 | 0.003 | . | . | . | . | . | . | . | . | . | . |
| logdha | rs142342833 | 0.015 | 0.339 | 0.187 | 0.049 | 4.80E+04 | 5.30E+05 | 0.325 | 7.00E-06 | 6.60E-05 | 0.206 | ####### |
| logdha | rs143489255 | 0.006 | . | . | . | . | . | . | . | . | . | . |
| logdha | rs138532518 | 0.006 | . | . | . | . | . | . | . | . | . | . |
| logdha | rs183768079 | 0.005 | . | . | . | . | . | . | . | . | . | . |
| logdha | rs117892272 | 0.012 | 0.288 | 0.167 | 0.032 | 0.811 | 2.15 | 0.937 | 4.87 | 7.82 | 0.325 | 1.04 |
| logdha | rs192993943 | 0.003 | . | . | . | . | . | . | . | . | . | . |
| logdha | rs182989043 | 0.004 | . | . | . | . | . | . | . | . | . | . |
| logdha | rs189703435 | 0.007 | . | . | . | . | . | . | . | . | . | . |
| logdha | rs188794040 | 0.007 | . | . | . | . | . | . | . | . | . | . |
| logdha | rs143920953 | 0.003 | . | . | . | . | . | . | . | . | . | . |
| logdha | rs191029121 | 0.006 | . | . | . | . | . | . | . | . | . | . |
| logdha | rs116875448 | 0.006 | . | . | . | . | . | . | . | . | . | . |
| logdha | rs190167877 | 0.002 | . | . | . | . | . | . | . | . | . | . |
| logdha | rs184993418 | 0.003 | . | . | . | . | . | . | . | . | . | . |
| logdha | rs143241158 | 0.01 | . | . | . | . | . | . | . | . | . | . |
| logdha | rs11239500 | 0.038 | 0.651 | 0.145 | 0.053 | 0.454 | 0.571 | 0.53 | 1.45 | 1.24 | 0.665 | 0.324 |

| logdha | rs140568994 | 0.001 | . | . | . | . | . | . | . | . | . | . |
| --- | --- | --- | --- | --- | --- | --- | --- | --- | --- | --- | --- | --- |
| logdha | rs187530317 | 0.003 | . | . | . | . | . | . | . | . | . | . |
| logdha | rs145189326 | 0.002 | . | . | . | . | . | . | . | . | . | . |
| logdha | rs186203332 | 0.009 | . | . | . | . | . | . | . | . | . | . |
| logdha | rs189921909 | 0.004 | . | . | . | . | . | . | . | . | . | . |
| logdha | rs187260989 | 0.01 | 0.242 | 0.144 | 0.017 | 6.10E-05 | 3.40E-04 | 0.084 | 970 | 3.30E+03 | 0.046 | 0.772 |
| logdha | rs114376586 | 0.037 | 0.654 | 0.145 | 0.056 | 0.471 | 0.605 | 0.558 | 1.38 | 1.21 | 0.712 | 0.301 |
| logdha | rs151070808 | 0.019 | 0.297 | 0.163 | 0.027 | 0.617 | 2.25 | 0.895 | 2.04 | 4.48 | 0.744 | 0.46 |
| logdha | rs140927225 | 0.038 | 0.654 | 0.146 | 0.057 | 0.493 | 0.619 | 0.573 | 1.36 | 1.16 | 0.723 | 0.29 |
| logdha | rs191555615 | 0.012 | 0.268 | 0.151 | 0.019 | 0.144 | 0.454 | 0.539 | 11.6 | 22.4 | 0.205 | 1.03 |
| logdha | rs80278898 | 0.012 | 0.655 | 0.146 | 0.058 | 0.094 | 0.243 | 0.361 | 2.46 | 4.16 | 0.595 | 0.402 |
| logdha | rs190849503 | 0.004 | . | . | . | . | . | . | . | . | . | . |
| logdha | rs150080315 | 0.006 | . | . | . | . | . | . | . | . | . | . |
| logdha | rs143623885 | 0.011 | 0.362 | 0.198 | 0.063 | 5.50E+27 | 2.80E+29 | 0.21 | 3.50E-27 | 1.70E-25 | 0.222 | ####### |
| logdha | rs187760040 | 0.002 | . | . | . | . | . | . | . | . | . | . |
| logdha | rs143917143 | 0.01 | 0.787 | 0.198 | 0.34 | 2.90E-03 | 0.01 | 0.104 | 45.1 | 92.6 | 0.063 | 0.312 |
| logdha | rs149851136 | 0.001 | . | . | . | . | . | . | . | . | . | . |
| logdha | rs181612054 | 0.001 | . | . | . | . | . | . | . | . | . | . |
| logdha | rs191006548 | 0.001 | . | . | . | . | . | . | . | . | . | . |
| logdha | rs117377631 | 0.012 | 0.667 | 0.149 | 0.07 | 0.537 | 0.614 | 0.587 | 0.648 | 0.519 | 0.588 | 0.028 |
| logdha | rs182107634 | 0.002 | . | . | . | . | . | . | . | . | . | . |
| logdha | rs190211867 | 0.002 | . | . | . | . | . | . | . | . | . | . |
| logdha | rs148293457 | 0.031 | 0.393 | 0.232 | 0.113 | 405 | 1.40E+03 | 0.083 | 0.013 | 0.034 | 0.11 | -403 |
| logdha | rs191811733 | 0.006 | . | . | . | . | . | . | . | . | . | . |
| logdha | rs146109272 | 0.008 | . | . | . | . | . | . | . | . | . | . |
| logdha | rs148397915 | 0.006 | . | . | . | . | . | . | . | . | . | . |
| logdha | rs142233290 | 0.007 | . | . | . | . | . | . | . | . | . | . |
| logdha | rs80226874 | 0.022 | 0.279 | 0.16 | 0.026 | 0.015 | 0.094 | 0.5 | 1.5 | 4.68 | 0.896 | 0.712 |
| logdha | rs144496203 | 0.004 | . | . | . | . | . | . | . | . | . | . |
| logdha | rs191062130 | 0.001 | . | . | . | . | . | . | . | . | . | . |
| logdha | rs185807817 | 0.004 | . | . | . | . | . | . | . | . | . | . |
| logdha | rs117846213 | 0.012 | 0.439 | 0.155 | 0.019 | 51.4 | 154 | 0.188 | 0.097 | 0.206 | 0.272 | -48.7 |
| logdha | rs190545473 | 0.002 | . | . | . | . | . | . | . | . | . | . |
| logdha | rs192364918 | 0.01 | 0.308 | 0.179 | 0.043 | 77.3 | 451 | 0.456 | 0.205 | 0.689 | 0.637 | -71.7 |
| logdha | rs182939241 | 0.003 | . | . | . | . | . | . | . | . | . | . |
| logdha | rs12571125 | 0.011 | 0.425 | 0.15 | 0.015 | 0.067 | 0.266 | 0.497 | 3.39 | 7.04 | 0.556 | 0.605 |
| logdha | rs192418941 | 0.002 | . | . | . | . | . | . | . | . | . | . |
| logdha | rs141270243 | 0.015 | 0.329 | 0.188 | 0.052 | 461 | 3.60E+03 | 0.429 | 0.035 | 0.181 | 0.515 | -455 |
| logdha | rs182615502 | 0.006 | . | . | . | . | . | . | . | . | . | . |
| logdha | rs146669856 | 0.01 | 0.673 | 0.146 | 0.069 | 4.42 | 13.8 | 0.635 | 0.149 | 0.345 | 0.411 | -3.65 |
| logdha | rs78290029 | 0.053 | 0.607 | 0.136 | 0.026 | 0.268 | 0.336 | 0.293 | 2.58 | 2.23 | 0.273 | 0.544 |
| logdha | rs139955565 | 0.004 | . | . | . | . | . | . | . | . | . | . |
| logdha | rs12267314 | 0.047 | 0.618 | 0.137 | 0.03 | 0.387 | 0.476 | 0.44 | 2.16 | 1.86 | 0.371 | 0.512 |
| logdha | rs74477197 | 0.004 | . | . | . | . | . | . | . | . | . | . |
| logdha | rs188147236 | 0.005 | . | . | . | . | . | . | . | . | . | . |
| logdha | rs2228065 | 0.046 | 0.618 | 0.137 | 0.03 | 0.382 | 0.482 | 0.445 | 2.18 | 1.91 | 0.374 | 0.515 |
| logdha | rs116961353 | 0.01 | . | . | . | . | . | . | . | . | . | . |
| logdha | rs190222272 | 0.007 | . | . | . | . | . | . | . | . | . | . |
| logdha | rs182719940 | 0.006 | . | . | . | . | . | . | . | . | . | . |
| logdha | rs184477413 | 0.004 | . | . | . | . | . | . | . | . | . | . |
| logdha | rs12265175 | 0.048 | 0.618 | 0.137 | 0.03 | 0.341 | 0.422 | 0.385 | 2.21 | 1.9 | 0.354 | 0.508 |
| logdha | rs182928276 | 0.003 | . | . | . | . | . | . | . | . | . | . |
| logdha | rs144409895 | 0.011 | 0.321 | 0.188 | 0.053 | 603 | 3.40E+03 | 0.251 | 0.061 | 0.199 | 0.391 | -591 |
| logdha | rs58998447 | 0.031 | 0.679 | 0.151 | 0.081 | 2.64 | 3.94 | 0.515 | 0.416 | 0.437 | 0.404 | -1.57 |
| logdha | rs188668832 | 0.005 | . | . | . | . | . | . | . | . | . | . |
| logdha | rs148641686 | 0.002 | . | . | . | . | . | . | . | . | . | . |
| logdha | rs75468791 | 0.054 | 0.277 | 0.162 | 0.028 | 0.017 | 0.049 | 0.159 | 8.06 | 11.4 | 0.141 | 0.744 |
| logdha | rs147327397 | 0.003 | . | . | . | . | . | . | . | . | . | . |
| logdha | rs77354597 | 0.007 | . | . | . | . | . | . | . | . | . | . |
| logdha | rs41365544 | 0.045 | 0.654 | 0.142 | 0.05 | 0.994 | 1.33 | 0.996 | 1.12 | 1.07 | 0.907 | 0.079 |
| logdha | rs187564188 | 0.016 | 0.237 | 0.15 | 0.023 | 5.10E-04 | 2.90E-03 | 0.189 | 154 | 640 | 0.225 | 0.781 |
| logdha | rs183511984 | 0.002 | . | . | . | . | . | . | . | . | . | . |
| logdha | rs12262783 | 0.038 | 0.653 | 0.141 | 0.048 | 0.875 | 1.3 | 0.928 | 1.16 | 1.24 | 0.887 | 0.137 |
| logdha | rs188817687 | 0.002 | . | . | . | . | . | . | . | . | . | . |
| logdha | rs3815968 | 0.005 | . | . | . | . | . | . | . | . | . | . |
| logdha | rs187822300 | 0.001 | . | . | . | . | . | . | . | . | . | . |
| logdha | rs188902172 | 0.008 | . | . | . | . | . | . | . | . | . | . |
| logdha | rs181190448 | 0.006 | . | . | . | . | . | . | . | . | . | . |
| logdha | rs182699093 | 0.001 | . | . | . | . | . | . | . | . | . | . |
| logdha | rs182631303 | 0.005 | . | . | . | . | . | . | . | . | . | . |
| logdha | rs187014172 | 0.003 | . | . | . | . | . | . | . | . | . | . |
| logdha | rs142575406 | 0.009 | . | . | . | . | . | . | . | . | . | . |
| logdha | rs180711033 | 0.007 | . | . | . | . | . | . | . | . | . | . |
| logdha | rs137894823 | 0.002 | . | . | . | . | . | . | . | . | . | . |
| logdha | rs189053273 | 0.001 | . | . | . | . | . | . | . | . | . | . |
| logdha | rs143499388 | 0.002 | . | . | . | . | . | . | . | . | . | . |
| logdha | rs182936001 | 0.003 | . | . | . | . | . | . | . | . | . | . |
| logdha | rs148456406 | 0.003 | . | . | . | . | . | . | . | . | . | . |
| logdha | rs182499531 | 0.005 | . | . | . | . | . | . | . | . | . | . |
| logdha | rs74884915 | 0.016 | 0.73 | 0.175 | 0.19 | 8.62 | 9.74 | 0.057 | 0.036 | 0.033 | 3.80E-04 | -8.12 |
| logdha | rs184046374 | 0.001 | . | . | . | . | . | . | . | . | . | . |
| logdha | rs74333993 | 0.019 | 0.687 | 0.164 | 0.116 | 0.381 | 0.714 | 0.607 | 2.24 | 2.77 | 0.516 | 0.517 |
| logdha | rs138889719 | 0.001 | . | . | . | . | . | . | . | . | . | . |
| logdha | rs75330330 | 0.013 | 0.701 | 0.166 | 0.133 | 0.167 | 0.562 | 0.595 | 2.74 | 6.27 | 0.659 | 0.453 |
| logdha | rs193051693 | 0 | . | . | . | . | . | . | . | . | . | . |
| logdha | rs145929633 | 0 | . | . | . | . | . | . | . | . | . | . |

| logdha | rs184076904 | 0.003 | . | . | . | . | . | . | . | . | . | . |
| --- | --- | --- | --- | --- | --- | --- | --- | --- | --- | --- | --- | --- |
| logdha | rs141802494 | 0.011 | 0.71 | 0.167 | 0.146 | 0.606 | 3.26 | 0.926 | 0.731 | 2.88 | 0.937 | -1.30E-03 |
| logdha | rs149519759 | 0.001 | . | . | . | . | . | . | . | . | . | . |
| logdha | rs145467260 | 0.008 | . | . | . | . | . | . | . | . | . | . |
| logdha | rs79620914 | 0.017 | 0.672 | 0.161 | 0.097 | 0.033 | 0.06 | 0.061 | 10.9 | 12.8 | 0.043 | 0.535 |
| logdha | rs140538633 | 0.011 | 0.73 | 0.175 | 0.189 | 25.5 | 35.7 | 0.021 | 0.022 | 0.022 | 1.10E-04 | -24.8 |
| logdha | rs140862301 | 0.005 | . | . | . | . | . | . | . | . | . | . |
| logdha | rs114615847 | 0.006 | . | . | . | . | . | . | . | . | . | . |
| logdha | rs141800259 | 0.016 | 0.699 | 0.166 | 0.131 | 0.145 | 0.485 | 0.564 | 2.72 | 6.24 | 0.662 | 0.433 |
| logdha | rs149779059 | 0.011 | 0.715 | 0.168 | 0.154 | 2.85 | 8.08 | 0.712 | 0.714 | 1.5 | 0.873 | -1.11 |
| logdha | rs116539253 | 0.004 | . | . | . | . | . | . | . | . | . | . |
| logdha | rs184979550 | 0.001 | . | . | . | . | . | . | . | . | . | . |
| logdha | rs190128940 | 0.003 | . | . | . | . | . | . | . | . | . | . |
| logdha | rs146594816 | 0.002 | . | . | . | . | . | . | . | . | . | . |
| logdha | rs141370766 | 0.013 | 0.701 | 0.166 | 0.133 | 0.175 | 0.577 | 0.598 | 2.7 | 6.09 | 0.66 | 0.455 |
| logdha | rs144670033 | 0.002 | . | . | . | . | . | . | . | . | . | . |
| logdha | rs187152174 | 0.005 | . | . | . | . | . | . | . | . | . | . |
| logdha | rs184180263 | 0.003 | . | . | . | . | . | . | . | . | . | . |
| logdha | rs140075861 | 0.007 | . | . | . | . | . | . | . | . | . | . |
| logdha | rs186483129 | 0 | . | . | . | . | . | . | . | . | . | . |
| logdha | rs186634282 | 0.008 | . | . | . | . | . | . | . | . | . | . |
| logdha | rs9988663 | 0.019 | 0.687 | 0.164 | 0.116 | 0.375 | 0.703 | 0.601 | 2.26 | 2.8 | 0.511 | 0.52 |
| logdha | rs138828529 | 0.013 | 0.797 | 0.188 | 0.335 | 55.2 | 93.8 | 0.018 | 0.085 | 0.134 | 0.118 | -51.2 |
| logdha | rs188777615 | 0.003 | . | . | . | . | . | . | . | . | . | . |
| logdha | rs192321882 | 0.005 | . | . | . | . | . | . | . | . | . | . |
| logdha | rs150812270 | 0.016 | 0.735 | 0.18 | 0.209 | 4.04 | 5.42 | 0.298 | 0.375 | 0.341 | 0.28 | -2.66 |
| logdha | rs185906603 | 0.001 | . | . | . | . | . | . | . | . | . | . |
| logdha | rs191070862 | 0.004 | . | . | . | . | . | . | . | . | . | . |
| logdha | rs182904978 | 0 | . | . | . | . | . | . | . | . | . | . |
| logdha | rs192679655 | 0.001 | . | . | . | . | . | . | . | . | . | . |
| logdha | rs189673086 | 0.005 | . | . | . | . | . | . | . | . | . | . |
| logdha | rs181141892 | 0.002 | . | . | . | . | . | . | . | . | . | . |
| logdha | rs186404123 | 0 | . | . | . | . | . | . | . | . | . | . |
| logdha | rs192117287 | 0.003 | . | . | . | . | . | . | . | . | . | . |
| logdha | rs182748835 | 0.001 | . | . | . | . | . | . | . | . | . | . |
| logdha | rs141854549 | 0.011 | 0.731 | 0.175 | 0.19 | 26.6 | 37 | 0.018 | 0.021 | 0.021 | 1.10E-04 | -25.9 |
| logdha | rs140163397 | 0.003 | . | . | . | . | . | . | . | . | . | . |
| logdha | rs146347300 | 0.005 | . | . | . | . | . | . | . | . | . | . |
| logdha | rs139362377 | 0.011 | 0.731 | 0.175 | 0.19 | 26.8 | 37.2 | 0.018 | 0.021 | 0.021 | 1.10E-04 | -26.1 |
| logdha | rs185087671 | 0.001 | . | . | . | . | . | . | . | . | . | . |
| logdha | rs190466574 | 0.003 | . | . | . | . | . | . | . | . | . | . |
| logdha | rs147012778 | 0.003 | . | . | . | . | . | . | . | . | . | . |
| logdha | rs138236960 | 0.006 | . | . | . | . | . | . | . | . | . | . |
| logdha | rs116310311 | 0.011 | 0.731 | 0.175 | 0.19 | 27 | 37.4 | 0.017 | 0.021 | 0.021 | 1.20E-04 | -26.3 |
| logdha | rs142868356 | 0.008 | . | . | . | . | . | . | . | . | . | . |
| logdha | rs144956240 | 0.002 | . | . | . | . | . | . | . | . | . | . |
| logdha | rs188527776 | 0.002 | . | . | . | . | . | . | . | . | . | . |
| logdha | rs183971165 | 0.005 | . | . | . | . | . | . | . | . | . | . |
| logdha | rs76748216 | 0.006 | . | . | . | . | . | . | . | . | . | . |
| logdha | rs148554990 | 0.001 | . | . | . | . | . | . | . | . | . | . |
| logdha | rs114446477 | 0.017 | 0.675 | 0.162 | 0.101 | 0.042 | 0.079 | 0.093 | 8.98 | 11.1 | 0.076 | 0.538 |
| logdha | rs185223933 | 0.004 | . | . | . | . | . | . | . | . | . | . |
| logdha | rs76869726 | 0.006 | . | . | . | . | . | . | . | . | . | . |
| logdha | rs151200830 | 0.011 | 0.732 | 0.175 | 0.192 | 34.4 | 45.4 | 7.30E-03 | 0.015 | 0.018 | 5.40E-04 | -33.7 |
| logdha | rs17157731 | 0.011 | 0.732 | 0.175 | 0.192 | 34.7 | 45.8 | 7.10E-03 | 0.015 | 0.018 | 5.40E-04 | -34.1 |
| logdha | rs181243469 | 0 | . | . | . | . | . | . | . | . | . | . |
| logdha | rs186890707 | 0 | . | . | . | . | . | . | . | . | . | . |
| logdha | rs184063360 | 0 | . | . | . | . | . | . | . | . | . | . |
| logdha | rs188550237 | 0.004 | . | . | . | . | . | . | . | . | . | . |
| logdha | rs140931068 | 0.01 | 0.699 | 0.165 | 0.129 | 0.603 | 2.31 | 0.895 | 2.82 | 7.94 | 0.712 | 0.888 |
| logdha | rs74867877 | 0.004 | . | . | . | . | . | . | . | . | . | . |
| logdha | rs11239495 | 0.036 | 0.674 | 0.16 | 0.097 | 0.316 | 0.446 | 0.414 | 2.37 | 2.3 | 0.373 | 0.515 |
| logdha | rs149745800 | 0 | . | . | . | . | . | . | . | . | . | . |
| logdha | rs145044458 | 0.01 | 0.731 | 0.175 | 0.191 | 34.6 | 45.5 | 7.10E-03 | 0.016 | 0.019 | 8.50E-04 | -33.9 |
| logdha | rs188067427 | 0.001 | . | . | . | . | . | . | . | . | . | . |
| logdha | rs146827800 | 0.001 | . | . | . | . | . | . | . | . | . | . |
| logdha | rs140619817 | 0.01 | 0.74 | 0.178 | 0.21 | 56 | 74.5 | 2.50E-03 | 9.20E-03 | 0.015 | 4.60E-03 | -55.4 |
| logdha | rs892689 | 0.997 | . | . | . | . | . | . | . | . | . | . |
| logdha | rs143642005 | 0.004 | . | . | . | . | . | . | . | . | . | . |
| logdha | rs181929304 | 0.004 | . | . | . | . | . | . | . | . | . | . |
| logdha | rs191086173 | 0.001 | . | . | . | . | . | . | . | . | . | . |
| logdha | rs114613116 | 0.012 | 0.746 | 0.18 | 0.226 | 54.9 | 71.1 | 2.00E-03 | 9.40E-03 | 0.013 | 5.70E-04 | -54.3 |
| logdha | rs141076768 | 0.002 | . | . | . | . | . | . | . | . | . | . |
| logdha | rs187316531 | 0 | . | . | . | . | . | . | . | . | . | . |
| logdha | rs11239499 | 0.046 | 0.703 | 0.17 | 0.145 | 0.517 | 0.646 | 0.598 | 1.34 | 1.16 | 0.733 | 0.268 |
| logdha | rs192726371 | 0.002 | . | . | . | . | . | . | . | . | . | . |
| logdha | rs74128481 | 0.012 | 0.726 | 0.171 | 0.175 | 63.2 | 101 | 9.50E-03 | 0.013 | 0.014 | 8.20E-05 | -62.4 |
| logdha | rs74128482 | 0.008 | . | . | . | . | . | . | . | . | . | . |
| logdha | rs11239502 | 0.033 | 0.68 | 0.163 | 0.107 | 0.251 | 0.434 | 0.424 | 2.44 | 2.83 | 0.44 | 0.486 |
| logdha | rs116330285 | 0.002 | . | . | . | . | . | . | . | . | . | . |
| logdha | rs189926729 | 0.002 | . | . | . | . | . | . | . | . | . | . |
| logdha | rs188975779 | 0.008 | . | . | . | . | . | . | . | . | . | . |
| logdha | rs143846038 | 0.002 | . | . | . | . | . | . | . | . | . | . |
| logdha | rs74128483 | 0.012 | 0.727 | 0.171 | 0.175 | 61.9 | 98.1 | 9.30E-03 | 0.013 | 0.014 | 8.10E-05 | -61 |
| logdha | rs139292055 | 0.045 | 0.708 | 0.171 | 0.154 | 0.531 | 0.689 | 0.626 | 1.26 | 1.12 | 0.792 | 0.236 |
| logdha | rs11239503 | 0.045 | 0.708 | 0.171 | 0.154 | 0.531 | 0.689 | 0.625 | 1.26 | 1.12 | 0.792 | 0.236 |

| logdha | rs58838686 | 0.008 | . | . | . | . | . | . | . | . | . | . |
| --- | --- | --- | --- | --- | --- | --- | --- | --- | --- | --- | --- | --- |
| logdha | rs185825373 | 0.005 | . | . | . | . | . | . | . | . | . | . |
| logdha | rs11239504 | 0.045 | 0.708 | 0.171 | 0.154 | 0.531 | 0.689 | 0.626 | 1.26 | 1.12 | 0.792 | 0.236 |
| logdha | rs150012354 | 0.003 | . | . | . | . | . | . | . | . | . | . |
| logdha | rs187763096 | 0.005 | . | . | . | . | . | . | . | . | . | . |
| logdha | rs193043982 | 0.005 | . | . | . | . | . | . | . | . | . | . |
| logdha | rs79186884 | 0.014 | 0.72 | 0.169 | 0.162 | 3.16 | 7.18 | 0.611 | 0.592 | 0.982 | 0.752 | -1.54 |
| logdha | rs74128484 | 0.008 | . | . | . | . | . | . | . | . | . | . |
| logdha | rs58461537 | 0.045 | 0.708 | 0.171 | 0.154 | 0.53 | 0.688 | 0.625 | 1.26 | 1.12 | 0.791 | 0.237 |
| logdha | rs188527474 | 0.005 | . | . | . | . | . | . | . | . | . | . |
| logdha | rs141659976 | 0.005 | . | . | . | . | . | . | . | . | . | . |
| logdha | rs115846407 | 0.045 | 0.708 | 0.171 | 0.154 | 0.53 | 0.688 | 0.625 | 1.26 | 1.12 | 0.792 | 0.236 |
| logdha | rs138378816 | 0.045 | 0.708 | 0.171 | 0.154 | 0.53 | 0.688 | 0.625 | 1.26 | 1.12 | 0.792 | 0.236 |
| logdha | rs146188136 | 0.033 | 0.68 | 0.163 | 0.107 | 0.248 | 0.43 | 0.421 | 2.46 | 2.84 | 0.437 | 0.487 |
| logdha | rs139383871 | 0.008 | . | . | . | . | . | . | . | . | . | . |
| logdha | rs182953869 | 0.004 | . | . | . | . | . | . | . | . | . | . |
| logdha | rs192430299 | 0.012 | 0.735 | 0.172 | 0.189 | 11.4 | 42.5 | 0.513 | 0.157 | 0.456 | 0.524 | -9.83 |
| logdha | rs144152844 | 0.045 | 0.708 | 0.171 | 0.154 | 0.528 | 0.685 | 0.623 | 1.27 | 1.13 | 0.789 | 0.238 |
| logdha | rs145987786 | 0.033 | 0.722 | 0.172 | 0.171 | 1.22 | 2.16 | 0.91 | 0.783 | 0.958 | 0.842 | -0.253 |
| logdha | rs181132926 | 0.002 | . | . | . | . | . | . | . | . | . | . |
| logdha | rs147882253 | 0.045 | 0.708 | 0.171 | 0.154 | 0.529 | 0.686 | 0.624 | 1.27 | 1.12 | 0.791 | 0.237 |
| logdha | rs141807621 | 0.015 | 0.795 | 0.188 | 0.332 | 39 | 63.9 | 0.025 | 0.085 | 0.131 | 0.109 | -36.1 |
| logdha | rs147116052 | 0.045 | 0.708 | 0.171 | 0.154 | 0.529 | 0.686 | 0.624 | 1.27 | 1.12 | 0.791 | 0.237 |
| logdha | rs147716618 | 0.045 | 0.708 | 0.171 | 0.154 | 0.53 | 0.686 | 0.624 | 1.27 | 1.12 | 0.791 | 0.237 |
| logdha | rs187198855 | 0.029 | 0.724 | 0.173 | 0.177 | 1.25 | 2.25 | 0.9 | 0.71 | 0.871 | 0.78 | -0.333 |
| logdha | rs189409638 | 0.011 | 0.746 | 0.179 | 0.221 | 129 | 174 | 3.20E-04 | 8.80E-03 | 9.20E-03 | 5.40E-06 | -128 |
| logdha | rs182647339 | 0.001 | . | . | . | . | . | . | . | . | . | . |
| logdha | rs187995988 | 0.004 | . | . | . | . | . | . | . | . | . | . |
| logdha | rs141034184 | 0.048 | 0.703 | 0.171 | 0.148 | 0.42 | 0.549 | 0.507 | 1.4 | 1.24 | 0.701 | 0.292 |
| logdha | rs187310430 | 0.005 | . | . | . | . | . | . | . | . | . | . |
| logdha | rs192608488 | 0.045 | 0.708 | 0.171 | 0.154 | 0.532 | 0.686 | 0.625 | 1.26 | 1.11 | 0.793 | 0.235 |
| logdha | rs148840366 | 0.006 | . | . | . | . | . | . | . | . | . | . |
| logdha | rs186785022 | 0.012 | 0.727 | 0.171 | 0.176 | 56.1 | 86 | 8.70E-03 | 0.015 | 0.016 | 7.80E-05 | -55.2 |
| logdha | rs192348728 | 0.008 | . | . | . | . | . | . | . | . | . | . |
| logdha | rs189656631 | 0.045 | 0.708 | 0.171 | 0.154 | 0.532 | 0.686 | 0.625 | 1.26 | 1.11 | 0.793 | 0.235 |
| logdha | rs185289750 | 0.004 | . | . | . | . | . | . | . | . | . | . |
| logdha | rs142382395 | 0.045 | 0.708 | 0.171 | 0.154 | 0.531 | 0.686 | 0.624 | 1.26 | 1.11 | 0.793 | 0.235 |
| logdha | rs144866696 | 0.012 | 0.727 | 0.171 | 0.176 | 55.7 | 85.3 | 8.60E-03 | 0.015 | 0.016 | 7.80E-05 | -54.8 |
| logdha | rs141371289 | 0.045 | 0.708 | 0.171 | 0.154 | 0.532 | 0.686 | 0.624 | 1.26 | 1.11 | 0.793 | 0.235 |
| logdha | rs114750008 | 0.045 | 0.708 | 0.171 | 0.154 | 0.532 | 0.686 | 0.625 | 1.26 | 1.11 | 0.793 | 0.235 |
| logdha | rs189063597 | 0.001 | . | . | . | . | . | . | . | . | . | . |
| logdha | rs139151571 | 0.041 | 0.7 | 0.169 | 0.141 | 0.385 | 0.551 | 0.505 | 1.55 | 1.51 | 0.656 | 0.332 |
| logdha | rs139846368 | 0.046 | 0.706 | 0.171 | 0.151 | 0.469 | 0.617 | 0.565 | 1.34 | 1.19 | 0.744 | 0.268 |
| logdha | rs182057653 | 0.012 | 0.738 | 0.177 | 0.207 | 19.5 | 28.4 | 0.042 | 0.04 | 0.035 | 2.10E-04 | -18.6 |
| logdha | rs148785420 | 0.045 | 0.708 | 0.171 | 0.154 | 0.531 | 0.686 | 0.624 | 1.26 | 1.11 | 0.793 | 0.235 |
| logdha | rs138383251 | 0.007 | . | . | . | . | . | . | . | . | . | . |
| logdha | rs116526705 | 0.012 | 0.75 | 0.181 | 0.233 | 43.2 | 58.9 | 5.70E-03 | 0.013 | 0.014 | 6.10E-05 | -42.5 |
| logdha | rs185946436 | 0 | . | . | . | . | . | . | . | . | . | . |
| logdha | rs190455427 | 0.004 | . | . | . | . | . | . | . | . | . | . |
| logdha | rs9282584 | 0.045 | 0.708 | 0.171 | 0.154 | 0.531 | 0.685 | 0.624 | 1.26 | 1.11 | 0.793 | 0.235 |
| logdha | rs17153289 | 0.045 | 0.708 | 0.171 | 0.154 | 0.531 | 0.685 | 0.624 | 1.26 | 1.11 | 0.793 | 0.235 |
| logdha | rs138359103 | 0.005 | . | . | . | . | . | . | . | . | . | . |
| logdha | rs150605268 | 0.002 | . | . | . | . | . | . | . | . | . | . |
| logdha | rs79242910 | 0.046 | 0.705 | 0.171 | 0.149 | 0.462 | 0.591 | 0.546 | 1.36 | 1.18 | 0.726 | 0.275 |
| logdha | rs139716904 | 0.013 | 0.735 | 0.175 | 0.195 | 20.8 | 35.7 | 0.078 | 0.044 | 0.059 | 0.02 | -19.8 |
| logdha | rs76238498 | 0.034 | 0.711 | 0.169 | 0.152 | 0.81 | 1.5 | 0.909 | 1.09 | 1.38 | 0.948 | 0.105 |
| logdha | rs184140639 | 0 | . | . | . | . | . | . | . | . | . | . |
| logdha | rs188517278 | 0.006 | . | . | . | . | . | . | . | . | . | . |
| logdha | rs139678913 | 0.002 | . | . | . | . | . | . | . | . | . | . |
| logdha | rs116697955 | 0.004 | . | . | . | . | . | . | . | . | . | . |
| logdha | rs79267994 | 0.007 | . | . | . | . | . | . | . | . | . | . |
| logdha | rs11239510 | 0.013 | 0.717 | 0.173 | 0.167 | 1.86 | 3.05 | 0.707 | 0.664 | 0.778 | 0.727 | -0.69 |
| logdha | rs75831645 | 0.012 | 0.75 | 0.181 | 0.233 | 43.4 | 59.1 | 5.60E-03 | 0.013 | 0.014 | 6.40E-05 | -42.8 |
| logdha | rs78452276 | 0.031 | 0.686 | 0.17 | 0.128 | 0.518 | 0.719 | 0.636 | 1.79 | 1.68 | 0.535 | 0.432 |
| logdha | rs144366924 | 0 | . | . | . | . | . | . | . | . | . | . |
| logdha | rs77519841 | 0.033 | 0.68 | 0.163 | 0.107 | 0.246 | 0.428 | 0.419 | 2.46 | 2.86 | 0.437 | 0.486 |
| logdha | rs76944454 | 0.045 | 0.709 | 0.172 | 0.155 | 0.533 | 0.687 | 0.625 | 1.26 | 1.11 | 0.795 | 0.233 |
| logdha | rs76443855 | 0.034 | 0.711 | 0.169 | 0.151 | 0.801 | 1.49 | 0.905 | 1.1 | 1.4 | 0.942 | 0.113 |
| logdha | rs146613174 | 0.001 | . | . | . | . | . | . | . | . | . | . |
| logdha | rs181424761 | 0.001 | . | . | . | . | . | . | . | . | . | . |
| logdha | rs142119241 | 0.001 | . | . | . | . | . | . | . | . | . | . |
| logdha | rs140137954 | 0.012 | 0.75 | 0.181 | 0.233 | 42.9 | 58.6 | 5.90E-03 | 0.013 | 0.014 | 5.70E-05 | -42.3 |
| logdha | rs187319549 | 0.002 | . | . | . | . | . | . | . | . | . | . |
| logdha | rs12241545 | 0.021 | 0.71 | 0.172 | 0.157 | 0.455 | 0.646 | 0.579 | 1.14 | 1.08 | 0.891 | 0.203 |
| logdha | rs10128228 | 0.045 | 0.709 | 0.172 | 0.155 | 0.534 | 0.688 | 0.626 | 1.26 | 1.11 | 0.796 | 0.233 |
| logdha | rs58580249 | 0.006 | . | . | . | . | . | . | . | . | . | . |
| logdha | rs193148863 | 0.001 | . | . | . | . | . | . | . | . | . | . |
| logdha | rs185638946 | 0.005 | . | . | . | . | . | . | . | . | . | . |
| logdha | rs117752921 | 0.001 | . | . | . | . | . | . | . | . | . | . |
| logdha | rs180841236 | 0.001 | . | . | . | . | . | . | . | . | . | . |
| logdha | rs73283155 | 0.006 | . | . | . | . | . | . | . | . | . | . |
| logdha | rs76560599 | 0.038 | 0.699 | 0.17 | 0.14 | 0.308 | 0.469 | 0.439 | 1.75 | 1.78 | 0.584 | 0.369 |
| logdha | rs76273620 | 0.038 | 0.699 | 0.17 | 0.14 | 0.308 | 0.469 | 0.439 | 1.75 | 1.78 | 0.584 | 0.369 |
| logdha | rs141432923 | 0.012 | 0.716 | 0.171 | 0.162 | 0.303 | 1.04 | 0.728 | 0.58 | 1.32 | 0.811 | 0.107 |
| logdha | rs113529322 | 0.012 | 0.728 | 0.171 | 0.177 | 54.3 | 82.1 | 8.30E-03 | 0.016 | 0.017 | 7.90E-05 | -53.4 |
| logdha | rs10128306 | 0.045 | 0.709 | 0.172 | 0.155 | 0.535 | 0.69 | 0.628 | 1.25 | 1.1 | 0.797 | 0.232 |

| logdha | rs149691688 | 0.001 | . | . | . | . | . | . | . | . | . | . |
| --- | --- | --- | --- | --- | --- | --- | --- | --- | --- | --- | --- | --- |
| logdha | rs75872495 | 0.045 | 0.709 | 0.172 | 0.156 | 0.541 | 0.697 | 0.633 | 1.25 | 1.1 | 0.803 | 0.227 |
| logdha | rs191873960 | 0.004 | . | . | . | . | . | . | . | . | . | . |
| logdha | rs80155622 | 0.045 | 0.709 | 0.172 | 0.156 | 0.543 | 0.699 | 0.635 | 1.24 | 1.09 | 0.803 | 0.227 |
| logdha | rs113914656 | 0.005 | . | . | . | . | . | . | . | . | . | . |
| logdha | rs12242840 | 0.045 | 0.71 | 0.172 | 0.157 | 0.56 | 0.716 | 0.65 | 1.23 | 1.07 | 0.815 | 0.218 |
| logdha | rs147824078 | 0.006 | . | . | . | . | . | . | . | . | . | . |
| logdha | rs59051493 | 0.045 | 0.71 | 0.172 | 0.157 | 0.571 | 0.722 | 0.658 | 1.22 | 1.06 | 0.818 | 0.214 |
| logdha | rs9282588 | 0.005 | . | . | . | . | . | . | . | . | . | . |
| logdha | rs150215207 | 0.006 | . | . | . | . | . | . | . | . | . | . |
| logdha | rs138772243 | 0.002 | . | . | . | . | . | . | . | . | . | . |
| logdha | rs149383244 | 0.014 | 0.731 | 0.179 | 0.201 | 3.84 | 3.92 | 0.188 | 0.544 | 0.404 | 0.412 | -2.04 |
| logdha | rs17522720 | 0.011 | 0.733 | 0.178 | 0.201 | 5.87 | 5.5 | 0.059 | 0.493 | 0.33 | 0.291 | -3.49 |
| logdha | rs79245105 | 0.045 | 0.709 | 0.172 | 0.156 | 0.568 | 0.719 | 0.655 | 1.23 | 1.06 | 0.813 | 0.217 |
| logdha | rs138222133 | 0.002 | . | . | . | . | . | . | . | . | . | . |
| logdha | rs12247314 | 0.05 | 0.717 | 0.174 | 0.172 | 0.74 | 0.867 | 0.797 | 1.05 | 0.848 | 0.956 | 0.098 |
| logdha | rs12263894 | 0.049 | 0.718 | 0.175 | 0.174 | 0.789 | 0.933 | 0.841 | 1.02 | 0.833 | 0.985 | 0.068 |
| logdha | rs149907880 | 0.005 | . | . | . | . | . | . | . | . | . | . |
| logdha | rs188153885 | 0.005 | . | . | . | . | . | . | . | . | . | . |
| logdha | rs73283159 | 0.003 | . | . | . | . | . | . | . | . | . | . |
| logdha | rs140476687 | 0.002 | . | . | . | . | . | . | . | . | . | . |
| logdha | rs186603148 | 0 | . | . | . | . | . | . | . | . | . | . |
| logdha | rs183296063 | 0.002 | . | . | . | . | . | . | . | . | . | . |
| logdha | rs145733694 | 0.006 | . | . | . | . | . | . | . | . | . | . |
| logdha | rs189807449 | 0 | . | . | . | . | . | . | . | . | . | . |
| logdha | rs12253223 | 0.038 | 0.688 | 0.165 | 0.12 | 0.412 | 0.631 | 0.563 | 1.8 | 1.87 | 0.574 | 0.409 |
| logdha | rs182113637 | 0 | . | . | . | . | . | . | . | . | . | . |
| logdha | rs142792099 | 0.01 | 0.713 | 0.168 | 0.152 | 0.966 | 4.55 | 0.994 | 0.572 | 2 | 0.873 | -0.285 |
| logdha | rs12249910 | 0.038 | 0.688 | 0.165 | 0.12 | 0.412 | 0.63 | 0.562 | 1.8 | 1.87 | 0.573 | 0.409 |
| logdha | rs149436339 | 0.001 | . | . | . | . | . | . | . | . | . | . |
| logdha | rs77632210 | 0 | . | . | . | . | . | . | . | . | . | . |
| logdha | rs74130818 | 0.008 | . | . | . | . | . | . | . | . | . | . |
| logdha | rs187583648 | 0 | . | . | . | . | . | . | . | . | . | . |
| logdha | rs191295987 | 0.004 | . | . | . | . | . | . | . | . | . | . |
| logdha | rs184200686 | 0.006 | . | . | . | . | . | . | . | . | . | . |
| logdha | rs76117263 | 0.033 | 0.712 | 0.17 | 0.154 | 0.845 | 1.62 | 0.93 | 1.05 | 1.38 | 0.97 | 0.075 |
| logdha | rs185393149 | 0.001 | . | . | . | . | . | . | . | . | . | . |
| logdha | rs144760014 | 0.003 | . | . | . | . | . | . | . | . | . | . |
| logdha | rs7922292 | 0.005 | . | . | . | . | . | . | . | . | . | . |
| logdha | rs115372844 | 0.006 | . | . | . | . | . | . | . | . | . | . |
| logdha | rs111460364 | 0.033 | 0.712 | 0.17 | 0.155 | 0.861 | 1.66 | 0.938 | 1.04 | 1.37 | 0.977 | 0.064 |
| logdha | rs182052255 | 0.001 | . | . | . | . | . | . | . | . | . | . |
| logdha | rs180983889 | 0.004 | . | . | . | . | . | . | . | . | . | . |
| logdha | rs139175327 | 0.006 | . | . | . | . | . | . | . | . | . | . |
| logdha | rs149959620 | 0.011 | 0.729 | 0.173 | 0.184 | 12.6 | 22.4 | 0.153 | 0.068 | 0.087 | 0.037 | -11.7 |
| logdha | rs114653117 | 0.005 | . | . | . | . | . | . | . | . | . | . |
| logdha | rs144270018 | 0.003 | . | . | . | . | . | . | . | . | . | . |
| logdha | rs182723204 | 0.005 | . | . | . | . | . | . | . | . | . | . |
| logdha | rs57204067 | 0.008 | . | . | . | . | . | . | . | . | . | . |
| logdha | rs144956226 | 0.038 | 0.686 | 0.165 | 0.117 | 0.394 | 0.6 | 0.541 | 1.88 | 1.94 | 0.541 | 0.428 |
| logdha | rs187842704 | 0.01 | 0.713 | 0.171 | 0.157 | 0.152 | 0.408 | 0.484 | 0.287 | 0.612 | 0.559 | 0.166 |
| logdha | rs184620511 | 0.008 | . | . | . | . | . | . | . | . | . | . |
| logdha | rs144031303 | 0.012 | 0.748 | 0.181 | 0.23 | 12 | 23.8 | 0.209 | 0.054 | 0.063 | 0.012 | -11.3 |
| logdha | rs187526473 | 0 | . | . | . | . | . | . | . | . | . | . |
| logdha | rs193002805 | 0.002 | . | . | . | . | . | . | . | . | . | . |
| logdha | rs138707426 | 0.014 | 0.731 | 0.179 | 0.202 | 4.06 | 4.07 | 0.162 | 0.536 | 0.387 | 0.388 | -2.2 |
| logdha | rs142754243 | 0.03 | 0.643 | 0.153 | 0.064 | 0.04 | 0.068 | 0.061 | 8.13 | 9.15 | 0.063 | 0.524 |
| logdha | rs181600331 | 0.005 | . | . | . | . | . | . | . | . | . | . |
| logdha | rs144421264 | 0.018 | 0.65 | 0.153 | 0.067 | 3.70E-03 | 0.011 | 0.051 | 33 | 58.5 | 0.049 | 0.425 |
| logdha | rs184414519 | 0.004 | . | . | . | . | . | . | . | . | . | . |
| logdha | rs150596349 | 0.031 | 0.703 | 0.166 | 0.136 | 0.619 | 1.23 | 0.809 | 1.37 | 1.83 | 0.816 | 0.272 |
| logdha | rs190753747 | 0.001 | . | . | . | . | . | . | . | . | . | . |
| logdha | rs139560940 | 0.004 | . | . | . | . | . | . | . | . | . | . |
| logdha | rs75640804 | 0.033 | 0.674 | 0.161 | 0.099 | 0.223 | 0.383 | 0.382 | 2.77 | 3.15 | 0.371 | 0.52 |
| logdha | rs73283175 | 0.052 | 0.69 | 0.168 | 0.127 | 0.322 | 0.394 | 0.355 | 1.71 | 1.43 | 0.522 | 0.368 |
| logdha | rs143337229 | 0.002 | . | . | . | . | . | . | . | . | . | . |
| logdha | rs73283177 | 0.03 | 0.645 | 0.153 | 0.065 | 0.037 | 0.063 | 0.053 | 8.37 | 9.28 | 0.055 | 0.518 |
| logdha | rs76053611 | 0.029 | 0.719 | 0.17 | 0.163 | 0.738 | 1.58 | 0.887 | 0.981 | 1.39 | 0.989 | 0.063 |
| logdha | rs116325983 | 0.004 | . | . | . | . | . | . | . | . | . | . |
| logdha | rs115451825 | 0.005 | . | . | . | . | . | . | . | . | . | . |
| logdha | rs183845246 | 0.001 | . | . | . | . | . | . | . | . | . | . |
| logdha | rs79756542 | 0.033 | 0.708 | 0.167 | 0.145 | 0.809 | 1.59 | 0.914 | 1.13 | 1.5 | 0.925 | 0.132 |
| logdha | rs187242860 | 0.002 | . | . | . | . | . | . | . | . | . | . |
| logdha | rs57318980 | 0.03 | 0.65 | 0.155 | 0.071 | 0.036 | 0.059 | 0.041 | 8 | 8.47 | 0.05 | 0.501 |
| logdha | rs59969732 | 0.028 | 0.656 | 0.155 | 0.075 | 0.016 | 0.03 | 0.031 | 11.9 | 14.7 | 0.044 | 0.45 |
| logdha | rs143811488 | 0.006 | . | . | . | . | . | . | . | . | . | . |
| logdha | rs189067644 | 0.006 | . | . | . | . | . | . | . | . | . | . |
| logdha | rs60449178 | 0.025 | 0.657 | 0.155 | 0.075 | 3.30E-03 | 8.50E-03 | 0.027 | 30.6 | 49.4 | 0.034 | 0.406 |
| logdha | rs59243389 | 0.025 | 0.657 | 0.155 | 0.075 | 3.30E-03 | 8.50E-03 | 0.027 | 30.6 | 49.4 | 0.034 | 0.406 |
| logdha | rs184946540 | 0.027 | 0.698 | 0.166 | 0.13 | 0.062 | 0.125 | 0.169 | 3.93 | 4.71 | 0.252 | 0.41 |
| logdha | rs188685881 | 0.001 | . | . | . | . | . | . | . | . | . | . |
| logdha | rs150335797 | 0.004 | . | . | . | . | . | . | . | . | . | . |
| logdha | rs143879260 | 0.004 | . | . | . | . | . | . | . | . | . | . |
| logdha | rs186044491 | 0.006 | . | . | . | . | . | . | . | . | . | . |
| logdha | rs11819084 | 0.018 | 0.699 | 0.169 | 0.139 | 1.38 | 2.02 | 0.824 | 1.27 | 1.28 | 0.81 | 0.149 |
| logdha | rs146915670 | 0.006 | . | . | . | . | . | . | . | . | . | . |

| logdha | rs113995695 | 0.017 | 0.697 | 0.169 | 0.136 | 1.31 | 1.97 | 0.856 | 1.32 | 1.36 | 0.789 | 0.196 |
| --- | --- | --- | --- | --- | --- | --- | --- | --- | --- | --- | --- | --- |
| logdha | rs143489451 | 0.003 | . | . | . | . | . | . | . | . | . | . |
| logdha | rs182653839 | 0 | . | . | . | . | . | . | . | . | . | . |
| logdha | rs141692564 | 0.004 | . | . | . | . | . | . | . | . | . | . |
| logdha | rs146281742 | 0.003 | . | . | . | . | . | . | . | . | . | . |
| logdha | rs192914353 | 0.003 | . | . | . | . | . | . | . | . | . | . |
| logdha | rs185182848 | 0.002 | . | . | . | . | . | . | . | . | . | . |
| logdha | rs28395858 | 0.017 | 0.695 | 0.168 | 0.134 | 1.26 | 1.91 | 0.881 | 1.36 | 1.43 | 0.767 | 0.24 |
| logdha | rs41429645 | 0.005 | . | . | . | . | . | . | . | . | . | . |
| logdha | rs28395874 | 0.018 | 0.695 | 0.168 | 0.133 | 1.25 | 1.9 | 0.886 | 1.37 | 1.45 | 0.763 | 0.248 |
| logdha | rs189663634 | 0.001 | . | . | . | . | . | . | . | . | . | . |
| logdha | rs77666700 | 0.006 | . | . | . | . | . | . | . | . | . | . |
| logdha | rs146711717 | 0.002 | . | . | . | . | . | . | . | . | . | . |
| logdha | rs144904251 | 0.001 | . | . | . | . | . | . | . | . | . | . |
| logdha | rs189893153 | 0.001 | . | . | . | . | . | . | . | . | . | . |
| logdha | rs150447740 | 0.006 | . | . | . | . | . | . | . | . | . | . |
| logdha | rs143099649 | 0.001 | . | . | . | . | . | . | . | . | . | . |
| logdha | rs191288436 | 0.007 | . | . | . | . | . | . | . | . | . | . |
| logdha | rs188577862 | 0.004 | . | . | . | . | . | . | . | . | . | . |
| logdha | rs192695334 | 0.004 | . | . | . | . | . | . | . | . | . | . |
| logdha | rs138612906 | 0.022 | 0.698 | 0.169 | 0.138 | 1.18 | 1.57 | 0.902 | 1.18 | 1.06 | 0.853 | 0.096 |
| logdha | rs191290051 | 0.022 | 0.693 | 0.163 | 0.119 | 0.105 | 0.27 | 0.381 | 3.65 | 6.06 | 0.436 | 0.467 |
| logdha | rs189631459 | 0.001 | . | . | . | . | . | . | . | . | . | . |
| logdha | rs114620874 | 0.005 | . | . | . | . | . | . | . | . | . | . |
| logdha | rs78246652 | 0.013 | 0.705 | 0.168 | 0.142 | 0.441 | 0.976 | 0.712 | 1.54 | 2.36 | 0.779 | 0.332 |
| logdha | rs79432451 | 0.005 | . | . | . | . | . | . | . | . | . | . |
| logdha | rs186022585 | 0.001 | . | . | . | . | . | . | . | . | . | . |
| logdha | rs142978891 | 0.005 | . | . | . | . | . | . | . | . | . | . |
| logdha | rs151086840 | 0.007 | . | . | . | . | . | . | . | . | . | . |
| logdha | rs183270259 | 0.006 | . | . | . | . | . | . | . | . | . | . |
| logdha | rs188544289 | 0.006 | . | . | . | . | . | . | . | . | . | . |
| logdha | rs188500808 | 0.001 | . | . | . | . | . | . | . | . | . | . |
| logdha | rs189424589 | 0.002 | . | . | . | . | . | . | . | . | . | . |
| logdha | rs78622303 | 0.025 | 0.659 | 0.157 | 0.079 | 6.50E-03 | 0.014 | 0.021 | 19 | 25.3 | 0.027 | 0.416 |
| logdha | rs143210835 | 0.008 | . | . | . | . | . | . | . | . | . | . |
| logdha | rs114761840 | 0.004 | . | . | . | . | . | . | . | . | . | . |
| logdha | rs185805402 | 0.003 | . | . | . | . | . | . | . | . | . | . |
| logdha | rs139273744 | 0.002 | . | . | . | . | . | . | . | . | . | . |
| logdha | rs184654150 | 0.004 | . | . | . | . | . | . | . | . | . | . |
| logdha | rs189069710 | 0 | . | . | . | . | . | . | . | . | . | . |
| logdha | rs114752115 | 0.018 | 0.717 | 0.171 | 0.162 | 1.6 | 3.02 | 0.805 | 0.595 | 0.831 | 0.71 | -0.633 |
| logdha | rs150162515 | 0.001 | . | . | . | . | . | . | . | . | . | . |
| logdha | rs180982454 | 0.004 | . | . | . | . | . | . | . | . | . | . |
| logdha | rs184932088 | 0.001 | . | . | . | . | . | . | . | . | . | . |
| logdha | rs181109625 | 0 | . | . | . | . | . | . | . | . | . | . |
| logdha | rs138121134 | 0.001 | . | . | . | . | . | . | . | . | . | . |
| logdha | rs183253861 | 0.003 | . | . | . | . | . | . | . | . | . | . |
| logdha | rs147073946 | 0.001 | . | . | . | . | . | . | . | . | . | . |
| logdha | rs139938489 | 0.005 | . | . | . | . | . | . | . | . | . | . |
| logdha | rs146362560 | 0.008 | . | . | . | . | . | . | . | . | . | . |
| logdha | rs193155956 | 0.004 | . | . | . | . | . | . | . | . | . | . |
| logdha | rs77663092 | 0.004 | . | . | . | . | . | . | . | . | . | . |
| logdha | rs73285319 | 0.021 | 0.694 | 0.168 | 0.132 | 0.833 | 1.16 | 0.895 | 1.52 | 1.46 | 0.663 | 0.351 |
| logdha | rs184019276 | 0.007 | . | . | . | . | . | . | . | . | . | . |
| logdha | rs28395863 | 0.017 | 0.699 | 0.169 | 0.138 | 2.02 | 3.27 | 0.663 | 1.01 | 1.14 | 0.991 | -0.289 |
| logdha | rs182121645 | 0.011 | 0.708 | 0.171 | 0.153 | 1.06 | 1.12 | 0.956 | 1.17 | 0.768 | 0.81 | 0.111 |
| logdha | rs73285327 | 0.016 | 0.696 | 0.168 | 0.132 | 0.33 | 0.564 | 0.517 | 2.33 | 2.63 | 0.456 | 0.508 |
| logdha | rs149589584 | 0.001 | . | . | . | . | . | . | . | . | . | . |
| logdha | rs148310335 | 0.005 | . | . | . | . | . | . | . | . | . | . |
| logdha | rs187246093 | 0.007 | . | . | . | . | . | . | . | . | . | . |
| logdha | rs184668894 | 0 | . | . | . | . | . | . | . | . | . | . |
| logdha | rs114269961 | 0.001 | . | . | . | . | . | . | . | . | . | . |
| logdha | rs149694374 | 0.02 | 0.7 | 0.169 | 0.14 | 1.37 | 2.17 | 0.842 | 1.15 | 1.27 | 0.902 | 0.03 |
| logdha | rs145284227 | 0.004 | . | . | . | . | . | . | . | . | . | . |
| logdha | rs189983820 | 0.004 | . | . | . | . | . | . | . | . | . | . |
| logdha | rs181200444 | 0 | . | . | . | . | . | . | . | . | . | . |
| logdha | rs185486385 | 0 | . | . | . | . | . | . | . | . | . | . |
| logdha | rs151198241 | 0.006 | . | . | . | . | . | . | . | . | . | . |
| logdha | rs189959341 | 0.006 | . | . | . | . | . | . | . | . | . | . |
| logdha | rs140549257 | 0.005 | . | . | . | . | . | . | . | . | . | . |
| logdha | rs186453139 | 0.016 | 0.692 | 0.168 | 0.129 | 1.11 | 1.4 | 0.933 | 1.47 | 1.29 | 0.659 | 0.329 |
| logdha | rs180922711 | 0.027 | 0.68 | 0.164 | 0.11 | 0.467 | 0.695 | 0.609 | 2.06 | 1.95 | 0.446 | 0.506 |
| logdha | rs56659903 | 0.004 | . | . | . | . | . | . | . | . | . | . |
| logdha | rs141202128 | 0.004 | . | . | . | . | . | . | . | . | . | . |
| logdha | rs59085314 | 0.033 | 0.686 | 0.166 | 0.12 | 0.093 | 0.189 | 0.242 | 3.86 | 5.05 | 0.303 | 0.467 |
| logdha | rs145564996 | 0.004 | . | . | . | . | . | . | . | . | . | . |
| logdha | rs114961009 | 0.006 | . | . | . | . | . | . | . | . | . | . |
| logdha | rs182294189 | 0.003 | . | . | . | . | . | . | . | . | . | . |
| logdha | rs187586636 | 0.005 | . | . | . | . | . | . | . | . | . | . |
| logdha | rs192254907 | 0.003 | . | . | . | . | . | . | . | . | . | . |
| logdha | rs149145663 | 0.001 | . | . | . | . | . | . | . | . | . | . |
| logdha | rs180713064 | 0.002 | . | . | . | . | . | . | . | . | . | . |
| logdha | rs183009023 | 0.004 | . | . | . | . | . | . | . | . | . | . |
| logdha | rs143385347 | 0.01 | 0.708 | 0.172 | 0.155 | 1.15 | 1.19 | 0.892 | 1.13 | 0.727 | 0.852 | 0.06 |
| logdha | rs185786329 | 0.004 | . | . | . | . | . | . | . | . | . | . |
| logdha | rs192341030 | 0.003 | . | . | . | . | . | . | . | . | . | . |

| logdha | rs74661429 | 0.009 | . | . | . | . | . | . | . | . | . | . |
| --- | --- | --- | --- | --- | --- | --- | --- | --- | --- | --- | --- | --- |
| logdha | rs188034853 | 0.001 | . | . | . | . | . | . | . | . | . | . |
| logdha | rs116808430 | 0.016 | 0.695 | 0.168 | 0.133 | 1.35 | 1.71 | 0.811 | 1.32 | 1.17 | 0.755 | 0.192 |
| logdha | rs148469010 | 0 | . | . | . | . | . | . | . | . | . | . |
| logdha | rs78621663 | 0.006 | . | . | . | . | . | . | . | . | . | . |
| logdha | rs192654886 | 0.001 | . | . | . | . | . | . | . | . | . | . |
| logdha | rs145996035 | 0.005 | . | . | . | . | . | . | . | . | . | . |
| logdha | rs139942226 | 0 | . | . | . | . | . | . | . | . | . | . |
| logdha | rs113645632 | 0.006 | . | . | . | . | . | . | . | . | . | . |
| logdha | rs74940661 | 0.016 | 0.705 | 0.17 | 0.147 | 1.8 | 2.84 | 0.709 | 1.02 | 1.14 | 0.983 | -0.206 |
| logdha | rs115355800 | 0.013 | 0.709 | 0.169 | 0.148 | 0.688 | 1.37 | 0.851 | 1.2 | 1.67 | 0.897 | 0.187 |
| logdha | rs150285898 | 0.005 | . | . | . | . | . | . | . | . | . | . |
| logdha | rs188117932 | 0.004 | . | . | . | . | . | . | . | . | . | . |
| logdha | rs139956566 | 0 | . | . | . | . | . | . | . | . | . | . |
| logdha | rs147673885 | 0 | . | . | . | . | . | . | . | . | . | . |
| logdha | rs189127928 | 0.005 | . | . | . | . | . | . | . | . | . | . |
| logdha | rs187558772 | 0.002 | . | . | . | . | . | . | . | . | . | . |
| logdha | rs181948587 | 0 | . | . | . | . | . | . | . | . | . | . |
| logdha | rs189983269 | 0.002 | . | . | . | . | . | . | . | . | . | . |
| logdha | rs187250069 | 0.002 | . | . | . | . | . | . | . | . | . | . |
| logdha | rs191485859 | 0.001 | . | . | . | . | . | . | . | . | . | . |
| logdha | rs185499555 | 0.002 | . | . | . | . | . | . | . | . | . | . |
| logdha | rs180862110 | 0.003 | . | . | . | . | . | . | . | . | . | . |
| logdha | rs190472603 | 0.005 | . | . | . | . | . | . | . | . | . | . |
| logdha | rs189226167 | 0.005 | . | . | . | . | . | . | . | . | . | . |
| logdha | rs181623256 | 0.002 | . | . | . | . | . | . | . | . | . | . |
| logdha | rs186079557 | 0.001 | . | . | . | . | . | . | . | . | . | . |
| logdha | rs147760994 | 0.002 | . | . | . | . | . | . | . | . | . | . |
| logdha | rs189176167 | 0 | . | . | . | . | . | . | . | . | . | . |
| logdha | rs117901000 | 0.001 | . | . | . | . | . | . | . | . | . | . |
| logdha | rs141984888 | 0 | . | . | . | . | . | . | . | . | . | . |
| logdha | rs192365061 | 0.007 | . | . | . | . | . | . | . | . | . | . |
| logdha | rs185976038 | 0 | . | . | . | . | . | . | . | . | . | . |
| logdha | rs184697303 | 0.002 | . | . | . | . | . | . | . | . | . | . |
| logdha | rs143383132 | 0.002 | . | . | . | . | . | . | . | . | . | . |
| logdha | rs141873881 | 0.004 | . | . | . | . | . | . | . | . | . | . |
| logdha | rs185507255 | 0 | . | . | . | . | . | . | . | . | . | . |
| logdha | rs191379425 | 0.001 | . | . | . | . | . | . | . | . | . | . |
| logdha | rs184199584 | 0.002 | . | . | . | . | . | . | . | . | . | . |
| logdha | rs183181360 | 0.002 | . | . | . | . | . | . | . | . | . | . |
| logdha | rs184495673 | 0.001 | . | . | . | . | . | . | . | . | . | . |
| logdha | rs182946297 | 0.001 | . | . | . | . | . | . | . | . | . | . |
| logdha | rs187743615 | 0 | . | . | . | . | . | . | . | . | . | . |
| logdha | rs183259862 | 0.001 | . | . | . | . | . | . | . | . | . | . |
| logdha | rs112433566 | 0 | . | . | . | . | . | . | . | . | . | . |
| logdha | rs182351085 | 0.001 | . | . | . | . | . | . | . | . | . | . |
| logdha | rs146248508 | 0.001 | . | . | . | . | . | . | . | . | . | . |
| logdha | rs188528439 | 0.001 | . | . | . | . | . | . | . | . | . | . |
| logdha | rs146167018 | 0.005 | . | . | . | . | . | . | . | . | . | . |
| logdha | rs192440980 | 0.004 | . | . | . | . | . | . | . | . | . | . |
| logdha | rs185601882 | 0.001 | . | . | . | . | . | . | . | . | . | . |
| logdha | rs117272563 | 0.002 | . | . | . | . | . | . | . | . | . | . |
| logdha | rs10430734 | 0.003 | . | . | . | . | . | . | . | . | . | . |
| logdha | rs181320952 | 0.001 | . | . | . | . | . | . | . | . | . | . |
| logdha | rs185480660 | 0.001 | . | . | . | . | . | . | . | . | . | . |
| logdha | rs188077917 | 0.001 | . | . | . | . | . | . | . | . | . | . |
| logdha | rs187562953 | 0.002 | . | . | . | . | . | . | . | . | . | . |
| logdha | rs182044631 | 0 | . | . | . | . | . | . | . | . | . | . |
| logdha | rs145455596 | 0.001 | . | . | . | . | . | . | . | . | . | . |
| logdha | rs181475877 | 0.004 | . | . | . | . | . | . | . | . | . | . |
| logdha | rs149413085 | 0.002 | . | . | . | . | . | . | . | . | . | . |
| logdha | rs12253656 | 0 | . | . | . | . | . | . | . | . | . | . |
| logdha | rs182718239 | 0 | . | . | . | . | . | . | . | . | . | . |
| logdha | rs192435695 | 0.002 | . | . | . | . | . | . | . | . | . | . |
| logdha | rs12248277 | 0 | . | . | . | . | . | . | . | . | . | . |
| logdha | rs143521710 | 0.001 | . | . | . | . | . | . | . | . | . | . |
| logdha | rs144454454 | 0 | . | . | . | . | . | . | . | . | . | . |
| logdha | rs190441610 | 0.004 | . | . | . | . | . | . | . | . | . | . |
| logdha | rs114690462 | 0 | . | . | . | . | . | . | . | . | . | . |
| logdha | rs184541357 | 0.001 | . | . | . | . | . | . | . | . | . | . |
| logdha | rs142657653 | 0 | . | . | . | . | . | . | . | . | . | . |
| logdha | rsurf1 | 2.146 | . | . | . | . | . | . | . | . | . | . |
| logdha | rsten1 | 2.304 | . | . | . | . | . | . | . | . | . | . |
| logdha | chr10:45852312:D | 0.086 | 0.743 | 0.117 | 0.059 | 1.56 | 0.659 | 0.292 | 0.753 | 0.237 | 0.366 | -0.431 |
| logdha | chr10:45853232:I | 0.1 | 0.739 | 0.117 | 0.057 | 1.32 | 0.55 | 0.507 | 0.811 | 0.246 | 0.489 | -0.268 |
| logdha | chr10:45853234:I | 0.168 | 0.746 | 0.124 | 0.078 | 1.09 | 0.354 | 0.78 | 0.858 | 0.212 | 0.535 | -0.14 |
| logdha | chr10:45854796:D | 0.013 | 0.718 | 0.109 | 0.028 | 1.74 | 1.86 | 0.605 | 0.862 | 0.745 | 0.863 | -0.382 |
| logdha | chr10:45854994:I | 0.019 | 0.716 | 0.108 | 0.027 | 1.29 | 1.45 | 0.82 | 0.878 | 0.747 | 0.878 | -0.196 |
| logdha | chr10:45858290:D | 0.045 | 0.761 | 0.119 | 0.081 | 2.88 | 1.94 | 0.117 | 0.458 | 0.241 | 0.137 | -1.64 |
| logdha | chr10:45858820:I | 0.015 | 0.757 | 0.117 | 0.073 | 8.82 | 8.87 | 0.03 | 0.152 | 0.135 | 0.034 | -7.56 |
| logdha | chr10:45860888:D | 0.081 | 0.696 | 0.11 | 0.022 | 0.7 | 0.381 | 0.513 | 1.2 | 0.481 | 0.647 | 0.189 |
| logdha | chr10:45863913:I | 0.165 | 0.743 | 0.123 | 0.072 | 1.1 | 0.351 | 0.769 | 0.871 | 0.213 | 0.571 | -0.131 |
| logdha | chr10:45866601:D | 0.036 | 0.777 | 0.125 | 0.116 | 1.53 | 1.59 | 0.682 | 0.584 | 0.436 | 0.471 | -0.614 |
| logdha | chr10:45867197:I | 0.006 | . | . | . | . | . | . | . | . | . | . |
| logdha | chr10:45867538:D | 0.171 | 0.721 | 0.119 | 0.047 | 0.978 | 0.308 | 0.943 | 0.971 | 0.229 | 0.9 | -0.014 |
| logdha | chr10:45868356:D | 0.161 | 0.746 | 0.123 | 0.076 | 1.13 | 0.366 | 0.71 | 0.852 | 0.209 | 0.515 | -0.157 |

| logdha | chr10:45871158:I | 0.167 | 0.742 | 0.123 | 0.071 | 1.1 | 0.347 | 0.767 | 0.875 | 0.21 | 0.578 | -0.127 |
| --- | --- | --- | --- | --- | --- | --- | --- | --- | --- | --- | --- | --- |
| logdha | chr10:45871637:D | 0.084 | 0.743 | 0.118 | 0.062 | 1.38 | 0.732 | 0.549 | 0.757 | 0.296 | 0.477 | -0.345 |
| logdha | chr10:45871638:I | 0.132 | 0.732 | 0.12 | 0.058 | 1.14 | 0.438 | 0.743 | 0.896 | 0.254 | 0.698 | -0.123 |
| logdha | chr10:45873629:I | 0.158 | 0.726 | 0.12 | 0.053 | 1.04 | 0.328 | 0.892 | 0.944 | 0.225 | 0.81 | -0.054 |
| logdha | chr10:45873701:D | 0.019 | 0.731 | 0.116 | 0.048 | 0.21 | 0.308 | 0.287 | 2.79 | 2.67 | 0.283 | 0.488 |
| logdha | chr10:45874997:I | 0.021 | 0.766 | 0.122 | 0.095 | 1.14 | 1.41 | 0.914 | 0.654 | 0.686 | 0.686 | -0.336 |
| logdha | chr10:45882030:I | 0.045 | 0.728 | 0.119 | 0.052 | 0.438 | 0.387 | 0.35 | 1.61 | 0.954 | 0.426 | 0.346 |
| logdha | chr10:45883774:D | 0.102 | 0.711 | 0.12 | 0.044 | 0.918 | 0.386 | 0.838 | 1.02 | 0.338 | 0.957 | 0.035 |
| logdha | chr10:45885169:D | 0.067 | 0.711 | 0.111 | 0.028 | 0.968 | 0.494 | 0.949 | 1.03 | 0.377 | 0.925 | 0.033 |
| logdha | chr10:45886451:D | 0.671 | 1.26 | 0.404 | 0.479 | 1.56 | 0.444 | 0.119 | 0.642 | 0.143 | 0.046 | -0.559 |
| logdha | chr10:45891800:I | 0.686 | 0.977 | 0.313 | 0.942 | 1.35 | 0.355 | 0.257 | 0.792 | 0.162 | 0.254 | -0.282 |
| logdha | chr10:45904771:I | 0.685 | 0.938 | 0.282 | 0.831 | 1.27 | 0.31 | 0.329 | 0.815 | 0.155 | 0.281 | -0.238 |
| logdha | chr10:45904959:I | 0.113 | 0.79 | 0.136 | 0.172 | 2.44 | 1.03 | 0.035 | 0.652 | 0.212 | 0.189 | -0.972 |
| logdha | chr10:45911028:I | 0.077 | 0.755 | 0.122 | 0.082 | 1.5 | 1.01 | 0.547 | 0.639 | 0.324 | 0.377 | -0.532 |
| logdha | chr10:45911031:I | 0.355 | 0.713 | 0.165 | 0.144 | 1.13 | 0.301 | 0.637 | 1.01 | 0.209 | 0.951 | -0.028 |
| logdha | chr10:45912154:D | 0.104 | 0.729 | 0.121 | 0.056 | 1.1 | 0.432 | 0.804 | 0.912 | 0.285 | 0.769 | -0.098 |
| logdha | chr10:45913885:I | 0.011 | 0.712 | 0.108 | 0.025 | 0.875 | 1.12 | 0.917 | 1.23 | 1.21 | 0.837 | 0.176 |
| logdha | chr10:45914753:D | 0.246 | 0.616 | 0.114 | 8.50E-03 | 0.818 | 0.254 | 0.519 | 1.32 | 0.304 | 0.228 | 0.231 |
| logdha | chr10:45919621 | 0.003 | . | . | . | . | . | . | . | . | . | . |
| logdha | chr10:45921739:I | 0.066 | 0.734 | 0.112 | 0.043 | 1.46 | 1.07 | 0.607 | 0.73 | 0.393 | 0.559 | -0.41 |
| logdha | chr10:45923527:I | 0.057 | 0.734 | 0.12 | 0.059 | 1.76 | 1.2 | 0.404 | 0.785 | 0.42 | 0.651 | -0.481 |
| logdha | chr10:45927001:I | 0.019 | 0.699 | 0.107 | 0.019 | 0.212 | 0.306 | 0.283 | 2.16 | 2.1 | 0.429 | 0.408 |
| logdha | chr10:45930354:I | 0.017 | 0.71 | 0.107 | 0.023 | 0.714 | 0.75 | 0.749 | 1.3 | 0.982 | 0.73 | 0.234 |
| logdha | chr10:45932864:I | 0.04 | 0.751 | 0.119 | 0.071 | 3.56 | 2.16 | 0.036 | 0.539 | 0.265 | 0.209 | -1.87 |
| logdha | chr10:45936163:I | 0.029 | 0.731 | 0.112 | 0.041 | 1.79 | 1.34 | 0.435 | 0.673 | 0.39 | 0.494 | -0.643 |
| logdha | chr10:45938095:D | 0.144 | 0.709 | 0.119 | 0.041 | 0.938 | 0.359 | 0.868 | 1.02 | 0.297 | 0.936 | 0.034 |
| logdha | chr10:45938886 | 0 | . | . | . | . | . | . | . | . | . | . |
| logdha | chr10:45939320 | 0.001 | . | . | . | . | . | . | . | . | . | . |
| logdha | chr10:45939659 | 0.002 | . | . | . | . | . | . | . | . | . | . |
| logdha | chr10:45941150:D | 0.012 | 0.713 | 0.108 | 0.025 | 0.977 | 1.13 | 0.984 | 1.07 | 0.942 | 0.939 | 0.055 |
| logdha | chr10:45947128:D | 0.058 | 0.65 | 0.105 | 7.70E-03 | 0.532 | 0.242 | 0.165 | 1.99 | 0.69 | 0.046 | 0.508 |
| logdha | chr10:45950990:I | 0.047 | 0.692 | 0.109 | 0.019 | 0.66 | 0.426 | 0.52 | 1.4 | 0.694 | 0.492 | 0.289 |
| logdha | chr10:45951854:I | 0.03 | 0.733 | 0.118 | 0.053 | 0.976 | 0.732 | 0.974 | 1.38 | 0.774 | 0.566 | 0.278 |
| logdha | chr10:45952670:I | 0.049 | 0.722 | 0.114 | 0.038 | 1.01 | 0.651 | 0.982 | 0.908 | 0.46 | 0.849 | -0.071 |
| logdha | chr10:45956741 | 0.001 | . | . | . | . | . | . | . | . | . | . |
| logdha | chr10:45957247:I | 0.22 | 0.705 | 0.129 | 0.056 | 1.03 | 0.287 | 0.918 | 1.03 | 0.228 | 0.889 | 0.014 |
| logdha | chr10:45958082:I | 0.216 | 0.697 | 0.128 | 0.049 | 0.998 | 0.287 | 0.994 | 1.06 | 0.242 | 0.809 | 0.04 |
| logdha | chr10:45905041:D | 0.053 | 0.668 | 0.145 | 0.064 | 1.05 | 1.13 | 0.967 | 0.858 | 0.678 | 0.847 | -0.114 |
| logdha | chr10:45938871 | 0.003 | . | . | . | . | . | . | . | . | . | . |
| logdha | chr10:45939160 | 0.004 | . | . | . | . | . | . | . | . | . | . |
| logdha | chr10:45869910 | 0.008 | . | . | . | . | . | . | . | . | . | . |
| logdha | chr10:45876214:D | 0.012 | 0.733 | 0.175 | 0.194 | 35.7 | 47.1 | 6.70E-03 | 0.015 | 0.018 | 5.50E-04 | -35.1 |
| logdha | chr10:45880237:I | 0.056 | 0.788 | 0.196 | 0.338 | 3.44 | 3.23 | 0.187 | 0.27 | 0.181 | 0.051 | -2.5 |
| logdha | chr10:45884371:I | 0.045 | 0.708 | 0.171 | 0.154 | 0.53 | 0.687 | 0.624 | 1.27 | 1.12 | 0.791 | 0.237 |
| logdha | chr10:45891535:D | 0.012 | 0.727 | 0.171 | 0.176 | 54.5 | 82.8 | 8.50E-03 | 0.016 | 0.017 | 7.70E-05 | -53.6 |
| logdha | chr10:45893516:D | 0.045 | 0.709 | 0.172 | 0.155 | 0.534 | 0.688 | 0.626 | 1.25 | 1.11 | 0.797 | 0.232 |
| logdha | chr10:45893782:D | 0.01 | 0.713 | 0.171 | 0.159 | 0.417 | 1.29 | 0.777 | 0.084 | 0.221 | 0.348 | -0.105 |
| logdha | chr10:45895254:I | 0.012 | 0.727 | 0.171 | 0.177 | 54 | 81.7 | 8.30E-03 | 0.016 | 0.017 | 7.70E-05 | -53.1 |
| logdha | chr10:45895848:D | 0.035 | 0.708 | 0.17 | 0.151 | 0.368 | 0.676 | 0.586 | 1.44 | 1.83 | 0.771 | 0.3 |
| logdha | chr10:45896316:D | 0.045 | 0.709 | 0.172 | 0.155 | 0.534 | 0.688 | 0.627 | 1.26 | 1.11 | 0.796 | 0.232 |
| logdha | chr10:45896317:D | 0.045 | 0.709 | 0.172 | 0.155 | 0.534 | 0.688 | 0.627 | 1.26 | 1.11 | 0.796 | 0.232 |
| logdha | chr10:45902683:I | 0.013 | 0.75 | 0.182 | 0.235 | 27.4 | 45.5 | 0.046 | 0.022 | 0.02 | 3.60E-05 | -26.7 |
| logdha | chr10:45905003:D | 0.006 | . | . | . | . | . | . | . | . | . | . |
| logdha | chr10:45927476:D | 0.021 | 0.699 | 0.169 | 0.139 | 1.17 | 1.76 | 0.915 | 1.26 | 1.31 | 0.826 | 0.159 |
| logdha | chr10:45935882 | 0.001 | . | . | . | . | . | . | . | . | . | . |
| logdha | chr10:45936100 | 0.001 | . | . | . | . | . | . | . | . | . | . |
| logdha | chr10:45939553 | 0.003 | . | . | . | . | . | . | . | . | . | . |
| logdha | chr10:45939631 | 0 | . | . | . | . | . | . | . | . | . | . |
| logdha | chr10:45944193:D | 0.008 | . | . | . | . | . | . | . | . | . | . |
| logdha | chr10:45954030 | 0 | . | . | . | . | . | . | . | . | . | . |

Supplemental table S37.

| fa | rs_id | freq | HR_logdha | se_logdha | p_logdha | HR_snp | se_snp | p_snp | HR_inter | se_inter | p_inter | RERI |
| --- | --- | --- | --- | --- | --- | --- | --- | --- | --- | --- | --- | --- |
| logdha | rs76035182 | 0.001 | . | . | . | . | . | . | . | . | . | . |
| logdha | rs12359547 | 0.105 | 0.826 | 0.188 | 0.401 | 0.794 | 0.558 | 0.743 | 1.13 | 0.638 | 0.829 | 0.121 |
| logdha | rs74595484 | 0 | . | . | . | . | . | . | . | . | . | . |
| logdha | rs1864415 | 0.605 | 0.863 | 0.385 | 0.741 | 1.14 | 0.405 | 0.715 | 0.984 | 0.295 | 0.958 | -0.034 |
| logdha | rs72788101 | 0.105 | 0.826 | 0.188 | 0.402 | 0.795 | 0.558 | 0.744 | 1.13 | 0.638 | 0.829 | 0.121 |
| logdha | rs11239481 | 0.183 | 1.01 | 0.248 | 0.953 | 1.72 | 0.702 | 0.183 | 0.546 | 0.2 | 0.099 | -0.782 |
| logdha | rs72788102 | 0.105 | 0.826 | 0.188 | 0.402 | 0.795 | 0.558 | 0.744 | 1.13 | 0.638 | 0.829 | 0.121 |
| logdha | rs72789705 | 0.105 | 0.826 | 0.188 | 0.402 | 0.795 | 0.558 | 0.744 | 1.13 | 0.638 | 0.829 | 0.121 |
| logdha | rs141529858 | 0.008 | . | . | . | . | . | . | . | . | . | . |
| logdha | rs145376292 | 0 | . | . | . | . | . | . | . | . | . | . |
| logdha | rs190418301 | 0.001 | . | . | . | . | . | . | . | . | . | . |
| logdha | rs11239482 | 0.105 | 0.826 | 0.188 | 0.402 | 0.796 | 0.559 | 0.745 | 1.13 | 0.637 | 0.829 | 0.121 |
| logdha | rs61855872 | 0.014 | 0.932 | 0.203 | 0.746 | 67.2 | 132 | 0.033 | 9.90E-03 | 0.023 | 0.049 | -66.5 |
| logdha | rs148991144 | 0.011 | 0.85 | 0.182 | 0.449 | 0.063 | 0.085 | 0.041 | 0.655 | 0.702 | 0.693 | 0.122 |
| logdha | rs191932221 | 0.007 | . | . | . | . | . | . | . | . | . | . |
| logdha | rs55978138 | 0.159 | 0.84 | 0.198 | 0.46 | 0.934 | 0.487 | 0.896 | 1.02 | 0.442 | 0.972 | 0.023 |
| logdha | rs73281167 | 0 | . | . | . | . | . | . | . | . | . | . |
| logdha | rs12768010 | 0.055 | 0.857 | 0.19 | 0.485 | 1.23 | 0.952 | 0.788 | 0.854 | 0.557 | 0.808 | -0.187 |
| logdha | rs11239484 | 0.605 | 0.86 | 0.384 | 0.736 | 1.14 | 0.403 | 0.722 | 0.987 | 0.296 | 0.965 | -0.032 |
| logdha | rs35906360 | 0.16 | 0.84 | 0.198 | 0.46 | 0.934 | 0.487 | 0.897 | 1.02 | 0.442 | 0.972 | 0.023 |
| logdha | rs186250512 | 0.004 | . | . | . | . | . | . | . | . | . | . |
| logdha | rs150810743 | 0 | . | . | . | . | . | . | . | . | . | . |
| logdha | rs55965812 | 0.16 | 0.84 | 0.198 | 0.46 | 0.935 | 0.487 | 0.897 | 1.02 | 0.442 | 0.972 | 0.023 |
| logdha | rs56180358 | 0.16 | 0.84 | 0.198 | 0.46 | 0.935 | 0.488 | 0.897 | 1.02 | 0.442 | 0.972 | 0.022 |
| logdha | rs55864145 | 0.055 | 0.857 | 0.19 | 0.485 | 1.23 | 0.952 | 0.788 | 0.854 | 0.557 | 0.808 | -0.187 |
| logdha | rs55986122 | 0.16 | 0.84 | 0.198 | 0.459 | 0.934 | 0.487 | 0.896 | 1.02 | 0.442 | 0.971 | 0.023 |
| logdha | rs7477350 | 0.605 | 0.859 | 0.383 | 0.733 | 1.13 | 0.403 | 0.725 | 0.988 | 0.296 | 0.968 | -0.03 |
| logdha | rs2099170 | 0.159 | 0.84 | 0.198 | 0.46 | 0.937 | 0.488 | 0.901 | 1.02 | 0.441 | 0.971 | 0.022 |
| logdha | rs2082108 | 0.16 | 0.84 | 0.198 | 0.459 | 0.934 | 0.487 | 0.896 | 1.02 | 0.442 | 0.971 | 0.023 |
| logdha | rs55902404 | 0 | . | . | . | . | . | . | . | . | . | . |
| logdha | rs58402662 | 0 | . | . | . | . | . | . | . | . | . | . |
| logdha | rs11511618 | 0.182 | 1.02 | 0.249 | 0.924 | 1.77 | 0.721 | 0.162 | 0.526 | 0.194 | 0.081 | -0.84 |
| logdha | rs1816492 | 0.605 | 0.858 | 0.383 | 0.732 | 1.13 | 0.403 | 0.726 | 0.989 | 0.296 | 0.97 | -0.03 |
| logdha | rs146611093 | 0.01 | 0.78 | 0.165 | 0.238 | 9.60E-04 | 2.80E-03 | 0.016 | 108 | 153 | 1.00E-03 | 0.3 |
| logdha | rs186897689 | 0 | . | . | . | . | . | . | . | . | . | . |
| logdha | rs10793616 | 0.605 | 0.858 | 0.383 | 0.731 | 1.13 | 0.402 | 0.727 | 0.989 | 0.297 | 0.971 | -0.029 |
| logdha | rs116700479 | 0.001 | . | . | . | . | . | . | . | . | . | . |
| logdha | rs192535936 | 0 | . | . | . | . | . | . | . | . | . | . |
| logdha | rs113363297 | 0.571 | 0.762 | 0.339 | 0.54 | 1.03 | 0.375 | 0.929 | 1.09 | 0.334 | 0.779 | 0.063 |
| logdha | rs12354994 | 0.152 | 0.85 | 0.201 | 0.492 | 0.98 | 0.553 | 0.971 | 0.974 | 0.452 | 0.955 | -0.019 |
| logdha | rs148068329 | 0.001 | . | . | . | . | . | . | . | . | . | . |
| logdha | rs12356109 | 0.16 | 0.84 | 0.198 | 0.459 | 0.934 | 0.487 | 0.896 | 1.02 | 0.442 | 0.971 | 0.023 |
| logdha | rs55908141 | 0 | . | . | . | . | . | . | . | . | . | . |
| logdha | rs12354712 | 0.159 | 0.84 | 0.198 | 0.46 | 0.936 | 0.488 | 0.899 | 1.02 | 0.441 | 0.973 | 0.022 |
| logdha | rs147282684 | 0.004 | . | . | . | . | . | . | . | . | . | . |
| logdha | rs12356140 | 0.16 | 0.839 | 0.198 | 0.456 | 0.93 | 0.485 | 0.89 | 1.02 | 0.443 | 0.962 | 0.028 |
| logdha | rs185476187 | 0 | . | . | . | . | . | . | . | . | . | . |
| logdha | rs145720057 | 0.039 | 0.878 | 0.192 | 0.55 | 2.07 | 2.12 | 0.478 | 0.534 | 0.474 | 0.479 | -0.976 |
| logdha | rs12354742 | 0.159 | 0.84 | 0.198 | 0.46 | 0.936 | 0.488 | 0.898 | 1.02 | 0.441 | 0.972 | 0.022 |
| logdha | rs180913842 | 0 | . | . | . | . | . | . | . | . | . | . |
| logdha | rs142976914 | 0.006 | . | . | . | . | . | . | . | . | . | . |
| logdha | rs11511619 | 0.163 | 0.836 | 0.198 | 0.449 | 0.911 | 0.476 | 0.859 | 1.03 | 0.448 | 0.938 | 0.041 |
| logdha | rs146702870 | 0 | . | . | . | . | . | . | . | . | . | . |
| logdha | rs150287496 | 0.002 | . | . | . | . | . | . | . | . | . | . |
| logdha | rs190917973 | 0.006 | . | . | . | . | . | . | . | . | . | . |
| logdha | rs11517281 | 0.16 | 0.84 | 0.198 | 0.46 | 0.935 | 0.487 | 0.897 | 1.02 | 0.441 | 0.971 | 0.023 |
| logdha | rs12359299 | 0.16 | 0.84 | 0.198 | 0.46 | 0.934 | 0.487 | 0.897 | 1.02 | 0.442 | 0.972 | 0.022 |
| logdha | rs182307932 | 0 | . | . | . | . | . | . | . | . | . | . |
| logdha | rs61855873 | 0.035 | 0.927 | 0.213 | 0.743 | 4.55 | 6.35 | 0.278 | 0.204 | 0.263 | 0.218 | -3.62 |
| logdha | rs12359302 | 0.156 | 0.85 | 0.201 | 0.491 | 0.995 | 0.54 | 0.992 | 0.977 | 0.439 | 0.958 | -0.019 |
| logdha | rs143208140 | 0.003 | . | . | . | . | . | . | . | . | . | . |
| logdha | rs9988764 | 0.595 | 0.887 | 0.403 | 0.792 | 1.17 | 0.429 | 0.665 | 0.963 | 0.298 | 0.903 | -0.058 |
| logdha | rs192875767 | 0.008 | . | . | . | . | . | . | . | . | . | . |
| logdha | rs147268846 | 0.015 | 0.889 | 0.191 | 0.583 | 9.73 | 11.8 | 0.061 | 0.016 | 0.022 | 2.50E-03 | -9.48 |
| logdha | rs11239485 | 0.111 | 0.771 | 0.177 | 0.258 | 0.576 | 0.405 | 0.433 | 1.56 | 0.879 | 0.432 | 0.345 |
| logdha | rs151317885 | 0.436 | 0.864 | 0.32 | 0.693 | 1.18 | 0.417 | 0.642 | 0.98 | 0.279 | 0.942 | -0.045 |
| logdha | rs11499085 | 0 | . | . | . | . | . | . | . | . | . | . |
| logdha | rs188130606 | 0 | . | . | . | . | . | . | . | . | . | . |
| logdha | rs11511620 | 0.16 | 0.84 | 0.198 | 0.46 | 0.935 | 0.487 | 0.897 | 1.02 | 0.441 | 0.971 | 0.023 |
| logdha | rs181498390 | 0 | . | . | . | . | . | . | . | . | . | . |
| logdha | rs186280701 | 0 | . | . | . | . | . | . | . | . | . | . |
| logdha | rs150059185 | 0.011 | 0.858 | 0.188 | 0.485 | 3.13 | 10.1 | 0.724 | 0.444 | 1.08 | 0.74 | -1.79 |
| logdha | rs183338707 | 0.004 | . | . | . | . | . | . | . | . | . | . |
| logdha | rs11499038 | 0.002 | . | . | . | . | . | . | . | . | . | . |
| logdha | rs190699595 | 0.008 | . | . | . | . | . | . | . | . | . | . |
| logdha | rs11523408 | 0.16 | 0.84 | 0.198 | 0.46 | 0.935 | 0.487 | 0.898 | 1.02 | 0.441 | 0.972 | 0.023 |
| logdha | rs188049317 | 0 | . | . | . | . | . | . | . | . | . | . |
| logdha | rs144948634 | 0.428 | 0.879 | 0.324 | 0.727 | 1.17 | 0.423 | 0.671 | 0.958 | 0.284 | 0.886 | -0.063 |
| logdha | rs143833576 | 0.001 | . | . | . | . | . | . | . | . | . | . |
| logdha | rs151239879 | 0.16 | 0.846 | 0.199 | 0.478 | 0.972 | 0.5 | 0.957 | 0.989 | 0.426 | 0.979 | -5.00E-03 |
| logdha | rs7073743 | 0.438 | 0.882 | 0.331 | 0.738 | 1.16 | 0.417 | 0.67 | 0.958 | 0.282 | 0.885 | -0.062 |
| logdha | rs142563349 | 0 | . | . | . | . | . | . | . | . | . | . |
| logdha | rs187246403 | 0 | . | . | . | . | . | . | . | . | . | . |
| logdha | rs150423942 | 0.158 | 0.847 | 0.198 | 0.478 | 0.953 | 0.505 | 0.927 | 0.989 | 0.435 | 0.98 | -1.50E-03 |

| logdha | rs184706102 | 0.002 | . | . | . | . | . | . | . | . | . | . |
| --- | --- | --- | --- | --- | --- | --- | --- | --- | --- | --- | --- | --- |
| logdha | rs189626288 | 0.031 | 0.84 | 0.19 | 0.441 | 1.17 | 1.09 | 0.863 | 1.35 | 1.11 | 0.718 | 0.314 |
| logdha | rs140749834 | 0 | . | . | . | . | . | . | . | . | . | . |
| logdha | rs7078822 | 0.131 | 0.835 | 0.194 | 0.439 | 0.891 | 0.545 | 0.851 | 1.05 | 0.524 | 0.925 | 0.054 |
| logdha | rs186227617 | 0.159 | 0.84 | 0.198 | 0.459 | 0.936 | 0.488 | 0.899 | 1.02 | 0.442 | 0.969 | 0.024 |
| logdha | rs188984368 | 0.025 | 0.832 | 0.181 | 0.399 | 0.221 | 0.397 | 0.401 | 1.69 | 2.38 | 0.709 | 0.257 |
| logdha | rs182078109 | 0.027 | 0.838 | 0.183 | 0.418 | 0.21 | 0.424 | 0.439 | 1.43 | 2.33 | 0.825 | 0.204 |
| logdha | rs141456274 | 0.002 | . | . | . | . | . | . | . | . | . | . |
| logdha | rs146976512 | 0.16 | 0.84 | 0.198 | 0.459 | 0.935 | 0.487 | 0.898 | 1.02 | 0.441 | 0.971 | 0.023 |
| logdha | rs138036816 | 0 | . | . | . | . | . | . | . | . | . | . |
| logdha | rs114879777 | 0.001 | . | . | . | . | . | . | . | . | . | . |
| logdha | rs149366475 | 0 | . | . | . | . | . | . | . | . | . | . |
| logdha | rs182463043 | 0 | . | . | . | . | . | . | . | . | . | . |
| logdha | rs144754632 | 0.001 | . | . | . | . | . | . | . | . | . | . |
| logdha | rs55717607 | 0.16 | 0.84 | 0.198 | 0.459 | 0.935 | 0.487 | 0.898 | 1.02 | 0.441 | 0.971 | 0.023 |
| logdha | rs187537973 | 0 | . | . | . | . | . | . | . | . | . | . |
| logdha | rs148085971 | 0 | . | . | . | . | . | . | . | . | . | . |
| logdha | rs11239491 | 0.16 | 0.84 | 0.198 | 0.459 | 0.936 | 0.487 | 0.898 | 1.02 | 0.441 | 0.971 | 0.023 |
| logdha | rs184412184 | 0.008 | . | . | . | . | . | . | . | . | . | . |
| logdha | rs4948998 | 0.607 | 0.865 | 0.388 | 0.747 | 1.15 | 0.409 | 0.703 | 0.983 | 0.296 | 0.954 | -0.037 |
| logdha | rs7076696 | 0.607 | 0.864 | 0.388 | 0.745 | 1.14 | 0.408 | 0.707 | 0.984 | 0.296 | 0.956 | -0.036 |
| logdha | rs11239492 | 0.16 | 0.84 | 0.198 | 0.459 | 0.934 | 0.486 | 0.895 | 1.02 | 0.442 | 0.969 | 0.024 |
| logdha | rs79462470 | 0 | . | . | . | . | . | . | . | . | . | . |
| logdha | rs187804654 | 0.003 | . | . | . | . | . | . | . | . | . | . |
| logdha | rs7093687 | 0.16 | 0.841 | 0.198 | 0.461 | 0.939 | 0.488 | 0.903 | 1.01 | 0.44 | 0.976 | 0.02 |
| logdha | rs7081004 | 0.16 | 0.84 | 0.198 | 0.46 | 0.936 | 0.487 | 0.899 | 1.02 | 0.441 | 0.972 | 0.022 |
| logdha | rs7084793 | 0.16 | 0.84 | 0.198 | 0.46 | 0.936 | 0.487 | 0.898 | 1.02 | 0.441 | 0.971 | 0.023 |
| logdha | rs61556080 | 0 | . | . | . | . | . | . | . | . | . | . |
| logdha | rs115552064 | . | . | . | . | . | . | . | . | . | . | . |
| logdha | rs7921503 | 0.16 | 0.84 | 0.198 | 0.46 | 0.936 | 0.487 | 0.898 | 1.02 | 0.441 | 0.971 | 0.023 |
| logdha | rs7909740 | 0.16 | 0.84 | 0.198 | 0.46 | 0.936 | 0.487 | 0.898 | 1.02 | 0.441 | 0.971 | 0.023 |
| logdha | rs7909768 | 0.16 | 0.84 | 0.198 | 0.46 | 0.936 | 0.487 | 0.898 | 1.02 | 0.441 | 0.971 | 0.023 |
| logdha | rs144168004 | 0 | . | . | . | . | . | . | . | . | . | . |
| logdha | rs75850448 | 0 | . | . | . | . | . | . | . | . | . | . |
| logdha | rs147777574 | 0 | . | . | . | . | . | . | . | . | . | . |
| logdha | rs7913488 | 0.16 | 0.84 | 0.198 | 0.46 | 0.936 | 0.487 | 0.899 | 1.02 | 0.441 | 0.972 | 0.023 |
| logdha | rs74390874 | 0.071 | 0.811 | 0.198 | 0.391 | 0.948 | 0.68 | 0.941 | 1.32 | 0.821 | 0.651 | 0.259 |
| logdha | rs141391142 | 0.009 | . | . | . | . | . | . | . | . | . | . |
| logdha | rs115294747 | 0.001 | . | . | . | . | . | . | . | . | . | . |
| logdha | rs148031781 | 0.003 | . | . | . | . | . | . | . | . | . | . |
| logdha | rs181340128 | 0.021 | 0.93 | 0.201 | 0.737 | 9.44 | 8.13 | 9.10E-03 | 0.032 | 0.031 | 3.90E-04 | -9.09 |
| logdha | rs189930007 | 0.002 | . | . | . | . | . | . | . | . | . | . |
| logdha | rs12242128 | 0 | . | . | . | . | . | . | . | . | . | . |
| logdha | rs78421750 | 0.002 | . | . | . | . | . | . | . | . | . | . |
| logdha | rs114370747 | 0.002 | . | . | . | . | . | . | . | . | . | . |
| logdha | rs12264166 | 0 | . | . | . | . | . | . | . | . | . | . |
| logdha | rs182964822 | 0.001 | . | . | . | . | . | . | . | . | . | . |
| logdha | rs67491417 | 0.15 | 0.86 | 0.203 | 0.525 | 1.1 | 0.599 | 0.867 | 0.93 | 0.422 | 0.873 | -0.079 |
| logdha | rs72789720 | 0.024 | 0.78 | 0.176 | 0.272 | 0.117 | 0.21 | 0.233 | 6.66 | 8.96 | 0.159 | 0.71 |
| logdha | rs6413416 | 0.16 | 0.844 | 0.2 | 0.473 | 0.98 | 0.503 | 0.969 | 0.999 | 0.426 | 0.999 | 2.60E-03 |
| logdha | rs4986832 | 0.16 | 0.84 | 0.198 | 0.46 | 0.936 | 0.487 | 0.899 | 1.02 | 0.441 | 0.972 | 0.022 |
| logdha | rs144866316 | 0.005 | . | . | . | . | . | . | . | . | . | . |
| logdha | rs187769540 | 0.001 | . | . | . | . | . | . | . | . | . | . |
| logdha | rs6593482 | 0.16 | 0.856 | 0.203 | 0.514 | 0.995 | 0.502 | 0.992 | 0.954 | 0.402 | 0.911 | -0.038 |
| logdha | rs7913948 | 0.16 | 0.841 | 0.198 | 0.463 | 0.94 | 0.489 | 0.905 | 1.01 | 0.439 | 0.979 | 0.019 |
| logdha | rs77185151 | 0.001 | . | . | . | . | . | . | . | . | . | . |
| logdha | rs12762303 | 0.16 | 0.84 | 0.198 | 0.459 | 0.934 | 0.487 | 0.896 | 1.02 | 0.441 | 0.971 | 0.023 |
| logdha | rs73283136 | 0.003 | . | . | . | . | . | . | . | . | . | . |
| logdha | rs187180218 | 0 | . | . | . | . | . | . | . | . | . | . |
| logdha | rs78137087 | 0.159 | 0.838 | 0.198 | 0.455 | 0.928 | 0.484 | 0.885 | 1.02 | 0.445 | 0.959 | 0.029 |
| logdha | rs72561472 | 0.158 | 0.842 | 0.199 | 0.466 | 0.911 | 0.479 | 0.859 | 1.01 | 0.448 | 0.983 | 0.022 |
| logdha | rs4987106 | 0.152 | 0.851 | 0.2 | 0.491 | 0.954 | 0.515 | 0.93 | 0.971 | 0.442 | 0.948 | -0.017 |
| logdha | rs4987105 | 0.155 | 0.85 | 0.199 | 0.487 | 0.935 | 0.499 | 0.899 | 0.974 | 0.44 | 0.954 | -0.011 |
| logdha | rs111916157 | 0.188 | 0.847 | 0.21 | 0.502 | 1.07 | 0.495 | 0.889 | 0.992 | 0.391 | 0.983 | -0.018 |
| logdha | rs111528152 | 0.16 | 0.84 | 0.198 | 0.459 | 0.933 | 0.486 | 0.894 | 1.02 | 0.442 | 0.97 | 0.024 |
| logdha | rs12763738 | 0.16 | 0.84 | 0.198 | 0.459 | 0.934 | 0.486 | 0.895 | 1.02 | 0.441 | 0.97 | 0.024 |
| logdha | rs191675886 | 0.006 | . | . | . | . | . | . | . | . | . | . |
| logdha | rs66489870 | 0.16 | 0.84 | 0.198 | 0.459 | 0.934 | 0.486 | 0.895 | 1.02 | 0.441 | 0.97 | 0.024 |
| logdha | rs71494775 | 0.16 | 0.84 | 0.198 | 0.459 | 0.934 | 0.486 | 0.895 | 1.02 | 0.441 | 0.97 | 0.024 |
| logdha | rs61855875 | 0.033 | 0.914 | 0.209 | 0.693 | 3.86 | 5.7 | 0.362 | 0.234 | 0.317 | 0.284 | -2.94 |
| logdha | rs116356837 | 0 | . | . | . | . | . | . | . | . | . | . |
| logdha | rs1864414 | 0.16 | 0.84 | 0.198 | 0.459 | 0.934 | 0.486 | 0.895 | 1.02 | 0.441 | 0.97 | 0.024 |
| logdha | rs1864413 | 0.16 | 0.839 | 0.198 | 0.457 | 0.932 | 0.485 | 0.892 | 1.02 | 0.443 | 0.964 | 0.026 |
| logdha | rs139497212 | 0 | . | . | . | . | . | . | . | . | . | . |
| logdha | rs892690 | 0.359 | 0.997 | 0.357 | 0.994 | 1.48 | 0.576 | 0.314 | 0.809 | 0.254 | 0.5 | -0.283 |
| logdha | rs1864412 | 0.16 | 0.84 | 0.198 | 0.458 | 0.933 | 0.486 | 0.893 | 1.02 | 0.442 | 0.968 | 0.025 |
| logdha | rs77827771 | 0.004 | . | . | . | . | . | . | . | . | . | . |
| logdha | rs145294963 | 0.004 | . | . | . | . | . | . | . | . | . | . |
| logdha | rs149081327 | 0.012 | 0.837 | 0.186 | 0.423 | 1.7 | 2.43 | 0.711 | 1.11 | 1.2 | 0.923 | 0.042 |
| logdha | rs67008983 | 0.16 | 0.839 | 0.198 | 0.455 | 0.927 | 0.483 | 0.884 | 1.02 | 0.444 | 0.96 | 0.029 |
| logdha | rs56150414 | 0.168 | 0.85 | 0.203 | 0.495 | 0.977 | 0.481 | 0.963 | 0.976 | 0.404 | 0.954 | -0.016 |
| logdha | rs55787481 | 0.16 | 0.839 | 0.198 | 0.458 | 0.929 | 0.484 | 0.887 | 1.02 | 0.444 | 0.965 | 0.026 |
| logdha | rs141270101 | 0.001 | . | . | . | . | . | . | . | . | . | . |
| logdha | rs114980075 | 0.002 | . | . | . | . | . | . | . | . | . | . |
| logdha | rs12783095 | 0.159 | 0.83 | 0.196 | 0.43 | 0.875 | 0.463 | 0.801 | 1.06 | 0.466 | 0.895 | 0.065 |
| logdha | rs182342688 | 0.005 | . | . | . | . | . | . | . | . | . | . |

| logdha | rs17157728 | 0.159 | 0.83 | 0.195 | 0.43 | 0.875 | 0.463 | 0.8 | 1.06 | 0.466 | 0.894 | 0.065 |
| --- | --- | --- | --- | --- | --- | --- | --- | --- | --- | --- | --- | --- |
| logdha | rs114835563 | 0 | . | . | . | . | . | . | . | . | . | . |
| logdha | rs3824612 | 0.359 | 0.989 | 0.353 | 0.975 | 1.46 | 0.566 | 0.33 | 0.818 | 0.256 | 0.522 | -0.267 |
| logdha | rs17157733 | 0.158 | 0.829 | 0.195 | 0.427 | 0.87 | 0.461 | 0.793 | 1.07 | 0.468 | 0.886 | 0.069 |
| logdha | rs74128478 | 0 | . | . | . | . | . | . | . | . | . | . |
| logdha | rs184973589 | 0.001 | . | . | . | . | . | . | . | . | . | . |
| logdha | rs150479461 | 0 | . | . | . | . | . | . | . | . | . | . |
| logdha | rs138310407 | 0.004 | . | . | . | . | . | . | . | . | . | . |
| logdha | rs187426603 | 0.001 | . | . | . | . | . | . | . | . | . | . |
| logdha | rs190843143 | 0.003 | . | . | . | . | . | . | . | . | . | . |
| logdha | rs191883449 | 0.003 | . | . | . | . | . | . | . | . | . | . |
| logdha | rs58238740 | 0.158 | 0.829 | 0.195 | 0.425 | 0.866 | 0.46 | 0.787 | 1.07 | 0.47 | 0.882 | 0.071 |
| logdha | rs3780894 | 0.158 | 0.829 | 0.195 | 0.425 | 0.866 | 0.459 | 0.786 | 1.07 | 0.47 | 0.881 | 0.072 |
| logdha | rs149646626 | 0.027 | 0.918 | 0.202 | 0.697 | 7.32 | 8.32 | 0.08 | 0.071 | 0.08 | 0.018 | -6.76 |
| logdha | rs79615212 | 0.023 | 0.996 | 0.217 | 0.984 | 236 | 495 | 9.10E-03 | 1.70E-03 | 4.00E-03 | 6.70E-03 | -236 |
| logdha | rs3780895 | 0.158 | 0.829 | 0.195 | 0.425 | 0.866 | 0.459 | 0.786 | 1.07 | 0.47 | 0.88 | 0.072 |
| logdha | rs3780896 | 0.158 | 0.829 | 0.195 | 0.425 | 0.866 | 0.459 | 0.786 | 1.07 | 0.47 | 0.88 | 0.072 |
| logdha | rs142747163 | 0.006 | . | . | . | . | . | . | . | . | . | . |
| logdha | rs189417584 | 0.005 | . | . | . | . | . | . | . | . | . | . |
| logdha | rs3780897 | 0.158 | 0.829 | 0.195 | 0.425 | 0.867 | 0.46 | 0.787 | 1.07 | 0.47 | 0.881 | 0.072 |
| logdha | rs117509491 | 0.004 | . | . | . | . | . | . | . | . | . | . |
| logdha | rs117811514 | 0.004 | . | . | . | . | . | . | . | . | . | . |
| logdha | rs116500932 | 0 | . | . | . | . | . | . | . | . | . | . |
| logdha | rs77584168 | 0.001 | . | . | . | . | . | . | . | . | . | . |
| logdha | rs2310776 | 0.158 | 0.828 | 0.195 | 0.424 | 0.864 | 0.459 | 0.784 | 1.07 | 0.471 | 0.879 | 0.073 |
| logdha | rs17157736 | 0.158 | 0.828 | 0.195 | 0.424 | 0.865 | 0.459 | 0.784 | 1.07 | 0.471 | 0.879 | 0.073 |
| logdha | rs72789735 | 0.105 | 0.825 | 0.188 | 0.4 | 0.798 | 0.56 | 0.748 | 1.13 | 0.638 | 0.824 | 0.123 |
| logdha | rs183747187 | 0.002 | . | . | . | . | . | . | . | . | . | . |
| logdha | rs7077891 | 0.158 | 0.828 | 0.195 | 0.424 | 0.864 | 0.459 | 0.783 | 1.07 | 0.471 | 0.878 | 0.073 |
| logdha | rs145063305 | 0.006 | . | . | . | . | . | . | . | . | . | . |
| logdha | rs3824613 | 0.159 | 0.829 | 0.195 | 0.425 | 0.866 | 0.459 | 0.786 | 1.07 | 0.47 | 0.881 | 0.072 |
| logdha | rs145543199 | 0.014 | 0.805 | 0.18 | 0.333 | 0.114 | 0.37 | 0.503 | 6.66 | 16.4 | 0.441 | 0.694 |
| logdha | rs140361993 | 0.005 | . | . | . | . | . | . | . | . | . | . |
| logdha | rs11239496 | 0.105 | 0.825 | 0.188 | 0.4 | 0.798 | 0.56 | 0.748 | 1.13 | 0.638 | 0.824 | 0.123 |
| logdha | rs11239497 | 0.158 | 0.828 | 0.195 | 0.424 | 0.864 | 0.459 | 0.783 | 1.07 | 0.471 | 0.878 | 0.073 |
| logdha | rs10900212 | 0.158 | 0.828 | 0.195 | 0.424 | 0.863 | 0.459 | 0.782 | 1.07 | 0.471 | 0.878 | 0.074 |
| logdha | rs186487136 | 0 | . | . | . | . | . | . | . | . | . | . |
| logdha | rs12774369 | 0.158 | 0.829 | 0.195 | 0.425 | 0.862 | 0.46 | 0.781 | 1.07 | 0.473 | 0.879 | 0.073 |
| logdha | rs60842538 | 0.158 | 0.829 | 0.195 | 0.425 | 0.861 | 0.46 | 0.779 | 1.07 | 0.475 | 0.88 | 0.073 |
| logdha | rs3780898 | 0.158 | 0.829 | 0.195 | 0.426 | 0.859 | 0.461 | 0.777 | 1.07 | 0.476 | 0.881 | 0.073 |
| logdha | rs3780899 | 0.158 | 0.829 | 0.195 | 0.426 | 0.858 | 0.461 | 0.776 | 1.07 | 0.477 | 0.881 | 0.073 |
| logdha | rs12252472 | 0 | . | . | . | . | . | . | . | . | . | . |
| logdha | rs185411011 | 0.003 | . | . | . | . | . | . | . | . | . | . |
| logdha | rs28395865 | 0.156 | 0.822 | 0.193 | 0.406 | 0.82 | 0.449 | 0.717 | 1.1 | 0.501 | 0.828 | 0.102 |
| logdha | rs191004074 | 0.024 | 0.807 | 0.176 | 0.327 | 0.211 | 0.549 | 0.55 | 2.82 | 5.71 | 0.61 | 0.462 |
| logdha | rs182401295 | 0.004 | . | . | . | . | . | . | . | . | . | . |
| logdha | rs2228064 | 0.002 | . | . | . | . | . | . | . | . | . | . |
| logdha | rs188284159 | 0.001 | . | . | . | . | . | . | . | . | . | . |
| logdha | rs2303887 | 0.001 | . | . | . | . | . | . | . | . | . | . |
| logdha | rs2303886 | 0.001 | . | . | . | . | . | . | . | . | . | . |
| logdha | rs192621006 | 0.003 | . | . | . | . | . | . | . | . | . | . |
| logdha | rs2303885 | 0.154 | 0.797 | 0.189 | 0.339 | 0.671 | 0.388 | 0.49 | 1.27 | 0.593 | 0.615 | 0.209 |
| logdha | rs114466168 | 0 | . | . | . | . | . | . | . | . | . | . |
| logdha | rs11239501 | 0.149 | 0.907 | 0.22 | 0.688 | 1.16 | 0.675 | 0.795 | 0.787 | 0.367 | 0.607 | -0.24 |
| logdha | rs745986 | 0.204 | 0.857 | 0.218 | 0.543 | 0.906 | 0.451 | 0.842 | 0.972 | 0.395 | 0.944 | -8.10E-03 |
| logdha | rs75262268 | 0 | . | . | . | . | . | . | . | . | . | . |
| logdha | rs71494776 | 0.049 | 0.824 | 0.181 | 0.379 | 0.52 | 0.44 | 0.439 | 1.44 | 0.927 | 0.572 | 0.273 |
| logdha | rs74386871 | 0.011 | 0.809 | 0.175 | 0.327 | 0.078 | 0.242 | 0.412 | 10.4 | 25.3 | 0.335 | 0.768 |
| logdha | rs76502040 | 0.01 | 0.819 | 0.177 | 0.354 | 0.379 | 1.07 | 0.732 | 3.63 | 8 | 0.559 | 0.929 |
| logdha | rs142213734 | 0.001 | . | . | . | . | . | . | . | . | . | . |
| logdha | rs4948671 | 0.604 | 0.794 | 0.349 | 0.6 | 1.04 | 0.363 | 0.908 | 1.05 | 0.312 | 0.864 | 0.034 |
| logdha | rs146286085 | 0.002 | . | . | . | . | . | . | . | . | . | . |
| logdha | rs144080623 | 0 | . | . | . | . | . | . | . | . | . | . |
| logdha | rs115074014 | 0 | . | . | . | . | . | . | . | . | . | . |
| logdha | rs17157756 | 0 | . | . | . | . | . | . | . | . | . | . |
| logdha | rs190459780 | 0.001 | . | . | . | . | . | . | . | . | . | . |
| logdha | rs12358647 | 0.204 | 0.853 | 0.217 | 0.531 | 0.897 | 0.445 | 0.827 | 0.984 | 0.398 | 0.967 | 2.50E-03 |
| logdha | rs147080556 | 0.011 | 0.835 | 0.18 | 0.403 | 0.608 | 0.86 | 0.725 | 1.74 | 1.93 | 0.617 | 0.441 |
| logdha | rs187543349 | 0.004 | . | . | . | . | . | . | . | . | . | . |
| logdha | rs114375595 | 0.004 | . | . | . | . | . | . | . | . | . | . |
| logdha | rs117211200 | 0.01 | . | . | . | . | . | . | . | . | . | . |
| logdha | rs192963047 | 0.001 | . | . | . | . | . | . | . | . | . | . |
| logdha | rs11239505 | 0.204 | 0.852 | 0.217 | 0.531 | 0.897 | 0.445 | 0.826 | 0.985 | 0.398 | 0.969 | 3.50E-03 |
| logdha | rs184434116 | 0.003 | . | . | . | . | . | . | . | . | . | . |
| logdha | rs4948999 | 0.604 | 0.789 | 0.347 | 0.59 | 1.04 | 0.361 | 0.917 | 1.06 | 0.313 | 0.85 | 0.039 |
| logdha | rs11239506 | 0.155 | 0.874 | 0.215 | 0.584 | 1.04 | 0.594 | 0.942 | 0.897 | 0.413 | 0.813 | -0.099 |
| logdha | rs190706620 | 0.006 | . | . | . | . | . | . | . | . | . | . |
| logdha | rs190770429 | 0.002 | . | . | . | . | . | . | . | . | . | . |
| logdha | rs181327133 | 0.006 | . | . | . | . | . | . | . | . | . | . |
| logdha | rs149251697 | 0.008 | . | . | . | . | . | . | . | . | . | . |
| logdha | rs118081602 | 0.001 | . | . | . | . | . | . | . | . | . | . |
| logdha | rs188233691 | 0.001 | . | . | . | . | . | . | . | . | . | . |
| logdha | rs183778794 | 0.005 | . | . | . | . | . | . | . | . | . | . |
| logdha | rs188040078 | 0.007 | . | . | . | . | . | . | . | . | . | . |
| logdha | rs184675372 | 0.003 | . | . | . | . | . | . | . | . | . | . |
| logdha | rs150326891 | 0.116 | 0.899 | 0.217 | 0.659 | 1.14 | 0.732 | 0.839 | 0.784 | 0.399 | 0.633 | -0.235 |

| logdha | rs185520021 | 0.002 | . | . | . | . | . | . | . | . | . | . |
| --- | --- | --- | --- | --- | --- | --- | --- | --- | --- | --- | --- | --- |
| logdha | rs7907779 | 0.155 | 0.873 | 0.215 | 0.582 | 1.04 | 0.592 | 0.944 | 0.899 | 0.414 | 0.817 | -0.097 |
| logdha | rs184962860 | 0.001 | . | . | . | . | . | . | . | . | . | . |
| logdha | rs149762784 | 0.025 | 0.816 | 0.181 | 0.359 | 0.114 | 0.228 | 0.279 | 2.38 | 3.87 | 0.595 | 0.291 |
| logdha | rs149203427 | 0.021 | 0.85 | 0.19 | 0.466 | 1.46 | 1.41 | 0.696 | 1.34 | 1.17 | 0.741 | 0.348 |
| logdha | rs60153083 | 0 | . | . | . | . | . | . | . | . | . | . |
| logdha | rs112001069 | 0.05 | 0.823 | 0.181 | 0.378 | 0.519 | 0.438 | 0.437 | 1.44 | 0.925 | 0.567 | 0.274 |
| logdha | rs182355382 | 0.021 | 0.82 | 0.179 | 0.364 | 0.244 | 0.388 | 0.375 | 2.57 | 3.34 | 0.469 | 0.449 |
| logdha | rs186740974 | 0.117 | 1.07 | 0.29 | 0.806 | 3.18 | 2.19 | 0.093 | 0.415 | 0.251 | 0.146 | -1.84 |
| logdha | rs191874916 | 0.014 | 0.836 | 0.182 | 0.41 | 0.61 | 1.28 | 0.814 | 1.39 | 2.11 | 0.828 | 0.263 |
| logdha | rs182989412 | 0.152 | 1.05 | 0.28 | 0.844 | 2.33 | 1.59 | 0.216 | 0.47 | 0.283 | 0.21 | -1.23 |
| logdha | rs188819476 | 0.002 | . | . | . | . | . | . | . | . | . | . |
| logdha | rs141217183 | 0.078 | 0.888 | 0.204 | 0.605 | 1.17 | 1.02 | 0.854 | 0.612 | 0.446 | 0.5 | -0.424 |
| logdha | rs192922652 | 0.045 | 0.818 | 0.181 | 0.363 | 0.479 | 0.427 | 0.409 | 1.58 | 1.07 | 0.499 | 0.323 |
| logdha | rs185092343 | 0.024 | 0.779 | 0.176 | 0.269 | 0.187 | 0.286 | 0.273 | 4.43 | 5.07 | 0.194 | 0.678 |
| logdha | rs145193311 | 0.051 | 0.812 | 0.181 | 0.35 | 0.408 | 0.377 | 0.332 | 1.74 | 1.21 | 0.424 | 0.357 |
| logdha | rs55789336 | 0.155 | 0.891 | 0.22 | 0.641 | 1.14 | 0.654 | 0.818 | 0.839 | 0.393 | 0.709 | -0.179 |
| logdha | rs55837212 | 0.152 | 0.884 | 0.219 | 0.62 | 1.12 | 0.654 | 0.841 | 0.86 | 0.403 | 0.747 | -0.154 |
| logdha | rs75408574 | 0.366 | 0.84 | 0.288 | 0.611 | 1.17 | 0.428 | 0.664 | 1.02 | 0.3 | 0.952 | -9.90E-03 |
| logdha | rs149306926 | 0.162 | 0.832 | 0.201 | 0.446 | 0.861 | 0.525 | 0.806 | 1.06 | 0.511 | 0.904 | 0.066 |
| logdha | rs35990892 | 0.598 | 0.707 | 0.292 | 0.401 | 1.01 | 0.352 | 0.967 | 1.15 | 0.327 | 0.616 | 0.105 |
| logdha | rs192356207 | 0.019 | 0.826 | 0.178 | 0.376 | 0.335 | 0.55 | 0.505 | 2.23 | 2.74 | 0.514 | 0.456 |
| logdha | rs183741163 | 0.129 | 0.848 | 0.208 | 0.502 | 0.894 | 0.602 | 0.868 | 1 | 0.531 | 0.999 | 0.017 |
| logdha | rs34737297 | 0.48 | 0.815 | 0.319 | 0.602 | 1.04 | 0.388 | 0.913 | 1.04 | 0.32 | 0.91 | 0.023 |
| logdha | rs181346933 | 0.026 | 0.722 | 0.157 | 0.134 | 0.014 | 0.029 | 0.037 | 24.3 | 31 | 0.012 | 0.508 |
| logdha | rs36093253 | 0.343 | 0.831 | 0.267 | 0.564 | 1.16 | 0.422 | 0.693 | 1.04 | 0.305 | 0.905 | 8.00E-03 |
| logdha | rs186307855 | 0.02 | 0.869 | 0.194 | 0.528 | 1.73 | 1.81 | 0.598 | 0.541 | 0.5 | 0.506 | -0.788 |
| logdha | rs189681865 | 0.003 | . | . | . | . | . | . | . | . | . | . |
| logdha | rs34942971 | 0.207 | 0.892 | 0.238 | 0.667 | 1.07 | 0.544 | 0.9 | 0.88 | 0.361 | 0.756 | -0.121 |
| logdha | rs181816554 | 0.019 | 0.92 | 0.203 | 0.705 | 12.2 | 16.4 | 0.061 | 0.104 | 0.136 | 0.082 | -11 |
| logdha | rs186846881 | 0.004 | . | . | . | . | . | . | . | . | . | . |
| logdha | rs191178220 | 0.024 | 0.919 | 0.202 | 0.702 | 13.4 | 24.8 | 0.163 | 0.089 | 0.163 | 0.185 | -12.2 |
| logdha | rs183305228 | 0 | . | . | . | . | . | . | . | . | . | . |
| logdha | rs139912761 | 0.15 | 0.899 | 0.225 | 0.671 | 1.21 | 0.689 | 0.737 | 0.816 | 0.376 | 0.66 | -0.221 |
| logdha | rs185499901 | 0.019 | 0.905 | 0.197 | 0.646 | 19.6 | 32.3 | 0.071 | 0.012 | 0.021 | 0.013 | -19.3 |
| logdha | rs189816197 | 0.061 | 0.832 | 0.187 | 0.412 | 0.746 | 0.539 | 0.685 | 1.15 | 0.658 | 0.805 | 0.137 |
| logdha | rs145656938 | 0.014 | 0.898 | 0.198 | 0.625 | 5.76 | 13.3 | 0.45 | 0.076 | 0.187 | 0.293 | -5.26 |
| logdha | rs35745197 | 0.05 | 0.823 | 0.181 | 0.377 | 0.517 | 0.434 | 0.432 | 1.45 | 0.927 | 0.562 | 0.276 |
| logdha | rs192856637 | 0.006 | . | . | . | . | . | . | . | . | . | . |
| logdha | rs2310775 | 0.402 | 0.846 | 0.286 | 0.621 | 1.18 | 0.398 | 0.625 | 1.01 | 0.276 | 0.972 | -0.018 |
| logdha | rs11239507 | 0.404 | 0.816 | 0.275 | 0.546 | 1.16 | 0.386 | 0.658 | 1.05 | 0.282 | 0.849 | 0.02 |
| logdha | rs74648467 | 0.049 | 0.823 | 0.181 | 0.378 | 0.519 | 0.437 | 0.436 | 1.44 | 0.925 | 0.567 | 0.274 |
| logdha | rs145815934 | 0.022 | 0.793 | 0.182 | 0.313 | 0.118 | 0.162 | 0.119 | 3.57 | 3.59 | 0.205 | 0.423 |
| logdha | rs145390687 | 0.029 | 0.833 | 0.187 | 0.413 | 1.11 | 0.916 | 0.895 | 1.53 | 1.12 | 0.564 | 0.468 |
| logdha | rs184354913 | 0.049 | 1.02 | 0.225 | 0.932 | 16.9 | 17.4 | 5.90E-03 | 0.028 | 0.029 | 7.50E-04 | -16.4 |
| logdha | rs181244920 | 0.007 | . | . | . | . | . | . | . | . | . | . |
| logdha | rs11499087 | 0 | . | . | . | . | . | . | . | . | . | . |
| logdha | rs148263794 | 0.05 | 0.823 | 0.181 | 0.377 | 0.516 | 0.434 | 0.432 | 1.45 | 0.928 | 0.563 | 0.276 |
| logdha | rs150758903 | 0.01 | 0.851 | 0.187 | 0.461 | 0.402 | 0.901 | 0.684 | 1.11 | 1.27 | 0.928 | 0.126 |
| logdha | rs147654382 | 0.183 | 0.871 | 0.22 | 0.585 | 1.04 | 0.537 | 0.937 | 0.915 | 0.38 | 0.831 | -0.082 |
| logdha | rs147495166 | 0 | . | . | . | . | . | . | . | . | . | . |
| logdha | rs35198778 | 0.092 | 0.832 | 0.196 | 0.434 | 0.68 | 0.511 | 0.608 | 1.12 | 0.693 | 0.86 | 0.119 |
| logdha | rs145207659 | 0.071 | 0.797 | 0.193 | 0.349 | 0.776 | 0.539 | 0.715 | 1.5 | 0.902 | 0.503 | 0.352 |
| logdha | rs186466464 | 0.003 | . | . | . | . | . | . | . | . | . | . |
| logdha | rs12778413 | 0.157 | 0.844 | 0.209 | 0.493 | 0.843 | 0.516 | 0.78 | 1.03 | 0.518 | 0.958 | 0.043 |
| logdha | rs7895552 | 0.532 | 0.708 | 0.294 | 0.405 | 0.99 | 0.34 | 0.976 | 1.17 | 0.349 | 0.595 | 0.123 |
| logdha | rs12783895 | 0.05 | 0.823 | 0.181 | 0.377 | 0.516 | 0.433 | 0.431 | 1.45 | 0.926 | 0.56 | 0.277 |
| logdha | rs34929604 | 0.05 | 0.823 | 0.181 | 0.377 | 0.516 | 0.433 | 0.43 | 1.45 | 0.926 | 0.56 | 0.277 |
| logdha | rs12761961 | 0.049 | 0.823 | 0.181 | 0.376 | 0.512 | 0.431 | 0.427 | 1.46 | 0.935 | 0.552 | 0.281 |
| logdha | rs35536930 | 0.198 | 0.861 | 0.221 | 0.56 | 0.952 | 0.487 | 0.924 | 0.956 | 0.394 | 0.913 | -0.029 |
| logdha | rs180951060 | 0 | . | . | . | . | . | . | . | . | . | . |
| logdha | rs148892128 | 0.022 | 0.793 | 0.182 | 0.313 | 0.118 | 0.162 | 0.119 | 3.57 | 3.59 | 0.205 | 0.423 |
| logdha | rs11239509 | 0.001 | . | . | . | . | . | . | . | . | . | . |
| logdha | rs41526545 | 0.155 | 0.871 | 0.215 | 0.575 | 1.04 | 0.588 | 0.947 | 0.907 | 0.415 | 0.832 | -0.089 |
| logdha | rs2029253 | 0.402 | 0.846 | 0.286 | 0.62 | 1.18 | 0.397 | 0.626 | 1.01 | 0.276 | 0.97 | -0.017 |
| logdha | rs17444064 | 0.155 | 0.871 | 0.215 | 0.575 | 1.04 | 0.589 | 0.945 | 0.907 | 0.415 | 0.832 | -0.089 |
| logdha | rs71494779 | 0.05 | 0.823 | 0.181 | 0.377 | 0.515 | 0.433 | 0.43 | 1.45 | 0.926 | 0.561 | 0.276 |
| logdha | rs74991420 | 0.001 | . | . | . | . | . | . | . | . | . | . |
| logdha | rs182736551 | 0.001 | . | . | . | . | . | . | . | . | . | . |
| logdha | rs78702074 | 0.001 | . | . | . | . | . | . | . | . | . | . |
| logdha | rs187791466 | 0.005 | . | . | . | . | . | . | . | . | . | . |
| logdha | rs35300270 | 0.054 | 0.814 | 0.18 | 0.352 | 0.433 | 0.361 | 0.316 | 1.64 | 1.03 | 0.425 | 0.333 |
| logdha | rs6593484 | 0.155 | 0.87 | 0.215 | 0.572 | 1.04 | 0.586 | 0.95 | 0.91 | 0.416 | 0.837 | -0.086 |
| logdha | rs12244601 | 0.004 | . | . | . | . | . | . | . | . | . | . |
| logdha | rs184429574 | 0.005 | . | . | . | . | . | . | . | . | . | . |
| logdha | rs7080897 | 0.05 | 0.823 | 0.181 | 0.377 | 0.515 | 0.433 | 0.43 | 1.45 | 0.926 | 0.561 | 0.276 |
| logdha | rs7097429 | 0.054 | 0.777 | 0.174 | 0.261 | 0.387 | 0.287 | 0.2 | 2.32 | 1.28 | 0.128 | 0.533 |
| logdha | rs77979229 | 0.001 | . | . | . | . | . | . | . | . | . | . |
| logdha | rs11239512 | 0.155 | 0.869 | 0.215 | 0.571 | 1.04 | 0.586 | 0.951 | 0.912 | 0.416 | 0.839 | -0.084 |
| logdha | rs60146827 | 0.05 | 0.823 | 0.181 | 0.377 | 0.516 | 0.433 | 0.431 | 1.45 | 0.926 | 0.562 | 0.276 |
| logdha | rs79032236 | 0 | . | . | . | . | . | . | . | . | . | . |
| logdha | rs140854206 | 0.009 | . | . | . | . | . | . | . | . | . | . |
| logdha | rs184049550 | 0 | . | . | . | . | . | . | . | . | . | . |
| logdha | rs114433607 | 0 | . | . | . | . | . | . | . | . | . | . |
| logdha | rs117653793 | 0.001 | . | . | . | . | . | . | . | . | . | . |
| logdha | rs117039533 | 0.001 | . | . | . | . | . | . | . | . | . | . |

| logdha | rs191660761 | 0.006 | . | . | . | . | . | . | . | . | . | . |
| --- | --- | --- | --- | --- | --- | --- | --- | --- | --- | --- | --- | --- |
| logdha | rs59514896 | 0.049 | 0.824 | 0.181 | 0.379 | 0.521 | 0.439 | 0.44 | 1.44 | 0.921 | 0.571 | 0.272 |
| logdha | rs184661876 | 0.002 | . | . | . | . | . | . | . | . | . | . |
| logdha | rs61854073 | 0.037 | 0.952 | 0.217 | 0.829 | 5.24 | 6.14 | 0.158 | 0.224 | 0.252 | 0.184 | -4.08 |
| logdha | rs2018518 | 0.315 | 0.925 | 0.307 | 0.814 | 1.52 | 0.583 | 0.272 | 0.892 | 0.272 | 0.706 | -0.192 |
| logdha | rs934187 | 0.608 | 0.779 | 0.348 | 0.576 | 1.06 | 0.375 | 0.864 | 1.07 | 0.318 | 0.826 | 0.042 |
| logdha | rs7918542 | 0.205 | 0.847 | 0.216 | 0.515 | 0.891 | 0.44 | 0.816 | 1 | 0.402 | 0.998 | 0.017 |
| logdha | rs144311920 | 0 | . | . | . | . | . | . | . | . | . | . |
| logdha | rs7099684 | 0.791 | 1.84 | 1.16 | 0.338 | 1.9 | 0.873 | 0.161 | 0.612 | 0.228 | 0.188 | -0.599 |
| logdha | rs150829171 | 0.002 | . | . | . | . | . | . | . | . | . | . |
| logdha | rs186060697 | 0 | . | . | . | . | . | . | . | . | . | . |
| logdha | rs11239513 | 0.009 | . | . | . | . | . | . | . | . | . | . |
| logdha | rs7917687 | 0.05 | 0.823 | 0.181 | 0.377 | 0.518 | 0.435 | 0.434 | 1.45 | 0.926 | 0.563 | 0.276 |
| logdha | rs61854076 | 0.014 | 0.931 | 0.203 | 0.744 | 64.4 | 126 | 0.034 | 0.011 | 0.025 | 0.05 | -63.7 |
| logdha | rs71494780 | 0.019 | 0.837 | 0.182 | 0.413 | 1.15 | 1.16 | 0.891 | 1.2 | 0.93 | 0.817 | 0.165 |
| logdha | rs7919239 | 0.205 | 0.848 | 0.217 | 0.519 | 0.897 | 0.443 | 0.826 | 0.997 | 0.4 | 0.994 | 0.013 |
| logdha | rs78334475 | 0.003 | . | . | . | . | . | . | . | . | . | . |
| logdha | rs3780900 | 0.011 | 0.832 | 0.181 | 0.399 | 0.435 | 1.47 | 0.805 | 1.89 | 4.54 | 0.791 | 0.417 |
| logdha | rs186729292 | 0.001 | . | . | . | . | . | . | . | . | . | . |
| logdha | rs12267438 | 0.001 | . | . | . | . | . | . | . | . | . | . |
| logdha | rs1369214 | 0.454 | 0.803 | 0.287 | 0.54 | 1.11 | 0.38 | 0.764 | 1.06 | 0.287 | 0.826 | 0.033 |
| logdha | rs9282589 | 0.011 | 0.859 | 0.187 | 0.486 | 3.43 | 10.9 | 0.696 | 0.338 | 1.04 | 0.723 | -2.3 |
| logdha | rs114522915 | 0 | . | . | . | . | . | . | . | . | . | . |
| logdha | rs2115819 | 0.452 | 0.835 | 0.299 | 0.615 | 1.13 | 0.391 | 0.728 | 1.02 | 0.281 | 0.946 | -3.30E-03 |
| logdha | rs117664603 | 0.001 | . | . | . | . | . | . | . | . | . | . |
| logdha | rs184979202 | 0.001 | . | . | . | . | . | . | . | . | . | . |
| logdha | rs190714671 | 0.001 | . | . | . | . | . | . | . | . | . | . |
| logdha | rs141538996 | 0 | . | . | . | . | . | . | . | . | . | . |
| logdha | rs150881741 | 0.003 | . | . | . | . | . | . | . | . | . | . |
| logdha | rs139076633 | 0 | . | . | . | . | . | . | . | . | . | . |
| logdha | rs12243649 | 0.002 | . | . | . | . | . | . | . | . | . | . |
| logdha | rs140348708 | 0.001 | . | . | . | . | . | . | . | . | . | . |
| logdha | rs79023256 | 0.001 | . | . | . | . | . | . | . | . | . | . |
| logdha | rs11239515 | 0.146 | 0.856 | 0.208 | 0.523 | 0.989 | 0.557 | 0.984 | 0.954 | 0.432 | 0.917 | -0.038 |
| logdha | rs55866086 | 0.148 | 0.835 | 0.203 | 0.457 | 0.942 | 0.525 | 0.914 | 1.04 | 0.459 | 0.937 | 0.038 |
| logdha | rs181340150 | 0.005 | . | . | . | . | . | . | . | . | . | . |
| logdha | rs11239516 | 0.146 | 0.856 | 0.208 | 0.521 | 0.984 | 0.553 | 0.978 | 0.957 | 0.433 | 0.922 | -0.034 |
| logdha | rs11239517 | 0.146 | 0.855 | 0.207 | 0.519 | 0.982 | 0.551 | 0.974 | 0.959 | 0.433 | 0.925 | -0.032 |
| logdha | rs11239519 | 0.135 | 0.874 | 0.211 | 0.578 | 1.08 | 0.623 | 0.888 | 0.872 | 0.409 | 0.771 | -0.131 |
| logdha | rs7909514 | 0.601 | 0.82 | 0.37 | 0.66 | 1.14 | 0.402 | 0.719 | 1.03 | 0.31 | 0.932 | -4.70E-05 |
| logdha | rs7894352 | 0.782 | 1.77 | 1.1 | 0.361 | 1.89 | 0.852 | 0.155 | 0.625 | 0.23 | 0.201 | -0.569 |
| logdha | rs189384146 | 0.004 | . | . | . | . | . | . | . | . | . | . |
| logdha | rs11239520 | 0.146 | 0.855 | 0.207 | 0.517 | 0.979 | 0.548 | 0.969 | 0.961 | 0.434 | 0.93 | -0.029 |
| logdha | rs12221233 | 0.405 | 0.865 | 0.292 | 0.668 | 1.27 | 0.417 | 0.474 | 0.987 | 0.262 | 0.959 | -0.051 |
| logdha | rs74523293 | 0.001 | . | . | . | . | . | . | . | . | . | . |
| logdha | rs79376040 | 0.001 | . | . | . | . | . | . | . | . | . | . |
| logdha | rs4949000 | 0.405 | 0.865 | 0.292 | 0.668 | 1.27 | 0.417 | 0.474 | 0.987 | 0.263 | 0.96 | -0.05 |
| logdha | rs7089406 | 0.018 | 0.773 | 0.167 | 0.233 | 0.044 | 0.058 | 0.019 | 11.6 | 10.7 | 7.50E-03 | 0.577 |
| logdha | rs138211056 | 0 | . | . | . | . | . | . | . | . | . | . |
| logdha | rs10900213 | 0.549 | 0.868 | 0.359 | 0.733 | 1.23 | 0.418 | 0.546 | 0.982 | 0.284 | 0.949 | -0.05 |
| logdha | rs145152827 | 0 | . | . | . | . | . | . | . | . | . | . |
| logdha | rs74443325 | 0.001 | . | . | . | . | . | . | . | . | . | . |
| logdha | rs188299446 | 0.016 | 0.915 | 0.2 | 0.686 | 9.68 | 10 | 0.029 | 0.201 | 0.184 | 0.079 | -7.81 |
| logdha | rs192699586 | 0.002 | . | . | . | . | . | . | . | . | . | . |
| logdha | rs72796464 | 0.145 | 0.85 | 0.206 | 0.503 | 0.954 | 0.529 | 0.932 | 0.978 | 0.437 | 0.961 | -0.011 |
| logdha | rs4949001 | 0.268 | 0.97 | 0.298 | 0.922 | 1.68 | 0.684 | 0.204 | 0.792 | 0.264 | 0.484 | -0.359 |
| logdha | rs147174641 | 0.013 | 0.813 | 0.173 | 0.33 | 5.30E-03 | 0.02 | 0.163 | 24.9 | 70.3 | 0.255 | 0.289 |
| logdha | rs190835238 | 0.001 | . | . | . | . | . | . | . | . | . | . |
| logdha | rs76698944 | 0.03 | 0.832 | 0.18 | 0.394 | 0.739 | 1.05 | 0.832 | 1.2 | 1.37 | 0.871 | 0.168 |
| logdha | rs56071381 | 0.142 | 0.838 | 0.201 | 0.461 | 0.876 | 0.499 | 0.816 | 1.04 | 0.476 | 0.939 | 0.046 |
| logdha | rs78130427 | 0.03 | 0.821 | 0.177 | 0.359 | 0.152 | 0.245 | 0.242 | 2.56 | 2.9 | 0.408 | 0.347 |
| logdha | rs10900215 | 0.146 | 0.843 | 0.203 | 0.479 | 0.908 | 0.5 | 0.861 | 1.01 | 0.448 | 0.984 | 0.021 |
| logdha | rs41491445 | 0 | . | . | . | . | . | . | . | . | . | . |
| logdha | rs144995222 | . | . | . | . | . | . | . | . | . | . | . |
| logdha | rs149097803 | 0.003 | . | . | . | . | . | . | . | . | . | . |
| logdha | rs139429646 | 0.007 | . | . | . | . | . | . | . | . | . | . |
| logdha | rs7075620 | 0.577 | 0.855 | 0.369 | 0.717 | 1.14 | 0.405 | 0.719 | 0.991 | 0.297 | 0.975 | -0.029 |
| logdha | rs148299213 | 0 | . | . | . | . | . | . | . | . | . | . |
| logdha | rs4949002 | 0.311 | 0.948 | 0.318 | 0.873 | 1.63 | 0.631 | 0.205 | 0.863 | 0.267 | 0.635 | -0.244 |
| logdha | rs150496007 | 0.011 | 0.854 | 0.186 | 0.468 | 0.39 | 0.801 | 0.646 | 0.356 | 0.659 | 0.577 | -0.125 |
| logdha | rs147303455 | 0.004 | . | . | . | . | . | . | . | . | . | . |
| logdha | rs189518433 | 0.005 | . | . | . | . | . | . | . | . | . | . |
| logdha | rs186281355 | 0.005 | . | . | . | . | . | . | . | . | . | . |
| logdha | rs115297114 | 0 | . | . | . | . | . | . | . | . | . | . |
| logdha | rs11239523 | 0.147 | 0.845 | 0.204 | 0.486 | 0.915 | 0.504 | 0.871 | 1 | 0.443 | 0.997 | 0.014 |
| logdha | rs143704897 | 0.004 | . | . | . | . | . | . | . | . | . | . |
| logdha | rs188024096 | 0.004 | . | . | . | . | . | . | . | . | . | . |
| logdha | rs11239524 | 0.134 | 0.818 | 0.197 | 0.406 | 0.815 | 0.482 | 0.73 | 1.13 | 0.54 | 0.8 | 0.119 |
| logdha | rs191656573 | 0.005 | . | . | . | . | . | . | . | . | . | . |
| logdha | rs193295875 | 0 | . | . | . | . | . | . | . | . | . | . |
| logdha | rs190458228 | 0.002 | . | . | . | . | . | . | . | . | . | . |
| logdha | rs139253993 | 0.002 | . | . | . | . | . | . | . | . | . | . |
| logdha | rs4948672 | 0.565 | 1.93 | 0.856 | 0.136 | 2.49 | 0.956 | 0.017 | 0.486 | 0.149 | 0.018 | -1.08 |
| logdha | rs180954921 | 0.004 | . | . | . | . | . | . | . | . | . | . |
| logdha | rs12264801 | 0.432 | 0.826 | 0.316 | 0.616 | 0.952 | 0.408 | 0.909 | 1.03 | 0.365 | 0.943 | 0.029 |
| logdha | rs73283184 | 0 | . | . | . | . | . | . | . | . | . | . |

| logdha | rs142746187 | 0.002 | . | . | . | . | . | . | . | . | . | . |
| --- | --- | --- | --- | --- | --- | --- | --- | --- | --- | --- | --- | --- |
| logdha | rs58704739 | 0 | . | . | . | . | . | . | . | . | . | . |
| logdha | rs61272335 | 0 | . | . | . | . | . | . | . | . | . | . |
| logdha | rs60959489 | 0 | . | . | . | . | . | . | . | . | . | . |
| logdha | rs58502629 | 0 | . | . | . | . | . | . | . | . | . | . |
| logdha | rs10900216 | 0.696 | 1.68 | 0.841 | 0.296 | 1.83 | 0.725 | 0.124 | 0.607 | 0.189 | 0.108 | -0.644 |
| logdha | rs181118141 | 0 | . | . | . | . | . | . | . | . | . | . |
| logdha | rs137882665 | 0.004 | . | . | . | . | . | . | . | . | . | . |
| logdha | rs149466736 | 0.004 | . | . | . | . | . | . | . | . | . | . |
| logdha | rs2099171 | 0.663 | 1.7 | 0.794 | 0.253 | 1.95 | 0.727 | 0.074 | 0.592 | 0.176 | 0.077 | -0.688 |
| logdha | rs141852280 | 0.009 | . | . | . | . | . | . | . | . | . | . |
| logdha | rs191539257 | 0.002 | . | . | . | . | . | . | . | . | . | . |
| logdha | rs17157771 | 0.033 | 0.805 | 0.174 | 0.314 | 0.162 | 0.218 | 0.177 | 2.98 | 2.79 | 0.241 | 0.422 |
| logdha | rs111339241 | 0.031 | 0.806 | 0.174 | 0.317 | 0.161 | 0.227 | 0.195 | 3.04 | 2.97 | 0.256 | 0.427 |
| logdha | rs116983255 | 0.013 | 0.85 | 0.184 | 0.451 | 0.371 | 0.595 | 0.537 | 0.648 | 0.437 | 0.52 | -0.017 |
| logdha | rs142324341 | 0.003 | . | . | . | . | . | . | . | . | . | . |
| logdha | rs187191219 | 0.009 | . | . | . | . | . | . | . | . | . | . |
| logdha | rs151283973 | . | . | . | . | . | . | . | . | . | . | . |
| logdha | rs139316218 | 0.005 | . | . | . | . | . | . | . | . | . | . |
| logdha | rs6593485 | 0.696 | 1.65 | 0.821 | 0.314 | 1.8 | 0.71 | 0.135 | 0.616 | 0.19 | 0.117 | -0.62 |
| logdha | rs6593486 | 0.696 | 1.65 | 0.821 | 0.314 | 1.8 | 0.71 | 0.134 | 0.616 | 0.19 | 0.117 | -0.62 |
| logdha | rs145196618 | 0.002 | . | . | . | . | . | . | . | . | . | . |
| logdha | rs142598335 | 0.006 | . | . | . | . | . | . | . | . | . | . |
| logdha | rs892691 | 0.704 | 1.78 | 0.89 | 0.249 | 1.91 | 0.752 | 0.1 | 0.586 | 0.18 | 0.082 | -0.696 |
| logdha | rs7080713 | 0.664 | 1.79 | 0.841 | 0.214 | 2.06 | 0.776 | 0.056 | 0.571 | 0.171 | 0.061 | -0.743 |
| logdha | rs7077173 | 0.057 | 0.791 | 0.173 | 0.282 | 0.33 | 0.243 | 0.133 | 2.18 | 1.1 | 0.125 | 0.447 |
| logdha | rs7080977 | 0.032 | 0.801 | 0.173 | 0.304 | 0.149 | 0.208 | 0.172 | 3.29 | 3.17 | 0.215 | 0.443 |
| logdha | rs3780901 | 0.663 | 1.81 | 0.847 | 0.206 | 2.07 | 0.783 | 0.053 | 0.567 | 0.169 | 0.057 | -0.756 |
| logdha | rs61854091 | 0.073 | 0.839 | 0.201 | 0.464 | 0.903 | 0.707 | 0.897 | 1.02 | 0.651 | 0.974 | 0.032 |
| logdha | rs77550209 | 0.003 | . | . | . | . | . | . | . | . | . | . |
| logdha | rs73283191 | 0.033 | 0.803 | 0.173 | 0.309 | 0.156 | 0.215 | 0.177 | 3.14 | 2.99 | 0.228 | 0.435 |
| logdha | rs191866099 | 0.01 | 0.898 | 0.193 | 0.616 | 228 | 680 | 0.068 | 1.20E-04 | 4.70E-04 | 0.022 | -228 |
| logdha | rs147998395 | . | . | . | . | . | . | . | . | . | . | . |
| logdha | rs7090328 | 0.699 | 1.7 | 0.835 | 0.28 | 1.84 | 0.711 | 0.116 | 0.603 | 0.184 | 0.098 | -0.652 |
| logdha | rs79814535 | 0.001 | . | . | . | . | . | . | . | . | . | . |
| logdha | rs7073082 | 0.699 | 1.7 | 0.835 | 0.281 | 1.84 | 0.711 | 0.116 | 0.604 | 0.184 | 0.098 | -0.652 |
| logdha | rs11239526 | 0.052 | 0.807 | 0.185 | 0.351 | 0.633 | 0.526 | 0.582 | 1.56 | 1.04 | 0.504 | 0.357 |
| logdha | rs141839841 | . | . | . | . | . | . | . | . | . | . | . |
| logdha | rs75697371 | 0.014 | 0.815 | 0.175 | 0.341 | 0.144 | 0.26 | 0.283 | 4.14 | 5.32 | 0.27 | 0.525 |
| logdha | rs75972854 | 0.016 | 0.837 | 0.182 | 0.413 | 0.697 | 0.923 | 0.785 | 1.21 | 1.36 | 0.865 | 0.172 |
| logdha | rs139291975 | 0.001 | . | . | . | . | . | . | . | . | . | . |
| logdha | rs144064711 | 0.009 | . | . | . | . | . | . | . | . | . | . |
| logdha | rs3780902 | 0.033 | 0.807 | 0.174 | 0.321 | 0.17 | 0.23 | 0.19 | 2.85 | 2.67 | 0.262 | 0.415 |
| logdha | rs190449801 | 0 | . | . | . | . | . | . | . | . | . | . |
| logdha | rs73283196 | 0.033 | 0.806 | 0.174 | 0.319 | 0.17 | 0.23 | 0.191 | 2.91 | 2.74 | 0.258 | 0.422 |
| logdha | rs28395867 | 0.005 | . | . | . | . | . | . | . | . | . | . |
| logdha | rs28395866 | 0.036 | 0.813 | 0.175 | 0.337 | 0.27 | 0.327 | 0.28 | 2.08 | 1.88 | 0.418 | 0.373 |
| logdha | rs55661611 | 0.032 | 0.806 | 0.174 | 0.318 | 0.169 | 0.232 | 0.195 | 2.94 | 2.8 | 0.26 | 0.424 |
| logdha | rs55684518 | 0.03 | 0.855 | 0.188 | 0.476 | 0.95 | 0.953 | 0.959 | 0.823 | 0.661 | 0.809 | -0.136 |
| logdha | rs78552493 | 0.057 | 0.768 | 0.172 | 0.239 | 0.45 | 0.379 | 0.343 | 2.16 | 1.41 | 0.239 | 0.529 |
| logdha | rs147665434 | 0.007 | . | . | . | . | . | . | . | . | . | . |
| logdha | rs34445001 | 0.014 | 0.815 | 0.175 | 0.341 | 0.143 | 0.257 | 0.279 | 4.16 | 5.33 | 0.265 | 0.527 |
| logdha | rs184019449 | 0.004 | . | . | . | . | . | . | . | . | . | . |
| logdha | rs3780903 | 0.72 | 1.53 | 0.856 | 0.449 | 1.77 | 0.736 | 0.171 | 0.668 | 0.229 | 0.24 | -0.491 |
| logdha | rs17157784 | 0.005 | . | . | . | . | . | . | . | . | . | . |
| logdha | rs3780904 | 0.001 | . | . | . | . | . | . | . | . | . | . |
| logdha | rs192064730 | 0 | . | . | . | . | . | . | . | . | . | . |
| logdha | rs12774534 | 0.014 | 0.815 | 0.175 | 0.34 | 0.142 | 0.254 | 0.275 | 4.19 | 5.34 | 0.261 | 0.528 |
| logdha | rs79179915 | 0 | . | . | . | . | . | . | . | . | . | . |
| logdha | rs117811571 | 0.016 | 0.912 | 0.2 | 0.674 | 9.03 | 9.24 | 0.031 | 0.225 | 0.202 | 0.096 | -7.09 |
| logdha | rs72796477 | 0.015 | 0.835 | 0.182 | 0.408 | 0.715 | 1 | 0.81 | 1.43 | 1.57 | 0.742 | 0.307 |
| logdha | rs55729342 | 0.005 | . | . | . | . | . | . | . | . | . | . |
| logdha | rs59317220 | 0.004 | . | . | . | . | . | . | . | . | . | . |
| logdha | rs3780905 | 0.042 | 0.927 | 0.207 | 0.736 | 2.55 | 1.53 | 0.118 | 0.324 | 0.162 | 0.024 | -1.71 |
| logdha | rs3780906 | 0.719 | 1.5 | 0.846 | 0.469 | 1.75 | 0.73 | 0.182 | 0.676 | 0.233 | 0.256 | -0.476 |
| logdha | rs149047975 | 0.006 | . | . | . | . | . | . | . | . | . | . |
| logdha | rs3824614 | 0.001 | . | . | . | . | . | . | . | . | . | . |
| logdha | rs140254404 | 0.003 | . | . | . | . | . | . | . | . | . | . |
| logdha | rs145262684 | 0.02 | 0.91 | 0.202 | 0.67 | 7.12 | 7.04 | 0.047 | 0.296 | 0.249 | 0.149 | -5.12 |
| logdha | rs76735482 | 0.001 | . | . | . | . | . | . | . | . | . | . |
| logdha | rs73283198 | 0.032 | 0.809 | 0.175 | 0.327 | 0.183 | 0.256 | 0.224 | 2.73 | 2.65 | 0.302 | 0.412 |
| logdha | rs74911239 | 0.052 | 0.803 | 0.181 | 0.331 | 0.249 | 0.247 | 0.161 | 1.8 | 1.37 | 0.439 | 0.309 |
| logdha | rs150551647 | 0.008 | . | . | . | . | . | . | . | . | . | . |
| logdha | rs115200794 | 0.004 | . | . | . | . | . | . | . | . | . | . |
| logdha | rs74130823 | 0.177 | 0.849 | 0.212 | 0.513 | 0.934 | 0.528 | 0.904 | 0.979 | 0.441 | 0.962 | -7.00E-03 |
| logdha | rs2279435 | 0.564 | 1.17 | 0.497 | 0.709 | 1.44 | 0.57 | 0.357 | 0.744 | 0.23 | 0.339 | -0.357 |
| logdha | rs147279673 | 0.02 | 0.896 | 0.197 | 0.62 | 8.61 | 11.9 | 0.118 | 0.29 | 0.37 | 0.332 | -6.27 |
| logdha | rs183525146 | 0.005 | . | . | . | . | . | . | . | . | . | . |
| logdha | rs3740107 | 0.728 | 1.47 | 0.81 | 0.485 | 1.53 | 0.648 | 0.317 | 0.68 | 0.232 | 0.258 | -0.471 |
| logdha | rs140697247 | 0.002 | . | . | . | . | . | . | . | . | . | . |
| logdha | rs1565096 | 0.745 | 1.4 | 0.764 | 0.537 | 1.43 | 0.589 | 0.391 | 0.705 | 0.236 | 0.297 | -0.419 |
| logdha | rs1565097 | 0.742 | 1.36 | 0.75 | 0.582 | 1.4 | 0.589 | 0.426 | 0.721 | 0.245 | 0.336 | -0.388 |
| logdha | rs144584548 | 0.03 | 0.854 | 0.188 | 0.475 | 0.985 | 0.968 | 0.988 | 0.831 | 0.651 | 0.813 | -0.14 |
| logdha | rs75811053 | 0.001 | . | . | . | . | . | . | . | . | . | . |
| logdha | rs147462967 | 0.01 | 0.853 | 0.181 | 0.454 | 2.2 | 9.81 | 0.86 | 3.80E-03 | 0.02 | 0.29 | -2.04 |
| logdha | rs74130824 | 0.004 | . | . | . | . | . | . | . | . | . | . |

| logdha | rs116883184 | 0.025 | 0.92 | 0.207 | 0.71 | 6.88 | 6.14 | 0.031 | 0.345 | 0.275 | 0.181 | -4.62 |
| --- | --- | --- | --- | --- | --- | --- | --- | --- | --- | --- | --- | --- |
| logdha | rs117258049 | 0.025 | 0.92 | 0.207 | 0.71 | 6.88 | 6.14 | 0.031 | 0.345 | 0.275 | 0.181 | -4.62 |
| logdha | rs3780908 | 0.171 | 0.819 | 0.205 | 0.425 | 0.814 | 0.46 | 0.715 | 1.1 | 0.484 | 0.823 | 0.103 |
| logdha | rs180938522 | 0.004 | . | . | . | . | . | . | . | . | . | . |
| logdha | rs76687372 | 0 | . | . | . | . | . | . | . | . | . | . |
| logdha | rs3780909 | 0.504 | 1.36 | 0.515 | 0.414 | 1.79 | 0.644 | 0.107 | 0.62 | 0.185 | 0.109 | -0.641 |
| logdha | rs78281156 | 0.001 | . | . | . | . | . | . | . | . | . | . |
| logdha | rs187840631 | 0.002 | . | . | . | . | . | . | . | . | . | . |
| logdha | rs76868242 | 0.001 | . | . | . | . | . | . | . | . | . | . |
| logdha | rs192794192 | 0.001 | . | . | . | . | . | . | . | . | . | . |
| logdha | rs12781248 | 0.015 | 0.818 | 0.175 | 0.349 | 0.277 | 0.409 | 0.384 | 2.84 | 3.19 | 0.353 | 0.549 |
| logdha | rs12762375 | 0.014 | 0.813 | 0.174 | 0.334 | 0.124 | 0.222 | 0.243 | 4.66 | 5.93 | 0.226 | 0.534 |
| logdha | rs192256986 | 0.006 | . | . | . | . | . | . | . | . | . | . |
| logdha | rs184411030 | 0 | . | . | . | . | . | . | . | . | . | . |
| logdha | rs189601483 | 0.001 | . | . | . | . | . | . | . | . | . | . |
| logdha | rs143254579 | 0.05 | 0.808 | 0.184 | 0.349 | 0.626 | 0.502 | 0.559 | 1.58 | 1.03 | 0.484 | 0.363 |
| logdha | rs12253903 | 0 | . | . | . | . | . | . | . | . | . | . |
| logdha | rs35489926 | 0.014 | 0.814 | 0.174 | 0.335 | 0.127 | 0.225 | 0.245 | 4.6 | 5.83 | 0.228 | 0.535 |
| logdha | rs71494783 | 0.015 | 0.81 | 0.173 | 0.324 | 0.115 | 0.2 | 0.213 | 5.02 | 6.12 | 0.186 | 0.543 |
| logdha | rs180711602 | 0.004 | . | . | . | . | . | . | . | . | . | . |
| logdha | rs117312402 | 0.001 | . | . | . | . | . | . | . | . | . | . |
| logdha | rs74831694 | 0.001 | . | . | . | . | . | . | . | . | . | . |
| logdha | rs140301943 | 0.004 | . | . | . | . | . | . | . | . | . | . |
| logdha | rs78553679 | 0.023 | 0.776 | 0.178 | 0.27 | 0.077 | 0.12 | 0.098 | 5.71 | 6.2 | 0.109 | 0.49 |
| logdha | rs78334250 | 0.001 | . | . | . | . | . | . | . | . | . | . |
| logdha | rs76000028 | 0.001 | . | . | . | . | . | . | . | . | . | . |
| logdha | rs145195254 | 0 | . | . | . | . | . | . | . | . | . | . |
| logdha | rs137888527 | 0 | . | . | . | . | . | . | . | . | . | . |
| logdha | rs149109592 | 0.005 | . | . | . | . | . | . | . | . | . | . |
| logdha | rs80280010 | 0.049 | 0.808 | 0.184 | 0.349 | 0.626 | 0.501 | 0.559 | 1.58 | 1.02 | 0.484 | 0.363 |
| logdha | rs189995374 | 0.001 | . | . | . | . | . | . | . | . | . | . |
| logdha | rs75467770 | 0.001 | . | . | . | . | . | . | . | . | . | . |
| logdha | rs1487562 | 0.178 | 0.797 | 0.199 | 0.362 | 0.746 | 0.415 | 0.598 | 1.19 | 0.514 | 0.686 | 0.165 |
| logdha | rs12266963 | 0.015 | 0.81 | 0.173 | 0.324 | 0.116 | 0.201 | 0.213 | 4.99 | 6.07 | 0.186 | 0.544 |
| logdha | rs7099874 | 0.693 | 1.55 | 0.784 | 0.389 | 1.59 | 0.636 | 0.244 | 0.638 | 0.214 | 0.181 | -0.568 |
| logdha | rs75687019 | 0.001 | . | . | . | . | . | . | . | . | . | . |
| logdha | rs148240462 | 0.056 | 0.813 | 0.185 | 0.363 | 0.309 | 0.299 | 0.225 | 1.57 | 1.19 | 0.55 | 0.272 |
| logdha | rs141278174 | 0.003 | . | . | . | . | . | . | . | . | . | . |
| logdha | rs76404852 | 0.001 | . | . | . | . | . | . | . | . | . | . |
| logdha | rs139112240 | 0.012 | 0.852 | 0.189 | 0.47 | 2.43 | 4.67 | 0.644 | 0.648 | 1.11 | 0.8 | -0.941 |
| logdha | rs12783037 | 0.015 | 0.81 | 0.173 | 0.324 | 0.117 | 0.202 | 0.213 | 4.98 | 6.04 | 0.185 | 0.546 |
| logdha | rs72796481 | 0.013 | 0.823 | 0.181 | 0.374 | 0.245 | 0.4 | 0.389 | 2.79 | 2.98 | 0.335 | 0.495 |
| logdha | rs76798749 | 0.001 | . | . | . | . | . | . | . | . | . | . |
| logdha | rs187732548 | 0.001 | . | . | . | . | . | . | . | . | . | . |
| logdha | rs75675474 | 0.001 | . | . | . | . | . | . | . | . | . | . |
| logdha | rs76552293 | 0.001 | . | . | . | . | . | . | . | . | . | . |
| logdha | rs147445993 | 0.005 | . | . | . | . | . | . | . | . | . | . |
| logdha | rs115902283 | 0.001 | . | . | . | . | . | . | . | . | . | . |
| logdha | rs4540799 | 0.692 | 1.54 | 0.784 | 0.392 | 1.59 | 0.636 | 0.245 | 0.639 | 0.215 | 0.183 | -0.565 |
| logdha | rs12765320 | 0.179 | 0.756 | 0.188 | 0.26 | 0.639 | 0.326 | 0.379 | 1.37 | 0.556 | 0.435 | 0.268 |
| logdha | rs12782256 | 0.014 | 0.814 | 0.174 | 0.335 | 0.131 | 0.229 | 0.245 | 4.54 | 5.68 | 0.226 | 0.54 |
| logdha | rs191727256 | 0 | . | . | . | . | . | . | . | . | . | . |
| logdha | rs2082111 | 0.165 | 0.82 | 0.202 | 0.421 | 0.838 | 0.466 | 0.751 | 1.1 | 0.478 | 0.829 | 0.097 |
| logdha | rs2082110 | 0.166 | 0.823 | 0.203 | 0.431 | 0.852 | 0.472 | 0.772 | 1.09 | 0.471 | 0.851 | 0.086 |
| logdha | rs2082109 | 0.166 | 0.823 | 0.203 | 0.431 | 0.852 | 0.472 | 0.773 | 1.08 | 0.471 | 0.851 | 0.086 |
| logdha | rs117316662 | 0.001 | . | . | . | . | . | . | . | . | . | . |
| logdha | rs12247163 | 0.181 | 0.784 | 0.195 | 0.328 | 0.729 | 0.389 | 0.553 | 1.24 | 0.521 | 0.612 | 0.194 |
| logdha | rs12355888 | 0.14 | 0.86 | 0.21 | 0.537 | 1.07 | 0.604 | 0.904 | 0.934 | 0.425 | 0.881 | -0.07 |
| logdha | rs143772290 | 0 | . | . | . | . | . | . | . | . | . | . |
| logdha | rs146849452 | 0.004 | . | . | . | . | . | . | . | . | . | . |
| logdha | rs140267121 | 0.006 | . | . | . | . | . | . | . | . | . | . |
| logdha | rs7080474 | 0.18 | 0.783 | 0.195 | 0.326 | 0.725 | 0.387 | 0.548 | 1.24 | 0.523 | 0.607 | 0.197 |
| logdha | rs144066203 | 0 | . | . | . | . | . | . | . | . | . | . |
| logdha | rs12765209 | 0.001 | . | . | . | . | . | . | . | . | . | . |
| logdha | rs150930735 | 0 | . | . | . | . | . | . | . | . | . | . |
| logdha | rs7068039 | 0.181 | 0.785 | 0.196 | 0.331 | 0.732 | 0.39 | 0.558 | 1.23 | 0.518 | 0.619 | 0.191 |
| logdha | rs117622652 | 0.001 | . | . | . | . | . | . | . | . | . | . |
| logdha | rs190191514 | 0.002 | . | . | . | . | . | . | . | . | . | . |
| logdha | rs74130835 | 0 | . | . | . | . | . | . | . | . | . | . |
| logdha | rs1811604 | 0.001 | . | . | . | . | . | . | . | . | . | . |
| logdha | rs146971256 | 0 | . | . | . | . | . | . | . | . | . | . |
| logdha | rs74336000 | 0 | . | . | . | . | . | . | . | . | . | . |
| logdha | rs186046492 | 0.009 | . | . | . | . | . | . | . | . | . | . |
| logdha | rs34971823 | 0.014 | 0.814 | 0.174 | 0.336 | 0.131 | 0.228 | 0.244 | 4.55 | 5.69 | 0.226 | 0.539 |
| logdha | rs75227446 | 0.001 | . | . | . | . | . | . | . | . | . | . |
| logdha | rs190457599 | 0.001 | . | . | . | . | . | . | . | . | . | . |
| logdha | rs79591917 | 0.001 | . | . | . | . | . | . | . | . | . | . |
| logdha | rs702365 | 0.51 | 1.38 | 0.529 | 0.408 | 1.76 | 0.649 | 0.123 | 0.612 | 0.186 | 0.106 | -0.654 |
| logdha | rs79643836 | 0.001 | . | . | . | . | . | . | . | . | . | . |
| logdha | rs702366 | 0.512 | 1.36 | 0.52 | 0.428 | 1.72 | 0.627 | 0.135 | 0.621 | 0.187 | 0.113 | -0.627 |
| logdha | rs1059696 | 0.166 | 0.82 | 0.203 | 0.423 | 0.838 | 0.467 | 0.751 | 1.1 | 0.481 | 0.831 | 0.097 |
| logdha | rs901681 | 0.515 | 1.36 | 0.519 | 0.423 | 1.72 | 0.626 | 0.138 | 0.62 | 0.186 | 0.111 | -0.631 |
| logdha | rs61854092 | 0.166 | 0.821 | 0.203 | 0.425 | 0.84 | 0.468 | 0.754 | 1.09 | 0.48 | 0.837 | 0.094 |
| logdha | rs148080753 | 0.001 | . | . | . | . | . | . | . | . | . | . |
| logdha | rs61854093 | 0.166 | 0.824 | 0.204 | 0.433 | 0.853 | 0.474 | 0.775 | 1.08 | 0.474 | 0.855 | 0.084 |
| logdha | rs141822774 | 0.001 | . | . | . | . | . | . | . | . | . | . |

| logdha | rs74130836 | 0 | . | . | . | . | . | . | . | . | . | . |
| --- | --- | --- | --- | --- | --- | --- | --- | --- | --- | --- | --- | --- |
| logdha | rs150620963 | 0.019 | 0.735 | 0.158 | 0.152 | 5.80E-03 | 0.011 | 4.70E-03 | 47.7 | 48.6 | 1.50E-04 | 0.462 |
| logdha | rs3780913 | 0.678 | 1.71 | 0.864 | 0.292 | 1.77 | 0.717 | 0.156 | 0.589 | 0.2 | 0.119 | -0.698 |
| logdha | rs142255723 | 0.002 | . | . | . | . | . | . | . | . | . | . |
| logdha | rs3780914 | 0.511 | 1.35 | 0.516 | 0.438 | 1.72 | 0.623 | 0.137 | 0.627 | 0.188 | 0.119 | -0.614 |
| logdha | rs182656507 | 0.002 | . | . | . | . | . | . | . | . | . | . |
| logdha | rs2291427 | 0.678 | 1.7 | 0.865 | 0.298 | 1.77 | 0.716 | 0.161 | 0.591 | 0.202 | 0.123 | -0.692 |
| logdha | rs10751382 | 0.678 | 1.71 | 0.865 | 0.292 | 1.78 | 0.719 | 0.153 | 0.589 | 0.2 | 0.12 | -0.697 |
| logdha | rs117610108 | 0.001 | . | . | . | . | . | . | . | . | . | . |
| logdha | rs144966941 | 0.031 | 0.878 | 0.191 | 0.55 | 2.46 | 2.49 | 0.373 | 0.537 | 0.449 | 0.457 | -1.18 |
| logdha | rs41502051 | 0.051 | 0.804 | 0.177 | 0.32 | 0.782 | 0.75 | 0.798 | 1.49 | 1.1 | 0.592 | 0.348 |
| logdha | rs41426046 | 0.001 | . | . | . | . | . | . | . | . | . | . |
| logdha | rs41519248 | 0.001 | . | . | . | . | . | . | . | . | . | . |
| logdha | rs189108245 | 0.008 | . | . | . | . | . | . | . | . | . | . |
| logdha | rs7896431 | 0.477 | 1.21 | 0.452 | 0.608 | 1.55 | 0.566 | 0.226 | 0.676 | 0.204 | 0.194 | -0.494 |
| logdha | rs183724547 | 0.001 | . | . | . | . | . | . | . | . | . | . |
| logdha | rs142044990 | 0.012 | 0.854 | 0.19 | 0.478 | 2.85 | 5.52 | 0.59 | 0.572 | 1 | 0.75 | -1.31 |
| logdha | rs188140929 | 0.002 | . | . | . | . | . | . | . | . | . | . |
| logdha | rs76819096 | 0 | . | . | . | . | . | . | . | . | . | . |
| logdha | rs10751383 | 0.561 | 1.26 | 0.525 | 0.579 | 1.54 | 0.586 | 0.253 | 0.696 | 0.209 | 0.228 | -0.45 |
| logdha | rs117819321 | 0.019 | 0.909 | 0.199 | 0.663 | 15 | 25.3 | 0.108 | 0.019 | 0.032 | 0.017 | -14.6 |
| logdha | rs2242332 | 0.676 | 1.73 | 0.884 | 0.287 | 1.82 | 0.74 | 0.141 | 0.585 | 0.201 | 0.118 | -0.708 |
| logdha | rs2242333 | 0.046 | 0.961 | 0.216 | 0.86 | 3.52 | 2.02 | 0.028 | 0.257 | 0.131 | 7.70E-03 | -2.61 |
| logdha | rs2242334 | 0.678 | 1.74 | 0.888 | 0.28 | 1.82 | 0.739 | 0.138 | 0.582 | 0.199 | 0.114 | -0.716 |
| logdha | rs189465041 | 0.001 | . | . | . | . | . | . | . | . | . | . |
| logdha | rs28395868 | 0.041 | 0.838 | 0.185 | 0.424 | 0.922 | 0.964 | 0.938 | 1.11 | 0.976 | 0.908 | 0.095 |
| logdha | rs56067550 | 0.03 | 0.853 | 0.187 | 0.468 | 0.931 | 0.938 | 0.944 | 0.865 | 0.689 | 0.855 | -0.097 |
| logdha | rs1051713 | 0.165 | 0.847 | 0.212 | 0.508 | 0.931 | 0.52 | 0.898 | 0.994 | 0.442 | 0.989 | 5.70E-03 |
| logdha | rs115074210 | 0 | . | . | . | . | . | . | . | . | . | . |
| logdha | rs7900977 | 0.74 | 1.49 | 0.834 | 0.478 | 1.53 | 0.643 | 0.311 | 0.677 | 0.233 | 0.257 | -0.476 |
| logdha | rs147613891 | 0.004 | . | . | . | . | . | . | . | . | . | . |
| logdha | rs2229136 | 0.063 | 0.773 | 0.176 | 0.259 | 0.429 | 0.316 | 0.25 | 2.06 | 1.19 | 0.209 | 0.482 |
| logdha | rs185482841 | 0 | . | . | . | . | . | . | . | . | . | . |
| logdha | rs2288619 | 0.063 | 0.773 | 0.176 | 0.259 | 0.428 | 0.315 | 0.249 | 2.06 | 1.19 | 0.208 | 0.482 |
| logdha | rs189891524 | 0.001 | . | . | . | . | . | . | . | . | . | . |
| logdha | rs56145077 | 0.164 | 0.847 | 0.212 | 0.507 | 0.935 | 0.521 | 0.904 | 0.993 | 0.441 | 0.988 | 4.60E-03 |
| logdha | rs143216970 | 0 | . | . | . | . | . | . | . | . | . | . |
| logdha | rs142246478 | 0.002 | . | . | . | . | . | . | . | . | . | . |
| logdha | rs13134 | 0.014 | 0.814 | 0.174 | 0.335 | 0.117 | 0.207 | 0.225 | 4.84 | 6.13 | 0.213 | 0.531 |
| logdha | rs41314980 | 0.034 | 0.861 | 0.192 | 0.503 | 1.35 | 0.995 | 0.685 | 0.675 | 0.444 | 0.55 | -0.426 |
| logdha | rs41304611 | 0.02 | 0.83 | 0.181 | 0.392 | 0.164 | 0.532 | 0.578 | 1.35 | 3.4 | 0.904 | 0.191 |
| logdha | rs193299617 | 0 | . | . | . | . | . | . | . | . | . | . |
| logdha | rs12772724 | 0.014 | 0.814 | 0.174 | 0.335 | 0.117 | 0.206 | 0.224 | 4.85 | 6.15 | 0.213 | 0.53 |
| logdha | rs139489537 | 0.048 | 0.802 | 0.182 | 0.332 | 0.56 | 0.45 | 0.47 | 1.73 | 1.12 | 0.401 | 0.413 |
| logdha | rs12776062 | 0.014 | 0.814 | 0.174 | 0.335 | 0.116 | 0.205 | 0.223 | 4.87 | 6.18 | 0.212 | 0.53 |
| logdha | rs752059 | 0.164 | 0.848 | 0.212 | 0.51 | 0.939 | 0.522 | 0.909 | 0.991 | 0.439 | 0.984 | 2.10E-03 |
| logdha | rs147649982 | 0.035 | 0.863 | 0.197 | 0.518 | 1.5 | 1.41 | 0.666 | 0.689 | 0.545 | 0.638 | -0.471 |
| logdha | rs17157798 | 0.063 | 0.771 | 0.176 | 0.254 | 0.414 | 0.306 | 0.233 | 2.13 | 1.23 | 0.192 | 0.494 |
| logdha | rs142298619 | 0.001 | . | . | . | . | . | . | . | . | . | . |
| logdha | rs4948673 | 0.721 | 1.29 | 0.688 | 0.63 | 1.37 | 0.557 | 0.442 | 0.74 | 0.248 | 0.369 | -0.352 |
| logdha | rs74231484 | 0.041 | 0.928 | 0.208 | 0.74 | 2.59 | 1.52 | 0.105 | 0.328 | 0.161 | 0.023 | -1.73 |
| logdha | rs72796489 | 0.004 | . | . | . | . | . | . | . | . | . | . |
| logdha | rs113447578 | 0.021 | 0.835 | 0.18 | 0.401 | 0.229 | 0.363 | 0.353 | 1.68 | 2.01 | 0.662 | 0.258 |
| logdha | rs34063374 | 0.014 | 0.813 | 0.174 | 0.333 | 0.108 | 0.193 | 0.213 | 5.09 | 6.49 | 0.202 | 0.526 |
| logdha | rs11239527 | 0.019 | 0.883 | 0.191 | 0.565 | 6.51 | 9.23 | 0.186 | 0.26 | 0.318 | 0.271 | -4.9 |
| logdha | rs34371883 | 0.014 | 0.813 | 0.174 | 0.335 | 0.114 | 0.202 | 0.221 | 4.93 | 6.27 | 0.209 | 0.529 |
| logdha | rs118168828 | 0.012 | 0.794 | 0.173 | 0.291 | 0.024 | 0.044 | 0.038 | 17.4 | 20.9 | 0.017 | 0.518 |
| logdha | rs34349618 | 0.014 | 0.813 | 0.174 | 0.335 | 0.114 | 0.202 | 0.221 | 4.93 | 6.27 | 0.209 | 0.529 |
| logdha | rs80041679 | 0.048 | 0.802 | 0.182 | 0.332 | 0.557 | 0.447 | 0.466 | 1.73 | 1.12 | 0.399 | 0.414 |
| logdha | rs149640607 | 0 | . | . | . | . | . | . | . | . | . | . |
| logdha | rs4427523 | 0.834 | 0.808 | 0.557 | 0.757 | 0.712 | 0.373 | 0.517 | 1.02 | 0.43 | 0.963 | 0.066 |
| logdha | rs185880109 | 0.002 | . | . | . | . | . | . | . | . | . | . |
| logdha | rs191523220 | 0.004 | . | . | . | . | . | . | . | . | . | . |
| logdha | rs144315284 | 0 | . | . | . | . | . | . | . | . | . | . |
| logdha | rs4948674 | 0.405 | 0.977 | 0.321 | 0.944 | 1.21 | 0.433 | 0.598 | 0.823 | 0.248 | 0.518 | -0.214 |
| logdha | rs182984298 | 0.01 | 0.866 | 0.187 | 0.505 | 2.82 | 4.23 | 0.488 | 0.216 | 0.253 | 0.191 | -2.16 |
| logdha | rs56749023 | 0 | . | . | . | . | . | . | . | . | . | . |
| logdha | rs112508957 | 0 | . | . | . | . | . | . | . | . | . | . |
| logdha | rs7089063 | 0.224 | 0.743 | 0.195 | 0.259 | 0.674 | 0.305 | 0.383 | 1.34 | 0.489 | 0.429 | 0.252 |
| logdha | rs145000779 | 0.001 | . | . | . | . | . | . | . | . | . | . |
| logdha | rs80201572 | 0.048 | 0.802 | 0.182 | 0.333 | 0.564 | 0.451 | 0.474 | 1.72 | 1.11 | 0.402 | 0.412 |
| logdha | rs140568227 | 0.009 | . | . | . | . | . | . | . | . | . | . |
| logdha | rs149756228 | 0.014 | 0.796 | 0.18 | 0.312 | 0.115 | 0.197 | 0.207 | 6.25 | 7.39 | 0.121 | 0.662 |
| logdha | rs112380685 | 0.009 | . | . | . | . | . | . | . | . | . | . |
| logdha | rs60481109 | 0 | . | . | . | . | . | . | . | . | . | . |
| logdha | rs138221843 | 0.004 | . | . | . | . | . | . | . | . | . | . |
| logdha | rs150283194 | 0 | . | . | . | . | . | . | . | . | . | . |
| logdha | rs138879661 | 0.003 | . | . | . | . | . | . | . | . | . | . |
| logdha | rs34960345 | 0.062 | 0.772 | 0.176 | 0.257 | 0.425 | 0.312 | 0.243 | 2.1 | 1.2 | 0.197 | 0.491 |
| logdha | rs147514068 | 0.004 | . | . | . | . | . | . | . | . | . | . |
| logdha | rs35410535 | 0.063 | 0.773 | 0.176 | 0.258 | 0.426 | 0.313 | 0.245 | 2.09 | 1.2 | 0.201 | 0.487 |
| logdha | rs191613635 | 0.005 | . | . | . | . | . | . | . | . | . | . |
| logdha | rs17157800 | 0.009 | . | . | . | . | . | . | . | . | . | . |
| logdha | rs17523413 | 0.062 | 0.772 | 0.176 | 0.256 | 0.422 | 0.31 | 0.24 | 2.1 | 1.21 | 0.196 | 0.491 |
| logdha | rs7897036 | 0.058 | 0.819 | 0.181 | 0.364 | 0.743 | 0.66 | 0.738 | 1.27 | 0.846 | 0.72 | 0.211 |
| logdha | rs76334323 | 0.001 | . | . | . | . | . | . | . | . | . | . |

| logdha | rs143535991 | 0.001 | . | . | . | . | . | . | . | . | . | . |
| --- | --- | --- | --- | --- | --- | --- | --- | --- | --- | --- | --- | --- |
| logdha | rs140344979 | 0.007 | . | . | . | . | . | . | . | . | . | . |
| logdha | rs36047811 | 0.063 | 0.773 | 0.176 | 0.259 | 0.429 | 0.314 | 0.248 | 2.07 | 1.19 | 0.204 | 0.485 |
| logdha | rs76493570 | 0.062 | 0.773 | 0.176 | 0.259 | 0.428 | 0.314 | 0.247 | 2.08 | 1.19 | 0.201 | 0.487 |
| logdha | rs192998920 | 0.003 | . | . | . | . | . | . | . | . | . | . |
| logdha | rs116840852 | 0.024 | 0.777 | 0.179 | 0.273 | 0.082 | 0.126 | 0.104 | 5.46 | 5.9 | 0.116 | 0.489 |
| logdha | rs184824101 | 0.001 | . | . | . | . | . | . | . | . | . | . |
| logdha | rs41301621 | 0.008 | . | . | . | . | . | . | . | . | . | . |
| logdha | rs3740109 | 0.063 | 0.775 | 0.176 | 0.263 | 0.436 | 0.319 | 0.256 | 2.04 | 1.17 | 0.213 | 0.479 |
| logdha | rs7899415 | 0.02 | 0.84 | 0.188 | 0.435 | 0.603 | 1.56 | 0.845 | 1.1 | 1.81 | 0.954 | 0.114 |
| logdha | rs184395284 | 0.001 | . | . | . | . | . | . | . | . | . | . |
| logdha | rs139209589 | 0.006 | . | . | . | . | . | . | . | . | . | . |
| logdha | rs149924163 | 0 | . | . | . | . | . | . | . | . | . | . |
| logdha | rs3740110 | 0.001 | . | . | . | . | . | . | . | . | . | . |
| logdha | rs144335113 | 0 | . | . | . | . | . | . | . | . | . | . |
| logdha | rs74743385 | 0 | . | . | . | . | . | . | . | . | . | . |
| logdha | rs146545829 | 0 | . | . | . | . | . | . | . | . | . | . |
| logdha | rs181842689 | 0 | . | . | . | . | . | . | . | . | . | . |
| logdha | rs3740111 | 0.062 | 0.774 | 0.176 | 0.261 | 0.433 | 0.317 | 0.253 | 2.06 | 1.18 | 0.208 | 0.483 |
| logdha | rs186261599 | 0 | . | . | . | . | . | . | . | . | . | . |
| logdha | rs73285348 | 0.012 | 0.819 | 0.176 | 0.352 | 0.085 | 0.168 | 0.214 | 5.77 | 9.02 | 0.263 | 0.496 |
| logdha | rs186356901 | 0 | . | . | . | . | . | . | . | . | . | . |
| logdha | rs3824618 | 0.048 | 0.802 | 0.182 | 0.333 | 0.565 | 0.451 | 0.475 | 1.72 | 1.11 | 0.403 | 0.411 |
| logdha | rs3802548 | 0.231 | 0.927 | 0.252 | 0.78 | 1.34 | 0.622 | 0.529 | 0.817 | 0.316 | 0.601 | -0.252 |
| logdha | rs3802547 | 0.001 | . | . | . | . | . | . | . | . | . | . |
| logdha | rs77677521 | 0.024 | 0.777 | 0.179 | 0.273 | 0.082 | 0.126 | 0.104 | 5.45 | 5.89 | 0.116 | 0.489 |
| logdha | rs3824616 | 0.063 | 0.777 | 0.177 | 0.268 | 0.448 | 0.326 | 0.27 | 2 | 1.14 | 0.227 | 0.47 |
| logdha | rs191073818 | 0 | . | . | . | . | . | . | . | . | . | . |
| logdha | rs147078004 | 0.002 | . | . | . | . | . | . | . | . | . | . |
| logdha | rs7908745 | 0.297 | 0.89 | 0.257 | 0.687 | 1.17 | 0.45 | 0.689 | 0.917 | 0.3 | 0.791 | -0.105 |
| logdha | rs17157811 | 0.009 | . | . | . | . | . | . | . | . | . | . |
| logdha | rs138506087 | 0.009 | . | . | . | . | . | . | . | . | . | . |
| logdha | rs77241269 | 0 | . | . | . | . | . | . | . | . | . | . |
| logdha | rs138374938 | 0.002 | . | . | . | . | . | . | . | . | . | . |
| logdha | rs2279433 | 0.062 | 0.768 | 0.175 | 0.245 | 0.37 | 0.279 | 0.188 | 2.26 | 1.33 | 0.166 | 0.505 |
| logdha | rs73285358 | 0 | . | . | . | . | . | . | . | . | . | . |
| logdha | rs11239528 | 0.232 | 0.942 | 0.257 | 0.825 | 1.41 | 0.646 | 0.454 | 0.791 | 0.303 | 0.541 | -0.301 |
| logdha | rs77453386 | 0.001 | . | . | . | . | . | . | . | . | . | . |
| logdha | rs2279434 | 0.069 | 0.784 | 0.182 | 0.294 | 0.488 | 0.34 | 0.303 | 1.73 | 0.952 | 0.317 | 0.391 |
| logdha | rs149236525 | 0.006 | . | . | . | . | . | . | . | . | . | . |
| logdha | rs12573609 | 0.052 | 0.686 | 0.157 | 0.099 | 0.333 | 0.321 | 0.254 | 4.61 | 3.39 | 0.037 | 1.03 |
| logdha | rs191716655 | 0 | . | . | . | . | . | . | . | . | . | . |
| logdha | rs17523660 | 0.014 | 0.817 | 0.175 | 0.346 | 0.129 | 0.23 | 0.25 | 4.33 | 5.62 | 0.259 | 0.51 |
| logdha | rs142671360 | 0.004 | . | . | . | . | . | . | . | . | . | . |
| logdha | rs3764990 | 0.062 | 0.766 | 0.174 | 0.241 | 0.356 | 0.271 | 0.175 | 2.32 | 1.38 | 0.156 | 0.511 |
| logdha | rs113682861 | 0.004 | . | . | . | . | . | . | . | . | . | . |
| logdha | rs117658270 | 0.001 | . | . | . | . | . | . | . | . | . | . |
| logdha | rs187309063 | 0 | . | . | . | . | . | . | . | . | . | . |
| logdha | rs73285360 | 0 | . | . | . | . | . | . | . | . | . | . |
| logdha | rs80133289 | 0.001 | . | . | . | . | . | . | . | . | . | . |
| logdha | rs186693257 | 0.001 | . | . | . | . | . | . | . | . | . | . |
| logdha | rs35735968 | 0.063 | 0.767 | 0.175 | 0.244 | 0.366 | 0.278 | 0.185 | 2.28 | 1.35 | 0.164 | 0.507 |
| logdha | rs17157825 | 0.048 | 0.793 | 0.18 | 0.308 | 0.465 | 0.379 | 0.348 | 1.94 | 1.28 | 0.317 | 0.456 |
| logdha | rs2291428 | 0.229 | 0.938 | 0.257 | 0.814 | 1.42 | 0.666 | 0.457 | 0.797 | 0.313 | 0.563 | -0.296 |
| logdha | rs2291429 | 0.231 | 0.945 | 0.258 | 0.835 | 1.42 | 0.652 | 0.439 | 0.786 | 0.3 | 0.529 | -0.311 |
| logdha | rs145714792 | 0.007 | . | . | . | . | . | . | . | . | . | . |
| logdha | rs12769244 | 0.062 | 0.766 | 0.174 | 0.242 | 0.358 | 0.273 | 0.178 | 2.31 | 1.37 | 0.158 | 0.51 |
| logdha | rs12771275 | 0.062 | 0.766 | 0.174 | 0.242 | 0.358 | 0.273 | 0.178 | 2.31 | 1.37 | 0.158 | 0.51 |
| logdha | rs146266174 | 0.002 | . | . | . | . | . | . | . | . | . | . |
| logdha | rs41301623 | 0.004 | . | . | . | . | . | . | . | . | . | . |
| logdha | rs2306814 | 0.001 | . | . | . | . | . | . | . | . | . | . |
| logdha | rs12244834 | 0 | . | . | . | . | . | . | . | . | . | . |
| logdha | rs184006011 | 0.004 | . | . | . | . | . | . | . | . | . | . |
| logdha | rs78829066 | 0.001 | . | . | . | . | . | . | . | . | . | . |
| logdha | rs34731408 | 0.062 | 0.766 | 0.174 | 0.242 | 0.358 | 0.273 | 0.178 | 2.31 | 1.37 | 0.158 | 0.51 |
| logdha | rs17451516 | 0.014 | 0.817 | 0.175 | 0.346 | 0.129 | 0.229 | 0.25 | 4.33 | 5.63 | 0.259 | 0.51 |
| logdha | rs139910133 | . | . | . | . | . | . | . | . | . | . | . |
| logdha | rs10900218 | 0.229 | 0.806 | 0.211 | 0.41 | 0.856 | 0.361 | 0.712 | 1.11 | 0.379 | 0.767 | 0.102 |
| logdha | rs146023999 | . | . | . | . | . | . | . | . | . | . | . |
| logdha | rs187296958 | . | . | . | . | . | . | . | . | . | . | . |
| logdha | rs142342833 | . | . | . | . | . | . | . | . | . | . | . |
| logdha | rs143489255 | . | . | . | . | . | . | . | . | . | . | . |
| logdha | rs138532518 | . | . | . | . | . | . | . | . | . | . | . |
| logdha | rs183768079 | . | . | . | . | . | . | . | . | . | . | . |
| logdha | rs117892272 | . | . | . | . | . | . | . | . | . | . | . |
| logdha | rs192993943 | . | . | . | . | . | . | . | . | . | . | . |
| logdha | rs182989043 | . | . | . | . | . | . | . | . | . | . | . |
| logdha | rs189703435 | . | . | . | . | . | . | . | . | . | . | . |
| logdha | rs188794040 | . | . | . | . | . | . | . | . | . | . | . |
| logdha | rs143920953 | . | . | . | . | . | . | . | . | . | . | . |
| logdha | rs191029121 | . | . | . | . | . | . | . | . | . | . | . |
| logdha | rs116875448 | . | . | . | . | . | . | . | . | . | . | . |
| logdha | rs190167877 | . | . | . | . | . | . | . | . | . | . | . |
| logdha | rs184993418 | . | . | . | . | . | . | . | . | . | . | . |
| logdha | rs143241158 | . | . | . | . | . | . | . | . | . | . | . |
| logdha | rs11239500 | . | . | . | . | . | . | . | . | . | . | . |

| logdha | rs140568994 | . | . | . | . | . | . | . | . | . | . | . |
| --- | --- | --- | --- | --- | --- | --- | --- | --- | --- | --- | --- | --- |
| logdha | rs187530317 | . | . | . | . | . | . | . | . | . | . | . |
| logdha | rs145189326 | . | . | . | . | . | . | . | . | . | . | . |
| logdha | rs186203332 | . | . | . | . | . | . | . | . | . | . | . |
| logdha | rs189921909 | . | . | . | . | . | . | . | . | . | . | . |
| logdha | rs187260989 | . | . | . | . | . | . | . | . | . | . | . |
| logdha | rs114376586 | . | . | . | . | . | . | . | . | . | . | . |
| logdha | rs151070808 | . | . | . | . | . | . | . | . | . | . | . |
| logdha | rs140927225 | . | . | . | . | . | . | . | . | . | . | . |
| logdha | rs191555615 | . | . | . | . | . | . | . | . | . | . | . |
| logdha | rs80278898 | . | . | . | . | . | . | . | . | . | . | . |
| logdha | rs190849503 | . | . | . | . | . | . | . | . | . | . | . |
| logdha | rs150080315 | . | . | . | . | . | . | . | . | . | . | . |
| logdha | rs143623885 | . | . | . | . | . | . | . | . | . | . | . |
| logdha | rs187760040 | . | . | . | . | . | . | . | . | . | . | . |
| logdha | rs143917143 | . | . | . | . | . | . | . | . | . | . | . |
| logdha | rs149851136 | . | . | . | . | . | . | . | . | . | . | . |
| logdha | rs181612054 | . | . | . | . | . | . | . | . | . | . | . |
| logdha | rs191006548 | . | . | . | . | . | . | . | . | . | . | . |
| logdha | rs117377631 | . | . | . | . | . | . | . | . | . | . | . |
| logdha | rs182107634 | . | . | . | . | . | . | . | . | . | . | . |
| logdha | rs190211867 | . | . | . | . | . | . | . | . | . | . | . |
| logdha | rs148293457 | . | . | . | . | . | . | . | . | . | . | . |
| logdha | rs191811733 | . | . | . | . | . | . | . | . | . | . | . |
| logdha | rs146109272 | . | . | . | . | . | . | . | . | . | . | . |
| logdha | rs148397915 | . | . | . | . | . | . | . | . | . | . | . |
| logdha | rs142233290 | . | . | . | . | . | . | . | . | . | . | . |
| logdha | rs80226874 | . | . | . | . | . | . | . | . | . | . | . |
| logdha | rs144496203 | . | . | . | . | . | . | . | . | . | . | . |
| logdha | rs191062130 | . | . | . | . | . | . | . | . | . | . | . |
| logdha | rs185807817 | . | . | . | . | . | . | . | . | . | . | . |
| logdha | rs117846213 | . | . | . | . | . | . | . | . | . | . | . |
| logdha | rs190545473 | . | . | . | . | . | . | . | . | . | . | . |
| logdha | rs192364918 | . | . | . | . | . | . | . | . | . | . | . |
| logdha | rs182939241 | . | . | . | . | . | . | . | . | . | . | . |
| logdha | rs12571125 | . | . | . | . | . | . | . | . | . | . | . |
| logdha | rs192418941 | . | . | . | . | . | . | . | . | . | . | . |
| logdha | rs141270243 | . | . | . | . | . | . | . | . | . | . | . |
| logdha | rs182615502 | . | . | . | . | . | . | . | . | . | . | . |
| logdha | rs146669856 | . | . | . | . | . | . | . | . | . | . | . |
| logdha | rs78290029 | . | . | . | . | . | . | . | . | . | . | . |
| logdha | rs139955565 | . | . | . | . | . | . | . | . | . | . | . |
| logdha | rs12267314 | . | . | . | . | . | . | . | . | . | . | . |
| logdha | rs74477197 | . | . | . | . | . | . | . | . | . | . | . |
| logdha | rs188147236 | . | . | . | . | . | . | . | . | . | . | . |
| logdha | rs2228065 | . | . | . | . | . | . | . | . | . | . | . |
| logdha | rs116961353 | . | . | . | . | . | . | . | . | . | . | . |
| logdha | rs190222272 | . | . | . | . | . | . | . | . | . | . | . |
| logdha | rs182719940 | . | . | . | . | . | . | . | . | . | . | . |
| logdha | rs184477413 | . | . | . | . | . | . | . | . | . | . | . |
| logdha | rs12265175 | . | . | . | . | . | . | . | . | . | . | . |
| logdha | rs182928276 | . | . | . | . | . | . | . | . | . | . | . |
| logdha | rs144409895 | . | . | . | . | . | . | . | . | . | . | . |
| logdha | rs58998447 | . | . | . | . | . | . | . | . | . | . | . |
| logdha | rs188668832 | . | . | . | . | . | . | . | . | . | . | . |
| logdha | rs148641686 | . | . | . | . | . | . | . | . | . | . | . |
| logdha | rs75468791 | . | . | . | . | . | . | . | . | . | . | . |
| logdha | rs147327397 | . | . | . | . | . | . | . | . | . | . | . |
| logdha | rs77354597 | . | . | . | . | . | . | . | . | . | . | . |
| logdha | rs41365544 | . | . | . | . | . | . | . | . | . | . | . |
| logdha | rs187564188 | . | . | . | . | . | . | . | . | . | . | . |
| logdha | rs183511984 | . | . | . | . | . | . | . | . | . | . | . |
| logdha | rs12262783 | . | . | . | . | . | . | . | . | . | . | . |
| logdha | rs188817687 | . | . | . | . | . | . | . | . | . | . | . |
| logdha | rs3815968 | . | . | . | . | . | . | . | . | . | . | . |
| logdha | rs187822300 | . | . | . | . | . | . | . | . | . | . | . |
| logdha | rs188902172 | . | . | . | . | . | . | . | . | . | . | . |
| logdha | rs181190448 | . | . | . | . | . | . | . | . | . | . | . |
| logdha | rs182699093 | . | . | . | . | . | . | . | . | . | . | . |
| logdha | rs182631303 | . | . | . | . | . | . | . | . | . | . | . |
| logdha | rs187014172 | . | . | . | . | . | . | . | . | . | . | . |
| logdha | rs142575406 | . | . | . | . | . | . | . | . | . | . | . |
| logdha | rs180711033 | . | . | . | . | . | . | . | . | . | . | . |
| logdha | rs137894823 | . | . | . | . | . | . | . | . | . | . | . |
| logdha | rs189053273 | . | . | . | . | . | . | . | . | . | . | . |
| logdha | rs143499388 | . | . | . | . | . | . | . | . | . | . | . |
| logdha | rs182936001 | . | . | . | . | . | . | . | . | . | . | . |
| logdha | rs148456406 | . | . | . | . | . | . | . | . | . | . | . |
| logdha | rs182499531 | . | . | . | . | . | . | . | . | . | . | . |
| logdha | rs74884915 | . | . | . | . | . | . | . | . | . | . | . |
| logdha | rs184046374 | . | . | . | . | . | . | . | . | . | . | . |
| logdha | rs74333993 | . | . | . | . | . | . | . | . | . | . | . |
| logdha | rs138889719 | . | . | . | . | . | . | . | . | . | . | . |
| logdha | rs75330330 | . | . | . | . | . | . | . | . | . | . | . |
| logdha | rs193051693 | . | . | . | . | . | . | . | . | . | . | . |
| logdha | rs145929633 | . | . | . | . | . | . | . | . | . | . | . |

| logdha | rs184076904 | . | . | . | . | . | . | . | . | . | . | . |
| --- | --- | --- | --- | --- | --- | --- | --- | --- | --- | --- | --- | --- |
| logdha | rs141802494 | . | . | . | . | . | . | . | . | . | . | . |
| logdha | rs149519759 | . | . | . | . | . | . | . | . | . | . | . |
| logdha | rs145467260 | . | . | . | . | . | . | . | . | . | . | . |
| logdha | rs79620914 | . | . | . | . | . | . | . | . | . | . | . |
| logdha | rs140538633 | . | . | . | . | . | . | . | . | . | . | . |
| logdha | rs140862301 | . | . | . | . | . | . | . | . | . | . | . |
| logdha | rs114615847 | . | . | . | . | . | . | . | . | . | . | . |
| logdha | rs141800259 | . | . | . | . | . | . | . | . | . | . | . |
| logdha | rs149779059 | . | . | . | . | . | . | . | . | . | . | . |
| logdha | rs116539253 | . | . | . | . | . | . | . | . | . | . | . |
| logdha | rs184979550 | . | . | . | . | . | . | . | . | . | . | . |
| logdha | rs190128940 | . | . | . | . | . | . | . | . | . | . | . |
| logdha | rs146594816 | . | . | . | . | . | . | . | . | . | . | . |
| logdha | rs141370766 | . | . | . | . | . | . | . | . | . | . | . |
| logdha | rs144670033 | . | . | . | . | . | . | . | . | . | . | . |
| logdha | rs187152174 | . | . | . | . | . | . | . | . | . | . | . |
| logdha | rs184180263 | . | . | . | . | . | . | . | . | . | . | . |
| logdha | rs140075861 | . | . | . | . | . | . | . | . | . | . | . |
| logdha | rs186483129 | . | . | . | . | . | . | . | . | . | . | . |
| logdha | rs186634282 | . | . | . | . | . | . | . | . | . | . | . |
| logdha | rs9988663 | . | . | . | . | . | . | . | . | . | . | . |
| logdha | rs138828529 | . | . | . | . | . | . | . | . | . | . | . |
| logdha | rs188777615 | . | . | . | . | . | . | . | . | . | . | . |
| logdha | rs192321882 | . | . | . | . | . | . | . | . | . | . | . |
| logdha | rs150812270 | . | . | . | . | . | . | . | . | . | . | . |
| logdha | rs185906603 | . | . | . | . | . | . | . | . | . | . | . |
| logdha | rs191070862 | . | . | . | . | . | . | . | . | . | . | . |
| logdha | rs182904978 | . | . | . | . | . | . | . | . | . | . | . |
| logdha | rs192679655 | . | . | . | . | . | . | . | . | . | . | . |
| logdha | rs189673086 | . | . | . | . | . | . | . | . | . | . | . |
| logdha | rs181141892 | . | . | . | . | . | . | . | . | . | . | . |
| logdha | rs186404123 | . | . | . | . | . | . | . | . | . | . | . |
| logdha | rs192117287 | . | . | . | . | . | . | . | . | . | . | . |
| logdha | rs182748835 | . | . | . | . | . | . | . | . | . | . | . |
| logdha | rs141854549 | . | . | . | . | . | . | . | . | . | . | . |
| logdha | rs140163397 | . | . | . | . | . | . | . | . | . | . | . |
| logdha | rs146347300 | . | . | . | . | . | . | . | . | . | . | . |
| logdha | rs139362377 | . | . | . | . | . | . | . | . | . | . | . |
| logdha | rs185087671 | . | . | . | . | . | . | . | . | . | . | . |
| logdha | rs190466574 | . | . | . | . | . | . | . | . | . | . | . |
| logdha | rs147012778 | . | . | . | . | . | . | . | . | . | . | . |
| logdha | rs138236960 | . | . | . | . | . | . | . | . | . | . | . |
| logdha | rs116310311 | . | . | . | . | . | . | . | . | . | . | . |
| logdha | rs142868356 | . | . | . | . | . | . | . | . | . | . | . |
| logdha | rs144956240 | . | . | . | . | . | . | . | . | . | . | . |
| logdha | rs188527776 | . | . | . | . | . | . | . | . | . | . | . |
| logdha | rs183971165 | . | . | . | . | . | . | . | . | . | . | . |
| logdha | rs76748216 | . | . | . | . | . | . | . | . | . | . | . |
| logdha | rs148554990 | . | . | . | . | . | . | . | . | . | . | . |
| logdha | rs114446477 | . | . | . | . | . | . | . | . | . | . | . |
| logdha | rs185223933 | . | . | . | . | . | . | . | . | . | . | . |
| logdha | rs76869726 | . | . | . | . | . | . | . | . | . | . | . |
| logdha | rs151200830 | . | . | . | . | . | . | . | . | . | . | . |
| logdha | rs17157731 | . | . | . | . | . | . | . | . | . | . | . |
| logdha | rs181243469 | . | . | . | . | . | . | . | . | . | . | . |
| logdha | rs186890707 | . | . | . | . | . | . | . | . | . | . | . |
| logdha | rs184063360 | . | . | . | . | . | . | . | . | . | . | . |
| logdha | rs188550237 | . | . | . | . | . | . | . | . | . | . | . |
| logdha | rs140931068 | . | . | . | . | . | . | . | . | . | . | . |
| logdha | rs74867877 | . | . | . | . | . | . | . | . | . | . | . |
| logdha | rs11239495 | . | . | . | . | . | . | . | . | . | . | . |
| logdha | rs149745800 | . | . | . | . | . | . | . | . | . | . | . |
| logdha | rs145044458 | . | . | . | . | . | . | . | . | . | . | . |
| logdha | rs188067427 | . | . | . | . | . | . | . | . | . | . | . |
| logdha | rs146827800 | . | . | . | . | . | . | . | . | . | . | . |
| logdha | rs140619817 | . | . | . | . | . | . | . | . | . | . | . |
| logdha | rs892689 | . | . | . | . | . | . | . | . | . | . | . |
| logdha | rs143642005 | . | . | . | . | . | . | . | . | . | . | . |
| logdha | rs181929304 | . | . | . | . | . | . | . | . | . | . | . |
| logdha | rs191086173 | . | . | . | . | . | . | . | . | . | . | . |
| logdha | rs114613116 | . | . | . | . | . | . | . | . | . | . | . |
| logdha | rs141076768 | . | . | . | . | . | . | . | . | . | . | . |
| logdha | rs187316531 | . | . | . | . | . | . | . | . | . | . | . |
| logdha | rs11239499 | . | . | . | . | . | . | . | . | . | . | . |
| logdha | rs192726371 | . | . | . | . | . | . | . | . | . | . | . |
| logdha | rs74128481 | . | . | . | . | . | . | . | . | . | . | . |
| logdha | rs74128482 | . | . | . | . | . | . | . | . | . | . | . |
| logdha | rs11239502 | . | . | . | . | . | . | . | . | . | . | . |
| logdha | rs116330285 | . | . | . | . | . | . | . | . | . | . | . |
| logdha | rs189926729 | . | . | . | . | . | . | . | . | . | . | . |
| logdha | rs188975779 | . | . | . | . | . | . | . | . | . | . | . |
| logdha | rs143846038 | . | . | . | . | . | . | . | . | . | . | . |
| logdha | rs74128483 | . | . | . | . | . | . | . | . | . | . | . |
| logdha | rs139292055 | . | . | . | . | . | . | . | . | . | . | . |
| logdha | rs11239503 | . | . | . | . | . | . | . | . | . | . | . |

| logdha | rs58838686 | . | . | . | . | . | . | . | . | . | . | . |
| --- | --- | --- | --- | --- | --- | --- | --- | --- | --- | --- | --- | --- |
| logdha | rs185825373 | . | . | . | . | . | . | . | . | . | . | . |
| logdha | rs11239504 | . | . | . | . | . | . | . | . | . | . | . |
| logdha | rs150012354 | . | . | . | . | . | . | . | . | . | . | . |
| logdha | rs187763096 | . | . | . | . | . | . | . | . | . | . | . |
| logdha | rs193043982 | . | . | . | . | . | . | . | . | . | . | . |
| logdha | rs79186884 | . | . | . | . | . | . | . | . | . | . | . |
| logdha | rs74128484 | . | . | . | . | . | . | . | . | . | . | . |
| logdha | rs58461537 | . | . | . | . | . | . | . | . | . | . | . |
| logdha | rs188527474 | . | . | . | . | . | . | . | . | . | . | . |
| logdha | rs141659976 | . | . | . | . | . | . | . | . | . | . | . |
| logdha | rs115846407 | . | . | . | . | . | . | . | . | . | . | . |
| logdha | rs138378816 | . | . | . | . | . | . | . | . | . | . | . |
| logdha | rs146188136 | . | . | . | . | . | . | . | . | . | . | . |
| logdha | rs139383871 | . | . | . | . | . | . | . | . | . | . | . |
| logdha | rs182953869 | . | . | . | . | . | . | . | . | . | . | . |
| logdha | rs192430299 | . | . | . | . | . | . | . | . | . | . | . |
| logdha | rs144152844 | . | . | . | . | . | . | . | . | . | . | . |
| logdha | rs145987786 | . | . | . | . | . | . | . | . | . | . | . |
| logdha | rs181132926 | . | . | . | . | . | . | . | . | . | . | . |
| logdha | rs147882253 | . | . | . | . | . | . | . | . | . | . | . |
| logdha | rs141807621 | . | . | . | . | . | . | . | . | . | . | . |
| logdha | rs147116052 | . | . | . | . | . | . | . | . | . | . | . |
| logdha | rs147716618 | . | . | . | . | . | . | . | . | . | . | . |
| logdha | rs187198855 | . | . | . | . | . | . | . | . | . | . | . |
| logdha | rs189409638 | . | . | . | . | . | . | . | . | . | . | . |
| logdha | rs182647339 | . | . | . | . | . | . | . | . | . | . | . |
| logdha | rs187995988 | . | . | . | . | . | . | . | . | . | . | . |
| logdha | rs141034184 | . | . | . | . | . | . | . | . | . | . | . |
| logdha | rs187310430 | . | . | . | . | . | . | . | . | . | . | . |
| logdha | rs192608488 | . | . | . | . | . | . | . | . | . | . | . |
| logdha | rs148840366 | . | . | . | . | . | . | . | . | . | . | . |
| logdha | rs186785022 | . | . | . | . | . | . | . | . | . | . | . |
| logdha | rs192348728 | . | . | . | . | . | . | . | . | . | . | . |
| logdha | rs189656631 | . | . | . | . | . | . | . | . | . | . | . |
| logdha | rs185289750 | . | . | . | . | . | . | . | . | . | . | . |
| logdha | rs142382395 | . | . | . | . | . | . | . | . | . | . | . |
| logdha | rs144866696 | . | . | . | . | . | . | . | . | . | . | . |
| logdha | rs141371289 | . | . | . | . | . | . | . | . | . | . | . |
| logdha | rs114750008 | . | . | . | . | . | . | . | . | . | . | . |
| logdha | rs189063597 | . | . | . | . | . | . | . | . | . | . | . |
| logdha | rs139151571 | . | . | . | . | . | . | . | . | . | . | . |
| logdha | rs139846368 | . | . | . | . | . | . | . | . | . | . | . |
| logdha | rs182057653 | . | . | . | . | . | . | . | . | . | . | . |
| logdha | rs148785420 | . | . | . | . | . | . | . | . | . | . | . |
| logdha | rs138383251 | . | . | . | . | . | . | . | . | . | . | . |
| logdha | rs116526705 | . | . | . | . | . | . | . | . | . | . | . |
| logdha | rs185946436 | . | . | . | . | . | . | . | . | . | . | . |
| logdha | rs190455427 | . | . | . | . | . | . | . | . | . | . | . |
| logdha | rs9282584 | . | . | . | . | . | . | . | . | . | . | . |
| logdha | rs17153289 | . | . | . | . | . | . | . | . | . | . | . |
| logdha | rs138359103 | . | . | . | . | . | . | . | . | . | . | . |
| logdha | rs150605268 | . | . | . | . | . | . | . | . | . | . | . |
| logdha | rs79242910 | . | . | . | . | . | . | . | . | . | . | . |
| logdha | rs139716904 | . | . | . | . | . | . | . | . | . | . | . |
| logdha | rs76238498 | . | . | . | . | . | . | . | . | . | . | . |
| logdha | rs184140639 | . | . | . | . | . | . | . | . | . | . | . |
| logdha | rs188517278 | . | . | . | . | . | . | . | . | . | . | . |
| logdha | rs139678913 | . | . | . | . | . | . | . | . | . | . | . |
| logdha | rs116697955 | . | . | . | . | . | . | . | . | . | . | . |
| logdha | rs79267994 | . | . | . | . | . | . | . | . | . | . | . |
| logdha | rs11239510 | . | . | . | . | . | . | . | . | . | . | . |
| logdha | rs75831645 | . | . | . | . | . | . | . | . | . | . | . |
| logdha | rs78452276 | . | . | . | . | . | . | . | . | . | . | . |
| logdha | rs144366924 | . | . | . | . | . | . | . | . | . | . | . |
| logdha | rs77519841 | . | . | . | . | . | . | . | . | . | . | . |
| logdha | rs76944454 | . | . | . | . | . | . | . | . | . | . | . |
| logdha | rs76443855 | . | . | . | . | . | . | . | . | . | . | . |
| logdha | rs146613174 | . | . | . | . | . | . | . | . | . | . | . |
| logdha | rs181424761 | . | . | . | . | . | . | . | . | . | . | . |
| logdha | rs142119241 | . | . | . | . | . | . | . | . | . | . | . |
| logdha | rs140137954 | . | . | . | . | . | . | . | . | . | . | . |
| logdha | rs187319549 | . | . | . | . | . | . | . | . | . | . | . |
| logdha | rs12241545 | . | . | . | . | . | . | . | . | . | . | . |
| logdha | rs10128228 | . | . | . | . | . | . | . | . | . | . | . |
| logdha | rs58580249 | . | . | . | . | . | . | . | . | . | . | . |
| logdha | rs193148863 | . | . | . | . | . | . | . | . | . | . | . |
| logdha | rs185638946 | . | . | . | . | . | . | . | . | . | . | . |
| logdha | rs117752921 | . | . | . | . | . | . | . | . | . | . | . |
| logdha | rs180841236 | . | . | . | . | . | . | . | . | . | . | . |
| logdha | rs73283155 | . | . | . | . | . | . | . | . | . | . | . |
| logdha | rs76560599 | . | . | . | . | . | . | . | . | . | . | . |
| logdha | rs76273620 | . | . | . | . | . | . | . | . | . | . | . |
| logdha | rs141432923 | . | . | . | . | . | . | . | . | . | . | . |
| logdha | rs113529322 | . | . | . | . | . | . | . | . | . | . | . |
| logdha | rs10128306 | . | . | . | . | . | . | . | . | . | . | . |

| logdha | rs149691688 | . | . | . | . | . | . | . | . | . | . | . |
| --- | --- | --- | --- | --- | --- | --- | --- | --- | --- | --- | --- | --- |
| logdha | rs75872495 | . | . | . | . | . | . | . | . | . | . | . |
| logdha | rs191873960 | . | . | . | . | . | . | . | . | . | . | . |
| logdha | rs80155622 | . | . | . | . | . | . | . | . | . | . | . |
| logdha | rs113914656 | . | . | . | . | . | . | . | . | . | . | . |
| logdha | rs12242840 | . | . | . | . | . | . | . | . | . | . | . |
| logdha | rs147824078 | . | . | . | . | . | . | . | . | . | . | . |
| logdha | rs59051493 | . | . | . | . | . | . | . | . | . | . | . |
| logdha | rs9282588 | . | . | . | . | . | . | . | . | . | . | . |
| logdha | rs150215207 | . | . | . | . | . | . | . | . | . | . | . |
| logdha | rs138772243 | . | . | . | . | . | . | . | . | . | . | . |
| logdha | rs149383244 | . | . | . | . | . | . | . | . | . | . | . |
| logdha | rs17522720 | . | . | . | . | . | . | . | . | . | . | . |
| logdha | rs79245105 | . | . | . | . | . | . | . | . | . | . | . |
| logdha | rs138222133 | . | . | . | . | . | . | . | . | . | . | . |
| logdha | rs12247314 | . | . | . | . | . | . | . | . | . | . | . |
| logdha | rs12263894 | . | . | . | . | . | . | . | . | . | . | . |
| logdha | rs149907880 | . | . | . | . | . | . | . | . | . | . | . |
| logdha | rs188153885 | . | . | . | . | . | . | . | . | . | . | . |
| logdha | rs73283159 | . | . | . | . | . | . | . | . | . | . | . |
| logdha | rs140476687 | . | . | . | . | . | . | . | . | . | . | . |
| logdha | rs186603148 | . | . | . | . | . | . | . | . | . | . | . |
| logdha | rs183296063 | . | . | . | . | . | . | . | . | . | . | . |
| logdha | rs145733694 | . | . | . | . | . | . | . | . | . | . | . |
| logdha | rs189807449 | . | . | . | . | . | . | . | . | . | . | . |
| logdha | rs12253223 | . | . | . | . | . | . | . | . | . | . | . |
| logdha | rs182113637 | . | . | . | . | . | . | . | . | . | . | . |
| logdha | rs142792099 | . | . | . | . | . | . | . | . | . | . | . |
| logdha | rs12249910 | . | . | . | . | . | . | . | . | . | . | . |
| logdha | rs149436339 | . | . | . | . | . | . | . | . | . | . | . |
| logdha | rs77632210 | . | . | . | . | . | . | . | . | . | . | . |
| logdha | rs74130818 | . | . | . | . | . | . | . | . | . | . | . |
| logdha | rs187583648 | . | . | . | . | . | . | . | . | . | . | . |
| logdha | rs191295987 | . | . | . | . | . | . | . | . | . | . | . |
| logdha | rs184200686 | . | . | . | . | . | . | . | . | . | . | . |
| logdha | rs76117263 | . | . | . | . | . | . | . | . | . | . | . |
| logdha | rs185393149 | . | . | . | . | . | . | . | . | . | . | . |
| logdha | rs144760014 | . | . | . | . | . | . | . | . | . | . | . |
| logdha | rs7922292 | . | . | . | . | . | . | . | . | . | . | . |
| logdha | rs115372844 | . | . | . | . | . | . | . | . | . | . | . |
| logdha | rs111460364 | . | . | . | . | . | . | . | . | . | . | . |
| logdha | rs182052255 | . | . | . | . | . | . | . | . | . | . | . |
| logdha | rs180983889 | . | . | . | . | . | . | . | . | . | . | . |
| logdha | rs139175327 | . | . | . | . | . | . | . | . | . | . | . |
| logdha | rs149959620 | . | . | . | . | . | . | . | . | . | . | . |
| logdha | rs114653117 | . | . | . | . | . | . | . | . | . | . | . |
| logdha | rs144270018 | . | . | . | . | . | . | . | . | . | . | . |
| logdha | rs182723204 | . | . | . | . | . | . | . | . | . | . | . |
| logdha | rs57204067 | . | . | . | . | . | . | . | . | . | . | . |
| logdha | rs144956226 | . | . | . | . | . | . | . | . | . | . | . |
| logdha | rs187842704 | . | . | . | . | . | . | . | . | . | . | . |
| logdha | rs184620511 | . | . | . | . | . | . | . | . | . | . | . |
| logdha | rs144031303 | . | . | . | . | . | . | . | . | . | . | . |
| logdha | rs187526473 | . | . | . | . | . | . | . | . | . | . | . |
| logdha | rs193002805 | . | . | . | . | . | . | . | . | . | . | . |
| logdha | rs138707426 | . | . | . | . | . | . | . | . | . | . | . |
| logdha | rs142754243 | . | . | . | . | . | . | . | . | . | . | . |
| logdha | rs181600331 | . | . | . | . | . | . | . | . | . | . | . |
| logdha | rs144421264 | . | . | . | . | . | . | . | . | . | . | . |
| logdha | rs184414519 | . | . | . | . | . | . | . | . | . | . | . |
| logdha | rs150596349 | . | . | . | . | . | . | . | . | . | . | . |
| logdha | rs190753747 | . | . | . | . | . | . | . | . | . | . | . |
| logdha | rs139560940 | . | . | . | . | . | . | . | . | . | . | . |
| logdha | rs75640804 | . | . | . | . | . | . | . | . | . | . | . |
| logdha | rs73283175 | . | . | . | . | . | . | . | . | . | . | . |
| logdha | rs143337229 | . | . | . | . | . | . | . | . | . | . | . |
| logdha | rs73283177 | . | . | . | . | . | . | . | . | . | . | . |
| logdha | rs76053611 | . | . | . | . | . | . | . | . | . | . | . |
| logdha | rs116325983 | . | . | . | . | . | . | . | . | . | . | . |
| logdha | rs115451825 | . | . | . | . | . | . | . | . | . | . | . |
| logdha | rs183845246 | . | . | . | . | . | . | . | . | . | . | . |
| logdha | rs79756542 | . | . | . | . | . | . | . | . | . | . | . |
| logdha | rs187242860 | . | . | . | . | . | . | . | . | . | . | . |
| logdha | rs57318980 | . | . | . | . | . | . | . | . | . | . | . |
| logdha | rs59969732 | . | . | . | . | . | . | . | . | . | . | . |
| logdha | rs143811488 | . | . | . | . | . | . | . | . | . | . | . |
| logdha | rs189067644 | . | . | . | . | . | . | . | . | . | . | . |
| logdha | rs60449178 | . | . | . | . | . | . | . | . | . | . | . |
| logdha | rs59243389 | . | . | . | . | . | . | . | . | . | . | . |
| logdha | rs184946540 | . | . | . | . | . | . | . | . | . | . | . |
| logdha | rs188685881 | . | . | . | . | . | . | . | . | . | . | . |
| logdha | rs150335797 | . | . | . | . | . | . | . | . | . | . | . |
| logdha | rs143879260 | . | . | . | . | . | . | . | . | . | . | . |
| logdha | rs186044491 | . | . | . | . | . | . | . | . | . | . | . |
| logdha | rs11819084 | . | . | . | . | . | . | . | . | . | . | . |
| logdha | rs146915670 | . | . | . | . | . | . | . | . | . | . | . |

| logdha | rs113995695 | . | . | . | . | . | . | . | . | . | . | . |
| --- | --- | --- | --- | --- | --- | --- | --- | --- | --- | --- | --- | --- |
| logdha | rs143489451 | . | . | . | . | . | . | . | . | . | . | . |
| logdha | rs182653839 | . | . | . | . | . | . | . | . | . | . | . |
| logdha | rs141692564 | . | . | . | . | . | . | . | . | . | . | . |
| logdha | rs146281742 | . | . | . | . | . | . | . | . | . | . | . |
| logdha | rs192914353 | . | . | . | . | . | . | . | . | . | . | . |
| logdha | rs185182848 | . | . | . | . | . | . | . | . | . | . | . |
| logdha | rs28395858 | . | . | . | . | . | . | . | . | . | . | . |
| logdha | rs41429645 | . | . | . | . | . | . | . | . | . | . | . |
| logdha | rs28395874 | . | . | . | . | . | . | . | . | . | . | . |
| logdha | rs189663634 | . | . | . | . | . | . | . | . | . | . | . |
| logdha | rs77666700 | . | . | . | . | . | . | . | . | . | . | . |
| logdha | rs146711717 | . | . | . | . | . | . | . | . | . | . | . |
| logdha | rs144904251 | . | . | . | . | . | . | . | . | . | . | . |
| logdha | rs189893153 | . | . | . | . | . | . | . | . | . | . | . |
| logdha | rs150447740 | . | . | . | . | . | . | . | . | . | . | . |
| logdha | rs143099649 | . | . | . | . | . | . | . | . | . | . | . |
| logdha | rs191288436 | . | . | . | . | . | . | . | . | . | . | . |
| logdha | rs188577862 | . | . | . | . | . | . | . | . | . | . | . |
| logdha | rs192695334 | . | . | . | . | . | . | . | . | . | . | . |
| logdha | rs138612906 | . | . | . | . | . | . | . | . | . | . | . |
| logdha | rs191290051 | . | . | . | . | . | . | . | . | . | . | . |
| logdha | rs189631459 | . | . | . | . | . | . | . | . | . | . | . |
| logdha | rs114620874 | . | . | . | . | . | . | . | . | . | . | . |
| logdha | rs78246652 | . | . | . | . | . | . | . | . | . | . | . |
| logdha | rs79432451 | . | . | . | . | . | . | . | . | . | . | . |
| logdha | rs186022585 | . | . | . | . | . | . | . | . | . | . | . |
| logdha | rs142978891 | . | . | . | . | . | . | . | . | . | . | . |
| logdha | rs151086840 | . | . | . | . | . | . | . | . | . | . | . |
| logdha | rs183270259 | . | . | . | . | . | . | . | . | . | . | . |
| logdha | rs188544289 | . | . | . | . | . | . | . | . | . | . | . |
| logdha | rs188500808 | . | . | . | . | . | . | . | . | . | . | . |
| logdha | rs189424589 | . | . | . | . | . | . | . | . | . | . | . |
| logdha | rs78622303 | . | . | . | . | . | . | . | . | . | . | . |
| logdha | rs143210835 | . | . | . | . | . | . | . | . | . | . | . |
| logdha | rs114761840 | . | . | . | . | . | . | . | . | . | . | . |
| logdha | rs185805402 | . | . | . | . | . | . | . | . | . | . | . |
| logdha | rs139273744 | . | . | . | . | . | . | . | . | . | . | . |
| logdha | rs184654150 | . | . | . | . | . | . | . | . | . | . | . |
| logdha | rs189069710 | . | . | . | . | . | . | . | . | . | . | . |
| logdha | rs114752115 | . | . | . | . | . | . | . | . | . | . | . |
| logdha | rs150162515 | . | . | . | . | . | . | . | . | . | . | . |
| logdha | rs180982454 | . | . | . | . | . | . | . | . | . | . | . |
| logdha | rs184932088 | . | . | . | . | . | . | . | . | . | . | . |
| logdha | rs181109625 | . | . | . | . | . | . | . | . | . | . | . |
| logdha | rs138121134 | . | . | . | . | . | . | . | . | . | . | . |
| logdha | rs183253861 | . | . | . | . | . | . | . | . | . | . | . |
| logdha | rs147073946 | . | . | . | . | . | . | . | . | . | . | . |
| logdha | rs139938489 | . | . | . | . | . | . | . | . | . | . | . |
| logdha | rs146362560 | . | . | . | . | . | . | . | . | . | . | . |
| logdha | rs193155956 | . | . | . | . | . | . | . | . | . | . | . |
| logdha | rs77663092 | . | . | . | . | . | . | . | . | . | . | . |
| logdha | rs73285319 | . | . | . | . | . | . | . | . | . | . | . |
| logdha | rs184019276 | . | . | . | . | . | . | . | . | . | . | . |
| logdha | rs28395863 | . | . | . | . | . | . | . | . | . | . | . |
| logdha | rs182121645 | . | . | . | . | . | . | . | . | . | . | . |
| logdha | rs73285327 | . | . | . | . | . | . | . | . | . | . | . |
| logdha | rs149589584 | . | . | . | . | . | . | . | . | . | . | . |
| logdha | rs148310335 | . | . | . | . | . | . | . | . | . | . | . |
| logdha | rs187246093 | . | . | . | . | . | . | . | . | . | . | . |
| logdha | rs184668894 | . | . | . | . | . | . | . | . | . | . | . |
| logdha | rs114269961 | . | . | . | . | . | . | . | . | . | . | . |
| logdha | rs149694374 | . | . | . | . | . | . | . | . | . | . | . |
| logdha | rs145284227 | . | . | . | . | . | . | . | . | . | . | . |
| logdha | rs189983820 | . | . | . | . | . | . | . | . | . | . | . |
| logdha | rs181200444 | . | . | . | . | . | . | . | . | . | . | . |
| logdha | rs185486385 | . | . | . | . | . | . | . | . | . | . | . |
| logdha | rs151198241 | . | . | . | . | . | . | . | . | . | . | . |
| logdha | rs189959341 | . | . | . | . | . | . | . | . | . | . | . |
| logdha | rs140549257 | . | . | . | . | . | . | . | . | . | . | . |
| logdha | rs186453139 | . | . | . | . | . | . | . | . | . | . | . |
| logdha | rs180922711 | . | . | . | . | . | . | . | . | . | . | . |
| logdha | rs56659903 | . | . | . | . | . | . | . | . | . | . | . |
| logdha | rs141202128 | . | . | . | . | . | . | . | . | . | . | . |
| logdha | rs59085314 | . | . | . | . | . | . | . | . | . | . | . |
| logdha | rs145564996 | . | . | . | . | . | . | . | . | . | . | . |
| logdha | rs114961009 | . | . | . | . | . | . | . | . | . | . | . |
| logdha | rs182294189 | . | . | . | . | . | . | . | . | . | . | . |
| logdha | rs187586636 | . | . | . | . | . | . | . | . | . | . | . |
| logdha | rs192254907 | . | . | . | . | . | . | . | . | . | . | . |
| logdha | rs149145663 | . | . | . | . | . | . | . | . | . | . | . |
| logdha | rs180713064 | . | . | . | . | . | . | . | . | . | . | . |
| logdha | rs183009023 | . | . | . | . | . | . | . | . | . | . | . |
| logdha | rs143385347 | . | . | . | . | . | . | . | . | . | . | . |
| logdha | rs185786329 | . | . | . | . | . | . | . | . | . | . | . |
| logdha | rs192341030 | . | . | . | . | . | . | . | . | . | . | . |

| logdha | rs74661429 | . | . | . | . | . | . | . | . | . | . | . |
| --- | --- | --- | --- | --- | --- | --- | --- | --- | --- | --- | --- | --- |
| logdha | rs188034853 | . | . | . | . | . | . | . | . | . | . | . |
| logdha | rs116808430 | . | . | . | . | . | . | . | . | . | . | . |
| logdha | rs148469010 | . | . | . | . | . | . | . | . | . | . | . |
| logdha | rs78621663 | . | . | . | . | . | . | . | . | . | . | . |
| logdha | rs192654886 | . | . | . | . | . | . | . | . | . | . | . |
| logdha | rs145996035 | . | . | . | . | . | . | . | . | . | . | . |
| logdha | rs139942226 | . | . | . | . | . | . | . | . | . | . | . |
| logdha | rs113645632 | . | . | . | . | . | . | . | . | . | . | . |
| logdha | rs74940661 | . | . | . | . | . | . | . | . | . | . | . |
| logdha | rs115355800 | . | . | . | . | . | . | . | . | . | . | . |
| logdha | rs150285898 | . | . | . | . | . | . | . | . | . | . | . |
| logdha | rs188117932 | . | . | . | . | . | . | . | . | . | . | . |
| logdha | rs139956566 | . | . | . | . | . | . | . | . | . | . | . |
| logdha | rs147673885 | . | . | . | . | . | . | . | . | . | . | . |
| logdha | rs189127928 | . | . | . | . | . | . | . | . | . | . | . |
| logdha | rs187558772 | . | . | . | . | . | . | . | . | . | . | . |
| logdha | rs181948587 | . | . | . | . | . | . | . | . | . | . | . |
| logdha | rs189983269 | . | . | . | . | . | . | . | . | . | . | . |
| logdha | rs187250069 | . | . | . | . | . | . | . | . | . | . | . |
| logdha | rs191485859 | . | . | . | . | . | . | . | . | . | . | . |
| logdha | rs185499555 | . | . | . | . | . | . | . | . | . | . | . |
| logdha | rs180862110 | . | . | . | . | . | . | . | . | . | . | . |
| logdha | rs190472603 | . | . | . | . | . | . | . | . | . | . | . |
| logdha | rs189226167 | . | . | . | . | . | . | . | . | . | . | . |
| logdha | rs181623256 | . | . | . | . | . | . | . | . | . | . | . |
| logdha | rs186079557 | . | . | . | . | . | . | . | . | . | . | . |
| logdha | rs147760994 | . | . | . | . | . | . | . | . | . | . | . |
| logdha | rs189176167 | . | . | . | . | . | . | . | . | . | . | . |
| logdha | rs117901000 | . | . | . | . | . | . | . | . | . | . | . |
| logdha | rs141984888 | . | . | . | . | . | . | . | . | . | . | . |
| logdha | rs192365061 | . | . | . | . | . | . | . | . | . | . | . |
| logdha | rs185976038 | . | . | . | . | . | . | . | . | . | . | . |
| logdha | rs184697303 | . | . | . | . | . | . | . | . | . | . | . |
| logdha | rs143383132 | . | . | . | . | . | . | . | . | . | . | . |
| logdha | rs141873881 | . | . | . | . | . | . | . | . | . | . | . |
| logdha | rs185507255 | . | . | . | . | . | . | . | . | . | . | . |
| logdha | rs191379425 | . | . | . | . | . | . | . | . | . | . | . |
| logdha | rs184199584 | . | . | . | . | . | . | . | . | . | . | . |
| logdha | rs183181360 | . | . | . | . | . | . | . | . | . | . | . |
| logdha | rs184495673 | . | . | . | . | . | . | . | . | . | . | . |
| logdha | rs182946297 | . | . | . | . | . | . | . | . | . | . | . |
| logdha | rs187743615 | . | . | . | . | . | . | . | . | . | . | . |
| logdha | rs183259862 | . | . | . | . | . | . | . | . | . | . | . |
| logdha | rs112433566 | . | . | . | . | . | . | . | . | . | . | . |
| logdha | rs182351085 | . | . | . | . | . | . | . | . | . | . | . |
| logdha | rs146248508 | . | . | . | . | . | . | . | . | . | . | . |
| logdha | rs188528439 | . | . | . | . | . | . | . | . | . | . | . |
| logdha | rs146167018 | . | . | . | . | . | . | . | . | . | . | . |
| logdha | rs192440980 | . | . | . | . | . | . | . | . | . | . | . |
| logdha | rs185601882 | . | . | . | . | . | . | . | . | . | . | . |
| logdha | rs117272563 | . | . | . | . | . | . | . | . | . | . | . |
| logdha | rs10430734 | . | . | . | . | . | . | . | . | . | . | . |
| logdha | rs181320952 | . | . | . | . | . | . | . | . | . | . | . |
| logdha | rs185480660 | . | . | . | . | . | . | . | . | . | . | . |
| logdha | rs188077917 | . | . | . | . | . | . | . | . | . | . | . |
| logdha | rs187562953 | . | . | . | . | . | . | . | . | . | . | . |
| logdha | rs182044631 | . | . | . | . | . | . | . | . | . | . | . |
| logdha | rs145455596 | . | . | . | . | . | . | . | . | . | . | . |
| logdha | rs181475877 | . | . | . | . | . | . | . | . | . | . | . |
| logdha | rs149413085 | . | . | . | . | . | . | . | . | . | . | . |
| logdha | rs12253656 | . | . | . | . | . | . | . | . | . | . | . |
| logdha | rs182718239 | . | . | . | . | . | . | . | . | . | . | . |
| logdha | rs192435695 | . | . | . | . | . | . | . | . | . | . | . |
| logdha | rs12248277 | . | . | . | . | . | . | . | . | . | . | . |
| logdha | rs143521710 | . | . | . | . | . | . | . | . | . | . | . |
| logdha | rs144454454 | . | . | . | . | . | . | . | . | . | . | . |
| logdha | rs190441610 | . | . | . | . | . | . | . | . | . | . | . |
| logdha | rs114690462 | . | . | . | . | . | . | . | . | . | . | . |
| logdha | rs184541357 | . | . | . | . | . | . | . | . | . | . | . |
| logdha | rs142657653 | . | . | . | . | . | . | . | . | . | . | . |
| logdha | rsurf1 | 2.02 | . | . | . | . | . | . | . | . | . | . |
| logdha | rsten1 | 2.196 | . | . | . | . | . | . | . | . | . | . |
| logdha | chr10:45852312:D | 0.055 | 0.857 | 0.19 | 0.486 | 1.23 | 0.957 | 0.785 | 0.852 | 0.556 | 0.806 | -0.19 |
| logdha | chr10:45853232:I | 0.071 | 0.814 | 0.185 | 0.364 | 0.779 | 0.516 | 0.706 | 1.31 | 0.709 | 0.622 | 0.236 |
| logdha | chr10:45853234:I | 0.157 | 0.806 | 0.192 | 0.364 | 0.762 | 0.411 | 0.614 | 1.19 | 0.524 | 0.7 | 0.16 |
| logdha | chr10:45854796:D | 0.001 | . | . | . | . | . | . | . | . | . | . |
| logdha | chr10:45854994:I | 0.001 | . | . | . | . | . | . | . | . | . | . |
| logdha | chr10:45858290:D | 0.04 | 0.867 | 0.188 | 0.51 | 2.31 | 2 | 0.331 | 0.729 | 0.529 | 0.663 | -0.719 |
| logdha | chr10:45858820:I | 0.015 | 0.881 | 0.196 | 0.567 | 3.8 | 4.91 | 0.3 | 0.233 | 0.246 | 0.168 | -2.9 |
| logdha | chr10:45860888:D | 0.067 | 0.796 | 0.178 | 0.307 | 0.515 | 0.415 | 0.411 | 1.58 | 0.991 | 0.469 | 0.335 |
| logdha | chr10:45863913:I | 0.159 | 0.841 | 0.198 | 0.461 | 0.937 | 0.489 | 0.901 | 1.01 | 0.441 | 0.975 | 0.021 |
| logdha | chr10:45866601:D | 0 | . | . | . | . | . | . | . | . | . | . |
| logdha | chr10:45867197:I | 0.008 | . | . | . | . | . | . | . | . | . | . |
| logdha | chr10:45867538:D | 0.167 | 0.794 | 0.188 | 0.329 | 0.779 | 0.401 | 0.628 | 1.22 | 0.512 | 0.635 | 0.182 |
| logdha | chr10:45868356:D | 0.151 | 0.85 | 0.198 | 0.485 | 0.942 | 0.511 | 0.912 | 0.974 | 0.446 | 0.954 | -0.012 |

| logdha | chr10:45871158:I | 0.16 | 0.839 | 0.198 | 0.456 | 0.928 | 0.484 | 0.887 | 1.02 | 0.443 | 0.962 | 0.028 |
| --- | --- | --- | --- | --- | --- | --- | --- | --- | --- | --- | --- | --- |
| logdha | chr10:45871637:D | 0.061 | 0.798 | 0.179 | 0.315 | 0.703 | 0.632 | 0.695 | 1.52 | 1.07 | 0.55 | 0.352 |
| logdha | chr10:45871638:I | 0.115 | 0.779 | 0.184 | 0.29 | 0.7 | 0.459 | 0.587 | 1.41 | 0.727 | 0.504 | 0.29 |
| logdha | chr10:45873629:I | 0.159 | 0.829 | 0.195 | 0.426 | 0.868 | 0.46 | 0.79 | 1.07 | 0.469 | 0.884 | 0.07 |
| logdha | chr10:45873701:D | 0.003 | . | . | . | . | . | . | . | . | . | . |
| logdha | chr10:45874997:I | 0.03 | 0.826 | 0.178 | 0.376 | 0.639 | 0.924 | 0.757 | 1.34 | 1.55 | 0.798 | 0.244 |
| logdha | chr10:45882030:I | 0.021 | 0.788 | 0.181 | 0.299 | 0.166 | 0.362 | 0.41 | 4.13 | 6.73 | 0.384 | 0.586 |
| logdha | chr10:45883774:D | 0.155 | 0.874 | 0.215 | 0.584 | 1.04 | 0.593 | 0.942 | 0.897 | 0.413 | 0.813 | -0.099 |
| logdha | chr10:45885169:D | 0.034 | 0.824 | 0.18 | 0.375 | 0.375 | 0.376 | 0.327 | 1.8 | 1.34 | 0.427 | 0.358 |
| logdha | chr10:45886451:D | 0.589 | 1.74 | 0.831 | 0.244 | 2.11 | 0.895 | 0.08 | 0.542 | 0.191 | 0.083 | -0.858 |
| logdha | chr10:45891800:I | 0.592 | 0.766 | 0.346 | 0.555 | 1.07 | 0.392 | 0.856 | 1.08 | 0.333 | 0.797 | 0.051 |
| logdha | chr10:45904771:I | 0.573 | 0.852 | 0.36 | 0.704 | 1.16 | 0.405 | 0.676 | 0.994 | 0.292 | 0.985 | -0.029 |
| logdha | chr10:45904959:I | 0.103 | 0.924 | 0.233 | 0.754 | 2.65 | 1.71 | 0.131 | 0.695 | 0.39 | 0.518 | -0.871 |
| logdha | chr10:45911028:I | 0.034 | 0.806 | 0.179 | 0.333 | 0.422 | 0.643 | 0.571 | 1.89 | 2.56 | 0.64 | 0.414 |
| logdha | chr10:45911031:I | 0.364 | 0.986 | 0.347 | 0.968 | 1.63 | 0.623 | 0.198 | 0.833 | 0.254 | 0.549 | -0.278 |
| logdha | chr10:45912154:D | 0.145 | 0.846 | 0.204 | 0.489 | 0.93 | 0.51 | 0.895 | 0.996 | 0.441 | 0.993 | 7.80E-03 |
| logdha | chr10:45913885:I | 0.001 | . | . | . | . | . | . | . | . | . | . |
| logdha | chr10:45914753:D | 0.243 | 0.707 | 0.193 | 0.205 | 0.663 | 0.297 | 0.358 | 1.44 | 0.52 | 0.316 | 0.304 |
| logdha | chr10:45919621 | 0.003 | . | . | . | . | . | . | . | . | . | . |
| logdha | chr10:45921739:I | 0.004 | . | . | . | . | . | . | . | . | . | . |
| logdha | chr10:45923527:I | 0.052 | 0.774 | 0.184 | 0.28 | 0.622 | 0.615 | 0.632 | 2.13 | 1.61 | 0.317 | 0.629 |
| logdha | chr10:45927001:I | 0.014 | 0.811 | 0.179 | 0.344 | 0.017 | 0.058 | 0.237 | 7.6 | 18.1 | 0.395 | 0.276 |
| logdha | chr10:45930354:I | 0.001 | . | . | . | . | . | . | . | . | . | . |
| logdha | chr10:45932864:I | 0.027 | 0.869 | 0.199 | 0.541 | 4.43 | 4.58 | 0.149 | 0.532 | 0.46 | 0.466 | -2.25 |
| logdha | chr10:45936163:I | 0.025 | 0.82 | 0.176 | 0.355 | 0.203 | 0.318 | 0.309 | 2.5 | 2.74 | 0.401 | 0.394 |
| logdha | chr10:45938095:D | 0.164 | 0.846 | 0.211 | 0.505 | 0.93 | 0.519 | 0.897 | 0.996 | 0.442 | 0.994 | 7.90E-03 |
| logdha | chr10:45938886 | 0 | . | . | . | . | . | . | . | . | . | . |
| logdha | chr10:45939320 | 0.001 | . | . | . | . | . | . | . | . | . | . |
| logdha | chr10:45939659 | 0.001 | . | . | . | . | . | . | . | . | . | . |
| logdha | chr10:45941150:D | 0.001 | . | . | . | . | . | . | . | . | . | . |
| logdha | chr10:45947128:D | 0.065 | 0.776 | 0.177 | 0.265 | 0.454 | 0.328 | 0.275 | 1.98 | 1.13 | 0.233 | 0.466 |
| logdha | chr10:45950990:I | 0.044 | 0.88 | 0.196 | 0.566 | 2.17 | 1.97 | 0.391 | 0.603 | 0.446 | 0.494 | -0.9 |
| logdha | chr10:45951854:I | 0.047 | 0.798 | 0.175 | 0.303 | 0.688 | 0.704 | 0.715 | 1.64 | 1.28 | 0.526 | 0.414 |
| logdha | chr10:45952670:I | 0.061 | 0.781 | 0.177 | 0.275 | 0.51 | 0.425 | 0.419 | 1.85 | 1.22 | 0.35 | 0.446 |
| logdha | chr10:45956741 | 0.001 | . | . | . | . | . | . | . | . | . | . |
| logdha | chr10:45957247:I | 0.233 | 0.942 | 0.257 | 0.826 | 1.4 | 0.644 | 0.458 | 0.791 | 0.302 | 0.541 | -0.299 |
| logdha | chr10:45958082:I | 0.233 | 0.913 | 0.25 | 0.74 | 1.31 | 0.61 | 0.563 | 0.844 | 0.326 | 0.662 | -0.213 |
| logdha | chr10:45905041:D | . | . | . | . | . | . | . | . | . | . | . |
| logdha | chr10:45938871 | . | . | . | . | . | . | . | . | . | . | . |
| logdha | chr10:45939160 | . | . | . | . | . | . | . | . | . | . | . |
| logdha | chr10:45869910 | . | . | . | . | . | . | . | . | . | . | . |
| logdha | chr10:45876214:D | . | . | . | . | . | . | . | . | . | . | . |
| logdha | chr10:45880237:I | . | . | . | . | . | . | . | . | . | . | . |
| logdha | chr10:45884371:I | . | . | . | . | . | . | . | . | . | . | . |
| logdha | chr10:45891535:D | . | . | . | . | . | . | . | . | . | . | . |
| logdha | chr10:45893516:D | . | . | . | . | . | . | . | . | . | . | . |
| logdha | chr10:45893782:D | . | . | . | . | . | . | . | . | . | . | . |
| logdha | chr10:45895254:I | . | . | . | . | . | . | . | . | . | . | . |
| logdha | chr10:45895848:D | . | . | . | . | . | . | . | . | . | . | . |
| logdha | chr10:45896316:D | . | . | . | . | . | . | . | . | . | . | . |
| logdha | chr10:45896317:D | . | . | . | . | . | . | . | . | . | . | . |
| logdha | chr10:45902683:I | . | . | . | . | . | . | . | . | . | . | . |
| logdha | chr10:45905003:D | . | . | . | . | . | . | . | . | . | . | . |
| logdha | chr10:45927476:D | . | . | . | . | . | . | . | . | . | . | . |
| logdha | chr10:45935882 | . | . | . | . | . | . | . | . | . | . | . |
| logdha | chr10:45936100 | . | . | . | . | . | . | . | . | . | . | . |
| logdha | chr10:45939553 | . | . | . | . | . | . | . | . | . | . | . |
| logdha | chr10:45939631 | . | . | . | . | . | . | . | . | . | . | . |
| logdha | chr10:45944193:D | . | . | . | . | . | . | . | . | . | . | . |
| logdha | chr10:45954030 | . | . | . | . | . | . | . | . | . | . | . |

Supplemental table S38.

| fa | rs_id | freq | HR_logdha | se_logdha | p_logdha | HR_snp | se_snp | p_snp | HR_inter | se_inter | p_inter | RERI |
| --- | --- | --- | --- | --- | --- | --- | --- | --- | --- | --- | --- | --- |
| logdha | rs76035182 | 0.068 | 0.424 | 0.196 | 0.064 | 0.624 | 0.87 | 0.735 | 1.64 | 1.55 | 0.602 | 0.386 |
| logdha | rs12359547 | 0.052 | 0.527 | 0.228 | 0.139 | 7.08 | 8.58 | 0.106 | 0.126 | 0.111 | 0.019 | -6.14 |
| logdha | rs74595484 | 0.12 | 0.459 | 0.215 | 0.096 | 0.621 | 0.776 | 0.703 | 1.11 | 0.998 | 0.911 | 0.235 |
| logdha | rs1864415 | 0.749 | 1.08 | 0.958 | 0.935 | 1.86 | 1.45 | 0.425 | 0.562 | 0.303 | 0.285 | -0.813 |
| logdha | rs72788101 | 0.064 | 0.541 | 0.233 | 0.153 | 8.35 | 9.79 | 0.07 | 0.103 | 0.088 | 8.10E-03 | -7.43 |
| logdha | rs11239481 | 0.081 | 0.528 | 0.246 | 0.17 | 3.36 | 3.72 | 0.273 | 0.549 | 0.458 | 0.473 | -1.92 |
| logdha | rs72788102 | 0.064 | 0.541 | 0.233 | 0.153 | 8.35 | 9.8 | 0.07 | 0.103 | 0.088 | 8.10E-03 | -7.43 |
| logdha | rs72789705 | 0.064 | 0.541 | 0.233 | 0.154 | 8.35 | 9.79 | 0.07 | 0.103 | 0.089 | 8.10E-03 | -7.42 |
| logdha | rs141529858 | 0.002 | . | . | . | . | . | . | . | . | . | . |
| logdha | rs145376292 | 0 | . | . | . | . | . | . | . | . | . | . |
| logdha | rs190418301 | 0 | . | . | . | . | . | . | . | . | . | . |
| logdha | rs11239482 | 0.064 | 0.541 | 0.233 | 0.154 | 8.35 | 9.8 | 0.071 | 0.103 | 0.089 | 8.10E-03 | -7.42 |
| logdha | rs61855872 | 0.004 | . | . | . | . | . | . | . | . | . | . |
| logdha | rs148991144 | 0.003 | . | . | . | . | . | . | . | . | . | . |
| logdha | rs191932221 | 0.002 | . | . | . | . | . | . | . | . | . | . |
| logdha | rs55978138 | 0.179 | 0.512 | 0.254 | 0.178 | 1.32 | 1.18 | 0.759 | 0.765 | 0.48 | 0.67 | -0.312 |
| logdha | rs73281167 | 0.045 | 0.361 | 0.149 | 0.013 | 0.01 | 0.025 | 0.059 | 19.3 | 30.3 | 0.058 | 0.7 |
| logdha | rs12768010 | 0.092 | 0.455 | 0.198 | 0.07 | 1.05 | 1.26 | 0.968 | 1.05 | 0.837 | 0.955 | -4.80E-03 |
| logdha | rs11239484 | 0.749 | 1.08 | 0.964 | 0.93 | 1.87 | 1.46 | 0.421 | 0.56 | 0.302 | 0.282 | -0.82 |
| logdha | rs35906360 | 0.18 | 0.514 | 0.251 | 0.173 | 1.35 | 1.22 | 0.739 | 0.755 | 0.473 | 0.654 | -0.341 |
| logdha | rs186250512 | 0.001 | . | . | . | . | . | . | . | . | . | . |
| logdha | rs150810743 | 0.007 | . | . | . | . | . | . | . | . | . | . |
| logdha | rs55965812 | 0.161 | 0.499 | 0.243 | 0.154 | 1.34 | 1.15 | 0.732 | 0.807 | 0.483 | 0.72 | -0.301 |
| logdha | rs56180358 | 0.181 | 0.513 | 0.251 | 0.173 | 1.35 | 1.21 | 0.742 | 0.756 | 0.474 | 0.656 | -0.337 |
| logdha | rs55864145 | 0.092 | 0.455 | 0.198 | 0.071 | 1.05 | 1.27 | 0.965 | 1.04 | 0.831 | 0.958 | -9.00E-03 |
| logdha | rs55986122 | 0.181 | 0.513 | 0.251 | 0.173 | 1.35 | 1.21 | 0.741 | 0.756 | 0.473 | 0.655 | -0.338 |
| logdha | rs7477350 | 0.749 | 1.08 | 0.966 | 0.928 | 1.87 | 1.46 | 0.419 | 0.559 | 0.301 | 0.281 | -0.822 |
| logdha | rs2099170 | 0.18 | 0.512 | 0.25 | 0.171 | 1.34 | 1.21 | 0.748 | 0.761 | 0.477 | 0.663 | -0.328 |
| logdha | rs2082108 | 0.181 | 0.513 | 0.251 | 0.173 | 1.35 | 1.21 | 0.741 | 0.756 | 0.473 | 0.654 | -0.338 |
| logdha | rs55902404 | 0.02 | 0.48 | 0.2 | 0.077 | 40.7 | 74.7 | 0.043 | 0.014 | 0.017 | 4.10E-04 | -39.9 |
| logdha | rs58402662 | 0.02 | 0.479 | 0.199 | 0.077 | 40 | 73.6 | 0.045 | 0.014 | 0.017 | 4.20E-04 | -39.2 |
| logdha | rs11511618 | 0.084 | 0.527 | 0.246 | 0.17 | 3.22 | 3.63 | 0.299 | 0.559 | 0.473 | 0.492 | -1.8 |
| logdha | rs1816492 | 0.747 | 1.09 | 0.971 | 0.927 | 1.89 | 1.47 | 0.416 | 0.559 | 0.302 | 0.282 | -0.828 |
| logdha | rs146611093 | 0.002 | . | . | . | . | . | . | . | . | . | . |
| logdha | rs186897689 | 0.006 | . | . | . | . | . | . | . | . | . | . |
| logdha | rs10793616 | 0.749 | 1.09 | 0.968 | 0.927 | 1.88 | 1.46 | 0.418 | 0.559 | 0.301 | 0.28 | -0.824 |
| logdha | rs116700479 | 0.052 | 0.441 | 0.19 | 0.057 | 0.34 | 0.675 | 0.587 | 1.87 | 2.46 | 0.637 | 0.499 |
| logdha | rs192535936 | 0.004 | . | . | . | . | . | . | . | . | . | . |
| logdha | rs113363297 | 0.723 | 0.76 | 0.636 | 0.743 | 1.34 | 0.994 | 0.691 | 0.7 | 0.36 | 0.488 | -0.388 |
| logdha | rs12354994 | 0.172 | 0.51 | 0.248 | 0.167 | 1.33 | 1.26 | 0.76 | 0.756 | 0.496 | 0.669 | -0.33 |
| logdha | rs148068329 | 0.025 | 0.434 | 0.182 | 0.046 | 0.071 | 0.201 | 0.35 | 6.46 | 12.3 | 0.326 | 0.694 |
| logdha | rs12356109 | 0.198 | 0.546 | 0.275 | 0.23 | 1.52 | 1.4 | 0.65 | 0.657 | 0.423 | 0.515 | -0.52 |
| logdha | rs55908141 | 0.02 | 0.479 | 0.199 | 0.077 | 38.8 | 71.6 | 0.048 | 0.015 | 0.018 | 4.30E-04 | -38 |
| logdha | rs12354712 | 0.161 | 0.499 | 0.244 | 0.154 | 1.34 | 1.15 | 0.729 | 0.805 | 0.479 | 0.715 | -0.303 |
| logdha | rs147282684 | 0.002 | . | . | . | . | . | . | . | . | . | . |
| logdha | rs12356140 | 0.191 | 0.548 | 0.272 | 0.225 | 1.72 | 1.5 | 0.532 | 0.642 | 0.393 | 0.468 | -0.664 |
| logdha | rs185476187 | 0.004 | . | . | . | . | . | . | . | . | . | . |
| logdha | rs145720057 | 0.012 | 0.478 | 0.205 | 0.085 | 3.55 | 5.47 | 0.411 | 0.3 | 0.421 | 0.391 | -2.52 |
| logdha | rs12354742 | 0.181 | 0.514 | 0.251 | 0.173 | 1.35 | 1.21 | 0.74 | 0.755 | 0.471 | 0.653 | -0.338 |
| logdha | rs180913842 | 0.02 | 0.479 | 0.199 | 0.077 | 38.5 | 71.1 | 0.048 | 0.015 | 0.018 | 4.30E-04 | -37.7 |
| logdha | rs142976914 | 0.002 | . | . | . | . | . | . | . | . | . | . |
| logdha | rs11511619 | 0.201 | 0.549 | 0.277 | 0.236 | 1.53 | 1.42 | 0.644 | 0.649 | 0.418 | 0.502 | -0.536 |
| logdha | rs146702870 | 0.003 | . | . | . | . | . | . | . | . | . | . |
| logdha | rs150287496 | 0.001 | . | . | . | . | . | . | . | . | . | . |
| logdha | rs190917973 | 0.002 | . | . | . | . | . | . | . | . | . | . |
| logdha | rs11517281 | 0.181 | 0.514 | 0.251 | 0.174 | 1.35 | 1.21 | 0.739 | 0.755 | 0.47 | 0.652 | -0.339 |
| logdha | rs12359299 | 0.181 | 0.514 | 0.251 | 0.174 | 1.35 | 1.21 | 0.74 | 0.755 | 0.47 | 0.652 | -0.338 |
| logdha | rs182307932 | 0.071 | 0.558 | 0.242 | 0.179 | 5.98 | 10.6 | 0.315 | 0.215 | 0.306 | 0.281 | -4.82 |
| logdha | rs61855873 | 0.011 | 0.449 | 0.192 | 0.06 | 4.45 | 28.4 | 0.815 | 1.43 | 5.93 | 0.93 | -1.03 |
| logdha | rs12359302 | 0.18 | 0.519 | 0.253 | 0.178 | 1.38 | 1.29 | 0.732 | 0.727 | 0.477 | 0.627 | -0.378 |
| logdha | rs143208140 | 0.028 | 0.428 | 0.181 | 0.045 | 0.072 | 0.193 | 0.328 | 6.2 | 11.2 | 0.313 | 0.69 |
| logdha | rs9988764 | 0.712 | 1.34 | 1.18 | 0.742 | 2.43 | 1.97 | 0.272 | 0.464 | 0.263 | 0.175 | -1.26 |
| logdha | rs192875767 | 0.002 | . | . | . | . | . | . | . | . | . | . |
| logdha | rs147268846 | 0.005 | . | . | . | . | . | . | . | . | . | . |
| logdha | rs11239485 | 0.109 | 0.541 | 0.263 | 0.206 | 2.29 | 2.07 | 0.362 | 0.518 | 0.336 | 0.311 | -1.19 |
| logdha | rs151317885 | 0.24 | 0.557 | 0.3 | 0.277 | 1.77 | 1.39 | 0.464 | 0.76 | 0.449 | 0.642 | -0.58 |
| logdha | rs11499085 | 0.322 | 0.525 | 0.283 | 0.231 | 0.96 | 0.91 | 0.966 | 0.816 | 0.548 | 0.763 | -0.074 |
| logdha | rs188130606 | 0.086 | 0.54 | 0.246 | 0.176 | 2.3 | 2.35 | 0.414 | 0.345 | 0.263 | 0.162 | -1.41 |
| logdha | rs11511620 | 0.161 | 0.5 | 0.244 | 0.155 | 1.35 | 1.14 | 0.727 | 0.804 | 0.475 | 0.712 | -0.305 |
| logdha | rs181498390 | 0.057 | 0.403 | 0.171 | 0.032 | 0.166 | 0.359 | 0.407 | 3.39 | 5.14 | 0.421 | 0.657 |
| logdha | rs186280701 | 0.02 | 0.479 | 0.199 | 0.077 | 36.5 | 67.9 | 0.053 | 0.016 | 0.019 | 4.60E-04 | -35.7 |
| logdha | rs150059185 | 0.034 | 0.624 | 0.265 | 0.267 | 29.7 | 50.1 | 0.044 | 0.103 | 0.157 | 0.136 | -27.5 |
| logdha | rs183338707 | 0.001 | . | . | . | . | . | . | . | . | . | . |
| logdha | rs11499038 | 0.306 | 0.494 | 0.265 | 0.189 | 0.87 | 0.853 | 0.887 | 0.906 | 0.632 | 0.887 | 0.026 |
| logdha | rs190699595 | 0.004 | . | . | . | . | . | . | . | . | . | . |
| logdha | rs11523408 | 0.175 | 0.508 | 0.248 | 0.166 | 1.32 | 1.2 | 0.761 | 0.775 | 0.489 | 0.687 | -0.308 |
| logdha | rs188049317 | 0.007 | . | . | . | . | . | . | . | . | . | . |
| logdha | rs144948634 | 0.214 | 0.487 | 0.249 | 0.16 | 1.33 | 1.07 | 0.721 | 0.952 | 0.574 | 0.935 | -0.202 |
| logdha | rs143833576 | . | . | . | . | . | . | . | . | . | . | . |
| logdha | rs151239879 | 0.175 | 0.519 | 0.254 | 0.179 | 1.44 | 1.29 | 0.687 | 0.731 | 0.457 | 0.616 | -0.41 |
| logdha | rs7073743 | 0.212 | 0.516 | 0.268 | 0.203 | 1.58 | 1.22 | 0.557 | 0.876 | 0.512 | 0.82 | -0.381 |
| logdha | rs142563349 | 0.092 | 0.549 | 0.252 | 0.19 | 2.53 | 2.46 | 0.339 | 0.332 | 0.241 | 0.128 | -1.62 |
| logdha | rs187246403 | 0.02 | 0.479 | 0.199 | 0.077 | 35.4 | 66.2 | 0.056 | 0.017 | 0.02 | 4.80E-04 | -34.6 |
| logdha | rs150423942 | 0.178 | 0.51 | 0.25 | 0.169 | 1.33 | 1.19 | 0.755 | 0.769 | 0.484 | 0.676 | -0.316 |

| logdha | rs184706102 | . | . | . | . | . | . | . | . | . | . | . |
| --- | --- | --- | --- | --- | --- | --- | --- | --- | --- | --- | --- | --- |
| logdha | rs189626288 | 0.007 | . | . | . | . | . | . | . | . | . | . |
| logdha | rs140749834 | 0.142 | 0.472 | 0.222 | 0.11 | 1.01 | 1.35 | 0.993 | 0.933 | 0.897 | 0.942 | -0.039 |
| logdha | rs7078822 | 0.136 | 0.461 | 0.222 | 0.107 | 0.931 | 0.877 | 0.94 | 1.03 | 0.669 | 0.965 | 0.05 |
| logdha | rs186227617 | 0.177 | 0.517 | 0.254 | 0.18 | 1.41 | 1.28 | 0.703 | 0.739 | 0.464 | 0.63 | -0.389 |
| logdha | rs188984368 | 0.018 | 0.42 | 0.183 | 0.047 | 0.049 | 0.231 | 0.522 | 11 | 34.1 | 0.439 | 0.757 |
| logdha | rs182078109 | 0.04 | 0.399 | 0.184 | 0.046 | 0.339 | 0.856 | 0.668 | 3.82 | 7.03 | 0.467 | 0.779 |
| logdha | rs141456274 | 0.001 | . | . | . | . | . | . | . | . | . | . |
| logdha | rs146976512 | 0.181 | 0.514 | 0.252 | 0.175 | 1.35 | 1.21 | 0.735 | 0.753 | 0.467 | 0.647 | -0.344 |
| logdha | rs138036816 | 0.305 | 0.495 | 0.266 | 0.191 | 0.878 | 0.863 | 0.895 | 0.9 | 0.63 | 0.881 | 0.018 |
| logdha | rs114879777 | 0.051 | 0.44 | 0.189 | 0.056 | 0.325 | 0.656 | 0.578 | 1.93 | 2.59 | 0.626 | 0.511 |
| logdha | rs149366475 | 0.307 | 0.499 | 0.268 | 0.196 | 0.878 | 0.877 | 0.897 | 0.891 | 0.631 | 0.87 | 0.013 |
| logdha | rs182463043 | 0.007 | . | . | . | . | . | . | . | . | . | . |
| logdha | rs144754632 | 0.025 | 0.434 | 0.182 | 0.046 | 0.07 | 0.199 | 0.348 | 6.5 | 12.3 | 0.324 | 0.694 |
| logdha | rs55717607 | 0.181 | 0.514 | 0.251 | 0.174 | 1.35 | 1.2 | 0.738 | 0.755 | 0.465 | 0.647 | -0.338 |
| logdha | rs187537973 | 0.001 | . | . | . | . | . | . | . | . | . | . |
| logdha | rs148085971 | 0.007 | . | . | . | . | . | . | . | . | . | . |
| logdha | rs11239491 | 0.181 | 0.514 | 0.251 | 0.174 | 1.35 | 1.2 | 0.738 | 0.755 | 0.464 | 0.647 | -0.338 |
| logdha | rs184412184 | 0.002 | . | . | . | . | . | . | . | . | . | . |
| logdha | rs4948998 | 0.798 | 0.506 | 0.458 | 0.452 | 0.875 | 0.673 | 0.863 | 0.947 | 0.513 | 0.92 | 0.038 |
| logdha | rs7076696 | 0.798 | 0.505 | 0.458 | 0.451 | 0.874 | 0.672 | 0.861 | 0.948 | 0.514 | 0.922 | 0.04 |
| logdha | rs11239492 | 0.198 | 0.548 | 0.276 | 0.232 | 1.53 | 1.41 | 0.64 | 0.652 | 0.415 | 0.501 | -0.535 |
| logdha | rs79462470 | 0.02 | 0.478 | 0.199 | 0.076 | 32.1 | 60.9 | 0.068 | 0.019 | 0.022 | 6.00E-04 | -31.3 |
| logdha | rs187804654 | . | . | . | . | . | . | . | . | . | . | . |
| logdha | rs7093687 | 0.161 | 0.5 | 0.243 | 0.154 | 1.34 | 1.13 | 0.728 | 0.804 | 0.469 | 0.709 | -0.301 |
| logdha | rs7081004 | 0.162 | 0.5 | 0.244 | 0.155 | 1.35 | 1.13 | 0.725 | 0.802 | 0.468 | 0.706 | -0.306 |
| logdha | rs7084793 | 0.181 | 0.514 | 0.251 | 0.173 | 1.34 | 1.19 | 0.74 | 0.756 | 0.463 | 0.648 | -0.334 |
| logdha | rs61556080 | 0.02 | 0.478 | 0.199 | 0.076 | 32 | 60.8 | 0.068 | 0.019 | 0.022 | 6.10E-04 | -31.2 |
| logdha | rs115552064 | 0.019 | 0.478 | 0.196 | 0.072 | 3.44 | 17 | 0.802 | 0.386 | 1.43 | 0.798 | -2.28 |
| logdha | rs7921503 | 0.18 | 0.525 | 0.257 | 0.188 | 1.45 | 1.27 | 0.669 | 0.709 | 0.428 | 0.569 | -0.437 |
| logdha | rs7909740 | 0.181 | 0.514 | 0.251 | 0.173 | 1.34 | 1.18 | 0.742 | 0.758 | 0.463 | 0.65 | -0.33 |
| logdha | rs7909768 | 0.181 | 0.514 | 0.251 | 0.173 | 1.34 | 1.18 | 0.742 | 0.758 | 0.462 | 0.65 | -0.33 |
| logdha | rs144168004 | 0.001 | . | . | . | . | . | . | . | . | . | . |
| logdha | rs75850448 | 0.02 | 0.478 | 0.199 | 0.076 | 32.3 | 61.2 | 0.067 | 0.019 | 0.022 | 6.00E-04 | -31.5 |
| logdha | rs147777574 | 0.016 | 0.466 | 0.198 | 0.073 | 1.21 | 1.75 | 0.895 | 0.551 | 0.516 | 0.525 | -0.366 |
| logdha | rs7913488 | 0.181 | 0.513 | 0.251 | 0.172 | 1.33 | 1.17 | 0.743 | 0.759 | 0.462 | 0.65 | -0.328 |
| logdha | rs74390874 | 0.019 | 0.462 | 0.203 | 0.079 | 0.72 | 1.61 | 0.883 | 1.13 | 1.54 | 0.931 | 0.192 |
| logdha | rs141391142 | 0.002 | . | . | . | . | . | . | . | . | . | . |
| logdha | rs115294747 | 0.017 | 0.496 | 0.212 | 0.101 | 129 | 224 | 5.10E-03 | 6.40E-03 | 9.10E-03 | 4.10E-04 | -128 |
| logdha | rs148031781 | 0.001 | . | . | . | . | . | . | . | . | . | . |
| logdha | rs181340128 | 0.007 | . | . | . | . | . | . | . | . | . | . |
| logdha | rs189930007 | 0 | . | . | . | . | . | . | . | . | . | . |
| logdha | rs12242128 | 0.304 | 0.501 | 0.269 | 0.198 | 0.902 | 0.897 | 0.917 | 0.882 | 0.623 | 0.859 | -4.40E-03 |
| logdha | rs78421750 | 0.016 | 0.518 | 0.22 | 0.121 | 837 | 1.60E+03 | 3.30E-04 | 4.30E-04 | 1.00E-03 | 9.90E-04 | -836 |
| logdha | rs114370747 | 0.007 | . | . | . | . | . | . | . | . | . | . |
| logdha | rs12264166 | 0.304 | 0.502 | 0.269 | 0.199 | 0.906 | 0.9 | 0.921 | 0.88 | 0.621 | 0.856 | -7.70E-03 |
| logdha | rs182964822 | 0 | . | . | . | . | . | . | . | . | . | . |
| logdha | rs67491417 | 0.178 | 0.509 | 0.246 | 0.163 | 1.24 | 1.14 | 0.816 | 0.764 | 0.491 | 0.676 | -0.266 |
| logdha | rs72789720 | 0.006 | . | . | . | . | . | . | . | . | . | . |
| logdha | rs6413416 | 0.182 | 0.512 | 0.25 | 0.17 | 1.32 | 1.15 | 0.751 | 0.765 | 0.461 | 0.656 | -0.314 |
| logdha | rs4986832 | 0.182 | 0.512 | 0.25 | 0.17 | 1.32 | 1.15 | 0.751 | 0.765 | 0.461 | 0.656 | -0.314 |
| logdha | rs144866316 | . | . | . | . | . | . | . | . | . | . | . |
| logdha | rs187769540 | 0.001 | . | . | . | . | . | . | . | . | . | . |
| logdha | rs6593482 | 0.182 | 0.512 | 0.25 | 0.169 | 1.31 | 1.14 | 0.755 | 0.767 | 0.462 | 0.659 | -0.309 |
| logdha | rs7913948 | 0.183 | 0.512 | 0.249 | 0.169 | 1.31 | 1.14 | 0.758 | 0.766 | 0.463 | 0.659 | -0.307 |
| logdha | rs77185151 | 0.025 | 0.434 | 0.182 | 0.046 | 0.07 | 0.198 | 0.348 | 6.51 | 12.4 | 0.324 | 0.694 |
| logdha | rs12762303 | 0.182 | 0.512 | 0.25 | 0.17 | 1.32 | 1.15 | 0.751 | 0.765 | 0.461 | 0.656 | -0.314 |
| logdha | rs73283136 | 0.008 | . | . | . | . | . | . | . | . | . | . |
| logdha | rs187180218 | 0.007 | . | . | . | . | . | . | . | . | . | . |
| logdha | rs78137087 | 0.182 | 0.51 | 0.248 | 0.167 | 1.29 | 1.12 | 0.769 | 0.774 | 0.467 | 0.671 | -0.291 |
| logdha | rs72561472 | 0.181 | 0.491 | 0.237 | 0.141 | 1.07 | 0.989 | 0.94 | 0.874 | 0.542 | 0.828 | -0.103 |
| logdha | rs4987106 | 0.18 | 0.505 | 0.243 | 0.155 | 1.14 | 1.04 | 0.887 | 0.793 | 0.5 | 0.713 | -0.188 |
| logdha | rs4987105 | 0.159 | 0.507 | 0.246 | 0.162 | 1.37 | 1.16 | 0.706 | 0.772 | 0.45 | 0.657 | -0.343 |
| logdha | rs111916157 | 0.187 | 0.557 | 0.281 | 0.247 | 1.8 | 1.58 | 0.502 | 0.602 | 0.365 | 0.402 | -0.755 |
| logdha | rs111528152 | 0.184 | 0.546 | 0.272 | 0.224 | 1.6 | 1.42 | 0.597 | 0.627 | 0.384 | 0.445 | -0.599 |
| logdha | rs12763738 | 0.178 | 0.516 | 0.252 | 0.175 | 1.37 | 1.19 | 0.718 | 0.745 | 0.45 | 0.626 | -0.359 |
| logdha | rs191675886 | 0.011 | 0.507 | 0.224 | 0.124 | 978 | 3.80E+03 | 0.076 | 9.80E-03 | 0.028 | 0.099 | -973 |
| logdha | rs66489870 | 0.182 | 0.512 | 0.25 | 0.17 | 1.32 | 1.15 | 0.75 | 0.764 | 0.46 | 0.655 | -0.315 |
| logdha | rs71494775 | 0.182 | 0.512 | 0.25 | 0.17 | 1.32 | 1.15 | 0.75 | 0.764 | 0.46 | 0.655 | -0.315 |
| logdha | rs61855875 | 0.01 | 0.444 | 0.189 | 0.057 | 1.66 | 12.5 | 0.946 | 2.7 | 13.2 | 0.839 | 0.886 |
| logdha | rs116356837 | 0.019 | 0.481 | 0.197 | 0.074 | 4.22 | 20.7 | 0.769 | 0.325 | 1.2 | 0.761 | -3.04 |
| logdha | rs1864414 | 0.179 | 0.515 | 0.251 | 0.174 | 1.38 | 1.22 | 0.716 | 0.749 | 0.462 | 0.639 | -0.362 |
| logdha | rs1864413 | 0.182 | 0.512 | 0.25 | 0.17 | 1.32 | 1.15 | 0.75 | 0.764 | 0.46 | 0.655 | -0.316 |
| logdha | rs139497212 | 0 | . | . | . | . | . | . | . | . | . | . |
| logdha | rs892690 | 0.104 | 0.43 | 0.215 | 0.091 | 0.876 | 1.09 | 0.915 | 1.5 | 1.39 | 0.665 | 0.258 |
| logdha | rs1864412 | 0.182 | 0.512 | 0.25 | 0.17 | 1.32 | 1.15 | 0.748 | 0.763 | 0.46 | 0.654 | -0.318 |
| logdha | rs77827771 | 0.087 | 0.541 | 0.247 | 0.178 | 2.34 | 2.38 | 0.404 | 0.337 | 0.257 | 0.153 | -1.45 |
| logdha | rs145294963 | . | . | . | . | . | . | . | . | . | . | . |
| logdha | rs149081327 | 0.003 | . | . | . | . | . | . | . | . | . | . |
| logdha | rs67008983 | 0.182 | 0.513 | 0.251 | 0.172 | 1.34 | 1.16 | 0.738 | 0.759 | 0.457 | 0.647 | -0.329 |
| logdha | rs56150414 | 0.182 | 0.503 | 0.248 | 0.164 | 1.22 | 1.06 | 0.817 | 0.81 | 0.485 | 0.725 | -0.227 |
| logdha | rs55787481 | 0.182 | 0.503 | 0.245 | 0.158 | 1.22 | 1.07 | 0.822 | 0.808 | 0.488 | 0.724 | -0.226 |
| logdha | rs141270101 | 0.001 | . | . | . | . | . | . | . | . | . | . |
| logdha | rs114980075 | 0.022 | 0.458 | 0.21 | 0.089 | 1.34 | 1.71 | 0.82 | 1.27 | 1.09 | 0.784 | -0.02 |
| logdha | rs12783095 | 0.181 | 0.514 | 0.252 | 0.174 | 1.36 | 1.18 | 0.723 | 0.755 | 0.453 | 0.64 | -0.346 |
| logdha | rs182342688 | 0.001 | . | . | . | . | . | . | . | . | . | . |

| logdha | rs17157728 | 0.181 | 0.514 | 0.252 | 0.174 | 1.36 | 1.18 | 0.722 | 0.755 | 0.453 | 0.639 | -0.347 |
| --- | --- | --- | --- | --- | --- | --- | --- | --- | --- | --- | --- | --- |
| logdha | rs114835563 | 0.086 | 0.543 | 0.247 | 0.18 | 2.43 | 2.45 | 0.377 | 0.33 | 0.25 | 0.143 | -1.54 |
| logdha | rs3824612 | 0.104 | 0.429 | 0.214 | 0.09 | 0.876 | 1.09 | 0.915 | 1.5 | 1.39 | 0.663 | 0.259 |
| logdha | rs17157733 | 0.181 | 0.515 | 0.252 | 0.176 | 1.37 | 1.19 | 0.713 | 0.752 | 0.45 | 0.634 | -0.357 |
| logdha | rs74128478 | 0.019 | 0.479 | 0.2 | 0.077 | 36.7 | 69.4 | 0.057 | 0.016 | 0.019 | 5.30E-04 | -35.9 |
| logdha | rs184973589 | 0.001 | . | . | . | . | . | . | . | . | . | . |
| logdha | rs150479461 | 0 | . | . | . | . | . | . | . | . | . | . |
| logdha | rs138310407 | . | . | . | . | . | . | . | . | . | . | . |
| logdha | rs187426603 | . | . | . | . | . | . | . | . | . | . | . |
| logdha | rs190843143 | 0.001 | . | . | . | . | . | . | . | . | . | . |
| logdha | rs191883449 | 0 | . | . | . | . | . | . | . | . | . | . |
| logdha | rs58238740 | 0.173 | 0.512 | 0.252 | 0.174 | 1.43 | 1.25 | 0.68 | 0.757 | 0.455 | 0.644 | -0.389 |
| logdha | rs3780894 | 0.173 | 0.512 | 0.252 | 0.174 | 1.43 | 1.25 | 0.68 | 0.757 | 0.455 | 0.644 | -0.389 |
| logdha | rs149646626 | 0.006 | . | . | . | . | . | . | . | . | . | . |
| logdha | rs79615212 | 0.006 | . | . | . | . | . | . | . | . | . | . |
| logdha | rs3780895 | 0.173 | 0.514 | 0.253 | 0.176 | 1.45 | 1.26 | 0.671 | 0.751 | 0.45 | 0.633 | -0.402 |
| logdha | rs3780896 | 0.173 | 0.512 | 0.252 | 0.174 | 1.43 | 1.25 | 0.679 | 0.757 | 0.455 | 0.644 | -0.389 |
| logdha | rs142747163 | 0.003 | . | . | . | . | . | . | . | . | . | . |
| logdha | rs189417584 | 0.01 | 0.487 | 0.203 | 0.085 | 2.90E+04 | 3.60E+05 | 0.412 | 3.40E-06 | 4.40E-05 | 0.322 | ####### |
| logdha | rs3780897 | 0.17 | 0.489 | 0.238 | 0.142 | 1.16 | 1.04 | 0.866 | 0.867 | 0.531 | 0.816 | -0.159 |
| logdha | rs117509491 | 0.002 | . | . | . | . | . | . | . | . | . | . |
| logdha | rs117811514 | 0.002 | . | . | . | . | . | . | . | . | . | . |
| logdha | rs116500932 | 0.086 | 0.543 | 0.247 | 0.179 | 2.4 | 2.41 | 0.386 | 0.336 | 0.254 | 0.148 | -1.5 |
| logdha | rs77584168 | . | . | . | . | . | . | . | . | . | . | . |
| logdha | rs2310776 | 0.173 | 0.512 | 0.252 | 0.174 | 1.43 | 1.25 | 0.679 | 0.757 | 0.455 | 0.643 | -0.389 |
| logdha | rs17157736 | 0.173 | 0.512 | 0.252 | 0.174 | 1.43 | 1.25 | 0.679 | 0.757 | 0.455 | 0.643 | -0.389 |
| logdha | rs72789735 | 0.036 | 0.515 | 0.223 | 0.125 | 7.34 | 9.79 | 0.135 | 0.133 | 0.131 | 0.041 | -6.35 |
| logdha | rs183747187 | 0.001 | . | . | . | . | . | . | . | . | . | . |
| logdha | rs7077891 | 0.173 | 0.512 | 0.252 | 0.174 | 1.43 | 1.25 | 0.679 | 0.757 | 0.455 | 0.643 | -0.39 |
| logdha | rs145063305 | 0.002 | . | . | . | . | . | . | . | . | . | . |
| logdha | rs3824613 | 0.174 | 0.5 | 0.247 | 0.161 | 1.35 | 1.14 | 0.725 | 0.814 | 0.477 | 0.725 | -0.298 |
| logdha | rs145543199 | 0.004 | . | . | . | . | . | . | . | . | . | . |
| logdha | rs140361993 | 0.001 | . | . | . | . | . | . | . | . | . | . |
| logdha | rs11239496 | 0.036 | 0.515 | 0.223 | 0.125 | 7.35 | 9.81 | 0.135 | 0.132 | 0.131 | 0.041 | -6.37 |
| logdha | rs11239497 | 0.154 | 0.5 | 0.246 | 0.159 | 1.48 | 1.25 | 0.645 | 0.791 | 0.461 | 0.688 | -0.392 |
| logdha | rs10900212 | 0.245 | 0.433 | 0.22 | 0.099 | 0.748 | 0.63 | 0.73 | 1.17 | 0.665 | 0.784 | 0.198 |
| logdha | rs186487136 | . | . | . | . | . | . | . | . | . | . | . |
| logdha | rs12774369 | 0.173 | 0.514 | 0.253 | 0.177 | 1.46 | 1.29 | 0.667 | 0.749 | 0.456 | 0.635 | -0.413 |
| logdha | rs60842538 | 0.173 | 0.516 | 0.255 | 0.18 | 1.5 | 1.34 | 0.652 | 0.739 | 0.458 | 0.625 | -0.441 |
| logdha | rs3780898 | 0.153 | 0.504 | 0.249 | 0.165 | 1.55 | 1.36 | 0.62 | 0.772 | 0.472 | 0.672 | -0.45 |
| logdha | rs3780899 | 0.172 | 0.519 | 0.257 | 0.186 | 1.55 | 1.42 | 0.629 | 0.723 | 0.459 | 0.609 | -0.49 |
| logdha | rs12252472 | 0.273 | 0.607 | 0.321 | 0.345 | 1.61 | 1.63 | 0.635 | 0.556 | 0.41 | 0.427 | -0.676 |
| logdha | rs185411011 | 0.001 | . | . | . | . | . | . | . | . | . | . |
| logdha | rs28395865 | 0.152 | 0.505 | 0.25 | 0.167 | 1.6 | 1.44 | 0.605 | 0.762 | 0.481 | 0.667 | -0.488 |
| logdha | rs191004074 | 0.034 | 0.492 | 0.219 | 0.112 | 4.88 | 10.1 | 0.441 | 0.362 | 0.502 | 0.464 | -3.51 |
| logdha | rs182401295 | . | . | . | . | . | . | . | . | . | . | . |
| logdha | rs2228064 | 0.277 | 0.585 | 0.313 | 0.316 | 1.47 | 1.46 | 0.695 | 0.607 | 0.439 | 0.49 | -0.535 |
| logdha | rs188284159 | . | . | . | . | . | . | . | . | . | . | . |
| logdha | rs2303887 | 0.003 | . | . | . | . | . | . | . | . | . | . |
| logdha | rs2303886 | 0.014 | 0.477 | 0.2 | 0.078 | 5.07 | 10.2 | 0.42 | 0.421 | 0.604 | 0.547 | -3.53 |
| logdha | rs192621006 | . | . | . | . | . | . | . | . | . | . | . |
| logdha | rs2303885 | 0.491 | 0.615 | 0.352 | 0.396 | 1.09 | 0.756 | 0.906 | 0.753 | 0.36 | 0.553 | -0.198 |
| logdha | rs114466168 | 0.086 | 0.544 | 0.248 | 0.182 | 2.45 | 2.49 | 0.38 | 0.329 | 0.252 | 0.147 | -1.55 |
| logdha | rs11239501 | 0.038 | 0.473 | 0.205 | 0.084 | 1.7 | 3.93 | 0.82 | 0.754 | 1.29 | 0.869 | -0.563 |
| logdha | rs745986 | 0.156 | 0.493 | 0.239 | 0.145 | 1.73 | 1.64 | 0.56 | 0.786 | 0.513 | 0.712 | -0.555 |
| logdha | rs75262268 | 0.004 | . | . | . | . | . | . | . | . | . | . |
| logdha | rs71494776 | 0.022 | 0.486 | 0.204 | 0.086 | 9.7 | 18.8 | 0.241 | 0.361 | 0.477 | 0.441 | -7.48 |
| logdha | rs74386871 | 0.006 | . | . | . | . | . | . | . | . | . | . |
| logdha | rs76502040 | 0.004 | . | . | . | . | . | . | . | . | . | . |
| logdha | rs142213734 | 0.001 | . | . | . | . | . | . | . | . | . | . |
| logdha | rs4948671 | 0.796 | 0.506 | 0.473 | 0.467 | 0.925 | 0.722 | 0.92 | 0.948 | 0.523 | 0.922 | 0.013 |
| logdha | rs146286085 | 0 | . | . | . | . | . | . | . | . | . | . |
| logdha | rs144080623 | 0 | . | . | . | . | . | . | . | . | . | . |
| logdha | rs115074014 | 0.133 | 0.488 | 0.23 | 0.128 | 1.22 | 1.63 | 0.879 | 0.814 | 0.793 | 0.833 | -0.226 |
| logdha | rs17157756 | 0.133 | 0.488 | 0.23 | 0.128 | 1.22 | 1.63 | 0.879 | 0.814 | 0.793 | 0.833 | -0.226 |
| logdha | rs190459780 | 0.001 | . | . | . | . | . | . | . | . | . | . |
| logdha | rs12358647 | 0.25 | 0.43 | 0.213 | 0.088 | 0.857 | 0.728 | 0.856 | 1.15 | 0.654 | 0.801 | 0.138 |
| logdha | rs147080556 | 0.009 | . | . | . | . | . | . | . | . | . | . |
| logdha | rs187543349 | 0.002 | . | . | . | . | . | . | . | . | . | . |
| logdha | rs114375595 | 0.024 | 0.437 | 0.184 | 0.049 | 0.128 | 0.356 | 0.46 | 4.24 | 8.07 | 0.447 | 0.672 |
| logdha | rs117211200 | 0.003 | . | . | . | . | . | . | . | . | . | . |
| logdha | rs192963047 | . | . | . | . | . | . | . | . | . | . | . |
| logdha | rs11239505 | 0.254 | 0.438 | 0.219 | 0.098 | 0.862 | 0.729 | 0.861 | 1.12 | 0.635 | 0.841 | 0.123 |
| logdha | rs184434116 | 0.001 | . | . | . | . | . | . | . | . | . | . |
| logdha | rs4948999 | 0.801 | 0.492 | 0.456 | 0.444 | 0.88 | 0.683 | 0.869 | 0.964 | 0.528 | 0.947 | 0.045 |
| logdha | rs11239506 | 0.078 | 0.493 | 0.235 | 0.137 | 1.83 | 2.29 | 0.63 | 0.704 | 0.648 | 0.703 | -0.688 |
| logdha | rs190706620 | 0.001 | . | . | . | . | . | . | . | . | . | . |
| logdha | rs190770429 | 0.001 | . | . | . | . | . | . | . | . | . | . |
| logdha | rs181327133 | 0.003 | . | . | . | . | . | . | . | . | . | . |
| logdha | rs149251697 | 0.057 | 0.379 | 0.159 | 0.021 | 0.082 | 0.15 | 0.169 | 5.84 | 7.06 | 0.144 | 0.721 |
| logdha | rs118081602 | 0.003 | . | . | . | . | . | . | . | . | . | . |
| logdha | rs188233691 | . | . | . | . | . | . | . | . | . | . | . |
| logdha | rs183778794 | 0.006 | . | . | . | . | . | . | . | . | . | . |
| logdha | rs188040078 | 0.003 | . | . | . | . | . | . | . | . | . | . |
| logdha | rs184675372 | 0.001 | . | . | . | . | . | . | . | . | . | . |
| logdha | rs150326891 | 0.03 | 0.451 | 0.192 | 0.061 | 0.415 | 1.72 | 0.832 | 1.93 | 5.7 | 0.825 | 0.494 |

| logdha | rs185520021 | . | . | . | . | . | . | . | . | . | . | . |
| --- | --- | --- | --- | --- | --- | --- | --- | --- | --- | --- | --- | --- |
| logdha | rs7907779 | 0.078 | 0.493 | 0.234 | 0.137 | 1.82 | 2.28 | 0.633 | 0.705 | 0.65 | 0.705 | -0.679 |
| logdha | rs184962860 | . | . | . | . | . | . | . | . | . | . | . |
| logdha | rs149762784 | 0.034 | 0.52 | 0.229 | 0.138 | 8.74 | 20.5 | 0.356 | 0.132 | 0.227 | 0.239 | -7.66 |
| logdha | rs149203427 | 0.005 | . | . | . | . | . | . | . | . | . | . |
| logdha | rs60153083 | 0.045 | 0.352 | 0.145 | 0.011 | 5.50E-03 | 0.014 | 0.035 | 27.9 | 43.2 | 0.032 | 0.697 |
| logdha | rs112001069 | 0.073 | 0.468 | 0.202 | 0.079 | 1.5 | 2.24 | 0.786 | 0.87 | 0.871 | 0.89 | -0.356 |
| logdha | rs182355382 | 0.057 | 0.56 | 0.245 | 0.184 | 9.15 | 17.4 | 0.246 | 0.192 | 0.28 | 0.258 | -7.72 |
| logdha | rs186740974 | 0.157 | 0.407 | 0.21 | 0.082 | 0.527 | 0.705 | 0.632 | 1.58 | 1.43 | 0.609 | 0.406 |
| logdha | rs191874916 | 0.06 | 0.656 | 0.272 | 0.309 | 24.1 | 41.2 | 0.063 | 0.1 | 0.147 | 0.116 | -22.1 |
| logdha | rs182989412 | 0.126 | 0.495 | 0.257 | 0.175 | 2.06 | 2.89 | 0.605 | 0.774 | 0.778 | 0.799 | -0.768 |
| logdha | rs188819476 | 0.003 | . | . | . | . | . | . | . | . | . | . |
| logdha | rs141217183 | 0.083 | 0.483 | 0.213 | 0.099 | 1.98 | 2.81 | 0.631 | 0.736 | 0.717 | 0.753 | -0.758 |
| logdha | rs192922652 | 0.081 | 0.476 | 0.207 | 0.088 | 1.63 | 2.4 | 0.739 | 0.797 | 0.794 | 0.82 | -0.489 |
| logdha | rs185092343 | 0.025 | 0.47 | 0.199 | 0.074 | 0.111 | 0.56 | 0.662 | 1.79 | 5.64 | 0.853 | 0.513 |
| logdha | rs145193311 | 0.082 | 0.452 | 0.195 | 0.066 | 1.14 | 1.66 | 0.926 | 1.05 | 1.03 | 0.96 | -0.053 |
| logdha | rs55789336 | 0.084 | 0.49 | 0.234 | 0.134 | 1.59 | 2.03 | 0.714 | 0.735 | 0.694 | 0.745 | -0.509 |
| logdha | rs55837212 | 0.083 | 0.49 | 0.233 | 0.133 | 1.61 | 2.1 | 0.716 | 0.731 | 0.713 | 0.748 | -0.522 |
| logdha | rs75408574 | 0.208 | 0.553 | 0.282 | 0.245 | 1.66 | 1.26 | 0.509 | 0.681 | 0.392 | 0.504 | -0.585 |
| logdha | rs149306926 | 0.089 | 0.469 | 0.226 | 0.117 | 1.24 | 1.72 | 0.876 | 0.928 | 0.92 | 0.94 | -0.17 |
| logdha | rs35990892 | 0.683 | 0.635 | 0.582 | 0.621 | 1.33 | 1.07 | 0.721 | 0.8 | 0.464 | 0.7 | -0.291 |
| logdha | rs192356207 | 0.128 | 0.419 | 0.184 | 0.048 | 0.314 | 0.453 | 0.422 | 1.8 | 1.83 | 0.565 | 0.503 |
| logdha | rs183741163 | 0.097 | 0.567 | 0.262 | 0.22 | 3.38 | 2.75 | 0.134 | 0.428 | 0.268 | 0.176 | -2.13 |
| logdha | rs34737297 | 0.591 | 0.298 | 0.226 | 0.11 | 0.523 | 0.49 | 0.489 | 1.46 | 0.996 | 0.58 | 0.406 |
| logdha | rs181346933 | 0.042 | 0.454 | 0.2 | 0.074 | 0.507 | 0.686 | 0.616 | 0.909 | 0.741 | 0.907 | 0.249 |
| logdha | rs36093253 | 0.24 | 0.415 | 0.215 | 0.089 | 0.75 | 0.639 | 0.736 | 1.24 | 0.769 | 0.734 | 0.22 |
| logdha | rs186307855 | 0.262 | 0.579 | 0.307 | 0.303 | 1.53 | 1.47 | 0.656 | 0.618 | 0.436 | 0.495 | -0.563 |
| logdha | rs189681865 | 0.096 | 0.466 | 0.213 | 0.095 | 0.905 | 1.17 | 0.939 | 1 | 0.941 | 1 | 0.051 |
| logdha | rs34942971 | 0.259 | 0.428 | 0.222 | 0.102 | 0.788 | 0.684 | 0.784 | 1.17 | 0.678 | 0.786 | 0.179 |
| logdha | rs181816554 | 0.064 | 0.613 | 0.271 | 0.268 | 12.6 | 22.7 | 0.159 | 0.159 | 0.24 | 0.224 | -11 |
| logdha | rs186846881 | 0.278 | 0.56 | 0.311 | 0.297 | 1.31 | 1.32 | 0.788 | 0.683 | 0.502 | 0.604 | -0.37 |
| logdha | rs191178220 | 0.008 | . | . | . | . | . | . | . | . | . | . |
| logdha | rs183305228 | 0.046 | 0.349 | 0.144 | 0.011 | 5.10E-03 | 0.013 | 0.034 | 28.7 | 44.6 | 0.031 | 0.697 |
| logdha | rs139912761 | 0.149 | 0.403 | 0.201 | 0.068 | 0.477 | 0.565 | 0.532 | 1.61 | 1.34 | 0.566 | 0.43 |
| logdha | rs185499901 | 0.018 | 0.414 | 0.186 | 0.049 | 0.037 | 0.085 | 0.155 | 11.8 | 18.8 | 0.121 | 0.729 |
| logdha | rs189816197 | 0.086 | 0.446 | 0.196 | 0.066 | 1.03 | 1.34 | 0.984 | 1.14 | 0.986 | 0.879 | 0.05 |
| logdha | rs145656938 | 0.058 | 0.493 | 0.211 | 0.099 | 1.3 | 2.67 | 0.9 | 0.599 | 0.898 | 0.733 | -0.406 |
| logdha | rs35745197 | 0.072 | 0.466 | 0.201 | 0.077 | 1.49 | 2.26 | 0.792 | 0.886 | 0.899 | 0.905 | -0.342 |
| logdha | rs192856637 | . | . | . | . | . | . | . | . | . | . | . |
| logdha | rs2310775 | 0.267 | 0.415 | 0.202 | 0.071 | 0.805 | 0.531 | 0.742 | 1.22 | 0.593 | 0.683 | 0.187 |
| logdha | rs11239507 | 0.538 | 0.484 | 0.323 | 0.278 | 0.98 | 0.697 | 0.978 | 0.954 | 0.481 | 0.925 | -0.012 |
| logdha | rs74648467 | 0.027 | 0.538 | 0.235 | 0.156 | 15.4 | 27.1 | 0.119 | 0.182 | 0.204 | 0.129 | -13.5 |
| logdha | rs145815934 | 0.005 | . | . | . | . | . | . | . | . | . | . |
| logdha | rs145390687 | 0.012 | 0.456 | 0.194 | 0.064 | 0.328 | 0.955 | 0.702 | 2.69 | 4.69 | 0.57 | 0.619 |
| logdha | rs184354913 | 0.012 | 0.478 | 0.208 | 0.089 | 4.25 | 13.6 | 0.651 | 0.28 | 0.706 | 0.613 | -3.16 |
| logdha | rs181244920 | 0.003 | . | . | . | . | . | . | . | . | . | . |
| logdha | rs11499087 | 0.167 | 0.479 | 0.229 | 0.124 | 1 | 1.09 | 1 | 0.892 | 0.71 | 0.885 | -0.052 |
| logdha | rs148263794 | 0.079 | 0.462 | 0.201 | 0.076 | 1.44 | 2.11 | 0.802 | 0.916 | 0.903 | 0.929 | -0.294 |
| logdha | rs150758903 | 0.089 | 0.456 | 0.204 | 0.079 | 0.578 | 0.955 | 0.74 | 1.18 | 1.42 | 0.888 | 0.278 |
| logdha | rs147654382 | 0.192 | 0.493 | 0.25 | 0.164 | 1.32 | 1.41 | 0.793 | 0.849 | 0.621 | 0.823 | -0.262 |
| logdha | rs147495166 | 0.077 | 0.536 | 0.246 | 0.175 | 2.57 | 2.67 | 0.362 | 0.346 | 0.274 | 0.181 | -1.63 |
| logdha | rs35198778 | 0.14 | 0.484 | 0.208 | 0.092 | 0.815 | 0.996 | 0.867 | 0.935 | 0.819 | 0.939 | 0.07 |
| logdha | rs145207659 | 0.02 | 0.459 | 0.201 | 0.076 | 0.589 | 1.56 | 0.841 | 1.37 | 2.24 | 0.849 | 0.322 |
| logdha | rs186466464 | 0.024 | 0.419 | 0.19 | 0.055 | 0.293 | 0.604 | 0.552 | 3.72 | 5.32 | 0.358 | 0.745 |
| logdha | rs12778413 | 0.199 | 0.508 | 0.257 | 0.181 | 1.38 | 1.23 | 0.722 | 0.802 | 0.501 | 0.724 | -0.323 |
| logdha | rs7895552 | 0.724 | 0.347 | 0.308 | 0.233 | 0.624 | 0.511 | 0.565 | 1.21 | 0.735 | 0.748 | 0.292 |
| logdha | rs12783895 | 0.076 | 0.464 | 0.201 | 0.076 | 1.6 | 2.42 | 0.755 | 0.871 | 0.887 | 0.892 | -0.419 |
| logdha | rs34929604 | 0.081 | 0.458 | 0.197 | 0.07 | 1.35 | 2 | 0.839 | 0.955 | 0.957 | 0.963 | -0.219 |
| logdha | rs12761961 | 0.072 | 0.465 | 0.2 | 0.075 | 1.37 | 2.24 | 0.849 | 0.911 | 1 | 0.932 | -0.253 |
| logdha | rs35536930 | 0.252 | 0.432 | 0.213 | 0.089 | 0.803 | 0.709 | 0.804 | 1.16 | 0.684 | 0.803 | 0.167 |
| logdha | rs180951060 | 0.006 | . | . | . | . | . | . | . | . | . | . |
| logdha | rs148892128 | 0.005 | . | . | . | . | . | . | . | . | . | . |
| logdha | rs11239509 | 0.255 | 0.568 | 0.295 | 0.277 | 1.44 | 1.16 | 0.65 | 0.616 | 0.375 | 0.426 | -0.505 |
| logdha | rs41526545 | 0.045 | 0.501 | 0.219 | 0.114 | 3.7 | 6.92 | 0.483 | 0.411 | 0.576 | 0.526 | -2.44 |
| logdha | rs2029253 | 0.286 | 0.436 | 0.2 | 0.071 | 0.901 | 0.561 | 0.868 | 1.11 | 0.517 | 0.823 | 0.099 |
| logdha | rs17444064 | 0.041 | 0.476 | 0.206 | 0.087 | 1.79 | 4.13 | 0.802 | 0.7 | 1.19 | 0.834 | -0.668 |
| logdha | rs71494779 | 0.084 | 0.456 | 0.197 | 0.069 | 1.23 | 1.76 | 0.886 | 1 | 0.974 | 0.997 | -0.122 |
| logdha | rs74991420 | 0.003 | . | . | . | . | . | . | . | . | . | . |
| logdha | rs182736551 | 0.003 | . | . | . | . | . | . | . | . | . | . |
| logdha | rs78702074 | 0.003 | . | . | . | . | . | . | . | . | . | . |
| logdha | rs187791466 | . | . | . | . | . | . | . | . | . | . | . |
| logdha | rs35300270 | 0.085 | 0.455 | 0.197 | 0.069 | 1.2 | 1.74 | 0.899 | 1.01 | 0.99 | 0.989 | -0.102 |
| logdha | rs6593484 | 0.078 | 0.492 | 0.234 | 0.135 | 1.78 | 2.22 | 0.643 | 0.713 | 0.656 | 0.714 | -0.648 |
| logdha | rs12244601 | 0.064 | 0.415 | 0.179 | 0.042 | 0.162 | 0.318 | 0.353 | 2.81 | 3.77 | 0.442 | 0.612 |
| logdha | rs184429574 | 0.006 | . | . | . | . | . | . | . | . | . | . |
| logdha | rs7080897 | 0.084 | 0.456 | 0.197 | 0.069 | 1.23 | 1.77 | 0.886 | 1 | 0.974 | 0.998 | -0.123 |
| logdha | rs7097429 | 0.085 | 0.452 | 0.196 | 0.067 | 1.12 | 1.57 | 0.933 | 1.06 | 0.999 | 0.951 | -0.038 |
| logdha | rs77979229 | . | . | . | . | . | . | . | . | . | . | . |
| logdha | rs11239512 | 0.079 | 0.492 | 0.233 | 0.134 | 1.74 | 2.17 | 0.654 | 0.717 | 0.658 | 0.717 | -0.622 |
| logdha | rs60146827 | 0.084 | 0.456 | 0.197 | 0.069 | 1.23 | 1.77 | 0.884 | 1 | 0.972 | 1 | -0.127 |
| logdha | rs79032236 | 0.105 | 0.567 | 0.253 | 0.204 | 2.73 | 3.29 | 0.406 | 0.338 | 0.311 | 0.238 | -1.77 |
| logdha | rs140854206 | . | . | . | . | . | . | . | . | . | . | . |
| logdha | rs184049550 | 0.012 | 0.52 | 0.223 | 0.127 | 398 | 596 | 6.30E-05 | 0.011 | 0.017 | 3.30E-03 | -395 |
| logdha | rs114433607 | 0.029 | 0.354 | 0.143 | 0.01 | 5.90E-04 | 1.70E-03 | 0.01 | 109 | 198 | 9.60E-03 | 0.668 |
| logdha | rs117653793 | 0.003 | . | . | . | . | . | . | . | . | . | . |
| logdha | rs117039533 | 0.003 | . | . | . | . | . | . | . | . | . | . |

| logdha | rs191660761 | 0.002 | . | . | . | . | . | . | . | . | . | . |
| --- | --- | --- | --- | --- | --- | --- | --- | --- | --- | --- | --- | --- |
| logdha | rs59514896 | 0.03 | 0.496 | 0.212 | 0.102 | 13 | 28.4 | 0.239 | 0.254 | 0.389 | 0.371 | -10.9 |
| logdha | rs184661876 | 0.001 | . | . | . | . | . | . | . | . | . | . |
| logdha | rs61854073 | 0.011 | 0.431 | 0.185 | 0.05 | 0.333 | 2.17 | 0.866 | 7.74 | 33.1 | 0.632 | 1.35 |
| logdha | rs2018518 | 0.09 | 0.423 | 0.201 | 0.07 | 0.638 | 0.756 | 0.704 | 1.69 | 1.42 | 0.53 | 0.396 |
| logdha | rs934187 | 0.802 | 0.521 | 0.482 | 0.481 | 0.928 | 0.724 | 0.923 | 0.931 | 0.511 | 0.896 | 1.10E-03 |
| logdha | rs7918542 | 0.269 | 0.406 | 0.2 | 0.067 | 0.715 | 0.589 | 0.684 | 1.28 | 0.7 | 0.649 | 0.251 |
| logdha | rs144311920 | 0.004 | . | . | . | . | . | . | . | . | . | . |
| logdha | rs7099684 | 0.883 | 1.56 | 2.3 | 0.765 | 2.75 | 3.18 | 0.383 | 0.509 | 0.407 | 0.398 | -1.13 |
| logdha | rs150829171 | 0.001 | . | . | . | . | . | . | . | . | . | . |
| logdha | rs186060697 | 0.006 | . | . | . | . | . | . | . | . | . | . |
| logdha | rs11239513 | 0.277 | 0.599 | 0.324 | 0.342 | 1.56 | 1.59 | 0.661 | 0.585 | 0.436 | 0.472 | -0.614 |
| logdha | rs7917687 | 0.084 | 0.457 | 0.198 | 0.071 | 1.26 | 1.82 | 0.873 | 0.982 | 0.959 | 0.985 | -0.151 |
| logdha | rs61854076 | 0.004 | . | . | . | . | . | . | . | . | . | . |
| logdha | rs71494780 | 0.007 | . | . | . | . | . | . | . | . | . | . |
| logdha | rs7919239 | 0.257 | 0.444 | 0.221 | 0.102 | 0.883 | 0.737 | 0.882 | 1.09 | 0.608 | 0.877 | 0.1 |
| logdha | rs78334475 | 0.004 | . | . | . | . | . | . | . | . | . | . |
| logdha | rs3780900 | 0.035 | 0.621 | 0.264 | 0.263 | 32.2 | 56.2 | 0.046 | 0.088 | 0.141 | 0.128 | -30.1 |
| logdha | rs186729292 | 0.001 | . | . | . | . | . | . | . | . | . | . |
| logdha | rs12267438 | 0.073 | 0.408 | 0.176 | 0.038 | 0.216 | 0.311 | 0.288 | 2.59 | 2.54 | 0.332 | 0.604 |
| logdha | rs1369214 | 0.564 | 0.587 | 0.398 | 0.431 | 1.23 | 0.884 | 0.778 | 0.81 | 0.416 | 0.682 | -0.229 |
| logdha | rs9282589 | 0.005 | . | . | . | . | . | . | . | . | . | . |
| logdha | rs114522915 | 0.078 | 0.548 | 0.251 | 0.188 | 3.47 | 4.25 | 0.309 | 0.275 | 0.257 | 0.168 | -2.5 |
| logdha | rs2115819 | 0.292 | 0.428 | 0.217 | 0.094 | 0.942 | 0.649 | 0.931 | 1.15 | 0.595 | 0.793 | 0.092 |
| logdha | rs117664603 | 0.003 | . | . | . | . | . | . | . | . | . | . |
| logdha | rs184979202 | 0.001 | . | . | . | . | . | . | . | . | . | . |
| logdha | rs190714671 | . | . | . | . | . | . | . | . | . | . | . |
| logdha | rs141538996 | . | . | . | . | . | . | . | . | . | . | . |
| logdha | rs150881741 | 0.001 | . | . | . | . | . | . | . | . | . | . |
| logdha | rs139076633 | . | . | . | . | . | . | . | . | . | . | . |
| logdha | rs12243649 | 0.156 | 0.393 | 0.174 | 0.035 | 0.355 | 0.367 | 0.317 | 1.88 | 1.32 | 0.368 | 0.515 |
| logdha | rs140348708 | . | . | . | . | . | . | . | . | . | . | . |
| logdha | rs79023256 | 0.003 | . | . | . | . | . | . | . | . | . | . |
| logdha | rs11239515 | 0.071 | 0.501 | 0.239 | 0.148 | 2.45 | 2.99 | 0.463 | 0.636 | 0.573 | 0.616 | -1.17 |
| logdha | rs55866086 | 0.24 | 0.416 | 0.212 | 0.086 | 0.671 | 0.594 | 0.652 | 1.29 | 0.785 | 0.677 | 0.273 |
| logdha | rs181340150 | 0.002 | . | . | . | . | . | . | . | . | . | . |
| logdha | rs11239516 | 0.158 | 0.426 | 0.215 | 0.092 | 0.69 | 0.754 | 0.734 | 1.31 | 1.01 | 0.728 | 0.268 |
| logdha | rs11239517 | 0.158 | 0.426 | 0.216 | 0.092 | 0.692 | 0.756 | 0.736 | 1.31 | 1 | 0.728 | 0.267 |
| logdha | rs11239519 | 0.128 | 0.433 | 0.217 | 0.095 | 0.688 | 0.928 | 0.781 | 1.31 | 1.24 | 0.777 | 0.269 |
| logdha | rs7909514 | 0.788 | 0.409 | 0.381 | 0.338 | 0.818 | 0.645 | 0.799 | 1.08 | 0.603 | 0.886 | 0.135 |
| logdha | rs7894352 | 0.885 | 1.47 | 2.14 | 0.792 | 2.53 | 2.91 | 0.421 | 0.525 | 0.417 | 0.417 | -1.05 |
| logdha | rs189384146 | 0.001 | . | . | . | . | . | . | . | . | . | . |
| logdha | rs11239520 | 0.107 | 0.613 | 0.284 | 0.29 | 4.69 | 4.29 | 0.091 | 0.382 | 0.274 | 0.179 | -3.2 |
| logdha | rs12221233 | 0.234 | 0.332 | 0.166 | 0.027 | 0.391 | 0.309 | 0.235 | 2.01 | 1.16 | 0.226 | 0.538 |
| logdha | rs74523293 | 0.066 | 0.43 | 0.183 | 0.048 | 0.343 | 0.632 | 0.562 | 2 | 2.56 | 0.586 | 0.523 |
| logdha | rs79376040 | 0.066 | 0.43 | 0.183 | 0.048 | 0.342 | 0.631 | 0.561 | 2.01 | 2.56 | 0.585 | 0.523 |
| logdha | rs4949000 | 0.234 | 0.332 | 0.166 | 0.027 | 0.391 | 0.31 | 0.236 | 2.01 | 1.15 | 0.227 | 0.537 |
| logdha | rs7089406 | 0.039 | 0.418 | 0.174 | 0.036 | 0.017 | 0.041 | 0.089 | 13.7 | 21.2 | 0.09 | 0.663 |
| logdha | rs138211056 | 0 | . | . | . | . | . | . | . | . | . | . |
| logdha | rs10900213 | 0.434 | 0.355 | 0.239 | 0.125 | 0.735 | 0.542 | 0.676 | 1.34 | 0.732 | 0.597 | 0.259 |
| logdha | rs145152827 | . | . | . | . | . | . | . | . | . | . | . |
| logdha | rs74443325 | 0.003 | . | . | . | . | . | . | . | . | . | . |
| logdha | rs188299446 | 0.004 | . | . | . | . | . | . | . | . | . | . |
| logdha | rs192699586 | . | . | . | . | . | . | . | . | . | . | . |
| logdha | rs72796464 | 0.041 | 0.478 | 0.209 | 0.091 | 2.12 | 4.66 | 0.732 | 0.649 | 1.05 | 0.79 | -0.941 |
| logdha | rs4949001 | 0.077 | 0.442 | 0.209 | 0.084 | 0.839 | 1.02 | 0.885 | 1.57 | 1.37 | 0.607 | 0.301 |
| logdha | rs147174641 | 0.003 | . | . | . | . | . | . | . | . | . | . |
| logdha | rs190835238 | 0.001 | . | . | . | . | . | . | . | . | . | . |
| logdha | rs76698944 | 0.013 | 0.491 | 0.204 | 0.087 | 123 | 1.60E+03 | 0.703 | 8.70E-06 | 9.10E-05 | 0.265 | -123 |
| logdha | rs56071381 | 0.129 | 0.652 | 0.327 | 0.393 | 4.86 | 4.53 | 0.09 | 0.358 | 0.255 | 0.149 | -3.38 |
| logdha | rs78130427 | 0.019 | 0.455 | 0.187 | 0.056 | 4.51 | 16 | 0.67 | 0.655 | 1.49 | 0.853 | -2.62 |
| logdha | rs10900215 | 0.249 | 0.372 | 0.199 | 0.065 | 0.648 | 0.592 | 0.635 | 1.48 | 0.975 | 0.553 | 0.336 |
| logdha | rs41491445 | 0.046 | 0.348 | 0.143 | 0.01 | 5.90E-03 | 0.014 | 0.025 | 27.5 | 40.2 | 0.023 | 0.703 |
| logdha | rs144995222 | 0.006 | . | . | . | . | . | . | . | . | . | . |
| logdha | rs149097803 | 0.002 | . | . | . | . | . | . | . | . | . | . |
| logdha | rs139429646 | 0.002 | . | . | . | . | . | . | . | . | . | . |
| logdha | rs7075620 | 0.764 | 0.418 | 0.41 | 0.374 | 0.756 | 0.607 | 0.727 | 1.06 | 0.607 | 0.915 | 0.162 |
| logdha | rs148299213 | 0.049 | 0.338 | 0.141 | 9.40E-03 | 0.01 | 0.02 | 0.019 | 21.8 | 27.6 | 0.015 | 0.729 |
| logdha | rs4949002 | 0.084 | 0.453 | 0.217 | 0.098 | 1.02 | 1.29 | 0.988 | 1.24 | 1.13 | 0.815 | 0.099 |
| logdha | rs150496007 | 0.023 | 0.534 | 0.24 | 0.163 | 25.6 | 50.5 | 0.1 | 0.05 | 0.065 | 0.022 | -24.5 |
| logdha | rs147303455 | 0.028 | 0.524 | 0.23 | 0.141 | 44.3 | 74.7 | 0.024 | 0.024 | 0.028 | 1.20E-03 | -43.3 |
| logdha | rs189518433 | . | . | . | . | . | . | . | . | . | . | . |
| logdha | rs186281355 | . | . | . | . | . | . | . | . | . | . | . |
| logdha | rs115297114 | 0.077 | 0.524 | 0.243 | 0.164 | 2.16 | 2.33 | 0.477 | 0.402 | 0.332 | 0.27 | -1.23 |
| logdha | rs11239523 | 0.106 | 0.607 | 0.278 | 0.276 | 4.56 | 4.16 | 0.096 | 0.386 | 0.276 | 0.183 | -3.1 |
| logdha | rs143704897 | 0.007 | . | . | . | . | . | . | . | . | . | . |
| logdha | rs188024096 | 0.001 | . | . | . | . | . | . | . | . | . | . |
| logdha | rs11239524 | 0.133 | 0.737 | 0.368 | 0.541 | 7.28 | 6.61 | 0.029 | 0.24 | 0.167 | 0.041 | -5.73 |
| logdha | rs191656573 | 0.002 | . | . | . | . | . | . | . | . | . | . |
| logdha | rs193295875 | 0.001 | . | . | . | . | . | . | . | . | . | . |
| logdha | rs190458228 | . | . | . | . | . | . | . | . | . | . | . |
| logdha | rs139253993 | 0.001 | . | . | . | . | . | . | . | . | . | . |
| logdha | rs4948672 | 0.497 | 0.451 | 0.403 | 0.373 | 1.22 | 1.08 | 0.82 | 1.03 | 0.642 | 0.964 | -0.106 |
| logdha | rs180954921 | 0.001 | . | . | . | . | . | . | . | . | . | . |
| logdha | rs12264801 | 0.383 | 0.33 | 0.214 | 0.087 | 0.684 | 0.729 | 0.721 | 1.53 | 1.16 | 0.571 | 0.332 |
| logdha | rs73283184 | 0.057 | 0.335 | 0.136 | 7.10E-03 | 2.90E-03 | 5.50E-03 | 2.40E-03 | 37.1 | 44.1 | 2.40E-03 | 0.697 |

| logdha | rs142746187 | 0.022 | 0.467 | 0.199 | 0.074 | 1.36 | 3.99 | 0.918 | 0.172 | 0.393 | 0.44 | -0.713 |
| --- | --- | --- | --- | --- | --- | --- | --- | --- | --- | --- | --- | --- |
| logdha | rs58704739 | 0.053 | 0.342 | 0.136 | 7.10E-03 | 6.90E-04 | 1.40E-03 | 4.10E-04 | 73.8 | 92.2 | 5.70E-04 | 0.675 |
| logdha | rs61272335 | 0.04 | 0.336 | 0.134 | 6.40E-03 | 1.50E-04 | 4.20E-04 | 1.80E-03 | 237 | 408 | 1.50E-03 | 0.676 |
| logdha | rs60959489 | 0.103 | 0.336 | 0.148 | 0.013 | 0.151 | 0.172 | 0.097 | 3.98 | 2.91 | 0.059 | 0.715 |
| logdha | rs58502629 | 0.04 | 0.336 | 0.134 | 6.30E-03 | 1.50E-04 | 4.10E-04 | 1.80E-03 | 239 | 412 | 1.50E-03 | 0.676 |
| logdha | rs10900216 | 0.744 | 0.171 | 0.196 | 0.123 | 0.444 | 0.42 | 0.391 | 1.92 | 1.32 | 0.345 | 0.53 |
| logdha | rs181118141 | 0.004 | . | . | . | . | . | . | . | . | . | . |
| logdha | rs137882665 | 0.019 | 0.48 | 0.202 | 0.081 | 0.023 | 0.077 | 0.256 | 1.76 | 3.85 | 0.796 | 0.516 |
| logdha | rs149466736 | 0.002 | . | . | . | . | . | . | . | . | . | . |
| logdha | rs2099171 | 0.553 | 0.586 | 0.501 | 0.531 | 1.48 | 1.11 | 0.604 | 0.822 | 0.439 | 0.714 | -0.351 |
| logdha | rs141852280 | 0.002 | . | . | . | . | . | . | . | . | . | . |
| logdha | rs191539257 | . | . | . | . | . | . | . | . | . | . | . |
| logdha | rs17157771 | 0.045 | 0.528 | 0.216 | 0.119 | 5.33 | 11.4 | 0.436 | 0.27 | 0.48 | 0.461 | -4.09 |
| logdha | rs111339241 | 0.041 | 0.531 | 0.217 | 0.122 | 6.41 | 13.5 | 0.378 | 0.254 | 0.444 | 0.433 | -5.08 |
| logdha | rs116983255 | 0.004 | . | . | . | . | . | . | . | . | . | . |
| logdha | rs142324341 | . | . | . | . | . | . | . | . | . | . | . |
| logdha | rs187191219 | 0.015 | 0.479 | 0.203 | 0.083 | 0.076 | 0.275 | 0.477 | 0.688 | 1.71 | 0.881 | 0.47 |
| logdha | rs151283973 | 0.005 | . | . | . | . | . | . | . | . | . | . |
| logdha | rs139316218 | 0.001 | . | . | . | . | . | . | . | . | . | . |
| logdha | rs6593485 | 0.697 | 0.278 | 0.272 | 0.19 | 0.613 | 0.524 | 0.567 | 1.44 | 0.903 | 0.564 | 0.354 |
| logdha | rs6593486 | 0.697 | 0.277 | 0.271 | 0.189 | 0.611 | 0.522 | 0.564 | 1.44 | 0.905 | 0.561 | 0.356 |
| logdha | rs145196618 | 0.036 | 0.457 | 0.194 | 0.065 | 0.324 | 0.679 | 0.591 | 1.54 | 2.24 | 0.766 | 0.447 |
| logdha | rs142598335 | 0.001 | . | . | . | . | . | . | . | . | . | . |
| logdha | rs892691 | 0.663 | 0.272 | 0.245 | 0.148 | 0.611 | 0.509 | 0.554 | 1.49 | 0.908 | 0.511 | 0.365 |
| logdha | rs7080713 | 0.597 | 0.263 | 0.236 | 0.137 | 0.529 | 0.434 | 0.437 | 1.6 | 0.991 | 0.444 | 0.431 |
| logdha | rs7077173 | 0.135 | 0.534 | 0.231 | 0.146 | 1.86 | 2.28 | 0.612 | 0.548 | 0.53 | 0.534 | -0.851 |
| logdha | rs7080977 | 0.088 | 0.558 | 0.232 | 0.16 | 4.07 | 5.34 | 0.285 | 0.27 | 0.307 | 0.25 | -3.01 |
| logdha | rs3780901 | 0.599 | 0.225 | 0.198 | 0.09 | 0.482 | 0.384 | 0.36 | 1.8 | 1.07 | 0.323 | 0.488 |
| logdha | rs61854091 | 0.021 | 0.41 | 0.183 | 0.045 | 0.253 | 1.02 | 0.734 | 6.72 | 18.4 | 0.487 | 1.03 |
| logdha | rs77550209 | 0.011 | 0.521 | 0.222 | 0.125 | 1.30E+03 | 2.40E+03 | 1.70E-04 | 2.80E-03 | 6.40E-03 | 9.70E-03 | ####### |
| logdha | rs73283191 | 0.097 | 0.556 | 0.232 | 0.16 | 3.59 | 4.72 | 0.33 | 0.306 | 0.338 | 0.283 | -2.54 |
| logdha | rs191866099 | 0.003 | . | . | . | . | . | . | . | . | . | . |
| logdha | rs147998395 | 0.005 | . | . | . | . | . | . | . | . | . | . |
| logdha | rs7090328 | 0.861 | 0.231 | 0.252 | 0.179 | 0.65 | 0.581 | 0.63 | 1.5 | 0.975 | 0.531 | 0.345 |
| logdha | rs79814535 | 0.003 | . | . | . | . | . | . | . | . | . | . |
| logdha | rs7073082 | 0.861 | 0.231 | 0.252 | 0.179 | 0.651 | 0.582 | 0.631 | 1.5 | 0.974 | 0.531 | 0.344 |
| logdha | rs11239526 | 0.012 | 0.46 | 0.197 | 0.069 | 0.933 | 1.97 | 0.974 | 1.31 | 1.95 | 0.854 | 0.171 |
| logdha | rs141839841 | 0.005 | . | . | . | . | . | . | . | . | . | . |
| logdha | rs75697371 | 0.005 | . | . | . | . | . | . | . | . | . | . |
| logdha | rs75972854 | 0.005 | . | . | . | . | . | . | . | . | . | . |
| logdha | rs139291975 | . | . | . | . | . | . | . | . | . | . | . |
| logdha | rs144064711 | 0.002 | . | . | . | . | . | . | . | . | . | . |
| logdha | rs3780902 | 0.098 | 0.557 | 0.233 | 0.161 | 3.68 | 5 | 0.337 | 0.298 | 0.338 | 0.286 | -2.63 |
| logdha | rs190449801 | 0.004 | . | . | . | . | . | . | . | . | . | . |
| logdha | rs73283196 | 0.096 | 0.558 | 0.233 | 0.162 | 3.73 | 5.07 | 0.332 | 0.296 | 0.337 | 0.285 | -2.67 |
| logdha | rs28395867 | 0.118 | 0.445 | 0.191 | 0.059 | 0.681 | 0.887 | 0.768 | 1.24 | 1.17 | 0.818 | 0.25 |
| logdha | rs28395866 | 0.264 | 0.589 | 0.278 | 0.262 | 1.8 | 1.81 | 0.556 | 0.606 | 0.464 | 0.513 | -0.748 |
| logdha | rs55661611 | 0.088 | 0.564 | 0.235 | 0.168 | 4.47 | 6.18 | 0.279 | 0.252 | 0.3 | 0.247 | -3.4 |
| logdha | rs55684518 | 0.008 | . | . | . | . | . | . | . | . | . | . |
| logdha | rs78552493 | 0.041 | 0.593 | 0.256 | 0.227 | 57.7 | 85.8 | 6.40E-03 | 0.043 | 0.054 | 0.011 | -55.8 |
| logdha | rs147665434 | 0.001 | . | . | . | . | . | . | . | . | . | . |
| logdha | rs34445001 | 0.005 | . | . | . | . | . | . | . | . | . | . |
| logdha | rs184019449 | 0.001 | . | . | . | . | . | . | . | . | . | . |
| logdha | rs3780903 | 0.645 | 0.182 | 0.179 | 0.083 | 0.438 | 0.378 | 0.339 | 2.03 | 1.3 | 0.27 | 0.542 |
| logdha | rs17157784 | 0.159 | 0.381 | 0.171 | 0.032 | 0.452 | 0.43 | 0.403 | 1.89 | 1.27 | 0.345 | 0.492 |
| logdha | rs3780904 | 0.01 | . | . | . | . | . | . | . | . | . | . |
| logdha | rs192064730 | 0 | . | . | . | . | . | . | . | . | . | . |
| logdha | rs12774534 | 0.005 | . | . | . | . | . | . | . | . | . | . |
| logdha | rs79179915 | 0.003 | . | . | . | . | . | . | . | . | . | . |
| logdha | rs117811571 | 0.004 | . | . | . | . | . | . | . | . | . | . |
| logdha | rs72796477 | 0.004 | . | . | . | . | . | . | . | . | . | . |
| logdha | rs55729342 | 0.172 | 0.438 | 0.209 | 0.084 | 0.866 | 1.06 | 0.906 | 1.18 | 1.04 | 0.849 | 0.144 |
| logdha | rs59317220 | 0.172 | 0.438 | 0.209 | 0.084 | 0.865 | 1.06 | 0.906 | 1.18 | 1.04 | 0.849 | 0.145 |
| logdha | rs3780905 | 0.018 | 0.435 | 0.179 | 0.043 | 0.059 | 0.296 | 0.572 | 8.08 | 27.5 | 0.539 | 0.714 |
| logdha | rs3780906 | 0.645 | 0.196 | 0.195 | 0.101 | 0.47 | 0.412 | 0.389 | 1.92 | 1.25 | 0.317 | 0.51 |
| logdha | rs149047975 | 0.003 | . | . | . | . | . | . | . | . | . | . |
| logdha | rs3824614 | 0.002 | . | . | . | . | . | . | . | . | . | . |
| logdha | rs140254404 | . | . | . | . | . | . | . | . | . | . | . |
| logdha | rs145262684 | 0.005 | . | . | . | . | . | . | . | . | . | . |
| logdha | rs76735482 | 0.002 | . | . | . | . | . | . | . | . | . | . |
| logdha | rs73283198 | 0.045 | 0.521 | 0.215 | 0.114 | 4.2 | 9.47 | 0.525 | 0.326 | 0.598 | 0.541 | -3 |
| logdha | rs74911239 | 0.013 | 0.493 | 0.218 | 0.109 | 0.237 | 1.68 | 0.839 | 0.046 | 0.242 | 0.559 | 0.275 |
| logdha | rs150551647 | 0.004 | . | . | . | . | . | . | . | . | . | . |
| logdha | rs115200794 | 0.143 | 0.491 | 0.221 | 0.113 | 1.26 | 1.78 | 0.871 | 0.794 | 0.827 | 0.825 | -0.259 |
| logdha | rs74130823 | 0.245 | 0.54 | 0.258 | 0.197 | 1.49 | 1.76 | 0.736 | 0.702 | 0.629 | 0.693 | -0.464 |
| logdha | rs2279435 | 0.604 | 0.188 | 0.184 | 0.088 | 0.484 | 0.421 | 0.404 | 2.03 | 1.33 | 0.281 | 0.513 |
| logdha | rs147279673 | 0.007 | . | . | . | . | . | . | . | . | . | . |
| logdha | rs183525146 | 0.002 | . | . | . | . | . | . | . | . | . | . |
| logdha | rs3740107 | 0.769 | 0.334 | 0.382 | 0.338 | 0.764 | 0.74 | 0.781 | 1.24 | 0.863 | 0.757 | 0.218 |
| logdha | rs140697247 | 0 | . | . | . | . | . | . | . | . | . | . |
| logdha | rs1565096 | 0.903 | 0.099 | 0.108 | 0.034 | 0.429 | 0.411 | 0.377 | 2.35 | 1.56 | 0.197 | 0.572 |
| logdha | rs1565097 | 0.766 | 0.189 | 0.234 | 0.178 | 0.536 | 0.548 | 0.542 | 1.78 | 1.33 | 0.441 | 0.455 |
| logdha | rs144584548 | 0.008 | . | . | . | . | . | . | . | . | . | . |
| logdha | rs75811053 | 0.001 | . | . | . | . | . | . | . | . | . | . |
| logdha | rs147462967 | 0.009 | . | . | . | . | . | . | . | . | . | . |
| logdha | rs74130824 | 0.121 | 0.498 | 0.226 | 0.125 | 1.54 | 2.42 | 0.784 | 0.735 | 0.837 | 0.787 | -0.474 |

| logdha | rs116883184 | 0.006 | . | . | . | . | . | . | . | . | . | . |
| --- | --- | --- | --- | --- | --- | --- | --- | --- | --- | --- | --- | --- |
| logdha | rs117258049 | 0.006 | . | . | . | . | . | . | . | . | . | . |
| logdha | rs3780908 | 0.129 | 0.551 | 0.238 | 0.167 | 2.35 | 2.82 | 0.474 | 0.476 | 0.441 | 0.423 | -1.29 |
| logdha | rs180938522 | 0.001 | . | . | . | . | . | . | . | . | . | . |
| logdha | rs76687372 | 0.024 | 0.447 | 0.188 | 0.056 | 0.218 | 0.527 | 0.528 | 2.64 | 4.41 | 0.563 | 0.592 |
| logdha | rs3780909 | 0.483 | 0.575 | 0.406 | 0.434 | 1.36 | 0.964 | 0.661 | 0.797 | 0.414 | 0.662 | -0.314 |
| logdha | rs78281156 | 0.001 | . | . | . | . | . | . | . | . | . | . |
| logdha | rs187840631 | . | . | . | . | . | . | . | . | . | . | . |
| logdha | rs76868242 | 0.001 | . | . | . | . | . | . | . | . | . | . |
| logdha | rs192794192 | 0 | . | . | . | . | . | . | . | . | . | . |
| logdha | rs12781248 | 0.005 | . | . | . | . | . | . | . | . | . | . |
| logdha | rs12762375 | 0.005 | . | . | . | . | . | . | . | . | . | . |
| logdha | rs192256986 | . | . | . | . | . | . | . | . | . | . | . |
| logdha | rs184411030 | 0.033 | 0.504 | 0.213 | 0.105 | 8.41 | 13.6 | 0.188 | 0.109 | 0.163 | 0.138 | -7.45 |
| logdha | rs189601483 | 0.001 | . | . | . | . | . | . | . | . | . | . |
| logdha | rs143254579 | 0.015 | 0.455 | 0.193 | 0.064 | 0.341 | 0.666 | 0.582 | 2.15 | 2.9 | 0.571 | 0.538 |
| logdha | rs12253903 | 0.081 | 0.299 | 0.126 | 4.20E-03 | 0.017 | 0.029 | 0.014 | 16.3 | 17.4 | 9.20E-03 | 0.769 |
| logdha | rs35489926 | 0.005 | . | . | . | . | . | . | . | . | . | . |
| logdha | rs71494783 | 0.095 | 0.324 | 0.144 | 0.011 | 0.1 | 0.132 | 0.08 | 5.37 | 4.95 | 0.068 | 0.75 |
| logdha | rs180711602 | 0.001 | . | . | . | . | . | . | . | . | . | . |
| logdha | rs117312402 | 0.001 | . | . | . | . | . | . | . | . | . | . |
| logdha | rs74831694 | 0.001 | . | . | . | . | . | . | . | . | . | . |
| logdha | rs140301943 | . | . | . | . | . | . | . | . | . | . | . |
| logdha | rs78553679 | 0.006 | . | . | . | . | . | . | . | . | . | . |
| logdha | rs78334250 | 0.001 | . | . | . | . | . | . | . | . | . | . |
| logdha | rs76000028 | 0.001 | . | . | . | . | . | . | . | . | . | . |
| logdha | rs145195254 | 0.005 | . | . | . | . | . | . | . | . | . | . |
| logdha | rs137888527 | 0.005 | . | . | . | . | . | . | . | . | . | . |
| logdha | rs149109592 | . | . | . | . | . | . | . | . | . | . | . |
| logdha | rs80280010 | 0.013 | 0.458 | 0.197 | 0.069 | 0.683 | 1.29 | 0.84 | 1.61 | 2.1 | 0.717 | 0.361 |
| logdha | rs189995374 | . | . | . | . | . | . | . | . | . | . | . |
| logdha | rs75467770 | 0.002 | . | . | . | . | . | . | . | . | . | . |
| logdha | rs1487562 | 0.242 | 0.718 | 0.346 | 0.492 | 3.37 | 3.52 | 0.244 | 0.37 | 0.306 | 0.23 | -2.19 |
| logdha | rs12266963 | 0.095 | 0.323 | 0.144 | 0.011 | 0.096 | 0.127 | 0.077 | 5.49 | 5.08 | 0.065 | 0.751 |
| logdha | rs7099874 | 0.857 | 0.408 | 0.418 | 0.381 | 0.917 | 0.855 | 0.926 | 1.08 | 0.715 | 0.907 | 0.079 |
| logdha | rs75687019 | 0.001 | . | . | . | . | . | . | . | . | . | . |
| logdha | rs148240462 | 0.014 | 0.491 | 0.219 | 0.11 | 0.035 | 0.271 | 0.662 | 0.151 | 0.817 | 0.727 | 0.476 |
| logdha | rs141278174 | 0.001 | . | . | . | . | . | . | . | . | . | . |
| logdha | rs76404852 | 0.001 | . | . | . | . | . | . | . | . | . | . |
| logdha | rs139112240 | 0.004 | . | . | . | . | . | . | . | . | . | . |
| logdha | rs12783037 | 0.118 | 0.307 | 0.137 | 8.10E-03 | 0.093 | 0.114 | 0.052 | 5.46 | 4.72 | 0.05 | 0.756 |
| logdha | rs72796481 | 0.003 | . | . | . | . | . | . | . | . | . | . |
| logdha | rs76798749 | 0.025 | 0.444 | 0.189 | 0.056 | 0.248 | 0.493 | 0.483 | 2.87 | 3.91 | 0.438 | 0.625 |
| logdha | rs187732548 | 0.001 | . | . | . | . | . | . | . | . | . | . |
| logdha | rs75675474 | 0.025 | 0.444 | 0.189 | 0.056 | 0.248 | 0.493 | 0.483 | 2.87 | 3.91 | 0.438 | 0.625 |
| logdha | rs76552293 | 0.025 | 0.444 | 0.189 | 0.056 | 0.248 | 0.493 | 0.483 | 2.87 | 3.91 | 0.438 | 0.625 |
| logdha | rs147445993 | 0.008 | . | . | . | . | . | . | . | . | . | . |
| logdha | rs115902283 | 0.025 | 0.444 | 0.189 | 0.056 | 0.248 | 0.494 | 0.484 | 2.87 | 3.91 | 0.44 | 0.624 |
| logdha | rs4540799 | 0.855 | 0.391 | 0.398 | 0.356 | 0.89 | 0.825 | 0.9 | 1.11 | 0.729 | 0.876 | 0.105 |
| logdha | rs12765320 | 0.35 | 0.461 | 0.234 | 0.126 | 0.953 | 0.868 | 0.958 | 1.01 | 0.68 | 0.992 | 0.029 |
| logdha | rs12782256 | 0.005 | . | . | . | . | . | . | . | . | . | . |
| logdha | rs191727256 | 0.007 | . | . | . | . | . | . | . | . | . | . |
| logdha | rs2082111 | 0.113 | 0.615 | 0.268 | 0.264 | 4.88 | 4.95 | 0.118 | 0.288 | 0.238 | 0.132 | -3.63 |
| logdha | rs2082110 | 0.257 | 0.656 | 0.315 | 0.379 | 2.56 | 2.46 | 0.329 | 0.482 | 0.367 | 0.338 | -1.41 |
| logdha | rs2082109 | 0.257 | 0.656 | 0.315 | 0.379 | 2.56 | 2.46 | 0.329 | 0.482 | 0.367 | 0.338 | -1.41 |
| logdha | rs117316662 | 0.001 | . | . | . | . | . | . | . | . | . | . |
| logdha | rs12247163 | 0.357 | 0.432 | 0.217 | 0.094 | 0.888 | 0.786 | 0.893 | 1.11 | 0.725 | 0.872 | 0.106 |
| logdha | rs12355888 | 0.037 | 0.468 | 0.204 | 0.081 | 1.63 | 3.61 | 0.826 | 0.834 | 1.35 | 0.911 | -0.46 |
| logdha | rs143772290 | 0.033 | 0.43 | 0.184 | 0.048 | 0.224 | 0.459 | 0.465 | 3.65 | 5.2 | 0.363 | 0.698 |
| logdha | rs146849452 | 0.009 | . | . | . | . | . | . | . | . | . | . |
| logdha | rs140267121 | 0.003 | . | . | . | . | . | . | . | . | . | . |
| logdha | rs7080474 | 0.352 | 0.446 | 0.224 | 0.108 | 0.941 | 0.833 | 0.946 | 1.06 | 0.695 | 0.929 | 0.058 |
| logdha | rs144066203 | 0.007 | . | . | . | . | . | . | . | . | . | . |
| logdha | rs12765209 | 0.005 | . | . | . | . | . | . | . | . | . | . |
| logdha | rs150930735 | 0.007 | . | . | . | . | . | . | . | . | . | . |
| logdha | rs7068039 | 0.365 | 0.442 | 0.227 | 0.112 | 0.929 | 0.819 | 0.933 | 1.07 | 0.701 | 0.912 | 0.07 |
| logdha | rs117622652 | 0.001 | . | . | . | . | . | . | . | . | . | . |
| logdha | rs190191514 | . | . | . | . | . | . | . | . | . | . | . |
| logdha | rs74130835 | 0.118 | 0.5 | 0.225 | 0.123 | 1.53 | 2.54 | 0.796 | 0.705 | 0.848 | 0.771 | -0.493 |
| logdha | rs1811604 | 0.114 | 0.504 | 0.227 | 0.129 | 1.69 | 2.67 | 0.739 | 0.682 | 0.783 | 0.739 | -0.614 |
| logdha | rs146971256 | . | . | . | . | . | . | . | . | . | . | . |
| logdha | rs74336000 | 0.033 | 0.43 | 0.184 | 0.048 | 0.221 | 0.452 | 0.461 | 3.69 | 5.27 | 0.36 | 0.7 |
| logdha | rs186046492 | 0.003 | . | . | . | . | . | . | . | . | . | . |
| logdha | rs34971823 | 0.009 | . | . | . | . | . | . | . | . | . | . |
| logdha | rs75227446 | 0.001 | . | . | . | . | . | . | . | . | . | . |
| logdha | rs190457599 | 0.001 | . | . | . | . | . | . | . | . | . | . |
| logdha | rs79591917 | 0.001 | . | . | . | . | . | . | . | . | . | . |
| logdha | rs702365 | 0.492 | 0.476 | 0.335 | 0.292 | 1.02 | 0.737 | 0.974 | 0.974 | 0.508 | 0.96 | -0.025 |
| logdha | rs79643836 | 0.004 | . | . | . | . | . | . | . | . | . | . |
| logdha | rs702366 | 0.493 | 0.476 | 0.334 | 0.291 | 1.02 | 0.736 | 0.974 | 0.974 | 0.507 | 0.96 | -0.025 |
| logdha | rs1059696 | 0.197 | 0.473 | 0.222 | 0.11 | 1.07 | 1.24 | 0.952 | 0.955 | 0.799 | 0.956 | -0.061 |
| logdha | rs901681 | 0.489 | 0.462 | 0.32 | 0.266 | 0.974 | 0.701 | 0.971 | 1.01 | 0.524 | 0.991 | 0.016 |
| logdha | rs61854092 | 0.196 | 0.473 | 0.222 | 0.11 | 1.07 | 1.24 | 0.955 | 0.954 | 0.802 | 0.956 | -0.059 |
| logdha | rs148080753 | . | . | . | . | . | . | . | . | . | . | . |
| logdha | rs61854093 | 0.315 | 0.521 | 0.259 | 0.189 | 1.27 | 1.24 | 0.808 | 0.826 | 0.6 | 0.792 | -0.243 |
| logdha | rs141822774 | 0 | . | . | . | . | . | . | . | . | . | . |

| logdha | rs74130836 | 0.118 | 0.5 | 0.224 | 0.123 | 1.52 | 2.53 | 0.8 | 0.707 | 0.852 | 0.774 | -0.485 |
| --- | --- | --- | --- | --- | --- | --- | --- | --- | --- | --- | --- | --- |
| logdha | rs150620963 | 0.006 | . | . | . | . | . | . | . | . | . | . |
| logdha | rs3780913 | 0.849 | 0.509 | 0.499 | 0.491 | 1.17 | 1.05 | 0.862 | 0.948 | 0.605 | 0.933 | -0.114 |
| logdha | rs142255723 | 0.001 | . | . | . | . | . | . | . | . | . | . |
| logdha | rs3780914 | 0.479 | 0.523 | 0.382 | 0.375 | 1.22 | 0.904 | 0.79 | 0.882 | 0.473 | 0.815 | -0.18 |
| logdha | rs182656507 | 0.001 | . | . | . | . | . | . | . | . | . | . |
| logdha | rs2291427 | 0.821 | 0.827 | 0.812 | 0.847 | 1.54 | 1.39 | 0.634 | 0.697 | 0.449 | 0.576 | -0.478 |
| logdha | rs10751382 | 0.816 | 0.819 | 0.736 | 0.824 | 1.56 | 1.31 | 0.596 | 0.7 | 0.418 | 0.551 | -0.485 |
| logdha | rs117610108 | 0.001 | . | . | . | . | . | . | . | . | . | . |
| logdha | rs144966941 | 0.009 | . | . | . | . | . | . | . | . | . | . |
| logdha | rs41502051 | 0.014 | 0.465 | 0.201 | 0.076 | 2.72 | 4.93 | 0.58 | 0.705 | 0.776 | 0.751 | -1.3 |
| logdha | rs41426046 | 0.001 | . | . | . | . | . | . | . | . | . | . |
| logdha | rs41519248 | 0.001 | . | . | . | . | . | . | . | . | . | . |
| logdha | rs189108245 | 0.001 | . | . | . | . | . | . | . | . | . | . |
| logdha | rs7896431 | 0.497 | 0.47 | 0.324 | 0.273 | 0.91 | 0.652 | 0.895 | 0.991 | 0.514 | 0.987 | 0.044 |
| logdha | rs183724547 | 0.009 | . | . | . | . | . | . | . | . | . | . |
| logdha | rs142044990 | 0.004 | . | . | . | . | . | . | . | . | . | . |
| logdha | rs188140929 | . | . | . | . | . | . | . | . | . | . | . |
| logdha | rs76819096 | 0.041 | 0.372 | 0.157 | 0.019 | 3.40E-03 | 9.00E-03 | 0.03 | 30 | 47.8 | 0.033 | 0.663 |
| logdha | rs10751383 | 0.58 | 0.318 | 0.277 | 0.189 | 0.691 | 0.581 | 0.661 | 1.39 | 0.844 | 0.591 | 0.296 |
| logdha | rs117819321 | 0.005 | . | . | . | . | . | . | . | . | . | . |
| logdha | rs2242332 | 0.681 | 0.413 | 0.358 | 0.307 | 0.923 | 0.666 | 0.912 | 1.09 | 0.6 | 0.873 | 0.08 |
| logdha | rs2242333 | 0.02 | 0.44 | 0.182 | 0.047 | 0.135 | 0.609 | 0.657 | 4.82 | 15.1 | 0.615 | 0.711 |
| logdha | rs2242334 | 0.681 | 0.412 | 0.357 | 0.307 | 0.921 | 0.665 | 0.91 | 1.09 | 0.6 | 0.872 | 0.081 |
| logdha | rs189465041 | . | . | . | . | . | . | . | . | . | . | . |
| logdha | rs28395868 | 0.009 | . | . | . | . | . | . | . | . | . | . |
| logdha | rs56067550 | 0.008 | . | . | . | . | . | . | . | . | . | . |
| logdha | rs1051713 | 0.182 | 0.533 | 0.249 | 0.178 | 1.78 | 2.04 | 0.613 | 0.702 | 0.581 | 0.669 | -0.649 |
| logdha | rs115074210 | 0.048 | 0.375 | 0.158 | 0.02 | 3.00E-03 | 8.60E-03 | 0.045 | 27.8 | 48.1 | 0.055 | 0.653 |
| logdha | rs7900977 | 0.893 | 0.054 | 0.068 | 0.021 | 0.215 | 0.206 | 0.108 | 3.47 | 2.53 | 0.088 | 0.772 |
| logdha | rs147613891 | 0.001 | . | . | . | . | . | . | . | . | . | . |
| logdha | rs2229136 | 0.161 | 0.263 | 0.116 | 2.60E-03 | 0.089 | 0.08 | 6.90E-03 | 5.52 | 3.32 | 4.50E-03 | 0.777 |
| logdha | rs185482841 | 0.009 | . | . | . | . | . | . | . | . | . | . |
| logdha | rs2288619 | 0.156 | 0.264 | 0.116 | 2.50E-03 | 0.089 | 0.08 | 7.30E-03 | 5.63 | 3.4 | 4.20E-03 | 0.779 |
| logdha | rs189891524 | . | . | . | . | . | . | . | . | . | . | . |
| logdha | rs56145077 | 0.176 | 0.468 | 0.208 | 0.088 | 1.05 | 1.11 | 0.967 | 0.982 | 0.764 | 0.981 | -0.033 |
| logdha | rs143216970 | 0.013 | 0.456 | 0.191 | 0.061 | 4.50E-03 | 0.011 | 0.026 | 15.4 | 18.5 | 0.023 | 0.571 |
| logdha | rs142246478 | 0 | . | . | . | . | . | . | . | . | . | . |
| logdha | rs13134 | 0.012 | 0.426 | 0.18 | 0.043 | 0.043 | 0.127 | 0.285 | 14.6 | 29.6 | 0.188 | 0.8 |
| logdha | rs41314980 | 0.012 | 0.473 | 0.204 | 0.083 | 4.72 | 12.4 | 0.556 | 0.559 | 1.13 | 0.774 | -2.94 |
| logdha | rs41304611 | 0.005 | . | . | . | . | . | . | . | . | . | . |
| logdha | rs193299617 | . | . | . | . | . | . | . | . | . | . | . |
| logdha | rs12772724 | 0.012 | 0.426 | 0.18 | 0.043 | 0.043 | 0.127 | 0.285 | 14.6 | 29.6 | 0.188 | 0.8 |
| logdha | rs139489537 | 0.011 | 0.455 | 0.195 | 0.066 | 0.629 | 1.11 | 0.793 | 2 | 2.5 | 0.579 | 0.488 |
| logdha | rs12776062 | 0.005 | . | . | . | . | . | . | . | . | . | . |
| logdha | rs752059 | 0.181 | 0.463 | 0.207 | 0.085 | 0.978 | 1.03 | 0.983 | 1 | 0.77 | 0.995 | 0.014 |
| logdha | rs147649982 | 0.013 | 0.51 | 0.221 | 0.12 | 144 | 320 | 0.025 | 5.90E-03 | 0.015 | 0.047 | -143 |
| logdha | rs17157798 | 0.113 | 0.294 | 0.125 | 3.80E-03 | 0.057 | 0.074 | 0.028 | 7.45 | 6.52 | 0.022 | 0.774 |
| logdha | rs142298619 | 0.009 | . | . | . | . | . | . | . | . | . | . |
| logdha | rs4948673 | 0.893 | 0.053 | 0.068 | 0.022 | 0.214 | 0.206 | 0.109 | 3.49 | 2.57 | 0.09 | 0.773 |
| logdha | rs74231484 | 0.045 | 0.431 | 0.191 | 0.057 | 0.499 | 0.724 | 0.632 | 1.9 | 1.93 | 0.528 | 0.479 |
| logdha | rs72796489 | 0.001 | . | . | . | . | . | . | . | . | . | . |
| logdha | rs113447578 | 0.006 | . | . | . | . | . | . | . | . | . | . |
| logdha | rs34063374 | 0.014 | 0.424 | 0.179 | 0.042 | 0.045 | 0.127 | 0.269 | 14.5 | 28.1 | 0.168 | 0.809 |
| logdha | rs11239527 | 0.006 | . | . | . | . | . | . | . | . | . | . |
| logdha | rs34371883 | 0.007 | . | . | . | . | . | . | . | . | . | . |
| logdha | rs118168828 | 0.004 | . | . | . | . | . | . | . | . | . | . |
| logdha | rs34349618 | 0.005 | . | . | . | . | . | . | . | . | . | . |
| logdha | rs80041679 | 0.011 | 0.454 | 0.195 | 0.065 | 0.627 | 1.1 | 0.79 | 2 | 2.49 | 0.576 | 0.489 |
| logdha | rs149640607 | 0.01 | 0.472 | 0.201 | 0.079 | 2.5 | 5.41 | 0.671 | 0.474 | 0.613 | 0.564 | -1.41 |
| logdha | rs4427523 | 0.932 | 0.08 | 0.147 | 0.168 | 0.267 | 0.402 | 0.381 | 2.57 | 2.7 | 0.369 | 0.708 |
| logdha | rs185880109 | . | . | . | . | . | . | . | . | . | . | . |
| logdha | rs191523220 | 0.001 | . | . | . | . | . | . | . | . | . | . |
| logdha | rs144315284 | 0.02 | 0.536 | 0.234 | 0.153 | 74.6 | 141 | 0.023 | 0.035 | 0.055 | 0.03 | -72.7 |
| logdha | rs4948674 | 0.329 | 0.606 | 0.389 | 0.436 | 1.68 | 1.56 | 0.577 | 0.649 | 0.435 | 0.519 | -0.625 |
| logdha | rs182984298 | 0.002 | . | . | . | . | . | . | . | . | . | . |
| logdha | rs56749023 | 0.071 | 0.346 | 0.141 | 9.10E-03 | 5.10E-03 | 0.015 | 0.068 | 24.6 | 44.2 | 0.074 | 0.692 |
| logdha | rs112508957 | 0.045 | 0.512 | 0.22 | 0.119 | 6.89 | 10 | 0.185 | 0.167 | 0.2 | 0.136 | -5.81 |
| logdha | rs7089063 | 0.313 | 0.263 | 0.139 | 0.012 | 0.297 | 0.255 | 0.157 | 2.46 | 1.55 | 0.154 | 0.632 |
| logdha | rs145000779 | . | . | . | . | . | . | . | . | . | . | . |
| logdha | rs80201572 | 0.013 | 0.451 | 0.193 | 0.063 | 0.377 | 0.644 | 0.568 | 2.67 | 3.25 | 0.419 | 0.626 |
| logdha | rs140568227 | 0.003 | . | . | . | . | . | . | . | . | . | . |
| logdha | rs149756228 | 0.004 | . | . | . | . | . | . | . | . | . | . |
| logdha | rs112380685 | 0.003 | . | . | . | . | . | . | . | . | . | . |
| logdha | rs60481109 | 0.067 | 0.341 | 0.139 | 8.20E-03 | 4.70E-03 | 0.013 | 0.062 | 27.3 | 48.9 | 0.065 | 0.697 |
| logdha | rs138221843 | . | . | . | . | . | . | . | . | . | . | . |
| logdha | rs150283194 | 0.009 | . | . | . | . | . | . | . | . | . | . |
| logdha | rs138879661 | 0.008 | . | . | . | . | . | . | . | . | . | . |
| logdha | rs34960345 | 0.067 | 0.385 | 0.18 | 0.041 | 0.37 | 0.404 | 0.363 | 2.91 | 2.31 | 0.18 | 0.659 |
| logdha | rs147514068 | . | . | . | . | . | . | . | . | . | . | . |
| logdha | rs35410535 | 0.138 | 0.283 | 0.124 | 4.10E-03 | 0.099 | 0.093 | 0.014 | 5.19 | 3.2 | 7.60E-03 | 0.763 |
| logdha | rs191613635 | 0.001 | . | . | . | . | . | . | . | . | . | . |
| logdha | rs17157800 | 0.004 | . | . | . | . | . | . | . | . | . | . |
| logdha | rs17523413 | 0.027 | 0.411 | 0.177 | 0.039 | 0.124 | 0.229 | 0.26 | 6.37 | 8.35 | 0.158 | 0.789 |
| logdha | rs7897036 | 0.013 | 0.481 | 0.201 | 0.08 | 5.08 | 12.1 | 0.495 | 0.145 | 0.228 | 0.219 | -4.21 |
| logdha | rs76334323 | 0.001 | . | . | . | . | . | . | . | . | . | . |

| logdha | rs143535991 | 0 | . | . | . | . | . | . | . | . | . | . |
| --- | --- | --- | --- | --- | --- | --- | --- | --- | --- | --- | --- | --- |
| logdha | rs140344979 | 0.002 | . | . | . | . | . | . | . | . | . | . |
| logdha | rs36047811 | 0.138 | 0.283 | 0.124 | 4.10E-03 | 0.1 | 0.093 | 0.014 | 5.19 | 3.2 | 7.60E-03 | 0.763 |
| logdha | rs76493570 | 0.057 | 0.382 | 0.175 | 0.035 | 0.308 | 0.357 | 0.309 | 3.41 | 2.83 | 0.141 | 0.711 |
| logdha | rs192998920 | . | . | . | . | . | . | . | . | . | . | . |
| logdha | rs116840852 | 0.007 | . | . | . | . | . | . | . | . | . | . |
| logdha | rs184824101 | 0 | . | . | . | . | . | . | . | . | . | . |
| logdha | rs41301621 | 0.002 | . | . | . | . | . | . | . | . | . | . |
| logdha | rs3740109 | 0.138 | 0.284 | 0.124 | 4.10E-03 | 0.099 | 0.093 | 0.014 | 5.19 | 3.2 | 7.60E-03 | 0.763 |
| logdha | rs7899415 | 0.032 | 0.422 | 0.183 | 0.047 | 0.017 | 0.079 | 0.392 | 10.9 | 36.6 | 0.474 | 0.638 |
| logdha | rs184395284 | 0.001 | . | . | . | . | . | . | . | . | . | . |
| logdha | rs139209589 | 0.002 | . | . | . | . | . | . | . | . | . | . |
| logdha | rs149924163 | 0.009 | . | . | . | . | . | . | . | . | . | . |
| logdha | rs3740110 | 0.001 | . | . | . | . | . | . | . | . | . | . |
| logdha | rs144335113 | 0.009 | . | . | . | . | . | . | . | . | . | . |
| logdha | rs74743385 | 0.045 | 0.512 | 0.22 | 0.119 | 6.89 | 10 | 0.185 | 0.167 | 0.2 | 0.136 | -5.81 |
| logdha | rs146545829 | 0.009 | . | . | . | . | . | . | . | . | . | . |
| logdha | rs181842689 | . | . | . | . | . | . | . | . | . | . | . |
| logdha | rs3740111 | 0.067 | 0.385 | 0.18 | 0.041 | 0.37 | 0.404 | 0.362 | 2.9 | 2.31 | 0.18 | 0.659 |
| logdha | rs186261599 | 0.009 | . | . | . | . | . | . | . | . | . | . |
| logdha | rs73285348 | 0.004 | . | . | . | . | . | . | . | . | . | . |
| logdha | rs186356901 | 0.009 | . | . | . | . | . | . | . | . | . | . |
| logdha | rs3824618 | 0.011 | 0.453 | 0.194 | 0.064 | 0.523 | 0.892 | 0.704 | 2.25 | 2.72 | 0.505 | 0.556 |
| logdha | rs3802548 | 0.201 | 0.556 | 0.26 | 0.209 | 1.98 | 1.82 | 0.455 | 0.658 | 0.447 | 0.537 | -0.814 |
| logdha | rs3802547 | 0.001 | . | . | . | . | . | . | . | . | . | . |
| logdha | rs77677521 | 0.007 | . | . | . | . | . | . | . | . | . | . |
| logdha | rs3824616 | 0.138 | 0.284 | 0.124 | 4.10E-03 | 0.099 | 0.093 | 0.014 | 5.19 | 3.2 | 7.60E-03 | 0.763 |
| logdha | rs191073818 | 0.009 | . | . | . | . | . | . | . | . | . | . |
| logdha | rs147078004 | . | . | . | . | . | . | . | . | . | . | . |
| logdha | rs7908745 | 0.342 | 0.247 | 0.144 | 0.016 | 0.346 | 0.284 | 0.196 | 2.39 | 1.44 | 0.15 | 0.611 |
| logdha | rs17157811 | 0.004 | . | . | . | . | . | . | . | . | . | . |
| logdha | rs138506087 | 0.002 | . | . | . | . | . | . | . | . | . | . |
| logdha | rs77241269 | 0.071 | 0.348 | 0.141 | 9.30E-03 | 5.30E-03 | 0.015 | 0.069 | 24.1 | 43.2 | 0.076 | 0.691 |
| logdha | rs138374938 | 0.001 | . | . | . | . | . | . | . | . | . | . |
| logdha | rs2279433 | 0.068 | 0.391 | 0.182 | 0.044 | 0.441 | 0.456 | 0.429 | 2.57 | 1.94 | 0.213 | 0.611 |
| logdha | rs73285358 | 0.071 | 0.348 | 0.141 | 9.40E-03 | 5.30E-03 | 0.015 | 0.07 | 24 | 43.1 | 0.076 | 0.691 |
| logdha | rs11239528 | 0.198 | 0.546 | 0.254 | 0.194 | 1.89 | 1.73 | 0.487 | 0.679 | 0.462 | 0.569 | -0.736 |
| logdha | rs77453386 | 0.001 | . | . | . | . | . | . | . | . | . | . |
| logdha | rs2279434 | 0.071 | 0.384 | 0.18 | 0.042 | 0.395 | 0.397 | 0.355 | 2.72 | 1.99 | 0.172 | 0.634 |
| logdha | rs149236525 | 0.003 | . | . | . | . | . | . | . | . | . | . |
| logdha | rs12573609 | 0.018 | 0.532 | 0.243 | 0.167 | 27.5 | 62.4 | 0.145 | 0.073 | 0.113 | 0.09 | -26 |
| logdha | rs191716655 | 0.006 | . | . | . | . | . | . | . | . | . | . |
| logdha | rs17523660 | 0.005 | . | . | . | . | . | . | . | . | . | . |
| logdha | rs142671360 | 0.001 | . | . | . | . | . | . | . | . | . | . |
| logdha | rs3764990 | 0.025 | 0.411 | 0.178 | 0.04 | 0.141 | 0.25 | 0.271 | 6.1 | 7.7 | 0.152 | 0.801 |
| logdha | rs113682861 | 0.001 | . | . | . | . | . | . | . | . | . | . |
| logdha | rs117658270 | 0.001 | . | . | . | . | . | . | . | . | . | . |
| logdha | rs187309063 | . | . | . | . | . | . | . | . | . | . | . |
| logdha | rs73285360 | 0.072 | 0.348 | 0.141 | 9.40E-03 | 5.30E-03 | 0.015 | 0.07 | 24 | 42.9 | 0.076 | 0.691 |
| logdha | rs80133289 | 0.002 | . | . | . | . | . | . | . | . | . | . |
| logdha | rs186693257 | . | . | . | . | . | . | . | . | . | . | . |
| logdha | rs35735968 | 0.081 | 0.376 | 0.172 | 0.033 | 0.333 | 0.346 | 0.29 | 2.83 | 2.03 | 0.148 | 0.645 |
| logdha | rs17157825 | 0.011 | 0.451 | 0.193 | 0.063 | 0.434 | 0.734 | 0.621 | 2.56 | 3.11 | 0.438 | 0.617 |
| logdha | rs2291428 | 0.183 | 0.556 | 0.257 | 0.204 | 2.16 | 2.03 | 0.414 | 0.645 | 0.449 | 0.528 | -0.939 |
| logdha | rs2291429 | 0.21 | 0.528 | 0.253 | 0.182 | 1.71 | 1.56 | 0.555 | 0.755 | 0.513 | 0.679 | -0.557 |
| logdha | rs145714792 | 0.002 | . | . | . | . | . | . | . | . | . | . |
| logdha | rs12769244 | 0.068 | 0.393 | 0.183 | 0.045 | 0.464 | 0.473 | 0.451 | 2.47 | 1.84 | 0.224 | 0.594 |
| logdha | rs12771275 | 0.068 | 0.393 | 0.183 | 0.045 | 0.464 | 0.473 | 0.451 | 2.47 | 1.84 | 0.224 | 0.594 |
| logdha | rs146266174 | 0.001 | . | . | . | . | . | . | . | . | . | . |
| logdha | rs41301623 | 0.001 | . | . | . | . | . | . | . | . | . | . |
| logdha | rs2306814 | 0.001 | . | . | . | . | . | . | . | . | . | . |
| logdha | rs12244834 | 0.08 | 0.416 | 0.176 | 0.038 | 0.543 | 0.805 | 0.681 | 1.8 | 1.94 | 0.585 | 0.448 |
| logdha | rs184006011 | 0.001 | . | . | . | . | . | . | . | . | . | . |
| logdha | rs78829066 | 0.001 | . | . | . | . | . | . | . | . | . | . |
| logdha | rs34731408 | 0.068 | 0.393 | 0.183 | 0.045 | 0.463 | 0.473 | 0.451 | 2.47 | 1.84 | 0.224 | 0.594 |
| logdha | rs17451516 | 0.012 | 0.425 | 0.18 | 0.043 | 0.044 | 0.128 | 0.282 | 14.5 | 29.1 | 0.182 | 0.804 |
| logdha | rs139910133 | 0.009 | . | . | . | . | . | . | . | . | . | . |
| logdha | rs10900218 | 0.319 | 0.236 | 0.123 | 5.60E-03 | 0.252 | 0.209 | 0.097 | 2.81 | 1.7 | 0.087 | 0.679 |
| logdha | rs146023999 | . | . | . | . | . | . | . | . | . | . | . |
| logdha | rs187296958 | 0 | . | . | . | . | . | . | . | . | . | . |
| logdha | rs142342833 | . | . | . | . | . | . | . | . | . | . | . |
| logdha | rs143489255 | . | . | . | . | . | . | . | . | . | . | . |
| logdha | rs138532518 | . | . | . | . | . | . | . | . | . | . | . |
| logdha | rs183768079 | 0.007 | . | . | . | . | . | . | . | . | . | . |
| logdha | rs117892272 | . | . | . | . | . | . | . | . | . | . | . |
| logdha | rs192993943 | . | . | . | . | . | . | . | . | . | . | . |
| logdha | rs182989043 | 0.001 | . | . | . | . | . | . | . | . | . | . |
| logdha | rs189703435 | 0.001 | . | . | . | . | . | . | . | . | . | . |
| logdha | rs188794040 | 0 | . | . | . | . | . | . | . | . | . | . |
| logdha | rs143920953 | . | . | . | . | . | . | . | . | . | . | . |
| logdha | rs191029121 | . | . | . | . | . | . | . | . | . | . | . |
| logdha | rs116875448 | . | . | . | . | . | . | . | . | . | . | . |
| logdha | rs190167877 | 0.003 | . | . | . | . | . | . | . | . | . | . |
| logdha | rs184993418 | . | . | . | . | . | . | . | . | . | . | . |
| logdha | rs143241158 | . | . | . | . | . | . | . | . | . | . | . |
| logdha | rs11239500 | 0.074 | 0.403 | 0.174 | 0.036 | 0.188 | 0.28 | 0.262 | 2.81 | 2.85 | 0.307 | 0.622 |

| logdha | rs140568994 | . | . | . | . | . | . | . | . | . | . | . |
| --- | --- | --- | --- | --- | --- | --- | --- | --- | --- | --- | --- | --- |
| logdha | rs187530317 | 0.001 | . | . | . | . | . | . | . | . | . | . |
| logdha | rs145189326 | 0.001 | . | . | . | . | . | . | . | . | . | . |
| logdha | rs186203332 | . | . | . | . | . | . | . | . | . | . | . |
| logdha | rs189921909 | . | . | . | . | . | . | . | . | . | . | . |
| logdha | rs187260989 | . | . | . | . | . | . | . | . | . | . | . |
| logdha | rs114376586 | 0.073 | 0.404 | 0.175 | 0.036 | 0.191 | 0.284 | 0.265 | 2.78 | 2.8 | 0.311 | 0.619 |
| logdha | rs151070808 | . | . | . | . | . | . | . | . | . | . | . |
| logdha | rs140927225 | 0.074 | 0.405 | 0.175 | 0.037 | 0.19 | 0.284 | 0.267 | 2.76 | 2.81 | 0.318 | 0.618 |
| logdha | rs191555615 | . | . | . | . | . | . | . | . | . | . | . |
| logdha | rs80278898 | 0.001 | . | . | . | . | . | . | . | . | . | . |
| logdha | rs190849503 | . | . | . | . | . | . | . | . | . | . | . |
| logdha | rs150080315 | 0.007 | . | . | . | . | . | . | . | . | . | . |
| logdha | rs143623885 | . | . | . | . | . | . | . | . | . | . | . |
| logdha | rs187760040 | . | . | . | . | . | . | . | . | . | . | . |
| logdha | rs143917143 | . | . | . | . | . | . | . | . | . | . | . |
| logdha | rs149851136 | 0.002 | . | . | . | . | . | . | . | . | . | . |
| logdha | rs181612054 | . | . | . | . | . | . | . | . | . | . | . |
| logdha | rs191006548 | . | . | . | . | . | . | . | . | . | . | . |
| logdha | rs117377631 | 0.003 | . | . | . | . | . | . | . | . | . | . |
| logdha | rs182107634 | . | . | . | . | . | . | . | . | . | . | . |
| logdha | rs190211867 | . | . | . | . | . | . | . | . | . | . | . |
| logdha | rs148293457 | . | . | . | . | . | . | . | . | . | . | . |
| logdha | rs191811733 | . | . | . | . | . | . | . | . | . | . | . |
| logdha | rs146109272 | . | . | . | . | . | . | . | . | . | . | . |
| logdha | rs148397915 | . | . | . | . | . | . | . | . | . | . | . |
| logdha | rs142233290 | . | . | . | . | . | . | . | . | . | . | . |
| logdha | rs80226874 | . | . | . | . | . | . | . | . | . | . | . |
| logdha | rs144496203 | 0 | . | . | . | . | . | . | . | . | . | . |
| logdha | rs191062130 | . | . | . | . | . | . | . | . | . | . | . |
| logdha | rs185807817 | . | . | . | . | . | . | . | . | . | . | . |
| logdha | rs117846213 | 0.001 | . | . | . | . | . | . | . | . | . | . |
| logdha | rs190545473 | . | . | . | . | . | . | . | . | . | . | . |
| logdha | rs192364918 | . | . | . | . | . | . | . | . | . | . | . |
| logdha | rs182939241 | . | . | . | . | . | . | . | . | . | . | . |
| logdha | rs12571125 | 0.001 | . | . | . | . | . | . | . | . | . | . |
| logdha | rs192418941 | 0 | . | . | . | . | . | . | . | . | . | . |
| logdha | rs141270243 | . | . | . | . | . | . | . | . | . | . | . |
| logdha | rs182615502 | . | . | . | . | . | . | . | . | . | . | . |
| logdha | rs146669856 | 0.02 | 0.485 | 0.203 | 0.084 | 7.15 | 21 | 0.503 | 0.113 | 0.255 | 0.334 | -6.25 |
| logdha | rs78290029 | 0.098 | 0.319 | 0.144 | 0.011 | 0.087 | 0.123 | 0.085 | 5.93 | 5.83 | 0.07 | 0.759 |
| logdha | rs139955565 | . | . | . | . | . | . | . | . | . | . | . |
| logdha | rs12267314 | 0.087 | 0.346 | 0.153 | 0.016 | 0.136 | 0.195 | 0.165 | 4.59 | 4.64 | 0.131 | 0.734 |
| logdha | rs74477197 | . | . | . | . | . | . | . | . | . | . | . |
| logdha | rs188147236 | . | . | . | . | . | . | . | . | . | . | . |
| logdha | rs2228065 | 0.086 | 0.345 | 0.152 | 0.016 | 0.131 | 0.189 | 0.159 | 4.71 | 4.76 | 0.126 | 0.737 |
| logdha | rs116961353 | . | . | . | . | . | . | . | . | . | . | . |
| logdha | rs190222272 | . | . | . | . | . | . | . | . | . | . | . |
| logdha | rs182719940 | . | . | . | . | . | . | . | . | . | . | . |
| logdha | rs184477413 | . | . | . | . | . | . | . | . | . | . | . |
| logdha | rs12265175 | 0.089 | 0.341 | 0.151 | 0.015 | 0.118 | 0.167 | 0.131 | 4.8 | 4.73 | 0.111 | 0.734 |
| logdha | rs182928276 | . | . | . | . | . | . | . | . | . | . | . |
| logdha | rs144409895 | . | . | . | . | . | . | . | . | . | . | . |
| logdha | rs58998447 | 0.003 | . | . | . | . | . | . | . | . | . | . |
| logdha | rs188668832 | . | . | . | . | . | . | . | . | . | . | . |
| logdha | rs148641686 | . | . | . | . | . | . | . | . | . | . | . |
| logdha | rs75468791 | . | . | . | . | . | . | . | . | . | . | . |
| logdha | rs147327397 | . | . | . | . | . | . | . | . | . | . | . |
| logdha | rs77354597 | 0.002 | . | . | . | . | . | . | . | . | . | . |
| logdha | rs41365544 | 0.082 | 0.408 | 0.174 | 0.035 | 0.476 | 0.723 | 0.625 | 1.96 | 2.16 | 0.54 | 0.497 |
| logdha | rs187564188 | . | . | . | . | . | . | . | . | . | . | . |
| logdha | rs183511984 | 0 | . | . | . | . | . | . | . | . | . | . |
| logdha | rs12262783 | 0.08 | 0.414 | 0.176 | 0.038 | 0.523 | 0.801 | 0.672 | 1.83 | 2.03 | 0.587 | 0.458 |
| logdha | rs188817687 | 0.002 | . | . | . | . | . | . | . | . | . | . |
| logdha | rs3815968 | . | . | . | . | . | . | . | . | . | . | . |
| logdha | rs187822300 | . | . | . | . | . | . | . | . | . | . | . |
| logdha | rs188902172 | 0.002 | . | . | . | . | . | . | . | . | . | . |
| logdha | rs181190448 | . | . | . | . | . | . | . | . | . | . | . |
| logdha | rs182699093 | . | . | . | . | . | . | . | . | . | . | . |
| logdha | rs182631303 | 0.001 | . | . | . | . | . | . | . | . | . | . |
| logdha | rs187014172 | . | . | . | . | . | . | . | . | . | . | . |
| logdha | rs142575406 | . | . | . | . | . | . | . | . | . | . | . |
| logdha | rs180711033 | . | . | . | . | . | . | . | . | . | . | . |
| logdha | rs137894823 | 0.004 | . | . | . | . | . | . | . | . | . | . |
| logdha | rs189053273 | 0.001 | . | . | . | . | . | . | . | . | . | . |
| logdha | rs143499388 | 0.002 | . | . | . | . | . | . | . | . | . | . |
| logdha | rs182936001 | 0.006 | . | . | . | . | . | . | . | . | . | . |
| logdha | rs148456406 | 0.006 | . | . | . | . | . | . | . | . | . | . |
| logdha | rs182499531 | 0.008 | . | . | . | . | . | . | . | . | . | . |
| logdha | rs74884915 | 0.026 | 0.485 | 0.209 | 0.094 | 6.8 | 9.6 | 0.174 | 0.051 | 0.049 | 2.00E-03 | -6.12 |
| logdha | rs184046374 | 0.001 | . | . | . | . | . | . | . | . | . | . |
| logdha | rs74333993 | 0.032 | 0.401 | 0.17 | 0.031 | 0.086 | 0.188 | 0.262 | 6.16 | 8.7 | 0.198 | 0.726 |
| logdha | rs138889719 | 0.001 | . | . | . | . | . | . | . | . | . | . |
| logdha | rs75330330 | 0.022 | 0.44 | 0.183 | 0.049 | 0.078 | 0.261 | 0.446 | 5.4 | 12.3 | 0.457 | 0.667 |
| logdha | rs193051693 | 0 | . | . | . | . | . | . | . | . | . | . |
| logdha | rs145929633 | 0 | . | . | . | . | . | . | . | . | . | . |

| logdha | rs184076904 | 0.004 | . | . | . | . | . | . | . | . | . | . |
| --- | --- | --- | --- | --- | --- | --- | --- | --- | --- | --- | --- | --- |
| logdha | rs141802494 | 0.018 | 0.457 | 0.19 | 0.059 | 0.288 | 1.49 | 0.81 | 1.41 | 5.26 | 0.926 | 0.441 |
| logdha | rs149519759 | 0.002 | . | . | . | . | . | . | . | . | . | . |
| logdha | rs145467260 | 0.013 | 0.432 | 0.181 | 0.045 | 0.016 | 0.046 | 0.16 | 17.7 | 33.1 | 0.123 | 0.672 |
| logdha | rs79620914 | 0.027 | 0.409 | 0.173 | 0.035 | 0.03 | 0.06 | 0.079 | 13 | 16.7 | 0.046 | 0.721 |
| logdha | rs140538633 | 0.017 | 0.49 | 0.209 | 0.094 | 89.3 | 161 | 0.013 | 9.30E-03 | 0.012 | 4.80E-04 | -88.4 |
| logdha | rs140862301 | 0.008 | . | . | . | . | . | . | . | . | . | . |
| logdha | rs114615847 | 0.009 | . | . | . | . | . | . | . | . | . | . |
| logdha | rs141800259 | 0.026 | 0.433 | 0.183 | 0.048 | 0.072 | 0.243 | 0.434 | 5.2 | 11.9 | 0.471 | 0.657 |
| logdha | rs149779059 | 0.019 | 0.458 | 0.191 | 0.061 | 1.64 | 4.67 | 0.862 | 1.12 | 2.37 | 0.956 | -0.254 |
| logdha | rs116539253 | 0.007 | . | . | . | . | . | . | . | . | . | . |
| logdha | rs184979550 | 0.001 | . | . | . | . | . | . | . | . | . | . |
| logdha | rs190128940 | 0.004 | . | . | . | . | . | . | . | . | . | . |
| logdha | rs146594816 | 0.002 | . | . | . | . | . | . | . | . | . | . |
| logdha | rs141370766 | 0.022 | 0.44 | 0.183 | 0.049 | 0.081 | 0.269 | 0.448 | 5.29 | 11.9 | 0.458 | 0.667 |
| logdha | rs144670033 | 0.002 | . | . | . | . | . | . | . | . | . | . |
| logdha | rs187152174 | 0.007 | . | . | . | . | . | . | . | . | . | . |
| logdha | rs184180263 | 0.005 | . | . | . | . | . | . | . | . | . | . |
| logdha | rs140075861 | 0.01 | 0.474 | 0.199 | 0.076 | 3.50E-32 | 2.90E-30 | 0.372 | 5.60E+12 | 2.10E+14 | 0.432 | 0.526 |
| logdha | rs186483129 | 0 | . | . | . | . | . | . | . | . | . | . |
| logdha | rs186634282 | 0.012 | 0.476 | 0.198 | 0.074 | 31.3 | 62.1 | 0.083 | 0.024 | 0.03 | 2.80E-03 | -30.4 |
| logdha | rs9988663 | 0.032 | 0.401 | 0.17 | 0.031 | 0.084 | 0.185 | 0.259 | 6.24 | 8.83 | 0.196 | 0.726 |
| logdha | rs138828529 | 0.02 | 0.564 | 0.237 | 0.173 | 18.8 | 35.9 | 0.124 | 0.192 | 0.331 | 0.339 | -16.3 |
| logdha | rs188777615 | 0.004 | . | . | . | . | . | . | . | . | . | . |
| logdha | rs192321882 | 0.008 | . | . | . | . | . | . | . | . | . | . |
| logdha | rs150812270 | 0.027 | 0.498 | 0.222 | 0.117 | 4.48 | 6.37 | 0.291 | 0.297 | 0.283 | 0.202 | -3.32 |
| logdha | rs185906603 | 0.001 | . | . | . | . | . | . | . | . | . | . |
| logdha | rs191070862 | 0.004 | . | . | . | . | . | . | . | . | . | . |
| logdha | rs182904978 | 0 | . | . | . | . | . | . | . | . | . | . |
| logdha | rs192679655 | 0.001 | . | . | . | . | . | . | . | . | . | . |
| logdha | rs189673086 | 0.007 | . | . | . | . | . | . | . | . | . | . |
| logdha | rs181141892 | 0.002 | . | . | . | . | . | . | . | . | . | . |
| logdha | rs186404123 | 0 | . | . | . | . | . | . | . | . | . | . |
| logdha | rs192117287 | 0.005 | . | . | . | . | . | . | . | . | . | . |
| logdha | rs182748835 | 0.001 | . | . | . | . | . | . | . | . | . | . |
| logdha | rs141854549 | 0.017 | 0.49 | 0.209 | 0.094 | 92.8 | 166 | 0.011 | 9.10E-03 | 0.012 | 4.80E-04 | -91.9 |
| logdha | rs140163397 | 0.005 | . | . | . | . | . | . | . | . | . | . |
| logdha | rs146347300 | 0.008 | . | . | . | . | . | . | . | . | . | . |
| logdha | rs139362377 | 0.017 | 0.49 | 0.209 | 0.094 | 93.3 | 167 | 0.011 | 9.10E-03 | 0.012 | 4.80E-04 | -92.4 |
| logdha | rs185087671 | 0.001 | . | . | . | . | . | . | . | . | . | . |
| logdha | rs190466574 | 0.005 | . | . | . | . | . | . | . | . | . | . |
| logdha | rs147012778 | 0.005 | . | . | . | . | . | . | . | . | . | . |
| logdha | rs138236960 | 0.009 | . | . | . | . | . | . | . | . | . | . |
| logdha | rs116310311 | 0.017 | 0.49 | 0.209 | 0.094 | 93.6 | 167 | 0.011 | 9.00E-03 | 0.012 | 4.90E-04 | -92.7 |
| logdha | rs142868356 | 0.012 | 0.471 | 0.193 | 0.066 | 3.78 | 22.4 | 0.823 | 0.463 | 2.08 | 0.864 | -2.43 |
| logdha | rs144956240 | 0.003 | . | . | . | . | . | . | . | . | . | . |
| logdha | rs188527776 | 0.002 | . | . | . | . | . | . | . | . | . | . |
| logdha | rs183971165 | 0.007 | . | . | . | . | . | . | . | . | . | . |
| logdha | rs76748216 | 0.009 | . | . | . | . | . | . | . | . | . | . |
| logdha | rs148554990 | 0.001 | . | . | . | . | . | . | . | . | . | . |
| logdha | rs114446477 | 0.027 | 0.411 | 0.174 | 0.036 | 0.032 | 0.065 | 0.092 | 12.3 | 16.4 | 0.058 | 0.718 |
| logdha | rs185223933 | 0.006 | . | . | . | . | . | . | . | . | . | . |
| logdha | rs76869726 | 0.009 | . | . | . | . | . | . | . | . | . | . |
| logdha | rs151200830 | 0.017 | 0.489 | 0.208 | 0.092 | 107 | 192 | 8.80E-03 | 7.40E-03 | 0.011 | 6.70E-04 | -106 |
| logdha | rs17157731 | 0.017 | 0.489 | 0.208 | 0.092 | 108 | 192 | 8.70E-03 | 7.40E-03 | 0.011 | 6.70E-04 | -107 |
| logdha | rs181243469 | 0 | . | . | . | . | . | . | . | . | . | . |
| logdha | rs186890707 | 0 | . | . | . | . | . | . | . | . | . | . |
| logdha | rs184063360 | 0 | . | . | . | . | . | . | . | . | . | . |
| logdha | rs188550237 | 0.007 | . | . | . | . | . | . | . | . | . | . |
| logdha | rs140931068 | 0.017 | 0.444 | 0.186 | 0.053 | 0.596 | 2.51 | 0.902 | 2.71 | 8.41 | 0.749 | 0.676 |
| logdha | rs74867877 | 0.007 | . | . | . | . | . | . | . | . | . | . |
| logdha | rs11239495 | 0.055 | 0.381 | 0.16 | 0.022 | 0.077 | 0.142 | 0.163 | 6.2 | 7.55 | 0.134 | 0.724 |
| logdha | rs149745800 | 0 | . | . | . | . | . | . | . | . | . | . |
| logdha | rs145044458 | 0.015 | 0.488 | 0.208 | 0.092 | 108 | 190 | 8.00E-03 | 7.50E-03 | 0.011 | 9.60E-04 | -107 |
| logdha | rs188067427 | 0.002 | . | . | . | . | . | . | . | . | . | . |
| logdha | rs146827800 | 0.001 | . | . | . | . | . | . | . | . | . | . |
| logdha | rs140619817 | 0.015 | 0.496 | 0.213 | 0.102 | 152 | 271 | 4.70E-03 | 5.30E-03 | 8.90E-03 | 1.80E-03 | -151 |
| logdha | rs892689 | 0.997 | . | . | . | . | . | . | . | . | . | . |
| logdha | rs143642005 | 0.008 | . | . | . | . | . | . | . | . | . | . |
| logdha | rs181929304 | 0.007 | . | . | . | . | . | . | . | . | . | . |
| logdha | rs191086173 | 0.002 | . | . | . | . | . | . | . | . | . | . |
| logdha | rs114613116 | 0.018 | 0.501 | 0.216 | 0.109 | 143 | 253 | 4.90E-03 | 5.60E-03 | 8.30E-03 | 4.70E-04 | -142 |
| logdha | rs141076768 | 0.002 | . | . | . | . | . | . | . | . | . | . |
| logdha | rs187316531 | 0 | . | . | . | . | . | . | . | . | . | . |
| logdha | rs11239499 | 0.075 | 0.403 | 0.175 | 0.036 | 0.183 | 0.279 | 0.265 | 2.85 | 2.96 | 0.314 | 0.624 |
| logdha | rs192726371 | 0.003 | . | . | . | . | . | . | . | . | . | . |
| logdha | rs74128481 | 0.019 | 0.488 | 0.203 | 0.085 | 51.8 | 88.1 | 0.02 | 0.018 | 0.021 | 6.90E-04 | -50.8 |
| logdha | rs74128482 | 0.012 | 0.477 | 0.198 | 0.075 | 50.4 | 107 | 0.066 | 0.017 | 0.024 | 3.40E-03 | -49.5 |
| logdha | rs11239502 | 0.055 | 0.377 | 0.158 | 0.02 | 0.063 | 0.122 | 0.151 | 6.77 | 8.56 | 0.13 | 0.721 |
| logdha | rs116330285 | 0.002 | . | . | . | . | . | . | . | . | . | . |
| logdha | rs189926729 | 0.003 | . | . | . | . | . | . | . | . | . | . |
| logdha | rs188975779 | 0.012 | 0.472 | 0.193 | 0.067 | 3.88 | 22.9 | 0.818 | 0.454 | 2.04 | 0.86 | -2.52 |
| logdha | rs143846038 | 0.002 | . | . | . | . | . | . | . | . | . | . |
| logdha | rs74128483 | 0.019 | 0.488 | 0.204 | 0.086 | 51 | 86 | 0.02 | 0.019 | 0.022 | 6.80E-04 | -50 |
| logdha | rs139292055 | 0.073 | 0.404 | 0.175 | 0.036 | 0.192 | 0.285 | 0.266 | 2.77 | 2.8 | 0.312 | 0.619 |
| logdha | rs11239503 | 0.073 | 0.404 | 0.175 | 0.036 | 0.191 | 0.284 | 0.266 | 2.77 | 2.8 | 0.312 | 0.619 |

| logdha | rs58838686 | 0.012 | 0.477 | 0.198 | 0.075 | 47.2 | 99 | 0.066 | 0.018 | 0.025 | 3.30E-03 | -46.3 |
| --- | --- | --- | --- | --- | --- | --- | --- | --- | --- | --- | --- | --- |
| logdha | rs185825373 | 0.008 | . | . | . | . | . | . | . | . | . | . |
| logdha | rs11239504 | 0.073 | 0.404 | 0.175 | 0.036 | 0.191 | 0.284 | 0.266 | 2.77 | 2.8 | 0.312 | 0.619 |
| logdha | rs150012354 | 0.006 | . | . | . | . | . | . | . | . | . | . |
| logdha | rs187763096 | 0.01 | . | . | . | . | . | . | . | . | . | . |
| logdha | rs193043982 | 0.008 | . | . | . | . | . | . | . | . | . | . |
| logdha | rs79186884 | 0.023 | 0.466 | 0.194 | 0.067 | 1.97 | 4.6 | 0.771 | 0.878 | 1.5 | 0.939 | -0.631 |
| logdha | rs74128484 | 0.012 | 0.477 | 0.198 | 0.075 | 46 | 96 | 0.066 | 0.019 | 0.025 | 3.20E-03 | -45.1 |
| logdha | rs58461537 | 0.073 | 0.404 | 0.175 | 0.036 | 0.191 | 0.284 | 0.265 | 2.78 | 2.8 | 0.311 | 0.619 |
| logdha | rs188527474 | 0.008 | . | . | . | . | . | . | . | . | . | . |
| logdha | rs141659976 | 0.008 | . | . | . | . | . | . | . | . | . | . |
| logdha | rs115846407 | 0.073 | 0.404 | 0.175 | 0.036 | 0.191 | 0.284 | 0.265 | 2.78 | 2.8 | 0.311 | 0.619 |
| logdha | rs138378816 | 0.073 | 0.404 | 0.175 | 0.036 | 0.191 | 0.284 | 0.265 | 2.78 | 2.8 | 0.311 | 0.619 |
| logdha | rs146188136 | 0.055 | 0.377 | 0.158 | 0.02 | 0.063 | 0.121 | 0.15 | 6.81 | 8.62 | 0.13 | 0.721 |
| logdha | rs139383871 | 0.014 | 0.49 | 0.207 | 0.091 | 5.01 | 11.3 | 0.475 | 0.547 | 1.03 | 0.748 | -3.16 |
| logdha | rs182953869 | 0.004 | . | . | . | . | . | . | . | . | . | . |
| logdha | rs192430299 | 0.019 | 0.481 | 0.197 | 0.074 | 4.23 | 20.7 | 0.768 | 0.329 | 1.21 | 0.763 | -3.04 |
| logdha | rs144152844 | 0.073 | 0.404 | 0.175 | 0.036 | 0.191 | 0.283 | 0.265 | 2.78 | 2.8 | 0.311 | 0.619 |
| logdha | rs145987786 | 0.051 | 0.438 | 0.188 | 0.054 | 0.345 | 0.741 | 0.62 | 1.97 | 2.86 | 0.64 | 0.515 |
| logdha | rs181132926 | 0.003 | . | . | . | . | . | . | . | . | . | . |
| logdha | rs147882253 | 0.073 | 0.404 | 0.175 | 0.036 | 0.191 | 0.283 | 0.265 | 2.78 | 2.8 | 0.31 | 0.619 |
| logdha | rs141807621 | 0.023 | 0.56 | 0.235 | 0.168 | 15.6 | 29.2 | 0.141 | 0.173 | 0.295 | 0.304 | -13.7 |
| logdha | rs147116052 | 0.073 | 0.404 | 0.175 | 0.036 | 0.191 | 0.283 | 0.265 | 2.78 | 2.8 | 0.31 | 0.619 |
| logdha | rs147716618 | 0.073 | 0.404 | 0.175 | 0.036 | 0.191 | 0.283 | 0.265 | 2.78 | 2.8 | 0.31 | 0.619 |
| logdha | rs187198855 | 0.046 | 0.443 | 0.19 | 0.058 | 0.349 | 0.741 | 0.62 | 1.82 | 2.6 | 0.675 | 0.49 |
| logdha | rs189409638 | 0.016 | 0.509 | 0.217 | 0.114 | 158 | 280 | 4.20E-03 | 9.80E-03 | 0.013 | 7.80E-04 | -157 |
| logdha | rs182647339 | 0.001 | . | . | . | . | . | . | . | . | . | . |
| logdha | rs187995988 | 0.004 | . | . | . | . | . | . | . | . | . | . |
| logdha | rs141034184 | 0.079 | 0.395 | 0.173 | 0.034 | 0.149 | 0.226 | 0.208 | 3.1 | 3.16 | 0.266 | 0.639 |
| logdha | rs187310430 | 0.008 | . | . | . | . | . | . | . | . | . | . |
| logdha | rs192608488 | 0.073 | 0.405 | 0.175 | 0.036 | 0.193 | 0.285 | 0.266 | 2.75 | 2.76 | 0.312 | 0.617 |
| logdha | rs148840366 | 0.009 | . | . | . | . | . | . | . | . | . | . |
| logdha | rs186785022 | 0.019 | 0.489 | 0.204 | 0.087 | 46.9 | 76.6 | 0.018 | 0.021 | 0.024 | 6.50E-04 | -46 |
| logdha | rs192348728 | 0.014 | 0.463 | 0.207 | 0.085 | 2.31 | 2.96 | 0.514 | 1.07 | 0.894 | 0.933 | -0.625 |
| logdha | rs189656631 | 0.073 | 0.405 | 0.175 | 0.036 | 0.193 | 0.285 | 0.266 | 2.75 | 2.76 | 0.312 | 0.617 |
| logdha | rs185289750 | 0.004 | . | . | . | . | . | . | . | . | . | . |
| logdha | rs142382395 | 0.073 | 0.405 | 0.175 | 0.036 | 0.193 | 0.285 | 0.265 | 2.76 | 2.76 | 0.312 | 0.618 |
| logdha | rs144866696 | 0.019 | 0.49 | 0.204 | 0.087 | 46.7 | 76.1 | 0.018 | 0.021 | 0.024 | 6.50E-04 | -45.7 |
| logdha | rs141371289 | 0.073 | 0.405 | 0.175 | 0.036 | 0.193 | 0.285 | 0.266 | 2.75 | 2.76 | 0.312 | 0.617 |
| logdha | rs114750008 | 0.073 | 0.405 | 0.175 | 0.036 | 0.193 | 0.285 | 0.266 | 2.75 | 2.76 | 0.312 | 0.617 |
| logdha | rs189063597 | 0.001 | . | . | . | . | . | . | . | . | . | . |
| logdha | rs139151571 | 0.067 | 0.393 | 0.169 | 0.03 | 0.123 | 0.2 | 0.197 | 3.7 | 4.04 | 0.232 | 0.663 |
| logdha | rs139846368 | 0.075 | 0.404 | 0.175 | 0.036 | 0.183 | 0.274 | 0.257 | 2.79 | 2.82 | 0.311 | 0.619 |
| logdha | rs182057653 | 0.019 | 0.498 | 0.215 | 0.106 | 13 | 20.5 | 0.102 | 0.057 | 0.057 | 4.60E-03 | -12.2 |
| logdha | rs148785420 | 0.073 | 0.405 | 0.175 | 0.036 | 0.193 | 0.285 | 0.265 | 2.76 | 2.76 | 0.312 | 0.618 |
| logdha | rs138383251 | 0.011 | 0.483 | 0.206 | 0.088 | 5.50E-27 | 2.90E-25 | 0.241 | 3.00E+09 | 7.50E+10 | 0.372 | 0.517 |
| logdha | rs116526705 | 0.018 | 0.502 | 0.217 | 0.111 | 116 | 216 | 0.011 | 7.10E-03 | 9.60E-03 | 2.70E-04 | -115 |
| logdha | rs185946436 | 0 | . | . | . | . | . | . | . | . | . | . |
| logdha | rs190455427 | 0.004 | . | . | . | . | . | . | . | . | . | . |
| logdha | rs9282584 | 0.073 | 0.405 | 0.175 | 0.036 | 0.193 | 0.285 | 0.265 | 2.76 | 2.76 | 0.312 | 0.618 |
| logdha | rs17153289 | 0.073 | 0.405 | 0.175 | 0.036 | 0.193 | 0.285 | 0.265 | 2.76 | 2.76 | 0.312 | 0.618 |
| logdha | rs138359103 | 0.008 | . | . | . | . | . | . | . | . | . | . |
| logdha | rs150605268 | 0.003 | . | . | . | . | . | . | . | . | . | . |
| logdha | rs79242910 | 0.075 | 0.403 | 0.174 | 0.036 | 0.182 | 0.272 | 0.255 | 2.82 | 2.85 | 0.304 | 0.622 |
| logdha | rs139716904 | 0.021 | 0.499 | 0.209 | 0.097 | 28.2 | 52.9 | 0.075 | 0.041 | 0.059 | 0.026 | -27.2 |
| logdha | rs76238498 | 0.053 | 0.421 | 0.179 | 0.042 | 0.212 | 0.466 | 0.48 | 2.88 | 4.26 | 0.475 | 0.624 |
| logdha | rs184140639 | 0 | . | . | . | . | . | . | . | . | . | . |
| logdha | rs188517278 | 0.009 | . | . | . | . | . | . | . | . | . | . |
| logdha | rs139678913 | 0.004 | . | . | . | . | . | . | . | . | . | . |
| logdha | rs116697955 | 0.004 | . | . | . | . | . | . | . | . | . | . |
| logdha | rs79267994 | 0.012 | 0.477 | 0.198 | 0.075 | 39.1 | 78.5 | 0.068 | 0.022 | 0.028 | 3.00E-03 | -38.2 |
| logdha | rs11239510 | 0.022 | 0.464 | 0.202 | 0.078 | 1.21 | 2.3 | 0.919 | 0.954 | 1.3 | 0.972 | -0.14 |
| logdha | rs75831645 | 0.018 | 0.502 | 0.217 | 0.111 | 117 | 219 | 0.01 | 7.00E-03 | 9.50E-03 | 2.80E-04 | -117 |
| logdha | rs78452276 | 0.052 | 0.429 | 0.194 | 0.062 | 0.296 | 0.543 | 0.507 | 2.29 | 2.86 | 0.507 | 0.566 |
| logdha | rs144366924 | 0 | . | . | . | . | . | . | . | . | . | . |
| logdha | rs77519841 | 0.056 | 0.377 | 0.158 | 0.02 | 0.062 | 0.119 | 0.149 | 6.84 | 8.67 | 0.129 | 0.721 |
| logdha | rs76944454 | 0.073 | 0.405 | 0.175 | 0.037 | 0.194 | 0.286 | 0.266 | 2.74 | 2.75 | 0.313 | 0.617 |
| logdha | rs76443855 | 0.053 | 0.42 | 0.179 | 0.042 | 0.21 | 0.463 | 0.479 | 2.9 | 4.3 | 0.473 | 0.626 |
| logdha | rs146613174 | 0.002 | . | . | . | . | . | . | . | . | . | . |
| logdha | rs181424761 | 0.002 | . | . | . | . | . | . | . | . | . | . |
| logdha | rs142119241 | 0.001 | . | . | . | . | . | . | . | . | . | . |
| logdha | rs140137954 | 0.018 | 0.502 | 0.217 | 0.111 | 115 | 215 | 0.011 | 7.10E-03 | 9.70E-03 | 2.70E-04 | -114 |
| logdha | rs187319549 | 0.002 | . | . | . | . | . | . | . | . | . | . |
| logdha | rs12241545 | 0.035 | 0.458 | 0.201 | 0.075 | 0.403 | 0.666 | 0.583 | 1.33 | 1.54 | 0.805 | 0.384 |
| logdha | rs10128228 | 0.073 | 0.405 | 0.175 | 0.037 | 0.194 | 0.286 | 0.266 | 2.74 | 2.74 | 0.313 | 0.616 |
| logdha | rs58580249 | 0.009 | . | . | . | . | . | . | . | . | . | . |
| logdha | rs193148863 | 0.001 | . | . | . | . | . | . | . | . | . | . |
| logdha | rs185638946 | 0.005 | . | . | . | . | . | . | . | . | . | . |
| logdha | rs117752921 | 0.001 | . | . | . | . | . | . | . | . | . | . |
| logdha | rs180841236 | 0.002 | . | . | . | . | . | . | . | . | . | . |
| logdha | rs73283155 | 0.009 | . | . | . | . | . | . | . | . | . | . |
| logdha | rs76560599 | 0.061 | 0.39 | 0.169 | 0.029 | 0.087 | 0.147 | 0.149 | 4.55 | 5.11 | 0.177 | 0.677 |
| logdha | rs76273620 | 0.061 | 0.39 | 0.169 | 0.029 | 0.087 | 0.147 | 0.149 | 4.55 | 5.11 | 0.177 | 0.677 |
| logdha | rs141432923 | 0.02 | 0.478 | 0.201 | 0.08 | 1.26 | 3.79 | 0.94 | 0.255 | 0.551 | 0.527 | -0.58 |
| logdha | rs113529322 | 0.019 | 0.49 | 0.205 | 0.088 | 45.4 | 72.8 | 0.017 | 0.022 | 0.025 | 6.40E-04 | -44.4 |
| logdha | rs10128306 | 0.073 | 0.405 | 0.175 | 0.037 | 0.194 | 0.287 | 0.267 | 2.74 | 2.74 | 0.314 | 0.616 |

| logdha | rs149691688 | 0.002 | . | . | . | . | . | . | . | . | . | . |
| --- | --- | --- | --- | --- | --- | --- | --- | --- | --- | --- | --- | --- |
| logdha | rs75872495 | 0.073 | 0.405 | 0.175 | 0.037 | 0.197 | 0.291 | 0.271 | 2.72 | 2.71 | 0.317 | 0.614 |
| logdha | rs191873960 | 0.007 | . | . | . | . | . | . | . | . | . | . |
| logdha | rs80155622 | 0.073 | 0.406 | 0.175 | 0.037 | 0.198 | 0.292 | 0.272 | 2.71 | 2.71 | 0.318 | 0.614 |
| logdha | rs113914656 | 0.008 | . | . | . | . | . | . | . | . | . | . |
| logdha | rs12242840 | 0.073 | 0.407 | 0.176 | 0.038 | 0.208 | 0.305 | 0.284 | 2.64 | 2.62 | 0.327 | 0.609 |
| logdha | rs147824078 | 0.009 | . | . | . | . | . | . | . | . | . | . |
| logdha | rs59051493 | 0.073 | 0.407 | 0.176 | 0.038 | 0.214 | 0.31 | 0.287 | 2.62 | 2.57 | 0.328 | 0.607 |
| logdha | rs9282588 | 0.008 | . | . | . | . | . | . | . | . | . | . |
| logdha | rs150215207 | 0.009 | . | . | . | . | . | . | . | . | . | . |
| logdha | rs138772243 | 0.003 | . | . | . | . | . | . | . | . | . | . |
| logdha | rs149383244 | 0.025 | 0.458 | 0.209 | 0.087 | 1.29 | 1.73 | 0.85 | 1.2 | 1.1 | 0.842 | -0.039 |
| logdha | rs17522720 | 0.016 | 0.462 | 0.206 | 0.084 | 2.06 | 2.81 | 0.598 | 1.11 | 1 | 0.907 | -0.463 |
| logdha | rs79245105 | 0.073 | 0.406 | 0.176 | 0.038 | 0.213 | 0.308 | 0.285 | 2.63 | 2.59 | 0.324 | 0.609 |
| logdha | rs138222133 | 0.004 | . | . | . | . | . | . | . | . | . | . |
| logdha | rs12247314 | 0.082 | 0.412 | 0.181 | 0.044 | 0.289 | 0.389 | 0.356 | 2.21 | 2.06 | 0.394 | 0.563 |
| logdha | rs12263894 | 0.08 | 0.412 | 0.182 | 0.044 | 0.302 | 0.406 | 0.373 | 2.18 | 2.03 | 0.401 | 0.558 |
| logdha | rs149907880 | 0.008 | . | . | . | . | . | . | . | . | . | . |
| logdha | rs188153885 | 0.007 | . | . | . | . | . | . | . | . | . | . |
| logdha | rs73283159 | 0.005 | . | . | . | . | . | . | . | . | . | . |
| logdha | rs140476687 | 0.002 | . | . | . | . | . | . | . | . | . | . |
| logdha | rs186603148 | 0 | . | . | . | . | . | . | . | . | . | . |
| logdha | rs183296063 | 0.003 | . | . | . | . | . | . | . | . | . | . |
| logdha | rs145733694 | 0.009 | . | . | . | . | . | . | . | . | . | . |
| logdha | rs189807449 | 0 | . | . | . | . | . | . | . | . | . | . |
| logdha | rs12253223 | 0.064 | 0.382 | 0.163 | 0.025 | 0.117 | 0.197 | 0.204 | 4.71 | 5.37 | 0.174 | 0.711 |
| logdha | rs182113637 | 0 | . | . | . | . | . | . | . | . | . | . |
| logdha | rs142792099 | 0.018 | 0.462 | 0.192 | 0.063 | 0.481 | 2.2 | 0.873 | 1.06 | 3.54 | 0.985 | 0.294 |
| logdha | rs12249910 | 0.064 | 0.382 | 0.163 | 0.025 | 0.117 | 0.197 | 0.204 | 4.71 | 5.36 | 0.174 | 0.711 |
| logdha | rs149436339 | 0.001 | . | . | . | . | . | . | . | . | . | . |
| logdha | rs77632210 | 0 | . | . | . | . | . | . | . | . | . | . |
| logdha | rs74130818 | 0.012 | 0.476 | 0.197 | 0.073 | 42.8 | 82 | 0.05 | 0.017 | 0.022 | 2.00E-03 | -42 |
| logdha | rs187583648 | 0 | . | . | . | . | . | . | . | . | . | . |
| logdha | rs191295987 | 0.008 | . | . | . | . | . | . | . | . | . | . |
| logdha | rs184200686 | 0.01 | . | . | . | . | . | . | . | . | . | . |
| logdha | rs76117263 | 0.052 | 0.423 | 0.181 | 0.044 | 0.23 | 0.52 | 0.516 | 2.74 | 4.16 | 0.508 | 0.613 |
| logdha | rs185393149 | 0.001 | . | . | . | . | . | . | . | . | . | . |
| logdha | rs144760014 | 0.006 | . | . | . | . | . | . | . | . | . | . |
| logdha | rs7922292 | 0.008 | . | . | . | . | . | . | . | . | . | . |
| logdha | rs115372844 | 0.009 | . | . | . | . | . | . | . | . | . | . |
| logdha | rs111460364 | 0.052 | 0.423 | 0.181 | 0.045 | 0.234 | 0.532 | 0.523 | 2.71 | 4.13 | 0.514 | 0.611 |
| logdha | rs182052255 | 0.002 | . | . | . | . | . | . | . | . | . | . |
| logdha | rs180983889 | 0.006 | . | . | . | . | . | . | . | . | . | . |
| logdha | rs139175327 | 0.009 | . | . | . | . | . | . | . | . | . | . |
| logdha | rs149959620 | 0.018 | 0.492 | 0.205 | 0.089 | 26.7 | 51.7 | 0.09 | 0.047 | 0.066 | 0.03 | -25.6 |
| logdha | rs114653117 | 0.009 | . | . | . | . | . | . | . | . | . | . |
| logdha | rs144270018 | 0.005 | . | . | . | . | . | . | . | . | . | . |
| logdha | rs182723204 | 0.007 | . | . | . | . | . | . | . | . | . | . |
| logdha | rs57204067 | 0.012 | 0.475 | 0.197 | 0.072 | 41.3 | 77.6 | 0.048 | 0.015 | 0.021 | 1.90E-03 | -40.5 |
| logdha | rs144956226 | 0.064 | 0.379 | 0.163 | 0.024 | 0.117 | 0.195 | 0.198 | 4.8 | 5.39 | 0.163 | 0.717 |
| logdha | rs187842704 | 0.019 | 0.459 | 0.193 | 0.063 | 3.60E-03 | 0.011 | 0.063 | 2.91 | 5.51 | 0.573 | 0.542 |
| logdha | rs184620511 | 0.013 | 0.432 | 0.183 | 0.048 | 6.20E-05 | 3.50E-04 | 0.081 | 170 | 493 | 0.077 | 0.572 |
| logdha | rs144031303 | 0.019 | 0.5 | 0.217 | 0.11 | 15.3 | 39 | 0.286 | 0.056 | 0.09 | 0.074 | -14.3 |
| logdha | rs187526473 | 0 | . | . | . | . | . | . | . | . | . | . |
| logdha | rs193002805 | 0.002 | . | . | . | . | . | . | . | . | . | . |
| logdha | rs138707426 | 0.024 | 0.459 | 0.209 | 0.088 | 1.37 | 1.82 | 0.81 | 1.17 | 1.06 | 0.86 | -0.094 |
| logdha | rs142754243 | 0.049 | 0.338 | 0.141 | 9.50E-03 | 0.01 | 0.019 | 0.017 | 22.2 | 28 | 0.014 | 0.728 |
| logdha | rs181600331 | 0.008 | . | . | . | . | . | . | . | . | . | . |
| logdha | rs144421264 | 0.029 | 0.356 | 0.144 | 0.011 | 5.40E-04 | 1.50E-03 | 7.20E-03 | 114 | 202 | 7.40E-03 | 0.665 |
| logdha | rs184414519 | 0.006 | . | . | . | . | . | . | . | . | . | . |
| logdha | rs150596349 | 0.048 | 0.414 | 0.175 | 0.037 | 0.192 | 0.431 | 0.463 | 3.31 | 4.95 | 0.423 | 0.657 |
| logdha | rs190753747 | 0.001 | . | . | . | . | . | . | . | . | . | . |
| logdha | rs139560940 | 0.004 | . | . | . | . | . | . | . | . | . | . |
| logdha | rs75640804 | 0.057 | 0.369 | 0.156 | 0.019 | 0.066 | 0.123 | 0.142 | 6.98 | 8.41 | 0.107 | 0.736 |
| logdha | rs73283175 | 0.082 | 0.382 | 0.168 | 0.028 | 0.129 | 0.182 | 0.146 | 3.58 | 3.47 | 0.188 | 0.665 |
| logdha | rs143337229 | 0.003 | . | . | . | . | . | . | . | . | . | . |
| logdha | rs73283177 | 0.048 | 0.34 | 0.142 | 9.80E-03 | 9.30E-03 | 0.018 | 0.013 | 23.1 | 28.5 | 0.011 | 0.724 |
| logdha | rs76053611 | 0.045 | 0.439 | 0.184 | 0.05 | 0.228 | 0.569 | 0.554 | 2.39 | 3.88 | 0.592 | 0.572 |
| logdha | rs116325983 | 0.004 | . | . | . | . | . | . | . | . | . | . |
| logdha | rs115451825 | 0.008 | . | . | . | . | . | . | . | . | . | . |
| logdha | rs183845246 | 0.002 | . | . | . | . | . | . | . | . | . | . |
| logdha | rs79756542 | 0.051 | 0.42 | 0.177 | 0.04 | 0.264 | 0.594 | 0.554 | 2.68 | 4.01 | 0.51 | 0.613 |
| logdha | rs187242860 | 0.002 | . | . | . | . | . | . | . | . | . | . |
| logdha | rs57318980 | 0.048 | 0.346 | 0.145 | 0.011 | 8.60E-03 | 0.015 | 7.60E-03 | 23 | 27 | 7.30E-03 | 0.714 |
| logdha | rs59969732 | 0.043 | 0.358 | 0.147 | 0.013 | 3.10E-03 | 6.30E-03 | 4.50E-03 | 36.9 | 48.4 | 5.90E-03 | 0.679 |
| logdha | rs143811488 | 0.009 | . | . | . | . | . | . | . | . | . | . |
| logdha | rs189067644 | 0.01 | . | . | . | . | . | . | . | . | . | . |
| logdha | rs60449178 | 0.038 | 0.362 | 0.148 | 0.013 | 5.00E-04 | 1.30E-03 | 2.80E-03 | 107 | 173 | 3.80E-03 | 0.657 |
| logdha | rs59243389 | 0.038 | 0.362 | 0.148 | 0.013 | 5.00E-04 | 1.30E-03 | 2.80E-03 | 107 | 173 | 3.80E-03 | 0.657 |
| logdha | rs184946540 | 0.043 | 0.418 | 0.172 | 0.034 | 0.016 | 0.035 | 0.056 | 9.85 | 12.7 | 0.075 | 0.633 |
| logdha | rs188685881 | 0.001 | . | . | . | . | . | . | . | . | . | . |
| logdha | rs150335797 | 0.006 | . | . | . | . | . | . | . | . | . | . |
| logdha | rs143879260 | 0.006 | . | . | . | . | . | . | . | . | . | . |
| logdha | rs186044491 | 0.009 | . | . | . | . | . | . | . | . | . | . |
| logdha | rs11819084 | 0.03 | 0.414 | 0.184 | 0.047 | 0.331 | 0.683 | 0.592 | 3.38 | 4.71 | 0.383 | 0.717 |
| logdha | rs146915670 | 0.006 | . | . | . | . | . | . | . | . | . | . |

| logdha | rs113995695 | 0.029 | 0.411 | 0.182 | 0.045 | 0.301 | 0.644 | 0.575 | 3.61 | 5.22 | 0.375 | 0.735 |
| --- | --- | --- | --- | --- | --- | --- | --- | --- | --- | --- | --- | --- |
| logdha | rs143489451 | 0.005 | . | . | . | . | . | . | . | . | . | . |
| logdha | rs182653839 | 0 | . | . | . | . | . | . | . | . | . | . |
| logdha | rs141692564 | 0.007 | . | . | . | . | . | . | . | . | . | . |
| logdha | rs146281742 | 0.005 | . | . | . | . | . | . | . | . | . | . |
| logdha | rs192914353 | 0.005 | . | . | . | . | . | . | . | . | . | . |
| logdha | rs185182848 | 0.003 | . | . | . | . | . | . | . | . | . | . |
| logdha | rs28395858 | 0.029 | 0.409 | 0.181 | 0.044 | 0.282 | 0.614 | 0.561 | 3.81 | 5.61 | 0.365 | 0.748 |
| logdha | rs41429645 | 0.001 | . | . | . | . | . | . | . | . | . | . |
| logdha | rs28395874 | 0.029 | 0.408 | 0.181 | 0.043 | 0.28 | 0.61 | 0.559 | 3.83 | 5.66 | 0.363 | 0.75 |
| logdha | rs189663634 | 0.001 | . | . | . | . | . | . | . | . | . | . |
| logdha | rs77666700 | 0.01 | . | . | . | . | . | . | . | . | . | . |
| logdha | rs146711717 | 0.003 | . | . | . | . | . | . | . | . | . | . |
| logdha | rs144904251 | 0.001 | . | . | . | . | . | . | . | . | . | . |
| logdha | rs189893153 | 0.001 | . | . | . | . | . | . | . | . | . | . |
| logdha | rs150447740 | 0.009 | . | . | . | . | . | . | . | . | . | . |
| logdha | rs143099649 | 0.001 | . | . | . | . | . | . | . | . | . | . |
| logdha | rs191288436 | 0.01 | 0.547 | 0.232 | 0.155 | 118 | 173 | 1.10E-03 | 0.027 | 0.034 | 4.10E-03 | -116 |
| logdha | rs188577862 | 0.006 | . | . | . | . | . | . | . | . | . | . |
| logdha | rs192695334 | 0.006 | . | . | . | . | . | . | . | . | . | . |
| logdha | rs138612906 | 0.036 | 0.415 | 0.184 | 0.047 | 0.379 | 0.609 | 0.546 | 2.58 | 2.73 | 0.371 | 0.611 |
| logdha | rs191290051 | 0.037 | 0.417 | 0.172 | 0.033 | 0.025 | 0.078 | 0.233 | 10.3 | 20.2 | 0.238 | 0.666 |
| logdha | rs189631459 | 0.001 | . | . | . | . | . | . | . | . | . | . |
| logdha | rs114620874 | 0.007 | . | . | . | . | . | . | . | . | . | . |
| logdha | rs78246652 | 0.023 | 0.448 | 0.189 | 0.057 | 0.237 | 0.566 | 0.547 | 2.53 | 4.18 | 0.574 | 0.584 |
| logdha | rs79432451 | 0.007 | . | . | . | . | . | . | . | . | . | . |
| logdha | rs186022585 | 0.001 | . | . | . | . | . | . | . | . | . | . |
| logdha | rs142978891 | 0.009 | . | . | . | . | . | . | . | . | . | . |
| logdha | rs151086840 | 0.011 | 0.463 | 0.193 | 0.065 | 0.699 | 1.21 | 0.836 | 0.383 | 0.486 | 0.45 | -0.038 |
| logdha | rs183270259 | 0.01 | . | . | . | . | . | . | . | . | . | . |
| logdha | rs188544289 | 0.01 | . | . | . | . | . | . | . | . | . | . |
| logdha | rs188500808 | 0.001 | . | . | . | . | . | . | . | . | . | . |
| logdha | rs189424589 | 0.003 | . | . | . | . | . | . | . | . | . | . |
| logdha | rs78622303 | 0.04 | 0.357 | 0.151 | 0.015 | 1.50E-03 | 3.30E-03 | 3.10E-03 | 54.1 | 73.1 | 3.10E-03 | 0.67 |
| logdha | rs143210835 | 0.013 | 0.431 | 0.183 | 0.048 | 3.80E-03 | 0.013 | 0.095 | 30.2 | 60.4 | 0.088 | 0.615 |
| logdha | rs114761840 | 0.007 | . | . | . | . | . | . | . | . | . | . |
| logdha | rs185805402 | 0.003 | . | . | . | . | . | . | . | . | . | . |
| logdha | rs139273744 | 0.002 | . | . | . | . | . | . | . | . | . | . |
| logdha | rs184654150 | 0.006 | . | . | . | . | . | . | . | . | . | . |
| logdha | rs189069710 | 0 | . | . | . | . | . | . | . | . | . | . |
| logdha | rs114752115 | 0.031 | 0.462 | 0.196 | 0.068 | 0.779 | 1.68 | 0.908 | 0.999 | 1.57 | 1 | 0.119 |
| logdha | rs150162515 | 0.002 | . | . | . | . | . | . | . | . | . | . |
| logdha | rs180982454 | 0.008 | . | . | . | . | . | . | . | . | . | . |
| logdha | rs184932088 | 0.001 | . | . | . | . | . | . | . | . | . | . |
| logdha | rs181109625 | 0 | . | . | . | . | . | . | . | . | . | . |
| logdha | rs138121134 | 0.001 | . | . | . | . | . | . | . | . | . | . |
| logdha | rs183253861 | 0.004 | . | . | . | . | . | . | . | . | . | . |
| logdha | rs147073946 | 0.001 | . | . | . | . | . | . | . | . | . | . |
| logdha | rs139938489 | 0.008 | . | . | . | . | . | . | . | . | . | . |
| logdha | rs146362560 | 0.013 | 0.517 | 0.223 | 0.126 | 312 | 755 | 0.017 | 7.80E-03 | 0.019 | 0.05 | -311 |
| logdha | rs193155956 | 0.007 | . | . | . | . | . | . | . | . | . | . |
| logdha | rs77663092 | 0.007 | . | . | . | . | . | . | . | . | . | . |
| logdha | rs73285319 | 0.033 | 0.424 | 0.189 | 0.054 | 0.55 | 0.913 | 0.719 | 2.48 | 2.85 | 0.429 | 0.604 |
| logdha | rs184019276 | 0.007 | . | . | . | . | . | . | . | . | . | . |
| logdha | rs28395863 | 0.027 | 0.419 | 0.184 | 0.047 | 0.506 | 1.21 | 0.776 | 2.63 | 4.28 | 0.552 | 0.633 |
| logdha | rs182121645 | 0.017 | 0.451 | 0.199 | 0.072 | 0.949 | 1.13 | 0.965 | 1.4 | 1.04 | 0.651 | 0.199 |
| logdha | rs73285327 | 0.025 | 0.43 | 0.187 | 0.053 | 0.228 | 0.445 | 0.448 | 3.75 | 4.93 | 0.314 | 0.71 |
| logdha | rs149589584 | 0.001 | . | . | . | . | . | . | . | . | . | . |
| logdha | rs148310335 | 0.005 | . | . | . | . | . | . | . | . | . | . |
| logdha | rs187246093 | 0.01 | 0.479 | 0.2 | 0.078 | 20.2 | 63.7 | 0.341 | 0.04 | 0.085 | 0.132 | -19.3 |
| logdha | rs184668894 | 0 | . | . | . | . | . | . | . | . | . | . |
| logdha | rs114269961 | 0.001 | . | . | . | . | . | . | . | . | . | . |
| logdha | rs149694374 | 0.034 | 0.42 | 0.184 | 0.047 | 0.321 | 0.701 | 0.603 | 3.01 | 4.49 | 0.46 | 0.665 |
| logdha | rs145284227 | 0.004 | . | . | . | . | . | . | . | . | . | . |
| logdha | rs189983820 | 0.004 | . | . | . | . | . | . | . | . | . | . |
| logdha | rs181200444 | 0 | . | . | . | . | . | . | . | . | . | . |
| logdha | rs185486385 | 0 | . | . | . | . | . | . | . | . | . | . |
| logdha | rs151198241 | 0.01 | 0.462 | 0.193 | 0.065 | 0.71 | 1.22 | 0.842 | 0.77 | 0.869 | 0.817 | 0.081 |
| logdha | rs189959341 | 0.01 | 0.462 | 0.193 | 0.065 | 0.71 | 1.22 | 0.842 | 0.77 | 0.869 | 0.817 | 0.081 |
| logdha | rs140549257 | 0.007 | . | . | . | . | . | . | . | . | . | . |
| logdha | rs186453139 | 0.027 | 0.423 | 0.188 | 0.053 | 0.631 | 1.01 | 0.775 | 2.55 | 2.85 | 0.404 | 0.626 |
| logdha | rs180922711 | 0.044 | 0.397 | 0.172 | 0.033 | 0.193 | 0.337 | 0.346 | 4.29 | 4.76 | 0.189 | 0.739 |
| logdha | rs56659903 | 0.005 | . | . | . | . | . | . | . | . | . | . |
| logdha | rs141202128 | 0.006 | . | . | . | . | . | . | . | . | . | . |
| logdha | rs59085314 | 0.056 | 0.428 | 0.183 | 0.048 | 0.02 | 0.054 | 0.153 | 9.25 | 16.3 | 0.208 | 0.63 |
| logdha | rs145564996 | 0.004 | . | . | . | . | . | . | . | . | . | . |
| logdha | rs114961009 | 0.01 | 0.462 | 0.194 | 0.065 | 0.76 | 1.3 | 0.873 | 0.773 | 0.87 | 0.819 | 0.049 |
| logdha | rs182294189 | 0.004 | . | . | . | . | . | . | . | . | . | . |
| logdha | rs187586636 | 0.008 | . | . | . | . | . | . | . | . | . | . |
| logdha | rs192254907 | 0.002 | . | . | . | . | . | . | . | . | . | . |
| logdha | rs149145663 | 0.001 | . | . | . | . | . | . | . | . | . | . |
| logdha | rs180713064 | 0.001 | . | . | . | . | . | . | . | . | . | . |
| logdha | rs183009023 | 0.007 | . | . | . | . | . | . | . | . | . | . |
| logdha | rs143385347 | 0.017 | 0.451 | 0.199 | 0.071 | 0.936 | 1.12 | 0.956 | 1.42 | 1.06 | 0.633 | 0.214 |
| logdha | rs185786329 | 0.007 | . | . | . | . | . | . | . | . | . | . |
| logdha | rs192341030 | 0.004 | . | . | . | . | . | . | . | . | . | . |

| logdha | rs74661429 | 0.017 | 0.455 | 0.19 | 0.06 | 4.10E-03 | 0.01 | 0.027 | 8.03 | 12.9 | 0.196 | 0.556 |
| --- | --- | --- | --- | --- | --- | --- | --- | --- | --- | --- | --- | --- |
| logdha | rs188034853 | 0.002 | . | . | . | . | . | . | . | . | . | . |
| logdha | rs116808430 | 0.027 | 0.43 | 0.191 | 0.058 | 0.891 | 1.43 | 0.943 | 2.07 | 2.35 | 0.52 | 0.474 |
| logdha | rs148469010 | 0 | . | . | . | . | . | . | . | . | . | . |
| logdha | rs78621663 | 0.01 | . | . | . | . | . | . | . | . | . | . |
| logdha | rs192654886 | 0.001 | . | . | . | . | . | . | . | . | . | . |
| logdha | rs145996035 | 0.008 | . | . | . | . | . | . | . | . | . | . |
| logdha | rs139942226 | 0.001 | . | . | . | . | . | . | . | . | . | . |
| logdha | rs113645632 | 0.01 | 0.528 | 0.218 | 0.122 | 372 | 896 | 0.014 | 4.40E-03 | 0.013 | 0.059 | -371 |
| logdha | rs74940661 | 0.027 | 0.428 | 0.187 | 0.052 | 0.44 | 1 | 0.719 | 2.65 | 4.15 | 0.532 | 0.632 |
| logdha | rs115355800 | 0.022 | 0.448 | 0.188 | 0.056 | 0.2 | 0.525 | 0.54 | 2.56 | 4.7 | 0.607 | 0.582 |
| logdha | rs150285898 | 0.008 | . | . | . | . | . | . | . | . | . | . |
| logdha | rs188117932 | 0.004 | . | . | . | . | . | . | . | . | . | . |
| logdha | rs139956566 | . | . | . | . | . | . | . | . | . | . | . |
| logdha | rs147673885 | . | . | . | . | . | . | . | . | . | . | . |
| logdha | rs189127928 | . | . | . | . | . | . | . | . | . | . | . |
| logdha | rs187558772 | . | . | . | . | . | . | . | . | . | . | . |
| logdha | rs181948587 | . | . | . | . | . | . | . | . | . | . | . |
| logdha | rs189983269 | . | . | . | . | . | . | . | . | . | . | . |
| logdha | rs187250069 | . | . | . | . | . | . | . | . | . | . | . |
| logdha | rs191485859 | . | . | . | . | . | . | . | . | . | . | . |
| logdha | rs185499555 | . | . | . | . | . | . | . | . | . | . | . |
| logdha | rs180862110 | . | . | . | . | . | . | . | . | . | . | . |
| logdha | rs190472603 | . | . | . | . | . | . | . | . | . | . | . |
| logdha | rs189226167 | . | . | . | . | . | . | . | . | . | . | . |
| logdha | rs181623256 | . | . | . | . | . | . | . | . | . | . | . |
| logdha | rs186079557 | . | . | . | . | . | . | . | . | . | . | . |
| logdha | rs147760994 | . | . | . | . | . | . | . | . | . | . | . |
| logdha | rs189176167 | . | . | . | . | . | . | . | . | . | . | . |
| logdha | rs117901000 | . | . | . | . | . | . | . | . | . | . | . |
| logdha | rs141984888 | . | . | . | . | . | . | . | . | . | . | . |
| logdha | rs192365061 | . | . | . | . | . | . | . | . | . | . | . |
| logdha | rs185976038 | . | . | . | . | . | . | . | . | . | . | . |
| logdha | rs184697303 | . | . | . | . | . | . | . | . | . | . | . |
| logdha | rs143383132 | . | . | . | . | . | . | . | . | . | . | . |
| logdha | rs141873881 | . | . | . | . | . | . | . | . | . | . | . |
| logdha | rs185507255 | . | . | . | . | . | . | . | . | . | . | . |
| logdha | rs191379425 | . | . | . | . | . | . | . | . | . | . | . |
| logdha | rs184199584 | . | . | . | . | . | . | . | . | . | . | . |
| logdha | rs183181360 | . | . | . | . | . | . | . | . | . | . | . |
| logdha | rs184495673 | . | . | . | . | . | . | . | . | . | . | . |
| logdha | rs182946297 | . | . | . | . | . | . | . | . | . | . | . |
| logdha | rs187743615 | . | . | . | . | . | . | . | . | . | . | . |
| logdha | rs183259862 | . | . | . | . | . | . | . | . | . | . | . |
| logdha | rs112433566 | . | . | . | . | . | . | . | . | . | . | . |
| logdha | rs182351085 | . | . | . | . | . | . | . | . | . | . | . |
| logdha | rs146248508 | . | . | . | . | . | . | . | . | . | . | . |
| logdha | rs188528439 | . | . | . | . | . | . | . | . | . | . | . |
| logdha | rs146167018 | . | . | . | . | . | . | . | . | . | . | . |
| logdha | rs192440980 | . | . | . | . | . | . | . | . | . | . | . |
| logdha | rs185601882 | . | . | . | . | . | . | . | . | . | . | . |
| logdha | rs117272563 | . | . | . | . | . | . | . | . | . | . | . |
| logdha | rs10430734 | . | . | . | . | . | . | . | . | . | . | . |
| logdha | rs181320952 | . | . | . | . | . | . | . | . | . | . | . |
| logdha | rs185480660 | . | . | . | . | . | . | . | . | . | . | . |
| logdha | rs188077917 | . | . | . | . | . | . | . | . | . | . | . |
| logdha | rs187562953 | . | . | . | . | . | . | . | . | . | . | . |
| logdha | rs182044631 | . | . | . | . | . | . | . | . | . | . | . |
| logdha | rs145455596 | . | . | . | . | . | . | . | . | . | . | . |
| logdha | rs181475877 | . | . | . | . | . | . | . | . | . | . | . |
| logdha | rs149413085 | . | . | . | . | . | . | . | . | . | . | . |
| logdha | rs12253656 | . | . | . | . | . | . | . | . | . | . | . |
| logdha | rs182718239 | . | . | . | . | . | . | . | . | . | . | . |
| logdha | rs192435695 | . | . | . | . | . | . | . | . | . | . | . |
| logdha | rs12248277 | . | . | . | . | . | . | . | . | . | . | . |
| logdha | rs143521710 | . | . | . | . | . | . | . | . | . | . | . |
| logdha | rs144454454 | . | . | . | . | . | . | . | . | . | . | . |
| logdha | rs190441610 | . | . | . | . | . | . | . | . | . | . | . |
| logdha | rs114690462 | . | . | . | . | . | . | . | . | . | . | . |
| logdha | rs184541357 | . | . | . | . | . | . | . | . | . | . | . |
| logdha | rs142657653 | . | . | . | . | . | . | . | . | . | . | . |
| logdha | rsurf1 | 2.117 | . | . | . | . | . | . | . | . | . | . |
| logdha | rsten1 | 2.28 | . | . | . | . | . | . | . | . | . | . |
| logdha | chr10:45852312:D | 0.092 | 0.455 | 0.198 | 0.07 | 1.04 | 1.26 | 0.972 | 1.05 | 0.845 | 0.95 | 4.00E-04 |
| logdha | chr10:45853232:I | 0.108 | 0.469 | 0.204 | 0.081 | 0.992 | 1.32 | 0.995 | 0.965 | 0.86 | 0.968 | -0.012 |
| logdha | chr10:45853234:I | 0.198 | 0.569 | 0.282 | 0.255 | 1.78 | 1.62 | 0.524 | 0.574 | 0.369 | 0.388 | -0.77 |
| logdha | chr10:45854796:D | 0.003 | . | . | . | . | . | . | . | . | . | . |
| logdha | chr10:45854994:I | 0.025 | 0.434 | 0.182 | 0.046 | 0.071 | 0.201 | 0.35 | 6.44 | 12.2 | 0.326 | 0.694 |
| logdha | chr10:45858290:D | 0.052 | 0.527 | 0.245 | 0.169 | 6.05 | 9.92 | 0.272 | 0.323 | 0.385 | 0.343 | -4.55 |
| logdha | chr10:45858820:I | 0.022 | 0.53 | 0.229 | 0.142 | 44.5 | 121 | 0.162 | 0.048 | 0.122 | 0.232 | -42.9 |
| logdha | chr10:45860888:D | 0.122 | 0.443 | 0.199 | 0.069 | 0.795 | 1.06 | 0.863 | 1.19 | 1.06 | 0.845 | 0.181 |
| logdha | chr10:45863913:I | 0.179 | 0.509 | 0.249 | 0.168 | 1.28 | 1.16 | 0.786 | 0.774 | 0.487 | 0.684 | -0.285 |
| logdha | chr10:45866601:D | 0.111 | 0.45 | 0.211 | 0.088 | 0.568 | 0.733 | 0.661 | 1.22 | 1.13 | 0.832 | 0.293 |
| logdha | chr10:45867197:I | 0.002 | . | . | . | . | . | . | . | . | . | . |
| logdha | chr10:45867538:D | 0.182 | 0.512 | 0.249 | 0.168 | 1.3 | 1.14 | 0.767 | 0.767 | 0.467 | 0.663 | -0.3 |
| logdha | chr10:45868356:D | 0.18 | 0.511 | 0.25 | 0.169 | 1.33 | 1.16 | 0.743 | 0.77 | 0.462 | 0.663 | -0.317 |

| logdha | chr10:45871158:I | 0.182 | 0.513 | 0.251 | 0.172 | 1.33 | 1.16 | 0.74 | 0.76 | 0.458 | 0.649 | -0.326 |
| --- | --- | --- | --- | --- | --- | --- | --- | --- | --- | --- | --- | --- |
| logdha | chr10:45871637:D | 0.127 | 0.478 | 0.228 | 0.122 | 0.97 | 1.05 | 0.977 | 0.906 | 0.704 | 0.899 | -0.028 |
| logdha | chr10:45871638:I | 0.164 | 0.485 | 0.231 | 0.129 | 1.03 | 1.01 | 0.977 | 0.884 | 0.617 | 0.86 | -0.073 |
| logdha | chr10:45873629:I | 0.154 | 0.5 | 0.246 | 0.158 | 1.47 | 1.24 | 0.648 | 0.792 | 0.461 | 0.689 | -0.388 |
| logdha | chr10:45873701:D | 0.055 | 0.381 | 0.162 | 0.023 | 0.097 | 0.176 | 0.198 | 5.49 | 6.5 | 0.151 | 0.725 |
| logdha | chr10:45874997:I | 0.011 | 0.489 | 0.206 | 0.089 | 2.16 | 32.7 | 0.959 | 6.50E-04 | 8.10E-03 | 0.555 | -1.65 |
| logdha | chr10:45882030:I | 0.096 | 0.397 | 0.18 | 0.042 | 0.342 | 0.409 | 0.37 | 2.16 | 1.78 | 0.351 | 0.554 |
| logdha | chr10:45883774:D | 0.078 | 0.493 | 0.235 | 0.137 | 1.83 | 2.29 | 0.63 | 0.704 | 0.648 | 0.703 | -0.687 |
| logdha | chr10:45885169:D | 0.075 | 0.445 | 0.191 | 0.06 | 0.928 | 1.37 | 0.96 | 1.2 | 1.17 | 0.856 | 0.121 |
| logdha | chr10:45886451:D | 0.736 | 0.425 | 0.415 | 0.381 | 0.842 | 0.774 | 0.852 | 1.06 | 0.698 | 0.931 | 0.112 |
| logdha | chr10:45891800:I | 0.768 | 0.557 | 0.531 | 0.539 | 0.982 | 0.801 | 0.982 | 0.883 | 0.509 | 0.829 | -0.056 |
| logdha | chr10:45904771:I | 0.772 | 0.452 | 0.439 | 0.414 | 0.805 | 0.66 | 0.792 | 1.01 | 0.595 | 0.983 | 0.112 |
| logdha | chr10:45904959:I | 0.156 | 0.493 | 0.256 | 0.173 | 1.95 | 1.92 | 0.499 | 0.801 | 0.55 | 0.747 | -0.673 |
| logdha | chr10:45911028:I | 0.161 | 0.512 | 0.266 | 0.197 | 1.34 | 1.82 | 0.826 | 0.725 | 0.687 | 0.734 | -0.358 |
| logdha | chr10:45911031:I | 0.259 | 0.51 | 0.305 | 0.26 | 1.3 | 1.37 | 0.806 | 0.829 | 0.606 | 0.797 | -0.259 |
| logdha | chr10:45912154:D | 0.104 | 0.609 | 0.279 | 0.279 | 4.66 | 4.23 | 0.091 | 0.384 | 0.274 | 0.179 | -3.18 |
| logdha | chr10:45913885:I | 0.003 | . | . | . | . | . | . | . | . | . | . |
| logdha | chr10:45914753:D | 0.351 | 0.357 | 0.209 | 0.078 | 0.729 | 0.712 | 0.747 | 1.41 | 0.961 | 0.61 | 0.282 |
| logdha | chr10:45919621 | . | . | . | . | . | . | . | . | . | . | . |
| logdha | chr10:45921739:I | 0.178 | 0.44 | 0.209 | 0.084 | 0.858 | 1.03 | 0.898 | 1.17 | 0.998 | 0.857 | 0.143 |
| logdha | chr10:45923527:I | 0.066 | 0.669 | 0.304 | 0.376 | 55.2 | 78.4 | 4.70E-03 | 0.033 | 0.04 | 5.40E-03 | -53.7 |
| logdha | chr10:45927001:I | 0.04 | 0.44 | 0.185 | 0.051 | 0.133 | 0.336 | 0.425 | 3 | 5.08 | 0.518 | 0.602 |
| logdha | chr10:45930354:I | 0.025 | 0.444 | 0.189 | 0.056 | 0.248 | 0.493 | 0.483 | 2.87 | 3.91 | 0.438 | 0.625 |
| logdha | chr10:45932864:I | 0.043 | 0.625 | 0.296 | 0.322 | 49.7 | 67 | 3.80E-03 | 0.108 | 0.126 | 0.057 | -46 |
| logdha | chr10:45936163:I | 0.038 | 0.573 | 0.241 | 0.185 | 16.4 | 32.2 | 0.152 | 0.124 | 0.212 | 0.224 | -14.9 |
| logdha | chr10:45938095:D | 0.179 | 0.459 | 0.205 | 0.081 | 0.947 | 0.994 | 0.958 | 1.03 | 0.788 | 0.971 | 0.041 |
| logdha | chr10:45938886 | . | . | . | . | . | . | . | . | . | . | . |
| logdha | chr10:45939320 | 0.001 | . | . | . | . | . | . | . | . | . | . |
| logdha | chr10:45939659 | 0.003 | . | . | . | . | . | . | . | . | . | . |
| logdha | chr10:45941150:D | 0.008 | . | . | . | . | . | . | . | . | . | . |
| logdha | chr10:45947128:D | 0.067 | 0.386 | 0.181 | 0.042 | 0.387 | 0.42 | 0.382 | 2.84 | 2.25 | 0.188 | 0.651 |
| logdha | chr10:45950990:I | 0.039 | 0.396 | 0.174 | 0.035 | 0.105 | 0.188 | 0.209 | 6.48 | 7.28 | 0.096 | 0.768 |
| logdha | chr10:45951854:I | 0.012 | 0.469 | 0.202 | 0.08 | 5.25 | 10.3 | 0.4 | 0.477 | 0.571 | 0.536 | -3.54 |
| logdha | chr10:45952670:I | 0.061 | 0.526 | 0.227 | 0.136 | 7.52 | 15 | 0.311 | 0.186 | 0.296 | 0.291 | -6.31 |
| logdha | chr10:45956741 | . | . | . | . | . | . | . | . | . | . | . |
| logdha | chr10:45957247:I | 0.198 | 0.547 | 0.255 | 0.195 | 1.9 | 1.74 | 0.482 | 0.677 | 0.46 | 0.566 | -0.746 |
| logdha | chr10:45958082:I | 0.188 | 0.569 | 0.261 | 0.22 | 2.3 | 2.31 | 0.407 | 0.583 | 0.44 | 0.474 | -1.1 |
| logdha | chr10:45905041:D | 0.068 | 0.421 | 0.18 | 0.044 | 0.271 | 0.507 | 0.485 | 2.32 | 2.98 | 0.513 | 0.572 |
| logdha | chr10:45938871 | . | . | . | . | . | . | . | . | . | . | . |
| logdha | chr10:45939160 | . | . | . | . | . | . | . | . | . | . | . |
| logdha | chr10:45869910 | 0.015 | 0.475 | 0.198 | 0.075 | 351 | 2.90E+03 | 0.471 | 6.00E-06 | 6.80E-05 | 0.284 | -351 |
| logdha | chr10:45876214:D | 0.017 | 0.489 | 0.208 | 0.093 | 108 | 193 | 9.00E-03 | 7.40E-03 | 0.011 | 6.80E-04 | -107 |
| logdha | chr10:45880237:I | 0.094 | 0.531 | 0.244 | 0.168 | 1.82 | 1.95 | 0.577 | 0.425 | 0.332 | 0.273 | -0.94 |
| logdha | chr10:45884371:I | 0.073 | 0.404 | 0.175 | 0.036 | 0.191 | 0.284 | 0.265 | 2.78 | 2.8 | 0.311 | 0.619 |
| logdha | chr10:45891535:D | 0.019 | 0.49 | 0.205 | 0.087 | 45.8 | 74 | 0.018 | 0.022 | 0.024 | 6.40E-04 | -44.8 |
| logdha | chr10:45893516:D | 0.073 | 0.405 | 0.175 | 0.037 | 0.194 | 0.286 | 0.266 | 2.74 | 2.74 | 0.313 | 0.617 |
| logdha | chr10:45893782:D | 0.018 | 0.457 | 0.193 | 0.063 | 7.80E-03 | 0.032 | 0.242 | 0.991 | 3.17 | 0.998 | 0.539 |
| logdha | chr10:45895254:I | 0.019 | 0.49 | 0.205 | 0.088 | 45.3 | 72.6 | 0.017 | 0.022 | 0.025 | 6.40E-04 | -44.3 |
| logdha | chr10:45895848:D | 0.057 | 0.411 | 0.175 | 0.036 | 0.105 | 0.222 | 0.287 | 3.66 | 5.21 | 0.361 | 0.642 |
| logdha | chr10:45896316:D | 0.073 | 0.405 | 0.175 | 0.037 | 0.194 | 0.286 | 0.266 | 2.74 | 2.74 | 0.313 | 0.616 |
| logdha | chr10:45896317:D | 0.073 | 0.405 | 0.175 | 0.037 | 0.194 | 0.286 | 0.266 | 2.74 | 2.74 | 0.313 | 0.616 |
| logdha | chr10:45902683:I | 0.019 | 0.503 | 0.217 | 0.112 | 54.9 | 121 | 0.07 | 0.016 | 0.021 | 2.10E-03 | -54 |
| logdha | chr10:45905003:D | 0.01 | . | . | . | . | . | . | . | . | . | . |
| logdha | chr10:45927476:D | 0.036 | 0.414 | 0.183 | 0.046 | 0.266 | 0.541 | 0.515 | 3.37 | 4.68 | 0.381 | 0.691 |
| logdha | chr10:45935882 | 0.001 | . | . | . | . | . | . | . | . | . | . |
| logdha | chr10:45936100 | 0.001 | . | . | . | . | . | . | . | . | . | . |
| logdha | chr10:45939553 | 0.001 | . | . | . | . | . | . | . | . | . | . |
| logdha | chr10:45939631 | 0 | . | . | . | . | . | . | . | . | . | . |
| logdha | chr10:45944193:D | 0.012 | 0.455 | 0.191 | 0.061 | 6.90E-03 | 0.016 | 0.029 | 13.2 | 15.5 | 0.028 | 0.58 |
| logdha | chr10:45954030 | 0 | . | . | . | . | . | . | . | . | . | . |

Supplemental table S39.

| fa | rs_id | freq | HR_logdha | se_logdha | p_logdha | HR_snp | se_snp | p_snp | HR_inter | se_inter | p_inter | RERI |
| --- | --- | --- | --- | --- | --- | --- | --- | --- | --- | --- | --- | --- |
| logdha | rs76035182 | 0.016 | 1.03 | 0.306 | 0.923 | 32.4 | 42.9 | 8.50E-03 | 0.033 | 0.041 | 6.60E-03 | -31.4 |
| logdha | rs12359547 | 0.065 | 0.977 | 0.296 | 0.939 | 0.483 | 0.516 | 0.495 | 1.06 | 0.871 | 0.945 | 0.039 |
| logdha | rs74595484 | 0.017 | 1.04 | 0.324 | 0.902 | 8.22 | 10.6 | 0.102 | 0.187 | 0.146 | 0.032 | -6.66 |
| logdha | rs1864415 | 0.722 | 1.19 | 0.875 | 0.81 | 1.05 | 0.582 | 0.925 | 0.855 | 0.367 | 0.716 | -0.171 |
| logdha | rs72788101 | 0.068 | 0.977 | 0.294 | 0.937 | 0.44 | 0.482 | 0.454 | 1.08 | 0.916 | 0.928 | 0.048 |
| logdha | rs11239481 | 0.128 | 0.922 | 0.289 | 0.795 | 0.825 | 0.622 | 0.799 | 1.27 | 0.766 | 0.692 | 0.219 |
| logdha | rs72788102 | 0.068 | 0.977 | 0.294 | 0.938 | 0.44 | 0.483 | 0.454 | 1.08 | 0.916 | 0.928 | 0.047 |
| logdha | rs72789705 | 0.068 | 0.977 | 0.294 | 0.938 | 0.44 | 0.483 | 0.454 | 1.08 | 0.915 | 0.928 | 0.047 |
| logdha | rs141529858 | 0.005 | . | . | . | . | . | . | . | . | . | . |
| logdha | rs145376292 | 0 | . | . | . | . | . | . | . | . | . | . |
| logdha | rs190418301 | 0.001 | . | . | . | . | . | . | . | . | . | . |
| logdha | rs11239482 | 0.068 | 0.977 | 0.294 | 0.938 | 0.44 | 0.483 | 0.455 | 1.08 | 0.914 | 0.929 | 0.047 |
| logdha | rs61855872 | 0.011 | 1.02 | 0.304 | 0.942 | 3.63 | 12 | 0.696 | 0.132 | 0.35 | 0.444 | -3.16 |
| logdha | rs148991144 | 0.005 | . | . | . | . | . | . | . | . | . | . |
| logdha | rs191932221 | 0.005 | . | . | . | . | . | . | . | . | . | . |
| logdha | rs55978138 | 0.144 | 1.07 | 0.341 | 0.821 | 1.43 | 0.989 | 0.604 | 0.588 | 0.345 | 0.365 | -0.602 |
| logdha | rs73281167 | 0.01 | . | . | . | . | . | . | . | . | . | . |
| logdha | rs12768010 | 0.073 | 1.03 | 0.316 | 0.923 | 2.17 | 2.05 | 0.41 | 0.534 | 0.466 | 0.472 | -1.01 |
| logdha | rs11239484 | 0.722 | 1.19 | 0.876 | 0.809 | 1.05 | 0.583 | 0.923 | 0.855 | 0.367 | 0.715 | -0.172 |
| logdha | rs35906360 | 0.144 | 1.08 | 0.342 | 0.816 | 1.45 | 0.993 | 0.583 | 0.584 | 0.335 | 0.349 | -0.616 |
| logdha | rs186250512 | 0.004 | . | . | . | . | . | . | . | . | . | . |
| logdha | rs150810743 | 0.001 | . | . | . | . | . | . | . | . | . | . |
| logdha | rs55965812 | 0.14 | 1.07 | 0.344 | 0.821 | 1.49 | 1.01 | 0.557 | 0.591 | 0.344 | 0.367 | -0.618 |
| logdha | rs56180358 | 0.144 | 1.08 | 0.342 | 0.816 | 1.45 | 0.993 | 0.583 | 0.584 | 0.335 | 0.349 | -0.617 |
| logdha | rs55864145 | 0.073 | 1.03 | 0.316 | 0.923 | 2.18 | 2.05 | 0.409 | 0.533 | 0.465 | 0.471 | -1.01 |
| logdha | rs55986122 | 0.144 | 1.08 | 0.342 | 0.816 | 1.45 | 0.993 | 0.583 | 0.584 | 0.335 | 0.348 | -0.617 |
| logdha | rs7477350 | 0.722 | 1.19 | 0.877 | 0.808 | 1.05 | 0.583 | 0.923 | 0.855 | 0.367 | 0.714 | -0.173 |
| logdha | rs2099170 | 0.143 | 1.08 | 0.342 | 0.812 | 1.47 | 1.01 | 0.57 | 0.579 | 0.333 | 0.342 | -0.633 |
| logdha | rs2082108 | 0.144 | 1.08 | 0.342 | 0.814 | 1.46 | 0.995 | 0.582 | 0.582 | 0.334 | 0.346 | -0.62 |
| logdha | rs55902404 | 0.004 | . | . | . | . | . | . | . | . | . | . |
| logdha | rs58402662 | 0.003 | . | . | . | . | . | . | . | . | . | . |
| logdha | rs11511618 | 0.129 | 0.926 | 0.291 | 0.806 | 0.841 | 0.637 | 0.819 | 1.24 | 0.756 | 0.72 | 0.201 |
| logdha | rs1816492 | 0.721 | 1.2 | 0.881 | 0.804 | 1.06 | 0.587 | 0.916 | 0.852 | 0.366 | 0.71 | -0.176 |
| logdha | rs146611093 | 0.006 | . | . | . | . | . | . | . | . | . | . |
| logdha | rs186897689 | 0.001 | . | . | . | . | . | . | . | . | . | . |
| logdha | rs10793616 | 0.722 | 1.19 | 0.877 | 0.808 | 1.05 | 0.582 | 0.923 | 0.855 | 0.367 | 0.714 | -0.173 |
| logdha | rs116700479 | 0.013 | 1.01 | 0.298 | 0.98 | 10.4 | 17.8 | 0.174 | 0.049 | 0.063 | 0.02 | -9.85 |
| logdha | rs192535936 | . | . | . | . | . | . | . | . | . | . | . |
| logdha | rs113363297 | 0.654 | 1.23 | 0.904 | 0.779 | 1.18 | 0.677 | 0.769 | 0.838 | 0.381 | 0.697 | -0.194 |
| logdha | rs12354994 | 0.136 | 1.06 | 0.336 | 0.863 | 1.36 | 0.966 | 0.669 | 0.633 | 0.378 | 0.443 | -0.506 |
| logdha | rs148068329 | 0.025 | 1.02 | 0.306 | 0.937 | 9.55 | 9.6 | 0.025 | 0.087 | 0.089 | 0.017 | -8.72 |
| logdha | rs12356109 | 0.149 | 1.08 | 0.344 | 0.807 | 1.42 | 0.976 | 0.613 | 0.58 | 0.333 | 0.343 | -0.61 |
| logdha | rs55908141 | 0.003 | . | . | . | . | . | . | . | . | . | . |
| logdha | rs12354712 | 0.14 | 1.07 | 0.343 | 0.828 | 1.46 | 0.998 | 0.58 | 0.599 | 0.35 | 0.38 | -0.594 |
| logdha | rs147282684 | 0.003 | . | . | . | . | . | . | . | . | . | . |
| logdha | rs12356140 | 0.145 | 1.08 | 0.342 | 0.815 | 1.42 | 0.984 | 0.61 | 0.585 | 0.34 | 0.357 | -0.604 |
| logdha | rs185476187 | 0.001 | . | . | . | . | . | . | . | . | . | . |
| logdha | rs145720057 | 0.018 | 0.992 | 0.293 | 0.979 | 1.37 | 1.81 | 0.81 | 0.185 | 0.236 | 0.186 | -1.11 |
| logdha | rs12354742 | 0.144 | 1.07 | 0.341 | 0.822 | 1.43 | 0.98 | 0.606 | 0.592 | 0.341 | 0.362 | -0.593 |
| logdha | rs180913842 | 0.003 | . | . | . | . | . | . | . | . | . | . |
| logdha | rs142976914 | 0.002 | . | . | . | . | . | . | . | . | . | . |
| logdha | rs11511619 | 0.15 | 1.1 | 0.349 | 0.775 | 1.49 | 1.01 | 0.552 | 0.554 | 0.315 | 0.299 | -0.683 |
| logdha | rs146702870 | 0.002 | . | . | . | . | . | . | . | . | . | . |
| logdha | rs150287496 | . | . | . | . | . | . | . | . | . | . | . |
| logdha | rs190917973 | 0.004 | . | . | . | . | . | . | . | . | . | . |
| logdha | rs11517281 | 0.144 | 1.07 | 0.341 | 0.822 | 1.42 | 0.978 | 0.606 | 0.592 | 0.34 | 0.361 | -0.593 |
| logdha | rs12359299 | 0.144 | 1.07 | 0.341 | 0.821 | 1.42 | 0.978 | 0.606 | 0.592 | 0.34 | 0.361 | -0.594 |
| logdha | rs182307932 | 0.014 | 0.99 | 0.301 | 0.973 | 6.70E-03 | 0.024 | 0.169 | 0.339 | 1.18 | 0.755 | 5.80E-03 |
| logdha | rs61855873 | 0.026 | 0.997 | 0.299 | 0.992 | 0.641 | 1.25 | 0.82 | 0.598 | 0.951 | 0.746 | -0.256 |
| logdha | rs12359302 | 0.143 | 1.06 | 0.341 | 0.846 | 1.39 | 0.98 | 0.639 | 0.619 | 0.365 | 0.417 | -0.539 |
| logdha | rs143208140 | 0.027 | 1.03 | 0.307 | 0.919 | 11.5 | 11.7 | 0.016 | 0.066 | 0.07 | 0.011 | -10.8 |
| logdha | rs9988764 | 0.708 | 1.12 | 0.807 | 0.873 | 1 | 0.548 | 0.999 | 0.888 | 0.384 | 0.783 | -0.126 |
| logdha | rs192875767 | 0.003 | . | . | . | . | . | . | . | . | . | . |
| logdha | rs147268846 | 0.014 | 0.924 | 0.276 | 0.79 | 0.085 | 0.466 | 0.653 | 7.77 | 29 | 0.582 | 0.601 |
| logdha | rs11239485 | 0.094 | 1.07 | 0.34 | 0.836 | 2.01 | 1.67 | 0.398 | 0.505 | 0.361 | 0.339 | -0.995 |
| logdha | rs151317885 | 0.476 | 0.908 | 0.498 | 0.861 | 0.987 | 0.488 | 0.979 | 1.09 | 0.437 | 0.833 | 0.081 |
| logdha | rs11499085 | 0.055 | 0.974 | 0.311 | 0.935 | 0.759 | 0.756 | 0.782 | 1.11 | 0.72 | 0.872 | 0.088 |
| logdha | rs188130606 | 0.012 | 1.01 | 0.31 | 0.962 | 0.985 | 1.87 | 0.994 | 0.183 | 0.26 | 0.233 | -0.817 |
| logdha | rs11511620 | 0.141 | 1.07 | 0.343 | 0.827 | 1.46 | 0.996 | 0.58 | 0.599 | 0.349 | 0.379 | -0.594 |
| logdha | rs181498390 | 0.012 | 0.997 | 0.298 | 0.991 | 1.41 | 2.71 | 0.858 | 0.531 | 0.496 | 0.498 | -0.66 |
| logdha | rs186280701 | 0.003 | . | . | . | . | . | . | . | . | . | . |
| logdha | rs150059185 | 0.02 | 1.05 | 0.334 | 0.876 | 4.83 | 10.3 | 0.458 | 0.213 | 0.276 | 0.232 | -3.8 |
| logdha | rs183338707 | . | . | . | . | . | . | . | . | . | . | . |
| logdha | rs11499038 | 0.052 | 0.972 | 0.312 | 0.929 | 0.868 | 0.88 | 0.889 | 1.06 | 0.731 | 0.931 | 0.056 |
| logdha | rs190699595 | 0.004 | . | . | . | . | . | . | . | . | . | . |
| logdha | rs11523408 | 0.143 | 1.07 | 0.34 | 0.829 | 1.42 | 0.974 | 0.612 | 0.598 | 0.345 | 0.373 | -0.58 |
| logdha | rs188049317 | 0.001 | . | . | . | . | . | . | . | . | . | . |
| logdha | rs144948634 | 0.435 | 0.937 | 0.485 | 0.9 | 1.04 | 0.485 | 0.925 | 1.06 | 0.41 | 0.871 | 0.06 |
| logdha | rs143833576 | 0.001 | . | . | . | . | . | . | . | . | . | . |
| logdha | rs151239879 | 0.143 | 1.08 | 0.343 | 0.813 | 1.45 | 1 | 0.588 | 0.582 | 0.338 | 0.351 | -0.62 |
| logdha | rs7073743 | 0.456 | 0.861 | 0.453 | 0.775 | 0.939 | 0.434 | 0.892 | 1.15 | 0.439 | 0.719 | 0.128 |
| logdha | rs142563349 | 0.013 | 1.02 | 0.314 | 0.957 | 4.27 | 7.15 | 0.386 | 0.24 | 0.224 | 0.126 | -3.25 |
| logdha | rs187246403 | 0.003 | . | . | . | . | . | . | . | . | . | . |
| logdha | rs150423942 | 0.134 | 1.07 | 0.339 | 0.82 | 1.45 | 1.02 | 0.601 | 0.58 | 0.345 | 0.36 | -0.62 |

| logdha | rs184706102 | . | . | . | . | . | . | . | . | . | . | . |
| --- | --- | --- | --- | --- | --- | --- | --- | --- | --- | --- | --- | --- |
| logdha | rs189626288 | 0.014 | 0.985 | 0.313 | 0.963 | 1.14 | 1.34 | 0.912 | 0.859 | 0.775 | 0.867 | -0.16 |
| logdha | rs140749834 | 0.027 | 0.897 | 0.278 | 0.726 | 0.096 | 0.127 | 0.076 | 5.03 | 4.65 | 0.081 | 0.44 |
| logdha | rs7078822 | 0.115 | 1.05 | 0.331 | 0.885 | 1.42 | 1.14 | 0.658 | 0.625 | 0.435 | 0.499 | -0.54 |
| logdha | rs186227617 | 0.143 | 1.07 | 0.341 | 0.824 | 1.43 | 0.98 | 0.602 | 0.594 | 0.341 | 0.364 | -0.592 |
| logdha | rs188984368 | 0.022 | 1 | 0.303 | 0.989 | 1.72 | 2.56 | 0.715 | 0.36 | 0.505 | 0.466 | -1.1 |
| logdha | rs182078109 | 0.027 | 1.02 | 0.312 | 0.946 | 2.03 | 2.97 | 0.626 | 0.352 | 0.386 | 0.341 | -1.32 |
| logdha | rs141456274 | 0.006 | . | . | . | . | . | . | . | . | . | . |
| logdha | rs146976512 | 0.144 | 1.08 | 0.341 | 0.82 | 1.43 | 0.98 | 0.601 | 0.59 | 0.338 | 0.357 | -0.598 |
| logdha | rs138036816 | 0.05 | 0.97 | 0.311 | 0.925 | 0.863 | 0.879 | 0.885 | 1.07 | 0.736 | 0.919 | 0.065 |
| logdha | rs114879777 | 0.013 | 1.01 | 0.298 | 0.983 | 10.2 | 17.5 | 0.176 | 0.05 | 0.065 | 0.022 | -9.71 |
| logdha | rs149366475 | 0.052 | 0.97 | 0.31 | 0.924 | 0.914 | 0.904 | 0.927 | 1.05 | 0.681 | 0.941 | 0.046 |
| logdha | rs182463043 | 0.001 | . | . | . | . | . | . | . | . | . | . |
| logdha | rs144754632 | 0.025 | 1.02 | 0.306 | 0.936 | 9.56 | 9.6 | 0.025 | 0.087 | 0.089 | 0.017 | -8.74 |
| logdha | rs55717607 | 0.144 | 1.07 | 0.341 | 0.82 | 1.43 | 0.977 | 0.603 | 0.59 | 0.338 | 0.357 | -0.596 |
| logdha | rs187537973 | . | . | . | . | . | . | . | . | . | . | . |
| logdha | rs148085971 | 0.001 | . | . | . | . | . | . | . | . | . | . |
| logdha | rs11239491 | 0.144 | 1.08 | 0.341 | 0.82 | 1.43 | 0.977 | 0.603 | 0.59 | 0.337 | 0.357 | -0.597 |
| logdha | rs184412184 | 0.002 | . | . | . | . | . | . | . | . | . | . |
| logdha | rs4948998 | 0.733 | 1.29 | 0.994 | 0.745 | 1.1 | 0.621 | 0.871 | 0.816 | 0.371 | 0.654 | -0.232 |
| logdha | rs7076696 | 0.733 | 1.29 | 0.994 | 0.745 | 1.1 | 0.62 | 0.871 | 0.816 | 0.37 | 0.653 | -0.233 |
| logdha | rs11239492 | 0.149 | 1.08 | 0.344 | 0.805 | 1.42 | 0.974 | 0.609 | 0.579 | 0.33 | 0.338 | -0.613 |
| logdha | rs79462470 | 0.003 | . | . | . | . | . | . | . | . | . | . |
| logdha | rs187804654 | 0.004 | . | . | . | . | . | . | . | . | . | . |
| logdha | rs7093687 | 0.141 | 1.07 | 0.344 | 0.825 | 1.46 | 0.993 | 0.574 | 0.598 | 0.345 | 0.374 | -0.598 |
| logdha | rs7081004 | 0.141 | 1.07 | 0.343 | 0.825 | 1.46 | 0.994 | 0.577 | 0.598 | 0.346 | 0.374 | -0.597 |
| logdha | rs7084793 | 0.145 | 1.08 | 0.341 | 0.82 | 1.43 | 0.977 | 0.603 | 0.59 | 0.337 | 0.356 | -0.597 |
| logdha | rs61556080 | 0.003 | . | . | . | . | . | . | . | . | . | . |
| logdha | rs115552064 | 0.004 | . | . | . | . | . | . | . | . | . | . |
| logdha | rs7921503 | 0.144 | 1.07 | 0.341 | 0.82 | 1.43 | 0.976 | 0.604 | 0.591 | 0.338 | 0.357 | -0.595 |
| logdha | rs7909740 | 0.145 | 1.08 | 0.341 | 0.82 | 1.43 | 0.977 | 0.603 | 0.59 | 0.337 | 0.356 | -0.597 |
| logdha | rs7909768 | 0.145 | 1.08 | 0.341 | 0.82 | 1.43 | 0.977 | 0.603 | 0.59 | 0.337 | 0.356 | -0.597 |
| logdha | rs144168004 | . | . | . | . | . | . | . | . | . | . | . |
| logdha | rs75850448 | 0.003 | . | . | . | . | . | . | . | . | . | . |
| logdha | rs147777574 | 0.003 | . | . | . | . | . | . | . | . | . | . |
| logdha | rs7913488 | 0.145 | 1.08 | 0.341 | 0.82 | 1.43 | 0.976 | 0.603 | 0.59 | 0.337 | 0.356 | -0.596 |
| logdha | rs74390874 | 0.042 | 0.977 | 0.313 | 0.942 | 1.4 | 1.27 | 0.711 | 1.09 | 0.841 | 0.907 | 0.12 |
| logdha | rs141391142 | 0.004 | . | . | . | . | . | . | . | . | . | . |
| logdha | rs115294747 | 0.006 | . | . | . | . | . | . | . | . | . | . |
| logdha | rs148031781 | 0.001 | . | . | . | . | . | . | . | . | . | . |
| logdha | rs181340128 | 0.025 | 0.864 | 0.26 | 0.627 | 0.084 | 0.225 | 0.354 | 11.2 | 21.6 | 0.212 | 0.864 |
| logdha | rs189930007 | 0.001 | . | . | . | . | . | . | . | . | . | . |
| logdha | rs12242128 | 0.049 | 0.969 | 0.311 | 0.922 | 0.868 | 0.882 | 0.889 | 1.08 | 0.736 | 0.914 | 0.068 |
| logdha | rs78421750 | 0.003 | . | . | . | . | . | . | . | . | . | . |
| logdha | rs114370747 | 0.002 | . | . | . | . | . | . | . | . | . | . |
| logdha | rs12264166 | 0.049 | 0.969 | 0.311 | 0.922 | 0.87 | 0.884 | 0.891 | 1.08 | 0.736 | 0.915 | 0.068 |
| logdha | rs182964822 | 0.001 | . | . | . | . | . | . | . | . | . | . |
| logdha | rs67491417 | 0.139 | 1.06 | 0.334 | 0.845 | 1.26 | 0.974 | 0.765 | 0.588 | 0.375 | 0.405 | -0.535 |
| logdha | rs72789720 | 0.008 | . | . | . | . | . | . | . | . | . | . |
| logdha | rs6413416 | 0.145 | 1.08 | 0.341 | 0.818 | 1.43 | 0.978 | 0.599 | 0.589 | 0.336 | 0.353 | -0.6 |
| logdha | rs4986832 | 0.145 | 1.08 | 0.341 | 0.819 | 1.43 | 0.976 | 0.602 | 0.59 | 0.336 | 0.355 | -0.597 |
| logdha | rs144866316 | 0.004 | . | . | . | . | . | . | . | . | . | . |
| logdha | rs187769540 | 0.007 | . | . | . | . | . | . | . | . | . | . |
| logdha | rs6593482 | 0.146 | 1.09 | 0.347 | 0.783 | 1.48 | 1 | 0.559 | 0.561 | 0.315 | 0.302 | -0.668 |
| logdha | rs7913948 | 0.146 | 1.07 | 0.34 | 0.837 | 1.36 | 0.937 | 0.652 | 0.609 | 0.348 | 0.386 | -0.544 |
| logdha | rs77185151 | 0.025 | 1.02 | 0.306 | 0.936 | 9.55 | 9.57 | 0.024 | 0.087 | 0.089 | 0.017 | -8.73 |
| logdha | rs12762303 | 0.145 | 1.08 | 0.341 | 0.818 | 1.43 | 0.976 | 0.598 | 0.589 | 0.334 | 0.351 | -0.601 |
| logdha | rs73283136 | 0.003 | . | . | . | . | . | . | . | . | . | . |
| logdha | rs187180218 | 0.001 | . | . | . | . | . | . | . | . | . | . |
| logdha | rs78137087 | 0.145 | 1.08 | 0.341 | 0.819 | 1.42 | 0.976 | 0.605 | 0.589 | 0.336 | 0.354 | -0.597 |
| logdha | rs72561472 | 0.146 | 1.13 | 0.358 | 0.708 | 1.78 | 1.2 | 0.391 | 0.466 | 0.266 | 0.181 | -0.973 |
| logdha | rs4987106 | 0.141 | 1.07 | 0.342 | 0.825 | 1.44 | 0.991 | 0.597 | 0.596 | 0.342 | 0.367 | -0.592 |
| logdha | rs4987105 | 0.139 | 1.07 | 0.343 | 0.833 | 1.46 | 0.993 | 0.58 | 0.606 | 0.353 | 0.39 | -0.582 |
| logdha | rs111916157 | 0.158 | 1.09 | 0.373 | 0.8 | 1.31 | 0.836 | 0.674 | 0.657 | 0.341 | 0.418 | -0.461 |
| logdha | rs111528152 | 0.162 | 1.05 | 0.34 | 0.869 | 1.21 | 0.853 | 0.782 | 0.683 | 0.394 | 0.509 | -0.394 |
| logdha | rs12763738 | 0.144 | 1.08 | 0.341 | 0.818 | 1.43 | 0.98 | 0.597 | 0.589 | 0.335 | 0.352 | -0.602 |
| logdha | rs191675886 | 0.004 | . | . | . | . | . | . | . | . | . | . |
| logdha | rs66489870 | 0.145 | 1.08 | 0.341 | 0.818 | 1.43 | 0.977 | 0.598 | 0.589 | 0.334 | 0.351 | -0.601 |
| logdha | rs71494775 | 0.145 | 1.08 | 0.341 | 0.818 | 1.43 | 0.977 | 0.598 | 0.589 | 0.334 | 0.351 | -0.601 |
| logdha | rs61855875 | 0.024 | 0.987 | 0.297 | 0.965 | 0.602 | 1.19 | 0.797 | 0.685 | 1.07 | 0.808 | -0.182 |
| logdha | rs116356837 | 0.004 | . | . | . | . | . | . | . | . | . | . |
| logdha | rs1864414 | 0.144 | 1.07 | 0.34 | 0.823 | 1.42 | 0.973 | 0.605 | 0.594 | 0.338 | 0.36 | -0.59 |
| logdha | rs1864413 | 0.145 | 1.07 | 0.342 | 0.823 | 1.44 | 0.97 | 0.59 | 0.6 | 0.337 | 0.362 | -0.586 |
| logdha | rs139497212 | 0 | . | . | . | . | . | . | . | . | . | . |
| logdha | rs892690 | 0.425 | 0.706 | 0.32 | 0.442 | 0.688 | 0.284 | 0.364 | 1.42 | 0.496 | 0.309 | 0.298 |
| logdha | rs1864412 | 0.145 | 1.08 | 0.341 | 0.817 | 1.43 | 0.978 | 0.597 | 0.588 | 0.334 | 0.35 | -0.603 |
| logdha | rs77827771 | 0.016 | 1.03 | 0.318 | 0.925 | 3.51 | 5.92 | 0.456 | 0.222 | 0.259 | 0.197 | -2.74 |
| logdha | rs145294963 | . | . | . | . | . | . | . | . | . | . | . |
| logdha | rs149081327 | 0.008 | . | . | . | . | . | . | . | . | . | . |
| logdha | rs67008983 | 0.145 | 1.08 | 0.342 | 0.816 | 1.44 | 0.984 | 0.593 | 0.586 | 0.334 | 0.349 | -0.608 |
| logdha | rs56150414 | 0.147 | 1.08 | 0.344 | 0.808 | 1.44 | 0.986 | 0.594 | 0.58 | 0.332 | 0.341 | -0.618 |
| logdha | rs55787481 | 0.145 | 1.08 | 0.342 | 0.813 | 1.45 | 0.99 | 0.585 | 0.583 | 0.332 | 0.344 | -0.617 |
| logdha | rs141270101 | 0.001 | . | . | . | . | . | . | . | . | . | . |
| logdha | rs114980075 | 0.003 | . | . | . | . | . | . | . | . | . | . |
| logdha | rs12783095 | 0.144 | 1.06 | 0.339 | 0.846 | 1.4 | 0.947 | 0.618 | 0.621 | 0.349 | 0.397 | -0.54 |
| logdha | rs182342688 | . | . | . | . | . | . | . | . | . | . | . |

| logdha | rs17157728 | 0.144 | 1.06 | 0.339 | 0.846 | 1.4 | 0.947 | 0.618 | 0.621 | 0.349 | 0.397 | -0.54 |
| --- | --- | --- | --- | --- | --- | --- | --- | --- | --- | --- | --- | --- |
| logdha | rs114835563 | 0.012 | 1.01 | 0.311 | 0.972 | 3.95 | 6.58 | 0.409 | 0.269 | 0.249 | 0.156 | -2.89 |
| logdha | rs3824612 | 0.425 | 0.702 | 0.319 | 0.436 | 0.685 | 0.283 | 0.359 | 1.43 | 0.501 | 0.302 | 0.303 |
| logdha | rs17157733 | 0.143 | 1.06 | 0.34 | 0.845 | 1.41 | 0.952 | 0.614 | 0.619 | 0.349 | 0.395 | -0.544 |
| logdha | rs74128478 | 0.003 | . | . | . | . | . | . | . | . | . | . |
| logdha | rs184973589 | 0.002 | . | . | . | . | . | . | . | . | . | . |
| logdha | rs150479461 | 0 | . | . | . | . | . | . | . | . | . | . |
| logdha | rs138310407 | 0.001 | . | . | . | . | . | . | . | . | . | . |
| logdha | rs187426603 | . | . | . | . | . | . | . | . | . | . | . |
| logdha | rs190843143 | 0.002 | . | . | . | . | . | . | . | . | . | . |
| logdha | rs191883449 | 0.001 | . | . | . | . | . | . | . | . | . | . |
| logdha | rs58238740 | 0.143 | 1.07 | 0.34 | 0.843 | 1.41 | 0.956 | 0.609 | 0.618 | 0.348 | 0.393 | -0.548 |
| logdha | rs3780894 | 0.143 | 1.07 | 0.34 | 0.843 | 1.41 | 0.957 | 0.608 | 0.618 | 0.348 | 0.392 | -0.549 |
| logdha | rs149646626 | 0.018 | 0.968 | 0.29 | 0.913 | 0.971 | 2.21 | 0.99 | 1.65 | 3.23 | 0.798 | 0.612 |
| logdha | rs79615212 | 0.015 | 0.985 | 0.301 | 0.96 | 1.65 | 5.17 | 0.874 | 0.604 | 1.64 | 0.853 | -0.653 |
| logdha | rs3780895 | 0.143 | 1.06 | 0.34 | 0.844 | 1.41 | 0.955 | 0.61 | 0.619 | 0.349 | 0.394 | -0.547 |
| logdha | rs3780896 | 0.143 | 1.07 | 0.34 | 0.843 | 1.41 | 0.957 | 0.608 | 0.618 | 0.348 | 0.392 | -0.549 |
| logdha | rs142747163 | 0.005 | . | . | . | . | . | . | . | . | . | . |
| logdha | rs189417584 | 0.005 | . | . | . | . | . | . | . | . | . | . |
| logdha | rs3780897 | 0.141 | 1.06 | 0.34 | 0.854 | 1.41 | 0.955 | 0.609 | 0.631 | 0.356 | 0.414 | -0.528 |
| logdha | rs117509491 | 0.025 | 0.986 | 0.293 | 0.962 | 0.704 | 0.935 | 0.792 | 0.567 | 0.532 | 0.545 | -0.296 |
| logdha | rs117811514 | 0.025 | 0.986 | 0.293 | 0.962 | 0.704 | 0.935 | 0.792 | 0.567 | 0.532 | 0.545 | -0.296 |
| logdha | rs116500932 | 0.012 | 1.01 | 0.311 | 0.97 | 4.07 | 6.7 | 0.394 | 0.263 | 0.244 | 0.15 | -3 |
| logdha | rs77584168 | . | . | . | . | . | . | . | . | . | . | . |
| logdha | rs2310776 | 0.143 | 1.07 | 0.34 | 0.843 | 1.42 | 0.957 | 0.608 | 0.618 | 0.348 | 0.392 | -0.549 |
| logdha | rs17157736 | 0.143 | 1.07 | 0.34 | 0.843 | 1.42 | 0.957 | 0.608 | 0.618 | 0.348 | 0.392 | -0.549 |
| logdha | rs72789735 | 0.063 | 1.01 | 0.304 | 0.978 | 0.675 | 0.701 | 0.705 | 0.691 | 0.574 | 0.656 | -0.213 |
| logdha | rs183747187 | 0.001 | . | . | . | . | . | . | . | . | . | . |
| logdha | rs7077891 | 0.143 | 1.07 | 0.34 | 0.843 | 1.42 | 0.958 | 0.607 | 0.618 | 0.348 | 0.392 | -0.55 |
| logdha | rs145063305 | 0.002 | . | . | . | . | . | . | . | . | . | . |
| logdha | rs3824613 | 0.143 | 1.07 | 0.341 | 0.836 | 1.42 | 0.957 | 0.6 | 0.612 | 0.341 | 0.378 | -0.561 |
| logdha | rs145543199 | 0.006 | . | . | . | . | . | . | . | . | . | . |
| logdha | rs140361993 | 0.003 | . | . | . | . | . | . | . | . | . | . |
| logdha | rs11239496 | 0.063 | 1.01 | 0.304 | 0.978 | 0.675 | 0.701 | 0.705 | 0.691 | 0.574 | 0.656 | -0.213 |
| logdha | rs11239497 | 0.139 | 1.06 | 0.342 | 0.851 | 1.44 | 0.97 | 0.585 | 0.629 | 0.36 | 0.418 | -0.541 |
| logdha | rs10900212 | 0.155 | 1.11 | 0.358 | 0.748 | 1.56 | 0.999 | 0.492 | 0.557 | 0.287 | 0.256 | -0.704 |
| logdha | rs186487136 | . | . | . | . | . | . | . | . | . | . | . |
| logdha | rs12774369 | 0.142 | 1.07 | 0.34 | 0.843 | 1.42 | 0.962 | 0.605 | 0.617 | 0.349 | 0.393 | -0.552 |
| logdha | rs60842538 | 0.142 | 1.07 | 0.341 | 0.843 | 1.42 | 0.966 | 0.603 | 0.617 | 0.349 | 0.394 | -0.553 |
| logdha | rs3780898 | 0.138 | 1.06 | 0.343 | 0.852 | 1.45 | 0.982 | 0.579 | 0.629 | 0.362 | 0.421 | -0.545 |
| logdha | rs3780899 | 0.142 | 1.06 | 0.34 | 0.844 | 1.43 | 0.973 | 0.599 | 0.617 | 0.351 | 0.396 | -0.555 |
| logdha | rs12252472 | 0.045 | 0.948 | 0.299 | 0.867 | 0.579 | 0.601 | 0.599 | 1.41 | 1.01 | 0.629 | 0.249 |
| logdha | rs185411011 | . | . | . | . | . | . | . | . | . | . | . |
| logdha | rs28395865 | 0.137 | 1.06 | 0.342 | 0.854 | 1.46 | 0.996 | 0.579 | 0.629 | 0.367 | 0.427 | -0.546 |
| logdha | rs191004074 | 0.038 | 0.902 | 0.282 | 0.743 | 0.25 | 0.421 | 0.41 | 2.59 | 3.79 | 0.515 | 0.433 |
| logdha | rs182401295 | 0.002 | . | . | . | . | . | . | . | . | . | . |
| logdha | rs2228064 | 0.068 | 0.977 | 0.311 | 0.941 | 0.991 | 0.881 | 0.992 | 0.977 | 0.724 | 0.975 | -0.022 |
| logdha | rs188284159 | . | . | . | . | . | . | . | . | . | . | . |
| logdha | rs2303887 | 0.022 | 1 | 0.298 | 0.999 | 4.66 | 5.26 | 0.172 | 0.219 | 0.241 | 0.168 | -3.64 |
| logdha | rs2303886 | 0.024 | 1.01 | 0.3 | 0.983 | 5.54 | 5.93 | 0.11 | 0.172 | 0.179 | 0.091 | -4.59 |
| logdha | rs192621006 | . | . | . | . | . | . | . | . | . | . | . |
| logdha | rs2303885 | 0.191 | 1.05 | 0.365 | 0.877 | 1.13 | 0.681 | 0.839 | 0.792 | 0.398 | 0.643 | -0.241 |
| logdha | rs114466168 | 0.012 | 1.01 | 0.311 | 0.972 | 3.87 | 6.43 | 0.416 | 0.274 | 0.252 | 0.159 | -2.81 |
| logdha | rs11239501 | 0.083 | 1.01 | 0.317 | 0.983 | 0.995 | 0.78 | 0.995 | 0.808 | 0.49 | 0.725 | -0.193 |
| logdha | rs745986 | 0.156 | 1.1 | 0.366 | 0.776 | 1.73 | 1.03 | 0.354 | 0.603 | 0.301 | 0.311 | -0.683 |
| logdha | rs75262268 | . | . | . | . | . | . | . | . | . | . | . |
| logdha | rs71494776 | 0.051 | 0.987 | 0.302 | 0.967 | 1.51 | 1.5 | 0.68 | 0.921 | 0.85 | 0.929 | -0.125 |
| logdha | rs74386871 | 0.03 | 0.997 | 0.296 | 0.992 | 2.31 | 3.35 | 0.562 | 0.516 | 0.704 | 0.628 | -1.12 |
| logdha | rs76502040 | 0.006 | . | . | . | . | . | . | . | . | . | . |
| logdha | rs142213734 | 0.001 | . | . | . | . | . | . | . | . | . | . |
| logdha | rs4948671 | 0.731 | 1.26 | 0.995 | 0.77 | 1.13 | 0.642 | 0.835 | 0.832 | 0.38 | 0.687 | -0.206 |
| logdha | rs146286085 | 0.001 | . | . | . | . | . | . | . | . | . | . |
| logdha | rs144080623 | . | . | . | . | . | . | . | . | . | . | . |
| logdha | rs115074014 | 0.023 | 0.902 | 0.278 | 0.738 | 0.154 | 0.211 | 0.173 | 4.12 | 3.77 | 0.121 | 0.516 |
| logdha | rs17157756 | 0.023 | 0.902 | 0.278 | 0.738 | 0.154 | 0.211 | 0.173 | 4.12 | 3.77 | 0.121 | 0.516 |
| logdha | rs190459780 | . | . | . | . | . | . | . | . | . | . | . |
| logdha | rs12358647 | 0.174 | 1.17 | 0.4 | 0.643 | 1.94 | 1.12 | 0.251 | 0.513 | 0.243 | 0.158 | -0.946 |
| logdha | rs147080556 | 0.024 | 0.925 | 0.288 | 0.803 | 0.765 | 1.25 | 0.87 | 2.75 | 3.36 | 0.408 | 1.26 |
| logdha | rs187543349 | 0.01 | 0.971 | 0.29 | 0.921 | 0.954 | 2.99 | 0.988 | 0.838 | 2.35 | 0.95 | -0.148 |
| logdha | rs114375595 | 0.006 | . | . | . | . | . | . | . | . | . | . |
| logdha | rs117211200 | 0.005 | . | . | . | . | . | . | . | . | . | . |
| logdha | rs192963047 | . | . | . | . | . | . | . | . | . | . | . |
| logdha | rs11239505 | 0.175 | 1.18 | 0.401 | 0.635 | 1.95 | 1.12 | 0.247 | 0.507 | 0.239 | 0.149 | -0.962 |
| logdha | rs184434116 | 0 | . | . | . | . | . | . | . | . | . | . |
| logdha | rs4948999 | 0.733 | 1.31 | 1.04 | 0.731 | 1.15 | 0.659 | 0.801 | 0.809 | 0.369 | 0.642 | -0.241 |
| logdha | rs11239506 | 0.092 | 1.06 | 0.337 | 0.862 | 1.4 | 1.01 | 0.646 | 0.599 | 0.348 | 0.377 | -0.57 |
| logdha | rs190706620 | 0.003 | . | . | . | . | . | . | . | . | . | . |
| logdha | rs190770429 | . | . | . | . | . | . | . | . | . | . | . |
| logdha | rs181327133 | 0.005 | . | . | . | . | . | . | . | . | . | . |
| logdha | rs149251697 | 0.011 | 1.01 | 0.305 | 0.974 | 12.3 | 21.3 | 0.147 | 0.138 | 0.134 | 0.042 | -10.6 |
| logdha | rs118081602 | 0.022 | 1 | 0.298 | 0.999 | 4.61 | 5.2 | 0.175 | 0.22 | 0.243 | 0.169 | -3.6 |
| logdha | rs188233691 | 0.002 | . | . | . | . | . | . | . | . | . | . |
| logdha | rs183778794 | 0.003 | . | . | . | . | . | . | . | . | . | . |
| logdha | rs188040078 | 0.007 | . | . | . | . | . | . | . | . | . | . |
| logdha | rs184675372 | 0 | . | . | . | . | . | . | . | . | . | . |
| logdha | rs150326891 | 0.067 | 0.998 | 0.317 | 0.994 | 1.15 | 0.937 | 0.865 | 0.833 | 0.524 | 0.772 | -0.191 |

| logdha | rs185520021 | 0.014 | 1.01 | 0.3 | 0.978 | 4.73 | 9.36 | 0.432 | 0.183 | 0.434 | 0.474 | -3.86 |
| --- | --- | --- | --- | --- | --- | --- | --- | --- | --- | --- | --- | --- |
| logdha | rs7907779 | 0.092 | 1.06 | 0.337 | 0.862 | 1.4 | 1.01 | 0.646 | 0.598 | 0.347 | 0.376 | -0.57 |
| logdha | rs184962860 | 0.001 | . | . | . | . | . | . | . | . | . | . |
| logdha | rs149762784 | 0.066 | 0.844 | 0.263 | 0.585 | 0.145 | 0.257 | 0.275 | 3.88 | 5.28 | 0.32 | 0.485 |
| logdha | rs149203427 | 0.008 | . | . | . | . | . | . | . | . | . | . |
| logdha | rs60153083 | 0.01 | 0.985 | 0.292 | 0.96 | 1.05 | 2.1 | 0.98 | 0.722 | 0.763 | 0.758 | -0.289 |
| logdha | rs112001069 | 0.062 | 1 | 0.309 | 0.988 | 1.82 | 1.75 | 0.533 | 0.731 | 0.649 | 0.724 | -0.488 |
| logdha | rs182355382 | 0.032 | 0.955 | 0.294 | 0.881 | 0.252 | 0.486 | 0.475 | 1.52 | 1.94 | 0.74 | 0.16 |
| logdha | rs186740974 | 0.111 | 0.93 | 0.347 | 0.846 | 0.96 | 1.14 | 0.972 | 1.18 | 1.21 | 0.871 | 0.164 |
| logdha | rs191874916 | 0.026 | 1.06 | 0.335 | 0.862 | 5.85 | 9.95 | 0.298 | 0.213 | 0.24 | 0.169 | -4.59 |
| logdha | rs182989412 | 0.168 | 1.05 | 0.389 | 0.898 | 0.889 | 0.669 | 0.876 | 0.754 | 0.462 | 0.646 | -0.234 |
| logdha | rs188819476 | 0.001 | . | . | . | . | . | . | . | . | . | . |
| logdha | rs141217183 | 0.08 | 0.976 | 0.311 | 0.94 | 1.42 | 1.48 | 0.736 | 1.01 | 0.96 | 0.987 | 0.011 |
| logdha | rs192922652 | 0.064 | 1 | 0.309 | 0.991 | 1.81 | 1.74 | 0.538 | 0.751 | 0.671 | 0.749 | -0.449 |
| logdha | rs185092343 | 0.014 | 1.04 | 0.316 | 0.903 | 1.65 | 3.91 | 0.834 | 0.16 | 0.31 | 0.345 | -1.41 |
| logdha | rs145193311 | 0.065 | 1 | 0.309 | 0.999 | 1.79 | 1.75 | 0.554 | 0.77 | 0.691 | 0.771 | -0.411 |
| logdha | rs55789336 | 0.093 | 1.06 | 0.337 | 0.86 | 1.38 | 1.02 | 0.663 | 0.596 | 0.349 | 0.377 | -0.567 |
| logdha | rs55837212 | 0.093 | 1.05 | 0.335 | 0.879 | 1.37 | 1.02 | 0.671 | 0.619 | 0.367 | 0.418 | -0.53 |
| logdha | rs75408574 | 0.406 | 0.931 | 0.436 | 0.879 | 0.923 | 0.412 | 0.857 | 1.04 | 0.386 | 0.923 | 0.037 |
| logdha | rs149306926 | 0.098 | 1.08 | 0.356 | 0.827 | 1.27 | 0.923 | 0.746 | 0.619 | 0.356 | 0.404 | -0.499 |
| logdha | rs35990892 | 0.706 | 1.06 | 0.86 | 0.946 | 1.07 | 0.625 | 0.907 | 0.946 | 0.439 | 0.905 | -0.057 |
| logdha | rs192356207 | 0.036 | 1.06 | 0.327 | 0.846 | 2.22 | 2.78 | 0.525 | 0.261 | 0.222 | 0.115 | -1.67 |
| logdha | rs183741163 | 0.092 | 1.1 | 0.367 | 0.777 | 2.17 | 1.65 | 0.305 | 0.483 | 0.293 | 0.23 | -1.12 |
| logdha | rs34737297 | 0.559 | 1.4 | 0.986 | 0.632 | 1.44 | 0.886 | 0.548 | 0.742 | 0.363 | 0.542 | -0.344 |
| logdha | rs181346933 | 0.022 | 0.956 | 0.287 | 0.882 | 0.273 | 1.05 | 0.736 | 2.04 | 5.62 | 0.796 | 0.303 |
| logdha | rs36093253 | 0.409 | 0.867 | 0.449 | 0.782 | 0.933 | 0.452 | 0.887 | 1.15 | 0.483 | 0.739 | 0.13 |
| logdha | rs186307855 | 0.059 | 0.921 | 0.295 | 0.797 | 0.363 | 0.388 | 0.343 | 1.84 | 1.39 | 0.418 | 0.332 |
| logdha | rs189681865 | 0.019 | 1.06 | 0.323 | 0.854 | 5.75 | 6.55 | 0.125 | 0.178 | 0.13 | 0.018 | -4.73 |
| logdha | rs34942971 | 0.176 | 1.16 | 0.406 | 0.668 | 2.06 | 1.2 | 0.213 | 0.543 | 0.261 | 0.204 | -0.922 |
| logdha | rs181816554 | 0.025 | 1.1 | 0.338 | 0.753 | 48 | 67.4 | 5.90E-03 | 0.026 | 0.029 | 8.10E-04 | -46.7 |
| logdha | rs186846881 | 0.049 | 0.976 | 0.313 | 0.939 | 0.943 | 1.02 | 0.956 | 1.01 | 0.772 | 0.993 | 8.00E-03 |
| logdha | rs191178220 | 0.016 | 0.828 | 0.247 | 0.526 | 1.10E-05 | 4.20E-05 | 2.30E-03 | 6.80E+03 | 1.70E+04 | 3.80E-04 | 0.235 |
| logdha | rs183305228 | 0.01 | 0.986 | 0.292 | 0.961 | 1.08 | 2.09 | 0.968 | 0.703 | 0.735 | 0.736 | -0.317 |
| logdha | rs139912761 | 0.102 | 1.09 | 0.354 | 0.787 | 1.56 | 1.1 | 0.529 | 0.549 | 0.296 | 0.266 | -0.717 |
| logdha | rs185499901 | 0.016 | 1.02 | 0.306 | 0.959 | 8.43 | 13.3 | 0.178 | 0.2 | 0.254 | 0.205 | -6.73 |
| logdha | rs189816197 | 0.072 | 1.03 | 0.319 | 0.931 | 1.99 | 1.99 | 0.493 | 0.606 | 0.563 | 0.59 | -0.776 |
| logdha | rs145656938 | 0.022 | 1.03 | 0.306 | 0.923 | 0.876 | 2.71 | 0.966 | 0.424 | 1.24 | 0.769 | -0.523 |
| logdha | rs35745197 | 0.062 | 1 | 0.309 | 0.99 | 1.81 | 1.76 | 0.543 | 0.739 | 0.661 | 0.735 | -0.471 |
| logdha | rs192856637 | 0.004 | . | . | . | . | . | . | . | . | . | . |
| logdha | rs2310775 | 0.492 | 0.784 | 0.406 | 0.638 | 0.8 | 0.356 | 0.615 | 1.22 | 0.457 | 0.6 | 0.18 |
| logdha | rs11239507 | 0.559 | 0.776 | 0.454 | 0.664 | 0.804 | 0.383 | 0.647 | 1.2 | 0.465 | 0.638 | 0.169 |
| logdha | rs74648467 | 0.053 | 0.989 | 0.303 | 0.972 | 1.52 | 1.54 | 0.679 | 0.897 | 0.838 | 0.907 | -0.161 |
| logdha | rs145815934 | 0.012 | 0.933 | 0.28 | 0.817 | 0.199 | 0.763 | 0.674 | 5.93 | 19.5 | 0.587 | 0.97 |
| logdha | rs145390687 | 0.015 | 0.962 | 0.305 | 0.902 | 1.12 | 1.31 | 0.923 | 1.14 | 1.05 | 0.885 | 0.149 |
| logdha | rs184354913 | 0.033 | 1.01 | 0.31 | 0.961 | 3.18 | 5.32 | 0.49 | 0.46 | 0.657 | 0.587 | -1.71 |
| logdha | rs181244920 | 0.007 | . | . | . | . | . | . | . | . | . | . |
| logdha | rs11499087 | 0.028 | 0.903 | 0.279 | 0.742 | 0.128 | 0.164 | 0.108 | 4.3 | 3.89 | 0.107 | 0.465 |
| logdha | rs148263794 | 0.064 | 1.01 | 0.31 | 0.981 | 1.87 | 1.79 | 0.515 | 0.709 | 0.621 | 0.695 | -0.54 |
| logdha | rs150758903 | 0.021 | 1.05 | 0.317 | 0.868 | 2 | 2.93 | 0.635 | 0.194 | 0.193 | 0.1 | -1.65 |
| logdha | rs147654382 | 0.152 | 1.16 | 0.389 | 0.665 | 1.91 | 1.25 | 0.325 | 0.481 | 0.263 | 0.181 | -1 |
| logdha | rs147495166 | 0.011 | 1.01 | 0.31 | 0.976 | 3.9 | 6.61 | 0.421 | 0.277 | 0.26 | 0.171 | -2.82 |
| logdha | rs35198778 | 0.081 | 1.08 | 0.349 | 0.803 | 2.37 | 2.34 | 0.382 | 0.396 | 0.309 | 0.235 | -1.44 |
| logdha | rs145207659 | 0.043 | 0.982 | 0.314 | 0.954 | 1.52 | 1.33 | 0.629 | 1.05 | 0.789 | 0.944 | 0.071 |
| logdha | rs186466464 | 0.007 | . | . | . | . | . | . | . | . | . | . |
| logdha | rs12778413 | 0.146 | 1.24 | 0.416 | 0.52 | 2.66 | 1.91 | 0.172 | 0.319 | 0.195 | 0.061 | -1.85 |
| logdha | rs7895552 | 0.632 | 1.66 | 1.29 | 0.513 | 1.56 | 0.964 | 0.475 | 0.67 | 0.325 | 0.409 | -0.485 |
| logdha | rs12783895 | 0.064 | 1.01 | 0.31 | 0.981 | 1.87 | 1.79 | 0.512 | 0.708 | 0.62 | 0.693 | -0.543 |
| logdha | rs34929604 | 0.065 | 1.01 | 0.311 | 0.975 | 1.9 | 1.79 | 0.494 | 0.69 | 0.591 | 0.665 | -0.588 |
| logdha | rs12761961 | 0.063 | 0.996 | 0.307 | 0.99 | 1.65 | 1.6 | 0.606 | 0.804 | 0.713 | 0.806 | -0.325 |
| logdha | rs35536930 | 0.172 | 1.16 | 0.397 | 0.661 | 1.94 | 1.13 | 0.256 | 0.524 | 0.249 | 0.173 | -0.919 |
| logdha | rs180951060 | 0 | . | . | . | . | . | . | . | . | . | . |
| logdha | rs148892128 | 0.012 | 0.933 | 0.28 | 0.817 | 0.199 | 0.762 | 0.673 | 5.94 | 19.5 | 0.587 | 0.97 |
| logdha | rs11239509 | 0.043 | 0.938 | 0.292 | 0.837 | 0.328 | 0.337 | 0.278 | 1.91 | 1.27 | 0.328 | 0.323 |
| logdha | rs41526545 | 0.088 | 1.01 | 0.319 | 0.964 | 0.973 | 0.758 | 0.972 | 0.785 | 0.474 | 0.688 | -0.213 |
| logdha | rs2029253 | 0.496 | 0.799 | 0.419 | 0.669 | 0.82 | 0.369 | 0.66 | 1.2 | 0.452 | 0.636 | 0.164 |
| logdha | rs17444064 | 0.086 | 1.01 | 0.318 | 0.978 | 0.989 | 0.765 | 0.988 | 0.804 | 0.481 | 0.715 | -0.196 |
| logdha | rs71494779 | 0.066 | 1.01 | 0.311 | 0.978 | 1.88 | 1.75 | 0.499 | 0.697 | 0.592 | 0.671 | -0.566 |
| logdha | rs74991420 | 0.022 | 1 | 0.298 | 0.999 | 4.48 | 5.14 | 0.191 | 0.24 | 0.267 | 0.2 | -3.41 |
| logdha | rs182736551 | 0.022 | 1 | 0.298 | 0.999 | 4.48 | 5.14 | 0.191 | 0.24 | 0.267 | 0.2 | -3.41 |
| logdha | rs78702074 | 0.021 | 1 | 0.298 | 0.995 | 5.01 | 5.74 | 0.16 | 0.209 | 0.235 | 0.163 | -3.96 |
| logdha | rs187791466 | 0.002 | . | . | . | . | . | . | . | . | . | . |
| logdha | rs35300270 | 0.068 | 1.01 | 0.313 | 0.97 | 1.88 | 1.73 | 0.494 | 0.68 | 0.574 | 0.647 | -0.599 |
| logdha | rs6593484 | 0.093 | 1.06 | 0.337 | 0.859 | 1.38 | 0.998 | 0.652 | 0.6 | 0.344 | 0.373 | -0.563 |
| logdha | rs12244601 | 0.013 | 1.02 | 0.307 | 0.948 | 4.12 | 6.07 | 0.337 | 0.147 | 0.141 | 0.045 | -3.52 |
| logdha | rs184429574 | 0.004 | . | . | . | . | . | . | . | . | . | . |
| logdha | rs7080897 | 0.066 | 1.01 | 0.311 | 0.978 | 1.88 | 1.75 | 0.498 | 0.697 | 0.592 | 0.671 | -0.566 |
| logdha | rs7097429 | 0.07 | 1.01 | 0.314 | 0.977 | 1.92 | 1.74 | 0.474 | 0.719 | 0.593 | 0.689 | -0.536 |
| logdha | rs77979229 | 0.001 | . | . | . | . | . | . | . | . | . | . |
| logdha | rs11239512 | 0.093 | 1.06 | 0.337 | 0.859 | 1.38 | 0.997 | 0.652 | 0.6 | 0.343 | 0.372 | -0.563 |
| logdha | rs60146827 | 0.066 | 1.01 | 0.311 | 0.978 | 1.87 | 1.74 | 0.501 | 0.7 | 0.593 | 0.674 | -0.559 |
| logdha | rs79032236 | 0.017 | 1.03 | 0.32 | 0.923 | 6.21 | 8.69 | 0.192 | 0.204 | 0.183 | 0.077 | -4.94 |
| logdha | rs140854206 | 0.005 | . | . | . | . | . | . | . | . | . | . |
| logdha | rs184049550 | 0.002 | . | . | . | . | . | . | . | . | . | . |
| logdha | rs114433607 | 0.007 | . | . | . | . | . | . | . | . | . | . |
| logdha | rs117653793 | 0.022 | 1 | 0.298 | 0.996 | 4.82 | 5.49 | 0.167 | 0.216 | 0.24 | 0.169 | -3.78 |
| logdha | rs117039533 | 0.022 | 1 | 0.298 | 0.996 | 4.76 | 5.43 | 0.171 | 0.219 | 0.243 | 0.172 | -3.72 |

| logdha | rs191660761 | 0.002 | . | . | . | . | . | . | . | . | . | . |
| --- | --- | --- | --- | --- | --- | --- | --- | --- | --- | --- | --- | --- |
| logdha | rs59514896 | 0.053 | 0.987 | 0.302 | 0.967 | 1.51 | 1.5 | 0.678 | 0.91 | 0.835 | 0.918 | -0.14 |
| logdha | rs184661876 | 0.001 | . | . | . | . | . | . | . | . | . | . |
| logdha | rs61854073 | 0.028 | 0.991 | 0.298 | 0.975 | 0.488 | 1.01 | 0.73 | 0.663 | 1.07 | 0.799 | -0.158 |
| logdha | rs2018518 | 0.391 | 0.705 | 0.296 | 0.405 | 0.63 | 0.257 | 0.257 | 1.44 | 0.491 | 0.289 | 0.303 |
| logdha | rs934187 | 0.735 | 1.27 | 1.02 | 0.762 | 1.14 | 0.652 | 0.812 | 0.83 | 0.378 | 0.683 | -0.208 |
| logdha | rs7918542 | 0.179 | 1.19 | 0.406 | 0.616 | 1.95 | 1.12 | 0.242 | 0.5 | 0.233 | 0.137 | -0.982 |
| logdha | rs144311920 | 0.002 | . | . | . | . | . | . | . | . | . | . |
| logdha | rs7099684 | 0.864 | 1.03 | 1.01 | 0.977 | 0.909 | 0.57 | 0.88 | 0.958 | 0.505 | 0.935 | -0.042 |
| logdha | rs150829171 | . | . | . | . | . | . | . | . | . | . | . |
| logdha | rs186060697 | 0 | . | . | . | . | . | . | . | . | . | . |
| logdha | rs11239513 | 0.054 | 0.958 | 0.305 | 0.893 | 0.532 | 0.549 | 0.541 | 1.38 | 1.08 | 0.681 | 0.212 |
| logdha | rs7917687 | 0.067 | 1.01 | 0.31 | 0.983 | 1.83 | 1.69 | 0.515 | 0.716 | 0.599 | 0.69 | -0.516 |
| logdha | rs61854076 | 0.01 | 1.02 | 0.303 | 0.944 | 3.75 | 12.7 | 0.696 | 0.132 | 0.352 | 0.447 | -3.27 |
| logdha | rs71494780 | 0.029 | 1.02 | 0.307 | 0.952 | 2.92 | 3.96 | 0.431 | 0.346 | 0.432 | 0.395 | -1.91 |
| logdha | rs7919239 | 0.186 | 1.12 | 0.38 | 0.739 | 1.63 | 0.871 | 0.364 | 0.62 | 0.275 | 0.281 | -0.618 |
| logdha | rs78334475 | 0.047 | 0.978 | 0.31 | 0.944 | 0.804 | 0.658 | 0.79 | 0.823 | 0.555 | 0.773 | -0.135 |
| logdha | rs3780900 | 0.02 | 1.01 | 0.317 | 0.981 | 1.93 | 4.81 | 0.793 | 0.429 | 0.577 | 0.529 | -1.1 |
| logdha | rs186729292 | 0.001 | . | . | . | . | . | . | . | . | . | . |
| logdha | rs12267438 | 0.015 | 1.01 | 0.304 | 0.982 | 5.28 | 5.56 | 0.114 | 0.266 | 0.169 | 0.037 | -3.87 |
| logdha | rs1369214 | 0.61 | 0.782 | 0.516 | 0.71 | 0.89 | 0.465 | 0.824 | 1.2 | 0.499 | 0.668 | 0.16 |
| logdha | rs9282589 | 0.134 | 0.833 | 0.266 | 0.566 | 0.548 | 0.352 | 0.35 | 2.05 | 1.05 | 0.162 | 0.555 |
| logdha | rs114522915 | 0.011 | 1.01 | 0.311 | 0.969 | 7.1 | 15.8 | 0.379 | 0.19 | 0.21 | 0.133 | -5.75 |
| logdha | rs2115819 | 0.542 | 0.806 | 0.465 | 0.709 | 0.9 | 0.434 | 0.828 | 1.19 | 0.475 | 0.654 | 0.161 |
| logdha | rs117664603 | 0.022 | 0.999 | 0.297 | 0.997 | 4.31 | 4.82 | 0.192 | 0.235 | 0.256 | 0.184 | -3.3 |
| logdha | rs184979202 | . | . | . | . | . | . | . | . | . | . | . |
| logdha | rs190714671 | 0.002 | . | . | . | . | . | . | . | . | . | . |
| logdha | rs141538996 | . | . | . | . | . | . | . | . | . | . | . |
| logdha | rs150881741 | . | . | . | . | . | . | . | . | . | . | . |
| logdha | rs139076633 | . | . | . | . | . | . | . | . | . | . | . |
| logdha | rs12243649 | 0.054 | 1.07 | 0.324 | 0.83 | 4.89 | 4.73 | 0.101 | 0.162 | 0.138 | 0.032 | -4.11 |
| logdha | rs140348708 | 0.001 | . | . | . | . | . | . | . | . | . | . |
| logdha | rs79023256 | 0.022 | 0.999 | 0.297 | 0.996 | 4.25 | 4.75 | 0.195 | 0.236 | 0.257 | 0.185 | -3.24 |
| logdha | rs11239515 | 0.081 | 1.02 | 0.317 | 0.961 | 0.983 | 0.824 | 0.984 | 0.75 | 0.492 | 0.661 | -0.25 |
| logdha | rs55866086 | 0.137 | 1.13 | 0.359 | 0.712 | 1.51 | 0.941 | 0.506 | 0.49 | 0.244 | 0.152 | -0.804 |
| logdha | rs181340150 | 0.001 | . | . | . | . | . | . | . | . | . | . |
| logdha | rs11239516 | 0.096 | 1.08 | 0.344 | 0.808 | 1.22 | 0.93 | 0.797 | 0.595 | 0.341 | 0.365 | -0.515 |
| logdha | rs11239517 | 0.096 | 1.08 | 0.344 | 0.808 | 1.22 | 0.931 | 0.798 | 0.595 | 0.341 | 0.365 | -0.515 |
| logdha | rs11239519 | 0.088 | 1.07 | 0.34 | 0.826 | 1.25 | 1 | 0.782 | 0.582 | 0.353 | 0.371 | -0.542 |
| logdha | rs7909514 | 0.722 | 1.21 | 0.918 | 0.804 | 1.05 | 0.583 | 0.925 | 0.854 | 0.374 | 0.72 | -0.174 |
| logdha | rs7894352 | 0.853 | 0.855 | 0.847 | 0.874 | 0.788 | 0.503 | 0.709 | 1.07 | 0.572 | 0.904 | 0.076 |
| logdha | rs189384146 | 0.002 | . | . | . | . | . | . | . | . | . | . |
| logdha | rs11239520 | 0.088 | 1.08 | 0.343 | 0.816 | 1.48 | 1.13 | 0.609 | 0.54 | 0.324 | 0.304 | -0.695 |
| logdha | rs12221233 | 0.483 | 0.783 | 0.386 | 0.62 | 0.782 | 0.339 | 0.571 | 1.22 | 0.438 | 0.584 | 0.18 |
| logdha | rs74523293 | 0.038 | 1.02 | 0.302 | 0.96 | 5.35 | 5.29 | 0.09 | 0.143 | 0.125 | 0.026 | -4.58 |
| logdha | rs79376040 | 0.038 | 1.02 | 0.302 | 0.96 | 5.34 | 5.28 | 0.091 | 0.143 | 0.125 | 0.026 | -4.57 |
| logdha | rs4949000 | 0.482 | 0.786 | 0.386 | 0.624 | 0.786 | 0.34 | 0.579 | 1.21 | 0.436 | 0.589 | 0.178 |
| logdha | rs7089406 | 0.023 | 0.949 | 0.285 | 0.861 | 0.455 | 0.779 | 0.646 | 1.75 | 2.17 | 0.653 | 0.35 |
| logdha | rs138211056 | 0.001 | . | . | . | . | . | . | . | . | . | . |
| logdha | rs10900213 | 0.586 | 1.05 | 0.613 | 0.931 | 0.954 | 0.424 | 0.915 | 0.924 | 0.347 | 0.834 | -0.078 |
| logdha | rs145152827 | . | . | . | . | . | . | . | . | . | . | . |
| logdha | rs74443325 | 0.022 | 0.996 | 0.296 | 0.989 | 4.1 | 4.59 | 0.207 | 0.238 | 0.261 | 0.19 | -3.12 |
| logdha | rs188299446 | 0.011 | 1 | 0.304 | 0.988 | 6.71 | 11.7 | 0.276 | 0.377 | 0.684 | 0.591 | -4.17 |
| logdha | rs192699586 | 0.005 | . | . | . | . | . | . | . | . | . | . |
| logdha | rs72796464 | 0.076 | 0.988 | 0.307 | 0.97 | 0.697 | 0.645 | 0.697 | 0.958 | 0.667 | 0.951 | -0.025 |
| logdha | rs4949001 | 0.366 | 0.709 | 0.276 | 0.377 | 0.573 | 0.239 | 0.182 | 1.48 | 0.5 | 0.241 | 0.321 |
| logdha | rs147174641 | 0.005 | . | . | . | . | . | . | . | . | . | . |
| logdha | rs190835238 | 0.001 | . | . | . | . | . | . | . | . | . | . |
| logdha | rs76698944 | 0.017 | 1.06 | 0.324 | 0.843 | 2.49 | 3.81 | 0.553 | 0.14 | 0.155 | 0.077 | -2.18 |
| logdha | rs56071381 | 0.091 | 1.09 | 0.35 | 0.782 | 1.5 | 1.16 | 0.598 | 0.508 | 0.307 | 0.263 | -0.762 |
| logdha | rs78130427 | 0.023 | 0.923 | 0.277 | 0.791 | 0.527 | 0.701 | 0.63 | 3.49 | 3.98 | 0.274 | 1.25 |
| logdha | rs10900215 | 0.118 | 1.14 | 0.37 | 0.697 | 1.63 | 1.06 | 0.456 | 0.52 | 0.252 | 0.178 | -0.802 |
| logdha | rs41491445 | 0.01 | 0.987 | 0.293 | 0.964 | 2 | 3.11 | 0.656 | 0.482 | 0.493 | 0.475 | -1.03 |
| logdha | rs144995222 | 0.002 | . | . | . | . | . | . | . | . | . | . |
| logdha | rs149097803 | 0.002 | . | . | . | . | . | . | . | . | . | . |
| logdha | rs139429646 | 0.005 | . | . | . | . | . | . | . | . | . | . |
| logdha | rs7075620 | 0.675 | 0.782 | 0.55 | 0.726 | 0.757 | 0.397 | 0.595 | 1.16 | 0.483 | 0.722 | 0.148 |
| logdha | rs148299213 | 0.01 | 0.989 | 0.293 | 0.969 | 2.07 | 3.29 | 0.647 | 0.451 | 0.465 | 0.44 | -1.14 |
| logdha | rs4949002 | 0.313 | 0.737 | 0.302 | 0.455 | 0.661 | 0.328 | 0.404 | 1.55 | 0.662 | 0.309 | 0.355 |
| logdha | rs150496007 | 0.036 | 0.903 | 0.28 | 0.742 | 0.183 | 0.291 | 0.287 | 2.61 | 2.81 | 0.372 | 0.345 |
| logdha | rs147303455 | 0.008 | . | . | . | . | . | . | . | . | . | . |
| logdha | rs189518433 | . | . | . | . | . | . | . | . | . | . | . |
| logdha | rs186281355 | . | . | . | . | . | . | . | . | . | . | . |
| logdha | rs115297114 | 0.014 | 0.996 | 0.303 | 0.99 | 0.54 | 1.23 | 0.786 | 0.822 | 1.04 | 0.877 | -0.094 |
| logdha | rs11239523 | 0.087 | 1.07 | 0.34 | 0.825 | 1.47 | 1.11 | 0.612 | 0.544 | 0.327 | 0.311 | -0.685 |
| logdha | rs143704897 | 0.005 | . | . | . | . | . | . | . | . | . | . |
| logdha | rs188024096 | 0.002 | . | . | . | . | . | . | . | . | . | . |
| logdha | rs11239524 | 0.087 | 1.09 | 0.351 | 0.779 | 1.6 | 1.28 | 0.554 | 0.49 | 0.301 | 0.245 | -0.837 |
| logdha | rs191656573 | 0.004 | . | . | . | . | . | . | . | . | . | . |
| logdha | rs193295875 | . | . | . | . | . | . | . | . | . | . | . |
| logdha | rs190458228 | . | . | . | . | . | . | . | . | . | . | . |
| logdha | rs139253993 | 0.002 | . | . | . | . | . | . | . | . | . | . |
| logdha | rs4948672 | 0.604 | 1.18 | 0.628 | 0.757 | 1.07 | 0.506 | 0.878 | 0.833 | 0.332 | 0.647 | -0.198 |
| logdha | rs180954921 | 0.002 | . | . | . | . | . | . | . | . | . | . |
| logdha | rs12264801 | 0.447 | 0.446 | 0.224 | 0.108 | 0.361 | 0.189 | 0.052 | 2.4 | 1.01 | 0.037 | 0.58 |
| logdha | rs73283184 | 0.014 | 1.01 | 0.299 | 0.975 | 2.01 | 2.81 | 0.619 | 0.298 | 0.355 | 0.31 | -1.41 |

| logdha | rs142746187 | 0.007 | . | . | . | . | . | . | . | . | . | . |
| --- | --- | --- | --- | --- | --- | --- | --- | --- | --- | --- | --- | --- |
| logdha | rs58704739 | 0.014 | 1.01 | 0.298 | 0.977 | 1.96 | 2.59 | 0.611 | 0.306 | 0.353 | 0.305 | -1.36 |
| logdha | rs61272335 | 0.011 | 0.998 | 0.295 | 0.995 | 2.28 | 3.32 | 0.571 | 0.336 | 0.361 | 0.309 | -1.51 |
| logdha | rs60959489 | 0.021 | 1.01 | 0.302 | 0.98 | 3.55 | 6.27 | 0.473 | 0.366 | 0.521 | 0.48 | -2.25 |
| logdha | rs58502629 | 0.011 | 0.998 | 0.295 | 0.995 | 2.28 | 3.31 | 0.57 | 0.336 | 0.36 | 0.309 | -1.51 |
| logdha | rs10900216 | 0.739 | 0.338 | 0.296 | 0.215 | 0.498 | 0.268 | 0.195 | 1.98 | 0.952 | 0.153 | 0.498 |
| logdha | rs181118141 | 0.001 | . | . | . | . | . | . | . | . | . | . |
| logdha | rs137882665 | 0.01 | 1.11 | 0.333 | 0.739 | 6.60E+03 | 1.90E+04 | 2.10E-03 | 2.90E-05 | 1.30E-04 | 0.016 | ####### |
| logdha | rs149466736 | 0.004 | . | . | . | . | . | . | . | . | . | . |
| logdha | rs2099171 | 0.675 | 0.46 | 0.289 | 0.217 | 0.532 | 0.229 | 0.143 | 1.71 | 0.619 | 0.137 | 0.427 |
| logdha | rs141852280 | 0.005 | . | . | . | . | . | . | . | . | . | . |
| logdha | rs191539257 | . | . | . | . | . | . | . | . | . | . | . |
| logdha | rs17157771 | 0.027 | 0.886 | 0.279 | 0.701 | 1.27 | 1.18 | 0.801 | 1.68 | 1.22 | 0.477 | 0.728 |
| logdha | rs111339241 | 0.025 | 0.923 | 0.289 | 0.799 | 2.17 | 2.14 | 0.433 | 1.02 | 0.753 | 0.983 | -0.058 |
| logdha | rs116983255 | 0.006 | . | . | . | . | . | . | . | . | . | . |
| logdha | rs142324341 | . | . | . | . | . | . | . | . | . | . | . |
| logdha | rs187191219 | 0.007 | . | . | . | . | . | . | . | . | . | . |
| logdha | rs151283973 | 0 | . | . | . | . | . | . | . | . | . | . |
| logdha | rs139316218 | 0.003 | . | . | . | . | . | . | . | . | . | . |
| logdha | rs6593485 | 0.748 | 0.333 | 0.289 | 0.205 | 0.476 | 0.263 | 0.179 | 2.01 | 0.946 | 0.139 | 0.509 |
| logdha | rs6593486 | 0.751 | 0.336 | 0.291 | 0.208 | 0.478 | 0.264 | 0.182 | 1.99 | 0.939 | 0.143 | 0.506 |
| logdha | rs145196618 | 0.011 | 1.05 | 0.314 | 0.88 | 1.40E+03 | 2.60E+03 | 1.90E-04 | 1.30E-04 | 4.80E-04 | 0.017 | ####### |
| logdha | rs142598335 | . | . | . | . | . | . | . | . | . | . | . |
| logdha | rs892691 | 0.745 | 0.423 | 0.356 | 0.306 | 0.578 | 0.308 | 0.304 | 1.74 | 0.802 | 0.232 | 0.424 |
| logdha | rs7080713 | 0.689 | 0.504 | 0.327 | 0.291 | 0.571 | 0.247 | 0.195 | 1.59 | 0.597 | 0.218 | 0.382 |
| logdha | rs7077173 | 0.068 | 0.882 | 0.284 | 0.696 | 0.685 | 0.449 | 0.565 | 1.55 | 0.689 | 0.324 | 0.37 |
| logdha | rs7080977 | 0.04 | 0.924 | 0.293 | 0.804 | 1.53 | 1.42 | 0.647 | 1.03 | 0.648 | 0.959 | 6.10E-03 |
| logdha | rs3780901 | 0.688 | 0.5 | 0.327 | 0.29 | 0.573 | 0.25 | 0.202 | 1.6 | 0.606 | 0.216 | 0.385 |
| logdha | rs61854091 | 0.046 | 0.957 | 0.294 | 0.885 | 0.982 | 1.08 | 0.987 | 1.16 | 1.1 | 0.872 | 0.155 |
| logdha | rs77550209 | 0.003 | . | . | . | . | . | . | . | . | . | . |
| logdha | rs73283191 | 0.062 | 0.937 | 0.296 | 0.836 | 1.11 | 0.894 | 0.902 | 1.15 | 0.651 | 0.798 | 0.154 |
| logdha | rs191866099 | . | . | . | . | . | . | . | . | . | . | . |
| logdha | rs147998395 | 0 | . | . | . | . | . | . | . | . | . | . |
| logdha | rs7090328 | 0.789 | 0.44 | 0.418 | 0.388 | 0.583 | 0.343 | 0.359 | 1.63 | 0.83 | 0.333 | 0.396 |
| logdha | rs79814535 | 0.021 | 0.978 | 0.289 | 0.939 | 1.81 | 2.29 | 0.637 | 0.412 | 0.402 | 0.363 | -1.06 |
| logdha | rs7073082 | 0.789 | 0.442 | 0.42 | 0.391 | 0.586 | 0.344 | 0.363 | 1.63 | 0.826 | 0.337 | 0.394 |
| logdha | rs11239526 | 0.033 | 0.981 | 0.312 | 0.952 | 1.38 | 1.09 | 0.682 | 1.06 | 0.701 | 0.931 | 0.073 |
| logdha | rs141839841 | 0 | . | . | . | . | . | . | . | . | . | . |
| logdha | rs75697371 | 0.006 | . | . | . | . | . | . | . | . | . | . |
| logdha | rs75972854 | 0.005 | . | . | . | . | . | . | . | . | . | . |
| logdha | rs139291975 | . | . | . | . | . | . | . | . | . | . | . |
| logdha | rs144064711 | 0.004 | . | . | . | . | . | . | . | . | . | . |
| logdha | rs3780902 | 0.062 | 0.932 | 0.295 | 0.824 | 1.09 | 0.879 | 0.914 | 1.18 | 0.665 | 0.773 | 0.174 |
| logdha | rs190449801 | 0.001 | . | . | . | . | . | . | . | . | . | . |
| logdha | rs73283196 | 0.061 | 0.936 | 0.296 | 0.834 | 1.11 | 0.901 | 0.896 | 1.16 | 0.656 | 0.797 | 0.156 |
| logdha | rs28395867 | 0.031 | 1.08 | 0.328 | 0.811 | 7.5 | 7.9 | 0.056 | 0.086 | 0.069 | 2.40E-03 | -6.89 |
| logdha | rs28395866 | 0.079 | 1.01 | 0.329 | 0.986 | 1.74 | 1.22 | 0.428 | 0.719 | 0.364 | 0.514 | -0.488 |
| logdha | rs55661611 | 0.04 | 0.924 | 0.293 | 0.804 | 1.55 | 1.46 | 0.64 | 1.03 | 0.653 | 0.969 | -5.40E-03 |
| logdha | rs55684518 | 0.016 | 1 | 0.305 | 0.992 | 4.91 | 15.4 | 0.612 | 0.348 | 1.19 | 0.758 | -3.2 |
| logdha | rs78552493 | 0.038 | 1.04 | 0.309 | 0.883 | 0.991 | 2.74 | 0.998 | 0.404 | 0.989 | 0.711 | -0.617 |
| logdha | rs147665434 | 0.001 | . | . | . | . | . | . | . | . | . | . |
| logdha | rs34445001 | 0.006 | . | . | . | . | . | . | . | . | . | . |
| logdha | rs184019449 | 0.003 | . | . | . | . | . | . | . | . | . | . |
| logdha | rs3780903 | 0.731 | 0.616 | 0.434 | 0.492 | 0.618 | 0.295 | 0.314 | 1.34 | 0.564 | 0.493 | 0.274 |
| logdha | rs17157784 | 0.058 | 1.08 | 0.334 | 0.792 | 3.47 | 3.53 | 0.221 | 0.267 | 0.237 | 0.138 | -2.55 |
| logdha | rs3780904 | 0.021 | 0.983 | 0.29 | 0.953 | 2.81 | 3.1 | 0.349 | 0.265 | 0.237 | 0.137 | -2.06 |
| logdha | rs192064730 | . | . | . | . | . | . | . | . | . | . | . |
| logdha | rs12774534 | 0.006 | . | . | . | . | . | . | . | . | . | . |
| logdha | rs79179915 | 0.02 | 0.977 | 0.289 | 0.938 | 1.93 | 2.25 | 0.57 | 0.411 | 0.399 | 0.36 | -1.14 |
| logdha | rs117811571 | 0.011 | 1 | 0.303 | 0.994 | 6.11 | 10.8 | 0.304 | 0.419 | 0.766 | 0.634 | -3.55 |
| logdha | rs72796477 | 0.009 | . | . | . | . | . | . | . | . | . | . |
| logdha | rs55729342 | 0.058 | 1.09 | 0.336 | 0.778 | 3.7 | 4.12 | 0.24 | 0.236 | 0.241 | 0.157 | -2.84 |
| logdha | rs59317220 | 0.058 | 1.09 | 0.335 | 0.779 | 3.69 | 4.11 | 0.241 | 0.237 | 0.241 | 0.158 | -2.83 |
| logdha | rs3780905 | 0.057 | 1.07 | 0.321 | 0.825 | 2.38 | 1.92 | 0.284 | 0.366 | 0.278 | 0.185 | -1.52 |
| logdha | rs3780906 | 0.731 | 0.646 | 0.456 | 0.535 | 0.647 | 0.312 | 0.367 | 1.3 | 0.55 | 0.543 | 0.248 |
| logdha | rs149047975 | 0.125 | 0.848 | 0.268 | 0.602 | 0.551 | 0.381 | 0.389 | 1.97 | 1.1 | 0.225 | 0.521 |
| logdha | rs3824614 | 0.02 | 0.976 | 0.288 | 0.934 | 1.82 | 2.13 | 0.607 | 0.423 | 0.413 | 0.379 | -1.05 |
| logdha | rs140254404 | . | . | . | . | . | . | . | . | . | . | . |
| logdha | rs145262684 | 0.013 | 1 | 0.305 | 0.993 | 5.26 | 9.12 | 0.339 | 0.457 | 0.798 | 0.654 | -2.85 |
| logdha | rs76735482 | 0.02 | 0.975 | 0.288 | 0.932 | 1.76 | 2.06 | 0.627 | 0.431 | 0.421 | 0.389 | -0.998 |
| logdha | rs73283198 | 0.027 | 0.948 | 0.297 | 0.863 | 2.71 | 2.75 | 0.326 | 0.783 | 0.567 | 0.735 | -0.647 |
| logdha | rs74911239 | 0.029 | 0.875 | 0.254 | 0.647 | 0.06 | 0.12 | 0.16 | 8.7 | 10.9 | 0.084 | 0.522 |
| logdha | rs150551647 | 0.005 | . | . | . | . | . | . | . | . | . | . |
| logdha | rs115200794 | 0.034 | 1.09 | 0.332 | 0.774 | 8.17 | 9.09 | 0.059 | 0.062 | 0.055 | 1.60E-03 | -7.71 |
| logdha | rs74130823 | 0.139 | 1.03 | 0.357 | 0.941 | 1.1 | 0.707 | 0.884 | 0.86 | 0.399 | 0.745 | -0.155 |
| logdha | rs2279435 | 0.662 | 0.763 | 0.435 | 0.635 | 0.746 | 0.32 | 0.495 | 1.18 | 0.433 | 0.654 | 0.162 |
| logdha | rs147279673 | 0.015 | 1.1 | 0.337 | 0.765 | 180 | 576 | 0.104 | 0.019 | 0.05 | 0.14 | -177 |
| logdha | rs183525146 | 0.004 | . | . | . | . | . | . | . | . | . | . |
| logdha | rs3740107 | 0.754 | 0.614 | 0.548 | 0.585 | 0.742 | 0.43 | 0.606 | 1.34 | 0.678 | 0.561 | 0.255 |
| logdha | rs140697247 | . | . | . | . | . | . | . | . | . | . | . |
| logdha | rs1565096 | 0.825 | 1.15 | 1.08 | 0.885 | 0.931 | 0.575 | 0.907 | 0.898 | 0.464 | 0.835 | -0.119 |
| logdha | rs1565097 | 0.793 | 0.742 | 0.681 | 0.745 | 0.786 | 0.475 | 0.69 | 1.18 | 0.599 | 0.749 | 0.158 |
| logdha | rs144584548 | 0.016 | 1 | 0.305 | 0.993 | 4.79 | 15.2 | 0.621 | 0.352 | 1.21 | 0.762 | -3.1 |
| logdha | rs75811053 | 0.02 | 0.978 | 0.289 | 0.94 | 2.01 | 2.32 | 0.545 | 0.383 | 0.379 | 0.332 | -1.24 |
| logdha | rs147462967 | 0.009 | . | . | . | . | . | . | . | . | . | . |
| logdha | rs74130824 | 0.051 | 1.09 | 0.331 | 0.782 | 5.38 | 4.45 | 0.042 | 0.109 | 0.073 | 8.70E-04 | -4.83 |

| logdha | rs116883184 | 0.017 | 0.996 | 0.308 | 0.99 | 4.12 | 6.98 | 0.402 | 0.548 | 0.834 | 0.693 | -1.87 |
| --- | --- | --- | --- | --- | --- | --- | --- | --- | --- | --- | --- | --- |
| logdha | rs117258049 | 0.017 | 0.996 | 0.308 | 0.99 | 4.12 | 6.98 | 0.402 | 0.548 | 0.834 | 0.693 | -1.87 |
| logdha | rs3780908 | 0.107 | 0.91 | 0.304 | 0.778 | 0.841 | 0.566 | 0.797 | 1.26 | 0.57 | 0.605 | 0.216 |
| logdha | rs180938522 | 0.002 | . | . | . | . | . | . | . | . | . | . |
| logdha | rs76687372 | 0.003 | . | . | . | . | . | . | . | . | . | . |
| logdha | rs3780909 | 0.606 | 0.845 | 0.408 | 0.727 | 0.796 | 0.339 | 0.592 | 1.1 | 0.384 | 0.775 | 0.102 |
| logdha | rs78281156 | 0.02 | 0.978 | 0.289 | 0.94 | 2.01 | 2.32 | 0.544 | 0.382 | 0.378 | 0.33 | -1.24 |
| logdha | rs187840631 | . | . | . | . | . | . | . | . | . | . | . |
| logdha | rs76868242 | 0.02 | 0.978 | 0.289 | 0.94 | 2.01 | 2.32 | 0.544 | 0.38 | 0.377 | 0.329 | -1.24 |
| logdha | rs192794192 | 0.001 | . | . | . | . | . | . | . | . | . | . |
| logdha | rs12781248 | 0.007 | . | . | . | . | . | . | . | . | . | . |
| logdha | rs12762375 | 0.006 | . | . | . | . | . | . | . | . | . | . |
| logdha | rs192256986 | 0.004 | . | . | . | . | . | . | . | . | . | . |
| logdha | rs184411030 | 0.01 | . | . | . | . | . | . | . | . | . | . |
| logdha | rs189601483 | 0.02 | 0.978 | 0.289 | 0.94 | 2 | 2.32 | 0.549 | 0.38 | 0.377 | 0.33 | -1.24 |
| logdha | rs143254579 | 0.033 | 0.999 | 0.317 | 0.998 | 1.56 | 1.22 | 0.569 | 0.864 | 0.571 | 0.825 | -0.212 |
| logdha | rs12253903 | 0.017 | 1.01 | 0.298 | 0.974 | 1.65 | 2.08 | 0.693 | 0.356 | 0.327 | 0.26 | -1.07 |
| logdha | rs35489926 | 0.006 | . | . | . | . | . | . | . | . | . | . |
| logdha | rs71494783 | 0.039 | 1.02 | 0.307 | 0.951 | 1.67 | 1.71 | 0.616 | 0.36 | 0.327 | 0.261 | -1.08 |
| logdha | rs180711602 | 0.002 | . | . | . | . | . | . | . | . | . | . |
| logdha | rs117312402 | 0.02 | 0.978 | 0.289 | 0.94 | 2.02 | 2.33 | 0.544 | 0.377 | 0.374 | 0.325 | -1.25 |
| logdha | rs74831694 | 0.004 | . | . | . | . | . | . | . | . | . | . |
| logdha | rs140301943 | 0.002 | . | . | . | . | . | . | . | . | . | . |
| logdha | rs78553679 | 0.013 | 0.945 | 0.286 | 0.851 | 0.63 | 2.4 | 0.904 | 2.54 | 8.65 | 0.784 | 0.938 |
| logdha | rs78334250 | 0.02 | 0.978 | 0.289 | 0.94 | 2.02 | 2.33 | 0.544 | 0.377 | 0.373 | 0.324 | -1.25 |
| logdha | rs76000028 | 0.02 | 0.978 | 0.289 | 0.94 | 2.02 | 2.33 | 0.544 | 0.377 | 0.373 | 0.324 | -1.25 |
| logdha | rs145195254 | 0.001 | . | . | . | . | . | . | . | . | . | . |
| logdha | rs137888527 | 0.001 | . | . | . | . | . | . | . | . | . | . |
| logdha | rs149109592 | 0.005 | . | . | . | . | . | . | . | . | . | . |
| logdha | rs80280010 | 0.032 | 0.995 | 0.316 | 0.987 | 1.54 | 1.19 | 0.574 | 0.926 | 0.607 | 0.907 | -0.116 |
| logdha | rs189995374 | . | . | . | . | . | . | . | . | . | . | . |
| logdha | rs75467770 | 0.021 | 0.979 | 0.29 | 0.943 | 2.08 | 2.39 | 0.523 | 0.361 | 0.359 | 0.306 | -1.33 |
| logdha | rs1487562 | 0.139 | 1.07 | 0.368 | 0.852 | 1.28 | 0.749 | 0.679 | 0.746 | 0.33 | 0.507 | -0.328 |
| logdha | rs12266963 | 0.039 | 1.02 | 0.307 | 0.951 | 1.67 | 1.71 | 0.618 | 0.359 | 0.328 | 0.262 | -1.08 |
| logdha | rs7099874 | 0.787 | 1.09 | 0.864 | 0.909 | 0.825 | 0.48 | 0.74 | 0.916 | 0.433 | 0.852 | -0.093 |
| logdha | rs75687019 | 0.02 | 0.978 | 0.289 | 0.94 | 2.02 | 2.34 | 0.544 | 0.376 | 0.372 | 0.322 | -1.25 |
| logdha | rs148240462 | 0.031 | 0.89 | 0.261 | 0.692 | 0.298 | 0.582 | 0.536 | 3.38 | 4.32 | 0.34 | 0.708 |
| logdha | rs141278174 | . | . | . | . | . | . | . | . | . | . | . |
| logdha | rs76404852 | 0.02 | 0.978 | 0.289 | 0.94 | 2.02 | 2.34 | 0.544 | 0.375 | 0.371 | 0.322 | -1.26 |
| logdha | rs139112240 | 0.006 | . | . | . | . | . | . | . | . | . | . |
| logdha | rs12783037 | 0.042 | 1.01 | 0.306 | 0.962 | 1.54 | 1.54 | 0.67 | 0.419 | 0.363 | 0.315 | -0.897 |
| logdha | rs72796481 | 0.006 | . | . | . | . | . | . | . | . | . | . |
| logdha | rs76798749 | 0.023 | 0.974 | 0.289 | 0.931 | 1.26 | 1.74 | 0.865 | 0.693 | 0.79 | 0.748 | -0.385 |
| logdha | rs187732548 | . | . | . | . | . | . | . | . | . | . | . |
| logdha | rs75675474 | 0.023 | 0.974 | 0.289 | 0.931 | 1.26 | 1.74 | 0.866 | 0.694 | 0.792 | 0.749 | -0.383 |
| logdha | rs76552293 | 0.023 | 0.974 | 0.289 | 0.93 | 1.26 | 1.74 | 0.869 | 0.699 | 0.8 | 0.755 | -0.375 |
| logdha | rs147445993 | 0.002 | . | . | . | . | . | . | . | . | . | . |
| logdha | rs115902283 | 0.023 | 0.974 | 0.289 | 0.929 | 1.22 | 1.71 | 0.886 | 0.722 | 0.835 | 0.779 | -0.336 |
| logdha | rs4540799 | 0.786 | 1.11 | 0.877 | 0.892 | 0.835 | 0.487 | 0.758 | 0.905 | 0.426 | 0.832 | -0.107 |
| logdha | rs12765320 | 0.172 | 1.06 | 0.375 | 0.865 | 1.23 | 0.628 | 0.685 | 0.773 | 0.325 | 0.54 | -0.283 |
| logdha | rs12782256 | 0.006 | . | . | . | . | . | . | . | . | . | . |
| logdha | rs191727256 | 0 | . | . | . | . | . | . | . | . | . | . |
| logdha | rs2082111 | 0.111 | 1 | 0.34 | 0.994 | 1.4 | 0.89 | 0.592 | 0.844 | 0.406 | 0.725 | -0.218 |
| logdha | rs2082110 | 0.157 | 1.05 | 0.366 | 0.885 | 1.33 | 0.759 | 0.622 | 0.761 | 0.348 | 0.551 | -0.316 |
| logdha | rs2082109 | 0.157 | 1.05 | 0.366 | 0.885 | 1.33 | 0.76 | 0.621 | 0.761 | 0.348 | 0.551 | -0.316 |
| logdha | rs117316662 | 0.02 | 0.978 | 0.289 | 0.939 | 2 | 2.33 | 0.55 | 0.376 | 0.372 | 0.322 | -1.24 |
| logdha | rs12247163 | 0.177 | 1.09 | 0.379 | 0.807 | 1.31 | 0.673 | 0.604 | 0.718 | 0.292 | 0.415 | -0.374 |
| logdha | rs12355888 | 0.075 | 1.02 | 0.323 | 0.945 | 1.38 | 1.09 | 0.681 | 0.709 | 0.424 | 0.566 | -0.402 |
| logdha | rs143772290 | 0.005 | . | . | . | . | . | . | . | . | . | . |
| logdha | rs146849452 | 0.003 | . | . | . | . | . | . | . | . | . | . |
| logdha | rs140267121 | 0.003 | . | . | . | . | . | . | . | . | . | . |
| logdha | rs7080474 | 0.155 | 1.08 | 0.376 | 0.824 | 1.36 | 0.714 | 0.564 | 0.721 | 0.297 | 0.428 | -0.379 |
| logdha | rs144066203 | 0.001 | . | . | . | . | . | . | . | . | . | . |
| logdha | rs12765209 | 0.021 | 0.979 | 0.29 | 0.943 | 2.17 | 2.49 | 0.5 | 0.341 | 0.344 | 0.286 | -1.42 |
| logdha | rs150930735 | 0.001 | . | . | . | . | . | . | . | . | . | . |
| logdha | rs7068039 | 0.177 | 1.09 | 0.381 | 0.801 | 1.31 | 0.679 | 0.597 | 0.712 | 0.29 | 0.405 | -0.384 |
| logdha | rs117622652 | 0.02 | 0.978 | 0.289 | 0.939 | 1.99 | 2.32 | 0.553 | 0.378 | 0.373 | 0.324 | -1.23 |
| logdha | rs190191514 | 0.001 | . | . | . | . | . | . | . | . | . | . |
| logdha | rs74130835 | 0.023 | 1.04 | 0.314 | 0.897 | 3.14 | 3.46 | 0.298 | 0.074 | 0.09 | 0.032 | -2.94 |
| logdha | rs1811604 | 0.022 | 1.03 | 0.311 | 0.925 | 2.63 | 2.93 | 0.387 | 0.099 | 0.105 | 0.03 | -2.39 |
| logdha | rs146971256 | . | . | . | . | . | . | . | . | . | . | . |
| logdha | rs74336000 | 0.005 | . | . | . | . | . | . | . | . | . | . |
| logdha | rs186046492 | 0.005 | . | . | . | . | . | . | . | . | . | . |
| logdha | rs34971823 | 0.006 | . | . | . | . | . | . | . | . | . | . |
| logdha | rs75227446 | 0.02 | 0.978 | 0.289 | 0.939 | 2.05 | 2.36 | 0.536 | 0.365 | 0.361 | 0.308 | -1.29 |
| logdha | rs190457599 | 0.003 | . | . | . | . | . | . | . | . | . | . |
| logdha | rs79591917 | 0.02 | 0.977 | 0.289 | 0.938 | 1.98 | 2.3 | 0.559 | 0.382 | 0.375 | 0.328 | -1.22 |
| logdha | rs702365 | 0.609 | 0.795 | 0.403 | 0.651 | 0.738 | 0.319 | 0.481 | 1.16 | 0.419 | 0.672 | 0.15 |
| logdha | rs79643836 | 0.021 | 0.977 | 0.289 | 0.937 | 1.92 | 2.22 | 0.574 | 0.387 | 0.38 | 0.334 | -1.17 |
| logdha | rs702366 | 0.61 | 0.834 | 0.418 | 0.718 | 0.761 | 0.331 | 0.529 | 1.12 | 0.399 | 0.759 | 0.113 |
| logdha | rs1059696 | 0.123 | 1.05 | 0.36 | 0.885 | 1.53 | 0.979 | 0.509 | 0.723 | 0.347 | 0.499 | -0.418 |
| logdha | rs901681 | 0.61 | 0.885 | 0.432 | 0.802 | 0.79 | 0.34 | 0.584 | 1.06 | 0.37 | 0.87 | 0.065 |
| logdha | rs61854092 | 0.122 | 1.05 | 0.36 | 0.884 | 1.55 | 0.992 | 0.496 | 0.721 | 0.345 | 0.495 | -0.426 |
| logdha | rs148080753 | 0.001 | . | . | . | . | . | . | . | . | . | . |
| logdha | rs61854093 | 0.145 | 1.1 | 0.386 | 0.779 | 1.5 | 0.907 | 0.503 | 0.652 | 0.307 | 0.364 | -0.524 |
| logdha | rs141822774 | 0.002 | . | . | . | . | . | . | . | . | . | . |

| logdha | rs74130836 | 0.023 | 1.04 | 0.315 | 0.895 | 3.13 | 3.45 | 0.299 | 0.071 | 0.091 | 0.039 | -2.94 |
| --- | --- | --- | --- | --- | --- | --- | --- | --- | --- | --- | --- | --- |
| logdha | rs150620963 | 0.01 | . | . | . | . | . | . | . | . | . | . |
| logdha | rs3780913 | 0.781 | 1.21 | 0.918 | 0.806 | 0.908 | 0.515 | 0.865 | 0.856 | 0.391 | 0.734 | -0.176 |
| logdha | rs142255723 | 0.002 | . | . | . | . | . | . | . | . | . | . |
| logdha | rs3780914 | 0.608 | 0.818 | 0.405 | 0.685 | 0.749 | 0.324 | 0.504 | 1.13 | 0.402 | 0.723 | 0.128 |
| logdha | rs182656507 | 0.002 | . | . | . | . | . | . | . | . | . | . |
| logdha | rs2291427 | 0.756 | 1.29 | 0.936 | 0.73 | 0.957 | 0.51 | 0.934 | 0.822 | 0.361 | 0.655 | -0.231 |
| logdha | rs10751382 | 0.755 | 1.29 | 0.939 | 0.729 | 0.961 | 0.513 | 0.941 | 0.822 | 0.361 | 0.655 | -0.232 |
| logdha | rs117610108 | 0.02 | 0.976 | 0.288 | 0.936 | 1.9 | 2.24 | 0.584 | 0.397 | 0.387 | 0.344 | -1.14 |
| logdha | rs144966941 | 0.037 | 1 | 0.308 | 0.993 | 1.76 | 1.87 | 0.593 | 0.642 | 0.536 | 0.596 | -0.631 |
| logdha | rs41502051 | 0.022 | 0.927 | 0.279 | 0.802 | 0.712 | 1.12 | 0.829 | 2.73 | 3.72 | 0.46 | 1.16 |
| logdha | rs41426046 | 0.02 | 0.976 | 0.288 | 0.934 | 1.79 | 2.14 | 0.624 | 0.425 | 0.412 | 0.377 | -1.03 |
| logdha | rs41519248 | 0.02 | 0.976 | 0.288 | 0.935 | 1.87 | 2.21 | 0.596 | 0.404 | 0.393 | 0.352 | -1.11 |
| logdha | rs189108245 | 0.002 | . | . | . | . | . | . | . | . | . | . |
| logdha | rs7896431 | 0.591 | 0.821 | 0.422 | 0.702 | 0.766 | 0.358 | 0.569 | 1.14 | 0.421 | 0.725 | 0.129 |
| logdha | rs183724547 | 0.003 | . | . | . | . | . | . | . | . | . | . |
| logdha | rs142044990 | 0.006 | . | . | . | . | . | . | . | . | . | . |
| logdha | rs188140929 | . | . | . | . | . | . | . | . | . | . | . |
| logdha | rs76819096 | 0.008 | . | . | . | . | . | . | . | . | . | . |
| logdha | rs10751383 | 0.654 | 0.95 | 0.534 | 0.928 | 0.91 | 0.374 | 0.818 | 1.01 | 0.348 | 0.982 | 0.011 |
| logdha | rs117819321 | 0.015 | 1.05 | 0.312 | 0.876 | 37.6 | 88.1 | 0.122 | 0.058 | 0.115 | 0.152 | -35.4 |
| logdha | rs2242332 | 0.75 | 1.09 | 0.752 | 0.904 | 0.885 | 0.481 | 0.821 | 0.912 | 0.389 | 0.828 | -0.095 |
| logdha | rs2242333 | 0.06 | 1.11 | 0.332 | 0.728 | 3.46 | 2.57 | 0.096 | 0.299 | 0.209 | 0.084 | -2.42 |
| logdha | rs2242334 | 0.752 | 1.07 | 0.749 | 0.921 | 0.867 | 0.476 | 0.795 | 0.92 | 0.397 | 0.847 | -0.083 |
| logdha | rs189465041 | 0.002 | . | . | . | . | . | . | . | . | . | . |
| logdha | rs28395868 | 0.014 | 1 | 0.304 | 0.996 | 4.89 | 7.42 | 0.295 | 0.413 | 0.599 | 0.542 | -2.87 |
| logdha | rs56067550 | 0.016 | 0.996 | 0.302 | 0.99 | 3.44 | 10.2 | 0.678 | 0.516 | 1.61 | 0.832 | -1.67 |
| logdha | rs1051713 | 0.134 | 1.06 | 0.353 | 0.861 | 1.46 | 0.977 | 0.571 | 0.699 | 0.348 | 0.472 | -0.439 |
| logdha | rs115074210 | 0.009 | . | . | . | . | . | . | . | . | . | . |
| logdha | rs7900977 | 0.822 | 1.36 | 1.15 | 0.714 | 0.991 | 0.569 | 0.988 | 0.803 | 0.375 | 0.638 | -0.269 |
| logdha | rs147613891 | 0.004 | . | . | . | . | . | . | . | . | . | . |
| logdha | rs2229136 | 0.063 | 1.05 | 0.34 | 0.892 | 1.41 | 1.01 | 0.633 | 0.662 | 0.402 | 0.496 | -0.48 |
| logdha | rs185482841 | 0.001 | . | . | . | . | . | . | . | . | . | . |
| logdha | rs2288619 | 0.063 | 1.04 | 0.34 | 0.892 | 1.41 | 1.01 | 0.634 | 0.662 | 0.403 | 0.498 | -0.478 |
| logdha | rs189891524 | . | . | . | . | . | . | . | . | . | . | . |
| logdha | rs56145077 | 0.115 | 1.05 | 0.349 | 0.888 | 1.5 | 1.08 | 0.573 | 0.707 | 0.378 | 0.517 | -0.436 |
| logdha | rs143216970 | 0.003 | . | . | . | . | . | . | . | . | . | . |
| logdha | rs142246478 | 0.001 | . | . | . | . | . | . | . | . | . | . |
| logdha | rs13134 | 0.008 | . | . | . | . | . | . | . | . | . | . |
| logdha | rs41314980 | 0.031 | 0.869 | 0.262 | 0.641 | 0.132 | 0.317 | 0.399 | 8.63 | 15 | 0.216 | 0.986 |
| logdha | rs41304611 | 0.009 | . | . | . | . | . | . | . | . | . | . |
| logdha | rs193299617 | . | . | . | . | . | . | . | . | . | . | . |
| logdha | rs12772724 | 0.008 | . | . | . | . | . | . | . | . | . | . |
| logdha | rs139489537 | 0.03 | 0.993 | 0.313 | 0.983 | 1.41 | 1.14 | 0.67 | 0.876 | 0.607 | 0.848 | -0.177 |
| logdha | rs12776062 | 0.006 | . | . | . | . | . | . | . | . | . | . |
| logdha | rs752059 | 0.116 | 1.05 | 0.349 | 0.89 | 1.48 | 1.06 | 0.585 | 0.71 | 0.381 | 0.524 | -0.426 |
| logdha | rs147649982 | 0.049 | 0.928 | 0.293 | 0.812 | 0.38 | 0.354 | 0.299 | 1.24 | 0.929 | 0.771 | 0.131 |
| logdha | rs17157798 | 0.055 | 1.05 | 0.335 | 0.876 | 1.47 | 1.12 | 0.615 | 0.604 | 0.397 | 0.442 | -0.587 |
| logdha | rs142298619 | 0.001 | . | . | . | . | . | . | . | . | . | . |
| logdha | rs4948673 | 0.819 | 1.76 | 1.43 | 0.487 | 1.22 | 0.68 | 0.727 | 0.686 | 0.311 | 0.406 | -0.507 |
| logdha | rs74231484 | 0.066 | 1.15 | 0.349 | 0.648 | 4.15 | 2.77 | 0.033 | 0.27 | 0.164 | 0.031 | -3.01 |
| logdha | rs72796489 | 0.002 | . | . | . | . | . | . | . | . | . | . |
| logdha | rs113447578 | 0.014 | 0.977 | 0.292 | 0.939 | 2.12 | 2.75 | 0.562 | 0.86 | 1.09 | 0.905 | -0.316 |
| logdha | rs34063374 | 0.008 | . | . | . | . | . | . | . | . | . | . |
| logdha | rs11239527 | 0.011 | 0.835 | 0.247 | 0.543 | 8.40E-11 | 1.30E-09 | 0.119 | 8.00E+07 | 8.90E+08 | 0.102 | 0.17 |
| logdha | rs34371883 | 0.007 | . | . | . | . | . | . | . | . | . | . |
| logdha | rs118168828 | 0.006 | . | . | . | . | . | . | . | . | . | . |
| logdha | rs34349618 | 0.006 | . | . | . | . | . | . | . | . | . | . |
| logdha | rs80041679 | 0.03 | 0.994 | 0.313 | 0.985 | 1.41 | 1.14 | 0.669 | 0.868 | 0.602 | 0.838 | -0.188 |
| logdha | rs149640607 | 0.001 | . | . | . | . | . | . | . | . | . | . |
| logdha | rs4427523 | 0.896 | 2.24 | 2.41 | 0.455 | 0.693 | 0.526 | 0.629 | 0.613 | 0.37 | 0.417 | -0.981 |
| logdha | rs185880109 | . | . | . | . | . | . | . | . | . | . | . |
| logdha | rs191523220 | 0.001 | . | . | . | . | . | . | . | . | . | . |
| logdha | rs144315284 | 0.002 | . | . | . | . | . | . | . | . | . | . |
| logdha | rs4948674 | 0.345 | 0.806 | 0.329 | 0.597 | 0.619 | 0.302 | 0.326 | 1.32 | 0.561 | 0.513 | 0.234 |
| logdha | rs182984298 | 0.003 | . | . | . | . | . | . | . | . | . | . |
| logdha | rs56749023 | 0.016 | 1.01 | 0.297 | 0.983 | 2.21 | 2.47 | 0.475 | 0.304 | 0.315 | 0.251 | -1.54 |
| logdha | rs112508957 | 0.01 | 0.943 | 0.278 | 0.842 | 0.012 | 0.022 | 0.017 | 14.1 | 14.8 | 0.012 | 0.204 |
| logdha | rs7089063 | 0.195 | 1.18 | 0.416 | 0.632 | 1.52 | 0.738 | 0.385 | 0.602 | 0.232 | 0.188 | -0.621 |
| logdha | rs145000779 | . | . | . | . | . | . | . | . | . | . | . |
| logdha | rs80201572 | 0.03 | 0.995 | 0.314 | 0.987 | 1.42 | 1.15 | 0.667 | 0.861 | 0.596 | 0.829 | -0.197 |
| logdha | rs140568227 | 0.002 | . | . | . | . | . | . | . | . | . | . |
| logdha | rs149756228 | 0.009 | . | . | . | . | . | . | . | . | . | . |
| logdha | rs112380685 | 0.008 | . | . | . | . | . | . | . | . | . | . |
| logdha | rs60481109 | 0.015 | 1 | 0.296 | 0.993 | 1.93 | 2.27 | 0.576 | 0.347 | 0.345 | 0.287 | -1.26 |
| logdha | rs138221843 | 0.002 | . | . | . | . | . | . | . | . | . | . |
| logdha | rs150283194 | 0.001 | . | . | . | . | . | . | . | . | . | . |
| logdha | rs138879661 | 0.004 | . | . | . | . | . | . | . | . | . | . |
| logdha | rs34960345 | 0.044 | 1.03 | 0.327 | 0.936 | 1.3 | 0.993 | 0.73 | 0.677 | 0.409 | 0.519 | -0.424 |
| logdha | rs147514068 | 0.004 | . | . | . | . | . | . | . | . | . | . |
| logdha | rs35410535 | 0.06 | 1.06 | 0.342 | 0.846 | 1.4 | 1.04 | 0.654 | 0.584 | 0.367 | 0.393 | -0.593 |
| logdha | rs191613635 | 0.001 | . | . | . | . | . | . | . | . | . | . |
| logdha | rs17157800 | 0.005 | . | . | . | . | . | . | . | . | . | . |
| logdha | rs17523413 | 0.039 | 1 | 0.317 | 0.994 | 1.27 | 1 | 0.76 | 0.785 | 0.515 | 0.712 | -0.274 |
| logdha | rs7897036 | 0.037 | 0.983 | 0.297 | 0.954 | 0.584 | 0.712 | 0.659 | 0.943 | 0.966 | 0.955 | -0.025 |
| logdha | rs76334323 | 0.02 | 0.975 | 0.288 | 0.932 | 1.81 | 2.17 | 0.619 | 0.411 | 0.398 | 0.358 | -1.06 |

| logdha | rs143535991 | 0.001 | . | . | . | . | . | . | . | . | . | . |
| --- | --- | --- | --- | --- | --- | --- | --- | --- | --- | --- | --- | --- |
| logdha | rs140344979 | 0.003 | . | . | . | . | . | . | . | . | . | . |
| logdha | rs36047811 | 0.06 | 1.06 | 0.342 | 0.846 | 1.4 | 1.04 | 0.654 | 0.584 | 0.367 | 0.393 | -0.593 |
| logdha | rs76493570 | 0.043 | 1.02 | 0.326 | 0.943 | 1.29 | 0.99 | 0.737 | 0.687 | 0.424 | 0.543 | -0.408 |
| logdha | rs192998920 | . | . | . | . | . | . | . | . | . | . | . |
| logdha | rs116840852 | 0.013 | 0.952 | 0.291 | 0.872 | 0.729 | 2.91 | 0.937 | 2 | 7.19 | 0.847 | 0.709 |
| logdha | rs184824101 | 0 | . | . | . | . | . | . | . | . | . | . |
| logdha | rs41301621 | 0.003 | . | . | . | . | . | . | . | . | . | . |
| logdha | rs3740109 | 0.06 | 1.06 | 0.342 | 0.846 | 1.4 | 1.04 | 0.654 | 0.584 | 0.367 | 0.393 | -0.593 |
| logdha | rs7899415 | 0.025 | 0.839 | 0.276 | 0.593 | 0.019 | 0.067 | 0.255 | 37.1 | 98.7 | 0.175 | 0.738 |
| logdha | rs184395284 | 0.003 | . | . | . | . | . | . | . | . | . | . |
| logdha | rs139209589 | 0.004 | . | . | . | . | . | . | . | . | . | . |
| logdha | rs149924163 | 0.001 | . | . | . | . | . | . | . | . | . | . |
| logdha | rs3740110 | 0.02 | 0.975 | 0.288 | 0.932 | 1.82 | 2.19 | 0.617 | 0.41 | 0.396 | 0.356 | -1.07 |
| logdha | rs144335113 | 0.001 | . | . | . | . | . | . | . | . | . | . |
| logdha | rs74743385 | 0.01 | 0.943 | 0.278 | 0.842 | 0.012 | 0.022 | 0.016 | 14.1 | 14.9 | 0.012 | 0.204 |
| logdha | rs146545829 | 0.001 | . | . | . | . | . | . | . | . | . | . |
| logdha | rs181842689 | 0.001 | . | . | . | . | . | . | . | . | . | . |
| logdha | rs3740111 | 0.044 | 1.03 | 0.327 | 0.936 | 1.3 | 0.994 | 0.73 | 0.677 | 0.409 | 0.518 | -0.424 |
| logdha | rs186261599 | 0.001 | . | . | . | . | . | . | . | . | . | . |
| logdha | rs73285348 | 0.008 | . | . | . | . | . | . | . | . | . | . |
| logdha | rs186356901 | 0.001 | . | . | . | . | . | . | . | . | . | . |
| logdha | rs3824618 | 0.03 | 0.995 | 0.314 | 0.987 | 1.42 | 1.15 | 0.666 | 0.861 | 0.596 | 0.828 | -0.199 |
| logdha | rs3802548 | 0.304 | 0.926 | 0.351 | 0.84 | 0.943 | 0.429 | 0.897 | 1.09 | 0.393 | 0.8 | 0.087 |
| logdha | rs3802547 | 0.02 | 0.975 | 0.288 | 0.933 | 1.83 | 2.2 | 0.616 | 0.41 | 0.4 | 0.361 | -1.07 |
| logdha | rs77677521 | 0.013 | 0.952 | 0.291 | 0.872 | 0.723 | 2.89 | 0.935 | 2.02 | 7.25 | 0.844 | 0.716 |
| logdha | rs3824616 | 0.06 | 1.06 | 0.342 | 0.846 | 1.4 | 1.04 | 0.654 | 0.584 | 0.367 | 0.392 | -0.593 |
| logdha | rs191073818 | 0.001 | . | . | . | . | . | . | . | . | . | . |
| logdha | rs147078004 | . | . | . | . | . | . | . | . | . | . | . |
| logdha | rs7908745 | 0.388 | 1.03 | 0.423 | 0.943 | 1.04 | 0.459 | 0.934 | 0.921 | 0.309 | 0.805 | -0.084 |
| logdha | rs17157811 | 0.005 | . | . | . | . | . | . | . | . | . | . |
| logdha | rs138506087 | 0.006 | . | . | . | . | . | . | . | . | . | . |
| logdha | rs77241269 | 0.016 | 1.01 | 0.297 | 0.983 | 2.08 | 2.33 | 0.515 | 0.321 | 0.311 | 0.24 | -1.41 |
| logdha | rs138374938 | 0.001 | . | . | . | . | . | . | . | . | . | . |
| logdha | rs2279433 | 0.044 | 1.03 | 0.326 | 0.934 | 1.3 | 0.991 | 0.727 | 0.675 | 0.398 | 0.505 | -0.426 |
| logdha | rs73285358 | 0.016 | 1.01 | 0.297 | 0.984 | 2.06 | 2.31 | 0.521 | 0.324 | 0.31 | 0.239 | -1.39 |
| logdha | rs11239528 | 0.303 | 0.923 | 0.349 | 0.831 | 0.938 | 0.427 | 0.889 | 1.1 | 0.395 | 0.782 | 0.095 |
| logdha | rs77453386 | 0.02 | 0.975 | 0.288 | 0.932 | 1.83 | 2.21 | 0.618 | 0.4 | 0.386 | 0.342 | -1.09 |
| logdha | rs2279434 | 0.049 | 1.05 | 0.334 | 0.868 | 1.68 | 1.18 | 0.462 | 0.601 | 0.337 | 0.364 | -0.669 |
| logdha | rs149236525 | 0.005 | . | . | . | . | . | . | . | . | . | . |
| logdha | rs12573609 | 0.037 | 0.86 | 0.267 | 0.628 | 0.141 | 0.232 | 0.234 | 3.9 | 4.48 | 0.236 | 0.472 |
| logdha | rs191716655 | . | . | . | . | . | . | . | . | . | . | . |
| logdha | rs17523660 | 0.006 | . | . | . | . | . | . | . | . | . | . |
| logdha | rs142671360 | 0.004 | . | . | . | . | . | . | . | . | . | . |
| logdha | rs3764990 | 0.038 | 1 | 0.316 | 0.989 | 1.28 | 1.01 | 0.752 | 0.771 | 0.497 | 0.687 | -0.293 |
| logdha | rs113682861 | 0.005 | . | . | . | . | . | . | . | . | . | . |
| logdha | rs117658270 | 0.02 | 0.975 | 0.288 | 0.932 | 1.83 | 2.21 | 0.618 | 0.4 | 0.386 | 0.342 | -1.09 |
| logdha | rs187309063 | . | . | . | . | . | . | . | . | . | . | . |
| logdha | rs73285360 | 0.016 | 1.01 | 0.297 | 0.984 | 2.05 | 2.3 | 0.524 | 0.325 | 0.31 | 0.239 | -1.38 |
| logdha | rs80133289 | 0.007 | . | . | . | . | . | . | . | . | . | . |
| logdha | rs186693257 | . | . | . | . | . | . | . | . | . | . | . |
| logdha | rs35735968 | 0.048 | 1.04 | 0.334 | 0.894 | 1.31 | 0.999 | 0.724 | 0.636 | 0.389 | 0.46 | -0.484 |
| logdha | rs17157825 | 0.031 | 0.999 | 0.315 | 0.999 | 1.45 | 1.19 | 0.647 | 0.817 | 0.572 | 0.773 | -0.266 |
| logdha | rs2291428 | 0.299 | 0.906 | 0.338 | 0.792 | 0.908 | 0.413 | 0.832 | 1.14 | 0.408 | 0.704 | 0.128 |
| logdha | rs2291429 | 0.305 | 0.935 | 0.354 | 0.86 | 0.949 | 0.427 | 0.907 | 1.07 | 0.38 | 0.838 | 0.07 |
| logdha | rs145714792 | 0.003 | . | . | . | . | . | . | . | . | . | . |
| logdha | rs12769244 | 0.044 | 1.03 | 0.326 | 0.931 | 1.3 | 0.993 | 0.728 | 0.669 | 0.394 | 0.495 | -0.434 |
| logdha | rs12771275 | 0.044 | 1.03 | 0.326 | 0.931 | 1.3 | 0.993 | 0.728 | 0.669 | 0.394 | 0.495 | -0.434 |
| logdha | rs146266174 | 0 | . | . | . | . | . | . | . | . | . | . |
| logdha | rs41301623 | 0.002 | . | . | . | . | . | . | . | . | . | . |
| logdha | rs2306814 | 0.021 | 0.978 | 0.289 | 0.939 | 1.92 | 2.19 | 0.566 | 0.407 | 0.371 | 0.324 | -1.14 |
| logdha | rs12244834 | 0.012 | 1.02 | 0.307 | 0.948 | 0.329 | 2.95 | 0.901 | 0.014 | 0.148 | 0.684 | -0.344 |
| logdha | rs184006011 | 0.002 | . | . | . | . | . | . | . | . | . | . |
| logdha | rs78829066 | 0.02 | 0.976 | 0.288 | 0.934 | 1.91 | 2.27 | 0.588 | 0.38 | 0.369 | 0.319 | -1.18 |
| logdha | rs34731408 | 0.044 | 1.03 | 0.326 | 0.932 | 1.3 | 0.992 | 0.729 | 0.671 | 0.395 | 0.498 | -0.432 |
| logdha | rs17451516 | 0.008 | . | . | . | . | . | . | . | . | . | . |
| logdha | rs139910133 | 0.002 | . | . | . | . | . | . | . | . | . | . |
| logdha | rs10900218 | 0.197 | 1.14 | 0.399 | 0.705 | 1.39 | 0.689 | 0.502 | 0.671 | 0.26 | 0.303 | -0.467 |
| logdha | rs146023999 | 0.001 | . | . | . | . | . | . | . | . | . | . |
| logdha | rs187296958 | . | . | . | . | . | . | . | . | . | . | . |
| logdha | rs142342833 | . | . | . | . | . | . | . | . | . | . | . |
| logdha | rs143489255 | . | . | . | . | . | . | . | . | . | . | . |
| logdha | rs138532518 | . | . | . | . | . | . | . | . | . | . | . |
| logdha | rs183768079 | 0.001 | . | . | . | . | . | . | . | . | . | . |
| logdha | rs117892272 | . | . | . | . | . | . | . | . | . | . | . |
| logdha | rs192993943 | . | . | . | . | . | . | . | . | . | . | . |
| logdha | rs182989043 | 0.001 | . | . | . | . | . | . | . | . | . | . |
| logdha | rs189703435 | . | . | . | . | . | . | . | . | . | . | . |
| logdha | rs188794040 | . | . | . | . | . | . | . | . | . | . | . |
| logdha | rs143920953 | . | . | . | . | . | . | . | . | . | . | . |
| logdha | rs191029121 | . | . | . | . | . | . | . | . | . | . | . |
| logdha | rs116875448 | . | . | . | . | . | . | . | . | . | . | . |
| logdha | rs190167877 | 0.001 | . | . | . | . | . | . | . | . | . | . |
| logdha | rs184993418 | . | . | . | . | . | . | . | . | . | . | . |
| logdha | rs143241158 | . | . | . | . | . | . | . | . | . | . | . |
| logdha | rs11239500 | 0.015 | 1.02 | 0.307 | 0.945 | 4.38 | 6.5 | 0.32 | 0.211 | 0.164 | 0.045 | -3.46 |

| logdha | rs140568994 | . | . | . | . | . | . | . | . | . | . | . |
| --- | --- | --- | --- | --- | --- | --- | --- | --- | --- | --- | --- | --- |
| logdha | rs187530317 | . | . | . | . | . | . | . | . | . | . | . |
| logdha | rs145189326 | . | . | . | . | . | . | . | . | . | . | . |
| logdha | rs186203332 | . | . | . | . | . | . | . | . | . | . | . |
| logdha | rs189921909 | . | . | . | . | . | . | . | . | . | . | . |
| logdha | rs187260989 | . | . | . | . | . | . | . | . | . | . | . |
| logdha | rs114376586 | 0.013 | 1.02 | 0.307 | 0.944 | 6.19 | 7.94 | 0.155 | 0.149 | 0.11 | 9.70E-03 | -5.27 |
| logdha | rs151070808 | . | . | . | . | . | . | . | . | . | . | . |
| logdha | rs140927225 | 0.015 | 1.03 | 0.308 | 0.934 | 6.89 | 8.57 | 0.121 | 0.135 | 0.105 | 0.01 | -5.96 |
| logdha | rs191555615 | . | . | . | . | . | . | . | . | . | . | . |
| logdha | rs80278898 | 0.005 | . | . | . | . | . | . | . | . | . | . |
| logdha | rs190849503 | . | . | . | . | . | . | . | . | . | . | . |
| logdha | rs150080315 | 0.003 | . | . | . | . | . | . | . | . | . | . |
| logdha | rs143623885 | . | . | . | . | . | . | . | . | . | . | . |
| logdha | rs187760040 | . | . | . | . | . | . | . | . | . | . | . |
| logdha | rs143917143 | 0.005 | . | . | . | . | . | . | . | . | . | . |
| logdha | rs149851136 | 0.001 | . | . | . | . | . | . | . | . | . | . |
| logdha | rs181612054 | . | . | . | . | . | . | . | . | . | . | . |
| logdha | rs191006548 | . | . | . | . | . | . | . | . | . | . | . |
| logdha | rs117377631 | 0.026 | 0.985 | 0.293 | 0.96 | 0.706 | 0.947 | 0.795 | 0.552 | 0.528 | 0.535 | -0.307 |
| logdha | rs182107634 | . | . | . | . | . | . | . | . | . | . | . |
| logdha | rs190211867 | . | . | . | . | . | . | . | . | . | . | . |
| logdha | rs148293457 | . | . | . | . | . | . | . | . | . | . | . |
| logdha | rs191811733 | . | . | . | . | . | . | . | . | . | . | . |
| logdha | rs146109272 | . | . | . | . | . | . | . | . | . | . | . |
| logdha | rs148397915 | . | . | . | . | . | . | . | . | . | . | . |
| logdha | rs142233290 | . | . | . | . | . | . | . | . | . | . | . |
| logdha | rs80226874 | . | . | . | . | . | . | . | . | . | . | . |
| logdha | rs144496203 | . | . | . | . | . | . | . | . | . | . | . |
| logdha | rs191062130 | . | . | . | . | . | . | . | . | . | . | . |
| logdha | rs185807817 | . | . | . | . | . | . | . | . | . | . | . |
| logdha | rs117846213 | . | . | . | . | . | . | . | . | . | . | . |
| logdha | rs190545473 | . | . | . | . | . | . | . | . | . | . | . |
| logdha | rs192364918 | . | . | . | . | . | . | . | . | . | . | . |
| logdha | rs182939241 | . | . | . | . | . | . | . | . | . | . | . |
| logdha | rs12571125 | . | . | . | . | . | . | . | . | . | . | . |
| logdha | rs192418941 | . | . | . | . | . | . | . | . | . | . | . |
| logdha | rs141270243 | . | . | . | . | . | . | . | . | . | . | . |
| logdha | rs182615502 | . | . | . | . | . | . | . | . | . | . | . |
| logdha | rs146669856 | 0.004 | . | . | . | . | . | . | . | . | . | . |
| logdha | rs78290029 | 0.014 | 1.04 | 0.314 | 0.895 | 4.86 | 8.28 | 0.353 | 0.03 | 0.056 | 0.063 | -4.75 |
| logdha | rs139955565 | . | . | . | . | . | . | . | . | . | . | . |
| logdha | rs12267314 | 0.013 | 1.03 | 0.311 | 0.926 | 5.41 | 8.09 | 0.259 | 0.029 | 0.054 | 0.056 | -5.27 |
| logdha | rs74477197 | . | . | . | . | . | . | . | . | . | . | . |
| logdha | rs188147236 | . | . | . | . | . | . | . | . | . | . | . |
| logdha | rs2228065 | 0.012 | 1.03 | 0.31 | 0.93 | 6.08 | 12.5 | 0.378 | 0.027 | 0.047 | 0.04 | -5.94 |
| logdha | rs116961353 | 0.007 | . | . | . | . | . | . | . | . | . | . |
| logdha | rs190222272 | . | . | . | . | . | . | . | . | . | . | . |
| logdha | rs182719940 | . | . | . | . | . | . | . | . | . | . | . |
| logdha | rs184477413 | . | . | . | . | . | . | . | . | . | . | . |
| logdha | rs12265175 | 0.012 | 1.04 | 0.314 | 0.901 | 8.26 | 17.7 | 0.323 | 0.017 | 0.039 | 0.072 | -8.15 |
| logdha | rs182928276 | . | . | . | . | . | . | . | . | . | . | . |
| logdha | rs144409895 | . | . | . | . | . | . | . | . | . | . | . |
| logdha | rs58998447 | 0.003 | . | . | . | . | . | . | . | . | . | . |
| logdha | rs188668832 | . | . | . | . | . | . | . | . | . | . | . |
| logdha | rs148641686 | . | . | . | . | . | . | . | . | . | . | . |
| logdha | rs75468791 | . | . | . | . | . | . | . | . | . | . | . |
| logdha | rs147327397 | . | . | . | . | . | . | . | . | . | . | . |
| logdha | rs77354597 | 0.001 | . | . | . | . | . | . | . | . | . | . |
| logdha | rs41365544 | 0.012 | 1.04 | 0.317 | 0.906 | 2.25 | 22.2 | 0.935 | 9.20E-04 | 0.012 | 0.593 | -2.28 |
| logdha | rs187564188 | . | . | . | . | . | . | . | . | . | . | . |
| logdha | rs183511984 | . | . | . | . | . | . | . | . | . | . | . |
| logdha | rs12262783 | 0.011 | 1.05 | 0.329 | 0.878 | 6.40E-07 | 3.30E-05 | 0.783 | 5.90E-05 | 1.90E-03 | 0.758 | -0.049 |
| logdha | rs188817687 | 0.001 | . | . | . | . | . | . | . | . | . | . |
| logdha | rs3815968 | . | . | . | . | . | . | . | . | . | . | . |
| logdha | rs187822300 | . | . | . | . | . | . | . | . | . | . | . |
| logdha | rs188902172 | 0.001 | . | . | . | . | . | . | . | . | . | . |
| logdha | rs181190448 | . | . | . | . | . | . | . | . | . | . | . |
| logdha | rs182699093 | . | . | . | . | . | . | . | . | . | . | . |
| logdha | rs182631303 | . | . | . | . | . | . | . | . | . | . | . |
| logdha | rs187014172 | 0.001 | . | . | . | . | . | . | . | . | . | . |
| logdha | rs142575406 | . | . | . | . | . | . | . | . | . | . | . |
| logdha | rs180711033 | . | . | . | . | . | . | . | . | . | . | . |
| logdha | rs137894823 | 0.001 | . | . | . | . | . | . | . | . | . | . |
| logdha | rs189053273 | 0 | . | . | . | . | . | . | . | . | . | . |
| logdha | rs143499388 | . | . | . | . | . | . | . | . | . | . | . |
| logdha | rs182936001 | 0.001 | . | . | . | . | . | . | . | . | . | . |
| logdha | rs148456406 | 0 | . | . | . | . | . | . | . | . | . | . |
| logdha | rs182499531 | 0.001 | . | . | . | . | . | . | . | . | . | . |
| logdha | rs74884915 | 0.005 | . | . | . | . | . | . | . | . | . | . |
| logdha | rs184046374 | . | . | . | . | . | . | . | . | . | . | . |
| logdha | rs74333993 | 0.004 | . | . | . | . | . | . | . | . | . | . |
| logdha | rs138889719 | . | . | . | . | . | . | . | . | . | . | . |
| logdha | rs75330330 | 0.003 | . | . | . | . | . | . | . | . | . | . |
| logdha | rs193051693 | . | . | . | . | . | . | . | . | . | . | . |
| logdha | rs145929633 | . | . | . | . | . | . | . | . | . | . | . |

| logdha | rs184076904 | 0.001 | . | . | . | . | . | . | . | . | . | . |
| --- | --- | --- | --- | --- | --- | --- | --- | --- | --- | --- | --- | --- |
| logdha | rs141802494 | 0.002 | . | . | . | . | . | . | . | . | . | . |
| logdha | rs149519759 | 0 | . | . | . | . | . | . | . | . | . | . |
| logdha | rs145467260 | 0.002 | . | . | . | . | . | . | . | . | . | . |
| logdha | rs79620914 | 0.006 | . | . | . | . | . | . | . | . | . | . |
| logdha | rs140538633 | 0.005 | . | . | . | . | . | . | . | . | . | . |
| logdha | rs140862301 | 0.001 | . | . | . | . | . | . | . | . | . | . |
| logdha | rs114615847 | 0.003 | . | . | . | . | . | . | . | . | . | . |
| logdha | rs141800259 | 0.004 | . | . | . | . | . | . | . | . | . | . |
| logdha | rs149779059 | 0.003 | . | . | . | . | . | . | . | . | . | . |
| logdha | rs116539253 | 0.001 | . | . | . | . | . | . | . | . | . | . |
| logdha | rs184979550 | 0 | . | . | . | . | . | . | . | . | . | . |
| logdha | rs190128940 | 0.001 | . | . | . | . | . | . | . | . | . | . |
| logdha | rs146594816 | . | . | . | . | . | . | . | . | . | . | . |
| logdha | rs141370766 | 0.003 | . | . | . | . | . | . | . | . | . | . |
| logdha | rs144670033 | . | . | . | . | . | . | . | . | . | . | . |
| logdha | rs187152174 | 0.003 | . | . | . | . | . | . | . | . | . | . |
| logdha | rs184180263 | 0.002 | . | . | . | . | . | . | . | . | . | . |
| logdha | rs140075861 | 0.002 | . | . | . | . | . | . | . | . | . | . |
| logdha | rs186483129 | . | . | . | . | . | . | . | . | . | . | . |
| logdha | rs186634282 | 0.003 | . | . | . | . | . | . | . | . | . | . |
| logdha | rs9988663 | 0.004 | . | . | . | . | . | . | . | . | . | . |
| logdha | rs138828529 | 0.004 | . | . | . | . | . | . | . | . | . | . |
| logdha | rs188777615 | 0.001 | . | . | . | . | . | . | . | . | . | . |
| logdha | rs192321882 | 0.001 | . | . | . | . | . | . | . | . | . | . |
| logdha | rs150812270 | 0.003 | . | . | . | . | . | . | . | . | . | . |
| logdha | rs185906603 | . | . | . | . | . | . | . | . | . | . | . |
| logdha | rs191070862 | . | . | . | . | . | . | . | . | . | . | . |
| logdha | rs182904978 | . | . | . | . | . | . | . | . | . | . | . |
| logdha | rs192679655 | . | . | . | . | . | . | . | . | . | . | . |
| logdha | rs189673086 | 0.003 | . | . | . | . | . | . | . | . | . | . |
| logdha | rs181141892 | 0.001 | . | . | . | . | . | . | . | . | . | . |
| logdha | rs186404123 | . | . | . | . | . | . | . | . | . | . | . |
| logdha | rs192117287 | 0.001 | . | . | . | . | . | . | . | . | . | . |
| logdha | rs182748835 | 0 | . | . | . | . | . | . | . | . | . | . |
| logdha | rs141854549 | 0.005 | . | . | . | . | . | . | . | . | . | . |
| logdha | rs140163397 | 0.001 | . | . | . | . | . | . | . | . | . | . |
| logdha | rs146347300 | 0.001 | . | . | . | . | . | . | . | . | . | . |
| logdha | rs139362377 | 0.005 | . | . | . | . | . | . | . | . | . | . |
| logdha | rs185087671 | 0 | . | . | . | . | . | . | . | . | . | . |
| logdha | rs190466574 | 0 | . | . | . | . | . | . | . | . | . | . |
| logdha | rs147012778 | 0.001 | . | . | . | . | . | . | . | . | . | . |
| logdha | rs138236960 | 0.003 | . | . | . | . | . | . | . | . | . | . |
| logdha | rs116310311 | 0.005 | . | . | . | . | . | . | . | . | . | . |
| logdha | rs142868356 | 0.003 | . | . | . | . | . | . | . | . | . | . |
| logdha | rs144956240 | 0.001 | . | . | . | . | . | . | . | . | . | . |
| logdha | rs188527776 | . | . | . | . | . | . | . | . | . | . | . |
| logdha | rs183971165 | 0.003 | . | . | . | . | . | . | . | . | . | . |
| logdha | rs76748216 | 0.003 | . | . | . | . | . | . | . | . | . | . |
| logdha | rs148554990 | . | . | . | . | . | . | . | . | . | . | . |
| logdha | rs114446477 | 0.007 | . | . | . | . | . | . | . | . | . | . |
| logdha | rs185223933 | 0.002 | . | . | . | . | . | . | . | . | . | . |
| logdha | rs76869726 | 0.003 | . | . | . | . | . | . | . | . | . | . |
| logdha | rs151200830 | 0.005 | . | . | . | . | . | . | . | . | . | . |
| logdha | rs17157731 | 0.005 | . | . | . | . | . | . | . | . | . | . |
| logdha | rs181243469 | . | . | . | . | . | . | . | . | . | . | . |
| logdha | rs186890707 | . | . | . | . | . | . | . | . | . | . | . |
| logdha | rs184063360 | . | . | . | . | . | . | . | . | . | . | . |
| logdha | rs188550237 | 0.001 | . | . | . | . | . | . | . | . | . | . |
| logdha | rs140931068 | 0.003 | . | . | . | . | . | . | . | . | . | . |
| logdha | rs74867877 | 0.001 | . | . | . | . | . | . | . | . | . | . |
| logdha | rs11239495 | 0.015 | 0.977 | 0.292 | 0.939 | 3.13 | 6.03 | 0.553 | 0.568 | 0.898 | 0.72 | -1.37 |
| logdha | rs149745800 | . | . | . | . | . | . | . | . | . | . | . |
| logdha | rs145044458 | 0.005 | . | . | . | . | . | . | . | . | . | . |
| logdha | rs188067427 | 0 | . | . | . | . | . | . | . | . | . | . |
| logdha | rs146827800 | . | . | . | . | . | . | . | . | . | . | . |
| logdha | rs140619817 | 0.005 | . | . | . | . | . | . | . | . | . | . |
| logdha | rs892689 | 0.998 | . | . | . | . | . | . | . | . | . | . |
| logdha | rs143642005 | 0.001 | . | . | . | . | . | . | . | . | . | . |
| logdha | rs181929304 | 0.001 | . | . | . | . | . | . | . | . | . | . |
| logdha | rs191086173 | 0.001 | . | . | . | . | . | . | . | . | . | . |
| logdha | rs114613116 | 0.005 | . | . | . | . | . | . | . | . | . | . |
| logdha | rs141076768 | . | . | . | . | . | . | . | . | . | . | . |
| logdha | rs187316531 | . | . | . | . | . | . | . | . | . | . | . |
| logdha | rs11239499 | 0.014 | 0.992 | 0.301 | 0.98 | 2.61 | 3.85 | 0.516 | 0.454 | 0.434 | 0.409 | -1.43 |
| logdha | rs192726371 | 0.001 | . | . | . | . | . | . | . | . | . | . |
| logdha | rs74128481 | 0.004 | . | . | . | . | . | . | . | . | . | . |
| logdha | rs74128482 | 0.003 | . | . | . | . | . | . | . | . | . | . |
| logdha | rs11239502 | 0.008 | . | . | . | . | . | . | . | . | . | . |
| logdha | rs116330285 | . | . | . | . | . | . | . | . | . | . | . |
| logdha | rs189926729 | 0.001 | . | . | . | . | . | . | . | . | . | . |
| logdha | rs188975779 | 0.003 | . | . | . | . | . | . | . | . | . | . |
| logdha | rs143846038 | . | . | . | . | . | . | . | . | . | . | . |
| logdha | rs74128483 | 0.004 | . | . | . | . | . | . | . | . | . | . |
| logdha | rs139292055 | 0.013 | 1.02 | 0.307 | 0.945 | 6.28 | 8.03 | 0.15 | 0.148 | 0.11 | 9.90E-03 | -5.35 |
| logdha | rs11239503 | 0.013 | 1.02 | 0.307 | 0.945 | 6.27 | 8.02 | 0.151 | 0.148 | 0.11 | 9.90E-03 | -5.35 |

| logdha | rs58838686 | 0.003 | . | . | . | . | . | . | . | . | . | . |
| --- | --- | --- | --- | --- | --- | --- | --- | --- | --- | --- | --- | --- |
| logdha | rs185825373 | 0.001 | . | . | . | . | . | . | . | . | . | . |
| logdha | rs11239504 | 0.013 | 1.02 | 0.307 | 0.945 | 6.28 | 8.02 | 0.15 | 0.148 | 0.11 | 9.80E-03 | -5.35 |
| logdha | rs150012354 | 0 | . | . | . | . | . | . | . | . | . | . |
| logdha | rs187763096 | 0.001 | . | . | . | . | . | . | . | . | . | . |
| logdha | rs193043982 | 0.002 | . | . | . | . | . | . | . | . | . | . |
| logdha | rs79186884 | 0.004 | . | . | . | . | . | . | . | . | . | . |
| logdha | rs74128484 | 0.003 | . | . | . | . | . | . | . | . | . | . |
| logdha | rs58461537 | 0.013 | 1.02 | 0.307 | 0.945 | 6.27 | 8 | 0.15 | 0.148 | 0.11 | 9.70E-03 | -5.34 |
| logdha | rs188527474 | 0.002 | . | . | . | . | . | . | . | . | . | . |
| logdha | rs141659976 | 0.002 | . | . | . | . | . | . | . | . | . | . |
| logdha | rs115846407 | 0.013 | 1.02 | 0.307 | 0.944 | 6.27 | 7.99 | 0.15 | 0.149 | 0.109 | 9.70E-03 | -5.34 |
| logdha | rs138378816 | 0.013 | 1.02 | 0.307 | 0.944 | 6.26 | 7.99 | 0.15 | 0.149 | 0.109 | 9.60E-03 | -5.33 |
| logdha | rs146188136 | 0.008 | . | . | . | . | . | . | . | . | . | . |
| logdha | rs139383871 | 0.002 | . | . | . | . | . | . | . | . | . | . |
| logdha | rs182953869 | . | . | . | . | . | . | . | . | . | . | . |
| logdha | rs192430299 | 0.004 | . | . | . | . | . | . | . | . | . | . |
| logdha | rs144152844 | 0.013 | 1.02 | 0.307 | 0.945 | 6.08 | 7.86 | 0.163 | 0.153 | 0.112 | 0.01 | -5.15 |
| logdha | rs145987786 | 0.013 | 0.993 | 0.295 | 0.98 | 6.43 | 10 | 0.232 | 0.116 | 0.118 | 0.034 | -5.68 |
| logdha | rs181132926 | 0.001 | . | . | . | . | . | . | . | . | . | . |
| logdha | rs147882253 | 0.013 | 1.02 | 0.307 | 0.944 | 6.25 | 7.97 | 0.151 | 0.149 | 0.109 | 9.50E-03 | -5.32 |
| logdha | rs141807621 | 0.005 | . | . | . | . | . | . | . | . | . | . |
| logdha | rs147116052 | 0.013 | 1.02 | 0.307 | 0.944 | 6.25 | 7.97 | 0.151 | 0.149 | 0.109 | 9.50E-03 | -5.32 |
| logdha | rs147716618 | 0.013 | 1.02 | 0.307 | 0.944 | 6.25 | 7.97 | 0.151 | 0.149 | 0.109 | 9.50E-03 | -5.32 |
| logdha | rs187198855 | 0.01 | 0.991 | 0.294 | 0.976 | 9.63 | 17.9 | 0.223 | 0.076 | 0.101 | 0.053 | -8.89 |
| logdha | rs189409638 | 0.005 | . | . | . | . | . | . | . | . | . | . |
| logdha | rs182647339 | . | . | . | . | . | . | . | . | . | . | . |
| logdha | rs187995988 | . | . | . | . | . | . | . | . | . | . | . |
| logdha | rs141034184 | 0.015 | 1.02 | 0.308 | 0.937 | 5.21 | 6.3 | 0.172 | 0.157 | 0.116 | 0.012 | -4.4 |
| logdha | rs187310430 | 0.002 | . | . | . | . | . | . | . | . | . | . |
| logdha | rs192608488 | 0.013 | 1.02 | 0.307 | 0.944 | 6.11 | 7.77 | 0.154 | 0.151 | 0.11 | 9.50E-03 | -5.19 |
| logdha | rs148840366 | 0.003 | . | . | . | . | . | . | . | . | . | . |
| logdha | rs186785022 | 0.004 | . | . | . | . | . | . | . | . | . | . |
| logdha | rs192348728 | 0.002 | . | . | . | . | . | . | . | . | . | . |
| logdha | rs189656631 | 0.013 | 1.02 | 0.307 | 0.944 | 6.11 | 7.77 | 0.155 | 0.151 | 0.11 | 9.50E-03 | -5.19 |
| logdha | rs185289750 | . | . | . | . | . | . | . | . | . | . | . |
| logdha | rs142382395 | 0.013 | 1.02 | 0.307 | 0.944 | 6.11 | 7.76 | 0.154 | 0.151 | 0.11 | 9.50E-03 | -5.19 |
| logdha | rs144866696 | 0.004 | . | . | . | . | . | . | . | . | . | . |
| logdha | rs141371289 | 0.013 | 1.02 | 0.307 | 0.944 | 6.11 | 7.76 | 0.155 | 0.151 | 0.11 | 9.40E-03 | -5.19 |
| logdha | rs114750008 | 0.013 | 1.02 | 0.307 | 0.944 | 6.11 | 7.76 | 0.154 | 0.151 | 0.11 | 9.40E-03 | -5.19 |
| logdha | rs189063597 | 0.002 | . | . | . | . | . | . | . | . | . | . |
| logdha | rs139151571 | 0.013 | 1.02 | 0.307 | 0.941 | 6.77 | 9.22 | 0.16 | 0.136 | 0.104 | 9.00E-03 | -5.85 |
| logdha | rs139846368 | 0.014 | 1.02 | 0.309 | 0.941 | 3.45 | 5.79 | 0.461 | 0.219 | 0.175 | 0.057 | -2.7 |
| logdha | rs182057653 | 0.004 | . | . | . | . | . | . | . | . | . | . |
| logdha | rs148785420 | 0.013 | 1.02 | 0.307 | 0.944 | 6.09 | 7.74 | 0.155 | 0.151 | 0.11 | 9.40E-03 | -5.17 |
| logdha | rs138383251 | 0.002 | . | . | . | . | . | . | . | . | . | . |
| logdha | rs116526705 | 0.006 | . | . | . | . | . | . | . | . | . | . |
| logdha | rs185946436 | . | . | . | . | . | . | . | . | . | . | . |
| logdha | rs190455427 | . | . | . | . | . | . | . | . | . | . | . |
| logdha | rs9282584 | 0.013 | 1.02 | 0.307 | 0.943 | 6.09 | 7.73 | 0.155 | 0.152 | 0.11 | 9.30E-03 | -5.17 |
| logdha | rs17153289 | 0.013 | 1.02 | 0.307 | 0.943 | 6.09 | 7.73 | 0.155 | 0.152 | 0.11 | 9.30E-03 | -5.17 |
| logdha | rs138359103 | 0.001 | . | . | . | . | . | . | . | . | . | . |
| logdha | rs150605268 | 0.001 | . | . | . | . | . | . | . | . | . | . |
| logdha | rs79242910 | 0.015 | 1.02 | 0.307 | 0.946 | 2.44 | 2.95 | 0.46 | 0.272 | 0.176 | 0.044 | -1.79 |
| logdha | rs139716904 | 0.004 | . | . | . | . | . | . | . | . | . | . |
| logdha | rs76238498 | 0.013 | 0.994 | 0.295 | 0.983 | 6.81 | 10.5 | 0.214 | 0.107 | 0.11 | 0.03 | -6.08 |
| logdha | rs184140639 | . | . | . | . | . | . | . | . | . | . | . |
| logdha | rs188517278 | 0.002 | . | . | . | . | . | . | . | . | . | . |
| logdha | rs139678913 | 0.001 | . | . | . | . | . | . | . | . | . | . |
| logdha | rs116697955 | . | . | . | . | . | . | . | . | . | . | . |
| logdha | rs79267994 | 0.003 | . | . | . | . | . | . | . | . | . | . |
| logdha | rs11239510 | 0.003 | . | . | . | . | . | . | . | . | . | . |
| logdha | rs75831645 | 0.006 | . | . | . | . | . | . | . | . | . | . |
| logdha | rs78452276 | 0.008 | . | . | . | . | . | . | . | . | . | . |
| logdha | rs144366924 | . | . | . | . | . | . | . | . | . | . | . |
| logdha | rs77519841 | 0.008 | . | . | . | . | . | . | . | . | . | . |
| logdha | rs76944454 | 0.013 | 1.02 | 0.307 | 0.943 | 6.06 | 7.71 | 0.157 | 0.151 | 0.11 | 9.10E-03 | -5.14 |
| logdha | rs76443855 | 0.013 | 0.993 | 0.295 | 0.982 | 6.63 | 10.3 | 0.225 | 0.11 | 0.112 | 0.03 | -5.89 |
| logdha | rs146613174 | 0.001 | . | . | . | . | . | . | . | . | . | . |
| logdha | rs181424761 | 0 | . | . | . | . | . | . | . | . | . | . |
| logdha | rs142119241 | 0 | . | . | . | . | . | . | . | . | . | . |
| logdha | rs140137954 | 0.006 | . | . | . | . | . | . | . | . | . | . |
| logdha | rs187319549 | . | . | . | . | . | . | . | . | . | . | . |
| logdha | rs12241545 | 0.006 | . | . | . | . | . | . | . | . | . | . |
| logdha | rs10128228 | 0.013 | 1.02 | 0.307 | 0.943 | 6.04 | 7.68 | 0.158 | 0.152 | 0.109 | 9.00E-03 | -5.12 |
| logdha | rs58580249 | 0.003 | . | . | . | . | . | . | . | . | . | . |
| logdha | rs193148863 | . | . | . | . | . | . | . | . | . | . | . |
| logdha | rs185638946 | . | . | . | . | . | . | . | . | . | . | . |
| logdha | rs117752921 | 0 | . | . | . | . | . | . | . | . | . | . |
| logdha | rs180841236 | 0 | . | . | . | . | . | . | . | . | . | . |
| logdha | rs73283155 | 0.003 | . | . | . | . | . | . | . | . | . | . |
| logdha | rs76560599 | 0.012 | 1.02 | 0.308 | 0.942 | 8.1 | 11.5 | 0.141 | 0.122 | 0.097 | 8.30E-03 | -7.11 |
| logdha | rs76273620 | 0.012 | 1.02 | 0.308 | 0.942 | 8.1 | 11.5 | 0.141 | 0.122 | 0.097 | 8.30E-03 | -7.11 |
| logdha | rs141432923 | 0.003 | . | . | . | . | . | . | . | . | . | . |
| logdha | rs113529322 | 0.004 | . | . | . | . | . | . | . | . | . | . |
| logdha | rs10128306 | 0.013 | 1.02 | 0.307 | 0.943 | 6.02 | 7.65 | 0.158 | 0.152 | 0.109 | 8.80E-03 | -5.1 |

| logdha | rs149691688 | 0.001 | . | . | . | . | . | . | . | . | . | . |
| --- | --- | --- | --- | --- | --- | --- | --- | --- | --- | --- | --- | --- |
| logdha | rs75872495 | 0.013 | 1.02 | 0.307 | 0.943 | 5.93 | 7.5 | 0.159 | 0.154 | 0.11 | 8.80E-03 | -5.02 |
| logdha | rs191873960 | 0.001 | . | . | . | . | . | . | . | . | . | . |
| logdha | rs80155622 | 0.013 | 1.02 | 0.307 | 0.943 | 5.88 | 7.43 | 0.161 | 0.155 | 0.11 | 8.90E-03 | -4.97 |
| logdha | rs113914656 | 0.001 | . | . | . | . | . | . | . | . | . | . |
| logdha | rs12242840 | 0.013 | 1.02 | 0.307 | 0.944 | 5.58 | 6.95 | 0.167 | 0.161 | 0.113 | 9.60E-03 | -4.69 |
| logdha | rs147824078 | 0.002 | . | . | . | . | . | . | . | . | . | . |
| logdha | rs59051493 | 0.013 | 1.02 | 0.307 | 0.945 | 5.44 | 6.67 | 0.168 | 0.163 | 0.116 | 0.01 | -4.55 |
| logdha | rs9282588 | 0.001 | . | . | . | . | . | . | . | . | . | . |
| logdha | rs150215207 | 0.003 | . | . | . | . | . | . | . | . | . | . |
| logdha | rs138772243 | 0.001 | . | . | . | . | . | . | . | . | . | . |
| logdha | rs149383244 | 0.002 | . | . | . | . | . | . | . | . | . | . |
| logdha | rs17522720 | 0.006 | . | . | . | . | . | . | . | . | . | . |
| logdha | rs79245105 | 0.013 | 1.02 | 0.307 | 0.945 | 5.45 | 6.68 | 0.167 | 0.163 | 0.116 | 0.011 | -4.56 |
| logdha | rs138222133 | 0.001 | . | . | . | . | . | . | . | . | . | . |
| logdha | rs12247314 | 0.015 | 1.02 | 0.308 | 0.936 | 5.02 | 6.16 | 0.188 | 0.163 | 0.115 | 9.90E-03 | -4.2 |
| logdha | rs12263894 | 0.014 | 1.03 | 0.309 | 0.931 | 7.99 | 10.2 | 0.102 | 0.118 | 0.086 | 3.30E-03 | -7.06 |
| logdha | rs149907880 | 0.002 | . | . | . | . | . | . | . | . | . | . |
| logdha | rs188153885 | 0.002 | . | . | . | . | . | . | . | . | . | . |
| logdha | rs73283159 | 0.002 | . | . | . | . | . | . | . | . | . | . |
| logdha | rs140476687 | . | . | . | . | . | . | . | . | . | . | . |
| logdha | rs186603148 | . | . | . | . | . | . | . | . | . | . | . |
| logdha | rs183296063 | 0.001 | . | . | . | . | . | . | . | . | . | . |
| logdha | rs145733694 | 0.003 | . | . | . | . | . | . | . | . | . | . |
| logdha | rs189807449 | . | . | . | . | . | . | . | . | . | . | . |
| logdha | rs12253223 | 0.009 | . | . | . | . | . | . | . | . | . | . |
| logdha | rs182113637 | . | . | . | . | . | . | . | . | . | . | . |
| logdha | rs142792099 | 0.002 | . | . | . | . | . | . | . | . | . | . |
| logdha | rs12249910 | 0.009 | . | . | . | . | . | . | . | . | . | . |
| logdha | rs149436339 | 0 | . | . | . | . | . | . | . | . | . | . |
| logdha | rs77632210 | . | . | . | . | . | . | . | . | . | . | . |
| logdha | rs74130818 | 0.003 | . | . | . | . | . | . | . | . | . | . |
| logdha | rs187583648 | . | . | . | . | . | . | . | . | . | . | . |
| logdha | rs191295987 | 0.001 | . | . | . | . | . | . | . | . | . | . |
| logdha | rs184200686 | 0.002 | . | . | . | . | . | . | . | . | . | . |
| logdha | rs76117263 | 0.013 | 0.993 | 0.295 | 0.982 | 5.41 | 9.42 | 0.332 | 0.11 | 0.114 | 0.033 | -4.81 |
| logdha | rs185393149 | 0.001 | . | . | . | . | . | . | . | . | . | . |
| logdha | rs144760014 | 0 | . | . | . | . | . | . | . | . | . | . |
| logdha | rs7922292 | 0.002 | . | . | . | . | . | . | . | . | . | . |
| logdha | rs115372844 | 0.003 | . | . | . | . | . | . | . | . | . | . |
| logdha | rs111460364 | 0.013 | 0.994 | 0.295 | 0.983 | 5.32 | 9.38 | 0.344 | 0.11 | 0.114 | 0.033 | -4.73 |
| logdha | rs182052255 | 0 | . | . | . | . | . | . | . | . | . | . |
| logdha | rs180983889 | 0.002 | . | . | . | . | . | . | . | . | . | . |
| logdha | rs139175327 | 0.002 | . | . | . | . | . | . | . | . | . | . |
| logdha | rs149959620 | 0.004 | . | . | . | . | . | . | . | . | . | . |
| logdha | rs114653117 | 0.002 | . | . | . | . | . | . | . | . | . | . |
| logdha | rs144270018 | 0 | . | . | . | . | . | . | . | . | . | . |
| logdha | rs182723204 | 0.002 | . | . | . | . | . | . | . | . | . | . |
| logdha | rs57204067 | 0.003 | . | . | . | . | . | . | . | . | . | . |
| logdha | rs144956226 | 0.009 | . | . | . | . | . | . | . | . | . | . |
| logdha | rs187842704 | 0.001 | . | . | . | . | . | . | . | . | . | . |
| logdha | rs184620511 | 0.003 | . | . | . | . | . | . | . | . | . | . |
| logdha | rs144031303 | 0.005 | . | . | . | . | . | . | . | . | . | . |
| logdha | rs187526473 | . | . | . | . | . | . | . | . | . | . | . |
| logdha | rs193002805 | . | . | . | . | . | . | . | . | . | . | . |
| logdha | rs138707426 | 0.002 | . | . | . | . | . | . | . | . | . | . |
| logdha | rs142754243 | 0.01 | 0.989 | 0.293 | 0.971 | 2.11 | 3.37 | 0.641 | 0.439 | 0.454 | 0.426 | -1.18 |
| logdha | rs181600331 | 0.002 | . | . | . | . | . | . | . | . | . | . |
| logdha | rs144421264 | 0.006 | . | . | . | . | . | . | . | . | . | . |
| logdha | rs184414519 | 0.002 | . | . | . | . | . | . | . | . | . | . |
| logdha | rs150596349 | 0.012 | 0.99 | 0.294 | 0.972 | 1.32 | 3.56 | 0.918 | 0.14 | 0.181 | 0.127 | -1.13 |
| logdha | rs190753747 | . | . | . | . | . | . | . | . | . | . | . |
| logdha | rs139560940 | . | . | . | . | . | . | . | . | . | . | . |
| logdha | rs75640804 | 0.008 | . | . | . | . | . | . | . | . | . | . |
| logdha | rs73283175 | 0.018 | 1.02 | 0.303 | 0.958 | 1.26 | 1.44 | 0.836 | 0.396 | 0.378 | 0.332 | -0.772 |
| logdha | rs143337229 | 0.001 | . | . | . | . | . | . | . | . | . | . |
| logdha | rs73283177 | 0.01 | 0.991 | 0.294 | 0.976 | 2.2 | 3.59 | 0.628 | 0.407 | 0.427 | 0.391 | -1.3 |
| logdha | rs76053611 | 0.011 | 0.989 | 0.293 | 0.97 | 1.12 | 3.09 | 0.968 | 0.176 | 0.243 | 0.208 | -0.913 |
| logdha | rs116325983 | . | . | . | . | . | . | . | . | . | . | . |
| logdha | rs115451825 | 0.001 | . | . | . | . | . | . | . | . | . | . |
| logdha | rs183845246 | 0 | . | . | . | . | . | . | . | . | . | . |
| logdha | rs79756542 | 0.013 | 0.99 | 0.294 | 0.972 | 0.726 | 2.51 | 0.926 | 0.251 | 0.44 | 0.431 | -0.535 |
| logdha | rs187242860 | . | . | . | . | . | . | . | . | . | . | . |
| logdha | rs57318980 | 0.01 | 0.995 | 0.295 | 0.986 | 2.53 | 4.19 | 0.574 | 0.337 | 0.367 | 0.318 | -1.68 |
| logdha | rs59969732 | 0.01 | 0.995 | 0.295 | 0.987 | 2.63 | 4.35 | 0.558 | 0.325 | 0.358 | 0.307 | -1.78 |
| logdha | rs143811488 | 0.003 | . | . | . | . | . | . | . | . | . | . |
| logdha | rs189067644 | 0.002 | . | . | . | . | . | . | . | . | . | . |
| logdha | rs60449178 | 0.01 | . | . | . | . | . | . | . | . | . | . |
| logdha | rs59243389 | 0.01 | . | . | . | . | . | . | . | . | . | . |
| logdha | rs184946540 | 0.009 | . | . | . | . | . | . | . | . | . | . |
| logdha | rs188685881 | 0 | . | . | . | . | . | . | . | . | . | . |
| logdha | rs150335797 | 0.002 | . | . | . | . | . | . | . | . | . | . |
| logdha | rs143879260 | 0.002 | . | . | . | . | . | . | . | . | . | . |
| logdha | rs186044491 | 0.003 | . | . | . | . | . | . | . | . | . | . |
| logdha | rs11819084 | 0.004 | . | . | . | . | . | . | . | . | . | . |
| logdha | rs146915670 | . | . | . | . | . | . | . | . | . | . | . |

| logdha | rs113995695 | 0.004 | . | . | . | . | . | . | . | . | . | . |
| --- | --- | --- | --- | --- | --- | --- | --- | --- | --- | --- | --- | --- |
| logdha | rs143489451 | 0.001 | . | . | . | . | . | . | . | . | . | . |
| logdha | rs182653839 | . | . | . | . | . | . | . | . | . | . | . |
| logdha | rs141692564 | 0.002 | . | . | . | . | . | . | . | . | . | . |
| logdha | rs146281742 | 0.001 | . | . | . | . | . | . | . | . | . | . |
| logdha | rs192914353 | 0.001 | . | . | . | . | . | . | . | . | . | . |
| logdha | rs185182848 | 0 | . | . | . | . | . | . | . | . | . | . |
| logdha | rs28395858 | 0.004 | . | . | . | . | . | . | . | . | . | . |
| logdha | rs41429645 | 0.01 | . | . | . | . | . | . | . | . | . | . |
| logdha | rs28395874 | 0.004 | . | . | . | . | . | . | . | . | . | . |
| logdha | rs189663634 | . | . | . | . | . | . | . | . | . | . | . |
| logdha | rs77666700 | 0.003 | . | . | . | . | . | . | . | . | . | . |
| logdha | rs146711717 | 0.001 | . | . | . | . | . | . | . | . | . | . |
| logdha | rs144904251 | . | . | . | . | . | . | . | . | . | . | . |
| logdha | rs189893153 | . | . | . | . | . | . | . | . | . | . | . |
| logdha | rs150447740 | 0.002 | . | . | . | . | . | . | . | . | . | . |
| logdha | rs143099649 | . | . | . | . | . | . | . | . | . | . | . |
| logdha | rs191288436 | 0.003 | . | . | . | . | . | . | . | . | . | . |
| logdha | rs188577862 | 0.001 | . | . | . | . | . | . | . | . | . | . |
| logdha | rs192695334 | 0.001 | . | . | . | . | . | . | . | . | . | . |
| logdha | rs138612906 | 0.007 | . | . | . | . | . | . | . | . | . | . |
| logdha | rs191290051 | 0.006 | . | . | . | . | . | . | . | . | . | . |
| logdha | rs189631459 | 0 | . | . | . | . | . | . | . | . | . | . |
| logdha | rs114620874 | 0.002 | . | . | . | . | . | . | . | . | . | . |
| logdha | rs78246652 | 0.003 | . | . | . | . | . | . | . | . | . | . |
| logdha | rs79432451 | 0.002 | . | . | . | . | . | . | . | . | . | . |
| logdha | rs186022585 | . | . | . | . | . | . | . | . | . | . | . |
| logdha | rs142978891 | 0.002 | . | . | . | . | . | . | . | . | . | . |
| logdha | rs151086840 | 0.003 | . | . | . | . | . | . | . | . | . | . |
| logdha | rs183270259 | 0.002 | . | . | . | . | . | . | . | . | . | . |
| logdha | rs188544289 | 0.002 | . | . | . | . | . | . | . | . | . | . |
| logdha | rs188500808 | 0 | . | . | . | . | . | . | . | . | . | . |
| logdha | rs189424589 | 0 | . | . | . | . | . | . | . | . | . | . |
| logdha | rs78622303 | 0.008 | . | . | . | . | . | . | . | . | . | . |
| logdha | rs143210835 | 0.002 | . | . | . | . | . | . | . | . | . | . |
| logdha | rs114761840 | 0.001 | . | . | . | . | . | . | . | . | . | . |
| logdha | rs185805402 | . | . | . | . | . | . | . | . | . | . | . |
| logdha | rs139273744 | 0.001 | . | . | . | . | . | . | . | . | . | . |
| logdha | rs184654150 | 0.001 | . | . | . | . | . | . | . | . | . | . |
| logdha | rs189069710 | . | . | . | . | . | . | . | . | . | . | . |
| logdha | rs114752115 | 0.004 | . | . | . | . | . | . | . | . | . | . |
| logdha | rs150162515 | 0 | . | . | . | . | . | . | . | . | . | . |
| logdha | rs180982454 | 0.001 | . | . | . | . | . | . | . | . | . | . |
| logdha | rs184932088 | . | . | . | . | . | . | . | . | . | . | . |
| logdha | rs181109625 | . | . | . | . | . | . | . | . | . | . | . |
| logdha | rs138121134 | . | . | . | . | . | . | . | . | . | . | . |
| logdha | rs183253861 | 0 | . | . | . | . | . | . | . | . | . | . |
| logdha | rs147073946 | . | . | . | . | . | . | . | . | . | . | . |
| logdha | rs139938489 | 0.002 | . | . | . | . | . | . | . | . | . | . |
| logdha | rs146362560 | 0.003 | . | . | . | . | . | . | . | . | . | . |
| logdha | rs193155956 | 0.001 | . | . | . | . | . | . | . | . | . | . |
| logdha | rs77663092 | 0.001 | . | . | . | . | . | . | . | . | . | . |
| logdha | rs73285319 | 0.007 | . | . | . | . | . | . | . | . | . | . |
| logdha | rs184019276 | . | . | . | . | . | . | . | . | . | . | . |
| logdha | rs28395863 | 0.004 | . | . | . | . | . | . | . | . | . | . |
| logdha | rs182121645 | 0.004 | . | . | . | . | . | . | . | . | . | . |
| logdha | rs73285327 | 0.006 | . | . | . | . | . | . | . | . | . | . |
| logdha | rs149589584 | . | . | . | . | . | . | . | . | . | . | . |
| logdha | rs148310335 | . | . | . | . | . | . | . | . | . | . | . |
| logdha | rs187246093 | 0.003 | . | . | . | . | . | . | . | . | . | . |
| logdha | rs184668894 | . | . | . | . | . | . | . | . | . | . | . |
| logdha | rs114269961 | . | . | . | . | . | . | . | . | . | . | . |
| logdha | rs149694374 | 0.005 | . | . | . | . | . | . | . | . | . | . |
| logdha | rs145284227 | . | . | . | . | . | . | . | . | . | . | . |
| logdha | rs189983820 | . | . | . | . | . | . | . | . | . | . | . |
| logdha | rs181200444 | . | . | . | . | . | . | . | . | . | . | . |
| logdha | rs185486385 | . | . | . | . | . | . | . | . | . | . | . |
| logdha | rs151198241 | 0.002 | . | . | . | . | . | . | . | . | . | . |
| logdha | rs189959341 | 0.002 | . | . | . | . | . | . | . | . | . | . |
| logdha | rs140549257 | 0.002 | . | . | . | . | . | . | . | . | . | . |
| logdha | rs186453139 | 0.005 | . | . | . | . | . | . | . | . | . | . |
| logdha | rs180922711 | 0.009 | . | . | . | . | . | . | . | . | . | . |
| logdha | rs56659903 | 0.002 | . | . | . | . | . | . | . | . | . | . |
| logdha | rs141202128 | 0.002 | . | . | . | . | . | . | . | . | . | . |
| logdha | rs59085314 | 0.008 | . | . | . | . | . | . | . | . | . | . |
| logdha | rs145564996 | . | . | . | . | . | . | . | . | . | . | . |
| logdha | rs114961009 | 0.002 | . | . | . | . | . | . | . | . | . | . |
| logdha | rs182294189 | 0.001 | . | . | . | . | . | . | . | . | . | . |
| logdha | rs187586636 | 0.002 | . | . | . | . | . | . | . | . | . | . |
| logdha | rs192254907 | 0.005 | . | . | . | . | . | . | . | . | . | . |
| logdha | rs149145663 | . | . | . | . | . | . | . | . | . | . | . |
| logdha | rs180713064 | 0.002 | . | . | . | . | . | . | . | . | . | . |
| logdha | rs183009023 | 0.001 | . | . | . | . | . | . | . | . | . | . |
| logdha | rs143385347 | 0.004 | . | . | . | . | . | . | . | . | . | . |
| logdha | rs185786329 | 0.001 | . | . | . | . | . | . | . | . | . | . |
| logdha | rs192341030 | 0.001 | . | . | . | . | . | . | . | . | . | . |

| logdha | rs74661429 | 0.002 | . | . | . | . | . | . | . | . | . | . |
| --- | --- | --- | --- | --- | --- | --- | --- | --- | --- | --- | --- | --- |
| logdha | rs188034853 | 0 | . | . | . | . | . | . | . | . | . | . |
| logdha | rs116808430 | 0.005 | . | . | . | . | . | . | . | . | . | . |
| logdha | rs148469010 | . | . | . | . | . | . | . | . | . | . | . |
| logdha | rs78621663 | 0.002 | . | . | . | . | . | . | . | . | . | . |
| logdha | rs192654886 | . | . | . | . | . | . | . | . | . | . | . |
| logdha | rs145996035 | 0.002 | . | . | . | . | . | . | . | . | . | . |
| logdha | rs139942226 | 0 | . | . | . | . | . | . | . | . | . | . |
| logdha | rs113645632 | 0.002 | . | . | . | . | . | . | . | . | . | . |
| logdha | rs74940661 | 0.005 | . | . | . | . | . | . | . | . | . | . |
| logdha | rs115355800 | 0.003 | . | . | . | . | . | . | . | . | . | . |
| logdha | rs150285898 | 0.001 | . | . | . | . | . | . | . | . | . | . |
| logdha | rs188117932 | . | . | . | . | . | . | . | . | . | . | . |
| logdha | rs139956566 | 0 | . | . | . | . | . | . | . | . | . | . |
| logdha | rs147673885 | 0 | . | . | . | . | . | . | . | . | . | . |
| logdha | rs189127928 | 0.005 | . | . | . | . | . | . | . | . | . | . |
| logdha | rs187558772 | 0.002 | . | . | . | . | . | . | . | . | . | . |
| logdha | rs181948587 | 0 | . | . | . | . | . | . | . | . | . | . |
| logdha | rs189983269 | 0.002 | . | . | . | . | . | . | . | . | . | . |
| logdha | rs187250069 | 0.002 | . | . | . | . | . | . | . | . | . | . |
| logdha | rs191485859 | 0.001 | . | . | . | . | . | . | . | . | . | . |
| logdha | rs185499555 | 0.002 | . | . | . | . | . | . | . | . | . | . |
| logdha | rs180862110 | 0.003 | . | . | . | . | . | . | . | . | . | . |
| logdha | rs190472603 | 0.005 | . | . | . | . | . | . | . | . | . | . |
| logdha | rs189226167 | 0.005 | . | . | . | . | . | . | . | . | . | . |
| logdha | rs181623256 | 0.002 | . | . | . | . | . | . | . | . | . | . |
| logdha | rs186079557 | 0.001 | . | . | . | . | . | . | . | . | . | . |
| logdha | rs147760994 | 0.002 | . | . | . | . | . | . | . | . | . | . |
| logdha | rs189176167 | 0 | . | . | . | . | . | . | . | . | . | . |
| logdha | rs117901000 | 0.001 | . | . | . | . | . | . | . | . | . | . |
| logdha | rs141984888 | 0 | . | . | . | . | . | . | . | . | . | . |
| logdha | rs192365061 | 0.007 | . | . | . | . | . | . | . | . | . | . |
| logdha | rs185976038 | 0 | . | . | . | . | . | . | . | . | . | . |
| logdha | rs184697303 | 0.002 | . | . | . | . | . | . | . | . | . | . |
| logdha | rs143383132 | 0.002 | . | . | . | . | . | . | . | . | . | . |
| logdha | rs141873881 | 0.004 | . | . | . | . | . | . | . | . | . | . |
| logdha | rs185507255 | 0 | . | . | . | . | . | . | . | . | . | . |
| logdha | rs191379425 | 0.001 | . | . | . | . | . | . | . | . | . | . |
| logdha | rs184199584 | 0.002 | . | . | . | . | . | . | . | . | . | . |
| logdha | rs183181360 | 0.002 | . | . | . | . | . | . | . | . | . | . |
| logdha | rs184495673 | 0.001 | . | . | . | . | . | . | . | . | . | . |
| logdha | rs182946297 | 0.001 | . | . | . | . | . | . | . | . | . | . |
| logdha | rs187743615 | 0 | . | . | . | . | . | . | . | . | . | . |
| logdha | rs183259862 | 0.001 | . | . | . | . | . | . | . | . | . | . |
| logdha | rs112433566 | 0 | . | . | . | . | . | . | . | . | . | . |
| logdha | rs182351085 | 0.001 | . | . | . | . | . | . | . | . | . | . |
| logdha | rs146248508 | 0.001 | . | . | . | . | . | . | . | . | . | . |
| logdha | rs188528439 | 0.001 | . | . | . | . | . | . | . | . | . | . |
| logdha | rs146167018 | 0.005 | . | . | . | . | . | . | . | . | . | . |
| logdha | rs192440980 | 0.004 | . | . | . | . | . | . | . | . | . | . |
| logdha | rs185601882 | 0.001 | . | . | . | . | . | . | . | . | . | . |
| logdha | rs117272563 | 0.002 | . | . | . | . | . | . | . | . | . | . |
| logdha | rs10430734 | 0.003 | . | . | . | . | . | . | . | . | . | . |
| logdha | rs181320952 | 0.001 | . | . | . | . | . | . | . | . | . | . |
| logdha | rs185480660 | 0.001 | . | . | . | . | . | . | . | . | . | . |
| logdha | rs188077917 | 0.001 | . | . | . | . | . | . | . | . | . | . |
| logdha | rs187562953 | 0.002 | . | . | . | . | . | . | . | . | . | . |
| logdha | rs182044631 | 0 | . | . | . | . | . | . | . | . | . | . |
| logdha | rs145455596 | 0.001 | . | . | . | . | . | . | . | . | . | . |
| logdha | rs181475877 | 0.004 | . | . | . | . | . | . | . | . | . | . |
| logdha | rs149413085 | 0.002 | . | . | . | . | . | . | . | . | . | . |
| logdha | rs12253656 | 0 | . | . | . | . | . | . | . | . | . | . |
| logdha | rs182718239 | 0 | . | . | . | . | . | . | . | . | . | . |
| logdha | rs192435695 | 0.002 | . | . | . | . | . | . | . | . | . | . |
| logdha | rs12248277 | 0 | . | . | . | . | . | . | . | . | . | . |
| logdha | rs143521710 | 0.001 | . | . | . | . | . | . | . | . | . | . |
| logdha | rs144454454 | 0 | . | . | . | . | . | . | . | . | . | . |
| logdha | rs190441610 | 0.004 | . | . | . | . | . | . | . | . | . | . |
| logdha | rs114690462 | 0 | . | . | . | . | . | . | . | . | . | . |
| logdha | rs184541357 | 0.001 | . | . | . | . | . | . | . | . | . | . |
| logdha | rs142657653 | 0 | . | . | . | . | . | . | . | . | . | . |
| logdha | rsurf1 | 2.225 | . | . | . | . | . | . | . | . | . | . |
| logdha | rsten1 | 2.374 | . | . | . | . | . | . | . | . | . | . |
| logdha | chr10:45852312:D | 0.072 | 1.03 | 0.316 | 0.924 | 2.18 | 2.06 | 0.41 | 0.534 | 0.467 | 0.473 | -1.01 |
| logdha | chr10:45853232:I | 0.086 | 1.07 | 0.329 | 0.833 | 2.31 | 2.19 | 0.376 | 0.43 | 0.367 | 0.322 | -1.32 |
| logdha | chr10:45853234:I | 0.141 | 1.07 | 0.34 | 0.838 | 1.41 | 0.952 | 0.614 | 0.615 | 0.35 | 0.393 | -0.55 |
| logdha | chr10:45854796:D | 0.022 | 1 | 0.298 | 0.997 | 4.85 | 5.49 | 0.163 | 0.216 | 0.238 | 0.165 | -3.8 |
| logdha | chr10:45854994:I | 0.025 | 1.02 | 0.305 | 0.938 | 9.51 | 9.56 | 0.025 | 0.088 | 0.09 | 0.017 | -8.68 |
| logdha | chr10:45858290:D | 0.045 | 1.09 | 0.339 | 0.779 | 2.55 | 6.27 | 0.703 | 0.175 | 0.335 | 0.362 | -2.15 |
| logdha | chr10:45858820:I | 0.012 | 1.01 | 0.295 | 0.984 | 8.87 | 17.6 | 0.271 | 0.181 | 0.315 | 0.326 | -7.26 |
| logdha | chr10:45860888:D | 0.072 | 1.12 | 0.35 | 0.713 | 2.42 | 2.71 | 0.433 | 0.26 | 0.232 | 0.131 | -1.83 |
| logdha | chr10:45863913:I | 0.144 | 1.07 | 0.34 | 0.827 | 1.41 | 0.971 | 0.617 | 0.598 | 0.344 | 0.371 | -0.579 |
| logdha | chr10:45866601:D | 0.016 | 1.04 | 0.324 | 0.905 | 10.2 | 14 | 0.09 | 0.169 | 0.14 | 0.032 | -8.46 |
| logdha | chr10:45867197:I | 0.005 | . | . | . | . | . | . | . | . | . | . |
| logdha | chr10:45867538:D | 0.149 | 1.02 | 0.323 | 0.946 | 1.04 | 0.762 | 0.957 | 0.797 | 0.476 | 0.705 | -0.214 |
| logdha | chr10:45868356:D | 0.14 | 1.08 | 0.342 | 0.814 | 1.51 | 1.07 | 0.561 | 0.572 | 0.34 | 0.347 | -0.656 |

| logdha | chr10:45871158:I | 0.145 | 1.08 | 0.342 | 0.816 | 1.44 | 0.982 | 0.594 | 0.587 | 0.334 | 0.349 | -0.606 |
| --- | --- | --- | --- | --- | --- | --- | --- | --- | --- | --- | --- | --- |
| logdha | chr10:45871637:D | 0.066 | 1.01 | 0.316 | 0.974 | 1.61 | 2.37 | 0.744 | 0.654 | 0.808 | 0.731 | -0.558 |
| logdha | chr10:45871638:I | 0.111 | 1.06 | 0.339 | 0.848 | 1.65 | 1.31 | 0.523 | 0.582 | 0.382 | 0.41 | -0.694 |
| logdha | chr10:45873629:I | 0.139 | 1.06 | 0.342 | 0.851 | 1.44 | 0.967 | 0.588 | 0.629 | 0.36 | 0.418 | -0.539 |
| logdha | chr10:45873701:D | 0.009 | . | . | . | . | . | . | . | . | . | . |
| logdha | chr10:45874997:I | 0.016 | 1.06 | 0.323 | 0.844 | 2.5 | 3.69 | 0.533 | 0.155 | 0.169 | 0.088 | -2.15 |
| logdha | chr10:45882030:I | 0.028 | 1.02 | 0.31 | 0.959 | 0.454 | 0.812 | 0.659 | 0.689 | 0.547 | 0.639 | -0.152 |
| logdha | chr10:45883774:D | 0.092 | 1.06 | 0.337 | 0.862 | 1.4 | 1.01 | 0.646 | 0.599 | 0.348 | 0.377 | -0.57 |
| logdha | chr10:45885169:D | 0.054 | 0.977 | 0.297 | 0.94 | 1.09 | 1.24 | 0.938 | 0.923 | 0.956 | 0.939 | -0.084 |
| logdha | chr10:45886451:D | 0.701 | 1.09 | 0.776 | 0.902 | 0.897 | 0.478 | 0.839 | 0.907 | 0.408 | 0.829 | -0.1 |
| logdha | chr10:45891800:I | 0.669 | 1.3 | 1.01 | 0.734 | 1.2 | 0.697 | 0.749 | 0.807 | 0.379 | 0.648 | -0.242 |
| logdha | chr10:45904771:I | 0.683 | 0.927 | 0.663 | 0.915 | 0.89 | 0.474 | 0.827 | 1.03 | 0.433 | 0.948 | 0.031 |
| logdha | chr10:45904959:I | 0.083 | 0.952 | 0.323 | 0.884 | 1.21 | 1.19 | 0.845 | 1.06 | 0.767 | 0.94 | 0.054 |
| logdha | chr10:45911028:I | 0.045 | 0.992 | 0.322 | 0.979 | 0.901 | 1.63 | 0.954 | 0.909 | 1.22 | 0.943 | -0.081 |
| logdha | chr10:45911031:I | 0.411 | 0.614 | 0.278 | 0.282 | 0.525 | 0.257 | 0.189 | 1.74 | 0.729 | 0.183 | 0.423 |
| logdha | chr10:45912154:D | 0.086 | 1.07 | 0.341 | 0.822 | 1.48 | 1.14 | 0.612 | 0.54 | 0.327 | 0.309 | -0.694 |
| logdha | chr10:45913885:I | 0.021 | 0.988 | 0.292 | 0.967 | 2.86 | 3.22 | 0.351 | 0.305 | 0.311 | 0.244 | -1.98 |
| logdha | chr10:45914753:D | 0.181 | 0.881 | 0.322 | 0.728 | 1.26 | 0.654 | 0.659 | 1.14 | 0.435 | 0.722 | 0.13 |
| logdha | chr10:45919621 | . | . | . | . | . | . | . | . | . | . | . |
| logdha | chr10:45921739:I | 0.06 | 1.09 | 0.335 | 0.783 | 3.39 | 3.75 | 0.269 | 0.249 | 0.25 | 0.167 | -2.56 |
| logdha | chr10:45923527:I | 0.054 | 0.891 | 0.285 | 0.719 | 0.786 | 1.04 | 0.856 | 1.77 | 1.55 | 0.515 | 0.564 |
| logdha | chr10:45927001:I | 0.011 | 0.971 | 0.29 | 0.922 | 1.89 | 4.48 | 0.789 | 0.842 | 2.1 | 0.945 | -0.315 |
| logdha | chr10:45930354:I | 0.023 | 0.975 | 0.289 | 0.931 | 1.27 | 1.75 | 0.864 | 0.691 | 0.788 | 0.746 | -0.388 |
| logdha | chr10:45932864:I | 0.046 | 0.993 | 0.309 | 0.983 | 0.909 | 1.41 | 0.951 | 0.504 | 0.623 | 0.579 | -0.447 |
| logdha | chr10:45936163:I | 0.025 | 0.966 | 0.3 | 0.913 | 2.06 | 2.16 | 0.491 | 0.975 | 0.837 | 0.976 | -0.085 |
| logdha | chr10:45938095:D | 0.116 | 1.05 | 0.349 | 0.888 | 1.48 | 1.07 | 0.585 | 0.71 | 0.379 | 0.521 | -0.427 |
| logdha | chr10:45938886 | . | . | . | . | . | . | . | . | . | . | . |
| logdha | chr10:45939320 | . | . | . | . | . | . | . | . | . | . | . |
| logdha | chr10:45939659 | 0.002 | . | . | . | . | . | . | . | . | . | . |
| logdha | chr10:45941150:D | 0.021 | 0.975 | 0.288 | 0.931 | 1.81 | 2.17 | 0.622 | 0.408 | 0.398 | 0.358 | -1.06 |
| logdha | chr10:45947128:D | 0.046 | 1.05 | 0.334 | 0.888 | 1.6 | 1.15 | 0.512 | 0.614 | 0.358 | 0.404 | -0.618 |
| logdha | chr10:45950990:I | 0.06 | 0.934 | 0.291 | 0.826 | 0.433 | 0.556 | 0.515 | 1.36 | 1.59 | 0.794 | 0.182 |
| logdha | chr10:45951854:I | 0.02 | 0.943 | 0.285 | 0.846 | 1.09 | 1.66 | 0.956 | 2.17 | 2.9 | 0.564 | 1.19 |
| logdha | chr10:45952670:I | 0.042 | 1.08 | 0.327 | 0.788 | 1.69 | 3.06 | 0.771 | 0.344 | 0.538 | 0.495 | -1.15 |
| logdha | chr10:45956741 | . | . | . | . | . | . | . | . | . | . | . |
| logdha | chr10:45957247:I | 0.304 | 0.919 | 0.349 | 0.824 | 0.929 | 0.425 | 0.871 | 1.11 | 0.4 | 0.771 | 0.1 |
| logdha | chr10:45958082:I | 0.302 | 0.918 | 0.348 | 0.822 | 0.908 | 0.427 | 0.837 | 1.11 | 0.415 | 0.781 | 0.099 |
| logdha | chr10:45905041:D | 0.039 | 1.02 | 0.302 | 0.955 | 5.74 | 5.7 | 0.078 | 0.124 | 0.115 | 0.025 | -5.04 |
| logdha | chr10:45938871 | . | . | . | . | . | . | . | . | . | . | . |
| logdha | chr10:45939160 | . | . | . | . | . | . | . | . | . | . | . |
| logdha | chr10:45869910 | 0.001 | . | . | . | . | . | . | . | . | . | . |
| logdha | chr10:45876214:D | 0.005 | . | . | . | . | . | . | . | . | . | . |
| logdha | chr10:45880237:I | 0.015 | 1.02 | 0.315 | 0.957 | 3.78 | 6.33 | 0.428 | 0.266 | 0.248 | 0.156 | -2.77 |
| logdha | chr10:45884371:I | 0.013 | 1.02 | 0.307 | 0.944 | 6.26 | 7.98 | 0.15 | 0.149 | 0.109 | 9.60E-03 | -5.33 |
| logdha | chr10:45891535:D | 0.004 | . | . | . | . | . | . | . | . | . | . |
| logdha | chr10:45893516:D | 0.013 | 1.02 | 0.307 | 0.943 | 6.13 | 7.75 | 0.152 | 0.15 | 0.109 | 8.90E-03 | -5.21 |
| logdha | chr10:45893782:D | 0.001 | . | . | . | . | . | . | . | . | . | . |
| logdha | chr10:45895254:I | 0.004 | . | . | . | . | . | . | . | . | . | . |
| logdha | chr10:45895848:D | 0.011 | 1.01 | 0.305 | 0.964 | 7.37 | 10.5 | 0.16 | 0.133 | 0.125 | 0.032 | -6.39 |
| logdha | chr10:45896316:D | 0.013 | 1.02 | 0.307 | 0.943 | 6.03 | 7.68 | 0.158 | 0.152 | 0.109 | 8.90E-03 | -5.12 |
| logdha | chr10:45896317:D | 0.013 | 1.02 | 0.307 | 0.943 | 6.03 | 7.68 | 0.158 | 0.152 | 0.109 | 8.90E-03 | -5.12 |
| logdha | chr10:45902683:I | 0.006 | . | . | . | . | . | . | . | . | . | . |
| logdha | chr10:45905003:D | 0.002 | . | . | . | . | . | . | . | . | . | . |
| logdha | chr10:45927476:D | 0.005 | . | . | . | . | . | . | . | . | . | . |
| logdha | chr10:45935882 | . | . | . | . | . | . | . | . | . | . | . |
| logdha | chr10:45936100 | . | . | . | . | . | . | . | . | . | . | . |
| logdha | chr10:45939553 | 0.004 | . | . | . | . | . | . | . | . | . | . |
| logdha | chr10:45939631 | . | . | . | . | . | . | . | . | . | . | . |
| logdha | chr10:45944193:D | 0.003 | . | . | . | . | . | . | . | . | . | . |
| logdha | chr10:45954030 | . | . | . | . | . | . | . | . | . | . | . |

Supplemental table S40.

| fa | rs_id | freq | HR_logdha | se_logdha | p_logdha | HR_snp | se_snp | p_snp | HR_inter | se_inter | p_inter | RERI |
| --- | --- | --- | --- | --- | --- | --- | --- | --- | --- | --- | --- | --- |
| logdha | rs76035182 | . | . | . | . | . | . | . | . | . | . | . |
| logdha | rs12359547 | 0.001 | . | . | . | . | . | . | . | . | . | . |
| logdha | rs74595484 | . | . | . | . | . | . | . | . | . | . | . |
| logdha | rs1864415 | 0.873 | 0.191 | 0.416 | 0.447 | 0.381 | 0.655 | 0.574 | 1.22 | 1.36 | 0.856 | 0.517 |
| logdha | rs72788101 | 0.001 | . | . | . | . | . | . | . | . | . | . |
| logdha | rs11239481 | . | . | . | . | . | . | . | . | . | . | . |
| logdha | rs72788102 | 0.001 | . | . | . | . | . | . | . | . | . | . |
| logdha | rs72789705 | 0.001 | . | . | . | . | . | . | . | . | . | . |
| logdha | rs141529858 | . | . | . | . | . | . | . | . | . | . | . |
| logdha | rs145376292 | . | . | . | . | . | . | . | . | . | . | . |
| logdha | rs190418301 | . | . | . | . | . | . | . | . | . | . | . |
| logdha | rs11239482 | 0.001 | . | . | . | . | . | . | . | . | . | . |
| logdha | rs61855872 | . | . | . | . | . | . | . | . | . | . | . |
| logdha | rs148991144 | . | . | . | . | . | . | . | . | . | . | . |
| logdha | rs191932221 | . | . | . | . | . | . | . | . | . | . | . |
| logdha | rs55978138 | 0.201 | 0.338 | 0.231 | 0.112 | 1.35 | 1.4 | 0.769 | 0.823 | 0.565 | 0.776 | -0.316 |
| logdha | rs73281167 | . | . | . | . | . | . | . | . | . | . | . |
| logdha | rs12768010 | 0.2 | 0.339 | 0.231 | 0.112 | 1.37 | 1.42 | 0.76 | 0.817 | 0.561 | 0.768 | -0.331 |
| logdha | rs11239484 | 0.873 | 0.192 | 0.419 | 0.449 | 0.383 | 0.658 | 0.576 | 1.22 | 1.36 | 0.858 | 0.515 |
| logdha | rs35906360 | 0.201 | 0.338 | 0.231 | 0.112 | 1.35 | 1.4 | 0.769 | 0.823 | 0.565 | 0.776 | -0.316 |
| logdha | rs186250512 | . | . | . | . | . | . | . | . | . | . | . |
| logdha | rs150810743 | . | . | . | . | . | . | . | . | . | . | . |
| logdha | rs55965812 | 0.201 | 0.338 | 0.231 | 0.112 | 1.35 | 1.4 | 0.769 | 0.823 | 0.565 | 0.776 | -0.316 |
| logdha | rs56180358 | 0.203 | 0.334 | 0.229 | 0.109 | 1.31 | 1.37 | 0.796 | 0.835 | 0.577 | 0.795 | -0.278 |
| logdha | rs55864145 | 0.2 | 0.339 | 0.231 | 0.112 | 1.37 | 1.42 | 0.76 | 0.817 | 0.561 | 0.768 | -0.331 |
| logdha | rs55986122 | 0.201 | 0.338 | 0.231 | 0.112 | 1.35 | 1.4 | 0.769 | 0.823 | 0.565 | 0.776 | -0.316 |
| logdha | rs7477350 | 0.873 | 0.193 | 0.419 | 0.449 | 0.383 | 0.659 | 0.577 | 1.22 | 1.36 | 0.859 | 0.514 |
| logdha | rs2099170 | 0.201 | 0.338 | 0.231 | 0.112 | 1.35 | 1.4 | 0.769 | 0.823 | 0.565 | 0.776 | -0.316 |
| logdha | rs2082108 | 0.201 | 0.338 | 0.231 | 0.112 | 1.35 | 1.4 | 0.769 | 0.823 | 0.565 | 0.776 | -0.316 |
| logdha | rs55902404 | . | . | . | . | . | . | . | . | . | . | . |
| logdha | rs58402662 | . | . | . | . | . | . | . | . | . | . | . |
| logdha | rs11511618 | . | . | . | . | . | . | . | . | . | . | . |
| logdha | rs1816492 | 0.873 | 0.193 | 0.421 | 0.45 | 0.384 | 0.661 | 0.578 | 1.22 | 1.35 | 0.86 | 0.513 |
| logdha | rs146611093 | . | . | . | . | . | . | . | . | . | . | . |
| logdha | rs186897689 | . | . | . | . | . | . | . | . | . | . | . |
| logdha | rs10793616 | 0.873 | 0.193 | 0.42 | 0.45 | 0.384 | 0.66 | 0.578 | 1.22 | 1.36 | 0.859 | 0.514 |
| logdha | rs116700479 | . | . | . | . | . | . | . | . | . | . | . |
| logdha | rs192535936 | . | . | . | . | . | . | . | . | . | . | . |
| logdha | rs113363297 | 0.852 | 0.337 | 0.705 | 0.603 | 0.657 | 1.13 | 0.806 | 0.904 | 0.998 | 0.927 | 0.206 |
| logdha | rs12354994 | 0.186 | 0.381 | 0.259 | 0.156 | 2.12 | 2.48 | 0.519 | 0.631 | 0.484 | 0.548 | -0.995 |
| logdha | rs148068329 | 0.059 | 0.381 | 0.178 | 0.039 | 144 | 870 | 0.412 | 0.028 | 0.128 | 0.429 | -141 |
| logdha | rs12356109 | 0.198 | 0.343 | 0.234 | 0.117 | 1.45 | 1.48 | 0.713 | 0.806 | 0.543 | 0.748 | -0.395 |
| logdha | rs55908141 | . | . | . | . | . | . | . | . | . | . | . |
| logdha | rs12354712 | 0.196 | 0.359 | 0.243 | 0.13 | 1.7 | 1.74 | 0.602 | 0.732 | 0.508 | 0.653 | -0.614 |
| logdha | rs147282684 | . | . | . | . | . | . | . | . | . | . | . |
| logdha | rs12356140 | 0.194 | 0.361 | 0.244 | 0.132 | 1.76 | 1.86 | 0.593 | 0.715 | 0.512 | 0.639 | -0.667 |
| logdha | rs185476187 | . | . | . | . | . | . | . | . | . | . | . |
| logdha | rs145720057 | . | . | . | . | . | . | . | . | . | . | . |
| logdha | rs12354742 | 0.196 | 0.359 | 0.243 | 0.13 | 1.7 | 1.74 | 0.602 | 0.732 | 0.508 | 0.653 | -0.614 |
| logdha | rs180913842 | . | . | . | . | . | . | . | . | . | . | . |
| logdha | rs142976914 | 0.001 | . | . | . | . | . | . | . | . | . | . |
| logdha | rs11511619 | 0.198 | 0.343 | 0.234 | 0.117 | 1.46 | 1.48 | 0.712 | 0.805 | 0.543 | 0.747 | -0.397 |
| logdha | rs146702870 | . | . | . | . | . | . | . | . | . | . | . |
| logdha | rs150287496 | 0.024 | 0.315 | 0.173 | 0.035 | 4.10E-06 | 8.20E-05 | 0.536 | 25 | 252 | 0.75 | 0.685 |
| logdha | rs190917973 | . | . | . | . | . | . | . | . | . | . | . |
| logdha | rs11517281 | 0.197 | 0.343 | 0.234 | 0.117 | 1.45 | 1.48 | 0.713 | 0.806 | 0.543 | 0.748 | -0.395 |
| logdha | rs12359299 | 0.208 | 0.345 | 0.241 | 0.128 | 1.48 | 1.52 | 0.7 | 0.804 | 0.545 | 0.748 | -0.417 |
| logdha | rs182307932 | . | . | . | . | . | . | . | . | . | . | . |
| logdha | rs61855873 | . | . | . | . | . | . | . | . | . | . | . |
| logdha | rs12359302 | 0.193 | 0.338 | 0.231 | 0.113 | 1.4 | 1.43 | 0.739 | 0.828 | 0.558 | 0.779 | -0.349 |
| logdha | rs143208140 | 0.054 | 0.374 | 0.177 | 0.038 | 123 | 697 | 0.395 | 0.034 | 0.144 | 0.42 | -121 |
| logdha | rs9988764 | 0.858 | 0.198 | 0.44 | 0.466 | 0.407 | 0.724 | 0.613 | 1.21 | 1.39 | 0.868 | 0.493 |
| logdha | rs192875767 | 0.015 | 0.343 | 0.207 | 0.076 | 1.40E+62 | 1.90E+64 | 0.282 | 1.10E-98 | 1.60E-96 | 0.137 | ####### |
| logdha | rs147268846 | . | . | . | . | . | . | . | . | . | . | . |
| logdha | rs11239485 | 0.1 | 0.453 | 0.301 | 0.233 | 14.1 | 27.3 | 0.171 | 0.276 | 0.371 | 0.338 | -11.8 |
| logdha | rs151317885 | 0.66 | 0.262 | 0.216 | 0.105 | 0.582 | 0.429 | 0.463 | 1.12 | 0.532 | 0.805 | 0.327 |
| logdha | rs11499085 | . | . | . | . | . | . | . | . | . | . | . |
| logdha | rs188130606 | . | . | . | . | . | . | . | . | . | . | . |
| logdha | rs11511620 | 0.196 | 0.342 | 0.233 | 0.116 | 1.45 | 1.47 | 0.716 | 0.81 | 0.545 | 0.754 | -0.388 |
| logdha | rs181498390 | 0.002 | . | . | . | . | . | . | . | . | . | . |
| logdha | rs186280701 | . | . | . | . | . | . | . | . | . | . | . |
| logdha | rs150059185 | 0.082 | 0.376 | 0.245 | 0.134 | 5.03 | 10.6 | 0.443 | 0.327 | 0.452 | 0.419 | -3.79 |
| logdha | rs183338707 | . | . | . | . | . | . | . | . | . | . | . |
| logdha | rs11499038 | 0.006 | . | . | . | . | . | . | . | . | . | . |
| logdha | rs190699595 | . | . | . | . | . | . | . | . | . | . | . |
| logdha | rs11523408 | 0.197 | 0.343 | 0.234 | 0.117 | 1.46 | 1.48 | 0.712 | 0.805 | 0.542 | 0.748 | -0.397 |
| logdha | rs188049317 | . | . | . | . | . | . | . | . | . | . | . |
| logdha | rs144948634 | 0.584 | 0.225 | 0.222 | 0.13 | 0.455 | 0.442 | 0.418 | 1.3 | 0.813 | 0.669 | 0.454 |
| logdha | rs143833576 | 0.016 | 0.32 | 0.181 | 0.044 | 10.7 | 23.2 | 0.274 | 0.333 | 0.499 | 0.463 | -8.87 |
| logdha | rs151239879 | 0.2 | 0.343 | 0.235 | 0.118 | 1.44 | 1.48 | 0.721 | 0.803 | 0.546 | 0.747 | -0.388 |
| logdha | rs7073743 | 0.61 | 0.18 | 0.188 | 0.1 | 0.365 | 0.352 | 0.296 | 1.58 | 0.983 | 0.459 | 0.559 |
| logdha | rs142563349 | . | . | . | . | . | . | . | . | . | . | . |
| logdha | rs187246403 | . | . | . | . | . | . | . | . | . | . | . |
| logdha | rs150423942 | 0.197 | 0.343 | 0.234 | 0.117 | 1.46 | 1.49 | 0.71 | 0.805 | 0.542 | 0.747 | -0.4 |

| logdha | rs184706102 | . | . | . | . | . | . | . | . | . | . | . |
| --- | --- | --- | --- | --- | --- | --- | --- | --- | --- | --- | --- | --- |
| logdha | rs189626288 | . | . | . | . | . | . | . | . | . | . | . |
| logdha | rs140749834 | . | . | . | . | . | . | . | . | . | . | . |
| logdha | rs7078822 | 0.163 | 0.303 | 0.205 | 0.078 | 1.09 | 1.13 | 0.935 | 1.02 | 0.71 | 0.972 | -0.053 |
| logdha | rs186227617 | 0.188 | 0.334 | 0.226 | 0.105 | 1.35 | 1.48 | 0.784 | 0.832 | 0.611 | 0.802 | -0.31 |
| logdha | rs188984368 | 0.024 | 0.277 | 0.168 | 0.034 | 0.508 | 1.53 | 0.822 | 5.73 | 10.8 | 0.353 | 1.02 |
| logdha | rs182078109 | . | . | . | . | . | . | . | . | . | . | . |
| logdha | rs141456274 | 0.023 | 0.306 | 0.152 | 0.017 | 3.5 | 19.5 | 0.822 | 0.605 | 2.48 | 0.902 | -2.16 |
| logdha | rs146976512 | 0.198 | 0.343 | 0.234 | 0.117 | 1.45 | 1.48 | 0.713 | 0.806 | 0.543 | 0.748 | -0.395 |
| logdha | rs138036816 | . | . | . | . | . | . | . | . | . | . | . |
| logdha | rs114879777 | . | . | . | . | . | . | . | . | . | . | . |
| logdha | rs149366475 | . | . | . | . | . | . | . | . | . | . | . |
| logdha | rs182463043 | . | . | . | . | . | . | . | . | . | . | . |
| logdha | rs144754632 | 0.055 | 0.374 | 0.177 | 0.038 | 121 | 688 | 0.4 | 0.034 | 0.145 | 0.422 | -119 |
| logdha | rs55717607 | 0.198 | 0.343 | 0.234 | 0.117 | 1.45 | 1.48 | 0.713 | 0.806 | 0.543 | 0.748 | -0.395 |
| logdha | rs187537973 | . | . | . | . | . | . | . | . | . | . | . |
| logdha | rs148085971 | . | . | . | . | . | . | . | . | . | . | . |
| logdha | rs11239491 | 0.198 | 0.343 | 0.234 | 0.117 | 1.45 | 1.48 | 0.713 | 0.806 | 0.543 | 0.748 | -0.395 |
| logdha | rs184412184 | . | . | . | . | . | . | . | . | . | . | . |
| logdha | rs4948998 | 0.868 | 0.239 | 0.511 | 0.503 | 0.478 | 0.805 | 0.661 | 1.08 | 1.17 | 0.943 | 0.407 |
| logdha | rs7076696 | 0.873 | 0.262 | 0.558 | 0.529 | 0.515 | 0.868 | 0.694 | 1.03 | 1.11 | 0.98 | 0.362 |
| logdha | rs11239492 | 0.197 | 0.343 | 0.234 | 0.117 | 1.46 | 1.49 | 0.71 | 0.805 | 0.542 | 0.747 | -0.4 |
| logdha | rs79462470 | . | . | . | . | . | . | . | . | . | . | . |
| logdha | rs187804654 | . | . | . | . | . | . | . | . | . | . | . |
| logdha | rs7093687 | 0.198 | 0.343 | 0.234 | 0.117 | 1.45 | 1.48 | 0.713 | 0.806 | 0.543 | 0.748 | -0.395 |
| logdha | rs7081004 | 0.198 | 0.343 | 0.234 | 0.117 | 1.45 | 1.48 | 0.713 | 0.806 | 0.543 | 0.748 | -0.395 |
| logdha | rs7084793 | 0.198 | 0.343 | 0.234 | 0.117 | 1.45 | 1.48 | 0.713 | 0.806 | 0.543 | 0.748 | -0.395 |
| logdha | rs61556080 | . | . | . | . | . | . | . | . | . | . | . |
| logdha | rs115552064 | . | . | . | . | . | . | . | . | . | . | . |
| logdha | rs7921503 | 0.198 | 0.343 | 0.234 | 0.117 | 1.45 | 1.48 | 0.713 | 0.806 | 0.543 | 0.748 | -0.395 |
| logdha | rs7909740 | 0.198 | 0.343 | 0.234 | 0.117 | 1.45 | 1.48 | 0.713 | 0.806 | 0.543 | 0.748 | -0.395 |
| logdha | rs7909768 | 0.198 | 0.343 | 0.234 | 0.117 | 1.45 | 1.48 | 0.713 | 0.806 | 0.543 | 0.748 | -0.395 |
| logdha | rs144168004 | . | . | . | . | . | . | . | . | . | . | . |
| logdha | rs75850448 | . | . | . | . | . | . | . | . | . | . | . |
| logdha | rs147777574 | . | . | . | . | . | . | . | . | . | . | . |
| logdha | rs7913488 | 0.198 | 0.343 | 0.234 | 0.117 | 1.45 | 1.48 | 0.713 | 0.806 | 0.543 | 0.748 | -0.395 |
| logdha | rs74390874 | 0.048 | 0.268 | 0.153 | 0.021 | 0.158 | 0.322 | 0.365 | 2.93 | 4.51 | 0.484 | 0.698 |
| logdha | rs141391142 | . | . | . | . | . | . | . | . | . | . | . |
| logdha | rs115294747 | . | . | . | . | . | . | . | . | . | . | . |
| logdha | rs148031781 | . | . | . | . | . | . | . | . | . | . | . |
| logdha | rs181340128 | . | . | . | . | . | . | . | . | . | . | . |
| logdha | rs189930007 | . | . | . | . | . | . | . | . | . | . | . |
| logdha | rs12242128 | . | . | . | . | . | . | . | . | . | . | . |
| logdha | rs78421750 | . | . | . | . | . | . | . | . | . | . | . |
| logdha | rs114370747 | . | . | . | . | . | . | . | . | . | . | . |
| logdha | rs12264166 | . | . | . | . | . | . | . | . | . | . | . |
| logdha | rs182964822 | . | . | . | . | . | . | . | . | . | . | . |
| logdha | rs67491417 | 0.188 | 0.299 | 0.198 | 0.069 | 0.905 | 0.96 | 0.925 | 1.02 | 0.709 | 0.972 | 0.073 |
| logdha | rs72789720 | . | . | . | . | . | . | . | . | . | . | . |
| logdha | rs6413416 | 0.198 | 0.343 | 0.234 | 0.117 | 1.45 | 1.48 | 0.713 | 0.806 | 0.543 | 0.748 | -0.395 |
| logdha | rs4986832 | 0.198 | 0.343 | 0.234 | 0.117 | 1.45 | 1.48 | 0.713 | 0.806 | 0.543 | 0.748 | -0.395 |
| logdha | rs144866316 | . | . | . | . | . | . | . | . | . | . | . |
| logdha | rs187769540 | . | . | . | . | . | . | . | . | . | . | . |
| logdha | rs6593482 | 0.198 | 0.339 | 0.233 | 0.116 | 1.39 | 1.42 | 0.744 | 0.826 | 0.559 | 0.777 | -0.343 |
| logdha | rs7913948 | 0.199 | 0.343 | 0.234 | 0.117 | 1.45 | 1.48 | 0.716 | 0.806 | 0.544 | 0.749 | -0.392 |
| logdha | rs77185151 | 0.055 | 0.374 | 0.177 | 0.038 | 121 | 688 | 0.4 | 0.034 | 0.145 | 0.422 | -119 |
| logdha | rs12762303 | 0.198 | 0.343 | 0.234 | 0.117 | 1.45 | 1.48 | 0.713 | 0.806 | 0.543 | 0.748 | -0.395 |
| logdha | rs73283136 | . | . | . | . | . | . | . | . | . | . | . |
| logdha | rs187180218 | . | . | . | . | . | . | . | . | . | . | . |
| logdha | rs78137087 | 0.198 | 0.343 | 0.234 | 0.117 | 1.45 | 1.48 | 0.713 | 0.806 | 0.543 | 0.748 | -0.395 |
| logdha | rs72561472 | 0.193 | 0.342 | 0.233 | 0.115 | 1.47 | 1.54 | 0.71 | 0.808 | 0.554 | 0.756 | -0.408 |
| logdha | rs4987106 | 0.193 | 0.348 | 0.235 | 0.118 | 1.49 | 1.54 | 0.699 | 0.776 | 0.531 | 0.711 | -0.437 |
| logdha | rs4987105 | 0.198 | 0.343 | 0.234 | 0.117 | 1.45 | 1.48 | 0.713 | 0.806 | 0.543 | 0.748 | -0.395 |
| logdha | rs111916157 | 0.199 | 0.347 | 0.24 | 0.126 | 1.49 | 1.52 | 0.698 | 0.796 | 0.543 | 0.738 | -0.424 |
| logdha | rs111528152 | 0.198 | 0.343 | 0.234 | 0.117 | 1.45 | 1.48 | 0.713 | 0.806 | 0.543 | 0.748 | -0.395 |
| logdha | rs12763738 | 0.197 | 0.343 | 0.234 | 0.117 | 1.46 | 1.48 | 0.713 | 0.805 | 0.543 | 0.748 | -0.396 |
| logdha | rs191675886 | . | . | . | . | . | . | . | . | . | . | . |
| logdha | rs66489870 | 0.198 | 0.343 | 0.234 | 0.117 | 1.45 | 1.48 | 0.713 | 0.806 | 0.543 | 0.748 | -0.395 |
| logdha | rs71494775 | 0.198 | 0.343 | 0.234 | 0.117 | 1.45 | 1.48 | 0.713 | 0.806 | 0.543 | 0.748 | -0.395 |
| logdha | rs61855875 | . | . | . | . | . | . | . | . | . | . | . |
| logdha | rs116356837 | . | . | . | . | . | . | . | . | . | . | . |
| logdha | rs1864414 | 0.198 | 0.343 | 0.234 | 0.117 | 1.45 | 1.48 | 0.713 | 0.806 | 0.543 | 0.748 | -0.395 |
| logdha | rs1864413 | 0.198 | 0.343 | 0.234 | 0.117 | 1.45 | 1.48 | 0.713 | 0.805 | 0.543 | 0.748 | -0.395 |
| logdha | rs139497212 | . | . | . | . | . | . | . | . | . | . | . |
| logdha | rs892690 | 0.504 | 0.161 | 0.133 | 0.027 | 0.202 | 0.171 | 0.059 | 2.23 | 1.26 | 0.156 | 0.71 |
| logdha | rs1864412 | 0.198 | 0.343 | 0.234 | 0.117 | 1.45 | 1.48 | 0.713 | 0.806 | 0.543 | 0.748 | -0.395 |
| logdha | rs77827771 | . | . | . | . | . | . | . | . | . | . | . |
| logdha | rs145294963 | . | . | . | . | . | . | . | . | . | . | . |
| logdha | rs149081327 | . | . | . | . | . | . | . | . | . | . | . |
| logdha | rs67008983 | 0.198 | 0.343 | 0.234 | 0.117 | 1.45 | 1.48 | 0.713 | 0.805 | 0.543 | 0.748 | -0.395 |
| logdha | rs56150414 | 0.198 | 0.343 | 0.234 | 0.117 | 1.45 | 1.48 | 0.715 | 0.807 | 0.544 | 0.75 | -0.392 |
| logdha | rs55787481 | 0.199 | 0.344 | 0.234 | 0.117 | 1.46 | 1.49 | 0.71 | 0.804 | 0.541 | 0.746 | -0.401 |
| logdha | rs141270101 | . | . | . | . | . | . | . | . | . | . | . |
| logdha | rs114980075 | . | . | . | . | . | . | . | . | . | . | . |
| logdha | rs12783095 | 0.198 | 0.343 | 0.234 | 0.117 | 1.45 | 1.48 | 0.713 | 0.805 | 0.543 | 0.748 | -0.396 |
| logdha | rs182342688 | . | . | . | . | . | . | . | . | . | . | . |

| logdha | rs17157728 | 0.198 | 0.343 | 0.234 | 0.117 | 1.45 | 1.48 | 0.713 | 0.805 | 0.543 | 0.748 | -0.396 |
| --- | --- | --- | --- | --- | --- | --- | --- | --- | --- | --- | --- | --- |
| logdha | rs114835563 | . | . | . | . | . | . | . | . | . | . | . |
| logdha | rs3824612 | 0.504 | 0.161 | 0.133 | 0.027 | 0.204 | 0.173 | 0.061 | 2.22 | 1.26 | 0.16 | 0.708 |
| logdha | rs17157733 | 0.196 | 0.338 | 0.228 | 0.108 | 1.35 | 1.4 | 0.775 | 0.815 | 0.565 | 0.768 | -0.314 |
| logdha | rs74128478 | . | . | . | . | . | . | . | . | . | . | . |
| logdha | rs184973589 | . | . | . | . | . | . | . | . | . | . | . |
| logdha | rs150479461 | . | . | . | . | . | . | . | . | . | . | . |
| logdha | rs138310407 | . | . | . | . | . | . | . | . | . | . | . |
| logdha | rs187426603 | . | . | . | . | . | . | . | . | . | . | . |
| logdha | rs190843143 | . | . | . | . | . | . | . | . | . | . | . |
| logdha | rs191883449 | . | . | . | . | . | . | . | . | . | . | . |
| logdha | rs58238740 | 0.198 | 0.343 | 0.234 | 0.117 | 1.45 | 1.48 | 0.713 | 0.805 | 0.543 | 0.748 | -0.396 |
| logdha | rs3780894 | 0.198 | 0.343 | 0.234 | 0.117 | 1.45 | 1.48 | 0.713 | 0.805 | 0.543 | 0.748 | -0.396 |
| logdha | rs149646626 | . | . | . | . | . | . | . | . | . | . | . |
| logdha | rs79615212 | . | . | . | . | . | . | . | . | . | . | . |
| logdha | rs3780895 | 0.197 | 0.343 | 0.234 | 0.117 | 1.46 | 1.49 | 0.71 | 0.804 | 0.542 | 0.747 | -0.401 |
| logdha | rs3780896 | 0.198 | 0.343 | 0.234 | 0.117 | 1.45 | 1.48 | 0.713 | 0.805 | 0.543 | 0.748 | -0.396 |
| logdha | rs142747163 | 0.003 | . | . | . | . | . | . | . | . | . | . |
| logdha | rs189417584 | . | . | . | . | . | . | . | . | . | . | . |
| logdha | rs3780897 | 0.197 | 0.343 | 0.234 | 0.117 | 1.45 | 1.48 | 0.713 | 0.805 | 0.543 | 0.748 | -0.396 |
| logdha | rs117509491 | . | . | . | . | . | . | . | . | . | . | . |
| logdha | rs117811514 | . | . | . | . | . | . | . | . | . | . | . |
| logdha | rs116500932 | . | . | . | . | . | . | . | . | . | . | . |
| logdha | rs77584168 | 0.014 | 0.323 | 0.18 | 0.043 | 5.31 | 13.4 | 0.509 | 0.72 | 1.28 | 0.853 | -3.4 |
| logdha | rs2310776 | 0.198 | 0.343 | 0.234 | 0.117 | 1.45 | 1.48 | 0.713 | 0.805 | 0.543 | 0.748 | -0.396 |
| logdha | rs17157736 | 0.198 | 0.343 | 0.234 | 0.117 | 1.45 | 1.48 | 0.713 | 0.805 | 0.543 | 0.748 | -0.396 |
| logdha | rs72789735 | 0.001 | . | . | . | . | . | . | . | . | . | . |
| logdha | rs183747187 | . | . | . | . | . | . | . | . | . | . | . |
| logdha | rs7077891 | 0.198 | 0.343 | 0.234 | 0.117 | 1.45 | 1.48 | 0.713 | 0.805 | 0.543 | 0.748 | -0.396 |
| logdha | rs145063305 | 0.001 | . | . | . | . | . | . | . | . | . | . |
| logdha | rs3824613 | 0.198 | 0.347 | 0.238 | 0.123 | 1.5 | 1.54 | 0.694 | 0.787 | 0.535 | 0.724 | -0.436 |
| logdha | rs145543199 | . | . | . | . | . | . | . | . | . | . | . |
| logdha | rs140361993 | . | . | . | . | . | . | . | . | . | . | . |
| logdha | rs11239496 | 0.001 | . | . | . | . | . | . | . | . | . | . |
| logdha | rs11239497 | 0.197 | 0.343 | 0.234 | 0.117 | 1.45 | 1.48 | 0.713 | 0.805 | 0.543 | 0.748 | -0.396 |
| logdha | rs10900212 | 0.198 | 0.343 | 0.234 | 0.117 | 1.45 | 1.48 | 0.713 | 0.805 | 0.543 | 0.748 | -0.396 |
| logdha | rs186487136 | . | . | . | . | . | . | . | . | . | . | . |
| logdha | rs12774369 | 0.197 | 0.343 | 0.234 | 0.117 | 1.45 | 1.48 | 0.714 | 0.807 | 0.543 | 0.75 | -0.394 |
| logdha | rs60842538 | 0.197 | 0.343 | 0.234 | 0.116 | 1.45 | 1.48 | 0.715 | 0.808 | 0.544 | 0.752 | -0.392 |
| logdha | rs3780898 | 0.197 | 0.342 | 0.233 | 0.116 | 1.45 | 1.47 | 0.716 | 0.81 | 0.545 | 0.754 | -0.389 |
| logdha | rs3780899 | 0.197 | 0.342 | 0.233 | 0.115 | 1.45 | 1.47 | 0.717 | 0.81 | 0.546 | 0.755 | -0.388 |
| logdha | rs12252472 | . | . | . | . | . | . | . | . | . | . | . |
| logdha | rs185411011 | . | . | . | . | . | . | . | . | . | . | . |
| logdha | rs28395865 | 0.193 | 0.343 | 0.231 | 0.113 | 1.47 | 1.53 | 0.713 | 0.802 | 0.549 | 0.747 | -0.407 |
| logdha | rs191004074 | . | . | . | . | . | . | . | . | . | . | . |
| logdha | rs182401295 | . | . | . | . | . | . | . | . | . | . | . |
| logdha | rs2228064 | 0.057 | 0.375 | 0.177 | 0.038 | 123 | 708 | 0.404 | 0.034 | 0.144 | 0.426 | -121 |
| logdha | rs188284159 | . | . | . | . | . | . | . | . | . | . | . |
| logdha | rs2303887 | 0.055 | 0.374 | 0.177 | 0.037 | 120 | 690 | 0.405 | 0.035 | 0.147 | 0.427 | -118 |
| logdha | rs2303886 | 0.055 | 0.374 | 0.177 | 0.037 | 120 | 690 | 0.405 | 0.035 | 0.147 | 0.428 | -118 |
| logdha | rs192621006 | . | . | . | . | . | . | . | . | . | . | . |
| logdha | rs2303885 | 0.195 | 0.34 | 0.231 | 0.112 | 1.43 | 1.45 | 0.724 | 0.82 | 0.549 | 0.767 | -0.372 |
| logdha | rs114466168 | . | . | . | . | . | . | . | . | . | . | . |
| logdha | rs11239501 | 0.003 | . | . | . | . | . | . | . | . | . | . |
| logdha | rs745986 | 0.196 | 0.342 | 0.232 | 0.114 | 1.44 | 1.47 | 0.719 | 0.806 | 0.543 | 0.749 | -0.387 |
| logdha | rs75262268 | . | . | . | . | . | . | . | . | . | . | . |
| logdha | rs71494776 | 0.192 | 0.341 | 0.231 | 0.112 | 1.45 | 1.48 | 0.714 | 0.813 | 0.544 | 0.757 | -0.39 |
| logdha | rs74386871 | 0.055 | 0.373 | 0.177 | 0.037 | 119 | 687 | 0.406 | 0.035 | 0.148 | 0.429 | -117 |
| logdha | rs76502040 | . | . | . | . | . | . | . | . | . | . | . |
| logdha | rs142213734 | . | . | . | . | . | . | . | . | . | . | . |
| logdha | rs4948671 | 0.869 | 0.2 | 0.413 | 0.436 | 0.407 | 0.671 | 0.585 | 1.2 | 1.26 | 0.864 | 0.49 |
| logdha | rs146286085 | . | . | . | . | . | . | . | . | . | . | . |
| logdha | rs144080623 | . | . | . | . | . | . | . | . | . | . | . |
| logdha | rs115074014 | . | . | . | . | . | . | . | . | . | . | . |
| logdha | rs17157756 | . | . | . | . | . | . | . | . | . | . | . |
| logdha | rs190459780 | . | . | . | . | . | . | . | . | . | . | . |
| logdha | rs12358647 | 0.195 | 0.337 | 0.226 | 0.105 | 1.34 | 1.4 | 0.782 | 0.816 | 0.566 | 0.769 | -0.305 |
| logdha | rs147080556 | 0.009 | . | . | . | . | . | . | . | . | . | . |
| logdha | rs187543349 | . | . | . | . | . | . | . | . | . | . | . |
| logdha | rs114375595 | 0.01 | . | . | . | . | . | . | . | . | . | . |
| logdha | rs117211200 | . | . | . | . | . | . | . | . | . | . | . |
| logdha | rs192963047 | . | . | . | . | . | . | . | . | . | . | . |
| logdha | rs11239505 | 0.196 | 0.342 | 0.232 | 0.114 | 1.44 | 1.47 | 0.72 | 0.806 | 0.544 | 0.749 | -0.386 |
| logdha | rs184434116 | . | . | . | . | . | . | . | . | . | . | . |
| logdha | rs4948999 | 0.869 | 0.201 | 0.415 | 0.437 | 0.41 | 0.674 | 0.588 | 1.19 | 1.26 | 0.867 | 0.487 |
| logdha | rs11239506 | 0.003 | . | . | . | . | . | . | . | . | . | . |
| logdha | rs190706620 | . | . | . | . | . | . | . | . | . | . | . |
| logdha | rs190770429 | . | . | . | . | . | . | . | . | . | . | . |
| logdha | rs181327133 | . | . | . | . | . | . | . | . | . | . | . |
| logdha | rs149251697 | . | . | . | . | . | . | . | . | . | . | . |
| logdha | rs118081602 | 0.055 | 0.373 | 0.177 | 0.037 | 119 | 687 | 0.407 | 0.035 | 0.148 | 0.429 | -117 |
| logdha | rs188233691 | . | . | . | . | . | . | . | . | . | . | . |
| logdha | rs183778794 | 0.032 | 0.348 | 0.204 | 0.072 | 140 | 483 | 0.152 | 0.067 | 0.156 | 0.244 | -136 |
| logdha | rs188040078 | . | . | . | . | . | . | . | . | . | . | . |
| logdha | rs184675372 | . | . | . | . | . | . | . | . | . | . | . |
| logdha | rs150326891 | 0.004 | . | . | . | . | . | . | . | . | . | . |

| logdha | rs185520021 | . | . | . | . | . | . | . | . | . | . | . |
| --- | --- | --- | --- | --- | --- | --- | --- | --- | --- | --- | --- | --- |
| logdha | rs7907779 | 0.003 | . | . | . | . | . | . | . | . | . | . |
| logdha | rs184962860 | . | . | . | . | . | . | . | . | . | . | . |
| logdha | rs149762784 | 0.027 | 0.245 | 0.145 | 0.018 | 0.051 | 0.232 | 0.512 | 13.7 | 38.9 | 0.359 | 0.876 |
| logdha | rs149203427 | . | . | . | . | . | . | . | . | . | . | . |
| logdha | rs60153083 | . | . | . | . | . | . | . | . | . | . | . |
| logdha | rs112001069 | 0.194 | 0.374 | 0.249 | 0.139 | 1.89 | 1.93 | 0.531 | 0.667 | 0.462 | 0.559 | -0.794 |
| logdha | rs182355382 | 0.012 | 0.273 | 0.152 | 0.019 | 1.00E-03 | 5.50E-03 | 0.205 | 385 | 1.40E+03 | 0.094 | 0.833 |
| logdha | rs186740974 | 0.109 | 0.371 | 0.248 | 0.137 | 1.71 | 2.99 | 0.759 | 0.336 | 0.398 | 0.357 | -0.866 |
| logdha | rs191874916 | 0.081 | 0.378 | 0.246 | 0.135 | 5.23 | 10.9 | 0.428 | 0.319 | 0.435 | 0.402 | -3.98 |
| logdha | rs182989412 | 0.122 | 0.161 | 0.099 | 3.10E-03 | 6.60E-03 | 0.016 | 0.045 | 21.5 | 30.4 | 0.03 | 0.855 |
| logdha | rs188819476 | . | . | . | . | . | . | . | . | . | . | . |
| logdha | rs141217183 | 0.186 | 0.329 | 0.228 | 0.109 | 1.26 | 1.38 | 0.83 | 0.854 | 0.611 | 0.826 | -0.238 |
| logdha | rs192922652 | 0.199 | 0.345 | 0.234 | 0.116 | 1.46 | 1.5 | 0.711 | 0.795 | 0.538 | 0.735 | -0.406 |
| logdha | rs185092343 | 0.012 | 0.275 | 0.159 | 0.025 | 0.012 | 0.048 | 0.279 | 49.2 | 122 | 0.117 | 0.871 |
| logdha | rs145193311 | 0.194 | 0.341 | 0.232 | 0.114 | 1.44 | 1.47 | 0.723 | 0.819 | 0.551 | 0.767 | -0.376 |
| logdha | rs55789336 | 0.004 | . | . | . | . | . | . | . | . | . | . |
| logdha | rs55837212 | 0.009 | . | . | . | . | . | . | . | . | . | . |
| logdha | rs75408574 | 0.565 | 0.229 | 0.246 | 0.171 | 0.424 | 0.481 | 0.45 | 1.3 | 0.935 | 0.72 | 0.473 |
| logdha | rs149306926 | 0.01 | 0.301 | 0.169 | 0.033 | 0.908 | 4.06 | 0.983 | 0.078 | 0.439 | 0.65 | -0.187 |
| logdha | rs35990892 | 0.843 | 0.321 | 0.64 | 0.569 | 0.544 | 0.981 | 0.736 | 0.918 | 1.05 | 0.941 | 0.295 |
| logdha | rs192356207 | 0.015 | 0.262 | 0.149 | 0.018 | 0.147 | 0.505 | 0.577 | 9.58 | 20.3 | 0.287 | 0.96 |
| logdha | rs183741163 | 0.049 | 0.209 | 0.135 | 0.015 | 2.90E-03 | 7.40E-03 | 0.023 | 50.3 | 75.8 | 9.30E-03 | 0.818 |
| logdha | rs34737297 | 0.719 | 0.52 | 0.904 | 0.707 | 1.12 | 2.14 | 0.951 | 0.659 | 0.765 | 0.719 | -0.259 |
| logdha | rs181346933 | . | . | . | . | . | . | . | . | . | . | . |
| logdha | rs36093253 | 0.576 | 0.128 | 0.126 | 0.038 | 0.23 | 0.221 | 0.125 | 2.23 | 1.3 | 0.172 | 0.708 |
| logdha | rs186307855 | . | . | . | . | . | . | . | . | . | . | . |
| logdha | rs189681865 | . | . | . | . | . | . | . | . | . | . | . |
| logdha | rs34942971 | 0.214 | 0.373 | 0.256 | 0.15 | 1.74 | 1.82 | 0.598 | 0.685 | 0.472 | 0.583 | -0.668 |
| logdha | rs181816554 | . | . | . | . | . | . | . | . | . | . | . |
| logdha | rs186846881 | . | . | . | . | . | . | . | . | . | . | . |
| logdha | rs191178220 | 0.003 | . | . | . | . | . | . | . | . | . | . |
| logdha | rs183305228 | . | . | . | . | . | . | . | . | . | . | . |
| logdha | rs139912761 | 0.004 | . | . | . | . | . | . | . | . | . | . |
| logdha | rs185499901 | . | . | . | . | . | . | . | . | . | . | . |
| logdha | rs189816197 | 0.194 | 0.338 | 0.231 | 0.113 | 1.37 | 1.44 | 0.762 | 0.823 | 0.579 | 0.782 | -0.33 |
| logdha | rs145656938 | 0.009 | . | . | . | . | . | . | . | . | . | . |
| logdha | rs35745197 | 0.193 | 0.339 | 0.229 | 0.109 | 1.42 | 1.45 | 0.731 | 0.82 | 0.553 | 0.769 | -0.364 |
| logdha | rs192856637 | 0.006 | . | . | . | . | . | . | . | . | . | . |
| logdha | rs2310775 | 0.617 | 0.179 | 0.189 | 0.104 | 0.369 | 0.351 | 0.295 | 1.58 | 0.972 | 0.46 | 0.556 |
| logdha | rs11239507 | 0.673 | 0.26 | 0.218 | 0.108 | 0.583 | 0.415 | 0.448 | 1.13 | 0.515 | 0.796 | 0.328 |
| logdha | rs74648467 | 0.192 | 0.339 | 0.229 | 0.109 | 1.42 | 1.45 | 0.732 | 0.819 | 0.553 | 0.768 | -0.363 |
| logdha | rs145815934 | . | . | . | . | . | . | . | . | . | . | . |
| logdha | rs145390687 | . | . | . | . | . | . | . | . | . | . | . |
| logdha | rs184354913 | . | . | . | . | . | . | . | . | . | . | . |
| logdha | rs181244920 | . | . | . | . | . | . | . | . | . | . | . |
| logdha | rs11499087 | . | . | . | . | . | . | . | . | . | . | . |
| logdha | rs148263794 | 0.207 | 0.386 | 0.273 | 0.178 | 1.96 | 2.1 | 0.531 | 0.68 | 0.482 | 0.586 | -0.83 |
| logdha | rs150758903 | . | . | . | . | . | . | . | . | . | . | . |
| logdha | rs147654382 | 0.187 | 0.421 | 0.295 | 0.217 | 3.17 | 4.53 | 0.42 | 0.495 | 0.456 | 0.445 | -1.93 |
| logdha | rs147495166 | . | . | . | . | . | . | . | . | . | . | . |
| logdha | rs35198778 | 0.131 | 0.352 | 0.221 | 0.096 | 1.84 | 2.18 | 0.605 | 0.703 | 0.539 | 0.646 | -0.739 |
| logdha | rs145207659 | 0.048 | 0.268 | 0.153 | 0.021 | 0.16 | 0.324 | 0.365 | 2.96 | 4.51 | 0.477 | 0.699 |
| logdha | rs186466464 | . | . | . | . | . | . | . | . | . | . | . |
| logdha | rs12778413 | 0.131 | 0.306 | 0.199 | 0.069 | 1.02 | 1.41 | 0.99 | 0.975 | 0.909 | 0.979 | -0.02 |
| logdha | rs7895552 | 0.833 | 0.41 | 0.731 | 0.617 | 0.789 | 1.16 | 0.871 | 0.804 | 0.764 | 0.818 | 0.061 |
| logdha | rs12783895 | 0.193 | 0.339 | 0.229 | 0.109 | 1.41 | 1.44 | 0.735 | 0.822 | 0.554 | 0.771 | -0.357 |
| logdha | rs34929604 | 0.193 | 0.339 | 0.228 | 0.109 | 1.41 | 1.44 | 0.737 | 0.823 | 0.554 | 0.773 | -0.354 |
| logdha | rs12761961 | 0.185 | 0.354 | 0.239 | 0.124 | 1.7 | 1.74 | 0.606 | 0.752 | 0.524 | 0.682 | -0.598 |
| logdha | rs35536930 | 0.197 | 0.343 | 0.233 | 0.115 | 1.45 | 1.48 | 0.716 | 0.8 | 0.54 | 0.741 | -0.395 |
| logdha | rs180951060 | . | . | . | . | . | . | . | . | . | . | . |
| logdha | rs148892128 | . | . | . | . | . | . | . | . | . | . | . |
| logdha | rs11239509 | . | . | . | . | . | . | . | . | . | . | . |
| logdha | rs41526545 | 0.003 | . | . | . | . | . | . | . | . | . | . |
| logdha | rs2029253 | 0.617 | 0.179 | 0.189 | 0.104 | 0.37 | 0.352 | 0.296 | 1.57 | 0.97 | 0.461 | 0.555 |
| logdha | rs17444064 | 0.003 | . | . | . | . | . | . | . | . | . | . |
| logdha | rs71494779 | 0.194 | 0.341 | 0.231 | 0.112 | 1.45 | 1.47 | 0.717 | 0.814 | 0.546 | 0.759 | -0.385 |
| logdha | rs74991420 | 0.05 | 0.377 | 0.179 | 0.04 | 139 | 765 | 0.371 | 0.034 | 0.139 | 0.408 | -136 |
| logdha | rs182736551 | 0.05 | 0.377 | 0.179 | 0.04 | 139 | 765 | 0.371 | 0.034 | 0.139 | 0.408 | -136 |
| logdha | rs78702074 | 0.055 | 0.374 | 0.177 | 0.037 | 125 | 723 | 0.406 | 0.034 | 0.145 | 0.429 | -122 |
| logdha | rs187791466 | . | . | . | . | . | . | . | . | . | . | . |
| logdha | rs35300270 | 0.194 | 0.341 | 0.231 | 0.112 | 1.45 | 1.47 | 0.717 | 0.814 | 0.546 | 0.759 | -0.385 |
| logdha | rs6593484 | 0.003 | . | . | . | . | . | . | . | . | . | . |
| logdha | rs12244601 | . | . | . | . | . | . | . | . | . | . | . |
| logdha | rs184429574 | . | . | . | . | . | . | . | . | . | . | . |
| logdha | rs7080897 | 0.194 | 0.341 | 0.231 | 0.112 | 1.45 | 1.47 | 0.717 | 0.814 | 0.546 | 0.759 | -0.385 |
| logdha | rs7097429 | 0.196 | 0.346 | 0.236 | 0.119 | 1.49 | 1.54 | 0.698 | 0.791 | 0.541 | 0.732 | -0.431 |
| logdha | rs77979229 | 0.016 | 0.319 | 0.184 | 0.047 | 9.87 | 24.4 | 0.354 | 0.431 | 0.759 | 0.633 | -7.83 |
| logdha | rs11239512 | 0.003 | . | . | . | . | . | . | . | . | . | . |
| logdha | rs60146827 | 0.195 | 0.341 | 0.231 | 0.112 | 1.44 | 1.46 | 0.72 | 0.814 | 0.546 | 0.759 | -0.381 |
| logdha | rs79032236 | . | . | . | . | . | . | . | . | . | . | . |
| logdha | rs140854206 | . | . | . | . | . | . | . | . | . | . | . |
| logdha | rs184049550 | . | . | . | . | . | . | . | . | . | . | . |
| logdha | rs114433607 | . | . | . | . | . | . | . | . | . | . | . |
| logdha | rs117653793 | 0.055 | 0.374 | 0.177 | 0.037 | 124 | 721 | 0.406 | 0.034 | 0.145 | 0.429 | -122 |
| logdha | rs117039533 | 0.055 | 0.374 | 0.177 | 0.037 | 124 | 721 | 0.406 | 0.034 | 0.145 | 0.429 | -122 |

| logdha | rs191660761 | 0.001 | . | . | . | . | . | . | . | . | . | . |
| --- | --- | --- | --- | --- | --- | --- | --- | --- | --- | --- | --- | --- |
| logdha | rs59514896 | 0.197 | 0.341 | 0.23 | 0.111 | 1.42 | 1.45 | 0.729 | 0.812 | 0.545 | 0.756 | -0.37 |
| logdha | rs184661876 | . | . | . | . | . | . | . | . | . | . | . |
| logdha | rs61854073 | . | . | . | . | . | . | . | . | . | . | . |
| logdha | rs2018518 | 0.5 | 0.162 | 0.135 | 0.029 | 0.213 | 0.18 | 0.067 | 2.18 | 1.22 | 0.165 | 0.7 |
| logdha | rs934187 | 0.872 | 0.197 | 0.405 | 0.429 | 0.404 | 0.661 | 0.58 | 1.21 | 1.27 | 0.854 | 0.495 |
| logdha | rs7918542 | 0.202 | 0.342 | 0.232 | 0.113 | 1.42 | 1.45 | 0.732 | 0.802 | 0.54 | 0.743 | -0.372 |
| logdha | rs144311920 | . | . | . | . | . | . | . | . | . | . | . |
| logdha | rs7099684 | 0.871 | 0.196 | 0.404 | 0.428 | 0.403 | 0.66 | 0.579 | 1.22 | 1.27 | 0.852 | 0.497 |
| logdha | rs150829171 | . | . | . | . | . | . | . | . | . | . | . |
| logdha | rs186060697 | . | . | . | . | . | . | . | . | . | . | . |
| logdha | rs11239513 | . | . | . | . | . | . | . | . | . | . | . |
| logdha | rs7917687 | 0.2 | 0.338 | 0.228 | 0.108 | 1.38 | 1.39 | 0.75 | 0.822 | 0.548 | 0.768 | -0.334 |
| logdha | rs61854076 | . | . | . | . | . | . | . | . | . | . | . |
| logdha | rs71494780 | . | . | . | . | . | . | . | . | . | . | . |
| logdha | rs7919239 | 0.235 | 0.323 | 0.219 | 0.095 | 1.13 | 0.919 | 0.884 | 0.894 | 0.475 | 0.832 | -0.124 |
| logdha | rs78334475 | 0.166 | 0.264 | 0.204 | 0.085 | 0.297 | 0.566 | 0.524 | 1.6 | 1.72 | 0.66 | 0.565 |
| logdha | rs3780900 | 0.068 | 0.355 | 0.226 | 0.104 | 3.42 | 6.67 | 0.527 | 0.489 | 0.616 | 0.571 | -2.18 |
| logdha | rs186729292 | . | . | . | . | . | . | . | . | . | . | . |
| logdha | rs12267438 | . | . | . | . | . | . | . | . | . | . | . |
| logdha | rs1369214 | 0.868 | 0.207 | 0.395 | 0.409 | 0.419 | 0.64 | 0.569 | 1.18 | 1.16 | 0.863 | 0.477 |
| logdha | rs9282589 | . | . | . | . | . | . | . | . | . | . | . |
| logdha | rs114522915 | . | . | . | . | . | . | . | . | . | . | . |
| logdha | rs2115819 | 0.812 | 0.099 | 0.211 | 0.28 | 0.249 | 0.375 | 0.357 | 1.88 | 2.02 | 0.558 | 0.699 |
| logdha | rs117664603 | 0.055 | 0.371 | 0.175 | 0.036 | 101 | 576 | 0.418 | 0.04 | 0.169 | 0.442 | -98.9 |
| logdha | rs184979202 | . | . | . | . | . | . | . | . | . | . | . |
| logdha | rs190714671 | . | . | . | . | . | . | . | . | . | . | . |
| logdha | rs141538996 | . | . | . | . | . | . | . | . | . | . | . |
| logdha | rs150881741 | . | . | . | . | . | . | . | . | . | . | . |
| logdha | rs139076633 | 0.011 | 0.308 | 0.175 | 0.039 | 8.63 | 52.9 | 0.725 | 0.357 | 1.14 | 0.746 | -6.99 |
| logdha | rs12243649 | 0.056 | 0.373 | 0.176 | 0.037 | 96.2 | 544 | 0.42 | 0.042 | 0.175 | 0.444 | -94 |
| logdha | rs140348708 | . | . | . | . | . | . | . | . | . | . | . |
| logdha | rs79023256 | 0.055 | 0.37 | 0.175 | 0.036 | 96.3 | 547 | 0.421 | 0.042 | 0.175 | 0.445 | -94.1 |
| logdha | rs11239515 | 0.003 | . | . | . | . | . | . | . | . | . | . |
| logdha | rs55866086 | 0.059 | 0.376 | 0.178 | 0.038 | 84.3 | 458 | 0.414 | 0.044 | 0.174 | 0.429 | -82.3 |
| logdha | rs181340150 | . | . | . | . | . | . | . | . | . | . | . |
| logdha | rs11239516 | 0.003 | . | . | . | . | . | . | . | . | . | . |
| logdha | rs11239517 | 0.003 | . | . | . | . | . | . | . | . | . | . |
| logdha | rs11239519 | 0.003 | . | . | . | . | . | . | . | . | . | . |
| logdha | rs7909514 | 0.872 | 0.192 | 0.398 | 0.426 | 0.383 | 0.637 | 0.564 | 1.23 | 1.31 | 0.844 | 0.516 |
| logdha | rs7894352 | 0.872 | 0.192 | 0.398 | 0.426 | 0.382 | 0.636 | 0.563 | 1.23 | 1.31 | 0.844 | 0.517 |
| logdha | rs189384146 | 0 | . | . | . | . | . | . | . | . | . | . |
| logdha | rs11239520 | 0.004 | . | . | . | . | . | . | . | . | . | . |
| logdha | rs12221233 | 0.617 | 0.189 | 0.2 | 0.115 | 0.399 | 0.379 | 0.334 | 1.51 | 0.927 | 0.503 | 0.526 |
| logdha | rs74523293 | 0.049 | 0.37 | 0.175 | 0.035 | 135 | 815 | 0.418 | 0.036 | 0.159 | 0.452 | -132 |
| logdha | rs79376040 | 0.049 | 0.37 | 0.175 | 0.035 | 134 | 809 | 0.419 | 0.036 | 0.16 | 0.452 | -131 |
| logdha | rs4949000 | 0.617 | 0.189 | 0.2 | 0.115 | 0.399 | 0.379 | 0.334 | 1.51 | 0.927 | 0.504 | 0.526 |
| logdha | rs7089406 | . | . | . | . | . | . | . | . | . | . | . |
| logdha | rs138211056 | . | . | . | . | . | . | . | . | . | . | . |
| logdha | rs10900213 | 0.621 | 0.189 | 0.203 | 0.12 | 0.394 | 0.38 | 0.334 | 1.5 | 0.938 | 0.512 | 0.529 |
| logdha | rs145152827 | 0.011 | 0.308 | 0.175 | 0.039 | 8.52 | 52.3 | 0.727 | 0.362 | 1.15 | 0.75 | -6.88 |
| logdha | rs74443325 | 0.048 | 0.368 | 0.174 | 0.034 | 130 | 788 | 0.42 | 0.037 | 0.163 | 0.454 | -128 |
| logdha | rs188299446 | . | . | . | . | . | . | . | . | . | . | . |
| logdha | rs192699586 | . | . | . | . | . | . | . | . | . | . | . |
| logdha | rs72796464 | 0.003 | . | . | . | . | . | . | . | . | . | . |
| logdha | rs4949001 | 0.455 | 0.21 | 0.16 | 0.04 | 0.278 | 0.225 | 0.114 | 1.8 | 0.958 | 0.268 | 0.617 |
| logdha | rs147174641 | . | . | . | . | . | . | . | . | . | . | . |
| logdha | rs190835238 | . | . | . | . | . | . | . | . | . | . | . |
| logdha | rs76698944 | . | . | . | . | . | . | . | . | . | . | . |
| logdha | rs56071381 | 0.003 | . | . | . | . | . | . | . | . | . | . |
| logdha | rs78130427 | 0.2 | 0.338 | 0.228 | 0.107 | 1.34 | 1.38 | 0.779 | 0.808 | 0.556 | 0.757 | -0.31 |
| logdha | rs10900215 | 0.007 | . | . | . | . | . | . | . | . | . | . |
| logdha | rs41491445 | . | . | . | . | . | . | . | . | . | . | . |
| logdha | rs144995222 | . | . | . | . | . | . | . | . | . | . | . |
| logdha | rs149097803 | . | . | . | . | . | . | . | . | . | . | . |
| logdha | rs139429646 | . | . | . | . | . | . | . | . | . | . | . |
| logdha | rs7075620 | 0.861 | 0.305 | 0.596 | 0.543 | 0.579 | 0.914 | 0.729 | 0.963 | 0.981 | 0.971 | 0.286 |
| logdha | rs148299213 | . | . | . | . | . | . | . | . | . | . | . |
| logdha | rs4949002 | 0.238 | 0.214 | 0.136 | 0.015 | 0.159 | 0.192 | 0.128 | 2.79 | 2.22 | 0.198 | 0.722 |
| logdha | rs150496007 | . | . | . | . | . | . | . | . | . | . | . |
| logdha | rs147303455 | . | . | . | . | . | . | . | . | . | . | . |
| logdha | rs189518433 | . | . | . | . | . | . | . | . | . | . | . |
| logdha | rs186281355 | . | . | . | . | . | . | . | . | . | . | . |
| logdha | rs115297114 | . | . | . | . | . | . | . | . | . | . | . |
| logdha | rs11239523 | 0.003 | . | . | . | . | . | . | . | . | . | . |
| logdha | rs143704897 | . | . | . | . | . | . | . | . | . | . | . |
| logdha | rs188024096 | . | . | . | . | . | . | . | . | . | . | . |
| logdha | rs11239524 | 0.003 | . | . | . | . | . | . | . | . | . | . |
| logdha | rs191656573 | . | . | . | . | . | . | . | . | . | . | . |
| logdha | rs193295875 | 0.011 | 0.307 | 0.168 | 0.031 | 3.90E-04 | 2.80E-03 | 0.28 | 16.2 | 57.7 | 0.434 | 0.695 |
| logdha | rs190458228 | . | . | . | . | . | . | . | . | . | . | . |
| logdha | rs139253993 | 0.002 | . | . | . | . | . | . | . | . | . | . |
| logdha | rs4948672 | 0.812 | 0.105 | 0.155 | 0.127 | 0.177 | 0.241 | 0.203 | 2.01 | 1.91 | 0.463 | 0.755 |
| logdha | rs180954921 | . | . | . | . | . | . | . | . | . | . | . |
| logdha | rs12264801 | 0.447 | 0.455 | 0.455 | 0.431 | 1.56 | 2.75 | 0.799 | 0.603 | 0.68 | 0.654 | -0.59 |
| logdha | rs73283184 | . | . | . | . | . | . | . | . | . | . | . |

| logdha | rs142746187 | . | . | . | . | . | . | . | . | . | . | . |
| --- | --- | --- | --- | --- | --- | --- | --- | --- | --- | --- | --- | --- |
| logdha | rs58704739 | . | . | . | . | . | . | . | . | . | . | . |
| logdha | rs61272335 | . | . | . | . | . | . | . | . | . | . | . |
| logdha | rs60959489 | . | . | . | . | . | . | . | . | . | . | . |
| logdha | rs58502629 | . | . | . | . | . | . | . | . | . | . | . |
| logdha | rs10900216 | 0.838 | 0.066 | 0.14 | 0.197 | 0.21 | 0.314 | 0.297 | 2.47 | 2.63 | 0.393 | 0.758 |
| logdha | rs181118141 | . | . | . | . | . | . | . | . | . | . | . |
| logdha | rs137882665 | . | . | . | . | . | . | . | . | . | . | . |
| logdha | rs149466736 | . | . | . | . | . | . | . | . | . | . | . |
| logdha | rs2099171 | 0.806 | 0.045 | 0.082 | 0.091 | 0.147 | 0.199 | 0.158 | 3.25 | 3.07 | 0.211 | 0.83 |
| logdha | rs141852280 | . | . | . | . | . | . | . | . | . | . | . |
| logdha | rs191539257 | 0.001 | . | . | . | . | . | . | . | . | . | . |
| logdha | rs17157771 | 0.029 | 0.338 | 0.195 | 0.06 | 15.6 | 38.6 | 0.265 | 0.148 | 0.264 | 0.284 | -14.2 |
| logdha | rs111339241 | 0.029 | 0.337 | 0.195 | 0.06 | 15.3 | 38.4 | 0.279 | 0.153 | 0.279 | 0.304 | -13.8 |
| logdha | rs116983255 | . | . | . | . | . | . | . | . | . | . | . |
| logdha | rs142324341 | 0.037 | 0.293 | 0.158 | 0.023 | 7.30E-08 | 5.40E-07 | 0.027 | 2.30E+03 | 8.40E+03 | 0.034 | 0.707 |
| logdha | rs187191219 | . | . | . | . | . | . | . | . | . | . | . |
| logdha | rs151283973 | . | . | . | . | . | . | . | . | . | . | . |
| logdha | rs139316218 | . | . | . | . | . | . | . | . | . | . | . |
| logdha | rs6593485 | 0.886 | 0.078 | 0.151 | 0.188 | 0.296 | 0.46 | 0.433 | 2.17 | 2.24 | 0.453 | 0.676 |
| logdha | rs6593486 | 0.886 | 0.078 | 0.151 | 0.188 | 0.296 | 0.46 | 0.434 | 2.17 | 2.23 | 0.453 | 0.676 |
| logdha | rs145196618 | . | . | . | . | . | . | . | . | . | . | . |
| logdha | rs142598335 | . | . | . | . | . | . | . | . | . | . | . |
| logdha | rs892691 | 0.885 | 0.016 | 0.027 | 0.012 | 0.122 | 0.166 | 0.123 | 5.05 | 4.51 | 0.069 | 0.872 |
| logdha | rs7080713 | 0.809 | 0.046 | 0.085 | 0.094 | 0.15 | 0.203 | 0.162 | 3.2 | 3.02 | 0.217 | 0.826 |
| logdha | rs7077173 | 0.065 | 0.368 | 0.211 | 0.081 | 37.6 | 88.5 | 0.123 | 0.054 | 0.104 | 0.126 | -36.3 |
| logdha | rs7080977 | 0.03 | 0.338 | 0.195 | 0.06 | 15.3 | 38.4 | 0.277 | 0.152 | 0.276 | 0.299 | -13.8 |
| logdha | rs3780901 | 0.809 | 0.046 | 0.084 | 0.093 | 0.149 | 0.202 | 0.16 | 3.22 | 3.04 | 0.216 | 0.827 |
| logdha | rs61854091 | . | . | . | . | . | . | . | . | . | . | . |
| logdha | rs77550209 | . | . | . | . | . | . | . | . | . | . | . |
| logdha | rs73283191 | 0.077 | 0.372 | 0.186 | 0.048 | 13.5 | 37.9 | 0.354 | 0.209 | 0.407 | 0.421 | -11.8 |
| logdha | rs191866099 | . | . | . | . | . | . | . | . | . | . | . |
| logdha | rs147998395 | . | . | . | . | . | . | . | . | . | . | . |
| logdha | rs7090328 | 0.885 | 0.076 | 0.148 | 0.185 | 0.291 | 0.455 | 0.429 | 2.2 | 2.27 | 0.447 | 0.682 |
| logdha | rs79814535 | 0.048 | 0.337 | 0.161 | 0.023 | 16.2 | 73.9 | 0.541 | 0.205 | 0.642 | 0.612 | -14.4 |
| logdha | rs7073082 | 0.885 | 0.076 | 0.147 | 0.185 | 0.29 | 0.453 | 0.429 | 2.2 | 2.28 | 0.446 | 0.683 |
| logdha | rs11239526 | 0.039 | 0.235 | 0.135 | 0.012 | 0.209 | 0.483 | 0.498 | 5.1 | 7.72 | 0.282 | 0.806 |
| logdha | rs141839841 | . | . | . | . | . | . | . | . | . | . | . |
| logdha | rs75697371 | . | . | . | . | . | . | . | . | . | . | . |
| logdha | rs75972854 | . | . | . | . | . | . | . | . | . | . | . |
| logdha | rs139291975 | . | . | . | . | . | . | . | . | . | . | . |
| logdha | rs144064711 | . | . | . | . | . | . | . | . | . | . | . |
| logdha | rs3780902 | 0.078 | 0.372 | 0.186 | 0.048 | 13.6 | 38.2 | 0.355 | 0.207 | 0.405 | 0.421 | -11.9 |
| logdha | rs190449801 | . | . | . | . | . | . | . | . | . | . | . |
| logdha | rs73283196 | 0.077 | 0.372 | 0.186 | 0.048 | 13.7 | 38.4 | 0.353 | 0.208 | 0.405 | 0.42 | -12 |
| logdha | rs28395867 | . | . | . | . | . | . | . | . | . | . | . |
| logdha | rs28395866 | 0.029 | 0.34 | 0.197 | 0.062 | 17.1 | 42.4 | 0.252 | 0.143 | 0.257 | 0.279 | -15.6 |
| logdha | rs55661611 | 0.028 | 0.339 | 0.196 | 0.062 | 17 | 42 | 0.251 | 0.144 | 0.259 | 0.28 | -15.5 |
| logdha | rs55684518 | . | . | . | . | . | . | . | . | . | . | . |
| logdha | rs78552493 | . | . | . | . | . | . | . | . | . | . | . |
| logdha | rs147665434 | . | . | . | . | . | . | . | . | . | . | . |
| logdha | rs34445001 | . | . | . | . | . | . | . | . | . | . | . |
| logdha | rs184019449 | . | . | . | . | . | . | . | . | . | . | . |
| logdha | rs3780903 | 0.808 | 0.04 | 0.075 | 0.083 | 0.13 | 0.178 | 0.137 | 3.48 | 3.31 | 0.191 | 0.848 |
| logdha | rs17157784 | 0.048 | 0.336 | 0.16 | 0.022 | 15.7 | 71 | 0.543 | 0.211 | 0.655 | 0.616 | -13.9 |
| logdha | rs3780904 | 0.048 | 0.336 | 0.16 | 0.022 | 15.7 | 71 | 0.543 | 0.211 | 0.655 | 0.616 | -13.9 |
| logdha | rs192064730 | . | . | . | . | . | . | . | . | . | . | . |
| logdha | rs12774534 | . | . | . | . | . | . | . | . | . | . | . |
| logdha | rs79179915 | 0.047 | 0.336 | 0.16 | 0.022 | 15.6 | 70.7 | 0.545 | 0.213 | 0.664 | 0.619 | -13.8 |
| logdha | rs117811571 | . | . | . | . | . | . | . | . | . | . | . |
| logdha | rs72796477 | . | . | . | . | . | . | . | . | . | . | . |
| logdha | rs55729342 | 0.057 | 0.342 | 0.165 | 0.026 | 15.2 | 63.5 | 0.515 | 0.225 | 0.637 | 0.598 | -13.4 |
| logdha | rs59317220 | 0.047 | 0.336 | 0.16 | 0.022 | 15.5 | 70.4 | 0.548 | 0.215 | 0.67 | 0.622 | -13.7 |
| logdha | rs3780905 | 0.562 | 0.238 | 0.266 | 0.199 | 0.479 | 0.526 | 0.503 | 1.27 | 0.965 | 0.749 | 0.429 |
| logdha | rs3780906 | 0.807 | 0.038 | 0.071 | 0.078 | 0.122 | 0.167 | 0.124 | 3.6 | 3.42 | 0.177 | 0.857 |
| logdha | rs149047975 | . | . | . | . | . | . | . | . | . | . | . |
| logdha | rs3824614 | 0.047 | 0.336 | 0.16 | 0.022 | 15.2 | 69.6 | 0.551 | 0.217 | 0.679 | 0.625 | -13.5 |
| logdha | rs140254404 | . | . | . | . | . | . | . | . | . | . | . |
| logdha | rs145262684 | . | . | . | . | . | . | . | . | . | . | . |
| logdha | rs76735482 | 0.047 | 0.335 | 0.16 | 0.022 | 15.1 | 68.9 | 0.553 | 0.219 | 0.686 | 0.628 | -13.3 |
| logdha | rs73283198 | 0.028 | 0.348 | 0.202 | 0.069 | 26.9 | 60 | 0.14 | 0.114 | 0.186 | 0.183 | -25.2 |
| logdha | rs74911239 | . | . | . | . | . | . | . | . | . | . | . |
| logdha | rs150551647 | . | . | . | . | . | . | . | . | . | . | . |
| logdha | rs115200794 | . | . | . | . | . | . | . | . | . | . | . |
| logdha | rs74130823 | 0.038 | 0.36 | 0.214 | 0.085 | 21.6 | 41.8 | 0.113 | 0.158 | 0.208 | 0.16 | -19.7 |
| logdha | rs2279435 | 0.754 | 0.059 | 0.077 | 0.03 | 0.135 | 0.163 | 0.096 | 3.15 | 2.41 | 0.135 | 0.831 |
| logdha | rs147279673 | 0.01 | . | . | . | . | . | . | . | . | . | . |
| logdha | rs183525146 | 0.006 | . | . | . | . | . | . | . | . | . | . |
| logdha | rs3740107 | 0.737 | 0.322 | 0.407 | 0.37 | 0.748 | 0.871 | 0.803 | 0.981 | 0.705 | 0.979 | 0.166 |
| logdha | rs140697247 | . | . | . | . | . | . | . | . | . | . | . |
| logdha | rs1565096 | 0.806 | 0.148 | 0.234 | 0.227 | 0.38 | 0.565 | 0.516 | 1.59 | 1.47 | 0.616 | 0.561 |
| logdha | rs1565097 | 0.806 | 0.147 | 0.233 | 0.225 | 0.377 | 0.56 | 0.511 | 1.6 | 1.48 | 0.613 | 0.564 |
| logdha | rs144584548 | . | . | . | . | . | . | . | . | . | . | . |
| logdha | rs75811053 | 0.044 | 0.334 | 0.16 | 0.022 | 13.7 | 61.7 | 0.56 | 0.242 | 0.741 | 0.643 | -12 |
| logdha | rs147462967 | . | . | . | . | . | . | . | . | . | . | . |
| logdha | rs74130824 | 0.044 | 0.334 | 0.16 | 0.022 | 13.8 | 62.1 | 0.559 | 0.241 | 0.738 | 0.642 | -12 |

| logdha | rs116883184 | . | . | . | . | . | . | . | . | . | . | . |
| --- | --- | --- | --- | --- | --- | --- | --- | --- | --- | --- | --- | --- |
| logdha | rs117258049 | . | . | . | . | . | . | . | . | . | . | . |
| logdha | rs3780908 | 0.029 | 0.357 | 0.208 | 0.077 | 36.4 | 78.2 | 0.094 | 0.09 | 0.143 | 0.128 | -34.6 |
| logdha | rs180938522 | . | . | . | . | . | . | . | . | . | . | . |
| logdha | rs76687372 | . | . | . | . | . | . | . | . | . | . | . |
| logdha | rs3780909 | 0.666 | 0.113 | 0.131 | 0.06 | 0.145 | 0.165 | 0.089 | 2.2 | 1.58 | 0.273 | 0.778 |
| logdha | rs78281156 | 0.044 | 0.334 | 0.16 | 0.022 | 13.7 | 61.7 | 0.56 | 0.242 | 0.741 | 0.643 | -12 |
| logdha | rs187840631 | . | . | . | . | . | . | . | . | . | . | . |
| logdha | rs76868242 | 0.044 | 0.334 | 0.16 | 0.022 | 13.7 | 61.7 | 0.56 | 0.242 | 0.741 | 0.643 | -12 |
| logdha | rs192794192 | . | . | . | . | . | . | . | . | . | . | . |
| logdha | rs12781248 | . | . | . | . | . | . | . | . | . | . | . |
| logdha | rs12762375 | . | . | . | . | . | . | . | . | . | . | . |
| logdha | rs192256986 | . | . | . | . | . | . | . | . | . | . | . |
| logdha | rs184411030 | . | . | . | . | . | . | . | . | . | . | . |
| logdha | rs189601483 | 0.049 | 0.332 | 0.161 | 0.023 | 11.2 | 45.4 | 0.553 | 0.301 | 0.82 | 0.66 | -9.37 |
| logdha | rs143254579 | 0.045 | 0.232 | 0.135 | 0.012 | 0.17 | 0.383 | 0.431 | 5.69 | 8.27 | 0.232 | 0.822 |
| logdha | rs12253903 | . | . | . | . | . | . | . | . | . | . | . |
| logdha | rs35489926 | . | . | . | . | . | . | . | . | . | . | . |
| logdha | rs71494783 | 0.073 | 0.38 | 0.187 | 0.05 | 32.6 | 96.7 | 0.241 | 0.138 | 0.276 | 0.322 | -30.3 |
| logdha | rs180711602 | . | . | . | . | . | . | . | . | . | . | . |
| logdha | rs117312402 | 0.044 | 0.334 | 0.16 | 0.022 | 13.8 | 61.7 | 0.559 | 0.242 | 0.74 | 0.643 | -12 |
| logdha | rs74831694 | 0.053 | 0.271 | 0.173 | 0.041 | 0.105 | 0.237 | 0.317 | 3.64 | 5.87 | 0.424 | 0.728 |
| logdha | rs140301943 | . | . | . | . | . | . | . | . | . | . | . |
| logdha | rs78553679 | . | . | . | . | . | . | . | . | . | . | . |
| logdha | rs78334250 | 0.044 | 0.334 | 0.16 | 0.022 | 13.8 | 61.7 | 0.559 | 0.242 | 0.74 | 0.643 | -12 |
| logdha | rs76000028 | 0.044 | 0.334 | 0.16 | 0.022 | 13.8 | 61.7 | 0.559 | 0.242 | 0.74 | 0.643 | -12 |
| logdha | rs145195254 | . | . | . | . | . | . | . | . | . | . | . |
| logdha | rs137888527 | . | . | . | . | . | . | . | . | . | . | . |
| logdha | rs149109592 | . | . | . | . | . | . | . | . | . | . | . |
| logdha | rs80280010 | 0.038 | 0.236 | 0.136 | 0.012 | 0.252 | 0.571 | 0.543 | 4.62 | 6.83 | 0.301 | 0.787 |
| logdha | rs189995374 | 0.005 | . | . | . | . | . | . | . | . | . | . |
| logdha | rs75467770 | 0.05 | 0.338 | 0.161 | 0.023 | 12.7 | 59.5 | 0.588 | 0.237 | 0.76 | 0.653 | -11 |
| logdha | rs1487562 | 0.029 | 0.357 | 0.208 | 0.077 | 37.2 | 79.5 | 0.091 | 0.089 | 0.141 | 0.126 | -35.3 |
| logdha | rs12266963 | 0.073 | 0.38 | 0.187 | 0.05 | 32.5 | 96.6 | 0.241 | 0.138 | 0.277 | 0.323 | -30.2 |
| logdha | rs7099874 | 0.769 | 0.245 | 0.34 | 0.311 | 0.441 | 0.668 | 0.589 | 1.13 | 1.03 | 0.895 | 0.436 |
| logdha | rs75687019 | 0.044 | 0.334 | 0.16 | 0.022 | 13.7 | 61.7 | 0.559 | 0.242 | 0.741 | 0.643 | -12 |
| logdha | rs148240462 | . | . | . | . | . | . | . | . | . | . | . |
| logdha | rs141278174 | . | . | . | . | . | . | . | . | . | . | . |
| logdha | rs76404852 | 0.044 | 0.334 | 0.16 | 0.022 | 13.7 | 61.7 | 0.559 | 0.242 | 0.741 | 0.643 | -12 |
| logdha | rs139112240 | . | . | . | . | . | . | . | . | . | . | . |
| logdha | rs12783037 | 0.073 | 0.38 | 0.187 | 0.05 | 32.5 | 96.6 | 0.241 | 0.138 | 0.277 | 0.323 | -30.2 |
| logdha | rs72796481 | . | . | . | . | . | . | . | . | . | . | . |
| logdha | rs76798749 | 0.044 | 0.334 | 0.16 | 0.022 | 13.7 | 61.6 | 0.559 | 0.242 | 0.741 | 0.643 | -12 |
| logdha | rs187732548 | . | . | . | . | . | . | . | . | . | . | . |
| logdha | rs75675474 | 0.044 | 0.334 | 0.16 | 0.022 | 13.7 | 61.6 | 0.559 | 0.242 | 0.741 | 0.643 | -12 |
| logdha | rs76552293 | 0.044 | 0.334 | 0.16 | 0.022 | 13.7 | 61.6 | 0.559 | 0.242 | 0.741 | 0.643 | -12 |
| logdha | rs147445993 | 0.001 | . | . | . | . | . | . | . | . | . | . |
| logdha | rs115902283 | 0.044 | 0.335 | 0.16 | 0.022 | 13.5 | 61.2 | 0.567 | 0.243 | 0.754 | 0.648 | -11.7 |
| logdha | rs4540799 | 0.759 | 0.243 | 0.323 | 0.286 | 0.42 | 0.638 | 0.568 | 1.11 | 1.01 | 0.912 | 0.45 |
| logdha | rs12765320 | 0.102 | 0.42 | 0.208 | 0.08 | 16.1 | 24.1 | 0.064 | 0.21 | 0.226 | 0.147 | -14.1 |
| logdha | rs12782256 | . | . | . | . | . | . | . | . | . | . | . |
| logdha | rs191727256 | . | . | . | . | . | . | . | . | . | . | . |
| logdha | rs2082111 | 0.028 | 0.357 | 0.208 | 0.077 | 36.8 | 78.9 | 0.092 | 0.09 | 0.142 | 0.127 | -35 |
| logdha | rs2082110 | 0.072 | 0.396 | 0.195 | 0.06 | 23 | 60.9 | 0.235 | 0.157 | 0.291 | 0.318 | -21 |
| logdha | rs2082109 | 0.072 | 0.396 | 0.195 | 0.06 | 23 | 60.9 | 0.235 | 0.157 | 0.291 | 0.318 | -21 |
| logdha | rs117316662 | 0.044 | 0.334 | 0.16 | 0.022 | 13.8 | 62 | 0.559 | 0.242 | 0.739 | 0.643 | -12 |
| logdha | rs12247163 | 0.102 | 0.42 | 0.207 | 0.079 | 16.4 | 24.4 | 0.059 | 0.202 | 0.211 | 0.125 | -14.5 |
| logdha | rs12355888 | 0.002 | . | . | . | . | . | . | . | . | . | . |
| logdha | rs143772290 | . | . | . | . | . | . | . | . | . | . | . |
| logdha | rs146849452 | . | . | . | . | . | . | . | . | . | . | . |
| logdha | rs140267121 | . | . | . | . | . | . | . | . | . | . | . |
| logdha | rs7080474 | 0.058 | 0.374 | 0.218 | 0.092 | 15.3 | 20.1 | 0.039 | 0.19 | 0.193 | 0.102 | -13.6 |
| logdha | rs144066203 | . | . | . | . | . | . | . | . | . | . | . |
| logdha | rs12765209 | 0.044 | 0.334 | 0.16 | 0.022 | 13.9 | 62.3 | 0.558 | 0.241 | 0.738 | 0.642 | -12.1 |
| logdha | rs150930735 | . | . | . | . | . | . | . | . | . | . | . |
| logdha | rs7068039 | 0.102 | 0.42 | 0.207 | 0.079 | 16.4 | 24.4 | 0.059 | 0.202 | 0.211 | 0.125 | -14.5 |
| logdha | rs117622652 | 0.043 | 0.334 | 0.16 | 0.022 | 13.9 | 62.4 | 0.558 | 0.241 | 0.738 | 0.642 | -12.1 |
| logdha | rs190191514 | . | . | . | . | . | . | . | . | . | . | . |
| logdha | rs74130835 | . | . | . | . | . | . | . | . | . | . | . |
| logdha | rs1811604 | . | . | . | . | . | . | . | . | . | . | . |
| logdha | rs146971256 | 0.001 | . | . | . | . | . | . | . | . | . | . |
| logdha | rs74336000 | . | . | . | . | . | . | . | . | . | . | . |
| logdha | rs186046492 | . | . | . | . | . | . | . | . | . | . | . |
| logdha | rs34971823 | . | . | . | . | . | . | . | . | . | . | . |
| logdha | rs75227446 | 0.043 | 0.334 | 0.16 | 0.022 | 14 | 63 | 0.558 | 0.24 | 0.736 | 0.642 | -12.2 |
| logdha | rs190457599 | . | . | . | . | . | . | . | . | . | . | . |
| logdha | rs79591917 | 0.043 | 0.334 | 0.16 | 0.022 | 14 | 63.1 | 0.558 | 0.24 | 0.736 | 0.642 | -12.2 |
| logdha | rs702365 | 0.647 | 0.152 | 0.155 | 0.065 | 0.187 | 0.194 | 0.107 | 1.69 | 1.12 | 0.428 | 0.709 |
| logdha | rs79643836 | 0.043 | 0.334 | 0.16 | 0.022 | 14 | 63.1 | 0.558 | 0.24 | 0.735 | 0.642 | -12.2 |
| logdha | rs702366 | 0.649 | 0.154 | 0.157 | 0.066 | 0.187 | 0.195 | 0.108 | 1.68 | 1.12 | 0.433 | 0.707 |
| logdha | rs1059696 | 0.059 | 0.373 | 0.217 | 0.09 | 14.5 | 18.9 | 0.041 | 0.199 | 0.199 | 0.106 | -12.8 |
| logdha | rs901681 | 0.648 | 0.154 | 0.157 | 0.066 | 0.187 | 0.195 | 0.108 | 1.68 | 1.12 | 0.433 | 0.707 |
| logdha | rs61854092 | 0.058 | 0.373 | 0.217 | 0.091 | 14.6 | 19.1 | 0.04 | 0.198 | 0.198 | 0.105 | -12.9 |
| logdha | rs148080753 | . | . | . | . | . | . | . | . | . | . | . |
| logdha | rs61854093 | 0.064 | 0.392 | 0.237 | 0.121 | 16.1 | 22 | 0.042 | 0.184 | 0.192 | 0.105 | -14.3 |
| logdha | rs141822774 | . | . | . | . | . | . | . | . | . | . | . |

| logdha | rs74130836 | . | . | . | . | . | . | . | . | . | . | . |
| --- | --- | --- | --- | --- | --- | --- | --- | --- | --- | --- | --- | --- |
| logdha | rs150620963 | . | . | . | . | . | . | . | . | . | . | . |
| logdha | rs3780913 | 0.749 | 0.305 | 0.371 | 0.329 | 0.533 | 0.746 | 0.653 | 0.924 | 0.791 | 0.926 | 0.313 |
| logdha | rs142255723 | . | . | . | . | . | . | . | . | . | . | . |
| logdha | rs3780914 | 0.647 | 0.155 | 0.158 | 0.067 | 0.189 | 0.197 | 0.11 | 1.66 | 1.11 | 0.446 | 0.705 |
| logdha | rs182656507 | . | . | . | . | . | . | . | . | . | . | . |
| logdha | rs2291427 | 0.703 | 0.276 | 0.334 | 0.287 | 0.465 | 0.545 | 0.513 | 0.982 | 0.735 | 0.981 | 0.385 |
| logdha | rs10751382 | 0.703 | 0.277 | 0.335 | 0.288 | 0.466 | 0.546 | 0.514 | 0.98 | 0.734 | 0.979 | 0.384 |
| logdha | rs117610108 | 0.043 | 0.335 | 0.16 | 0.022 | 14.4 | 65.1 | 0.555 | 0.235 | 0.726 | 0.639 | -12.6 |
| logdha | rs144966941 | 0.002 | . | . | . | . | . | . | . | . | . | . |
| logdha | rs41502051 | 0.007 | . | . | . | . | . | . | . | . | . | . |
| logdha | rs41426046 | 0.047 | 0.333 | 0.159 | 0.021 | 13.1 | 56.5 | 0.551 | 0.261 | 0.762 | 0.645 | -11.3 |
| logdha | rs41519248 | 0.043 | 0.335 | 0.16 | 0.022 | 14.5 | 65.7 | 0.554 | 0.234 | 0.723 | 0.638 | -12.7 |
| logdha | rs189108245 | . | . | . | . | . | . | . | . | . | . | . |
| logdha | rs7896431 | 0.668 | 0.192 | 0.205 | 0.122 | 0.272 | 0.281 | 0.208 | 1.34 | 0.903 | 0.66 | 0.606 |
| logdha | rs183724547 | . | . | . | . | . | . | . | . | . | . | . |
| logdha | rs142044990 | . | . | . | . | . | . | . | . | . | . | . |
| logdha | rs188140929 | 0.021 | 0.248 | 0.139 | 0.013 | 1.18 | 4.96 | 0.969 | 2.12 | 5.58 | 0.776 | 0.192 |
| logdha | rs76819096 | . | . | . | . | . | . | . | . | . | . | . |
| logdha | rs10751383 | 0.681 | 0.117 | 0.131 | 0.055 | 0.187 | 0.19 | 0.099 | 2.06 | 1.34 | 0.269 | 0.741 |
| logdha | rs117819321 | . | . | . | . | . | . | . | . | . | . | . |
| logdha | rs2242332 | 0.744 | 0.307 | 0.375 | 0.334 | 0.548 | 0.768 | 0.668 | 0.903 | 0.776 | 0.905 | 0.297 |
| logdha | rs2242333 | 0.476 | 0.259 | 0.261 | 0.18 | 0.417 | 0.519 | 0.482 | 1.17 | 0.957 | 0.851 | 0.45 |
| logdha | rs2242334 | 0.744 | 0.308 | 0.377 | 0.335 | 0.551 | 0.772 | 0.67 | 0.899 | 0.773 | 0.902 | 0.294 |
| logdha | rs189465041 | . | . | . | . | . | . | . | . | . | . | . |
| logdha | rs28395868 | . | . | . | . | . | . | . | . | . | . | . |
| logdha | rs56067550 | . | . | . | . | . | . | . | . | . | . | . |
| logdha | rs1051713 | 0.1 | 0.422 | 0.209 | 0.082 | 16.8 | 24.9 | 0.056 | 0.2 | 0.208 | 0.121 | -14.8 |
| logdha | rs115074210 | . | . | . | . | . | . | . | . | . | . | . |
| logdha | rs7900977 | 0.782 | 0.252 | 0.344 | 0.313 | 0.531 | 0.709 | 0.635 | 1.11 | 0.937 | 0.902 | 0.365 |
| logdha | rs147613891 | . | . | . | . | . | . | . | . | . | . | . |
| logdha | rs2229136 | 0.038 | 0.235 | 0.135 | 0.012 | 0.236 | 0.54 | 0.527 | 4.79 | 7.11 | 0.292 | 0.795 |
| logdha | rs185482841 | . | . | . | . | . | . | . | . | . | . | . |
| logdha | rs2288619 | 0.038 | 0.235 | 0.135 | 0.012 | 0.236 | 0.539 | 0.527 | 4.79 | 7.11 | 0.292 | 0.795 |
| logdha | rs189891524 | 0.003 | . | . | . | . | . | . | . | . | . | . |
| logdha | rs56145077 | 0.058 | 0.374 | 0.218 | 0.091 | 14.8 | 19.4 | 0.04 | 0.197 | 0.197 | 0.105 | -13.1 |
| logdha | rs143216970 | . | . | . | . | . | . | . | . | . | . | . |
| logdha | rs142246478 | . | . | . | . | . | . | . | . | . | . | . |
| logdha | rs13134 | . | . | . | . | . | . | . | . | . | . | . |
| logdha | rs41314980 | . | . | . | . | . | . | . | . | . | . | . |
| logdha | rs41304611 | . | . | . | . | . | . | . | . | . | . | . |
| logdha | rs193299617 | 0.004 | . | . | . | . | . | . | . | . | . | . |
| logdha | rs12772724 | . | . | . | . | . | . | . | . | . | . | . |
| logdha | rs139489537 | 0.038 | 0.237 | 0.136 | 0.012 | 0.245 | 0.559 | 0.538 | 4.67 | 6.94 | 0.3 | 0.789 |
| logdha | rs12776062 | . | . | . | . | . | . | . | . | . | . | . |
| logdha | rs752059 | 0.059 | 0.373 | 0.218 | 0.091 | 14.6 | 19.1 | 0.04 | 0.199 | 0.198 | 0.105 | -12.9 |
| logdha | rs147649982 | 0.015 | 0.271 | 0.152 | 0.02 | 5.06 | 50 | 0.87 | 2.40E-06 | 2.70E-05 | 0.241 | -4.33 |
| logdha | rs17157798 | 0.038 | 0.237 | 0.136 | 0.012 | 0.245 | 0.558 | 0.537 | 4.67 | 6.95 | 0.299 | 0.789 |
| logdha | rs142298619 | . | . | . | . | . | . | . | . | . | . | . |
| logdha | rs4948673 | 0.777 | 0.273 | 0.37 | 0.337 | 0.603 | 0.781 | 0.696 | 1.05 | 0.863 | 0.955 | 0.297 |
| logdha | rs74231484 | 0.476 | 0.276 | 0.273 | 0.193 | 0.485 | 0.574 | 0.541 | 1.07 | 0.839 | 0.932 | 0.382 |
| logdha | rs72796489 | . | . | . | . | . | . | . | . | . | . | . |
| logdha | rs113447578 | 0.026 | 0.354 | 0.206 | 0.074 | 34 | 70 | 0.087 | 0.104 | 0.158 | 0.135 | -32.1 |
| logdha | rs34063374 | . | . | . | . | . | . | . | . | . | . | . |
| logdha | rs11239527 | . | . | . | . | . | . | . | . | . | . | . |
| logdha | rs34371883 | . | . | . | . | . | . | . | . | . | . | . |
| logdha | rs118168828 | 0.002 | . | . | . | . | . | . | . | . | . | . |
| logdha | rs34349618 | . | . | . | . | . | . | . | . | . | . | . |
| logdha | rs80041679 | 0.038 | 0.237 | 0.136 | 0.012 | 0.245 | 0.559 | 0.538 | 4.67 | 6.94 | 0.3 | 0.789 |
| logdha | rs149640607 | . | . | . | . | . | . | . | . | . | . | . |
| logdha | rs4427523 | 0.957 | 0.02 | 0.087 | 0.364 | 0.024 | 0.086 | 0.298 | 4.2 | 9.55 | 0.527 | 0.958 |
| logdha | rs185880109 | . | . | . | . | . | . | . | . | . | . | . |
| logdha | rs191523220 | . | . | . | . | . | . | . | . | . | . | . |
| logdha | rs144315284 | . | . | . | . | . | . | . | . | . | . | . |
| logdha | rs4948674 | 0.156 | 0.3 | 0.177 | 0.042 | 0.78 | 0.874 | 0.825 | 0.951 | 0.691 | 0.945 | 0.142 |
| logdha | rs182984298 | . | . | . | . | . | . | . | . | . | . | . |
| logdha | rs56749023 | . | . | . | . | . | . | . | . | . | . | . |
| logdha | rs112508957 | . | . | . | . | . | . | . | . | . | . | . |
| logdha | rs7089063 | 0.138 | 0.354 | 0.18 | 0.041 | 5.05 | 6.36 | 0.199 | 0.565 | 0.489 | 0.51 | -3.39 |
| logdha | rs145000779 | . | . | . | . | . | . | . | . | . | . | . |
| logdha | rs80201572 | 0.038 | 0.236 | 0.136 | 0.012 | 0.244 | 0.555 | 0.536 | 4.69 | 6.96 | 0.298 | 0.79 |
| logdha | rs140568227 | . | . | . | . | . | . | . | . | . | . | . |
| logdha | rs149756228 | . | . | . | . | . | . | . | . | . | . | . |
| logdha | rs112380685 | . | . | . | . | . | . | . | . | . | . | . |
| logdha | rs60481109 | . | . | . | . | . | . | . | . | . | . | . |
| logdha | rs138221843 | . | . | . | . | . | . | . | . | . | . | . |
| logdha | rs150283194 | . | . | . | . | . | . | . | . | . | . | . |
| logdha | rs138879661 | . | . | . | . | . | . | . | . | . | . | . |
| logdha | rs34960345 | 0.038 | 0.236 | 0.136 | 0.012 | 0.242 | 0.55 | 0.533 | 4.7 | 6.97 | 0.296 | 0.791 |
| logdha | rs147514068 | . | . | . | . | . | . | . | . | . | . | . |
| logdha | rs35410535 | 0.038 | 0.236 | 0.136 | 0.012 | 0.242 | 0.55 | 0.533 | 4.7 | 6.97 | 0.296 | 0.791 |
| logdha | rs191613635 | . | . | . | . | . | . | . | . | . | . | . |
| logdha | rs17157800 | . | . | . | . | . | . | . | . | . | . | . |
| logdha | rs17523413 | 0.038 | 0.236 | 0.136 | 0.012 | 0.243 | 0.553 | 0.534 | 4.7 | 6.97 | 0.297 | 0.79 |
| logdha | rs7897036 | 0.001 | . | . | . | . | . | . | . | . | . | . |
| logdha | rs76334323 | 0.042 | 0.335 | 0.16 | 0.022 | 14.9 | 67.3 | 0.55 | 0.23 | 0.71 | 0.634 | -13.1 |

| logdha | rs143535991 | . | . | . | . | . | . | . | . | . | . | . |
| --- | --- | --- | --- | --- | --- | --- | --- | --- | --- | --- | --- | --- |
| logdha | rs140344979 | . | . | . | . | . | . | . | . | . | . | . |
| logdha | rs36047811 | 0.038 | 0.236 | 0.136 | 0.012 | 0.242 | 0.55 | 0.533 | 4.7 | 6.97 | 0.296 | 0.791 |
| logdha | rs76493570 | 0.038 | 0.236 | 0.136 | 0.012 | 0.242 | 0.55 | 0.533 | 4.7 | 6.97 | 0.296 | 0.791 |
| logdha | rs192998920 | . | . | . | . | . | . | . | . | . | . | . |
| logdha | rs116840852 | . | . | . | . | . | . | . | . | . | . | . |
| logdha | rs184824101 | . | . | . | . | . | . | . | . | . | . | . |
| logdha | rs41301621 | . | . | . | . | . | . | . | . | . | . | . |
| logdha | rs3740109 | 0.038 | 0.236 | 0.136 | 0.012 | 0.242 | 0.55 | 0.533 | 4.7 | 6.97 | 0.296 | 0.791 |
| logdha | rs7899415 | 0.089 | 0.397 | 0.254 | 0.148 | 8.17 | 16.9 | 0.309 | 0.229 | 0.292 | 0.248 | -6.82 |
| logdha | rs184395284 | 0.004 | . | . | . | . | . | . | . | . | . | . |
| logdha | rs139209589 | . | . | . | . | . | . | . | . | . | . | . |
| logdha | rs149924163 | . | . | . | . | . | . | . | . | . | . | . |
| logdha | rs3740110 | 0.042 | 0.335 | 0.16 | 0.022 | 14.9 | 67.3 | 0.55 | 0.23 | 0.71 | 0.634 | -13.1 |
| logdha | rs144335113 | . | . | . | . | . | . | . | . | . | . | . |
| logdha | rs74743385 | . | . | . | . | . | . | . | . | . | . | . |
| logdha | rs146545829 | . | . | . | . | . | . | . | . | . | . | . |
| logdha | rs181842689 | . | . | . | . | . | . | . | . | . | . | . |
| logdha | rs3740111 | 0.038 | 0.236 | 0.136 | 0.012 | 0.242 | 0.55 | 0.533 | 4.7 | 6.97 | 0.296 | 0.791 |
| logdha | rs186261599 | . | . | . | . | . | . | . | . | . | . | . |
| logdha | rs73285348 | 0.013 | 0.346 | 0.201 | 0.068 | 76.6 | 208 | 0.111 | 0.083 | 0.171 | 0.228 | -73.8 |
| logdha | rs186356901 | . | . | . | . | . | . | . | . | . | . | . |
| logdha | rs3824618 | 0.038 | 0.236 | 0.136 | 0.012 | 0.244 | 0.555 | 0.536 | 4.69 | 6.96 | 0.298 | 0.79 |
| logdha | rs3802548 | 0.065 | 0.383 | 0.222 | 0.098 | 18.4 | 23.5 | 0.023 | 0.161 | 0.155 | 0.058 | -16.6 |
| logdha | rs3802547 | 0.04 | 0.336 | 0.16 | 0.022 | 18.3 | 86.3 | 0.537 | 0.207 | 0.669 | 0.626 | -16.4 |
| logdha | rs77677521 | . | . | . | . | . | . | . | . | . | . | . |
| logdha | rs3824616 | 0.038 | 0.236 | 0.136 | 0.012 | 0.242 | 0.55 | 0.533 | 4.7 | 6.97 | 0.296 | 0.791 |
| logdha | rs191073818 | . | . | . | . | . | . | . | . | . | . | . |
| logdha | rs147078004 | . | . | . | . | . | . | . | . | . | . | . |
| logdha | rs7908745 | 0.145 | 0.362 | 0.183 | 0.045 | 5.59 | 6.89 | 0.163 | 0.516 | 0.437 | 0.434 | -3.91 |
| logdha | rs17157811 | . | . | . | . | . | . | . | . | . | . | . |
| logdha | rs138506087 | . | . | . | . | . | . | . | . | . | . | . |
| logdha | rs77241269 | . | . | . | . | . | . | . | . | . | . | . |
| logdha | rs138374938 | . | . | . | . | . | . | . | . | . | . | . |
| logdha | rs2279433 | 0.038 | 0.236 | 0.136 | 0.012 | 0.24 | 0.547 | 0.531 | 4.73 | 7.02 | 0.294 | 0.792 |
| logdha | rs73285358 | . | . | . | . | . | . | . | . | . | . | . |
| logdha | rs11239528 | 0.065 | 0.383 | 0.222 | 0.098 | 18.5 | 23.7 | 0.023 | 0.16 | 0.155 | 0.058 | -16.7 |
| logdha | rs77453386 | 0.042 | 0.335 | 0.16 | 0.022 | 14.9 | 67.2 | 0.551 | 0.231 | 0.712 | 0.634 | -13.1 |
| logdha | rs2279434 | 0.04 | 0.234 | 0.135 | 0.012 | 0.225 | 0.518 | 0.517 | 4.92 | 7.37 | 0.287 | 0.8 |
| logdha | rs149236525 | . | . | . | . | . | . | . | . | . | . | . |
| logdha | rs12573609 | 0.13 | 0.273 | 0.174 | 0.042 | 1.15 | 1.77 | 0.927 | 1.32 | 1.24 | 0.769 | -9.80E-03 |
| logdha | rs191716655 | . | . | . | . | . | . | . | . | . | . | . |
| logdha | rs17523660 | . | . | . | . | . | . | . | . | . | . | . |
| logdha | rs142671360 | . | . | . | . | . | . | . | . | . | . | . |
| logdha | rs3764990 | 0.037 | 0.236 | 0.136 | 0.012 | 0.241 | 0.549 | 0.532 | 4.73 | 7.02 | 0.295 | 0.792 |
| logdha | rs113682861 | . | . | . | . | . | . | . | . | . | . | . |
| logdha | rs117658270 | 0.042 | 0.335 | 0.16 | 0.022 | 14.9 | 67.2 | 0.551 | 0.231 | 0.712 | 0.634 | -13.1 |
| logdha | rs187309063 | . | . | . | . | . | . | . | . | . | . | . |
| logdha | rs73285360 | . | . | . | . | . | . | . | . | . | . | . |
| logdha | rs80133289 | 0.1 | 0.297 | 0.201 | 0.073 | 0.484 | 0.931 | 0.706 | 1.13 | 1.52 | 0.93 | 0.381 |
| logdha | rs186693257 | 0.003 | . | . | . | . | . | . | . | . | . | . |
| logdha | rs35735968 | 0.05 | 0.21 | 0.13 | 0.011 | 0.136 | 0.279 | 0.332 | 6.69 | 9.06 | 0.16 | 0.845 |
| logdha | rs17157825 | 0.038 | 0.236 | 0.136 | 0.012 | 0.243 | 0.554 | 0.535 | 4.7 | 6.98 | 0.297 | 0.791 |
| logdha | rs2291428 | 0.065 | 0.383 | 0.222 | 0.098 | 18.5 | 23.7 | 0.023 | 0.16 | 0.155 | 0.058 | -16.8 |
| logdha | rs2291429 | 0.065 | 0.383 | 0.222 | 0.098 | 18.5 | 23.8 | 0.023 | 0.16 | 0.155 | 0.058 | -16.8 |
| logdha | rs145714792 | . | . | . | . | . | . | . | . | . | . | . |
| logdha | rs12769244 | 0.038 | 0.236 | 0.136 | 0.012 | 0.24 | 0.546 | 0.53 | 4.74 | 7.02 | 0.294 | 0.792 |
| logdha | rs12771275 | 0.038 | 0.236 | 0.136 | 0.012 | 0.24 | 0.546 | 0.53 | 4.74 | 7.02 | 0.294 | 0.792 |
| logdha | rs146266174 | . | . | . | . | . | . | . | . | . | . | . |
| logdha | rs41301623 | . | . | . | . | . | . | . | . | . | . | . |
| logdha | rs2306814 | 0.043 | 0.321 | 0.156 | 0.019 | 5.04 | 25.2 | 0.746 | 0.432 | 1.46 | 0.804 | -3.66 |
| logdha | rs12244834 | 0.03 | 0.339 | 0.194 | 0.059 | 49.8 | 138 | 0.16 | 0.101 | 0.193 | 0.23 | -47.4 |
| logdha | rs184006011 | 0 | . | . | . | . | . | . | . | . | . | . |
| logdha | rs78829066 | 0.042 | 0.334 | 0.159 | 0.021 | 14.3 | 66 | 0.565 | 0.238 | 0.75 | 0.649 | -12.5 |
| logdha | rs34731408 | 0.038 | 0.236 | 0.136 | 0.012 | 0.24 | 0.545 | 0.53 | 4.74 | 7.03 | 0.294 | 0.793 |
| logdha | rs17451516 | . | . | . | . | . | . | . | . | . | . | . |
| logdha | rs139910133 | . | . | . | . | . | . | . | . | . | . | . |
| logdha | rs10900218 | 0.138 | 0.356 | 0.193 | 0.057 | 7.33 | 11.8 | 0.215 | 0.486 | 0.514 | 0.495 | -5.42 |
| logdha | rs146023999 | 0.025 | 0.29 | 0.159 | 0.024 | 7.10E-10 | 8.60E-09 | 0.081 | 3.50E+04 | 2.00E+05 | 0.073 | 0.71 |
| logdha | rs187296958 | 0.007 | . | . | . | . | . | . | . | . | . | . |
| logdha | rs142342833 | 0.015 | 0.339 | 0.187 | 0.049 | 4.80E+04 | 5.30E+05 | 0.325 | 7.00E-06 | 6.60E-05 | 0.206 | ####### |
| logdha | rs143489255 | 0.006 | . | . | . | . | . | . | . | . | . | . |
| logdha | rs138532518 | 0.006 | . | . | . | . | . | . | . | . | . | . |
| logdha | rs183768079 | 0.007 | . | . | . | . | . | . | . | . | . | . |
| logdha | rs117892272 | 0.012 | 0.288 | 0.167 | 0.032 | 0.811 | 2.15 | 0.937 | 4.87 | 7.82 | 0.325 | 1.04 |
| logdha | rs192993943 | 0.003 | . | . | . | . | . | . | . | . | . | . |
| logdha | rs182989043 | 0.015 | 0.267 | 0.159 | 0.026 | 0.014 | 0.054 | 0.259 | 23.5 | 40.3 | 0.065 | 0.809 |
| logdha | rs189703435 | 0.021 | 0.348 | 0.203 | 0.071 | 876 | 4.00E+03 | 0.136 | 0.017 | 0.053 | 0.196 | -870 |
| logdha | rs188794040 | 0.02 | 0.35 | 0.205 | 0.073 | 1.40E+03 | 5.70E+03 | 0.078 | 0.013 | 0.038 | 0.139 | ####### |
| logdha | rs143920953 | 0.003 | . | . | . | . | . | . | . | . | . | . |
| logdha | rs191029121 | 0.006 | . | . | . | . | . | . | . | . | . | . |
| logdha | rs116875448 | 0.006 | . | . | . | . | . | . | . | . | . | . |
| logdha | rs190167877 | 0.002 | . | . | . | . | . | . | . | . | . | . |
| logdha | rs184993418 | 0.003 | . | . | . | . | . | . | . | . | . | . |
| logdha | rs143241158 | 0.01 | . | . | . | . | . | . | . | . | . | . |
| logdha | rs11239500 | 0.007 | . | . | . | . | . | . | . | . | . | . |

| logdha | rs140568994 | 0.001 | . | . | . | . | . | . | . | . | . | . |
| --- | --- | --- | --- | --- | --- | --- | --- | --- | --- | --- | --- | --- |
| logdha | rs187530317 | 0.008 | . | . | . | . | . | . | . | . | . | . |
| logdha | rs145189326 | 0.003 | . | . | . | . | . | . | . | . | . | . |
| logdha | rs186203332 | 0.009 | . | . | . | . | . | . | . | . | . | . |
| logdha | rs189921909 | 0.004 | . | . | . | . | . | . | . | . | . | . |
| logdha | rs187260989 | 0.01 | 0.242 | 0.144 | 0.017 | 6.10E-05 | 3.40E-04 | 0.084 | 970 | 3.30E+03 | 0.046 | 0.772 |
| logdha | rs114376586 | 0.006 | . | . | . | . | . | . | . | . | . | . |
| logdha | rs151070808 | 0.019 | 0.297 | 0.163 | 0.027 | 0.617 | 2.25 | 0.895 | 2.04 | 4.48 | 0.744 | 0.46 |
| logdha | rs140927225 | 0.006 | . | . | . | . | . | . | . | . | . | . |
| logdha | rs191555615 | 0.012 | 0.268 | 0.151 | 0.019 | 0.144 | 0.454 | 0.539 | 11.6 | 22.4 | 0.205 | 1.03 |
| logdha | rs80278898 | 0.047 | 0.273 | 0.175 | 0.043 | 0.054 | 0.169 | 0.349 | 3.87 | 8.28 | 0.526 | 0.73 |
| logdha | rs190849503 | 0.004 | . | . | . | . | . | . | . | . | . | . |
| logdha | rs150080315 | 0.009 | . | . | . | . | . | . | . | . | . | . |
| logdha | rs143623885 | 0.011 | 0.362 | 0.198 | 0.063 | 5.50E+27 | 2.80E+29 | 0.21 | 3.50E-27 | 1.70E-25 | 0.222 | ####### |
| logdha | rs187760040 | 0.002 | . | . | . | . | . | . | . | . | . | . |
| logdha | rs143917143 | 0.02 | 0.297 | 0.163 | 0.027 | 0.599 | 2.19 | 0.889 | 2.06 | 4.53 | 0.741 | 0.471 |
| logdha | rs149851136 | 0.002 | . | . | . | . | . | . | . | . | . | . |
| logdha | rs181612054 | 0.001 | . | . | . | . | . | . | . | . | . | . |
| logdha | rs191006548 | 0.001 | . | . | . | . | . | . | . | . | . | . |
| logdha | rs117377631 | 0.006 | . | . | . | . | . | . | . | . | . | . |
| logdha | rs182107634 | 0.002 | . | . | . | . | . | . | . | . | . | . |
| logdha | rs190211867 | 0.002 | . | . | . | . | . | . | . | . | . | . |
| logdha | rs148293457 | 0.031 | 0.393 | 0.232 | 0.113 | 405 | 1.40E+03 | 0.083 | 0.013 | 0.034 | 0.11 | -403 |
| logdha | rs191811733 | 0.006 | . | . | . | . | . | . | . | . | . | . |
| logdha | rs146109272 | 0.008 | . | . | . | . | . | . | . | . | . | . |
| logdha | rs148397915 | 0.006 | . | . | . | . | . | . | . | . | . | . |
| logdha | rs142233290 | 0.007 | . | . | . | . | . | . | . | . | . | . |
| logdha | rs80226874 | 0.022 | 0.279 | 0.16 | 0.026 | 0.015 | 0.094 | 0.5 | 1.5 | 4.68 | 0.896 | 0.712 |
| logdha | rs144496203 | 0.012 | 0.331 | 0.181 | 0.043 | 3.10E+89 | 4.00E+91 | 0.108 | 5.00E-102 | 7.00E-100 | 0.104 | ####### |
| logdha | rs191062130 | 0.001 | . | . | . | . | . | . | . | . | . | . |
| logdha | rs185807817 | 0.004 | . | . | . | . | . | . | . | . | . | . |
| logdha | rs117846213 | 0.032 | 0.355 | 0.208 | 0.077 | 106 | 342 | 0.146 | 0.061 | 0.135 | 0.208 | -104 |
| logdha | rs190545473 | 0.002 | . | . | . | . | . | . | . | . | . | . |
| logdha | rs192364918 | 0.01 | 0.308 | 0.179 | 0.043 | 77.3 | 451 | 0.456 | 0.205 | 0.689 | 0.637 | -71.7 |
| logdha | rs182939241 | 0.003 | . | . | . | . | . | . | . | . | . | . |
| logdha | rs12571125 | 0.031 | 0.285 | 0.166 | 0.031 | 0.053 | 0.217 | 0.472 | 5.58 | 11.7 | 0.411 | 0.746 |
| logdha | rs192418941 | 0.005 | . | . | . | . | . | . | . | . | . | . |
| logdha | rs141270243 | 0.015 | 0.329 | 0.188 | 0.052 | 461 | 3.60E+03 | 0.429 | 0.035 | 0.181 | 0.515 | -455 |
| logdha | rs182615502 | 0.006 | . | . | . | . | . | . | . | . | . | . |
| logdha | rs146669856 | 0.001 | . | . | . | . | . | . | . | . | . | . |
| logdha | rs78290029 | 0.034 | 0.348 | 0.201 | 0.068 | 72.4 | 220 | 0.158 | 0.073 | 0.153 | 0.214 | -69.9 |
| logdha | rs139955565 | 0.004 | . | . | . | . | . | . | . | . | . | . |
| logdha | rs12267314 | 0.03 | 0.347 | 0.2 | 0.067 | 74 | 201 | 0.113 | 0.088 | 0.161 | 0.184 | -71.1 |
| logdha | rs74477197 | 0.004 | . | . | . | . | . | . | . | . | . | . |
| logdha | rs188147236 | 0.005 | . | . | . | . | . | . | . | . | . | . |
| logdha | rs2228065 | 0.03 | 0.343 | 0.198 | 0.064 | 57.6 | 151 | 0.123 | 0.103 | 0.182 | 0.198 | -54.9 |
| logdha | rs116961353 | 0.015 | 0.291 | 0.169 | 0.034 | 0.116 | 0.705 | 0.723 | 1.97 | 5.49 | 0.807 | 0.659 |
| logdha | rs190222272 | 0.007 | . | . | . | . | . | . | . | . | . | . |
| logdha | rs182719940 | 0.006 | . | . | . | . | . | . | . | . | . | . |
| logdha | rs184477413 | 0.004 | . | . | . | . | . | . | . | . | . | . |
| logdha | rs12265175 | 0.03 | 0.339 | 0.195 | 0.06 | 51.7 | 143 | 0.155 | 0.097 | 0.187 | 0.224 | -49.3 |
| logdha | rs182928276 | 0.003 | . | . | . | . | . | . | . | . | . | . |
| logdha | rs144409895 | 0.011 | 0.321 | 0.188 | 0.053 | 603 | 3.40E+03 | 0.251 | 0.061 | 0.199 | 0.391 | -591 |
| logdha | rs58998447 | 0.136 | 0.311 | 0.19 | 0.056 | 0.916 | 1.31 | 0.951 | 0.769 | 0.782 | 0.797 | -8.00E-03 |
| logdha | rs188668832 | 0.005 | . | . | . | . | . | . | . | . | . | . |
| logdha | rs148641686 | 0.002 | . | . | . | . | . | . | . | . | . | . |
| logdha | rs75468791 | 0.054 | 0.277 | 0.162 | 0.028 | 0.017 | 0.049 | 0.159 | 8.06 | 11.4 | 0.141 | 0.744 |
| logdha | rs147327397 | 0.003 | . | . | . | . | . | . | . | . | . | . |
| logdha | rs77354597 | 0.029 | 0.341 | 0.196 | 0.062 | 69.9 | 209 | 0.156 | 0.081 | 0.168 | 0.226 | -67.3 |
| logdha | rs41365544 | 0.03 | 0.339 | 0.194 | 0.059 | 49.5 | 137 | 0.16 | 0.102 | 0.194 | 0.231 | -47.1 |
| logdha | rs187564188 | 0.016 | 0.237 | 0.15 | 0.023 | 5.10E-04 | 2.90E-03 | 0.189 | 154 | 640 | 0.225 | 0.781 |
| logdha | rs183511984 | 0.007 | . | . | . | . | . | . | . | . | . | . |
| logdha | rs12262783 | 0.003 | . | . | . | . | . | . | . | . | . | . |
| logdha | rs188817687 | 0.003 | . | . | . | . | . | . | . | . | . | . |
| logdha | rs3815968 | 0.005 | . | . | . | . | . | . | . | . | . | . |
| logdha | rs187822300 | 0.001 | . | . | . | . | . | . | . | . | . | . |
| logdha | rs188902172 | 0.032 | 0.335 | 0.195 | 0.06 | 28.5 | 80.4 | 0.235 | 0.175 | 0.333 | 0.36 | -26.1 |
| logdha | rs181190448 | 0.006 | . | . | . | . | . | . | . | . | . | . |
| logdha | rs182699093 | 0.001 | . | . | . | . | . | . | . | . | . | . |
| logdha | rs182631303 | 0.014 | 0.356 | 0.204 | 0.071 | 175 | 821 | 0.27 | 0.032 | 0.125 | 0.38 | -173 |
| logdha | rs187014172 | 0.006 | . | . | . | . | . | . | . | . | . | . |
| logdha | rs142575406 | 0.009 | . | . | . | . | . | . | . | . | . | . |
| logdha | rs180711033 | 0.007 | . | . | . | . | . | . | . | . | . | . |
| logdha | rs137894823 | . | . | . | . | . | . | . | . | . | . | . |
| logdha | rs189053273 | . | . | . | . | . | . | . | . | . | . | . |
| logdha | rs143499388 | . | . | . | . | . | . | . | . | . | . | . |
| logdha | rs182936001 | . | . | . | . | . | . | . | . | . | . | . |
| logdha | rs148456406 | . | . | . | . | . | . | . | . | . | . | . |
| logdha | rs182499531 | . | . | . | . | . | . | . | . | . | . | . |
| logdha | rs74884915 | . | . | . | . | . | . | . | . | . | . | . |
| logdha | rs184046374 | . | . | . | . | . | . | . | . | . | . | . |
| logdha | rs74333993 | . | . | . | . | . | . | . | . | . | . | . |
| logdha | rs138889719 | . | . | . | . | . | . | . | . | . | . | . |
| logdha | rs75330330 | . | . | . | . | . | . | . | . | . | . | . |
| logdha | rs193051693 | . | . | . | . | . | . | . | . | . | . | . |
| logdha | rs145929633 | . | . | . | . | . | . | . | . | . | . | . |

| logdha | rs184076904 | . | . | . | . | . | . | . | . | . | . | . |
| --- | --- | --- | --- | --- | --- | --- | --- | --- | --- | --- | --- | --- |
| logdha | rs141802494 | . | . | . | . | . | . | . | . | . | . | . |
| logdha | rs149519759 | . | . | . | . | . | . | . | . | . | . | . |
| logdha | rs145467260 | . | . | . | . | . | . | . | . | . | . | . |
| logdha | rs79620914 | . | . | . | . | . | . | . | . | . | . | . |
| logdha | rs140538633 | . | . | . | . | . | . | . | . | . | . | . |
| logdha | rs140862301 | . | . | . | . | . | . | . | . | . | . | . |
| logdha | rs114615847 | . | . | . | . | . | . | . | . | . | . | . |
| logdha | rs141800259 | . | . | . | . | . | . | . | . | . | . | . |
| logdha | rs149779059 | . | . | . | . | . | . | . | . | . | . | . |
| logdha | rs116539253 | . | . | . | . | . | . | . | . | . | . | . |
| logdha | rs184979550 | . | . | . | . | . | . | . | . | . | . | . |
| logdha | rs190128940 | . | . | . | . | . | . | . | . | . | . | . |
| logdha | rs146594816 | . | . | . | . | . | . | . | . | . | . | . |
| logdha | rs141370766 | . | . | . | . | . | . | . | . | . | . | . |
| logdha | rs144670033 | . | . | . | . | . | . | . | . | . | . | . |
| logdha | rs187152174 | . | . | . | . | . | . | . | . | . | . | . |
| logdha | rs184180263 | . | . | . | . | . | . | . | . | . | . | . |
| logdha | rs140075861 | . | . | . | . | . | . | . | . | . | . | . |
| logdha | rs186483129 | . | . | . | . | . | . | . | . | . | . | . |
| logdha | rs186634282 | . | . | . | . | . | . | . | . | . | . | . |
| logdha | rs9988663 | . | . | . | . | . | . | . | . | . | . | . |
| logdha | rs138828529 | . | . | . | . | . | . | . | . | . | . | . |
| logdha | rs188777615 | . | . | . | . | . | . | . | . | . | . | . |
| logdha | rs192321882 | . | . | . | . | . | . | . | . | . | . | . |
| logdha | rs150812270 | . | . | . | . | . | . | . | . | . | . | . |
| logdha | rs185906603 | . | . | . | . | . | . | . | . | . | . | . |
| logdha | rs191070862 | . | . | . | . | . | . | . | . | . | . | . |
| logdha | rs182904978 | . | . | . | . | . | . | . | . | . | . | . |
| logdha | rs192679655 | . | . | . | . | . | . | . | . | . | . | . |
| logdha | rs189673086 | . | . | . | . | . | . | . | . | . | . | . |
| logdha | rs181141892 | . | . | . | . | . | . | . | . | . | . | . |
| logdha | rs186404123 | . | . | . | . | . | . | . | . | . | . | . |
| logdha | rs192117287 | . | . | . | . | . | . | . | . | . | . | . |
| logdha | rs182748835 | . | . | . | . | . | . | . | . | . | . | . |
| logdha | rs141854549 | . | . | . | . | . | . | . | . | . | . | . |
| logdha | rs140163397 | . | . | . | . | . | . | . | . | . | . | . |
| logdha | rs146347300 | . | . | . | . | . | . | . | . | . | . | . |
| logdha | rs139362377 | . | . | . | . | . | . | . | . | . | . | . |
| logdha | rs185087671 | . | . | . | . | . | . | . | . | . | . | . |
| logdha | rs190466574 | . | . | . | . | . | . | . | . | . | . | . |
| logdha | rs147012778 | . | . | . | . | . | . | . | . | . | . | . |
| logdha | rs138236960 | . | . | . | . | . | . | . | . | . | . | . |
| logdha | rs116310311 | . | . | . | . | . | . | . | . | . | . | . |
| logdha | rs142868356 | . | . | . | . | . | . | . | . | . | . | . |
| logdha | rs144956240 | . | . | . | . | . | . | . | . | . | . | . |
| logdha | rs188527776 | . | . | . | . | . | . | . | . | . | . | . |
| logdha | rs183971165 | . | . | . | . | . | . | . | . | . | . | . |
| logdha | rs76748216 | . | . | . | . | . | . | . | . | . | . | . |
| logdha | rs148554990 | . | . | . | . | . | . | . | . | . | . | . |
| logdha | rs114446477 | . | . | . | . | . | . | . | . | . | . | . |
| logdha | rs185223933 | . | . | . | . | . | . | . | . | . | . | . |
| logdha | rs76869726 | . | . | . | . | . | . | . | . | . | . | . |
| logdha | rs151200830 | . | . | . | . | . | . | . | . | . | . | . |
| logdha | rs17157731 | . | . | . | . | . | . | . | . | . | . | . |
| logdha | rs181243469 | . | . | . | . | . | . | . | . | . | . | . |
| logdha | rs186890707 | . | . | . | . | . | . | . | . | . | . | . |
| logdha | rs184063360 | . | . | . | . | . | . | . | . | . | . | . |
| logdha | rs188550237 | . | . | . | . | . | . | . | . | . | . | . |
| logdha | rs140931068 | . | . | . | . | . | . | . | . | . | . | . |
| logdha | rs74867877 | . | . | . | . | . | . | . | . | . | . | . |
| logdha | rs11239495 | . | . | . | . | . | . | . | . | . | . | . |
| logdha | rs149745800 | . | . | . | . | . | . | . | . | . | . | . |
| logdha | rs145044458 | . | . | . | . | . | . | . | . | . | . | . |
| logdha | rs188067427 | . | . | . | . | . | . | . | . | . | . | . |
| logdha | rs146827800 | . | . | . | . | . | . | . | . | . | . | . |
| logdha | rs140619817 | . | . | . | . | . | . | . | . | . | . | . |
| logdha | rs892689 | . | . | . | . | . | . | . | . | . | . | . |
| logdha | rs143642005 | . | . | . | . | . | . | . | . | . | . | . |
| logdha | rs181929304 | . | . | . | . | . | . | . | . | . | . | . |
| logdha | rs191086173 | . | . | . | . | . | . | . | . | . | . | . |
| logdha | rs114613116 | . | . | . | . | . | . | . | . | . | . | . |
| logdha | rs141076768 | . | . | . | . | . | . | . | . | . | . | . |
| logdha | rs187316531 | . | . | . | . | . | . | . | . | . | . | . |
| logdha | rs11239499 | . | . | . | . | . | . | . | . | . | . | . |
| logdha | rs192726371 | . | . | . | . | . | . | . | . | . | . | . |
| logdha | rs74128481 | . | . | . | . | . | . | . | . | . | . | . |
| logdha | rs74128482 | . | . | . | . | . | . | . | . | . | . | . |
| logdha | rs11239502 | . | . | . | . | . | . | . | . | . | . | . |
| logdha | rs116330285 | . | . | . | . | . | . | . | . | . | . | . |
| logdha | rs189926729 | . | . | . | . | . | . | . | . | . | . | . |
| logdha | rs188975779 | . | . | . | . | . | . | . | . | . | . | . |
| logdha | rs143846038 | . | . | . | . | . | . | . | . | . | . | . |
| logdha | rs74128483 | . | . | . | . | . | . | . | . | . | . | . |
| logdha | rs139292055 | . | . | . | . | . | . | . | . | . | . | . |
| logdha | rs11239503 | . | . | . | . | . | . | . | . | . | . | . |

| logdha | rs58838686 | . | . | . | . | . | . | . | . | . | . | . |
| --- | --- | --- | --- | --- | --- | --- | --- | --- | --- | --- | --- | --- |
| logdha | rs185825373 | . | . | . | . | . | . | . | . | . | . | . |
| logdha | rs11239504 | . | . | . | . | . | . | . | . | . | . | . |
| logdha | rs150012354 | . | . | . | . | . | . | . | . | . | . | . |
| logdha | rs187763096 | . | . | . | . | . | . | . | . | . | . | . |
| logdha | rs193043982 | . | . | . | . | . | . | . | . | . | . | . |
| logdha | rs79186884 | . | . | . | . | . | . | . | . | . | . | . |
| logdha | rs74128484 | . | . | . | . | . | . | . | . | . | . | . |
| logdha | rs58461537 | . | . | . | . | . | . | . | . | . | . | . |
| logdha | rs188527474 | . | . | . | . | . | . | . | . | . | . | . |
| logdha | rs141659976 | . | . | . | . | . | . | . | . | . | . | . |
| logdha | rs115846407 | . | . | . | . | . | . | . | . | . | . | . |
| logdha | rs138378816 | . | . | . | . | . | . | . | . | . | . | . |
| logdha | rs146188136 | . | . | . | . | . | . | . | . | . | . | . |
| logdha | rs139383871 | . | . | . | . | . | . | . | . | . | . | . |
| logdha | rs182953869 | . | . | . | . | . | . | . | . | . | . | . |
| logdha | rs192430299 | . | . | . | . | . | . | . | . | . | . | . |
| logdha | rs144152844 | . | . | . | . | . | . | . | . | . | . | . |
| logdha | rs145987786 | . | . | . | . | . | . | . | . | . | . | . |
| logdha | rs181132926 | . | . | . | . | . | . | . | . | . | . | . |
| logdha | rs147882253 | . | . | . | . | . | . | . | . | . | . | . |
| logdha | rs141807621 | . | . | . | . | . | . | . | . | . | . | . |
| logdha | rs147116052 | . | . | . | . | . | . | . | . | . | . | . |
| logdha | rs147716618 | . | . | . | . | . | . | . | . | . | . | . |
| logdha | rs187198855 | . | . | . | . | . | . | . | . | . | . | . |
| logdha | rs189409638 | . | . | . | . | . | . | . | . | . | . | . |
| logdha | rs182647339 | . | . | . | . | . | . | . | . | . | . | . |
| logdha | rs187995988 | . | . | . | . | . | . | . | . | . | . | . |
| logdha | rs141034184 | . | . | . | . | . | . | . | . | . | . | . |
| logdha | rs187310430 | . | . | . | . | . | . | . | . | . | . | . |
| logdha | rs192608488 | . | . | . | . | . | . | . | . | . | . | . |
| logdha | rs148840366 | . | . | . | . | . | . | . | . | . | . | . |
| logdha | rs186785022 | . | . | . | . | . | . | . | . | . | . | . |
| logdha | rs192348728 | . | . | . | . | . | . | . | . | . | . | . |
| logdha | rs189656631 | . | . | . | . | . | . | . | . | . | . | . |
| logdha | rs185289750 | . | . | . | . | . | . | . | . | . | . | . |
| logdha | rs142382395 | . | . | . | . | . | . | . | . | . | . | . |
| logdha | rs144866696 | . | . | . | . | . | . | . | . | . | . | . |
| logdha | rs141371289 | . | . | . | . | . | . | . | . | . | . | . |
| logdha | rs114750008 | . | . | . | . | . | . | . | . | . | . | . |
| logdha | rs189063597 | . | . | . | . | . | . | . | . | . | . | . |
| logdha | rs139151571 | . | . | . | . | . | . | . | . | . | . | . |
| logdha | rs139846368 | . | . | . | . | . | . | . | . | . | . | . |
| logdha | rs182057653 | . | . | . | . | . | . | . | . | . | . | . |
| logdha | rs148785420 | . | . | . | . | . | . | . | . | . | . | . |
| logdha | rs138383251 | . | . | . | . | . | . | . | . | . | . | . |
| logdha | rs116526705 | . | . | . | . | . | . | . | . | . | . | . |
| logdha | rs185946436 | . | . | . | . | . | . | . | . | . | . | . |
| logdha | rs190455427 | . | . | . | . | . | . | . | . | . | . | . |
| logdha | rs9282584 | . | . | . | . | . | . | . | . | . | . | . |
| logdha | rs17153289 | . | . | . | . | . | . | . | . | . | . | . |
| logdha | rs138359103 | . | . | . | . | . | . | . | . | . | . | . |
| logdha | rs150605268 | . | . | . | . | . | . | . | . | . | . | . |
| logdha | rs79242910 | . | . | . | . | . | . | . | . | . | . | . |
| logdha | rs139716904 | . | . | . | . | . | . | . | . | . | . | . |
| logdha | rs76238498 | . | . | . | . | . | . | . | . | . | . | . |
| logdha | rs184140639 | . | . | . | . | . | . | . | . | . | . | . |
| logdha | rs188517278 | . | . | . | . | . | . | . | . | . | . | . |
| logdha | rs139678913 | . | . | . | . | . | . | . | . | . | . | . |
| logdha | rs116697955 | . | . | . | . | . | . | . | . | . | . | . |
| logdha | rs79267994 | . | . | . | . | . | . | . | . | . | . | . |
| logdha | rs11239510 | . | . | . | . | . | . | . | . | . | . | . |
| logdha | rs75831645 | . | . | . | . | . | . | . | . | . | . | . |
| logdha | rs78452276 | . | . | . | . | . | . | . | . | . | . | . |
| logdha | rs144366924 | . | . | . | . | . | . | . | . | . | . | . |
| logdha | rs77519841 | . | . | . | . | . | . | . | . | . | . | . |
| logdha | rs76944454 | . | . | . | . | . | . | . | . | . | . | . |
| logdha | rs76443855 | . | . | . | . | . | . | . | . | . | . | . |
| logdha | rs146613174 | . | . | . | . | . | . | . | . | . | . | . |
| logdha | rs181424761 | . | . | . | . | . | . | . | . | . | . | . |
| logdha | rs142119241 | . | . | . | . | . | . | . | . | . | . | . |
| logdha | rs140137954 | . | . | . | . | . | . | . | . | . | . | . |
| logdha | rs187319549 | . | . | . | . | . | . | . | . | . | . | . |
| logdha | rs12241545 | . | . | . | . | . | . | . | . | . | . | . |
| logdha | rs10128228 | . | . | . | . | . | . | . | . | . | . | . |
| logdha | rs58580249 | . | . | . | . | . | . | . | . | . | . | . |
| logdha | rs193148863 | . | . | . | . | . | . | . | . | . | . | . |
| logdha | rs185638946 | . | . | . | . | . | . | . | . | . | . | . |
| logdha | rs117752921 | . | . | . | . | . | . | . | . | . | . | . |
| logdha | rs180841236 | . | . | . | . | . | . | . | . | . | . | . |
| logdha | rs73283155 | . | . | . | . | . | . | . | . | . | . | . |
| logdha | rs76560599 | . | . | . | . | . | . | . | . | . | . | . |
| logdha | rs76273620 | . | . | . | . | . | . | . | . | . | . | . |
| logdha | rs141432923 | . | . | . | . | . | . | . | . | . | . | . |
| logdha | rs113529322 | . | . | . | . | . | . | . | . | . | . | . |
| logdha | rs10128306 | . | . | . | . | . | . | . | . | . | . | . |

| logdha | rs149691688 | . | . | . | . | . | . | . | . | . | . | . |
| --- | --- | --- | --- | --- | --- | --- | --- | --- | --- | --- | --- | --- |
| logdha | rs75872495 | . | . | . | . | . | . | . | . | . | . | . |
| logdha | rs191873960 | . | . | . | . | . | . | . | . | . | . | . |
| logdha | rs80155622 | . | . | . | . | . | . | . | . | . | . | . |
| logdha | rs113914656 | . | . | . | . | . | . | . | . | . | . | . |
| logdha | rs12242840 | . | . | . | . | . | . | . | . | . | . | . |
| logdha | rs147824078 | . | . | . | . | . | . | . | . | . | . | . |
| logdha | rs59051493 | . | . | . | . | . | . | . | . | . | . | . |
| logdha | rs9282588 | . | . | . | . | . | . | . | . | . | . | . |
| logdha | rs150215207 | . | . | . | . | . | . | . | . | . | . | . |
| logdha | rs138772243 | . | . | . | . | . | . | . | . | . | . | . |
| logdha | rs149383244 | . | . | . | . | . | . | . | . | . | . | . |
| logdha | rs17522720 | . | . | . | . | . | . | . | . | . | . | . |
| logdha | rs79245105 | . | . | . | . | . | . | . | . | . | . | . |
| logdha | rs138222133 | . | . | . | . | . | . | . | . | . | . | . |
| logdha | rs12247314 | . | . | . | . | . | . | . | . | . | . | . |
| logdha | rs12263894 | . | . | . | . | . | . | . | . | . | . | . |
| logdha | rs149907880 | . | . | . | . | . | . | . | . | . | . | . |
| logdha | rs188153885 | . | . | . | . | . | . | . | . | . | . | . |
| logdha | rs73283159 | . | . | . | . | . | . | . | . | . | . | . |
| logdha | rs140476687 | . | . | . | . | . | . | . | . | . | . | . |
| logdha | rs186603148 | . | . | . | . | . | . | . | . | . | . | . |
| logdha | rs183296063 | . | . | . | . | . | . | . | . | . | . | . |
| logdha | rs145733694 | . | . | . | . | . | . | . | . | . | . | . |
| logdha | rs189807449 | . | . | . | . | . | . | . | . | . | . | . |
| logdha | rs12253223 | . | . | . | . | . | . | . | . | . | . | . |
| logdha | rs182113637 | . | . | . | . | . | . | . | . | . | . | . |
| logdha | rs142792099 | . | . | . | . | . | . | . | . | . | . | . |
| logdha | rs12249910 | . | . | . | . | . | . | . | . | . | . | . |
| logdha | rs149436339 | . | . | . | . | . | . | . | . | . | . | . |
| logdha | rs77632210 | . | . | . | . | . | . | . | . | . | . | . |
| logdha | rs74130818 | . | . | . | . | . | . | . | . | . | . | . |
| logdha | rs187583648 | . | . | . | . | . | . | . | . | . | . | . |
| logdha | rs191295987 | . | . | . | . | . | . | . | . | . | . | . |
| logdha | rs184200686 | . | . | . | . | . | . | . | . | . | . | . |
| logdha | rs76117263 | . | . | . | . | . | . | . | . | . | . | . |
| logdha | rs185393149 | . | . | . | . | . | . | . | . | . | . | . |
| logdha | rs144760014 | . | . | . | . | . | . | . | . | . | . | . |
| logdha | rs7922292 | . | . | . | . | . | . | . | . | . | . | . |
| logdha | rs115372844 | . | . | . | . | . | . | . | . | . | . | . |
| logdha | rs111460364 | . | . | . | . | . | . | . | . | . | . | . |
| logdha | rs182052255 | . | . | . | . | . | . | . | . | . | . | . |
| logdha | rs180983889 | . | . | . | . | . | . | . | . | . | . | . |
| logdha | rs139175327 | . | . | . | . | . | . | . | . | . | . | . |
| logdha | rs149959620 | . | . | . | . | . | . | . | . | . | . | . |
| logdha | rs114653117 | . | . | . | . | . | . | . | . | . | . | . |
| logdha | rs144270018 | . | . | . | . | . | . | . | . | . | . | . |
| logdha | rs182723204 | . | . | . | . | . | . | . | . | . | . | . |
| logdha | rs57204067 | . | . | . | . | . | . | . | . | . | . | . |
| logdha | rs144956226 | . | . | . | . | . | . | . | . | . | . | . |
| logdha | rs187842704 | . | . | . | . | . | . | . | . | . | . | . |
| logdha | rs184620511 | . | . | . | . | . | . | . | . | . | . | . |
| logdha | rs144031303 | . | . | . | . | . | . | . | . | . | . | . |
| logdha | rs187526473 | . | . | . | . | . | . | . | . | . | . | . |
| logdha | rs193002805 | . | . | . | . | . | . | . | . | . | . | . |
| logdha | rs138707426 | . | . | . | . | . | . | . | . | . | . | . |
| logdha | rs142754243 | . | . | . | . | . | . | . | . | . | . | . |
| logdha | rs181600331 | . | . | . | . | . | . | . | . | . | . | . |
| logdha | rs144421264 | . | . | . | . | . | . | . | . | . | . | . |
| logdha | rs184414519 | . | . | . | . | . | . | . | . | . | . | . |
| logdha | rs150596349 | . | . | . | . | . | . | . | . | . | . | . |
| logdha | rs190753747 | . | . | . | . | . | . | . | . | . | . | . |
| logdha | rs139560940 | . | . | . | . | . | . | . | . | . | . | . |
| logdha | rs75640804 | . | . | . | . | . | . | . | . | . | . | . |
| logdha | rs73283175 | . | . | . | . | . | . | . | . | . | . | . |
| logdha | rs143337229 | . | . | . | . | . | . | . | . | . | . | . |
| logdha | rs73283177 | . | . | . | . | . | . | . | . | . | . | . |
| logdha | rs76053611 | . | . | . | . | . | . | . | . | . | . | . |
| logdha | rs116325983 | . | . | . | . | . | . | . | . | . | . | . |
| logdha | rs115451825 | . | . | . | . | . | . | . | . | . | . | . |
| logdha | rs183845246 | . | . | . | . | . | . | . | . | . | . | . |
| logdha | rs79756542 | . | . | . | . | . | . | . | . | . | . | . |
| logdha | rs187242860 | . | . | . | . | . | . | . | . | . | . | . |
| logdha | rs57318980 | . | . | . | . | . | . | . | . | . | . | . |
| logdha | rs59969732 | . | . | . | . | . | . | . | . | . | . | . |
| logdha | rs143811488 | . | . | . | . | . | . | . | . | . | . | . |
| logdha | rs189067644 | . | . | . | . | . | . | . | . | . | . | . |
| logdha | rs60449178 | . | . | . | . | . | . | . | . | . | . | . |
| logdha | rs59243389 | . | . | . | . | . | . | . | . | . | . | . |
| logdha | rs184946540 | . | . | . | . | . | . | . | . | . | . | . |
| logdha | rs188685881 | . | . | . | . | . | . | . | . | . | . | . |
| logdha | rs150335797 | . | . | . | . | . | . | . | . | . | . | . |
| logdha | rs143879260 | . | . | . | . | . | . | . | . | . | . | . |
| logdha | rs186044491 | . | . | . | . | . | . | . | . | . | . | . |
| logdha | rs11819084 | . | . | . | . | . | . | . | . | . | . | . |
| logdha | rs146915670 | . | . | . | . | . | . | . | . | . | . | . |

| logdha | rs113995695 | . | . | . | . | . | . | . | . | . | . | . |
| --- | --- | --- | --- | --- | --- | --- | --- | --- | --- | --- | --- | --- |
| logdha | rs143489451 | . | . | . | . | . | . | . | . | . | . | . |
| logdha | rs182653839 | . | . | . | . | . | . | . | . | . | . | . |
| logdha | rs141692564 | . | . | . | . | . | . | . | . | . | . | . |
| logdha | rs146281742 | . | . | . | . | . | . | . | . | . | . | . |
| logdha | rs192914353 | . | . | . | . | . | . | . | . | . | . | . |
| logdha | rs185182848 | . | . | . | . | . | . | . | . | . | . | . |
| logdha | rs28395858 | . | . | . | . | . | . | . | . | . | . | . |
| logdha | rs41429645 | . | . | . | . | . | . | . | . | . | . | . |
| logdha | rs28395874 | . | . | . | . | . | . | . | . | . | . | . |
| logdha | rs189663634 | . | . | . | . | . | . | . | . | . | . | . |
| logdha | rs77666700 | . | . | . | . | . | . | . | . | . | . | . |
| logdha | rs146711717 | . | . | . | . | . | . | . | . | . | . | . |
| logdha | rs144904251 | . | . | . | . | . | . | . | . | . | . | . |
| logdha | rs189893153 | . | . | . | . | . | . | . | . | . | . | . |
| logdha | rs150447740 | . | . | . | . | . | . | . | . | . | . | . |
| logdha | rs143099649 | . | . | . | . | . | . | . | . | . | . | . |
| logdha | rs191288436 | . | . | . | . | . | . | . | . | . | . | . |
| logdha | rs188577862 | . | . | . | . | . | . | . | . | . | . | . |
| logdha | rs192695334 | . | . | . | . | . | . | . | . | . | . | . |
| logdha | rs138612906 | . | . | . | . | . | . | . | . | . | . | . |
| logdha | rs191290051 | . | . | . | . | . | . | . | . | . | . | . |
| logdha | rs189631459 | . | . | . | . | . | . | . | . | . | . | . |
| logdha | rs114620874 | . | . | . | . | . | . | . | . | . | . | . |
| logdha | rs78246652 | . | . | . | . | . | . | . | . | . | . | . |
| logdha | rs79432451 | . | . | . | . | . | . | . | . | . | . | . |
| logdha | rs186022585 | . | . | . | . | . | . | . | . | . | . | . |
| logdha | rs142978891 | . | . | . | . | . | . | . | . | . | . | . |
| logdha | rs151086840 | . | . | . | . | . | . | . | . | . | . | . |
| logdha | rs183270259 | . | . | . | . | . | . | . | . | . | . | . |
| logdha | rs188544289 | . | . | . | . | . | . | . | . | . | . | . |
| logdha | rs188500808 | . | . | . | . | . | . | . | . | . | . | . |
| logdha | rs189424589 | . | . | . | . | . | . | . | . | . | . | . |
| logdha | rs78622303 | . | . | . | . | . | . | . | . | . | . | . |
| logdha | rs143210835 | . | . | . | . | . | . | . | . | . | . | . |
| logdha | rs114761840 | . | . | . | . | . | . | . | . | . | . | . |
| logdha | rs185805402 | . | . | . | . | . | . | . | . | . | . | . |
| logdha | rs139273744 | . | . | . | . | . | . | . | . | . | . | . |
| logdha | rs184654150 | . | . | . | . | . | . | . | . | . | . | . |
| logdha | rs189069710 | . | . | . | . | . | . | . | . | . | . | . |
| logdha | rs114752115 | . | . | . | . | . | . | . | . | . | . | . |
| logdha | rs150162515 | . | . | . | . | . | . | . | . | . | . | . |
| logdha | rs180982454 | . | . | . | . | . | . | . | . | . | . | . |
| logdha | rs184932088 | . | . | . | . | . | . | . | . | . | . | . |
| logdha | rs181109625 | . | . | . | . | . | . | . | . | . | . | . |
| logdha | rs138121134 | . | . | . | . | . | . | . | . | . | . | . |
| logdha | rs183253861 | . | . | . | . | . | . | . | . | . | . | . |
| logdha | rs147073946 | . | . | . | . | . | . | . | . | . | . | . |
| logdha | rs139938489 | . | . | . | . | . | . | . | . | . | . | . |
| logdha | rs146362560 | . | . | . | . | . | . | . | . | . | . | . |
| logdha | rs193155956 | . | . | . | . | . | . | . | . | . | . | . |
| logdha | rs77663092 | . | . | . | . | . | . | . | . | . | . | . |
| logdha | rs73285319 | . | . | . | . | . | . | . | . | . | . | . |
| logdha | rs184019276 | . | . | . | . | . | . | . | . | . | . | . |
| logdha | rs28395863 | . | . | . | . | . | . | . | . | . | . | . |
| logdha | rs182121645 | . | . | . | . | . | . | . | . | . | . | . |
| logdha | rs73285327 | . | . | . | . | . | . | . | . | . | . | . |
| logdha | rs149589584 | . | . | . | . | . | . | . | . | . | . | . |
| logdha | rs148310335 | . | . | . | . | . | . | . | . | . | . | . |
| logdha | rs187246093 | . | . | . | . | . | . | . | . | . | . | . |
| logdha | rs184668894 | . | . | . | . | . | . | . | . | . | . | . |
| logdha | rs114269961 | . | . | . | . | . | . | . | . | . | . | . |
| logdha | rs149694374 | . | . | . | . | . | . | . | . | . | . | . |
| logdha | rs145284227 | . | . | . | . | . | . | . | . | . | . | . |
| logdha | rs189983820 | . | . | . | . | . | . | . | . | . | . | . |
| logdha | rs181200444 | . | . | . | . | . | . | . | . | . | . | . |
| logdha | rs185486385 | . | . | . | . | . | . | . | . | . | . | . |
| logdha | rs151198241 | . | . | . | . | . | . | . | . | . | . | . |
| logdha | rs189959341 | . | . | . | . | . | . | . | . | . | . | . |
| logdha | rs140549257 | . | . | . | . | . | . | . | . | . | . | . |
| logdha | rs186453139 | . | . | . | . | . | . | . | . | . | . | . |
| logdha | rs180922711 | . | . | . | . | . | . | . | . | . | . | . |
| logdha | rs56659903 | . | . | . | . | . | . | . | . | . | . | . |
| logdha | rs141202128 | . | . | . | . | . | . | . | . | . | . | . |
| logdha | rs59085314 | . | . | . | . | . | . | . | . | . | . | . |
| logdha | rs145564996 | . | . | . | . | . | . | . | . | . | . | . |
| logdha | rs114961009 | . | . | . | . | . | . | . | . | . | . | . |
| logdha | rs182294189 | . | . | . | . | . | . | . | . | . | . | . |
| logdha | rs187586636 | . | . | . | . | . | . | . | . | . | . | . |
| logdha | rs192254907 | . | . | . | . | . | . | . | . | . | . | . |
| logdha | rs149145663 | . | . | . | . | . | . | . | . | . | . | . |
| logdha | rs180713064 | . | . | . | . | . | . | . | . | . | . | . |
| logdha | rs183009023 | . | . | . | . | . | . | . | . | . | . | . |
| logdha | rs143385347 | . | . | . | . | . | . | . | . | . | . | . |
| logdha | rs185786329 | . | . | . | . | . | . | . | . | . | . | . |
| logdha | rs192341030 | . | . | . | . | . | . | . | . | . | . | . |

| logdha | rs74661429 | . | . | . | . | . | . | . | . | . | . | . |
| --- | --- | --- | --- | --- | --- | --- | --- | --- | --- | --- | --- | --- |
| logdha | rs188034853 | . | . | . | . | . | . | . | . | . | . | . |
| logdha | rs116808430 | . | . | . | . | . | . | . | . | . | . | . |
| logdha | rs148469010 | . | . | . | . | . | . | . | . | . | . | . |
| logdha | rs78621663 | . | . | . | . | . | . | . | . | . | . | . |
| logdha | rs192654886 | . | . | . | . | . | . | . | . | . | . | . |
| logdha | rs145996035 | . | . | . | . | . | . | . | . | . | . | . |
| logdha | rs139942226 | . | . | . | . | . | . | . | . | . | . | . |
| logdha | rs113645632 | . | . | . | . | . | . | . | . | . | . | . |
| logdha | rs74940661 | . | . | . | . | . | . | . | . | . | . | . |
| logdha | rs115355800 | . | . | . | . | . | . | . | . | . | . | . |
| logdha | rs150285898 | . | . | . | . | . | . | . | . | . | . | . |
| logdha | rs188117932 | . | . | . | . | . | . | . | . | . | . | . |
| logdha | rs139956566 | . | . | . | . | . | . | . | . | . | . | . |
| logdha | rs147673885 | . | . | . | . | . | . | . | . | . | . | . |
| logdha | rs189127928 | . | . | . | . | . | . | . | . | . | . | . |
| logdha | rs187558772 | . | . | . | . | . | . | . | . | . | . | . |
| logdha | rs181948587 | . | . | . | . | . | . | . | . | . | . | . |
| logdha | rs189983269 | . | . | . | . | . | . | . | . | . | . | . |
| logdha | rs187250069 | . | . | . | . | . | . | . | . | . | . | . |
| logdha | rs191485859 | . | . | . | . | . | . | . | . | . | . | . |
| logdha | rs185499555 | . | . | . | . | . | . | . | . | . | . | . |
| logdha | rs180862110 | . | . | . | . | . | . | . | . | . | . | . |
| logdha | rs190472603 | . | . | . | . | . | . | . | . | . | . | . |
| logdha | rs189226167 | . | . | . | . | . | . | . | . | . | . | . |
| logdha | rs181623256 | . | . | . | . | . | . | . | . | . | . | . |
| logdha | rs186079557 | . | . | . | . | . | . | . | . | . | . | . |
| logdha | rs147760994 | . | . | . | . | . | . | . | . | . | . | . |
| logdha | rs189176167 | . | . | . | . | . | . | . | . | . | . | . |
| logdha | rs117901000 | . | . | . | . | . | . | . | . | . | . | . |
| logdha | rs141984888 | . | . | . | . | . | . | . | . | . | . | . |
| logdha | rs192365061 | . | . | . | . | . | . | . | . | . | . | . |
| logdha | rs185976038 | . | . | . | . | . | . | . | . | . | . | . |
| logdha | rs184697303 | . | . | . | . | . | . | . | . | . | . | . |
| logdha | rs143383132 | . | . | . | . | . | . | . | . | . | . | . |
| logdha | rs141873881 | . | . | . | . | . | . | . | . | . | . | . |
| logdha | rs185507255 | . | . | . | . | . | . | . | . | . | . | . |
| logdha | rs191379425 | . | . | . | . | . | . | . | . | . | . | . |
| logdha | rs184199584 | . | . | . | . | . | . | . | . | . | . | . |
| logdha | rs183181360 | . | . | . | . | . | . | . | . | . | . | . |
| logdha | rs184495673 | . | . | . | . | . | . | . | . | . | . | . |
| logdha | rs182946297 | . | . | . | . | . | . | . | . | . | . | . |
| logdha | rs187743615 | . | . | . | . | . | . | . | . | . | . | . |
| logdha | rs183259862 | . | . | . | . | . | . | . | . | . | . | . |
| logdha | rs112433566 | . | . | . | . | . | . | . | . | . | . | . |
| logdha | rs182351085 | . | . | . | . | . | . | . | . | . | . | . |
| logdha | rs146248508 | . | . | . | . | . | . | . | . | . | . | . |
| logdha | rs188528439 | . | . | . | . | . | . | . | . | . | . | . |
| logdha | rs146167018 | . | . | . | . | . | . | . | . | . | . | . |
| logdha | rs192440980 | . | . | . | . | . | . | . | . | . | . | . |
| logdha | rs185601882 | . | . | . | . | . | . | . | . | . | . | . |
| logdha | rs117272563 | . | . | . | . | . | . | . | . | . | . | . |
| logdha | rs10430734 | . | . | . | . | . | . | . | . | . | . | . |
| logdha | rs181320952 | . | . | . | . | . | . | . | . | . | . | . |
| logdha | rs185480660 | . | . | . | . | . | . | . | . | . | . | . |
| logdha | rs188077917 | . | . | . | . | . | . | . | . | . | . | . |
| logdha | rs187562953 | . | . | . | . | . | . | . | . | . | . | . |
| logdha | rs182044631 | . | . | . | . | . | . | . | . | . | . | . |
| logdha | rs145455596 | . | . | . | . | . | . | . | . | . | . | . |
| logdha | rs181475877 | . | . | . | . | . | . | . | . | . | . | . |
| logdha | rs149413085 | . | . | . | . | . | . | . | . | . | . | . |
| logdha | rs12253656 | . | . | . | . | . | . | . | . | . | . | . |
| logdha | rs182718239 | . | . | . | . | . | . | . | . | . | . | . |
| logdha | rs192435695 | . | . | . | . | . | . | . | . | . | . | . |
| logdha | rs12248277 | . | . | . | . | . | . | . | . | . | . | . |
| logdha | rs143521710 | . | . | . | . | . | . | . | . | . | . | . |
| logdha | rs144454454 | . | . | . | . | . | . | . | . | . | . | . |
| logdha | rs190441610 | . | . | . | . | . | . | . | . | . | . | . |
| logdha | rs114690462 | . | . | . | . | . | . | . | . | . | . | . |
| logdha | rs184541357 | . | . | . | . | . | . | . | . | . | . | . |
| logdha | rs142657653 | . | . | . | . | . | . | . | . | . | . | . |
| logdha | rsurf1 | 2.462 | . | . | . | . | . | . | . | . | . | . |
| logdha | rsten1 | 2.569 | . | . | . | . | . | . | . | . | . | . |
| logdha | chr10:45852312:D | 0.198 | 0.355 | 0.24 | 0.126 | 1.61 | 1.67 | 0.645 | 0.741 | 0.523 | 0.671 | -0.543 |
| logdha | chr10:45853232:I | 0.2 | 0.339 | 0.231 | 0.112 | 1.37 | 1.42 | 0.761 | 0.818 | 0.561 | 0.769 | -0.329 |
| logdha | chr10:45853234:I | 0.194 | 0.339 | 0.231 | 0.112 | 1.4 | 1.5 | 0.751 | 0.818 | 0.576 | 0.776 | -0.354 |
| logdha | chr10:45854796:D | 0.053 | 0.374 | 0.177 | 0.038 | 123 | 696 | 0.395 | 0.035 | 0.144 | 0.419 | -121 |
| logdha | chr10:45854994:I | 0.054 | 0.374 | 0.177 | 0.038 | 123 | 697 | 0.395 | 0.034 | 0.144 | 0.419 | -121 |
| logdha | chr10:45858290:D | 0.048 | 0.379 | 0.22 | 0.094 | 17.3 | 76.4 | 0.518 | 0.089 | 0.265 | 0.416 | -16.1 |
| logdha | chr10:45858820:I | 0.005 | . | . | . | . | . | . | . | . | . | . |
| logdha | chr10:45860888:D | 0.063 | 0.321 | 0.189 | 0.054 | 1.72 | 3.59 | 0.795 | 0.659 | 0.797 | 0.73 | -0.678 |
| logdha | chr10:45863913:I | 0.195 | 0.349 | 0.236 | 0.12 | 1.5 | 1.55 | 0.695 | 0.772 | 0.527 | 0.705 | -0.444 |
| logdha | chr10:45866601:D | . | . | . | . | . | . | . | . | . | . | . |
| logdha | chr10:45867197:I | . | . | . | . | . | . | . | . | . | . | . |
| logdha | chr10:45867538:D | 0.198 | 0.339 | 0.23 | 0.111 | 1.4 | 1.43 | 0.742 | 0.823 | 0.554 | 0.772 | -0.349 |
| logdha | chr10:45868356:D | 0.197 | 0.343 | 0.234 | 0.117 | 1.46 | 1.48 | 0.713 | 0.805 | 0.543 | 0.748 | -0.396 |

| logdha | chr10:45871158:I | 0.198 | 0.343 | 0.234 | 0.117 | 1.45 | 1.48 | 0.713 | 0.805 | 0.543 | 0.748 | -0.395 |
| --- | --- | --- | --- | --- | --- | --- | --- | --- | --- | --- | --- | --- |
| logdha | chr10:45871637:D | 0.1 | 0.463 | 0.276 | 0.197 | 13.9 | 27.9 | 0.19 | 0.15 | 0.188 | 0.13 | -12.4 |
| logdha | chr10:45871638:I | 0.161 | 0.464 | 0.318 | 0.262 | 5.33 | 8.64 | 0.301 | 0.353 | 0.361 | 0.309 | -3.92 |
| logdha | chr10:45873629:I | 0.198 | 0.343 | 0.234 | 0.117 | 1.45 | 1.48 | 0.713 | 0.805 | 0.543 | 0.748 | -0.396 |
| logdha | chr10:45873701:D | . | . | . | . | . | . | . | . | . | . | . |
| logdha | chr10:45874997:I | . | . | . | . | . | . | . | . | . | . | . |
| logdha | chr10:45882030:I | . | . | . | . | . | . | . | . | . | . | . |
| logdha | chr10:45883774:D | 0.003 | . | . | . | . | . | . | . | . | . | . |
| logdha | chr10:45885169:D | 0.176 | 0.295 | 0.194 | 0.064 | 0.893 | 0.991 | 0.919 | 1.06 | 0.769 | 0.935 | 0.091 |
| logdha | chr10:45886451:D | 0.744 | 0.749 | 1.14 | 0.85 | 1.17 | 1.7 | 0.913 | 0.469 | 0.446 | 0.426 | -0.509 |
| logdha | chr10:45891800:I | 0.85 | 0.341 | 0.677 | 0.588 | 0.699 | 1.14 | 0.826 | 0.901 | 0.934 | 0.92 | 0.175 |
| logdha | chr10:45904771:I | 0.87 | 0.198 | 0.412 | 0.437 | 0.396 | 0.663 | 0.58 | 1.21 | 1.29 | 0.857 | 0.501 |
| logdha | chr10:45904959:I | 0.11 | 0.323 | 0.212 | 0.085 | 1.08 | 2.42 | 0.974 | 0.685 | 1.06 | 0.808 | -0.161 |
| logdha | chr10:45911028:I | 0.105 | 0.441 | 0.308 | 0.241 | 8.25 | 19.6 | 0.374 | 0.161 | 0.303 | 0.332 | -7.1 |
| logdha | chr10:45911031:I | 0.419 | 0.242 | 0.192 | 0.074 | 0.442 | 0.441 | 0.413 | 1.41 | 1.02 | 0.635 | 0.467 |
| logdha | chr10:45912154:D | 0.003 | . | . | . | . | . | . | . | . | . | . |
| logdha | chr10:45913885:I | 0.043 | 0.353 | 0.166 | 0.027 | 69.6 | 410 | 0.472 | 0.075 | 0.311 | 0.532 | -67.1 |
| logdha | chr10:45914753:D | 0.159 | 0.251 | 0.175 | 0.047 | 0.976 | 1.39 | 0.987 | 1.5 | 1.37 | 0.66 | 0.139 |
| logdha | chr10:45919621 | . | . | . | . | . | . | . | . | . | . | . |
| logdha | chr10:45921739:I | 0.048 | 0.336 | 0.16 | 0.022 | 15.6 | 71.2 | 0.547 | 0.213 | 0.665 | 0.621 | -13.8 |
| logdha | chr10:45923527:I | 0.062 | 0.325 | 0.209 | 0.08 | 2.37 | 15.1 | 0.893 | 0.521 | 2.57 | 0.895 | -1.29 |
| logdha | chr10:45927001:I | 0.005 | . | . | . | . | . | . | . | . | . | . |
| logdha | chr10:45930354:I | 0.044 | 0.334 | 0.16 | 0.022 | 13.7 | 61.6 | 0.559 | 0.242 | 0.741 | 0.643 | -12 |
| logdha | chr10:45932864:I | 0.065 | 0.361 | 0.175 | 0.036 | 17.9 | 84 | 0.539 | 0.174 | 0.566 | 0.591 | -16.1 |
| logdha | chr10:45936163:I | 0.027 | 0.354 | 0.206 | 0.074 | 34.5 | 71.8 | 0.088 | 0.102 | 0.155 | 0.135 | -32.6 |
| logdha | chr10:45938095:D | 0.058 | 0.374 | 0.218 | 0.091 | 14.8 | 19.4 | 0.04 | 0.197 | 0.197 | 0.105 | -13.1 |
| logdha | chr10:45938886 | . | . | . | . | . | . | . | . | . | . | . |
| logdha | chr10:45939320 | . | . | . | . | . | . | . | . | . | . | . |
| logdha | chr10:45939659 | . | . | . | . | . | . | . | . | . | . | . |
| logdha | chr10:45941150:D | 0.042 | 0.335 | 0.16 | 0.022 | 14.8 | 67 | 0.551 | 0.231 | 0.713 | 0.635 | -13 |
| logdha | chr10:45947128:D | 0.038 | 0.237 | 0.136 | 0.012 | 0.245 | 0.555 | 0.535 | 4.66 | 6.89 | 0.297 | 0.789 |
| logdha | chr10:45950990:I | 0.052 | 0.346 | 0.165 | 0.026 | 16.3 | 84.4 | 0.589 | 0.189 | 0.677 | 0.642 | -14.6 |
| logdha | chr10:45951854:I | . | . | . | . | . | . | . | . | . | . | . |
| logdha | chr10:45952670:I | 0.001 | . | . | . | . | . | . | . | . | . | . |
| logdha | chr10:45956741 | . | . | . | . | . | . | . | . | . | . | . |
| logdha | chr10:45957247:I | 0.065 | 0.383 | 0.222 | 0.099 | 18.6 | 23.9 | 0.023 | 0.159 | 0.154 | 0.058 | -16.9 |
| logdha | chr10:45958082:I | 0.065 | 0.383 | 0.223 | 0.099 | 18.4 | 23.7 | 0.023 | 0.161 | 0.156 | 0.059 | -16.7 |
| logdha | chr10:45905041:D | 0.05 | 0.364 | 0.171 | 0.032 | 84.3 | 493 | 0.448 | 0.052 | 0.219 | 0.481 | -82.1 |
| logdha | chr10:45938871 | 0.003 | . | . | . | . | . | . | . | . | . | . |
| logdha | chr10:45939160 | 0.004 | . | . | . | . | . | . | . | . | . | . |
| logdha | chr10:45869910 | . | . | . | . | . | . | . | . | . | . | . |
| logdha | chr10:45876214:D | . | . | . | . | . | . | . | . | . | . | . |
| logdha | chr10:45880237:I | . | . | . | . | . | . | . | . | . | . | . |
| logdha | chr10:45884371:I | . | . | . | . | . | . | . | . | . | . | . |
| logdha | chr10:45891535:D | . | . | . | . | . | . | . | . | . | . | . |
| logdha | chr10:45893516:D | . | . | . | . | . | . | . | . | . | . | . |
| logdha | chr10:45893782:D | . | . | . | . | . | . | . | . | . | . | . |
| logdha | chr10:45895254:I | . | . | . | . | . | . | . | . | . | . | . |
| logdha | chr10:45895848:D | . | . | . | . | . | . | . | . | . | . | . |
| logdha | chr10:45896316:D | . | . | . | . | . | . | . | . | . | . | . |
| logdha | chr10:45896317:D | . | . | . | . | . | . | . | . | . | . | . |
| logdha | chr10:45902683:I | . | . | . | . | . | . | . | . | . | . | . |
| logdha | chr10:45905003:D | . | . | . | . | . | . | . | . | . | . | . |
| logdha | chr10:45927476:D | . | . | . | . | . | . | . | . | . | . | . |
| logdha | chr10:45935882 | . | . | . | . | . | . | . | . | . | . | . |
| logdha | chr10:45936100 | . | . | . | . | . | . | . | . | . | . | . |
| logdha | chr10:45939553 | . | . | . | . | . | . | . | . | . | . | . |
| logdha | chr10:45939631 | . | . | . | . | . | . | . | . | . | . | . |
| logdha | chr10:45944193:D | . | . | . | . | . | . | . | . | . | . | . |
| logdha | chr10:45954030 | . | . | . | . | . | . | . | . | . | . | . |
